# CD96, a new immune checkpoint, correlates with immune profile and clinical outcome of glioma

**DOI:** 10.1038/s41598-020-66806-z

**Published:** 2020-07-01

**Authors:** Fangkun Liu, Jing Huang, Fengqiong He, Xiaodong Ma, Fan Fan, Ming Meng, Yang Zhuo, Liyang Zhang

**Affiliations:** 1 Department of Neurosurgery, Xiangya Hospital, Central South University, Central South University, 87 Xiangya Road, Changsha, Hunan 410008 China; 2 Clinical Diagnosis and Therapy Center for Glioma of Xiangya Hospital, Central South University, 87 Xiangya Road, Changsha, Hunan 410008 China; 3 Department of Psychiatry, The Second Xiangya Hospital, Central South University, Changsha, Hunan 410011 China; 4 Mental Health Institute of the Second Xiangya Hospital, Central South University, Chinese National Clinical Research Center on Mental Disorders (xiangya), Chinese National Technology Institute on Mental Disorders, Hunan Key Laboratory of Psychiatry and Mental Health, Changsha, Hunan 410011 China; 5 Director and Training and Exchange Cooperation Center, Orient Science & Technology College, Hunan Agricultural University, Changsha, Hunan 410000 China

**Keywords:** Cancer, Immunology, Biomarkers

## Abstract

CD96 is a promising candidate for immunotherapy. However, its role and importance in glioma remains unknown. We thus aimed to genetically and clinically characterize CD96 expression in gliomas. For this, we extracted RNA-seq data of 699 glioma samples from the TCGA dataset and validated these findings using the CGGA dataset comprising 325 glioma samples. Clinical and isocitrate dehydrogenase (IDH) mutation status were also analyzed. Various packages in R language were mainly used for statistical analysis. CD96 expression was significantly up-regulated in high-grade, IDH-wildtype, and mesenchymal-molecular subtype gliomas based on TCGA data, which was validated using the CGGA dataset. Subsequent gene ontology analysis of both datasets suggested that genes relevant to CD96 are mainly involved in immune functions in glioma as such genes were positively correlated with CD96 expression. To further explore the relationship between CD96 and immune responses, we selected seven immune-related metagenes and found that CD96 expression was positively correlated with HCK, LCK, and MHC II in the CGGA and TCGA cohorts but negatively associated with IgG. Further, Pearson correlation analysis showed that CD96 is associated with TIGIT, CD226, CRTAM, TIM-3, PD-L1, CTLA-4, and STAT3, indicating the additive antitumoral effects of these checkpoint proteins. CD96 was also suggested to play an important role in immune responses and positively collaborate with other checkpoint members. These findings show that CD96 is promising candidate for immunotherapy, and that such agents could complement current immunotherapy strategies for glioma.

## Introduction

Glioma, especially high-grade glioma (HGG) is the most common primary brain tumor. The highly infiltrative and aggressive features make it hardly be eliminated with current treatment strategies through surgery, radiotherapy and chemotherapy^1^. With the continuous exploration of alternative treatment options, people are raising their interest in glioblastoma(GBM) immunotherapy, especially checkpoint inhibitors targeting programmed cell death protein 1 (PD-1) and cytotoxic T lymphocyte-associated antigen-4 (CTLA-4)^2^. Constant progress has achieved in checkpoint inhibitor therapies in glioma, however, many patients respond poorly to single checkpoint inhibitor, thus exploring novel immune checkpoints are necessary for additive or synergetic anti-tumor purposes^2,3^.

Special attention has been accorded to CD96 as a novel immune checkpoint receptor target^4,5^. CD96 (TACTILE) is a member of the immunoglobulin (Ig) superfamily that interact with nectin and nectin-like proteins^6^. Other family members include CD226 (DNAM-1)^7^, T cell immunoglobulin and ITM domain (TIGIT)^8,9^, and class-I restricted T cell-associated molecule (CRTAM)^10^. Similar to the CD28/CTLA-4 pathway, CD96 and TIGIT act as co-inhibitory receptors together with the co-stimulatory receptor CD226, which supports the contention that CD96 and TIGIT could be targeted for cancer immunotherapy^11^. Recent studies have also found that CD96 promotes NK cell–target cell adhesion and inhibits cytokine responses induced by NK cells by competing with CD226 for CD155 binding^12^. It was also shown that CD96^−/−^ mice are more susceptible to lipopolysaccharide (LPS)-induced innate inflammation and exhibit increases in NK cell-mediated control of metastasis^13^. The authors further found that CD96 blockade can be more effective when combined with other therapies including anti-CTLA-4, anti-PD-1, or doxorubicin chemotherapy. Moreover, researchers found higher intratumoral CD96 expression of human hepatocellular carcinoma exhibit poorer clinical outcomes^14^. Another group found that CD96 controls cytokine and colitis-inducing potential of Th9 cells, indicating blockade of CD96-mediated immune inhibition would be a promising approach in reinforcement of Th9-mediated immune control of tumors^15^. Therefore, CD96 shows potential as a new checkpoint target to uncover drugs that could complement existing immune checkpoint blockade therapies.

As a promising candidate for immunotherapy, CD96 plays an important role in anti-tumor immune responses. In addition to its function in the immune response, CD96 was also reported to be a cancer stem cell marker that is presented on acute myeloid leukemia stem cells (LSC), which makes it a potential LSC-specific therapeutic target^16^. However, upon review of the related literature, we could not find any comprehensive reports of CD96 expression in glioma. Therefore, we analyzed RNA-seq data of 699 glioma samples from The Cancer Genome Atlas (TCGA) dataset to investigate its expression in glioma. To overcome the limitations of individual studies, we validated our findings using the Chinese Glioma Genome Atlas (CGGA) dataset, which includes 325 glioma samples. To the best of our knowledge, this is the first integrative study to molecularly and clinically characterize CD96 expression in gliomas. Moreover, we believe that a better understanding of CD96 in glioma will help to further develop cancer immunotherapies.

## Results

### CD96 expression is significantly up-regulated in WHO grade IV and IDH-wildtype glioma

**Figure 1:**
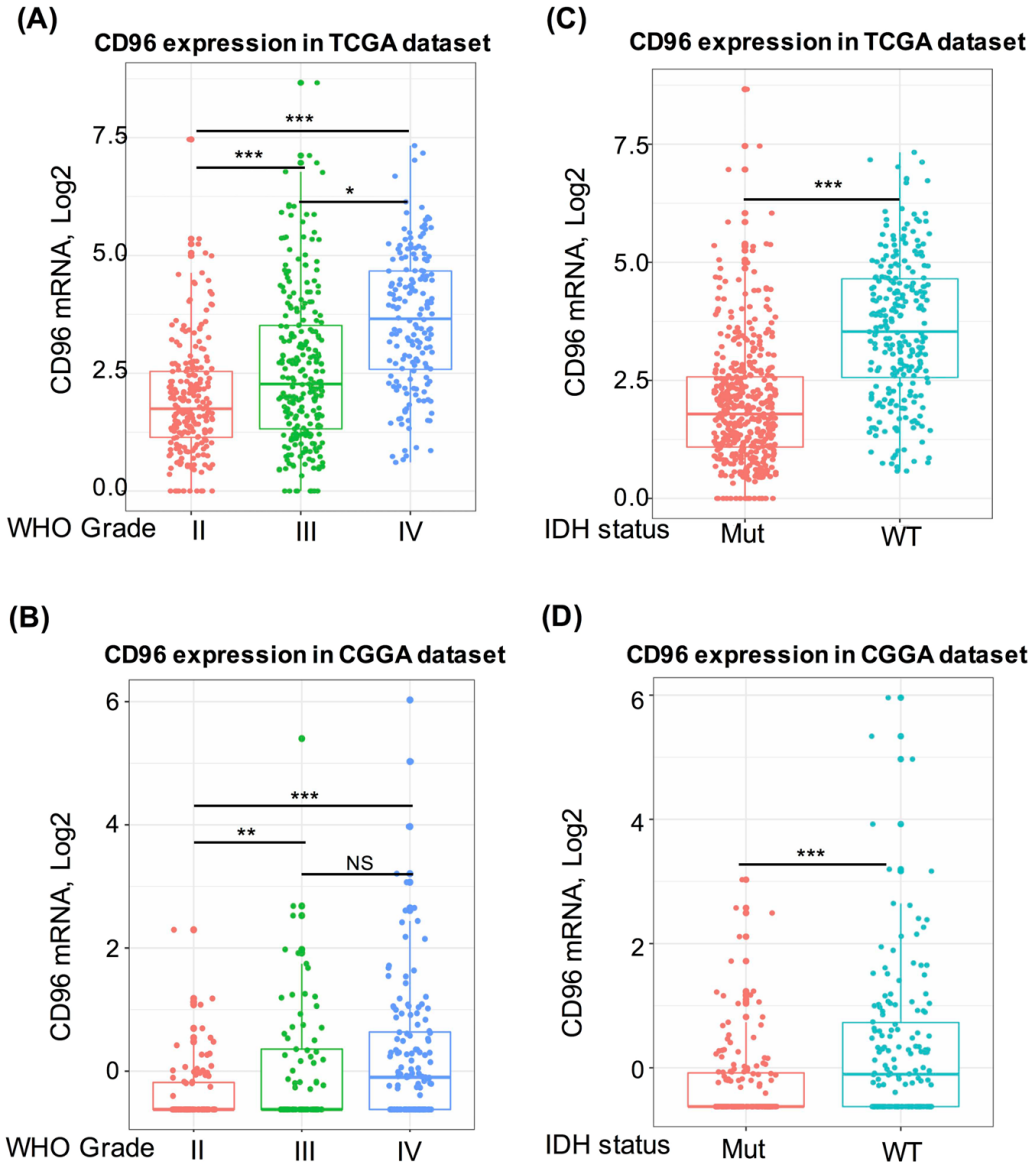
The association of CD96 expression with clinical glioma parameters. CD96 expression in the TCGA dataset according to WHO grade (**A**) and isocitrate dehydrogenase (IDH) status (**C**); CD96 expression in CGGA dataset according to WHO grade (**B**) and IDH status (**D**). *Indicates p value < 0.05, **Indicates p value < 0.01, ***Indicates p value < 0.001.

We extracted and analyze RNA-seq data from TCGA and CGGA datasets to find out the expression pattern of CD96 in gliomas. We found that CD96 expression was significantly higher in higher grade gliomas; when compared to that in WHO grade II and grade III gliomas, WHO grade IV glioma (glioblastoma) showed the highest expression, and the results were consistent in both TCGA dataset and CGGA dataset (Fig. 1A,B). The recent identification of mutations in IDHs has contributed to the development of new molecular markers for the classification of different subtypes of gliomas^17^. Therefore, we analyzed the expression pattern of CD96 based on IDH mutation status. This marker was significantly up-regulated in IDH-wildtype gliomas based on both TCGA and CGGA datasets (Fig. 1C,D). Our results also indicated that CD96 checkpoint-related immune responses were more prevalent in IDH-wildtype glioma.

### CD96 expression is higher in mesenchymal-molecular subtype glioma

**Figure 2:**
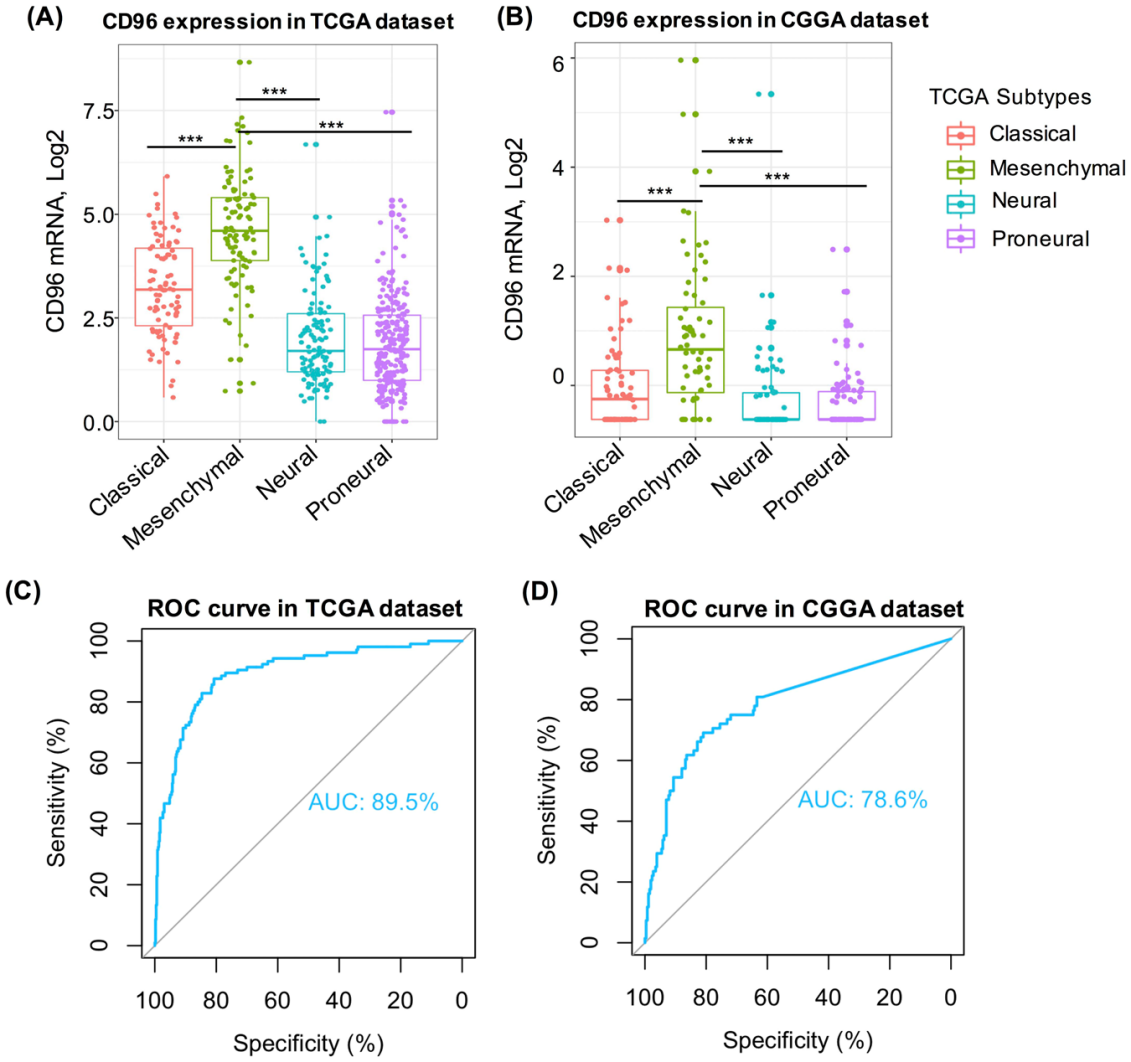
Relationship between CD96 expression and glioma molecular subtypes. CD96 expression pattern in different molecular subtypes of gliomas based on TCGA (**A**) and CGGA (**B**) datasets. ROC curve analysis revealed the predictive value of CD96 in the mesenchymal-subtype based on both datasets (**C**,**D**).

We further analyzed the transcript levels of *CD96* in different glioma subtypes. Recent studies have demonstrated that different subtypes of glioma are linked with prognoses and treatment responses^18^. Based on the TCGA dataset and CGGA dataset, our results showed that *CD96* expression was much higher in mesenchymal-molecular subtype glioma, compared to that in the other three subtypes (Fig. 2A,B). We also next conducted receiver operating characteristic curves (ROC curves) for CD96 and the mesenchymal subtype for these two datasets. The area under the curve (AUC) was 89.5% and 78.6% for TCGA and CGGA datasets, respectively (Fig. 2C,D) and these results indicated that CD96 can serve as a potential biomarker for the mesenchymal-molecular subtype of glioma.

### CD96 is closely related to immune functions in glioma

**Figure 3:**
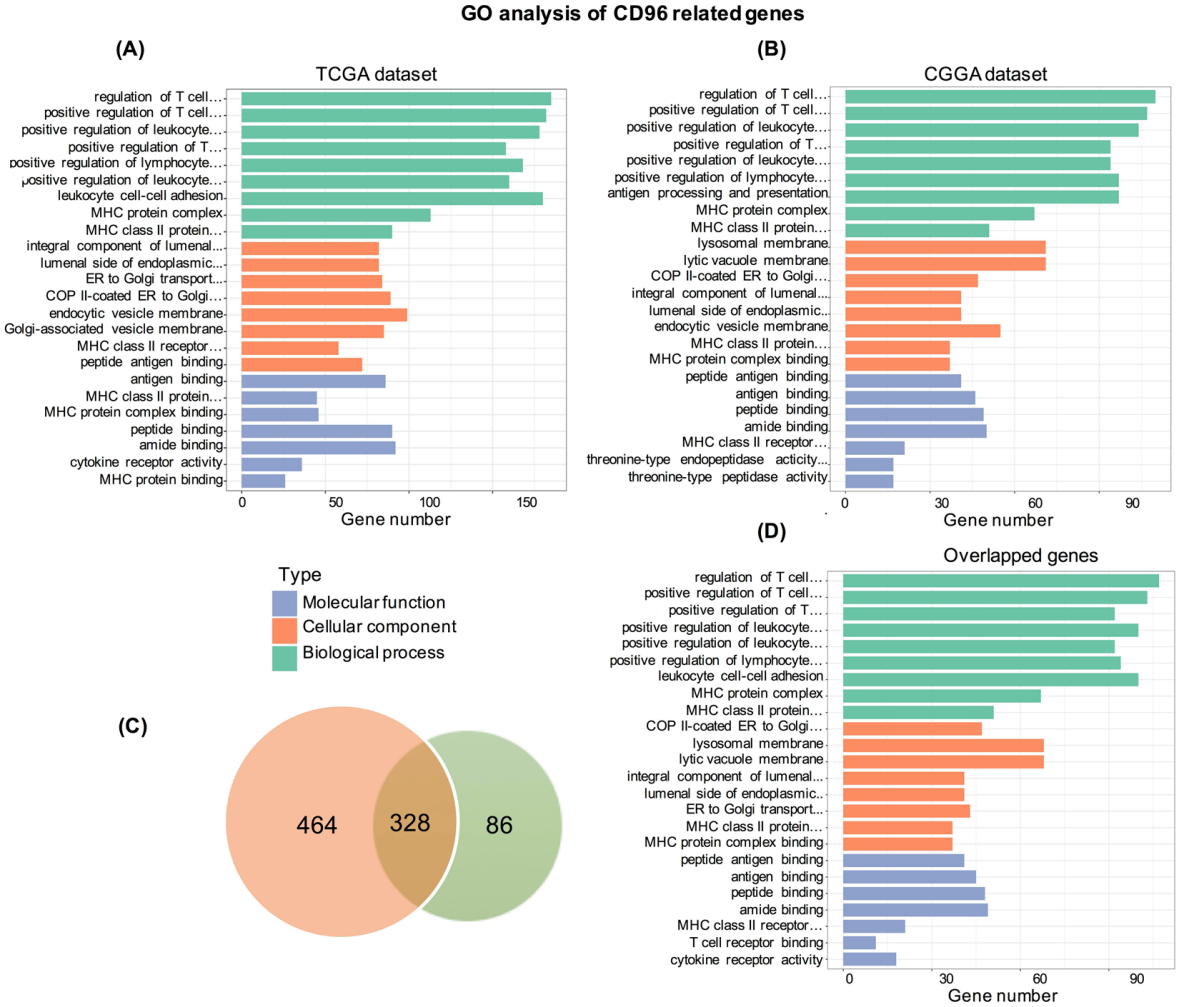
Gene ontology (GO) analysis of CD96-related signatures in gliomas. Results were based on in TCGA (**A**) and CGGA (**B**) datasets; 328 genes common to both datasets (**C**,**D**) were used for GO analysis and validation.

To investigate the biological signatures associated with CD96 in gliomas, we ranked the related genes based on Spearman correlation analysis (|R| > 0.4 and p < 0.05). A total of 792 and 414 genes were identified in TCGA and CGGA datasets, respectively (Table S1). Then, we performed GO functional analysis of these genes to identify enriched biological processes and functions using the DAVID website. This showed consistent results for TCGA and CGGA datasets (Fig. 3A,B), and genes most related to CD96 were mainly involved in the regulation of T cells, leukocyte and lymphocyte regulation, leukocyte cell–cell adhesion, the MHC protein complex, and MHC class II proteins. In terms of biological process, genes positively related to CD96 were significantly enriched in antigen binding. We then explored the function of 328 genes shared by the two datasets which found similar results (Fig. 3C,D). These findings suggested that CD96 plays an important role in immune functions in gliomas. Moreover, we produced a gene-concept network based on the GO analysis results, which provided a detailed list of genes included in the top five significantly-enriched biological process terms. Supplement Fig. 1 depicts the linkage of immune genes and biological terms related to CD96 expression as a network.

**Figure 4:**
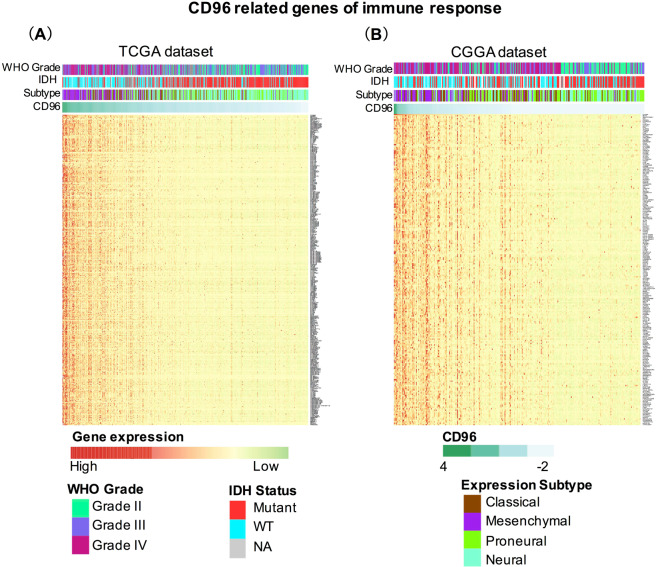
Heatmap analysis of the relationship between CD96 and immune function-related genes in glioma. Gene sets of immune system were downloaded from the AmiGO 2 website for TCGA (**A**) and CGGA (**B**) datasets.

To find out the immune functions of CD96 in gliomas, we downloaded gene sets of immune system in AmiGO 2 website (http://amigo.geneontology.org/amigo). We selected genes from TCGA and CGGA which are significantly related to CD96 (|R| > 0.4 and p < 0.05). In total, we found out 368 genes in TCGA and 251 immune-related genes in CGGA datasets which are closely linked to immune functions for heatmap analysis. These genes were positively correlated with CD96 expression (Fig. 4A,B). Table S2concluded the list of these genes from TCGA and CGGA datasets. Our results demonstrate that CD96 expression was significantly linked with immune functions in glioma.

### CD96-related inflammatory activities

**Figure 5:**
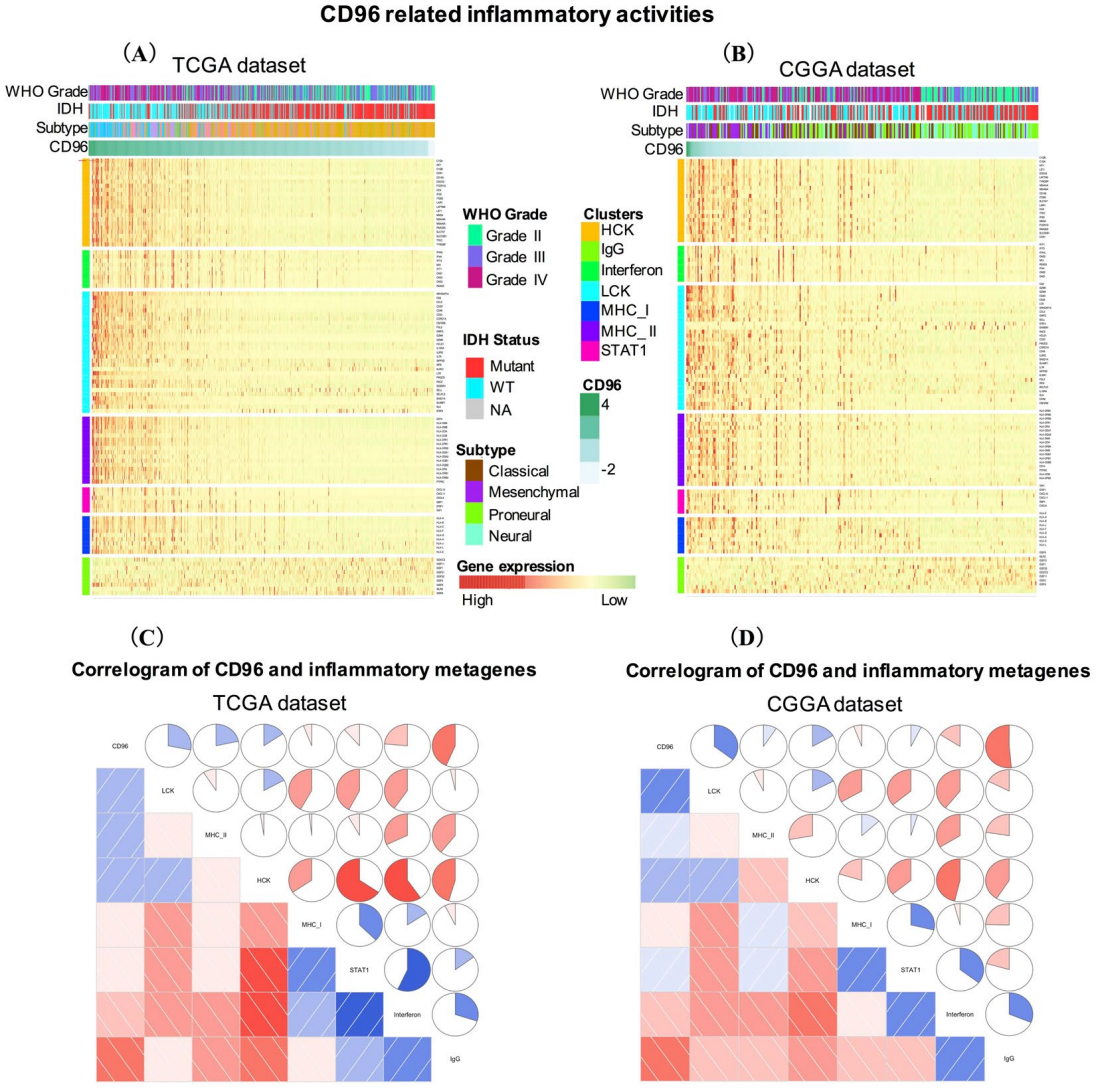
CD96-related inflammatory activities in gliomas. The relationship between CD96 and glioma grade, isocitrate dehydrogenase (IDH) status, molecular subtypes, and seven metagenes are presented as a heatmap (**A**,**B**). Correlogram show the correlation between CD96 and seven immune-related metagenes based on TCGA (**C**) and CGGA (**D**) datasets.

To further find out the role of CD96 in inflammatory activities, we include 104 inflammation and immune responses related-genes to generate seven metagenes^19^. Table S3 summarized the detailed list of these genes. Based on TCGA and CGGA datasets, most of the metagenes were positively associated with CD96 expression (Fig. 5A,B). To validate clustering results, we applied GSVA to transform the expression data of seven metagenes into enrichment scores. Then, correlograms were derived using R language according to the Pearson correlation between CD96 and the seven metagenes (Fig. 5C,D). GSVA results and heatmap analysis were highly consistent. Specifically, CD96 expression was positively correlated with HCK, LCK, and MHC II in the CGGA and TCGA cohorts but negatively associated with IgG, a marker for B cells. *CD96* transcript levels were found to be negatively associated with IgG and interferon levels.

Relationships between CD96 and tumor infiltrating immune cells including CD8+ T cells, neutrophils, myeloid-derived suppressor cells (MDSCs), regulatory T cells (Tregs), Macrophages (Macro), and natural killer (NK) cells were also analyzed by examining the correlation of CD96 expression with immune cell-specific marker genes expression1. As shown in Supplement Fig. 2, strong positive correlations between CD96 and the following immune cells were found: CD8+ T cells (r = 0.95 in TCGA dataset, r = 0.70 in CGGA dataset), Tregs (r = 0.95 in TCGA dataset, r = 0.73 in CGGA dataset), and macrophages (r = 0.76 in TCGA dataset, r = 0.62 in CGGA dataset). These results demonstrated that higher CD96 expression in gliomas have more immune cells infiltration compared with gliomas with low CD96 expression.

### Correlation between CD96 and immune checkpoints

**Figure 6:**
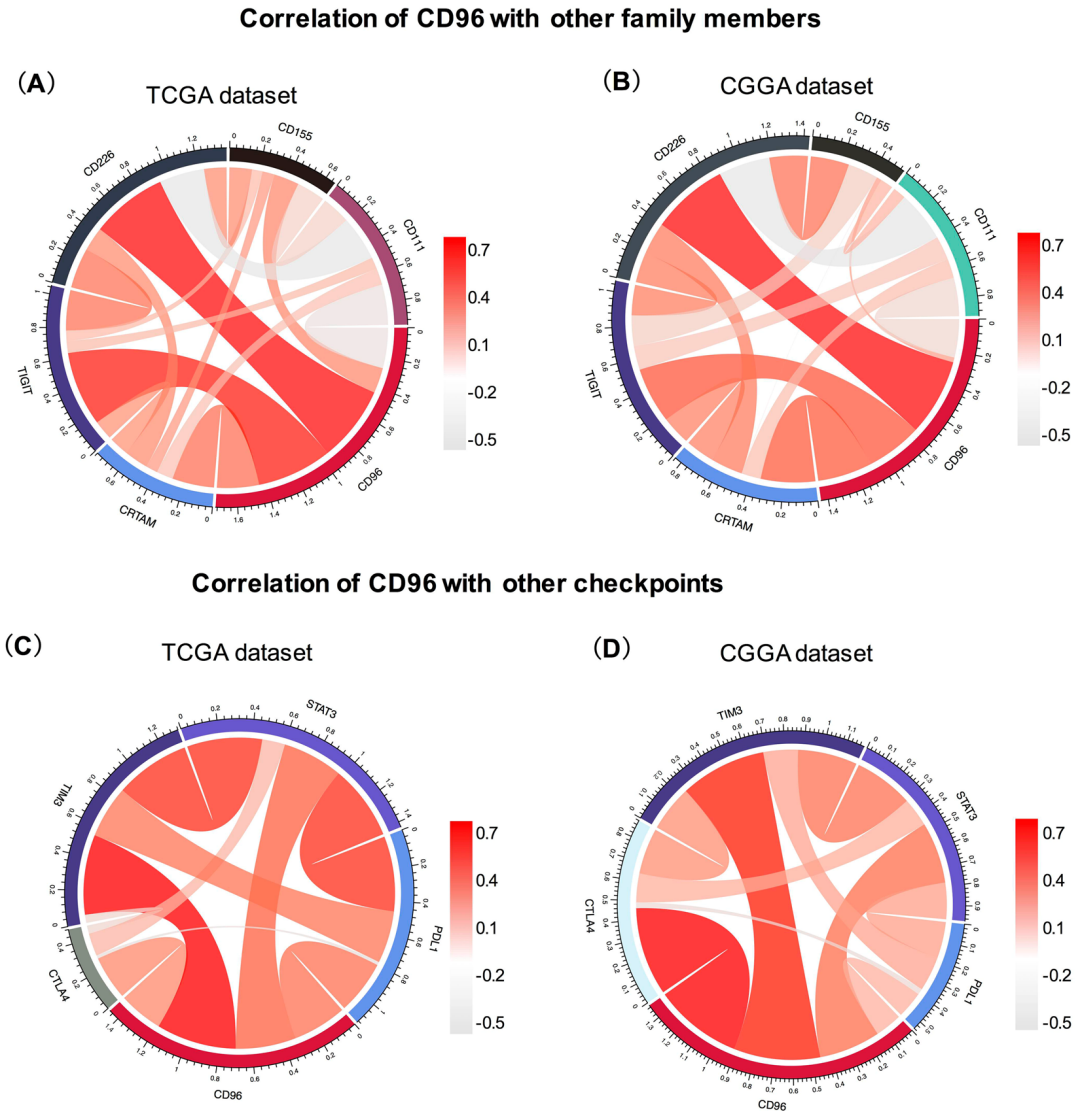
Association between CD96 and immune checkpoint markers in glioma. The correlations between CD96 and immunoglobulin family members including TIGIT, CD226, and CRTAM based on TCGA (**A**) and CGGA (**B**) datasets are presented. Ligands CD111 and CD115, which bind CD96, were also included in the analysis. Correlation between CD96 and other immune checkpoint markers that have been examined in clinical trials or clinical situations, including PD-L1, CTLA-4, TIM-3, and STAT3, were also subjected to the analysis (**C**,**D**).

As a transmembrane Ig superfamily receptor, CD96 is mainly expressed on various types of T cells and NK cells. Members in this family are gaining increasing attention as immune checkpoint receptor targets for cancer immunotherapy^11,20,21^. Therefore, we explored the relationship among CD96, TIGIT, CD226, and CRTAM. The ligands CD111 and CD115, which bind CD96, were also included in the analysis. Then, we performed Pearson correlation analysis of these six genes using both TCGA and CGGA datasets. CD96 showed strong positive concordance with TIGIT, CD226, and CRTAM in both datasets. However, it was only weakly associated with CD111, as indicated by the results (Fig. 6A,B). Moreover, we analyzed the relationship between CD96 and other immune checkpoint markers that have been examined in clinical trials or clinical situations. Specifically, PD-L1, CTLA-4, TIM-3, and STAT3 were subjected to this analysis^22,23^. Circos plots demonstrated that CD96 was tightly associated with TIM-3, PD-L1, CTLA-4, and STAT3 based on both TCGA and CGGA datasets (Fig. 6C,D), indicating the synergistic effects of CD96 with these checkpoint members.

### CD96 predicts worse survival in glioma patients

**Figure 7:**
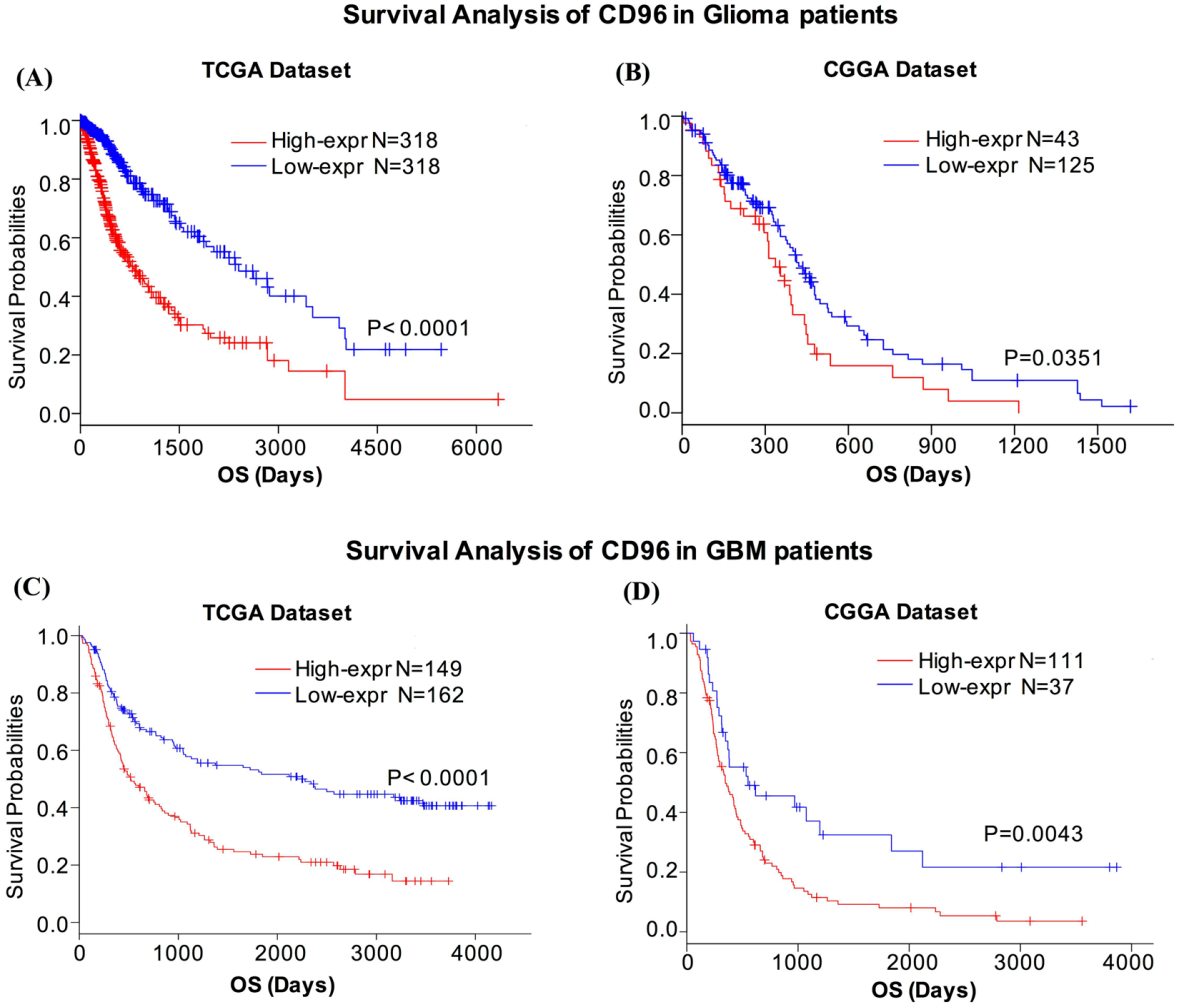
CD96 as a malignant biological indicator in glioma. Results show that higher CD96 expression predicted worse overall survival in patients with glioma (**A**,**C**) and GBM (**B**,**D**) for both TCGA and CGGA datasets.

As we discussed above, CD96 expressed higher in higher grades of glioma in TCGA and CGGA datasets, indicating CD96 could be a potential malignant biological indicator in glioma. Therefore, the prognostic value of CD96 were analyzed through these two glioma datasets. The Kaplan–Meier curve of the overall survival (OS) of patients with gliomas was depicted in Fig. 7. As shown, higher CD96 expression predicted worse overall survival for both TCGA and CGGA datasets (Fig. 7A,B). We also detected the prognostic value of CD96 expression in glioblastoma (GBM). Similarly, a strong association was observed between higher expression of CD96 and shorter patient OS for both datasets (Fig. 7C,D). These findings showed that CD96 is a negative prognostic marker in glioma.

We then performed a Cox multivariate analysis of survival to isolate important prognostic factors such as WHO grade, IDH status, and molecular subtype. The results showed that higher CD96 expression predicted worse overall survival in CGGA dataset after set these factors as covariates (Supplement Fig. 3).

## Discussion

In this study, we analyzed immune profile and clinical outcome of CD96 from both TCGA and CGGA datasets. The results demonstrated higher CD96 expression in higher WHO grade, IDH-wildtype, and mesenchymal-subtype gliomas. Previous studies have found highest expression patterns of distinct glioma antigens and immune genes in mesenchymal subtype glioma, which were consistent with our findings^24,25^. Similar expression patterns were also reported for other immune checkpoints such as PD-L1^26^, B7-H3^27^, TIM-3^28^, and PTPN2^29^ in glioma. Moreover, CD96 was found to predict worse survival for glioma and GBM patients. These results indicated a malignant biological property for CD96 in glioma.

Subsequent GO analysis suggested that genes most relevant to CD96 were mainly involved in immune functions in gliomas and that immune genes were significantly positively correlated with CD96 expression. Emerging data has demonstrated a critical role for CD96 in tumor immunology. Specifically, it can modulate NK and T cell activity in cancers and regulate tumor immune surveillance^30^. CD96 was also shown to negatively control cytokine responses mediated by NK cells^13^ and immunological stress led to NK cell High reactivity in CD96-deficient mice^5^. In the analysis of CD96 and immune metagenes, we found that CD96 expression was correlated with T-cell- and macrophage-related, but not B cell-related immune responses. Our results show that CD96 is a negative prognostic marker in glioma and plays an important role in the immune response.

As a member of the immunoglobulin family, CD96 shares its ligand CD155 with CD226 and TIGIT; however, the relationship between CD96 and other family members is unknown. Therefore, we analyzed the relationship between CD96 and other family members. We observed that CD96 was tightly associated with TIGIT, CD226, and CRTAM. TIGIT and CD226 are well-studied receptors that bind CD155 (necl-5) and CD112 (nectin-2). Moreover, CD96 shares its ligand CD155 with CD226 and TIGIT, but also binds CD111 (nectin-1)^11^. CD115 and CD111, which bind CD96, were also included in our analysis. Compared to its relationship with CD115, the correlation between CD96 and CD111 was very weak, which could be accounted for by noise. This is consistent with previous studies that demonstrated some differences between mouse CD96 and human CD96; specifically, CD96 binding to nectin 1 (CD111) has only been observed in mouse models^11,31^.

Clinical trials of checkpoint inhibitors for gliomas, have demonstrated the profound clinical benefits of immunotherapies that target combined checkpoint inhibitory pathways, compared to that using monotherapy treatments^32,33^. Crucially, combination treatment approaches were more effective and associated with significantly better clinical outcome than monotherapy^32^. One study using mice bearing B16F10 lung metastases found that an anti-CD96 mAb alone was more effective than either anti-CTLA-4 or anti-PD-1 to control both experimental and spontaneous metastases. Moreover, the combination of anti-CD96 with anti-CTLA-4 or anti-PD-1 showed enhanced anti-metastatic activity and survival compared to those with each monotherapy^5^. Our results showed that CD96 is tightly associated with the checkpoint proteins TIM-3, PD-L1, CTLA-4, and STAT3, indicating the potential synergistic effects of these markers.

There are several limitations of this study. Firstly, the expression level of CD96 in gliomas can be affected by tumor purity. The non-tumor cells in tumor tissues dilute the purity of tumor cells and play important roles in tumor biology. In this study, we extracted and analyze RNA-seq data from TCGA and CGGA databases and didn’t account for tumor purity. Several statistical methods have analyzed the DNA methylation microarray data from TCGA and CGGA datasets to analyze tumor purity. Distribution of estimated tumor purities from InfiniumPurify for gliomas were 0.7–0.8 in both datasets, which suggested good purity in these samples^34,35^. Besides, Zhang *et al*.^35^ found immune cells such as macrophage, microglia, and neutrophils were enriched in low purity glioma and indicate poor prognosis, which suggested the possibility of high expression of CD96 may partly due to relatively lower purity. Because higher expression of CD96 was associated with more immune cell responses and poorer prognosis. Moreover, it will be necessary to further explore our hypothesis by examine CD96 protein level using large sample size to confirm the important role of CD96 in gliomas.

## Conclusions

In summary, our study is the first to explore the expression and clinical characteristics of CD96 in gliomas. Moreover, CD96 is a promising immunotherapy target that positively collaborates with other checkpoint proteins in glioma. Future research is needed to further explore multi-checkpoint blockade combined with CD96 inhibitors for glioma treatment.

## Materials and Methods

### Sample and data collection

From the TCGA dataset, the clinicopathological characteristics and RNA-seq data from 697 glioma samples of all grades, ranging from WHO grade II to grade IV, were analyzed in our study (http://cancergenome.nih.gov). To maintain consistency, we also validated our findings using 325 glioma samples from the CGGA dataset. The transcriptome sequencing data from CGGA were generated using the Illumina Hiseq platform which can be downloaded from public datasets (http://www.cgga.org.cn/).

### Related signature identification

Gene expression profiling data were log-transformed for further analysis. Gene ontology (GO) analysis was performed to analyze related genes and biological functions using the DAVID website (http://david.ncifcrf.gov/)^36^. The gene set enrichment analysis (GSVA) package of R language was employed to determine the enrichment status of inflammatory response-associated metagenes^37^.

### Statistical analysis

R language was mainly used to perform statistical analysis^38^. A Student’s t test was performed to evaluate CD96 expression differences between grades, isocitrate dehydrogenase (IDH) mutation statuses, and subtypes of gliomas. Pearson correlation and correlograms were performed using the “circlize” package^39^ and “corrgram” package, respectively, and ROC curves were derived using the “pROC” package^40^. The prognostic value of CD96 was investigated by Kaplan–Meier analysis using R language (survival package)^41^. A heatmap was generated by clustering (using the R package “pheatmap”)^42^ based on p-values < 0·05 between two groups. All statistical tests were two-sided and p < 0.05 was considered a significant difference.

## Supplementary information

Supplementary Information.

Supplementary Information.

Supplementary Information.

Supplementary Information.

Supplementary Information.

Supplementary Information.

Supplementary Information.

Supplementary Information.
