# Supplementary material for: CD96, a new immune checkpoint, correlates with immune profile and clinical outcome of glioma
Source: Sci Rep. 2020 Jul 1;10:10768. doi: 10.1038/s41598-020-66806-z (PMC7330044; doi:10.1038/s41598-020-66806-z)
Supplement: Supplementary file 4 — Supplementary Information. [file 41598_2020_66806_MOESM4_ESM.pdf]

CD96, a new immune checkpoint, correlates with immune profile and clinical outcome of glioma

Fangkun Liu[1, 2 †], Jing Huang[3, 4], Fengqiong He[1, 2], Xiaodong Ma[5], Fan Fan[1, 2], Ming Meng[1, 2], Yang Zhuo[1, 2], and Liyang Zhang[1, 2 *]

[1] Department of Neurosurgery, Xiangya Hospital, Central South University, Central South University; 87 Xiangya Road; Changsha, Hunan, 410008. China;

[2] Clinical Diagnosis and Therapy Center for Glioma of Xiangya Hospital, Central South University; 87 Xiangya Road; Changsha, Hunan, 410008. China;

[3] Department of Psychiatry, The Second Xiangya Hospital, Central South University, Changsha, Hunan 410011, China;

[4] Mental Health Institute of the Second Xiangya Hospital, Central South University, Chinese National Clinical Research Center on Mental Disorders (xiangya), Chinese National Technology Institute on Mental Disorders, Hunan Key Laboratory of Psychiatry and Mental Health, Changsha, Hunan 410011, China;

[5] Director and Training and Exchange Cooperation Center, Orient Science & Technology College, Hunan Agricultural University, Changsha, Hunan 410000, China.

[†]The first author;

[*]Corresponding Author:

Dr. Liyang Zhang MD, Ph. D

Department of Neurosurgery, Xiangya Hospital, Central South University

87 Xiangya Rd, Changsha, Hunan, 410008, China,

Email: zhangliyang@csu.edu.cn

gene	cor	t.value	p.value	FDR	significant
CD2	0.777018014	22.18459697	6.71E-67	1.44E-62	yes
ICOS	0.731387712	19.27475281	1.22E-55	1.31E-51	yes
CCL5	0.730427927	19.22048863	1.99E-55	1.42E-51	yes
CD3G	0.729309148	19.15754316	3.50E-55	1.88E-51	yes
TRAT1	0.710687967	18.15564051	2.90E-51	1.24E-47	yes
CD3D	0.709311417	18.08479656	5.48E-51	1.96E-47	yes
CD5	0.704516793	17.84126183	4.93E-50	1.51E-46	yes
GZMK	0.702751583	17.75283319	1.09E-49	2.94E-46	yes
CD3E	0.697066928	17.47241395	1.37E-48	3.27E-45	yes
CXCR6	0.666781761	16.07982138	3.76E-43	8.09E-40	yes
ANO9	0.663241203	15.92706187	1.48E-42	2.89E-39	yes
CD27	0.653857694	15.53131006	5.09E-41	9.12E-38	yes
TBC1D10C	0.65286613	15.49023705	7.35E-41	1.21E-37	yes
SIT1	0.647185684	15.25758452	5.84E-40	8.97E-37	yes
CD6	0.643993041	15.12875552	1.84E-39	2.64E-36	yes
IL2RG	0.6408432	15.00297737	5.62E-39	7.55E-36	yes
CD52	0.632402678	14.67219678	1.06E-37	1.33E-34	yes
CST7	0.62811363	14.50748039	4.52E-37	5.40E-34	yes
CXCL9	0.626226466	14.4357015	8.52E-37	9.63E-34	yes
P2RY10	0.616079382	14.05678082	2.38E-35	2.56E-32	yes
NKG7	0.614286621	13.99102854	4.24E-35	4.34E-32	yes
DENND2D	0.61031419	13.84656737	1.50E-34	1.46E-31	yes
IL2RB	0.607664507	13.75113736	3.45E-34	3.22E-31	yes
KLRB1	0.599581556	13.46447099	4.17E-33	3.73E-30	yes
CCR7	0.599229281	13.45212608	4.64E-33	3.99E-30	yes
GZMH	0.590323023	13.14399938	6.65E-32	5.49E-29	yes
TRAF3IP3	0.589205547	13.1058673	9.23E-32	7.35E-29	yes
ARHGAP15	0.587470331	13.04688387	1.53E-31	1.18E-28	yes
PTPLAD2	0.586529496	13.0150181	2.02E-31	1.49E-28	yes
LTB	0.584994926	12.96321486	3.14E-31	2.25E-28	yes
TNFSF8	0.584892121	12.95975201	3.24E-31	2.25E-28	yes
EMB	0.578379874	12.74230511	2.08E-30	1.40E-27	yes
CD80	0.574626047	12.61863583	5.96E-30	3.88E-27	yes
B2M	0.573940004	12.59616357	7.22E-30	4.56E-27	yes
SH2D1A	0.57185523	12.52811596	1.29E-29	7.90E-27	yes
PDCD1	0.569694147	12.45795829	2.33E-29	1.39E-26	yes
HLA-DRA	0.568682817	12.42525807	3.08E-29	1.79E-26	yes
GZMA	0.567812399	12.39718076	3.90E-29	2.21E-26	yes
DAPP1	0.566629748	12.35912997	5.38E-29	2.96E-26	yes
GPR171	0.565617598	12.32665427	7.08E-29	3.80E-26	yes
IL9R	0.565221442	12.31396563	7.88E-29	4.13E-26	yes
CD48	0.562926654	12.24071013	1.46E-28	7.47E-26	yes
LOC10050677б	0.561633224	12.19960352	2.06E-28	1.03E-25	yes
P2RY8	0.558091855	12.08772062	5.28E-28	2.58E-25	yes
CTSS	0.557583707	12.07174581	6.03E-28	2.88E-25	yes
LCP1	0.55720493	12.05985089	6.66E-28	3.11E-25	yes
RASSF5	0.555066517	11.99290134	1.17E-27	5.33E-25	yes
LCK	0.554138126	11.96394251	1.49E-27	6.65E-25	yes
HLA-DOA	0.552649812	11.91765252	2.19E-27	9.58E-25	yes
GBP4	0.551105601	11.86979749	3.26E-27	1.40E-24	yes

LY9	0.549212326	11.81136387	5.29E-27	2.23E-24	yes
CCR2	0.549095124	11.80775514	5.45E-27	2.25E-24	yes
LINC00426	0.547777239	11.76724532	7.63E-27	3.09E-24	yes
ZAP70	0.545184618	11.68791636	1.47E-26	5.86E-24	yes
GBP5	0.541068185	11.56293938	4.13E-26	1.61E-23	yes
HSH2D	0.540995452	11.56074183	4.21E-26	1.61E-23	yes
PTGER4	0.540748712	11.55328953	4.47E-26	1.69E-23	yes
SLA2	0.540443306	11.54407115	4.83E-26	1.79E-23	yes
RAC2	0.54007328	11.53291095	5.29E-26	1.93E-23	yes
FPR3	0.539377495	11.51195121	6.29E-26	2.25E-23	yes
APOBEC3G	0.538937899	11.49872603	7.01E-26	2.47E-23	yes
SASH3	0.537504593	11.455697	9.98E-26	3.46E-23	yes
LOC100130231	0.534691467	11.37164955	1.99E-25	6.78E-23	yes
CSTA	0.534275632	11.35927079	2.20E-25	7.38E-23	yes
GVINP1	0.532953587	11.31999219	3.03E-25	1.00E-22	yes
SP100	0.530313819	11.24190898	5.73E-25	1.87E-22	yes
FASLG	0.529529977	11.21881115	6.91E-25	2.22E-22	yes
ACAP1	0.527004831	11.14467222	1.26E-24	3.99E-22	yes
NFAM1	0.525876781	11.11168479	1.65E-24	5.14E-22	yes
TLR1	0.525354745	11.09644642	1.87E-24	5.73E-22	yes
ELK2AP	0.522592508	11.01610283	3.57E-24	1.08E-21	yes
GRAP2	0.52234695	11.00898363	3.79E-24	1.13E-21	yes
STX11	0.52184395	10.99441246	4.26E-24	1.25E-21	yes
GPR65	0.519331286	10.92185935	7.64E-24	2.22E-21	yes
DOCK2	0.518872712	10.90866005	8.49E-24	2.43E-21	yes
PDCD1LG2	0.517386081	10.86595826	1.20E-23	3.38E-21	yes
MLKL	0.516546291	10.84189569	1.45E-23	4.05E-21	yes
LYZ	0.515736228	10.81872532	1.75E-23	4.81E-21	yes
HLA-DMB	0.515092761	10.80034832	2.02E-23	5.51E-21	yes
PLSCR1	0.514176263	10.77421661	2.49E-23	6.70E-21	yes
CTBS	0.513976524	10.76852821	2.61E-23	6.93E-21	yes
IL12RB1	0.512151505	10.71666306	3.95E-23	1.04E-20	yes
GAPT	0.512038171	10.71344874	4.05E-23	1.05E-20	yes
ITGAL	0.511982442	10.71186846	4.10E-23	1.05E-20	yes
CD247	0.511369975	10.69451308	4.71E-23	1.18E-20	yes
HLA-DMA	0.511349838	10.69394285	4.73E-23	1.18E-20	yes
LAPTM5	0.509608968	10.6447344	7.00E-23	1.73E-20	yes
SIRPG	0.508544505	10.6147328	8.89E-23	2.17E-20	yes
LAP3	0.508166751	10.60410169	9.67E-23	2.33E-20	yes
HLA-E	0.508066902	10.60129303	9.89E-23	2.36E-20	yes
RNASE6	0.507577447	10.58753343	1.10E-22	2.60E-20	yes
CD72	0.507323181	10.58039094	1.17E-22	2.73E-20	yes
RIPK3	0.505051416	10.51674013	1.93E-22	4.46E-20	yes
CMAHP	0.503136685	10.46332084	2.94E-22	6.73E-20	yes
CD7	0.502118045	10.43498589	3.68E-22	8.33E-20	yes
NMI	0.501847235	10.42746268	3.91E-22	8.69E-20	yes
TFEC	0.501825943	10.42687136	3.92E-22	8.69E-20	yes
ARHGDIB	0.500808123	10.39863383	4.90E-22	1.07E-19	yes
CYP1B1	0.500536669	10.39111257	5.20E-22	1.13E-19	yes
ADAP2	0.500418616	10.38784289	5.33E-22	1.15E-19	yes
CD86	0.500271524	10.38377004	5.51E-22	1.17E-19	yes

ITGB7	0.499838367	10.37178329	6.05E-22	1.27E-19	yes
ARHGAP30	0.499787444	10.37037477	6.12E-22	1.28E-19	yes
CREG1	0.499738245	10.36901407	6.18E-22	1.28E-19	yes
FGL2	0.499568253	10.36431364	6.42E-22	1.31E-19	yes
SCPEP1	0.498454477	10.33355622	8.17E-22	1.66E-19	yes
SLAMF1	0.498128461	10.32456603	8.76E-22	1.76E-19	yes
37135	0.497781842	10.3150141	9.44E-22	1.88E-19	yes
HLA-DPB1	0.497729283	10.31356628	9.55E-22	1.88E-19	yes
38961	0.497459738	10.3061436	1.01E-21	1.98E-19	yes
ABHD15	0.49666003	10.28414478	1.20E-21	2.33E-19	yes
PIK3AP1	0.496604463	10.2826175	1.22E-21	2.33E-19	yes
CD69	0.493686888	10.20266159	2.27E-21	4.32E-19	yes
PLEK	0.493104279	10.18675003	2.57E-21	4.84E-19	yes
RASAL3	0.493051057	10.18529741	2.60E-21	4.86E-19	yes
TPRG1	0.492663586	10.17472638	2.82E-21	5.23E-19	yes
CYBB	0.491883354	10.15346418	3.33E-21	6.11E-19	yes
IGJ	0.490963362	10.12843484	4.04E-21	7.36E-19	yes
CCDC69	0.49072682	10.1220067	4.25E-21	7.68E-19	yes
FYB	0.489191291	10.08034945	5.87E-21	1.05E-18	yes
MAP3K8	0.488920683	10.07302094	6.21E-21	1.10E-18	yes
IFITM3	0.48888884	10.07215884	6.25E-21	1.10E-18	yes
RUNX3	0.4887614	10.06870908	6.42E-21	1.12E-18	yes
SNX20	0.488226521	10.05423936	7.18E-21	1.24E-18	yes
ADPRH	0.487871907	10.04465443	7.74E-21	1.32E-18	yes
THEMIS2	0.487870545	10.04461765	7.74E-21	1.32E-18	yes
GIMAP2	0.487701956	10.0400631	8.02E-21	1.34E-18	yes
CSF2RB	0.487686436	10.03964389	8.04E-21	1.34E-18	yes
IL7R	0.487684434	10.03958984	8.04E-21	1.34E-18	yes
CD40LG	0.487353106	10.03064342	8.62E-21	1.43E-18	yes
TXK	0.48626977	10.00143114	1.08E-20	1.76E-18	yes
SCIMP	0.486267054	10.00135799	1.08E-20	1.76E-18	yes
TMC8	0.486058923	9.995752666	1.13E-20	1.82E-18	yes
BTK	0.485796629	9.98869181	1.19E-20	1.91E-18	yes
CD1D	0.485425801	9.978715293	1.29E-20	2.05E-18	yes
MOB1A	0.485178842	9.972075202	1.35E-20	2.13E-18	yes
FAM26F	0.485158599	9.971531042	1.36E-20	2.13E-18	yes
C1orf162	0.484855052	9.963374002	1.45E-20	2.25E-18	yes
TMEM106A	0.48482795	9.962645947	1.46E-20	2.25E-18	yes
GIMAP4	0.484251943	9.947180972	1.64E-20	2.52E-18	yes
CYTIP	0.484120961	9.943666678	1.68E-20	2.55E-18	yes
SH2B3	0.484116203	9.943539021	1.69E-20	2.55E-18	yes
EVI2B	0.483542742	9.928163159	1.90E-20	2.85E-18	yes
PTPRC	0.483273901	9.920960638	2.01E-20	2.99E-18	yes
MSR1	0.483158516	9.917870483	2.05E-20	3.04E-18	yes
STAT5A	0.48196353	9.885906814	2.63E-20	3.86E-18	yes
C1S	0.48021819	9.839351159	3.75E-20	5.48E-18	yes
MCOLN2	0.480051543	9.834913956	3.88E-20	5.60E-18	yes
TLR2	0.480046131	9.834769868	3.88E-20	5.60E-18	yes
NCF4	0.47994016	9.831948974	3.97E-20	5.68E-18	yes
CD226	0.479913043	9.831227237	3.99E-20	5.68E-18	yes
TMEM109	0.479692488	9.825358279	4.17E-20	5.90E-18	yes

BIN2	0.479317619	9.815388589	4.50E-20	6.31E-18	yes
TYMP	0.479297114	9.814843473	4.52E-20	6.31E-18	yes
RCSD1	0.478617031	9.796775072	5.19E-20	7.20E-18	yes
LPXN	0.478496251	9.793568603	5.32E-20	7.33E-18	yes
SLC15A3	0.478195958	9.785599506	5.65E-20	7.74E-18	yes
PTGER2	0.477776816	9.774483865	6.15E-20	8.37E-18	yes
AIM1	0.477728726	9.773209091	6.21E-20	8.40E-18	yes
BATF	0.476437983	9.739035922	8.06E-20	1.08E-17	yes
DTX3L	0.47613365	9.730990433	8.57E-20	1.14E-17	yes
CD4	0.475843259	9.723317714	9.08E-20	1.20E-17	yes
NAGA	0.475131635	9.704532559	1.05E-19	1.38E-17	yes
GPRIN3	0.474867013	9.697553453	1.10E-19	1.45E-17	yes
ATP2A3	0.473794431	9.669300052	1.37E-19	1.78E-17	yes
GPR174	0.473591767	9.663967839	1.42E-19	1.84E-17	yes
USP15	0.472784766	9.642754696	1.67E-19	2.15E-17	yes
CD300C	0.472634113	9.638798041	1.72E-19	2.20E-17	yes
PTPRCAP	0.472384984	9.632257439	1.81E-19	2.30E-17	yes
TRIM38	0.472358348	9.631558328	1.82E-19	2.30E-17	yes
SAMHD1	0.47231564	9.630437425	1.84E-19	2.31E-17	yes
IL13RA1	0.472157414	9.626285455	1.89E-19	2.37E-17	yes
S1PR4	0.471853064	9.618302461	2.01E-19	2.50E-17	yes
PRKCH	0.470349384	9.578926213	2.71E-19	3.33E-17	yes
C16orf54	0.470344542	9.578799595	2.71E-19	3.33E-17	yes
LOC100506585	0.470237021	9.575988124	2.77E-19	3.38E-17	yes
LY75	0.470137134	9.573376769	2.82E-19	3.43E-17	yes
VAMP8	0.469630649	9.560142879	3.12E-19	3.77E-17	yes
HLA-B	0.469342076	9.552608214	3.30E-19	3.96E-17	yes
FCGR2A	0.468989057	9.543396218	3.54E-19	4.23E-17	yes
SERPINB9	0.467825137	9.513065245	4.45E-19	5.28E-17	yes
PARP9	0.467605187	9.507340627	4.64E-19	5.45E-17	yes
LAMP3	0.467601476	9.507244076	4.64E-19	5.45E-17	yes
ARL11	0.467424464	9.502638676	4.81E-19	5.62E-17	yes
PIK3CD	0.466255289	9.472256389	6.04E-19	7.01E-17	yes
LOC100505812	0.466063556	9.467280065	6.27E-19	7.24E-17	yes
TNFRSF1B	0.46585634	9.461903799	6.53E-19	7.50E-17	yes
TLR8	0.464924677	9.437756125	7.82E-19	8.94E-17	yes
B4GALT1	0.464286322	9.421233775	8.85E-19	1.01E-16	yes
TEC	0.46269127	9.380031213	1.20E-18	1.36E-16	yes
MS4A6A	0.462576405	9.377068571	1.23E-18	1.38E-16	yes
PARP14	0.462524646	9.375733769	1.24E-18	1.39E-16	yes
SKAP1	0.461785814	9.356693575	1.43E-18	1.59E-16	yes
CD28	0.46123311	9.342466206	1.59E-18	1.76E-16	yes
ARPC1B	0.461133924	9.339914482	1.62E-18	1.79E-16	yes
FCER1G	0.461023338	9.337070004	1.66E-18	1.82E-16	yes
LY96	0.460707258	9.328942872	1.76E-18	1.92E-16	yes
XCL1	0.460527582	9.324325028	1.82E-18	1.98E-16	yes
CMTM7	0.460041887	9.31184941	2.00E-18	2.16E-16	yes
MFSD1	0.459810354	9.305905979	2.09E-18	2.24E-16	yes
SRGN	0.459671368	9.302339362	2.14E-18	2.29E-16	yes
MYO1G	0.459288344	9.292514822	2.31E-18	2.45E-16	yes
PML	0.458746569	9.278629541	2.56E-18	2.71E-16	yes

EPHA1	0.458444568	9.270895176	2.71E-18	2.85E-16	yes
HLA-DQA1	0.458328456	9.267922589	2.77E-18	2.90E-16	yes
GPR18	0.458287498	9.26687417	2.79E-18	2.91E-16	yes
CD300LB	0.458038392	9.260499251	2.92E-18	3.03E-16	yes
FERMT3	0.457975956	9.258901876	2.96E-18	3.06E-16	yes
TRIM21	0.457938426	9.2579418	2.98E-18	3.06E-16	yes
CTLA4	0.4578467	9.255595518	3.03E-18	3.10E-16	yes
SLAMF8	0.457763366	9.253464241	3.08E-18	3.14E-16	yes
CARD11	0.457608787	9.249511674	3.17E-18	3.21E-16	yes
MNDA	0.457482464	9.246282392	3.25E-18	3.28E-16	yes
MZB1	0.457076904	9.235919583	3.51E-18	3.52E-16	yes
GRN	0.456850973	9.230149765	3.66E-18	3.66E-16	yes
LAIR1	0.456772876	9.228155873	3.71E-18	3.69E-16	yes
FUCA1	0.456498414	9.221150668	3.91E-18	3.87E-16	yes
CCR4	0.456398264	9.218595322	3.98E-18	3.93E-16	yes
CCR1	0.456318799	9.216568089	4.04E-18	3.97E-16	yes
MFSD7	0.455692012	9.20058779	4.55E-18	4.44E-16	yes
WDFY4	0.455545866	9.196864186	4.68E-18	4.55E-16	yes
NPC2	0.45508072	9.185019186	5.10E-18	4.94E-16	yes
LCP2	0.454100568	9.160090466	6.13E-18	5.91E-16	yes
TLR6	0.454038304	9.158508302	6.20E-18	5.95E-16	yes
IFITM2	0.453149244	9.135935022	7.32E-18	6.99E-16	yes
HCLS1	0.452658106	9.123479739	8.02E-18	7.63E-16	yes
GPR160	0.452463304	9.118542436	8.32E-18	7.88E-16	yes
RNF149	0.451658642	9.098165487	9.66E-18	9.11E-16	yes
CFI	0.451127161	9.084721763	1.07E-17	1.00E-15	yes
PLBD1	0.450799567	9.07644138	1.13E-17	1.06E-15	yes
IL16	0.450569904	9.070639077	1.18E-17	1.10E-15	yes
IFI30	0.450523772	9.069473851	1.19E-17	1.10E-15	yes
RNF144B	0.450460141	9.067866788	1.21E-17	1.11E-15	yes
C3	0.450191981	9.06109599	1.27E-17	1.16E-15	yes
SP140L	0.450130007	9.059531643	1.28E-17	1.17E-15	yes
CORO1A	0.45009094	9.058545616	1.29E-17	1.18E-15	yes
APOBEC3C	0.449075934	9.032949859	1.56E-17	1.41E-15	yes
FCGR3B	0.448998295	9.030993806	1.58E-17	1.43E-15	yes
TCIRG1	0.44881866	9.026469059	1.63E-17	1.47E-15	yes
CXCR4	0.448736017	9.024387852	1.66E-17	1.48E-15	yes
HAVCR2	0.448719177	9.023963811	1.66E-17	1.48E-15	yes
MYO1F	0.448584843	9.020581587	1.70E-17	1.51E-15	yes
CD79A	0.447434036	8.99163818	2.10E-17	1.85E-15	yes
GALM	0.447432985	8.991611786	2.10E-17	1.85E-15	yes
SAMD9L	0.447128523	8.98396377	2.22E-17	1.95E-15	yes
IL10RB	0.446526092	8.96884235	2.48E-17	2.17E-15	yes
SP140	0.446481412	8.967721463	2.50E-17	2.18E-15	yes
LHFPL2	0.445944799	8.95426595	2.76E-17	2.39E-15	yes
MILR1	0.445475644	8.942511822	3.01E-17	2.59E-15	yes
TRIM14	0.445355367	8.939499901	3.07E-17	2.64E-15	yes
IFITM1	0.445256605	8.937027224	3.13E-17	2.68E-15	yes
SECTM1	0.444940003	8.929103203	3.31E-17	2.82E-15	yes
IL18	0.444889069	8.927828796	3.34E-17	2.84E-15	yes
SAMSN1	0.444844989	8.926725987	3.37E-17	2.85E-15	yes

GZMB	0.443975846	8.904997699	3.94E-17	3.32E-15	yes
HLA-H	0.443915417	8.903488147	3.99E-17	3.35E-15	yes
RASSF3	0.443145017	8.88425652	4.58E-17	3.83E-15	yes
DPYD	0.442953774	8.879486263	4.74E-17	3.95E-15	yes
IGLL5	0.442889809	8.877891116	4.80E-17	3.98E-15	yes
SIGLEC7	0.442773661	8.874995038	4.90E-17	4.05E-15	yes
C3AR1	0.442395006	8.86555736	5.24E-17	4.32E-15	yes
SPATS2L	0.442185607	8.860340807	5.45E-17	4.47E-15	yes
TNFRSF9	0.442058029	8.85716345	5.57E-17	4.55E-15	yes
EPSTI1	0.441649395	8.846990856	6.00E-17	4.88E-15	yes
GBP3	0.441546547	8.844431621	6.11E-17	4.95E-15	yes
IGFLR1	0.440539793	8.819402702	7.31E-17	5.91E-15	yes
GMFG	0.440439104	8.816901755	7.45E-17	5.99E-15	yes
GZMM	0.439829272	8.801763328	8.30E-17	6.66E-15	yes
CYTH4	0.439737683	8.799491027	8.44E-17	6.74E-15	yes
HLA-DRB1	0.439223016	8.786728599	9.25E-17	7.36E-15	yes
COLEC12	0.438954995	8.780086616	9.70E-17	7.69E-15	yes
IL10RA	0.438695154	8.773650127	1.02E-16	8.03E-15	yes
TNFRSF14	0.438607104	8.771469666	1.03E-16	8.13E-15	yes
KLHDC7B	0.438459811	8.767822824	1.06E-16	8.31E-15	yes
CD40	0.438265712	8.763018424	1.10E-16	8.57E-15	yes
MAN2B1	0.438228134	8.762088483	1.10E-16	8.60E-15	yes
RGS18	0.437957546	8.75539376	1.16E-16	8.99E-15	yes
TLR7	0.437831225	8.75226941	1.18E-16	9.16E-15	yes
KIAA0247	0.436701887	8.724365469	1.45E-16	1.11E-14	yes
EHBP1L1	0.436184834	8.711607053	1.58E-16	1.22E-14	yes
GPR141	0.436145972	8.710648537	1.60E-16	1.22E-14	yes
CD79B	0.435693189	8.699485443	1.73E-16	1.32E-14	yes
MPEG1	0.435563481	8.69628907	1.77E-16	1.34E-14	yes
PLA2G2D	0.435333601	8.690625837	1.84E-16	1.39E-14	yes
SH2D2A	0.435079319	8.68436385	1.92E-16	1.45E-14	yes
C1RL	0.43458254	8.672137531	2.10E-16	1.58E-14	yes
RGS1	0.434405817	8.667790533	2.17E-16	1.62E-14	yes
UNC93B1	0.434085743	8.659920539	2.29E-16	1.71E-14	yes
DOK3	0.433914629	8.655714831	2.36E-16	1.76E-14	yes
PCED1B	0.433736746	8.651343969	2.43E-16	1.80E-14	yes
PLCG2	0.433679978	8.649949367	2.46E-16	1.82E-14	yes
CAPZA1	0.433448909	8.64427403	2.56E-16	1.88E-14	yes
C1QA	0.432941898	8.63182858	2.80E-16	2.05E-14	yes
DEF6	0.432770486	8.627623277	2.88E-16	2.11E-14	yes
CXorf21	0.432145761	8.612306387	3.21E-16	2.34E-14	yes
C1R	0.431809408	8.604066054	3.41E-16	2.47E-14	yes
RARRES3	0.431794882	8.603710302	3.42E-16	2.47E-14	yes
CTSZ	0.43152384	8.597073396	3.58E-16	2.58E-14	yes
ZC3H12D	0.431061913	8.585768945	3.88E-16	2.79E-14	yes
PPM1M	0.430852572	8.580648623	4.02E-16	2.88E-14	yes
CLDN23	0.43056097	8.573519082	4.23E-16	3.02E-14	yes
RAB20	0.430492886	8.571854936	4.28E-16	3.05E-14	yes
HCP5	0.430392542	8.56940258	4.35E-16	3.08E-14	yes
RAB32	0.430388674	8.569308073	4.36E-16	3.08E-14	yes
CD37	0.430061315	8.561310396	4.61E-16	3.25E-14	yes

CIDEB	0.429673572	8.551842836	4.93E-16	3.46E-14	yes
RPS6KA1	0.429298839	8.542698457	5.26E-16	3.68E-14	yes
SLC37A2	0.429217346	8.540710549	5.33E-16	3.72E-14	yes
TMEM173	0.429147923	8.539017283	5.40E-16	3.75E-14	yes
USP30-AS1	0.429096021	8.537751478	5.45E-16	3.78E-14	yes
TLR3	0.428511688	8.523507695	6.02E-16	4.16E-14	yes
JAK3	0.428212869	8.516228722	6.34E-16	4.37E-14	yes
PSD4	0.426643129	8.478047229	8.29E-16	5.69E-14	yes
LRRC25	0.426611808	8.477286358	8.34E-16	5.71E-14	yes
APOL6	0.426346905	8.470852561	8.72E-16	5.95E-14	yes
PARP12	0.426070063	8.464131672	9.14E-16	6.21E-14	yes
PARP15	0.426057997	8.463838815	9.16E-16	6.21E-14	yes
CARD6	0.425983133	8.462021866	9.28E-16	6.27E-14	yes
TRPV2	0.424764414	8.43247349	1.14E-15	7.69E-14	yes
KMO	0.424583154	8.428083551	1.18E-15	7.91E-14	yes
LTBR	0.424363064	8.422754824	1.22E-15	8.18E-14	yes
LYN	0.424345298	8.422324775	1.23E-15	8.18E-14	yes
MGAT4A	0.423927545	8.412215639	1.32E-15	8.75E-14	yes
CD2AP	0.423773322	8.408485265	1.35E-15	8.96E-14	yes
PCED1B-AS1	0.423574442	8.403676068	1.40E-15	9.24E-14	yes
CTSC	0.422984235	8.389412683	1.54E-15	1.02E-13	yes
SLC46A3	0.422941705	8.388385386	1.55E-15	1.02E-13	yes
PROCR	0.422810132	8.385207672	1.59E-15	1.04E-13	yes
TRIM22	0.422576376	8.379563666	1.65E-15	1.08E-13	yes
GCH1	0.422537911	8.378635146	1.66E-15	1.08E-13	yes
LAX1	0.422430359	8.376039155	1.69E-15	1.10E-13	yes
TNFRSF10A	0.422137705	8.368977525	1.78E-15	1.15E-13	yes
SLC7A7	0.421757658	8.359811881	1.90E-15	1.22E-13	yes
CMTM6	0.421023822	8.342129043	2.15E-15	1.38E-13	yes
ELK3	0.420935547	8.340003268	2.18E-15	1.40E-13	yes
RGS19	0.420424241	8.327696065	2.37E-15	1.52E-13	yes
KCNK6	0.419798133	8.312638666	2.63E-15	1.68E-13	yes
NOD1	0.419697984	8.310231489	2.68E-15	1.70E-13	yes
DENND1C	0.419463034	8.30458571	2.79E-15	1.77E-13	yes
STAP1	0.419174966	8.297666285	2.92E-15	1.85E-13	yes
KLHL6	0.419029066	8.294162929	2.99E-15	1.89E-13	yes
ZMYM6NB	0.418924688	8.29165706	3.05E-15	1.91E-13	yes
MAN1C1	0.418667187	8.285476801	3.18E-15	1.99E-13	yes
ICAM3	0.418571198	8.283173573	3.23E-15	2.02E-13	yes
TYROBP	0.418536057	8.282330471	3.25E-15	2.02E-13	yes
C1QB	0.418428136	8.279741505	3.31E-15	2.05E-13	yes
PSMB9	0.418379244	8.278568753	3.34E-15	2.07E-13	yes
SERPING1	0.41831648	8.277063388	3.37E-15	2.08E-13	yes
PSMB10	0.418208413	8.274471781	3.43E-15	2.11E-13	yes
FMNL1	0.417428815	8.255788474	3.90E-15	2.40E-13	yes
C2	0.417171085	8.249616766	4.08E-15	2.50E-13	yes
OSTF1	0.416427856	8.231832558	4.61E-15	2.81E-13	yes
GNA15	0.416426079	8.231790056	4.61E-15	2.81E-13	yes
PARVG	0.416266642	8.227977625	4.73E-15	2.87E-13	yes
PSMB8	0.416040085	8.222561797	4.91E-15	2.97E-13	yes
SLC2A9	0.415847251	8.217953554	5.07E-15	3.06E-13	yes

HEXB	0.415770259	8.216114033	5.14E-15	3.09E-13	yes
TMEM37	0.41562994	8.212761993	5.26E-15	3.15E-13	yes
RUNX1	0.415623105	8.212598731	5.26E-15	3.15E-13	yes
CD300LF	0.415307812	8.205069489	5.54E-15	3.31E-13	yes
ARHGAP9	0.415138952	8.201038561	5.70E-15	3.39E-13	yes
HMHA1	0.414512492	8.186092999	6.31E-15	3.75E-13	yes
DDX60L	0.413802653	8.169175229	7.09E-15	4.20E-13	yes
SPI1	0.413695823	8.166630681	7.22E-15	4.26E-13	yes
RTP4	0.412789527	8.145060254	8.37E-15	4.93E-13	yes
ADAM28	0.412389557	8.13554999	8.93E-15	5.25E-13	yes
ARHGAP18	0.412128063	8.129335412	9.32E-15	5.46E-13	yes
NBEAL2	0.412105639	8.128802602	9.36E-15	5.46E-13	yes
NOD2	0.411988829	8.126027397	9.54E-15	5.55E-13	yes
GYPC	0.411959298	8.125325878	9.58E-15	5.57E-13	yes
CD300A	0.411784733	8.12117961	9.86E-15	5.71E-13	yes
UBD	0.411281113	8.109223646	1.07E-14	6.18E-13	yes
LOC284751	0.41073031	8.096157796	1.17E-14	6.74E-13	yes
SERPINA1	0.40989673	8.076404327	1.34E-14	7.69E-13	yes
FCRL5	0.409677283	8.071208115	1.39E-14	7.95E-13	yes
TPP1	0.409031397	8.055924122	1.54E-14	8.80E-13	yes
ZNRF2	0.408831981	8.051208165	1.59E-14	9.06E-13	yes
DRAM1	0.408432344	8.04176138	1.69E-14	9.63E-13	yes
IFI35	0.408364481	8.040157772	1.71E-14	9.71E-13	yes
EDEM2	0.407967936	8.030790486	1.83E-14	1.03E-12	yes
CD180	0.407816025	8.027203447	1.87E-14	1.06E-12	yes
LGALS3BP	0.407737184	8.025342125	1.89E-14	1.07E-12	yes
DOK2	0.407528776	8.020422927	1.96E-14	1.10E-12	yes
F13A1	0.406707033	8.001041348	2.23E-14	1.25E-12	yes
IL1R1	0.406667102	8.000100112	2.25E-14	1.26E-12	yes
FTL	0.406260429	7.99051753	2.40E-14	1.34E-12	yes
CD68	0.406141115	7.98770717	2.45E-14	1.36E-12	yes
MGAT1	0.405332494	7.968673514	2.78E-14	1.54E-12	yes
FAM78A	0.405332235	7.968667424	2.78E-14	1.54E-12	yes
HCST	0.405232192	7.966314131	2.83E-14	1.55E-12	yes
A4GALT	0.40523165	7.966301377	2.83E-14	1.55E-12	yes
ZNF600	0.405137735	7.964092555	2.87E-14	1.57E-12	yes
ST8SIA4	0.405107848	7.963389687	2.88E-14	1.58E-12	yes
BST2	0.404775581	7.955577694	3.04E-14	1.66E-12	yes
C5AR1	0.404392309	7.946571192	3.23E-14	1.76E-12	yes
ETV6	0.404067336	7.938938582	3.40E-14	1.85E-12	yes
ITGB2	0.403857138	7.934003602	3.51E-14	1.90E-12	yes
KIAA0125	0.403170036	7.917882427	3.92E-14	2.12E-12	yes
PECAM1	0.402380725	7.899382896	4.44E-14	2.39E-12	yes
MAN1A1	0.402293013	7.897328432	4.50E-14	2.42E-12	yes
PIGB	0.401904582	7.888233423	4.78E-14	2.56E-12	yes
MR1	0.401866075	7.887332078	4.81E-14	2.57E-12	yes
EBI3	0.401827321	7.886424993	4.84E-14	2.58E-12	yes
IFNG	0.401807529	7.885961735	4.86E-14	2.58E-12	yes
ALPK1	0.401326066	7.874697027	5.24E-14	2.78E-12	yes
PLA2G15	0.401202043	7.871796555	5.34E-14	2.83E-12	yes
TMEM154	0.401034079	7.867869262	5.48E-14	2.90E-12	yes

AIF1	0.400983478	7.866686297	5.53E-14	2.91E-12	yes
CTSW	0.400798847	7.862370711	5.69E-14	2.99E-12	yes
SWAP70	0.400781112	7.861956226	5.70E-14	2.99E-12	yes
CREBL2	0.400716924	7.86045619	5.76E-14	3.01E-12	yes
PLK3	0.400433074	7.853824446	6.02E-14	3.14E-12	yes
TRAM1	0.400200665	7.84839653	6.25E-14	3.25E-12	yes
IL15RA	0.399914852	7.841723849	6.53E-14	3.39E-12	no
CD33	0.399660047	7.8357774	6.80E-14	3.52E-12	no
SNX9	0.399063968	7.821874965	7.46E-14	3.85E-12	no
MB21D1	0.398964696	7.819560773	7.58E-14	3.90E-12	no
CTSD	0.398864259	7.817219764	7.69E-14	3.96E-12	no
LY86	0.398837301	7.816591476	7.73E-14	3.96E-12	no
STAT1	0.398058111	7.798441959	8.72E-14	4.46E-12	no
IRF8	0.397740791	7.791056418	9.16E-14	4.68E-12	no
SIRPB2	0.397255294	7.779763063	9.88E-14	5.03E-12	no
ITGA4	0.397181395	7.778044745	9.99E-14	5.08E-12	no
CFLAR	0.397087116	7.775852812	1.01E-13	5.14E-12	no
CD58	0.396992233	7.773647128	1.03E-13	5.20E-12	no
TLR5	0.396839819	7.770104674	1.05E-13	5.31E-12	no
RIN3	0.396793061	7.769018056	1.06E-13	5.34E-12	no
SLFN12	0.3967729	7.768549561	1.06E-13	5.34E-12	no
RNF19B	0.396232272	7.755991462	1.16E-13	5.80E-12	no
CASP4	0.396081177	7.752483414	1.18E-13	5.92E-12	no
PRSS23	0.396015372	7.750955846	1.20E-13	5.96E-12	no
SH3TC1	0.395645963	7.742383055	1.27E-13	6.29E-12	no
MS4A4A	0.395637008	7.742175295	1.27E-13	6.29E-12	no
FAM20A	0.395473149	7.738374143	1.30E-13	6.44E-12	no
EHD4	0.395385159	7.736333325	1.32E-13	6.51E-12	no
ARHGAP4	0.395367412	7.735921739	1.32E-13	6.52E-12	no
TBC1D2B	0.395342787	7.735350655	1.33E-13	6.52E-12	no
C1QC	0.394999789	7.72739817	1.40E-13	6.86E-12	no
SLC25A43	0.394783208	7.722378664	1.45E-13	7.08E-12	no
IFNGR2	0.394716424	7.720831177	1.46E-13	7.12E-12	no
RAB27A	0.394707535	7.720625205	1.46E-13	7.12E-12	no
FCGR1A	0.394699947	7.720449394	1.46E-13	7.12E-12	no
CXCL10	0.394528687	7.7164818	1.50E-13	7.29E-12	no
FUT4	0.394418629	7.713932583	1.53E-13	7.40E-12	no
MX2	0.394209172	7.70908212	1.58E-13	7.63E-12	no
CTSB	0.393909912	7.702154514	1.65E-13	7.97E-12	no
LSP1	0.393577899	7.694472102	1.74E-13	8.36E-12	no
RBM47	0.393466077	7.691885447	1.77E-13	8.49E-12	no
ST14	0.393429973	7.691050401	1.78E-13	8.51E-12	no
RNF135	0.393188737	7.685471842	1.85E-13	8.81E-12	no
MAFB	0.392741683	7.675138741	1.98E-13	9.42E-12	no
IFI16	0.392402133	7.667294773	2.08E-13	9.89E-12	no
LAIR2	0.391972536	7.657375913	2.22E-13	1.05E-11	no
SLC24A6	0.391558684	7.647826202	2.37E-13	1.12E-11	no
PION	0.39151633	7.646849189	2.38E-13	1.12E-11	no
HEXA	0.391375441	7.643599592	2.43E-13	1.15E-11	no
CD14	0.391358637	7.643212035	2.44E-13	1.15E-11	no
GLIPR1	0.391203939	7.63964474	2.50E-13	1.17E-11	no

CXCL16	0.390988017	7.63466693	2.58E-13	1.21E-11	no
DAB2	0.390909909	7.632866606	2.61E-13	1.22E-11	no
DDX60	0.390811817	7.630605951	2.65E-13	1.24E-11	no
CLEC10A	0.390618448	7.626150403	2.73E-13	1.27E-11	no
STAB1	0.390414287	7.621447508	2.81E-13	1.31E-11	no
BST1	0.390366398	7.620344561	2.83E-13	1.31E-11	no
M6PR	0.390295728	7.618717079	2.86E-13	1.32E-11	no
PYCARD	0.390201168	7.616539662	2.91E-13	1.34E-11	no
TRANK1	0.390104621	7.614316787	2.95E-13	1.36E-11	no
SQRDL	0.389977772	7.611396685	3.01E-13	1.38E-11	no
DSC2	0.389643386	7.603701515	3.16E-13	1.45E-11	no
UTS2	0.389459662	7.599475008	3.25E-13	1.49E-11	no
HAAO	0.389334693	7.596600736	3.31E-13	1.51E-11	no
BTN3A2	0.388437288	7.575975154	3.79E-13	1.73E-11	no
CD163	0.388026016	7.56653112	4.03E-13	1.83E-11	no
PIK3R6	0.387938626	7.564525074	4.08E-13	1.85E-11	no
C7	0.387541274	7.555406834	4.34E-13	1.96E-11	no
IRF1	0.387530985	7.555170795	4.34E-13	1.96E-11	no
GAL3ST4	0.387328739	7.550531724	4.48E-13	2.02E-11	no
S100A11	0.387265103	7.549072321	4.52E-13	2.03E-11	no
TMBIM1	0.387208679	7.547778436	4.56E-13	2.04E-11	no
WAS	0.386360472	7.528339602	5.17E-13	2.32E-11	no
GPM6A	-0.386102601	-7.522434276	5.38E-13	2.40E-11	no
CD8B	0.386093753	7.522231694	5.38E-13	2.40E-11	no
F11R	0.386083122	7.52198829	5.39E-13	2.40E-11	no
BAK1	0.385395691	7.506256481	5.97E-13	2.65E-11	no
RNASE2	0.385388992	7.506103226	5.98E-13	2.65E-11	no
LINC00601	0.385297514	7.504010897	6.06E-13	2.68E-11	no
OAS2	0.385056716	7.498504461	6.28E-13	2.77E-11	no
ATP6V0E1	0.384114879	7.476984264	7.22E-13	3.18E-11	no
TBC1D8B	0.383886564	7.471771588	7.47E-13	3.28E-11	no
RILPL2	0.383857904	7.471117371	7.50E-13	3.29E-11	no
PQLC3	0.38346941	7.462251651	7.95E-13	3.48E-11	no
EMBP1	0.382882292	7.448862035	8.66E-13	3.78E-11	no
MYL12A	0.382786311	7.446674119	8.79E-13	3.83E-11	no
DNASE2	0.382767965	7.446255959	8.81E-13	3.83E-11	no
GBP1	0.38259951	7.44241676	9.03E-13	3.92E-11	no
MS4A1	0.382535196	7.440951242	9.12E-13	3.95E-11	no
CFD	0.382122697	7.431554624	9.69E-13	4.19E-11	no
NTAN1	0.382010563	7.429001153	9.85E-13	4.25E-11	no
SPPL2A	0.381851991	7.425390846	1.01E-12	4.34E-11	no
STK10	0.381824381	7.424762323	1.01E-12	4.35E-11	no
LOC283143	0.381559935	7.418743469	1.05E-12	4.51E-11	no
C19orf38	0.381396763	7.415030678	1.08E-12	4.61E-11	no
MAPKAPK2	0.381123564	7.408816201	1.12E-12	4.79E-11	no
RPS6KA3	0.380483064	7.394255598	1.23E-12	5.25E-11	no
GGA2	0.380285971	7.389777543	1.27E-12	5.40E-11	no
GNGT2	0.380166297	7.38705907	1.29E-12	5.48E-11	no
TGFB1	0.380144951	7.386574221	1.29E-12	5.49E-11	no
GLA	0.380106668	7.385704717	1.30E-12	5.51E-11	no
HLA-DRB3	0.380012161	7.383558389	1.32E-12	5.57E-11	no

NLRC5	0.379751182	7.377632803	1.37E-12	5.78E-11	no
ACP5	0.379692402	7.376298474	1.38E-12	5.82E-11	no
LINC00324	0.379552954	7.373133341	1.41E-12	5.92E-11	no
CCRL2	0.37954912	7.37304633	1.41E-12	5.92E-11	no
GCLM	0.379387873	7.369387204	1.45E-12	6.05E-11	no
ARHGAP29	0.379333717	7.368158426	1.46E-12	6.08E-11	no
NFE2L3	0.37922043	7.365588266	1.48E-12	6.17E-11	no
ISG20	0.37904559	7.361622438	1.52E-12	6.32E-11	no
LTBP2	0.378725628	7.354367229	1.59E-12	6.61E-11	no
LINC00525	0.378233797	7.34322085	1.71E-12	7.08E-11	no
LOC100132987	0.378051255	7.339085742	1.76E-12	7.26E-11	no
CD55	0.377870429	7.334990502	1.80E-12	7.44E-11	no
LGALS9	0.377575666	7.328316973	1.88E-12	7.74E-11	no
PSAP	0.377127244	7.318169535	2.01E-12	8.25E-11	no
REEP4	0.376929476	7.313696105	2.07E-12	8.47E-11	no
HLA-DQB1	0.376794426	7.310641986	2.11E-12	8.62E-11	no
CLEC7A	0.376679957	7.308053747	2.14E-12	8.75E-11	no
SLFN5	0.376281906	7.299056483	2.27E-12	9.25E-11	no
TGFBR2	0.376098306	7.294908113	2.33E-12	9.48E-11	no
UBA7	0.375987464	7.292404154	2.37E-12	9.61E-11	no
C20orf197	0.375829078	7.288826812	2.42E-12	9.82E-11	no
FAM129A	0.375444506	7.280143866	2.56E-12	1.04E-10	no
SIGLEC9	0.374985359	7.269782871	2.73E-12	1.10E-10	no
UCP2	0.374650587	7.262232425	2.87E-12	1.16E-10	no
A2M	0.374496741	7.258763683	2.93E-12	1.18E-10	no
TXNDC11	0.374208756	7.252272424	3.05E-12	1.23E-10	no
CXCL12	0.373977221	7.247055323	3.16E-12	1.27E-10	no
PARP4	0.373806191	7.243202586	3.24E-12	1.29E-10	no
SIGIRR	0.373340268	7.232711213	3.46E-12	1.38E-10	no
ARRDC2	0.37331104	7.23205329	3.47E-12	1.38E-10	no
RIPK1	0.373297165	7.231740952	3.48E-12	1.38E-10	no
ALOX5	0.373243396	7.230530695	3.51E-12	1.39E-10	no
NCF1	0.373106049	7.227439608	3.58E-12	1.42E-10	no
TNFRSF11B	0.37300407	7.225144853	3.63E-12	1.44E-10	no
SAT1	0.372687042	7.218012966	3.80E-12	1.50E-10	no
MSN	0.37268216	7.217903168	3.80E-12	1.50E-10	no
DENND3	0.372627641	7.216676997	3.83E-12	1.51E-10	no
CPQ	0.372352128	7.210481897	3.98E-12	1.56E-10	no
PILRA	0.372086482	7.204510762	4.13E-12	1.62E-10	no
LHFPL4	-0.37206114	-7.203941226	4.15E-12	1.62E-10	no
SP110	0.371838517	7.198938899	4.28E-12	1.67E-10	no
CSF1R	0.371680946	7.195399163	4.38E-12	1.71E-10	no
APOBEC3F	0.371172716	7.183986978	4.71E-12	1.83E-10	no
LST1	0.370685476	7.173053144	5.04E-12	1.96E-10	no
NCKAP1L	0.370430653	7.167337566	5.23E-12	2.02E-10	no
DGKA	0.37042857	7.167290853	5.23E-12	2.02E-10	no
OASL	0.370369602	7.16596849	5.27E-12	2.04E-10	no
BHLHE40	0.370274999	7.163847219	5.34E-12	2.06E-10	no
LOC283194	0.370188501	7.161907931	5.41E-12	2.08E-10	no
SSH1	0.370071867	7.15929331	5.50E-12	2.11E-10	no
HLA-DPB2	0.369912682	7.155725467	5.62E-12	2.16E-10	no

RFTN1	0.369875692	7.154896483	5.65E-12	2.17E-10	no
SEL1L3	0.369854249	7.154415976	5.67E-12	2.17E-10	no
SHKBP1	0.369718775	7.151380353	5.78E-12	2.21E-10	no
IL2RA	0.369003907	7.135370799	6.39E-12	2.44E-10	no
HK3	0.368979857	7.134832444	6.41E-12	2.44E-10	no
GIMAP6	0.368472758	7.123485146	6.89E-12	2.61E-10	no
B3GNT2	0.368149145	7.116247551	7.21E-12	2.73E-10	no
LOC283587	0.368062958	7.114320475	7.29E-12	2.75E-10	no
GPR183	0.368062635	7.114313263	7.29E-12	2.75E-10	no
INPP5D	0.367935761	7.111476846	7.42E-12	2.80E-10	no
SYNGR2	0.36763605	7.104778295	7.74E-12	2.91E-10	no
FCGR1C	0.367193457	7.094891015	8.24E-12	3.09E-10	no
TOR4A	0.36702933	7.091225941	8.43E-12	3.16E-10	no
TLN1	0.36682209	7.08659919	8.67E-12	3.25E-10	no
CLECL1	0.366620702	7.082104259	8.92E-12	3.33E-10	no
ZNFX1	0.366590054	7.081420302	8.96E-12	3.34E-10	no
CPVL	0.366313506	7.07524994	9.31E-12	3.47E-10	no
C10orf11	0.366217092	7.073099256	9.44E-12	3.51E-10	no
FAP	0.366076364	7.069960532	9.62E-12	3.57E-10	no
TNFAIP8L2	0.365961862	7.06740716	9.78E-12	3.62E-10	no
SEC24D	0.36574445	7.062559912	1.01E-11	3.73E-10	no
RNPEPL1	0.365517153	7.057493733	1.04E-11	3.84E-10	no
CYBASC3	0.365484478	7.056765556	1.04E-11	3.85E-10	no
LRMP	0.364998558	7.04594028	1.12E-11	4.11E-10	no
KIAA1217	0.364992914	7.045814584	1.12E-11	4.11E-10	no
GNS	0.364968491	7.04527066	1.12E-11	4.12E-10	no
HNRNPUL2	-0.364885145	-7.043414631	1.14E-11	4.16E-10	no
GPX8	0.364861883	7.042896646	1.14E-11	4.16E-10	no
SOCS1	0.364854095	7.042723218	1.14E-11	4.16E-10	no
HLA-DOB	0.364796189	7.041433873	1.15E-11	4.19E-10	no
IFIH1	0.36465663	7.038326816	1.17E-11	4.26E-10	no
ZNF217	0.364230035	7.028832748	1.24E-11	4.51E-10	no
CARD16	0.364144316	7.026925664	1.26E-11	4.55E-10	no
OLFML3	0.364140099	7.026831839	1.26E-11	4.55E-10	no
KIAA1551	0.364099039	7.025918411	1.27E-11	4.57E-10	no
APOBEC3H	0.363734555	7.017812019	1.33E-11	4.80E-10	no
ACSL5	0.363567695	7.01410217	1.36E-11	4.90E-10	no
FPR1	0.363366609	7.009632412	1.40E-11	5.03E-10	no
ZFYVE20	-0.363265118	-7.007376887	1.42E-11	5.10E-10	no
BRSK2	-0.363085077	-7.003376366	1.46E-11	5.22E-10	no
SLC12A7	0.363054818	7.002704115	1.46E-11	5.23E-10	no
RNASET2	0.362820488	6.997498858	1.51E-11	5.39E-10	no
CYP2S1	0.362700955	6.994844216	1.54E-11	5.47E-10	no
PUS10	0.362663397	6.994010195	1.54E-11	5.49E-10	no
MICB	0.362546292	6.991409966	1.57E-11	5.57E-10	no
CLEC2B	0.362518427	6.99079131	1.57E-11	5.58E-10	no
GPX1	0.362187865	6.983453788	1.65E-11	5.83E-10	no
IQGAP1	0.362153314	6.982687042	1.66E-11	5.85E-10	no
FKBP15	0.362125375	6.982067033	1.66E-11	5.85E-10	no
IL4R	0.362120707	6.981963451	1.66E-11	5.85E-10	no
NAIP	0.362112866	6.981789452	1.66E-11	5.85E-10	no

HHEX	0.362083915	6.981147038	1.67E-11	5.87E-10	no
RHOG	0.361918116	6.977468395	1.71E-11	5.99E-10	no
GXYLT2	0.361677644	6.972134321	1.77E-11	6.18E-10	no
ZBP1	0.361510168	6.968420364	1.81E-11	6.32E-10	no
GPR55	0.361381572	6.965569122	1.84E-11	6.42E-10	no
SIGLEC17P	0.361350445	6.96487905	1.85E-11	6.44E-10	no
ADPGK	0.360797516	6.952625218	1.99E-11	6.93E-10	no
TES	0.360534777	6.946805436	2.07E-11	7.17E-10	no
POU2AF1	0.360315256	6.941944399	2.13E-11	7.38E-10	no
42248	0.360144888	6.938172727	2.18E-11	7.54E-10	no
ZC3HAV1	0.36000697	6.935120014	2.22E-11	7.67E-10	no
RGS10	0.359878613	6.9322794	2.26E-11	7.79E-10	no
AOAH	0.359771855	6.929917137	2.29E-11	7.89E-10	no
APOL3	0.359737043	6.929146906	2.30E-11	7.92E-10	no
ATG4A	0.359470708	6.92325525	2.39E-11	8.19E-10	no
TMEM150A	0.359464052	6.923108032	2.39E-11	8.19E-10	no
GMIP	0.359256852	6.91852588	2.46E-11	8.41E-10	no
PLXDC2	0.359081679	6.914652939	2.52E-11	8.60E-10	no
ITGAM	0.358994899	6.912734592	2.55E-11	8.69E-10	no
ASCL2	0.358656654	6.905259387	2.67E-11	9.08E-10	no
ASPN	0.358558636	6.903093771	2.70E-11	9.19E-10	no
C1RL-AS1	0.35838429	6.899242415	2.77E-11	9.40E-10	no
LAT2	0.358273703	6.896799953	2.81E-11	9.52E-10	no
IL17RA	0.358243946	6.896142777	2.82E-11	9.55E-10	no
BTN2A2	0.357814418	6.886659561	2.99E-11	1.01E-09	no
CYBA	0.357681938	6.883735642	3.04E-11	1.03E-09	no
METRNL	0.357424606	6.878057518	3.15E-11	1.06E-09	no
CASP8	0.357325803	6.875877894	3.19E-11	1.07E-09	no
CD74	0.357324161	6.875841659	3.19E-11	1.07E-09	no
ODF3B	0.357170535	6.872453122	3.26E-11	1.09E-09	no
S100A4	0.357161301	6.872249468	3.27E-11	1.09E-09	no
SOCS3	0.357037513	6.869519569	3.32E-11	1.11E-09	no
PLXND1	0.356998023	6.868648794	3.34E-11	1.11E-09	no
LGMN	0.356896411	6.866408358	3.38E-11	1.13E-09	no
HLA-F-AS1	0.356848373	6.865349263	3.41E-11	1.13E-09	no
DNAJC5B	0.35669963	6.86207036	3.47E-11	1.15E-09	no
GIT2	0.356584838	6.859540256	3.53E-11	1.17E-09	no
ARHGAP25	0.356529943	6.858330475	3.56E-11	1.18E-09	no
TSPAN4	0.356370408	6.854815035	3.63E-11	1.20E-09	no
CD93	0.356291747	6.853081963	3.67E-11	1.21E-09	no
CFB	0.356233229	6.851792785	3.70E-11	1.22E-09	no
S100Z	0.355218107	6.829443912	4.24E-11	1.40E-09	no
HPS3	0.355154363	6.828041459	4.28E-11	1.41E-09	no
SERPINA3	0.354717889	6.818441303	4.53E-11	1.49E-09	no
VMO1	0.354606896	6.816000861	4.60E-11	1.51E-09	no
NQO1	0.354364571	6.810673901	4.75E-11	1.55E-09	no
LOC100505622	0.354082884	6.804483651	4.93E-11	1.61E-09	no
HLA-A	0.354015718	6.803007942	4.98E-11	1.62E-09	no
HDAC7	0.353942338	6.801395843	5.03E-11	1.63E-09	no
TTC12	0.353941976	6.801387882	5.03E-11	1.63E-09	no
TAP2	0.353859063	6.799566531	5.08E-11	1.65E-09	no

TNFRSF1A	0.353853615	6.799446865	5.09E-11	1.65E-09	no
HTRA4	0.35374782	6.797123145	5.16E-11	1.67E-09	no
ARL14EPL	0.353609424	6.7940838	5.26E-11	1.70E-09	no
OSMR	0.353585108	6.793549849	5.27E-11	1.70E-09	no
C1orf54	0.353567752	6.793168746	5.29E-11	1.70E-09	no
SAMD3	0.353433809	6.79022782	5.38E-11	1.73E-09	no
AMIGO2	0.353134844	6.783665346	5.60E-11	1.80E-09	no
AMICA1	0.353063597	6.782101783	5.65E-11	1.81E-09	no
HLA-DQB2	0.35304317	6.781653512	5.67E-11	1.82E-09	no
TMSB4X	0.352479766	6.769294237	6.11E-11	1.95E-09	no
VASN	0.352233696	6.763898901	6.31E-11	2.02E-09	no
ITGB5	0.352122346	6.761457958	6.41E-11	2.04E-09	no
RUNX2	0.351799228	6.754376646	6.69E-11	2.13E-09	no
FYCO1	0.351696916	6.752134987	6.78E-11	2.15E-09	no
TTC7A	0.351549173	6.748898424	6.91E-11	2.19E-09	no
C17orf96	-0.351322945	-6.743943634	7.12E-11	2.26E-09	no
SIGLEC1	0.351284854	6.743109503	7.16E-11	2.26E-09	no
TIFAB	0.35071389	6.730610965	7.72E-11	2.44E-09	no
PODNL1	0.350594398	6.727996343	7.84E-11	2.47E-09	no
IGSF6	0.350506724	6.726078161	7.93E-11	2.50E-09	no
ACP2	0.350485975	6.725624225	7.95E-11	2.50E-09	no
GNG10	0.350326226	6.722129777	8.12E-11	2.55E-09	no
DRAM2	0.350324023	6.722081597	8.12E-11	2.55E-09	no
SPN	0.350318585	6.721962648	8.13E-11	2.55E-09	no
DNAJC18	-0.349792351	-6.710456274	8.71E-11	2.73E-09	no
VENTX	0.349742032	6.709356411	8.77E-11	2.74E-09	no
KCTD9	0.349704382	6.708533509	8.81E-11	2.75E-09	no
TNFRSF10D	0.349695724	6.708344267	8.82E-11	2.75E-09	no
KBTBD6	-0.349680592	-6.708013534	8.84E-11	2.75E-09	no
BAI3	-0.349616971	-6.706623109	8.91E-11	2.77E-09	no
NNMT	0.349614993	6.70657988	8.92E-11	2.77E-09	no
LRCH1	0.349588643	6.706004045	8.95E-11	2.77E-09	no
BCL3	0.349581421	6.705846211	8.96E-11	2.77E-09	no
C10orf10	0.349506364	6.704206072	9.05E-11	2.79E-09	no
AHR	0.349453673	6.703054734	9.11E-11	2.81E-09	no
TIFA	0.349419208	6.702301699	9.15E-11	2.82E-09	no
NAPSB	0.349382576	6.701501366	9.19E-11	2.83E-09	no
TIGIT	0.349286788	6.6994087	9.31E-11	2.86E-09	no
PPCS	0.349213355	6.697804603	9.40E-11	2.88E-09	no
SOD2	0.349151179	6.69644652	9.48E-11	2.90E-09	no
ICAM1	0.349046205	6.694153814	9.61E-11	2.94E-09	no
TNFSF13	0.349039469	6.694006724	9.62E-11	2.94E-09	no
GAS6	0.348787774	6.688510785	9.94E-11	3.03E-09	no
CCL22	0.348765149	6.688016825	9.97E-11	3.03E-09	no
FUCA2	0.34868939	6.686362952	1.01E-10	3.06E-09	no
TNFAIP3	0.348568166	6.683716829	1.02E-10	3.11E-09	no
ZNF519	-0.348225258	-6.676233793	1.07E-10	3.24E-09	no
CREB3L2	0.348203067	6.67574964	1.07E-10	3.25E-09	no
GIMAP7	0.348135707	6.674280106	1.08E-10	3.27E-09	no
RNF213	0.348111879	6.673760291	1.09E-10	3.28E-09	no
MANBA	0.347912878	6.669419617	1.11E-10	3.36E-09	no

CXXC4	-0.347882039	-6.668747049	1.12E-10	3.37E-09	no
OLFML2B	0.347611728	6.6628528	1.16E-10	3.48E-09	no
CLIC2	0.347465727	6.659669974	1.18E-10	3.55E-09	no
GIMAP8	0.347444257	6.659201977	1.18E-10	3.55E-09	no
FAM57B	-0.347356642	-6.657292263	1.20E-10	3.59E-09	no
CRB2	0.347028281	6.650136912	1.25E-10	3.74E-09	no
TM6SF1	0.34696307	6.648716224	1.26E-10	3.76E-09	no
UBASH3A	0.346885205	6.647020015	1.27E-10	3.80E-09	no
RAB38	0.346720215	6.64342636	1.30E-10	3.87E-09	no
CLEC6A	0.346716097	6.643336667	1.30E-10	3.87E-09	no
ICMT	0.346474749	6.638081144	1.34E-10	3.99E-09	no
TBKBP1	-0.346327233	-6.634869612	1.37E-10	4.06E-09	no
SCAMP2	0.346241823	6.633010435	1.39E-10	4.10E-09	no
AMPD3	0.346173518	6.631523729	1.40E-10	4.13E-09	no
LOC10013047€	0.346158404	6.631194774	1.40E-10	4.13E-09	no
SRSF12	-0.346127468	-6.630521494	1.41E-10	4.15E-09	no
ACVR2B	-0.345928724	-6.626196594	1.44E-10	4.25E-09	no
IFI44	0.345757529	6.622472014	1.48E-10	4.34E-09	no
GBP2	0.34555089	6.617977293	1.52E-10	4.45E-09	no
TMEM86A	0.345505884	6.616998486	1.52E-10	4.47E-09	no
HGF	0.345178417	6.609878191	1.59E-10	4.66E-09	no
CCDC109B	0.345100877	6.608192592	1.61E-10	4.70E-09	no
UBQLN4	-0.345027546	-6.606598632	1.62E-10	4.73E-09	no
PODXL2	-0.34499009	-6.605784539	1.63E-10	4.75E-09	no
SIPA1	0.344934111	6.604567897	1.64E-10	4.78E-09	no
MIR155HG	0.344471294	6.59451217	1.74E-10	5.07E-09	no
JMY	-0.344289937	-6.590573269	1.78E-10	5.18E-09	no
TMCO4	0.344149472	6.587523073	1.82E-10	5.27E-09	no
EDEM1	0.344120741	6.586899243	1.82E-10	5.28E-09	no
CRTAM	0.34404651	6.585287582	1.84E-10	5.32E-09	no
PDIA3	0.343972171	6.583673708	1.86E-10	5.37E-09	no
LIMK2	0.343940677	6.582990033	1.87E-10	5.38E-09	no
APBB1IP	0.343916291	6.582460671	1.87E-10	5.39E-09	no
STARD5	0.343825018	6.580479507	1.89E-10	5.45E-09	no
PRDM1	0.343738557	6.57860297	1.91E-10	5.50E-09	no
SNAP23	0.343731443	6.578448571	1.92E-10	5.50E-09	no
TNFSF10	0.343600988	6.575617587	1.95E-10	5.58E-09	no
PIP4K2B	-0.343531327	-6.574106071	1.97E-10	5.63E-09	no
SLC2A5	0.343471777	6.57281403	1.98E-10	5.66E-09	no
WHSC1	-0.343356868	-6.570321131	2.01E-10	5.74E-09	no
IL32	0.343347778	6.570123935	2.01E-10	5.74E-09	no
BTN3A3	0.343310752	6.569320768	2.02E-10	5.76E-09	no
JAK2	0.343197066	6.566854864	2.05E-10	5.83E-09	no
LOC10050746З	0.343112646	6.565023983	2.07E-10	5.89E-09	no
RNF166	0.343004373	6.562676029	2.10E-10	5.97E-09	no
IL4I1	0.342768175	6.557554992	2.17E-10	6.14E-09	no
SLC11A1	0.342682752	6.555703262	2.19E-10	6.20E-09	no
CD99P1	0.342574247	6.553351461	2.22E-10	6.28E-09	no
INHBA	0.342481677	6.551345283	2.25E-10	6.35E-09	no
CXorf38	0.342434257	6.550317688	2.26E-10	6.38E-09	no
GIMAP1	0.342361172	6.548734008	2.28E-10	6.43E-09	no

LBX2-AS1	0.342145049	6.544051687	2.35E-10	6.60E-09	no
C11orf75	0.341984851	6.540581731	2.40E-10	6.73E-09	no
FCGR2C	0.341934323	6.539487401	2.41E-10	6.76E-09	no
MBOAT1	0.341888308	6.53849088	2.43E-10	6.79E-09	no
CEACAM21	0.341873975	6.538180483	2.43E-10	6.80E-09	no
LOC389641	0.341718249	6.53480845	2.48E-10	6.92E-09	no
RELB	0.341390184	6.527706651	2.59E-10	7.21E-09	no
PRPS2	0.341271503	6.525138162	2.63E-10	7.31E-09	no
RARRES1	0.341040497	6.520139746	2.70E-10	7.52E-09	no
CCR6	0.341007752	6.519431336	2.72E-10	7.54E-09	no
CHPF2	0.340749674	6.513848946	2.81E-10	7.78E-09	no
ARID3A	0.340698902	6.512750917	2.83E-10	7.82E-09	no
DSE	0.340597683	6.510562074	2.86E-10	7.91E-09	no
CLEC2D	0.340593251	6.510466228	2.86E-10	7.91E-09	no
TNFSF12	0.340537008	6.509250103	2.88E-10	7.96E-09	no
EMR1	0.3404393	6.507137575	2.92E-10	8.05E-09	no
PYGL	0.340341708	6.505027799	2.96E-10	8.14E-09	no
TNFAIP2	0.340186881	6.501681156	3.02E-10	8.29E-09	no
VNN2	0.340078642	6.499341911	3.06E-10	8.39E-09	no
VAMP3	0.339877931	6.495004909	3.14E-10	8.59E-09	no
LOC286442	0.339872941	6.4948971	3.14E-10	8.59E-09	no
LPCAT2	0.339691458	6.490976488	3.21E-10	8.78E-09	no
CALHM2	0.33942662	6.485256577	3.32E-10	9.07E-09	no
FBP1	0.339358976	6.483795915	3.35E-10	9.13E-09	no
LRRC8C	0.339126128	6.478768754	3.45E-10	9.38E-09	no
PRDM11	-0.339125373	-6.478752465	3.45E-10	9.38E-09	no
TMOD3	0.339121631	6.478671685	3.45E-10	9.38E-09	no
FSD1	-0.339024642	-6.476578111	3.49E-10	9.48E-09	no
FXYD5	0.33893546	6.474653264	3.53E-10	9.58E-09	no
PRICKLE3	0.338506623	6.465400296	3.73E-10	1.01E-08	no
APOBEC3D	0.337543857	6.444643346	4.21E-10	1.14E-08	no
RNPEP	0.337194977	6.437127239	4.40E-10	1.19E-08	no
LOC100506548	-0.337187254	-6.436960892	4.40E-10	1.19E-08	no
PDZD4	-0.33698601	-6.432626794	4.52E-10	1.22E-08	no
EVA1C	0.336941373	6.431665585	4.54E-10	1.22E-08	no
KCNK13	0.336564672	6.423555826	4.76E-10	1.28E-08	no
LOC100128420	0.336407109	6.42016478	4.86E-10	1.30E-08	no
FAM214B	0.336290104	6.417647017	4.93E-10	1.32E-08	no
LILRB1	0.336280216	6.417434258	4.94E-10	1.32E-08	no
KDELC2	0.336205735	6.415831721	4.98E-10	1.33E-08	no
ARID5A	0.336203628	6.415786398	4.98E-10	1.33E-08	no
HP	0.336075224	6.413023993	5.06E-10	1.35E-08	no
CP	0.335737666	6.405763905	5.28E-10	1.41E-08	no
NUDT16P1	0.335715461	6.405286425	5.30E-10	1.41E-08	no
VSIG4	0.335666583	6.404235424	5.33E-10	1.42E-08	no
CHSY1	0.335601446	6.402834918	5.37E-10	1.43E-08	no
C5orf20	0.335535556	6.401418319	5.42E-10	1.44E-08	no
TMEM145	-0.33544732	-6.399521473	5.48E-10	1.45E-08	no
CCDC88B	0.335055606	6.391102878	5.75E-10	1.52E-08	no
SMPDL3A	0.335038446	6.39073416	5.76E-10	1.52E-08	no
XCL2	0.334682219	6.383081622	6.03E-10	1.59E-08	no

PABPC4	0.33449838	6.379133577	6.17E-10	1.62E-08	no
PHF11	0.334436815	6.37781162	6.21E-10	1.63E-08	no
SSR3	0.334427693	6.377615746	6.22E-10	1.63E-08	no
SOBP	-0.334391087	-6.376829779	6.25E-10	1.64E-08	no
ZNF101	0.334299418	6.374861668	6.32E-10	1.66E-08	no
STAT2	0.334228161	6.373331932	6.38E-10	1.67E-08	no
MBD2	0.334142619	6.371495721	6.45E-10	1.69E-08	no
CEBPD	0.333841955	6.365043129	6.69E-10	1.75E-08	no
LYL1	0.33376531	6.363398582	6.75E-10	1.76E-08	no
SAMD9	0.333732025	6.362684455	6.78E-10	1.77E-08	no
MTMR9	-0.333463068	-6.356914905	7.01E-10	1.82E-08	no
NLGN2	-0.3333618	-6.354742996	7.10E-10	1.85E-08	no
RGS2	0.333108736	6.349316586	7.33E-10	1.90E-08	no
KCNJ8	0.333042309	6.347892469	7.39E-10	1.92E-08	no
PLAUR	0.332939048	6.34567887	7.48E-10	1.94E-08	no
TRADD	0.332900229	6.344846766	7.52E-10	1.94E-08	no
TNFSF14	0.33289948	6.344830719	7.52E-10	1.94E-08	no
RHBDF2	0.332848946	6.34374756	7.57E-10	1.95E-08	no
ITGB2-AS1	0.332776473	6.342194274	7.64E-10	1.97E-08	no
RAP2B	0.332732219	6.341245859	7.68E-10	1.98E-08	no
TTC38	0.332712845	6.340830662	7.70E-10	1.98E-08	no
BCL10	0.332534999	6.337019756	7.87E-10	2.02E-08	no
HECTD4	-0.332475667	-6.33574856	7.92E-10	2.03E-08	no
TMEM194B	0.332423294	6.334626518	7.98E-10	2.04E-08	no
ERP29	0.332283982	6.331642226	8.11E-10	2.08E-08	no
THBD	0.332271968	6.33138488	8.13E-10	2.08E-08	no
SFRP4	0.332251408	6.330944504	8.15E-10	2.08E-08	no
DDB2	0.33197159	6.324951989	8.43E-10	2.15E-08	no
MAP2K3	0.331959521	6.324693548	8.45E-10	2.15E-08	no
HLA-DQA2	0.331945657	6.324396701	8.46E-10	2.15E-08	no
CRHR1-IT1	-0.331804923	-6.321383564	8.61E-10	2.19E-08	no
IL10	0.331767301	6.320578155	8.65E-10	2.19E-08	no
FGD2	0.331702062	6.319181603	8.72E-10	2.21E-08	no
FRRS1	0.331641459	6.317884374	8.78E-10	2.22E-08	no
HLA-DRB5	0.331537474	6.315658744	8.90E-10	2.25E-08	no
SOCS7	-0.331408363	-6.312895707	9.04E-10	2.28E-08	no
TREM2	0.331197023	6.308373788	9.28E-10	2.34E-08	no
FOSL2	0.330976318	6.30365261	9.53E-10	2.40E-08	no
GPR77	0.330942002	6.302918665	9.57E-10	2.41E-08	no
ORMDL2	0.330853821	6.301032757	9.68E-10	2.43E-08	no
UGCG	0.330653022	6.296738995	9.92E-10	2.49E-08	no
HSD3B7	0.330342763	6.290106513	1.03E-09	2.58E-08	no
DNASE1L1	0.330295811	6.289103005	1.04E-09	2.60E-08	no
C22orf39	-0.330268596	-6.288521359	1.04E-09	2.60E-08	no
LOC100506385	0.330261215	6.288363608	1.04E-09	2.60E-08	no
GLT25D1	0.330102086	6.284963067	1.06E-09	2.65E-08	no
SS18L1	-0.329789961	-6.278294779	1.10E-09	2.75E-08	no
TMEM255B	0.329688851	6.276135145	1.12E-09	2.78E-08	no
ADORA3	0.329462555	6.27130252	1.15E-09	2.85E-08	no
MAST1	-0.329360721	-6.269128216	1.16E-09	2.89E-08	no
OAS3	0.329228893	6.26631384	1.18E-09	2.93E-08	no

ELF1	0.329110375	6.263783983	1.20E-09	2.97E-08	no
DTX2	0.329001037	6.261450386	1.21E-09	3.01E-08	no
FCRL1	0.328985099	6.261110237	1.22E-09	3.01E-08	no
IL6R	0.328922912	6.259783121	1.23E-09	3.03E-08	no
LAG3	0.328607302	6.253049204	1.27E-09	3.14E-08	no
RNASE3	0.328260314	6.245648513	1.33E-09	3.28E-08	no
HMGCL	0.327910149	6.238182936	1.39E-09	3.41E-08	no
PDIA5	0.327541707	6.230330786	1.45E-09	3.57E-08	no
FAH	0.32749405	6.229315374	1.46E-09	3.58E-08	no
MEI1	0.327426944	6.227885629	1.47E-09	3.61E-08	no
OAS1	0.327253876	6.224198825	1.50E-09	3.68E-08	no
COL8A2	0.327109866	6.221131558	1.53E-09	3.74E-08	no
PARP10	0.326944689	6.217614057	1.56E-09	3.81E-08	no
OSTC	0.326659632	6.211545152	1.61E-09	3.94E-08	no
ATP6V1G3	0.326612495	6.210541788	1.62E-09	3.96E-08	no
KCNE3	0.326581232	6.209876348	1.63E-09	3.97E-08	no
HERC5	0.326490942	6.207954615	1.65E-09	4.01E-08	no
LOC100272216	0.326288062	6.203637232	1.69E-09	4.10E-08	no
CFH	0.326250912	6.202846765	1.70E-09	4.12E-08	no
ZBTB7B	0.326230391	6.202410131	1.70E-09	4.12E-08	no
MVP	0.325985481	6.19719994	1.75E-09	4.24E-08	no
APOL1	0.325836822	6.194038069	1.78E-09	4.31E-08	no
ELL2	0.324866018	6.173402337	2.00E-09	4.84E-08	no
TNFRSF10C	0.32479732	6.171942902	2.02E-09	4.88E-08	no
DNAJC3	0.32468357	6.169526595	2.05E-09	4.94E-08	no
KYNU	0.32464575	6.168723287	2.06E-09	4.96E-08	no
L2HGDH	-0.324430529	-6.164152529	2.11E-09	5.08E-08	no
NME8	0.324424436	6.164023153	2.11E-09	5.08E-08	no
TRPM2	0.324340005	6.16223034	2.13E-09	5.13E-08	no
C21orf88	0.324131656	6.157806987	2.19E-09	5.25E-08	no
C1orf140	0.324015486	6.155341078	2.22E-09	5.32E-08	no
SLC50A1	0.323900001	6.152890007	2.25E-09	5.39E-08	no
MYOF	0.323711145	6.148882381	2.30E-09	5.50E-08	no
DUSP23	0.32370404	6.14873162	2.30E-09	5.50E-08	no
TRPC2	0.323591499	6.146343849	2.33E-09	5.57E-08	no
ACRBP	0.32330887	6.140348585	2.42E-09	5.75E-08	no
CHRNB2	-0.323180503	-6.137626229	2.45E-09	5.84E-08	no
LAPTM4A	0.323107602	6.136080328	2.47E-09	5.88E-08	no
SOWAHD	0.323070498	6.135293563	2.48E-09	5.90E-08	no
FMO4	0.322711423	6.127681324	2.59E-09	6.15E-08	no
RASL10B	-0.32262802	-6.125913653	2.62E-09	6.21E-08	no
ITGAD	0.322543857	6.124130012	2.65E-09	6.26E-08	no
CCDC85C	-0.322419513	-6.121495147	2.69E-09	6.35E-08	no
LRRC33	0.322281026	6.118560992	2.73E-09	6.45E-08	no
SPIRE2	-0.322098922	-6.114703378	2.79E-09	6.58E-08	no
RUNDC3A	-0.322027967	-6.113200512	2.81E-09	6.63E-08	no
APOBR	0.322008047	6.112778616	2.82E-09	6.64E-08	no
COPZ2	0.321404818	6.100006731	3.03E-09	7.12E-08	no
ZNF385A	0.321341774	6.09867243	3.05E-09	7.17E-08	no
NCOA7	0.321163036	6.094889948	3.12E-09	7.32E-08	no
FBLN2	0.321147969	6.094571125	3.12E-09	7.32E-08	no

CD244	0.320948808	6.090357376	3.20E-09	7.49E-08	no
FAIM3	0.320845375	6.088169344	3.24E-09	7.57E-08	no
SERPINB6	0.320799109	6.087190697	3.26E-09	7.61E-08	no
ZFP36L2	0.320770201	6.086579242	3.27E-09	7.62E-08	no
DYRK4	0.320638679	6.083797602	3.32E-09	7.73E-08	no
CYLD	0.320630471	6.083624004	3.32E-09	7.73E-08	no
RSAD2	0.320372231	6.078163494	3.42E-09	7.97E-08	no
RNASE4	0.320357849	6.077859427	3.43E-09	7.97E-08	no
ARNT2	-0.320154006	-6.07355028	3.51E-09	8.16E-08	no
CLEC4A	0.319927192	6.068756637	3.61E-09	8.37E-08	no
ZFP36	0.319903284	6.068251414	3.62E-09	8.38E-08	no
LOC100132273	-0.319789598	-6.065849199	3.67E-09	8.49E-08	no
PGM2	0.319535312	6.06047712	3.78E-09	8.74E-08	no
IKBIP	0.319317666	6.055880249	3.88E-09	8.95E-08	no
ABI3	0.319064098	6.050526039	4.00E-09	9.21E-08	no
FSTL1	0.319043165	6.05008409	4.01E-09	9.23E-08	no
TAPBPL	0.318849093	6.045987224	4.10E-09	9.43E-08	no
SFT2D2	0.318640249	6.041579472	4.20E-09	9.65E-08	no
CHCHD6	-0.31853702	-6.039401134	4.25E-09	9.76E-08	no
DAP	0.318430511	6.037153839	4.31E-09	9.87E-08	no
CLIC1	0.318307244	6.03455327	4.37E-09	1.00E-07	no
ANXA2	0.318255143	6.033454194	4.39E-09	1.01E-07	no
FAM105A	0.318060587	6.02935054	4.50E-09	1.03E-07	no
TMEM140	0.317977019	6.027588166	4.54E-09	1.04E-07	no
BMP2K	0.317889612	6.025744995	4.59E-09	1.05E-07	no
RNF130	0.317854625	6.02500726	4.61E-09	1.05E-07	no
MYH9	0.317810649	6.024080017	4.63E-09	1.05E-07	no
ACCS	0.317760009	6.023012323	4.66E-09	1.06E-07	no
NCF1C	0.317625614	6.020179019	4.73E-09	1.07E-07	no
SQSTM1	0.317571754	6.019043666	4.76E-09	1.08E-07	no
GBP1P1	0.317504512	6.017626314	4.80E-09	1.09E-07	no
LOC646719	-0.317144143	-6.010031995	5.01E-09	1.13E-07	no
ARF6	0.316980454	6.006583431	5.10E-09	1.15E-07	no
LRP10	0.316931269	6.005547321	5.13E-09	1.16E-07	no
LILRA6	0.316855605	6.003953538	5.18E-09	1.17E-07	no
OSM	0.316794341	6.002663149	5.21E-09	1.18E-07	no
CSNK1D	0.316771353	6.002178981	5.23E-09	1.18E-07	no
KHNYN	0.316743832	6.001599364	5.24E-09	1.18E-07	no
FAM114A1	0.316708482	6.000854883	5.27E-09	1.18E-07	no
CEACAM4	0.316633518	5.999276205	5.31E-09	1.19E-07	no
CXCR3	0.316498445	5.996432005	5.40E-09	1.21E-07	no
ANKRD22	0.3164956	5.996372097	5.40E-09	1.21E-07	no
ABRACL	0.316394426	5.994241978	5.46E-09	1.22E-07	no
TMEM87B	0.316171821	5.989556013	5.61E-09	1.25E-07	no
CD1C	0.31616502	5.989412864	5.61E-09	1.25E-07	no
ADORA2A	0.316117613	5.988415082	5.64E-09	1.26E-07	no
VSTM1	0.316039386	5.986768723	5.69E-09	1.27E-07	no
RUFY3	-0.316039204	-5.986764892	5.69E-09	1.27E-07	no
PI4K2A	0.315953527	5.984961879	5.75E-09	1.28E-07	no
APEX2	0.315937004	5.98461418	5.76E-09	1.28E-07	no
DCBLD2	0.315843168	5.982639707	5.83E-09	1.29E-07	no

DERL3	0.315841616	5.982607046	5.83E-09	1.29E-07	no
CELSR3	-0.315744491	-5.980563582	5.89E-09	1.31E-07	no
ABCD1	0.315735689	5.980378401	5.90E-09	1.31E-07	no
ANXA4	0.315549283	5.976457097	6.03E-09	1.33E-07	no
STAT3	0.315515971	5.975756409	6.05E-09	1.34E-07	no
MACC1	0.315497403	5.975365882	6.06E-09	1.34E-07	no
PHLPP1	-0.315492474	-5.975262196	6.07E-09	1.34E-07	no
GNG5	0.31532243	5.971685988	6.19E-09	1.36E-07	no
BTLA	0.31528947	5.970992878	6.21E-09	1.37E-07	no
MCMDC2	-0.315178054	-5.968650099	6.29E-09	1.38E-07	no
TBC1D22A	0.315130174	5.967643383	6.33E-09	1.39E-07	no
LINC00619	0.315014773	5.965217234	6.41E-09	1.41E-07	no
C16orf45	-0.314979718	-5.964480294	6.44E-09	1.41E-07	no
CRISPLD2	0.314867274	5.962116686	6.52E-09	1.43E-07	no
REL	0.31457364	5.955945674	6.75E-09	1.48E-07	no
IRF2	0.314540582	5.955251043	6.78E-09	1.48E-07	no
NLRC4	0.314516841	5.954752199	6.79E-09	1.48E-07	no
CA13	0.314481631	5.954012405	6.82E-09	1.49E-07	no
SPP1	0.31434489	5.951139593	6.93E-09	1.51E-07	no
DPAGT1	0.314212065	5.948349438	7.04E-09	1.53E-07	no
VAMP5	0.313726769	5.938158471	7.44E-09	1.62E-07	no
KIF3A	-0.313706734	-5.93773786	7.46E-09	1.62E-07	no
GPSM1	-0.313395901	-5.931213393	7.73E-09	1.68E-07	no
NAGK	0.313344506	5.930134789	7.78E-09	1.69E-07	no
NRXN1	-0.313300555	-5.929212479	7.82E-09	1.69E-07	no
DDX58	0.313293934	5.929073528	7.83E-09	1.69E-07	no
CEP68	-0.313079626	-5.92457684	8.02E-09	1.73E-07	no
ADAMTS12	0.313015997	5.923241956	8.08E-09	1.74E-07	no
SPIB	0.312989397	5.922683936	8.10E-09	1.75E-07	no
PLB1	0.312839974	5.919549592	8.24E-09	1.78E-07	no
USB1	0.312813965	5.919004067	8.27E-09	1.78E-07	no
ACOT9	0.312642619	5.915410543	8.43E-09	1.81E-07	no
HPGDS	0.312613876	5.914807793	8.46E-09	1.82E-07	no
TWF2	0.312561073	5.913700562	8.51E-09	1.83E-07	no
FPGT	0.312517322	5.912783179	8.56E-09	1.83E-07	no
ATP9A	-0.312379961	-5.909903244	8.69E-09	1.86E-07	no
AIDA	0.312289109	5.907998647	8.78E-09	1.88E-07	no
PNMA1	-0.312237975	-5.906926754	8.84E-09	1.89E-07	no
AAED1	0.312235436	5.90687354	8.84E-09	1.89E-07	no
ZDHHC12	0.31216524	5.905402179	8.91E-09	1.90E-07	no
LOC730102	0.312165105	5.905399347	8.91E-09	1.90E-07	no
CPA3	0.312092914	5.903886268	8.98E-09	1.91E-07	no
CD164	0.31207962	5.903607641	9.00E-09	1.91E-07	no
LOXL3	0.311950578	5.900903337	9.13E-09	1.94E-07	no
PLEKHA4	0.311928073	5.900431748	9.16E-09	1.94E-07	no
C14orf132	-0.311902918	-5.89990463	9.18E-09	1.95E-07	no
PFN1	0.311687384	5.895388761	9.41E-09	1.99E-07	no
MACROD1	-0.311417901	-5.889743969	9.71E-09	2.05E-07	no
MAF	0.31131997	5.887693028	9.82E-09	2.07E-07	no
RALGPS1	-0.311205073	-5.885287029	9.95E-09	2.10E-07	no
HOXB-AS3	0.311183252	5.884830117	9.97E-09	2.10E-07	no

AXL	0.311069615	5.88245085	1.01E-08	2.13E-07	no
LILRB3	0.311054213	5.882128391	1.01E-08	2.13E-07	no
CACNA2D4	0.310936402	5.879662054	1.03E-08	2.16E-07	no
CDK5R1	-0.310772119	-5.876223343	1.04E-08	2.20E-07	no
CYBRD1	0.310666457	5.874011967	1.06E-08	2.22E-07	no
SVIL	0.31065477	5.873767397	1.06E-08	2.22E-07	no
HLX	0.310527694	5.871108213	1.07E-08	2.25E-07	no
EPHA2	0.310467433	5.869847304	1.08E-08	2.26E-07	no
FAM26E	0.310423334	5.868924631	1.09E-08	2.27E-07	no
IL21R	0.310373858	5.867889509	1.09E-08	2.28E-07	no
OR2A9P	0.310314373	5.866645037	1.10E-08	2.30E-07	no
DPY19L1	0.310313832	5.86663373	1.10E-08	2.30E-07	no
PSTPIP2	0.310230712	5.864894931	1.11E-08	2.31E-07	no
SUSD1	0.310191784	5.86408065	1.12E-08	2.32E-07	no
PLEKHO2	0.309677226	5.853320303	1.18E-08	2.46E-07	no
TNFAIP8	0.309638785	5.852516676	1.19E-08	2.47E-07	no
DPEP2	0.309504934	5.84971864	1.21E-08	2.50E-07	no
ANKRD13B	-0.309491977	-5.84944781	1.21E-08	2.51E-07	no
TAB1	-0.309287419	-5.84517254	1.24E-08	2.56E-07	no
CREM	0.309262124	5.844643922	1.24E-08	2.57E-07	no
PRF1	0.309198799	5.843320645	1.25E-08	2.58E-07	no
CKB	-0.309023474	-5.839657378	1.27E-08	2.63E-07	no
USP11	-0.308955236	-5.838231779	1.28E-08	2.65E-07	no
GALNT12	0.308929823	5.837700895	1.29E-08	2.66E-07	no
FLJ42627	-0.308630751	-5.831454158	1.33E-08	2.74E-07	no
KLHL25	-0.308569649	-5.830178156	1.34E-08	2.76E-07	no
CLEC17A	0.308543347	5.829628922	1.35E-08	2.77E-07	no
SLCO2B1	0.308530704	5.829364908	1.35E-08	2.77E-07	no
RINL	0.308472014	5.828139389	1.36E-08	2.78E-07	no
YY2	0.308217376	5.822823123	1.40E-08	2.86E-07	no
BDKRB2	0.308204207	5.822548228	1.40E-08	2.86E-07	no
EPB41L4A-AS1	-0.308186833	-5.822185565	1.40E-08	2.87E-07	no
FAM109B	0.30810628	5.820504141	1.41E-08	2.89E-07	no
SLC2A10	0.30801359	5.818569535	1.43E-08	2.92E-07	no
VRK2	0.307549189	5.808879474	1.51E-08	3.07E-07	no
CLIC3	0.307423217	5.806251756	1.53E-08	3.11E-07	no
VEGFC	0.307345952	5.804640216	1.54E-08	3.14E-07	no
CIRBP	-0.30728907	-5.803453897	1.55E-08	3.15E-07	no
ARL3	-0.307225308	-5.80212417	1.56E-08	3.17E-07	no
PTRF	0.307056525	5.798604683	1.59E-08	3.23E-07	no
ADCY7	0.307033767	5.798130183	1.60E-08	3.24E-07	no
OLR1	0.306925973	5.795882823	1.62E-08	3.27E-07	no
CD47	0.306809909	5.793463307	1.64E-08	3.31E-07	no
AKNA	0.306723685	5.791666042	1.65E-08	3.34E-07	no
NDRG2	-0.306661518	-5.79037032	1.66E-08	3.36E-07	no
ESYT1	0.306635546	5.789829014	1.67E-08	3.37E-07	no
SLC25A24	0.306598104	5.789048673	1.68E-08	3.38E-07	no
HSPA7	0.306414294	5.785218326	1.71E-08	3.45E-07	no
CTSL1	0.306297381	5.782782382	1.73E-08	3.49E-07	no
TMED7-TICAM2	0.306212812	5.781020517	1.75E-08	3.52E-07	no
TAB2	0.306102226	5.778716877	1.77E-08	3.56E-07	no

CASC3	-0.305811967	-5.772671622	1.83E-08	3.67E-07	no
HMOX1	0.305374795	5.763569958	1.92E-08	3.86E-07	no
DUSP26	-0.305325479	-5.762543475	1.93E-08	3.87E-07	no
LPAR6	0.305235628	5.760673436	1.95E-08	3.91E-07	no
RPAIN	-0.305102867	-5.757910607	1.98E-08	3.96E-07	no
TMEM178B	-0.304996069	-5.755688353	2.01E-08	4.01E-07	no
DNAJC22	0.304744964	5.750464305	2.06E-08	4.12E-07	no
PALMD	0.304712864	5.749796592	2.07E-08	4.13E-07	no
MIR22HG	0.304652294	5.748536718	2.08E-08	4.15E-07	no
SEPHS2	0.304649272	5.74847387	2.09E-08	4.15E-07	no
COTL1	0.304620881	5.747883346	2.09E-08	4.16E-07	no
SLIT1	-0.304610313	-5.747663547	2.09E-08	4.16E-07	no
SLC35F6	0.304517537	5.745734008	2.12E-08	4.20E-07	no
BNIP2	0.304483724	5.745030816	2.12E-08	4.21E-07	no
ERP44	0.304451252	5.744355542	2.13E-08	4.22E-07	no
IL18RAP	0.304324248	5.74171461	2.16E-08	4.28E-07	no
SLC25A39	0.304256786	5.740311929	2.18E-08	4.31E-07	no
IRAK1	0.304174179	5.738594492	2.20E-08	4.34E-07	no
ADAM12	0.30408037	5.736644318	2.22E-08	4.38E-07	no
HIST1H2BK	0.303885995	5.732604104	2.27E-08	4.48E-07	no
TC2N	0.303848797	5.731831015	2.28E-08	4.49E-07	no
TNFRSF17	0.303686418	5.728456597	2.32E-08	4.57E-07	no
IGFBP7	0.303501846	5.724621643	2.37E-08	4.66E-07	no
CHI3L2	0.303479725	5.724162066	2.38E-08	4.67E-07	no
FCRL6	0.303333786	5.721130379	2.41E-08	4.74E-07	no
ZNF552	0.303291982	5.720262046	2.43E-08	4.76E-07	no
SRPR	0.303203666	5.718427694	2.45E-08	4.80E-07	no
BLVRB	0.30320004	5.718352385	2.45E-08	4.80E-07	no
FAM167B	0.303152727	5.717369749	2.46E-08	4.82E-07	no
IFI27	0.30311665	5.716620511	2.47E-08	4.83E-07	no
HLA-DRB4	0.30310224	5.716321243	2.48E-08	4.83E-07	no
TOM1L1	0.303033469	5.714893106	2.50E-08	4.87E-07	no
TMEM176B	0.303021698	5.714648671	2.50E-08	4.87E-07	no
FAM115C	0.303004037	5.714281936	2.50E-08	4.87E-07	no
LATS2	0.302781107	5.709653248	2.57E-08	4.99E-07	no
MICAL3	-0.302378249	-5.701291345	2.68E-08	5.21E-07	no
STEAP3	0.30219507	5.69749032	2.74E-08	5.32E-07	no
ARAP1	0.302153894	5.696636004	2.75E-08	5.34E-07	no
CYP27A1	0.302022003	5.693899745	2.79E-08	5.41E-07	no
WIPI1	0.301792596	5.689141284	2.86E-08	5.54E-07	no
MAGEH1	-0.301686853	-5.686948267	2.90E-08	5.60E-07	no
TMEM170B	-0.301457004	-5.682182201	2.97E-08	5.74E-07	no
ANXA1	0.301446057	5.681955247	2.97E-08	5.74E-07	no
SIGLEC5	0.301409284	5.681192839	2.99E-08	5.76E-07	no
NADK	0.301095672	5.674691971	3.09E-08	5.96E-07	no
HDGFRP3	-0.3010012	-5.67273407	3.12E-08	6.02E-07	no
IFNAR2	0.300963871	5.671960481	3.14E-08	6.04E-07	no
MAP3K14	0.300923651	5.671127011	3.15E-08	6.06E-07	no
TEX261	0.300290397	5.658008693	3.38E-08	6.49E-07	no
HLA-DRB6	0.300273851	5.657666046	3.38E-08	6.49E-07	no
MAGT1	0.300261544	5.65741119	3.39E-08	6.50E-07	no

FAM96A	0.300254867	5.657272907	3.39E-08	6.50E-07	no
TDO2	0.300108304	5.654238032	3.45E-08	6.59E-07	no
VNN1	0.300084077	5.653736401	3.46E-08	6.61E-07	no
TAP1	0.300045649	5.652940768	3.47E-08	6.63E-07	no
IMPA2	0.300029111	5.652598354	3.48E-08	6.63E-07	no
SCIN	0.299973236	5.651441564	3.50E-08	6.67E-07	no
DDX51	-0.299956047	-5.651085718	3.50E-08	6.67E-07	no
KIAA0895L	-0.299955797	-5.651080535	3.50E-08	6.67E-07	no
MAP4K1	0.299811817	5.648100014	3.56E-08	6.77E-07	no
GPR82	0.299757446	5.646974593	3.58E-08	6.80E-07	no
ITPK1	-0.299626529	-5.644265003	3.63E-08	6.90E-07	no
CXCR2P1	0.299599741	5.643710632	3.64E-08	6.91E-07	no
PREB	0.299592114	5.643552783	3.65E-08	6.91E-07	no
SERPINB1	0.299389386	5.639357762	3.73E-08	7.06E-07	no
LINGO1	-0.299356073	-5.638668501	3.74E-08	7.08E-07	no
TTF2	0.299310944	5.637734804	3.76E-08	7.11E-07	no
REXO2	0.29928671	5.637233412	3.77E-08	7.12E-07	no
CNTFR	-0.299205483	-5.635553001	3.80E-08	7.18E-07	no
PRAM1	0.299155836	5.634525979	3.82E-08	7.21E-07	no
FUOM	0.298856497	5.628334749	3.95E-08	7.44E-07	no
C6orf62	0.298665124	5.624377554	4.04E-08	7.59E-07	no
NCF1B	0.29847998	5.620549852	4.12E-08	7.73E-07	no
SLC29A1	0.298477401	5.620496536	4.12E-08	7.73E-07	no
FHOD1	0.298476024	5.62046807	4.12E-08	7.73E-07	no
IL27	0.298448793	5.619905164	4.13E-08	7.75E-07	no
GPR84	0.298427464	5.619464247	4.14E-08	7.76E-07	no
TET1	-0.298306811	-5.61697036	4.20E-08	7.85E-07	no
SLAMF6	0.29804821	5.611626079	4.32E-08	8.07E-07	no
DUSP1	0.297903051	5.60862679	4.38E-08	8.19E-07	no
KLRF1	0.297854244	5.607618451	4.41E-08	8.23E-07	no
TGFBR1	0.297819229	5.606895062	4.42E-08	8.25E-07	no
RPRD1A	-0.297796905	-5.606433875	4.44E-08	8.27E-07	no
ACAA2	0.297769818	5.605874309	4.45E-08	8.28E-07	no
NAALADL1	0.297760275	5.605677172	4.45E-08	8.28E-07	no
ARHGEF35	0.297754852	5.605565135	4.46E-08	8.28E-07	no
PI4K2B	0.297744364	5.605348481	4.46E-08	8.29E-07	no
CCR5	0.297593758	5.602237592	4.53E-08	8.41E-07	no
LYRM7	-0.297573966	-5.601828813	4.54E-08	8.43E-07	no
CCL2	0.297387331	5.597974431	4.64E-08	8.59E-07	no
37865	-0.297380641	-5.597836273	4.64E-08	8.59E-07	no
RPN2	0.297357097	5.597350095	4.65E-08	8.60E-07	no
FCGBP	0.297224258	5.594607213	4.72E-08	8.72E-07	no
DDX25	-0.297214266	-5.59440091	4.72E-08	8.72E-07	no
PICALM	0.297179897	5.593691315	4.74E-08	8.75E-07	no
MYEF2	-0.296958478	-5.589120403	4.86E-08	8.95E-07	no
CD302	0.296800594	5.585861703	4.94E-08	9.10E-07	no
RNLS	0.296788966	5.585621729	4.95E-08	9.10E-07	no
LRRC16B	-0.296763521	-5.585096594	4.96E-08	9.12E-07	no
NXF3	0.296633457	5.582412571	5.03E-08	9.24E-07	no
DHX58	0.296499633	5.579651294	5.11E-08	9.37E-07	no
CECR2	-0.296474962	-5.579142289	5.12E-08	9.39E-07	no

LSAMP	-0.296402554	-5.577648432	5.16E-08	9.45E-07	no
THBS1	0.296212536	5.573728674	5.27E-08	9.64E-07	no
PURG	-0.296116855	-5.571755203	5.32E-08	9.73E-07	no
FOLR2	0.295581302	5.560712547	5.64E-08	1.03E-06	no
ITGB4	0.295540524	5.559871972	5.66E-08	1.03E-06	no
SEC22B	0.295505241	5.559144709	5.68E-08	1.04E-06	no
RRAS	0.295474423	5.558509486	5.70E-08	1.04E-06	no
KBTBD7	-0.29542583	-5.557507914	5.73E-08	1.04E-06	no
EMP1	0.295353704	5.55602141	5.78E-08	1.05E-06	no
CAT	0.295342323	5.555786859	5.78E-08	1.05E-06	no
HTR7	0.295306176	5.555041925	5.81E-08	1.05E-06	no
UNC13A	-0.295267736	-5.554249754	5.83E-08	1.06E-06	no
HPS1	0.295079676	5.550374642	5.95E-08	1.08E-06	no
SCHIP1	-0.295020618	-5.549157869	5.99E-08	1.09E-06	no
OSTM1	0.294986522	5.548455412	6.01E-08	1.09E-06	no
KLHDC3	-0.29485155	-5.545674914	6.10E-08	1.10E-06	no
CNRIP1	-0.294809003	-5.544798505	6.13E-08	1.11E-06	no
BTBD19	0.294672528	5.541987535	6.22E-08	1.12E-06	no
RPS6KA4	0.294596714	5.540426153	6.27E-08	1.13E-06	no
LRRK1	0.294433306	5.537061177	6.38E-08	1.15E-06	no
CSTB	0.294339977	5.535139535	6.44E-08	1.16E-06	no
CYP4V2	0.294200937	5.532277027	6.54E-08	1.18E-06	no
RAB43	0.293989762	5.527930174	6.69E-08	1.20E-06	no
ZNF664-FAM10	-0.293956267	-5.527240786	6.71E-08	1.21E-06	no
ENTPD1	0.293843099	5.524911754	6.79E-08	1.22E-06	no
IER3	0.293766558	5.523336671	6.85E-08	1.23E-06	no
NAMPT	0.293367898	5.515134766	7.15E-08	1.28E-06	no
ZCCHC14	-0.293252386	-5.512758862	7.24E-08	1.30E-06	no
STX3	0.293234182	5.512384455	7.25E-08	1.30E-06	no
IFNGR1	0.293175883	5.51118544	7.30E-08	1.30E-06	no
FCER1A	0.293172142	5.511108514	7.30E-08	1.30E-06	no
GSTA4	-0.293068808	-5.508983469	7.38E-08	1.32E-06	no
GCSH	-0.2929229	-5.505983249	7.50E-08	1.34E-06	no
SLA	0.29261266	5.499605386	7.75E-08	1.38E-06	no
MIS18BP1	0.292430274	5.495856822	7.90E-08	1.41E-06	no
EPS8	0.292321735	5.493626333	7.99E-08	1.42E-06	no
CELF5	-0.292146972	-5.490035423	8.14E-08	1.45E-06	no
SMPD3	-0.292119319	-5.489467278	8.17E-08	1.45E-06	no
LOC100133445	0.291672794	5.480295364	8.56E-08	1.52E-06	no
SLC10A3	0.291667186	5.480180198	8.57E-08	1.52E-06	no
ACTL6B	-0.291665323	-5.480141944	8.57E-08	1.52E-06	no
NALCN	-0.2916559	-5.47994843	8.58E-08	1.52E-06	no
TNFRSF13B	0.291554713	5.477870547	8.67E-08	1.53E-06	no
PDP2	-0.291284386	-5.472320382	8.92E-08	1.58E-06	no
MMP11	0.291263562	5.471892913	8.94E-08	1.58E-06	no
MPZL2	0.291251518	5.471645662	8.96E-08	1.58E-06	no
BACE2	0.291170516	5.469982914	9.03E-08	1.59E-06	no
ITPRIPL2	0.291126074	5.469070706	9.08E-08	1.60E-06	no
PRR13	0.291105422	5.468646809	9.10E-08	1.60E-06	no
FHL3	0.291045817	5.467423445	9.15E-08	1.61E-06	no
SAA2	0.291037327	5.467249196	9.16E-08	1.61E-06	no

HEG1	0.290899305	5.464416655	9.30E-08	1.63E-06	no
FGR	0.290852206	5.463450137	9.34E-08	1.64E-06	no
PRDX6	0.290703704	5.460403065	9.49E-08	1.66E-06	no
FASN	-0.290679206	-5.459900449	9.51E-08	1.67E-06	no
TSC22D1	-0.29060655	-5.458409822	9.59E-08	1.68E-06	no
GDF5	0.290509464	5.45641817	9.69E-08	1.69E-06	no
SYTL1	0.290455414	5.455309444	9.74E-08	1.70E-06	no
ZNF554	-0.290414986	-5.454480172	9.78E-08	1.71E-06	no
LRRC4	-0.290228178	-5.450648775	9.98E-08	1.74E-06	no
CAST	0.290159899	5.449248553	1.01E-07	1.75E-06	no
SIGLEC14	0.29015208	5.449088226	1.01E-07	1.75E-06	no
HEBP2	0.290145313	5.448949456	1.01E-07	1.75E-06	no
THAP10	-0.290112805	-5.448282831	1.01E-07	1.76E-06	no
LITAF	0.290102222	5.448065822	1.01E-07	1.76E-06	no
GGT5	0.290073291	5.447472593	1.01E-07	1.76E-06	no
BGN	0.289925792	5.444448344	1.03E-07	1.79E-06	no
PNRC1	0.289743635	5.440714049	1.05E-07	1.82E-06	no
PKD2	0.289713821	5.440102925	1.05E-07	1.82E-06	no
ZDHHC22	-0.289568701	-5.437128451	1.07E-07	1.85E-06	no
GLUD1P7	-0.289546072	-5.436664674	1.07E-07	1.85E-06	no
MMP14	0.289474533	5.43519856	1.08E-07	1.87E-06	no
UBE2O	-0.289362603	-5.432904856	1.09E-07	1.89E-06	no
LOC100129794	-0.289342081	-5.432484339	1.10E-07	1.89E-06	no
BRMS1L	-0.289240132	-5.430395436	1.11E-07	1.91E-06	no
LOC257358	0.289162433	5.428803528	1.12E-07	1.92E-06	no
B3GNT8	0.289022236	5.425931477	1.13E-07	1.95E-06	no
GCNT1	0.288972442	5.424911473	1.14E-07	1.96E-06	no
PRRX1	0.288963639	5.424731177	1.14E-07	1.96E-06	no
GNL1	-0.288962453	-5.424706865	1.14E-07	1.96E-06	no
ATL3	0.288851845	5.422441365	1.15E-07	1.98E-06	no
GNAO1	-0.288759126	-5.420542449	1.16E-07	2.00E-06	no
CRY2	-0.288653028	-5.418369729	1.18E-07	2.02E-06	no
ALDOC	-0.288469617	-5.414614275	1.20E-07	2.05E-06	no
CD53	0.288404277	5.41327654	1.21E-07	2.07E-06	no
BNC2	0.288380369	5.412787083	1.21E-07	2.07E-06	no
SERP2	-0.287955364	-5.404088078	1.27E-07	2.16E-06	no
NEU1	0.287869693	5.402334984	1.28E-07	2.18E-06	no
LGALS2	0.28770368	5.39893824	1.30E-07	2.22E-06	no
SNX10	0.287667717	5.398202481	1.31E-07	2.22E-06	no
POU6F1	-0.287641183	-5.397659648	1.31E-07	2.23E-06	no
LINC00488	0.287526319	5.395309893	1.33E-07	2.25E-06	no
LOC115110	0.287397706	5.3926792	1.34E-07	2.28E-06	no
CPED1	0.2873939	5.392601338	1.34E-07	2.28E-06	no
MALT1	0.287370524	5.392123242	1.35E-07	2.29E-06	no
MORN4	-0.287345903	-5.391619686	1.35E-07	2.29E-06	no
C19orf66	0.287305242	5.390788084	1.36E-07	2.30E-06	no
LINC00641	-0.287282623	-5.390325492	1.36E-07	2.30E-06	no
CACNG2	-0.287142863	-5.387467453	1.38E-07	2.33E-06	no
RPS6KL1	-0.287123674	-5.387075073	1.38E-07	2.34E-06	no
ATP1B3	0.286958255	5.383692868	1.41E-07	2.37E-06	no
INA	-0.286953025	-5.383585946	1.41E-07	2.37E-06	no

YPEL1	-0.28695155	-5.383555773	1.41E-07	2.37E-06	no
TGOLN2	0.286943645	5.383394172	1.41E-07	2.37E-06	no
EVA1B	0.286890281	5.3823032	1.42E-07	2.38E-06	no
FP588	-0.286823741	-5.380942941	1.43E-07	2.40E-06	no
SOAT1	0.286760445	5.379649058	1.44E-07	2.41E-06	no
SPTBN2	-0.286689572	-5.37820039	1.45E-07	2.43E-06	no
HIRA	-0.286669564	-5.377791449	1.45E-07	2.43E-06	no
C15orf59	-0.286640527	-5.37719796	1.45E-07	2.44E-06	no
PLAU	0.286612156	5.376618108	1.46E-07	2.44E-06	no
MICALL2	0.286587094	5.376105889	1.46E-07	2.45E-06	no
MSRB3	0.286532328	5.374986615	1.47E-07	2.46E-06	no
TEP1	0.286522297	5.374781623	1.47E-07	2.46E-06	no
SHQ1	0.286342966	5.371117042	1.50E-07	2.50E-06	no
RRAGB	-0.286316062	-5.370567326	1.50E-07	2.51E-06	no
EMILIN2	0.286278766	5.369805295	1.51E-07	2.52E-06	no
ADCK2	0.286238035	5.368973113	1.52E-07	2.53E-06	no
ESPNL	0.28612731	5.366710992	1.53E-07	2.55E-06	no
PROS1	0.285948978	5.363068194	1.56E-07	2.60E-06	no
TPST1	0.285934494	5.362772341	1.56E-07	2.60E-06	no
KDELR3	0.285781791	5.359653586	1.59E-07	2.64E-06	no
SNX32	-0.285761899	-5.359247342	1.59E-07	2.64E-06	no
ATP8B1	0.285709541	5.358178122	1.60E-07	2.66E-06	no
MXRA5	0.285653308	5.357029833	1.61E-07	2.67E-06	no
ZNF529	-0.285557631	-5.355076202	1.63E-07	2.69E-06	no
GALNT5	0.285518042	5.3542679	1.63E-07	2.70E-06	no
IRF5	0.285287582	5.349563039	1.67E-07	2.77E-06	no
LRP4	-0.285242183	-5.348636333	1.68E-07	2.78E-06	no
TCTN1	0.285239815	5.348587997	1.68E-07	2.78E-06	no
COL15A1	0.284779333	5.339190684	1.76E-07	2.91E-06	no
GRIA4	-0.284758379	-5.338763156	1.77E-07	2.91E-06	no
GSDMD	0.284713024	5.337837807	1.78E-07	2.92E-06	no
DCUN1D5	-0.284708568	-5.337746885	1.78E-07	2.92E-06	no
FXR2	-0.284432838	-5.332122209	1.83E-07	3.01E-06	no
SYK	0.284418869	5.331837279	1.83E-07	3.01E-06	no
TOR3A	0.284228127	5.327947189	1.87E-07	3.06E-06	no
IBSP	0.284092847	5.325188636	1.89E-07	3.11E-06	no
XKR8	0.284068809	5.324698505	1.90E-07	3.11E-06	no
GATAD2B	-0.284020556	-5.323714644	1.91E-07	3.12E-06	no
FAM168B	-0.284019236	-5.323687735	1.91E-07	3.12E-06	no
LOC10050610C	0.284017324	5.32364875	1.91E-07	3.12E-06	no
FOSL1	0.283998554	5.323266054	1.91E-07	3.12E-06	no
NGFRAP1	-0.283937015	-5.322011417	1.92E-07	3.14E-06	no
COX20	-0.28392481	-5.321762586	1.93E-07	3.14E-06	no
MICA	0.28388927	5.321038042	1.93E-07	3.15E-06	no
CYB5R1	0.283828562	5.319800468	1.95E-07	3.17E-06	no
SH3BP1	0.283746524	5.318128171	1.96E-07	3.19E-06	no
THRA	-0.283521308	-5.31353791	2.01E-07	3.27E-06	no
RSF1	-0.2834824	-5.312745003	2.02E-07	3.28E-06	no
SMOC1	-0.283474142	-5.312576716	2.02E-07	3.28E-06	no
LOC400027	-0.283452982	-5.312145524	2.02E-07	3.28E-06	no
WHAMMP1	0.283396958	5.311003884	2.03E-07	3.30E-06	no

C17orf100	-0.28326806	-5.308377476	2.06E-07	3.34E-06	no
CXADR	0.283233222	5.307667665	2.07E-07	3.35E-06	no
CISH	0.282813715	5.299122289	2.16E-07	3.49E-06	no
DNAJC27-AS1	-0.282725838	-5.297332668	2.18E-07	3.52E-06	no
RIC3	-0.282576726	-5.294296275	2.21E-07	3.57E-06	no
MT1A	0.282482329	5.292374282	2.23E-07	3.61E-06	no
GPR173	-0.282395661	-5.290609807	2.25E-07	3.64E-06	no
ASTN1	-0.282347698	-5.28963339	2.26E-07	3.65E-06	no
LOC100287846	-0.282301296	-5.288688775	2.28E-07	3.67E-06	no
GLUD1	-0.282198195	-5.286590118	2.30E-07	3.70E-06	no
CXCL13	0.282118421	5.284966412	2.32E-07	3.73E-06	no
ARMCX4	-0.282083381	-5.284253241	2.33E-07	3.74E-06	no
ZNF445	-0.282055413	-5.283684029	2.33E-07	3.75E-06	no
SPTLC3	0.281988144	5.282315024	2.35E-07	3.77E-06	no
KDM3B	-0.281962654	-5.281796305	2.36E-07	3.78E-06	no
ERMAP	0.281937037	5.281274986	2.36E-07	3.79E-06	no
FBXO6	0.281930306	5.281138024	2.36E-07	3.79E-06	no
NINJ1	0.28191143	5.280753896	2.37E-07	3.79E-06	no
ISG15	0.281754735	5.277565512	2.41E-07	3.85E-06	no
RFTN2	-0.281740966	-5.277285367	2.41E-07	3.85E-06	no
TMEM214	0.281701387	5.276480101	2.42E-07	3.86E-06	no
ITGA5	0.281700334	5.276458687	2.42E-07	3.86E-06	no
CRLF3	0.281684731	5.276141237	2.42E-07	3.86E-06	no
EMR2	0.281616549	5.274754129	2.44E-07	3.89E-06	no
LILRA2	0.281555853	5.273519388	2.46E-07	3.91E-06	no
ACTN1	0.281371896	5.269777551	2.50E-07	3.98E-06	no
ACSS1	-0.281359902	-5.269533587	2.51E-07	3.98E-06	no
UBE2Z	0.281335236	5.269031919	2.51E-07	3.99E-06	no
SGMS2	0.281260951	5.267521126	2.53E-07	4.02E-06	no
CCL19	0.281194191	5.266163472	2.55E-07	4.04E-06	no
LRRTM3	-0.281154185	-5.265349924	2.56E-07	4.05E-06	no
DEAF1	-0.281062955	-5.263494839	2.58E-07	4.09E-06	no
UNC79	-0.281054886	-5.263330778	2.58E-07	4.09E-06	no
PTGIR	0.281000825	5.262231567	2.60E-07	4.11E-06	no
TNK2	-0.280990607	-5.262023808	2.60E-07	4.11E-06	no
ZDHHC24	0.280772799	5.257595771	2.66E-07	4.20E-06	no
RAC3	-0.280689084	-5.255894079	2.68E-07	4.23E-06	no
FAXC	-0.280391526	-5.249846622	2.76E-07	4.36E-06	no
NFE2L2	0.280343568	5.248872103	2.78E-07	4.38E-06	no
CDH11	0.280309582	5.248181518	2.79E-07	4.39E-06	no
C5orf30	-0.280199788	-5.245950667	2.82E-07	4.44E-06	no
TMEM234	0.280182872	5.245607001	2.82E-07	4.44E-06	no
NTNG2	-0.280113754	-5.244202772	2.84E-07	4.47E-06	no
CAMSAP3	-0.27998843	-5.24165686	2.88E-07	4.52E-06	no
CDH20	-0.279922082	-5.240309138	2.90E-07	4.55E-06	no
PSME2	0.279878734	5.239428678	2.91E-07	4.57E-06	no
CSF2RA	0.279860253	5.239053289	2.92E-07	4.57E-06	no
RENBP	0.279766194	5.237142943	2.95E-07	4.61E-06	no
ADAM22	-0.279578525	-5.233331849	3.00E-07	4.70E-06	no
DUSP22	0.279567856	5.233115207	3.01E-07	4.70E-06	no
EML5	-0.279514388	-5.232029532	3.02E-07	4.72E-06	no

AGA	0.279490968	5.23155399	3.03E-07	4.73E-06	no
OPHN1	-0.27947034	-5.231135172	3.04E-07	4.73E-06	no
MEDAG	0.279436993	5.2304581	3.05E-07	4.75E-06	no
BCL7A	-0.279384192	-5.229386106	3.06E-07	4.77E-06	no
DOCK11	0.27918624	5.225367592	3.12E-07	4.86E-06	no
SMIM13	-0.279113695	-5.223895081	3.15E-07	4.89E-06	no
TBC1D15	0.279009739	5.221785162	3.18E-07	4.94E-06	no
HOXB9	0.27888772	5.219308871	3.22E-07	5.00E-06	no
LMF1	-0.278837922	-5.218298338	3.24E-07	5.02E-06	no
CD44	0.278802012	5.217569669	3.25E-07	5.04E-06	no
RAB34	0.278738165	5.216274153	3.27E-07	5.07E-06	no
LOC100506190	0.278621637	5.213909875	3.31E-07	5.12E-06	no
HIST1H2AC	0.278524123	5.211931585	3.34E-07	5.17E-06	no
C17orf75	-0.278367264	-5.2087497	3.39E-07	5.25E-06	no
PAR-SN	-0.27831458	-5.207681116	3.41E-07	5.27E-06	no
S1PR3	0.278252654	5.206425125	3.43E-07	5.30E-06	no
GALNT2	0.278249803	5.206367302	3.43E-07	5.30E-06	no
IL7	0.278083453	5.20299376	3.49E-07	5.38E-06	no
SIGLEC15	0.278073965	5.202801345	3.50E-07	5.39E-06	no
RPL39L	0.278052266	5.202361348	3.50E-07	5.39E-06	no
FOXP3	0.277994229	5.201184506	3.52E-07	5.42E-06	no
TMEM8A	0.277989634	5.201091335	3.53E-07	5.42E-06	no
BCAN	-0.277917816	-5.199635162	3.55E-07	5.45E-06	no
MAPK12	-0.277911816	-5.199513502	3.55E-07	5.45E-06	no
TREML3P	0.277865198	5.198568343	3.57E-07	5.48E-06	no
TMEM59	0.277825288	5.197759212	3.58E-07	5.49E-06	no
VASP	0.277776339	5.19676686	3.60E-07	5.52E-06	no
FAM110C	0.277752033	5.196274117	3.61E-07	5.53E-06	no
MYH7B	-0.277746591	-5.196163807	3.61E-07	5.53E-06	no
LOXL1	0.277678499	5.194783463	3.64E-07	5.56E-06	no
STK38	0.277661768	5.194444327	3.64E-07	5.56E-06	no
PPM1L	-0.27765788	-5.194365512	3.64E-07	5.56E-06	no
KIF1B	-0.277558968	-5.192360597	3.68E-07	5.61E-06	no
LRRC2	0.277537865	5.19193287	3.69E-07	5.62E-06	no
ZNF25	-0.277476915	-5.190697544	3.71E-07	5.65E-06	no
CBS	-0.277450381	-5.190159786	3.72E-07	5.66E-06	no
SMIM18	-0.27726607	-5.186424722	3.79E-07	5.77E-06	no
GNAZ	-0.277192273	-5.184929394	3.82E-07	5.81E-06	no
S100A10	0.277166218	5.184401475	3.83E-07	5.81E-06	no
HIRIP3	-0.277165846	-5.184393923	3.83E-07	5.81E-06	no
LOC100289511	0.277165284	5.184382544	3.83E-07	5.81E-06	no
ZFP2	-0.277095263	-5.182963855	3.86E-07	5.85E-06	no
SMIM3	0.277040359	5.181851524	3.88E-07	5.87E-06	no
RHOQ	0.277018437	5.181407397	3.89E-07	5.88E-06	no
C2orf29	0.276822437	5.177437006	3.96E-07	5.99E-06	no
TAPBP	0.276793047	5.176841724	3.98E-07	6.01E-06	no
LOC100132707	0.276592477	5.172779592	4.06E-07	6.13E-06	no
RNU6-64	0.276502753	5.170962661	4.09E-07	6.18E-06	no
NLRP1	0.276495665	5.170819128	4.10E-07	6.18E-06	no
STK4	0.276342993	5.167727833	4.16E-07	6.26E-06	no
C7orf31	0.276341519	5.167698	4.16E-07	6.26E-06	no

C1orf186	0.276257584	5.165998674	4.19E-07	6.31E-06	no
KLHL28	-0.276164114	-5.16410644	4.23E-07	6.37E-06	no
SAA1	0.276140866	5.163635824	4.24E-07	6.38E-06	no
TERF2	-0.276127935	-5.163374069	4.25E-07	6.38E-06	no
LOC728730	-0.276060649	-5.162012062	4.28E-07	6.42E-06	no
GPR123	-0.27605252	-5.16184752	4.28E-07	6.42E-06	no
TCEAL2	-0.276037052	-5.161534439	4.29E-07	6.43E-06	no
PALD1	0.276030626	5.161404379	4.29E-07	6.43E-06	no
MFSD12	0.276010202	5.160990978	4.30E-07	6.43E-06	no
FAM20C	0.275927058	5.159308156	4.34E-07	6.48E-06	no
KLRK1	0.275922208	5.159209996	4.34E-07	6.48E-06	no
JUNB	0.27589521	5.158663588	4.35E-07	6.49E-06	no
FAM91A1	0.275808883	5.156916539	4.39E-07	6.55E-06	no
AXIN2	-0.275798075	-5.156697819	4.39E-07	6.55E-06	no
BHLHE41	0.275740583	5.155534408	4.42E-07	6.58E-06	no
PCK2	0.275700676	5.154726878	4.43E-07	6.60E-06	no
GTSF1	0.275670278	5.154111779	4.45E-07	6.62E-06	no
TMEM151B	-0.27565504	-5.15380346	4.45E-07	6.62E-06	no
SLC16A3	0.275629465	5.153285976	4.47E-07	6.64E-06	no
PSTK	-0.275604434	-5.152779507	4.48E-07	6.65E-06	no
ZKSCAN2	-0.275572363	-5.152130624	4.49E-07	6.67E-06	no
CTNNB1	0.275563259	5.151946436	4.50E-07	6.67E-06	no
ZMYND11	-0.275543622	-5.151549134	4.50E-07	6.68E-06	no
LOC728431	0.275486383	5.150391092	4.53E-07	6.71E-06	no
ADAMDEC1	0.275468865	5.150036694	4.54E-07	6.71E-06	no
FCGRT	0.275465481	5.149968232	4.54E-07	6.71E-06	no
39692	-0.275438316	-5.149418683	4.55E-07	6.73E-06	no
KSR2	-0.275299319	-5.146606917	4.62E-07	6.82E-06	no
HTRA3	0.275247784	5.145564507	4.64E-07	6.85E-06	no
ECRP	0.27520175	5.144633416	4.66E-07	6.87E-06	no
C3orf38	0.27519937	5.14458527	4.66E-07	6.87E-06	no
CRTAP	0.275176271	5.144118092	4.67E-07	6.88E-06	no
NFKBIE	0.275157409	5.143736602	4.68E-07	6.89E-06	no
TPSB2	0.275157201	5.143732386	4.68E-07	6.89E-06	no
NFKBIZ	0.275005906	5.140672656	4.75E-07	6.99E-06	no
LAMC3	0.274849223	5.137504378	4.83E-07	7.09E-06	no
SLC9A9	0.27482855	5.137086373	4.84E-07	7.10E-06	no
BATF2	0.274755212	5.135603607	4.87E-07	7.14E-06	no
HTATIP2	0.274752553	5.135549847	4.87E-07	7.14E-06	no
USP49	-0.274752091	-5.135540498	4.87E-07	7.14E-06	no
NMRK1	0.274555841	5.131573106	4.97E-07	7.27E-06	no
LNP1	-0.274520277	-5.130854213	4.99E-07	7.30E-06	no
CXorf65	0.274506373	5.130573176	4.99E-07	7.30E-06	no
HGSNAT	0.274482772	5.130096117	5.01E-07	7.31E-06	no
C5orf15	0.27433291	5.127067177	5.08E-07	7.42E-06	no
RAB42	0.274315731	5.12671999	5.09E-07	7.42E-06	no
LGALS7B	0.274224394	5.124874157	5.14E-07	7.49E-06	no
MARCO	0.274178941	5.123955653	5.16E-07	7.52E-06	no
TUBA1C	0.274155434	5.12348064	5.17E-07	7.53E-06	no
LINC00461	-0.27414944	-5.123359513	5.17E-07	7.53E-06	no
HLA-F	0.274100911	5.122378921	5.20E-07	7.56E-06	no

LINC00672	-0.274047754	-5.121304843	5.23E-07	7.59E-06	no
HSD11B1L	-0.273841119	-5.117130147	5.33E-07	7.75E-06	no
EPS15L1	0.273777605	5.115847117	5.37E-07	7.79E-06	no
TXNDC5	0.27373794	5.115045897	5.39E-07	7.81E-06	no
CC2D1B	0.273681058	5.113896943	5.42E-07	7.85E-06	no
TMEM71	0.273679391	5.113863267	5.42E-07	7.85E-06	no
CLEC4C	0.273678299	5.113841201	5.42E-07	7.85E-06	no
EBF4	-0.273649652	-5.113262593	5.44E-07	7.86E-06	no
STK40	0.273579162	5.111838879	5.47E-07	7.91E-06	no
LOC100630918	-0.273537809	-5.111003707	5.50E-07	7.94E-06	no
RARRES2	0.273345933	5.107128914	5.60E-07	8.09E-06	no
TMEM176A	0.273319065	5.106586382	5.62E-07	8.10E-06	no
PFN2	-0.273295224	-5.106104995	5.63E-07	8.12E-06	no
ABCA13	0.273196614	5.104113998	5.69E-07	8.19E-06	no
KDM4B	-0.273172238	-5.103621857	5.70E-07	8.20E-06	no
HEATR3	0.273041112	5.100974654	5.77E-07	8.30E-06	no
ISLR	0.272878826	5.097698813	5.87E-07	8.43E-06	no
LRRTM2	-0.272849642	-5.097109761	5.88E-07	8.45E-06	no
CDV3	0.27283291	5.096772057	5.89E-07	8.46E-06	no
AGPAT2	0.272745256	5.095002976	5.94E-07	8.53E-06	no
NRXN2	-0.272637314	-5.092824622	6.01E-07	8.61E-06	no
NIPAL2	0.272510141	5.09025842	6.08E-07	8.71E-06	no
USP27X	-0.272505325	-5.090161248	6.09E-07	8.71E-06	no
GPHN	-0.272388393	-5.087801989	6.16E-07	8.81E-06	no
STEAP1	0.27236453	5.087320535	6.17E-07	8.82E-06	no
CHI3L1	0.272290581	5.085828677	6.22E-07	8.88E-06	no
PHYHIPL	-0.27227466	-5.085507479	6.23E-07	8.89E-06	no
FAM117B	-0.272214207	-5.084287978	6.26E-07	8.94E-06	no
B3GNT5	0.272191572	5.083831376	6.28E-07	8.95E-06	no
ASF1A	-0.272145787	-5.082907821	6.31E-07	8.99E-06	no
AIFM1	-0.272139618	-5.082783395	6.31E-07	8.99E-06	no
SLC31A1	0.272075984	5.08149988	6.35E-07	9.04E-06	no
FCRL3	0.271868843	5.077322255	6.48E-07	9.22E-06	no
SLC17A9	0.271848932	5.076920731	6.49E-07	9.23E-06	no
KCNIP2	-0.271763534	-5.075198665	6.55E-07	9.30E-06	no
SCAPER	-0.271582283	-5.071544168	6.66E-07	9.46E-06	no
MAPK13	0.271293956	5.065731919	6.86E-07	9.73E-06	no
CAMSAP2	-0.271260766	-5.065062957	6.88E-07	9.74E-06	no
GDPD1	-0.27125975	-5.06504248	6.88E-07	9.74E-06	no
ZNF737	-0.271221683	-5.064275233	6.90E-07	9.77E-06	no
ZNF711	-0.27121371	-5.064114529	6.91E-07	9.77E-06	no
ENTPD7	0.271212248	5.064085067	6.91E-07	9.77E-06	no
GDI1	-0.271133068	-5.062489299	6.96E-07	9.84E-06	no
DBIL5P	-0.27105825	-5.060981526	7.02E-07	9.91E-06	no
CASKIN1	-0.271033605	-5.060484897	7.03E-07	9.92E-06	no
CCDC144C	-0.271033529	-5.060483352	7.03E-07	9.92E-06	no
TGFBI	0.27098762	5.059558236	7.06E-07	9.96E-06	no
YLPM1	-0.270970401	-5.059211274	7.08E-07	9.97E-06	no
DCAF7	-0.270870273	-5.057193765	7.15E-07	1.01E-05	no
CLEC1A	0.270815086	5.056081863	7.18E-07	1.01E-05	no
LINC00634	-0.270802475	-5.055827769	7.19E-07	1.01E-05	no

PPP1R26	-0.27075667	-5.054904948	7.23E-07	1.01E-05	no
BCRP2	-0.270713108	-5.054027348	7.26E-07	1.02E-05	no
HMP19	-0.270644808	-5.052651443	7.31E-07	1.02E-05	no
RPGRIP1	0.270609869	5.051947625	7.33E-07	1.03E-05	no
AKT3	-0.270558966	-5.050922246	7.37E-07	1.03E-05	no
JPH3	-0.270551077	-5.050763343	7.37E-07	1.03E-05	no
CLNK	0.270528735	5.050313317	7.39E-07	1.03E-05	no
PAK7	-0.270479799	-5.049327656	7.42E-07	1.04E-05	no
ITGA11	0.270467554	5.049081025	7.43E-07	1.04E-05	no
DPP6	-0.270459957	-5.048928014	7.44E-07	1.04E-05	no
GUSB	0.270442994	5.048586361	7.45E-07	1.04E-05	no
SCRT1	-0.270432686	-5.048378744	7.46E-07	1.04E-05	no
SHISA7	-0.270366817	-5.04705212	7.51E-07	1.05E-05	no
MAGI1	-0.270284048	-5.045385252	7.57E-07	1.05E-05	no
PCSK7	0.270187884	5.043448779	7.64E-07	1.06E-05	no
SNAI1	0.270154682	5.042780215	7.66E-07	1.07E-05	no
COQ3	-0.270081861	-5.041313954	7.72E-07	1.07E-05	no
SNTB2	0.27007803	5.041236816	7.72E-07	1.07E-05	no
ICA1L	-0.270004345	-5.039753246	7.78E-07	1.08E-05	no
TBC1D1	0.269922515	5.038105794	7.84E-07	1.09E-05	no
RBPMS	0.26986479	5.036943728	7.88E-07	1.09E-05	no
TMED9	0.269748598	5.034604813	7.97E-07	1.10E-05	no
RCOR2	-0.269690723	-5.033439889	8.02E-07	1.11E-05	no
CHGB	-0.269664941	-5.032920967	8.04E-07	1.11E-05	no
WASF3	-0.269621811	-5.032052896	8.07E-07	1.12E-05	no
MAN2B2	0.26961703	5.031956668	8.08E-07	1.12E-05	no
FAM192A	-0.269566205	-5.030933777	8.12E-07	1.12E-05	no
TBX21	0.269547175	5.030550782	8.13E-07	1.12E-05	no
PRKCD	0.269337152	5.026324416	8.30E-07	1.14E-05	no
NMNAT3	0.269135928	5.022275844	8.46E-07	1.17E-05	no
TPTE2P1	-0.269127537	-5.022107044	8.47E-07	1.17E-05	no
TXLNA	0.269110624	5.021766791	8.48E-07	1.17E-05	no
GSN	0.269094631	5.021445045	8.50E-07	1.17E-05	no
RGS12	-0.269081235	-5.021175559	8.51E-07	1.17E-05	no
SLC16A10	0.269043448	5.020415401	8.54E-07	1.17E-05	no
CDH10	-0.268824296	-5.016007297	8.72E-07	1.20E-05	no
BANF1	-0.268663261	-5.012768697	8.86E-07	1.22E-05	no
MCM3AP-AS1	-0.268611007	-5.011717913	8.91E-07	1.22E-05	no
SLC41A2	0.268604144	5.0115799	8.91E-07	1.22E-05	no
HS2ST1	0.268509109	5.009668964	8.99E-07	1.23E-05	no
FAM69B	-0.268502409	-5.009534246	9.00E-07	1.23E-05	no
BEND3P3	-0.268487569	-5.009235864	9.01E-07	1.23E-05	no
KIAA0195	-0.268385527	-5.007184236	9.10E-07	1.24E-05	no
FRA10AC1	-0.268318258	-5.005831829	9.16E-07	1.25E-05	no
CHD7	-0.268299491	-5.005454543	9.18E-07	1.25E-05	no
DHX32	0.268295298	5.005370254	9.18E-07	1.25E-05	no
LPAR5	0.26829128	5.005289485	9.19E-07	1.25E-05	no
NYAP1	-0.26821468	-5.003749622	9.25E-07	1.26E-05	no
CPNE5	-0.268202316	-5.003501094	9.26E-07	1.26E-05	no
GREB1	-0.268189159	-5.003236622	9.28E-07	1.26E-05	no
C11orf35	0.268010886	4.999653355	9.44E-07	1.28E-05	no

HR	-0.267992707	-4.999287988	9.45E-07	1.29E-05	no
FAM157A	0.26797565	4.998945181	9.47E-07	1.29E-05	no
RNF157	-0.26793056	-4.998038982	9.51E-07	1.29E-05	no
ARSA	0.267867096	4.996763592	9.57E-07	1.30E-05	no
ZMPSTE24	0.267846768	4.996355091	9.59E-07	1.30E-05	no
LOC100652772	-0.267840363	-4.996226366	9.59E-07	1.30E-05	no
IFI6	0.267831872	4.996055751	9.60E-07	1.30E-05	no
C11orf21	0.267782347	4.995060558	9.65E-07	1.31E-05	no
C6orf201	0.267733154	4.994072068	9.69E-07	1.31E-05	no
CFDP1	-0.267686788	-4.99314043	9.74E-07	1.32E-05	no
ETHE1	0.267571199	4.990818049	9.85E-07	1.33E-05	no
INMT	0.267454874	4.988481135	9.96E-07	1.34E-05	no
PLCB1	-0.267387624	-4.987130209	1.00E-06	1.35E-05	no
TMEM61	0.267385313	4.987083774	1.00E-06	1.35E-05	no
KLHL11	-0.267324728	-4.985866808	1.01E-06	1.36E-05	no
ZNF775	-0.267226645	-4.983896755	1.02E-06	1.37E-05	no
HELZ2	0.267191851	4.983197939	1.02E-06	1.37E-05	no
SKAP2	0.267099168	4.981336558	1.03E-06	1.39E-05	no
SLIT2	0.267038536	4.980118948	1.04E-06	1.39E-05	no
TUT1	-0.267015813	-4.979662644	1.04E-06	1.40E-05	no
ZNFX1-AS1	-0.266851371	-4.976360711	1.06E-06	1.42E-05	no
TTBK1	-0.266839935	-4.97613111	1.06E-06	1.42E-05	no
GIMAP5	0.266778919	4.97490605	1.06E-06	1.42E-05	no
OMG	-0.266774428	-4.974815888	1.06E-06	1.42E-05	no
MN1	-0.266755575	-4.97443738	1.07E-06	1.43E-05	no
TNFSF4	0.266719625	4.973715639	1.07E-06	1.43E-05	no
VSIG10L	0.266697863	4.973278753	1.07E-06	1.43E-05	no
SOX8	-0.266487754	-4.969061087	1.09E-06	1.46E-05	no
LRRN1	-0.26647383	-4.968781602	1.09E-06	1.46E-05	no
CLASP2	-0.266330745	-4.965909825	1.11E-06	1.48E-05	no
CAMSAP1	-0.266325189	-4.965798329	1.11E-06	1.48E-05	no
CPPED1	0.266302158	4.965336125	1.11E-06	1.48E-05	no
PCYT1A	0.266279117	4.964873726	1.12E-06	1.49E-05	no
GPRIN1	-0.266141808	-4.962118317	1.13E-06	1.50E-05	no
PARP2	-0.266102048	-4.961320516	1.13E-06	1.51E-05	no
RCAN3	0.266092769	4.96113433	1.14E-06	1.51E-05	no
ENHO	-0.266066618	-4.960609608	1.14E-06	1.51E-05	no
C12orf76	-0.266007038	-4.959414186	1.14E-06	1.52E-05	no
ZAK	0.265989073	4.959053753	1.15E-06	1.52E-05	no
TMEM119	0.265964269	4.958556116	1.15E-06	1.52E-05	no
TMEM26	0.265832568	4.955913959	1.16E-06	1.54E-05	no
KIDINS220	-0.265752705	-4.954311904	1.17E-06	1.55E-05	no
SEC61A1	0.265628747	4.951825534	1.19E-06	1.57E-05	no
INPP5A	-0.265470596	-4.948653712	1.21E-06	1.59E-05	no
LMTK3	-0.265457726	-4.948395615	1.21E-06	1.60E-05	no
FAM171A2	-0.265427588	-4.947791223	1.21E-06	1.60E-05	no
QSOX1	0.265373526	4.946707115	1.22E-06	1.61E-05	no
NUCB1	0.265319943	4.945632643	1.22E-06	1.61E-05	no
PHF20L1	-0.26531745	-4.945582658	1.22E-06	1.61E-05	no
COA5	-0.265302895	-4.945290813	1.22E-06	1.61E-05	no
DDOST	0.265302337	4.945279619	1.22E-06	1.61E-05	no

LDOC1L	-0.265156104	-4.942347616	1.24E-06	1.64E-05	no
TSPYL4	-0.265087391	-4.940970021	1.25E-06	1.64E-05	no
ST3GAL1	0.265085212	4.940926335	1.25E-06	1.64E-05	no
STC1	0.264875522	4.9367229	1.28E-06	1.68E-05	no
SLC25A19	0.264862353	4.936458947	1.28E-06	1.68E-05	no
PDE6G	0.264841521	4.936041391	1.28E-06	1.68E-05	no
NAGLU	0.264833785	4.935886322	1.28E-06	1.68E-05	no
NUDT19	0.264796216	4.935133326	1.29E-06	1.69E-05	no
SPATC1	0.264738884	4.933984265	1.29E-06	1.69E-05	no
PIGK	0.264734075	4.933887875	1.29E-06	1.69E-05	no
LRRC37A6P	0.264731648	4.933839236	1.29E-06	1.69E-05	no
ARRB2	0.264726661	4.933739295	1.29E-06	1.69E-05	no
PSMC2	0.264713355	4.933472613	1.30E-06	1.69E-05	no
TMEM200B	0.264681626	4.932836733	1.30E-06	1.70E-05	no
TERF2IP	-0.264675179	-4.932707533	1.30E-06	1.70E-05	no
TARSL2	-0.264638854	-4.931979561	1.30E-06	1.70E-05	no
IFI44L	0.264627022	4.931742443	1.31E-06	1.70E-05	no
ANKRD36BP1	0.264532807	4.929854462	1.32E-06	1.72E-05	no
PHGDH	-0.264527265	-4.929743404	1.32E-06	1.72E-05	no
FCRL2	0.264444562	4.928086259	1.33E-06	1.73E-05	no
ALCAM	-0.264394566	-4.92708452	1.34E-06	1.74E-05	no
PTPN9	0.264362637	4.926444794	1.34E-06	1.74E-05	no
RBMS1	0.264354141	4.926274564	1.34E-06	1.74E-05	no
FAM177B	0.264316597	4.925522384	1.35E-06	1.75E-05	no
ACSM3	0.264278592	4.924760986	1.35E-06	1.75E-05	no
STARD10	-0.264226358	-4.923714551	1.36E-06	1.76E-05	no
MADD	-0.264090306	-4.920989171	1.37E-06	1.78E-05	no
PIM3	0.26408747	4.920932351	1.38E-06	1.78E-05	no
RNASEL	0.264031934	4.919819961	1.38E-06	1.79E-05	no
CHST2	0.264020444	4.919589819	1.38E-06	1.79E-05	no
EMR4P	0.264003985	4.919260157	1.39E-06	1.79E-05	no
EFR3B	-0.263997822	-4.919136706	1.39E-06	1.79E-05	no
PLA2G4A	0.26399348	4.919049754	1.39E-06	1.79E-05	no
SLC2A3	0.263979796	4.918775665	1.39E-06	1.79E-05	no
FAM155B	-0.263865664	-4.916489848	1.40E-06	1.81E-05	no
SMOC2	0.263809569	4.915366455	1.41E-06	1.82E-05	no
CRYBB1	0.26379037	4.914981979	1.41E-06	1.82E-05	no
SEC31A	0.263766941	4.9145128	1.42E-06	1.83E-05	no
EOGT	0.263732816	4.913829454	1.42E-06	1.83E-05	no
FXYD6-FXYD2	-0.263700495	-4.91318224	1.43E-06	1.84E-05	no
SH2D3C	0.263692973	4.913031622	1.43E-06	1.84E-05	no
TADA2A	-0.263676618	-4.91270414	1.43E-06	1.84E-05	no
GPM6B	-0.26361534	-4.911477156	1.44E-06	1.85E-05	no
EIF2AK4	0.263580165	4.910772869	1.44E-06	1.85E-05	no
CADM2	-0.263560196	-4.910373055	1.45E-06	1.85E-05	no
KCNK3	-0.263539661	-4.909961916	1.45E-06	1.86E-05	no
CCL23	0.263520738	4.909583047	1.45E-06	1.86E-05	no
HLA-L	0.263520565	4.909579595	1.45E-06	1.86E-05	no
CYB5R4	0.263511077	4.909389634	1.45E-06	1.86E-05	no
TNFAIP8L2-SC	0.263439539	4.907957414	1.46E-06	1.87E-05	no
CARD9	0.263387588	4.906917396	1.47E-06	1.88E-05	no

MAPK8IP3	-0.263327798	-4.905720504	1.48E-06	1.89E-05	no
LOC100505549	0.263285954	4.904882905	1.48E-06	1.89E-05	no
GABBR1	-0.263266337	-4.904490219	1.49E-06	1.90E-05	no
ZBTB47	-0.263187225	-4.902906705	1.50E-06	1.91E-05	no
UNC13D	0.263163878	4.90243941	1.50E-06	1.91E-05	no
HERC6	0.263106262	4.901286261	1.51E-06	1.92E-05	no
SBNO2	0.263045436	4.900068916	1.52E-06	1.93E-05	no
TMEM246	-0.2630205	-4.89956989	1.52E-06	1.94E-05	no
FOXRED1	-0.262951656	-4.898192176	1.53E-06	1.95E-05	no
BID	-0.262872313	-4.896604466	1.54E-06	1.96E-05	no
FCGR1B	0.26284773	4.896112562	1.55E-06	1.96E-05	no
TTC3	-0.262847718	-4.896112325	1.55E-06	1.96E-05	no
KCNB1	-0.262836588	-4.895889623	1.55E-06	1.96E-05	no
PFKM	-0.262803755	-4.895232667	1.55E-06	1.97E-05	no
ANXA5	0.262769883	4.894554925	1.56E-06	1.97E-05	no
RBP5	0.262727542	4.893707768	1.56E-06	1.98E-05	no
TMED3	0.262696917	4.893095058	1.57E-06	1.99E-05	no
ALDH16A1	0.262640801	4.891972354	1.58E-06	1.99E-05	no
NEAT1	0.262589316	4.890942374	1.59E-06	2.00E-05	no
GLRX	0.262553254	4.89022095	1.59E-06	2.01E-05	no
CD300E	0.262513097	4.889417637	1.60E-06	2.01E-05	no
CYB561D2	0.262513052	4.889416741	1.60E-06	2.01E-05	no
IFNAR1	0.262481437	4.888784317	1.60E-06	2.02E-05	no
SLC39A8	0.262395796	4.887071275	1.61E-06	2.03E-05	no
ADAMTS14	0.262306682	4.885288872	1.63E-06	2.05E-05	no
C11orf63	0.262286725	4.884889743	1.63E-06	2.05E-05	no
RER1	0.262212928	4.883413826	1.64E-06	2.07E-05	no
DEGS1	0.262131852	4.881792446	1.66E-06	2.08E-05	no
APC2	-0.262117266	-4.88150078	1.66E-06	2.08E-05	no
TSPO	0.262074914	4.880653862	1.66E-06	2.09E-05	no
GADD45B	0.261990738	4.878970691	1.68E-06	2.10E-05	no
S100A9	0.261897234	4.877101145	1.69E-06	2.12E-05	no
RHOD	0.261895586	4.87706819	1.69E-06	2.12E-05	no
ZXDC	-0.261868376	-4.876524182	1.70E-06	2.13E-05	no
SPNS3	0.261792041	4.874998048	1.71E-06	2.14E-05	no
ACTL10	0.261770389	4.874565189	1.71E-06	2.14E-05	no
KCNE1	0.261616079	4.871480513	1.74E-06	2.17E-05	no
MGC21881	-0.261597914	-4.871117419	1.74E-06	2.18E-05	no
LOC541471	0.261577486	4.870709106	1.74E-06	2.18E-05	no
IL3RA	0.26157651	4.870689591	1.74E-06	2.18E-05	no
SH3BGRL3	0.261556222	4.870284077	1.75E-06	2.18E-05	no
GSTK1	0.261493919	4.869038804	1.76E-06	2.19E-05	no
SLC7A1	-0.261475279	-4.868666258	1.76E-06	2.19E-05	no
POLR2F	-0.26136718	-4.866505849	1.78E-06	2.22E-05	no
LOC100131047	0.261330752	4.865777866	1.79E-06	2.22E-05	no
SARM1	-0.261229689	-4.863758289	1.80E-06	2.24E-05	no
C9orf64	0.261112925	4.861425208	1.82E-06	2.27E-05	no
ZNF292	-0.261025987	-4.859688216	1.84E-06	2.28E-05	no
TSPAN7	-0.260982592	-4.858821234	1.84E-06	2.29E-05	no
SLC35D1	0.260970236	4.858574401	1.85E-06	2.29E-05	no
DLG3	-0.260910725	-4.857385518	1.86E-06	2.30E-05	no

GCAT	-0.260862838	-4.856428896	1.87E-06	2.31E-05	no
NFKB2	0.260806408	4.855301664	1.88E-06	2.32E-05	no
TMEM179B	0.260713282	4.853441536	1.89E-06	2.34E-05	no
MYO10	-0.260664527	-4.852467741	1.90E-06	2.35E-05	no
ASB1	-0.26064097	-4.851997247	1.91E-06	2.36E-05	no
LOC389906	-0.260590672	-4.850992679	1.91E-06	2.36E-05	no
GCA	0.260588932	4.85095794	1.91E-06	2.36E-05	no
GDAP1L1	-0.26055139	-4.850208167	1.92E-06	2.37E-05	no
BEX1	-0.260535627	-4.849893374	1.92E-06	2.37E-05	no
SREBF2	-0.260509456	-4.849370732	1.93E-06	2.38E-05	no
NCF2	0.260382022	4.846825985	1.95E-06	2.41E-05	no
WWTR1	0.260375252	4.84669079	1.95E-06	2.41E-05	no
SLC25A41	-0.26020379	-4.843267296	1.98E-06	2.44E-05	no
CD83	0.260182907	4.842850365	1.99E-06	2.45E-05	no
C4orf32	0.260175535	4.842703188	1.99E-06	2.45E-05	no
SAMD1	-0.260111375	-4.841422282	2.00E-06	2.46E-05	no
RHOA	0.260084599	4.84088775	2.01E-06	2.46E-05	no
MIB1	-0.260082461	-4.840845058	2.01E-06	2.46E-05	no
ANKRD36B	-0.259938949	-4.8379803	2.03E-06	2.50E-05	no
FUT7	0.259867851	4.836561167	2.05E-06	2.51E-05	no
ANKRD16	-0.259769605	-4.834600299	2.07E-06	2.53E-05	no
PHLPP2	-0.259750243	-4.834213882	2.07E-06	2.54E-05	no
LOC100144595	-0.259726207	-4.833734191	2.08E-06	2.54E-05	no
SPIN3	-0.259703948	-4.83328996	2.08E-06	2.54E-05	no
PIM2	0.25966531	4.832518892	2.09E-06	2.55E-05	no
KIAA2013	0.259623996	4.831694433	2.10E-06	2.56E-05	no
KPNA5	-0.259489331	-4.829007298	2.12E-06	2.59E-05	no
CTTNBP2NL	0.259481948	4.828859981	2.12E-06	2.59E-05	no
KCNJ11	-0.25946373	-4.82849648	2.13E-06	2.59E-05	no
GPLD1	-0.259436627	-4.827955692	2.13E-06	2.60E-05	no
OPN3	0.259402343	4.827271662	2.14E-06	2.61E-05	no
VPS37D	-0.259392571	-4.827076697	2.14E-06	2.61E-05	no
COL10A1	0.259328039	4.825789216	2.15E-06	2.62E-05	no
NBEA	-0.259326972	-4.825767933	2.15E-06	2.62E-05	no
SV2A	-0.259296618	-4.825162357	2.16E-06	2.63E-05	no
TMEM51	0.259271872	4.824668671	2.17E-06	2.63E-05	no
LINC00515	-0.259197385	-4.823182737	2.18E-06	2.65E-05	no
SLC24A3	-0.259196252	-4.823160143	2.18E-06	2.65E-05	no
ESRG	-0.259178196	-4.822799955	2.18E-06	2.65E-05	no
IDO1	0.259116173	4.821562757	2.20E-06	2.66E-05	no
KIAA1549	-0.25909764	-4.821193081	2.20E-06	2.67E-05	no
FTH1	0.259078861	4.820818504	2.20E-06	2.67E-05	no
MLLT6	-0.258787684	-4.81501128	2.27E-06	2.74E-05	no
C14orf169	0.258715407	4.813570008	2.28E-06	2.76E-05	no
CHD3	-0.258695229	-4.813167655	2.29E-06	2.76E-05	no
LINC00294	-0.258653668	-4.812338956	2.29E-06	2.77E-05	no
PLEKHG1	0.25863595	4.811985663	2.30E-06	2.77E-05	no
SLC6A1	-0.258600679	-4.811282399	2.31E-06	2.78E-05	no
CCL21	0.258317614	4.80563919	2.37E-06	2.85E-05	no
FAM161B	-0.258304469	-4.805377166	2.37E-06	2.86E-05	no
ZRANB1	-0.258190512	-4.803105717	2.39E-06	2.89E-05	no

DSCAML1	-0.258103793	-4.801377319	2.41E-06	2.91E-05	no
CTNNA1	0.258084986	4.8010025	2.42E-06	2.91E-05	no
C3orf52	0.258063233	4.80056897	2.42E-06	2.91E-05	no
SLC22A17	-0.258018617	-4.799679802	2.43E-06	2.92E-05	no
ARPC2	0.257980341	4.798917027	2.44E-06	2.93E-05	no
FLRT1	-0.257964197	-4.798595313	2.45E-06	2.94E-05	no
BRD3	-0.257959434	-4.79850038	2.45E-06	2.94E-05	no
PIR	0.257915464	4.797624176	2.46E-06	2.95E-05	no
C2orf40	0.257881665	4.796950674	2.46E-06	2.95E-05	no
GSDMA	0.257857596	4.796471063	2.47E-06	2.96E-05	no
TMEM50A	0.257798835	4.795300214	2.48E-06	2.97E-05	no
BEX4	-0.257684079	-4.793013788	2.51E-06	3.00E-05	no
DHTKD1	-0.257381118	-4.786978574	2.58E-06	3.09E-05	no
MEIS3P1	0.25725631	4.784492746	2.61E-06	3.12E-05	no
CAMLG	-0.257253735	-4.78444145	2.61E-06	3.12E-05	no
KCNQ1	0.257249969	4.784366439	2.61E-06	3.12E-05	no
MAN2A1	0.257192507	4.78322205	2.63E-06	3.13E-05	no
SHF	-0.257190262	-4.783177344	2.63E-06	3.13E-05	no
FAM171A1	-0.257138509	-4.782146707	2.64E-06	3.15E-05	no
CCDC125	0.25709841	4.781348165	2.65E-06	3.16E-05	no
C1orf61	-0.257073866	-4.780859419	2.66E-06	3.16E-05	no
DOPEY1	-0.257053312	-4.780450116	2.66E-06	3.17E-05	no
ADHFE1	-0.257036908	-4.78012348	2.67E-06	3.17E-05	no
SLC40A1	0.256992057	4.77923039	2.68E-06	3.18E-05	no
GM2A	0.256982495	4.779039997	2.68E-06	3.18E-05	no
FRMPD1	-0.25694962	-4.778385406	2.69E-06	3.19E-05	no
SLITRK5	-0.256948492	-4.778362935	2.69E-06	3.19E-05	no
TSPAN5	-0.256932134	-4.778037248	2.69E-06	3.19E-05	no
NRSN1	-0.256872828	-4.776856441	2.71E-06	3.21E-05	no
ABCA3	-0.256855694	-4.7765153	2.71E-06	3.21E-05	no
SLC16A13	0.256838879	4.776180529	2.71E-06	3.21E-05	no
TBC1D8	0.256821187	4.775828284	2.72E-06	3.22E-05	no
OMP	0.25680135	4.775433356	2.72E-06	3.22E-05	no
RAMP2-AS1	-0.256785205	-4.775111934	2.73E-06	3.22E-05	no
ATCAY	-0.256725039	-4.773914137	2.74E-06	3.24E-05	no
EPB41L4A	-0.256697603	-4.773367977	2.75E-06	3.25E-05	no
ZNF510	-0.256643287	-4.772286715	2.76E-06	3.26E-05	no
DESI1	-0.256447762	-4.768394886	2.81E-06	3.32E-05	no
C22orf32	-0.2563792	-4.76703034	2.83E-06	3.34E-05	no
LAMC1	0.25637592	4.766965051	2.83E-06	3.34E-05	no
ALDH5A1	-0.256288087	-4.765217089	2.86E-06	3.36E-05	no
TIGD3	-0.256270113	-4.764859412	2.86E-06	3.37E-05	no
STOX2	-0.256254181	-4.764542361	2.86E-06	3.37E-05	no
MGAT2	0.256216138	4.763785326	2.88E-06	3.38E-05	no
SELP	0.256200447	4.763473082	2.88E-06	3.38E-05	no
TMEM248	0.256111939	4.761711937	2.90E-06	3.41E-05	no
TRIM61	0.256110535	4.761684	2.90E-06	3.41E-05	no
GKAP1	-0.256100732	-4.761488952	2.91E-06	3.41E-05	no
TMEM110	0.256048435	4.760448416	2.92E-06	3.42E-05	no
EMP3	0.25597219	4.758931444	2.94E-06	3.45E-05	no
EMILIN1	0.255880477	4.757106879	2.97E-06	3.47E-05	no

FAS-AS1	0.255817389	4.755851859	2.98E-06	3.49E-05	no
COL5A1	0.255799647	4.755498924	2.99E-06	3.50E-05	no
WDR59	-0.255698314	-4.753483265	3.02E-06	3.53E-05	no
ZNF609	-0.255677622	-4.753071684	3.02E-06	3.53E-05	no
DNAJC1	0.255621265	4.751950756	3.04E-06	3.55E-05	no
GCSHP3	-0.255567952	-4.750890404	3.05E-06	3.56E-05	no
MAP3K2	0.255546964	4.750472999	3.06E-06	3.57E-05	no
ORAI1	0.255536195	4.750258816	3.06E-06	3.57E-05	no
EML4	0.255411273	4.747774482	3.10E-06	3.61E-05	no
DTD1	-0.25537897	-4.747132124	3.11E-06	3.62E-05	no
KBTBD11	-0.255378788	-4.747128493	3.11E-06	3.62E-05	no
AKR1B1	0.255349012	4.746536401	3.11E-06	3.62E-05	no
KIF2A	-0.255254505	-4.744657207	3.14E-06	3.65E-05	no
C8orf42	-0.25524086	-4.744385886	3.14E-06	3.66E-05	no
CASKIN2	-0.255121327	-4.742009303	3.18E-06	3.69E-05	no
ARPP21	-0.255070843	-4.741005637	3.19E-06	3.71E-05	no
PPT1	0.255022653	4.740047623	3.21E-06	3.72E-05	no
SEMA6B	-0.25492492	-4.738104788	3.24E-06	3.75E-05	no
SBK1	-0.254907311	-4.737754765	3.24E-06	3.76E-05	no
CDC42BPB	-0.254843696	-4.736490264	3.26E-06	3.78E-05	no
ENOPH1	-0.254838112	-4.736379283	3.26E-06	3.78E-05	no
RCN3	0.254829585	4.736209793	3.27E-06	3.78E-05	no
SSR4P1	0.254730833	4.73424703	3.30E-06	3.81E-05	no
PHF2	-0.254707355	-4.733780405	3.30E-06	3.82E-05	no
ALG2	0.25464294	4.732500219	3.32E-06	3.84E-05	no
CLVS1	-0.25459028	-4.731453699	3.34E-06	3.85E-05	no
PTCHD2	-0.254550368	-4.730660559	3.35E-06	3.87E-05	no
FAM155A	-0.254540102	-4.730456562	3.35E-06	3.87E-05	no
ZDHHC18	0.254532178	4.73029909	3.36E-06	3.87E-05	no
FURIN	0.254470846	4.729080349	3.37E-06	3.89E-05	no
PPIB	0.254429579	4.728260363	3.39E-06	3.90E-05	no
TMEM39A	0.254427978	4.728228544	3.39E-06	3.90E-05	no
TTLL12	-0.2544215	-4.728099831	3.39E-06	3.90E-05	no
TPSAB1	0.254377222	4.727220052	3.40E-06	3.91E-05	no
PSMD9	0.25437362	4.727148482	3.41E-06	3.91E-05	no
IFIT2	0.254318102	4.726045402	3.42E-06	3.93E-05	no
LOC150568	-0.254317123	-4.726025962	3.42E-06	3.93E-05	no
ARHGEF26-AS1	-0.254290183	-4.725490707	3.43E-06	3.94E-05	no
ZNF681	-0.25413729	-4.722453249	3.48E-06	3.99E-05	no
CLN6	0.254076655	4.721248753	3.50E-06	4.01E-05	no
ZC3H12B	-0.25405204	-4.720759796	3.51E-06	4.02E-05	no
PDGFRB	0.254044404	4.720608107	3.51E-06	4.02E-05	no
TRMT5	-0.254036099	-4.720443148	3.51E-06	4.02E-05	no
KGFLP2	-0.254019669	-4.720116787	3.52E-06	4.02E-05	no
TRMT2B	0.254003127	4.71978821	3.52E-06	4.03E-05	no
HIST1H2BJ	0.253937326	4.718481232	3.54E-06	4.05E-05	no
MDGA2	-0.253931345	-4.718362427	3.55E-06	4.05E-05	no
SLC35F5	0.253924904	4.718234498	3.55E-06	4.05E-05	no
TMEM106A-AS1	0.253909622	4.717930974	3.55E-06	4.05E-05	no
ARMC2	0.253757994	4.714919593	3.60E-06	4.11E-05	no
IFITM4P	0.253713703	4.714040022	3.62E-06	4.12E-05	no

TNFSF13B	0.253691604	4.713601175	3.62E-06	4.13E-05	no
C11orf24	0.253673442	4.713240502	3.63E-06	4.13E-05	no
GPNMB	0.253646462	4.712704746	3.64E-06	4.14E-05	no
RAI14	0.253608989	4.711960648	3.65E-06	4.15E-05	no
HIATL1	0.253570958	4.711205496	3.66E-06	4.16E-05	no
RAB33A	-0.253455047	-4.708904047	3.70E-06	4.21E-05	no
FUT9	-0.253396874	-4.707749098	3.72E-06	4.22E-05	no
NAAA	0.253395603	4.707723853	3.72E-06	4.22E-05	no
SDC2	0.253357934	4.706976014	3.74E-06	4.24E-05	no
HSD17B1	-0.253329415	-4.706409841	3.75E-06	4.25E-05	no
VMP1	0.253297991	4.705786011	3.76E-06	4.26E-05	no
TMTC3	0.253292072	4.70566852	3.76E-06	4.26E-05	no
PTPRN2	-0.253276096	-4.705351371	3.76E-06	4.26E-05	no
CAMK2N2	-0.253225	-4.704337061	3.78E-06	4.28E-05	no
SLC30A9	-0.253205636	-4.70395267	3.79E-06	4.28E-05	no
TRAF3IP2-AS1	-0.25316913	-4.703228025	3.80E-06	4.29E-05	no
ZC3H7B	-0.253126801	-4.702387837	3.82E-06	4.31E-05	no
DYNC1H1	-0.253118379	-4.702220663	3.82E-06	4.31E-05	no
HERC2	-0.253112341	-4.702100817	3.82E-06	4.31E-05	no
SLC25A23	-0.253072663	-4.701313279	3.83E-06	4.32E-05	no
COL6A1	0.253017175	4.700211964	3.85E-06	4.34E-05	no
PVRL2	0.252940547	4.698691165	3.88E-06	4.37E-05	no
NAP1L5	-0.252898906	-4.697864761	3.90E-06	4.39E-05	no
COQ10B	0.252804495	4.695991224	3.93E-06	4.42E-05	no
FRY	-0.252780934	-4.695523692	3.94E-06	4.43E-05	no
TRIM25	0.252662073	4.693165183	3.98E-06	4.47E-05	no
DOCK8	0.252640441	4.692735973	3.99E-06	4.48E-05	no
C8orf44	-0.252637633	-4.692680267	3.99E-06	4.48E-05	no
CSRNP3	-0.252601483	-4.691963015	4.00E-06	4.49E-05	no
VIM	0.252505506	4.690058856	4.04E-06	4.53E-05	no
CELF3	-0.252399048	-4.687946921	4.08E-06	4.57E-05	no
MORC2-AS1	-0.25238633	-4.687694648	4.08E-06	4.57E-05	no
SLC22A18	0.252345521	4.686885116	4.10E-06	4.59E-05	no
SNORA8	-0.252269673	-4.685380605	4.12E-06	4.62E-05	no
TTC28-AS1	-0.252264243	-4.685272905	4.13E-06	4.62E-05	no
HINFP	-0.252254231	-4.685074317	4.13E-06	4.62E-05	no
MAP2	-0.252231639	-4.684626219	4.14E-06	4.62E-05	no
RHBDL3	-0.25219735	-4.683946102	4.15E-06	4.64E-05	no
FLJ10038	-0.252010578	-4.680241942	4.22E-06	4.71E-05	no
FAM211B	-0.251883447	-4.677720922	4.27E-06	4.77E-05	no
HDAC4	-0.251846466	-4.676987649	4.29E-06	4.78E-05	no
CDKL3	-0.251775864	-4.675587748	4.31E-06	4.81E-05	no
FFAR3	0.251630829	4.67271226	4.37E-06	4.87E-05	no
ASH1L	-0.251556589	-4.67124051	4.40E-06	4.90E-05	no
HABP4	-0.251550319	-4.671116212	4.40E-06	4.90E-05	no
CLN5	0.251536439	4.670841065	4.41E-06	4.90E-05	no
PLEK2	0.251424513	4.668622402	4.45E-06	4.95E-05	no
MEIS3	-0.251313667	-4.666425343	4.50E-06	5.00E-05	no
ST8SIA3	-0.251294576	-4.666046967	4.50E-06	5.00E-05	no
TOR1B	0.25113092	4.66280358	4.57E-06	5.07E-05	no
SAA2-SAA4	0.251129464	4.662774711	4.57E-06	5.07E-05	no

C20orf118	0.251105924	4.662308235	4.58E-06	5.08E-05	no
CDR1	-0.251086162	-4.661916625	4.59E-06	5.09E-05	no
PAR5	-0.251042829	-4.661057923	4.61E-06	5.10E-05	no
PDGFB	0.251042421	4.661049842	4.61E-06	5.10E-05	no
TBXAS1	0.251039514	4.660992252	4.61E-06	5.10E-05	no
TCN2	0.250962193	4.659460132	4.64E-06	5.13E-05	no
SEZ6L	-0.250921884	-4.658661453	4.66E-06	5.15E-05	no
PNP	0.250866777	4.657569604	4.68E-06	5.17E-05	no
FAM216A	-0.250793467	-4.656117177	4.71E-06	5.20E-05	no
CRAMP1L	-0.250769271	-4.655637805	4.72E-06	5.21E-05	no
PDLIM1	0.250758231	4.655419097	4.73E-06	5.22E-05	no
GATA6	0.250709456	4.654452834	4.75E-06	5.24E-05	no
PMS2L2	0.250701569	4.654296579	4.75E-06	5.24E-05	no
B3GALT4	0.250666455	4.653600986	4.77E-06	5.25E-05	no
GFOD2	-0.250521321	-4.650726096	4.83E-06	5.32E-05	no
TRAK1	-0.250416108	-4.648642201	4.88E-06	5.37E-05	no
SOGA3	-0.250382865	-4.647983798	4.89E-06	5.38E-05	no
LRP4-AS1	-0.250358541	-4.647502071	4.90E-06	5.39E-05	no
CACNG7	-0.250346467	-4.64726296	4.91E-06	5.39E-05	no
PPP1R12B	-0.250346393	-4.647261477	4.91E-06	5.39E-05	no
LOC10050681C	0.250333511	4.647006367	4.91E-06	5.39E-05	no
38596	-0.250313285	-4.646605798	4.92E-06	5.40E-05	no
C8orf48	0.250294755	4.646238839	4.93E-06	5.40E-05	no
TXNDC12	0.250214012	4.644639876	4.97E-06	5.44E-05	no
HEBP1	0.250176696	4.643900948	4.98E-06	5.46E-05	no
FCHSD1	0.250139037	4.64315524	5.00E-06	5.47E-05	no
CBR4	-0.250096359	-4.642310177	5.02E-06	5.49E-05	no
CDKN1B	-0.250048124	-4.641355101	5.04E-06	5.51E-05	no
TCAP	-0.250023917	-4.640875817	5.05E-06	5.52E-05	no
EPHB1	-0.249975062	-4.639908535	5.07E-06	5.54E-05	no
GADD45G	-0.249945698	-4.639327183	5.09E-06	5.55E-05	no
LOC388152	-0.249919599	-4.638810473	5.10E-06	5.56E-05	no
PPP1R18	0.249916726	4.638753583	5.10E-06	5.56E-05	no
SMCR5	-0.249907649	-4.638573893	5.10E-06	5.56E-05	no
CTNS	0.249906769	4.638556465	5.10E-06	5.56E-05	no
RIPPLY2	-0.249883968	-4.638105062	5.11E-06	5.57E-05	no
CHST13	0.249688592	4.634237461	5.20E-06	5.67E-05	no
AKAP6	-0.249678264	-4.634033039	5.21E-06	5.67E-05	no
ZNF197	-0.249654949	-4.633571539	5.22E-06	5.68E-05	no
LPPR3	-0.249634483	-4.633166444	5.23E-06	5.69E-05	no
TOMM20	-0.249625227	-4.632983236	5.23E-06	5.69E-05	no
TPK1	0.249622891	4.632937004	5.24E-06	5.69E-05	no
PAFAH2	0.249524315	4.630985962	5.28E-06	5.73E-05	no
KIAA1244	-0.249499948	-4.630503708	5.29E-06	5.74E-05	no
HNRNPA1	-0.249415232	-4.62882714	5.33E-06	5.78E-05	no
RAPGEFL1	-0.249389218	-4.628312338	5.35E-06	5.79E-05	no
HCG26	0.249375796	4.628046716	5.35E-06	5.80E-05	no
TUBG2	-0.249294356	-4.626435137	5.39E-06	5.84E-05	no
RNFT2	-0.249255465	-4.62566559	5.41E-06	5.85E-05	no
ERI1	0.249254318	4.625642886	5.41E-06	5.85E-05	no
PHTF1	0.249233873	4.625238341	5.42E-06	5.86E-05	no

PCGF2	-0.249219929	-4.624962428	5.43E-06	5.87E-05	no
GPR157	0.249183037	4.624232471	5.45E-06	5.88E-05	no
GAREML	-0.249169151	-4.623957729	5.45E-06	5.89E-05	no
MUC1	0.249146388	4.623507328	5.46E-06	5.89E-05	no
COL6A2	0.249141603	4.623412665	5.47E-06	5.89E-05	no
TMEM70	0.249128622	4.623155837	5.47E-06	5.90E-05	no
PPIC	0.249096413	4.622518567	5.49E-06	5.91E-05	no
ZNF704	-0.249092648	-4.622444075	5.49E-06	5.91E-05	no
FRS3	-0.249064809	-4.62189331	5.50E-06	5.92E-05	no
SYDE1	0.249057498	4.621748665	5.51E-06	5.92E-05	no
CYSLTR1	0.249001389	4.620638626	5.54E-06	5.95E-05	no
ENDOG	-0.248961108	-4.619841741	5.56E-06	5.97E-05	no
TSC22D1-AS1	-0.248924346	-4.619114504	5.57E-06	5.99E-05	no
LRAT	0.248886427	4.618364396	5.59E-06	6.00E-05	no
FFAR4	0.248847465	4.617593675	5.61E-06	6.02E-05	no
C17orf76-AS1	-0.248805155	-4.616756765	5.63E-06	6.04E-05	no
ENPEP	0.248779232	4.616244012	5.65E-06	6.05E-05	no
CD248	0.248737589	4.615420338	5.67E-06	6.07E-05	no
SLC1A5	0.248675409	4.614190497	5.70E-06	6.10E-05	no
TRIM5	0.248431632	4.609369512	5.82E-06	6.23E-05	no
APLNR	0.248418416	4.609108172	5.83E-06	6.24E-05	no
LOC285033	0.2484066	4.608874519	5.84E-06	6.24E-05	no
SBF1	-0.248350188	-4.607759071	5.87E-06	6.27E-05	no
SHD	-0.248209767	-4.604982676	5.94E-06	6.35E-05	no
MRPL35	-0.248135582	-4.603516011	5.98E-06	6.38E-05	no
HAMP	0.248107877	4.602968298	5.99E-06	6.40E-05	no
MMP19	0.248028566	4.601400441	6.04E-06	6.44E-05	no
ATXN7L3B	-0.24797458	-4.600333262	6.07E-06	6.47E-05	no
PCDH18	0.247934082	4.599532736	6.09E-06	6.49E-05	no
NIP7	0.247844472	4.59776153	6.14E-06	6.54E-05	no
ZNF517	-0.247806913	-4.597019175	6.16E-06	6.55E-05	no
STAT6	0.24778609	4.596607624	6.17E-06	6.56E-05	no
CNPY3	0.247780286	4.596492921	6.17E-06	6.56E-05	no
LRFN1	-0.247761215	-4.596115996	6.18E-06	6.57E-05	no
CASQ1	-0.247743041	-4.595756822	6.19E-06	6.58E-05	no
NFKB1	0.247739469	4.595686222	6.19E-06	6.58E-05	no
HBEGF	0.247707044	4.595045391	6.21E-06	6.59E-05	no
KIAA1161	-0.247678383	-4.594478981	6.23E-06	6.61E-05	no
FCN1	0.247563872	4.592216052	6.29E-06	6.67E-05	no
CXCL11	0.247558855	4.592116921	6.30E-06	6.67E-05	no
RABGAP1	-0.247523576	-4.591419798	6.31E-06	6.69E-05	no
OR2A7	0.247458513	4.590134177	6.35E-06	6.72E-05	no
HEY1	-0.247426595	-4.589503507	6.37E-06	6.74E-05	no
RARB	0.247420066	4.589374521	6.37E-06	6.74E-05	no
SERPINF1	0.247311502	4.587229553	6.43E-06	6.80E-05	no
POLR3H	-0.247309044	-4.58718098	6.44E-06	6.80E-05	no
LINC00526	-0.247214282	-4.585308869	6.49E-06	6.85E-05	no
RASSF8	0.247114324	4.583334253	6.55E-06	6.91E-05	no
CLNS1A	-0.247070219	-4.582463027	6.57E-06	6.94E-05	no
UBN2	-0.247042387	-4.581913275	6.59E-06	6.95E-05	no
CMPK2	0.247009369	4.581261083	6.61E-06	6.97E-05	no

KCNC1	-0.246992482	-4.580927544	6.62E-06	6.97E-05	no
TCEA3	0.246990763	4.580893574	6.62E-06	6.97E-05	no
TMEM132B	-0.246939758	-4.579886167	6.65E-06	7.00E-05	no
UQCRC2	-0.246916819	-4.579433099	6.66E-06	7.01E-05	no
F2RL1	0.246916608	4.579428931	6.66E-06	7.01E-05	no
TMEM43	0.246888391	4.57887164	6.68E-06	7.02E-05	no
BTNL9	-0.246880673	-4.578719212	6.69E-06	7.02E-05	no
CUEDC2	-0.246775582	-4.57664373	6.75E-06	7.08E-05	no
AARS	-0.246752106	-4.576180135	6.76E-06	7.10E-05	no
HNRNPH3	-0.246660551	-4.574372153	6.82E-06	7.15E-05	no
NFE2L1	0.246595537	4.573088378	6.86E-06	7.19E-05	no
YWHAE	-0.246578029	-4.572742687	6.87E-06	7.20E-05	no
RWDD2A	-0.246541001	-4.572011571	6.89E-06	7.22E-05	no
EFS	-0.246465601	-4.570522849	6.94E-06	7.26E-05	no
KLHL22	-0.246431754	-4.569854598	6.96E-06	7.28E-05	no
ZNF682	-0.246348937	-4.568219583	7.01E-06	7.33E-05	no
TRPM7	0.246323886	4.567725041	7.02E-06	7.34E-05	no
NF1	-0.246320396	-4.567656139	7.03E-06	7.34E-05	no
LRRC32	0.246317814	4.567605179	7.03E-06	7.34E-05	no
DPYSL4	-0.246300564	-4.567264649	7.04E-06	7.35E-05	no
OST4	0.246272287	4.566706436	7.06E-06	7.36E-05	no
GALNT13	-0.24626002	-4.566464277	7.06E-06	7.37E-05	no
DCTN6	-0.246232182	-4.565914738	7.08E-06	7.38E-05	no
CAV1	0.246221842	4.565710638	7.09E-06	7.38E-05	no
GRIA2	-0.246209408	-4.565465194	7.09E-06	7.39E-05	no
PFKFB3	0.24619693	4.565218882	7.10E-06	7.39E-05	no
AATK	-0.246117774	-4.563656427	7.15E-06	7.44E-05	no
MPZL1	0.246117242	4.563645933	7.15E-06	7.44E-05	no
PTPN2	0.246114023	4.563582403	7.15E-06	7.44E-05	no
SLC25A4	-0.246028743	-4.561899187	7.21E-06	7.49E-05	no
EIF4G2	0.245940626	4.560160091	7.27E-06	7.54E-05	no
WDR25	-0.245878538	-4.558934788	7.31E-06	7.58E-05	no
POLD4	0.245870099	4.558768247	7.31E-06	7.58E-05	no
ASIC1	-0.2458651	-4.558669603	7.31E-06	7.58E-05	no
ELAVL3	-0.245826647	-4.557910782	7.34E-06	7.60E-05	no
TNFRSF4	0.245794803	4.557282378	7.36E-06	7.62E-05	no
38047	-0.245755191	-4.556500733	7.39E-06	7.65E-05	no
ANK1	-0.245731474	-4.556032737	7.40E-06	7.66E-05	no
KIR3DL2	0.245699093	4.555393798	7.42E-06	7.68E-05	no
FAM159A	0.245689668	4.555207821	7.43E-06	7.68E-05	no
GATS	-0.245649437	-4.554414014	7.45E-06	7.70E-05	no
CLEC12A	0.245625283	4.553937433	7.47E-06	7.71E-05	no
HRSP12	-0.24557462	-4.552937853	7.50E-06	7.75E-05	no
GNG4	-0.245529634	-4.552050299	7.53E-06	7.77E-05	no
S100A8	0.245494592	4.551358965	7.56E-06	7.79E-05	no
PTPN22	0.245454672	4.550571406	7.58E-06	7.82E-05	no
LINC00599	-0.24544332	-4.550347453	7.59E-06	7.82E-05	no
BANK1	0.245430117	4.550086987	7.60E-06	7.83E-05	no
SLC46A1	0.245357736	4.548659134	7.65E-06	7.87E-05	no
CNN2	0.245337261	4.548255249	7.66E-06	7.88E-05	no
TRIM34	0.245218381	4.545910317	7.74E-06	7.96E-05	no

VWA1	0.245137074	4.54430663	7.80E-06	8.02E-05	no
C10orf54	0.245087343	4.543325819	7.83E-06	8.05E-05	no
CCDC71L	0.245082885	4.543237896	7.84E-06	8.05E-05	no
LOC100131289	0.245050328	4.54259581	7.86E-06	8.07E-05	no
ARHGEF9	-0.245042416	-4.542439765	7.86E-06	8.07E-05	no
LPHN1	-0.245029131	-4.542177764	7.87E-06	8.07E-05	no
SEMA4G	-0.245002543	-4.541653417	7.89E-06	8.09E-05	no
FHDC1	-0.244999739	-4.541598129	7.89E-06	8.09E-05	no
MYL9	0.244897501	4.539581987	7.96E-06	8.15E-05	no
NAP1L3	-0.244811962	-4.537895257	8.02E-06	8.21E-05	no
DGKB	-0.244809537	-4.537847448	8.03E-06	8.21E-05	no
MYT1	-0.244809399	-4.537844725	8.03E-06	8.21E-05	no
TMEM196	-0.244763644	-4.536942555	8.06E-06	8.23E-05	no
GPR39	0.244759601	4.536862839	8.06E-06	8.23E-05	no
ATP5G1	-0.244685263	-4.535397141	8.11E-06	8.28E-05	no
TLR10	0.244667331	4.535043582	8.13E-06	8.29E-05	no
PVRL1	-0.244602299	-4.533761459	8.17E-06	8.34E-05	no
PHLDA3	0.244567398	4.533073415	8.20E-06	8.36E-05	no
GCKR	0.244546732	4.532666008	8.21E-06	8.37E-05	no
HDAC3	0.24444121	4.530585828	8.29E-06	8.44E-05	no
PTPN18	0.24443714	4.530505608	8.29E-06	8.44E-05	no
OSBPL10	0.244431861	4.530401549	8.30E-06	8.44E-05	no
LOC254559	-0.24440225	-4.529817853	8.32E-06	8.46E-05	no
ZNF425	-0.24436563	-4.529096015	8.35E-06	8.48E-05	no
SUCLG2	0.244346672	4.528722346	8.36E-06	8.49E-05	no
LOC389765	-0.244342877	-4.528647543	8.36E-06	8.49E-05	no
MTMR7	-0.244275002	-4.527309697	8.41E-06	8.54E-05	no
CLEC12B	0.244233825	4.526498129	8.44E-06	8.57E-05	no
MSL1	-0.244228792	-4.526398926	8.45E-06	8.57E-05	no
CDK13	0.244175709	4.525352738	8.49E-06	8.60E-05	no
MDN1	-0.244163498	-4.525112091	8.49E-06	8.61E-05	no
C22orf29	-0.244135187	-4.524554132	8.52E-06	8.62E-05	no
DGKI	-0.24407783	-4.523423787	8.56E-06	8.66E-05	no
C2orf80	-0.244047761	-4.522831238	8.58E-06	8.68E-05	no
TNKS	-0.244007441	-4.522036687	8.61E-06	8.71E-05	no
SOX2	-0.243969691	-4.521292809	8.64E-06	8.73E-05	no
DGCR10	-0.24395732	-4.521049056	8.65E-06	8.74E-05	no
NUB1	0.243941502	4.520737354	8.66E-06	8.74E-05	no
BTN2A3P	0.243940534	4.52071828	8.66E-06	8.74E-05	no
HOXB6	0.243916899	4.520252567	8.68E-06	8.76E-05	no
P2RX1	0.243881348	4.519552077	8.71E-06	8.78E-05	no
TSPAN2	0.243844902	4.518833954	8.73E-06	8.80E-05	no
CHMP4C	0.243713562	4.51624628	8.84E-06	8.90E-05	no
ANKRD23	-0.243681926	-4.51562303	8.86E-06	8.92E-05	no
RAB8A	0.243672615	4.515439584	8.87E-06	8.93E-05	no
PTP4A2	0.243631853	4.514636577	8.90E-06	8.95E-05	no
SIL1	0.243559798	4.513217129	8.96E-06	9.01E-05	no
ANO1	0.243500296	4.512045049	9.00E-06	9.05E-05	no
CCNI2	-0.243457788	-4.511207738	9.04E-06	9.08E-05	no
SULF1	0.243401335	4.510095811	9.08E-06	9.12E-05	no
MPP4	0.243366494	4.509409575	9.11E-06	9.14E-05	no

WDFY3-AS2	-0.243346473	-4.509015248	9.12E-06	9.15E-05	no
NUDT18	0.243258845	4.507289432	9.19E-06	9.22E-05	no
CSMD1	-0.243244517	-4.507007243	9.21E-06	9.23E-05	no
PEA15	-0.24306781	-4.503527421	9.35E-06	9.37E-05	no
PRR3	-0.243027773	-4.502739062	9.38E-06	9.39E-05	no
GDAP2	0.242920302	4.500622971	9.47E-06	9.48E-05	no
DENND5B	-0.242891181	-4.500049612	9.49E-06	9.50E-05	no
DACT3	-0.242882799	-4.499884575	9.50E-06	9.50E-05	no
KCNH8	-0.242877161	-4.499773583	9.50E-06	9.50E-05	no
FAM3B	0.242869003	4.49961296	9.51E-06	9.50E-05	no
HDHD3	0.242855241	4.499342017	9.52E-06	9.51E-05	no
ZNF827	-0.242821291	-4.498673609	9.55E-06	9.53E-05	no
ELF4	0.242777647	4.497814381	9.59E-06	9.57E-05	no
ADAMTS1	0.242670772	4.495710454	9.68E-06	9.65E-05	no
PLBD2	0.242616162	4.494635467	9.72E-06	9.69E-05	no
ELFN2	-0.242589246	-4.494105641	9.75E-06	9.71E-05	no
PLXNB2	0.242558843	4.493507192	9.77E-06	9.73E-05	no
ZNF37A	-0.242536723	-4.493071806	9.79E-06	9.75E-05	no
MLEC	0.242469821	4.491754983	9.85E-06	9.80E-05	no
MTTP	0.242402049	4.490421125	9.91E-06	9.85E-05	no
EHMT2	-0.242386534	-4.490115773	9.92E-06	9.86E-05	no
FAM181B	-0.242379367	-4.489974733	9.93E-06	9.86E-05	no
SETD5	-0.242332868	-4.489059605	9.97E-06	9.89E-05	no
SLITRK1	-0.242330514	-4.489013273	9.97E-06	9.89E-05	no
ATRIP	-0.242312532	-4.488659397	9.98E-06	9.91E-05	no
ARL4C	0.242257614	4.487578651	1.00E-05	9.95E-05	no
FBLL1	-0.24218808	-4.48621032	1.01E-05	0.000100042	no
MEFV	0.242178124	4.48601441	1.01E-05	0.000100082	no
TMEM59L	-0.242169466	-4.485844039	1.01E-05	0.000100111	no
C1orf112	0.242157667	4.48561187	1.01E-05	0.000100168	no
ZNF397	-0.24208714	-4.484224118	1.02E-05	0.000100737	no
C1QTNF6	0.242080859	4.484100525	1.02E-05	0.000100746	no
PPP2R4	0.242069376	4.483874582	1.02E-05	0.0001008	no
WIBG	0.241965748	4.481835672	1.03E-05	0.000101593	no
POMT1	-0.241965474	-4.481830291	1.03E-05	0.000101593	no
MYL12B	0.241963177	4.481785103	1.03E-05	0.000101593	no
YIPF1	0.241900128	4.480544669	1.03E-05	0.000102075	no
PRDX2	-0.241898022	-4.480503239	1.03E-05	0.000102075	no
DUSP8	-0.241856623	-4.479688783	1.04E-05	0.000102396	no
PATL2	0.24183681	4.479299014	1.04E-05	0.000102525	no
ANGPT1	0.241681305	4.476240031	1.05E-05	0.000103869	no
ARL6IP5	0.241651852	4.475660689	1.06E-05	0.000104087	no
MTSS1L	-0.241555573	-4.473766976	1.07E-05	0.000104911	no
TNRC6C	-0.241543872	-4.473536841	1.07E-05	0.000104969	no
ABAT	-0.241516631	-4.473001063	1.07E-05	0.000105169	no
USP22	-0.241493035	-4.472536979	1.07E-05	0.000105336	no
RTN2	-0.241468349	-4.472051481	1.07E-05	0.000105465	no
EZH1	-0.241467895	-4.472042553	1.07E-05	0.000105465	no
KCNH7	-0.241463101	-4.471948278	1.07E-05	0.000105465	no
P4HB	0.241426369	4.471225875	1.08E-05	0.000105753	no
VAX2	-0.241407133	-4.470847568	1.08E-05	0.000105881	no

FLJ37035	-0.241387616	-4.470463751	1.08E-05	0.000105975	no
LPHN3	-0.241386332	-4.4704385	1.08E-05	0.000105975	no
LINC00840	0.241306117	4.468861073	1.09E-05	0.000106664	no
GRK5	0.241262615	4.468005642	1.09E-05	0.000107018	no
CCNYL1	0.241195216	4.466680354	1.10E-05	0.000107595	no
ANPEP	0.241172302	4.466229806	1.10E-05	0.000107759	no
VWF	0.241158465	4.465957722	1.10E-05	0.000107839	no
ZFP1	-0.241094516	-4.464700374	1.11E-05	0.000108388	no
CDCP1	0.241026977	4.463372501	1.12E-05	0.000108973	no
ITPKC	0.240884551	4.460572528	1.13E-05	0.000110273	no
HCK	0.240682661	4.456604041	1.15E-05	0.000112161	no
DOCK3	-0.240645391	-4.455871508	1.15E-05	0.000112472	no
LOC285696	-0.240639872	-4.45576305	1.15E-05	0.000112474	no
SUZ12P1	-0.240573614	-4.454460801	1.16E-05	0.000113068	no
RBM43	0.24050736	4.453158722	1.17E-05	0.000113664	no
SUB1	-0.240480066	-4.452622338	1.17E-05	0.00011388	no
HINT3	-0.240442191	-4.451878026	1.17E-05	0.000114201	no
TRIM6	0.240295784	4.449001078	1.19E-05	0.000115592	no
DGCR9	-0.240291279	-4.44891255	1.19E-05	0.000115592	no
SCARNA9	-0.240281627	-4.448722903	1.19E-05	0.000115636	no
HESX1	0.240267939	4.448453951	1.19E-05	0.00011572	no
ZCCHC18	-0.240257393	-4.448246725	1.19E-05	0.00011575	no
GUSBP4	-0.240254441	-4.448188733	1.19E-05	0.00011575	no
GPR137	0.240189002	4.446902966	1.20E-05	0.000116352	no
NUFIP1	-0.240068927	-4.444543884	1.21E-05	0.000117508	no
BAG6	-0.240056756	-4.444304765	1.21E-05	0.000117578	no
TSG1	-0.240007136	-4.443329978	1.22E-05	0.000118028	no
HSPG2	0.239998208	4.443154589	1.22E-05	0.000118066	no
GPR114	0.239969611	4.442592803	1.22E-05	0.000118303	no
MB21D2	-0.239922177	-4.441661017	1.23E-05	0.000118734	no
ZNF24	-0.239857937	-4.440399135	1.23E-05	0.000119338	no
CDIP1	-0.239819198	-4.439638201	1.24E-05	0.000119682	no
MMP24	-0.239773775	-4.438746021	1.24E-05	0.000120079	no
SCYL2	0.239770285	4.43867748	1.24E-05	0.000120079	no
USP32P1	-0.239759724	-4.438470036	1.24E-05	0.000120134	no
RAB3C	-0.239710855	-4.437510204	1.25E-05	0.000120586	no
MESDC1	0.23970324	4.43736064	1.25E-05	0.00012061	no
ATRN	-0.239658599	-4.436483897	1.26E-05	0.00012102	no
BAZ2B	-0.239589133	-4.435119649	1.26E-05	0.00012169	no
RNF208	-0.239579475	-4.434929974	1.26E-05	0.000121736	no
NFKBID	0.239556379	4.434476403	1.27E-05	0.000121923	no
AGPAT4	-0.239500326	-4.433375655	1.27E-05	0.000122457	no
ALG1	0.239483781	4.433050758	1.27E-05	0.000122529	no
NOVA2	-0.239483034	-4.433036081	1.27E-05	0.000122529	no
NOS3	0.239476735	4.432912393	1.28E-05	0.000122541	no
RTKN	-0.239406019	-4.43152378	1.28E-05	0.000123232	no
SLC35A4	0.2392832	4.429112217	1.30E-05	0.000124482	no
CDHR1	-0.239234752	-4.428160998	1.30E-05	0.000124945	no
LRRC37A4P	-0.239223818	-4.427946332	1.30E-05	0.000125006	no
FAM66C	-0.2391702	-4.42689366	1.31E-05	0.000125526	no
FAM46C	0.239124459	4.425995665	1.31E-05	0.000125963	no

ZFP14	-0.239112296	-4.42575689	1.32E-05	0.000126038	no
ZDHHC1	0.239106357	4.4256403	1.32E-05	0.000126046	no
LXN	0.239094963	4.425416623	1.32E-05	0.000126113	no
CHST6	0.239081048	4.425143454	1.32E-05	0.000126208	no
LMAN1	0.239067213	4.424871863	1.32E-05	0.000126301	no
SPTY2D1	0.23903481	4.424235762	1.32E-05	0.000126596	no
BMF	0.239016113	4.423868739	1.33E-05	0.000126743	no
ST6GAL2	-0.238989788	-4.423351982	1.33E-05	0.000126973	no
WASF1	-0.238978838	-4.423137033	1.33E-05	0.000127036	no
ACOT12	0.238955651	4.422681886	1.33E-05	0.000127232	no
CHPT1	0.238926291	4.422105593	1.34E-05	0.000127495	no
CD19	0.238903297	4.421654239	1.34E-05	0.00012769	no
MOB3A	0.238845753	4.420524768	1.35E-05	0.000128234	no
HMGCS1	-0.238843283	-4.42047629	1.35E-05	0.000128234	no
KLF12	-0.238827061	-4.420157907	1.35E-05	0.00012831	no
XKR7	-0.238826066	-4.420138369	1.35E-05	0.00012831	no
RPS6KA6	-0.238787989	-4.419391025	1.35E-05	0.000128672	no
RPS3	-0.238745217	-4.418551569	1.36E-05	0.000129038	no
SOX6	-0.238744512	-4.418537729	1.36E-05	0.000129038	no
KCNJ5	0.238736678	4.418383994	1.36E-05	0.000129067	no
RAB11FIP1	0.238706587	4.417793424	1.36E-05	0.000129343	no
FZD4	0.238612434	4.415945697	1.37E-05	0.0001303	no
SLC2A13	-0.238607147	-4.415841939	1.37E-05	0.0001303	no
MEX3A	-0.238604943	-4.415798689	1.37E-05	0.0001303	no
SPTBN4	-0.238581904	-4.415346571	1.38E-05	0.000130499	no
TADA1	-0.238470864	-4.413167657	1.39E-05	0.000131666	no
ACO2	-0.238467493	-4.413101521	1.39E-05	0.000131666	no
RPN1	0.238453807	4.412832979	1.39E-05	0.000131762	no
MAOB	0.238438023	4.412523272	1.39E-05	0.000131844	no
TMEM62	0.238436293	4.412489321	1.39E-05	0.000131844	no
EHHADH	0.238350463	4.410805272	1.40E-05	0.000132756	no
LTC4S	0.238307232	4.409957085	1.41E-05	0.000133189	no
FAM110B	-0.238230449	-4.408450668	1.42E-05	0.000134006	no
SPAG9	-0.238155472	-4.406979785	1.43E-05	0.000134808	no
LPCAT3	0.238123201	4.406346726	1.43E-05	0.00013512	no
SCG3	-0.23806559	-4.405216615	1.44E-05	0.000135727	no
MRPS25	-0.23803183	-4.404554379	1.44E-05	0.000136059	no
SLC10A7	0.238020996	4.404341874	1.44E-05	0.000136125	no
PLA2G4F	0.237941081	4.402774363	1.45E-05	0.000136973	no
MAPKAPK3	0.23793791	4.402712169	1.46E-05	0.000136973	no
RCAN1	0.237916339	4.402289064	1.46E-05	0.000137165	no
LRRC37B	-0.237899709	-4.401962894	1.46E-05	0.0001373	no
PAPSS2	0.237879066	4.401558013	1.46E-05	0.000137482	no
CLEC18B	0.237847675	4.400942355	1.47E-05	0.00013779	no
LOC10050580€	0.237840758	4.400806698	1.47E-05	0.000137811	no
NREP	-0.237832334	-4.40064148	1.47E-05	0.00013785	no
PAMR1	0.237785735	4.39972757	1.47E-05	0.000138338	no
FAM65C	0.2377743	4.399503312	1.48E-05	0.000138412	no
TICAM2	0.237738564	4.39880247	1.48E-05	0.000138774	no
LOC100128993	0.237732815	4.398689739	1.48E-05	0.000138781	no
ATF5	0.237713008	4.398301294	1.48E-05	0.000138955	no

PC	-0.237689879	-4.397847733	1.49E-05	0.000139169	no
GRID2	-0.237658766	-4.39723759	1.49E-05	0.000139477	no
DGCR6	-0.237637764	-4.396825755	1.49E-05	0.000139666	no
LOC730091	0.237583623	4.395764099	1.50E-05	0.000140251	no
LYSMD1	-0.237550486	-4.395114324	1.50E-05	0.000140586	no
SERPINE1	0.237529859	4.394709864	1.51E-05	0.000140772	no
MLL	-0.237508125	-4.394283699	1.51E-05	0.000140971	no
CLIC4	0.23750086	4.394141264	1.51E-05	0.000140997	no
LOC100507266	-0.237383559	-4.391841366	1.53E-05	0.00014235	no
ELL	0.237350265	4.391188623	1.53E-05	0.000142692	no
HCN3	-0.237339701	-4.390981504	1.53E-05	0.000142759	no
HDAC1	0.237218291	4.388601357	1.55E-05	0.000144178	no
ABCC8	-0.237191065	-4.388067642	1.55E-05	0.000144449	no
IRF9	0.237184342	4.387935841	1.55E-05	0.000144469	no
NRP1	0.237121996	4.386713722	1.56E-05	0.000145174	no
TNR	-0.237106297	-4.386405979	1.56E-05	0.000145305	no
P2RX4	0.237092998	4.386145304	1.56E-05	0.000145406	no
WNT4	0.237075133	4.385795132	1.57E-05	0.000145564	no
CCDC94	-0.237046507	-4.385234022	1.57E-05	0.000145856	no
METTL24	0.236971715	4.383768071	1.58E-05	0.000146722	no
LOC100506472	-0.236944063	-4.383226106	1.58E-05	0.000146942	no
MAP3K6	0.236943937	4.383223638	1.58E-05	0.000146942	no
DYNLT3	0.236933852	4.383025982	1.58E-05	0.000146981	no
PLIN2	0.236930651	4.382963236	1.59E-05	0.000146981	no
TRIM9	-0.236906096	-4.382482	1.59E-05	0.000147224	no
ARVCF	-0.236889864	-4.382163858	1.59E-05	0.000147363	no
ZNF207	0.236882233	4.382014318	1.59E-05	0.000147395	no
AKR1B10	0.236803888	4.380478918	1.60E-05	0.000148315	no
NSUN6	-0.236796229	-4.380328814	1.60E-05	0.000148348	no
GUF1	-0.236786031	-4.380128975	1.60E-05	0.000148412	no
DYRK3	0.236773835	4.379889963	1.61E-05	0.000148502	no
VAMP2	-0.236746258	-4.379349552	1.61E-05	0.000148786	no
RGS13	0.236684415	4.378137684	1.62E-05	0.000149504	no
PMM2	0.236614568	4.376769017	1.63E-05	0.000150328	no
GDAP1	-0.236587455	-4.37623776	1.63E-05	0.00015061	no
DNPEP	0.236576511	4.376023323	1.63E-05	0.000150685	no
EFNA3	-0.236560552	-4.375710633	1.64E-05	0.000150824	no
TMEM35	-0.236447849	-4.373502463	1.65E-05	0.000152207	no
TMEM102	0.236420446	4.372965588	1.66E-05	0.000152495	no
FLJ39639	0.236405616	4.372675046	1.66E-05	0.000152621	no
CABP4	0.236377204	4.372118414	1.66E-05	0.000152862	no
PKIA	-0.236376909	-4.372112637	1.66E-05	0.000152862	no
FAM156A	-0.236335978	-4.371310757	1.67E-05	0.000153272	no
PRKCE	-0.236335218	-4.371295882	1.67E-05	0.000153272	no
NT5M	-0.236323061	-4.371057714	1.67E-05	0.000153364	no
GNPDA1	0.236259745	4.369817381	1.68E-05	0.000154059	no
CYP4F11	0.236259571	4.369813957	1.68E-05	0.000154059	no
CTIF	-0.236216474	-4.368969741	1.68E-05	0.00015454	no
SNX8	0.236212665	4.368895138	1.68E-05	0.00015454	no
FBN1	0.236150523	4.367677878	1.69E-05	0.000155289	no
KLHL23	-0.236132624	-4.367327283	1.70E-05	0.000155458	no

PIEZO1	0.236090091	4.36649418	1.70E-05	0.000155952	no
SLC16A14	-0.236075865	-4.366215556	1.70E-05	0.000156073	no
GOLGA7B	-0.236050114	-4.365711183	1.71E-05	0.000156347	no
HM13	0.23604442	4.365599648	1.71E-05	0.000156356	no
PARVA	0.235997658	4.364683784	1.72E-05	0.000156909	no
GRIPAP1	-0.235978283	-4.364304317	1.72E-05	0.000157099	no
COQ2	0.235943553	4.363624133	1.72E-05	0.000157494	no
ULK4	0.235935624	4.363468856	1.72E-05	0.000157533	no
SYT15	-0.235816259	-4.361131253	1.74E-05	0.000159034	no
CUL7	0.235813421	4.361075677	1.74E-05	0.000159034	no
RRP7B	-0.23580554	-4.360921348	1.74E-05	0.000159072	no
MAPK8IP1	-0.235737609	-4.359591108	1.75E-05	0.00015992	no
KAT6B	-0.235717101	-4.359189539	1.76E-05	0.000160129	no
YPEL4	-0.235705561	-4.35896358	1.76E-05	0.000160218	no
CPLX1	-0.235611782	-4.357127335	1.77E-05	0.000161368	no
CLEC16A	-0.235610795	-4.357108006	1.77E-05	0.000161368	no
STX1B	-0.235569401	-4.356297546	1.78E-05	0.000161865	no
PRR16	0.235561306	4.356139043	1.78E-05	0.000161907	no
MAPK8IP2	-0.235549483	-4.355907574	1.78E-05	0.000162	no
LENG9	0.235473416	4.3544183	1.79E-05	0.000162917	no
FEM1B	-0.235472558	-4.354401507	1.79E-05	0.000162917	no
CLCF1	0.235464351	4.354240844	1.79E-05	0.000162961	no
GPR31	0.235451377	4.35398683	1.80E-05	0.000163071	no
ALG12	0.235363494	4.352266371	1.81E-05	0.000164215	no
GABPB1-AS1	-0.235333374	-4.351676745	1.81E-05	0.000164563	no
KLHDC1	-0.235315915	-4.351334972	1.82E-05	0.000164736	no
NCAPH2	-0.235309379	-4.351207036	1.82E-05	0.000164758	no
HTR7P1	0.235300753	4.351038173	1.82E-05	0.000164808	no
FOXS1	0.235268988	4.350416379	1.82E-05	0.00016518	no
BECN1	0.235257077	4.350183218	1.83E-05	0.000165277	no
GPBP1L1	0.235244292	4.349932966	1.83E-05	0.000165385	no
PPFIA3	-0.235192822	-4.348925494	1.84E-05	0.000166035	no
COL5A2	0.235157704	4.348238111	1.84E-05	0.000166457	no
RNF146	-0.235147392	-4.34803627	1.84E-05	0.000166531	no
CLVS2	-0.2351301	-4.347697821	1.85E-05	0.000166704	no
PXN-AS1	-0.235115811	-4.347418148	1.85E-05	0.000166835	no
GRID2IP	-0.235081211	-4.346740942	1.85E-05	0.000167252	no
CLPTM1L	0.23503309	4.345799142	1.86E-05	0.00016786	no
ZNF91	-0.234968869	-4.34454228	1.87E-05	0.0001687	no
DAGLA	-0.234962345	-4.344414616	1.87E-05	0.000168722	no
HSPB1	0.234836459	4.341951101	1.89E-05	0.000170448	no
FN3K	-0.234784582	-4.340935974	1.90E-05	0.000171122	no
C19orf40	0.234727209	4.339813346	1.91E-05	0.000171844	no
NDUFA10	-0.234724554	-4.339761387	1.91E-05	0.000171844	no
DICER1-AS1	-0.234689401	-4.33907357	1.92E-05	0.000172227	no
LOC100128288	-0.234684955	-4.338986576	1.92E-05	0.000172227	no
PLEKHA7	0.234683164	4.338951541	1.92E-05	0.000172227	no
CAMP	0.234673325	4.338759032	1.92E-05	0.000172298	no
LRRC1	-0.234629468	-4.337900939	1.93E-05	0.000172862	no
WDR17	-0.234525441	-4.335865676	1.94E-05	0.000174307	no
FAM86B1	0.234465129	4.334685764	1.95E-05	0.00017512	no

RAB6B	-0.234458391	-4.334553947	1.95E-05	0.000175146	no
TRAPPC3	0.234423883	4.333878884	1.96E-05	0.000175581	no
MX1	0.234318734	4.331821998	1.98E-05	0.000177065	no
LRPPRC	-0.234312659	-4.331703171	1.98E-05	0.000177081	no
PPP1R9A	-0.234248293	-4.330444139	1.99E-05	0.000177967	no
MTHFD2L	-0.234241037	-4.330302219	1.99E-05	0.000178001	no
DISP2	-0.234224582	-4.329980359	1.99E-05	0.000178173	no
PDLIM4	0.234156543	4.328649598	2.00E-05	0.000179118	no
SCD5	-0.234141476	-4.328354898	2.01E-05	0.00017927	no
CGN	-0.234133733	-4.328203476	2.01E-05	0.000179312	no
PPM1E	-0.234125943	-4.328051107	2.01E-05	0.000179355	no
CCDC122	0.234036213	4.326296214	2.02E-05	0.000180635	no
GTF2F2	-0.234000353	-4.325594918	2.03E-05	0.000181104	no
C1orf85	0.233985713	4.325308616	2.03E-05	0.000181224	no
OXA1L	0.233982246	4.325240828	2.03E-05	0.000181224	no
ATG7	0.233976048	4.325119606	2.03E-05	0.000181224	no
SFXN1	-0.233972669	-4.325053533	2.03E-05	0.000181224	no
ITGB3	0.23396386	4.324881274	2.04E-05	0.000181282	no
LRRC8A	-0.233949568	-4.324601775	2.04E-05	0.000181424	no
TRAF3IP1	0.233936611	4.324348407	2.04E-05	0.000181546	no
TPD52L2	0.233868414	4.323014817	2.05E-05	0.000182512	no
POPDC2	0.233811699	4.321905816	2.06E-05	0.000183305	no
TOP2B	-0.233786738	-4.321417748	2.07E-05	0.000183613	no
FAM129B	0.233748578	4.320671602	2.07E-05	0.000184125	no
SLC30A7	0.233711727	4.319951095	2.08E-05	0.000184617	no
CTHRC1	0.233693498	4.319594673	2.08E-05	0.000184823	no
KIF3C	-0.233556407	-4.316914434	2.11E-05	0.000186879	no
WNT3	-0.233491462	-4.315644797	2.12E-05	0.00018782	no
HRASLS5	0.23347939	4.315408808	2.12E-05	0.000187932	no
SAP18	-0.233451318	-4.314860051	2.13E-05	0.000188296	no
SACS	-0.233445088	-4.314738255	2.13E-05	0.000188317	no
BRWD1	-0.233431821	-4.314478909	2.13E-05	0.000188448	no
RAP2A	-0.233418141	-4.314211499	2.13E-05	0.000188586	no
LOC100507632	0.23338857	4.313633463	2.14E-05	0.000188975	no
LAMB2	0.233253757	4.310998348	2.16E-05	0.000191039	no
BICC1	0.233222341	4.310384324	2.17E-05	0.000191462	no
TRIM56	0.233120182	4.308387706	2.19E-05	0.000192952	no
TRIP4	0.233119693	4.308378147	2.19E-05	0.000192952	no
GRB2	0.232976741	4.305584508	2.21E-05	0.000195189	no
RBM8A	-0.232915567	-4.304389111	2.22E-05	0.000196108	no
MYCBP	0.23286558	4.303412366	2.23E-05	0.000196846	no
SELPLG	0.232726614	4.300697128	2.26E-05	0.000199011	no
SVOP	-0.232724596	-4.300657704	2.26E-05	0.000199011	no
RNPS1	-0.23269201	-4.300021047	2.26E-05	0.000199471	no
DOK1	0.232655073	4.299299414	2.27E-05	0.000200004	no
ARSD	0.23254366	4.297122842	2.29E-05	0.000201749	no
PLP2	0.232541015	4.297071183	2.29E-05	0.000201749	no
OXER1	0.232458258	4.295454557	2.31E-05	0.000203061	no
FUS	-0.232406546	-4.294444432	2.32E-05	0.000203854	no
PEX19	-0.232364516	-4.293623471	2.33E-05	0.000204479	no
ZNF785	-0.232359997	-4.293535199	2.33E-05	0.000204479	no

LOXL2	0.232344419	4.293230932	2.33E-05	0.000204598	no
CKAP4	0.232343186	4.293206846	2.33E-05	0.000204598	no
ARHGDIA	0.232260023	4.291582536	2.35E-05	0.000205935	no
ERRFI1	0.232245501	4.291298903	2.35E-05	0.000206099	no
ELF3	0.232230491	4.291005747	2.35E-05	0.000206273	no
C10orf128	0.232182621	4.290070835	2.36E-05	0.000207011	no
SHC2	-0.232103183	-4.288519456	2.38E-05	0.000208217	no
A2M-AS1	0.232099668	4.288450809	2.38E-05	0.000208217	no
FOXC1	0.232098103	4.288420241	2.38E-05	0.000208217	no
CFLAR-AS1	0.232074158	4.287952624	2.38E-05	0.000208547	no
RPL38	-0.232068447	-4.287841107	2.39E-05	0.000208561	no
ILF3-AS1	-0.23202942	-4.287078974	2.39E-05	0.000209154	no
DKFZP434H168	-0.231946423	-4.285458278	2.41E-05	0.000210516	no
SMIM8	-0.231927049	-4.285079989	2.41E-05	0.000210769	no
C1orf127	0.231875322	4.284069971	2.42E-05	0.000211591	no
LINC00607	0.231850865	4.283592423	2.43E-05	0.000211935	no
ZBTB3	-0.231789506	-4.282394407	2.44E-05	0.000212932	no
ARHGEF26	-0.231721776	-4.281072057	2.46E-05	0.000214045	no
GABBR2	-0.231655955	-4.279787038	2.47E-05	0.000215131	no
SPINT1	0.231545808	4.277636798	2.49E-05	0.000216931	no
PPFIA1	-0.231545765	-4.277635946	2.49E-05	0.000216931	no
MAPT-IT1	-0.231517447	-4.277083168	2.50E-05	0.000217353	no
ELL3	0.231506745	4.276874256	2.50E-05	0.000217457	no
GAD1	-0.2314354	-4.275481634	2.51E-05	0.000218659	no
HPSE	0.231385384	4.274505377	2.52E-05	0.000219479	no
ADAMTS2	0.231310002	4.273034069	2.54E-05	0.000220765	no
PCBP4	-0.231279749	-4.272443605	2.55E-05	0.00022123	no
AP2B1	-0.231109063	-4.269112523	2.58E-05	0.000224288	no
RND1	-0.231023553	-4.267443894	2.60E-05	0.000225725	no
SHANK2	-0.231022165	-4.267416798	2.60E-05	0.000225725	no
MTMR4	-0.23101068	-4.267192703	2.60E-05	0.00022581	no
FGF20	0.231007852	4.267137505	2.60E-05	0.00022581	no
IL1RAPL1	-0.230935921	-4.265733961	2.62E-05	0.000227067	no
PTPRJ	0.230889156	4.264821486	2.63E-05	0.000227785	no
DCAF16	-0.230885453	-4.264749241	2.63E-05	0.000227785	no
SLC9A1	0.230883142	4.264704137	2.63E-05	0.000227785	no
CAMK2G	-0.230837404	-4.263811745	2.64E-05	0.000228556	no
SLC25A48	-0.230804142	-4.26316278	2.65E-05	0.000229072	no
THNSL1	-0.230796006	-4.263004049	2.65E-05	0.000229072	no
UBR3	-0.23079551	-4.26299437	2.65E-05	0.000229072	no
WDR20	-0.230785906	-4.262806998	2.65E-05	0.000229107	no
ZNF333	-0.230782766	-4.262745732	2.65E-05	0.000229107	no
MMD2	-0.230779089	-4.262674001	2.65E-05	0.000229107	no
PTPN4	-0.230746421	-4.262036666	2.66E-05	0.000229634	no
C11orf95	-0.230718502	-4.261491985	2.67E-05	0.000230072	no
CDS2	-0.230711056	-4.261346718	2.67E-05	0.000230122	no
C8orf46	-0.230694248	-4.261018809	2.67E-05	0.000230273	no
CCDC90A	-0.23069341	-4.261002473	2.67E-05	0.000230273	no
LOC728377	0.230632126	4.259806933	2.69E-05	0.000231349	no
GTPBP1	-0.230615929	-4.259490963	2.69E-05	0.000231566	no
AMER2	-0.230499121	-4.257212416	2.72E-05	0.000233717	no

NLE1	-0.230443466	-4.256126845	2.73E-05	0.000234699	no
LOC729852	0.230438389	4.25602781	2.73E-05	0.000234704	no
DOK6	-0.230433528	-4.255933008	2.73E-05	0.000234704	no
HAS3	0.230365746	4.254610955	2.75E-05	0.000235926	no
GCHFR	0.230323149	4.253780148	2.76E-05	0.000236662	no
TM2D2	0.230303662	4.253400099	2.76E-05	0.000236948	no
ANKEF1	0.23029244	4.253181235	2.76E-05	0.000237	no
ZNF418	-0.230291356	-4.253160091	2.76E-05	0.000237	no
THOP1	-0.230228422	-4.25193272	2.78E-05	0.000238063	no
ZMYM6	0.230227425	4.251913279	2.78E-05	0.000238063	no
ATP5S	-0.230219871	-4.251765955	2.78E-05	0.000238063	no
GPR34	0.230217765	4.25172489	2.78E-05	0.000238063	no
RPRD2	-0.230096472	-4.249359553	2.81E-05	0.000240359	no
MRPS28	-0.230069499	-4.248833578	2.81E-05	0.000240798	no
LOC93622	-0.230022374	-4.247914676	2.83E-05	0.000241639	no
AKAP11	-0.229921019	-4.245938407	2.85E-05	0.000243568	no
NCAM1	-0.229808812	-4.243750718	2.88E-05	0.000245731	no
PAFAH1B1	-0.229768892	-4.242972453	2.89E-05	0.000246441	no
GIN1	-0.229756283	-4.242726627	2.89E-05	0.000246599	no
PTPRA	-0.229734222	-4.242296553	2.89E-05	0.000246949	no
KIAA1549L	-0.229711729	-4.241858056	2.90E-05	0.000247308	no
SALL2	-0.229658445	-4.240819313	2.91E-05	0.000248296	no
TFE3	0.22959496	4.239581773	2.93E-05	0.000249497	no
CDH22	-0.229547575	-4.238658112	2.94E-05	0.000250372	no
TNFRSF12A	0.229505503	4.237838044	2.95E-05	0.00025114	no
ORAI3	0.229454795	4.23684968	2.96E-05	0.000252089	no
TBX19	0.229396094	4.235705557	2.97E-05	0.000253207	no
CUX2	-0.229374604	-4.235286714	2.98E-05	0.000253554	no
PON1	-0.229277861	-4.233401262	3.00E-05	0.000255476	no
PSEN2	0.22921667	4.232208762	3.02E-05	0.000256661	no
CPXM2	0.229102469	4.229983355	3.05E-05	0.000258896	no
SSTR2	-0.229101398	-4.229962477	3.05E-05	0.000258896	no
TOP1P1	0.229072874	4.229406667	3.05E-05	0.0002594	no
APC	-0.229063841	-4.229230647	3.06E-05	0.00025949	no
LOC100506314	0.229051199	4.228984322	3.06E-05	0.000259657	no
MLL5	-0.228968022	-4.227363637	3.08E-05	0.000261331	no
PTPN1	0.228959177	4.227191289	3.08E-05	0.000261418	no
NMT2	-0.22894402	-4.226895975	3.09E-05	0.00026164	no
NKIRAS1	-0.228904205	-4.226120233	3.10E-05	0.000262392	no
ATOH8	-0.228899354	-4.22602572	3.10E-05	0.000262393	no
TCEAL5	-0.228878999	-4.225629144	3.10E-05	0.000262727	no
TPBG	0.228862615	4.225309945	3.11E-05	0.000262977	no
CCDC101	-0.228826542	-4.224607151	3.12E-05	0.000263652	no
GALNS	0.228798796	4.224066615	3.12E-05	0.000264148	no
NID2	0.228789388	4.223883332	3.13E-05	0.000264248	no
NPRL3	-0.22878223	-4.223743876	3.13E-05	0.000264299	no
POM121L9P	0.228760902	4.223328386	3.13E-05	0.000264657	no
B3GNT1	-0.228741406	-4.222948575	3.14E-05	0.000264976	no
APBA1	-0.228718768	-4.222507569	3.14E-05	0.000265363	no
PLEKHM3	-0.228682651	-4.221803991	3.15E-05	0.000266045	no
KSR1	-0.228610305	-4.220394727	3.17E-05	0.000267521	no

DHRS3	0.228593264	4.220062779	3.18E-05	0.000267789	no
TMEFF2	-0.228492039	-4.218091096	3.20E-05	0.000269912	no
ARL10	-0.228481792	-4.217891518	3.21E-05	0.000270032	no
PLS3	0.228476472	4.217787898	3.21E-05	0.000270044	no
CBX3P2	-0.228461504	-4.217496369	3.21E-05	0.000270269	no
WDFY3	-0.228455159	-4.21737279	3.21E-05	0.000270303	no
CEP112	0.228427621	4.216836427	3.22E-05	0.000270807	no
BPIFB4	0.228414004	4.216571222	3.22E-05	0.000271003	no
CCDC8	0.22838253	4.21595822	3.23E-05	0.000271595	no
ARF4	0.228346763	4.215261635	3.24E-05	0.000272251	no
SCFD1	-0.228338974	-4.215109954	3.24E-05	0.000272251	no
FAM19A5	-0.228338723	-4.215105065	3.24E-05	0.000272251	no
GRID1	-0.228219977	-4.21279255	3.28E-05	0.0002748	no
RP1-177G6.2	-0.228184193	-4.212095717	3.29E-05	0.000275497	no
QPRT	0.228173839	4.211894097	3.29E-05	0.000275622	no
ZNF436	0.228050099	4.209484612	3.32E-05	0.00027831	no
NOVA1	-0.228045537	-4.209395791	3.32E-05	0.00027831	no
HRH3	-0.228022711	-4.208951345	3.33E-05	0.000278664	no
SGSH	0.228020417	4.208906678	3.33E-05	0.000278664	no
PDIA4	0.228008806	4.208680598	3.33E-05	0.00027882	no
GJD3	0.227962352	4.207776125	3.35E-05	0.00027977	no
MIR600HG	-0.227953044	-4.207594895	3.35E-05	0.00027978	no
ADCYAP1R1	-0.227952407	-4.207582491	3.35E-05	0.00027978	no
RPP30	-0.2279412	-4.207364299	3.35E-05	0.000279927	no
EPHA10	-0.227894294	-4.206451068	3.36E-05	0.000280892	no
LINC00671	-0.22784528	-4.205496825	3.38E-05	0.000281908	no
LAMB1	0.227820299	4.205010493	3.38E-05	0.000282373	no
XAB2	-0.227788193	-4.204385452	3.39E-05	0.000283004	no
KIAA1147	-0.2277257	-4.203168892	3.41E-05	0.00028434	no
PDZD8	-0.227686315	-4.202402217	3.42E-05	0.00028483	no
EFNB2	0.227684856	4.202373811	3.42E-05	0.00028483	no
SNURF	-0.227683854	-4.20235431	3.42E-05	0.00028483	no
B3GALT2	-0.227683218	-4.20234193	3.42E-05	0.00028483	no
LEPREL1	0.227680821	4.202295264	3.42E-05	0.00028483	no
MOAP1	-0.227654745	-4.201787677	3.43E-05	0.000285326	no
NUDT10	-0.227627793	-4.201263045	3.44E-05	0.000285843	no
EPB41L2	0.227615822	4.201030023	3.44E-05	0.000286011	no
KLF15	-0.22755105	-4.199769259	3.46E-05	0.000287414	no
SAA4	0.227466928	4.198131938	3.48E-05	0.000289279	no
ACSF2	0.22744444	4.197694263	3.49E-05	0.000289697	no
TAGLN	0.227420606	4.197230384	3.50E-05	0.000290143	no
LINC00630	-0.227416069	-4.197142077	3.50E-05	0.000290143	no
HAPLN3	0.227392007	4.19667379	3.50E-05	0.0002906	no
MTHFR	0.227374999	4.196342784	3.51E-05	0.000290891	no
JMJD6	-0.227292517	-4.194737573	3.53E-05	0.000292738	no
VASH1	0.227277354	4.1944425	3.54E-05	0.000292903	no
BRI3	0.227276119	4.194418467	3.54E-05	0.000292903	no
TCEB3C	0.227219943	4.193325285	3.55E-05	0.000294132	no
AKAP8L	-0.227214836	-4.193225905	3.56E-05	0.000294141	no
MRFAP1L1	-0.227204168	-4.193018306	3.56E-05	0.000294283	no
TAGLN2	0.22718992	4.192741052	3.56E-05	0.000294511	no

SPSB1	0.227145977	4.191885975	3.58E-05	0.000295452	no
TMEM156	0.227121436	4.191408448	3.58E-05	0.000295867	no
RGMB	-0.227119287	-4.191366627	3.58E-05	0.000295867	no
BSN	-0.227104416	-4.191077263	3.59E-05	0.000296111	no
DDX6	-0.227065306	-4.190316286	3.60E-05	0.00029694	no
SPRN	-0.227017902	-4.189393936	3.61E-05	0.000297869	no
SCAMP1	-0.227017466	-4.189385443	3.61E-05	0.000297869	no
FAN1	-0.226999855	-4.189042806	3.62E-05	0.000298181	no
NFIX	-0.226988936	-4.18883035	3.62E-05	0.000298332	no
NUDT11	-0.226918293	-4.187455941	3.64E-05	0.000299934	no
GPR125	-0.226913062	-4.18735416	3.64E-05	0.000299947	no
TLX1NB	-0.226898229	-4.187065584	3.65E-05	0.000300194	no
PLAC8	0.226885853	4.186824801	3.65E-05	0.000300381	no
LDHA	-0.226839762	-4.185928129	3.67E-05	0.000301392	no
HSD17B11	0.226827162	4.185683003	3.67E-05	0.000301585	no
CAMTA1	-0.226785858	-4.184879482	3.68E-05	0.000302483	no
PET112	-0.226777684	-4.18472047	3.68E-05	0.000302568	no
LACTB2	0.226768981	4.184551172	3.69E-05	0.000302667	no
IL13RA2	0.226748612	4.184154929	3.69E-05	0.000303052	no
ACVRL1	0.226730402	4.183800684	3.70E-05	0.000303385	no
SPATA20	0.226713707	4.18347592	3.70E-05	0.00030368	no
ITGAX	0.226704873	4.18330408	3.71E-05	0.000303782	no
GALNT10	0.226660527	4.182441452	3.72E-05	0.000304761	no
CCNDBP1	0.226648542	4.182208333	3.72E-05	0.000304942	no
LAMTOR2	0.226639212	4.182026845	3.73E-05	0.000305057	no
CLEC4D	0.22658844	4.181039278	3.74E-05	0.000306199	no
MYO1E	0.226524046	4.179786786	3.76E-05	0.000307686	no
IRAK2	0.226489668	4.179118147	3.77E-05	0.000308428	no
ULBP2	0.226458243	4.178506971	3.78E-05	0.000309037	no
PELO	0.226455955	4.178462468	3.78E-05	0.000309037	no
COL3A1	0.226410501	4.177578452	3.80E-05	0.000309949	no
PRPF6	-0.226410248	-4.177573525	3.80E-05	0.000309949	no
GNG11	0.226399609	4.17736661	3.80E-05	0.000310098	no
C6orf136	-0.226341981	-4.176245872	3.82E-05	0.000311432	no
TOB1-AS1	-0.226326254	-4.175940042	3.82E-05	0.000311711	no
TMEM189	0.226313317	4.17568844	3.83E-05	0.00031192	no
PAWR	0.226287551	4.175187389	3.83E-05	0.000312453	no
SPIRE1	-0.226220929	-4.173891835	3.85E-05	0.000314025	no
USP54	-0.22620095	-4.173503348	3.86E-05	0.000314415	no
ALS2CL	0.226139761	4.172313517	3.88E-05	0.000315857	no
DERL2	0.226089778	4.171341653	3.90E-05	0.000317018	no
HSD17B7	0.226082406	4.171198298	3.90E-05	0.000317087	no
ADAM1A	-0.22605304	-4.170627335	3.91E-05	0.000317634	no
NEK6	0.226051777	4.17060277	3.91E-05	0.000317634	no
ANKRD13D	0.226036564	4.170306987	3.91E-05	0.000317905	no
FAM43A	0.226015096	4.169889584	3.92E-05	0.000318337	no
IRF4	0.225969732	4.169007591	3.93E-05	0.000319387	no
ZNF438	0.225926022	4.168157789	3.95E-05	0.000320398	no
SH3BGRL	0.225880645	4.167275591	3.96E-05	0.000321455	no
GNAL	-0.225862743	-4.166927575	3.97E-05	0.000321799	no
SEMA6A	-0.225721042	-4.164172939	4.01E-05	0.000325384	no

CCDC73	-0.225640342	-4.162604277	4.04E-05	0.00032739	no
MORF4L2-AS1	-0.225628111	-4.162366544	4.04E-05	0.00032759	no
SLC15A4	0.225572041	4.161276721	4.06E-05	0.000328953	no
KIAA1143	-0.225566747	-4.161173811	4.06E-05	0.00032897	no
SYNGAP1	-0.225539204	-4.160638474	4.07E-05	0.000329578	no
CLLU1OS	0.22549629	4.159804399	4.09E-05	0.000330598	no
UNC5A	-0.225434934	-4.158611935	4.11E-05	0.000332115	no
OMA1	0.225391716	4.157772022	4.12E-05	0.00033315	no
LOC283761	-0.22535897	-4.157135643	4.13E-05	0.000333906	no
TAGLN3	-0.225353281	-4.157025085	4.13E-05	0.000333934	no
LSM14B	-0.225346351	-4.156890407	4.14E-05	0.000333995	no
FADD	0.225330912	4.156590377	4.14E-05	0.000334286	no
FAIM2	-0.22525711	-4.155156186	4.17E-05	0.000336063	no
MANEA	0.22525589	4.155132483	4.17E-05	0.000336063	no
TMLHE	-0.225200301	-4.154052281	4.19E-05	0.000337446	no
UBASH3B	0.225142605	4.152931196	4.21E-05	0.000338892	no
AADAT	-0.225131926	-4.15272369	4.21E-05	0.000338961	no
LOC389791	-0.225130784	-4.152701497	4.21E-05	0.000338961	no
ACBD7	-0.225112705	-4.152350225	4.22E-05	0.000339328	no
MCTP2	0.22505622	4.151252725	4.23E-05	0.000340749	no
GTDC2	-0.225018368	-4.150517284	4.25E-05	0.000341601	no
PCDHB9	-0.22501595	-4.1504703	4.25E-05	0.000341601	no
KCNN2	-0.224970483	-4.149586938	4.26E-05	0.000342726	no
FAS	0.224961924	4.149420656	4.27E-05	0.000342834	no
RNU6ATAC	-0.224951482	-4.149217791	4.27E-05	0.000342945	no
CSDC2	-0.22494865	-4.149162763	4.27E-05	0.000342945	no
CD34	0.224916784	4.14854369	4.28E-05	0.000343697	no
HERC1	-0.224898023	-4.148179197	4.29E-05	0.000344053	no
CRTC1	-0.224894666	-4.148113994	4.29E-05	0.000344053	no
CCL8	0.224794618	4.146170394	4.33E-05	0.000346706	no
TEF	-0.224728919	-4.144894162	4.35E-05	0.000348415	no
HOXB5	0.224649622	4.143353869	4.38E-05	0.000350469	no
LRRC55	0.224646567	4.143294522	4.38E-05	0.000350469	no
SERTAD3	0.224606079	4.142508095	4.39E-05	0.000351481	no
KLHDC5	-0.224483488	-4.140127084	4.43E-05	0.000354831	no
CLEC4E	0.224418293	4.138860912	4.46E-05	0.000356562	no
PPP2R2B	-0.22432476	-4.137044493	4.49E-05	0.000359118	no
ATP5G3	0.224312829	4.13681281	4.50E-05	0.000359328	no
HIAT1	0.224269856	4.135978307	4.51E-05	0.000360437	no
RGS16	0.224212378	4.134862201	4.53E-05	0.000361969	no
LOC100129034	0.224138152	4.133420926	4.56E-05	0.000363997	no
NUP37	0.223969407	4.130144611	4.62E-05	0.000368761	no
FGFR1	0.223966796	4.130093933	4.62E-05	0.000368761	no
WHSC1L1	-0.223932807	-4.129434044	4.64E-05	0.000369631	no
ZDHHC7	0.223895957	4.128718652	4.65E-05	0.000370587	no
TECTA	-0.223828254	-4.127404332	4.67E-05	0.000372465	no
RAPGEF2	-0.223775962	-4.126389219	4.69E-05	0.000373891	no
P2RX5-TAX1BF	-0.223738777	-4.125667408	4.71E-05	0.000374867	no
CYR61	0.22366659	4.124266198	4.74E-05	0.000376901	no
CYSLTR2	0.223641318	4.123775656	4.74E-05	0.000377525	no
HBQ1	-0.223623207	-4.123424117	4.75E-05	0.000377933	no

NANS	0.223580994	4.122604795	4.77E-05	0.000379072	no
TCTEX1D4	0.223502384	4.121079068	4.80E-05	0.000381324	no
PPP3CA	-0.223463408	-4.120322627	4.81E-05	0.000382373	no
PFDN6	-0.223443336	-4.11993309	4.82E-05	0.000382846	no
GPR137C	-0.223433498	-4.119742167	4.82E-05	0.000383006	no
MYO7A	0.223378607	4.118676906	4.85E-05	0.00038455	no
MEX3B	-0.223312479	-4.117393628	4.87E-05	0.000386446	no
LOC643837	0.223261215	4.116398829	4.89E-05	0.00038789	no
PMVK	-0.223226904	-4.115733044	4.90E-05	0.000388811	no
SLC24A1	0.223212157	4.115446883	4.91E-05	0.000389126	no
ATG13	-0.223203908	-4.11528683	4.91E-05	0.00038924	no
ZNF781	-0.223189145	-4.115000363	4.92E-05	0.000389555	no
FBLN1	0.223148973	4.114220882	4.94E-05	0.000390664	no
AKAP2	0.223114917	4.113560093	4.95E-05	0.000391584	no
EHD2	0.223108836	4.113442096	4.95E-05	0.00039163	no
UBE3C	0.223090871	4.113093528	4.96E-05	0.000392048	no
COL1A1	0.22300533	4.111433879	4.99E-05	0.000394577	no
FAM126B	-0.223001147	-4.111352715	4.99E-05	0.000394577	no
FAM84B	-0.222980327	-4.110948794	5.00E-05	0.000395088	no
FAM151B	0.22297512	4.110847757	5.00E-05	0.000395107	no
PCBP2	-0.222945526	-4.110273629	5.02E-05	0.000395897	no
HIST2H2BF	0.222903832	4.109464739	5.03E-05	0.00039707	no
DNAJC10	0.222892185	4.109238792	5.04E-05	0.000397294	no
GAS6-AS1	0.222860975	4.108633343	5.05E-05	0.000398138	no
SYNPO	0.222850451	4.108429184	5.05E-05	0.000398327	no
USP53	0.222711606	4.105735825	5.11E-05	0.000402615	no
DUSP2	0.222671971	4.104967027	5.13E-05	0.000403741	no
SLC30A5	0.222622077	4.10399925	5.15E-05	0.000405202	no
WDR1	0.222591502	4.103406229	5.16E-05	0.000405983	no
MMRN1	0.222588734	4.103352538	5.16E-05	0.000405983	no
ABCC3	0.222514252	4.101907943	5.19E-05	0.00040825	no
BAX	0.222506918	4.101765716	5.19E-05	0.000408339	no
ZDHHC17	-0.222487777	-4.101394488	5.20E-05	0.000408813	no
UBC	0.222466721	4.100986121	5.21E-05	0.000409349	no
CALML4	0.222443845	4.100542465	5.22E-05	0.000409946	no
SMPD1	0.222423566	4.100149182	5.23E-05	0.000410459	no
NKAIN4	-0.222312209	-4.097989679	5.28E-05	0.000413963	no
RILP	0.22228015	4.097367998	5.29E-05	0.000414869	no
IL18R1	0.222165416	4.095143232	5.34E-05	0.000418521	no
TMEM63C	-0.222158592	-4.095010908	5.34E-05	0.000418595	no
SPTAN1	-0.22214153	-4.094680083	5.35E-05	0.000419011	no
HUS1	0.222133482	4.094524045	5.35E-05	0.000419126	no
SPATS2	-0.222093775	-4.093754146	5.37E-05	0.000420245	no
MUT	-0.222090794	-4.093696359	5.37E-05	0.000420245	no
M1AP	0.222078847	4.09346471	5.37E-05	0.000420492	no
LOC100287314	0.222068508	4.093264255	5.38E-05	0.000420587	no
HIPK3	0.222066849	4.093232096	5.38E-05	0.000420587	no
SRGAP3	-0.222011767	-4.092164149	5.40E-05	0.000422279	no
SIGLEC11	0.22200047	4.09194512	5.41E-05	0.000422505	no
APLF	0.221958223	4.091126062	5.43E-05	0.000423771	no
KCNIP3	-0.221942391	-4.09081913	5.43E-05	0.000424151	no

NOG	-0.22182219	-4.088488915	5.49E-05	0.000428065	no
TECPR2	-0.221771951	-4.087515038	5.51E-05	0.00042962	no
ARRDC3	0.221733256	4.086764961	5.52E-05	0.000430786	no
CLEC11A	0.221725976	4.086623853	5.53E-05	0.000430855	no
ASGR1	-0.221722084	-4.086548409	5.53E-05	0.000430855	no
IMPDH1	0.221716491	4.086439994	5.53E-05	0.00043089	no
FAT3	-0.221707085	-4.086257674	5.54E-05	0.000431056	no
IL17D	-0.221645002	-4.085054291	5.56E-05	0.000432928	no
MROH8	-0.221643356	-4.085022388	5.56E-05	0.000432928	no
RHOF	0.221618071	4.084532307	5.58E-05	0.00043364	no
JPH4	-0.221551002	-4.083232361	5.60E-05	0.000435712	no
TCEA2	-0.221548885	-4.083191331	5.61E-05	0.000435712	no
TUB	-0.221454514	-4.081362345	5.65E-05	0.000438826	no
MANF	0.221399929	4.0803045	5.67E-05	0.00044057	no
TTC33	-0.221363157	-4.079591881	5.69E-05	0.000441696	no
KIAA1211	-0.221332938	-4.079006273	5.70E-05	0.000442575	no
CD84	0.221328952	4.078929038	5.70E-05	0.000442575	no
TMEM198	-0.2213234	-4.078821435	5.71E-05	0.000442583	no
TRIP6	0.221319637	4.078748513	5.71E-05	0.000442583	no
GK5	-0.221277974	-4.077941161	5.73E-05	0.000443828	no
HIC2	-0.221275059	-4.077884675	5.73E-05	0.000443828	no
EPHX3	0.221266765	4.077723966	5.73E-05	0.000443873	no
ECHDC3	0.221264695	4.07768385	5.73E-05	0.000443873	no
BHLHE40-AS1	0.221251403	4.077426275	5.74E-05	0.000444181	no
ARHGAP33	-0.221211857	-4.076659999	5.76E-05	0.000445414	no
SPHKAP	-0.221149959	-4.07546062	5.79E-05	0.000447441	no
GABRB3	-0.221104888	-4.074587341	5.81E-05	0.000448879	no
SART1	-0.22094126	-4.071417129	5.88E-05	0.000454566	no
LOC100288346	-0.220935504	-4.071305621	5.88E-05	0.000454609	no
FAM114A2	0.220898991	4.070598266	5.90E-05	0.00045576	no
LHPP	-0.220814163	-4.068954961	5.94E-05	0.000458663	no
MCF2L2	-0.220793158	-4.068548065	5.95E-05	0.00045926	no
EPM2AIP1	-0.220775513	-4.068206257	5.96E-05	0.000459736	no
ATP8A1	-0.220740962	-4.067536975	5.98E-05	0.000460828	no
SUV420H1	-0.220684773	-4.066448573	6.00E-05	0.000462712	no
AVEN	0.22063542	4.065492632	6.03E-05	0.00046424	no
FLII	0.220633985	4.065464847	6.03E-05	0.00046424	no
ITGA1	0.220591662	4.064645096	6.05E-05	0.000465627	no
KIAA1467	-0.220580849	-4.064435656	6.05E-05	0.000465858	no
ZSCAN2	-0.220518227	-4.063222796	6.08E-05	0.000468	no
KRT86	0.220511552	4.063093509	6.09E-05	0.000468079	no
RIOK3	0.220497819	4.062827541	6.09E-05	0.000468419	no
TMEM41A	0.220477962	4.062442976	6.10E-05	0.000468986	no
ATP8B3	0.220441484	4.061736508	6.12E-05	0.00047017	no
SLC9A3R2	0.220348988	4.059945229	6.16E-05	0.000473445	no
CHRM4	-0.220251446	-4.058056366	6.21E-05	0.000476931	no
ASXL3	-0.220226319	-4.057569805	6.22E-05	0.000477539	no
BRPF3	-0.220226235	-4.057568183	6.22E-05	0.000477539	no
EVC	0.220215817	4.057366446	6.23E-05	0.000477761	no
PHF17	-0.220210605	-4.057265536	6.23E-05	0.000477786	no
ANKMY1	0.220198674	4.057034497	6.24E-05	0.000477901	no

CHRNA6	0.220198508	4.057031296	6.24E-05	0.000477901	no
TFAP4	-0.220185107	-4.05677181	6.24E-05	0.000478236	no
ZC3H6	-0.220165293	-4.056388144	6.25E-05	0.000478813	no
GAB3	0.220118845	4.055488793	6.28E-05	0.000480398	no
SUGP1	-0.220112872	-4.055373151	6.28E-05	0.000480453	no
NDUFB9	-0.220096466	-4.055055487	6.29E-05	0.000480903	no
TMPRSS5	-0.220055024	-4.054253114	6.31E-05	0.000482304	no
MTRNR2L2	-0.220032875	-4.053824271	6.32E-05	0.0004828	no
FAM196B	-0.220029084	-4.053750868	6.32E-05	0.0004828	no
LOC100506384	-0.220028452	-4.053738633	6.32E-05	0.0004828	no
HMGN5	-0.2200158	-4.053493694	6.33E-05	0.00048311	no
HIST1H1C	0.219974251	4.052689275	6.35E-05	0.0004845	no
ZADH2	-0.219970282	-4.052612433	6.35E-05	0.0004845	no
RTCA	0.219898718	4.051226975	6.39E-05	0.000487065	no
LMTK2	-0.219844973	-4.050186518	6.41E-05	0.000488957	no
RP2	0.219801377	4.049342579	6.44E-05	0.000490426	no
PRR15	0.219797842	4.049274159	6.44E-05	0.000490426	no
ANKRD36	-0.219738157	-4.048118802	6.47E-05	0.00049256	no
EYA1	-0.219706519	-4.047506386	6.48E-05	0.000493612	no
LOC439990	0.219687209	4.047132607	6.49E-05	0.000494188	no
LAMA2	0.219652896	4.046468432	6.51E-05	0.000495348	no
LOC100507433	-0.219603657	-4.04551539	6.54E-05	0.000497093	no
DIRAS3	0.219471148	4.04295075	6.61E-05	0.00050212	no
GABRA3	-0.219461306	-4.04276027	6.61E-05	0.000502331	no
ZNF721	-0.21943301	-4.042212658	6.62E-05	0.000503192	no
G6PC3	0.219430501	4.042164094	6.63E-05	0.000503192	no
FAM78B	0.219417984	4.041921868	6.63E-05	0.000503509	no
RUFY2	-0.219363421	-4.040865938	6.66E-05	0.000505493	no
SH3GL2	-0.219328966	-4.040199165	6.68E-05	0.000506684	no
NEDD9	0.219312972	4.039889643	6.69E-05	0.000507142	no
GID8	-0.219306727	-4.039768796	6.69E-05	0.000507211	no
CD276	0.219298132	4.03960248	6.70E-05	0.000507375	no
SCARA3	0.219259622	4.038857273	6.72E-05	0.000508732	no
CTTNBP2	-0.219219414	-4.038079236	6.74E-05	0.00051016	no
PLOD1	0.219107554	4.035914797	6.80E-05	0.000514416	no
PAM	0.219104545	4.035856587	6.80E-05	0.000514416	no
MOB3B	-0.219079837	-4.035378514	6.81E-05	0.000515233	no
FAM157B	0.219046518	4.034733863	6.83E-05	0.000516399	no
MCOLN1	0.219006165	4.033953128	6.85E-05	0.000517854	no
SPTBN5	0.218998604	4.033806845	6.85E-05	0.000517975	no
HIST1H2BC	0.218994203	4.033721703	6.86E-05	0.000517975	no
SPATA13-AS1	0.218981638	4.033478591	6.86E-05	0.000518304	no
ENPP1	0.218962594	4.033110149	6.87E-05	0.000518896	no
ATP1A3	-0.218891168	-4.031728341	6.91E-05	0.000521626	no
BRSK1	-0.218869201	-4.031303368	6.92E-05	0.000522341	no
PXN	0.218827356	4.030493874	6.95E-05	0.000523872	no
MYL6B	-0.218803452	-4.030031438	6.96E-05	0.00052467	no
PDZK1IP1	0.21877654	4.029510854	6.97E-05	0.000525593	no
LAMB2P1	-0.218772036	-4.029423717	6.98E-05	0.000525594	no
COL4A2	0.218731395	4.028637565	7.00E-05	0.000527085	no
SYT11	-0.218703933	-4.028106362	7.01E-05	0.000528035	no

ADARB2	-0.218683297	-4.027707193	7.03E-05	0.000528704	no
RASL10A	-0.218668891	-4.027428528	7.03E-05	0.000529115	no
THSD7A	-0.218585461	-4.025814818	7.08E-05	0.000532395	no
CRMP1	-0.218539038	-4.024916924	7.11E-05	0.000534146	no
ZNF157	-0.218487577	-4.023921632	7.13E-05	0.000536113	no
AGXT2L1	-0.21847131	-4.02360703	7.14E-05	0.000536571	no
VPS53	-0.218467728	-4.023537752	7.15E-05	0.000536571	no
TBC1D5	-0.218457011	-4.023330478	7.15E-05	0.000536833	no
LOC100289495	-0.218430713	-4.022821886	7.17E-05	0.000537751	no
SART3	-0.218409979	-4.022420891	7.18E-05	0.000538435	no
KBTBD8	0.218397407	4.022177749	7.19E-05	0.000538601	no
PPP1R13B	-0.218395209	-4.022135255	7.19E-05	0.000538601	no
CPLX2	-0.218392645	-4.022085665	7.19E-05	0.000538601	no
NMNAT2	-0.218384488	-4.021927923	7.19E-05	0.000538757	no
SMAD9	-0.218357119	-4.021398636	7.21E-05	0.000539723	no
FLNC	0.218283129	4.019967794	7.25E-05	0.000542665	no
SRRM3	-0.218254802	-4.019420034	7.27E-05	0.000543678	no
NXPH1	-0.218168457	-4.017750393	7.31E-05	0.000547139	no
CMKLR1	0.218164659	4.017676955	7.32E-05	0.000547139	no
PIGT	0.218157353	4.017535692	7.32E-05	0.000547261	no
MS4A6E	0.218127111	4.016950924	7.34E-05	0.000548364	no
CALR	0.218117099	4.01675734	7.34E-05	0.000548599	no
OBFC1	0.218112722	4.01667271	7.35E-05	0.000548599	no
BCR	-0.218061994	-4.015691891	7.38E-05	0.000550585	no
PORCN	-0.218048144	-4.015424105	7.38E-05	0.000550989	no
LUZP2	-0.218037165	-4.01521183	7.39E-05	0.00055127	no
SNCAIP	-0.218009128	-4.014669754	7.41E-05	0.000552287	no
COL8A1	0.217962708	4.013772276	7.43E-05	0.000554099	no
AFP	0.217931981	4.013178229	7.45E-05	0.000555236	no
HLA-DPA1	0.217919585	4.012938565	7.46E-05	0.000555581	no
INSRR	0.217899412	4.012548576	7.47E-05	0.000556263	no
DTX2P1-UPK3E	0.217888718	4.012341834	7.48E-05	0.000556535	no
PLEKHS1	0.217881003	4.012192678	7.48E-05	0.000556677	no
STARD3	0.217813207	4.010882052	7.52E-05	0.000559434	no
PSD2	-0.217750321	-4.009666408	7.56E-05	0.00056199	no
SLC39A6	-0.217696929	-4.008634333	7.59E-05	0.000564138	no
PIK3R1	-0.217664454	-4.008006601	7.61E-05	0.000565372	no
CRYZ	0.217569331	4.006167973	7.66E-05	0.000569381	no
MUM1	-0.217559988	-4.005987393	7.67E-05	0.000569542	no
NPL	0.217556842	4.005926576	7.67E-05	0.000569542	no
MAPKAPK5-AS1	-0.217538376	-4.005569674	7.68E-05	0.000570164	no
CCDC121	0.217519686	4.005208435	7.69E-05	0.000570798	no
KCTD10	0.217495794	4.004746673	7.71E-05	0.000571609	no
PCDH17	-0.217492595	-4.004684835	7.71E-05	0.000571609	no
RANBP17	-0.217470788	-4.004263379	7.72E-05	0.000572329	no
CD109	0.217467558	4.00420095	7.73E-05	0.000572329	no
TRAM2	0.217418541	4.003253615	7.76E-05	0.000574319	no
COL1A2	0.217344054	4.001814104	7.80E-05	0.00057746	no
FSIP1	0.217282189	4.000618566	7.84E-05	0.000580046	no
PNO1	0.217243839	3.999877484	7.86E-05	0.000581579	no
ANKRD26	-0.217219553	-3.999408189	7.88E-05	0.000582478	no

ATR	0.217200389	3.999037875	7.89E-05	0.000583146	no
GBP6	0.217089247	3.996890329	7.96E-05	0.000588004	no
MKRN3	-0.216951695	-3.994232696	8.04E-05	0.000594118	no
BTG1	0.216909773	3.993422766	8.07E-05	0.000595851	no
RNF217	0.216832763	3.99193501	8.12E-05	0.000599218	no
LINC00152	0.216804167	3.991382593	8.13E-05	0.000600344	no
CBWD1	0.216755558	3.990443575	8.17E-05	0.000602406	no
AP2S1	0.216737261	3.990090114	8.18E-05	0.000603055	no
USP40	0.216681468	3.989012362	8.21E-05	0.000605463	no
SNTG1	-0.216676072	-3.988908135	8.22E-05	0.000605509	no
PTPRT	-0.216613295	-3.987695536	8.26E-05	0.000608256	no
ZFP36L1	0.216562468	3.986713808	8.29E-05	0.000610449	no
PDXP	-0.21654967	-3.986466614	8.30E-05	0.000610846	no
EML3	0.216534073	3.986165363	8.31E-05	0.000611295	no
PARP8	0.216531351	3.986112784	8.31E-05	0.000611295	no
DIP2B	-0.216494796	-3.985406753	8.33E-05	0.00061282	no
TRIB1	0.216467973	3.984888698	8.35E-05	0.000613885	no
BCHE	-0.216416079	-3.98388644	8.38E-05	0.000616148	no
SLC17A5	0.216410554	3.983779746	8.39E-05	0.000616201	no
NPTXR	-0.216377249	-3.983136531	8.41E-05	0.000617582	no
TOM1L2	-0.216362493	-3.98285157	8.42E-05	0.000618077	no
HMGA1P7	-0.216335248	-3.982325407	8.44E-05	0.000619171	no
BMP2	-0.216307903	-3.981797327	8.45E-05	0.000620272	no
SYNM	0.216284787	3.981350917	8.47E-05	0.000621171	no
KCNK4	-0.216257732	-3.980828467	8.49E-05	0.000622262	no
CDR2	0.216244911	3.980580884	8.49E-05	0.000622667	no
TSPYL2	-0.216231103	-3.980314231	8.50E-05	0.000623121	no
PIWIL4	0.216082664	3.977447932	8.60E-05	0.000630106	no
ALG3	0.216056195	3.976936857	8.62E-05	0.000631182	no
MT1DP	0.2160382	3.976589417	8.63E-05	0.000631846	no
MRGPRF	0.215992398	3.975705087	8.66E-05	0.000633872	no
IFNE	0.215970102	3.975274612	8.68E-05	0.00063475	no
CASS4	0.215921978	3.974345474	8.71E-05	0.000636899	no
MGAT4B	0.215867004	3.973284137	8.75E-05	0.000639394	no
ATP11C	0.215861078	3.973169737	8.75E-05	0.000639469	no
RPLP0P2	0.215804604	3.972079486	8.79E-05	0.000642048	no
ZC3H12A	0.21576373	3.971290426	8.82E-05	0.000643738	no
FLJ42709	-0.215761773	-3.971252643	8.82E-05	0.000643738	no
MTA1	-0.215745139	-3.970931538	8.83E-05	0.000644347	no
TGDS	0.215687986	3.969828279	8.87E-05	0.000646978	no
TREML1	0.215642103	3.968942587	8.90E-05	0.000648878	no
GABARAPL2	-0.215641213	-3.968925415	8.90E-05	0.000648878	no
TRAP1	-0.215628137	-3.968673006	8.91E-05	0.000649313	no
H2AFY2	-0.215605895	-3.968243685	8.93E-05	0.000650209	no
MYH10	-0.215568728	-3.967526282	8.95E-05	0.000651642	no
FSTL3	0.215568596	3.967523722	8.95E-05	0.000651642	no
HMGB3	-0.215549264	-3.967150575	8.96E-05	0.000652394	no
SERINC5	-0.215527701	-3.966734393	8.98E-05	0.000653259	no
AGSK1	-0.215517068	-3.966529153	8.99E-05	0.000653574	no
FGF9	-0.215360439	-3.963506189	9.10E-05	0.00066109	no
AGAP3	-0.215360052	-3.963498721	9.10E-05	0.00066109	no

OLIG2	-0.215303354	-3.962404522	9.14E-05	0.00066362	no
ATP6V1G2	-0.215301749	-3.962373552	9.14E-05	0.00066362	no
FLJ37201	-0.215265712	-3.961678102	9.16E-05	0.000665241	no
ITSN2	0.215249142	3.961358325	9.17E-05	0.000665866	no
MTPAP	-0.215214779	-3.960695215	9.20E-05	0.000667406	no
TWSG1	0.215187154	3.960162129	9.22E-05	0.000668603	no
TRABD	0.215090377	3.958294691	9.29E-05	0.000673378	no
PLA2G5	0.215051637	3.957547191	9.31E-05	0.000675162	no
LRRC4B	-0.215024282	-3.957019371	9.33E-05	0.000676259	no
HES6	-0.215021804	-3.956971564	9.34E-05	0.000676259	no
RFPL3-AS1	-0.215004034	-3.956628706	9.35E-05	0.000676956	no
PAPPA2	0.214993262	3.956420872	9.36E-05	0.00067729	no
MYD88	0.214965497	3.955885169	9.38E-05	0.00067851	no
GJA4	0.214856567	3.953783545	9.46E-05	0.000683991	no
COL4A1	0.214821141	3.953100096	9.48E-05	0.000685459	no
TBPL1	-0.214819946	-3.953077029	9.48E-05	0.000685459	no
PTPN6	0.214791265	3.952523719	9.50E-05	0.000686742	no
HSP90B1	0.214702044	3.950802542	9.57E-05	0.000691237	no
PKI55	-0.214677417	-3.950327492	9.59E-05	0.000692314	no
PM20D2	-0.214661524	-3.950020908	9.60E-05	0.000692822	no
EPT1	-0.214659126	-3.949974657	9.60E-05	0.000692822	no
GPR158	-0.214642649	-3.949656806	9.61E-05	0.000693467	no
VPS33B	-0.21461627	-3.949147978	9.63E-05	0.000694641	no
RMND5A	-0.214538462	-3.947647128	9.69E-05	0.000698572	no
ITGB1	0.214506737	3.947035218	9.71E-05	0.000699956	no
PARVB	0.214503963	3.946981707	9.72E-05	0.000699956	no
ACBD6	-0.214437065	-3.945691421	9.77E-05	0.000703325	no
IL1B	0.214422412	3.94540881	9.78E-05	0.000703881	no
ANKRD45	0.214351002	3.944031552	9.83E-05	0.000707298	no
ECSIT	-0.214349375	-3.944000177	9.83E-05	0.000707298	no
MYO1C	0.214346227	3.943939471	9.83E-05	0.000707298	no
RIMS2	-0.214334305	-3.943709547	9.84E-05	0.000707688	no
MGC57346	-0.214326041	-3.943550162	9.85E-05	0.000707688	no
DKFZp686D085	0.214325969	3.943548769	9.85E-05	0.000707688	no
40603	-0.214244481	-3.941977265	9.91E-05	0.000711889	no
TXNRD2	-0.214222277	-3.941549073	9.93E-05	0.000712596	no
KIF1A	-0.214218544	-3.941477079	9.93E-05	0.000712596	no
TPM4	0.214218469	3.941475639	9.93E-05	0.000712596	no
HSP90AA1	-0.214183664	-3.94080446	9.96E-05	0.000714262	no
BRE-AS1	0.214171451	3.940568952	9.97E-05	0.000714693	no
SPON1	-0.214151073	-3.940175986	9.98E-05	0.000715572	no
CKMT1B	-0.214093769	-3.939070985	0.000100262	0.000718483	no
SIGLEC10	0.214084871	3.93889942	0.00010033	0.000718733	no
LOC400940	-0.214074244	-3.938694505	0.000100412	0.000719079	no
CIT	-0.214066449	-3.938544197	0.000100472	0.000719269	no
PRPSAP2	-0.214048761	-3.938203122	0.000100608	0.000720005	no
RPS6KA5	-0.213983261	-3.936940175	0.000101115	0.000723388	no
VPS4A	-0.213858787	-3.934540271	0.000102083	0.000730009	no
FAM117A	0.213855619	3.934479188	0.000102108	0.000730009	no
CLIP2	-0.213794935	-3.933309248	0.000102584	0.000733164	no
RAB41	-0.213780063	-3.933022554	0.0001027	0.000733755	no

ZNF853	-0.213724166	-3.931944944	0.000103141	0.000736524	no
ALDH2	-0.213722166	-3.931906385	0.000103157	0.000736524	no
NUP98	0.213671453	3.930928777	0.000103558	0.000738908	no
GABRG2	-0.213671253	-3.930924921	0.000103559	0.000738908	no
ZNF74	-0.213661158	-3.930730322	0.000103639	0.000739234	no
VPS13B	-0.213625784	-3.930048424	0.00010392	0.000740991	no
KIAA0040	0.213539622	3.92838756	0.000104607	0.000745642	no
ACTB	0.21352015	3.928012225	0.000104763	0.000746506	no
CPB2-AS1	-0.213509546	-3.927807834	0.000104848	0.000746863	no
SUPT16H	-0.213485617	-3.927346611	0.00010504	0.000747983	no
TMIE	0.21348002	3.92723873	0.000105085	0.000748055	no
TMEM107	0.213452631	3.926710803	0.000105305	0.000749375	no
C12orf29	-0.213447383	-3.926609661	0.000105347	0.000749427	no
RAB11FIP4	-0.21343784	-3.926425732	0.000105424	0.000749726	no
ATXN1	0.213425886	3.926195319	0.000105521	0.000750163	no
SSH3	0.213417737	3.926038257	0.000105586	0.000750382	no
EIF3L	-0.213409923	-3.92588766	0.000105649	0.000750582	no
ADCY3	0.213377444	3.925261664	0.000105912	0.000752198	no
ZNF138	-0.213369775	-3.925113864	0.000105974	0.00075239	no
CNTNAP2	-0.213361119	-3.924947026	0.000106044	0.000752639	no
PCIF1	-0.213339609	-3.924532477	0.000106218	0.000753628	no
MYO1B	0.213318766	3.924130761	0.000106388	0.00075458	no
ASCL1	-0.213303523	-3.923836999	0.000106512	0.00075521	no
FBXO45	-0.213288034	-3.92353849	0.000106638	0.000755854	no
ZNF843	-0.213263124	-3.923058429	0.000106841	0.000757044	no
KCTD12	0.213228421	3.922389629	0.000107124	0.0007588	no
VCAM1	0.213224147	3.92230727	0.000107159	0.0007588	no
DYNLRB1	-0.213205087	-3.921939962	0.000107315	0.000759655	no
LAMTOR3	-0.213152439	-3.920925391	0.000107747	0.000762462	no
ABCA7	0.213111602	3.920138455	0.000108084	0.000764591	no
SMCR7L	-0.213066064	-3.919260953	0.00010846	0.000766999	no
NKAIN3	-0.21304262	-3.918809202	0.000108654	0.00076812	no
C20orf203	-0.213021312	-3.918398617	0.000108831	0.000769116	no
DDHD2	-0.212995626	-3.917903691	0.000109044	0.000770371	no
LIPH	0.212958777	3.917193677	0.000109351	0.000772285	no
ARHGAP32	-0.21289896	-3.916041139	0.000109851	0.000775561	no
CDK5R2	-0.212847291	-3.915045653	0.000110284	0.000778365	no
NCR3	0.212839177	3.914889324	0.000110353	0.000778591	no
NANOS3	-0.212824707	-3.914610535	0.000110474	0.000779051	no
TDP2	0.212822818	3.914574135	0.00011049	0.000779051	no
MLLT11	-0.21280354	-3.914202725	0.000110653	0.00077994	no
LOC440461	0.212769913	3.913554872	0.000110937	0.000781685	no
SLC34A2	0.212707195	3.912346606	0.000111468	0.000785171	no
SRPX2	0.212660457	3.911446227	0.000111865	0.000787712	no
CACNG8	-0.21263609	-3.910976823	0.000112073	0.000788917	no
BOD1L1	-0.21261137	-3.910500617	0.000112284	0.000790144	no
FAM222A	-0.212571502	-3.90973263	0.000112626	0.000792287	no
AMDHD1	0.212518794	3.908717337	0.000113078	0.000795211	no
C2CD5	0.212481554	3.908000013	0.000113399	0.000797207	no
PDE4A	-0.212456054	-3.907508852	0.000113619	0.000798494	no
SERINC4	-0.212431923	-3.907044051	0.000113828	0.000799699	no

EFEMP2	0.212374575	3.90593949	0.000114326	0.000802932	no
VWA5B2	-0.212367571	-3.905804578	0.000114387	0.000803098	no
DZIP3	-0.212346916	-3.905406773	0.000114566	0.000804097	no
CLINT1	0.212333717	3.905152561	0.000114681	0.000804642	no
FAM81A	-0.212323663	-3.904958905	0.000114769	0.000804995	no
GPBAR1	0.212253625	3.903610037	0.000115382	0.000809028	no
TRIP10	0.212248817	3.903517431	0.000115424	0.00080906	no
MRPL30	-0.212131351	-3.901255268	0.000116459	0.000816047	no
TLX1	-0.212105849	-3.900764165	0.000116685	0.000817363	no
QSOX2	-0.212068437	-3.900043733	0.000117017	0.000819204	no
PCDH20	-0.212067639	-3.900028373	0.000117024	0.000819204	no
REXO1L2P	0.212009837	3.898915324	0.000117538	0.000822539	no
CXorf40A	0.211971804	3.898182973	0.000117878	0.000824392	no
DNMT3A	0.211971619	3.898179406	0.00011788	0.000824392	no
LOC150622	-0.211783892	-3.894564908	0.000119571	0.000835946	no
UNC80	-0.21173972	-3.893714475	0.000119972	0.000838478	no
VPS18	0.211720809	3.8933504	0.000120144	0.000839408	no
BCL2L1	0.211688896	3.892736017	0.000120435	0.000841168	no
NRBP1	0.211675007	3.89246863	0.000120562	0.000841689	no
HSPA5	0.211672157	3.892413768	0.000120588	0.000841689	no
SESTD1	-0.211659565	-3.892171352	0.000120703	0.000842219	no
OPA3	0.211630243	3.891606872	0.000120972	0.000843819	no
LMO2	0.211603563	3.891093256	0.000121217	0.000845253	no
C14orf119	0.211514886	3.889386231	0.000122034	0.000850674	no
AIM2	0.211504859	3.889193206	0.000122127	0.000851044	no
TCF12	-0.211498296	-3.889066876	0.000122187	0.000851192	no
CXCR7	0.211493249	3.888969722	0.000122234	0.000851241	no
PAAF1	-0.211425786	-3.887671155	0.00012286	0.000855324	no
NDUFV3	0.211413202	3.887428927	0.000122977	0.000855862	no
MSL3	0.211397204	3.887121003	0.000123126	0.000856621	no
TMEM254-AS1	-0.211391837	-3.88701771	0.000123176	0.000856692	no
NRIP1	0.21138488	3.886883797	0.000123241	0.000856866	no
E2F4	0.211352465	3.886259891	0.000123544	0.000858475	no
IGFBP4	0.211351559	3.886242449	0.000123552	0.000858475	no
AHSA1	-0.211327539	-3.885780137	0.000123777	0.000859759	no
RAD21	-0.211309653	-3.885435878	0.000123945	0.000860645	no
LRRC14	-0.211276298	-3.884793907	0.000124258	0.000862541	no
NAGPA-AS1	-0.211268914	-3.884651788	0.000124327	0.000862744	no
GRIK2	-0.211244912	-3.884189854	0.000124553	0.000863742	no
SLC1A4	-0.211243163	-3.884156179	0.00012457	0.000863742	no
DIRAS1	-0.211236618	-3.884030221	0.000124632	0.000863742	no
SYCE2	-0.211236571	-3.884029324	0.000124632	0.000863742	no
ADAMTSL4	0.211214768	3.88360971	0.000124838	0.000864889	no
FABP5	0.211161669	3.882587799	0.00012534	0.000868089	no
CCDC50	0.211130597	3.881989834	0.000125635	0.000869851	no
CEP97	-0.211104236	-3.881482521	0.000125885	0.000871306	no
RSL24D1	-0.211080893	-3.881033311	0.000126108	0.000872564	no
PLEKHA3	0.21102913	3.880037209	0.000126602	0.000875702	no
ASIC4	-0.211003036	-3.87953508	0.000126852	0.000877148	no
PRTG	-0.210991281	-3.879308888	0.000126965	0.000877645	no
VKORC1	0.210962927	3.878763276	0.000127237	0.000879245	no

MVD	-0.210921104	-3.877958507	0.00012764	0.000881743	no
RNF141	-0.210891694	-3.877392613	0.000127923	0.000883179	no
JMJD1C	-0.210891054	-3.877380294	0.00012793	0.000883179	no
TBC1D10A	-0.210884671	-3.877257489	0.000127991	0.000883321	no
FCHSD2	-0.21087811	-3.87713124	0.000128055	0.000883475	no
NAT1	0.210853871	3.876664852	0.000128289	0.00088481	no
SYT4	-0.210840307	-3.876403865	0.000128421	0.000885432	no
ZHX2	-0.210829632	-3.876198485	0.000128524	0.000885862	no
LOC100505495	0.210816802	3.875951612	0.000128649	0.000886436	no
ANK3	-0.210761321	-3.874884163	0.000129189	0.000889871	no
TTC19	-0.21075144	-3.874694042	0.000129285	0.000889965	no
CASD1	-0.210751432	-3.874693885	0.000129285	0.000889965	no
KCNH4	-0.210740156	-3.874476939	0.000129395	0.000890438	no
DLEU7-AS1	0.210726342	3.874211183	0.00012953	0.000891081	no
CCR10	0.210691431	3.873539522	0.000129872	0.000893146	no
ZNF561	0.210676467	3.873251626	0.000130019	0.000893869	no
SLC35C1	0.210654207	3.872823385	0.000130237	0.000895085	no
NIF3L1	-0.210634064	-3.872435858	0.000130435	0.000896159	no
TM2D3	-0.210604936	-3.871875489	0.000130722	0.000897843	no
SAMD4A	0.210562363	3.871056501	0.000131143	0.000900443	no
EPS8L2	0.210520907	3.870259004	0.000131553	0.000902774	no
PTBP3	0.210519603	3.870233928	0.000131566	0.000902774	no
GPR124	0.210344798	3.866871425	0.000133311	0.000914453	no
TSPAN3	-0.210335001	-3.866683001	0.000133409	0.000914836	no
NCR3LG1	-0.210207444	-3.864229607	0.000134697	0.000923372	no
BANF2	0.210177099	3.863646007	0.000135005	0.000924922	no
TMEM217	0.210176678	3.863637912	0.000135009	0.000924922	no
ZNF462	-0.210127387	-3.862689946	0.000135511	0.000928064	no
CORO6	-0.210119179	-3.862532084	0.000135595	0.000928342	no
LOC100131434	0.210110701	3.862369045	0.000135681	0.000928638	no
LRRC4C	-0.210104506	-3.862249891	0.000135744	0.000928776	no
NEURL4	-0.210060619	-3.861405902	0.000136193	0.000931551	no
KDR	0.210052106	3.861242194	0.000136281	0.000931851	no
BEX2	-0.210028934	-3.860796567	0.000136518	0.00093318	no
PCDHGC3	-0.209964065	-3.859549129	0.000137186	0.000937402	no
ABCB6	-0.209960447	-3.859479566	0.000137223	0.000937402	no
PPP1R15A	0.209917285	3.85864959	0.000137669	0.000940151	no
NGDN	-0.209893249	-3.858187393	0.000137918	0.000941552	no
DLG4	-0.209867015	-3.857682951	0.000138191	0.000943111	no
CMTM4	-0.209854878	-3.857449577	0.000138317	0.000943672	no
SLC52A1	0.209836447	3.857095195	0.000138508	0.00094468	no
DERL1	0.209812202	3.856628999	0.000138761	0.000946102	no
RPS10-NUDT3	-0.209786544	-3.856135656	0.000139029	0.000947627	no
RDH10	0.209757704	3.855581151	0.00013933	0.000949382	no
MARVELD1	0.209713455	3.854730385	0.000139794	0.000952241	no
CEP72	-0.209688753	-3.854255448	0.000140054	0.000953707	no
PIH1D2	0.209661432	3.853730183	0.000140342	0.000955134	no
APOBEC3B	0.209656797	3.853641069	0.00014039	0.000955134	no
TICAM1	0.20965619	3.853629392	0.000140397	0.000955134	no
MOB3C	0.209647429	3.853460969	0.000140489	0.00095546	no
PLAGL1	0.209605359	3.852652143	0.000140934	0.000958179	no

SRRM4	-0.209547797	-3.851545538	0.000141544	0.000962024	no
CD97	0.20952064	3.851023463	0.000141833	0.000963681	no
TWISTNB	0.209407037	3.848839622	0.000143047	0.000971622	no
FAM212B	-0.209386274	-3.848440502	0.000143269	0.000972829	no
C5orf42	-0.209357731	-3.847891849	0.000143576	0.000974605	no
RHOC	0.209318271	3.847133367	0.000144002	0.000977184	no
NDRG3	-0.209196029	-3.844783776	0.000145327	0.000985867	no
CNPY4	0.209045286	3.84188664	0.000146977	0.000996746	no
ZNF493	-0.20901457	-3.841296337	0.000147316	0.000998726	no
CHD6	-0.208953683	-3.840126254	0.000147989	0.001002972	no
PLD4	0.208949416	3.840044265	0.000148036	0.001002976	no
TMEFF1	-0.208929686	-3.839665106	0.000148255	0.001004141	no
NUCKS1	-0.208919941	-3.839477846	0.000148363	0.001004557	no
C11orf73	-0.208905205	-3.839194672	0.000148526	0.001005348	no
DISP1	0.208877919	3.838670339	0.00014883	0.001007086	no
DCLK2	-0.20886653	-3.838451496	0.000148957	0.001007627	no
EMP2	0.208833383	3.837814557	0.000149327	0.001009811	no
TBC1D17	-0.208778591	-3.836761727	0.00014994	0.001013638	no
AP1G2	0.20869755	3.835204577	0.000150851	0.001019478	no
TAOK2	-0.208667944	-3.834635756	0.000151185	0.001021415	no
SDHA	-0.20863675	-3.834036405	0.000151538	0.001023477	no
FLJ20021	-0.208610152	-3.833525386	0.00015184	0.001025191	no
LOC653786	0.208544164	3.832257621	0.00015259	0.001029934	no
ACTR3C	0.208526753	3.831923132	0.000152788	0.001030951	no
LIPN	0.208503041	3.831467585	0.000153059	0.001032454	no
SVEP1	0.208483941	3.831100647	0.000153278	0.001033603	no
NFATC3	0.208477886	3.83098433	0.000153347	0.001033746	no
LRP1B	-0.208436009	-3.830179837	0.000153827	0.001036446	no
ALOX15B	0.208434549	3.830151802	0.000153844	0.001036446	no
KIAA1737	-0.20842711	-3.830008892	0.00015393	0.001036697	no
EFCAB4B	0.208407999	3.829641773	0.000154149	0.001037852	no
HAR1A	-0.208388433	-3.829265897	0.000154375	0.001039043	no
SGK494	-0.208363018	-3.828777687	0.000154668	0.00104069	no
PXDN	0.20835006	3.828528776	0.000154817	0.001041371	no
CTGF	0.208311344	3.827785067	0.000155265	0.001044057	no
DOK4	-0.208301145	-3.827589173	0.000155384	0.001044512	no
NUDT16	0.208297126	3.827511967	0.00015543	0.001044512	no
GOLPH3	0.20827521	3.827090986	0.000155684	0.001045894	no
PDK3	0.208266888	3.82693114	0.000155781	0.001046216	no
USP34	-0.208257829	-3.826757135	0.000155886	0.001046597	no
MYT1L	-0.208240827	-3.826430561	0.000156084	0.001047377	no
FN1	0.208232828	3.826276904	0.000156177	0.001047377	no
GYG1	0.208232199	3.826264835	0.000156185	0.001047377	no
LEPROT	0.208231094	3.82624361	0.000156198	0.001047377	no
RFX8	0.208200837	3.825662448	0.00015655	0.001049416	no
TAF10	0.208144821	3.824586527	0.000157206	0.00105348	no
PLEKHA8P1	0.208078157	3.823306163	0.000157989	0.001058398	no
PHACTR3	-0.208053272	-3.822828223	0.000158282	0.001060032	no
ZSCAN29	-0.208034268	-3.822463243	0.000158507	0.001061204	no
POM121L10P	0.207978274	3.821387883	0.000159169	0.001065086	no
GMDS	0.20797689	3.821361296	0.000159186	0.001065086	no

RPL9	-0.207969813	-3.82122539	0.00015927	0.001065316	no
SHPRH	-0.20794769	-3.820800519	0.000159532	0.001066741	no
SYNPO2	0.207926851	3.820400342	0.00015978	0.001068066	no
HECTD3	0.207919536	3.820259856	0.000159867	0.001068316	no
SLC37A4	-0.207855106	-3.819022566	0.000160636	0.00107312	no
PMAIP1	0.207843984	3.818808991	0.000160769	0.001073675	no
SH2D4A	0.207768156	3.817352904	0.000161679	0.001079417	no
EEF2	-0.207760559	-3.817207031	0.00016177	0.001079692	no
WWC2-AS2	0.207745252	3.816913113	0.000161955	0.00108045	no
DIP2C	-0.207742784	-3.816865711	0.000161985	0.00108045	no
MLL3	-0.207718666	-3.816402623	0.000162276	0.001082055	no
PJA1	-0.20770533	-3.81614655	0.000162437	0.00108273	no
DNASE1L2	-0.207701943	-3.816081508	0.000162478	0.00108273	no
GLCCI1	-0.207694042	-3.81592981	0.000162573	0.001083031	no
LINC00319	-0.207656326	-3.81520563	0.00016303	0.001085738	no
MARCKS	-0.207630096	-3.814701989	0.000163348	0.001087521	no
CCDC81	0.207611054	3.81433638	0.00016358	0.001088725	no
OPLAH	0.207606881	3.814256258	0.000163631	0.001088726	no
CAV2	0.207543368	3.813036833	0.000164405	0.001093541	no
ITK	0.207538934	3.812951703	0.000164459	0.001093563	no
UPP1	0.207509946	3.812395158	0.000164814	0.001095158	no
ASH1L-AS1	-0.207507027	-3.812339126	0.00016485	0.001095158	no
FAM120AOS	0.207506851	3.812335749	0.000164852	0.001095158	no
RPL34	-0.207467472	-3.811579725	0.000165335	0.001098028	no
KIAA1279	-0.207424947	-3.810763325	0.000165859	0.001101163	no
AASDHPPT	-0.207389616	-3.810085054	0.000166294	0.001103717	no
SMIM10	0.207373153	3.80976901	0.000166498	0.001104313	no
PIGG	0.207372904	3.80976424	0.000166501	0.001104313	no
IER2	0.207369875	3.809706092	0.000166538	0.001104313	no
SEC14L6	0.207314457	3.808642249	0.000167225	0.001108526	no
C7orf29	0.207223909	3.806904103	0.000168353	0.001115369	no
SNHG6	-0.207223261	-3.806891665	0.000168362	0.001115369	no
BTN3A1	0.2072182	3.806794516	0.000168425	0.001115445	no
CD46	0.207180998	3.806080433	0.000168891	0.001118184	no
TAPT1	-0.207147176	-3.805431229	0.000169315	0.001120649	no
LOC730811	-0.207085677	-3.804250829	0.000170089	0.001125428	no
C1orf87	0.207020633	3.803002437	0.000170912	0.001130522	no
UBE2QL1	-0.207011562	-3.802828329	0.000171027	0.001130935	no
AMY2A	-0.207000298	-3.802612155	0.00017117	0.001131358	no
ANKFN1	-0.206998225	-3.802572369	0.000171196	0.001131358	no
HLA-G	0.206930413	3.801270914	0.000172059	0.001136711	no
GYG2	0.206921295	3.801095917	0.000172175	0.00113713	no
TTBK2	-0.206881467	-3.800331579	0.000172684	0.001140141	no
SMARCB1	-0.206846282	-3.799656347	0.000173135	0.001142767	no
CYFIP1	0.206838809	3.799512942	0.000173231	0.001143048	no
KDELR2	0.206701896	3.796885637	0.000174997	0.001154345	no
NTHL1	-0.206676763	-3.796403361	0.000175323	0.001156141	no
FBXL17	-0.206591859	-3.794774232	0.000176428	0.001162944	no
PATZ1	-0.206589214	-3.794723484	0.000176463	0.001162944	no
GPR61	-0.20656657	-3.794289011	0.000176759	0.001164537	no
ARHGEF5	0.206558382	3.794131913	0.000176866	0.001164886	no

ZNF275	-0.206530418	-3.793595362	0.000177232	0.001166752	no
TEAD3	0.206528473	3.79355804	0.000177258	0.001166752	no
TTYH1	-0.20648825	-3.79278631	0.000177786	0.001169871	no
CSNK1G2	0.206479332	3.792615217	0.000177903	0.001170284	no
SULT1B1	0.206473688	3.792506936	0.000177978	0.001170415	no
TREM1	0.206465997	3.79235938	0.000178079	0.001170722	no
TXN2	-0.206412065	-3.791324657	0.000178791	0.001175042	no
CH25H	0.206322496	3.789606318	0.000179978	0.001182487	no
CIAO1	-0.206308539	-3.789338568	0.000180164	0.001183346	no
FAAH	-0.206294578	-3.789070741	0.00018035	0.001184206	no
RRBP1	0.20626235	3.788452502	0.00018078	0.001186667	no
PAN3-AS1	-0.206214211	-3.787529037	0.000181424	0.001190438	no
RPL31P11	0.206211142	3.787470163	0.000181466	0.001190438	no
RAMP3	0.206191144	3.787086561	0.000181734	0.001191834	no
NDRG4	-0.206143454	-3.786171772	0.000182375	0.001195316	no
ATP2B2	-0.206143393	-3.786170595	0.000182376	0.001195316	no
TGM2	0.206122883	3.785777184	0.000182652	0.001196635	no
TMEM243	0.206120198	3.785725686	0.000182689	0.001196635	no
UNC5B	0.20605248	3.78442676	0.000183604	0.001202267	no
SEC31B	-0.206022156	-3.783845141	0.000184016	0.001204594	no
DENND1B	0.205969095	3.78282743	0.000184738	0.001208592	no
RRAGD	-0.205968999	-3.782825597	0.000184739	0.001208592	no
LARS	-0.205961671	-3.782685043	0.000184839	0.001208878	no
LOC100507032	-0.205883615	-3.781187992	0.000185906	0.001215457	no
CYFIP2	-0.205879852	-3.781115831	0.000185958	0.001215457	no
NCSTN	0.205856343	3.78066497	0.000186281	0.001217197	no
PTCD3	-0.20583396	-3.780235708	0.000186588	0.001218837	no
HDDC2	-0.205824668	-3.780057499	0.000186716	0.001219302	no
PIN1	-0.205778696	-3.779175858	0.00018735	0.00122307	no
MPPED2	-0.20576074	-3.778831528	0.000187599	0.001224318	no
PTPRZ1	-0.205748162	-3.778590311	0.000187773	0.001224719	no
MDK	0.205748068	3.778588514	0.000187774	0.001224719	no
PLXNB3	-0.205738396	-3.778403026	0.000187908	0.00122522	no
INPP5B	0.205715631	3.777966484	0.000188223	0.001226906	no
EPHA3	0.205690901	3.777492248	0.000188567	0.001228771	no
RRN3	-0.205679124	-3.777266401	0.00018873	0.001229465	no
PTPRS	-0.205649887	-3.776705752	0.000189137	0.001231465	no
XPNPEP3	-0.205648841	-3.776685708	0.000189152	0.001231465	no
LOC100289509	-0.205643534	-3.776583933	0.000189226	0.001231574	no
CXXC11	-0.205630428	-3.776332617	0.000189409	0.00123239	no
GIT1	-0.205594887	-3.775651109	0.000189905	0.001235247	no
ZNF559-ZNF17	-0.205583588	-3.775434462	0.000190064	0.001235902	no
TEX22	-0.205571506	-3.775202788	0.000190233	0.001236547	no
SDF4	0.205559495	3.774972474	0.000190401	0.001236547	no
HIP1R	-0.205557613	-3.774936401	0.000190427	0.001236547	no
BTBD2	-0.205548528	-3.774762195	0.000190555	0.001236547	no
LOC100288432	0.20554585	3.774710851	0.000190593	0.001236547	no
KIF5C	-0.20554166	-3.774630506	0.000190651	0.001236547	no
KIRREL	0.205541546	3.774628314	0.000190653	0.001236547	no
RPL10	-0.205541441	-3.774626305	0.000190654	0.001236547	no
ACPP	0.205539575	3.774590518	0.000190681	0.001236547	no

MRPL17	0.205512441	3.774070246	0.000191062	0.001238283	no
LDHB	-0.205508512	-3.773994917	0.000191117	0.001238283	no
ASRGL1	-0.205508232	-3.77398954	0.000191121	0.001238283	no
GMPPB	0.205502722	3.773883909	0.000191199	0.001238412	no
GABRE	0.205462802	3.773118486	0.000191762	0.001241683	no
OR2A20P	0.205407681	3.772061645	0.000192541	0.001246356	no
IL11	0.205376906	3.771471607	0.000192978	0.001248476	no
SLAIN2	0.205376399	3.771461902	0.000192985	0.001248476	no
CEP44	-0.205371401	-3.771366066	0.000193056	0.00124856	no
FDFT1	-0.205362975	-3.771204526	0.000193176	0.001248958	no
SCARNA7	-0.205313367	-3.770253449	0.000193882	0.001253148	no
MKNK2	0.20529774	3.769953861	0.000194105	0.001254212	no
PDZRN4	-0.205279747	-3.769608898	0.000194362	0.001255495	no
ZNF675	-0.205258327	-3.769198258	0.000194668	0.001257097	no
DPH3	0.205241052	3.768867092	0.000194916	0.001258153	no
EIF4E2	0.205238738	3.768822735	0.000194949	0.001258153	no
VN1R1	-0.205188211	-3.767854102	0.000195675	0.001261979	no
ATP6V0B	0.205185599	3.767804031	0.000195712	0.001261979	no
RRAGC	0.205185195	3.767796286	0.000195718	0.001261979	no
IRF7	0.205154369	3.76720537	0.000196162	0.001264463	no
PCDHGC4	-0.205126351	-3.766668281	0.000196566	0.001266689	no
HECTD1	-0.205106167	-3.766281373	0.000196858	0.00126819	no
TTC28	-0.205097292	-3.766111238	0.000196987	0.001268421	no
RMND5B	-0.205095529	-3.766077455	0.000197012	0.001268421	no
CA2	0.205083779	3.765852219	0.000197182	0.001269136	no
PTPN7	0.20507919	3.765764252	0.000197249	0.001269184	no
CSPG5	-0.205066154	-3.765514366	0.000197438	0.00127002	no
CARD8	0.205047799	3.765162532	0.000197704	0.001271354	no
TOMM70A	-0.205042095	-3.765053201	0.000197787	0.001271506	no
PMEPA1	0.205032806	3.76487514	0.000197922	0.001271994	no
SLC46A2	0.205007361	3.764387413	0.000198293	0.001273993	no
DTNB	0.204967003	3.763613852	0.000198881	0.001277063	no
ZBTB5	-0.20496645	-3.763603241	0.000198889	0.001277063	no
GDF9	-0.204939202	-3.76308099	0.000199288	0.001279239	no
LRRC24	-0.204930258	-3.762909555	0.000199419	0.001279697	no
TUBB6	0.204902475	3.762377051	0.000199826	0.001281927	no
RPL15	-0.20488316	-3.762006846	0.000200109	0.001283363	no
B3GAT3	0.204875087	3.761852124	0.000200228	0.001283741	no
CD1A	0.204854742	3.761462191	0.000200527	0.001285276	no
DCC	-0.204828289	-3.760955204	0.000200917	0.00128739	no
LAS1L	-0.204792185	-3.760263259	0.00020145	0.001290421	no
ARL1	0.204772909	3.759893833	0.000201735	0.001291862	no
RNF144A	-0.204764201	-3.759726939	0.000201864	0.001292303	no
MANEAL	-0.204733037	-3.759129694	0.000202326	0.001294802	no
C17orf51	-0.204729744	-3.759066573	0.000202375	0.001294802	no
UBAP1L	-0.204720074	-3.758881262	0.000202519	0.001295336	no
C2orf27A	-0.204715651	-3.758796498	0.000202584	0.00129537	no
B4GALNT4	-0.204687308	-3.758253329	0.000203006	0.00129768	no
LOC100131551	0.204675765	3.75803212	0.000203178	0.001298393	no
PMFBP1	0.204666884	3.757861943	0.00020331	0.001298853	no
CHERP	-0.204627416	-3.757105593	0.0002039	0.00130223	no

ZC3H13	-0.204584536	-3.756283892	0.000204542	0.001305942	no
ZNF84	-0.204570619	-3.756017209	0.00020475	0.001306887	no
HAS2	0.204503448	3.754730081	0.000205761	0.001312947	no
HMGB1	-0.204457666	-3.753852835	0.000206452	0.001316967	no
RB1	0.204410318	3.752945612	0.000207169	0.001321151	no
ACTA2	0.204395062	3.752653289	0.000207401	0.001322236	no
TMEM167B	0.204379169	3.752348786	0.000207643	0.001323383	no
ERGIC1	0.204353556	3.751858045	0.000208032	0.001325475	no
HLF	-0.204340762	-3.751612915	0.000208227	0.001326324	no
GPA33	0.20432439	3.751299227	0.000208477	0.001327521	no
TXLNB	0.204310428	3.751031724	0.00020869	0.001328485	no
RUSC2	-0.204277593	-3.750402654	0.000209193	0.001330935	no
GRAP	0.204277171	3.750394551	0.000209199	0.001330935	no
PLA2G6	-0.20425889	-3.750044318	0.000209479	0.001332323	no
RTN4R	-0.204249189	-3.749858458	0.000209628	0.001332875	no
KCNN1	-0.204239487	-3.749672579	0.000209777	0.001333427	no
TMED10	0.204219225	3.749284412	0.000210088	0.001335011	no
TNFAIP6	0.204206509	3.749040788	0.000210284	0.001335859	no
HMGCR	-0.20417469	-3.748431209	0.000210774	0.001338577	no
ZNF10	-0.204075067	-3.746522738	0.000212316	0.001347969	no
CNTN3	-0.204062898	-3.746289617	0.000212505	0.00134877	no
SERPINH1	0.204037462	3.745802369	0.0002129	0.001350882	no
TLE6	0.204011837	3.745311513	0.000213299	0.001353016	no
BDP1	-0.203987754	-3.744850183	0.000213675	0.001355	no
NIPSNAP3A	0.203959656	3.744311964	0.000214115	0.001357385	no
SH3KBP1	0.203944061	3.744013242	0.000214359	0.001358533	no
NCAM2	-0.203923565	-3.743620659	0.00021468	0.001360168	no
NECAB2	-0.203919038	-3.743533936	0.000214751	0.001360217	no
NOP10	0.203906192	3.743287888	0.000214953	0.001361093	no
SIRT1	-0.20388769	-3.742933495	0.000215244	0.001362533	no
FLOT1	0.203842865	3.742074932	0.00021595	0.0013666	no
DUSP3	0.203819401	3.741625499	0.00021632	0.001368432	no
AGPAT5	-0.203816468	-3.741569322	0.000216366	0.001368432	no
METTL2A	-0.203802331	-3.741298572	0.00021659	0.001369325	no
MUL1	0.203799473	3.741243831	0.000216635	0.001369325	no
PSPC1	-0.203648817	-3.738358448	0.000219031	0.001384062	no
USP18	0.203640807	3.738205058	0.000219159	0.001384465	no
RBMS2	0.203633357	3.738062371	0.000219278	0.001384539	no
CHD9	-0.20363202	-3.738036782	0.0002193	0.001384539	no
KDELR1	0.203625988	3.737921262	0.000219396	0.001384742	no
ANGPTL4	0.203444225	3.734440541	0.000222324	0.001402811	no
DCXR	-0.203409216	-3.733770173	0.000222893	0.001405983	no
PCDH12	0.203374038	3.733096588	0.000223465	0.001409179	no
BTBD17	-0.203362129	-3.732868557	0.000223659	0.001409989	no
TMOD2	-0.20335277	-3.732689342	0.000223811	0.001410537	no
ELTD1	0.203316672	3.731998172	0.000224401	0.001413187	no
EVA1A	0.203312741	3.731922903	0.000224465	0.001413187	no
RNF112	-0.203311985	-3.731908422	0.000224478	0.001413187	no
C11orf45	0.203310926	3.731888157	0.000224495	0.001413187	no
TMEM132E	0.203303952	3.73175462	0.000224609	0.001413491	no
B3GNT7	0.20327684	3.73123551	0.000225053	0.001415872	no

COX7A2	-0.203247167	-3.730667389	0.00022554	0.00141852	no
PGAP1	-0.203236307	-3.730459459	0.000225719	0.001419227	no
SMIM17	-0.203216553	-3.730081242	0.000226044	0.001420855	no
KDM3A	-0.203199126	-3.729747599	0.000226331	0.001422243	no
ZNF492	-0.203188582	-3.729545725	0.000226505	0.00142292	no
SETBP1	-0.20307896	-3.727447027	0.000228319	0.001433902	no
SLC16A9	-0.20304287	-3.726756112	0.00022892	0.001437253	no
LIMS1	0.203026538	3.72644346	0.000229192	0.001438542	no
DCX	-0.202999963	-3.725934721	0.000229636	0.001440905	no
IGFN1	-0.202942877	-3.724841934	0.000230591	0.001446479	no
OLIG1	-0.202929993	-3.724595307	0.000230807	0.001447413	no
ASB8	-0.20292192	-3.724440763	0.000230943	0.00144784	no
SUCNR1	0.202916032	3.724328056	0.000231042	0.001448038	no
MT1L	0.202905903	3.724134157	0.000231212	0.001448683	no
VPS52	-0.202891128	-3.723851337	0.000231461	0.001449712	no
OXCT1	-0.202888117	-3.723793698	0.000231511	0.001449712	no
CLEC5A	0.20288291	3.723694029	0.000231599	0.001449839	no
RIMS1	-0.202836325	-3.722802336	0.000232385	0.001454334	no
RBKS	0.202821339	3.722515473	0.000232638	0.00145547	no
BACE1-AS	-0.202817572	-3.722443379	0.000232702	0.00145547	no
PSIP1	-0.202809331	-3.722285634	0.000232841	0.001455919	no
CALN1	-0.202766105	-3.721458265	0.000233574	0.001459827	no
LOC728743	-0.202763195	-3.721402559	0.000233623	0.001459827	no
DLGAP3	-0.202760444	-3.721349904	0.00023367	0.001459827	no
BLZF1	0.202720585	3.720587001	0.000234348	0.00146336	no
MSC	0.202719176	3.720560038	0.000234372	0.00146336	no
FAM19A2	-0.202686764	-3.719939683	0.000234924	0.001466384	no
ARID4A	-0.202675561	-3.71972526	0.000235116	0.001467152	no
RGS3	0.202656999	3.719370006	0.000235433	0.001468705	no
ABHD14B	0.202645881	3.719157208	0.000235623	0.001469465	no
ZNF761	0.2025987	3.718254232	0.000236432	0.00147408	no
MGEA5	-0.202553372	-3.717386742	0.000237211	0.001478511	no
JAZF1	0.202504945	3.71645996	0.000238047	0.001483287	no
SLC25A18	-0.202498994	-3.716346074	0.000238149	0.001483498	no
SLC7A14	-0.20245036	-3.715415378	0.000238991	0.001488239	no
TRAPPC9	-0.202447039	-3.715351827	0.000239049	0.001488239	no
SMYD5	-0.202400196	-3.714455433	0.000239863	0.001492745	no
HS1BP3	0.202397392	3.714401778	0.000239912	0.001492745	no
PAPPA	0.202355893	3.713607656	0.000240635	0.001496812	no
CRBN	-0.202336532	-3.713237185	0.000240973	0.001498481	no
C9orf66	0.202327179	3.713058206	0.000241137	0.001499064	no
NT5C1A	-0.202278182	-3.712120666	0.000241995	0.001503965	no
AMPD1	0.202262729	3.711824974	0.000242266	0.001505215	no
P2RY13	0.20222375	3.711079163	0.000242952	0.001509038	no
ASPHD1	-0.202181418	-3.710269183	0.000243698	0.001513199	no
ZNF627	-0.202177787	-3.710199717	0.000243762	0.001513199	no
OR7E14P	0.202104003	3.708788024	0.000245069	0.001520871	no
PPARG	0.202032676	3.707423391	0.000246338	0.001528307	no
IFT57	0.202012352	3.70703458	0.000246701	0.001530116	no
MKNK1	0.201985703	3.706524742	0.000247178	0.001532629	no
SLC36A1	0.201980462	3.706424485	0.000247271	0.001532769	no

THSD4	-0.201943598	-3.705719255	0.000247932	0.001536421	no
EMC3	0.20189495	3.7047886	0.000248807	0.001541396	no
ZNF429	-0.201834693	-3.703635927	0.000249894	0.001547686	no
IL12B	0.20181202	3.703202205	0.000250304	0.00154978	no
GGACT	0.201729904	3.70163146	0.000251795	0.001558563	no
THAP9	-0.201638135	-3.699876182	0.000253471	0.001568486	no
EEPD1	-0.201630127	-3.699723012	0.000253618	0.001568942	no
NADSYN1	0.201604951	3.699241485	0.00025408	0.001571347	no
CDK2	0.201566863	3.698513006	0.00025478	0.001575225	no
TRUB2	-0.20155138	-3.698216876	0.000255065	0.001576203	no
BRD1	-0.201550305	-3.698196316	0.000255085	0.001576203	no
GRIN3A	-0.201520972	-3.697635307	0.000255626	0.001579093	no
CAMK4	-0.201506399	-3.697356592	0.000255896	0.001580302	no
TGFB2	0.201488536	3.697014963	0.000256226	0.001581888	no
CES1P1	0.201482538	3.696900266	0.000256337	0.001582119	no
IRAK3	0.201472996	3.696717768	0.000256514	0.001582755	no
SORCS3	-0.201463768	-3.696541292	0.000256685	0.001583355	no
CDKL1	-0.201454643	-3.696366782	0.000256854	0.001583945	no
EVI5L	-0.201422215	-3.695746618	0.000257456	0.001587203	no
SLC16A4	0.201380048	3.694940223	0.000258241	0.001591585	no
ARHGAP5-AS1	-0.201371945	-3.69478526	0.000258392	0.00159206	no
IL1RN	0.201298418	3.6933792	0.000259767	0.001600073	no
CPZ	0.201257215	3.692591295	0.00026054	0.001604377	no
NKAIN1	-0.201237421	-3.69221279	0.000260913	0.001606209	no
FAM206A	0.201198325	3.691465208	0.00026165	0.001610284	no
TOR1A	0.201178336	3.691082995	0.000262027	0.001611308	no
UG0898H09	-0.201178062	-3.691077748	0.000262032	0.001611308	no
GDPD5	0.201175362	3.691026127	0.000262083	0.001611308	no
SLC33A1	0.201173642	3.690993231	0.000262116	0.001611308	no
LRIG3	0.201095077	3.689491025	0.000263605	0.001619998	no
MLLT4	-0.201082463	-3.689249836	0.000263845	0.001621009	no
TRPM8	0.20106204	3.68885936	0.000264233	0.001622933	no
ZSCAN23	-0.201050523	-3.688639146	0.000264453	0.001623609	no
TIGD1	-0.201048329	-3.688597209	0.000264495	0.001623609	no
KDM5B	-0.201010846	-3.687880553	0.00026521	0.001627536	no
SNAI3	0.200981365	3.687316894	0.000265774	0.001630352	no
HCN4	-0.200978937	-3.687270486	0.000265821	0.001630352	no
DGCR14	-0.200923282	-3.686206452	0.000266889	0.001636435	no
ZNF709	-0.200909214	-3.685937489	0.000267159	0.001637627	no
TREML2	0.200891587	3.685600496	0.000267499	0.001639239	no
KCTD15	-0.200870096	-3.68518965	0.000267913	0.00164131	no
PTK2	-0.200832761	-3.684475902	0.000268634	0.00164526	no
MICU1	-0.200825267	-3.684332643	0.000268779	0.001645679	no
BTRC	-0.200797589	-3.683803526	0.000269315	0.001648492	no
VRK3	0.200762091	3.683124919	0.000270004	0.001652011	no
BBIP1	-0.200760047	-3.68308585	0.000270044	0.001652011	no
PTPN23	-0.200741879	-3.682738556	0.000270397	0.001653702	no
LOC256880	-0.200658965	-3.681153605	0.000272015	0.001663125	no
HOXB8	0.200653838	3.68105561	0.000272116	0.001663265	no
GPR162	-0.200647897	-3.680942054	0.000272232	0.001663503	no
LOC646762	-0.200603381	-3.68009114	0.000273105	0.001668363	no

YTHDF2	0.200597872	3.679985852	0.000273213	0.00166855	no
FOXL1	0.200567951	3.679413928	0.000273802	0.001671669	no
YDJC	0.200547224	3.679017758	0.00027421	0.001673442	no
TANC2	-0.20054531	-3.678981183	0.000274248	0.001673442	no
VPS72	-0.200520916	-3.678514933	0.000274729	0.001675904	no
SMAGP	0.200516004	3.678421035	0.000274826	0.00167602	no
SC5DL	-0.200497777	-3.678072667	0.000275187	0.001677741	no
CCDC28A	0.200466174	3.677468641	0.000275813	0.00168108	no
UBE2V2	-0.200461908	-3.677387101	0.000275897	0.001681119	no
ATAD1	-0.200457058	-3.677294414	0.000275993	0.001681228	no
NEU4	-0.200423291	-3.67664904	0.000276664	0.001684835	no
LMO3	-0.200384257	-3.675903024	0.000277441	0.001689089	no
PDHA1	-0.200367962	-3.675591605	0.000277766	0.001690588	no
YAF2	-0.200324656	-3.674763985	0.000278631	0.001695375	no
SUPT20H	-0.200283297	-3.673973567	0.00027946	0.001699937	no
ANXA11	0.200270768	3.673734148	0.000279712	0.001700986	no
ADAMTSL1	0.20025087	3.673353881	0.000280112	0.001702936	no
GPR4	0.200205646	3.672489673	0.000281023	0.001707991	no
ZBTB38	0.200192712	3.672242508	0.000281284	0.001709094	no
TTC9B	-0.200149471	-3.671416202	0.000282158	0.001713922	no
RNFT1	0.200034328	3.669216027	0.000284498	0.001727361	no
SEMA3C	0.200032725	3.669185403	0.000284531	0.001727361	no
CAPG	0.200015065	3.668847962	0.000284892	0.001729062	no
NHP2L1	-0.199982904	-3.668233469	0.000285549	0.001732565	no
NDST4	-0.199954588	-3.667692435	0.00028613	0.001735596	no
HRK	-0.199940961	-3.667432082	0.000286409	0.001736802	no
SPAG4	0.199905477	3.666754119	0.000287139	0.001740734	no
FAM5B	-0.199854771	-3.665785347	0.000288184	0.001746579	no
FXYD6	-0.199850602	-3.665705686	0.00028827	0.001746608	no
CLDN1	0.199831427	3.665339342	0.000288667	0.001748517	no
BIRC3	0.199771439	3.664193291	0.00028991	0.001755553	no
RFX7	-0.199733921	-3.663476544	0.00029069	0.001759781	no
ZFR	-0.199710999	-3.663038643	0.000291168	0.001762176	no
LOC100130691	-0.199690142	-3.662640201	0.000291603	0.001764313	no
MCM7	-0.199640165	-3.661685495	0.000292648	0.001770139	no
CCDC102A	0.199594813	3.660819154	0.0002936	0.001775395	no
CSTF2T	-0.19955605	-3.660078712	0.000294415	0.001779826	no
NPHP3-AS1	0.199451516	3.658081981	0.000296625	0.001792681	no
MAML2	-0.199428907	-3.657650142	0.000297105	0.001794674	no
ZFR2	-0.199428122	-3.657635147	0.000297122	0.001794674	no
LINC00563	0.199422788	3.657533259	0.000297235	0.001794854	no
GNRHR2	-0.199410744	-3.657303216	0.000297492	0.001795896	no
DNAJB2	-0.199394202	-3.656987272	0.000297844	0.001797288	no
PLSCR2	0.199392055	3.656946258	0.000297889	0.001797288	no
TMEM138	0.199384039	3.656793153	0.00029806	0.001797613	no
TM4SF1	0.199381672	3.656747945	0.000298111	0.001797613	no
UBE2E2	-0.199364155	-3.656413384	0.000298484	0.001799361	no
AP3S2	0.199333431	3.655826571	0.00029914	0.001802812	no
LINC00609	-0.199289816	-3.654993574	0.000300074	0.001807933	no
PNMA3	-0.19928368	-3.654876385	0.000300206	0.001808219	no
HTATSF1	-0.199267287	-3.654563307	0.000300558	0.001809832	no

WSCD2	-0.199222975	-3.653717029	0.000301511	0.001815062	no
WDR6	-0.199109285	-3.651545879	0.000303969	0.001829348	no
PCDHB10	-0.199048861	-3.65039202	0.000305283	0.001836539	no
B4GALT5	0.199046491	3.650346767	0.000305335	0.001836539	no
MGST1	0.199005219	3.649558648	0.000306236	0.001841441	no
RSC1A1	0.19892834	3.648090667	0.00030792	0.001851053	no
TMEM97	-0.198914614	-3.647828579	0.000308222	0.001852348	no
ZNF285	-0.198870021	-3.646977117	0.000309204	0.00185773	no
KLF6	0.1988483	3.646562392	0.000309683	0.00186009	no
DNAJB1	0.198791305	3.645474185	0.000310944	0.001867142	no
MRPL23-AS1	-0.198778749	-3.645234451	0.000311223	0.001868292	no
ECM2	0.198754595	3.644773292	0.000311759	0.001870989	no
TRIM37	-0.19869088	-3.643556862	0.000313178	0.00187898	no
ACADS	0.198674032	3.643235206	0.000313554	0.001880712	no
MRPL47	0.19864918	3.642760757	0.00031411	0.001883519	no
USO1	0.198633851	3.642468108	0.000314453	0.001885051	no
MOK	0.198595556	3.641737041	0.000315312	0.001889673	no
AMER3	-0.198577372	-3.641389897	0.000315721	0.001891595	no
CDH18	-0.198564096	-3.641136452	0.000316019	0.001892856	no
C6orf48	-0.198557788	-3.641016041	0.000316161	0.001893179	no
C7orf55	-0.198547613	-3.64082179	0.00031639	0.001894024	no
SDF2L1	0.198496363	3.639843463	0.000317547	0.001900418	no
RTN3	-0.198485573	-3.639637487	0.000317791	0.001901349	no
TMEM2	0.198445546	3.638873426	0.000318698	0.001906243	no
DDR2	0.198440589	3.638778797	0.00031881	0.001906385	no
SCN3B	-0.198385001	-3.637717714	0.000320074	0.001913409	no
FLJ22447	0.198351961	3.637087059	0.000320827	0.001917379	no
TST	-0.198341027	-3.636878368	0.000321077	0.001918337	no
WDR63	0.198318652	3.636451288	0.000321588	0.001920456	no
AEBP1	0.198317696	3.636433042	0.00032161	0.001920456	no
FKBP10	0.19830615	3.636212664	0.000321874	0.001921074	no
SLIT2-IT1	0.198305364	3.636197656	0.000321892	0.001921074	no
EPN1	0.198291225	3.635927791	0.000322216	0.001922472	no
MAFK	0.198266375	3.635453478	0.000322786	0.001925338	no
ZNF572	-0.198221812	-3.634602936	0.00032381	0.001930913	no
ZMIZ1	-0.198211482	-3.634405784	0.000324048	0.001931795	no
ARRDC1	0.198175558	3.633720146	0.000324877	0.001936198	no
ASPDH	-0.198044853	-3.631225701	0.000327908	0.001953722	no
KIAA1755	-0.198038635	-3.631107035	0.000328053	0.001954044	no
OCIAD1	-0.197963777	-3.629678484	0.000329802	0.001963719	no
MESP1	-0.197961286	-3.629630961	0.00032986	0.001963719	no
MGAT4C	-0.19794131	-3.629249761	0.000330328	0.001965961	no
VDR	0.197910082	3.628653858	0.000331061	0.001969779	no
NPLOC4	0.197890474	3.628279687	0.000331523	0.001971977	no
LOC145820	0.197885032	3.628175836	0.000331651	0.001972193	no
FGD5	0.197826811	3.627064896	0.000333024	0.001979322	no
SYNGR1	-0.197826394	-3.627056927	0.000333034	0.001979322	no
HOOK3	-0.197780329	-3.626177973	0.000334124	0.001985252	no
SLC9A3	0.197763348	3.625853954	0.000334527	0.001987096	no
PDGFRL	0.197733487	3.625284199	0.000335236	0.001990759	no
MAPT-AS1	-0.197724307	-3.62510905	0.000335454	0.001991505	no

CCDC124	-0.197713644	-3.624905587	0.000335708	0.001992462	no
ZNF696	-0.197665132	-3.623980002	0.000336865	0.001998325	no
STBD1	0.197662406	3.623927981	0.00033693	0.001998325	no
TOB2	-0.197660526	-3.623892117	0.000336975	0.001998325	no
PHKA2-AS1	0.197636794	3.623439328	0.000337542	0.002001138	no
GINM1	0.197627958	3.623270756	0.000337754	0.00200184	no
DUSP6	0.197596145	3.622663802	0.000338516	0.002005418	no
ADRA1A	-0.197594052	-3.622623857	0.000338567	0.002005418	no
UACA	0.197591093	3.622567403	0.000338638	0.002005418	no
PCNT	-0.197575452	-3.622268999	0.000339013	0.00200709	no
RPS6KB2	0.197548699	3.621758615	0.000339657	0.002010345	no
PIANP	-0.197515101	-3.621117629	0.000340466	0.002014074	no
CDKN1A	0.19751477	3.621111308	0.000340474	0.002014074	no
PF4V1	0.197479225	3.620433216	0.000341333	0.002018597	no
HIST1H4H	0.197455099	3.619972949	0.000341917	0.002021493	no
LHFP	0.197448195	3.61984125	0.000342084	0.002021926	no
ZNF253	-0.197431224	-3.619517487	0.000342495	0.002023801	no
ZNF578	-0.197407278	-3.61906068	0.000343076	0.002026679	no
KGFLP1	-0.197395633	-3.618838537	0.00034336	0.002027793	no
GPR98	-0.197388168	-3.618696135	0.000343541	0.002028308	no
LMF2	0.197370164	3.618352692	0.000343979	0.00202962	no
HSF2	-0.197368194	-3.618315117	0.000344027	0.00202962	no
RPRM	-0.197367398	-3.618299929	0.000344047	0.00202962	no
SELM	0.197352208	3.618010169	0.000344417	0.002031246	no
C19orf35	0.197345508	3.617882358	0.00034458	0.002031653	no
LOC729737	0.19733983	3.617774062	0.000344719	0.002031912	no
CTTN	0.197326771	3.617524939	0.000345038	0.002033234	no
FAM169A	-0.197303555	-3.617082101	0.000345605	0.002036019	no
FAM69C	-0.197266225	-3.616370025	0.000346519	0.002040846	no
CLUH	-0.197251048	-3.616080525	0.000346892	0.002042479	no
LINC00612	0.197168662	3.614509102	0.000348919	0.002053856	no
ATP6AP2	0.197102776	3.613252437	0.000350549	0.002062883	no
TMEM69	0.197078596	3.612791268	0.000351149	0.002065847	no
ZNF98	-0.19703613	-3.611981345	0.000352205	0.002071491	no
TRIM23	-0.197030224	-3.611868712	0.000352352	0.002071789	no
LOC100507557	-0.196982216	-3.610953118	0.000353549	0.002078262	no
LPPR5	-0.196951026	-3.610358275	0.000354329	0.002082278	no
GEM	0.196927495	3.609909519	0.000354919	0.002085172	no
RAB2A	-0.196881803	-3.609038151	0.000356066	0.002091342	no
MAGEE1	-0.196833931	-3.608125236	0.000357272	0.002097851	no
ABL2	0.196826452	3.607982625	0.000357461	0.002098386	no
GDF15	0.196789303	3.607274224	0.0003584	0.002103323	no
LOC339803	0.19677013	3.6069086	0.000358885	0.002105597	no
LRRFIP2	-0.196762407	-3.606761343	0.000359081	0.002105752	no
ZNF177	-0.19676135	-3.606741189	0.000359108	0.002105752	no
PDZK1P1	-0.19675506	-3.606621244	0.000359267	0.002106113	no
F3	0.196748905	3.606503878	0.000359423	0.002106351	no
ARSJ	0.196745734	3.606443402	0.000359504	0.002106351	no
NCAN	-0.196739492	-3.606324377	0.000359662	0.002106705	no
PAM16	-0.196719393	-3.605941113	0.000360173	0.002109121	no
OAZ1	0.196713063	3.605820414	0.000360334	0.002109488	no

STMN2	-0.196620088	-3.604047612	0.000362705	0.002122794	no
HES5	-0.196585296	-3.60338423	0.000363597	0.002127431	no
UBL7	-0.196543708	-3.602591284	0.000364664	0.002133099	no
FZD1	0.196533558	3.602397774	0.000364926	0.002133548	no
ARMC5	-0.196530123	-3.602332275	0.000365014	0.002133548	no
LRIG1	-0.196529145	-3.602313623	0.000365039	0.002133548	no
SHC3	-0.196477645	-3.601331745	0.000366367	0.002140422	no
CBR3-AS1	0.196475804	3.601296643	0.000366415	0.002140422	no
SPSB3	-0.19647173	-3.601218961	0.00036652	0.002140456	no
GTF2H5	-0.196464503	-3.601081177	0.000366707	0.002140964	no
CHST14	0.19643854	3.600586193	0.000367378	0.002144303	no
FAM106CP	-0.196426558	-3.600357769	0.000367689	0.002145532	no
DNAL4	-0.196392052	-3.599699911	0.000368584	0.002150172	no
PRLHR	-0.196356648	-3.599024961	0.000369504	0.002154957	no
POLR3B	-0.196334564	-3.598603957	0.00037008	0.002157727	no
GGTLC1	0.196316447	3.59825857	0.000370552	0.002159896	no
TREML4	0.196259908	3.597180759	0.000372031	0.002167731	no
ZFHX2	-0.196257337	-3.597131752	0.000372098	0.002167731	no
JRK	-0.196247893	-3.596951729	0.000372346	0.002168585	no
HSPA1L	-0.196227861	-3.596569854	0.000372871	0.002171058	no
UBAP2	-0.196217282	-3.596368189	0.000373149	0.002171996	no
LOC100130155	-0.196214031	-3.596306231	0.000373234	0.002171996	no
WHAMM	0.196174122	3.59554548	0.000374284	0.002177517	no
DNAJC6	-0.196149488	-3.595075909	0.000374934	0.002180705	no
TFPI2	0.196127296	3.594652884	0.00037552	0.002183523	no
DNAJC12	-0.196120323	-3.594519986	0.000375704	0.002184003	no
MBOAT2	-0.196075802	-3.593671354	0.000376883	0.002190263	no
BTG2	0.196048741	3.593155569	0.000377601	0.002193844	no
ALDH6A1	-0.195902319	-3.590364812	0.000381509	0.00221595	no
SLC9B1	-0.195845409	-3.589280207	0.000383038	0.00222423	no
GRIA3	-0.195817543	-3.588749127	0.000383789	0.002227988	no
DCTD	0.195807406	3.588555944	0.000384063	0.002228973	no
ABCC1	0.195798971	3.588395201	0.00038429	0.002229466	no
MGST2	0.195796576	3.588349551	0.000384355	0.002229466	no
ANKH	0.195785539	3.588139219	0.000384653	0.002230593	no
CKMT1A	-0.195773044	-3.58790109	0.000384991	0.00223195	no
GPR35	0.195738904	3.587250493	0.000385915	0.002236706	no
FLJ35946	-0.195710916	-3.586717131	0.000386675	0.002240502	no
GUCY2C	0.195706027	3.586623958	0.000386807	0.002240668	no
TMEM204	0.195629476	3.585165207	0.000388892	0.002252137	no
WDR89	-0.195597477	-3.584555441	0.000389767	0.002256593	no
GATA3	0.195569644	3.584025092	0.000390529	0.002260397	no
ZNF503-AS2	0.195544009	3.583536613	0.000391232	0.002263857	no
SNHG1	-0.195488729	-3.582483308	0.000392752	0.00227145	no
PTGS1	0.195488614	3.582481115	0.000392755	0.00227145	no
SULT1C2	0.195462026	3.581974505	0.000393489	0.002275078	no
MAP3K11	0.195429106	3.581347263	0.000394398	0.002279724	no
CD151	0.195407166	3.580929234	0.000395006	0.00228262	no
CN5H6.4	-0.195378195	-3.580377261	0.000395809	0.002286647	no
DR1	0.195339571	3.579641381	0.000396882	0.002292231	no
LBP	0.195315924	3.579190852	0.00039754	0.002295417	no

SCML2	-0.19530596	-3.57900102	0.000397818	0.002296403	no
ZNF653	-0.195299954	-3.578886599	0.000397986	0.002296753	no
PPARGC1A	-0.195197781	-3.576940091	0.000400845	0.002312634	no
RIMS4	-0.195040552	-3.573944931	0.000405283	0.00233761	no
TMEM240	-0.195034482	-3.57382931	0.000405455	0.002337976	no
GALNT4	0.19502881	3.573721264	0.000405616	0.002338277	no
CNNM2	-0.195018905	-3.573532588	0.000405897	0.002339271	no
FBXO34	-0.194959166	-3.572394659	0.000407598	0.002348441	no
BAP1	-0.194943108	-3.572088793	0.000408056	0.002350104	no
VIPR2	-0.194941391	-3.572056084	0.000408105	0.002350104	no
DBC1	-0.194907401	-3.571408677	0.000409077	0.002355067	no
RNF103-CHMP3	-0.194850096	-3.570317183	0.00041072	0.002363892	no
ADAM11	-0.194838174	-3.570090127	0.000411062	0.002365084	no
C1orf95	-0.19483523	-3.570034056	0.000411147	0.002365084	no
EGR2	0.194778446	3.568952539	0.000412782	0.002373858	no
MMP10	0.194759164	3.5685853	0.000413339	0.002376424	no
PPME1	-0.194755302	-3.568511738	0.000413451	0.002376429	no
LOC440300	-0.194749317	-3.568397752	0.000413624	0.002376788	no
CAND2	-0.194716356	-3.567770011	0.000414578	0.002380718	no
MRPL37	0.194716254	3.567768073	0.000414581	0.002380718	no
MATR3	-0.194714208	-3.567729106	0.00041464	0.002380718	no
HIVEP3	0.194692675	3.567319002	0.000415264	0.002383666	no
BMP7	-0.194598423	-3.565524051	0.000418008	0.002398773	no
TMEM220	0.194566465	3.564915472	0.000418942	0.002403491	no
FOLR3	0.194545078	3.564508188	0.000419568	0.002406441	no
PANK2	0.194533224	3.56428246	0.000419915	0.002407791	no
MYO9A	-0.194513152	-3.563900236	0.000420504	0.002410525	no
ZNF433	-0.194499963	-3.563649079	0.000420892	0.002412102	no
SMARCD3	-0.194475656	-3.563186215	0.000421606	0.002415554	no
HDAC6	-0.194443207	-3.562568331	0.000422562	0.002420386	no
HUWE1	-0.19442533	-3.562227917	0.00042309	0.002422762	no
FKBP9	0.194412172	3.56197736	0.000423479	0.002424323	no
OSGEPL1	-0.194408464	-3.561906765	0.000423588	0.002424323	no
RBM4	-0.194401515	-3.561774447	0.000423794	0.002424853	no
IL18BP	0.194372437	3.561220766	0.000424654	0.002429131	no
CNTNAP5	-0.194358654	-3.560958321	0.000425063	0.002430821	no
C11orf70	0.194351883	3.560829398	0.000425264	0.002431323	no
SESN2	0.194345785	3.560713282	0.000425445	0.00243171	no
PPM1K	-0.194337325	-3.560552196	0.000425696	0.002432444	no
SLC1A1	-0.194333837	-3.560485782	0.000425799	0.002432444	no
OTUD5	0.194314566	3.560118848	0.000426372	0.002434919	no
C11orf71	-0.19431164	-3.560063138	0.000426459	0.002434919	no
SLC8A2	-0.194230218	-3.558512877	0.000428887	0.002448133	no
NLGN1	-0.194163896	-3.557250178	0.000430875	0.002458268	no
MIPEP	0.19415958	3.557168003	0.000431005	0.002458268	no
ATP5J	-0.194159525	-3.557166957	0.000431006	0.002458268	no
FLNA	0.194140219	3.556799402	0.000431586	0.002460925	no
JAKMIP2-AS1	0.194126409	3.556536489	0.000432002	0.002462641	no
MERTK	0.194121229	3.556437871	0.000432158	0.002462876	no
TRIM67	-0.194094907	-3.555936761	0.000432951	0.002466743	no
LINC00278	0.194023944	3.554585803	0.000435097	0.002478311	no

PPP2R5C	-0.193979839	-3.553746191	0.000436435	0.002485276	no
LINC00239	0.193918654	3.552581462	0.000438298	0.002495224	no
DOCK7	0.193878281	3.551812941	0.000439532	0.002501583	no
RNF181	0.193817981	3.550665131	0.00044138	0.002511438	no
ERAP2	0.1937696	3.549744223	0.000442868	0.002518755	no
PIKFYVE	-0.193765837	-3.549672607	0.000442984	0.002518755	no
MCAM	0.19376475	3.549651909	0.000443018	0.002518755	no
POLR2L	0.193745642	3.549288218	0.000443607	0.002521437	no
RNF150	-0.193695757	-3.548338729	0.000445149	0.002529531	no
ANKRD18B	-0.193678785	-3.548015697	0.000445674	0.002531849	no
LOC152225	0.193655615	3.547574707	0.000446393	0.00253526	no
ZBTB7C	0.1936095	3.54669702	0.000447826	0.002542729	no
KIAA1033	0.193591388	3.546352302	0.00044839	0.002545259	no
MOCS2	-0.193578963	-3.546115838	0.000448777	0.002546785	no
ZNF804A	-0.193434855	-3.543373306	0.000453293	0.002571731	no
NKD1	-0.193397012	-3.542653155	0.000454486	0.002577818	no
AARSD1	-0.193389463	-3.542509497	0.000454724	0.002577946	no
DCN	0.193387431	3.54247084	0.000454788	0.002577946	no
CCDC23	0.193384895	3.54242258	0.000454868	0.002577946	no
C5orf47	-0.193355935	-3.541871487	0.000455784	0.002582453	no
HIC1	0.193343035	3.541625998	0.000456192	0.002584086	no
CELA1	0.193308385	3.540966643	0.00045729	0.002589625	no
LOC729732	-0.193265639	-3.540153257	0.000458649	0.002596634	no
STRN3	-0.193239044	-3.539647207	0.000459496	0.002600745	no
BRD9	-0.193224836	-3.539376847	0.000459949	0.002602625	no
RABL5	0.193184642	3.538612056	0.000461233	0.002609204	no
MXI1	-0.193149211	-3.537937897	0.000462368	0.002614399	no
ORM2	0.193148379	3.537922067	0.000462395	0.002614399	no
SOGA1	-0.193141324	-3.537787834	0.000462621	0.00261499	no
KCND2	-0.193132737	-3.537624457	0.000462897	0.00261574	no
CYP39A1	0.193129603	3.537564836	0.000462997	0.00261574	no
KLRD1	0.193045307	3.535960992	0.000465711	0.00263038	no
C4B	0.193031299	3.535694471	0.000466163	0.002632129	no
NOTCH3	0.193028134	3.535634259	0.000466266	0.002632129	no
CCPG1	0.193016072	3.535404776	0.000466656	0.002633639	no
COMMD8	0.192982755	3.534770909	0.000467734	0.002639033	no
THEMIS	0.192966022	3.534452571	0.000468277	0.002641401	no
FADS1	-0.192955179	-3.53424628	0.000468629	0.002642693	no
TAS2R4	-0.192945884	-3.534069453	0.000468931	0.002643701	no
PET117	-0.1929275	-3.533719706	0.000469528	0.002646376	no
FRMD6-AS1	-0.192919434	-3.533566251	0.000469791	0.002647161	no
MRPL43	-0.192905749	-3.533305909	0.000470236	0.002648977	no
MGME1	0.192819197	3.531659363	0.000473063	0.002664204	no
RASSF2	-0.192806656	-3.531420796	0.000473474	0.002665819	no
COL13A1	0.192798758	3.531270561	0.000473733	0.002666579	no
FAM131B	-0.192726245	-3.529891181	0.000476117	0.002679294	no
SEMA3A	0.192606412	3.527611782	0.00048008	0.002700892	no
ARSE	0.192575308	3.527020172	0.000481114	0.002706	no
BLOC1S3	0.192500905	3.52560503	0.000483595	0.002719244	no
IBA57	-0.192495352	-3.525499402	0.000483781	0.002719576	no
FARSA	-0.192486979	-3.52534015	0.000484061	0.002720439	no

POP4	0.192434654	3.52434499	0.000485815	0.002729581	no
TPRA1	0.192405054	3.523782033	0.00048681	0.002734455	no
LSM10	0.192390999	3.523514722	0.000487283	0.002736396	no
LIX1L	-0.19236826	-3.523082267	0.000488049	0.002739983	no
TMEM19	0.192347959	3.52269619	0.000488734	0.002743111	no
NTSR2	-0.192337389	-3.522495182	0.00048909	0.002744398	no
CRKL	-0.192320787	-3.522179449	0.000489652	0.002746829	no
RPL34-AS1	-0.192305415	-3.5218871	0.000490172	0.002749029	no
IQSEC3	-0.19229872	-3.521759784	0.000490398	0.002749583	no
LINC00339	0.19228063	3.521415762	0.000491011	0.002752302	no
FUBP3	-0.192269401	-3.521202223	0.000491392	0.002753719	no
CCDC120	-0.192258803	-3.521000685	0.000491752	0.002755016	no
PQBP1	-0.192251971	-3.520870768	0.000491984	0.002755597	no
C17orf103	0.192223036	3.52032052	0.000492968	0.002760388	no
MYADML2	-0.192201928	-3.519919118	0.000493687	0.002763693	no
ZBTB44	-0.192172383	-3.519357294	0.000494694	0.002768613	no
CPNE8	0.192107845	3.518130077	0.000496902	0.002780246	no
THTPA	-0.192097241	-3.517928439	0.000497266	0.002781557	no
ZFP37	-0.192068416	-3.51738035	0.000498256	0.002786368	no
CUL9	-0.192044165	-3.516919225	0.00049909	0.002790306	no
PLOD2	0.192033347	3.516713523	0.000499462	0.002790663	no
SNAI3-AS1	-0.192033134	-3.516709481	0.00049947	0.002790663	no
DHRS7	0.192031	3.516668902	0.000499543	0.002790663	no
SOX4	-0.191974444	-3.515593557	0.000501495	0.002800839	no
HNRNPF	0.191968446	3.515479506	0.000501702	0.00280127	no
ANAPC13	-0.191952433	-3.51517505	0.000502257	0.002803636	no
DUSP9	-0.191946058	-3.515053846	0.000502477	0.002803855	no
EIF2S3	0.191942286	3.514982115	0.000502608	0.002803855	no
TMEM230	0.191940002	3.514938687	0.000502687	0.002803855	no
PEAK1	-0.191905454	-3.51428184	0.000503886	0.00280981	no
CRIP3	-0.191891465	-3.514015862	0.000504372	0.002811791	no
C11orf48	0.191849506	3.513218126	0.000505832	0.002819185	no
LOC400043	0.191842933	3.51309316	0.000506061	0.002819185	no
RAB3IL1	0.191837769	3.51299498	0.000506241	0.002819185	no
PSME4	0.191836986	3.512980091	0.000506269	0.002819185	no
EDC3	-0.191834542	-3.512933622	0.000506354	0.002819185	no
PLK1S1	-0.191758757	-3.511492841	0.000509004	0.002833207	no
PRPF40B	-0.191722608	-3.510805622	0.000510273	0.002839534	no
DKFZP434I071	-0.191689113	-3.510168853	0.000511451	0.002845353	no
LOC286186	-0.191676512	-3.509929303	0.000511895	0.002846428	no
EFNA1	0.19167611	3.509921663	0.000511909	0.002846428	no
ABHD6	-0.191668626	-3.509779396	0.000512173	0.002847158	no
C16orf96	-0.191655247	-3.509525054	0.000512645	0.002849045	no
SRPK2	-0.191623287	-3.508917517	0.000513774	0.002854581	no
ARL2BP	0.191602359	3.508519682	0.000514515	0.00285758	no
ZNF738	-0.191600517	-3.508484657	0.00051458	0.00285758	no
C16orf72	0.191580569	3.508105464	0.000515287	0.002860766	no
UXS1	0.191560983	3.507733152	0.000515982	0.002863885	no
REEP6	-0.191543467	-3.507400211	0.000516604	0.002866598	no
KLC1	-0.191521435	-3.506981409	0.000517387	0.002870205	no
LAMA4	0.191506255	3.506692867	0.000517928	0.002871925	no

NFYA	-0.19150522	-3.506673181	0.000517965	0.002871925	no
TCTN3	0.19149788	3.506533677	0.000518226	0.002872634	no
SLC37A1	0.191416296	3.504982944	0.000521142	0.002888052	no
CYP27C1	-0.19139734	-3.504622654	0.000521822	0.002891072	no
MRPS21	-0.191384961	-3.504387353	0.000522266	0.002892788	no
ATF1	0.191380928	3.504310704	0.000522411	0.002892845	no
SLC4A4	-0.191357745	-3.503870075	0.000523244	0.002896712	no
LEPROTL1	-0.191324725	-3.503242484	0.000524433	0.002902546	no
EIF4A2	-0.191297681	-3.502728491	0.000525408	0.002907196	no
PIFO	0.19127538	3.50230464	0.000526214	0.002910193	no
FAM167A	-0.191275196	-3.502301132	0.000526221	0.002910193	no
TET2	-0.191264789	-3.502103343	0.000526597	0.002911109	no
ERAS	-0.191263126	-3.502071741	0.000526657	0.002911109	no
ARPP19	-0.191212927	-3.501117702	0.000528477	0.002920414	no
CSMD3	-0.191152775	-3.499974536	0.000530664	0.002931749	no
COL27A1	0.191133872	3.499615309	0.000531353	0.002934802	no
AHNAK2	0.191129443	3.499531131	0.000531515	0.00293494	no
PXDNL	0.191123053	3.499409692	0.000531748	0.002935474	no
PLEKHG3	-0.191112145	-3.499202405	0.000532147	0.002936919	no
UPF3A	-0.191102636	-3.499021703	0.000532494	0.002938082	no
ACADSB	-0.191065496	-3.498315912	0.000533853	0.002944826	no
RAB33B	0.191054448	3.49810596	0.000534258	0.002946303	no
LOC731424	0.191048169	3.49798664	0.000534489	0.002946817	no
SLC25A42	-0.191007438	-3.497212645	0.000535985	0.002954307	no
STK32C	-0.190964259	-3.496392136	0.000537575	0.002962099	no
ROM1	0.190961569	3.496341028	0.000537674	0.002962099	no
CMTM5	-0.190955808	-3.496231549	0.000537886	0.002962511	no
LRTOMT	-0.190947056	-3.496065238	0.000538209	0.002963162	no
APBB3	-0.190944763	-3.496021666	0.000538294	0.002963162	no
LOC100287042	-0.190941398	-3.495957741	0.000538418	0.002963162	no
ZBTB18	-0.190924009	-3.495627308	0.000539061	0.002965939	no
TNFRSF13C	-0.190865844	-3.4945221	0.000541215	0.002976423	no
HFE	0.19086509	3.494507777	0.000541243	0.002976423	no
SEPT5-GP1BB	-0.190855364	-3.494322973	0.000541604	0.002977035	no
LOC728407	-0.190854629	-3.494309005	0.000541632	0.002977035	no
TRPC3	-0.190821993	-3.493688907	0.000542845	0.002982941	no
PRMT8	-0.19081085	-3.493477184	0.00054326	0.002984322	no
LPP-AS2	0.190807782	3.493418892	0.000543374	0.002984322	no
IGDCC3	-0.190790103	-3.493082973	0.000544033	0.002987177	no
TSNARE1	-0.190748114	-3.492285193	0.000545601	0.002995021	no
THAP2	-0.190729177	-3.491925391	0.000546309	0.002998144	no
PLEKHG7	0.190711823	3.491595677	0.000546959	0.003000274	no
MYBPH	0.190709714	3.491555609	0.000547038	0.003000274	no
LETM1	-0.190707636	-3.491516129	0.000547116	0.003000274	no
LOC284395	-0.190674638	-3.490889207	0.000548354	0.003005838	no
OXR1	-0.190673142	-3.490860783	0.000548411	0.003005838	no
SLC38A1	-0.190667966	-3.490762444	0.000548605	0.003006137	no
SF3B2	-0.19065747	-3.490563029	0.000549	0.003006874	no
C6orf141	0.190656948	3.490553112	0.000549019	0.003006874	no
CSPG4P1Y	-0.190606503	-3.489594752	0.000550919	0.003016511	no
SUSD2	0.190600025	3.489471683	0.000551164	0.003017081	no

FAM134A	-0.190565042	-3.488807081	0.000552485	0.003023015	no
CSNK1G3	0.19056389	3.488785193	0.000552529	0.003023015	no
ACBD5	-0.190534063	-3.48821855	0.000553658	0.003028423	no
CCRN4L	0.190513194	3.487822107	0.00055445	0.00303198	no
PLA2G10	-0.190477339	-3.487140977	0.000555812	0.003038656	no
CPEB3	-0.190471448	-3.487029074	0.000556036	0.003039109	no
GGCT	0.19044784	3.486580598	0.000556935	0.003043248	no
HSF2BP	-0.190406705	-3.485799194	0.000558505	0.003051049	no
ERH	-0.190374154	-3.48518086	0.00055975	0.003057073	no
LINC00346	0.190367222	3.4850492	0.000560015	0.003057175	no
LOC644656	-0.190366238	-3.485030494	0.000560053	0.003057175	no
OSBPL3	0.190311654	3.48399368	0.000562147	0.003067829	no
PTOV1	-0.190304314	-3.483854245	0.00056243	0.00306859	no
PLOD3	0.190287836	3.483541264	0.000563064	0.00307127	no
CYP2A6	-0.190281097	-3.483413255	0.000563323	0.003071905	no
MKRN2	-0.190272596	-3.483251792	0.000563651	0.003072911	no
LINC00588	0.190267649	3.483157818	0.000563841	0.003073171	no
TSC2	-0.190245697	-3.482740867	0.000564688	0.003077006	no
NOTCH1	-0.190229972	-3.482442191	0.000565295	0.003079534	no
PHOSPHO2-KLH	-0.190225158	-3.482350742	0.000565481	0.003079767	no
TEX40	-0.190219534	-3.482243924	0.000565699	0.003080171	no
ZNF727	-0.19021038	-3.482070053	0.000566053	0.003081318	no
IFRD2	0.190191073	3.481703351	0.0005668	0.003084604	no
GPR75-ASB3	-0.190167972	-3.481264589	0.000567696	0.003088695	no
ZC2HC1A	-0.190161968	-3.481150557	0.000567929	0.003088948	no
KLHL21	0.190159368	3.481101165	0.000568029	0.003088948	no
DMRT3	0.190134766	3.480633915	0.000568985	0.003093361	no
VAV1	0.190122636	3.480403523	0.000569457	0.003095143	no
DUSP11	0.190116193	3.480281161	0.000569707	0.003095619	no
AKIRIN1	0.190112982	3.480220168	0.000569832	0.003095619	no
CTNND2	-0.190075178	-3.479502186	0.000571306	0.003102838	no
GIPC3	0.190037088	3.478778788	0.000572794	0.003110134	no
FEZ1	-0.190001127	-3.478095828	0.000574202	0.003116992	no
JDP2	0.189969479	3.477494816	0.000575444	0.003122945	no
LRFN5	-0.189942494	-3.476982337	0.000576505	0.003127913	no
FBXL22	-0.18989076	-3.475999894	0.000578544	0.003138183	no
GABRD	-0.189886283	-3.475914888	0.00057872	0.003138349	no
ELMOD2	0.189880699	3.475808842	0.000578941	0.003138753	no
DLL3	-0.189859058	-3.475397886	0.000579796	0.003142598	no
NR4A2	0.18982921	3.474831093	0.000580978	0.003147624	no
BCL2A1	0.189828242	3.474812698	0.000581016	0.003147624	no
POLDIP3	-0.189771174	-3.473729047	0.000583282	0.003159103	no
APBA2	-0.189759963	-3.473516148	0.000583728	0.003160384	no
RPL4	-0.18975784	-3.473475836	0.000583813	0.003160384	no
PPP2R2C	-0.189750766	-3.473341528	0.000584095	0.003161112	no
PSAT1	-0.189735154	-3.473045075	0.000584717	0.003163682	no
LRP11	-0.189711569	-3.47259724	0.000585658	0.003167208	no
ACTR2	0.189711435	3.472594692	0.000585663	0.003167208	no
RNF187	-0.189655632	-3.471535117	0.000587895	0.003178478	no
ERP27	0.189646158	3.471355234	0.000588275	0.003179731	no
FEM1A	-0.189632542	-3.471096705	0.000588821	0.003181883	no

CNTN1	-0.189622507	-3.47090617	0.000589224	0.003183259	no
EP400	-0.189610041	-3.470669492	0.000589724	0.003185163	no
C1QTNF4	-0.189603633	-3.47054782	0.000589982	0.003185754	no
ARFIP1	0.189570924	3.469926783	0.000591298	0.003192059	no
TMEM121	-0.189505683	-3.468688112	0.000593932	0.003204823	no
CTSK	0.189504961	3.468674403	0.000593961	0.003204823	no
PABPC5	-0.189501113	-3.468601357	0.000594116	0.003204858	no
USH1C	-0.189478196	-3.468166264	0.000595044	0.003209058	no
C15orf52	0.189422199	3.467103153	0.000597317	0.003220508	no
CMTM3	0.189398784	3.466658617	0.00059827	0.003224836	no
ZBTB8OS	0.189337282	3.465491064	0.000600779	0.003236915	no
ATL1	-0.189334014	-3.465429015	0.000600913	0.003236915	no
CDK17	-0.189332797	-3.465405911	0.000600963	0.003236915	no
NGRN	-0.189326007	-3.465277017	0.000601241	0.003237093	no
MRPL48	-0.189324626	-3.465250803	0.000601297	0.003237093	no
FOXN3-AS1	0.189313487	3.465039347	0.000601753	0.003237761	no
ALPK3	0.189312739	3.465025147	0.000601784	0.003237761	no
CRYL1	-0.189310553	-3.464983645	0.000601873	0.003237761	no
LOC100129518	-0.189302664	-3.464833878	0.000602196	0.003238688	no
DEPDC5	-0.18925607	-3.463949374	0.000604108	0.003247451	no
TREX2	0.189255589	3.463940254	0.000604128	0.003247451	no
HECW1	-0.189194384	-3.462778421	0.000606648	0.003260181	no
MYLK-AS1	-0.189176569	-3.46244026	0.000607383	0.003263316	no
GALNT7	0.189163875	3.4621993	0.000607907	0.003265317	no
APOC1P1	0.189159113	3.46210892	0.000608104	0.003265558	no
RAB9B	-0.189109829	-3.461173416	0.000610145	0.003275699	no
C6orf203	-0.189078495	-3.46057867	0.000611445	0.003281862	no
ING5	-0.189071999	-3.460455363	0.000611715	0.003282491	no
RASGRF1	-0.189061233	-3.460251023	0.000612163	0.003284073	no
ZNF563	0.18903148	3.459686291	0.000613402	0.003289898	no
ARMCX1	-0.189008257	-3.459245509	0.00061437	0.00329427	no
MTMR11	0.188957913	3.458289991	0.000616475	0.00330473	no
MFSD6	-0.188937306	-3.457898891	0.000617338	0.003308533	no
UBL3	-0.188896879	-3.457131612	0.000619035	0.003316801	no
R3HCC1	-0.188886131	-3.456927633	0.000619487	0.003317685	no
FBLN5	0.18888339	3.456875607	0.000619602	0.003317685	no
SLCO3A1	0.188881939	3.456848065	0.000619664	0.003317685	no
KLHDC2	-0.188873692	-3.456691561	0.000620011	0.003318263	no
SLC25A45	0.188872036	3.456660125	0.00062008	0.003318263	no
ME2	0.188855392	3.456344243	0.000620781	0.003321188	no
GALNT9	-0.188848608	-3.456215507	0.000621067	0.00332189	no
IL17RE	0.188803337	3.455356341	0.000622978	0.003331284	no
PCBD1	0.188763111	3.454592945	0.000624681	0.003339558	no
ZNF354C	-0.188757124	-3.454479326	0.000624935	0.003340084	no
USP32P2	-0.188689294	-3.453192129	0.000627817	0.003354654	no
CPT2	0.188683776	3.453087411	0.000628052	0.003355076	no
C22orf34	0.188655035	3.452542011	0.000629278	0.003360786	no
MBLAC2	-0.188640689	-3.452269779	0.00062989	0.003362741	no
NR3C2	-0.188637899	-3.452216837	0.000630009	0.003362741	no
CAHM	-0.188635472	-3.452170775	0.000630113	0.003362741	no
ZP3	0.188584389	3.451201449	0.000632299	0.003373569	no

SEPN1	0.188569516	3.45091922	0.000632937	0.003376134	no
ZNF236	-0.188561204	-3.450761514	0.000633293	0.003377198	no
KRT7	0.188491747	3.449443582	0.000636281	0.003392291	no
PLVAP	0.188457318	3.44879032	0.000637767	0.00339937	no
RBM4B	-0.188439095	-3.448444553	0.000638555	0.003402725	no
MORC2	-0.188435214	-3.448370915	0.000638723	0.003402776	no
PVRL3-AS1	-0.188372719	-3.447185161	0.000641432	0.003416362	no
PYGB	-0.188351916	-3.446790478	0.000642337	0.00342033	no
DAZAP1	0.188338105	3.446528444	0.000642937	0.003422682	no
LTF	0.188334334	3.446456891	0.000643102	0.003422709	no
FBXL20	-0.188273122	-3.445295557	0.000645772	0.00343607	no
TMED4	0.188269155	3.445220306	0.000645945	0.003436141	no
RREB1	0.188247497	3.444809408	0.000646893	0.003440001	no
C11orf91	0.188245256	3.44476689	0.000646991	0.003440001	no
ADRA2B	0.18822794	3.444438387	0.00064775	0.003443182	no
LOC100505648	-0.188224244	-3.44436827	0.000647912	0.003443192	no
TEX9	0.188208313	3.444066033	0.00064861	0.003446053	no
TMCC2	-0.188106823	-3.442140712	0.000653078	0.003468935	no
ABHD14A	-0.188089272	-3.441807762	0.000653854	0.003472196	no
USP9X	0.188045169	3.440971135	0.000655807	0.003481705	no
RTF1	-0.188031193	-3.440706029	0.000656427	0.003484136	no
DUSP5	0.187996603	3.44004989	0.000657963	0.003491429	no
RAB3B	-0.187942025	-3.439014613	0.000660395	0.003502849	no
GPR56	-0.187940979	-3.438994782	0.000660441	0.003502849	no
ACTR1A	-0.187882708	-3.437889497	0.000663046	0.003515798	no
ENG	0.187873299	3.437711027	0.000663468	0.003517166	no
DNAJC4	0.187852208	3.437310998	0.000664414	0.003521311	no
UQCRC1	-0.187831448	-3.436917228	0.000665346	0.003525382	no
LOC100130451	-0.187810255	-3.43651527	0.000666299	0.003529561	no
HEATR2	0.187792733	3.436182938	0.000667088	0.003532868	no
ZNF185	0.187784064	3.436018506	0.000667478	0.003534066	no
KIAA1328	-0.187764933	-3.435655663	0.000668341	0.003537432	no
LINC00263	-0.187762667	-3.435612683	0.000668443	0.003537432	no
BAHCC1	-0.187745055	-3.435278653	0.000669238	0.003540629	no
ZNF714	-0.18774199	-3.43522052	0.000669377	0.003540629	no
ZNF830	-0.1877098	-3.434610005	0.000670833	0.003547458	no
TAF4	-0.187702019	-3.434462445	0.000671185	0.003548448	no
NAP1L2	-0.187625445	-3.433010196	0.000674662	0.003565953	no
CBR3	0.18761748	3.432859135	0.000675025	0.003566799	no
GPT	-0.18761417	-3.432796377	0.000675175	0.003566799	no
CTCF	-0.187610994	-3.432736146	0.00067532	0.003566799	no
PRRT1	-0.187533881	-3.431273754	0.000678842	0.003584517	no
HIST2H2BA	0.187507606	3.430775476	0.000680045	0.003589991	no
FBXL16	-0.187465059	-3.429968655	0.000681999	0.003599419	no
LOC283174	-0.187460645	-3.42988495	0.000682202	0.003599606	no
KCNJ9	-0.187452215	-3.429725094	0.000682589	0.003600768	no
MOSPD1	0.187394679	3.428634069	0.000685241	0.00361387	no
SYP	-0.187380571	-3.428366546	0.000685893	0.00361642	no
PDCL	-0.187374945	-3.428259878	0.000686153	0.003616904	no
ERAL1	-0.187369912	-3.428164431	0.000686386	0.003617243	no
FXYD2	0.187350418	3.427794797	0.000687288	0.003621109	no

AFF3	-0.187326598	-3.427343126	0.000688392	0.003626035	no
VAT1	0.187316496	3.427151583	0.00068886	0.003627614	no
KCNK7	-0.187310911	-3.427045685	0.000689119	0.00362809	no
BCCIP	-0.187291864	-3.42668453	0.000690004	0.003631858	no
PQLC1	0.187276539	3.426393945	0.000690717	0.003634718	no
CRTAC1	-0.187255527	-3.425995538	0.000691695	0.003638523	no
OTUD7A	-0.187253731	-3.425961489	0.000691778	0.003638523	no
KRBA2	-0.187248892	-3.425869746	0.000692004	0.003638818	no
NKX3-1	0.187173978	3.424449356	0.000695503	0.003656322	no
MTRNR2L6	-0.187168501	-3.424345512	0.000695759	0.003656776	no
SLC30A1	0.187135009	3.423710521	0.000697329	0.003664132	no
LMAN2	0.187122733	3.423477781	0.000697905	0.003666263	no
MBD5	-0.187116291	-3.423355635	0.000698208	0.003666957	no
CTSH	0.187098462	3.423017619	0.000699046	0.003670462	no
UBXN11	0.187079369	3.422655637	0.000699945	0.003674282	no
NRBF2	0.187044435	3.42199335	0.000701591	0.003681702	no
MIR143HG	0.187039054	3.421891331	0.000701845	0.003681702	no
TMEM60	0.187038489	3.421880618	0.000701872	0.003681702	no
KIAA2026	-0.186969228	-3.420567571	0.000705149	0.003697989	no
RGN	0.186949818	3.420199616	0.00070607	0.003701915	no
TWF1	-0.186936503	-3.419947203	0.000706702	0.003704326	no
HEXIM2	-0.186912465	-3.419491497	0.000707845	0.003709413	no
IMMP1L	-0.186900862	-3.419271555	0.000708397	0.003711402	no
TP53INP1	0.186892888	3.419120381	0.000708777	0.003712487	no
TIMD4	0.186869858	3.418683822	0.000709875	0.003717331	no
ZMYM3	-0.186848925	-3.418287013	0.000710874	0.003721108	no
TNFSF15	0.186847498	3.41825995	0.000710943	0.003721108	no
C5orf56	0.186746323	3.416342114	0.000715792	0.003745575	no
HS6ST1	0.186704023	3.415540333	0.000717828	0.003755316	no
ZSWIM5	-0.186695657	-3.415381752	0.000718231	0.003756511	no
UAP1	0.186665434	3.414808906	0.00071969	0.003763226	no
NR2C2	-0.186627635	-3.41409245	0.000721519	0.00377187	no
C1GALT1C1	0.186614785	3.4138489	0.000722141	0.003774206	no
EIF4B	-0.186600622	-3.413580456	0.000722828	0.003776877	no
LOC284454	0.186571807	3.41303432	0.000724227	0.003783268	no
WBP5	0.186557351	3.412760337	0.00072493	0.003786019	no
ZNF280B	-0.186545375	-3.41253335	0.000725513	0.003788143	no
HDAC10	0.186499999	3.41167336	0.000727725	0.003798769	no
WARS2	0.186486526	3.411418017	0.000728383	0.00380128	no
NYAP2	-0.186457695	-3.410871622	0.000729793	0.003807713	no
PRR14L	-0.186452778	-3.410778421	0.000730033	0.003808045	no
NR1H3	-0.186398095	-3.409742094	0.000732715	0.003821106	no
LSM11	-0.186381161	-3.409421182	0.000733547	0.003824519	no
CD27-AS1	-0.18636954	-3.409200951	0.000734119	0.003826571	no
PLEKHA2	0.186356448	3.40895285	0.000734764	0.003829002	no
DNAJC21	0.186352722	3.408882233	0.000734947	0.00382903	no
CPD	0.186340337	3.408647529	0.000735558	0.003831281	no
MEG3	-0.186333586	-3.408519591	0.00073589	0.003831672	no
HIGD1A	-0.186331424	-3.408478632	0.000735997	0.003831672	no
ZBTB12	-0.186327972	-3.408413218	0.000736167	0.003831672	no
RSRC2	-0.186269704	-3.407309027	0.000739048	0.003845731	no

PELI2	-0.186263647	-3.407194236	0.000739348	0.003846361	no
FOXC2	0.186259946	3.40712411	0.000739531	0.003846384	no
NUAK2	0.186256323	3.40705545	0.00073971	0.003846387	no
TPM1	-0.186247902	-3.406895878	0.000740128	0.003847626	no
PRKG1-AS1	0.186238085	3.406709862	0.000740615	0.003849226	no
ARHGAP1	0.186208949	3.406157737	0.000742062	0.003854947	no
ARFGAP2	-0.186208697	-3.406152973	0.000742074	0.003854947	no
HID1	-0.186201103	-3.406009079	0.000742452	0.003855976	no
PANK1	-0.186189561	-3.405790354	0.000743026	0.003858026	no
C12orf73	-0.186159876	-3.405227873	0.000744505	0.00386477	no
YEATS2	-0.186145092	-3.404947738	0.000745242	0.003867663	no
PACRGL	-0.186135993	-3.404775324	0.000745696	0.003869086	no
LOC10013235€	-0.186129656	-3.404655243	0.000746013	0.003869794	no
FAM106A	-0.186077714	-3.403671055	0.000748611	0.003881755	no
SH2D3A	0.186076351	3.40364522	0.00074868	0.003881755	no
FGD1	-0.186034263	-3.402847761	0.000750792	0.003891766	no
STXBP5L	-0.186007354	-3.402337907	0.000752145	0.00389784	no
C6orf170	-0.185990149	-3.402011939	0.000753011	0.003901389	no
ELP5	-0.18596271	-3.40149205	0.000754395	0.003907615	no
SPATA17	0.185932313	3.400916139	0.00075593	0.003914626	no
FOXO3B	-0.185925247	-3.400782279	0.000756288	0.003915533	no
C5orf54	-0.185898379	-3.400273239	0.000757648	0.00392163	no
ARHGAP35	-0.18587936	-3.399912907	0.000758612	0.003925676	no
DROSHA	-0.185870831	-3.399751317	0.000759045	0.00392697	no
URM1	-0.185864713	-3.399635416	0.000759355	0.003927631	no
ANGPTL5	0.185784918	3.398123706	0.000763417	0.003947689	no
38777	-0.185767227	-3.397788543	0.00076432	0.003951409	no
MALL	0.185736307	3.397202793	0.000765901	0.003958629	no
XRN2	0.185704711	3.396604258	0.00076752	0.003966041	no
C18orf32	-0.185647922	-3.39552848	0.000770437	0.003979203	no
TUBB4A	-0.185646259	-3.395496978	0.000770523	0.003979203	no
SPATA25	-0.185640319	-3.395384455	0.000770828	0.003979203	no
MBP	0.185638383	3.395347785	0.000770928	0.003979203	no
IGIP	-0.185637128	-3.395324004	0.000770993	0.003979203	no
MEGF11	-0.185620879	-3.395016197	0.00077183	0.003982567	no
TMEM178A	-0.18561051	-3.394819788	0.000772364	0.003984369	no
DSEL	-0.185581759	-3.394275177	0.000773849	0.003991068	no
TTPA	-0.185536935	-3.393426107	0.000776168	0.004002069	no
AQP9	0.18550563	3.392833137	0.000777791	0.004009342	no
PAFAH1B3	-0.185502545	-3.392774713	0.000777952	0.004009342	no
PSMB2	0.185467469	3.392110331	0.000779775	0.004017775	no
HDHD2	-0.18545816	-3.391934013	0.000780259	0.004019308	no
MAPK11	-0.185447096	-3.391724452	0.000780836	0.004021312	no
ADRBK2	-0.185439221	-3.391575284	0.000781246	0.004022462	no
LARS2	-0.185423623	-3.39127985	0.000782059	0.004025501	no
OR51E1	0.185420721	3.391224885	0.000782211	0.004025501	no
SDS	0.18540356	3.390899841	0.000783107	0.004026794	no
NRP2	0.185403515	3.390898996	0.000783109	0.004026794	no
TCF20	-0.185402849	-3.390886373	0.000783144	0.004026794	no
NCALD	-0.185401554	-3.390861861	0.000783212	0.004026794	no
PHAX	-0.185376811	-3.390393215	0.000784505	0.004031724	no

CRB1	-0.185376041	-3.39037863	0.000784546	0.004031724	no
POLB	-0.185358922	-3.390054395	0.000785442	0.004035365	no
HTR1A	-0.185350813	-3.389900806	0.000785867	0.004036018	no
ARRDC4	0.185349331	3.389872739	0.000785945	0.004036018	no
TNFRSF10B	0.18531095	3.38914582	0.000787959	0.004045394	no
AP1S3	0.185264221	3.388260826	0.000790417	0.004057048	no
GNE	-0.185242319	-3.387846035	0.000791572	0.004062005	no
GAN	-0.185209333	-3.387221332	0.000793314	0.004069973	no
COL6A3	0.185202161	3.387085492	0.000793694	0.004070947	no
HOMER1	-0.185178171	-3.386631168	0.000794963	0.004076487	no
PHF6	-0.185116686	-3.385466802	0.000798226	0.004091363	no
TM9SF2	0.185114291	3.385421439	0.000798354	0.004091363	no
FAM199X	-0.185109978	-3.385339777	0.000798583	0.004091363	no
MS4A2	0.185109176	3.385324572	0.000798626	0.004091363	no
HSDL1	-0.185073114	-3.38464168	0.000800546	0.004100224	no
SETD5-AS1	-0.185063089	-3.384451839	0.000801081	0.004101985	no
SCN3A	-0.185012226	-3.383488668	0.000803799	0.004114921	no
PEG3	-0.184997615	-3.383211992	0.000804581	0.004117944	no
PLD6	-0.184990313	-3.383073732	0.000804972	0.004118966	no
CECR6	-0.184979903	-3.382876614	0.00080553	0.00412084	no
CX3CR1	0.184945325	3.382221855	0.000807386	0.004129352	no
NOL12	-0.184939949	-3.382120062	0.000807675	0.004129847	no
CYP17A1	-0.184924796	-3.381833143	0.00080849	0.00413303	no
DNM3	-0.184898795	-3.3813408	0.00080989	0.004138413	no
CSK	0.184898088	3.381327423	0.000809928	0.004138413	no
LOC254128	-0.184856397	-3.380538013	0.000812178	0.004148922	no
FAM198B	0.184840478	3.3802366	0.000813038	0.00415233	no
LOC100506343	-0.184824474	-3.379933577	0.000813904	0.004155765	no
LOC442497	0.184793035	3.379338323	0.000815608	0.004163475	no
TPMT	0.184789073	3.379263307	0.000815823	0.004163583	no
EMID1	0.184737987	3.378296066	0.000818599	0.004176758	no
PFAS	-0.184620579	-3.376073239	0.000825012	0.004208248	no
C9orf129	-0.184617836	-3.376021312	0.000825162	0.004208248	no
RPS23	-0.184604975	-3.375777831	0.000825867	0.004210846	no
POU4F1-AS1	-0.18458051	-3.375314682	0.000827211	0.004216695	no
BCAR1	-0.184547127	-3.374682692	0.000829047	0.004224768	no
LOC100302640	-0.184543152	-3.374607446	0.000829266	0.004224768	no
ETV1	-0.184541005	-3.374566795	0.000829384	0.004224768	no
UBE2Q2P2	-0.184527425	-3.374309716	0.000830133	0.004227579	no
MGAT5B	-0.184511676	-3.374011571	0.000831001	0.004231	no
ZNF501	-0.18450145	-3.373817992	0.000831566	0.004232242	no
ASCL5	-0.184500122	-3.373792854	0.000831639	0.004232242	no
ZNF720	-0.184418244	-3.372242902	0.000836172	0.004253767	no
MRPS10	0.184415868	3.372197917	0.000836304	0.004253767	no
CSRP2BP	-0.184413011	-3.37214384	0.000836463	0.004253767	no
NAV1	-0.184375523	-3.371434218	0.000838546	0.004263355	no
LINC00663	-0.184316805	-3.370322741	0.00084182	0.004278846	no
UBQLN2	-0.18431018	-3.370197338	0.00084219	0.004278846	no
HCG4B	0.184310164	3.37019704	0.000842191	0.004278846	no
RRP36	0.184287188	3.369762152	0.000843475	0.00428436	no
JAKMIP1	-0.184245416	-3.368971489	0.000845815	0.004295231	no

SLC25A20	0.184233249	3.368741199	0.000846498	0.004297683	no
NDUFA6	-0.184225611	-3.368596621	0.000846927	0.004298356	no
ACER2	0.184223762	3.368561629	0.000847031	0.004298356	no
CASK	-0.184050421	-3.365280872	0.000856819	0.004346999	no
FAM200B	-0.184023972	-3.364780302	0.000858321	0.004353595	no
ELAVL2	-0.184015346	-3.364617058	0.000858812	0.004355055	no
LRRC29	0.183976439	3.363880739	0.000861028	0.004365263	no
TNKS1BP1	0.183969074	3.363741353	0.000861448	0.004366362	no
ITM2B	0.18395072	3.363394024	0.000862495	0.004370641	no
NECAP2	0.183935006	3.363096634	0.000863393	0.00437416	no
EXOC3L2	0.183885142	3.362152995	0.000866248	0.00438759	no
LOC344887	0.183809917	3.360729484	0.000870572	0.004408449	no
SNHG8	-0.183779781	-3.360159227	0.000872309	0.004416208	no
BEND7	-0.183773601	-3.360042289	0.000872666	0.004416973	no
CCDC144B	-0.183756592	-3.359720435	0.000873649	0.004420905	no
LOC652276	-0.183696849	-3.358589972	0.000877108	0.004436897	no
PCDH7	-0.183694882	-3.358552747	0.000877222	0.004436897	no
GFRA1	-0.183678968	-3.358251618	0.000878146	0.004440524	no
FPR2	0.183673153	3.358141594	0.000878483	0.004441186	no
SLC6A8	-0.183668889	-3.358060917	0.000878731	0.004441394	no
ACVR2B-AS1	-0.18365896	-3.357873034	0.000879308	0.004443266	no
BRWD3	-0.183639613	-3.357506969	0.000880434	0.004447907	no
LOC100128750	0.183635834	3.357435463	0.000880654	0.004447973	no
SAMM50	-0.183615707	-3.357054645	0.000881826	0.004452849	no
CLTA	-0.183578176	-3.356344533	0.000884016	0.00446286	no
SND1-IT1	-0.183566787	-3.356129036	0.000884682	0.004464852	no
CBX1	-0.183564317	-3.356082306	0.000884826	0.004464852	no
RPL23AP82	0.183442901	3.353785143	0.000891953	0.004499755	no
WBP4	-0.183401246	-3.352997081	0.00089441	0.004510601	no
LOC643733	0.183399338	3.352960984	0.000894522	0.004510601	no
SNAP91	-0.183362864	-3.352270954	0.000896679	0.004520416	no
APOC1	0.183341243	3.351861932	0.00089796	0.004525811	no
DPP10	-0.183301283	-3.351105977	0.000900331	0.0045367	no
C8orf44-SGK3	-0.183291784	-3.350926274	0.000900896	0.004538481	no
LPIN3	0.18325809	3.350288872	0.000902901	0.004547517	no
DLC1	0.183238244	3.349913442	0.000904085	0.004552409	no
GREB1L	-0.183230179	-3.34976088	0.000904566	0.004553765	no
GLUD1P3	-0.183204277	-3.34927091	0.000906113	0.004559017	no
TMED2	0.183202709	3.349241241	0.000906206	0.004559017	no
FRMD8	0.183202058	3.34922893	0.000906245	0.004559017	no
FRRS1L	-0.183191357	-3.34902651	0.000906885	0.004561168	no
ANAPC2	-0.183141889	-3.348090767	0.000909849	0.004574807	no
GLB1	0.183138991	3.348035939	0.000910023	0.004574807	no
DKFZp434J022	-0.183120975	-3.347695152	0.000911105	0.004579174	no
CLEC4F	-0.183111903	-3.347523547	0.00091165	0.004580843	no
NEGR1	-0.183100361	-3.347305222	0.000912344	0.004583258	no
CAPZA2	0.183077597	3.346874636	0.000913714	0.004589069	no
FBRSL1	-0.183025317	-3.345885769	0.000916868	0.004603178	no
ZNF691	0.183023937	3.345859676	0.000916951	0.004603178	no
FLJ22184	-0.182999706	-3.345401349	0.000918417	0.004609457	no
KLF3	0.182924455	3.343978066	0.000922982	0.004631285	no

STXBP1	-0.182882843	-3.343191035	0.000925515	0.004642912	no
SRCIN1	-0.182843246	-3.342442148	0.000927931	0.004653948	no
DDAH1	-0.182783905	-3.34131988	0.000931563	0.004671075	no
ADIPOR1	0.182768913	3.341036349	0.000932483	0.004674596	no
PIK3R2	-0.18275177	-3.340712135	0.000933536	0.004678782	no
LOC728606	-0.182746911	-3.340620255	0.000933834	0.004679187	no
IFIT1	0.182710807	3.339937462	0.000936056	0.004689224	no
HOXB7	0.182682016	3.339393	0.00093783	0.00469702	no
PCDH15	-0.182620637	-3.338232288	0.000941625	0.004714924	no
FXYD7	-0.182594239	-3.337733104	0.000943261	0.004722016	no
SLC8A1	0.182511692	3.33617217	0.000948394	0.004746607	no
ACVR1	0.182497542	3.335904608	0.000949276	0.004748822	no
DGCR5	-0.182497509	-3.335903983	0.000949278	0.004748822	no
KCTD4	-0.182370689	-3.333506044	0.000957222	0.004787444	no
PPFIBP1	0.182354618	3.333202187	0.000958233	0.004791385	no
HECTD2	-0.182343951	-3.333000493	0.000958904	0.004792001	no
LINC00617	-0.182340889	-3.332942607	0.000959097	0.004792001	no
LOC338651	-0.182339381	-3.332914086	0.000959192	0.004792001	no
KCNA2	-0.1823385	-3.332897425	0.000959247	0.004792001	no
PIK3C2B	-0.182314404	-3.332441846	0.000960767	0.004798474	no
FAM46A	0.182303603	3.332237634	0.000961448	0.004800763	no
KLRG1	0.182297578	3.332123727	0.000961829	0.004801547	no
FAM86C1	0.18227862	3.331765289	0.000963027	0.004806411	no
PCOLCE	0.182271514	3.33163093	0.000963476	0.004807537	no
LINC00032	-0.182262245	-3.33145569	0.000964062	0.004808608	no
LCORL	-0.182261048	-3.331433055	0.000964138	0.004808608	no
ZFYVE21	-0.182252947	-3.331279908	0.000964651	0.00481005	no
PPP1R9B	-0.182237792	-3.33099338	0.000965611	0.004813721	no
DTX1	-0.182216089	-3.330583049	0.000966988	0.004819465	no
RPA3	0.182187523	3.330042994	0.000968802	0.004827389	no
MYH15	-0.182181228	-3.329923992	0.000969202	0.004827517	no
C19orf24	0.182180054	3.329901781	0.000969277	0.004827517	no
OLFML2A	0.182145975	3.329257515	0.000971447	0.004837203	no
PLD1	0.182134773	3.32904573	0.000972161	0.004839638	no
MED12L	-0.182114522	-3.328662893	0.000973454	0.004844949	no
DNAJC13	0.182101802	3.328422429	0.000974266	0.004847871	no
TMEM209	0.182051055	3.327463072	0.000977514	0.004862303	no
ZNF638	-0.182049243	-3.327428822	0.00097763	0.004862303	no
NLRP6	0.182045891	3.327365465	0.000977845	0.004862303	no
TBC1D19	0.182039484	3.327244341	0.000978256	0.004863221	no
KCNJ10	-0.182016481	-3.32680949	0.000979733	0.004869435	no
C19orf10	0.181978121	3.326084358	0.0009822	0.004879686	no
MLLT4-AS1	-0.181977349	-3.326069758	0.000982249	0.004879686	no
VDAC3	-0.181957917	-3.325702433	0.000983501	0.004884493	no
MSANTD3-TMEF	-0.181955277	-3.325652523	0.000983672	0.004884493	no
LCTL	0.181916528	3.324920057	0.000986173	0.004895724	no
SPARCL1	-0.181913183	-3.32485683	0.000986389	0.004895724	no
PLCD1	0.181906516	3.324730801	0.00098682	0.004896733	no
RASL11A	0.181881678	3.3242613	0.000988427	0.004903577	no
DET1	-0.18187153	-3.324069472	0.000989085	0.004905706	no
PTCH1	-0.181808415	-3.322876492	0.000993183	0.004924893	no

CCSAP	-0.181790026	-3.3225289	0.000994379	0.004929691	no
LOC646268	0.18176427	3.322042086	0.000996058	0.004936873	no
SPAG11B	0.181722243	3.321247735	0.000998803	0.004949335	no
C4A	0.181718579	3.321178481	0.000999042	0.004949381	no
SYT16	-0.181640973	-3.319711723	0.00100413	0.004973439	no
COG1	-0.181622363	-3.319359987	0.001005353	0.004978352	no
CACNG1	-0.181615743	-3.319234886	0.001005789	0.004979361	no
LOC100130872	0.181589993	3.318748223	0.001007485	0.004986608	no
LOC100499183	0.1815653	3.318281543	0.001009113	0.00499352	no
DVL2	-0.181512681	-3.317287104	0.001012592	0.005009582	no
WNK2	-0.181505906	-3.317159072	0.001013041	0.005010648	no
CRYZL1	-0.181494862	-3.316950367	0.001013773	0.005013114	no
LOC728218	-0.18147403	-3.316556669	0.001015155	0.005018793	no
RND2	-0.181466372	-3.316411947	0.001015663	0.005020151	no
SAMD10	-0.181461666	-3.316323015	0.001015976	0.005020542	no
NDN	-0.181431461	-3.315752209	0.001017984	0.005029309	no
SLC23A2	-0.181423317	-3.315598314	0.001018526	0.00503083	no
ZBTB6	-0.18141247	-3.315393338	0.001019248	0.005033241	no
FOXRED2	-0.181361862	-3.314436993	0.001022625	0.005048492	no
RARS	0.181359148	3.314385697	0.001022806	0.005048492	no
NTRK2	-0.181350319	-3.314218856	0.001023397	0.005049624	no
GJB3	0.181348688	3.314188042	0.001023506	0.005049624	no
CD63	0.181333615	3.313903209	0.001024514	0.005053439	no
MAGED1	-0.181327547	-3.313788555	0.00102492	0.005054283	no
KIAA1109	-0.181321047	-3.313665716	0.001025356	0.00505527	no
SUCLG1	-0.181303387	-3.313332021	0.001026539	0.005059944	no
PDS5B	-0.181240313	-3.312140186	0.001030777	0.005079668	no
LOC100506388	0.181233232	3.312006386	0.001031254	0.005080852	no
PCCB	-0.181170385	-3.310818881	0.001035494	0.005100574	no
CACYBP	-0.181162822	-3.310675981	0.001036006	0.005101924	no
DMRTC1	0.181155041	3.310528954	0.001036532	0.005103346	no
FAM22A	-0.181145324	-3.310345353	0.00103719	0.005105401	no
EEF1D	-0.181141857	-3.310279859	0.001037424	0.005105401	no
ANAPC1P1	0.181074777	3.309012424	0.001041976	0.005126625	no
PAR1	-0.181021296	-3.308001997	0.001045618	0.005143366	no
ANKS1B	-0.181005285	-3.307699491	0.00104671	0.005147562	no
LTBP3	0.18099876	3.307576208	0.001047156	0.005148576	no
LRRCC1	0.180991549	3.307439977	0.001047648	0.005149819	no
MAP3K10	-0.180964471	-3.306928401	0.0010495	0.005157742	no
TMEM257	-0.180952346	-3.306699324	0.00105033	0.005160641	no
FGFBP3	-0.180945364	-3.306567416	0.001050808	0.005161811	no
LRRC15	0.180925945	3.30620056	0.00105214	0.00516717	no
H1F0	-0.180910722	-3.305912957	0.001053185	0.005171119	no
ZSCAN18	-0.180899765	-3.305705963	0.001053937	0.005172641	no
GPR179	-0.180899201	-3.305695297	0.001053976	0.005172641	no
GLRB	-0.180882925	-3.30538782	0.001055095	0.00517695	no
ZIM2	-0.180875507	-3.305247687	0.001055605	0.00517759	no
ECSCR	0.18087061	3.305155168	0.001055942	0.00517759	no
CACNG4	-0.180870524	-3.305153538	0.001055948	0.00517759	no
CHKA	-0.180843506	-3.304643128	0.001057809	0.005185532	no
BOP1	-0.180821804	-3.304233163	0.001059306	0.005191395	no

CNNM1	-0.180819168	-3.304183351	0.001059488	0.005191395	no
KHDC1L	-0.180813231	-3.304071195	0.001059898	0.00519222	no
SLC43A3	0.180783071	3.303501451	0.001061983	0.005201247	no
CLDN2	0.180742909	3.30274278	0.001064765	0.005213685	no
FHL1	-0.180706112	-3.302047688	0.00106732	0.005225004	no
ZNF483	-0.180699077	-3.301914788	0.001067809	0.005226208	no
MRPS26	-0.180677372	-3.301504791	0.001069319	0.005232409	no
DLEU7	0.180669715	3.301360152	0.001069852	0.005233826	no
GABRB1	-0.180658314	-3.301144784	0.001070647	0.005236521	no
PRKX	-0.180617432	-3.300372566	0.0010735	0.005249282	no
KAT2A	-0.180526358	-3.298652317	0.001079882	0.005279286	no
FGF14	-0.180459778	-3.297394781	0.001084569	0.005300995	no
SEZ6	-0.180429982	-3.296832026	0.001086673	0.00531007	no
SEC24C	-0.180413131	-3.296513764	0.001087864	0.005314684	no
MMP15	-0.180409029	-3.296436286	0.001088155	0.005314893	no
TPPP2	-0.180368555	-3.295671874	0.001091022	0.005327687	no
CUL1	0.180356094	3.295436529	0.001091906	0.005330794	no
NPAS3	-0.180274768	-3.293900631	0.001097693	0.005357828	no
TUBA3FP	-0.18025785	-3.293581125	0.0010989	0.005362503	no
PNMA2	-0.180248455	-3.293403715	0.001099571	0.005364559	no
SFTA1P	0.180187717	3.292256678	0.001103918	0.005384545	no
NETO1	-0.180158842	-3.29171141	0.00110599	0.005392246	no
CPE	-0.180158723	-3.291709162	0.001105999	0.005392246	no
SHCBP1	0.180130304	3.2911725	0.001108042	0.005400981	no
ITIH2	0.180121178	3.291000153	0.001108699	0.005402452	no
LOC100128573	-0.180116838	-3.290918215	0.001109011	0.005402452	no
STAG2	-0.180115634	-3.290895463	0.001109098	0.005402452	no
TNFSF12-TNFS	0.180080547	3.29023291	0.001111627	0.0054134	no
HARS2	0.180077468	3.290174763	0.001111849	0.0054134	no
TOX3	-0.180036696	-3.289404868	0.001114795	0.005426516	no
TNRC6B	-0.180020446	-3.289098027	0.001115972	0.005431011	no
MTRNR2L4	-0.180011645	-3.288931838	0.001116609	0.005432884	no
ZNF765	0.18000309	3.288770291	0.001117229	0.00543467	no
SYTL4	-0.179954367	-3.287850298	0.001120767	0.005450086	no
VCP	0.179952456	3.287814218	0.001120905	0.005450086	no
VSTM2B	-0.179929402	-3.287378914	0.001122583	0.005457008	no
ALG1L9P	-0.179923754	-3.287272275	0.001122994	0.005457773	no
MGC39372	0.179919948	3.287200419	0.001123272	0.005457886	no
NEMF	-0.17990851	-3.286984444	0.001124105	0.005460702	no
GTF2E2	0.179880191	3.286449749	0.001126172	0.005469504	no
SATB1	-0.179874581	-3.286343824	0.001126581	0.005470257	no
KIAA0087	-0.179860865	-3.286084851	0.001127584	0.005473888	no
TP53I13	0.179839055	3.285673063	0.001129179	0.005480396	no
SNX27	-0.179832282	-3.28554517	0.001129675	0.005481566	no
TRNAU1AP	0.179805174	3.285033365	0.001131662	0.005489967	no
ASB3	-0.179773569	-3.284436655	0.001133983	0.005499984	no
ARHGDIG	-0.179766485	-3.284302916	0.001134504	0.005501268	no
TERF1	-0.179742249	-3.283845338	0.001136287	0.005508672	no
CD163L1	0.179728876	3.28359287	0.001137272	0.005512204	no
MTMR1	-0.17971346	-3.283301821	0.001138409	0.005516469	no
SCRT2	-0.179709937	-3.283235297	0.001138668	0.005516484	no

AMH	-0.179623343	-3.281600503	0.001145073	0.005546264	no
DUSP27	0.179604388	3.281242668	0.00114648	0.005551825	no
ZNRD1-AS1	-0.179566064	-3.280519174	0.001149328	0.005564365	no
CGB8	0.179540437	3.280035405	0.001151237	0.005572348	no
RPS25	-0.17951299	-3.279517277	0.001153284	0.005581	no
MGMT	0.179448456	3.278299071	0.00115811	0.005603094	no
THRB	-0.179442472	-3.278186117	0.001158559	0.005604003	no
CCBL1	-0.179437574	-3.278093649	0.001158926	0.005604518	no
KBTBD4	-0.179423657	-3.277830957	0.00115997	0.005608304	no
ZNF525	0.179411123	3.277594354	0.001160911	0.005611591	no
CSDA	0.179370244	3.276822729	0.001163984	0.005625183	no
PRKCZ	-0.179333233	-3.276124112	0.001166774	0.005637396	no
RGS7BP	-0.179326178	-3.275990954	0.001167306	0.0056387	no
SGSM3	-0.179310413	-3.275693383	0.001168497	0.005643183	no
TCEB2	-0.1792524	-3.274598399	0.001172887	0.005663113	no
REPS2	-0.179242913	-3.274419334	0.001173607	0.005665314	no
SEMA3F	0.179233216	3.274236314	0.001174342	0.005667593	no
PRMT7	-0.179203054	-3.273667034	0.001176634	0.005677376	no
TAS2R19	-0.179151532	-3.272694613	0.001180557	0.005695029	no
FAM13A	-0.179134993	-3.272382472	0.001181819	0.005699837	no
RAB3A	-0.179084005	-3.271420176	0.001185718	0.005717165	no
NFYB	-0.179081049	-3.271364374	0.001185944	0.005717165	no
GUCA1B	-0.179069918	-3.271154308	0.001186797	0.005719993	no
MRC1	0.179058052	3.270930366	0.001187706	0.005722428	no
GNAQ	-0.179056381	-3.270898823	0.001187835	0.005722428	no
PHF16	-0.179052813	-3.270831492	0.001188108	0.005722464	no
DENND5B-AS1	-0.179033745	-3.27047164	0.001189572	0.00572823	no
SF3A2	-0.179023945	-3.270286688	0.001190325	0.005730571	no
EID2B	-0.179003999	-3.269910273	0.001191858	0.00573667	no
NKPD1	-0.178913718	-3.268206522	0.001198823	0.005768901	no
ADAMTS6	-0.178859096	-3.267175773	0.001203055	0.00578797	no
ZSCAN21	-0.178830406	-3.266634381	0.001205284	0.005797392	no
PKP4	-0.17877988	-3.265680968	0.001209217	0.005815011	no
ADAMTS19	-0.178758214	-3.26527213	0.001210907	0.005821837	no
FAM171B	-0.178738545	-3.264901008	0.001212444	0.00582792	no
LOC728558	-0.178726123	-3.2646666	0.001213415	0.005831285	no
ZNF812	0.178708481	3.264333726	0.001214796	0.005836614	no
ZNF512	-0.178701666	-3.264205138	0.001215329	0.005837873	no
TNIP1	0.178601023	3.262306168	0.001223236	0.005874542	no
RANBP9	0.178491102	3.26023227	0.001231926	0.005914953	no
CNTNAP3	0.178448533	3.25942916	0.001235307	0.005929859	no
H3F3B	-0.178444074	-3.259345034	0.001235661	0.005930237	no
FAM163B	-0.178437884	-3.259228248	0.001236154	0.005931276	no
LRRC37A2	-0.178411647	-3.25873326	0.001238243	0.005939974	no
SNAI2	0.178396149	3.25844088	0.001239479	0.005944574	no
ZNF8	-0.178351035	-3.257589784	0.001243082	0.005960524	no
LOC441666	-0.17834192	-3.25741783	0.001243811	0.00596269	no
TEX26	0.178310491	3.256824936	0.001246328	0.005972332	no
LOC84989	-0.178309869	-3.256813204	0.001246378	0.005972332	no
SH3GL3	-0.178296614	-3.256563146	0.001247441	0.005976093	no
SKIV2L2	-0.17828605	-3.25636386	0.001248289	0.005978822	no

RPS10	-0.178279139	-3.256233486	0.001248844	0.005980147	no
TDRKH	-0.178201266	-3.254764494	0.001255112	0.006008576	no
HIF3A	-0.17819845	-3.25471137	0.00125534	0.006008576	no
ZNF346	-0.178175064	-3.254270237	0.001257228	0.006016275	no
MMP2	0.17816234	3.25403022	0.001258257	0.006019857	no
LINC00693	-0.178155475	-3.253900726	0.001258812	0.006021173	no
SNRNP200	-0.178105647	-3.252960829	0.001262849	0.00603914	no
FAM126A	0.178085427	3.252579427	0.001264491	0.006045645	no
SNAP47	-0.178078	-3.252439334	0.001265094	0.006047185	no
BRCC3	0.178020375	3.251352413	0.001269786	0.00606826	no
FDX1	0.178012034	3.251195099	0.001270466	0.006070162	no
SRXN1	0.178000826	3.250983686	0.001271381	0.006073183	no
WDR77	0.177977074	3.250535702	0.001273322	0.006081101	no
ALG14	0.177967816	3.250361085	0.001274079	0.006083365	no
DNAJB7	-0.177954159	-3.250103501	0.001275196	0.006087349	no
OTUD4	0.177893054	3.248951013	0.001280208	0.006109916	no
MIR5093	0.17786307	3.248385514	0.001282674	0.006120325	no
TUG1	-0.17784385	-3.248023027	0.001284257	0.006126518	no
GGCX	0.177811112	3.247405589	0.001286957	0.006138037	no
MARK4	-0.177711184	-3.245521044	0.001295232	0.006176133	no
PURA	-0.17767471	-3.244833212	0.001298264	0.006189219	no
RPUSD4	-0.177641561	-3.244208089	0.001301026	0.006201009	no
AKTIP	-0.177604989	-3.243518435	0.001304079	0.006214183	no
SMC5	0.177592158	3.243276475	0.001305152	0.006217916	no
NAALADL2	0.177564549	3.242755849	0.001307463	0.006227545	no
KREMEN1	0.177559237	3.242655676	0.001307908	0.006228285	no
KHDRBS2	-0.177545017	-3.24238753	0.0013091	0.006232581	no
PEX2	-0.177520679	-3.241928612	0.001311143	0.006239814	no
USP2	-0.177519082	-3.241898491	0.001311277	0.006239814	no
ALKBH3	0.177516538	3.241850524	0.00131149	0.006239814	no
SOX12	-0.177412651	-3.239891631	0.001320245	0.006280075	no
FBXO41	-0.177394181	-3.239543374	0.001321807	0.006286113	no
CERS3	-0.177387814	-3.239423326	0.001322345	0.006287284	no
CYSTM1	0.177371318	3.239112289	0.001323742	0.006292522	no
ZNF485	-0.177367102	-3.2390328	0.0013241	0.006292522	no
DENND5A	-0.177364439	-3.238982583	0.001324325	0.006292522	no
NTN1	0.177322429	3.238190516	0.001327891	0.006308068	no
BFAR	0.177301151	3.237789334	0.0013297	0.006315266	no
HIST1H3E	0.177297132	3.237713543	0.001330042	0.006315495	no
SLAIN1	-0.177239801	-3.236632637	0.001334929	0.0063373	no
FADS2	-0.177235276	-3.23654733	0.001335315	0.006337734	no
HIST3H2A	-0.177185407	-3.235607133	0.001339581	0.006356576	no
CHRM1	-0.177174892	-3.23540889	0.001340482	0.006359447	no
LY75-CD302	0.177170702	3.235329902	0.001340841	0.006359747	no
IL1A	0.17714127	3.23477503	0.001343366	0.00637032	no
FAM188A	-0.177135633	-3.234668756	0.001343851	0.00637121	no
LOC100499484	0.177118087	3.234337979	0.001345359	0.006376953	no
ROBO1	-0.177093562	-3.233875611	0.00134747	0.006384342	no
RFPL1-AS1	-0.177093072	-3.233866388	0.001347512	0.006384342	no
ZNF521	-0.177082988	-3.23367627	0.00134838	0.00638705	no
TRAF3IP2	0.177066108	3.233358058	0.001349836	0.006392534	no

LOC440297	-0.177029181	-3.232661935	0.001353025	0.006406224	no
VANGL2	-0.177005956	-3.232224099	0.001355034	0.006414324	no
MNF1	-0.176984248	-3.231814883	0.001356914	0.006421811	no
LINC00704	0.176959631	3.231350828	0.001359049	0.0064305	no
ADH4	0.176946211	3.231097854	0.001360215	0.006432499	no
TAF13	0.17694112	3.231001884	0.001360657	0.006432499	no
RAB18	-0.176940435	-3.230988989	0.001360717	0.006432499	no
TCN1	0.176939928	3.230979421	0.001360761	0.006432499	no
UNKL	-0.176937538	-3.230934373	0.001360968	0.006432499	no
DDIT4L	0.176912786	3.230467791	0.001363121	0.006440197	no
STAR	-0.176911922	-3.230451504	0.001363196	0.006440197	no
GPN2	0.176875656	3.229767894	0.001366357	0.006453707	no
IGBP1	-0.17686254	-3.229520659	0.001367501	0.006457694	no
ABCA1	0.176759889	3.22758577	0.001376489	0.006498711	no
TJP2	-0.176735468	-3.227125466	0.001378636	0.006507414	no
DSTYK	-0.176728614	-3.226996279	0.001379239	0.006508831	no
MARCKSL1	-0.176666261	-3.225821061	0.001384735	0.006533333	no
RGR	-0.176651205	-3.225537292	0.001386065	0.006538173	no
HPN	0.176646211	3.225443168	0.001386506	0.00653882	no
ZNF624	-0.176597181	-3.224519073	0.001390847	0.006557854	no
CWC27	-0.17658775	-3.224341345	0.001391684	0.006560358	no
PACSIN2	-0.176582824	-3.224248504	0.001392121	0.006560979	no
DNAJB5	-0.176566469	-3.22394026	0.001393573	0.006565607	no
BEND5	-0.176564882	-3.22391035	0.001393714	0.006565607	no
LOC148709	-0.17654673	-3.223568256	0.001395327	0.006571767	no
MYCBP2	-0.176470868	-3.222138541	0.001402088	0.006602164	no
ETS1	0.176419119	3.221163325	0.001406717	0.006622512	no
SDR39U1	-0.176406249	-3.220920791	0.001407871	0.00662649	no
WIPF2	-0.176401281	-3.220827171	0.001408316	0.006627136	no
LOC729506	-0.17638981	-3.220610993	0.001409346	0.006630527	no
ZFYVE16	-0.176318355	-3.219264457	0.001415772	0.006659303	no
TMEM115	0.17630735	3.219057079	0.001416764	0.006662511	no
PRDM16	0.176266186	3.218281393	0.001420481	0.006678221	no
JUP	0.17626347	3.218230227	0.001420726	0.006678221	no
ATP6V0A1	-0.176245353	-3.21788884	0.001422365	0.006684462	no
TUBGCP6	-0.176208568	-3.217195685	0.001425698	0.00669866	no
CEND1	-0.17617371	-3.216538865	0.001428863	0.006712063	no
ABHD3	0.176134639	3.215802673	0.001432418	0.006727293	no
CES1	0.17610738	3.215289065	0.001434903	0.006737491	no
DNMBP-AS1	-0.176087832	-3.21492074	0.001436687	0.006744397	no
MTRNR2L8	-0.176073125	-3.214643648	0.001438031	0.006749232	no
PUS7L	-0.176068008	-3.214547236	0.001438499	0.006749953	no
EEF1A2	-0.176033498	-3.213897016	0.001441658	0.006761715	no
AQP3	0.176031893	3.213866769	0.001441805	0.006761715	no
LOC96610	0.176028746	3.21380749	0.001442094	0.006761715	no
DNM1L	0.176026887	3.213772459	0.001442264	0.006761715	no
HTR2A	-0.175999616	-3.213258645	0.001444766	0.006771967	no
LPIN1	-0.175970383	-3.21270788	0.001447452	0.006783078	no
ZNF286B	-0.175949176	-3.212308333	0.001449404	0.006790743	no
HNF1A	-0.175924178	-3.211837352	0.001451708	0.006800054	no
PNISR	-0.175914302	-3.211651305	0.001452619	0.00680253	no

SYN3	-0.175911587	-3.211600138	0.001452869	0.00680253	no
IGSF11	-0.175901287	-3.21140609	0.00145382	0.006805499	no
P4HA3	0.175852345	3.210484058	0.001458346	0.006825198	no
TIGD4	0.175843966	3.210326188	0.001459122	0.006827309	no
MYADM	0.175840616	3.210263081	0.001459432	0.006827309	no
TMEM8B	-0.17582214	-3.209915011	0.001461145	0.006833836	no
KANK1	-0.175717083	-3.207935911	0.001470921	0.00687806	no
EVL	-0.175675161	-3.207146191	0.001474839	0.00689488	no
IL27RA	0.175630223	3.206299692	0.001479049	0.006912832	no
CELSR2	-0.17562731	-3.206244819	0.001479322	0.006912832	no
ZC3H4	-0.175619492	-3.20609754	0.001480056	0.006914757	no
ZNF740	-0.17560121	-3.205753166	0.001481773	0.006921274	no
GNAI3	0.175594423	3.205625333	0.001482411	0.006922749	no
PKD1P1	-0.175577145	-3.205299872	0.001484036	0.006928832	no
SUV39H2	-0.175557995	-3.204939145	0.001485839	0.00693504	no
GTF3A	0.175556171	3.204904784	0.001486011	0.00693504	no
POR	0.175530443	3.204420183	0.001488437	0.006944852	no
ZNF687	-0.175483414	-3.203534348	0.00149288	0.006963268	no
MRPS31P5	-0.175481814	-3.203504213	0.001493031	0.006963268	no
BET1	0.175468252	3.203248766	0.001494315	0.006967744	no
FTO	-0.175457592	-3.203047976	0.001495325	0.00697094	no
TSPAN33	0.175439911	3.202714955	0.001497001	0.00697724	no
BPTF	-0.175428454	-3.202499172	0.001498088	0.006980793	no
STRA6	0.175394404	3.201857848	0.001501324	0.006994352	no
NUBP1	0.175390359	3.201781647	0.001501708	0.006994628	no
CA4	-0.175361732	-3.20124249	0.001504434	0.007005804	no
DIRAS2	-0.175310987	-3.200286756	0.001509276	0.007026421	no
TMEM180	-0.175308488	-3.200239692	0.001509515	0.007026421	no
POGK	-0.175290366	-3.199898375	0.001511248	0.007032965	no
COMMD9	0.175267939	3.199476011	0.001513395	0.007041433	no
FAM105B	0.175246327	3.19906899	0.001515467	0.007048612	no
CELF4	-0.175245002	-3.199044032	0.001515594	0.007048612	no
ADCY5	-0.175240377	-3.198956918	0.001516038	0.007049151	no
RNF121	-0.175234644	-3.198848949	0.001516588	0.007050184	no
DDHD1	0.175231134	3.198782861	0.001516925	0.007050225	no
HRH1	0.175209027	3.198366517	0.001519049	0.007058569	no
FAM213A	-0.175202412	-3.198241943	0.001519685	0.007059998	no
HIST2H3D	0.175107638	3.196457133	0.001528825	0.007100923	no
ARHGAP24	0.175094558	3.196210825	0.00153009	0.007104639	no
IMPDH2	-0.175092536	-3.196172738	0.001530286	0.007104639	no
PYROXD2	0.175070809	3.195763591	0.00153239	0.007112496	no
ASCC2	0.175068229	3.195715001	0.00153264	0.007112496	no
GPATCH2	0.175039172	3.195167834	0.001535459	0.007124037	no
BIRC6	-0.17502668	-3.194932589	0.001536672	0.007128128	no
C9orf114	-0.175017194	-3.19475396	0.001537594	0.007130865	no
SNX33	0.175001951	3.194466933	0.001539076	0.0071362	no
C14orf80	-0.174912639	-3.192785184	0.001547788	0.007175047	no
JUN	0.174875156	3.192079383	0.001551458	0.007189518	no
LEF1	0.174870651	3.191994567	0.0015519	0.007189518	no
CACNB1	-0.174870509	-3.191991897	0.001551914	0.007189518	no
EBF2	0.174861471	3.191821715	0.0015528	0.007192074	no

BLK	0.174820341	3.191047281	0.001556839	0.007208348	no
KIAA2022	-0.174818866	-3.191019498	0.001556984	0.007208348	no
SYTL3	0.174785046	3.190382731	0.001560313	0.007222203	no
SFTPA2	-0.174775911	-3.190210724	0.001561213	0.007224814	no
PID1	-0.174767013	-3.190043189	0.001562091	0.007227318	no
ZNF844	-0.17476181	-3.189945237	0.001562604	0.007228137	no
ANKRD19P	-0.174709503	-3.188960401	0.001567772	0.007250483	no
IGF2R	0.174677369	3.188355407	0.001570955	0.00726364	no
LOC100129480	-0.174667548	-3.188170487	0.001571929	0.00726658	no
SLU7	-0.174611721	-3.187119441	0.001577476	0.007290653	no
CDK14	-0.174560145	-3.186148466	0.001582616	0.007312838	no
RAB1B	0.174549666	3.185951176	0.001583663	0.007316099	no
SOX11	-0.174522104	-3.185432306	0.001586418	0.007327251	no
NPEPPS	-0.174477208	-3.184587115	0.001590915	0.007346443	no
C3orf37	-0.174461637	-3.184293993	0.001592477	0.007352078	no
SNORA69	0.174427549	3.183652285	0.001595902	0.007366309	no
SEMA6C	-0.174418006	-3.183472638	0.001596862	0.007369158	no
ATOX1	0.174398416	3.18310387	0.001598835	0.007376409	no
PNCK	-0.174395586	-3.183050595	0.00159912	0.007376409	no
LOC101055625	0.174379946	3.182756183	0.001600697	0.007382098	no
UNK	-0.174364095	-3.182457803	0.001602296	0.007387889	no
ZNF708	-0.174323417	-3.181692077	0.001606408	0.007405257	no
CYP2D7P1	-0.174319397	-3.181616399	0.001606815	0.007405544	no
KLC2	-0.174314356	-3.181521521	0.001607325	0.007406307	no
ANKRD12	-0.174305895	-3.181362251	0.001608182	0.007408667	no
HSPA12A	-0.174275622	-3.180792404	0.001611251	0.007421215	no
HSPBAP1	0.17423614	3.180049238	0.001615262	0.007438094	no
PTPRD	-0.174208517	-3.179529303	0.001618073	0.007449444	no
GPR85	-0.174163306	-3.178678334	0.001622684	0.007467873	no
EPX	-0.174162459	-3.17866239	0.001622771	0.007467873	no
CYP46A1	-0.174133007	-3.178108043	0.001625781	0.007480126	no
GFPT2	0.174080193	3.177114012	0.001631193	0.007503417	no
MAP3K14-AS1	0.174067995	3.176884425	0.001632445	0.00750757	no
LRRFIP1	-0.174061222	-3.176756957	0.001633141	0.007507729	no
POM121L4P	0.174060856	3.176750059	0.001633178	0.007507729	no
MYH14	-0.173982338	-3.175272307	0.001641263	0.007543281	no
FHL2	0.173966992	3.174983482	0.001642847	0.007548949	no
IPW	-0.173943995	-3.174550684	0.001645224	0.007558255	no
KIF7	-0.173924045	-3.174175229	0.001647289	0.007566122	no
RASGEF1C	-0.173887953	-3.173495997	0.00165103	0.007581684	no
LURAP1L	0.173866397	3.173090329	0.001653268	0.00759034	no
KHDRBS3	-0.173853989	-3.17285684	0.001654557	0.007593039	no
FGF12	-0.173853941	-3.172855933	0.001654562	0.007593039	no
ACTR3B	-0.17383786	-3.17255331	0.001656235	0.007599092	no
PNMAL1	-0.173790089	-3.171654318	0.001661213	0.007619508	no
PM20D1	0.173788356	3.171621711	0.001661394	0.007619508	no
ACSS3	0.17376513	3.171184638	0.001663819	0.007629003	no
SYT3	-0.173760886	-3.17110477	0.001664263	0.00762941	no
AIP	-0.173717605	-3.170290332	0.001668792	0.007648542	no
IKBKE	0.173714139	3.1702251	0.001669155	0.007648576	no
RAB11B	-0.173694823	-3.169861626	0.001671181	0.007656225	no

COA3	-0.173640325	-3.168836136	0.001676908	0.007680824	no
SRFBP1	0.17361135	3.168290922	0.00167996	0.007693164	no
FGF14-IT1	-0.173592759	-3.167941114	0.001681921	0.007700504	no
MED8	0.173588462	3.167860262	0.001682375	0.007700939	no
DACH2	-0.173572926	-3.167567922	0.001684015	0.007706809	no
C7orf41	-0.173543338	-3.167011206	0.001687144	0.007719484	no
AAGAB	0.173522433	3.166617859	0.001689358	0.007727968	no
SH2B1	-0.173491066	-3.166027682	0.001692685	0.007741538	no
COL18A1	0.173474503	3.165716035	0.001694444	0.007746502	no
TCF4	-0.17347406	-3.165707697	0.001694491	0.007746502	no
TP53TG5	-0.173457193	-3.16539035	0.001696284	0.00775305	no
VWC2L	-0.173452263	-3.165297602	0.001696809	0.007753393	no
ZNF676	-0.173449705	-3.165249469	0.001697081	0.007753393	no
NPSR1	0.173420199	3.16469431	0.001700223	0.007766099	no
RAB6A	-0.17341575	-3.164610612	0.001700697	0.007766614	no
EEF1DP3	-0.173395521	-3.164230008	0.001702855	0.007774817	no
PCDHA2	-0.173379569	-3.163929891	0.001704559	0.007780282	no
ATRAID	0.173377534	3.163891601	0.001704776	0.007780282	no
XPO7	-0.173361854	-3.16359661	0.001706452	0.007786278	no
SCARNA17	-0.173348934	-3.163353521	0.001707835	0.007790931	no
PLA2R1	0.173329504	3.162987983	0.001709915	0.007797688	no
CSF3R	0.173328326	3.162965805	0.001710042	0.007797688	no
ZNF32-AS3	-0.173294524	-3.162329882	0.001713667	0.007812562	no
GRHL1	-0.173288097	-3.16220896	0.001714357	0.007814051	no
NAA30	-0.173274178	-3.161947113	0.001715853	0.007819209	no
HEPACAM	-0.173210698	-3.160752875	0.001722688	0.007848695	no
MTCP1	0.173135305	3.15933457	0.001730839	0.007884159	no
EDNRB	-0.173122812	-3.159099562	0.001732193	0.007888654	no
SLC25A36	-0.173114025	-3.158934261	0.001733146	0.007890027	no
MSANTD1	-0.173113263	-3.158919935	0.001733229	0.007890027	no
LOC100505715	-0.173094681	-3.158570388	0.001735245	0.007896876	no
AREG	0.173092631	3.158531825	0.001735468	0.007896876	no
PI4KAP2	-0.173066175	-3.158034143	0.001738344	0.007908286	no
LIN7B	-0.173057861	-3.157877762	0.001739248	0.007910726	no
IGLON5	-0.172973545	-3.156291735	0.001748446	0.007949709	no
LOC645212	-0.172972517	-3.156272394	0.001748559	0.007949709	no
PRKAG1	-0.17281378	-3.153286667	0.001765997	0.008027293	no
AGFG1	-0.172808401	-3.153185503	0.001766591	0.008028293	no
PTGFRN	0.172777118	3.152597127	0.001770047	0.008038691	no
CISD3	0.172776577	3.152586943	0.001770107	0.008038691	no
SPTB	-0.172776466	-3.15258487	0.001770119	0.008038691	no
SUCLA2	-0.172774154	-3.152541381	0.001770375	0.008038691	no
KCNH3	-0.172670223	-3.150586703	0.001781904	0.008089334	no
PSMD13	0.172661141	3.150415886	0.001782915	0.008092214	no
TRAPPC12	-0.172619958	-3.149641374	0.001787505	0.008111233	no
RPAP2	0.172616776	3.149581526	0.00178786	0.008111233	no
KIAA1967	-0.172605724	-3.14937368	0.001789094	0.008115117	no
FLT3LG	0.172596332	3.149197048	0.001790143	0.008118163	no
LOC100128239	-0.172586374	-3.149009789	0.001791256	0.008121496	no
SPIN1	-0.172577472	-3.148842376	0.001792252	0.008124296	no
MAFG	0.172543669	3.148206669	0.001796037	0.008139735	no

LYPLA2	0.172524494	3.14784608	0.001798187	0.008146972	no
ROR2	0.172522669	3.147811755	0.001798392	0.008146972	no
PNMA6C	-0.172511447	-3.147600729	0.001799651	0.008150959	no
MMRN2	0.172505261	3.147484388	0.001800346	0.008152388	no
HMX1	-0.172501663	-3.147416726	0.00180075	0.0081525	no
FNDC3B	0.172478381	3.146978902	0.001803367	0.00816263	no
DHRSX	0.172436107	3.146183951	0.001808128	0.008182352	no
ERBB4	-0.172432937	-3.146124333	0.001808486	0.008182352	no
SELK	-0.172412421	-3.145738547	0.001810801	0.008191103	no
MREG	0.17240523	3.145603319	0.001811613	0.008193052	no
IL17RD	-0.172401224	-3.145527997	0.001812066	0.008193374	no
SSC5D	0.172380456	3.14513746	0.001814414	0.008202265	no
TSPY26P	-0.172374917	-3.145033307	0.001815041	0.008203372	no
ACAT1	-0.17236777	-3.14489892	0.001815849	0.008205303	no
LOC389895	-0.17235936	-3.14474078	0.001816802	0.00820788	no
CCNG2	-0.172308451	-3.143783498	0.001822576	0.008232236	no
SYNC	0.172246719	3.142622762	0.0018296	0.008262226	no
GPC4	0.172232095	3.142347783	0.001831268	0.00826802	no
CELSR1	0.172209899	3.141930448	0.001833802	0.008277721	no
MPHOSPH8	-0.172090797	-3.13969111	0.001847452	0.008335676	no
SH3BP2	0.172090317	3.13968209	0.001847508	0.008335676	no
LOC100289473	-0.172085055	-3.139583168	0.001848113	0.008335676	no
FKBP6	0.172084366	3.139570209	0.001848192	0.008335676	no
DAGLB	0.172026979	3.138491278	0.001854805	0.008363209	no
ZDHHC5	0.172024639	3.138447295	0.001855075	0.008363209	no
KCP	0.171814266	3.134492386	0.001879506	0.008470783	no
KLHDC4	-0.171812394	-3.134457197	0.001879725	0.008470783	no
IL9	-0.171806944	-3.134354743	0.001880362	0.008471876	no
AMPD2	0.171769483	3.133650539	0.001884745	0.008489844	no
DNAAF2	-0.171757621	-3.133427571	0.001886134	0.008494323	no
CEBPE	0.171725507	3.132823895	0.001889902	0.008509507	no
THBS2	0.17171201	3.132570191	0.001891487	0.008514861	no
TSSC1	-0.17170044	-3.132352696	0.001892847	0.008519199	no
C17orf62	0.171676023	3.131893726	0.00189572	0.008530343	no
LINC00667	-0.171670106	-3.131782507	0.001896417	0.008531692	no
SCAI	-0.171664927	-3.131685165	0.001897027	0.008532651	no
SFTPC	-0.171642115	-3.131256361	0.001899717	0.00854296	no
WDR81	0.171629463	3.131018555	0.00190121	0.008547886	no
REPIN1	-0.171579756	-3.130084249	0.001907086	0.008572514	no
GPC2	-0.1715714	-3.129927176	0.001908076	0.008575169	no
ZNF202	-0.171548943	-3.129505082	0.001910738	0.008585336	no
HNRNPA1L2	-0.171493485	-3.128462727	0.001917325	0.008613135	no
ATP13A3	0.171485997	3.12832199	0.001918216	0.008615337	no
WHSC2	-0.171450601	-3.127656709	0.001922434	0.008632474	no
BRAP	-0.17144625	-3.127574947	0.001922952	0.008633	no
FSTL5	-0.171417384	-3.127032411	0.001926399	0.008646666	no
MSRA	-0.171412653	-3.126943494	0.001926964	0.008647398	no
LOC399744	-0.171404108	-3.126782901	0.001927986	0.008650176	no
PLAT	0.171382438	3.126375635	0.001930579	0.008660001	no
RPH3A	-0.171321152	-3.125223828	0.001937929	0.008691158	no
SCARNA9L	-0.171315357	-3.125114924	0.001938625	0.008692449	no

SLC24A5	-0.171312025	-3.125052312	0.001939026	0.008692449	no
DAAM2	-0.171270858	-3.124278639	0.00194398	0.008712841	no
EDNRA	0.171262962	3.124130256	0.001944931	0.008715288	no
ENDOD1	0.171228811	3.123488462	0.001949052	0.008731932	no
SLC25A30	0.171215917	3.123246143	0.00195061	0.00873709	no
BRD8	-0.171202191	-3.122988208	0.001952269	0.008742701	no
CHD5	-0.171179802	-3.122567462	0.001954979	0.008753012	no
SH2B2	-0.171176313	-3.122501903	0.001955402	0.00875308	no
ZNF385C	-0.171172464	-3.12242957	0.001955868	0.008753344	no
DVL1	-0.17115237	-3.122051971	0.001958304	0.008762421	no
SLC5A12	-0.171147096	-3.121952852	0.001958944	0.00876346	no
STATH	0.171125352	3.121544257	0.001961584	0.008773443	no
IER5L	0.171095789	3.120988725	0.001965178	0.00878769	no
SMC3	-0.171076339	-3.120623225	0.001967546	0.008795186	no
AP3M2	-0.171075299	-3.120603698	0.001967673	0.008795186	no
FAM83G	0.171057211	3.120263803	0.001969877	0.00880321	no
S100A16	0.171016087	3.119491044	0.001974898	0.008823813	no
KLHDC8B	0.171005162	3.119285762	0.001976234	0.008827664	no
NDUFB10	-0.17100232	-3.119232361	0.001976582	0.008827664	no
CAMKV	-0.170998132	-3.119153675	0.001977094	0.008828118	no
GNG12	0.170977186	3.118760087	0.001979659	0.008837732	no
PGPEP1	0.170972272	3.118667748	0.001980261	0.008838584	no
KCNQ2	-0.170967091	-3.118570409	0.001980896	0.008839582	no
CALM3	-0.170927087	-3.117818741	0.001985805	0.008859649	no
PNMAL2	-0.170912243	-3.117539825	0.001987629	0.008865947	no
LRRC39	0.170902718	3.117360866	0.0019888	0.008869332	no
ANGPTL6	0.170831565	3.116023971	0.001997571	0.008906598	no
ACOX2	0.170823273	3.115868174	0.001998596	0.008909318	no
KCNK15	0.170802806	3.11548362	0.002001126	0.008918749	no
PARK7	-0.170790148	-3.115245799	0.002002693	0.008923881	no
RAB36	0.170754468	3.11457544	0.002007115	0.00894173	no
TAS2R31	-0.170749077	-3.114474153	0.002007784	0.008942856	no
HIST1H4F	0.170744759	3.114393034	0.00200832	0.008943389	no
BAG3	0.170735814	3.114224983	0.00200943	0.008946481	no
MED13	-0.170724765	-3.1140174	0.002010803	0.008950737	no
MNAT1	-0.170711624	-3.113770504	0.002012436	0.008955415	no
ZC2HC1B	-0.170709608	-3.113732636	0.002012687	0.008955415	no
KCTD1	-0.170683325	-3.113238839	0.002015958	0.008968114	no
ZNF793	-0.170676838	-3.113116982	0.002016766	0.008969852	no
GNB5	-0.170634101	-3.112314081	0.002022098	0.008990233	no
ATP6V1H	-0.1706334	-3.112300903	0.002022185	0.008990233	no
PHF7	-0.170628509	-3.112209013	0.002022796	0.008991089	no
PRUNE	0.170605619	3.11177899	0.002025658	0.009001947	no
PPP2CB	-0.170599549	-3.111664972	0.002026417	0.00900346	no
NAGPA	0.170571835	3.111144329	0.002029888	0.009017018	no
ANKRD28	-0.170561411	-3.110948503	0.002031195	0.009020959	no
KIAA1107	-0.170552475	-3.110780622	0.002032316	0.009024073	no
INSL3	0.170500096	3.109796647	0.002038899	0.009051429	no
PPP1R3B	0.170470177	3.109234614	0.002042667	0.009066286	no
RPL3	-0.170375955	-3.107464704	0.002054576	0.009117261	no
LTB4R	0.17036703	3.107297056	0.002055707	0.009120398	no

BZRAP1	-0.170292547	-3.105897993	0.002065171	0.009160495	no
C5orf45	-0.170264675	-3.105374469	0.002068723	0.009174355	no
PCDH10	-0.170260128	-3.105289059	0.002069303	0.009175034	no
HTR2B	0.170251764	3.10513195	0.00207037	0.009177873	no
GDF10	-0.170244026	-3.104986609	0.002071358	0.009180358	no
CLSTN2	-0.170232374	-3.104767758	0.002072846	0.009185059	no
CDK19	-0.170211286	-3.104371673	0.002075542	0.009195108	no
NAPG	-0.170202482	-3.104206308	0.002076668	0.009198202	no
TM7SF3	0.170142176	3.103073626	0.002084399	0.009228754	no
TSPYL1	-0.170141977	-3.10306989	0.002084425	0.009228754	no
TUBE1	-0.170131356	-3.102870407	0.002085789	0.009232892	no
NSFL1C	-0.17011029	-3.10247476	0.002088497	0.009242977	no
C6orf164	-0.170085311	-3.102005625	0.002091713	0.009255301	no
MEIS2	-0.170055877	-3.101452817	0.002095508	0.009270184	no
MORC4	0.169999926	3.100402015	0.002102739	0.009300259	no
RBM7	0.169987405	3.100166863	0.00210436	0.009305514	no
TRIM69	0.169973563	3.099906919	0.002106154	0.009310467	no
LINC00320	-0.169972076	-3.099878988	0.002106346	0.009310467	no
SYN1	-0.169914825	-3.098803818	0.002113781	0.009341074	no
TIMM21	-0.169911028	-3.098732521	0.002114274	0.009341074	no
LUST	-0.169908721	-3.098689183	0.002114575	0.009341074	no
CYB5D1	0.169852995	3.097642701	0.002121836	0.009371225	no
TASP1	0.169816655	3.096960281	0.002126584	0.009388152	no
SNAPC4	-0.169815751	-3.096943292	0.002126702	0.009388152	no
RAB11FIP3	-0.169813633	-3.096903521	0.002126979	0.009388152	no
ANGEL2	-0.169798307	-3.096615728	0.002128985	0.009395074	no
AURKAPS1	0.169778217	3.096238462	0.002131616	0.009404756	no
MT2A	0.169773014	3.096140776	0.002132298	0.009405834	no
RABL3	0.169678346	3.094363115	0.002144742	0.009458785	no
SARDH	0.169674219	3.094285626	0.002145286	0.009459243	no
SPEN	-0.169659088	-3.094001504	0.002147282	0.0094661	no
FKBP8	-0.169654695	-3.093919027	0.002147862	0.009466713	no
PTGES	0.16965122	3.093853774	0.00214832	0.009466793	no
ZER1	-0.169621199	-3.093290065	0.002152286	0.009482326	no
OLFM1	-0.169546246	-3.091882739	0.002162218	0.009524127	no
RGS22	0.169520506	3.091399438	0.002165638	0.009537238	no
CHRNA4	-0.169500891	-3.091031158	0.002168247	0.009545274	no
PLXNC1	0.169500115	3.091016578	0.002168351	0.009545274	no
FRAT1	-0.169487821	-3.090785758	0.002169988	0.009550525	no
NXPH3	-0.16947353	-3.090517443	0.002171892	0.009556949	no
KIAA1875	-0.16943702	-3.089831958	0.002176765	0.009576429	no
KLF11	0.169406078	3.089251046	0.002180902	0.009592666	no
TM2D1	0.169367602	3.088528683	0.002186057	0.009613371	no
GGA3	-0.169349057	-3.088180513	0.002188545	0.009622346	no
EEA1	0.16929207	3.08711065	0.002196208	0.009654062	no
PROX1	-0.169263322	-3.086570954	0.002200083	0.009669118	no
SNRPD1	-0.169239107	-3.086116371	0.002203352	0.009681504	no
MAD1L1	-0.169217597	-3.085712562	0.002206259	0.009690085	no
GABRG1	-0.169215035	-3.08566447	0.002206606	0.009690085	no
LOC100128252	-0.169214653	-3.085657287	0.002206657	0.009690085	no
ISCA1	-0.16917062	-3.084830681	0.002212621	0.009714287	no

TGFB3	0.169155191	3.084541044	0.002214714	0.009721491	no
MESTIT1	-0.169097849	-3.083464639	0.002222509	0.009752745	no
ADO	-0.169096136	-3.083432487	0.002222742	0.009752745	no
DYNLL2	-0.169066555	-3.082877209	0.002226773	0.009768439	no
MARK2P9	0.169054057	3.082642611	0.002228478	0.009773924	no
SAFB	-0.169033775	-3.082261881	0.002231248	0.009783948	no
LANCL1	-0.169030655	-3.082203332	0.002231674	0.009783948	no
SDCBP	0.169002302	3.081671117	0.002235552	0.009798951	no
WBP2	-0.168941378	-3.080527545	0.002243906	0.009833561	no
CTSO	0.168936517	3.080436305	0.002244574	0.009834482	no
TSHZ1	-0.168925735	-3.08023394	0.002246055	0.009838968	no
MTCH1	-0.168907418	-3.079890125	0.002248575	0.009847997	no
PLEKHF1	0.168797094	3.077819444	0.002263803	0.009911459	no
MBTD1	-0.168795763	-3.077794455	0.002263987	0.009911459	no
KRCC1	0.168757641	3.077078965	0.002269272	0.009932569	no
DPRXP4	0.168751395	3.076961743	0.002270138	0.00993273	no
CD5L	0.168750716	3.076948996	0.002270233	0.00993273	no
MASP1	-0.168732383	-3.076604923	0.002272779	0.009940626	no
GPR19	-0.168730184	-3.076563657	0.002273084	0.009940626	no
MYH7	-0.168727733	-3.076517665	0.002273425	0.009940626	no
DUS2L	-0.168719601	-3.076365035	0.002274556	0.009943547	no
CLDN9	-0.168652976	-3.07511465	0.002283838	0.009982093	no
POLR3A	-0.168624692	-3.074583836	0.002287788	0.009997328	no
NIN	-0.168618384	-3.074465457	0.00228867	0.009999149	no
MT1M	0.168588006	3.073895374	0.002292922	0.010015689	no
VWA8-AS1	-0.168543836	-3.073066448	0.002299117	0.010040709	no
ADSS	0.168535758	3.072914866	0.002300252	0.010043622	no
TSN	-0.16846845	-3.071651783	0.002309725	0.010082939	no
KCTD16	-0.168464748	-3.071582311	0.002310248	0.01008317	no
GATSL3	-0.168453255	-3.071366637	0.002311869	0.010088199	no
PKD1	-0.168428636	-3.070904661	0.002315346	0.01010132	no
STK17A	0.168420031	3.070743194	0.002316563	0.010104576	no
KAT6A	-0.168383216	-3.070052363	0.002321773	0.01012525	no
SHMT1	0.16831543	3.068780413	0.002331396	0.010165151	no
TMEM72-AS1	-0.168306204	-3.068607304	0.002332708	0.01016881	no
HMSD	0.168300866	3.068507129	0.002333468	0.010170059	no
BTBD11	0.168279435	3.068105013	0.00233652	0.010181297	no
HDAC5	-0.168247854	-3.06751244	0.002341025	0.010198857	no
ATP6AP1	0.168234335	3.067258789	0.002342955	0.010205199	no
HELQ	0.168216868	3.066931052	0.002345452	0.010212405	no
LOC100506083	-0.168216112	-3.066916875	0.00234556	0.010212405	no
SLC9A2	-0.168205733	-3.066722133	0.002347045	0.0102168	no
AHCYL1	-0.168183855	-3.066311644	0.002350177	0.010228364	no
BAIAP2-AS1	-0.168178681	-3.066214563	0.002350919	0.010229519	no
ECE1	0.168175129	3.066147921	0.002351428	0.010229662	no
TIMP1	0.168171293	3.066075951	0.002351978	0.010229984	no
SPCS3	0.168156098	3.065790848	0.002354157	0.010237391	no
MMS19	-0.168145795	-3.065597546	0.002355636	0.010241749	no
NEDD1	0.168137557	3.065442986	0.002356819	0.01024482	no
RPL23AP7	0.168131946	3.065337705	0.002357625	0.010245067	no
PLXDC1	0.168130512	3.065310794	0.002357831	0.010245067	no

ZFP41	-0.168127208	-3.065248804	0.002358306	0.010245067	no
MAPK1	-0.168065575	-3.064092467	0.002367178	0.010281535	no
SLC25A27	-0.168039244	-3.063598461	0.002370978	0.010295958	no
TMX2-CTNND1	-0.167994073	-3.062751014	0.00237751	0.010319137	no
MYO6	-0.167992097	-3.062713926	0.002377796	0.010319137	no
NPDC1	-0.167991148	-3.062696135	0.002377934	0.010319137	no
CSF1	0.167989056	3.062656881	0.002378237	0.010319137	no
DLG2	-0.167984861	-3.062578192	0.002378844	0.01031969	no
LOC100130357	-0.167952478	-3.061970658	0.00238354	0.010336896	no
GALNT3	0.167950872	3.061940535	0.002383773	0.010336896	no
TSPAN10	0.167924097	3.061438224	0.002387661	0.01035167	no
ZNF726	-0.167914542	-3.061258977	0.00238905	0.010355502	no
CEP170	-0.167911352	-3.061199123	0.002389514	0.010355502	no
FLJ16779	-0.167908078	-3.061137711	0.002389991	0.010355502	no
SPRY2	0.16790094	3.061003805	0.002391029	0.010357914	no
GPSM2	-0.167891952	-3.060835192	0.002392338	0.010360113	no
CBLN3	0.16789083	3.060814139	0.002392501	0.010360113	no
ADAM9	0.167835755	3.059780953	0.002400533	0.010392573	no
ADAM8	0.167832802	3.059725569	0.002400964	0.010392573	no
TMEM245	-0.167829176	-3.059657543	0.002401494	0.010392773	no
LOC283070	-0.167817861	-3.059445293	0.002403148	0.010397836	no
PAIP2B	-0.167752396	-3.058217245	0.002412737	0.010437223	no
CLTB	-0.167734831	-3.057887752	0.002415315	0.010446275	no
MPDZ	-0.167688833	-3.057024919	0.00242208	0.010473425	no
LOC100506730	-0.167657398	-3.056435281	0.002426713	0.010491348	no
STXBP6	-0.167627876	-3.05588152	0.002431071	0.010506122	no
TIMM23	-0.167622107	-3.055773315	0.002431924	0.010506122	no
NMT1	0.167622005	3.055771402	0.002431939	0.010506122	no
TAF6	-0.167621012	-3.055752776	0.002432086	0.010506122	no
FHAD1	0.167605386	3.055459674	0.002434396	0.01051399	no
SFXN2	0.167596937	3.055301201	0.002435647	0.010517276	no
NICN1	-0.167576897	-3.054925316	0.002438614	0.010527975	no
TMEM123	0.167551189	3.054443112	0.002442426	0.010542314	no
TRAPPC2	-0.16745035	-3.052551782	0.002457431	0.010604948	no
NARFL	-0.167443157	-3.052416875	0.002458504	0.01060745	no
EFHA2	-0.167437072	-3.052302754	0.002459412	0.01060924	no
DPF1	-0.167395462	-3.051522348	0.002465633	0.010633938	no
FAM5C	-0.167370074	-3.051046201	0.002469435	0.010646193	no
FKBP3	-0.167369875	-3.051042457	0.002469465	0.010646193	no
KCNH1	-0.167357418	-3.050808833	0.002471333	0.010651046	no
SLC4A7	0.167355757	3.050777683	0.002471582	0.010651046	no
MTF1	0.167339373	3.050470409	0.00247404	0.010659503	no
ZBTB8A	-0.167321549	-3.050136137	0.002476718	0.010668899	no
SIPA1L2	0.167309838	3.04991651	0.002478478	0.010674343	no
LGALS1	0.167291447	3.049571601	0.002481245	0.010684118	no
SGCG	-0.16728532	-3.049456685	0.002482167	0.01068589	no
METTL16	-0.16728211	-3.0493965	0.002482651	0.01068589	no
CNDP2	0.167275478	3.049272116	0.00248365	0.010688049	no
PCDH11X	-0.167250323	-3.048800376	0.002487442	0.010702226	no
RBFOX3	-0.167245468	-3.048709327	0.002488175	0.010703235	no
SAT2	-0.167228728	-3.048395383	0.002490702	0.010710121	no

ZNF252P-AS1	-0.167228265	-3.048386703	0.002490772	0.010710121	no
AJAP1	-0.167224205	-3.048310566	0.002491386	0.010710615	no
ZNF250	-0.167217805	-3.048190538	0.002492353	0.01071263	no
ARMCX5-GPRAS	-0.167170425	-3.047302032	0.002499523	0.010741302	no
UBE2Q2P3	-0.167164333	-3.047187794	0.002500446	0.010743121	no
NAV2	-0.167125889	-3.046466873	0.00250628	0.010766034	no
ZBTB42	0.167116195	3.046285082	0.002507753	0.010770208	no
EIF2D	0.167108812	3.046146627	0.002508876	0.010772876	no
ZNF821	-0.167043914	-3.044929679	0.002518761	0.010813163	no
MFNG	0.166995204	3.044016288	0.002526204	0.010842951	no
MFHAS1	0.166982002	3.043768741	0.002528225	0.010849457	no
TACSTD2	0.166970151	3.043546534	0.00253004	0.010855079	no
TEX29	-0.166911625	-3.042449133	0.002539022	0.010891364	no
GALNTL6	-0.16690635	-3.042350236	0.002539833	0.010891364	no
PPP2R5B	-0.166905149	-3.042327712	0.002540018	0.010891364	no
ZFP3	-0.166895138	-3.042140002	0.002541557	0.010895793	no
DEGS2	-0.166852935	-3.041348699	0.002548058	0.010921483	no
FAM181A-AS1	0.166845668	3.04121244	0.002549179	0.010924109	no
C8orf4	0.166835018	3.041012758	0.002550823	0.010928973	no
SCN2A	-0.166821934	-3.040767441	0.002552843	0.010935354	no
NUCB2	0.166816267	3.040661183	0.002553719	0.010935354	no
ARHGAP27	0.16681549	3.040646629	0.002553838	0.010935354	no
LIN52	-0.166801496	-3.040384235	0.002556002	0.010942438	no
WDR5B	-0.166741457	-3.039258577	0.002565302	0.010979807	no
FBXW11	-0.166738555	-3.03920417	0.002565753	0.010979807	no
LCN2	0.166709251	3.038654758	0.002570304	0.010996457	no
RTBDN	-0.166706916	-3.038610991	0.002570667	0.010996457	no
ZNF441	-0.166700841	-3.038497098	0.002571611	0.010998309	no
GPD1L	-0.166669886	-3.03791675	0.002576429	0.011016722	no
CDH5	0.166644987	3.037449932	0.002580311	0.011031124	no
LRP6	-0.166638768	-3.037333351	0.002581281	0.011033076	no
COBRA1	-0.166580314	-3.036237491	0.002590417	0.011069924	no
CDC5L	-0.166575408	-3.036145523	0.002591185	0.011071005	no
NKX1-2	-0.166547303	-3.035618634	0.002595589	0.011087619	no
PIRT	0.166541302	3.035506129	0.002596531	0.011088441	no
SERPINA5	0.166539497	3.035472298	0.002596814	0.011088441	no
FBXO21	-0.166506771	-3.034858802	0.002601953	0.011108179	no
EFEMP1	0.166501281	3.034755889	0.002602816	0.011108834	no
SPANXE	0.166499218	3.034717215	0.00260314	0.011108834	no
TDRD7	0.166485864	3.034466873	0.002605241	0.011115591	no
ANKRD6	-0.166467636	-3.034125171	0.002608111	0.011125627	no
EXOC5	-0.166450603	-3.033805865	0.002610795	0.011134867	no
WDR48	-0.166442664	-3.033657038	0.002612047	0.011137997	no
CC2D2B	0.166398781	3.032834426	0.002618977	0.011165332	no
SLC9A8	0.166381963	3.032519177	0.002621638	0.011173631	no
FGD5-AS1	-0.166379903	-3.032480565	0.002621964	0.011173631	no
EXOC6	-0.166365673	-3.032213812	0.002624217	0.011181016	no
NDUFAF2	-0.166352279	-3.031962755	0.002626339	0.011187833	no
FAM108B1	-0.166349006	-3.031901405	0.002626858	0.011187833	no
ESR1	0.166322452	3.031403654	0.002631071	0.011203557	no
CBR1	0.166283916	3.030681323	0.002637196	0.011225901	no

STMN1	-0.166280332	-3.03061414	0.002637767	0.011225901	no
RUNDC3B	-0.166278817	-3.030585745	0.002638008	0.011225901	no
LINC00574	-0.166276303	-3.030538624	0.002638408	0.011225901	no
TTYH2	-0.166244937	-3.0299507	0.002643405	0.011244937	no
ZNF524	0.166211367	3.029321468	0.002648763	0.011265499	no
LYPD5	-0.166202611	-3.029157348	0.002650162	0.011269219	no
GLDC	-0.166181531	-3.028762234	0.002653533	0.011281322	no
RHPN2	0.166167619	3.028501489	0.00265576	0.011288556	no
BAI1	-0.166162211	-3.028400119	0.002656626	0.011290005	no
BAIAP2	-0.166128348	-3.027765429	0.002662056	0.011310842	no
STAMBP	-0.166077228	-3.026807312	0.002670271	0.011343505	no
SLC35G6	-0.166062758	-3.026536113	0.0026726	0.011351157	no
SLFN11	0.166049271	3.026283346	0.002674773	0.011358141	no
OAT	-0.166026756	-3.025861359	0.002678405	0.011370686	no
CHTF18	-0.166024394	-3.025817092	0.002678786	0.011370686	no
NELL1	-0.166006602	-3.025483658	0.002681659	0.011380633	no
ARHGEF4	-0.165978238	-3.024952061	0.002686245	0.011397844	no
LOC154761	0.165972018	3.024835488	0.002687251	0.011399865	no
PPP2R2D	-0.165944049	-3.024311321	0.002691782	0.01141683	no
IFIT3	0.165937695	3.024192246	0.002692812	0.011418946	no
BATF3	0.165914672	3.02376078	0.002696548	0.011432532	no
PRKACA	0.165882117	3.023150684	0.002701838	0.011452703	no
WASH3P	-0.165857069	-3.022681273	0.002705915	0.011467724	no
RPL13A	-0.165834829	-3.022264487	0.00270954	0.011480822	no
LOC439994	-0.165817243	-3.021934938	0.002712409	0.011490714	no
PTGER4P2-CDK	-0.165785402	-3.021338253	0.002717612	0.011506764	no
FRZB	0.165784332	3.021318201	0.002717787	0.011506764	no
FKBP14	0.16578301	3.021293417	0.002718003	0.011506764	no
BCO2	0.16578095	3.021254815	0.00271834	0.011506764	no
METTL10	-0.16573195	-3.020336609	0.002726365	0.011538462	no
CTAGE4	0.165674765	3.019265028	0.002735758	0.011575936	no
SLC38A6	0.165663542	3.019054741	0.002737605	0.01158021	no
RAET1G	0.165662079	3.01902732	0.002737846	0.01158021	no
ZNF835	-0.165650948	-3.018818741	0.002739679	0.011585683	no
SLC6A1-AS1	-0.16563195	-3.018462749	0.00274281	0.011596642	no
PITPNM3	-0.165622262	-3.018281217	0.002744408	0.011601116	no
ADAM6	-0.165594984	-3.017770089	0.002748912	0.01161787	no
DKFZp779M065	-0.165576681	-3.017427134	0.002751938	0.011628371	no
SRP19	-0.165558903	-3.01709403	0.00275488	0.011638513	no
GRIN1	-0.165528407	-3.016522617	0.002759933	0.01165757	no
FAM19A1	-0.165498031	-3.015953472	0.002764974	0.01167657	no
BAIAP2L2	-0.165469492	-3.01541875	0.002769719	0.011694308	no
LOC650293	-0.165463662	-3.015309512	0.002770689	0.011696106	no
LOC100128593	-0.165426675	-3.014616516	0.002776851	0.011719815	no
KNDC1	-0.165423163	-3.014550719	0.002777436	0.011719985	no
MAPKAP1	0.165419607	3.01448409	0.00277803	0.011720187	no
NFIL3	0.165405275	3.014215582	0.002780421	0.011727975	no
IPO7	-0.165334158	-3.012883154	0.002792318	0.011775689	no
MITF	0.165329261	3.012791412	0.002793139	0.011775689	no
CALM1	-0.165327841	-3.012764805	0.002793377	0.011775689	no
PSMA2	0.165315804	3.012539299	0.002795396	0.011781888	no

ZFP30	-0.165304741	-3.012332032	0.002797252	0.011787401	no
PNN	-0.165290056	-3.012056914	0.002799718	0.011795481	no
PGGT1B	0.165248846	3.011284869	0.00280665	0.011822366	no
PLIN5	-0.165242958	-3.011174564	0.002807641	0.011824225	no
ZXDB	-0.165219853	-3.010741714	0.002811536	0.011838305	no
ARHGAP19-SLI	-0.165195162	-3.010279147	0.002815703	0.011853529	no
ANKRD33B	-0.165175448	-3.009909839	0.002819034	0.011865228	no
CBLN2	-0.165160044	-3.009621269	0.002821639	0.011873868	no
RASSF9	0.165135204	3.009155932	0.002825845	0.011889239	no
GPRASP2	-0.165110535	-3.0086938	0.002830027	0.011904506	no
SLPI	0.165106609	3.008620248	0.002830694	0.011904978	no
UBXN7	-0.165067682	-3.007891045	0.002837306	0.011930454	no
RAB11FIP2	-0.165050492	-3.007569039	0.002840231	0.011940415	no
TMEM9	-0.165036015	-3.007297841	0.002842696	0.011948442	no
BICD2	-0.165019674	-3.006991733	0.002845481	0.011957809	no
PDE2A	-0.16498707	-3.006380994	0.002851045	0.011978849	no
FGF13	-0.164950275	-3.00569178	0.002857336	0.012002934	no
C20orf24	0.164916114	3.005051895	0.002863188	0.012025167	no
PGBD5	-0.164879041	-3.004357479	0.002869551	0.012049537	no
FAM46B	0.164870516	3.0041978	0.002871016	0.012050509	no
STK36	0.164869771	3.004183846	0.002871145	0.012050509	no
FLJ16341	-0.164867906	-3.004148912	0.002871465	0.012050509	no
CACNA2D3	-0.164847236	-3.00376175	0.002875021	0.012063075	no
CEP250	-0.164828796	-3.003416371	0.002878196	0.01207404	no
TCF7L1	-0.164811695	-3.003096063	0.002881143	0.012084047	no
PMM1	-0.164805154	-3.002973537	0.002882272	0.012085946	no
LHFPL3	-0.164802549	-3.002924749	0.002882721	0.012085946	no
SLC15A2	-0.16479407	-3.002765936	0.002884184	0.012089722	no
LOC440040	-0.164706568	-3.001127066	0.002899324	0.012148662	no
AMZ2	-0.164706268	-3.001121438	0.002899376	0.012148662	no
LRRC37BP1	-0.16469288	-3.0008707	0.002901699	0.012156025	no
GNA14	0.164644177	2.999958561	0.002910163	0.012189107	no
FAM124B	0.164619752	2.999501107	0.002914416	0.012204543	no
ZNF248	-0.164583881	-2.998829319	0.002920673	0.012228361	no
TOE1	0.164571721	2.998601575	0.002922797	0.01223487	no
TBX18	0.16453861	2.997981483	0.002928586	0.012256719	no
SHISA4	-0.164513936	-2.99751941	0.002932908	0.012272414	no
LOC10013132C	-0.164502428	-2.997303891	0.002934925	0.012278466	no
ATG2B	-0.164486324	-2.997002315	0.00293775	0.012287893	no
LOC730183	-0.164463963	-2.996583557	0.002941678	0.012300382	no
SECISBP2L	-0.164462807	-2.996561907	0.002941881	0.012300382	no
UBE2J2	0.164450538	2.996332157	0.002944038	0.012307005	no
KIF16B	0.164434632	2.996034297	0.002946836	0.012316307	no
CCDC74A	0.164416808	2.995700517	0.002949975	0.012327029	no
NEURL	-0.164409273	-2.995559399	0.002951303	0.01233018	no
EIF3I	0.164386361	2.995130355	0.002955343	0.012344662	no
DLL1	-0.164297214	-2.99346102	0.002971113	0.012408123	no
SYCE1	-0.164286033	-2.993251649	0.002973097	0.012413993	no
SDHC	0.164244096	2.992466382	0.002980546	0.012442681	no
ATP10D	0.164229316	2.992189634	0.002983176	0.01245124	no
GTPBP6	-0.164215937	-2.991939113	0.002985558	0.012458762	no

SLC16A5	0.164212403	2.991872939	0.002986188	0.01245897	no
ZNF358	-0.164153899	-2.990777491	0.002996626	0.012500096	no
KLRC3	-0.16405438	-2.988914164	0.003014459	0.012572044	no
CDC42EP5	0.164007849	2.988042969	0.00302283	0.01260451	no
REXO1	-0.163998076	-2.987860011	0.003024591	0.012609405	no
PXMP2	-0.163973095	-2.987392304	0.003029096	0.012624138	no
GIPC2	0.163971968	2.987371209	0.0030293	0.012624138	no
PSMB7	-0.163953808	-2.987031211	0.003032579	0.012635353	no
LOC100270804	-0.163929459	-2.986575343	0.00303698	0.01265124	no
RHOJ	0.163887999	2.98579915	0.003044489	0.01268006	no
PALLD	0.163835796	2.984821857	0.003053966	0.012717069	no
KCNB2	-0.163795321	-2.984064141	0.003061333	0.012743544	no
TVP23A	0.16379435	2.98404595	0.00306151	0.012743544	no
LRRN4CL	0.163777352	2.983727748	0.003064609	0.012752946	no
SCNN1G	0.163773809	2.983661427	0.003065255	0.012752946	no
PCDH9	-0.163772197	-2.983631245	0.003065549	0.012752946	no
NAV2-AS4	-0.163734816	-2.98293146	0.003072376	0.012778618	no
VWCE	-0.163731897	-2.982876826	0.00307291	0.012778618	no
ACAP3	-0.163708869	-2.982445747	0.003077123	0.012792909	no
CD200	-0.163706607	-2.982403397	0.003077537	0.012792909	no
HTR6	-0.163674472	-2.981801852	0.003083425	0.012814908	no
LIPG	0.163646233	2.981273244	0.003088608	0.012833967	no
SLC25A37	0.163567111	2.979792179	0.003103173	0.012891994	no
HLA-J	0.163553492	2.97953724	0.003105686	0.012899942	no
MYCN	-0.163498682	-2.978511322	0.003115819	0.01293953	no
ERBB2IP	0.163494216	2.978427728	0.003116646	0.012940463	no
LOC100130417	-0.163458133	-2.977752344	0.003123335	0.012965731	no
ALOX12B	-0.163443333	-2.977475328	0.003126082	0.01297463	no
CUL3	-0.163424194	-2.977117108	0.003129638	0.012986881	no
MTRNR2L1	-0.163379804	-2.976286259	0.0031379	0.013018651	no
ATP2A2	-0.163374217	-2.976181695	0.003138941	0.013020457	no
FERMT1	-0.163334729	-2.975442614	0.00314631	0.013048503	no
FAM13C	-0.163313956	-2.97505383	0.003150192	0.013062083	no
SENP3	-0.1633101	-2.974981662	0.003150913	0.013062381	no
SNORD22	-0.163307073	-2.974925009	0.003151479	0.013062381	no
HPS5	0.163270299	2.97423676	0.003158366	0.013088399	no
SHANK1	-0.163252726	-2.973907874	0.003161661	0.013099529	no
LZTS1	0.163242733	2.973720845	0.003163537	0.013104774	no
NPEPL1	0.163231622	2.9735129	0.003165623	0.013110889	no
APBB1	-0.163222691	-2.97334576	0.003167301	0.013115311	no
ZNF410	0.163211554	2.973137335	0.003169394	0.013121452	no
DLG5	-0.163158093	-2.972136818	0.003179462	0.013160029	no
TOM1	-0.163155571	-2.972089628	0.003179937	0.013160029	no
YJEFN3	-0.163149414	-2.971974396	0.003181099	0.013162199	no
FAM188B	0.163146299	2.971916096	0.003181686	0.013162199	no
MXRA7	0.163093891	2.970935325	0.00319159	0.013200626	no
POU3F3	-0.163078499	-2.970647282	0.003194504	0.013210136	no
AMDHD2	0.163033233	2.969800209	0.003203087	0.013243081	no
FKBP7	0.163016184	2.969481166	0.003206325	0.01325392	no
STAP2	0.163000526	2.969188146	0.003209302	0.013263674	no
HOXB2	0.162977511	2.968757478	0.003213681	0.013279221	no

LOC441081	0.162937743	2.968013316	0.003221262	0.013307986	no
SNN	-0.162923892	-2.967754122	0.003223906	0.013312795	no
WBSCR27	0.16292214	2.967721338	0.003224241	0.013312795	no
SORBS3	0.162921912	2.967717077	0.003224284	0.013312795	no
C12orf70	0.162884982	2.967026034	0.003231345	0.01333649	no
DNAJC16	0.162882245	2.96697482	0.003231868	0.01333649	no
ATP5G2	-0.162882158	-2.966973194	0.003231885	0.01333649	no
UCMA	0.162867697	2.966702598	0.003234654	0.013343143	no
UCK2	-0.162867254	-2.966694309	0.003234739	0.013343143	no
CYB561	0.162859867	2.966556087	0.003236154	0.013346419	no
HPD	0.162850489	2.966380621	0.003237951	0.013351269	no
YKT6	0.162802201	2.96547708	0.003247221	0.013386924	no
METTL7B	0.162762354	2.964731507	0.003254889	0.013414423	no
PNPLA4	0.162760757	2.964701618	0.003255197	0.013414423	no
DPF2	-0.162757811	-2.964646507	0.003255764	0.013414423	no
TNF	0.162739749	2.96430855	0.003259246	0.013423814	no
ARF3	-0.162739507	-2.96430403	0.003259293	0.013423814	no
DHX38	-0.162729102	-2.964109331	0.0032613	0.013429508	no
MAMLD1	-0.162724032	-2.96401448	0.003262279	0.013430696	no
EIF4E1B	-0.162721132	-2.963960218	0.003262839	0.013430696	no
PLA2G2C	0.162716834	2.963879794	0.003263668	0.01343154	no
PLA2G12A	-0.162641892	-2.962477634	0.003278169	0.013488633	no
BEND4	-0.162634619	-2.962341558	0.003279579	0.013491596	no
LOX	0.162631706	2.962287055	0.003280145	0.013491596	no
NTRK1	0.162574754	2.961221534	0.003291209	0.013534516	no
FYN	-0.162505032	-2.959917128	0.003304801	0.013585811	no
ST3GAL6	-0.162504284	-2.959903135	0.003304947	0.013585811	no
ODF2L	0.162500813	2.95983819	0.003305625	0.013586	no
DPP9	0.162480288	2.959454207	0.003309637	0.013599888	no
CENPT	-0.162466188	-2.959190423	0.003312396	0.013608621	no
WASF2	0.162455085	2.9589827	0.003314569	0.013614949	no
LRRC61	0.162428508	2.958485505	0.003319778	0.013632583	no
GALNT6	0.162426706	2.9584518	0.003320131	0.013632583	no
CRLF2	0.16241279	2.958191463	0.003322861	0.013641187	no
LRFN4	-0.162389477	-2.957755345	0.00332744	0.013657375	no
PPT2-EGFL8	-0.162353527	-2.957082823	0.003334512	0.013683787	no
SOGA2	-0.162345729	-2.956936951	0.003336048	0.013687476	no
ATL2	-0.162327666	-2.956599044	0.003339607	0.013699466	no
RIMS3	-0.162323732	-2.956525448	0.003340383	0.013700033	no
QPCT	0.162261355	2.955358594	0.003352705	0.013747947	no
PCDH19	-0.162251537	-2.955174956	0.003354648	0.013751854	no
POU2F1	-0.162250076	-2.955147608	0.003354938	0.013751854	no
AFF1	0.162241553	2.954988188	0.003356626	0.013756148	no
B3GAT1	-0.162228895	-2.954751404	0.003359134	0.013763803	no
IFFO2	0.162196722	2.954149594	0.003365517	0.013787327	no
NDUFA2	0.162170383	2.953656913	0.00337075	0.013806135	no
EXT1	0.162137636	2.953044374	0.003377267	0.013830192	no
RPL37A	-0.162125927	-2.952825364	0.0033796	0.013837109	no
ZNF770	-0.162035246	-2.951129227	0.003397718	0.013908639	no
MAPKBP1	-0.162029925	-2.951029699	0.003398784	0.013910269	no
CROCCP3	-0.162026796	-2.950971174	0.003399411	0.013910269	no

GATSL1	-0.162012945	-2.950712123	0.003402187	0.013918979	no
GUCY2GP	0.161970811	2.94992406	0.003410645	0.013950927	no
LOC728978	0.161948651	2.949509597	0.003415101	0.013964509	no
CDK2AP2	0.161947837	2.949494372	0.003415265	0.013964509	no
FRMD4B	0.161940207	2.949351665	0.003416801	0.01396813	no
PTGR2	-0.161931373	-2.949186445	0.003418579	0.013972743	no
N4BP1	0.161922245	2.949015722	0.003420418	0.013977601	no
METTL21A	-0.161901618	-2.948629946	0.003424576	0.013986965	no
HNF4G	0.161901562	2.948628897	0.003424588	0.013986965	no
PAXBP1-AS1	-0.161901194	-2.948622006	0.003424662	0.013986965	no
NAA16	-0.161800183	-2.94673288	0.003445093	0.014066598	no
MAML3	0.161798328	2.946698191	0.003445469	0.014066598	no
KDM4D	-0.161766835	-2.946109207	0.003451862	0.014090022	no
PRPF39	-0.161725196	-2.945330514	0.003460331	0.01412191	no
IKBKG	0.161718181	2.945199332	0.00346176	0.014125057	no
ASTN2	-0.161695257	-2.944770617	0.003466432	0.014139455	no
LOC646903	-0.161694414	-2.944754862	0.003466604	0.014139455	no
GSX1	-0.161675762	-2.944406057	0.00347041	0.014152293	no
IQCG	0.161669047	2.94428048	0.003471781	0.014155199	no
ILK	0.161662043	2.944149494	0.003473212	0.014158346	no
KIAA1199	0.161657995	2.944073804	0.003474039	0.014158612	no
JAKMIP3	-0.161655275	-2.944022942	0.003474595	0.014158612	no
WNT5A	0.161633559	2.943616849	0.003479036	0.014174021	no
YTHDC1	-0.161623106	-2.943421377	0.003481175	0.014180049	no
GRID1-AS1	-0.161596734	-2.942928206	0.003486578	0.014199366	no
NNT	-0.161579297	-2.942602144	0.003490155	0.014211239	no
TSR2	-0.161558655	-2.94221614	0.003494393	0.014225801	no
RNASEH2C	-0.161528866	-2.941659115	0.003500518	0.014248035	no
PCSK2	-0.161524737	-2.941581894	0.003501368	0.014248796	no
THAP5	-0.161500438	-2.941127536	0.003506372	0.01426532	no
COL12A1	0.161498575	2.941092692	0.003506756	0.01426532	no
ARHGAP31	-0.161468956	-2.940538865	0.003512865	0.014283784	no
FBXW7	-0.16146754	-2.940512386	0.003513157	0.014283784	no
LONRF2	-0.161465362	-2.94047166	0.003513606	0.014283784	no
PTBP2	-0.161463684	-2.940440278	0.003513953	0.014283784	no
GHITM	-0.161432967	-2.939865923	0.0035203	0.014304296	no
TFR2	-0.161432822	-2.939863205	0.00352033	0.014304296	no
RIMKLA	-0.161349054	-2.938296909	0.003537694	0.014372132	no
ALPK2	0.1613209	2.937770512	0.003543547	0.014393189	no
AGAP6	-0.161294298	-2.937273118	0.003549085	0.014412962	no
PRRC2B	-0.161275665	-2.936924744	0.003552969	0.014426008	no
ADRBK1	0.161268034	2.936782067	0.003554561	0.014429745	no
TCEAL3	-0.161234383	-2.936152899	0.003561588	0.014455542	no
ORC4	-0.16116525	-2.934860381	0.003576064	0.014511555	no
STX1A	-0.161121428	-2.934041095	0.003585268	0.014546157	no
LOC100505687	-0.161115811	-2.933936088	0.003586449	0.014548203	no
RAP1GAP	-0.161106547	-2.933762886	0.003588398	0.014553362	no
TXN	0.161091536	2.933482264	0.003591558	0.014563429	no
SUSD4	-0.161077791	-2.933225299	0.003594454	0.014572422	no
MOB4	-0.161062517	-2.932939755	0.003597674	0.014582727	no
SLC44A2	-0.161044291	-2.932599022	0.00360152	0.014595564	no

RPL17	-0.161019347	-2.932132692	0.00360679	0.014614166	no
GJD2	-0.161004814	-2.931861003	0.003609864	0.014622517	no
ACTN4	0.161003167	2.931830221	0.003610212	0.014622517	no
KIAA1984	-0.160962161	-2.931063646	0.003618898	0.014654936	no
CNOT6	-0.160935552	-2.930566218	0.003624545	0.014675037	no
ADA	0.160931364	2.930487925	0.003625434	0.014675873	no
HIF1A-AS2	0.160925082	2.9303705	0.003626769	0.01467851	no
MAGI2	-0.160919115	-2.930258958	0.003628037	0.014680877	no
TIMM13	-0.160893461	-2.929779397	0.003633493	0.014700188	no
GPR21	-0.160875853	-2.92945025	0.003637242	0.014712586	no
40057	0.160838416	2.928750432	0.003645225	0.014742103	no
ZNF404	-0.160790815	-2.927860637	0.003655398	0.014780464	no
CFL1	0.160782751	2.927709911	0.003657124	0.014782667	no
C7orf49	0.160781842	2.927692924	0.003657319	0.014782667	no
TMOD1	0.160768413	2.927441902	0.003660195	0.01479151	no
MOCOS	0.16075064	2.927109692	0.003664004	0.014804121	no
ABHD14A-ACY1	-0.160722103	-2.926576271	0.003670129	0.014826078	no
LOC100130744	0.160714559	2.926435257	0.003671749	0.014829837	no
MIR3911	-0.160692881	-2.926030058	0.00367641	0.014845869	no
HLA-C	0.160678692	2.925764853	0.003679463	0.014855407	no
SSR1	0.160664181	2.92549363	0.003682587	0.01486523	no
AMZ1	-0.160654008	-2.925303487	0.00368478	0.014871286	no
FAM98B	-0.160631515	-2.924883055	0.003689631	0.014888069	no
PMCH	0.160601945	2.924330376	0.003696017	0.014909538	no
RNF115	0.160600455	2.924302527	0.003696339	0.014909538	no
NDUFAF4	-0.160571965	-2.923770029	0.003702502	0.014931595	no
TXNIP	0.160562921	2.923601003	0.00370446	0.014936689	no
ANO8	-0.160519586	-2.922791066	0.003713857	0.014971768	no
SP4	-0.160510921	-2.922629124	0.003715738	0.014976543	no
GNAI2	0.160468583	2.921837839	0.003724943	0.01501083	no
TMEM254	-0.160437401	-2.921255059	0.003731736	0.015032809	no
CD300LG	-0.160437126	-2.921249924	0.003731796	0.015032809	no
TCF7L2	-0.160417447	-2.920882147	0.003736089	0.015047281	no
SUGT1P3	-0.160384635	-2.920268928	0.003743257	0.015073325	no
ESRRB	0.160365465	2.919910669	0.00374745	0.015087383	no
MRPL24	-0.1603153	-2.918973166	0.003758444	0.01512881	no
ZNF32	-0.16029154	-2.918529149	0.003763661	0.015146973	no
LLGL1	-0.160288023	-2.91846343	0.003764434	0.015147247	no
MRPS31	-0.160277908	-2.918274394	0.003766657	0.015153356	no
CLEC2L	-0.160244843	-2.917656505	0.003773933	0.015179787	no
ARHGAP10	0.160146283	2.915814714	0.003795699	0.015264476	no
TPRN	-0.160133989	-2.915584989	0.003798421	0.015272568	no
CDC42EP3	0.160120807	2.915338671	0.003801343	0.015281455	no
HTT	-0.160089927	-2.914761636	0.003808195	0.015306137	no
TMEM64	0.160072839	2.914442337	0.003811991	0.01531853	no
HSPB11	0.160049453	2.91400536	0.003817192	0.015336563	no
CDA	0.159989794	2.912890604	0.003830489	0.015387112	no
CATSPER1	0.159960268	2.912338915	0.003837086	0.01541073	no
LOC283038	-0.159956164	-2.912262243	0.003838004	0.015411535	no
TAF3	-0.159884798	-2.910928804	0.003853994	0.015472852	no
CES2	-0.159871411	-2.910678679	0.003857	0.015482029	no

LOC100132077	-0.15985848	-2.910437088	0.003859906	0.015490799	no
FLT3	0.159844336	2.910172825	0.003863086	0.015499102	no
KCNAB1	-0.159842679	-2.910141858	0.003863459	0.015499102	no
HLCS	0.159839661	2.910085474	0.003864138	0.015499102	no
PAPOLG	0.159830086	2.909906582	0.003866293	0.015504852	no
MAD2L1BP	-0.15981354	-2.90959744	0.00387002	0.015515078	no
ABLIM1	-0.159812354	-2.909575283	0.003870287	0.015515078	no
ZNF683	0.159796768	2.90928408	0.0038738	0.015526267	no
SOX15	-0.159784554	-2.90905589	0.003876556	0.015534414	no
PLEKHN1	0.159777849	2.908930611	0.003878069	0.015537581	no
ZNF287	-0.159772869	-2.908837581	0.003879193	0.015538572	no
C11orf30	-0.159770349	-2.908790502	0.003879762	0.015538572	no
CMYA5	0.159766624	2.908720897	0.003880604	0.015539046	no
NCOA2	-0.159737835	-2.908183046	0.003887112	0.015562205	no
CBY3	-0.159729577	-2.908028767	0.00388898	0.015566786	no
THAP6	0.159721696	2.907881533	0.003890764	0.015571026	no
TXNDC9	0.159686256	2.90721942	0.003898796	0.015600264	no
POLR1D	0.159674808	2.907005557	0.003901393	0.015607752	no
SLC1A2	-0.159638909	-2.906334881	0.003909549	0.015637469	no
DYNC1LI1	-0.159625441	-2.90608328	0.003912613	0.015646811	no
SPECC1	-0.159581254	-2.905257799	0.00392268	0.015684152	no
LRTM2	-0.159532808	-2.904352784	0.003933744	0.015725464	no
HNRNPH2	-0.159512086	-2.903965676	0.003938485	0.015739664	no
LOH12CR2	-0.159510881	-2.903943174	0.003938761	0.015739664	no
RAB6C	-0.159484645	-2.90345306	0.003944772	0.015760753	no
ZNF518B	0.159455855	2.902915269	0.003951377	0.015784208	no
ATG14	-0.159426375	-2.902364581	0.003958151	0.015808329	no
PNMA5	-0.159402616	-2.90192077	0.003963618	0.015826703	no
NTRK3	-0.159399982	-2.901871571	0.003964224	0.015826703	no
TCTE3	-0.159391009	-2.901703963	0.003966291	0.015826859	no
SUMO2	-0.159390401	-2.901692591	0.003966431	0.015826859	no
GPAA1	0.15939022	2.901689218	0.003966473	0.015826859	no
ADAM17	0.159363053	2.901181747	0.003972736	0.015848909	no
TP73-AS1	0.159326776	2.90050413	0.003981114	0.015877909	no
TEAD4	0.159325179	2.90047431	0.003981483	0.015877909	no
C7orf60	-0.15930993	-2.900189461	0.00398501	0.015889026	no
ANP32A	-0.159270999	-2.899462298	0.003994026	0.015922022	no
TRERF1	-0.159263783	-2.899327509	0.0039957	0.015925739	no
RTN4RL2	-0.15925168	-2.899101451	0.003998508	0.015933975	no
ELOVL4	-0.159218116	-2.89847454	0.004006304	0.015962082	no
ST8SIA1	-0.159186331	-2.897880862	0.004013699	0.015986493	no
CTAGE15P	0.159185391	2.897863306	0.004013918	0.015986493	no
DHRS4L2	0.159177819	2.897721882	0.004015682	0.015990554	no
TBC1D14	-0.159174047	-2.897651419	0.004016561	0.015991091	no
MECP2	-0.159156492	-2.89732354	0.004020653	0.01600442	no
MST1	-0.159144176	-2.897093509	0.004023527	0.016012892	no
GSTM3	-0.159121997	-2.896679282	0.004028706	0.016030535	no
B3GALNT2	0.159097871	2.896228673	0.004034347	0.016050009	no
HAUS4	0.159089645	2.896075039	0.004036272	0.016054695	no
ASGR2	0.159081209	2.895917478	0.004038247	0.01605691	no
LUM	0.159080883	2.895911404	0.004038323	0.01605691	no

TRPC5	-0.159038052	-2.895111459	0.004048364	0.016093856	no
LINC00657	-0.158978404	-2.893997475	0.004062384	0.016146606	no
STK39	-0.158928312	-2.893061978	0.004074192	0.016190545	no
CCL4	0.15891635	2.892838598	0.004077017	0.016193821	no
MURC	-0.158915301	-2.892819001	0.004077264	0.016193821	no
ADAT1	0.158915247	2.892817986	0.004077277	0.016193821	no
LRRC41	0.158871569	2.892002301	0.004087606	0.016231844	no
NIM1	-0.158855338	-2.891699204	0.00409145	0.016244107	no
MSX2P1	-0.158834089	-2.891302382	0.004096488	0.016261104	no
PRH1-PRR4	-0.158825837	-2.891148291	0.004098445	0.01626587	no
HAGH	-0.158815329	-2.890952054	0.00410094	0.016270493	no
LY6E	0.158813646	2.890920631	0.004101339	0.016270493	no
ZKSCAN7	-0.158811363	-2.890877999	0.004101881	0.016270493	no
KIAA0825	0.1588063	2.890783453	0.004103084	0.01627226	no
C16orf93	0.158798808	2.890643538	0.004104864	0.016276316	no
C14orf159	-0.158777519	-2.890245979	0.004109926	0.016293382	no
VWA5A	0.158766866	2.890047061	0.004112461	0.016300425	no
KCNE4	0.15874549	2.889647891	0.004117552	0.016313963	no
DPYS	0.158743234	2.889605756	0.00411809	0.016313963	no
BASP1	-0.158742966	-2.889600752	0.004118154	0.016313963	no
ZNF780B	-0.158739607	-2.889538028	0.004118955	0.016314127	no
LINC00475	0.158730874	2.889374953	0.004121037	0.016319367	no
FRS2	-0.158710931	-2.889002559	0.004125795	0.016335201	no
ADAM10	0.158704558	2.888883545	0.004127317	0.016338216	no
CSNK1G1	0.158684988	2.888518116	0.004131993	0.016353714	no
EPM2A	-0.158675822	-2.888346949	0.004134185	0.016359377	no
C1orf106	-0.15866828	-2.888206119	0.004135989	0.016363503	no
TNFRSF6B	0.158622239	2.887346423	0.004147019	0.016404119	no
KCTD6	-0.158574029	-2.88644623	0.004158596	0.016446889	no
LOC158696	-0.158552498	-2.886044209	0.004163776	0.016464346	no
DOPEY2	0.158373111	2.882694863	0.004207162	0.016629931	no
STUB1	-0.158372953	-2.882691914	0.0042072	0.016629931	no
CLGN	-0.158356146	-2.882378131	0.004211286	0.016643019	no
MCTS1	0.158344528	2.882161213	0.004214112	0.016651128	no
N6AMT1	-0.15833674	-2.882015811	0.004216008	0.016655555	no
C16orf87	-0.158331075	-2.881910041	0.004217387	0.016657943	no
C12orf54	-0.158292523	-2.881190303	0.004226784	0.016691993	no
SFRP2	-0.158282183	-2.880997249	0.004229308	0.016698891	no
ZIC3	-0.158276888	-2.880898397	0.004230601	0.016700928	no
MPPED1	-0.1582611	-2.880603647	0.004234458	0.016712012	no
SLITRK4	-0.158257757	-2.88054123	0.004235276	0.016712012	no
GPR3	0.158255849	2.880505613	0.004235742	0.016712012	no
ASIC2	-0.158239404	-2.880198614	0.004239764	0.01672481	no
RAPGEF1	0.158223265	2.879897303	0.004243715	0.016737323	no
FBXW4	-0.158152864	-2.878583023	0.004260987	0.016801574	no
NMNAT1	0.158147935	2.878491011	0.004262198	0.016801574	no
WARS	0.158147316	2.878479452	0.004262351	0.016801574	no
AMMECR1	-0.15812278	-2.878021417	0.004268387	0.016822282	no
ZMYM2	-0.15810612	-2.8777104	0.00427249	0.016835366	no
RETN	0.158096648	2.877533592	0.004274824	0.016841476	no
KIF22	-0.15808998	-2.877409104	0.004276469	0.01684333	no

C10orf88	-0.158088382	-2.877379273	0.004276863	0.01684333	no
C4B_2	0.158074667	2.877123256	0.004280246	0.016853567	no
LOC642361	-0.158061254	-2.876872865	0.004283557	0.016863516	no
AEN	0.158052515	2.876709726	0.004285716	0.016867024	no
SLC17A3	0.158051292	2.87668691	0.004286018	0.016867024	no
WSB1	-0.158044918	-2.876567925	0.004287594	0.016868417	no
PRR24	0.158043509	2.87654161	0.004287942	0.016868417	no
TRABD2B	0.158033125	2.876347783	0.004290509	0.016875102	no
ZBTB39	-0.158030285	-2.876294764	0.004291212	0.016875102	no
ACE	0.158007008	2.875860254	0.004296973	0.016894667	no
SNCB	-0.157993757	-2.875612894	0.004300256	0.016904483	no
ME1	0.157977223	2.875304254	0.004304356	0.016917503	no
HMG20A	-0.157949001	-2.874777466	0.004311361	0.016941938	no
C20orf26	0.157907204	2.873997273	0.004321755	0.016979678	no
ADM	0.157882968	2.873544885	0.004327793	0.017000291	no
RNF185	-0.157865772	-2.873223899	0.004332081	0.017014027	no
BBS10	-0.157836174	-2.872671454	0.004339471	0.017039936	no
L3HYPDH	0.157832693	2.872606476	0.004340341	0.017040238	no
PRSS35	-0.157818474	-2.87234108	0.004343896	0.01705108	no
GNG13	-0.157798206	-2.87196277	0.004348967	0.017067806	no
KLB	-0.1577951	-2.871904793	0.004349745	0.017067806	no
CGREF1	-0.157774307	-2.871516694	0.004354955	0.017085128	no
PELI3	-0.157749736	-2.871058094	0.004361118	0.017106185	no
TRAPPC6B	-0.157732796	-2.870741905	0.004365372	0.017119746	no
IQCH	0.157721924	2.870538985	0.004368104	0.017127335	no
ACVR2A	-0.157695944	-2.870054092	0.004374639	0.017148086	no
LEPRE1	0.157694539	2.870027872	0.004374992	0.017148086	no
FAM181A	0.157689915	2.869941573	0.004376156	0.017149521	no
C7orf63	0.157669499	2.869560519	0.004381299	0.017163581	no
SENP8	-0.157669333	-2.869557424	0.004381341	0.017163581	no
APOC2	0.157639502	2.869000668	0.004388866	0.017188365	no
ZFP62	-0.157637911	-2.868970971	0.004389268	0.017188365	no
APBB2	-0.157582716	-2.867940864	0.004403222	0.017239869	no
SNRNP25	-0.157540583	-2.867154535	0.004413901	0.017278532	no
SYT5	-0.157510226	-2.866587998	0.00442161	0.017305557	no
DRG1	-0.157505718	-2.866503876	0.004422755	0.017306889	no
CHP2	-0.157471885	-2.865872478	0.004431363	0.017335242	no
GLRX5	-0.15746794	-2.86579885	0.004432368	0.017335242	no
MYBPC2	0.157467732	2.865794962	0.004432421	0.017335242	no
PSMC4	0.15742826	2.865058356	0.004442484	0.017371439	no
VSTM2L	-0.157425009	-2.864997676	0.004443314	0.017371524	no
LRSAM1	-0.157419217	-2.864889598	0.004444793	0.017374144	no
GLTSCR1L	-0.157387508	-2.864297862	0.004452896	0.017402653	no
RTN1	-0.157377545	-2.864111943	0.004455445	0.017408041	no
CHURC1-FNTB	-0.157375787	-2.864079128	0.004455895	0.017408041	no
PAQR9	-0.157355238	-2.863695663	0.004461156	0.017425428	no
C2orf66	0.15734968	2.863591944	0.00446258	0.017427824	no
GTF2I	-0.157339182	-2.863396048	0.004465271	0.017435164	no
CTSA	0.15731662	2.862975018	0.004471059	0.017454594	no
RILPL1	-0.15730269	-2.862715078	0.004474637	0.017465386	no
RELL1	0.157296513	2.862599818	0.004476223	0.017468407	no

DCTN1	-0.157268653	-2.862079934	0.004483388	0.017493189	no
MTX1	0.157256512	2.861853387	0.004486513	0.017501039	no
BRD7	-0.157254511	-2.861816051	0.004487028	0.017501039	no
MAFF	0.157239815	2.861541815	0.004490814	0.017512628	no
PCBP3	-0.157200873	-2.860815159	0.004500861	0.017548621	no
LOC643723	-0.157161019	-2.860071515	0.004511163	0.017585599	no
SMPD2	0.157125959	2.859417333	0.004520244	0.017617803	no
LOC10050554C	-0.157120959	-2.859324041	0.00452154	0.01761966	no
SATB2-AS1	-0.157024992	-2.857533448	0.004546487	0.017713662	no
CCNB3	-0.156999538	-2.857058526	0.004553125	0.017736308	no
UTP14C	-0.156992703	-2.856930996	0.004554909	0.017739398	no
APOM	-0.15698996	-2.856879814	0.004555625	0.017739398	no
ZNF32-AS1	-0.156987013	-2.85682483	0.004556395	0.017739398	no
LGALS17A	0.156943506	2.856013108	0.004567767	0.017780455	no
SLC25A3	-0.156927913	-2.855722176	0.00457185	0.017791169	no
LOC144486	-0.156926669	-2.855698979	0.004572176	0.017791169	no
HSPB6	0.156919125	2.855558222	0.004574152	0.017795637	no
LOC494558	0.15690776	2.855346195	0.004577131	0.017802281	no
NDUFS2	-0.156906289	-2.855318745	0.004577516	0.017802281	no
LPPR2	-0.15689772	-2.855158868	0.004579764	0.017807799	no
MYO16	-0.15686436	-2.854536487	0.004588522	0.017838627	no
ARPC5	0.15685304	2.854325287	0.004591498	0.017846967	no
SMG6	-0.156834962	-2.853988029	0.004596253	0.01786222	no
DDB1	-0.156817028	-2.853653444	0.004600975	0.017877337	no
SOD3	0.156808664	2.853497408	0.004603179	0.017882667	no
DBF4B	-0.156769662	-2.852769784	0.004613467	0.017919397	no
SIRT3	-0.156716058	-2.851769774	0.004627641	0.017971204	no
STOX1	-0.156698896	-2.851449619	0.004632188	0.017985609	no
CNNM3	-0.156691616	-2.851313807	0.004634117	0.017989851	no
ARL8A	-0.156675946	-2.851021489	0.004638273	0.018002734	no
KIAA1045	-0.156660792	-2.850738786	0.004642296	0.018015093	no
PROC	0.156646092	2.850464575	0.004646201	0.018026991	no
HMGN4	0.156635468	2.850266386	0.004649025	0.018033365	no
LRGUK	0.1566336	2.850231533	0.004649522	0.018033365	no
EIF3E	-0.156624577	-2.850063225	0.004651922	0.018039417	no
MYO18A	-0.156613564	-2.849857786	0.004654852	0.018047525	no
CACNA1B	-0.156593774	-2.849488616	0.004660123	0.018064701	no
TP53TG1	0.156558767	2.848835586	0.00466946	0.01809763	no
TYK2	0.15654414	2.848562744	0.004673366	0.018109503	no
AMOTL2	-0.156495177	-2.847649408	0.004686463	0.01815698	no
CASP6	0.156474957	2.847272256	0.004691881	0.018174694	no
DBN1	-0.156467809	-2.847138918	0.004693797	0.018176949	no
NISCH	-0.156466479	-2.847114107	0.004694154	0.018176949	no
ZNF608	-0.156424511	-2.846331292	0.004705423	0.018217301	no
LOC10024073€	0.156412172	2.84610114	0.00470874	0.018226862	no
FAM49B	0.156405945	2.845984993	0.004710416	0.018230063	no
VNN3	0.156376066	2.845427668	0.004718461	0.018257912	no
ZNF800	0.156356847	2.845069192	0.004723642	0.01827239	no
LRRC19	-0.156355881	-2.845051183	0.004723903	0.01827239	no
CCDC144A	-0.156323584	-2.844448788	0.004732622	0.018302822	no
EAPP	-0.156303584	-2.844075742	0.004738029	0.018320436	no

TAOK1	-0.156295136	-2.843918183	0.004740314	0.018325975	no
DGKK	-0.156266494	-2.843383971	0.00474807	0.018352658	no
SEC24A	0.156238799	2.842867425	0.004755581	0.018378383	no
GBAP1	-0.156208921	-2.842310184	0.004763695	0.018406432	no
KCNQ5	-0.156189986	-2.841957042	0.004768844	0.018423014	no
HTR1D	0.156178739	2.841747271	0.004771905	0.018431526	no
TCEAL1	-0.156174002	-2.841658919	0.004773195	0.018433194	no
CCNB1IP1	-0.156148957	-2.841191836	0.004780018	0.018454386	no
GSE1	-0.156147558	-2.841165748	0.0047804	0.018454386	no
BTG3	0.156143328	2.841086849	0.004781553	0.018455524	no
NUBPL	-0.156107806	-2.840424369	0.004791249	0.018489628	no
LOC284578	-0.156072263	-2.839761498	0.004800969	0.018521334	no
FLVCR1	-0.156071459	-2.839746509	0.004801189	0.018521334	no
SHANK2-AS1	-0.156064153	-2.839610263	0.004803189	0.018525724	no
GFER	-0.156001921	-2.838449681	0.004820257	0.018588218	no
LOC150935	-0.155984719	-2.838128885	0.004824984	0.018600278	no
GAS5	-0.155984243	-2.838120013	0.004825115	0.018600278	no
SUGT1P1	-0.155958705	-2.83764377	0.004832141	0.018624023	no
MARVELD3	-0.155936772	-2.83723476	0.004838183	0.018643965	no
EFNA2	-0.155932287	-2.837151108	0.00483942	0.018645387	no
C1orf220	-0.155919899	-2.836920104	0.004842836	0.018655204	no
RNF165	-0.155829312	-2.835230868	0.004867883	0.018748328	no
KIAA0020	0.155816693	2.834995557	0.004871381	0.018758414	no
C2orf68	-0.15581357	-2.834937327	0.004872247	0.018758414	no
GPIHBP1	-0.15580983	-2.83486758	0.004873285	0.018759048	no
ZNF154	-0.155770832	-2.83414039	0.004884115	0.018797369	no
PPM1H	-0.155764469	-2.83402174	0.004885884	0.01880081	no
FADS3	0.155719285	2.833179226	0.004898462	0.018845838	no
UBE2K	-0.155699355	-2.832807598	0.00490402	0.018863843	no
SLC25A15	-0.155689931	-2.83263188	0.00490665	0.018870581	no
PARP6	-0.155678874	-2.832425714	0.004909737	0.018879075	no
TCL1A	0.155658878	2.832052868	0.004915324	0.01889443	no
TBCD	-0.155658291	-2.832041929	0.004915488	0.01889443	no
SNAP25	-0.155650451	-2.831895744	0.004917681	0.018899477	no
KLHL13	-0.155639477	-2.831691119	0.004920751	0.018907896	no
LOC729080	-0.155602705	-2.831005496	0.004931051	0.018944087	no
DUSP18	0.155572904	2.83044985	0.004939413	0.018972043	no
NUDT21	-0.155570481	-2.83040468	0.004940094	0.018972043	no
FNDC8	-0.155566467	-2.83032983	0.004941221	0.018972983	no
EXOC3L1	0.155515619	2.829381794	0.004955523	0.019024498	no
C6orf57	-0.155476833	-2.828658654	0.004966457	0.01906307	no
PGBD4	-0.155466922	-2.828473874	0.004969254	0.019070401	no
FLJ45445	0.155459514	2.828335759	0.004971346	0.019075023	no
ZNF169	-0.155433567	-2.827851996	0.00497868	0.019097582	no
BCDIN3D-AS1	-0.155432424	-2.827830692	0.004979003	0.019097582	no
USP51	-0.155418662	-2.82757412	0.004982897	0.019109106	no
KIAA1522	0.15540302	2.827282492	0.004987326	0.019122679	no
SCN2B	-0.155382349	-2.826897125	0.004993184	0.019141726	no
DNM1P41	-0.155377854	-2.826813308	0.004994459	0.019143199	no
SGSM2	-0.155344381	-2.82618928	0.00500396	0.019176197	no
YIPF2	0.155327293	2.825870705	0.005008817	0.019189951	no

EMX2OS	-0.155325472	-2.825836749	0.005009335	0.019189951	no
RIN1	0.155300348	2.825368377	0.005016485	0.019213914	no
FAM3D	0.155282633	2.825038119	0.005021531	0.019226903	no
E2F7	0.155282163	2.82502936	0.005021665	0.019226903	no
HTATSF1P2	0.155233514	2.824122447	0.005035548	0.019273602	no
DOT1L	-0.155233137	-2.824115409	0.005035656	0.019273602	no
LOC284950	-0.155207215	-2.823632178	0.005043067	0.019298532	no
LRG1	0.155200368	2.823504533	0.005045027	0.019302593	no
LOC100506963	0.155189615	2.823304091	0.005048105	0.019310933	no
BCKDHA	-0.155183564	-2.823191297	0.005049838	0.019314124	no
C4orf52	0.155175054	2.823032644	0.005052276	0.019318713	no
GLIS3	0.155173102	2.822996267	0.005052835	0.019318713	no
CANT1	0.155159144	2.822736056	0.005056837	0.019330575	no
CNTD1	-0.155151989	-2.822602688	0.00505889	0.019334981	no
EGLN1	-0.155121681	-2.822037703	0.005067592	0.019364799	no
NRARP	-0.155101165	-2.821655269	0.005073491	0.019383892	no
YWHAQ	-0.155075674	-2.821180104	0.005080828	0.019408475	no
AMER1	-0.155064339	-2.820968825	0.005084094	0.019417498	no
NLGN3	-0.155036577	-2.820451317	0.005092101	0.019444623	no
TSHZ3	0.155017434	2.820094486	0.005097628	0.019462272	no
RNF38	-0.155005671	-2.819875228	0.005101027	0.019470281	no
KIAA1598	-0.155003904	-2.819842303	0.005101538	0.019470281	no
NKTR	-0.154999072	-2.819752234	0.005102935	0.019472155	no
UPF2	-0.154980237	-2.819401145	0.005108384	0.019489487	no
LOXL1-AS1	0.154961485	2.819051611	0.005113814	0.019506742	no
NPTX1	-0.15495569	-2.818943594	0.005115493	0.019509684	no
RNF5	-0.154949586	-2.818829831	0.005117262	0.019512968	no
CD82	0.154944714	2.818739018	0.005118675	0.019514881	no
CHD2	-0.154941592	-2.818680824	0.00511958	0.019514881	no
XCR1	0.154935797	2.818572801	0.005121261	0.019517827	no
GAD2	-0.154923037	-2.81833498	0.005124964	0.019528475	no
SLC6A17	-0.15491984	-2.818275383	0.005125892	0.019528549	no
RBM25	-0.154897301	-2.81785527	0.00513244	0.019550028	no
MRPL28	-0.15486277	-2.817211655	0.005142485	0.019584822	no
NFIB	-0.154827625	-2.81655661	0.005152728	0.019620353	no
RNF215	-0.154823771	-2.816484779	0.005153852	0.019621157	no
LOC400927	-0.154810547	-2.816238306	0.005157711	0.019632373	no
CPA4	0.154803048	2.816098538	0.005159901	0.01963723	no
EGFEM1P	-0.15476173	-2.815328447	0.005171981	0.019679719	no
PCSK1N	-0.154702143	-2.814217892	0.005189447	0.019742683	no
EIF2S2	0.154686884	2.813933522	0.005193928	0.019756233	no
IGFBP6	0.154652364	2.813290169	0.005204079	0.019791341	no
TPTE2P6	-0.154610657	-2.812512883	0.005216367	0.019834562	no
RAD52	-0.15459369	-2.812196679	0.005221373	0.019850086	no
VPS51	-0.154574657	-2.81184198	0.005226994	0.019867941	no
KCNT1	-0.154554241	-2.81146152	0.00523303	0.019887364	no
COX6C	-0.154539902	-2.811194297	0.005237272	0.019899969	no
SAMD8	-0.154535533	-2.811112878	0.005238566	0.019901364	no
RALYL	-0.154514458	-2.810720117	0.005244809	0.01992156	no
LOC339894	0.154471973	2.809928398	0.005257414	0.01996591	no
VPS26B	-0.154459338	-2.809692937	0.005261168	0.019973445	no

UTP23	-0.154459039	-2.809687373	0.005261257	0.019973445	no
RNF103	-0.154439891	-2.809330543	0.005266951	0.01999153	no
PNKP	0.15442891	2.809125915	0.005270219	0.020000401	no
CDKN2AIPNL	-0.154412164	-2.808813867	0.005275205	0.020012453	no
NLK	-0.154411992	-2.808810653	0.005275257	0.020012453	no
ZYX	0.154393645	2.808468775	0.005280725	0.020029662	no
RIMKLB	-0.154375506	-2.808130755	0.005286137	0.020046651	no
RBM26-AS1	-0.154351925	-2.807691346	0.00529318	0.020069817	no
LOC100130673	-0.154331656	-2.807313652	0.00529924	0.020088339	no
ELAVL4	-0.154329335	-2.807270411	0.005299934	0.020088339	no
SYNPO2L	-0.154323871	-2.807168584	0.005301569	0.020090993	no
CCDC177	-0.154297984	-2.806686222	0.005309321	0.020116823	no
NAA35	-0.154285087	-2.806445897	0.005313187	0.020127922	no
SCD	-0.154280585	-2.806362015	0.005314537	0.02012856	no
COQ10A	-0.15427828	-2.806319058	0.005315229	0.02012856	no
CLCA2	0.154260394	2.805985784	0.005320596	0.020145336	no
AGPAT4-IT1	-0.154216225	-2.805162771	0.005333872	0.020192044	no
CNTNAP3B	0.154209809	2.805043232	0.005335802	0.020195796	no
CALY	-0.154201432	-2.804887134	0.005338324	0.020201784	no
CCSER1	-0.154197958	-2.804822405	0.005339371	0.020202186	no
B4GALT7	0.154180853	2.804503691	0.005344525	0.020218127	no
HSD52	0.154148955	2.803909357	0.005354148	0.020248778	no
PRDX4	0.15414775	2.803886908	0.005354511	0.020248778	no
PFDN2	-0.154136795	-2.803682788	0.00535782	0.020257726	no
DARS	-0.154129053	-2.803538536	0.00536016	0.020263006	no
CDAN1	-0.154120118	-2.803372061	0.005362861	0.020269651	no
UPK2	-0.154087397	-2.802762401	0.005372763	0.020303507	no
CDH9	-0.154057053	-2.802197038	0.00538196	0.020334688	no
TSC22D3	0.154049229	2.802051266	0.005384334	0.020340081	no
MTL5	0.15403534	2.801792492	0.00538855	0.020352431	no
ARPC4	0.153940571	2.800026863	0.005417398	0.020457793	no
SNCG	-0.153920274	-2.799648713	0.005423594	0.020474851	no
LOC646214	-0.153919534	-2.799634933	0.00542382	0.020474851	no
EPHB4	0.153916412	2.799576778	0.005424774	0.020474854	no
GPS1	-0.153891265	-2.799108273	0.005432462	0.020500269	no
FAM66D	-0.153876215	-2.798827894	0.005437067	0.020514046	no
SRF	0.153870455	2.798720592	0.00543883	0.020517098	no
APCDD1	-0.15386331	-2.798587482	0.005441019	0.020520174	no
ATP6V0E2	-0.153861558	-2.798554839	0.005441556	0.020520174	no
DGKG	-0.153839701	-2.798147657	0.005448255	0.020540514	no
ADCY1	-0.153837726	-2.798110861	0.005448861	0.020540514	no
TIMM17B	-0.153824138	-2.797857723	0.00545303	0.020552625	no
ARID2	-0.153814463	-2.797677469	0.005456001	0.020560216	no
CAMKK1	-0.153795655	-2.797327091	0.005461779	0.020578382	no
SULT4A1	-0.153786	-2.797147224	0.005464747	0.020585957	no
SEMA3D	-0.153765043	-2.796756831	0.005471195	0.020603705	no
HDAC11	-0.153764457	-2.796745898	0.005471376	0.020603705	no
DNAJC5G	-0.153752125	-2.796516173	0.005475174	0.020614394	no
NQO2	0.153745525	2.796393216	0.005477207	0.020618438	no
GALR3	-0.153706653	-2.795669089	0.005489198	0.020659956	no
EMC2	-0.15369034	-2.795365201	0.005494237	0.020669803	no

SDC4P	0.153690231	2.795363175	0.00549427	0.020669803	no
FSTL4	-0.153688842	-2.795337305	0.005494699	0.020669803	no
MYO9B	0.153646556	2.794549603	0.005507781	0.020715389	no
CTAGE5	0.153619755	2.794050352	0.005516088	0.020742999	no
FNDC1	0.153579804	2.793306156	0.00552849	0.020786	no
ARHGAP44	-0.153569238	-2.793109338	0.005531774	0.02079471	no
ACTN2	-0.153564084	-2.793013348	0.005533376	0.020797096	no
SASH1	-0.153558252	-2.792904708	0.005535191	0.020800276	no
FKBP11	0.153554911	2.792842466	0.00553623	0.020800546	no
HIPK2	-0.153540454	-2.79257319	0.00554073	0.020813812	no
CECR1	0.153519014	2.792173817	0.005547409	0.020835262	no
PEX11G	0.153489346	2.791621214	0.005556663	0.020863267	no
SEC62	-0.153488886	-2.791612641	0.005556807	0.020863267	no
PSD	-0.153402605	-2.79000556	0.005583801	0.020960954	no
LOC143666	-0.153398019	-2.789920138	0.005585239	0.020962691	no
USP32	-0.153371405	-2.789424428	0.005593591	0.020990373	no
MPG	0.153353106	2.7890836	0.00559934	0.021008279	no
PRMT6	0.15332375	2.788536844	0.005608574	0.021039251	no
COL4A6	0.153297883	2.788055081	0.005616722	0.021066138	no
LETMD1	-0.15329362	-2.787975674	0.005618066	0.021067502	no
TSPAN32	0.153290118	2.787910454	0.00561917	0.021067966	no
LOC619207	-0.153262554	-2.787397089	0.005627867	0.021096895	no
YY1AP1	-0.153231169	-2.786812556	0.005637786	0.021130389	no
OLFM4	-0.15322587	-2.786713856	0.005639462	0.021132986	no
ZMYND19	-0.153195672	-2.786151451	0.005649022	0.021161494	no
TTC7B	-0.153195619	-2.786150465	0.005649039	0.021161494	no
SIGLEC8	0.153189268	2.786032191	0.005651051	0.021165343	no
FAM111A	0.153184869	2.785950262	0.005652445	0.021166877	no
TDH	-0.153180104	-2.785861508	0.005653956	0.021168846	no
KCNC2	-0.153139463	-2.785104641	0.005666855	0.021213445	no
NIPAL1	0.153128672	2.784903674	0.005670285	0.021222587	no
PODXL	0.153113599	2.784622962	0.005675078	0.021236829	no
FAM153C	-0.153092639	-2.784232618	0.00568175	0.021258094	no
ADAMTS4	0.153061934	2.783660805	0.005691536	0.021291002	no
ONECUT2	-0.153056243	-2.783554812	0.005693352	0.021294088	no
BCL11A	-0.153036488	-2.783186925	0.005699658	0.021313964	no
HOXA1	0.152933066	2.781260996	0.005732774	0.021434073	no
ANKRD24	-0.152910785	-2.780846079	0.005739931	0.0214571	no
CKAP5	-0.152889285	-2.780445719	0.005746845	0.021479209	no
SNORD89	0.15287072	2.780100015	0.005752821	0.021497806	no
SERF2	0.15283328	2.779402855	0.00576489	0.02153916	no
MLXIP	0.152809487	2.778959809	0.005772572	0.021564112	no
ELOVL1	0.15277335	2.778286917	0.005784256	0.021604005	no
PRPF3	-0.15273972	-2.777660727	0.005795148	0.021640927	no
HS3ST1	0.152731506	2.777507779	0.005797812	0.021647112	no
CXCL14	0.15272075	2.777307504	0.005801301	0.021656377	no
SIM2	-0.152630539	-2.775627838	0.005830639	0.021762116	no
OPTN	0.152614721	2.775333313	0.005835797	0.021776371	no
ARNT	0.152612613	2.775294065	0.005836485	0.021776371	no
FLJ38576	-0.1525984	-2.775029435	0.005841123	0.021787449	no
ADAM32	0.152597303	2.775009014	0.005841482	0.021787449	no

IGSF11-AS1	-0.152584755	-2.774775393	0.00584558	0.02179895	no
LOC731275	-0.15257558	-2.774604556	0.005848578	0.021806348	no
NOX4	0.152555807	2.77423643	0.005855044	0.021826669	no
MIMT1	-0.15254247	-2.773988116	0.005859409	0.021839153	no
SERTAD1	0.152535708	2.773862218	0.005861623	0.021843617	no
C1orf35	-0.152502463	-2.77324326	0.00587252	0.021880432	no
ATOH7	-0.152411629	-2.771552157	0.005902386	0.021987361	no
SMCR8	0.152408963	2.771502515	0.005903265	0.021987361	no
IFNA22P	0.152398065	2.771299638	0.005906858	0.02199693	no
CORO1C	-0.152375768	-2.770884527	0.005914215	0.022020513	no
LGALS12	0.152368002	2.770739945	0.00591678	0.022026245	no
H2BFXP	-0.15236441	-2.770673082	0.005917966	0.022026845	no
EHBP1	-0.152331827	-2.770066505	0.005928738	0.022063117	no
RET	-0.152297112	-2.769420238	0.005940234	0.022102072	no
MRPS15	0.152287924	2.769249185	0.00594328	0.022109578	no
MAGED2	-0.152273393	-2.768978673	0.0059481	0.02212368	no
TEN1-CDK3	-0.152231527	-2.76819932	0.005962007	0.02217157	no
SUMO1P1	0.152205288	2.76771086	0.005970739	0.022200198	no
CCDC18	0.152187172	2.767373626	0.005976774	0.022218792	no
RBCK1	0.152178214	2.767206879	0.00597976	0.022226048	no
MAP1A	-0.152151231	-2.766704584	0.005988762	0.022255662	no
ATHL1	0.15214073	2.766509125	0.005992269	0.022264844	no
BRF1	-0.15210031	-2.765756706	0.006005785	0.022311207	no
PDK2	-0.152085964	-2.765489666	0.006010589	0.022325193	no
LOC100506668	0.15206391	2.765079149	0.00601798	0.022348784	no
GRWD1	0.152026125	2.764375829	0.006030663	0.022392013	no
LOC100507600	0.151993428	2.76376722	0.006041657	0.022428959	no
CHMP1B	0.151972221	2.763372491	0.006048797	0.022451588	no
FNDC5	-0.151964559	-2.763229878	0.006051378	0.022457291	no
FLJ46257	-0.151960573	-2.763155679	0.006052722	0.022458399	no
LINC00568	-0.151952658	-2.76300836	0.00605539	0.022464421	no
ENOX1	-0.151938019	-2.762735883	0.006060328	0.022478861	no
DKKL1	0.151911342	2.762239335	0.006069337	0.022508389	no
RNMT	-0.151907718	-2.7621719	0.006070561	0.022509046	no
PDCD11	-0.151904231	-2.762106992	0.00607174	0.022509532	no
FAM178A	-0.151843627	-2.760979004	0.006092256	0.022581696	no
PQLC2	0.15183361	2.760792558	0.006095653	0.022587109	no
C9orf37	-0.151833123	-2.760783499	0.006095818	0.022587109	no
CPLX3	-0.151821779	-2.760572357	0.006099668	0.022597475	no
EIF1B	-0.15177301	-2.75966469	0.00611624	0.022654967	no
LOC100129917	-0.15176519	-2.75951915	0.006118901	0.022657156	no
GCSAML-AS1	0.151765078	2.759517055	0.00611894	0.022657156	no
CPSF3L	-0.151753761	-2.75930643	0.006122793	0.022667517	no
USE1	-0.151739597	-2.759042816	0.006127618	0.022681475	no
RPL22	-0.151732913	-2.758918421	0.006129897	0.022686001	no
UFSP1	0.151623892	2.75688947	0.006167165	0.022819996	no
PDE9A	-0.151617216	-2.756765217	0.006169454	0.022821335	no
PYGO1	-0.151616642	-2.756754548	0.00616965	0.022821335	no
PER3	-0.151604469	-2.756527998	0.006173826	0.02283285	no
NUP155	-0.15159643	-2.756378392	0.006176585	0.022839123	no
LANCL3	0.151590351	2.756265255	0.006178672	0.022842909	no

FTSJ2	0.151580379	2.756079679	0.006182096	0.02285164	no
LOC729987	0.151576626	2.756009848	0.006183385	0.022852474	no
CACNG3	-0.151555496	-2.755616619	0.006190649	0.022875386	no
ZNF76	-0.151548206	-2.755480948	0.006193157	0.022880719	no
OR2L13	-0.151541952	-2.755364571	0.006195309	0.022883958	no
C2CD2	0.15153947	2.755318385	0.006196163	0.022883958	no
SRPK1	-0.151524173	-2.755033701	0.006201431	0.022899478	no
PTGDR2	-0.151507887	-2.754730636	0.006207044	0.022914947	no
FMNL2	-0.15150583	-2.75469235	0.006207753	0.022914947	no
DCUN1D3	0.151493137	2.754456154	0.006212131	0.022924645	no
BCL2L12	0.151492027	2.7544355	0.006212514	0.022924645	no
KCNK17	0.151477723	2.754169306	0.006217451	0.022938925	no
EGF	0.15147121	2.754048109	0.0062197	0.022943284	no
UBE2R2	-0.151462902	-2.75389351	0.00622257	0.022949932	no
ANKRD55	-0.151449171	-2.75363799	0.006227316	0.022963496	no
LUC7L3	-0.151435619	-2.753385805	0.006232004	0.022976839	no
SLC12A3	0.151431542	2.753309941	0.006233415	0.022978098	no
ANAPC11	-0.151375864	-2.752273854	0.00625271	0.023045274	no
38412	-0.151362364	-2.752022657	0.006257396	0.023058592	no
N4BP2L2	-0.151306892	-2.750990439	0.006276687	0.023123436	no
WNT7B	-0.151305575	-2.750965944	0.006277145	0.023123436	no
CCDC152	0.151285661	2.750595396	0.006284084	0.023145028	no
NEUROD2	-0.151269392	-2.750292671	0.006289757	0.023161956	no
ZNF43	-0.151249625	-2.749924868	0.006296657	0.023183391	no
GPR45	-0.151243544	-2.749811717	0.006298781	0.023187238	no
PRCP	0.151236945	2.749688924	0.006301086	0.023191753	no
GDF3	0.151219706	2.749368163	0.006307113	0.023209959	no
TMEM132D	-0.151173561	-2.748509551	0.00632327	0.023265434	no
DLL4	0.151169126	2.748427038	0.006324825	0.023267171	no
PTPN14	0.151165368	2.748357114	0.006326143	0.023268036	no
DPH1	-0.151132645	-2.747748274	0.006337627	0.023306288	no
MSRB1	0.151123481	2.747577772	0.006340847	0.023314138	no
DDX46	-0.151112515	-2.747373735	0.006344702	0.023323609	no
EIF3H	-0.151109979	-2.747326555	0.006345593	0.023323609	no
MEF2A	0.15109311	2.747012688	0.006351528	0.02334143	no
MYLK	0.151075262	2.746680622	0.006357812	0.023360529	no
LIG3	-0.151028106	-2.745803269	0.006374442	0.023417631	no
LIF	0.151007672	2.745423096	0.006381661	0.023440142	no
DNTTIP2	0.150966466	2.744656461	0.00639624	0.023489676	no
AIRN	-0.15094921	-2.744335414	0.006402354	0.023508113	no
USP30	-0.150937811	-2.744123357	0.006406395	0.023518934	no
MTHFS	0.150933595	2.744044919	0.006407891	0.023520406	no
KCNJ4	-0.150917171	-2.74373935	0.006413719	0.023537781	no
CHD8	-0.150888064	-2.743197847	0.006424061	0.023571707	no
NCKAP1	-0.150876608	-2.742984727	0.006428135	0.02358263	no
LACC1	0.150845238	2.742401126	0.006439303	0.023619571	no
FOS	0.150833203	2.742177232	0.006443592	0.023631271	no
PSMD3	-0.150823379	-2.74199447	0.006447096	0.023640084	no
P4HA2	0.150806069	2.741672445	0.006453273	0.023658697	no
LEMD3	-0.150796073	-2.741486485	0.006456842	0.023667745	no
KIF13A	-0.150740873	-2.740459616	0.006476584	0.023736063	no

FZD5	0.15071424	2.739964165	0.006486129	0.023766991	no
CDH8	-0.150700934	-2.739716646	0.006490902	0.023780427	no
CADPS2	0.150692781	2.739564986	0.006493829	0.023787092	no
ST6GALNAC4	0.150675976	2.739252384	0.006499864	0.023803686	no
ECE2	0.150674001	2.73921563	0.006500574	0.023803686	no
ATP11B	0.150632977	2.738452517	0.00651533	0.023853654	no
HERC2P7	-0.150554201	-2.736987164	0.006543749	0.023950913	no
EVC2	0.150553165	2.736967903	0.006544124	0.023950913	no
CDK8	-0.150525546	-2.736454161	0.006554114	0.023983394	no
NDUFS4	-0.150483487	-2.735671833	0.006569355	0.024035071	no
LSS	-0.150460356	-2.735241586	0.00657775	0.02406169	no
LOC441454	-0.150452686	-2.735098941	0.006580536	0.024063967	no
SLFN13	0.150452476	2.735095019	0.006580612	0.024063967	no
EAF1	0.150415716	2.734411286	0.006593979	0.024107396	no
C9orf172	-0.150413647	-2.734372799	0.006594732	0.024107396	no
C2orf69	-0.150389435	-2.733922464	0.00660355	0.024135526	no
SCMH1	0.150381769	2.733779879	0.006606344	0.024141632	no
CXXC1	-0.1503717	-2.733592601	0.006610016	0.024150943	no
CGRRF1	-0.150365486	-2.733477033	0.006612282	0.024153579	no
ZNF883	-0.150363561	-2.733441223	0.006612985	0.024153579	no
USP16	-0.150355688	-2.733294802	0.006615858	0.024159967	no
C11orf49	-0.150340345	-2.733009418	0.006621461	0.02417632	no
UPRT	0.15032963	2.73281013	0.006625376	0.024186506	no
CIAPIN1	-0.150315057	-2.732539087	0.006630705	0.024201847	no
RS1	-0.150282173	-2.731927492	0.006642742	0.024241665	no
CCDC28B	-0.150264852	-2.731605343	0.00664909	0.024260713	no
FLOT2	0.150247252	2.731278005	0.006655547	0.024280148	no
CCBE1	-0.150236613	-2.731080143	0.006659452	0.024290272	no
OPCML	-0.150228447	-2.730928272	0.006662451	0.024297087	no
IGSF21	-0.150175423	-2.72994212	0.006681954	0.024364077	no
ANKRD20A9P	-0.150169354	-2.72982926	0.006684189	0.024368093	no
JAKMIP2	0.150164456	2.729738157	0.006685994	0.024370539	no
TUBA8	-0.15013169	-2.729128802	0.006698077	0.024404937	no
ANO3	-0.150125976	-2.729022525	0.006700187	0.024404937	no
UBXN4	0.150125244	2.72900892	0.006700457	0.024404937	no
ITPKA	-0.150123312	-2.728972994	0.00670117	0.024404937	no
CERS2	0.150121441	2.728938191	0.006701861	0.024404937	no
CAPN5	0.150117484	2.728864595	0.006703323	0.024404937	no
CXCL1	0.150117328	2.728861705	0.00670338	0.024404937	no
CYP1A1	-0.150099625	-2.728532466	0.006709922	0.024424127	no
C20orf112	-0.150096913	-2.728482046	0.006710924	0.024424127	no
BZW1	0.150089095	2.728336643	0.006713815	0.024430513	no
ZNF729	-0.150073834	-2.72805284	0.006719462	0.02444692	no
AP5Z1	0.150046258	2.727540007	0.006729676	0.024479937	no
LCA5L	0.150041645	2.72745423	0.006731386	0.024482012	no
MDC1	-0.150011123	-2.726886626	0.00674271	0.024518669	no
TBL2	0.150008331	2.726834702	0.006743746	0.024518669	no
RXRB	-0.150003188	-2.726739058	0.006745656	0.024518808	no
ZNF513	-0.150002084	-2.726718533	0.006746066	0.024518808	no
TCEB1	-0.14999668	-2.726618037	0.006748074	0.024521958	no
PRMT3	-0.149929219	-2.725363529	0.006773181	0.024609034	no

TSPAN11	-0.149914967	-2.725098517	0.006778496	0.02462418	no
LNPEP	-0.149879883	-2.724446116	0.006791595	0.024667596	no
RNF139	-0.149847163	-2.723837686	0.006803832	0.024707866	no
LOC100507391	0.149822318	2.723375687	0.006813137	0.024737478	no
RIMBP2	-0.14981503	-2.723240169	0.006815869	0.024743216	no
RPS29	-0.149802951	-2.723015558	0.006820399	0.024755478	no
ANGPT4	-0.149793465	-2.722839183	0.006823957	0.024764213	no
C9orf89	0.149790229	2.722778998	0.006825172	0.02476444	no
BEND3	-0.149772136	-2.722442577	0.006831966	0.02478407	no
TVP23B	0.14976968	2.722396914	0.006832889	0.02478407	no
ERC2	-0.149684167	-2.720806877	0.006865086	0.024893545	no
SLMO1	-0.149683368	-2.720792031	0.006865387	0.024893545	no
SLC52A2	0.149664612	2.720443288	0.006872468	0.024915014	no
FBXL19	-0.149649342	-2.720159363	0.006878237	0.024931724	no
ACD	-0.149645547	-2.720088806	0.006879671	0.024932718	no
GLTPD2	-0.149642011	-2.720023062	0.006881008	0.024933358	no
ACTG2	0.149629678	2.719793759	0.006885672	0.024946053	no
LOC100128946	-0.149620779	-2.719628298	0.00688904	0.024954046	no
MBIP	-0.149611297	-2.719451995	0.00689263	0.024962841	no
TSPAN9	0.149564464	2.718581247	0.006910384	0.025022924	no
TMEM54	0.149556828	2.718439267	0.006913282	0.025029204	no
HAT1	0.149511628	2.7175989	0.006930463	0.025087178	no
TET3	-0.149495517	-2.717299362	0.006936596	0.02510515	no
LOC550643	0.149459415	2.716628157	0.006950356	0.025150717	no
GGT7	-0.149436531	-2.71620271	0.006959091	0.025178086	no
FAM159B	-0.149418715	-2.715871479	0.006965899	0.025198473	no
FATE1	0.149414133	2.715786301	0.00696765	0.025200568	no
ASB6	0.149403849	2.715595104	0.006971583	0.02521055	no
TEX21P	-0.149387076	-2.715283269	0.006978002	0.025229517	no
NLRP3	0.149379244	2.715137664	0.006981002	0.025236115	no
FLI1-AS1	0.149362658	2.714829325	0.006987356	0.025250663	no
ABCA6	0.149362607	2.714828382	0.006987376	0.025250663	no
ZDHHC21	-0.149332512	-2.714268879	0.00699892	0.02528813	no
MIR10B	0.149317777	2.713994961	0.007004579	0.025302015	no
FAM229B	-0.149316374	-2.713968867	0.007005118	0.025302015	no
LRCH4	0.149282647	2.713341874	0.007018086	0.025339116	no
QKI	-0.149281316	-2.713317128	0.007018598	0.025339116	no
NHP2	-0.149280461	-2.713301246	0.007018927	0.025339116	no
NRF1	-0.149239631	-2.712542217	0.007034656	0.025391636	no
FZD7	0.149235225	2.712460308	0.007036356	0.025393504	no
FMR1	-0.149218883	-2.712156516	0.007042661	0.025411994	no
GTF2IP1	0.149175031	2.711341325	0.007059608	0.025468864	no
SPRNP1	-0.149154018	-2.710950718	0.00706774	0.025493926	no
NR1H2	0.149129328	2.710491758	0.007077307	0.025524151	no
TNFAIP8L3	0.149112134	2.710172133	0.007083977	0.025543917	no
DTNA	0.149084392	2.709656451	0.007094749	0.025578469	no
THEM6	-0.149078595	-2.709548705	0.007097002	0.025582299	no
OIP5-AS1	-0.14906044	-2.709211234	0.007104061	0.025603452	no
MRVI1	-0.149054887	-2.709107999	0.007106222	0.025605334	no
DNAJB11	0.149050116	2.709019314	0.007108079	0.025605334	no
LOC149373	-0.149049913	-2.709015546	0.007108158	0.025605334	no

LRP2BP	-0.149026354	-2.708577628	0.007117333	0.025634089	no
GNG2	-0.148974499	-2.707613755	0.007137566	0.025699359	no
ZNF491	-0.148973778	-2.707600356	0.007137847	0.025699359	no
SLC25A5-AS1	-0.148969516	-2.707521143	0.007139512	0.025701048	no
SPOCK3	-0.148963006	-2.707400135	0.007142057	0.025705902	no
NEXN	0.148951579	2.707187737	0.007146525	0.025717676	no
LOC553103	-0.148941583	-2.707001937	0.007150435	0.0257262	no
FOXG1	-0.148939404	-2.706961446	0.007151287	0.0257262	no
MMADHC	0.148935251	2.706884248	0.007152913	0.025727186	no
PEAR1	0.148932587	2.706834727	0.007153956	0.025727186	no
DDX49	-0.148920645	-2.706612752	0.007158632	0.025739697	no
HCN1	-0.148912741	-2.706465849	0.007161729	0.025746522	no
JHDM1D	-0.148909678	-2.706408916	0.007162929	0.025746531	no
MSL3P1	0.148897242	2.706177773	0.007167804	0.025759746	no
OTUB2	-0.148893008	-2.706099081	0.007169465	0.025760791	no
BCRP3	-0.148890388	-2.706050381	0.007170492	0.025760791	no
ACAT2	-0.148878335	-2.705826343	0.007175222	0.025773474	no
BMP4	0.148862852	2.705538581	0.007181301	0.025791	no
MRO	-0.148839954	-2.705112983	0.007190301	0.025819007	no
TRPC6	0.148819809	2.704738556	0.007198227	0.025843149	no
ZNF14	-0.148736918	-2.703197965	0.007230921	0.025956193	no
NEFL	-0.148713842	-2.702769079	0.007240047	0.02598461	no
SLC25A53	-0.148667501	-2.701907833	0.007258403	0.026046142	no
LOC100129858	-0.148662421	-2.701813423	0.007260418	0.026049022	no
COL26A1	-0.148647103	-2.70152874	0.007266497	0.026066479	no
RHBDF1	0.148628598	2.701184828	0.007273846	0.026088488	no
PHEX	0.148556025	2.6998361	0.007302732	0.026187721	no
C8orf58	0.148541029	2.699557417	0.007308713	0.026204798	no
TMX3	0.148531548	2.69938122	0.007312498	0.026213993	no
ZNF658	-0.148499667	-2.698788759	0.007325235	0.026255273	no
HAPLN4	-0.148461467	-2.698078875	0.007340522	0.02630568	no
PMP22	0.148432972	2.697549354	0.007351944	0.02634222	no
LOC285629	0.148409888	2.697120378	0.00736121	0.026371021	no
C22orf24	-0.1483927	-2.696800988	0.007368115	0.026391359	no
SYPL1	0.148374126	2.696455843	0.007375583	0.026413707	no
FZD6	0.148367305	2.696329089	0.007378328	0.026419133	no
PAQR7	0.148343984	2.695895725	0.007387718	0.02644835	no
AP1S2	0.14832707	2.695581429	0.007394535	0.026468346	no
CCL26	0.148260778	2.694349609	0.007421307	0.026559752	no
JARID2	-0.148208144	-2.693371596	0.007442625	0.026631612	no
PELP1	-0.148201666	-2.693251234	0.007445253	0.026636579	no
KIRREL3	-0.148189233	-2.693020219	0.007450298	0.026650192	no
UBAC1	-0.148158541	-2.692449943	0.007462766	0.026690348	no
FSD1L	-0.148148863	-2.692270116	0.007466701	0.026699979	no
C3orf70	-0.148139977	-2.692105006	0.007470316	0.026708461	no
C2CD4C	-0.148117773	-2.691692439	0.007479356	0.026736333	no
STAC	0.148086449	2.691110449	0.007492124	0.026777523	no
CMC1	0.148079033	2.690972651	0.00749515	0.026783884	no
EXOSC7	-0.148072845	-2.690857682	0.007497676	0.026788455	no
BBS9	0.14806231	2.69066195	0.007501978	0.026799369	no
HENMT1	0.14805101	2.690451992	0.007506594	0.026811404	no

MFSD4	-0.147953421	-2.688638863	0.007546569	0.026949704	no
SYNGR3	-0.147949582	-2.688567524	0.007548146	0.026950856	no
LIX1	-0.147921203	-2.68804028	0.007559808	0.026988014	no
PTGES2	-0.147895689	-2.68756628	0.007570307	0.027021004	no
RNF19A	0.147872479	2.687135079	0.007579869	0.027050642	no
ARHGEF2	-0.147840711	-2.686544883	0.007592974	0.027092913	no
FGFRL1	0.147827451	2.686298544	0.007598451	0.027107952	no
ZNF300	-0.14782388	-2.686232198	0.007599926	0.027108715	no
FKBP4	-0.147806088	-2.685901655	0.007607281	0.027130447	no
GPX7	0.147792936	2.685657328	0.007612721	0.027145345	no
NEUROD1	-0.147765754	-2.685152368	0.007623977	0.027180969	no
CHRD	-0.147748124	-2.684824834	0.007631285	0.027202513	no
L1CAM	-0.147730604	-2.684499369	0.007638554	0.027223907	no
SARS2	-0.147717573	-2.684257286	0.007643964	0.027238673	no
MED13L	-0.147687513	-2.683698869	0.007656458	0.02727867	no
C19orf26	-0.147670675	-2.683386085	0.007663464	0.027299106	no
ATRNL1	-0.147635109	-2.682725406	0.007678281	0.027347356	no
KRT222	-0.147624482	-2.682527995	0.007682714	0.027356928	no
APOLD1	-0.147622563	-2.68249234	0.007683515	0.027356928	no
LRRC70	0.14761779	2.682403669	0.007685507	0.027359487	no
ZNF626	-0.147571197	-2.681538166	0.007704973	0.027422019	no
SLC25A1	-0.147567118	-2.681462396	0.00770668	0.027422019	no
CEP350	-0.14756659	-2.681452598	0.0077069	0.027422019	no
SAMD14	-0.147504909	-2.680306849	0.007732745	0.027509421	no
LYVE1	0.14749222	2.68007114	0.007738071	0.027523814	no
ZNF649	-0.147471915	-2.67969398	0.007746601	0.027549594	no
SOCS6	0.147452661	2.679336353	0.007754697	0.027567714	no
SLC39A7	0.147452471	2.679332814	0.007754777	0.027567714	no
PPP3CB	-0.147450647	-2.679298936	0.007755544	0.027567714	no
PRKCA	-0.147422988	-2.678785184	0.007767189	0.02760454	no
RPL7	-0.147403694	-2.678426805	0.007775321	0.027628873	no
NPPA-AS1	0.147366063	2.677727855	0.007791204	0.027677958	no
CCL3	0.147364864	2.677705575	0.00779171	0.027677958	no
RABGGTA	-0.147347414	-2.677381464	0.007799085	0.027699578	no
ULBP3	0.147334606	2.677143569	0.007804503	0.027714238	no
C1orf63	0.147328801	2.677035746	0.007806959	0.027718381	no
C7orf43	0.14730888	2.676665749	0.007815394	0.027743745	no
CNKSR2	-0.147278578	-2.676102936	0.00782824	0.027782318	no
TMEM120A	0.147277151	2.676076432	0.007828845	0.027782318	no
RAP1B	0.14727156	2.675972582	0.007831218	0.027786149	no
KIAA0430	-0.147267542	-2.675897969	0.007832923	0.027787611	no
PPCDC	0.147240002	2.675386468	0.007844621	0.027820343	no
MTHFSD	0.147239725	2.675381315	0.007844739	0.027820343	no
COX4I1	-0.147201404	-2.674669593	0.007861041	0.027873559	no
CCR8	0.147193545	2.674523636	0.007864389	0.027880827	no
UNC13C	-0.147183107	-2.674329762	0.007868836	0.027891995	no
ELOVL5	0.147159873	2.673898262	0.007878744	0.027922509	no
ST13	-0.147151831	-2.673748912	0.007882176	0.027930065	no
FEZF2	-0.147136817	-2.673470054	0.007888587	0.027948176	no
DESI2	0.14712809	2.673307981	0.007892316	0.027952246	no
ZNF613	0.147128039	2.673307033	0.007892338	0.027952246	no

ISY1	0.147119892	2.673155736	0.00789582	0.02795949	no
UBTD1	0.147114558	2.673056666	0.0078981	0.02795949	no
FOXK2	-0.147114124	-2.673048609	0.007898286	0.02795949	no
UBXN10	0.147105814	2.672894279	0.00790184	0.027967465	no
C1orf200	0.147098267	2.672754118	0.007905069	0.027974287	no
SPNS2	-0.147087177	-2.67254815	0.007909817	0.027986479	no
DTD2	-0.147080129	-2.67241726	0.007912835	0.02799255	no
STT3B	0.147074215	2.672307437	0.007915368	0.027994354	no
RHOBTB3	-0.147072857	-2.672282216	0.00791595	0.027994354	no
PPBPP2	-0.147012964	-2.67116992	0.00794165	0.02808062	no
HCFC1R1	-0.146999884	-2.670927018	0.007947273	0.028095877	no
ZNF428	-0.146994973	-2.670835808	0.007949385	0.028098722	no
TMEM183A	-0.146986999	-2.670687735	0.007952815	0.028106223	no
SPOCD1	0.146948069	2.669964779	0.00796958	0.028158166	no
LARGE	-0.146946787	-2.669940974	0.007970133	0.028158166	no
RALBP1	0.146934334	2.669709721	0.007975503	0.028172506	no
ATF7IP2	0.146929662	2.669622945	0.007977519	0.028174996	no
WDR7	-0.14692538	-2.66954343	0.007979366	0.028176891	no
MAGEF1	-0.146918196	-2.66941003	0.007982467	0.028183209	no
DDN	-0.14689577	-2.668993572	0.007992153	0.028212773	no
C19orf57	-0.146868168	-2.668481008	0.008004089	0.028250267	no
GOSR2	0.146838594	2.667931834	0.008016896	0.028286744	no
RAP1GAP2	-0.146838223	-2.667924937	0.008017056	0.028286744	no
ZNF41	-0.146829184	-2.667757085	0.008020974	0.028295923	no
SLC45A1	-0.14682528	-2.66768459	0.008022667	0.028297249	no
AUP1	0.146817258	2.66753564	0.008026146	0.028304874	no
DNER	-0.146782808	-2.666895923	0.008041103	0.028352967	no
RPS27A	-0.146745082	-2.666195385	0.00805751	0.028406159	no
DNAJC7	-0.14672622	-2.665845152	0.008065724	0.028430454	no
DNAJB4	0.146719558	2.665721437	0.008068627	0.028432121	no
LRP3	-0.146719063	-2.665712244	0.008068843	0.028432121	no
PRKCG	-0.146709125	-2.665527723	0.008073175	0.028442723	no
ALDH3A2	0.146678995	2.664968253	0.008086323	0.028484375	no
ZNF345	0.146639676	2.66423817	0.00810351	0.028540237	no
GPATCH8	-0.14662637	-2.663991108	0.008109333	0.028551583	no
NDUFS3	-0.146626244	-2.663988769	0.008109388	0.028551583	no
ICAM2	0.146615848	2.663795754	0.00811394	0.028562931	no
ENO2	-0.146606153	-2.663615736	0.008118188	0.028573203	no
WDR24	-0.146572045	-2.662982429	0.008133147	0.028618167	no
PACS2	-0.146570953	-2.662962161	0.008133626	0.028618167	no
FLJ31306	-0.146537821	-2.662346992	0.008148182	0.028664687	no
ARHGAP5	-0.146534414	-2.662283735	0.00814968	0.028665265	no
EMX1	-0.146517935	-2.661977761	0.008156929	0.028686069	no
C16orf52	-0.146465909	-2.661011827	0.008179853	0.028761981	no
GNB4	0.146397512	2.659741951	0.008210079	0.028863537	no
DAPK1	-0.14638695	-2.659545856	0.008214755	0.028875254	no
SPON2	0.146371755	2.659263761	0.008221486	0.028894189	no
LOC283177	-0.146364065	-2.659120984	0.008224895	0.028896432	no
STXBP5-AS1	-0.1463629	-2.659099359	0.008225412	0.028896432	no
LRCH2	-0.146361216	-2.659068089	0.008226159	0.028896432	no
TMEM104	0.146355412	2.658960344	0.008228732	0.028900749	no

SHROOM2	-0.146325378	-2.658402737	0.008242063	0.02894284	no
LOC100506713	-0.146318307	-2.658271477	0.008245204	0.028949139	no
TRHDE-AS1	-0.146309502	-2.658108003	0.008249117	0.028958148	no
PKN2	0.146292261	2.657787916	0.008256784	0.028980329	no
SLC16A11	0.146285593	2.657664134	0.008259751	0.028986008	no
GLIS2	-0.146281541	-2.657588903	0.008261555	0.028987604	no
FAM182B	-0.146274997	-2.657467428	0.008264468	0.028993091	no
IFNLR1	0.146265049	2.657282734	0.008268898	0.0290039	no
C1orf198	-0.146255726	-2.65710966	0.008273052	0.029013734	no
RBFOX1	-0.14623287	-2.656685354	0.008283243	0.029044736	no
C10orf55	0.146225183	2.656542651	0.008286674	0.029052023	no
DKFZP586I1420	0.146221056	2.656466034	0.008288516	0.029053742	no
SURF2	-0.146186083	-2.655816786	0.008304141	0.029103765	no
SAFB2	-0.14617862	-2.655678238	0.008307479	0.029110716	no
ARL5A	0.146156959	2.65527612	0.008317173	0.029139935	no
LOC100507217	-0.146124835	-2.65467978	0.008331568	0.029185612	no
MPP5	-0.146112475	-2.654450335	0.008337113	0.029191901	no
ATP13A4	-0.14611074	-2.654418128	0.008337891	0.029191901	no
TTYH3	0.146110723	2.654417814	0.008337899	0.029191901	no
TMEM100	-0.146108723	-2.654380679	0.008338797	0.029191901	no
LIMD2	0.146103287	2.654279768	0.008341237	0.029195687	no
BLOC1S1	0.146089873	2.654030773	0.00834726	0.029212013	no
RPS14	-0.146016173	-2.652662666	0.008380426	0.029323306	no
HMBOX1	-0.146002569	-2.652410144	0.008386561	0.029339994	no
LLPH	0.145997625	2.652318372	0.008388792	0.029343021	no
RASA4	-0.145983153	-2.652049738	0.008395324	0.02936109	no
VCL	0.145951058	2.651453983	0.008409826	0.029406864	no
TNFRSF11A	0.145947049	2.651379572	0.008411639	0.029406864	no
PGAM1	-0.14594361	-2.651315726	0.008413195	0.029406864	no
MIR3936	-0.145942084	-2.651287402	0.008413885	0.029406864	no
ADRB1	-0.145890488	-2.650329699	0.008437256	0.029482235	no
TBL3	-0.145888421	-2.650291319	0.008438194	0.029482235	no
ZNF536	-0.145861398	-2.649789738	0.008450459	0.029520287	no
ACTR5	-0.145856154	-2.649692406	0.00845284	0.029523808	no
LOC100131067	-0.145826787	-2.649147317	0.00846619	0.029565632	no
TMEM238	0.145810086	2.648837318	0.008473791	0.029587367	no
SNED1	0.145785227	2.648375924	0.008485115	0.029622094	no
IRX2	-0.145743485	-2.647601158	0.008504161	0.029683762	no
NANP	0.145737893	2.647497381	0.008506715	0.029685887	no
PACSIN1	-0.145736104	-2.647464174	0.008507532	0.029685887	no
PRPSAP1	-0.145718267	-2.647133116	0.008515685	0.029709512	no
EFHC1	0.145693903	2.646680909	0.008526833	0.029743572	no
SLC17A6	-0.145690882	-2.646624846	0.008528216	0.029743572	no
FAM90A2P	0.145679649	2.646416349	0.008533361	0.029756687	no
LOC151009	-0.14561077	-2.645137991	0.008564968	0.029862057	no
PEX5	-0.145586502	-2.644687588	0.008576129	0.02989612	no
TAOK3	-0.145577935	-2.644528598	0.008580072	0.029903562	no
P2RY2	0.145575817	2.6444893	0.008581046	0.029903562	no
DRD5	-0.14557238	-2.644425506	0.008582629	0.029904228	no
ADCY2	-0.145562918	-2.644249893	0.008586987	0.029906161	no
CCDC82	-0.14555461	-2.644095715	0.008590815	0.029906161	no

GSTZ1	-0.145552883	-2.644063658	0.008591611	0.029906161	no
LRRC69	0.145552281	2.64405249	0.008591889	0.029906161	no
NHSL1	-0.145550573	-2.644020783	0.008592676	0.029906161	no
HS3ST4	-0.145550093	-2.644011885	0.008592897	0.029906161	no
CASP7	0.145550033	2.644010765	0.008592925	0.029906161	no
MMP12	0.145543071	2.643881556	0.008596135	0.029912489	no
MZT2A	-0.145535569	-2.643742334	0.008599594	0.029919683	no
GINS3	-0.145507082	-2.64321366	0.008612744	0.029960582	no
GOLPH3L	0.145483569	2.642777297	0.008623611	0.02999353	no
BABAM1	-0.145472262	-2.642567452	0.008628841	0.030006865	no
MIOS	0.145458302	2.642308392	0.008635302	0.030024474	no
SUMO1	-0.14545207	-2.642192738	0.008638188	0.030029649	no
TXNDC16	-0.145447326	-2.642104696	0.008640385	0.03003243	no
NAA38	-0.145424299	-2.64167736	0.008651058	0.030062479	no
STEAP4	0.145422638	2.641646543	0.008651828	0.030062479	no
ID4	-0.145366255	-2.640600207	0.008678012	0.030148588	no
CECR3	-0.145345159	-2.640208722	0.008687827	0.030177808	no
PPM1N	0.145314076	2.639631921	0.008702307	0.030219628	no
ARL17A	-0.145313278	-2.639617106	0.008702679	0.030219628	no
SMAD4	-0.145303802	-2.639441257	0.008707098	0.030230087	no
TMED5	0.145296049	2.639297398	0.008710714	0.030237758	no
NTM	-0.145284273	-2.639078872	0.00871621	0.030251951	no
C1GALT1	0.145272568	2.638861667	0.008721676	0.030266034	no
FBRS	0.145244214	2.638335523	0.008734929	0.030307131	no
STXBP3	0.145239658	2.638250979	0.008737061	0.030309632	no
LAMP1	0.145234426	2.638153897	0.008739509	0.030313231	no
CHRDL2	0.145219122	2.637869912	0.008746673	0.030333185	no
MAP3K3	0.145153246	2.636647532	0.008777571	0.030435426	no
PDIA2	-0.145131167	-2.636237847	0.008787948	0.030465366	no
KRT33A	-0.145128845	-2.636194745	0.00878904	0.030465366	no
SLFN12L	0.14509875	2.635636341	0.008803204	0.030494836	no
LINC00087	-0.145098591	-2.635633389	0.008803279	0.030494836	no
ZNF818P	0.145096755	2.635599325	0.008804144	0.030494836	no
PGK1	0.145095888	2.63558322	0.008804553	0.030494836	no
GNAI1	-0.145095708	-2.635579894	0.008804637	0.030494836	no
ZNF514	-0.145070571	-2.635113477	0.008816485	0.030530951	no
LINC00338	-0.145061971	-2.634953901	0.008820542	0.030540079	no
L3MBTL1	-0.145054532	-2.634815882	0.008824052	0.030547311	no
SLMO2	0.144987065	2.633564054	0.008855947	0.030652786	no
CMSS1	-0.144963919	-2.633134599	0.008866912	0.030685799	no
LOC728040	0.144940513	2.632700332	0.008878013	0.030719269	no
FAM45B	-0.144931943	-2.632541329	0.008882081	0.030728396	no
PLEKHO1	0.144925635	2.6324243	0.008885075	0.030733809	no
TPTE2P3	-0.144914296	-2.632213923	0.008890461	0.030743282	no
RAI1	-0.144913847	-2.632205579	0.008890675	0.030743282	no
TRIM58	0.144902395	2.631993103	0.008896118	0.030755626	no
RBM12B	-0.144900315	-2.631954511	0.008897107	0.030755626	no
AMPH	-0.144894021	-2.631837745	0.0089001	0.030761023	no
VCAN	-0.144878862	-2.631556488	0.008907312	0.030781	no
FAM228A	-0.144874893	-2.63148285	0.008909201	0.030782579	no
ZBTB24	-0.144848757	-2.630997959	0.00892165	0.030820636	no

SOS2	-0.144830999	-2.630668489	0.008930117	0.030844929	no
NSL1	0.14481921	2.630449759	0.008935743	0.0308594	no
GNL2	0.144800769	2.630107641	0.008944548	0.030884846	no
LOC100132111	0.144787175	2.629855431	0.008951044	0.030902311	no
LOC100132832	0.144768464	2.629508294	0.008959992	0.030928235	no
VEZF1	-0.144706634	-2.628361223	0.008989617	0.031025512	no
TCP10L	-0.144664885	-2.627586707	0.00900967	0.031089727	no
BACH2	-0.144648428	-2.627281408	0.009017585	0.031112045	no
TBC1D26	-0.144644834	-2.627214739	0.009019315	0.031113017	no
H2AFY	0.144577337	2.625962584	0.00905185	0.031220239	no
MMP9	0.144571254	2.625849741	0.009054787	0.031225359	no
GPR133	0.144564983	2.62573342	0.009057816	0.031228224	no
THEGL	0.144563517	2.625706219	0.009058524	0.031228224	no
E4F1	-0.144544427	-2.625352096	0.009067751	0.031255018	no
SSTR5-AS1	-0.144541169	-2.625291653	0.009069327	0.031255436	no
MRPL46	-0.144509537	-2.62470487	0.009084636	0.031303176	no
ERBB3	-0.144496317	-2.62445963	0.009091041	0.031320225	no
SP6	0.144487619	2.62429829	0.009095257	0.031329728	no
AUTS2	-0.144479898	-2.624155055	0.009099002	0.031337604	no
HDGFRP2	-0.144475095	-2.62406597	0.009101331	0.031338375	no
CHPF	0.144473424	2.624034971	0.009102142	0.031338375	no
PITPNM1	0.144462122	2.623825323	0.009107627	0.031352236	no
CAPNS1	-0.14445502	-2.623693578	0.009111075	0.031359083	no
C3orf45	-0.144439713	-2.623409628	0.009118511	0.031376436	no
ADAMTS13	-0.144438631	-2.623389567	0.009119037	0.031376436	no
IFFO1	0.144435091	2.623323903	0.009120757	0.031377332	no
ZBTB26	-0.144398618	-2.62264735	0.0091385	0.031433341	no
NRAS	0.144387838	2.622447387	0.009143751	0.031446367	no
FCGR3A	0.144339137	2.621544029	0.009167503	0.031523008	no
WISP2	0.144320828	2.621204408	0.009176446	0.031548714	no
ZNF22	-0.14431432	-2.621083701	0.009179627	0.031554601	no
IRF2BPL	0.144302582	2.620865971	0.009185367	0.031569282	no
UROD	0.144297051	2.620763392	0.009188072	0.031573531	no
LOC100506178	0.14426163	2.620106394	0.009205416	0.031628074	no
MDFIC	0.144250034	2.619891299	0.009211101	0.031642546	no
TMCO1	0.14422344	2.61939804	0.009224149	0.031681515	no
FGD6	0.144219847	2.619331389	0.009225913	0.031681515	no
MPV17L2	0.144217904	2.619295356	0.009226867	0.031681515	no
XKR4	-0.144213379	-2.619211418	0.00922909	0.031684084	no
ST8SIA2	-0.144201089	-2.618983472	0.009235128	0.03169975	no
GREM2	-0.144197099	-2.618909476	0.009237089	0.031701418	no
HSDL2	-0.144175585	-2.618510428	0.00924767	0.031732665	no
SH2D5	-0.144146409	-2.617969299	0.009262036	0.031776887	no
WRB	-0.144123972	-2.617553159	0.009273098	0.031809759	no
CPNE2	-0.144118442	-2.617450598	0.009275826	0.031814039	no
ARCN1	0.144092482	2.616969107	0.009288642	0.031852914	no
CACNA1I	-0.144084786	-2.616826368	0.009292445	0.03186087	no
DBP	-0.144074145	-2.616629021	0.009297704	0.031873818	no
PHC1	-0.144043733	-2.616064988	0.009312752	0.03192031	no
AQP11	-0.144035508	-2.615912434	0.009316825	0.031927967	no
CXorf57	-0.144033224	-2.615870073	0.009317957	0.031927967	no

CYYR1	0.144019702	2.615619303	0.009324657	0.031944876	no
RAG1	0.144017266	2.615574126	0.009325865	0.031944876	no
UBQLNL	0.144005527	2.615356397	0.009331686	0.031959724	no
ANKRD34A	-0.143989896	-2.615066514	0.009339442	0.031981191	no
NAT9	-0.143985346	-2.614982135	0.009341701	0.031983829	no
KLHL24	-0.143963513	-2.614577225	0.009352547	0.032015862	no
RAB14	-0.143921385	-2.613795937	0.009373506	0.032082499	no
DPP7	0.14391584	2.613693089	0.009376268	0.032086843	no
HPCA	-0.143904947	-2.613491078	0.009381695	0.032100305	no
ALG8	0.143888814	2.613191887	0.009389739	0.032117648	no
KCNV1	-0.143888786	-2.613191364	0.009389753	0.032117648	no
ATP5A1	-0.143862084	-2.61269618	0.009403079	0.032157027	no
CHL1	0.143859723	2.612652403	0.009404258	0.032157027	no
CYC1	-0.143847172	-2.612419646	0.009410529	0.03217335	no
RBM19	-0.143794039	-2.611434309	0.009437116	0.032259114	no
CDC42	-0.143748066	-2.610581781	0.009460173	0.032332789	no
ADCY10P1	-0.143724016	-2.610135792	0.009472255	0.032366085	no
SH3BP5	0.143722679	2.610111013	0.009472927	0.032366085	no
STON2	-0.143699875	-2.609688138	0.009484397	0.032400122	no
C1orf174	0.143686823	2.609446109	0.009490967	0.032417414	no
TALDO1	0.143676957	2.609263154	0.009495936	0.032429233	no
GCC1	0.14366664	2.609071846	0.009501135	0.032439655	no
LOC415056	-0.14366491	-2.609039766	0.009502007	0.032439655	no
ZNF454	-0.143641161	-2.608599385	0.009513984	0.032475387	no
SCAP	-0.14362494	-2.608298586	0.009522173	0.032493105	no
KCTD8	-0.143624889	-2.608297644	0.009522199	0.032493105	no
SLC12A5	-0.14362025	-2.60821163	0.009524542	0.03249594	no
SSTR3	-0.143566485	-2.60721468	0.009551734	0.032583542	no
TMF1	0.143563019	2.607150411	0.009553489	0.032584358	no
ZKSCAN1	-0.143543244	-2.606783732	0.00956351	0.032613359	no
KPNA4	0.143537511	2.606677435	0.009566416	0.032618095	no
NDUFA8	-0.143530155	-2.606541023	0.009570148	0.032625641	no
CTF1	0.143519169	2.606337334	0.009575721	0.032639465	no
FAM219B	-0.143489383	-2.605785041	0.009590849	0.03268142	no
FANCE	-0.143488946	-2.605776935	0.009591071	0.03268142	no
CLIP3	-0.143478849	-2.60558972	0.009596204	0.032693727	no
40422	0.143466943	2.60536895	0.009602261	0.032709174	no
SLC39A10	-0.143448246	-2.605022278	0.009611777	0.032736404	no
HFM1	-0.143419043	-2.604480825	0.009626658	0.03278189	no
MAGIX	-0.143403479	-2.604192242	0.009634598	0.032803729	no
TCERG1	-0.143391609	-2.603972155	0.009640657	0.032819159	no
LOC399829	-0.143386385	-2.603875292	0.009643324	0.032823042	no
WIPF1	0.143368276	2.603539541	0.009652577	0.032849331	no
C14orf28	-0.143352534	-2.603247674	0.009660626	0.032871518	no
SST	-0.143348498	-2.60317284	0.009662691	0.032873339	no
SEC63	-0.143294683	-2.602175091	0.009690258	0.032961908	no
NSMCE1	-0.143273041	-2.601773833	0.009701364	0.032994464	no
ZCCHC24	-0.143215093	-2.600699479	0.009731157	0.033090554	no
RASGRP3	0.143205157	2.600515278	0.009736274	0.033102715	no
C1orf233	-0.143197214	-2.60036801	0.009740366	0.03311139	no
FRMPD4	-0.143189893	-2.600232291	0.009744139	0.033118976	no

RPL32	-0.143185958	-2.600159336	0.009746167	0.033120632	no
GNLY	0.143164041	2.59975301	0.009757472	0.033153807	no
GOT1	-0.143156783	-2.599618462	0.009761218	0.033161292	no
TMEM169	-0.143127677	-2.599078861	0.009776254	0.033207125	no
HERC2P9	-0.143109492	-2.598741718	0.009785659	0.033232686	no
C21orf49	-0.143107147	-2.598698259	0.009786872	0.033232686	no
EFCAB14	0.143095615	2.598484471	0.009792841	0.033247701	no
H2AFJ	0.143045313	2.597551934	0.009818915	0.033329006	no
SYT13	-0.143043433	-2.597517081	0.00981989	0.033329006	no
RABL6	-0.143036379	-2.597386316	0.009823552	0.033336169	no
LOC606724	0.143029613	2.597260896	0.009827065	0.033342825	no
PRSS3	-0.1430163	-2.597014097	0.009833981	0.033361024	no
DNALI1	0.142962004	2.596007557	0.009862232	0.033451584	no
C1orf21	-0.142918111	-2.595193908	0.009885123	0.033523935	no
PLCL1	-0.142913835	-2.595114646	0.009887355	0.033526215	no
ZNF625-ZNF20	-0.14288813	-2.594638142	0.009900786	0.033566459	no
CNNM4	0.142855428	2.594031951	0.009917895	0.03361916	no
CST1	0.14283328	2.593621395	0.009929497	0.03365315	no
TTLL11	-0.142828945	-2.593541039	0.00993177	0.03365315	no
LACTB	0.142827323	2.593510986	0.00993262	0.03365315	no
MAPRE2	-0.142809968	-2.59318927	0.009941723	0.033678682	no
PRPF8	-0.142770595	-2.592459453	0.0099624	0.033739557	no
SLC17A1	0.142769774	2.592444225	0.009962832	0.033739557	no
TPT1-AS1	-0.142751936	-2.592113597	0.009972213	0.033766004	no
HEYL	0.14274283	2.591944803	0.009977005	0.033776909	no
SCP2	-0.142720712	-2.591534821	0.009988653	0.033811017	no
NSF	-0.142715355	-2.59143553	0.009991476	0.033815246	no
ARMC9	0.142709478	2.591326597	0.009994574	0.033820404	no
SLC26A9	-0.142702935	-2.591205318	0.009998024	0.033826752	no
CDC34	-0.142647858	-2.590184448	0.010027105	0.033919804	no
ZFPM2	-0.142596923	-2.589240381	0.010054066	0.034005655	no
SYAP1	0.142593726	2.589181125	0.010055761	0.034006035	no
PAPOLA	0.142557642	2.588512319	0.010074903	0.034065408	no
WLS	0.142550602	2.588381853	0.010078641	0.034070802	no
TOX4	-0.142548667	-2.588345975	0.010079669	0.034070802	no
CCT3	-0.142531035	-2.588019188	0.010089038	0.034097107	no
NECAP1	-0.142517196	-2.587762697	0.010096397	0.034116613	no
LOC100130539	0.142506707	2.5875683	0.010101977	0.034130104	no
IPO5	-0.14249886	-2.587422852	0.010106154	0.03413885	no
CCDC167	-0.142480379	-2.587080341	0.010115997	0.034161735	no
ANGPT2	0.142480171	2.587076481	0.010116108	0.034161735	no
C3orf49	-0.142450014	-2.586517564	0.010132189	0.034210663	no
ARL6IP6	-0.142435848	-2.586255023	0.01013975	0.034230816	no
GABRA1	-0.142393905	-2.58547768	0.010162168	0.034301108	no
SQLE	-0.142369396	-2.585023464	0.010175288	0.034336159	no
LOC100287015	-0.142368537	-2.585007547	0.010175748	0.034336159	no
SERINC1	-0.142334045	-2.584368311	0.010194238	0.03439315	no
GPRC5A	0.142326528	2.584229003	0.010198271	0.034393601	no
LIFR	-0.142325284	-2.584205946	0.010198939	0.034393601	no
ZNF594	-0.142324849	-2.584197882	0.010199173	0.034393601	no
CCKBR	-0.14231298	-2.583977926	0.010205545	0.03440969	no

PCDHB2	-0.142308898	-2.583902277	0.010207737	0.034411683	no
LOC729911	-0.142285479	-2.583468271	0.010220323	0.034448708	no
ZNF32-AS2	-0.14227432	-2.583261475	0.010226325	0.034463532	no
SMARCC1	-0.142270397	-2.583188777	0.010228435	0.034465241	no
LOC283335	-0.142266776	-2.58312166	0.010230384	0.034466404	no
ARMCX2	0.142233142	2.582498366	0.0102485	0.034522023	no
LINC00578	0.142168713	2.581304398	0.010283281	0.034633755	no
ADAM20P1	-0.142146131	-2.580885927	0.010295496	0.034669463	no
LOC285484	-0.142111503	-2.58024424	0.010314253	0.034724864	no
DTX4	-0.142109792	-2.580212546	0.01031518	0.034724864	no
XRCC3	-0.142057434	-2.579242331	0.010343599	0.034815081	no
KHSRP	-0.142043553	-2.578985103	0.010351146	0.034835026	no
LOC100133612	0.142033408	2.578797123	0.010356664	0.034848139	no
FLJ43315	0.142016493	2.578483678	0.010365871	0.034873657	no
B4GALT6	-0.141996662	-2.578116224	0.010376673	0.034904536	no
LOC285419	0.141974545	2.577706394	0.010388733	0.034939634	no
NBR1	-0.141960962	-2.577454713	0.010396145	0.034959093	no
PARK2	-0.14195405	-2.577326629	0.01039992	0.034966313	no
NIPSNAP1	-0.141949816	-2.577248179	0.010402232	0.034968616	no
CELF6	-0.141940017	-2.577066606	0.010407585	0.034981141	no
ST7-OT3	-0.141928499	-2.57685318	0.010413881	0.034996828	no
PLSCR3	0.141921036	2.576714908	0.010417961	0.035005067	no
SIK3	-0.141914493	-2.576593669	0.010421541	0.035011619	no
RETSAT	0.141899326	2.576312632	0.010429841	0.035034029	no
BIK	0.141893373	2.576202331	0.010433101	0.035039501	no
DNAJC2	0.141874191	2.575846914	0.01044361	0.035069315	no
NCK2	0.141866035	2.575695785	0.010448081	0.035078849	no
C16orf80	-0.141852443	-2.575443956	0.010455536	0.035098394	no
CACNA2D1	-0.141837196	-2.575161446	0.010463904	0.035121001	no
SSPO	-0.14181677	-2.574782988	0.010475124	0.03515317	no
WNT9B	-0.141806802	-2.574598296	0.010480604	0.035166067	no
TEX10	-0.141734823	-2.573264666	0.010520246	0.03529357	no
ZBTB43	-0.141695659	-2.572539056	0.010541871	0.035360598	no
MTERFD3	-0.141673977	-2.57213735	0.01055386	0.035395289	no
CYP4X1	-0.141663225	-2.571938143	0.01055981	0.035409717	no
NECAB1	-0.141635611	-2.571426534	0.010575104	0.035450253	no
CTR9	0.141631733	2.571354686	0.010577254	0.035450253	no
ZNF845	0.141629589	2.571314977	0.010578442	0.035450253	no
SGK196	-0.141627238	-2.571271418	0.010579745	0.035450253	no
TOX	-0.141626515	-2.571258029	0.010580146	0.035450253	no
ZNF534	-0.14161366	-2.571019858	0.010587276	0.035464891	no
LOC100287879	-0.141611986	-2.570988844	0.010588205	0.035464891	no
NBL1	0.141609713	2.570946739	0.010589466	0.035464891	no
SDHB	0.141595777	2.570688546	0.010597201	0.035485268	no
PSORS1C1	0.141582119	2.570435511	0.010604787	0.035502782	no
POU2F2	0.141580412	2.570403888	0.010605735	0.035502782	no
SOHLH1	-0.141577025	-2.570341133	0.010607618	0.035503553	no
LCMT1	-0.14156414	-2.570102434	0.01061478	0.035518751	no
ZNF37BP	-0.141560657	-2.570037893	0.010616717	0.035518751	no
CGB	0.141558797	2.570003444	0.010617751	0.035518751	no
TSIX	-0.141556967	-2.569969536	0.010618769	0.035518751	no

GRM5	-0.141553108	-2.569898041	0.010620916	0.035520403	no
ARNTL2	0.141548786	2.569817982	0.010623321	0.035522916	no
USP3	-0.141522089	-2.569323381	0.010638186	0.035567089	no
PLTP	0.141505613	2.569018148	0.010647368	0.035592253	no
XYLT2	0.141446429	2.567921731	0.010680413	0.035697161	no
FAM153A	-0.141436312	-2.567734308	0.01068607	0.035710517	no
VPRBP	-0.1414295	-2.56760811	0.010689881	0.035717699	no
SEPT7L	-0.141414268	-2.567325949	0.010698407	0.035740628	no
SPRYD7	-0.141370264	-2.566510762	0.010723072	0.035814856	no
RPS2P32	0.141368682	2.566481471	0.010723959	0.035814856	no
ZFHX4	-0.141362776	-2.566372055	0.010727273	0.035820359	no
CALCRL	-0.141325357	-2.565678881	0.010748294	0.035882816	no
EXOSC3	0.141323537	2.565645173	0.010749317	0.035882816	no
QSER1	-0.14130862	-2.565368842	0.010757707	0.035905248	no
KCNA1	-0.141276084	-2.564766142	0.010776028	0.035960811	no
SLX4	-0.141269928	-2.564652104	0.010779498	0.035966805	no
CD274	0.141265488	2.564569863	0.010782001	0.035969572	no
RGCC	-0.141206462	-2.563476476	0.010815325	0.036071787	no
TMED7	0.141205274	2.563454471	0.010815997	0.036071787	no
PATL1	0.141195217	2.563268194	0.010821684	0.036085153	no
NRGN	-0.141143155	-2.562303827	0.010851168	0.036177854	no
LOC100289230	-0.141114944	-2.56178129	0.010867174	0.036225598	no
RPF1	0.14110987	2.561687304	0.010870055	0.036229582	no
LOC100505679	-0.141097928	-2.56146611	0.010876838	0.036246569	no
ARRDC5	0.14108507	2.561227952	0.010884146	0.036264646	no
NAA50	0.141081126	2.561154887	0.010886389	0.036264646	no
AIFM2	0.141079479	2.561124389	0.010887325	0.036264646	no
LOC145837	-0.141075404	-2.561048913	0.010889642	0.036266745	no
TMEM186	-0.141067932	-2.560910499	0.010893893	0.036275281	no
MED12	-0.141063199	-2.560822837	0.010896587	0.036278628	no
C10orf111	-0.141054493	-2.560661591	0.010901542	0.036289503	no
AMOT	-0.141039449	-2.560382941	0.01091011	0.036312149	no
GABRA4	-0.141036616	-2.560330471	0.010911724	0.036312149	no
AGMAT	0.141031599	2.560237538	0.010914583	0.03631604	no
SPAG1	0.141024495	2.560105965	0.010918632	0.036323889	no
LRP8	-0.141005354	-2.559751426	0.01092955	0.036354583	no
VPREB3	0.140984939	2.559373311	0.010941204	0.036382599	no
H2AFV	-0.140984669	-2.559368317	0.010941358	0.036382599	no
DNM1	-0.14094987	-2.558723788	0.01096125	0.036443105	no
RERE	-0.140945443	-2.558641781	0.010963784	0.036445889	no
TMEM27	-0.140906359	-2.557917899	0.010986168	0.03651465	no
DHX30	-0.140890135	-2.557617412	0.010995471	0.036539922	no
KLHL35	-0.140874407	-2.557326123	0.011004497	0.036564262	no
ALDH3A1	0.140868129	2.557209847	0.011008102	0.036570585	no
EBAG9	0.14083278	2.556555167	0.011028417	0.036632413	no
DBI	-0.14082798	-2.556466263	0.011031178	0.036635923	no
LIPE	-0.140795687	-2.555868191	0.011049771	0.036689519	no
PABPN1	-0.140794021	-2.555837329	0.011050731	0.036689519	no
MSH5	-0.140770615	-2.555403859	0.011064225	0.036728645	no
GTF2H2B	-0.140730841	-2.554667253	0.011087189	0.036799192	no
PHIP	-0.140691719	-2.553942736	0.011109817	0.036863789	no

TRHDE	-0.140691263	-2.553934276	0.011110082	0.036863789	no
ZNF606	-0.140678288	-2.553693998	0.011117595	0.036883026	no
KIR2DL4	0.140655033	2.553263333	0.011131074	0.036922043	no
CCL13	0.140643739	2.553054182	0.011137625	0.036938072	no
BHMT2	0.140628404	2.552770191	0.011146526	0.036956992	no
BCDIN3D	-0.140627985	-2.552762436	0.011146769	0.036956992	no
ZNF724P	-0.140618699	-2.552590475	0.011152162	0.036969168	no
PBRM1	-0.140615173	-2.55252517	0.011154211	0.036970257	no
CCT7	-0.14061089	-2.552445851	0.011156699	0.036972803	no
GRIN2B	-0.140602998	-2.552299705	0.011161286	0.036982301	no
RPL30	-0.140595823	-2.552166833	0.011165458	0.03699042	no
SLC4A10	-0.140592488	-2.552105074	0.011167397	0.036991143	no
ATP2B3	-0.140583142	-2.551931995	0.011172834	0.037003448	no
IPP	0.140567443	2.55164128	0.011181971	0.037028004	no
RNASEK	0.140551183	2.551340176	0.011191442	0.037053657	no
EPHA1-AS1	0.140542636	2.551181906	0.011196423	0.037064439	no
LOC728024	-0.140528175	-2.550914112	0.011204856	0.037086641	no
HES7	-0.140500179	-2.550395685	0.011221197	0.037135008	no
SLC2A4	-0.140492405	-2.550251727	0.011225738	0.037144317	no
WDR55	0.140486787	2.550147696	0.011229021	0.037144459	no
KIF9-AS1	-0.140486416	-2.550140825	0.011229238	0.037144459	no
GRHPR	-0.140467782	-2.549795764	0.011240133	0.037174777	no
LGI3	-0.140463207	-2.549711052	0.011242809	0.037177906	no
PKNOX2	-0.140451902	-2.549501705	0.011249425	0.037194061	no
SHISA8	-0.140447247	-2.549415505	0.01125215	0.037197349	no
INHBB	0.140440397	2.549288668	0.011256161	0.037204885	no
SNHG7	-0.140428571	-2.549069679	0.01126309	0.03722206	no
CYS1	-0.140419214	-2.548896418	0.011268574	0.037234458	no
PREPL	-0.140397049	-2.548485983	0.011281575	0.037271686	no
SPPL3	-0.140386992	-2.548299761	0.011287478	0.037285457	no
STOM	0.140379661	2.548164003	0.011291783	0.037293946	no
C8orf82	-0.140371227	-2.548007837	0.011296737	0.037304575	no
DEFB109P1B	-0.140366416	-2.547918755	0.011299564	0.037306906	no
PPYR1	0.140364117	2.547876192	0.011300915	0.037306906	no
CLCN3	-0.140339213	-2.547415049	0.01131556	0.037349516	no
C1orf51	-0.140296602	-2.546626048	0.011340657	0.037426606	no
LOC286184	-0.140289956	-2.546502984	0.011344576	0.037433791	no
MESDC2	0.140262983	2.54600355	0.011360493	0.037480557	no
TMED8	-0.140243253	-2.545638237	0.011372148	0.037510702	no
U2SURP	-0.140241607	-2.54560776	0.011373121	0.037510702	no
FAM131A	-0.140226592	-2.545329741	0.011381998	0.037530503	no
TCEAL7	-0.140225546	-2.545310387	0.011382617	0.037530503	no
SLC27A3	0.140198291	2.544805749	0.011398748	0.037577924	no
COMT	-0.14018104	-2.54448633	0.011408968	0.03760585	no
MTHFD1L	0.140174754	2.54436994	0.011412695	0.037612363	no
IL11RA	-0.140171279	-2.544305605	0.011414755	0.037613385	no
HTR5A	-0.140134199	-2.543619066	0.01143676	0.03768012	no
DCP1B	0.140115951	2.543281201	0.011447604	0.037709948	no
LINC00176	-0.140113058	-2.543227649	0.011449324	0.037709948	no
TCERG1L	-0.14008695	-2.542744262	0.011464855	0.037755318	no
NTNG1	0.140063078	2.542302285	0.011479073	0.037796348	no

ZFP69B	-0.140046217	-2.541990122	0.011489124	0.037823648	no
COLEC11	0.140030984	2.541708101	0.011498212	0.037847767	no
DNM1P35	0.140023801	2.541575109	0.0115025	0.037856082	no
LYPD8	-0.140011026	-2.541338599	0.011510128	0.03787033	no
FDCSP	0.14001065	2.541331623	0.011510353	0.03787033	no
THAP4	0.140000442	2.54114265	0.011516452	0.037880994	no
MYOM2	-0.139999325	-2.541121963	0.011517119	0.037880994	no
LOC645638	0.139971929	2.540614757	0.011533503	0.037929077	no
USP10	-0.139928116	-2.539803637	0.011559747	0.038009566	no
OAF	0.13991948	2.539643751	0.011564926	0.038020779	no
TCF25	-0.139895466	-2.539199173	0.011579339	0.03806234	no
FCHO2	0.139881204	2.538935145	0.011587906	0.038084675	no
VGLL3	0.139871902	2.538762936	0.011593497	0.038097224	no
MMP17	-0.139868302	-2.538696294	0.011595661	0.03809851	no
SLC6A10P	-0.139848128	-2.538322813	0.011607797	0.038132552	no
TAF5	-0.139836737	-2.53811195	0.011614653	0.038149245	no
CEP170P1	-0.139802973	-2.537486897	0.011634999	0.038205914	no
GRIP1	-0.139802206	-2.53747269	0.011635462	0.038205914	no
EVI5	0.139778696	2.537037477	0.011649647	0.03824665	no
LOC440434	-0.13975214	-2.536545871	0.01166569	0.038293468	no
ZNF467	0.139748206	2.536473044	0.011668068	0.038295425	no
EXOC6B	-0.139737851	-2.536281347	0.01167433	0.038310127	no
WDR85	-0.139733237	-2.536195936	0.011677121	0.038313435	no
CASP9	-0.139688917	-2.535375511	0.01170396	0.038395636	no
LRFN2	-0.139681558	-2.535239281	0.011708422	0.038404412	no
SIK1	0.1396737	2.535093818	0.011713188	0.038408779	no
FBXW4P1	-0.13967347	-2.535089557	0.011713328	0.038408779	no
ZSCAN5A	0.139644072	2.534545363	0.011731174	0.038461428	no
S100A13	0.139628421	2.534255655	0.011740685	0.038486737	no
UBALD1	-0.13958156	-2.533388219	0.011769201	0.038573619	no
CYP21A2	0.139578971	2.533340305	0.011770778	0.038573619	no
PEMT	-0.139566478	-2.533109054	0.011778392	0.038592685	no
VBP1	-0.139535454	-2.532534788	0.011797318	0.038648805	no
EPDR1	0.139532128	2.532473228	0.011799348	0.038649565	no
ZFP64	0.139479972	2.53150782	0.011831231	0.038747107	no
CNTN6	-0.139477519	-2.531462413	0.011832733	0.038747107	no
CA8	-0.139447611	-2.530908831	0.011851052	0.038801181	no
PIGZ	-0.139438624	-2.530742485	0.011856561	0.038813306	no
SNHG12	0.139406307	2.530144319	0.011876392	0.038872303	no
RBM12B-AS1	-0.139364471	-2.52936999	0.011902107	0.038950538	no
H6PD	0.139347311	2.529052376	0.011912669	0.038979167	no
ADORA1	0.139319602	2.528539515	0.011929742	0.039029088	no
ZNF702P	0.139293874	2.528063327	0.011945613	0.039075063	no
IGHMBP2	-0.139269122	-2.527605224	0.011960899	0.039119112	no
OGDHL	-0.139230402	-2.526888588	0.011984847	0.039191472	no
EPS15	0.139218592	2.526670024	0.011992159	0.039208759	no
NRG3	-0.139215972	-2.526621523	0.011993783	0.039208759	no
PTPN11	-0.13919134	-2.526165658	0.012009049	0.039252695	no
SLCO2A1	0.139171482	2.525798129	0.012021369	0.03928699	no
ZNF611	-0.139160113	-2.525587732	0.012028427	0.03930408	no
CTDSP1	0.139126169	2.524959522	0.012049524	0.039367029	no

QPCTL	0.139113681	2.524728405	0.012057293	0.039384017	no
VSTM5	-0.139111921	-2.52469583	0.012058389	0.039384017	no
PRRX2	0.139106028	2.524586772	0.012062057	0.039390011	no
STAT5B	-0.139075605	-2.524023752	0.01208101	0.039445909	no
TTLL1	-0.139041348	-2.523389767	0.012102383	0.039509693	no
RPL31	-0.139031924	-2.523215362	0.012108268	0.039522903	no
GABRA5	-0.139014292	-2.522889064	0.012119287	0.039550178	no
WDR37	-0.139012662	-2.522858914	0.012120305	0.039550178	no
RNF20	-0.138961181	-2.521906204	0.012152529	0.03964889	no
DHRS11	-0.138958442	-2.521855528	0.012154245	0.03964889	no
CLPX	-0.138945786	-2.521621307	0.01216218	0.039668753	no
WISP1	0.138941821	2.521547944	0.012164666	0.039670841	no
PLIN1	-0.138927018	-2.521273997	0.012173955	0.039690404	no
FAM150A	-0.138926374	-2.521262086	0.012174359	0.039690404	no
OSBPL11	-0.138917316	-2.52109446	0.012180045	0.03970292	no
CABP1	-0.138906222	-2.520889175	0.012187013	0.039719607	no
NDUFC2	-0.138881685	-2.520435103	0.012202437	0.039763846	no
KIF5A	-0.138863846	-2.520105001	0.012213661	0.039791863	no
CYB5B	0.138862135	2.520073346	0.012214737	0.039791863	no
SDSL	0.138815164	2.519204163	0.012244337	0.03987897	no
SIAH1	-0.138813816	-2.519179211	0.012245188	0.03987897	no
ATP5D	-0.138808389	-2.519078785	0.012248612	0.039884078	no
FONG	0.13880445	2.519005907	0.012251097	0.039886128	no
S100A6	0.138790346	2.518744915	0.012260002	0.039909074	no
USP7	-0.138782401	-2.518597899	0.012265021	0.039919364	no
HNRNPA3	-0.138774513	-2.518451944	0.012270005	0.039929539	no
VSIG1	0.138716503	2.517378524	0.012306716	0.040042942	no
CLDND1	-0.13871273	-2.517308714	0.012309107	0.040044659	no
TPP2	-0.138704672	-2.517159612	0.012314215	0.040055213	no
CECR5-AS1	-0.138673716	-2.516586813	0.012333855	0.040113027	no
FAM131C	-0.138652964	-2.516202825	0.012347037	0.040149823	no
BPIFA1	0.138647482	2.516101389	0.012350522	0.040155077	no
CEP192	-0.138637984	-2.51592566	0.01235656	0.040168632	no
RPLP0	-0.13863307	-2.515834735	0.012359685	0.040172716	no
MACROD2	-0.138626859	-2.515719815	0.012363636	0.040179481	no
EXD3	-0.138622563	-2.515640327	0.01236637	0.040182289	no
EPB41L4B	-0.138610947	-2.515425381	0.012373765	0.040200238	no
TRIAP1	0.138583228	2.514912516	0.012391424	0.040251527	no
HNRNPA1P10	-0.13857779	-2.514811891	0.012394892	0.040256706	no
CTAGE10P	-0.138571788	-2.51470085	0.012398719	0.040263052	no
ADCK1	-0.138561413	-2.514508885	0.012405339	0.040274767	no
IL12A	-0.138560259	-2.514487532	0.012406075	0.040274767	no
ANKRD34C	-0.138528874	-2.513906838	0.012426119	0.040333743	no
MAGEL2	-0.138517773	-2.513701434	0.012433215	0.040350684	no
RAB39B	-0.138498367	-2.513342379	0.012445629	0.040384874	no
LOC100505783	-0.13848198	-2.513039194	0.01245612	0.040412814	no
TATDN3	0.138421075	2.511912355	0.012495181	0.040533423	no
TRPV6	-0.138392841	-2.511389984	0.012513325	0.040581108	no
GNG3	-0.138392324	-2.511380426	0.012513657	0.040581108	no
NEK8	0.138368184	2.510933808	0.012529189	0.040625347	no
TMEM130	-0.138337857	-2.510372735	0.012548726	0.040682556	no

LOC100271702	-0.138333312	-2.510288637	0.012551656	0.040685919	no
LRRC7	-0.138278557	-2.50927565	0.012587005	0.040794347	no
ZBTB8B	-0.138241234	-2.508585172	0.01261115	0.040866438	no
KCNS2	-0.138213464	-2.508071439	0.012629141	0.040918568	no
HK2	0.138209372	2.507995734	0.012631794	0.040920995	no
PCDHGA12	0.138201501	2.507850128	0.012636898	0.040931361	no
APCDD1L	0.138195359	2.507736495	0.012640883	0.040938098	no
GLT1D1	-0.138186309	-2.507569065	0.012646756	0.040950948	no
BCKDK	0.138171448	2.507294151	0.012656405	0.040976018	no
SPDEF	-0.138153707	-2.50696596	0.012667933	0.041007161	no
CBX4	-0.138137199	-2.506660567	0.012678668	0.04103573	no
ASPH	-0.138133179	-2.5065862	0.012681283	0.041038015	no
POU3F2	-0.138130003	-2.506527442	0.01268335	0.041038523	no
DONSON	0.138115818	2.506265039	0.012692583	0.04105675	no
CCNI	-0.138115479	-2.506258775	0.012692804	0.04105675	no
GRM2	-0.138099205	-2.505957724	0.012703405	0.041079164	no
ST3GAL2	0.138096335	2.50590462	0.012705275	0.041079164	no
TOLLIP	-0.13809604	-2.505899164	0.012705468	0.041079164	no
SATB2	-0.138084029	-2.505676975	0.012713298	0.041092443	no
LOC728190	-0.138082914	-2.505656345	0.012714025	0.041092443	no
LOC100133985	-0.138080942	-2.505619872	0.012715311	0.041092443	no
C9orf9	0.138073793	2.505487631	0.012719974	0.041101331	no
LOC100268168	-0.138067567	-2.505372451	0.012724036	0.041108278	no
TCTA	0.13806227	2.505274464	0.012727493	0.041113266	no
EDARADD	0.13804828	2.505015679	0.012736628	0.041136589	no
AKR1B15	0.138045233	2.5049593	0.012738618	0.041136837	no
ADAT3	0.138023255	2.504552749	0.012752982	0.041174882	no
SLC7A8	0.138021344	2.504517403	0.012754232	0.041174882	no
KLF1	-0.13801244	-2.504352689	0.012760056	0.041187497	no
LAMP2	0.138000742	2.504136297	0.01276771	0.041206017	no
SLC25A21	-0.137975979	-2.50367823	0.012783928	0.041252162	no
ZNF33BP1	-0.137962225	-2.503423817	0.012792943	0.041275056	no
HTT-AS1	-0.137951085	-2.503217756	0.012800249	0.041292429	no
ADCY8	-0.137939576	-2.503004859	0.012807802	0.041310591	no
RFC5	-0.137916374	-2.502575686	0.012823038	0.041353528	no
LOC340017	-0.137908871	-2.502436896	0.012827969	0.041363223	no
LOC389023	-0.137904224	-2.502350931	0.012831024	0.041366867	no
GPR26	-0.137885391	-2.502002587	0.012843409	0.041400587	no
LINC00669	0.137871617	2.501747791	0.012852476	0.041423598	no
MCCC1	-0.137865637	-2.501637185	0.012856413	0.041430075	no
GATA3-AS1	0.137858451	2.501504266	0.012861146	0.041439114	no
C15orf38-AP3	-0.137852566	-2.501395421	0.012865023	0.041445392	no
NDUFV1	-0.13782616	-2.500906981	0.012882434	0.041490912	no
ARHGEF10L	-0.13782528	-2.50089071	0.012883014	0.041490912	no
SIX5	0.137775346	2.499967111	0.012915995	0.041590897	no
ANKHD1-EIF4E	0.137720026	2.498943889	0.012952621	0.041702587	no
RAPGEF3	-0.13769564	-2.498492854	0.012968795	0.041748407	no
UTRN	0.137670474	2.498027393	0.012985505	0.041795938	no
AGRN	0.137652759	2.497699753	0.012997278	0.041827569	no
EPB41L3	-0.13761906	-2.497076478	0.013019702	0.041893459	no
TLR4	0.137594938	2.496630344	0.013035774	0.041938894	no

ISOC2	0.137577326	2.496304606	0.013047519	0.041970399	no
LOC100128264	-0.137569337	-2.496156852	0.01305285	0.041981263	no
NID1	0.137505698	2.494979879	0.013095384	0.04211176	no
INPP1	-0.13749999	-2.494874327	0.013099204	0.042117744	no
GLP1R	-0.137493783	-2.494759518	0.013103361	0.042124806	no
IPMK	-0.137484887	-2.49459501	0.013109319	0.042137657	no
CAB39L	-0.13744273	-2.493815359	0.013137589	0.04222221	no
LIPT2	0.137425919	2.493504462	0.013148877	0.042248031	no
FRK	0.13742491	2.493485793	0.013149555	0.042248031	no
STK38L	0.137407282	2.493159789	0.013161401	0.042274717	no
TECPR1	-0.137406696	-2.493148953	0.013161795	0.042274717	no
TADA2B	-0.137399909	-2.49302344	0.013166358	0.042276229	no
SHANK3	-0.137398771	-2.493002396	0.013167124	0.042276229	no
ARMC10	0.137396322	2.492957109	0.013168771	0.042276229	no
TBC1D10B	-0.137394295	-2.492919625	0.013170134	0.042276229	no
TMEM18	-0.137384003	-2.492729287	0.013177059	0.042292141	no
ARSK	0.137377004	2.49259985	0.01318177	0.042297341	no
EGFLAM-AS4	0.137375748	2.49257663	0.013182615	0.042297341	no
VPS13D	-0.137325877	-2.49165435	0.013216229	0.042393205	no
LOC643339	0.137325566	2.491648604	0.013216438	0.042393205	no
ZNF365	-0.137311634	-2.491390958	0.013225842	0.042417038	no
ZNF99	-0.137290815	-2.491005964	0.013239905	0.04245383	no
ACACA	-0.137288803	-2.490968758	0.013241265	0.04245383	no
CDH6	0.137284252	2.490884598	0.013244341	0.042457359	no
SLC22A6	-0.137272668	-2.490670374	0.013252174	0.042470238	no
CDK20	0.137271701	2.490652502	0.013252828	0.042470238	no
IKBKAP	-0.137269544	-2.490612609	0.013254287	0.042470238	no
SUMF1	0.137258493	2.490408245	0.013261765	0.042484839	no
FLJ30838	-0.137256967	-2.490380028	0.013262798	0.042484839	no
TAF1D	-0.137252847	-2.490303839	0.013265586	0.042487372	no
TWIST1	0.137249957	2.49025041	0.013267542	0.042487372	no
HERC2P3	-0.137243759	-2.490135785	0.01327174	0.042494482	no
P2RX6	-0.137193328	-2.489203218	0.013305932	0.042597615	no
PHF21B	-0.137167456	-2.488724796	0.013323504	0.042647516	no
C14orf37	-0.137131884	-2.488067015	0.013347697	0.042718593	no
TMX1	0.137100035	2.487478102	0.01336939	0.042781649	no
KANSL1	-0.137094263	-2.487371371	0.013373325	0.04278787	no
MIER1	-0.137086417	-2.487226279	0.013378675	0.042798618	no
MPP1	0.137057867	2.486698372	0.01339816	0.04285457	no
OLFML1	0.137028551	2.486156298	0.013418193	0.042912261	no
FAM217B	-0.137016322	-2.485930167	0.013426558	0.042932624	no
AIFM3	-0.136983789	-2.485328624	0.013448833	0.042997453	no
SLC26A4-AS1	-0.136977446	-2.485211346	0.01345318	0.042998975	no
COQ6	-0.136974799	-2.485162406	0.013454994	0.042998975	no
PRAF2	0.136974335	2.48515383	0.013455312	0.042998975	no
NOA1	-0.136951211	-2.484726256	0.013471172	0.043043257	no
CS	-0.136918508	-2.484121596	0.013493628	0.043108601	no
FAM184B	-0.136910524	-2.483973968	0.013499116	0.043119723	no
FN3KRP	-0.136906287	-2.483895631	0.013502028	0.043120598	no
ANKRD42	0.136904289	2.483858684	0.013503402	0.043120598	no
LOC100506368	0.136885823	2.483517252	0.013516106	0.043154751	no

LRRC58	-0.136880122	-2.483411842	0.01352003	0.043155006	no
MCF2L	-0.136879872	-2.483407221	0.013520202	0.043155006	no
NPR2	0.136868062	2.483188872	0.013528333	0.043170967	no
ZNF667	-0.136866775	-2.483165069	0.01352922	0.043170967	no
STH	-0.136851237	-2.482877785	0.013539926	0.043198716	no
H2BFM	-0.136801626	-2.481960528	0.013574159	0.043301507	no
FST	0.136793339	2.481807322	0.013579884	0.043313342	no
NEFM	-0.136780293	-2.481566106	0.013588903	0.043335675	no
AATK-AS1	-0.136757777	-2.481149824	0.01360448	0.043378912	no
EPHA6	-0.136750283	-2.481011262	0.013609668	0.043389018	no
TBR1	-0.136702372	-2.480125485	0.013642876	0.043487012	no
D2HGDH	-0.136698819	-2.480059787	0.013645342	0.043487012	no
PARG	-0.136695143	-2.479991824	0.013647893	0.043487012	no
SGTB	-0.136694269	-2.479975682	0.013648499	0.043487012	no
FAM122A	0.136672035	2.47956462	0.01366394	0.043529756	no
ADIPOR2	-0.136657415	-2.479294323	0.013674102	0.043549805	no
ODF3L1	0.13665715	2.479289418	0.013674286	0.043549805	no
CNIH3	0.136652396	2.479201527	0.013677592	0.043552472	no
LOC731223	0.136650117	2.479159401	0.013679177	0.043552472	no
CAMK2A	-0.136638136	-2.478937906	0.013687511	0.043572553	no
PSMA5	0.136628819	2.478765651	0.013693996	0.043586741	no
PDIK1L	-0.136619039	-2.478584841	0.013700806	0.043601959	no
NGB	-0.136615738	-2.478523825	0.013703105	0.043602818	no
SLC27A5	-0.136604389	-2.478314001	0.013711013	0.043621522	no
COL4A2-AS1	0.136582975	2.47791813	0.013725943	0.043662559	no
C11orf1	-0.136540731	-2.477137156	0.01375544	0.043749914	no
MINPP1	-0.136522997	-2.476809312	0.01376784	0.043782871	no
BIVM	-0.136518216	-2.476720936	0.013771184	0.043787026	no
MEF2BNB-MEF2	-0.136511737	-2.476601158	0.013775717	0.043791431	no
DDX52	-0.136510412	-2.476576665	0.013776645	0.043791431	no
NAT8L	-0.136485382	-2.476113955	0.013794171	0.043840659	no
SPAST	-0.136480924	-2.476031534	0.013797295	0.043844104	no
SPRY1	0.136452459	2.475505336	0.013817255	0.043901039	no
TIMM50	-0.136422626	-2.474953836	0.013838202	0.043961094	no
ZNF670	-0.136416174	-2.474834568	0.013842736	0.043963385	no
SLC32A1	-0.136415777	-2.474827233	0.013843015	0.043963385	no
IL6	0.136405263	2.474632874	0.013850406	0.043980359	no
C10orf95	-0.136388726	-2.474327177	0.013862038	0.044010793	no
RGS9	-0.136382524	-2.474212521	0.013866403	0.044018148	no
ATAT1	-0.136378011	-2.474129103	0.013869579	0.044021729	no
TRPC1	-0.136363524	-2.473861295	0.013879782	0.044043645	no
PDE4DIP	-0.136362387	-2.473840276	0.013880583	0.044043645	no
SIX4	-0.136341852	-2.473460681	0.013895056	0.04408306	no
GGN	0.136333056	2.473298086	0.01390126	0.044096232	no
RMND1	-0.136318584	-2.473030579	0.013911471	0.044122112	no
EP300	-0.136310435	-2.472879942	0.013917224	0.044133845	no
SNAPC1	0.13621421	2.471101247	0.013985317	0.044343236	no
CHMP2B	0.136189602	2.470646396	0.014002778	0.044392048	no
F2RL2	0.136145887	2.469838373	0.014033843	0.044483969	no
SUSD5	-0.136125389	-2.469459496	0.01404843	0.04452364	no
PHACTR1	-0.136116802	-2.469300769	0.014054545	0.044536453	no

FLJ37505	0.136105523	2.469092312	0.01406258	0.044555344	no
TRMT1	-0.136094273	-2.468884359	0.014070599	0.044574181	no
EEF1G	-0.136018077	-2.467476028	0.014125015	0.044739971	no
SNORA6	-0.135995964	-2.467067323	0.014140842	0.044783501	no
COL24A1	0.135980942	2.466789676	0.014151603	0.044810976	no
ATP7A	0.135975979	2.466697961	0.014155159	0.044813769	no
SIRT2	-0.135973892	-2.46665937	0.014156655	0.044813769	no
PIAS2	-0.135964447	-2.466484819	0.014163426	0.044824795	no
G0S2	0.135963215	2.466462049	0.01416431	0.044824795	no
ZMYND12	0.135958815	2.46638072	0.014167466	0.044827708	no
KRT18	0.135956085	2.466330269	0.014169424	0.044827708	no
ZDHHC6	0.135953208	2.466277089	0.014171488	0.044827708	no
METTL21EP	-0.135920598	-2.46567438	0.014194901	0.04489516	no
TAF15	-0.135903633	-2.465360839	0.014207094	0.044927114	no
ATP8A2	-0.135891962	-2.465145146	0.014215488	0.044947043	no
FBXO40	-0.135882649	-2.464973028	0.014222189	0.044961616	no
APITD1	0.135858096	2.464519242	0.01423987	0.045010889	no
HEXA-AS1	0.135809095	2.46361363	0.014275213	0.04511597	no
HAPLN1	-0.135800936	-2.463462858	0.014281104	0.045127393	no
MAPRE3	-0.135798275	-2.463413677	0.014283027	0.045127393	no
GPC6	0.135775291	2.462988907	0.014299639	0.045173238	no
AZI1	-0.135750256	-2.462526241	0.014317753	0.045223813	no
ZNF219	-0.13573908	-2.462319697	0.014325846	0.045242725	no
RCBTB1	-0.135730534	-2.462161769	0.014332036	0.045255626	no
GLRA3	-0.135724772	-2.462055288	0.014336212	0.04526216	no
RAPGEF4	-0.135721345	-2.461991943	0.014338696	0.045263355	no
ZSWIM1	-0.135693423	-2.461475947	0.014358948	0.045312553	no
PPA1	-0.135693014	-2.46146838	0.014359245	0.045312553	no
ZNF81	-0.135691138	-2.461433715	0.014360607	0.045312553	no
EIF2A	-0.135685383	-2.461327351	0.014364785	0.045316514	no
KIF6	-0.1356836	-2.461294403	0.014366079	0.045316514	no
SFXN4	-0.135674616	-2.461128391	0.014372603	0.04533044	no
ZNF876P	-0.135659149	-2.46084255	0.014383842	0.04535923	no
ANKRA2	-0.135644646	-2.460574546	0.014394387	0.045385824	no
AKAP5	-0.135633335	-2.460365518	0.014402616	0.045405109	no
CCDC174	-0.135613008	-2.459989878	0.014417415	0.045445096	no
VARS	-0.135606875	-2.459876536	0.014421883	0.045452513	no
HIPK4	-0.135602129	-2.459788838	0.014425341	0.045456745	no
ICAM5	-0.135592898	-2.459618253	0.014432069	0.045471279	no
TMEM159	0.135587862	2.45952519	0.014435741	0.045476181	no
FBXO17	-0.135575421	-2.459295288	0.014444815	0.045497991	no
CHD4	-0.135570375	-2.459202045	0.014448497	0.045497991	no
NDUFAF3	0.135569664	2.459188908	0.014449015	0.045497991	no
SNORA18	-0.135550759	-2.458839548	0.014462818	0.045526348	no
CCNL1	0.135547589	2.458780975	0.014465133	0.045526348	no
ZNF131	-0.135546634	-2.458763329	0.014465831	0.045526348	no
REV3L	-0.135545726	-2.458746538	0.014466494	0.045526348	no
CA11	-0.135535683	-2.458560959	0.014473833	0.045542772	no
LAT	0.135525491	2.458372627	0.014481283	0.045559319	no
EMR3	0.135520952	2.458288754	0.014484602	0.045559319	no
CRYM	-0.135518835	-2.458249625	0.014486151	0.045559319	no

JPX	-0.135516876	-2.458213424	0.014487584	0.045559319	no
JRKL	-0.135513995	-2.458160196	0.014489691	0.045559319	no
RWDD1	-0.135507537	-2.458040844	0.014494417	0.045567511	no
DNASE1	-0.135468269	-2.457315223	0.014523177	0.04565125	no
FADS6	-0.135459682	-2.457156565	0.014529472	0.045664359	no
ACBD3	0.135433392	2.456670757	0.014548763	0.045718302	no
NUPR1	0.135429763	2.45660371	0.014551427	0.045719989	no
NOL8	0.135419599	2.456415892	0.014558893	0.045736758	no
MBNL3	0.135414827	2.456327711	0.014562399	0.045741086	no
RYR2	-0.135408715	-2.456214775	0.01456689	0.045748508	no
PDZD7	-0.135394435	-2.455950907	0.014577389	0.045774791	no
DNAJC25-GNG1	0.135306291	2.454322219	0.014642341	0.045972032	no
DOCK4	0.13529792	2.454167543	0.014648523	0.045984722	no
LINC00520	0.135294507	2.454104476	0.014651044	0.04598592	no
LHFPL1	-0.135281432	-2.453862897	0.014660705	0.046009524	no
PLCB4	-0.135216928	-2.452671053	0.014708451	0.046152625	no
COX5B	-0.135205823	-2.452465886	0.014716684	0.046171717	no
PEX12	-0.135197265	-2.452307748	0.014723033	0.046184892	no
ATP1A1	0.13518827	2.452141553	0.014729707	0.046199087	no
KCNMB1	0.135162685	2.45166885	0.014748707	0.046251927	no
SETX	0.135158657	2.451594421	0.0147517	0.046254565	no
BTAF1	-0.135101957	-2.450546833	0.01479389	0.046380087	no
FAM133A	-0.135080548	-2.450151294	0.014809848	0.046423343	no
EIF3CL	-0.135061202	-2.449793866	0.014824281	0.046461808	no
MRPS5	-0.135054624	-2.449672341	0.014829191	0.04647042	no
SKOR1	-0.13504822	-2.449554014	0.014833973	0.046478629	no
EPB49	-0.135027633	-2.449173674	0.014849354	0.046520039	no
SLC6A7	-0.135014379	-2.448928796	0.014859264	0.046544301	no
KCNJ12	-0.134994313	-2.448558087	0.014874278	0.046579227	no
VPS36	-0.134993684	-2.448546462	0.014874749	0.046579227	no
PPFIA4	-0.134980256	-2.448298378	0.014884805	0.046603924	no
UBA5	-0.134954096	-2.447815083	0.014904411	0.046658512	no
STRIP2	-0.13490607	-2.446927825	0.014940464	0.046764566	no
SLC39A13	0.134890005	2.446631034	0.014952541	0.046795553	no
NAV2-AS5	-0.134883017	-2.446501942	0.014957797	0.046805185	no
PES1	-0.134879645	-2.446439656	0.014960333	0.046806307	no
SHROOM3	0.134856798	2.446017574	0.014977532	0.046853295	no
UTP20	0.134815913	2.445262277	0.015008351	0.046942872	no
ZNF559	-0.134810146	-2.44515575	0.015012702	0.046949649	no
MIER3	-0.134805772	-2.445074944	0.015016004	0.046953141	no
LOC389332	-0.134798276	-2.444936462	0.015021663	0.0469579	no
RERGL	-0.134797968	-2.444930773	0.015021896	0.0469579	no
CYP4F22	0.134791487	2.444811048	0.01502679	0.046966368	no
FBXL13	0.134787754	2.444742078	0.01502961	0.046968351	no
QARS	0.134766035	2.444340861	0.015046025	0.047012812	no
GABARAPL1	-0.134754643	-2.444130412	0.015054641	0.047032897	no
BMS1P1	-0.134705503	-2.443222652	0.015091857	0.047142312	no
PLK5	-0.134692403	-2.442980674	0.015101792	0.047166488	no
RPAP1	-0.134682778	-2.442802862	0.015109095	0.047178223	no
FLYWCH1	-0.134681666	-2.442782323	0.015109939	0.047178223	no
RTN4RL1	-0.134668097	-2.442531674	0.015120241	0.047199223	no

ZNF860	0.134667021	2.442511797	0.015121058	0.047199223	no
CBX6	-0.134663053	-2.442438504	0.015124071	0.047201774	no
DUS3L	-0.134622726	-2.441693562	0.015154731	0.047290594	no
POLE2	-0.134619535	-2.441634633	0.015157159	0.047291303	no
CYP4B1	-0.134612734	-2.441508998	0.015162335	0.047300588	no
LINC00229	0.134606987	2.441402833	0.015166711	0.047307372	no
PTGES3L	-0.134599992	-2.441273627	0.015172038	0.04731712	no
HNRNPA0	-0.134562624	-2.440583384	0.015200524	0.047399082	no
CNOT10	-0.134554846	-2.440439712	0.01520646	0.04741071	no
C19orf73	-0.134540441	-2.440173629	0.015217457	0.047438116	no
GLI3	0.134533013	2.440036418	0.015223131	0.047448921	no
SLC18A2	-0.134519369	-2.439784384	0.015233558	0.047474535	no
FAM98A	0.134514418	2.439692945	0.015237342	0.047479444	no
KIAA1715	-0.134500116	-2.439428759	0.015248281	0.047506641	no
EXT2	0.134485377	2.439156512	0.015259561	0.04753294	no
ANK2	-0.134483306	-2.439118274	0.015261146	0.04753294	no
SCO2	0.134404581	2.437664159	0.015321524	0.047707167	no
FBXO4	0.134402906	2.437633216	0.015322811	0.047707167	no
MMP16	-0.134401692	-2.437610795	0.015323744	0.047707167	no
PLIN3	0.134396431	2.437513621	0.015327786	0.047712842	no
LYPD1	0.134380594	2.43722111	0.015339962	0.047743827	no
GLB1L	0.134364937	2.436931919	0.015352008	0.047774399	no
ANKFY1	-0.134318185	-2.436068419	0.015388025	0.04787955	no
SLCO5A1	-0.134303567	-2.435798436	0.015399302	0.047907701	no
RPL21P28	-0.13426838	-2.435148542	0.015426476	0.047985295	no
OSBPL2	-0.134265443	-2.435094297	0.015428747	0.047985411	no
CAMK1D	-0.134257145	-2.434941033	0.015435162	0.047998418	no
TUFM	-0.134248153	-2.434774961	0.015442116	0.048004546	no
IFT74	0.134246488	2.4347442	0.015443405	0.048004546	no
RCN1	0.134245933	2.43473396	0.015443834	0.048004546	no
RDBP	-0.134212149	-2.434109999	0.01546999	0.048078894	no
CHFR	0.13420653	2.434006228	0.015474344	0.048085472	no
TDRD3	-0.134166977	-2.43327572	0.015505024	0.048173842	no
PANK3	-0.134162756	-2.43319777	0.015508301	0.048177058	no
TMEM127	0.134114709	2.432310425	0.015545647	0.048286095	no
SNX29P2	0.134108644	2.432198402	0.015550367	0.048290419	no
SLC6A9	-0.134107146	-2.432170743	0.015551533	0.048290419	no
LCNL1	-0.134099753	-2.432034202	0.015557288	0.048301312	no
IL22RA1	0.134077215	2.431617985	0.015574845	0.048348836	no
CCDC64	-0.134069117	-2.431468429	0.015581158	0.048361447	no
LOC100130950	-0.134053406	-2.431178269	0.015593412	0.048392492	no
LOC642852	-0.134039152	-2.430915045	0.015604535	0.048420022	no
VILL	0.134008178	2.430343017	0.015628733	0.048488106	no
B4GALT3	0.133999458	2.430181986	0.015635551	0.048502257	no
TPD52	-0.133996377	-2.430125097	0.015637961	0.048502729	no
BAI2	-0.133993309	-2.430068438	0.015640361	0.048503173	no
SPATA18	0.133936774	2.429024392	0.015684641	0.048627183	no
RAPGEF6	-0.133932945	-2.428953681	0.015687644	0.048627183	no
PYGO2	0.133932232	2.428940521	0.015688203	0.048627183	no
DDX19A	0.133930706	2.428912334	0.0156894	0.048627183	no
LOC100288069	-0.13391512	-2.428624516	0.015701629	0.04865807	no

COL20A1	-0.133872516	-2.427837757	0.015735103	0.048754771	no
PNRC2	0.133844244	2.427315695	0.015757349	0.048816662	no
PPARA	-0.133813095	-2.426740491	0.015781892	0.04888565	no
EXOC7	0.133800456	2.426507098	0.01579186	0.048909477	no
NMRAL1	0.133793605	2.426380588	0.015797265	0.048919169	no
LRRC26	-0.133777305	-2.426079602	0.015810132	0.04895196	no
BBX	-0.13374982	-2.425572071	0.01583185	0.049012142	no
LOC440900	0.133744164	2.425467622	0.015836323	0.049018928	no
TBRG4	-0.133739399	-2.425379639	0.015840092	0.049023532	no
AIRE	0.133722324	2.425064343	0.015853603	0.049056549	no
RSBN1L	-0.133720148	-2.425024169	0.015855325	0.049056549	no
ZNF502	-0.133689627	-2.424460597	0.015879503	0.049124285	no
LACE1	-0.133683037	-2.4243389	0.015884729	0.049133377	no
ISG20L2	0.133675905	2.424207212	0.015890385	0.049143799	no
SNORA47	-0.133659114	-2.423897169	0.015903708	0.049177927	no
ZCRB1	-0.133651488	-2.423756354	0.015909763	0.049189571	no
B3GAT2	-0.133643982	-2.423617767	0.015915723	0.049200922	no
TRIM26	0.133574652	2.422337623	0.015970876	0.049364317	no
ERLEC1	0.133570417	2.422259421	0.015974251	0.049367647	no
FBLN7	0.133555037	2.421975448	0.01598651	0.049398431	no
METTL3	-0.133541718	-2.421729539	0.015997133	0.04942415	no
ERN1	0.133509171	2.421128594	0.01602312	0.049497321	no
VN1R2	-0.133465417	-2.420320745	0.016058112	0.049598287	no
UGT8	-0.133449398	-2.420024967	0.01607094	0.049630777	no
LOC100288637	0.133441755	2.419883869	0.016077063	0.049642553	no
LOC100128714	-0.133399774	-2.419108769	0.016110736	0.049727207	no
EPB41L5	-0.133398931	-2.419093196	0.016111413	0.049727207	no
TLN2	-0.133398921	-2.419093012	0.016111421	0.049727207	no
CWF19L2	-0.133394449	-2.419010451	0.016115011	0.049731147	no
LAPTM4B	-0.13338154	-2.418772116	0.01612538	0.049749837	no
OSGIN1	0.13338005	2.418744614	0.016126577	0.049749837	no
CTSF	-0.133378264	-2.418711629	0.016128012	0.049749837	no
CHST1	-0.133340076	-2.418006578	0.016158725	0.049837421	no
WDR18	-0.133333305	-2.417881569	0.016164175	0.049847079	no
ZNRF1	-0.133307701	-2.417408868	0.016184801	0.049902196	no
CXorf36	0.133305355	2.417365558	0.016186692	0.049902196	no
DMTF1	-0.13326912	-2.416696581	0.016215926	0.049985149	no
HRH2	0.13323724	2.416108026	0.016241683	0.050057366	no
MTHFD1	-0.133186359	-2.415168694	0.016282867	0.050177099	no
TNC	0.133172994	2.414921953	0.0162937	0.050203284	no
SAPCD2	-0.133169108	-2.414850224	0.01629685	0.050205793	no
SSBP3	-0.133161197	-2.414704176	0.016303267	0.050218361	no
MDFI	-0.133157291	-2.41463207	0.016306435	0.050220923	no
COLQ	0.133138646	2.414287867	0.016321569	0.050260329	no
STK16	-0.133111648	-2.413789459	0.016343504	0.050320666	no
C12orf5	0.133106636	2.413696927	0.016347579	0.050326003	no
C9orf43	-0.133086616	-2.413327344	0.016363865	0.050368924	no
C15orf48	0.133077804	2.413164679	0.016371038	0.050379114	no
LHFPL5	-0.133076789	-2.413145942	0.016371864	0.050379114	no
ALDH1L2	-0.133067804	-2.412980066	0.016379181	0.050394415	no
EDC4	-0.133063133	-2.412893841	0.016382986	0.050398906	no

HCAR3	0.133048333	2.412620638	0.016395047	0.05042834	no
DKFZp451B082	-0.133045634	-2.412570813	0.016397247	0.05042834	no
METAP1D	-0.133021963	-2.412133836	0.016416556	0.050480499	no
PLD3	0.133013973	2.411986349	0.016423078	0.050493328	no
SEZ6L2	-0.132990528	-2.411553553	0.016442228	0.050544976	no
CASC2	0.132967503	2.41112852	0.016461055	0.050595613	no
NPPA	-0.132930995	-2.410454608	0.016490944	0.050680233	no
FLT4	0.132925198	2.410347601	0.016495694	0.050687584	no
SNX22	-0.132920142	-2.410254267	0.016499838	0.05069307	no
HIST1H4E	-0.132883827	-2.40958393	0.016529631	0.050777343	no
SPSB2	0.132873048	2.409384959	0.016538483	0.050797275	no
GOLGA4	0.132849646	2.408952977	0.016557716	0.050849081	no
CYP2B7P1	-0.132829408	-2.408579421	0.016574364	0.050892934	no
SESN1	-0.132803414	-2.4080996	0.016595769	0.05095138	no
IL1RAPL2	-0.132789672	-2.407845953	0.016607094	0.050978868	no
SLC6A12	-0.132776686	-2.407606248	0.016617803	0.051004456	no
GPR27	-0.132760002	-2.407298299	0.01663157	0.05103942	no
ASPSCR1	-0.132750518	-2.407123243	0.0166394	0.05105616	no
MGC16275	-0.132744117	-2.407005096	0.016644686	0.05106509	no
SYT9	-0.132738242	-2.406896662	0.01664954	0.051071493	no
MYCNOS	-0.132735838	-2.406852283	0.016651526	0.051071493	no
MAL2	-0.132697252	-2.406140065	0.016683438	0.051162067	no
GPR108	0.132687931	2.405968033	0.016691154	0.051178427	no
APEX1	-0.132681207	-2.405843929	0.016696723	0.051188198	no
TSTD2	-0.132675099	-2.405731183	0.016701783	0.051193246	no
EWSR1	-0.132670148	-2.405639793	0.016705886	0.051193246	no
ACSM2B	0.13266817	2.405603286	0.016707525	0.051193246	no
ERC2-IT1	-0.13266772	-2.405594982	0.016707898	0.051193246	no
ERCC6L2	-0.132659539	-2.405443991	0.016714679	0.051206722	no
FBXO39	0.132623949	2.404787097	0.016744208	0.051288113	no
KBTBD3	-0.132621768	-2.404746848	0.016746019	0.051288113	no
RNASE1	0.13261646	2.404648868	0.016750428	0.051294306	no
SIRPB1	0.132595416	2.404260456	0.016767915	0.051340542	no
ARHGAP22	0.132584003	2.404049821	0.016777406	0.051362282	no
IFITM10	-0.132566489	-2.403726564	0.016791979	0.051399576	no
ATPAF1	-0.13252948	-2.403043515	0.016822811	0.051486617	no
SEMA5B	-0.132498564	-2.40247292	0.016848604	0.051558216	no
MAMDC2	0.13249528	2.402412321	0.016851346	0.051559264	no
LAMB3	0.132491632	2.402344979	0.016854392	0.051561245	no
RAPGEF4-AS1	-0.132486773	-2.402255311	0.01685845	0.051566319	no
ELMOD3	0.13248338	2.402192691	0.016861285	0.051566925	no
FBXW5	0.132480792	2.402144915	0.016863447	0.051566925	no
SLC25A11	-0.13244388	-2.401463673	0.016894312	0.051653957	no
ZNF408	-0.132434136	-2.401283848	0.016902468	0.051671542	no
SMAD6	0.132424588	2.40110764	0.016910463	0.05168863	no
LOC100128164	-0.132407697	-2.400795897	0.016924616	0.051724533	no
MAGEA12	0.132379214	2.40027025	0.016948503	0.05178555	no
MIR181A2HG	-0.132378145	-2.400250518	0.0169494	0.05178555	no
HNRPDL	-0.132368183	-2.400066671	0.016957762	0.051803734	no
SGCZ	-0.132358779	-2.399893104	0.01696566	0.051817456	no
TRIOBP	-0.132357092	-2.39986198	0.016967076	0.051817456	no

AKR1E2	0.132339072	2.399529424	0.016982219	0.051851508	no
PRKAB2	-0.132338081	-2.399511139	0.016983052	0.051851508	no
DSCAM-IT1	-0.132333285	-2.399422629	0.016987084	0.051856331	no
RAB1A	0.132330463	2.399370548	0.016989457	0.051856331	no
DLGAP1-AS1	0.132285449	2.398539824	0.017027349	0.051964608	no
WTH3DI	-0.13226392	-2.398142511	0.017045499	0.05201261	no
PPM1F	0.132255948	2.397995406	0.017052223	0.052025741	no
DSCAM	-0.132245541	-2.397803354	0.017061005	0.052045146	no
HMGXB4	-0.132225842	-2.397439813	0.017077639	0.052088497	no
PCP4L1	-0.132218983	-2.397313249	0.017083434	0.052097576	no
HNRNPK	-0.13221658	-2.397268908	0.017085464	0.052097576	no
LOC79015	-0.132176628	-2.396531637	0.017119258	0.052193214	no
KIFC2	-0.132138168	-2.39582191	0.017151844	0.052285146	no
ST7-AS1	-0.132111376	-2.395327502	0.017174576	0.05233943	no
EPN3	-0.132107053	-2.39524773	0.017178247	0.05233943	no
RUNX1T1	-0.132106998	-2.395246723	0.017178293	0.05233943	no
NXPH2	-0.132105703	-2.395222816	0.017179393	0.05233943	no
EXOSC5	-0.132076751	-2.394688568	0.017203993	0.052406948	no
SKINTL	0.132073752	2.394633229	0.017206543	0.052407287	no
AKAP9	-0.132047657	-2.394151698	0.017228745	0.052466516	no
LOC154092	0.132045159	2.394105605	0.017230872	0.052466516	no
GRIN2D	-0.132035896	-2.393934684	0.01723876	0.052483097	no
TBCC	-0.132018433	-2.393612453	0.017253639	0.052515688	no
FAM27B	-0.132017597	-2.393597016	0.017254352	0.052515688	no
LOC100507373	-0.132011814	-2.393490305	0.017259282	0.052523255	no
TINAGL1	0.132007558	2.393411774	0.017262911	0.052526861	no
LCAT	-0.131989848	-2.393084992	0.017278019	0.052565388	no
MTRNR2L5	-0.131956808	-2.392475324	0.017306236	0.052643782	no
CISD1	-0.131948816	-2.392327847	0.017313068	0.052657111	no
CRH	-0.131878903	-2.391037837	0.017372929	0.052831698	no
CLDN14	0.131867598	2.390829248	0.017382625	0.052853706	no
KIAA1704	-0.131864167	-2.390765935	0.017385569	0.05285484	no
MED24	-0.131861431	-2.390715456	0.017387917	0.05285484	no
PCDHAC2	-0.131842094	-2.390358669	0.017404517	0.052895122	no
PDE10A	-0.131840262	-2.390324862	0.017406091	0.052895122	no
GCLC	-0.131813269	-2.389826808	0.01742929	0.052958133	no
RFFL	0.131776463	2.389147712	0.017460966	0.053046878	no
CD59	0.131756228	2.388774354	0.017478402	0.053092346	no
CDNF	-0.131745183	-2.388570585	0.017487925	0.053113123	no
PUM2	-0.131742563	-2.388522237	0.017490185	0.053113123	no
SALL3	-0.131731156	-2.388311771	0.017500027	0.053135502	no
RRP8	0.131710297	2.387926915	0.017518036	0.053182669	no
SGIP1	-0.1317052	-2.387832885	0.017522439	0.053188521	no
ZBED3	-0.131684118	-2.387443918	0.017540661	0.053236314	no
PABPC1L2B	-0.131633892	-2.386517246	0.01758414	0.05336074	no
KATNBL1	-0.131615627	-2.386180274	0.017599975	0.053398469	no
KIF21A	-0.131613819	-2.386146914	0.017601543	0.053398469	no
SLC5A2	-0.131601236	-2.385914758	0.01761246	0.053422535	no
TMEM65	-0.131598946	-2.385872512	0.017614447	0.053422535	no
ALG6	0.131581027	2.385541912	0.017630005	0.053462177	no
LOC440894	-0.131560408	-2.38516152	0.017647922	0.053508153	no

SCO1	0.131557849	2.385114313	0.017650147	0.053508153	no
LOC100506233	-0.13151818	-2.384382463	0.017684666	0.05360524	no
RBM41	-0.13150649	-2.384166789	0.01769485	0.053628545	no
ZHX3	-0.131492085	-2.383901037	0.017707405	0.053653336	no
ZC3H18	-0.131491377	-2.383887978	0.017708023	0.053653336	no
DDX26B	0.131479697	2.383672501	0.017718209	0.053676633	no
TP53	-0.13146735	-2.38344471	0.017728983	0.053701703	no
MIR3942	0.131456155	2.383238179	0.017738757	0.053715485	no
IGSF1	-0.131454073	-2.383199781	0.017740575	0.053715485	no
GPR152	0.13145355	2.383190127	0.017741032	0.053715485	no
CPXCR1	0.131437415	2.382892464	0.017755128	0.053750593	no
NTN4	-0.131415363	-2.382485641	0.01777441	0.053789464	no
SERPINI1	-0.131414794	-2.382475141	0.017774908	0.053789464	no
C1orf115	-0.131414143	-2.38246314	0.017775477	0.053789464	no
PCDHGA1	-0.131388914	-2.381997707	0.01779756	0.053848707	no
C7orf73	0.131313455	2.380605684	0.017863751	0.054041368	no
ERO1LB	-0.131304779	-2.380445641	0.017871375	0.054054033	no
SETDB1	-0.131302967	-2.380412213	0.017872968	0.054054033	no
SNORA57	-0.131254874	-2.379525037	0.017915286	0.054174393	no
OR2H2	-0.131220973	-2.378899678	0.017945168	0.05425712	no
PDCD7	-0.131195047	-2.378421425	0.017968051	0.054318663	no
FITM2	-0.13117737	-2.37809535	0.017983667	0.054358225	no
ACTR1B	-0.131160265	-2.377779826	0.017998789	0.054396283	no
APOD	0.131140036	2.377406681	0.018016687	0.054442719	no
INSM1	-0.131084526	-2.376382763	0.018065881	0.054583697	no
RNF114	0.131073903	2.376186821	0.018075308	0.054604504	no
FNBP1L	0.131068338	2.376084155	0.018080249	0.054611755	no
RPRML	-0.131061488	-2.375957819	0.018086331	0.054622449	no
POLRMT	-0.131014897	-2.37509843	0.018127753	0.054739853	no
TAAR3	-0.131007408	-2.374960292	0.018134419	0.054750539	no
LOC158376	0.131002536	2.374870431	0.018138756	0.054750539	no
C1QBP	-0.131002336	-2.374866737	0.018138934	0.054750539	no
SPTSSA	0.130986658	2.374577568	0.018152898	0.054782526	no
ZNF777	-0.130984715	-2.374541717	0.01815463	0.054782526	no
FANCF	0.130975909	2.374379305	0.018162478	0.054798512	no
FLJ37453	-0.130967841	-2.374230495	0.018169671	0.05481252	no
C3orf18	-0.130957088	-2.374032149	0.018179262	0.054833757	no
SOWAHA	-0.130949566	-2.373893417	0.018185973	0.054846302	no
SNX2	0.130944858	2.373806567	0.018190176	0.054851279	no
TNPO3	-0.130908552	-2.37313693	0.018222607	0.054941364	no
PLXNB1	-0.130904909	-2.373069741	0.018225864	0.054943475	no
VTN	-0.130873916	-2.372498106	0.018253593	0.055011973	no
CSGALNACT2	0.130873791	2.372495794	0.018253705	0.055011973	no
PPP1R1A	-0.13086536	-2.37234029	0.018261255	0.05502701	no
WBP1	-0.13083941	-2.371861674	0.018284509	0.055089358	no
CEP170B	-0.130829142	-2.371672301	0.018293718	0.055109376	no
SMARCA4	-0.130779602	-2.37075861	0.018338203	0.055233916	no
NKX2-2	-0.130776473	-2.370700897	0.018341016	0.055233916	no
ESAM	0.130774523	2.370664923	0.01834277	0.055233916	no
CHGA	-0.130737264	-2.369977758	0.018376296	0.055327119	no
C14orf166	0.130687084	2.369052297	0.018421533	0.05545555	no

NUFIP2	0.130678908	2.368901506	0.018428914	0.055469997	no
SNHG10	-0.130672897	-2.368790656	0.01843434	0.055473315	no
PHF3	-0.13067197	-2.368773547	0.018435178	0.055473315	no
PDHB	-0.130615372	-2.367729759	0.018486351	0.05561951	no
ETV7	0.130605342	2.367544774	0.018495433	0.055639046	no
LCN9	-0.130591335	-2.367286457	0.018508122	0.055669425	no
GRM3	-0.130556714	-2.366647995	0.018539517	0.055755054	no
REST	0.130553309	2.36658519	0.018542607	0.055755054	no
CREBBP	-0.130551363	-2.366549313	0.018544373	0.055755054	no
YIPF5	0.130548173	2.366490477	0.018547269	0.055755961	no
FCN3	0.130534611	2.366240373	0.018559585	0.055778778	no
NUDT2	-0.130534099	-2.366230927	0.01856005	0.055778778	no
CRYGA	-0.130515258	-2.365883476	0.018577172	0.055822427	no
C17orf104	-0.130503883	-2.365673699	0.018587516	0.055845701	no
ZDHHC20	0.130478369	2.365203197	0.018610734	0.055907645	no
TMEM108	-0.130466722	-2.364988412	0.018621342	0.055928262	no
XAF1	0.130465119	2.364958858	0.018622802	0.055928262	no
SLC29A2	-0.130442902	-2.364549156	0.018643053	0.055981255	no
DSTN	0.130432655	2.364360177	0.0186524	0.056000834	no
ZNF623	-0.130430041	-2.364311982	0.018654784	0.056000834	no
PYDC1	-0.130418336	-2.364096133	0.018665467	0.05602015	no
TNIK	0.130417279	2.364076637	0.018666432	0.05602015	no
DNAJC14	0.130411057	2.363961905	0.018672113	0.056029374	no
SH3BGRL2	-0.130406892	-2.363885103	0.018675916	0.056032963	no
FAM101A	-0.130383508	-2.363453878	0.018697284	0.056089243	no
MRPS33	-0.130367626	-2.363161015	0.018711809	0.056120144	no
NCOA1	-0.130366534	-2.363140881	0.018712808	0.056120144	no
ZZZ3	0.130336196	2.362581444	0.018740581	0.056195596	no
GMFB	-0.130317554	-2.362237672	0.018757666	0.05623898	no
CEP55	0.130308244	2.362066003	0.018766203	0.056256726	no
SHISA9	-0.130298192	-2.361880644	0.018775424	0.05627652	no
PANK4	-0.130286362	-2.361662512	0.018786281	0.056301209	no
DPM3	-0.130276047	-2.361472302	0.018795753	0.056321741	no
ATXN10	-0.130264075	-2.361251533	0.018806752	0.056346842	no
DDO	0.130255404	2.361091659	0.01881472	0.056362859	no
ARL4D	-0.130214448	-2.360336451	0.018852401	0.056467867	no
PALM	-0.130207141	-2.360201708	0.018859131	0.05647477	no
RPS3A	-0.130206239	-2.360185089	0.018859961	0.05647477	no
PDPR	-0.13018758	-2.359841036	0.018877156	0.056518383	no
GPR161	-0.130167264	-2.359466426	0.018895893	0.056566602	no
CNOT8	-0.130159192	-2.359317591	0.018903342	0.056581019	no
CDC27	0.130150913	2.359164929	0.018910985	0.056594646	no
EPN2	-0.130148556	-2.359121474	0.018913161	0.056594646	no
HOXB3	0.13012019	2.358598444	0.01893937	0.056665183	no
ZXDA	-0.130114591	-2.358495208	0.018944547	0.056672782	no
PTER	0.13007561	2.357776461	0.018980625	0.056772806	no
SCARB1	0.130043914	2.357192047	0.019010004	0.056852769	no
PAQR6	-0.130006552	-2.356503178	0.019044686	0.05694477	no
SV2B	-0.130005066	-2.35647578	0.019046066	0.05694477	no
SERTM1	-0.129994967	-2.356289579	0.019055451	0.056961181	no
SRRD	-0.129993455	-2.356261701	0.019056856	0.056961181	no

IQCE	0.129984037	2.35608805	0.019065612	0.056979429	no
ZW10	0.129971199	2.355851349	0.019077553	0.057007189	no
KRT8	0.129948734	2.355437165	0.019098464	0.057060593	no
TMEM41B	-0.129946295	-2.355392198	0.019100736	0.057060593	no
DHDDS	0.129941915	2.355311446	0.019104815	0.057064848	no
OPRD1	-0.129924167	-2.35498421	0.019121355	0.057106316	no
PTBP1	0.129909584	2.354715357	0.019134954	0.057138988	no
EFCAB5	-0.129905929	-2.354647965	0.019138364	0.057141231	no
DLGAP2	-0.129875435	-2.35408574	0.019166833	0.057218281	no
TMIGD2	0.129849403	2.35360581	0.019191164	0.05728296	no
CD99L2	-0.129781524	-2.352354376	0.019254737	0.057462776	no
LSM3	-0.129776787	-2.352267042	0.01925918	0.057462776	no
LOC283683	-0.129776523	-2.352262167	0.019259428	0.057462776	no
FGF17	-0.129773174	-2.352200422	0.019262571	0.057464173	no
PPP1R3D	0.129765926	2.352066813	0.019269371	0.057476483	no
AQP1	0.129755935	2.351882623	0.01927875	0.057489107	no
SUPT5H	-0.129755718	-2.351878621	0.019278954	0.057489107	no
NOMO3	-0.129747389	-2.35172506	0.019286776	0.057504454	no
USP37	-0.129732884	-2.35145766	0.019300404	0.057537103	no
HTR1E	-0.129721047	-2.351239431	0.019311532	0.057558483	no
SFPQ	-0.129717638	-2.351176591	0.019314737	0.057558483	no
NHLRC4	0.12971671	2.351159474	0.01931561	0.057558483	no
PON3	-0.129697195	-2.3507997	0.019333972	0.057605211	no
DYNC1I1	-0.129680856	-2.350498494	0.019349356	0.057643056	no
EIF5B	-0.129654616	-2.350014745	0.019374086	0.057708729	no
RBBP6	-0.129636299	-2.349677084	0.019391364	0.05775219	no
LOC100507377	-0.129631789	-2.34959394	0.019395621	0.057756864	no
TBC1D4	-0.129614163	-2.349268999	0.019412264	0.057791317	no
MRM1	-0.12961384	-2.349263051	0.019412569	0.057791317	no
LINC00643	-0.129572554	-2.348501952	0.019451602	0.057896545	no
PPP6R3	-0.129570755	-2.348468791	0.019453304	0.057896545	no
BCMO1	0.129554332	2.348166046	0.01946885	0.057934791	no
OSBP	-0.129508933	-2.34732915	0.019511883	0.058054808	no
C3orf33	-0.129482761	-2.346846699	0.019536729	0.058120686	no
GALNTL1	-0.129477541	-2.34675048	0.019541687	0.05812739	no
LOC100506474	0.129473215	2.346670727	0.019545798	0.058131572	no
C12orf49	0.129467643	2.346568008	0.019551093	0.058139276	no
HNRNPL	-0.129463515	-2.346491926	0.019555016	0.058142897	no
KIAA0586	-0.129457692	-2.346384573	0.019560553	0.058151314	no
CBLN1	-0.129446534	-2.346178891	0.019571165	0.058174815	no
CADM4	-0.129427115	-2.345820938	0.019589646	0.058218331	no
EFCAB2	-0.129425458	-2.345790404	0.019591223	0.058218331	no
C18orf42	-0.129393011	-2.345192284	0.019622139	0.058302142	no
TP53RK	0.129378991	2.34493385	0.01963551	0.058327431	no
MGC2889	-0.129378395	-2.344922875	0.019636078	0.058327431	no
MICALCL	0.12933573	2.344136426	0.01967682	0.058440372	no
SYT7	-0.129327123	-2.343977781	0.019685047	0.058456729	no
ZNF582-AS1	-0.129312643	-2.34371087	0.019698896	0.058489773	no
GJA5	0.129308677	2.343637763	0.019702691	0.058492959	no
WAC	-0.129269155	-2.342909278	0.019740539	0.058597227	no
KCNK12	-0.129260869	-2.342756553	0.019748482	0.058612709	no

LOC100170939	-0.129239527	-2.34236318	0.019768953	0.058665365	no
EAF2	0.129226604	2.342124985	0.019781358	0.058694072	no
DCAKD	-0.12918184	-2.341299893	0.01982438	0.058813605	no
LINC00471	-0.12917779	-2.341225257	0.019828276	0.058817043	no
S100A12	0.129160173	2.340900535	0.019845233	0.05885922	no
DCDC2B	0.129137868	2.340489422	0.01986672	0.058914817	no
CBX7	-0.129134921	-2.340435108	0.01986956	0.05891511	no
KIAA1239	-0.12912537	-2.340259071	0.019878768	0.058934282	no
NR2F1	-0.129111457	-2.340002634	0.019892188	0.058965934	no
HIP1	-0.129101612	-2.339821194	0.019901689	0.058985959	no
ZNF394	0.129093957	2.339680092	0.019909079	0.058999727	no
C1QTNF9B-AS1	0.129082831	2.339475042	0.019919824	0.05902343	no
PPP3R1	-0.129075655	-2.339342776	0.019926757	0.059035834	no
EMX2	-0.129069807	-2.339234997	0.019932409	0.059044438	no
PERP	0.12905501	2.338962263	0.019946716	0.059078675	no
COQ5	-0.129050837	-2.33888536	0.019950752	0.059082486	no
NME7	0.129042883	2.338738764	0.019958447	0.059084959	no
38231	-0.129042432	-2.338730444	0.019958884	0.059084959	no
USF1	0.129041449	2.338712332	0.019959835	0.059084959	no
TIGD6	0.129021828	2.338350702	0.01997883	0.059133044	no
CPNE1	-0.129018721	-2.338293441	0.019981839	0.059133806	no
IGFBP3	0.129014124	2.338208717	0.019986292	0.059138841	no
LINC00160	0.129001443	2.337975009	0.01999858	0.059163973	no
LOC100506801	0.128999678	2.337942476	0.020000292	0.059163973	no
TFAP2E	-0.128981169	-2.337601353	0.020018241	0.059208921	no
ENTPD3-AS1	-0.12897176	-2.337427945	0.020027371	0.059227773	no
SMARCD2	0.128966092	2.33732347	0.020032873	0.059235894	no
ZBTB16	-0.128961585	-2.337240418	0.020037248	0.05924068	no
SLC25A33	-0.128937539	-2.336797241	0.020060608	0.059299883	no
PCDHA1	-0.128935293	-2.336755849	0.020062791	0.059299883	no
FAM86DP	0.128928578	2.336632096	0.020069319	0.059303399	no
RAD51B	0.128928392	2.336628672	0.0200695	0.059303399	no
LRRTM1	-0.128923344	-2.336535633	0.020074409	0.059305664	no
IPO9	-0.128921929	-2.336509547	0.020075785	0.059305664	no
LILRB5	0.128875847	2.335660288	0.020120647	0.059430019	no
NUP62CL	0.128856667	2.335306805	0.020139345	0.059477073	no
PAX8	0.128852703	2.335233758	0.020143211	0.059480316	no
AMZ2P1	-0.12883222	-2.334856263	0.020163199	0.05953116	no
LINC00507	-0.128802031	-2.334299916	0.02019269	0.05961004	no
VAV3-AS1	0.128777194	2.333842202	0.02021698	0.059672244	no
IDI2	-0.128774809	-2.333798246	0.020219314	0.059672244	no
DHX35	-0.128741473	-2.333183912	0.02025196	0.059760384	no
PHYHIP	-0.128729285	-2.332959301	0.020263908	0.05978743	no
LOC100289341	-0.128722908	-2.332841784	0.020270161	0.05979767	no
LRRC59	0.128714807	2.3326925	0.020278107	0.059812902	no
ATP5F1	0.128708642	2.332578883	0.020284157	0.059822535	no
OCEL1	0.12869992	2.332418157	0.020292717	0.05983957	no
ZDHHC19	0.128692668	2.33228452	0.020299837	0.059850592	no
CASP8AP2	-0.128690441	-2.332243475	0.020302025	0.059850592	no
OLFM3	-0.128677709	-2.332008849	0.020314532	0.05987925	no
FGD3	-0.128655456	-2.331598781	0.020336408	0.059935511	no

VSNL1	-0.128649492	-2.331488872	0.020342275	0.059944581	no
FAHD1	-0.12863832	-2.331283001	0.020353268	0.059968753	no
LINC00446	0.128621256	2.330968549	0.020370069	0.060003699	no
PSMD5	0.128620603	2.330956509	0.020370713	0.060003699	no
SOX18	0.128588799	2.330370455	0.020402059	0.060087797	no
RARG	0.128580655	2.330220381	0.020410093	0.060103221	no
37500	-0.128573876	-2.330095453	0.020416783	0.060107086	no
ALG5	0.128573657	2.330091415	0.020416999	0.060107086	no
BCL6B	0.128519335	2.329090423	0.020470671	0.060256841	no
RPS20	-0.128511903	-2.32895348	0.020478024	0.060270228	no
AANAT	0.128503597	2.328800432	0.020486243	0.060279755	no
SNX31	-0.128502964	-2.328788762	0.02048687	0.060279755	no
LINC00837	0.128474923	2.328272054	0.020514643	0.06035321	no
ODC1	-0.128466683	-2.328120219	0.020522811	0.060368974	no
PRPF38A	-0.128413646	-2.327142948	0.020575447	0.060515525	no
RAVER2	-0.12839681	-2.326832735	0.020592181	0.060531931	no
TPPP	-0.128395962	-2.326817105	0.020593024	0.060531931	no
CTXN2	-0.128394504	-2.32679025	0.020594473	0.060531931	no
SAMD15	0.128394069	2.32678223	0.020594906	0.060531931	no
SPAG7	-0.128393865	-2.326778476	0.020595109	0.060531931	no
LINC00582	0.128386816	2.326648586	0.020602119	0.060544256	no
CCDC9	0.128373998	2.326412401	0.020614873	0.060573452	no
TRAF7	0.128365081	2.326248093	0.020623749	0.060591249	no
ZFP82	-0.128361192	-2.326176445	0.020627621	0.060594341	no
KCNJ3	-0.128351935	-2.326005876	0.02063684	0.060613138	no
C6orf1	0.128346101	2.325898377	0.020642653	0.060613983	no
TSPAN6	0.128345984	2.325896229	0.020642769	0.060613983	no
FAM53C	-0.128325655	-2.32552165	0.020663033	0.060665197	no
C14orf2	-0.128313187	-2.325291931	0.020675469	0.060693418	no
NAT6	-0.128292086	-2.324903128	0.020696533	0.060746953	no
PDXDC2P	-0.12828071	-2.32469353	0.020707896	0.060772004	no
PGD	0.128247035	2.324073062	0.020741565	0.060858307	no
LDB1	-0.128245634	-2.324047243	0.020742967	0.060858307	no
REEP5	-0.128229845	-2.323756338	0.02075877	0.060892087	no
ISM1	-0.128228469	-2.323730993	0.020760147	0.060892087	no
C17orf50	-0.128225149	-2.323669825	0.020763472	0.060893528	no
TOMM22	-0.1282162	-2.32350494	0.020772435	0.060911503	no
MPV17	0.128189813	2.323018772	0.020798885	0.060980741	no
NEUROD6	-0.12817446	-2.322735889	0.020814288	0.06101094	no
SLAMF9	0.128173887	2.322725342	0.020814863	0.06101094	no
COLEC10	0.128165873	2.322577695	0.020822906	0.061024431	no
KIF12	-0.128163644	-2.322536625	0.020825144	0.061024431	no
CYP2E1	-0.128151133	-2.322306109	0.020837709	0.061044936	no
SGCA	0.12815102	2.322304028	0.020837823	0.061044936	no
CAMK1G	-0.128129752	-2.32191219	0.020859197	0.061099223	no
PRTFDC1	-0.128120276	-2.321737605	0.020868726	0.061118805	no
PIPOX	0.128113046	2.321604406	0.020875999	0.061128402	no
CTPS2	-0.128111364	-2.321573416	0.020877692	0.061128402	no
FOXA3	0.128084742	2.321082938	0.020904494	0.061195929	no
COBLL1	0.128082801	2.321047171	0.02090645	0.061195929	no
THNSL2	0.128074464	2.320893573	0.02091485	0.06121218	no

RABEP2	0.128061697	2.320658363	0.020927719	0.061239657	no
PRR5	0.128059498	2.320617838	0.020929937	0.061239657	no
LOC100505702	0.128040565	2.320269039	0.020949036	0.061287195	no
KRT83	-0.128017776	-2.319849184	0.020972046	0.061345819	no
GTF2H1	0.128015066	2.319799257	0.020974784	0.061345819	no
FBXO10	-0.12799929	-2.319508606	0.020990727	0.061384096	no
ZNF586	0.127986747	2.319277529	0.021003411	0.06141283	no
KIAA1731	-0.127977376	-2.319104892	0.021012891	0.061432191	no
MAPK10	-0.127971627	-2.318998974	0.021018709	0.061440842	no
SCYL1	0.127967626	2.318925258	0.021022759	0.061444324	no
CORT	-0.127963709	-2.318853111	0.021026724	0.061447554	no
RAD51C	-0.127949524	-2.318591769	0.02104109	0.061481178	no
MCL1	0.127927239	2.318181232	0.021063676	0.061538805	no
HMGA2	0.127921631	2.318077914	0.021069363	0.061547054	no
PANX2	-0.127913576	-2.317929519	0.021077534	0.061562555	no
SMA4	-0.127906504	-2.317799246	0.021084709	0.061565564	no
GNPDA2	-0.127906054	-2.317790941	0.021085167	0.061565564	no
AMN1	-0.127904092	-2.317754797	0.021087158	0.061565564	no
DDX42	-0.127896985	-2.317623866	0.021094373	0.061578262	no
SFTPB	0.127874762	2.317214476	0.021116945	0.061635782	no
FAM179B	-0.127869673	-2.317120724	0.021122117	0.061642506	no
LOC148145	-0.127865873	-2.31705073	0.021125979	0.061645406	no
RFPL2	-0.127824586	-2.316290138	0.021167987	0.0617596	no
THAP7-AS1	-0.127821158	-2.316226985	0.021171479	0.061761401	no
IFT140	-0.127817402	-2.31615779	0.021175304	0.061764178	no
PHF1	-0.127808709	-2.315997651	0.021184161	0.061781626	no
GABRB2	-0.127778259	-2.315436702	0.02121521	0.061863783	no
INTS10	0.127773249	2.315344411	0.021220322	0.061870296	no
TSGA13	0.12775734	2.315051357	0.021236563	0.061909247	no
ZNF267	0.127753801	2.314986153	0.021240177	0.061911387	no
EDAR	-0.12774424	-2.314810031	0.021249944	0.061931455	no
PAK3	-0.127699337	-2.313982869	0.021295867	0.062055461	no
TIAF1	-0.127696991	-2.31393966	0.021298268	0.062055461	no
UNC5CL	-0.1276687	-2.313418508	0.021327249	0.062131478	no
SELV	-0.127646654	-2.31301241	0.021349856	0.062188907	no
HACE1	-0.127612094	-2.312375795	0.021385338	0.062283817	no
KIAA0319L	0.127604339	2.312232956	0.021393306	0.062298582	no
PCM1	-0.127593318	-2.312029933	0.021404636	0.06232313	no
MCFD2	0.127583831	2.311855186	0.021414392	0.062341464	no
FAM20B	-0.127581554	-2.311813246	0.021416734	0.062341464	no
ASB13	-0.127572286	-2.311642524	0.02142627	0.062360776	no
CSGALNACT1	-0.127550631	-2.311243641	0.021448565	0.062417212	no
SPHK2	-0.127533263	-2.310923735	0.021466461	0.062460832	no
ATP9B	-0.127520385	-2.310686526	0.021479738	0.062485577	no
PMS2P1	0.127519375	2.310667925	0.02148078	0.062485577	no
IDI1	-0.127499731	-2.310306079	0.021501049	0.062536073	no
AQP7	-0.127482766	-2.309993592	0.021518567	0.062578555	no
ZNF100	-0.127438072	-2.309170358	0.021564776	0.062704452	no
NPHP1	0.127431988	2.309058312	0.021571072	0.062714274	no
C8orf34	0.127398291	2.308437645	0.021605978	0.062807259	no
GPR22	-0.127383586	-2.308166799	0.021621225	0.062843082	no

LOC100652791	-0.127353585	-2.30761422	0.021652362	0.062923511	no
EIF4EBP2	-0.127351283	-2.30757182	0.021654753	0.062923511	no
RPL8	-0.127346141	-2.307477109	0.021660094	0.062930523	no
HIST1H3H	0.12734216	2.307403796	0.021664229	0.06293403	no
ABCA9	0.127313893	2.306883168	0.021693616	0.06300586	no
OPALIN	-0.127312736	-2.306861859	0.021694819	0.06300586	no
CCDC71	0.127288991	2.306424506	0.021719533	0.063069111	no
LPHN2	0.127285754	2.306364896	0.021722904	0.063070376	no
TRIM29	0.127271057	2.306094208	0.021738214	0.063106304	no
SLC17A7	-0.12725015	-2.305709138	0.021760011	0.063161047	no
LOC727924	-0.127239864	-2.305519702	0.021770741	0.063176149	no
MED27	-0.127239527	-2.305513485	0.021771093	0.063176149	no
CADPS	-0.12723262	-2.305386278	0.021778301	0.063188533	no
TMEM191A	-0.127212672	-2.305018893	0.021799129	0.063240428	no
SNORA76	-0.127206519	-2.304905567	0.021805558	0.063250539	no
TCP1	-0.127179265	-2.304403625	0.021834051	0.06332464	no
SLC30A3	-0.127132561	-2.303543476	0.021882952	0.063457905	no
EXD2	-0.127124975	-2.303403758	0.021890905	0.063472401	no
C11orf65	-0.127116903	-2.303255107	0.021899368	0.063488376	no
TNPO2	-0.127101798	-2.302976922	0.021915215	0.063525746	no
SGTA	-0.127085036	-2.30266822	0.021932811	0.063567912	no
TMEM87A	0.127080236	2.302579821	0.021937853	0.063567912	no
CTBP2	-0.127079491	-2.302566102	0.021938635	0.063567912	no
KIRREL2	0.127060453	2.302215492	0.02195864	0.063610078	no
MYOD1	-0.127060009	-2.302207314	0.021959107	0.063610078	no
HS3ST3A1	0.127046519	2.301958865	0.021973293	0.063642592	no
CACNB2	-0.127041204	-2.301860996	0.021978883	0.063650206	no
ZNF792	0.127029017	2.301636558	0.021991708	0.063678764	no
RELN	-0.127019219	-2.3014561	0.022002024	0.063700054	no
ATP1A2	-0.126971348	-2.300574524	0.022052482	0.06383754	no
GFI1	0.126957185	2.300313692	0.022067431	0.063871968	no
ACTG1	0.126954448	2.300263303	0.022070319	0.063871968	no
ATXN7L2	-0.126949542	-2.300172944	0.022075501	0.06387836	no
UBE3D	-0.126934711	-2.299899823	0.022091168	0.06391509	no
PAF1	-0.126931859	-2.299847312	0.022094181	0.063915204	no
LOC100133286	-0.126905876	-2.299368826	0.022121656	0.06398607	no
COX6A1	-0.126888954	-2.299057196	0.022139565	0.064029255	no
MSI2	-0.126877709	-2.298850129	0.022151473	0.064055072	no
SIRPD	0.126834234	2.298049536	0.022197563	0.064179716	no
HERC2P4	-0.126829888	-2.297969501	0.022202175	0.064184416	no
SNRK	-0.126823389	-2.297849827	0.022209074	0.064192232	no
MIR137HG	-0.126821713	-2.297818963	0.022210853	0.064192232	no
WDR82	-0.126800311	-2.297424854	0.022233585	0.064247154	no
SYT17	-0.126798193	-2.297385858	0.022235835	0.064247154	no
PTMA	0.126766742	2.296806707	0.022269279	0.064335137	no
SMS	0.126761506	2.296710273	0.022274853	0.064342588	no
ALKBH5	0.126743675	2.296381935	0.022293837	0.064388772	no
KCNIP4-IT1	-0.126740822	-2.296329406	0.022296875	0.064388894	no
MAP4K3	-0.126722148	-2.295985535	0.022316775	0.064437703	no
WBSCR17	-0.126687866	-2.29535428	0.022353347	0.064534629	no
ZNF620	-0.126661198	-2.29486323	0.022381832	0.06459939	no

PRKCB	-0.126660199	-2.294844823	0.0223829	0.06459939	no
ST6GALNAC1	-0.126657773	-2.294800163	0.022385493	0.06459939	no
PTF1A	-0.126654234	-2.294734998	0.022389276	0.06459939	no
TMEM155	-0.126652801	-2.294708601	0.022390808	0.06459939	no
EZR	-0.126627508	-2.294242865	0.022417863	0.064668766	no
KLHL17	-0.126586175	-2.293481799	0.022462136	0.064787783	no
ZNF506	-0.126558517	-2.292972536	0.022491804	0.064864648	no
VIP	-0.126551049	-2.292835039	0.02249982	0.064875974	no
TCEANC2	0.126549234	2.292801615	0.022501769	0.064875974	no
LOC100507387	-0.126540264	-2.292636444	0.022511402	0.064895042	no
LRP5	-0.126535159	-2.292542448	0.022516886	0.064902145	no
ARPC1A	0.126511104	2.292099538	0.022542741	0.064967955	no
B3GNT4	-0.126500161	-2.291898052	0.022554511	0.064993162	no
MATN3	0.126477853	2.291487316	0.022578522	0.065053629	no
LOC729739	0.126466463	2.291277587	0.022590791	0.065080254	no
PEX26	-0.126448833	-2.290952982	0.022609792	0.065126262	no
ZNF141	-0.126428197	-2.290573042	0.02263205	0.065181637	no
SRR	-0.12641221	-2.290278699	0.022649306	0.065222595	no
CHSY3	0.126373931	2.289573911	0.022690671	0.065332961	no
LOC283856	-0.126364202	-2.289394783	0.022701195	0.065354507	no
39508	-0.126359085	-2.289300585	0.022706731	0.065361688	no
ZNF155	0.126336371	2.288882377	0.022731323	0.065423714	no
SLC26A2	0.126303561	2.288278323	0.022766885	0.06551729	no
CCDC86	0.126299207	2.288198149	0.022771608	0.06552211	no
NRN1L	-0.126283398	-2.28790709	0.022788764	0.065562694	no
OXTR	0.12627709	2.287790962	0.022795612	0.065573617	no
KLF10	0.126258876	2.287455633	0.022815396	0.065621744	no
POU3F4	-0.126238604	-2.287082404	0.022837433	0.065676339	no
ZNF471	-0.126204777	-2.286459642	0.022874246	0.065773405	no
RCC1	0.126134008	2.285156775	0.022951429	0.06598651	no
POP5	0.126113912	2.284786812	0.022973388	0.066040807	no
RRAD	0.126109829	2.284711652	0.022977851	0.066044803	no
ITPR3	0.126098473	2.284502583	0.02299027	0.066071662	no
VPS37C	0.126012831	2.282925985	0.023084111	0.066332482	no
FOXO4	-0.125996439	-2.282624234	0.02310211	0.066372594	no
CPNE6	-0.125994493	-2.282588412	0.023104247	0.066372594	no
APOL2	-0.125989097	-2.282489079	0.023110175	0.066380751	no
SLC38A11	0.125963029	2.282009187	0.023138834	0.066454186	no
SSTR1	-0.125958533	-2.281926431	0.023143779	0.066459507	no
NCOR1	-0.125954809	-2.281857868	0.023147877	0.066462393	no
ZNF786	-0.125927175	-2.281349181	0.023178299	0.066540852	no
LOC100507173	-0.125922457	-2.281262318	0.023183497	0.066546885	no
SYN2	-0.125896655	-2.280787351	0.023211939	0.06661963	no
MAST3	-0.125882086	-2.280519163	0.023228013	0.06665686	no
LOC729177	-0.125863945	-2.280185232	0.02324804	0.066705424	no
OR5T1	0.125793794	2.278893923	0.023325626	0.066919108	no
SLC7A5P1	-0.125780397	-2.27864732	0.023340469	0.066952752	no
ALPL	-0.125742758	-2.277954481	0.023382213	0.067063546	no
FGF11	-0.125739847	-2.277900904	0.023385444	0.067063862	no
SYT1	-0.125715033	-2.277444162	0.023413002	0.067133934	no
SRPRB	0.125703898	2.277239198	0.023425378	0.06716046	no

HAGHL	-0.125692329	-2.277026253	0.023438242	0.067188378	no
TTC14	-0.125687491	-2.276937203	0.023443624	0.067194841	no
PRND	0.125667718	2.276573248	0.023465629	0.067248944	no
FEM1C	0.125647732	2.276205376	0.023487889	0.067303763	no
WDR11-AS1	-0.125639874	-2.27606073	0.023496647	0.06731961	no
SDHAP3	-0.125637149	-2.276010574	0.023499684	0.06731961	no
PIK3CG	0.125631055	2.27589841	0.023506478	0.067330097	no
TTI2	-0.12561762	-2.275651133	0.023521461	0.067364036	no
CPNE9	-0.125613026	-2.275566562	0.023526588	0.06736974	no
PRKXP1	-0.125604486	-2.27540938	0.023536118	0.067388052	no
CLRN1-AS1	-0.125589212	-2.275128247	0.023553173	0.067427899	no
NXT1	0.125528125	2.274003888	0.023621488	0.067614464	no
BCAM	0.125492739	2.273352608	0.023661138	0.06771894	no
PRKCDBP	0.125471929	2.272969589	0.023684484	0.067776729	no
C15orf37	0.125445945	2.272491351	0.023713661	0.067846589	no
FAM73B	-0.125444566	-2.272465976	0.02371521	0.067846589	no
C5orf46	0.125440106	2.272383882	0.023720222	0.067851895	no
SFN	0.125424201	2.272091153	0.023738101	0.067894002	no
QRICH2	-0.125396798	-2.271586821	0.023768932	0.067970122	no
CKLF	0.125394925	2.271552345	0.023771041	0.067970122	no
SOWAHB	-0.125382317	-2.271320297	0.023785239	0.068001673	no
IP6K1	-0.125325982	-2.270283501	0.023848768	0.068174233	no
KIF20B	0.125311184	2.270011141	0.023865482	0.068212936	no
PTGS2	0.125308303	2.269958131	0.023868736	0.068213165	no
PABPC1L2A	-0.12530377	-2.269874702	0.023873858	0.068216598	no
GPR12	-0.125301622	-2.269835169	0.023876286	0.068216598	no
KATNB1	-0.125277072	-2.269383357	0.023904044	0.068286829	no
TM9SF1	0.125254977	2.268976731	0.023929051	0.06834918	no
KCNA5	-0.125252002	-2.268921983	0.023932419	0.068349718	no
ZNF212	-0.12523724	-2.268650303	0.023949142	0.068388388	no
LINC00656	0.125224108	2.26840863	0.023964026	0.068421799	no
ZNF319	-0.125201396	-2.26799065	0.023989787	0.068486253	no
PLA2G4B	-0.125197215	-2.267913712	0.023994532	0.0684907	no
BDNF-AS	-0.125178516	-2.267569589	0.024015763	0.068542199	no
C10orf25	-0.125174074	-2.267487844	0.024020808	0.068547496	no
TGIF1	0.125133316	2.266737776	0.02406715	0.068670621	no
PTGDR	0.125127523	2.266631173	0.024073743	0.068680313	no
AGAP9	-0.125122975	-2.266547485	0.024078919	0.068685185	no
C5orf22	0.125120408	2.266500237	0.024081842	0.068685185	no
PSMB4	0.12510272	2.266174735	0.024101987	0.068733521	no
HCAR2	0.125089321	2.26592815	0.024117258	0.068767945	no
NELL2	-0.125075994	-2.265682909	0.024132455	0.068802147	no
POLR2M	-0.12506761	-2.265528618	0.024142019	0.06881635	no
ESM1	0.125066014	2.265499247	0.02414384	0.06881635	no
AATF	-0.125054443	-2.265286325	0.024157046	0.068841986	no
MANSC1	0.12505252	2.265250941	0.024159241	0.068841986	no
EDEM3	0.125033013	2.264891961	0.024181522	0.068896339	no
PDGFD	0.125001713	2.264315982	0.024217308	0.068982599	no
NCS1	-0.124999029	-2.264266589	0.024220379	0.068982599	no
PRRG2	0.124998112	2.264249725	0.024221427	0.068982599	no
MGP	0.124984425	2.263997852	0.024237093	0.069017076	no

CIDECP	-0.124981925	-2.263951845	0.024239956	0.069017076	no
KPNA6	0.124961153	2.263569622	0.024263748	0.069075668	no
FLJ46361	-0.124951377	-2.263389722	0.024274954	0.069098415	no
TCTN2	0.124945069	2.263273639	0.024282187	0.069109849	no
WNK3	-0.124941914	-2.263215592	0.024285804	0.069110993	no
CENPJ	-0.124926898	-2.262939269	0.024303031	0.069145715	no
UHRF1BP1L	-0.12492567	-2.262916669	0.02430444	0.069145715	no
SENP3-EIF4A1	0.124903155	2.262502369	0.02433029	0.069210096	no
SNRPF	-0.124897482	-2.262397985	0.024336807	0.069219471	no
LOC286177	-0.124882334	-2.262119244	0.024354217	0.069259821	no
LHX5	-0.124873587	-2.261958284	0.024364275	0.069279257	no
PRDM2	-0.124865689	-2.261812952	0.024373359	0.069295921	no
INHBA-AS1	-0.124858584	-2.261682216	0.024381534	0.069309993	no
RBM6	-0.124852832	-2.261576382	0.024388153	0.069319641	no
TGFBR3L	-0.124829545	-2.261147888	0.02441497	0.069386685	no
HOMER3	0.124822228	2.261013247	0.024423401	0.06940147	no
ZUFSP	-0.124779577	-2.260228447	0.024472597	0.069532071	no
PRKD2	0.124727007	2.259261165	0.024533351	0.069695472	no
RFESD	0.124706765	2.258888714	0.024556779	0.069752808	no
MGAM	0.124691928	2.258615712	0.024573964	0.069792397	no
PBDC1	0.124656512	2.257964075	0.024615026	0.069899778	no
PRKAR1B	-0.124645123	-2.257754524	0.024628243	0.069928071	no
TOMM34	0.124637043	2.257605851	0.024637624	0.069945466	no
SLC4A3	0.124625907	2.25740097	0.024650557	0.069972939	no
PLS1	-0.124612722	-2.257158366	0.024665879	0.070007184	no
CDH12	-0.124602561	-2.25697142	0.024677691	0.070028972	no
C14orf1	-0.124600513	-2.256933741	0.024680072	0.070028972	no
LOC728716	-0.124567325	-2.256323124	0.024718692	0.070129295	no
HMGCLL1	-0.124531407	-2.255662277	0.024760548	0.070238771	no
GIGYF1	-0.124504845	-2.25517357	0.02479154	0.070317407	no
HS6ST2	-0.12449623	-2.255015079	0.024801599	0.070336653	no
SDAD1	0.124486497	2.254835999	0.024812968	0.070359611	no
ZBTB46	0.124417254	2.253562071	0.024893977	0.070580008	no
SERPINB2	0.124414318	2.253508066	0.024897416	0.070580448	no
NET1	-0.124411494	-2.253456103	0.024900726	0.07058052	no
RPS15A	-0.124401069	-2.253264301	0.024912945	0.070605843	no
DCBLD1	0.124385759	2.252982638	0.024930899	0.070642889	no
ANO5	-0.124384234	-2.252954584	0.024932688	0.070642889	no
LOC152217	-0.124381515	-2.252904563	0.024935878	0.070642889	no
RANGAP1	-0.124374863	-2.252782183	0.024943684	0.070655688	no
SCN8A	-0.12435252	-2.252371137	0.024969917	0.070720674	no
LOC339524	0.124342338	2.252183801	0.02498188	0.070745235	no
ANO6	0.124335043	2.252049594	0.024990454	0.070757197	no
MZT1	-0.124333141	-2.252014616	0.024992689	0.070757197	no
SLC9A4	-0.124329911	-2.251955193	0.024996487	0.070758627	no
HSPBP1	0.124319017	2.251754769	0.025009298	0.070785569	no
CCNG1	0.12430166	2.251435459	0.025029721	0.070834045	no
PRR12	-0.124286684	-2.251159934	0.025047355	0.070874616	no
GREM1	0.124261701	2.250700339	0.025076794	0.070948576	no
ETFB	-0.124249658	-2.250478785	0.025090996	0.070979413	no
SLC43A2	0.124222248	2.249974536	0.025123346	0.071061573	no

EIF3K	0.124216152	2.249862388	0.025130546	0.071072583	no
FAM149B1	-0.124210521	-2.249758806	0.025137197	0.07107498	no
LOC202781	-0.124208768	-2.249726548	0.025139269	0.07107498	no
TACR2	0.124207036	2.249694695	0.025141315	0.07107498	no
MED1	-0.124125282	-2.248190747	0.025238075	0.071339139	no
SEMA3G	-0.124116929	-2.248037082	0.02524798	0.071357751	no
AQP6	-0.124102742	-2.247776112	0.025264809	0.071395925	no
C11orf68	-0.124058385	-2.24696014	0.02531749	0.071535391	no
LOC100287534	0.12405096	2.246823557	0.025326318	0.071550927	no
GPR83	-0.124020247	-2.246258596	0.02536286	0.071644747	no
OVCA2	0.123972401	2.245378476	0.025419879	0.071790479	no
NR2E3	-0.123971351	-2.245359161	0.025421132	0.071790479	no
DIRC1	0.123962878	2.245203305	0.025431241	0.071809592	no
PIK3CB	-0.123959724	-2.245145282	0.025435006	0.071810786	no
KIAA0947	-0.12394536	-2.244881072	0.025452153	0.07184976	no
STK17B	0.123932996	2.24465365	0.025466921	0.071882007	no
ZNF705B	0.123878527	2.243651736	0.025532071	0.072056432	no
NSDHL	-0.123873758	-2.243564017	0.025537781	0.072063086	no
NPM1	0.123847529	2.24308155	0.025569212	0.072142304	no
ENOX2	0.123840639	2.242954816	0.025577473	0.072154385	no
CYTH3	0.123838358	2.24291287	0.025580208	0.072154385	no
SNORA40	-0.12377169	-2.24168661	0.025660274	0.072370728	no
WTAPP1	0.123742638	2.241152258	0.025695231	0.07245981	no
LOC100289361	-0.123738774	-2.241081175	0.025699885	0.072463424	no
PACRG-AS1	-0.123729513	-2.240910846	0.025711038	0.072485362	no
CTAGE11P	0.123704522	2.240451199	0.025741157	0.072560757	no
ZNF560	-0.123688105	-2.240149235	0.025760961	0.072607057	no
ENO4	0.123675381	2.239915206	0.025776318	0.072640513	no
SLC30A10	-0.12367267	-2.239865342	0.025779591	0.072640513	no
NXN	-0.1236497	-2.239442866	0.025807338	0.072709163	no
CLEC4GP1	-0.123645063	-2.239357592	0.025812941	0.072715418	no
MIR4500HG	-0.123640068	-2.239265724	0.025818979	0.072722895	no
MTMR3	-0.123617913	-2.238858229	0.025845777	0.072788835	no
LINC00593	-0.123610576	-2.238723289	0.025854656	0.072804301	no
FLJ34503	-0.123603958	-2.238601581	0.025862667	0.072817317	no
KCTD11	0.123581608	2.238190507	0.02588974	0.072883994	no
ZNF318	-0.123572221	-2.238017868	0.025901117	0.072906472	no
ZBTB20	-0.123560586	-2.237803876	0.025915226	0.072936632	no
CRIPT	-0.123542602	-2.237473121	0.025937046	0.072988483	no
LOC100287177	0.123517098	2.237004067	0.025968016	0.073066069	no
SLC6A13	-0.123499793	-2.236685804	0.025989049	0.073115675	no
DCDC1	0.123493196	2.236564483	0.02599707	0.073128669	no
KALRN	-0.123488966	-2.236486683	0.026002215	0.07313357	no
KCNJ2	0.123459267	2.235940486	0.026038361	0.07322565	no
LOC648987	0.123451091	2.235790118	0.02604832	0.073244071	no
LOC338799	-0.123445619	-2.235689484	0.026054986	0.073253233	no
ZNF19	-0.123437263	-2.235535812	0.026065169	0.073272277	no
RB1CC1	-0.123431626	-2.235432129	0.026072042	0.073282011	no
IGSF8	-0.123428657	-2.23537753	0.026075662	0.073282601	no
LINC00467	0.123391683	2.234697556	0.026120778	0.073392928	no
DHRS2	-0.123390888	-2.234682927	0.026121749	0.073392928	no

KITLG	0.123384892	2.23457267	0.026129071	0.073403238	no
RAD17	-0.12338229	-2.234524819	0.02613225	0.073403238	no
C19orf55	0.123310423	2.233203155	0.026220169	0.073640571	no
SUFU	-0.123297798	-2.23297098	0.02623564	0.073674394	no
UBE2D1	0.123272487	2.232505512	0.026266681	0.073751925	no
GTF2H2	-0.123247817	-2.232051837	0.026296966	0.073827314	no
FGF8	-0.123201996	-2.231209214	0.026353295	0.073975792	no
CCK	-0.123152618	-2.230301202	0.026414113	0.07413683	no
LRRC73	-0.123148161	-2.230219237	0.026419609	0.074142572	no
MLL2	-0.123126078	-2.229813166	0.026446852	0.074209334	no
RABEP1	-0.123096939	-2.229277328	0.026482838	0.074300609	no
C20orf195	0.123075386	2.228881009	0.026509481	0.074365651	no
PEX6	-0.123044211	-2.228307744	0.026548061	0.074464158	no
MFSD3	-0.123036585	-2.228167521	0.026557505	0.074480927	no
SLC19A3	0.123031289	2.228070129	0.026564066	0.074489608	no
NAE1	-0.123015636	-2.227782309	0.026583465	0.074534278	no
LPCAT4	-0.12299628	-2.227426398	0.02660747	0.074591851	no
GBA	0.122986404	2.22724479	0.026619725	0.074616476	no
LOC388692	0.122979516	2.227118145	0.026628275	0.074617403	no
TRUB1	-0.122978913	-2.227107049	0.026629024	0.074617403	no
ZBTB11	-0.122977747	-2.227085603	0.026630472	0.074617403	no
NT5E	0.122962959	2.226813698	0.026648838	0.074659128	no
KLHL7	-0.12295715	-2.226706887	0.026656055	0.074669614	no
SNIP1	0.122945888	2.226499794	0.026670053	0.074699089	no
RGS9BP	-0.122929141	-2.226191864	0.026690879	0.074743686	no
LOC100505933	-0.122927491	-2.226161531	0.026692932	0.074743686	no
ZDHHC11	-0.122920775	-2.226038033	0.026701289	0.074757347	no
NFRKB	-0.122907099	-2.225786564	0.026718312	0.074795265	no
CYB561D1	0.122891403	2.225497973	0.026737861	0.074835067	no
ASAH1	0.122890092	2.225473857	0.026739495	0.074835067	no
SLC9B2	-0.12287087	-2.225120425	0.026763454	0.074892367	no
PI4KA	-0.122866627	-2.225042398	0.026768745	0.074897423	no
ZNF221	-0.122837993	-2.224515912	0.026804476	0.074987632	no
GLOD4	-0.122833198	-2.22442775	0.026810463	0.074989582	no
NDUFA13	-0.122831846	-2.22440289	0.026812152	0.074989582	no
SPINK8	0.122821928	2.224220535	0.02682454	0.075014469	no
MPST	-0.122817451	-2.224138207	0.026830135	0.075020352	no
DNMT3B	-0.12280882	-2.22397952	0.026840921	0.075040749	no
IDO2	0.122804591	2.223901763	0.026846208	0.075045767	no
GAL3ST3	-0.122799843	-2.223814467	0.026852145	0.075052599	no
CEP78	-0.122786812	-2.223574862	0.026868444	0.075088392	no
RRH	-0.122761822	-2.223115393	0.026899725	0.075166037	no
SOD1	-0.12274969	-2.222892331	0.026914923	0.075198726	no
ANP32A-IT1	-0.122737136	-2.222661514	0.026930656	0.075232904	no
PI4KAP1	-0.122727584	-2.222485882	0.026942634	0.075256582	no
TMEM220-AS1	0.122699638	2.221972081	0.026977699	0.075344735	no
C16orf46	-0.122653475	-2.221123342	0.02703571	0.075496939	no
FLVCR1-AS1	0.122647474	2.221013009	0.027043259	0.075508208	no
CSMD2	-0.122641829	-2.22090922	0.027050362	0.07551823	no
CACNA1E	-0.122632562	-2.220738836	0.027062026	0.07554098	no
CASP1	0.122618883	2.220487359	0.02707925	0.075579242	no

FLJ34208	0.122613526	2.220388862	0.027085999	0.075588261	no
GPRC5B	-0.122608725	-2.220300588	0.027092048	0.075595326	no
PRKAG2-AS1	-0.122602897	-2.220193448	0.027099392	0.075606002	no
C19orf59	0.122584845	2.219861561	0.027122152	0.075651501	no
BBS2	-0.122584379	-2.219852982	0.02712274	0.075651501	no
SH3PXD2B	0.122581362	2.219797527	0.027126545	0.075652294	no
SPRR2G	-0.122576969	-2.219716755	0.027132088	0.075657323	no
C10orf67	0.122574352	2.219668649	0.027135389	0.075657323	no
NECAB3	-0.12257119	-2.219610514	0.027139379	0.075658632	no
LRP1	0.122545727	2.219142384	0.027171528	0.075738433	no
RXRG	-0.122528927	-2.21883351	0.027192759	0.07578778	no
LYPD6B	-0.122499255	-2.218288007	0.027230288	0.075882538	no
NR4A3	0.122489546	2.218109503	0.027242579	0.075906945	no
RECQL	0.12248667	2.218056644	0.02724622	0.075907247	no
LOC440243	-0.12247081	-2.217765062	0.027266309	0.075953368	no
PCBP1	0.122465472	2.217666922	0.027273073	0.075962365	no
ZNF274	-0.122458905	-2.217546195	0.027281396	0.075975701	no
PLA2G4C	0.122428956	2.21699561	0.027319383	0.076071633	no
PCDHB8	-0.12242523	-2.216927111	0.027324112	0.076074944	no
UBTF	-0.122377492	-2.216049505	0.027384765	0.076233936	no
MTDH	0.122366605	2.215849362	0.027398614	0.076262609	no
RPL24	-0.122358935	-2.215708359	0.027408374	0.076279897	no
CSRNP1	0.122312742	2.214859183	0.027467217	0.076433764	no
GSTM4	0.122305414	2.214724464	0.027476562	0.076440998	no
NOP14-AS1	-0.122304023	-2.214698907	0.027478335	0.076440998	no
DDX55	-0.122301378	-2.214650281	0.027481709	0.076440998	no
UBA6	0.122298713	2.214601282	0.02748511	0.076440998	no
ZNF184	-0.12229676	-2.214565384	0.027487601	0.076440998	no
POLD1	-0.122277487	-2.214211097	0.027512199	0.076497417	no
AGT	-0.122271645	-2.214103694	0.02751966	0.076497417	no
PRELID2	0.122270588	2.214084274	0.027521009	0.076497417	no
RHBDD2	0.122269713	2.214068188	0.027522127	0.076497417	no
AZIN1	-0.122262092	-2.213928087	0.027531862	0.076514581	no
MRPL45	-0.122228254	-2.213306046	0.027575124	0.0766143	no
TRAF3	-0.122226578	-2.213275246	0.027577267	0.0766143	no
WHAMMP3	0.122225663	2.21325842	0.027578439	0.0766143	no
RAB26	-0.122221446	-2.213180893	0.027583835	0.076619387	no
RYR1	-0.122173648	-2.21230226	0.027645059	0.076779524	no
FAM215A	-0.122150894	-2.211883983	0.027674246	0.076850655	no
CSAD	-0.122143063	-2.211740049	0.027684296	0.076868631	no
MVK	-0.122104563	-2.21103234	0.027733756	0.076996015	no
CCDC89	0.122100041	2.210949208	0.027739571	0.077002211	no
LY6D	-0.122087336	-2.210715683	0.027755911	0.077037619	no
KLHDC7A	0.122083813	2.210650914	0.027760445	0.077040252	no
CLDND2	-0.122068638	-2.210371975	0.027779976	0.077084502	no
AOX2P	0.122061228	2.210235772	0.027789518	0.077101023	no
RFXAP	-0.122053847	-2.210100097	0.027799025	0.077117445	no
B9D2	0.122046572	2.209966369	0.027808398	0.077133491	no
RBM45	0.122026673	2.209600611	0.02783405	0.077194679	no
HPS4	-0.121984448	-2.208824463	0.027888551	0.077335852	no
HSBP1L1	0.121951842	2.208225136	0.027930699	0.077442737	no

WDR86-AS1	-0.121947962	-2.208153821	0.027935718	0.077446661	no
SCRG1	-0.121939437	-2.207997132	0.027946748	0.077467247	no
TMSB10	0.121932986	2.207878544	0.027955099	0.077480401	no
BOLA1	-0.121925981	-2.207749806	0.027964166	0.077495538	no
NFS1	-0.121896554	-2.207208909	0.028002292	0.077591189	no
TIMP4	-0.121891099	-2.207108649	0.028009364	0.077592406	no
PFKP	-0.121890645	-2.207100309	0.028009952	0.077592406	no
C2orf72	-0.121878149	-2.20687062	0.02802616	0.077627297	no
LYRM9	-0.121871357	-2.206745787	0.028034972	0.077641698	no
ADD3	-0.121813231	-2.205677428	0.028110485	0.077840797	no
WDR44	0.12180807	2.205582573	0.028117198	0.077849355	no
GPATCH2L	-0.121786681	-2.205189448	0.028145034	0.077916389	no
SHISA6	-0.121773531	-2.204947758	0.02816216	0.077953758	no
MYL6	0.121735494	2.204248672	0.028211746	0.078080958	no
LOC100507346	-0.121710705	-2.203793062	0.028244103	0.078160446	no
FAM149A	-0.12169701	-2.203541367	0.028261992	0.078199882	no
BACH1	0.121691615	2.203442209	0.028269043	0.07820932	no
RNF44	-0.121660298	-2.202866637	0.028309997	0.078312542	no
NPAS2	0.121646864	2.202619744	0.02832758	0.078351096	no
LINC00441	-0.121638537	-2.2024667	0.028338484	0.078365376	no
TCEA1	-0.121637352	-2.202444931	0.028340035	0.078365376	no
MCHR2	-0.121626466	-2.202244857	0.028354296	0.078394723	no
ZDHHC3	0.121595435	2.20167455	0.028394982	0.078497112	no
ULBP1	0.121591869	2.201609019	0.02839966	0.078499947	no
PUSL1	0.121578917	2.201370983	0.028416658	0.078536832	no
INTS6	0.12153545	2.200572143	0.028473769	0.078684554	no
ACYP2	-0.121511381	-2.200129812	0.028505435	0.078761933	no
FAM72B	0.121470274	2.199374355	0.028559588	0.078901416	no
LOC389831	-0.121439544	-2.198809629	0.028600127	0.079003257	no
MYLIP	0.121433244	2.198693861	0.028608444	0.079016074	no
ERG	0.121421069	2.198470119	0.028624523	0.079050325	no
RGL4	-0.121415775	-2.198372826	0.028631518	0.079059482	no
F2RL3	0.121411429	2.198292964	0.02863726	0.079065179	no
LOC100506804	-0.121390622	-2.197910589	0.028664768	0.079130961	no
PPP4R4	-0.121371545	-2.197560015	0.028690008	0.079190466	no
CEP95	-0.121349415	-2.19715333	0.028719313	0.079261172	no
MAP4K5	-0.121339479	-2.196970737	0.028732478	0.079287325	no
PRR18	-0.12133128	-2.196820075	0.028743345	0.07930473	no
TMEM181	-0.121329152	-2.196780976	0.028746166	0.07930473	no
MLX	0.121311785	2.196461835	0.028769199	0.079358086	no
MTMR14	0.121302022	2.196282414	0.028782155	0.079383636	no
REM2	-0.121296285	-2.196176987	0.02878977	0.07939445	no
LOC729970	0.121290853	2.196077171	0.028796982	0.079404148	no
TRIM2	-0.121267677	-2.195651292	0.028827769	0.079477904	no
PRELID1	0.121265151	2.195604864	0.028831127	0.079477904	no
MTX3	-0.12124989	-2.195324439	0.028851416	0.079523635	no
ADAMTS20	-0.121231284	-2.194982543	0.02887617	0.079581658	no
AGAP5	-0.121225462	-2.194875558	0.02888392	0.079592809	no
DSCR3	0.121184778	2.194127962	0.028938124	0.079731951	no
C1QTNF1	0.121142821	2.193356983	0.028994115	0.079849565	no
EPC2	-0.121141411	-2.193331073	0.028995998	0.079849565	no

UTP15	0.121141271	2.193328506	0.028996185	0.079849565	no
ZBTB14	-0.121140074	-2.193306504	0.028997784	0.079849565	no
ASL	0.121138873	2.193284437	0.028999388	0.079849565	no
LEFTY2	0.121123963	2.193010458	0.029019311	0.079894187	no
PTPRR	-0.121111954	-2.192789795	0.029035366	0.079928148	no
SCRN2	-0.1211066	-2.192691426	0.029042526	0.079937617	no
MIR223	0.121102361	2.19261352	0.029048197	0.079942989	no
XPNPEP2	0.121098181	2.192536727	0.029053788	0.079948138	no
GUSBP11	-0.12107138	-2.192044263	0.029089665	0.08002708	no
WDR26	0.121071187	2.192040716	0.029089924	0.08002708	no
LOXL4	0.121037381	2.191419541	0.029135233	0.080141468	no
WNT10B	-0.121014993	-2.191008175	0.029165272	0.080213829	no
EEF1A1	-0.120992871	-2.190601691	0.029194981	0.080285264	no
ZNF829	-0.120985943	-2.190474403	0.02920429	0.080292383	no
ELFN1	-0.120985383	-2.190464114	0.029205042	0.080292383	no
ARHGAP26	0.120926088	2.189374626	0.029284823	0.080501423	no
TCF23	0.120909181	2.189063989	0.029307605	0.080553745	no
EGFL7	0.120893832	2.188781957	0.029328302	0.08059142	no
UBXN1	-0.120893453	-2.188775005	0.029328812	0.08059142	no
MPPE1	0.120887119	2.188658623	0.029337357	0.080604594	no
GNAT2	0.120882477	2.188573335	0.02934362	0.080609851	no
RNF39	-0.120879166	-2.188512507	0.029348087	0.080609851	no
NTPCR	-0.120877362	-2.188479352	0.029350523	0.080609851	no
STRADA	-0.120856535	-2.188096692	0.029378643	0.080676772	no
ATP6AP1L	-0.120838579	-2.187766787	0.029402905	0.080733083	no
CCDC3	0.120814827	2.187330379	0.029435027	0.080810956	no
LOC158434	-0.120805566	-2.187160239	0.029447558	0.080822163	no
SAMD12	-0.120805283	-2.187155027	0.029447942	0.080822163	no
POLE4	0.120803473	2.18712178	0.029450391	0.080822163	no
PEX7	-0.120767636	-2.186463359	0.029498933	0.080938592	no
PDCL3P4	-0.120764076	-2.186397949	0.029503759	0.080938592	no
ZBTB4	-0.120763814	-2.186393133	0.029504115	0.080938592	no
UNC119B	0.120760889	2.186339401	0.02950808	0.080939138	no
RASGRF2	-0.120739481	-2.185946082	0.029537118	0.081008448	no
DPY19L4	-0.12071402	-2.185478298	0.029571686	0.081092905	no
BET3L	0.120709103	2.185387959	0.029578366	0.081095528	no
MRPL45P2	-0.120707761	-2.185363301	0.029580189	0.081095528	no
AAMP	-0.12069946	-2.185210785	0.02959147	0.081116107	no
GAA	0.120686042	2.184964276	0.029609711	0.081155757	no
PBXIP1	0.120623998	2.183824403	0.029694184	0.081376908	no
PGAM4	0.120609559	2.183559141	0.029713872	0.081420479	no
DIS3L	-0.120601373	-2.183408749	0.029725039	0.081440695	no
CCDC75	-0.120598491	-2.183355802	0.029728971	0.081441086	no
PPP1R16B	-0.120592318	-2.183242397	0.029737395	0.081449371	no
KIAA1383	0.120590721	2.183213052	0.029739576	0.081449371	no
DNAH9	0.120587405	2.183152139	0.029744102	0.081451387	no
ZFHX4-AS1	-0.120538039	-2.182245229	0.029811557	0.081625706	no
SNRNP35	-0.120509039	-2.181712454	0.029851246	0.081712742	no
HES4	-0.120507991	-2.181693215	0.02985268	0.081712742	no
ABCB4	0.120506479	2.181665436	0.029854751	0.081712742	no
MUM1L1	-0.120493962	-2.181435484	0.029871897	0.08174926	no

GLT25D2	-0.120486205	-2.181292972	0.029882527	0.08176794	no
RIOK2	-0.120475328	-2.181093163	0.029897437	0.081798323	no
FGFR1OP	0.120454754	2.180715201	0.029925659	0.081865114	no
DAB2IP	-0.120425515	-2.180178076	0.029965805	0.081964504	no
TNFRSF18	0.120406329	2.17982562	0.029992173	0.082026188	no
DHPS	-0.120370966	-2.179176004	0.030040825	0.082148795	no
FAM201A	-0.120360328	-2.178980573	0.030055475	0.082178399	no
ZNF532	-0.120349022	-2.178772879	0.030071051	0.082207574	no
LINC00476	-0.12034703	-2.178736289	0.030073796	0.082207574	no
KANK3	-0.12032635	-2.178356413	0.030102305	0.082275039	no
TCF3	-0.120315441	-2.178156013	0.030117354	0.082305704	no
EMCN	0.120291392	2.177714251	0.030150551	0.08238595	no
NHS	0.120284455	2.177586818	0.030160133	0.082401655	no
CCDC12	0.120281489	2.177532328	0.030164231	0.082402376	no
ANKRD34B	0.120255979	2.17706373	0.030199493	0.082488219	no
NAP1L1	-0.120250127	-2.176956241	0.030207587	0.082499841	no
LYRM1	-0.120244841	-2.176859131	0.0302149	0.08250933	no
TSNAX	0.120239346	2.176758202	0.030222503	0.082519606	no
LPIN2	0.120235293	2.176683747	0.030228113	0.082524438	no
XPO5	-0.120227154	-2.176534232	0.030239381	0.082540751	no
CATSPER2	-0.120225428	-2.176502541	0.03024177	0.082540751	no
FLJ35024	-0.120196758	-2.175975892	0.03028149	0.082632646	no
HS3ST3B1	0.120195576	2.175954182	0.030283129	0.082632646	no
SPRYD3	-0.120189671	-2.17584572	0.030291315	0.082644491	no
GUSBP2	-0.120168605	-2.175458764	0.030320537	0.082713717	no
PRORSD1P	0.120148468	2.175088865	0.030348493	0.082773082	no
MFSD5	0.120147381	2.175068915	0.030350002	0.082773082	no
N4BP2L1	0.120120687	2.174578582	0.030387096	0.082863734	no
LOC254100	-0.120102989	-2.174253494	0.030411711	0.082920336	no
TUBB2B	-0.120099574	-2.174190779	0.030416462	0.082922769	no
HUNK	-0.120092899	-2.174068158	0.030425752	0.082937575	no
MPP3	-0.120047621	-2.173236493	0.030488828	0.083098973	no
OGFOD1	0.120023428	2.172792134	0.030522575	0.083180404	no
LINC00086	-0.120006867	-2.172487941	0.030545696	0.083232859	no
FRAT2	-0.119979329	-2.171982136	0.030584175	0.083317433	no
EMC9	-0.119979105	-2.171978022	0.030584488	0.083317433	no
XPO4	-0.119974037	-2.171884932	0.030591574	0.083326175	no
TBC1D24	-0.119970064	-2.171811966	0.03059713	0.083330746	no
PCDH8	-0.119960892	-2.171643494	0.03060996	0.083355125	no
MLF2	-0.119950691	-2.171456136	0.030624234	0.08338343	no
AMY1A	-0.119917895	-2.170853768	0.030670165	0.083497911	no
FAM225B	0.11988604	2.170268691	0.030714834	0.083608929	no
ZYG11B	-0.119881116	-2.170178255	0.030721744	0.083617147	no
AKR7A2	0.119861807	2.169823619	0.030748852	0.083680331	no
ST3GAL5	-0.119858735	-2.169767199	0.030753166	0.083681476	no
USPL1	-0.119840795	-2.16943769	0.030778375	0.083739469	no
TIPARP	0.119808673	2.168847733	0.030823554	0.083851773	no
CYGB	0.119790843	2.168520272	0.030848656	0.083909438	no
DDX17	-0.119749536	-2.16776163	0.030906878	0.084057165	no
ZNF664	-0.119708059	-2.166999875	0.030965434	0.084205763	no
FLJ21408	0.11969083	2.166683456	0.030989786	0.084261321	no

SOX3	-0.119677001	-2.166429477	0.031009343	0.084303833	no
SIVA1	0.119660388	2.166124372	0.031032852	0.084357075	no
PROKR2	-0.119636383	-2.165683515	0.031066849	0.084428128	no
LOC285000	0.11963595	2.165675557	0.031067462	0.084428128	no
FOXN2	-0.11963361	-2.1656326	0.031070777	0.084428128	no
CCDC154	-0.119618232	-2.165350173	0.031092575	0.084476679	no
TARBP1	-0.119604639	-2.165100535	0.031111854	0.084518372	no
SNX15	-0.119597862	-2.164976075	0.031121469	0.084533808	no
LRRC10B	-0.119586457	-2.164766636	0.031137655	0.084567086	no
PVALB	-0.119580569	-2.164658492	0.031146016	0.084579104	no
NOC3L	0.119577635	2.164604616	0.031150182	0.084579729	no
PAQR8	-0.119563988	-2.164353995	0.031169567	0.084621674	no
PGLS	0.119557744	2.164239326	0.03117844	0.08462655	no
DUSP5P1	-0.119557182	-2.164229005	0.031179239	0.08462655	no
MAPK4	0.119529306	2.163717087	0.031218878	0.084723439	no
SNORA52	-0.119503618	-2.16324534	0.031255446	0.084811967	no
DEPDC7	-0.119493061	-2.163051471	0.031270484	0.084842062	no
TMEM175	-0.119485847	-2.162918986	0.031280764	0.084859241	no
NUDT12	-0.119480134	-2.16281407	0.031288908	0.084870619	no
PTGIS	0.119472658	2.162676778	0.031299566	0.084888817	no
ZNF500	-0.119467307	-2.162578522	0.031307197	0.084898797	no
TSPEAR	0.119457344	2.162395569	0.031321408	0.08492662	no
CREB3L4	-0.119447783	-2.162219981	0.031335053	0.084952899	no
PTAFR	0.119437349	2.162028368	0.031349949	0.084982563	no
CDC37L1	-0.11943431	-2.161972578	0.031354287	0.084983603	no
STK24	-0.119405021	-2.161434714	0.031396138	0.085086307	no
ALKBH6	0.119396676	2.161281467	0.031408071	0.085107914	no
RAB37	-0.119388134	-2.16112462	0.031420289	0.085130285	no
SMARCAD1	-0.119373664	-2.160858896	0.031440996	0.085175652	no
CAMKK2	-0.11936795	-2.160753975	0.031449176	0.085187073	no
KIAA1644	-0.119350725	-2.160437654	0.031473847	0.085243156	no
LY86-AS1	-0.119322541	-2.159920124	0.031514247	0.08534182	no
ISCU	0.119306132	2.159618807	0.03153779	0.08539481	no
PEX11A	-0.119303244	-2.159565771	0.031541935	0.08539481	no
FOPNL	0.119300597	2.159517156	0.031545735	0.08539481	no
MASP2	-0.11926973	-2.158950357	0.031590071	0.08548911	no
STAMBPL1	0.119269115	2.158939066	0.031590955	0.08548911	no
ANGPTL2	-0.119268037	-2.15891927	0.031592504	0.08548911	no
TBP	-0.119263402	-2.158834163	0.031599167	0.085496373	no
STPG2	0.119251988	2.158624568	0.031615579	0.085530011	no
YBEY	-0.119232664	-2.158269733	0.031643381	0.08559445	no
MCF2	-0.119218213	-2.158004388	0.031664186	0.085639946	no
CEPT1	0.119192092	2.157524734	0.031701823	0.085725258	no
GPKOW	-0.119190785	-2.157500744	0.031703706	0.085725258	no
CAPS	0.119176337	2.157235451	0.031724541	0.08577075	no
NLGN4X	-0.119173584	-2.157184893	0.031728512	0.08577075	no
ZNF629	-0.119156217	-2.156865996	0.031753575	0.085827704	no
NAGS	0.119132216	2.156425301	0.031788237	0.08591059	no
PLEC	-0.119116396	-2.156134828	0.031811102	0.085958765	no
NUP133	-0.119114349	-2.156097234	0.031814063	0.085958765	no
TMEM206	-0.119101854	-2.1558678	0.031832134	0.085996781	no

VIPR1	-0.11909475	-2.155737362	0.031842412	0.086013736	no
TMEM132A	0.119087987	2.155613194	0.031852199	0.086027076	no
PPP4R1	-0.119085806	-2.155573136	0.031855357	0.086027076	no
KIF5B	-0.119075455	-2.155383084	0.031870342	0.086054657	no
NARS2	-0.11907322	-2.155342053	0.031873578	0.086054657	no
TGFBR3	0.119061841	2.155133107	0.031890062	0.086088347	no
LOC100289019	-0.119046521	-2.154851813	0.031912266	0.086137465	no
CAPRIN2	0.119036713	2.154671739	0.031926486	0.086165028	no
ZNF614	-0.11903305	-2.154604486	0.031931799	0.086168544	no
POLR2J2	-0.118988019	-2.153777672	0.031997172	0.086334116	no
ZNF33B	-0.118977877	-2.153591447	0.032011913	0.086363045	no
PART1	-0.118974176	-2.1535235	0.032017292	0.086366716	no
ZNF300P1	-0.118961915	-2.153298374	0.032035122	0.086403967	no
DRP2	-0.118955785	-2.153185823	0.032044039	0.086416719	no
SPHK1	0.118953136	2.153137199	0.032047892	0.086416719	no
TIMM8A	-0.118931067	-2.152731993	0.032080016	0.086476189	no
JAZF1-AS1	-0.118931057	-2.152731809	0.032080031	0.086476189	no
FAM120C	-0.118929692	-2.152706755	0.032082018	0.086476189	no
SYT10	-0.11892132	-2.152553046	0.032094212	0.086492061	no
KCNT2	-0.118920123	-2.15253107	0.032095956	0.086492061	no
LOC100133161	0.118905003	2.152253459	0.03211799	0.086539081	no
COPS2	0.118902624	2.152209781	0.032121458	0.086539081	no
DENND6A	0.118880529	2.151804103	0.032153683	0.086615042	no
LYSMD3	0.118854012	2.151317262	0.032192392	0.086708448	no
FBXO27	-0.118850754	-2.151257436	0.032197152	0.0867104	no
SGK3	-0.118790254	-2.150146692	0.03228563	0.086937787	no
FOXO3	-0.118785965	-2.15006795	0.03229191	0.086943804	no
SMU1	-0.118781315	-2.149982563	0.032298722	0.086947674	no
OPTC	-0.118779459	-2.149948503	0.032301439	0.086947674	no
RTEL1-TNFRSF	-0.118776357	-2.14989155	0.032305983	0.086948729	no
GSPT2	-0.118773668	-2.149842181	0.032309923	0.086948729	no
EPHA8	-0.118763558	-2.149656563	0.032324739	0.086977708	no
DIEXF	0.118719181	2.148841842	0.032389837	0.087134835	no
LOC643201	-0.118718224	-2.148824269	0.032391243	0.087134835	no
DNAH5	0.118710501	2.148682481	0.032402584	0.087154434	no
INTS1	-0.118701163	-2.148511054	0.0324163	0.087169247	no
FAM101B	0.118700744	2.148503364	0.032416916	0.087169247	no
PCDH11Y	-0.118696544	-2.148426259	0.032423087	0.087169247	no
HPSE2	-0.118695709	-2.148410914	0.032424315	0.087169247	no
PCMT1	0.118657373	2.147707129	0.032480692	0.087309888	no
PVRL4	0.118652322	2.147614391	0.032488127	0.087318953	no
SMPDL3B	0.11863649	2.14732375	0.032511438	0.087370679	no
KIAA1671	-0.118633603	-2.147270735	0.032515692	0.087371185	no
CITED2	0.118625139	2.14711535	0.032528162	0.087393766	no
ZNF774	-0.118622336	-2.147063894	0.032532292	0.087393937	no
EPHB3	-0.118576762	-2.146227253	0.032599513	0.087563572	no
H1FX	-0.118552357	-2.145779219	0.03263556	0.087649441	no
FAM24B	-0.118543071	-2.145608753	0.032649284	0.087675343	no
RNF128	0.118529621	2.145361829	0.032669173	0.087714521	no
RDH11	-0.118527684	-2.145326283	0.032672037	0.087714521	no
SMIM15	0.118517078	2.145131574	0.032687728	0.087745685	no

TPH2	-0.118514183	-2.145078423	0.032692012	0.087746226	no
BICD1	0.118495961	2.144743913	0.032718988	0.087807663	no
PEX5L	-0.118485549	-2.144552778	0.03273441	0.087838083	no
TJP1	-0.118478846	-2.14442974	0.032744341	0.08785139	no
SCAND2P	-0.118476684	-2.144390052	0.032747545	0.08785139	no
FAM3A	-0.118472446	-2.144312247	0.032753827	0.087857275	no
PAIP2	-0.118463296	-2.144144274	0.032767392	0.087882694	no
MITD1	0.118457373	2.144035554	0.032776175	0.08789528	no
ATP6V1B1	-0.118405746	-2.143087828	0.032852821	0.088089828	no
TRIM13	-0.118363682	-2.142315668	0.032915382	0.088245647	no
ENOSF1	0.118359953	2.142247224	0.032920932	0.088245647	no
SURF6	-0.118358396	-2.142218631	0.032923251	0.088245647	no
TUBB2A	-0.118263652	-2.140479487	0.033064563	0.088613359	no
SCARNA16	-0.118259217	-2.140398088	0.03307119	0.088620067	no
GTPBP4	-0.118247131	-2.140176227	0.033089257	0.088657428	no
SPINK5	-0.118228287	-2.139830343	0.033117442	0.088721883	no
LOC339535	0.118224715	2.139764763	0.033122788	0.088725145	no
DIAPH1	0.118197177	2.139259284	0.03316402	0.088824521	no
GSTT2B	0.118183285	2.139004291	0.033184837	0.088855187	no
ADAT2	-0.118182063	-2.138981871	0.033186668	0.088855187	no
KLKB1	0.118181259	2.138967106	0.033187873	0.088855187	no
LOC100009676	0.11813186	2.138060374	0.033261992	0.089042535	no
ARHGAP23	-0.118128923	-2.138006462	0.033266403	0.089043253	no
SCML4	0.118124264	2.137920949	0.033273402	0.089045452	no
ACAD8	-0.118122859	-2.137895167	0.033275512	0.089045452	no
IARS2	0.118119128	2.137826682	0.033281118	0.089049365	no
FAM76B	-0.118095981	-2.137401815	0.033315914	0.08913137	no
CD36	0.118091023	2.137310827	0.033323369	0.089140221	no
RPTOR	-0.118070852	-2.136940584	0.033353723	0.089210313	no
BAMBI	0.118060649	2.136753317	0.033369085	0.089240294	no
ACSS2	-0.118055433	-2.136657569	0.033376941	0.089250199	no
RAB15	-0.118043816	-2.136444353	0.033394443	0.08928129	no
CLUHP3	-0.118042201	-2.136414703	0.033396877	0.08928129	no
LINC-ROR	0.118035895	2.136298972	0.033406381	0.089295588	no
LOC339788	-0.118020505	-2.136016493	0.033429586	0.089346504	no
LYST	0.118015549	2.135925527	0.033437062	0.089355373	no
CES4A	-0.11800843	-2.135794856	0.033447804	0.089372964	no
ANKRD20A8P	-0.117985116	-2.135366956	0.033482999	0.089455884	no
SPG11	0.11796646	2.135024542	0.033511186	0.089520062	no
LOC643406	-0.117954035	-2.134796497	0.033529969	0.089555814	no
MYOM3	0.117952095	2.134760883	0.033532904	0.089555814	no
TBXA2R	0.117944396	2.134619583	0.033544548	0.089575779	no
NEUROD4	-0.117939461	-2.134529007	0.033552013	0.089584584	no
ZNF503	0.11792797	2.134318107	0.033569403	0.08961988	no
TRMT11	-0.117894646	-2.13370649	0.033619876	0.089739698	no
MRPL38	-0.117892826	-2.133673075	0.033622635	0.089739698	no
ITGA7	0.117887833	2.133581448	0.033630203	0.08974875	no
OTUB1	-0.11785847	-2.133042524	0.033674742	0.089856455	no
ZNF205	-0.117849301	-2.132874256	0.03368866	0.089882431	no
ANKRD27	0.117844921	2.132793866	0.03369531	0.089889016	no
COPG1	0.117833605	2.132586187	0.033712496	0.089923701	no

PRAMEF20	0.117809756	2.132148478	0.033748744	0.089998045	no
ADIPOQ	0.117809756	2.132148478	0.033748744	0.089998045	no
T	0.117798771	2.131946866	0.03376545	0.090031426	no
ATN1	-0.117762154	-2.13127484	0.03382119	0.090168863	no
GATA5	-0.11775639	-2.131169056	0.033829972	0.090181086	no
CDK9	-0.117747919	-2.131013593	0.03384288	0.090204309	no
GYG2P1	-0.117741204	-2.130890353	0.033853116	0.090220402	no
ITGA8	0.117731797	2.130717715	0.03386746	0.090236368	no
TPI1P2	0.117731767	2.130717176	0.033867505	0.090236368	no
C1orf168	-0.117720397	-2.130508499	0.033884849	0.090271389	no
TRIM71	-0.117710937	-2.130334885	0.033899286	0.090298655	no
SLC20A2	-0.11770321	-2.130193083	0.033911081	0.090318879	no
FAM86B3P	0.117696805	2.130075532	0.033920861	0.090333733	no
DCLRE1A	-0.117691456	-2.129977368	0.03392903	0.090344293	no
PRPF19	-0.117688298	-2.129919417	0.033933854	0.090345943	no
DSTNP2	0.117682151	2.129806596	0.033943246	0.090359755	no
PSME3	-0.117673058	-2.12963973	0.033957142	0.09038555	no
MIR448	0.117652544	2.129263245	0.033988511	0.090457844	no
WNT5B	-0.117648943	-2.129197172	0.033994019	0.090458796	no
MANBAL	-0.117646807	-2.12915797	0.033997287	0.090458796	no
LOC100506023	0.11760808	2.12844726	0.034056585	0.090605357	no
PCDHB19P	-0.11758842	-2.128086471	0.034086722	0.090674308	no
MTR	0.117579599	2.127924596	0.034100251	0.09069907	no
ACOT11	0.117560076	2.127566319	0.03413021	0.090767522	no
HIST3H2BB	-0.117546914	-2.127324779	0.034150421	0.090810034	no
QTRT1	-0.117542507	-2.127243909	0.03415719	0.090816796	no
ECHDC2	0.117538015	2.127161467	0.034164092	0.09082391	no
CPEB4	-0.117518797	-2.126808796	0.03419363	0.090891193	no
SCAF1	-0.117515932	-2.126756213	0.034198036	0.090891662	no
ASH2L	-0.117497482	-2.126417654	0.034226415	0.09094605	no
ORC6	-0.117497127	-2.126411127	0.034226963	0.09094605	no
ADPRHL2	0.117492062	2.126318178	0.034234758	0.090949044	no
FRMD6-AS2	-0.117488268	-2.126248553	0.034240598	0.090949044	no
KLF2	0.117484659	2.12618234	0.034246153	0.090949044	no
ADRA2C	-0.117483213	-2.126155804	0.034248379	0.090949044	no
BBC3	0.117482648	2.12614543	0.034249249	0.090949044	no
TSC22D4	-0.117473921	-2.125985287	0.034262688	0.09097349	no
HMBS	-0.117431547	-2.125207701	0.034328005	0.09113566	no
OTUD1	0.117407125	2.124759539	0.0343657	0.091224463	no
LOC10028965	-0.117401283	-2.124652335	0.034374722	0.091237143	no
CATSPER2P1	-0.117396999	-2.124573719	0.034381339	0.091243438	no
HIF1A	0.117379804	2.124258191	0.034407909	0.091302677	no
MGC15885	0.117375817	2.124185037	0.034414072	0.091307756	no
HEXIM1	0.117370369	2.12408506	0.034422496	0.091308325	no
CHML	-0.117370183	-2.124081643	0.034422784	0.091308325	no
DHRS7B	0.117361497	2.123922267	0.034436216	0.091332683	no
ATP6V1A	-0.117343313	-2.12358859	0.034464354	0.091396031	no
CALHM1	-0.117327168	-2.12329232	0.034489354	0.091451043	no
ZBED4	-0.117297958	-2.122756328	0.034534621	0.091559776	no
ZNF449	0.117291033	2.122629266	0.03454536	0.091576949	no
TGM1	-0.117286367	-2.122543637	0.034552598	0.091584841	no

MT1E	0.117279996	2.122426739	0.034562482	0.091599742	no
SYT2	-0.117267548	-2.12219832	0.034581802	0.091629122	no
NAT2	0.117267359	2.122194857	0.034582095	0.091629122	no
RAD23A	-0.11726085	-2.122075424	0.034592201	0.091644599	no
FARS2	0.117254426	2.121957536	0.034602178	0.091659733	no
SEC16B	0.117248688	2.121852246	0.034611091	0.091672044	no
CCND2	-0.117230583	-2.121520048	0.034639226	0.091735257	no
COX10	-0.117196316	-2.120891282	0.034692532	0.091865107	no
C12orf10	-0.117184903	-2.120681854	0.034710303	0.09190084	no
LOC728724	0.117170965	2.120426111	0.034732014	0.091946996	no
FAM115A	-0.117136206	-2.119788332	0.034786209	0.092079124	no
GULP1	-0.117122813	-2.119542588	0.03480711	0.092123103	no
RBBP8	0.117109635	2.119300792	0.034827686	0.0921605	no
COMMD1	0.117108271	2.119275762	0.034829816	0.0921605	no
CSNK1E	-0.117097653	-2.119080945	0.034846403	0.092193038	no
GTPBP3	-0.117078893	-2.118736729	0.034875726	0.092244542	no
C16orf59	-0.117076191	-2.118687153	0.034879951	0.092244542	no
WEE2	-0.117075987	-2.118683406	0.034880271	0.092244542	no
ST20	0.117073958	2.11864619	0.034883442	0.092244542	no
APRT	0.117071472	2.118600567	0.034887331	0.092244542	no
BPIFB3	0.117012644	2.1175212	0.034979444	0.092476716	no
SMG1	-0.117007145	-2.117420307	0.034988065	0.09248813	no
TTN-AS1	0.117001842	2.117323001	0.034996381	0.092498736	no
DHX33	-0.11697725	-2.116871806	0.035034963	0.092589326	no
MON1A	-0.116972786	-2.116789902	0.035041971	0.092596459	no
OGDH	-0.116944181	-2.116265078	0.035086903	0.092703791	no
SV2C	-0.11693839	-2.116158825	0.035096006	0.092716443	no
FTSJD1	0.11691162	2.115667669	0.03513811	0.092816264	no
AGAP2	-0.116905309	-2.115551885	0.035148042	0.092831088	no
LOC100507472	-0.116890119	-2.115273186	0.035171958	0.09288284	no
ITPR1	-0.116873538	-2.114968984	0.035198079	0.092940399	no
CCL18	0.116867946	2.114866383	0.035206892	0.092947691	no
NPAS1	-0.116863621	-2.114787041	0.035213709	0.092947691	no
LOC387895	0.116863554	2.114785814	0.035213815	0.092947691	no
TOB2P1	0.116851227	2.114559648	0.035233253	0.092987578	no
COPG2	-0.116813423	-2.113866075	0.035292921	0.093133616	no
TMEM53	0.11680989	2.113801263	0.035298501	0.093136905	no
STYK1	-0.116799422	-2.113609204	0.035315042	0.093169109	no
POLR3K	-0.116795483	-2.113536943	0.035321267	0.093170193	no
TUBGCP4	-0.116789575	-2.113428555	0.035330605	0.093170193	no
FAM200A	-0.116786951	-2.113380413	0.035334754	0.093170193	no
ACTG1P4	-0.116786471	-2.113371609	0.035335513	0.093170193	no
ABCA11P	-0.116785449	-2.113352853	0.035337129	0.093170193	no
KIAA1841	-0.116767148	-2.1130171	0.035366076	0.093235076	no
SNRNP48	-0.116758698	-2.112862081	0.035379448	0.093249695	no
TMEM161B-AS1	-0.11675816	-2.112852211	0.035380299	0.093249695	no
NTRK3-AS1	-0.116735737	-2.112440838	0.035415806	0.093331831	no
ACBD4	-0.116731828	-2.112369123	0.035421999	0.093336707	no
CLTCL1	-0.116728729	-2.112312272	0.035426909	0.0933382	no
C20orf111	0.116696045	2.111712653	0.035478732	0.093463279	no
RC3H2	-0.11669286	-2.111654215	0.035483786	0.093465136	no

BCAP31	0.116684543	2.111501642	0.035496984	0.093488442	no
LOC100505912	-0.116620515	-2.110327024	0.035598736	0.093733897	no
ADI1	0.116620409	2.11032507	0.035598905	0.093733897	no
FLYWCH2	-0.116605129	-2.110044766	0.035623223	0.093786438	no
PPP5D1	-0.116575157	-2.109494931	0.035670967	0.09390063	no
RQCD1	0.116564218	2.109294247	0.035688406	0.093935032	no
RRN3P1	-0.116514584	-2.108383728	0.035767622	0.094132007	no
USP12	-0.116503322	-2.108177143	0.035785616	0.094167832	no
TMEM182	0.11649589	2.108040798	0.035797497	0.094187561	no
PCSK5	0.116486628	2.107870893	0.035812306	0.094214991	no
LOC100507634	0.116474137	2.107641767	0.035832285	0.094256015	no
SPATA31C2	-0.116460971	-2.107400241	0.035853356	0.094299899	no
MAP7D2	-0.11644385	-2.107086177	0.035880771	0.094360456	no
TPSD1	0.116394627	2.106183225	0.035959691	0.094554124	no
IKZF3	0.116392432	2.106142968	0.035963213	0.094554124	no
ERO1L	0.116347059	2.105310663	0.036036096	0.094726305	no
ARHGEF12	-0.116346175	-2.10529445	0.036037517	0.094726305	no
FBXO5	-0.116318637	-2.104789305	0.036081814	0.094816022	no
NFYC	0.116317574	2.104769816	0.036083524	0.094816022	no
BRE	0.116316729	2.104754314	0.036084884	0.094816022	no
RRP1	-0.116294835	-2.104352712	0.036120138	0.094897051	no
C6orf163	-0.116271069	-2.10391677	0.036158439	0.094983348	no
RPP25L	-0.11626897	-2.103878261	0.036161824	0.094983348	no
DLGAP1-AS3	-0.116237248	-2.103296391	0.036213004	0.095106156	no
LCMT2	-0.116209228	-2.102782437	0.036258262	0.095213382	no
AMBP	0.116145746	2.101618035	0.036360978	0.095471444	no
TTC1	-0.116133445	-2.101392403	0.03638091	0.095512111	no
SLITRK3	-0.116127697	-2.101286983	0.036390226	0.095524899	no
LIMCH1	-0.116081679	-2.100442926	0.03646489	0.095709202	no
MEAF6	0.11607528	2.100325559	0.036475282	0.095724788	no
ANKRD20A12P	0.116056277	2.099977006	0.03650616	0.095794126	no
HERC3	-0.116042801	-2.099729835	0.036528071	0.095839918	no
TBC1D16	0.116029719	2.099489908	0.036549349	0.095878017	no
LOC644669	-0.116028389	-2.099465507	0.036551514	0.095878017	no
BVES	0.11602494	2.099402248	0.036557127	0.095881036	no
PFDN4	-0.116003652	-2.09901179	0.036591785	0.095960226	no
ZKSCAN5	-0.115996249	-2.098876025	0.036603843	0.095973796	no
SPAG17	0.115994992	2.098852962	0.036605891	0.095973796	no
NDUFA12	-0.115967208	-2.098343379	0.036651181	0.096080815	no
ATG9A	-0.115956179	-2.098141093	0.036669172	0.096110735	no
LOC401134	0.115954729	2.098114499	0.036671538	0.096110735	no
TEKT3	0.115948746	2.09800476	0.036681302	0.096124603	no
CALM2	-0.115921129	-2.097498257	0.036726397	0.096231042	no
CARD14	-0.115899703	-2.097105281	0.036761417	0.096311059	no
CALML6	0.115829356	2.095815105	0.036876593	0.09660103	no
FAM208B	-0.11581687	-2.095586109	0.036897068	0.096642841	no
ZFP92	-0.115814139	-2.095536023	0.036901547	0.096642841	no
FAM153B	-0.115805069	-2.095369676	0.036916428	0.096670033	no
RNF152	0.115798199	2.09524368	0.036927703	0.096687776	no
THAP11	-0.115779294	-2.094896968	0.036958744	0.096757262	no
IL8	0.115765226	2.094638968	0.036981856	0.096805978	no

UBAP1	-0.115737471	-2.094129954	0.037027493	0.096913634	no
MIR218-1	0.115725962	2.093918888	0.03704643	0.096941872	no
AS3MT	-0.115725432	-2.093909159	0.037047303	0.096941872	no
TMEM223	0.115722379	2.09385318	0.037052327	0.096943215	no
TRIM46	-0.115695612	-2.093362295	0.037096409	0.097046735	no
C6orf52	-0.115671935	-2.092928082	0.037135439	0.097137016	no
TMEM120B	-0.115654256	-2.092603864	0.037164605	0.097201475	no
ZSCAN12	-0.115627489	-2.09211298	0.037208801	0.097297524	no
LOC100507564	0.115626393	2.092092884	0.037210611	0.097297524	no
HMHB1	0.11562379	2.092045153	0.037214911	0.097297524	no
HEATR6	-0.1156125	-2.0918381	0.037233569	0.097334464	no
ERCC1	0.115575569	2.091160846	0.037294653	0.097482289	no
RBM27	-0.115571294	-2.091082451	0.037301729	0.097488928	no
LINC00251	0.115565868	2.090982941	0.037310713	0.09750055	no
FAHD2A	-0.11554886	-2.090671044	0.037338883	0.097553443	no
ASB9	0.115548166	2.090658328	0.037340032	0.097553443	no
IGF2BP2	0.115543061	2.090564708	0.037348491	0.097563684	no
PIP5KL1	-0.115515114	-2.090052208	0.037394829	0.09766378	no
RNF220	-0.115513512	-2.090022839	0.037397486	0.09766378	no
CRHR1	-0.115511731	-2.089990163	0.037400443	0.09766378	no
WDR53	0.115506278	2.089890166	0.037409491	0.09767554	no
MALAT1	-0.115498255	-2.089743044	0.037422807	0.097698437	no
C4orf21	-0.115476411	-2.089342484	0.037459081	0.09778126	no
TCTEX1D1	0.115457438	2.088994549	0.037490614	0.097851687	no
PTTG1IP	0.115453446	2.088921353	0.037497251	0.097857124	no
LMBRD2	-0.115433632	-2.088558008	0.03753021	0.097928197	no
COIL	-0.115430631	-2.088502989	0.037535203	0.097928197	no
SNORD67	0.115428858	2.088470462	0.037538155	0.097928197	no
ZNF610	-0.115385073	-2.087667562	0.037611087	0.09809691	no
GOLGA6L5	-0.115384551	-2.087658004	0.037611956	0.09809691	no
SNRNP70	-0.115380859	-2.087590293	0.037618112	0.098101061	no
ZNF555	-0.115376342	-2.087507468	0.037625643	0.098108797	no
MYCL1	-0.115358707	-2.087184095	0.037655061	0.098164763	no
CCDC84	0.115357999	2.087171113	0.037656243	0.098164763	no
NUDT1	-0.115345558	-2.086942972	0.037677009	0.098206987	no
KLRC2	-0.115325298	-2.086571468	0.037710847	0.098283266	no
PAFAH1B2	-0.115322028	-2.086511514	0.03771631	0.098285584	no
OR2L3	-0.115306341	-2.086223873	0.03774253	0.098341987	no
THSD7B	0.115303445	2.086170764	0.037747373	0.098342683	no
PTPLAD1	-0.115287184	-2.085872597	0.037774572	0.098396691	no
ZBTB49	-0.115285578	-2.085843136	0.03777726	0.098396691	no
RPL18A	-0.115282331	-2.085783607	0.037782693	0.098398915	no
OR4N4	-0.115272302	-2.0855997	0.03779948	0.098430708	no
VHLL	-0.115257811	-2.085333985	0.037823747	0.098481965	no
ZNF23	-0.115239878	-2.085005171	0.037853794	0.098548261	no
OTC	-0.115224312	-2.084719746	0.037879893	0.098604262	no
FBXL15	-0.115207074	-2.084403663	0.037908814	0.098667593	no
ZNF174	-0.115193612	-2.084156832	0.037931411	0.098714452	no
TAGAP	0.115188536	2.084063754	0.037939935	0.098724681	no
STARD3NL	0.115171842	2.083757657	0.037967979	0.098785695	no
SLC43A1	0.115166365	2.083657231	0.037977184	0.098797683	no

UCN	-0.115158323	-2.083509784	0.037990702	0.098820888	no
SLC47A2	0.11513432	2.083069672	0.038031076	0.098912173	no
SH3BP4	-0.115131988	-2.083026916	0.038035	0.098912173	no
CLEC14A	0.115124663	2.082892606	0.03804733	0.098932265	no
DOLK	0.115104275	2.08251878	0.038081665	0.099009564	no
PRR19	-0.115062192	-2.081747184	0.038152617	0.099182036	no
STS	0.115059163	2.081691632	0.03815773	0.09918333	no
ASAH2B	-0.115017571	-2.080929046	0.038227973	0.099353895	no
NSMF	-0.114993949	-2.080495945	0.038267915	0.099445679	no
PSG8	0.114988022	2.080387275	0.038277943	0.099448413	no
TM4SF18	0.114987857	2.080384247	0.038278222	0.099448413	no
ORMDL3	-0.114960401	-2.079880867	0.038324702	0.099557134	no
LUZP1	0.114951023	2.079708925	0.038340589	0.099586367	no
ZSWIM6	-0.114899385	-2.078762179	0.038428169	0.099801785	no
ZNF527	-0.11488015	-2.078409517	0.038460836	0.099874555	no
LOC400657	-0.114868874	-2.078202794	0.038479995	0.099912236	no
GRAPL	0.114854719	2.077943264	0.038504061	0.099962644	no
TAS2R5	-0.114851113	-2.077877158	0.038510193	0.099966487	no
UBE2Q2P1	-0.114835987	-2.077599854	0.038535924	0.1000212	no
CRX	-0.114831025	-2.077508868	0.03854437	0.100031041	no
RGAG4	-0.114808715	-2.077099859	0.038582356	0.100117534	no
ALMS1	-0.11479981	-2.076936592	0.038597528	0.100144812	no
DBIL5P2	-0.114796824	-2.076881854	0.038602616	0.100145923	no
DHX9	-0.114789917	-2.076755229	0.038614388	0.100155025	no
USP13	-0.114789297	-2.076743856	0.038615445	0.100155025	no
ZNF286A	-0.114785357	-2.076671615	0.038622163	0.10016036	no
FMO3	0.114779978	2.076572999	0.038631335	0.100172058	no
FBXO3	-0.114759923	-2.076205332	0.038665546	0.100248672	no
RLTPR	-0.114730907	-2.075673371	0.03871509	0.10035622	no
DYNC1I2	-0.114730159	-2.075659675	0.038716366	0.10035622	no
ANXA13	0.114720283	2.075478607	0.038733243	0.100387858	no
RASSF6	0.114703382	2.075168759	0.038762137	0.100450631	no
CHST7	0.114698821	2.075085159	0.038769936	0.100456826	no
RPP21	-0.114696518	-2.075042928	0.038773876	0.100456826	no
LOC375295	0.114667175	2.074504996	0.038824097	0.100574814	no
PPP2R1A	-0.114642574	-2.074053997	0.038866244	0.100671863	no
PSMG4	0.114614493	2.073539215	0.038914399	0.100784449	no
MSL2	-0.114590675	-2.073102566	0.038955286	0.100878184	no
MEST	-0.114584336	-2.072986364	0.038966173	0.100894219	no
RBP4	-0.114574151	-2.072799652	0.038983671	0.100927368	no
CTXN3	-0.114551884	-2.07239146	0.03902195	0.101014302	no
CDC40	-0.114544407	-2.072254386	0.039034812	0.101035426	no
TAF9B	-0.114539799	-2.072169913	0.03904274	0.101043776	no
PRKRIP1	0.114535569	2.072092371	0.039050018	0.101046528	no
GPRASP1	-0.114533716	-2.07205841	0.039053206	0.101046528	no
DNM2	0.114508986	2.071605057	0.039095787	0.101139514	no
PIGP	0.114507378	2.071575579	0.039098557	0.101139514	no
LOC100130705	-0.114475494	-2.070991096	0.039153515	0.101269491	no
PSCA	-0.114457503	-2.070661309	0.039184554	0.101337576	no
NSUN5	0.114449304	2.070511002	0.039198708	0.101358758	no
NKRF	-0.114447294	-2.070474161	0.039202177	0.101358758	no

37226	0.114444117	2.070415923	0.039207663	0.101360746	no
PHEX-AS1	0.114430282	2.070162306	0.039231559	0.101410324	no
NOL3	0.114426053	2.070084791	0.039238865	0.101417011	no
PGS1	0.114400948	2.069624597	0.039282264	0.101511462	no
ZNF44	-0.114397781	-2.069566549	0.039287741	0.101511462	no
MIPOL1	-0.114396719	-2.069547069	0.039289579	0.101511462	no
TOP3B	-0.114383384	-2.069302627	0.039312651	0.101558864	no
ZNF337	-0.114377256	-2.069190297	0.039323258	0.101574055	no
PSENEN	0.114367219	2.069006324	0.039340634	0.101599567	no
C3orf17	0.11436609	2.068985625	0.03934259	0.101599567	no
LINC00094	-0.114341997	-2.068543987	0.039384332	0.101684133	no
GALNT1	0.114341727	2.068539038	0.039384799	0.101684133	no
EIF4ENIF1	-0.114338129	-2.068473083	0.039391037	0.10168802	no
LINC00652	-0.11432444	-2.068222145	0.039414775	0.101737079	no
C10orf62	-0.114303746	-2.067842834	0.039450679	0.101808395	no
EIF3A	-0.11429873	-2.067750874	0.039459388	0.101808395	no
CALCOCO1	-0.114298396	-2.067744762	0.039459967	0.101808395	no
MOGS	0.114297598	2.067730133	0.039461353	0.101808395	no
ERAP1	0.114288599	2.067565185	0.039476979	0.101827695	no
PSIMCT-1	-0.114287833	-2.067551133	0.03947831	0.101827695	no
MPRIP	0.11427556	2.067326164	0.039499631	0.101870463	no
NEU3	-0.11426872	-2.067200786	0.039511518	0.101888892	no
SLC30A6	0.114245616	2.066777305	0.039551689	0.101980246	no
NDUFS1	0.114237992	2.066637556	0.039564953	0.102002208	no
KDM2B	-0.114212011	-2.06616132	0.039610184	0.102106568	no
LINC00629	-0.114208096	-2.066089561	0.039617003	0.102111898	no
PHF20	-0.114186003	-2.065684618	0.039655502	0.102198873	no
KCNJ15	0.114177949	2.065536991	0.039669546	0.102222806	no
NADKD1	-0.114170752	-2.065405074	0.039682098	0.102242893	no
ZNF385B	-0.114148745	-2.065001696	0.039720503	0.102329576	no
MRPL32	0.114127209	2.064606947	0.039758116	0.102414201	no
PSKH1	0.114120786	2.064489223	0.03976934	0.102426284	no
TYRO3	-0.114119069	-2.064457764	0.039772339	0.102426284	no
CACNB4	-0.11406286	-2.063427513	0.03987068	0.10264441	no
DCTN1-AS1	-0.11406284	-2.063427137	0.039870715	0.10264441	no
PPP4R1L	0.114062468	2.063420323	0.039871367	0.10264441	no
BIN3	0.113995371	2.062190537	0.039989026	0.102934982	no
CNTN4	-0.113986213	-2.062022686	0.040005108	0.102964045	no
C6orf147	0.113970126	2.061727843	0.040033371	0.103024449	no
CNOT7	-0.113936619	-2.061113729	0.040092292	0.103163728	no
DACT3-AS1	-0.113925509	-2.060910098	0.040111846	0.103193629	no
DANCR	-0.11392456	-2.060892712	0.040113516	0.103193629	no
FARSB	-0.113913382	-2.060687835	0.040133199	0.103217831	no
LUC7L	-0.113912129	-2.060664867	0.040135406	0.103217831	no
RPIA	0.113908961	2.060606802	0.040140986	0.103217831	no
PP14571	-0.113908308	-2.060594838	0.040142135	0.103217831	no
VPS26A	0.113851821	2.059559573	0.040241737	0.103461559	no
CASP5	0.113845643	2.059446344	0.040252644	0.103477221	no
RBMX	-0.11382648	-2.059095133	0.040286489	0.10355184	no
HS6ST3	-0.113814318	-2.058872242	0.040307981	0.103587332	no
ST6GALNAC2	0.113813211	2.058851961	0.040309937	0.103587332	no

ARHGEF17	-0.113767049	-2.05800595	0.040391605	0.10376136	no
NSUN7	0.113765232	2.05797264	0.040394823	0.10376136	no
KLC4	-0.113764951	-2.057967503	0.04039532	0.10376136	no
SLC35B4	0.113764019	2.057950417	0.040396971	0.10376136	no
DGCR6L	-0.113720699	-2.057156499	0.040473747	0.103946139	no
LOC100652768	0.113708679	2.056936217	0.040495071	0.103988478	no
SH3GL1P2	-0.113676455	-2.05634566	0.040552287	0.104122964	no
CAPN13	-0.11366916	-2.056211972	0.040565249	0.104134184	no
MUSTN1	-0.113668541	-2.056200642	0.040566348	0.104134184	no
ULK2	-0.113664556	-2.0561276	0.040573432	0.104139928	no
THRAP3	0.113652435	2.055905464	0.040594981	0.104182795	no
WDR12	-0.113628734	-2.055471118	0.040637144	0.104275543	no
LOC100129620	-0.113626666	-2.055433227	0.040640824	0.104275543	no
TMSB15A	-0.113617437	-2.05526409	0.040657254	0.104298145	no
ZNF652	-0.113616265	-2.055242627	0.040659339	0.104298145	no
CHN1	-0.113605116	-2.055038308	0.040679195	0.104336626	no
ZMAT2	-0.113570989	-2.054412913	0.040740024	0.104480173	no
UEVLD	0.11351735	2.053429949	0.040835787	0.104713267	no
ZFC3H1	-0.1135006	-2.053123007	0.040865729	0.104777546	no
HYAL1	-0.113492884	-2.052981593	0.040879531	0.104800428	no
WWOX	-0.113483767	-2.052814533	0.04089584	0.104829735	no
PSMB3	0.113453331	2.052256798	0.040950329	0.104950954	no
PSMD11	0.113451898	2.052230535	0.040952897	0.104950954	no
KIN	-0.113428277	-2.05179768	0.04099523	0.105046918	no
ZNF431	-0.113405143	-2.051373758	0.041036727	0.105140712	no
FBL	-0.113384487	-2.050995258	0.041073807	0.105223171	no
TRPM4	0.113364665	2.050632029	0.041109418	0.105301848	no
NAT16	-0.113346316	-2.050295795	0.041142406	0.105350267	no
TTTY18	0.11334627	2.050294953	0.041142488	0.105350267	no
ARPC4-TTLL3	0.113345036	2.050272337	0.041144708	0.105350267	no
HIST1H4C	-0.113343245	-2.05023952	0.041147929	0.105350267	no
SOS1	-0.11332597	-2.049922978	0.041179009	0.105417282	no
FAM47E	-0.113319467	-2.049803813	0.041190715	0.105434689	no
LOC286189	0.113303741	2.049515646	0.041219033	0.105494609	no
POTEA	0.113289878	2.049261625	0.041244009	0.105545964	no
DNAJB6	0.113251944	2.048566535	0.041312419	0.105708442	no
CST6	0.113237889	2.048308987	0.041337791	0.10576077	no
C1orf145	-0.113215272	-2.047894572	0.041378645	0.105852691	no
IZUMO1	-0.113210093	-2.047799669	0.041388005	0.105864035	no
ZNF415	-0.113201436	-2.047641054	0.041403654	0.105891458	no
GRIA1	-0.113166476	-2.047000479	0.041466902	0.1060406	no
SFT2D1	0.1130955	2.045699993	0.041595562	0.106356957	no
SRSF7	0.113044994	2.044774599	0.04168732	0.106570382	no
BIVM-ERCC5	-0.113044098	-2.04475818	0.041688949	0.106570382	no
FLJ27352	0.113040492	2.044692117	0.041695506	0.106574468	no
ASAP3	0.113030374	2.044506725	0.041713913	0.106608836	no
JMJD7	-0.113010452	-2.044141715	0.041750172	0.106672574	no
NDUFS7	-0.113007455	-2.044086806	0.041755629	0.106672574	no
CCDC85B	-0.113006295	-2.044065552	0.041757742	0.106672574	no
ABHD11-AS1	0.113005765	2.044055841	0.041758707	0.106672574	no
PTX3	0.112999434	2.043939837	0.041770238	0.106683856	no

MRPL10	-0.112995194	-2.04386216	0.041777961	0.106683856	no
DDIT3	0.112994335	2.043846419	0.041779526	0.106683856	no
GRIK5	-0.11299244	-2.043811688	0.04178298	0.106683856	no
ATXN3	-0.112974086	-2.043475421	0.04181643	0.106756581	no
FAM8A1	-0.112971076	-2.043420262	0.041821919	0.106757913	no
SORCS2	-0.112945248	-2.042947063	0.041869035	0.10686549	no
MRPL1	-0.112930105	-2.042669603	0.041896682	0.106923358	no
SAP130	-0.112922836	-2.042536438	0.041909957	0.106944535	no
FOXD4	-0.11290627	-2.042232919	0.041940226	0.107009071	no
ABCC4	0.112896121	2.042046966	0.04195878	0.107043703	no
ZNF85	-0.112885334	-2.041849335	0.041978508	0.107081318	no
SEC23A	0.112876011	2.041678536	0.041995563	0.10711211	no
IRAK1BP1	-0.112871223	-2.041590806	0.042004325	0.107121746	no
RPRD1B	0.112866545	2.041505097	0.042012887	0.107130869	no
C1orf74	0.112839691	2.041013108	0.042062065	0.107243544	no
ESYT2	0.112828949	2.040816307	0.04208175	0.107281007	no
NCMAP	0.112786275	2.040034482	0.04216003	0.107467823	no
LOC283710	0.112782993	2.039974361	0.042166055	0.107470433	no
POMT2	-0.112778892	-2.039899219	0.042173586	0.107472179	no
ARSB	0.112777173	2.039867735	0.042176742	0.107472179	no
DOC2GP	-0.112762335	-2.039595887	0.042203999	0.107528884	no
NPRL2	-0.112749814	-2.039366506	0.042227009	0.107574758	no
PLA2G16	0.112743544	2.039251636	0.042238537	0.107591371	no
RPS24	-0.112738426	-2.039157865	0.042247949	0.107602592	no
INPP4A	0.112724916	2.038910359	0.0422728	0.107650111	no
PGM5P2	-0.112721471	-2.03884725	0.042279139	0.107650111	no
ATP1B1	-0.112720115	-2.038822411	0.042281634	0.107650111	no
MMP7	0.112714504	2.038719609	0.042291961	0.107663651	no
GNB1L	-0.112704519	-2.038536672	0.042310344	0.107697692	no
MIR7-3HG	-0.11269938	-2.038442538	0.042319806	0.10770902	no
ELSPBP1	0.112659765	2.037716781	0.042392818	0.107882067	no
BACE1	-0.112634939	-2.037261973	0.042438627	0.107985854	no
IWS1	-0.112621934	-2.037023729	0.042462639	0.108034164	no
C7orf13	-0.112572354	-2.036115445	0.042554292	0.108254532	no
PAPSS1	-0.112564575	-2.035972948	0.042568686	0.108278333	no
DBNDD2	-0.112549845	-2.0357031	0.042595956	0.108334875	no
PAX1	-0.112533208	-2.035398325	0.042626774	0.108400425	no
ZNRF3	-0.112507876	-2.034934278	0.042673732	0.108507001	no
EEFSEC	-0.112491896	-2.034641538	0.042703378	0.108560013	no
NLN	0.112491193	2.034628653	0.042704683	0.108560013	no
LDOC1	-0.11248573	-2.034528587	0.042714821	0.108572942	no
UROS	-0.112466262	-2.034171953	0.04275097	0.108651974	no
UBL5	-0.11244439	-2.033771288	0.042791612	0.108742407	no
ZSCAN31	-0.112423217	-2.03338343	0.042830987	0.108829597	no
LRRC8E	0.11241749	2.033278528	0.042841641	0.108843801	no
CWC15	-0.112365446	-2.032325163	0.042938576	0.10907718	no
NDUFAF4P1	-0.112342288	-2.031900958	0.042981768	0.109173995	no
GRIP2	-0.112329603	-2.03166861	0.043005441	0.109221215	no
LRRC42	0.112291696	2.030974235	0.043076253	0.109381797	no
SLC12A9	0.112290308	2.030948804	0.043078848	0.109381797	no
ZNF474	0.112263578	2.03045919	0.043128841	0.109495798	no

MYOZ1	-0.112249073	-2.030193492	0.043155991	0.109551785	no
PEX16	-0.112212223	-2.029518501	0.04322503	0.109714081	no
RRN3P2	0.112169937	2.028743959	0.043304367	0.109902473	no
GNB3	-0.11215778	-2.028521275	0.043327199	0.109947436	no
LOC440518	-0.112143043	-2.028251347	0.043354889	0.110004713	no
ARHGAP39	-0.112122967	-2.027883618	0.043392636	0.110087492	no
ALDOB	-0.112115994	-2.027755896	0.043405753	0.110107772	no
TBX15	0.11210379	2.027532363	0.043428718	0.1101491	no
C1QL3	-0.112101889	-2.027497549	0.043432296	0.1101491	no
ZMYND10	0.112053997	2.026620339	0.043522523	0.110358288	no
YIPF7	0.112052657	2.026595797	0.04352505	0.110358288	no
DNAJC28	-0.112047437	-2.026500196	0.043534893	0.110370225	no
ARG2	0.112040848	2.026379508	0.043547323	0.110388713	no
ALKBH4	-0.112034322	-2.026259978	0.043559635	0.110406902	no
NAA25	-0.112007679	-2.025771984	0.043609934	0.110521356	no
PCLO	-0.112002724	-2.025681229	0.043619294	0.110532042	no
FIS1	-0.111985575	-2.025367127	0.043651702	0.110601122	no
ZNF233	-0.11197584	-2.025188837	0.043670106	0.110634709	no
BMPR2	-0.111954844	-2.024804278	0.043709824	0.110722281	no
EPN2-AS1	-0.111929701	-2.02434378	0.043757427	0.110829801	no
SEC23IP	0.111909045	2.023965454	0.043796568	0.110915866	no
AP2A1	-0.11190262	-2.023847781	0.043808748	0.11093364	no
ETF1	0.111879885	2.023431394	0.043851871	0.111029756	no
TPM3P9	0.111874237	2.023327941	0.043862591	0.111043815	no
ANO4	-0.111867439	-2.023203442	0.043875495	0.111055917	no
SPDYE7P	-0.111866274	-2.023182103	0.043877707	0.111055917	no
RSRC1	-0.11186092	-2.023084053	0.043887872	0.111068242	no
CDSN	0.111856853	2.023009555	0.043895596	0.111068242	no
GID4	-0.111855545	-2.022985597	0.043898081	0.111068242	no
STRA13	-0.111850591	-2.022894874	0.043907489	0.11106952	no
RMDN3	-0.111849837	-2.022881063	0.043908922	0.11106952	no
GARS	0.111846306	2.022816383	0.043915631	0.111073418	no
PTP4A3	0.111786276	2.021716962	0.044029807	0.111339272	no
LGI2	0.111785594	2.021704477	0.044031105	0.111339272	no
TMEM45A	0.111780023	2.021602445	0.044041714	0.111352997	no
FAM196A	-0.111761104	-2.021255952	0.044077758	0.111431019	no
RAF1	-0.111757539	-2.02119066	0.044084553	0.111435089	no
BCL2L2	-0.111724724	-2.020589689	0.044147136	0.11158016	no
LRRC28	0.111720123	2.020505426	0.044155917	0.111586823	no
PCDHA13	-0.111717902	-2.020464747	0.044160157	0.111586823	no
RNF219	-0.111695589	-2.02005611	0.044202765	0.111681357	no
PAX6	-0.111671976	-2.01962367	0.044247893	0.111782234	no
HERC2P2	-0.111659185	-2.019389421	0.044272355	0.111830885	no
LOC283332	-0.111646786	-2.01916235	0.044296078	0.111877659	no
DAP3	0.111594397	2.01820292	0.044396432	0.112117947	no
MSMO1	-0.111582375	-2.01798277	0.044419487	0.112162988	no
ASMTL	0.111559729	2.017568051	0.044462945	0.112259532	no
ARRB1	-0.111545967	-2.017316027	0.044489371	0.11231306	no
TFDP2	-0.111506033	-2.016584727	0.044566129	0.112493621	no
TAF5L	-0.1114947	-2.01637718	0.044587934	0.112535442	no
NARS	-0.111484104	-2.016183148	0.044608327	0.112573692	no

ARID1B	-0.111459915	-2.015740177	0.044654914	0.112678027	no
SIAH2	0.111427808	2.01515223	0.044716811	0.112813766	no
C2orf91	0.111423485	2.015073067	0.044725151	0.112813766	no
CCDC66	-0.111423318	-2.015070015	0.044725472	0.112813766	no
GABRQ	-0.111421124	-2.015029836	0.044729706	0.112813766	no
PHC2	0.111403812	2.01471282	0.044763118	0.112884789	no
LHB	0.111400472	2.014651659	0.044769567	0.112887805	no
ARFGAP3	0.111394482	2.014541965	0.044781135	0.112903727	no
C16orf13	-0.111374963	-2.014184527	0.044818846	0.112985551	no
HAUS3	-0.111335162	-2.013455711	0.044895823	0.113166331	no
KCNH5	-0.111330386	-2.013368261	0.044905067	0.113176357	no
CFL2	-0.111319817	-2.013174725	0.044925531	0.11321379	no
AOX1	0.111317275	2.013128161	0.044930455	0.11321379	no
ACYP1	-0.111312679	-2.013044002	0.044939357	0.113222945	no
LAYN	0.111277094	2.012392394	0.045008331	0.113383246	no
DIS3L2	-0.111274411	-2.012343275	0.045013534	0.113383246	no
HSPA9	-0.111269576	-2.012254743	0.045022913	0.113385804	no
FAU	-0.111267876	-2.012223611	0.045026211	0.113385804	no
ZDHHC14	0.111265729	2.012184295	0.045030377	0.113385804	no
USP25	0.111250662	2.011908404	0.04505962	0.113446145	no
TGFB1I1	0.111246556	2.011833208	0.045067593	0.113452927	no
CDADC1	-0.111222952	-2.011400997	0.045113444	0.11355505	no
LOC283692	-0.111203862	-2.011051454	0.045150554	0.113631526	no
RAB7L1	0.111201884	2.011015227	0.045154401	0.113631526	no
ZNF788	0.111195299	2.010894655	0.045167209	0.113650449	no
RPL21	-0.111189099	-2.010781131	0.045179271	0.11366749	no
LRRN2	-0.111184689	-2.010700381	0.045187852	0.113675772	no
TBC1D30	-0.111135534	-2.009800321	0.045283594	0.113903292	no
ABI2	-0.111116406	-2.009450084	0.045320897	0.113983778	no
PLA2G4E	-0.111106105	-2.009261461	0.045340997	0.1140201	no
FUT1	-0.111103567	-2.009214996	0.045345949	0.1140201	no
C14orf142	0.111089766	2.0089623	0.045372892	0.114074499	no
SDR16C5	-0.111074808	-2.00868842	0.045402108	0.114134601	no
RASSF7	0.111049527	2.008225518	0.045451525	0.114245464	no
LIPA	0.111034179	2.0079445	0.045481547	0.114298798	no
SLTM	-0.11103144	-2.007894353	0.045486906	0.114298798	no
PHLDB3	0.111030524	2.007877579	0.045488698	0.114298798	no
SETD1A	-0.11101134	-2.00752632	0.045526253	0.11437904	no
FICD	0.111008774	2.007479341	0.045531278	0.11437904	no
ZNF430	-0.110999091	-2.007302052	0.045550244	0.114413312	no
LOC100131626	-0.110992988	-2.00719031	0.045562202	0.114427939	no
LRRC57	-0.110990684	-2.007148129	0.045566716	0.114427939	no
KIAA0226L	0.110987308	2.007086311	0.045573333	0.114431184	no
MUC6	-0.110978776	-2.0069301	0.045590057	0.114459804	no
MIR497HG	-0.110962398	-2.00663022	0.045622178	0.114527067	no
SPRED1	0.110954272	2.006481451	0.04563812	0.114553706	no
CHCHD7	0.110936766	2.006160929	0.045672482	0.114615413	no
TUBGCP2	-0.110936314	-2.006152649	0.04567337	0.114615413	no
ACSBG1	-0.110931941	-2.006072574	0.045681958	0.114623581	no
C4BPA	0.110914394	2.005751307	0.045716429	0.114696682	no
F7	-0.110899109	-2.005471453	0.045746474	0.114748843	no

CNN1	0.110898384	2.005458187	0.045747899	0.114748843	no
LOC100132735	0.110865627	2.004858454	0.045812344	0.11489708	no
SRSF1	-0.110860467	-2.00476397	0.045822504	0.114906925	no
NDUFA4L2	0.110857084	2.004702025	0.045829166	0.114906925	no
DARS2	0.110855487	2.004672795	0.045832309	0.114906925	no
MVB12B	-0.110850595	-2.004583233	0.045841944	0.114912331	no
HRH4	0.110848962	2.004553339	0.04584516	0.114912331	no
AKAP3	-0.110830035	-2.004206805	0.045882455	0.114992157	no
RPS21	-0.11082737	-2.004158006	0.045887708	0.114992157	no
SLC7A4	-0.110815888	-2.003947792	0.045910347	0.115035474	no
NT5DC3	-0.110810683	-2.003852509	0.045920611	0.11504778	no
HOTAIRM1	0.110803701	2.003724677	0.045934385	0.115068874	no
MPV17L	0.110771706	2.003138899	0.045997547	0.115213668	no
SLC35F1	-0.1107608	-2.002939237	0.046019092	0.115235723	no
KCNAB3	-0.110759442	-2.002914363	0.046021777	0.115235723	no
KIAA0907	-0.110757123	-2.002871904	0.04602636	0.115235723	no
MLIP	0.110756394	2.002858565	0.0460278	0.115235723	no
RBM5	-0.110753317	-2.002802224	0.046033882	0.115237526	no
TTC5	-0.110734907	-2.002465176	0.046070282	0.115315214	no
SLC26A11	-0.110728388	-2.002345825	0.046083177	0.115334059	no
CLSTN1	-0.110708746	-2.001986234	0.046122048	0.115400709	no
WNT7A	-0.110708562	-2.00198287	0.046122412	0.115400709	no
DEFB131	-0.110706792	-2.001950452	0.046125917	0.115400709	no
MTRNR2L10	-0.110700709	-2.001839085	0.046137962	0.115417407	no
TUBB3	-0.110682578	-2.001507163	0.046173877	0.115493806	no
POLI	-0.110674887	-2.001366346	0.046189121	0.115518491	no
RASD2	-0.110624629	-2.00044626	0.046288827	0.115736245	no
CLCN6	0.110620328	2.000367524	0.046297368	0.115736245	no
DMXL1	-0.110620245	-2.000366005	0.046297533	0.115736245	no
BHLHB9	-0.110620146	-2.000364195	0.046297729	0.115736245	no
KIAA0930	-0.11060136	-2.000020271	0.046335052	0.115816074	no
PHLDA2	0.110576889	1.999572285	0.046383706	0.11592212	no
CAPN2	0.110574597	1.999530313	0.046388266	0.11592212	no
GPR64	0.110562806	1.999314469	0.046411726	0.115967259	no
STRAP	-0.110537629	-1.998853559	0.046461854	0.116072336	no
CECR5	-0.11053626	-1.998828498	0.046464581	0.116072336	no
FAM50B	0.110515617	1.998450589	0.046505718	0.116161597	no
RAB17	0.110512434	1.998392319	0.046512064	0.116163946	no
SMPX	-0.110507438	-1.998300856	0.046522026	0.116175325	no
CREG2	-0.11049402	-1.998055225	0.046548788	0.116228652	no
CYP2C9	0.110487057	1.997927754	0.046562682	0.116248721	no
SRP68	-0.11048457	-1.997882232	0.046567644	0.116248721	no
NKAP	0.11045699	1.99737734	0.046622714	0.116368647	no
GP1BA	-0.110455088	-1.997342517	0.046626514	0.116368647	no
WDR74	-0.110439168	-1.99705108	0.046658329	0.116434528	no
CDHR4	0.110413305	1.996577641	0.046710052	0.116545513	no
TMC3	-0.110411507	-1.996544725	0.04671365	0.116545513	no
TMEM14C	0.110403567	1.996399373	0.04672954	0.116571624	no
SLC16A8	-0.110367349	-1.995736376	0.046802079	0.116739029	no
NR5A2	0.110360103	1.995603743	0.046816601	0.116761701	no
ATE1	-0.11035652	-1.995538141	0.046823786	0.116766069	no

LEPREL2	0.110328703	1.995028936	0.046879585	0.116887822	no
RRP7A	-0.110326758	-1.99499334	0.046883487	0.116887822	no
COL5A3	0.11032098	1.994887578	0.046895085	0.116903174	no
FNIP1	-0.110312681	-1.994735651	0.046911748	0.116931151	no
LAMP5	-0.110300807	-1.99451829	0.046935598	0.11697703	no
PINK1	-0.110297536	-1.994458418	0.046942169	0.116979841	no
SOST	0.110283701	1.994205173	0.046969971	0.117035554	no
NARG2	-0.110278108	-1.994102796	0.046981215	0.117049999	no
SNRPN	-0.110244224	-1.99348254	0.047049383	0.117206247	no
PROM2	-0.110241266	-1.993428392	0.047055338	0.117207496	no
LOC100128554	-0.110228403	-1.993192938	0.04708124	0.117258423	no
MIR3940	0.110208873	1.992835451	0.047120589	0.117334168	no
PCOLCE-AS1	0.110207889	1.992817441	0.047122573	0.117334168	no
WDR45B	-0.110180432	-1.992314858	0.047177942	0.117458428	no
CLCN4	-0.110157607	-1.991897047	0.047224014	0.117558465	no
HNRNPUL1	-0.110155107	-1.991851283	0.047229063	0.117558465	no
ADPRHL1	-0.110146931	-1.991701625	0.047245576	0.11758595	no
SLC35F3	-0.110134913	-1.991481651	0.047269857	0.117632759	no
HCFC1	-0.110113834	-1.991095816	0.047312472	0.117725175	no
ABT1	0.11009198	1.990695795	0.047356688	0.117821554	no
C5orf24	-0.110068997	-1.990275127	0.047403224	0.117923681	no
EIF3F	-0.110066263	-1.990225086	0.047408762	0.117923808	no
ZSWIM7	-0.110021953	-1.989414042	0.0474986	0.118133597	no
ZNF280D	0.110010426	1.989203053	0.047521994	0.118178105	no
AJUBA	0.109986324	1.988761917	0.047570939	0.118286134	no
OR14I1	-0.109968326	-1.988432498	0.047607516	0.118363389	no
SLIRP	-0.109962932	-1.988333768	0.047618484	0.118376961	no
TRAF5	0.109958418	1.988251151	0.047627663	0.118386086	no
ATF3	0.10994929	1.988084068	0.047646231	0.118418543	no
ZNF831	0.109919308	1.987535307	0.047707257	0.118542954	no
TMX4	0.109919277	1.987534745	0.04770732	0.118542954	no
HDAC9	-0.109889876	-1.986996626	0.047767227	0.11867809	no
CAPN11	0.109879944	1.986814837	0.04778748	0.118714683	no
QRICH1	-0.109869929	-1.986631541	0.047807907	0.118751703	no
PPP6R2	-0.109857344	-1.986401197	0.047833589	0.118801763	no
SNX7	0.109835313	1.985997975	0.047878573	0.118899746	no
TULP4	-0.109826794	-1.985842065	0.047895976	0.118929222	no
PRRT2	-0.109787232	-1.985117988	0.047976869	0.119116323	no
TTC29	0.109763681	1.98468696	0.048025078	0.119209856	no
RASA2	0.109762897	1.98467261	0.048026684	0.119209856	no
RIT2	-0.109760611	-1.984630774	0.048031365	0.119209856	no
PPFIA2	-0.109757992	-1.984582843	0.048036729	0.119209856	no
SDHAP2	-0.10975059	-1.984447371	0.048051893	0.119233718	no
VMAC	0.109742506	1.984299424	0.048068458	0.119261051	no
MAP1B	0.109736324	1.98418628	0.048081129	0.119278718	no
KIRREL3-AS3	-0.109721666	-1.983918001	0.048111185	0.119327108	no
RPS13	-0.109721395	-1.983913053	0.048111739	0.119327108	no
GJC3	-0.109667944	-1.982934808	0.048221471	0.119585465	no
POLE	-0.109660062	-1.98279057	0.048237669	0.11961183	no
CUBN	0.109641205	1.98244546	0.048276442	0.119694163	no
MRPS36	-0.109615307	-1.9819715	0.048329734	0.119807843	no

SOX5	-0.109613506	-1.981938536	0.048333442	0.119807843	no
LOC645166	0.109602008	1.981728099	0.048357121	0.119852715	no
MIR222	0.109599023	1.981673472	0.04836327	0.119854131	no
RAC1	0.109576933	1.981269211	0.048408791	0.119944091	no
CHRDL1	-0.109575991	-1.981251974	0.048410732	0.119944091	no
ST3GAL4	0.109568662	1.981117853	0.048425843	0.1199677	no
ZNF420	-0.109549419	-1.980765687	0.04846554	0.120052203	no
LNX1-AS2	0.109531596	1.980439509	0.048502331	0.120129491	no
MAP7	-0.109521986	-1.980263651	0.048522177	0.120164796	no
ENO1	0.109513225	1.980103312	0.048540277	0.120195771	no
FAM86A	0.109488732	1.979655085	0.048590907	0.120307279	no
PPIAL4G	0.109483856	1.979565851	0.048600992	0.120314385	no
MAGI3	-0.109481931	-1.97953062	0.048604974	0.120314385	no
C2orf73	0.109473334	1.979373294	0.04862276	0.12034455	no
PTCHD4	0.109449367	1.978934691	0.048672373	0.120453473	no
COPZ1	-0.109446297	-1.978878521	0.04867873	0.120455334	no
SPINT2	0.109436525	1.978699684	0.048698974	0.120491554	no
SLC44A4	0.109433798	1.978649786	0.048704623	0.120491661	no
HTR3B	-0.109389837	-1.9778453	0.048795786	0.120703295	no
HCG17	-0.109384899	-1.977754946	0.048806033	0.12071475	no
TFG	0.109381177	1.977686841	0.048813759	0.120719964	no
SSFA2	0.109365926	1.977407747	0.048845429	0.120784387	no
FLJ40194	0.109357192	1.977247917	0.048863573	0.12079233	no
ZNF83	0.109356577	1.977236659	0.048864851	0.12079233	no
GALNT8	-0.109356263	-1.977230922	0.048865502	0.12079233	no
GNAS-AS1	0.109344431	1.977014391	0.048890093	0.120839218	no
LINGO4	-0.109332511	-1.976796263	0.048914876	0.120873042	no
ZNF660	-0.10933242	-1.976794598	0.048915066	0.120873042	no
SF3A3	0.109329734	1.976745446	0.048920651	0.120873042	no
BEND6	-0.10932082	-1.976582329	0.048939193	0.120904954	no
MED18	0.109305575	1.976303369	0.048970916	0.12096942	no
ADAR	-0.109299894	-1.976199405	0.048982743	0.12098473	no
PIGA	0.109282174	1.975875148	0.049019647	0.121053397	no
TAT	-0.109281136	-1.97585615	0.04902181	0.121053397	no
P4HA1	0.109269454	1.975642384	0.049046152	0.121099593	no
AKR1C6P	0.109254881	1.97537571	0.049076533	0.121160687	no
VSTM2A	-0.109237345	-1.975054817	0.049113113	0.121237068	no
PCP2	0.109232297	1.974962448	0.049123647	0.121249143	no
SYNCRIP	-0.109224835	-1.974825899	0.049139222	0.121273659	no
LRCOL1	0.109195313	1.97428569	0.049200882	0.121406087	no
SLC38A10	0.109193236	1.97424769	0.049205221	0.121406087	no
PPP6R1	0.109191032	1.974207353	0.049209828	0.121406087	no
PPIL3	-0.109184563	-1.97408898	0.04922335	0.121425507	no
CRHBP	-0.109180862	-1.97402126	0.049231088	0.121430656	no
ZNF808	0.109177544	1.97396055	0.049238025	0.121433829	no
MRPL18	-0.109157969	-1.973602361	0.04927897	0.121520867	no
GJB6	-0.109143803	-1.97334315	0.049308619	0.12158003	no
APCDD1L-AS1	0.109136064	1.973201535	0.049324824	0.121606033	no
CDK12	0.109129016	1.973072575	0.049339584	0.121628471	no
KRTAP4-7	-0.109125197	-1.973002698	0.049347584	0.121634239	no
IAH1	0.109102725	1.972591499	0.04939468	0.121736361	no

C11orf42	-0.109096979	-1.97248637	0.049406727	0.121752089	no
LOC100505666	0.109092533	1.972405012	0.049416052	0.121761106	no
PPARD	0.109084448	1.972257069	0.049433011	0.121781459	no
LGALS4	-0.109082489	-1.972221231	0.049437121	0.121781459	no
GAS2L1	-0.109080437	-1.97218368	0.049441426	0.121781459	no
C17orf78	-0.109077791	-1.972135268	0.049446978	0.121781459	no
SMARCA1	0.109074494	1.972074928	0.049453898	0.121784546	no
DSP	0.109056203	1.971740257	0.049492296	0.12186514	no
SLC6A15	-0.109044524	-1.971526552	0.049516828	0.121911577	no
SFXN3	0.108983167	1.970403877	0.049645873	0.122201624	no
C15orf27	-0.108983102	-1.970402699	0.049646009	0.122201624	no
MRPL27	-0.108949225	-1.969782839	0.04971738	0.122363286	no
BTN2A1	0.108887707	1.968657246	0.049847202	0.122668753	no
TFCP2L1	0.108853458	1.968030616	0.049919599	0.122832849	no
C9orf156	-0.108835821	-1.967707931	0.049956915	0.122910596	no
C7orf10	0.108825765	1.967523941	0.049978202	0.122948895	no
FLJ46906	-0.108808873	-1.967214877	0.050013978	0.123022822	no
PIP5K1B	-0.108802708	-1.96710208	0.05002704	0.123040869	no
DNAH17	-0.108792781	-1.966920457	0.050048078	0.123078526	no
FAM163A	-0.108785935	-1.966795203	0.050062591	0.123100131	no
RPL6	-0.108783217	-1.966745469	0.050068355	0.123100219	no
ITGA6	0.108765077	1.966413581	0.050106831	0.123174469	no
CABLES1	-0.108763575	-1.966386106	0.050110017	0.123174469	no
NPHS1	-0.108756748	-1.966261204	0.050124505	0.123182357	no
MYCBP2-AS1	-0.108756661	-1.966259608	0.05012469	0.123182357	no
FGF1	0.108743991	1.966027794	0.050151588	0.123234367	no
GPT2	-0.108706834	-1.965347979	0.050230537	0.123414254	no
MYH3	-0.108682725	-1.964906908	0.050281817	0.123521289	no
DSG2	0.108679721	1.964851945	0.05028821	0.123521289	no
AEBP2	0.108676513	1.964793249	0.050295038	0.123521289	no
NKX6-3	-0.108673909	-1.964745609	0.05030058	0.123521289	no
LOC339862	-0.108672848	-1.964726194	0.050302839	0.123521289	no
SAMSN1-AS1	0.108651712	1.964339509	0.050347848	0.123617684	no
ANKK1	0.108614775	1.963663741	0.050426585	0.123796864	no
HSD3BP4	-0.108606389	-1.963510314	0.050444476	0.123826641	no
COL11A2	-0.108598647	-1.963368688	0.050460996	0.123853046	no
C2CD2L	-0.108580056	-1.963028569	0.050500688	0.123936311	no
LOC100130987	-0.108571801	-1.962877547	0.05051832	0.123965427	no
USP31	-0.108568556	-1.96281817	0.050525254	0.123968288	no
AMIGO1	-0.108547232	-1.962428064	0.050570829	0.124065947	no
PLCH2	-0.108532484	-1.96215825	0.050602372	0.12412916	no
MPC1	-0.108491298	-1.96140478	0.050690543	0.124331256	no
YARS2	0.108458053	1.960796585	0.050761809	0.124491844	no
LTV1	0.108425148	1.960194619	0.050832427	0.124650809	no
SLIT3	0.108411839	1.959951158	0.050861012	0.124699678	no
PARP1	-0.108410467	-1.95992605	0.050863961	0.124699678	no
XBP1	0.108407427	1.959870437	0.050870492	0.124701466	no
FAM182A	-0.108397324	-1.959685629	0.050892203	0.124740458	no
UPK3A	0.108374925	1.95927587	0.050940368	0.124844275	no
CST2	0.108367355	1.959137386	0.050956655	0.124869951	no
RPL28	0.108350407	1.95882734	0.050993134	0.124945098	no

POLR1B	0.108338202	1.958604068	0.051019418	0.124995248	no
KIR2DS2	0.108291112	1.957742652	0.05112093	0.125229672	no
NEFH	-0.108268561	-1.957330121	0.051169605	0.125334622	no
KHDC3L	-0.108253379	-1.957052406	0.051202394	0.125400644	no
POLR2I	-0.108247708	-1.956948653	0.051214648	0.125416364	no
LINC00309	0.108227614	1.956581081	0.051258083	0.125508427	no
CCDC110	0.108216351	1.95637505	0.051282442	0.125553767	no
LOC646938	0.108199209	1.956061479	0.051319535	0.125630269	no
TRAPPC11	0.108190824	1.955908106	0.051337686	0.125660389	no
PIGC	0.10816203	1.95538139	0.051400062	0.125798738	no
C16orf88	-0.10814933	-1.955149079	0.051427593	0.125851787	no
FLJ30403	-0.108145604	-1.955080922	0.051435672	0.125857228	no
TSTA3	0.108138973	1.954959624	0.051450054	0.125878087	no
ABCA17P	-0.108130191	-1.954798996	0.051469105	0.125910362	no
TTC21B	-0.108123829	-1.95468261	0.051482912	0.125929804	no
SLC51A	0.108106312	1.954362198	0.051520938	0.126008477	no
C15orf61	-0.108102382	-1.954290314	0.051529473	0.126015009	no
KIAA0317	-0.108077228	-1.953830199	0.051584129	0.126134317	no
ZMAT5	-0.108069538	-1.953689542	0.051600847	0.126160842	no
KRTCAP2	0.108054107	1.953407277	0.05163441	0.12622854	no
SPAG5-AS1	-0.10803847	-1.953121257	0.051668437	0.12629736	no
LOC647012	-0.108034135	-1.953041955	0.051677875	0.126306064	no
LOC100128398	-0.108008512	-1.952573275	0.051733684	0.126415198	no
ATF6	0.108008233	1.95256817	0.051734292	0.126415198	no
PLEKHA6	-0.107999544	-1.952409239	0.051753229	0.126447094	no
ZCCHC2	0.107984236	1.952129238	0.051786605	0.126503029	no
MYO5C	0.107983645	1.952118417	0.051787895	0.126503029	no
INPPL1	0.107960267	1.951690813	0.051838901	0.126613232	no
LOC653653	0.107953802	1.951572567	0.051853014	0.126633308	no
DRD1	-0.10792802	-1.951100986	0.051909328	0.126756432	no
MGC32805	-0.107923885	-1.95102536	0.051918363	0.126764093	no
PXDC1	0.107902996	1.950643276	0.051964034	0.126861191	no
WDR78	0.107898031	1.950552461	0.051974894	0.126873291	no
FRMPD2P1	-0.107853581	-1.94973945	0.052072204	0.127092492	no
RHPN1-AS1	0.107851611	1.949703424	0.05207652	0.127092492	no
AKAP13	0.10783377	1.949377109	0.052115622	0.12717348	no
LOC100130238	-0.107828075	-1.949272945	0.05212811	0.12718951	no
RFPL4A	0.107818803	1.949103348	0.052148446	0.127214664	no
PFKL	0.107815004	1.949033873	0.052156779	0.127214664	no
ERBB2	-0.107812763	-1.948992872	0.052161697	0.127214664	no
UBB	-0.107811114	-1.948962721	0.052165314	0.127214664	no
CCL17	0.107809684	1.94893657	0.052168452	0.127214664	no
GMNN	-0.107807185	-1.948890863	0.052173935	0.127214664	no
RNF145	0.107803478	1.948823061	0.052182071	0.127218579	no
HORMAD1	0.107801059	1.948778818	0.05218738	0.127218579	no
ATP6V1G2-DDX	-0.107793705	-1.948644303	0.052203525	0.127236409	no
TRIM3	-0.107792334	-1.948619224	0.052206536	0.127236409	no
MYO18B	-0.107769301	-1.948197952	0.052257127	0.127345269	no
PLAC8L1	-0.107736812	-1.947603734	0.052328559	0.127504882	no
DCLRE1B	0.10772639	1.947413115	0.052351491	0.127546298	no
SNORA51	-0.107715965	-1.947222454	0.052374436	0.127587736	no

ADAM19	0.107687117	1.946694837	0.052437977	0.127728048	no
PLEKHG2	0.107668079	1.946346652	0.052479944	0.127807336	no
SDCBP2-AS1	0.107666956	1.9463261	0.052482422	0.127807336	no
GEMIN2	-0.107660964	-1.946216517	0.052495637	0.127825032	no
LOC92249	-0.107627853	-1.945610947	0.052568713	0.127988468	no
MSS51	-0.107618954	-1.945448182	0.052588369	0.128021821	no
ROBO4	0.107603415	1.945164006	0.052622701	0.128090891	no
EEF2K	0.107562701	1.944419395	0.052712751	0.128295552	no
RANBP6	-0.107548799	-1.94416514	0.052743528	0.128355924	no
EXOSC8	-0.107522148	-1.943677737	0.052802571	0.12848506	no
C12orf44	0.107516049	1.943566188	0.052816092	0.12850341	no
ZNF331	-0.107509059	-1.943438354	0.05283159	0.128526567	no
UBR5	-0.107453898	-1.942429558	0.052954025	0.128797127	no
CACTIN-AS1	0.107453553	1.942423255	0.052954791	0.128797127	no
ERCC3	-0.10744551	-1.942276157	0.052972664	0.128826019	no
KDM1A	-0.107409337	-1.941614638	0.053053105	0.128982317	no
LOC100294362	-0.107408836	-1.941605464	0.053054221	0.128982317	no
TTC13	-0.107408513	-1.941599565	0.053054939	0.128982317	no
NDFIP2	-0.107386786	-1.941202222	0.053103306	0.129085301	no
IPO11	-0.107370908	-1.940911858	0.053138675	0.129156668	no
ST18	-0.107351776	-1.940561978	0.053181319	0.1292457	no
LINC00592	-0.107333325	-1.940224563	0.053222471	0.129331086	no
ZNF879	-0.107319043	-1.93996339	0.053254343	0.129393904	no
CINP	-0.107303157	-1.939672882	0.053289813	0.129465451	no
LOC148413	0.107282676	1.939298344	0.053335573	0.129561976	no
CETN2	0.107274337	1.939145846	0.053354214	0.129592611	no
LOC286437	-0.107269122	-1.939050472	0.053365875	0.129606287	no
NUDCD2	-0.107260294	-1.938889048	0.053385617	0.129639582	no
PRSS21	0.107255079	1.938793668	0.053397285	0.129653265	no
LINC00341	0.107247658	1.938657966	0.053413889	0.129678929	no
ZNF93	-0.107219447	-1.938142075	0.05347705	0.129817608	no
FAM129C	0.107214084	1.938044009	0.053489064	0.129830761	no
ZNF771	-0.107211635	-1.937999224	0.053494551	0.129830761	no
FAM212A	0.107199171	1.937771309	0.053522482	0.129883883	no
AFF4	-0.107195028	-1.937695544	0.05353177	0.129891755	no
HOOK1	-0.107190926	-1.937620538	0.053540966	0.129898793	no
NR2F2	0.107188343	1.937573293	0.05354676	0.129898793	no
HHLA3	0.107181599	1.937449978	0.053561883	0.129920816	no
GNA12	0.107178544	1.937394111	0.053568736	0.129922774	no
PRKRA	-0.107168152	-1.937204069	0.053592052	0.129964657	no
ZNF670-ZNF69	-0.107141375	-1.936714429	0.053652166	0.130095757	no
OTUD6A	-0.107131546	-1.936534682	0.053674248	0.130126398	no
MMP3	0.10713036	1.936513	0.053676912	0.130126398	no
MTIF2	0.107126077	1.936434676	0.053686537	0.130135051	no
SYNPR	-0.107114846	-1.936229305	0.053711781	0.130181559	no
TSLP	0.107090916	1.935791724	0.053765601	0.130297308	no
NACA	-0.107054259	-1.935121433	0.053848131	0.130482601	no
PITPNA-AS1	-0.107050192	-1.935047063	0.053857295	0.130490092	no
LIMD1-AS1	-0.107041265	-1.934883828	0.053877412	0.130524119	no
PCF11	-0.107032371	-1.934721201	0.053897461	0.130546975	no
USP46	-0.107031691	-1.934708754	0.053898995	0.130546975	no

SLC4A5	-0.107012851	-1.934364267	0.053941486	0.130635166	no
LOC440700	-0.107004977	-1.934220291	0.053959252	0.130663469	no
HCN2	-0.106995648	-1.934049698	0.05398031	0.130699735	no
SACM1L	0.106978946	1.933744308	0.054018024	0.130776316	no
C7orf55-LUC7	-0.106937564	-1.932987621	0.054111566	0.130988023	no
RPL7A	-0.106928488	-1.932821668	0.0541321	0.13102297	no
TUBA4A	-0.106922457	-1.932711401	0.054145746	0.131041243	no
ZNF813	0.106918829	1.932645057	0.054153959	0.131046361	no
GSTT2	-0.106889102	-1.932101516	0.054221279	0.131191118	no
MPP7	-0.106887025	-1.932063525	0.054225988	0.131191118	no
CHMP4B	0.106872991	1.931806931	0.054257795	0.131241173	no
LINC00222	-0.106872508	-1.931798092	0.054258891	0.131241173	no
COL16A1	0.106867879	1.931713456	0.054269386	0.131251786	no
NRXN3	-0.106819927	-1.930836689	0.054378207	0.131500175	no
MYO5A	-0.106792824	-1.930341119	0.054439797	0.131634302	no
CCDC62	-0.106759427	-1.929730502	0.054515765	0.131803161	no
WDR41	0.106687924	1.928423151	0.054678713	0.132182252	no
JPH2	0.106665511	1.928013378	0.054729871	0.132291043	no
ILDR1	0.106659973	1.927912119	0.054742519	0.132296655	no
BNIP3	-0.106659104	-1.927896224	0.054744505	0.132296655	no
FBXO44	-0.106648768	-1.927707249	0.054768116	0.132338833	no
ZNF277	-0.106628891	-1.927343835	0.054813547	0.132433719	no
BBS1	-0.106615248	-1.927094394	0.054844747	0.132494207	no
KCNMB3	0.106589214	1.926618432	0.054904324	0.132608973	no
C10orf32	-0.106589096	-1.926616271	0.054904595	0.132608973	no
PRR22	-0.106576342	-1.926383085	0.054933802	0.132664608	no
ANKRD54	-0.106557976	-1.9260473	0.054975884	0.132744551	no
ILDR2	-0.106556504	-1.926020385	0.054979259	0.132744551	no
DNAI1	0.106548502	1.925874103	0.0549976	0.13277392	no
ALOX5AP	0.106532084	1.925573928	0.055035255	0.132836969	no
BCAT2	0.106531725	1.92556736	0.055036079	0.132836969	no
APLN	0.106527666	1.925493161	0.05504539	0.132844523	no
CYP2D6	-0.106522777	-1.925403781	0.055056608	0.132856677	no
PBLD	-0.106519204	-1.925338456	0.055064808	0.132861546	no
NAB1	-0.106510479	-1.925178935	0.055084836	0.13289495	no
SLC9A7P1	0.106500175	1.924990557	0.055108496	0.132937106	no
LOC400548	0.106488724	1.924781207	0.055134799	0.13298563	no
ACADVL	0.106408398	1.923312678	0.055319604	0.133416407	no
DIABLO	-0.106396583	-1.923096689	0.055346828	0.133467088	no
MAK	0.10638766	1.922933545	0.0553674	0.133501715	no
ANKUB1	0.106349913	1.922243484	0.055454481	0.133696686	no
ATXN2	-0.106344333	-1.922141469	0.055467365	0.133712747	no
C2orf82	-0.106324469	-1.92177832	0.055513247	0.133808343	no
KIAA1257	0.106292143	1.921187373	0.055587978	0.133973449	no
UQCRH	-0.106280179	-1.920968648	0.05561566	0.134025134	no
TCHP	-0.106261837	-1.920633345	0.055658118	0.134112412	no
COX16	-0.106216171	-1.919798529	0.055763944	0.134352141	no
KLHL26	0.106213512	1.919749923	0.055770111	0.134352141	no
LOC154449	-0.106207734	-1.919644303	0.055783513	0.134354659	no
MROH6	0.106207447	1.919639049	0.05578418	0.134354659	no
MEMO1	0.106204977	1.919593888	0.055789911	0.134354659	no

C2orf50	0.10617858	1.919111343	0.055851182	0.134478273	no
FLJ44635	0.10617482	1.919042606	0.055859914	0.134478273	no
KCNG3	-0.106172327	-1.918997041	0.055865704	0.134478273	no
RBM33	-0.106172083	-1.918992579	0.055866271	0.134478273	no
PSMD10	-0.106154296	-1.918667419	0.055907599	0.134562684	no
SUCO	0.106133142	1.918280717	0.055956782	0.134665981	no
ZNF488	-0.106124008	-1.918113749	0.05597803	0.134702031	no
METTL18	0.106090062	1.917493214	0.056057055	0.134869895	no
CDC73	0.106086584	1.917429632	0.056065157	0.134869895	no
LINC00487	0.106085957	1.917418183	0.056066616	0.134869895	no
PTPRB	0.106034851	1.916483972	0.05618578	0.135132753	no
TBCE	-0.106033701	-1.916462951	0.056188464	0.135132753	no
C17orf80	-0.106024686	-1.916298159	0.056209507	0.135168235	no
ICK	-0.106020076	-1.9162139	0.056220269	0.135178989	no
MRPS18A	0.106001983	1.915883166	0.056262529	0.135256483	no
LOC100188947	-0.106000889	-1.915863161	0.056265086	0.135256483	no
CLCC1	0.105972117	1.915337231	0.056332344	0.135390379	no
BCYRN1	-0.105971672	-1.915329096	0.056333384	0.135390379	no
AFAP1-AS1	-0.105959977	-1.915115321	0.056360743	0.135440984	no
PRRG3	-0.105949185	-1.914918054	0.056385998	0.135486525	no
LOC254896	0.105938833	1.914728827	0.056410233	0.135529604	no
LOC100288911	-0.105887844	-1.91379681	0.056529726	0.135801513	no
ABTB2	-0.105847388	-1.913057331	0.056624684	0.136014429	no
UTP11L	0.105833937	1.912811459	0.056656287	0.136063898	no
LOC100505835	-0.105833233	-1.912798591	0.056657941	0.136063898	no
ADRB2	0.105820622	1.912568086	0.056687583	0.136119871	no
VPS11	-0.10580605	-1.912301738	0.05672185	0.136186937	no
ZMYM1	0.105801702	1.912222265	0.056732078	0.136196278	no
INADL	0.105784764	1.911912665	0.056771937	0.136276745	no
LRIF1	0.105774513	1.911725296	0.056796071	0.136305821	no
ATP6V0E2-AS1	-0.105771798	-1.911675661	0.056802466	0.136305821	no
TXNRD3NB	-0.105771538	-1.911670915	0.056803077	0.136305821	no
DIRC3	0.105762362	1.91150319	0.056824691	0.136342462	no
NEDD8	-0.10574479	-1.911182019	0.056866097	0.136426577	no
TMEM168	0.105732207	1.91095202	0.056895764	0.136482516	no
PDSS2	0.10572157	1.910757612	0.05692085	0.13651858	no
UBR4	-0.105717947	-1.910691391	0.056929398	0.13651858	no
LOC645355	-0.105717754	-1.91068785	0.056929855	0.13651858	no
MIPEPP3	0.105712681	1.91059513	0.056941824	0.136532048	no
NUMA1	0.105702133	1.910402332	0.05696672	0.136576504	no
XKR5	-0.105699388	-1.910352169	0.056973199	0.136576801	no
TMEM114	0.105660339	1.909638452	0.057065448	0.136772352	no
SYTL2	0.10565947	1.909622569	0.057067502	0.136772352	no
EFHC2	0.105652884	1.909502186	0.057083074	0.136794419	no
BMPR1A	-0.105624541	-1.90898416	0.057150125	0.136939831	no
CXCL2	0.105611955	1.908754117	0.057179922	0.136995954	no
UBE2L6	0.105606986	1.908663292	0.05719169	0.137008874	no
LOC100288198	-0.105576586	-1.908107687	0.057263722	0.137166144	no
COL4A3BP	0.105559588	1.90779701	0.057304033	0.137247405	no
INSM2	-0.10554595	-1.907547748	0.057336392	0.137309606	no
C3orf36	0.105487027	1.906470845	0.057476372	0.137629495	no

SELL	-0.105450072	-1.905795435	0.05756431	0.137815534	no
PMF1	-0.105448988	-1.905775626	0.057566891	0.137815534	no
ATP5I	-0.105399949	-1.904879397	0.057683757	0.138065575	no
RALB	0.105399771	1.904876127	0.057684184	0.138065575	no
LOC100288748	-0.105383545	-1.904579598	0.057722894	0.138142842	no
ASTE1	0.105377604	1.90447102	0.057737074	0.138161392	no
MIR5047	0.10535508	1.904059382	0.057790859	0.138274699	no
RPL19	-0.105344434	-1.903864805	0.057816297	0.138320164	no
CEP89	0.105337708	1.903741895	0.05783237	0.138343218	no
LOC100996455	0.105325603	1.903520662	0.057861311	0.138397044	no
BCAS3	-0.105317677	-1.903375808	0.057880267	0.138426977	no
TARS	-0.105301884	-1.903087194	0.057918051	0.138501928	no
ZNF280C	-0.105291628	-1.902899764	0.057942599	0.138545216	no
LOC284260	0.105272666	1.902553223	0.05798801	0.138638372	no
JTB	0.105261871	1.902355948	0.058013874	0.13868478	no
USP4	0.105243285	1.902016289	0.058058429	0.138775806	no
CCDC74B	0.105240601	1.901967226	0.058064867	0.138775806	no
FBXL12	0.105234509	1.901855907	0.058079476	0.138795288	no
LCN15	-0.105224483	-1.901672684	0.05810353	0.138837331	no
B3GNT9	0.105212884	1.901460718	0.058131366	0.138888404	no
NRIP3	-0.105186655	-1.900981385	0.058194357	0.139023446	no
DDX47	0.105180472	1.900868385	0.058209215	0.139043485	no
HAP1	0.105166759	1.900617786	0.058242176	0.139089196	no
PP12613	-0.105166436	-1.900611885	0.058242953	0.139089196	no
CCDC176	0.105164433	1.900575292	0.058247767	0.139089196	no
MAN2C1	-0.105160386	-1.900501331	0.058257499	0.13909698	no
TMCO5B	0.105155759	1.900416764	0.058268628	0.139108097	no
LIN54	-0.105152739	-1.900361589	0.05827589	0.139109981	no
EYA4	0.105119863	1.899760793	0.058355015	0.139283389	no
MOXD2P	0.105085484	1.899132548	0.058437851	0.139465615	no
KCTD2	-0.105077522	-1.898987046	0.05845705	0.139495944	no
GFAP	0.105060156	1.898669696	0.058498943	0.139580413	no
LPAL2	-0.105006462	-1.897688503	0.058628625	0.139864612	no
SLITRK2	-0.105005451	-1.897670034	0.058631068	0.139864612	no
ZNF581	0.104984511	1.897287396	0.058681708	0.139966415	no
LOC493754	0.104982419	1.897249162	0.05868677	0.139966415	no
ROBO2	-0.104969158	-1.897006837	0.058718861	0.140027412	no
BRWD1-IT2	-0.10495181	-1.896689838	0.058760863	0.140112028	no
RARA	-0.104943585	-1.896539529	0.058780788	0.140143988	no
ZNF833P	-0.104938047	-1.896438338	0.058794205	0.140160427	no
NEK7	0.104933727	1.896359394	0.058804673	0.140169836	no
FBXO32	0.104921223	1.896130908	0.058834982	0.140226528	no
PLEKHA1	-0.104917254	-1.896058379	0.058844606	0.140233913	no
SLC22A4	0.10488664	1.895498972	0.058918877	0.14039534	no
CYP1B1-AS1	0.104875724	1.895299507	0.058945378	0.140442917	no
IL1RL2	0.10485453	1.894912225	0.058996861	0.140549999	no
TVP23C	0.104845192	1.894741598	0.059019555	0.140588479	no
FLVCR2	0.104823021	1.894336481	0.059073467	0.140701304	no
ZNF251	-0.104806323	-1.894031368	0.059114097	0.140782476	no
KATNAL2	0.10479958	1.89390815	0.059130512	0.140805965	no
DIO3	0.104787086	1.893679853	0.059160935	0.140862803	no

PCDH1	-0.104776077	-1.893478703	0.059187752	0.140905138	no
PACS1	0.104774404	1.893448134	0.059191828	0.140905138	no
LOC644554	-0.104767358	-1.893319377	0.059209	0.140930404	no
TRIM17	-0.104744586	-1.892903291	0.05926452	0.141046933	no
SLC26A1	-0.104737057	-1.892765727	0.059282885	0.141075018	no
CSAG1	0.104692576	1.891952975	0.059391487	0.141317809	no
ATXN7L3	-0.104687016	-1.891851384	0.059405074	0.141333828	no
FAM127A	-0.104684439	-1.891804294	0.059411372	0.141333828	no
SERF2-C15ORF	0.104678542	1.891696547	0.059425786	0.14135247	no
LOC283914	0.10466454	1.891440703	0.059460023	0.141418256	no
LOC10050715€	0.104629368	1.890798066	0.059546094	0.141607293	no
MKS1	-0.10461681	-1.890568601	0.059576852	0.141664764	no
CORO7-PAM16	-0.104603497	-1.890325359	0.059609472	0.141726647	no
SF3A1	-0.104584876	-1.889985134	0.059655122	0.141819494	no
LOC729444	-0.104546661	-1.889286902	0.059748899	0.142026722	no
MRPL36	0.104542512	1.889211104	0.059759087	0.142035228	no
KAT8	-0.104536894	-1.889108455	0.059772886	0.142048412	no
XRCC6	-0.104534872	-1.889071506	0.059777853	0.142048412	no
HERPUD1	0.104519558	1.888791716	0.059815481	0.142122111	no
DNAJC27	-0.104511463	-1.888643804	0.05983538	0.142153677	no
TAS1R1	0.104486497	1.888187657	0.059896784	0.142283828	no
LCOR	-0.104482211	-1.888109354	0.05990733	0.142293152	no
ZNF326	-0.104451649	-1.887550977	0.059982578	0.142456138	no
ZNF607	-0.10441405	-1.886864024	0.060075262	0.142660491	no
CXorf31	-0.104383689	-1.886309339	0.060150186	0.142822633	no
GALK1	0.104378909	1.886221993	0.060161992	0.142834883	no
INF2	0.10437178	1.886091753	0.060179598	0.1428506	no
RAB25	-0.104370846	-1.886074688	0.060181905	0.1428506	no
ACY3	-0.104347838	-1.885654346	0.060238761	0.142969763	no
LOC647323	0.104316697	1.8850854	0.060315787	0.143136768	no
SNX25	-0.104299343	-1.884768356	0.060358745	0.143216256	no
LINC00115	-0.104297783	-1.884739843	0.06036261	0.143216256	no
YIPF4	0.104244219	1.883761268	0.060495371	0.143515401	no
NCOA5	-0.104238153	-1.883650443	0.060510422	0.143525605	no
KIAA1429	-0.104237102	-1.883631245	0.060513029	0.143525605	no
VAV3	0.104219541	1.883310424	0.060556618	0.14361314	no
MSRB2	-0.104213017	-1.883191241	0.060572817	0.143635707	no
NUP210	0.104201101	1.882973539	0.060602417	0.143690042	no
PEBP1	-0.104196347	-1.882886702	0.060614227	0.14370219	no
TP53BP2	-0.104191922	-1.882805857	0.060625224	0.143712408	no
PTPRH	0.104172035	1.882442542	0.060674664	0.143813743	no
CLU	0.104139748	1.88185271	0.060755	0.143988279	no
PKM	-0.10412254	-1.881538336	0.060797855	0.144073955	no
HEXDC	-0.104116869	-1.881434734	0.060811983	0.144091547	no
SPTSSB	0.104111223	1.881331601	0.06082605	0.144108989	no
NR1D2	-0.104101999	-1.881163087	0.060849041	0.144147568	no
TLCD2	0.104095526	1.881044845	0.060865177	0.144169901	no
TRIM7	-0.10407089	-1.880594784	0.060926628	0.144290182	no
GSDMB	-0.104069786	-1.880574606	0.060929385	0.144290182	no
PPP1R3G	0.104048275	1.880181646	0.060983083	0.144401437	no
ZNF815P	-0.104042868	-1.880082878	0.060996587	0.144417499	no

RGPD3	-0.104021765	-1.879697355	0.061049317	0.144526424	no
LOC100294145	0.104006554	1.879419482	0.061087347	0.144600527	no
SLC38A8	-0.103972018	-1.878788588	0.061173766	0.144789141	no
DCTN4	-0.103968467	-1.878723724	0.061182657	0.144794238	no
PKD1L1	0.103957629	1.878525739	0.0612098	0.144842526	no
FAM69A	-0.103952387	-1.878429978	0.061222933	0.144857651	no
NAB2	0.1039493	1.878373589	0.061230667	0.144860002	no
LINC00359	0.103936068	1.878131884	0.061263828	0.144922501	no
LINC00511	0.103928743	1.877998067	0.061282193	0.14494999	no
RSU1P2	0.103923821	1.877908154	0.061294536	0.144963229	no
C21orf7	0.103892425	1.877334632	0.061373313	0.145123846	no
KRT18P55	-0.103891372	-1.877315411	0.061375954	0.145123846	no
GPR97	0.103888004	1.877253885	0.061384411	0.145127874	no
AMACR	0.103859693	1.876736714	0.061455531	0.145272394	no
UQCRB	-0.103858289	-1.876711078	0.061459058	0.145272394	no
MIR548H3	0.103847068	1.876506104	0.061487265	0.14530841	no
LINC00260	-0.10383918	-1.876362008	0.061507102	0.14530841	no
MAP2K4	-0.103838838	-1.87635577	0.061507961	0.14530841	no
ITGB1BP2	-0.10383881	-1.876355252	0.061508032	0.14530841	no
ZNF816	0.103837071	1.876323497	0.061512404	0.14530841	no
FASTKD5	0.103836094	1.876305643	0.061514863	0.14530841	no
LPL	-0.103832282	-1.876236016	0.06152445	0.145315085	no
PPP2R5A	-0.103826605	-1.876132311	0.061538733	0.145332848	no
PNPLA6	-0.103796165	-1.875576265	0.061615362	0.145497828	no
B4GALT4	0.103782289	1.875322812	0.061650316	0.145564375	no
RBX1	-0.1037656	-1.875017955	0.061692382	0.145647696	no
HDC	0.103758606	1.874890195	0.061710018	0.14567333	no
RANGRF	0.103754179	1.874809335	0.061721182	0.145683682	no
KCTD14	0.103746616	1.874671193	0.061740259	0.145712706	no
CMTM8	0.103735609	1.874470136	0.061768033	0.145735273	no
PNLIPRP3	0.103735132	1.874461422	0.061769237	0.145735273	no
MPC2	0.103734765	1.874454708	0.061770164	0.145735273	no
CCDC30	0.10368174	1.873486151	0.061904109	0.146035259	no
ZNF592	-0.10367358	-1.873337099	0.061924744	0.146067903	no
LMBRD1	0.103647826	1.872866677	0.061989905	0.146181127	no
KCNS1	-0.10364736	-1.872858172	0.061991083	0.146181127	no
CA10	-0.103646525	-1.872842912	0.061993198	0.146181127	no
HIVEP2	-0.103642481	-1.872769047	0.062003435	0.146181127	no
FABP6	-0.103638256	-1.872691881	0.062014132	0.146181127	no
ATG16L2	0.10363702	1.872669304	0.062017261	0.146181127	no
TM4SF20	-0.103635797	-1.872646965	0.062020358	0.146181127	no
NUDC	-0.103610018	-1.872176101	0.062085665	0.146319006	no
GLRA2	-0.103607278	-1.872126058	0.062092609	0.146319326	no
ZNF133	-0.103599497	-1.871983928	0.062112334	0.146349762	no
NME6	0.103567387	1.871397432	0.062193787	0.146503258	no
ITPR2	0.103565679	1.871366226	0.062198124	0.146503258	no
SNORD8	-0.103563459	-1.871325686	0.062203758	0.146503258	no
LINC00462	0.103563069	1.871318562	0.062204748	0.146503258	no
RRM2B	0.103558373	1.871232786	0.062216669	0.14651528	no
CLMP	0.103531638	1.870744458	0.062284577	0.146659126	no
ZEB2	0.103518774	1.870509498	0.062317273	0.146718188	no

SUV420H2	-0.103516398	-1.870466097	0.062323314	0.146718188	no
CDK7	0.103505025	1.870258379	0.062352233	0.146770191	no
TRIM8	-0.103492172	-1.870023616	0.062384931	0.146831076	no
EYS	-0.103475217	-1.86971394	0.062428085	0.146916554	no
FREM3	-0.1034476	-1.869209524	0.062498429	0.147065995	no
DPP4	0.103444741	1.869157299	0.062505716	0.147067039	no
PSORS1C2	0.103433804	1.868957549	0.062533593	0.147116524	no
LOC100335030	-0.103422879	-1.868757999	0.062561453	0.147151213	no
CA12	0.103422652	1.86875385	0.062562032	0.147151213	no
NOP9	-0.103412522	-1.868568838	0.062587871	0.147195879	no
SUGP2	-0.103394816	-1.868245442	0.062633059	0.147282453	no
TONSL	-0.103392728	-1.868207313	0.062638389	0.147282453	no
ZSCAN26	-0.103389817	-1.868154154	0.06264582	0.147283811	no
STARD4-AS1	-0.103383369	-1.868036375	0.062662287	0.14730641	no
ANKRD26P3	-0.103359501	-1.867600455	0.062723264	0.147433629	no
SLC38A7	0.103344915	1.867334057	0.062760553	0.147505144	no
LINC00158	-0.103338803	-1.867222425	0.062776183	0.147525747	no
SAMD13	-0.103334658	-1.867146713	0.062786787	0.147534532	no
HIST2H2BE	0.10331119	1.866718104	0.06284684	0.147659499	no
GPR75	-0.103302559	-1.866560464	0.062868939	0.147695274	no
GAS1	-0.10328551	-1.866249096	0.062912608	0.147781709	no
LHX2	-0.103267721	-1.865924194	0.062958202	0.147872647	no
MED21	-0.103262431	-1.865827586	0.062971764	0.147888339	no
DOLPP1	-0.103255864	-1.865707648	0.062988605	0.147897416	no
ZNF416	0.103255557	1.86570204	0.062989393	0.147897416	no
SNX30	-0.103214817	-1.864957995	0.063093952	0.148126735	no
CCT6P3	-0.10320585	-1.864794219	0.063116987	0.148159182	no
CCDC85A	-0.103204069	-1.8647617	0.063121561	0.148159182	no
KHK	0.103193339	1.864565724	0.063149135	0.148207718	no
LDLRAP1	0.103186774	1.864445835	0.063166009	0.148231131	no
PIN4P1	-0.103180238	-1.864326473	0.063182812	0.148254374	no
RSBN1	-0.103173998	-1.864212513	0.063198858	0.148275836	no
FBXO8	0.103166741	1.864079969	0.063217525	0.148297838	no
NLGN4Y	-0.103164987	-1.864047939	0.063222037	0.148297838	no
UROC1	-0.103157793	-1.863916554	0.063240546	0.148325066	no
MFN1	0.103132794	1.86345999	0.063304901	0.148459803	no
C2orf88	-0.103109292	-1.863030797	0.063365448	0.148585174	no
ZNF605	-0.103106677	-1.862983039	0.063372188	0.148585174	no
PHKA2	0.10310073	1.862874422	0.06338752	0.148601545	no
SCAMP4	0.103098604	1.862835606	0.063393	0.148601545	no
FAM66E	-0.103088532	-1.862651651	0.063418975	0.14864622	no
NUMBL	0.10306675	1.862253863	0.063475174	0.148761718	no
LOC401074	-0.103044327	-1.861844367	0.06353307	0.148881169	no
INTS4	-0.103013017	-1.861272571	0.063613985	0.149054531	no
CCBP2	0.102956549	1.860241358	0.06376013	0.149380678	no
CRIM1	0.102942486	1.859984542	0.06379657	0.149449759	no
CACTIN	-0.102921094	-1.859593884	0.063852034	0.149563385	no
HTR1F	0.102875467	1.858760672	0.063970463	0.149822238	no
DNPH1	-0.102873148	-1.858718323	0.063976487	0.149822238	no
SLC14A2	-0.102865841	-1.858584886	0.063995471	0.149850368	no
SRGAP1	-0.102844848	-1.858201532	0.064050038	0.149961801	no

GLYCTK	0.102839767	1.858108747	0.064063251	0.149976398	no
SNRNP40	0.102827866	1.857891431	0.064094207	0.150032523	no
TRIL	-0.102799516	-1.857373719	0.064168002	0.150173275	no
EXTL2	0.102799397	1.857371547	0.064168311	0.150173275	no
TNIP3	0.102755808	1.85657559	0.064281906	0.150414083	no
DTYMK	0.102754543	1.856552486	0.064285206	0.150414083	no
NCKAP5	-0.102748867	-1.856448838	0.064300011	0.150428499	no
DKFZP434L187	-0.102746814	-1.856411349	0.064305366	0.150428499	no
OTOGL	-0.102740919	-1.856303705	0.064320746	0.150448101	no
FER1L5	0.102736029	1.856214417	0.064333505	0.15046157	no
LIN28A	-0.102716902	-1.855865146	0.064383437	0.150556653	no
ZNF259	0.102715089	1.85583204	0.064388172	0.150556653	no
CWF19L1	-0.102709858	-1.855736522	0.064401833	0.150572215	no
SEC14L5	-0.102702926	-1.855609942	0.064419941	0.150598168	no
BAG4	-0.102669412	-1.854997962	0.064507549	0.15078657	no
ROR1	0.102656932	1.854770078	0.064540196	0.150846477	no
ZNF680	-0.102643283	-1.854520843	0.064575919	0.150913556	no
LSM2	-0.102634161	-1.85435427	0.064599802	0.150952957	no
ORAI2	0.102629173	1.854263197	0.064612863	0.150962831	no
RPS6KA2	-0.102627183	-1.854226852	0.064618077	0.150962831	no
NDUFB1	-0.102597778	-1.853689933	0.064695129	0.151126415	no
TMEM30A	-0.102587628	-1.853504597	0.064721744	0.151172155	no
CDKL5	-0.10257713	-1.853312891	0.064749283	0.151220045	no
BRMS1	0.102567567	1.853138275	0.064774376	0.15126221	no
GBE1	0.102556889	1.852943306	0.064802403	0.151310952	no
NEGR1-IT1	0.102554251	1.852895131	0.06480933	0.151310952	no
PTDSS1	0.102537741	1.852593681	0.064852687	0.151395731	no
SLC7A5P2	-0.102507323	-1.85203826	0.064932636	0.151565905	no
AACSP1	-0.102456799	-1.851115748	0.065065606	0.151859789	no
LSM7	-0.102453514	-1.851055754	0.065074261	0.151863497	no
PCA3	-0.102447514	-1.850946215	0.065090067	0.151877066	no
RASGRP4	0.102445942	1.850917508	0.065094209	0.151877066	no
LOC643714	0.102408344	1.850231015	0.065193342	0.152091849	no
SNPH	-0.102401316	-1.850102679	0.065211888	0.152117544	no
LOC100507254	0.102397565	1.8500342	0.065221786	0.152117544	no
CDX1	0.102393531	1.84996054	0.065232434	0.152117544	no
EFNA4	0.102393442	1.849958915	0.065232669	0.152117544	no
TLR9	0.10237679	1.849654877	0.065276635	0.152203556	no
KIF27	-0.102363188	-1.849406533	0.065312566	0.152254421	no
ANGEL1	-0.102363168	-1.849406165	0.065312619	0.152254421	no
PCDHGA10	-0.102305939	-1.848361258	0.065463978	0.15259071	no
SCAND1	-0.102272988	-1.847759631	0.065551257	0.15277758	no
C19orf45	-0.102264418	-1.847603174	0.06557397	0.152813945	no
KRTDAP	-0.102254472	-1.847421576	0.065600342	0.152858826	no
SNAPC3	-0.102246664	-1.847279018	0.06562105	0.152890501	no
HELZ	-0.102233357	-1.847036066	0.065656354	0.152956174	no
ZNF439	-0.102195065	-1.846336937	0.065758034	0.15317179	no
PIGF	0.102192598	1.8462919	0.065764589	0.15317179	no
PPP4C	0.102190453	1.846252737	0.065770289	0.15317179	no
SLC39A4	-0.102182201	-1.846102085	0.06579222	0.153206262	no
IL23A	0.102172018	1.845916165	0.065819293	0.153248731	no

LRRC37A	-0.102169977	-1.845878912	0.065824719	0.153248731	no
SMIM14	0.102155159	1.845608367	0.065864135	0.153323887	no
CYTL1	0.102123379	1.845028148	0.065948733	0.153504193	no
AHSG	-0.10211418	-1.844860206	0.065973236	0.153544598	no
SDPR	-0.102106346	-1.844717192	0.065994108	0.153576543	no
MRPL12	-0.102086525	-1.844355318	0.066046946	0.153682863	no
C20orf196	-0.102074697	-1.84413938	0.066078492	0.153739621	no
TMCO3	0.102038629	1.843480894	0.066174767	0.15394695	no
GOLGA8A	-0.102021579	-1.843169615	0.066220319	0.154036245	no
PRSS27	-0.102016198	-1.84307137	0.066234701	0.154053026	no
LINC00457	0.10201122	1.842980501	0.066248006	0.154067297	no
GADD45A	0.101994927	1.842683048	0.066291573	0.154145023	no
MIR497	0.101993357	1.842654385	0.066295773	0.154145023	no
HSD17B6	-0.10197559	-1.842330018	0.066343312	0.15423887	no
TMEM150C	0.101953047	1.84191848	0.066403667	0.154362489	no
AP2M1	0.101933228	1.841556661	0.066456768	0.15446922	no
FOCAD	0.101917786	1.841274757	0.066498166	0.154548727	no
ARSI	0.101879626	1.840578109	0.066600559	0.154767405	no
SMTNL1	0.101877354	1.840536626	0.06660666	0.154767405	no
UNC5D	-0.101855793	-1.840143024	0.066664573	0.154885226	no
ACAN	0.101850862	1.840053013	0.066677823	0.154899265	no
PLG	-0.101811697	-1.839338024	0.066783149	0.155126265	no
MLLT3	-0.10180916	-1.839291722	0.066789974	0.155126265	no
DHODH	-0.101802797	-1.839175568	0.066807099	0.155149273	no
MATK	-0.101799302	-1.839111766	0.066816507	0.155154356	no
TIGD5	-0.101784551	-1.838842476	0.066856229	0.15522982	no
TMEM136	-0.101778264	-1.838727702	0.066873164	0.15524732	no
FMOD	0.101773831	1.838646777	0.066885107	0.15524732	no
PYCR2	0.101773708	1.83864454	0.066885438	0.15524732	no
ANKRD62P1-PA	-0.101758079	-1.838359223	0.066927559	0.155328313	no
VCX	0.101746626	1.83815015	0.066958439	0.1553832	no
AK5	-0.101730535	-1.83785642	0.067001843	0.155464456	no
NUPR1L	-0.10172538	-1.837762312	0.067015753	0.155464456	no
ARL13B	-0.101725191	-1.837758853	0.067016265	0.155464456	no
SIRT4	-0.101722921	-1.837717422	0.06702239	0.155464456	no
SKIL	0.101716553	1.837601181	0.067039577	0.155487541	no
LOC100288123	-0.101668761	-1.836728745	0.067168691	0.155770189	no
SLC35D3	-0.101643355	-1.836264978	0.067237409	0.155912727	no
ADPGK-AS1	0.101632504	1.836066899	0.067266776	0.155963997	no
LPPR1	-0.101612959	-1.835710124	0.067319699	0.156069866	no
JMJD7-PLA2G4	-0.101595074	-1.835383649	0.067368158	0.156165363	no
GTF2F1	-0.101569639	-1.834919353	0.067437123	0.15630837	no
NFX1	-0.10154357	-1.834443502	0.067507865	0.156455464	no
SPATA5L1	-0.101537249	-1.834328104	0.067525029	0.15647837	no
SPEF2	0.101526013	1.834123013	0.067555544	0.156525804	no
NRN1	-0.101524349	-1.834092636	0.067560065	0.156525804	no
MSH2	-0.101497702	-1.833606227	0.067632486	0.156676701	no
LSM12	0.101480991	1.833301194	0.067677935	0.156765089	no
RNF182	-0.101475671	-1.833204091	0.067692408	0.156776628	no
TIGD7	-0.101473797	-1.833169891	0.067697506	0.156776628	no
WNT9A	0.101423437	1.832250643	0.067834657	0.157071803	no

COX5A	-0.101421629	-1.832217654	0.067839583	0.157071803	no
CA7	-0.101418614	-1.832162614	0.067847802	0.157073912	no
LOC100288181	-0.10137648	-1.831393548	0.06796274	0.157323056	no
C5orf27	-0.101373239	-1.831334396	0.067971587	0.15732659	no
CTAGE7P	-0.101360385	-1.831099768	0.068006689	0.157390884	no
TSACC	0.101350265	1.830915042	0.068034335	0.157437913	no
SMC2	-0.101342459	-1.830772561	0.068055665	0.157470317	no
SLC9A9-AS1	0.101329155	1.830529726	0.068092032	0.157537502	no
TTPAL	-0.101320559	-1.83037283	0.068115537	0.157574919	no
FAM122C	0.101296916	1.829941291	0.068180221	0.15770758	no
FAM136A	0.101272978	1.829504376	0.068245763	0.157842197	no
RASGRP2	-0.101225381	-1.828635615	0.068376241	0.158126955	no
CLCN2	-0.101190195	-1.82799341	0.068472825	0.158333279	no
XPR1	0.101180828	1.827822452	0.068498556	0.158375735	no
OR10V2P	0.101156104	1.827371189	0.068566512	0.158515803	no
ARHGEF15	0.101112516	1.826575641	0.06868645	0.158776002	no
UBE2G2	-0.101107244	-1.826479413	0.068700969	0.158792485	no
ATXN7	-0.101101063	-1.826366605	0.068717993	0.158814753	no
HSPB9	-0.1010953	-1.826261436	0.068733867	0.158823813	no
REP15	0.101094275	1.826242711	0.068736694	0.158823813	no
SPIN2A	-0.101061683	-1.825647877	0.068826539	0.159014315	no
GIGYF2	-0.101058555	-1.825590789	0.068835167	0.159017153	no
HEATR5B	-0.101038423	-1.825223362	0.068890718	0.159128378	no
SPDYE3	0.101024981	1.824978026	0.068927831	0.159187368	no
ZFP69	-0.101023808	-1.824956614	0.068931071	0.159187368	no
RBM10	-0.100991417	-1.824365451	0.06902057	0.159376928	no
C15orf53	0.100980569	1.824167483	0.069050563	0.159428251	no
POP7	-0.100978013	-1.824120828	0.069057633	0.159428251	no
ANKRD7	0.100954443	1.823690655	0.069122848	0.159561667	no
PIGQ	0.100949649	1.823603172	0.069136117	0.159575156	no
TCEB3	0.100918105	1.823027482	0.069223486	0.159759657	no
RCBTB2	0.100862774	1.822017683	0.069376957	0.160083164	no
DKK1	0.100862197	1.822007158	0.069378558	0.160083164	no
ZNF330	0.100854113	1.821859619	0.069401005	0.160117767	no
RAB13	0.100843053	1.821657766	0.069431725	0.160171448	no
LRRC47	-0.100838384	-1.821572566	0.069444696	0.160184175	no
LANCL2	0.100819965	1.821236419	0.069495887	0.160285052	no
PLEKHH1	-0.100810255	-1.821059216	0.069522886	0.160327489	no
ZDHHC23	0.100807982	1.821017735	0.069529207	0.160327489	no
GTF3C4	-0.100778263	-1.820475385	0.0696119	0.160500949	no
CDK2AP1	-0.100771624	-1.820354222	0.069630385	0.160526346	no
WBP1L	-0.100757208	-1.820091129	0.069670537	0.160601685	no
ZNF263	-0.100732915	-1.819647797	0.069738239	0.160740508	no
GTF2IRD2B	-0.100727395	-1.819547074	0.069753629	0.160758738	no
LMCD1-AS1	-0.100719395	-1.81940108	0.06977594	0.160792915	no
ZNF70	-0.100709751	-1.819225075	0.069802845	0.160837669	no
MTMR8	0.100670882	1.818515757	0.069911363	0.161070443	no
LOC286083	-0.100640071	-1.817953499	0.069997481	0.161251565	no
RTKN2	-0.100637078	-1.817898871	0.070005852	0.161253566	no
DUSP28	-0.100623694	-1.817654636	0.070043291	0.161322513	no
LOC100130301	-0.100620117	-1.817589366	0.070053299	0.161328274	no

MYO1D	0.100601828	1.817255602	0.070104495	0.161428876	no
LOC100506124	-0.100586609	-1.81697788	0.070147118	0.161509719	no
HCFC2	-0.100576853	-1.816799863	0.07017445	0.161555341	no
LOC100506123	-0.10049509	-1.815307833	0.070403877	0.162048462	no
DIO2	0.100492805	1.815266129	0.070410299	0.162048462	no
SIGLEC16	0.100490197	1.815218542	0.070417627	0.162048462	no
XPA	-0.100489777	-1.815210878	0.070418807	0.162048462	no
LYNX1	-0.100477347	-1.814984061	0.070453745	0.162111502	no
ZBED2	0.100465057	1.814759787	0.070488304	0.162173659	no
FAM127C	0.100453714	1.8145528	0.070520213	0.162229704	no
EXOSC10	0.100448695	1.814461227	0.070534333	0.16224482	no
LINC00307	0.100434588	1.814203803	0.07057404	0.162318781	no
SLC35G2	0.100429181	1.814105146	0.070589262	0.162336418	no
NMRK2	-0.100422134	-1.813976548	0.070609108	0.162364685	no
PLA2G7	0.100411852	1.813788925	0.070638072	0.162396593	no
ZNF773	0.100411843	1.813788757	0.070638098	0.162396593	no
HNF1A-AS1	-0.10040185	-1.813606415	0.070666255	0.162443951	no
EDF1	-0.100397144	-1.813520545	0.070679519	0.162457063	no
VSX2	-0.1003909	-1.813406607	0.070697121	0.162480144	no
NDEL1	0.100362491	1.812888219	0.070777251	0.162645205	no
MMP28	-0.100360072	-1.81284408	0.070784077	0.162645205	no
KEAP1	-0.10035009	-1.812661931	0.070812253	0.162692552	no
ABCF1	-0.100346258	-1.812592006	0.070823072	0.162700015	no
SLC8A1-AS1	0.100342351	1.812520724	0.070834102	0.162707962	no
SCML1	0.100322499	1.812158487	0.070890177	0.162819364	no
COL9A1	-0.100296687	-1.811687488	0.070963142	0.162969533	no
FBXL8	0.100260825	1.811033135	0.071064615	0.163185131	no
ODF2	0.100245207	1.810748147	0.071108847	0.163269254	no
LOC649352	-0.100238113	-1.810618719	0.071128942	0.163296579	no
DMXL2	-0.100235642	-1.810573626	0.071135944	0.163296579	no
BHLHA9	-0.100219352	-1.810276395	0.071182114	0.163369518	no
UST	-0.100218629	-1.810263206	0.071184164	0.163369518	no
WBP11	-0.100216386	-1.81022228	0.071190523	0.163369518	no
ASCC3	0.100209722	1.810100678	0.071209421	0.163390362	no
ARL9	0.100207821	1.810065994	0.071214812	0.163390362	no
KCNMB2	-0.100194833	-1.809829006	0.071251656	0.163457444	no
ATP13A2	-0.100191941	-1.809776249	0.07125986	0.163458817	no
LOC100289137	-0.100159528	-1.809184835	0.071351883	0.163652436	no
CRIP1	0.100126683	1.808585538	0.071445233	0.163849055	no
PRRC2A	-0.10011432	-1.808359967	0.071480395	0.163912203	no
EFHD2	0.100099131	1.808082821	0.071523616	0.163993815	no
PLEKHF2	0.100094567	1.807999548	0.071536607	0.164006104	no
PPP2CA	-0.100062082	-1.807406841	0.071629126	0.164194769	no
LRRC37A11P	0.100060308	1.807374485	0.071634179	0.164194769	no
TM4SF19	0.100035957	1.806930185	0.071703602	0.164336366	no
SFMBT1	0.100031098	1.806841527	0.071717461	0.164350603	no
SCARNA3	-0.100023841	-1.806709125	0.071738163	0.164380516	no
LOC401127	-0.100018082	-1.806604056	0.071754595	0.164385304	no
LRRC34	0.100017747	1.806597943	0.071755551	0.164385304	no
MRPL34	0.100011856	1.806490455	0.071772364	0.164406297	no
ALG10B	-0.100007853	-1.806417411	0.071783792	0.164414949	no

PRRC1	0.100003847	1.806344329	0.071795226	0.164423616	no
GUSBP1	-0.099984351	-1.805988619	0.071850905	0.164533595	no
ANKS6	-0.099978477	-1.805881446	0.071867687	0.164554492	no
RAB11A	0.099962068	1.805582062	0.071914585	0.164644333	no
CDON	-0.099954637	-1.805446491	0.071935831	0.164668958	no
C8orf37	-0.099952946	-1.805415641	0.071940666	0.164668958	no
RPL14	-0.099920999	-1.804832762	0.072032073	0.164860626	no
C1orf227	0.099901166	1.804470913	0.072088866	0.16497304	no
ANKRD18A	-0.09988743	-1.804220305	0.072128221	0.165045528	no
SYMPK	-0.099855123	-1.803630887	0.072220852	0.16522412	no
PPAP2A	0.099853953	1.803609542	0.072224209	0.16522412	no
TBC1D28	-0.099852167	-1.803576953	0.072229333	0.16522412	no
HHLA2	0.09983285	1.803224536	0.07228477	0.165333333	no
RBMX2	-0.09981573	-1.802912186	0.072333934	0.165428176	no
ZNF16	-0.099795246	-1.802538476	0.072392791	0.165545167	no
SPC24	-0.099789617	-1.802435783	0.072408972	0.165564551	no
CD24	-0.099764549	-1.801978444	0.072481067	0.165711768	no
RAP1GDS1	-0.099736825	-1.801472648	0.07256087	0.165876574	no
PEX13	0.09972986	1.801345582	0.07258093	0.165904783	no
PDCL2	0.099726172	1.801278292	0.072591555	0.165911422	no
DNAJC25	0.099720857	1.80118134	0.072606865	0.165928768	no
SCNN1D	-0.09971688	-1.801108774	0.072618326	0.165937315	no
KLHDC10	-0.099702987	-1.800855322	0.072658368	0.166011163	no
LOC100288122	-0.099694685	-1.800703865	0.072682305	0.166048201	no
HSF4	-0.099691576	-1.80064714	0.072691272	0.166051034	no
LGALS8	0.099681224	1.800458293	0.07272113	0.166069344	no
ZNF862	-0.099679041	-1.800418466	0.072727429	0.166069344	no
LOC653160	0.099678919	1.800416243	0.07272778	0.166069344	no
RGS7	-0.099678082	-1.80040096	0.072730197	0.166069344	no
NCEH1	0.099637879	1.79966754	0.072846265	0.166316698	no
ART3	-0.099560446	-1.798254925	0.073070248	0.166810357	no
HDAC2	-0.099508602	-1.797309156	0.073220524	0.167135665	no
ATP6V1E2	-0.099502389	-1.797195818	0.07323855	0.167155824	no
DHCR7	-0.099500197	-1.797155825	0.073244911	0.167155824	no
MEX3D	-0.099494505	-1.797051981	0.073261431	0.167171647	no
TNKS2	-0.099492447	-1.797014453	0.073267402	0.167171647	no
FAM90A25P	0.09947287	1.796657318	0.073324245	0.167283582	no
HSPB3	-0.099466398	-1.79653926	0.073343043	0.167308708	no
GOLT1B	0.099452992	1.796294705	0.073381996	0.1673798	no
CCDC113	-0.09943565	-1.795978335	0.073432413	0.167477023	no
SPATA31D4	0.099432203	1.795915456	0.073442437	0.16748211	no
KRT81	0.099428987	1.795856799	0.073451788	0.167485664	no
OGFR	0.099424694	1.795778489	0.073464275	0.167496365	no
PLCD3	-0.099417182	-1.795641456	0.073486129	0.167528419	no
TXNL4A	-0.099412112	-1.795548963	0.073500883	0.167544281	no
FLJ42875	-0.099400586	-1.795338707	0.073534431	0.167602976	no
SOX9	-0.099395647	-1.795248613	0.07354881	0.167617973	no
UBAP2L	-0.099387514	-1.795100247	0.073572495	0.167654171	no
CBLN4	-0.099363206	-1.794656829	0.073643317	0.167797765	no
TNRC6A	-0.099344419	-1.794314122	0.073698092	0.167904769	no
ATP6V1C1	-0.099341103	-1.794253632	0.073707763	0.167909004	no

TUBA3D	-0.099311927	-1.793721424	0.073792902	0.168085136	no
ZG16B	0.099307964	1.793649123	0.073804474	0.168093679	no
GPANK1	-0.099296001	-1.793430913	0.07383941	0.168155426	no
PIK3R5	0.099291565	1.793349981	0.07385237	0.168167122	no
RPSAP52	0.099272478	1.793001821	0.073908147	0.168276299	no
ZRANB2-AS1	0.09926878	1.792934357	0.073918959	0.168283088	no
CPSF7	-0.099259037	-1.79275663	0.073947448	0.168330115	no
ARHGEF33	-0.099252308	-1.792633902	0.073967127	0.168357077	no
XKRX	0.099247787	1.792551433	0.073980352	0.168369348	no
EIF1AX	-0.099224633	-1.792129082	0.074048115	0.168505723	no
SPO11	0.099220848	1.792060026	0.0740592	0.168513103	no
KRTAP5-7	-0.099213628	-1.791928332	0.074080342	0.168543365	no
FBXO25	-0.099201969	-1.79171566	0.074114495	0.168603219	no
FLJ41278	-0.099188497	-1.791469919	0.074153975	0.168675177	no
USP43	-0.099182709	-1.791364351	0.07417094	0.168686032	no
RBM18	-0.099181514	-1.791342543	0.074174445	0.168686032	no
MSMP	0.099151062	1.790787092	0.074263766	0.168871293	no
SPSB4	-0.099139289	-1.79057235	0.074298321	0.168931996	no
BRWD1-AS1	-0.09913605	-1.790513268	0.074307831	0.168935745	no
SNX16	-0.099129125	-1.79038694	0.074328168	0.168964105	no
ATPBD4-AS1	0.099118988	1.790202042	0.074357941	0.16901391	no
FKBP1B	-0.0991116	-1.790067283	0.074379647	0.169043476	no
ABCC12	-0.099109206	-1.790023624	0.074386681	0.169043476	no
RRNAD1	-0.099092865	-1.789725547	0.074434715	0.16913475	no
ZNF677	-0.099090017	-1.789673612	0.074443087	0.16913589	no
PARN	0.099077853	1.789451724	0.074478864	0.169189688	no
MOXD1	0.099076613	1.789429108	0.074482511	0.169189688	no
PTGER3	-0.099049408	-1.788932901	0.074562572	0.16935365	no
KIR3DL1	0.099026442	1.788513998	0.074630216	0.16948717	no
ING3	-0.099024095	-1.788471193	0.074637131	0.16948717	no
ATP6V1F	0.09901947	1.788386831	0.07465076	0.16950021	no
KRT36	0.099005237	1.788127215	0.074692717	0.169561834	no
TIPRL	-0.09900491	-1.788121256	0.07469368	0.169561834	no
PPP1R1B	-0.098953658	-1.787186441	0.074844919	0.169887216	no
PGRMC2	-0.098925792	-1.786678188	0.074927252	0.170056139	no
CYP4F24P	-0.098919742	-1.786567834	0.074945139	0.170078772	no
SNORA44	0.098911346	1.7864147	0.074969964	0.170117148	no
TRAPPC6A	-0.098907188	-1.786338865	0.074982261	0.170127088	no
RPL5	-0.098870043	-1.785661369	0.075092192	0.170358524	no
OBSCN	-0.09886587	-1.785585259	0.07510455	0.170368575	no
G2E3	-0.09884835	-1.785265715	0.075156452	0.170455641	no
ZEB1-AS1	-0.09884756	-1.785251296	0.075158795	0.170455641	no
OR51E2	0.098795571	1.7843031	0.075312983	0.170787309	no
WBSCR16	0.098783371	1.784080583	0.075349205	0.170851421	no
HYAL2	0.098780276	1.784024134	0.075358396	0.170854235	no
ZNF451	-0.098777228	-1.783968541	0.075367449	0.170856734	no
CXCL3	0.098761814	1.783687406	0.075413242	0.170937349	no
DLGAP1	-0.098759904	-1.783652576	0.075418917	0.170937349	no
MDM1	0.098749499	1.783462818	0.075449841	0.170989405	no
LOC284757	0.098731518	1.783134872	0.075503309	0.171092535	no
PDE12	0.098726167	1.783037266	0.075519228	0.171110568	no

FAM65B	-0.098712298	-1.782784323	0.075560496	0.171186024	no
ULK1	-0.09869687	-1.782502947	0.075606425	0.171272022	no
SEMA4A	-0.098690709	-1.782390591	0.075624771	0.171293783	no
PBOV1	-0.098688291	-1.782346496	0.075631972	0.171293783	no
MIR1250	-0.098679752	-1.782190752	0.075657411	0.171333342	no
GPR25	0.098675079	1.782105521	0.075671335	0.171346819	no
TMEM161B	-0.098659829	-1.781827396	0.075716787	0.171431677	no
THBS3	0.098625395	1.781199401	0.075819499	0.171629938	no
OSGEP	-0.098625118	-1.781194348	0.075820326	0.171629938	no
WDR11	0.098609354	1.780906855	0.075867386	0.171714735	no
PPIE	-0.098607217	-1.780867884	0.075873767	0.171714735	no
CHRNE	0.098601799	1.780769071	0.075889948	0.171733271	no
SFR1	-0.098588552	-1.780527472	0.075929523	0.171796059	no
NPY	-0.09858716	-1.78050209	0.075933682	0.171796059	no
VOPP1	0.098567754	1.780148174	0.075991689	0.171894844	no
ETFDH	0.098567201	1.78013809	0.075993342	0.171894844	no
PWWP2B	-0.098546271	-1.779756383	0.076055946	0.172018346	no
ZSCAN16	-0.098531994	-1.779496009	0.076098674	0.172096874	no
CAMK2B	-0.098513796	-1.779164138	0.076153163	0.17220198	no
LOC100287036	0.09850287	1.77896488	0.076185894	0.172257869	no
ZNF444	-0.098482964	-1.778601845	0.076245558	0.172374635	no
SS18L2	-0.098473976	-1.778437944	0.076272507	0.17239996	no
GMPR	0.098473877	1.77843614	0.076272803	0.17239996	no
ASAP1-IT1	-0.098470532	-1.778375133	0.076282836	0.172404505	no
PDLIM3	0.09846351	1.778247068	0.076303901	0.172433979	no
LINC00662	-0.098431369	-1.777660922	0.076400374	0.172633838	no
AUH	-0.098422172	-1.777493213	0.076427995	0.172660078	no
FAM110D	0.098422153	1.77749285	0.076428055	0.172660078	no
CLCNKB	0.098417599	1.777409812	0.076441734	0.17267283	no
LTA	0.09841048	1.77727999	0.076463124	0.172702995	no
TXNL1	-0.098405277	-1.777185109	0.07647876	0.172720159	no
ZNF565	-0.098398727	-1.777065656	0.076498449	0.172746472	no
IMP3	-0.098394751	-1.776993149	0.076510403	0.172755312	no
PCDHGB7	-0.098386841	-1.776848893	0.076534189	0.172790866	no
PDIA6	0.09836401	1.776432545	0.076602874	0.172927769	no
EREG	0.098359451	1.776349394	0.076616597	0.172940583	no
FA2H	-0.098330782	-1.775826585	0.076702929	0.17311727	no
AGAP7	0.098310522	1.775457125	0.076763986	0.173236882	no
NCOA6	-0.098294649	-1.77516768	0.076811848	0.173326693	no
TNNI3K	-0.09828048	-1.774909293	0.076854595	0.173404945	no
BTBD3	-0.098272963	-1.774772209	0.076877281	0.173437923	no
GLI1	0.098268167	1.774684749	0.076891758	0.173447276	no
PPWD1	0.098264291	1.774614076	0.076903458	0.173447276	no
ADAMTS8	-0.098263569	-1.774600903	0.076905639	0.173447276	no
KANK4	-0.098190068	-1.773260577	0.077127812	0.173930095	no
RAD21-AS1	-0.098172003	-1.772931149	0.077182498	0.174035156	no
SPRR2B	0.098159539	1.772703884	0.077220244	0.174096115	no
SORBS1	-0.098157191	-1.772661052	0.077227359	0.174096115	no
CADM1	0.098155053	1.772622071	0.077233836	0.174096115	no
LAMB4	0.098149651	1.772523575	0.077250201	0.174114743	no
C1orf56	-0.098139662	-1.772341414	0.077280476	0.174164714	no

SNORA49	-0.098088467	-1.771407888	0.077435779	0.174496417	no
SMIM2-IT1	-0.09803635	-1.770457548	0.077594141	0.174834944	no
C7orf61	-0.098014998	-1.770068208	0.077659097	0.174953652	no
IDE	0.09801368	1.770044176	0.077663107	0.174953652	no
LOC100506136	-0.097991944	-1.769647843	0.077729278	0.17507586	no
LAMC2	0.097989281	1.769599279	0.077737389	0.17507586	no
ARMC4	0.097987834	1.769572896	0.077741796	0.17507586	no
SLC5A6	0.097978258	1.769398292	0.077770966	0.1751232	no
MALSU1	0.09795856	1.769039108	0.077831	0.17523354	no
TNS3	0.09795683	1.769007561	0.077836274	0.17523354	no
GTF3C1	0.097947841	1.768843663	0.077863682	0.175276883	no
GCNT2	-0.097930229	-1.768522523	0.077917409	0.17537152	no
URB1	0.097925172	1.768430321	0.077932839	0.17537152	no
TAL2	-0.09792485	-1.768424449	0.077933822	0.17537152	no
SPRED3	0.097923362	1.768397312	0.077938364	0.17537152	no
HOTTIP	-0.097865803	-1.767347798	0.078114196	0.175748763	no
ST6GAL1	0.097858588	1.767216241	0.07813626	0.175780001	no
MRC2	0.097843121	1.76693423	0.078183573	0.175868031	no
TMEM211	-0.097838378	-1.766847743	0.078198088	0.175882271	no
ZC4H2	-0.097819084	-1.766495958	0.078257149	0.175996691	no
LIN9	-0.097813138	-1.766387534	0.078275359	0.176019226	no
UTP14A	0.097792404	1.766009492	0.078338882	0.176143639	no
HOXA13	-0.097749819	-1.765233045	0.07846948	0.176409812	no
PI16	-0.097746643	-1.765175128	0.078479229	0.176409812	no
DOCK6	0.097745777	1.765159342	0.078481886	0.176409812	no
CNKSR1	-0.09773692	-1.764997852	0.078509074	0.176452469	no
RING1	-0.097730378	-1.764878582	0.078529159	0.176479156	no
ALDH1B1	-0.097708473	-1.764479178	0.07859645	0.176608765	no
MRPS7	-0.097706254	-1.764438725	0.078603268	0.176608765	no
41883	-0.097678989	-1.763941613	0.078687092	0.176778623	no
CSNK2A1	-0.097661032	-1.763614217	0.078742338	0.176882277	no
GALNT14	-0.097658643	-1.763570654	0.078749692	0.176882277	no
TRRAP	-0.097641664	-1.763261084	0.078801963	0.176981188	no
RSAD1	-0.097630149	-1.76305115	0.078837427	0.177042335	no
ANKRD50	-0.097592679	-1.762367988	0.078952923	0.177279887	no
HIST1H2BD	0.097590479	1.76232788	0.078959708	0.177279887	no
CAP1	0.09757923	1.76212279	0.078994409	0.177339272	no
DUSP14	0.097566461	1.761889976	0.079033818	0.177392439	no
PRSS54	-0.097566207	-1.76188535	0.079034601	0.177392439	no
GGA1	-0.097559874	-1.761769879	0.079054152	0.177417794	no
ZPLD1	0.097548679	1.761565784	0.07908872	0.177476838	no
DIAPH2	0.097528637	1.761200386	0.079150638	0.177597239	no
FAM92A1P2	-0.097522107	-1.761081336	0.07917082	0.1776217	no
HIST1H2AG	0.097519762	1.761038578	0.079178069	0.1776217	no
YWHAG	-0.097499661	-1.760672094	0.079240229	0.17774259	no
CDIPT	0.097490747	1.76050959	0.079267804	0.177785887	no
MGC16025	-0.09748769	-1.760453843	0.079277265	0.177788553	no
CCL11	0.097467273	1.760081618	0.079340463	0.177911717	no
COL18A1-AS2	-0.097433271	-1.759461716	0.079445804	0.178129345	no
ZNF322	-0.097403024	-1.758910282	0.079539606	0.17832106	no
HMGN3	-0.097399588	-1.758847647	0.079550267	0.178326356	no

ZFYVE28	0.097388629	1.758647846	0.07958428	0.178383996	no
GUSBP3	-0.09738589	-1.75859792	0.079592781	0.178384446	no
LOC554206	-0.097374401	-1.758388467	0.079628453	0.178445786	no
PGAP2	-0.097362714	-1.758175397	0.079664755	0.17849094	no
GTF2IRD1P1	0.09736243	1.758170225	0.079665636	0.17849094	no
C20orf96	0.097359893	1.758123972	0.079673518	0.17849094	no
TRDN	0.097311746	1.757246214	0.079823222	0.178801145	no
URGCP	-0.09731001	-1.757214559	0.079828625	0.178801145	no
LOC158572	0.097293308	1.756910076	0.079880612	0.178898941	no
MGC27345	-0.097277544	-1.756622703	0.079929703	0.178990231	no
RESP18	-0.097253456	-1.756183565	0.080004767	0.179139659	no
DMRTB1	-0.097219406	-1.755562834	0.080110969	0.179341262	no
TTC30B	0.097219238	1.755559769	0.080111494	0.179341262	no
ACP6	0.097203003	1.755263804	0.080162172	0.179436022	no
RSPO2	-0.097196664	-1.755148238	0.080181968	0.17946164	no
PIGH	-0.097178644	-1.754819732	0.08023826	0.179553881	no
YTHDF1	0.097177571	1.754800181	0.080241611	0.179553881	no
SH3RF1	-0.097175448	-1.754761484	0.080248245	0.179553881	no
MIR5193	0.097154654	1.754382406	0.080313249	0.179664815	no
CYP21A1P	0.09715424	1.754374855	0.080314545	0.179664815	no
PNKD	-0.097121688	-1.753781447	0.080416391	0.179873923	no
RORB	-0.097118792	-1.753728657	0.080425456	0.179875479	no
ATP6V1C2	-0.097099924	-1.753384703	0.080484542	0.179988897	no
CDC45	-0.097096384	-1.753320177	0.080495631	0.179994964	no
GADD45GIP1	-0.097091681	-1.75323444	0.080510366	0.180009185	no
NOXA1	-0.097087264	-1.753153915	0.080524208	0.180021404	no
ZCCHC16	-0.097072095	-1.752877409	0.080571753	0.18010144	no
SH3BGR	0.097070495	1.752848242	0.080576769	0.18010144	no
ABCE1	-0.097063945	-1.752728836	0.080597309	0.180128615	no
S100P	0.097058638	1.752632093	0.080613953	0.18014708	no
ANKRD49	0.097051009	1.752493029	0.080637884	0.180176828	no
DMRTA1	0.097047827	1.752435008	0.08064787	0.180176828	no
CDKL2	-0.097046378	-1.752408592	0.080652417	0.180176828	no
H1FX-AS1	-0.097036125	-1.7522217	0.080684591	0.180229971	no
LOC100505875	-0.097022943	-1.751981396	0.080725977	0.180303676	no
THUMPD1	-0.097010468	-1.751753996	0.080765156	0.180372437	no
SPATA12	0.09700612	1.751674742	0.080778814	0.180384195	no
COX15	-0.096997861	-1.75152419	0.080804765	0.180423398	no
MAST4	0.096994412	1.751461317	0.080815605	0.180428855	no
KCNS3	0.096982232	1.751239289	0.080853893	0.180495586	no
LOC286114	0.096971624	1.751045921	0.08088725	0.180551298	no
SLMAP	0.096927548	1.750242469	0.081025973	0.180842163	no
NAF1	-0.096915808	-1.750028479	0.081062953	0.18090591	no
TEX15	-0.096902633	-1.749788311	0.081104473	0.180979776	no
CAPN12	0.09688025	1.749380307	0.081175048	0.181118455	no
TMCO6	0.09687343	1.749255988	0.081196562	0.181147651	no
POMP	0.096865164	1.749105316	0.081222643	0.181187028	no
SLC5A11	-0.096859645	-1.749004716	0.081240061	0.181207073	no
OTOA	0.096855636	1.748931638	0.081252715	0.181216491	no
NME5	0.096848742	1.74880597	0.08127448	0.181246223	no
BEX5	-0.096840682	-1.748659055	0.081299931	0.181284168	no

MRP63	-0.096829034	-1.748446741	0.081336722	0.18134739	no
SGMS1	-0.096821337	-1.748306434	0.081361043	0.181382798	no
CWC25	-0.096801331	-1.747941779	0.08142428	0.181491052	no
PDE6H	-0.096800632	-1.747929031	0.081426491	0.181491052	no
KCNH6	-0.096788008	-1.747698924	0.081466417	0.181561212	no
IGSF22	-0.096759978	-1.747187996	0.081555125	0.181738396	no
INTS2	-0.096757543	-1.747143627	0.081562832	0.181738396	no
LPP	0.096751921	1.747041149	0.081580636	0.181759221	no
SNX11	0.096743489	1.746887445	0.081607344	0.181762623	no
JOSD2	0.096743295	1.746883911	0.081607958	0.181762623	no
GGNBP1	-0.096743196	-1.746882107	0.081608272	0.181762623	no
MIS12	-0.096740759	-1.746837678	0.081615993	0.181762623	no
ZC3H8	-0.096728409	-1.746612571	0.081655126	0.18183093	no
PHF21A	-0.09672077	-1.746473337	0.081679338	0.181866001	no
TDRD1	0.096692578	1.745959471	0.081768746	0.182046215	no
ARMC1	-0.096688613	-1.745887212	0.081781325	0.18205536	no
WAC-AS1	-0.096683422	-1.745792578	0.081797801	0.182073179	no
MAN2A2	-0.096675437	-1.745647048	0.081823144	0.182110728	no
CHID1	0.096620236	1.74464089	0.081998533	0.182482186	no
NONO	-0.096587101	-1.744036948	0.082103957	0.182697881	no
F8A1	0.096575085	1.743817933	0.082142215	0.182739115	no
SPG7	-0.096572194	-1.743765251	0.08215142	0.182739115	no
DMAP1	-0.096571786	-1.743757802	0.082152722	0.182739115	no
KCNE2	-0.096570599	-1.743736181	0.0821565	0.182739115	no
DAND5	-0.096551827	-1.743394019	0.082216307	0.182853219	no
PRKACB	-0.09654355	-1.743243173	0.082242685	0.182892958	no
MYCBPAP	0.096524046	1.742887685	0.082304876	0.183012322	no
ZSCAN1	-0.096513523	-1.742695879	0.082338448	0.18306803	no
SNORD97	-0.096497066	-1.742395944	0.082390967	0.18316585	no
ALMS1P	-0.096477058	-1.742031267	0.082454859	0.183288931	no
PRKG1	0.096459482	1.741710936	0.082511016	0.183394791	no
POLDIP2	-0.096436229	-1.741287128	0.08258536	0.183541052	no
PPIF	-0.096431483	-1.741200618	0.082600542	0.183555811	no
C5orf4	-0.096409727	-1.740804094	0.08267016	0.183691523	no
IQSEC2	-0.096402465	-1.740671744	0.082693408	0.183724183	no
TIMM10	-0.096384808	-1.740349944	0.082749954	0.183830812	no
SLC47A1	0.096379112	1.740246129	0.082768203	0.183852347	no
LINC00638	0.096375597	1.740182056	0.082779468	0.183858366	no
TMEM151A	-0.096346332	-1.739648689	0.082873288	0.184047725	no
VWA8	-0.096333477	-1.739414401	0.082914527	0.184120283	no
HIST1H4G	-0.096330786	-1.739365358	0.082923162	0.184120432	no
FOXO1	0.096326471	1.739286714	0.082937009	0.184132155	no
APPL1	-0.096282157	-1.73847909	0.083079325	0.184429064	no
MFI2	0.0962673	1.738208312	0.083127085	0.184516027	no
BET1L	0.096260801	1.738089869	0.083147983	0.184528212	no
AP4B1	0.096260252	1.738079872	0.083149747	0.184528212	no
SMYD3	-0.096238348	-1.737680681	0.083220212	0.184665522	no
NMBR	-0.096225333	-1.737443481	0.083262106	0.184731983	no
RDH16	-0.096223703	-1.73741377	0.083267355	0.184731983	no
SUPV3L1	-0.096109298	-1.735328815	0.083636353	0.185531468	no
HCRTR1	-0.096102065	-1.735196991	0.083659728	0.185564167	no

USP20	-0.096094205	-1.735053745	0.083685134	0.185601364	no
C1orf216	-0.096074159	-1.734688426	0.083749956	0.185725963	no
LINC00635	0.096069616	1.734605634	0.083764653	0.185739388	no
SNORA11	-0.096065906	-1.734538023	0.083776656	0.185746839	no
PLAG1	0.096054463	1.734329494	0.083813685	0.185809769	no
SLC35G5	-0.096044151	-1.734141572	0.083847066	0.185864601	no
KLHL30	0.096038366	1.734036145	0.083865799	0.185886951	no
KLK7	-0.096033463	-1.733946802	0.083881676	0.185902969	no
KRT31	-0.096021378	-1.73372657	0.083920823	0.185970552	no
TMEM177	-0.09601261	-1.733566784	0.083949236	0.186005431	no
GLRA1	-0.09601118	-1.733540709	0.083953873	0.186005431	no
ADAP1	-0.095997828	-1.733297389	0.083997156	0.186082143	no
OR2M3	-0.095977457	-1.73292617	0.084063225	0.186209314	no
EIF2AK3	0.095936416	1.732178269	0.084196464	0.186485231	no
LOC642846	-0.095928999	-1.7320431	0.084220562	0.186519384	no
FRMPD2	-0.095917793	-1.731838886	0.084256981	0.186550256	no
PTEN	0.095917153	1.73182723	0.08425906	0.186550256	no
ZEB1	-0.095916695	-1.73181888	0.08426055	0.186550256	no
FAM83H	0.095914027	1.731770256	0.084269224	0.186550256	no
ATP1A4	-0.095903433	-1.731577211	0.084303667	0.186607282	no
HS3ST5	-0.095900718	-1.731527727	0.084312497	0.186607609	no
SPIN2B	-0.095892377	-1.731375737	0.084339626	0.186648429	no
RASEF	0.095825165	1.730150938	0.084558497	0.187113535	no
SPG21	0.095791317	1.729534154	0.08466889	0.187338528	no
NDE1	0.095782992	1.729382445	0.084696062	0.187379356	no
WFIKKN1	-0.095774381	-1.729225536	0.084724172	0.187422252	no
TICRR	-0.09577047	-1.729154274	0.084736941	0.187431206	no
PAK6	-0.095755502	-1.728881509	0.08478583	0.187520045	no
UBE2D2	0.095743396	1.728660915	0.084825386	0.187588224	no
NT5C3L	-0.095740305	-1.728604587	0.084835488	0.187591262	no
C15orf54	0.095734906	1.728506209	0.084853135	0.18761098	no
GNRH1	0.095725976	1.728343486	0.084882331	0.187656226	no
ILKAP	-0.095721852	-1.728268339	0.084895817	0.187666734	no
FBXO11	-0.095710028	-1.728052881	0.084934491	0.187732917	no
FBXO22	0.095704837	1.727958287	0.084951476	0.187751148	no
RBP2	-0.095683821	-1.727575333	0.085020263	0.187878651	no
IRGQ	-0.095679284	-1.727492675	0.085035116	0.187878651	no
CC2D2A	-0.0956792	-1.72749113	0.085035393	0.187878651	no
SLC38A3	-0.095672268	-1.727364824	0.085058094	0.187892773	no
DNAJC8	0.095671909	1.727358277	0.085059271	0.187892773	no
SPOCK1	-0.095657935	-1.727103655	0.085105049	0.187974574	no
EPHA7	-0.09565461	-1.727043067	0.085115945	0.18797932	no
CYP11B1	-0.095620116	-1.726414526	0.085229046	0.18820905	no
FAM86EP	0.095617545	1.726367683	0.08523748	0.18820905	no
LOC100130275	-0.095579387	-1.725672378	0.085362747	0.188466283	no
KCNA4	-0.095576551	-1.725620702	0.085372063	0.188467489	no
WWTR1-AS1	0.095525159	1.724684294	0.085541019	0.188821081	no
SKA2	-0.095521302	-1.724614002	0.085553713	0.188829706	no
PPA2	0.095503066	1.724281734	0.085613736	0.188942782	no
G6PD	0.095473875	1.723749849	0.085709892	0.189135566	no
MCM10	-0.09546577	-1.72360216	0.085736607	0.189175094	no

CPSF6	-0.095453825	-1.723384523	0.085775987	0.189242555	no
FNDC7	0.095435502	1.723050667	0.085836424	0.189338232	no
HHATL	-0.095435336	-1.723047636	0.085836973	0.189338232	no
TMEM55B	-0.095425901	-1.722875736	0.085868106	0.189387465	no
SLC24A4	-0.095419471	-1.722758576	0.08588933	0.189399855	no
TRIM32	-0.095418859	-1.722747429	0.085891349	0.189399855	no
FDX1L	-0.09541353	-1.722650318	0.085908945	0.18941922	no
TRNP1	-0.095408212	-1.722553435	0.085926502	0.189434588	no
SMAD5	-0.09540608	-1.722514578	0.085933544	0.189434588	no
HAVCR1P1	-0.095383572	-1.722104479	0.0860079	0.189579054	no
ICT1	-0.095367968	-1.72182018	0.086059477	0.189673287	no
AQP4	-0.09535871	-1.721651497	0.086090091	0.189721303	no
LRRN4	-0.09535344	-1.72155547	0.086107523	0.189737632	no
STYX	0.095351132	1.721513415	0.086115158	0.189737632	no
DCUN1D1	-0.095342572	-1.721357445	0.086143479	0.189780576	no
BCL2L15	-0.095337674	-1.72126821	0.086159686	0.189796824	no
GNA11	-0.095331101	-1.721148456	0.08618144	0.189825287	no
PARD6A	-0.095316437	-1.72088127	0.086229991	0.189903459	no
MAGEA10	0.09531419	1.720840332	0.086237432	0.189903459	no
SLC27A1	0.095311368	1.720788915	0.086246778	0.189903459	no
LYPD3	0.095309708	1.720758673	0.086252276	0.189903459	no
PNMA6A	-0.095293149	-1.720456963	0.086307139	0.190004786	no
ADCY4	0.095267521	1.719990038	0.0863921	0.190172347	no
MSX2	-0.095252882	-1.71972331	0.086440664	0.190259762	no
SCT	-0.095242548	-1.719535028	0.086474959	0.190315754	no
COMMD10	0.095234938	1.719396385	0.086500219	0.190351853	no
TTC23	0.095190676	1.718589955	0.086647265	0.19065592	no
WWP1	0.095176325	1.718328496	0.086694983	0.190741389	no
FKRP	0.095166971	1.718158072	0.086726099	0.190790315	no
BTBD9	-0.0951571	-1.717978225	0.086758944	0.190843037	no
LOC100129427	-0.095151508	-1.717876341	0.086777556	0.190864441	no
EMC8	0.095146687	1.717788519	0.086793601	0.190880197	no
LPAR1	0.095108104	1.717085569	0.08692212	0.191130257	no
SLC22A9	-0.095107212	-1.717069328	0.086925091	0.191130257	no
C2orf27B	-0.095098628	-1.716912938	0.086953705	0.191169567	no
AASS	-0.095096512	-1.716874388	0.08696076	0.191169567	no
HTR2C	-0.095085978	-1.716682458	0.086995889	0.191227233	no
C11orf87	-0.095080917	-1.716590264	0.087012768	0.191244773	no
ZNF672	0.095075841	1.716497785	0.087029701	0.191262431	no
MORN3	0.095036077	1.715773332	0.087162446	0.191534573	no
GOLGA6L6	-0.095023156	-1.715537942	0.087205612	0.191609837	no
LOR	-0.095004973	-1.715206672	0.087266391	0.19172378	no
LOC100132287	-0.094991112	-1.714954149	0.087312746	0.191806012	no
DTNBP1	0.094974613	1.714653568	0.087367948	0.191907662	no
KIAA1456	-0.094954097	-1.714279807	0.087436628	0.192038894	no
ALOXE3	-0.094943349	-1.714083993	0.087472628	0.192098329	no
SEC24B-AS1	-0.094911975	-1.713512437	0.087577775	0.192309589	no
FBXO31	-0.094904592	-1.713377934	0.087602533	0.192344303	no
LINC00311	-0.094873378	-1.712809275	0.087707273	0.192551482	no
NPAT	-0.094871132	-1.712768367	0.087714811	0.192551482	no
TACR3	-0.094867757	-1.712706876	0.087726144	0.19255669	no

PIAS4	-0.094854642	-1.712467965	0.087770186	0.192633687	no
LINGO2	-0.094845252	-1.712296887	0.087801734	0.192658578	no
MED15	0.094844002	1.712274128	0.087805932	0.192658578	no
MIP	-0.094843262	-1.712260635	0.087808421	0.192658578	no
LOC100505865	0.094833564	1.712083964	0.087841012	0.192710412	no
RECQL4	-0.094823218	-1.711895495	0.087875791	0.192767033	no
FMNL3	0.094782907	1.711161132	0.088011412	0.19304483	no
ANKRD53	0.094768856	1.710905172	0.088058722	0.193128889	no
CSNK1A1	0.094762532	1.710789956	0.088080024	0.193155898	no
GRIK3	-0.094757353	-1.710695614	0.08809747	0.193174445	no
SERTAD2	0.094747413	1.710514537	0.088130964	0.193228172	no
MST02P	-0.094730767	-1.710211306	0.088187075	0.193331473	no
CNTRL	0.094721328	1.710039353	0.088218907	0.193369142	no
RPL23AP64	-0.094720336	-1.710021279	0.088222253	0.193369142	no
SETD3	-0.094716954	-1.709959684	0.088233658	0.193374418	no
MUCL1	0.094697857	1.709611788	0.088298097	0.193495912	no
MUC12	0.094686242	1.709400208	0.088337306	0.193562097	no
CCDC116	-0.094682225	-1.709327029	0.08835087	0.193570297	no
DRD2	-0.094679799	-1.709282837	0.088359063	0.193570297	no
SERPINF2	0.094669483	1.709094914	0.088393906	0.193626892	no
AFAP1L1	0.094657112	1.708869549	0.088435707	0.193698713	no
TUSC1	0.094639489	1.70854853	0.088495277	0.193809436	no
C14orf182	0.094619347	1.708181605	0.088563405	0.193921452	no
MEF2B	0.094619031	1.708175865	0.088564471	0.193921452	no
CACNG6	-0.094595835	-1.707753307	0.088642982	0.194073587	no
LINC00381	-0.094591378	-1.707672131	0.088658071	0.19408685	no
PDCD2L	-0.094542169	-1.70677573	0.088824832	0.194432111	no
KCTD3	-0.094533865	-1.706624463	0.088852998	0.19445878	no
FLJ31662	-0.094533241	-1.706613106	0.088855113	0.19445878	no
LOC285972	-0.094530504	-1.706563239	0.088864399	0.194459302	no
ANKRD29	-0.094516508	-1.706308298	0.088911891	0.194543417	no
STARD8	0.094511569	1.706218325	0.088928656	0.194560292	no
SSBP4	0.094504293	1.706085788	0.088953357	0.194594523	no
LOC645434	-0.09449676	-1.705948578	0.088978935	0.194630666	no
NBPF24	-0.094492563	-1.705872121	0.08899319	0.194642037	no
FAM63A	-0.094472215	-1.705501466	0.089062325	0.194773422	no
NMUR1	0.094444964	1.705005089	0.089154977	0.194956207	no
TDRD6	-0.094439998	-1.704914632	0.08917187	0.194973308	no
ECH1	-0.094434902	-1.704821795	0.08918921	0.194991384	no
C1D	-0.094420831	-1.70456549	0.089237096	0.195076231	no
ZNF763	-0.09439862	-1.704160919	0.089312726	0.195221703	no
CNTLN	0.094380074	1.703823098	0.089375917	0.195338246	no
SLC6A4	-0.094377637	-1.703778716	0.089384222	0.195338246	no
CHRM3	-0.094373579	-1.703704795	0.089398055	0.195348612	no
C5orf63	0.09436996	1.703638869	0.089410394	0.19535571	no
SMIM12	0.094348383	1.703245851	0.089483978	0.195496613	no
DDX1	-0.094318828	-1.702707515	0.089584851	0.195697095	no
IDH3A	-0.094304678	-1.702449776	0.089633178	0.195782765	no
ENTPD3	-0.094301542	-1.702392644	0.089643893	0.195786271	no
CNTF	0.094286921	1.702126343	0.089693852	0.195875478	no
CRYBA4	0.094270045	1.70181895	0.089751549	0.195979733	no

VAMP1	-0.094261825	-1.701669233	0.089779661	0.195979733	no
LEFTY1	-0.094261484	-1.701663017	0.089780829	0.195979733	no
LOC100130000	-0.09426048	-1.701644728	0.089784263	0.195979733	no
MDP1	-0.094259625	-1.701629155	0.089787188	0.195979733	no
CENPH	-0.094237311	-1.701222724	0.089863544	0.196126478	no
GSTA7P	0.09422192	1.700942383	0.089916243	0.196221565	no
LOC574538	-0.094204955	-1.700633381	0.089974357	0.196328451	no
TPTE2	-0.094201558	-1.700571511	0.089985997	0.196333915	no
ZBTB37	-0.09419661	-1.700481383	0.090002955	0.196350981	no
COL22A1	0.094178991	1.700160461	0.09006336	0.196462816	no
RUFY4	0.094139467	1.69944058	0.090198976	0.196738678	no
ZNF92	-0.094128037	-1.699232395	0.090238226	0.196804315	no
RMI1	-0.094121283	-1.69910939	0.090261424	0.196834932	no
ENPP5	0.094072003	1.698211812	0.090430842	0.197157266	no
GNB2	0.094071715	1.698206578	0.09043183	0.197157266	no
KIAA1462	-0.094070283	-1.698180502	0.090436756	0.197157266	no
HEATR1	0.09406705	1.698121609	0.090447882	0.19716152	no
TAF6L	-0.094062386	-1.698036653	0.090463933	0.19717651	no
MS4A8B	-0.094058394	-1.697963955	0.09047767	0.197181692	no
SLC9A6	-0.094056363	-1.697926962	0.090484661	0.197181692	no
ORMDL1	0.094024427	1.697345301	0.09059464	0.19738918	no
BRD7P3	-0.094023381	-1.697326246	0.090598245	0.19738918	no
DNA2	-0.094008829	-1.697061212	0.090648394	0.197478422	no
EID1	-0.094006082	-1.697011177	0.090657864	0.197479034	no
EPB41	-0.094001443	-1.696926691	0.090673856	0.197493853	no
CPNE7	-0.093972381	-1.696397382	0.090774101	0.197677746	no
EGR1	0.093971633	1.69638376	0.090776682	0.197677746	no
GRTP1	0.093946428	1.695924711	0.090863695	0.19784718	no
NDST3	-0.093931214	-1.69564762	0.09091625	0.197941559	no
BASP1P1	0.093909491	1.695251979	0.090991333	0.198084962	no
FAM219A	-0.093901749	-1.695110976	0.091018104	0.198123172	no
ACSL6	-0.093885659	-1.69481794	0.091073761	0.198204597	no
MAGEB1	0.093885602	1.694816912	0.091073956	0.198204597	no
SEC61A2	-0.093854387	-1.69424841	0.091182011	0.198419664	no
NDFIP1	-0.09383935	-1.693974554	0.0912341	0.198512912	no
RIT1	0.093828076	1.693769222	0.09127317	0.198576012	no
LINC00598	0.093820925	1.693638989	0.091297958	0.198576012	no
PCNX	-0.093820407	-1.693629563	0.091299752	0.198576012	no
SLCO1C1	-0.093820319	-1.69362795	0.09130006	0.198576012	no
GPAM	-0.093812657	-1.693488407	0.091326626	0.198613693	no
ERCC6	-0.09379608	-1.693186506	0.091384124	0.198718628	no
ZNF391	-0.093781154	-1.692914685	0.091435918	0.19881114	no
RAB7A	0.093772386	1.692754997	0.091466356	0.198856059	no
OPRL1	0.093769873	1.692709224	0.091475083	0.198856059	no
PRKAB1	0.093766711	1.692651647	0.09148606	0.198859808	no
LOC285758	-0.093757668	-1.692486953	0.091517467	0.198907958	no
EFHD1	-0.093753254	-1.692406577	0.091532798	0.198921161	no
ECD	-0.093738977	-1.692146559	0.091582408	0.19900885	no
DNAJB12	-0.093700273	-1.691441696	0.091716999	0.199267764	no
CALD1	0.093699381	1.691425455	0.091720102	0.199267764	no
UBA1	-0.093682107	-1.691110865	0.091780226	0.19937823	no

MKL2	0.093675618	1.69099269	0.091802819	0.199407155	no
PPP1R3C	-0.093641666	-1.690374391	0.091921103	0.199643902	no
FOXN4	-0.093628626	-1.690136906	0.091966567	0.199717398	no
SFTA3	-0.09362663	-1.690100556	0.091973528	0.199717398	no
LOC339505	0.093606461	1.689733268	0.092043882	0.199849977	no
ACTL8	0.093589897	1.689431615	0.092101697	0.199955305	no
LINC00092	0.093571734	1.689100852	0.092165124	0.200052968	no
RPL23AP53	-0.093571684	-1.689099941	0.092165299	0.200052968	no
SKI	0.093563351	1.688948183	0.092194412	0.200095951	no
KRIT1	-0.0935433	-1.688583053	0.092264488	0.200227821	no
CCDC141	-0.093540382	-1.688529907	0.092274692	0.200229745	no
EIF4EBP3	0.093508747	1.687953813	0.092385354	0.200439039	no
DEPDC4	-0.093507478	-1.687930697	0.092389797	0.200439039	no
KIAA1468	-0.093499728	-1.687789564	0.092416924	0.200477654	no
NEDD4	0.093486382	1.687546534	0.092463653	0.200558777	no
GP9	-0.093474412	-1.687328553	0.092505582	0.200629474	no
PPBP	0.093469379	1.687236903	0.092523215	0.200644776	no
MCM5	0.093467069	1.687194837	0.09253131	0.200644776	no
STARD4	0.093453967	1.686956254	0.092577229	0.200724095	no
GPAT2	0.093442475	1.686746969	0.092617525	0.200791205	no
ASNS	-0.09343374	-1.686587905	0.09264816	0.200837362	no
CNP	-0.09342266	-1.686386149	0.09268703	0.200901357	no
CLPTM1	-0.093376688	-1.685549003	0.092848452	0.201230949	no
ACTR8	-0.093363918	-1.685316463	0.092893332	0.201307916	no
SLC7A11-AS1	-0.093316389	-1.684450969	0.093060525	0.201649902	no
WASH2P	-0.093297369	-1.68410463	0.093127497	0.201774678	no
TMEM190	0.093293038	1.684025764	0.093142753	0.201787389	no
STEAP1B	0.093276346	1.683721806	0.093201569	0.201894459	no
TIMP2	0.093269869	1.683603874	0.093224397	0.201923556	no
INO80D	-0.093232955	-1.682931699	0.093354597	0.20218519	no
ARHGEF10	0.093220992	1.68271385	0.093396826	0.202256266	no
CUL2	-0.093188133	-1.682115538	0.093512884	0.202486913	no
RUNDC1	-0.093185504	-1.682067655	0.093522177	0.202486913	no
PRKD1	-0.093163669	-1.681670076	0.093599368	0.202633627	no
CLSTN3	-0.093153284	-1.681480979	0.0936361	0.202671569	no
ZNF232	-0.093149072	-1.68140427	0.093651003	0.202671569	no
GPR158-AS1	-0.09314639	-1.681355446	0.093660491	0.202671569	no
CCL1	-0.093145823	-1.681345111	0.093662499	0.202671569	no
SREK1IP1	-0.093145385	-1.681337146	0.093664047	0.202671569	no
ZNF268	0.09313918	1.681224147	0.093686007	0.202698679	no
UBL4A	0.093121709	1.680906028	0.093747854	0.202812073	no
F2R	0.093115903	1.680800321	0.093768412	0.20283613	no
MAU2	-0.093098684	-1.680486786	0.093829411	0.202947652	no
BRAF	-0.093090943	-1.68034583	0.093856845	0.202969437	no
DBT	-0.093090512	-1.680337983	0.093858372	0.202969437	no
WWP2	0.093087757	1.680287823	0.093868136	0.202970129	no
FAT1	-0.093084878	-1.680235391	0.093878343	0.202971778	no
FAM213B	-0.093081585	-1.680175445	0.093890014	0.202976591	no
LRRC6	0.093052047	1.679637604	0.093994781	0.203164011	no
NEK4	-0.093051812	-1.679633324	0.093995615	0.203164011	no
DPH2	0.093028873	1.679215651	0.094077039	0.203319554	no

MTHFD2	-0.093013252	-1.678931216	0.094132522	0.203396804	no
POLG2	-0.09301153	-1.678899863	0.094138639	0.203396804	no
KCNA7	-0.093010816	-1.678886862	0.094141176	0.203396804	no
POLR3D	-0.093004745	-1.678776328	0.094162746	0.203422956	no
KANK2	0.092998758	1.678667321	0.094184021	0.203448467	no
LINC00340	0.092989827	1.678504704	0.094215767	0.203496589	no
MT1JP	0.092970368	1.678150391	0.094284966	0.203624716	no
APOBEC2	-0.092967818	-1.678103958	0.094294038	0.203624716	no
LOC100287632	-0.09296242	-1.678005681	0.094313241	0.203645721	no
FAM189A1	-0.092917713	-1.67719168	0.094472413	0.203968919	no
PPP3CC	0.092901336	1.676893491	0.094530776	0.204074425	no
RUNX1-IT1	0.092891982	1.676723172	0.094564124	0.204125913	no
MAP6D1	-0.09287412	-1.67639795	0.094627829	0.204242912	no
HIST1H3D	0.092865386	1.676238937	0.094658989	0.204272501	no
DEDD	-0.092864949	-1.676230984	0.094660548	0.204272501	no
GPR153	-0.092848864	-1.675938111	0.094717962	0.204375875	no
ADCK3	0.092838072	1.675741623	0.094756496	0.204438497	no
GART	-0.092807033	-1.675176498	0.094867398	0.204657222	no
TSSK4	-0.092799015	-1.67503051	0.094896063	0.204698514	no
SMIM4	0.092784449	1.674765314	0.094948155	0.204775717	no
ROGDI	-0.092783678	-1.674751286	0.094950911	0.204775717	no
WDR47	-0.092751288	-1.674161558	0.095066833	0.205005149	no
LIPC	0.092745037	1.674047753	0.095089217	0.205032844	no
RAB24	-0.092729152	-1.673758544	0.095146119	0.205134956	no
LY6G5B	-0.092721583	-1.673620733	0.095173243	0.205172852	no
CLUL1	0.09271224	1.673450641	0.095206729	0.205224455	no
DNAJC11	-0.092699667	-1.673221722	0.095251811	0.205301041	no
BPIFB6	-0.092692069	-1.673083386	0.095279063	0.205339185	no
GPR63	-0.092687058	-1.672992166	0.095297037	0.205357326	no
AXIN1	-0.092678261	-1.672832	0.095328602	0.20540475	no
MFI2-AS1	-0.092673551	-1.672746242	0.095345506	0.205420578	no
RAB40AL	-0.092631704	-1.671984371	0.095495788	0.205720168	no
EGR3	0.092629501	1.671944265	0.095503705	0.205720168	no
C2orf48	-0.092623818	-1.671840805	0.095524129	0.205743541	no
VPS37A	0.092620697	1.671783991	0.095535346	0.205747081	no
ATP5C1	-0.092594271	-1.671302878	0.095630378	0.205920905	no
ADAMTS7	0.092592925	1.671278364	0.095635222	0.205920905	no
SMAD2	0.092568467	1.670833092	0.095723245	0.206076463	no
PPIL2	-0.092567523	-1.670815899	0.095726645	0.206076463	no
BRCA2	0.092540671	1.670327042	0.095823362	0.206264011	no
FAM86HP	0.09252728	1.670083259	0.095871623	0.206347225	no
TMEM231	-0.092515005	-1.669859784	0.09591588	0.206421808	no
CENPO	-0.092506973	-1.669713564	0.095944846	0.206463472	no
KLF13	-0.092503142	-1.669643809	0.095958668	0.20647254	no
TPTEP1	-0.092494049	-1.669478271	0.095991473	0.20652245	no
PLXNA4	-0.09248999	-1.669404385	0.096006119	0.206533283	no
SRMS	-0.092476548	-1.669159668	0.096054638	0.206616978	no
TJAP1	-0.092437229	-1.668443862	0.096196673	0.206901791	no
NACAD	-0.0924282	-1.668279492	0.096229312	0.20695128	no
TMEM213	-0.092384078	-1.667476255	0.096388939	0.207273834	no
KRT80	0.092375184	1.667314332	0.096421144	0.207322342	no

RPL23	-0.092358902	-1.667017927	0.096480118	0.207428394	no
OACYLP	0.092351334	1.666880147	0.096507542	0.207466598	no
BRICD5	-0.092339978	-1.666673416	0.096548701	0.207534319	no
EXPH5	-0.092325234	-1.66640502	0.096602158	0.20762846	no
KIAA1609	0.09232072	1.666322831	0.096618532	0.207642887	no
LOC339442	0.092317315	1.666260853	0.096630881	0.207648662	no
ARID3C	-0.092297493	-1.665900004	0.096702807	0.207782446	no
RAB35	0.092290979	1.665781426	0.096726452	0.207812474	no
LOC100506085	-0.092283983	-1.665654075	0.096751851	0.207846265	no
ARPC3	0.092278722	1.665558287	0.096770959	0.207866534	no
ELF2	-0.092224051	-1.664563052	0.096969668	0.20827255	no
TBX6	-0.092192553	-1.663989673	0.097084298	0.208497916	no
RHO	-0.092168243	-1.663547142	0.097172843	0.208667223	no
UXT	-0.092147047	-1.663161298	0.097250099	0.208801528	no
C9orf78	-0.092145753	-1.663137737	0.097254818	0.208801528	no
LOC283663	0.092142466	1.663077909	0.097266802	0.208806397	no
KLHL1	-0.092138962	-1.663014113	0.097279582	0.208812974	no
GAB1	-0.092135049	-1.662942887	0.097293852	0.208822748	no
FAM13A-AS1	-0.09207546	-1.661858174	0.097511382	0.209268734	no
CBWD3	0.092041435	1.661238818	0.097635763	0.209514747	no
RBM11	-0.092037402	-1.661165399	0.097650516	0.209525483	no
SCARNA21	-0.092005315	-1.660581328	0.097767942	0.209736994	no
TFAP2A	-0.092005133	-1.660578007	0.09776861	0.209736994	no
USF2	0.091998134	1.660450605	0.097794239	0.209771035	no
TAF7	-0.091986558	-1.660239895	0.097836639	0.209841039	no
FLT1	0.091962116	1.659794973	0.097926217	0.210012206	no
ACSM5	0.09191642	1.658963194	0.098093857	0.210350737	no
CAPNS2	0.091904697	1.658749819	0.098136899	0.210422039	no
SNHG4	0.091889144	1.658466714	0.09819403	0.210523533	no
JSRP1	0.091885536	1.658401037	0.098207287	0.210530954	no
LINC00673	-0.091877541	-1.658255517	0.098236667	0.210572932	no
CEP85	-0.091860246	-1.657940708	0.098300249	0.210688208	no
LOC728554	-0.091854065	-1.657828199	0.098322981	0.210715914	no
ACOX1	0.091841252	1.657594977	0.098370115	0.210795906	no
RBM20	0.091829969	1.657389605	0.098411636	0.210863855	no
POLE3	-0.09182199	-1.657244368	0.098441007	0.210905761	no
AARS2	-0.091802488	-1.656889399	0.098512823	0.211038584	no
BAG5	-0.091776287	-1.656412491	0.098609375	0.211224367	no
C15orf62	-0.091762939	-1.65616953	0.098658592	0.211300443	no
RAB5B	-0.091760522	-1.656125552	0.098667503	0.211300443	no
CCDC65	0.091758656	1.656091581	0.098674387	0.211300443	no
PPM1B	-0.091752383	-1.655977397	0.098697527	0.211328938	no
SYTL5	-0.091698077	-1.654988959	0.098898025	0.211737145	no
TFAP2C	0.091668293	1.654446842	0.099008129	0.211951757	no
LPA	-0.091650989	-1.654131905	0.099072137	0.212067659	no
DIRC2	-0.091636961	-1.653876578	0.099124055	0.212157659	no
CXorf22	0.091629572	1.653742096	0.099151409	0.212195073	no
P2RX2	0.091624325	1.653646589	0.099170839	0.212196894	no
TMEM221	0.09162401	1.653640848	0.099172007	0.212196894	no
MRPS9	-0.091615926	-1.653493725	0.099201944	0.21222233	no
CXCR2	0.091615467	1.653485368	0.099203645	0.21222233	no

C6orf211	0.091608063	1.653350608	0.099231073	0.212259878	no
RINT1	0.091602209	1.653244064	0.099252762	0.212285143	no
BLCAP	-0.091597648	-1.653161051	0.099269665	0.212300165	no
NT5C2	-0.091588748	-1.652999055	0.099302655	0.212347951	no
KRTAP19-7	-0.091584904	-1.652929102	0.099316903	0.212347951	no
SENP2	-0.091583624	-1.652905792	0.099321651	0.212347951	no
TRPS1	0.091579665	1.652833747	0.099336328	0.212350206	no
TIMM44	-0.09157801	-1.652803613	0.099342467	0.212350206	no
CDH3	0.091571431	1.652683881	0.099366864	0.21238123	no
ALOX15P1	-0.0915459	-1.652219188	0.099461595	0.212562564	no
CHST5	-0.09153647	-1.652047572	0.099496599	0.212616228	no
CBX2	-0.091527751	-1.651888881	0.099528975	0.212664267	no
CEP104	0.091523391	1.651809525	0.099545168	0.212677722	no
STRC	-0.0915103	-1.651571253	0.099593802	0.212760478	no
CYP51A1	-0.091502546	-1.651430136	0.099622615	0.212800877	no
UTP6	0.091495826	1.651307833	0.099647592	0.212814354	no
SLC8A3	-0.09149552	-1.651302264	0.099648729	0.212814354	no
HN1L	0.091487039	1.651147909	0.099680259	0.212844513	no
LINC00622	-0.091486394	-1.651136162	0.099682659	0.212844513	no
EHMT1	-0.091481541	-1.651047847	0.099700703	0.212856986	no
CD160	0.091479496	1.651010621	0.09970831	0.212856986	no
AVIL	0.091475582	1.650939388	0.099722866	0.212866917	no
PCNXL4	-0.091470698	-1.650850493	0.099741035	0.212884554	no
AP5B1	0.091459018	1.650637924	0.09978449	0.212944184	no
TNNT2	-0.091457863	-1.650616897	0.099788789	0.212944184	no
C21orf128	-0.091440364	-1.650298413	0.099853928	0.213048565	no
C11orf80	-0.091439396	-1.650280802	0.099857531	0.213048565	no
LOC150776	-0.091405055	-1.649655795	0.099985464	0.213300338	no
PSMD14	0.091399482	1.64955437	0.100006237	0.21331043	no
FCGR2B	0.091398461	1.649535777	0.100010045	0.21331043	no
HIST1H3G	0.091392635	1.649429758	0.100031764	0.21333558	no
HECW2	-0.091378787	-1.649177728	0.100083408	0.213424541	no
LOC100129148	-0.091364938	-1.648925674	0.100135079	0.213504328	no
SLC4A9	-0.091362206	-1.648875948	0.100145275	0.213504328	no
LOC220729	-0.091359324	-1.648823502	0.10015603	0.213504328	no
AKAP7	-0.09135811	-1.648801406	0.100160562	0.213504328	no
SYNDIG1L	-0.091346884	-1.648597097	0.100202469	0.213572475	no
ZNF865	0.091341028	1.648490533	0.100224333	0.213593943	no
RHOU	-0.0913364	-1.648406304	0.100241617	0.213593943	no
WSCD1	-0.091336202	-1.648402693	0.100242358	0.213593943	no
BCL7C	0.091324152	1.648183401	0.100287368	0.213660109	no
TAX1BP3	0.091321191	1.648129496	0.100298435	0.213660109	no
UBE2G1	-0.091319906	-1.648106113	0.100303236	0.213660109	no
MBLAC1	-0.091306707	-1.64786591	0.100352563	0.213743998	no
C8orf12	-0.0912986	-1.647718366	0.100382872	0.213787366	no
TSKS	0.09129439	1.647641744	0.100398615	0.213796624	no
HMMR	0.091292117	1.647600374	0.100407116	0.213796624	no
ARHGAP21	-0.091285353	-1.647477278	0.100432413	0.21381345	no
PHOSPHO1	-0.091284684	-1.6474651	0.100434916	0.21381345	no
STRN	-0.091281298	-1.64740348	0.100447581	0.213819233	no
TRAPPC4	-0.091276658	-1.647319041	0.100464939	0.213835001	no

LOC255512	-0.091267316	-1.647149025	0.100499896	0.213888223	no
MADCAM1	-0.091238387	-1.646622537	0.10060821	0.214067123	no
GTF2B	0.091237093	1.646598994	0.100613055	0.214067123	no
OR1F1	-0.091236884	-1.646595187	0.100613839	0.214067123	no
LOC283788	-0.091220291	-1.646293207	0.10067601	0.214172507	no
EIF2B5	-0.091218345	-1.646257798	0.100683302	0.214172507	no
TMEM11	0.09120309	1.645980179	0.100740487	0.214272942	no
IDH3B	-0.091163575	-1.645261042	0.100888741	0.214567039	no
RDH5	0.091153842	1.645083921	0.100925282	0.214623514	no
LOC284344	-0.091148683	-1.644990041	0.100944654	0.214643471	no
LOC81691	-0.091138749	-1.644809262	0.100981967	0.214701567	no
MPHOSPH10	-0.091134972	-1.644740523	0.100996157	0.214710496	no
EFHB	-0.091128864	-1.644629358	0.101019109	0.214738049	no
C20orf194	-0.091109301	-1.644273349	0.101092643	0.214873107	no
GTF3C2	-0.091087125	-1.64386978	0.101176051	0.215029126	no
C1orf226	0.091083463	1.643803146	0.101189829	0.215037141	no
LOC100130557	-0.091048746	-1.643171364	0.101320529	0.215279529	no
METAP1	-0.091046629	-1.64313283	0.101328505	0.215279529	no
LTA4H	0.091045186	1.643106571	0.10133394	0.215279529	no
DGKE	-0.091035173	-1.642924362	0.101371664	0.215338384	no
C1orf50	0.091016139	1.642577973	0.10144341	0.215469492	no
BAHD1	-0.091003137	-1.642341363	0.101492441	0.215531122	no
GALNTL2	0.091003125	1.642341159	0.101492483	0.215531122	no
HDAC8	0.090997556	1.642239806	0.101513491	0.215554436	no
SMIM1	0.090987453	1.642055957	0.101551609	0.21561407	no
GBP7	0.090971422	1.641764235	0.101612114	0.215721224	no
GAPDH	0.09096278	1.641606963	0.101644746	0.215769186	no
LINC00173	-0.090952948	-1.641428038	0.101681881	0.215826696	no
PTCHD1	0.090946493	1.641310577	0.101706265	0.215857133	no
LOC100506013	0.09094308	1.641248473	0.101719159	0.215863182	no
PPP1R21	0.090922421	1.640872541	0.101797239	0.216007549	no
FANCG	-0.090912238	-1.640687238	0.101835743	0.216058729	no
FGF18	-0.090910726	-1.640659708	0.101841465	0.216058729	no
PCGF3	-0.090900892	-1.640480755	0.101878663	0.21611631	no
PSMB5	0.090892448	1.640327102	0.10191061	0.216162744	no
TTF1	-0.09088937	-1.640271098	0.101922256	0.216166112	no
ATAD5	-0.090879724	-1.640095566	0.101958766	0.216222207	no
C19orf80	-0.090866151	-1.639848571	0.102010158	0.216309847	no
AGPS	0.090860902	1.639753051	0.102030038	0.216330657	no
C19orf12	0.09085822	1.639704247	0.102040196	0.216330853	no
FLJ31485	-0.090837776	-1.639332237	0.102117656	0.216464282	no
BOLA3	0.090836293	1.639305251	0.102123277	0.216464282	no
SLC2A6	0.090831109	1.639210921	0.102142927	0.21648458	no
SETDB2	0.090796931	1.638588987	0.102272557	0.216720274	no
REPS1	-0.090795821	-1.638568775	0.102276772	0.216720274	no
LOC401321	-0.090793814	-1.638532265	0.102284386	0.216720274	no
PCDHA5	-0.090762829	-1.637968446	0.102402028	0.216948144	no
LOC388813	0.090747528	1.637690015	0.102460163	0.217049912	no
CCBL2	0.090737757	1.637512207	0.102497302	0.217107186	no
FLJ46300	-0.090730307	-1.637376653	0.102525623	0.217145773	no
MRPS22	-0.090725914	-1.637296711	0.102542327	0.217159751	no

GPD2	-0.090712212	-1.637047392	0.10259444	0.217248704	no
FAM35DP	-0.090682533	-1.636507344	0.102707392	0.217466459	no
MEGF9	-0.090677896	-1.636422958	0.102725051	0.217482422	no
SH3TC2	-0.090667592	-1.636235471	0.102764293	0.217520262	no
ZNF282	-0.090666197	-1.636210097	0.102769605	0.217520262	no
C4orf27	-0.09066523	-1.6361925	0.102773288	0.217520262	no
SLC7A6OS	-0.090630897	-1.635567773	0.102904143	0.217775769	no
CD38	-0.0906213	-1.635393148	0.102940743	0.21782599	no
KLF5	0.090619302	1.635356795	0.102948364	0.21782599	no
CCDC164	0.090616703	1.635309492	0.102958281	0.21782599	no
METTL5	-0.090586755	-1.634764578	0.103072574	0.218032427	no
MLF1	0.090584915	1.634731097	0.1030796	0.218032427	no
ARHGEF18	-0.090583163	-1.63469921	0.103086292	0.218032427	no
MIR31HG	0.090547374	1.634048001	0.103223028	0.218300146	no
SDC4	0.090526006	1.633659214	0.103304731	0.218444445	no
TSHR	-0.090524214	-1.633626595	0.103311588	0.218444445	no
RBM22	-0.09050929	-1.63335506	0.103368685	0.21854367	no
YAP1	0.090455663	1.63237931	0.103574068	0.218956354	no
RIPPLY1	0.090452953	1.632329991	0.103584457	0.218956779	no
HSPB7	0.090448266	1.632244713	0.103602424	0.21897322	no
AK3	0.090440464	1.632102752	0.103632339	0.218987002	no
C21orf59	0.090439219	1.632080095	0.103637114	0.218987002	no
SEC23B	-0.090438593	-1.632068708	0.103639514	0.218987002	no
DPY19L2P4	-0.090422601	-1.63177774	0.103700853	0.219095067	no
SPATA19	-0.09041142	-1.631574299	0.103743757	0.219164169	no
HDX	-0.090398777	-1.631344265	0.103792287	0.219245139	no
MIR210	0.090394162	1.631260303	0.103810005	0.219261014	no
LINC00332	0.090379352	1.630990827	0.103866887	0.219359598	no
ATG16L1	-0.090368533	-1.630793978	0.103908454	0.219425822	no
MSMB	-0.090361361	-1.630663493	0.103936015	0.219462458	no
MEGF8	0.090339018	1.630256973	0.104021917	0.219604406	no
TRPV4	0.090336871	1.630217916	0.104030173	0.219604406	no
CCDC43	-0.090335904	-1.630200308	0.104033896	0.219604406	no
PADI2	0.090332666	1.630141396	0.10404635	0.219609126	no
MRGPRE	0.090327474	1.630046941	0.104066322	0.219629708	no
STAU2-AS1	-0.09032464	-1.629995376	0.104077226	0.219631152	no
ANAPC10	0.09027427	1.629078924	0.104271172	0.220018828	no
SCARNA2	-0.090259198	-1.628804712	0.104329259	0.220119783	no
SOX21	-0.09024818	-1.628604237	0.104371742	0.2201878	no
TMSB15B	-0.090233236	-1.628332355	0.10442938	0.220287771	no
ZNF782	-0.090174668	-1.62726678	0.104655521	0.220743135	no
ZNF593	0.090167932	1.627144222	0.104681556	0.22075963	no
ERICH1	0.090167329	1.627133255	0.104683886	0.22075963	no
C6	0.090157185	1.626948701	0.104723101	0.220820659	no
PODN	-0.090135039	-1.626545777	0.104808757	0.220979593	no
GFOD1	-0.09011259	-1.626137357	0.104895638	0.22114108	no
ARSG	0.09010937	1.626078769	0.104908106	0.22114567	no
DCUN1D2	-0.090105493	-1.626008236	0.104923118	0.221155622	no
LOC100506421	-0.090095676	-1.625829638	0.104961136	0.221214061	no
TBK1	0.090085085	1.625636947	0.105002167	0.221278836	no
GGT1	0.090068282	1.625331254	0.105067286	0.221385734	no

FAM86B2	0.09006668	1.625302113	0.105073495	0.221385734	no
AAMDC	-0.090055922	-1.625106397	0.105115206	0.221451905	no
MARK3	-0.090043287	-1.624876517	0.105164214	0.221533437	no
PSMD8	0.090034976	1.624725308	0.10519646	0.221579646	no
RNF144A-AS1	-0.090016163	-1.624383058	0.105269476	0.221710394	no
KAT2B	-0.090013667	-1.624337651	0.105279167	0.221710394	no
PTPRE	0.090007337	1.62422248	0.105303748	0.221740433	no
CLIP1	0.089986846	1.623849692	0.105383346	0.221886303	no
LOC100505839	-0.089980002	-1.623725192	0.10540994	0.221920555	no
ATP13A5	-0.089969883	-1.623541086	0.105449276	0.221981623	no
INO80C	-0.089952388	-1.623222813	0.105517306	0.222103077	no
GLIPR1L2	-0.089949232	-1.623165398	0.105529582	0.222107163	no
BTBD1	-0.089942704	-1.62304664	0.105554977	0.222138858	no
NKAPP1	0.08991807	1.622598485	0.105650855	0.222318861	no
IL1RL1	0.089913655	1.622518156	0.105668048	0.22233327	no
ZNF735	0.089906879	1.622394895	0.105694434	0.222367017	no
CHADL	-0.089865273	-1.62163798	0.105856579	0.222646046	no
NPR1	0.089865105	1.621634936	0.105857231	0.222646046	no
PNPLA3	-0.089864872	-1.621630694	0.10585814	0.222646046	no
FOLH1	-0.089836337	-1.621111581	0.10596946	0.222858367	no
SMG7-AS1	-0.089833588	-1.621061575	0.105980188	0.222859121	no
UBL4B	0.089821536	1.620842317	0.106027237	0.222936245	no
RAPH1	0.089817357	1.620766297	0.106043554	0.22294874	no
ITGA2B	-0.08981103	-1.620651196	0.106068263	0.222978875	no
HIST1H2BO	0.089803132	1.620507517	0.106099113	0.223021913	no
ALDOA	0.089796539	1.620387582	0.10612487	0.223054238	no
DEFB134	-0.089776218	-1.620017911	0.106204292	0.22319934	no
PSG3	0.089765835	1.619829021	0.106244893	0.223262833	no
LOC151171	-0.089762463	-1.619767687	0.106258079	0.223268711	no
OR4K15	0.089742665	1.619407525	0.106335535	0.223409618	no
SCG5	-0.089721027	-1.619013895	0.10642024	0.223565725	no
RNF138P1	-0.089710202	-1.618816975	0.106462635	0.223632928	no
RAB40A	-0.089696742	-1.618572125	0.106515368	0.223721831	no
DSCR9	-0.089694052	-1.618523191	0.106525909	0.223722106	no
C6orf165	0.089677594	1.6182238	0.106590422	0.22383572	no
CEP76	-0.089674393	-1.618165572	0.106602973	0.223840204	no
VPS45	-0.089665784	-1.618008958	0.106636735	0.223889222	no
DENND6B	-0.089650701	-1.617734577	0.106695906	0.223991572	no
EGLN2	0.089638321	1.617509377	0.106744491	0.22407168	no
FAM76A	0.089628341	1.617327833	0.106783671	0.224132031	no
ADAMTS16	0.089601056	1.616831482	0.106890847	0.224335077	no
INTS4L2	-0.089593615	-1.61669614	0.106920086	0.224374531	no
LMBR1	-0.089585776	-1.616553537	0.106950901	0.224400249	no
RBPJ	-0.089585185	-1.616542784	0.106953224	0.224400249	no
DSCAM-AS1	-0.089565219	-1.616179581	0.107031741	0.224543065	no
PRR4	0.08955928	1.616071563	0.107055101	0.224564039	no
DYNC2H1	-0.089557365	-1.616036718	0.107062638	0.224564039	no
SBSPON	0.089538374	1.615691267	0.107137377	0.224698874	no
DVL3	-0.089501035	-1.615012054	0.107284447	0.224985367	no
ABCD4	0.08949224	1.614852059	0.107319113	0.225036108	no
METTL21C	-0.089487559	-1.614766915	0.107337566	0.225052842	no

PABPC1L	0.089473328	1.614508053	0.107393681	0.225118137	no
KRTAP9-8	0.089471031	1.61446627	0.107402741	0.225118137	no
C7orf57	0.089469311	1.614434985	0.107409525	0.225118137	no
AKT1	-0.089469037	-1.614429992	0.107410608	0.225118137	no
LINC00343	-0.089464285	-1.614343565	0.107429351	0.225135464	no
PRKAR2A	0.089451287	1.614107132	0.107480639	0.225194074	no
TMC6	0.089450706	1.614096553	0.107482934	0.225194074	no
RASAL1	-0.089449231	-1.614069728	0.107488754	0.225194074	no
BAD	-0.089445473	-1.614001358	0.10750359	0.225203201	no
MATN1-AS1	0.089441299	1.61392544	0.107520065	0.225215762	no
PDE1B	-0.089423275	-1.613597577	0.107591239	0.225342882	no
NUP35	-0.089414434	-1.613436771	0.107626161	0.225394057	no
PLCXD3	0.089408991	1.613337766	0.107647666	0.225417128	no
SYVN1	0.089403929	1.61324569	0.107667669	0.225424975	no
PP7080	-0.089402734	-1.613223954	0.107672392	0.225424975	no
FAM9B	0.089399974	1.61317374	0.107683302	0.225425857	no
TTC32	-0.089390872	-1.613008178	0.107719282	0.225471605	no
MLC1	-0.089389138	-1.612976633	0.107726138	0.225471605	no
TNRC18	-0.089374657	-1.612713226	0.107783404	0.225569493	no
WASH1	-0.089364475	-1.612528023	0.107823682	0.225631816	no
PRPH2	-0.089360751	-1.612460287	0.107838416	0.225640678	no
CT60	-0.089351721	-1.612296032	0.107874153	0.225693479	no
LOC100506274	-0.089344026	-1.612156059	0.107904614	0.225714463	no
ACTR10	-0.089343881	-1.61215342	0.107905188	0.225714463	no
SAGE1	0.089314867	1.611625676	0.108020097	0.225932837	no
SIGLEC6	0.089303622	1.611421146	0.108064657	0.225978276	no
ARAF	-0.089300079	-1.611356684	0.108078704	0.225978276	no
PP2D1	-0.089298818	-1.611333763	0.108083699	0.225978276	no
PRKD3	0.089298772	1.611332927	0.108083881	0.225978276	no
WBSCR22	-0.089260016	-1.610627974	0.1082376	0.226277651	no
LOC100132891	0.089246013	1.610373272	0.108293181	0.226371827	no
SLC26A4	-0.089240127	-1.610266212	0.108316551	0.226398657	no
GTF3C3	-0.089220756	-1.609913876	0.108393489	0.226537438	no
NXF4	-0.089215014	-1.609809437	0.108416303	0.226563085	no
FAM35BP	-0.089201186	-1.60955792	0.108471262	0.226646136	no
AZGP1P1	-0.089199623	-1.609529491	0.108477475	0.226646136	no
LOC728175	0.089197055	1.609482786	0.108487683	0.226646136	no
GINS4	0.089194138	1.609429729	0.108499281	0.226648332	no
SLC22A10	-0.089186801	-1.609296267	0.108528458	0.22668434	no
TFPI	0.089182809	1.609223657	0.108544335	0.22668434	no
DND1	-0.089181847	-1.609206158	0.108548162	0.22668434	no
LOC100134368	-0.08916495	-1.608898821	0.108615385	0.226802686	no
KRT16	0.089155121	1.608720047	0.108654503	0.226862327	no
ZNF311	-0.089123074	-1.608137161	0.108782125	0.227106727	no
MCM4	-0.08910284	-1.607769132	0.108862765	0.227253006	no
ABCC9	0.089092996	1.607590087	0.108902014	0.227312857	no
SLC25A12	-0.089084439	-1.607434449	0.10893614	0.227362008	no
ANKS3	-0.089040339	-1.606632357	0.109112149	0.227707244	no
RPS6KA2-IT1	-0.08903095	-1.606461585	0.109149652	0.227763391	no
SELO	-0.089021245	-1.60628507	0.109188427	0.227822182	no
ANP32E	-0.089018175	-1.606229233	0.109200695	0.22782566	no

TUBBP5	0.089003187	1.605956634	0.109260603	0.22792852	no
B3GALT1	-0.088995726	-1.605820932	0.109290435	0.227961264	no
FKBP2	-0.088993956	-1.60578874	0.109297513	0.227961264	no
MIR320B2	0.088957997	1.605134739	0.109441386	0.228239188	no
NOL4	-0.088933208	-1.604683885	0.109540657	0.228424049	no
STX16	0.088914029	1.604335061	0.109617511	0.228562135	no
DGKH	0.088910797	1.604276286	0.109630465	0.228566969	no
RNF4	0.088892785	1.603948685	0.109702689	0.228695362	no
DMRTA2	0.088889777	1.603893986	0.109714752	0.228698325	no
ATP5E	0.088876715	1.603656416	0.109767155	0.228785369	no
PRICKLE2-AS3	-0.088851854	-1.603204267	0.109866946	0.228971154	no
EIF2B2	-0.088842417	-1.603032629	0.109904846	0.229027931	no
MGAT3	-0.088836043	-1.602916708	0.109930449	0.229041746	no
RDH12	0.08883546	1.602906106	0.109932791	0.229041746	no
INTS12	-0.088824554	-1.602707755	0.109976611	0.229110833	no
SPC25	-0.088801288	-1.602284621	0.110070138	0.229283448	no
FMN2	-0.088787979	-1.602042569	0.110123667	0.229358935	no
FEV	-0.088786971	-1.602024244	0.110127721	0.229358935	no
CD70	0.088760013	1.601533953	0.110236215	0.229562645	no
MAN1B1	-0.088748004	-1.601315565	0.110284569	0.229641087	no
WTIP	0.088726176	1.60091858	0.110372508	0.229801935	no
KLK9	-0.088719541	-1.600797917	0.110399248	0.229835343	no
METTL25	0.088714649	1.600708954	0.110418967	0.229854128	no
LOC400456	0.088703092	1.600498767	0.110465565	0.229928859	no
FRAS1	-0.088693551	-1.600325256	0.110504045	0.229986677	no
SWI5	0.088670912	1.599913521	0.110595397	0.230129971	no
DKK3	0.088669724	1.599891921	0.110600191	0.230129971	no
RPSA	-0.088668528	-1.599870167	0.110605019	0.230129971	no
ATP10B	0.088657844	1.599675875	0.110648151	0.230167612	no
LOC728752	-0.0886558	-1.599638694	0.110656407	0.230167612	no
LOC100505619	0.088655152	1.59962692	0.110659021	0.230167612	no
MRPL4	-0.088653436	-1.599595715	0.11066595	0.230167612	no
PTK7	0.088650296	1.599538609	0.110678631	0.230171712	no
VPS54	0.088615362	1.598903293	0.110819792	0.230442975	no
GBA2	-0.088609267	-1.598792444	0.110844436	0.23047192	no
ZNF486	-0.088599756	-1.598619477	0.110882899	0.23052959	no
FLJ40292	-0.088593759	-1.598510422	0.110907155	0.230557716	no
FAM180B	0.088581749	1.598292011	0.110955747	0.23063642	no
MRPS2	-0.088562817	-1.597947706	0.111032381	0.230773396	no
EFHA1	-0.08854776	-1.597673885	0.111093358	0.230877803	no
MAPK6	-0.088528884	-1.59733062	0.111169837	0.231014404	no
PCDHGB2	-0.08850576	-1.596910101	0.111263584	0.23118686	no
ITGA10	0.088500245	1.596809802	0.111285953	0.231210984	no
IFNA17	0.088497125	1.596753072	0.111298607	0.231214922	no
ZNF507	-0.088480037	-1.596442323	0.111367941	0.231336596	no
NPC1L1	0.088467194	1.59620876	0.111420075	0.231422523	no
PSMA3	-0.088439916	-1.595712697	0.111530868	0.231610961	no
TMEM256	-0.088438909	-1.595694396	0.111534957	0.231610961	no
PDIA3P	0.088436661	1.595653516	0.111544092	0.231610961	no
ALKBH2	-0.088434245	-1.595609583	0.111553909	0.231610961	no
RTN4	-0.088419822	-1.595347287	0.111612536	0.231710299	no

SMIM6	-0.088412587	-1.595215723	0.111641952	0.23174898	no
EIF4A1	0.088402897	1.595039515	0.111681359	0.231808391	no
C1QL2	-0.088385792	-1.594728458	0.11175095	0.231912196	no
PSMC6	-0.0883853	-1.594719511	0.111752953	0.231912196	no
NHEG1	-0.088374335	-1.594520112	0.111797582	0.231982412	no
SNX17	0.088367941	1.594403849	0.11182361	0.232014019	no
SLC31A2	0.088352848	1.59412938	0.111885076	0.232119141	no
ZNF512B	-0.088344416	-1.59397604	0.111919428	0.232167995	no
ALS2	-0.088333238	-1.593772775	0.111964977	0.232212189	no
TRIO	-0.088331004	-1.593732144	0.111974083	0.232212189	no
HRASLS2	0.088328732	1.593690845	0.11198334	0.232212189	no
TRAM1L1	-0.088325947	-1.593640201	0.111994692	0.232212189	no
OR7E37P	-0.088325931	-1.593639908	0.111994758	0.232212189	no
PRKAR2B	-0.088315715	-1.593454125	0.112036411	0.232263238	no
CCT2	-0.088312859	-1.593402198	0.112048055	0.232263238	no
ACSF3	0.088311941	1.593385495	0.112051801	0.232263238	no
ZNF227	-0.088295967	-1.593095014	0.112116959	0.232375886	no
PRNP	-0.088288141	-1.592952702	0.112148892	0.232419656	no
GPRC5C	-0.088282074	-1.592842379	0.112173652	0.232448553	no
FOXK1	-0.088276364	-1.592738551	0.112196958	0.232458428	no
CLP1	0.088275606	1.592724777	0.11220005	0.232458428	no
PLCXD1	-0.088266874	-1.592565983	0.112235703	0.232509879	no
SNORA74B	-0.088244084	-1.592151572	0.112328789	0.23268029	no
SLC25A22	-0.088239277	-1.592064157	0.112348433	0.232698551	no
SNORA66	0.088208968	1.591513009	0.112472346	0.232932753	no
LSG1	0.088195933	1.591275982	0.112525669	0.233020731	no
CLTC	0.088170269	1.590809309	0.112630713	0.233215788	no
LOC100129924	-0.088150547	-1.590450691	0.112711489	0.233360559	no
NS3BP	-0.088131436	-1.590103182	0.112789805	0.233500212	no
DIP2A	-0.088126992	-1.590022375	0.112808022	0.233515431	no
STX17	0.088122542	1.589941466	0.112826265	0.2335307	no
TMEM128	-0.088106932	-1.589657611	0.112890285	0.233606951	no
SERBP1	0.088105934	1.589639473	0.112894376	0.233606951	no
SDCCAG8	0.088104981	1.589622135	0.112898288	0.233606951	no
MUS81	-0.088102959	-1.589585361	0.112906584	0.233606951	no
MINOS1	0.088089901	1.589347925	0.112960163	0.23367393	no
AKAP1	-0.088089769	-1.589345533	0.112960703	0.23367393	no
INSIG2	0.088073919	1.589057332	0.113025764	0.233767214	no
MATN2	0.088073484	1.589049414	0.113027552	0.233767214	no
ING4	-0.088050923	-1.588639177	0.113120215	0.233936349	no
LOC100129534	0.088034502	1.588340597	0.113187695	0.234053378	no
FLJ45340	-0.088028009	-1.588222527	0.113214388	0.234080038	no
SLC12A6	-0.088026067	-1.588187217	0.113222372	0.234080038	no
ALG11	-0.088010455	-1.587903338	0.113286575	0.234190244	no
GTDC1	-0.088000533	-1.58772293	0.113327391	0.234252089	no
SPINK7	-0.087990234	-1.587535668	0.113369771	0.234317152	no
CDH13	0.087976464	1.587285286	0.113426455	0.234411765	no
SLC25A13	0.0879676	1.587124108	0.113462956	0.234464652	no
CCDC157	-0.087944216	-1.586698925	0.113559289	0.234641157	no
HSPA6	0.087932349	1.586483161	0.113608199	0.23471965	no
KLHL3	-0.08791783	-1.586219157	0.113668066	0.234820765	no

LOC283922	-0.08790228	-1.585936425	0.113732209	0.23493069	no
LOC100287225	-0.087861359	-1.585192379	0.113901145	0.235239078	no
MINOS1P1	-0.087860386	-1.585174683	0.113905165	0.235239078	no
CCDC158	0.087858165	1.5851343	0.11391434	0.235239078	no
EGOT	0.087853043	1.585041171	0.113935501	0.23526017	no
PDXK	-0.087843796	-1.584873044	0.113973711	0.235316459	no
LDHD	-0.08782587	-1.584547116	0.114047813	0.235446835	no
LOC642426	-0.087821629	-1.584469988	0.114065354	0.235460429	no
RNF214	-0.087818112	-1.584406059	0.114079895	0.235467828	no
VRK1	-0.087812303	-1.584300437	0.114103922	0.235494804	no
RPL19P12	-0.087803465	-1.584139734	0.114140488	0.235532729	no
IGF1R	-0.087802466	-1.584121565	0.114144622	0.235532729	no
PSMD4	-0.087799915	-1.584075188	0.114155177	0.235532729	no
GPR137B	-0.087775438	-1.583630144	0.114256497	0.23571915	no
HNMT	0.087770874	1.583547167	0.114275396	0.235735509	no
PRPF38B	0.087751695	1.58319845	0.114354847	0.235876764	no
TBC1D7	-0.087741527	-1.583013592	0.114396982	0.23594103	no
SLC17A8	-0.087722741	-1.582672031	0.114474867	0.23607901	no
UBA52	-0.087671167	-1.581734321	0.114688906	0.236497723	no
BCL7B	0.087631249	1.581008564	0.114854782	0.236808371	no
MAMDC4	-0.087624305	-1.58088232	0.114883655	0.236808371	no
PRKCSH	-0.087623186	-1.580861964	0.114888311	0.236808371	no
SCN1A	-0.087622153	-1.580843186	0.114892607	0.236808371	no
CCDC38	-0.087621662	-1.58083426	0.114894648	0.236808371	no
TMTC4	-0.087614639	-1.580706581	0.114923858	0.23684586	no
WNT2	0.087606396	1.580556712	0.114958151	0.236893818	no
SATL1	-0.087591197	-1.58028037	0.115021405	0.237001441	no
CHD1L	-0.087588492	-1.58023119	0.115032666	0.237001919	no
KCNK10	-0.087583767	-1.580145291	0.115052335	0.237019721	no
LRIT3	-0.087572608	-1.579942408	0.115098802	0.237066708	no
STARD9	-0.08757145	-1.579921354	0.115103625	0.237066708	no
ADARB2-AS1	-0.087570343	-1.579901227	0.115108236	0.237066708	no
EFNB1	0.087552892	1.579583962	0.115180935	0.237193701	no
ARG1	0.087545827	1.579455512	0.115210378	0.237231603	no
DHFRL1	-0.087531501	-1.579195058	0.115270098	0.237331834	no
MYOM1	-0.087526965	-1.579112578	0.115289016	0.237348044	no
TOMM20L	-0.087521861	-1.579019795	0.115310299	0.237369122	no
DUSP7	-0.087512882	-1.57885654	0.115347755	0.237423484	no
C17orf49	0.087434421	1.577430088	0.115675438	0.238075162	no
TBC1D25	-0.087423211	-1.577226288	0.115722315	0.238148834	no
ZNF490	-0.087413939	-1.577057723	0.115761099	0.238205838	no
VMA21	-0.087405593	-1.576905984	0.11579602	0.238246157	no
FDXACB1	-0.087403957	-1.576876246	0.115802864	0.238246157	no
ZNF284	-0.087388206	-1.576589883	0.115868793	0.238358977	no
PFN4	0.08738069	1.576453254	0.11590026	0.238400888	no
LEMD2	0.087341287	1.575736912	0.116065347	0.238717617	no
TFIP11	-0.087335677	-1.57563491	0.116088869	0.238743148	no
THY1	0.087326857	1.575474572	0.116125852	0.238796353	no
STK35	0.087316884	1.575293257	0.116167685	0.238859521	no
SMARCA5	-0.087305623	-1.575088542	0.11621493	0.238933804	no
DDX12P	-0.087278251	-1.574590934	0.116329834	0.239147165	no

SNAPC2	0.08723884	1.57387447	0.116495433	0.239445216	no
FRMD6	0.087238444	1.57386726	0.1164971	0.239445216	no
PMS2P4	0.087232752	1.573763782	0.116521033	0.239471504	no
ZSCAN30	-0.087228231	-1.573681594	0.116540044	0.239487674	no
NIPA1	-0.087208844	-1.573329162	0.116621596	0.239614313	no
LOC401010	-0.08720828	-1.57331891	0.116623969	0.239614313	no
CBX8	-0.087192429	-1.57303075	0.116690683	0.239728464	no
TM9SF3	-0.087183896	-1.57287563	0.116726608	0.239779347	no
CRLF1	-0.087166403	-1.572557612	0.116800287	0.239907767	no
CCR9	0.087144798	1.572164862	0.116891331	0.240071827	no
HGS	-0.087117399	-1.571666772	0.117006875	0.240286168	no
FAM90A7P	-0.087111134	-1.571552886	0.117033306	0.240317482	no
LCE1C	0.087101713	1.571381633	0.11707306	0.240376147	no
CD81	0.087070177	1.570808344	0.117206218	0.240610976	no
RBP1	0.087069259	1.570791653	0.117210097	0.240610976	no
SERF1A	-0.087066673	-1.570744653	0.117221019	0.240610976	no
WDR13	-0.087060492	-1.570632287	0.117247135	0.240641599	no
ZNF2	-0.087053536	-1.570505842	0.117276529	0.240678942	no
ZNF728	-0.087048417	-1.570412787	0.117298164	0.240700358	no
TANK	0.087025725	1.570000275	0.117394112	0.240874247	no
CIDEA	-0.087018608	-1.569870911	0.117424214	0.240913011	no
SIDT1	-0.087003039	-1.569587891	0.117490092	0.24102516	no
PPP1R36	0.086974499	1.569069084	0.117610929	0.241250022	no
DACH1	-0.086949186	-1.568608936	0.117718186	0.241427785	no
RTEL1	-0.086947293	-1.568574535	0.117726208	0.241427785	no
STK4-AS1	0.086943896	1.568512785	0.117740608	0.241427785	no
TTC39C	0.086943444	1.568504562	0.117742525	0.241427785	no
KIAA0895	-0.086918329	-1.56804802	0.117849035	0.241623126	no
FAM73A	-0.086913066	-1.567952352	0.117871364	0.241645853	no
HDHD1	-0.086900813	-1.567729624	0.117923361	0.241729392	no
MIR100HG	-0.086893736	-1.567600978	0.117953402	0.241767912	no
PMEL	0.086858709	1.566964272	0.118102174	0.242049763	no
ADSSL1	0.086844306	1.566702473	0.118163388	0.242152129	no
IL1RAP	-0.086830496	-1.566451428	0.118222112	0.242249371	no
TMEM200A	0.08681352	1.566142852	0.118294324	0.242374232	no
SLC22A16	0.08678191	1.565568279	0.118428877	0.242620823	no
ABCC10	0.086779943	1.565532519	0.118437255	0.242620823	no
TRIM47	0.086772296	1.565393514	0.118469827	0.242643994	no
FAM58A	-0.086771922	-1.565386729	0.118471417	0.242643994	no
UGT2B15	-0.086769335	-1.565339705	0.118482438	0.242643994	no
PSMD12	0.086729688	1.564619032	0.118651435	0.242945229	no
MAGEA10-MAGE	0.086729523	1.564616031	0.118652139	0.242945229	no
WWC3	0.086726692	1.564564573	0.118664213	0.242946805	no
LOC149086	0.086715925	1.564368864	0.118710144	0.243017689	no
HNRNPA3P1	0.086700625	1.564090754	0.118775436	0.243118564	no
TMEM242	-0.086699076	-1.564062614	0.118782045	0.243118564	no
NPBWR2	-0.086695815	-1.564003334	0.118795966	0.243123903	no
TRAPPC10	0.086688711	1.563874208	0.118826295	0.243162817	no
CTU2	-0.086675519	-1.563634413	0.118882634	0.243254946	no
PHF12	-0.086639787	-1.562984927	0.119035335	0.243522019	no
SEC11C	0.086637095	1.562936005	0.119046843	0.243522019	no

PWWP2A	0.086637023	1.562934694	0.119047151	0.243522019	no
CIRBP-AS1	0.086625441	1.562724168	0.119096685	0.24358971	no
ZNF45	0.086623985	1.56269771	0.119102911	0.24358971	no
RBM38	0.086612936	1.562496885	0.11915018	0.243663194	no
LOC158435	-0.086608707	-1.562420008	0.119168278	0.243669084	no
CCM2	0.086606964	1.562388333	0.119175736	0.243669084	no
WNK1	-0.086595818	-1.562185738	0.119223444	0.243723278	no
LOC100506054	-0.086595473	-1.562179456	0.119224923	0.243723278	no
LZTFL1	0.086574438	1.561797135	0.119314996	0.243873773	no
FAM45A	-0.086571775	-1.561748731	0.119326403	0.243873773	no
ANKLE1	-0.086570332	-1.561722499	0.119332586	0.243873773	no
CNPPD1	0.086565807	1.561640253	0.119351971	0.243890198	no
PAK4	0.086539843	1.561168324	0.119463256	0.244094394	no
MIR3180-4	-0.086531574	-1.561018022	0.119498715	0.244143635	no
GFRA4	-0.086518414	-1.560778836	0.119555162	0.244224632	no
TNMD	0.086517033	1.560753726	0.119561089	0.244224632	no
ZDHHC8	-0.086495266	-1.5603581	0.119654503	0.244392219	no
AP3M1	-0.086480917	-1.560097303	0.119716114	0.244494821	no
HECA	0.086476289	1.560013181	0.119735992	0.244512182	no
CUX1	-0.08645304	-1.55959061	0.119835886	0.244692923	no
ACTN1-AS1	0.086445541	1.559454314	0.119868119	0.244735489	no
ERVV-1	-0.086436034	-1.55928152	0.119908995	0.244795688	no
LOC642236	-0.086400447	-1.558634714	0.120062097	0.245084966	no
CCDC74B-AS1	-0.086397781	-1.558586257	0.120073573	0.245085114	no
GPR149	-0.086373401	-1.558143139	0.120178558	0.245255879	no
RABGAP1L	0.086371409	1.558106945	0.120187136	0.245255879	no
KCNAB2	-0.086370403	-1.558088655	0.120191472	0.245255879	no
FUT5	-0.086367066	-1.558027998	0.120205849	0.24526193	no
LRRC20	-0.086363558	-1.557964248	0.120220962	0.245269479	no
ACO1	-0.086342794	-1.557586859	0.120310456	0.245428762	no
ANKS4B	-0.086338489	-1.557508613	0.120329018	0.24544333	no
ESYT3	-0.086305072	-1.556901259	0.120473173	0.245695259	no
MOB2	-0.086304537	-1.556891538	0.120475482	0.245695259	no
USP5	-0.086301909	-1.556843776	0.120486824	0.245695259	no
C1orf27	0.086294083	1.55670154	0.120520606	0.245740699	no
FAM193A	-0.08629145	-1.556653673	0.120531977	0.245740699	no
LOC90834	-0.086286292	-1.556559938	0.120554245	0.245762786	no
ZFAND1	-0.086264737	-1.556168183	0.12064735	0.24592926	no
LINC00642	-0.086260838	-1.556097319	0.120664197	0.245940275	no
SNORA5C	-0.086256689	-1.556021913	0.120682127	0.245953493	no
ZNF678	-0.086247219	-1.555849794	0.120723059	0.246013585	no
ZNF571	-0.086237555	-1.555674162	0.120764839	0.246075391	no
LOC100132078	-0.08622646	-1.555472503	0.120812824	0.246149829	no
SPINK6	0.086194361	1.554889121	0.120951724	0.246409471	no
NUP85	0.086188321	1.554779355	0.120977873	0.246426109	no
CALB1	-0.086187178	-1.554758574	0.120982824	0.246426109	no
SPTBN1	-0.086155761	-1.5541876	0.121118919	0.246679937	no
COQ9	-0.086150182	-1.554086215	0.121143098	0.246705801	no
DOC2A	-0.086145671	-1.554004223	0.121162654	0.246722247	no
DHX57	-0.086138418	-1.55387241	0.121194099	0.246762896	no
RPUSD3	-0.08611349	-1.55341937	0.121302222	0.246959648	no

PLCE1	0.086104908	1.553263394	0.121339465	0.246998924	no
PCOLCE2	0.086103748	1.553242311	0.1213445	0.246998924	no
NOSIP	-0.086087507	-1.552947157	0.121415002	0.247099213	no
IL36B	0.086085599	1.552912476	0.121423288	0.247099213	no
PRPS1	-0.086084453	-1.552891656	0.121428263	0.247099213	no
LOC643529	0.086073007	1.552683634	0.121477975	0.247173109	no
MCOLN3	0.086070796	1.552643457	0.121487579	0.247173109	no
JMJD8	0.08605165	1.552295491	0.121570776	0.247318965	no
ACTR3	0.086023756	1.551788563	0.121692061	0.247542269	no
PCDH9-AS2	-0.086019298	-1.551707543	0.121711454	0.247542641	no
CUL4A	0.086018419	1.551691558	0.12171528	0.247542641	no
KLC3	-0.086007063	-1.551485193	0.121764688	0.247619693	no
CBWD5	0.086002129	1.551395529	0.121786161	0.247639927	no
SULT1A1	0.085994941	1.551264897	0.12181745	0.247680114	no
LEP	0.085967071	1.55075839	0.121938827	0.247903446	no
ZNF347	-0.085953246	-1.550507151	0.121999068	0.248002456	no
PCGF1	0.085948643	1.550423496	0.122019131	0.248019781	no
IFNL2	-0.085936778	-1.550207871	0.122070858	0.248085957	no
PSMG3-AS1	0.085935879	1.550191545	0.122074775	0.248085957	no
TOPBP1	-0.085925746	-1.550007387	0.122118968	0.24811143	no
MAGEA8	0.0859255	1.550002917	0.122120041	0.24811143	no
LOC284009	-0.085923585	-1.549968117	0.122128394	0.24811143	no
LHX1	-0.085920999	-1.549921132	0.122139672	0.24811143	no
PPP1R14C	-0.085919317	-1.549890552	0.122147012	0.24811143	no
PRG1	-0.085917124	-1.549850699	0.122156579	0.24811143	no
MGC2752	0.085912313	1.549763275	0.122177568	0.24813061	no
SNRPE	-0.085899675	-1.549533603	0.122232722	0.248219165	no
EXOSC9	-0.085871311	-1.549018149	0.122356574	0.248447197	no
FLJ12825	-0.085868651	-1.548969811	0.122368193	0.248447316	no
PLEKHG4	0.085848968	1.548612107	0.122454206	0.248598464	no
RPL36A	-0.085830037	-1.548268092	0.122536972	0.248742993	no
HSP90AB4P	-0.08581358	-1.547969013	0.122608963	0.248865623	no
IL22RA2	0.08580162	1.547751681	0.122661297	0.248948336	no
CCDC178	-0.085766968	-1.54712197	0.122813032	0.249232755	no
ZNF461	-0.085740692	-1.546644468	0.12292819	0.249442897	no
LOC100505761	-0.08573455	-1.546532853	0.12295512	0.24945097	no
PNPLA2	0.08573449	1.546531764	0.122955382	0.24945097	no
ASNA1	-0.08570901	-1.546068736	0.123067149	0.249654154	no
HAUS2	0.085703044	1.545960329	0.123093328	0.249683549	no
ABCG4	-0.085700413	-1.545912517	0.123104876	0.249683549	no
PARD6G	0.085686824	1.545665578	0.123164529	0.249780966	no
MRPL11	0.085670802	1.54537442	0.123234894	0.249879049	no
COX18	-0.085670517	-1.545369237	0.123236147	0.249879049	no
KIF26A	-0.085664346	-1.5452571	0.123263256	0.249909787	no
CDC42EP4	-0.085661772	-1.545210333	0.123274564	0.249909787	no
GPX3	0.085645754	1.544919261	0.123344957	0.250028907	no
ASCC1	-0.085633131	-1.544689876	0.123400454	0.250094642	no
CMBL	-0.085629938	-1.54463186	0.123414494	0.250094642	no
FAM107A	-0.085628873	-1.54461249	0.123419181	0.250094642	no
LOC100133669	-0.085627792	-1.544592851	0.123423934	0.250094642	no
LOC100505738	-0.085624979	-1.544541737	0.123436305	0.250096128	no

LSMD1	-0.085611937	-1.544304735	0.123493679	0.250188787	no
GDPGP1	0.085603689	1.544154859	0.123529972	0.25021775	no
CREB5	-0.085603396	-1.544149536	0.123531261	0.25021775	no
GALC	0.085581139	1.5437451	0.123629239	0.250392609	no
DDRGK1	0.085576295	1.543657077	0.123650571	0.250412215	no
KIAA1324	0.085568645	1.543518061	0.123684268	0.250433156	no
ZNF646	-0.085567165	-1.543491163	0.123690788	0.250433156	no
HOXD3	-0.085566011	-1.543470199	0.12369587	0.250433156	no
NIT2	0.085547971	1.543142384	0.123775365	0.250570494	no
TPCN1	0.085525992	1.542743005	0.123872267	0.250743043	no
PSRC1	0.085516307	1.542567012	0.123914987	0.250805895	no
GATM	0.085495002	1.542179871	0.124009002	0.250972546	no
PGM5	0.085491807	1.542121819	0.124023105	0.250977452	no
SCUBE1	-0.085477019	-1.541853112	0.124088398	0.251085938	no
LOC100507537	-0.085462797	-1.541594683	0.124151218	0.251182637	no
CNBD2	-0.085460084	-1.541545384	0.124163205	0.251182637	no
SRBD1	0.085458263	1.541512299	0.12417125	0.251182637	no
CIR1	-0.085444083	-1.541254629	0.124233919	0.251285756	no
CPA2	-0.085433447	-1.54106137	0.124280939	0.251357204	no
ZNF717	-0.085418628	-1.540792101	0.124346474	0.251466085	no
KIAA0226	-0.085409231	-1.540621337	0.12438805	0.251526494	no
THEM4	-0.085401217	-1.540475719	0.124423512	0.251574531	no
LOC100216545	-0.08539496	-1.540362038	0.124451201	0.251606846	no
NUDCD3	-0.085389665	-1.540265816	0.124474642	0.251630565	no
NANOG	-0.085383651	-1.540156539	0.124501268	0.2516531	no
ZNF12	0.085381857	1.540123949	0.124509209	0.2516531	no
SNORA63	-0.085363821	-1.539796215	0.124589093	0.251771796	no
KCTD13	-0.085362337	-1.539769259	0.124595665	0.251771796	no
FOLH1B	-0.085360663	-1.539738842	0.124603082	0.251771796	no
PCDHB3	-0.085353934	-1.539616565	0.124632899	0.25180837	no
MTAP	0.085342609	1.539410783	0.124683092	0.2518861	no
EFCAB4A	0.085321722	1.53903127	0.124775701	0.252031567	no
MTRNR2L3	-0.085321079	-1.539019589	0.124778553	0.252031567	no
LOC100996307	-0.08531647	-1.538935838	0.124798997	0.252049173	no
TMEM17	0.085311364	1.53884305	0.124821651	0.252071237	no
FAM111B	0.085293803	1.538523969	0.124899578	0.252204908	no
KLRF2	0.085288044	1.538419331	0.124925141	0.252232827	no
DDX24	-0.085260683	-1.537922188	0.125046651	0.252454446	no
RGPD2	-0.085249706	-1.537722728	0.125095428	0.252522572	no
POLG	-0.085245417	-1.537644796	0.12511449	0.252522572	no
CCDC64B	-0.085245157	-1.537640069	0.125115646	0.252522572	no
CFHR3	0.085231254	1.53738745	0.125177452	0.25261369	no
C4orf45	-0.085229713	-1.537359464	0.125184301	0.25261369	no
SLC25A44	-0.085226687	-1.537304473	0.125197759	0.252617128	no
FRMD4A	-0.085216001	-1.53711032	0.125245282	0.252683843	no
SIK2	-0.085213965	-1.53707333	0.125254338	0.252683843	no
LOC440895	0.085210744	1.53701479	0.125268671	0.252689038	no
SMNDC1	0.085206741	1.536942067	0.125286478	0.252701239	no
NTN3	-0.085202315	-1.536861649	0.125306172	0.252717243	no
LRRC10	-0.085197386	-1.536772079	0.125328109	0.252726747	no
PCDHA6	-0.085195971	-1.536746382	0.125334404	0.252726747	no

PSMA7	0.085171458	1.536300993	0.125443538	0.252923078	no
PDE6D	-0.085168787	-1.536252459	0.125455435	0.252923336	no
FAM222B	-0.085151846	-1.535944646	0.125530908	0.253051754	no
UBA3	-0.08512755	-1.535503206	0.125639208	0.253241821	no
GIPC1	-0.085125408	-1.535464278	0.125648762	0.253241821	no
PLIN4	-0.085117163	-1.535314468	0.125685534	0.253292179	no
PCDHA4	-0.085105366	-1.535100124	0.125738161	0.253374478	no
VWA9	-0.085099086	-1.534986031	0.12576618	0.25340718	no
BSG	-0.085077966	-1.534602299	0.125860456	0.253573363	no
MSI1	-0.085048803	-1.534072424	0.125990727	0.253812028	no
POU5F1B	-0.085040968	-1.533930071	0.126025743	0.253855346	no
SLC12A1	-0.085035056	-1.533822659	0.126052169	0.253855346	no
DCAF8	-0.085034656	-1.533815391	0.126053957	0.253855346	no
UGT2B11	-0.085033421	-1.533792953	0.126059478	0.253855346	no
DAXX	-0.085022136	-1.533587921	0.126109936	0.253933161	no
BLMH	-0.085009631	-1.533360727	0.126165866	0.254007771	no
CCZ1	0.085008567	1.533341388	0.126170627	0.254007771	no
CHST9	-0.084994137	-1.53307922	0.126235193	0.254102362	no
KDM1B	-0.084992782	-1.533054592	0.12624126	0.254102362	no
N4BP2L2-IT2	-0.084955255	-1.532372783	0.126409301	0.254416771	no
SNX3	0.084925158	1.531825963	0.126544198	0.254664422	no
NRM	0.084908386	1.531521242	0.12661942	0.254791943	no
FOXJ3	0.084886911	1.531131091	0.126715781	0.254961975	no
ZNF639	-0.084865995	-1.530751072	0.126809695	0.255127051	no
LOC150381	-0.08485948	-1.53063271	0.126838957	0.255162036	no
MAP1LC3C	0.084848839	1.530439391	0.126886762	0.255234313	no
HSD17B7P2	-0.084839636	-1.530272191	0.126928119	0.255275212	no
MAL	-0.084839028	-1.530261147	0.126930851	0.255275212	no
ADH1B	-0.084822578	-1.529962269	0.127004806	0.255400046	no
MAZ	-0.084813028	-1.529788773	0.127047752	0.255462504	no
LONP1	-0.084805671	-1.529655109	0.127080846	0.255505142	no
LOC728012	0.084796395	1.529486585	0.12712258	0.255558896	no
ECHDC1	0.084794443	1.52945112	0.127131365	0.255558896	no
PPP1R1C	0.084779328	1.529176515	0.127199396	0.255671738	no
CORIN	0.084758024	1.528789477	0.127295331	0.255840639	no
CLRN3	0.084749457	1.528633829	0.127333927	0.25589428	no
IDH2	-0.084743659	-1.528528496	0.127360052	0.25592285	no
FGF16	0.084720269	1.528103555	0.127465488	0.256110772	no
LOC100133315	-0.084710796	-1.527931454	0.12750821	0.256172659	no
SNORA4	0.084667893	1.527152035	0.127701828	0.256528986	no
RGS14	0.084666207	1.527121402	0.127709442	0.256528986	no
DST	0.084653818	1.526896329	0.127765398	0.2566174	no
FBXO15	-0.084641712	-1.5266764	0.127820094	0.256703266	no
LINC00842	-0.084637081	-1.526592252	0.127841027	0.256721314	no
CSNK1A1L	-0.084628648	-1.526439053	0.127879143	0.256773863	no
RNF126	-0.084623504	-1.526345613	0.127902395	0.256796559	no
NPHP3-ACAD11	-0.084613052	-1.526155735	0.127949656	0.256867451	no
MED14	0.084596309	1.525851565	0.128025393	0.25699549	no
MMP25	0.084592935	1.525790267	0.12804066	0.257002131	no
ARHGAP17	0.084585569	1.525656454	0.128073993	0.257032653	no
DDX19B	-0.084584289	-1.525633201	0.128079786	0.257032653	no

GOLGA6L7P	-0.084576846	-1.525497976	0.128113479	0.257067306	no
MAGEC2	0.08457519	1.525467885	0.128120977	0.257067306	no
RGL2	-0.084515857	-1.52439002	0.128389798	0.257582632	no
SLC13A1	-0.084510704	-1.524296401	0.128413168	0.257605469	no
LOC100505768	-0.084507053	-1.52423007	0.128429727	0.257609251	no
ASIP	0.084505003	1.524192824	0.128439027	0.257609251	no
SRG7	-0.084489307	-1.523907695	0.128510233	0.257728016	no
LPAR4	-0.084471632	-1.523586607	0.128590457	0.257864842	no
MGC34034	0.084461987	1.523411381	0.128634254	0.25790849	no
LIMS3	0.084461553	1.523403496	0.128636225	0.25790849	no
CRSP8P	-0.084457281	-1.5233259	0.128655623	0.257923321	no
PDCD6	-0.08445074	-1.523207082	0.128685332	0.25794695	no
MIR5010	-0.084449401	-1.523182755	0.128691415	0.25794695	no
CXCR1	0.084429437	1.522820076	0.128782132	0.25810471	no
FCHO1	-0.084417545	-1.522604045	0.128836193	0.258188979	no
BRS3	-0.084386418	-1.52203859	0.128977777	0.258448616	no
CDHR2	-0.084382135	-1.521960796	0.128997265	0.258463568	no
FTCD	-0.084374344	-1.521819258	0.129032728	0.258510521	no
NFKBIA	0.084370847	1.521755732	0.129048647	0.258518315	no
PIAS3	0.084364973	1.521649027	0.129075391	0.258547788	no
CCL20	0.084355026	1.52146833	0.129120688	0.258614417	no
PRELP	-0.08434681	-1.521319087	0.129158109	0.258637024	no
ZNF577	-0.084345322	-1.52129205	0.12916489	0.258637024	no
ERCC8	-0.084344622	-1.521279332	0.129168079	0.258637024	no
CEP41	0.084336156	1.521125546	0.129206651	0.258668764	no
LY6H	-0.084335859	-1.521120158	0.129208002	0.258668764	no
DNAH10	-0.084302387	-1.520512111	0.129360599	0.258950134	no
SLC24A2	-0.084292415	-1.520330974	0.129406085	0.25899448	no
OR7E156P	-0.084291776	-1.520319355	0.129409003	0.25899448	no
FAM203A	0.084289605	1.520279923	0.129418907	0.25899448	no
CLEC18C	0.084282934	1.520158744	0.129449346	0.259031274	no
SSTR5	-0.084272887	-1.519976231	0.129495201	0.259098908	no
ZMAT1	-0.084249005	-1.519542404	0.12960425	0.259292956	no
C19orf43	-0.084244762	-1.519465328	0.129623631	0.259307592	no
RHCE	0.084234386	1.519276846	0.129671037	0.259378281	no
SH2D1B	0.084229969	1.519196623	0.129691218	0.259394506	no
ITPK1-AS1	-0.084221796	-1.519048145	0.129728576	0.259433828	no
IQGAP2	0.084220385	1.519022531	0.129735022	0.259433828	no
CENPV	-0.084212119	-1.51887237	0.129772813	0.259485256	no
CYB5A	0.084206501	1.518770317	0.129798502	0.259493903	no
MIR199B	-0.084200661	-1.518664235	0.129825209	0.259493903	no
HELB	0.084200051	1.518653151	0.129828	0.259493903	no
MSANTD3	0.084199429	1.518641855	0.129830844	0.259493903	no
ZNF230	0.084197971	1.518615379	0.129837511	0.259493903	no
LOC100128568	-0.084168321	-1.518076778	0.129973185	0.259726277	no
SPANXA2-OT1	0.08416728	1.518057874	0.129977949	0.259726277	no
LOC339666	-0.084132301	-1.51742248	0.130138153	0.260022225	no
WDR88	-0.084101478	-1.516862587	0.130279448	0.260280341	no
LOC284080	0.084083283	1.516532086	0.13036291	0.260422876	no
SLC35E2	-0.084078598	-1.51644699	0.130384406	0.260441609	no
FLJ42102	-0.084071097	-1.516310739	0.13041883	0.26048616	no

SMCHD1	-0.084060396	-1.516116354	0.130467954	0.26056006	no
MLST8	-0.084042884	-1.515798263	0.130548372	0.260696437	no
DZIP1L	0.084022705	1.515431735	0.130641083	0.260857336	no
EFNA5	-0.084014536	-1.515283353	0.13067863	0.260908066	no
ZNF148	-0.083989455	-1.514827773	0.130793963	0.261114079	no
MCU	-0.083984002	-1.514728722	0.130819049	0.261139902	no
KLHL15	-0.083953174	-1.514168772	0.130960934	0.261373921	no
HCG18	-0.083951802	-1.514143837	0.130967255	0.261373921	no
TFB2M	0.083950604	1.514122093	0.130972768	0.261373921	no
SNORA75	-0.083939467	-1.513919796	0.131024061	0.261452005	no
PLCH1	-0.0839317	-1.513778724	0.131059839	0.261499118	no
SNCA	-0.083924763	-1.51365272	0.131091803	0.261538612	no
TOB1	0.083918615	1.51354105	0.131120135	0.261570855	no
ZNF146	-0.083908368	-1.51335492	0.13116737	0.261640796	no
BCAS2	0.083900523	1.513212425	0.13120354	0.261688656	no
AAK1	-0.083895694	-1.513124718	0.131225807	0.26170878	no
SBF2-AS1	0.083883049	1.51289503	0.131284134	0.261800808	no
PRMT1	-0.083877527	-1.512794735	0.131309609	0.261827315	no
ATP12A	-0.083865681	-1.512579561	0.131364276	0.261905235	no
DCAF11	-0.083863777	-1.512544994	0.13137306	0.261905235	no
LARP4B	-0.083852526	-1.512340632	0.131425	0.26198448	no
YES1	-0.083846277	-1.51222712	0.131453857	0.2620177	no
CILP	0.083828794	1.511909569	0.13153461	0.262154346	no
SPINK1	0.083815552	1.511669064	0.131595796	0.262243494	no
ABI1	-0.083804557	-1.511469352	0.131646621	0.262243494	no
PTPRQ	0.083804144	1.511461859	0.131648528	0.262243494	no
PLCD4	-0.083803803	-1.511455661	0.131650106	0.262243494	no
MAPK9	-0.083798649	-1.511362054	0.131673934	0.262243494	no
RAB27B	-0.083796764	-1.511327807	0.131682652	0.262243494	no
NRDE2	-0.083795339	-1.511301927	0.131689241	0.262243494	no
TOMM40L	0.083794231	1.511281799	0.131694366	0.262243494	no
PPP1R10	-0.083792867	-1.51125703	0.131700672	0.262243494	no
CHKB-CPT1B	-0.083792717	-1.51125431	0.131701365	0.262243494	no
GJB2	0.083765384	1.510757844	0.131827818	0.262470969	no
GPC1	0.083752532	1.510524417	0.131887306	0.262565086	no
AFF2	-0.083748947	-1.510459311	0.131903902	0.262573801	no
DDX20	0.083738736	1.510273834	0.13195119	0.262643607	no
LOC389247	0.083705906	1.509677549	0.132103304	0.262922032	no
TP53I3	0.083685675	1.509310099	0.132197109	0.263084366	no
ZNF542	0.083678656	1.509182625	0.132229663	0.263124787	no
DTHD1	0.083663593	1.508909033	0.132299555	0.26323949	no
HSPD1	-0.083659021	-1.508826005	0.132320771	0.263257331	no
RECK	0.083645924	1.50858813	0.132381569	0.263353911	no
ZMAT4	-0.083605321	-1.507850685	0.13257019	0.263704735	no
DUSP10	0.083591312	1.507596236	0.132635321	0.263809873	no
SMAD5-AS1	0.083587926	1.507534749	0.132651063	0.263816769	no
LOC100507351	-0.083561983	-1.507063567	0.132771747	0.264032352	no
ERVK13-1	-0.083555458	-1.506945061	0.132802114	0.264068304	no
C16orf82	0.083552606	1.506893254	0.132815391	0.264070271	no
SETD2	-0.083546084	-1.506774804	0.13284575	0.2641062	no
PHF10	-0.083542503	-1.506709777	0.13286242	0.264114907	no

HSPA4L	0.083525696	1.506404524	0.132940691	0.264234876	no
MAGI2-AS2	0.083524265	1.506378524	0.13294736	0.264234876	no
ZNF663	-0.083515628	-1.506221666	0.132987596	0.264290405	no
C19orf25	-0.083508909	-1.506099635	0.133018906	0.264328183	no
FAM134B	0.083505533	1.506038316	0.13303464	0.264335009	no
GATM-AS1	-0.083492493	-1.505801485	0.133095426	0.264431339	no
MYCT1	0.083457833	1.505172001	0.133257095	0.264728066	no
C11orf83	-0.083447044	-1.504976068	0.133307447	0.264787875	no
DPP8	-0.083446103	-1.504958967	0.133311842	0.264787875	no
SYBU	-0.083442004	-1.504884526	0.133330977	0.264801407	no
ELP4	-0.083434744	-1.504752669	0.133364875	0.264844256	no
SLC20A1	0.083429364	1.504654968	0.133389997	0.264869669	no
TACR1	-0.083404561	-1.5042045	0.133505873	0.26507527	no
CYB5RL	0.08337743	1.503711768	0.133632711	0.265302595	no
BMP6	-0.083362762	-1.503445393	0.13370132	0.265414286	no
GP6	0.083354993	1.503304296	0.133737672	0.265461929	no
RUFY1	0.083339178	1.503017076	0.133811696	0.265584333	no
FEZF1-AS1	0.083332398	1.502893941	0.13384344	0.265622807	no
SLC35A5	-0.083304773	-1.502392236	0.133972842	0.265855065	no
G6PC2	-0.083293848	-1.502193835	0.134024041	0.265932109	no
DDX11	-0.083280196	-1.501945901	0.134088045	0.266034543	no
NUP205	0.083271953	1.501796197	0.134126702	0.266086674	no
DIMT1	-0.083253969	-1.501469608	0.134211064	0.266215967	no
CDC123	-0.083252779	-1.501447995	0.134216649	0.266215967	no
LINC00472	-0.083247365	-1.501349673	0.134242056	0.266241788	no
RSPH10B2	-0.083236153	-1.501146057	0.134294683	0.266321586	no
N4BP2	-0.083233256	-1.501093437	0.134308286	0.266323987	no
OSBPL9	0.083228688	1.501010477	0.134329734	0.266341943	no
PIWIL2	-0.083220105	-1.500854609	0.134370039	0.26639728	no
MED31	0.083213327	1.500731525	0.134401874	0.266435815	no
RFT1	0.083179328	1.500114079	0.134561658	0.266724477	no
SLC22A18AS	0.083175926	1.500052305	0.134577652	0.266724477	no
COX7C	-0.083174423	-1.500025007	0.13458472	0.266724477	no
ECI1	-0.083150786	-1.49959575	0.134695906	0.266920214	no
CCDC111	0.083120367	1.499043339	0.134839096	0.26717933	no
FOXN3	-0.083113522	-1.498919042	0.134871331	0.267218566	no
TMEM74B	-0.083093706	-1.498559172	0.134964694	0.267378894	no
CTXN1	-0.083079178	-1.498295356	0.135033169	0.267489891	no
FBXW9	0.08305674	1.497887883	0.135138983	0.267674829	no
MINK1	-0.083037001	-1.497529431	0.135232121	0.267834625	no
ATP5L	-0.083018701	-1.497197102	0.135318516	0.267981038	no
SLC48A1	-0.083004099	-1.496931937	0.135387481	0.26809291	no
ELP2	-0.083000655	-1.496869393	0.135403751	0.268100426	no
ELMO2	-0.082990948	-1.496693115	0.135449618	0.268166534	no
PDZD2	-0.082964591	-1.496214495	0.135574212	0.268388485	no
ADD2	-0.08295993	-1.496129854	0.135596255	0.268407398	no
LOC374443	0.08295218	1.495989119	0.135632913	0.268455234	no
QDPR	-0.082947328	-1.495901005	0.135655868	0.268475942	no
LMO7	-0.082937667	-1.495725568	0.135701581	0.268494814	no
GSK3A	-0.082935148	-1.495679829	0.135713502	0.268494814	no
DISC1	-0.082935119	-1.4956793	0.135713639	0.268494814	no

EGLN3	-0.082934752	-1.495672634	0.135715377	0.268494814	no
MAP2K7	-0.082926499	-1.495522769	0.135754439	0.268547372	no
EPHA4	-0.082898835	-1.495020406	0.135885445	0.268781785	no
SGPP2	-0.082875392	-1.494594705	0.135996536	0.268971022	no
HIST2H2BC	-0.082873364	-1.494557888	0.136006147	0.268971022	no
VENTXP7	-0.082864936	-1.494404833	0.136046107	0.269025294	no
SEMA5A	-0.082858568	-1.494289199	0.136076304	0.269060249	no
KRT6C	0.082853819	1.494202975	0.136098824	0.269066047	no
FAM72A	0.082851391	1.494158873	0.136110343	0.269066047	no
TRIM51	-0.08285003	-1.49413417	0.136116796	0.269066047	no
CES5AP1	-0.082829933	-1.493769225	0.136212153	0.269229779	no
LRIG2	-0.082819001	-1.493570717	0.136264043	0.269307574	no
HMGXB3	0.082815374	1.493504855	0.136281263	0.269316839	no
SLC44A3	0.082799792	1.493221909	0.13635526	0.269438294	no
MAP4K4	-0.082772091	-1.492718903	0.136486883	0.269673587	no
FLJ35390	-0.082765822	-1.492605053	0.136516688	0.269707681	no
STAG3L3	-0.082747906	-1.492279742	0.136601881	0.269851183	no
CAPS2	0.082740708	1.492149036	0.136636121	0.269894015	no
SCN9A	0.08273766	1.492093693	0.136650622	0.26989785	no
STYXL1	0.082732717	1.492003924	0.136674144	0.269919503	no
ZNF583	-0.082708292	-1.491560414	0.136790404	0.270124283	no
EIF2S1	-0.08269317	-1.491285831	0.136862421	0.270241665	no
MSTN	-0.08268859	-1.491202655	0.136884242	0.270259921	no
MRPL13	0.082673986	1.490937474	0.136953829	0.270354122	no
NDUFAF5	-0.08267109	-1.490884898	0.136967629	0.270354122	no
RPS10P7	0.082670657	1.490877034	0.136969693	0.270354122	no
IGF2BP1	0.082662491	1.490728761	0.137008617	0.270406116	no
VPS13A-AS1	-0.082626447	-1.490074271	0.137180535	0.270720559	no
CYP11A1	0.082609241	1.489761844	0.137262661	0.270857759	no
SUMO3	0.082592788	1.489463097	0.137341226	0.270987715	no
SMO	0.082590169	1.489415534	0.137353738	0.270987715	no
CNST	-0.082559516	-1.488858965	0.137500209	0.271251789	no
37681	0.08254853	1.488659472	0.137552739	0.27133051	no
ALKBH7	-0.082533651	-1.488389317	0.137623899	0.271424034	no
CPA5	0.082533335	1.488383581	0.137625411	0.271424034	no
SYNRG	-0.08252216	-1.488180675	0.137678877	0.271504564	no
NGEF	-0.082509604	-1.487952681	0.137738973	0.271598152	no
GLTSCR2	-0.082503514	-1.487842116	0.137768124	0.27163071	no
FOXQ1	-0.082495279	-1.487692588	0.137807554	0.271683529	no
BFSP1	0.082486799	1.487538612	0.137848168	0.271738669	no
KRT33B	-0.082468455	-1.487205537	0.137936052	0.271886975	no
EGFL8	-0.082457137	-1.487000035	0.137990297	0.271968954	no
SNX24	0.082454314	1.486948781	0.138003829	0.271970682	no
STRN4	-0.082440929	-1.486705744	0.138068008	0.272072213	no
SLED1	0.082428493	1.48647994	0.138127657	0.2721648	no
LINC00689	-0.082423924	-1.486396993	0.138149573	0.272181863	no
HIGD1C	-0.082420805	-1.486340352	0.13816454	0.272181863	no
KLF4	0.082418769	1.486303379	0.138174311	0.272181863	no
GJC1	0.082393499	1.485844557	0.138295608	0.272395831	no
TENC1	0.08238657	1.485718752	0.138328881	0.27241479	no
PCMTD2	-0.082386215	-1.48571231	0.138330585	0.27241479	no

RANBP3	-0.08238302	-1.485654296	0.138345931	0.272420047	no
BYSL	-0.082371676	-1.485448322	0.138400425	0.272502386	no
GDE1	0.082361122	1.485256699	0.138451138	0.272577263	no
HSFX2	-0.082343685	-1.484940109	0.138534954	0.272717295	no
KLHL31	-0.082338074	-1.484838234	0.138561934	0.272745422	no
LRRC8B	-0.082319569	-1.484502237	0.138650945	0.272895636	no
CTNNA2	-0.082304427	-1.484227311	0.13872381	0.273014047	no
CCDC41	-0.082298779	-1.484124765	0.138750996	0.273032616	no
DENND4B	-0.082297188	-1.484095879	0.138758654	0.273032616	no
ARL16	-0.082289141	-1.483949787	0.138797393	0.273083839	no
CLEC19A	0.082282756	1.483833853	0.138828141	0.273100889	no
C1QL4	-0.082282064	-1.483821288	0.138831474	0.273100889	no
ANKRD52	0.082278832	1.483762614	0.138847038	0.273106506	no
SLC16A6	0.082257872	1.483382061	0.138948016	0.273280115	no
ISCA2	0.082226943	1.482820503	0.139097127	0.273548348	no
TBX2	0.082215555	1.482613749	0.139152057	0.273631336	no
HSPA13	-0.082206144	-1.482442877	0.139197468	0.273695588	no
IRF2BP1	-0.082194762	-1.482236231	0.1392524	0.27377855	no
ANKRD32	0.082177866	1.481929466	0.139333978	0.273913879	no
FAM135B	-0.082169244	-1.481772926	0.139375621	0.273970683	no
KCNJ6	-0.082154474	-1.481504768	0.139446979	0.274085882	no
ADAMTS9-AS2	0.082142807	1.481292953	0.139503364	0.274171633	no
ANKRD26P1	0.082133549	1.481124859	0.139548123	0.27423452	no
ASIC3	-0.082122698	-1.480927845	0.139600597	0.274312556	no
CCDC105	-0.082117343	-1.480830631	0.139626495	0.274338362	no
GRIK1-AS1	-0.08208873	-1.480311146	0.13976495	0.274585296	no
ZC3HAV1L	0.082083523	1.480216616	0.139790157	0.274609712	no
CCT6P1	-0.082074425	-1.480051444	0.139834207	0.27467114	no
ZNF77	-0.082069249	-1.479957461	0.139859277	0.27467323	no
PTPRK	-0.082068928	-1.47995163	0.139860832	0.27467323	no
SNF8	0.082062893	1.479842061	0.139890064	0.274705534	no
MRAP2	-0.082021655	-1.479093392	0.140089928	0.275072876	no
CTSG	0.081981989	1.478373249	0.140282384	0.275425608	no
NBPF3	0.081973587	1.478220704	0.140323177	0.275480534	no
PPAPDC1A	0.081970676	1.478167859	0.140337311	0.275483116	no
TMEM14A	-0.081950168	-1.477795536	0.140436924	0.275653478	no
PROSER1	-0.081942508	-1.47765648	0.140474141	0.275701349	no
ENPP3	-0.081910828	-1.477081338	0.140628156	0.275978421	no
MGC45922	-0.081899155	-1.476869416	0.140684938	0.276064646	no
PROB1	0.081893188	1.476761096	0.140713968	0.276096402	no
ILVBL	-0.081889184	-1.476688405	0.140733452	0.276109423	no
KLRC4	-0.081855366	-1.476074459	0.140898097	0.276407211	no
FAM208A	0.08182748	1.475568205	0.141033973	0.27657626	no
IP6K2	-0.081826528	-1.475550914	0.141038615	0.27657626	no
GRAMD3	-0.081824917	-1.475521667	0.141046468	0.27657626	no
ZNF124	0.081823922	1.475503605	0.141051318	0.27657626	no
OTOF	-0.081822544	-1.475478592	0.141058035	0.27657626	no
UPF3B	-0.081821836	-1.475465742	0.141061486	0.27657626	no
SCARNA8	0.081816364	1.475366405	0.141088163	0.276603331	no
MIRLET7BHG	-0.081795917	-1.47499521	0.141187884	0.276773585	no
FGL1	0.081787921	1.474850047	0.141226896	0.276824718	no

PITX3	-0.081785291	-1.474802303	0.14123973	0.276824718	no
KIAA0368	0.081775532	1.474625126	0.14128736	0.27689282	no
MIR769	-0.081730599	-1.473809422	0.141506808	0.277297604	no
ATP6V1D	-0.081724393	-1.47369677	0.141537136	0.277331745	no
C2orf76	-0.081690481	-1.473081141	0.141702959	0.277631351	no
ARL5B	-0.081683709	-1.472958198	0.141736093	0.277658529	no
RNF6	0.081682365	1.472933794	0.14174267	0.277658529	no
LOC399815	-0.081667218	-1.472658822	0.1418168	0.277772462	no
DOHH	-0.081665199	-1.472622174	0.141826682	0.277772462	no
GRIK4	-0.081642867	-1.472216767	0.141936036	0.277961305	no
CCDC115	-0.08161928	-1.471788585	0.142051605	0.278162282	no
MTCH2	0.081596277	1.47137101	0.14216438	0.278357753	no
SERINC3	0.081583357	1.471136467	0.142227754	0.278436677	no
RFWD3	0.081582776	1.471125932	0.142230601	0.278436677	no
BRF2	-0.081579229	-1.471061534	0.142248006	0.278445386	no
ZNF671	-0.081573508	-1.470957676	0.142276078	0.278474972	no
EXTL1	-0.081551367	-1.470555757	0.142384756	0.278662308	no
ZDHHC13	0.081543053	1.470404824	0.142425585	0.278716832	no
CPSF4	0.081540353	1.470355818	0.142438843	0.278717398	no
ENC1	-0.081527313	-1.470119104	0.142502899	0.278817353	no
FMN1	0.081521174	1.470007656	0.142533065	0.278836913	no
LOC727896	-0.081518589	-1.469960734	0.142545767	0.278836913	no
CTNNBL1	-0.081516448	-1.469921881	0.142556285	0.278836913	no
QRFP	0.081514717	1.46989045	0.142564795	0.278836913	no
FAM189A2	-0.081494395	-1.469521552	0.142664698	0.278988513	no
HELLS	0.081493669	1.46950837	0.142668269	0.278988513	no
KIRREL3-AS2	-0.081458254	-1.468865498	0.1428425	0.27930381	no
KRTAP5-1	-0.081451458	-1.468742135	0.142875953	0.279343805	no
LOC10013425S	0.08143881	1.468512532	0.142938231	0.279440146	no
C22orf26	0.08143372	1.468420144	0.142963296	0.27944489	no
BBS7	-0.081433037	-1.468407734	0.142966664	0.27944489	no
LYSMD4	-0.08142609	-1.468281646	0.143000878	0.279486347	no
FLJ33630	0.081419496	1.46816194	0.143033367	0.27950025	no
MECR	0.0814176	1.46812752	0.14304271	0.27950025	no
PMF1-BGLAP	-0.081416727	-1.468111685	0.143047008	0.27950025	no
PHF8	0.081362439	1.467126229	0.1433147	0.279997838	no
LEPREL4	0.081331487	1.466564382	0.143467495	0.280245612	no
LSR	-0.081331465	-1.466563991	0.143467601	0.280245612	no
MRPL44	-0.081326453	-1.466473004	0.143492357	0.280268494	no
LINC00583	0.081300473	1.466001415	0.14362072	0.28049372	no
TMEM229B	0.081295365	1.46590869	0.143645969	0.28051754	no
ZNF569	-0.08127833	-1.465599484	0.143730192	0.280656511	no
SRCAP	-0.081248774	-1.465062996	0.143876414	0.280916508	no
HSPA4	0.081239567	1.464895866	0.143921989	0.280979965	no
CYCSP52	0.081198296	1.464146746	0.144126405	0.281353489	no
WDR45	0.081185003	1.46390546	0.144192293	0.281456547	no
SPOP	-0.081176208	-1.463745819	0.1442359	0.281516095	no
RABEPK	-0.081171692	-1.463663839	0.144258297	0.281534241	no
TMEM86B	0.081164073	1.46352555	0.144296083	0.281582414	no
CHD1	0.08116113	1.463472118	0.144310686	0.281585341	no
UBR7	-0.081153299	-1.463329982	0.144349534	0.281635573	no

ANXA2R	0.081133306	1.462967092	0.144448756	0.281800309	no
LOC152578	-0.081131003	-1.462925277	0.144460193	0.281800309	no
FARP1	-0.081123552	-1.462790038	0.144497186	0.281846889	no
PAH	-0.081106554	-1.462481506	0.144581608	0.281973417	no
RASGRP1	-0.081105208	-1.462457075	0.144588295	0.281973417	no
PER2	-0.08109994	-1.462361464	0.144614465	0.281989185	no
LOC154872	0.081098298	1.462331663	0.144622623	0.281989185	no
DHRS4-AS1	-0.081093716	-1.462248483	0.144645395	0.282008	no
C4orf36	0.081018789	1.460888507	0.1450181	0.282708997	no
MAGEC1	0.08100504	1.460638953	0.145086571	0.282816824	no
EFCAB12	0.080997073	1.460494351	0.145126257	0.282868528	no
STX16-NPEPL1	-0.080959552	-1.459813326	0.145313278	0.283207369	no
EIF3M	0.080928904	1.45925706	0.145466176	0.283436273	no
MGA	-0.080926235	-1.459208615	0.145479498	0.283436273	no
KCNQ4	0.08092558	1.459196722	0.145482768	0.283436273	no
ESX1	-0.080925106	-1.459188119	0.145485134	0.283436273	no
FAM172A	-0.080921512	-1.459122891	0.145503073	0.283436273	no
BEST3	-0.080920153	-1.459098215	0.14550986	0.283436273	no
RELT	0.080906042	1.458842108	0.145580312	0.283547806	no
MRAS	-0.080898748	-1.458709721	0.145616741	0.283593057	no
MYB	-0.080882143	-1.458408341	0.145699697	0.283728905	no
OR13J1	-0.080876993	-1.458314855	0.145725437	0.283753318	no
C12orf65	-0.080848366	-1.457795292	0.145868554	0.28400626	no
IL17RB	0.08084369	1.457710413	0.145891945	0.284026071	no
B4GALT2	-0.080835546	-1.457562603	0.145932685	0.284077907	no
ST5	-0.080833083	-1.457517899	0.145945008	0.284077907	no
KDSR	0.080828769	1.457439596	0.145966595	0.284094195	no
DEXI	-0.080808257	-1.457067315	0.146069262	0.284262015	no
LOC145845	0.080805165	1.457011191	0.146084745	0.284262015	no
VWA7	-0.080802106	-1.456955679	0.14610006	0.284262015	no
MID1	0.080800974	1.456935131	0.146105729	0.284262015	no
TTC21A	0.080789254	1.456722425	0.146164425	0.284349132	no
IDH1-AS1	-0.080786751	-1.456676981	0.146176967	0.284349132	no
APOC4	0.080756749	1.456132474	0.146327315	0.284615833	no
SNORA71D	-0.080718749	-1.45544279	0.14651792	0.28496078	no
TCEAL4	-0.080715955	-1.45539209	0.146531939	0.284962257	no
FAM227A	-0.080704247	-1.455179595	0.146590708	0.285050751	no
WRAP73	0.080696846	1.455045276	0.146627865	0.285097209	no
CHDH	-0.080689251	-1.45490742	0.146666009	0.285145576	no
POSTN	0.080675458	1.454657093	0.146735291	0.285232191	no
LOC201617	-0.080675097	-1.454650544	0.146737104	0.285232191	no
TSHB	0.080670914	1.454574617	0.146758124	0.285247249	no
PPP1R27	-0.080661129	-1.454397031	0.146807295	0.285317017	no
FAF2	0.080654656	1.454279561	0.146839828	0.285354439	no
BCAT1	0.080650323	1.454200923	0.14686161	0.285370963	no
OR52I1	-0.080641279	-1.454036781	0.146907082	0.285433515	no
MAP2K5	0.080636409	1.453948389	0.146931575	0.285442114	no
ANKRD65	0.080635117	1.453924941	0.146938072	0.285442114	no
ERV3-1	0.080617997	1.45361423	0.147024193	0.285583599	no
ANKRD39	-0.080612981	-1.453523202	0.147049432	0.285606808	no
TXLNG	-0.080607635	-1.453426185	0.147076333	0.285633244	no

TMEM132C	-0.08060206	-1.453324991	0.147104398	0.28565329	no
KPNA3	-0.080597702	-1.453245914	0.147126331	0.28565329	no
SYNE1	-0.080597663	-1.453245197	0.14712653	0.28565329	no
SLC35E1	0.08058573	1.453028624	0.147186614	0.28574413	no
DHRS9	0.080582427	1.452968678	0.147203248	0.28575061	no
LINC00538	-0.080563005	-1.452616202	0.147301084	0.285887173	no
FNDC3A	-0.080560757	-1.452575409	0.14731241	0.285887173	no
RP9P	-0.08056054	-1.452571462	0.147313506	0.285887173	no
ANKRD17	0.080503134	1.45152963	0.147602995	0.286413718	no
NTSR1	-0.080500399	-1.451479993	0.147616798	0.286413718	no
ERMN	-0.080498613	-1.451447595	0.147625808	0.286413718	no
RPPH1	-0.080496171	-1.451403271	0.147638136	0.286413718	no
SLC1A3	-0.080488758	-1.451268727	0.147675559	0.286460461	no
LOC349196	-0.080482689	-1.451158596	0.147706198	0.286494034	no
POU3F1	-0.080478823	-1.451088436	0.147725719	0.28650604	no
KIAA1586	-0.080448974	-1.450546719	0.147876512	0.286772616	no
PCGF5	0.080442699	1.45043285	0.147908224	0.286808233	no
C14orf39	-0.080433636	-1.450268371	0.14795404	0.286859638	no
RAB40B	-0.080432174	-1.450241844	0.14796143	0.286859638	no
CBFA2T2	-0.080427346	-1.450154219	0.147985843	0.286881089	no
PANX1	0.080404647	1.449742281	0.148100655	0.287035452	no
PROSC	0.08040464	1.449742162	0.148100688	0.287035452	no
MLL4	-0.080403682	-1.449724765	0.148105538	0.287035452	no
EFTUD1P1	-0.080396148	-1.449588036	0.148143663	0.287058381	no
BMS1	-0.080396064	-1.449586526	0.148144084	0.287058381	no
CHRM5	-0.080392983	-1.449530607	0.148159677	0.287062714	no
RNF25	0.08038276	1.44934508	0.148211424	0.287137088	no
PVT1	0.080374274	1.449191073	0.14825439	0.287194438	no
QTRTD1	0.080369349	1.449101695	0.14827933	0.287216861	no
LOC100190940	-0.080329677	-1.448381749	0.148480337	0.287580292	no
EMD	0.080313161	1.448082029	0.148564079	0.287716557	no
MRPS34	-0.080309777	-1.448020603	0.148581246	0.287723875	no
SNORA38	-0.080299797	-1.447839499	0.14863187	0.287795974	no
LCE3B	-0.080293509	-1.44772538	0.148663775	0.28783182	no
ART5	0.080290755	1.447675402	0.14867775	0.287832946	no
BTG4	0.08025678	1.447058844	0.148850234	0.288129982	no
DEF8	-0.080255251	-1.44703111	0.148857996	0.288129982	no
LOC440896	-0.080245526	-1.446854632	0.148907396	0.288199644	no
KIF19	-0.080236496	-1.446690752	0.148953281	0.288262492	no
PDGFA	0.080229843	1.446570014	0.148987094	0.288301093	no
AUNIP	-0.080227292	-1.446523728	0.149000058	0.288301093	no
POLD2	-0.080217991	-1.446354938	0.149047341	0.288366618	no
USP44	-0.080211683	-1.446240477	0.149079411	0.288402702	no
PLGLB2	0.080208085	1.446175184	0.149097707	0.288412135	no
FBXO28	-0.080190374	-1.445853777	0.149187797	0.28855351	no
GOLGA6L4	-0.080188438	-1.445818648	0.149197646	0.28855351	no
ADCYAP1	-0.080180731	-1.445678781	0.149236865	0.28860339	no
JKAMP	0.080165573	1.445403717	0.149314018	0.288726612	no
CA14	-0.080151964	-1.445156763	0.149383312	0.288834616	no
ZNF890P	-0.080141396	-1.444964984	0.149437141	0.288912702	no
LIMS2	-0.080136077	-1.444868448	0.149464243	0.288939106	no

MOSPD3	-0.080113306	-1.444455237	0.149580291	0.289137438	no
USP39	0.080107784	1.44435503	0.149608444	0.289165849	no
LOC100505716	-0.080101956	-1.444249267	0.149638162	0.28919728	no
B3GALT6	0.080093604	1.444097707	0.149680757	0.289253588	no
HCG27	0.080085269	1.443946467	0.149723271	0.289263748	no
FAM19A4	-0.080082832	-1.443902244	0.149735704	0.289263748	no
STON1-GTF2A1	0.080082259	1.443891852	0.149738626	0.289263748	no
ASF1B	0.080082019	1.443887485	0.149739854	0.289263748	no
ELOVL2-AS1	-0.080069406	-1.44365861	0.149804215	0.289362069	no
LOC286059	0.080053421	1.443368529	0.149885817	0.289493673	no
NKX3-2	-0.080045391	-1.443222815	0.149926821	0.289546846	no
TP53I11	0.080040562	1.443135198	0.14995148	0.289568448	no
WDR92	0.080020398	1.442769295	0.150054495	0.289741344	no
FSD2	0.07998335	1.442097018	0.150243907	0.290081018	no
RHOT2	-0.079957357	-1.441625345	0.150376909	0.290311727	no
RALGDS	-0.079949864	-1.441489381	0.150415265	0.290359692	no
LMX1A	0.079935835	1.441234812	0.150487099	0.290466126	no
LOC400685	-0.079933818	-1.4411982	0.150497432	0.290466126	no
MMP1	0.079922544	1.440993641	0.150555177	0.290520223	no
PITRM1-AS1	-0.079922472	-1.44099233	0.150555547	0.290520223	no
GOLGA8S	-0.079920429	-1.440955253	0.150566016	0.290520223	no
MIR210HG	0.079914081	1.440840062	0.150598542	0.290535529	no
EDIL3	-0.079913604	-1.440831406	0.150600986	0.290535529	no
ABCB5	0.079899987	1.440584317	0.150670776	0.290644075	no
LOC100507412	0.07987604	1.44014979	0.150793566	0.29084349	no
TBCB	-0.079874548	-1.440122716	0.150801219	0.29084349	no
ZC3H12C	-0.079864523	-1.439940804	0.15085265	0.290916574	no
CHM	0.079861804	1.439891464	0.150866601	0.290917374	no
CRYGS	0.079857302	1.439809775	0.150889702	0.290924418	no
ZBTB33	-0.079855816	-1.439782811	0.150897328	0.290924418	no
DNAJC9-AS1	-0.079833571	-1.439379175	0.151011518	0.291118456	no
C2orf83	-0.079826555	-1.439251853	0.151047552	0.291161803	no
SIDT2	0.079823141	1.439189914	0.151065084	0.291169481	no
GTF3C5	-0.07980823	-1.438919353	0.151141684	0.291291	no
PPP1R35	0.079783877	1.438477459	0.151266856	0.291506097	no
TEAD1	-0.079779968	-1.438406527	0.151286956	0.291518691	no
PSMC3	-0.079764364	-1.438123395	0.151367206	0.291647178	no
CNFN	0.079759561	1.438036246	0.151391914	0.291648623	no
HAPLN2	-0.079758943	-1.438025017	0.151395098	0.291648623	no
CCL25	0.07975047	1.437871275	0.151438694	0.291706459	no
NAPEPLD	-0.079740911	-1.437697841	0.151487885	0.291761101	no
ARFIP2	-0.079739682	-1.437675535	0.151494213	0.291761101	no
COPS3	-0.07972689	-1.437443426	0.151560068	0.291861776	no
LOC100506050	-0.079721993	-1.437354575	0.151585283	0.291884178	no
MEPCE	-0.079674876	-1.436499636	0.15182807	0.292325484	no
GAMT	-0.079672142	-1.436450022	0.151842168	0.292326439	no
GNG12-AS1	0.079659595	1.436222377	0.15190687	0.292424807	no
SRP14	0.079643853	1.435936745	0.151988083	0.292554939	no
PPP2R2B-IT1	-0.079634458	-1.435766277	0.152036568	0.292622056	no
DGAT1	-0.079625932	-1.435611567	0.15208058	0.292680554	no
CYP2F1	-0.079590989	-1.434977555	0.152261049	0.292996804	no

PRODH	-0.079586512	-1.434896313	0.152284186	0.292996804	no
TOR2A	-0.079586198	-1.434890619	0.152285808	0.292996804	no
LOC100129213	-0.079551475	-1.434260599	0.152465325	0.293293857	no
PCDHGB5	-0.079551055	-1.434252981	0.152467497	0.293293857	no
TPRKB	-0.079545984	-1.434160971	0.152493728	0.293318062	no
GCN1L1	-0.079520615	-1.433700662	0.152625009	0.293544305	no
TSPAN13	-0.079507144	-1.433456249	0.152694751	0.293652161	no
BMPR1B	-0.079501756	-1.433358496	0.152722651	0.293657437	no
GNRHR	-0.079500396	-1.433333812	0.152729697	0.293657437	no
CCDC53	0.079498699	1.433303017	0.152738487	0.293657437	no
DDX53	0.079489306	1.433132598	0.152787141	0.293724702	no
SLC1A7	-0.079482965	-1.433017543	0.152819995	0.293761584	no
ZNF135	-0.079473565	-1.432846994	0.152868705	0.293816213	no
ZNF574	-0.079470865	-1.432798007	0.152882698	0.293816213	no
CCS	-0.079469566	-1.432774447	0.152889429	0.293816213	no
RNF125	0.079459545	1.432592613	0.152941381	0.293889772	no
SIAH3	-0.0794528	-1.432470241	0.152976351	0.293928832	no
GYS2	-0.079450349	-1.43242577	0.152989061	0.293928832	no
CFL1P1	-0.079441088	-1.43225775	0.15303709	0.293994824	no
HOXD1	-0.079399664	-1.431506167	0.153252071	0.294381503	no
PPAN	-0.079390697	-1.431343477	0.153298637	0.294444633	no
TMEM81	-0.079387324	-1.431282277	0.153316156	0.294451967	no
DIS3	-0.079381062	-1.431168655	0.153348687	0.294473749	no
IFNA2	-0.079379865	-1.431146949	0.153354902	0.294473749	no
BMS1P4	-0.079375799	-1.431073165	0.15337603	0.294488008	no
PROK2	0.079363391	1.430848042	0.153440509	0.29458549	no
CR1L	0.079358268	1.430755094	0.153467136	0.294610293	no
POLR2K	-0.079349886	-1.430603018	0.15351071	0.294653411	no
CCT4	-0.079348673	-1.430581006	0.153517018	0.294653411	no
NLRC3	0.079344223	1.43050027	0.153540156	0.294671503	no
FIGNL2	-0.07932349	-1.430124117	0.153647991	0.294852128	no
RAB3D	0.079308971	1.429860685	0.153723546	0.294970779	no
FAM154B	0.079305092	1.429790319	0.153743732	0.294979436	no
MORC1	0.07930283	1.429749271	0.153755509	0.294979436	no
WASL	-0.079280393	-1.429342191	0.153872338	0.295158639	no
NOTUM	-0.079279615	-1.429328092	0.153876385	0.295158639	no
HIST1H4B	-0.07926873	-1.429130597	0.15393309	0.295241057	no
C9orf69	0.079257478	1.428926446	0.153991723	0.295327157	no
IDH1	0.079252787	1.428841353	0.154016167	0.29534768	no
ZNF648	0.079213874	1.428135349	0.15421909	0.295710428	no
LOC730101	-0.079204453	-1.427964437	0.154268246	0.295778291	no
RNF123	-0.079183311	-1.427580867	0.154378605	0.29596348	no
DCAF12L2	-0.079174918	-1.427428592	0.154422434	0.296017876	no
IQCK	-0.079172602	-1.427386583	0.154434527	0.296017876	no
S100A1	-0.079167953	-1.427302237	0.15445881	0.296038017	no
FIBCD1	-0.079137672	-1.426752862	0.154617043	0.296298298	no
NOL6	0.079134839	1.426701465	0.154631852	0.296298298	no
C4orf3	-0.079131206	-1.426635552	0.154650846	0.296298298	no
STIM1	0.079129832	1.426610617	0.154658032	0.296298298	no
TXNRD3	0.079128777	1.42659148	0.154663548	0.296298298	no
GNMT	-0.079124163	-1.42650778	0.154687672	0.296318099	no

YIF1B	0.07911557	1.426351886	0.154732611	0.296377766	no
SLC5A5	-0.079107445	-1.426204472	0.154775115	0.296432759	no
CATSPERB	0.079104537	1.426151707	0.154790331	0.296435483	no
SCEL	-0.079097857	-1.426030515	0.154825283	0.296476001	no
H1FNT	-0.07909225	-1.4259288	0.154854623	0.296505765	no
INO80B-WBP1	-0.079063594	-1.425408922	0.155004649	0.296766585	no
MCRS1	-0.079050238	-1.425166621	0.155074611	0.296861708	no
C5orf38	-0.079048836	-1.425141172	0.15508196	0.296861708	no
COX19	0.079045586	1.425082216	0.155098987	0.29686786	no
GLRX3	-0.079038382	-1.424951519	0.155136738	0.296913674	no
LOC100130264	-0.079027585	-1.424755643	0.15519333	0.296995534	no
TSPAN17	0.079007986	1.424400093	0.155296094	0.297165733	no
DZIP1	-0.079001015	-1.424273611	0.155332663	0.297209246	no
LOC100272217	-0.078983337	-1.423952915	0.155425414	0.297360239	no
OBSL1	-0.078974641	-1.423795143	0.15547106	0.297421092	no
KLK1	0.078967186	1.423659898	0.155510197	0.297469483	no
C5orf58	-0.078961458	-1.423555984	0.155540272	0.297500533	no
RAD21L1	-0.078956043	-1.423457743	0.155568709	0.297528445	no
SLC28A2	-0.078939582	-1.423159122	0.155655175	0.297667322	no
DEDD2	0.078917638	1.42276102	0.155770502	0.297861363	no
ZMIZ2	-0.078911157	-1.422643456	0.155804572	0.297900004	no
IAPP	-0.078901151	-1.422461938	0.155857186	0.297974094	no
OVOL2	-0.07886422	-1.421791953	0.156051505	0.298313917	no
PTN	-0.078860697	-1.42172804	0.156070051	0.298313917	no
TRIM41	-0.078859458	-1.421705559	0.156076576	0.298313917	no
SLC25A35	0.078854832	1.421621648	0.156100929	0.298333931	no
UCKL1-AS1	-0.078843588	-1.421417673	0.156160139	0.298420555	no
PRKAA1	0.078831959	1.421206712	0.156221396	0.298511071	no
RAB9A	0.078825929	1.421097307	0.156253171	0.298532532	no
OR8B2	-0.078824555	-1.421072388	0.156260409	0.298532532	no
TBC1D13	-0.078785575	-1.420365248	0.156465914	0.298898573	no
ASB16	-0.078776359	-1.420198067	0.156514529	0.298964869	no
LARP1	-0.078771381	-1.42010776	0.156540794	0.298988465	no
HEPH	0.0787582	1.419868652	0.156610354	0.299094741	no
ZZEF1	-0.078754445	-1.41980054	0.156630173	0.29910506	no
KIT	-0.078747847	-1.419680832	0.15666501	0.29910506	no
FAM90A27P	0.078741022	1.419557028	0.156701045	0.29910506	no
HRNR	0.078740679	1.419550806	0.156702856	0.29910506	no
CCDC78	-0.078740073	-1.419539817	0.156706055	0.29910506	no
TAS2R20	-0.078739146	-1.419523005	0.156710949	0.29910506	no
ANKDD1A	-0.078737682	-1.419496447	0.156718681	0.29910506	no
FTX	-0.078736088	-1.419467531	0.156727099	0.29910506	no
WDR38	0.07873123	1.419379398	0.156752759	0.299127467	no
TRIM35	-0.078720794	-1.419190076	0.15680789	0.299206105	no
CALCR	0.078688693	1.418607756	0.156977556	0.299503255	no
KCNQ3	-0.078684423	-1.418530297	0.157000135	0.299519744	no
DFNB59	-0.078677793	-1.418410014	0.157035203	0.299560052	no
ZNF383	0.078667093	1.418215921	0.157091801	0.299641422	no
LOC100506321	0.078662762	1.418137352	0.157114717	0.299658536	no
ZNF302	-0.078658747	-1.41806452	0.157135961	0.299672459	no
ARHGEF28	0.078644883	1.417813019	0.157209339	0.299785794	no

LOC100506060	-0.078641713	-1.417755513	0.157226121	0.299791195	no
TMA7	-0.078619582	-1.417354053	0.157343314	0.299988037	no
SFTPD	-0.078607983	-1.417143656	0.157404759	0.300064076	no
LOC400084	0.078606782	1.417121876	0.157411121	0.300064076	no
LOC100130480	-0.078591676	-1.416847844	0.15749118	0.300190061	no
CSRP2	0.07858332	1.416696267	0.157535477	0.300247865	no
POU5F1P4	-0.07856795	-1.416417462	0.157616979	0.300376563	no
GXYLT1	0.078564518	1.416355196	0.157635186	0.300384623	no
LOC389634	-0.078558385	-1.416243953	0.157667717	0.300419976	no
MYO15B	0.078551101	1.41611181	0.157706367	0.30046698	no
PPP2R3C	-0.07853904	-1.415893041	0.15777037	0.300562274	no
SH3D21	0.078533139	1.415785987	0.157801696	0.300595308	no
MTUS2	-0.07852367	-1.415614225	0.157851968	0.30066442	no
SEC1P	-0.078510353	-1.415372659	0.157922691	0.30077247	no
PAPD5	-0.078478554	-1.414795836	0.158091664	0.301046027	no
MBOAT7	-0.078478051	-1.414786706	0.15809434	0.301046027	no
CBLL1	-0.078464341	-1.414538021	0.158167232	0.301158146	no
SHARPIN	0.07845454	1.41436024	0.158219358	0.301221625	no
APOO	-0.078452802	-1.41432871	0.158228604	0.301221625	no
SFI1	-0.078443416	-1.414158461	0.158278535	0.301289991	no
WFIKKN2	-0.078432804	-1.41396596	0.158335007	0.301370795	no
PAG1	-0.078425538	-1.413834164	0.15837368	0.301417707	no
CARS	-0.078417653	-1.413691123	0.15841566	0.301470907	no
TMA16	-0.078413634	-1.413618225	0.158437058	0.30148493	no
MRPL42	-0.078393084	-1.413245471	0.158546506	0.301666486	no
VPS28	-0.078378751	-1.412985478	0.158622879	0.301763688	no
APPBP2	-0.078375973	-1.412935083	0.158637686	0.301763688	no
BCLAF1	0.078375592	1.412928172	0.158639717	0.301763688	no
EFNB3	-0.078350373	-1.412470738	0.158774167	0.301943951	no
PSMB1	0.07835032	1.412469776	0.15877445	0.301943951	no
CDC14A	0.078347864	1.412425215	0.158787552	0.301943951	no
ANXA3	-0.078347277	-1.412414574	0.158790681	0.301943951	no
FAM218A	-0.078328698	-1.412077562	0.1588898	0.302105697	no
SULT1C4	-0.078312091	-1.411776334	0.158978435	0.30223201	no
THUMPD2	-0.078308708	-1.411714967	0.158996496	0.30223201	no
FAM178B	-0.078308347	-1.411708422	0.158998423	0.30223201	no
LOC389705	-0.078286514	-1.4113124	0.159115018	0.302426891	no
ANKRD2	-0.078282625	-1.411241853	0.159135795	0.302439633	no
LINC00684	-0.078279514	-1.411185436	0.159152412	0.302444468	no
RBM14-RBM4	0.078276778	1.411135801	0.159167032	0.302445508	no
C12orf57	-0.078271184	-1.41103434	0.159196922	0.302475559	no
TCF21	0.07826266	1.410879726	0.159242478	0.30253537	no
COX6A2	0.078249135	1.410634395	0.159314784	0.302628066	no
RAMP2	-0.078248265	-1.410618625	0.159319433	0.302628066	no
SSX8	0.07823482	1.410374752	0.159391336	0.302737887	no
CLN8	-0.078226291	-1.410220037	0.159436964	0.302797791	no
INPP5E	-0.078222263	-1.410146985	0.159458512	0.302811954	no
PTRH1	0.078206363	1.409858575	0.159543605	0.302942598	no
UPK1B	-0.07820414	-1.409818265	0.159555501	0.302942598	no
STXBP2	0.078167157	1.409147461	0.15975356	0.303291852	no
LOC100131060	0.07815705	1.408964126	0.159807723	0.303367881	no

HPR	-0.078153085	-1.408892207	0.159828975	0.303381425	no
CHDC2	0.078149201	1.408821767	0.159849791	0.30339414	no
SNX5	0.078108354	1.408080886	0.160068858	0.3037831	no
KTN1-AS1	-0.078094338	-1.407826651	0.160144084	0.30389903	no
BNIPL	-0.078069059	-1.407368161	0.160279815	0.304129747	no
C1orf64	-0.078058796	-1.407181998	0.160334952	0.304207508	no
XIAP	-0.07802923	-1.406645747	0.160493856	0.30448212	no
FAM221A	0.078022726	1.406527786	0.160528826	0.304521582	no
LOC100216546	-0.07800973	-1.406292063	0.160598726	0.304627292	no
LOC100129055	-0.078003671	-1.406182169	0.160631321	0.304662229	no
MID2	0.077994684	1.406019176	0.160679675	0.304727046	no
DGCR2	-0.077982326	-1.405795034	0.160746187	0.304826273	no
MIR548I1	-0.077978248	-1.405721064	0.160768142	0.304826273	no
LOC286094	-0.077977058	-1.405699484	0.160774547	0.304826273	no
RGL3	0.077959946	1.405389105	0.160866697	0.304949118	no
TPCN2	0.077959756	1.405385662	0.160867719	0.304949118	no
MTFP1	0.077952012	1.40524521	0.160909432	0.305001287	no
SIX1	-0.077935374	-1.404943441	0.160999081	0.305103123	no
FNBP4	-0.077933256	-1.404905034	0.161010494	0.305103123	no
C17orf89	-0.077932204	-1.404885957	0.161016163	0.305103123	no
SEMA4D	-0.077931503	-1.404873232	0.161019944	0.305103123	no
LOC113230	-0.077915233	-1.404578136	0.161107657	0.30524241	no
CCDC160	-0.077911493	-1.404510306	0.161127824	0.305253707	no
PKMYT1	-0.077906231	-1.40441487	0.161156201	0.305280556	no
ATP6V0D1	0.077902968	1.404355697	0.161173797	0.30528698	no
PTGR1	0.07789755	1.404257421	0.161203026	0.305315433	no
JAK1	0.077893231	1.404179101	0.161226321	0.305332647	no
ZFAND2A	0.077885925	1.404046579	0.161265745	0.305380397	no
KLHL34	-0.07787908	-1.403922441	0.161302681	0.30542343	no
ADAMTSL2	-0.077874485	-1.403839097	0.161327484	0.305443481	no
NCDN	-0.077858541	-1.403549931	0.161413558	0.305579525	no
C9orf3	0.077839398	1.403202726	0.161516954	0.305748336	no
PCAT1	-0.077827892	-1.402994039	0.161579124	0.305839084	no
LOC100129961	-0.07782213	-1.402889539	0.161610263	0.305865971	no
LINC00654	0.077819996	1.402850845	0.161621794	0.305865971	no
SETD9	0.077810531	1.402679185	0.161672957	0.305912189	no
CARD17	0.077810212	1.402673391	0.161674684	0.305912189	no
PSMD2	0.07780457	1.402571057	0.161705191	0.305942976	no
CD177	0.077800283	1.402493314	0.16172837	0.305959894	no
AQP12A	0.077796443	1.402423663	0.161749138	0.30597225	no
PNPLA5	-0.077784287	-1.402203201	0.161814889	0.306044253	no
FERMT2	0.07778414	1.402200537	0.161815683	0.306044253	no
SCARNA12	-0.077771616	-1.401973384	0.161883451	0.30614548	no
CTNNA3	-0.077754082	-1.401655387	0.161978356	0.306298007	no
GATAD1	0.077746606	1.401519801	0.162018834	0.306339704	no
CHRFAM7A	0.077744744	1.401486036	0.162028915	0.306339704	no
GRM7	-0.07773296	-1.401272313	0.162092739	0.306433414	no
NRBP2	-0.077728775	-1.401196417	0.162115408	0.306449313	no
ALYREF	-0.077722152	-1.401076297	0.162151292	0.306490186	no
RABIF	0.077717679	1.40099517	0.16217553	0.306492144	no
ZNF222	-0.077716697	-1.400977363	0.162180851	0.306492144	no

SDF2	-0.077713596	-1.400921124	0.162197656	0.30649695	no
MYO3A	0.077705628	1.400776616	0.162240842	0.306523218	no
ALK	0.07769796	1.400637536	0.162282414	0.306523218	no
LOC401320	0.077696725	1.400615152	0.162289106	0.306523218	no
CXorf23	-0.077693234	-1.400551826	0.162308037	0.306523218	no
LOC255167	-0.077691566	-1.400521582	0.16231708	0.306523218	no
ZNF790	0.077691084	1.400512841	0.162319693	0.306523218	no
USP6	-0.077689547	-1.400484961	0.162328029	0.306523218	no
ITGB8	0.077688737	1.400470276	0.16233242	0.306523218	no
BCOR	-0.077687354	-1.400445187	0.162339922	0.306523218	no
NUPL1	-0.077645422	-1.3996847	0.162567442	0.306925846	no
MRVI1-AS1	-0.077635476	-1.399504321	0.162621443	0.307000829	no
GGT3P	-0.077613534	-1.399106396	0.16274062	0.307198828	no
KRT74	-0.077610653	-1.399054134	0.162756277	0.3072014	no
LOC643542	0.077584923	1.398587504	0.162896124	0.30743836	no
REC8	-0.077581933	-1.398533277	0.162912382	0.307442044	no
PRDX5	-0.077566842	-1.398259601	0.16299445	0.307569912	no
CNTN5	-0.077538577	-1.397747004	0.163148249	0.307833101	no
EIF5A	-0.077535255	-1.397686751	0.163166335	0.307834442	no
MKKS	-0.077533184	-1.397649197	0.163177607	0.307834442	no
RPAP3	0.077510625	1.397240073	0.163300456	0.308024715	no
WDR61	0.077509399	1.397217842	0.163307133	0.308024715	no
NEDD4L	-0.077488818	-1.39684459	0.163419275	0.308171175	no
RRS1	-0.077488261	-1.396834493	0.16342231	0.308171175	no
LOC441601	0.077487253	1.396816219	0.163427802	0.308171175	no
LINC00648	-0.077484054	-1.396758193	0.163445241	0.30817702	no
PSMC5	-0.077438201	-1.395926639	0.163695318	0.308621464	no
LOC90499	-0.077424254	-1.395673723	0.163771435	0.308737888	no
PDE5A	0.077411281	1.395438459	0.163842265	0.308844322	no
WDR33	-0.07740464	-1.395318026	0.163878532	0.308885592	no
MECOM	0.077391395	1.395077827	0.163950883	0.308994863	no
NPY1R	0.077379701	1.394865756	0.164014781	0.309088185	no
RPS15	-0.077371806	-1.394722573	0.164057934	0.309142399	no
LINC00423	-0.077338756	-1.394123222	0.164238661	0.309455819	no
TPRX1	-0.077330281	-1.393969528	0.16428503	0.30951605	no
OR4N3P	-0.077326985	-1.393909762	0.164303064	0.309522892	no
CDCA7	-0.077284463	-1.393138647	0.164535875	0.309934306	no
IMP4	-0.077279047	-1.393040423	0.164565548	0.309963032	no
SPP2	0.077267378	1.392828818	0.164629487	0.310053914	no
STAC2	-0.077264976	-1.392785253	0.164642653	0.310053914	no
ZNF256	-0.077252177	-1.39255316	0.164712809	0.310158852	no
CLIC5	-0.077249494	-1.392504497	0.164727521	0.31015938	no
LOC284023	-0.077244032	-1.39240546	0.164757467	0.310188587	no
FLAD1	-0.077234812	-1.392238259	0.164808031	0.310229781	no
LOC100287792	-0.077234778	-1.392237642	0.164808218	0.310229781	no
RPS2	0.077225443	1.392068355	0.164859426	0.310298994	no
RCE1	0.077208054	1.391753027	0.164954842	0.310451397	no
RGS6	-0.077204643	-1.391691168	0.164973565	0.310459446	no
CDHR3	-0.077184399	-1.391324067	0.165084709	0.310641404	no
LNX2	0.077170558	1.391073067	0.165160734	0.310757253	no
ZBTB45	-0.077154242	-1.390777202	0.165250383	0.310898711	no

ARHGEF19	0.077134653	1.390421975	0.165358068	0.311074075	no
KIAA1430	-0.077101082	-1.389813201	0.165542736	0.311394219	no
PRM1	-0.077089534	-1.389603797	0.165606294	0.311479576	no
RBL1	0.077087571	1.389568198	0.165617101	0.311479576	no
ICA1	-0.077073077	-1.389305371	0.165696904	0.311602394	no
RPP14	-0.077042162	-1.388744786	0.165867212	0.311895377	no
ZCCHC9	0.077036312	1.388638708	0.165899454	0.311928712	no
C3orf27	-0.077028433	-1.388495829	0.165942889	0.311983084	no
GABPB2	0.076983609	1.387683013	0.166190147	0.312420613	no
TMTC1	0.076976971	1.387562656	0.166226783	0.31243652	no
SNORA60	-0.076976808	-1.387559695	0.166227685	0.31243652	no
DENND1A	0.076971989	1.387472318	0.166254286	0.312437372	no
CCDC58	-0.076971459	-1.387462699	0.166257214	0.312437372	no
C11orf57	-0.076967053	-1.387382801	0.166281542	0.312455768	no
GON4L	-0.076963388	-1.387316343	0.166301779	0.312466474	no
VPS9D1	-0.076948157	-1.387040169	0.166385897	0.312587174	no
PPP2R3B	-0.076946178	-1.387004282	0.16639683	0.312587174	no
DSN1	0.076943857	1.386962191	0.166409653	0.312587174	no
STOML3	0.076930087	1.386712513	0.166485737	0.312702759	no
QRSL1	-0.076912243	-1.386388949	0.166584374	0.312860682	no
PAGE2B	0.076892797	1.386036334	0.166691918	0.313035303	no
FKBP5	0.076883044	1.385859488	0.166745874	0.313109269	no
TTC8	0.076878164	1.385770998	0.166772878	0.313132615	no
COPE	-0.076869314	-1.385610514	0.166821859	0.31318946	no
WDR27	-0.076867428	-1.385576312	0.166832299	0.31318946	no
TMEM255A	0.076856971	1.385386703	0.166890186	0.313270766	no
KRTAP21-3	-0.076843232	-1.385137589	0.166966263	0.313381136	no
PPM1A	-0.076841086	-1.38509868	0.166978148	0.313381136	no
C11orf88	0.076837824	1.385039524	0.166996219	0.313387682	no
PRKY	0.076835168	1.384991372	0.167010929	0.313387922	no
LOC100130899	-0.076831435	-1.384923677	0.167031611	0.313399367	no
LOC646813	0.076820865	1.38473202	0.167090176	0.313481884	no
MTMR2	-0.076817326	-1.384667837	0.167109792	0.313491319	no
LOC100506229	0.076802792	1.384404299	0.167190355	0.313615076	no
P2RX7	-0.076768021	-1.383773833	0.167383206	0.313949422	no
ABCA2	-0.076744936	-1.383355241	0.16751134	0.31414854	no
ZNF414	-0.076743628	-1.383331524	0.167518602	0.31414854	no
TPH1	0.076723341	1.382963691	0.167631263	0.314332385	no
ABHD12B	0.076696714	1.382480887	0.167779224	0.314576737	no
PPEF1	0.076694623	1.38244297	0.167790849	0.314576737	no
ZWINT	-0.076690846	-1.382374476	0.167811849	0.314588664	no
MNS1	0.076644783	1.381539286	0.168068074	0.315036411	no
RASA1	-0.076642639	-1.381500403	0.16808001	0.315036411	no
GNL3	-0.076635928	-1.381378713	0.168117369	0.315078955	no
ZFPL1	-0.076623513	-1.381153624	0.168186489	0.315181011	no
TSC1	-0.076611024	-1.380927177	0.168256048	0.315283871	no
PYCR1	-0.076604454	-1.380808047	0.16829265	0.315324965	no
HADHA	-0.07657567	-1.380286143	0.168453074	0.315567855	no
SLC2A4RG	0.076575128	1.380276322	0.168456094	0.315567855	no
TRIM44	0.076573291	1.38024302	0.168466335	0.315567855	no
NLRP11	-0.076567617	-1.38014014	0.168497974	0.315599613	no

LOC284933	-0.076563641	-1.380068038	0.16852015	0.315613643	no
TMEM44-AS1	0.076538591	1.379613854	0.168659894	0.315847838	no
FLJ33534	-0.076527976	-1.379421384	0.16871914	0.315931257	no
GDF6	-0.076514457	-1.379176277	0.168794611	0.316045042	no
PRRT3-AS1	0.076507085	1.379042616	0.168835778	0.316094581	no
PPP1R42	0.076498463	1.378886294	0.168883933	0.316157196	no
C9orf116	0.076495243	1.378827903	0.168901923	0.316163334	no
FAM138D	-0.076485863	-1.37865783	0.168954331	0.31623389	no
CRABP1	-0.076471826	-1.378403327	0.169032778	0.316353168	no
DLEU1	-0.076464299	-1.378266849	0.169074857	0.316404367	no
ITPKB	0.076437474	1.37778049	0.169224875	0.316657535	no
SCAF11	0.076433079	1.377700809	0.169249462	0.31667597	no
MDM4	-0.07643017	-1.377648074	0.169265736	0.316678849	no
NDUFAB1	-0.076420061	-1.377464777	0.169322311	0.316757118	no
LOC10019098€	-0.076413681	-1.377349114	0.169358017	0.31679634	no
PPP1R37	-0.076383284	-1.376797983	0.169528237	0.317087148	no
LAMTOR1	0.076367633	1.376514226	0.169615926	0.317223555	no
LOC643623	-0.076344082	-1.376087235	0.169747944	0.317442836	no
NWD1	0.07634003	1.376013768	0.169770667	0.317457705	no
STK31	0.076328728	1.375808857	0.169834056	0.317548607	no
GOPC	-0.076309384	-1.375458145	0.169942589	0.317721192	no
NOTCH2	0.076307007	1.375415057	0.169955927	0.317721192	no
PRSS46	0.076295584	1.37520794	0.17002005	0.317790355	no
LRRIQ3	0.076295148	1.375200037	0.170022498	0.317790355	no
DDX11L2	-0.076288987	-1.375088337	0.170057088	0.317827366	no
LOC389033	-0.07628313	-1.374982149	0.170089977	0.317858592	no
SYCP2L	-0.076280744	-1.374938889	0.170103376	0.317858592	no
LOC100130331	-0.076270509	-1.374753332	0.170160862	0.317938366	no
ASPG	-0.076264575	-1.374645756	0.170194196	0.317973004	no
CYP4Z1	-0.076229518	-1.37401016	0.170391243	0.318313474	no
POLR1C	-0.076209857	-1.373653716	0.170501823	0.318492367	no
MNX1	-0.076205743	-1.373579121	0.170524971	0.318507923	no
SNORA20	-0.076196531	-1.373412115	0.170576806	0.318577052	no
CEP19	0.076157167	1.372698456	0.17079844	0.318949051	no
RBM39	-0.076155884	-1.372675192	0.170805669	0.318949051	no
LOC338963	-0.076135913	-1.372313127	0.170918199	0.319131451	no
S100G	-0.076133094	-1.372262017	0.170934088	0.319133393	no
TMEM125	-0.076101053	-1.371681132	0.171114756	0.319417759	no
PCDP1	0.07610081	1.37167673	0.171116126	0.319417759	no
NR4A1	0.076078907	1.371279638	0.171239713	0.319620695	no
BTBD8	-0.076057839	-1.370897684	0.171358653	0.319804594	no
PRKRIR	-0.076056184	-1.370867673	0.171368001	0.319804594	no
LCE1F	0.076041637	1.370603959	0.17145016	0.319930138	no
CERS6	-0.076030564	-1.370403209	0.171512723	0.319995153	no
NEK1	-0.076028112	-1.370358751	0.171526581	0.319995153	no
ANAPC4	0.076027565	1.370348838	0.171529671	0.319995153	no
SPATA31A4	-0.076009966	-1.370029787	0.171629145	0.320152934	no
FBXO48	0.076002839	1.369900578	0.171669442	0.320200311	no
EIF4E	-0.07597306	-1.369360714	0.17183789	0.320486687	no
ABR	-0.075956209	-1.369055221	0.171933265	0.32063674	no
EID2	-0.075950742	-1.368956117	0.171964213	0.32066663	no

LIG1	-0.075938258	-1.368729787	0.172034908	0.320770624	no
PSMD7	0.075921049	1.368417826	0.172132387	0.320924536	no
DDI2	0.07591598	1.368325928	0.17216111	0.320950244	no
MLANA	-0.07591002	-1.368217879	0.172194886	0.320970822	no
APOH	0.075907167	1.368166158	0.172211056	0.320970822	no
C21orf37	-0.075906127	-1.368147294	0.172216954	0.320970822	no
LIMA1	-0.075899568	-1.368028394	0.172254131	0.321012272	no
FBXW2	0.075894657	1.367939372	0.17228197	0.321017893	no
GOLGA6L1	-0.075893766	-1.367923219	0.172287021	0.321017893	no
PARPBP	0.075876575	1.367611568	0.17238451	0.321171695	no
USHBP1	-0.075870636	-1.367503901	0.172418199	0.321206616	no
RBM12	-0.075866068	-1.367421096	0.172444113	0.321227046	no
ANXA2P1	-0.075863358	-1.367371954	0.172459493	0.321227852	no
HAR1B	-0.075840011	-1.366948717	0.172591997	0.321418939	no
ZBED5	-0.075839488	-1.366939232	0.172594967	0.321418939	no
ZDHHC15	-0.075837377	-1.366900967	0.172606951	0.321418939	no
TMPRSS11GP	-0.075828565	-1.366741218	0.172656987	0.321475749	no
PA2G4	-0.075826736	-1.366708054	0.172667376	0.321475749	no
AMY2B	-0.07579511	-1.366134735	0.172847047	0.321782388	no
MYPOP	-0.075781428	-1.365886702	0.172924821	0.321877581	no
CSNK2A2	-0.075779328	-1.365848635	0.17293676	0.321877581	no
LOC100129931	-0.07577821	-1.365828381	0.172943112	0.321877581	no
POLR1A	-0.075769103	-1.365663284	0.172994899	0.321946084	no
ZNF223	-0.075758066	-1.365463203	0.173057676	0.322035025	no
FNDC4	0.075738078	1.365100866	0.173171404	0.322218757	no
ZNF484	-0.075730481	-1.364963148	0.173214645	0.322271313	no
MED22	0.075727501	1.36490912	0.173231611	0.322274978	no
LOC400655	-0.075721136	-1.364793736	0.173267848	0.322314492	no
ZNF320	-0.075709779	-1.364587863	0.173332519	0.322406886	no
HSPE1-MOB4	0.075700752	1.364424223	0.173383936	0.322422902	no
ZNF546	-0.07569879	-1.364388647	0.173395115	0.322422902	no
GK3P	-0.075698644	-1.36438601	0.173395944	0.322422902	no
CDT1	-0.075697732	-1.364369475	0.17340114	0.322422902	no
LOC644936	-0.075682903	-1.364100652	0.173485637	0.322552109	no
THAP7	-0.075675608	-1.36396842	0.173527212	0.322601498	no
CENPQ	-0.075643779	-1.363391425	0.173708712	0.322888449	no
AZU1	-0.07564327	-1.36338221	0.173711612	0.322888449	no
C14orf93	-0.075636345	-1.363256676	0.173751119	0.322933953	no
MGC72080	0.075631716	1.363172765	0.173777531	0.322954119	no
PTCD1	-0.075628313	-1.363111061	0.173796954	0.322954119	no
DLX1	-0.075626543	-1.363078988	0.173807051	0.322954119	no
REEP3	0.075606011	1.362706781	0.173924258	0.323143964	no
TMEM184C	0.075594204	1.362492767	0.173991677	0.323214789	no
LCE5A	-0.075594068	-1.362490292	0.173992457	0.323214789	no
BCKDHB	-0.075589207	-1.362402184	0.174020219	0.32323842	no
LOC152742	-0.075552049	-1.361728612	0.174232563	0.323604875	no
EID3	0.075541189	1.361531745	0.174294662	0.323666427	no
DEFB113	0.075540986	1.361528061	0.174295824	0.323666427	no
PAN2	-0.075518209	-1.361115185	0.174426116	0.323880391	no
SEMA6D	-0.075484843	-1.360510351	0.174617115	0.324207033	no
SETD4	-0.075480269	-1.360427449	0.174643306	0.324227651	no

FAM193B	-0.075475512	-1.360341215	0.174670554	0.324250227	no
SUOX	-0.075471246	-1.360263894	0.174694988	0.324267575	no
LOC100507584	-0.075453143	-1.359935734	0.174798717	0.324406866	no
MRPS12	0.075452881	1.359930982	0.174800219	0.324406866	no
MAD2L1	-0.075432926	-1.359569274	0.174914607	0.324591125	no
GYS1	0.075428079	1.359481404	0.174942404	0.324600119	no
ANKRD10	0.075426813	1.359458463	0.174949661	0.324600119	no
PGP	-0.075406382	-1.359088118	0.175066856	0.324789521	no
SAR1B	0.075392856	1.358842941	0.175144474	0.324905472	no
CACNA2D2	-0.075383859	-1.358679852	0.175196119	0.324965686	no
RASL12	-0.075381933	-1.358644943	0.175207175	0.324965686	no
LINC00085	0.075377004	1.358555599	0.175235474	0.324990125	no
TOX2	-0.075356699	-1.358187532	0.17535209	0.325178339	no
BMP3	-0.075325978	-1.35763068	0.175528631	0.325477636	no
TMEM105	-0.075266176	-1.356546697	0.175872672	0.326087446	no
FBXO2	-0.075262283	-1.356476141	0.175895082	0.326100864	no
KIR2DL3	0.075255005	1.356344221	0.17593699	0.326150424	no
WBSCR28	0.075243006	1.356126726	0.1760061	0.326248679	no
TPM2	0.075240532	1.356081874	0.176020354	0.326248679	no
AQP7P3	-0.075224573	-1.355792611	0.176112305	0.326390959	no
SLC14A1	-0.075213246	-1.355587291	0.176177594	0.326483805	no
VSTM4	0.07519835	1.355317286	0.17626348	0.3266148	no
C12orf39	-0.075193284	-1.355225466	0.176292694	0.32664077	no
CCNJ	-0.075180981	-1.355002475	0.176363657	0.326744083	no
LRPAP1	0.075172533	1.354849349	0.1764124	0.326806214	no
C21orf33	-0.07515056	-1.354451073	0.176539225	0.32701297	no
SRP9	-0.075145936	-1.354367265	0.176565921	0.327034233	no
HBZ	-0.075137899	-1.35422159	0.176612331	0.327092003	no
TMC2	-0.075129185	-1.354063638	0.176662663	0.327157026	no
MTM1	0.075115004	1.3538066	0.176744591	0.327273693	no
GGT8P	-0.0751093	-1.353703207	0.176777555	0.327273693	no
LOC100506714	-0.07510906	-1.353698869	0.176778938	0.327273693	no
MED4	-0.075106674	-1.353655623	0.176792727	0.327273693	no
RCOR1	-0.075105104	-1.353627157	0.176801804	0.327273693	no
IQCB1	0.075072753	1.353040791	0.176988855	0.327591723	no
RANBP2	0.07505531	1.35272464	0.177089769	0.327734767	no
NEIL1	-0.075054123	-1.352703126	0.177096638	0.327734767	no
SMURF1	-0.075026405	-1.35220073	0.177257093	0.327979541	no
YBX2	-0.075025798	-1.352189732	0.177260607	0.327979541	no
BTF3	-0.075023367	-1.352145656	0.17727469	0.327979541	no
IER5	0.075004039	1.351795349	0.177386643	0.328156213	no
FGGY	0.07500161	1.351751313	0.17740072	0.328156213	no
RASL11B	-0.074997465	-1.351676192	0.177424737	0.328170399	no
PLAA	0.074995016	1.3516318	0.17743893	0.328170399	no
LIPJ	0.074984551	1.351442133	0.17749958	0.328254322	no
KRTAP23-1	-0.074981165	-1.351380752	0.177519212	0.32826238	no
RXFP1	-0.074976181	-1.351290426	0.177548104	0.328274921	no
AMD1	0.074974726	1.351264041	0.177556544	0.328274921	no
C1orf146	-0.074969975	-1.351177934	0.17758409	0.328297608	no
NAALAD2	0.074962437	1.351041304	0.177627806	0.32832978	no
AACS	-0.074961705	-1.351028048	0.177632048	0.32832978	no

GSTP1	0.074954103	1.350890268	0.177676141	0.328383037	no
MZT2B	-0.074948873	-1.350795462	0.177706486	0.328410878	no
TTLL2	-0.074938262	-1.350603156	0.17776805	0.328496403	no
WRN	-0.074915711	-1.350194411	0.177898957	0.328686495	no
CAGE1	-0.074913706	-1.350158073	0.177910599	0.328686495	no
PDE6A	-0.074912637	-1.350138709	0.177916802	0.328686495	no
ZNF587B	0.074897779	1.349869412	0.178003095	0.328817647	no
NDUFB3	-0.07482588	-1.348566292	0.178421103	0.329561489	no
GPR1	0.074811537	1.348306335	0.178504579	0.329682271	no
SMC1A	-0.074809373	-1.34826712	0.178517174	0.329682271	no
CENPP	0.074794833	1.348003589	0.178601831	0.329810273	no
RBBP8NL	-0.074778559	-1.347708629	0.178696621	0.329956961	no
TMPRSS9	-0.074769412	-1.347542855	0.178749911	0.330027004	no
SUN2	-0.074754438	-1.347271464	0.178837178	0.330159763	no
PAQR3	0.074746095	1.347120262	0.178885812	0.330182393	no
PTRH2	-0.074745157	-1.347103259	0.178891282	0.330182393	no
HK1	0.074744429	1.347090062	0.178895528	0.330182393	no
ASIC5	0.07473002	1.34682893	0.178979547	0.330309098	no
SIGLEC12	0.07471612	1.346577007	0.179060631	0.330430365	no
ZNF684	0.07470718	1.346414966	0.179112801	0.330498257	no
ILF2	-0.074673448	-1.345803625	0.179309725	0.330833216	no
SRSF4	0.074662169	1.345599213	0.179375606	0.330926358	no
NBEAP1	-0.074650939	-1.345395686	0.17944122	0.331018991	no
DKFZp566F094	-0.074639271	-1.345184215	0.179509413	0.331110819	no
KISS1R	0.074635888	1.345122901	0.179529189	0.331110819	no
KCNJ2-AS1	-0.074634514	-1.345098005	0.179537219	0.331110819	no
OCLM	-0.074621955	-1.344870391	0.179610649	0.331196068	no
PROP1	-0.074621336	-1.344859183	0.179614265	0.331196068	no
NHSL2	0.074606432	1.344589069	0.179701435	0.331328376	no
NCL	-0.074588986	-1.344272883	0.179803515	0.331488147	no
PINX1	-0.074582528	-1.344155844	0.179841311	0.331529389	no
PLEKHH2	-0.074561203	-1.343769376	0.179966158	0.331731084	no
GBAS	-0.074557806	-1.343707809	0.179986054	0.331739304	no
LOC648691	-0.074523767	-1.343090911	0.180185491	0.332074609	no
PUS3	-0.074520214	-1.343026518	0.180206319	0.332074609	no
PLEKHB2	0.074518847	1.343001751	0.18021433	0.332074609	no
EVX1	-0.074500385	-1.342667162	0.180322582	0.332245593	no
CHRNA7	-0.07447877	-1.342275439	0.18044938	0.332450718	no
C1orf229	-0.074469292	-1.342103669	0.180505002	0.332482383	no
HIF1AN	-0.074467217	-1.342066073	0.180517178	0.332482383	no
LOC100128822	-0.074467128	-1.342064458	0.180517701	0.332482383	no
ITM2C	0.074465297	1.342031268	0.18052845	0.332482383	no
CCDC63	-0.07444162	-1.341602187	0.180667463	0.332709892	no
HIST1H2AI	0.07443564	1.341493817	0.180702585	0.332746059	no
LOC100288846	-0.074423892	-1.341280916	0.1807716	0.332844624	no
CTSL2	-0.074414208	-1.341105415	0.180828506	0.332920879	no
CHIT1	0.074407771	1.340988749	0.180866342	0.332962014	no
SUDS3	-0.074351923	-1.339976647	0.181194826	0.333538159	no
POLR2B	-0.074327585	-1.339535605	0.181338108	0.333755685	no
NDUFAF7	0.074326575	1.339517297	0.181344057	0.333755685	no
ZBED1	0.074319039	1.339380723	0.181388445	0.333808791	no

TRMT10A	-0.074306464	-1.339152837	0.181462527	0.333914498	no
DAZAP2	0.074304014	1.339108448	0.18147696	0.333914498	no
FOXN3-AS2	-0.074290238	-1.338858798	0.181558148	0.334035284	no
RPP25	0.074280184	1.338676585	0.181617422	0.334115734	no
MCMBP	0.07427452	1.338573944	0.181650817	0.334148567	no
CPAMD8	-0.074263604	-1.338376134	0.18171519	0.334212418	no
F5	-0.07426336	-1.338371709	0.18171663	0.334212418	no
PBX1	-0.074258939	-1.338291596	0.181742706	0.334231773	no
LOC388942	0.074244975	1.338038535	0.181825093	0.334346147	no
TRAK2	0.074238968	1.337929682	0.181860541	0.334346147	no
SPRR1A	-0.074238187	-1.337915526	0.181865151	0.334346147	no
KLHL4	0.074237852	1.337909453	0.181867129	0.334346147	no
PRRT3	-0.074231562	-1.337795475	0.181904251	0.334385789	no
WNT6	-0.07422653	-1.337704277	0.181933958	0.334411794	no
PCDHA11	-0.074194302	-1.337120258	0.182124284	0.334733002	no
MFSD9	0.074188203	1.337009724	0.182160323	0.334770609	no
BOK	-0.074179102	-1.336844808	0.182214102	0.33484081	no
SMYD1	-0.074145194	-1.336230348	0.182414582	0.335180556	no
LOC100506195	-0.074139051	-1.336119016	0.182450924	0.335199554	no
FLJ25363	-0.074138173	-1.336103114	0.182456116	0.335199554	no
FBXL18	-0.074132488	-1.33600009	0.182489751	0.33523269	no
PLEKHM1	0.074107871	1.335553998	0.182635445	0.335459932	no
ZBTB21	0.074105084	1.335503501	0.182651943	0.335459932	no
NARF	-0.074103676	-1.335477978	0.182660282	0.335459932	no
TMEM187	0.074079901	1.335047155	0.182801086	0.33565012	no
POLR3GL	-0.074078919	-1.33502935	0.182806907	0.33565012	no
CCDC61	0.074078279	1.335017761	0.182810695	0.33565012	no
LOC283440	-0.074061975	-1.334722318	0.182907304	0.335774005	no
ANKRD20A11P	-0.074061619	-1.33471586	0.182909417	0.335774005	no
CXCL5	0.074049536	1.334496908	0.182981038	0.335876793	no
LOC100128176	-0.07404578	-1.334428835	0.18300331	0.335888986	no
RUVBL2	-0.074024423	-1.33404183	0.183129966	0.33609275	no
TNFAIP8L1	0.074021419	1.3339874	0.183147785	0.33609675	no
SH3GL1P1	-0.074016123	-1.333891418	0.183179209	0.336125716	no
FGFBP2	0.074012714	1.333829659	0.183199431	0.336134123	no
STAM	-0.074009798	-1.333776811	0.183216737	0.336137178	no
PSMD6	0.074006907	1.333724418	0.183233895	0.336139961	no
EIF2AK2	0.074000323	1.333605108	0.183272972	0.336182949	no
PHF14	-0.073942677	-1.332560524	0.18361536	0.336772902	no
TSPY1	-0.073940898	-1.332528298	0.18362593	0.336772902	no
PRR14	-0.073935961	-1.332438833	0.183655278	0.336797985	no
IDH3G	-0.073920953	-1.33216687	0.183744513	0.336932878	no
DUPD1	-0.073887941	-1.331568688	0.183940899	0.337264215	no
PREX1	0.073878454	1.331396769	0.18399737	0.337338976	no
GORASP2	-0.073871762	-1.331275524	0.184037204	0.337383225	no
ZNF28	0.073826572	1.330456669	0.184306395	0.337847897	no
TEX264	0.073816963	1.330282543	0.184363675	0.337924072	no
DDT	-0.073809643	-1.330149903	0.184407317	0.337975239	no
SEC22A	0.073806484	1.330092666	0.184426152	0.337980936	no
THG1L	0.073791709	1.329824945	0.184514269	0.338105568	no
MIR17HG	-0.073789805	-1.32979045	0.184525625	0.338105568	no

PARD3B	-0.073786584	-1.329732082	0.184544841	0.338111951	no
APLP1	-0.073772716	-1.329480794	0.184627588	0.33823472	no
CWH43	0.073757885	1.329212045	0.184716116	0.338368057	no
PALM2	-0.073745808	-1.328993221	0.184788221	0.338471291	no
KDM6B	-0.073732657	-1.328754924	0.184866766	0.338586303	no
RSPH4A	0.073706724	1.328285027	0.185021723	0.338841231	no
CNTNAP4	-0.073698744	-1.328140439	0.185069422	0.338899707	no
MRPL21	-0.073684619	-1.327884497	0.18515388	0.339025478	no
PRSS50	-0.07366756	-1.327575392	0.185255919	0.339153204	no
LCE2D	0.073665332	1.327535032	0.185269246	0.339153204	no
DQX1	-0.073665043	-1.327529783	0.185270979	0.339153204	no
LINC00469	0.073655726	1.32736097	0.185326727	0.33922636	no
ATXN7L1	0.073650605	1.327268184	0.185357374	0.339245208	no
NDUFS6	-0.073648731	-1.327234214	0.185368595	0.339245208	no
APEH	0.073646016	1.327185035	0.185384841	0.33924605	no
SHE	0.073642704	1.327125008	0.185404671	0.339253452	no
NDUFAF1	-0.073624047	-1.326786964	0.185516378	0.339428953	no
LGALS14	0.073614231	1.326609103	0.185575173	0.339507622	no
TTC17	-0.073591999	-1.326206281	0.185708382	0.339695808	no
SCRIB	-0.073591788	-1.326202454	0.185709648	0.339695808	no
PDE8B	-0.073576158	-1.32591925	0.185803344	0.339838269	no
KRTAP20-1	0.073563905	1.325697247	0.185876816	0.33992395	no
FAM195A	-0.073562527	-1.325672265	0.185885085	0.33992395	no
AKNAD1	-0.073560433	-1.325634338	0.18589764	0.33992395	no
MST1L	-0.073555771	-1.325549856	0.185925607	0.339946165	no
SULF2	-0.073547593	-1.325401681	0.185974668	0.340006941	no
TLX3	-0.073531692	-1.325113564	0.186070091	0.34015246	no
SPACA4	-0.073524456	-1.324982463	0.186113522	0.340202918	no
NOL10	-0.073514567	-1.324803282	0.186172895	0.340239745	no
ZNF79	0.073513943	1.324791974	0.186176642	0.340239745	no
PPP1CA	0.07351319	1.324778329	0.186181164	0.340239745	no
PGAP3	-0.073505835	-1.324645061	0.186225333	0.340291526	no
FLRT3	-0.073493037	-1.324413182	0.186302204	0.340397958	no
LOC151475	0.073490864	1.324373817	0.186315256	0.340397958	no
REM1	-0.073477721	-1.324135685	0.186394229	0.340506387	no
RGS8	-0.073475714	-1.324099314	0.186406293	0.340506387	no
OPN1SW	-0.073468144	-1.323962155	0.186451793	0.340560554	no
PNMT	-0.073460795	-1.32382901	0.186495969	0.340612295	no
PCDHA3	-0.073434659	-1.323355445	0.186653156	0.34087041	no
PTENP1	-0.073429923	-1.323269646	0.186681646	0.34089347	no
SLCO4C1	0.073419696	1.323084352	0.186743183	0.340949931	no
BTBD6	-0.073417727	-1.323048677	0.186755033	0.340949931	no
NDUFA4	-0.073416875	-1.323033242	0.186760159	0.340949931	no
INS	-0.07341142	-1.322934401	0.186792993	0.340965985	no
PCDHB16	-0.073409442	-1.322898559	0.186804901	0.340965985	no
PIR-FIGF	0.073407507	1.322863496	0.18681655	0.340965985	no
SIN3B	-0.073388042	-1.322510833	0.186933747	0.341150913	no
CYP2W1	-0.073374548	-1.322266344	0.187015028	0.341265944	no
SH2D6	-0.073372306	-1.322225715	0.187028537	0.341265944	no
CCL7	0.073359579	1.321995122	0.187105226	0.341376893	no
COA4	-0.073350222	-1.321825586	0.187161624	0.341394351	no

SEPP1	0.073350097	1.321823322	0.187162378	0.341394351	no
ZBTB22	-0.073348133	-1.321787734	0.187174218	0.341394351	no
GTF2IRD2	-0.073347449	-1.321775355	0.187178337	0.341394351	no
IMPAD1	-0.073341395	-1.321665666	0.187214835	0.341431943	no
KCNK1	-0.073335997	-1.321567858	0.187247384	0.341462328	no
LOC285847	-0.073318362	-1.321248349	0.187353743	0.341627295	no
DGCR11	-0.073310265	-1.321101651	0.187402591	0.341687375	no
TMEM99	-0.073303576	-1.320980457	0.187442954	0.341703955	no
PRSS8	-0.073303488	-1.320978863	0.187443485	0.341703955	no
SPRR2F	0.073284202	1.320629429	0.187559897	0.341887172	no
LOC253039	-0.073268315	-1.320341596	0.187655828	0.342016585	no
SSBP2	0.073267174	1.320320914	0.187662723	0.342016585	no
LOC100303749	-0.073257425	-1.320144293	0.187721608	0.342077677	no
MON1B	-0.073256354	-1.320124888	0.187728078	0.342077677	no
LOC100131257	-0.073247182	-1.319958708	0.187783496	0.342149649	no
SOAT2	0.073235405	1.319745343	0.187854667	0.342250309	no
SPAG16	-0.073221523	-1.319493829	0.187938589	0.342374181	no
DDX59	0.073209873	1.319282747	0.188009041	0.342473496	no
ZFP42	-0.073202873	-1.319155928	0.188051379	0.342521585	no
TRNT1	-0.073180475	-1.318750141	0.188186895	0.342710827	no
SPCS1	0.07318043	1.318749319	0.18818717	0.342710827	no
NOTCH4	0.073169357	1.318548707	0.188254193	0.342803835	no
ZNF442	-0.07314455	-1.318099265	0.188404412	0.343048312	no
FAM95B1	-0.073119703	-1.317649093	0.188554965	0.343293355	no
ZBTB48	-0.073108663	-1.317449092	0.188621881	0.343386094	no
CHMP4A	0.073103509	1.317355707	0.188653131	0.343413895	no
UHRF1	-0.073084187	-1.317005652	0.188770308	0.343598094	no
SLC35F2	0.073070204	1.316752312	0.188855145	0.343723401	no
CARTPT	-0.073046466	-1.316322252	0.188999224	0.343956502	no
TULP2	0.07303999	1.316204929	0.189038544	0.343998929	no
ARIH2OS	-0.073033556	-1.316088366	0.189077615	0.344025271	no
NXT2	-0.073029571	-1.316016172	0.189101817	0.344025271	no
LETM2	-0.073028063	-1.315988842	0.18911098	0.344025271	no
PRRG4	0.073027063	1.315970733	0.189117051	0.344025271	no
TAF4B	-0.073022035	-1.315879637	0.189147595	0.344051711	no
VTI1B	-0.073003262	-1.315539539	0.189261659	0.344230054	no
ANKRD46	-0.072996869	-1.315423715	0.189300517	0.344271592	no
SNORA14A	-0.072993315	-1.315359326	0.189322121	0.344281748	no
ZDBF2	-0.072990484	-1.31530804	0.18933933	0.344283911	no
DHX36	-0.072930259	-1.314216952	0.189705721	0.344920952	no
KIAA0100	0.072899849	1.313666037	0.189890919	0.34522847	no
ZNF706	0.072894172	1.313563198	0.189925505	0.345232334	no
TDGF1	-0.072893762	-1.313555763	0.189928005	0.345232334	no
PIDD	-0.072889727	-1.313482662	0.189952593	0.345232334	no
C2orf81	0.072888954	1.313468666	0.189957301	0.345232334	no
TMEM92	-0.072864752	-1.313030213	0.190104827	0.345464192	no
ISLR2	-0.072862752	-1.312993971	0.190117025	0.345464192	no
DDX56	0.072846199	1.312694104	0.190217975	0.345618406	no
GYPE	0.072823779	1.312287935	0.190354775	0.345837727	no
BAGE	0.072802574	1.311903786	0.190484225	0.346043658	no
HN1	-0.072788724	-1.311652871	0.190568813	0.346168064	no

SDHAP1	-0.072756619	-1.311071255	0.190764994	0.346493652	no
SLC10A1	-0.072754115	-1.311025901	0.190780298	0.346493652	no
COL9A3	-0.072751477	-1.310978117	0.190796423	0.346493657	no
TCF15	-0.072729151	-1.310573665	0.190932951	0.346712298	no
RECQL5	-0.072724613	-1.310491447	0.190960713	0.346733414	no
TANC1	0.072712714	1.310275887	0.191033515	0.346816176	no
APOA1BP	0.072711888	1.310260925	0.191038569	0.346816176	no
C4orf47	0.07270804	1.310191208	0.19106212	0.346829632	no
MIR1248	0.072704189	1.310121454	0.191085685	0.346843113	no
TNFSF9	0.072693652	1.309930567	0.191150185	0.346901682	no
C22orf46	-0.072693643	-1.309930415	0.191150236	0.346901682	no
BPIFA4P	0.072685839	1.309789029	0.19119802	0.346959101	no
CHCHD1	-0.072668979	-1.309483602	0.191301274	0.347117162	no
OPRK1	-0.072617642	-1.308553609	0.191615925	0.347658746	no
OR3A3	-0.072608541	-1.308388742	0.191671746	0.347730668	no
LINC00499	-0.072593704	-1.308119974	0.191762771	0.34786644	no
PCP4	-0.072587823	-1.308013435	0.191798862	0.347902544	no
DEFA9P	-0.072573444	-1.307752952	0.191887124	0.348033267	no
LIPT1	-0.072566386	-1.307625104	0.191930455	0.348082481	no
RBM3	0.072550978	1.30734599	0.192025079	0.348224704	no
NAT10	-0.072543501	-1.307210541	0.19207101	0.34827861	no
FAM99B	-0.072529109	-1.306949818	0.192159446	0.348409572	no
SPA17	0.07252277	1.306834989	0.192198405	0.348450812	no
IKBKB	0.072515535	1.306703931	0.192242877	0.348502039	no
PITHD1	-0.072500685	-1.306434922	0.192334184	0.348638154	no
FAM13B	0.072486394	1.306176043	0.192422084	0.348768069	no
CCDC170	0.072457788	1.305657851	0.192598118	0.349057696	no
LMO1	-0.072450563	-1.305526976	0.192642597	0.349077358	no
ZNF18	0.07244902	1.305499026	0.192652097	0.349077358	no
AHCTF1	-0.07244811	-1.305482552	0.192657696	0.349077358	no
POGLUT1	0.072438506	1.305308573	0.192716838	0.349155081	no
TMEM47	-0.072385981	-1.304357107	0.193040517	0.349712025	no
DHX8	0.072381504	1.304276014	0.193068123	0.349728547	no
ARGLU1	-0.072379224	-1.304234711	0.193082184	0.349728547	no
NUP50	-0.072358339	-1.303856393	0.193211016	0.349932407	no
NDUFA11	-0.072349122	-1.30368943	0.193267893	0.350005923	no
GPN1	-0.072339854	-1.303521551	0.193325095	0.350080015	no
SLC38A2	0.072333493	1.303406335	0.19336436	0.350100064	no
C9orf57	-0.072332783	-1.303393462	0.193368748	0.350100064	no
LOC283585	0.07232567	1.30326462	0.193412664	0.350150078	no
PCCA	-0.072319175	-1.303146961	0.193452775	0.350193194	no
KRBOX1	0.072312685	1.303029405	0.193492858	0.350236251	no
KIF9	0.072271622	1.302285596	0.193746611	0.350666027	no
TMX2	-0.072246001	-1.301821499	0.193905064	0.350923261	no
RAET1K	0.072227337	1.30148342	0.194020551	0.3511027	no
DPPA3	-0.072220511	-1.301359778	0.1940628	0.351149586	no
MPND	-0.072210616	-1.301180538	0.194124058	0.351230859	no
LINC00674	-0.072202324	-1.301030336	0.194175404	0.351294183	no
DHRS1	0.072177015	1.30057191	0.194332175	0.351548213	no
NDUFA7	-0.072173192	-1.300502659	0.194355865	0.351561476	no
NDUFA1	-0.072134218	-1.299796695	0.194597493	0.351968922	no

TPM3	-0.072126152	-1.299650603	0.194647523	0.352029784	no
B4GALNT2	-0.072109928	-1.299356736	0.194748188	0.352182204	no
KRI1	-0.072105938	-1.299284453	0.194772955	0.352197356	no
CEACAM8	-0.072091937	-1.299030848	0.194859867	0.35232487	no
DNAH1	-0.072087258	-1.298946095	0.194888919	0.352347755	no
PPM1J	0.072080978	1.298832347	0.194927915	0.35237502	no
CUL4B	-0.072079548	-1.298806454	0.194936792	0.35237502	no
ZNF747	0.072075824	1.298738991	0.194959924	0.352387193	no
ZCCHC3	-0.072066358	-1.298567543	0.195018719	0.35246382	no
ASS1	0.072055375	1.298368598	0.19508696	0.352546913	no
MRPL54	-0.072053678	-1.298337865	0.195097503	0.352546913	no
TMUB1	0.072044267	1.298167408	0.195155988	0.352599141	no
PLEKHJ1	-0.072043748	-1.298157993	0.195159219	0.352599141	no
TUBGCP5	-0.07203993	-1.298088842	0.195182949	0.352612371	no
LOC401242	-0.072035299	-1.29800497	0.195211734	0.35263473	no
NOMO2	-0.072026156	-1.297839351	0.195268584	0.35268802	no
SCNN1B	0.072025276	1.297823411	0.195274056	0.35268802	no
KRTAP9-9	-0.07202094	-1.297744879	0.195301018	0.352707073	no
PSMC3IP	-0.072015205	-1.297641004	0.195336684	0.352741843	no
BRCA1	0.072005009	1.297456323	0.195400108	0.352826728	no
LINC00442	-0.071997687	-1.297323693	0.195445665	0.35287934	no
NPIPL3	-0.071970864	-1.296837865	0.19561261	0.353151092	no
LOC442132	-0.071938037	-1.296243283	0.195817069	0.353473998	no
ZCCHC7	-0.071934609	-1.296181179	0.195838434	0.353473998	no
XG	0.071932025	1.296134387	0.195854532	0.353473998	no
NDUFC2-KCTD1	-0.071931588	-1.296126462	0.195857259	0.353473998	no
LOC100126784	0.071928058	1.296062529	0.195879257	0.353484012	no
CAPN9	-0.071922833	-1.295967896	0.19591182	0.353513092	no
MRPL52	-0.071908114	-1.295701292	0.196003581	0.353648977	no
ISPD	0.071891258	1.295395986	0.196108702	0.353808941	no
CDKN2AIP	-0.071882232	-1.2952325	0.196165009	0.35388082	no
ZSCAN32	-0.071878726	-1.295169009	0.19618688	0.353890568	no
CCDC19	0.071868849	1.294990103	0.196248516	0.353927577	no
LOC340107	-0.071864323	-1.294908128	0.196276763	0.353927577	no
MAP4	-0.071863643	-1.29489582	0.196281005	0.353927577	no
IRX3	-0.071863603	-1.294895094	0.196281255	0.353927577	no
C1orf173	0.071862244	1.294870473	0.196289739	0.353927577	no
TMEM116	-0.07185583	-1.294754308	0.196329775	0.353944262	no
UBE2C	-0.071855485	-1.294748048	0.196331932	0.353944262	no
OCA2	-0.071818791	-1.294083454	0.196561095	0.354327671	no
PIGN	0.071806985	1.293869609	0.196634874	0.354406038	no
LOC100505659	-0.071806557	-1.293861854	0.19663755	0.354406038	no
A4GNT	0.071802802	1.293793845	0.196661019	0.354418612	no
OSBPL10-AS1	0.071780229	1.293385016	0.19680214	0.354643199	no
SNORA16A	0.071720308	1.292299731	0.197177125	0.355289142	no
RAPGEF5	-0.071679914	-1.291568123	0.197430205	0.355715336	no
OR2M1P	-0.071656135	-1.291137444	0.197579298	0.35595412	no
FAM83E	-0.07164551	-1.290945009	0.197645942	0.356044337	no
KCNJ1	-0.071639363	-1.290833686	0.197684504	0.356083955	no
SMAP2	0.071632754	1.29071399	0.197725971	0.3561288	no
ZBBX	0.071627825	1.290624709	0.197756906	0.356154668	no

KRTAP4-1	-0.071616897	-1.290426793	0.197825493	0.356213922	no
NDUFB6	-0.071615666	-1.290404491	0.197833223	0.356213922	no
HOXD-AS1	-0.071614661	-1.290386292	0.197839531	0.356213922	no
SRSF5	-0.07160144	-1.290146837	0.197922542	0.356333531	no
ZNF705A	-0.071597121	-1.290068618	0.197949664	0.356352507	no
SLC9A5	-0.071582802	-1.289809288	0.198039603	0.356484555	no
ATPIF1	-0.071571386	-1.289602524	0.198111333	0.356583806	no
WFDC3	-0.071562815	-1.289447302	0.198165194	0.356650883	no
FBXO42	0.071539093	1.289017673	0.198314331	0.356889406	no
RIC8B	-0.071531116	-1.288873197	0.198364501	0.356949803	no
LINC00254	-0.071508863	-1.288470168	0.198504505	0.357165104	no
AGR3	0.071506816	1.288433094	0.198517387	0.357165104	no
MIF	0.071501543	1.288337592	0.198550574	0.35719491	no
TMEM235	-0.07149244	-1.288172727	0.198607876	0.357268089	no
MS4A15	-0.071487972	-1.288091822	0.198636	0.357288775	no
KATNA1	0.071482708	1.287996472	0.198669149	0.357318494	no
RAD51AP1	0.071447211	1.2873536	0.198892756	0.357690729	no
C11orf86	0.071438349	1.287193108	0.198948608	0.357761236	no
ARHGEF6	0.07142658	1.286979953	0.199022804	0.357864716	no
FLJ36777	0.071406347	1.28661353	0.199150399	0.358064186	no
LOC644248	-0.071398515	-1.286471696	0.199199804	0.358123054	no
MAP2K2	-0.071389249	-1.28630388	0.199258271	0.358190036	no
VEPH1	-0.071387328	-1.286269084	0.199270395	0.358190036	no
TSSK1B	-0.071384183	-1.286212127	0.199290243	0.358195752	no
LOC100128292	-0.071366465	-1.285891256	0.199402083	0.358366797	no
C16orf95	-0.07136354	-1.285838269	0.199420555	0.358370027	no
LOC728613	-0.071346084	-1.28552215	0.199530791	0.358538147	no
FAAH2	-0.071329701	-1.285225437	0.199634301	0.358694153	no
CDKN2D	-0.071322791	-1.285100306	0.199677965	0.358742614	no
SLC25A29	-0.071316578	-1.284987776	0.199717238	0.358783178	no
LSP1P3	-0.071311651	-1.284898547	0.199748383	0.358783887	no
NFIC	0.071311233	1.284890988	0.199751022	0.358783887	no
ARHGEF25	-0.071305168	-1.284781147	0.199789366	0.358822771	no
RPUSD2	-0.071299002	-1.284669485	0.199828353	0.3588628	no
NDRG1	0.071284858	1.284413322	0.199917811	0.358993456	no
LOC728084	-0.07124844	-1.283753792	0.200148272	0.359377267	no
ATP2A1	-0.071233687	-1.28348662	0.200241685	0.359514959	no
B9D1	0.071223333	1.28329911	0.200307265	0.359602659	no
ARGFX	-0.07120825	-1.283025972	0.20040282	0.359744154	no
ARHGAP19	0.071187157	1.28264397	0.200536517	0.359941352	no
NPBWR1	-0.071185634	-1.282616396	0.200546171	0.359941352	no
SEC24B	-0.071170882	-1.282349242	0.200639714	0.360079172	no
LMOD1	0.071165596	1.282253515	0.20067324	0.360109269	no
MCPH1	0.071158586	1.282126558	0.200717711	0.360151667	no
LOC387723	-0.071156588	-1.282090384	0.200730383	0.360151667	no
LOC400752	-0.071153322	-1.282031228	0.200751108	0.360158782	no
KIAA0196	0.071146934	1.281915546	0.20079164	0.360173444	no
CUTC	-0.071146751	-1.281912239	0.200792799	0.360173444	no
C8orf33	-0.071125881	-1.281534288	0.200925266	0.360351678	no
CBFB	0.071125813	1.281533058	0.200925697	0.360351678	no
KIAA0513	-0.071113361	-1.281307556	0.201004764	0.3604634	no

FAM209A	0.071096217	1.280997098	0.201113655	0.360601693	no
LGALS3	0.071095936	1.280992015	0.201115438	0.360601693	no
TTLL5	-0.071088789	-1.280862587	0.201160848	0.360653022	no
C19orf60	0.07107536	1.28061939	0.201246193	0.360757666	no
EPN2-IT1	0.071074322	1.2806006	0.201252788	0.360757666	no
GFPT1	0.07105747	1.280295423	0.201359923	0.360919609	no
UBE2E3	0.071047006	1.280105925	0.201426469	0.360995594	no
TNIP2	0.071045522	1.280079042	0.201435911	0.360995594	no
UGT3A2	0.071026132	1.279727921	0.201559262	0.361186534	no
APOBEC1	-0.071021921	-1.279651655	0.201586062	0.361187846	no
TSR3	-0.071020735	-1.279630183	0.201593607	0.361187846	no
OR10AD1	0.071016053	1.279545389	0.201623408	0.361211126	no
LOC400958	-0.071005384	-1.279352193	0.201691318	0.361271545	no
SGOL1-AS1	0.071004335	1.279333197	0.201697996	0.361271545	no
RBM15	0.071002832	1.279305981	0.201707564	0.361271545	no
ANKRD63	0.070999512	1.279245855	0.201728704	0.361277606	no
MPP6	-0.07099702	-1.279200732	0.20174457	0.361277606	no
SMG1P1	-0.070986873	-1.279016978	0.201809189	0.361363213	no
ANKRD35	-0.070975296	-1.278807322	0.201882935	0.361454192	no
OSBPL7	-0.070973616	-1.278776906	0.201893636	0.361454192	no
UGT2B10	-0.070962549	-1.278576501	0.201964149	0.361549221	no
WEE1	0.070960005	1.278530432	0.201980362	0.361549221	no
CHRNA5	0.070935155	1.278080413	0.202138778	0.361781715	no
WDR31	-0.070934349	-1.278065832	0.202143913	0.361781715	no
LIN28B	-0.0708913	-1.277286275	0.202418554	0.36224308	no
UFD1L	-0.070885351	-1.277178534	0.202456533	0.362280879	no
C3orf58	0.070835005	1.276266859	0.202778111	0.362826109	no
GDNF	-0.070825936	-1.276102635	0.202836078	0.362899614	no
SPDYC	-0.070797859	-1.2755942	0.20301562	0.363190602	no
BZRAP1-AS1	-0.070789453	-1.27544199	0.203069392	0.36325656	no
COMMD7	0.070779517	1.275262059	0.203132971	0.363339193	no
PTGER1	0.070776949	1.275215572	0.2031494	0.363339193	no
ZCCHC12	-0.070774138	-1.275164658	0.203167394	0.363341138	no
ASPA	-0.070761048	-1.274927631	0.203251179	0.363460734	no
CXorf40B	0.07075166	1.274757621	0.203311292	0.363537979	no
TMTC2	0.070731374	1.274390287	0.203441217	0.363740033	no
CSF2	0.07072245	1.274228704	0.203498388	0.363811984	no
SAP30	0.070714554	1.274085722	0.203548988	0.363872176	no
GPR155	0.070707781	1.273963063	0.203592403	0.363919515	no
C19orf47	-0.070684886	-1.273548492	0.203739189	0.364151606	no
LOC150197	0.070679862	1.273457513	0.203771412	0.364178912	no
KDELC1	0.070661267	1.273120803	0.203890701	0.364331877	no
DARC	0.070661235	1.273120222	0.203890907	0.364331877	no
PTPRN	-0.070657593	-1.273054288	0.203914272	0.364343334	no
PABPC1P2	-0.070635834	-1.272660274	0.204053939	0.364559232	no
BRPF1	0.070633483	1.272617707	0.204069032	0.364559232	no
CGNL1	0.070614984	1.272282742	0.204187829	0.364741138	no
AOAH-IT1	0.070597975	1.271974741	0.204297108	0.364906013	no
LINC00189	0.070589894	1.271828414	0.20434904	0.364968438	no
FAM210B	0.07057619	1.271580282	0.204437125	0.365095417	no
TTC23L	0.070568575	1.271442398	0.204486084	0.365152508	no

BCS1L	-0.070549938	-1.271104925	0.20460595	0.365329611	no
PKIB	-0.070547869	-1.271067459	0.20461926	0.365329611	no
LRTM1	0.070538077	1.270890159	0.204682258	0.365411731	no
RNMTL1	-0.070522676	-1.270611294	0.204781373	0.365542467	no
WDR34	0.070521413	1.270588412	0.204789507	0.365542467	no
CRIP2	-0.07050203	-1.270237442	0.204914303	0.365714013	no
TRIM6-TRIM34	0.0705012	1.270222415	0.204919648	0.365714013	no
GSC	0.070491026	1.270038204	0.204985172	0.365800575	no
ZNF215	0.070482682	1.269887121	0.205038923	0.365866116	no
GTF2H4	-0.070468766	-1.269635143	0.205128594	0.365995734	no
LMAN1L	0.070463137	1.269533211	0.205164877	0.366030082	no
XAGE5	-0.070426163	-1.26886373	0.205403293	0.366425017	no
RNF11	-0.070417974	-1.26871546	0.205456123	0.366462664	no
RPS16	-0.070415704	-1.268674359	0.205470769	0.366462664	no
KPNA2	0.070414963	1.268660935	0.205475553	0.366462664	no
RAB2B	0.070411696	1.268601783	0.205496633	0.366469849	no
MED23	-0.070392305	-1.268250671	0.205621794	0.366633758	no
EEF1E1	-0.07039217	-1.268248229	0.205622665	0.366633758	no
GAL3ST1	-0.070385796	-1.268132816	0.205663819	0.366676715	no
LMBR1L	-0.070358996	-1.267647571	0.205836911	0.366954878	no
LOC100506655	-0.070353522	-1.267548457	0.205872279	0.366987488	no
DECR1	-0.070346753	-1.267425885	0.205916024	0.367035024	no
TNS4	0.070332646	1.267170469	0.206007203	0.367146672	no
PRDX1	0.070331776	1.26715471	0.206012829	0.367146672	no
ARHGEF1	-0.070321414	-1.266967084	0.206079828	0.36723562	no
FANCD2	-0.070314401	-1.266840109	0.206125178	0.367270181	no
LINC00284	-0.070313129	-1.266817087	0.206133401	0.367270181	no
PVRL3	0.07030679	1.266702303	0.206174405	0.367288558	no
RAX2	-0.07030625	-1.266692531	0.206177896	0.367288558	no
SOX13	-0.070293781	-1.266466758	0.206258565	0.367401809	no
SLC2A8	0.07028377	1.266285495	0.206323348	0.367486746	no
LOC151484	0.070279178	1.266202359	0.206353065	0.367509217	no
ZNF584	0.070272287	1.266077587	0.206397672	0.3675582	no
C19orf48	-0.070265857	-1.265961156	0.206439302	0.36757974	no
IGFL2	0.070265135	1.26594809	0.206443975	0.36757974	no
MIR212	-0.070262128	-1.265893642	0.206463445	0.367583953	no
ANKZF1	-0.070254729	-1.265759674	0.206511358	0.367636713	no
MKRN9P	-0.070252268	-1.265715126	0.206527292	0.367636713	no
TMCC1	-0.070248252	-1.265642408	0.206553304	0.367652564	no
NFIA	-0.070245517	-1.265592882	0.206571021	0.36765365	no
TMLHE-AS1	0.070228523	1.265285192	0.206681119	0.367785366	no
TBX5	-0.070225932	-1.265238284	0.206697907	0.367785366	no
IL26	0.07022447	1.265211802	0.206707385	0.367785366	no
C1orf111	-0.070223529	-1.26519477	0.206713482	0.367785366	no
ACER3	0.070213105	1.265006043	0.206781041	0.367860157	no
ISM2	-0.070211762	-1.264981712	0.206789752	0.367860157	no
FKBP9L	0.070188584	1.264562052	0.20694004	0.368097038	no
PTAR1	0.070183307	1.26446651	0.206974267	0.36812745	no
SLC38A4	0.070178574	1.264380829	0.207004965	0.368151581	no
FABP3	-0.070162123	-1.264082965	0.207111709	0.368280532	no
U2AF1	-0.070162118	-1.264082864	0.207111745	0.368280532	no

MYBL2	-0.070152415	-1.263907198	0.207174716	0.368362028	no
ICAM4	0.070148204	1.263830955	0.207202052	0.368380153	no
KLHL36	0.070142528	1.263728188	0.207238901	0.368415189	no
GAS2L3	0.070132011	1.263537764	0.207307193	0.368503481	no
BBS4	-0.070129599	-1.263494087	0.20732286	0.368503481	no
POLR2J3	-0.070122094	-1.263358222	0.207371599	0.368559629	no
LOC100131094	0.07011347	1.263202076	0.207427623	0.368628715	no
RBM44	0.07010003	1.262958731	0.207514956	0.368753425	no
GPER	0.070085384	1.262693568	0.20761015	0.368892082	no
ANKRD20A4	-0.070080118	-1.262598222	0.207644387	0.368922414	no
CDC26	0.070075695	1.26251814	0.207673146	0.368943009	no
LOC729059	-0.070071799	-1.262447607	0.207698479	0.368957514	no
CASP12	0.070041479	1.261898652	0.207895716	0.369226888	no
SNORD15B	-0.07003761	-1.261828591	0.207920898	0.369226888	no
KRTAP12-4	-0.07003736	-1.261824074	0.207922522	0.369226888	no
C10orf99	-0.07003629	-1.261804701	0.207929486	0.369226888	no
TXNRD1	0.070033036	1.261745774	0.207950669	0.369226888	no
STX6	-0.070032646	-1.261738729	0.207953202	0.369226888	no
IPO4	0.070029535	1.261682395	0.207973454	0.369232342	no
MRE11A	0.070022412	1.261553425	0.208019825	0.369284161	no
CCDC149	-0.069970944	-1.260621606	0.208355082	0.369848771	no
REREP3	-0.069958529	-1.260396817	0.208436017	0.369949996	no
RAB4A	-0.069955743	-1.260346376	0.208454181	0.369949996	no
ABHD8	-0.069954275	-1.260319806	0.20846375	0.369949996	no
CDK5RAP1	0.069948182	1.260209485	0.208503483	0.369989956	no
C6orf120	0.06993163	1.259909813	0.208611441	0.370150965	no
FBXL14	-0.069927179	-1.259829242	0.208640474	0.370171917	no
EIF1AD	0.06991935	1.259687487	0.208691561	0.370231991	no
WDR16	0.069906607	1.25945678	0.208774725	0.370348958	no
CHMP1A	-0.069901573	-1.259365653	0.208807581	0.370376669	no
FAM102B	0.069896371	1.259271466	0.208841544	0.370406339	no
FUNDC2	-0.069886848	-1.259099046	0.208903727	0.370461626	no
MIR204	-0.069886317	-1.259089441	0.208907191	0.370461626	no
PIP4K2A	-0.069881891	-1.259009304	0.208936098	0.370482317	no
HOXD-AS2	-0.069877682	-1.258933112	0.208963584	0.370500485	no
CYP2B6	-0.069869575	-1.258786339	0.209016539	0.37055819	no
LINC00620	-0.069867421	-1.258747331	0.209030615	0.37055819	no
TESPA1	-0.069847566	-1.25838787	0.209160355	0.370737867	no
KLHL40	-0.06984663	-1.258370927	0.209166472	0.370737867	no
DEFB1	0.069841449	1.258277127	0.209200337	0.370767313	no
ETFA	-0.06982856	-1.258043766	0.209284607	0.37088608	no
HTR1B	-0.069820254	-1.257893396	0.209338921	0.370951744	no
LOC344595	0.069817579	1.257844972	0.209356414	0.370952155	no
LOC400680	-0.069812611	-1.257755036	0.209388906	0.370979141	no
SNORA16B	0.069790064	1.257346831	0.209536427	0.371209905	no
USP14	0.069785949	1.257272326	0.209563361	0.371227019	no
BEGAIN	0.069775489	1.257082967	0.209631826	0.371317692	no
GLIS3-AS1	-0.069771094	-1.257003395	0.2096606	0.371338055	no
SLC19A2	-0.069763357	-1.256863323	0.209711261	0.371397174	no
COX8A	-0.069758583	-1.256776893	0.209742524	0.371403321	no
UTS2R	-0.069757549	-1.256758174	0.209749296	0.371403321	no

CCDC162P	0.069740976	1.256458124	0.209857859	0.371564941	no
ZNF317	-0.069737803	-1.256400677	0.209878649	0.371571139	no
TERC	-0.069730433	-1.25626726	0.209926939	0.371626017	no
C5orf55	0.069724432	1.256158613	0.209966269	0.371630099	no
C18orf54	-0.0697231	-1.256134498	0.209974999	0.371630099	no
SRD5A3-AS1	-0.069722166	-1.256117586	0.209981122	0.371630099	no
KCNN3	0.069712931	1.255950396	0.210041658	0.371689193	no
RPL32P3	-0.069711795	-1.255929839	0.210049102	0.371689193	no
CCNE1	0.069700621	1.255727532	0.210122372	0.371758566	no
PXMP4	0.06970054	1.255726067	0.210122903	0.371758566	no
ARL14EP	-0.069690941	-1.255552296	0.210185853	0.371839329	no
CRTC2	0.069684835	1.255441753	0.210225905	0.371879572	no
LEF1-AS1	0.069660293	1.254997451	0.210386942	0.372133808	no
NMUR2	-0.069648024	-1.254775323	0.210467486	0.372245637	no
TAC3	-0.069635078	-1.254540959	0.21055249	0.372365337	no
VEGFB	0.06960069	1.253918415	0.210778411	0.372705839	no
EFCAB6	0.069600492	1.253914834	0.210779711	0.372705839	no
OR10G3	-0.06959035	-1.253731216	0.210846379	0.372793051	no
LOC388906	0.069583816	1.253612924	0.210889337	0.372838331	no
LINC00293	-0.069576183	-1.25347474	0.210939527	0.372896388	no
ADRA1B	-0.069570193	-1.253366308	0.210978917	0.372911559	no
SH3RF3	-0.069569601	-1.253355582	0.210982814	0.372911559	no
CARS2	0.069565721	1.253285347	0.211008331	0.37292182	no
PRICKLE4	-0.069563441	-1.253244082	0.211023324	0.37292182	no
POTEE	-0.06955414	-1.253075686	0.211084517	0.372999289	no
RHEB	-0.069542442	-1.252863918	0.211161488	0.373104624	no
SLC6A11	-0.069535481	-1.252737908	0.211207299	0.373154888	no
LOC100128076	0.069525793	1.252562522	0.211271072	0.373236877	no
LOC100506305	-0.069521419	-1.252483341	0.211299868	0.373257067	no
MKNK1-AS1	0.069516155	1.252388037	0.211334532	0.373287616	no
BTBD18	-0.069495293	-1.25201036	0.211471939	0.373499625	no
LINC00264	0.069486651	1.25185392	0.211528874	0.373569483	no
TLE2	-0.069473957	-1.251624124	0.211612527	0.373661049	no
SCARNA4	-0.069473507	-1.251615969	0.211615496	0.373661049	no
SMEK2	0.069469208	1.25153814	0.211643834	0.373680384	no
MED19	-0.069466563	-1.251490264	0.211661267	0.373680465	no
TIMM9	-0.069452473	-1.251235195	0.211754164	0.373813762	no
LOC257396	-0.06944113	-1.251029851	0.211828972	0.373885269	no
CDH7	-0.069441055	-1.251028498	0.211829465	0.373885269	no
AFAP1	-0.069420317	-1.25065308	0.211966282	0.374096032	no
LRRC23	0.069386849	1.250047215	0.212187219	0.374455208	no
VARS2	-0.069358777	-1.249539028	0.212372665	0.374751699	no
DMBT1	-0.06934694	-1.249324745	0.212450896	0.374858965	no
LOC100131138	0.069331475	1.249044784	0.212553136	0.375008574	no
BREA2	-0.069326832	-1.248960734	0.212583837	0.375031952	no
LINC00299	0.069316361	1.248771181	0.212653088	0.375123329	no
ATP8B2	0.069300871	1.248490782	0.212755559	0.375273286	no
NEUROG2	-0.069293229	-1.248352442	0.212806128	0.375331677	no
PTCSC3	-0.069248517	-1.247543053	0.213102167	0.375822967	no
LOC100507206	-0.069234209	-1.247284051	0.213196962	0.375959294	no
PGC	-0.069227606	-1.247164508	0.213240725	0.376005614	no

MYH2	-0.069223116	-1.247083229	0.213270484	0.376027236	no
ASB16-AS1	0.069192919	1.246536607	0.213470699	0.376349367	no
WDR73	0.069181672	1.246333012	0.213545305	0.376450017	no
GSK3B	-0.069177917	-1.246265045	0.213570216	0.37646305	no
OR7E91P	-0.069163157	-1.245997856	0.213668163	0.376604814	no
BSX	-0.069159579	-1.24593308	0.213691914	0.376615789	no
LINC00323	-0.0691547	-1.245844777	0.213724294	0.376630893	no
GMPPA	0.069153007	1.245814127	0.213735534	0.376630893	no
ITGA9	0.069126653	1.245337067	0.213910538	0.376908369	no
ZNF239	-0.069111711	-1.245066598	0.214009803	0.377002156	no
SNORA3	0.069111575	1.24506413	0.214010709	0.377002156	no
EPHX1	-0.06911072	-1.245048645	0.214016393	0.377002156	no
MGC12916	0.069092872	1.244725572	0.21413501	0.37718019	no
FABP4	0.06907818	1.244459626	0.214232689	0.377321317	no
KTI12	0.069068948	1.244292504	0.214294087	0.377398527	no
PDZK1IP1-AS1	-0.069033262	-1.24364654	0.214531525	0.377785726	no
ZNF114	-0.069023487	-1.243469593	0.214596599	0.377832613	no
ARHGEF38	-0.069021014	-1.243424834	0.214613062	0.377832613	no
ZNF90	-0.069019778	-1.243402452	0.214621294	0.377832613	no
TMEM52B	0.069018699	1.243382933	0.214628474	0.377832613	no
HOXB4	0.069003788	1.24311302	0.214727775	0.377946387	no
PRSS48	-0.069003713	-1.243111659	0.214728276	0.377946387	no
TTC4	0.068997504	1.242999268	0.214769635	0.377988225	no
TRAIP	-0.068991294	-1.242886852	0.214811008	0.378030084	no
SKIDA1	-0.068979341	-1.242670494	0.214890653	0.37813928	no
ZSCAN25	-0.068973573	-1.242566089	0.214929094	0.378175958	no
DNAJA4	0.068945936	1.242065825	0.215113354	0.378469185	no
BCL2L14	0.068932288	1.241818787	0.215204387	0.378577286	no
BIRC2	0.068931443	1.241803482	0.215210028	0.378577286	no
OARD1	-0.068896973	-1.241179537	0.215440078	0.378949646	no
CNPY2	0.068894443	1.241133745	0.215456969	0.378949646	no
UQCR10	0.068878161	1.240839025	0.215565701	0.379109859	no
EIF2B4	-0.06886622	-1.240622895	0.215645464	0.379219104	no
DNAH7	-0.068857112	-1.240458024	0.215706324	0.379295092	no
LOC100144603	0.068850245	1.240333727	0.215752215	0.379344748	no
GLTPD1	0.068838445	1.240120141	0.215831089	0.379452383	no
FANK1	0.068832866	1.240019154	0.215868388	0.379486915	no
ZNF630	-0.068829627	-1.239960528	0.215890044	0.379493942	no
FZR1	-0.068812696	-1.239654075	0.21600327	0.379661919	no
DEFA8P	0.06879896	1.239405429	0.21609517	0.379792386	no
SOX1	-0.06878866	-1.239219008	0.21616409	0.379882447	no
MLH1	-0.068778292	-1.239031329	0.21623349	0.37997334	no
TMEM79	-0.068748384	-1.23848999	0.21643376	0.380290523	no
RAD54L2	-0.068746052	-1.238447778	0.216449382	0.380290523	no
LOC256021	-0.068741201	-1.238359981	0.216481877	0.380307663	no
USP35	-0.068739313	-1.238325797	0.21649453	0.380307663	no
LINGO3	0.068724547	1.23805853	0.216593476	0.380411344	no
NUF2	-0.068717613	-1.237933026	0.21663995	0.380411344	no
LOC284648	0.068715981	1.237903483	0.216650891	0.380411344	no
CTDSPL2	-0.068715135	-1.237888176	0.21665656	0.380411344	no
TBC1D3C	-0.06871343	-1.237857318	0.216667988	0.380411344	no

ADNP2	-0.068712049	-1.237832318	0.216677247	0.380411344	no
RNF222	-0.068711248	-1.237817817	0.216682618	0.380411344	no
PRR26	-0.068709377	-1.237783957	0.21669516	0.380411344	no
TCP11	-0.068706515	-1.237732145	0.216714351	0.380413961	no
COG3	-0.068696859	-1.237557376	0.216779097	0.380496535	no
GRINA	0.068688838	1.237412187	0.216832895	0.380559881	no
KCNA3	-0.068648827	-1.236687996	0.217101377	0.380918682	no
LOC100128682	-0.068647557	-1.236664993	0.217109909	0.380918682	no
GLT8D1	0.068646741	1.236650222	0.217115388	0.380918682	no
POM121L8P	0.068644321	1.236606424	0.217131634	0.380918682	no
MYSM1	0.068642486	1.236573217	0.217143952	0.380918682	no
LOC100130581	-0.068641772	-1.236560288	0.217148748	0.380918682	no
PTPRG	-0.068639887	-1.236526176	0.217161402	0.380918682	no
UBE2B	0.068632562	1.236393596	0.21721059	0.380968307	no
DCK	0.068630395	1.236354365	0.217225147	0.380968307	no
TBX3	-0.068611313	-1.236008995	0.217353325	0.381153739	no
PSMC1	0.068609375	1.235973907	0.21736635	0.381153739	no
PEX10	0.0685987	1.235780695	0.217438084	0.381248419	no
KDM8	-0.068582439	-1.235486381	0.217547388	0.38140895	no
LOC100127888	0.068577761	1.235401702	0.217578844	0.381432982	no
METTL21D	-0.068565979	-1.235188453	0.217658074	0.381539062	no
DDX21	-0.068563483	-1.235143276	0.217674861	0.381539062	no
C1orf151-NBL	-0.068551321	-1.234923141	0.217756677	0.381651341	no
MTERFD1	0.068522748	1.234405983	0.21794897	0.381957214	no
AKR1C3	-0.0685194	-1.234345384	0.217971511	0.381965569	no
MRPS16	-0.068514396	-1.234254816	0.218005201	0.381993459	no
PAGE5	0.068508101	1.23414088	0.21804759	0.382036586	no
CTNND1	0.068503943	1.234065622	0.218075593	0.382054501	no
PCDHA9	-0.068493021	-1.233867943	0.218149159	0.382127222	no
SLC9C1	0.068490337	1.233819359	0.218167242	0.382127222	no
TSPAN12	-0.068489499	-1.233804197	0.218172885	0.382127222	no
RRM2	-0.068482665	-1.2336805	0.218218931	0.382127222	no
DOC2B	-0.068481458	-1.233658663	0.218227061	0.382127222	no
CCKAR	0.06848002	1.233632636	0.218236751	0.382127222	no
NDNF	0.068479305	1.233619699	0.218241567	0.382127222	no
NR2E1	0.068445049	1.232999691	0.218472488	0.382500387	no
SNORA38B	-0.068441906	-1.232942805	0.218493685	0.382506335	no
C5orf28	0.068439186	1.232893564	0.218512033	0.382507298	no
KLK8	-0.068434631	-1.232811125	0.218542754	0.382529918	no
HNRNPUL2-BSC	-0.068428853	-1.23270654	0.218581733	0.382566987	no
DYRK1A	-0.068421051	-1.232565343	0.218634366	0.382627944	no
LMX1B	-0.068396289	-1.232117166	0.218801488	0.382889242	no
FAM214A	-0.068376249	-1.231754465	0.218936804	0.383048244	no
JAM2	0.068375223	1.2317359	0.218943732	0.383048244	no
OR10G7	-0.068374914	-1.231730303	0.21894582	0.383048244	no
IPPK	0.06835849	1.23143304	0.219056772	0.383211158	no
PTPN12	0.068336655	1.231037853	0.219204335	0.383438088	no
TMEM30B	0.068325871	1.230842673	0.219277241	0.383534402	no
CXCR5	0.068316852	1.230679448	0.219338225	0.383558493	no
ARAP3	0.068316189	1.230667447	0.219342709	0.383558493	no
GOLGA5	0.068315916	1.230662502	0.219344557	0.383558493	no

GFM1	-0.068300242	-1.230378824	0.219450576	0.383712662	no
ACOXL	0.068293919	1.230264383	0.219493356	0.383756241	no
PNPLA1	-0.068282552	-1.23005866	0.219570274	0.383843974	no
PROSER2-AS1	-0.068280862	-1.230028072	0.219581713	0.383843974	no
CRYBB2	-0.068278586	-1.229986879	0.219597118	0.383843974	no
MS4A10	-0.0682742	-1.229907496	0.219626806	0.383864646	no
PPDPF	0.068264124	1.229725146	0.219695015	0.383952635	no
ZNF295-AS1	0.068260087	1.229652079	0.21972235	0.383969184	no
SIN3A	-0.068247865	-1.229430877	0.219805119	0.384070757	no
ETV3	0.068244106	1.229362852	0.219830577	0.384070757	no
ROCK2	0.068243588	1.22935347	0.219834088	0.384070757	no
HIST4H4	0.068225148	1.229019743	0.219959016	0.38425778	no
CATSPER3	-0.06822116	-1.228947551	0.219986047	0.384273766	no
DDX41	-0.068207616	-1.228702437	0.220077844	0.384402873	no
HIST1H2BG	0.068204556	1.228647055	0.220098589	0.384407864	no
FLJ41649	-0.068178125	-1.228168697	0.220277829	0.384689649	no
CIB4	-0.068174569	-1.228104335	0.220301954	0.384700519	no
PNPO	0.068169258	1.228008212	0.220337986	0.384732179	no
SLCO1A2	-0.068157871	-1.227802133	0.220415251	0.384835825	no
PPAN-P2RY11	-0.06812173	-1.227148047	0.220660617	0.385232927	no
SUV39H1	-0.068110571	-1.226946094	0.220736416	0.385333954	no
FABP7	0.068105055	1.226846274	0.220773887	0.385368065	no
LOC100272228	0.068098823	1.226733481	0.220816235	0.38541068	no
LOC284412	-0.068092397	-1.226617181	0.220859905	0.385455596	no
TSPAN16	-0.068086052	-1.226502353	0.220903028	0.385499552	no
CA9	0.068081778	1.226425006	0.220932079	0.385518944	no
SLC36A3	-0.068074735	-1.22629754	0.220979961	0.385571189	no
LHX9	-0.068067449	-1.226165671	0.221029504	0.385626325	no
ODF3L2	-0.068056704	-1.225971223	0.221102572	0.385722493	no
ERCC5	-0.068052791	-1.225900399	0.221129191	0.385737616	no
CDK10	0.068031336	1.225512104	0.221275167	0.385960929	no
GSR	0.068024712	1.225392235	0.221320245	0.386008227	no
HEMGN	0.067969958	1.224401326	0.221693138	0.386627219	no
SETD7	0.06796203	1.224257849	0.221747167	0.386659995	no
MYO7B	0.067961921	1.224255863	0.221747915	0.386659995	no
SLC6A18	-0.067956681	-1.224161046	0.221783626	0.38669089	no
RHOT1	-0.06794755	-1.22399579	0.221845877	0.386768049	no
S1PR1	-0.067938905	-1.223839333	0.221904824	0.386839437	no
UBR2	-0.067898015	-1.223099344	0.222183779	0.387294313	no
PRSS36	0.067884707	1.222858512	0.22227462	0.387396468	no
MMP23B	0.067884149	1.222848399	0.222278435	0.387396468	no
CCRL1	0.067878768	1.222751027	0.222315171	0.387429074	no
DNAJA1P5	0.067875676	1.222695068	0.222336285	0.387434453	no
ADK	-0.067867688	-1.222550507	0.222390837	0.387498093	no
SLC5A3	0.067861148	1.222432151	0.222435507	0.387544506	no
PAR4	-0.067851069	-1.222249764	0.222504356	0.387633035	no
AQP8	-0.067842056	-1.222086644	0.222565946	0.387700928	no
PRR5L	-0.067840086	-1.222050995	0.222579408	0.387700928	no
LHX6	-0.067832144	-1.221907276	0.222633684	0.387764041	no
AKR7L	-0.067816688	-1.221627571	0.222739344	0.387886563	no
LOC100271832	-0.067816574	-1.221625499	0.222740127	0.387886563	no

ZNF707	-0.067803363	-1.221386418	0.22283047	0.388012448	no
ING1	-0.067799464	-1.221315864	0.222857135	0.388026782	no
H2BFWT	-0.06779688	-1.221269095	0.222874813	0.388026782	no
GPR62	-0.067786371	-1.221078914	0.222946707	0.388085374	no
TMEM101	0.067785067	1.221055321	0.222955627	0.388085374	no
LOC440173	-0.067784042	-1.221036769	0.222962641	0.388085374	no
KRT19	-0.067719788	-1.219873999	0.223402586	0.388799118	no
FANCA	-0.067718869	-1.219857369	0.223408883	0.388799118	no
SNRNP27	-0.067703723	-1.219583272	0.223512683	0.388874106	no
SRL	-0.067701682	-1.219546342	0.223526672	0.388874106	no
EPC1	-0.067701602	-1.219544891	0.223527221	0.388874106	no
LOC646471	-0.067700018	-1.21951623	0.223538078	0.388874106	no
SLC41A1	-0.067699381	-1.219504698	0.223542446	0.388874106	no
FBXO33	-0.067691338	-1.219359138	0.223597588	0.388938549	no
TCFL5	-0.067686235	-1.219266804	0.223632573	0.38896792	no
39873	-0.06767686	-1.219097152	0.223696862	0.389048252	no
TLE1	-0.067647402	-1.218564057	0.223898963	0.389368232	no
CHIC1	-0.067640429	-1.218437879	0.223946817	0.389419941	no
MED29	-0.067624242	-1.218144958	0.22405794	0.389581649	no
FAM184A	-0.067612757	-1.217937129	0.224136805	0.389659351	no
LOC730441	-0.067612454	-1.217931633	0.224138891	0.389659351	no
MPO	0.067602363	1.217749028	0.224208202	0.389720084	no
VPS37B	-0.067602087	-1.217744044	0.224210094	0.389720084	no
TMEM160	-0.067596931	-1.217650727	0.22424552	0.389750138	no
PRRT4	-0.067590445	-1.217533369	0.224290078	0.389769109	no
NOP56	-0.067590063	-1.217526445	0.224292707	0.389769109	no
ZNF385D	0.067581766	1.217376299	0.224349725	0.389795525	no
FASTK	0.067579896	1.217342468	0.224362573	0.389795525	no
FHL5	0.067578312	1.217313805	0.22437346	0.389795525	no
TENM3	-0.067577294	-1.217295377	0.224380459	0.389795525	no
LOC646626	-0.067570755	-1.217177052	0.224425404	0.389842092	no
ASNSD1	-0.067556508	-1.216919242	0.224523354	0.389980717	no
FOXJ1	0.067542308	1.216662275	0.224621015	0.390118816	no
GNG8	0.06753623	1.216552289	0.224662824	0.3901599	no
UGT2B7	-0.067522752	-1.216308385	0.224755561	0.39027612	no
MAS1	-0.067521225	-1.216280758	0.224766067	0.39027612	no
CGB7	0.067503233	1.215955176	0.224889906	0.390459603	no
TH1L	-0.067500134	-1.215899111	0.224911236	0.390465091	no
ZNF674-AS1	-0.067494632	-1.215799531	0.224949124	0.390499324	no
ITPRIPL1	0.067485057	1.215626278	0.225015055	0.390582227	no
SCARNA10	-0.06747585	-1.215459662	0.225078474	0.390644238	no
LOC100652739	-0.067474593	-1.215436912	0.225087134	0.390644238	no
SCN5A	-0.067465267	-1.215268157	0.225151382	0.390724189	no
EBF3	-0.067457947	-1.215135709	0.225201816	0.390780156	no
ASB17	-0.067444232	-1.214887524	0.225296343	0.390912619	no
INCA1	-0.067432016	-1.214666474	0.225380559	0.391000185	no
NKAIN2	-0.067431634	-1.214659549	0.225383197	0.391000185	no
PKD2L1	-0.067413853	-1.214337798	0.22550582	0.391181337	no
FAM60A	-0.067381305	-1.213748825	0.225730407	0.39153932	no
C12orf4	0.067375182	1.213638034	0.225772672	0.391581026	no
TMEM88B	-0.067361518	-1.213390776	0.225867017	0.39171231	no

C10orf116	-0.067356318	-1.213296676	0.225902929	0.39171231	no
DLEU2L	-0.067356301	-1.213296369	0.225903047	0.39171231	no
IFNA6	-0.067340243	-1.213005813	0.226013961	0.391873017	no
KIAA0232	-0.067334896	-1.212909043	0.22605091	0.391905463	no
THRB-AS1	-0.067324677	-1.212724141	0.226121522	0.391996259	no
C16orf89	0.067319709	1.212634244	0.226155858	0.392008404	no
ATP4A	-0.067318385	-1.212610288	0.226165009	0.392008404	no
RGAG1	-0.067309586	-1.212451072	0.226225833	0.392082207	no
LINC00485	-0.067294129	-1.212171364	0.226332715	0.392235817	no
TULP3	0.067290756	1.212110336	0.22635604	0.39224461	no
PCBD2	-0.067286129	-1.21202661	0.226388043	0.392251166	no
LOC399715	0.067283359	1.211976482	0.226407205	0.392251166	no
SEC13	0.067282293	1.211957192	0.226414579	0.392251166	no
DLX2	-0.067263542	-1.211617902	0.226544311	0.392444284	no
KRTAP10-4	-0.067238633	-1.211167181	0.226716733	0.392689022	no
SAYSD1	-0.067236104	-1.211121409	0.226734248	0.392689022	no
FAM74A2	-0.067235214	-1.211105315	0.226740406	0.392689022	no
SCARF1	0.067230917	1.211027562	0.226770162	0.392708909	no
CHAF1A	-0.067214137	-1.210723927	0.226886389	0.392853273	no
TSPAN14	0.067213603	1.210714274	0.226890085	0.392853273	no
CAPN1	-0.067206139	-1.210579215	0.226941798	0.392911156	no
LOC339593	-0.067198138	-1.210434435	0.226997242	0.39296727	no
SPCS2	-0.067196185	-1.21039909	0.227010779	0.39296727	no
GFI1B	-0.067181673	-1.210136509	0.227111366	0.393109726	no
RIOK1	-0.067178396	-1.210077217	0.227134083	0.393117385	no
FPGT-TNNI3K	-0.067172359	-1.20996798	0.22717594	0.393146607	no
ADAMTS9	0.067170685	1.209937675	0.227187554	0.393146607	no
LOC643802	0.067144151	1.209457568	0.227371593	0.393433406	no
QRFPR	-0.067129123	-1.209185631	0.227475882	0.393563676	no
PER1	-0.067128024	-1.209165761	0.227483504	0.393563676	no
LRRC17	0.067122414	1.209064244	0.227522446	0.393599363	no
ASAH2	0.067118942	1.209001424	0.227546546	0.393603291	no
SPPL2C	-0.06711681	-1.208962848	0.227561346	0.393603291	no
HILPDA	0.067080018	1.208297121	0.227816869	0.394013547	no
GPR78	-0.067075655	-1.208218173	0.227847185	0.394034268	no
SENP5	-0.067064307	-1.20801284	0.227926046	0.394138933	no
METTL2B	-0.067036612	-1.207511718	0.22811859	0.39444015	no
FOXA2	0.06703008	1.207393537	0.228164016	0.394486956	no
ADAM15	0.06702631	1.207325315	0.228190241	0.394500561	no
PDE4D	-0.067011675	-1.207060512	0.228292056	0.394644834	no
MBTPS1	0.067005033	1.206940332	0.228338274	0.394692983	no
SEPSECS	0.06699332	1.206728402	0.228419795	0.394777606	no
ATXN2L	-0.066990192	-1.206671811	0.228441567	0.394777606	no
LTN1	-0.066990081	-1.206669803	0.228442339	0.394777606	no
IL17RC	0.066922692	1.205450474	0.228911801	0.395557088	no
FMO9P	0.066901686	1.205070399	0.229058277	0.395778375	no
NR2C1	-0.066890573	-1.204869327	0.229135794	0.395838294	no
ACTN3	-0.066890287	-1.204864151	0.22913779	0.395838294	no
ERCC2	-0.066888793	-1.204837122	0.229148212	0.395838294	no
SHISA2	-0.066884079	-1.204751836	0.229181098	0.395863284	no
RPF2	0.066873713	1.204564274	0.229253435	0.395956406	no

STX10	-0.066861604	-1.204345185	0.229337951	0.396070548	no
ASB18	-0.066858416	-1.204287496	0.229360209	0.396077159	no
OVGP1	-0.06684358	-1.204019069	0.229463795	0.396224202	no
TTLL4	-0.066837556	-1.203910074	0.229505866	0.396265008	no
PTPN3	-0.066833537	-1.203837355	0.229533937	0.39628164	no
TMEM185B	0.066805743	1.203334482	0.229728129	0.396578602	no
PRAC	-0.066803637	-1.203296372	0.22974285	0.396578602	no
NME9	-0.066799169	-1.203215521	0.229774084	0.396600662	no
SLC13A5	0.066795282	1.203145202	0.229801252	0.396615701	no
ATP5H	0.066788831	1.203028477	0.229846354	0.396661688	no
POGZ	0.066773279	1.202747097	0.229955104	0.3968175	no
KCNIP4	-0.066745096	-1.202237174	0.230152277	0.397125861	no
DDA1	-0.066731535	-1.201991819	0.230247192	0.39725774	no
ABL1	-0.066725261	-1.201878315	0.23029111	0.397301618	no
ANKRD11	0.066713348	1.201662759	0.230374532	0.397391288	no
HNRNPU	-0.06671059	-1.201612872	0.230393842	0.397391288	no
LCN12	-0.066709917	-1.201600684	0.23039856	0.397391288	no
RAB21	0.066704885	1.201509641	0.230433803	0.397418167	no
TMEM141	-0.066702411	-1.201464889	0.230451128	0.397418167	no
TTC30A	0.066691805	1.201272999	0.230525427	0.397485912	no
LOC100652999	-0.066691523	-1.201267896	0.230527403	0.397485912	no
ADAM20	-0.066687012	-1.201186282	0.230559009	0.397508516	no
LOC643355	-0.066669447	-1.200868476	0.230682111	0.397688854	no
MRS2P2	-0.066652926	-1.200569566	0.230797938	0.39785662	no
MRPL50	-0.066638028	-1.200300029	0.230902418	0.398004801	no
CPNE4	-0.066623954	-1.200045387	0.231001155	0.398129378	no
GLYR1	-0.066622445	-1.200018089	0.231011742	0.398129378	no
MT1H	0.066614735	1.199878596	0.231065844	0.398190687	no
SSX2IP	0.066611087	1.199812589	0.231091449	0.39820288	no
MAP7D1	0.066599003	1.199593968	0.231176266	0.398317096	no
SLC9A3R1	0.066581478	1.199276894	0.23129932	0.398440937	no
LINC00221	0.066581368	1.199274911	0.23130009	0.398440937	no
RHBG	-0.066580846	-1.199265454	0.231303761	0.398440937	no
TBCCD1	-0.066575674	-1.199171894	0.23134008	0.398471561	no
RNU11	-0.066555163	-1.198800799	0.231484177	0.398658391	no
KRTAP5-5	-0.066554954	-1.198797011	0.231485648	0.398658391	no
CCDC13-AS1	-0.066550745	-1.198720872	0.231515221	0.398677373	no
TRIM52	-0.066546667	-1.198647078	0.231543885	0.398694787	no
PLEKHM1P	0.066543762	1.198594521	0.231564302	0.398697998	no
GTPBP2	0.066531577	1.198374071	0.231649954	0.39881352	no
C18orf61	-0.066525815	-1.198269832	0.231690463	0.398845246	no
RRM1	0.066523677	1.19823114	0.2317055	0.398845246	no
CRCP	-0.06651183	-1.198016814	0.231788809	0.398956696	no
CHRND	0.066505609	1.197904247	0.231832573	0.398970484	no
PKIG	-0.066505413	-1.197900707	0.231833949	0.398970484	no
RALGAPA2	0.066492189	1.197661457	0.231926984	0.399098632	no
BPIFB2	-0.066482093	-1.1974788	0.23199803	0.399188925	no
SEL1L	0.066468527	1.197233373	0.232093516	0.399321251	no
C6orf106	0.066445794	1.19682208	0.232253596	0.399536776	no
NCAPH	-0.066444976	-1.196807292	0.232259353	0.399536776	no
CYP2G1P	-0.066442818	-1.196768242	0.232274556	0.399536776	no

SETD6	-0.066416048	-1.196283927	0.23246317	0.399829211	no
ZEB2-AS1	-0.066408653	-1.196150131	0.232515296	0.399882023	no
CHRNB3	-0.066406412	-1.196109596	0.232531089	0.399882023	no
NMB	-0.06640048	-1.196002272	0.23257291	0.39992194	no
MMEL1	0.066393874	1.195882754	0.232619488	0.39997003	no
CYP4Z2P	-0.0663775	-1.195586532	0.232734959	0.400136561	no
LOC100132352	-0.066349522	-1.195080364	0.232932365	0.40044392	no
TK1	0.066346191	1.195020097	0.232955877	0.400452308	no
GLP2R	-0.066329748	-1.194722622	0.233071956	0.400603195	no
RHPN1	-0.066328254	-1.194695581	0.23308251	0.400603195	no
C12orf61	0.06632358	1.19461103	0.233115512	0.400603195	no
TMEM208	0.066323197	1.194604104	0.233118215	0.400603195	no
OR5M10	-0.066316486	-1.194482683	0.233165614	0.400642328	no
LOC728739	-0.066314194	-1.194441226	0.233181799	0.400642328	no
LOC284889	-0.066310634	-1.194376822	0.233206945	0.400642328	no
LOC100507066	-0.066309415	-1.194354766	0.233215556	0.400642328	no
DRD4	-0.066305314	-1.194280567	0.233244529	0.400660073	no
A1BG-AS1	0.066300296	1.194189792	0.233279978	0.400673194	no
RAB23	0.066298955	1.194165525	0.233289455	0.400673194	no
TSEN54	-0.066282684	-1.19387117	0.233404434	0.400838636	no
ZAR1L	-0.066269628	-1.193634966	0.233496727	0.400965095	no
BTBD7	-0.066242504	-1.193144267	0.233688544	0.401262424	no
ANAPC7	-0.066228516	-1.192891205	0.233787511	0.401400288	no
STPG1	0.066207961	1.192519336	0.233932996	0.401596864	no
RAB4B	0.06620706	1.19250303	0.233939376	0.401596864	no
MIR5006	-0.066175785	-1.191937248	0.234160852	0.401944958	no
PDF	0.06616187	1.1916855	0.234259447	0.402082084	no
ARL17B	-0.066155102	-1.191563074	0.234307405	0.402132282	no
CPS1-IT1	-0.066143047	-1.19134499	0.234392852	0.402223511	no
DYDC1	-0.066142322	-1.191331871	0.234397992	0.402223511	no
DYRK2	0.066130871	1.191124706	0.234479183	0.402330709	no
LOC100422737	-0.066113069	-1.190802664	0.234605435	0.402508889	no
LOC284837	0.066110948	1.190764282	0.234620486	0.402508889	no
BHLHE22	-0.066106101	-1.190676612	0.234654865	0.402520843	no
PSG1	0.066100486	1.190575021	0.234694709	0.402520843	no
DHX16	0.066099736	1.190561455	0.23470003	0.402520843	no
FAM160A2	0.066099406	1.190555483	0.234702372	0.402520843	no
OR2C1	-0.066093624	-1.190450887	0.2347434	0.40254935	no
SPDL1	-0.066090997	-1.190403356	0.234762046	0.40254935	no
ROPN1L	0.066089145	1.190369858	0.234775187	0.40254935	no
CSF3	0.066075537	1.190123685	0.234871778	0.402682839	no
CDYL	-0.066061435	-1.189868574	0.234971906	0.40282237	no
PFN1P2	-0.066046093	-1.189591031	0.235080873	0.402977031	no
TMEM237	-0.06604282	-1.189531825	0.235104122	0.402980578	no
ANP32B	-0.066039754	-1.189476353	0.235125907	0.402980578	no
FRG2B	0.066035821	1.189405195	0.235153854	0.402980578	no
CDKN3	0.066032123	1.189338306	0.235180127	0.402980578	no
MAP2K1	-0.066029769	-1.189295712	0.235196858	0.402980578	no
SDHAF2	0.066028591	1.189274418	0.235205223	0.402980578	no
ABCG8	-0.066027328	-1.189251565	0.2352142	0.402980578	no
ACOT2	-0.066024432	-1.189199169	0.235234784	0.402983717	no

KIF17	-0.066021354	-1.18914349	0.235256658	0.402989068	no
VIMP	0.066009408	1.188927388	0.235341573	0.403102394	no
OR10Q1	-0.065993196	-1.188634107	0.235456848	0.403253112	no
C20orf202	-0.065991756	-1.188608046	0.235467093	0.403253112	no
TBC1D3	-0.065982904	-1.188447922	0.23553005	0.403305115	no
POU2F3	0.065980473	1.188403943	0.235547343	0.403305115	no
LOC392196	-0.065979571	-1.188387631	0.235553758	0.403305115	no
ZNF662	-0.065968174	-1.188181441	0.235634849	0.403411817	no
GPBP1	-0.065878065	-1.186551387	0.236276626	0.404478331	no
LOC202181	0.065865321	1.186320842	0.236367495	0.404601659	no
POLH	0.065862434	1.18626862	0.236388081	0.404604671	no
FBXW8	-0.065857154	-1.186173094	0.236425743	0.404636907	no
CYCS	-0.065852323	-1.186085718	0.236460194	0.404651975	no
NCOR2	-0.06585064	-1.186055258	0.236472205	0.404651975	no
LOC644189	-0.065845412	-1.185960695	0.236509496	0.404683565	no
MIXL1	0.065833814	1.185750888	0.236592248	0.404771824	no
LONRF1	-0.065832903	-1.185734416	0.236598746	0.404771824	no
CNKSR3	-0.06581526	-1.18541525	0.236724674	0.404955023	no
PDZRN3	-0.065792817	-1.18500927	0.236884922	0.405180378	no
PRSS12	-0.06579153	-1.184985983	0.236894116	0.405180378	no
SMIM16	-0.065783928	-1.184848472	0.236948414	0.405240997	no
KIAA1656	-0.065775894	-1.184703144	0.237005807	0.405306899	no
GPR146	0.065765562	1.184516246	0.237079633	0.40540089	no
PHF5A	-0.065737876	-1.184015417	0.237277542	0.405707029	no
SNORA12	-0.065723091	-1.183747966	0.237383276	0.405855529	no
GBF1	-0.065708718	-1.183487962	0.237486099	0.405999027	no
ZIC5	0.065705861	1.183436296	0.237506535	0.406001666	no
IFT20	0.065670559	1.182797711	0.237759224	0.406350569	no
TUBD1	0.06566974	1.182782892	0.23776509	0.406350569	no
GAB2	-0.065669423	-1.182777149	0.237767363	0.406350569	no
LOC642366	-0.065663005	-1.182661052	0.237813324	0.406390631	no
LARS2-AS1	-0.065660868	-1.182622407	0.237828625	0.406390631	no
TM4SF5	-0.065649739	-1.1824211	0.237908338	0.406494521	no
CDH15	-0.065638388	-1.182215759	0.237989668	0.406601157	no
ZNF384	-0.065628835	-1.182042952	0.238058128	0.406685789	no
HOMER2	0.06562573	1.181986785	0.238080382	0.406691478	no
LAMA1	-0.065619523	-1.181874518	0.238124868	0.406735141	no
ZAR1	0.065614736	1.181787928	0.238159184	0.406761426	no
ZNF713	-0.065601874	-1.18155527	0.238251404	0.406886596	no
CLDN11	0.065593859	1.181410275	0.23830889	0.406952431	no
EXOSC2	-0.065584033	-1.181232551	0.238379364	0.407040436	no
C4orf22	0.065578714	1.181136323	0.238417529	0.407073259	no
PRR23C	-0.065569885	-1.180976623	0.238480876	0.407149071	no
CD99	0.065562907	1.180850392	0.238530956	0.407168566	no
LOC100289187	-0.065561486	-1.180824689	0.238541154	0.407168566	no
LINC00483	-0.065560374	-1.18080458	0.238549133	0.407168566	no
LYSMD2	-0.065550021	-1.180617302	0.238623449	0.407263068	no
ZNF271	0.065537175	1.180384947	0.238715676	0.407388121	no
SEC61B	0.065494661	1.179615921	0.239021102	0.407876965	no
CBFA2T3	-0.06547582	-1.179275125	0.23915654	0.408075681	no
CRISPLD1	0.065449805	1.178804548	0.239343645	0.40833631	no

MYLK4	0.065449299	1.178795397	0.239347285	0.40833631	no
FAM27L	-0.065440244	-1.178631614	0.239412432	0.408415031	no
AFAP1L2	-0.065427187	-1.178395437	0.239506396	0.408542896	no
C4BPB	0.06541757	1.178221486	0.23957562	0.408628543	no
LINC00552	-0.06541069	-1.178097034	0.239625154	0.408680595	no
ECT2	-0.065402652	-1.177951649	0.239683029	0.408746864	no
C17orf82	0.06539406	1.177796226	0.239744911	0.408805685	no
LCLAT1	-0.065392581	-1.177769471	0.239755565	0.408805685	no
RHOXF1	-0.065378883	-1.177521709	0.239854239	0.408941488	no
RIMBP3C	0.065363666	1.17724645	0.239963898	0.409095996	no
ORM1	0.06535298	1.177053175	0.240040917	0.40919484	no
PRIMA1	0.065345575	1.176919221	0.240094306	0.409236519	no
FAM26D	-0.065341296	-1.176841834	0.240125155	0.409236519	no
ZNF440	-0.065335429	-1.176735711	0.240167462	0.409236519	no
AGPHD1	0.065334755	1.176723517	0.240172323	0.409236519	no
EEF1B2	-0.065334596	-1.176720636	0.240173472	0.409236519	no
ALDH1L1-AS1	-0.065331481	-1.176664293	0.240195936	0.409236519	no
RBBP5	-0.065330333	-1.176643529	0.240204216	0.409236519	no
ATP2C2	-0.065328462	-1.1766097	0.240217705	0.409236519	no
ZNF767	-0.065303548	-1.176159055	0.240397445	0.409510264	no
E2F2	-0.065281554	-1.175761235	0.240556195	0.409730804	no
SLC9C2	0.065280329	1.175739075	0.240565041	0.409730804	no
ST7-OT4	-0.065268215	-1.175519957	0.240652514	0.409835858	no
DYNC1LI2	-0.065266505	-1.175489033	0.240664861	0.409835858	no
ZNF350	0.065251895	1.175224771	0.24077039	0.409983079	no
MIR1272	0.065246821	1.175132986	0.24080705	0.410013018	no
LCE4A	-0.065240244	-1.175014026	0.24085457	0.410061441	no
TTC34	0.065235243	1.174923577	0.240890706	0.410090476	no
EGFLAM	0.065227469	1.174782959	0.240946893	0.410153637	no
NRL	-0.065217272	-1.174598525	0.241020601	0.410246613	no
COX10-AS1	-0.065209425	-1.174456585	0.241077338	0.410310689	no
TPST2	0.065187153	1.174053755	0.241238409	0.410550127	no
CHKB	0.065177892	1.173886245	0.241305411	0.410550127	no
MMD	0.065177552	1.173880093	0.241307872	0.410550127	no
MC2R	-0.065177487	-1.17387891	0.241308345	0.410550127	no
KAZALD1	0.065176769	1.173865933	0.241313536	0.410550127	no
SLC16A2	-0.065173332	-1.17380376	0.241338408	0.410559941	no
LZTS1-AS1	0.065146027	1.173309898	0.241536041	0.410833783	no
LOC100506710	-0.06514581	-1.173305969	0.241537614	0.410833783	no
PTPRG-AS1	0.065134348	1.173098646	0.241620614	0.410915552	no
SLC22A8	-0.06513389	-1.17309037	0.241623928	0.410915552	no
BCL9	-0.065129215	-1.173005804	0.241657789	0.410940619	no
MAATS1	0.06509462	1.172380091	0.241908439	0.411334304	no
MYO16-AS1	-0.065084676	-1.172200235	0.241980521	0.411400358	no
SLC22A31	0.06508398	1.172187632	0.241985572	0.411400358	no
CDH24	-0.065076551	-1.172053274	0.24203943	0.411449728	no
RYK	0.065072922	1.171987627	0.242065747	0.411449728	no
CCDC114	0.065072053	1.171971914	0.242072047	0.411449728	no
EIF4E3	0.065062326	1.171795989	0.242142586	0.411537076	no
C18orf25	0.065011618	1.170878832	0.242510568	0.412129891	no
PPM1D	-0.064991922	-1.170522603	0.2426536	0.412340358	no

ARSH	-0.064988708	-1.170464462	0.24267695	0.412347433	no
HGFAC	-0.064981883	-1.170341034	0.242726526	0.412399065	no
SREBF1	0.064977434	1.17026056	0.242758853	0.412421383	no
ZSCAN12P1	0.06494917	1.169749355	0.242964278	0.412726715	no
GSG1L	-0.064947422	-1.169717742	0.242976986	0.412726715	no
C1orf192	0.064925337	1.169318299	0.243137591	0.412947131	no
OSGIN2	-0.064924294	-1.169299436	0.243145177	0.412947131	no
ETV5	0.064866626	1.168256433	0.243564904	0.413627289	no
HRASLS	-0.064858474	-1.168109002	0.243624274	0.413695423	no
FFAR2	0.06483988	1.167772707	0.243759738	0.413828763	no
SCRN1	-0.064839839	-1.167771971	0.243760035	0.413828763	no
EXOSC1	0.064839767	1.167770655	0.243760565	0.413828763	no
RPLP2	-0.064828782	-1.167571992	0.243840615	0.413931963	no
HAUS1	0.064822722	1.167462384	0.243884788	0.41397425	no
LOC100128675	-0.06481533	-1.167328692	0.243938675	0.414033018	no
IDS	0.064809724	1.167227305	0.243979547	0.414069687	no
DHFR	-0.06479482	-1.166957743	0.244088238	0.414221441	no
SGK223	0.064786817	1.166813007	0.244146612	0.414260967	no
PLXNA1	0.064785007	1.166780266	0.244159818	0.414260967	no
H2AFX	-0.064783699	-1.166756615	0.244169358	0.414260967	no
PPP1R3F	-0.064772191	-1.166548484	0.244253322	0.414370709	no
IFT27	-0.064759259	-1.166314601	0.2443477	0.414494767	no
CDC23	-0.064756886	-1.166271678	0.244365023	0.414494767	no
FLJ38109	0.064738657	1.165942	0.244498106	0.414661421	no
RNF34	-0.064738143	-1.165932696	0.244501863	0.414661421	no
C1orf109	0.064708968	1.16540504	0.244714973	0.414990097	no
THYN1	-0.064698252	-1.165211242	0.244793278	0.415090132	no
TUBB1	-0.064680037	-1.164881807	0.244926427	0.415283144	no
LOC494141	-0.064670365	-1.164706889	0.244997146	0.41537028	no
FNDC9	-0.064653956	-1.164410125	0.245117159	0.415540969	no
KCNQ1OT1	-0.064646169	-1.164269282	0.245174131	0.415604768	no
SPAM1	-0.064609216	-1.163600973	0.245444594	0.416030426	no
LECT2	-0.06460031	-1.163439913	0.245509806	0.416108143	no
MIR5188	-0.064590054	-1.163254434	0.245584921	0.416189199	no
CDKN2B-AS1	0.064588492	1.163226184	0.245596362	0.416189199	no
CAV3	-0.064560498	-1.162719904	0.245801482	0.416462369	no
JAG1	0.064560133	1.162713297	0.24580416	0.416462369	no
POM121L1P	-0.064558559	-1.162684829	0.245815697	0.416462369	no
RSPH9	0.064541099	1.162369077	0.245943691	0.416646371	no
TEKT4P2	0.064528672	1.162144325	0.246034825	0.416767569	no
HYMAI	-0.064526055	-1.162096998	0.246054019	0.416767569	no
ZNF341	-0.06451499	-1.161896885	0.246135186	0.416872196	no
WIF1	-0.064508961	-1.161787849	0.246179421	0.416914258	no
MRPL20	0.064494231	1.161521454	0.246287516	0.417042504	no
SCARNA5	-0.064488448	-1.161416878	0.246329959	0.417042504	no
IMPA1	0.064488302	1.161414234	0.246331032	0.417042504	no
SSUH2	-0.064488065	-1.161409955	0.246332769	0.417042504	no
KCND3	-0.064472531	-1.161129016	0.246446817	0.417202721	no
MSH5-SAPCD1	-0.06445881	-1.160880878	0.246547581	0.417340426	no
TMEM39B	0.064454966	1.160811366	0.246575813	0.417347739	no
CACNA1S	-0.064452934	-1.160774616	0.24659074	0.417347739	no

GABRA2	-0.064449231	-1.160707636	0.246617947	0.417353564	no
GPR116	0.064447179	1.160670527	0.246633022	0.417353564	no
LOC100288524	0.064424074	1.160252679	0.246802807	0.417607994	no
GPD1	0.064418647	1.160154542	0.246842696	0.417642606	no
LYPD4	-0.064404818	-1.159904452	0.246944366	0.417781735	no
ZNF140	-0.064397031	-1.15976362	0.247001633	0.417845725	no
ETAA1	0.064382657	1.159503675	0.247107359	0.417991676	no
SLC6A5	-0.064371128	-1.159295177	0.247192183	0.41806618	no
C3orf67	0.064369432	1.159264505	0.247204663	0.41806618	no
USMG5	-0.064366972	-1.159220024	0.247222762	0.41806618	no
ANKRD40	-0.064366095	-1.159204165	0.247229216	0.41806618	no
PAGE2	0.064341573	1.158760697	0.247409721	0.418338498	no
AWAT2	-0.064330993	-1.15856936	0.247487629	0.418437309	no
LOC100131635	-0.0643283	-1.158520658	0.247507462	0.418437923	no
AGFG2	0.064314167	1.158265069	0.247611566	0.418580993	no
KCNG2	0.06431123	1.158211955	0.247633203	0.418584645	no
FLJ13197	-0.064297205	-1.157958339	0.24773654	0.418726385	no
PTS	-0.064294007	-1.157900489	0.247760115	0.4187333	no
E2F1	-0.064285172	-1.157740722	0.247825233	0.418810418	no
TMEM189-UBE2	-0.064268352	-1.157436556	0.247949238	0.418977513	no
CCDC88C	-0.064266472	-1.157402559	0.247963101	0.418977513	no
ATAD3B	-0.064255372	-1.157201814	0.248044969	0.419082895	no
DEFB135	-0.064242288	-1.156965202	0.248141489	0.419213013	no
SHROOM1	0.064223898	1.156632642	0.248277193	0.419386544	no
UBOX5-AS1	-0.064220911	-1.156578617	0.248299243	0.419386544	no
SLC35F4	-0.064220436	-1.156570028	0.248302749	0.419386544	no
NBR2	-0.064210922	-1.15639799	0.248372976	0.419472191	no
TTC26	0.064207034	1.156327665	0.248401688	0.419487716	no
C17orf74	-0.064190162	-1.156022557	0.248526279	0.419665143	no
CTNNAL1	0.064157089	1.155424478	0.248770635	0.420044761	no
MTF2	0.064147441	1.155250008	0.248841949	0.420128644	no
RHOBTB1	0.064145079	1.15520729	0.248859412	0.420128644	no
MAX	-0.064134239	-1.155011256	0.248939562	0.420230942	no
RNASEH2A	-0.064126775	-1.154876291	0.248994754	0.420291098	no
METTL12	-0.064115291	-1.15466861	0.249079699	0.420375636	no
TFCP2	-0.064114715	-1.154658197	0.249083958	0.420375636	no
SHBG	-0.064109659	-1.154566765	0.249121362	0.420405747	no
SPRED2	0.064091152	1.15423209	0.249258308	0.420603824	no
ADARB1	0.064086201	1.15414257	0.249294948	0.420632624	no
SCARNA11	-0.064081457	-1.154056775	0.249330067	0.420658853	no
C10orf53	-0.064057278	-1.153619534	0.249509098	0.42088875	no
TECTB	0.064055406	1.153585685	0.249522961	0.42088875	no
TBC1D9B	0.06405512	1.153580504	0.249525083	0.42088875	no
KIAA0355	0.064037111	1.153254841	0.249658493	0.421080731	no
ARHGEF7	-0.064028917	-1.153106677	0.249719205	0.421150078	no
NCOA3	0.064023621	1.153010915	0.249758451	0.42118294	no
OR4K14	-0.064020999	-1.152963494	0.249777887	0.42118294	no
FAM222A-AS1	-0.063993958	-1.152474501	0.249978367	0.421487924	no
KRTAP5-2	-0.063969215	-1.15202707	0.250161905	0.421764298	no
FIP1L1	-0.063960973	-1.151878039	0.25022306	0.421834309	no
PLXNA3	0.063945493	1.151598098	0.250337961	0.42199491	no

C19orf77	-0.063919983	-1.151136803	0.250527379	0.42228109	no
SEH1L	-0.063914164	-1.151031579	0.2505706	0.422299313	no
SAC3D1	-0.063913236	-1.151014807	0.25057749	0.422299313	no
FAT2	0.063900297	1.150780835	0.250673616	0.422428188	no
LINC00665	-0.063873823	-1.150302102	0.250870382	0.422726625	no
HTR3E	0.063841765	1.14972241	0.251108788	0.423076495	no
RNH1	0.063840609	1.149701506	0.251117388	0.423076495	no
LOC728989	-0.063837894	-1.149652419	0.251137583	0.423077353	no
LOC646168	0.063817281	1.149279684	0.251290972	0.423301896	no
PKDREJ	-0.063814689	-1.149232814	0.251310264	0.423301896	no
CCDC134	0.063802691	1.149015848	0.251399585	0.42341916	no
CRYGD	-0.063795381	-1.148883673	0.25145401	0.423449918	no
KCNIP1	0.063794945	1.148875795	0.251457254	0.423449918	no
ACSBG2	-0.063788948	-1.148767342	0.251501918	0.423491947	no
FDPSL2A	-0.063785301	-1.148701404	0.251529076	0.423504494	no
ARPC5L	0.06376422	1.148320202	0.25168612	0.423735716	no
MXD3	-0.063755075	-1.148154846	0.251754264	0.42381724	no
TCP11L1	-0.063715436	-1.147438077	0.252049795	0.424281519	no
TCF19	0.063700978	1.147176647	0.252157646	0.424405048	no
APBA3	0.063700304	1.147164457	0.252162675	0.424405048	no
C1orf158	0.063672931	1.146669501	0.252366957	0.424715605	no
MYH6	-0.063665493	-1.14653501	0.252422485	0.42477579	no
PCDHB12	-0.063661929	-1.146470557	0.252449099	0.424787315	no
CDCA5	-0.063657646	-1.146393107	0.252481082	0.424807871	no
ZNF121	-0.063628489	-1.145865906	0.252698868	0.425141019	no
NCLN	0.063623192	1.145770124	0.25273845	0.425174327	no
C8orf69	0.06361297	1.145585277	0.252814849	0.425269562	no
BVES-AS1	0.063595179	1.145263596	0.252947842	0.425459973	no
CSTF2	-0.063584032	-1.145062039	0.253031198	0.425566871	no
ZNF622	-0.063578413	-1.144960424	0.253073228	0.425573103	no
TMEM50B	-0.063578241	-1.144957331	0.253074508	0.425573103	no
SERPINE2	0.063572629	1.144855849	0.253116488	0.4255812	no
CNGA3	0.063572303	1.144849951	0.253118929	0.4255812	no
LOC100129027	-0.063566107	-1.144737918	0.25316528	0.425625834	no
ZNF579	-0.063555347	-1.144543359	0.253245789	0.425727883	no
C6orf132	0.063546964	1.144391789	0.253308522	0.425800034	no
NT5DC2	-0.063536283	-1.144198659	0.253388471	0.425901113	no
RAB5A	-0.063529678	-1.144079231	0.253437919	0.425950912	no
NSUN3	0.063512166	1.143762576	0.253569059	0.426133284	no
AGBL4	-0.063509893	-1.143721473	0.253586086	0.426133284	no
C9orf170	0.063495496	1.143461165	0.253693931	0.426281178	no
AP3B1	0.063485984	1.143289172	0.253765205	0.426367604	no
AP1B1	0.06346733	1.142951885	0.253905018	0.426569164	no
PIPSL	-0.06346345	-1.142881722	0.253934109	0.42658469	no
C20orf166-AS	-0.063457661	-1.142777045	0.253977515	0.426624259	no
PLGRKT	0.063438418	1.142429109	0.254121827	0.426833309	no
KCNMB2-IT1	0.063434395	1.142356371	0.254152004	0.426842424	no
MTO1	-0.063432399	-1.142320282	0.254166977	0.426842424	no
STAB2	-0.063425998	-1.142204554	0.254214996	0.426889707	no
NPB	-0.063402083	-1.141772141	0.254394472	0.427157716	no
PRR23A	-0.063373135	-1.141248728	0.254611838	0.427485337	no

LOC645513	-0.063368527	-1.1411654	0.254646455	0.427485337	no
NMU	-0.063368153	-1.141158643	0.254649262	0.427485337	no
TTC9C	0.06335532	1.140926613	0.254745672	0.427613781	no
FKTN	-0.063336973	-1.14059488	0.254883553	0.427811812	no
CHRNA2	-0.063309945	-1.140106196	0.255086765	0.428090619	no
TBCEL	-0.063309581	-1.140099617	0.255089501	0.428090619	no
TESK2	-0.063294365	-1.139824486	0.255203961	0.428187482	no
TMEM184A	0.06329427	1.139822783	0.255204669	0.428187482	no
TRAF1	0.063293962	1.139817202	0.255206992	0.428187482	no
YOD1	-0.06327384	-1.139453385	0.255358402	0.428408074	no
CELF2	-0.063253123	-1.139078804	0.255514359	0.428591676	no
GUK1	-0.063251352	-1.139046783	0.255527694	0.428591676	no
CNTN2	-0.063250546	-1.139032216	0.25553376	0.428591676	no
LOC284379	-0.063248706	-1.138998955	0.255547612	0.428591676	no
C12orf45	0.063245622	1.138943196	0.255570835	0.428597177	no
SPR	0.063228827	1.138639524	0.255697337	0.428775863	no
TSFM	-0.063221279	-1.138503064	0.255754197	0.42880986	no
SPATA7	-0.063220839	-1.138495097	0.255757516	0.42880986	no
TNFSF11	0.063213373	1.138360106	0.255813773	0.428870723	no
RNF2	-0.063209392	-1.13828813	0.255843773	0.42888756	no
FAM49A	-0.063206095	-1.138228525	0.255868617	0.428895753	no
EME2	0.063196299	1.138051403	0.255942456	0.428963022	no
PMS2	-0.063195475	-1.138036503	0.255948668	0.428963022	no
GRIK1-AS2	0.063177995	1.137720453	0.256080463	0.429150439	no
FSBP	0.06316359	1.137460012	0.256189104	0.429299029	no
ATP2C1	-0.063159976	-1.137394666	0.256216368	0.429311239	no
NAA60	-0.063156627	-1.137334118	0.256241631	0.429320098	no
LOC100133331	0.063133605	1.136917876	0.256415355	0.429577673	no
FOXD4L6	-0.063121483	-1.136698705	0.256506862	0.42969748	no
PRB2	-0.063116255	-1.136604186	0.256546332	0.429730103	no
BDNF	-0.063109253	-1.136477598	0.2565992	0.429785162	no
GSTCD	-0.063102164	-1.136349429	0.256652736	0.429841331	no
MAPK3	0.0630981	1.136275944	0.256683435	0.429859245	no
KRTAP19-4	-0.063092354	-1.136172059	0.256726837	0.42989843	no
ELMO3	-0.063086682	-1.136069501	0.256769689	0.429936688	no
IGFBP2	0.063065606	1.135688458	0.256928948	0.430169837	no
PPIP5K2	0.063052088	1.135444039	0.25703114	0.430307411	no
HIST2H2AB	-0.063045593	-1.135326615	0.257080245	0.430356096	no
UBE2DNL	-0.063042587	-1.135272264	0.257102977	0.430360625	no
ETV4	-0.063035149	-1.13513779	0.257159223	0.430421252	no
IGF1	0.063029936	1.13504355	0.257198647	0.430453712	no
WDR60	-0.06301116	-1.134704069	0.257340696	0.430640971	no
CEP70	-0.063009849	-1.134680377	0.257350611	0.430640971	no
RNU4ATAC	-0.063006892	-1.134626918	0.257372986	0.43064488	no
COX4I2	0.062998025	1.134466599	0.257440093	0.430691156	no
WIZ	-0.062997942	-1.134465093	0.257440724	0.430691156	no
LOC100128787	0.062994659	1.13440575	0.257465567	0.43069919	no
CHUK	0.062986019	1.134249533	0.257530973	0.430750702	no
CADM2-AS2	-0.062985296	-1.134236462	0.257536447	0.430750702	no
C8orf47	-0.062980585	-1.134151297	0.25757211	0.430776826	no
ZNF703	0.062967749	1.13391921	0.257669314	0.430905862	no

TRIM36	-0.062961974	-1.133814802	0.257713052	0.430945471	no
RFC3	-0.062933652	-1.133302752	0.257927629	0.431238843	no
OR2T29	-0.062930694	-1.133249281	0.257950043	0.431238843	no
SOCS5	-0.062928317	-1.133206308	0.257968058	0.431238843	no
ASXL1	-0.062926401	-1.133171672	0.257982579	0.431238843	no
SH2D7	-0.062925577	-1.133156776	0.257988824	0.431238843	no
CERK	-0.062912142	-1.132913865	0.258090677	0.431368956	no
C2orf74	-0.062910014	-1.1328754	0.258106808	0.431368956	no
UBE2D3	0.062888341	1.13248356	0.258271174	0.431591412	no
NDUFAF6	-0.062883044	-1.1323878	0.258311353	0.431591412	no
FAM43B	0.062882884	1.132384907	0.258312568	0.431591412	no
CCNO	0.062877447	1.132286609	0.258353817	0.431591412	no
LINC00639	0.062875736	1.132255664	0.258366803	0.431591412	no
LSM4	-0.062875274	-1.13224732	0.258370305	0.431591412	no
LOC645249	-0.062873932	-1.132223052	0.25838049	0.431591412	no
HSPA2	-0.062847794	-1.131750496	0.25857887	0.431889212	no
CAMK2D	0.062841488	1.131636496	0.258626743	0.431935602	no
TMEM191C	-0.062836033	-1.131537868	0.258668166	0.431971214	no
NLRP4	0.062826873	1.131372274	0.258737724	0.432045348	no
SLC29A4	-0.062824893	-1.13133647	0.258752765	0.432045348	no
TMEM183B	0.062781373	1.130549678	0.259083451	0.432563894	no
CYP26B1	-0.062771149	-1.130364834	0.259161183	0.432660063	no
HMCN1	0.062764846	1.130250872	0.259209116	0.432706471	no
AGTPBP1	-0.062758326	-1.130133007	0.259258696	0.432755622	no
RPS12	-0.06274576	-1.129905825	0.259354279	0.432881549	no
MIR181A2	-0.062734039	-1.129693931	0.259443453	0.432996757	no
PPIH	0.06272594	1.129547512	0.259505084	0.433065986	no
FANCM	-0.062696357	-1.129012677	0.259730296	0.433408167	no
AMFR	0.062687464	1.128851915	0.259798017	0.433487514	no
LEMD1	-0.06267797	-1.128680275	0.259870334	0.433574517	no
GLI4	-0.062675059	-1.128627638	0.259892515	0.433577863	no
ARID1A	-0.062629564	-1.127805158	0.260239266	0.434122647	no
LOC148696	-0.062607806	-1.127411805	0.260405214	0.43436576	no
C7orf50	0.062594025	1.127162663	0.26051036	0.434507423	no
ITGB3BP	0.062586192	1.127021064	0.260570133	0.43454155	no
PCDHGB1	-0.062586044	-1.127018394	0.26057126	0.43454155	no
FSCN2	0.062580394	1.126916242	0.260614387	0.434579748	no
SNORA41	-0.062575319	-1.126824504	0.260653122	0.434610617	no
OVOL3	-0.062569325	-1.126716134	0.260698884	0.434653198	no
C10orf82	-0.062562052	-1.126584657	0.260754412	0.434712052	no
PJA2	-0.062556915	-1.126491784	0.26079364	0.434743726	no
DEFB103B	0.062547614	1.126323645	0.260864671	0.434817144	no
LRRC45	-0.062544497	-1.126267296	0.260888479	0.434817144	no
PLLP	0.062543201	1.126243864	0.260898379	0.434817144	no
GNRH2	0.062527285	1.125956122	0.261019978	0.434963236	no
GPRIN2	-0.062526429	-1.125940655	0.261026516	0.434963236	no
IL13	0.06252172	1.125855522	0.261062501	0.434981739	no
CRYAA	-0.06251746	-1.125778515	0.261095055	0.434981739	no
NSRP1	-0.06251703	-1.125770743	0.26109834	0.434981739	no
PMPCB	-0.062512029	-1.125680323	0.261136568	0.435011702	no
ANKRD20A3	-0.062504571	-1.125545511	0.26119357	0.435044754	no

MIR219-1	-0.062504137	-1.125537649	0.261196895	0.435044754	no
SMYD4	0.062501327	1.125486863	0.261218371	0.435046808	no
PLA2G2A	0.062496753	1.125404166	0.261253344	0.435071338	no
SPATA31A6	-0.062491291	-1.12530542	0.261295109	0.435107175	no
TARDBP	-0.062482218	-1.125141409	0.261364488	0.435188984	no
DCAF4	0.062473089	1.124976367	0.261434316	0.435244208	no
NEUROG1	-0.062472586	-1.124967285	0.261438159	0.435244208	no
C4orf26	-0.062467317	-1.124872023	0.261478471	0.4352776	no
LINC00314	0.062458182	1.124706887	0.26154836	0.435333973	no
PDK4	-0.062457596	-1.124696282	0.261552848	0.435333973	no
SLC36A2	-0.062443761	-1.124446177	0.261658724	0.435446423	no
NEK11	0.062443472	1.12444096	0.261660933	0.435446423	no
PCDHGA11	-0.062439645	-1.124371775	0.261690226	0.435457788	no
LINC00398	0.062437286	1.124329121	0.261708287	0.435457788	no
C9orf142	-0.062428861	-1.124176827	0.26177278	0.435531378	no
ZNF791	-0.062420522	-1.12402607	0.261836632	0.435603891	no
SERP1	0.062415079	1.123927675	0.261878313	0.43563951	no
TMPRSS6	0.062402645	1.12370289	0.261973552	0.43576421	no
LOC440970	-0.062391503	-1.123501474	0.262058909	0.435846051	no
PGBD2	0.062390928	1.123491082	0.262063313	0.435846051	no
LOC153910	-0.062380447	-1.123301611	0.262143627	0.435923743	no
PCED1A	-0.062379538	-1.123285173	0.262150596	0.435923743	no
LOC285692	-0.062372943	-1.123165962	0.262201137	0.435974053	no
ZNF850	-0.062348288	-1.122720247	0.262390165	0.436248582	no
NFXL1	-0.062346113	-1.122680937	0.262406841	0.436248582	no
GNPTAB	0.062338743	1.122547698	0.262463368	0.436308806	no
VHL	-0.062311903	-1.122062505	0.262669286	0.436604943	no
LOC100192426	-0.062308683	-1.122004301	0.262693995	0.436604943	no
KRT17	-0.062307581	-1.121984368	0.262702458	0.436604943	no
MFAP3	-0.062300114	-1.121849396	0.262759765	0.436666418	no
SKP2	0.062291675	1.121696838	0.26282455	0.436740308	no
ZNF595	-0.062288193	-1.121633887	0.262851286	0.436750965	no
C3orf80	-0.062284189	-1.121561516	0.262882025	0.436768272	no
PDCL3	0.062269493	1.121295843	0.262994888	0.436911859	no
SNORA72	-0.062267641	-1.121262377	0.263009107	0.436911859	no
GSG1	-0.062264507	-1.12120571	0.263033186	0.436918086	no
CCDC137	0.062249067	1.1209266	0.263151806	0.437080233	no
PRICKLE2-AS2	-0.062246507	-1.120880322	0.263171477	0.437080233	no
MIR205	0.062221514	1.120428519	0.263363579	0.43736548	no
SLC22A23	0.062197896	1.120001579	0.263545198	0.437627787	no
LOC100505633	-0.062195678	-1.119961491	0.263562256	0.437627787	no
SP9	-0.062174613	-1.119580702	0.263724324	0.43786306	no
RNF169	-0.06216402	-1.119389197	0.263805856	0.437964593	no
DNAJA3	-0.062143151	-1.119011967	0.263966511	0.438197458	no
MARS2	-0.062137728	-1.118913934	0.264008273	0.438232934	no
ZNF229	-0.062119484	-1.118584135	0.264148799	0.438421482	no
TMPRSS11A	0.062117684	1.118551607	0.264162662	0.438421482	no
MAPK14	-0.062105253	-1.118326894	0.264258445	0.438546582	no
HHIPL1	-0.062068281	-1.117658561	0.26454346	0.438956878	no
CYP20A1	-0.062067883	-1.117651363	0.26454653	0.438956878	no
CHCHD2	-0.06206357	-1.1175734	0.264579793	0.438965662	no

MOV10L1	-0.0620619	-1.117543206	0.264592675	0.438965662	no
LOC100505989	-0.06205903	-1.117491327	0.264614811	0.438968499	no
RGL1	0.062027034	1.116912949	0.264861682	0.43934412	no
RPS15AP10	-0.062011085	-1.116624654	0.264984796	0.439514413	no
TMPRSS7	0.062001635	1.116453831	0.265057763	0.439601511	no
CCDC104	-0.06197057	-1.115892299	0.265297719	0.439965528	no
MFAP5	0.061938163	1.115306505	0.265548203	0.440305007	no
CLK4	-0.06193678	-1.1152815	0.265558898	0.440305007	no
POMC	0.061936135	1.115269836	0.265563887	0.440305007	no
DOM3Z	-0.061918401	-1.114949285	0.265701029	0.440498404	no
ARMCX6	0.061908299	1.114766686	0.265779173	0.440593968	no
GLIS1	-0.061905158	-1.114709894	0.26580348	0.440600277	no
GSTA3	-0.06190032	-1.114622451	0.265840909	0.440628334	no
C15orf39	-0.061897288	-1.114567636	0.265864374	0.440633243	no
SLURP1	-0.061886815	-1.114378334	0.265945421	0.440733578	no
WNT11	-0.061878665	-1.114231007	0.266008509	0.440804138	no
CCDC60	-0.061874682	-1.114159026	0.266039336	0.44082123	no
SDHAF1	-0.061868537	-1.114047946	0.266086912	0.440863101	no
TRPC4AP	0.06186612	1.114004243	0.266105632	0.440863101	no
LOC100131234	-0.061841623	-1.113561439	0.266295358	0.441143416	no
AP4B1-AS1	-0.061832038	-1.113388196	0.266369611	0.441232412	no
CCT5	-0.061816389	-1.113105317	0.266490887	0.44139928	no
POM121C	0.061797569	1.11276514	0.266636778	0.441586175	no
NOX3	-0.061796532	-1.112746396	0.266644818	0.441586175	no
SNORD17	-0.061778633	-1.112422855	0.266783628	0.441782013	no
TLK2	-0.061773031	-1.112321596	0.266827082	0.441819927	no
ZNF321P	0.061764763	1.112172154	0.266891222	0.441892085	no
TSGA10IP	0.061761551	1.112114088	0.266916147	0.441894713	no
PRDM8	-0.061759258	-1.112072654	0.266933933	0.441894713	no
CCDC39	0.06175431	1.111983206	0.266972333	0.441913756	no
PLEKHG6	0.061752476	1.111950064	0.266986562	0.441913756	no
MLYCD	-0.061746272	-1.111837915	0.267034715	0.441959419	no
EIF2B3	0.06174317	1.11178185	0.267058789	0.441965227	no
INIP	-0.061739003	-1.111706526	0.267091136	0.441984724	no
APOL5	0.06173562	1.111645377	0.267117398	0.441994148	no
BAGE3	-0.06171711	-1.111310797	0.267261121	0.442197918	no
SPAG5	-0.061713938	-1.111253467	0.267285753	0.442204628	no
RUSC1-AS1	-0.061691009	-1.110839015	0.267463872	0.44246525	no
MYL3	-0.061681753	-1.110671719	0.267535793	0.442550164	no
LOC100129316	0.061655661	1.110200087	0.267738623	0.442851594	no
AMMECR1L	-0.061649648	-1.110091404	0.267785379	0.442894842	no
B3GALTL	-0.061646687	-1.110037893	0.267808401	0.442898834	no
LOC643486	-0.061638415	-1.109888369	0.267872739	0.442971148	no
GPR52	-0.06163117	-1.109757415	0.267929096	0.443004821	no
CETN3	-0.061628554	-1.10971014	0.267949442	0.443004821	no
SMA5	0.061627847	1.109697361	0.267954943	0.443004821	no
LHX3	-0.06161927	-1.109542327	0.268021677	0.443080016	no
SYCN	-0.061616702	-1.109495912	0.268041659	0.443080016	no
FES	0.061603172	1.109251356	0.268146956	0.443219985	no
ABCC5	-0.061586385	-1.108947933	0.26827764	0.443401889	no
CHRM2	-0.061576332	-1.108766225	0.268355922	0.443497164	no

CYB5D2	-0.061548287	-1.108259311	0.268574391	0.443824086	no
ITGAV	-0.061537617	-1.108066459	0.268657538	0.443927353	no
C12orf68	-0.06152214	-1.107786703	0.268778185	0.444092564	no
ARID5B	0.061514212	1.10764341	0.268839996	0.444160543	no
CSRP1	-0.061495507	-1.107305332	0.268985868	0.444367383	no
FANCD2OS	-0.061488363	-1.107176206	0.269041596	0.444425284	no
RPL13	-0.061474446	-1.106924667	0.26915018	0.44457048	no
SLC4A2	0.061467739	1.106803436	0.269202523	0.444622766	no
SNORA36B	-0.061457378	-1.106616164	0.269283394	0.444722157	no
C17orf67	0.061442342	1.106344389	0.269400787	0.444881844	no
ATP5J2-PTCD1	0.061432443	1.106165483	0.269478085	0.444968654	no
KCNN4	0.061428705	1.106097905	0.269507286	0.444968654	no
SGPP1	0.061427657	1.106078966	0.26951547	0.444968654	no
PATE2	-0.061411522	-1.105787335	0.269641516	0.445142558	no
ATF2	-0.061408779	-1.105737763	0.269662946	0.445143741	no
GAB4	-0.061395307	-1.105494268	0.269768223	0.445283325	no
EVPLL	-0.061384205	-1.10529361	0.269855002	0.445392354	no
LOC283299	-0.061376526	-1.105154816	0.269915037	0.44545723	no
ACOT7	-0.061361189	-1.104877606	0.270034971	0.445620945	no
DPEP1	0.061352719	1.104724516	0.270101221	0.445696048	no
SMG8	-0.061333162	-1.104371037	0.270254232	0.445914295	no
PPIAP30	-0.0613272	-1.104263287	0.270300885	0.445957034	no
LOC643669	-0.061322265	-1.104174089	0.270339511	0.445983859	no
LINC00571	0.06131532	1.104048567	0.270393872	0.445983859	no
TRPC7	-0.061310362	-1.103958954	0.270432686	0.445983859	no
RPL37	-0.061309707	-1.10394712	0.270437812	0.445983859	no
RPL36	-0.061309428	-1.103942083	0.270439994	0.445983859	no
PRKAA2	-0.061309216	-1.103938244	0.270441657	0.445983859	no
ABHD12	0.061299012	1.103753825	0.270521549	0.446081378	no
MS4A3	0.061286872	1.103534402	0.270616625	0.446180629	no
RIBC2	0.061286025	1.10351909	0.270623261	0.446180629	no
TRPM6	-0.06128302	-1.103464783	0.270646796	0.446185202	no
ARGFXP2	-0.061277656	-1.103367836	0.270688814	0.446220243	no
LINC00597	-0.061274731	-1.103314958	0.270711734	0.446223798	no
ANKRD30BP3	-0.061262593	-1.103095588	0.270806834	0.44634632	no
C15orf56	0.061250627	1.102879311	0.270900615	0.446466651	no
CDC42BPA	-0.061247797	-1.102828172	0.270922793	0.446468964	no
SRD5A1P1	0.061240328	1.102693175	0.270981345	0.446531213	no
UGDH	0.06122568	1.102428428	0.271096198	0.446686222	no
ADAMTS17	-0.06122261	-1.102372946	0.271120271	0.44669164	no
FMR1NB	0.06121846	1.102297929	0.271152823	0.446711025	no
BUB3	-0.061211372	-1.102169833	0.271208414	0.44676836	no
TRMT61A	-0.061202507	-1.102009609	0.271277959	0.446834779	no
DPRX	-0.061200932	-1.101981141	0.271290316	0.446834779	no
C10orf105	0.061185085	1.101694725	0.271414669	0.447005337	no
LPO	0.061174205	1.101498077	0.27150007	0.447111724	no
MRPL33	0.061161353	1.1012658	0.271600967	0.447243612	no
SPRY3	-0.061127151	-1.10064765	0.271869608	0.447651681	no
ESRRG	-0.061114173	-1.100413098	0.271971589	0.447785292	no
OSR1	0.061085388	1.099892851	0.272197882	0.448123539	no
DUSP13	0.06105982	1.099430762	0.272398987	0.44842027	no

TAF1	-0.061045263	-1.09916768	0.272513528	0.448574465	no
IFNA10	-0.061040691	-1.099085034	0.272549517	0.448585185	no
REEP1	-0.061039132	-1.099056861	0.272561786	0.448585185	no
RNF151	-0.061025876	-1.098817287	0.272666133	0.448704906	no
PLSCR4	0.061024586	1.098793979	0.272676287	0.448704906	no
MIR3653	-0.061020976	-1.098728735	0.272704709	0.448717319	no
RLIM	-0.061005541	-1.098449778	0.272826257	0.448882949	no
MC1R	0.061002073	1.098387098	0.272853573	0.448888082	no
LOC100507140	-0.060999841	-1.098346765	0.272871151	0.448888082	no
CHODL	0.060993673	1.098235282	0.272919742	0.448933653	no
OR2L1P	0.060975235	1.097902049	0.273065021	0.44913825	no
PPP1R12A	-0.060959766	-1.097622477	0.273186947	0.449288006	no
ZNF20	-0.060958379	-1.097597411	0.273197881	0.449288006	no
DUSP16	-0.060947709	-1.097404581	0.273282001	0.449358668	no
CLK3	-0.060945198	-1.097359208	0.273301797	0.449358668	no
FGF21	-0.060944973	-1.097355131	0.273303576	0.449358668	no
TSG101	-0.060913771	-1.096791227	0.27354969	0.449728915	no
CEP290	-0.060901286	-1.096565597	0.273648208	0.44985647	no
LINC00029	0.060894177	1.096437108	0.273704321	0.4499143	no
TP53BP1	-0.060886216	-1.096293234	0.273767163	0.449983181	no
FAM166A	0.060873258	1.096059054	0.273869471	0.450043709	no
FAM96B	-0.060872609	-1.096047327	0.273874594	0.450043709	no
NASP	0.060872609	1.096047317	0.273874599	0.450043709	no
PHB2	-0.060870943	-1.096017214	0.273887752	0.450043709	no
ZCCHC10	-0.060850176	-1.0956419	0.274051779	0.450278807	no
TRIM16L	-0.06084343	-1.09551998	0.274105078	0.45033195	no
FAM22F	-0.060816704	-1.095036979	0.274316297	0.450626107	no
ZNF573	-0.060811998	-1.094951939	0.274353497	0.450626107	no
TXNDC17	0.060811698	1.09494651	0.274355871	0.450626107	no
FAM169B	-0.060810164	-1.094918794	0.274367996	0.450626107	no
CHAF1B	0.060804972	1.094824957	0.27440905	0.450659093	no
COL25A1	-0.060797689	-1.09469334	0.27446664	0.450719229	no
RPL13P5	0.060790165	1.094557352	0.27452615	0.450782511	no
TAF12	0.060787305	1.094505668	0.27454877	0.450785211	no
ITFG1	-0.060775729	-1.094296464	0.274640344	0.450888599	no
H19	0.060774041	1.094265958	0.274653699	0.450888599	no
NAPA	0.060765218	1.094106502	0.274723513	0.450968761	no
CT49	0.060746822	1.093774062	0.274869104	0.451115945	no
RNF113A	-0.060746799	-1.093773631	0.274869292	0.451115945	no
CPN1	-0.060745933	-1.09375798	0.274876148	0.451115945	no
TIRAP	0.060741412	1.09367628	0.274911937	0.451140229	no
KIAA2018	-0.060733029	-1.093524788	0.274978307	0.45121469	no
SLC30A4	-0.060725975	-1.093397299	0.275034169	0.451271899	no
RPL26L1	0.060711407	1.093134041	0.275149547	0.451426744	no
LOC643401	0.060708195	1.093075988	0.275174994	0.451426854	no
LOC100527964	-0.060706096	-1.093038049	0.275191625	0.451426854	no
LOC440600	-0.060700219	-1.092931845	0.275238186	0.451468772	no
TGIF2	0.060691907	1.092781633	0.275304048	0.451542342	no
STIM2	-0.060686822	-1.092689725	0.275344352	0.451571897	no
NSMCE4A	-0.060684331	-1.092644712	0.275364093	0.451571897	no
LOC284385	-0.06067975	-1.092561931	0.275400399	0.451596977	no

RGS5	-0.060674532	-1.092467632	0.275441761	0.451603469	no
GJC2	-0.060673949	-1.09245709	0.275446386	0.451603469	no
POMGNT1	0.060668384	1.092356516	0.275490505	0.45164135	no
SORD	-0.060663552	-1.0922692	0.275528813	0.451669696	no
MOB1B	0.060651535	1.092052035	0.275624104	0.451769359	no
LONRF3	0.060650584	1.092034837	0.275631652	0.451769359	no
VIT	0.060642737	1.09189303	0.27569389	0.451836909	no
GALK2	0.060636644	1.091782931	0.275742218	0.451843107	no
C20orf78	-0.060635528	-1.091762755	0.275751075	0.451843107	no
SPATA22	-0.060634309	-1.091740725	0.275760746	0.451843107	no
WDPCP	-0.060623208	-1.091540102	0.275848829	0.451945669	no
LOC100499484	-0.060621119	-1.091502366	0.275865399	0.451945669	no
AP1AR	0.060617367	1.091434555	0.275895177	0.45196	no
PPP1R14B	-0.060593428	-1.091001944	0.2760852	0.452236817	no
DPY19L3	-0.060587383	-1.09089269	0.276133204	0.452280977	no
NXF1	0.060581649	1.090789078	0.276178735	0.452321078	no
LASP1	0.060563625	1.090463344	0.276321905	0.452487655	no
G6PC	-0.060563543	-1.09046187	0.276322553	0.452487655	no
PDSS1	-0.060552437	-1.09026117	0.276410793	0.452597665	no
PDLIM5	0.060548396	1.090188136	0.276442908	0.452615765	no
FGD5P1	-0.060540784	-1.090050582	0.2765034	0.452680321	no
ZSCAN20	0.060535732	1.089959273	0.276543561	0.452711584	no
FOLR1	0.060526963	1.089800808	0.276613268	0.452761654	no
FLJ30679	-0.060526584	-1.089793957	0.276616282	0.452761654	no
AP5S1	0.060522757	1.089724802	0.276646707	0.452776969	no
LOC441204	-0.060518367	-1.089645478	0.276681608	0.452799607	no
AGAP4	-0.060494342	-1.089211297	0.276872694	0.453032567	no
MKRN1	-0.060484129	-1.089026742	0.276953945	0.453032567	no
LOC100505978	-0.060483865	-1.089021971	0.276956046	0.453032567	no
GPR17	-0.060483275	-1.089011311	0.276960739	0.453032567	no
PDAP1	-0.060482983	-1.089006039	0.27696306	0.453032567	no
ATP6V0D2	0.060482591	1.088998947	0.276966183	0.453032567	no
CTBP1	-0.060480828	-1.088967087	0.276980212	0.453032567	no
FBXO46	-0.060479271	-1.088938957	0.276992599	0.453032567	no
LINC00518	-0.060469324	-1.08875919	0.277071765	0.453127563	no
HSBP1	-0.060465778	-1.08869511	0.277099989	0.453139237	no
STT3A	0.060461666	1.088620818	0.277132713	0.453158269	no
CAPSL	0.060447825	1.088370683	0.27724291	0.453284929	no
ARHGEF3	0.060446638	1.088349245	0.277252356	0.453284929	no
ARID3B	0.060416809	1.08781019	0.277489946	0.453638858	no
ALG1L	-0.060395152	-1.087418833	0.277662525	0.453886463	no
LOC400794	-0.060388995	-1.087307567	0.277711604	0.453932164	no
SARS	-0.06037941	-1.08713435	0.277788021	0.454003125	no
MIR218-2	-0.06037825	-1.087113392	0.277797268	0.454003125	no
BBOX1	-0.06037052	-1.086973708	0.277858903	0.454037844	no
HOXD10	0.060370287	1.086969487	0.277860766	0.454037844	no
ACMSD	0.060340457	1.086430443	0.278098708	0.454392103	no
LOC146513	-0.06033256	-1.08628773	0.278161727	0.454435737	no
LRRC3-AS1	-0.060331811	-1.086274195	0.278167704	0.454435737	no
COPS5	-0.060327852	-1.086202663	0.278199295	0.454452801	no
A1CF	-0.06032103	-1.086079382	0.278253746	0.454507202	no

PPP1R13L	0.060318139	1.08602714	0.278276823	0.454510351	no
TMEM126B	-0.060292272	-1.085559708	0.278483357	0.454813119	no
SCNN1A	0.060263783	1.085044887	0.278710951	0.455150232	no
SNAR-E	0.060254557	1.084878172	0.278784681	0.455236044	no
MIR3935	-0.060251193	-1.084817381	0.278811569	0.45524536	no
MAGI1-AS1	-0.06024316	-1.084672222	0.27887578	0.455315611	no
R3HDM1	-0.060237286	-1.08456608	0.278922739	0.455343777	no
FRG1B	0.060235702	1.084537448	0.278935407	0.455343777	no
TSPAN1	0.060228729	1.084411445	0.27899116	0.455400199	no
LINC00516	-0.06020004	-1.083893024	0.279220632	0.455712349	no
C9orf47	0.060199519	1.083883607	0.279224802	0.455712349	no
OR5H1	-0.06019636	-1.083826526	0.279250076	0.45571899	no
UNG	0.060190104	1.08371348	0.279300135	0.455766075	no
CA1	0.060176856	1.083474081	0.279406165	0.455871891	no
IBTK	-0.060176701	-1.083471282	0.279407405	0.455871891	no
WDR83OS	0.06013941	1.082797419	0.27970601	0.456324442	no
PSG7	-0.060127206	-1.082576896	0.279803777	0.456449292	no
PHF13	0.060110634	1.082277435	0.279936577	0.456580642	no
TEX19	-0.060110023	-1.082266397	0.279941472	0.456580642	no
SYNJ1	-0.060108523	-1.082239302	0.27995349	0.456580642	no
LOC100128531	-0.060106554	-1.082203715	0.279969275	0.456580642	no
CRABP2	0.060099465	1.082075611	0.280026103	0.45658726	no
MAGOH	0.060098832	1.082064185	0.280031171	0.45658726	no
VENTXP1	-0.060097272	-1.082035996	0.280043678	0.45658726	no
ADH1C	0.060095446	1.082003002	0.280058316	0.45658726	no
MIR766	-0.060091263	-1.081927412	0.280091854	0.456607299	no
PATE1	-0.060077815	-1.081684403	0.280199692	0.456724391	no
SIX6	-0.060077006	-1.081669775	0.280206184	0.456724391	no
HMGN2P46	0.060061609	1.081391568	0.280329678	0.456891029	no
FLJ11235	0.060021069	1.080659006	0.280655035	0.457386621	no
DLST	-0.0600102	-1.080462614	0.280742304	0.457475079	no
CDH23	0.060009007	1.080441049	0.280751888	0.457475079	no
LOC100288079	-0.059990952	-1.0801148	0.280896903	0.457676676	no
ZSWIM2	-0.059967565	-1.079692203	0.281084821	0.457948139	no
MAGEB2	0.059963223	1.079613757	0.281119714	0.457970267	no
GRB7	0.059953395	1.079436173	0.281198713	0.458020828	no
MAK16	0.059950628	1.079386168	0.281220961	0.458020828	no
LOC283214	-0.059949552	-1.07936672	0.281229614	0.458020828	no
CCDC92	-0.059947943	-1.079337656	0.281242546	0.458020828	no
CDC14C	-0.059942487	-1.079239061	0.281286418	0.458020828	no
CYP2R1	0.059939462	1.079184399	0.281310742	0.458020828	no
SNAR-F	-0.059938687	-1.079170395	0.281316974	0.458020828	no
TANGO6	-0.059935659	-1.07911568	0.281341325	0.458020828	no
OR1L8	0.059932527	1.079059097	0.281366508	0.458020828	no
CYP4A11	-0.059931597	-1.079042283	0.281373992	0.458020828	no
MIR202	-0.059930205	-1.079017136	0.281385184	0.458020828	no
MXD4	-0.059911482	-1.078678822	0.281535795	0.458231277	no
LOC100130452	-0.059901233	-1.078493625	0.281618265	0.458330794	no
INTS8	0.059891453	1.078316912	0.281696972	0.458424173	no
RBP3	-0.059882261	-1.078150813	0.281770965	0.458509866	no
CEACAM22P	-0.05987304	-1.077984202	0.281845199	0.45859594	no

BMS1P5	-0.059844034	-1.077460084	0.28207881	0.458909555	no
HOPX	0.059843806	1.07745596	0.282080649	0.458909555	no
NHLH1	-0.059832426	-1.077250327	0.28217234	0.459023977	no
SAMD11	-0.059829289	-1.077193659	0.282197612	0.459030342	no
MRPL2	-0.059798769	-1.076642175	0.282443633	0.459395755	no
ACHE	0.059780567	1.07631329	0.282590421	0.459599723	no
LOC643923	0.059761512	1.075968988	0.282744145	0.459814939	no
SNAR-G1	-0.059754487	-1.075842056	0.282800832	0.459872328	no
LOC116437	-0.059746379	-1.075695544	0.282866273	0.459921593	no
HIST1H4A	-0.059745058	-1.075671682	0.282876932	0.459921593	no
CLDN5	-0.059742779	-1.075630498	0.28289533	0.459921593	no
HTRA2	-0.059733509	-1.075463	0.282970162	0.459982533	no
HMX2	-0.059732833	-1.075450783	0.282975621	0.459982533	no
KCNC4	-0.059726205	-1.075331034	0.283029129	0.460034716	no
IGFBP1	0.059721556	1.075247037	0.283066667	0.460060934	no
EIF2C3	0.059709136	1.075022607	0.283166979	0.460175407	no
ANGPTL7	0.059706767	1.074979814	0.283186109	0.460175407	no
C21orf91-OT1	-0.059704882	-1.074945749	0.283201337	0.460175407	no
TIMM17A	0.059695748	1.074780713	0.283275124	0.460260504	no
CHTOP	-0.059689056	-1.074659787	0.283329198	0.460313561	no
RGS4	-0.05967401	-1.074387938	0.283450784	0.460476287	no
HIST1H4K	0.059633422	1.07365457	0.283778966	0.460974584	no
TPR	-0.059623293	-1.073471544	0.28386091	0.461072845	no
SLCO6A1	-0.059596914	-1.072994934	0.284074373	0.4613847	no
NPSR1-AS1	0.059579777	1.072685293	0.284213112	0.46156	no
RHNO1	-0.059578277	-1.072658189	0.284225259	0.46156	no
C2orf62	0.059567061	1.072455527	0.284316092	0.461672621	no
ATP2B1	-0.0595488	-1.072125583	0.284464017	0.461877923	no
SLC11A2	0.059544041	1.072039601	0.284502574	0.461879241	no
CRB3	-0.059543394	-1.072027919	0.284507812	0.461879241	no
RFX4	-0.059532514	-1.071831326	0.284595985	0.461975834	no
LRRIQ1	0.059530747	1.071799404	0.284610304	0.461975834	no
RABL2B	-0.059516454	-1.071541145	0.284726166	0.462111019	no
FAM195B	-0.059515167	-1.071517907	0.284736593	0.462111019	no
RIPK4	0.059509431	1.071414253	0.284783105	0.462151606	no
SNORA14B	-0.059483008	-1.070936847	0.284997397	0.462464441	no
MIR187	-0.059479922	-1.070881086	0.285022433	0.462466933	no
AGER	-0.059477513	-1.070837574	0.285041971	0.462466933	no
DEPDC1	0.059461163	1.070542151	0.285174645	0.462647264	no
MPL	-0.059442079	-1.070197345	0.285329551	0.46286363	no
FLJ22763	0.059438859	1.070139176	0.285355689	0.462871093	no
PSG6	-0.059414984	-1.069707806	0.285549576	0.463150637	no
MCM6	0.059412208	1.069657652	0.285572124	0.463152255	no
NR6A1	-0.059406673	-1.06955763	0.285617096	0.463190236	no
NEDD8-MDP1	0.0594008	1.069451528	0.285664807	0.463232654	no
MBTPS2	-0.05938874	-1.069233629	0.285762806	0.46332911	no
SLC35G3	-0.05938655	-1.069194054	0.285780608	0.46332911	no
ARSF	0.059385521	1.069175471	0.285788967	0.46332911	no
NF2	-0.059372429	-1.068938925	0.285895385	0.463435625	no
CCDC93	-0.059372132	-1.068933569	0.285897795	0.463435625	no
POLR3C	0.059364764	1.068800438	0.285957701	0.463495431	no

HNRNPM	-0.059362289	-1.068755721	0.285977824	0.463495431	no
IRAK4	0.059354847	1.068621267	0.286038337	0.463558548	no
TMEM42	-0.059321905	-1.068026076	0.286306314	0.463957849	no
DCAF12L1	-0.059312477	-1.067855737	0.286383038	0.464047189	no
WDR96	0.059303789	1.067698771	0.286453751	0.464126486	no
LMNB1	-0.059301158	-1.067651236	0.286475168	0.464126486	no
DEFA5	-0.059287861	-1.067410998	0.286583424	0.464266875	no
SNORA70E	-0.059284182	-1.067344529	0.286613381	0.464280408	no
LINC00333	0.059270853	1.067103702	0.286721937	0.464421251	no
C20orf201	-0.059260789	-1.066921867	0.286803921	0.464519034	no
OR2T12	-0.059243209	-1.066604256	0.28694716	0.464716007	no
NAA15	0.059235844	1.066471181	0.287007189	0.464778201	no
MB	-0.059228698	-1.066342076	0.287065436	0.464837499	no
SYT6	0.059219344	1.066173072	0.287141696	0.464925954	no
INO80E	-0.059214303	-1.066082006	0.287182793	0.464948519	no
GNB2L1	-0.059212328	-1.066046315	0.287198901	0.464948519	no
PGCP1	0.059177902	1.065424337	0.287479713	0.465368071	no
MOGAT1	-0.059168669	-1.065257521	0.287555059	0.46545498	no
DNAH2	-0.059164855	-1.065188617	0.287586185	0.465470304	no
GOT1L1	-0.059158856	-1.065080241	0.287635146	0.465514491	no
LINC00841	0.059147568	1.06487629	0.287727301	0.465628571	no
LOC100134317	-0.059122257	-1.064419002	0.287933997	0.465927982	no
EYA2	0.059112714	1.064246592	0.288011954	0.46601904	no
MMP8	0.059109122	1.064181693	0.288041301	0.466031439	no
TFDP3	-0.059075517	-1.06357456	0.288315953	0.466429654	no
TBL1X	-0.059073697	-1.063541677	0.288330834	0.466429654	no
HIST2H2AC	-0.059069528	-1.063466371	0.288364914	0.466449473	no
HMGB4	0.059066889	1.063418689	0.288386494	0.466449473	no
DPPA2	0.059062344	1.063336572	0.288423662	0.466474481	no
AKD1	-0.059053656	-1.063179603	0.288494717	0.466503883	no
PARP3	-0.059053397	-1.063174939	0.288496829	0.466503883	no
ERGIC3	0.059052159	1.063152557	0.288506962	0.466503883	no
ERMP1	-0.059044502	-1.06301423	0.28856959	0.466570047	no
RNF167	0.059038923	1.062913431	0.288615233	0.46660874	no
RPL13AP3	-0.059033554	-1.062816445	0.288659155	0.466634725	no
STAG3L1	0.059031651	1.06278205	0.288674732	0.466634725	no
MTA3	-0.059023367	-1.062632384	0.288742522	0.466709201	no
ANKRD9	-0.059019389	-1.062560517	0.288775077	0.466726719	no
PTPRU	-0.059007521	-1.062346109	0.288872216	0.466848609	no
TFAP2D	-0.059004376	-1.062289293	0.288897961	0.466855108	no
KRTAP5-6	-0.058989335	-1.062017562	0.289021111	0.466993531	no
TSPAN18	-0.058988607	-1.062004396	0.289027079	0.466993531	no
GPX5	-0.058952951	-1.06136022	0.289319169	0.467430331	no
EPRS	-0.058942495	-1.061171321	0.289404859	0.467510072	no
LOC100506241	-0.058941619	-1.061155509	0.289412033	0.467510072	no
PLEKHM2	0.05893841	1.061097535	0.289438336	0.46751742	no
FER	-0.058929903	-1.060943846	0.289508072	0.467537633	no
SNORA70F	0.058929115	1.060929597	0.289514538	0.467537633	no
SMARCD1	-0.058928922	-1.060926123	0.289516115	0.467537633	no
FAM90A10P	-0.05891972	-1.060759865	0.289591569	0.467615666	no
TCEAL6	-0.058917722	-1.060723767	0.289607953	0.467615666	no

ONECUT1	-0.058901085	-1.060423208	0.289744396	0.467800828	no
FBXL21	-0.058896744	-1.060344779	0.289780007	0.467823177	no
THUMPD3	-0.05889234	-1.060265222	0.289816134	0.467846356	no
MST4	0.058877918	1.06000466	0.289934475	0.468002238	no
ZNF518A	-0.058875199	-1.059955546	0.289956785	0.468003099	no
MED30	-0.058855873	-1.0596064	0.290115419	0.468223976	no
PITX2	-0.05884634	-1.059434174	0.29019369	0.468315131	no
ABCC6	0.058824523	1.059040029	0.290372872	0.468569108	no
TMEM199	-0.058816246	-1.058890504	0.290440867	0.468643642	no
ZCCHC6	0.058806691	1.058717891	0.290519374	0.468735125	no
SLFNL1-AS1	-0.058793222	-1.058474551	0.290630074	0.468844206	no
KIAA1751	-0.058793156	-1.058473364	0.290630614	0.468844206	no
RXRA	0.058785855	1.058341461	0.290690631	0.468905828	no
KIF15	-0.058758142	-1.057840819	0.290918503	0.469228515	no
RRP15	-0.058755107	-1.057785982	0.29094347	0.469228515	no
LINC00595	0.058753562	1.057758074	0.290956177	0.469228515	no
CASR	-0.058741139	-1.057533643	0.291058377	0.469358114	no
RASAL2	0.058721554	1.057179823	0.291219547	0.469560383	no
IQSEC1	0.058720587	1.057162351	0.291227507	0.469560383	no
KNTC1	0.058715054	1.057062398	0.291273049	0.469598582	no
B3GNT6	-0.058707359	-1.056923378	0.291336399	0.469665482	no
ATP5O	-0.058699917	-1.056788938	0.29139767	0.469729023	no
ITGBL1	-0.058687178	-1.056558803	0.291502576	0.469862885	no
AKAP14	0.058663942	1.056139041	0.291693987	0.470136153	no
LMNA	0.058641005	1.055724666	0.291883025	0.470405556	no
FAM57A	0.058633946	1.055597151	0.291941213	0.470448987	no
OR7E12P	-0.058632425	-1.05556967	0.291953755	0.470448987	no
ZNF732	-0.058623474	-1.055407968	0.292027558	0.470532632	no
MAGEC3	-0.058602568	-1.055030292	0.292199983	0.470775158	no
APOF	-0.058593495	-1.054866403	0.292274827	0.470860443	no
LOC441025	-0.058586513	-1.054740271	0.292332437	0.470917952	no
RPL3L	-0.058575658	-1.054544164	0.292422022	0.471026959	no
ZNF585A	0.058570402	1.054449212	0.292465405	0.471061532	no
FAR2	0.058565964	1.054369045	0.292502036	0.471085227	no
RBM14	-0.058555979	-1.054188658	0.292584472	0.471182682	no
SLK	0.058544128	1.053974576	0.292682327	0.471304953	no
TRIM73	-0.058524363	-1.053617518	0.292845584	0.471532513	no
ZNF540	-0.058510328	-1.053363988	0.292961543	0.471683887	no
PRHOXNB	-0.058482742	-1.052865648	0.293189561	0.472015647	no
NRADDP	-0.058457993	-1.05241855	0.293394236	0.472275268	no
HSPB2-C11orf	-0.058457927	-1.052417373	0.293394775	0.472275268	no
POLM	0.058454107	1.052348358	0.293426378	0.472290764	no
C10orf35	-0.058445145	-1.052186463	0.29350052	0.472374722	no
ERI2	-0.05844084	-1.052108699	0.293536137	0.47239667	no
LOC100270746	-0.058421966	-1.051767738	0.293692339	0.472597971	no
RBMXL1	0.058420412	1.051739666	0.293705202	0.472597971	no
OCM	-0.058415547	-1.051651795	0.293745468	0.472627376	no
PRMT10	-0.058409104	-1.051535402	0.29379881	0.472651612	no
ZNF273	-0.058408415	-1.05152295	0.293804517	0.472651612	no
LOC100130298	0.058398009	1.051334965	0.293890684	0.472722332	no
CETP	0.058394133	1.051264958	0.293922777	0.472722332	no

GRK7	-0.058393132	-1.051246863	0.293931073	0.472722332	no
GCSAM	0.058392481	1.051235105	0.293936463	0.472722332	no
SLC44A1	-0.05837851	-1.050982725	0.294052185	0.472873056	no
LOC100289092	-0.058371502	-1.050856127	0.294110245	0.472931034	no
TECR	-0.058363379	-1.05070939	0.29417755	0.47300387	no
LOC100134868	-0.058354601	-1.050550835	0.294250288	0.47308543	no
MYEOV2	-0.058328721	-1.050083327	0.294464829	0.473394947	no
SNORA70G	-0.058323578	-1.049990421	0.294507477	0.473394997	no
TBX20	-0.058323405	-1.049987287	0.294508916	0.473394997	no
CCDC68	-0.0583123	-1.049786694	0.29460101	0.473507614	no
RPL36AL	-0.058306006	-1.049672989	0.294653222	0.473556117	no
ZNF589	-0.058302008	-1.049600769	0.294686388	0.473574005	no
DNAJC9	-0.058293942	-1.049455068	0.294753306	0.473633149	no
CRP	-0.058292211	-1.049423802	0.294767667	0.473633149	no
TAF8	0.058289196	1.049369333	0.294792688	0.473633149	no
LIN37	-0.058283803	-1.049271922	0.294837437	0.473633149	no
C19orf21	-0.058282905	-1.049255697	0.294844891	0.473633149	no
MC4R	-0.058279551	-1.049195109	0.294872727	0.473633149	no
AP1B1P1	-0.058278981	-1.049184803	0.294877462	0.473633149	no
ZNF432	0.058273151	1.049079489	0.294925851	0.47367547	no
POC1B	-0.058265273	-1.048937186	0.294991244	0.473745093	no
LOC340094	0.058254589	1.048744196	0.295079945	0.473852134	no
IP6K3	-0.058251211	-1.04868317	0.295107997	0.473861773	no
TMEM185A	0.058237485	1.048435216	0.295221995	0.474009405	no
EGFL6	0.058209114	1.047922722	0.295457709	0.474352429	no
SLC16A12	0.05819719	1.047707342	0.295556807	0.474386678	no
HCG22	0.058194894	1.047665866	0.295575894	0.474386678	no
FBXW10	-0.058193274	-1.047636601	0.295589361	0.474386678	no
TAF1C	-0.058192563	-1.04762375	0.295595275	0.474386678	no
AKR1C2	0.058191793	1.047609845	0.295601674	0.474386678	no
LOC100507547	-0.058190613	-1.04758853	0.295611483	0.474386678	no
DPM1	0.058156275	1.046968252	0.29589704	0.474797921	no
C17orf99	-0.058154485	-1.046935913	0.295911933	0.474797921	no
PPP1R14D	-0.058149844	-1.046852084	0.295950541	0.474813479	no
GDPD4	0.058148008	1.046818918	0.295965816	0.474813479	no
NLRX1	0.058144396	1.04675367	0.29599587	0.474826248	no
LOC389043	-0.058141325	-1.046698197	0.296021423	0.474831796	no
TAS2R30	-0.05812601	-1.046421559	0.296148874	0.47500078	no
PIP5K1C	-0.058118889	-1.046292921	0.296208153	0.475060403	no
LOC155060	-0.058106087	-1.046061675	0.296314734	0.475185605	no
CHRNG	-0.058104201	-1.046027603	0.296330439	0.475185605	no
MYO1H	-0.058099534	-1.0459433	0.296369302	0.4751897	no
RP9	-0.058098584	-1.045926136	0.296377215	0.4751897	no
C9orf123	-0.058089755	-1.045766664	0.296450741	0.475272128	no
EPS8L3	-0.058081455	-1.045616722	0.296519885	0.475341661	no
SLC22A12	0.058079238	1.045576686	0.296538348	0.475341661	no
PMCHL2	-0.058075398	-1.045507317	0.296570342	0.475342544	no
NEURL2	0.058073744	1.045477434	0.296584125	0.475342544	no
NR5A1	-0.058070803	-1.045424319	0.296608624	0.475342544	no
C3orf55	0.058068553	1.045383675	0.296627372	0.475342544	no
ZNF547	-0.058051629	-1.045077965	0.296768413	0.475533102	no

FAM24B-CUZD1	-0.058048777	-1.045026449	0.296792185	0.475535737	no
LOC100506469	-0.058043965	-1.044939523	0.296832299	0.475564554	no
STK32A	0.058035049	1.044778479	0.296906626	0.475648178	no
GM140	-0.058018427	-1.044478229	0.297045236	0.475834762	no
SMURF2	-0.058000326	-1.044151269	0.297196225	0.476041147	no
LPCAT1	0.057994907	1.044053372	0.297241444	0.476078094	no
IFNW1	-0.057990006	-1.043964853	0.297282334	0.476108104	no
SPOPL	0.057965512	1.043522413	0.297486774	0.476400019	no
DCAF6	0.057945937	1.043168834	0.297650221	0.476603464	no
RPL23AP32	-0.057941752	-1.043093223	0.297685181	0.476603464	no
SLC15A5	-0.057941601	-1.043090501	0.29768644	0.476603464	no
ROBO3	-0.057939675	-1.043055721	0.297702522	0.476603464	no
ZNF276	-0.057936106	-1.042991239	0.29773234	0.476615696	no
CCNC	-0.057926712	-1.042821569	0.297810808	0.476688979	no
ZNF214	0.057925315	1.042796333	0.29782248	0.476688979	no
FGF22	-0.057915455	-1.042618233	0.297904864	0.476743386	no
COASY	0.05791397	1.042591413	0.297917272	0.476743386	no
PHACTR4	0.05791195	1.042554924	0.297934153	0.476743386	no
HGD	0.057910628	1.042531035	0.297945206	0.476743386	no
ZFYVE27	-0.057892328	-1.042200494	0.298098159	0.476952616	no
C12orf74	-0.057880761	-1.041991544	0.298194875	0.477071843	no
PCDHGA4	-0.057876012	-1.041905777	0.29823458	0.477095433	no
ZNF211	-0.057871569	-1.041825519	0.298271738	0.477095433	no
CFC1B	-0.057871033	-1.041815839	0.298276219	0.477095433	no
KIFC1	-0.057854199	-1.041511771	0.298417026	0.477285132	no
FAM71E1	-0.057827266	-1.041025282	0.2986424	0.477610048	no
TTR	0.057818783	1.040872057	0.298713408	0.477688061	no
SLC23A3	0.057763005	1.039864553	0.299180588	0.478399555	no
AQP7P1	-0.057755949	-1.039737118	0.299239715	0.4784585	no
COX7A2L	-0.057739267	-1.0394358	0.299379549	0.478646473	no
RNASE8	-0.0577342	-1.039344269	0.299422035	0.478678789	no
RNF207	-0.057723574	-1.039152346	0.299511134	0.478785613	no
HSPE1	-0.057705421	-1.038824454	0.299663396	0.478993384	no
KLF8	0.057701571	1.038754909	0.299695697	0.479009388	no
ZFP57	-0.057692087	-1.038583614	0.299775267	0.479100934	no
FLRT2	-0.057682375	-1.038408186	0.299856771	0.479195558	no
DENR	0.057678023	1.038329579	0.299893297	0.479218295	no
NOM1	0.05765959	1.03799664	0.300048035	0.479409649	no
C15orf55	-0.057658444	-1.037975934	0.300057661	0.479409649	no
SCCPDH	-0.057647003	-1.037769292	0.300153729	0.47952749	no
ADAMTS15	0.057618347	1.037251712	0.300394445	0.479876386	no
POTEM	-0.05761454	-1.037182932	0.300426443	0.479884025	no
MIR1289-2	-0.057612464	-1.037145439	0.300443886	0.479884025	no
STXBP5	-0.057608082	-1.037066293	0.300480711	0.479907176	no
CBWD2	0.057586442	1.036675438	0.30066261	0.480162008	no
PLEKHD1	-0.057581097	-1.036578896	0.300707551	0.480198096	no
CDC42SE2	-0.057574252	-1.036455268	0.300765107	0.480198962	no
STX19	-0.057571845	-1.036411785	0.300785352	0.480198962	no
BAGE5	0.057571513	1.036405785	0.300788146	0.480198962	no
GJA8	-0.05756965	-1.036372133	0.300803815	0.480198962	no
EFCAB7	-0.057564637	-1.036281594	0.300845974	0.480198962	no

FUT2	-0.057564472	-1.036278616	0.300847361	0.480198962	no
PEPD	-0.057562434	-1.036241808	0.300864502	0.480198962	no
TRIM62	-0.057549202	-1.03600281	0.300975815	0.480340951	no
ESPL1	-0.057528413	-1.035627316	0.301150757	0.480584459	no
ATP1B2	0.057517756	1.035434833	0.30124046	0.480691916	no
DPH5	-0.057502996	-1.035168251	0.301364726	0.480854505	no
SPATA5	-0.05748961	-1.034926472	0.30147746	0.48099867	no
SNAP25-AS1	-0.0574838	-1.034821541	0.301526394	0.481013326	no
APIP	0.057483205	1.034810787	0.30153141	0.481013326	no
LOC391322	-0.057475416	-1.0346701	0.301597028	0.481052866	no
CHEK2P2	-0.057474949	-1.034661662	0.301600964	0.481052866	no
ATP6V0A4	-0.057465095	-1.0344837	0.301683983	0.481149571	no
USP21	-0.057452115	-1.034249261	0.301793371	0.481288316	no
CA3	0.057446041	1.034139546	0.301844573	0.481314344	no
RAD1	0.05744296	1.03408389	0.301870548	0.481314344	no
CCDC147	0.057442208	1.034070324	0.30187688	0.481314344	no
AMBRA1	-0.057439353	-1.03401875	0.301900953	0.481317016	no
TMEM88	-0.057425556	-1.033769564	0.302017279	0.481466756	no
PLGLB1	-0.057420517	-1.033678552	0.302059772	0.481482548	no
NOP14	0.057417704	1.033627731	0.302083503	0.481482548	no
PAX7	-0.057416412	-1.033604401	0.302094397	0.481482548	no
RCL1	0.05739259	1.033174138	0.302295359	0.481734495	no
RNF157-AS1	-0.057392359	-1.033169971	0.302297306	0.481734495	no
DPYSL5	-0.057387431	-1.033080967	0.302338888	0.481746626	no
RIIAD1	-0.057386144	-1.033057717	0.30234975	0.481746626	no
FAM9A	-0.057381387	-1.03297181	0.302389891	0.48174745	no
KLHL20	-0.05738077	-1.03296066	0.3023951	0.48174745	no
WDR72	0.057368282	1.032735114	0.302500505	0.481879649	no
PRODH2	-0.057360384	-1.032592454	0.302567187	0.481950149	no
NKX2-4	-0.057351749	-1.032436497	0.302640095	0.482030555	no
INE1	-0.057334393	-1.032123034	0.302786671	0.482228275	no
ELMSAN1	0.057326603	1.031982328	0.302852481	0.482265404	no
ZNF516	-0.05732632	-1.031977232	0.302854864	0.482265404	no
ARHGEF11	-0.057299017	-1.031484099	0.303085585	0.48258405	no
RXFP4	-0.057297238	-1.031451972	0.303100621	0.48258405	no
LOC644172	-0.057294383	-1.031400406	0.303124754	0.48258405	no
DLX6	-0.057292013	-1.0313576	0.303144789	0.48258405	no
RFPL3	-0.057279371	-1.031129268	0.303251672	0.482718442	no
CNTROB	-0.057260911	-1.030795863	0.303407785	0.482847655	no
LUC7L2	-0.057260763	-1.030793197	0.303409034	0.482847655	no
HES1	0.057258085	1.030744819	0.303431691	0.482847655	no
ZNF618	0.057254167	1.030674063	0.30346483	0.482847655	no
TMED6	0.057254116	1.030673147	0.303465259	0.482847655	no
MASTL	0.057253833	1.030668041	0.303467651	0.482847655	no
AMBN	-0.057233044	-1.030292559	0.303643553	0.483091768	no
FLJ33581	-0.057206404	-1.029811417	0.303869054	0.483414749	no
SPATA31D3	0.057186025	1.029443356	0.304041632	0.483632819	no
HELT	-0.057184902	-1.029423084	0.304051138	0.483632819	no
BSN-AS2	-0.057156862	-1.028916669	0.3042887	0.48397487	no
SBF1P1	-0.057136618	-1.028551048	0.304460291	0.484211953	no
CCDC166	-0.05713052	-1.028440911	0.304511993	0.484258342	no

LOC146880	0.057122848	1.028302346	0.304577047	0.484325958	no
MTNR1A	-0.057111692	-1.028100867	0.304671656	0.484440556	no
SPATA21	-0.057106751	-1.02801162	0.30471357	0.484451778	no
LOC650226	-0.057105545	-1.027989844	0.304723797	0.484451778	no
RAI2	-0.057094065	-1.027782519	0.304821183	0.484570756	no
SSX6	-0.057090206	-1.027712822	0.304853926	0.484586962	no
CENPB	0.057087277	1.027659925	0.304878778	0.484590624	no
LOC100132247	-0.057071099	-1.027367743	0.305016075	0.484741028	no
OR4N2	-0.057070812	-1.027362551	0.305018516	0.484741028	no
OR5R1	0.057057296	1.027118442	0.305133255	0.484887517	no
PLCL2	-0.057044993	-1.026896248	0.305237719	0.485017658	no
TCP10L2	-0.057039806	-1.026802569	0.305281769	0.48505179	no
GUSBP10	-0.057029659	-1.026619312	0.305367953	0.485152856	no
RXFP3	-0.057014901	-1.026352785	0.305493328	0.485292109	no
CNIH2	-0.057014026	-1.026336976	0.305500765	0.485292109	no
LTB4R2	0.057003903	1.026154161	0.305586783	0.485357306	no
NDOR1	-0.057002977	-1.026137436	0.305594653	0.485357306	no
DEFA6	-0.057001223	-1.026105756	0.30560956	0.485357306	no
ZNF496	-0.056995217	-1.025997296	0.305660603	0.485402499	no
SLC25A5	-0.056948311	-1.025150166	0.306059465	0.485999997	no
LIG4	-0.056941596	-1.025028892	0.306116594	0.486054801	no
COL28A1	-0.056928658	-1.024795236	0.306226683	0.48619368	no
NT5C3	-0.056905638	-1.024379486	0.306422633	0.486404008	no
INS-IGF2	-0.056905564	-1.02437815	0.306423263	0.486404008	no
MIA2	-0.056905118	-1.024370104	0.306427056	0.486404008	no
UBP1	0.056889664	1.024091003	0.306558649	0.486576952	no
ISL1	-0.056875324	-1.023832035	0.306680783	0.486734857	no
CTBP1-AS1	-0.056836197	-1.023125416	0.307014202	0.487201965	no
AP5M1	-0.056835466	-1.023112207	0.307020437	0.487201965	no
C6orf7	-0.056826566	-1.02295148	0.307096311	0.487286387	no
APOOL	-0.056814099	-1.022726321	0.307202622	0.487370852	no
ZFP112	-0.056813903	-1.022722785	0.307204292	0.487370852	no
NUDT8	-0.056812345	-1.022694654	0.307217576	0.487370852	no
HLTF	-0.056804921	-1.02256058	0.307280894	0.487435319	no
PLA1A	-0.056799695	-1.022466204	0.307325469	0.487470047	no
TG	-0.056781042	-1.022129333	0.307484615	0.487686484	no
RAB11FIP5	0.056769393	1.02191896	0.307584027	0.487808157	no
NBPF16	-0.056757114	-1.021697205	0.307688842	0.487933703	no
ABCC2	-0.0567548	-1.021655412	0.307708598	0.487933703	no
MAP3K13	-0.056744572	-1.021470698	0.307795926	0.488034203	no
SNTA1	-0.056742057	-1.021425295	0.307817395	0.488034203	no
TAS2R14	-0.056733526	-1.021271225	0.307890251	0.488113706	no
PCDHGB6	-0.056717139	-1.020975293	0.308030224	0.488293722	no
RGPD4	0.056713522	1.020909965	0.308061129	0.488293722	no
LCE2C	-0.056711145	-1.020867045	0.308081434	0.488293722	no
WDFY2	0.056708211	1.02081406	0.308106503	0.488293722	no
AMT	0.056706935	1.020791016	0.308117406	0.488293722	no
CNGA4	-0.056685276	-1.020399862	0.308302517	0.488526171	no
DEFA3	0.056684454	1.020385012	0.308309546	0.488526171	no
EIF2C4	-0.056680522	-1.020314015	0.308343154	0.488543403	no
ZMYM4	0.056659131	1.019927714	0.308526057	0.488797161	no

LOC643770	-0.056648595	-1.019737442	0.308616172	0.488903888	no
CLYBL	0.056644885	1.019670444	0.308647907	0.488918123	no
COX17	0.056640636	1.019593706	0.308684259	0.488939667	no
SLC15A1	-0.056625085	-1.019312873	0.308817316	0.489114374	no
LYZL4	-0.056611581	-1.01906901	0.308932888	0.489251326	no
PTH1R	0.056609662	1.019034351	0.308949316	0.489251326	no
RIC8A	0.056593643	1.018745055	0.309086462	0.489432446	no
SERGEF	0.056586658	1.018618927	0.309146268	0.489491081	no
OR1D5	-0.056579447	-1.018488699	0.309208026	0.489552798	no
FAM3C	-0.05655182	-1.017989786	0.309444701	0.489748656	no
FKBP1A-SDCBF	-0.056550027	-1.017957403	0.309460067	0.489748656	no
SEC14L2	-0.056547298	-1.017908117	0.309483454	0.489748656	no
TRIM63	0.05654711	1.017904724	0.309485065	0.489748656	no
IFI27L2	-0.056546646	-1.017896352	0.309489038	0.489748656	no
NDUFB7	-0.056541813	-1.017809068	0.30953046	0.489748656	no
SNRPC	0.056541728	1.017807545	0.309531182	0.489748656	no
UBLCP1	0.056541351	1.017800722	0.309534421	0.489748656	no
UNC45A	0.05654107	1.017795648	0.309536829	0.489748656	no
APITD1-CORT	-0.056525604	-1.017516354	0.3096694	0.489916345	no
NR1H4	-0.056523387	-1.017476317	0.309688407	0.489916345	no
SS18	0.056517223	1.017365001	0.309741257	0.489944911	no
HIST1H2BE	-0.05651342	-1.017296336	0.30977386	0.489944911	no
MRGPRX3	-0.056513304	-1.017294238	0.309774857	0.489944911	no
KIAA0753	-0.056509206	-1.017220234	0.309809998	0.489964432	no
TRIM53AP	-0.056486972	-1.016818721	0.310000705	0.49022996	no
XKR3	0.056473454	1.016574603	0.310116692	0.490377296	no
LOC646736	-0.05646985	-1.016509516	0.310147621	0.490390121	no
CHRNA10	-0.056465737	-1.016435244	0.310182918	0.49040985	no
ARMS2	-0.056456808	-1.016274006	0.310259553	0.490450107	no
CSH2	-0.05645586	-1.016256882	0.310267693	0.490450107	no
DYNLRB2	0.056454794	1.01623763	0.310276844	0.490450107	no
HCG25	-0.056450686	-1.016163444	0.310312109	0.490469776	no
PPP2R5D	-0.056407498	-1.015383544	0.310683009	0.491019896	no
RELL2	-0.056391169	-1.015088664	0.310823322	0.491205531	no
SH3BP5L	-0.056363985	-1.01459777	0.311056998	0.491538673	no
C15orf41	0.056351854	1.014378701	0.311161318	0.491627473	no
KCNAB1-AS2	0.056351747	1.014376777	0.311162234	0.491627473	no
TINCR	-0.056349469	-1.014335649	0.311181821	0.491627473	no
CSAG4	0.056345642	1.01426653	0.311214742	0.491643341	no
TBC1D9	0.056334969	1.014073799	0.311306548	0.491717298	no
EML2	-0.05633488	-1.014072185	0.311307317	0.491717298	no
PPP6C	0.056331155	1.014004928	0.311339359	0.491731768	no
TRIM42	-0.056325198	-1.013897358	0.311390612	0.491776575	no
KRTAP4-6	-0.056313887	-1.013693091	0.31148795	0.491894154	no
DFFA	0.056299493	1.013433167	0.311611841	0.492053642	no
ZFAND2B	-0.05629382	-1.013330727	0.311660677	0.492067469	no
HSF5	-0.056291292	-1.013285079	0.31168244	0.492067469	no
CD320	0.05628927	1.013248564	0.31169985	0.492067469	no
UNC13B	0.056287838	1.013222697	0.311712183	0.492067469	no
DCAF13P3	0.056280514	1.013090445	0.311775246	0.492130379	no
NEK3	-0.056277891	-1.01304308	0.311797833	0.492130379	no

LOC84931	0.056246577	1.012477612	0.312067577	0.49251996	no
OTUD3	-0.056227311	-1.012129725	0.312233606	0.49271538	no
ITIH4	0.056226654	1.012117846	0.312239276	0.49271538	no
GRK1	-0.056224229	-1.012074059	0.312260178	0.49271538	no
ZNF225	-0.056221041	-1.012016493	0.312287658	0.492722565	no
LOC339807	0.056208453	1.01178918	0.312396187	0.492857617	no
ITIH3	0.056188	1.01141985	0.312572574	0.493099699	no
MYO19	0.056168512	1.011067936	0.312740705	0.493328721	no
GAGE12J	-0.056165441	-1.011012485	0.312767203	0.493334308	no
CDK4	-0.056146017	-1.010661747	0.31293484	0.493562502	no
ZNF80	0.056131485	1.010399323	0.313060307	0.493711894	no
KAZN	-0.056129725	-1.010367538	0.313075506	0.493711894	no
NLRP7	0.056124306	1.010269686	0.313122299	0.493749456	no
LRRC8D	0.05611268	1.010059759	0.313222704	0.493838224	no
UNC50	0.056112284	1.010052613	0.313226122	0.493838224	no
TNNC2	-0.056109806	-1.010007858	0.31324753	0.493838224	no
NAPSA	0.056094867	1.009738099	0.313376589	0.494005448	no
KAT7	-0.056090164	-1.009653169	0.313417229	0.494033275	no
GOLGA1	-0.056080453	-1.009477828	0.313501142	0.494123019	no
CDRT15P1	0.056078254	1.00943812	0.313520147	0.494123019	no
TMED11P	-0.056072049	-1.009326061	0.313573785	0.494171315	no
CA6	0.056066799	1.009231262	0.313619167	0.494206593	no
HNRNPH1	-0.056062947	-1.009161707	0.313652466	0.494222828	no
BPI	0.056051605	1.008956904	0.313750529	0.494341101	no
RNF111	0.056041078	1.008766826	0.31384156	0.49441825	no
LOC100132781	-0.056040622	-1.008758586	0.313845506	0.49441825	no
MIR181D	-0.056011346	-1.008229947	0.314098771	0.494780964	no
FCAR	-0.0559906	-1.007855323	0.314278331	0.49502753	no
MYBPC1	0.055984395	1.007743284	0.314332046	0.495075852	no
FLJ23867	-0.055980766	-1.007677759	0.314363463	0.49508905	no
CREBZF	-0.055922216	-1.006620525	0.314870659	0.495851494	no
GTF3C6	-0.055913738	-1.00646744	0.314944145	0.495930878	no
KCNJ14	-0.055903022	-1.006273942	0.315037046	0.496040821	no
ZNF175	-0.055891134	-1.00605929	0.315140125	0.496166771	no
CYP3A7-CYP3A	-0.055878767	-1.005835968	0.315247391	0.496299295	no
OR2T8	-0.055875609	-1.005778958	0.315274778	0.496306054	no
ACTRT3	0.055867705	1.005636226	0.315343351	0.496377643	no
PLK4	0.055858338	1.005467096	0.31542462	0.496469204	no
NOP16	0.055845812	1.005240912	0.315533325	0.496603932	no
C1orf65	-0.055813657	-1.004660316	0.315812477	0.497006879	no
RNF26	0.055806519	1.004531419	0.315874473	0.497068045	no
NUAK1	-0.055784472	-1.004133332	0.316065992	0.49733301	no
TRIM72	-0.055773812	-1.003940858	0.316158619	0.497442337	no
NR1D1	-0.055763138	-1.003748125	0.316251388	0.497551873	no
HOOK2	-0.055743237	-1.003388771	0.316424406	0.497787637	no
LOC100505545	-0.055736754	-1.003271713	0.31648078	0.497831925	no
LOC100129726	-0.055732351	-1.003192215	0.316519068	0.497831925	no
ERC1	-0.055732008	-1.00318602	0.316522052	0.497831925	no
EOMES	-0.055729119	-1.003133857	0.316547177	0.497835008	no
HPCAL4	-0.055714888	-1.0028769	0.316670964	0.497993245	no
LINC00163	0.055708989	1.002770386	0.316722285	0.498020114	no

INTS7	-0.055707597	-1.002745252	0.316734397	0.498020114	no
MYO1A	-0.055696425	-1.002543531	0.316831609	0.498062023	no
NUS1	0.055695734	1.002531057	0.316837621	0.498062023	no
FOXB2	-0.055695617	-1.002528937	0.316838643	0.498062023	no
MAPK7	-0.055693881	-1.00249759	0.316853752	0.498062023	no
POU4F3	-0.055679051	-1.002229822	0.31698283	0.498228479	no
SNX13	0.055672919	1.002119107	0.31703621	0.498275939	no
SULT1C3	-0.055664325	-1.001963926	0.317111039	0.498300718	no
MOG	0.055663655	1.001951833	0.317116871	0.498300718	no
TDRD9	-0.055663119	-1.001942161	0.317121535	0.498300718	no
LOC349160	-0.055652343	-1.001747573	0.317215385	0.498411745	no
RPL29	-0.055645845	-1.001630259	0.317271975	0.498430283	no
FAM228B	-0.055645662	-1.001626954	0.317273569	0.498430283	no
KRT27	-0.055629155	-1.001328892	0.317417378	0.498619755	no
PLP1	-0.055622928	-1.001216461	0.317471635	0.498668267	no
ZNF134	0.055617728	1.001122566	0.317516951	0.498668267	no
C1QTNF9B	-0.055617622	-1.00112066	0.317517871	0.498668267	no
BAAT	-0.055605284	-1.000897891	0.317625403	0.498775504	no
CELA3B	-0.055604462	-1.000883047	0.317632569	0.498775504	no
LOC100505876	0.055591896	1.000656161	0.317742115	0.498911068	no
AURKC	0.055574288	1.00033822	0.317895665	0.499115703	no
MT1IP	0.055570284	1.000265924	0.317930587	0.499134068	no
SLC2A1-AS1	0.055550927	0.999916431	0.318099445	0.499362686	no
RIMBP3B	0.055544077	0.999792746	0.318159217	0.499420038	no
CCDC146	0.055536474	0.999655473	0.318225565	0.499487702	no
SMTNL2	0.05552931	0.999526112	0.318288097	0.499549368	no
OR6W1P	-0.05552144	-0.999384024	0.31835679	0.499589206	no
C17orf58	0.055521076	0.999377442	0.318359973	0.499589206	no
ARMC7	0.055506937	0.999122153	0.318483419	0.499746434	no
GRPEL1	-0.055478821	-0.998614502	0.31872899	0.500095256	no
DGUOK	-0.055467856	-0.998416527	0.318824793	0.500209054	no
C1orf86	0.055464851	0.998362281	0.318851046	0.500213725	no
GDF11	-0.055448045	-0.998058843	0.318997927	0.500407625	no
HTR3A	-0.055429295	-0.997720291	0.319161859	0.50062824	no
PLEKHA8	-0.055424604	-0.997635599	0.319202877	0.500656037	no
CD22	-0.055421729	-0.997583686	0.31922802	0.500656707	no
SERAC1	0.05541894	0.997533341	0.319252406	0.500656707	no
AAAS	0.055416564	0.99749043	0.319273192	0.500656707	no
LINC00605	0.055407501	0.997326798	0.319352463	0.500702601	no
UCHL5	-0.055406648	-0.9973114	0.319359923	0.500702601	no
C13orf45	-0.055405227	-0.997285744	0.319372353	0.500702601	no
DNAJC15	0.055395099	0.997102884	0.319460958	0.500804978	no
BEND2	-0.055385676	-0.996932737	0.319543417	0.500897708	no
LHX4	-0.055367957	-0.996612828	0.319698494	0.501104247	no
FUT11	0.055350158	0.996291457	0.319854329	0.501248012	no
C19orf69	0.055348311	0.996258112	0.319870501	0.501248012	no
TRPV1	-0.055347376	-0.99624123	0.319878689	0.501248012	no
CCDC97	0.055346825	0.996231292	0.319883509	0.501248012	no
SLC3A2	-0.055327525	-0.995882813	0.320052555	0.501476339	no
SZRD1	0.055317042	0.995693557	0.320144388	0.501544566	no
UGT1A5	-0.055315208	-0.995660434	0.320160462	0.501544566	no

AGTRAP	0.055314563	0.995648793	0.320166111	0.501544566	no
GPR6	-0.055309641	-0.995559926	0.32020924	0.501575566	no
LYRM2	-0.055298299	-0.99535514	0.320308641	0.501694701	no
PGM2L1	0.055294251	0.99528206	0.320344118	0.501713703	no
DCPS	0.05527722	0.994974567	0.32049342	0.501910959	no
PROZ	-0.055273771	-0.994912296	0.320523661	0.501921743	no
NHEJ1	0.055268962	0.99482547	0.32056583	0.501926134	no
SORT1	0.055268124	0.994810346	0.320573176	0.501926134	no
TMEM253	-0.055252438	-0.994527126	0.320710756	0.502104965	no
FPGS	-0.055248647	-0.994458678	0.320744011	0.502120451	no
MAP7D3	-0.055234361	-0.994200748	0.320869348	0.502280076	no
PHOX2B	0.055228629	0.994097263	0.320919644	0.502322219	no
ARHGAP26-AS1	-0.055212813	-0.993811709	0.321058456	0.502502897	no
GYPA	-0.055204187	-0.99365597	0.321134179	0.502584813	no
ACVR1C	-0.055197893	-0.993542336	0.321189438	0.502634691	no
AGPAT9	0.055189575	0.99339216	0.321262476	0.502712383	no
LINC00558	0.055180621	0.993230494	0.321341114	0.502798827	no
UGT1A8	-0.05517781	-0.993179744	0.321365803	0.50280085	no
IGF2-AS	-0.055157996	-0.992822018	0.321539864	0.503036559	no
PET100	-0.05513614	-0.99242742	0.321731937	0.503298449	no
LOC1720	-0.055133619	-0.992381895	0.321754101	0.503298449	no
CATSPER4	-0.055129296	-0.992303845	0.321792104	0.503321259	no
C9orf173	0.055112159	0.991994455	0.321942772	0.503520276	no
ZNF204P	-0.055102499	-0.991820037	0.322027732	0.503616503	no
HIST1H1D	0.055088075	0.99155962	0.322154609	0.503778266	no
SDK2	-0.055084517	-0.991495387	0.32218591	0.503790555	no
AKT2	0.055072144	0.991271997	0.32229478	0.503903053	no
APP	-0.055071011	-0.991251544	0.322304749	0.503903053	no
CORO2A	0.055062936	0.991105761	0.322375812	0.503977491	no
ZSWIM8	-0.055052525	-0.990917799	0.32246745	0.504084083	no
CCDC25	-0.054987782	-0.989748926	0.323037701	0.504926528	no
LBX1	-0.054986006	-0.989716849	0.32305336	0.504926528	no
KRTAP5-9	0.054970694	0.989440421	0.32318832	0.505100734	no
HOXA4	0.054963409	0.989308882	0.323252554	0.505164387	no
WFDC8	-0.054940189	-0.988889683	0.323457316	0.505447624	no
LNX1-AS1	-0.054926563	-0.988643673	0.323577522	0.505579998	no
KRTAP5-4	-0.054925253	-0.988620026	0.323589078	0.505579998	no
NUDT4	-0.054893056	-0.98803874	0.323873229	0.505987174	no
ITGA3	0.054878444	0.987774947	0.324002234	0.506151923	no
TIAL1	-0.054872944	-0.98767565	0.324050802	0.506167733	no
LINC00313	0.054870354	0.987628891	0.324073675	0.506167733	no
ZNF576	0.054869297	0.987609804	0.324083012	0.506167733	no
FAM120B	-0.054857529	-0.987397353	0.324186949	0.506293273	no
INCENP	-0.054847429	-0.987215007	0.324276176	0.506395822	no
HDGFL1	-0.054840227	-0.987084996	0.324339804	0.506458383	no
TMBIM6	0.054832415	0.986943948	0.324408842	0.506529383	no
ZNF260	-0.054815854	-0.986644979	0.32455521	0.506721105	no
LINC00608	-0.054796998	-0.986304557	0.324721924	0.506913332	no
C19orf81	0.054796593	0.986297245	0.324725506	0.506913332	no
OR6S1	-0.054780049	-0.985998567	0.324871824	0.507104908	no
TRIM33	-0.054776521	-0.985934877	0.324903031	0.507116786	no

GOLGB1	0.054755262	0.985551077	0.325091124	0.507373518	no
WDR62	-0.054749537	-0.985447731	0.325141784	0.507415734	no
DNAJA2	-0.054723469	-0.984977111	0.325372547	0.507738992	no
AGAP11	-0.05470801	-0.984698034	0.32550944	0.507915731	no
STAM2	0.054696681	0.984493501	0.325609791	0.508035431	no
ABCC6P2	-0.054691897	-0.984407145	0.325652167	0.508064662	no
HS3ST6	-0.054675642	-0.98411369	0.325796194	0.508245075	no
CAND1	-0.054673509	-0.984075167	0.325815104	0.508245075	no
LOC100289673	0.054656709	0.983771884	0.325964004	0.508440442	no
CBY1	-0.054610505	-0.982937764	0.326373754	0.509022848	no
IFNA16	0.054609264	0.98291537	0.326384759	0.509022848	no
RTL1	0.054605973	0.982855945	0.326413964	0.509028636	no
LOC440117	-0.054602618	-0.98279538	0.326443731	0.509028636	no
RFC1	-0.054600838	-0.982763244	0.326459527	0.509028636	no
TMEM170A	-0.054585433	-0.982485142	0.326596238	0.509204858	no
CTRC	-0.054577858	-0.982348392	0.326663477	0.509272744	no
PROM1	0.054546906	0.981789623	0.326938311	0.509664242	no
NUP93	-0.054530497	-0.981493404	0.32708407	0.509845018	no
CRAT	-0.05452851	-0.981457535	0.327101722	0.509845018	no
KPTN	-0.054523459	-0.981366342	0.327146605	0.509860543	no
FUT8	-0.054522049	-0.981340893	0.327159131	0.509860543	no
SDCCAG3	0.054502576	0.980989358	0.32733219	0.510080828	no
SH3GL1	-0.054500803	-0.980957351	0.327347949	0.510080828	no
BRD2	-0.054497002	-0.980888724	0.327381742	0.5100965	no
EPCAM	-0.054485226	-0.980676131	0.32748644	0.510157316	no
SCARNA6	-0.054484959	-0.980671325	0.327488807	0.510157316	no
HTR4	0.054484601	0.980664864	0.327491989	0.510157316	no
NPFFR1	-0.054469832	-0.980398235	0.32762333	0.510324925	no
SAP25	0.05446685	0.980344413	0.327649848	0.510329242	no
LOC100506422	-0.054462512	-0.980266103	0.327688431	0.510347192	no
OR6B3	-0.054460215	-0.980224631	0.327708866	0.510347192	no
IGSF3	-0.054448687	-0.980016514	0.327811426	0.510446812	no
NTS	0.054447685	0.97999843	0.327820339	0.510446812	no
ARL2-SNX15	-0.054444348	-0.979938184	0.327850032	0.510456063	no
LGALSL	-0.054441315	-0.979883434	0.327877018	0.510461098	no
KLK14	-0.054428969	-0.979660556	0.32798689	0.510595165	no
PLRG1	0.054421344	0.979522915	0.328054754	0.510663822	no
RBBP7	-0.054418532	-0.979472153	0.328079785	0.510665797	no
PAOX	0.054415699	0.979421015	0.328105002	0.510668062	no
NEIL2	-0.054405594	-0.979238594	0.328194968	0.510771096	no
HEMK1	0.054398776	0.97911551	0.32825568	0.51082859	no
FAM92B	0.054385387	0.978873815	0.328374919	0.510977147	no
AKAP10	0.054379849	0.978773832	0.328424253	0.511015204	no
DIAPH3	0.054377303	0.978727873	0.328446932	0.511015204	no
CCDC83	-0.054363596	-0.978480428	0.328569053	0.511168201	no
MFSD6L	0.054333673	0.977940249	0.328835751	0.511546082	no
RPL36A-HNRNF	-0.054325296	-0.977789036	0.328910433	0.511625226	no
FEZF1	0.054310511	0.977522144	0.329042275	0.511758823	no
PF4	0.054306361	0.977447217	0.329079294	0.511758823	no
SNRK-AS1	-0.054305728	-0.977435793	0.329084939	0.511758823	no
DGKQ	-0.054304984	-0.977422371	0.32909157	0.511758823	no

CPB2	-0.054296018	-0.97726051	0.329171553	0.511819651	no
MAB21L2	-0.05429526	-0.977246822	0.329178317	0.511819651	no
POFUT1	0.054288878	0.97713161	0.329235257	0.51187115	no
THOC3	-0.054260646	-0.976621977	0.329487201	0.512189402	no
ACRV1	-0.054260599	-0.976621126	0.329487622	0.512189402	no
ZBTB17	-0.054243036	-0.976304084	0.32964442	0.512364478	no
RPSAP9	0.054242342	0.976291557	0.329650616	0.512364478	no
BTBD16	-0.054238819	-0.976227955	0.329682078	0.512364478	no
ISL2	-0.054237303	-0.976200599	0.329695611	0.512364478	no
EIF4G3	0.054232382	0.976111768	0.329739557	0.512371477	no
ZCWPW2	0.054231445	0.97609485	0.329747926	0.512371477	no
ZSWIM4	-0.05422879	-0.976046924	0.329771638	0.512371477	no
C9orf16	-0.054224458	-0.97596871	0.329810337	0.51239456	no
PNPLA8	-0.054216694	-0.975828557	0.32987969	0.512465261	no
LOC375196	-0.054210764	-0.975721518	0.329932664	0.512510508	no
DNAH6	0.054182788	0.975216501	0.33018267	0.512861792	no
XYLB	0.054161698	0.974835791	0.330371219	0.513117574	no
RACGAP1	-0.054154867	-0.974712485	0.330432303	0.513175359	no
WNT8B	-0.054149078	-0.974607977	0.33048408	0.513192234	no
TNXA	0.054148312	0.974594157	0.330490927	0.513192234	no
CLEC18A	0.054131971	0.974299177	0.330637101	0.513382121	no
MRPS17	-0.054119999	-0.974083068	0.330744218	0.513491158	no
NOL7	-0.054118782	-0.974061094	0.330755111	0.513491158	no
LOC388849	-0.054111686	-0.973932998	0.330818616	0.513552649	no
SCUBE2	0.054102336	0.973764223	0.330902299	0.513645454	no
KRT85	0.054087204	0.973491077	0.331037761	0.513818614	no
PLA2G4D	-0.054081769	-0.973392957	0.331086431	0.513857044	no
AADACL3	0.054069875	0.973178251	0.331192947	0.513985241	no
H2AFZ	-0.054037963	-0.972602199	0.331478836	0.514373528	no
ZNF768	-0.054036604	-0.97257767	0.331491013	0.514373528	no
SSRP1	-0.054003538	-0.971980795	0.331787413	0.514761268	no
RBM34	-0.054003383	-0.971978003	0.331788799	0.514761268	no
SELENBP1	0.053986863	0.971679799	0.331936948	0.514937698	no
SLC12A4	-0.053985359	-0.971652649	0.331950438	0.514937698	no
TPRXL	-0.053981403	-0.971581226	0.331985929	0.514955582	no
C10orf107	0.053974829	0.971462571	0.332044895	0.515009875	no
GLT8D2	0.053949458	0.971004598	0.332272548	0.51532578	no
NRG2	-0.053940465	-0.970842264	0.332353267	0.515413773	no
OTX1	-0.053934777	-0.970739586	0.33240433	0.515455765	no
DUT	-0.053926694	-0.97059368	0.332476898	0.515517963	no
NEK10	0.053923183	0.970530308	0.33250842	0.515517963	no
STRIP1	-0.053922294	-0.970514261	0.332516402	0.515517963	no
CCDC127	-0.053905065	-0.970203258	0.33267113	0.515720642	no
DES	0.0538963	0.97004505	0.332749859	0.515768828	no
OR2T34	-0.053896261	-0.970044341	0.332750211	0.515768828	no
POTEC	-0.053879489	-0.969741587	0.332900903	0.515965189	no
KLHL8	-0.053861428	-0.969415585	0.333063217	0.516149112	no
MBNL1-AS1	0.053860231	0.969393966	0.333073982	0.516149112	no
BBS5	0.053858268	0.969358546	0.333091621	0.516149112	no
S100A3	0.053832462	0.968892728	0.333323649	0.516471416	no
IGFL1	-0.053808259	-0.968455845	0.333541359	0.516771491	no

ADH7	-0.053799732	-0.968301935	0.333618079	0.516853095	no
SPICE1	0.053785542	0.968045786	0.333745786	0.517013673	no
SCN4B	0.053768932	0.967745972	0.333895304	0.517208013	no
C16orf90	-0.053763837	-0.967654013	0.333941172	0.517214011	no
SCGB3A1	-0.05376197	-0.967620319	0.33395798	0.517214011	no
SNORA28	-0.053760483	-0.967593466	0.333971376	0.517214011	no
SIRT7	0.053747485	0.967358853	0.334088425	0.517358001	no
GSTTP1	-0.053739208	-0.967209451	0.334162976	0.517422935	no
LOC170425	-0.053737484	-0.967178325	0.334178509	0.517422935	no
WIPF3	0.053720396	0.966869888	0.334332458	0.517571841	no
SETMAR	0.053718421	0.966834232	0.334350257	0.517571841	no
DNAH14	0.053717089	0.966810199	0.334362255	0.517571841	no
TMEM131	0.053716117	0.966792655	0.334371013	0.517571841	no
ANKRD20A5P	-0.053711521	-0.966709696	0.334412431	0.517586689	no
IREB2	-0.053709708	-0.966676964	0.334428773	0.517586689	no
PGLYRP1	-0.053702296	-0.966543183	0.334495573	0.517652795	no
DUSP19	-0.053699217	-0.966487596	0.334523332	0.517658477	no
EFR3A	0.053688931	0.96630193	0.334616058	0.517764685	no
SBK2	0.05368321	0.966198668	0.334667637	0.517807213	no
PRDM12	-0.053674356	-0.966038861	0.33474747	0.517871042	no
APMAP	0.053672845	0.966011571	0.334761105	0.517871042	no
ACOT8	-0.053670618	-0.965971386	0.334781182	0.517871042	no
LOC100131089	0.05364724	0.9655494	0.334992062	0.518159955	no
ZNF257	-0.053640465	-0.965427112	0.335053189	0.518217207	no
GLRA4	-0.053633934	-0.965309235	0.335112119	0.518258555	no
NBPF6	-0.053632157	-0.965277164	0.335128153	0.518258555	no
KIAA0408	-0.053612752	-0.9649269	0.335303303	0.518464034	no
CRIPAK	-0.053612091	-0.964914961	0.335309274	0.518464034	no
39326	-0.053602542	-0.964742611	0.33539548	0.518560019	no
DLX3	-0.053594673	-0.964600572	0.335466537	0.518632569	no
SSX5	-0.053578609	-0.964310618	0.335611619	0.518819544	no
LINC00290	-0.053560658	-0.963986599	0.335773794	0.519032914	no
RNPC3	-0.053555814	-0.963899168	0.335817563	0.519053699	no
SNORA31	0.053550484	0.963802973	0.335865723	0.519053699	no
ZGLP1	-0.053550222	-0.963798238	0.335868094	0.519053699	no
RPLP1	0.053548479	0.963766771	0.335883849	0.519053699	no
TOPORS	-0.053536275	-0.963546499	0.33599415	0.519186819	no
LINC00226	0.053501896	0.962925972	0.336305004	0.519629796	no
TOR1AIP2	0.053475573	0.962450847	0.336543144	0.519960367	no
HAND2	-0.053453735	-0.96205669	0.336740784	0.520228322	no
SNORA46	-0.053450502	-0.961998332	0.336770053	0.520236141	no
E2F3	-0.053445465	-0.961907417	0.336815653	0.520269187	no
AP4M1	0.053440261	0.96181348	0.336862773	0.520304575	no
NCRUPAR	-0.053411033	-0.961285928	0.33712748	0.52067601	no
EFCAB10	0.053404508	0.961168169	0.337186586	0.520724728	no
SVOPL	0.053402202	0.961126535	0.337207484	0.520724728	no
PKNOX1	0.053390751	0.960919863	0.337311238	0.520847522	no
LOC100144602	0.053378393	0.960696814	0.337423236	0.520983028	no
DCTN5	-0.053368282	-0.960514316	0.33751489	0.521087105	no
PRAMEF10	-0.053362597	-0.960411697	0.337566434	0.521129247	no
CEACAM19	-0.053357171	-0.960313766	0.337615629	0.521167755	no

TRPV3	-0.053351717	-0.960215315	0.33766509	0.521184793	no
UCN3	-0.053350605	-0.960195253	0.337675169	0.521184793	no
NDUFB2	-0.053329583	-0.959815816	0.33786584	0.521441635	no
DDX4	-0.053314838	-0.959549684	0.337999615	0.521610637	no
MAP3K5	-0.053307704	-0.959420926	0.33806435	0.521658326	no
FDPS	-0.053306083	-0.959391662	0.338079064	0.521658326	no
MED16	-0.053295521	-0.95920103	0.338174924	0.521768776	no
TAF9	0.053283584	0.95898558	0.338283284	0.521898497	no
UBE2NL	0.05326642	0.958675797	0.338439129	0.522101452	no
UBTD2	-0.053263675	-0.958626245	0.338464062	0.522102437	no
KIF18A	0.053259945	0.958558915	0.338497942	0.522117222	no
PITRM1	-0.053244711	-0.958283973	0.338636313	0.522259864	no
ZNF252P	-0.053244413	-0.958278593	0.338639021	0.522259864	no
LOC100505918	-0.053232833	-0.958069575	0.338744239	0.522368515	no
P2RX3	-0.053229303	-0.95800587	0.338776312	0.522368515	no
CCDC150	-0.053228634	-0.957993797	0.338782391	0.522368515	no
LYZL6	-0.053224168	-0.957913186	0.338822978	0.522393617	no
LOC285577	-0.053218367	-0.957808491	0.338875697	0.522437418	no
FLJ41200	-0.053207766	-0.957617157	0.338972056	0.522548487	no
BCL2L10	-0.053197435	-0.957430698	0.339065977	0.522655782	no
CHIA	-0.053185158	-0.957209106	0.339177617	0.522787192	no
TREX1	0.05318271	0.957164922	0.339199879	0.522787192	no
RNF122	-0.053172141	-0.956974163	0.339296008	0.522897849	no
NT5C1B-RDH14	-0.053166119	-0.956865481	0.339350784	0.522944765	no
ZNF473	-0.053156085	-0.956684379	0.339442072	0.523021771	no
NUGGC	0.053155277	0.956669786	0.339449428	0.523021771	no
TESK1	0.053145556	0.956494336	0.339537883	0.523120557	no
CEP57	-0.053140866	-0.956409698	0.33958056	0.523148803	no
CABP2	-0.053135579	-0.956314262	0.339628685	0.52318544	no
GUCY1B3	-0.053130999	-0.956231608	0.339670368	0.523212148	no
CHORDC1	0.053120252	0.956037649	0.339768197	0.52332533	no
C12orf75	0.053086284	0.955424567	0.340077542	0.523744272	no
RIF1	-0.053083142	-0.955367868	0.340106159	0.523744272	no
C9orf96	-0.053082358	-0.95535371	0.340113306	0.523744272	no
VWA3B	0.053078658	0.955286941	0.340147009	0.523758642	no
CDK11A	0.053075635	0.955232379	0.340174551	0.523763525	no
KIAA1211L	-0.053071845	-0.955163967	0.340209088	0.523779175	no
FGF13-AS1	-0.053060433	-0.954958005	0.340313077	0.523871149	no
HCAR1	-0.053059939	-0.954949087	0.34031758	0.523871149	no
ADIG	0.053053133	0.954826245	0.340379613	0.523929112	no
MCCD1	-0.053047988	-0.954733389	0.340426508	0.523963767	no
SMCR7	-0.053038427	-0.954560829	0.340513668	0.524060386	no
C1orf204	-0.053018926	-0.95420887	0.340691485	0.524296506	no
MDH1	-0.053006173	-0.953978699	0.340807804	0.524437958	no
LOC286367	0.052998012	0.953831411	0.340882252	0.524514962	no
CCDC14	-0.052989057	-0.953669789	0.340963956	0.52460312	no
PCNXL2	-0.052984556	-0.953588552	0.341005029	0.524628754	no
EFCC1	0.052979742	0.953501676	0.341048955	0.524658776	no
DCD	-0.05293503	-0.952694701	0.341457158	0.525249143	no
C12orf23	0.052930109	0.952605887	0.341502103	0.525280682	no
IL36RN	0.052919709	0.952418188	0.341597102	0.525367991	no

ELK4	0.052918543	0.952397138	0.341607757	0.525367991	no
PADI3	0.052907195	0.95219234	0.341711432	0.52548983	no
ZNF281	-0.052900258	-0.95206714	0.341774822	0.525549706	no
BHLHE23	-0.052842501	-0.95102476	0.342302882	0.526300677	no
PCDHB18	-0.052841486	-0.951006433	0.342312171	0.526300677	no
GTPBP5	-0.052831582	-0.950827694	0.342402772	0.526402316	no
CACUL1	0.052814534	0.950520004	0.342558775	0.52660448	no
TMEM198B	0.052793422	0.950138994	0.342752014	0.526863854	no
PTP4A1	-0.052786431	-0.950012812	0.342816027	0.526924562	no
TRMT6	0.052778604	0.949871565	0.34288769	0.526997022	no
DPPA2P3	0.052770639	0.94972781	0.342960637	0.527071442	no
CDC16	0.052754416	0.949435033	0.343109232	0.527262102	no
AP3S1	0.052734038	0.949067257	0.343295951	0.527511317	no
LOC284100	0.052726475	0.948930759	0.343365267	0.527580106	no
SLC25A46	-0.052723246	-0.948872498	0.343394856	0.52758785	no
CENPBD1	0.05268994	0.94827141	0.343700223	0.527966173	no
CYP24A1	-0.05268988	-0.948270324	0.343700775	0.527966173	no
TATDN2	-0.052688351	-0.948242729	0.343714798	0.527966173	no
PRB3	-0.052661901	-0.947765383	0.343957433	0.528301114	no
SNTB1	0.05265891	0.947711401	0.343984878	0.528305512	no
ORC5	0.052652931	0.947603493	0.344039746	0.528323693	no
TM6SF2	0.052651825	0.947583536	0.344049894	0.528323693	no
PPRC1	-0.052649583	-0.947543079	0.344070467	0.528323693	no
AP1S1	-0.052643768	-0.947438123	0.344123842	0.528367899	no
SNORA48	-0.05262291	-0.9470617	0.344315314	0.528624118	no
DIXDC1	-0.052605327	-0.946744372	0.34447678	0.528834236	no
PSG11	0.052601589	0.94667691	0.344511112	0.528849165	no
YIF1A	-0.052588629	-0.946443033	0.344630155	0.528994119	no
C15orf26	0.05257834	0.946257338	0.344724691	0.529101438	no
EDA2R	0.052572791	0.946157204	0.344775676	0.529141902	no
NPNT	0.052563491	0.945989365	0.344861144	0.529235279	no
GMEB1	0.052553328	0.945805954	0.344954558	0.529340836	no
FOLR4	-0.052541161	-0.945586371	0.345066416	0.529474678	no
TTTY5	0.052531499	0.945412004	0.345155257	0.529573187	no
RAD18	0.052515064	0.945115401	0.345306412	0.529767282	no
KCTD18	-0.052497614	-0.944800482	0.345466947	0.529975741	no
NKX2-5	0.052494005	0.944735355	0.345500152	0.529988849	no
HSPB2	0.052486991	0.944608777	0.345564695	0.530050022	no
KLK10	0.052476425	0.944418099	0.345661937	0.530122016	no
TAPT1-AS1	0.052475814	0.944407079	0.345667558	0.530122016	no
ALG10	0.052473851	0.94437164	0.345685633	0.530122016	no
IL6ST	-0.052470399	-0.944309342	0.345717409	0.530132917	no
KIAA0922	-0.052466793	-0.944244268	0.345750603	0.530145991	no
KRT5	-0.052461	-0.94413973	0.345803931	0.530189933	no
TAF1B	0.052453731	0.944008541	0.345870863	0.530254723	no
MUC4	-0.052444871	-0.943848652	0.345952448	0.530341969	no
PCYT2	-0.052438863	-0.943740233	0.346007778	0.530365599	no
SEPW1	-0.052437838	-0.943721732	0.346017219	0.530365599	no
RWDD4	0.052425439	0.943497983	0.346131425	0.530460257	no
NOTO	-0.052424354	-0.943478401	0.346141421	0.530460257	no
SRRM2	-0.052423095	-0.943455671	0.346153024	0.530460257	no

GRAMD1A	0.052413861	0.943289028	0.3462381	0.5305528	no
SLC39A3	0.052383326	0.942737983	0.346519521	0.530946173	no
CCIN	0.05236339	0.942378205	0.34670334	0.531176449	no
SNX29	0.052361665	0.942347086	0.346719242	0.531176449	no
LOC646498	-0.052351755	-0.942168248	0.34681064	0.53126631	no
C16orf62	-0.052349945	-0.942135578	0.346827338	0.53126631	no
LGALS7	0.052344371	0.94203498	0.346878759	0.531307206	no
UQCRHL	-0.052335697	-0.941878458	0.346958775	0.531391892	no
LOC401164	0.052322927	0.941647995	0.347076612	0.531534488	no
TRMT10C	-0.05231663	-0.941534373	0.347134717	0.531585593	no
PEX11B	-0.052311594	-0.941443485	0.347181201	0.531618895	no
TENM2	-0.052279971	-0.940872804	0.34747316	0.532028048	no
TMEM9B-AS1	0.05225831	0.940481915	0.347673228	0.532294848	no
SEMA3B	-0.052255743	-0.940435583	0.347696947	0.532294848	no
TIMM8B	-0.052238079	-0.940116829	0.347860156	0.532506775	no
SMCP	-0.052234799	-0.940057634	0.347890471	0.532515249	no
UBE2N	-0.052228997	-0.939952924	0.347944098	0.532559405	no
GH2	-0.052218804	-0.939768982	0.348038317	0.532646424	no
DCAF17	0.052217484	0.93974516	0.34805052	0.532646424	no
TMED1	0.052210376	0.939616887	0.348116236	0.532709059	no
OR2AE1	0.05220769	0.939568422	0.348141066	0.532709125	no
LARP6	0.052196331	0.939363438	0.348246103	0.53283191	no
MRGBP	0.052190341	0.939255343	0.3483015	0.532878732	no
NFE2	0.052174905	0.938976784	0.348444285	0.533059234	no
DEFB124	-0.052169614	-0.938881303	0.348493235	0.533096171	no
C1orf53	0.052164308	0.938785551	0.348542328	0.533133322	no
CCL15	0.052156708	0.938648409	0.348612651	0.533202938	no
CDC25A	-0.052150989	-0.938545198	0.348665581	0.533245943	no
IFT46	0.05213836	0.938317308	0.348782468	0.533386751	no
ALDH1L1	-0.052118382	-0.937956781	0.348967437	0.533631649	no
IRS4	-0.052108638	-0.937780948	0.349057671	0.533731655	no
ELP6	-0.052102627	-0.937672484	0.34911334	0.533778799	no
LOC100379224	-0.052099292	-0.93761229	0.349144237	0.533788063	no
CARHSP1	-0.052044119	-0.936616671	0.349655532	0.534500121	no
KRTAP2-1	-0.0520408	-0.936556771	0.349686308	0.534500121	no
SPDYE5	-0.052038945	-0.936523295	0.349703509	0.534500121	no
ZFYVE9	-0.052038302	-0.936511695	0.349709469	0.534500121	no
AICDA	-0.052034472	-0.936442587	0.34974498	0.534504495	no
OLIG3	-0.052032629	-0.936409325	0.349762073	0.534504495	no
CAMK2N1	-0.052018144	-0.936147942	0.34989641	0.534671768	no
SOX30	0.052007401	0.935954077	0.349996068	0.534766441	no
LOC440288	0.0520035	0.935883689	0.350032256	0.534766441	no
MTIF3	-0.052003418	-0.935882212	0.350033015	0.534766441	no
NXNL2	0.051994263	0.935716995	0.350117966	0.534844176	no
STAU2	-0.05199257	-0.935686455	0.35013367	0.534844176	no
MDS2	-0.051982074	-0.935497046	0.350231079	0.534954948	no
BIRC7	0.051977583	0.93541601	0.350272759	0.534980589	no
ARHGAP11A	-0.05193826	-0.934706406	0.350637874	0.535489515	no
FAM91A2	0.051936329	0.934671565	0.350655807	0.535489515	no
HMGB2	-0.051927712	-0.934516069	0.35073585	0.535573692	no
MIR498	-0.051921925	-0.934411641	0.350789611	0.535598624	no

LOC100288974	-0.051920589	-0.934387537	0.350802021	0.535598624	no
SLITRK6	-0.051902105	-0.934053989	0.350973777	0.535822793	no
LINC00661	-0.051887573	-0.933791757	0.351108848	0.535990926	no
CTSE	0.051881564	0.933683326	0.351164708	0.536038124	no
MARVELD2	0.051876921	0.933599543	0.351207875	0.53606594	no
NOC4L	-0.05186352	-0.93335773	0.351332479	0.536218047	no
NLRP13	-0.051849081	-0.933097168	0.351466777	0.536384925	no
KANSL2	-0.051846089	-0.933043175	0.35149461	0.536389311	no
DOCK5	0.051838521	0.932906611	0.351565014	0.536458656	no
ZKSCAN4	0.051834649	0.932836754	0.351601031	0.536475525	no
PTPN13	0.051831664	0.932782879	0.35162881	0.536479821	no
HMG20B	0.051807349	0.93234412	0.351855093	0.536786953	no
OGFOD2	0.051789089	0.932014635	0.352025081	0.536997175	no
CORO1B	0.05178718	0.931980172	0.352042864	0.536997175	no
NCK1	0.051769769	0.931666003	0.352205004	0.537206369	no
MTRNR2L7	-0.051765157	-0.93158278	0.352247962	0.537233763	no
ZNF692	-0.051756435	-0.931425393	0.352329213	0.537319551	no
POTEF	0.051743971	0.93120049	0.352445338	0.537427892	no
VSIG8	0.051743442	0.931190944	0.352450268	0.537427892	no
C14orf183	-0.051718625	-0.930743131	0.352681564	0.537742426	no
ZNF3	-0.051704956	-0.930496472	0.352809006	0.537847647	no
GAGE6	0.05170461	0.930490241	0.352812225	0.537847647	no
LRP5L	-0.05170317	-0.930464255	0.352825654	0.537847647	no
CTCFL	-0.051697249	-0.9303574	0.352880873	0.537893669	no
ZSCAN9	-0.051690685	-0.930238961	0.352942085	0.53794882	no
HCG11	0.05168473	0.930131517	0.352997621	0.537974132	no
P2RY1	0.051683536	0.930109972	0.353008758	0.537974132	no
URGCP-MRPS24	-0.051660678	-0.929697501	0.353222011	0.538260954	no
CGGBP1	0.051656398	0.929620268	0.35326195	0.538283648	no
PACRG	0.051635036	0.929234821	0.35346132	0.538549255	no
EPHB2	0.051625884	0.929069676	0.353546762	0.538617192	no
STC2	-0.051624891	-0.929051756	0.353556034	0.538617192	no
KIAA0319	-0.051606718	-0.928723835	0.353725734	0.538837522	no
ZFP28	-0.051602425	-0.92864637	0.353765831	0.538860406	no
CEMP1	0.051598575	0.928576901	0.35380179	0.538876986	no
EXOSC6	-0.05159243	-0.928466022	0.35385919	0.538926217	no
RBMY2FP	0.05158357	0.928306148	0.353941963	0.539014083	no
ELMO1	0.051575112	0.928153521	0.354020997	0.539088734	no
ANKRD18DP	-0.051572955	-0.928114601	0.354041152	0.539088734	no
ANKRD37	0.051542971	0.927573566	0.354321411	0.539477254	no
SMOX	0.051533257	0.927398301	0.354412229	0.539577303	no
LOC100506874	0.05152699	0.927285215	0.354470836	0.539628301	no
RAVER1	-0.051518901	-0.92713925	0.35454649	0.539705243	no
MET	0.051512028	0.927015246	0.354610771	0.539741961	no
RBM28	-0.051510952	-0.926995821	0.354620841	0.539741961	no
TUBA3E	-0.051501836	-0.926831326	0.354706124	0.539833532	no
GALT	0.051496207	0.926729759	0.354758788	0.53987545	no
TROAP	-0.051491497	-0.926644782	0.354802854	0.539904279	no
HIPK1	-0.051486305	-0.926551089	0.354851443	0.539931122	no
RFC4	-0.051484243	-0.926513879	0.354870741	0.539931122	no
CEBPZ	-0.051472574	-0.926303341	0.354979946	0.540059042	no

CNGB1	0.051464106	0.926150541	0.355059216	0.540141404	no
ENTHD1	0.051456435	0.926012125	0.355131034	0.540202126	no
ZNF530	0.051454473	0.925976723	0.355149404	0.540202126	no
PPP1CB	-0.051447291	-0.92584713	0.355216654	0.540266179	no
GABRA6	0.051443667	0.925781735	0.355250593	0.540279562	no
SAP30L	0.051438844	0.925694721	0.355295755	0.54031001	no
NABP2	-0.051431254	-0.925557768	0.355366842	0.540379878	no
UGT2A3	-0.051424355	-0.925433283	0.355431467	0.540439908	no
NAA11	-0.051404283	-0.925071102	0.35561953	0.540687606	no
CDCP2	-0.051390844	-0.924828618	0.355745475	0.540805033	no
GLS	-0.051390672	-0.924825506	0.355747092	0.540805033	no
UBFD1	0.051385248	0.924727651	0.355797925	0.540810846	no
MIR181B1	0.051384894	0.924721261	0.355801245	0.540810846	no
TRMT12	-0.051373882	-0.924522559	0.355904481	0.540929505	no
UNC119	-0.051329792	-0.923727016	0.356317998	0.541519702	no
NR3C1	-0.051320856	-0.923565787	0.356401841	0.541608823	no
LINC00577	-0.051313679	-0.92343628	0.356469197	0.541672878	no
COMTD1	0.051309154	0.923354634	0.356511664	0.541699108	no
DPY19L2P3	0.051302291	0.923230817	0.356576073	0.541718693	no
TBC1D3G	-0.051301236	-0.923211767	0.356585983	0.541718693	no
LRRC40	-0.051299724	-0.923184489	0.356600175	0.541718693	no
FLCN	0.051294947	0.923098308	0.356645012	0.541748512	no
FAM47A	-0.051288015	-0.922973229	0.356710092	0.541809074	no
RPA2	0.05128352	0.922892117	0.3567523	0.541834889	no
ANXA9	-0.051276424	-0.922764082	0.356818932	0.541897792	no
DDX5	0.051253108	0.922343389	0.357037922	0.542171699	no
C4orf48	-0.05125185	-0.922320682	0.357049744	0.542171699	no
LOC402779	-0.051249075	-0.922270611	0.357075815	0.542172979	no
C7orf33	0.051232212	0.921966353	0.357234259	0.542375236	no
VWC2	0.051224243	0.921822576	0.357309148	0.542450614	no
MIR196A2	-0.051221443	-0.921772055	0.357335465	0.542452248	no
SF1	-0.051208579	-0.921539938	0.357456393	0.542597496	no
RRP9	-0.05119296	-0.921258124	0.357603246	0.542781797	no
SLC39A14	0.051190293	0.921210013	0.357628321	0.542781797	no
ZNF804B	-0.051186672	-0.921144668	0.35766238	0.542795156	no
IGFBPL1	-0.051183462	-0.921086761	0.357692563	0.542802632	no
ZNF587	0.051161655	0.920693293	0.357897696	0.543075577	no
MTPN	0.051148054	0.920447883	0.358025677	0.54323142	no
PPEF2	-0.051136119	-0.920232544	0.358138001	0.543363486	no
TRIM55	-0.051132356	-0.920164653	0.358173418	0.543378859	no
GMPS	0.051129651	0.920115833	0.358198888	0.543379141	no
AQP5	0.051123752	0.920009402	0.358254417	0.54342502	no
WDR52	-0.051115902	-0.919867766	0.358328324	0.543498765	no
BOLA3-AS1	-0.051100762	-0.919594609	0.358470885	0.543676626	no
LOC729950	-0.051097665	-0.919538725	0.358500055	0.543682499	no
CYP19A1	0.051092033	0.919437108	0.358553102	0.543684824	no
LOC641367	-0.051089658	-0.919394252	0.358575475	0.543684824	no
ZNF487P	0.051089445	0.919390405	0.358577484	0.543684824	no
KIR2DL5B	-0.051082847	-0.919271359	0.358639637	0.543740701	no
CRYBB2P1	-0.051070612	-0.919050604	0.35875491	0.543870097	no
LOC100507003	-0.051067365	-0.918992019	0.358785506	0.543870097	no

OTUD6B	-0.05106573	-0.918962535	0.358800904	0.543870097	no
GAGE12D	0.051031854	0.918351311	0.359120219	0.544315723	no
NOXO1	-0.051023791	-0.918205841	0.359196242	0.544392555	no
FRG1	-0.051004773	-0.917862699	0.359375609	0.544625993	no
SLC28A1	0.050999029	0.917759063	0.359429792	0.544669698	no
VPS41	0.050995118	0.917688507	0.359466683	0.544687195	no
UBE2T	-0.050988472	-0.917568584	0.359529393	0.54473989	no
DCHS2	0.050986059	0.917525059	0.359552154	0.54473989	no
AP1M2	-0.0509798	-0.917412117	0.359611222	0.544790975	no
SLC10A5	-0.050971921	-0.917269973	0.35968557	0.54482722	no
PRSS37	-0.050971892	-0.917269439	0.35968585	0.54482722	no
PRSS16	0.050965133	0.917147492	0.359749642	0.544885443	no
ZNF814	-0.050957442	-0.917008728	0.35982224	0.544956995	no
PA2G4P4	0.050924231	0.916409531	0.360135832	0.545365933	no
KDM5A	0.050921403	0.916358496	0.36016255	0.545365933	no
GPR101	-0.050919398	-0.916322326	0.360181486	0.545365933	no
CREB3L1	0.050918099	0.916298885	0.360193758	0.545365933	no
TRIM11	-0.050915155	-0.916245777	0.360221563	0.545369611	no
SNRPD2	-0.05088248	-0.915656238	0.360530316	0.545798607	no
C22orf43	-0.050872129	-0.915469491	0.360628153	0.545908266	no
CTDNEP1	0.050863642	0.915316359	0.360708393	0.545991271	no
KRTAP8-1	-0.050859936	-0.915249495	0.360743432	0.546005852	no
PCDHA12	0.050822969	0.914582545	0.361093057	0.546496541	no
IL20RB	-0.05081672	-0.914469793	0.361152184	0.546547538	no
GLCE	0.050810825	0.914363429	0.361207967	0.546584564	no
ZNF710	-0.050808759	-0.914326156	0.361227517	0.546584564	no
GGNBP2	-0.050787596	-0.913944328	0.36142782	0.54683583	no
LTBP1	0.050785837	0.913912607	0.361444463	0.54683583	no
SALL1	-0.050740839	-0.913100745	0.361870602	0.547370398	no
TMEM216	0.05074077	0.913099514	0.361871249	0.547370398	no
LOC100133920	-0.050740458	-0.913093877	0.361874209	0.547370398	no
LOC286359	-0.0507165	-0.912661639	0.362101217	0.547675223	no
AVPI1	-0.050711323	-0.912568238	0.362150282	0.547710888	no
MED11	-0.050705329	-0.912460095	0.362207097	0.547758266	no
FLJ40852	-0.050701522	-0.912391409	0.362243185	0.547774296	no
TELO2	-0.050696668	-0.912303829	0.362289204	0.547805339	no
MID1IP1	-0.050691771	-0.912215485	0.362335628	0.547826635	no
LINC00838	0.050687383	0.912136306	0.362377239	0.547826635	no
SPOCK2	0.050687117	0.912131506	0.362379761	0.547826635	no
GPS2	-0.050667718	-0.911781526	0.362563723	0.548066186	no
TMEM202	-0.050664232	-0.911718625	0.362596792	0.548077624	no
LOC100129175	0.050650751	0.911475408	0.362724677	0.54819797	no
YY1	0.050650461	0.911470179	0.362727427	0.54819797	no
PCID2	-0.050643978	-0.911353213	0.362788939	0.548252379	no
ARAP2	-0.050620356	-0.910927047	0.363013113	0.548552582	no
HINT1	0.050615447	0.910838467	0.36305972	0.548580151	no
LOC100507205	0.050613057	0.910795344	0.36308241	0.548580151	no
EIF3D	-0.050591022	-0.910397812	0.363291626	0.548820736	no
GTF2H2D	0.050590907	0.910395739	0.363292717	0.548820736	no
LOC100093698	-0.050581358	-0.91022345	0.363383414	0.548919164	no
TCTE1	-0.050571034	-0.910037194	0.36348148	0.54902871	no

HOXC6	-0.050567375	-0.909971188	0.363516237	0.549040012	no
LOC653513	-0.050564868	-0.909925953	0.363540058	0.549040012	no
PLSCR5	-0.050547333	-0.909609597	0.363706677	0.549229217	no
IFT88	-0.05054245	-0.909521508	0.363753081	0.549229217	no
POU6F2-AS1	-0.05054233	-0.90951934	0.363754223	0.549229217	no
GUCY1A2	-0.050540926	-0.909494019	0.363767562	0.549229217	no
CDC20B	0.050530727	0.909310015	0.363864506	0.549336994	no
C9orf50	0.050518435	0.909088251	0.363981367	0.549456031	no
PPIL6	-0.050517055	-0.909063356	0.363994487	0.549456031	no
PKHD1	0.050514082	0.909009703	0.364022764	0.549460122	no
C12orf69	0.050510918	0.908952636	0.364052841	0.54946693	no
CXXC1P1	0.050502233	0.908795943	0.364135436	0.549549647	no
NCKIPSD	-0.050498698	-0.908732167	0.364169057	0.549549647	no
CALU	0.050497089	0.90870314	0.364184359	0.549549647	no
LINC00514	-0.050488663	-0.90855112	0.364264509	0.549631999	no
WDFY1	0.050473728	0.908281671	0.364406598	0.549791851	no
SLC6A2	0.050472149	0.908253198	0.364421615	0.549791851	no
DDX23	-0.050466945	-0.908159307	0.364471136	0.549827964	no
MIR3666	-0.050457195	-0.907983412	0.36456392	0.549929333	no
CCDC179	-0.050453536	-0.907917385	0.364598753	0.549943276	no
VTCN1	-0.05044278	-0.907723342	0.364701133	0.550059096	no
COX7B	0.050428734	0.907469933	0.364834864	0.550222179	no
CBLC	0.050412463	0.907176404	0.364989805	0.550417226	no
ENPP2	-0.050407707	-0.907090591	0.365035109	0.550446922	no
CELA2B	-0.050396773	-0.906893331	0.365139266	0.550565352	no
GOLGA3	0.050393897	0.906841444	0.365166666	0.550568038	no
MKX	-0.050382553	-0.906636785	0.365274753	0.550692369	no
TNFRSF8	0.050366726	0.906351265	0.36542558	0.550796459	no
LOC339529	-0.050364827	-0.906317	0.365443684	0.550796459	no
TMEM184B	-0.050364378	-0.906308901	0.365447963	0.550796459	no
ZNF480	0.050363741	0.906297402	0.365454038	0.550796459	no
UBE2L3	-0.050361862	-0.906263515	0.365471942	0.550796459	no
INGX	-0.050352573	-0.90609592	0.3655605	0.550891291	no
WDYHV1	-0.050295389	-0.905064285	0.366105916	0.551674537	no
SPANXN4	0.050289392	0.904956097	0.366163144	0.551722086	no
GPC3	-0.050286188	-0.9048983	0.366193719	0.551729473	no
HIATL2	-0.050281067	-0.904805913	0.366242595	0.551764431	no
EML6	-0.050275815	-0.904711165	0.366292725	0.551801272	no
LOC100506207	-0.050267854	-0.904567542	0.366368722	0.551877073	no
OPA1	-0.05026101	-0.904444079	0.366434059	0.551900863	no
CNIH	-0.050259624	-0.904419069	0.366447295	0.551900863	no
SFRP5	-0.050255481	-0.904344321	0.366486857	0.551900863	no
SAMD4B	0.050255441	0.904343602	0.366487237	0.551900863	no
ENTPD4	0.050228747	0.903862041	0.366742175	0.552246082	no
UCN2	0.050221907	0.903738631	0.366807526	0.55230579	no
FOXP2	-0.050218579	-0.903678595	0.36683932	0.552314966	no
RNF14	0.050212113	0.903561959	0.366901094	0.552369276	no
HIST1H2BH	0.050200838	0.903358546	0.367008843	0.552439902	no
GBX1	-0.050199807	-0.903339939	0.3670187	0.552439902	no
FOXE3	-0.050199135	-0.903327816	0.367025123	0.552439902	no
BRIP1	-0.050194033	-0.903235789	0.367073878	0.552469373	no

RBAK-LOC3894	0.050191707	0.903193815	0.367096117	0.552469373	no
BTC	0.050179739	0.902977909	0.367210522	0.552568548	no
TPO	-0.050179434	-0.902972407	0.367213438	0.552568548	no
RNF10	0.050172225	0.902842365	0.367282357	0.552633559	no
GRSF1	0.050169281	0.90278925	0.367310508	0.552637225	no
SLC25A2	-0.050144856	-0.902348616	0.367544103	0.55289372	no
TULP1	-0.050144673	-0.902345316	0.367545852	0.55289372	no
LOC340515	-0.050143386	-0.902322091	0.367558168	0.55289372	no
ZNF407	-0.050138488	-0.902233742	0.367605017	0.552925491	no
GEMIN4	-0.050131819	-0.902113426	0.367668823	0.552982761	no
CARM1	0.050126837	0.902023546	0.367716492	0.55299106	no
PI3	0.050125864	0.902005991	0.367725804	0.55299106	no
DNAJC30	0.050111699	0.90175046	0.367861354	0.553156196	no
NFASC	-0.05008445	-0.901258887	0.368122203	0.553509709	no
UBE2MP1	-0.050074822	-0.901085187	0.368214404	0.553590002	no
PFKFB4	0.050073494	0.901061229	0.368227122	0.553590002	no
SLC5A7	-0.050061006	-0.900835958	0.36834672	0.553700176	no
KDM4A	0.050059692	0.900812239	0.368359314	0.553700176	no
MMAA	0.050057772	0.900777615	0.368377698	0.553700176	no
RBBP4	0.050052562	0.90068363	0.368427606	0.553736463	no
PCCA-AS1	-0.050045288	-0.900552396	0.3684973	0.553786401	no
LOC100289656	0.050041705	0.900487769	0.368531624	0.553786401	no
TSTD3	-0.050041026	-0.900475507	0.368538137	0.553786401	no
LOC285889	-0.050037546	-0.900412741	0.368571475	0.553797075	no
SNX12	-0.050034906	-0.900365105	0.368596778	0.553797075	no
MEG8	-0.050019826	-0.900093061	0.368741303	0.553975486	no
C13orf35	-0.050006369	-0.899850314	0.368870293	0.554130537	no
ITSN1	0.049998688	0.899711735	0.368943942	0.554202437	no
DNAJC5	-0.049983132	-0.899431108	0.369093115	0.554387099	no
KCTD17	-0.049980488	-0.89938342	0.369118468	0.554387099	no
TMEM110-MUST	-0.049969135	-0.899178622	0.36922736	0.5545003	no
IZUMO3	-0.049967251	-0.899144619	0.369245442	0.5545003	no
ANKRD30B	-0.049958541	-0.898987502	0.369328998	0.554568543	no
LINC00379	-0.049957134	-0.898962125	0.369342495	0.554568543	no
TBC1D29	-0.049954335	-0.898911623	0.369369356	0.554570129	no
NIPBL	-0.049945348	-0.898749502	0.369455592	0.554647477	no
LOC344967	-0.049943587	-0.898717736	0.36947249	0.554647477	no
BMP8A	0.049935589	0.898573453	0.36954925	0.55472396	no
PTGDS	-0.049930395	-0.898479759	0.369599101	0.554760043	no
SLC3A1	-0.04992595	-0.898399569	0.369641771	0.554785342	no
GLB1L3	-0.04990684	-0.898054836	0.369825242	0.555021946	no
MMP13	0.049892059	0.897788191	0.369967192	0.555167929	no
LINC00502	0.049889715	0.897745901	0.369989709	0.555167929	no
OR4C45	0.049888642	0.897726551	0.370000012	0.555167929	no
TMPRSS3	0.049876961	0.897515826	0.370112223	0.555252971	no
FAM198A	-0.049876383	-0.8975054	0.370117775	0.555252971	no
MTMR10	-0.049874673	-0.897474562	0.370134198	0.555252971	no
FH	0.049863624	0.897275231	0.370240366	0.555373469	no
FAM98C	-0.049858236	-0.897178037	0.37029214	0.555408124	no
OTOP1	-0.049853613	-0.897094648	0.370336564	0.555408124	no
AGL	0.049853152	0.897086322	0.370340999	0.555408124	no

HSF1	-0.049839887	-0.896847046	0.370468489	0.555560554	no
HVCN1	-0.049822624	-0.896535625	0.370634459	0.55576227	no
POLR1E	-0.049820517	-0.896497612	0.370654721	0.55576227	no
DHRS4	0.049811122	0.896328138	0.370745064	0.555858949	no
ESPNP	-0.04980815	-0.896274527	0.370773646	0.555863023	no
DUS1L	0.049801042	0.896146305	0.370842011	0.555926734	no
GEMIN6	0.049753205	0.89528336	0.371302316	0.556577951	no
HHIP	-0.049743826	-0.895114164	0.371392609	0.55664004	no
MGC70870	0.049742099	0.89508302	0.371409231	0.55664004	no
C7orf71	0.049739608	0.895038082	0.371433215	0.55664004	no
TMEM95	-0.049738141	-0.895011617	0.371447341	0.55664004	no
ADH5	0.049732633	0.894912258	0.371500376	0.556680699	no
AFMID	0.049711978	0.894539663	0.371699299	0.556939945	no
OR51G1	-0.049707439	-0.89445779	0.371743018	0.556966621	no
MC5R	0.049702909	0.894376065	0.371786662	0.55699318	no
CCDC70	0.049667834	0.893743356	0.372124658	0.557460687	no
PCDHB7	0.049664321	0.893679972	0.372158528	0.557472567	no
TUBA3C	-0.049651315	-0.893445369	0.372283909	0.557621515	no
NDST2	0.049648619	0.89339674	0.372309902	0.557621584	no
METTL23	0.049635379	0.893157892	0.372437585	0.557773947	no
C9orf139	0.049621753	0.8929121	0.372569008	0.55793189	no
POLA1	-0.049616506	-0.89281746	0.372619619	0.557961066	no
ID3	0.049614351	0.892778574	0.372640416	0.557961066	no
PKP2	0.049600987	0.892537513	0.372769352	0.55811524	no
P4HTM	-0.049581608	-0.892187937	0.372956381	0.558356362	no
CDK5RAP2	0.049576073	0.89208809	0.373009811	0.558397454	no
CDH1	-0.049573078	-0.892034073	0.373038718	0.558401832	no
XK	-0.049562459	-0.891842519	0.373141242	0.558502121	no
DCLRE1C	-0.049560756	-0.891811786	0.373157692	0.558502121	no
MIR1257	-0.049555979	-0.891725621	0.373203816	0.558532257	no
PABPC3	0.04955136	0.891642306	0.373248418	0.55856011	no
FLJ44511	0.049545302	0.89153302	0.373306928	0.558599447	no
LRRC18	0.049543256	0.891496114	0.373326688	0.558599447	no
ASCL4	0.049539882	0.89143525	0.373359277	0.558609317	no
TEX11	-0.049533659	-0.891322994	0.373419388	0.55866036	no
L1TD1	0.049527834	0.891217931	0.373475653	0.558705643	no
CHCHD4	-0.049519586	-0.891069151	0.373555339	0.558785953	no
USP17L1P	-0.049514528	-0.890977901	0.373604218	0.558820173	no
BUD31	0.049508353	0.890866514	0.373663888	0.558865207	no
ABLIM3	-0.04950603	-0.890824615	0.373686335	0.558865207	no
LINC00633	-0.049500187	-0.890719222	0.373742802	0.558910761	no
ZNF799	0.049495362	0.890632191	0.373789434	0.558941604	no
OR4A47	-0.049474309	-0.890252419	0.373992966	0.559207043	no
C1orf172	-0.049466446	-0.89011058	0.374069	0.559274048	no
ZNF764	0.049464292	0.890071734	0.374089825	0.559274048	no
CEACAM5	-0.049459494	-0.889985181	0.374136229	0.559304514	no
DLG1	-0.04944734	-0.889765936	0.374253789	0.559441342	no
AFG3L2	-0.049428697	-0.889429654	0.374434148	0.559672018	no
CERKL	-0.049421403	-0.889298079	0.374504731	0.55973859	no
SLC22A13	-0.049414509	-0.889173723	0.374571449	0.559773167	no
FAM63B	-0.049413629	-0.889157861	0.374579959	0.559773167	no

CTRB2	-0.049390752	-0.888745203	0.37480141	0.560065158	no
TMEM239	-0.049383072	-0.888606669	0.374875771	0.560137329	no
LOC285819	-0.049376425	-0.888486764	0.37494014	0.56019456	no
SMARCA2	-0.049356233	-0.888122538	0.375135712	0.560444778	no
WBP2NL	-0.04935375	-0.888077746	0.375159768	0.560444778	no
CDCA7L	0.04934802	0.887974394	0.375215277	0.560488741	no
SEMA3E	0.049330184	0.887652673	0.375388101	0.56068607	no
CCDC103	0.049329002	0.887631351	0.375399556	0.56068607	no
ZNF766	0.049309696	0.887283102	0.37558669	0.560926585	no
PPAT	-0.049304356	-0.887186775	0.375638463	0.560964922	no
PICK1	0.049301474	0.887134787	0.375666406	0.560967672	no
SLC52A3	-0.049294115	-0.887002058	0.375737753	0.561035229	no
SLC37A3	-0.049290736	-0.886941098	0.375770525	0.561045182	no
GABRR3	-0.049278359	-0.886717852	0.375890554	0.561139505	no
EXD1	-0.049277948	-0.886710438	0.375894541	0.561139505	no
OR1B1	-0.049271922	-0.886601738	0.375952993	0.561139505	no
RBM17	0.0492718	0.886599542	0.375954174	0.561139505	no
SNAR-D	-0.04926944	-0.886556976	0.375977065	0.561139505	no
MIR3939	-0.04926741	-0.886520358	0.375996758	0.561139505	no
GEMIN5	-0.049265378	-0.8864837	0.376016473	0.561139505	no
ACPL2	0.049260192	0.886390152	0.376066787	0.561175623	no
PRSS3P2	0.049256259	0.886319213	0.376104944	0.561193595	no
LOC554223	-0.049251856	-0.886239784	0.37614767	0.561218382	no
CAPN3	-0.049239574	-0.886018253	0.376266852	0.561348678	no
AKR7A3	-0.049237473	-0.885980361	0.376287239	0.561348678	no
MIR3689D2	-0.049227181	-0.885794719	0.376387135	0.561409124	no
SLC22A7	0.049225907	0.885771731	0.376399505	0.561409124	no
LCN1	-0.049225225	-0.885759428	0.376406127	0.561409124	no
SPTLC2	0.049217991	0.88562894	0.376476355	0.561474903	no
IRS1	0.049213678	0.88555115	0.376518225	0.561498382	no
C10orf32-AS3	0.049210635	0.885496265	0.376547768	0.561503476	no
SCGB1A1	-0.049196272	-0.885237181	0.376687248	0.561672493	no
SPEM1	-0.049178829	-0.884922554	0.376856671	0.561854397	no
LOC100132354	-0.049173002	-0.884817446	0.376913281	0.561854397	no
CDH4	-0.049171675	-0.884793512	0.376926173	0.561854397	no
NOX1	-0.049171366	-0.884787934	0.376929177	0.561854397	no
CCNJL	-0.049170256	-0.884767914	0.37693996	0.561854397	no
SNORA35	-0.04916241	-0.884626386	0.377016198	0.561890854	no
C6orf10	-0.049159398	-0.884572064	0.377045462	0.561890854	no
C1QTNF8	-0.049159222	-0.884568887	0.377047174	0.561890854	no
DOK5	-0.049156976	-0.884528372	0.377069001	0.561890854	no
KRTAP9-2	-0.049152135	-0.884441066	0.377116039	0.561921986	no
ATP7B	-0.049145106	-0.884314268	0.377184361	0.561984824	no
TRIB3	0.049140423	0.884229799	0.377229879	0.56201368	no
ABP1	0.049133316	0.884101619	0.377298959	0.562065535	no
FNTB	0.049131461	0.88406816	0.377316992	0.562065535	no
TEX14	-0.049115644	-0.883782859	0.377470781	0.562255653	no
FAM64A	-0.049110478	-0.883689676	0.377521019	0.562268668	no
MAGOHB	-0.04910649	-0.883617735	0.377559808	0.562268668	no
LIMK1	0.049106039	0.88360961	0.377564188	0.562268668	no
FUK	0.049101444	0.88352673	0.377608878	0.562268668	no

TCHH	0.049100457	0.883508924	0.37761848	0.562268668	no
LOC286370	-0.049098605	-0.883475512	0.377636497	0.562268668	no
MIR216A	-0.049089969	-0.883319739	0.377720505	0.562354788	no
DPEP3	0.049082186	0.883179358	0.377796221	0.562389886	no
FOXF1-AS1	-0.049082166	-0.883178996	0.377796416	0.562389886	no
SPDYE2	-0.049072788	-0.883009835	0.377887668	0.562486762	no
OCIAD2	-0.049067042	-0.882906207	0.377943575	0.562531018	no
MIR545	-0.049058019	-0.882743452	0.378031392	0.562622759	no
BPESC1	-0.049049268	-0.882585604	0.378116574	0.562710566	no
ZNF395	-0.049042543	-0.882464306	0.378182039	0.562769021	no
CASC4	0.049035924	0.882344914	0.378246483	0.562825948	no
OR5D13	0.049008433	0.881849059	0.378514201	0.563185315	no
HSD17B4	-0.04900217	-0.881736094	0.378575209	0.563237093	no
LOC100505841	-0.048988004	-0.881480574	0.378713227	0.563370963	no
PGA5	-0.048987553	-0.881472448	0.378717617	0.563370963	no
MAEL	-0.048972227	-0.881195998	0.378866976	0.563472	no
FSHB	-0.048968993	-0.881137683	0.378898487	0.563472	no
ERGIC2	0.048968608	0.881130734	0.378902242	0.563472	no
MRPL19	-0.048967767	-0.881115555	0.378910444	0.563472	no
ELF5	-0.0489642	-0.881051217	0.378945212	0.563472	no
SLC18A1	-0.048962975	-0.881029132	0.378957147	0.563472	no
GPR20	0.048961752	0.88100707	0.37896907	0.563472	no
SYT8	-0.048953399	-0.880856409	0.379050498	0.563521395	no
ZNF566	-0.048952965	-0.880848571	0.379054734	0.563521395	no
TMEM144	-0.048933236	-0.880492733	0.379247097	0.563768373	no
BRDT	-0.048928544	-0.880408099	0.379292859	0.563797401	no
GINS1	-0.048924182	-0.880329427	0.3793354	0.563821639	no
LOC649330	0.048915223	0.880167829	0.379422791	0.563912531	no
C1orf131	0.048908742	0.880050934	0.379486014	0.563967494	no
PPP1R11	-0.048900263	-0.879897999	0.37956874	0.564051431	no
CCT8L2	-0.048896056	-0.879822107	0.379609796	0.564073438	no
PRH2	-0.048886972	-0.879658267	0.379698439	0.564166148	no
HMX3	0.048877964	0.879495796	0.379786355	0.564257764	no
GJD4	-0.048873728	-0.879419389	0.379827704	0.564267533	no
CCDC79	-0.048871911	-0.879386613	0.379845442	0.564267533	no
KIAA1683	-0.048867972	-0.879315562	0.379883896	0.564285652	no
HIST1H2BI	0.04885655	0.879109544	0.379995411	0.564412287	no
PPP1R15B	-0.048852122	-0.879029678	0.380038647	0.564437496	no
KIF14	0.048832232	0.87867094	0.380232889	0.564686961	no
ATF4	0.048824389	0.878529483	0.380309499	0.564761709	no
SWSAP1	0.048796982	0.878035152	0.380577293	0.565080182	no
HIBCH	-0.048794762	-0.877995109	0.380598991	0.565080182	no
DEFB109P1	-0.048790591	-0.877919871	0.380639761	0.565080182	no
ETV3L	0.048789684	0.877903513	0.380648625	0.565080182	no
DDR1	-0.048786838	-0.877852183	0.380676442	0.565080182	no
KCNMA1	0.048786298	0.877842442	0.380681722	0.565080182	no
CRYM-AS1	-0.048782586	-0.877775487	0.380718008	0.565095014	no
C9orf163	-0.048779455	-0.877719013	0.380748616	0.565101416	no
RANBP10	0.048773012	0.8776028	0.380811606	0.565113395	no
C9orf91	-0.048772049	-0.877585435	0.380821019	0.565113395	no
SCAMP3	0.04877056	0.877558586	0.380835573	0.565113395	no

ATF6B	-0.048764827	-0.877455183	0.380891628	0.565157552	no
LOC100499405	0.048746208	0.877119352	0.381073717	0.565388695	no
LATS1	0.048715954	0.876573688	0.381369692	0.565788765	no
SNORA32	-0.048701324	-0.876309811	0.381512873	0.565962115	no
SRRM2-AS1	-0.048695188	-0.876199139	0.381572934	0.566012143	no
PCDHGB8P	-0.04868062	-0.875936395	0.381715547	0.566184611	no
TAC1	-0.04867511	-0.875837017	0.381769497	0.566225552	no
NSUN2	0.048663678	0.875630827	0.381881446	0.566352506	no
C6orf15	-0.048653778	-0.875452265	0.381978412	0.566457221	no
SMC6	0.048649088	0.875367671	0.382024354	0.566486262	no
ATAD2	-0.04864538	-0.875300797	0.382060676	0.566501034	no
TMEM165	0.048628329	0.874993265	0.382227733	0.566681565	no
ZSCAN4	-0.048627571	-0.874979584	0.382235166	0.566681565	no
CTC1	-0.048616041	-0.874771624	0.382348162	0.566809985	no
MAP4K2	0.048603018	0.874536754	0.382475803	0.566943685	no
NUDT9P1	-0.048601457	-0.874508588	0.382491112	0.566943685	no
HEY2	-0.048581948	-0.87415672	0.382682391	0.567156029	no
BOLL	-0.048581462	-0.874147964	0.382687151	0.567156029	no
SFRP1	-0.048568883	-0.873921084	0.382810518	0.56729974	no
C1orf105	-0.048563098	-0.873816747	0.382867259	0.567312135	no
GOLGA6L10	0.048562648	0.873808624	0.382871677	0.567312135	no
DHX34	0.048539773	0.873396057	0.383096096	0.567605528	no
TRIM4	0.048534619	0.873303094	0.383146674	0.567641333	no
SNORD114-4	-0.048522824	-0.873090366	0.38326243	0.567740536	no
MKRN7P	-0.048522412	-0.873082944	0.38326647	0.567740536	no
LOC643387	-0.048513576	-0.872923572	0.383353206	0.567829882	no
CHMP7	-0.048508867	-0.872838652	0.383399429	0.567859209	no
MIR133B	0.04850226	0.872719483	0.383464298	0.567916148	no
LOC644145	-0.048492492	-0.872543315	0.383560207	0.568006878	no
CXorf66	-0.048490638	-0.872509864	0.38357842	0.568006878	no
FER1L6	0.048482151	0.872356792	0.38366177	0.56809116	no
UBALD2	0.048469552	0.872129559	0.383785521	0.568235249	no
C4orf40	-0.048458099	-0.871923009	0.38389803	0.568362673	no
PPT2	-0.04843755	-0.871552387	0.384099961	0.56862246	no
ARID4B	-0.048432175	-0.871455451	0.384152787	0.568637383	no
NSA2	-0.048429062	-0.871399308	0.384183384	0.568637383	no
FIZ1	-0.048428448	-0.871388236	0.384189419	0.568637383	no
C9orf53	-0.048418064	-0.871200945	0.384291502	0.568749306	no
YWHAH	-0.048414074	-0.871128987	0.384330727	0.56876819	no
DACT1	0.048410597	0.871066274	0.384364916	0.568776654	no
DFNB31	0.048408109	0.871021403	0.384389378	0.568776654	no
CCP110	-0.0483974	-0.870828269	0.38449468	0.568893299	no
CXCL6	0.048371474	0.870360671	0.384749702	0.569231436	no
BNIP3L	-0.048352299	-0.870014851	0.384938374	0.56947137	no
TLL1	-0.048339828	-0.869789928	0.385061118	0.569613745	no
F13B	-0.048316633	-0.869371586	0.385289477	0.569912324	no
ASTL	0.048312362	0.869294571	0.385331527	0.569935295	no
TMEM72	-0.048294962	-0.868980759	0.385502893	0.570149519	no
FLJ26850	0.048266403	0.868465688	0.385784263	0.570526397	no
EVPL	-0.048246267	-0.868102536	0.38598272	0.570780612	no
RAG2	0.048241142	0.868010088	0.386033251	0.570816059	no

GPR135	-0.048233413	-0.867870712	0.386109441	0.570889439	no
RFNG	-0.048228045	-0.86777389	0.386162373	0.570928424	no
ENY2	0.048216054	0.867557627	0.386280622	0.571030294	no
SRP54	0.048215217	0.867542531	0.386288876	0.571030294	no
RFX3	-0.048210689	-0.867460868	0.386333534	0.571030294	no
KLF16	0.048210281	0.867453509	0.386337558	0.571030294	no
TMEM74	-0.048174141	-0.866801737	0.3866941	0.571514675	no
HIST1H2AE	0.048170318	0.86673279	0.386731827	0.571514675	no
NKX2-1	-0.048166796	-0.866669261	0.386766593	0.571514675	no
MAGEB18	-0.048166284	-0.866660028	0.386771646	0.571514675	no
NENF	0.048149177	0.866351509	0.386940507	0.571724883	no
AZI2	-0.048135561	-0.866105952	0.387074939	0.571824355	no
SLC22A5	-0.048133407	-0.8660671	0.387096212	0.571824355	no
EME1	-0.048132399	-0.866048918	0.387106167	0.571824355	no
BLVRA	0.04813158	0.86603414	0.387114259	0.571824355	no
OXSM	-0.048118432	-0.865797031	0.387244102	0.571976838	no
RPL27	-0.048111136	-0.865665441	0.387316173	0.572043974	no
EPHX4	-0.048096856	-0.865407905	0.387457248	0.57221301	no
TACO1	-0.048089735	-0.865279478	0.387527611	0.572277598	no
ALAD	0.048077844	0.865065036	0.387645117	0.572402471	no
ARMC12	-0.048075788	-0.865027951	0.38766544	0.572402471	no
LOC100129138	-0.048067103	-0.86487133	0.387751278	0.572489882	no
GRM4	-0.048058928	-0.86472389	0.387832096	0.572569869	no
FOXP4	-0.048051335	-0.864586943	0.387907171	0.572641367	no
C17orf72	0.048042016	0.864418892	0.38799931	0.572738043	no
LINC00331	-0.048036273	-0.864315311	0.388056108	0.572760049	no
KRTAP4-4	-0.048035119	-0.8642945	0.38806752	0.572760049	no
NUP214	-0.048014016	-0.863913917	0.388276258	0.573028778	no
R3HDM2	-0.047994413	-0.863560393	0.388470216	0.573243524	no
KRT40	-0.047993918	-0.863551466	0.388475114	0.573243524	no
SLX1A-SULT1A	0.04798791	0.863443114	0.388534573	0.573251554	no
ZNF543	-0.047987209	-0.86343047	0.388541512	0.573251554	no
RPL18	-0.047985283	-0.863395728	0.388560578	0.573251554	no
ZC3H3	-0.047974588	-0.863202848	0.38866644	0.573368373	no
C10orf137	-0.047962335	-0.862981887	0.388787736	0.573507944	no
PDDC1	-0.047958855	-0.862919126	0.388822193	0.573519406	no
RNU6-79	-0.047950842	-0.862774616	0.388901538	0.573597074	no
ZNF35	0.047938992	0.862560907	0.389018896	0.573730792	no
ZNRF2P1	-0.047922307	-0.862260004	0.389184173	0.573935159	no
LOC100507424	-0.047917116	-0.862166388	0.389235603	0.573971617	no
RERG	0.047914281	0.862115263	0.38926369	0.573973652	no
CNOT3	0.047897413	0.861811053	0.389430849	0.574180733	no
INHBC	-0.047893416	-0.861738982	0.389470457	0.574199739	no
AMOTL1	-0.047885003	-0.861587247	0.389553854	0.574283295	no
C6orf58	-0.047865023	-0.861226931	0.389751937	0.574535899	no
GYPB	-0.047844707	-0.860860552	0.389953415	0.574786921	no
ALG1L2	-0.047841254	-0.860798279	0.389987667	0.574786921	no
LOC654342	-0.047839763	-0.860771383	0.390002461	0.574786921	no
NLRP14	-0.047826771	-0.860537094	0.390131344	0.574937441	no
SCARNA1	-0.047816837	-0.860357934	0.390229918	0.575043278	no
ALG13	0.047808548	0.860208462	0.390312169	0.575068325	no

UNCX	-0.04780749	-0.860189382	0.390322669	0.575068325	no
DEFB136	-0.047807035	-0.860181165	0.390327191	0.575068325	no
IVL	-0.047798672	-0.860030348	0.390410195	0.575139911	no
HMGB3P1	-0.047796302	-0.859987618	0.390433714	0.575139911	no
DNAJC19	0.047794051	0.859947012	0.390456065	0.575139911	no
LOC100129250	0.047786894	0.859817947	0.390527111	0.575205136	no
TDRD5	-0.047774128	-0.859587724	0.390653861	0.575352394	no
TMC7	0.047749346	0.859140811	0.390899982	0.575675428	no
OLAH	0.047743532	0.859035964	0.390957737	0.575721032	no
ATP10A	-0.047708548	-0.858405075	0.391305369	0.576193469	no
PTK2B	0.047684274	0.857967321	0.391546691	0.576509313	no
GABPB1	0.047673965	0.857781413	0.391649204	0.576586653	no
MYL2	0.047673596	0.857774755	0.391652876	0.576586653	no
CCDC171	-0.047665373	-0.857626458	0.391734662	0.576667554	no
PIWIL1	-0.04765483	-0.857436345	0.391839525	0.576782413	no
LOC401497	0.047646767	0.857290938	0.39191974	0.576860978	no
KIF20A	0.047641187	0.857190311	0.391975258	0.576903183	no
DHX37	0.047630043	0.856989334	0.392086155	0.577026882	no
CRYBB3	-0.047617231	-0.856758285	0.39221367	0.577175019	no
COPS7A	-0.047604713	-0.856532552	0.392338275	0.577273768	no
PRIM1	-0.047600879	-0.856463418	0.392376442	0.577273768	no
LOC100505658	-0.047600263	-0.856452299	0.392382581	0.577273768	no
RPL23A	-0.047599697	-0.856442086	0.392388219	0.577273768	no
THADA	-0.047596101	-0.856377253	0.392424014	0.577286911	no
OR1K1	-0.047553017	-0.855600287	0.392853142	0.577878636	no
GSTM2	-0.047548493	-0.855518716	0.392898211	0.577905376	no
LOC729609	-0.047542103	-0.855403481	0.392961886	0.577953458	no
POC1A	-0.047539815	-0.855362221	0.392984686	0.577953458	no
C1orf114	0.047531302	0.855208696	0.393069531	0.578038681	no
LINC00534	-0.047478699	-0.854260107	0.393594011	0.578751161	no
SHOX	-0.047476198	-0.854214993	0.393618965	0.578751161	no
TAS2R43	-0.047474609	-0.854186348	0.393634811	0.578751161	no
PNOC	-0.047465699	-0.854025677	0.393723694	0.578823212	no
CXorf28	0.047462144	0.853961558	0.393759168	0.578823212	no
DLGAP4	-0.047461598	-0.853951712	0.393764616	0.578823212	no
NAP1L4	-0.047457692	-0.853881276	0.393803587	0.578840907	no
C11orf16	-0.047434932	-0.853470842	0.394030724	0.579108363	no
AKAP17A	0.047432017	0.853418277	0.39405982	0.579108363	no
ABI3BP	0.047431359	0.853406414	0.394066386	0.579108363	no
SLC39A5	-0.047419604	-0.85319444	0.394183732	0.579214943	no
MACC1-AS1	-0.047418694	-0.853178036	0.394192814	0.579214943	no
WDR43	0.047414273	0.8530983	0.39423696	0.579240208	no
CHST10	-0.047408756	-0.852998812	0.394292047	0.579281541	no
C6orf47	0.047400228	0.852845026	0.394377208	0.57936705	no
EXOC2	-0.047397033	-0.852787416	0.394409113	0.579374316	no
CDRT7	-0.047379007	-0.852462345	0.394589171	0.579599198	no
SDK1	0.047367727	0.852258943	0.394701861	0.579725102	no
XKR9	-0.047344403	-0.85183835	0.394934943	0.580027805	no
TRIM77	-0.047332798	-0.851629074	0.39505095	0.580158533	no
MRPL16	-0.047326815	-0.851521185	0.395110764	0.580206725	no
ORC3	-0.047312548	-0.851263914	0.395253417	0.580376549	no

SCARNA22	-0.047304354	-0.851116148	0.395335365	0.58045722	no
C1orf228	0.047294355	0.850935839	0.395435375	0.580564397	no
GRK6	0.047288392	0.850828321	0.395495018	0.580612299	no
RNU6-53	-0.047284001	-0.850749139	0.395538946	0.580637123	no
JOSD1	0.047273764	0.850564531	0.395641372	0.580747813	no
LOC728342	-0.047260753	-0.850329917	0.395771567	0.58081452	no
ZNF674	-0.047259825	-0.850313175	0.395780859	0.58081452	no
SSU72	0.047258936	0.850297149	0.395789754	0.58081452	no
CLRN1	-0.04725842	-0.850287838	0.395794921	0.58081452	no
TWIST2	0.047230097	0.849777113	0.396078439	0.581165108	no
CAP2	-0.047229151	-0.849760051	0.396087913	0.581165108	no
ARHGEF3-AS1	-0.047225443	-0.849693185	0.396125041	0.581179906	no
LOC649395	0.047221003	0.84961312	0.396169502	0.581205459	no
ZFYVE26	0.047200295	0.849239708	0.396376901	0.581470032	no
LOC284551	0.047193222	0.849112161	0.396447757	0.581534281	no
TRIML1	-0.047189349	-0.849042326	0.396486556	0.581551499	no
MRPL40	-0.04716406	-0.848586293	0.396739975	0.581883491	no
ZDHHC16	-0.047159212	-0.84849887	0.396788567	0.581915047	no
LOC284865	-0.047156208	-0.848444717	0.396818669	0.581919482	no
MIR221	0.047152091	0.848370464	0.396859946	0.581940304	no
OR10H1	-0.04714673	-0.848273799	0.396913685	0.581979396	no
PPP1R12C	0.047135432	0.848070063	0.397026964	0.582071297	no
RPS28	-0.047135076	-0.848063648	0.397030531	0.582071297	no
SF3B3	-0.047113524	-0.847675016	0.397246668	0.582327536	no
LRRC38	-0.047109485	-0.847602192	0.397287177	0.582327536	no
OR4F5	-0.047107732	-0.84757058	0.397304762	0.582327536	no
OAZ3	0.047105732	0.847534513	0.397324826	0.582327536	no
FRMD3	0.04710414	0.847505812	0.397340793	0.582327536	no
FAM81B	0.047100959	0.847448439	0.397372712	0.582334602	no
GPR89C	-0.047096414	-0.847366483	0.397418309	0.582361713	no
IFNL1	-0.04708058	-0.847080972	0.397577183	0.582554799	no
ATIC	-0.047076875	-0.84701415	0.397614372	0.582569571	no
OR7A5	-0.047070417	-0.846897714	0.397679178	0.582616546	no
ZNF30	-0.047068278	-0.846859133	0.397700653	0.582616546	no
LOC339874	0.047060091	0.846711514	0.397782826	0.582660728	no
LMNB2	-0.047059871	-0.846707545	0.397785036	0.582660728	no
DLX5	-0.04704906	-0.846512603	0.397893569	0.582736596	no
IFT52	0.047047168	0.846478479	0.397912569	0.582736596	no
NIT1	-0.047046609	-0.846468407	0.397918177	0.582736596	no
SLC10A6	0.047039432	0.846338979	0.397990248	0.582802427	no
SPRY4	0.04703397	0.84624049	0.398045096	0.58284303	no
RNU6-33	-0.047026107	-0.846098707	0.398124062	0.582918941	no
LINC00351	0.047018134	0.845954944	0.398204141	0.58299647	no
DEFB119	-0.047004189	-0.845703475	0.398344237	0.583161852	no
TRIM28	-0.046994061	-0.845520848	0.398445999	0.583232568	no
CST4	0.046989947	0.845446666	0.398487339	0.583232568	no
AKAP4	0.046988918	0.845428125	0.398497672	0.583232568	no
TNNI1	-0.046988578	-0.845421983	0.398501095	0.583232568	no
CLPSL1	-0.046984879	-0.845355282	0.398538269	0.583247254	no
CPSF1	-0.046980344	-0.845273511	0.398583844	0.583274233	no
FBXO38	-0.046959049	-0.844889518	0.398797907	0.58354775	no

ATF7	-0.04693767	-0.84450403	0.399012872	0.583770883	no
ALLC	-0.046936709	-0.844486689	0.399022544	0.583770883	no
SHMT2	-0.04693578	-0.844469938	0.399031887	0.583770883	no
LINC00839	-0.046932652	-0.844413532	0.399063348	0.583777171	no
NPY6R	-0.046916314	-0.844118934	0.399227689	0.583913805	no
TSPAN19	0.046914668	0.844089254	0.399244248	0.583913805	no
LOC100133461	-0.046912876	-0.844056944	0.399262276	0.583913805	no
GABPA	-0.046910464	-0.844013451	0.399286543	0.583913805	no
BCL6	0.046909862	0.844002594	0.399292601	0.583913805	no
MICAL1	-0.046906912	-0.843949395	0.399322285	0.583917481	no
CPS1	-0.04689423	-0.843720724	0.399449895	0.584064341	no
C9orf72	0.046867811	0.843244345	0.399715818	0.584404733	no
SCAMP5	0.046865674	0.843205814	0.399737332	0.584404733	no
LEAP2	-0.046859792	-0.843099761	0.399796549	0.584404733	no
NAA40	-0.04685622	-0.843035342	0.399832521	0.584404733	no
HSPA1B	0.046854045	0.842996133	0.399854417	0.584404733	no
SSR2	0.046848804	0.842901629	0.399907195	0.584404733	no
ZNF668	-0.046848285	-0.842892258	0.399912428	0.584404733	no
LOC100506035	-0.046847709	-0.842881885	0.399918222	0.584404733	no
ACTA1	-0.046846795	-0.842865397	0.39992743	0.584404733	no
MYOT	-0.046799016	-0.842003875	0.400408773	0.585068326	no
TPPP3	0.046791871	0.841875044	0.400480783	0.585133762	no
GPR87	0.046784633	0.841744534	0.400553738	0.585164977	no
UBXN2A	0.046784349	0.841739408	0.400556604	0.585164977	no
BANCR	-0.046767609	-0.841437564	0.400725368	0.585371729	no
PBX3	0.046764282	0.841377569	0.400758917	0.585380947	no
UGT2B4	-0.046761205	-0.841322095	0.400789939	0.585386474	no
LOC401109	-0.04674638	-0.841054767	0.400939456	0.585531627	no
NCKAP5L	-0.046745948	-0.841046982	0.40094381	0.585531627	no
HRAS	-0.046722107	-0.840617104	0.401184313	0.58584091	no
PEBP4	-0.04671955	-0.840570999	0.401210112	0.58584091	no
CORO7	-0.046706806	-0.840341207	0.401338713	0.585988877	no
SLC6A3	0.046686424	0.839973696	0.40154444	0.586249427	no
EBF1	0.046683281	0.839917022	0.401576171	0.586255926	no
LOC442028	0.046677727	0.83981688	0.401632243	0.586297957	no
LINC00200	-0.046659833	-0.839494239	0.401812928	0.58652188	no
LOC100506746	0.046647803	0.839277323	0.401934433	0.586643463	no
SAG	-0.046646181	-0.839248078	0.401950816	0.586643463	no
AP3D1	-0.046638732	-0.839113764	0.402026065	0.586713443	no
SFT2D3	-0.046628627	-0.838931559	0.402128158	0.586822587	no
MYH13	-0.046609805	-0.838592169	0.402318366	0.587060293	no
DDX18	-0.046596301	-0.838348685	0.402454858	0.587182103	no
ZNF616	-0.04659614	-0.838345778	0.402456488	0.587182103	no
LOH12CR1	-0.046583041	-0.838109603	0.402588909	0.587335432	no
CABYR	-0.046577605	-0.838011591	0.402643871	0.587375742	no
HPVC1	0.046551677	0.837544075	0.402906102	0.58771839	no
SIRPA	-0.046530149	-0.837155907	0.403123906	0.58796946	no
FLJ43663	-0.046527731	-0.837112313	0.403148371	0.58796946	no
TAAR5	-0.046526552	-0.837091061	0.403160298	0.58796946	no
PDX1	-0.046515862	-0.836898304	0.403268487	0.588063906	no
MEN1	-0.046514745	-0.836878178	0.403279784	0.588063906	no

PUS1	-0.04650406	-0.836685515	0.40338794	0.58818171	no
LSM14A	-0.04649994	-0.836611225	0.403429649	0.588202618	no
SLC7A6	0.046486754	0.836373475	0.403563147	0.5883428	no
ATXN8OS	-0.046480809	-0.836266285	0.403623344	0.5883428	no
DICER1	-0.046479709	-0.836246456	0.403634481	0.5883428	no
HNRNPKP3	0.046479628	0.836244996	0.403635301	0.5883428	no
RHBDD1	0.046473537	0.836135169	0.403696985	0.588392805	no
DUXA	-0.046464846	-0.83597846	0.403785011	0.588481192	no
KDM4C	0.046461264	0.835913867	0.403821296	0.588482222	no
FBP2	-0.04645937	-0.835879716	0.403840482	0.588482222	no
PDE7A	0.046454187	0.835786267	0.403892984	0.588518823	no
PCDHB6	-0.046437388	-0.835483378	0.40406318	0.588713835	no
KIR2DS4	0.04643557	0.835450593	0.404081605	0.588713835	no
MED26	-0.046426938	-0.835294962	0.404169075	0.588801355	no
HCRT	-0.046399923	-0.834807869	0.404442912	0.589133855	no
KRTAP4-3	-0.046395344	-0.834725302	0.404489341	0.589133855	no
SLC38A9	0.046394579	0.834711513	0.404497095	0.589133855	no
KAL1	-0.046393606	-0.834693964	0.404506964	0.589133855	no
LINC00272	0.046364856	0.834175596	0.404798533	0.589518552	no
SMG7	-0.046359642	-0.834081586	0.404851425	0.589555628	no
SYNE3	-0.046352075	-0.833945155	0.404928191	0.589627465	no
ABCB11	0.046343079	0.833782943	0.405019475	0.589720429	no
TBCK	-0.046338657	-0.833703219	0.405064343	0.589745803	no
RHOV	-0.046335502	-0.833646336	0.405096359	0.589752462	no
C12orf71	-0.046319925	-0.833365474	0.405254459	0.589942667	no
DCTPP1	-0.046316199	-0.833298293	0.405292282	0.589957764	no
SLCO1B3	0.046303633	0.833071726	0.405419853	0.590012327	no
GDEP	-0.046301975	-0.833041842	0.405436682	0.590012327	no
C8orf31	-0.04630191	-0.833040669	0.405437342	0.590012327	no
ZCCHC5	0.046299991	0.833006061	0.405456831	0.590012327	no
AGBL5	0.046298986	0.832987942	0.405467035	0.590012327	no
HKR1	-0.046292231	-0.83286615	0.405535626	0.590061398	no
CHEK1	0.046290257	0.832830561	0.40555567	0.590061398	no
DNAJB8-AS1	0.046262255	0.832325675	0.405840097	0.590435251	no
KRT35	-0.04625577	-0.832208753	0.405905982	0.590491129	no
ALDH7A1	-0.046250447	-0.832112778	0.405960068	0.590529837	no
SMAD7	-0.046237705	-0.83188305	0.406089547	0.590678203	no
METTL8	0.046230569	0.831754385	0.406162076	0.590743717	no
DKK4	0.046222953	0.831617071	0.406239489	0.590816013	no
POLR2J	-0.04622027	-0.831568692	0.406266765	0.590816013	no
CIB3	-0.046204477	-0.831283948	0.406427329	0.590987131	no
ADAM5	0.046203287	0.83126249	0.40643943	0.590987131	no
UBE2I	0.046177725	0.830801617	0.406699396	0.591266358	no
MEX3C	-0.046177568	-0.830798788	0.406700992	0.591266358	no
C8orf86	-0.04617629	-0.830775733	0.406714	0.591266358	no
SPTA1	0.046171586	0.830690928	0.406761848	0.59127871	no
RPS6KC1	-0.046170045	-0.830663149	0.406777522	0.59127871	no
RRP1B	0.046160072	0.830483339	0.406878987	0.591386197	no
THOC2	-0.046151247	-0.830324218	0.406968789	0.591446901	no
LOC284798	-0.046150559	-0.830311808	0.406975793	0.591446901	no
ANKRD30BP2	-0.046130193	-0.829944619	0.407183071	0.591708119	no

KRT77	-0.046119326	-0.829748685	0.4072937	0.591828865	no
SDCBP2	-0.046104299	-0.829477755	0.407446705	0.592011165	no
LOC100652770	0.046097539	0.829355875	0.407515547	0.592071161	no
HES3	-0.046089291	-0.829207172	0.407599548	0.592153173	no
C17orf53	-0.046074964	-0.828948855	0.407745494	0.59232516	no
ADAD1	-0.046071845	-0.828892621	0.40777727	0.592331282	no
NCOA4	-0.046063172	-0.828736256	0.407865634	0.592417452	no
MIB2	-0.046059187	-0.828664401	0.407906244	0.592417452	no
RBMXL2	-0.046057906	-0.828641319	0.40791929	0.592417452	no
NPY5R	0.046044101	0.828392405	0.408059989	0.592581744	no
SNORA80B	-0.046035926	-0.828245024	0.40814331	0.592626515	no
DSC1	0.046035665	0.82824032	0.40814597	0.592626515	no
GOLGA8EP	-0.046029033	-0.828120738	0.408213583	0.59265948	no
DIDO1	-0.046028028	-0.82810262	0.408223828	0.59265948	no
MST1R	-0.046024996	-0.828047966	0.408254732	0.59266431	no
SLC30A8	-0.046020736	-0.827971146	0.408298173	0.592687338	no
KIR2DS5	-0.046003575	-0.827661758	0.408473157	0.592901194	no
NSD1	-0.046000877	-0.827613113	0.408500674	0.592901194	no
POU4F1	-0.045987273	-0.82736784	0.408639433	0.593062537	no
ARL6IP1	-0.045980335	-0.827242757	0.408710207	0.593086294	no
KIF25	-0.045980258	-0.827241363	0.408710996	0.593086294	no
GPR107	0.045971869	0.827090118	0.408796584	0.59317044	no
PITPNC1	-0.045968494	-0.827029258	0.408831027	0.593175228	no
PIK3R4	-0.045966136	-0.826986747	0.408855086	0.593175228	no
IGSF5	0.04595877	0.826853951	0.408930248	0.593244226	no
SNORA55	-0.045929871	-0.826332922	0.409225231	0.593598512	no
LHFPL3-AS1	0.045926417	0.826270646	0.409260498	0.593598512	no
ZSCAN22	-0.045925445	-0.826253115	0.409270426	0.593598512	no
G3BP1	-0.045924023	-0.826227477	0.409284945	0.593598512	no
PRKCQ-AS1	-0.045920289	-0.826160165	0.409323066	0.59361374	no
MED10	0.045916131	0.82608519	0.40936553	0.593635264	no
UHRF1BP1	-0.045909183	-0.825959938	0.409436476	0.593698084	no
FBXL2	-0.045904697	-0.825879056	0.409482293	0.59372446	no
CCDC144NL	-0.04590172	-0.825825377	0.409512702	0.593728495	no
CYP2U1	0.04589505	0.825705129	0.409580827	0.593787207	no
SEC22C	-0.045884799	-0.825520309	0.409685549	0.593898963	no
C11orf85	0.045861366	0.825097838	0.409924987	0.594205982	no
BNC1	0.045853984	0.824964736	0.41000044	0.594250834	no
RFX5	0.045852928	0.824945701	0.410011231	0.594250834	no
METTL4	0.045837074	0.824659867	0.410173297	0.594445636	no
DNMT3L	0.045819581	0.824344497	0.410352154	0.594664743	no
COPB2	0.045816616	0.824291029	0.410382482	0.594668595	no
MEGF6	0.045807808	0.824132232	0.410472563	0.594722624	no
ARFGEF1	-0.045807558	-0.824127734	0.410475115	0.594722624	no
LINC00312	0.045797654	0.823949174	0.41057642	0.594829301	no
LOC727915	-0.045794322	-0.823889102	0.410610505	0.594838583	no
OR3A4P	-0.045785767	-0.823734863	0.410698029	0.594888612	no
EXOC3L4	0.045785535	0.823730683	0.410700402	0.594888612	no
RSG1	-0.045775892	-0.823556828	0.41079907	0.594991429	no
OTX2-AS1	-0.045766062	-0.823379595	0.410899671	0.59509703	no
XIST	-0.045756484	-0.823206931	0.410997692	0.595165503	no

SPRR3	-0.045754191	-0.823165578	0.41102117	0.595165503	no
UBE2M	-0.045753325	-0.823149972	0.411030031	0.595165503	no
CT64	-0.045737274	-0.822860582	0.411194356	0.595312965	no
PDCD4	0.045737242	0.822860023	0.411194673	0.595312965	no
SLC26A5	-0.045734516	-0.822810866	0.41122259	0.595312965	no
LOC647859	-0.045732555	-0.822775505	0.411242673	0.595312965	no
UBXN6	0.045720586	0.822559721	0.411365237	0.595450279	no
ZCCHC11	-0.045704351	-0.82226704	0.411531511	0.595650843	no
HIBADH	0.045695109	0.822100405	0.411626197	0.595747767	no
ZNF446	-0.045679877	-0.821825797	0.411782262	0.595933508	no
OR5K2	-0.045672904	-0.821700077	0.411853723	0.595977155	no
CEP63	-0.045671522	-0.821675165	0.411867885	0.595977155	no
RASA4CP	-0.045667366	-0.821600238	0.411910478	0.595998659	no
ITGAE	0.045658953	0.821448574	0.411996703	0.596078554	no
LRRC72	-0.045653991	-0.821359121	0.412047565	0.596078554	no
ASPHD2	-0.045653861	-0.821356766	0.412048904	0.596078554	no
FFAR1	-0.045639375	-0.821095608	0.412197415	0.596253258	no
ALB	-0.04562004	-0.82074703	0.412395689	0.596499916	no
LYPD6	0.045615001	0.820656181	0.412447374	0.596534526	no
SYNJ2-IT1	-0.045600251	-0.820390256	0.412598684	0.596713211	no
LOC100288814	-0.045568872	-0.819824546	0.412920679	0.597138707	no
FXYD3	-0.045560427	-0.819672293	0.413007365	0.597223879	no
FLJ43879	0.045557447	0.819618573	0.413037954	0.597227927	no
TAZ	0.04555285	0.819535709	0.41308514	0.59724351	no
GRIN2A	-0.045550983	-0.819502041	0.413104312	0.59724351	no
USP17L6P	-0.045533942	-0.819194819	0.413279289	0.597456289	no
DGCR8	-0.045518394	-0.818914513	0.413438973	0.597646935	no
FAM124A	-0.045511099	-0.81878301	0.413513901	0.597677536	no
SLC4A11	0.045510918	0.818779741	0.413515764	0.597677536	no
SAMD12-AS1	-0.045500233	-0.818587103	0.413625539	0.597765871	no
TBC1D3B	0.045499554	0.818574871	0.41363251	0.597765871	no
HBP1	-0.045490729	-0.818415774	0.413723186	0.597848515	no
GSTA5	0.045486718	0.818343467	0.4137644	0.597848515	no
HCG4	0.045485867	0.818328114	0.413773152	0.597848515	no
CGB5	-0.04548275	-0.81827192	0.413805184	0.597854603	no
TDGF1P3	-0.045478501	-0.818195328	0.413848847	0.597877492	no
SMC4	-0.045466088	-0.817971541	0.413976435	0.598021616	no
SLC25A6	-0.045452513	-0.817726814	0.414115989	0.598144757	no
ANKRD31	-0.045452381	-0.817724438	0.414117344	0.598144757	no
CCDC106	-0.045449072	-0.817664772	0.414151372	0.598153706	no
SERPINE3	-0.045442104	-0.817539155	0.414223019	0.598216982	no
SPATA24	-0.045438773	-0.817479103	0.414257273	0.59822625	no
CATSPERD	-0.045431915	-0.817355467	0.4143278	0.5982286	no
FZD2	0.045427487	0.817275634	0.414373345	0.5982286	no
KRTAP10-3	-0.045426204	-0.817252513	0.414386535	0.5982286	no
TAS2R50	-0.045425531	-0.817240377	0.414393459	0.5982286	no
GSTM2P1	-0.045425082	-0.817232275	0.414398082	0.5982286	no
LINC00159	0.04541776	0.817100288	0.414473389	0.598297125	no
FOXJ2	-0.045413145	-0.817017086	0.414520865	0.598325469	no
FAM27C	0.045395274	0.816694899	0.414704739	0.598499604	no
CTDP1	0.04539473	0.816685107	0.414710328	0.598499604	no

DKK2	0.0453933	0.81665931	0.414725053	0.598499604	no
HAS1	0.045387231	0.816549905	0.414787504	0.598549536	no
KLHL10	0.045383801	0.816488065	0.414822806	0.598560288	no
ARMCX5	0.045379832	0.816416515	0.414863653	0.598579038	no
COL4A5	0.045374286	0.816316536	0.414920735	0.598621209	no
VCPIP1	-0.045362402	-0.816102301	0.415043065	0.598757502	no
CSH1	-0.045349429	-0.815868427	0.415176634	0.59890999	no
HAX1	-0.045342961	-0.815751824	0.415243237	0.598965864	no
SCAF8	-0.045337201	-0.81564798	0.415302557	0.599011225	no
D21S2088E	0.045332838	0.815569317	0.415347496	0.599029556	no
LOC645752	0.045330554	0.815528154	0.415371013	0.599029556	no
CCDC36	0.045317156	0.815286604	0.415509031	0.599188391	no
MIA-RAB4B	-0.045311096	-0.815177361	0.415571459	0.599211104	no
EIF1	0.045310214	0.815161462	0.415580546	0.599211104	no
NNAT	-0.045299302	-0.814964749	0.415692975	0.599333003	no
LINC00366	-0.045296282	-0.814910309	0.415724094	0.59933766	no
MIIP	-0.045292526	-0.814842593	0.415762802	0.599353259	no
LINC00330	-0.045275788	-0.814540846	0.415935315	0.599561732	no
HPCAL1	0.04525383	0.814144998	0.416161693	0.599847816	no
FAM174A	-0.045239945	-0.813894684	0.41630488	0.600013222	no
TIMP3	-0.045237288	-0.813846778	0.416332287	0.600013222	no
STRBP	-0.045213771	-0.813422831	0.416574873	0.600291482	no
RSPH1	0.045213155	0.813411728	0.416581227	0.600291482	no
ZNF117	-0.045210287	-0.81336003	0.416610815	0.600293867	no
FLJ42351	0.045182785	0.812864225	0.41689464	0.600625504	no
AKIRIN2	-0.045182569	-0.81286033	0.41689687	0.600625504	no
SNTN	-0.045172709	-0.812682582	0.416998651	0.600726451	no
HAO1	0.04516949	0.812624554	0.417031881	0.600726451	no
CCDC42	0.045167658	0.812591527	0.417050795	0.600726451	no
UBE2Q1	0.045160327	0.812459365	0.417126488	0.600795212	no
AWAT1	-0.045138714	-0.812069754	0.417349676	0.601076389	no
NVL	0.045126575	0.811850913	0.417475069	0.601216693	no
COL9A2	-0.045105111	-0.811463981	0.417696831	0.601495752	no
HMOX2	-0.045101686	-0.811402238	0.417732225	0.601506415	no
RABAC1	0.045065871	0.810756592	0.418102438	0.601977857	no
BMX	0.045064593	0.810733553	0.418115652	0.601977857	no
KRTAP10-6	-0.045044876	-0.810378125	0.418319541	0.602231059	no
WDR66	0.04499649	0.809505867	0.418820155	0.602911377	no
OGN	0.04498597	0.809316224	0.418929043	0.603027733	no
OS9	-0.044979858	-0.809206045	0.418992313	0.603051726	no
OLA1	-0.044978939	-0.809189469	0.419001832	0.603051726	no
PRAMEF13	-0.044976026	-0.809136958	0.419031988	0.603054742	no
TTC24	0.044971255	0.809050963	0.419081378	0.603085435	no
OXCT2	0.044958791	0.808826268	0.419210441	0.603230773	no
HTRA1	0.044955242	0.808762289	0.419247195	0.603243268	no
LOC338817	-0.044947683	-0.808626025	0.41932548	0.603315517	no
C1orf189	0.044931534	0.808334909	0.419492758	0.603482908	no
UBA2	-0.044931029	-0.808325815	0.419497983	0.603482908	no
SCARNA18	-0.044928079	-0.808272623	0.419528553	0.603486488	no
SNHG5	-0.044919736	-0.80812224	0.419614984	0.603566629	no
LOC144742	0.04491728	0.808077962	0.419640434	0.603566629	no

RPS6	-0.044893015	-0.807640545	0.419891904	0.603887901	no
OTOS	-0.044884923	-0.807494664	0.419975791	0.603968129	no
KIAA1324L	-0.044880576	-0.807416314	0.420020848	0.603988335	no
SLC39A11	-0.044878146	-0.807372494	0.42004605	0.603988335	no
PHLDA1	-0.044850033	-0.806865723	0.420337566	0.604367072	no
CACNG5	0.044841205	0.806706582	0.420429135	0.604458291	no
BAZ1B	0.044828716	0.806481449	0.420558695	0.604604115	no
PRAMEF17	-0.044821396	-0.806349496	0.420634644	0.604672851	no
CCDC88A	-0.04481379	-0.806212391	0.420713565	0.604745852	no
NYX	-0.044806071	-0.806073233	0.420793678	0.604820555	no
KTN1	0.044801805	0.805996337	0.42083795	0.604843736	no
SNORA29	0.044796216	0.805895591	0.420895959	0.604886656	no
C17orf105	0.044790981	0.805801223	0.420950299	0.604924298	no
CPXM1	0.044783038	0.805658029	0.421032762	0.60497262	no
TMEM106B	-0.044782294	-0.805644618	0.421040486	0.60497262	no
TRIM49B	-0.044779607	-0.805596194	0.421068375	0.60497262	no
TNK1	0.044769354	0.805411368	0.421174834	0.605085123	no
IZUMO4	-0.044762518	-0.805288138	0.421245822	0.605139717	no
DCT	-0.044760272	-0.805247645	0.42126915	0.605139717	no
ZDHHC8P1	-0.044747968	-0.80502585	0.42139694	0.605282826	no
LCE3A	-0.04471774	-0.804480959	0.421710983	0.605683457	no
SPINK13	0.044713972	0.804413037	0.421750138	0.605683457	no
PPHLN1	0.044712984	0.804395223	0.421760408	0.605683457	no
SIX3	-0.044701569	-0.804189443	0.421879052	0.605813357	no
CXCL17	0.044689177	0.803966079	0.422007856	0.605957829	no
SMCR9	0.044683369	0.80386138	0.422068239	0.606004043	no
KEL	-0.044670971	-0.803637886	0.422197151	0.606114837	no
EDDM3A	0.044669243	0.803606731	0.422215124	0.606114837	no
WDR49	0.044667811	0.803580919	0.422230014	0.606114837	no
ATM	-0.044664969	-0.803529687	0.42225957	0.606116779	no
LCN8	-0.044659902	-0.803438352	0.422312264	0.606145293	no
TF	-0.044657634	-0.803397482	0.422335844	0.606145293	no
MAEA	0.044650754	0.803273461	0.422407404	0.606207513	no
INSIG1	-0.044643433	-0.803141482	0.422483563	0.606276326	no
SULT2B1	-0.044639896	-0.803077733	0.422520353	0.606288636	no
MGARP	0.044610064	0.802539983	0.422830766	0.606642256	no
PTPDC1	-0.044609731	-0.802533975	0.422834234	0.606642256	no
IGBP1P1	-0.044608075	-0.802504119	0.422851472	0.606642256	no
KBTBD2	0.044588568	0.802152489	0.423054527	0.606893054	no
TBC1D23	0.044583353	0.802058476	0.423108826	0.606902688	no
40787	0.044582498	0.802043073	0.423117723	0.606902688	no
DYX1C1-CCPG1	-0.044561676	-0.80166773	0.423334552	0.607173176	no
LINC00698	-0.04455101	-0.801475481	0.423445638	0.607291972	no
LINC00271	0.044545347	0.801373384	0.423504638	0.607335258	no
NMS	-0.044542687	-0.80132545	0.42353234	0.607335258	no
JAM3	0.044537084	0.801224443	0.423590717	0.607365222	no
IFT43	-0.044535256	-0.801191499	0.423609758	0.607365222	no
LOC100505718	0.044528626	0.80107198	0.423678842	0.607423749	no
CHAC2	-0.044507155	-0.800684947	0.423902601	0.607704009	no
RAB9BP1	0.044500098	0.800557751	0.423976153	0.60776891	no
PDE3A	0.04448636	0.800310113	0.424119373	0.607933665	no

TAF2	-0.044478043	-0.800160192	0.424206093	0.608017417	no
CAB39	0.044470345	0.800021431	0.424286367	0.608091918	no
C1QTNF3-AMAC	0.044466213	0.799946935	0.424329467	0.608113135	no
TMED10P1	-0.044459264	-0.79982168	0.424401939	0.60817644	no
ZRANB2	0.044420959	0.799131221	0.424801568	0.608708528	no
CDK3	-0.044417252	-0.799064397	0.424840257	0.608723379	no
EIF5A2	0.04439944	0.798743315	0.42502618	0.608949176	no
NDUFS5	-0.044394457	-0.798653501	0.425078196	0.608983102	no
LAGE3	0.044384073	0.798466327	0.425186609	0.609097815	no
CLIC6	0.044357978	0.797995961	0.425459123	0.609447578	no
RSPH6A	-0.044354416	-0.797931754	0.42549633	0.609460252	no
TBX10	-0.04433688	-0.797615662	0.425679529	0.609682024	no
CCDC169-SOHL	-0.044319284	-0.79729848	0.425863407	0.609904737	no
ZCWPW1	-0.044301873	-0.796984648	0.426045388	0.610117875	no
PPP2R3A	-0.044299305	-0.796938357	0.426072235	0.610117875	no
ADCY6	-0.044296722	-0.796891794	0.42609924	0.610117875	no
SCARF2	0.044294183	0.796846022	0.426125788	0.610117875	no
HAVCR1	-0.04429095	-0.796787753	0.426159585	0.610125617	no
FAM86FP	0.044285916	0.796697019	0.426212216	0.61016032	no
SNORA26	-0.044282828	-0.796641346	0.426244511	0.610165909	no
PRSS38	-0.044274603	-0.796493099	0.426330514	0.610248373	no
PREP	-0.044267929	-0.796372795	0.426400314	0.610307635	no
ARL15	0.044249266	0.796036401	0.426595524	0.610529576	no
USP17L8	-0.044247592	-0.796006215	0.426613044	0.610529576	no
IQCF5	-0.044243099	-0.795925235	0.426660045	0.610529576	no
PLEKHB1	-0.044242242	-0.795909791	0.42666901	0.610529576	no
GALE	0.044225608	0.795609958	0.426843065	0.610732843	no
SFTA2	0.044223235	0.795567184	0.4268679	0.610732843	no
RAB39A	0.044218587	0.795483405	0.426916543	0.610761778	no
P2RY4	-0.044214546	-0.795410561	0.42695884	0.610771447	no
KRTAP10-7	-0.044211879	-0.795362485	0.426986757	0.610771447	no
LINC00707	0.044206608	0.795267481	0.427041927	0.610771447	no
PENK	-0.044205437	-0.795246373	0.427054185	0.610771447	no
FAM220A	0.04420298	0.795202082	0.427079908	0.610771447	no
C1orf110	0.044201651	0.795178125	0.427093821	0.610771447	no
CC2D1A	0.04418574	0.794891333	0.427260402	0.610969014	no
LYZL2	-0.044178451	-0.794759955	0.427336725	0.611037496	no
NEBL-AS1	0.04417466	0.794691614	0.42737643	0.611053613	no
RNF133	0.044167685	0.794565899	0.427449475	0.611108394	no
CLDN20	-0.044165571	-0.794527795	0.427471615	0.611108394	no
DZANK1-AS1	0.044158133	0.794393712	0.427549532	0.611179127	no
UFSP2	-0.044153934	-0.794318041	0.427593509	0.611201337	no
RARS2	0.044134126	0.793961003	0.42780104	0.611457313	no
LOC100505782	0.044114887	0.793614212	0.428002671	0.611704822	no
SCFD2	-0.044089593	-0.793158306	0.428267828	0.612025828	no
THAP1	0.044086494	0.793102437	0.428300329	0.612025828	no
ACACB	0.044085313	0.793081154	0.42831271	0.612025828	no
SNORA2A	-0.044078126	-0.792951606	0.428388078	0.612060048	no
AQP2	-0.044077598	-0.792942084	0.428393618	0.612060048	no
PDE1C	0.044072062	0.792842305	0.428451673	0.6121023	no
ANKRD66	0.044046938	0.792389461	0.42871521	0.612405228	no

CGB2	-0.044046415	-0.792380023	0.428720704	0.612405228	no
SNORA24	0.044028629	0.792059443	0.428907327	0.612615144	no
DEFB122	-0.044026976	-0.792029657	0.42892467	0.612615144	no
ASB14	-0.044022102	-0.791941805	0.428975822	0.612647487	no
HMGN2	-0.044001939	-0.791578374	0.429187468	0.612909022	no
PCDHGA9	0.04398777	0.791322974	0.429336238	0.613041874	no
MED20	-0.043987645	-0.791320725	0.429337548	0.613041874	no
OR2T4	-0.04396787	-0.790964298	0.429545217	0.613297653	no
SCUBE3	-0.043954116	-0.790716378	0.4296897	0.613463187	no
SYNGR4	-0.043943169	-0.790519064	0.429804712	0.613586625	no
SLC22A2	-0.04393898	-0.790443558	0.429848727	0.613608701	no
U2AF2	-0.043933135	-0.790338212	0.429910143	0.613655611	no
RNASE9	-0.043926297	-0.790214966	0.429982001	0.613717419	no
LMOD3	0.043919934	0.790100282	0.430048872	0.613768236	no
ADCK4	0.043907705	0.78987986	0.430177416	0.613768236	no
FSCB	-0.043905279	-0.789836131	0.43020292	0.613768236	no
PDHX	-0.043903429	-0.789802781	0.430222372	0.613768236	no
MAP6	-0.043902767	-0.789790851	0.43022933	0.613768236	no
GPI	0.043897862	0.789702442	0.430280898	0.613768236	no
AP2A2	0.04389663	0.789680245	0.430293846	0.613768236	no
LINC00523	0.043893511	0.789624012	0.430326648	0.613768236	no
ACAD11	-0.043890553	-0.789570703	0.430357746	0.613768236	no
CR2	0.043889451	0.789550843	0.430369331	0.613768236	no
LOC100996291	0.043888121	0.789526867	0.430383319	0.613768236	no
DHRS12	-0.043888087	-0.78952625	0.430383679	0.613768236	no
TMPRSS13	0.043887592	0.789517342	0.430388875	0.613768236	no
POLL	-0.043880188	-0.789383877	0.430466742	0.613838547	no
USP1	-0.043875684	-0.789302704	0.430514104	0.613865354	no
KCNF1	-0.04385788	-0.7889818	0.430701372	0.614091633	no
GTPBP8	0.043847514	0.788794961	0.430810426	0.614206373	no
PCDHGA6	-0.043821312	-0.788322692	0.431086153	0.614534151	no
FECH	0.043819439	0.788288928	0.431105869	0.614534151	no
DSPP	-0.043817515	-0.788254254	0.431126117	0.614534151	no
PSMB11	-0.043807267	-0.788069543	0.431233992	0.614647149	no
MRRF	-0.043798631	-0.787913883	0.431324911	0.614735969	no
GATA2	0.04379135	0.787782657	0.431401569	0.614790007	no
CHRNA9	0.043789596	0.787751037	0.431420041	0.614790007	no
KRBOX4	-0.043762647	-0.787265304	0.431703863	0.615147911	no
GSTA2	0.043760313	0.787223248	0.431728442	0.615147911	no
TAF11	0.043742227	0.786897257	0.431918992	0.615378616	no
PFDN1	-0.043729932	-0.786675665	0.432048545	0.615497938	no
H1FOO	0.043728843	0.78665604	0.43206002	0.615497938	no
TYW5	-0.043717939	-0.786459502	0.432174947	0.615620851	no
FIBP	0.043703855	0.786205647	0.432323416	0.615759909	no
LOC440028	0.043703242	0.78619461	0.432329871	0.615759909	no
IFNA13	-0.043695742	-0.786059432	0.432408944	0.615831718	no
ZNF718	-0.043659846	-0.785412437	0.432787522	0.61633004	no
MT4	0.04364662	0.785174057	0.432927055	0.616469384	no
C1orf52	-0.043643675	-0.785120987	0.432958123	0.616469384	no
C11orf53	-0.043642415	-0.785098265	0.432971424	0.616469384	no
HSFY1P1	-0.043639008	-0.785036875	0.433007365	0.616479713	no

FLJ14107	-0.043622598	-0.784741106	0.433180544	0.616685418	no
TREH	-0.04361932	-0.784682011	0.43321515	0.616693833	no
LOC400620	0.043611969	0.784549534	0.433292735	0.616763424	no
BARHL1	-0.043593255	-0.784212239	0.433490308	0.616990576	no
FBXO9	0.043591416	0.784179079	0.433509734	0.616990576	no
ADPRM	-0.04358823	-0.784121672	0.433543367	0.616997584	no
GZF1	-0.04356334	-0.783673052	0.433806249	0.617330824	no
GRPR	0.043559289	0.783600053	0.433849034	0.617330944	no
HP07349	0.043557893	0.783574887	0.433863784	0.617330944	no
PCBP1-AS1	-0.043532914	-0.783124685	0.434127707	0.617628149	no
ROCK1P1	-0.043530649	-0.783083863	0.434151642	0.617628149	no
JMJD4	-0.043529965	-0.783071522	0.434158878	0.617628149	no
OR2T3	-0.043524528	-0.782973533	0.434216337	0.617669004	no
DRAP1	-0.043518779	-0.782869914	0.434277102	0.617694219	no
SAE1	0.043517412	0.782845283	0.434291547	0.617694219	no
MOS	-0.043503727	-0.78259863	0.434436214	0.617859088	no
UTP3	0.043439271	0.781436929	0.43511795	0.618787709	no
ZNF568	-0.043434014	-0.781342175	0.435173583	0.618825877	no
GPCPD1	0.043430372	0.781276534	0.435212126	0.618839737	no
ATAD2B	-0.043420603	-0.78110046	0.43531552	0.618945805	no
IFNA8	-0.043394534	-0.780630618	0.435591492	0.619258476	no
TMEM30C	-0.043394386	-0.780627955	0.435593057	0.619258476	no
CCDC17	0.043389932	0.780547679	0.435640219	0.619270418	no
ASB2	-0.04338815	-0.780515565	0.435659087	0.619270418	no
CPM	0.04337745	0.780322721	0.435772398	0.619390516	no
SLC51B	-0.043367775	-0.780148343	0.435874873	0.619495198	no
ATMIN	-0.043360273	-0.780013124	0.435954346	0.619567177	no
CCL3L1	0.043351524	0.779855455	0.436047023	0.619599675	no
TMEM249	-0.043349633	-0.779821362	0.436067065	0.619599675	no
RAP1A	-0.043348149	-0.779794616	0.436082787	0.619599675	no
ACADL	0.043347229	0.779778034	0.436092536	0.619599675	no
GOLGA6B	-0.043330188	-0.779470907	0.436273108	0.619815255	no
CITED4	0.043320218	0.779291231	0.436378768	0.619924385	no
PRRG1	0.043311534	0.779134705	0.436470825	0.620012337	no
CELP	0.043308934	0.77908786	0.436498378	0.620012337	no
C3orf22	-0.043303902	-0.778997163	0.436551727	0.620047133	no
ZBTB25	-0.043298085	-0.778892322	0.436613401	0.620093748	no
CCM2L	-0.043292776	-0.778796641	0.436669689	0.62013271	no
CHRNA1	0.043288242	0.778714924	0.436717767	0.620145264	no
FBXL3	-0.0432865	-0.778683524	0.436736242	0.620145264	no
ANG	-0.043269469	-0.778376589	0.436916856	0.620360739	no
ESR2	0.043266509	0.778323243	0.436948252	0.620364331	no
SLC5A10	-0.043251494	-0.778052623	0.437107539	0.620531408	no
ENKUR	-0.043249973	-0.778025205	0.437123679	0.620531408	no
XDH	0.043239089	0.777829043	0.437239163	0.62065435	no
CNOT6L	0.04323564	0.77776689	0.437275758	0.6206653	no
FAM53A	-0.043231306	-0.777688774	0.437321754	0.620689592	no
SRSF6	-0.043209699	-0.777299362	0.437551086	0.6209534	no
SCGB2A2	0.043208349	0.777275038	0.437565413	0.6209534	no
LOC285548	-0.043174176	-0.776659145	0.437928273	0.621424452	no
LARP4	0.043171643	0.776613488	0.437955179	0.621424452	no

ACTRT1	-0.043158047	-0.77636846	0.438099593	0.621588325	no
CDX4	-0.043144769	-0.776129149	0.438240665	0.621739077	no
NRIP2	-0.0431426	-0.776090069	0.438263705	0.621739077	no
CLDN18	-0.04313842	-0.776014732	0.438308122	0.621761046	no
AKAP12	0.043131573	0.775891332	0.438380882	0.621823215	no
PYCRL	-0.043091191	-0.775163559	0.438810137	0.622391015	no
DPY19L2P1	0.04306143	0.774627192	0.439126652	0.622798844	no
DFNA5	-0.043034504	-0.774141918	0.439413131	0.623164022	no
CASZ1	0.043027107	0.774008617	0.439491843	0.623234523	no
LUZP4	0.043006445	0.773636237	0.439711771	0.623505257	no
PRUNE2	0.042970821	0.772994207	0.440091103	0.623987132	no
GPATCH4	0.042969077	0.772962789	0.440109671	0.623987132	no
HYOU1	-0.042965064	-0.772890459	0.440152419	0.624006573	no
DPM2	0.042950082	0.772620454	0.440312016	0.624191658	no
HYAL3	0.042942923	0.772491431	0.440388292	0.624258609	no
DYNLT1	0.042929677	0.772252715	0.440529436	0.624417498	no
PLN	-0.042918523	-0.772051699	0.44064831	0.624544801	no
PON2	-0.042912929	-0.771950887	0.440707933	0.624561035	no
LINC00645	-0.042911995	-0.77193406	0.440717886	0.624561035	no
UOX	0.04290669	0.771838442	0.440774442	0.624599996	no
PROCA1	-0.042891944	-0.771572699	0.440931648	0.624781568	no
C16orf58	-0.042864722	-0.771082105	0.441221953	0.625117439	no
LOC731779	0.042864262	0.771073815	0.441226859	0.625117439	no
NLRP5	-0.042848561	-0.770790849	0.441394353	0.625313517	no
TSPAN8	-0.042833527	-0.770519916	0.441554759	0.625475953	no
PLCXD2	-0.042830165	-0.770459318	0.441590641	0.625475953	no
BAALC	-0.042829632	-0.770449717	0.441596326	0.625475953	no
NEU2	-0.042826681	-0.770396541	0.441627814	0.62547933	no
RPL23P8	-0.042797052	-0.769862566	0.441944082	0.625875568	no
DLAT	-0.042795015	-0.769825858	0.441965828	0.625875568	no
UGT2A1	-0.042775308	-0.769470708	0.442176258	0.62610964	no
TAC4	0.042774079	0.769448553	0.442189387	0.62610964	no
SLC35C2	-0.04277093	-0.769391815	0.44222301	0.626115997	no
RGS17	0.042759287	0.769181973	0.442347378	0.626250823	no
ID1	-0.042751074	-0.769033975	0.442435105	0.626333762	no
SNORD12B	-0.04272582	-0.768578855	0.442704942	0.626674477	no
LOC730227	0.042720891	0.768490023	0.442757621	0.626686378	no
CYP1A2	-0.042719576	-0.768466334	0.44277167	0.626686378	no
KCTD21	0.04270616	0.768224543	0.442915077	0.626848068	no
HTN3	0.042693998	0.768005368	0.443045093	0.626975973	no
FTSJ3	-0.042692248	-0.767973838	0.443063799	0.626975973	no
PARP16	-0.042679278	-0.767740098	0.443202483	0.62713093	no
SOLH	0.042673928	0.767643691	0.443259691	0.627170585	no
GPR68	0.04265594	0.767319511	0.443452091	0.627360976	no
SNORA36A	-0.042655889	-0.767318594	0.443452636	0.627360976	no
KRT19P2	-0.04263309	-0.766907733	0.44369655	0.627664728	no
UNQ6494	-0.042622913	-0.76672433	0.443805454	0.62775301	no
TMEM38B	0.042621296	0.766695191	0.443822759	0.62775301	no
TTC39B	-0.042616764	-0.766613508	0.443871268	0.62775301	no
THBS4	0.042616341	0.766605883	0.443875796	0.62775301	no
ST8SIA6	-0.042602025	-0.766347891	0.444029033	0.627928403	no

TCEANC	-0.042585171	-0.766044173	0.444209466	0.628142232	no
LSM1	0.042580879	0.765966829	0.444255422	0.628153339	no
ATG10	-0.042578978	-0.76593257	0.444275779	0.628153339	no
TNNT3	-0.042575453	-0.765869042	0.444313528	0.628165385	no
PLEKHG4B	0.042562398	0.765633778	0.444453342	0.62832172	no
RNASE10	0.042558596	0.76556526	0.444494067	0.628337959	no
ABCA10	0.042545831	0.765335209	0.444630814	0.628489925	no
AFG3L1P	-0.042513932	-0.764760363	0.444972622	0.628900956	no
UBAC2	-0.042512868	-0.764741181	0.44498403	0.628900956	no
SRGAP2	0.042510501	0.764698538	0.445009392	0.628900956	no
DAB1	-0.042498073	-0.764474568	0.445142613	0.629047863	no
ETNK2	0.042490034	0.764329688	0.445228802	0.629128292	no
VGLL2	0.042484533	0.764230568	0.445287774	0.629170254	no
RFPL4B	-0.042467505	-0.763923698	0.445470377	0.629351084	no
RSL1D1	-0.042467137	-0.763917066	0.445474324	0.629351084	no
SMIM11	-0.042464398	-0.763867711	0.445503697	0.629351209	no
LOC643648	0.042459179	0.763773651	0.445559678	0.629385575	no
ABCC6P1	0.042453509	0.763671485	0.445620488	0.629385575	no
MIEN1	-0.042451028	-0.763626776	0.445647101	0.629385575	no
EDN1	0.042448798	0.763586589	0.445671023	0.629385575	no
POLR3E	0.042448479	0.763580826	0.445674453	0.629385575	no
DIP2A-IT1	0.04244459	0.763510756	0.445716165	0.629403122	no
FAM162B	0.042441305	0.763451561	0.445751405	0.629411527	no
CX3CL1	-0.042428982	-0.763229478	0.44588363	0.629556869	no
EZH2	-0.04242112	-0.763087805	0.445967992	0.629634615	no
MAP3K4	-0.042407851	-0.76284868	0.446110403	0.629794303	no
ANKHD1	-0.042398554	-0.762681137	0.446210199	0.629798494	no
PWRN2	-0.042396606	-0.762646044	0.446231104	0.629798494	no
ELAVL1	-0.042396132	-0.762637504	0.446236192	0.629798494	no
SLC25A28	-0.042395088	-0.762618692	0.446247398	0.629798494	no
SPRR2D	-0.042393924	-0.762597709	0.446259898	0.629798494	no
CSE1L	0.042389429	0.762516708	0.446308154	0.629804192	no
C20orf173	-0.042388089	-0.76249255	0.446322546	0.629804192	no
PRAMEF4	0.042367137	0.762114991	0.446547517	0.630080276	no
TRIM31	-0.042351687	-0.76183657	0.446713457	0.630273037	no
ZNF780A	-0.042345975	-0.761733628	0.44677482	0.630281859	no
PPY2	-0.042345645	-0.761727681	0.446778365	0.630281859	no
KCNK18	-0.042342441	-0.761669942	0.446812785	0.630289042	no
NPFF	-0.042333569	-0.761510066	0.4469081	0.630377229	no
LOC100507384	-0.042331162	-0.761466685	0.446933965	0.630377229	no
LDLR	0.042320915	0.761282039	0.447044065	0.630491141	no
RDM1	-0.042312557	-0.761131412	0.447133892	0.630564214	no
ISOC1	0.042310634	0.76109676	0.447154559	0.630564214	no
SNAP29	-0.042293013	-0.760779222	0.447343963	0.630744972	no
EVX2	-0.042291895	-0.760759079	0.447355979	0.630744972	no
FAM47C	-0.042287882	-0.76068676	0.447399123	0.630744972	no
GUCA1C	-0.042287787	-0.76068506	0.447400137	0.630744972	no
FAM74A4	-0.042282239	-0.760585073	0.447459791	0.630787692	no
OR1E2	-0.042278503	-0.760517758	0.447499954	0.630802933	no
LINC00304	-0.042266847	-0.760307702	0.447625298	0.630938236	no
FREM2	-0.042259665	-0.760178278	0.447702537	0.630983622	no

C19orf71	0.042255402	0.760101455	0.447748389	0.630983622	no
CLPP	0.042254658	0.760088057	0.447756385	0.630983622	no
CEL	-0.042252933	-0.760056971	0.447774939	0.630983622	no
C20orf85	-0.042234604	-0.759726676	0.447972109	0.631196863	no
CACNA1F	0.042232392	0.759686809	0.447995912	0.631196863	no
SPATA9	-0.042230676	-0.759655883	0.448014376	0.631196863	no
MRPL39	-0.04222636	-0.759578105	0.448060816	0.631200994	no
MTFMT	-0.042222688	-0.759511947	0.448100319	0.631200994	no
LAD1	0.042222214	0.759503406	0.448105419	0.631200994	no
HEATR5A	-0.04220927	-0.759270134	0.448244725	0.631355838	no
NDUFA9	0.042204595	0.759185903	0.448295032	0.631385316	no
LOC400654	-0.042190136	-0.758925341	0.448450674	0.631563135	no
CABP5	-0.042184436	-0.75882263	0.448512035	0.631608161	no
STK3	0.042163181	0.758439612	0.448740898	0.631858294	no
GUCY1A3	0.042162479	0.758426959	0.448748459	0.631858294	no
GAGE10	-0.042158006	-0.758346348	0.448796636	0.631861973	no
PGF	-0.042156777	-0.758324196	0.448809875	0.631861973	no
LSM6	-0.042153357	-0.758262577	0.448846703	0.631872429	no
SLMO2-ATP5E	0.042149208	0.758187816	0.448891389	0.631893943	no
MGAT5	0.042142821	0.758072715	0.44896019	0.631949401	no
TRPV5	-0.042130104	-0.757843543	0.449097196	0.632099971	no
ZNF329	-0.042127431	-0.757795391	0.449125986	0.632099971	no
CYTH2	0.042124534	0.757743172	0.449157208	0.632102519	no
NUDT7	-0.042119088	-0.757645033	0.449215891	0.632114425	no
C4orf33	0.042118289	0.757630644	0.449224495	0.632114425	no
SUMF2	-0.042105602	-0.757402029	0.449361213	0.632265408	no
CKS2	-0.042096868	-0.757244635	0.449455354	0.632341152	no
UGP2	0.042095148	0.757213641	0.449473893	0.632341152	no
EPGN	-0.042082537	-0.756986383	0.449609843	0.632454428	no
OGT	-0.042080972	-0.756958195	0.449626707	0.632454428	no
FAM173B	-0.0420783	-0.756910047	0.449655514	0.632454428	no
MTFR1L	0.04207676	0.756882283	0.449672126	0.632454428	no
ZNF695	-0.042072484	-0.756805241	0.449718222	0.632477869	no
SYNE2	-0.042064316	-0.756658042	0.449806304	0.632522188	no
OR8D2	0.042064103	0.756654208	0.449808599	0.632522188	no
DLEC1	0.042040412	0.756227302	0.45006411	0.632826269	no
B4GALNT3	-0.042036843	-0.756162991	0.450102608	0.632826269	no
HIST3H3	-0.042035863	-0.756145332	0.45011318	0.632826269	no
OR5B12	-0.042027601	-0.75599645	0.450202313	0.63291018	no
LOC100144597	0.04202	0.755859482	0.450284323	0.632984065	no
MRPS18B	-0.04201185	-0.755712605	0.450372275	0.633066294	no
MSH6	-0.04199169	-0.755349324	0.450589855	0.633290108	no
USP45	-0.041991636	-0.755348356	0.450590435	0.633290108	no
LDB2	-0.041985064	-0.755229937	0.450661373	0.633348389	no
OCSTAMP	0.041971535	0.754986147	0.450807433	0.63351223	no
ZFAT-AS1	-0.041955316	-0.754693879	0.450982572	0.633708381	no
LOC728392	0.041953148	0.754654809	0.451005988	0.633708381	no
LZTR1	-0.041947651	-0.754555756	0.451065355	0.633750363	no
SHISA3	-0.041944768	-0.754503804	0.451096494	0.633752681	no
LINC00701	-0.041933797	-0.754306119	0.451214995	0.633877726	no
TADA3	-0.041913522	-0.753940761	0.451434052	0.634110881	no

ERCC6L	0.041912974	0.753930885	0.451439974	0.634110881	no
PURB	-0.041908176	-0.753844438	0.451491814	0.634117504	no
IQCF2	-0.04190534	-0.753793334	0.451522461	0.634117504	no
HNRPLL	-0.04190092	-0.753713686	0.451570229	0.634117504	no
ESPN	-0.041900638	-0.753708606	0.451573276	0.634117504	no
LOC100508120	0.041898886	0.75367702	0.45159222	0.634117504	no
FXYD1	-0.041880784	-0.75335084	0.451787877	0.634314765	no
CLEC2A	-0.041878912	-0.753317099	0.451808119	0.634314765	no
LOC100499489	0.041876239	0.753268942	0.45183701	0.634314765	no
HSD17B13	-0.041874967	-0.75324602	0.451850763	0.634314765	no
LOC441455	-0.041872136	-0.753195011	0.451881367	0.634316294	no
ANXA7	0.041866507	0.753093564	0.451942236	0.634360303	no
GDI2	0.041855787	0.7529004	0.452058149	0.634481563	no
TKT	0.041848795	0.752774404	0.452133766	0.634546253	no
PARD6G-AS1	0.041798742	0.751872478	0.452675267	0.635264735	no
LOC100130964	0.041794519	0.751796386	0.452720968	0.635287386	no
CEBPA-AS1	0.041786523	0.751652304	0.452807511	0.635367343	no
ADAMTSL5	-0.041776328	-0.751468596	0.45291787	0.635480704	no
PPIP5K1	0.041771351	0.751378918	0.452971747	0.635514807	no
WRAP53	-0.041764484	-0.751255175	0.453046097	0.635577627	no
MBD3L2	0.041758445	0.751146349	0.453111489	0.63558483	no
OIP5	0.041756816	0.751116992	0.45312913	0.63558483	no
JAGN1	0.041755816	0.751098979	0.453139955	0.63558483	no
ODAM	0.041750435	0.751002012	0.453198228	0.635625081	no
LOC91450	0.041747205	0.750943821	0.4532332	0.635632649	no
ST6GALNAC3	0.041736033	0.750742514	0.453354196	0.635731004	no
RWDD3	-0.041734357	-0.750712308	0.453372354	0.635731004	no
SNRPB	0.041732536	0.750679499	0.453392075	0.635731004	no
NBAS	-0.041728501	-0.750606784	0.453435788	0.635750816	no
LY6G6F	-0.041722077	-0.750491034	0.453505374	0.635806902	no
PINLYP	0.041716238	0.750385816	0.453568635	0.635854112	no
EGR4	-0.041705534	-0.750192934	0.453684616	0.635973928	no
ZNF841	-0.041702888	-0.750145255	0.453713288	0.635973928	no
ZNF304	-0.04169094	-0.749929969	0.453842764	0.636113927	no
ACTC1	-0.041666424	-0.749488204	0.454108514	0.636413913	no
NBLA00301	0.041665732	0.749475731	0.454116019	0.636413913	no
ZNF17	0.041657657	0.749330228	0.454203568	0.636495102	no
RPS19BP1	-0.041652016	-0.749228595	0.454264727	0.6365393	no
CADM3	0.041629062	0.748814988	0.454513666	0.636846603	no
MUC2	-0.041621612	-0.748680741	0.454594482	0.636918314	no
GNG7	-0.041617563	-0.748607785	0.454638405	0.636938329	no
H3F3C	-0.041610328	-0.74847742	0.454716897	0.637006768	no
INO80	0.04160218	0.748330599	0.454805306	0.63708909	no
CXorf48	0.041595926	0.748217913	0.454873166	0.63714262	no
SGCD	0.04159299	0.748164997	0.454905035	0.637145731	no
SERPINB4	0.041580218	0.747934858	0.455043651	0.637298345	no
HNRNPU-AS1	-0.04156583	-0.747675614	0.455199826	0.637475528	no
SYCE1L	0.041560954	0.747587741	0.455252769	0.637508129	no
NPM3	0.041548083	0.747355825	0.455392515	0.637662272	no
DDX3X	0.041530155	0.747032788	0.455587209	0.637820188	no
MTNR1B	-0.04152983	-0.747026925	0.455590744	0.637820188	no

MAMSTR	-0.0415295	-0.747020978	0.455594328	0.637820188	no
LIPK	0.041506456	0.746605765	0.455844648	0.638122646	no
ARIH2	-0.041504145	-0.74656412	0.455869759	0.638122646	no
IFT81	-0.041498813	-0.746468044	0.455927693	0.638162176	no
HIST1H4J	-0.041495029	-0.746399863	0.455968809	0.638170663	no
GRM6	-0.04149279	-0.746359508	0.455993146	0.638170663	no
FAM53B	0.041482575	0.746175446	0.456104157	0.638214813	no
SYS1	-0.041481251	-0.746151603	0.456118538	0.638214813	no
DDX27	0.041478694	0.746105518	0.456146335	0.638214813	no
YPEL3	-0.041476272	-0.746061883	0.456172656	0.638214813	no
C7orf25	0.041476224	0.746061019	0.456173177	0.638214813	no
LINC00207	-0.04146956	-0.745940931	0.456245618	0.638274612	no
RPS4X	-0.04145144	-0.745614435	0.456442606	0.638508628	no
HBE1	-0.041425405	-0.745145334	0.456725716	0.638840424	no
CRYGC	-0.041423296	-0.745107323	0.456748661	0.638840424	no
PNLIPRP2	-0.041421428	-0.745073677	0.456768971	0.638840424	no
ALDH1A3	0.041411556	0.744895786	0.456876363	0.638945041	no
TTTY13	0.041409086	0.74485128	0.456903233	0.638945041	no
TNFRSF25	0.041400771	0.744701458	0.456993694	0.63899366	no
TMEM89	0.041400424	0.74469521	0.456997467	0.63899366	no
FGF7	0.041396536	0.744625156	0.457039768	0.639011233	no
C1orf213	0.041384759	0.744412951	0.45716792	0.639148827	no
VCX3A	0.041373932	0.744217864	0.457285752	0.639271976	no
PCDHGC5	-0.041351779	-0.743818694	0.457526902	0.639561051	no
OR7G2	-0.041348499	-0.743759593	0.457562613	0.639561051	no
OR2A5	-0.041346735	-0.74372782	0.457581812	0.639561051	no
RPS16P5	0.041339997	0.743606414	0.457655176	0.639621993	no
F10	-0.041334642	-0.74350992	0.457713491	0.639661895	no
LNX1	-0.04132429	-0.743323384	0.457826233	0.639777852	no
TRIM24	-0.041315765	-0.743169793	0.457919075	0.639865985	no
HHIP-AS1	-0.041310605	-0.743076805	0.45797529	0.639902929	no
ZRSR2	0.041304983	0.742975513	0.458036529	0.639946889	no
GUCY1B2	-0.041292055	-0.742742576	0.458177374	0.640102058	no
GSTTP2	-0.041286245	-0.742637886	0.458240683	0.64014889	no
SLC25A26	-0.041282469	-0.742569846	0.458281831	0.64016476	no
MIR3648	-0.041279473	-0.742515875	0.458314473	0.640168746	no
COL14A1	0.041251654	0.742014627	0.458617687	0.64055064	no
HORMAD2	0.041239252	0.741791154	0.458752906	0.640697862	no
C14orf166B	-0.041210015	-0.741264363	0.459071747	0.641082801	no
LINC00628	-0.041208184	-0.74123137	0.459091719	0.641082801	no
SRM	0.041204297	0.741161342	0.459134115	0.641082801	no
PRAMEF11	0.041203038	0.741138653	0.459147851	0.641082801	no
PRTN3	-0.041194867	-0.740991416	0.459236997	0.641165614	no
NFAT5	-0.041175965	-0.740650849	0.459443233	0.641411882	no
FITM1	-0.041170254	-0.74054794	0.459505561	0.641457228	no
MIR1284	-0.041156423	-0.740298741	0.459656513	0.641626274	no
GAREM	0.041142481	0.740047526	0.459808714	0.641797043	no
SNHG3	0.041134335	0.739900768	0.459897641	0.641879478	no
CDK5	-0.041117999	-0.739606428	0.460076025	0.642086456	no
WASH7P	-0.04110941	-0.739451668	0.460169832	0.642086456	no
CELF1	-0.041109111	-0.739446273	0.460173103	0.642086456	no

PHF15	0.04110721	0.739412032	0.46019386	0.642086456	no
MRGPRG	-0.041107076	-0.739409618	0.460195323	0.642086456	no
KCNV2	-0.041085255	-0.739016441	0.460433702	0.64237735	no
NGF	0.041069122	0.738725765	0.46060998	0.64258157	no
SKP1	-0.041062996	-0.738615391	0.460676926	0.642633248	no
DNAJB13	0.041055212	0.738475128	0.460762008	0.642710217	no
FETUB	0.041048752	0.738358735	0.460832617	0.64276699	no
PGAM5	-0.041041542	-0.738228842	0.460911424	0.642835188	no
PTCD2	0.041038521	0.738174399	0.460944457	0.642839541	no
SPIN4	-0.041021524	-0.737868158	0.461130291	0.643016371	no
PVRIG	-0.041021451	-0.737866837	0.461131093	0.643016371	no
SLC13A4	-0.041012395	-0.737703671	0.461230123	0.643112735	no
AHCYL2	0.041006336	0.7375945	0.461296389	0.643163403	no
PCDHB5	-0.040995765	-0.737404041	0.461412009	0.643237825	no
RNF216-IT1	0.040994877	0.737388048	0.461421718	0.643237825	no
CCDC153	0.040991971	0.737335686	0.461453508	0.643237825	no
KRTAP19-5	-0.040989051	-0.737283066	0.461485456	0.643237825	no
SNORA70B	0.040987774	0.737260068	0.46149942	0.643237825	no
RGS20	-0.040983493	-0.737182919	0.461546263	0.643261397	no
PSD3	-0.040974994	-0.737029796	0.461639244	0.643349264	no
GABRR2	0.040967725	0.736898827	0.461718782	0.643418385	no
SERPINA6	0.040960625	0.736770906	0.461796475	0.643484928	no
KRTAP2-2	-0.040957833	-0.736720596	0.461827034	0.643485787	no
LRRC36	0.04094862	0.736554595	0.46192787	0.643584561	no
C16orf70	0.040944231	0.736475517	0.46197591	0.643585556	no
SNORA15	0.040943082	0.73645483	0.461988478	0.643585556	no
POTEG	-0.040928088	-0.736184662	0.462152628	0.643736184	no
PHLDB1	0.040927733	0.736178272	0.462156511	0.643736184	no
NIPA2	0.040921829	0.7360719	0.462221151	0.643784494	no
SCGB2A1	0.040917736	0.735998147	0.462265971	0.643805196	no
MICALL1	0.040906568	0.735796939	0.46238826	0.643933781	no
EHHADH-AS1	-0.040898557	-0.735652586	0.462476006	0.644010446	no
LOC339975	-0.04089607	-0.735607779	0.462503244	0.644010446	no
C16orf11	0.040892624	0.735545703	0.462540981	0.644021265	no
LOC10013149€	-0.040864805	-0.735044467	0.462845756	0.644374698	no
NMD3	0.040863981	0.735029618	0.462854786	0.644374698	no
SPANXD	-0.040853604	-0.734842653	0.4629685	0.644491257	no
SULT1E1	0.040834281	0.734494519	0.463180279	0.644732418	no
RPL13AP17	-0.040831705	-0.734448096	0.463208523	0.644732418	no
ANKRD20A1	-0.040829555	-0.734409364	0.463232089	0.644732418	no
GNA13	0.040826851	0.734360637	0.463261737	0.644732418	no
FLJ36000	-0.040820299	-0.734242603	0.46333356	0.64479062	no
SMG5	0.040814301	0.734134527	0.463399328	0.644814592	no
ARHGAP11B	0.040813255	0.734115687	0.463410794	0.644814592	no
TRPC4	-0.040810379	-0.73406387	0.463442329	0.644816722	no
ANAPC5	-0.040807359	-0.73400946	0.463475443	0.644821049	no
KXD1	-0.040787421	-0.73365023	0.463694107	0.645071548	no
FBXO18	0.04078492	0.733605164	0.463721543	0.645071548	no
DDX31	-0.040778815	-0.73349518	0.463788504	0.645071548	no
KIF18B	-0.040776994	-0.733462362	0.463808486	0.645071548	no
GPRC6A	0.040776147	0.733447111	0.463817771	0.645071548	no

WDR69	0.040774523	0.733417849	0.463835589	0.645071548	no
OTOP2	-0.040752861	-0.733027553	0.464073269	0.645360335	no
NAV3	-0.04073607	-0.732725041	0.464257537	0.645574812	no
MOCS3	-0.04072922	-0.732601608	0.464332735	0.645637604	no
C17orf77	-0.040718595	-0.732410183	0.464449369	0.645723336	no
ARHGAP28	-0.040718129	-0.732401789	0.464454484	0.645723336	no
GOLM1	-0.040694526	-0.731976531	0.464713651	0.646041858	no
CLDN15	-0.04068384	-0.731784001	0.464831012	0.646163213	no
SIRT6	-0.040677985	-0.731678509	0.464895324	0.646210815	no
TMEM25	-0.04066298	-0.731408181	0.46506015	0.646357217	no
PLCG1	-0.040662921	-0.731407115	0.4650608	0.646357217	no
EIF6	0.040650967	0.73119174	0.465192142	0.646446575	no
MAFG-AS1	-0.040649408	-0.731163657	0.46520927	0.646446575	no
MAP1S	-0.040648857	-0.731153715	0.465215333	0.646446575	no
GFM2	-0.040641727	-0.73102527	0.465293676	0.646511428	no
GPR89B	-0.040639135	-0.730978554	0.465322171	0.646511428	no
ZNF189	-0.040622002	-0.730669876	0.465510478	0.646731248	no
FOXR1	-0.040602999	-0.7303275	0.465719392	0.646979668	no
UVSSA	-0.040589989	-0.730093107	0.465862447	0.647136568	no
KRT71	-0.040576504	-0.729850154	0.466010751	0.647300741	no
CLCA4	-0.040569862	-0.729730492	0.466083806	0.647360375	no
ADAMTS3	0.040565214	0.729646743	0.466134939	0.647389555	no
EVI2A	0.040561955	0.729588022	0.466170793	0.647397513	no
CYP4F8	-0.040551027	-0.729391146	0.466291014	0.647467329	no
SSTR4	-0.040550029	-0.729373171	0.466301991	0.647467329	no
LINC00521	-0.04054917	-0.729357686	0.466311447	0.647467329	no
ARMC8	-0.040533528	-0.729075872	0.466483568	0.647591568	no
ALDH3B1	0.040533236	0.729070619	0.466486777	0.647591568	no
PPAPDC3	-0.040532823	-0.729063174	0.466491325	0.647591568	no
SPEF1	0.040514615	0.728735121	0.466691734	0.647827933	no
ADNP	-0.040506677	-0.728592117	0.466779111	0.647907374	no
LINC00238	0.040501784	0.728503954	0.466832983	0.647940303	no
FOXH1	0.040493933	0.728362516	0.466919417	0.648018418	no
GTF2H2C	-0.040446991	-0.727516778	0.467436444	0.64865781	no
DRG2	0.040443998	0.727462858	0.467469418	0.64865781	no
LOC145474	0.04044154	0.727418575	0.467496499	0.64865781	no
SLC26A6	0.040439859	0.727388279	0.467515028	0.64865781	no
OR11L1	-0.040438406	-0.727362105	0.467531035	0.64865781	no
GINS2	-0.040422221	-0.727070508	0.467709391	0.648826267	no
AQP4-AS1	-0.040419617	-0.727023608	0.467738081	0.648826267	no
TMEM8C	-0.040419169	-0.727015526	0.467743025	0.648826267	no
NXPE4	-0.040413801	-0.726918818	0.467802188	0.648866453	no
PDE1A	-0.040407491	-0.726805138	0.467871739	0.648921042	no
SMLR1	-0.040394696	-0.726574621	0.468012789	0.649074784	no
LINC00668	-0.040390822	-0.726504812	0.468055509	0.649092144	no
FAM47E-STBD1	0.040388037	0.726454648	0.468086209	0.649092832	no
TACC1	0.040379356	0.726298242	0.468181934	0.649183685	no
NEURL1B	0.040360271	0.72595441	0.468392407	0.64939374	no
HIST1H2AD	0.04036014	0.725952042	0.468393856	0.64939374	no
ACY1	0.040347043	0.72571609	0.468538323	0.649552128	no
MLH3	-0.040336042	-0.725517899	0.468659688	0.649678472	no

FMO5	-0.040327126	-0.725357258	0.468758072	0.649772944	no
FAF1	0.040307302	0.725000108	0.468976848	0.650034275	no
COG2	-0.040276542	-0.724445933	0.469316426	0.65038589	no
NACC2	0.040275846	0.724433405	0.469324105	0.65038589	no
SNORD11B	0.040269923	0.724326691	0.469389511	0.65038589	no
VASH2	0.040267618	0.724285165	0.469414965	0.65038589	no
CLCA3P	0.040266966	0.724273405	0.469422173	0.65038589	no
VAMP7	-0.040265695	-0.724250518	0.469436202	0.65038589	no
ENKD1	-0.040265137	-0.724240459	0.469442368	0.65038589	no
BTF3L4	0.04025831	0.724117475	0.469517758	0.650448407	no
LOC100131691	0.040248918	0.723948256	0.469621501	0.650550192	no
MIR211	-0.040234913	-0.723695951	0.469776205	0.650722554	no
CCDC7	0.040225107	0.723519278	0.469884551	0.650822966	no
TK2	0.040220605	0.723438168	0.469934298	0.650822966	no
PRDX3	0.04022013	0.723429611	0.469939546	0.650822966	no
RAB40C	-0.04020685	-0.723190362	0.4700863	0.650950977	no
COG5	-0.040206283	-0.72318016	0.470092558	0.650950977	no
MAGI2-AS3	-0.040197853	-0.723028287	0.470185729	0.651038046	no
VGLL1	-0.040190303	-0.722892252	0.470269194	0.651075449	no
F8	0.040188201	0.722854384	0.470292428	0.651075449	no
RNF113B	-0.040187188	-0.722836132	0.470303628	0.651075449	no
RPL29P2	-0.040166588	-0.722465019	0.470531374	0.651346846	no
LRRC52	-0.040163974	-0.722417913	0.470560287	0.651346846	no
C5orf48	-0.040149919	-0.722164703	0.470715718	0.651514789	no
CUL5	-0.04014752	-0.722121489	0.470742248	0.651514789	no
IGDCC4	0.040140579	0.721996437	0.470819023	0.651527336	no
POU5F1P3	-0.040139034	-0.7219686	0.470836115	0.651527336	no
OPN1MW	-0.040138478	-0.721958587	0.470842262	0.651527336	no
SYNJ2BP	-0.040119855	-0.72162308	0.471048286	0.651770456	no
NRG4	-0.040116436	-0.721561485	0.471086115	0.651780834	no
HPX	0.04008981	0.721081802	0.471380772	0.652129691	no
ZNF716	-0.040088168	-0.721052221	0.471398946	0.652129691	no
COL23A1	-0.040085339	-0.721001251	0.471430263	0.652131036	no
ISM1-AS1	-0.040082293	-0.720946389	0.471463971	0.652135689	no
SNORD90	0.040078344	0.720875237	0.471507691	0.652154189	no
MIR548K	-0.040070689	-0.720737331	0.471592435	0.652164045	no
NBPF14	0.040070586	0.720735476	0.471593574	0.652164045	no
LOC100506071	-0.040069477	-0.720715493	0.471605855	0.652164045	no
MCM2	-0.040062313	-0.720586433	0.471685172	0.652231761	no
LINC00466	-0.040053755	-0.720432249	0.471779939	0.65232083	no
TNS1	0.040044873	0.720272243	0.471878295	0.652414851	no
HOXA5	0.040031021	0.720022682	0.472031724	0.652584997	no
TMEM9B	-0.040025323	-0.719920042	0.472094835	0.652630265	no
BCORP1	0.040012053	0.719680969	0.472241852	0.652791513	no
MIR4524B	-0.039994667	-0.71936776	0.472434498	0.653015809	no
ACTL9	-0.039988153	-0.719250409	0.472506689	0.65307359	no
PGAM2	0.039957469	0.718697619	0.472846829	0.653501685	no
KRT26	-0.039954652	-0.718646863	0.472878067	0.653502832	no
SLC25A38	-0.039950403	-0.718570325	0.472925174	0.653525908	no
MIR98	-0.03993661	-0.718321847	0.473078124	0.653673915	no
GLUD2	0.039935259	0.718297501	0.473093112	0.653673915	no

CRADD	-0.03993152	-0.718230135	0.473134585	0.653689191	no
GNAS	-0.039924364	-0.718101229	0.473213949	0.653756812	no
LOC100133957	-0.039911889	-0.717876492	0.473352331	0.653905956	no
FAM160B2	-0.03988405	-0.717374952	0.473661236	0.654235085	no
ANKRD20A19P	-0.039882649	-0.717349726	0.473676776	0.654235085	no
KRT6B	0.039882187	0.717341393	0.47368191	0.654235085	no
RNF8	-0.039878838	-0.717281062	0.473719076	0.654244372	no
FAM150B	0.039866999	0.71706779	0.473850476	0.654383792	no
ATG3	-0.039843974	-0.716652978	0.474106104	0.654669999	no
LOC255654	-0.039842844	-0.716632626	0.474118648	0.654669999	no
NBN	-0.039838729	-0.716558486	0.474164345	0.654691034	no
ZNF423	-0.039824271	-0.716298029	0.474324903	0.654814547	no
ZNF778	-0.039824218	-0.716297075	0.474325491	0.654814547	no
ADC	-0.039822442	-0.716265092	0.474345208	0.654814547	no
PRMT2	0.039815819	0.71614577	0.474418776	0.654874038	no
FUT8-AS1	-0.039802167	-0.715899826	0.47457043	0.655041304	no
ZFAND5	-0.039789298	-0.715668001	0.474713402	0.65514213	no
ZNF208	-0.03978878	-0.71565867	0.474719158	0.65514213	no
LOC100133050	-0.039787361	-0.715633097	0.474734931	0.65514213	no
PDCD5	0.039773436	0.715382245	0.474889669	0.655313591	no
ZNF654	0.039752144	0.714998666	0.475126333	0.655530459	no
HNRNPCL1	-0.039747792	-0.714920273	0.475174709	0.655530459	no
DBNDD1	0.039747369	0.714912643	0.475179417	0.655530459	no
AMIGO3	0.039745072	0.714871262	0.475204955	0.655530459	no
NOV	0.039743472	0.714842447	0.475222737	0.655530459	no
PPY	0.039742833	0.714830934	0.475229842	0.655530459	no
LILRA4	0.039738152	0.714746607	0.475281886	0.65556017	no
SOSTDC1	-0.039731769	-0.714631617	0.475352859	0.655615986	no
PXT1	-0.039728548	-0.714573591	0.475388676	0.655623309	no
FAM90A1	-0.039704863	-0.714146904	0.475652094	0.655907409	no
TMEM45B	-0.039701395	-0.714084427	0.475690671	0.655907409	no
HOXC12	-0.039700264	-0.714064053	0.475703251	0.655907409	no
C3orf62	-0.039695497	-0.71397819	0.475756273	0.655907409	no
ABCG2	-0.039693576	-0.713943572	0.47577765	0.655907409	no
C9orf135	0.039692031	0.713915734	0.475794841	0.655907409	no
RASIP1	0.039690819	0.713893916	0.475808316	0.655907409	no
SCGB2B2	-0.039682227	-0.713739128	0.475903912	0.655972457	no
THOC1	-0.039681091	-0.713718668	0.475916549	0.655972457	no
FOSB	0.03967768	0.713657206	0.475954511	0.65598271	no
CCDC112	-0.039674585	-0.71360145	0.47598895	0.655988106	no
CCDC24	0.039666672	0.713458906	0.476077004	0.656067386	no
MUC21	-0.03965438	-0.713237464	0.476213811	0.656213837	no
MIR1247	-0.039627858	-0.712759681	0.47650906	0.656578587	no
PCDHGB3	0.039612323	0.712479838	0.476682038	0.656774823	no
COG4	0.039602375	0.712300618	0.476792837	0.656867967	no
TIMMDC1	0.039600765	0.712271611	0.476810771	0.656867967	no
ITIH1	-0.039595658	-0.712179613	0.476867654	0.6568684	no
PDLIM7	0.039595248	0.712172235	0.476872216	0.6568684	no
CLK2P	-0.039577067	-0.71184471	0.477074755	0.657105272	no
ANXA10	0.039543326	0.711236886	0.477450755	0.657581017	no
NLGN4Y-AS1	-0.039538461	-0.711149241	0.477504986	0.657613563	no

SPRR2A	-0.039530185	-0.711000159	0.477597239	0.657687941	no
GPR37L1	-0.039527003	-0.710942827	0.477632719	0.657687941	no
COPS4	0.039520154	0.71081946	0.47770907	0.657687941	no
SLC5A8	-0.039520075	-0.71081803	0.477709954	0.657687941	no
PIGL	0.039519891	0.710814712	0.477712008	0.657687941	no
MTERFD2	-0.039514206	-0.7107123	0.477775395	0.657733074	no
LINC00624	-0.039509471	-0.710627014	0.477828186	0.65775839	no
DAD1	0.039504875	0.710544213	0.477879441	0.65775839	no
KRTAP10-12	-0.039504323	-0.71053426	0.477885603	0.65775839	no
LRRC66	0.039497438	0.710410239	0.477962381	0.657821936	no
ABHD16B	0.039488898	0.710256397	0.478057629	0.657910893	no
SRCRB4D	0.039484621	0.710179349	0.478105337	0.657934417	no
C20orf166	-0.039458642	-0.709711358	0.478395165	0.658291106	no
BMI1	-0.039424878	-0.709103124	0.47877199	0.658767453	no
USP17L10	-0.03942094	-0.709032187	0.478815949	0.65878576	no
NOS1AP	-0.039416316	-0.708948894	0.478867568	0.658814602	no
RNF216P1	0.039409093	0.708818772	0.478948214	0.658876299	no
MED28	-0.039406808	-0.708777605	0.47897373	0.658876299	no
LOC100507117	-0.039401276	-0.708677958	0.479035495	0.658919087	no
REXO4	-0.039396223	-0.708586925	0.479091924	0.65895453	no
FUNDC1	0.039387154	0.708423555	0.479193203	0.659010845	no
ATPAF2	0.039387065	0.708421954	0.479194196	0.659010845	no
IQCJ	-0.039374282	-0.708191682	0.479336971	0.659165014	no
FAM71E2	-0.039350032	-0.707754838	0.479607888	0.659495369	no
DPY19L2	-0.039334957	-0.707483276	0.479776346	0.659645522	no
ALPPL2	-0.039334767	-0.707479847	0.479778473	0.659645522	no
TMPPE	0.039329668	0.707388002	0.479835455	0.659681663	no
ABCF3	-0.03932577	-0.707317777	0.479879025	0.659699362	no
ART1	-0.039298494	-0.706826427	0.480183941	0.660050416	no
CCNE2	-0.039295423	-0.70677112	0.48021827	0.660050416	no
LOC100506990	-0.039294685	-0.706757815	0.480226528	0.660050416	no
STX8	-0.039281806	-0.706525828	0.480370535	0.660206125	no
LYPLAL1	0.039262723	0.706182059	0.480583976	0.660431551	no
NEIL3	0.039260356	0.706139418	0.480610454	0.660431551	no
ERLIN2	0.039258899	0.706113178	0.480626749	0.660431551	no
UTY	-0.039249532	-0.705944437	0.480731542	0.660533314	no
AGR2	0.039239934	0.705771546	0.480838924	0.660602859	no
RCCD1	0.039239513	0.705763965	0.480843633	0.660602859	no
TUSC5	-0.039224092	-0.705486166	0.481016203	0.6607977	no
SLC2A11	0.039211013	0.705250568	0.481162583	0.660956541	no
FBN3	0.039201591	0.705080837	0.481268055	0.661022396	no
OR2M7	0.039201235	0.705074424	0.481272041	0.661022396	no
C17orf61-PLS	-0.039182976	-0.704745526	0.481476456	0.661103727	no
EXOSC4	-0.039182933	-0.704744739	0.481476945	0.661103727	no
ARL5C	-0.039181996	-0.704727857	0.481487439	0.661103727	no
RIBC1	0.039181708	0.704722674	0.481490661	0.661103727	no
TIPARP-AS1	-0.039181487	-0.704718696	0.481493134	0.661103727	no
ATG2A	-0.039177357	-0.704644306	0.481539376	0.661103727	no
LOC338758	-0.039176713	-0.704632702	0.481546589	0.661103727	no
KRTAP3-1	-0.039167238	-0.704462027	0.481652693	0.661146591	no
GSC2	-0.039163337	-0.704391745	0.48169639	0.661146591	no

CCND1	-0.039162501	-0.704376683	0.481705755	0.661146591	no
TMEM129	0.039160995	0.704349555	0.481722622	0.661146591	no
DSG3	-0.03915758	-0.70428804	0.48176087	0.661146591	no
TXNDC15	0.039157444	0.704285589	0.481762394	0.661146591	no
EI24	0.039148405	0.704122762	0.481863645	0.661243317	no
C10orf114	0.039135346	0.703887526	0.482009941	0.661401842	no
LOC100132831	-0.039128036	-0.703755845	0.482091846	0.661471995	no
ATP6V1B2	0.039103566	0.703315053	0.482366071	0.661806003	no
NRCAM	0.039089329	0.703058611	0.482525648	0.661982682	no
UFC1	0.039079109	0.702874508	0.482640228	0.662052841	no
ELOVL7	0.039077427	0.7028442	0.482659092	0.662052841	no
M1	-0.039076525	-0.702827951	0.482669206	0.662052841	no
APOC4-APOC2	0.03907171	0.702741222	0.48272319	0.662084631	no
DKFZp686K168	0.039068959	0.702691662	0.48275404	0.662084689	no
HOXC5	0.039065632	0.702631731	0.482791348	0.662093603	no
IGFALS	-0.039054335	-0.70242824	0.482918033	0.662185838	no
LINC00421	-0.03905408	-0.702423644	0.482920895	0.662185838	no
EML1	-0.039051392	-0.702375225	0.482951041	0.662185838	no
PRAME	-0.039048097	-0.702315881	0.482987991	0.662194253	no
CRELD2	-0.039042815	-0.70222072	0.483047246	0.662211647	no
MAP9	-0.039040183	-0.702173317	0.483076764	0.662211647	no
RIMBP3	0.039038725	0.702147055	0.483093118	0.662211647	no
CLDN25	-0.039016078	-0.701739096	0.483347202	0.662486352	no
TEKT4	0.039015367	0.7017263	0.483355172	0.662486352	no
HRCT1	0.039010728	0.701642723	0.483407235	0.662515457	no
GLDN	0.038980794	0.70110352	0.483743195	0.662933618	no
LOC100506834	0.038973947	0.700980184	0.483820059	0.662996677	no
BMPER	-0.038965038	-0.700819714	0.483920076	0.663091453	no
LOC101054525	0.038953777	0.700616867	0.484046521	0.663222428	no
DCAF13	-0.038948241	-0.700517135	0.484108696	0.663265331	no
FAM226B	-0.038945249	-0.700463251	0.48414229	0.663269074	no
FOXI2	-0.038931358	-0.700213026	0.484298311	0.663440527	no
AKR7A2P1	-0.038924131	-0.700082849	0.484379489	0.66350944	no
DEPDC1B	-0.038904372	-0.699726918	0.484601487	0.663771227	no
CDRT15	-0.038889834	-0.699465051	0.484764851	0.663943498	no
NFU1	-0.038886099	-0.699397776	0.484806825	0.663943498	no
CD2BP2	0.038880499	0.699296888	0.484869774	0.663943498	no
FAM22G	-0.038877851	-0.699249204	0.484899529	0.663943498	no
LOC284581	-0.038877374	-0.699240609	0.484904892	0.663943498	no
FANCL	0.038876687	0.699228222	0.484912621	0.663943498	no
CAPN8	-0.038873751	-0.699175349	0.484945615	0.663946373	no
DUOX1	-0.03886606	-0.699036798	0.485032079	0.664022449	no
DGKD	-0.03885914	-0.698912154	0.485109871	0.664086645	no
MYO15A	-0.038855287	-0.698842742	0.485153195	0.66410365	no
MFSD8	0.03885198	0.69878319	0.485190367	0.664112233	no
NUDT17	0.03883512	0.698479491	0.485379956	0.664329425	no
PKP3	-0.038818392	-0.698178164	0.485568103	0.664530264	no
OR14J1	-0.038816575	-0.698145442	0.485588537	0.664530264	no
VAMP4	0.038809002	0.698009016	0.485673737	0.664604538	no
CES3	-0.038792166	-0.697705762	0.48586315	0.664790597	no
UBXN2B	-0.038791418	-0.697692283	0.48587157	0.664790597	no

MS4A5	-0.038787758	-0.697626349	0.485912759	0.664804628	no
CD300LD	-0.038781663	-0.697516572	0.485981339	0.664856131	no
PSMB6	-0.038774561	-0.69738864	0.486061269	0.664923152	no
STX2	-0.038770141	-0.697309023	0.486111016	0.664948879	no
KRTAP1-1	-0.038762908	-0.697178733	0.48619243	0.664973831	no
MAGEA6	-0.038761946	-0.697161403	0.48620326	0.664973831	no
OR4C46	-0.038760273	-0.697131282	0.486222083	0.664973831	no
USP47	-0.03875156	-0.696974327	0.486320173	0.665065659	no
CCDC107	0.038746951	0.696891309	0.486372059	0.665094294	no
GPN3	0.038727559	0.69654201	0.486590407	0.665323552	no
CCDC67	-0.038724637	-0.696489374	0.486623314	0.665323552	no
CRCT1	-0.038723814	-0.696474544	0.486632586	0.665323552	no
LOC100507577	0.038700082	0.696047072	0.486899884	0.665646655	no
VPS39	-0.038695325	-0.695961395	0.486953468	0.665677564	no
LOC100216479	-0.038691122	-0.695885686	0.48700082	0.66569995	no
CENPL	-0.038682975	-0.695738933	0.487092614	0.665747613	no
PIK3C2G	-0.038682529	-0.69573089	0.487097644	0.665747613	no
PCDHB1	0.03867435	0.695583578	0.487189797	0.66583122	no
VPS29	0.03866804	0.695469903	0.487260915	0.665886069	no
MYH8	-0.038663258	-0.695383767	0.487314807	0.665917372	no
ZSWIM3	-0.038658374	-0.695295794	0.487369852	0.665929866	no
DYRK1B	-0.038656948	-0.695270111	0.487385922	0.665929866	no
SNX29P1	-0.038644053	-0.695037838	0.487531275	0.665990715	no
XPO1	-0.038642609	-0.695011842	0.487547544	0.665990715	no
KIF4A	0.038640654	0.694976611	0.487569594	0.665990715	no
RPL11	-0.038637714	-0.694923672	0.487602727	0.665990715	no
KIR2DS3	0.038637195	0.694914316	0.487608582	0.665990715	no
ARHGEF38-IT1	-0.038636502	-0.694901837	0.487616393	0.665990715	no
CIZ1	-0.038631448	-0.69481079	0.48767338	0.666026221	no
IK	-0.038626921	-0.694729249	0.487724421	0.666032776	no
UPK1A	-0.038625525	-0.694704102	0.487740162	0.666032776	no
CACNA1G	-0.038617315	-0.694556232	0.487832731	0.666101785	no
PRG3	-0.038611903	-0.694458737	0.487893768	0.666101785	no
TNFRSF19	0.038611724	0.694455523	0.487895781	0.666101785	no
COL17A1	-0.038610049	-0.694425345	0.487914676	0.666101785	no
ZFP90	-0.038600339	-0.694250447	0.488024185	0.666195355	no
PRLR	-0.038598475	-0.694216867	0.488045213	0.666195355	no
VSX1	-0.038576166	-0.693815025	0.488296878	0.666496551	no
ADAD2	-0.038566642	-0.69364348	0.488404334	0.666600885	no
HFE2	-0.038560018	-0.693524175	0.488479075	0.666660557	no
SLC22A11	0.038551033	0.693362329	0.488580476	0.666756604	no
LDLRAD2	0.038542584	0.693210133	0.488675841	0.666844403	no
STX7	-0.038535431	-0.693081305	0.488756572	0.666887282	no
TAAR2	-0.038534302	-0.693060954	0.488769326	0.666887282	no
C1orf101	0.038527019	0.692929781	0.488851535	0.666957107	no
ELOVL6	-0.038519326	-0.692791208	0.48893839	0.667033259	no
FAM189B	0.03850554	0.692542901	0.489094045	0.66719529	no
FAIM	0.038503308	0.692502698	0.48911925	0.66719529	no
CALHM3	-0.038497587	-0.69239965	0.489183857	0.667204853	no
IPO8	-0.038497189	-0.692392481	0.489188352	0.667204853	no
TXLNG2P	0.038486344	0.692197131	0.489310841	0.667329565	no

IFNA4	0.038481594	0.692111577	0.489364491	0.667360383	no
PXK	-0.038475744	-0.6920062	0.489430576	0.667408154	no
TCP10	-0.038470888	-0.691918731	0.489485434	0.66744061	no
PNLIPRP1	-0.03846767	-0.69186077	0.489521788	0.667447832	no
KRTAP4-9	-0.038437857	-0.691323782	0.489858658	0.667864773	no
XAGE2	0.038434649	0.691265991	0.489894919	0.667871842	no
SSX3	-0.03843013	-0.691184597	0.489945994	0.667885058	no
MTBP	-0.038428292	-0.69115149	0.489966769	0.667885058	no
PISD	-0.038424877	-0.691089973	0.490005373	0.667895317	no
C16orf71	0.038407498	0.690776956	0.490201828	0.668120718	no
FXR1	-0.038402194	-0.690681413	0.490261801	0.668160085	no
GPR32	-0.038393615	-0.690526888	0.490358806	0.668228786	no
LOC613037	0.038389839	0.690458868	0.49040151	0.668228786	no
RABGEF1	0.038389487	0.690452526	0.490405491	0.668228786	no
TP73	-0.038366607	-0.690040411	0.490664266	0.668539006	no
SUMO1P3	-0.038351499	-0.689768287	0.490835177	0.668729479	no
CGB1	-0.038348045	-0.689706073	0.490874256	0.668736945	no
GHRH	-0.038345514	-0.689660487	0.490902892	0.668736945	no
PCNXL3	0.038331096	0.689400794	0.491066038	0.668916792	no
ENO1-AS1	-0.038312815	-0.689071516	0.491272942	0.669146733	no
FZD10	0.038310679	0.68903305	0.491297115	0.669146733	no
AGGF1	0.038303373	0.688901458	0.491379817	0.669216962	no
OR11G2	-0.038290024	-0.68866101	0.491530952	0.669380374	no
PSG10P	0.038286245	0.688592956	0.491573732	0.669396215	no
HOGA1	-0.038280421	-0.688488052	0.49163968	0.669420121	no
SLC5A9	0.038279193	0.688465935	0.491653585	0.669420121	no
OR5H6	-0.038262997	-0.688174214	0.491837005	0.669627436	no
DEFA10P	-0.038257529	-0.688075723	0.49189894	0.669669335	no
MGC27382	0.038222656	0.687447604	0.492294024	0.670164747	no
KRTAP10-2	-0.03820606	-0.687148676	0.492482108	0.670352651	no
SNRPG	-0.038204972	-0.687129079	0.49249444	0.670352651	no
LGI4	0.038192251	0.686899957	0.49263863	0.670505869	no
LOC147646	0.038189454	0.686849579	0.492670338	0.670505869	no
RAB44	0.038186784	0.68680149	0.492700604	0.670505869	no
CXADRP3	-0.038154723	-0.686224024	0.49306414	0.67095811	no
PPP4R2	0.038150432	0.686146736	0.493112807	0.670981849	no
ATP4B	0.038145974	0.686066441	0.493163369	0.671008165	no
SNORD15A	-0.038129688	-0.6857731	0.493348114	0.671203359	no
TLL2	0.038127821	0.685739475	0.493369292	0.671203359	no
MIR663B	-0.038113179	-0.685475748	0.493535422	0.671386868	no
CYP11B2	-0.038107232	-0.685368631	0.493602906	0.67143617	no
CHRNA3	-0.038086859	-0.685001697	0.493834115	0.671708162	no
C4orf29	-0.038078678	-0.684854343	0.493926981	0.671791959	no
IRF6	0.038075178	0.684791307	0.49396671	0.671803479	no
KRT4	-0.038071208	-0.684719794	0.494011784	0.671822265	no
LSAMP-AS3	-0.038060674	-0.684530062	0.494131383	0.671942391	no
OVOL1	-0.038041689	-0.684188118	0.494346967	0.672181944	no
PCNP	-0.038039652	-0.684151432	0.494370099	0.672181944	no
MIR548I3	-0.038035076	-0.684069005	0.494422075	0.672210085	no
VANGL1	-0.038012618	-0.683664522	0.494677175	0.67251437	no
C1QTNF7	0.038002869	0.683488926	0.494787942	0.672586873	no

RAD54B	0.038002415	0.683480753	0.494793097	0.672586873	no
GPX4	-0.037990819	-0.683271889	0.494924868	0.672723442	no
C1orf194	0.037979718	0.68307194	0.495051032	0.672852374	no
FLNB	-0.03797513	-0.682989311	0.495103175	0.672880689	no
PRDM13	0.037965859	0.682822338	0.495208551	0.672981343	no
OR6A2	-0.03795775	-0.682676272	0.495300742	0.673039983	no
LINC00161	0.037956554	0.682654736	0.495314335	0.673039983	no
NHLH2	-0.037948618	-0.682511808	0.495404556	0.673054157	no
RWDD2B	0.037946455	0.682472849	0.49542915	0.673054157	no
PHOSPHO2	-0.037944004	-0.682428691	0.495457027	0.673054157	no
PTPRM	-0.037942394	-0.682399691	0.495475334	0.673054157	no
MTMR6	0.037941864	0.682390153	0.495481356	0.673054157	no
RALGPS2	0.037938162	0.682323467	0.495523457	0.673068804	no
CCNK	-0.037909643	-0.681809827	0.495847799	0.673455365	no
IDNK	-0.037907628	-0.681773531	0.495870723	0.673455365	no
OR10V1	-0.037889069	-0.681439253	0.496081871	0.673699557	no
OR4M1	-0.037882263	-0.681316678	0.496159308	0.673762144	no
BLOC1S5-TXND	-0.037879505	-0.681267006	0.496190691	0.673762187	no
CASC1	0.037872524	0.681141276	0.49627013	0.673827481	no
RORA	-0.037859283	-0.680902782	0.496420837	0.673989526	no
PTCHD3P1	0.037854316	0.680813332	0.496477367	0.674023696	no
EMC1	0.037849689	0.680729997	0.496530036	0.674052619	no
PSG2	0.037846791	0.680677799	0.496563028	0.674054827	no
ZNF132	0.03783759	0.680512071	0.496667783	0.674154444	no
CALR3	-0.03782491	-0.680283695	0.496812157	0.674307823	no
SBNO1	-0.037804961	-0.679924407	0.497039336	0.674538353	no
LOC729683	-0.037803571	-0.679899371	0.497055169	0.674538353	no
C17orf112	-0.037801728	-0.679866167	0.497076167	0.674538353	no
FAR1	-0.037796122	-0.679765199	0.497140021	0.674582409	no
ZNF497	-0.037783824	-0.679543713	0.497280111	0.674716117	no
DEFB103A	0.03778196	0.679510137	0.497301349	0.674716117	no
PCDHA7	0.037773911	0.679365167	0.497393056	0.67479794	no
MIR215	-0.0377486	-0.678909296	0.497681494	0.675079987	no
SLC7A9	0.037747531	0.678890049	0.497693674	0.675079987	no
LOC100128361	-0.037747398	-0.678887655	0.497695189	0.675079987	no
SPINT4	0.037744041	0.678827194	0.497733451	0.675089277	no
IQCA1	-0.037734122	-0.678648536	0.497846522	0.675200026	no
C9orf117	0.037723428	0.678455941	0.497968429	0.675313255	no
UHMK1	-0.037716852	-0.678337488	0.498043415	0.675313255	no
TSC22D2	0.037713971	0.678285612	0.498076256	0.675313255	no
ASPRV1	-0.037713264	-0.678272872	0.498084322	0.675313255	no
CROCCP2	-0.037711913	-0.67824855	0.49809972	0.675313255	no
TMEM91	-0.037710262	-0.678218811	0.498118548	0.675313255	no
TTTY22	-0.037684311	-0.677751415	0.49841451	0.675671875	no
COL18A1-AS1	0.037669427	0.677483339	0.498584303	0.67585942	no
UBE2E1	-0.037649087	-0.677117016	0.498816372	0.676056644	no
MLN	-0.037648852	-0.677112771	0.498819061	0.676056644	no
MIR1236	-0.037648404	-0.677104711	0.498824168	0.676056644	no
MRAP	0.037636249	0.676885791	0.498962884	0.676202002	no
ALDH9A1	-0.037628506	-0.676746341	0.499051256	0.676279119	no
SNORA5A	-0.037623278	-0.676652179	0.499110933	0.676317344	no

LINC00551	0.037617554	0.676549084	0.499176276	0.676363239	no
CDX2	-0.037606089	-0.67634259	0.499307168	0.676481746	no
LRRC37A3	0.037604378	0.676311791	0.499326692	0.676481746	no
ZNF733P	0.037596656	0.676172714	0.499414862	0.676558547	no
GPR115	0.037589409	0.676042181	0.499497623	0.676628012	no
DNAJB14	-0.037585961	-0.67598008	0.499536999	0.676638702	no
FAM183A	0.037570363	0.675699162	0.499715139	0.676837339	no
STARD7	-0.037567187	-0.675641953	0.499751422	0.676843824	no
FBLIM1	-0.037543319	-0.675212089	0.50002409	0.677170441	no
CALCB	-0.037532994	-0.67502613	0.500142071	0.67728754	no
SND1	0.03752743	0.67492592	0.500205656	0.677330965	no
PGA3	-0.037521858	-0.674825569	0.500269334	0.677374512	no
EBP	-0.037501237	-0.674454192	0.500505029	0.677650953	no
FOXD4L1	-0.037497828	-0.674392785	0.500544006	0.677661033	no
KLHL9	-0.037453261	-0.673590134	0.501053633	0.67830826	no
S100A7A	0.037435137	0.673263713	0.501260967	0.678546198	no
SNORA70D	-0.037429435	-0.673161022	0.501326202	0.678591762	no
AOC2	-0.037424448	-0.673071199	0.501383268	0.678626263	no
IL2	0.037416514	0.672928309	0.501474054	0.678706397	no
ATAD3A	-0.037401409	-0.67265628	0.501646912	0.678897594	no
ATP5L2	-0.037398374	-0.672601604	0.50168166	0.678901867	no
LIMD1	-0.037389377	-0.672439578	0.501784637	0.678998466	no
SPRR2C	-0.037373088	-0.672146208	0.50197112	0.679208043	no
SLN	0.037362029	0.671947048	0.502097739	0.679293927	no
C15orf43	-0.037362023	-0.671946936	0.502097809	0.679293927	no
GALR1	0.03735546	0.671828738	0.502172964	0.679352837	no
PRAP1	-0.037347185	-0.671679703	0.502267733	0.679430495	no
LOC100506895	-0.03734283	-0.671601276	0.502317608	0.679430495	no
ASMT	-0.037340206	-0.67155401	0.502347667	0.679430495	no
C2CD4A	0.037336765	0.67149204	0.502387079	0.679430495	no
ZNF165	0.037336646	0.671489898	0.502388441	0.679430495	no
GPSM3	-0.037327993	-0.671334061	0.502487559	0.67952178	no
C17orf64	0.037322012	0.671226334	0.502556084	0.679571685	no
WFDC13	-0.037312495	-0.671054939	0.502665116	0.679676357	no
MTG1	-0.037298751	-0.670807424	0.502822596	0.679846518	no
GPR143	0.037284481	0.670550417	0.502986141	0.679987351	no
PRSS22	-0.037284141	-0.670544293	0.502990039	0.679987351	no
PCDHB14	-0.037279704	-0.670464384	0.503040895	0.680013327	no
MAGEA4	-0.0372726	-0.670336443	0.503122325	0.680080626	no
HACL1	0.037259644	0.670103116	0.503270848	0.680169837	no
SRPK3	-0.037251154	-0.669950198	0.503368199	0.680169837	no
FAM210A	-0.037251019	-0.669947783	0.503369737	0.680169837	no
GFRA3	-0.037250873	-0.669945152	0.503371412	0.680169837	no
LOC400558	-0.037250865	-0.669944996	0.503371511	0.680169837	no
PHKG2	0.037246847	0.669872642	0.503417578	0.680169837	no
AADACL2	-0.037245285	-0.669844501	0.503435495	0.680169837	no
ANKRD61	-0.037242739	-0.669798662	0.503464681	0.680169837	no
WWC1	-0.037242	-0.66978534	0.503473164	0.680169837	no
SNX14	-0.037217533	-0.669344705	0.50375377	0.680434008	no
PIGS	0.03721495	0.66929819	0.503783397	0.680434008	no
SPRR4	-0.037214216	-0.66928497	0.503791817	0.680434008	no

FAM161A	0.037213908	0.669279419	0.503795353	0.680434008	no
IMMT	0.037206016	0.669137289	0.503885886	0.680434733	no
COX6B2	-0.037205588	-0.669129582	0.503890796	0.680434733	no
NUDT15	0.037205581	0.66912946	0.503890874	0.680434733	no
U2AF1L4	-0.037198267	-0.66899773	0.503974791	0.680505292	no
BHMT	-0.037192605	-0.668895759	0.504039755	0.680523753	no
GLIPR1L1	-0.037190329	-0.66885478	0.504065864	0.680523753	no
TAS2R3	-0.037184822	-0.668755593	0.50412906	0.680523753	no
BOK-AS1	-0.037184226	-0.668744866	0.504135895	0.680523753	no
SNORA64	-0.037183277	-0.668727768	0.50414679	0.680523753	no
VTA1	-0.037174914	-0.66857715	0.504242766	0.680610556	no
SLC7A2	-0.037167332	-0.668440599	0.504329786	0.68064444	no
ZNF235	-0.037167207	-0.668438364	0.504331211	0.68064444	no
WDR93	0.037162767	0.668358401	0.504382173	0.680670473	no
ABHD16A	-0.037151357	-0.668152905	0.504513153	0.680777925	no
CELA2A	-0.037150312	-0.668134082	0.504525151	0.680777925	no
AQP12B	-0.037140644	-0.667959977	0.504636138	0.680884935	no
CNEP1R1	0.037135789	0.667872537	0.504691884	0.680917401	no
FTH1P3	0.037123963	0.667659548	0.504827684	0.681023805	no
DEFA11P	-0.037123401	-0.667649441	0.504834128	0.681023805	no
POLK	0.037110659	0.667419965	0.504980463	0.681156434	no
TMEM31	-0.037108322	-0.667377877	0.505007304	0.681156434	no
CCNF	-0.037106368	-0.667342681	0.505029751	0.681156434	no
ETV2	-0.037103802	-0.66729647	0.505059224	0.681156434	no
P2RX6P	-0.037093843	-0.667117108	0.505173626	0.681207555	no
CHMP3	-0.037093032	-0.667102506	0.50518294	0.681207555	no
ZNF205-AS1	-0.037092224	-0.667087957	0.505192221	0.681207555	no
UVRAG	0.037082428	0.666911533	0.505304765	0.681288465	no
CETN1	-0.037079932	-0.66686658	0.505333444	0.681288465	no
MIR365B	0.037078615	0.66684287	0.50534857	0.681288465	no
TMEM38A	-0.037075964	-0.666795128	0.505379029	0.681288465	no
CELF2-AS2	-0.037058791	-0.666485852	0.505576369	0.681495293	no
TRIM51HP	0.037057094	0.666455285	0.505595875	0.681495293	no
ZSCAN5B	-0.037051362	-0.666352056	0.505661753	0.681541345	no
CCDC77	0.037044059	0.666220531	0.505745695	0.681592615	no
RNF5P1	-0.037042534	-0.666193069	0.505763224	0.681592615	no
CDRT1	0.037030894	0.665983441	0.50589703	0.68173019	no
LOC100128054	0.037022719	0.665836219	0.505991014	0.681814088	no
CSTF3	-0.037005689	-0.665529517	0.506186837	0.682002393	no
OSR2	0.037005046	0.665517941	0.506194229	0.682002393	no
PREX2	-0.037001748	-0.665458553	0.506232152	0.682010731	no
TNP2	-0.036993344	-0.665307197	0.506328809	0.682094932	no
PSKH2	0.036990795	0.665261288	0.506358128	0.682094932	no
GLTSCR1	-0.036982486	-0.665111655	0.506453698	0.68218091	no
HIST1H4L	-0.036961831	-0.664739686	0.506691312	0.682385424	no
PHYH	0.036961668	0.664736743	0.506693193	0.682385424	no
KBTBD13	-0.036961008	-0.664724857	0.506700786	0.682385424	no
NPAP1	-0.036951337	-0.664550695	0.506812063	0.682492515	no
HOXD11	0.036928948	0.664147486	0.507069732	0.682796717	no
MUC5B	-0.036923982	-0.664058054	0.507126893	0.682830903	no
RPL22L1	0.036915502	0.663905343	0.507224506	0.68291955	no

TUSC3	-0.036911095	-0.66382598	0.507275239	0.682945071	no
GHDC	-0.036886132	-0.663376411	0.507562677	0.68325618	no
TSNAX-DISC1	-0.036885504	-0.663365103	0.507569909	0.68325618	no
IRX6	-0.036876267	-0.66319876	0.507676285	0.683313492	no
TM4SF19-TCTE	-0.036874668	-0.663169958	0.507694706	0.683313492	no
TFRC	-0.036873525	-0.663149374	0.50770787	0.683313492	no
MPC1L	-0.036861996	-0.662941756	0.507840663	0.683449414	no
TYSND1	-0.036857983	-0.662869483	0.507886893	0.68346883	no
LINC00626	0.036853812	0.662794374	0.507934939	0.683490688	no
TENM4	0.036847716	0.662684576	0.50800518	0.683542408	no
ACTR3BP2	0.036839203	0.662531274	0.508103261	0.683631577	no
RD3L	-0.036811689	-0.662035788	0.508420335	0.683978904	no
C1QL1	-0.036810834	-0.662020386	0.508430193	0.683978904	no
TSPYL6	-0.036808518	-0.661978678	0.508456888	0.683978904	no
CRYAB	-0.036803347	-0.661885553	0.508516495	0.683981289	no
DMRTC2	-0.036800188	-0.66182866	0.508552912	0.683981289	no
TTC16	0.036799924	0.661823911	0.508555952	0.683981289	no
LOC401177	0.036794893	0.661733307	0.508613951	0.683981289	no
GPATCH3	-0.03679456	-0.661727306	0.508617793	0.683981289	no
GTF2A2	-0.036779984	-0.661464804	0.508785851	0.68413644	no
FLJ43681	-0.036778292	-0.661434333	0.508805361	0.68413644	no
LYG2	0.03677627	0.661397935	0.508828666	0.68413644	no
C8orf87	-0.036773002	-0.661339068	0.508866359	0.684144318	no
FTHL17	-0.036769835	-0.661282039	0.508902877	0.684150615	no
RGP1	0.036744946	0.660833823	0.509189935	0.684493707	no
SLC26A10	-0.036727907	-0.660526965	0.50938651	0.684715129	no
BCL2L2-PABPN	0.036705066	0.66011564	0.509650069	0.685026558	no
IQCF1	-0.036700796	-0.660038746	0.509699348	0.685049949	no
C1orf116	0.036693934	0.659915171	0.509778547	0.685113549	no
POLR2C	-0.036690634	-0.659855748	0.509816634	0.685121891	no
OTOR	-0.036676715	-0.659605073	0.509977318	0.685294976	no
TMPRSS11F	0.036673449	0.659546271	0.510015014	0.68530278	no
SAMD5	-0.036667306	-0.65943563	0.510085947	0.685355242	no
CDC42SE1	0.036658125	0.659270309	0.510191946	0.685444685	no
HKDC1	0.036656016	0.659232319	0.510216306	0.685444685	no
ANKRD1	0.036638103	0.658909741	0.51042317	0.685679732	no
EPPIN	-0.03660218	-0.658262826	0.510838161	0.686129834	no
OR1F2P	-0.036601319	-0.658247308	0.510848118	0.686129834	no
ZC3H11A	-0.036600809	-0.65823813	0.510854007	0.686129834	no
PTPLA	0.036597764	0.658183292	0.510889194	0.686134212	no
LOXHD1	-0.036592782	-0.658093577	0.510946762	0.686150188	no
LRRC43	0.036591209	0.658065243	0.510964944	0.686150188	no
C18orf8	0.036586409	0.657978805	0.511020414	0.686181801	no
DEK	-0.03657593	-0.657790094	0.511141527	0.686293149	no
SP5	-0.036573708	-0.657750084	0.511167207	0.686293149	no
SH3RF2	0.036566507	0.657620405	0.511250444	0.686362025	no
OR4M2	-0.036559065	-0.657486382	0.511336478	0.686395997	no
LOC283731	-0.036558792	-0.657481477	0.511339626	0.686395997	no
SNORA22	-0.03654389	-0.657213109	0.511511923	0.686554007	no
TFB1M	0.036543085	0.657198613	0.51152123	0.686554007	no
KRTAP1-5	0.036540019	0.65714341	0.511556676	0.686558703	no

CDKN2A	0.036534089	0.657036622	0.511625247	0.686607854	no
AGTR1	0.036527347	0.656915209	0.511703215	0.686632204	no
GAS5-AS1	-0.036523462	-0.656845237	0.511748152	0.686632204	no
GDF2	-0.036522435	-0.656826755	0.511760022	0.686632204	no
LRRC3C	-0.03652147	-0.656809365	0.51177119	0.686632204	no
ATP6V1E1	-0.03651691	-0.656727245	0.511823933	0.6866601	no
SUN1	-0.03651342	-0.656664408	0.511864292	0.686662601	no
MTRF1	-0.036511224	-0.656624852	0.5118897	0.686662601	no
GRXCR2	-0.036502061	-0.656459847	0.511995692	0.686761915	no
ZNF396	0.03649494	0.656331617	0.512078069	0.686808138	no
TBX22	-0.036493534	-0.656306301	0.512094333	0.686808138	no
TRIM64C	-0.036490795	-0.656256971	0.512126026	0.686808138	no
CTH	-0.03647999	-0.656062392	0.512251047	0.686932937	no
TH	-0.036459632	-0.655695789	0.512486639	0.687205987	no
ENTPD5	0.036439031	0.655324798	0.512725109	0.687472676	no
C17orf102	-0.036436924	-0.655286856	0.512749501	0.687472676	no
REN	-0.036423465	-0.655044479	0.512905334	0.687638711	no
PPARGC1B	0.036412415	0.654845502	0.513033283	0.687767343	no
TEKT1	0.036392522	0.654487267	0.51326368	0.688033293	no
LRRC14B	-0.036350604	-0.653732408	0.513749344	0.688588718	no
TRIM49D2P	-0.036345476	-0.653640066	0.513808771	0.688588718	no
TNFSF18	0.036345209	0.653635251	0.51381187	0.688588718	no
CXADRP2	-0.036343538	-0.653605158	0.513831238	0.688588718	no
ABCF2	0.036342935	0.653594304	0.513838224	0.688588718	no
DEFB116	-0.036336515	-0.653478691	0.513912635	0.688641662	no
LOC728819	0.036333997	0.653433351	0.513941818	0.688641662	no
FGFR3	-0.036329869	-0.653359008	0.513989672	0.688662845	no
SPINK14	0.036323923	0.65325193	0.5140586	0.68871226	no
RNF175	-0.036292713	-0.652689913	0.514420462	0.689130277	no
CCDC135	0.036290602	0.652651907	0.514444937	0.689130277	no
TCF7	0.03628794	0.652603966	0.514475811	0.689130277	no
AMELX	-0.036283464	-0.652523363	0.514527723	0.689130277	no
RGPD5	-0.036283035	-0.65251564	0.514532697	0.689130277	no
RYR3	0.0362796	0.652453774	0.514572543	0.689130277	no
MMP23A	-0.036275799	-0.652385339	0.514616621	0.689130277	no
TMEM56	-0.036274893	-0.652369012	0.514627138	0.689130277	no
NDUFC1	0.036266743	0.652222246	0.514721676	0.689213928	no
MIR3689B	-0.036253283	-0.651979876	0.514877819	0.689356061	no
SLC26A3	0.036252063	0.651957899	0.514891978	0.689356061	no
LOC100287704	-0.036242682	-0.651788974	0.51500082	0.689436887	no
ZFAND3	-0.03624133	-0.651764628	0.515016508	0.689436887	no
TRIQK	0.036202202	0.651060013	0.515470646	0.689978892	no
KRTAP16-1	-0.036200913	-0.651036812	0.515485603	0.689978892	no
DNMT1	-0.036183086	-0.650715796	0.515692575	0.690212938	no
RNF216	0.036179256	0.650646812	0.515737058	0.690229488	no
LOC100240734	-0.036173071	-0.650535443	0.515808876	0.690252699	no
TGM6	-0.03617223	-0.650520306	0.515818638	0.690252699	no
ABHD2	-0.036158491	-0.650272902	0.5159782	0.690409994	no
LOC253044	-0.036156577	-0.650238431	0.516000434	0.690409994	no
TSEN2	0.036151138	0.650140477	0.516063618	0.690426135	no
ZNF48	-0.036150007	-0.650120119	0.51607675	0.690426135	no

SNORA19	-0.036136075	-0.649869231	0.516238601	0.690599674	no
POU4F2	-0.036120207	-0.649583493	0.516422967	0.690803311	no
LRBA	0.036108804	0.649378162	0.516555473	0.690937554	no
LINC00265	0.036104917	0.649308171	0.516600645	0.69095497	no
ZRANB2-AS2	0.036095853	0.649144942	0.516705999	0.691052873	no
PSME1	0.036088225	0.649007592	0.516794658	0.691128438	no
PPP3R2	-0.036063731	-0.648566505	0.517079433	0.69146625	no
PAIP1	0.036059523	0.648490738	0.517128358	0.691488648	no
SCGN	-0.036033151	-0.648015846	0.517435065	0.69185572	no
PWP1	-0.036017863	-0.64774055	0.517612908	0.692050454	no
ZNF182	-0.036010405	-0.647606255	0.517699674	0.692116108	no
OR4D10	-0.036008106	-0.647564856	0.517726423	0.692116108	no
OR4K1	-0.035989451	-0.647228929	0.517943501	0.692363238	no
ZNF615	-0.035978959	-0.647040007	0.518065603	0.692448168	no
PIGX	-0.035978108	-0.647024682	0.518075509	0.692448168	no
IL15	0.035973878	0.646948506	0.518124747	0.692448168	no
TPRG1L	-0.03597099	-0.646896506	0.51815836	0.692448168	no
UCK1	-0.035967891	-0.646840698	0.518194436	0.692448168	no
UBE2D4	-0.035967382	-0.646831537	0.518200358	0.692448168	no
VDAC1	0.035963683	0.646764931	0.518243416	0.692462649	no
SPATA31C1	-0.035956993	-0.646644456	0.518321302	0.692523662	no
KIR3DL3	-0.035945114	-0.646430557	0.518459601	0.692665379	no
ATP1B4	0.035938126	0.64630471	0.518540979	0.692718204	no
BTN1A1	-0.035934993	-0.646248308	0.518577453	0.692718204	no
OSCP1	0.035930711	0.646171196	0.518627321	0.692718204	no
TMEM194A	0.035930647	0.646170034	0.518628073	0.692718204	no
ROCK1	0.035914473	0.645878803	0.518816434	0.692926729	no
FBXL19-AS1	0.035899065	0.645601341	0.518995924	0.693123378	no
ZNF548	-0.035895729	-0.645541274	0.519034786	0.693132206	no
LINC00210	0.035884308	0.645335619	0.51916785	0.693212305	no
OR4A5	0.035883344	0.645318256	0.519179085	0.693212305	no
HMGCS2	-0.035879541	-0.64524977	0.519223401	0.693212305	no
LY6G5C	-0.035879507	-0.645249169	0.51922379	0.693212305	no
SOX14	-0.035872875	-0.645129749	0.51930107	0.693272413	no
YWHAB	0.03586032	0.644903665	0.519447392	0.693387004	no
ITGA2	-0.035859973	-0.64489742	0.519451434	0.693387004	no
KIR3DX1	-0.035837848	-0.64449901	0.519709338	0.69368818	no
BLM	0.035822081	0.64421509	0.51989317	0.693846871	no
ATG4B	0.035821031	0.644196195	0.519905405	0.693846871	no
PRKACG	-0.035819343	-0.644165804	0.519925085	0.693846871	no
TRIM64B	-0.035798599	-0.643792272	0.520166998	0.694126572	no
CSRP3	-0.035793319	-0.643697179	0.520228593	0.694126572	no
STAT4	-0.035793063	-0.643692581	0.520231571	0.694126572	no
FLJ46284	-0.035789366	-0.643626012	0.520274692	0.694141011	no
YAE1D1	-0.035769332	-0.643265253	0.520508412	0.694409727	no
LINC00174	0.035765919	0.643203808	0.520548226	0.694419734	no
ST7	0.035758409	0.643068573	0.520635856	0.694459332	no
MYOG	-0.035757837	-0.643058263	0.520642537	0.694459332	no
SIRT5	-0.035748021	-0.64288151	0.520757083	0.694569011	no
CMIP	-0.03573567	-0.642659121	0.520901222	0.694718143	no
KIF26B	0.035732661	0.642604929	0.520936349	0.694721879	no

LPPR4	-0.035712627	-0.642244182	0.521170215	0.694990636	no
NBPF11	0.035696123	0.64194701	0.521362908	0.695204458	no
CLK2	-0.035655405	-0.641213811	0.521838487	0.695795442	no
OR5AK2	0.035645223	0.641030482	0.521957436	0.695869199	no
FRMD7	-0.035644938	-0.64102534	0.521960772	0.695869199	no
FLJ25758	-0.035642302	-0.640977868	0.521991576	0.695869199	no
EXOG	-0.035639585	-0.640928945	0.522023322	0.695869199	no
LOC285084	-0.035634715	-0.640841256	0.522080225	0.695901887	no
PCDHGA8	-0.035631056	-0.640775382	0.522122975	0.695915707	no
ANXA2P3	-0.035627556	-0.640712354	0.522163879	0.695927066	no
KRTAP17-1	-0.035623744	-0.640643707	0.522208432	0.695942972	no
WDR76	-0.035620287	-0.640581473	0.522248825	0.695942972	no
OBP2B	-0.035618222	-0.640544282	0.522272963	0.695942972	no
HIST1H2AA	-0.03559058	-0.640046553	0.522596075	0.696297438	no
KRT13	0.035589922	0.640034697	0.522603773	0.696297438	no
IGFBP5	0.035582689	0.639904465	0.522688333	0.696349177	no
C2CD3	0.035581058	0.639875088	0.522707409	0.696349177	no
CRNKL1	-0.035574692	-0.639760464	0.522781842	0.696405168	no
SUPT3H	-0.035571451	-0.639702104	0.522819742	0.696412487	no
PYY2	0.035559721	0.639490883	0.522956922	0.696552043	no
METAP2	-0.035548976	-0.639297413	0.52308259	0.696676248	no
LOC255025	-0.035542428	-0.63917951	0.523159181	0.696735078	no
GPR139	-0.035532987	-0.639009506	0.523269627	0.696838986	no
CPN2	0.035520142	0.63877821	0.523419913	0.696995932	no
KCNJ13	-0.035487985	-0.638199187	0.523796233	0.697438188	no
LCE1E	-0.035486216	-0.638167338	0.523816937	0.697438188	no
EN2	0.035476503	0.637992448	0.523930631	0.697546351	no
LOC283050	-0.035472123	-0.637913571	0.523981913	0.697571411	no
MIR3907	-0.035459786	-0.637691426	0.524126354	0.697720482	no
MRGPRD	-0.035436735	-0.637276372	0.52439628	0.698036571	no
OR2K2	-0.035428672	-0.637131195	0.524490711	0.698100633	no
FAM103A1	0.0354262	0.637086688	0.524519663	0.698100633	no
TRIP12	-0.035424306	-0.637052572	0.524541856	0.698100633	no
MBNL1	-0.035413418	-0.63685653	0.524669393	0.698184076	no
CSN1S1	-0.035411529	-0.636822505	0.52469153	0.698184076	no
GPR176	-0.035410633	-0.636806389	0.524702015	0.698184076	no
TMCO5A	0.035401255	0.636637521	0.52481189	0.698287044	no
FAM160A1	0.035396938	0.636559787	0.524862472	0.698311112	no
PLEKHG5	0.035392206	0.636474577	0.524917922	0.698341653	no
NFATC4	0.035380619	0.636265946	0.5250537	0.69847905	no
LCE2A	-0.035372449	-0.636118843	0.525149446	0.69856318	no
ATP5B	-0.035369136	-0.636059181	0.525188281	0.698569207	no
ECT2L	-0.035362981	-0.635948353	0.525260425	0.698569207	no
NOBOX	-0.035362768	-0.635944516	0.525262923	0.698569207	no
NR2F6	0.03536097	0.635912142	0.525283998	0.698569207	no
DEFB133	-0.035345775	-0.635638554	0.525462117	0.698762846	no
CLUAP1	-0.035342799	-0.635584957	0.525497015	0.698766016	no
METTL1	-0.035336492	-0.635471397	0.52557096	0.698821104	no
SNORA50	0.035330247	0.635358958	0.52564418	0.698875221	no
EIF2B1	0.035324236	0.635250711	0.525714675	0.698889452	no
LOC100505795	-0.035323788	-0.635242653	0.525719923	0.698889452	no

GNPNAT1	0.035313064	0.635049565	0.525845684	0.698992015	no
FABP9	-0.035311663	-0.635024326	0.525862123	0.698992015	no
IRGC	-0.035301658	-0.634844186	0.525979466	0.69910475	no
PWP2	0.035294671	0.63471838	0.526061424	0.699111844	no
HERPUD2	0.035293968	0.634705714	0.526069675	0.699111844	no
DDX11-AS1	-0.035292884	-0.634686192	0.526082394	0.699111844	no
LOC100507501	0.035285131	0.634546596	0.526173345	0.699150503	no
TTLL7	-0.035282209	-0.634493983	0.526207626	0.699150503	no
TRIM60	-0.035282085	-0.634491748	0.526209082	0.699150503	no
ZG16	-0.035276087	-0.634383749	0.526279455	0.699200777	no
ATOH1	-0.035272526	-0.634319641	0.52632123	0.699213053	no
C22orf23	-0.035249026	-0.633896496	0.526597011	0.699536182	no
ANAPC16	-0.035243171	-0.63379107	0.526665733	0.69958423	no
C4orf6	-0.035236593	-0.633672642	0.526742935	0.699643537	no
CNGA1	0.035193752	0.632901254	0.527245942	0.70020656	no
FAM224A	0.035193243	0.632892083	0.527251924	0.70020656	no
ZNF689	-0.035192166	-0.632872703	0.527264564	0.70020656	no
LOC100507299	0.035188884	0.632813593	0.52730312	0.700214493	no
TBC1D22B	0.035165164	0.632386503	0.527581738	0.700499675	no
C8A	0.03516505	0.632384463	0.527583069	0.700499675	no
CCDC117	0.035147883	0.632075349	0.527784771	0.700704659	no
CMTM2	-0.03514636	-0.632047934	0.527802662	0.700704659	no
CLDN12	-0.035116865	-0.631516866	0.528149292	0.701121529	no
BRI3BP	-0.035106636	-0.631332685	0.528269534	0.701227845	no
METTL13	-0.035104501	-0.631294237	0.528294637	0.701227845	no
AGPAT1	-0.035079244	-0.630839481	0.528591592	0.701544638	no
C10orf120	-0.035078649	-0.630828764	0.528598591	0.701544638	no
CYP3A5	-0.035071616	-0.630702136	0.528681294	0.701546731	no
TECRL	-0.0350713	-0.630696443	0.528685013	0.701546731	no
MS4A13	-0.035066505	-0.63061011	0.528741403	0.701546731	no
NOTCH2NL	-0.035060981	-0.630510634	0.528806381	0.701546731	no
FAM66A	-0.03505851	-0.630466156	0.528835436	0.701546731	no
ASXL2	0.035057381	0.630445823	0.528848718	0.701546731	no
TPTE	0.03505601	0.630421131	0.528864848	0.701546731	no
APOPT1	-0.035053715	-0.630379812	0.528891841	0.701546731	no
SOCS2	0.035052	0.630348931	0.528912015	0.701546731	no
CDC7	-0.03504994	-0.630311839	0.528936247	0.701546731	no
OR2A25	-0.035047984	-0.630276631	0.52895925	0.701546731	no
SMUG1	0.035038349	0.630103137	0.529072604	0.701653769	no
LOC100130992	-0.035025355	-0.629869188	0.529225476	0.701813199	no
HS3ST2	-0.03501565	-0.629694451	0.529339672	0.701921323	no
INPP5J	-0.035010823	-0.629607527	0.529396483	0.701953346	no
SERPINB5	-0.035004025	-0.629485137	0.529476481	0.702016105	no
MYL1	-0.034996678	-0.629352853	0.529562952	0.70208744	no
IFRD1	0.034982547	0.629098406	0.529729298	0.702161613	no
SAMD7	-0.034980806	-0.629067061	0.529749792	0.702161613	no
OR52N1	0.034976395	0.628987641	0.52980172	0.702161613	no
CACNA1D	-0.034976199	-0.62898412	0.529804022	0.702161613	no
ZBED6	-0.034975941	-0.628979463	0.529807067	0.702161613	no
RASA3	-0.034972677	-0.62892071	0.529845484	0.702161613	no
MAML1	-0.034959162	-0.628677368	0.530004613	0.702161613	no

LHX8	-0.034951469	-0.628538841	0.530095212	0.702161613	no
HDLBP	0.034929273	0.628139202	0.530356625	0.702161613	no
CSTL1	-0.034929157	-0.628137113	0.530357991	0.702161613	no
CLEC3B	-0.034916548	-0.627910095	0.530506518	0.702161613	no
TMEM241	0.034906694	0.627732675	0.530622611	0.702161613	no
SMPD4	-0.034902173	-0.627651266	0.530675884	0.702161613	no
LINC00235	0.034897321	0.627563898	0.53073306	0.702161613	no
FLJ45974	-0.034891276	-0.627455057	0.530804292	0.702161613	no
WFDC1	-0.034889611	-0.627425085	0.530823908	0.702161613	no
CHRNB4	0.034868983	0.627053672	0.531067026	0.702161613	no
TVP23C-CDRT4	0.034867055	0.627018958	0.531089752	0.702161613	no
PZP	0.034845454	0.626630031	0.5313444	0.702161613	no
KCTD7	-0.034840585	-0.626542371	0.531401804	0.702161613	no
SPDYE4	-0.034818677	-0.626147928	0.531660141	0.702161613	no
PHKA1	0.034818611	0.626146736	0.531660922	0.702161613	no
NODAL	-0.034818122	-0.626137924	0.531666694	0.702161613	no
MIR3944	0.034805527	0.625911151	0.531815247	0.702161613	no
VPS25	0.034800289	0.625816849	0.531877029	0.702161613	no
TRAPPC1	-0.034791572	-0.625659896	0.531979863	0.702161613	no
SPATA31A7	-0.034791572	-0.625659896	0.531979863	0.702161613	no
OR11H1	-0.034791572	-0.625659896	0.531979863	0.702161613	no
LOC100505776	-0.034791572	-0.625659896	0.531979863	0.702161613	no
RNU6-6	-0.034791572	-0.625659896	0.531979863	0.702161613	no
GGTLC2	-0.034791572	-0.625659896	0.531979863	0.702161613	no
GAGE12I	-0.034791572	-0.625659896	0.531979863	0.702161613	no
TRIM40	-0.034791572	-0.625659896	0.531979863	0.702161613	no
H2AFB3	-0.034791572	-0.625659896	0.531979863	0.702161613	no
OR4F21	-0.034791572	-0.625659896	0.531979863	0.702161613	no
RHOXF2B	-0.034791572	-0.625659896	0.531979863	0.702161613	no
RHOXF2	-0.034791572	-0.625659896	0.531979863	0.702161613	no
TTTY7B	-0.034791572	-0.625659896	0.531979863	0.702161613	no
TISP43	-0.034791572	-0.625659896	0.531979863	0.702161613	no
CSN1S2BP	-0.034791572	-0.625659896	0.531979863	0.702161613	no
LINC00112	-0.034791572	-0.625659896	0.531979863	0.702161613	no
LOC100288570	-0.034791572	-0.625659896	0.531979863	0.702161613	no
NRG1-IT3	-0.034791572	-0.625659896	0.531979863	0.702161613	no
MIR1253	-0.034791572	-0.625659896	0.531979863	0.702161613	no
PRAMEF21	-0.034791572	-0.625659896	0.531979863	0.702161613	no
MIR7-3	-0.034791572	-0.625659896	0.531979863	0.702161613	no
OR2T35	-0.034791572	-0.625659896	0.531979863	0.702161613	no
LOC100288966	-0.034791572	-0.625659896	0.531979863	0.702161613	no
IRX4	-0.034791572	-0.625659896	0.531979863	0.702161613	no
SLC2A2	-0.034791572	-0.625659896	0.531979863	0.702161613	no
SPANXA2	-0.034791572	-0.625659896	0.531979863	0.702161613	no
TTTY19	-0.034791572	-0.625659896	0.531979863	0.702161613	no
NR1I3	-0.034791572	-0.625659896	0.531979863	0.702161613	no
RNU6-16	-0.034791572	-0.625659896	0.531979863	0.702161613	no
TSPY2	-0.034791572	-0.625659896	0.531979863	0.702161613	no
MIR548J	-0.034791572	-0.625659896	0.531979863	0.702161613	no
MIR3689A	-0.034791572	-0.625659896	0.531979863	0.702161613	no
MIR3910-1	-0.034791572	-0.625659896	0.531979863	0.702161613	no

LOC255411	-0.034791572	-0.625659896	0.531979863	0.702161613	no
MIR5000	-0.034791572	-0.625659896	0.531979863	0.702161613	no
MIR5100	-0.034791572	-0.625659896	0.531979863	0.702161613	no
LOC100130954	-0.034791572	-0.625659896	0.531979863	0.702161613	no
MIR7-2	-0.034791572	-0.625659896	0.531979863	0.702161613	no
FAM223B	-0.034791572	-0.625659896	0.531979863	0.702161613	no
PRAMEF7	-0.034791572	-0.625659896	0.531979863	0.702161613	no
MIR4330	-0.034791572	-0.625659896	0.531979863	0.702161613	no
MAGEA5	-0.034791572	-0.625659896	0.531979863	0.702161613	no
MIR5186	-0.034791572	-0.625659896	0.531979863	0.702161613	no
MUC7	-0.034791572	-0.625659896	0.531979863	0.702161613	no
ZPBP2	-0.034791572	-0.625659896	0.531979863	0.702161613	no
MIR518C	-0.034791572	-0.625659896	0.531979863	0.702161613	no
CST8	-0.034791572	-0.625659896	0.531979863	0.702161613	no
LINC00493	0.034789528	0.625623089	0.53200398	0.702161613	no
TYW1	0.034785502	0.625550611	0.532051472	0.702164877	no
LINC00303	-0.034783014	-0.625505812	0.532080828	0.702164877	no
PIGR	0.03478101	0.625469732	0.532104471	0.702164877	no
MSLN	0.034769381	0.625260356	0.532241684	0.70230282	no
SIGLECL1	-0.034724445	-0.62445129	0.532772071	0.702922939	no
UBE2J1	0.034724023	0.624443685	0.532777058	0.702922939	no
FLJ35282	0.034719689	0.62436566	0.532828222	0.702947288	no
RSPH3	0.034691438	0.623857005	0.533161828	0.703314973	no
MAP3K19	0.034690098	0.623832885	0.53317765	0.703314973	no
DAOA-AS1	-0.034687774	-0.623791036	0.533205102	0.703314973	no
ZNF280A	0.034674648	0.623554702	0.533360147	0.703476306	no
PRAMEF1	-0.034655179	-0.62320417	0.533590152	0.703714835	no
FGG	-0.034652326	-0.623152801	0.533623862	0.703714835	no
LOC645949	-0.034651025	-0.623129386	0.533639229	0.703714835	no
NLRP8	-0.034634503	-0.622831919	0.533834463	0.703886076	no
NPPC	-0.034634493	-0.622831727	0.533834589	0.703886076	no
ECEL1	-0.034622922	-0.622623399	0.533971341	0.703939777	no
ZNF846	-0.034621644	-0.622600397	0.533986441	0.703939777	no
RBL2	-0.034620603	-0.622581643	0.533998754	0.703939777	no
COX6B1	0.03461691	0.62251515	0.534042406	0.703939777	no
DMPK	-0.034616471	-0.622507261	0.534047585	0.703939777	no
LINC00424	-0.034614419	-0.622470306	0.534071847	0.703939777	no
CREB3	0.03461061	0.622401737	0.534116866	0.70395594	no
CACNA1G-AS1	-0.034605237	-0.622304986	0.53418039	0.7039712	no
OBP2A	-0.034601544	-0.622238506	0.534224042	0.7039712	no
MAS1L	-0.034601318	-0.622234438	0.534226713	0.7039712	no
SCGB1B2P	-0.034588155	-0.62199743	0.534382352	0.704091477	no
KATNAL1	0.034585165	0.621943604	0.534417701	0.704091477	no
VWDE	0.03458388	0.621920473	0.534432892	0.704091477	no
SEC14L4	-0.034582515	-0.62189589	0.534449038	0.704091477	no
RASGEF1A	-0.034574582	-0.621753068	0.534542842	0.704171891	no
TIMELESS	-0.034565896	-0.621596677	0.534645569	0.704249152	no
CXorf58	0.034564081	0.621564005	0.534667031	0.704249152	no
PPIA	0.034556985	0.621436232	0.534750969	0.704316545	no
SPNS1	0.03454685	0.621253761	0.534870851	0.70443127	no
GCG	-0.034539189	-0.621115828	0.534961481	0.704507457	no

FEN1	-0.034523378	-0.620831161	0.535148549	0.704710629	no
LOC441242	-0.034511406	-0.62061561	0.535290219	0.704817176	no
KRTAP4-5	-0.034510997	-0.620608258	0.535295051	0.704817176	no
CCZ1B	0.034503375	0.620471031	0.535385254	0.704887622	no
KRTAP10-8	-0.034500934	-0.62042707	0.535414152	0.704887622	no
VCX2	-0.034497699	-0.620368837	0.535452433	0.704894839	no
ALDH8A1	0.034493012	0.620284439	0.535507917	0.704924699	no
LACRT	-0.034474897	-0.619958297	0.535722353	0.705139451	no
KIAA0101	-0.034473687	-0.619936512	0.535736678	0.705139451	no
ATRX	-0.034462689	-0.619738497	0.535866895	0.705267648	no
TERT	-0.034443197	-0.619387562	0.536097712	0.705473432	no
WDR87	-0.034441247	-0.61935245	0.536120808	0.705473432	no
C2orf57	-0.034440161	-0.619332902	0.536133668	0.705473432	no
NKX6-2	-0.034438397	-0.619301147	0.536154556	0.705473432	no
COA6	0.034435367	0.619246579	0.536190453	0.705477472	no
BCL9L	-0.034426666	-0.61908993	0.53629351	0.70556987	no
FAM102A	0.034411278	0.618812876	0.536475804	0.705719289	no
LOC283688	-0.034409476	-0.618780433	0.536497152	0.705719289	no
ECI2	-0.034408763	-0.618767602	0.536505595	0.705719289	no
SNORA5B	-0.034401334	-0.618633843	0.536593618	0.705791109	no
GOLIM4	0.034398611	0.618584826	0.536625877	0.705791109	no
TMEM56-RWDD3	-0.034395386	-0.61852677	0.536664085	0.705798168	no
GPR182	-0.034387763	-0.618389515	0.536754423	0.70587378	no
SEPHS1	0.034373603	0.618134582	0.536922233	0.706051258	no
FAM172BP	0.034370371	0.618076388	0.536960543	0.706058433	no
FAT4	-0.034347642	-0.617667169	0.537229977	0.706326926	no
C10orf76	0.034347601	0.617666428	0.537230465	0.706326926	no
DSC3	-0.034335662	-0.61745149	0.53737201	0.706453552	no
LINC00550	-0.034333932	-0.617420347	0.537392521	0.706453552	no
GPR128	0.034320149	0.61717219	0.537555968	0.706625195	no
SIPA1L3	-0.034316729	-0.617110612	0.53759653	0.706635292	no
SPG20OS	-0.034305851	-0.616914766	0.537725546	0.706761648	no
SF3B14	-0.034294471	-0.616709878	0.537860535	0.706864107	no
MRPL15	-0.034293733	-0.616696602	0.537869282	0.706864107	no
LINC00281	-0.034277028	-0.61639584	0.538067469	0.707081325	no
IL31	-0.034263867	-0.616158896	0.53822363	0.707228039	no
OXGR1	0.034262072	0.616126579	0.538244931	0.707228039	no
CACHD1	0.034244406	0.615808511	0.538454597	0.707425259	no
GCOM1	-0.034243878	-0.615799008	0.538460862	0.707425259	no
THEM5	0.034235185	0.61564251	0.538564039	0.707459966	no
GPR89A	0.034234866	0.61563676	0.538567829	0.707459966	no
OR6M1	0.034233332	0.615609146	0.538586036	0.707459966	no
KLHL41	-0.034219747	-0.61536456	0.538747312	0.707628559	no
LOC729603	-0.034210145	-0.61519169	0.538861314	0.707735042	no
SMARCE1	-0.034205258	-0.615103699	0.538919346	0.707768006	no
TMC4	-0.034175735	-0.614572176	0.539269963	0.708148572	no
ST7L	0.034175309	0.614564506	0.539275023	0.708148572	no
MYBPHL	-0.034167528	-0.614424414	0.539367453	0.708226672	no
IMPG1	-0.0341644	-0.61436811	0.539404604	0.708232181	no
MTX2	-0.034155061	-0.614199979	0.539515549	0.708297608	no
ZNF746	-0.034154513	-0.6141901	0.539522068	0.708297608	no

RAB31	-0.034149252	-0.614095384	0.539584574	0.708297608	no
LECT1	-0.034148493	-0.614081717	0.539593593	0.708297608	no
GNAT1	0.034145304	0.614024311	0.53963148	0.708297608	no
YARS	-0.034141733	-0.613960017	0.539673913	0.708297608	no
XRCC5	0.034140788	0.613943008	0.539685139	0.708297608	no
TMEM139	-0.034117667	-0.613526736	0.539959917	0.70861496	no
PALB2	-0.034110988	-0.613406492	0.540039303	0.708675866	no
MCAT	-0.034090395	-0.613035745	0.540284108	0.708911215	no
RPE65	-0.034090352	-0.613034968	0.540284621	0.708911215	no
GLOD5	0.034083358	0.612909049	0.540367778	0.70897704	no
CHN2	0.034077178	0.612797798	0.540441254	0.709030156	no
GORASP1	-0.03407019	-0.612671984	0.540524354	0.709068175	no
SCARA5	-0.034069192	-0.61265402	0.54053622	0.709068175	no
SP2	-0.034061049	-0.612507416	0.540633061	0.709130644	no
CYP26A1	-0.034059086	-0.612472069	0.540656411	0.709130644	no
BAZ2A	-0.034056864	-0.612432075	0.540682832	0.709130644	no
FLJ41350	-0.034052639	-0.612356	0.54073309	0.70913483	no
FAM227B	-0.034048081	-0.61227395	0.540787298	0.70913483	no
PCDHAC1	-0.034046507	-0.612245601	0.540806028	0.70913483	no
POC1B-GALNT4	0.034045499	0.612227464	0.540818011	0.70913483	no
TUBG1	-0.034040643	-0.612140033	0.540875778	0.709167308	no
WDR75	-0.034036287	-0.612061617	0.540927592	0.709191977	no
OSTN	-0.034025118	-0.611860537	0.541060468	0.709246177	no
TAB3	0.034025024	0.611858838	0.541061591	0.709246177	no
OR51I2	-0.034023661	-0.611834304	0.541077805	0.709246177	no
OR6K3	-0.034021717	-0.611799295	0.541100941	0.709246177	no
KLLN	0.033980796	0.611062582	0.541587929	0.709841201	no
ABHD5	0.03396788	0.610830048	0.541741686	0.709999425	no
SNORA37	-0.033961738	-0.610719485	0.541814801	0.710051947	no
FAM194A	-0.03395758	-0.610644613	0.541864315	0.710073537	no
KIAA1024	-0.033946102	-0.610437969	0.542000988	0.71020933	no
B3GNTL1	0.033941938	0.610363015	0.542050566	0.71023099	no
SPANXN3	-0.033932403	-0.610191345	0.542164124	0.710336474	no
COQ7	0.033926989	0.610093872	0.542228607	0.710377652	no
OR4K2	-0.033921665	-0.609998033	0.542292013	0.71038846	no
CLDN24	0.033920745	0.609981476	0.542302968	0.71038846	no
SDR9C7	-0.033913239	-0.609846329	0.542392386	0.710462289	no
RPL13AP6	0.033897824	0.609568813	0.542576025	0.71062626	no
MOSPD2	0.033894894	0.609516061	0.542610937	0.71062626	no
PDE6B	0.033894404	0.609507252	0.542616766	0.71062626	no
BMP8B	0.033888319	0.609397697	0.542689273	0.710677909	no
BCAS1	-0.033883361	-0.609308437	0.542748351	0.710711968	no
FAM86JP	0.033875794	0.609172204	0.542838526	0.710786739	no
FKBP1A	0.033865119	0.608980019	0.542965748	0.71086201	no
DKC1	0.033863357	0.608948291	0.542986753	0.71086201	no
PRH1	-0.033862644	-0.608935467	0.542995243	0.71086201	no
FRYL	-0.033854355	-0.608786243	0.543094039	0.710948042	no
GLI2	-0.033845324	-0.608623649	0.543201698	0.710981175	no
CELA3A	-0.033842497	-0.608572753	0.5432354	0.710981175	no
PTH2	-0.033841747	-0.608559257	0.543244337	0.710981175	no
KRT6A	-0.033840846	-0.608543028	0.543255083	0.710981175	no

HSD17B10	0.033838103	0.608493652	0.543287781	0.710981175	no
LINC00326	-0.033835581	-0.60844825	0.543317847	0.710981175	no
FAM21B	-0.033823943	-0.608238721	0.543456613	0.711119464	no
METTL20	0.033817928	0.608130439	0.543528333	0.711170009	no
MAB21L1	-0.033811568	-0.608015933	0.543604179	0.711225948	no
SNAPIN	0.033799668	0.607801695	0.543746102	0.711360126	no
ZAN	-0.033794013	-0.607699893	0.543813547	0.711360126	no
LOC727982	-0.033792807	-0.607678172	0.543827938	0.711360126	no
CSN1S2AP	0.033789312	0.607615266	0.543869617	0.711360126	no
FAM74A1	-0.033789093	-0.607611313	0.543872236	0.711360126	no
C10orf90	0.033784522	0.607529024	0.543926759	0.71138175	no
CTAGE9	0.033782157	0.607486447	0.543954972	0.71138175	no
SLC27A6	0.033772541	0.607313337	0.544069684	0.711454615	no
RFX6	-0.033771937	-0.607302453	0.544076897	0.711454615	no
ZNF226	0.033765093	0.607179244	0.54415855	0.711482057	no
SERTAD4-AS1	-0.033764312	-0.607165181	0.54416787	0.711482057	no
FGFR4	-0.033761853	-0.607120925	0.544197202	0.711482057	no
PSTPIP1	0.033755371	0.607004216	0.544274555	0.711488083	no
YPEL2	0.033753777	0.606975531	0.544293569	0.711488083	no
TATDN1	0.033751212	0.606929352	0.544324178	0.711488083	no
PRSS56	-0.0337468	-0.60684992	0.54437683	0.711488083	no
AK8	0.033745874	0.606833251	0.54438788	0.711488083	no
SEMA7A	0.033744821	0.60681429	0.544400448	0.711488083	no
RAD51L3-RFFL	0.033734401	0.606626698	0.544524809	0.711602524	no
CCNB1	0.033731935	0.606582311	0.544554237	0.711602524	no
SULT6B1	-0.033712119	-0.606225562	0.544790782	0.71183014	no
TBL1XR1	-0.033711794	-0.606219706	0.544794665	0.71183014	no
SGK110	0.033706681	0.606127657	0.544855708	0.711866619	no
BTF3P11	-0.03369149	-0.60585418	0.545037084	0.712060303	no
LCE3D	-0.033686168	-0.605758362	0.545100641	0.712100047	no
OR10G4	0.033662866	0.605338862	0.545378938	0.712420298	no
GCNT4	-0.033651564	-0.605135391	0.545513946	0.712553347	no
ATP6V1G1	0.033645431	0.605024986	0.545587211	0.712570075	no
FXN	-0.033644941	-0.605016162	0.545593066	0.712570075	no
SLC22A14	-0.033616148	-0.604497804	0.545937111	0.712967852	no
NUP210L	-0.033613899	-0.604457326	0.545963982	0.712967852	no
EXO1	-0.033605126	-0.604299388	0.546068832	0.713061446	no
TNPO1	-0.033589456	-0.604017278	0.546256143	0.713231408	no
RFX2	0.033586968	0.603972491	0.546285883	0.713231408	no
PDE8A	-0.033584967	-0.603936472	0.546309801	0.713231408	no
E2F5	-0.033581266	-0.603869842	0.546354047	0.713231408	no
IQCH-AS1	0.033580356	0.603853457	0.546364928	0.713231408	no
PTTG2	0.033572165	0.603705994	0.546462861	0.713273362	no
GOLGA2	0.033572116	0.603705114	0.546463445	0.713273362	no
MIR548H4	-0.033551508	-0.603334123	0.546709864	0.713551664	no
C17orf66	0.033544591	0.603209604	0.546792584	0.713616289	no
DCLK3	0.033525457	0.602865145	0.547021447	0.713867327	no
C9orf85	-0.033522956	-0.602820113	0.54705137	0.713867327	no
FLJ13224	-0.033519059	-0.602749955	0.547097991	0.713884817	no
TRPT1	0.03351096	0.602604152	0.547194886	0.713947007	no
GAPDHS	-0.033509071	-0.602570144	0.547217487	0.713947007	no

PCDHB11	-0.033506745	-0.602528274	0.547245314	0.713947007	no
TRAF6	-0.033494932	-0.602315615	0.54738666	0.714047826	no
DCAF12	0.033494733	0.602312029	0.547389044	0.714047826	no
TNN	0.03348309	0.602102421	0.547528379	0.714186235	no
LOC100128505	-0.033476792	-0.601989049	0.547603751	0.714236174	no
LOC100131825	-0.033474337	-0.601944853	0.547633134	0.714236174	no
ALOX15	-0.033458368	-0.601657366	0.547824286	0.714442122	no
RPL27A	-0.033450768	-0.601520551	0.547915267	0.714517416	no
PRR25	-0.033446462	-0.601443033	0.54796682	0.714541285	no
ZNF526	0.033443403	0.601387957	0.548003448	0.714545693	no
PPOX	-0.033433529	-0.601210209	0.548121671	0.714656484	no
HOXC13	-0.033422723	-0.601015664	0.548251078	0.714781844	no
SH3D19	-0.033418536	-0.600940297	0.548301216	0.714792081	no
KRT73	-0.033416512	-0.600903868	0.54832545	0.714792081	no
MPZ	-0.033391401	-0.60045181	0.548626231	0.715135328	no
NANOGNB	-0.033388181	-0.600393842	0.548664806	0.715135328	no
DEFB118	-0.033386196	-0.600358107	0.548688587	0.715135328	no
TDRG1	0.033369431	0.600056286	0.548889463	0.715330401	no
FGB	0.033367511	0.600021728	0.548912465	0.715330401	no
LOC100499227	0.033365371	0.599983197	0.548938113	0.715330401	no
CXorf56	-0.033360172	-0.599889605	0.549000413	0.715368209	no
PDE4B	-0.033345853	-0.599631837	0.549172015	0.715548428	no
ESRP2	-0.033338237	-0.599494723	0.549263306	0.715606209	no
MUSK	-0.033335566	-0.599446641	0.549295321	0.715606209	no
SRRM1	0.033333819	0.599415203	0.549316255	0.715606209	no
CYP3A4	-0.033296803	-0.598748824	0.549760061	0.716140954	no
CXorf27	-0.033277218	-0.598396252	0.549994944	0.7164035	no
EDA	0.033273189	0.598323732	0.550043263	0.716423016	no
KLK11	-0.033243273	-0.59778517	0.550402165	0.716840617	no
PILRB	-0.033240903	-0.59774252	0.550430592	0.716840617	no
SERPINC1	0.033216251	0.597298725	0.550726435	0.71718244	no
UQCRFS1	-0.033201851	-0.597039507	0.550899271	0.71736168	no
WBP11P1	-0.033199222	-0.596992175	0.550930833	0.71736168	no
TAS2R10	-0.033186733	-0.596767348	0.551080765	0.717451208	no
KRT25	-0.033184242	-0.596722496	0.551110679	0.717451208	no
GLB1L2	0.033182742	0.596695509	0.551128677	0.717451208	no
AP4E1	0.033182372	0.59668884	0.551133125	0.717451208	no
CDC14B	-0.033178823	-0.596624949	0.551175738	0.717454146	no
MARK2	-0.033176623	-0.59658535	0.55120215	0.717454146	no
FCAMR	-0.033164112	-0.596360125	0.551352384	0.71760623	no
SERPINA11	-0.033146887	-0.596050034	0.55155926	0.717832014	no
TGIF2LX	-0.033143417	-0.595987577	0.551600932	0.717842777	no
KRT24	-0.033138437	-0.595897922	0.551660754	0.717877158	no
EPHX2	0.03311881	0.595544605	0.551896536	0.718140497	no
OR9A4	0.033114953	0.595475166	0.551942881	0.71815732	no
PRDM5	-0.033099998	-0.595205957	0.552122575	0.718347637	no
GOLGA8F	0.033095224	0.595120008	0.552179951	0.718378796	no
SPDYE1	-0.033088376	-0.594996737	0.552262247	0.71844237	no
FAM19A3	0.033076646	0.59478558	0.55240323	0.718582278	no
LRRC16A	0.033071434	0.594691744	0.552465887	0.718620287	no
TBC1D12	-0.033063078	-0.594541324	0.552566334	0.718707443	no

SCRN3	-0.033049552	-0.594297835	0.552728949	0.718808551	no
SPATA6L	-0.033048287	-0.594275074	0.552744152	0.718808551	no
SYS1-DBNDD2	-0.033046771	-0.594247777	0.552762384	0.718808551	no
CARNS1	-0.033045484	-0.594224612	0.552777857	0.718808551	no
GRAMD1C	-0.033041933	-0.594160683	0.552820558	0.718820584	no
STAG1	-0.033039094	-0.594109578	0.552854694	0.71882148	no
FAM86C2P	0.033029888	0.593943857	0.552965398	0.718921922	no
ARV1	-0.033026535	-0.593883496	0.553005722	0.718926767	no
NKX2-6	-0.033024015	-0.593838131	0.55303603	0.718926767	no
CYP2C19	-0.033000562	-0.593415937	0.553318128	0.719229094	no
CYP27B1	-0.032999115	-0.593389899	0.553335528	0.719229094	no
DYM	-0.032988826	-0.593204678	0.553459312	0.719346482	no
CTSL1P8	0.032981475	0.593072342	0.55354776	0.719388447	no
RNF180	-0.032980578	-0.593056203	0.553558548	0.719388447	no
HAS2-AS1	0.03297542	0.592963342	0.553620617	0.719425607	no
SYCP3	0.032960641	0.592697292	0.553798468	0.71961321	no
HSD11B2	-0.032948078	-0.592471148	0.553949665	0.719766158	no
LBX2	0.032939605	0.592318612	0.55405166	0.719855161	no
GK2	-0.032922588	-0.592012282	0.554256518	0.720077792	no
PIP	0.032918615	0.591940777	0.554304343	0.720079554	no
CDC20	0.032916909	0.591910062	0.554324887	0.720079554	no
FAM223A	-0.032902962	-0.591658987	0.554492831	0.720254182	no
DSCR8	-0.032894503	-0.591506726	0.554594691	0.720342953	no
SFMBT2	-0.032890641	-0.59143719	0.554641213	0.720359842	no
DIO2-AS1	-0.032877736	-0.591204885	0.554796645	0.720518171	no
EGFR	-0.032860849	-0.590900906	0.555000065	0.7207388	no
NDUFV2	-0.032856321	-0.590819389	0.555054623	0.720766096	no
ANLN	-0.032847255	-0.59065619	0.555163854	0.720840164	no
MIR496	-0.032846019	-0.590633945	0.555178744	0.720840164	no
DCTN2	-0.032837799	-0.590485977	0.555277792	0.720925212	no
ZFX	0.032822396	0.590208698	0.555463423	0.721122654	no
SERPINI2	0.032814603	0.590068413	0.555557351	0.721201029	no
CKS1B	-0.032810983	-0.590003241	0.55560099	0.721201333	no
SNORA25	0.032809016	0.589967829	0.555624702	0.721201333	no
GCDH	0.032804637	0.589889017	0.555677478	0.721226276	no
ELOVL2	-0.032773043	-0.589320272	0.556058404	0.721642359	no
RBM23	-0.032768414	-0.589236944	0.556114225	0.721642359	no
VRTN	-0.032767665	-0.589223462	0.556123257	0.721642359	no
EED	0.032766909	0.58920986	0.556132369	0.721642359	no
C7orf26	0.032762975	0.589139042	0.556179813	0.721660349	no
THOC7	-0.032735729	-0.588648589	0.556508439	0.722043158	no
C2CD4B	-0.032718617	-0.588340553	0.556714887	0.722192436	no
HEPACAM2	-0.03271259	-0.588232051	0.556787614	0.722192436	no
PCMTD1	-0.032710802	-0.588199862	0.556809191	0.722192436	no
DNAJC24	-0.032709979	-0.588185058	0.556819114	0.722192436	no
CNGB3	-0.032708679	-0.588161655	0.556834802	0.722192436	no
DLG1-AS1	-0.032708327	-0.588155316	0.556839052	0.722192436	no
MSX1	0.032706697	0.588125968	0.556858725	0.722192436	no
OR8B3	0.03269283	0.587876347	0.557026071	0.722323392	no
IQCF4	-0.032692759	-0.587875078	0.557026922	0.722323392	no
PGR	-0.032689368	-0.587814037	0.557067847	0.722332877	no

PROX2	0.032685582	0.587745886	0.557113542	0.722341067	no
EHD1	0.032683275	0.587704359	0.557141386	0.722341067	no
EXOC3	-0.032672559	-0.587511458	0.557270737	0.722432325	no
DNASE1L3	-0.032671874	-0.587499129	0.557279005	0.722432325	no
GIF	-0.032665498	-0.587384351	0.557355977	0.722488528	no
TP53INP2	0.032651998	0.587141345	0.557518959	0.722614684	no
DAZ2	-0.032651867	-0.587138977	0.557520547	0.722614684	no
RPL10L	-0.032633977	-0.586816938	0.557736573	0.722851085	no
COPB1	0.032630742	0.586758709	0.557775637	0.722858121	no
MIA3	-0.03262694	-0.586690278	0.557821548	0.722860079	no
DDX54	-0.032625047	-0.586656188	0.557844419	0.722860079	no
ST8SIA5	-0.032620692	-0.586577794	0.557897018	0.722884651	no
SFSWAP	-0.032614555	-0.586467328	0.557971139	0.722937104	no
WFDC10A	-0.032597257	-0.586155953	0.558180092	0.723057456	no
SEBOX	-0.03259607	-0.586134574	0.55819444	0.723057456	no
IFT172	-0.032592959	-0.586078586	0.558232016	0.723057456	no
NBPF7	-0.032592753	-0.586074871	0.55823451	0.723057456	no
RPL10A	-0.032590279	-0.586030345	0.558264394	0.723057456	no
NRAP	-0.032590155	-0.586028108	0.558265896	0.723057456	no
NES	0.032585852	0.585950642	0.558317891	0.723081221	no
LOC100507091	-0.032573453	-0.58572745	0.558467709	0.723225762	no
F9	0.032571045	0.585684114	0.558496801	0.723225762	no
ZHX1-C8ORF76	-0.032560571	-0.585495567	0.558623382	0.723346093	no
CD101	0.032547394	0.58525837	0.558782644	0.723466043	no
TUBB	-0.032547336	-0.585257327	0.558783344	0.723466043	no
LINC00111	-0.032533275	-0.585004224	0.558953311	0.723642507	no
NANOS2	-0.032521507	-0.584792393	0.559095582	0.723783095	no
AHI1	0.032513332	0.584645233	0.559194429	0.723800901	no
MFSD10	0.032509912	0.58458367	0.559235782	0.723800901	no
FLJ27354	-0.032508656	-0.584561063	0.559250969	0.723800901	no
PANX3	-0.032506198	-0.58451682	0.55928069	0.723800901	no
LOC286238	0.0325046	0.584488054	0.559300014	0.723800901	no
FAM65A	0.032503658	0.584471087	0.559311413	0.723800901	no
MAGEB10	-0.032497606	-0.58436216	0.559384591	0.723826897	no
MRPL23	0.032496426	0.58434092	0.559398861	0.723826897	no
ATP6V0A2	-0.032492904	-0.584277505	0.559441467	0.723838444	no
TOP1P2	-0.032482866	-0.584096813	0.559562874	0.723879321	no
LZIC	0.032482222	0.584085234	0.559570655	0.723879321	no
CXXC5	-0.032481937	-0.584080095	0.559574108	0.723879321	no
OR6C70	0.032473782	0.583933303	0.559672749	0.723963346	no
C9orf174	0.032463395	0.583746334	0.559798399	0.724082299	no
RNF224	0.032459808	0.583681759	0.559841799	0.724094855	no
DNTTIP1	0.032456104	0.583615095	0.559886605	0.724109228	no
APH1B	0.032448246	0.583473647	0.55998168	0.724188608	no
SMIM5	0.032445229	0.583419339	0.560018185	0.724192239	no
SEC16A	0.032439724	0.583320235	0.560084806	0.724233172	no
LY6G6E	-0.032436249	-0.583257694	0.56012685	0.724233172	no
ORC2	0.032427794	0.583105482	0.560229182	0.724233172	no
RALA	0.032427668	0.583103214	0.560230706	0.724233172	no
OR13G1	-0.032422378	-0.583008006	0.560294719	0.724233172	no
SRC	-0.032420832	-0.582980167	0.560313437	0.724233172	no

PLEKHH3	0.032419444	0.582955192	0.56033023	0.724233172	no
RBAK	0.032415447	0.58288324	0.560378611	0.724233172	no
TMEM147	-0.032414857	-0.582872615	0.560385755	0.724233172	no
AGMO	-0.032412725	-0.582834231	0.560411565	0.724233172	no
RAPSN	-0.032411984	-0.582820897	0.560420532	0.724233172	no
MRPS14	-0.032386006	-0.582353281	0.560735019	0.724596012	no
EPAS1	0.03238191	0.582279555	0.56078461	0.724616525	no
IKZF1	0.032376847	0.582188419	0.560845914	0.72465217	no
NKAPL	0.032370339	0.582071267	0.560924724	0.724710427	no
DUSP12	-0.032367032	-0.58201174	0.56096477	0.724718598	no
ZNF826P	-0.032359671	-0.581879232	0.561053919	0.724790201	no
CCDC151	0.032337145	0.581473756	0.56132676	0.72509908	no
ZNF790-AS1	-0.032331779	-0.581377176	0.561391758	0.725139456	no
NEK5	-0.032313586	-0.581049693	0.561612177	0.72538057	no
CTDSP2	0.032308874	0.580964873	0.561669273	0.725410718	no
SLC13A3	0.03230161	0.580834118	0.561757297	0.725480805	no
TOR1AIP1	-0.032291537	-0.580652802	0.561879369	0.725594852	no
TRAFD1	-0.032284545	-0.580526937	0.561964116	0.725628725	no
LOC100507489	-0.032283801	-0.580513552	0.561973128	0.725628725	no
SNORA77	-0.032279042	-0.580427883	0.562030815	0.725659612	no
TMPRSS11E	0.032275509	0.580364298	0.562073632	0.725659732	no
LOC100505474	-0.032271852	-0.580298466	0.562117964	0.725659732	no
PLEKHA5	-0.032270677	-0.58027732	0.562132205	0.725659732	no
NIPSNAP3B	0.032264961	0.580174429	0.562201498	0.725688117	no
LINC00623	0.032263293	0.580144392	0.562221727	0.725688117	no
PTCRA	-0.032253632	-0.579970497	0.56233885	0.725795701	no
FOXE1	-0.03225002	-0.579905487	0.562382638	0.725808629	no
C10orf71	-0.032237895	-0.579687233	0.56252966	0.725954778	no
ANTXR2	0.032231709	0.579575882	0.562604676	0.726007992	no
ZP2	0.032221866	0.5793987	0.562724052	0.726118439	no
KRTAP19-1	-0.032219034	-0.579347721	0.562758402	0.726119165	no
PHOX2A	0.032208415	0.579156573	0.562887204	0.726241755	no
SIM1	-0.032200307	-0.579010633	0.562985555	0.726314711	no
LOC728463	0.032198181	0.578972367	0.563011343	0.726314711	no
SERINC2	0.032176007	0.578573231	0.563280371	0.726618155	no
CDCA2	0.032167814	0.578425754	0.563379791	0.726685071	no
SEMA4B	-0.03216379	-0.578353327	0.563428619	0.726685071	no
SFXN5	-0.032162356	-0.578327506	0.563446028	0.726685071	no
IL1F10	0.032160586	0.578295659	0.563467499	0.726685071	no
MIR3973	-0.032150098	-0.578106866	0.563594793	0.726779705	no
ADAM3A	-0.032148104	-0.578070978	0.563618992	0.726779705	no
OFD1	-0.032146181	-0.578036365	0.563642331	0.726779705	no
CMC4	0.03213362	0.577810261	0.563794807	0.726887132	no
OR7A17	-0.032132915	-0.577797576	0.563803361	0.726887132	no
HGC6.3	0.032130959	0.577762357	0.563827113	0.726887132	no
TRMT2A	-0.032124969	-0.577654541	0.56389983	0.726911501	no
DDX39B	-0.032123829	-0.577634029	0.563913664	0.726911501	no
LRP2	-0.032118426	-0.577536776	0.56397926	0.726952455	no
LGSN	-0.032102437	-0.57724897	0.564173404	0.727159088	no
TUSC2	-0.032097309	-0.577156669	0.564235674	0.727195735	no
TCEB3B	-0.032092598	-0.577071868	0.564292887	0.727198504	no

IGSF9	-0.03209156	-0.577053178	0.564305498	0.727198504	no
GUCY2F	-0.032079096	-0.576828823	0.564456879	0.727328851	no
SPATA16	0.032077659	0.576802956	0.564474334	0.727328851	no
KRAS	-0.032071786	-0.576697248	0.564545667	0.727377154	no
TMEM205	-0.032066837	-0.576608168	0.564605784	0.727411	no
TMSB4Y	-0.032051469	-0.576331538	0.564792488	0.727572863	no
SLC12A8	-0.032050922	-0.5763217	0.564799129	0.727572863	no
NCBP2	-0.032038387	-0.576096074	0.564951432	0.727725439	no
CEACAM6	-0.032028926	-0.575925776	0.565066401	0.727829909	no
PTPRF	-0.032022256	-0.575805721	0.565147458	0.727890688	no
OR52E4	0.032018529	0.575738622	0.565192763	0.727905415	no
ZNRF4	-0.032006559	-0.575523165	0.565338251	0.728049158	no
C7orf53	0.032001393	0.575430188	0.565401039	0.728086388	no
POLR2E	-0.031989161	-0.57521001	0.565549741	0.728234242	no
LINC00559	-0.031985184	-0.575138423	0.565598093	0.72823714	no
SCP2D1	-0.031983402	-0.575106341	0.565619764	0.72823714	no
DDX28	0.031967439	0.574819018	0.565813853	0.728443391	no
CLK1	-0.03194849	-0.574477936	0.5660443	0.728672765	no
EPG5	-0.031947213	-0.574454953	0.56605983	0.728672765	no
TTC31	0.031920154	0.573967897	0.566388985	0.729052808	no
TMEM80	-0.031915011	-0.573875325	0.566451556	0.729089681	no
DTX3	-0.03191137	-0.573809784	0.566495859	0.729103036	no
TMEM212	0.031893114	0.573481181	0.566718002	0.729345266	no
ACTR3BP5	0.031875766	0.573168928	0.566929132	0.729573292	no
APLP2	0.031870449	0.57307323	0.566993846	0.729612882	no
GCGR	-0.031866558	-0.573003195	0.567041208	0.72963014	no
SLC17A4	-0.031856726	-0.572826212	0.567160903	0.729740463	no
STAU1	-0.031843709	-0.57259192	0.567319376	0.729859486	no
SALL4	-0.031841752	-0.572556695	0.567343203	0.729859486	no
LOC100507118	-0.031840759	-0.572538824	0.567355292	0.729859486	no
SNORA23	-0.031835774	-0.57244909	0.567415994	0.729893885	no
FAM194B	-0.031827428	-0.572298862	0.567517627	0.729980926	no
YTHDC2	0.031817489	0.572119971	0.567638661	0.730092912	no
RNF17	-0.031809595	-0.571977887	0.567734801	0.730172867	no
LOC729013	-0.031789988	-0.57162496	0.567973641	0.730436331	no
PLD2	0.031786935	0.571570008	0.568010834	0.730440452	no
SSSCA1	-0.0317835	-0.571508191	0.568052675	0.730450549	no
GSX2	0.03177915	0.571429884	0.568105678	0.730474999	no
PRKAG2	0.031768216	0.571233083	0.568238897	0.730602581	no
PDYN	-0.031756386	-0.571020145	0.568383057	0.730744214	no
TRMT13	0.031743168	0.570782221	0.568544152	0.730900733	no
DKFZp6860132	0.031738224	0.570693235	0.56860441	0.730900733	no
RCAN2	0.031738025	0.570689662	0.568606829	0.730900733	no
PEX14	-0.031732998	-0.570599167	0.568668112	0.730935789	no
NRD1	0.031727105	0.570493106	0.568739939	0.730984393	no
ZNRF2P2	-0.031721598	-0.570393979	0.568807075	0.731026961	no
PIK3R3	-0.031714966	-0.570274608	0.568887926	0.73108715	no
LOC100862671	-0.03169794	-0.569968162	0.56909551	0.731310189	no
ANKMY2	0.031694894	0.569913333	0.569132654	0.731314192	no
RALGAPA1	-0.031691942	-0.569860197	0.569168652	0.731316723	no
USP28	-0.031679584	-0.569637761	0.569319361	0.731466634	no

LINC00685	-0.031675218	-0.569559181	0.569372606	0.731491313	no
KRTAP22-1	-0.031666608	-0.569404208	0.569477622	0.731564008	no
LOC100873065	-0.031664998	-0.569375213	0.569497271	0.731564008	no
GBX2	-0.031653786	-0.569173418	0.569634032	0.731695952	no
OR52H1	0.031646545	0.569043073	0.569722377	0.731728331	no
DPP3	-0.031646138	-0.569035758	0.569727336	0.731728331	no
ADAL	-0.031639719	-0.568920216	0.569805654	0.731752685	no
NDUFB11	-0.031638959	-0.568906532	0.569814929	0.731752685	no
TPTE2P5	-0.031636212	-0.568857089	0.569848445	0.731752685	no
YEATS4	-0.031622822	-0.56861609	0.570011825	0.731898433	no
PVR	0.031621328	0.568589197	0.570030058	0.731898433	no
ACIN1	-0.031610266	-0.56839008	0.570165064	0.731992041	no
CPT1C	-0.031608791	-0.568363537	0.570183062	0.731992041	no
CKLF-CMTM1	-0.031606982	-0.568330971	0.570205144	0.731992041	no
PGPEP1L	-0.031600052	-0.568206244	0.570289722	0.732056888	no
OR7D4	-0.031595203	-0.568118965	0.570348911	0.732089138	no
ELAC2	0.031588671	0.568001394	0.570428646	0.732147756	no
TAS2R7	0.031581998	0.567881284	0.570510109	0.732208584	no
COX8C	-0.031578059	-0.567810397	0.570558189	0.732226564	no
TSPO2	-0.031570352	-0.56767167	0.570652289	0.732303598	no
SLC41A3	-0.031566914	-0.56760979	0.570694266	0.732313737	no
SKA3	-0.031552073	-0.567342658	0.570875491	0.732483915	no
SAV1	0.031550471	0.567313826	0.570895053	0.732483915	no
MRPS11	0.03153509	0.56703699	0.571082895	0.73268008	no
WDHD1	-0.031532369	-0.566988017	0.571116128	0.73268008	no
INTS5	-0.03152313	-0.566821711	0.571228989	0.732765327	no
UCHL3	0.031521346	0.566789617	0.57125077	0.732765327	no
GEN1	-0.031516075	-0.566694744	0.57131516	0.732804183	no
DNAJB3	-0.031508543	-0.566559175	0.571407176	0.732878469	no
ZNF324	-0.031499184	-0.566390711	0.57152153	0.732981392	no
ENPP6	-0.031488146	-0.566192038	0.571656403	0.733084534	no
KRTAP20-2	-0.031487019	-0.566171754	0.571670174	0.733084534	no
H3F3AP4	0.031481119	0.566065565	0.57174227	0.733133241	no
NOS1	0.031477581	0.566001887	0.571785505	0.733138804	no
LINC00433	-0.031475181	-0.565958689	0.571814836	0.733138804	no
JAG2	-0.031463094	-0.565741135	0.571962564	0.733227259	no
DKFZP434A062	-0.03146203	-0.565721975	0.571975575	0.733227259	no
MAOA	-0.031461162	-0.565706358	0.57198618	0.733227259	no
AMELY	-0.031453928	-0.565576157	0.572074603	0.733263556	no
KCNQ1DN	-0.031453263	-0.565564183	0.572082735	0.733263556	no
AGK	0.03143545	0.565243571	0.572300498	0.733498927	no
RND3	0.031428092	0.565111143	0.572390457	0.733556665	no
CCDC126	0.031423431	0.565027243	0.572447453	0.733556665	no
SGCB	-0.031423391	-0.565026516	0.572447947	0.733556665	no
GABARAP	0.031418693	0.564941959	0.572505393	0.733586537	no
ADTRP	0.031414317	0.564863198	0.572558904	0.733611364	no
USH1G	-0.031406072	-0.564714808	0.572659727	0.733696804	no
LINC00489	-0.031394347	-0.564503762	0.572803137	0.733795187	no
PLAGL2	0.031391548	0.564453387	0.572837369	0.733795187	no
LCE3E	-0.031391419	-0.564451063	0.572838949	0.733795187	no
DMKN	-0.031380467	-0.564253948	0.572972911	0.733923044	no

BCL2L11	0.031371799	0.564097921	0.573078961	0.734015135	no
ADAM23	-0.031349539	-0.563697267	0.573351322	0.734320218	no
IQCF3	-0.031335592	-0.563446256	0.573521989	0.734495028	no
GUCY2EP	-0.031289251	-0.562612168	0.574089272	0.735177723	no
CYB5R2	0.031283701	0.562512281	0.574157226	0.735220934	no
NR0B1	-0.0312668	-0.562208082	0.574364197	0.735442145	no
C5orf64	-0.031260705	-0.562098391	0.574438838	0.735467801	no
MC3R	-0.031259575	-0.562078052	0.574452678	0.735467801	no
G3BP2	0.031209493	0.561176639	0.575066236	0.736209476	no
ZNF564	-0.03120157	-0.561034042	0.575163325	0.73628991	no
SLC39A2	0.031195189	0.560919192	0.575241527	0.736346159	no
BCL2	0.031174819	0.56055257	0.575491198	0.736476194	no
AKR1D1	-0.03117181	-0.560498405	0.575528089	0.736476194	no
GAS8	0.031171039	0.560484535	0.575537536	0.736476194	no
SPDYA	0.031170762	0.56047955	0.575540931	0.736476194	no
ATP2B4	-0.031170205	-0.56046951	0.575547769	0.736476194	no
KRTAP9-1	-0.0311687	-0.560442439	0.575566208	0.736476194	no
VEZT	0.031167331	0.560417792	0.575582995	0.736476194	no
PABPC4L	0.031146845	0.560049076	0.575834163	0.736739214	no
GUSBP9	-0.031143234	-0.559984083	0.575878441	0.736739214	no
TARBP2	0.031142177	0.559965064	0.575891399	0.736739214	no
PI15	0.031139037	0.559908542	0.575929908	0.736744622	no
XYLT1	-0.031132968	-0.559799317	0.576004327	0.736795965	no
ZC3H14	0.031124942	0.559654864	0.576102756	0.73687801	no
GABRP	0.031119395	0.559555021	0.576170793	0.736921176	no
KRT14	-0.031112834	-0.559436926	0.576251272	0.736939856	no
CXorf61	-0.031112613	-0.559432953	0.57625398	0.736939856	no
SLC35E3	-0.031109009	-0.559368099	0.576298178	0.736952526	no
OR10H5	-0.031100358	-0.559212393	0.576404301	0.737000253	no
OR5K1	-0.031100058	-0.559206984	0.576407988	0.737000253	no
LOC100129216	-0.031096983	-0.55915164	0.57644571	0.737000253	no
ZNF398	-0.031094785	-0.55911208	0.576472675	0.737000253	no
COX11	0.0310877	0.558984573	0.57655959	0.737050193	no
MLIP-IT1	-0.031083392	-0.558907028	0.576612451	0.737050193	no
GJA10	-0.031083215	-0.55890384	0.576614624	0.737050193	no
NTN5	-0.031074424	-0.558745622	0.576722487	0.737129365	no
CCNA1	-0.031072576	-0.558712363	0.576745162	0.737129365	no
LGALS16	-0.031061108	-0.558505962	0.57688589	0.737265381	no
TMEM252	0.031049158	0.558290879	0.577032554	0.737408968	no
UNC93A	-0.031046013	-0.558234263	0.577071163	0.737414459	no
WDR70	-0.031040534	-0.558135651	0.577138414	0.737456547	no
TPBGL	0.031032682	0.55799434	0.577234792	0.737535845	no
SPATA4	0.03102458	0.557848517	0.577334254	0.737619075	no
PAPL	-0.031021353	-0.557790434	0.577373874	0.737625843	no
KRTAP21-1	-0.0310144	-0.557665294	0.577459239	0.737673494	no
RP1L1	-0.031011018	-0.557604434	0.577500757	0.737673494	no
TIA1	0.031009928	0.557584805	0.577514148	0.737673494	no
RXFP2	-0.031000736	-0.557419372	0.577627012	0.73777381	no
TGS1	0.030990622	0.557237339	0.577751214	0.737888593	no
RIN2	0.030977926	0.557008836	0.577907141	0.738043877	no
BCAR4	-0.030965749	-0.556789672	0.578056714	0.738191029	no

KLF17	0.030944144	0.556400821	0.578322137	0.738430597	no
APTX	-0.030943196	-0.556383753	0.578333789	0.738430597	no
INTS9	-0.03094036	-0.556332713	0.578368632	0.738430597	no
MIDN	-0.030939292	-0.556313495	0.578381752	0.738430597	no
LOC90784	0.030928916	0.556126747	0.57850925	0.738549501	no
OR2H1	-0.03091359	-0.555850905	0.578697599	0.738735989	no
LOC100130093	-0.030909237	-0.555772557	0.578751102	0.738735989	no
NBPF22P	-0.030908639	-0.555761797	0.57875845	0.738735989	no
LLGL2	0.0308872	0.555375946	0.579021976	0.739028464	no
CUTA	-0.030879194	-0.555231851	0.579120404	0.739110197	no
HYLS1	0.030874591	0.555149012	0.579176993	0.739122765	no
NSMAF	-0.030872671	-0.555114456	0.5792006	0.739122765	no
DDX43	0.030870001	0.555066401	0.579233428	0.739122765	no
HOXC8	-0.030858342	-0.554856557	0.579376795	0.739195637	no
ZNF749	-0.030855451	-0.554804518	0.579412351	0.739195637	no
LZTS2	-0.030854928	-0.554795111	0.579418778	0.739195637	no
CCDC54	-0.030854168	-0.554781441	0.579428118	0.739195637	no
UCA1	-0.03084771	-0.554665208	0.57950754	0.739242732	no
CYLC1	-0.030845573	-0.554626734	0.579533831	0.739242732	no
PRDM7	-0.030839326	-0.55451431	0.579610656	0.739296849	no
ANKRD44	-0.030829431	-0.554336218	0.579732366	0.739408206	no
MYNN	-0.030826285	-0.554279601	0.579771061	0.739408524	no
OR10K1	-0.030823817	-0.554235173	0.579801426	0.739408524	no
DUSP4	0.03081792	0.554129043	0.579873966	0.739457153	no
DNLZ	-0.030807763	-0.553946237	0.579998924	0.739572616	no
MTA2	-0.030803947	-0.553877573	0.580045864	0.739588588	no
LINC00640	-0.030792319	-0.55366828	0.580188948	0.73972714	no
LOC157273	0.03078538	0.553543394	0.580274335	0.739789712	no
NOL9	0.030779714	0.553441425	0.580344057	0.739789712	no
SPINT3	-0.030778104	-0.553412453	0.580363868	0.739789712	no
MMACHC	-0.030777141	-0.553395124	0.580375718	0.739789712	no
DNAJB9	0.030767624	0.553223833	0.580492852	0.739895136	no
TMEM200C	-0.030763629	-0.553151926	0.580542028	0.739913932	no
SRSF2	-0.030755942	-0.553013575	0.580636648	0.739990643	no
C17orf107	0.030745204	0.552820323	0.580768829	0.740115211	no
SNORD10	-0.030729331	-0.552534643	0.580964255	0.740315824	no
FAM71C	-0.030723685	-0.552433038	0.581033767	0.740315824	no
KLK12	0.030722545	0.552412518	0.581047807	0.740315824	no
GUCA2B	-0.030721227	-0.552388789	0.581064042	0.740315824	no
GMNC	0.030711593	0.55221541	0.58118267	0.74042307	no
PAPOLB	0.030696027	0.551935249	0.581374384	0.740623408	no
POM121L2	-0.030683325	-0.551706638	0.581530844	0.740778814	no
ID2	-0.030675304	-0.551562287	0.581629648	0.740833384	no
SIMC1	0.030674251	0.551543327	0.581642627	0.740833384	no
FOXD3	-0.030661303	-0.551310308	0.581802138	0.740992637	no
USP38	0.030655739	0.551210157	0.581870703	0.741036046	no
SSPN	-0.030650954	-0.551124054	0.581929653	0.741036242	no
CEBPG	0.030650129	0.551109205	0.581939819	0.741036242	no
SEC14L1	-0.030643413	-0.550988321	0.582022587	0.741097726	no
APOL4	0.030636014	0.550855161	0.582113766	0.741169912	no
POLR2J4	0.03062422	0.550642906	0.582259119	0.741311062	no

LOC100506497	-0.030619199	-0.550552532	0.582321012	0.741344954	no
ABCC11	-0.030616463	-0.550503301	0.58235473	0.741344954	no
SGCE	0.030606107	0.550316914	0.582482392	0.741463549	no
OR10S1	-0.030597936	-0.550169851	0.582583129	0.741520992	no
MIA	-0.030596849	-0.550150294	0.582596526	0.741520992	no
PAEP	-0.030575141	-0.549759597	0.582864194	0.741730913	no
ZIC2	-0.030573569	-0.549731316	0.582883571	0.741730913	no
NUDT14	0.030573047	0.549721918	0.582890011	0.741730913	no
PRR7-AS1	0.030572277	0.549708053	0.582899511	0.741730913	no
OR11A1	0.030568773	0.549644987	0.582942724	0.741741982	no
GRXCR1	-0.03056552	-0.549586453	0.582982833	0.741749101	no
PCDHB17	0.030551498	0.549334087	0.583155777	0.741925219	no
RNF212	-0.03054384	-0.54919626	0.583250238	0.742001472	no
L3MBTL3	0.030539791	0.549123394	0.583300181	0.742021084	no
NEUROG3	-0.030532027	-0.548983667	0.583395955	0.742036847	no
TIMM10B	0.030528684	0.548923494	0.583437203	0.742036847	no
SPANXN2	-0.030525453	-0.54886535	0.583477061	0.742036847	no
SPINK2	0.0305246	0.54884999	0.583487591	0.742036847	no
CIC	-0.030523051	-0.548822108	0.583506704	0.742036847	no
LOC100129046	0.03051772	0.548726167	0.583572476	0.742036847	no
MCCC2	-0.03051739	-0.548720236	0.583576542	0.742036847	no
RPS11	-0.030514032	-0.548659804	0.583617973	0.742036847	no
ASB9P1	-0.030513599	-0.548652001	0.583623323	0.742036847	no
FAM179A	0.030501585	0.548435794	0.583771562	0.742181414	no
HIST1H2AJ	0.030497409	0.548360622	0.583823106	0.742187128	no
CMAS	-0.030495214	-0.548321134	0.583850184	0.742187128	no
LAMA5	0.030492826	0.548278149	0.58387966	0.742187128	no
CAMTA2	0.030485619	0.548148449	0.583968602	0.742252861	no
DLX6-AS1	-0.030483039	-0.548102013	0.584000448	0.742252861	no
SNAR-H	-0.03046692	-0.547811903	0.584199422	0.742442187	no
POU5F1	0.030465374	0.547784086	0.584218502	0.742442187	no
VAC14	0.030461166	0.547708358	0.584270446	0.742464295	no
FAM187B	-0.030457925	-0.547650033	0.584310455	0.742471234	no
RAD50	-0.030454828	-0.547594287	0.584348695	0.742475926	no
SH3RF3-AS1	-0.030443306	-0.547386921	0.584490955	0.742595913	no
EARS2	-0.030441582	-0.547355902	0.584512236	0.742595913	no
DNAL1	-0.030436661	-0.547267333	0.584573003	0.742629214	no
KLHL7-AS1	-0.030426957	-0.547092693	0.584692832	0.742721986	no
ST6GALNAC5	-0.03042515	-0.547060169	0.58471515	0.742721986	no
PTDSS2	-0.030411447	-0.546813556	0.584884385	0.742893045	no
ZC3H7A	0.030407048	0.546734387	0.584938719	0.742896315	no
PRCD	-0.030405642	-0.546709069	0.584956095	0.742896315	no
C11orf54	-0.030395107	-0.546519475	0.585086225	0.742973454	no
MIR5194	0.030393962	0.54649887	0.585100368	0.742973454	no
ZFP91-CNTF	-0.030392328	-0.54646947	0.585120548	0.742973454	no
NTF4	0.030386095	0.54635728	0.58519756	0.74302734	no
METTL17	-0.030373919	-0.546138164	0.585347983	0.743174425	no
INPP5F	-0.030358797	-0.545866005	0.585534845	0.743367755	no
MED7	0.03035096	0.545724956	0.585631699	0.743446798	no
LOC441155	-0.030335673	-0.545449831	0.58582064	0.743642727	no
COPS8	-0.030318212	-0.545135586	0.586036481	0.743869748	no

DDX10	-0.030315605	-0.545088673	0.586068706	0.743869748	no
FILIP1	0.030311945	0.545022797	0.58611396	0.743876684	no
ACSL4	-0.030309563	-0.544979945	0.586143398	0.743876684	no
N6AMT2	0.030301728	0.544838926	0.586240278	0.743955702	no
PIEZO2	0.030293884	0.544697764	0.586337264	0.744034845	no
RASD1	0.030288764	0.54460561	0.586400582	0.744043387	no
EDN3	-0.03028774	-0.544587193	0.586413237	0.744043387	no
EGFR-AS1	-0.030273106	-0.544323829	0.586594213	0.744229071	no
ZNF701	0.030254417	0.543987482	0.586825379	0.744478408	no
C7orf34	-0.030242524	-0.543773435	0.586972512	0.744621112	no
NRSN2	-0.030236972	-0.543673521	0.587041198	0.744629529	no
LINC00162	0.030236335	0.54366206	0.587049077	0.744629529	no
F2	-0.030229339	-0.543536154	0.587135636	0.744629529	no
PGM5-AS1	-0.030228327	-0.543517947	0.587148154	0.744629529	no
SHH	-0.030226266	-0.543480849	0.58717366	0.744629529	no
SEC14L3	0.030225185	0.543461391	0.587187038	0.744629529	no
LOC100128338	0.030220074	0.543369411	0.58725028	0.744665787	no
IFNB1	0.030210638	0.543199588	0.587367051	0.744769916	no
LOC283693	-0.030178851	-0.542627532	0.587760482	0.745224809	no
ROMO1	-0.030164908	-0.542376601	0.587933099	0.745399695	no
MIR1256	0.030125998	0.541676346	0.58841493	0.745966568	no
SUZ12	-0.030120924	-0.541585036	0.588477773	0.74600223	no
C12orf40	-0.030117885	-0.541530343	0.588515415	0.746005945	no
EMC6	0.030114414	0.541467865	0.588558417	0.746016452	no
ZCCHC13	-0.030102383	-0.541251346	0.588707454	0.746161352	no
FGFR1OP2	0.030093956	0.541099705	0.588811843	0.746221436	no
UBAC2-AS1	-0.030088082	-0.540993985	0.588884626	0.746221436	no
PRR23B	-0.030087926	-0.54099118	0.588886557	0.746221436	no
MRPS18C	-0.030087346	-0.540980734	0.588893749	0.746221436	no
KLF7	-0.030081185	-0.540869866	0.588970081	0.746236676	no
TSNAXIP1	-0.03008077	-0.540862398	0.588975222	0.746236676	no
FAM89A	0.03005994	0.540487523	0.589233356	0.746491632	no
ITLN2	-0.030058926	-0.54046928	0.589245919	0.746491632	no
ST8SIA6-AS1	0.03004772	0.540267611	0.589384808	0.746623572	no
PLA2G2F	0.030038275	0.540097636	0.589501881	0.746727863	no
KCTD20	-0.030032627	-0.539995983	0.589571902	0.746772543	no
REG1B	-0.030024419	-0.539848266	0.589673659	0.746814185	no
ADGB	0.030022338	0.539810809	0.589699463	0.746814185	no
SIGMAR1	0.030021566	0.539796926	0.589709028	0.746814185	no
TSSK3	-0.030012682	-0.539637044	0.589819177	0.746909666	no
OPA1-AS1	-0.030007307	-0.539540312	0.589885824	0.746943737	no
SLC7A13	-0.030001822	-0.539441593	0.589953845	0.746943737	no
MYO3B	0.030001124	0.539429035	0.589962498	0.746943737	no
TEFM	0.0299993	0.539396223	0.589985107	0.746943737	no
PTMS	-0.029976811	-0.53899149	0.590264023	0.747239314	no
SEMA4F	0.029974869	0.538956538	0.590288113	0.747239314	no
ABCG5	-0.029971319	-0.538892647	0.590332149	0.747251043	no
RNF31	0.02996667	0.538808992	0.59038981	0.747280016	no
CDC37	0.02995453	0.538590512	0.590540412	0.74742662	no
41153	-0.029943708	-0.538395759	0.590674675	0.747552525	no
FUT6	-0.02993779	-0.538289253	0.590748106	0.747599241	no

KCNG1	-0.029935126	-0.538241313	0.59078116	0.747599241	no
WDSUB1	-0.029926702	-0.538089713	0.590885691	0.747655112	no
SOCS2-AS1	0.029924909	0.538057437	0.590907947	0.747655112	no
PCSK1	0.029920159	0.537971957	0.590966892	0.747655112	no
KISS1	-0.02991802	-0.537933462	0.590993438	0.747655112	no
TAX1BP1	-0.029917551	-0.537925023	0.590999258	0.747655112	no
ASAP2	-0.029891131	-0.537449563	0.591327181	0.748025926	no
TRIM49C	-0.029885295	-0.537344543	0.591399624	0.748047522	no
ARHGEF16	0.029884148	0.537323894	0.591413869	0.748047522	no
CEP120	0.029876242	0.537181611	0.591512024	0.748090446	no
AK7	0.029875807	0.537173785	0.591517423	0.748090446	no
PSMA1	0.029871771	0.537101152	0.591567533	0.748100163	no
HHAT	-0.029868385	-0.537040209	0.591609579	0.748100163	no
DPYD-AS1	-0.029866777	-0.537011283	0.591629537	0.748100163	no
OR10T2	-0.029862556	-0.536935314	0.591681952	0.748120214	no
CDK11B	-0.029858743	-0.536866693	0.5917293	0.748120214	no
C17orf97	-0.02985709	-0.536836945	0.591749826	0.748120214	no
INO80B	0.029850981	0.536727003	0.59182569	0.748167577	no
SPACA3	0.029848466	0.536681752	0.591856916	0.748167577	no
NINJ2	0.029835504	0.536448484	0.592017898	0.748327058	no
LILRP2	0.029830817	0.536364135	0.592076113	0.748356628	no
SNORD9	-0.029825376	-0.536266212	0.5921437	0.748398039	no
HSPA1A	0.02982027	0.536174322	0.592207127	0.748434188	no
RNF148	0.029803127	0.535865814	0.592420095	0.748588531	no
CENPI	0.029802657	0.535857359	0.592425932	0.748588531	no
LINC00536	-0.029802028	-0.535846034	0.59243375	0.748588531	no
KCNK2	-0.02979057	-0.535639844	0.592576108	0.74872439	no
SLC10A2	-0.029785618	-0.535550723	0.592637644	0.748758119	no
UQCRBP1	-0.029772455	-0.535313835	0.592801223	0.748920762	no
TMC5	-0.0297495	-0.534900735	0.593086533	0.749237167	no
UBE2W	-0.02972586	-0.534475322	0.593380412	0.749564359	no
LOC146481	-0.029720287	-0.534375014	0.593449716	0.749607843	no
SSBP1	-0.029713092	-0.534245548	0.59353917	0.749676774	no
SNORA74A	0.029702569	0.534056168	0.593670034	0.749797996	no
RNF183	-0.029690326	-0.533835851	0.593822292	0.749946222	no
CABIN1	-0.029661304	-0.533313556	0.594183313	0.750358067	no
GRAMD4	0.029650052	0.533111078	0.594323298	0.750471179	no
CLCN7	-0.029648491	-0.533082982	0.594342723	0.750471179	no
LOC100129722	-0.029637661	-0.532888092	0.594477478	0.750597232	no
BAGE4	0.029617819	0.532531021	0.594724408	0.750849115	no
SET	-0.029611275	-0.532413245	0.594805865	0.750849115	no
GLYAT	0.029610785	0.532404423	0.594811966	0.750849115	no
ZBTB32	0.029610403	0.532397547	0.594816722	0.750849115	no
RCN2	-0.029588128	-0.531996706	0.595093997	0.751155003	no
RAD9A	-0.029585317	-0.531946118	0.595128994	0.75115506	no
ZBED3-AS1	-0.02957606	-0.531779522	0.595244254	0.751256417	no
OSBPL8	-0.029565042	-0.531581249	0.595381443	0.751385436	no
LGALS9B	0.029544999	0.531220564	0.595631046	0.751656302	no
GSPT1	0.029541899	0.531164769	0.595669662	0.751660895	no
RPS5	-0.029532431	-0.530994378	0.595787597	0.751730142	no
HPYR1	-0.029531877	-0.530984411	0.595794496	0.751730142	no

CHTF8	-0.029524031	-0.530843224	0.595892226	0.751809313	no
C1orf68	-0.029518704	-0.530747352	0.595958593	0.751818719	no
C14orf101	0.0295167	0.5307113	0.595983551	0.751818719	no
LOC10012877C	0.029515008	0.53068085	0.596004631	0.751818719	no
MFAP4	0.029507109	0.5305387	0.596103044	0.751898727	no
RCC2	-0.029496134	-0.530341195	0.596239792	0.752027077	no
ANHX	-0.029488121	-0.530197002	0.596339637	0.752108869	no
EIF4G1	-0.029482314	-0.530092489	0.596412011	0.752156008	no
CCL4L2	0.029479124	0.530035097	0.596451756	0.752161993	no
CDKN1C	-0.029473654	-0.529936647	0.596519937	0.752203835	no
MUC22	-0.029470141	-0.529873431	0.596563719	0.752214907	no
DENND4A	-0.02945283	-0.529561917	0.596779487	0.752442826	no
ABHD13	-0.029430892	-0.529167123	0.59705299	0.752738669	no
GEMIN8P4	-0.02942839	-0.529122108	0.597084179	0.752738669	no
LOC100131564	0.029412916	0.528843632	0.597277139	0.752937763	no
PCSK9	-0.029393212	-0.528489045	0.597522879	0.753160696	no
ARF5	-0.029393116	-0.52848732	0.597524074	0.753160696	no
REG3A	-0.029388535	-0.528404893	0.597581205	0.753188533	no
KIAA1407	0.029380642	0.528262854	0.597679659	0.753268447	no
LINC00301	-0.029375449	-0.528169402	0.59774444	0.753305915	no
ERVV-2	0.029371182	0.528092605	0.597797678	0.753328832	no
ACE2	-0.029347164	-0.527660396	0.598097337	0.753662262	no
PDE11A	-0.029340351	-0.527537791	0.598182354	0.753725198	no
ARL2	0.029322101	0.527209383	0.598410107	0.753967967	no
KRT23	-0.029302714	-0.526860497	0.598652104	0.754207924	no
S1PR5	-0.029301221	-0.526833626	0.598670744	0.754207924	no
SNORA62	-0.02929285	-0.526682993	0.598775243	0.754295355	no
POLR2G	0.029275183	0.526365064	0.598995827	0.754529003	no
HNRNPAB	-0.029261916	-0.526126335	0.599161485	0.754685286	no
OR2AT4	-0.029259623	-0.526085062	0.599190128	0.754685286	no
NCR2	-0.029240741	-0.525745285	0.599425946	0.754938058	no
MORF4L2	0.029232484	0.525596683	0.599529095	0.75502372	no
AFM	0.029214223	0.525268078	0.599757218	0.755266751	no
LRWD1	0.02921074	0.525205401	0.599800733	0.755277293	no
OR5AU1	-0.029205878	-0.525117912	0.599861478	0.755309528	no
LINC00348	-0.029199287	-0.5249993	0.599943837	0.755368973	no
OR1M1	-0.029180531	-0.52466178	0.600178222	0.75561049	no
PAK1IP1	0.02917831	0.524621815	0.600205978	0.75561049	no
ANKAR	-0.029168416	-0.524443766	0.600329641	0.755659752	no
LCE2B	-0.029167814	-0.524432936	0.600337163	0.755659752	no
CDHR5	-0.029165196	-0.52438582	0.60036989	0.755659752	no
GHRLOS	-0.029163926	-0.524362979	0.600385755	0.755659752	no
VIPAS39	-0.029151864	-0.524145923	0.600536532	0.75580526	no
TTC36	-0.029147481	-0.524067048	0.600591327	0.755829959	no
ATG12	0.029137933	0.52389523	0.600710697	0.755935916	no
CER1	-0.02912975	-0.523747966	0.600813016	0.756020407	no
UGT2A2	-0.029117376	-0.523525297	0.600967742	0.756170829	no
DAZL	0.02910801	0.523356764	0.601084863	0.756253338	no
AKAP8	-0.029106504	-0.523329665	0.601103696	0.756253338	no
PDPK1	-0.029102875	-0.523264353	0.601149087	0.75626295	no
MIR205HG	0.029100266	0.523217408	0.601181715	0.75626295	no

PCDH9-AS3	0.029094862	0.523120161	0.601249306	0.756267308	no
WDR19	0.029094362	0.523111164	0.601255559	0.756267308	no
STX5	0.029085415	0.522950158	0.601367473	0.756363806	no
OR8U8	-0.029075404	-0.522770006	0.601492706	0.756447992	no
ACSM1	0.029074437	0.522752605	0.601504803	0.756447992	no
OR2M2	-0.029071569	-0.522701005	0.601540676	0.75644884	no
IL33	0.02906547	0.522591245	0.601616985	0.756500533	no
OR5M3	-0.029043301	-0.522192319	0.601894368	0.756805047	no
OR2AK2	-0.029026914	-0.521897437	0.602099444	0.757018613	no
DEFB4A	-0.029018659	-0.521748892	0.602202761	0.757056547	no
KRTAP9-3	-0.029016715	-0.521713908	0.602227095	0.757056547	no
ASMTL-AS1	0.02901606	0.521702119	0.602235295	0.757056547	no
WDR67	-0.029012897	-0.521645197	0.602274889	0.757062037	no
MAVS	0.029005374	0.521509834	0.602369051	0.757130395	no
HM13-AS1	-0.029002924	-0.521465731	0.602399731	0.757130395	no
BLOC1S6	-0.028994938	-0.521322041	0.602499695	0.757211751	no
CDC25B	-0.028991471	-0.521259649	0.602543102	0.757222022	no
PPTC7	-0.028984978	-0.52114281	0.602624394	0.7572799	no
IRX5	-0.028971066	-0.520892457	0.602798595	0.757443224	no
FMR1-AS1	-0.028968969	-0.520854725	0.602824852	0.757443224	no
SLC22A3	-0.028955368	-0.520609982	0.602995176	0.757612939	no
COPS6	-0.028930564	-0.520163632	0.603305859	0.757958975	no
OR2S2	-0.028922124	-0.520011755	0.60341159	0.758047494	no
PBX4	-0.028917038	-0.519920234	0.603475307	0.758070091	no
OR2L8	-0.028914682	-0.519877842	0.603504822	0.758070091	no
FRMD8P1	-0.028912242	-0.519833925	0.603535399	0.758070091	no
TBCA	-0.0288951	-0.519525465	0.603750183	0.758295552	no
PPAP2C	0.028890734	0.519446901	0.603804894	0.758319949	no
DCST1	-0.028869129	-0.519058129	0.604075659	0.758615672	no
MRGPRG-AS1	-0.028866163	-0.519004746	0.604112843	0.758618039	no
FOXB1	0.028860494	0.518902736	0.604183901	0.75866294	no
PDCD6IP	0.028847369	0.518666565	0.604348426	0.758798486	no
CTAGE6P	0.028846249	0.518646416	0.604362463	0.758798486	no
MEF2C	0.028826709	0.518294786	0.60460746	0.759061744	no
CCT6B	0.028813665	0.518060079	0.604771017	0.759222731	no
PLCZ1	-0.028802765	-0.51786393	0.60490772	0.759349989	no
CCDC175	0.028791345	0.51765843	0.605050954	0.759453299	no
IFNL3	-0.028788568	-0.517608468	0.60508578	0.759453299	no
SRD5A1	-0.028787612	-0.517591258	0.605097776	0.759453299	no
LDLRAD3	-0.028784934	-0.517543065	0.605131371	0.759453299	no
BLOC1S4	0.028778034	0.517418906	0.605217923	0.759507975	no
COL7A1	0.028775826	0.517379178	0.605245618	0.759507975	no
SYNPR-AS1	-0.028766973	-0.517219877	0.605356679	0.75959765	no
SYNJ2BP-COX1	-0.02876185	-0.51712768	0.60542096	0.75959765	no
ZFAT	-0.028761678	-0.517124591	0.605423114	0.75959765	no
IHH	-0.028742477	-0.516779083	0.605664036	0.759855563	no
CEACAM16	-0.028737484	-0.516689236	0.605726693	0.759889811	no
PAXBP1	-0.028730772	-0.51656845	0.605810931	0.759951127	no
BMP5	0.028727904	0.516516845	0.605846922	0.759951918	no
DCHS1	-0.028718041	-0.516339369	0.60597071	0.760046819	no
MBD3L1	-0.028716241	-0.516306967	0.605993311	0.760046819	no

KCNJ18	-0.028700231	-0.516018873	0.60619428	0.760254509	no
PLA2G12B	-0.028688868	-0.515814416	0.606336923	0.760354547	no
UBE2V1	0.02868824	0.515803118	0.606344806	0.760354547	no
RNA45S5	-0.028674116	-0.515548959	0.606522148	0.760532556	no
ADORA2A-AS1	-0.028664989	-0.515384729	0.606636753	0.760554351	no
PDZD3	-0.028663886	-0.515364867	0.606650614	0.760554351	no
SKP1P2	-0.028660126	-0.515297212	0.606697829	0.760554351	no
FAM133B	0.02865902	0.51527732	0.606711712	0.760554351	no
ZNF75A	-0.028658085	-0.515260489	0.606723458	0.760554351	no
ATAD3C	0.028655823	0.515219785	0.606751866	0.760554351	no
S100A2	0.028651012	0.515133219	0.606812284	0.760585722	no
LOC541473	0.028640949	0.51495213	0.606938682	0.760699785	no
TBRG1	-0.028627853	-0.514716478	0.607103181	0.760861585	no
BRAT1	0.028622712	0.514623978	0.607167757	0.760898144	no
AURKAIP1	0.028616996	0.514521126	0.607239564	0.760943759	no
NDUFB2-AS1	0.028607147	0.514343895	0.607363308	0.761054448	no
ABHD11	0.028600749	0.514228769	0.607443696	0.761110801	no
FGF14-AS2	-0.028580635	-0.513866822	0.607696459	0.761380987	no
IGSF23	-0.028577951	-0.513818529	0.607730187	0.761380987	no
RPL12	0.028573719	0.513742381	0.607783373	0.761403233	no
MIR34A	-0.02857028	-0.513680507	0.607826589	0.761412989	no
LRIT2	-0.028562483	-0.513540192	0.6079246	0.761450416	no
LOC401980	-0.028562265	-0.513536284	0.60792733	0.761450416	no
CYP4F30P	-0.02855837	-0.513466184	0.607976298	0.761451022	no
SRRM5	-0.028552371	-0.513358246	0.6080517	0.761451022	no
OR2B3	0.028551643	0.513345137	0.608060858	0.761451022	no
COCH	-0.028550952	-0.513332712	0.608069538	0.761451022	no
STMND1	0.028546003	0.513243653	0.608131756	0.761484564	no
KRTAP1-3	-0.02852122	-0.512797702	0.608443348	0.761830342	no
TTTY6	0.02850134	0.512439975	0.608693349	0.762078235	no
HBS1L	-0.028499837	-0.51241293	0.608712251	0.762078235	no
PKN3	-0.028493516	-0.512299188	0.608791752	0.762117615	no
MYL5	0.028490599	0.51224671	0.608828433	0.762117615	no
CBX5	-0.028488086	-0.51220149	0.608860041	0.762117615	no
C4orf17	-0.028483785	-0.512124095	0.608914142	0.762117615	no
TCL1B	-0.028483238	-0.51211426	0.608921017	0.762117615	no
GPR88	-0.028461863	-0.511729623	0.60918992	0.762342013	no
ARHGAP6	0.028459313	0.511683751	0.609221993	0.762342013	no
BAG2	0.028458962	0.511677423	0.609226418	0.762342013	no
LOC340074	-0.028456461	-0.511632424	0.609257881	0.762342013	no
SPACA5	-0.028454888	-0.511604122	0.60927767	0.762342013	no
SBSN	-0.02844709	-0.511463798	0.609375791	0.762382934	no
LOC221122	-0.02844665	-0.511455886	0.609381324	0.762382934	no
LOC10085993C	0.02844076	0.511349899	0.60945544	0.762431275	no
FAHD2CP	-0.028431745	-0.511187689	0.609568881	0.762488285	no
C21orf119	-0.0284315	-0.51118327	0.609571971	0.762488285	no
CYP7B1	0.028426118	0.511086431	0.609639699	0.762528621	no
BIN1	0.028422187	0.5110157	0.60968917	0.762534546	no
TXNL4B	0.028420103	0.5109782	0.609715399	0.762534546	no
GHR	-0.02841434	-0.5108745	0.609787934	0.762577713	no
LGI1	-0.028411722	-0.510827397	0.609820882	0.762577713	no

CASP14	-0.028403357	-0.510676875	0.609926176	0.762590164	no
ZNF142	-0.028400185	-0.510619792	0.609966109	0.762590164	no
FAM83A	-0.028397574	-0.510572807	0.609998979	0.762590164	no
R3HDML	-0.028395485	-0.510535222	0.610025274	0.762590164	no
LBH	0.028394043	0.510509278	0.610043424	0.762590164	no
CDC42EP1	-0.028393627	-0.510501793	0.610048661	0.762590164	no
THOC5	-0.028391199	-0.510458102	0.610079228	0.762590164	no
PGM3	0.028376684	0.510196913	0.610261976	0.762774231	no
ABCA4	0.028363224	0.509954728	0.610431451	0.762941687	no
CTU1	-0.028352959	-0.509770019	0.610560718	0.763058874	no
EIF5AL1	-0.02834993	-0.509715501	0.610598875	0.763062187	no
SUPT20HL2	-0.028332025	-0.509393322	0.610824386	0.76329962	no
GOLGA8CP	-0.028328962	-0.50933821	0.610862965	0.763303447	no
KIF11	-0.02831916	-0.509161843	0.610986434	0.76341334	no
GGT6	-0.028291099	-0.508656905	0.611339985	0.763810686	no
KRTAP13-4	-0.02828545	-0.508555261	0.611411166	0.763855212	no
KIF23	-0.028279686	-0.508451555	0.611483794	0.763901542	no
ACAA1	0.028269508	0.508268416	0.611612061	0.764005428	no
MED17	-0.028267446	-0.508231308	0.611638053	0.764005428	no
IL3	-0.02825607	-0.508026608	0.611781438	0.764128496	no
RPL41	-0.028253987	-0.507989134	0.611807689	0.764128496	no
BDKRB1	0.028247114	0.507865464	0.611894324	0.76419229	no
C9orf84	0.028239331	0.507725408	0.611992445	0.764227461	no
CCDC163P	-0.028239239	-0.50772375	0.611993606	0.764227461	no
C1orf170	0.028232304	0.507598965	0.612081035	0.764292227	no
SNORA65	-0.028226747	-0.507498988	0.612151086	0.764321084	no
LINC00317	-0.028224829	-0.507464468	0.612175274	0.764321084	no
ADRB3	-0.028217159	-0.507326446	0.612271989	0.764392079	no
KANSL1-AS1	-0.028214678	-0.507281804	0.612303273	0.764392079	no
LCE3C	-0.028208557	-0.507171665	0.612380457	0.76443263	no
OR4D2	-0.028205559	-0.507117731	0.612418255	0.76443263	no
ZNF705G	-0.028203639	-0.507083186	0.612442465	0.76443263	no
SHCBP1L	-0.028198817	-0.506996422	0.612503274	0.76446413	no
DNM3OS	0.028184064	0.506730956	0.612689343	0.764645698	no
LOC200772	-0.028181641	-0.506687351	0.612719909	0.764645698	no
JPH1	-0.028174826	-0.506564732	0.612805865	0.764658213	no
C16orf3	-0.028173026	-0.506532341	0.612828572	0.764658213	no
HCG9	0.028172383	0.506520777	0.612836679	0.764658213	no
CABS1	0.028151477	0.506144599	0.613100419	0.76494288	no
LOC93432	0.028130131	0.505760515	0.613369755	0.765164764	no
OR51A4	0.028122719	0.50562713	0.613463301	0.765164764	no
C4orf46	-0.028117658	-0.505536065	0.613527172	0.765164764	no
LINC00273	-0.028117358	-0.505530679	0.613530949	0.765164764	no
SDC3	-0.028114065	-0.505471426	0.61357251	0.765164764	no
SERF1B	-0.028112062	-0.50543539	0.613597786	0.765164764	no
ISX	-0.028109864	-0.505395829	0.613625535	0.765164764	no
ACTL7B	-0.028108604	-0.505373163	0.613641434	0.765164764	no
C11orf92	-0.028108089	-0.505363885	0.613647942	0.765164764	no
CDKN2B	0.028106704	0.505338979	0.613665413	0.765164764	no
SUN5	0.028106349	0.505332579	0.613669902	0.765164764	no
METRN	0.028094197	0.505113931	0.613823283	0.765311608	no

TINAG	0.028088557	0.505012435	0.613894489	0.765333501	no
GMEB2	-0.028087164	-0.504987381	0.613912066	0.765333501	no
BAGE2	0.028083581	0.504922903	0.613957303	0.765345499	no
AP4S1	-0.028076292	-0.50479176	0.614049317	0.765415804	no
LINC00261	-0.028067261	-0.50462926	0.61416334	0.765513534	no
SLC23A1	0.028051202	0.504340304	0.614366118	0.765715168	no
PRDM15	-0.028048807	-0.504297202	0.614396368	0.765715168	no
LOC10012952C	-0.028033033	-0.504013369	0.614595585	0.765919033	no
IGFL4	-0.02802557	-0.503879084	0.614689847	0.765992085	no
PCDHB13	-0.028022737	-0.503828116	0.614725625	0.765992255	no
A2MP1	-0.028012256	-0.503639519	0.614858026	0.766112815	no
GOLGA8DP	0.028006991	0.503544789	0.614924533	0.766151264	no
CDK16	0.027991614	0.503268105	0.615118805	0.766344604	no
NOB1	0.027989064	0.503222216	0.615151029	0.766344604	no
LOC100506844	-0.027983987	-0.503130872	0.615215173	0.766345144	no
DPYSL2	-0.027983385	-0.503120039	0.615222781	0.766345144	no
CT45A2	-0.027980096	-0.503060855	0.615264343	0.766352498	no
C6orf25	-0.027954776	-0.502605266	0.615584324	0.766706619	no
POU1F1	-0.027945303	-0.502434816	0.615704058	0.766811307	no
AMN	0.027942103	0.502377249	0.615744499	0.766817235	no
DAPK2	-0.027934342	-0.502237601	0.615842606	0.766894974	no
KRT37	-0.027920549	-0.501989409	0.616016986	0.767067678	no
TRIM48	0.027909975	0.501799154	0.616150674	0.767109636	no
KIAA1210	0.027909874	0.501797342	0.616151947	0.767109636	no
C10orf12	-0.027904947	-0.501708676	0.616214256	0.767109636	no
ING2	0.027904421	0.501699222	0.616220899	0.767109636	no
RAE1	-0.027903768	-0.501687476	0.616229154	0.767109636	no
RPL26	-0.027871509	-0.501107035	0.61663712	0.767573029	no
OR2D3	0.027868423	0.501051494	0.616676163	0.76757717	no
ABCB10	0.027860867	0.500915549	0.616771733	0.767649347	no
TRAF4	-0.027858191	-0.500867391	0.61680559	0.767649347	no
BPIFC	-0.027847717	-0.500678942	0.616938083	0.767769781	no
PER4	-0.027843865	-0.500609619	0.616986826	0.76778598	no
NSUN5P1	0.02783179	0.500392355	0.6171396	0.767931628	no
B3GALNT1	-0.027828432	-0.500331946	0.617182081	0.767940024	no
PADI6	0.027803129	0.49987665	0.617502295	0.768293974	no
C20orf141	-0.027797513	-0.499775602	0.617573373	0.768337928	no
DPY19L2P2	-0.027790688	-0.499652801	0.617659757	0.768400917	no
ENGASE	0.027781873	0.499494192	0.617771339	0.768495244	no
TTC22	0.027772472	0.499325051	0.617890338	0.768594744	no
C21orf58	-0.027769904	-0.499278842	0.617922851	0.768594744	no
TEN1	0.02776518	0.499193841	0.617982659	0.76862465	no
ABCA12	-0.027761787	-0.499132783	0.618025622	0.768633602	no
ALDH4A1	-0.027750946	-0.49893773	0.618162879	0.768716281	no
PITX1	0.027750886	0.498936649	0.618163639	0.768716281	no
ZHX1	-0.027746274	-0.498853675	0.618222031	0.768744412	no
KIR2DL2	-0.027740676	-0.498752948	0.618292919	0.768788077	no
SLC27A4	-0.027730797	-0.498575193	0.618418027	0.76889915	no
C10orf131	0.027726013	0.498489115	0.618478614	0.768901016	no
TMCO2	0.027725029	0.4984714	0.618491083	0.768901016	no
KC6	0.027716244	0.498313334	0.618602348	0.768994855	no

PCK1	-0.027706827	-0.498143902	0.618721624	0.769082194	no
IL17F	0.027705047	0.498111864	0.618744178	0.769082194	no
ACOT4	0.027699665	0.49801504	0.618812345	0.76912244	no
IFI27L1	-0.027692868	-0.49789273	0.61889846	0.769184987	no
TRIM16	0.027689829	0.497838051	0.618936959	0.769188353	no
PRAMEF2	-0.027673839	-0.497550358	0.619139541	0.769395622	no
GAS2	0.027660134	0.497303762	0.619313207	0.769566934	no
GPR180	0.027657103	0.497249212	0.619351627	0.769570179	no
MEG9	-0.027644595	-0.497024172	0.619510135	0.769722629	no
SLC7A3	-0.02763393	-0.496832273	0.619645314	0.769846077	no
SH3BP5-AS1	0.027622858	0.496633056	0.619785662	0.769975933	no
MYO5B	0.027609152	0.496386448	0.619959415	0.770147272	no
VN1R4	-0.027604114	-0.496295791	0.620023295	0.770168656	no
PCYOX1	0.027602141	0.496260301	0.620048304	0.770168656	no
MAP3K12	0.027593193	0.496099302	0.620161758	0.77026506	no
CPA1	-0.027567699	-0.495640593	0.620485056	0.770608712	no
PHKB	-0.02756572	-0.495604984	0.620510156	0.770608712	no
TEDDM1	-0.027546996	-0.495268096	0.620747646	0.770785231	no
COL21A1	0.027546571	0.495260441	0.620753042	0.770785231	no
GSTM1	0.027546031	0.495250729	0.620759889	0.770785231	no
MIR5196	0.027542374	0.495184934	0.620806277	0.770798295	no
CLIP4	-0.027537597	-0.495098978	0.62086688	0.770829008	no
C2orf49	-0.027534386	-0.495041205	0.620907615	0.77083505	no
CTRL	-0.027529395	-0.494951407	0.620970932	0.770869125	no
CRY1	-0.027518959	-0.494763638	0.621103338	0.770988958	no
FREM1	-0.027498994	-0.494404407	0.621356685	0.771258896	no
DUX4L4	-0.027479247	-0.494049106	0.621607305	0.771525417	no
BAIAP3	0.027455727	0.493625918	0.621905869	0.77185141	no
IL36G	-0.027443014	-0.493397194	0.622067262	0.771938074	no
OR14A16	0.027441686	0.493373299	0.622084124	0.771938074	no
TOP2A	-0.027438695	-0.493319471	0.622122109	0.771938074	no
SLC9A7	-0.027437889	-0.493304972	0.622132341	0.771938074	no
LOC100507331	-0.02743556	-0.493263069	0.622161912	0.771938074	no
PPAP2B	-0.027433253	-0.493221552	0.622191211	0.771938074	no
CCDC136	-0.027412081	-0.492840622	0.622460068	0.772187384	no
CHRNB1	0.02741177	0.492835024	0.622464019	0.772187384	no
PRX	-0.027406857	-0.492746637	0.62252641	0.772220206	no
STRADB	-0.027399278	-0.492610264	0.622622677	0.772221737	no
LOC399753	-0.027398753	-0.492600827	0.622629339	0.772221737	no
GTF2A1L	0.027398273	0.492592184	0.622635441	0.772221737	no
AK4	0.027380676	0.492275571	0.622858969	0.772407391	no
WDR3	0.027378634	0.492238828	0.622884912	0.772407391	no
C3orf43	-0.027378001	-0.492227437	0.622892955	0.772407391	no
ABCC13	-0.027374746	-0.492168883	0.622934299	0.772414091	no
SERPINB8	-0.027367395	-0.492036606	0.623027702	0.772480814	no
NOSTRIN	-0.027364853	-0.491990869	0.623059999	0.772480814	no
LOC339622	-0.027357027	-0.491850067	0.62315943	0.772559522	no
APOB	0.027347267	0.491674459	0.62328345	0.772667868	no
C18orf56	-0.027344491	-0.491624507	0.62331873	0.772667868	no
HAUS7	0.027341536	0.491571351	0.623356274	0.77266984	no
SAP30BP	-0.027322537	-0.491229509	0.623597737	0.772924561	no

NANOS1	-0.027292436	-0.490687925	0.623980373	0.773314087	no
PLAC1	-0.027290332	-0.490650064	0.624007127	0.773314087	no
AGPAT6	0.027289323	0.490631906	0.624019957	0.773314087	no
CLDN3	-0.027286207	-0.490575849	0.624059569	0.773318584	no
PRSS58	-0.027278278	-0.490433193	0.62416038	0.773398912	no
SERTAD4	-0.027274126	-0.490358489	0.624213174	0.773419736	no
ADAM33	-0.027255401	-0.490021578	0.624451296	0.773670172	no
TBPL2	-0.027248888	-0.489904389	0.624534132	0.77368551	no
CASP2	-0.027247903	-0.489886676	0.624546653	0.77368551	no
C10orf91	-0.027245936	-0.489851275	0.624571677	0.77368551	no
LALBA	0.027232623	0.489611746	0.62474101	0.773850665	no
AHRR	0.027217381	0.48933752	0.624934896	0.77400289	no
SNRPA	-0.02721641	-0.489320054	0.624947246	0.77400289	no
TRPA1	0.027213075	0.48926005	0.624989674	0.77400289	no
PBK	-0.027210901	-0.489220935	0.625017332	0.77400289	no
CCHCR1	-0.027207732	-0.489163913	0.625057654	0.77400289	no
DUOXA2	-0.027205269	-0.489119599	0.625088991	0.77400289	no
ALS2CR8	-0.027203146	-0.489081392	0.625116009	0.77400289	no
IL25	-0.027200179	-0.489028009	0.62515376	0.774005038	no
SNORA61	0.027196222	0.488956811	0.625204111	0.774022786	no
KRT9	-0.02718629	-0.488778121	0.625330488	0.774134649	no
NSMCE2	0.02717237	0.488527677	0.62550763	0.774298445	no
RETNLB	-0.027167538	-0.488440739	0.625569127	0.774298445	no
PRSS55	-0.0271674	-0.488438254	0.625570885	0.774298445	no
LOC728228	0.027155536	0.488224794	0.625721892	0.774401781	no
FAM108A1	-0.027154077	-0.488198537	0.625740468	0.774401781	no
LYG1	-0.027152109	-0.488163138	0.625765512	0.774401781	no
KIFC3	-0.027149517	-0.488116501	0.625798508	0.774401781	no
PTRHD1	-0.027145558	-0.488045263	0.625848909	0.774419559	no
LOC100131655	-0.027141513	-0.487972486	0.625900402	0.774438686	no
DDX11L1	0.027118301	0.487554856	0.626195925	0.774759737	no
NFATC2IP	-0.02711228	-0.48744652	0.626272596	0.774809991	no
FAM71D	-0.027097063	-0.487172737	0.626466374	0.775005114	no
LRRC46	0.027093327	0.487105527	0.626513947	0.775019354	no
SCGB3A2	-0.027089427	-0.487035364	0.626563613	0.775036181	no
WASH5P	-0.027078056	-0.486830767	0.626708449	0.775113508	no
INSR	-0.027077845	-0.48682698	0.62671113	0.775113508	no
CARD18	-0.027076024	-0.486794213	0.626734327	0.775113508	no
OR8S1	-0.027060007	-0.486506023	0.626938367	0.775321237	no
NAT8	-0.027051554	-0.486353944	0.627046052	0.77535398	no
P2RY12	-0.027050323	-0.48633179	0.62706174	0.77535398	no
ANKRD30BL	-0.027049433	-0.486315777	0.627073078	0.77535398	no
ELOVL3	0.027042719	0.486194985	0.627158617	0.775415132	no
TMPO	0.027030146	0.485968776	0.627318818	0.775568586	no
FAM107B	0.027018118	0.48575237	0.627472094	0.775713459	no
SORL1	-0.026997142	-0.485374968	0.627739438	0.775961107	no
DNMBP	-0.026996735	-0.485367642	0.627744628	0.775961107	no
ZNF816-ZNF32	-0.026988616	-0.485221576	0.627848112	0.776044388	no
NXPE2	-0.026981697	-0.485097092	0.627936312	0.776094869	no
KIF4B	-0.026979747	-0.485061998	0.627961178	0.776094869	no
GPR148	-0.026972906	-0.48493891	0.628048394	0.776145256	no

RLN3	-0.026969298	-0.484874003	0.628094388	0.776145256	no
VCX3B	-0.02696805	-0.484851559	0.628110293	0.776145256	no
SULT2A1	-0.026956316	-0.484640443	0.628259903	0.776285492	no
KCNK16	-0.026948051	-0.484491731	0.628365298	0.776371084	no
SPERT	-0.0269208	-0.484001445	0.62871283	0.776755817	no
C3orf20	-0.026912515	-0.483852376	0.628818512	0.776841724	no
LOC729020	-0.026880529	-0.483276896	0.629226567	0.777301152	no
RBM42	0.026863092	0.482963175	0.629449065	0.777531317	no
PRLH	0.026856635	0.482847008	0.629531462	0.777588404	no
PDGFRA	-0.026852157	-0.482766434	0.629588615	0.777614306	no
GDPD2	0.026843838	0.482616764	0.629694786	0.777700745	no
OR7G3	-0.026840196	-0.482551229	0.629741277	0.777713469	no
RANBP3L	-0.026832124	-0.48240601	0.629844301	0.777796005	no
GKN1	0.026820574	0.482198201	0.629991742	0.777933379	no
ZNF143	0.026813967	0.48207934	0.630076081	0.777946705	no
TMEM218	-0.026813826	-0.482076797	0.630077886	0.777946705	no
GCM2	-0.026811222	-0.482029947	0.63011113	0.777946705	no
FRG2C	0.026804406	0.48190731	0.630198155	0.778000742	no
LOC100131726	0.026800884	0.481843954	0.630243116	0.778000742	no
H2AFB2	-0.026797682	-0.481786336	0.630284006	0.778000742	no
DPYSL3	0.026796452	0.481764218	0.630299703	0.778000742	no
MED9	-0.026793607	-0.481713017	0.63033604	0.778000909	no
NACAP1	-0.026783847	-0.481537433	0.630460658	0.778064195	no
THSD1	0.026777157	0.481417058	0.630546098	0.778064195	no
FABP1	-0.026776913	-0.481412671	0.630549212	0.778064195	no
WFDC12	0.026771971	0.481323753	0.630612328	0.778064195	no
RPA1	0.026770454	0.481296465	0.630631698	0.778064195	no
ARL4A	-0.026770214	-0.481292149	0.630634762	0.778064195	no
AKR1CL1	-0.026769746	-0.481283722	0.630640744	0.778064195	no
KCMF1	0.026763357	0.481168775	0.630722341	0.778120198	no
NRK	0.026757562	0.481064518	0.630796354	0.778166836	no
SLC16A7	-0.026753708	-0.480995188	0.630845575	0.778182886	no
C14orf105	-0.026748356	-0.480898884	0.630913947	0.778222559	no
IGF2BP3	-0.02674191	-0.480782908	0.63099629	0.778279458	no
SLC6A19	-0.026734887	-0.480656558	0.631086004	0.778345441	no
FZD8	-0.026729173	-0.480553765	0.631158996	0.77836061	no
DMRT1	0.026728051	0.480533571	0.631173336	0.77836061	no
TRA2A	0.026725419	0.480486226	0.631206957	0.77836061	no
KRT79	-0.02671113	-0.480229141	0.63138953	0.778514201	no
SON	0.026710001	0.480208823	0.631403961	0.778514201	no
CD8A	-0.026706486	-0.480145582	0.631448876	0.778524915	no
S100A7L2	-0.026698901	-0.480009117	0.631545802	0.778599749	no
NCAPD3	-0.026690384	-0.479855897	0.631654636	0.778689256	no
CAPN7	-0.02668364	-0.479734553	0.631740834	0.778750847	no
SLC29A3	-0.026677074	-0.479616415	0.63182476	0.778808072	no
EIF5	0.02666985	0.479486446	0.631917096	0.778808072	no
TEX13A	-0.026669638	-0.479482646	0.631919795	0.778808072	no
PCDHB15	-0.026668667	-0.47946517	0.631932212	0.778808072	no
RNU6-35	0.0266593	0.479296636	0.632051955	0.778910979	no
USP19	0.02665108	0.479148762	0.632157028	0.778995797	no
CYP8B1	-0.026635953	-0.478876595	0.632350438	0.779189454	no

TAS2R42	-0.02662767	-0.47872758	0.632456342	0.77927527	no
UBR1	-0.026620353	-0.478595941	0.632549905	0.779342805	no
GSS	0.026617667	0.478547602	0.632584263	0.779342805	no
GTSF1L	-0.026610999	-0.478427639	0.632669533	0.779342805	no
ZNF367	0.026608041	0.478374425	0.63270736	0.779342805	no
DUSP21	-0.026607914	-0.47837214	0.632708984	0.779342805	no
DDX11L9	-0.026604162	-0.47830463	0.632756974	0.779342805	no
CDC6	0.026603534	0.478293341	0.632764999	0.779342805	no
DNM1P46	0.026597747	0.478189221	0.632839017	0.779344936	no
HSCB	0.026597728	0.478188884	0.632839257	0.779344936	no
CFC1	0.026586053	0.477978833	0.632988592	0.779466364	no
MAFA	-0.026584348	-0.477948163	0.633010397	0.779466364	no
LOC390660	-0.026571698	-0.477720571	0.633172222	0.779595417	no
SRD5A3	0.026570484	0.477698729	0.633187753	0.779595417	no
ECM1	0.026549136	0.477314656	0.633460883	0.779887021	no
LOC100127983	0.026542673	0.477198379	0.633543583	0.779944157	no
DKFZp434L192	-0.026536393	-0.477085392	0.633623946	0.779986184	no
ASPM	0.026534333	0.47704833	0.633650309	0.779986184	no
TTTY1B	0.026526045	0.476899219	0.633756374	0.780028142	no
ZBTB7A	0.026524626	0.476873693	0.633774533	0.780028142	no
MAN1A2	0.026523161	0.476847336	0.633793282	0.780028142	no
COL2A1	-0.026515095	-0.476702211	0.633896522	0.780085713	no
RFX1	0.026513834	0.476679532	0.633912656	0.780085713	no
ZNRF3-AS1	-0.026508461	-0.476582852	0.633981438	0.780125685	no
ODF4	-0.026502475	-0.476475163	0.634058056	0.780175294	no
SUMO4	0.026465185	0.475804275	0.634535464	0.780682284	no
DOK7	-0.026463546	-0.475774792	0.634556448	0.780682284	no
PROKR1	-0.026461779	-0.475743006	0.634579071	0.780682284	no
CA5A	0.026453133	0.475587454	0.634689788	0.780747307	no
PSAPL1	-0.026451978	-0.475566668	0.634704584	0.780747307	no
ZNF469	0.026430709	0.475184017	0.634976979	0.781037675	no
DEFB110	0.026427662	0.475129195	0.63501601	0.781040981	no
FBXO43	0.026424054	0.475064286	0.635062222	0.781053119	no
FAM174B	-0.026416283	-0.474924479	0.635161764	0.781120929	no
BCAS4	-0.026414075	-0.474884753	0.63519005	0.781120929	no
KLRC4-KLRK1	-0.026408964	-0.474792804	0.635255522	0.781156744	no
NUP54	0.026403627	0.474696781	0.635323898	0.781196126	no
KRT38	-0.026399898	-0.4746297	0.635371666	0.781210165	no
UFL1	-0.026394082	-0.47452505	0.635446191	0.781250717	no
SNORA39	-0.02639165	-0.474481295	0.635477352	0.781250717	no
HIST1H1A	-0.026377805	-0.474232214	0.63565475	0.781414607	no
FAM47B	-0.02637557	-0.474192015	0.635683382	0.781414607	no
CACNA1H	0.026364432	0.473991617	0.635826126	0.781506119	no
UPP2	-0.026364086	-0.473985398	0.635830556	0.781506119	no
CTNNBIP1	0.026357455	0.473866103	0.635915536	0.781565869	no
ADCK5	-0.026354464	-0.473812291	0.635953872	0.781568288	no
OR4C13	0.026347297	0.473683344	0.636045736	0.781636488	no
IQGAP3	0.02633953	0.473543622	0.636145282	0.78166658	no
LINC00649	0.026335888	0.473478098	0.636191968	0.78166658	no
CRK	0.026331732	0.473403327	0.636245244	0.78166658	no
ARIH1	0.026331561	0.473400238	0.636247445	0.78166658	no

TCTEX1D2	0.026331029	0.473390682	0.636254254	0.78166658	no
METTL11B	0.026328362	0.473342686	0.636288453	0.78166658	no
ADRA2A	-0.026322724	-0.473241257	0.636360729	0.781710684	no
CKMT2	-0.02631752	-0.473147633	0.636427445	0.781747955	no
B3GNT3	-0.026313948	-0.473083376	0.636473236	0.78175952	no
PPP1R2P3	-0.026309463	-0.473002689	0.636530739	0.781779727	no
CLHC1	0.026306991	0.472958205	0.636562442	0.781779727	no
SSX9	-0.026282158	-0.472511442	0.636880876	0.782126108	no
OR5H14	-0.026273474	-0.47235521	0.636992247	0.782193133	no
CRYBA1	0.026272227	0.472332768	0.637008246	0.782193133	no
CYP4A22	-0.026265373	-0.472209472	0.637096146	0.782256372	no
RAD54L	-0.026261759	-0.472144443	0.637142508	0.782268604	no
CCR3	-0.026257024	-0.472059268	0.637203236	0.782285506	no
TMEM135	-0.02625501	-0.472023026	0.637229076	0.782285506	no
KDM5D	-0.026246476	-0.471869488	0.637338552	0.782338123	no
DOCK10	0.026241879	0.471786786	0.637397524	0.782338123	no
MYLPF	-0.026240078	-0.471754396	0.637420621	0.782338123	no
TTTY15	-0.026236821	-0.471695802	0.637462405	0.782338123	no
L3MBTL2	-0.026235746	-0.471676458	0.637476199	0.782338123	no
SOX17	0.026234643	0.471656608	0.637490354	0.782338123	no
THAP3	-0.026229092	-0.471556742	0.637561573	0.782380846	no
BFSP2	-0.026213522	-0.471276635	0.637761346	0.782581312	no
LINC00347	-0.026197154	-0.47098215	0.637971403	0.782794371	no
PIH1D1	0.026189334	0.470841472	0.638071759	0.782851488	no
NEB	-0.02618785	-0.470814773	0.638090806	0.782851488	no
HBG1	0.026181706	0.470704237	0.638169665	0.78288129	no
PATE3	-0.026180281	-0.470678602	0.638187954	0.78288129	no
MFSD11	0.026167069	0.470440898	0.638357555	0.783044646	no
OSBPL1A	-0.026159995	-0.470313637	0.638448362	0.783111337	no
DAPK3	0.026156688	0.47025415	0.638490811	0.783118709	no
LOC388948	0.026148031	0.47009839	0.638601965	0.783210342	no
COL6A6	-0.026120035	-0.469594725	0.638961447	0.783588801	no
RLN1	0.02611832	0.469563873	0.638983469	0.783588801	no
HAL	0.026106315	0.469347907	0.63913764	0.783691745	no
CTAG2	-0.026106104	-0.469344113	0.639140348	0.783691745	no
FOXD4L5	-0.026100845	-0.469249485	0.639207905	0.783729866	no
AIMP2	0.026097574	0.469190651	0.639249909	0.783736653	no
MANSC4	0.026084595	0.468957152	0.639416625	0.783896332	no
GABARAPL3	-0.02608093	-0.468891209	0.639463712	0.783909339	no
NME3	-0.026075312	-0.468790144	0.639535879	0.783909805	no
IL21	-0.026075222	-0.468788512	0.639537044	0.783909805	no
KIAA0391	-0.026068038	-0.468659271	0.639629336	0.78393511	no
VPS35	0.026067936	0.468657442	0.639630643	0.78393511	no
C6orf195	0.026060536	0.468524313	0.639725717	0.784006922	no
SCNM1	-0.02605293	-0.468387483	0.639823441	0.784081974	no
PLD5	-0.0260287	-0.467951569	0.640134813	0.784408387	no
SLC35G1	0.026025119	0.467887135	0.640180843	0.784408387	no
GJB7	0.026023683	0.467861302	0.640199298	0.784408387	no
RNASEH2B	-0.026019023	-0.467777481	0.640259181	0.784437036	no
CCDC37	0.026005043	0.46752596	0.640438885	0.784612477	no
KIAA1804	0.025988916	0.467235841	0.640646192	0.784811525	no

KRTCAP3	-0.025986723	-0.467196376	0.640674394	0.784811525	no
IGSF10	0.02597803	0.467039999	0.640786149	0.784903683	no
TCEAL8	0.025968995	0.466877455	0.640902319	0.78499205	no
RRN3P3	-0.025966738	-0.466836847	0.640931343	0.78499205	no
EIF3G	-0.025941671	-0.466385881	0.641253701	0.785294817	no
DDI1	-0.025941101	-0.466375631	0.641261029	0.785294817	no
ARMC6	-0.025938991	-0.466337667	0.641288169	0.785294817	no
ALX3	-0.025928535	-0.466149554	0.641422659	0.785365735	no
GAP43	0.025928481	0.466148584	0.641423352	0.785365735	no
MYL10	-0.025925965	-0.466103322	0.641455714	0.785365735	no
OCRL	0.025922819	0.466046717	0.641496186	0.785370544	no
MRPS35	-0.025918878	-0.465975818	0.64154688	0.785373852	no
TFAMP1	0.025916927	0.46594072	0.641571976	0.785373852	no
AGBL3	0.025900593	0.465646868	0.641782107	0.78556878	no
DNAAF1	0.025898866	0.465615808	0.64180432	0.78556878	no
CDH17	0.025893202	0.465513899	0.641877201	0.785613243	no
LOC401463	-0.025888355	-0.465426707	0.641939561	0.785644824	no
PSMD1	-0.025879663	-0.465270331	0.642051407	0.785736962	no
MYADML	-0.025873222	-0.465154451	0.642134294	0.785793652	no
ZNF567	-0.025852912	-0.464789074	0.642395672	0.786068746	no
MYEOV	-0.025843681	-0.464623018	0.642514477	0.786150927	no
PRPF31	0.025839389	0.464545802	0.642569725	0.786150927	no
GOLGA8I	-0.025839168	-0.46454182	0.642572574	0.786150927	no
HOXA11	-0.025832004	-0.46441294	0.642664791	0.78616863	no
LOC90246	-0.025830963	-0.464394206	0.642678197	0.78616863	no
SMIM7	-0.025827935	-0.464339731	0.642717177	0.78616863	no
TAAR9	-0.025823889	-0.464266955	0.642769254	0.78616863	no
PRR5-ARHGAP8	0.025823835	0.464265983	0.64276995	0.78616863	no
SPATA2	-0.025812908	-0.464069397	0.642910633	0.786295949	no
CEP152	0.025797083	0.463784709	0.643114388	0.786500388	no
LOC286467	-0.025766699	-0.463238094	0.643505683	0.786934143	no
BCORL1	-0.02575604	-0.463046326	0.643642984	0.787011679	no
MMP20	0.025754926	0.463026288	0.643657331	0.787011679	no
C6orf226	-0.025753248	-0.462996097	0.643678948	0.787011679	no
IGLL1	-0.025741773	-0.462789674	0.643826759	0.787113768	no
CMPK1	0.02574108	0.462777194	0.643835696	0.787113768	no
PIGM	-0.025725132	-0.462490293	0.644041158	0.787320166	no
DHX29	0.025715774	0.462321947	0.64416173	0.787422771	no
ANXA6	-0.025708923	-0.462198688	0.644250016	0.7874859	no
USP50	0.025704839	0.462125217	0.644302644	0.787505438	no
NACA2	-0.025699542	-0.462029934	0.644370898	0.787544072	no
ALKBH8	0.025691402	0.461883495	0.644475802	0.78758646	no
AVPR1B	-0.025691164	-0.461879206	0.644478874	0.78758646	no
SNHG11	0.02568474	0.461763651	0.644561659	0.787642839	no
FAM225A	0.025672264	0.461539205	0.644722468	0.787714428	no
KCNQ5-AS1	-0.025671932	-0.461533228	0.64472675	0.787714428	no
RUVBL1	0.025671664	0.461528409	0.644730203	0.787714428	no
CTSL1P2	-0.025662299	-0.461359933	0.644850922	0.78776386	no
PRB1	-0.025659115	-0.461302648	0.644891971	0.78776386	no
NUP62	-0.025657629	-0.461275911	0.644911131	0.78776386	no
PABPN1L	-0.025654967	-0.461228035	0.644945439	0.78776386	no

FGF19	-0.025654308	-0.461216173	0.64495394	0.78776386	no
FOXN1	-0.025650514	-0.461147925	0.645002847	0.787778824	no
ZFX-AS1	-0.02563292	-0.460831399	0.645229698	0.788004826	no
OR2V1	-0.02562751	-0.460734076	0.645299455	0.788004826	no
SCARNA14	-0.025625709	-0.46070168	0.645322676	0.788004826	no
PRIM2	0.025623478	0.460661547	0.645351442	0.788004826	no
LINC00547	0.025621944	0.460633952	0.645371223	0.788004826	no
OR5T2	-0.025608734	-0.460396303	0.64554158	0.788151251	no
LOC100506451	-0.025606958	-0.460364344	0.645564491	0.788151251	no
SCTR	-0.025597818	-0.460199929	0.645682363	0.788227347	no
S100A7	-0.025596438	-0.460175087	0.645700174	0.788227347	no
VGF	-0.025591949	-0.460094339	0.645758068	0.788253246	no
FOXL2	0.025581621	0.459908532	0.645891295	0.78833397	no
POU5F2	-0.025581135	-0.459899789	0.645897564	0.78833397	no
OR5H15	-0.025573929	-0.459770153	0.645990522	0.788402652	no
ANP32AP1	-0.025556474	-0.459456147	0.646215709	0.788632697	no
ENTPD8	-0.02554172	-0.459190725	0.64640608	0.78882023	no
SOX7	0.025525837	0.458904997	0.646611041	0.788944098	no
ZNF789	0.025522203	0.458839628	0.646657936	0.788944098	no
FLJ33360	0.025521587	0.45882853	0.646665898	0.788944098	no
DUS4L	0.025521441	0.458825918	0.646667771	0.788944098	no
C7orf62	0.025519631	0.458793349	0.646691137	0.788944098	no
LOC729966	-0.025512147	-0.458658724	0.646787722	0.788979932	no
OR8B4	-0.025511666	-0.458650065	0.646793935	0.788979932	no
PI4KB	0.025504825	0.458526988	0.64688224	0.789042864	no
ELMOD1	0.025497055	0.45838721	0.646982534	0.789120411	no
LINC00443	-0.025485572	-0.458180633	0.64713077	0.789237786	no
INPP4B	-0.025483911	-0.458150749	0.647152215	0.789237786	no
CLDN7	0.025478777	0.458058389	0.647218496	0.78927383	no
C21orf91	-0.02547305	-0.45795537	0.64729243	0.789319202	no
TIMM22	0.025464954	0.457809721	0.647396965	0.789401882	no
CLPS	0.025459344	0.457708806	0.647469397	0.789407674	no
ZIM3	-0.025458896	-0.457700751	0.647475179	0.789407674	no
LBR	-0.025449196	-0.457526241	0.647600442	0.789458326	no
C5orf60	-0.02544915	-0.457525419	0.647601032	0.789458326	no
AOC4	0.025447145	0.457489347	0.647626927	0.789458326	no
LINC00308	-0.025438967	-0.457342235	0.647732533	0.789532692	no
DEPTOR	-0.025434108	-0.457254825	0.647795285	0.789532692	no
CDR2L	0.025433067	0.457236088	0.647808737	0.789532692	no
SMR3B	-0.025427697	-0.45713948	0.647878096	0.789532692	no
LONP2	-0.025423239	-0.457059287	0.647935673	0.789532692	no
FLJ39739	-0.025422563	-0.457047127	0.647944403	0.789532692	no
SF3B5	0.025421579	0.457029432	0.647957109	0.789532692	no
FGF23	-0.025419665	-0.456994994	0.647981835	0.789532692	no
TRAPPC2L	-0.025415678	-0.456923261	0.64803334	0.789550684	no
GOLT1A	0.025411374	0.456845846	0.648088927	0.789562324	no
UQCRQ	-0.02540925	-0.456807625	0.648116372	0.789562324	no
REG3G	-0.025402914	-0.456693653	0.648198214	0.789617267	no
HP1BP3	0.025397565	0.456597418	0.648267323	0.789656693	no
SP3	0.025391228	0.456483423	0.648349189	0.789711653	no
ZNF69	0.025382469	0.456325852	0.648462357	0.789804733	no

DYX1C1	0.025378668	0.456257473	0.648511468	0.789819787	no
MMS22L	-0.025365862	-0.456027103	0.64867694	0.789976547	no
LOC285074	0.025362207	0.45596135	0.648724173	0.789989303	no
FAM134C	-0.025358072	-0.455886958	0.648777613	0.790009614	no
CHST4	0.025335939	0.455488794	0.649063669	0.790313162	no
SLC35E2B	-0.025323031	-0.455256589	0.649230517	0.790460277	no
MIR765	0.025320901	0.455218269	0.649258053	0.790460277	no
REG1A	-0.025306364	-0.454956764	0.649445979	0.790616138	no
LY6G6C	0.025305307	0.454937744	0.649459648	0.790616138	no
PDE6C	-0.025297683	-0.454800595	0.649558218	0.790691343	no
ALAS2	0.025288322	0.454632188	0.649679261	0.790776038	no
OR2Y1	-0.025286611	-0.454601406	0.649701387	0.790776038	no
MTRR	0.025281207	0.454504198	0.649771261	0.790816296	no
MGLL	0.025267052	0.45424955	0.649954318	0.790994295	no
TRAF2	0.025257549	0.454078609	0.650077214	0.79109906	no
SMG9	0.025242967	0.453816288	0.650265824	0.79128378	no
LINC00327	0.025234553	0.453664911	0.650374676	0.791338662	no
LENG8	0.025231528	0.453610496	0.650413806	0.791338662	no
FBXO47	0.025230941	0.453599947	0.650421392	0.791338662	no
PPL	-0.025225968	-0.453510479	0.650485731	0.791367424	no
CLASP1	-0.025221743	-0.453434476	0.65054039	0.791367424	no
LINC00244	0.025220575	0.453413463	0.650555502	0.791367424	no
SPATA31E1	-0.025213544	-0.453286981	0.650646468	0.791433283	no
MAGEB5	-0.025205694	-0.45314576	0.65074804	0.791512034	no
OSBPL5	0.025197227	0.452993445	0.650857599	0.791600491	no
EIF3C	-0.025157306	-0.452275296	0.651374261	0.792145579	no
GOT2	-0.025156902	-0.452268026	0.651379492	0.792145579	no
WDR64	0.0251425	0.452008946	0.651565925	0.792327466	no
IST1	0.025123819	0.45167289	0.651807783	0.792576726	no
HSD17B2	-0.025117655	-0.451561998	0.651887599	0.792615423	no
MLNR	-0.025115664	-0.451526196	0.651913369	0.792615423	no
TOP1MT	0.025106214	0.451356184	0.652035748	0.792719368	no
DCP1A	-0.025102414	-0.451287829	0.652084954	0.792734345	no
DEFB115	-0.025081088	-0.450904192	0.652361149	0.792996558	no
LOC100130855	-0.025080061	-0.450885728	0.652374442	0.792996558	no
OR2Z1	-0.025066422	-0.450640363	0.652551117	0.793166453	no
TYW1B	0.025061771	0.450556695	0.652611366	0.793194823	no
OR10A7	-0.025056382	-0.45045975	0.652681178	0.793234813	no
ABLIM2	-0.025049935	-0.450343787	0.652764691	0.793291448	no
PRPF40A	0.025040808	0.450179585	0.65288295	0.793390301	no
SLC45A4	0.025024739	0.449890522	0.653091157	0.793598443	no
OR1L6	-0.02502025	-0.449809778	0.65314932	0.793624246	no
EMCN-IT3	0.025007279	0.449576442	0.653317414	0.793751946	no
PDZK1	0.025006441	0.449561352	0.653328285	0.793751946	no
C15orf40	-0.025002421	-0.449489041	0.653380381	0.793770366	no
OR52R1	-0.024997225	-0.449395573	0.653447723	0.793807304	no
MRPL14	-0.024992707	-0.449314307	0.653506275	0.793833561	no
KCNU1	-0.024989779	-0.449261624	0.653544234	0.793834801	no
TMEM161A	-0.024982549	-0.449131567	0.653637948	0.793878484	no
LOC649133	-0.024976841	-0.449028888	0.653711937	0.793878484	no
SMR3A	-0.024976351	-0.449020072	0.65371829	0.793878484	no

KRBA1	0.024975605	0.449006657	0.653727958	0.793878484	no
LINC00202-1	-0.02497075	-0.448919307	0.653790904	0.793910064	no
PMS1	-0.024967384	-0.448858768	0.653834532	0.793918183	no
EIF4A3	-0.024959791	-0.448722164	0.653932981	0.793992864	no
ROS1	-0.024954623	-0.448629198	0.653999983	0.794029356	no
ACTL6A	0.024947597	0.448502806	0.654091082	0.794095098	no
C4orf19	0.024937204	0.448315851	0.65422584	0.794213835	no
DMC1	-0.024924281	-0.448083385	0.65439342	0.794372401	no
PEX3	0.024920347	0.448012619	0.654444437	0.794380121	no
SRSF3	0.02491809	0.44797202	0.654473706	0.794380121	no
CHMP5	0.024915028	0.447916933	0.654513422	0.794383461	no
NAA20	0.024897201	0.447596234	0.654744653	0.79461923	no
TM4SF4	-0.024891453	-0.447492846	0.654819205	0.794664833	no
LOC441461	0.02488772	0.447425686	0.654867635	0.794678732	no
NPAS4	-0.024873837	-0.447175951	0.655047735	0.794852401	no
UMPS	-0.02486778	-0.447066983	0.655126326	0.794872523	no
LOC401397	0.024866857	0.447050394	0.655138291	0.794872523	no
RASSF4	0.024863313	0.446986635	0.655184277	0.794883443	no
WDR46	0.02484708	0.446694622	0.655394911	0.795094103	no
FCER2	-0.024842937	-0.446620089	0.655448678	0.795114447	no
C3orf35	0.02482895	0.446368479	0.655630196	0.795289753	no
NDUFB4	0.024815802	0.446131961	0.655800846	0.795451856	no
LOC728323	0.02480765	0.445985326	0.655906654	0.795505238	no
API5	0.024806708	0.445968371	0.655918888	0.795505238	no
LINC00570	0.024803101	0.445903492	0.655965705	0.795517125	no
LOC283403	-0.024798164	-0.44581468	0.656029795	0.79553982	no
CPSF4L	0.02479551	0.445766931	0.656064253	0.79553982	no
RBM46	0.024793105	0.445723673	0.656095471	0.79553982	no
OR51B6	0.024789268	0.445654649	0.656145285	0.795542427	no
WNT2B	0.024787044	0.445614638	0.656174161	0.795542427	no
LHFPL3-AS2	-0.024784385	-0.445566819	0.656208674	0.795542427	no
LCE6A	-0.024772995	-0.445361922	0.65635656	0.79567683	no
ST20-MTHFS	0.02475796	0.445091455	0.656551793	0.795804578	no
SNORA36C	0.024756638	0.445067676	0.656568959	0.795804578	no
C15orf57	0.024754559	0.445030284	0.656595952	0.795804578	no
PGM1	0.024750205	0.44495196	0.656652495	0.795804578	no
EMC10	-0.024749716	-0.444943166	0.656658844	0.795804578	no
ZNF324B	0.02474777	0.444908157	0.656684118	0.795804578	no
CCNH	-0.024738325	-0.444738255	0.656806782	0.795904179	no
XKR6	-0.024731583	-0.444616962	0.656894357	0.795904179	no
DIO3OS	0.024728702	0.444565148	0.656931769	0.795904179	no
C8orf22	-0.024727501	-0.44454354	0.656947371	0.795904179	no
HABP2	0.024727185	0.444537851	0.656951479	0.795904179	no
OR3A2	-0.024712622	-0.44427588	0.657140648	0.796088482	no
ATXN3L	0.024704474	0.444129321	0.657246489	0.796171822	no
CABLES2	-0.024699523	-0.444040257	0.657310811	0.796179193	no
HIST1H1E	-0.024697112	-0.443996881	0.657342139	0.796179193	no
TRMT1L	0.024695451	0.443967006	0.657363715	0.796179193	no
GTF2A1	0.024686711	0.443809776	0.657477278	0.796240181	no
ZBTB40	-0.024685872	-0.443794696	0.65748817	0.796240181	no
GANC	-0.024680314	-0.443694706	0.657560394	0.796282776	no

NAALADL2-AS3	-0.024671211	-0.443530962	0.657678675	0.796340441	no
FAM204A	-0.024668236	-0.443477443	0.657717338	0.796340441	no
LOC284688	0.024667195	0.443458723	0.657730861	0.796340441	no
FAM71F1	0.024665243	0.443423604	0.657756231	0.796340441	no
DHRS7C	-0.024662004	-0.443365337	0.657798325	0.796346541	no
DYNLL1	-0.024648848	-0.443128686	0.657969299	0.796508658	no
KRT10	-0.024631858	-0.442823054	0.658190137	0.79669931	no
C1orf122	0.024631027	0.442808113	0.658200933	0.79669931	no
SLC2A1	0.024623199	0.442667292	0.658302696	0.796744542	no
TENM1	-0.024620862	-0.442625256	0.658333074	0.796744542	no
PIP5K1P1	-0.024619597	-0.442602497	0.658349522	0.796744542	no
FAM118B	0.024616229	0.442541917	0.658393302	0.796752658	no
COL6A5	0.024597401	0.442203222	0.658638096	0.797004015	no
PPFIBP2	-0.024590114	-0.442072145	0.658732841	0.797073785	no
CPA6	0.024580848	0.441905463	0.658853333	0.797174697	no
UGT1A3	-0.024576732	-0.441831416	0.658906863	0.797194583	no
NT5DC1	0.024537471	0.441125167	0.659417509	0.79776749	no
TRIT1	-0.024532087	-0.441028327	0.659487541	0.797770647	no
LSM5	0.024527658	0.44094865	0.659545164	0.797770647	no
LINC00602	-0.024526553	-0.440928772	0.659559539	0.797770647	no
C11orf84	-0.024523762	-0.440878561	0.659595853	0.797770647	no
TKTL1	-0.024521175	-0.44083203	0.659629506	0.797770647	no
KCTD19	-0.024520149	-0.440813586	0.659642846	0.797770647	no
MATN1	-0.024514511	-0.440712165	0.659716201	0.797775524	no
LOC440335	-0.024514133	-0.440705362	0.659721121	0.797775524	no
NF1P2	0.024511012	0.440649219	0.65976173	0.797779741	no
ESCO2	-0.024506036	-0.440559707	0.659826475	0.797813141	no
FAM151A	-0.024496849	-0.440394449	0.659946016	0.797876313	no
NDP	-0.024496314	-0.440384833	0.659952973	0.797876313	no
SLC18B1	-0.024491438	-0.440297121	0.660016424	0.797899165	no
GEMIN7	0.024488555	0.440245245	0.660053952	0.797899165	no
FOXI1	0.024486303	0.44020474	0.660083256	0.797899165	no
KIFAP3	0.02448227	0.440132188	0.660135744	0.797917732	no
OR7C1	0.024476388	0.440026384	0.660212292	0.797965376	no
CPLX4	0.024466024	0.439839965	0.660347173	0.798083516	no
LOC100505676	-0.024456559	-0.439669704	0.660470372	0.798187526	no
ARHGEF37	-0.024447754	-0.439511316	0.660584989	0.798281151	no
OR10P1	-0.024436183	-0.439303167	0.660735627	0.798418294	no
PPP1R17	0.024431004	0.439210001	0.660803056	0.798454879	no
FEZ2	-0.024427012	-0.439138188	0.660855032	0.798472789	no
METTL9	-0.024423277	-0.439071011	0.660903654	0.798486645	no
ELOF1	-0.024406059	-0.43876128	0.661127856	0.798712618	no
FNIP2	-0.024380062	-0.438293648	0.661466414	0.79903383	no
TTC25	0.024379933	0.438291323	0.661468097	0.79903383	no
BROX	0.024372973	0.438166123	0.661558752	0.799071886	no
CERS4	-0.024371805	-0.438145114	0.661573964	0.799071886	no
CCDC140	0.024364952	0.438021852	0.661663221	0.799134781	no
IL31RA	0.024361358	0.437957193	0.661710045	0.799146422	no
KRTAP19-2	-0.024356786	-0.437874945	0.661769607	0.799164222	no
ZNF503-AS1	0.024354517	0.437834142	0.661799156	0.799164222	no
PRDM9	-0.024343857	-0.437642379	0.661938037	0.799287019	no

GNL3L	0.024330372	0.43739982	0.662113723	0.799429087	no
PMPCA	-0.024328096	-0.437358875	0.662143382	0.799429087	no
SLC25A51P1	-0.024326262	-0.437325872	0.662167288	0.799429087	no
OR6N1	-0.024321758	-0.437244866	0.662225967	0.799455019	no
CACNA2D3-AS1	-0.024302824	-0.436904275	0.662472706	0.799707966	no
NUSAP1	-0.024298292	-0.43682276	0.662531765	0.799731901	no
SLFNL1	0.024295592	0.436774186	0.662566958	0.799731901	no
FARP2	-0.024279215	-0.436479594	0.662780416	0.799944621	no
LINC00705	-0.024271956	-0.43634901	0.662875044	0.799994937	no
ZIC4	0.024270306	0.436319329	0.662896553	0.799994937	no
LOC100505817	0.024264252	0.436210442	0.662975464	0.800045241	no
SCGB1D4	-0.024259785	-0.436130082	0.663033703	0.800070596	no
TESC	0.024238993	0.435756075	0.663304785	0.800327174	no
GPR142	-0.024236927	-0.435718913	0.663331722	0.800327174	no
SENP1	-0.024230614	-0.435605362	0.663414035	0.800327174	no
SNRPB2	-0.024229261	-0.435581014	0.663431685	0.800327174	no
RD3	-0.024229196	-0.435579841	0.663432535	0.800327174	no
NPHP3	-0.024224078	-0.435487794	0.663499263	0.800362745	no
CLDN16	0.024201613	0.435083679	0.663792251	0.800671228	no
OR10H2	-0.024193254	-0.434933316	0.663901279	0.800757795	no
SNORD23	-0.024189731	-0.434869945	0.663947232	0.800768278	no
TGFA	-0.024172895	-0.434567102	0.664166851	0.80095853	no
LINC00113	-0.024171924	-0.434549639	0.664179515	0.80095853	no
IGLL3P	-0.024156005	-0.434263287	0.664387204	0.801164033	no
HDDC3	-0.024145218	-0.434069247	0.664527954	0.801191101	no
HPDL	-0.024144668	-0.434059351	0.664535132	0.801191101	no
KLF9	-0.024144629	-0.434058666	0.664535629	0.801191101	no
DKFZP434K028	-0.024138218	-0.433943338	0.66461929	0.801191101	no
MYC	-0.0241354	-0.433892642	0.664656067	0.801191101	no
LOC644961	0.024134258	0.433872105	0.664670965	0.801191101	no
IL23R	-0.024133074	-0.433850812	0.664686413	0.801191101	no
OPN1LW	-0.024131428	-0.433821203	0.664707893	0.801191101	no
VAPB	-0.024117409	-0.433569028	0.66489085	0.801356102	no
DYNC2LI1	-0.024113895	-0.433505811	0.664936718	0.801356102	no
CEP164	0.024112367	0.43347834	0.66495665	0.801356102	no
SETD8	0.024094371	0.433154614	0.665191557	0.801594244	no
RPS17	0.024080768	0.432909936	0.665369125	0.801763267	no
CCDC42B	0.024077029	0.432842675	0.665417941	0.801777135	no
SH2D4B	-0.024057137	-0.432484857	0.66567766	0.802045108	no
MXD1	0.024049426	0.43234616	0.665778342	0.802121446	no
OR4C16	-0.024043796	-0.43224489	0.665851859	0.802165049	no
INSC	-0.024025729	-0.431919908	0.666087804	0.802404315	no
CYP4F35P	-0.0240213	-0.431840232	0.666145655	0.802429027	no
TRIM59	0.024010635	0.431648402	0.666284949	0.802551835	no
KRTAP13-2	-0.024003396	-0.431518187	0.666379508	0.802620748	no
FAM207A	-0.023998193	-0.431424585	0.666447483	0.802657636	no
TTC9	-0.023987171	-0.431226328	0.666591469	0.802745661	no
ZNF366	0.023986879	0.431221087	0.666595275	0.802745661	no
FAM50A	0.023980979	0.431114949	0.666672365	0.802793511	no
CLDN10-AS1	-0.023969889	-0.430915465	0.666817262	0.802923004	no
HPRT1	-0.023959751	-0.430733111	0.666949726	0.803012286	no

ACVR1B	-0.023957819	-0.430698356	0.666974975	0.803012286	no
PAQR4	-0.023954571	-0.430639933	0.667017416	0.803012286	no
FLI1	-0.023952776	-0.43060765	0.667040869	0.803012286	no
YIPF6	-0.023947078	-0.43050515	0.667115336	0.803013263	no
LDHC	-0.023946996	-0.430503669	0.667116412	0.803013263	no
WFDC5	-0.023934802	-0.430284331	0.667275773	0.803160102	no
WHAMMP2	0.02392686	0.430141476	0.667379572	0.803240053	no
FGA	0.023921596	0.430046794	0.667448372	0.803277872	no
PRKCQ	-0.023911245	-0.429860603	0.667583676	0.803374946	no
MYOZ3	-0.023909706	-0.429832918	0.667603795	0.803374946	no
TNFRSF21	-0.023902539	-0.429703994	0.66769749	0.803442707	no
PTCHD3	-0.023879931	-0.429297328	0.667993068	0.803753375	no
MRPL42P5	0.02387067	0.429130745	0.668114161	0.803854072	no
OR2T10	0.023861609	0.428967766	0.668232642	0.803951616	no
HOXA9	-0.023839358	-0.428567522	0.668523644	0.804256697	no
DLD	-0.023830046	-0.428400027	0.668645438	0.804358193	no
KLK6	-0.023820801	-0.428233729	0.668766371	0.80444935	no
ESD	0.02381853	0.428192878	0.668796079	0.80444935	no
RSPO1	-0.023807936	-0.428002326	0.668934662	0.804509776	no
PDE3B	0.023807008	0.427985634	0.668946802	0.804509776	no
KNCN	-0.023806105	-0.427969385	0.66895862	0.804509776	no
NCAPG	0.023802703	0.42790819	0.669003128	0.804518282	no
PDE7B	0.023796736	0.42780087	0.669081187	0.804567133	no
WFDC11	-0.023792863	-0.427731194	0.669131867	0.804583056	no
MRPL53	0.023789813	0.427676331	0.669171774	0.804586025	no
BNIP1	-0.023783915	-0.427570247	0.669248942	0.804633791	no
DEFA1	-0.023779034	-0.427482455	0.669312806	0.804649811	no
NFKBIB	0.023777174	0.427448992	0.669337149	0.804649811	no
NRG1	-0.023773634	-0.427385315	0.669383473	0.804660489	no
LINC00691	-0.023764939	-0.42722891	0.669497261	0.804752258	no
MYF5	-0.023752115	-0.426998237	0.669665093	0.804896863	no
KIF3B	-0.023749269	-0.426947057	0.669702332	0.804896863	no
KRTAP12-3	-0.023747161	-0.426909143	0.66972992	0.804896863	no
ARF1	0.023738342	0.426750504	0.669845357	0.804990581	no
FBXW12	0.023730367	0.426607062	0.669949741	0.805071007	no
RABGGTB	-0.023722102	-0.426458397	0.670057933	0.805155999	no
VAX1	-0.023717397	-0.426373757	0.670119533	0.805184999	no
TEX35	0.023713316	0.426300351	0.67017296	0.805204175	no
PSORS1C3	0.023684749	0.425786506	0.670546993	0.805574266	no
ZNF283	0.023684064	0.425774194	0.670555956	0.805574266	no
PDILT	-0.02367895	-0.425682198	0.67062293	0.805609691	no
CTSL3P	0.023664253	0.425417834	0.670815403	0.805788389	no
MROH1	0.023661865	0.425374888	0.670846673	0.805788389	no
OR51A2	0.023649337	0.425149535	0.671010764	0.805928747	no
NME2	-0.023647217	-0.425111409	0.671038528	0.805928747	no
SPRYD4	0.023641812	0.425014183	0.67110933	0.80596874	no
NUP210P1	-0.023614228	-0.424518024	0.671470685	0.806357649	no
KRTAP4-8	0.023605773	0.424365948	0.671581459	0.806440408	no
IL1R2	0.02360324	0.424320384	0.671614649	0.806440408	no
HTR3C	-0.023598796	-0.424240439	0.671672886	0.806464969	no
OR1L3	0.02359398	0.424153816	0.671735989	0.806464969	no

DNAI2	0.023593088	0.424137767	0.671747681	0.806464969	no
IL19	-0.023583769	-0.423970154	0.671869791	0.806518858	no
HIST1H3F	0.023579127	0.423886656	0.671930625	0.806518858	no
OR7E2P	0.023578795	0.423880678	0.671934981	0.806518858	no
HYI	0.023578207	0.423870109	0.671942681	0.806518858	no
KRTAP10-9	-0.023573212	-0.423780267	0.67200814	0.806552382	no
BSND	-0.023564971	-0.423632026	0.672116153	0.806607204	no
EFCAB1	-0.023562021	-0.423578964	0.672154819	0.806607204	no
LOC727677	-0.023561136	-0.42356305	0.672166414	0.806607204	no
LIME1	-0.023557487	-0.423497416	0.672214241	0.806619556	no
GCSAML	0.023550077	0.423364136	0.672311365	0.806691058	no
ZFHX3	0.023539253	0.423169436	0.672453257	0.806816265	no
KCNMB4	-0.023531827	-0.423035867	0.672550605	0.806888017	no
GPC5	0.023514733	0.422728396	0.672774718	0.807058694	no
OR5W2	-0.023514352	-0.422721541	0.672779714	0.807058694	no
HOXC11	-0.02351161	-0.422672209	0.672815675	0.807058694	no
OR52I2	0.02350952	0.422634615	0.672843079	0.807058694	no
FAHD2B	-0.023503992	-0.422535187	0.672915561	0.807099139	no
POTEH	0.02350122	0.422485328	0.672951908	0.807099139	no
ASCL3	-0.023493629	-0.422348789	0.67305145	0.807173477	no
CEP57L1	0.023480266	0.422108427	0.673226696	0.807335995	no
DNAAF3	0.023476703	0.422044341	0.673273423	0.807335995	no
POLR3G	0.02347354	0.421987454	0.673314903	0.807335995	no
TLCD1	-0.023470819	-0.421938505	0.673350596	0.807335995	no
CLEC9A	0.023468974	0.421905318	0.673374795	0.807335995	no
TFF2	-0.023458979	-0.421725542	0.673505891	0.807430652	no
STON1	-0.023457226	-0.421694009	0.673528887	0.807430652	no
RLBP1	-0.023439336	-0.421372214	0.673763574	0.807666943	no
LOC729121	-0.023423306	-0.421083887	0.67397388	0.807873984	no
NR1I2	-0.023418196	-0.420991973	0.674040927	0.807891895	no
PIN1P1	0.023414998	0.420934447	0.674082892	0.807891895	no
C6orf223	-0.023413572	-0.420908803	0.674101599	0.807891895	no
ERCC4	0.02340981	0.420841144	0.674150957	0.807897869	no
C7orf76	0.023407462	0.420798909	0.674181768	0.807897869	no
RHOBTB2	-0.023392086	-0.420522335	0.674383549	0.808017241	no
HNF1B	-0.023390488	-0.420493592	0.674404521	0.808017241	no
TCP11L2	0.023389029	0.420467342	0.674423674	0.808017241	no
DERA	0.023385765	0.420408642	0.674466504	0.808017241	no
DSCC1	-0.023383693	-0.420371378	0.674493694	0.808017241	no
LINC00478	0.023378655	0.420280757	0.674559818	0.808017241	no
LOC254099	0.023374776	0.42021098	0.674610734	0.808017241	no
DMWD	-0.023371743	-0.42015642	0.674650549	0.808017241	no
LRRC3B	-0.023368931	-0.420105849	0.674687452	0.808017241	no
DCST2	0.023367227	0.420075188	0.674709827	0.808017241	no
TTTY12	-0.023366914	-0.42006957	0.674713926	0.808017241	no
UBIAD1	-0.023365495	-0.420044039	0.674732558	0.808017241	no
ZNF443	-0.023358008	-0.419909374	0.674830835	0.808089902	no
LINC00565	0.02335165	0.419795006	0.674914303	0.808139279	no
RSU1	0.023349138	0.419749827	0.674947277	0.808139279	no
CCER1	-0.023345065	-0.419676574	0.675000742	0.80815827	no
FAM109A	-0.023339482	-0.419576154	0.675074038	0.808201	no

SPAG11A	-0.023330106	-0.419407506	0.675197139	0.808268718	no
THRSP	0.02332839	0.419376644	0.675219668	0.808268718	no
DCUN1D4	-0.02332658	-0.419344091	0.675243431	0.808268718	no
ZCCHC4	0.023322799	0.419276072	0.675293084	0.808283134	no
NDST1	-0.023317615	-0.419182824	0.675361157	0.808319593	no
ELK1	0.02330409	0.418939564	0.675538753	0.808487127	no
ADCY9	0.02329918	0.41885124	0.675603239	0.808519279	no
LCN6	-0.023284551	-0.418588117	0.675795364	0.808685016	no
NT5C	-0.023282905	-0.418558504	0.675816988	0.808685016	no
FBXL5	0.023273756	0.418393948	0.675937154	0.808783775	no
ACOX3	-0.023266083	-0.418255938	0.676037942	0.808859337	no
ZFYVE19	-0.023262799	-0.418196873	0.676081079	0.808865917	no
IFNA14	-0.02325438	-0.418045437	0.676191681	0.808919749	no
GPATCH1	-0.023251758	-0.41799827	0.676226131	0.808919749	no
C5orf44	-0.023250779	-0.417980659	0.676238994	0.808919749	no
LOC100286793	-0.02324624	-0.417899017	0.676298626	0.808946055	no
CCDC138	-0.023242935	-0.417839576	0.676342044	0.808952964	no
CCDC11	0.023238338	0.417756881	0.676402448	0.808980189	no
DMRT2	-0.023226594	-0.417545644	0.676556756	0.809119714	no
OIT3	-0.023216409	-0.417362463	0.676690581	0.80921638	no
STRA8	-0.023214711	-0.417331924	0.676712893	0.80921638	no
SOCS4	0.023207105	0.417195115	0.676812848	0.809290876	no
CBLB	-0.023202223	-0.41710729	0.676877018	0.809308864	no
HIST1H2APS1	-0.02320023	-0.417071446	0.676903207	0.809308864	no
RNASEH2B-AS1	0.023191953	0.416922583	0.677011982	0.809393886	no
FBF1	0.023182563	0.416753685	0.677135403	0.809496409	no
CRNN	-0.023170065	-0.416528883	0.67729969	0.809618297	no
CACNA1C-IT3	-0.023166254	-0.416460327	0.677349794	0.809618297	no
C11orf96	0.023166209	0.416459528	0.677350378	0.809618297	no
GRIK1	-0.023154193	-0.41624339	0.677508353	0.809762083	no
C16orf86	-0.023145628	-0.416089336	0.677620959	0.809851631	no
HCCS	0.023142639	0.416035586	0.677660249	0.809853551	no
SEMA4C	0.023131115	0.415828299	0.677811781	0.809950474	no
UBE3B	-0.023130739	-0.415821533	0.677816727	0.809950474	no
CST3	0.023123882	0.415698213	0.677906884	0.810013168	no
FBN2	0.023117854	0.415589787	0.677986154	0.810062848	no
APOA1	-0.023106429	-0.415384283	0.678136411	0.810197331	no
NCBP1	-0.023101951	-0.415303739	0.678195305	0.810222652	no
TMEM143	0.0230918	0.415121157	0.678328817	0.810287285	no
PDLIM2	-0.023090854	-0.415104139	0.678341261	0.810287285	no
SNORA78	0.023089238	0.415075074	0.678362516	0.810287285	no
PUM1	-0.023084001	-0.414980873	0.678431405	0.810324533	no
ATG4C	-0.023079345	-0.414897138	0.678492643	0.810352639	no
XRCC1	-0.023066051	-0.414658028	0.678667522	0.810501137	no
TSPY4	0.02306416	0.414624008	0.678692405	0.810501137	no
ARR3	0.023050178	0.414372523	0.678876356	0.810650338	no
CYLC2	0.02304893	0.414350067	0.678892782	0.810650338	no
CCDC142	-0.023031878	-0.41404336	0.679117155	0.810873202	no
NBPF15	-0.023006757	-0.413591527	0.679447746	0.811201094	no
EPS8L1	0.023005274	0.413564858	0.67946726	0.811201094	no
GPR37	0.023002062	0.413507074	0.679509544	0.811206511	no

LOC339822	-0.02299874	-0.413447333	0.679553261	0.811209934	no
RNF126P1	-0.022996108	-0.413399991	0.679587905	0.811209934	no
GML	0.022986881	0.413234026	0.679709361	0.811309851	no
LINC00479	0.022973645	0.412995961	0.679883596	0.811472751	no
RASAL2-AS1	0.022967678	0.412888632	0.679962154	0.811521444	no
CCDC6	-0.022960889	-0.412766529	0.680051529	0.811583041	no
EFCAB3	0.022948157	0.412537516	0.680219171	0.811738031	no
KLRAP1	-0.022939075	-0.412374164	0.680338757	0.81183566	no
PYDC2	0.02292936	0.412199429	0.680466687	0.811943232	no
NUP188	0.022925337	0.41212707	0.680519666	0.811945022	no
OR7D2	-0.022919326	-0.41201896	0.680598823	0.811945022	no
DNAJC17	-0.022917306	-0.411982628	0.680625426	0.811945022	no
PRR15L	-0.022912826	-0.41190204	0.680684435	0.811945022	no
GALR2	0.022912049	0.411888068	0.680694666	0.811945022	no
LENEP	-0.022912033	-0.41188779	0.68069487	0.811945022	no
ANO7	0.022902954	0.411724488	0.680814452	0.811998061	no
HOXB1	-0.02290292	-0.411723873	0.680814902	0.811998061	no
PAXIP1	-0.022891123	-0.411511694	0.680970288	0.812138317	no
LOC284294	-0.022873492	-0.411194579	0.681202547	0.812370232	no
TEX13B	-0.022862222	-0.410991875	0.681351026	0.81248396	no
MTMR9LP	-0.022860515	-0.410961162	0.681373524	0.81248396	no
TMEM158	0.022855948	0.410879029	0.68143369	0.812510621	no
PLXNA2	-0.022838982	-0.410573856	0.681657261	0.812732105	no
LOC285547	-0.022820353	-0.410238804	0.681902753	0.8129757	no
TOMM6	-0.022816778	-0.410174507	0.681949867	0.8129757	no
MYBPC3	0.022814861	0.41014002	0.681975138	0.8129757	no
DHRS13	-0.022809957	-0.410051815	0.682039774	0.8129757	no
PSG9	0.022809127	0.410036888	0.682050713	0.8129757	no
PSMA4	0.022800259	0.409877384	0.682167603	0.813069932	no
RLF	0.022771653	0.409362868	0.68254471	0.813474288	no
OR56A5	-0.022767475	-0.409287723	0.682599793	0.813494823	no
DSG1	-0.022763007	-0.409207356	0.682658706	0.81351992	no
OR2L2	-0.022760071	-0.409154556	0.682697412	0.813520936	no
MDGA1	-0.022756602	-0.409092159	0.682743154	0.813530335	no
ATP5SL	0.022747154	0.408922234	0.68286773	0.813633663	no
CFTR	-0.022734915	-0.408702099	0.683029128	0.813780851	no
POTEB	0.022731562	0.408641787	0.68307335	0.813788423	no
MORN1	0.022725866	0.408539338	0.68314847	0.813832803	no
LINC00589	0.022721828	0.408466715	0.683201723	0.813851129	no
KLKP1	0.022711777	0.408285929	0.683334295	0.813963935	no
LOC100128881	-0.022706648	-0.408193687	0.68340194	0.813999396	no
SDC1	0.02270245	0.408118171	0.683457321	0.814020245	no
SRSF9	0.022694597	0.407976929	0.68356091	0.814098505	no
IFIT5	0.022686247	0.407826748	0.68367106	0.81418457	no
VAT1L	-0.022683189	-0.407771744	0.683711405	0.8141875	no
LOC145783	0.022679999	0.40771437	0.683753489	0.814192499	no
PCTP	-0.022673559	-0.407598543	0.683838451	0.814248554	no
OR8G5	0.022667312	0.407486176	0.683920878	0.814289228	no
LRRC30	-0.022665227	-0.407448673	0.68394839	0.814289228	no
UGT2B17	0.022660896	0.407370785	0.684005529	0.814312144	no
TEX101	-0.022648985	-0.407156551	0.6841627	0.814410525	no

IL17C	-0.02264889	-0.407154836	0.684163958	0.814410525	no
SLC36A4	-0.022639361	-0.40698346	0.684289697	0.814446802	no
KY	0.022637305	0.40694648	0.68431683	0.814446802	no
ITPRIP	0.022635434	0.406912827	0.684341523	0.814446802	no
OR10A2	-0.022635093	-0.406906696	0.684346022	0.814446802	no
LOC100507651	-0.022624355	-0.406713555	0.684487745	0.814570359	no
KRTAP20-4	-0.022614386	-0.406534257	0.684619319	0.814634827	no
KRT12	-0.022607921	-0.406417973	0.684704658	0.814634827	no
HOXA10	-0.022606267	-0.406388225	0.68472649	0.814634827	no
KRTAP5-11	-0.022606233	-0.406387615	0.684726937	0.814634827	no
CYP2A7	0.022605892	0.406381471	0.684731447	0.814634827	no
KRT16P1	0.022583455	0.405977934	0.685027629	0.814935334	no
BOC	-0.022578782	-0.405893874	0.685089334	0.814935334	no
SNORA79	-0.022578141	-0.405882349	0.685097793	0.814935334	no
C6orf123	-0.022575142	-0.405828416	0.685137384	0.814937321	no
AKR1C4	0.022558682	0.405532361	0.685354723	0.815108258	no
RAB28	-0.022558513	-0.405529326	0.68535695	0.815108258	no
H3F3A	-0.02255549	-0.40547495	0.685396872	0.815110629	no
SNORA42	-0.022540434	-0.40520415	0.685595698	0.815278529	no
MRPL51	-0.022534091	-0.405090074	0.685679461	0.815278529	no
CXorf64	-0.022530943	-0.405033443	0.685721046	0.815278529	no
KRT16P2	0.022530776	0.405030446	0.685723246	0.815278529	no
OR9Q2	-0.022527756	-0.404976129	0.685763132	0.815278529	no
UGT3A1	-0.022527564	-0.404972676	0.685765668	0.815278529	no
CDRT15L2	0.022520783	0.404850706	0.685855236	0.815339909	no
ARFGEF2	-0.022517105	-0.404784558	0.685903813	0.815352556	no
NPW	-0.022503422	-0.404538455	0.686084556	0.815494504	no
CD1B	0.022502319	0.40451863	0.686099117	0.815494504	no
TUBA1A	-0.022498263	-0.404445664	0.686152709	0.8155131	no
XRN1	0.022494544	0.404378789	0.686201828	0.815526377	no
RPS27L	0.022491303	0.404320494	0.686244646	0.815532166	no
MAP3K15	-0.022487881	-0.404258938	0.686289862	0.815540803	no
OR11H4	-0.022481685	-0.404147499	0.68637172	0.815588057	no
ZYG11A	-0.022474346	-0.404015511	0.686468677	0.815588057	no
GDPD3	0.022472992	0.403991157	0.686486568	0.815588057	no
PFDN5	-0.022471376	-0.403962085	0.686507925	0.815588057	no
RNF40	0.022470509	0.403946495	0.686519378	0.815588057	no
LOC100288255	0.022453314	0.403637224	0.686746593	0.815812892	no
OR5A1	-0.022449557	-0.403569654	0.686796239	0.815826773	no
SETD1B	-0.022445621	-0.403498853	0.68684826	0.815843473	no
AQPEP	-0.022402205	-0.402717984	0.687422108	0.816479967	no
PRPF4B	-0.022392551	-0.402544355	0.687549729	0.816561834	no
MS4A12	0.022391243	0.402520824	0.687567026	0.816561834	no
TRMT112	0.022380192	0.402322072	0.687713126	0.816677401	no
AR	-0.022378134	-0.402285055	0.687740338	0.816677401	no
FKSG29	-0.022371201	-0.402160366	0.687832002	0.816734458	no
HMGN1	-0.022368751	-0.402116305	0.687864394	0.816734458	no
YBX1	-0.022358407	-0.401930259	0.688001176	0.816851736	no
OVCH1	-0.022346412	-0.40171451	0.688159809	0.816994942	no
CNGA2	-0.022330905	-0.40143561	0.688364894	0.817177916	no
CRYBA2	0.022324309	0.401316985	0.68845213	0.817177916	no

OR8H1	0.022321864	0.401273008	0.688484472	0.817177916	no
KRR1	0.022321462	0.401265771	0.688489794	0.817177916	no
SLC25A31	0.022320384	0.401246389	0.688504049	0.817177916	no
ZNF75D	0.022315516	0.401158828	0.688568445	0.817209214	no
HOXA3	0.022303463	0.400942056	0.688727879	0.817353297	no
HTN1	0.022289313	0.400687562	0.688915075	0.817491832	no
ZWILCH	0.022288889	0.400679928	0.688920691	0.817491832	no
CDK15	0.022248584	0.399955017	0.689454015	0.818040558	no
TMPRSS11B	-0.022248189	-0.399947909	0.689459245	0.818040558	no
CLEC1B	-0.022237006	-0.399746786	0.689607241	0.818169786	no
TAAR1	-0.022234206	-0.399696425	0.689644301	0.818169786	no
ZNF195	-0.022227177	-0.39957001	0.689737331	0.818234985	no
PSPN	-0.022220172	-0.399444012	0.689830059	0.818299817	no
SLC35B3	0.022207898	0.399223268	0.689992526	0.818447366	no
SRSF10	-0.022183953	-0.398792602	0.690309538	0.818777559	no
CLRN2	-0.022179456	-0.398711715	0.690369084	0.818777559	no
SULT1A2	-0.022175768	-0.398645389	0.690417913	0.818777559	no
ERLIN1	-0.022175362	-0.398638085	0.69042329	0.818777559	no
TAAR8	-0.022167114	-0.398489732	0.690532511	0.818861899	no
ZNF528	-0.022163745	-0.398429155	0.690577112	0.818869604	no
RMDN2	0.022157092	0.398309485	0.690665222	0.818919254	no
C12orf60	-0.022154829	-0.398268781	0.690695193	0.818919254	no
MAGEA11	-0.022151736	-0.398213156	0.690736151	0.818922636	no
PTGES3L-AARS	-0.022147923	-0.398144576	0.690786649	0.818923368	no
CLPB	0.022145935	0.398108819	0.690812979	0.818923368	no
KCNE1L	-0.022132261	-0.397862889	0.690994081	0.819054781	no
PROL1	-0.022131809	-0.397854773	0.691000058	0.819054781	no
RPL35A	-0.022125593	-0.397742971	0.691082395	0.819106429	no
OR4D11	-0.022122765	-0.397692102	0.691119858	0.819106429	no
CDK1	0.022115666	0.397564431	0.691213888	0.819172696	no
OR9A2	0.022111684	0.397492802	0.691266645	0.819190045	no
BIRC8	-0.022106474	-0.397399113	0.691335652	0.819207643	no
NXNL1	-0.022104808	-0.397369137	0.691357732	0.819207643	no
ENTPD6	-0.02210156	-0.397310724	0.691400758	0.819213457	no
LOC100129935	0.022091836	0.397135828	0.69152959	0.819305299	no
GAL3ST2	0.022087119	0.397050992	0.691592084	0.819305299	no
CCNY	0.022087077	0.397050237	0.69159264	0.819305299	no
KLHL5	-0.022068921	-0.396723702	0.691833205	0.819545112	no
HIST1H2AH	0.022060554	0.396573209	0.691944086	0.819605222	no
CPEB1	-0.022052491	-0.396428209	0.692050927	0.819605222	no
KRTAP4-12	-0.02204928	-0.396370452	0.692093486	0.819605222	no
HBD	0.022048579	0.396357845	0.692102776	0.819605222	no
CAPZB	-0.022047439	-0.396337339	0.692117886	0.819605222	no
LOC550112	0.022045783	0.396307559	0.69213983	0.819605222	no
SNRPA1	-0.022042798	-0.396253863	0.692179399	0.819605222	no
PRR7	-0.022039514	-0.396194813	0.692222914	0.819605222	no
HINT2	0.022039192	0.39618902	0.692227183	0.819605222	no
ZNF136	-0.022021973	-0.395879323	0.69245542	0.819716724	no
TFF3	0.02201781	0.395804453	0.6925106	0.819716724	no
STK11IP	0.02201674	0.395785213	0.692524781	0.819716724	no
RNF24	0.022014083	0.395737421	0.692560006	0.819716724	no

KLHL38	0.022011032	0.395682546	0.692600452	0.819716724	no
TAS2R46	-0.02200921	-0.395649786	0.692624599	0.819716724	no
ENSA	0.022009173	0.39564911	0.692625097	0.819716724	no
HBG2	0.022009067	0.395647215	0.692626494	0.819716724	no
CIB2	-0.02200087	-0.395499775	0.692735173	0.819779567	no
PAX3	-0.021999307	-0.395471677	0.692755884	0.819779567	no
LOC285593	0.021995287	0.395399367	0.692809187	0.819782868	no
LOC10050730C	-0.021993342	-0.395364398	0.692834965	0.819782868	no
ANTXR1	0.021989717	0.395299197	0.69288303	0.819794604	no
WDR83	-0.021982681	-0.39517264	0.692976327	0.819859854	no
KIAA0556	0.021979413	0.395113868	0.693019656	0.819865981	no
OR2T33	-0.021973026	-0.394998996	0.693104346	0.819878957	no
ADM5	0.021972831	0.3949955	0.693106923	0.819878957	no
C22orf28	-0.02196973	-0.394939726	0.693148044	0.819882471	no
IFITM5	-0.021954103	-0.394658671	0.693355274	0.820082455	no
MT3	-0.021950371	-0.394591546	0.693404771	0.820095863	no
OR4X1	-0.021945736	-0.394508192	0.693466236	0.820123425	no
PRDM14	-0.021923127	-0.394101553	0.693766121	0.820432934	no
CCDC148-AS1	-0.02191379	-0.393933625	0.693889978	0.820534253	no
RPS18	-0.021908415	-0.393836961	0.693961277	0.820573414	no
WDR36	0.021904795	0.393771852	0.694009303	0.820585053	no
OR4K13	-0.021901882	-0.393719455	0.694047953	0.820585605	no
RIPK2	0.021888464	0.393478141	0.694225965	0.820750919	no
C19orf33	0.021881911	0.393360287	0.694312909	0.820808555	no
HIST1H2AB	-0.021869182	-0.393131347	0.694481817	0.820963076	no
SLC7A5	0.021857743	0.392925627	0.694633607	0.821097312	no
LEKR1	0.021852662	0.39283424	0.69470104	0.821097312	no
MFSD2A	0.021851988	0.392822109	0.694709991	0.821097312	no
RAD51D	-0.02182647	-0.392363174	0.695048672	0.821452432	no
RBM24	0.02181374	0.392134228	0.695217651	0.821595125	no
SLC25A40	0.021810464	0.392075295	0.69526115	0.821595125	no
CKM	-0.021808735	-0.392044206	0.695284098	0.821595125	no
SPHAR	-0.021761603	-0.391196545	0.695909887	0.822289387	no
HOXA7	0.021748462	0.390960202	0.696084405	0.822418699	no
LOC10050741C	-0.0217476	-0.390944689	0.69609586	0.822418699	no
CLDN17	-0.021741185	-0.390829323	0.696181054	0.822474137	no
LOC149950	-0.021731001	-0.390646169	0.696316315	0.822588717	no
DAAM1	-0.021701535	-0.39011622	0.696707742	0.82295906	no
PEF1	-0.021699912	-0.390087026	0.696729308	0.82295906	no
UBE2H	-0.021698754	-0.390066204	0.696744688	0.82295906	no
PRDM6	0.021691769	0.389940574	0.696837494	0.823023444	no
DCDC5	-0.021672771	-0.38959891	0.697089912	0.823276326	no
CETN4P	-0.021666799	-0.389491494	0.697169276	0.823324811	no
SLC34A1	-0.021661857	-0.389402619	0.697234945	0.823357119	no
RGPD1	-0.021656602	-0.389308097	0.697304788	0.823394352	no
NXPE1	0.021640207	0.389013249	0.69752267	0.823606379	no
LOC440356	-0.021616705	-0.388590567	0.697835061	0.823929969	no
ABCA5	-0.021610901	-0.388486181	0.697912217	0.823975799	no
WDR54	0.021603556	0.388354085	0.69800986	0.824041227	no
SLC25A16	-0.021600964	-0.388307463	0.698044323	0.824041227	no
PDC	0.021595186	0.388203549	0.698121139	0.824056876	no

CAPRIN1	0.021593244	0.388168624	0.698146957	0.824056876	no
C7orf66	-0.021591315	-0.38813392	0.698172612	0.824056876	no
CSN2	-0.021564912	-0.387659085	0.698523671	0.824393977	no
MEIG1	-0.021564065	-0.38764385	0.698534936	0.824393977	no
SCAND3	-0.021557738	-0.387530063	0.698619072	0.824447998	no
C7orf72	0.021545561	0.387311055	0.698781021	0.824553849	no
FAM221B	-0.021545224	-0.387304994	0.698785503	0.824553849	no
GPR50	-0.021532186	-0.387070518	0.698958906	0.824713179	no
UGDH-AS1	-0.021525359	-0.386947724	0.699049722	0.82471978	no
INSL6	0.021520089	0.386852957	0.699119813	0.82471978	no
FAM166B	-0.021519132	-0.386835734	0.699132552	0.82471978	no
CST9L	-0.021516795	-0.386793706	0.699163637	0.82471978	no
ZNF619	-0.021515862	-0.386776928	0.699176048	0.82471978	no
PRKDC	-0.021514456	-0.386751641	0.699194751	0.82471978	no
MACROD2-AS1	-0.021507313	-0.386623169	0.699289779	0.8247866	no
CHRAC1	0.021501299	0.386515012	0.699369784	0.824817021	no
ABRA	-0.021499604	-0.386484532	0.69939233	0.824817021	no
PIK3IP1	0.021496104	0.386421579	0.699438899	0.824826678	no
NSG1	-0.021479443	-0.386121943	0.699660569	0.825042814	no
C11orf34	-0.021463802	-0.38584064	0.699868699	0.825193121	no
IFNA1	0.021462073	0.385809546	0.699891706	0.825193121	no
EPB41L1	0.021461207	0.385793978	0.699903225	0.825193121	no
MROH5	-0.021456202	-0.385703956	0.699969836	0.825226384	no
MSANTD2	-0.02143832	-0.385382368	0.700207813	0.825432273	no
ZNF549	0.021437308	0.385364155	0.700221291	0.825432273	no
GIMD1	-0.021433584	-0.385297179	0.700270858	0.825445426	no
HOMEZ	0.021428573	0.385207059	0.700337555	0.825478769	no
PGLYRP4	-0.021415644	-0.384974546	0.700509645	0.825552232	no
NSUN4	0.021415409	0.384970311	0.70051278	0.825552232	no
RSPO3	0.021415233	0.384967146	0.700515122	0.825552232	no
FLJ46066	-0.021385525	-0.38443286	0.700910624	0.825973034	no
C14orf79	0.021378493	0.3843064	0.701004248	0.826038068	no
DPPA5	-0.02137012	-0.384155807	0.701115744	0.826124154	no
LOC150185	-0.021364003	-0.384045812	0.701197186	0.82617482	no
CYP2J2	0.021350008	0.38379412	0.701383556	0.826307954	no
OR1G1	0.021348128	0.383760296	0.701408602	0.826307954	no
PAPD7	0.021346857	0.383737441	0.701425527	0.826307954	no
ITPA	0.021342416	0.383657574	0.701484671	0.826332332	no
LAMTOR4	0.021337257	0.383564793	0.70155338	0.826367974	no
IMPACT	0.021329978	0.383433882	0.701650331	0.826436878	no
CD3EAP	-0.021314264	-0.383151266	0.701859649	0.826605465	no
LOC388499	-0.021313457	-0.383136767	0.701870389	0.826605465	no
PPP1R2	0.021304402	0.382973903	0.701991024	0.826694115	no
PROSER2	0.021302032	0.382931282	0.702022595	0.826694115	no
C22orf15	0.021296107	0.382824735	0.702101522	0.826741757	no
ZFAND6	0.021292581	0.382761316	0.702148502	0.826751778	no
LGALS13	-0.021278436	-0.382506925	0.702336963	0.826905724	no
GCC2	-0.021276992	-0.38248096	0.7023562	0.826905724	no
PCDHGB4	-0.021252216	-0.382035376	0.702686351	0.827249103	no
SPATA2L	-0.021248047	-0.381960393	0.702741914	0.827269198	no
LOC286190	-0.021235882	-0.381741621	0.702904036	0.827414726	no

NOC2L	-0.021218731	-0.381433173	0.703132637	0.827599324	no
C8orf74	-0.021215894	-0.381382156	0.70317045	0.827599324	no
COX14	-0.02121318	-0.38133333	0.703206639	0.827599324	no
FAM170B	-0.02121256	-0.381322194	0.703214893	0.827599324	no
GPR15	-0.021205807	-0.381200743	0.703304915	0.827659946	no
FAM205A	-0.021193082	-0.380971886	0.703474561	0.827814257	no
LINC00474	-0.021188657	-0.380892305	0.703533555	0.827836189	no
R3HDM4	0.021185905	0.380842823	0.703570238	0.827836189	no
FGF2	0.021176827	0.380679561	0.703691275	0.827917471	no
WT1-AS	0.021174945	0.380645717	0.703716367	0.827917471	no
CARD10	-0.021163781	-0.380444927	0.703865237	0.828047286	no
GOLGA6A	-0.021149039	-0.380179814	0.704061816	0.828227899	no
SLC6A14	0.021146488	0.38013393	0.704095841	0.828227899	no
GH1	-0.021130566	-0.379847592	0.704308184	0.828432335	no
ZNF776	0.021112529	0.379523196	0.704548779	0.828669976	no
FBXO36	0.0211039	0.37936801	0.704663885	0.828714068	no
VPREB1	-0.021101462	-0.379324163	0.704696409	0.828714068	no
POLN	0.021100573	0.379308175	0.704708269	0.828714068	no
DUOXA1	-0.021098156	-0.37926471	0.704740511	0.828714068	no
FAM177A1	0.021071226	0.378780398	0.7050998	0.829091197	no
LCT	-0.021058101	-0.378544359	0.705274931	0.829251753	no
PTOV1-AS1	0.021041931	0.378253563	0.705490711	0.82943315	no
KIAA1009	0.021040756	0.378232424	0.705506397	0.82943315	no
CFHR1	-0.021026472	-0.377975544	0.705697032	0.829611887	no
KRTAP12-1	-0.021005247	-0.377593819	0.705980349	0.829832506	no
C11orf82	0.021002838	0.377550502	0.706012502	0.829832506	no
MEF2D	0.020998387	0.377470457	0.706071918	0.829832506	no
LGR6	-0.020998147	-0.377466137	0.706075125	0.829832506	no
RNU5E-1	-0.020997949	-0.377462582	0.706077763	0.829832506	no
FANCC	0.020970217	0.376963852	0.706448003	0.830222236	no
LIPI	-0.02096496	-0.376869297	0.706518205	0.830259336	no
LOC100129550	0.020950914	0.376616689	0.706705766	0.830434338	no
RAET1L	0.020932348	0.376282814	0.706953695	0.830680254	no
USP42	-0.020921778	-0.376092707	0.707094878	0.830800723	no
OR51S1	-0.020913029	-0.375935375	0.707211728	0.830892589	no
DTWD2	0.020900378	0.375707849	0.707380724	0.831045708	no
LHCGR	0.020895151	0.375613855	0.707450543	0.831082301	no
SERHL	-0.020891202	-0.375542831	0.707503302	0.83109885	no
RNF41	-0.020886746	-0.375462705	0.707562822	0.831123339	no
DECR2	-0.020879923	-0.375339991	0.707653983	0.831184989	no
COQ4	0.020869054	0.375144529	0.707799195	0.831272665	no
APOC3	0.020868546	0.375135385	0.707805989	0.831272665	no
ZMYM5	-0.020857472	-0.374936243	0.707953946	0.831400997	no
RNF168	-0.020849464	-0.374792218	0.70806096	0.831481236	no
SPAG6	-0.020824111	-0.374336278	0.708399771	0.831763796	no
DTL	-0.020822906	-0.374314602	0.708415881	0.831763796	no
FGF5	0.020822655	0.374310092	0.708419233	0.831763796	no
OR8G1	-0.020819875	-0.374260094	0.70845639	0.831763796	no
LIN7A	0.020809907	0.374080835	0.708589618	0.831874767	no
KIF24	0.02079942	0.373892236	0.708729798	0.831969639	no
LAMTOR5	0.020798069	0.373867942	0.708747855	0.831969639	no

DLGAP1-AS5	-0.020793046	-0.373777599	0.708815008	0.832003023	no
OR4F13P	0.020788955	0.373704026	0.708869698	0.832021774	no
CNTD2	-0.020785432	-0.373640687	0.708916781	0.832031595	no
PLA2G1B	0.020780436	0.373550824	0.708983583	0.832064558	no
TAF1A	-0.020776136	-0.373473492	0.709041072	0.832069993	no
ZFP106	0.020774297	0.373440433	0.709065649	0.832069993	no
AVP	0.020767693	0.373321663	0.709153946	0.832122837	no
SPANXN5	0.020765137	0.373275698	0.70918812	0.832122837	no
OR2W1	-0.020749501	-0.372994498	0.709397192	0.832322709	no
GNPTG	-0.020721313	-0.37248758	0.709774144	0.832719518	no
GPC6-AS2	0.020703619	0.37216936	0.710010813	0.832951711	no
SEMG1	0.020697896	0.372066442	0.710087362	0.832960513	no
PROK1	0.020695378	0.37202117	0.710121036	0.832960513	no
HDGF	0.020694365	0.372002945	0.710134592	0.832960513	no
KIAA1024L	0.020686361	0.371859004	0.710241661	0.833040634	no
COMMD2	-0.020681215	-0.371766469	0.710310495	0.833065059	no
TMEM219	0.020676579	0.371683085	0.710372524	0.833065059	no
C3P1	-0.020672335	-0.371606767	0.710429298	0.833065059	no
C8orf76	0.020671824	0.371597576	0.710436136	0.833065059	no
OR5M9	0.020670317	0.371570467	0.710456303	0.833065059	no
CKAP2	-0.020663964	-0.371456219	0.710541298	0.833074744	no
TBC1D2	-0.020663905	-0.371455156	0.71054209	0.833074744	no
ZNF599	-0.020651625	-0.371234326	0.710706387	0.833221917	no
MTUS1	-0.020629902	-0.370843664	0.710997074	0.833517244	no
OR6B2	-0.020625453	-0.370763651	0.711056615	0.833541576	no
ESCO1	-0.020600012	-0.370306132	0.711397112	0.83389524	no
BCAR3	-0.020589728	-0.370121185	0.711534771	0.834011113	no
SNX21	-0.020566421	-0.369702034	0.711846786	0.83433133	no
LRRIQ4	-0.020562555	-0.369632522	0.711898535	0.834332164	no
SLC17A2	0.020560568	0.369596783	0.711925142	0.834332164	no
MCIN	0.020555191	0.369500084	0.711997135	0.834371036	no
ABCB1	-0.020546979	-0.369352406	0.712107086	0.834454382	no
GP2	-0.020524672	-0.368951249	0.71240579	0.834758892	no
FAM27A	-0.020518895	-0.368847347	0.712483163	0.834804038	no
TYW3	-0.02051556	-0.368787378	0.712527822	0.83481085	no
DEFB114	0.020505862	0.368612969	0.71265771	0.834826575	no
C3orf30	-0.020505683	-0.368609753	0.712660105	0.834826575	no
KRT42P	-0.020503409	-0.368568867	0.712690555	0.834826575	no
LINC00167	-0.020501929	-0.368542249	0.71271038	0.834826575	no
LRIT1	-0.020500056	-0.36850856	0.712735471	0.834826575	no
MIR1307	-0.020497115	-0.368455676	0.712774859	0.83482721	no
LEPR	-0.02048902	-0.368310094	0.712883291	0.834892725	no
KPNA1	0.020487138	0.36827626	0.712908491	0.834892725	no
PRSS41	-0.020483098	-0.368203599	0.712962613	0.83491061	no
CROT	0.020455247	0.367702732	0.713335728	0.835248345	no
LOC441009	-0.020452894	-0.367660417	0.713367253	0.835248345	no
GTSE1	-0.020452493	-0.367653219	0.713372616	0.835248345	no
C1orf159	-0.020449966	-0.367607768	0.713406478	0.835248345	no
CCDC130	-0.020440311	-0.367434141	0.71353584	0.835354292	no
AGBL2	0.020434052	0.367321576	0.713619711	0.835387729	no
FAM104A	0.020432378	0.36729147	0.713642144	0.835387729	no

C11orf40	-0.020426877	-0.367192551	0.713715852	0.835428506	no
RNU6-19	-0.020420093	-0.367070552	0.713806761	0.835460547	no
TLR8-AS1	0.020419032	0.367051477	0.713820975	0.835460547	no
DLEU2	-0.020403795	-0.36677745	0.714025186	0.835654048	no
DPPA4	0.020377163	0.366298521	0.714382144	0.835972839	no
WFS1	0.020376258	0.366282244	0.714394277	0.835972839	no
COX7A1	-0.020374766	-0.366255417	0.714414274	0.835972839	no
KRT2	-0.020358419	-0.365961434	0.714633421	0.836183746	no
IDI2-AS1	-0.02033412	-0.36552447	0.714959197	0.836447986	no
TSEN15	0.020331175	0.365471499	0.714998692	0.836447986	no
ZFY	-0.020330782	-0.365464442	0.715003954	0.836447986	no
LOC100507194	0.020329964	0.365449716	0.715014934	0.836447986	no
LOC100507244	0.020325035	0.36536109	0.715081017	0.836479759	no
OR2T27	-0.020317576	-0.365226954	0.715181038	0.836551227	no
SLC26A7	-0.020294044	-0.364803757	0.715496633	0.836786588	no
SNORA1	0.020291675	0.364761161	0.715528402	0.836786588	no
RMDN1	0.020287705	0.364689768	0.715581648	0.836786588	no
FAM132A	-0.020286384	-0.364666002	0.715599373	0.836786588	no
C6orf99	-0.020284107	-0.364625066	0.715629906	0.836786588	no
FTLP10	-0.020282603	-0.36459802	0.715650077	0.836786588	no
PDS5A	0.020281225	0.364573226	0.71566857	0.836786588	no
DCAF4L1	-0.020279348	-0.364539473	0.715693745	0.836786588	no
SLC25A3P1	-0.020262868	-0.364243114	0.715914803	0.836968553	no
PDZD11	-0.020261528	-0.36421901	0.715932784	0.836968553	no
LINC00242	0.020259036	0.364174197	0.715966213	0.836968553	no
SEL1L2	0.020239465	0.363822247	0.716228774	0.837229947	no
HIST1H2BB	-0.02023047	-0.3636605	0.716349452	0.837325469	no
KCTD5	-0.02022668	-0.363592338	0.716400309	0.837339372	no
HLTF-AS1	-0.020219286	-0.363459377	0.716499518	0.837372139	no
PHPT1	-0.020216477	-0.363408853	0.716537217	0.837372139	no
PLGLA	-0.020215879	-0.363398108	0.716545235	0.837372139	no
ERN2	-0.020195611	-0.363033625	0.716817223	0.837644443	no
WNT1	-0.020182834	-0.362803848	0.716988709	0.83779928	no
ZNF354B	0.020178501	0.362725921	0.71704687	0.837821687	no
SERPIND1	-0.020172111	-0.362611016	0.717132633	0.837876341	no
TACC2	-0.020160637	-0.36240467	0.717286655	0.837933614	no
RAP2C	0.020160555	0.362403202	0.71728775	0.837933614	no
EFTUD1	0.020159745	0.362388637	0.717298623	0.837933614	no
RTTN	0.020153127	0.362269622	0.717387464	0.8379747	no
CYP26C1	-0.020146711	-0.362154244	0.717473595	0.8379747	no
LOC100506082	0.020146361	0.362147952	0.717478292	0.8379747	no
AP1M1	0.020142302	0.36207495	0.717532789	0.8379747	no
PCDHGA2	-0.020140111	-0.36203556	0.717562196	0.8379747	no
MCHR1	0.020139698	0.362028128	0.717567745	0.8379747	no
ADSL	-0.020112767	-0.361543829	0.717929332	0.838332064	no
RNF7	0.020111096	0.361513769	0.717951777	0.838332064	no
FAM118A	0.020107576	0.361450471	0.717999042	0.838341704	no
FIG4	-0.020103566	-0.361378366	0.718052884	0.838353643	no
ZNF645	0.020100113	0.36131627	0.718099253	0.838353643	no
FAM162A	-0.0200981	-0.361280057	0.718126296	0.838353643	no
DLX4	-0.020089009	-0.36111657	0.718248384	0.838413888	no

GRHL2	-0.020088447	-0.361106473	0.718255925	0.838413888	no
NOMO1	-0.02008552	-0.361053843	0.718295229	0.838414228	no
BLID	0.020073654	0.36084044	0.718454609	0.838504664	no
RPL7L1	-0.020072461	-0.36081899	0.71847063	0.838504664	no
MEA1	-0.020071037	-0.360793378	0.718489758	0.838504664	no
THAP9-AS1	-0.020065147	-0.360687467	0.718568864	0.838551447	no
AA06	-0.020059984	-0.360594627	0.718638209	0.838586835	no
CLEC3A	-0.020056295	-0.360528271	0.718687773	0.838599139	no
SRA1	-0.020047269	-0.36036596	0.718809017	0.838695074	no
PPP1R14A	-0.020036239	-0.360167614	0.718957187	0.838822416	no
ASUN	-0.02002223	-0.359915692	0.719145396	0.838996455	no
ZNHIT6	-0.020016655	-0.359815424	0.71922031	0.839027596	no
SASS6	-0.020014432	-0.359775459	0.71925017	0.839027596	no
PPP1R3A	0.02001093	0.359712481	0.719297226	0.839036945	no
INVS	0.020007871	0.359657466	0.719338332	0.839039354	no
KRT84	-0.019999685	-0.359510255	0.71944833	0.839122114	no
ZPBP	-0.019985884	-0.359262074	0.719633788	0.839292871	no
DDIT4	0.019974486	0.359057107	0.719786966	0.839411138	no
RC3H1	0.019972526	0.359021857	0.719813311	0.839411138	no
OPN4	0.019967239	0.358926783	0.719884367	0.83944845	no
LOC730668	-0.019959327	-0.358784505	0.719990707	0.839500339	no
DLK1	-0.019956776	-0.358738635	0.720024992	0.839500339	no
RSPH10B	-0.019955209	-0.358710456	0.720046054	0.839500339	no
HOXA6	-0.019916688	-0.358017723	0.720563901	0.840049729	no
C19orf54	0.019914341	0.357975529	0.720595447	0.840049729	no
C21orf54	-0.019902716	-0.357766481	0.720751747	0.840139474	no
TRDMT1	-0.019902243	-0.357757975	0.720758107	0.840139474	no
FCRLA	0.019899893	0.357715711	0.720789709	0.840139474	no
KDM5B-AS1	-0.019892896	-0.35758988	0.720883796	0.840203572	no
TAS2R9	0.019881209	0.357379716	0.721040953	0.840341167	no
WIPI2	0.019871049	0.357197006	0.721177589	0.840454833	no
TEX37	-0.01986315	-0.357054957	0.721283824	0.840533059	no
LOC10061653C	-0.019856257	-0.356931	0.721376533	0.840595514	no
DHH	-0.019844998	-0.356728535	0.721527967	0.840618043	no
LOC100505624	-0.019844	-0.356710586	0.721541393	0.840618043	no
LOC100499194	-0.019843206	-0.356696315	0.721552068	0.840618043	no
TMEM191B	0.019843187	0.356695969	0.721552326	0.840618043	no
CALML3	-0.019824456	-0.356359145	0.721804282	0.840851705	no
RNF13	0.01982246	0.356323239	0.721831143	0.840851705	no
MORF4L1	-0.019806105	-0.356029134	0.722051171	0.841062424	no
ADCY10	-0.019792461	-0.35578378	0.722234745	0.841160658	no
LINC00548	0.019790517	0.355748816	0.722260907	0.841160658	no
APPL2	-0.019790217	-0.355743418	0.722264946	0.841160658	no
IL4	0.019788201	0.355707173	0.722292066	0.841160658	no
MUC13	0.019773708	0.355446539	0.722487096	0.841342193	no
SHB	0.019755491	0.355118947	0.722732255	0.841582081	no
FAM205B	-0.019747529	-0.354975769	0.722839413	0.841661258	no
OR52K1	0.019739663	0.354834317	0.722945286	0.841738929	no
NUDCD1	0.019732765	0.354710285	0.723038124	0.841801415	no
SURF1	-0.019724172	-0.35455576	0.723153793	0.841890475	no
TMEM63B	-0.01970294	-0.354173941	0.723439627	0.842094052	no

RHD	-0.019697019	-0.354067471	0.723519338	0.842094052	no
ASB7	0.01969698	0.354066767	0.723519865	0.842094052	no
POM121	-0.019696826	-0.354063994	0.723521942	0.842094052	no
RGMA	-0.01969663	-0.354060475	0.723524576	0.842094052	no
TNFAIP1	0.019691203	0.35396289	0.723597638	0.84213348	no
ITGB1BP1	-0.019687071	-0.353888571	0.723653283	0.842152635	no
PRR9	-0.019675615	-0.353682569	0.723807531	0.842247521	no
TCOF1	-0.019675194	-0.353674999	0.723813199	0.842247521	no
NRTN	-0.019659223	-0.353387799	0.724028264	0.842452161	no
CFHR5	-0.019652453	-0.353266051	0.724119439	0.84247322	no
SLC22A20	-0.019648845	-0.353201171	0.724168029	0.84247322	no
OR4C12	0.019647508	0.353177129	0.724186035	0.84247322	no
AURKA	0.019644428	0.353121746	0.724227513	0.84247322	no
SPATA31D1	-0.019643325	-0.353101911	0.724242369	0.84247322	no
SYNDIG1	-0.019638395	-0.353013265	0.72430876	0.842504848	no
SNORA11B	0.0196202	0.352686059	0.724553842	0.842744311	no
FLJ31813	-0.019607371	-0.352455357	0.724726659	0.842899698	no
CAD	-0.01957815	-0.351929894	0.725120329	0.843311921	no
POLA2	0.019569946	0.351782359	0.725230873	0.843394843	no
MPI	-0.019565454	-0.351701588	0.725291396	0.843419587	no
SRPX	-0.019558735	-0.351580773	0.725381927	0.843479222	no
GHRLOS2	-0.019548403	-0.351394966	0.725521166	0.843509399	no
LOC100134229	-0.019548122	-0.351389921	0.725524946	0.843509399	no
GPR156	0.019546135	0.351354192	0.725551722	0.843509399	no
PCDHGA3	-0.019545159	-0.351336639	0.725564876	0.843509399	no
ATP1A1OS	0.019527116	0.351012175	0.725808048	0.843746458	no
CCNT2	0.019507044	0.350651236	0.726078589	0.843994866	no
PAQR5	0.019505435	0.350622302	0.726100278	0.843994866	no
NR2C2AP	0.019501874	0.350558258	0.726148286	0.84400502	no
EPO	0.019488222	0.350312769	0.726332318	0.84412416	no
TYR	-0.019487997	-0.350308726	0.726335348	0.84412416	no
ANGPTL1	0.019481931	0.350199644	0.726417127	0.84412416	no
SLC16A1	-0.019479238	-0.350151205	0.726453443	0.84412416	no
CYP7A1	0.019479099	0.350148709	0.726455314	0.84412416	no
BMP15	-0.019471991	-0.350020892	0.726551145	0.84412416	no
STXBP4	0.01947026	0.349989766	0.726574482	0.84412416	no
GGTA1P	-0.01946814	-0.349951646	0.726603063	0.84412416	no
FKBP1AP1	-0.019464052	-0.349878128	0.726658186	0.84412416	no
LOC284801	-0.019462822	-0.349856018	0.726674764	0.84412416	no
FAM83D	0.019462223	0.349845236	0.726682848	0.84412416	no
RBBP9	-0.019454514	-0.349706613	0.726786791	0.844199272	no
RBMXL3	-0.019447379	-0.349578307	0.726883003	0.844265396	no
EQTN	-0.019439858	-0.349443073	0.726984414	0.844301718	no
KRTAP20-3	0.019439233	0.349431828	0.726992847	0.844301718	no
PPP5C	-0.019418813	-0.349064628	0.727268234	0.844532201	no
FAM209B	0.01941869	0.349062406	0.727269901	0.844532201	no
FAM83C	-0.019415043	-0.348996826	0.727319087	0.844543684	no
C6orf222	-0.019399135	-0.348710761	0.727533655	0.844747192	no
MLK7-AS1	-0.019396192	-0.34865784	0.727573352	0.844747645	no
CLDN19	-0.019390494	-0.348555392	0.727650201	0.8447527	no
MDH1B	0.019390041	0.348547235	0.72765632	0.8447527	no

ALAS1	-0.019380601	-0.348377486	0.727783661	0.844794251	no
LOC100287944	-0.019377644	-0.348324304	0.727823558	0.844794251	no
TPI1	0.019376478	0.348303345	0.727839281	0.844794251	no
RLN2	0.019375732	0.348289926	0.727849349	0.844794251	no
HBA2	0.019360522	0.348016409	0.728054555	0.844943609	no
OR10C1	-0.019360365	-0.348013599	0.728056664	0.844943609	no
LOC100130015	0.019349671	0.347821297	0.72820095	0.845065426	no
SYCE3	0.019336664	0.347587394	0.728376464	0.845148703	no
C19orf6	0.019336583	0.347585934	0.728377559	0.845148703	no
TRIM54	0.019330365	0.34747413	0.728461459	0.845148703	no
GRM5-AS1	-0.019328319	-0.347437336	0.72848907	0.845148703	no
ST3GAL3	0.019327532	0.347423181	0.728499693	0.845148703	no
OCM2	-0.019326867	-0.347411225	0.728508665	0.845148703	no
GOLGA8B	0.019316403	0.347223058	0.728649879	0.845266898	no
KIAA1377	-0.019312702	-0.347156499	0.728699831	0.845276695	no
OR7E5P	-0.019309949	-0.347106991	0.728736988	0.845276695	no
LOC494127	-0.019293086	-0.346803765	0.728964579	0.845423127	no
KIF2B	-0.019292046	-0.346785071	0.728978611	0.845423127	no
RAB22A	-0.01928459	-0.346650994	0.729079253	0.845423127	no
OR5J2	-0.019283344	-0.346628588	0.729096072	0.845423127	no
FXYD4	-0.019283314	-0.346628039	0.729096484	0.845423127	no
OR1D4	0.019281929	0.346603137	0.729115177	0.845423127	no
LOC100130700	-0.019280194	-0.346571933	0.7291386	0.845423127	no
SRD5A2	-0.019262234	-0.346248976	0.729381046	0.845658614	no
ABCD2	-0.019251473	-0.346055475	0.729526322	0.84578142	no
IRX1	-0.01921945	-0.345479619	0.729958717	0.846202779	no
FAM32A	0.019218724	0.345466574	0.729968513	0.846202779	no
ZNF511	-0.019207996	-0.345273666	0.730113383	0.846243513	no
TAS2R41	-0.019207419	-0.345263286	0.730121178	0.846243513	no
AQP10	-0.019207374	-0.345262482	0.730121782	0.846243513	no
DTWD1	-0.019203902	-0.345200042	0.730168676	0.846252225	no
GLT6D1	-0.019194644	-0.345033558	0.730293712	0.846351497	no
AGXT	-0.019181923	-0.344804805	0.730465528	0.846499009	no
SERPINB11	-0.019178104	-0.344736136	0.730517107	0.846499009	no
XIRP1	0.019174214	0.34466618	0.730569655	0.846499009	no
LINC00477	0.019173555	0.344654338	0.73057855	0.846499009	no
SCN11A	-0.019161025	-0.34442903	0.7307478	0.846649467	no
DAK	-0.019153495	-0.344293613	0.730849531	0.846682983	no
ENDOU	0.019151913	0.344265173	0.730870897	0.846682983	no
MACF1	-0.019150135	-0.344233197	0.730894919	0.846682983	no
RTP1	0.019146609	0.344169793	0.730942553	0.846692525	no
LYRM5	-0.019141796	-0.344083247	0.731007575	0.846722205	no
KIAA1524	0.019138545	0.344024791	0.731051494	0.846727441	no
SPATC1L	-0.019128465	-0.343843517	0.731187695	0.846839553	no
DEFA4	0.019115788	0.343615568	0.731358976	0.846992281	no
KRTAP10-1	0.019111335	0.343535498	0.731419145	0.847013653	no
ZNF772	-0.019108589	-0.343486112	0.731456256	0.847013653	no
KRTAP25-1	0.019103506	0.343394717	0.731524937	0.847039358	no
CCDC87	-0.019100844	-0.343346835	0.73156092	0.847039358	no
MEF2BNB	-0.019098196	-0.343299232	0.731596695	0.847039358	no
FLJ40288	-0.019095009	-0.343241916	0.731639769	0.847043596	no

MIR182	-0.019087264	-0.343102638	0.731744443	0.847093886	no
SF3B4	0.019085961	0.343079225	0.73176204	0.847093886	no
TEX2	0.019080164	0.342974978	0.73184039	0.847138954	no
PIN4	-0.019069373	-0.342780921	0.731986247	0.847235499	no
FANCI	0.01906816	0.342759112	0.732002641	0.847235499	no
GLYATL2	0.019049485	0.342423305	0.732255066	0.84748202	no
LOC286135	-0.019036657	-0.342192626	0.732428484	0.847637079	no
FASTKD1	-0.019028381	-0.342043805	0.73254037	0.847685688	no
OR4K5	-0.019027715	-0.34203183	0.732549374	0.847685688	no
GMCL1P1	0.019024195	0.341968542	0.732596957	0.847695106	no
LYPD2	-0.019014499	-0.341794192	0.732728047	0.847787698	no
TJP3	-0.019012441	-0.341757184	0.732755874	0.847787698	no
NXPH4	-0.019006544	-0.341651142	0.73283561	0.847824087	no
LOC284661	0.019001038	0.341552137	0.732910057	0.847824087	no
LOC400684	0.019000944	0.341550447	0.732911327	0.847824087	no
C16orf78	-0.018998445	-0.3415055	0.732945127	0.847824087	no
NGLY1	0.018995202	0.341447186	0.732988978	0.847829178	no
TAS1R2	-0.018987165	-0.341302675	0.733097651	0.847909242	no
OR6N2	0.018967675	0.340952204	0.733361232	0.848168455	no
PHYHD1	-0.018964208	-0.340889865	0.733408119	0.848177038	no
SIX2	-0.018960526	-0.340823644	0.733457927	0.848188996	no
DBNL	0.018957261	0.340764944	0.733502079	0.848191118	no
CITED1	0.018952143	0.340672903	0.73357131	0.848191118	no
PMS2P3	-0.018951636	-0.340663792	0.733578163	0.848191118	no
ACSM2A	-0.018940807	-0.340469072	0.733724636	0.848314835	no
TSTD1	0.018935997	0.340382574	0.733789704	0.848318536	no
RRAS2	0.018934735	0.340359871	0.733806783	0.848318536	no
OR7E24	-0.018924946	-0.34018386	0.733939195	0.848425972	no
CST11	-0.018921917	-0.340129384	0.733980179	0.848427712	no
POC5	-0.018918101	-0.340060771	0.734031799	0.848441746	no
MYOCD	0.018899294	0.339722576	0.734286255	0.848690217	no
MBNL2	-0.018890473	-0.339563971	0.734405598	0.848782506	no
NCR1	-0.018878254	-0.339344252	0.734570937	0.848897347	no
SYT14	0.018877292	0.339326943	0.734583963	0.848897347	no
C2orf54	-0.018870372	-0.339202516	0.734677601	0.848959905	no
GHSR	-0.018860257	-0.339020635	0.734814484	0.849072427	no
TBC1D20	0.018845019	0.338746619	0.735020722	0.849265071	no
C8orf56	0.018834515	0.33855774	0.735162893	0.849355317	no
ARHGAP40	-0.018833409	-0.338537843	0.73517787	0.849355317	no
ZNF621	0.018828598	0.338451341	0.735242984	0.849384883	no
MICAL2	0.018808143	0.338083525	0.735519879	0.849659091	no
GUSBP5	-0.018788656	-0.337733121	0.735783698	0.849918165	no
CCT6A	-0.018772292	-0.337438869	0.736005265	0.850113699	no
SNRPD2P2	-0.018770311	-0.337403249	0.736032088	0.850113699	no
ADAM30	0.018743585	0.33692266	0.736394015	0.850415576	no
SPINK9	-0.018742768	-0.336907969	0.736405079	0.850415576	no
OR7A10	-0.018742245	-0.336898558	0.736412167	0.850415576	no
TMPRSS12	-0.018717373	-0.336451319	0.736749034	0.850758878	no
CCDC91	0.018705473	0.336237352	0.736910214	0.850899281	no
SWT1	-0.018698338	-0.336109051	0.737006869	0.850965165	no
TTTY16	-0.018686971	-0.335904645	0.737160865	0.851093624	no

BTBD10	-0.018684279	-0.335856246	0.73719733	0.851093624	no
LINC00336	-0.01866782	-0.33556028	0.737420328	0.851305343	no
TIAM1	-0.018664785	-0.335505701	0.737461453	0.85130709	no
TMEM167A	-0.01866033	-0.335425601	0.737521811	0.851331036	no
CES1P2	-0.018653642	-0.335305342	0.737612431	0.851389911	no
AIMP1	-0.018647055	-0.335186898	0.737701688	0.851447206	no
KPRP	-0.018637183	-0.335009384	0.737835465	0.851555877	no
USP48	0.018630849	0.334895493	0.737921299	0.851609206	no
PISRT1	-0.018618552	-0.334674369	0.738087959	0.851716276	no
METTL22	-0.018618155	-0.334667232	0.738093338	0.851716276	no
SLCO1B1	-0.018598733	-0.334317982	0.738356591	0.851893621	no
TUBA1B	0.018598192	0.334308262	0.738363918	0.851893621	no
TOMM5	-0.018598043	-0.334305576	0.738365943	0.851893621	no
SMIM9	-0.018592279	-0.334201938	0.738444069	0.851895459	no
OTX2	-0.018592076	-0.334198294	0.738446815	0.851895459	no
SLC35A3	0.018581517	0.334008412	0.738589961	0.85201486	no
RPL21P44	-0.01857766	-0.333939058	0.738642247	0.852029442	no
LTK	0.018567442	0.333755324	0.73878077	0.852132187	no
SEC61G	0.018561846	0.333654715	0.738856625	0.852132187	no
OR10A5	0.018552853	0.333492993	0.738978564	0.852132187	no
IFNK	0.018552609	0.333488609	0.738981869	0.852132187	no
SNORA71B	0.018548491	0.333414556	0.739037707	0.852132187	no
PASK	-0.018545739	-0.333365087	0.739075009	0.852132187	no
ACER1	-0.018543589	-0.333326424	0.739104163	0.852132187	no
DISC2	0.01854301	0.333316011	0.739112016	0.852132187	no
CYTH1	-0.018539178	-0.333247098	0.739163981	0.852132187	no
MAP3K1	-0.018538678	-0.333238108	0.73917076	0.852132187	no
CIRH1A	-0.018536788	-0.333204134	0.73919638	0.852132187	no
ACR	-0.018535996	-0.333189882	0.739207127	0.852132187	no
LOC253573	-0.018506849	-0.332665774	0.73960239	0.852504917	no
KCNA6	-0.018506302	-0.33265595	0.739609799	0.852504917	no
ZC3H15	-0.018495811	-0.332467305	0.739752085	0.852598502	no
RFK	0.018494466	0.33244311	0.739770335	0.852598502	no
IL24	0.018485185	0.332276237	0.739896208	0.852679823	no
GNAT3	0.018483413	0.33224437	0.739920246	0.852679823	no
ARC	-0.018472968	-0.332056543	0.740061935	0.852782148	no
STX12	0.018471016	0.332021459	0.740088402	0.852782148	no
GLYATL3	-0.018455508	-0.331742594	0.740298785	0.852978833	no
TMEM67	0.018451764	0.331675274	0.740349576	0.852991623	no
MUC15	-0.018442328	-0.33150561	0.740477587	0.853093375	no
C1orf43	-0.018417472	-0.331058652	0.74081485	0.853382038	no
SLC39A9	-0.01841616	-0.331035074	0.740832643	0.853382038	no
TOP1	0.018413096	0.330979979	0.740874221	0.853382038	no
FCF1	0.018412156	0.330963072	0.740886979	0.853382038	no
DDTL	-0.018407413	-0.330877791	0.740951338	0.853410429	no
TBC1D3P1-DHX	-0.01840184	-0.330777572	0.741026972	0.853451804	no
BPIFA2	-0.018394576	-0.330646958	0.741125549	0.853519596	no
SCGB1D2	0.018386857	0.330508164	0.741230304	0.853594495	no
IRGM	0.018378457	0.330357117	0.741344312	0.853680042	no
FUNDC2P2	-0.01837373	-0.330272125	0.741408466	0.853708173	no
KIR3DS1	-0.018369807	-0.330201577	0.741461718	0.853723749	no

HYAL4	0.018365928	0.330131831	0.741514366	0.853738629	no
TRO	0.018361858	0.330058642	0.741569615	0.8537565	no
DHDH	-0.018351501	-0.329872414	0.741710199	0.85387261	no
PRM3	-0.018348534	-0.329819056	0.741750481	0.853873244	no
FUT3	-0.018326325	-0.329419712	0.742051982	0.854174566	no
SLC13A2	-0.018320865	-0.329321531	0.742126114	0.854214146	no
CANX	-0.01829772	-0.32890536	0.742440372	0.854382987	no
BBS12	0.018292714	0.32881535	0.742508346	0.854382987	no
LOC389834	0.018291147	0.328787165	0.742529631	0.854382987	no
LOC100506394	-0.018291117	-0.328786637	0.74253003	0.854382987	no
GADL1	-0.01829062	-0.328777697	0.742536782	0.854382987	no
FCRL4	-0.018289825	-0.328763401	0.742547578	0.854382987	no
DCAF8L2	-0.018285653	-0.328688385	0.742604231	0.854382987	no
RCOR3	-0.01828378	-0.328654709	0.742629664	0.854382987	no
VKORC1L1	-0.018283711	-0.328653471	0.742630599	0.854382987	no
MIR490	0.018274434	0.328486648	0.742756592	0.854482197	no
PTX4	-0.018249071	-0.328030596	0.743101063	0.854795491	no
TMEM201	-0.018248526	-0.328020787	0.743108472	0.854795491	no
OR13C2	-0.018238711	-0.327844315	0.743241781	0.854903079	no
INMT-FAM188E	-0.018222919	-0.327560358	0.743456302	0.854921851	no
TMPRSS4-AS1	0.018222396	0.32755094	0.743463417	0.854921851	no
ANO10	-0.018220983	-0.327525543	0.743482604	0.854921851	no
ACTRT2	-0.018220951	-0.327524958	0.743483047	0.854921851	no
OR10G9	-0.018220016	-0.327508149	0.743495746	0.854921851	no
AHSP	-0.018219939	-0.327506774	0.743496785	0.854921851	no
ZNF181	0.018208626	0.327303354	0.743650477	0.855052828	no
DDC	-0.01820297	-0.327201653	0.74372732	0.855095433	no
BLOC1S1-RDH5	-0.018187429	-0.326922192	0.743938489	0.855292466	no
ST13P4	-0.018182894	-0.326840655	0.744000104	0.85530155	no
MIER2	0.018180989	0.326806405	0.744025986	0.85530155	no
NPC1	0.018158867	0.32640862	0.744326608	0.855601366	no
SPRR1B	-0.018141188	-0.326090731	0.744566878	0.85583178	no
RFXANK	0.018136425	0.326005086	0.744631615	0.855860415	no
LINC00700	-0.01812923	-0.325875713	0.744729409	0.85592704	no
OR8D1	-0.018126203	-0.325821294	0.744770546	0.855928545	no
TMEM225	-0.018114567	-0.325612056	0.744928721	0.855961608	no
FABP5P3	-0.018114251	-0.325606384	0.744933009	0.855961608	no
NUDT3	0.018114171	0.325604944	0.744934098	0.855961608	no
BEST1	0.0181116	0.325558712	0.744969049	0.855961608	no
HEATR4	0.018107951	0.325493096	0.745018655	0.855961608	no
HOXD13	-0.018105514	-0.325449271	0.745051788	0.855961608	no
CYP3A7	0.018103577	0.325414447	0.745078116	0.855961608	no
HIST1H4I	0.018095803	0.325274665	0.745183799	0.856037258	no
SHPK	0.018086504	0.32510746	0.745310221	0.8560663	no
COL11A1	0.018085249	0.325084898	0.74532728	0.8560663	no
OR52J3	0.018080492	0.324999359	0.745391958	0.8560663	no
HAUS8	0.01807935	0.324978831	0.74540748	0.8560663	no
LOC728875	-0.018079294	-0.324977815	0.745408249	0.8560663	no
SLC6A20	-0.018062668	-0.32467887	0.745634305	0.856190802	no
CHIC2	-0.018056517	-0.324568272	0.745717942	0.856190802	no
ESF1	0.018052219	0.32449098	0.745776394	0.856190802	no

HIST1H3C	0.018051423	0.324476661	0.745787223	0.856190802	no
LILRA3	0.01804773	0.324410263	0.745837438	0.856190802	no
ITFG2	0.018047508	0.324406267	0.74584046	0.856190802	no
OR4F6	-0.018046716	-0.324392035	0.745851224	0.856190802	no
IL22	0.018045713	0.324373992	0.745864869	0.856190802	no
LINC00575	-0.018044952	-0.324360315	0.745875213	0.856190802	no
TTC39A	0.018035329	0.324187282	0.746006081	0.856295289	no
MAGEB6	-0.018024883	-0.323999452	0.746148148	0.856412617	no
ARMCX3	0.017985509	0.323291468	0.746683717	0.856937507	no
CPB1	-0.017985399	-0.323289501	0.746685205	0.856937507	no
TAS1R3	0.017977366	0.323145054	0.74679449	0.85697935	no
LINC00184	0.017976857	0.323135898	0.746801417	0.85697935	no
CDCA4	-0.017969118	-0.322996754	0.746906695	0.857026955	no
CLEC4G	-0.017967945	-0.322975656	0.746922659	0.857026955	no
CPSF3	0.01795042	0.322660536	0.747161101	0.857254777	no
C10orf118	-0.017939183	-0.32245848	0.747314005	0.857384438	no
MYH1	-0.01793557	-0.322393531	0.747363156	0.857395056	no
UBE2F-SCLY	0.017916461	0.32204992	0.747623207	0.857614672	no
EXTL3	-0.017915638	-0.322035133	0.747634399	0.857614672	no
FLJ45513	-0.017911592	-0.321962376	0.747689467	0.857627161	no
MCTP1	0.017908974	0.321915298	0.7477251	0.857627161	no
DKFZP686I152	-0.017902301	-0.321795314	0.747815917	0.857685552	no
C19orf70	-0.017896378	-0.321688825	0.747896522	0.857732225	no
SNORA68	0.017889541	0.32156588	0.747989587	0.857784482	no
GNN	-0.017886077	-0.321503603	0.74803673	0.857784482	no
ANKRD13C	0.017884233	0.321470447	0.748061829	0.857784482	no
ISYNA1	-0.017874592	-0.321297091	0.748193064	0.857889192	no
VPS13A	-0.017870211	-0.321218315	0.748252701	0.857911801	no
CCL14	0.017866299	0.32114797	0.748305957	0.857927091	no
DZANK1	0.017863107	0.321090576	0.74834941	0.857931141	no
C9orf41	-0.017854779	-0.320940827	0.748462787	0.858015349	no
PRPF18	-0.0178446	-0.320757806	0.748601362	0.858128434	no
OR5L1	-0.017832732	-0.320544417	0.748762941	0.858267874	no
AVL9	-0.017828147	-0.32046197	0.748825373	0.858293658	no
RCAN3AS	0.017815672	0.320237651	0.748995244	0.858442578	no
ALPP	-0.017786349	-0.319710402	0.749394565	0.858854447	no
NSUN5P2	0.017783318	0.31965591	0.749435839	0.858855949	no
GOLGA8T	0.017776785	0.319538446	0.749524813	0.858889037	no
CCDC47	0.017773591	0.319481012	0.749568319	0.858889037	no
MRI1	-0.017771698	-0.319446972	0.749594103	0.858889037	no
PARD3	0.017769461	0.31940675	0.749624571	0.858889037	no
TAS2R8	-0.01776442	-0.319316107	0.749693235	0.858921916	no
NEBL	0.017756902	0.319180935	0.749795634	0.858993441	no
RNU5F-1	-0.017753096	-0.319112491	0.749847484	0.859007052	no
TAS2R13	0.017737203	0.318826725	0.750063984	0.859209268	no
PFN3	-0.017710334	-0.318343602	0.750430048	0.859582782	no
RABL2A	-0.017706984	-0.318283366	0.750475694	0.859589252	no
OR4S1	-0.017702685	-0.318206065	0.750534271	0.859610531	no
PCGF6	-0.017691349	-0.318002239	0.750688734	0.859741625	no
MBOAT4	0.017664674	0.317522617	0.751052241	0.86007684	no
KRT15	-0.017663873	-0.317508211	0.75106316	0.86007684	no

LOC100130776	0.017661061	0.317457642	0.751101491	0.86007684	no
BSPH1	-0.017644718	-0.317163793	0.751324231	0.860212279	no
TEX36	-0.017643252	-0.317137438	0.75134421	0.860212279	no
HES2	-0.017641165	-0.317099913	0.751372655	0.860212279	no
KRT72	0.017636796	0.317021348	0.751432214	0.860212279	no
TAS2R16	0.017635908	0.317005379	0.751444319	0.860212279	no
PIGW	0.017634763	0.316984788	0.751459929	0.860212279	no
THAP8	-0.01762758	-0.316855639	0.751557838	0.860273902	no
CSPG4	0.017624627	0.316802551	0.751598085	0.860273902	no
RPS27	0.017622004	0.316755377	0.75163385	0.860273902	no
RAMP1	0.017611975	0.316575059	0.751770561	0.860361329	no
RHCG	-0.017610527	-0.316549022	0.751790302	0.860361329	no
FNTA	-0.017587463	-0.316134318	0.752104749	0.860675355	no
ACOT13	0.017578955	0.315981335	0.752220759	0.860762277	no
ZNF705D	0.017571087	0.315839868	0.75232804	0.860839202	no
CFP	0.017559036	0.315623184	0.752492371	0.860924097	no
MAGEA1	0.01755654	0.315578305	0.752526408	0.860924097	no
HIST1H3I	-0.017555249	-0.315555092	0.752544014	0.860924097	no
LY6G6D	-0.017553896	-0.315530755	0.752562472	0.860924097	no
OR4F15	0.017531753	0.315132625	0.752864449	0.861223711	no
DAPL1	-0.017526522	-0.315038571	0.752935793	0.861259481	no
S100A14	-0.017521358	-0.314945713	0.753006232	0.861267803	no
CHAD	-0.017510754	-0.314755042	0.753150876	0.861267803	no
SMAD3	-0.017508724	-0.314718541	0.753178566	0.861267803	no
DEFB121	0.017508688	0.314717897	0.753179055	0.861267803	no
ZNF787	0.017505574	0.314661906	0.753221532	0.861267803	no
OR5P3	0.017500632	0.314573044	0.753288948	0.861267803	no
CMA1	0.017497869	0.314523368	0.753326636	0.861267803	no
RAD23B	-0.017497845	-0.314522937	0.753326963	0.861267803	no
DNTT	0.017497039	0.314508437	0.753337964	0.861267803	no
ZNF658B	0.017495024	0.314472212	0.753365447	0.861267803	no
C8orf40	-0.017493671	-0.314447888	0.753383902	0.861267803	no
AHNAK	0.017488935	0.314362725	0.753448516	0.861295855	no
CBL	-0.017482909	-0.314254382	0.75353072	0.861344008	no
FAM89B	0.01746565	0.313944065	0.753766183	0.861560835	no
XRCC4	0.017461332	0.313866414	0.753825106	0.861560835	no
EIF3IP1	0.017459518	0.313833794	0.75384986	0.861560835	no
UPK3BL	-0.017457253	-0.313793069	0.753880765	0.861560835	no
SIAE	-0.0174516	-0.313691422	0.753957901	0.861603172	no
NT5C1B	-0.017443451	-0.3135449	0.754069096	0.861684423	no
IFNA7	-0.017438665	-0.313458854	0.754134399	0.861713226	no
GP5	0.017430249	0.313307531	0.754249246	0.861798636	no
GATA1	-0.017427164	-0.313252054	0.754291352	0.861800927	no
SNX18	-0.017420703	-0.313135899	0.754379513	0.861832595	no
SI	-0.017419256	-0.313109866	0.754399273	0.861832595	no
SERPINA13P	-0.017415323	-0.313039163	0.754452938	0.861848089	no
IL17REL	-0.017399121	-0.312747838	0.754674074	0.862054881	no
YWHAZ	-0.01738497	-0.312493397	0.754867229	0.86222969	no
GLYCAM1	-0.017377472	-0.312358583	0.754969578	0.862300765	no
PRL	-0.017373377	-0.312284957	0.755025475	0.86231878	no
FLG	0.017362362	0.312086906	0.755175844	0.862444684	no

OR1E1	-0.017348611	-0.311839644	0.755363588	0.862613256	no
AADACL4	-0.017345346	-0.311780952	0.755408154	0.862618312	no
GC	0.017336092	0.311614549	0.755534512	0.862710467	no
ATPBD4	-0.017333555	-0.311568948	0.755569141	0.862710467	no
FAM71A	0.017325134	0.31141752	0.755684136	0.862795929	no
ZNF417	0.017319022	0.311307625	0.755767594	0.862845377	no
HHLA1	0.01730353	0.311029081	0.755979143	0.862892577	no
OR13A1	-0.017301875	-0.310999327	0.756001742	0.862892577	no
ZNF160	0.017299253	0.310952174	0.756037555	0.862892577	no
HSD11B1	-0.017297729	-0.310924781	0.756058361	0.862892577	no
C9orf62	-0.017296485	-0.310902415	0.756075349	0.862892577	no
REV1	-0.017296249	-0.310898161	0.75607858	0.862892577	no
BOD1L2	-0.017295413	-0.310883131	0.756089996	0.862892577	no
HRC	0.017272776	0.31047611	0.756399168	0.863176362	no
HSD17B12	0.017271325	0.310450021	0.756418986	0.863176362	no
RALY	0.017263708	0.310313076	0.756523019	0.86324924	no
EPPIN-WFDC6	0.01725418	0.310141763	0.756653166	0.863351908	no
HIGD2A	-0.017239409	-0.309876162	0.756854957	0.863536308	no
ZNF334	0.017230942	0.309723935	0.75697062	0.863583057	no
ZNF750	-0.017230527	-0.309716462	0.756976299	0.863583057	no
NBEAL1	-0.017215106	-0.309439206	0.757186973	0.863736896	no
ACAP2	0.017213087	0.309402902	0.75721456	0.863736896	no
LOC285878	-0.017211832	-0.309380323	0.757231718	0.863736896	no
PPP1R32	-0.017204676	-0.309251658	0.757329493	0.863802576	no
TMEM63A	-0.017200091	-0.309169218	0.757392142	0.863828187	no
LINC00114	-0.017167339	-0.308580337	0.757839705	0.864252359	no
DCLK1	-0.01716667	-0.308568317	0.757848841	0.864252359	no
CHCHD10	0.01716119	0.308469783	0.757923738	0.864252359	no
CES5A	-0.017161105	-0.308468243	0.757924908	0.864252359	no
C9orf24	-0.017151224	-0.308290589	0.75805995	0.864337038	no
SCLT1	0.017149786	0.30826473	0.758079607	0.864337038	no
LOC285540	-0.017143711	-0.308155499	0.758162642	0.864385853	no
KRTAP12-2	-0.017134601	-0.307991702	0.758287162	0.864481958	no
C19orf52	0.017130398	0.307916137	0.75834461	0.864501592	no
KRT82	-0.017126967	-0.307854442	0.758391514	0.864509204	no
MEOX2	0.017120921	0.307745732	0.758474163	0.86455756	no
CKAP2L	0.017116711	0.307670033	0.758531717	0.864577307	no
TNNI3	0.017092078	0.307227136	0.758868477	0.864915275	no
OR51I1	0.017065843	0.306755438	0.759227186	0.865278224	no
DBH	0.017062095	0.306688046	0.75927844	0.865279028	no
MIR492	-0.017059903	-0.30664863	0.759308417	0.865279028	no
SREK1	0.017048732	0.306447768	0.759461186	0.865407229	no
TMEM5	-0.017030308	-0.306116506	0.759713154	0.865648449	no
TMEM51-AS1	0.017024703	0.306015731	0.759789812	0.86565042	no
CHODL-AS1	0.017024291	0.306008327	0.759795444	0.86565042	no
LINC00659	0.017017817	0.305891919	0.759883996	0.865665834	no
SLX4IP	-0.017016201	-0.305862868	0.759906096	0.865665834	no
GATA4	0.017014467	0.305831691	0.759929814	0.865665834	no
KIAA1432	-0.017003616	-0.305636581	0.760078245	0.865753507	no
CCDC159	0.01700295	0.305624618	0.760087346	0.865753507	no
HOXD8	-0.016992764	-0.305441459	0.760226694	0.865823505	no

TBX1	-0.016992567	-0.305437933	0.760229377	0.865823505	no
FLJ42393	-0.016981878	-0.305245742	0.760375605	0.865916526	no
NPY2R	0.016980706	0.305224671	0.760391638	0.865916526	no
GUCA1A	0.016967544	0.304988004	0.760571719	0.866075706	no
COL4A4	-0.016955533	-0.304772061	0.760736042	0.866216927	no
GPR119	-0.01694935	-0.304660885	0.760820646	0.866260151	no
LINC00282	0.016944228	0.304568794	0.76089073	0.866260151	no
KCNG4	0.01694218	0.304531963	0.760918759	0.866260151	no
VTI1A	-0.016939636	-0.304486228	0.760953566	0.866260151	no
HSP90B2P	-0.01693803	-0.304457351	0.760975543	0.866260151	no
MEP1B	-0.01692144	-0.304159071	0.76120256	0.866472681	no
LINC00202-2	0.016909931	0.303952139	0.761360066	0.866606068	no
C18orf21	0.016887647	0.303551468	0.761665064	0.866868601	no
ACN9	0.016887185	0.303543162	0.761671388	0.866868601	no
LOC285441	0.016882938	0.303466794	0.761729524	0.866888859	no
HNRNPA2B1	-0.016875515	-0.303333336	0.761831127	0.866942116	no
WDR5	-0.016873625	-0.303299349	0.761857001	0.866942116	no
KLK2	0.016855458	0.302972708	0.762105694	0.867159562	no
L3MBTL4	0.01685377	0.302942375	0.762128789	0.867159562	no
ZBTB1	-0.01685044	-0.302882494	0.762174383	0.867165529	no
HSD3B2	-0.016840571	-0.302705047	0.762309498	0.867228103	no
BPGM	-0.016837175	-0.302643992	0.76235599	0.867228103	no
ZNF641	-0.01683526	-0.30260956	0.762382209	0.867228103	no
RAD51	-0.016832072	-0.302552237	0.76242586	0.867228103	no
HJURP	-0.016831686	-0.302545294	0.762431147	0.867228103	no
DIAPH3-AS2	-0.016824854	-0.302422457	0.762524689	0.867255903	no
C2orf15	-0.016824006	-0.302407215	0.762536296	0.867255903	no
FBXL7	-0.016813426	-0.302216983	0.762681168	0.867329185	no
HIST1H2BL	-0.016813406	-0.302216619	0.762681445	0.867329185	no
CCDC172	-0.016809825	-0.30215225	0.762730468	0.867339038	no
CBWD6	-0.016793892	-0.301865774	0.762948654	0.867473488	no
OR2F2	-0.016792701	-0.301844348	0.762964973	0.867473488	no
AGAP1	-0.016792348	-0.301838017	0.762969795	0.867473488	no
GJA3	0.016789326	0.301783682	0.76301118	0.867474648	no
C7orf69	0.01678239	0.301658959	0.763106181	0.867536761	no
SYPL2	-0.016757099	-0.301204237	0.763452567	0.86788464	no
ROPN1B	-0.016744713	-0.30098155	0.763622218	0.868031582	no
OR3A1	0.016736526	0.300834342	0.763734372	0.868113153	no
KRTAP4-11	0.016722177	0.300576345	0.763930946	0.868255447	no
FAM133DP	0.01672149	0.300563992	0.763940358	0.868255447	no
CLCA1	-0.016712771	-0.300407231	0.764059806	0.868288565	no
CCL24	-0.016712267	-0.300398169	0.76406671	0.868288565	no
FAM186A	-0.016710516	-0.30036668	0.764090705	0.868288565	no
ANKIB1	-0.016690082	-0.299999295	0.764370668	0.86856078	no
CCDC40	0.01668449	0.299898756	0.764447288	0.868601918	no
TTTY14	-0.016673532	-0.299701719	0.764597456	0.868691344	no
MIR506	0.016672847	0.299689415	0.764606833	0.868691344	no
MNT	-0.016658203	-0.299426115	0.764807517	0.868858474	no
NUPL2	-0.016655339	-0.299374621	0.764846767	0.868858474	no
ANGPTL3	-0.016653181	-0.299335826	0.764876338	0.868858474	no
LOC442459	-0.016650312	-0.299284246	0.764915654	0.868858474	no

CST13P	0.016641324	0.299122642	0.765038839	0.86895247	no
MCEE	-0.016638244	-0.299067255	0.765081061	0.868954501	no
OR5D14	-0.016630573	-0.298929341	0.765186194	0.86900397	no
ZNF296	-0.016629166	-0.298904033	0.765205488	0.86900397	no
LOC389458	0.016623154	0.29879595	0.765287884	0.869051621	no
CHAT	-0.016612263	-0.298600137	0.765437169	0.869175219	no
GTF2IRD1	-0.016603281	-0.298438635	0.765560301	0.86926911	no
LOC282997	-0.016600129	-0.298381966	0.765603509	0.869272242	no
POP1	-0.016596313	-0.298313358	0.76565582	0.869285711	no
SNW1	-0.016586851	-0.298143227	0.765785542	0.869328266	no
LOC407835	-0.0165859	-0.298126126	0.765798582	0.869328266	no
EPB42	-0.016584727	-0.298105049	0.765814654	0.869328266	no
KRTAP1-4	0.016579919	0.298018596	0.765880576	0.869331115	no
SCAF4	0.016578643	0.297995661	0.765898065	0.869331115	no
ATP11A	0.016569069	0.297823527	0.766029328	0.869434185	no
SLC6A6	0.016548626	0.297455962	0.766309642	0.869706406	no
TMEM258	-0.016545004	-0.297390842	0.766359307	0.869716844	no
OR11H12	-0.01653936	-0.29728937	0.766436698	0.869758744	no
KCNK5	0.016535154	0.297213747	0.766494378	0.869778271	no
SSB	0.016529402	0.297110316	0.766573267	0.869821863	no
HOXC-AS5	-0.016522359	-0.296983688	0.766669854	0.86988553	no
SCN4A	0.016518627	0.296916588	0.766721037	0.869897677	no
SRSF8	-0.016500368	-0.296588296	0.766971466	0.870091832	no
NKX2-8	-0.016500246	-0.296586107	0.766973136	0.870091832	no
SBF2	-0.016494754	-0.296487362	0.767048466	0.870131358	no
LARP1B	0.016484289	0.296299209	0.767192009	0.870248256	no
NOP58	-0.016481204	-0.296243738	0.76723433	0.870250329	no
TRIM49	-0.016476669	-0.296162204	0.767296536	0.870274955	no
USP33	-0.016468196	-0.29600987	0.767412763	0.870338236	no
PITPNM2	-0.016466697	-0.295982922	0.767433324	0.870338236	no
LOC201651	0.016452925	0.295735305	0.767622263	0.870506572	no
C5orf49	0.016445203	0.29559646	0.767728211	0.870534772	no
OR4D6	-0.01644429	-0.295580046	0.767740737	0.870534772	no
PALM3	0.016442255	0.295543467	0.76776865	0.870534772	no
ZNF597	-0.016426187	-0.295254558	0.767989126	0.870720808	no
ZNF783	-0.016424391	-0.295222283	0.768013757	0.870720808	no
IGFL3	0.01641658	0.295081839	0.768120942	0.87079639	no
CALML5	-0.016398265	-0.294752541	0.768372275	0.871035371	no
AMHR2	-0.016391624	-0.294633139	0.768463414	0.871066718	no
LOC338739	0.016390343	0.294610112	0.768480991	0.871066718	no
RNF43	-0.016386685	-0.294544341	0.768531195	0.871077681	no
OMD	0.016376854	0.294367585	0.76866612	0.871129068	no
XXYLT1	-0.016374893	-0.294332319	0.768693042	0.871129068	no
OR8K5	-0.016373343	-0.294304462	0.768714307	0.871129068	no
ORC1	-0.016371568	-0.294272546	0.768738671	0.871129068	no
TRPM1	0.016365663	0.294166371	0.768819725	0.871160264	no
CTRB1	-0.016363656	-0.294130286	0.768847272	0.871160264	no
MORN2	0.016353338	0.293944776	0.768988898	0.8712748	no
UBE2U	0.016344891	0.293792901	0.769104851	0.871326719	no
TRIB2	-0.016344092	-0.293778549	0.769115809	0.871326719	no
CASQ2	-0.016338218	-0.293672939	0.769196443	0.871372134	no

METTL14	-0.016321535	-0.293372979	0.769425479	0.871507322	no
ZNRD1	-0.016319623	-0.293338602	0.769451729	0.871507322	no
ENAM	-0.016318889	-0.293325412	0.769461801	0.871507322	no
TNXB	-0.01631771	-0.293304214	0.769477988	0.871507322	no
LOC283867	0.016299861	0.292983304	0.769723047	0.871738933	no
PKD1L3	0.016294613	0.292888948	0.769795105	0.871769569	no
LINC00208	-0.016291983	-0.292841651	0.769831226	0.871769569	no
ZDHHC2	0.016269548	0.292438296	0.770139289	0.872072473	no
PUS7	0.016262495	0.292311473	0.770236159	0.872106783	no
OR4Q3	-0.016261432	-0.292292371	0.770250749	0.872106783	no
CSPP1	0.016256554	0.292204672	0.770317737	0.872136681	no
RDH14	0.0162478	0.292047276	0.770437967	0.872226853	no
ZNF596	-0.016237914	-0.291869529	0.770573748	0.87233462	no
HBBP1	-0.016221872	-0.291581109	0.770794089	0.872485587	no
SERPINB12	0.016218915	0.291527948	0.770834704	0.872485587	no
LOC650368	0.01621745	0.29150161	0.770854827	0.872485587	no
FDXR	0.016213868	0.291437202	0.770904035	0.872485587	no
CABP7	-0.016213427	-0.291429272	0.770910093	0.872485587	no
MAGEA2	0.01621032	0.291373407	0.770952776	0.872487947	no
PPAPDC2	-0.016202178	-0.29122702	0.771064623	0.872538179	no
R3HCC1L	0.016201178	0.291209038	0.771078363	0.872538179	no
LY6K	0.016192612	0.29105503	0.771196039	0.872625392	no
MIR2682	-0.016184923	-0.29091679	0.771301671	0.872698969	no
ZNF419	-0.01617983	-0.290825232	0.771371636	0.872706207	no
LOC284276	0.016178546	0.290802136	0.771389284	0.872706207	no
TMEM57	-0.016151225	-0.290310933	0.771764671	0.873084937	no
CDC42BPG	0.016134764	0.290014963	0.771990882	0.873294875	no
SPATA13	-0.01613	-0.289929315	0.772056347	0.873322962	no
MBD3L5	0.016119854	0.2897469	0.772195781	0.873434712	no
WDR91	-0.016108598	-0.289544532	0.772350475	0.873563711	no
RGPD8	0.01610213	0.289428238	0.772439377	0.873618285	no
DSCR4	-0.016089658	-0.289203993	0.772610811	0.873755915	no
LRP12	-0.016087361	-0.289162699	0.772642381	0.873755915	no
DMP1	-0.016069771	-0.288846455	0.77288417	0.873942844	no
C11orf58	-0.016069419	-0.288840125	0.772889009	0.873942844	no
HOXA11-AS	-0.016049046	-0.288473829	0.773169094	0.874176519	no
VGLL4	0.016043605	0.288376003	0.773243901	0.874176519	no
AGPAT3	-0.016042767	-0.288360948	0.773255413	0.874176519	no
ZMAT3	-0.016042552	-0.288357081	0.77325837	0.874176519	no
KCNAB1-AS1	0.01603408	0.288204751	0.773374861	0.874262224	no
OR5D18	-0.01602688	-0.288075307	0.773473854	0.874319055	no
C6orf118	0.016022767	0.28800136	0.773530406	0.874319055	no
TLX2	-0.016021547	-0.287979424	0.773547183	0.874319055	no
MYRF	-0.016004425	-0.287671589	0.773782622	0.874539171	no
EBLN1	-0.015996955	-0.287537282	0.77388535	0.874609279	no
NR0B2	-0.015985463	-0.287330663	0.774043395	0.874741894	no
LINC00240	0.015958948	0.286853941	0.77440808	0.875108006	no
HIST1H2BN	-0.015955148	-0.286785635	0.774460337	0.875121043	no
C15orf32	0.01594392	0.286583767	0.774614782	0.875249541	no
SPATA31A3	-0.015937503	-0.286468386	0.774703061	0.875303269	no
TCL6	0.015917602	0.28611059	0.774976833	0.87556656	no

FTMT	0.015900064	0.285795269	0.775218128	0.875764686	no
ODF1	-0.015897177	-0.285743363	0.77525785	0.875764686	no
SLC5A1	-0.015895971	-0.285721675	0.775274448	0.875764686	no
CCDC13	-0.015892928	-0.285666965	0.775316317	0.87576595	no
ACOT1	-0.015883051	-0.285489395	0.775452214	0.875873418	no
PBX2	0.01587329	0.285313906	0.775586526	0.875979085	no
FAM122B	0.015864118	0.285148998	0.775712745	0.876075601	no
LOC285501	0.015845854	0.284820631	0.775964093	0.876276766	no
CCDC33	0.01584525	0.284809762	0.775972413	0.876276766	no
ZNF382	0.015836499	0.284652434	0.776092848	0.876296331	no
COMMD3	0.015835232	0.284629649	0.776110291	0.876296331	no
TMC1	0.015835103	0.284627333	0.776112064	0.876296331	no
CASP10	0.015822039	0.284392468	0.776291865	0.876411435	no
SKIV2L	0.01582177	0.28438763	0.776295569	0.876411435	no
LINC00606	0.015817648	0.284313507	0.776352317	0.876429461	no
TPD52L3	0.015811521	0.284203365	0.776436642	0.876478615	no
ACLY	-0.015792671	-0.283864447	0.776696136	0.87671831	no
CGA	0.015790169	0.28381948	0.776730568	0.87671831	no
ZNF839	0.015781315	0.283660284	0.776852467	0.876809851	no
ZNF644	0.015773152	0.283513516	0.776964855	0.876890647	no
PRAMEF3	0.015765206	0.283370664	0.777074249	0.876938982	no
ZNF679	0.015764113	0.28335102	0.777089292	0.876938982	no
TSPAN31	-0.015760153	-0.283279812	0.777143824	0.876954472	no
ACP1	0.015744357	0.282995823	0.777361317	0.877153842	no
CENPA	0.015719642	0.282551483	0.777701651	0.877491793	no
MIR5004	-0.015713134	-0.282434466	0.777791285	0.877546855	no
TP53AIP1	-0.015705303	-0.282293667	0.777899139	0.877584227	no
TUBA4B	-0.015704799	-0.282284609	0.777906078	0.877584227	no
PLA2G3	-0.015693824	-0.282087288	0.778057237	0.877669835	no
LOC503519	-0.015690496	-0.282027464	0.778103067	0.877669835	no
KRTAP21-2	-0.015690394	-0.282025622	0.778104479	0.877669835	no
FOXD2-AS1	0.01568538	0.281935482	0.778173535	0.877695315	no
CTAGE1	-0.015680773	-0.281852659	0.778236987	0.877695315	no
BHLHA15	0.015679858	0.281836211	0.778249589	0.877695315	no
TTL	0.015673859	0.281728345	0.77833223	0.877719226	no
OR11H2	-0.015672389	-0.281701923	0.778352473	0.877719226	no
WNT16	0.01566056	0.281489243	0.778515426	0.877842331	no
RNASE13	-0.015658534	-0.281452817	0.778543336	0.877842331	no
ITLN1	-0.015641577	-0.28114796	0.778776932	0.878059653	no
MT1B	0.015636239	0.281051977	0.778850483	0.878096513	no
GRIN3B	0.01562129	0.280783218	0.779056442	0.878280837	no
OR2M5	-0.01561351	-0.280643344	0.779163638	0.878280837	no
ADORA2B	0.015613494	0.280643058	0.779163857	0.878280837	no
PDCD10	-0.015612508	-0.28062533	0.779177443	0.878280837	no
WFDC9	-0.015594896	-0.280308695	0.779420122	0.878475377	no
UBE4B	-0.01559405	-0.280293478	0.779431785	0.878475377	no
SLC10A4	-0.015577658	-0.279998779	0.779657672	0.878683886	no
MAP1LC3B2	-0.015573357	-0.279921441	0.779716954	0.878704618	no
AK2	0.015564075	0.279754558	0.779844881	0.878802702	no
OR2B11	-0.015551064	-0.279520644	0.780024201	0.878880162	no
OR1D2	-0.015549846	-0.279498755	0.780040981	0.878880162	no

AGXT2	-0.015549689	-0.279495927	0.78004315	0.878880162	no
PIP4K2C	-0.01554579	-0.279425828	0.780096891	0.878880162	no
HEPHL1	0.015544252	0.279398171	0.780118095	0.878880162	no
LINC00616	-0.015539024	-0.27930418	0.780190154	0.87891527	no
ZNF878	0.015528287	0.279111148	0.780338152	0.879035917	no
SLC22A25	-0.01551573	-0.278885392	0.780511249	0.879184824	no
SHC4	0.015511536	0.278809974	0.780569077	0.879203881	no
CLDN8	-0.015501041	-0.2786213	0.780713754	0.879234525	no
OTP	-0.015499609	-0.27859556	0.780733492	0.879234525	no
CECR7	-0.015495891	-0.278528712	0.780784754	0.879234525	no
C14orf177	-0.015494558	-0.278504743	0.780803135	0.879234525	no
OR2M4	-0.015492902	-0.278474961	0.780825973	0.879234525	no
CSRNP2	-0.015491757	-0.278454382	0.780841754	0.879234525	no
ERI3	-0.015484289	-0.278320119	0.780944717	0.879304391	no
ADAM21	-0.015460486	-0.27789218	0.781272919	0.879627844	no
ARHGAP36	0.015454609	0.277786505	0.781353971	0.879640928	no
OR13D1	-0.015453707	-0.277770299	0.781366401	0.879640928	no
FLJ20518	-0.015441995	-0.277559723	0.781527919	0.879776676	no
MIR7-1	0.015437424	0.277477546	0.781590954	0.87980155	no
FAM71B	-0.015424408	-0.277243532	0.781770465	0.879957527	no
KRTAP19-6	0.015403947	0.276875683	0.782052664	0.880200635	no
ZFPM1	-0.015402809	-0.276855225	0.78206836	0.880200635	no
ZNF71	-0.01539557	-0.276725067	0.782168219	0.880266924	no
OLFM2	-0.015379291	-0.276432392	0.782392777	0.880473539	no
ZNF558	0.015376084	0.276374741	0.782437012	0.880477214	no
TCF24	0.015370215	0.276269223	0.782517979	0.88052222	no
MIR181C	0.015366702	0.276206058	0.782566447	0.880530655	no
C3orf14	-0.015353545	-0.275969529	0.782747951	0.880688771	no
OR5AS1	0.015337464	0.27568041	0.782969827	0.880857678	no
OR1C1	0.015333878	0.275615946	0.783019301	0.880857678	no
GPHA2	-0.015329842	-0.275543383	0.783074991	0.880857678	no
ADH1A	0.01532835	0.275516544	0.78309559	0.880857678	no
SLCO4A1	0.015327812	0.275506875	0.78310301	0.880857678	no
SPATA31D5P	0.015324563	0.275448462	0.783147842	0.880862002	no
SLC7A10	-0.015320643	-0.275377995	0.783201926	0.880876732	no
SPACA1	-0.01530932	-0.275174417	0.783358181	0.881006367	no
OR10H3	-0.015303852	-0.275076112	0.783433637	0.881045122	no
APCS	-0.015297849	-0.274968194	0.783516474	0.881092174	no
S1PR2	0.015287037	0.274773798	0.783665697	0.88112746	no
NETO2	-0.01528475	-0.274732681	0.78369726	0.88112746	no
LOC10029268C	0.015284066	0.274720387	0.783706698	0.88112746	no
ARL8B	-0.015281132	-0.274667642	0.783747188	0.88112746	no
ZKSCAN3	-0.015280722	-0.274660264	0.783752852	0.88112746	no
OR51M1	-0.01526612	-0.274397753	0.783954381	0.881281436	no
IL5RA	-0.015264856	-0.27437503	0.783971825	0.881281436	no
YIPF3	0.015259256	0.274274341	0.784049129	0.881322236	no
TRPC5OS	0.015255192	0.274201283	0.78410522	0.881339188	no
AADAC	-0.015249466	-0.274098339	0.784184258	0.881369212	no
COG6	-0.015247315	-0.274059663	0.784213953	0.881369212	no
OR2B6	0.015242527	0.273973583	0.784280045	0.881397398	no
DENND2A	0.015231227	0.273770432	0.784436032	0.881526603	no

SLC25A51	0.015227617	0.273705532	0.784485866	0.88153651	no
C16orf55	-0.015215098	-0.273480449	0.784658706	0.881679375	no
STAG3L2	0.015212465	0.273433116	0.784695054	0.881679375	no
WNT10A	0.015193468	0.273091576	0.784957345	0.881922254	no
OR2G6	-0.015190864	-0.273044772	0.78499329	0.881922254	no
HIGD2B	-0.015180121	-0.272851617	0.785141639	0.88204281	no
CLOCK	-0.015167859	-0.272631174	0.785310956	0.882186909	no
SLC34A3	-0.015151805	-0.272342541	0.785532662	0.882389841	no
TIGD2	-0.015148582	-0.272284608	0.785577164	0.882393709	no
CD200R1L	-0.015142741	-0.272179584	0.785657842	0.882438208	no
BEST2	-0.015133128	-0.272006768	0.7857906	0.88248919	no
OR5I1	-0.015130401	-0.271957741	0.785828265	0.88248919	no
SNORA58	0.015130119	0.271952673	0.785832158	0.88248919	no
ARL6IP4	0.015127562	0.271906688	0.785867485	0.88248919	no
LOC339298	0.015124054	0.271843621	0.785915937	0.882497487	no
SNORA54	-0.0151194	-0.27175995	0.785980219	0.882523558	no
VLDLR	0.015105755	0.271514641	0.786168691	0.882607923	no
CASC5	-0.015105551	-0.271510976	0.786171507	0.882607923	no
C21orf2	-0.01510504	-0.271501794	0.786178562	0.882607923	no
RAN	0.015092832	0.27128231	0.786347203	0.88270832	no
CRISP3	-0.015089834	-0.271228404	0.786388624	0.88270832	no
FSCN1	0.015089647	0.271225039	0.78639121	0.88270832	no
RAB10	0.01507951	0.271042804	0.786531241	0.882819392	no
PHKG1	-0.015073659	-0.270937609	0.786612077	0.882864015	no
GJB1	-0.015063841	-0.270761096	0.786747722	0.882948332	no
OSTCP1	0.015062274	0.270732924	0.786769372	0.882948332	no
CDRT4	-0.0150553	-0.270607547	0.786865725	0.883010354	no
EPOR	0.01504605	0.27044124	0.786993538	0.883047416	no
DSCR10	-0.01504441	-0.270411766	0.787016191	0.883047416	no
LOC200726	0.015042269	0.270373271	0.787045777	0.883047416	no
ABCB9	-0.015041015	-0.270350721	0.787063108	0.883047416	no
STX18	0.015035987	0.270260336	0.787132576	0.883079254	no
LCE1A	-0.015018211	-0.269940747	0.787378221	0.883289648	no
NUDT4P1	-0.015016468	-0.269909405	0.787402312	0.883289648	no
CNR2	0.015010994	0.269810994	0.787477958	0.883297691	no
TMEM207	-0.015010001	-0.269793139	0.787491683	0.883297691	no
ELANE	-0.014994811	-0.269520058	0.787701605	0.883458078	no
TSPYL5	0.014991297	0.269456883	0.787750171	0.883458078	no
NPTX2	-0.014989454	-0.269423745	0.787775646	0.883458078	no
TGIF2LY	-0.014985524	-0.269353098	0.787829958	0.883458078	no
PTPMT1	-0.014984085	-0.269327224	0.78784985	0.883458078	no
CDC25C	0.014981808	0.269286284	0.787881323	0.883458078	no
PTTG3P	-0.014966258	-0.269006726	0.788096254	0.883652976	no
EIF4EBP1	0.014953792	0.268782606	0.788268575	0.883759072	no
TRIM39-RPP21	-0.014953463	-0.268776693	0.788273122	0.883759072	no
OR52N5	0.014946747	0.268655942	0.788365969	0.88381706	no
PITPNA	-0.014936524	-0.268472152	0.788507293	0.883912313	no
FMO6P	-0.01493465	-0.268438469	0.788533194	0.883912313	no
XRCC2	-0.01493083	-0.268369795	0.788586003	0.883921876	no
CD164L2	-0.014925667	-0.268276977	0.78865738	0.883921876	no
FAM21C	-0.014925108	-0.268266918	0.788665115	0.883921876	no

LDB3	-0.014903863	-0.267884963	0.788958856	0.884204984	no
PTGFR	-0.014892407	-0.267679021	0.789117247	0.88433638	no
MMAB	0.014849804	0.266913094	0.789706403	0.88495048	no
APOA4	-0.014818861	-0.266356798	0.790134385	0.885383913	no
RPP38	-0.014807161	-0.266146445	0.790296236	0.885465654	no
IVD	0.014804414	0.266097059	0.790334235	0.885465654	no
SLC12A2	-0.014803617	-0.266082735	0.790345257	0.885465654	no
PTPLB	-0.014801526	-0.266045149	0.790374178	0.885465654	no
TEX26-AS1	0.014796888	0.265961754	0.790438348	0.885465654	no
ARNTL	0.014795717	0.265940709	0.790454542	0.885465654	no
BUB1B	-0.014784936	-0.265746879	0.790603695	0.885545731	no
BRIX1	-0.014784593	-0.265740716	0.790608438	0.885545731	no
BSCL2	0.014780115	0.26566022	0.790670383	0.88556896	no
ZNF570	-0.014771066	-0.265497536	0.790795577	0.885663023	no
C21orf90	-0.014764102	-0.265372325	0.790891939	0.885724787	no
KARS	-0.014736129	-0.264869422	0.791278999	0.886074371	no
STARD6	0.014735583	0.264859607	0.791286554	0.886074371	no
TEAD2	-0.014727593	-0.26471596	0.791397123	0.886106891	no
BIRC5	-0.014727525	-0.264714744	0.791398058	0.886106891	no
PTTG1	0.014715045	0.264490374	0.791570769	0.886211543	no
LIMS3-LOC440	0.014711725	0.26443069	0.791616714	0.886211543	no
RSPRY1	0.014710754	0.264413239	0.791630147	0.886211543	no
PDE4C	-0.014708715	-0.264376581	0.791658366	0.886211543	no
HIST1H1B	-0.014705873	-0.264325478	0.791697706	0.886211543	no
NDUFS8	-0.014699783	-0.264215997	0.791781988	0.886225359	no
OR12D3	0.014696435	0.264155806	0.791828325	0.886225359	no
CST5	0.014696042	0.264148746	0.791833761	0.886225359	no
RAX	0.014683131	0.263916636	0.792012457	0.886318946	no
OR4N5	-0.014678618	-0.263835488	0.792074933	0.886318946	no
RDH8	0.014678138	0.26382686	0.792081576	0.886318946	no
MIR10A	0.014677426	0.263814065	0.792091427	0.886318946	no
OR13C3	0.014672973	0.263734004	0.792153068	0.886318946	no
BLOC1S5	-0.014672123	-0.26371873	0.792164828	0.886318946	no
KRTAP10-5	-0.014666372	-0.263615344	0.79224443	0.88634022	no
CHCHD3	0.01466401	0.263572866	0.792277137	0.88634022	no
SERPINB10	-0.014661811	-0.263533342	0.792307569	0.88634022	no
CCNB2	-0.014656175	-0.263432018	0.792385587	0.886381359	no
ACTR6	0.014622457	0.262825831	0.792852388	0.886834834	no
OR51L1	-0.014620932	-0.26279841	0.792873505	0.886834834	no
MTRF1L	0.01461695	0.262726819	0.79292864	0.886840479	no
CRELD1	-0.014613472	-0.262664307	0.792976783	0.886840479	no
MPLKIP	0.014611626	0.262631111	0.793002349	0.886840479	no
KIR2DL1	0.014606555	0.262539947	0.793072561	0.886857029	no
OR5T3	0.014604308	0.262499555	0.79310367	0.886857029	no
GALP	0.014601616	0.262451155	0.793140948	0.886857029	no
RACGAP1P	0.014586732	0.262183566	0.793347052	0.887041334	no
MRPS6	-0.014575451	-0.261980754	0.793503272	0.887167923	no
LOC653486	-0.014572594	-0.261929399	0.793542831	0.887167923	no
SNORA67	0.014564551	0.261784806	0.793654213	0.887204222	no
MBD1	-0.014564288	-0.261780065	0.793657865	0.887204222	no
NREP-AS1	0.01455649	0.261639891	0.793765848	0.887252503	no

USP6NL	0.014553139	0.261579641	0.793812262	0.887252503	no
PAX2	0.014550688	0.261535582	0.793846205	0.887252503	no
ZBTB9	-0.014549245	-0.261509634	0.793866194	0.887252503	no
EBNA1BP2	-0.014545682	-0.261445573	0.793915547	0.88726152	no
OR52B2	0.014531516	0.261190897	0.794111756	0.887434649	no
GMCL1	-0.014516398	-0.260919117	0.794321157	0.887529532	no
MAPT	-0.014509701	-0.260798717	0.794413927	0.887529532	no
C2orf71	0.014508724	0.260781151	0.794427462	0.887529532	no
BAZ1A	0.014505987	0.260731937	0.794465384	0.887529532	no
OR56A1	0.014505614	0.260725241	0.794470543	0.887529532	no
ZFP91	0.014497547	0.260580207	0.794582301	0.887529532	no
SAA3P	0.014496843	0.260567552	0.794592053	0.887529532	no
FBXO30	-0.014496345	-0.260558598	0.794598952	0.887529532	no
CATSPERG	0.014495366	0.260541004	0.79461251	0.887529532	no
SNORA30	-0.014495187	-0.260537789	0.794614987	0.887529532	no
KPNA7	-0.01449189	-0.260478501	0.794660674	0.887529532	no
PRRC2C	-0.014489612	-0.260437548	0.794692233	0.887529532	no
EFCAB13	-0.014484379	-0.260343485	0.794764718	0.887564361	no
MIR203	-0.014481045	-0.260283547	0.794810909	0.887569823	no
OR4C6	-0.014469779	-0.260081007	0.794966997	0.887669312	no
CAPN6	-0.014468653	-0.26006075	0.794982608	0.887669312	no
C19orf53	-0.014459587	-0.25989777	0.795108217	0.88776344	no
ANKRD30A	-0.014431402	-0.259391072	0.795498761	0.888153352	no
OR10R2	-0.014428193	-0.259333369	0.79554324	0.888156871	no
KRT75	-0.014416953	-0.259131299	0.795699005	0.888269377	no
RPS26P11	-0.014413794	-0.259074518	0.795742775	0.888269377	no
OR4L1	-0.014410338	-0.259012376	0.795790679	0.888269377	no
THEG	0.014408991	0.258988167	0.795809342	0.888269377	no
C5orf52	-0.014402725	-0.258875519	0.795896184	0.88832017	no
ZC2HC1C	-0.014386914	-0.258591269	0.796115325	0.888518615	no
CD9	0.014370172	0.258290288	0.796347382	0.888702963	no
FZD3	0.014366644	0.258226859	0.796396289	0.888702963	no
C1orf123	-0.014361878	-0.258141171	0.796462359	0.888702963	no
CRTC3	-0.014357644	-0.258065063	0.796521044	0.888702963	no
PRKCI	-0.01435731	-0.25805906	0.796525673	0.888702963	no
C14orf178	-0.014352761	-0.257977273	0.796588737	0.888702963	no
NOL11	0.014351321	0.257951383	0.796608701	0.888702963	no
TACC3	0.014351132	0.257947987	0.79661132	0.888702963	no
AK1	-0.014339276	-0.257734852	0.796775674	0.888840177	no
RBP7	-0.014335159	-0.257660839	0.796832749	0.888857709	no
TRIM43B	0.014328413	0.257539557	0.796926278	0.8889159	no
ALOX12	-0.01432489	-0.257476225	0.796975119	0.888924242	no
DMD	-0.014312747	-0.257257918	0.797143482	0.889026735	no
OR8G2	-0.01430394	-0.257099589	0.797265594	0.889026735	no
ZNF544	0.014299993	0.25702862	0.797320331	0.889026735	no
DNAJB8	-0.01429805	-0.256993702	0.797347263	0.889026735	no
PGK2	-0.01429074	-0.256862283	0.797448627	0.889026735	no
FBXO7	0.014290703	0.256861614	0.797449143	0.889026735	no
DCTN3	-0.014290506	-0.256858068	0.797451878	0.889026735	no
UTP18	0.014287937	0.256811881	0.797487503	0.889026735	no
HMGA1	0.014286518	0.256786376	0.797507176	0.889026735	no

STK32B	0.014284813	0.256755729	0.797530816	0.889026735	no
POT1	0.014283273	0.256728036	0.797552176	0.889026735	no
ABHD1	0.014282293	0.256710424	0.797565761	0.889026735	no
DBX2	0.014277226	0.256619321	0.797636034	0.889026735	no
RHOB	-0.014276496	-0.256606201	0.797646155	0.889026735	no
OR9G9	0.014244891	0.256038013	0.798084469	0.889469135	no
PCDHA10	-0.014237602	-0.255906987	0.798185556	0.88949163	no
RHBDD3	0.014236405	0.255885457	0.798202166	0.88949163	no
PDZD9	0.014233601	0.255835049	0.798241057	0.88949163	no
C5	0.014231499	0.255797263	0.79827021	0.88949163	no
UGT1A7	-0.014214247	-0.255487103	0.798509517	0.889682319	no
RPS8	-0.014213192	-0.255468153	0.798524138	0.889682319	no
DNAH3	0.014204033	0.255303482	0.798651201	0.889755769	no
STK11	0.014202471	0.255275406	0.798672865	0.889755769	no
AXDND1	0.014175667	0.254793538	0.799044713	0.890123882	no
IRG1	0.014154307	0.254409532	0.799341076	0.890407872	no
CROCC	0.014146733	0.254273377	0.799446163	0.890478775	no
C12orf36	0.01414008	0.254153769	0.799538482	0.890534344	no
OPRM1	0.014137165	0.25410137	0.799578927	0.890534344	no
TBATA	-0.014129052	-0.253955512	0.799691511	0.890613581	no
LOC100093631	0.014114908	0.253701231	0.799887795	0.890741452	no
MIR192	-0.014112512	-0.25365816	0.799921044	0.890741452	no
SRP72	-0.014111818	-0.253645691	0.79993067	0.890741452	no
METTL21B	-0.01410806	-0.253578129	0.799982825	0.890753375	no
SESN3	-0.01409177	-0.253285268	0.800208913	0.890958955	no
MMP21	-0.014086842	-0.253196684	0.800277302	0.890986123	no
TMPRSS4	-0.014084038	-0.253146262	0.800316231	0.890986123	no
ISY1-RAB43	-0.01407998	-0.253073314	0.800372551	0.891002667	no
MAGEB4	-0.01407339	-0.252954849	0.800464015	0.891058331	no
SBDS	-0.014068558	-0.252867976	0.80053109	0.891086841	no
LENG1	-0.014060729	-0.252727228	0.800639764	0.891161651	no
OAZ2	-0.014050228	-0.252538445	0.800785533	0.891271384	no
C9orf171	-0.014047652	-0.252492134	0.800821294	0.891271384	no
SDR42E1	0.014033187	0.252232083	0.801022106	0.891441368	no
FGFBP1	0.014030674	0.252186915	0.801056987	0.891441368	no
PDHA2	-0.014020982	-0.252012684	0.801191538	0.891509248	no
RBM48	-0.014019336	-0.251983085	0.801214397	0.891509248	no
C17orf59	0.014017317	0.251946782	0.801242433	0.891509248	no
PRPF4	0.014011976	0.251850773	0.80131658	0.891515774	no
SCG2	0.014010919	0.251831759	0.801331264	0.891515774	no
BZW2	0.013998828	0.251614404	0.801499135	0.891656378	no
KDM4A-AS1	0.013979194	0.251261424	0.801771771	0.891892716	no
NUP88	0.013977551	0.251231898	0.801794578	0.891892716	no
FCRLB	0.013968071	0.251061461	0.801926231	0.891950373	no
AES	0.013967841	0.251057336	0.801929417	0.891950373	no
TMEM247	-0.013961788	-0.250948508	0.802013483	0.891951701	no
IL17A	0.013960358	0.250922817	0.80203333	0.891951701	no
MBL2	-0.013958789	-0.250894607	0.802055121	0.891951701	no
LOC147093	0.013940132	0.250559204	0.802314228	0.89215138	no
RAD51AP2	0.013937243	0.250507265	0.802354354	0.89215138	no
OR51B4	0.013936893	0.250500975	0.802359214	0.89215138	no

POM121L12	-0.013928826	-0.25035594	0.802471266	0.892191914	no
LINC00670	-0.013928291	-0.25034632	0.802478698	0.892191914	no
KL	-0.013911052	-0.250036422	0.802718134	0.892341406	no
ZNF837	0.013910795	0.250031789	0.802721713	0.892341406	no
PRR11	0.013909642	0.25001107	0.802737723	0.892341406	no
TMEM44	0.013892935	0.249710711	0.802969807	0.892511374	no
MIR499A	0.013892053	0.249694868	0.80298205	0.892511374	no
GLE1	0.013889666	0.249651951	0.803015213	0.892511374	no
UPB1	0.013882208	0.249517868	0.803118825	0.892569524	no
TRPM5	-0.013879921	-0.249476755	0.803150596	0.892569524	no
DDX11L10	0.013867865	0.249260019	0.803318086	0.892709499	no
DPCR1	0.013857477	0.249073283	0.803462401	0.892788167	no
NYNRIN	0.013855928	0.249045435	0.803483923	0.892788167	no
PTPN21	0.013853799	0.249007159	0.803513505	0.892788167	no
SLBP	0.013845244	0.248853349	0.803632381	0.892835292	no
LOC100507462	-0.013844767	-0.248844776	0.803639007	0.892835292	no
LOC149134	0.013831098	0.248599056	0.803828927	0.893000127	no
OR51B5	0.01382345	0.248461553	0.803935209	0.893072035	no
LOC100129617	-0.013819208	-0.248385291	0.803994157	0.893091355	no
UBTFL1	0.013811404	0.248244995	0.804102605	0.893165655	no
MIR548I2	-0.013803051	-0.248094842	0.804218676	0.893248414	no
GRHL3	-0.013799197	-0.248025554	0.804272239	0.893261741	no
GOLGA7	0.013789447	0.24785027	0.804407744	0.893323716	no
LRRC27	-0.013785588	-0.2477809	0.804461374	0.893323716	no
CASP3	0.013783548	0.247744219	0.804489732	0.893323716	no
ZNF362	-0.013783218	-0.247738297	0.804494309	0.893323716	no
OR51A7	-0.013774774	-0.247586498	0.804611668	0.893383766	no
FIBIN	0.013772417	0.247544116	0.804644435	0.893383766	no
RAB3GAP1	-0.013766968	-0.247446171	0.804720161	0.893383766	no
TAS2R60	0.013762282	0.24736193	0.804785293	0.893383766	no
KRTAP4-2	-0.013760254	-0.247325469	0.804813484	0.893383766	no
SPINK4	0.01375789	0.24728297	0.804846343	0.893383766	no
RFWD2	0.013757252	0.24727149	0.80485522	0.893383766	no
COL4A3	0.013754545	0.247222833	0.804892841	0.893383766	no
C2CD4D	-0.01375241	-0.24718445	0.804922519	0.893383766	no
OR6Q1	-0.013727864	-0.246743185	0.805263728	0.893716318	no
DNAH12	-0.013724586	-0.246684245	0.805309305	0.893720748	no
DMGDH	0.013720857	0.24661721	0.805361145	0.893725284	no
KRT20	0.013718309	0.246571407	0.805396565	0.893725284	no
C21orf15	-0.013713689	-0.246488355	0.805460792	0.893750406	no
OR5C1	-0.013705845	-0.246347338	0.805569848	0.893809381	no
CLCN1	-0.013703883	-0.246312075	0.80559712	0.893809381	no
PLCB3	-0.01369667	-0.246182396	0.805697412	0.89386588	no
FIGN	0.013694238	0.246138671	0.805731229	0.89386588	no
HOXA-AS4	0.013687287	0.246013715	0.805827872	0.89392695	no
SNORA11C	-0.013670122	-0.245705142	0.806066541	0.894112591	no
ZNF557	0.013669268	0.245689778	0.806078426	0.894112591	no
APAF1	0.013663713	0.245589917	0.806155668	0.894115188	no
SLC18A3	-0.013663115	-0.245579177	0.806163975	0.894115188	no
BCL2L13	0.013657509	0.245478393	0.806241935	0.894155508	no
ITCH	-0.013628161	-0.244950797	0.806650075	0.894527999	no

PAN3	-0.013627372	-0.244936611	0.80666105	0.894527999	no
ZNF805	-0.013616761	-0.244745848	0.806808634	0.894586154	no
MRGPRX4	-0.013615583	-0.244724675	0.806825015	0.894586154	no
SPACA7	-0.013614623	-0.244707413	0.806838371	0.894586154	no
RELA	0.01359657	0.24438287	0.807089473	0.8948184	no
TMEM68	-0.01358691	-0.244209216	0.80722384	0.894921204	no
ARHGAP42	-0.013581741	-0.244116289	0.807295745	0.894954754	no
EPYC	-0.013572882	-0.24395704	0.807418973	0.895033086	no
SYF2	0.013570674	0.243917333	0.807449699	0.895033086	no
LOC641746	-0.013556739	-0.243666826	0.807643555	0.895194288	no
GAR1	0.013550086	0.243547232	0.807736108	0.895194288	no
MEIS1	0.013548861	0.243525208	0.807753152	0.895194288	no
OR10AG1	-0.013548244	-0.243514106	0.807761744	0.895194288	no
CDKAL1	0.013541665	0.243395833	0.807853278	0.895216166	no
NAT8B	0.013540581	0.243376357	0.807868351	0.895216166	no
NUDT16L1	-0.01353683	-0.243308919	0.807920543	0.895216166	no
CPT1A	0.013534849	0.243273306	0.807948107	0.895216166	no
POMZP3	-0.013518644	-0.242981987	0.808173581	0.895391768	no
MFN2	0.013513553	0.242890469	0.808244417	0.895391768	no
TMEM233	0.013512563	0.242872667	0.808258197	0.895391768	no
LEMD1-AS1	-0.013507204	-0.242776326	0.808332769	0.895391768	no
LOC10013109€	0.01350596	0.242753961	0.80835008	0.895391768	no
SNORD94	-0.013503043	-0.242701535	0.808390661	0.895391768	no
NFATC1	-0.013502499	-0.242691749	0.808398236	0.895391768	no
TKTL2	-0.013488826	-0.242445955	0.808588501	0.895513301	no
C11orf93	-0.013488625	-0.242442341	0.808591299	0.895513301	no
OR52A5	-0.013477449	-0.242241416	0.80874684	0.895639408	no
DGKZ	0.013466902	0.24205182	0.808893618	0.895740761	no
C16orf74	0.013464497	0.242008587	0.808927089	0.895740761	no
KRT1	-0.013461427	-0.241953399	0.808969815	0.895740761	no
PPP2R1B	0.013458894	0.241907849	0.80900508	0.895740761	no
PAK2	-0.013452245	-0.241788332	0.809097613	0.895797063	no
APOBEC3A	0.013437911	0.241530641	0.809297131	0.895956765	no
TSHZ2	0.013435892	0.241494342	0.809325237	0.895956765	no
PGRMC1	-0.013432797	-0.241438703	0.809368318	0.895958305	no
HSFX1	0.01342941	0.241377827	0.809415455	0.895964334	no
FBXO24	-0.013421119	-0.241228773	0.809530871	0.895966824	no
IPO13	-0.013420855	-0.241224032	0.809534542	0.895966824	no
CLCNKA	0.013419492	0.241199529	0.809553515	0.895966824	no
FAM9C	-0.013417269	-0.24115956	0.809584465	0.895966824	no
RBPMS2	0.013409566	0.241021084	0.809691696	0.896039353	no
LINC00535	-0.013395883	-0.240775105	0.809882181	0.896204004	no
ZNF580	0.013389836	0.240666387	0.809966376	0.896205593	no
MST1P2	-0.01338938	-0.240658199	0.809972717	0.896205593	no
NDC80	0.013385599	0.240590221	0.810025363	0.896205593	no
IL17B	-0.013381907	-0.240523863	0.810076755	0.896205593	no
DDX11L5	0.013380803	0.240504017	0.810092125	0.896205593	no
VDAC2	-0.013375416	-0.240407167	0.810167133	0.896242439	no
FAM170B-AS1	0.013366007	0.240238015	0.810298142	0.89630822	no
PCSK4	-0.013360472	-0.240138527	0.810375199	0.89630822	no
ZNF625	-0.013360166	-0.240133026	0.81037946	0.89630822	no

AIF1L	-0.013359164	-0.240115	0.810393422	0.89630822	no
RNF32	-0.013349014	-0.239932539	0.810534749	0.896389937	no
PCDHGA7	-0.01334715	-0.239899034	0.810560702	0.896389937	no
TAS2R40	-0.013344871	-0.239858065	0.810592436	0.896389937	no
MAGEE2	-0.013338316	-0.239740216	0.810683722	0.896415998	no
TUFT1	0.0133321	0.239628483	0.810770273	0.896415998	no
DFFB	0.013331373	0.239615404	0.810780405	0.896415998	no
PRKAR1A	0.013331198	0.239612251	0.810782847	0.896415998	no
COPRS	-0.013327463	-0.239545112	0.810834856	0.896427383	no
OXT	0.013321916	0.239445401	0.810912098	0.896437182	no
GABRG3	0.013320836	0.239425979	0.810927144	0.896437182	no
C9orf152	0.013308065	0.239196394	0.811105002	0.896587676	no
NACC1	-0.013304213	-0.239127158	0.811158641	0.896600852	no
ASB11	-0.013291484	-0.238898315	0.811335937	0.896692048	no
OR2T2	-0.013290871	-0.238887308	0.811344465	0.896692048	no
SNORA34	-0.013285316	-0.23878744	0.811421841	0.896692048	no
UGT1A9	-0.01328124	-0.238714173	0.811478608	0.896692048	no
ZNF697	-0.013280821	-0.238706633	0.81148445	0.896692048	no
INSL4	0.013280315	0.238697545	0.811491491	0.896692048	no
C14orf23	-0.013258366	-0.238302968	0.811797227	0.896962716	no
MYPN	-0.013256738	-0.23827369	0.811819914	0.896962716	no
NKIRAS2	-0.013247163	-0.238101557	0.811953301	0.897063973	no
TIPIN	0.013241117	0.237992879	0.812037519	0.897110899	no
TPRG1-AS2	-0.013224011	-0.237685369	0.812275829	0.897328048	no
MYL7	0.01321899	0.237595104	0.812345785	0.897338976	no
DHCR24	-0.013217307	-0.237564853	0.81236923	0.897338976	no
ABCB7	-0.013197933	-0.237216563	0.812639172	0.897591019	no
SEC14L1P1	0.013167118	0.236662604	0.813068564	0.898019145	no
AHSA2	-0.013157607	-0.236491629	0.813201104	0.898119376	no
SMAD1	-0.013153008	-0.236408942	0.813265205	0.898144014	no
ADAM21P1	-0.013139192	-0.236160578	0.81345775	0.898269969	no
PGLYRP3	-0.013135855	-0.236100595	0.813504254	0.898269969	no
MORC3	-0.013134107	-0.236069172	0.813528616	0.898269969	no
CREB1	-0.013132828	-0.236046174	0.813546447	0.898269969	no
YRDC	-0.013127874	-0.235957119	0.813615491	0.898300052	no
RSPO4	0.013124607	0.235898385	0.813661029	0.89830418	no
LOC440563	0.013119006	0.235797694	0.813739097	0.898344221	no
LOC91948	0.013108832	0.235614812	0.813880897	0.898454611	no
PRAMEF12	0.013083872	0.235166108	0.814228831	0.898767265	no
COMMD6	0.013082514	0.235141695	0.814247762	0.898767265	no
UNQ6975	0.013068801	0.234895177	0.814438933	0.898920358	no
UBE2A	0.013066565	0.234854971	0.814470114	0.898920358	no
PAGR1	-0.013056662	-0.234676949	0.814608175	0.898925769	no
PPM1G	0.01305475	0.23464259	0.814634822	0.898925769	no
NBPF10	-0.013048665	-0.234533194	0.814719666	0.898925769	no
GFRA2	0.013044875	0.23446506	0.814772509	0.898925769	no
ARHGAP8	0.013039761	0.234373122	0.814843816	0.898925769	no
HBA1	-0.013038208	-0.234345211	0.814865464	0.898925769	no
LINC00615	-0.013036239	-0.234309815	0.814892918	0.898925769	no
FAM108C1	0.013034847	0.234284786	0.81491233	0.898925769	no
NDUFA3	0.01303455	0.234279447	0.814916471	0.898925769	no

CENPE	-0.013033286	-0.234256723	0.814934097	0.898925769	no
ZNF180	-0.013032001	-0.234233635	0.814952005	0.898925769	no
SGOL1	-0.013030212	-0.23420147	0.814976953	0.898925769	no
B3GALT5	-0.013019122	-0.234002098	0.815131597	0.899036852	no
SPAG8	0.013016989	0.233963768	0.815161328	0.899036852	no
TTC37	0.013012788	0.233888235	0.815219917	0.899055332	no
CLCN5	-0.012984263	-0.233375446	0.815617708	0.899447874	no
IQCF6	-0.012969345	-0.233107283	0.815825751	0.899630432	no
SHOC2	0.012964198	0.233014743	0.815897548	0.899630432	no
USP29	-0.012963388	-0.233000196	0.815908834	0.899630432	no
GPR33	-0.012957389	-0.232892341	0.815992515	0.899653894	no
UBQLN3	-0.01295586	-0.23286486	0.816013837	0.899653894	no
MLF1IP	0.012952782	0.232809521	0.816056773	0.899655079	no
HBB	0.01294571	0.232682406	0.816155402	0.899682235	no
TRIML2	0.012945013	0.232669866	0.816165132	0.899682235	no
BEST4	0.012938392	0.23255085	0.816257479	0.899692859	no
C12orf66	0.012938319	0.232549538	0.816258497	0.899692859	no
NUDT6	-0.012932746	-0.232449341	0.816336244	0.899732408	no
FAM104B	-0.012921009	-0.23223836	0.81649996	0.899850764	no
OR8B12	-0.012919044	-0.232203035	0.816527372	0.899850764	no
TMEM126A	0.012915737	0.232143587	0.816573504	0.899855459	no
GHRL	0.012908293	0.232009761	0.816677355	0.899889271	no
C9orf92	0.01290207	0.231897896	0.816764167	0.899889271	no
CRYBG3	-0.012890416	-0.231688387	0.816926761	0.899889271	no
CRYGB	-0.012889699	-0.231675513	0.816936753	0.899889271	no
GJB5	-0.012888116	-0.231647055	0.816958839	0.899889271	no
GFRAL	0.012884136	0.231575502	0.817014371	0.899889271	no
UTF1	-0.012882533	-0.231546676	0.817036744	0.899889271	no
ZDHHC9	0.012880145	0.231503751	0.817070059	0.899889271	no
CNDP1	0.012879808	0.231497703	0.817074753	0.899889271	no
ST7-AS2	-0.012879206	-0.23148687	0.817083161	0.899889271	no
ZNF57	-0.012877841	-0.231462333	0.817102204	0.899889271	no
RASSF10	0.012875624	0.231422479	0.817133137	0.899889271	no
OTOL1	0.012873905	0.231391581	0.817157118	0.899889271	no
CENPVP1	-0.012871519	-0.231348692	0.817190406	0.899889271	no
GLO1	-0.01285898	-0.231123283	0.817365362	0.900006532	no
OGG1	-0.012857885	-0.23110359	0.817380647	0.900006532	no
ANKS1A	0.01283793	0.230744878	0.81765909	0.900266997	no
LIN7C	0.012834733	0.230687398	0.81770371	0.900270001	no
EIF2C2	-0.012826875	-0.230546132	0.817813372	0.900330278	no
OR56B4	0.012824806	0.230508939	0.817842245	0.900330278	no
PMCHL1	0.012821118	0.230442646	0.817893709	0.900340814	no
LOC100507389	0.012812866	0.230294296	0.818008877	0.90041651	no
RMRP	-0.012810186	-0.230246133	0.818046268	0.90041651	no
MEPE	0.012795731	0.229986274	0.818248016	0.900592446	no
OR6C65	-0.012788263	-0.229852018	0.818352254	0.900658067	no
CPSF2	-0.012785454	-0.22980153	0.818391454	0.900658067	no
FAM25A	0.012770884	0.229539607	0.818594825	0.900800687	no
GPX2	-0.012770164	-0.22952666	0.818604878	0.900800687	no
MIS18A	-0.012753318	-0.229223829	0.818840028	0.901013314	no
LOC641515	-0.012748103	-0.229130087	0.818912822	0.90104728	no

OR12D2	-0.012742473	-0.229028872	0.818991422	0.901087628	no
DENND4C	-0.012729105	-0.228788569	0.819178038	0.901173177	no
SERPINB3	-0.012728592	-0.228779341	0.819185205	0.901173177	no
TOMM40	0.012721852	0.228658175	0.819279305	0.901173177	no
OR8H3	-0.012721007	-0.228642992	0.819291097	0.901173177	no
ZNF880	-0.012720953	-0.228642016	0.819291855	0.901173177	no
PRR21	0.012718881	0.228604783	0.819320772	0.901173177	no
ACPT	-0.012712671	-0.228493147	0.819407474	0.901222417	no
LOC732275	0.012707542	0.22840093	0.819479096	0.901251665	no
PIK3C2A	-0.012701772	-0.228297208	0.819559656	0.901251665	no
KRTAP22-2	0.012701756	0.228296925	0.819559876	0.901251665	no
ODF3	0.012697152	0.228214159	0.819624161	0.901261939	no
HIVEP1	-0.01269508	-0.22817691	0.819653092	0.901261939	no
LINC00651	-0.012691085	-0.228105098	0.81970887	0.901277158	no
OR1J2	0.012660835	0.227561299	0.820131283	0.901695472	no
ALX1	0.012640647	0.227198392	0.820413211	0.901959296	no
LOC100133123	-0.012634854	-0.227094266	0.820494107	0.902002089	no
TMEM98	0.012625599	0.226927894	0.820623366	0.90208599	no
C3orf72	-0.012623379	-0.226887981	0.820654376	0.90208599	no
CCDC155	0.012594892	0.226375884	0.821052272	0.902382352	no
NAIF1	-0.012594855	-0.226375216	0.82105279	0.902382352	no
IFT122	0.012593899	0.226358031	0.821066145	0.902382352	no
ATP13A1	-0.012592052	-0.226324833	0.821091941	0.902382352	no
LOC100271836	-0.012582818	-0.226158827	0.821220937	0.902446837	no
CNOT2	-0.012581839	-0.226141245	0.8212346	0.902446837	no
TBX4	-0.012562511	-0.225793796	0.821504606	0.902671741	no
FAM99A	-0.012561176	-0.225769781	0.82152327	0.902671741	no
ZNF234	0.012554702	0.225653415	0.821613704	0.902682478	no
DENND2C	0.012554463	0.225649113	0.821617047	0.902682478	no
ANKRD20A2	-0.012549472	-0.225559387	0.82168678	0.902712943	no
TFDP1	-0.012545738	-0.225492263	0.821738948	0.902724108	no
ACOT6	0.012538339	0.225359263	0.821842316	0.902791516	no
GRP	-0.012516542	-0.224967436	0.822146865	0.903079901	no
OR5D16	0.012504389	0.224748964	0.822316683	0.903220272	no
NDNL2	0.012486167	0.224421386	0.822571327	0.903453794	no
CD207	0.01247486	0.224218133	0.822729336	0.903581161	no
OR6P1	0.012467058	0.224077876	0.822838376	0.903624857	no
LOC63930	0.012461519	0.223978304	0.822915789	0.903624857	no
PTH2R	-0.012460988	-0.223968764	0.822923205	0.903624857	no
LOC100652909	-0.012459979	-0.223950624	0.822937308	0.903624857	no
MAFIP	-0.012450777	-0.223785215	0.823065911	0.903683452	no
CAMK1	-0.012450143	-0.22377382	0.82307477	0.903683452	no
APOBEC4	0.012440438	0.223599351	0.823210421	0.903786215	no
NDFIP2-AS1	0.012430239	0.223416013	0.823352975	0.903874701	no
C18orf62	0.012428654	0.223387513	0.823375135	0.903874701	no
MSH3	0.012419315	0.223219646	0.823505664	0.903931519	no
MGC45800	0.012418933	0.223212765	0.823511015	0.903931519	no
SLC25A25	-0.012406784	-0.222994376	0.823680837	0.904007019	no
CFHR4	0.012404985	0.222962029	0.823705991	0.904007019	no
TTTY7	0.012404985	0.222962029	0.823705991	0.904007019	no
SPZ1	-0.012397104	-0.22282037	0.823816152	0.904036683	no

STOML1	-0.012394704	-0.222777225	0.823849704	0.904036683	no
SMARCC2	-0.012394024	-0.222764993	0.823859217	0.904036683	no
NUP160	-0.012388946	-0.222673713	0.823930203	0.904068416	no
SENP6	-0.01238165	-0.222542556	0.824032204	0.904134175	no
VWA5B1	-0.012354046	-0.222046336	0.82441814	0.904429538	no
RAB5C	0.012349295	0.221960933	0.824484567	0.904429538	no
EHF	0.012342598	0.221840541	0.82457821	0.904429538	no
GAS2L2	-0.012341324	-0.221817641	0.824596022	0.904429538	no
EIF4H	-0.012337968	-0.221757308	0.824642951	0.904429538	no
MRPS24	-0.012334967	-0.22170337	0.824684907	0.904429538	no
N4BP3	-0.012334701	-0.221698595	0.824688621	0.904429538	no
OR4C15	-0.012332371	-0.221656712	0.824721201	0.904429538	no
TGM7	-0.012331954	-0.221649214	0.824727033	0.904429538	no
KLK5	0.012326162	0.221545096	0.824808024	0.904429538	no
SP1	0.012323609	0.221499202	0.824843724	0.904429538	no
PAX4	-0.012322462	-0.221478579	0.824859766	0.904429538	no
SNAPC5	0.012311612	0.221283536	0.825011492	0.904429538	no
CSTF1	-0.012311376	-0.2212793	0.825014787	0.904429538	no
SNORA71A	0.012309847	0.221251798	0.825036182	0.904429538	no
SELE	-0.012309379	-0.221243395	0.825042719	0.904429538	no
OR2W3	-0.012306551	-0.221192557	0.825082268	0.904429538	no
SSR4	0.0123057	0.22117725	0.825094176	0.904429538	no
GSTO1	0.012305212	0.221168482	0.825100997	0.904429538	no
MGC39584	0.012300393	0.221081857	0.825168387	0.904438558	no
OR2T11	-0.012296617	-0.221013984	0.82522119	0.904438558	no
CCDC27	-0.01229224	-0.22093529	0.825282413	0.904438558	no
RPS7	-0.012290996	-0.22091294	0.825299801	0.904438558	no
SCARNA20	-0.012287583	-0.22085158	0.825347539	0.904438558	no
MAPRE1	0.01228509	0.220806759	0.82538241	0.904438558	no
FOXP1	-0.012283559	-0.220779243	0.825403817	0.904438558	no
CDH19	-0.012258007	-0.220319921	0.825761194	0.904760659	no
OR2T1	-0.012255217	-0.220269754	0.825800229	0.904760659	no
LOC100506599	-0.012250045	-0.22017679	0.825872565	0.904760659	no
FAM185A	-0.012248993	-0.220157875	0.825887284	0.904760659	no
SNORA21	-0.012247493	-0.220130906	0.825908269	0.904760659	no
HADH	-0.01224374	-0.220063451	0.825960757	0.904772039	no
GET4	-0.012239762	-0.219991947	0.826016398	0.904786871	no
PCDHA8	-0.012230106	-0.219818363	0.826151475	0.904888708	no
OR14C36	-0.012221884	-0.219670559	0.826266495	0.904968568	no
SPRR2E	-0.012199151	-0.219261905	0.826584525	0.905256472	no
USP17L7	0.012197072	0.21922454	0.826613606	0.905256472	no
CHST11	0.012193851	0.219166633	0.826658674	0.905259698	no
TTLL10	-0.01218836	-0.219067923	0.8267355	0.905297698	no
TRIM43	0.012179415	0.218907136	0.826860644	0.905388603	no
SMTN	-0.012168699	-0.2187145	0.827010583	0.905506645	no
IL12RB2	0.012163239	0.218616344	0.827086985	0.905521225	no
C9orf131	-0.01216019	-0.218561541	0.827129643	0.905521225	no
ATP6V0CP3	-0.012158714	-0.218534998	0.827150304	0.905521225	no
LINC00305	0.012149121	0.218362549	0.827284541	0.905610415	no
OR6C74	0.012144552	0.218280418	0.827348474	0.905610415	no
SERPINA12	0.012143857	0.218267935	0.827358191	0.905610415	no

ZNF426	-0.012136493	-0.218135548	0.827461249	0.905677092	no
GOLGA2P5	0.012133089	0.21807437	0.827508875	0.905683093	no
TEK	0.012124005	0.217911066	0.827636005	0.905776105	no
PAPD4	0.012116609	0.217778108	0.827739515	0.905803411	no
SERPINA4	-0.012116199	-0.217770741	0.827745251	0.905803411	no
BEAN1	-0.012109787	-0.217655485	0.827834982	0.90584295	no
MIR183	0.012104342	0.217557611	0.827911182	0.90584295	no
OR1S1	0.012103221	0.217537455	0.827926875	0.90584295	no
BANP	0.01210157	0.217507776	0.827949982	0.90584295	no
OR11H6	-0.012095782	-0.21740373	0.82803099	0.905885462	no
MME	0.012089527	0.217291282	0.828118543	0.905935129	no
CPNE3	-0.012080011	-0.217120223	0.828251733	0.906034714	no
CCDC148	-0.01207418	-0.217015407	0.828333348	0.906077873	no
SLC2A7	-0.012069747	-0.216935718	0.828395399	0.90609963	no
INTS3	0.012063798	0.216828778	0.828478671	0.906105948	no
MRPL3	-0.01206331	-0.216820009	0.8284855	0.906105948	no
PTH	0.012050809	0.216595285	0.828660494	0.906251218	no
RAB12	-0.012040436	-0.216408828	0.828805696	0.906363893	no
PDP1	-0.012023492	-0.216104228	0.829042914	0.906577178	no
RRAGA	0.012019917	0.216039976	0.829092955	0.906585769	no
OR52M1	-0.012003711	-0.215748649	0.829319854	0.906787738	no
RBFA	-0.012000627	-0.215693203	0.82936304	0.906788822	no
TFAM	0.011987297	0.215453596	0.82954967	0.906946735	no
OR52L1	0.01197623	0.215254642	0.829704643	0.90702599	no
CCNA2	-0.011975117	-0.215234645	0.829720219	0.90702599	no
FAM41C	-0.011973078	-0.215197984	0.829748777	0.90702599	no
FAM160B1	-0.011952782	-0.214833143	0.830032986	0.907290519	no
OR4K17	-0.011948715	-0.214760044	0.830089933	0.90730109	no
OXLD1	-0.011946062	-0.214712345	0.830127092	0.90730109	no
ZBTB20-AS1	-0.011918768	-0.214221711	0.830509339	0.907672711	no
AHCY	0.011915281	0.214159027	0.830558178	0.907679929	no
ETS2	-0.01191225	-0.214104534	0.830600636	0.907680172	no
RADIL	-0.011899853	-0.213881697	0.830774263	0.90782375	no
OR5M1	-0.011877464	-0.21347922	0.83108788	0.908077401	no
UBE2S	-0.011873893	-0.213415025	0.831137904	0.908077401	no
LINC00637	-0.011873855	-0.213414354	0.831138427	0.908077401	no
PPP1R2P9	-0.011871216	-0.213366906	0.831175402	0.908077401	no
STK19	-0.011859868	-0.213162913	0.831334371	0.908204909	no
ZNF736	-0.011855831	-0.213090346	0.831390924	0.908220523	no
EIF1AY	0.011844741	0.212891005	0.831546276	0.908316604	no
LINC00460	-0.011843519	-0.212869024	0.831563408	0.908316604	no
C1orf94	0.011836468	0.212742278	0.831662188	0.908378334	no
RAB19	-0.011827074	-0.212573414	0.831793799	0.908475913	no
NCRNA00185	0.011821635	0.212475647	0.83187	0.908512968	no
OR1S2	-0.011806742	-0.212207926	0.832078673	0.908654	no
UGT1A1	-0.011806383	-0.212201481	0.832083697	0.908654	no
PRICKLE2-AS1	-0.011796972	-0.212032308	0.832215563	0.908736124	no
PCNA	-0.011794981	-0.211996508	0.832243469	0.908736124	no
RASGEF1B	0.01178905	0.211889889	0.832326579	0.9087807	no
KRTAP10-10	-0.011753635	-0.211253276	0.832822863	0.909244407	no
LOC390705	-0.011752705	-0.211236564	0.832835892	0.909244407	no

GCM1	0.01174539	0.211105076	0.832938405	0.909310132	no
TMEM14B	0.011741245	0.211030562	0.832996499	0.909327361	no
C20orf27	-0.011735742	-0.210931644	0.833073622	0.90936536	no
POLR2D	-0.011732216	-0.21086825	0.83312305	0.909373125	no
LOC100506122	0.011727522	0.210783868	0.833188842	0.909398751	no
LOC440354	0.011722159	0.210687471	0.833264003	0.909402332	no
SNX1	-0.011719498	-0.210639635	0.833301302	0.909402332	no
ALOX12P2	0.011717999	0.210612695	0.833322307	0.909402332	no
INSL5	-0.011715211	-0.210562578	0.833361385	0.909402332	no
USP9Y	-0.011705315	-0.210384693	0.833500091	0.909442239	no
TRAPPC5	0.011701923	0.210323715	0.83354764	0.909442239	no
OR8J3	0.011700304	0.210294604	0.83357034	0.909442239	no
FOXR2	-0.011697752	-0.210248741	0.833606103	0.909442239	no
PGLYRP2	-0.011697507	-0.210244331	0.833609542	0.909442239	no
RPGR	0.011679278	0.209916649	0.833865073	0.909674837	no
FBXL6	0.011672964	0.209803138	0.833953595	0.909725227	no
ZP1	0.011667581	0.209706379	0.834029054	0.909761364	no
LOC255130	0.011641261	0.209233255	0.834398051	0.910117673	no
FLG2	-0.011627362	-0.208983414	0.83459292	0.910277607	no
ZNF264	-0.011624761	-0.208936656	0.834629391	0.910277607	no
LOC402160	0.011620785	0.208865179	0.834685144	0.910292217	no
CCDC80	-0.011611335	-0.208695317	0.834817641	0.910390517	no
COG8	-0.011602907	-0.208543821	0.834935816	0.910458051	no
TMEM48	0.011599489	0.208482367	0.834983755	0.910458051	no
MRPL41	-0.011597294	-0.208442919	0.835014527	0.910458051	no
MCM9	0.011594834	0.208398693	0.835049027	0.910458051	no
HIST1H3J	0.011591335	0.208335804	0.835098086	0.910465349	no
CD209	-0.011572316	-0.20799391	0.835364807	0.910709942	no
C11orf94	0.011564792	0.207858663	0.835470323	0.910778772	no
TMOD4	0.011558875	0.207752307	0.8355533	0.910821179	no
PAGE1	0.011555974	0.207700159	0.835593986	0.910821179	no
LINC00482	0.011540774	0.207426913	0.83580718	0.91099921	no
C16orf92	0.011537585	0.207369593	0.835851904	0.91099921	no
IER3IP1	0.011533044	0.207287974	0.835915588	0.91099921	no
UTS2D	-0.01153224	-0.207273512	0.835926872	0.91099921	no
IRS2	0.011518843	0.207032688	0.836114786	0.911126618	no
CCDC168	0.01151786	0.207015019	0.836128573	0.911126618	no
GABRR1	0.01150998	0.20687338	0.836239098	0.911185445	no
TCHHL1	-0.011507966	-0.206837169	0.836267354	0.911185445	no
ZNF107	-0.011500298	-0.206699332	0.836374916	0.911256443	no
LELP1	-0.011484953	-0.206423501	0.836590171	0.911425437	no
KLK4	-0.011481671	-0.206364498	0.836636219	0.911425437	no
FUZ	-0.011480172	-0.206337545	0.836657253	0.911425437	no
SVIP	0.011471669	0.206184703	0.836776536	0.911509176	no
GOLGA6D	-0.01146694	-0.206099692	0.836842884	0.911532167	no
HIST1H2AM	0.011464118	0.206048969	0.836882471	0.911532167	no
EMG1	0.011458593	0.205949663	0.836959978	0.911567073	no
ZNF224	0.011455787	0.205899216	0.836999351	0.911567073	no
TAF7L	0.01145162	0.205824306	0.837057818	0.911584553	no
TIE1	0.011443713	0.205682182	0.837168749	0.911645179	no
TNP1	-0.011440005	-0.205615521	0.837220781	0.911645179	no

RPL35	-0.011436913	-0.20555994	0.837264164	0.911645179	no
SNTG2	0.011434375	0.205514316	0.837299776	0.911645179	no
MBL1P	0.011432335	0.205477657	0.837328391	0.911645179	no
EMC7	0.011429512	0.205426904	0.837368006	0.911645179	no
KRTAP2-4	0.01141628	0.205189049	0.837553673	0.911758452	no
KIF13B	0.011413809	0.205144624	0.837588352	0.911758452	no
LYAR	-0.011413027	-0.205130566	0.837599326	0.911758452	no
GLTP	-0.01140656	-0.205014323	0.837690069	0.911811045	no
ZNF582	0.011403298	0.204955692	0.837735839	0.911814683	no
KIAA1919	0.011395366	0.204813108	0.837847148	0.911856044	no
SELRC1	-0.01139377	-0.204784412	0.83786955	0.911856044	no
PRSS1	0.01139152	0.204743962	0.837901129	0.911856044	no
MGC34796	0.011377603	0.204493801	0.838096429	0.911992129	no
GLRX2	-0.011376561	-0.204475076	0.838111049	0.911992129	no
MFSD2B	0.011362775	0.204227262	0.838304528	0.912156479	no
TAAR6	-0.011343508	-0.203880913	0.838574954	0.912404533	no
PSG5	0.011335065	0.203729156	0.83869345	0.912447981	no
LRRC71	-0.011334542	-0.203719747	0.838700798	0.912447981	no
LOC284998	0.011328306	0.203607654	0.838788326	0.912447981	no
LOC285762	-0.011326065	-0.203567367	0.838819785	0.912447981	no
LOC653712	-0.011325538	-0.203557906	0.838827172	0.912447981	no
C18orf63	0.011320253	0.203462904	0.838901357	0.912482491	no
KRT39	0.011293029	0.202973532	0.83928352	0.91284223	no
ARFRP1	-0.011286055	-0.202848163	0.83938143	0.91284223	no
HUS1B	-0.01128531	-0.202834778	0.839391883	0.91284223	no
LGALS8-AS1	0.011282887	0.20279122	0.839425902	0.91284223	no
MELK	-0.011281565	-0.202767451	0.839444465	0.91284223	no
ANP32D	0.011277656	0.202697188	0.839499341	0.912846837	no
SNORA70	-0.011275212	-0.202653255	0.839533653	0.912846837	no
GALNTL5	0.011266865	0.202503215	0.839650838	0.912914734	no
NBPF4	-0.011264713	-0.202464527	0.839681055	0.912914734	no
FIGF	-0.011250865	-0.202215604	0.839875479	0.913037612	no
FLJ26245	0.011250611	0.202211039	0.839879045	0.913037612	no
LOC340508	-0.011247449	-0.202154207	0.839923436	0.913039685	no
SORBS2	-0.011240992	-0.202038124	0.840014108	0.913092064	no
ALS2CR11	0.01123759	0.201976979	0.84006187	0.91309248	no
TMEM52	0.011234913	0.20192885	0.840099465	0.91309248	no
SLC6A16	0.011228032	0.201805171	0.840196076	0.913151303	no
URI1	-0.011207449	-0.201435181	0.840485104	0.913406624	no
GOLGA8G	-0.011205249	-0.201395629	0.840516002	0.913406624	no
RTDR1	0.011199579	0.201293699	0.840595632	0.91344697	no
OR10Z1	-0.011195512	-0.201220603	0.840652737	0.913462836	no
RNASE12	-0.01118457	-0.201023915	0.8408064	0.913548633	no
MED4-AS1	-0.011178341	-0.200911948	0.840893878	0.913548633	no
SYNJ2	-0.01117779	-0.200902041	0.840901619	0.913548633	no
MCM3	0.011177782	0.200901899	0.840901729	0.913548633	no
SH3PXD2A	-0.011171128	-0.200782278	0.840995189	0.913603984	no
RAET1E	0.01114895	0.200383626	0.841306673	0.913854039	no
HAO2	0.011144952	0.200311752	0.841362834	0.913854039	no
TAL1	-0.011140535	-0.200232352	0.841424876	0.913854039	no
OGFRL1	0.011139167	0.200207768	0.841444086	0.913854039	no

TNNC1	0.011138094	0.200188485	0.841459154	0.913854039	no
SLFN14	0.01113601	0.200151025	0.841488425	0.913854039	no
FAM21A	-0.011131999	-0.200078911	0.841544776	0.913854039	no
OR2W5	-0.011130519	-0.200052323	0.841565553	0.913854039	no
COG7	0.011110253	0.199688016	0.841850242	0.914116994	no
MAST2	-0.011101758	-0.19953532	0.841969572	0.914200378	no
SKA1	-0.011091076	-0.199343298	0.842119642	0.914317128	no
CHMP6	-0.011077682	-0.199102548	0.842307801	0.914475219	no
NCCRP1	0.01107238	0.199007239	0.842382292	0.914509893	no
MPDU1	0.011056826	0.198727642	0.842600828	0.914662932	no
UBN1	0.011056288	0.198717978	0.842608382	0.914662932	no
TMPRSS2	-0.011042332	-0.198467102	0.84280448	0.914811518	no
KRTAP9-4	0.011037571	0.198381528	0.842871372	0.914811518	no
AQR	0.011037458	0.19837949	0.842872964	0.914811518	no
GLMN	0.011033838	0.198314418	0.842923831	0.914820526	no
CNOT1	-0.011014153	-0.197960574	0.843200439	0.915004471	no
KRTAP6-1	0.011013933	0.197956626	0.843203525	0.915004471	no
CTDSPL	-0.01100621	-0.197817805	0.84331205	0.915004471	no
MVB12A	-0.011005514	-0.197805291	0.843321833	0.915004471	no
KLHL18	-0.011004563	-0.197788192	0.843335201	0.915004471	no
RPS4Y1	0.01100055	0.197716058	0.843391594	0.915004471	no
TARM1	-0.010998087	-0.197671786	0.843426206	0.915004471	no
SPPL2B	-0.010997216	-0.197656125	0.843438449	0.915004471	no
GCFC2	0.010994508	0.197607448	0.843476505	0.915004471	no
PRKAG3	-0.010975271	-0.19726166	0.843746853	0.915228518	no
CYP3A43	-0.010966108	-0.19709695	0.843875635	0.915228518	no
LPAR3	-0.010964826	-0.197073901	0.843893657	0.915228518	no
PRICKLE2	0.010961424	0.197012755	0.843941467	0.915228518	no
TFAP2B	0.010959392	0.196976216	0.843970037	0.915228518	no
HNRNPA1P33	-0.010958705	-0.196963874	0.843979687	0.915228518	no
CRISP1	-0.010958601	-0.19696201	0.843981144	0.915228518	no
40238	-0.010953808	-0.196875845	0.844048518	0.915255396	no
PGBD1	-0.010949891	-0.196805434	0.844103574	0.915268916	no
ZRANB3	-0.010928051	-0.196412858	0.844410553	0.915555582	no
FAM186B	-0.010910038	-0.196089064	0.844663764	0.915783925	no
FGD4	0.010899713	0.19590347	0.844808909	0.915869527	no
HARBI1	-0.010898359	-0.195879122	0.844827951	0.915869527	no
ERF	0.010890515	0.195738129	0.844938219	0.915942863	no
CLDN4	-0.010883703	-0.195615676	0.84503399	0.916000478	no
GTPBP10	0.010879651	0.195542848	0.84509095	0.916016019	no
NKX6-1	0.010874842	0.195456398	0.845158565	0.916020623	no
WDR4	0.010871576	0.195397691	0.845204483	0.916020623	no
NTF3	-0.01086748	-0.195324061	0.845262073	0.916020623	no
CCNT1	-0.01086523	-0.19528363	0.845293696	0.916020623	no
PPIL4	0.010862413	0.195232977	0.845333315	0.916020623	no
LMOD2	0.010861159	0.195210446	0.845350938	0.916020623	no
FAM156B	0.010851628	0.195039127	0.845484942	0.91606132	no
ARL6	0.010850402	0.195017088	0.845502181	0.91606132	no
KRTAP29-1	-0.010849393	-0.194998947	0.845516371	0.91606132	no
SLC2A12	-0.010844996	-0.194919909	0.845578195	0.916082119	no
MIF4GD	-0.010835519	-0.19474956	0.845711448	0.916180297	no

EXOC1	-0.010822459	-0.194514794	0.845895097	0.916236686	no
FGF3	0.010821384	0.194495465	0.845910217	0.916236686	no
MAP2K6	-0.010821243	-0.194492936	0.845912196	0.916236686	no
CCND3	-0.01081969	-0.19446502	0.845934034	0.916236686	no
KERA	0.010812215	0.19433065	0.846039152	0.91630436	no
ATP5EP2	-0.010795207	-0.194024925	0.846278331	0.916517215	no
XAGE3	-0.010787666	-0.193889389	0.84638437	0.916585865	no
REG1P	-0.010774153	-0.193646489	0.846574413	0.916745476	no
LOC729176	0.010754294	0.193289504	0.846853733	0.916942023	no
FRMD1	-0.010752965	-0.193265622	0.84687242	0.916942023	no
KLHDC8A	0.010752148	0.193250932	0.846883915	0.916942023	no
UGGT1	-0.01074878	-0.19319039	0.846931287	0.916947118	no
AMTN	0.010718798	0.192651462	0.847353011	0.917323725	no
IFNA5	-0.010717981	-0.192636774	0.847364506	0.917323725	no
KIAA0141	0.010705997	0.19242135	0.847533093	0.917460016	no
EIF2C1	0.0106932	0.19219132	0.847713118	0.917541831	no
C12orf77	-0.010689152	-0.192118554	0.847770067	0.917541831	no
ZNF556	0.010686233	0.192066094	0.847811125	0.917541831	no
HCG23	-0.010684468	-0.192034367	0.847835957	0.917541831	no
PRG2	0.010680924	0.191970656	0.847885821	0.917541831	no
UCKL1	0.010680137	0.191956507	0.847896894	0.917541831	no
SLC4A1AP	-0.010675521	-0.191873543	0.847961828	0.917541831	no
PARM1	0.010673536	0.191837856	0.84798976	0.917541831	no
OR1L4	-0.010673311	-0.191833819	0.84799292	0.917541831	no
TMEM232	0.010654402	0.191493914	0.848258968	0.917783491	no
CXorf30	0.010651306	0.191438275	0.848302519	0.917784405	no
OOEP	0.010639473	0.191225574	0.848469014	0.917887332	no
CHCHD5	0.010638474	0.191207613	0.848483073	0.917887332	no
DCAF15	0.010630863	0.191070799	0.84859017	0.917956981	no
CLC	-0.010625252	-0.190969951	0.848669115	0.917996172	no
UIMC1	0.010608046	0.19066066	0.848911241	0.918211861	no
SNORA9	0.010592514	0.190381464	0.849129819	0.91840206	no
PSMG3	-0.01056579	-0.189901094	0.84950592	0.918762605	no
BTNL8	0.010562231	0.189837117	0.849556013	0.918770545	no
LTBP4	0.010551624	0.189646459	0.849705299	0.918885754	no
KNSTRN	-0.010545892	-0.189543431	0.849785972	0.918926755	no
DEFB108B	-0.010534606	-0.189340567	0.849944824	0.919028548	no
TMEM163	0.010527926	0.18922049	0.850038853	0.919028548	no
SLC22A1	-0.010527884	-0.189219738	0.850039442	0.919028548	no
TMEM133	0.010524319	0.189155653	0.850089625	0.919028548	no
PMS2CL	-0.010524014	-0.189150164	0.850093923	0.919028548	no
GNGT1	-0.010507014	-0.188844594	0.850333219	0.919241006	no
TMUB2	0.010497876	0.188680336	0.850461858	0.919333825	no
LOC644838	-0.010484057	-0.188431939	0.850656397	0.919413034	no
CCDC22	0.010483906	0.188429223	0.850658524	0.919413034	no
BOLA2	0.010483554	0.188422899	0.850663476	0.919413034	no
VWA3A	0.010474394	0.188258236	0.850792442	0.919506179	no
PTPRVP	0.010468378	0.1881501	0.850877138	0.919537873	no
ARTN	-0.010466233	-0.188111537	0.850907341	0.919537873	no
SPTLC1	-0.010454307	-0.187897167	0.851075248	0.919673077	no
CALCOCO2	0.010449838	0.187816837	0.851138169	0.919694826	no

CDK18	0.010443661	0.187705813	0.851225134	0.919742551	no
PCYT1B	-0.010427998	-0.187424263	0.851445678	0.919934597	no
DCAF8L1	-0.010421011	-0.187298676	0.851544058	0.919994638	no
LOC646278	0.0104165	0.187217588	0.85160758	0.920017016	no
FUBP1	0.010408585	0.187075314	0.851719035	0.920030215	no
MIR454	-0.010405872	-0.187026552	0.851757235	0.920030215	no
EMILIN3	0.010404852	0.187008219	0.851771598	0.920030215	no
UPF1	-0.010403472	-0.186983404	0.851791037	0.920030215	no
SLC22A15	0.010394336	0.186819178	0.851919696	0.920083604	no
MRPL55	-0.010393881	-0.186811014	0.851926092	0.920083604	no
C8orf59	-0.010385647	-0.186663003	0.85204205	0.920126257	no
LOC100129345	-0.010384996	-0.186651307	0.852051214	0.920126257	no
SNORA84	-0.010378565	-0.186535692	0.852141794	0.920171748	no
RPUSD1	0.010375925	0.186488239	0.852178972	0.920171748	no
RAB3GAP2	0.010367718	0.186340732	0.852294544	0.920179818	no
GKN2	-0.01036493	-0.186290607	0.852333816	0.920179818	no
MFAP3L	0.010363173	0.186259029	0.852358559	0.920179818	no
A2ML1	-0.010362574	-0.186248257	0.852366999	0.920179818	no
LOC339568	-0.010359587	-0.18619457	0.852409063	0.920179818	no
APH1A	-0.010357153	-0.186150813	0.852443348	0.920179818	no
LOC100505964	0.010343691	0.18590884	0.852632946	0.920338254	no
FASTKD3	-0.010338947	-0.185823555	0.852699773	0.920356682	no
RTP3	-0.010336398	-0.185777746	0.852735668	0.920356682	no
POLQ	-0.010308208	-0.185271019	0.853132751	0.920718593	no
TEPP	-0.010306509	-0.185240493	0.853156673	0.920718593	no
SLC27A2	0.010299922	0.18512208	0.85324947	0.920756223	no
RBMS3	0.010297707	0.185082266	0.853280672	0.920756223	no
OR52E8	0.010294561	0.185025717	0.853324989	0.920756223	no
XPC	0.010291868	0.18497732	0.853362917	0.920756223	no
ZP4	0.010288361	0.184914271	0.853412329	0.92076331	no
SHOX2	0.010283341	0.184824044	0.853483041	0.920793376	no
FAM24A	-0.010273376	-0.184644925	0.853623424	0.920859987	no
C6orf89	-0.010272875	-0.184635922	0.85363048	0.920859987	no
ABCA8	0.010266367	0.184518932	0.853722173	0.920912674	no
OR10H4	-0.010260323	-0.184410297	0.853807318	0.920925934	no
PFKFB2	-0.010259411	-0.184393902	0.853820168	0.920925934	no
ACRC	-0.010248881	-0.184204614	0.853968533	0.921009697	no
PSMA8	-0.01024638	-0.184159672	0.854003759	0.921009697	no
CERS1	0.010244774	0.184130794	0.854026394	0.921009697	no
DYNAP	-0.010239912	-0.1840434	0.854094896	0.921014616	no
VIL1	0.010238367	0.184015625	0.854116667	0.921014616	no
TRIM45	0.01021963	0.183678837	0.854380664	0.921226694	no
MIR508	0.010218324	0.183655354	0.854399072	0.921226694	no
TRIM65	0.010213455	0.183567834	0.854467679	0.921230931	no
LRCH3	0.010211961	0.183540976	0.854488734	0.921230931	no
OR6T1	-0.010204086	-0.18339943	0.854599694	0.921265698	no
ACTL7A	-0.010203588	-0.183390471	0.854606717	0.921265698	no
ALPI	0.010195858	0.183251535	0.854715635	0.921268093	no
SERPINB13	0.010194726	0.183231186	0.854731588	0.921268093	no
PRPH	0.010194304	0.183223592	0.854737541	0.921268093	no
C9orf169	0.010190347	0.183152471	0.854793297	0.92128119	no

POFUT2	0.010187357	0.183098729	0.854835429	0.92128119	no
DEFB128	0.010172006	0.1828228	0.855051756	0.921438735	no
OR5AN1	0.010169217	0.182772659	0.855091067	0.921438735	no
C2orf70	-0.010164925	-0.182695519	0.855151547	0.921438735	no
ARHGEF39	-0.010164814	-0.182693521	0.855153114	0.921438735	no
ENPP4	0.01015338	0.182487985	0.855314264	0.921566171	no
TRIM74	-0.010147985	-0.182391019	0.855390292	0.921601883	no
PAICS	0.010138248	0.182215997	0.855527525	0.921693531	no
CAPN14	-0.010133359	-0.182128113	0.855596436	0.921693531	no
SPATA3	-0.010129549	-0.182059633	0.855650132	0.921693531	no
OR5M11	0.010127753	0.182027338	0.855675455	0.921693531	no
HIST1H2AK	-0.010126735	-0.182009054	0.855689792	0.921693531	no
WFDC2	-0.010118617	-0.181863132	0.855804215	0.921748591	no
C9	-0.010117023	-0.181834472	0.85582669	0.921748591	no
KRTAP6-2	-0.010110029	-0.181708754	0.855925273	0.921808572	no
TMEM164	0.01009085	0.181364023	0.85619561	0.92203015	no
ADRA1D	-0.010089346	-0.181336976	0.856216821	0.92203015	no
TMEM236	0.010084975	0.181258411	0.856278435	0.922050298	no
OR2A4	-0.010079208	-0.181154757	0.856359725	0.92209163	no
ZNF335	0.010028822	0.180249062	0.857070077	0.922729676	no
UPK1A-AS1	0.010025662	0.180192266	0.857114627	0.922729676	no
C3orf65	0.010025348	0.180186614	0.85711906	0.922729676	no
LRRC3	-0.010017491	-0.180045385	0.857229841	0.922729676	no
LOC340073	-0.010014759	-0.179996288	0.857268353	0.922729676	no
BGLAP	0.010011103	0.179930569	0.857319904	0.922729676	no
TRIM10	-0.010010549	-0.179920611	0.857327716	0.922729676	no
APOA2	0.010008796	0.179889105	0.85735243	0.922729676	no
CAMKMT	0.010008323	0.179880595	0.857359106	0.922729676	no
PRSS30P	-0.010003907	-0.179801219	0.857421371	0.922729676	no
NHLRC2	-0.010003679	-0.17979713	0.857424579	0.922729676	no
LOC646329	-0.009987008	-0.179497467	0.857659654	0.922926918	no
KLHL33	0.009976082	0.179301065	0.857813732	0.922926918	no
KRTAP5-3	-0.009975172	-0.179284713	0.857826561	0.922926918	no
MIOX	-0.009974028	-0.179264144	0.857842697	0.922926918	no
TRIM64	-0.009973299	-0.179251045	0.857852974	0.922926918	no
RNF138	-0.009970243	-0.179196111	0.857896071	0.922926918	no
LINC00675	-0.009969363	-0.1791803	0.857908475	0.922926918	no
PKN1	-0.009960746	-0.179025417	0.858029988	0.923001091	no
MKI67	-0.009958383	-0.178982932	0.85806332	0.923001091	no
SLC5A4	0.009942062	0.178689563	0.858293492	0.923202474	no
GATC	0.009931069	0.178491961	0.858448533	0.923320821	no
LOC10023315€	-0.009927949	-0.178435885	0.858492532	0.923320821	no
IPCEF1	-0.009925122	-0.178385065	0.858532408	0.923320821	no
FUT10	-0.009920533	-0.178302591	0.858597121	0.923344211	no
SPIC	-0.009909871	-0.178110943	0.858747502	0.923459723	no
COA1	0.009896503	0.177870641	0.858936066	0.923580439	no
PTCH2	0.009895819	0.177858352	0.85894571	0.923580439	no
PADI1	-0.009889681	-0.177748033	0.85903228	0.923623522	no
PNLDC1	-0.009882589	-0.177620541	0.859132328	0.923623522	no
ENDOV	-0.009881305	-0.177597472	0.859150432	0.923623522	no
PHGR1	-0.009880791	-0.177588228	0.859157686	0.923623522	no

SULT1C2P1	-0.009871214	-0.17741608	0.859292782	0.923722548	no
UPK3B	0.009866654	0.177334113	0.859357109	0.923745492	no
PRNT	0.009861022	0.177232885	0.859436552	0.923784682	no
NCAPD2	0.009856598	0.177153356	0.859498968	0.923805567	no
MAGOH2	-0.009849847	-0.177032013	0.859594201	0.9238161	no
OR10A4	-0.009849809	-0.177031326	0.85959474	0.9238161	no
C2orf78	-0.009830047	-0.176676121	0.859873526	0.924032425	no
WDR52-AS1	0.0098291	0.176659101	0.859886884	0.924032425	no
OR2B2	-0.009824336	-0.176573465	0.859954099	0.924032425	no
ATG9B	0.00982335	0.17655574	0.859968012	0.924032425	no
NAT14	-0.009813155	-0.176372489	0.860111848	0.924140772	no
PHLDB2	0.009809588	0.176308363	0.860162182	0.924148651	no
INTU	0.009802835	0.176186987	0.860257456	0.92418773	no
NXPE3	-0.009797955	-0.176099265	0.860326314	0.92418773	no
GJA1	-0.009797769	-0.176095921	0.860328938	0.92418773	no
GORAB	-0.009792234	-0.175996428	0.860407037	0.92418773	no
TCAM1P	-0.009791771	-0.175988102	0.860413573	0.92418773	no
KLK13	0.009757089	0.175364706	0.860902953	0.924667168	no
CDPF1	0.009737752	0.175017124	0.861175836	0.924754746	no
OR2AG1	-0.009736345	-0.174991835	0.861195691	0.924754746	no
DPH3P1	-0.009735095	-0.17496937	0.861213328	0.924754746	no
CCDC59	-0.009735047	-0.174968518	0.861213997	0.924754746	no
FAM133CP	0.009734702	0.174962312	0.861218869	0.924754746	no
PRSS45	0.009733016	0.174931996	0.861242671	0.924754746	no
MAG	0.009720054	0.174699021	0.861425589	0.924904943	no
TTTY10	-0.009705325	-0.174434266	0.861633468	0.925036474	no
OR1A2	-0.009705275	-0.174433361	0.861634179	0.925036474	no
GRPEL2	-0.009701884	-0.17437242	0.86168203	0.925041636	no
FMO2	0.009692384	0.174201649	0.861816122	0.925139374	no
CCDC108	0.009687623	0.174116068	0.861883322	0.925140968	no
MSTO1	0.009686179	0.174090115	0.861903702	0.925140968	no
TUBB4B	-0.009674833	-0.173886186	0.862063838	0.92526664	no
WFDC10B	-0.009664507	-0.173700564	0.862209604	0.925338277	no
CDH26	-0.009662748	-0.17366895	0.862234429	0.925338277	no
BTNL2	-0.009660954	-0.173636704	0.862259752	0.925338277	no
SMYD2	0.009651123	0.173459992	0.862398527	0.925440992	no
LOC392232	-0.00961533	-0.172816626	0.862903807	0.925936974	no
MIR181A1	0.009605414	0.172638388	0.863043801	0.926040957	no
ITM2A	-0.009599298	-0.172528451	0.86313015	0.926087372	no
ME3	-0.009591927	-0.172395961	0.863234216	0.92615279	no
OR8H2	0.009565739	0.171925244	0.863603966	0.926435237	no
KLHL12	-0.009562609	-0.171868989	0.863648157	0.926435237	no
MLLT10P1	0.009562285	0.171863165	0.863652732	0.926435237	no
PLAC4	0.009559715	0.171816972	0.863689018	0.926435237	no
GPHB5	-0.009558016	-0.171786425	0.863713015	0.926435237	no
LOC440704	-0.009551786	-0.171674455	0.863800974	0.926473043	no
ALDH1A2	-0.009549413	-0.171631802	0.863834481	0.926473043	no
OR5AC2	0.009540258	0.171467243	0.863963756	0.926565451	no
KRT28	-0.009536307	-0.171396226	0.864019547	0.926579047	no
VPS8	0.009525456	0.171201176	0.864172782	0.926697134	no
RCVRN	-0.009513478	-0.170985872	0.864341936	0.926751343	no

C2orf53	-0.009513064	-0.170978426	0.864347786	0.926751343	no
ERVFRD-1	-0.009512715	-0.170972168	0.864352702	0.926751343	no
LOC100129361	-0.009501681	-0.170773831	0.864508531	0.92687218	no
GRB14	0.009493186	0.170621135	0.864628505	0.926954565	no
STL	-0.009487521	-0.170519301	0.864708517	0.926994101	no
GPR110	-0.009460334	-0.170030629	0.865092496	0.927294555	no
OXSR1	0.009457882	0.169986554	0.86512713	0.927294555	no
RMI2	-0.009457587	-0.169981257	0.865131292	0.927294555	no
OR51D1	0.009455457	0.169942973	0.865161375	0.927294555	no
ADAM7	0.00944516	0.169757888	0.865306818	0.927362943	no
LRRN3	-0.00944483	-0.169751951	0.865311483	0.927362943	no
TUBB8	-0.009432825	-0.169536161	0.86548106	0.927498427	no
LMAN2L	-0.009419987	-0.169305402	0.865662407	0.927646512	no
SYNE4	0.009416364	0.169240284	0.865713583	0.927655097	no
TTLL6	0.009402874	0.168997813	0.865904144	0.927789575	no
XGPY2	0.009399152	0.168930917	0.86595672	0.927789575	no
PIWIL3	0.009397782	0.168906276	0.865976086	0.927789575	no
MIR124-2	-0.00939285	-0.168817627	0.86604576	0.927789575	no
TPI1P3	-0.0093922	-0.16880595	0.866054938	0.927789575	no
TPSG1	-0.009383791	-0.168654804	0.866173733	0.927870586	no
PLK2	0.009370952	0.168424021	0.866355126	0.927920059	no
EFCAB6-AS1	-0.009370129	-0.168409229	0.866366752	0.927920059	no
KRTAP3-3	-0.009369965	-0.168406285	0.866369066	0.927920059	no
TBC1D3P2	0.009366295	0.168340317	0.866420918	0.927920059	no
RGSL1	-0.009365241	-0.168321381	0.866435802	0.927920059	no
USP17L2	-0.009359999	-0.168227146	0.866509873	0.927953144	no
MAP1LC3B	-0.009344317	-0.167945277	0.866731437	0.928144168	no
OR1I1	-0.009340814	-0.16788231	0.866780933	0.928150924	no
TSKU	0.009335382	0.167784677	0.86685768	0.928186857	no
UCP3	-0.009323899	-0.167578277	0.867019933	0.928310393	no
RNASE11	-0.009321103	-0.167528016	0.867059444	0.928310393	no
MTOR-AS1	-0.009305935	-0.16725537	0.867273782	0.928493616	no
OR13F1	0.009299476	0.167139279	0.867365049	0.92854507	no
EXO5	-0.009286957	-0.166914253	0.867541962	0.9286882	no
EDDM3B	0.009279543	0.166780991	0.867646735	0.928724093	no
C12orf50	-0.009278468	-0.166761676	0.86766192	0.928724093	no
OR4C3	0.009268251	0.166578015	0.867806322	0.928832395	no
LOC100130894	-0.009261197	-0.166451232	0.867906005	0.928892827	no
WWC2	0.009257113	0.166377823	0.867963725	0.928908342	no
SNX4	0.009245902	0.166176308	0.868122174	0.928997246	no
METTL6	-0.009242924	-0.16612278	0.868164263	0.928997246	no
OR2AG2	-0.00924206	-0.166107246	0.868176478	0.928997246	no
KCNC3	0.009238826	0.166049114	0.868222187	0.928999902	no
TRAPPC8	-0.009222145	-0.165749297	0.868457946	0.929180445	no
C9orf40	-0.009216466	-0.165647221	0.868538214	0.929180445	no
TRH	0.009206482	0.16546775	0.868679347	0.929180445	no
POLR2A	0.009199383	0.165340152	0.868779691	0.929180445	no
SGK1	-0.009197383	-0.165304207	0.868807958	0.929180445	no
GSTA1	0.009191465	0.165197824	0.86889162	0.929180445	no
KANSL3	0.009187854	0.165132919	0.868942663	0.929180445	no
EFTUD2	0.009186638	0.165111075	0.868959842	0.929180445	no

RBM15B	-0.009185286	-0.165086777	0.868978951	0.929180445	no
MYLK2	-0.009185051	-0.165082538	0.868982285	0.929180445	no
NCOR1P1	0.009183272	0.165050574	0.869007423	0.929180445	no
GPRC5D	0.009182431	0.165035451	0.869019317	0.929180445	no
ADIPOQ-AS1	0.009181278	0.165014719	0.869035621	0.929180445	no
RNF170	-0.00917894	-0.164972698	0.869068669	0.929180445	no
TSSC4	-0.009178783	-0.164969885	0.869070881	0.929180445	no
OR9G4	-0.009177948	-0.164954868	0.869082691	0.929180445	no
ECEL1P2	-0.009171868	-0.164845589	0.869168636	0.929226104	no
ERVMER34-1	-0.009168067	-0.164777267	0.86922237	0.929237325	no
MCC	-0.009159145	-0.164616898	0.869348499	0.929258545	no
CDKN2C	0.009159002	0.164614326	0.869350523	0.929258545	no
HIST1H2BF	0.009153432	0.164514203	0.86942927	0.929258545	no
OR6C68	-0.009151941	-0.16448741	0.869450343	0.929258545	no
LOC730159	-0.00915063	-0.164463851	0.869468873	0.929258545	no
ZNF847P	-0.009144806	-0.164359169	0.869551209	0.929258545	no
IL21R-AS1	-0.009144493	-0.164353545	0.869555632	0.929258545	no
PIGV	-0.009141299	-0.164296119	0.8696008	0.929258545	no
NPIP	-0.009139136	-0.164257248	0.869631374	0.929258545	no
KRTAP5-10	0.009134803	0.164179371	0.869692629	0.929277795	no
KIF1C	0.00913141	0.164118374	0.869740607	0.929282857	no
RAB30	-0.009127683	-0.164051379	0.869793304	0.92929296	no
MROH2B	0.009122717	0.163962119	0.869863514	0.929298544	no
SPATS1	-0.009120709	-0.163926037	0.869891896	0.929298544	no
P2RY14	-0.009118138	-0.163879815	0.869928254	0.929298544	no
FLJ16171	-0.009111246	-0.163755933	0.870025701	0.929356446	no
OR8U1	-0.009107431	-0.163687369	0.870079635	0.929367864	no
ZNF266	0.009102028	0.163590255	0.870156028	0.92940327	no
IL5	0.009096175	0.163485039	0.870238795	0.92944548	no
RPS4Y2	0.009090619	0.163385175	0.870317354	0.929483191	no
PELI1	0.009079474	0.163184851	0.870474945	0.929544755	no
SNORA27	-0.009076096	-0.163124143	0.870522703	0.929544755	no
LINC00353	0.009074876	0.163102218	0.870539951	0.929544755	no
TMEM192	0.009074307	0.163091975	0.87054801	0.929544755	no
LOC340357	0.009056777	0.162776883	0.8707959	0.929705554	no
FTSJ1	0.009053287	0.162714166	0.870845242	0.929705554	no
LOC100630923	0.009051435	0.162680878	0.870871431	0.929705554	no
HIST1H2BM	0.00905142	0.162680608	0.870871644	0.929705554	no
RHAG	0.009042777	0.162525251	0.870993873	0.929776521	no
TAMM41	0.009040601	0.162486136	0.871024648	0.929776521	no
SPTY2D1-AS1	0.009036565	0.162413599	0.871081719	0.929791259	no
CCDC173	-0.009032966	-0.162348895	0.871132628	0.929799419	no
LOC100144604	0.009028875	0.162275369	0.871190478	0.929814986	no
NPR3	-0.009009479	-0.161926742	0.871464788	0.930061566	no
NDUFB5	-0.009000054	-0.161757327	0.871598094	0.930157644	no
CDYL2	-0.008994433	-0.1616563	0.87167759	0.930196291	no
PHF19	-0.008986829	-0.161519624	0.87178514	0.930264869	no
CACNB3	-0.008981292	-0.161420096	0.871863459	0.93030225	no
MIR3976	-0.008960704	-0.161050042	0.87215467	0.930563176	no
OR4X2	-0.008957882	-0.160999309	0.872194595	0.930563176	no
OR5K3	-0.008942727	-0.160726913	0.872408967	0.930745687	no

PPP1CC	-0.008939318	-0.160665631	0.872457197	0.930750937	no
FOXF2	0.008916764	0.160260251	0.872776246	0.931010699	no
DAG1	-0.008915981	-0.160246166	0.872787332	0.931010699	no
C14orf64	-0.00891097	-0.160156096	0.872858224	0.931040107	no
PKDCC	0.008899	0.159940945	0.873027567	0.931174522	no
ACTBL2	0.008891922	0.159813724	0.873127704	0.931235111	no
VPS4B	-0.008878742	-0.159576827	0.873314174	0.931342995	no
EPPK1	0.008876918	0.159544032	0.873339989	0.931342995	no
EMC4	-0.008875583	-0.159520051	0.873358865	0.931342995	no
LOC388796	-0.008849997	-0.159060145	0.873720897	0.93161368	no
BAIAP2L1	0.008849689	0.159054607	0.873725256	0.93161368	no
GALNT11	-0.008848453	-0.159032391	0.873742745	0.93161368	no
IL37	0.008836246	0.158812989	0.873915465	0.931652964	no
CDC42EP2	0.008833515	0.158763908	0.873954104	0.931652964	no
CRLS1	0.00883285	0.158751949	0.873963519	0.931652964	no
PCSK6	0.008832605	0.158747545	0.873966987	0.931652964	no
USP24	0.008828716	0.158677636	0.874022023	0.931652964	no
DALRD3	0.008827467	0.15865519	0.874039694	0.931652964	no
SCGB1C1	0.008818019	0.158485367	0.874173392	0.931664303	no
OXNAD1	0.008815832	0.158446058	0.874204339	0.931664303	no
CSNK2A3	-0.008815239	-0.158435402	0.874212728	0.931664303	no
ZNF598	0.008814461	0.158421419	0.874223737	0.931664303	no
MAD2L2	-0.008808128	-0.15830758	0.874313363	0.931668725	no
LOC100507334	0.008807403	0.158294553	0.874323619	0.931668725	no
LDHAL6B	0.008799795	0.158157804	0.874431284	0.931668725	no
LOC100498859	0.008799057	0.158144547	0.874441721	0.931668725	no
KRTAP13-1	-0.008796773	-0.158103486	0.87447405	0.931668725	no
SLC22A24	-0.008795787	-0.158085773	0.874487996	0.931668725	no
SLC28A3	0.008787646	0.15793943	0.874603219	0.931745292	no
EHD3	0.008764488	0.157523185	0.874930962	0.932048246	no
LINC00052	0.008747186	0.157212195	0.875175844	0.932185422	no
CPO	-0.008746394	-0.157197965	0.875187049	0.932185422	no
MGC16142	-0.008743074	-0.157138282	0.875234047	0.932185422	no
NPS	0.008740637	0.157094476	0.875268542	0.932185422	no
DPT	-0.008740067	-0.157084231	0.87527661	0.932185422	no
PNLIP	-0.008733864	-0.156972745	0.875364402	0.932229372	no
CACNA1C	0.008729025	0.156885771	0.875432892	0.932229372	no
LOC642929	-0.008727957	-0.156866573	0.87544801	0.932229372	no
MPHOSPH9	-0.0087245	-0.156804439	0.87549694	0.932235284	no
IL34	-0.00871381	-0.156612283	0.875648264	0.93235022	no
MFAP2	0.008707935	0.156506686	0.875731425	0.932392572	no
HOXA2	0.008699403	0.15635333	0.875852199	0.932448168	no
SCYL3	0.008698116	0.156330196	0.875870418	0.932448168	no
SCARNA27	0.00868963	0.156177678	0.875990536	0.93252985	no
RORC	-0.008683155	-0.156061283	0.876082207	0.932581242	no
MT1F	0.008676806	0.15594717	0.876172082	0.932630719	no
SLC35A1	-0.008672985	-0.155878495	0.876226171	0.932642099	no
AVPR1A	0.00866378	0.155713032	0.876356492	0.932734617	no
LOC100506795	0.008637254	0.155236262	0.876732025	0.933088098	no
NBPF1	0.008631798	0.155138191	0.876809275	0.933124103	no
SP7	0.008626796	0.155048278	0.8768801	0.933153268	no

C11orf31	-0.008622118	-0.154964193	0.876946335	0.933177545	no
CDRT15P2	-0.008618126	-0.154892445	0.877002854	0.93319148	no
CLLU1	-0.008606855	-0.154689851	0.877162446	0.933297976	no
CHST3	0.008601794	0.154598889	0.877234102	0.933297976	no
TGFBRAP1	-0.008599144	-0.154551256	0.877271626	0.933297976	no
NIPAL3	-0.008596356	-0.154501142	0.877311105	0.933297976	no
MKLN1	-0.008590642	-0.154398444	0.877392009	0.933297976	no
UQCC	0.008590225	0.154390938	0.877397922	0.933297976	no
KRT78	-0.008586271	-0.154319874	0.877453906	0.933297976	no
DEFB127	-0.008584708	-0.154291782	0.877476036	0.933297976	no
FAM35A	-0.008580643	-0.154218714	0.877533601	0.933297976	no
UBE4A	-0.008580388	-0.154214131	0.877537211	0.933297976	no
LRRC2-AS1	-0.008575617	-0.154128371	0.877604774	0.933323644	no
OR6C3	0.008570772	0.154041295	0.877673375	0.933350413	no
HOXD12	-0.008539723	-0.153483218	0.878113067	0.933758182	no
TMEM134	-0.008537559	-0.153444319	0.878143716	0.933758182	no
PRAMEF5	0.008531664	0.153338359	0.878227204	0.933800754	no
COPA	0.008524866	0.153216176	0.878323475	0.933851326	no
AP1G1	-0.008522169	-0.153167699	0.878361672	0.933851326	no
OR5K4	-0.00851778	-0.153088811	0.878423831	0.933871212	no
GPR112	-0.008505899	-0.152875263	0.8785921	0.934003899	no
OR6K6	-0.008498669	-0.152745301	0.878694508	0.934066561	no
GLG1	0.008486037	0.152518247	0.878873428	0.934210547	no
OR10A3	0.00848283	0.152460613	0.878918845	0.934212616	no
FER1L6-AS1	-0.008476875	-0.152353567	0.879003201	0.934256072	no
TMEM55A	-0.008470999	-0.152247963	0.879086421	0.934271615	no
ANKRD13A	-0.008469704	-0.15222468	0.879104769	0.934271615	no
TCEB3CL2	0.008462593	0.152096868	0.879205493	0.934312665	no
OR8K1	0.008459081	0.152033739	0.879255243	0.934312665	no
MOGAT2	-0.008455933	-0.15197715	0.87929984	0.934312665	no
PPP2R2A	-0.008452198	-0.151910023	0.879352743	0.934312665	no
KLRG2	-0.008451631	-0.151899839	0.879360768	0.934312665	no
ARHGAP20	0.008439183	0.15167609	0.879537107	0.934420846	no
NPHS2	0.008438305	0.151660307	0.879549546	0.934420846	no
LOC100132215	-0.00842764	-0.151468622	0.879700619	0.934484578	no
PIBF1	0.008425221	0.151425139	0.87973489	0.934484578	no
TTC18	-0.008421386	-0.151356209	0.879789218	0.934484578	no
CWC22	-0.008419939	-0.151330197	0.879809719	0.934484578	no
CNBD1	0.008418723	0.151308337	0.879826949	0.934484578	no
SNORA10	0.008405246	0.151066113	0.880017865	0.934641163	no
OR56B1	0.008397876	0.150933632	0.880122287	0.934667397	no
OR9K2	-0.008397363	-0.15092442	0.880129548	0.934667397	no
CMC2	-0.008380452	-0.150620456	0.880369141	0.934755608	no
METTL7A	-0.008377561	-0.150568483	0.880410109	0.934755608	no
CHP1	0.008375101	0.150524279	0.880444953	0.934755608	no
FOXI3	0.008372984	0.15048623	0.880474945	0.934755608	no
KDM5C	0.008372099	0.150470309	0.880487495	0.934755608	no
FSCN3	-0.008371961	-0.150467832	0.880489448	0.934755608	no
PPP1R16A	0.008370011	0.150432779	0.880517079	0.934755608	no
DEFB123	-0.008353287	-0.150132183	0.880754035	0.934900799	no
CYP4F3	0.008352058	0.150110101	0.880771442	0.934900799	no

SLC25A52	0.008351147	0.150093727	0.88078435	0.934900799	no
NXF5	-0.008337524	-0.149848864	0.880977382	0.93500829	no
GRAMD2	-0.008336999	-0.149839435	0.880984815	0.93500829	no
PRSS33	-0.008334789	-0.149799699	0.88101614	0.93500829	no
C1QTNF3	0.008326436	0.149649564	0.8811345	0.935079726	no
HULC	0.008323897	0.149603936	0.881170472	0.935079726	no
SNORA33	-0.008308356	-0.149324588	0.881390705	0.935267251	no
CYP2A13	-0.008305012	-0.149264497	0.881438081	0.935271344	no
TTI1	-0.008286257	-0.148927379	0.881703874	0.935419578	no
ABTB1	0.008285031	0.148905343	0.881721249	0.935419578	no
PRICKLE1	-0.008283716	-0.148881724	0.881739871	0.935419578	no
NEK9	0.008282869	0.148866484	0.881751887	0.935419578	no
GIP	-0.008277082	-0.148762477	0.881833893	0.935460397	no
LOC285740	0.008270575	0.148645511	0.881926117	0.935512053	no
AGBL1	0.008261566	0.148483594	0.882053788	0.935601301	no
GDF7	0.008244599	0.148178624	0.882294262	0.935810186	no
SF3B1	0.008239746	0.148091389	0.88236305	0.935836959	no
ZBTB34	0.008223497	0.147799332	0.882593356	0.936008483	no
MAF1	-0.00822219	-0.147775842	0.88261188	0.936008483	no
LOC100270679	-0.008216685	-0.147676891	0.882689911	0.936026096	no
GPR113	-0.008214872	-0.147644319	0.882715597	0.936026096	no
LOC401324	0.008199129	0.147361341	0.882938758	0.936216541	no
AURKB	-0.008181309	-0.147041053	0.883191353	0.936376306	no
DDX3Y	0.008179554	0.147009499	0.883216239	0.936376306	no
NAPA-AS1	-0.008179278	-0.147004549	0.883220143	0.936376306	no
GCNT3	-0.008175249	-0.146932124	0.883277263	0.936390669	no
C17orf98	-0.00815773	-0.146617235	0.883525616	0.936607754	no
OR2T6	-0.008147137	-0.146426848	0.883675779	0.936687939	no
AVPR2	-0.008146245	-0.146410814	0.883688426	0.936687939	no
CSNK1A1P1	0.008139359	0.146287049	0.883786046	0.936725809	no
PCNAP1	0.008137576	0.146254996	0.883811327	0.936725809	no
SLC39A1	-0.00812259	-0.145985633	0.884023794	0.936904792	no
TMEM203	0.008107461	0.145713714	0.884238286	0.937085902	no
BDH2	0.008101718	0.145610485	0.884319716	0.937123801	no
OR2A2	0.008098072	0.145544943	0.884371418	0.937123801	no
DBF4	-0.008092911	-0.145452177	0.884444596	0.937123801	no
HOXA-AS3	-0.008092637	-0.145447267	0.884448469	0.937123801	no
C10orf129	-0.008089264	-0.145386633	0.8844963	0.937128278	no
LINC00277	0.00808116	0.145240981	0.884611201	0.937203812	no
RPS9	0.008075196	0.145133783	0.884695767	0.937247202	no
UNC5C	-0.008068343	-0.145010604	0.884792943	0.937296399	no
GRASP	-0.00806577	-0.14496435	0.884829433	0.937296399	no
DEFB132	-0.008043749	-0.144568555	0.885141688	0.937552521	no
DMBX1	-0.008042566	-0.144547286	0.885158468	0.937552521	no
ZBTB10	-0.008034018	-0.144393636	0.885279693	0.937634709	no
TRHR	0.00800245	0.143826252	0.885727362	0.938062623	no
PCDHGA5	-0.007982676	-0.14347083	0.886007811	0.938313402	no
FAM110A	-0.00797954	-0.143414466	0.886052287	0.938314265	no
CACNA1C-AS4	-0.007973645	-0.143308509	0.886135897	0.938356568	no
LGR4	0.007969847	0.143240233	0.886189774	0.938367384	no
DNAH8	-0.007961995	-0.143099112	0.886301134	0.938439063	no

TEX12	-0.00795666	-0.143003213	0.88637681	0.938472954	no
LOC100631378	-0.007949739	-0.142878816	0.886474977	0.938530653	no
TBX5-AS1	-0.007943177	-0.142760878	0.886568048	0.938547055	no
FAM135A	0.007942489	0.142748506	0.886577812	0.938547055	no
STARD13	0.007936709	0.142644613	0.886659801	0.938587616	no
SARNP	0.007930134	0.142526441	0.886753059	0.938640102	no
IMPG2	-0.007921467	-0.142370656	0.886876004	0.938724006	no
ABCD3	0.00791572	0.142267373	0.886957516	0.938764047	no
RHEBL1	-0.007907564	-0.142120763	0.887073224	0.938808344	no
OR56A3	-0.007906611	-0.142103643	0.887086736	0.938808344	no
SLC25A34	0.007896857	0.141928326	0.887225103	0.938908543	no
NKD2	-0.007869509	-0.141436772	0.887613078	0.939272867	no
OR5B17	0.007862752	0.141315317	0.887708944	0.939325565	no
FLJ45079	0.007856066	0.141195158	0.887803789	0.939325565	no
PNPLA7	-0.007855524	-0.141185412	0.887811481	0.939325565	no
ECHS1	-0.007853675	-0.141152185	0.887837709	0.939325565	no
LOC339166	0.007834591	0.14080916	0.888108479	0.939565784	no
ORAOV1	-0.007826241	-0.140659075	0.888226954	0.939618442	no
GRM8	-0.00782492	-0.140635332	0.888245697	0.939618442	no
OR51G2	-0.007818828	-0.140525837	0.888332132	0.939663624	no
OR8J1	-0.007804733	-0.140272502	0.888532122	0.939828911	no
IQCC	-0.007794004	-0.140079654	0.888684365	0.939943683	no
RMST	-0.007775629	-0.139749396	0.888945096	0.940173182	no
C1QTNF9	0.00777041	0.139655587	0.889019158	0.940205243	no
LOC646862	0.007753479	0.139351272	0.889259423	0.940413062	no
LARP7	-0.007748007	-0.139252922	0.889337074	0.940448903	no
MEP1A	0.007720552	0.138759437	0.889726719	0.940814649	no
MCF2L-AS1	0.007698366	0.138360684	0.890041586	0.94110129	no
XRCC6BP1	0.007680952	0.138047676	0.890288757	0.941316328	no
OR5AK4P	0.007673476	0.137913315	0.890394861	0.941382199	no
CAPN10	0.007668065	0.137816059	0.890471665	0.941417087	no
KRTAP10-11	-0.00766246	-0.137715316	0.890551223	0.941454884	no
REXO1L1	-0.007649598	-0.137484131	0.890733797	0.941601576	no
OC90	0.007639604	0.137304499	0.890875663	0.941705222	no
HOXD9	0.007628801	0.137110335	0.891029008	0.941820993	no
TAF1L	-0.007621102	-0.136971958	0.891138297	0.941890186	no
PECR	0.007609906	0.136770713	0.891297243	0.941948234	no
FZD9	0.007609297	0.13675977	0.891305886	0.941948234	no
OR2J3	-0.00760652	-0.136709855	0.89134531	0.941948234	no
LOC654433	0.007604884	0.136680448	0.891368537	0.941948234	no
LIAS	0.007597287	0.136543916	0.891476375	0.941950704	no
CCL28	0.007595562	0.136512905	0.891500869	0.941950704	no
LOC392364	-0.007589552	-0.136404877	0.891586196	0.941950704	no
PTK6	0.007588316	0.136382661	0.891603743	0.941950704	no
LINC00266-1	-0.007587377	-0.136365782	0.891617075	0.941950704	no
C17orf85	0.007586195	0.136344539	0.891633855	0.941950704	no
KHDC1	0.007569986	0.136053205	0.891863975	0.942147497	no
SPECC1L	0.007565917	0.135980065	0.891921748	0.942162216	no
CTPS1	0.007543696	0.13558068	0.892237234	0.942419343	no
ZNF836	0.007542595	0.13556089	0.892252868	0.942419343	no
KCNRG	0.007527184	0.135283901	0.89247168	0.942604132	no

HAND1	-0.007520719	-0.135167696	0.89256348	0.942628474	no
C10orf113	0.007517071	0.13510213	0.892615277	0.942628474	no
SPANXC	0.007515842	0.135080031	0.892632736	0.942628474	no
OR1N2	-0.00751099	-0.134992826	0.892701628	0.942628474	no
WNT3A	-0.007510116	-0.134977122	0.892714035	0.942628474	no
PRDM4	0.007494245	0.134691865	0.892939397	0.942820113	no
C21orf62	0.0074805	0.134444809	0.893134585	0.942948634	no
TGM3	-0.007478157	-0.134402695	0.893167859	0.942948634	no
S100B	-0.007476404	-0.134371193	0.893192748	0.942948634	no
FAM83B	-0.007470832	-0.134271034	0.893271882	0.942984482	no
TGIF2-C20orf	-0.007467833	-0.134217143	0.893314461	0.942984482	no
FABP2	0.007463901	0.134146458	0.893370309	0.942997118	no
SPARC	-0.007459788	-0.134072531	0.89342872	0.943012456	no
SYCP2	-0.007449997	-0.133896555	0.893567762	0.943112895	no
NALCN-AS1	-0.007446767	-0.133838506	0.893613628	0.943114987	no
C5orf34	-0.007407814	-0.133138381	0.894166849	0.943613968	no
DCAF10	-0.007404308	-0.13307535	0.894216657	0.943613968	no
HILS1	0.007404203	0.133073471	0.894218141	0.943613968	no
MGST3	0.007390932	0.132834947	0.894406631	0.943669595	no
FAM229A	-0.007390261	-0.132822889	0.894416159	0.943669595	no
BPIFB1	-0.007389982	-0.132817859	0.894420134	0.943669595	no
ALKBH1	0.007388126	0.1327845	0.894446496	0.943669595	no
PMS2P5	0.007374249	0.132535088	0.894643596	0.94376298	no
OR51T1	-0.007372891	-0.132510672	0.894662892	0.94376298	no
FNBP1	0.007372619	0.132505787	0.894666753	0.94376298	no
FAM175A	0.007364474	0.132359393	0.894782445	0.943807454	no
RPSAP58	0.007362584	0.13232543	0.894809286	0.943807454	no
FOXM1	-0.007360375	-0.132285728	0.894840663	0.943807454	no
GAK	-0.00734301	-0.131973609	0.895087336	0.944005163	no
FANCB	0.007340995	0.131937384	0.895115966	0.944005163	no
OR2L5	-0.007332665	-0.131787671	0.89523429	0.944083622	no
CFHR2	0.007322249	0.131600455	0.895382259	0.944158164	no
DUSP15	0.007321504	0.131587066	0.895392841	0.944158164	no
MOGAT3	-0.007307836	-0.131341412	0.895587003	0.944316568	no
MORN5	0.007294102	0.131094547	0.895782128	0.944445096	no
PSPH	0.00729307	0.131075996	0.895796791	0.944445096	no
NLRP10	-0.007287046	-0.130967738	0.895882361	0.944445447	no
CEP128	-0.00728686	-0.13096438	0.895885016	0.944445447	no
ATP8B5P	-0.007283517	-0.130904295	0.89593251	0.944449187	no
MAP2K4P1	0.007270974	0.130678865	0.896110702	0.944572148	no
OR2AP1	-0.007269119	-0.130645522	0.896137059	0.944572148	no
SEMG2	0.00726435	0.130559805	0.896204816	0.944597239	no
WFDC6	0.007236067	0.130051457	0.896606669	0.944902911	no
DOCK1	0.007234088	0.130015879	0.896634794	0.944902911	no
TRIM66	0.007232614	0.129989388	0.896655736	0.944902911	no
C8G	0.007230295	0.12994771	0.896688685	0.944902911	no
SNUPN	-0.007228467	-0.129914846	0.896714665	0.944902911	no
UAP1L1	0.007208157	0.129549819	0.897003243	0.945092658	no
PATE4	0.007207491	0.129537848	0.897012707	0.945092658	no
ZNF137P	0.007206505	0.129520119	0.897026723	0.945092658	no
IKZF2	0.007203414	0.129464569	0.897070641	0.945092658	no

MKI67IP	-0.007194381	-0.129302202	0.897199008	0.945132952	no
USP26	0.007192052	0.129260339	0.897232106	0.945132952	no
NAP1L6	-0.007191438	-0.129249315	0.897240821	0.945132952	no
ZNF585B	-0.007185615	-0.129144646	0.897323575	0.945173795	no
ACSL3	0.007165782	0.128788184	0.89760541	0.945424322	no
PIGO	0.007158559	0.128658355	0.897708062	0.945486104	no
CIDEC	0.007135345	0.12824112	0.898037968	0.945787218	no
GGPS1	0.00712835	0.128115386	0.898137389	0.945845573	no
BARX2	0.007058917	0.126867434	0.899124262	0.946838471	no
OR6C4	-0.007040182	-0.126530699	0.899390577	0.947072512	no
CT47B1	0.007031455	0.126373847	0.899514631	0.947131317	no
PSG4	0.007030053	0.126348645	0.899534564	0.947131317	no
LOC153684	0.00702252	0.126213246	0.899641653	0.947197666	no
USH2A	0.007011687	0.126018547	0.899795647	0.94731339	no
LCN10	-0.007006793	-0.125930589	0.899865217	0.947340226	no
OR5M8	0.007002162	0.125847348	0.899931057	0.947363132	no
XPOT	-0.006991184	-0.125650029	0.90008713	0.94748102	no
OR8K3	-0.006980988	-0.125466771	0.900232085	0.947587194	no
CEACAM1	-0.006970664	-0.12528121	0.900378865	0.947695279	no
SYT14L	-0.006965691	-0.125191828	0.900449568	0.94772328	no
LOC100133091	-0.006957066	-0.125036811	0.900572192	0.947778546	no
BSPRY	0.006955794	0.125013946	0.900590279	0.947778546	no
MAGEA3	0.006948915	0.124890317	0.900688075	0.947835052	no
YPEL5	-0.006943978	-0.124801576	0.900758275	0.947851443	no
THOC6	0.006941616	0.12475912	0.90079186	0.947851443	no
OR5L2	-0.006929513	-0.124541581	0.90096395	0.947986108	no
SGK2	-0.006920873	-0.124386303	0.901086791	0.948068942	no
OR7C2	-0.006916232	-0.124302876	0.90115279	0.948091967	no
LOC10012926[illegible]	0.006905258	0.124105635	0.901308832	0.948168981	no
SCARB2	-0.006904878	-0.124098813	0.901314229	0.948168981	no
MESP2	-0.006899307	-0.123998675	0.901393452	0.948205907	no
MCM8	-0.006888848	-0.123810695	0.901542173	0.948283463	no
GJB4	0.006887916	0.123793941	0.901555428	0.948283463	no
GGH	-0.00687919	-0.123637118	0.901679502	0.948367551	no
IYD	0.006869711	0.123466744	0.901814299	0.94846291	no
FAM170A	-0.006863529	-0.123355629	0.901902214	0.948470973	no
SNORA80	-0.006862965	-0.123345495	0.901910232	0.948470973	no
CNPY1	0.006855199	0.123205911	0.902020672	0.948476482	no
CA5BP1	-0.006852715	-0.123161264	0.902055998	0.948476482	no
MLLT10	0.006849546	0.123104298	0.902101071	0.948476482	no
GIPR	-0.006847163	-0.123061467	0.902134961	0.948476482	no
PARL	-0.00684708	-0.123059977	0.90213614	0.948476482	no
MAT2B	0.006840881	0.122948571	0.902224288	0.948496386	no
AKR1C1	-0.006838486	-0.12290552	0.902258352	0.948496386	no
ZGPAT	0.006836438	0.122868715	0.902287474	0.948496386	no
RSBN1L-AS1	0.006829458	0.122743249	0.902386751	0.948554349	no
TTTY11	-0.006817519	-0.12252867	0.902556542	0.948640118	no
FAM72D	0.006817143	0.122521916	0.902561887	0.948640118	no
HHIPL2	0.006811888	0.12242745	0.902636636	0.948640118	no
MRPL49	0.006809164	0.122378503	0.902675368	0.948640118	no
KCNJ16	-0.006808202	-0.12236121	0.902689052	0.948640118	no

RNF223	0.006784192	0.121929672	0.903030538	0.948923028	no
GSG2	0.00678259	0.121900865	0.903053334	0.948923028	no
FAM216B	-0.006779961	-0.121853622	0.903090721	0.948923028	no
KLF14	-0.00677454	-0.12175618	0.903167832	0.948957655	no
CA5B	0.006771066	0.121693745	0.903217241	0.948963174	no
PKD2L2	-0.006762401	-0.121538016	0.903340481	0.949046259	no
LOC100287010	-0.006743316	-0.121194984	0.903611956	0.949216655	no
LOC100506134	0.006742447	0.12117937	0.903624313	0.949216655	no
PKHD1L1	0.006739014	0.121117665	0.903673148	0.949216655	no
MFGE8	-0.006738579	-0.121109837	0.903679343	0.949216655	no
PLAC9	0.006733252	0.121014107	0.903755106	0.949247501	no
SPRTN	0.006730304	0.120961113	0.903797048	0.949247501	no
SGPL1	0.006721273	0.120798796	0.903925513	0.949289663	no
TPD52L1	-0.006721271	-0.120798768	0.903925535	0.949289663	no
LINC00028	-0.006715558	-0.120696082	0.904006806	0.949312123	no
ZIC1	0.006713558	0.120660124	0.904035266	0.949312123	no
CAAP1	-0.006707583	-0.120552747	0.904120252	0.949348275	no
FJX1	-0.006704927	-0.120505001	0.904158042	0.949348275	no
LINC00696	-0.006698726	-0.120393551	0.904246254	0.949391907	no
PSMF1	0.006691953	0.120271817	0.904342606	0.949391907	no
KDM2A	0.006690189	0.120240118	0.904367696	0.949391907	no
FOXA1	-0.006689584	-0.120229244	0.904376303	0.949391907	no
ABCG1	-0.006678873	-0.120036727	0.904528683	0.94946349	no
UCHL1	-0.006675241	-0.119971451	0.904580351	0.94946349	no
CENPM	0.006672459	0.119921449	0.904619929	0.94946349	no
SAAL1	0.006672369	0.119919831	0.90462121	0.94946349	no
UNC45B	-0.006656018	-0.119625935	0.904853844	0.949541496	no
TTC40	0.006655774	0.119621548	0.904857316	0.949541496	no
NEK2	-0.006651523	-0.119545158	0.904917784	0.949541496	no
LINC00692	-0.006651465	-0.119544102	0.90491862	0.949541496	no
KLK15	-0.006650996	-0.119535671	0.904925294	0.949541496	no
PHC3	0.006646329	0.1194518	0.904991684	0.949541496	no
TRIM39	0.006645406	0.119435214	0.905004814	0.949541496	no
MAT2A	0.006625649	0.119080112	0.905285913	0.949790058	no
NUDT9	-0.006597894	-0.11858126	0.905680825	0.950116579	no
OR4C11	-0.006593677	-0.118505457	0.905740835	0.950116579	no
MYF6	-0.006592248	-0.118479785	0.905761159	0.950116579	no
IRF3	-0.006589767	-0.118435191	0.905796463	0.950116579	no
OR4A16	-0.006588241	-0.118407755	0.905818183	0.950116579	no
F12	-0.006581065	-0.118278789	0.905920284	0.950177298	no
IDUA	-0.006559235	-0.117886422	0.906230925	0.950418192	no
BUD13	-0.006558709	-0.117876974	0.906238406	0.950418192	no
SLC25A47	-0.006546519	-0.117657883	0.906411869	0.950553724	no
LINC00636	0.006541527	0.117568149	0.906482916	0.95055568	no
TRIM68	-0.006540172	-0.117543801	0.906502194	0.95055568	no
OR10W1	0.006531151	0.117381664	0.90663057	0.95064391	no
MFAP1	-0.006522028	-0.117217691	0.906760401	0.95069283	no
KIR2DL5A	-0.006521656	-0.117211	0.906765699	0.95069283	no
AGTR2	-0.006518431	-0.117153034	0.906811596	0.95069457	no
GPC5-AS1	0.006505318	0.116917354	0.906998209	0.950809373	no
SELT	0.006504519	0.116902989	0.907009584	0.950809373	no

COMMD5	0.006499642	0.116815344	0.907078983	0.950835744	no
TAS2R38	-0.006489486	-0.116632805	0.907223524	0.950940875	no
RPGRIP1L	0.006481939	0.116497163	0.907330933	0.95096908	no
RNASEK-C17or	-0.006481377	-0.116487061	0.907338932	0.95096908	no
OR5E1P	0.006465882	0.116208566	0.907559465	0.951153831	no
HBM	0.006452897	0.115975181	0.907744282	0.951301134	no
HYDIN	-0.006448077	-0.11588854	0.907812894	0.951326648	no
GPR150	-0.006441013	-0.115761577	0.907913438	0.951342097	no
RBMY1A3P	0.006440821	0.115758127	0.90791617	0.951342097	no
TSPAN15	0.006434882	0.115651378	0.908000708	0.951377112	no
ALS2CR12	-0.006431446	-0.115589628	0.90804961	0.951377112	no
TMEM215	0.006424742	0.115469144	0.908145027	0.951377112	no
OR52N4	-0.006423347	-0.115444057	0.908164896	0.951377112	no
EXOC4	-0.006422923	-0.115436437	0.90817093	0.951377112	no
RNASE7	-0.006416097	-0.115313762	0.908268084	0.951432511	no
CHMP2A	0.00641265	0.115251809	0.908317149	0.951437533	no
AHDC1	-0.006407343	-0.115156415	0.908392699	0.951470295	no
MAP1LC3A	-0.006402526	-0.115069843	0.908461263	0.951495736	no
LOC401052	-0.006379587	-0.114657554	0.908787799	0.951771529	no
OVCH2	-0.006377806	-0.114625539	0.908813156	0.951771529	no
TMPO-AS1	0.006369516	0.114476541	0.908931168	0.951848735	no
KIR2DS1	-0.006360395	-0.114312612	0.909061008	0.95193832	no
ZNHIT2	-0.006356559	-0.114243665	0.909115619	0.951949121	no
RBPJL	-0.006344714	-0.114030767	0.90928425	0.952079309	no
SNHG9	0.006339046	0.113928897	0.90936494	0.952117408	no
SCLY	-0.006326251	-0.113698933	0.909547096	0.952236426	no
METTL15	-0.006324837	-0.113673514	0.909567231	0.952236426	no
C12orf52	-0.006318638	-0.11356209	0.909655492	0.952275342	no
SLC4A1	0.0063151	0.113498505	0.909705859	0.952275342	no
ZNF26	-0.006309468	-0.11339729	0.909786035	0.952275342	no
CENPC1	-0.006304695	-0.113311501	0.909853994	0.952275342	no
SPEG	-0.006302664	-0.113274988	0.909882917	0.952275342	no
HOXC10	-0.00630195	-0.113262155	0.909893082	0.952275342	no
GIMAP1-GIMAF	0.00630044	0.113235023	0.909914576	0.952275342	no
HOXC9	-0.006294444	-0.113127251	0.909999948	0.952281565	no
RTN4IP1	-0.006293798	-0.113115643	0.910009144	0.952281565	no
ZNF823	0.006289791	0.113043627	0.910066192	0.952294894	no
NINL	-0.006274993	-0.112777646	0.910276899	0.952469002	no
NPVF	-0.006268202	-0.112655601	0.910373583	0.952492986	no
OR13H1	-0.006267157	-0.11263682	0.910388462	0.952492986	no
TOPAZ1	-0.006258868	-0.112487836	0.91050649	0.952570099	no
ANXA2P2	0.006248013	0.11229273	0.910661059	0.952685432	no
SOX10	-0.006243533	-0.112212217	0.910724845	0.952705785	no
ADAMTS10	-0.006234433	-0.112048659	0.910854425	0.952789164	no
VAPA	-0.006228123	-0.111935247	0.910944277	0.952789164	no
FIGLA	-0.006223699	-0.111855732	0.911007276	0.952789164	no
SHFM1	0.006223614	0.111854202	0.911008487	0.952789164	no
PYROXD1	0.006222368	0.111831819	0.911026221	0.952789164	no
RNGTT	-0.00621418	-0.111684652	0.91114282	0.952796474	no
FBXO16	-0.006205486	-0.111528387	0.911266629	0.952796474	no
TDG	0.006204126	0.111503951	0.91128599	0.952796474	no

KBTBD12	0.006202063	0.111466861	0.911315377	0.952796474	no
MAGEB3	-0.00620011	-0.111431767	0.911343182	0.952796474	no
ZNF784	0.006196366	0.111364467	0.911396506	0.952796474	no
PIK3CA	-0.006188535	-0.111223722	0.911508022	0.952796474	no
KRT76	-0.006183429	-0.111131956	0.911580732	0.952796474	no
TDP1	0.006183164	0.111127196	0.911584503	0.952796474	no
TRIM27	-0.006181652	-0.111100007	0.911606046	0.952796474	no
CPOX	-0.006177623	-0.111027605	0.911663414	0.952796474	no
ZMYND15	0.006172624	0.110937754	0.911734608	0.952796474	no
OGFOD3	0.006172167	0.110929533	0.911741122	0.952796474	no
SUPT4H1	-0.006172045	-0.110927344	0.911742857	0.952796474	no
REG4	-0.006171814	-0.110923198	0.911746142	0.952796474	no
GEMIN8	-0.006169036	-0.110873257	0.911785713	0.952796474	no
KRTAP19-3	-0.006168952	-0.110871759	0.9117869	0.952796474	no
CAPZA3	0.00614499	0.110441076	0.912128167	0.953084011	no
PACSIN3	-0.006143404	-0.110412568	0.912150757	0.953084011	no
MOCS1	-0.006131913	-0.11020605	0.912314406	0.953208658	no
GLUL	0.006118243	0.109960348	0.912509108	0.953350155	no
CYB5R3	-0.006113419	-0.109873649	0.912577814	0.953350155	no
KIAA1614	0.006113061	0.109867213	0.912582913	0.953350155	no
GHRHR	-0.006099195	-0.109617997	0.912780409	0.953510124	no
NGFR	0.006091442	0.109478643	0.912890844	0.953579136	no
LOC100130348	-0.006083953	-0.10934404	0.912997517	0.953644211	no
C12orf56	-0.00607727	-0.109223937	0.913092699	0.953697278	no
VAV2	-0.006067178	-0.109042556	0.913236447	0.953709363	no
HIST1H4D	-0.006066024	-0.109021813	0.913252887	0.953709363	no
ASZ1	-0.006057577	-0.108869982	0.913373218	0.953709363	no
LOC100131347	0.006056252	0.108846181	0.913392081	0.953709363	no
SNORA7B	-0.00605172	-0.108764725	0.913456639	0.953709363	no
LMO4	-0.006050327	-0.10873969	0.91347648	0.953709363	no
TEKT5	-0.006049881	-0.10873167	0.913482837	0.953709363	no
CLMN	0.006045345	0.108650131	0.913547461	0.953709363	no
PRAMEF22	-0.006039894	-0.10855216	0.91362511	0.953709363	no
GAS7	-0.006038516	-0.108527394	0.913644738	0.953709363	no
OR4D9	-0.006037691	-0.10851258	0.91365648	0.953709363	no
LOC440910	0.006037573	0.108510446	0.913658171	0.953709363	no
OR5F1	-0.006035958	-0.108481425	0.913681172	0.953709363	no
TRABD2A	0.006019044	0.108177425	0.913922119	0.953914535	no
OR52B4	-0.006013186	-0.108072139	0.914005569	0.953930942	no
CSNK2B	-0.006010171	-0.108017945	0.914048524	0.953930942	no
KNG1	0.006008593	0.107989587	0.914071002	0.953930942	no
ASAP1	0.005988668	0.107631476	0.914354852	0.954180836	no
STAG3L4	0.00597602	0.107404155	0.91453504	0.954221075	no
LOC100507053	-0.005968396	-0.107267118	0.914643665	0.954221075	no
IFT80	0.0059683	0.107265397	0.914645029	0.954221075	no
LYRM4	0.005968292	0.10726526	0.914645138	0.954221075	no
FAM71F2	0.005964166	0.1071911	0.914703924	0.954221075	no
MRPS30	-0.005960448	-0.107124269	0.914756899	0.954221075	no
OR52N2	-0.005959753	-0.107111786	0.914766795	0.954221075	no
CPT1B	-0.005958711	-0.107093046	0.91478165	0.954221075	no
STK33	-0.005956576	-0.107054675	0.914812066	0.954221075	no

PRDM10	-0.005954615	-0.107019442	0.914839996	0.954221075	no
FLJ41941	0.00595168	0.106966677	0.914881823	0.954221075	no
MCM3AP	-0.005944475	-0.106837181	0.914984476	0.954281829	no
ADM2	0.005941295	0.106780038	0.915029774	0.954282761	no
INPP5K	-0.005924988	-0.106486948	0.915262116	0.954478753	no
WAPAL	0.005918543	0.106371098	0.915353956	0.954515134	no
FAM74A3	0.005914842	0.106304596	0.915406676	0.954515134	no
LOC100288842	-0.005913188	-0.106274865	0.915430246	0.954515134	no
INHBE	-0.00590817	-0.10618467	0.91550175	0.954543378	no
HSPA8	0.005899391	0.106026881	0.915626841	0.954576382	no
CYP2C8	0.005896069	0.10596717	0.91567418	0.954576382	no
ATP6V0C	0.00589394	0.105928912	0.91570451	0.954576382	no
DPY19L1P1	-0.005889298	-0.105845477	0.915770657	0.954576382	no
ALX4	0.005886128	0.105788507	0.915815823	0.954576382	no
LOC100271722	0.005881151	0.105699051	0.915886745	0.954576382	no
BDH1	0.005875396	0.105595625	0.915968742	0.954576382	no
SRY	0.005875317	0.1055942	0.915969872	0.954576382	no
XPO6	-0.005873197	-0.105556098	0.916000081	0.954576382	no
ENTHD2	0.005871691	0.105529025	0.916021544	0.954576382	no
SNORA53	-0.005871659	-0.105528455	0.916021996	0.954576382	no
CRYGN	-0.005864923	-0.105407386	0.916117984	0.954620077	no
SP8	-0.005862482	-0.105363515	0.916152766	0.954620077	no
LPGAT1	0.00585896	0.105300221	0.916202948	0.954626082	no
IZUMO2	-0.005849757	-0.105134808	0.916334097	0.954655691	no
UQCR11	0.005848911	0.105119601	0.916346154	0.954655691	no
PKLR	0.005847345	0.105091447	0.916368476	0.954655691	no
TFEB	-0.005844497	-0.105040272	0.916409051	0.954655691	no
MRPS23	0.005837046	0.104906357	0.916515229	0.954720022	no
OSBPL6	-0.00583069	-0.104792109	0.916605814	0.954768105	no
KIAA1191	-0.005821026	-0.104618419	0.916743532	0.954865276	no
SSNA1	-0.005810572	-0.104430522	0.916892517	0.954974173	no
RASSF1	0.005804983	0.10433007	0.916972168	0.955010849	no
OR2A14	0.005798371	0.104211244	0.917066389	0.955057963	no
OR2J2	-0.005795572	-0.104160929	0.917106285	0.955057963	no
SLC45A2	-0.005784283	-0.103958029	0.917267175	0.955179225	no
C2orf61	-0.005775788	-0.103805351	0.917388243	0.955259011	no
LINC00116	0.005768523	0.103674772	0.917491789	0.955320544	no
KLHL14	-0.005758912	-0.103502035	0.917628767	0.955342916	no
CDCA3	0.005757439	0.10347556	0.917649762	0.955342916	no
LOC440905	0.005755324	0.103437548	0.917679905	0.955342916	no
LOC728537	0.005754539	0.103423448	0.917691087	0.955342916	no
MTUS2-AS1	0.005739161	0.103147055	0.91791027	0.955468477	no
SNORA45	-0.005732981	-0.103035975	0.91799836	0.955468477	no
RYBP	-0.005731796	-0.103014675	0.918015251	0.955468477	no
NPFFR2	-0.005731539	-0.103010068	0.918018905	0.955468477	no
KRT32	-0.005727383	-0.102935363	0.918078149	0.955468477	no
FAM180A	-0.005727361	-0.102934979	0.918078454	0.955468477	no
LEUTX	0.005721199	0.102824213	0.918166296	0.955513624	no
NAA10	0.005717861	0.102764234	0.918213863	0.955516857	no
UBXN8	0.005711063	0.102642037	0.918310772	0.955553818	no
TUBAL3	0.005709131	0.102607317	0.918338307	0.955553818	no

HSD3B1	-0.005694903	-0.102351605	0.918541107	0.955718564	no
LCA5	-0.005688939	-0.102244411	0.918626122	0.955760747	no
CDCA8	-0.005673171	-0.101961001	0.918850897	0.955948329	no
LOC157381	-0.005658185	-0.101691671	0.919064511	0.956039767	no
MIR217	0.005658068	0.101689556	0.919066189	0.956039767	no
C7orf65	0.005656563	0.101662517	0.919087635	0.956039767	no
ART4	0.005654522	0.101625835	0.919116729	0.956039767	no
OR5A2	0.005644494	0.101445597	0.919259686	0.956142189	no
CISD2	-0.005640008	-0.101364976	0.919323631	0.956162424	no
LCE1B	-0.005636825	-0.101307756	0.919369017	0.956163354	no
SH3GLB1	-0.005630714	-0.101197918	0.919456138	0.956205233	no
TFPT	-0.005616273	-0.100938384	0.919661999	0.956205233	no
LRRC56	-0.005615375	-0.100922229	0.919674813	0.956205233	no
IL36A	0.005615279	0.100920516	0.919676171	0.956205233	no
UBE2Q2	0.005613257	0.100884165	0.919705006	0.956205233	no
USP8	-0.005612755	-0.100875155	0.919712152	0.956205233	no
RNU12	-0.005612153	-0.100864331	0.919720738	0.956205233	no
LOC595101	0.005605997	0.100753687	0.919808502	0.956250218	no
SNORD127	0.005589896	0.100464298	0.920038054	0.956442598	no
C5orf51	0.005581129	0.10030673	0.920163045	0.956526265	no
VN1R10P	-0.005565523	-0.100026247	0.920385542	0.956643437	no
HERC2P10	0.00556125	0.099949447	0.920446466	0.956643437	no
OR8B8	-0.005556501	-0.099864102	0.920514169	0.956643437	no
CLDN6	0.005554171	0.09982221	0.920547401	0.956643437	no
LOC285768	-0.005553652	-0.099812882	0.920554802	0.956643437	no
BLNK	0.005551559	0.099775263	0.920584644	0.956643437	no
CYP4F12	-0.005545489	-0.099666174	0.920671185	0.956643437	no
C2orf42	-0.005544517	-0.099648713	0.920685037	0.956643437	no
PNPT1	0.005543535	0.099631047	0.920699051	0.956643437	no
LYZL1	-0.005542002	-0.099603506	0.9207209	0.956643437	no
PSEN1	0.005534325	0.099465525	0.920830362	0.956710917	no
TSSK6	0.005530872	0.099403456	0.920879603	0.956715825	no
FOXD2	-0.005520836	-0.099223085	0.921022697	0.956818233	no
CYP4F2	-0.005514138	-0.099102703	0.921118202	0.956871195	no
CSN3	0.005493618	0.098733887	0.921410807	0.957128893	no
OR1L1	0.005481357	0.098513533	0.921585633	0.957225668	no
IGF2	-0.005480837	-0.098504182	0.921593053	0.957225668	no
DAOA	-0.005474314	-0.098386947	0.921686067	0.957276013	no
OR52K2	0.005455463	0.098048135	0.921954887	0.957483112	no
WNT8A	-0.005450039	-0.097950654	0.922032232	0.957483112	no
OR51V1	-0.005449669	-0.097944008	0.922037505	0.957483112	no
PCGEM1	-0.005447834	-0.097911023	0.922063677	0.957483112	no
SCGB1D1	0.005440949	0.097787276	0.922161864	0.957538803	no
WDTC1	-0.005396391	-0.096986429	0.922797323	0.958152346	no
OR6C2	-0.00539282	-0.096922256	0.922848245	0.958158928	no
ASB5	-0.005370486	-0.096520845	0.923166779	0.958437419	no
TRMT61B	0.005367759	0.096471838	0.923205668	0.958437419	no
SERPINB7	-0.005357205	-0.096282148	0.923356199	0.95854739	no
OR51F1	-0.005351019	-0.09617096	0.923444435	0.958592685	no
INHA	0.005342552	0.096018788	0.923565196	0.958671736	no
SCN10A	-0.005336802	-0.095915444	0.923647209	0.958710561	no

ZNF665	-0.005328433	-0.09576502	0.923766586	0.958717539	no
DGAT2L6	0.005327608	0.095750192	0.923778354	0.958717539	no
TXNDC2	0.005326948	0.095738337	0.923787762	0.958717539	no
HOXD4	-0.005322309	-0.095654962	0.923853929	0.95873991	no
OR9I1	0.00531474	0.095518921	0.923961895	0.958805653	no
OR10G2	0.005307769	0.095393642	0.92406132	0.958862528	no
SLC45A3	-0.005296203	-0.095185759	0.924226306	0.958939998	no
ADAMTS18	-0.005296042	-0.095182865	0.924228602	0.958939998	no
MEGF10	0.00528917	0.095059356	0.924326626	0.958939998	no
ZIK1	0.005289089	0.095057905	0.924327778	0.958939998	no
FTH1P18	0.005286895	0.095018464	0.92435908	0.958939998	no
TREML5P	0.005246682	0.094295716	0.92493272	0.959488782	no
AAR2	0.005234327	0.094073656	0.925108975	0.95961623	no
VPS16	-0.00522917	-0.093980975	0.92518254	0.95961623	no
FERD3L	-0.005226756	-0.093937594	0.925216973	0.95961623	no
NUDT5	0.00522555	0.093915909	0.925234186	0.95961623	no
KRTAP19-8	0.005216533	0.09375386	0.925362813	0.959676139	no
LOC388553	-0.00521524	-0.093730622	0.925381258	0.959676139	no
TMEM82	-0.005209469	-0.093626894	0.925463594	0.959685264	no
CRHR2	0.005208363	0.093607023	0.925479368	0.959685264	no
UGGT2	-0.005195775	-0.093380768	0.925658964	0.95979732	no
OCLN	-0.005194528	-0.09335836	0.925676751	0.95979732	no
HAUS6	0.005184971	0.093186587	0.925813104	0.959857845	no
OR1A1	0.005184175	0.093172292	0.925824451	0.959857845	no
OR5B2	0.005180066	0.093098428	0.925883085	0.959872329	no
GSKIP	0.00517425	0.092993903	0.925966058	0.959903945	no
RAB3IP	0.005171667	0.092947476	0.926002913	0.959903945	no
TEKT2	-0.005144599	-0.092460998	0.926389098	0.960244027	no
GNPAT	-0.005138748	-0.092355827	0.926472589	0.960244027	no
OR4D5	-0.005135816	-0.092303128	0.926514425	0.960244027	no
EBLN2	0.005133068	0.092253743	0.92655363	0.960244027	no
C2orf44	0.005133015	0.092252785	0.92655439	0.960244027	no
CDO1	0.005121977	0.09205441	0.926711876	0.960351105	no
GOLGA6C	0.005114735	0.091924256	0.926815205	0.960351105	no
XRRA1	-0.005113814	-0.091907687	0.926828359	0.960351105	no
PAGE3	0.005113246	0.091897487	0.926836456	0.960351105	no
SYT12	-0.005083374	-0.091360602	0.927262698	0.96074644	no
FLJ35424	0.005054378	0.090839448	0.927676471	0.961128817	no
CENPK	0.005048495	0.090733716	0.92776042	0.961141729	no
TMCC3	-0.005046933	-0.09070564	0.927782712	0.961141729	no
C3orf79	-0.005044102	-0.090654768	0.927823104	0.961141729	no
OR6B1	-0.005022294	-0.090262806	0.928134322	0.96129805	no
LOC341056	-0.005020545	-0.090231377	0.928159277	0.96129805	no
DEFB112	0.005020452	0.09022971	0.928160601	0.96129805	no
NUP43	-0.005020389	-0.090228581	0.928161497	0.96129805	no
NOP2	0.005017856	0.090183042	0.928197657	0.96129805	no
TP53TG3D	0.004998113	0.08982822	0.928479397	0.961481932	no
MEIOB	-0.0049963	-0.089795629	0.928505276	0.961481932	no
CCDC96	-0.00499601	-0.089790407	0.928509423	0.961481932	no
OR10A6	0.00498459	0.089585161	0.9286724	0.961593146	no
OR8A1	0.004982214	0.089542455	0.928706311	0.961593146	no

AFTPH	0.004978097	0.089468472	0.928765059	0.961607645	no
LOC100506172	-0.00496761	-0.089279978	0.928914738	0.961716285	no
DIO1	-0.004938733	-0.088760982	0.929326875	0.962071605	no
KRTAP26-1	-0.004935567	-0.088704086	0.929372058	0.962071605	no
ITIH6	-0.004934153	-0.088678674	0.929392238	0.962071605	no
OR2V2	-0.00492647	-0.088540585	0.9295019	0.962138779	no
MSANTD4	-0.004921222	-0.088446257	0.929576809	0.962169976	no
CIB1	0.004900355	0.088071217	0.92987465	0.962431907	no
TYRO3P	-0.004893869	-0.087954657	0.92996722	0.962481364	no
ZACN	-0.00488962	-0.087878282	0.930027875	0.962497788	no
SIKE1	-0.004871169	-0.087546664	0.930291245	0.962699058	no
OR6X1	-0.004867083	-0.087473232	0.930349566	0.962699058	no
ITIH5	-0.004864772	-0.087431688	0.93038256	0.962699058	no
USP17L15	-0.004858979	-0.087327571	0.930465252	0.962699058	no
OR10J5	0.004857499	0.087300976	0.930486375	0.962699058	no
TMEM174	0.004854535	0.087247699	0.930528688	0.962699058	no
KRTAP13-3	-0.004854028	-0.08723859	0.930535923	0.962699058	no
LRRK2	-0.004848383	-0.087137129	0.930616508	0.962736082	no
ARL14	-0.00480364	-0.086332975	0.931255218	0.96317115	no
ROPN1	0.004802515	0.086312762	0.931271273	0.96317115	no
VN1R5	-0.004800026	-0.086268027	0.931306806	0.96317115	no
HSD17B3	0.004793096	0.08614347	0.931405742	0.96317115	no
FABP12	0.004791017	0.086106115	0.931435414	0.96317115	no
PLAC1L	0.004789499	0.086078822	0.931457093	0.96317115	no
A1BG	-0.004788781	-0.086065928	0.931467334	0.96317115	no
MT1G	0.004787881	0.086049752	0.931480183	0.96317115	no
MAPK15	0.004786519	0.086025271	0.931499628	0.96317115	no
MYH4	0.004786353	0.086022283	0.931502002	0.96317115	no
HCRTR2	-0.004784388	-0.08598697	0.931530052	0.96317115	no
TTK	-0.004777584	-0.085864678	0.93162719	0.963223494	no
GLYATL1	0.00476951	0.085719566	0.931742458	0.963223494	no
UBE3A	0.004769476	0.085718949	0.931742948	0.963223494	no
KDM4E	-0.004766275	-0.08566142	0.931788645	0.963223494	no
GALNTL4	0.004765145	0.085641112	0.931804776	0.963223494	no
OR2D2	0.004741163	0.085210085	0.932147164	0.963531081	no
OR10G8	-0.004729873	-0.085007175	0.932308351	0.963651346	no
GPR111	0.004718953	0.08481091	0.932464262	0.963720932	no
DEFB125	0.004716066	0.084759024	0.93250548	0.963720932	no
COMP	0.004715735	0.084753079	0.932510203	0.963720932	no
SUPT6H	-0.004711951	-0.084685061	0.932564237	0.963730429	no
C4orf51	0.004695599	0.084391171	0.932797708	0.963925352	no
LRR1	0.004684447	0.084190742	0.932956934	0.964043537	no
PYY	-0.004674996	-0.084020881	0.93309188	0.964107174	no
LOC286297	-0.004673388	-0.08399199	0.933114832	0.964107174	no
FAM175B	-0.004670708	-0.083943818	0.933153102	0.964107174	no
C2orf47	-0.00466008	-0.083752806	0.933304854	0.964175203	no
CARKD	-0.004659813	-0.083747995	0.933308676	0.964175203	no
ENTPD2	-0.004643752	-0.083459337	0.933538009	0.964365763	no
ZNF460	0.00463628	0.083325051	0.933644697	0.964429617	no
CACNA1C-AS1	0.004630086	0.083213728	0.933733143	0.964474622	no
CILP2	-0.004603135	-0.082729343	0.934117997	0.964719831	no

APOE	0.00460172	0.082703905	0.934138208	0.964719831	no
GTF2E1	-0.004601552	-0.082700889	0.934140604	0.964719831	no
MYL4	0.004599557	0.082665029	0.934169097	0.964719831	no
ZNHIT1	0.004597744	0.082632448	0.934194984	0.964719831	no
PAGE4	-0.004590866	-0.082508837	0.934293198	0.964774895	no
FLJ39051	0.004575247	0.082228126	0.934516239	0.964895558	no
ASB10	-0.004571273	-0.082156701	0.934572992	0.964895558	no
GDA	0.004569292	0.082121084	0.934601292	0.964895558	no
C20orf144	-0.004565224	-0.082047972	0.934659385	0.964895558	no
SNHG15	-0.004564955	-0.082043144	0.934663221	0.964895558	no
LOC348761	0.004560802	0.08196851	0.934722524	0.964895558	no
LOC401557	0.004560676	0.081966235	0.934724332	0.964895558	no
SPESP1	-0.004553068	-0.081829498	0.934832982	0.964952477	no
GATAD2A	0.004550527	0.081783826	0.934869272	0.964952477	no
SNORA13	-0.00454562	-0.081695643	0.934939343	0.964978456	no
ARX	-0.004533496	-0.081477743	0.935112488	0.965110814	no
TTC27	-0.004526052	-0.081343951	0.935218803	0.965174188	no
C1orf185	-0.004518975	-0.081216753	0.935319878	0.965232148	no
FSIP2	0.004512355	0.081097775	0.935414422	0.965261034	no
CNTN4-AS2	0.004510725	0.081068484	0.935437698	0.965261034	no
OR6C76	-0.004490449	-0.080704071	0.935727281	0.965485981	no
DCSTAMP	-0.004489171	-0.080681088	0.935745545	0.965485981	no
HIST1H2AL	0.004483976	0.080587721	0.93581974	0.965516181	no
TAS2R39	-0.004475633	-0.080437787	0.93593889	0.965592755	no
MATN4	-0.004460492	-0.080165651	0.936155155	0.96576951	no
OR4A15	0.004449669	0.079971141	0.936309733	0.965844501	no
PIGU	-0.004449109	-0.079961079	0.93631773	0.965844501	no
CERCAM	0.004442325	0.079839144	0.936414633	0.965872323	no
RPA4	-0.004437531	-0.079752992	0.936483101	0.965872323	no
ANAPC1	-0.004435569	-0.079717724	0.936511129	0.965872323	no
OR5P2	0.004434635	0.079700933	0.936524474	0.965872323	no
PPP1R8	0.004428098	0.079583445	0.936617845	0.965883927	no
FGF6	-0.004426481	-0.07955438	0.936640945	0.965883927	no
OR10X1	-0.004424408	-0.079517121	0.936670556	0.965883927	no
ESRP1	-0.004412608	-0.079305051	0.936839098	0.966011374	no
MYOZ2	-0.004409123	-0.079242408	0.936888884	0.966016361	no
SCN1B	0.004403116	0.079134458	0.936974679	0.966058474	no
MROH9	0.004399588	0.079071039	0.937025083	0.966064095	no
ATXN1L	-0.00439317	-0.078955699	0.937116752	0.966086659	no
DBX1	0.004391761	0.07893038	0.937136875	0.966086659	no
DSCR6	0.004388338	0.07886885	0.937185778	0.966090731	no
CHST12	-0.004377445	-0.078673066	0.937341385	0.966204792	no
GUCA2A	-0.004369871	-0.078536948	0.937449572	0.966269964	no
CHEK2	-0.004360229	-0.078363649	0.937587312	0.966348087	no
SGOL2	-0.004356508	-0.078296783	0.937640458	0.966348087	no
ACSM4	0.004355122	0.078271869	0.93766026	0.966348087	no
DHX40	-0.004344655	-0.078083746	0.937809785	0.966441532	no
GYLTL1B	0.004342479	0.078044637	0.93784087	0.966441532	no
FOXD1	0.0043328	0.077870678	0.93797914	0.966537672	no
ACCSL	-0.004320367	-0.077647229	0.93815675	0.96667434	no
COMMD3-BMI1	-0.004268795	-0.076720342	0.938893521	0.967383332	no

GSDMC	-0.004265008	-0.076652274	0.93894763	0.967383332	no
COL6A4P2	0.004262751	0.076611722	0.938979866	0.967383332	no
TM9SF4	0.004248768	0.076360398	0.939179651	0.967542777	no
FLJ12334	-0.00424537	-0.076299321	0.939228203	0.967546416	no
ILF3	-0.00423063	-0.076034419	0.939438788	0.967716962	no
ACAD10	-0.004217577	-0.075799815	0.93962529	0.967784244	no
DPCD	0.004216214	0.075775325	0.939644758	0.967784244	no
HOXB13	-0.004216103	-0.075773328	0.939646346	0.967784244	no
FLJ42289	-0.004213452	-0.075725672	0.939684232	0.967784244	no
ADAMTS5	0.004207916	0.075626173	0.939763331	0.967819329	no
FOXD4L3	-0.004190382	-0.075311046	0.940013855	0.968030943	no
LOC339240	-0.004181754	-0.075155985	0.94013713	0.968035868	no
MMP27	0.004181591	0.075153043	0.940139469	0.968035868	no
TBC1D3F	-0.00418059	-0.075135056	0.940153768	0.968035868	no
FGFR2	0.004173185	0.07500198	0.940259566	0.968081219	no
SNORA56	0.004171202	0.074966335	0.940287905	0.968081219	no
EFCAB9	-0.004162495	-0.074809841	0.940412322	0.968162932	no
LINS	0.004155842	0.074690279	0.940507378	0.968214412	no
ZNF628	0.004128419	0.074197401	0.940899242	0.968529306	no
PRB4	0.004128128	0.074192179	0.940903395	0.968529306	no
ZSCAN10	-0.004121969	-0.074081481	0.940991408	0.968533059	no
PADI4	0.004116018	0.073974519	0.94107645	0.968533059	no
OR1Q1	0.004115569	0.073966463	0.941082856	0.968533059	no
SYDE2	0.004115258	0.073960864	0.941087308	0.968533059	no
AGRP	-0.004108887	-0.073846362	0.941178346	0.968580369	no
CENPF	0.004079381	0.073316073	0.941599982	0.968959203	no
SLC39A12	0.00407511	0.073239309	0.941661019	0.968959203	no
RPL13AP5	0.00407169	0.073177835	0.941709899	0.968959203	no
LINC00051	0.004070506	0.073156566	0.94172681	0.968959203	no
NHLRC1	0.004064139	0.073042122	0.941817808	0.96900644	no
S100PBP	-0.004052831	-0.072838887	0.94197941	0.969121396	no
TMEM33	-0.00405001	-0.072788182	0.942019727	0.969121396	no
KAAG1	-0.004039176	-0.072593468	0.942174555	0.969211052	no
HIST1H3A	-0.004036903	-0.072552628	0.94220703	0.969211052	no
OR52W1	-0.004033758	-0.072496107	0.942251974	0.969211052	no
KIAA1984-AS1	-0.004031289	-0.072451719	0.94228727	0.969211052	no
LOC100653515	0.00402505	0.072339595	0.942376428	0.969256368	no
CHAMP1	0.004014919	0.072157507	0.942521221	0.969313968	no
EN1	0.00401482	0.072155727	0.942522637	0.969313968	no
PRSS2	-0.004006144	-0.0719998	0.942646628	0.969382475	no
KRT3	-0.004003846	-0.071958509	0.942679463	0.969382475	no
NUP107	-0.003986469	-0.071646187	0.942927824	0.969591477	no
RUSC1	0.003976016	0.071458331	0.943077212	0.969698692	no
SDE2	-0.003971615	-0.071379216	0.943140126	0.969716986	no
LRFN3	0.003964115	0.071244429	0.943247313	0.969780797	no
TPX2	0.003958852	0.071149844	0.943322532	0.969811736	no
RHBDL2	-0.003946942	-0.070935783	0.943492764	0.969940348	no
SNORA71C	0.003928204	0.070599015	0.943760585	0.970126926	no
OR9G1	-0.003927928	-0.070594046	0.943764536	0.970126926	no
OR4P4	-0.003913876	-0.070341501	0.943965381	0.970286972	no
OR51B2	0.003894446	0.069992289	0.94424311	0.970526026	no

FTSJD2	-0.003881884	-0.069766516	0.944422671	0.970619685	no
OR52B6	-0.003881751	-0.06976414	0.944424561	0.970619685	no
KDM6A	-0.003876678	-0.069672952	0.944497085	0.970632438	no
CSHL1	0.003874003	0.069624879	0.944535319	0.970632438	no
IKZF4	-0.003871404	-0.069578176	0.944572463	0.970632438	no
CCDC57	0.00386785	0.069514295	0.94462327	0.970638235	no
MRTO4	0.003860548	0.06938306	0.944727647	0.970665084	no
RRP12	-0.003859703	-0.069367866	0.944739731	0.970665084	no
DCAF4L2	-0.003849425	-0.069183152	0.944886644	0.970769618	no
SHROOM4	-0.003836457	-0.068950091	0.945072012	0.970913648	no
WNK4	0.003829363	0.068822577	0.945173432	0.970971426	no
MEOX1	-0.003825125	-0.068746417	0.945234008	0.970976411	no
MBD6	-0.003820635	-0.068665729	0.945298187	0.970976411	no
CACNA1A	-0.003817635	-0.068611799	0.945341082	0.970976411	no
NPPB	0.003816381	0.068589261	0.945359007	0.970976411	no
RDX	0.003806154	0.068405458	0.945505204	0.971080159	no
LOC100129083	-0.003802258	-0.06833543	0.945560904	0.971090958	no
LOC729678	-0.003795823	-0.068219792	0.945652883	0.971139012	no
MSGN1	-0.003781146	-0.067956008	0.9458627	0.97130807	no
MAPKAPK5	0.003766683	0.067696071	0.946069462	0.971473974	no
ZNF575	0.003757831	0.067536966	0.946196021	0.971520569	no
HSD17B14	0.003757185	0.067525364	0.94620525	0.971520569	no
HPGD	0.003752321	0.067437937	0.946274793	0.971545556	no
WDR90	-0.003730033	-0.067037376	0.946593425	0.971826269	no
PAX9	-0.003726496	-0.066973792	0.946644004	0.971831771	no
IFNA21	-0.003705848	-0.066602704	0.946939199	0.972088384	no
DDAH2	0.003693762	0.066385489	0.947111994	0.972216696	no
OPN5	-0.003690777	-0.066331845	0.947154668	0.972216696	no
GJA9-MYCBP	-0.003675459	-0.066056534	0.947373682	0.972395062	no
LCE1D	0.003662308	0.065820175	0.947561713	0.97248786	no
OR2F1	0.00365824	0.06574706	0.947619879	0.97248786	no
COX7B2	0.003657512	0.06573398	0.947630285	0.97248786	no
LOC644649	-0.003655897	-0.06570496	0.947653372	0.97248786	no
FAM211A	0.003645078	0.065510509	0.947808066	0.97248786	no
FAM84A	-0.003643254	-0.065477723	0.947834149	0.97248786	no
GPR151	-0.003630963	-0.065256823	0.948009889	0.97248786	no
SERPINA10	0.003630626	0.065250778	0.948014698	0.97248786	no
PCYOX1L	-0.003630006	-0.06523962	0.948023575	0.97248786	no
TYRP1	0.003627176	0.065188757	0.94806404	0.97248786	no
TMPRSS11D	-0.003626428	-0.065175322	0.948074728	0.97248786	no
ABCC5-AS1	0.003626145	0.065170236	0.948078774	0.97248786	no
PPIL1	-0.003625988	-0.065167419	0.948081016	0.97248786	no
OR13C8	-0.003621039	-0.065078463	0.948151787	0.97248786	no
CDH2	0.003619184	0.065045123	0.948178312	0.97248786	no
GAL	-0.003618048	-0.06502471	0.948194552	0.97248786	no
ALDH1L1-AS2	-0.003612176	-0.064919182	0.948278508	0.97248786	no
LINC00544	0.003612169	0.064919054	0.948278609	0.97248786	no
CALCA	-0.003608672	-0.064856198	0.948328617	0.972492738	no
OR6K2	-0.003602663	-0.064748204	0.948414536	0.972507438	no
PAPLN	0.003597402	0.064653649	0.948489764	0.972507438	no
TROVE2	0.00358973	0.064515765	0.948599464	0.972507438	no

TBC1D3P5	0.003589433	0.064510421	0.948603716	0.972507438	no
CLDN10	0.003582632	0.064388202	0.948700954	0.972507438	no
VSIG2	-0.003582448	-0.06438489	0.948703589	0.972507438	no
MND1	-0.003576994	-0.064286874	0.948781572	0.972507438	no
OTUD7B	0.003576406	0.064276289	0.948789994	0.972507438	no
LOC100505478	-0.003575619	-0.064262146	0.948801246	0.972507438	no
HOXA10-HOXA9	-0.003574	-0.064233047	0.948824398	0.972507438	no
KLHL29	0.003572858	0.064212529	0.948840722	0.972507438	no
SCARNA23	0.003560933	0.063998201	0.949011247	0.97263583	no
OR9Q1	-0.003552922	-0.063854229	0.949125797	0.972706843	no
STEAP2	0.003542758	0.06367156	0.949271135	0.972809402	no
MAT1A	-0.003533539	-0.063505867	0.94940297	0.972824608	no
SERPINA7	0.003532873	0.063493903	0.949412489	0.972824608	no
URB2	-0.003528575	-0.063416645	0.94947396	0.972824608	no
TOP3A	0.003528296	0.063411632	0.949477949	0.972824608	no
BARX1	-0.003524389	-0.06334142	0.949533814	0.972824608	no
C10orf68	-0.003522728	-0.063311559	0.949557573	0.972824608	no
OR2G3	-0.003517768	-0.063222424	0.949628495	0.972850891	no
FLJ42969	-0.003502612	-0.062950029	0.949845233	0.973026546	no
MAPK1IP1L	-0.003498367	-0.06287374	0.949905936	0.973042348	no
SPDYE8P	-0.003491204	-0.062745006	0.950008368	0.973072409	no
RPL39	0.003489983	0.06272305	0.950025838	0.973072409	no
VPS33A	-0.003460598	-0.062194939	0.950446061	0.973443718	no
ZNF699	-0.003458299	-0.062153615	0.950478943	0.973443718	no
TRAPPC2P1	0.003452886	0.062056334	0.950556353	0.973476606	no
DGAT2	0.003440109	0.061826697	0.950739082	0.973617346	no
LOC100130880	0.00343558	0.061745295	0.950803857	0.973637285	no
PRSS42	0.00342587	0.061570784	0.950942724	0.97373309	no
LFNG	0.003418919	0.061445859	0.951042134	0.973788485	no
MYH16	0.003407194	0.061235133	0.951209822	0.973913783	no
DEFB126	-0.003402047	-0.061142629	0.951283434	0.973942751	no
NOS2	0.003393121	0.0609822	0.951411101	0.974027056	no
C1orf177	0.003383997	0.060818217	0.951541595	0.974053636	no
SPG20	0.003383516	0.060809586	0.951548464	0.974053636	no
LOC440925	0.003381799	0.06077871	0.951573035	0.974053636	no
MTOR	-0.003377265	-0.06069723	0.951637876	0.974073614	no
PAK1	0.003343108	0.060083348	0.95212641	0.9745192	no
GUCY2D	0.003340488	0.060036252	0.95216389	0.9745192	no
CHAC1	0.003334626	0.059930895	0.952247736	0.974558603	no
TIAM2	0.003325656	0.059769688	0.952376031	0.974643489	no
SNORA2B	-0.003304445	-0.059388465	0.952679427	0.974907555	no
OR5AP2	0.003298766	0.059286416	0.952760643	0.974944242	no
CLSPN	0.003285949	0.059056045	0.952943988	0.975085428	no
OR6C1	0.003275309	0.058864826	0.953096175	0.975171215	no
FAM173A	-0.003272661	-0.058817239	0.953134049	0.975171215	no
SEPT7P2	-0.003270571	-0.058779664	0.953163954	0.975171215	no
NPHP4	0.003258429	0.058561447	0.953337632	0.975271742	no
ANKRD33	-0.003256344	-0.058523984	0.953367448	0.975271742	no
OR52D1	-0.003253254	-0.058468452	0.953411646	0.975271742	no
KANSL1L	-0.003251011	-0.058428135	0.953443735	0.975271742	no
XIRP2	-0.00324756	-0.058366105	0.953493105	0.975275823	no

PPAPDC1B	0.003240183	0.058233525	0.953598626	0.975337335	no
FGF10	-0.003230424	-0.058058123	0.953738232	0.9754337	no
DCDC2	0.0032162	0.057802499	0.953941689	0.975595358	no
RNF186	-0.003207232	-0.057641308	0.954069987	0.97566711	no
LINC00473	-0.003202793	-0.05756153	0.954133486	0.97566711	no
PSMG2	0.003201775	0.057543238	0.954148045	0.97566711	no
TRIP13	-0.003197582	-0.057467882	0.954208025	0.975677387	no
C17orf70	0.003194725	0.057416535	0.954248894	0.975677387	no
ZNF562	-0.003181427	-0.057177541	0.954439123	0.975825461	no
DUX4L	0.003174246	0.057048481	0.95454185	0.975884064	no
OR6C75	0.003168537	0.056945868	0.954623527	0.97592114	no
PDRG1	0.003158072	0.056757795	0.954773228	0.976027753	no
TPGS1	0.00315332	0.056672379	0.954841218	0.976050828	no
LOC613038	-0.003149966	-0.056612106	0.954889194	0.976053445	no
HSPA12B	-0.003129768	-0.056249099	0.955178146	0.976300795	no
PKP1	-0.0031267	-0.056193958	0.955222038	0.976300795	no
LEO1	0.00312147	0.056099952	0.955296868	0.976330844	no
ZNF551	-0.003117255	-0.056024203	0.955357165	0.976346038	no
SCOC	0.003109714	0.055888673	0.955465049	0.976409861	no
FAM83F	-0.003104653	-0.055797714	0.955537454	0.976437422	no
MRPL9	0.003098842	0.05569327	0.955620594	0.976449854	no
C2orf16	0.003094615	0.055617309	0.955681062	0.976449854	no
CUZD1	-0.003088951	-0.055515517	0.955762092	0.976449854	no
PIH1D3	0.003088759	0.055512069	0.955764836	0.976449854	no
SLC4A8	0.003087923	0.055497044	0.955776796	0.976449854	no
SPANXN1	-0.003081694	-0.055385089	0.955865917	0.976449945	no
LOC100169752	-0.003078429	-0.055326398	0.955912638	0.976449945	no
CYP2C18	0.00307839	0.055325703	0.955913191	0.976449945	no
TRIP11	-0.003068806	-0.055153466	0.9560503	0.976518404	no
EIF2AK1	-0.003067354	-0.055127354	0.956071087	0.976518404	no
C1QTNF2	-0.003057357	-0.054947686	0.956214113	0.976618073	no
UGT1A6	0.003033093	0.054511603	0.956561265	0.976910673	no
OR1J1	0.003030815	0.054470669	0.956593852	0.976910673	no
CENPN	0.003027802	0.054416507	0.956636969	0.976910673	no
TNNI2	-0.00302249	-0.054321052	0.95671296	0.976941852	no
RAB11B-AS1	-0.003012792	-0.054146743	0.956851726	0.977037129	no
C21orf67	0.003003254	0.053975327	0.95698819	0.977090958	no
ETNK1	0.003002752	0.053966304	0.956995374	0.977090958	no
BSDC1	0.002998995	0.053898784	0.957049126	0.977099419	no
SCGB2B3P	-0.002982067	-0.05359454	0.957291339	0.977300278	no
SRSF11	-0.002975128	-0.053469842	0.957390614	0.97731024	no
UHRF2	0.002973306	0.053437083	0.957416694	0.97731024	no
LOC100288778	-0.002968346	-0.053347944	0.95748766	0.97731024	no
SNX19	0.002966725	0.053318818	0.957510848	0.97731024	no
IARS	0.002965494	0.05329668	0.957528473	0.97731024	no
HIST1H1T	0.002962116	0.05323598	0.957576798	0.977313149	no
UCP1	0.002955238	0.053112368	0.957675211	0.977367174	no
DYDC2	-0.002949563	-0.053010371	0.957756415	0.977403633	no
CALB2	0.002942077	0.052875824	0.957863534	0.97741124	no
OR5AR1	-0.002940288	-0.052843681	0.957889125	0.97741124	no
OR4D1	0.002939507	0.052829633	0.957900308	0.97741124	no

PRCC	-0.002924512	-0.052560143	0.958114866	0.977583752	no
EP400NL	0.002918252	0.052447628	0.958204446	0.977628738	no
LINC00494	-0.002900334	-0.052125604	0.958460833	0.977843901	no
LOC100132146	-0.002894915	-0.052028211	0.958538376	0.977876589	no
LIM2	0.00288891	0.051920282	0.958624308	0.977917834	no
BRK1	-0.002885295	-0.051855325	0.958676026	0.977924173	no
SLC25A10	0.002866389	0.051515533	0.958946569	0.978153719	no
FLJ39080	0.002856395	0.051335907	0.959089589	0.978253173	no
TRPM3	0.002845912	0.05114751	0.959239593	0.978321107	no
NHLRC3	0.002845378	0.051137911	0.959247237	0.978321107	no
BTNL3	-0.002830385	-0.050868453	0.959461786	0.978493487	no
MGC16121	0.002813006	0.050556117	0.959710481	0.978700671	no
DDX39A	0.002790846	0.05015784	0.96002761	0.978977622	no
LIPM	0.002762851	0.049654704	0.960428243	0.979308378	no
MTFR2	-0.002761813	-0.049636045	0.960443101	0.979308378	no
ZBTB2	-0.002745109	-0.049335831	0.960682158	0.979505658	no
C9orf106	0.002735205	0.04915783	0.9608239	0.979603702	no
KLK3	0.002711804	0.048737261	0.961158803	0.979838831	no
RPTN	-0.00271152	-0.048732162	0.961162864	0.979838831	no
IL20	0.002703898	0.048595184	0.961271942	0.979838831	no
ABO	-0.002702831	-0.04857599	0.961287227	0.979838831	no
P2RY11	-0.002700239	-0.04852941	0.96132432	0.979838831	no
TINF2	-0.002699976	-0.048524688	0.961328079	0.979838831	no
CEP135	-0.002693631	-0.048410649	0.961418892	0.979876162	no
CREBRF	0.002691045	0.048364183	0.961455895	0.979876162	no
RPL13AP20	0.002675801	0.04809021	0.96167407	0.980052041	no
E2F8	0.002667094	0.047933713	0.961798696	0.98013257	no
FLJ14186	0.002663764	0.047873873	0.961846349	0.980134656	no
FASTKD2	0.002655404	0.047723624	0.961966001	0.980165691	no
LURAP1	-0.002651272	-0.047649365	0.962025137	0.980165691	no
OR51F2	0.002649492	0.047617372	0.962050615	0.980165691	no
YME1L1	0.002648594	0.047601222	0.962063476	0.980165691	no
SLC26A8	0.002640849	0.047462034	0.962174321	0.980165691	no
LRRD1	-0.002639792	-0.047443041	0.962189446	0.980165691	no
SLC30A2	-0.00263933	-0.047434732	0.962196063	0.980165691	no
MIR5191	-0.002627849	-0.047228389	0.962360389	0.980277903	no
SORCS1	0.00262526	0.047181859	0.962397445	0.980277903	no
RAB8B	0.002620312	0.047092929	0.962468267	0.980303579	no
ZNF354A	-0.00260902	-0.046889996	0.962629879	0.980415461	no
OR6Y1	0.002606262	0.046840431	0.962669353	0.980415461	no
LDLRAD4	0.002599962	0.046727194	0.962759533	0.980460841	no
LOC285627	-0.00259556	-0.046648082	0.962822538	0.980478543	no
OR10J1	0.002587137	0.046496708	0.962943092	0.980554844	no
MYOC	0.002578131	0.04633485	0.963071996	0.980639642	no
FOXF1	-0.002564628	-0.046092168	0.963265272	0.980760151	no
RPS19	-0.002563486	-0.046071643	0.963281618	0.980760151	no
HSPA14	0.002547639	0.045786836	0.963508445	0.980880174	no
LOC100500773	0.002546162	0.045760289	0.963529589	0.980880174	no
POU6F2	-0.002544695	-0.045733915	0.963550593	0.980880174	no
NLRP9	-0.002542496	-0.045694396	0.963582068	0.980880174	no
LOC100130849	-0.002529568	-0.045462048	0.963767119	0.981003424	no

PABPC1	0.002527659	0.045427744	0.963794439	0.981003424	no
FAM217A	-0.002503657	-0.044996363	0.964138013	0.98126988	no
KRT8P41	-0.002502991	-0.044984402	0.96414754	0.98126988	no
HYPK	-0.002497982	-0.04489437	0.964219246	0.981296388	no
C19orf44	0.002493164	0.044807777	0.964288215	0.981320107	no
DCP2	0.002477363	0.044523807	0.964514388	0.981503799	no
CCDC15	-0.002439816	-0.04384899	0.965051871	0.98200425	no
IFLTD1	-0.002431233	-0.043694743	0.965174729	0.982082766	no
OR52E2	0.002422633	0.04354018	0.965297839	0.982161531	no
KIF2C	-0.002417066	-0.043440118	0.96537754	0.98218903	no
UGT1A4	0.002412729	0.043362179	0.965439619	0.98218903	no
WISP3	-0.002405299	-0.043228643	0.965545983	0.98218903	no
FCN2	-0.00239934	-0.043121549	0.965631286	0.98218903	no
CYHR1	-0.002398689	-0.043109842	0.965640611	0.98218903	no
TMPRSS15	-0.002398347	-0.043103699	0.965645504	0.98218903	no
UGT2B28	-0.002395854	-0.043058889	0.965681196	0.98218903	no
OR10J3	-0.002395205	-0.043047229	0.965690484	0.98218903	no
CHIAP2	-0.002372614	-0.04264121	0.966013891	0.982449067	no
NOXRED1	-0.002370959	-0.042611467	0.966037583	0.982449067	no
POLD3	-0.002361504	-0.042441543	0.966172935	0.982540224	no
PSMD6-AS2	0.002338291	0.042024356	0.966505247	0.982778762	no
PPID	0.002336961	0.042000442	0.966524296	0.982778762	no
MAB21L3	0.00233172	0.041906261	0.966599318	0.982778762	no
POPDC3	0.00232735	0.041827715	0.966661884	0.982778762	no
OR8D4	-0.002323339	-0.041755626	0.966719309	0.982778762	no
LOC400891	0.0023202	0.041699208	0.966764249	0.982778762	no
HIST1H2BA	0.002319938	0.041694502	0.966767998	0.982778762	no
ULK4P3	0.002319565	0.041687799	0.966773338	0.982778762	no
OR13C5	-0.002308131	-0.041482304	0.96693703	0.982822832	no
TMEM244	0.002307588	0.041472553	0.966944797	0.982822832	no
LOC729041	0.002303668	0.041402092	0.967000925	0.982822832	no
TMEM251	0.002303088	0.041391678	0.967009221	0.982822832	no
OR5B3	0.002295103	0.041248157	0.967123547	0.982822832	no
MUC16	-0.002293334	-0.041216366	0.967148871	0.982822832	no
LOC100506393	-0.002291013	-0.04117465	0.967182102	0.982822832	no
DDX50	0.002284379	0.041055426	0.967277075	0.982822832	no
BPIFA3	-0.00228375	-0.041044118	0.967286083	0.982822832	no
OR6V1	-0.002282657	-0.041024474	0.967301731	0.982822832	no
HPS6	0.002281399	0.041001866	0.967319741	0.982822832	no
PRSS53	-0.002253973	-0.040508961	0.96771239	0.983175293	no
OR2C3	0.002250175	0.040440706	0.967766763	0.983184055	no
TMEM171	0.00224597	0.040365127	0.967826971	0.983198744	no
CD1E	0.002224712	0.039983075	0.968131322	0.983396094	no
COBL	-0.002223746	-0.0399657	0.968145163	0.983396094	no
KIAA0754	0.002222813	0.039948944	0.968158511	0.983396094	no
ABHD4	0.002201501	0.03956591	0.96846365	0.983659513	no
CCL27	0.002198306	0.039508494	0.96850939	0.983659513	no
PSMG1	-0.0021922	-0.039398752	0.968596815	0.983678104	no
NEO1	-0.002190634	-0.039370606	0.968619238	0.983678104	no
HSPB8	-0.002184393	-0.039258442	0.968708593	0.983722363	no
TRMT44	0.002179585	0.039172026	0.968777437	0.98374579	no

CEP85L	-0.002166994	-0.038945739	0.96895771	0.983846496	no
LINC00410	0.002166263	0.038932609	0.96896817	0.983846496	no
RPS26	-0.002155991	-0.038747991	0.969115248	0.983923828	no
HYALP1	-0.002148216	-0.038608253	0.969226573	0.983923828	no
HIST1H3B	0.00214804	0.038605097	0.969229088	0.983923828	no
RTP2	-0.002142982	-0.038514189	0.969301512	0.983923828	no
OR4B1	0.002141055	0.038479566	0.969329095	0.983923828	no
COL6A4P1	0.002138748	0.038438102	0.969362129	0.983923828	no
STARD13-AS2	0.002138561	0.038434732	0.969364814	0.983923828	no
HSFY1	0.002135347	0.038376968	0.969410833	0.983924067	no
CCDC129	0.00212949	0.038271704	0.969494694	0.983962714	no
SLC44A5	-0.002124588	-0.038183609	0.969564878	0.983977375	no
CCL16	-0.002122085	-0.038138632	0.969600711	0.983977375	no
IQCD	-0.0021118	-0.03795377	0.969747988	0.984036614	no
PPIG	-0.002109627	-0.037914721	0.969779098	0.984036614	no
SERPINA9	-0.002108129	-0.037887806	0.969800541	0.984036614	no
IFIT1B	0.002105217	0.03783547	0.969842237	0.984036614	no
ZNF213	0.002079525	0.03737373	0.970210106	0.9843299	no
SMKR1	0.002076589	0.037320954	0.970252154	0.9843299	no
PARS2	-0.002075395	-0.037299501	0.970269245	0.9843299	no
ALDH18A1	-0.002069451	-0.037192676	0.970354354	0.9843299	no
PRSS57	-0.002069036	-0.03718521	0.970360302	0.9843299	no
CDKL4	0.002064912	0.03711109	0.970419355	0.98434334	no
SLC35E4	-0.002053237	-0.036901273	0.97058652	0.984466438	no
PMP2	-0.001967331	-0.035357331	0.971816649	0.985605211	no
LINC00658	0.001966063	0.03533455	0.9718348	0.985605211	no
MFF	0.001965224	0.035319463	0.97184682	0.985605211	no
DYTN	-0.001961033	-0.035244155	0.971906824	0.985619552	no
WRNIP1	0.00192564	0.034608057	0.972413654	0.986066757	no
CHURC1	0.00192383	0.034575529	0.972439572	0.986066757	no
HTR3D	-0.001917672	-0.03446485	0.97252776	0.986109653	no
DNHD1	-0.001909894	-0.034325054	0.972639148	0.986151295	no
CST9	-0.001905397	-0.034244243	0.972703538	0.986151295	no
BRD4	0.001903095	0.034202873	0.972736502	0.986151295	no
SUPT20HL1	-0.001898539	-0.03412098	0.972801754	0.986151295	no
KRTAP27-1	-0.001897331	-0.034099283	0.972819043	0.986151295	no
ZNF470	-0.001892991	-0.034021284	0.972881193	0.986151295	no
TDRD10	-0.001891585	-0.033996002	0.972901338	0.986151295	no
LOC284632	0.00188917	0.033952598	0.972935922	0.986151295	no
NRON	-0.001869946	-0.033607105	0.973211214	0.986383805	no
MIR3909	-0.001862008	-0.033464437	0.973324895	0.986452502	no
BOD1	0.001856288	0.033361636	0.973406809	0.986489	no
OR13C9	-0.001852949	-0.033301634	0.97345462	0.986490934	no
FAM183B	-0.001840791	-0.033083119	0.973628739	0.986546025	no
OR5B21	0.001840148	0.03307156	0.973637949	0.986546025	no
FSHR	-0.001839537	-0.033060581	0.973646698	0.986546025	no
MUTYH	-0.001801001	-0.032367996	0.974198576	0.987058677	no
SAR1A	0.001791695	0.032200751	0.974331846	0.987147167	no
ST3GAL6-AS1	0.001786245	0.032102803	0.974409896	0.987179704	no
KLHL2	0.001776805	0.031933148	0.974545087	0.98726953	no
ONECUT3	0.001771061	0.031829918	0.974627347	0.98726953	no

CNTNAP1	-0.001770431	-0.031818588	0.974636375	0.98726953	no
SPATA8	-0.001760283	-0.03163621	0.974781705	0.987345962	no
ENAH	0.001758746	0.031608591	0.974803714	0.987345962	no
MZF1	0.001750341	0.031457523	0.974924096	0.98740714	no
OR2G2	0.001748113	0.03141748	0.974956005	0.98740714	no
TOMM7	0.001704929	0.030641367	0.975574476	0.987889328	no
GCK	0.001704614	0.030635704	0.975578988	0.987889328	no
ASB4	-0.001703472	-0.030615187	0.975595338	0.987889328	no
LOC643441	-0.001701953	-0.030587892	0.975617089	0.987889328	no
SMC1B	-0.001698821	-0.030531597	0.975661951	0.987889328	no
CSDAP1	0.00169306	0.030428055	0.975744463	0.987926329	no
NUTF2	-0.001678878	-0.030173183	0.975947571	0.98793877	no
RP1	-0.001678621	-0.030168563	0.975951252	0.98793877	no
OR1N1	-0.00167478	-0.03009953	0.976006265	0.98793877	no
CCDC34	0.001673547	0.030077356	0.976023935	0.98793877	no
MOV10	0.001670762	0.030027307	0.97606382	0.98793877	no
WDR65	-0.001669187	-0.029999004	0.976086375	0.98793877	no
LOC285626	0.001665764	0.02993748	0.976135403	0.98793877	no
KRTAP2-3	0.001662109	0.0298718	0.976187745	0.98793877	no
TEX33	-0.001658167	-0.029800946	0.976244209	0.98793877	no
ADAM18	-0.001657839	-0.029795049	0.976248909	0.98793877	no
CCDC51	0.001656895	0.029778095	0.976262419	0.98793877	no
DEFB129	0.001643451	0.029536475	0.97645497	0.98807736	no
OR4E2	-0.001638187	-0.029441858	0.976530372	0.98807736	no
C1orf210	0.00163658	0.029412989	0.976553379	0.98807736	no
AARD	0.001630747	0.029308152	0.976636926	0.98807736	no
MYLK3	-0.001630347	-0.029300955	0.976642661	0.98807736	no
UBOX5	-0.001628073	-0.029260087	0.97667523	0.98807736	no
FGF4	-0.001624787	-0.02920104	0.976722286	0.988078453	no
LOC100289561	-0.001620637	-0.029126455	0.976781724	0.988091015	no
DNASE2B	0.00161481	0.029021728	0.976865185	0.988091015	no
SOWAHC	0.001613388	0.028996165	0.976885557	0.988091015	no
KRT34	-0.00161108	-0.028954686	0.976918612	0.988091015	no
AIM1L	0.001604989	0.028845225	0.977005845	0.988132741	no
OR52E6	0.001595868	0.028681295	0.977136487	0.988218365	no
FAM127B	0.00158791	0.028538268	0.977250471	0.988287134	no
PASD1	-0.001584283	-0.028473095	0.977302411	0.988293154	no
CDK6	0.001572404	0.028259592	0.977472561	0.988418708	no
CBX3	0.001559565	0.028028852	0.977656449	0.988551835	no
OR52A1	0.001553941	0.027927769	0.977737007	0.988551835	no
SSX1	0.001553578	0.027921243	0.977742209	0.988551835	no
PIP5K1A	-0.001544894	-0.027765169	0.977866592	0.988629122	no
LDLRAD1	0.001541817	0.027709881	0.977910655	0.988629122	no
ENO3	-0.001528276	-0.027466507	0.978104615	0.988704368	no
TDRD12	0.001527139	0.027446084	0.978120892	0.988704368	no
LOC151174	0.001526985	0.027443311	0.978123101	0.988704368	no
PRPS1L1	0.001523737	0.02738493	0.978169629	0.988704896	no
MYBL1	0.001508034	0.027102723	0.97839454	0.98888572	no
LGALS9C	0.001502347	0.027000509	0.978476002	0.988921546	no
FIGNL1	-0.001485605	-0.026699618	0.978715806	0.989117394	no
MAGEB16	0.00148169	0.026629259	0.978771881	0.989127551	no

AZGP1	-0.001459505	-0.026230541	0.979089655	0.989376577	no
P2RY6	0.001458058	0.026204544	0.979110373	0.989376577	no
RPS7P5	-0.001446698	-0.026000378	0.979273093	0.989494477	no
CHST8	-0.001436754	-0.025821665	0.979415527	0.989570987	no
ANKLE2	0.001433827	0.025769045	0.979457465	0.989570987	no
TAS2R1	-0.001431595	-0.025728931	0.979489436	0.989570987	no
CCDC132	-0.001428554	-0.025674277	0.979532996	0.989570987	no
PRC1	-0.001420508	-0.025529689	0.979648233	0.989607843	no
TSEN34	-0.001417027	-0.025467127	0.979698095	0.989607843	no
CEACAM7	-0.001415287	-0.025435841	0.97972303	0.989607843	no
PRADC1	-0.001413148	-0.025397401	0.979753667	0.989607843	no
MTMR12	0.001405808	0.025265493	0.9798588	0.989667519	no
SCN7A	0.001402514	0.02520629	0.979905985	0.989668665	no
TTLL3	0.001394598	0.025064011	0.980019384	0.989700416	no
OR8I2	-0.001391624	-0.025010576	0.980061972	0.989700416	no
NIPAL4	0.001388254	0.024950011	0.980110244	0.989700416	no
KRT16P3	-0.00138746	-0.024935725	0.98012163	0.989700416	no
PLK1	-0.001371702	-0.02465252	0.98034735	0.989881832	no
ZDHHC4	0.001363687	0.024508471	0.980462161	0.989909469	no
LINC00470	0.001362732	0.024491308	0.980475841	0.989909469	no
MPHOSPH6	0.001360144	0.024444803	0.980512906	0.989909469	no
DAO	0.00135132	0.024286224	0.980639298	0.98995783	no
EIF1B-AS1	0.001350368	0.024269114	0.980652935	0.98995783	no
DPY30	0.001336847	0.024026102	0.980846624	0.990106848	no
SZT2	-0.001330734	-0.023916235	0.980934192	0.990148736	no
BARHL2	0.001322419	0.023766794	0.981053303	0.990167197	no
LOC643037	-0.001319444	-0.023713335	0.981095912	0.990167197	no
WDR35	-0.001318418	-0.023694889	0.981110614	0.990167197	no
PFKFB1	-0.001314018	-0.023615816	0.981173639	0.990167197	no
EXOC8	-0.001307996	-0.023507584	0.981259905	0.990167197	no
LOC100129636	0.001305382	0.023460608	0.981297347	0.990167197	no
MEIS1-AS3	0.001304061	0.023436859	0.981316276	0.990167197	no
AIPL1	0.001303217	0.023421692	0.981328364	0.990167197	no
SSX7	-0.001299844	-0.023361079	0.981376676	0.990167197	no
TEX38	-0.001297293	-0.023315233	0.981413218	0.990167197	no
CCT8	-0.00129183	-0.023217041	0.981491482	0.990199673	no
KCNA10	-0.001267161	-0.022773689	0.981844858	0.990509686	no
S100A5	-0.001259703	-0.022639662	0.981951686	0.990570957	no
PIK3C3	-0.001251733	-0.022496407	0.982065869	0.990639643	no
LOC100507250	-0.001248094	-0.022431022	0.982117985	0.990645715	no
MIR3180-5	-0.001242506	-0.022330587	0.982198039	0.990679967	no
GRB10	0.001231695	0.022136287	0.98235291	0.9907406	no
ADH6	-0.001226871	-0.022049593	0.982422011	0.9907406	no
NAPRT1	-0.001226589	-0.022044514	0.982426059	0.9907406	no
C7orf45	-0.001225437	-0.02202382	0.982442554	0.9907406	no
KRTAP6-3	-0.001210612	-0.021757378	0.982654928	0.990864178	no
MED25	-0.001210446	-0.02175439	0.982657309	0.990864178	no
SPATA32	-0.001200467	-0.021575048	0.982800259	0.990961827	no
SLC7A11	0.001186263	0.021319768	0.983003739	0.991120495	no
ZCCHC8	0.001173406	0.021088701	0.98318792	0.991230375	no
HSD17B8	-0.001172216	-0.021067316	0.983204965	0.991230375	no

SLCO1B7	-0.001166296	-0.020960925	0.983289769	0.991269369	no
OR10K2	0.001153564	0.020732094	0.983472169	0.991406744	no
MUC17	-0.001149825	-0.020664903	0.983525726	0.99141423	no
VWC2L-IT1	-0.001144089	-0.020561808	0.983607902	0.991450562	no
TTC3P1	-0.001110373	-0.01995586	0.984090906	0.991854467	no
CCDC102B	0.001107	0.019895246	0.984139222	0.991854467	no
ACADM	-0.001105742	-0.019872633	0.984157247	0.991854467	no
C19orf18	-0.001103231	-0.019827502	0.984193221	0.991854467	no
SNX6	0.001095342	0.019685716	0.98430624	0.991921851	no
PYGM	0.001084862	0.019497374	0.98445637	0.992026626	no
HS6ST2-AS1	-0.001075157	-0.019322948	0.984595408	0.992077603	no
RFPL1	-0.001074886	-0.019318087	0.984599283	0.992077603	no
LRRC49	-0.001064233	-0.019126615	0.984751909	0.992155245	no
OR13C4	0.001060136	0.019052992	0.984810595	0.992155245	no
CORO2B	0.00105984	0.01904767	0.984814838	0.992155245	no
TLK1	0.001055902	0.018976886	0.984871262	0.992165578	no
C17orf47	0.001047765	0.018830652	0.984987828	0.992236496	no
CNR1	-0.001038823	-0.018669941	0.985115935	0.992319032	no
NKX2-3	-0.001034903	-0.018599487	0.985172096	0.992329093	no
PLA2G2E	-0.001024261	-0.018408238	0.985324546	0.992436135	no
SMARCAL1	-0.001017694	-0.018290219	0.985418623	0.992484377	no
CCNL2	0.001013637	0.018217302	0.985476748	0.992492253	no
HOXC4	0.001010701	0.018164539	0.985518807	0.992492253	no
UBQLN1	0.001004119	0.018046245	0.985613104	0.99251515	no
PWRN1	-0.001001967	-0.018007553	0.985643946	0.99251515	no
CT62	-0.000999444	-0.017962209	0.985680091	0.99251515	no
C10orf40	-0.000993854	-0.01786176	0.985760163	0.992516031	no
GSTT1	-0.00099143	-0.017818189	0.985794896	0.992516031	no
KCND1	-0.000989712	-0.017787304	0.985819515	0.992516031	no
MMGT1	-0.000978045	-0.017577624	0.98598666	0.992637809	no
MSH4	-0.000959194	-0.017238841	0.98625672	0.99286318	no
TSR1	-0.000947486	-0.017028414	0.986424462	0.992864358	no
VSIG10	-0.000947483	-0.017028369	0.986424497	0.992864358	no
JRKL-AS1	-0.000946524	-0.017011131	0.986438238	0.992864358	no
EFCAB14-AS1	-0.000945736	-0.016996964	0.986449532	0.992864358	no
HOTAIR	-0.000941351	-0.016918164	0.986512347	0.992864358	no
CNBP	-0.000939764	-0.016889641	0.986535085	0.992864358	no
EEF1E1-MUTED	-0.000916096	-0.016464267	0.986874174	0.993159114	no
TMIGD1	-0.000891814	-0.016027858	0.987222063	0.993416463	no
FAM66B	0.000891794	0.016027505	0.987222344	0.993416463	no
LINC00310	-0.000881645	-0.015845116	0.987367738	0.993495082	no
SPDYE6	-0.000876928	-0.015760323	0.987435332	0.993495082	no
LOC340113	-0.00087666	-0.015755524	0.987439158	0.993495082	no
PCDHB4	-0.000849504	-0.015267463	0.987828226	0.993755658	no
ASB12	0.000847337	0.015228522	0.987859269	0.993755658	no
MTERF	-0.000845739	-0.015199799	0.987882166	0.993755658	no
GAST	0.000845673	0.015198617	0.987883108	0.993755658	no
SLC19A1	0.000834323	0.014994618	0.988045732	0.993806581	no
TTN	-0.000833918	-0.014987353	0.988051523	0.993806581	no
C22orf42	0.000832457	0.014961091	0.988072459	0.993806581	no
ARRDC3-AS1	-0.000820333	-0.014743189	0.988246165	0.993913732	no

ZNF669	-0.000814979	-0.014646964	0.988322874	0.993913732	no
IVNS1ABP	-0.000812165	-0.014596389	0.988363192	0.993913732	no
OR56A4	-0.000812109	-0.014595396	0.988363983	0.993913732	no
ADAMTSL3	0.000800601	0.014388571	0.988528861	0.993961851	no
TGM4	0.000796598	0.014316618	0.988586221	0.993961851	no
RGS21	0.000794513	0.014279158	0.988616083	0.993961851	no
MTVR2	0.000793789	0.014266145	0.988626457	0.993961851	no
KRTAP5-8	0.000790702	0.014210659	0.988670689	0.993961851	no
EIF3B	0.000789401	0.014187271	0.988689334	0.993961851	no
OR5V1	0.000783236	0.014076476	0.988777658	0.994004147	no
GOSR1	0.000769842	0.013835751	0.988969562	0.994150562	no
TMEM66	-0.000735958	-0.013226785	0.989455026	0.994531801	no
STAC3	0.000734403	0.01319885	0.989477296	0.994531801	no
DBR1	-0.000733681	-0.013185868	0.989487645	0.994531801	no
OR1J4	-0.000722799	-0.012990292	0.989643558	0.994641992	no
ANKRD36BP2	0.000717651	0.01289777	0.989717317	0.994669606	no
MRGPRX1	0.000713306	0.012819684	0.989779567	0.994685652	no
SLC35B1	-0.000709459	-0.012750536	0.989834692	0.994694537	no
USP36	0.000704881	0.012668263	0.98990028	0.994713935	no
KRTAP11-1	0.000698027	0.012545091	0.989998473	0.994743314	no
LRRC37A5P	0.000695594	0.01250136	0.990033336	0.994743314	no
MTFR1	0.000693148	0.012457405	0.990068377	0.994743314	no
DRAXIN	0.000677253	0.012171735	0.990296115	0.994925614	no
C22orf31	0.00066612	0.011971642	0.990455631	0.995039101	no
C15orf60	-0.000660932	-0.011878405	0.99052996	0.995039101	no
TMEM14E	0.000654753	0.011767362	0.990618485	0.995039101	no
ASB15	-0.000653913	-0.011752256	0.990630528	0.995039101	no
ZNF688	-0.000653212	-0.011739652	0.990640575	0.995039101	no
DNAJA1	0.000649591	0.011674591	0.990692443	0.995044693	no
CRNDE	-0.00064374	-0.011569434	0.990776276	0.995082388	no
OR6C6	-0.000636616	-0.011441401	0.990878345	0.995108802	no
HTA	-0.000635441	-0.011420281	0.990895183	0.995108802	no
HAUS5	0.0006195	0.011133778	0.991123588	0.99529167	no
LOC100506025	-0.000610674	-0.010975156	0.991250044	0.995372147	no
GSTM5	-0.000601631	-0.010812642	0.991379603	0.99541213	no
PDGFC	-0.000595772	-0.010707334	0.991463557	0.99541213	no
DHX15	-0.000594573	-0.01068579	0.991480733	0.99541213	no
DSG4	-0.000594139	-0.010677993	0.991486948	0.99541213	no
TRA2B	0.000590375	0.010610344	0.991540879	0.99541213	no
SNORA81	0.000588499	0.010576617	0.991567767	0.99541213	no
ZC3H10	0.000582539	0.010469517	0.99165315	0.995451345	no
KRTAP24-1	0.0005683	0.010213606	0.991857169	0.995609641	no
ZBTB41	0.000562911	0.010116755	0.991934381	0.995640642	no
TMEM229A	-0.000539618	-0.009698121	0.992268127	0.99592912	no
ALDH1A1	0.00051831	0.009315167	0.99257343	0.996126719	no
TYMS	0.00051678	0.009287678	0.992595345	0.996126719	no
TFF1	0.000516172	0.009276757	0.992604052	0.996126719	no
CT45A5	-0.000485571	-0.008726776	0.993042516	0.996453318	no
LOC550113	0.000485207	0.008720236	0.99304773	0.996453318	no
KRTAP3-2	0.000483751	0.008694065	0.993068595	0.996453318	no
GPX6	0.000476414	0.008562214	0.993173711	0.996500596	no

MIR941-1	-0.00047399	-0.008518642	0.993208448	0.996500596	no
LDHAL6A	-0.000464282	-0.008344168	0.993347545	0.996593628	no
DLGAP5	-0.000447987	-0.008051321	0.993581015	0.996781327	no
ZNF700	0.000440676	0.007919925	0.99368577	0.996839886	no
PIAS1	0.000434234	0.007804147	0.993778073	0.996885948	no
MLLT1	-0.000427902	-0.007690337	0.993868807	0.99690395	no
LPAR2	0.000426507	0.007665269	0.993888792	0.99690395	no
IRF2BP2	0.000414163	0.00744343	0.994065652	0.997034813	no
ACAD9	0.000398539	0.007162619	0.994289527	0.997212816	no
ENPP7	0.00038062	0.006840573	0.994546278	0.997272107	no
TSGA10	-0.000380212	-0.006833255	0.994552112	0.997272107	no
OR5H2	-0.000378114	-0.006795542	0.994582179	0.997272107	no
MT1X	0.000375764	0.0067533	0.994615856	0.997272107	no
MMP26	-0.000375376	-0.006746333	0.994621411	0.997272107	no
TBC1D21	-0.000374981	-0.006739236	0.994627069	0.997272107	no
KPNB1	-0.000364875	-0.006557614	0.994771867	0.997370758	no
TGM5	-0.000354362	-0.00636866	0.994922511	0.99747526	no
LOC653501	-0.000340616	-0.00612162	0.995119463	0.997626179	no
HEATR8-TTC4	-0.000322084	-0.005788557	0.995384999	0.997845835	no
MYBBP1A	0.000310943	0.005588323	0.995544637	0.997922878	no
OR6F1	-0.000310239	-0.005575675	0.99555472	0.997922878	no
SMEK3P	0.000301862	0.005425131	0.995674742	0.997960845	no
OR2A12	-0.000301114	-0.005411675	0.99568547	0.997960845	no
BCAP29	0.000292011	0.005248076	0.995815901	0.997993494	no
KRTAP7-1	-0.000291802	-0.005244323	0.995818893	0.997993494	no
CYMP	0.000289117	0.005196076	0.995857358	0.997993494	no
LINC00486	0.000281599	0.00506095	0.995965088	0.998054915	no
GCNT7	0.000262277	0.004713694	0.99624194	0.99819743	no
MRGPRX2	0.000260177	0.00467595	0.996272032	0.99819743	no
OR7G1	0.000259191	0.004658228	0.996286161	0.99819743	no
ZNF479	-0.000254796	-0.00457924	0.996349135	0.99819743	no
LINC00297	0.000253025	0.004547417	0.996374506	0.99819743	no
SUGT1	0.000249007	0.004475213	0.996432071	0.99819743	no
HRG	-0.000248982	-0.004474757	0.996432435	0.99819743	no
F11	0.000240309	0.004318883	0.996556707	0.998275388	no
LOC100286922	-0.000235095	-0.004225183	0.99663141	0.998303688	no
C1orf100	-0.000217492	-0.003908817	0.996883636	0.998509798	no
COL19A1	-0.000211566	-0.003802313	0.996968547	0.998535609	no
UGT1A10	0.000209209	0.003759939	0.997002331	0.998535609	no
IMMP2L	0.000203527	0.003657834	0.997083736	0.998570603	no
TBL1Y	-0.000199253	-0.003581013	0.997144981	0.998585405	no
THPO	-0.000194523	-0.003496002	0.997212758	0.998606746	no
EDN2	-0.000188487	-0.003387532	0.997299237	0.998646813	no
ELAC1	0.000184226	0.003310955	0.997360288	0.998661415	no
FAM138B	-0.000172016	-0.003091504	0.997535249	0.998748119	no
PPIEL	0.000171697	0.003085764	0.997539825	0.998748119	no
SLC38A5	-0.00016175	-0.002906998	0.997682349	0.99881621	no
CSNK1G2-AS1	-0.000156067	-0.00280487	0.997763772	0.99881621	no
DUOX2	0.000153306	0.002755243	0.997803338	0.99881621	no
FKBPL	-0.000153076	-0.002751121	0.997806624	0.99881621	no
BMP10	-0.000150658	-0.002707652	0.997841281	0.99881621	no

MRPL22	0.000147489	0.002650696	0.99788669	0.99881621	no
TRIM15	-0.00012637	-0.002271156	0.998189284	0.999026738	no
KRTAP15-1	-0.00012351	-0.002219749	0.998230268	0.999026738	no
OR51Q1	-0.000121661	-0.002186515	0.998256765	0.999026738	no
POF1B	-0.000119832	-0.002153652	0.998282965	0.999026738	no
CDS1	-0.000116378	-0.002091568	0.998332463	0.999029752	no
TNNT1	9.05E-05	0.00162616	0.998703517	0.999253685	no
TTLL9	-8.99E-05	-0.00161575	0.998711816	0.999253685	no
RAB4B-EGLN2	8.89E-05	0.00159854	0.998725537	0.999253685	no
RDH13	8.49E-05	0.001525139	0.998784057	0.999253685	no
FHIT	8.30E-05	0.001491183	0.998811129	0.999253685	no
ANP32C	-8.13E-05	-0.001460969	0.998835218	0.999253685	no
CCL3L3	7.45E-05	0.001338276	0.998933037	0.999305027	no
XPNPEP1	6.85E-05	0.001230361	0.999019074	0.999344578	no
AKT1S1	-5.90E-05	-0.001059615	0.999155204	0.999407994	no
LINC00102	5.75E-05	0.001034188	0.999175476	0.999407994	no
OR4S2	-4.36E-05	-0.000784179	0.9993748	0.999560842	no
C1orf180	-3.09E-05	-0.000555477	0.999557136	0.999696687	no
PARD6B	2.52E-05	0.000452296	0.999639399	0.999732437	no
FYTTD1	1.97E-05	0.000354522	0.999717351	0.999763871	no
MARS	-8.49E-07	-1.53E-05	0.999987828	0.999987828	no
