# Supplementary material for: CD96, a new immune checkpoint, correlates with immune profile and clinical outcome of glioma
Source: Sci Rep. 2020 Jul 1;10:10768. doi: 10.1038/s41598-020-66806-z (PMC7330044; doi:10.1038/s41598-020-66806-z)
Supplement: Supplementary file 5 — Supplementary Information. [file 41598_2020_66806_MOESM5_ESM.pdf]

CD96, a new immune checkpoint, correlates with immune profile and clinical outcome of glioma

Fangkun Liu[1, 2 †], Jing Huang[3, 4], Fengqiong He[1, 2], Xiaodong Ma[5], Fan Fan[1, 2], Ming Meng[1, 2], Yang Zhuo[1, 2], and Liyang Zhang[1, 2 *]

[1] Department of Neurosurgery, Xiangya Hospital, Central South University, Central South University; 87 Xiangya Road; Changsha, Hunan, 410008. China;

[2] Clinical Diagnosis and Therapy Center for Glioma of Xiangya Hospital, Central South University; 87 Xiangya Road; Changsha, Hunan, 410008. China;

[3] Department of Psychiatry, The Second Xiangya Hospital, Central South University, Changsha, Hunan 410011, China;

[4] Mental Health Institute of the Second Xiangya Hospital, Central South University, Chinese National Clinical Research Center on Mental Disorders (xiangya), Chinese National Technology Institute on Mental Disorders, Hunan Key Laboratory of Psychiatry and Mental Health, Changsha, Hunan 410011, China;

[5] Director and Training and Exchange Cooperation Center, Orient Science & Technology College, Hunan Agricultural University, Changsha, Hunan 410000, China.

[†]The first author;

[*]Corresponding Author:

Dr. Liyang Zhang MD, Ph. D

Department of Neurosurgery, Xiangya Hospital, Central South University

87 Xiangya Rd, Changsha, Hunan, 410008, China,

Email: zhangliyang@csu.edu.cn

gene	cor	t.value	p.value	FDR	significant
LCK	0.957297036	87.4189774	0	0	yes
CD3G	0.951261179	81.4368469	0	0	yes
CD2	0.947214404	78.00098355	0	0	yes
CD3E	0.944886702	76.1939995	0	0	yes
ICOS	0.943504476	75.17277117	0	0	yes
PYHIN1	0.940047741	72.77082753	0	0	yes
SIRPG	0.939364704	72.31995251	0	0	yes
P2RY10	0.922382418	63.04163324	3.84E-290	9.71E-287	yes
CD5	0.921228008	62.51844298	5.35E-288	1.20E-284	yes
TRAT1	0.92079764	62.32621012	3.31E-287	6.69E-284	yes
SIT1	0.91743398	60.87378756	3.55E-281	6.53E-278	yes
SH2D1A	0.90670004	56.75421311	1.60E-263	2.70E-260	yes
GZMK	0.900437282	54.65057236	3.49E-254	5.43E-251	yes
SLAMF6	0.898665556	54.0893428	1.18E-251	1.70E-248	yes
CXCR6	0.892954527	52.37107505	8.26E-244	1.11E-240	yes
CD27	0.892232011	52.16294555	7.55E-243	9.54E-240	yes
SLA2	0.888913376	51.23171864	1.60E-238	1.90E-235	yes
IL2RB	0.888844082	51.21269419	1.96E-238	2.21E-235	yes
CXCR3	0.887798657	50.92769355	4.23E-237	4.50E-234	yes
CCR7	0.876391153	48.04275554	2.34E-223	2.37E-220	yes
CD247	0.87241658	47.12397754	6.93E-219	6.67E-216	yes
IL2RG	0.86983872	46.54919178	4.58E-216	4.21E-213	yes
CCR4	0.867720758	46.08871029	8.58E-214	7.55E-211	yes
CD40LG	0.864825576	45.4756109	9.49E-211	7.99E-208	yes
TBC1D10C	0.863505756	45.20214954	2.19E-209	1.77E-206	yes
GPR171	0.863099545	45.1187232	5.73E-209	4.46E-206	yes
LY9	0.862943315	45.08672863	8.28E-209	6.20E-206	yes
SLAMF1	0.861509844	44.79551279	2.38E-207	1.72E-204	yes
CD3D	0.855157376	43.55358059	4.48E-201	3.12E-198	yes
CCL5	0.846976862	42.06090926	2.00E-193	1.35E-190	yes
CD6	0.832029156	39.59786696	1.53E-180	1.00E-177	yes
GVIN1	0.826687792	38.78923131	3.05E-176	1.93E-173	yes
TXK	0.823802471	38.36636669	5.57E-174	3.41E-171	yes
IKZF3	0.823311887	38.29540143	1.34E-173	7.95E-171	yes
ACAP1	0.820080087	37.83448183	4.01E-171	2.31E-168	yes
GRAP2	0.818194135	37.57064752	1.06E-169	5.95E-167	yes
GZMA	0.797315653	34.87581747	5.52E-155	3.01E-152	yes
EOMES	0.79432709	34.52044687	5.09E-153	2.71E-150	yes
GPR18	0.791799118	34.22515799	2.21E-151	1.14E-148	yes
FASLG	0.790148006	34.03484825	2.52E-150	1.27E-147	yes
CD8A	0.787608674	33.74599704	1.02E-148	5.02E-146	yes
CCR8	0.784375162	33.38472043	1.05E-146	5.07E-144	yes
ZNF683	0.776699713	32.55501331	4.65E-142	2.19E-139	yes
PARP15	0.774998818	32.37620907	4.70E-141	2.16E-138	yes
ZC3H12D	0.770135172	31.87453585	3.14E-138	1.41E-135	yes
BTLA	0.766790149	31.53747965	2.51E-136	1.10E-133	yes
LTB	0.761263569	30.99416291	2.97E-133	1.28E-130	yes
IL7R	0.759800019	30.8530104	1.87E-132	7.89E-130	yes
SLAMF8	0.755393584	30.4346636	4.44E-130	1.83E-127	yes
ZAP70	0.751962282	30.11557825	2.89E-128	1.17E-125	yes

FCRL3	0.750058543	29.94098705	2.85E-127	1.13E-124	yes
HLA-DQA1	0.749481076	29.88836559	5.68E-127	2.21E-124	yes
ANO9	0.748433096	29.79326583	1.98E-126	7.55E-124	yes
CTSW	0.74451839	29.44246856	1.98E-124	7.41E-122	yes
CCR5	0.7422304	29.24061374	2.81E-123	1.03E-120	yes
GFI1	0.741628341	29.18787838	5.62E-123	2.03E-120	yes
CXCR5	0.74128896	29.15822059	8.30E-123	2.94E-120	yes
ZBED2	0.740618175	29.09974795	1.79E-122	6.24E-120	yes
GZMH	0.730596601	28.24844707	1.33E-117	4.55E-115	yes
CCR2	0.729354941	28.14577407	5.14E-117	1.73E-114	yes
C16orf54	0.723044051	27.63297267	4.47E-114	1.48E-111	yes
NKG7	0.715780565	27.06066925	8.57E-111	2.80E-108	yes
FCRL6	0.714493948	26.96120549	3.19E-110	1.02E-107	yes
SLAMF7	0.711228514	26.71126102	8.68E-109	2.74E-106	yes
HLA-DPB1	0.709276699	26.56354147	6.12E-108	1.90E-105	yes
CD7	0.707127316	26.402291	5.16E-107	1.58E-104	yes
DAPP1	0.706625782	26.36487672	8.45E-107	2.55E-104	yes
EMB	0.705271842	26.26426848	3.20E-106	9.50E-104	yes
GCET2	0.704401359	26.19988725	7.49E-106	2.19E-103	yes
PTPN22	0.699914813	25.87174464	5.73E-104	1.65E-101	yes
S1PR4	0.699721685	25.85775582	6.89E-104	1.96E-101	yes
CD79A	0.696632911	25.63552414	1.30E-102	3.65E-100	yes
XCL2	0.695442051	25.5505876	3.99E-102	1.11E-99	yes
PDCD1	0.694214018	25.46342663	1.26E-101	3.45E-99	yes
XCR1	0.692262765	25.32581553	7.78E-101	2.10E-98	yes
HLA-DPA1	0.690538517	25.20510211	3.83E-100	1.02E-97	yes
PTPRC	0.690077742	25.17298305	5.86E-100	1.54E-97	yes
CD28	0.687834422	25.01744011	4.57E-99	1.18E-96	yes
CNR2	0.687066904	24.96453706	9.18E-99	2.35E-96	yes
CST7	0.685210512	24.83723327	4.93E-98	1.25E-95	yes
HLA-DOA	0.685161238	24.83386674	5.15E-98	1.29E-95	yes
LOC100188949	0.680763195	24.53594076	2.62E-96	6.47E-94	yes
RAC2	0.676392127	24.24474314	1.22E-94	2.97E-92	yes
FOXP3	0.675172277	24.16432721	3.51E-94	8.46E-92	yes
CD52	0.671836252	23.94625774	6.21E-93	1.48E-90	yes
GPR141	0.67128949	23.91077214	9.90E-93	2.33E-90	yes
CD8B	0.670781385	23.87785909	1.53E-92	3.55E-90	yes
DENND2D	0.670056434	23.83100541	2.83E-92	6.50E-90	yes
FCRL1	0.668805438	23.750444	8.16E-92	1.85E-89	yes
TLR8	0.668249572	23.71476479	1.30E-91	2.93E-89	yes
TRAF3IP3	0.668032779	23.70086899	1.57E-91	3.48E-89	yes
ARHGAP30	0.666874136	23.62678789	4.14E-91	9.11E-89	yes
MS4A1	0.663011767	23.38205057	1.03E-89	2.24E-87	yes
1-Sep	0.659579539	23.16736816	1.73E-88	3.71E-86	yes
IL16	0.656193076	22.95806289	2.68E-87	5.70E-85	yes
CSF2RB	0.654870839	22.87700473	7.75E-87	1.63E-84	yes
HLA-DRA	0.65295673	22.76031328	3.57E-86	7.43E-84	yes
APOBEC3D	0.65257559	22.73716861	4.83E-86	9.88E-84	yes
TNFRSF13B	0.65257326	22.73702722	4.84E-86	9.88E-84	yes
PSTPIP2	0.6504711	22.60991281	2.55E-85	5.15E-83	yes
LY75	0.649448438	22.54840053	5.69E-85	1.14E-82	yes

CD74	0.648440818	22.48799992	1.25E-84	2.48E-82	yes
HLA-DMA	0.644754338	22.26874543	2.19E-83	4.30E-81	yes
IL10RA	0.644405327	22.24812674	2.87E-83	5.57E-81	yes
KLRB1	0.644174989	22.23453203	3.42E-83	6.59E-81	yes
CYTIP	0.643478012	22.19345884	5.84E-83	1.11E-80	yes
FCRL2	0.643272801	22.18138358	6.84E-83	1.29E-80	yes
CTSS	0.640882877	22.04135094	4.23E-82	7.93E-80	yes
PTPN7	0.640836468	22.03864252	4.39E-82	8.14E-80	yes
TFEC	0.640482739	22.01801248	5.74E-82	1.05E-79	yes
CLEC12A	0.638197102	21.88528063	3.22E-81	5.87E-79	yes
LAIR1	0.637082368	21.82090082	7.44E-81	1.34E-78	yes
LAX1	0.635424283	21.72556634	2.57E-80	4.59E-78	yes
SH2D3A	0.634951114	21.69845336	3.65E-80	6.47E-78	yes
P2RY8	0.634896097	21.69530352	3.80E-80	6.68E-78	yes
PTGER4	0.631901965	21.52471019	3.47E-79	6.05E-77	yes
LCP1	0.631409741	21.49681973	4.99E-79	8.61E-77	yes
CCR6	0.630678965	21.45549203	8.51E-79	1.46E-76	yes
FAIM3	0.630322094	21.43534435	1.11E-78	1.88E-76	yes
POU2AF1	0.628134867	21.31235159	5.43E-78	9.14E-76	yes
PTPRCAP	0.627742058	21.2903518	7.21E-78	1.21E-75	yes
RHOH	0.627070643	21.2528104	1.17E-77	1.94E-75	yes
CCL21	0.626925633	21.24471258	1.30E-77	2.14E-75	yes
HLA-DRB1	0.626714995	21.23295637	1.51E-77	2.47E-75	yes
GPR132	0.626307165	21.21021626	2.03E-77	3.29E-75	yes
SLFN12L	0.626092693	21.198269	2.37E-77	3.80E-75	yes
MAP4K1	0.626004876	21.1933794	2.53E-77	4.02E-75	yes
FCRL5	0.6239904	21.08157735	1.07E-76	1.69E-74	yes
SAMD3	0.623044808	21.02933553	2.10E-76	3.29E-74	yes
GIMAP4	0.621994468	20.97148332	4.42E-76	6.88E-74	yes
CD48	0.621651112	20.95261154	5.64E-76	8.71E-74	yes
ITGAL	0.619559916	20.83809708	2.46E-75	3.77E-73	yes
TLR2	0.617231201	20.71142308	1.26E-74	1.91E-72	yes
LOC647121	0.615015288	20.59170177	5.85E-74	8.82E-72	yes
FGL2	0.613821084	20.52750745	1.33E-73	1.99E-71	yes
CETN1	0.613388607	20.5043156	1.79E-73	2.67E-71	yes
TGFBR2	0.612243924	20.44307361	3.93E-73	5.80E-71	yes
PIK3R5	0.612062133	20.43336651	4.45E-73	6.52E-71	yes
CD4	0.611634827	20.41057007	5.96E-73	8.67E-71	yes
FCGR2C	0.609968348	20.32193667	1.85E-72	2.68E-70	yes
DOK3	0.609158448	20.27901663	3.21E-72	4.60E-70	yes
CD79B	0.608732183	20.25646757	4.28E-72	6.09E-70	yes
C17orf87	0.606207745	20.12349577	2.34E-71	3.31E-69	yes
FERMT3	0.605712622	20.09752906	3.26E-71	4.57E-69	yes
BATF	0.605532602	20.08809704	3.67E-71	5.12E-69	yes
SASH3	0.605037582	20.06218595	5.11E-71	7.08E-69	yes
RCSD1	0.604018269	20.00894699	1.01E-70	1.39E-68	yes
CECR1	0.60396616	20.0062295	1.04E-70	1.42E-68	yes
JAK3	0.602450163	19.92734592	2.85E-70	3.86E-68	yes
GPR82	0.601646444	19.88566265	4.84E-70	6.52E-68	yes
RIPK3	0.601492153	19.8776715	5.36E-70	7.17E-68	yes
HCLS1	0.601293407	19.86738301	6.11E-70	8.12E-68	yes

APOBEC3H	0.600781552	19.84091245	8.55E-70	1.13E-67	yes
ALOX5	0.600267684	19.81437622	1.20E-69	1.57E-67	yes
ITGB2	0.598793853	19.73847964	3.14E-69	4.10E-67	yes
TNFSF8	0.597925734	19.69392141	5.53E-69	7.17E-67	yes
LHFPL2	0.597764665	19.68566604	6.14E-69	7.91E-67	yes
GIMAP6	0.597568155	19.67559918	6.98E-69	8.93E-67	yes
AOAH	0.595675816	19.57893959	2.37E-68	3.02E-66	yes
EPHA1	0.59496233	19.54262665	3.76E-68	4.75E-66	yes
RUNX3	0.59379161	19.48319751	7.98E-68	1.00E-65	yes
NFAM1	0.593683162	19.47770206	8.55E-68	1.07E-65	yes
LCP2	0.592870193	19.43655821	1.44E-67	1.78E-65	yes
ATP2A3	0.590939695	19.33922314	4.92E-67	6.06E-65	yes
CD200R1	0.590248506	19.30449809	7.62E-67	9.34E-65	yes
FAM113B	0.589578632	19.27090607	1.16E-66	1.42E-64	yes
GAPT	0.589487753	19.26635345	1.23E-66	1.49E-64	yes
MFSD1	0.588010193	19.19249187	3.13E-66	3.76E-64	yes
CD163	0.587144951	19.14937587	5.38E-66	6.43E-64	yes
FCGR3A	0.586952611	19.13980499	6.07E-66	7.21E-64	yes
SPN	0.586588292	19.12168991	7.62E-66	9.01E-64	yes
MS4A6A	0.585940832	19.08953984	1.14E-65	1.34E-63	yes
SIRPB2	0.585552565	19.07028688	1.45E-65	1.70E-63	yes
PLEK	0.585399822	19.0627183	1.60E-65	1.86E-63	yes
CXCL9	0.585210179	19.05332562	1.80E-65	2.08E-63	yes
AMICA1	0.584875649	19.03676845	2.21E-65	2.54E-63	yes
MS4A7	0.5836774	18.97758362	4.66E-65	5.32E-63	yes
SP140	0.583504385	18.96905352	5.18E-65	5.89E-63	yes
CASP8	0.582482623	18.91875746	9.74E-65	1.10E-62	yes
ALPK1	0.58243584	18.91645787	1.00E-64	1.13E-62	yes
FPR3	0.581638646	18.87731532	1.64E-64	1.83E-62	yes
HLA-DMB	0.58111826	18.85180852	2.25E-64	2.50E-62	yes
DOK2	0.579564688	18.77586724	5.83E-64	6.44E-62	yes
LILRB2	0.579242367	18.76015031	7.09E-64	7.79E-62	yes
IL15	0.578759145	18.73661241	9.51E-64	1.04E-61	yes
TEC	0.578691254	18.73330779	9.92E-64	1.08E-61	yes
C1QA	0.578531739	18.72554566	1.09E-63	1.18E-61	yes
IKZF1	0.577578339	18.67921965	1.95E-63	2.10E-61	yes
CIITA	0.577467402	18.67383666	2.08E-63	2.23E-61	yes
TLR1	0.576983269	18.65036321	2.79E-63	2.97E-61	yes
FAM26F	0.576008659	18.60319796	5.03E-63	5.32E-61	yes
CD53	0.57590404	18.59814214	5.36E-63	5.64E-61	yes
GPR31	0.575658488	18.58628084	6.21E-63	6.51E-61	yes
ARPC1B	0.574971748	18.55314812	9.39E-63	9.78E-61	yes
CD68	0.574815014	18.54559448	1.03E-62	1.07E-60	yes
DOCK2	0.574680421	18.53911033	1.12E-62	1.15E-60	yes
NCR1	0.574540462	18.53237007	1.22E-62	1.25E-60	yes
MYO1F	0.574343897	18.52290778	1.37E-62	1.39E-60	yes
NCKAP1L	0.574340518	18.52274517	1.37E-62	1.39E-60	yes
GPR65	0.573994658	18.50610789	1.69E-62	1.70E-60	yes
OBFC2A	0.573153352	18.46569927	2.79E-62	2.80E-60	yes
RNASE6	0.572928318	18.45490542	3.19E-62	3.19E-60	yes
TXNDC3	0.571681108	18.39519506	6.69E-62	6.67E-60	yes

LILRB1	0.571429987	18.38319557	7.77E-62	7.70E-60	yes
TMEM106A	0.570279486	18.32831825	1.54E-61	1.51E-59	yes
TMEM150B	0.570096973	18.31962734	1.71E-61	1.68E-59	yes
LYZ	0.569977524	18.31394157	1.83E-61	1.79E-59	yes
PIK3CG	0.567691851	18.20547388	7.03E-61	6.84E-59	yes
EMR2	0.567468284	18.1948979	8.02E-61	7.75E-59	yes
FAM177B	0.567147669	18.17974135	9.67E-61	9.31E-59	yes
SIGLEC10	0.567073483	18.17623607	1.01E-60	9.68E-59	yes
LRRC25	0.567036541	18.17449077	1.03E-60	9.84E-59	yes
GBP5	0.566278703	18.13872357	1.61E-60	1.52E-58	yes
DSC2	0.565878645	18.11986961	2.03E-60	1.91E-58	yes
C15orf53	0.565838189	18.11796404	2.08E-60	1.95E-58	yes
CD1C	0.565750089	18.11381504	2.18E-60	2.04E-58	yes
MGAT4A	0.56536585	18.0957301	2.73E-60	2.54E-58	yes
SCPEP1	0.56508354	18.0824537	3.22E-60	2.98E-58	yes
PLBD1	0.565056802	18.08119677	3.27E-60	3.02E-58	yes
DSE	0.565020827	18.07950574	3.34E-60	3.07E-58	yes
TRIM38	0.563482287	18.00732649	8.13E-60	7.44E-58	yes
ADAP2	0.563468412	18.00667683	8.19E-60	7.46E-58	yes
CYBB	0.56275014	17.97307504	1.24E-59	1.12E-57	yes
PLB1	0.562354148	17.95457547	1.56E-59	1.41E-57	yes
EVI2B	0.562049281	17.9403453	1.86E-59	1.67E-57	yes
EMR1	0.561910761	17.93388318	2.01E-59	1.80E-57	yes
KCNK6	0.561439899	17.91193343	2.63E-59	2.34E-57	yes
ARL11	0.560306296	17.85919329	5.03E-59	4.46E-57	yes
TIFAB	0.558634031	17.78165903	1.30E-58	1.15E-56	yes
SLC2A9	0.558283428	17.76544348	1.59E-58	1.40E-56	yes
ARHGDIB	0.558256636	17.76420487	1.62E-58	1.41E-56	yes
SYTL1	0.558240259	17.76344781	1.63E-58	1.42E-56	yes
CYTH4	0.557728251	17.73979453	2.18E-58	1.89E-56	yes
CCL19	0.556515228	17.68387346	4.33E-58	3.74E-56	yes
FYB	0.556473209	17.68193928	4.43E-58	3.81E-56	yes
GPSM3	0.555883294	17.65480593	6.17E-58	5.29E-56	yes
GIMAP2	0.555502694	17.63732054	7.65E-58	6.52E-56	yes
CD84	0.555423932	17.63370413	7.99E-58	6.79E-56	yes
ARHGAP9	0.555372923	17.63136232	8.22E-58	6.96E-56	yes
ARHGAP25	0.554042289	17.57037575	1.73E-57	1.46E-55	yes
PARVG	0.554019502	17.56933304	1.76E-57	1.47E-55	yes
TES	0.553643676	17.55214408	2.17E-57	1.81E-55	yes
IL4R	0.553350545	17.53874805	2.55E-57	2.12E-55	yes
CASP10	0.552718124	17.50987852	3.63E-57	3.01E-55	yes
KIAA0125	0.552542178	17.50185445	4.00E-57	3.30E-55	yes
ELF4	0.552337865	17.49254095	4.48E-57	3.68E-55	yes
CFLAR	0.551849676	17.47030545	5.88E-57	4.81E-55	yes
IL12RB1	0.551726168	17.46468414	6.29E-57	5.13E-55	yes
C1QB	0.551589035	17.45844463	6.79E-57	5.51E-55	yes
CMAH	0.550317543	17.40068891	1.37E-56	1.11E-54	yes
MLKL	0.550209625	17.3957949	1.46E-56	1.17E-54	yes
VNN1	0.548210285	17.30535148	4.37E-56	3.51E-54	yes
FES	0.547929397	17.29267912	5.10E-56	4.08E-54	yes
DENND1C	0.547850875	17.28913809	5.33E-56	4.24E-54	yes

ICAM3	0.547530379	17.27469169	6.35E-56	5.03E-54	yes
NCF4	0.54704781	17.25296029	8.26E-56	6.52E-54	yes
HLA-E	0.546962916	17.24913983	8.65E-56	6.81E-54	yes
C1QC	0.546940689	17.24813969	8.76E-56	6.86E-54	yes
ITGB7	0.546795174	17.24159323	9.48E-56	7.40E-54	yes
SPI1	0.546665192	17.23574747	1.02E-55	7.91E-54	yes
CLEC7A	0.546071947	17.20908954	1.41E-55	1.09E-53	yes
CD72	0.545914178	17.20200627	1.53E-55	1.18E-53	yes
SLA	0.544647534	17.14523279	3.05E-55	2.34E-53	yes
HLA-DQB2	0.544478361	17.13766279	3.34E-55	2.56E-53	yes
FAM78A	0.54438102	17.13330839	3.52E-55	2.69E-53	yes
TNFRSF1B	0.543268155	17.08359604	6.42E-55	4.88E-53	yes
MAN2B1	0.543251331	17.08284548	6.48E-55	4.90E-53	yes
SH2D2A	0.542786263	17.06210931	8.32E-55	6.28E-53	yes
MSR1	0.542651204	17.05609156	8.95E-55	6.73E-53	yes
SOAT1	0.541234749	16.99309196	1.91E-54	1.43E-52	yes
SLCO2B1	0.541064098	16.98551576	2.10E-54	1.56E-52	yes
OSCAR	0.540559031	16.96311025	2.75E-54	2.04E-52	yes
TMPRSS13	0.540194806	16.94696877	3.34E-54	2.47E-52	yes
GBGT1	0.539903619	16.93407386	3.90E-54	2.88E-52	yes
CTSC	0.539333843	16.90886666	5.28E-54	3.88E-52	yes
GIMAP7	0.539284906	16.90670317	5.42E-54	3.97E-52	yes
GIMAP1	0.539184485	16.90226442	5.71E-54	4.17E-52	yes
TMC8	0.539052525	16.89643309	6.13E-54	4.46E-52	yes
KLRD1	0.538947742	16.89180397	6.48E-54	4.70E-52	yes
MS4A4A	0.538251155	16.86105812	9.38E-54	6.77E-52	yes
IL15RA	0.53781779	16.84195491	1.18E-53	8.49E-52	yes
NOD2	0.537754571	16.83916972	1.22E-53	8.75E-52	yes
CSTA	0.536601952	16.78845949	2.24E-53	1.60E-51	yes
CD33	0.536583384	16.78764366	2.26E-53	1.61E-51	yes
NAGA	0.53555662	16.74258358	3.89E-53	2.76E-51	yes
APOBEC3C	0.535263879	16.72975559	4.53E-53	3.20E-51	yes
VAV1	0.535195322	16.72675258	4.70E-53	3.31E-51	yes
C2	0.535097337	16.72246141	4.95E-53	3.47E-51	yes
TNFSF13	0.534924962	16.71491468	5.41E-53	3.79E-51	yes
CALHM2	0.53464687	16.7027457	6.26E-53	4.37E-51	yes
BIN2	0.533887577	16.66955861	9.32E-53	6.47E-51	yes
CLEC10A	0.53374174	16.66319086	1.01E-52	6.96E-51	yes
ITGAM	0.53281248	16.62266482	1.63E-52	1.13E-50	yes
RAB27A	0.532422818	16.60569622	2.00E-52	1.37E-50	yes
SYK	0.532016993	16.58803948	2.47E-52	1.69E-50	yes
C3AR1	0.531568728	16.56855474	3.11E-52	2.13E-50	yes
DAB2	0.530846615	16.53720747	4.52E-52	3.08E-50	yes
PILRA	0.530263883	16.51194739	6.11E-52	4.15E-50	yes
APOBEC3G	0.529632108	16.48459815	8.47E-52	5.72E-50	yes
ARHGAP15	0.529617932	16.48398491	8.53E-52	5.75E-50	yes
MS4A14	0.529391049	16.47417288	9.59E-52	6.44E-50	yes
LPXN	0.52912672	16.46274761	1.10E-51	7.35E-50	yes
SLC7A7	0.528564477	16.43846755	1.47E-51	9.78E-50	yes
TBC1D2B	0.528285792	16.42644383	1.69E-51	1.12E-49	yes
CD86	0.527969394	16.41280199	1.99E-51	1.32E-49	yes

CLEC6A	0.527055645	16.37345774	3.17E-51	2.10E-49	yes
PDCD1LG2	0.526964841	16.36955222	3.32E-51	2.19E-49	yes
LYN	0.526874311	16.36565924	3.48E-51	2.28E-49	yes
TLR5	0.526859545	16.36502435	3.51E-51	2.29E-49	yes
HK3	0.526723556	16.35917818	3.76E-51	2.45E-49	yes
OLFML3	0.526519717	16.35041838	4.17E-51	2.71E-49	yes
IL9R	0.526390472	16.34486622	4.45E-51	2.89E-49	yes
EHBP1L1	0.525931802	16.32517509	5.63E-51	3.63E-49	yes
LILRB3	0.52588273	16.32306958	5.77E-51	3.71E-49	yes
MAP3K8	0.525794457	16.3192826	6.03E-51	3.87E-49	yes
MYO1G	0.525656002	16.31334425	6.47E-51	4.14E-49	yes
LAPTM5	0.525207615	16.29412518	8.13E-51	5.18E-49	yes
STAT5A	0.524984193	16.28455571	9.10E-51	5.79E-49	yes
HLA-DQA2	0.524568704	16.26677209	1.12E-50	7.12E-49	yes
PLA2G2D	0.524400169	16.25956312	1.22E-50	7.73E-49	yes
GRN	0.524322842	16.25625635	1.27E-50	8.01E-49	yes
KYNU	0.523783905	16.23322515	1.67E-50	1.05E-48	yes
C11orf45	0.523536721	16.22267084	1.89E-50	1.18E-48	yes
PIK3AP1	0.523450474	16.2189896	1.98E-50	1.23E-48	yes
C3	0.523333697	16.21400632	2.10E-50	1.30E-48	yes
MGAT1	0.522948846	16.19759236	2.55E-50	1.58E-48	yes
C5orf20	0.522934914	16.19699841	2.56E-50	1.58E-48	yes
CCDC69	0.522644696	16.18462992	2.97E-50	1.83E-48	yes
ASGR2	0.522639934	16.18442702	2.97E-50	1.83E-48	yes
STXBP2	0.522620571	16.18360211	3.00E-50	1.84E-48	yes
SP100	0.522014224	16.1577872	4.07E-50	2.49E-48	yes
IFI30	0.52156555	16.1387068	5.10E-50	3.11E-48	yes
KLHL6	0.521139118	16.12058935	6.32E-50	3.83E-48	yes
PTPN6	0.520834326	16.10765003	7.36E-50	4.45E-48	yes
HLA-DQB1	0.520375441	16.08818498	9.25E-50	5.58E-48	yes
ADPRH	0.519196398	16.03825973	1.66E-49	1.00E-47	yes
TNFAIP8	0.518948621	16.02778386	1.88E-49	1.13E-47	yes
PLCG2	0.518658907	16.01554193	2.17E-49	1.30E-47	yes
TNFSF12-TNFS	0.518197939	15.99607917	2.73E-49	1.63E-47	yes
APOBEC3F	0.51748364	15.96595804	3.89E-49	2.31E-47	yes
PTAFR	0.516802787	15.93728971	5.45E-49	3.23E-47	yes
HCK	0.516699723	15.93295364	5.73E-49	3.39E-47	yes
PTPLAD2	0.516458226	15.92279716	6.46E-49	3.81E-47	yes
FRRS1	0.515750088	15.89304537	9.15E-49	5.38E-47	yes
FCGR2A	0.515203853	15.87012614	1.20E-48	7.01E-47	yes
DEF6	0.515107879	15.86610193	1.25E-48	7.33E-47	yes
WAS	0.514779831	15.85235292	1.47E-48	8.59E-47	yes
RNF135	0.5145523	15.84282234	1.65E-48	9.57E-47	yes
MAFB	0.514387299	15.83591374	1.79E-48	1.03E-46	yes
CCR1	0.514240469	15.82976802	1.92E-48	1.11E-46	yes
NCF2	0.514093157	15.82360402	2.06E-48	1.19E-46	yes
RGS18	0.514003064	15.81983516	2.16E-48	1.24E-46	yes
PLXDC2	0.513845379	15.81324049	2.33E-48	1.33E-46	yes
NLRC4	0.513738211	15.80875977	2.45E-48	1.40E-46	yes
CTSZ	0.513724998	15.80820737	2.47E-48	1.41E-46	yes
SAMSN1	0.513667844	15.80581821	2.54E-48	1.44E-46	yes

FXYD5	0.513275738	15.78943513	3.07E-48	1.74E-46	yes
BTK	0.511676369	15.72274849	6.69E-48	3.78E-46	yes
C1S	0.511544937	15.7172782	7.13E-48	4.02E-46	yes
CLIC2	0.511097477	15.69866589	8.86E-48	4.97E-46	yes
CD300C	0.510675483	15.68112867	1.09E-47	6.08E-46	yes
CRLF3	0.510522835	15.67478874	1.17E-47	6.53E-46	yes
CD226	0.510299807	15.66552927	1.30E-47	7.26E-46	yes
LPAR6	0.510181251	15.66060891	1.38E-47	7.66E-46	yes
SLC46A3	0.510053801	15.65532077	1.47E-47	8.12E-46	yes
CMTM7	0.509584563	15.63586323	1.84E-47	1.02E-45	yes
TLR6	0.509400171	15.62822237	2.01E-47	1.11E-45	yes
FCER1G	0.50931873	15.62484852	2.09E-47	1.15E-45	yes
TLR7	0.509316107	15.62473985	2.09E-47	1.15E-45	yes
WIPF1	0.508969375	15.61038224	2.47E-47	1.35E-45	yes
WDFY4	0.508869319	15.60624102	2.59E-47	1.41E-45	yes
SH2B3	0.508745807	15.60113012	2.75E-47	1.50E-45	yes
RNF144B	0.508348663	15.58470517	3.33E-47	1.81E-45	yes
NAAA	0.507991363	15.56993958	3.95E-47	2.14E-45	yes
GPR120	0.507765764	15.56062217	4.41E-47	2.37E-45	yes
TNFRSF11A	0.507763508	15.560529	4.41E-47	2.37E-45	yes
IL4I1	0.507617381	15.55449618	4.73E-47	2.54E-45	yes
IL18	0.507417774	15.54625836	5.20E-47	2.78E-45	yes
RASSF5	0.507391538	15.54517584	5.27E-47	2.81E-45	yes
ST14	0.507231099	15.5385573	5.69E-47	3.03E-45	yes
HLA-DOB	0.507214253	15.53786247	5.74E-47	3.04E-45	yes
SLFN5	0.506375998	15.50331863	8.56E-47	4.53E-45	yes
FLI1	0.505863719	15.48223713	1.09E-46	5.76E-45	yes
GALM	0.505731651	15.47680582	1.16E-46	6.12E-45	yes
HHEX	0.504952826	15.4448061	1.68E-46	8.84E-45	yes
MYOF	0.504745584	15.43629962	1.86E-46	9.73E-45	yes
ITPRIPL2	0.504731964	15.43574072	1.87E-46	9.77E-45	yes
GIMAP5	0.504230283	15.41516409	2.37E-46	1.24E-44	yes
FMNL1	0.503041664	15.36649596	4.16E-46	2.16E-44	yes
GNGT2	0.502801935	15.3566944	4.65E-46	2.41E-44	yes
HLA-F	0.501559181	15.30595903	8.34E-46	4.31E-44	yes
CD1E	0.501461779	15.30198796	8.73E-46	4.50E-44	yes
NCF1	0.500796061	15.27486747	1.19E-45	6.14E-44	yes
TIGIT	0.50065875	15.26927813	1.27E-45	6.53E-44	yes
MNDA	0.500441273	15.26042867	1.41E-45	7.21E-44	yes
TYROBP	0.500360448	15.25714075	1.46E-45	7.47E-44	yes
EBI3	0.49987935	15.23758107	1.83E-45	9.32E-44	yes
A2M	0.49910134	15.20598975	2.63E-45	1.34E-43	yes
STAB1	0.49848474	15.18098736	3.50E-45	1.78E-43	yes
CREG1	0.49790821	15.15763748	4.58E-45	2.31E-43	yes
PSD4	0.497719822	15.15001344	5.00E-45	2.52E-43	yes
CD37	0.497590002	15.14476126	5.30E-45	2.67E-43	yes
CD302	0.497271685	15.1318888	6.15E-45	3.08E-43	yes
TBXAS1	0.496394301	15.09645006	9.22E-45	4.61E-43	yes
MOBKL1B	0.49575472	15.07065525	1.24E-44	6.18E-43	yes
CASP1	0.495372259	15.0552458	1.48E-44	7.35E-43	yes
TPK1	0.495130341	15.04550489	1.65E-44	8.19E-43	yes

CCRL2	0.495075846	15.04331125	1.69E-44	8.38E-43	yes
OSTF1	0.494772458	15.03110306	1.94E-44	9.61E-43	yes
RAP2B	0.494564816	15.02275186	2.14E-44	1.05E-42	yes
NBEAL2	0.494373869	15.0150751	2.33E-44	1.15E-42	yes
LSP1	0.494311551	15.0125703	2.40E-44	1.18E-42	yes
GIMAP8	0.493897166	14.99592249	2.90E-44	1.42E-42	yes
LACTB	0.493807432	14.99231923	3.02E-44	1.48E-42	yes
MR1	0.493746079	14.98985597	3.11E-44	1.51E-42	yes
HLA-DPB2	0.493539499	14.98156414	3.42E-44	1.66E-42	yes
ANTXR2	0.493531565	14.98124575	3.43E-44	1.66E-42	yes
DPEP2	0.493311073	14.97239938	3.79E-44	1.83E-42	yes
CD300LF	0.493260844	14.97038468	3.88E-44	1.87E-42	yes
NCF1B	0.492305942	14.93212071	6.00E-44	2.89E-42	yes
CD80	0.492173028	14.92680035	6.37E-44	3.06E-42	yes
TCIRG1	0.492145844	14.92571239	6.45E-44	3.09E-42	yes
C11orf21	0.492043339	14.92161043	6.76E-44	3.23E-42	yes
VNN2	0.491807159	14.91216224	7.52E-44	3.59E-42	yes
SLC37A2	0.491786926	14.91135305	7.59E-44	3.61E-42	yes
SCIN	0.491767415	14.91057276	7.66E-44	3.63E-42	yes
CYBA	0.491703531	14.90801807	7.88E-44	3.73E-42	yes
HMHA1	0.491048097	14.88182606	1.06E-43	5.01E-42	yes
HAVCR2	0.4902387	14.84952739	1.53E-43	7.21E-42	yes
IL21R	0.489981207	14.83926285	1.72E-43	8.08E-42	yes
CASP4	0.489591627	14.82374256	2.05E-43	9.61E-42	yes
FUCA1	0.489118152	14.8048958	2.54E-43	1.19E-41	yes
TNFRSF10A	0.489010787	14.80062449	2.66E-43	1.24E-41	yes
CD40	0.488684496	14.78764909	3.08E-43	1.44E-41	yes
SQRDL	0.488414062	14.77690112	3.48E-43	1.62E-41	yes
CFI	0.488281735	14.77164401	3.69E-43	1.71E-41	yes
CASS4	0.488211973	14.76887302	3.81E-43	1.76E-41	yes
HLA-DRB6	0.487622156	14.74546013	4.97E-43	2.29E-41	yes
TYMP	0.487381665	14.73592141	5.53E-43	2.55E-41	yes
ASCL2	0.487253737	14.73084911	5.86E-43	2.69E-41	yes
ARHGAP18	0.48709658	14.72461961	6.28E-43	2.88E-41	yes
IKBKE	0.486786356	14.71232823	7.22E-43	3.30E-41	yes
C1orf38	0.486607195	14.70523302	7.82E-43	3.57E-41	yes
PTPN18	0.485818963	14.67404598	1.11E-42	5.06E-41	yes
GPR160	0.485370695	14.65633079	1.36E-42	6.16E-41	yes
GCNT1	0.484946046	14.63956295	1.64E-42	7.43E-41	yes
VDR	0.484666208	14.62852057	1.85E-42	8.39E-41	yes
ARHGAP4	0.484627058	14.62697619	1.89E-42	8.52E-41	yes
IL12B	0.484275474	14.61311208	2.21E-42	9.93E-41	yes
C17orf60	0.483680566	14.58967396	2.87E-42	1.29E-40	yes
PIK3R6	0.483667563	14.58916196	2.89E-42	1.29E-40	yes
SLC12A3	0.483579405	14.58569106	3.00E-42	1.34E-40	yes
VENTX	0.483560457	14.58494513	3.03E-42	1.35E-40	yes
C1orf162	0.483277861	14.57382324	3.43E-42	1.53E-40	yes
PLXND1	0.483067753	14.56555802	3.76E-42	1.67E-40	yes
IRF5	0.482645233	14.54894693	4.53E-42	2.01E-40	yes
RNF149	0.482618024	14.54787768	4.59E-42	2.03E-40	yes
PLEKHO2	0.482472787	14.54217112	4.89E-42	2.16E-40	yes

RGS19	0.482429499	14.5404706	4.98E-42	2.20E-40	yes
SERPINB1	0.482168498	14.53022031	5.59E-42	2.46E-40	yes
CASP5	0.48150694	14.50426151	7.48E-42	3.28E-40	yes
ACRBP	0.481296802	14.49602267	8.20E-42	3.59E-40	yes
TNFRSF14	0.481045616	14.48617876	9.15E-42	4.00E-40	yes
GNA15	0.480888015	14.48000478	9.81E-42	4.27E-40	yes
C14orf72	0.480510003	14.46520371	1.16E-41	5.03E-40	yes
RBMS1	0.479892906	14.44106377	1.52E-41	6.58E-40	yes
SIGLEC9	0.479407024	14.42207638	1.87E-41	8.11E-40	yes
HCST	0.47937958	14.42100443	1.90E-41	8.19E-40	yes
PLA2G15	0.478856404	14.40057997	2.38E-41	1.03E-39	yes
C19orf35	0.478831349	14.39960231	2.41E-41	1.04E-39	yes
RPGRIP1	0.477373253	14.34278667	4.53E-41	1.95E-39	yes
CD244	0.476653886	14.31481266	6.19E-41	2.65E-39	yes
STEAP4	0.476555619	14.31099424	6.46E-41	2.76E-39	yes
DNASE1L1	0.47647642	14.30791728	6.68E-41	2.85E-39	yes
TRPV2	0.475905414	14.28574636	8.55E-41	3.64E-39	yes
GIPC2	0.475885935	14.28499044	8.62E-41	3.66E-39	yes
SPATC1	0.475829326	14.28279379	8.83E-41	3.74E-39	yes
CLEC4A	0.475730998	14.27897882	9.21E-41	3.90E-39	yes
POU2F2	0.475455933	14.26831042	1.04E-40	4.38E-39	yes
TNFSF15	0.475268349	14.26103806	1.12E-40	4.73E-39	yes
RASAL3	0.475032258	14.25188874	1.24E-40	5.23E-39	yes
B2M	0.474643207	14.23682038	1.47E-40	6.17E-39	yes
FAM129A	0.474448314	14.22927599	1.60E-40	6.69E-39	yes
RPS6KA1	0.474352461	14.22556647	1.67E-40	6.96E-39	yes
SIPA1	0.474066756	14.2145136	1.88E-40	7.85E-39	yes
SNAP23	0.473361163	14.18724152	2.55E-40	1.06E-38	yes
TREML2	0.473342549	14.18652254	2.57E-40	1.07E-38	yes
FGD2	0.473115627	14.17775948	2.83E-40	1.17E-38	yes
FCGR2B	0.472399858	14.15014245	3.84E-40	1.59E-38	yes
HSPA7	0.472207769	14.1427371	4.16E-40	1.72E-38	yes
CEACAM4	0.47216386	14.14104467	4.24E-40	1.74E-38	yes
OLFML2B	0.47216202	14.14097376	4.25E-40	1.74E-38	yes
SFT2D2	0.471934601	14.13221043	4.68E-40	1.92E-38	yes
ARHGAP27	0.471780092	14.12625868	4.99E-40	2.04E-38	yes
APOL3	0.471532312	14.11671761	5.55E-40	2.27E-38	yes
PDE6G	0.471245966	14.10569682	6.27E-40	2.55E-38	yes
RASSF3	0.471152102	14.10208545	6.52E-40	2.65E-38	yes
LILRB4	0.47098952	14.09583168	6.99E-40	2.84E-38	yes
RAB43	0.470562627	14.07941989	8.37E-40	3.39E-38	yes
SLC15A3	0.470249808	14.0674017	9.56E-40	3.86E-38	yes
VSIG4	0.47005591	14.05995576	1.04E-39	4.19E-38	yes
TPM3	0.469964572	14.05644916	1.08E-39	4.34E-38	yes
TMPRSS11D	0.469939978	14.05550505	1.09E-39	4.38E-38	yes
CEACAM21	0.469934684	14.05530182	1.09E-39	4.38E-38	yes
CARD6	0.469793435	14.0498805	1.16E-39	4.64E-38	yes
ITGA4	0.469781005	14.04940348	1.16E-39	4.65E-38	yes
IL1R1	0.469623304	14.04335251	1.25E-39	4.97E-38	yes
ADPGK	0.469529853	14.0397676	1.30E-39	5.15E-38	yes
RASGRP4	0.4690925	14.02299816	1.56E-39	6.19E-38	yes

CXorf65	0.468522154	14.00114929	1.98E-39	7.85E-38	yes
C1orf127	0.468273948	13.99164799	2.20E-39	8.70E-38	yes
CD2AP	0.467664555	13.96833847	2.84E-39	1.12E-37	yes
ACSL1	0.467351245	13.95636414	3.24E-39	1.28E-37	yes
MEI1	0.467050918	13.94489237	3.67E-39	1.44E-37	yes
SERPING1	0.466403253	13.92017405	4.81E-39	1.89E-37	yes
NFKB1	0.466375671	13.91912201	4.87E-39	1.91E-37	yes
SP140L	0.465806685	13.89743118	6.17E-39	2.41E-37	yes
DENND3	0.465376249	13.88103681	7.38E-39	2.88E-37	yes
TNFSF10	0.464637756	13.85293841	1.00E-38	3.91E-37	yes
FKBP15	0.464391147	13.84356358	1.11E-38	4.32E-37	yes
NUDT16P1	0.464335562	13.84145109	1.14E-38	4.41E-37	yes
STAC3	0.46426054	13.83860023	1.17E-38	4.55E-37	yes
SERPINA1	0.463967476	13.82746734	1.33E-38	5.12E-37	yes
IL6R	0.463737333	13.81872876	1.46E-38	5.62E-37	yes
IL10	0.4636796	13.8165372	1.49E-38	5.75E-37	yes
C1RL	0.463581829	13.81282625	1.55E-38	5.98E-37	yes
FGR	0.463483353	13.80908919	1.62E-38	6.21E-37	yes
TICAM2	0.463375806	13.80500866	1.69E-38	6.48E-37	yes
MPEG1	0.463192001	13.79803654	1.83E-38	6.98E-37	yes
C1orf85	0.463083859	13.79393558	1.91E-38	7.29E-37	yes
C12orf35	0.46297729	13.789895	2.00E-38	7.60E-37	yes
BANK1	0.462539764	13.77331414	2.39E-38	9.08E-37	yes
MYD88	0.46225825	13.76265241	2.68E-38	1.02E-36	yes
IL13RA1	0.461931233	13.75027402	3.07E-38	1.16E-36	yes
CD19	0.460744728	13.70542163	5.00E-38	1.89E-36	yes
IDO2	0.460236816	13.68625002	6.16E-38	2.32E-36	yes
SIGLECP3	0.459872994	13.67252769	7.14E-38	2.69E-36	yes
LOC100233209	0.459850355	13.67167413	7.21E-38	2.71E-36	yes
CTSB	0.459770364	13.66865839	7.45E-38	2.79E-36	yes
DOCK8	0.459516519	13.65909096	8.26E-38	3.09E-36	yes
KLHDC7B	0.45943183	13.6559	8.55E-38	3.20E-36	yes
GAB3	0.458818576	13.63280738	1.10E-37	4.10E-36	yes
BTN2A2	0.457783238	13.59387681	1.67E-37	6.23E-36	yes
ROD1	0.45772707	13.5917668	1.71E-37	6.36E-36	yes
VAMP8	0.457693012	13.59048747	1.74E-37	6.44E-36	yes
CD69	0.457101954	13.5682975	2.20E-37	8.16E-36	yes
GMIP	0.455785432	13.51895316	3.75E-37	1.39E-35	yes
MYH9	0.455715024	13.51631735	3.86E-37	1.42E-35	yes
C1R	0.454724737	13.47927892	5.75E-37	2.12E-35	yes
NCR3	0.454646924	13.47637125	5.93E-37	2.18E-35	yes
GCH1	0.454525031	13.4718172	6.23E-37	2.29E-35	yes
TRANK1	0.454124161	13.45684706	7.32E-37	2.68E-35	yes
UNC93B1	0.453778909	13.44396216	8.41E-37	3.07E-35	yes
CSF3R	0.453537659	13.43496315	9.26E-37	3.38E-35	yes
MBNL1	0.453250806	13.42426794	1.04E-36	3.78E-35	yes
TRPM2	0.453187394	13.42190435	1.06E-36	3.87E-35	yes
GMFG	0.452993529	13.41467994	1.15E-36	4.18E-35	yes
ANKRD58	0.452824984	13.40840099	1.23E-36	4.46E-35	yes
TMEM37	0.45265683	13.40213846	1.32E-36	4.76E-35	yes
RUNX1	0.452520099	13.39704751	1.39E-36	5.02E-35	yes

TMEM86A	0.451926353	13.37495413	1.76E-36	6.35E-35	yes
C9orf167	0.451906013	13.3741977	1.78E-36	6.39E-35	yes
IRF8	0.451780545	13.36953205	1.87E-36	6.70E-35	yes
CD93	0.451634012	13.36408431	1.98E-36	7.09E-35	yes
CEBPA	0.451114415	13.34477798	2.43E-36	8.70E-35	yes
EDEM1	0.450847401	13.33486334	2.70E-36	9.66E-35	yes
RBM47	0.450827247	13.33411519	2.73E-36	9.71E-35	yes
CMTM6	0.450824039	13.33399609	2.73E-36	9.71E-35	yes
MACC1	0.450702831	13.32949712	2.86E-36	1.02E-34	yes
VASP	0.449848802	13.29782364	4.01E-36	1.42E-34	yes
CD14	0.449780384	13.2952882	4.12E-36	1.46E-34	yes
GAL3ST4	0.449693984	13.29208676	4.27E-36	1.51E-34	yes
ST8SIA4	0.449173178	13.27279907	5.24E-36	1.85E-34	yes
LOC115110	0.448714138	13.25581291	6.28E-36	2.21E-34	yes
AIF1	0.448366363	13.24295269	7.20E-36	2.53E-34	yes
RUNX2	0.448345972	13.2421989	7.26E-36	2.55E-34	yes
GLT25D1	0.448320818	13.24126907	7.33E-36	2.57E-34	yes
NCF1C	0.447977781	13.2285924	8.39E-36	2.94E-34	yes
TRIM34	0.447691816	13.21803037	9.39E-36	3.28E-34	yes
PRKCH	0.447667601	13.21713625	9.48E-36	3.30E-34	yes
PLTP	0.447155738	13.19824417	1.16E-35	4.03E-34	yes
LY96	0.446799446	13.18510354	1.33E-35	4.63E-34	yes
MAN1A1	0.446761634	13.18370945	1.35E-35	4.69E-34	yes
P2RX4	0.446742952	13.18302068	1.36E-35	4.71E-34	yes
ZNRF2	0.446550695	13.17593387	1.47E-35	5.07E-34	yes
GPBAR1	0.446447038	13.17211391	1.53E-35	5.27E-34	yes
TMEM109	0.446104974	13.15951285	1.75E-35	6.02E-34	yes
RILPL2	0.445690948	13.14427045	2.05E-35	7.06E-34	yes
MCOLN2	0.445152227	13.12445316	2.53E-35	8.70E-34	yes
GPR174	0.44494804	13.11694664	2.74E-35	9.40E-34	yes
MANBA	0.444788716	13.11109116	2.92E-35	9.98E-34	yes
ALDH3B1	0.444716267	13.10842904	3.00E-35	1.02E-33	yes
GPRIN3	0.444695523	13.10766688	3.03E-35	1.03E-33	yes
HLA-DRB5	0.444349855	13.09497021	3.46E-35	1.18E-33	yes
PYCARD	0.444260189	13.09167789	3.58E-35	1.22E-33	yes
C1orf200	0.444095758	13.08564167	3.82E-35	1.30E-33	yes
NAIP	0.444016908	13.0827477	3.94E-35	1.33E-33	yes
NPC2	0.443881134	13.07776537	4.15E-35	1.40E-33	yes
CREBL2	0.443613427	13.0679449	4.60E-35	1.55E-33	yes
SNX9	0.443542581	13.06534677	4.73E-35	1.59E-33	yes
CAST	0.443168982	13.05165068	5.47E-35	1.84E-33	yes
PTPN2	0.442964895	13.04417248	5.92E-35	1.99E-33	yes
C3orf64	0.442470877	13.02608098	7.16E-35	2.40E-33	yes
IL10RB	0.442192788	13.01590354	7.97E-35	2.67E-33	yes
IRAK3	0.442148534	13.01428437	8.11E-35	2.71E-33	yes
KCNE3	0.442000138	13.00885574	8.58E-35	2.86E-33	yes
LAP3	0.441944887	13.00683486	8.77E-35	2.92E-33	yes
ITGB1	0.441690503	12.99753286	9.67E-35	3.21E-33	yes
CD180	0.441686683	12.9973932	9.68E-35	3.21E-33	yes
XCL1	0.441600515	12.99424323	1.00E-34	3.32E-33	yes
CPVL	0.441377631	12.98609747	1.09E-34	3.61E-33	yes

MYL12A	0.44118802	12.97917012	1.17E-34	3.87E-33	yes
KCTD12	0.441114764	12.97649431	1.21E-34	3.98E-33	yes
ENG	0.440603975	12.9578458	1.47E-34	4.83E-33	yes
ABI3	0.440536615	12.95538769	1.51E-34	4.95E-33	yes
ZDHHC5	0.440197428	12.94301427	1.71E-34	5.63E-33	yes
ISG20	0.440149045	12.94124981	1.75E-34	5.72E-33	yes
S100A11	0.440121823	12.94025712	1.77E-34	5.77E-33	yes
CXCL16	0.439666819	12.92367156	2.10E-34	6.86E-33	yes
ELK3	0.439570027	12.92014495	2.18E-34	7.11E-33	yes
MAN2A1	0.439256019	12.90870789	2.46E-34	8.00E-33	yes
LRRFIP1	0.438870415	12.89467111	2.85E-34	9.25E-33	yes
DPYD	0.438697817	12.88839106	3.04E-34	9.87E-33	yes
ATP8B4	0.438537729	12.88256776	3.23E-34	1.05E-32	yes
RREB1	0.437169041	12.83284273	5.43E-34	1.76E-32	yes
FAM114A1	0.437158965	12.83247708	5.46E-34	1.76E-32	yes
GZMM	0.436987807	12.82626671	5.82E-34	1.88E-32	yes
TRIM22	0.43688715	12.82261525	6.05E-34	1.95E-32	yes
LITAF	0.436587804	12.81175953	6.77E-34	2.18E-32	yes
CAPZA1	0.436341635	12.80283626	7.43E-34	2.39E-32	yes
LILRA6	0.436316822	12.80193701	7.50E-34	2.40E-32	yes
ZNF217	0.436303184	12.80144275	7.54E-34	2.41E-32	yes
SYNGR2	0.435947472	12.78855576	8.63E-34	2.75E-32	yes
CORO1A	0.435808735	12.78353147	9.09E-34	2.90E-32	yes
B4GALT1	0.435456342	12.77077484	1.04E-33	3.31E-32	yes
LGMN	0.435302043	12.76519152	1.10E-33	3.50E-32	yes
STX11	0.433952347	12.71641154	1.83E-33	5.80E-32	yes
HSH2D	0.433792708	12.71064892	1.94E-33	6.15E-32	yes
SLC11A1	0.432826988	12.67582009	2.78E-33	8.81E-32	yes
CTBS	0.432564459	12.66636122	3.07E-33	9.70E-32	yes
TMEM173	0.432237855	12.65459928	3.47E-33	1.09E-31	yes
CYP2S1	0.431645134	12.63326937	4.32E-33	1.36E-31	yes
PRKCD	0.431511633	12.62846793	4.54E-33	1.43E-31	yes
CLEC4C	0.431481659	12.62739003	4.60E-33	1.44E-31	yes
ADORA3	0.431075808	12.6128003	5.34E-33	1.67E-31	yes
PRDM1	0.430886911	12.60601295	5.73E-33	1.79E-31	yes
FCN1	0.430824625	12.60377533	5.87E-33	1.83E-31	yes
RGL4	0.430344646	12.58653983	7.01E-33	2.19E-31	yes
ABHD15	0.430221375	12.58211539	7.34E-33	2.29E-31	yes
LOC96610	0.430116245	12.5783428	7.63E-33	2.37E-31	yes
SIGLEC7	0.430037455	12.57551581	7.85E-33	2.44E-31	yes
SLC10A3	0.429926024	12.57151826	8.18E-33	2.54E-31	yes
IFNGR2	0.429486656	12.55576294	9.63E-33	2.98E-31	yes
AIM1	0.429322358	12.5498742	1.02E-32	3.16E-31	yes
IQGAP1	0.42916361	12.54418581	1.08E-32	3.35E-31	yes
MYO7A	0.429007323	12.538587	1.15E-32	3.54E-31	yes
TMEM140	0.42899967	12.53831288	1.15E-32	3.55E-31	yes
REEP4	0.427781273	12.49471294	1.80E-32	5.54E-31	yes
UCP2	0.427439861	12.4825106	2.04E-32	6.27E-31	yes
WIPI1	0.427048065	12.46851557	2.36E-32	7.23E-31	yes
CARD11	0.426983788	12.4662204	2.42E-32	7.39E-31	yes
PQLC3	0.426767456	12.45849741	2.62E-32	7.99E-31	yes

NRP1	0.426743496	12.4576422	2.64E-32	8.05E-31	yes
HFE	0.426469901	12.44787908	2.92E-32	8.88E-31	yes
B3GNT8	0.426435988	12.4466692	2.95E-32	8.98E-31	yes
NLRP1	0.426038072	12.43247793	3.42E-32	1.04E-30	yes
KCNQ1	0.425969724	12.43004123	3.50E-32	1.06E-30	yes
HEXA	0.425933407	12.4287466	3.55E-32	1.07E-30	yes
ACPP	0.425739955	12.42185166	3.81E-32	1.15E-30	yes
SEC24D	0.425574145	12.41594356	4.04E-32	1.22E-30	yes
TM6SF1	0.425509699	12.41364763	4.14E-32	1.25E-30	yes
NFATC2	0.425504081	12.41344752	4.15E-32	1.25E-30	yes
BTN3A2	0.42502869	12.3965188	4.93E-32	1.48E-30	yes
INPP5D	0.424763366	12.38707608	5.43E-32	1.63E-30	yes
ST3GAL1	0.424701215	12.38486472	5.56E-32	1.66E-30	yes
TNFAIP8L2	0.424371915	12.37315163	6.26E-32	1.87E-30	yes
FTL	0.424168247	12.36591022	6.74E-32	2.01E-30	yes
TGFBR1	0.423903192	12.35648965	7.43E-32	2.21E-30	yes
PRSS36	0.423823996	12.35367561	7.64E-32	2.28E-30	yes
ACSL5	0.423749756	12.35103799	7.85E-32	2.33E-30	yes
MBD2	0.423702202	12.34934865	7.99E-32	2.37E-30	yes
IGSF6	0.423512058	12.34259504	8.56E-32	2.54E-30	yes
ADAMDEC1	0.423337618	12.33640094	9.11E-32	2.70E-30	yes
HPS3	0.423254623	12.33345452	9.39E-32	2.78E-30	yes
LRG1	0.42309209	12.32768549	9.96E-32	2.94E-30	yes
IL17RA	0.423023688	12.32525802	1.02E-31	3.01E-30	yes
STAT6	0.422840606	12.31876203	1.09E-31	3.21E-30	yes
PLCB2	0.422687258	12.31332247	1.15E-31	3.39E-30	yes
SLC24A6	0.422525531	12.30758707	1.22E-31	3.59E-30	yes
C8orf80	0.422323247	12.30041537	1.31E-31	3.85E-30	yes
BTN3A1	0.422070446	12.29145584	1.44E-31	4.21E-30	yes
FCGRT	0.421708257	12.27862553	1.64E-31	4.79E-30	yes
RHBDF2	0.421166663	12.2594533	1.99E-31	5.82E-30	yes
ELF1	0.421010711	12.25393559	2.11E-31	6.14E-30	yes
DTX2	0.420510705	12.23625392	2.52E-31	7.34E-30	yes
SLC2A5	0.420408332	12.2326354	2.62E-31	7.60E-30	yes
SMPDL3A	0.419961436	12.21684578	3.07E-31	8.91E-30	yes
LGALS9	0.419561	12.20270685	3.54E-31	1.03E-29	yes
CXCL10	0.419146353	12.18807529	4.11E-31	1.19E-29	yes
TNFAIP3	0.419040339	12.18433586	4.27E-31	1.23E-29	yes
FAM91A1	0.418835163	12.17710044	4.59E-31	1.32E-29	yes
CARD16	0.418144063	12.15274577	5.87E-31	1.69E-29	yes
SPATS2L	0.418064361	12.14993869	6.04E-31	1.74E-29	yes
TGFB1	0.418001135	12.14771215	6.18E-31	1.77E-29	yes
FHOD1	0.417792799	12.1403769	6.65E-31	1.91E-29	yes
ARRDC5	0.41777938	12.13990453	6.68E-31	1.91E-29	yes
TPRG1	0.417729605	12.13815238	6.80E-31	1.95E-29	yes
MYO1C	0.417522349	12.13085818	7.32E-31	2.09E-29	yes
DPRXP4	0.417322474	12.12382592	7.86E-31	2.24E-29	yes
SRPR	0.417196793	12.11940515	8.22E-31	2.34E-29	yes
CXCR4	0.416893874	12.10875353	9.15E-31	2.60E-29	yes
CARD9	0.416730026	12.10299413	9.69E-31	2.75E-29	yes
NLRP12	0.416673449	12.10100574	9.89E-31	2.80E-29	yes

SUMF1	0.416616415	12.09900147	1.01E-30	2.86E-29	yes
VRK2	0.416440256	12.092812	1.07E-30	3.04E-29	yes
MBD4	0.416380102	12.09069883	1.10E-30	3.10E-29	yes
IL22RA2	0.416359701	12.08998218	1.11E-30	3.12E-29	yes
CAPG	0.416074269	12.07995805	1.22E-30	3.44E-29	yes
GPR183	0.415511295	12.06019949	1.49E-30	4.19E-29	yes
ARPC2	0.415446401	12.05792302	1.53E-30	4.28E-29	yes
EMR4P	0.415436261	12.0575673	1.53E-30	4.29E-29	yes
HLA-B	0.415381995	12.05566385	1.56E-30	4.37E-29	yes
FUT7	0.415181813	12.04864352	1.67E-30	4.68E-29	yes
FAM20A	0.415080201	12.04508085	1.74E-30	4.85E-29	yes
CLDN23	0.414799496	12.03524165	1.92E-30	5.34E-29	yes
SPPL2A	0.414555396	12.02668891	2.09E-30	5.81E-29	yes
LTBR	0.414000912	12.00727264	2.54E-30	7.05E-29	yes
ESR1	0.413714802	11.99726029	2.80E-30	7.78E-29	yes
RNF130	0.413325629	11.98364815	3.21E-30	8.91E-29	yes
HTRA4	0.413251679	11.98106248	3.30E-30	9.13E-29	yes
SERPINB8	0.413165779	11.97805935	3.40E-30	9.39E-29	yes
TIFA	0.413090818	11.97543896	3.49E-30	9.63E-29	yes
PECAM1	0.412874402	11.96787545	3.76E-30	1.04E-28	yes
C11orf75	0.412704033	11.96192295	3.99E-30	1.10E-28	yes
LAT2	0.412364473	11.95006358	4.49E-30	1.24E-28	yes
EPSTI1	0.412341709	11.94926876	4.53E-30	1.24E-28	yes
LAMP3	0.412031159	11.93842822	5.05E-30	1.38E-28	yes
DTX3L	0.410827208	11.89644859	7.67E-30	2.10E-28	yes
EFCAB4B	0.410693242	11.89178207	8.03E-30	2.20E-28	yes
CREB3L2	0.410552188	11.88686966	8.44E-30	2.30E-28	yes
C12orf59	0.4103364	11.8793565	9.09E-30	2.48E-28	yes
CXorf21	0.410289532	11.87772499	9.24E-30	2.52E-28	yes
APBB1IP	0.410112125	11.87155037	9.82E-30	2.67E-28	yes
MVP	0.410078898	11.87039413	9.94E-30	2.70E-28	yes
PGCP	0.410020599	11.86836547	1.01E-29	2.75E-28	yes
HEXB	0.409680273	11.85652666	1.14E-29	3.09E-28	yes
AICDA	0.409499694	11.85024732	1.21E-29	3.29E-28	yes
PPM1M	0.408661575	11.82112498	1.62E-29	4.38E-28	yes
FCGR1A	0.408658471	11.82101719	1.62E-29	4.38E-28	yes
GAA	0.408460058	11.8141282	1.74E-29	4.68E-28	yes
HCP5	0.40841996	11.8127362	1.76E-29	4.74E-28	yes
FBP1	0.408327661	11.80953242	1.82E-29	4.89E-28	yes
IRF2	0.40814234	11.80310103	1.94E-29	5.20E-28	yes
TLR3	0.408068348	11.80053369	1.99E-29	5.33E-28	yes
AHR	0.407154696	11.76885529	2.72E-29	7.28E-28	yes
ACTR3	0.40687239	11.75907567	3.00E-29	8.01E-28	yes
FMO4	0.406684182	11.75255804	3.20E-29	8.53E-28	yes
PARP14	0.406584582	11.7491096	3.31E-29	8.82E-28	yes
DOK1	0.406377295	11.74193437	3.55E-29	9.45E-28	yes
MSN	0.406371343	11.7417284	3.56E-29	9.46E-28	yes
ETV6	0.406294891	11.73908257	3.65E-29	9.70E-28	yes
CTSA	0.406204998	11.735972	3.76E-29	9.99E-28	yes
JAK2	0.406142638	11.73381438	3.84E-29	1.02E-27	yes
PPARG	0.405865464	11.72422669	4.23E-29	1.12E-27	yes

NMI	0.405563331	11.71378007	4.68E-29	1.24E-27	yes
SH2D4A	0.405520039	11.71228357	4.75E-29	1.25E-27	yes
ADAM6	0.405443163	11.70962636	4.88E-29	1.29E-27	yes
LPAR5	0.405388397	11.70773359	4.97E-29	1.31E-27	yes
GGCX	0.404593902	11.68029183	6.51E-29	1.71E-27	yes
CAP1	0.404584751	11.67997593	6.54E-29	1.72E-27	yes
CYBASC3	0.404183968	11.66614512	7.49E-29	1.96E-27	yes
F13A1	0.404045804	11.66137903	7.85E-29	2.05E-27	yes
KIAA1949	0.403931649	11.65744187	8.16E-29	2.13E-27	yes
TRIM21	0.403921963	11.65710782	8.18E-29	2.14E-27	yes
MGST2	0.403730073	11.65049115	8.73E-29	2.28E-27	yes
NAT1	0.40361835	11.64663959	9.07E-29	2.36E-27	yes
ERMAP	0.403471401	11.64157464	9.53E-29	2.48E-27	yes
KMO	0.403354283	11.63753863	9.92E-29	2.58E-27	yes
SIGLEC5	0.40317073	11.63121459	1.06E-28	2.74E-27	yes
FILIP1L	0.403139214	11.63012892	1.07E-28	2.76E-27	yes
CLEC4D	0.40273034	11.61604847	1.22E-28	3.17E-27	yes
S100Z	0.40241375	11.6051517	1.36E-28	3.52E-27	yes
SAT1	0.402398339	11.60462139	1.37E-28	3.54E-27	yes
PLK3	0.402143615	11.59585783	1.49E-28	3.85E-27	yes
IFI16	0.402141616	11.59578906	1.49E-28	3.85E-27	yes
BTN3A3	0.401831983	11.58514072	1.66E-28	4.26E-27	yes
CSF1R	0.401709192	11.58091924	1.73E-28	4.44E-27	yes
RIN3	0.401564064	11.57593076	1.81E-28	4.65E-27	yes
CD1D	0.401301919	11.56692274	1.98E-28	5.07E-27	yes
BMP2K	0.40044752	11.53758667	2.64E-28	6.75E-27	yes
CLEC2B	0.400189957	11.5287502	2.88E-28	7.35E-27	yes
UBASH3A	0.399390832	11.5013545	3.75E-28	9.59E-27	no
SWAP70	0.399306873	11.49847801	3.86E-28	9.85E-27	no
DOCK11	0.399292992	11.49800248	3.88E-28	9.88E-27	no
ARF6	0.398991651	11.48768141	4.29E-28	1.09E-26	no
PARP9	0.398841145	11.48252819	4.51E-28	1.15E-26	no
RNASE2	0.398461198	11.46952392	5.12E-28	1.30E-26	no
MFSD7	0.39825021	11.46230559	5.49E-28	1.39E-26	no
TCN2	0.397787951	11.44649825	6.40E-28	1.62E-26	no
H6PD	0.397678422	11.44275434	6.64E-28	1.68E-26	no
CXCL12	0.397443342	11.43472083	7.18E-28	1.81E-26	no
TCL1A	0.396776472	11.41194602	8.95E-28	2.26E-26	no
CD300LB	0.396569557	11.40488387	9.58E-28	2.41E-26	no
TGOLN2	0.39653161	11.40358894	9.70E-28	2.44E-26	no
ANXA2	0.396287104	11.39524688	1.05E-27	2.64E-26	no
TNFRSF9	0.396117495	11.38946185	1.11E-27	2.79E-26	no
SLC22A16	0.396012713	11.38588865	1.15E-27	2.89E-26	no
SKAP1	0.396008961	11.3857607	1.15E-27	2.89E-26	no
MKNK1	0.395525452	11.36927925	1.35E-27	3.38E-26	no
NTAN1	0.395153113	11.35659492	1.53E-27	3.82E-26	no
C5AR1	0.395120858	11.35549642	1.54E-27	3.85E-26	no
DRAM1	0.394985143	11.35087492	1.62E-27	4.02E-26	no
TMEM149	0.394958384	11.34996382	1.63E-27	4.05E-26	no
B3GNT2	0.394662281	11.33988409	1.80E-27	4.46E-26	no
SIGLEC1	0.394652282	11.33954376	1.80E-27	4.47E-26	no

GPR34	0.39449457	11.33417684	1.90E-27	4.70E-26	no
TNFAIP2	0.394307992	11.3278291	2.02E-27	4.99E-26	no
SLFN14	0.394289148	11.32718808	2.03E-27	5.02E-26	no
GIT2	0.394232967	11.32527709	2.07E-27	5.10E-26	no
SECTM1	0.394099557	11.32073972	2.16E-27	5.33E-26	no
TRIM14	0.394011073	11.31773081	2.22E-27	5.48E-26	no
DARC	0.393750802	11.30888237	2.42E-27	5.96E-26	no
IL2	0.393682344	11.30655552	2.48E-27	6.08E-26	no
SRGN	0.393133803	11.2879191	2.96E-27	7.27E-26	no
BNIP2	0.393038846	11.28469441	3.06E-27	7.49E-26	no
HSD3B7	0.392995979	11.28323881	3.10E-27	7.59E-26	no
SLC25A24	0.392865243	11.27880007	3.23E-27	7.91E-26	no
TEP1	0.392768765	11.27552497	3.34E-27	8.15E-26	no
GPNMB	0.392196437	11.25610546	4.02E-27	9.81E-26	no
APOL6	0.39175467	11.24112653	4.64E-27	1.13E-25	no
MICB	0.391726238	11.24016282	4.69E-27	1.14E-25	no
PICALM	0.39137923	11.22840377	5.25E-27	1.27E-25	no
MAPKAPK2	0.390655417	11.20389415	6.63E-27	1.61E-25	no
TNFRSF1A	0.390505919	11.19883491	6.96E-27	1.69E-25	no
CD300A	0.390389429	11.19489347	7.23E-27	1.75E-25	no
ESYT1	0.390166675	11.18735832	7.77E-27	1.88E-25	no
DNASE2	0.389918048	11.17895069	8.42E-27	2.03E-25	no
LOXL3	0.389592068	11.16793166	9.35E-27	2.26E-25	no
TERF2	-0.389355925	-11.15995247	1.01E-26	2.43E-25	no
LY86	0.388968006	11.14685043	1.14E-26	2.75E-25	no
FPR1	0.388840854	11.14255735	1.19E-26	2.86E-25	no
AMPD1	0.38854217	11.13247577	1.31E-26	3.15E-25	no
SDCBP	0.388521335	11.13177265	1.32E-26	3.17E-25	no
GLIPR1	0.388486039	11.1305816	1.34E-26	3.20E-25	no
PLAUR	0.388384801	11.12716571	1.38E-26	3.30E-25	no
REL	0.388194923	11.12076019	1.47E-26	3.51E-25	no
ZNF600	0.388111936	11.11796119	1.51E-26	3.60E-25	no
FGF20	0.387850674	11.1091513	1.64E-26	3.91E-25	no
C14orf182	0.387723667	11.1048697	1.71E-26	4.06E-25	no
BTBD19	0.387510358	11.09768039	1.83E-26	4.35E-25	no
TBC1D8	0.387348918	11.09224067	1.92E-26	4.57E-25	no
LRRK1	0.387344317	11.09208564	1.93E-26	4.57E-25	no
FAM109B	0.38645076	11.06199908	2.56E-26	6.08E-25	no
S100A4	0.386357177	11.05885023	2.64E-26	6.25E-25	no
TMEM110	0.38633399	11.05807009	2.66E-26	6.29E-25	no
SLC4A7	0.38564761	11.03498785	3.31E-26	7.82E-25	no
DRAM2	0.385454463	11.02849638	3.52E-26	8.31E-25	no
FAM105A	0.38522516	11.02079197	3.79E-26	8.93E-25	no
FOLR2	0.385129554	11.01758041	3.90E-26	9.19E-25	no
NUMB	0.384172768	10.98546313	5.29E-26	1.24E-24	no
CHST13	0.384123625	10.98381462	5.37E-26	1.26E-24	no
RHOG	0.38411804	10.98362727	5.38E-26	1.26E-24	no
RELB	0.384063604	10.98180136	5.47E-26	1.28E-24	no
PALLD	0.38400778	10.97992903	5.57E-26	1.30E-24	no
MFSD6L	0.38376121	10.97166079	6.02E-26	1.41E-24	no
POU6F1	-0.383621084	-10.96696315	6.30E-26	1.47E-24	no

LOC283314	0.383467635	10.96181989	6.61E-26	1.54E-24	no
CEBPB	0.383432679	10.96064841	6.68E-26	1.56E-24	no
CTSD	0.383152139	10.95124855	7.30E-26	1.70E-24	no
KCNK13	0.382423549	10.92685276	9.19E-26	2.14E-24	no
CDH11	0.38239548	10.9259134	9.27E-26	2.15E-24	no
GLB1	0.38230484	10.92288025	9.54E-26	2.21E-24	no
SLC30A7	0.382289496	10.92236682	9.58E-26	2.22E-24	no
SP110	0.382275574	10.92190094	9.63E-26	2.23E-24	no
HLA-H	0.38224861	10.92099874	9.71E-26	2.24E-24	no
HGF	0.382232479	10.92045901	9.76E-26	2.25E-24	no
PYGL	0.382064786	10.91484888	1.03E-25	2.37E-24	no
NLRC5	0.381824933	10.90682684	1.11E-25	2.55E-24	no
SLC17A9	0.381731878	10.90371525	1.14E-25	2.63E-24	no
KIF16B	0.381559929	10.89796662	1.21E-25	2.77E-24	no
TRAM1	0.381341783	10.89067541	1.29E-25	2.96E-24	no
IL7	0.381082459	10.88201068	1.40E-25	3.21E-24	no
C10orf11	0.380842784	10.87400514	1.51E-25	3.45E-24	no
PION	0.380840131	10.87391653	1.51E-25	3.45E-24	no
TMEM51	0.380745939	10.87077108	1.56E-25	3.55E-24	no
SHKBP1	0.380610799	10.8662589	1.62E-25	3.70E-24	no
IRF1	0.38052752	10.86347869	1.67E-25	3.80E-24	no
MDFIC	0.380362398	10.85796715	1.75E-25	3.99E-24	no
IL18R1	0.37971743	10.83645064	2.15E-25	4.88E-24	no
PRR16	0.379584527	10.83201923	2.24E-25	5.08E-24	no
GALNT2	0.379555159	10.83104012	2.26E-25	5.12E-24	no
CTSL1	0.379359015	10.8245017	2.40E-25	5.44E-24	no
RFTN1	0.379125922	10.81673384	2.58E-25	5.84E-24	no
TMEM176B	0.378985778	10.8120647	2.69E-25	6.09E-24	no
SLC25A43	0.378964242	10.81134725	2.71E-25	6.13E-24	no
SULT1B1	0.378915664	10.80972902	2.75E-25	6.21E-24	no
GLA	0.378753609	10.80433144	2.90E-25	6.53E-24	no
COL14A1	0.378745952	10.80407645	2.90E-25	6.53E-24	no
PLSCR1	0.378689799	10.80220644	2.95E-25	6.64E-24	no
NAPSB	0.37865612	10.80108492	2.98E-25	6.70E-24	no
LRCH1	0.378633927	10.80034593	3.01E-25	6.74E-24	no
KCTD9	0.378214355	10.78637879	3.42E-25	7.67E-24	no
ITGA5	0.378122862	10.78333409	3.52E-25	7.89E-24	no
SIGLEC6	0.378062771	10.78133462	3.59E-25	8.03E-24	no
TIMD4	0.377983311	10.77869087	3.68E-25	8.22E-24	no
TMEM194B	0.377954629	10.77773665	3.71E-25	8.28E-24	no
UBA7	0.37786857	10.77487378	3.81E-25	8.49E-24	no
IFITM2	0.377642496	10.76735464	4.09E-25	9.10E-24	no
SAMD9L	0.377468831	10.76158016	4.31E-25	9.59E-24	no
TLN1	0.377093836	10.74911579	4.84E-25	1.08E-23	no
NLRP6	0.377070926	10.7483545	4.88E-25	1.08E-23	no
ABP1	0.377020922	10.74669293	4.95E-25	1.10E-23	no
AIM2	0.376980794	10.74535964	5.02E-25	1.11E-23	no
ALOX5AP	0.37695489	10.74449895	5.06E-25	1.12E-23	no
LRMP	0.376940366	10.74401643	5.08E-25	1.12E-23	no
EHD4	0.376918724	10.74329739	5.11E-25	1.13E-23	no
TTF2	0.376776838	10.73858397	5.34E-25	1.18E-23	no

SH3TC1	0.376673246	10.73514325	5.51E-25	1.21E-23	no
MAPK13	0.376651207	10.73441127	5.55E-25	1.22E-23	no
RCAN3	0.376629438	10.73368833	5.59E-25	1.23E-23	no
MGC29506	0.37657989	10.73204287	5.68E-25	1.25E-23	no
ZNF267	0.376552698	10.7311399	5.72E-25	1.26E-23	no
GZMB	0.376457338	10.72797349	5.89E-25	1.29E-23	no
CD164	0.376209214	10.71973645	6.36E-25	1.39E-23	no
LUM	0.376205443	10.71961129	6.37E-25	1.39E-23	no
ICAM1	0.376203354	10.71954197	6.37E-25	1.39E-23	no
FNDC3B	0.376039538	10.71410522	6.70E-25	1.46E-23	no
MAPT	-0.375588544	-10.69914368	7.70E-25	1.68E-23	no
PLAU	0.375573048	10.69862976	7.74E-25	1.68E-23	no
TRADD	0.375471117	10.69524954	7.99E-25	1.74E-23	no
RASL11A	0.375326337	10.69044914	8.35E-25	1.81E-23	no
RNF19B	0.37504782	10.68121703	9.09E-25	1.97E-23	no
FAH	0.374880148	10.6756608	9.57E-25	2.07E-23	no
TRIM6	0.374801832	10.67306597	9.81E-25	2.12E-23	no
SHC1	0.374791672	10.67272938	9.84E-25	2.13E-23	no
STARD5	0.374016119	10.6470482	1.25E-24	2.69E-23	no
RNASET2	0.373954659	10.64501414	1.27E-24	2.74E-23	no
CCR3	0.373646881	10.63483058	1.40E-24	3.01E-23	no
CACNA2D4	0.372867874	10.60907354	1.77E-24	3.81E-23	no
BCL7A	-0.372810786	-10.60718702	1.80E-24	3.87E-23	no
TBC1D1	0.372687823	10.60312406	1.87E-24	4.02E-23	no
TMBIM1	0.372600112	10.60022629	1.92E-24	4.12E-23	no
PTRF	0.372535397	10.59808849	1.96E-24	4.20E-23	no
TWF2	0.372371654	10.59268016	2.06E-24	4.41E-23	no
IL18BP	0.372283131	10.58975677	2.12E-24	4.53E-23	no
GPR84	0.372274767	10.58948057	2.12E-24	4.53E-23	no
FYCO1	0.37217321	10.58612717	2.19E-24	4.67E-23	no
PARP4	0.372036253	10.58160561	2.28E-24	4.86E-23	no
FSTL1	0.371832088	10.5748667	2.43E-24	5.17E-23	no
STAT3	0.371706154	10.57071086	2.52E-24	5.36E-23	no
PDK3	0.371624069	10.56800242	2.58E-24	5.49E-23	no
ZC3HAV1	0.371514534	10.56438869	2.67E-24	5.67E-23	no
CASP6	0.371499716	10.56389986	2.68E-24	5.69E-23	no
ARID3A	0.371483048	10.56334998	2.70E-24	5.71E-23	no
DERL3	0.371456476	10.56247345	2.72E-24	5.75E-23	no
S100A9	0.37122358	10.55479211	2.92E-24	6.17E-23	no
OSMR	0.371051809	10.54912828	3.07E-24	6.49E-23	no
SPINT1	0.370924572	10.54493368	3.19E-24	6.74E-23	no
PRICKLE3	0.370870667	10.54315681	3.25E-24	6.84E-23	no
RIPK1	0.370852747	10.54256611	3.26E-24	6.87E-23	no
HPS5	0.370738511	10.53880105	3.38E-24	7.11E-23	no
NHLRC3	0.370598437	10.53418517	3.52E-24	7.41E-23	no
ANKRD22	0.370289996	10.52402394	3.87E-24	8.12E-23	no
SPIB	0.370099977	10.51776602	4.10E-24	8.59E-23	no
SSH1	0.369212407	10.48855575	5.35E-24	1.12E-22	no
KIAA0247	0.36910729	10.48509853	5.52E-24	1.16E-22	no
TMEM154	0.368995189	10.4814121	5.71E-24	1.19E-22	no
C10orf125	0.368953827	10.48005204	5.78E-24	1.21E-22	no

ANXA2P2	0.368885797	10.47781528	5.90E-24	1.23E-22	no
TMOD3	0.368831246	10.47602182	6.00E-24	1.25E-22	no
ARSA	0.368590081	10.46809461	6.45E-24	1.34E-22	no
SYTL3	0.368545015	10.46661353	6.54E-24	1.36E-22	no
ACCS	0.36843633	10.463042	6.75E-24	1.40E-22	no
GCA	0.368007548	10.44895652	7.68E-24	1.59E-22	no
REXO2	0.367562688	10.43435104	8.77E-24	1.82E-22	no
CFP	0.367146159	10.42068324	9.94E-24	2.06E-22	no
LOC151534	0.367120142	10.41982977	1.00E-23	2.07E-22	no
FUT4	0.366924806	10.4134228	1.06E-23	2.19E-22	no
ATL3	0.366611704	10.40315645	1.17E-23	2.41E-22	no
LTBP2	0.366445354	10.39770362	1.22E-23	2.53E-22	no
LGALS8	0.366048552	10.38470147	1.38E-23	2.84E-22	no
MOBKL2C	0.365707471	10.37353034	1.52E-23	3.14E-22	no
FLII	0.36553589	10.3679125	1.60E-23	3.30E-22	no
NEXN	0.365408824	10.36375296	1.67E-23	3.42E-22	no
LOC100272216	0.365204894	10.35707864	1.77E-23	3.63E-22	no
SLC39A1	0.365184891	10.3564241	1.78E-23	3.65E-22	no
PTPN1	0.365151884	10.355344	1.80E-23	3.68E-22	no
CCL17	0.365114885	10.35413334	1.82E-23	3.72E-22	no
GNRHR2	-0.365017992	-10.35096315	1.87E-23	3.82E-22	no
TREML1	0.364849125	10.34543901	1.97E-23	4.02E-22	no
RPS6KA3	0.364797747	10.34375853	2.00E-23	4.07E-22	no
STK10	0.364526822	10.33489879	2.16E-23	4.41E-22	no
COLEC12	0.364094883	10.32077985	2.46E-23	5.00E-22	no
FCGR1C	0.363910386	10.31475148	2.60E-23	5.28E-22	no
HTR7	0.363841285	10.31249399	2.65E-23	5.38E-22	no
ADAM12	0.363549598	10.30296693	2.89E-23	5.86E-22	no
EPS8	0.363388855	10.29771824	3.03E-23	6.14E-22	no
SLC43A3	0.363261613	10.29356418	3.14E-23	6.37E-22	no
C6orf150	0.363017086	10.28558303	3.38E-23	6.83E-22	no
ALG2	0.36289088	10.28146471	3.50E-23	7.08E-22	no
FCGR1B	0.362734515	10.27636317	3.67E-23	7.41E-22	no
FAS	0.362704657	10.27538913	3.70E-23	7.47E-22	no
ZBTB7B	0.362618575	10.27258119	3.80E-23	7.65E-22	no
APEX2	0.362323859	10.26296996	4.14E-23	8.34E-22	no
ZDHHC12	0.362315257	10.26268946	4.15E-23	8.35E-22	no
ATP8B1	0.362112513	10.25607975	4.40E-23	8.85E-22	no
PUS10	0.361879858	10.24849694	4.71E-23	9.47E-22	no
SH3BGRL3	0.361848358	10.24747043	4.76E-23	9.54E-22	no
C10orf128	0.36139428	10.23267782	5.43E-23	1.09E-21	no
ZAK	0.361289787	10.22927491	5.60E-23	1.12E-21	no
LRRC8C	0.361246777	10.22787439	5.67E-23	1.13E-21	no
BCL2A1	0.361219424	10.22698375	5.72E-23	1.14E-21	no
CLDN7	0.361025262	10.2206624	6.05E-23	1.21E-21	no
DUSP23	0.360896117	10.21645868	6.29E-23	1.25E-21	no
SMARCC2	-0.360871504	-10.2156576	6.33E-23	1.26E-21	no
SLFN12	0.36077458	10.21250321	6.51E-23	1.30E-21	no
ACOT9	0.360604019	10.20695326	6.85E-23	1.36E-21	no
C14orf106	0.36058443	10.20631593	6.88E-23	1.37E-21	no
MAPKAPK3	0.360240962	10.1951435	7.61E-23	1.51E-21	no

LPCAT2	0.360188171	10.19342672	7.73E-23	1.53E-21	no
RAB8A	0.360171059	10.19287027	7.77E-23	1.54E-21	no
CYFIP1	0.360155609	10.19236787	7.80E-23	1.54E-21	no
CPPED1	0.359903605	10.18417453	8.39E-23	1.66E-21	no
NLGN2	-0.359864743	-10.18291124	8.49E-23	1.68E-21	no
HLA-A	0.359813434	10.18124344	8.62E-23	1.70E-21	no
BIRC3	0.359763122	10.17960813	8.74E-23	1.72E-21	no
BST1	0.359574094	10.17346506	9.24E-23	1.82E-21	no
TMEM50A	0.359529594	10.1720191	9.36E-23	1.84E-21	no
CFH	0.359523851	10.17183249	9.37E-23	1.84E-21	no
CMKLR1	0.359235502	10.16246501	1.02E-22	2.00E-21	no
SVIL	0.358773758	10.14747148	1.17E-22	2.29E-21	no
C9orf66	0.358758755	10.14698445	1.17E-22	2.29E-21	no
ACP5	0.358722163	10.14579666	1.18E-22	2.32E-21	no
MAP2K3	0.358669958	10.14410212	1.20E-22	2.35E-21	no
GSTK1	0.358606599	10.1420457	1.22E-22	2.39E-21	no
YY2	0.358482493	10.13801808	1.27E-22	2.47E-21	no
GSDMD	0.358451669	10.13701783	1.28E-22	2.49E-21	no
NFE2L3	0.358437275	10.13655077	1.28E-22	2.50E-21	no
RAP1A	0.35835005	10.13372058	1.32E-22	2.56E-21	no
C6orf115	0.358138676	10.12686337	1.40E-22	2.72E-21	no
BCL3	0.3581012	10.12564778	1.42E-22	2.75E-21	no
BNC2	0.357832223	10.1169249	1.53E-22	2.97E-21	no
MYOM3	0.35773939	10.11391499	1.57E-22	3.05E-21	no
NOD1	0.357700035	10.11263908	1.59E-22	3.08E-21	no
GPR77	0.357603911	10.109523	1.63E-22	3.16E-21	no
TNFRSF10C	0.357593649	10.10919035	1.64E-22	3.17E-21	no
NFKBIZ	0.357573045	10.10852247	1.65E-22	3.18E-21	no
DNAJC10	0.357542973	10.10754774	1.66E-22	3.21E-21	no
P2RY6	0.357225594	10.09726251	1.82E-22	3.51E-21	no
GPA33	0.357123072	10.09394098	1.88E-22	3.61E-21	no
FN1	0.357101546	10.09324361	1.89E-22	3.63E-21	no
CLNK	0.35692137	10.08740737	1.99E-22	3.82E-21	no
SCML4	0.356917298	10.0872755	1.99E-22	3.82E-21	no
XBP1	0.356702541	10.08032082	2.12E-22	4.06E-21	no
CHSY1	0.356677838	10.07952098	2.13E-22	4.09E-21	no
NPL	0.356421933	10.0712364	2.30E-22	4.40E-21	no
PNMA1	-0.356324863	-10.06809459	2.36E-22	4.52E-21	no
APOB48R	0.356193593	10.06384644	2.45E-22	4.69E-21	no
ANXA4	0.356048743	10.05915957	2.56E-22	4.88E-21	no
ACTR2	0.355974401	10.05675445	2.61E-22	4.98E-21	no
IER3	0.35553984	10.04269982	2.96E-22	5.64E-21	no
SNX2	0.355392383	10.03793243	3.09E-22	5.87E-21	no
ST6GAL1	0.355387458	10.03777321	3.09E-22	5.88E-21	no
FAM129C	0.355348435	10.03651173	3.13E-22	5.94E-21	no
FAM70B	0.355264408	10.03379559	3.20E-22	6.08E-21	no
CHRNA6	0.35500637	10.02545642	3.45E-22	6.54E-21	no
POLD4	0.354707606	10.01580436	3.75E-22	7.11E-21	no
TMEM92	0.354684636	10.01506242	3.78E-22	7.15E-21	no
TMEM2	0.35464399	10.01374958	3.82E-22	7.23E-21	no
DDX60L	0.354356223	10.00445681	4.15E-22	7.84E-21	no

MMP14	0.354305114	10.00280673	4.21E-22	7.95E-21	no
CLEC17A	0.354163985	9.998250769	4.38E-22	8.27E-21	no
ALOX15B	0.353568657	9.979040846	5.19E-22	9.78E-21	no
SCAMP2	0.35356571	9.978945804	5.20E-22	9.78E-21	no
TNNI2	0.353434791	9.974723216	5.39E-22	1.01E-20	no
UBQLN4	-0.353382469	-9.973035823	5.47E-22	1.03E-20	no
LST1	0.353283275	9.969837118	5.63E-22	1.06E-20	no
SIGLEC14	0.353266759	9.969304559	5.66E-22	1.06E-20	no
KIAA0649	-0.353128001	-9.964830778	5.89E-22	1.10E-20	no
DDB2	0.353084927	9.963442153	5.96E-22	1.12E-20	no
NCAM1	-0.353031429	-9.961717604	6.05E-22	1.13E-20	no
HTR2B	0.352539297	9.945858341	6.96E-22	1.30E-20	no
STX3	0.352465444	9.943479208	7.10E-22	1.33E-20	no
PPIC	0.352143153	9.933099232	7.78E-22	1.45E-20	no
IKBIP	0.352100273	9.931718526	7.88E-22	1.47E-20	no
C7	0.352097097	9.931616235	7.88E-22	1.47E-20	no
DIO3	0.352030459	9.929470665	8.03E-22	1.49E-20	no
GPX8	0.351872764	9.924393958	8.40E-22	1.56E-20	no
RNF213	0.351765606	9.920944761	8.66E-22	1.61E-20	no
F11R	0.351504742	9.912549946	9.32E-22	1.73E-20	no
NAGK	0.351297808	9.90589248	9.88E-22	1.83E-20	no
TPM4	0.3512259	9.903579449	1.01E-21	1.87E-20	no
DCTD	0.351073132	9.898666125	1.05E-21	1.95E-20	no
CARD17	0.351039274	9.897577287	1.06E-21	1.96E-20	no
CALD1	0.351004558	9.896460934	1.07E-21	1.98E-20	no
IRAK4	0.350867555	9.892055747	1.12E-21	2.06E-20	no
ANXA1	0.350866314	9.892015858	1.12E-21	2.06E-20	no
TNPO2	-0.350671687	-9.885759105	1.18E-21	2.17E-20	no
LSAMP	-0.350577821	-9.882742073	1.21E-21	2.23E-20	no
LATS2	0.35047413	9.87940965	1.25E-21	2.29E-20	no
CASP7	0.3501996	9.870588792	1.35E-21	2.47E-20	no
HGSNAT	0.350049837	9.865778026	1.40E-21	2.58E-20	no
ERAP1	0.349868628	9.859958274	1.48E-21	2.71E-20	no
EMILIN1	0.349839246	9.859014766	1.49E-21	2.73E-20	no
GBP1	0.349772805	9.856881319	1.52E-21	2.78E-20	no
NFKB2	0.349366358	9.843833862	1.70E-21	3.11E-20	no
COPZ2	0.349342601	9.843071416	1.71E-21	3.13E-20	no
SNX20	0.349104595	9.835434267	1.83E-21	3.34E-20	no
KIR2DL4	0.348977802	9.831366639	1.90E-21	3.46E-20	no
CD44	0.348946752	9.830370597	1.91E-21	3.48E-20	no
HPSE	0.348893276	9.82865529	1.94E-21	3.53E-20	no
BLK	0.348878186	9.828171267	1.95E-21	3.54E-20	no
BACE2	0.348814106	9.826116013	1.98E-21	3.61E-20	no
MARCO	0.348727811	9.823348453	2.03E-21	3.69E-20	no
SOBP	-0.348687666	-9.822061068	2.06E-21	3.73E-20	no
TNFSF13B	0.348532875	9.817097768	2.15E-21	3.89E-20	no
USP11	-0.348462615	-9.814845214	2.19E-21	3.96E-20	no
MEFV	0.348118022	9.803800198	2.41E-21	4.36E-20	no
TMEM119	0.348096115	9.803098168	2.43E-21	4.38E-20	no
ADCY7	0.348031406	9.801024674	2.47E-21	4.46E-20	no
ENOX2	0.34801264	9.800423374	2.48E-21	4.48E-20	no

S100A8	0.34797387	9.799181118	2.51E-21	4.52E-20	no
SRPX2	0.347625968	9.788036533	2.76E-21	4.98E-20	no
SLC25A45	0.347486752	9.783578213	2.87E-21	5.17E-20	no
COL5A2	0.347318041	9.778176343	3.01E-21	5.41E-20	no
FAM192A	-0.347237224	-9.775589065	3.08E-21	5.53E-20	no
SERPINB9	0.347149857	9.77279239	3.16E-21	5.66E-20	no
PARP12	0.347139745	9.77246872	3.17E-21	5.67E-20	no
MPZL3	0.346945044	9.766237342	3.34E-21	5.98E-20	no
PFN1	0.346881379	9.764200043	3.40E-21	6.08E-20	no
CLIC1	0.346797082	9.761502784	3.48E-21	6.22E-20	no
LASS2	0.346784884	9.761112498	3.49E-21	6.24E-20	no
FAM105B	0.346421202	9.749478921	3.86E-21	6.89E-20	no
PIGB	0.346258433	9.744273848	4.04E-21	7.21E-20	no
CCNYL1	0.345898041	9.732752654	4.47E-21	7.96E-20	no
SMARCAL1	0.345639895	9.724503101	4.80E-21	8.54E-20	no
PGM2	0.34563212	9.724254681	4.81E-21	8.55E-20	no
ATP7A	0.345552578	9.721713289	4.91E-21	8.73E-20	no
UBE2Z	0.345539106	9.721282879	4.93E-21	8.76E-20	no
SLC16A3	0.345427909	9.71773058	5.09E-21	9.02E-20	no
VNN3	0.345423601	9.717592965	5.09E-21	9.02E-20	no
CLECL1	0.34505267	9.705746601	5.64E-21	9.98E-20	no
MSLN	0.345050904	9.705690192	5.64E-21	9.98E-20	no
SNTB2	0.345024811	9.704857057	5.68E-21	1.00E-19	no
CFD	0.344821496	9.698366209	6.01E-21	1.06E-19	no
CR1	0.344727456	9.695364513	6.17E-21	1.09E-19	no
ABHD14B	0.344620253	9.691943043	6.36E-21	1.12E-19	no
ADAMTSL4	0.344602951	9.691390898	6.39E-21	1.12E-19	no
GCLM	0.344588199	9.690920113	6.41E-21	1.13E-19	no
SLC1A5	0.344515731	9.688607578	6.54E-21	1.15E-19	no
LPHN1	-0.344445581	-9.686369191	6.67E-21	1.17E-19	no
CALCR	0.343981499	9.671565658	7.58E-21	1.33E-19	no
CYP1B1	0.343954217	9.670695646	7.63E-21	1.34E-19	no
LOC387646	0.343929931	9.669921224	7.68E-21	1.35E-19	no
TRIM56	0.343876054	9.66820324	7.80E-21	1.37E-19	no
C10orf55	0.34367869	9.661910858	8.23E-21	1.44E-19	no
SLC16A13	0.34349462	9.656043584	8.66E-21	1.51E-19	no
DDR2	0.343273175	9.64898668	9.20E-21	1.61E-19	no
CFB	0.343265524	9.648742889	9.22E-21	1.61E-19	no
CALHM1	0.343200615	9.646674762	9.39E-21	1.64E-19	no
TNFSF14	0.342896011	9.636971565	1.02E-20	1.78E-19	no
AQP9	0.342541524	9.625683696	1.12E-20	1.96E-19	no
SELP	0.342423397	9.621923228	1.16E-20	2.02E-19	no
COL1A2	0.34239921	9.621153329	1.17E-20	2.03E-19	no
TXNDC5	0.342248251	9.616348582	1.22E-20	2.11E-19	no
VIM	0.342178982	9.61414414	1.24E-20	2.15E-19	no
ANO6	0.342118538	9.61222073	1.26E-20	2.19E-19	no
TBC1D22A	0.342062004	9.610421836	1.28E-20	2.22E-19	no
TTC7A	0.341905462	9.605441352	1.34E-20	2.31E-19	no
TREM2	0.341890567	9.604967505	1.34E-20	2.32E-19	no
FKBP5	0.341888239	9.604893454	1.34E-20	2.32E-19	no
C1orf186	0.341660727	9.59765676	1.43E-20	2.47E-19	no

SLC35A2	0.341222478	9.583722369	1.61E-20	2.78E-19	no
C17orf69	-0.341114047	-9.580275829	1.66E-20	2.86E-19	no
BACH1	0.341028763	9.577565335	1.70E-20	2.92E-19	no
TPP1	0.34091834	9.574056254	1.75E-20	3.01E-19	no
CCL22	0.34091075	9.573815082	1.75E-20	3.01E-19	no
CABP4	0.340889492	9.573139584	1.76E-20	3.03E-19	no
SLC25A30	0.34046276	9.559583412	1.98E-20	3.40E-19	no
C1GALT1C1	0.340272816	9.553551509	2.08E-20	3.57E-19	no
FUCA2	0.340263388	9.553252149	2.09E-20	3.58E-19	no
HERPUD1	0.34015663	9.549862565	2.15E-20	3.68E-19	no
WASF2	0.340132149	9.549085344	2.17E-20	3.70E-19	no
SNX6	0.340129712	9.549007967	2.17E-20	3.70E-19	no
TRAF5	0.34007991	9.547426914	2.20E-20	3.75E-19	no
TNFSF12	0.340037611	9.546084153	2.22E-20	3.79E-19	no
LAMB1	0.339928948	9.542634965	2.29E-20	3.90E-19	no
PDLIM1	0.339848678	9.540087322	2.34E-20	3.98E-19	no
STAP1	0.339680055	9.534736206	2.45E-20	4.16E-19	no
PIP4K2B	-0.339638432	-9.533415522	2.48E-20	4.21E-19	no
PHLPP1	-0.339606848	-9.532413375	2.50E-20	4.24E-19	no
CLDN14	0.339380018	9.525217409	2.65E-20	4.50E-19	no
ATP8B3	0.338983398	9.512639493	2.95E-20	5.01E-19	no
TBKBP1	-0.33894875	-9.511540981	2.98E-20	5.05E-19	no
KIAA0427	-0.338946425	-9.511467264	2.98E-20	5.05E-19	no
UBASH3B	0.338714015	9.504099887	3.18E-20	5.37E-19	no
GPHN	-0.338708944	-9.503939136	3.18E-20	5.37E-19	no
TNFRSF11B	0.338660207	9.502394432	3.22E-20	5.44E-19	no
PPCS	0.338624775	9.501271486	3.25E-20	5.49E-19	no
LGALS3BP	0.338578809	9.499814763	3.30E-20	5.55E-19	no
GPR25	0.338387957	9.493767173	3.47E-20	5.84E-19	no
IL2RA	0.338284608	9.49049285	3.57E-20	6.00E-19	no
GAS6	0.338220497	9.488461882	3.63E-20	6.10E-19	no
FURIN	0.338194153	9.487627379	3.65E-20	6.14E-19	no
FGL1	0.33818927	9.487472703	3.66E-20	6.14E-19	no
GSDMA	0.338131774	9.485651474	3.72E-20	6.23E-19	no
RHOQ	0.338131362	9.485638442	3.72E-20	6.23E-19	no
TAP2	0.338089714	9.484319284	3.76E-20	6.29E-19	no
SERTAD3	0.337934425	9.479401256	3.92E-20	6.55E-19	no
PATZ1	-0.337537512	-9.466834904	4.36E-20	7.29E-19	no
TMEM71	0.337495575	9.465507475	4.41E-20	7.36E-19	no
LHFPL4	-0.337457244	-9.464294274	4.45E-20	7.43E-19	no
RAG1AP1	0.337441403	9.463792917	4.47E-20	7.46E-19	no
FCGBP	0.337374035	9.461660843	4.56E-20	7.59E-19	no
FAM157B	0.337362075	9.461282339	4.57E-20	7.60E-19	no
GBP2	0.337320455	9.459965244	4.62E-20	7.68E-19	no
TMEM127	0.337230279	9.457111738	4.73E-20	7.87E-19	no
TMEM214	0.33722738	9.457020016	4.74E-20	7.87E-19	no
MYO1E	0.337211375	9.456513579	4.76E-20	7.89E-19	no
GSTA4	-0.3370829	-9.452448759	4.93E-20	8.16E-19	no
TMEM145	-0.337074528	-9.452183901	4.94E-20	8.17E-19	no
NEAT1	0.337012024	9.450206581	5.02E-20	8.31E-19	no
ALDH5A1	-0.336836672	-9.444660041	5.26E-20	8.70E-19	no

RUFY3	-0.336660951	-9.439102952	5.51E-20	9.11E-19	no
ZNF821	-0.336451873	-9.432492387	5.83E-20	9.62E-19	no
ELMOD2	0.336446479	9.432321867	5.84E-20	9.63E-19	no
GDI1	-0.336350323	-9.429282198	5.99E-20	9.87E-19	no
THRA	-0.336347495	-9.429192787	6.00E-20	9.87E-19	no
CTSK	0.336071237	9.420461629	6.46E-20	1.06E-18	no
PKD2	0.335581831	9.405000582	7.36E-20	1.21E-18	no
MPP1	0.335531773	9.403419677	7.45E-20	1.22E-18	no
SLC16A5	0.335511651	9.402784213	7.49E-20	1.23E-18	no
SLC40A1	0.335291918	9.39584587	7.95E-20	1.30E-18	no
EMILIN2	0.33516377	9.391800244	8.22E-20	1.35E-18	no
ZFP36L2	0.33501253	9.38702635	8.56E-20	1.40E-18	no
ICOSLG	0.334884034	9.382971032	8.86E-20	1.45E-18	no
IGFBP4	0.334827616	9.381190673	8.99E-20	1.47E-18	no
USP15	0.334808342	9.380582487	9.04E-20	1.48E-18	no
CRTC1	-0.334750753	-9.378765308	9.18E-20	1.50E-18	no
CNRIP1	-0.334721397	-9.377839068	9.25E-20	1.51E-18	no
CEBPE	0.334718826	9.377757936	9.25E-20	1.51E-18	no
MYCBP	0.334547955	9.37236718	9.68E-20	1.58E-18	no
TRIM5	0.334483676	9.370339538	9.85E-20	1.60E-18	no
LAMC1	0.334443844	9.369083132	9.96E-20	1.62E-18	no
TMEM39A	0.33438581	9.36725268	1.01E-19	1.64E-18	no
APOL1	0.334352503	9.366202202	1.02E-19	1.66E-18	no
TNS4	0.333921502	9.352612265	1.14E-19	1.85E-18	no
TSPO	0.333751991	9.347269227	1.20E-19	1.94E-18	no
PRPS2	0.333697076	9.345538513	1.21E-19	1.96E-18	no
ANXA2P1	0.333670351	9.344696265	1.22E-19	1.98E-18	no
MUM1	-0.333555488	-9.341076642	1.26E-19	2.04E-18	no
FAM167B	0.333553832	9.341024487	1.26E-19	2.04E-18	no
BGN	0.333503005	9.339422949	1.28E-19	2.06E-18	no
LAPTM4A	0.333430755	9.337146546	1.30E-19	2.10E-18	no
NEURL4	-0.333397613	-9.336102405	1.31E-19	2.12E-18	no
BCL10	0.333332045	9.334036754	1.34E-19	2.15E-18	no
FAM171A2	-0.333303209	-9.333128381	1.35E-19	2.17E-18	no
LOC606724	0.333135042	9.327831371	1.41E-19	2.26E-18	no
TGM2	0.333021943	9.324269497	1.45E-19	2.33E-18	no
DIO1	0.332950452	9.322018218	1.48E-19	2.37E-18	no
FKBP11	0.332939914	9.321686405	1.48E-19	2.38E-18	no
CD55	0.332921559	9.321108422	1.49E-19	2.39E-18	no
KDELC2	0.332896756	9.320327451	1.50E-19	2.40E-18	no
CCL13	0.33286122	9.319208555	1.51E-19	2.42E-18	no
APOBEC3A	0.332829128	9.318198103	1.53E-19	2.44E-18	no
CDCP1	0.332752645	9.315790176	1.56E-19	2.49E-18	no
AMPD3	0.332741375	9.315435366	1.56E-19	2.49E-18	no
ALG12	0.332696587	9.314025418	1.58E-19	2.52E-18	no
ATG7	0.332634225	9.312062326	1.61E-19	2.56E-18	no
ZFYVE20	-0.332621758	-9.311669892	1.61E-19	2.57E-18	no
SOD2	0.3325842	9.310487698	1.63E-19	2.59E-18	no
AXL	0.332542277	9.309168167	1.65E-19	2.62E-18	no
PARP2	-0.332489431	-9.307504918	1.67E-19	2.65E-18	no
GNG12	0.332477654	9.307134248	1.67E-19	2.66E-18	no

DCN	0.332342342	9.302876021	1.74E-19	2.75E-18	no
LILRA1	0.332267647	9.300525647	1.77E-19	2.80E-18	no
KDELR3	0.332235609	9.299517594	1.78E-19	2.83E-18	no
ANKRD13B	-0.332183554	-9.297879802	1.81E-19	2.86E-18	no
BRSK2	-0.332145803	-9.296692098	1.83E-19	2.89E-18	no
PHF11	0.33197782	9.291407759	1.91E-19	3.02E-18	no
PROCR	0.331581352	9.278939783	2.12E-19	3.35E-18	no
AP2B1	-0.331312455	-9.270486727	2.27E-19	3.59E-18	no
TAPBPL	0.331262272	9.268909469	2.30E-19	3.63E-18	no
ARHGAP29	0.331126997	9.264658163	2.39E-19	3.76E-18	no
CD300E	0.331099691	9.263800097	2.41E-19	3.78E-18	no
RGS1	0.330998055	9.260606504	2.47E-19	3.88E-18	no
FPR2	0.33089349	9.257321225	2.54E-19	3.99E-18	no
FOSL2	0.330843628	9.255754776	2.57E-19	4.04E-18	no
MAN1C1	0.330740777	9.252523916	2.64E-19	4.14E-18	no
PROS1	0.33071284	9.251646384	2.66E-19	4.17E-18	no
MITF	0.33044931	9.243370076	2.85E-19	4.47E-18	no
RNASE3	0.330302532	9.238761471	2.96E-19	4.64E-18	no
C9orf95	0.330288652	9.238325717	2.97E-19	4.65E-18	no
BLNK	0.330259779	9.237419227	3.00E-19	4.68E-18	no
C1orf84	0.330169868	9.234596674	3.07E-19	4.79E-18	no
WARS	0.330037054	9.230427799	3.18E-19	4.95E-18	no
PPP1R3B	0.330022681	9.229976666	3.19E-19	4.97E-18	no
DUSP26	-0.330000402	-9.229277439	3.21E-19	4.99E-18	no
DPYSL4	-0.329899881	-9.226122729	3.29E-19	5.12E-18	no
HPS1	0.329727198	9.220704121	3.44E-19	5.36E-18	no
SLC33A1	0.3296352	9.217817766	3.53E-19	5.48E-18	no
RNPEP	0.329631308	9.217695653	3.53E-19	5.48E-18	no
HEATR3	0.329575407	9.215941959	3.58E-19	5.56E-18	no
FXR2	-0.329528996	-9.21448606	3.63E-19	5.62E-18	no
C20orf197	0.329466269	9.212518432	3.69E-19	5.71E-18	no
DNAJC5B	0.329412907	9.210844696	3.74E-19	5.79E-18	no
SHISA7	-0.32931375	-9.207734828	3.84E-19	5.93E-18	no
LILRA3	0.329140688	9.202307862	4.01E-19	6.20E-18	no
PTCHD3	0.329089623	9.200706756	4.07E-19	6.28E-18	no
IFITM3	0.328882567	9.194215532	4.29E-19	6.62E-18	no
FAM120A	0.328757306	9.19028931	4.43E-19	6.84E-18	no
LCTL	0.328615462	9.185843959	4.60E-19	7.09E-18	no
VPS4A	-0.328580265	-9.184741017	4.64E-19	7.15E-18	no
ITGB3	0.328530882	9.18319359	4.70E-19	7.23E-18	no
DNAJC3	0.328328461	9.176851568	4.95E-19	7.62E-18	no
KLF11	0.328278432	9.175284346	5.02E-19	7.71E-18	no
BAK1	0.327999737	9.166555418	5.40E-19	8.28E-18	no
FAP	0.327947891	9.164931856	5.47E-19	8.39E-18	no
C4orf32	0.327934342	9.164507601	5.49E-19	8.41E-18	no
SS18L1	-0.327898207	-9.163376092	5.54E-19	8.48E-18	no
GLRX	0.32776638	9.15924856	5.73E-19	8.77E-18	no
IL18RAP	0.327709664	9.157472983	5.82E-19	8.90E-18	no
ARFIP1	0.327397376	9.147698197	6.31E-19	9.64E-18	no
CATSPER1	0.327327694	9.145517577	6.42E-19	9.80E-18	no
KBTBD6	-0.32731715	-9.145187598	6.44E-19	9.82E-18	no

PRR13	0.327157065	9.140178617	6.71E-19	1.02E-17	no
OLR1	0.327097585	9.138317712	6.81E-19	1.04E-17	no
ADAM9	0.326894736	9.131972329	7.18E-19	1.09E-17	no
WDR1	0.326874024	9.131324512	7.22E-19	1.10E-17	no
PIGT	0.32684831	9.130520253	7.27E-19	1.10E-17	no
ARPC5	0.326805186	9.129171538	7.35E-19	1.12E-17	no
C17orf66	0.32673578	9.127000977	7.48E-19	1.14E-17	no
KCNJ5	0.326687192	9.12548154	7.58E-19	1.15E-17	no
EHMT2	-0.326672413	-9.125019384	7.60E-19	1.15E-17	no
SLC2A10	0.326607111	9.122977463	7.73E-19	1.17E-17	no
SIGLEC11	0.326526353	9.120452429	7.90E-19	1.19E-17	no
TOR1B	0.326491454	9.119361334	7.97E-19	1.20E-17	no
SRGAP3	-0.326325248	-9.114165512	8.32E-19	1.26E-17	no
YIPF1	0.326322686	9.114085445	8.32E-19	1.26E-17	no
CD274	0.326221323	9.110917181	8.54E-19	1.29E-17	no
VAMP3	0.326219726	9.110867267	8.54E-19	1.29E-17	no
MORN4	-0.32620206	-9.11031514	8.58E-19	1.29E-17	no
KHNYN	0.32603502	9.105094908	8.96E-19	1.35E-17	no
AKAP2	0.325974918	9.103216886	9.10E-19	1.37E-17	no
LIMS1	0.325947515	9.102360651	9.16E-19	1.38E-17	no
ACVR1	0.325872544	9.100018251	9.34E-19	1.40E-17	no
CAPZB	0.325850914	9.099342473	9.40E-19	1.41E-17	no
CSF2RA	0.325830926	9.098718014	9.44E-19	1.42E-17	no
HTATIP2	0.325724464	9.095392197	9.71E-19	1.45E-17	no
ZCCHC6	0.325686075	9.094193043	9.80E-19	1.47E-17	no
3-Sep	-0.325616199	-9.092010438	9.98E-19	1.49E-17	no
YIPF5	0.325288722	9.081783804	1.09E-18	1.62E-17	no
SOAT2	0.32525544	9.080744663	1.09E-18	1.64E-17	no
SLC22A17	-0.325246363	-9.080461269	1.10E-18	1.64E-17	no
HLA-G	0.325055569	9.074505009	1.15E-18	1.72E-17	no
IL27	0.324919037	9.070243477	1.19E-18	1.78E-17	no
ACTN1	0.32491628	9.070157435	1.19E-18	1.78E-17	no
GNAO1	-0.324605571	-9.060461769	1.29E-18	1.93E-17	no
GMPPB	0.324589086	9.059947451	1.30E-18	1.93E-17	no
PRR15	0.324533549	9.058214787	1.32E-18	1.96E-17	no
C6orf134	-0.324523261	-9.057893851	1.32E-18	1.96E-17	no
IFNGR1	0.324389627	9.053725186	1.37E-18	2.03E-17	no
HLA-C	0.324380957	9.05345474	1.37E-18	2.03E-17	no
SH3KBP1	0.324302972	9.051022335	1.40E-18	2.07E-17	no
TNFRSF4	0.324295954	9.050803452	1.40E-18	2.07E-17	no
CACNG7	-0.324264128	-9.049810842	1.41E-18	2.09E-17	no
MTA3	-0.32416511	-9.046722823	1.45E-18	2.14E-17	no
DPP6	-0.324136837	-9.045841131	1.46E-18	2.15E-17	no
VPREB3	0.324047118	9.043043498	1.49E-18	2.20E-17	no
GPX1	0.324016005	9.042073403	1.50E-18	2.22E-17	no
C15orf48	0.324004469	9.041713723	1.51E-18	2.22E-17	no
HCG26	0.323967509	9.040561329	1.52E-18	2.24E-17	no
PDIA3	0.323909931	9.038766218	1.54E-18	2.27E-17	no
ZNF101	0.323873114	9.037618438	1.56E-18	2.29E-17	no
ARRB2	0.323848417	9.036848523	1.57E-18	2.30E-17	no
TSPAN4	0.323847631	9.036824027	1.57E-18	2.30E-17	no

AGTRAP	0.323846794	9.036797924	1.57E-18	2.30E-17	no
DYRK3	0.323679021	9.031568265	1.64E-18	2.40E-17	no
ZNF711	-0.323631018	-9.030072126	1.66E-18	2.43E-17	no
TBX19	0.323506754	9.026199496	1.71E-18	2.51E-17	no
COL18A1	0.323492161	9.025744731	1.72E-18	2.52E-17	no
C17orf44	0.323327249	9.020606213	1.79E-18	2.62E-17	no
FAM20C	0.323327237	9.020605828	1.79E-18	2.62E-17	no
CSNK1E	-0.322925542	-9.00809319	1.99E-18	2.90E-17	no
USO1	0.32290303	9.007392111	2.00E-18	2.91E-17	no
SBNO2	0.322844037	9.005555005	2.03E-18	2.96E-17	no
ABCD1	0.322799996	9.004183577	2.05E-18	2.99E-17	no
SEC22B	0.322751972	9.00268821	2.07E-18	3.02E-17	no
PSAP	0.322748305	9.002574041	2.08E-18	3.02E-17	no
FCHSD1	0.32266731	9.000052236	2.12E-18	3.08E-17	no
IL21	0.322666742	9.000034531	2.12E-18	3.08E-17	no
PODXL	0.322434419	8.992802271	2.25E-18	3.27E-17	no
TAPBP	0.32232541	8.989409414	2.31E-18	3.36E-17	no
MATR3	-0.322236015	-8.986627319	2.36E-18	3.43E-17	no
CIDEB	0.322170531	8.98458957	2.40E-18	3.48E-17	no
SERPINH1	0.322149559	8.983936971	2.42E-18	3.50E-17	no
GATS	-0.321983629	-8.97877424	2.52E-18	3.65E-17	no
PSMB9	0.321963569	8.978150142	2.53E-18	3.66E-17	no
SLC39A8	0.3218559	8.974800705	2.60E-18	3.76E-17	no
PDIA3P	0.321781727	8.972493508	2.65E-18	3.83E-17	no
EDEM2	0.321724311	8.970707658	2.69E-18	3.89E-17	no
SFRP4	0.321689953	8.969639049	2.72E-18	3.92E-17	no
ARPC3	0.321529845	8.964659891	2.83E-18	4.08E-17	no
FAM82A1	0.321410769	8.960957364	2.91E-18	4.20E-17	no
DDX60	0.32138431	8.960134704	2.93E-18	4.22E-17	no
CDH10	-0.321315667	-8.958000585	2.99E-18	4.29E-17	no
OSTM1	0.321078381	8.950624586	3.17E-18	4.56E-17	no
MAP2	-0.32104432	-8.949565936	3.20E-18	4.59E-17	no
C17orf100	-0.321010932	-8.948528265	3.22E-18	4.63E-17	no
C5orf15	0.320992229	8.947947005	3.24E-18	4.65E-17	no
SLC12A7	0.320938466	8.946276207	3.28E-18	4.71E-17	no
THBD	0.32086183	8.943894741	3.35E-18	4.79E-17	no
HSPG2	0.320639284	8.936980239	3.54E-18	5.07E-17	no
BEX1	-0.320432038	-8.930542605	3.73E-18	5.34E-17	no
A4GALT	0.32033257	8.927453342	3.83E-18	5.47E-17	no
SUMO1P1	0.320327889	8.927307987	3.83E-18	5.47E-17	no
BAI3	-0.320264288	-8.925332849	3.89E-18	5.55E-17	no
SSH2	0.320187463	8.922947247	3.97E-18	5.66E-17	no
RAB32	0.320159843	8.922089607	4.00E-18	5.69E-17	no
PVRL2	0.320126519	8.92105493	4.03E-18	5.74E-17	no
CMTM3	0.320118693	8.920811913	4.04E-18	5.74E-17	no
ITGAX	0.319995615	8.916990718	4.16E-18	5.92E-17	no
TUB	-0.319923167	-8.914741685	4.24E-18	6.03E-17	no
RIC3	-0.319914328	-8.914467307	4.25E-18	6.04E-17	no
PALM2-AKAP2	0.319824091	8.911666264	4.35E-18	6.17E-17	no
FOXL1	0.3196842	8.907324493	4.50E-18	6.39E-17	no
ZG16B	0.319590311	8.904410825	4.61E-18	6.53E-17	no

GPSM1	-0.319462739	-8.900452356	4.76E-18	6.74E-17	no
PARP8	0.319331172	8.896370504	4.92E-18	6.96E-17	no
FAM57B	-0.319238227	-8.893487207	5.04E-18	7.12E-17	no
RALB	0.319177772	8.891611988	5.11E-18	7.23E-17	no
LILRP2	0.319122624	8.889901469	5.19E-18	7.32E-17	no
TNFRSF6B	0.319099185	8.889174489	5.22E-18	7.36E-17	no
LYSMD3	0.319022439	8.886794304	5.32E-18	7.50E-17	no
GLB1L	0.31884243	8.881212271	5.56E-18	7.84E-17	no
FXYD6	-0.318817863	-8.880450531	5.60E-18	7.88E-17	no
PSMB10	0.318808241	8.880152203	5.61E-18	7.89E-17	no
FAM49B	0.318748683	8.878305616	5.69E-18	8.00E-17	no
CHPF2	0.31874822	8.878291283	5.70E-18	8.00E-17	no
PCYT1A	0.31872759	8.877651691	5.72E-18	8.04E-17	no
TNRC6C	-0.318711895	-8.87716509	5.75E-18	8.06E-17	no
ZNF853	-0.318629349	-8.874606084	5.87E-18	8.23E-17	no
DCBLD2	0.3185128	8.870993331	6.04E-18	8.46E-17	no
LEPROT	0.318503814	8.870714785	6.05E-18	8.48E-17	no
RAB11FIP1	0.318489553	8.870272761	6.08E-18	8.50E-17	no
IFITM1	0.318418927	8.868083787	6.18E-18	8.65E-17	no
KIAA0020	0.318354627	8.866091035	6.28E-18	8.78E-17	no
ZMPSTE24	0.318349269	8.86592499	6.29E-18	8.79E-17	no
TMSL3	0.318329447	8.865310706	6.32E-18	8.82E-17	no
GUSB	0.3181222	8.858888877	6.66E-18	9.29E-17	no
MX2	0.317980918	8.854511856	6.90E-18	9.61E-17	no
PML	0.317908872	8.852280082	7.02E-18	9.78E-17	no
DDX51	-0.317908476	-8.852267815	7.03E-18	9.78E-17	no
HDAC7	0.317898134	8.851947453	7.04E-18	9.79E-17	no
MYADM	0.317883088	8.851481397	7.07E-18	9.82E-17	no
MAST1	-0.317755743	-8.847537155	7.30E-18	1.01E-16	no
SLC6A6	0.31770043	8.845824084	7.40E-18	1.03E-16	no
BCAN	-0.317613302	-8.843125934	7.56E-18	1.05E-16	no
C8orf4	0.317546435	8.841055385	7.69E-18	1.07E-16	no
MTSS1L	-0.317536305	-8.840741729	7.71E-18	1.07E-16	no
GPM6A	-0.317488996	-8.839276902	7.80E-18	1.08E-16	no
SHMT1	0.317480835	8.839024234	7.82E-18	1.08E-16	no
DAP	0.317271383	8.832539914	8.23E-18	1.14E-16	no
LMNA	0.317221735	8.831003106	8.34E-18	1.15E-16	no
RNLS	0.317216163	8.830830625	8.35E-18	1.15E-16	no
HTRA3	0.317133493	8.828271824	8.52E-18	1.18E-16	no
FBN1	0.31705131	8.825728335	8.70E-18	1.20E-16	no
ATG4A	0.317032523	8.825146943	8.74E-18	1.20E-16	no
NOL4	-0.317024903	-8.824911103	8.76E-18	1.21E-16	no
RRAS	0.317017195	8.824672558	8.77E-18	1.21E-16	no
ABI2	-0.316935894	-8.822156677	8.95E-18	1.23E-16	no
KDELR2	0.316782182	8.817400596	9.30E-18	1.28E-16	no
FGD3	0.316758249	8.81666013	9.36E-18	1.28E-16	no
DCUN1D2	-0.316679457	-8.814222545	9.54E-18	1.31E-16	no
TMEM43	0.316583136	8.811242949	9.77E-18	1.34E-16	no
ITSN2	0.316573122	8.810933187	9.80E-18	1.34E-16	no
CYP27A1	0.316537387	8.809827848	9.88E-18	1.35E-16	no
HSD17B11	0.316422169	8.806264273	1.02E-17	1.39E-16	no

COL3A1	0.316375495	8.804820783	1.03E-17	1.41E-16	no
SHQ1	0.316363811	8.804459451	1.03E-17	1.41E-16	no
STK3	0.316353166	8.80413026	1.03E-17	1.41E-16	no
CELA1	0.316248021	8.80087882	1.06E-17	1.45E-16	no
PAFAH2	0.316080351	8.795694669	1.11E-17	1.51E-16	no
NR1I2	0.316076741	8.795583065	1.11E-17	1.51E-16	no
ZNF554	-0.316073774	-8.795491335	1.11E-17	1.51E-16	no
KIAA1274	0.316035376	8.794304247	1.12E-17	1.52E-16	no
TMCO4	0.315996419	8.79309992	1.13E-17	1.54E-16	no
PCOLCE	0.315903413	8.790224928	1.16E-17	1.57E-16	no
SFRS13B	-0.315719661	-8.784545632	1.21E-17	1.64E-16	no
PANK2	0.31562569	8.78164166	1.24E-17	1.68E-16	no
FAM114A2	0.315575895	8.780102982	1.25E-17	1.70E-16	no
CSRP2BP	-0.315560955	-8.77964134	1.26E-17	1.71E-16	no
TPD52L2	0.315480549	8.77715696	1.28E-17	1.74E-16	no
SLC38A9	0.315479571	8.777126741	1.28E-17	1.74E-16	no
C3orf52	0.315302207	8.771647312	1.34E-17	1.81E-16	no
C22orf39	-0.315237151	-8.769637753	1.36E-17	1.84E-16	no
LRRC37B	-0.315019116	-8.762903687	1.44E-17	1.94E-16	no
RALGPS1	-0.315016184	-8.762813136	1.44E-17	1.94E-16	no
LRCH2	-0.314893661	-8.759029704	1.48E-17	2.00E-16	no
CD1B	0.314875177	8.758458949	1.49E-17	2.01E-16	no
HAMP	0.314783562	8.755630293	1.53E-17	2.05E-16	no
FBLIM1	0.31469593	8.752924876	1.56E-17	2.10E-16	no
TSPAN7	-0.314662536	-8.75189398	1.57E-17	2.11E-16	no
SOCS1	0.314639966	8.751197238	1.58E-17	2.12E-16	no
SPIRE1	-0.314564639	-8.748872028	1.61E-17	2.16E-16	no
TMEM150A	0.314532019	8.74786517	1.62E-17	2.18E-16	no
SEPHS2	0.314479172	8.746234044	1.64E-17	2.21E-16	no
FSD1	-0.314459842	-8.745637437	1.65E-17	2.21E-16	no
TNFRSF10B	0.31443536	8.744881848	1.66E-17	2.23E-16	no
LILRA5	0.314313063	8.741107684	1.71E-17	2.29E-16	no
MYBPC3	0.314292756	8.74048104	1.72E-17	2.30E-16	no
SIGLEC15	0.314239172	8.738827585	1.74E-17	2.33E-16	no
PTPN12	0.314227491	8.738467123	1.75E-17	2.34E-16	no
CPD	0.314122476	8.735226973	1.80E-17	2.40E-16	no
PHF21B	-0.314101471	-8.734578909	1.80E-17	2.41E-16	no
ETF1	0.313972942	8.730613789	1.86E-17	2.48E-16	no
TTC38	0.313817828	8.725829269	1.93E-17	2.58E-16	no
ACVR2B	-0.313711501	-8.722550021	1.99E-17	2.65E-16	no
RRAGB	-0.313607098	-8.719330453	2.04E-17	2.71E-16	no
TMEM170B	-0.313508971	-8.716304768	2.09E-17	2.78E-16	no
CR2	0.313426055	8.713748319	2.13E-17	2.83E-16	no
PLA2G4A	0.313409539	8.713239134	2.14E-17	2.84E-16	no
KIR3DL1	0.313099137	8.703671084	2.31E-17	3.06E-16	no
CRMP1	-0.312981039	-8.700031558	2.38E-17	3.15E-16	no
OLIG2	-0.3129739	-8.699811565	2.38E-17	3.16E-16	no
TRIP4	0.312936769	8.698667362	2.40E-17	3.18E-16	no
FZD1	0.312798691	8.694412854	2.48E-17	3.29E-16	no
C2orf18	0.312745742	8.692781543	2.52E-17	3.33E-16	no
GPC6	0.312725586	8.692160579	2.53E-17	3.34E-16	no

NFE2L2	0.312720834	8.692014192	2.53E-17	3.35E-16	no
CASC3	-0.312718882	-8.691954036	2.53E-17	3.35E-16	no
KDM4B	-0.312691534	-8.691111529	2.55E-17	3.37E-16	no
RNASEL	0.312621067	8.688940759	2.59E-17	3.42E-16	no
KLF6	0.312563636	8.687171694	2.63E-17	3.47E-16	no
RFTN2	-0.312394062	-8.681948825	2.74E-17	3.61E-16	no
CD248	0.312324011	8.679791539	2.79E-17	3.67E-16	no
PCK2	0.312305853	8.679232372	2.80E-17	3.69E-16	no
TTBK1	-0.312282475	-8.678512467	2.82E-17	3.70E-16	no
KRT36	0.31217208	8.675113211	2.90E-17	3.80E-16	no
FAM19A5	-0.312119968	-8.673508715	2.93E-17	3.85E-16	no
LRRC20	-0.311989767	-8.669500293	3.03E-17	3.97E-16	no
FAM3B	0.311952469	8.66835212	3.05E-17	4.01E-16	no
MBNL3	0.311895474	8.666597713	3.10E-17	4.06E-16	no
TTC39B	0.311879117	8.666094209	3.11E-17	4.07E-16	no
MGAT2	0.311809286	8.663944839	3.16E-17	4.14E-16	no
ZCCHC14	-0.311732969	-8.661596033	3.22E-17	4.21E-16	no
ZNF552	0.311716349	8.661084534	3.24E-17	4.23E-16	no
CLCF1	0.311669668	8.659647928	3.27E-17	4.27E-16	no
CISH	0.311651712	8.659095354	3.29E-17	4.29E-16	no
CRY2	-0.311649653	-8.659031986	3.29E-17	4.29E-16	no
C21orf96	0.311647463	8.658964616	3.29E-17	4.29E-16	no
PODXL2	-0.311645229	-8.658895862	3.29E-17	4.29E-16	no
LGALS1	0.311611156	8.657847338	3.32E-17	4.32E-16	no
FCGR3B	0.311563773	8.65638929	3.36E-17	4.37E-16	no
COL1A1	0.311488352	8.654068636	3.42E-17	4.45E-16	no
SLC30A6	0.311333659	8.649309398	3.55E-17	4.62E-16	no
ARL5A	0.311297918	8.648209888	3.58E-17	4.65E-16	no
MXRA8	0.311273139	8.647447644	3.60E-17	4.68E-16	no
STEAP3	0.31121183	8.645561765	3.66E-17	4.75E-16	no
ABCC1	0.311126493	8.64293696	3.74E-17	4.84E-16	no
KIAA1543	-0.311093839	-8.641932621	3.77E-17	4.88E-16	no
TMEM87B	0.311030118	8.639972921	3.82E-17	4.95E-16	no
ICMT	0.310851677	8.63448571	3.99E-17	5.17E-16	no
IFNAR2	0.310631953	8.627730402	4.21E-17	5.45E-16	no
SERP2	-0.310420542	-8.62123211	4.43E-17	5.73E-16	no
HIVEP3	0.310370288	8.61968762	4.49E-17	5.80E-16	no
SLAIN2	0.310316109	8.61802261	4.55E-17	5.87E-16	no
SLC46A2	0.310315474	8.61800308	4.55E-17	5.87E-16	no
ARHGAP33	-0.310199051	-8.614425511	4.68E-17	6.03E-16	no
PRAM1	0.310074862	8.610609771	4.82E-17	6.21E-16	no
PTPRS	-0.310065866	-8.610333375	4.83E-17	6.22E-16	no
RCN3	0.30996784	8.607321849	4.95E-17	6.36E-16	no
FAM65C	0.309956793	8.606982506	4.96E-17	6.38E-16	no
SSR3	0.309905437	8.605404897	5.02E-17	6.45E-16	no
VCL	0.309875217	8.604476625	5.06E-17	6.50E-16	no
RASL10B	-0.30987341	-8.60442111	5.06E-17	6.50E-16	no
LRRC4	-0.309548009	-8.594427429	5.48E-17	7.02E-16	no
CLIC4	0.309472625	8.592112713	5.58E-17	7.15E-16	no
ZNF74	-0.309432366	-8.590876617	5.63E-17	7.21E-16	no
C16orf45	-0.309422889	-8.590585654	5.65E-17	7.22E-16	no

TTC26	0.309418917	8.590463698	5.65E-17	7.23E-16	no
HSDL1	-0.30938489	-8.589419003	5.70E-17	7.28E-16	no
TMEM176A	0.309261067	8.585617675	5.87E-17	7.49E-16	no
GTSF1L	0.309260692	8.585606163	5.87E-17	7.49E-16	no
IGFBP6	0.309215377	8.584215107	5.94E-17	7.57E-16	no
LAMA4	0.309189629	8.583424767	5.97E-17	7.61E-16	no
ACP2	0.309176304	8.583015733	5.99E-17	7.63E-16	no
CD59	0.309096049	8.58055242	6.11E-17	7.78E-16	no
C13orf18	0.308942722	8.575846804	6.34E-17	8.07E-16	no
QTRTD1	0.308917475	8.575072031	6.38E-17	8.11E-16	no
SOCS3	0.308802132	8.571532726	6.56E-17	8.33E-16	no
CD276	0.30863761	8.566485081	6.82E-17	8.67E-16	no
SLC25A23	-0.308499685	-8.562254099	7.05E-17	8.95E-16	no
GATA3	0.308490827	8.561982394	7.07E-17	8.97E-16	no
MRC2	0.308477178	8.561563723	7.09E-17	8.99E-16	no
RAB11FIP3	-0.308375268	-8.558437984	7.27E-17	9.21E-16	no
TSC2	-0.308244381	-8.554423961	7.50E-17	9.50E-16	no
PABPC4	0.308152891	8.5516185	7.67E-17	9.70E-16	no
RALGAPA1	-0.307765037	-8.539728101	8.42E-17	1.06E-15	no
TXLNA	0.307739689	8.538951173	8.47E-17	1.07E-15	no
ANKRD16	-0.307652304	-8.53627294	8.65E-17	1.09E-15	no
NAP1L3	-0.307561875	-8.533501677	8.84E-17	1.12E-15	no
SPTY2D1	0.307446551	8.529967865	9.09E-17	1.15E-15	no
RGS10	0.307410508	8.528863496	9.16E-17	1.16E-15	no
SEMA4G	-0.307320303	-8.526099759	9.36E-17	1.18E-15	no
TTC3	-0.307243837	-8.523757171	9.54E-17	1.20E-15	no
ITPRIP	0.307233012	8.523425549	9.56E-17	1.20E-15	no
PHTF1	0.307111744	8.519710846	9.84E-17	1.24E-15	no
GALNT12	0.306986125	8.515863317	1.01E-16	1.27E-15	no
TRAP1	-0.306921668	-8.513889296	1.03E-16	1.29E-15	no
FLNA	0.30664514	8.505421968	1.10E-16	1.38E-15	no
DPAGT1	0.30662589	8.504832616	1.11E-16	1.39E-15	no
C1orf54	0.30655501	8.502662673	1.12E-16	1.41E-15	no
RAB38	0.306515716	8.501459786	1.14E-16	1.42E-15	no
NUDT10	-0.306468768	-8.50002267	1.15E-16	1.44E-15	no
STAT2	0.306357462	8.496615731	1.18E-16	1.48E-15	no
PFKM	-0.306283436	-8.494350126	1.20E-16	1.50E-15	no
SCAMP5	-0.306277929	-8.494181571	1.20E-16	1.50E-15	no
TNC	0.306223144	8.492504969	1.22E-16	1.52E-15	no
C22orf25	0.306142662	8.490042084	1.24E-16	1.55E-15	no
ACTB	0.305991745	8.485424333	1.29E-16	1.60E-15	no
MPZL2	0.305980665	8.485085328	1.29E-16	1.61E-15	no
ATP10D	0.305957327	8.484371325	1.30E-16	1.62E-15	no
CTNNA1	0.305878103	8.481947598	1.32E-16	1.65E-15	no
SAMD9	0.305830639	8.480495614	1.34E-16	1.66E-15	no
GRIA3	-0.305740606	-8.477741574	1.37E-16	1.70E-15	no
C14orf34	0.305332823	8.465270949	1.51E-16	1.87E-15	no
AFF1	0.305247287	8.462655781	1.54E-16	1.91E-15	no
PRPSAP2	-0.305225303	-8.46198369	1.54E-16	1.92E-15	no
KCNJ2	0.305216678	8.461720006	1.55E-16	1.92E-15	no
SLC31A1	0.305080682	8.457562668	1.60E-16	1.98E-15	no

SIL1	0.305029639	8.456002445	1.62E-16	2.00E-15	no
GKAP1	-0.304843505	-8.450313661	1.69E-16	2.09E-15	no
RTN3	-0.304718918	-8.446506485	1.74E-16	2.15E-15	no
EHD2	0.304659832	8.444701116	1.77E-16	2.18E-15	no
POSTN	0.304649886	8.444397212	1.77E-16	2.19E-15	no
C4orf38	0.304592907	8.442656308	1.79E-16	2.22E-15	no
UGCG	0.304592208	8.442634961	1.79E-16	2.22E-15	no
PDIA5	0.304567506	8.441880269	1.81E-16	2.23E-15	no
NRXN2	-0.304491072	-8.439545161	1.84E-16	2.27E-15	no
CLP1	0.304488302	8.439460541	1.84E-16	2.27E-15	no
LYL1	0.304421878	8.437431415	1.87E-16	2.30E-15	no
TMEM8B	-0.304401412	-8.436806258	1.88E-16	2.31E-15	no
CCDC109B	0.304367389	8.435766989	1.89E-16	2.33E-15	no
SLC25A19	0.304351576	8.435283979	1.90E-16	2.34E-15	no
APC2	-0.3040625	-8.426455453	2.03E-16	2.50E-15	no
ZNF80	0.303988721	8.424202614	2.07E-16	2.54E-15	no
PSMB8	0.303967689	8.423560404	2.08E-16	2.55E-15	no
TMEM49	0.303961957	8.423385407	2.08E-16	2.55E-15	no
ZC3H12A	0.303913362	8.421901697	2.11E-16	2.58E-15	no
RAP2C	0.303884373	8.421016612	2.12E-16	2.60E-15	no
PLA2G6	-0.303825608	-8.419222544	2.15E-16	2.63E-15	no
OSTC	0.303521613	8.409943354	2.31E-16	2.83E-15	no
M6PR	0.303476911	8.408579096	2.34E-16	2.86E-15	no
TAGLN2	0.303280538	8.402586738	2.45E-16	2.99E-15	no
CBS	-0.303167334	-8.399132848	2.51E-16	3.07E-15	no
DEAF1	-0.303145137	-8.398455648	2.53E-16	3.08E-15	no
ARL6IP5	0.302741151	8.386133346	2.78E-16	3.39E-15	no
HIATL1	0.302714223	8.38531216	2.80E-16	3.41E-15	no
C9orf21	0.302708133	8.385126465	2.80E-16	3.41E-15	no
GTPBP1	-0.302707566	-8.385109151	2.80E-16	3.41E-15	no
CLASP2	-0.302681023	-8.384299769	2.82E-16	3.43E-15	no
HSPA5	0.302609889	8.382130688	2.87E-16	3.49E-15	no
NMNAT1	0.302607403	8.382054869	2.87E-16	3.49E-15	no
CEACAM3	0.302560302	8.380618716	2.90E-16	3.52E-15	no
KLHL25	-0.302513341	-8.379186901	2.93E-16	3.56E-15	no
GATAD2B	-0.302436431	-8.376842115	2.99E-16	3.62E-15	no
LSM14B	-0.302403097	-8.375825911	3.01E-16	3.65E-15	no
NFE2	0.302375113	8.374972801	3.03E-16	3.67E-15	no
NUCB1	0.302187524	8.36925479	3.17E-16	3.83E-15	no
FPGT	0.301729212	8.355289187	3.53E-16	4.27E-15	no
PRR3	-0.301720222	-8.355015304	3.53E-16	4.27E-15	no
FNDC1	0.30167903	8.353760428	3.57E-16	4.31E-15	no
PSMC2	0.301667928	8.353422229	3.58E-16	4.32E-15	no
TIMP1	0.30150991	8.348608883	3.71E-16	4.48E-15	no
RCOR2	-0.301299396	-8.342197684	3.90E-16	4.70E-15	no
PLEKHA9	0.300984029	8.332595677	4.20E-16	5.06E-15	no
GSR	0.300945633	8.331426842	4.24E-16	5.10E-15	no
TERF2IP	-0.300930274	-8.330959288	4.25E-16	5.12E-15	no
GRIA2	-0.300906121	-8.330224063	4.28E-16	5.15E-15	no
VAMP5	0.300847929	8.328452763	4.33E-16	5.21E-15	no
RARRES3	0.300801522	8.327040221	4.38E-16	5.26E-15	no

RAB42	0.300800828	8.327019102	4.38E-16	5.26E-15	no
RPN2	0.300590129	8.320606769	4.60E-16	5.53E-15	no
LOC374443	0.300493171	8.317656461	4.71E-16	5.65E-15	no
EBF4	-0.300476005	-8.317134148	4.73E-16	5.67E-15	no
MAP3K2	0.300427482	8.31565777	4.78E-16	5.73E-15	no
CSTB	0.300395459	8.314683468	4.82E-16	5.77E-15	no
SCAPER	-0.300356277	-8.313491383	4.86E-16	5.82E-15	no
SH3GLB1	0.30029365	8.311586121	4.93E-16	5.90E-15	no
C12orf53	-0.300286798	-8.311377677	4.94E-16	5.91E-15	no
FBLN5	0.300260466	8.310576627	4.97E-16	5.94E-15	no
VMO1	0.300201585	8.30878549	5.04E-16	6.02E-15	no
MOBKL2A	0.30017675	8.308030046	5.07E-16	6.05E-15	no
CYB5R4	0.300171813	8.307879888	5.08E-16	6.05E-15	no
VPS52	-0.300134317	-8.30673935	5.12E-16	6.10E-15	no
MAF	0.300000626	8.302673178	5.28E-16	6.29E-15	no
SARM1	-0.299905986	-8.299795071	5.40E-16	6.43E-15	no
AEBP1	0.299897559	8.299538793	5.41E-16	6.44E-15	no
TOM1L2	-0.299873428	-8.298805013	5.44E-16	6.47E-15	no
STK4	0.299709923	8.293833403	5.65E-16	6.72E-15	no
FKBP9	0.299564529	8.289413186	5.85E-16	6.95E-15	no
FHL3	0.299547306	8.288889603	5.87E-16	6.97E-15	no
ZNF213	-0.299519102	-8.288032236	5.91E-16	7.01E-15	no
C9orf64	0.299511553	8.28780276	5.92E-16	7.02E-15	no
SOX8	-0.299496014	-8.287330424	5.94E-16	7.04E-15	no
TCEAL2	-0.29926861	-8.280418687	6.26E-16	7.42E-15	no
MAN2B2	0.29924826	8.27980024	6.29E-16	7.45E-15	no
RNF165	-0.299215611	-8.278808051	6.34E-16	7.50E-15	no
AP1S3	0.299076942	8.274594315	6.55E-16	7.74E-15	no
MSC	0.299014023	8.272682585	6.64E-16	7.85E-15	no
APBA2	-0.298882678	-8.268692163	6.85E-16	8.09E-15	no
FRY	-0.298823282	-8.266887811	6.95E-16	8.20E-15	no
GRIA4	-0.298683743	-8.262649284	7.17E-16	8.46E-15	no
AARS	-0.298637076	-8.26123189	7.25E-16	8.55E-15	no
BST2	0.298582263	8.259567163	7.34E-16	8.65E-15	no
PRF1	0.298567183	8.259109187	7.37E-16	8.68E-15	no
JPH4	-0.2985352	-8.258137899	7.43E-16	8.74E-15	no
HLA-L	0.298469495	8.256142575	7.54E-16	8.87E-15	no
ZNF519	-0.298412626	-8.254415685	7.64E-16	8.98E-15	no
ERAP2	0.298389033	8.253699305	7.68E-16	9.02E-15	no
CCBL1	-0.298245874	-8.249352657	7.94E-16	9.32E-15	no
BTN2A3	0.29802221	8.242562931	8.36E-16	9.81E-15	no
ZDHHC22	-0.297988193	-8.241530438	8.43E-16	9.88E-15	no
LOC400027	-0.297952341	-8.240442254	8.50E-16	9.96E-15	no
LINGO1	-0.297938889	-8.240033977	8.52E-16	9.98E-15	no
DNM3	-0.297884601	-8.238386323	8.63E-16	1.01E-14	no
PTCHD2	-0.297810323	-8.236132113	8.78E-16	1.03E-14	no
UBE2O	-0.297759742	-8.234597149	8.88E-16	1.04E-14	no
ALDOC	-0.297729276	-8.233672664	8.95E-16	1.05E-14	no
OLIG1	-0.297676954	-8.232084997	9.06E-16	1.06E-14	no
TGFBI	0.297651498	8.231312608	9.11E-16	1.06E-14	no
ZRANB1	-0.297579195	-8.229118816	9.26E-16	1.08E-14	no

C11orf24	0.297495417	8.226577072	9.44E-16	1.10E-14	no
CCDC125	0.297399849	8.223677898	9.65E-16	1.12E-14	no
PMM2	0.297296083	8.22053032	9.89E-16	1.15E-14	no
CECR6	-0.297262894	-8.21952367	9.96E-16	1.16E-14	no
CD46	0.297132195	8.215559702	1.03E-15	1.19E-14	no
ZNFX1	0.297123754	8.215303689	1.03E-15	1.20E-14	no
HDGFRP3	-0.297081453	-8.214020879	1.04E-15	1.21E-14	no
TAB1	-0.297016096	-8.212038965	1.05E-15	1.22E-14	no
TMEM70	0.296995952	8.211428116	1.06E-15	1.23E-14	no
NECAP2	0.296892837	8.20830153	1.09E-15	1.26E-14	no
TRH	0.296858481	8.207259883	1.09E-15	1.27E-14	no
LOC100130872	0.296813468	8.205895158	1.11E-15	1.28E-14	no
LIPA	0.296775562	8.204745979	1.11E-15	1.29E-14	no
C7orf58	0.296745381	8.20383099	1.12E-15	1.30E-14	no
CCL23	0.296740581	8.203685484	1.12E-15	1.30E-14	no
SEC31A	0.296699214	8.202431451	1.13E-15	1.31E-14	no
IFI44	0.296675113	8.201700852	1.14E-15	1.32E-14	no
COQ2	0.296674281	8.201675643	1.14E-15	1.32E-14	no
RAB20	0.296497341	8.196312447	1.19E-15	1.37E-14	no
KLC4	-0.296448081	-8.194819502	1.20E-15	1.39E-14	no
TRPC2	0.296389527	8.193044976	1.22E-15	1.40E-14	no
CALU	0.296300179	8.190337387	1.24E-15	1.43E-14	no
PNRC1	0.296271527	8.189469175	1.25E-15	1.44E-14	no
NADK	0.296229121	8.188184226	1.26E-15	1.45E-14	no
HAAO	0.296225261	8.188067284	1.27E-15	1.45E-14	no
METRNL	0.296225137	8.188063528	1.27E-15	1.45E-14	no
PHACTR3	-0.296223067	-8.188000804	1.27E-15	1.45E-14	no
GPR173	-0.296205066	-8.187455381	1.27E-15	1.46E-14	no
PDZD4	-0.296193517	-8.187105438	1.27E-15	1.46E-14	no
IMPA2	0.296075342	8.183525043	1.31E-15	1.50E-14	no
SLC22A18	0.296063153	8.183155755	1.31E-15	1.50E-14	no
EIF4ENIF1	-0.29582469	-8.175932264	1.39E-15	1.59E-14	no
SF3B2	-0.295807979	-8.175426118	1.39E-15	1.59E-14	no
FZD6	0.295773182	8.174372221	1.40E-15	1.61E-14	no
GPBP1L1	0.295670133	8.171251333	1.44E-15	1.64E-14	no
SPON2	0.295595472	8.168990395	1.46E-15	1.67E-14	no
SMPD3	-0.295553009	-8.167704583	1.48E-15	1.69E-14	no
CLIP3	-0.295446814	-8.164489139	1.51E-15	1.73E-14	no
SLC35D2	0.295443111	8.164376999	1.51E-15	1.73E-14	no
LOC257358	0.295408291	8.163322764	1.53E-15	1.74E-14	no
GIT1	-0.295316409	-8.160541077	1.56E-15	1.78E-14	no
COL6A3	0.295275868	8.159313772	1.57E-15	1.79E-14	no
ZDHHC18	0.295088108	8.153630381	1.64E-15	1.87E-14	no
SPIRE2	-0.294983793	-8.150473289	1.68E-15	1.91E-14	no
GNG4	-0.294949019	-8.149420911	1.69E-15	1.93E-14	no
SPP1	0.2948176	8.145444073	1.75E-15	1.98E-14	no
ARSD	0.294810766	8.145237287	1.75E-15	1.99E-14	no
HORMAD1	0.294760641	8.143720616	1.77E-15	2.01E-14	no
SLC25A27	-0.294652001	-8.140433665	1.81E-15	2.06E-14	no
PHYHIPL	-0.294585966	-8.13843591	1.84E-15	2.09E-14	no
TWIST1	0.294572294	8.138022291	1.85E-15	2.09E-14	no

PLCB1	-0.294305323	-8.129947029	1.96E-15	2.22E-14	no
FAM120AOS	0.294289528	8.129469349	1.97E-15	2.23E-14	no
SEC61A1	0.294240008	8.127971726	1.99E-15	2.25E-14	no
ANKRD46	-0.294213658	-8.127174886	2.00E-15	2.27E-14	no
TMEM64	0.294087512	8.123360308	2.06E-15	2.33E-14	no
BRD9	-0.293981653	-8.120159563	2.11E-15	2.39E-14	no
ABAT	-0.293904473	-8.117826171	2.15E-15	2.43E-14	no
CYBRD1	0.293890879	8.117415192	2.16E-15	2.43E-14	no
C20orf11	-0.293842212	-8.115943962	2.18E-15	2.46E-14	no
ZNF436	0.293829252	8.115552163	2.19E-15	2.46E-14	no
UBE2F	0.293826581	8.115471421	2.19E-15	2.47E-14	no
H2AFJ	0.293634231	8.10965725	2.29E-15	2.57E-14	no
FAM168B	-0.29358835	-8.108270569	2.31E-15	2.60E-14	no
IDO1	0.293488834	8.105263062	2.36E-15	2.66E-14	no
NXF3	0.293338046	8.100706603	2.45E-15	2.75E-14	no
PLXNB2	0.293326637	8.100361854	2.45E-15	2.75E-14	no
MYO1B	0.293295771	8.099429254	2.47E-15	2.77E-14	no
FASN	-0.293262658	-8.098428779	2.49E-15	2.79E-14	no
RELL1	0.293257241	8.098265106	2.49E-15	2.79E-14	no
ARNT2	-0.293183341	-8.096032448	2.53E-15	2.84E-14	no
ZNF248	-0.293146292	-8.094913154	2.55E-15	2.86E-14	no
LRRC16B	-0.293000219	-8.090500625	2.64E-15	2.96E-14	no
LMAN1	0.29291948	8.08806194	2.69E-15	3.01E-14	no
ANG	0.29280794	8.084693226	2.76E-15	3.08E-14	no
B3GNT5	0.292792144	8.084216189	2.77E-15	3.09E-14	no
SBK1	-0.29264579	-8.079796644	2.86E-15	3.20E-14	no
PSME2	0.292541854	8.0766584	2.93E-15	3.27E-14	no
SHF	-0.292451128	-8.073919295	2.99E-15	3.34E-14	no
ARL4C	0.292445205	8.073740466	2.99E-15	3.34E-14	no
TUBAL3	0.292423226	8.073076929	3.01E-15	3.35E-14	no
C4orf34	0.292390622	8.07209267	3.03E-15	3.38E-14	no
RB1	0.292302936	8.069445726	3.09E-15	3.44E-14	no
GABBR1	-0.292201134	-8.066372916	3.16E-15	3.52E-14	no
TANK	0.292082749	8.062799942	3.25E-15	3.61E-14	no
HNRPDL	-0.292047961	-8.061750093	3.28E-15	3.64E-14	no
CDC42EP5	0.2919662	8.05928279	3.34E-15	3.71E-14	no
MOCOS	0.291938866	8.058457982	3.36E-15	3.73E-14	no
C6orf168	-0.291868683	-8.056340272	3.41E-15	3.78E-14	no
MICAL3	-0.291618109	-8.048780618	3.61E-15	4.00E-14	no
CD109	0.291612124	8.04860008	3.61E-15	4.01E-14	no
PDIA4	0.291590722	8.04795449	3.63E-15	4.02E-14	no
SLC35F5	0.291568019	8.047269648	3.65E-15	4.04E-14	no
GLUD1	-0.291507729	-8.045451115	3.70E-15	4.09E-14	no
COL15A1	0.291428812	8.043070857	3.77E-15	4.16E-14	no
ARL3	-0.291427277	-8.043024558	3.77E-15	4.16E-14	no
ADAMTS2	0.291366278	8.041184862	3.82E-15	4.22E-14	no
RUNDC3A	-0.291338914	-8.040359639	3.84E-15	4.24E-14	no
HIRA	-0.291181706	-8.035618938	3.98E-15	4.39E-14	no
LOC339674	-0.291150668	-8.034683058	4.01E-15	4.42E-14	no
SIGLEC16	0.291093701	8.032965447	4.06E-15	4.48E-14	no
ARFGAP2	-0.29106976	-8.032243595	4.08E-15	4.50E-14	no

C12orf70	0.291023516	8.030849377	4.13E-15	4.54E-14	no
CCDC46	0.290917856	8.027664045	4.23E-15	4.65E-14	no
TLR10	0.290884899	8.02667056	4.26E-15	4.68E-14	no
DSCAM	-0.290859649	-8.025909404	4.28E-15	4.71E-14	no
MRC1	0.290822438	8.024787742	4.32E-15	4.74E-14	no
CEPT1	0.290759086	8.022878189	4.38E-15	4.81E-14	no
MALL	0.290677438	8.020417333	4.46E-15	4.89E-14	no
PURG	-0.290631902	-8.019044951	4.51E-15	4.94E-14	no
ASAH1	0.290519168	8.015647651	4.62E-15	5.07E-14	no
TNFRSF17	0.29044565	8.013432316	4.70E-15	5.15E-14	no
ZNF25	-0.290400854	-8.012082563	4.75E-15	5.20E-14	no
IFIH1	0.290306322	8.009234373	4.85E-15	5.31E-14	no
MAP7D3	0.290280799	8.00846542	4.87E-15	5.33E-14	no
ZKSCAN2	-0.290245491	-8.007401705	4.91E-15	5.37E-14	no
FAM110B	-0.290174386	-8.005259651	4.99E-15	5.46E-14	no
CTHRC1	0.290144519	8.004359914	5.03E-15	5.49E-14	no
NRXN1	-0.2901358	-8.004097291	5.04E-15	5.50E-14	no
OMG	-0.289937977	-7.998138798	5.26E-15	5.74E-14	no
DHX30	-0.289891361	-7.996734855	5.32E-15	5.80E-14	no
ENTPD1	0.289888542	7.996649979	5.32E-15	5.80E-14	no
LEPREL1	0.289866381	7.995982586	5.35E-15	5.83E-14	no
IL32	0.289783157	7.993476352	5.45E-15	5.93E-14	no
SMOC2	0.289776466	7.993274874	5.46E-15	5.94E-14	no
DHRS7	0.289765107	7.992932817	5.47E-15	5.95E-14	no
ODF2L	0.289697704	7.990903207	5.56E-15	6.04E-14	no
CCDC85C	-0.2896544	-7.989599329	5.61E-15	6.09E-14	no
SPTBN2	-0.289592759	-7.987743391	5.69E-15	6.18E-14	no
EFEMP2	0.289543466	7.986259343	5.75E-15	6.24E-14	no
TMEM198	-0.289483914	-7.984466509	5.83E-15	6.32E-14	no
AIDA	0.289315766	7.979404903	6.05E-15	6.56E-14	no
ATP6V0E1	0.289016236	7.970390408	6.47E-15	7.01E-14	no
PLOD3	0.288999906	7.96989903	6.49E-15	7.03E-14	no
OAS2	0.288948752	7.968359789	6.57E-15	7.11E-14	no
ZNF816A	0.2888514	7.965430684	6.71E-15	7.26E-14	no
SPATA1	0.288780312	7.963291964	6.82E-15	7.37E-14	no
KLHL23	-0.288628112	-7.958713421	7.05E-15	7.62E-14	no
SHISA5	0.288623513	7.958575107	7.06E-15	7.63E-14	no
SLC29A3	0.288538922	7.956030695	7.20E-15	7.77E-14	no
C4BPB	0.28848763	7.954487988	7.28E-15	7.85E-14	no
LGALS2	0.288458663	7.953616792	7.32E-15	7.90E-14	no
GALNT7	0.288428275	7.952702874	7.37E-15	7.95E-14	no
MGST1	0.288205893	7.946015542	7.75E-15	8.35E-14	no
SLC30A5	0.288167079	7.944848525	7.82E-15	8.41E-14	no
CKAP4	0.288151108	7.944368327	7.84E-15	8.44E-14	no
HNRNPH3	-0.28810289	-7.942918586	7.93E-15	8.52E-14	no
CNN2	0.288101806	7.942886017	7.93E-15	8.52E-14	no
CXCR2P1	0.288070686	7.941950397	7.99E-15	8.58E-14	no
ANPEP	0.288029459	7.940710947	8.06E-15	8.65E-14	no
PORCN	-0.287989282	-7.939503112	8.13E-15	8.73E-14	no
HAPLN3	0.287938993	7.937991365	8.22E-15	8.82E-14	no
APOC2	0.287917251	7.937337808	8.26E-15	8.86E-14	no

CAV1	0.287888351	7.936469065	8.32E-15	8.91E-14	no
TMED9	0.28779027	7.933520975	8.50E-15	9.10E-14	no
MESDC1	0.287740162	7.932014956	8.59E-15	9.20E-14	no
HNRNPUL2	-0.287731942	-7.931767911	8.61E-15	9.21E-14	no
MRFAP1	-0.287674621	-7.93004518	8.72E-15	9.32E-14	no
ABCC3	0.287621767	7.928456784	8.82E-15	9.43E-14	no
ATF7IP2	0.287504633	7.924936922	9.05E-15	9.67E-14	no
ANGPT1	0.287457905	7.923532845	9.15E-15	9.77E-14	no
SURF4	0.287420698	7.922414916	9.22E-15	9.84E-14	no
GALNT5	0.287412194	7.922159417	9.24E-15	9.86E-14	no
ISLR	0.287349604	7.920278901	9.37E-15	9.99E-14	no
FRS3	-0.287346245	-7.920177991	9.38E-15	9.99E-14	no
ITPR3	0.287322313	7.91945899	9.43E-15	1.00E-13	no
KCNMB1	0.287319843	7.91938477	9.43E-15	1.00E-13	no
ILDR1	0.287319547	7.91937588	9.43E-15	1.00E-13	no
MYEF2	-0.287282148	-7.918252318	9.51E-15	1.01E-13	no
PLEKHA4	0.287250564	7.917303472	9.58E-15	1.02E-13	no
PLK1S1	-0.287247272	-7.917204579	9.59E-15	1.02E-13	no
STEAP1	0.287224894	7.916532351	9.63E-15	1.02E-13	no
GDAP1	-0.287162621	-7.91466168	9.77E-15	1.04E-13	no
TTC19	-0.287135534	-7.913848043	9.83E-15	1.04E-13	no
IBSP	0.287128116	7.913625229	9.84E-15	1.04E-13	no
PTGS1	0.287058793	7.911542991	1.00E-14	1.06E-13	no
CDK5R1	-0.286981853	-7.90923211	1.02E-14	1.08E-13	no
PCDH12	0.286958987	7.908545373	1.02E-14	1.08E-13	no
GXYLT2	0.286875108	7.906026342	1.04E-14	1.10E-13	no
GYPC	0.286781311	7.903209686	1.06E-14	1.12E-13	no
LOC100132707	0.286723298	7.901467744	1.08E-14	1.14E-13	no
SGSM2	-0.286697438	-7.900691273	1.08E-14	1.14E-13	no
LOC254559	-0.286694558	-7.900604772	1.08E-14	1.14E-13	no
IL1R2	0.286687812	7.900402225	1.09E-14	1.14E-13	no
GPR123	-0.286653862	-7.899382885	1.09E-14	1.15E-13	no
FAM111A	0.286640471	7.898980828	1.10E-14	1.16E-13	no
GMPPA	0.286543862	7.896080373	1.12E-14	1.18E-13	no
LEF1	0.286530929	7.89569212	1.12E-14	1.18E-13	no
OLFML2A	0.2864915	7.894508449	1.13E-14	1.19E-13	no
ZMYND11	-0.28648874	-7.894425593	1.13E-14	1.19E-13	no
NDRG2	-0.286431862	-7.892718174	1.15E-14	1.21E-13	no
SSC5D	0.286427124	7.892575975	1.15E-14	1.21E-13	no
UPP1	0.2863932	7.891557638	1.16E-14	1.22E-13	no
LILRA2	0.286329422	7.889643272	1.17E-14	1.23E-13	no
USP20	-0.286328755	-7.889623271	1.17E-14	1.23E-13	no
FCHO2	0.286236795	7.886863194	1.20E-14	1.26E-13	no
SUZ12P	-0.286220223	-7.886365807	1.20E-14	1.26E-13	no
MAPK8IP3	-0.286209612	-7.886047363	1.21E-14	1.26E-13	no
ZNF84	-0.286184644	-7.885298021	1.21E-14	1.27E-13	no
WASF3	-0.286183493	-7.885263495	1.21E-14	1.27E-13	no
FAM198B	0.286060814	7.88158197	1.25E-14	1.30E-13	no
RNASEN	-0.2860478	-7.881191439	1.25E-14	1.31E-13	no
MTA1	-0.286041005	-7.880987565	1.25E-14	1.31E-13	no
CD58	0.286039627	7.880946216	1.25E-14	1.31E-13	no

RGS3	0.285963277	7.878655247	1.27E-14	1.33E-13	no
RIPPLY2	-0.285957168	-7.878471943	1.27E-14	1.33E-13	no
APOL2	0.285880101	7.876159651	1.30E-14	1.35E-13	no
AGA	0.285842899	7.875043526	1.31E-14	1.36E-13	no
TRIB1	0.285577616	7.867085606	1.39E-14	1.44E-13	no
S100A10	0.285543925	7.866075071	1.40E-14	1.45E-13	no
NLRC3	0.285519127	7.865331308	1.40E-14	1.46E-13	no
GBP6	0.285514188	7.865183179	1.41E-14	1.46E-13	no
BZRAP1	-0.285433734	-7.862770258	1.43E-14	1.49E-13	no
CTNNB1	0.285277172	7.858075301	1.48E-14	1.54E-13	no
CEP68	-0.285254128	-7.857384312	1.49E-14	1.54E-13	no
RNASE1	0.285207061	7.855973031	1.50E-14	1.56E-13	no
ZNF185	0.285177291	7.855080439	1.51E-14	1.57E-13	no
G6PD	0.285119951	7.853361265	1.53E-14	1.59E-13	no
C20orf141	0.28509097	7.852492369	1.54E-14	1.60E-13	no
GCSH	-0.28507795	-7.852102035	1.55E-14	1.60E-13	no
LAMA2	0.28491201	7.84712751	1.60E-14	1.66E-13	no
EVI5L	-0.284779137	-7.8431448	1.65E-14	1.71E-13	no
BCAS4	0.284771561	7.842917742	1.65E-14	1.71E-13	no
COL6A2	0.284745653	7.842141244	1.66E-14	1.72E-13	no
SKAP2	0.284742569	7.842048819	1.67E-14	1.72E-13	no
C22orf26	0.28473306	7.841763823	1.67E-14	1.72E-13	no
ANKRD5	0.284687239	7.840390583	1.69E-14	1.74E-13	no
EVC2	0.284624152	7.838499971	1.71E-14	1.76E-13	no
CEBPD	0.284614261	7.838203538	1.71E-14	1.77E-13	no
SEPN1	0.284608492	7.838030675	1.71E-14	1.77E-13	no
HS2ST1	0.284571294	7.836915988	1.73E-14	1.78E-13	no
PEA15	-0.284560561	-7.836594338	1.73E-14	1.78E-13	no
CSDAP1	0.284544368	7.836109109	1.74E-14	1.79E-13	no
STT3A	0.284455834	7.833456267	1.77E-14	1.82E-13	no
ARHGEF7	-0.284431798	-7.832736087	1.78E-14	1.83E-13	no
TRPM4	0.284330718	7.829707655	1.82E-14	1.87E-13	no
CHST2	0.284298286	7.828736036	1.84E-14	1.88E-13	no
COL8A2	0.284293369	7.828588721	1.84E-14	1.88E-13	no
MLX	0.284279562	7.828175088	1.84E-14	1.89E-13	no
FUT9	-0.284197863	-7.825727668	1.88E-14	1.92E-13	no
COPB1	0.284132372	7.823765897	1.90E-14	1.95E-13	no
TMEM185B	0.284104123	7.822919744	1.92E-14	1.96E-13	no
GDPD1	-0.284095986	-7.822675999	1.92E-14	1.96E-13	no
EML4	0.284084412	7.822329346	1.92E-14	1.97E-13	no
SREBF2	-0.283967239	-7.818819898	1.97E-14	2.02E-13	no
RILP	0.283861375	7.815649516	2.02E-14	2.06E-13	no
TIGD3	-0.283816914	-7.814318104	2.04E-14	2.08E-13	no
LRRN4CL	0.283736437	7.811908282	2.08E-14	2.12E-13	no
HNRNPF	0.283666761	7.809822071	2.11E-14	2.15E-13	no
CRTAM	0.283650311	7.809329552	2.12E-14	2.16E-13	no
CRIP1	0.283571506	7.80697017	2.15E-14	2.19E-13	no
ANO1	0.283567222	7.806841921	2.15E-14	2.19E-13	no
GALNT4	0.28355855	7.80658228	2.16E-14	2.20E-13	no
DSCAML1	-0.28355598	-7.806505345	2.16E-14	2.20E-13	no
P4HA3	0.283495844	7.80470504	2.19E-14	2.22E-13	no

C15orf34	0.283415389	7.802296637	2.23E-14	2.26E-13	no
TARSL2	-0.283409341	-7.802115574	2.23E-14	2.26E-13	no
SDR39U1	-0.283376748	-7.801139958	2.25E-14	2.28E-13	no
BLZF1	0.283351914	7.800396634	2.26E-14	2.29E-13	no
PRSS23	0.283298603	7.79880096	2.28E-14	2.32E-13	no
ASTN1	-0.283265654	-7.797814774	2.30E-14	2.33E-13	no
HCN3	-0.283235431	-7.796910216	2.32E-14	2.35E-13	no
CDH20	-0.283203059	-7.795941369	2.33E-14	2.36E-13	no
MGEA5	-0.283179877	-7.795247592	2.34E-14	2.37E-13	no
LRRC33	0.283161412	7.79469498	2.35E-14	2.38E-13	no
ELAVL3	-0.283147999	-7.794293572	2.36E-14	2.39E-13	no
TMED7-TICAM2	0.283103431	7.792959839	2.38E-14	2.41E-13	no
SSH3	0.283102305	7.792926126	2.38E-14	2.41E-13	no
PJA1	-0.283099919	-7.792854737	2.38E-14	2.41E-13	no
EVC	0.283056098	7.791543413	2.41E-14	2.43E-13	no
TRIP6	0.282930787	7.787793808	2.47E-14	2.50E-13	no
MED8	0.282902596	7.786950334	2.49E-14	2.51E-13	no
WBSCR26	0.282839348	7.785058008	2.52E-14	2.54E-13	no
P4HB	0.282588743	7.777561274	2.67E-14	2.69E-13	no
LOXL2	0.282545063	7.776254771	2.69E-14	2.71E-13	no
C12orf76	-0.282519577	-7.775492503	2.71E-14	2.72E-13	no
SNX8	0.282500365	7.774917889	2.72E-14	2.73E-13	no
BRWD1	-0.282433049	-7.772904631	2.76E-14	2.77E-13	no
FBXL19	-0.282429081	-7.772785939	2.76E-14	2.77E-13	no
GBP3	0.282417884	7.772451087	2.77E-14	2.78E-13	no
IL1RN	0.2823889	7.77158429	2.78E-14	2.79E-13	no
FLRT1	-0.282327981	-7.769762527	2.82E-14	2.83E-13	no
STAT1	0.282280887	7.768354264	2.85E-14	2.86E-13	no
PIGK	0.282270396	7.768040535	2.86E-14	2.86E-13	no
GALNT13	-0.282143087	-7.764233926	2.94E-14	2.94E-13	no
AKR1B1	0.282113409	7.763346616	2.96E-14	2.96E-13	no
ORMDL2	0.282112298	7.763313397	2.96E-14	2.96E-13	no
CXCL11	0.282077617	7.762276494	2.98E-14	2.98E-13	no
GNG10	0.282064229	7.761876244	2.99E-14	2.99E-13	no
VEGFC	0.282060434	7.761762794	2.99E-14	2.99E-13	no
C19orf40	0.282009601	7.760243096	3.02E-14	3.02E-13	no
RARS	0.282006659	7.76015514	3.02E-14	3.02E-13	no
KIAA1432	0.281947247	7.758379049	3.06E-14	3.06E-13	no
HSP90B1	0.281920047	7.75756596	3.08E-14	3.07E-13	no
CP	0.281911404	7.75730761	3.09E-14	3.08E-13	no
CASKIN1	-0.28173119	-7.751921001	3.21E-14	3.20E-13	no
APBB1	-0.281717463	-7.751510737	3.22E-14	3.21E-13	no
TSHZ1	-0.281576227	-7.747289876	3.32E-14	3.31E-13	no
IQGAP2	0.281519187	7.745585362	3.36E-14	3.35E-13	no
STMN3	-0.281464567	-7.743953258	3.40E-14	3.38E-13	no
ATP6V1G2	-0.281364928	-7.740976172	3.48E-14	3.46E-13	no
DERL2	0.281352201	7.740595911	3.49E-14	3.46E-13	no
CBARA1	-0.281300048	-7.73903777	3.53E-14	3.50E-13	no
COPB2	0.28115454	7.734690903	3.64E-14	3.61E-13	no
HTATSF1	-0.281086421	-7.732656114	3.69E-14	3.66E-13	no
HMOX1	0.281049638	7.731557432	3.72E-14	3.69E-13	no

EMP3	0.280808459	7.724354503	3.92E-14	3.89E-13	no
DHX38	-0.280768363	-7.723157146	3.95E-14	3.92E-13	no
EIF2AK4	0.280748541	7.722565233	3.97E-14	3.93E-13	no
UBD	0.280742403	7.722381967	3.98E-14	3.94E-13	no
TCF7	0.280651501	7.719667711	4.06E-14	4.01E-13	no
NDRG3	-0.280611552	-7.718474933	4.09E-14	4.04E-13	no
VPS33B	-0.280550885	-7.716663652	4.14E-14	4.10E-13	no
JMY	-0.280471558	-7.714295388	4.22E-14	4.16E-13	no
MCFD2	0.280426955	7.712963895	4.26E-14	4.20E-13	no
SLITRK5	-0.280385241	-7.711718669	4.30E-14	4.24E-13	no
SMAGP	0.280361578	7.711012319	4.32E-14	4.26E-13	no
RNASE4	0.280286109	7.70875963	4.39E-14	4.33E-13	no
CNPY4	0.280243118	7.707476459	4.43E-14	4.36E-13	no
ADCK2	0.280233665	7.707194311	4.44E-14	4.37E-13	no
CTTNBP2NL	0.280211667	7.706537757	4.46E-14	4.39E-13	no
LMTK3	-0.280163484	-7.705099717	4.51E-14	4.43E-13	no
C2orf29	0.280111457	7.703547029	4.56E-14	4.48E-13	no
SH2B1	-0.280089644	-7.702896053	4.58E-14	4.50E-13	no
CD209	0.279939991	7.698430322	4.73E-14	4.64E-13	no
WWTR1	0.27992039	7.697845455	4.75E-14	4.66E-13	no
TMEM102	0.279862614	7.696121579	4.81E-14	4.72E-13	no
MTMR9	-0.279700568	-7.69128703	4.98E-14	4.88E-13	no
LAMB2L	-0.279637353	-7.689401251	5.05E-14	4.95E-13	no
CADM2	-0.279596842	-7.688192832	5.09E-14	4.99E-13	no
TRIP12	0.279468697	7.684370559	5.23E-14	5.12E-13	no
C20orf26	0.279404053	7.682442569	5.30E-14	5.19E-13	no
PARVB	0.279366467	7.681321602	5.35E-14	5.23E-13	no
BMF	0.279274222	7.6785707	5.45E-14	5.34E-13	no
TMEM136	-0.279260943	-7.678174704	5.47E-14	5.35E-13	no
LOC90110	-0.279242573	-7.677626935	5.49E-14	5.37E-13	no
SOX6	-0.279227914	-7.6771898	5.51E-14	5.38E-13	no
PLSCR3	0.279171989	7.675522198	5.58E-14	5.44E-13	no
CXCR2	0.279119076	7.673944502	5.64E-14	5.50E-13	no
CLEC4E	0.279086578	7.67297553	5.68E-14	5.54E-13	no
ASB13	-0.279082858	-7.67286463	5.68E-14	5.54E-13	no
PRRG4	0.279033379	7.671389405	5.74E-14	5.60E-13	no
EDNRA	0.278952101	7.668966266	5.84E-14	5.69E-13	no
ZCCHC9	0.278798753	7.664395	6.04E-14	5.88E-13	no
TTLL12	-0.27878696	-7.664043489	6.06E-14	5.89E-13	no
FRMD8	0.278784986	7.663984642	6.06E-14	5.89E-13	no
NOVA2	-0.278621607	-7.659115106	6.27E-14	6.10E-13	no
DDOST	0.27861976	7.659060082	6.28E-14	6.10E-13	no
JPH3	-0.278579047	-7.657846733	6.33E-14	6.15E-13	no
AP1B1	0.278490769	7.655216006	6.45E-14	6.26E-13	no
OBFC1	0.27842927	7.653383415	6.54E-14	6.34E-13	no
INPP5A	-0.27834547	-7.650886467	6.66E-14	6.45E-13	no
AP3B2	-0.278302122	-7.64959492	6.72E-14	6.51E-13	no
TMED5	0.278292493	7.64930804	6.73E-14	6.52E-13	no
HSPC157	0.278238815	7.647708784	6.81E-14	6.59E-13	no
MAGEF1	-0.278176361	-7.645848172	6.90E-14	6.68E-13	no
HNRNPA1L2	-0.278169776	-7.645651986	6.91E-14	6.68E-13	no

ECM1	0.278164188	7.64548554	6.92E-14	6.69E-13	no
SHANK2	-0.278109308	-7.643850669	7.00E-14	6.76E-13	no
ATP1B3	0.278046581	7.641982129	7.09E-14	6.85E-13	no
XRN2	0.278016863	7.641096925	7.14E-14	6.89E-13	no
CKM	0.277980027	7.63999972	7.20E-14	6.94E-13	no
SEPP1	0.277979812	7.639993311	7.20E-14	6.94E-13	no
ICAM4	0.277975428	7.639862728	7.20E-14	6.94E-13	no
TMOD2	-0.277921789	-7.638265104	7.29E-14	7.02E-13	no
OSM	0.277917381	7.638133827	7.29E-14	7.02E-13	no
FAM176B	0.277779149	7.634016996	7.51E-14	7.23E-13	no
GGA2	0.277753429	7.633251043	7.55E-14	7.27E-13	no
CHCHD7	0.277743474	7.632954596	7.57E-14	7.28E-13	no
TMBIM4	0.277727862	7.632489682	7.59E-14	7.30E-13	no
KLF12	-0.277713946	-7.6320753	7.62E-14	7.32E-13	no
SAMD14	-0.277646004	-7.630052151	7.73E-14	7.42E-13	no
C17orf91	0.27758564	7.62825475	7.83E-14	7.51E-13	no
KAT5	-0.277568127	-7.627733302	7.86E-14	7.54E-13	no
C8orf42	-0.277491776	-7.625460063	7.99E-14	7.66E-13	no
ACCN2	-0.277485504	-7.625273335	8.00E-14	7.67E-13	no
AMIGO2	0.277459561	7.624500968	8.04E-14	7.70E-13	no
NMUR1	0.277451054	7.624247706	8.05E-14	7.71E-13	no
IL23R	0.277444847	7.624062909	8.07E-14	7.72E-13	no
C1QTNF6	0.277391463	7.622473658	8.16E-14	7.81E-13	no
MALT1	0.277319232	7.620323399	8.28E-14	7.92E-13	no
DERL1	0.277274975	7.619006002	8.36E-14	7.99E-13	no
LEPRE1	0.277236877	7.617871979	8.43E-14	8.06E-13	no
C8orf45	-0.277185566	-7.616344696	8.52E-14	8.14E-13	no
MBOAT1	0.277183218	7.616274805	8.53E-14	8.14E-13	no
ARHGEF19	0.277142278	7.615056307	8.60E-14	8.21E-13	no
DEGS1	0.277101013	7.613828127	8.68E-14	8.28E-13	no
CD63	0.277076901	7.613110537	8.72E-14	8.31E-13	no
IL27RA	0.277012499	7.611193901	8.84E-14	8.43E-13	no
TSPYL4	-0.277003378	-7.610922452	8.86E-14	8.44E-13	no
ARHGAP32	-0.276925197	-7.608595918	9.01E-14	8.57E-13	no
MMRN1	0.276913806	7.608256942	9.03E-14	8.59E-13	no
CCL14	0.276875794	7.607125856	9.10E-14	8.66E-13	no
HIRIP3	-0.276851354	-7.606398612	9.15E-14	8.70E-13	no
BBS12	0.27677612	7.604160091	9.30E-14	8.83E-13	no
CDC42	0.276738485	7.603040342	9.37E-14	8.90E-13	no
GM2A	0.276716552	7.602387794	9.42E-14	8.94E-13	no
NALCN	-0.276653881	-7.600523276	9.54E-14	9.05E-13	no
CCL2	0.276533477	7.596941449	9.79E-14	9.28E-13	no
ELFN2	-0.276469512	-7.595038754	9.92E-14	9.41E-13	no
RNF112	-0.276410007	-7.59326881	1.00E-13	9.52E-13	no
OPHN1	-0.276353462	-7.591587001	1.02E-13	9.63E-13	no
SMOC1	-0.276341431	-7.591229174	1.02E-13	9.65E-13	no
USP30	-0.276274225	-7.589230431	1.03E-13	9.79E-13	no
LOC284900	-0.276179102	-7.586401604	1.06E-13	9.98E-13	no
TAOK2	-0.276174383	-7.586261267	1.06E-13	9.99E-13	no
POM121L4P	0.276112829	7.584430873	1.07E-13	1.01E-12	no
MSL3	0.276076128	7.583339564	1.08E-13	1.02E-12	no

MUTED	0.275800652	7.575149418	1.14E-13	1.08E-12	no
FLT3	0.275774138	7.574361226	1.15E-13	1.08E-12	no
PCIF1	-0.275752132	-7.573707068	1.16E-13	1.09E-12	no
TMEM35	-0.275739873	-7.573342655	1.16E-13	1.09E-12	no
B3GAT1	-0.275675077	-7.571416617	1.17E-13	1.11E-12	no
C12orf51	-0.275664531	-7.571103154	1.18E-13	1.11E-12	no
PXN	0.275653717	7.570781714	1.18E-13	1.11E-12	no
PTPN9	0.275645766	7.570545378	1.18E-13	1.11E-12	no
UBE2L6	0.275606498	7.569378252	1.19E-13	1.12E-12	no
ANXA11	0.275585179	7.568744616	1.20E-13	1.12E-12	no
CA13	0.275555857	7.567873109	1.20E-13	1.13E-12	no
CXorf1	-0.275479259	-7.565596642	1.22E-13	1.15E-12	no
TWISTNB	0.275453158	7.564820978	1.23E-13	1.16E-12	no
MSL1	-0.275437391	-7.56435241	1.23E-13	1.16E-12	no
SERPINA9	0.275281338	7.559715222	1.28E-13	1.20E-12	no
LXN	0.275249607	7.558772379	1.28E-13	1.20E-12	no
MLLT6	-0.275232029	-7.558250109	1.29E-13	1.21E-12	no
NPAS3	-0.275212072	-7.557657128	1.29E-13	1.21E-12	no
FBXO45	-0.27508122	-7.553769553	1.33E-13	1.25E-12	no
SNAI1	0.275077346	7.553654452	1.33E-13	1.25E-12	no
FBLN1	0.275069679	7.553426674	1.33E-13	1.25E-12	no
ARPP21	-0.27506336	-7.55323896	1.34E-13	1.25E-12	no
C3orf55	0.275060028	7.553139969	1.34E-13	1.25E-12	no
XKR8	0.275041963	7.552603301	1.34E-13	1.25E-12	no
BRMS1L	-0.274950924	-7.549898921	1.37E-13	1.28E-12	no
MIR155HG	0.274927375	7.549199413	1.37E-13	1.28E-12	no
SIGLEC8	0.274905584	7.548552135	1.38E-13	1.29E-12	no
PELO	0.274858452	7.547152166	1.40E-13	1.30E-12	no
SNAI2	0.274815142	7.545865806	1.41E-13	1.31E-12	no
BDKRB2	0.274815087	7.545864174	1.41E-13	1.31E-12	no
ICA1L	-0.274775854	-7.544698919	1.42E-13	1.32E-12	no
NTNG2	-0.274734251	-7.54346334	1.43E-13	1.33E-12	no
L2HGDH	-0.274706523	-7.542639852	1.44E-13	1.34E-12	no
APBB2	-0.274693224	-7.542244901	1.44E-13	1.34E-12	no
CTLA4	0.274691921	7.542206201	1.44E-13	1.34E-12	no
DKK1	0.274684073	7.541973125	1.45E-13	1.34E-12	no
ASB9	0.274643823	7.540777818	1.46E-13	1.35E-12	no
ZNF346	-0.274525019	-7.53724995	1.50E-13	1.39E-12	no
TCF25	-0.274512668	-7.536883192	1.50E-13	1.39E-12	no
OAF	0.274463239	7.535415525	1.52E-13	1.41E-12	no
ETHE1	0.274322491	7.531236729	1.56E-13	1.45E-12	no
PAPOLG	0.274232683	7.528570613	1.59E-13	1.47E-12	no
DPF1	-0.274211241	-7.527934087	1.60E-13	1.48E-12	no
NUMA1	-0.274187687	-7.527234912	1.61E-13	1.49E-12	no
FHAD1	0.274169355	7.526690741	1.61E-13	1.49E-12	no
VSIG10L	0.274054636	7.523285586	1.65E-13	1.53E-12	no
ATG10	0.274024095	7.522379107	1.66E-13	1.54E-12	no
C7orf42	0.273995683	7.521535839	1.67E-13	1.54E-12	no
FAM86C	0.27391049	7.519007461	1.70E-13	1.57E-12	no
MTRR	0.273894761	7.518540679	1.71E-13	1.58E-12	no
RABGAP1	-0.273858048	-7.517451179	1.72E-13	1.59E-12	no

SPTAN1	-0.273828071	-7.516561586	1.73E-13	1.60E-12	no
SQSTM1	0.27380423	7.515854122	1.74E-13	1.60E-12	no
MYST2	-0.2737868	-7.515336893	1.75E-13	1.61E-12	no
ATCAY	-0.273777386	-7.515057548	1.75E-13	1.61E-12	no
TNK2	-0.273756669	-7.514442791	1.76E-13	1.62E-12	no
ARVCF	-0.273710728	-7.513079622	1.78E-13	1.63E-12	no
RAB34	0.273696033	7.512643588	1.78E-13	1.64E-12	no
KCNIP3	-0.273681808	-7.512221509	1.79E-13	1.64E-12	no
COQ10B	0.273629488	7.510669158	1.81E-13	1.66E-12	no
KIF3A	-0.27360151	-7.509839073	1.82E-13	1.67E-12	no
MAP3K6	0.273552927	7.508397669	1.84E-13	1.68E-12	no
CARD8	0.273476106	7.506118663	1.87E-13	1.71E-12	no
APLF	0.273449206	7.505320637	1.88E-13	1.72E-12	no
SAMD1	-0.273424747	-7.504595092	1.89E-13	1.73E-12	no
IGFBP7	0.273413438	7.504259604	1.89E-13	1.73E-12	no
CBX1	-0.273291828	-7.500652362	1.94E-13	1.78E-12	no
FAM96A	0.273287446	7.500522383	1.94E-13	1.78E-12	no
PAAF1	-0.273271007	-7.500034792	1.95E-13	1.78E-12	no
BTG1	0.273156158	7.496628497	1.99E-13	1.82E-12	no
SGSM1	-0.27311874	-7.495518807	2.01E-13	1.84E-12	no
NAMPT	0.273072436	7.494145621	2.03E-13	1.85E-12	no
MICA	0.273011056	7.492325422	2.06E-13	1.88E-12	no
TNFRSF10D	0.272996057	7.491880657	2.06E-13	1.88E-12	no
GPR162	-0.272967837	-7.491043872	2.07E-13	1.89E-12	no
GABRB3	-0.272961794	-7.490864666	2.08E-13	1.89E-12	no
RAB5B	-0.272938126	-7.490162864	2.09E-13	1.90E-12	no
CRYBB1	0.272883347	7.488538614	2.11E-13	1.92E-12	no
MTDH	0.272794009	7.485889835	2.15E-13	1.96E-12	no
ACACA	-0.272785006	-7.485622922	2.16E-13	1.96E-12	no
APOC1	0.272692609	7.4828837	2.20E-13	2.00E-12	no
LAMP1	0.272669136	7.482187835	2.21E-13	2.01E-12	no
NCK1	0.272645245	7.481479593	2.22E-13	2.02E-12	no
AGPAT2	0.272524292	7.477894281	2.28E-13	2.07E-12	no
CLN6	0.272516715	7.477669689	2.28E-13	2.07E-12	no
SPIN3	-0.272506681	-7.47737228	2.28E-13	2.07E-12	no
ERI1	0.272408356	7.47445801	2.33E-13	2.12E-12	no
SELPLG	0.272323353	7.471938802	2.37E-13	2.15E-12	no
CLEC1A	0.272269667	7.470347842	2.40E-13	2.18E-12	no
HAS2	0.272207895	7.46851732	2.43E-13	2.20E-12	no
PTP4A2	0.272193882	7.468102086	2.44E-13	2.21E-12	no
DNAJC18	-0.272082199	-7.464792836	2.50E-13	2.26E-12	no
BRI3	0.272057867	7.46407192	2.51E-13	2.27E-12	no
TEX261	0.271948576	7.46083394	2.57E-13	2.32E-12	no
SLC35C1	0.271939775	7.4605732	2.57E-13	2.32E-12	no
SERPINB6	0.271900007	7.459395066	2.59E-13	2.34E-12	no
FAM69B	-0.271800699	-7.456453273	2.65E-13	2.39E-12	no
RAB33B	0.271790453	7.456149755	2.65E-13	2.40E-12	no
GTF2E2	0.271751205	7.454987184	2.67E-13	2.41E-12	no
ENTPD7	0.271657471	7.452210837	2.73E-13	2.46E-12	no
SLC9A9	0.271439601	7.44575855	2.85E-13	2.57E-12	no
DDX25	-0.271431123	-7.445507512	2.86E-13	2.58E-12	no

TRAF1	0.2713714	7.443739029	2.89E-13	2.61E-12	no
IMPDH1	0.271360381	7.443412754	2.90E-13	2.61E-12	no
TOP2B	-0.271342723	-7.442889887	2.91E-13	2.62E-12	no
KIAA1279	-0.271277572	-7.440960821	2.95E-13	2.66E-12	no
BMP1	0.271226499	7.439448673	2.98E-13	2.68E-12	no
HDAC4	-0.271173168	-7.437869763	3.02E-13	2.71E-12	no
SATB1	-0.271153318	-7.437282074	3.03E-13	2.72E-12	no
RIOK3	0.271129222	7.436568731	3.04E-13	2.73E-12	no
PRPF40B	-0.271097183	-7.435620243	3.06E-13	2.75E-12	no
CREM	0.271056756	7.434423486	3.09E-13	2.77E-12	no
PCGF2	-0.271029863	-7.433627409	3.11E-13	2.79E-12	no
CCDC120	-0.270984097	-7.432272663	3.14E-13	2.81E-12	no
SCARF1	0.270972824	7.431938996	3.14E-13	2.82E-12	no
FAM78B	0.270963978	7.431677138	3.15E-13	2.82E-12	no
HINT3	-0.270803036	-7.426913578	3.26E-13	2.92E-12	no
MKRN3	-0.270761455	-7.425682986	3.28E-13	2.94E-12	no
GNAI2	0.27075959	7.425627785	3.29E-13	2.94E-12	no
MGC57346	-0.270751203	-7.425379554	3.29E-13	2.94E-12	no
PDGFRL	0.270678591	7.423230714	3.34E-13	2.99E-12	no
P2RY13	0.270629961	7.421791641	3.37E-13	3.02E-12	no
CSMD3	-0.270596257	-7.420794307	3.40E-13	3.03E-12	no
MAGEH1	-0.270572411	-7.420088709	3.42E-13	3.05E-12	no
SLITRK1	-0.270499645	-7.417935628	3.47E-13	3.09E-12	no
COTL1	0.270463065	7.416853327	3.49E-13	3.12E-12	no
TPST2	0.270354507	7.413641544	3.57E-13	3.19E-12	no
LRP10	0.270296499	7.411925474	3.62E-13	3.22E-12	no
PLOD2	0.270249369	7.410531268	3.65E-13	3.25E-12	no
SBF1	-0.270238523	-7.410210442	3.66E-13	3.26E-12	no
C11orf2	-0.270220963	-7.409690974	3.67E-13	3.27E-12	no
TCTEX1D4	0.270188603	7.408733771	3.70E-13	3.29E-12	no
CELSR1	0.270174551	7.40831812	3.71E-13	3.30E-12	no
MFAP3	0.270162249	7.40795422	3.72E-13	3.30E-12	no
IFT57	0.270107464	7.406333743	3.76E-13	3.34E-12	no
CAMSAP1L1	-0.270032518	-7.404117088	3.82E-13	3.39E-12	no
DACT3	-0.270016679	-7.403648635	3.83E-13	3.40E-12	no
IKBKB	0.270009413	7.403433724	3.84E-13	3.40E-12	no
SH3PXD2B	0.269987501	7.402785691	3.85E-13	3.42E-12	no
ECHDC3	0.269981025	7.402594151	3.86E-13	3.42E-12	no
TOR3A	0.269952964	7.401764292	3.88E-13	3.44E-12	no
ZBP1	0.269910393	7.400505306	3.92E-13	3.47E-12	no
CDC42BPB	-0.269813215	-7.397631605	4.00E-13	3.54E-12	no
TUBA1C	0.269811406	7.39757811	4.00E-13	3.54E-12	no
SYDE1	0.269797528	7.397167752	4.01E-13	3.55E-12	no
GTF2F2	-0.26974123	-7.395503057	4.06E-13	3.59E-12	no
RFX8	0.269727693	7.395102803	4.07E-13	3.59E-12	no
FCHSD2	-0.269721975	-7.394933725	4.07E-13	3.60E-12	no
ITGB5	0.269653893	7.392920736	4.13E-13	3.65E-12	no
DGKA	0.269606971	7.391533457	4.17E-13	3.68E-12	no
TRAK1	-0.269601478	-7.391371053	4.17E-13	3.68E-12	no
PTGIR	0.269588529	7.390988219	4.19E-13	3.69E-12	no
PPP1R9A	-0.269574935	-7.390586343	4.20E-13	3.70E-12	no

SLC35B3	0.269565401	7.390304461	4.21E-13	3.70E-12	no
IP6K1	-0.269497593	-7.388299866	4.26E-13	3.76E-12	no
CHRNB2	-0.269494872	-7.38821942	4.27E-13	3.76E-12	no
SAFB	-0.26943972	-7.386589042	4.32E-13	3.80E-12	no
BCR	-0.269343646	-7.383749158	4.40E-13	3.87E-12	no
FHL2	0.269315869	7.382928117	4.43E-13	3.89E-12	no
ALDH16A1	0.269297759	7.382392835	4.44E-13	3.90E-12	no
LOC100240735	0.269235489	7.380552383	4.50E-13	3.95E-12	no
OSBPL3	0.269168036	7.378558827	4.56E-13	4.01E-12	no
LDLRAD2	0.269160939	7.378349097	4.57E-13	4.01E-12	no
TMEM138	0.269085647	7.376124047	4.64E-13	4.07E-12	no
PVRL1	-0.268997496	-7.373519144	4.73E-13	4.14E-12	no
PELP1	-0.268939846	-7.371815656	4.78E-13	4.19E-12	no
C20orf72	0.268911644	7.370982356	4.81E-13	4.21E-12	no
MED24	-0.268891956	-7.370400638	4.83E-13	4.23E-12	no
SUSD1	0.268827866	7.368507058	4.89E-13	4.28E-12	no
TBX21	0.26879825	7.367632073	4.92E-13	4.31E-12	no
BEX4	-0.268626172	-7.362548541	5.10E-13	4.46E-12	no
PAPPA2	0.268598377	7.361727492	5.13E-13	4.49E-12	no
TMEM8A	0.268477254	7.358149842	5.26E-13	4.60E-12	no
PIGN	0.26841943	7.356441974	5.32E-13	4.65E-12	no
PLEKHA2	0.268414716	7.356302749	5.33E-13	4.65E-12	no
CCL11	0.268377801	7.355212511	5.37E-13	4.68E-12	no
HMP19	-0.268373296	-7.355079465	5.37E-13	4.69E-12	no
ATP6V0A1	-0.268313755	-7.353321092	5.44E-13	4.74E-12	no
HSFX2	-0.268284473	-7.352456332	5.47E-13	4.77E-12	no
KIF1A	-0.268266529	-7.351926429	5.49E-13	4.78E-12	no
LRRC15	0.268181155	7.349405409	5.59E-13	4.87E-12	no
UAP1	0.268134861	7.348038427	5.64E-13	4.91E-12	no
UBE3C	0.26811207	7.347365504	5.67E-13	4.93E-12	no
GNAZ	-0.268060862	-7.345853521	5.73E-13	4.98E-12	no
RGS12	-0.268041633	-7.345285799	5.75E-13	5.00E-12	no
TNFRSF18	0.268033446	7.34504407	5.76E-13	5.01E-12	no
DRP2	-0.268002016	-7.344116138	5.80E-13	5.04E-12	no
EDC4	-0.267993326	-7.343859574	5.81E-13	5.04E-12	no
GPRIN1	-0.26786961	-7.340207227	5.96E-13	5.17E-12	no
CSPG5	-0.267819567	-7.338729969	6.02E-13	5.22E-12	no
NAP1L5	-0.267740842	-7.336406175	6.12E-13	5.30E-12	no
RIT1	0.267691225	7.334941641	6.18E-13	5.36E-12	no
STARD10	-0.267686248	-7.334794743	6.19E-13	5.36E-12	no
PLP2	0.267678263	7.334559069	6.20E-13	5.37E-12	no
TRAM2	0.267660141	7.334024173	6.22E-13	5.38E-12	no
DYNC1H1	-0.267514047	-7.329712495	6.41E-13	5.54E-12	no
CIRBP	-0.26750984	-7.329588336	6.41E-13	5.54E-12	no
YIPF4	0.267509057	7.329565229	6.41E-13	5.54E-12	no
MXI1	-0.267461439	-7.328159986	6.48E-13	5.60E-12	no
TMEM59L	-0.267400535	-7.326362779	6.56E-13	5.66E-12	no
BCL2L12	0.267384397	7.325886574	6.58E-13	5.68E-12	no
TSKS	0.267366376	7.325354814	6.60E-13	5.70E-12	no
ZFP62	-0.267339924	-7.324574276	6.64E-13	5.73E-12	no
DLGAP1	-0.267167986	-7.319501312	6.88E-13	5.93E-12	no

KLHDC3	-0.267157496	-7.319191836	6.89E-13	5.94E-12	no
C5orf62	0.267095552	7.317364392	6.98E-13	6.01E-12	no
PDZD9	-0.266918741	-7.312148742	7.24E-13	6.23E-12	no
CALR	0.26689839	7.311548497	7.27E-13	6.25E-12	no
MYST1	-0.26687728	-7.310925853	7.30E-13	6.28E-12	no
FBLL1	-0.266746219	-7.307060391	7.49E-13	6.45E-12	no
FAM133A	-0.266703479	-7.305799938	7.56E-13	6.50E-12	no
PAPSS2	0.266678789	7.305071846	7.60E-13	6.53E-12	no
HDAC5	-0.266659107	-7.304491408	7.63E-13	6.55E-12	no
CD97	0.266635416	7.303792793	7.67E-13	6.58E-12	no
SLC1A6	-0.26649556	-7.299668818	7.89E-13	6.77E-12	no
LOC221710	-0.266459719	-7.298612048	7.95E-13	6.82E-12	no
CXXC4	-0.266427287	-7.297655825	8.00E-13	6.86E-12	no
MFNG	0.266413046	7.297235961	8.02E-13	6.87E-12	no
ULK4	0.266374563	7.296101359	8.08E-13	6.93E-12	no
IL1F6	0.266158488	7.289731569	8.45E-13	7.23E-12	no
MUC1	0.266151865	7.289536357	8.46E-13	7.24E-12	no
FBXO32	0.26601252	7.285429169	8.70E-13	7.45E-12	no
PIM3	0.266007946	7.285294362	8.71E-13	7.45E-12	no
NUDT11	-0.265959995	-7.283881133	8.79E-13	7.52E-12	no
BRD3	-0.265883819	-7.281636144	8.93E-13	7.63E-12	no
SLIT1	-0.265855448	-7.280800074	8.98E-13	7.67E-12	no
CYB561D2	0.265796315	7.279057512	9.09E-13	7.76E-12	no
ATP9A	-0.265760512	-7.278002515	9.16E-13	7.82E-12	no
SCAI	-0.265736918	-7.277307282	9.20E-13	7.85E-12	no
NRBF2	0.265711334	7.276553412	9.25E-13	7.89E-12	no
FBXW11	-0.265700119	-7.276222951	9.27E-13	7.90E-12	no
SPCS3	0.265694673	7.276062487	9.28E-13	7.91E-12	no
KCNN4	0.265693123	7.276016833	9.28E-13	7.91E-12	no
CAMSAP1	-0.265576744	-7.272587889	9.51E-13	8.09E-12	no
PDLIM7	0.265474514	7.269576115	9.71E-13	8.26E-12	no
THBS2	0.265459115	7.269122459	9.74E-13	8.28E-12	no
HEBP1	0.265433916	7.268380119	9.79E-13	8.32E-12	no
PLEK2	0.265404644	7.267517829	9.84E-13	8.37E-12	no
VASN	0.26539205	7.26714683	9.87E-13	8.38E-12	no
ZNF48	-0.265271068	-7.263583185	1.01E-12	8.59E-12	no
KIAA1919	0.265225621	7.262244604	1.02E-12	8.66E-12	no
RPS6KA4	0.265221275	7.262116601	1.02E-12	8.67E-12	no
ZADH2	-0.265114383	-7.258968439	1.04E-12	8.85E-12	no
ATR	0.265044258	7.256903284	1.06E-12	8.97E-12	no
FTSJD1	0.265043478	7.256880323	1.06E-12	8.97E-12	no
C20orf30	0.264945956	7.254008554	1.08E-12	9.15E-12	no
CELF5	-0.264944852	-7.253976032	1.08E-12	9.15E-12	no
CRAMP1L	-0.264943907	-7.253948222	1.08E-12	9.15E-12	no
CLEC11A	0.264912196	7.253014445	1.09E-12	9.20E-12	no
PTGR1	0.26491029	7.252958322	1.09E-12	9.20E-12	no
LOC219347	-0.264870345	-7.251782152	1.10E-12	9.27E-12	no
STBD1	0.264812415	7.250076505	1.11E-12	9.38E-12	no
C14orf132	-0.264744047	-7.248063618	1.13E-12	9.50E-12	no
FLJ35220	-0.264728761	-7.24761357	1.13E-12	9.53E-12	no
DIRAS3	0.264653592	7.245400603	1.15E-12	9.67E-12	no

CD151	0.264639773	7.244993807	1.15E-12	9.69E-12	no
EZH1	-0.264638876	-7.24496738	1.15E-12	9.69E-12	no
MGP	0.264584479	7.243366063	1.16E-12	9.79E-12	no
TSPAN2	0.264577742	7.243167755	1.16E-12	9.80E-12	no
C20orf134	0.264572201	7.243004637	1.16E-12	9.81E-12	no
HABP4	-0.264432181	-7.238883149	1.20E-12	1.01E-11	no
S100A6	0.264410124	7.23823392	1.20E-12	1.01E-11	no
TUBG2	-0.264395619	-7.237807019	1.21E-12	1.02E-11	no
SHD	-0.264376306	-7.237238596	1.21E-12	1.02E-11	no
CYSLTR1	0.264344423	7.236300221	1.22E-12	1.02E-11	no
NRBP1	0.264342035	7.236229954	1.22E-12	1.03E-11	no
BICC1	0.264337763	7.23610422	1.22E-12	1.03E-11	no
GNPDA1	0.264295584	7.234862873	1.23E-12	1.03E-11	no
TXNRD1	0.264254799	7.233662584	1.24E-12	1.04E-11	no
PTPRT	-0.264242314	-7.233295176	1.25E-12	1.04E-11	no
RIMBP3C	0.264236341	7.233119389	1.25E-12	1.04E-11	no
BTRC	-0.264222371	-7.232708295	1.25E-12	1.05E-11	no
DHRS3	0.264193415	7.231856165	1.26E-12	1.05E-11	no
ZFP2	-0.264139289	-7.230263432	1.27E-12	1.06E-11	no
C10orf4	-0.264063745	-7.228040556	1.29E-12	1.08E-11	no
YTHDC1	-0.263896523	-7.22312056	1.33E-12	1.12E-11	no
KIF21B	-0.263860214	-7.222052355	1.34E-12	1.12E-11	no
GRIPAP1	-0.263753029	-7.218899234	1.37E-12	1.15E-11	no
C6orf174	-0.26371026	-7.217641156	1.39E-12	1.16E-11	no
CCDC134	0.263709578	7.2176211	1.39E-12	1.16E-11	no
TMSB10	0.263649421	7.215851591	1.40E-12	1.17E-11	no
AKAP6	-0.263627068	-7.215194124	1.41E-12	1.18E-11	no
S1PR3	0.26359115	7.214137685	1.42E-12	1.18E-11	no
OASL	0.263588928	7.21407232	1.42E-12	1.18E-11	no
NEK6	0.263584928	7.21395469	1.42E-12	1.18E-11	no
MAPK8IP2	-0.263527465	-7.212264616	1.44E-12	1.20E-11	no
ECE1	0.263503096	7.211547921	1.44E-12	1.20E-11	no
ZBTB47	-0.263494772	-7.211303117	1.45E-12	1.20E-11	no
RAG1	0.263480346	7.210878849	1.45E-12	1.21E-11	no
SUV420H1	-0.26341738	-7.209027098	1.47E-12	1.22E-11	no
EFEMP1	0.263345823	7.206922818	1.49E-12	1.24E-11	no
MAGT1	0.263337045	7.206664678	1.49E-12	1.24E-11	no
A2LD1	0.263304083	7.205695437	1.50E-12	1.25E-11	no
LOC92973	-0.263279415	-7.204970079	1.51E-12	1.25E-11	no
NFATC3	0.263254744	7.204244637	1.52E-12	1.26E-11	no
SFXN1	-0.263119731	-7.200274957	1.56E-12	1.29E-11	no
TBC1D19	0.263033864	7.197750526	1.59E-12	1.32E-11	no
GALNT3	0.26300579	7.196925202	1.60E-12	1.32E-11	no
PSME4	0.262978297	7.196117007	1.61E-12	1.33E-11	no
TMED2	0.262968045	7.195815614	1.61E-12	1.33E-11	no
WDR59	-0.262958283	-7.195528645	1.61E-12	1.33E-11	no
BRF1	-0.262934913	-7.19484165	1.62E-12	1.34E-11	no
GLIS3	0.262894706	7.193659763	1.63E-12	1.35E-11	no
MEIS3	-0.262880866	-7.193252951	1.64E-12	1.35E-11	no
SCG3	-0.262807724	-7.191103019	1.66E-12	1.37E-11	no
THOP1	-0.2628028	-7.190958298	1.66E-12	1.37E-11	no

TXNDC16	-0.262772915	-7.19007993	1.67E-12	1.38E-11	no
RHOBTB1	0.262771784	7.190046682	1.67E-12	1.38E-11	no
ZMYM6	0.262732843	7.188902161	1.69E-12	1.39E-11	no
CPSF7	-0.262694075	-7.187762753	1.70E-12	1.40E-11	no
BRD8	-0.262615142	-7.185442984	1.73E-12	1.42E-11	no
15-Sep	0.262609145	7.185266743	1.73E-12	1.42E-11	no
DPP9	0.262585229	7.184563932	1.74E-12	1.43E-11	no
FBLN2	0.262579712	7.184401798	1.74E-12	1.43E-11	no
PLIN3	0.262554813	7.183670093	1.75E-12	1.44E-11	no
MYLK	0.262547183	7.183445862	1.75E-12	1.44E-11	no
KLF3	0.262510016	7.182353695	1.76E-12	1.45E-11	no
SPAST	-0.262497181	-7.181976551	1.77E-12	1.45E-11	no
ADAMTS12	0.262490994	7.181794744	1.77E-12	1.45E-11	no
PSMA8	0.262476722	7.181375363	1.78E-12	1.46E-11	no
ACAA2	0.262249152	7.174688982	1.86E-12	1.53E-11	no
ZNF839	-0.262211793	-7.173591431	1.87E-12	1.54E-11	no
PATL2	0.262157386	7.171993094	1.89E-12	1.55E-11	no
NGFRAP1	-0.262054823	-7.168980277	1.93E-12	1.58E-11	no
FAR2	0.26204799	7.168779585	1.93E-12	1.59E-11	no
LYSMD1	-0.262025107	-7.16810742	1.94E-12	1.59E-11	no
ETV7	0.262023041	7.168046739	1.94E-12	1.59E-11	no
AK2	0.261989846	7.167071707	1.96E-12	1.60E-11	no
HK2	0.26195064	7.165920153	1.97E-12	1.61E-11	no
STARD8	0.26193699	7.165519237	1.98E-12	1.62E-11	no
MTMR1	-0.261922711	-7.16509985	1.98E-12	1.62E-11	no
XAF1	0.2617836	7.161014296	2.04E-12	1.67E-11	no
FAM83G	0.26177732	7.160829879	2.04E-12	1.67E-11	no
BRD7	-0.261701322	-7.158598092	2.07E-12	1.69E-11	no
CTF1	0.261684106	7.158092557	2.08E-12	1.70E-11	no
PAFAH1B1	-0.261671225	-7.157714316	2.09E-12	1.70E-11	no
GRID1	-0.261650359	-7.157101594	2.09E-12	1.71E-11	no
RBKS	0.261624362	7.156338222	2.10E-12	1.72E-11	no
COL6A1	0.261599337	7.15560341	2.12E-12	1.72E-11	no
DBNL	0.261548034	7.154097059	2.14E-12	1.74E-11	no
HDAC1	0.261489412	7.15237586	2.16E-12	1.76E-11	no
RBMS2	0.261445133	7.151075868	2.18E-12	1.78E-11	no
C15orf59	-0.261440305	-7.150934123	2.18E-12	1.78E-11	no
AKAP1	-0.26142797	-7.150571976	2.19E-12	1.78E-11	no
RHOA	0.261422264	7.15040446	2.19E-12	1.78E-11	no
C22orf36	-0.261410436	-7.150057217	2.20E-12	1.79E-11	no
SERPINE1	0.261261046	7.145671685	2.26E-12	1.84E-11	no
LRRTM4	-0.261250538	-7.145363241	2.27E-12	1.84E-11	no
PPFIBP1	0.261153211	7.142506404	2.31E-12	1.88E-11	no
NGRN	-0.261132979	-7.141912563	2.32E-12	1.88E-11	no
C10orf2	-0.261122215	-7.141596642	2.33E-12	1.89E-11	no
USP27X	-0.261096076	-7.140829431	2.34E-12	1.90E-11	no
HSD11B1L	-0.261061893	-7.13982618	2.35E-12	1.91E-11	no
FAM117B	-0.261053103	-7.139568189	2.36E-12	1.91E-11	no
PIGP	0.260973588	7.137234586	2.40E-12	1.94E-11	no
SH3BGRL	0.260957842	7.136772475	2.40E-12	1.95E-11	no
CDC42EP3	0.260945945	7.136423332	2.41E-12	1.95E-11	no

SLC24A3	-0.260852704	-7.133687147	2.45E-12	1.99E-11	no
MADD	-0.260831721	-7.133071409	2.46E-12	1.99E-11	no
PCDH18	0.26077689	7.131462518	2.49E-12	2.01E-11	no
CPA4	0.260768541	7.131217527	2.50E-12	2.02E-11	no
SYNGAP1	-0.260762183	-7.13103097	2.50E-12	2.02E-11	no
YPEL1	-0.260721873	-7.129848217	2.52E-12	2.03E-11	no
FLJ10038	-0.260674637	-7.128462276	2.54E-12	2.05E-11	no
ATG4C	0.260666002	7.128208945	2.55E-12	2.05E-11	no
SEPHS1	-0.260643791	-7.127557285	2.56E-12	2.06E-11	no
SLC9A1	0.260548469	7.124760719	2.61E-12	2.10E-11	no
PPP2R2D	-0.260458916	-7.122133624	2.65E-12	2.14E-11	no
VWF	0.260453853	7.121985118	2.66E-12	2.14E-11	no
PLBD2	0.260452777	7.121953529	2.66E-12	2.14E-11	no
NOVA1	-0.26034216	-7.118708805	2.72E-12	2.19E-11	no
TGIF1	0.260340131	7.118649283	2.72E-12	2.19E-11	no
RPS6KL1	-0.260307646	-7.117696463	2.73E-12	2.20E-11	no
JAKMIP2	-0.26019988	-7.11453575	2.79E-12	2.25E-11	no
ILF3	-0.260135435	-7.112645728	2.83E-12	2.27E-11	no
RARRES2	0.260127943	7.112426009	2.83E-12	2.28E-11	no
REPS1	-0.260114295	-7.112025762	2.84E-12	2.28E-11	no
PHF21A	-0.260093616	-7.111419331	2.85E-12	2.29E-11	no
ARF4	0.260062617	7.110510288	2.87E-12	2.30E-11	no
ECSIT	-0.2600003	-7.108682915	2.91E-12	2.33E-11	no
PMS2L11	0.25998795	7.108320767	2.91E-12	2.34E-11	no
RNF126P1	0.259927048	7.106534962	2.95E-12	2.36E-11	no
ZC3H12B	-0.259867898	-7.104800649	2.98E-12	2.39E-11	no
B3GALT4	0.259831089	7.103721432	3.01E-12	2.41E-11	no
MYST4	-0.2598219	-7.103452006	3.01E-12	2.41E-11	no
RTCD1	0.259777287	7.102144032	3.04E-12	2.43E-11	no
MARCKSL1	-0.25977571	-7.102097806	3.04E-12	2.43E-11	no
TPTE2P1	-0.259736963	-7.100961845	3.06E-12	2.45E-11	no
FBRSL1	-0.259661002	-7.098734968	3.11E-12	2.48E-11	no
USP43	-0.259652097	-7.098473927	3.11E-12	2.49E-11	no
SIRPD	0.2596486	7.098371402	3.12E-12	2.49E-11	no
6-Sep	0.259627717	7.097759226	3.13E-12	2.50E-11	no
PCDH7	-0.259463546	-7.092947032	3.23E-12	2.58E-11	no
C7orf49	0.259437861	7.09219419	3.25E-12	2.59E-11	no
PDIA6	0.25942346	7.091772107	3.26E-12	2.60E-11	no
ITGAV	0.259396107	7.090970434	3.28E-12	2.61E-11	no
RAB36	0.259383446	7.090599346	3.28E-12	2.62E-11	no
C9orf89	0.259377297	7.09041915	3.29E-12	2.62E-11	no
RPN1	0.259345727	7.089493899	3.31E-12	2.63E-11	no
GPR15	0.259333765	7.089143317	3.32E-12	2.64E-11	no
TRMT2B	0.259322103	7.08880156	3.32E-12	2.64E-11	no
SH3YL1	-0.259314847	-7.088588916	3.33E-12	2.65E-11	no
USP10	-0.259303522	-7.088256993	3.34E-12	2.65E-11	no
KIAA1967	-0.259261967	-7.0870392	3.36E-12	2.67E-11	no
KDELR1	0.259233386	7.086201647	3.38E-12	2.69E-11	no
HES6	-0.259226787	-7.086008269	3.39E-12	2.69E-11	no
CLIC3	0.259212175	7.08558007	3.40E-12	2.70E-11	no
ARMC9	0.259208343	7.085467771	3.40E-12	2.70E-11	no

RTP4	0.25915394	7.083873581	3.44E-12	2.72E-11	no
AGAP3	-0.259108053	-7.082529022	3.47E-12	2.75E-11	no
ITGA1	0.259081268	7.081744186	3.49E-12	2.76E-11	no
MYL12B	0.259067544	7.081342068	3.49E-12	2.77E-11	no
DAP3	0.259067475	7.081340047	3.50E-12	2.77E-11	no
EFR3B	-0.259049575	-7.080815559	3.51E-12	2.78E-11	no
HSPB1	0.259001509	7.079407257	3.54E-12	2.80E-11	no
TFAP4	-0.258998341	-7.079314426	3.54E-12	2.80E-11	no
FAM190B	-0.25890601	-7.076609361	3.61E-12	2.85E-11	no
INPP1	-0.258901596	-7.076480037	3.61E-12	2.85E-11	no
PLOD1	0.258835236	7.074535986	3.66E-12	2.89E-11	no
BET1L	0.258813859	7.073909743	3.67E-12	2.90E-11	no
C6orf136	-0.258788333	-7.073162	3.69E-12	2.91E-11	no
MAPK8IP1	-0.258745988	-7.07192157	3.72E-12	2.94E-11	no
UXS1	0.258725964	7.071335014	3.74E-12	2.95E-11	no
DGKB	-0.258725005	-7.071306915	3.74E-12	2.95E-11	no
SLC35D1	0.258709322	7.070847553	3.75E-12	2.96E-11	no
ADD1	-0.258669683	-7.069686474	3.78E-12	2.98E-11	no
THBS1	0.258662834	7.069485847	3.79E-12	2.98E-11	no
PRKAA1	0.258616407	7.068125992	3.82E-12	3.01E-11	no
C17orf96	-0.258555946	-7.06635517	3.87E-12	3.04E-11	no
CASKIN2	-0.25847131	-7.063876448	3.93E-12	3.09E-11	no
AP3B1	0.258465221	7.063698133	3.94E-12	3.09E-11	no
LOC100270710	0.258462295	7.06361242	3.94E-12	3.09E-11	no
DDX42	-0.258441181	-7.062994104	3.95E-12	3.11E-11	no
ZNF275	-0.258385224	-7.061355411	4.00E-12	3.14E-11	no
PAK7	-0.258379438	-7.061185994	4.00E-12	3.14E-11	no
SOX2	-0.258375168	-7.061060941	4.01E-12	3.14E-11	no
GFOD2	-0.258283559	-7.058378388	4.08E-12	3.20E-11	no
TM9SF2	0.258277281	7.058194561	4.08E-12	3.20E-11	no
ARID5A	0.258267528	7.057908981	4.09E-12	3.21E-11	no
C19orf28	0.258194834	7.055780491	4.15E-12	3.25E-11	no
ATF5	0.258187515	7.055566202	4.16E-12	3.25E-11	no
CITED2	0.258176422	7.055241411	4.17E-12	3.26E-11	no
RXRB	-0.258145025	-7.054322168	4.19E-12	3.28E-11	no
IRAK1	0.258089157	7.05268649	4.24E-12	3.31E-11	no
VPS37D	-0.258075378	-7.052283092	4.25E-12	3.32E-11	no
GOLPH3L	0.258061292	7.051870716	4.26E-12	3.33E-11	no
FUS	-0.257948133	-7.04855804	4.36E-12	3.40E-11	no
CFDP1	-0.257880868	-7.046589016	4.41E-12	3.45E-11	no
REST	0.257823502	7.044909869	4.46E-12	3.48E-11	no
C13orf29	0.257725164	7.04203161	4.55E-12	3.55E-11	no
CYB561	0.257641666	7.039587892	4.63E-12	3.61E-11	no
FARSA	-0.257598476	-7.038323914	4.67E-12	3.64E-11	no
HEG1	0.25754486	7.036754886	4.71E-12	3.67E-11	no
GBP4	0.257506853	7.035642686	4.75E-12	3.70E-11	no
COL4A2	0.257504911	7.035585862	4.75E-12	3.70E-11	no
PRPF19	-0.257499632	-7.035431388	4.76E-12	3.70E-11	no
MTMR7	-0.257488478	-7.035104981	4.77E-12	3.71E-11	no
LIF	0.257458951	7.034240978	4.79E-12	3.73E-11	no
GPR109B	0.257454258	7.034103665	4.80E-12	3.73E-11	no

RTN1	-0.257431289	-7.033431567	4.82E-12	3.75E-11	no
PIM1	0.257430648	7.033412811	4.82E-12	3.75E-11	no
ATP6AP2	0.257377732	7.031864486	4.87E-12	3.78E-11	no
DYNLT3	0.257359917	7.031343228	4.89E-12	3.80E-11	no
RAB40C	-0.257355896	-7.031225569	4.89E-12	3.80E-11	no
DYSF	0.257337167	7.030677584	4.91E-12	3.81E-11	no
USP54	-0.257253466	-7.028228706	4.99E-12	3.87E-11	no
PLA1A	0.257224216	7.027372967	5.02E-12	3.89E-11	no
HELQ	0.257196136	7.026551481	5.05E-12	3.91E-11	no
HMGCL	0.25716834	7.025738329	5.08E-12	3.93E-11	no
ADAMTS14	0.257135132	7.024766855	5.11E-12	3.96E-11	no
SBSN	0.257119392	7.024306399	5.12E-12	3.97E-11	no
NFATC1	0.257030976	7.02172004	5.21E-12	4.03E-11	no
RUSC2	-0.257011586	-7.021152889	5.23E-12	4.05E-11	no
LRIT2	-0.257011471	-7.021149526	5.23E-12	4.05E-11	no
AMACR	0.257008712	7.021068818	5.24E-12	4.05E-11	no
ACVRL1	0.256932398	7.01883666	5.32E-12	4.11E-11	no
PHLDB2	0.256907954	7.018121706	5.34E-12	4.12E-11	no
C1GALT1	0.256907329	7.018103427	5.34E-12	4.12E-11	no
SALL2	-0.256820279	-7.015557483	5.43E-12	4.19E-11	no
SNX32	-0.256715684	-7.012498632	5.55E-12	4.28E-11	no
CDHR1	-0.256710995	-7.012361522	5.55E-12	4.28E-11	no
FBXO6	0.256710483	7.012346543	5.55E-12	4.28E-11	no
GALNS	0.256664557	7.011003541	5.60E-12	4.32E-11	no
ZDHHC7	0.256594594	7.008957715	5.68E-12	4.37E-11	no
C7orf54	-0.256578844	-7.008497194	5.70E-12	4.39E-11	no
RASSF1	0.256544702	7.007498885	5.73E-12	4.41E-11	no
SERPINA3	0.256528642	7.007029305	5.75E-12	4.42E-11	no
LAYN	0.256489818	7.005894148	5.80E-12	4.46E-11	no
KIAA0895L	-0.256473595	-7.00541984	5.81E-12	4.47E-11	no
C1orf61	-0.256420686	-7.003872935	5.87E-12	4.51E-11	no
SORCS3	-0.25641088	-7.003586248	5.89E-12	4.52E-11	no
CPNE5	-0.256325158	-7.00108015	5.98E-12	4.60E-11	no
VPS26B	-0.256268059	-6.999410943	6.05E-12	4.64E-11	no
CELF3	-0.25622564	-6.99817097	6.10E-12	4.68E-11	no
ADHFE1	-0.25620997	-6.99771289	6.12E-12	4.69E-11	no
DEF8	-0.256198337	-6.997372843	6.13E-12	4.70E-11	no
ACTL6B	-0.256189919	-6.997126788	6.14E-12	4.71E-11	no
SFRS14	-0.256188347	-6.997080837	6.15E-12	4.71E-11	no
MARVELD1	0.256176512	6.996734883	6.16E-12	4.72E-11	no
PAM	0.256171502	6.996588451	6.17E-12	4.72E-11	no
WASF1	-0.256063557	-6.993433308	6.30E-12	4.82E-11	no
KIAA1199	0.256051335	6.993076089	6.31E-12	4.83E-11	no
WARS2	0.256039872	6.992741083	6.33E-12	4.84E-11	no
NFIX	-0.255983274	-6.991086903	6.40E-12	4.89E-11	no
NCRNA00219	-0.255806767	-6.985928733	6.62E-12	5.06E-11	no
ITPK1	-0.255802428	-6.985801938	6.63E-12	5.06E-11	no
MRFAP1L1	-0.255785879	-6.985318351	6.65E-12	5.08E-11	no
AGXT2L2	0.255759763	6.984555214	6.68E-12	5.10E-11	no
ANGPTL5	0.255744412	6.984106662	6.70E-12	5.11E-11	no
C13orf26	0.255729574	6.983673089	6.72E-12	5.13E-11	no

EFNA4	0.255713606	6.983206515	6.74E-12	5.14E-11	no
PEX5	-0.255683846	-6.982336973	6.78E-12	5.17E-11	no
HIST1H2BK	0.255662161	6.981703354	6.81E-12	5.19E-11	no
MDGA2	-0.255660255	-6.981647668	6.81E-12	5.19E-11	no
PHF2	-0.255638653	-6.981016519	6.84E-12	5.21E-11	no
FCER1A	0.25561317	6.980271955	6.87E-12	5.23E-11	no
C1orf144	0.25556759	6.978940287	6.94E-12	5.28E-11	no
TBXA2R	0.255532932	6.977927735	6.98E-12	5.31E-11	no
TCEA2	-0.255503276	-6.977061354	7.02E-12	5.34E-11	no
MYO10	-0.255448422	-6.975458856	7.10E-12	5.39E-11	no
ALDH1A3	0.255427551	6.974849169	7.13E-12	5.41E-11	no
MAP3K3	0.255414234	6.974460137	7.15E-12	5.42E-11	no
DYNLL2	-0.255404676	-6.97418093	7.16E-12	5.43E-11	no
WDR6	-0.255376496	-6.973357749	7.20E-12	5.46E-11	no
DNAJC12	-0.255375543	-6.97332992	7.20E-12	5.46E-11	no
SNAPC4	-0.255302851	-6.971206553	7.30E-12	5.53E-11	no
DGCR2	-0.255299701	-6.971114525	7.31E-12	5.54E-11	no
GPR126	0.255292911	6.970916213	7.32E-12	5.54E-11	no
FAM123C	-0.2551624	-6.967104269	7.50E-12	5.68E-11	no
DHTKD1	-0.255156947	-6.966945007	7.51E-12	5.69E-11	no
ZCCHC24	-0.255134726	-6.96629604	7.54E-12	5.71E-11	no
LOC400759	0.255122416	6.965936528	7.56E-12	5.72E-11	no
TM9SF1	0.255096284	6.965173338	7.60E-12	5.75E-11	no
LRRC4C	-0.255061299	-6.964151644	7.65E-12	5.78E-11	no
HCCS	0.255059545	6.964100398	7.65E-12	5.78E-11	no
TMEM61	0.255052857	6.963905092	7.66E-12	5.79E-11	no
RRBP1	0.25500775	6.962587843	7.73E-12	5.84E-11	no
ARSK	0.255005219	6.962513921	7.74E-12	5.84E-11	no
EML5	-0.254982045	-6.961837206	7.77E-12	5.86E-11	no
SUGT1L1	-0.254935248	-6.960470666	7.84E-12	5.91E-11	no
DCAF11	-0.254928297	-6.960267685	7.85E-12	5.92E-11	no
CELSR3	-0.25486958	-6.958553156	7.94E-12	5.98E-11	no
RNPEPL1	0.254809244	6.956791443	8.03E-12	6.05E-11	no
SDF4	0.25479164	6.956277466	8.06E-12	6.07E-11	no
C11orf63	0.254731984	6.954535718	8.16E-12	6.14E-11	no
TGFB3	0.254728232	6.954426172	8.16E-12	6.14E-11	no
ATF1	0.254689245	6.95328794	8.22E-12	6.19E-11	no
GRIK4	-0.254681592	-6.953064498	8.24E-12	6.19E-11	no
KIRREL	0.254629787	6.951552114	8.32E-12	6.25E-11	no
COG1	-0.254490544	-6.947487341	8.55E-12	6.42E-11	no
TTC33	-0.254475695	-6.947053904	8.57E-12	6.44E-11	no
RENBP	0.254438094	6.945956358	8.63E-12	6.48E-11	no
NANP	0.254436209	6.945901346	8.64E-12	6.48E-11	no
KANK2	0.254376919	6.944170776	8.74E-12	6.55E-11	no
ASL	0.254371572	6.944014709	8.74E-12	6.56E-11	no
PARP10	0.254362984	6.943764063	8.76E-12	6.57E-11	no
ZNF831	0.254237441	6.940100029	8.97E-12	6.73E-11	no
GPR114	0.254164718	6.937977719	9.10E-12	6.82E-11	no
LRRC14	-0.25408808	-6.935741303	9.24E-12	6.92E-11	no
ORAI3	0.254048713	6.934592592	9.31E-12	6.97E-11	no
PSTK	-0.254018577	-6.933713251	9.36E-12	7.01E-11	no

PDGFRB	0.254003359	6.933269212	9.39E-12	7.02E-11	no
TMEM60	0.253986643	6.932781447	9.42E-12	7.04E-11	no
GALNT10	0.253975772	6.932464259	9.44E-12	7.06E-11	no
CALCOCO1	-0.253965192	-6.93215557	9.46E-12	7.07E-11	no
SUSD3	0.253927343	6.93105123	9.53E-12	7.12E-11	no
ADAM8	0.253912072	6.930605688	9.56E-12	7.13E-11	no
ZNF238	-0.253895447	-6.930120615	9.59E-12	7.16E-11	no
EFS	-0.253860941	-6.929113883	9.65E-12	7.20E-11	no
LDHB	-0.253843041	-6.928591661	9.68E-12	7.22E-11	no
MARK1	-0.253823514	-6.928021975	9.72E-12	7.25E-11	no
KDM3B	-0.253776999	-6.926664958	9.81E-12	7.31E-11	no
NIPA2	0.253769453	6.926444813	9.82E-12	7.32E-11	no
WNT2B	0.253722913	6.925087127	9.91E-12	7.38E-11	no
ERP44	0.253720683	6.925022091	9.92E-12	7.38E-11	no
MGAT4B	0.253666664	6.923446291	1.00E-11	7.46E-11	no
GLUD2	-0.253508208	-6.918824344	1.03E-11	7.68E-11	no
LOC100128542	0.253449554	6.917113648	1.04E-11	7.77E-11	no
HPS4	-0.253416071	-6.916137114	1.05E-11	7.82E-11	no
LDOC1L	-0.253363945	-6.914616909	1.06E-11	7.89E-11	no
KBTBD8	0.25335334	6.914307647	1.06E-11	7.91E-11	no
PRPF6	-0.253348047	-6.914153261	1.07E-11	7.91E-11	no
SDC2	0.253333814	6.913738197	1.07E-11	7.93E-11	no
HDHD3	0.253279474	6.91215353	1.08E-11	8.01E-11	no
ZBTB38	0.253255506	6.911454596	1.08E-11	8.04E-11	no
CXCL13	0.253195759	6.909712373	1.10E-11	8.13E-11	no
DIRC1	0.252938751	6.902218967	1.15E-11	8.54E-11	no
HTR7P1	0.252890888	6.900823625	1.16E-11	8.62E-11	no
ACD	-0.252843946	-6.899455203	1.17E-11	8.69E-11	no
LZTS1	0.252746368	6.89661083	1.20E-11	8.85E-11	no
ARHGEF9	-0.252739925	-6.896423026	1.20E-11	8.86E-11	no
RRAGC	0.25272755	6.896062303	1.20E-11	8.88E-11	no
HAL	0.252641508	6.893554407	1.22E-11	9.02E-11	no
FADD	0.252612407	6.892706231	1.23E-11	9.07E-11	no
HM13	0.252555082	6.891035513	1.24E-11	9.17E-11	no
HIC1	0.252549777	6.890880904	1.24E-11	9.17E-11	no
AARSD1	-0.252530925	-6.890331467	1.25E-11	9.20E-11	no
LOC100129716	0.252482778	6.888928326	1.26E-11	9.29E-11	no
JSRP1	0.252435589	6.887553148	1.27E-11	9.37E-11	no
OMA1	0.252416411	6.886994268	1.27E-11	9.40E-11	no
YLPM1	-0.252321466	-6.884227602	1.30E-11	9.57E-11	no
RND1	-0.252316678	-6.884088082	1.30E-11	9.57E-11	no
LOC728643	-0.252278841	-6.882985578	1.31E-11	9.64E-11	no
NKAIN4	-0.252276895	-6.882928878	1.31E-11	9.64E-11	no
CHGB	-0.252274398	-6.882856134	1.31E-11	9.64E-11	no
ZNF540	-0.25222796	-6.881503057	1.32E-11	9.72E-11	no
SNAP91	-0.25210003	-6.877775818	1.35E-11	9.96E-11	no
ZC4H2	-0.252094803	-6.877623523	1.36E-11	9.97E-11	no
SEMA3C	0.25201527	6.875306547	1.38E-11	1.01E-10	no
AP3S1	0.251963049	6.873785311	1.39E-11	1.02E-10	no
MTHFS	0.251950008	6.873405416	1.39E-11	1.02E-10	no
DUSP10	0.251941738	6.873164496	1.40E-11	1.02E-10	no

KIAA0664	-0.251912323	-6.872307656	1.40E-11	1.03E-10	no
PID1	-0.251892054	-6.87171725	1.41E-11	1.03E-10	no
VANGL2	-0.251762883	-6.867954875	1.44E-11	1.06E-10	no
KIR2DS4	0.251617828	6.863730329	1.48E-11	1.09E-10	no
FBXO21	-0.251572341	-6.862405664	1.50E-11	1.10E-10	no
DISP1	0.251526497	6.861070647	1.51E-11	1.11E-10	no
ITPKC	0.251508312	6.86054112	1.52E-11	1.11E-10	no
F2RL2	0.25144899	6.858813718	1.53E-11	1.12E-10	no
TAS2R4	-0.251408009	-6.857620448	1.55E-11	1.13E-10	no
RINL	0.251389702	6.857087414	1.55E-11	1.13E-10	no
SNX10	0.251352158	6.855994268	1.56E-11	1.14E-10	no
FKBP1A	0.251351148	6.855964859	1.56E-11	1.14E-10	no
ADD2	-0.251293305	-6.854280733	1.58E-11	1.15E-10	no
ZNF721	-0.251290684	-6.854204442	1.58E-11	1.15E-10	no
TWSG1	0.251283518	6.853995812	1.58E-11	1.16E-10	no
TNFRSF12A	0.251225963	6.852320176	1.60E-11	1.17E-10	no
SAMD10	-0.251209448	-6.85183936	1.60E-11	1.17E-10	no
TAPT1	-0.251192716	-6.851352261	1.61E-11	1.17E-10	no
ZNF687	-0.251169997	-6.85069086	1.62E-11	1.18E-10	no
TUBB6	0.25116528	6.850553549	1.62E-11	1.18E-10	no
BAX	0.251163781	6.850509892	1.62E-11	1.18E-10	no
MIB1	-0.251136731	-6.849722446	1.63E-11	1.19E-10	no
CC2D1B	0.251136533	6.849716681	1.63E-11	1.19E-10	no
B3GNT1	-0.251128523	-6.849483508	1.63E-11	1.19E-10	no
MOV10	0.251101995	6.848711271	1.64E-11	1.19E-10	no
NINJ1	0.251090457	6.848375391	1.64E-11	1.19E-10	no
PPM1E	-0.2510445	-6.847037623	1.66E-11	1.20E-10	no
CTNS	0.250881961	6.842306638	1.71E-11	1.24E-10	no
LRIG1	-0.250877904	-6.842188535	1.71E-11	1.24E-10	no
MINA	0.250819553	6.840490302	1.73E-11	1.26E-10	no
DISP2	-0.250686767	-6.836625972	1.77E-11	1.29E-10	no
OAZ1	0.250652546	6.835630142	1.78E-11	1.30E-10	no
PPAP2A	0.250605447	6.83425961	1.80E-11	1.31E-10	no
HMGN5	-0.250591109	-6.833842374	1.81E-11	1.31E-10	no
COL4A1	0.250578965	6.833489007	1.81E-11	1.31E-10	no
NSUN6	-0.250519531	-6.831759657	1.83E-11	1.33E-10	no
CPEB3	-0.250471675	-6.830367258	1.85E-11	1.34E-10	no
IFI35	0.250471205	6.830353584	1.85E-11	1.34E-10	no
PPIB	0.250430333	6.829164418	1.86E-11	1.35E-10	no
MYT1	-0.250415608	-6.828735984	1.87E-11	1.35E-10	no
ZNF394	0.250401563	6.828327373	1.87E-11	1.35E-10	no
KSR1	-0.250357796	-6.827054053	1.89E-11	1.37E-10	no
SETD1A	-0.250335978	-6.826419303	1.90E-11	1.37E-10	no
BLVRB	0.250335238	6.826397783	1.90E-11	1.37E-10	no
UNC5A	-0.25033146	-6.826287861	1.90E-11	1.37E-10	no
ZNF785	-0.250330734	-6.826266761	1.90E-11	1.37E-10	no
5-Mar	-0.250197606	-6.822393987	1.95E-11	1.40E-10	no
LOC283267	-0.250161918	-6.821355888	1.96E-11	1.41E-10	no
SART3	-0.250078542	-6.818930714	1.99E-11	1.44E-10	no
SETD5	-0.25005495	-6.81824452	2.00E-11	1.44E-10	no
C9orf6	0.250010544	6.816952966	2.02E-11	1.45E-10	no

TNFSF11	0.249996726	6.816551079	2.02E-11	1.46E-10	no
TPST1	0.249966023	6.8156581	2.03E-11	1.46E-10	no
KLHL28	-0.249937378	-6.81482502	2.04E-11	1.47E-10	no
TRIM23	-0.249899324	-6.813718307	2.06E-11	1.48E-10	no
SUCLG2	0.249847191	6.812202219	2.08E-11	1.50E-10	no
KIF3C	-0.249807147	-6.811037728	2.10E-11	1.51E-10	no
TC2N	0.249780578	6.810265115	2.11E-11	1.51E-10	no
CUL7	0.249775098	6.810105755	2.11E-11	1.52E-10	no
TMEM26	0.249752123	6.809437658	2.12E-11	1.52E-10	no
CSMD1	-0.249743685	-6.809192287	2.12E-11	1.52E-10	no
GBA	0.249712516	6.80828598	2.13E-11	1.53E-10	no
RECQL	0.249692629	6.807707691	2.14E-11	1.54E-10	no
PCDH15	-0.249602948	-6.805100109	2.18E-11	1.56E-10	no
IDI1	-0.249597629	-6.804945468	2.18E-11	1.56E-10	no
METTL10	-0.249536902	-6.803179887	2.21E-11	1.58E-10	no
PHLPP2	-0.249533853	-6.803091229	2.21E-11	1.58E-10	no
COL8A1	0.249485943	6.801698345	2.23E-11	1.60E-10	no
RNFT1	0.249480008	6.801525801	2.23E-11	1.60E-10	no
CHERP	-0.249477347	-6.801448432	2.23E-11	1.60E-10	no
LIMK2	0.249347121	6.797662686	2.29E-11	1.64E-10	no
SMARCA4	-0.249339238	-6.797433517	2.29E-11	1.64E-10	no
C13orf31	0.24924001	6.794549188	2.33E-11	1.67E-10	no
FAM176A	0.249213926	6.793791005	2.34E-11	1.68E-10	no
BEND7	-0.249158506	-6.792180195	2.37E-11	1.69E-10	no
EXOC3L2	0.249085582	6.790060746	2.40E-11	1.72E-10	no
DKFZP434H168	-0.249083486	-6.789999824	2.40E-11	1.72E-10	no
SNX15	-0.249082301	-6.789965376	2.40E-11	1.72E-10	no
SPAG9	-0.249070437	-6.789620588	2.41E-11	1.72E-10	no
EEF2	-0.249004047	-6.787691153	2.44E-11	1.74E-10	no
CMTM4	-0.248880914	-6.784112945	2.50E-11	1.78E-10	no
DUSP22	0.248755454	6.78046746	2.56E-11	1.82E-10	no
LOC642852	-0.24874338	-6.780116625	2.56E-11	1.83E-10	no
USP22	-0.248726057	-6.779613305	2.57E-11	1.83E-10	no
ARNTL2	0.248623677	6.776638812	2.62E-11	1.87E-10	no
FAM134A	-0.248583501	-6.77547161	2.64E-11	1.88E-10	no
HLCS	0.248542537	6.774281552	2.66E-11	1.89E-10	no
ANKHD1-EIF4E	0.248501713	6.773095627	2.68E-11	1.91E-10	no
PRSSL1	0.248501641	6.773093541	2.68E-11	1.91E-10	no
CORO1B	0.248452615	6.771669377	2.71E-11	1.92E-10	no
TSNARE1	-0.248441217	-6.771338286	2.71E-11	1.93E-10	no
FAM155A	-0.248422767	-6.770802343	2.72E-11	1.93E-10	no
TAX1BP3	0.248402816	6.770222823	2.73E-11	1.94E-10	no
TSC1	-0.248386789	-6.769757283	2.74E-11	1.95E-10	no
TEF	-0.248378885	-6.769527679	2.74E-11	1.95E-10	no
KCNE4	0.248375986	6.769443474	2.75E-11	1.95E-10	no
RAC3	-0.248359468	-6.768963694	2.75E-11	1.95E-10	no
KLHL22	-0.248264055	-6.766192409	2.80E-11	1.99E-10	no
YAP1	0.248261601	6.766121123	2.81E-11	1.99E-10	no
NUFIP1	-0.24826123	-6.766110336	2.81E-11	1.99E-10	no
DGCR9	-0.248234393	-6.76533091	2.82E-11	2.00E-10	no
TDO2	0.248226098	6.765089971	2.82E-11	2.00E-10	no

CRB2	0.248144675	6.762725283	2.87E-11	2.03E-10	no
BMP2	-0.248097838	-6.76136509	2.89E-11	2.05E-10	no
C2orf65	0.248093205	6.761230531	2.90E-11	2.05E-10	no
TM2D2	0.248033281	6.759490371	2.93E-11	2.07E-10	no
LRRC32	0.248023036	6.759192882	2.93E-11	2.07E-10	no
C12orf34	-0.24800724	-6.75873417	2.94E-11	2.08E-10	no
LPP	0.247971184	6.757687208	2.96E-11	2.09E-10	no
HPGDS	0.247958437	6.757317061	2.97E-11	2.10E-10	no
VDAC2	-0.247879484	-6.755024551	3.01E-11	2.13E-10	no
C17orf62	0.247837662	6.753810278	3.04E-11	2.14E-10	no
TTC39A	0.247808495	6.752963438	3.06E-11	2.16E-10	no
C6orf211	0.247791419	6.752467669	3.06E-11	2.16E-10	no
NET1	-0.247741144	-6.751008032	3.09E-11	2.18E-10	no
MAPK12	-0.247705654	-6.7499777	3.11E-11	2.19E-10	no
OPA3	0.247705255	6.749966117	3.12E-11	2.19E-10	no
LUZP2	-0.247690332	-6.749532882	3.12E-11	2.20E-10	no
FAIM2	-0.247624068	-6.747609244	3.16E-11	2.23E-10	no
FZD5	0.24758509	6.746477737	3.19E-11	2.24E-10	no
USP7	-0.24756628	-6.745931713	3.20E-11	2.25E-10	no
CASK	-0.247537107	-6.745084904	3.21E-11	2.26E-10	no
LTA4H	0.24752038	6.744599346	3.23E-11	2.27E-10	no
PRKCDBP	0.247507843	6.744235436	3.23E-11	2.27E-10	no
CTCF	-0.247491325	-6.743755973	3.24E-11	2.28E-10	no
GHRL	0.247438262	6.742215792	3.28E-11	2.30E-10	no
MAPK10	-0.24739383	-6.740926176	3.30E-11	2.32E-10	no
CHIT1	0.247331413	6.739114613	3.34E-11	2.34E-10	no
ZDHHC17	-0.247330874	-6.739098972	3.34E-11	2.34E-10	no
RICTOR	-0.247326597	-6.738974831	3.34E-11	2.35E-10	no
AASDHPPT	-0.24721232	-6.735658366	3.42E-11	2.40E-10	no
CCDC28A	0.247179293	6.734699945	3.44E-11	2.41E-10	no
LOC283050	0.24715323	6.733943607	3.45E-11	2.42E-10	no
NF1	-0.247101835	-6.732452235	3.49E-11	2.44E-10	no
GPBP1	-0.247080727	-6.731839748	3.50E-11	2.45E-10	no
HNRNPA1	-0.247069582	-6.731516344	3.51E-11	2.46E-10	no
SOD3	0.247040553	6.730674035	3.53E-11	2.47E-10	no
GNG5	0.247009596	6.729775789	3.55E-11	2.48E-10	no
TNIP1	0.246962573	6.728411443	3.58E-11	2.50E-10	no
WFIKKN1	-0.24680842	-6.723939069	3.69E-11	2.58E-10	no
MMP2	0.246754435	6.722372965	3.72E-11	2.60E-10	no
PLIN2	0.246751046	6.722274642	3.72E-11	2.60E-10	no
ZNF737	-0.246711067	-6.721114896	3.75E-11	2.62E-10	no
MUL1	0.246678827	6.720179676	3.78E-11	2.63E-10	no
RMND5B	-0.246642705	-6.719131884	3.80E-11	2.65E-10	no
ZNF34	-0.246596925	-6.717803963	3.83E-11	2.67E-10	no
WNT3	-0.246593588	-6.717707161	3.84E-11	2.67E-10	no
RBM4B	-0.246555569	-6.716604403	3.86E-11	2.69E-10	no
RAB39B	-0.246553911	-6.716556326	3.86E-11	2.69E-10	no
PDGFB	0.246513447	6.715382672	3.89E-11	2.71E-10	no
CHMP4C	0.24650583	6.715161762	3.90E-11	2.72E-10	no
GLIS2	-0.246485333	-6.714567289	3.91E-11	2.72E-10	no
KIAA1549	-0.246460711	-6.71385315	3.93E-11	2.74E-10	no

RNF125	0.246372825	6.711304325	4.00E-11	2.78E-10	no
SEMA3A	0.24637141	6.711263284	4.00E-11	2.78E-10	no
SLC15A4	0.246365197	6.711083106	4.00E-11	2.78E-10	no
PCBP4	-0.246334306	-6.710187253	4.03E-11	2.80E-10	no
C7orf51	-0.246325257	-6.709924818	4.03E-11	2.80E-10	no
INA	-0.246300575	-6.709209068	4.05E-11	2.81E-10	no
CLNS1A	-0.246282029	-6.708671264	4.07E-11	2.82E-10	no
PPT1	0.246179113	6.705686929	4.14E-11	2.88E-10	no
GSN	0.246175665	6.705586961	4.15E-11	2.88E-10	no
SLC36A1	0.246175517	6.705582662	4.15E-11	2.88E-10	no
NAALADL2	0.246172701	6.705501025	4.15E-11	2.88E-10	no
MMP19	0.246031804	6.701415756	4.26E-11	2.95E-10	no
TAF12	0.246030408	6.701375293	4.26E-11	2.95E-10	no
PTPN23	-0.246028049	-6.701306909	4.26E-11	2.95E-10	no
UNC13A	-0.24600893	-6.700752574	4.28E-11	2.96E-10	no
DVL1	-0.245971344	-6.699662899	4.31E-11	2.98E-10	no
NOTCH1	-0.245958214	-6.69928222	4.32E-11	2.99E-10	no
LRRTM3	-0.245948691	-6.699006136	4.33E-11	2.99E-10	no
ZMIZ1	-0.245894115	-6.697423964	4.37E-11	3.02E-10	no
ASRGL1	-0.24589049	-6.697318873	4.37E-11	3.02E-10	no
MARCKS	-0.245813246	-6.69507967	4.44E-11	3.07E-10	no
MTCP1	0.245791442	6.694447598	4.46E-11	3.08E-10	no
KCNK4	-0.245739146	-6.692931699	4.50E-11	3.11E-10	no
TMCC2	-0.24571964	-6.692366298	4.52E-11	3.12E-10	no
STYX	0.245668469	6.690883088	4.56E-11	3.14E-10	no
DNAJC21	0.245652936	6.690432873	4.57E-11	3.15E-10	no
LAMB2	0.245640048	6.690059326	4.58E-11	3.16E-10	no
GPR55	0.245617538	6.689406916	4.60E-11	3.17E-10	no
LRRC1	-0.245614487	-6.689318486	4.60E-11	3.17E-10	no
FAM126A	0.245597983	6.688840143	4.62E-11	3.18E-10	no
KIF1B	-0.245579987	-6.688318561	4.63E-11	3.19E-10	no
OSBPL2	-0.245503255	-6.68609471	4.70E-11	3.24E-10	no
NPRL3	-0.245497428	-6.685925852	4.71E-11	3.24E-10	no
PCSK5	0.245495196	6.685861168	4.71E-11	3.24E-10	no
MTMR11	0.245476262	6.685312443	4.72E-11	3.25E-10	no
GALC	0.245463374	6.684938954	4.74E-11	3.25E-10	no
SLC25A37	0.245431587	6.684017776	4.76E-11	3.27E-10	no
ABCA3	-0.245419975	-6.683681268	4.77E-11	3.28E-10	no
SMARCB1	-0.245401785	-6.68315414	4.79E-11	3.29E-10	no
WTAP	0.2453544	6.681781021	4.83E-11	3.32E-10	no
HRH2	0.245340415	6.681375765	4.85E-11	3.32E-10	no
PMEPA1	0.245330203	6.681079835	4.85E-11	3.33E-10	no
DMRTC1B	-0.245323186	-6.680876512	4.86E-11	3.33E-10	no
SHC2	-0.245185407	-6.676884325	4.99E-11	3.42E-10	no
FLNC	0.24517293	6.67652283	5.00E-11	3.42E-10	no
CAND2	-0.245166253	-6.67632935	5.00E-11	3.43E-10	no
RAP2A	-0.245165207	-6.676299071	5.01E-11	3.43E-10	no
CLINT1	0.245144738	6.67570602	5.02E-11	3.44E-10	no
DUSP18	0.245091686	6.674168969	5.07E-11	3.47E-10	no
CHD3	-0.245040883	-6.672697145	5.12E-11	3.50E-10	no
MCL1	0.244799404	6.665702058	5.36E-11	3.66E-10	no

CHST14	0.244777039	6.665054246	5.38E-11	3.68E-10	no
MAOB	0.244744756	6.664119204	5.41E-11	3.70E-10	no
RNF146	-0.244687737	-6.662467765	5.47E-11	3.74E-10	no
RUFY2	-0.24465144	-6.661416508	5.51E-11	3.76E-10	no
C10orf41	0.244602259	6.659992181	5.56E-11	3.79E-10	no
CAMLG	-0.244545375	-6.658344833	5.62E-11	3.83E-10	no
FGF12	-0.244536822	-6.658097138	5.62E-11	3.84E-10	no
NMNAT2	-0.244524273	-6.65773373	5.64E-11	3.84E-10	no
RDH10	0.244479853	6.656447418	5.68E-11	3.87E-10	no
ZNF681	-0.244414543	-6.654556218	5.75E-11	3.92E-10	no
SCRT1	-0.244395066	-6.653992227	5.77E-11	3.93E-10	no
LOC400804	0.244386321	6.653739028	5.78E-11	3.94E-10	no
HEATR7B2	0.244351743	6.652737811	5.82E-11	3.96E-10	no
CACNG2	-0.244275298	-6.650524419	5.90E-11	4.02E-10	no
PPP2R2B	-0.244266579	-6.650271967	5.91E-11	4.02E-10	no
NAA30	-0.244256003	-6.649965757	5.92E-11	4.03E-10	no
FERMT1	-0.244227097	-6.649128876	5.96E-11	4.05E-10	no
ACBD5	-0.244124228	-6.646150727	6.07E-11	4.13E-10	no
LTBP1	0.244121052	6.646058758	6.07E-11	4.13E-10	no
TCTN2	0.244103642	6.645554755	6.09E-11	4.14E-10	no
KCNJ15	0.244085283	6.645023275	6.11E-11	4.15E-10	no
UNC80	-0.244057976	-6.644232792	6.15E-11	4.17E-10	no
NIPSNAP3A	0.244024627	6.643267412	6.18E-11	4.19E-10	no
MBTD1	-0.244024259	-6.643256748	6.18E-11	4.19E-10	no
RAB7L1	0.2439701	6.641689021	6.25E-11	4.23E-10	no
FAM171A1	-0.243960641	-6.641415203	6.26E-11	4.24E-10	no
TMEM159	0.243949251	6.641085508	6.27E-11	4.25E-10	no
SEMA6B	-0.243935829	-6.640696995	6.29E-11	4.26E-10	no
LOC201651	0.243920823	6.64026264	6.30E-11	4.27E-10	no
TTC12	0.243841772	6.637974591	6.40E-11	4.33E-10	no
FOSL1	0.243829108	6.63760804	6.41E-11	4.34E-10	no
TMEM123	0.243823131	6.637435059	6.42E-11	4.34E-10	no
RAB1A	0.243803684	6.636872199	6.44E-11	4.36E-10	no
SECISBP2	-0.243800801	-6.63678875	6.44E-11	4.36E-10	no
NRIP1	0.243781765	6.636237824	6.47E-11	4.37E-10	no
LOC144571	0.243730879	6.634765102	6.53E-11	4.41E-10	no
ZNF445	-0.243711215	-6.634196009	6.55E-11	4.42E-10	no
SPHKAP	-0.243651122	-6.632456925	6.62E-11	4.47E-10	no
PSMD5	0.243481071	6.627536132	6.84E-11	4.61E-10	no
TBK1	0.243479092	6.627478859	6.84E-11	4.61E-10	no
LNP1	-0.243473139	-6.627306606	6.85E-11	4.62E-10	no
SURF6	-0.243472406	-6.627285403	6.85E-11	4.62E-10	no
CSDA	0.243469638	6.627205303	6.85E-11	4.62E-10	no
IDH1	0.243469003	6.627186922	6.85E-11	4.62E-10	no
SLC25A41	-0.243406452	-6.625377059	6.93E-11	4.67E-10	no
HSPBAP1	0.243380542	6.624627409	6.96E-11	4.69E-10	no
C9orf125	-0.243367301	-6.624244314	6.98E-11	4.70E-10	no
HIAT1	0.243350818	6.623767413	7.00E-11	4.71E-10	no
PIGG	0.24334412	6.623573626	7.01E-11	4.72E-10	no
MMS19	-0.24332573	-6.62304155	7.03E-11	4.73E-10	no
ZNF276	-0.243260703	-6.621160265	7.12E-11	4.79E-10	no

RFX5	0.243213685	6.619800047	7.18E-11	4.83E-10	no
FBXO11	-0.243141672	-6.617716809	7.28E-11	4.89E-10	no
LPPR1	-0.243090616	-6.616239892	7.35E-11	4.93E-10	no
UCK1	-0.243062835	-6.615436296	7.38E-11	4.96E-10	no
PLA2R1	0.243024047	6.614314352	7.44E-11	4.99E-10	no
SYPL1	0.243015319	6.614061875	7.45E-11	5.00E-10	no
MAGED4B	-0.242995839	-6.613498449	7.47E-11	5.01E-10	no
DENND5A	-0.242935277	-6.61174675	7.56E-11	5.07E-10	no
SF1	-0.242922278	-6.611370783	7.58E-11	5.08E-10	no
CHSY3	0.242864138	6.609689266	7.66E-11	5.13E-10	no
INSRR	0.242852283	6.609346397	7.67E-11	5.14E-10	no
AKR1A1	0.242842472	6.609062643	7.69E-11	5.15E-10	no
TOMM20	-0.242806012	-6.608008208	7.74E-11	5.18E-10	no
PALMD	0.242796101	6.607721593	7.75E-11	5.19E-10	no
SERPINF2	0.242654522	6.603627344	7.96E-11	5.32E-10	no
ZDHHC24	0.242612378	6.602408693	8.02E-11	5.36E-10	no
P4HA2	0.242582178	6.60153544	8.07E-11	5.39E-10	no
ADO	-0.242575296	-6.601336453	8.08E-11	5.40E-10	no
LOC145820	0.242569308	6.601163321	8.08E-11	5.40E-10	no
LIPN	0.242467915	6.59823166	8.24E-11	5.50E-10	no
RTF1	-0.242429187	-6.597111957	8.29E-11	5.54E-10	no
PIK3R1	-0.242427604	-6.597066193	8.30E-11	5.54E-10	no
SYT11	-0.242415619	-6.596719688	8.32E-11	5.55E-10	no
FLYWCH1	-0.242368267	-6.595350688	8.39E-11	5.59E-10	no
DARS2	0.242351938	6.594878618	8.41E-11	5.61E-10	no
IL23A	0.242297375	6.593301228	8.50E-11	5.66E-10	no
CLEC12B	0.242231733	6.591403644	8.60E-11	5.73E-10	no
TET1	-0.242221306	-6.591102241	8.62E-11	5.74E-10	no
C10orf75	-0.242201428	-6.590527627	8.65E-11	5.76E-10	no
SCYL2	0.242177105	6.589824536	8.69E-11	5.78E-10	no
COMMD8	0.24216407	6.589447752	8.71E-11	5.79E-10	no
RAMP3	0.242159108	6.589304317	8.72E-11	5.80E-10	no
ATXN7L3B	-0.242156452	-6.589227558	8.72E-11	5.80E-10	no
SNX24	0.242106867	6.587794299	8.80E-11	5.85E-10	no
KSR2	-0.242034253	-6.585695462	8.92E-11	5.92E-10	no
VPS72	-0.242031876	-6.585626757	8.92E-11	5.93E-10	no
WNT4	0.241983115	6.58421746	9.00E-11	5.98E-10	no
CAT	0.241982295	6.584193764	9.00E-11	5.98E-10	no
NMT2	-0.241964279	-6.583673072	9.03E-11	5.99E-10	no
TREML4	0.241922019	6.582451735	9.10E-11	6.04E-10	no
HIP1R	-0.241918604	-6.582353024	9.11E-11	6.04E-10	no
GPR124	0.241911281	6.582141381	9.12E-11	6.05E-10	no
BCCIP	-0.24187897	-6.581207602	9.17E-11	6.08E-10	no
NUB1	0.241864347	6.58078503	9.20E-11	6.09E-10	no
ADAMTSL1	0.241804283	6.579049266	9.30E-11	6.16E-10	no
VPS53	-0.241788512	-6.578593504	9.33E-11	6.18E-10	no
CTBP2	-0.241704746	-6.576172973	9.47E-11	6.27E-10	no
PHF16	-0.241696275	-6.575928205	9.49E-11	6.28E-10	no
LOC100129034	0.24167907	6.57543105	9.52E-11	6.29E-10	no
PIK3CD	0.241624842	6.573864163	9.61E-11	6.35E-10	no
ATMIN	-0.241617747	-6.573659156	9.62E-11	6.36E-10	no

H2AFY2	-0.241612751	-6.573514806	9.63E-11	6.36E-10	no
LOC388152	-0.241542346	-6.571480609	9.76E-11	6.44E-10	no
NRSN1	-0.241542252	-6.5714779	9.76E-11	6.44E-10	no
CRTAP	0.241533058	6.571212277	9.77E-11	6.45E-10	no
CLEC5A	0.241470525	6.569405641	9.89E-11	6.52E-10	no
CXorf38	0.241350053	6.565925296	1.01E-10	6.67E-10	no
FBXO22	0.241322622	6.565132895	1.02E-10	6.70E-10	no
AP1S2	0.24128262	6.563977374	1.02E-10	6.74E-10	no
KLRC3	-0.241265318	-6.563477589	1.03E-10	6.76E-10	no
C12orf5	0.241221228	6.562204045	1.03E-10	6.82E-10	no
STK40	0.241212847	6.561961948	1.04E-10	6.82E-10	no
TCF12	-0.241211346	-6.56191859	1.04E-10	6.82E-10	no
LOC399744	-0.241189873	-6.561298352	1.04E-10	6.85E-10	no
EMP1	0.241146676	6.560050676	1.05E-10	6.90E-10	no
KLRF1	0.241105825	6.558870783	1.06E-10	6.95E-10	no
CCDC88B	0.240979115	6.555211295	1.08E-10	7.11E-10	no
FKBP10	0.240896218	6.552817345	1.10E-10	7.21E-10	no
TSHZ3	0.240895566	6.552798525	1.10E-10	7.21E-10	no
DUSP1	0.240863712	6.55187867	1.10E-10	7.25E-10	no
LAG3	0.240859101	6.551745504	1.11E-10	7.26E-10	no
NCSTN	0.240835688	6.551069431	1.11E-10	7.29E-10	no
KIAA1409	-0.240815052	-6.550473538	1.11E-10	7.31E-10	no
B3GALT2	-0.240805455	-6.55019641	1.12E-10	7.32E-10	no
CCDC102A	0.240786534	6.549650079	1.12E-10	7.34E-10	no
KATNB1	-0.240760086	-6.548886381	1.13E-10	7.38E-10	no
ZFP41	-0.240704863	-6.547291886	1.14E-10	7.45E-10	no
CECR2	-0.240658887	-6.54596441	1.15E-10	7.51E-10	no
DLL3	-0.240638513	-6.545376189	1.15E-10	7.54E-10	no
ELTD1	0.24063088	6.545155793	1.15E-10	7.54E-10	no
CIZ1	-0.240593666	-6.544081371	1.16E-10	7.59E-10	no
RASL10A	-0.240592901	-6.544059298	1.16E-10	7.59E-10	no
TLK2	-0.24055858	-6.543068434	1.17E-10	7.64E-10	no
GPR133	0.240557272	6.543030672	1.17E-10	7.64E-10	no
MYBPC2	0.240508999	6.54163704	1.18E-10	7.70E-10	no
C2orf27A	-0.240507609	-6.541596906	1.18E-10	7.70E-10	no
BDKRB1	0.24045321	6.5400265	1.19E-10	7.77E-10	no
MICALL2	0.240413526	6.538880921	1.20E-10	7.83E-10	no
KCTD10	0.240397668	6.53842317	1.20E-10	7.85E-10	no
RAI14	0.240396894	6.538400805	1.20E-10	7.85E-10	no
ENHO	-0.240386879	-6.538111707	1.20E-10	7.86E-10	no
TRPC1	-0.240371431	-6.537665803	1.21E-10	7.88E-10	no
FAM86D	0.24035107	6.537078047	1.21E-10	7.90E-10	no
ZNF775	-0.240342847	-6.536840691	1.21E-10	7.91E-10	no
NISCH	-0.24026709	-6.534654019	1.23E-10	8.02E-10	no
RNF133	0.240241424	6.533913185	1.24E-10	8.06E-10	no
GTF2F1	-0.240185996	-6.532313393	1.25E-10	8.13E-10	no
DDX58	0.240163375	6.531660531	1.25E-10	8.17E-10	no
EDARADD	0.240156134	6.531451534	1.26E-10	8.17E-10	no
HIST1H2AC	0.240146185	6.531164395	1.26E-10	8.19E-10	no
BAT2	-0.24013891	-6.530954435	1.26E-10	8.19E-10	no
MFI2	0.240067909	6.528905349	1.28E-10	8.30E-10	no

FADS1	-0.240050837	-6.528412647	1.28E-10	8.32E-10	no
MMP1	0.24002297	6.527608468	1.29E-10	8.36E-10	no
DZIP1L	0.239981788	6.526420036	1.30E-10	8.42E-10	no
DNAJC25	0.239979409	6.526351375	1.30E-10	8.42E-10	no
GNAL	-0.239960551	-6.525807176	1.30E-10	8.45E-10	no
PARD6A	-0.239940778	-6.525236619	1.31E-10	8.47E-10	no
DPH1	-0.239847533	-6.522545963	1.33E-10	8.62E-10	no
PTPRA	-0.239778592	-6.520556763	1.35E-10	8.72E-10	no
PHGDH	-0.239758356	-6.519972916	1.35E-10	8.75E-10	no
GPR152	0.239687893	6.517939942	1.37E-10	8.86E-10	no
DPH3	0.239664651	6.517269373	1.37E-10	8.89E-10	no
MPRIP	-0.239664555	-6.517266614	1.37E-10	8.89E-10	no
TBC1D24	-0.239660087	-6.517137717	1.37E-10	8.90E-10	no
GPR109A	0.239659328	6.517115806	1.37E-10	8.90E-10	no
DNPEP	0.239590693	6.515135716	1.39E-10	9.00E-10	no
LAIR2	0.239565239	6.514401416	1.40E-10	9.04E-10	no
DHH	0.239486858	6.512140322	1.42E-10	9.17E-10	no
CLASP1	-0.239483743	-6.512050485	1.42E-10	9.17E-10	no
PRDM11	-0.239424646	-6.510345783	1.43E-10	9.27E-10	no
ENPEP	0.239393764	6.509454993	1.44E-10	9.32E-10	no
ZNF506	-0.239391826	-6.509399095	1.44E-10	9.32E-10	no
C21orf63	0.23936258	6.508555508	1.45E-10	9.36E-10	no
LOC653653	0.23935391	6.508305429	1.45E-10	9.37E-10	no
OPN3	0.239346827	6.508101128	1.45E-10	9.38E-10	no
S100A12	0.239309194	6.507015684	1.46E-10	9.44E-10	no
CKB	-0.239290313	-6.506471117	1.47E-10	9.47E-10	no
FYN	-0.239130432	-6.501860075	1.51E-10	9.75E-10	no
GFPT2	0.239102803	6.501063291	1.52E-10	9.79E-10	no
TACC2	-0.239099992	-6.500982225	1.52E-10	9.80E-10	no
UNKL	-0.239058687	-6.499791093	1.53E-10	9.87E-10	no
GRAP	0.239055467	6.499698233	1.53E-10	9.87E-10	no
NUAK2	0.239009869	6.498383359	1.55E-10	9.95E-10	no
ANKRD26	-0.238963236	-6.497038656	1.56E-10	1.00E-09	no
CHRNA4	-0.238951169	-6.496690717	1.56E-10	1.00E-09	no
DEDD2	0.23885478	6.493911459	1.59E-10	1.02E-09	no
LOC147727	-0.238812842	-6.492702297	1.60E-10	1.03E-09	no
AAMP	-0.238810201	-6.492626143	1.60E-10	1.03E-09	no
MCF2L2	-0.23866217	-6.488358422	1.65E-10	1.06E-09	no
PDHA1	-0.238603701	-6.486672873	1.66E-10	1.07E-09	no
ERGIC1	0.238560432	6.485425576	1.68E-10	1.08E-09	no
PPFIA3	-0.238546983	-6.485037898	1.68E-10	1.08E-09	no
MEIS3P1	0.238543454	6.484936164	1.68E-10	1.08E-09	no
ALPK3	0.238454159	6.482362263	1.71E-10	1.10E-09	no
DMC1	0.238361904	6.479703216	1.74E-10	1.11E-09	no
SDC1	0.238357611	6.479579478	1.74E-10	1.11E-09	no
IQCG	0.238264874	6.476906735	1.77E-10	1.13E-09	no
ACTN4	0.238187969	6.474690403	1.79E-10	1.15E-09	no
FSTL3	0.238117087	6.472647784	1.82E-10	1.16E-09	no
GTF2E1	0.238116913	6.472642777	1.82E-10	1.16E-09	no
TMEM188	0.238116666	6.472635658	1.82E-10	1.16E-09	no
LOC92249	-0.23809435	-6.471992574	1.82E-10	1.17E-09	no

CTTNBP2	-0.23806933	-6.471271597	1.83E-10	1.17E-09	no
THBS3	0.238049874	6.470710977	1.84E-10	1.17E-09	no
UBTF	-0.238048182	-6.470662226	1.84E-10	1.17E-09	no
TSSC1	-0.238026801	-6.47004613	1.85E-10	1.18E-09	no
SLC6A1	-0.237982773	-6.468777507	1.86E-10	1.19E-09	no
IGF1	0.237976906	6.46860847	1.86E-10	1.19E-09	no
REPS2	-0.237969967	-6.468408534	1.86E-10	1.19E-09	no
GLRX2	0.237875508	6.465686958	1.90E-10	1.21E-09	no
LGALS12	0.237872437	6.465598474	1.90E-10	1.21E-09	no
CD2BP2	-0.237865982	-6.465412485	1.90E-10	1.21E-09	no
IRAK2	0.237801496	6.463554643	1.92E-10	1.23E-09	no
ATP8A1	-0.237687633	-6.46027441	1.96E-10	1.25E-09	no
COL22A1	0.237654497	6.459319874	1.97E-10	1.26E-09	no
MAMLD1	-0.237645834	-6.459070324	1.98E-10	1.26E-09	no
PAR-SN	-0.237623503	-6.458427062	1.98E-10	1.26E-09	no
HSF2	-0.237600343	-6.457759913	1.99E-10	1.27E-09	no
RBM43	0.237573033	6.45697326	2.00E-10	1.27E-09	no
ARHGEF4	-0.237562497	-6.456669768	2.01E-10	1.28E-09	no
IKZF5	-0.237525839	-6.455613871	2.02E-10	1.28E-09	no
C10orf81	0.237450722	6.453450301	2.05E-10	1.30E-09	no
PVT1	0.237422793	6.452645884	2.06E-10	1.31E-09	no
POLR1D	0.237387186	6.451620385	2.07E-10	1.32E-09	no
SDHA	-0.237364182	-6.450957844	2.08E-10	1.32E-09	no
CYFIP2	-0.237331131	-6.450006008	2.09E-10	1.33E-09	no
TOR1A	0.237257562	6.447887303	2.12E-10	1.34E-09	no
GDAP1L1	-0.237216943	-6.446717602	2.13E-10	1.35E-09	no
C5orf53	-0.237181774	-6.445704839	2.15E-10	1.36E-09	no
LMAN2	0.237163233	6.445170936	2.16E-10	1.37E-09	no
AGPAT9	0.237144792	6.444639919	2.16E-10	1.37E-09	no
C22orf27	-0.237129242	-6.444192144	2.17E-10	1.37E-09	no
NCK2	0.237111792	6.443689686	2.18E-10	1.38E-09	no
PANK1	-0.237057175	-6.442117045	2.20E-10	1.39E-09	no
PC	-0.237056819	-6.442106802	2.20E-10	1.39E-09	no
ZNF321	0.237030871	6.441359669	2.21E-10	1.40E-09	no
EPHB4	0.237018778	6.441011471	2.21E-10	1.40E-09	no
TMEM107	0.237005155	6.440619239	2.22E-10	1.40E-09	no
MMP24	-0.236964909	-6.439460468	2.23E-10	1.41E-09	no
LIX1L	-0.236949262	-6.439009976	2.24E-10	1.42E-09	no
CCIN	0.236939227	6.438721072	2.24E-10	1.42E-09	no
SLC1A4	-0.236910928	-6.437906316	2.25E-10	1.42E-09	no
MMP9	0.236856454	6.436338036	2.28E-10	1.44E-09	no
BDNFOS	-0.236831343	-6.435615097	2.29E-10	1.44E-09	no
KCNQ2	-0.236804943	-6.4348551	2.30E-10	1.45E-09	no
HRH1	0.236803516	6.434814029	2.30E-10	1.45E-09	no
HIST1H3E	0.23676543	6.433717627	2.31E-10	1.46E-09	no
ACAP3	-0.236762672	-6.433638235	2.32E-10	1.46E-09	no
NKIRAS1	-0.236752511	-6.433345712	2.32E-10	1.46E-09	no
KIAA0495	0.236735588	6.432858561	2.33E-10	1.47E-09	no
TMEM151B	-0.23668577	-6.431424501	2.35E-10	1.48E-09	no
WHSC2	-0.236662158	-6.43074484	2.36E-10	1.48E-09	no
PLEKHF2	0.236632524	6.429891816	2.37E-10	1.49E-09	no

ZNF292	-0.236626125	-6.429707641	2.37E-10	1.49E-09	no
SCD5	-0.236556145	-6.427693377	2.40E-10	1.51E-09	no
DBN1	-0.236480203	-6.425507635	2.44E-10	1.53E-09	no
FAM46A	0.236445012	6.424494808	2.45E-10	1.54E-09	no
TRAPPC9	-0.236425938	-6.423945864	2.46E-10	1.55E-09	no
SGSH	0.236408937	6.423456584	2.47E-10	1.55E-09	no
MCM3APAS	-0.236387796	-6.422848151	2.48E-10	1.56E-09	no
ARL13B	0.236360085	6.422050671	2.49E-10	1.56E-09	no
KLHDC2	-0.236345636	-6.421634875	2.49E-10	1.57E-09	no
C3orf59	-0.236274045	-6.419574658	2.53E-10	1.59E-09	no
LOC644538	0.23626271	6.419248494	2.53E-10	1.59E-09	no
TP53INP1	0.236249172	6.418858918	2.54E-10	1.59E-09	no
PNRC2	0.236232129	6.418368485	2.55E-10	1.60E-09	no
COQ3	-0.236216747	-6.417925873	2.55E-10	1.60E-09	no
CD163L1	0.23618717	6.417074801	2.57E-10	1.61E-09	no
EPYC	0.23617973	6.416860706	2.57E-10	1.61E-09	no
PIGC	0.236162271	6.416358332	2.58E-10	1.61E-09	no
G0S2	0.236144308	6.41584147	2.59E-10	1.62E-09	no
SACM1L	0.236085423	6.41414716	2.61E-10	1.63E-09	no
CAPN1	0.236085048	6.414136375	2.61E-10	1.63E-09	no
QSOX2	-0.236073163	-6.413794423	2.62E-10	1.64E-09	no
SLC9A6	-0.236041583	-6.412885803	2.63E-10	1.65E-09	no
RAP1B	0.235997654	6.411621938	2.65E-10	1.66E-09	no
ZNF696	-0.235994125	-6.411520404	2.66E-10	1.66E-09	no
FEM1A	-0.235964075	-6.410655851	2.67E-10	1.67E-09	no
FCAR	0.235934797	6.409813533	2.68E-10	1.68E-09	no
LEP	0.235910671	6.409119463	2.70E-10	1.68E-09	no
DIP2C	-0.235881335	-6.408275512	2.71E-10	1.69E-09	no
PF4V1	0.235808458	6.406179059	2.75E-10	1.71E-09	no
FBXO22OS	0.235807582	6.406153841	2.75E-10	1.71E-09	no
GDAP2	0.235772019	6.405130848	2.76E-10	1.72E-09	no
NRARP	-0.235759036	-6.404757386	2.77E-10	1.73E-09	no
SLC26A2	0.235734297	6.404045751	2.78E-10	1.73E-09	no
TUT1	-0.235723871	-6.403745848	2.79E-10	1.74E-09	no
AAGAB	0.235661304	6.401946188	2.82E-10	1.75E-09	no
ATP13A3	0.235649653	6.401611055	2.82E-10	1.76E-09	no
ALG1	0.235609791	6.400464511	2.84E-10	1.77E-09	no
MAP1LC3C	0.235593145	6.399985742	2.85E-10	1.77E-09	no
TMEM59	0.235590989	6.399923731	2.85E-10	1.77E-09	no
MXRA5	0.235536335	6.398351841	2.88E-10	1.79E-09	no
LRP4	-0.23553542	-6.398325501	2.88E-10	1.79E-09	no
CLEC16A	-0.23552578	-6.398048253	2.89E-10	1.79E-09	no
PPP1R3E	-0.235513129	-6.397684408	2.89E-10	1.80E-09	no
MANF	0.23551145	6.397636136	2.89E-10	1.80E-09	no
DENND5B	-0.235485416	-6.396887385	2.91E-10	1.80E-09	no
C11orf95	-0.235448811	-6.395834678	2.93E-10	1.82E-09	no
FBXL13	0.235369456	6.393552586	2.97E-10	1.84E-09	no
OLFML1	0.235343406	6.39280347	2.98E-10	1.85E-09	no
PGLYRP4	0.235323431	6.392229081	2.99E-10	1.86E-09	no
WDR24	-0.235280113	-6.39098344	3.02E-10	1.87E-09	no
TMED10	0.235268429	6.390647461	3.02E-10	1.87E-09	no

ATP6V0E2	-0.235192689	-6.388469634	3.06E-10	1.90E-09	no
FOXO4	-0.235159732	-6.387522039	3.08E-10	1.91E-09	no
NUDT19	0.235141698	6.387003504	3.09E-10	1.91E-09	no
DPY19L1	0.235133985	6.386781739	3.10E-10	1.92E-09	no
GGT5	0.235117769	6.386315501	3.10E-10	1.92E-09	no
ZNF423	-0.235085278	-6.385381363	3.12E-10	1.93E-09	no
C7orf23	0.235074592	6.385074122	3.13E-10	1.93E-09	no
RNF145	0.235044487	6.384208597	3.14E-10	1.94E-09	no
B4GALT5	0.235020868	6.383529536	3.16E-10	1.95E-09	no
TAGAP	0.235006471	6.383115633	3.17E-10	1.96E-09	no
LOX	0.234898105	6.380000345	3.23E-10	1.99E-09	no
PROK2	0.234830209	6.378048587	3.27E-10	2.02E-09	no
ITGBL1	0.234816868	6.377665088	3.27E-10	2.02E-09	no
RAB6B	-0.234805178	-6.37732905	3.28E-10	2.02E-09	no
ZNF197	-0.234776439	-6.376502974	3.30E-10	2.03E-09	no
ZC3H7B	-0.234707615	-6.374524714	3.34E-10	2.06E-09	no
C14orf169	0.234692055	6.374077486	3.35E-10	2.06E-09	no
NGDN	-0.234680814	-6.3737544	3.35E-10	2.07E-09	no
TCTN1	0.234611813	6.371771212	3.40E-10	2.09E-09	no
ELP2P	-0.234585894	-6.371026281	3.41E-10	2.10E-09	no
KCNB1	-0.234584066	-6.370973753	3.41E-10	2.10E-09	no
ZBTB12	-0.234519188	-6.369109173	3.45E-10	2.12E-09	no
TSC22D1	-0.234427935	-6.366486787	3.51E-10	2.16E-09	no
PTCD3	-0.234360182	-6.36453981	3.55E-10	2.18E-09	no
PPP1CB	0.234313365	6.363194539	3.58E-10	2.20E-09	no
FMO1	0.234297277	6.362732279	3.59E-10	2.21E-09	no
EMP2	0.234294718	6.362658726	3.59E-10	2.21E-09	no
TRPM7	0.23424256	6.361160063	3.62E-10	2.23E-09	no
BCAR1	-0.234223875	-6.360623184	3.64E-10	2.23E-09	no
ZNF91	-0.234180998	-6.359391235	3.66E-10	2.25E-09	no
TRIP10	0.234143947	6.358326732	3.69E-10	2.26E-09	no
FGGY	0.234064399	6.356041319	3.74E-10	2.30E-09	no
GPR45	-0.234056929	-6.35582672	3.75E-10	2.30E-09	no
CLN5	0.234034868	6.355192948	3.76E-10	2.31E-09	no
TSEN2	-0.234012749	-6.354557503	3.77E-10	2.31E-09	no
FLJ90757	-0.234009941	-6.354476817	3.78E-10	2.31E-09	no
DCAF7	-0.234000506	-6.354205788	3.78E-10	2.32E-09	no
PLS3	0.233982086	6.353676623	3.80E-10	2.32E-09	no
REPIN1	-0.233967771	-6.353265377	3.80E-10	2.33E-09	no
ASB1	-0.233963204	-6.353134183	3.81E-10	2.33E-09	no
SPOCD1	0.233944505	6.352597048	3.82E-10	2.34E-09	no
UBE2D3	0.233901597	6.351364462	3.85E-10	2.35E-09	no
PNMAL1	-0.233886866	-6.350941312	3.86E-10	2.36E-09	no
MEF2A	0.233880824	6.350767745	3.86E-10	2.36E-09	no
ZNF425	-0.233839755	-6.349588057	3.89E-10	2.38E-09	no
NR1H3	0.233827915	6.349247953	3.90E-10	2.38E-09	no
ATP1A3	-0.233823969	-6.349134614	3.90E-10	2.38E-09	no
MTMR14	0.23382341	6.349118567	3.90E-10	2.38E-09	no
TM2D3	-0.233799002	-6.348417457	3.92E-10	2.39E-09	no
NDUFV1	-0.233778587	-6.347831099	3.93E-10	2.40E-09	no
EFNB2	0.233753418	6.347108155	3.95E-10	2.41E-09	no

TBC1D16	0.233720564	6.346164517	3.97E-10	2.42E-09	no
ITGA9	0.233717666	6.346081297	3.98E-10	2.42E-09	no
LBXCOR1	-0.233690922	-6.345313172	4.00E-10	2.44E-09	no
FRMD4B	0.233679813	6.344994102	4.00E-10	2.44E-09	no
TMCC1	-0.233590855	-6.342439238	4.07E-10	2.48E-09	no
NQO1	0.233566423	6.341737586	4.08E-10	2.49E-09	no
SAFB2	-0.233562823	-6.341634216	4.09E-10	2.49E-09	no
CCDC152	0.233511113	6.340149203	4.12E-10	2.51E-09	no
NLGN3	-0.233458068	-6.338625931	4.16E-10	2.53E-09	no
MFSD11	0.233427235	6.337740533	4.19E-10	2.55E-09	no
SYT17	-0.233391234	-6.336706751	4.21E-10	2.56E-09	no
FSIP1	0.233370184	6.33610232	4.23E-10	2.57E-09	no
TTYH1	-0.233331888	-6.335002688	4.26E-10	2.59E-09	no
CAMK2N2	-0.233316623	-6.334564391	4.27E-10	2.59E-09	no
NBEA	-0.233287238	-6.333720663	4.29E-10	2.61E-09	no
POM121L8P	0.23326566	6.333101095	4.31E-10	2.61E-09	no
2-Mar	0.233258179	6.332886327	4.31E-10	2.62E-09	no
RFXAP	-0.233226067	-6.331964322	4.34E-10	2.63E-09	no
ABCB4	0.233217113	6.331707248	4.34E-10	2.63E-09	no
C2orf85	-0.233171127	-6.330386959	4.38E-10	2.66E-09	no
VAMP2	-0.233133971	-6.32932024	4.41E-10	2.67E-09	no
NUP37	0.233123727	6.329026149	4.42E-10	2.68E-09	no
NOS3	0.233122024	6.328977239	4.42E-10	2.68E-09	no
TRIM37	-0.233109804	-6.328626416	4.43E-10	2.68E-09	no
KLF15	-0.233109046	-6.32860466	4.43E-10	2.68E-09	no
MANEAL	-0.233047605	-6.326840798	4.47E-10	2.71E-09	no
SUSD4	-0.233019216	-6.326025845	4.50E-10	2.72E-09	no
AKAP13	0.232976843	6.324809457	4.53E-10	2.74E-09	no
C1orf226	0.232934682	6.323599204	4.56E-10	2.76E-09	no
C19orf10	0.232862084	6.321515349	4.62E-10	2.80E-09	no
GRIK2	-0.232796616	-6.319636222	4.68E-10	2.83E-09	no
B4GALNT4	-0.232777166	-6.319077972	4.69E-10	2.84E-09	no
GPM6B	-0.232753211	-6.318390428	4.71E-10	2.85E-09	no
NR5A2	0.232734814	6.317862425	4.73E-10	2.85E-09	no
HIPK2	-0.232733199	-6.317816076	4.73E-10	2.85E-09	no
TM7SF4	0.232727844	6.31766239	4.73E-10	2.86E-09	no
KIAA0195	-0.232685778	-6.316455071	4.77E-10	2.88E-09	no
FOXO3B	-0.232665033	-6.31585972	4.79E-10	2.89E-09	no
ZNF250	-0.232597879	-6.313932487	4.84E-10	2.92E-09	no
KLHDC1	-0.232548835	-6.312525046	4.88E-10	2.94E-09	no
LMLN	0.232526631	6.311887875	4.90E-10	2.96E-09	no
CNIH4	0.232457862	6.309914493	4.96E-10	2.99E-09	no
FAM84B	-0.232438782	-6.309366996	4.98E-10	3.00E-09	no
CDKN1B	-0.232362764	-6.307185779	5.05E-10	3.04E-09	no
SIGLEC12	0.232352597	6.306894061	5.06E-10	3.04E-09	no
C21orf88	0.232332473	6.306316649	5.07E-10	3.05E-09	no
CMTM8	0.232308959	6.305641986	5.09E-10	3.06E-09	no
C19orf38	0.232239524	6.303649808	5.16E-10	3.10E-09	no
POGZ	-0.232203763	-6.30262383	5.19E-10	3.12E-09	no
ACADS	0.232191913	6.302283876	5.20E-10	3.13E-09	no
SNRPN	-0.232106108	-6.29982227	5.28E-10	3.17E-09	no

POP4	0.23210211	6.299707565	5.28E-10	3.17E-09	no
SPTBN4	-0.232100917	-6.299673345	5.28E-10	3.17E-09	no
FTO	-0.232095151	-6.299507942	5.29E-10	3.17E-09	no
FAM151B	0.232069288	6.298765999	5.31E-10	3.19E-09	no
TBC1D17	-0.232044185	-6.298045884	5.34E-10	3.20E-09	no
CROT	0.232006309	6.296959375	5.37E-10	3.22E-09	no
TNFAIP6	0.231977355	6.29612882	5.40E-10	3.24E-09	no
CKLF	0.231959606	6.295619705	5.42E-10	3.25E-09	no
AKNA	0.231944309	6.295180942	5.43E-10	3.25E-09	no
MAP3K14	0.231931267	6.294806824	5.44E-10	3.26E-09	no
GTF3C5	-0.231872246	-6.293113932	5.50E-10	3.29E-09	no
TSPAN3	-0.231870622	-6.293067357	5.50E-10	3.29E-09	no
NTRK3	-0.231842182	-6.292251627	5.53E-10	3.31E-09	no
MRPS31	-0.231789039	-6.290727413	5.58E-10	3.34E-09	no
SV2A	-0.231767273	-6.290103142	5.60E-10	3.35E-09	no
RP2	0.231758616	6.28985485	5.61E-10	3.35E-09	no
LDHA	0.231703399	6.288271269	5.66E-10	3.39E-09	no
MTMR15	-0.231652956	-6.286824626	5.71E-10	3.41E-09	no
ALDH1A2	0.231631312	6.286203943	5.74E-10	3.43E-09	no
KIAA0652	-0.231561161	-6.28419223	5.81E-10	3.47E-09	no
APLN	0.231542657	6.283661605	5.83E-10	3.48E-09	no
CGREF1	-0.231531452	-6.283340317	5.84E-10	3.48E-09	no
SLC25A36	-0.231519639	-6.283001562	5.85E-10	3.49E-09	no
ProSAPiP1	-0.231495809	-6.282318258	5.87E-10	3.50E-09	no
FRMPD1	-0.231451348	-6.281043374	5.92E-10	3.53E-09	no
ARMCX1	-0.231406332	-6.279752606	5.97E-10	3.56E-09	no
PALM	-0.231401284	-6.279607883	5.97E-10	3.56E-09	no
NKAIN1	-0.231384326	-6.279121642	5.99E-10	3.57E-09	no
GPR39	0.231334229	6.277685269	6.04E-10	3.60E-09	no
MLEC	0.231326646	6.277467857	6.05E-10	3.60E-09	no
MBOAT2	-0.23132482	-6.277415511	6.05E-10	3.60E-09	no
ZSWIM5	-0.231285223	-6.276280243	6.09E-10	3.63E-09	no
NLGN1	-0.23115497	-6.272546044	6.23E-10	3.71E-09	no
ZNF510	-0.231091054	-6.270713786	6.30E-10	3.75E-09	no
CACNA2D2	-0.231050233	-6.269543623	6.35E-10	3.77E-09	no
SLC25A42	-0.231032832	-6.269044819	6.37E-10	3.78E-09	no
CXorf50B	-0.231031628	-6.269010298	6.37E-10	3.78E-09	no
FAM26E	0.231028108	6.268909404	6.37E-10	3.79E-09	no
EGF	0.230919075	6.265784114	6.49E-10	3.86E-09	no
C17orf57	0.230908173	6.265471635	6.51E-10	3.86E-09	no
NOG	-0.230856514	-6.263990997	6.57E-10	3.90E-09	no
PLXDC1	0.230779276	6.261777336	6.65E-10	3.95E-09	no
TAF13	0.230726424	6.260262653	6.72E-10	3.98E-09	no
OST4	0.230677933	6.258872985	6.77E-10	4.02E-09	no
LOC285419	0.230657955	6.258300478	6.80E-10	4.03E-09	no
BTBD2	-0.230636749	-6.257692784	6.82E-10	4.04E-09	no
JARID2	-0.230587815	-6.256290524	6.88E-10	4.08E-09	no
ZYX	0.23058534	6.256219602	6.88E-10	4.08E-09	no
DCAF16	-0.230541632	-6.254967148	6.94E-10	4.11E-09	no
LMO2	0.23052598	6.254518653	6.95E-10	4.12E-09	no
ASF1A	-0.230510745	-6.254082101	6.97E-10	4.13E-09	no

TRMT11	-0.230463387	-6.252725134	7.03E-10	4.16E-09	no
GPRC5A	0.230460702	6.252648194	7.03E-10	4.16E-09	no
SMYD5	-0.230447464	-6.252268907	7.05E-10	4.17E-09	no
8-Sep	-0.230446388	-6.252238053	7.05E-10	4.17E-09	no
C16orf5	-0.230431825	-6.251820811	7.07E-10	4.18E-09	no
POM121L9P	0.230351486	6.249518966	7.17E-10	4.24E-09	no
PTGR2	-0.230332784	-6.248983158	7.19E-10	4.25E-09	no
NAALADL1	0.230263149	6.24698813	7.28E-10	4.30E-09	no
GJA4	0.230247195	6.246531084	7.30E-10	4.31E-09	no
RCAN1	0.23023688	6.246235565	7.31E-10	4.32E-09	no
SCO1	0.230203566	6.245281205	7.35E-10	4.34E-09	no
AGMAT	0.230193099	6.244981342	7.37E-10	4.35E-09	no
TRAPPC3	0.230138341	6.243412701	7.44E-10	4.39E-09	no
DGCR14	-0.230074785	-6.241592104	7.52E-10	4.43E-09	no
CNTFR	-0.230070573	-6.241471465	7.53E-10	4.43E-09	no
C11orf49	-0.230069203	-6.241432229	7.53E-10	4.43E-09	no
KCNE1	0.229999789	6.239443918	7.62E-10	4.49E-09	no
UTP15	0.229958059	6.238248675	7.67E-10	4.52E-09	no
HMGCR	-0.22994948	-6.238002933	7.69E-10	4.52E-09	no
ZNF740	-0.229938188	-6.237679518	7.70E-10	4.53E-09	no
GPT2	-0.22989522	-6.236448841	7.76E-10	4.56E-09	no
MDK	0.229863594	6.235543053	7.80E-10	4.59E-09	no
TXNDC12	0.229854544	6.235283846	7.81E-10	4.59E-09	no
CNTNAP3	0.229842239	6.234931427	7.83E-10	4.60E-09	no
PLVAP	0.229835524	6.234739132	7.84E-10	4.61E-09	no
TFPI	0.229762552	6.232649281	7.94E-10	4.66E-09	no
FOXC1	0.22974852	6.232247427	7.96E-10	4.67E-09	no
PRMT7	-0.229746068	-6.232177208	7.96E-10	4.67E-09	no
WAC	-0.229745328	-6.232156011	7.96E-10	4.67E-09	no
B3GNT3	0.229668739	6.229962723	8.07E-10	4.73E-09	no
CPA3	0.229617886	6.228506508	8.14E-10	4.77E-09	no
MAML3	0.229612488	6.228351921	8.15E-10	4.78E-09	no
PCCA	-0.229572803	-6.227215546	8.20E-10	4.81E-09	no
TXN	0.229564047	6.226964824	8.22E-10	4.82E-09	no
C17orf86	-0.229527246	-6.225911067	8.27E-10	4.84E-09	no
VKORC1	0.229507127	6.22533502	8.30E-10	4.86E-09	no
SCD	-0.229485642	-6.224719826	8.33E-10	4.88E-09	no
ING5	-0.229473582	-6.224374526	8.35E-10	4.89E-09	no
CNTN3	-0.229451737	-6.223749056	8.38E-10	4.90E-09	no
TMED8	-0.229433571	-6.223228936	8.40E-10	4.92E-09	no
LY6E	0.229403847	6.22237789	8.45E-10	4.94E-09	no
EPHB1	-0.229306933	-6.219603278	8.59E-10	5.02E-09	no
PSME1	0.22930645	6.219589449	8.59E-10	5.02E-09	no
KIAA1033	0.229299899	6.219401887	8.60E-10	5.03E-09	no
C21orf71	-0.229230155	-6.217405277	8.71E-10	5.09E-09	no
OTUD5	-0.229203323	-6.216637145	8.75E-10	5.11E-09	no
BAIAP2L1	0.229045725	6.212125889	8.99E-10	5.25E-09	no
ELAVL4	-0.229037758	-6.211897859	9.00E-10	5.25E-09	no
SART1	-0.229017057	-6.211305325	9.03E-10	5.27E-09	no
TMEM165	0.229014727	6.211238644	9.04E-10	5.27E-09	no
BAP1	-0.228897447	-6.207881912	9.22E-10	5.38E-09	no

TMED3	0.228859981	6.206809638	9.28E-10	5.41E-09	no
MPP2	-0.228857599	-6.206741466	9.28E-10	5.41E-09	no
LRRN1	-0.228849624	-6.206513218	9.30E-10	5.42E-09	no
SNAI3	0.228816938	6.205577781	9.35E-10	5.45E-09	no
IGHMBP2	-0.228796364	-6.204988992	9.38E-10	5.46E-09	no
KREMEN1	0.228793985	6.204920883	9.39E-10	5.46E-09	no
TMEM100	-0.22875434	-6.203786354	9.45E-10	5.50E-09	no
FRZB	0.228722363	6.202871254	9.50E-10	5.53E-09	no
NKRF	-0.228684767	-6.201795399	9.56E-10	5.56E-09	no
ACTR6	-0.22863698	-6.200427946	9.64E-10	5.61E-09	no
CNNM3	-0.228594709	-6.199218389	9.71E-10	5.65E-09	no
DHX58	0.228592753	6.199162421	9.72E-10	5.65E-09	no
ZNF493	-0.22856378	-6.198333385	9.77E-10	5.67E-09	no
IRF7	0.228542452	6.197723122	9.80E-10	5.69E-09	no
PPT2	-0.228515991	-6.196966025	9.85E-10	5.72E-09	no
SPATA18	0.228511726	6.19684399	9.85E-10	5.72E-09	no
FABP5	0.228491415	6.196262833	9.89E-10	5.74E-09	no
MGC21881	-0.228462734	-6.195442242	9.94E-10	5.76E-09	no
DOPEY1	-0.228396576	-6.193549414	1.01E-09	5.83E-09	no
ZNF860	0.228395188	6.193509687	1.01E-09	5.83E-09	no
SPG7	-0.2283847	-6.193209638	1.01E-09	5.84E-09	no
XAB2	-0.228354862	-6.192356001	1.01E-09	5.87E-09	no
CSF1	0.22834421	6.192051232	1.01E-09	5.88E-09	no
HDGFRP2	-0.228331364	-6.191683727	1.02E-09	5.89E-09	no
SNHG1	-0.228296098	-6.190674849	1.02E-09	5.92E-09	no
DPF2	-0.22821437	-6.188336841	1.04E-09	6.00E-09	no
SFRS18	-0.22816982	-6.187062451	1.05E-09	6.05E-09	no
ZDHHC1	0.228135661	6.186085344	1.05E-09	6.08E-09	no
TMEM63C	-0.228035395	-6.183217374	1.07E-09	6.19E-09	no
LOC285830	0.227897273	6.179266934	1.10E-09	6.33E-09	no
STX1B	-0.227858225	-6.178150188	1.10E-09	6.37E-09	no
ZNF534	-0.227708835	-6.173878043	1.13E-09	6.54E-09	no
ABCA9	0.227698092	6.173570843	1.13E-09	6.55E-09	no
RNF39	-0.227696933	-6.173537691	1.13E-09	6.55E-09	no
TMEM19	0.227606546	6.170953123	1.15E-09	6.65E-09	no
TMEM156	0.227596679	6.170670976	1.15E-09	6.66E-09	no
DR1	0.227542292	6.169115913	1.16E-09	6.72E-09	no
KLHDC5	-0.227536988	-6.168964242	1.17E-09	6.72E-09	no
HAVCR1	0.227512007	6.168250002	1.17E-09	6.75E-09	no
NID1	0.227498927	6.167876026	1.17E-09	6.76E-09	no
PDE9A	-0.227495494	-6.167777867	1.17E-09	6.76E-09	no
CNPY3	0.227452663	6.166553302	1.18E-09	6.81E-09	no
TSPYL2	-0.227427158	-6.165824088	1.19E-09	6.84E-09	no
HUS1	0.227416356	6.165515269	1.19E-09	6.85E-09	no
DHDDS	0.227399026	6.165019816	1.19E-09	6.87E-09	no
IL1A	0.227282659	6.161693075	1.22E-09	7.00E-09	no
ATP7B	-0.22727503	-6.161474984	1.22E-09	7.01E-09	no
PTGFRN	0.227269837	6.161326559	1.22E-09	7.01E-09	no
WBP2	-0.22725929	-6.161025052	1.22E-09	7.03E-09	no
CCL18	0.227257184	6.160964829	1.22E-09	7.03E-09	no
KIAA1737	-0.227143629	-6.157718848	1.25E-09	7.16E-09	no

TMEFF2	-0.227133205	-6.157420863	1.25E-09	7.17E-09	no
LPHN3	-0.227101957	-6.156527682	1.26E-09	7.21E-09	no
HYAL2	0.227046166	6.154933035	1.27E-09	7.28E-09	no
GABRA3	-0.226964199	-6.152590329	1.29E-09	7.38E-09	no
PSMD9	0.226943309	6.151993263	1.29E-09	7.40E-09	no
MOAP1	-0.226940969	-6.151926403	1.29E-09	7.40E-09	no
NLE1	-0.226937459	-6.15182609	1.29E-09	7.40E-09	no
RHBDD1	0.226928596	6.151572771	1.29E-09	7.41E-09	no
CHD7	-0.226824978	-6.148611519	1.32E-09	7.54E-09	no
RNF187	-0.22680117	-6.147931133	1.32E-09	7.57E-09	no
TRO	-0.226773874	-6.147151101	1.33E-09	7.61E-09	no
GPN1	0.226762847	6.146835986	1.33E-09	7.62E-09	no
APITD1	0.226730793	6.145920029	1.34E-09	7.66E-09	no
RASSF8	0.226675699	6.144345721	1.35E-09	7.73E-09	no
FUBP3	-0.226584103	-6.141728474	1.37E-09	7.85E-09	no
RBPMS	0.226576851	6.141521257	1.37E-09	7.85E-09	no
TEAD3	0.226488487	6.138996555	1.39E-09	7.97E-09	no
HMGCS1	-0.226464689	-6.138316652	1.40E-09	8.00E-09	no
FAM120C	-0.226436158	-6.137501529	1.41E-09	8.04E-09	no
KIAA1147	-0.226364779	-6.135462309	1.42E-09	8.13E-09	no
TUBGCP2	-0.226361929	-6.135380879	1.42E-09	8.14E-09	no
CLIP1	0.226329243	6.134447102	1.43E-09	8.18E-09	no
SAPS2	-0.226310839	-6.133921349	1.44E-09	8.20E-09	no
THADA	0.226308599	6.133857374	1.44E-09	8.20E-09	no
DAGLB	0.226265065	6.132613748	1.45E-09	8.26E-09	no
ST8SIA3	-0.226260366	-6.132479531	1.45E-09	8.27E-09	no
ZEB1	-0.226239253	-6.131876406	1.45E-09	8.29E-09	no
RAB13	0.226236886	6.131808786	1.45E-09	8.30E-09	no
FLJ35776	0.226228307	6.13156374	1.46E-09	8.31E-09	no
SLC17A5	0.226170659	6.12991702	1.47E-09	8.38E-09	no
SEC16B	0.22616468	6.129746249	1.47E-09	8.39E-09	no
PAMR1	0.226127407	6.128681585	1.48E-09	8.44E-09	no
SIK3	-0.226030392	-6.125910621	1.51E-09	8.58E-09	no
EEPD1	-0.226013987	-6.12544207	1.51E-09	8.60E-09	no
LRRC47	-0.22600482	-6.125180243	1.51E-09	8.61E-09	no
FAM188B	0.225977144	6.124389809	1.52E-09	8.65E-09	no
SPSB3	-0.225914703	-6.122606533	1.54E-09	8.74E-09	no
SH3GL2	-0.225870866	-6.121354597	1.55E-09	8.80E-09	no
AKAP8L	-0.225854204	-6.120878753	1.55E-09	8.83E-09	no
ARPC4	0.225848994	6.120729976	1.55E-09	8.83E-09	no
LOC647979	-0.225799517	-6.119317052	1.57E-09	8.90E-09	no
CXCR1	0.225798945	6.119300718	1.57E-09	8.90E-09	no
C9orf25	-0.225797808	-6.119268255	1.57E-09	8.90E-09	no
NAGLU	0.225736802	6.117526181	1.58E-09	8.99E-09	no
TCEAL5	-0.225724853	-6.117184975	1.59E-09	9.01E-09	no
PRRX1	0.225711154	6.1167938	1.59E-09	9.03E-09	no
ZNF609	-0.225663581	-6.115435364	1.60E-09	9.10E-09	no
C19orf59	0.225628086	6.114421856	1.61E-09	9.15E-09	no
TOX3	-0.225615132	-6.114051987	1.62E-09	9.17E-09	no
ACTR1A	-0.225574098	-6.112880381	1.63E-09	9.23E-09	no
HELB	0.225556582	6.112380246	1.63E-09	9.25E-09	no

SLC2A4RG	0.225546881	6.11210328	1.64E-09	9.27E-09	no
SMC5	0.225458175	6.109570667	1.66E-09	9.40E-09	no
CLCN6	-0.225443683	-6.109156912	1.67E-09	9.42E-09	no
C1orf174	0.225420387	6.108491848	1.67E-09	9.46E-09	no
CRTAC1	-0.225414049	-6.108310915	1.67E-09	9.47E-09	no
KCNJ9	-0.225411239	-6.108230672	1.67E-09	9.47E-09	no
KHSRP	-0.225399556	-6.107897156	1.68E-09	9.48E-09	no
PDP2	-0.225370336	-6.107062974	1.69E-09	9.53E-09	no
DUSP6	0.225367951	6.106994869	1.69E-09	9.53E-09	no
MTHFD1L	0.225329642	6.105901257	1.70E-09	9.59E-09	no
RGS16	0.22524918	6.10360438	1.72E-09	9.72E-09	no
TOM1L1	0.225184238	6.101750609	1.74E-09	9.82E-09	no
FGFR1	0.225176443	6.101528103	1.74E-09	9.83E-09	no
C1orf91	0.225111743	6.099681364	1.76E-09	9.94E-09	no
WRNIP1	-0.225078163	-6.098722922	1.77E-09	9.99E-09	no
STK25	-0.22506441	-6.098330377	1.78E-09	1.00E-08	no
EDC3	-0.225018997	-6.097034225	1.79E-09	1.01E-08	no
TMEM220	0.224949771	6.095058527	1.81E-09	1.02E-08	no
PSD	-0.224933361	-6.094590187	1.82E-09	1.02E-08	no
ZFP36	0.224928787	6.094459646	1.82E-09	1.02E-08	no
ARSJ	0.224925195	6.094357148	1.82E-09	1.02E-08	no
C5orf30	-0.224913397	-6.094020441	1.82E-09	1.03E-08	no
KIAA1539	0.224893306	6.093447067	1.83E-09	1.03E-08	no
CMPK2	0.224868534	6.09274013	1.84E-09	1.03E-08	no
DNAJC7	-0.224859649	-6.092486566	1.84E-09	1.03E-08	no
STK38	0.224836563	6.091827752	1.85E-09	1.04E-08	no
JUNB	0.224829211	6.091617968	1.85E-09	1.04E-08	no
PBX1	-0.224823691	-6.091460425	1.85E-09	1.04E-08	no
TSPYL3	-0.224814321	-6.091193036	1.85E-09	1.04E-08	no
C15orf17	-0.224810439	-6.091082256	1.85E-09	1.04E-08	no
OSBPL10	0.224768577	6.089887688	1.87E-09	1.05E-08	no
HOXA1	0.224721997	6.088558514	1.88E-09	1.06E-08	no
RPAP1	-0.224708045	-6.088160399	1.89E-09	1.06E-08	no
LOC100133545	-0.224690146	-6.087649684	1.89E-09	1.06E-08	no
RECK	0.224680921	6.087386434	1.89E-09	1.06E-08	no
GANAB	0.224643562	6.08632046	1.91E-09	1.07E-08	no
ADAM17	0.22461639	6.085545153	1.92E-09	1.07E-08	no
FBXO48	0.224606323	6.08525792	1.92E-09	1.08E-08	no
GLRB	-0.224600697	-6.085097401	1.92E-09	1.08E-08	no
LOC644172	-0.224571332	-6.084259575	1.93E-09	1.08E-08	no
ZNF638	-0.22456983	-6.084216717	1.93E-09	1.08E-08	no
NPIP	-0.224543518	-6.083465999	1.94E-09	1.09E-08	no
SLC8A3	-0.224532774	-6.083159448	1.94E-09	1.09E-08	no
DGKI	-0.224493579	-6.082041211	1.96E-09	1.09E-08	no
PELI3	-0.224476482	-6.081553426	1.96E-09	1.10E-08	no
CCPG1	0.224368323	6.078467785	2.00E-09	1.12E-08	no
CSNK1G1	0.224366662	6.078420401	2.00E-09	1.12E-08	no
FFAR2	0.224335491	6.077531179	2.01E-09	1.12E-08	no
TMEM25	-0.224151435	-6.072280968	2.07E-09	1.16E-08	no
FDFT1	-0.2241512	-6.072274279	2.07E-09	1.16E-08	no
ZFYVE27	-0.223966136	-6.06699601	2.14E-09	1.19E-08	no

FAM86B2	0.223917369	6.06560522	2.16E-09	1.20E-08	no
MAGI1	-0.223866433	-6.064152639	2.17E-09	1.21E-08	no
RRN3	-0.223833686	-6.063218772	2.19E-09	1.22E-08	no
PCYT1B	-0.223786751	-6.061880378	2.20E-09	1.23E-08	no
KIAA0430	-0.22373138	-6.060301431	2.22E-09	1.24E-08	no
CHI3L2	0.223694507	6.05925002	2.24E-09	1.25E-08	no
CCDC89	0.223683885	6.058947172	2.24E-09	1.25E-08	no
PMFBP1	0.223658456	6.058222089	2.25E-09	1.25E-08	no
SNX27	-0.223611512	-6.056883574	2.27E-09	1.26E-08	no
FTH1	0.223529572	6.054547358	2.30E-09	1.28E-08	no
SMAP2	0.223495176	6.053566703	2.31E-09	1.29E-08	no
GDF9	-0.223485917	-6.053302737	2.32E-09	1.29E-08	no
SSBP3	-0.223415982	-6.051308963	2.35E-09	1.31E-08	no
PABPC4L	0.223391742	6.050617909	2.35E-09	1.31E-08	no
HEY1	-0.223387487	-6.050496625	2.36E-09	1.31E-08	no
ASCL1	-0.223385332	-6.050435177	2.36E-09	1.31E-08	no
ARNT	0.223364243	6.049834	2.37E-09	1.32E-08	no
PRKD3	0.223340545	6.049158415	2.38E-09	1.32E-08	no
EPX	-0.223329675	-6.048848558	2.38E-09	1.32E-08	no
LRRC28	0.223271905	6.047201763	2.40E-09	1.33E-08	no
L3MBTL	-0.223259397	-6.046845227	2.41E-09	1.34E-08	no
RTKN	-0.223222261	-6.045786655	2.42E-09	1.35E-08	no
ZBTB8OS	0.223218016	6.045665671	2.42E-09	1.35E-08	no
POTEE	0.223138828	6.043408526	2.46E-09	1.36E-08	no
VWA1	0.223115782	6.042751657	2.47E-09	1.37E-08	no
SH3BP1	0.223077535	6.041661524	2.48E-09	1.38E-08	no
TAP1	0.223068618	6.041407386	2.49E-09	1.38E-08	no
FOXRED1	-0.223060862	-6.041186316	2.49E-09	1.38E-08	no
SPHK1	0.22303398	6.040420157	2.50E-09	1.39E-08	no
PTCRA	0.223000036	6.039452739	2.52E-09	1.39E-08	no
FN3K	-0.222921436	-6.0372127	2.55E-09	1.41E-08	no
DCUN1D3	0.222868319	6.035698985	2.57E-09	1.42E-08	no
CBLN1	-0.22279895	-6.033722195	2.60E-09	1.44E-08	no
PMP22	0.222743258	6.032135248	2.63E-09	1.45E-08	no
TCEAL3	-0.222678811	-6.030298863	2.65E-09	1.47E-08	no
ROR1	0.222672405	6.030116345	2.66E-09	1.47E-08	no
SPRY1	0.222611813	6.028389899	2.68E-09	1.48E-08	no
PPP1R3F	-0.222568259	-6.027148957	2.70E-09	1.49E-08	no
LRRTM2	-0.222556527	-6.026814702	2.71E-09	1.50E-08	no
UPF3A	-0.222547285	-6.026551405	2.71E-09	1.50E-08	no
CCDC101	-0.22250616	-6.025379712	2.73E-09	1.51E-08	no
LIMD2	0.222488232	6.024868961	2.74E-09	1.51E-08	no
MTFR1	0.222466302	6.024244189	2.75E-09	1.52E-08	no
RBP5	0.222393299	6.022164433	2.78E-09	1.54E-08	no
CHD8	-0.222366977	-6.021414585	2.80E-09	1.54E-08	no
KLF10	0.222300642	6.019524906	2.83E-09	1.56E-08	no
SMCR5	-0.222285212	-6.019085374	2.84E-09	1.56E-08	no
ALDH2	-0.222251068	-6.018112762	2.85E-09	1.57E-08	no
FAM82A2	-0.222234796	-6.01764926	2.86E-09	1.58E-08	no
FAM193A	-0.222229227	-6.017490619	2.86E-09	1.58E-08	no
LYRM7	-0.222193047	-6.016460072	2.88E-09	1.59E-08	no

TANC2	-0.222186605	-6.016276565	2.88E-09	1.59E-08	no
C7orf25	0.222166482	6.015703412	2.89E-09	1.59E-08	no
ST13	-0.222154692	-6.015367581	2.90E-09	1.59E-08	no
ERLIN1	0.222085803	6.013405472	2.93E-09	1.61E-08	no
ARHGAP17	0.221956619	6.009726254	3.00E-09	1.65E-08	no
LOC400696	0.221912053	6.008457061	3.02E-09	1.66E-08	no
TPBG	0.221857452	6.006902148	3.05E-09	1.67E-08	no
ARFGAP3	0.221846321	6.006585165	3.05E-09	1.68E-08	no
TDP2	0.221826424	6.006018559	3.06E-09	1.68E-08	no
RNF208	-0.221800096	-6.005268852	3.07E-09	1.69E-08	no
RUFY1	0.221753284	6.00393585	3.10E-09	1.70E-08	no
DPP4	0.221750293	6.003850685	3.10E-09	1.70E-08	no
NHLRC4	0.221734799	6.003409477	3.11E-09	1.71E-08	no
FAM13C	-0.221675834	-6.001730526	3.14E-09	1.72E-08	no
SPRN	-0.22166619	-6.001455923	3.14E-09	1.72E-08	no
RAB3D	0.221606055	5.999743699	3.18E-09	1.74E-08	no
NLN	0.221603293	5.999665058	3.18E-09	1.74E-08	no
MFSD5	0.221549231	5.998125844	3.21E-09	1.76E-08	no
C20orf203	-0.221516233	-5.997186366	3.22E-09	1.77E-08	no
FOXK2	-0.221510568	-5.997025081	3.23E-09	1.77E-08	no
BIK	0.221443089	5.995103964	3.26E-09	1.79E-08	no
LARS	-0.221429665	-5.994721821	3.27E-09	1.79E-08	no
EPB41L4A	-0.221353584	-5.992555952	3.31E-09	1.81E-08	no
SPAG1	0.221340451	5.992182078	3.32E-09	1.82E-08	no
LILRB5	0.221333111	5.991973136	3.32E-09	1.82E-08	no
MIPEP	0.221277223	5.990382216	3.35E-09	1.83E-08	no
DDHD1	-0.221209474	-5.98845374	3.39E-09	1.85E-08	no
DGCR6	-0.221195807	-5.988064721	3.40E-09	1.86E-08	no
MRGPRF	0.221144861	5.986614625	3.43E-09	1.87E-08	no
PREB	0.221104528	5.985466653	3.45E-09	1.89E-08	no
KCND2	-0.221064829	-5.98433674	3.47E-09	1.90E-08	no
NNMT	0.221050493	5.983928722	3.48E-09	1.90E-08	no
C3orf39	-0.221048142	-5.983861822	3.48E-09	1.90E-08	no
ALG3	0.221014123	5.982893617	3.50E-09	1.91E-08	no
THTPA	-0.220984727	-5.982056983	3.52E-09	1.92E-08	no
REEP2	-0.220904621	-5.979777245	3.57E-09	1.95E-08	no
TCERG1	-0.220786277	-5.976409539	3.64E-09	1.98E-08	no
DBC1	-0.22078605	-5.976403076	3.64E-09	1.98E-08	no
PDE2A	-0.22076253	-5.975733786	3.65E-09	1.99E-08	no
CAV2	0.220746806	5.975286354	3.66E-09	2.00E-08	no
NLRP3	0.220732698	5.974884924	3.67E-09	2.00E-08	no
ZFHX3	0.220718757	5.97448824	3.68E-09	2.00E-08	no
GARNL3	-0.220672836	-5.973181587	3.71E-09	2.02E-08	no
LACTB2	0.220527611	5.969049629	3.80E-09	2.07E-08	no
LTC4S	0.22052759	5.969049033	3.80E-09	2.07E-08	no
TBL2	0.220505331	5.968415747	3.81E-09	2.07E-08	no
PDPN	0.220498934	5.968233761	3.82E-09	2.08E-08	no
HGS	-0.220491325	-5.968017261	3.82E-09	2.08E-08	no
TFG	0.220429053	5.966245663	3.86E-09	2.10E-08	no
SLC35A4	0.220391611	5.965180513	3.89E-09	2.11E-08	no
DCLK2	-0.22035953	-5.964267867	3.91E-09	2.12E-08	no

LOC645323	-0.220342349	-5.963779128	3.92E-09	2.13E-08	no
NFIB	-0.220288664	-5.962251972	3.95E-09	2.15E-08	no
SMPD2	0.220282548	5.962077979	3.96E-09	2.15E-08	no
MBD1	-0.220270034	-5.961722009	3.96E-09	2.15E-08	no
MFF	-0.220206756	-5.959922069	4.01E-09	2.17E-08	no
ZNF777	-0.220180648	-5.959179466	4.02E-09	2.18E-08	no
FLJ45244	-0.220174909	-5.959016208	4.03E-09	2.18E-08	no
SLFN11	0.220124606	5.957585443	4.06E-09	2.20E-08	no
YPEL4	-0.220075948	-5.956201496	4.09E-09	2.22E-08	no
PHF10	-0.220036355	-5.955075398	4.12E-09	2.23E-08	no
ZNF512	-0.220013857	-5.954435556	4.14E-09	2.24E-08	no
LUC7L3	-0.219999397	-5.954024291	4.15E-09	2.25E-08	no
PNO1	0.219995772	5.953921183	4.15E-09	2.25E-08	no
C9orf3	0.219958594	5.952863859	4.17E-09	2.26E-08	no
PHC1	-0.219934496	-5.952178517	4.19E-09	2.27E-08	no
LOC284441	0.219887552	5.950843495	4.22E-09	2.28E-08	no
IFNAR1	0.219831605	5.949252521	4.26E-09	2.31E-08	no
MOSC1	-0.219814834	-5.948775584	4.28E-09	2.31E-08	no
PEX13	0.219761887	5.947269995	4.31E-09	2.33E-08	no
RGMB	-0.219745565	-5.946805864	4.32E-09	2.34E-08	no
RHBDL3	-0.219722222	-5.946142113	4.34E-09	2.34E-08	no
HR	-0.21968968	-5.945216797	4.36E-09	2.36E-08	no
ZMYND12	0.21963355	5.943620817	4.41E-09	2.38E-08	no
MERTK	0.219624045	5.943350543	4.41E-09	2.38E-08	no
CNDP2	0.219594725	5.942516899	4.43E-09	2.39E-08	no
TASP1	0.219508966	5.940078643	4.50E-09	2.43E-08	no
NDRG4	-0.21942046	-5.937562435	4.56E-09	2.46E-08	no
RPAP3	0.219401804	5.937032065	4.58E-09	2.47E-08	no
MLPH	0.219346718	5.935466086	4.62E-09	2.49E-08	no
LOC650623	-0.219331532	-5.935034395	4.63E-09	2.49E-08	no
FAM159A	0.21933108	5.935021548	4.63E-09	2.49E-08	no
GRID2IP	-0.219329634	-5.934980436	4.63E-09	2.49E-08	no
ARHGEF1	0.219323623	5.93480956	4.64E-09	2.50E-08	no
RIN1	0.219314338	5.934545615	4.64E-09	2.50E-08	no
SPIN2B	-0.219304277	-5.934259623	4.65E-09	2.50E-08	no
ELAVL2	-0.219203125	-5.931384313	4.73E-09	2.54E-08	no
AMZ1	-0.219189611	-5.931000173	4.74E-09	2.55E-08	no
FOXRED2	-0.219188962	-5.930981735	4.74E-09	2.55E-08	no
ARMC5	-0.219175762	-5.930606516	4.75E-09	2.55E-08	no
PHF1	-0.219135113	-5.929451128	4.78E-09	2.57E-08	no
POGK	-0.219119215	-5.928999248	4.80E-09	2.58E-08	no
IFFO1	0.219114732	5.928871839	4.80E-09	2.58E-08	no
BTN2A1	0.219084164	5.928003007	4.82E-09	2.59E-08	no
THNSL2	0.219048123	5.926978648	4.85E-09	2.60E-08	no
OAS1	0.219046246	5.926925313	4.85E-09	2.60E-08	no
LOC339290	-0.219020158	-5.926183844	4.87E-09	2.62E-08	no
RNF14	0.218987165	5.925246176	4.90E-09	2.63E-08	no
RAPGEFL1	-0.218974291	-5.924880287	4.91E-09	2.63E-08	no
BMP7	-0.218970178	-5.924763401	4.91E-09	2.63E-08	no
NEU1	0.218952094	5.92424946	4.93E-09	2.64E-08	no
HRASLS5	0.218933991	5.923734972	4.94E-09	2.65E-08	no

GALR3	-0.21892142	-5.923377703	4.95E-09	2.65E-08	no
RAPGEF4	-0.218911637	-5.92309968	4.96E-09	2.66E-08	no
ZNF792	0.218897799	5.922706428	4.97E-09	2.66E-08	no
PPM1B	-0.218876379	-5.9220977	4.99E-09	2.67E-08	no
RAB11B	-0.218838108	-5.921010118	5.02E-09	2.69E-08	no
GPR172B	0.218837438	5.920991092	5.02E-09	2.69E-08	no
THAP10	-0.218835602	-5.920938903	5.02E-09	2.69E-08	no
C19orf66	0.21881883	5.920462294	5.04E-09	2.69E-08	no
HIPK3	0.218811958	5.920267004	5.04E-09	2.70E-08	no
ZNF10	-0.218766448	-5.918973786	5.08E-09	2.72E-08	no
MMAA	0.218664518	5.916077421	5.17E-09	2.76E-08	no
CANT1	0.218607427	5.91445527	5.22E-09	2.79E-08	no
HBP1	0.218565536	5.913265052	5.25E-09	2.80E-08	no
LOC152225	0.218522835	5.912051822	5.29E-09	2.82E-08	no
TMEM179B	0.218469422	5.910534336	5.34E-09	2.85E-08	no
LARP4	0.218459591	5.910255022	5.34E-09	2.85E-08	no
DTD1	-0.218408342	-5.908799072	5.39E-09	2.88E-08	no
ITPRIPL1	0.218363509	5.907525409	5.43E-09	2.90E-08	no
SLC20A1	0.218286353	5.905333623	5.50E-09	2.93E-08	no
LSM11	-0.21827358	-5.904970768	5.51E-09	2.94E-08	no
ACE	0.218263752	5.90469161	5.52E-09	2.94E-08	no
LOC93622	-0.218262178	-5.904646895	5.52E-09	2.94E-08	no
CXCL6	0.218221035	5.903478173	5.56E-09	2.96E-08	no
EWSR1	-0.218216836	-5.903358916	5.56E-09	2.96E-08	no
ARAP1	0.218206188	5.903056466	5.57E-09	2.97E-08	no
PCNT	-0.218161919	-5.901799013	5.61E-09	2.99E-08	no
SLC24A1	0.218145787	5.901340795	5.63E-09	2.99E-08	no
AMD1	0.218082143	5.899533078	5.69E-09	3.02E-08	no
CX3CR1	0.218046511	5.898521066	5.72E-09	3.04E-08	no
TMEM168	0.218015775	5.89764811	5.75E-09	3.06E-08	no
WDR78	0.218008434	5.897439602	5.76E-09	3.06E-08	no
MAMDC2	0.218000951	5.897227082	5.76E-09	3.06E-08	no
SLC10A6	0.217934332	5.895335057	5.83E-09	3.09E-08	no
CILP	0.217933617	5.895314761	5.83E-09	3.09E-08	no
HESX1	0.217925711	5.895090237	5.83E-09	3.10E-08	no
ASS1	0.217906429	5.894542639	5.85E-09	3.11E-08	no
ATP6V0D2	0.217898428	5.894315417	5.86E-09	3.11E-08	no
CHEK2	0.217876226	5.893684906	5.88E-09	3.12E-08	no
QKI	-0.217843968	-5.89276883	5.91E-09	3.14E-08	no
UCHL3	0.217827253	5.892294153	5.93E-09	3.14E-08	no
BID	-0.217812956	-5.891888151	5.94E-09	3.15E-08	no
PPP2R5C	-0.217802166	-5.891581759	5.95E-09	3.15E-08	no
FBXW4	-0.217688515	-5.888354501	6.06E-09	3.21E-08	no
ABHD14A	-0.217668135	-5.887775798	6.09E-09	3.22E-08	no
APOBEC3B	0.217611002	5.88615358	6.14E-09	3.25E-08	no
TP53I13	0.217600609	5.885858469	6.15E-09	3.26E-08	no
PARK2	-0.217599596	-5.885829703	6.15E-09	3.26E-08	no
APOC4	0.217583302	5.885367068	6.17E-09	3.26E-08	no
AMOTL2	-0.217576352	-5.885169733	6.18E-09	3.27E-08	no
CSDC2	-0.21757151	-5.885032258	6.18E-09	3.27E-08	no
ZNF843	-0.217567583	-5.88492078	6.19E-09	3.27E-08	no

FAM161B	-0.217540679	-5.884156887	6.21E-09	3.28E-08	no
MCF2L	-0.217529938	-5.883851934	6.22E-09	3.29E-08	no
MFAP4	0.217448821	5.881548932	6.31E-09	3.33E-08	no
HAT1	0.217441573	5.88134317	6.31E-09	3.34E-08	no
PCBP3	-0.217419057	-5.880703931	6.34E-09	3.35E-08	no
ILK	0.217376572	5.879497801	6.38E-09	3.37E-08	no
ZHX2	-0.217362846	-5.879108154	6.40E-09	3.38E-08	no
LRRC2	0.217341194	5.878493464	6.42E-09	3.39E-08	no
SIRT1	-0.217340207	-5.878465463	6.42E-09	3.39E-08	no
GAD1	-0.217295826	-5.87720557	6.47E-09	3.41E-08	no
FAM38A	0.217282428	5.87682524	6.48E-09	3.42E-08	no
ZNF500	-0.217244211	-5.875740378	6.52E-09	3.44E-08	no
C5orf32	0.217197467	5.874413517	6.57E-09	3.46E-08	no
PI4K2A	0.217188999	5.874173157	6.58E-09	3.47E-08	no
ACCN4	-0.217178003	-5.873861021	6.59E-09	3.47E-08	no
RBM17	-0.2171375	-5.872711366	6.64E-09	3.49E-08	no
DLG3	-0.217120233	-5.87222125	6.66E-09	3.50E-08	no
SEZ6L	-0.217103284	-5.871740162	6.67E-09	3.51E-08	no
CUEDC2	-0.217095802	-5.871527805	6.68E-09	3.52E-08	no
E2F4	0.217094171	5.871481492	6.68E-09	3.52E-08	no
C1orf66	-0.21708398	-5.871192259	6.69E-09	3.52E-08	no
TMEM217	0.21706087	5.870536306	6.72E-09	3.53E-08	no
OR10J1	0.217028755	5.869624819	6.76E-09	3.55E-08	no
EID2B	-0.217025399	-5.869529564	6.76E-09	3.55E-08	no
MLL	-0.217009943	-5.869090879	6.78E-09	3.56E-08	no
EPHA10	-0.216997832	-5.868747167	6.79E-09	3.57E-08	no
EFCAB5	-0.216987872	-5.868464492	6.80E-09	3.57E-08	no
HERC2	-0.216969073	-5.867930946	6.82E-09	3.58E-08	no
SRD5A3	0.216950935	5.867416153	6.84E-09	3.59E-08	no
SIAH1	-0.216906342	-5.866150616	6.89E-09	3.62E-08	no
LRRC27	-0.216874041	-5.865233921	6.93E-09	3.63E-08	no
ALDH6A1	-0.216831845	-5.864036459	6.98E-09	3.66E-08	no
BLCAP	-0.216829574	-5.863972009	6.98E-09	3.66E-08	no
POLDIP3	-0.216766924	-5.862194126	7.05E-09	3.69E-08	no
TBCD	-0.216760959	-5.862024874	7.06E-09	3.70E-08	no
MTPAP	-0.216757286	-5.861920648	7.06E-09	3.70E-08	no
TXNDC11	0.216748526	5.861672062	7.07E-09	3.70E-08	no
RPRM	-0.216747045	-5.861630025	7.07E-09	3.70E-08	no
C18orf18	-0.216678626	-5.859688548	7.15E-09	3.74E-08	no
DGCR10	-0.216669906	-5.859441122	7.16E-09	3.75E-08	no
PPP1R15A	0.216644814	5.858729138	7.19E-09	3.76E-08	no
C1orf151	0.216642734	5.858670124	7.19E-09	3.76E-08	no
PRG4	0.216616371	5.857922095	7.22E-09	3.78E-08	no
SPG21	0.216613308	5.857835189	7.23E-09	3.78E-08	no
HEBP2	0.216584401	5.85701496	7.26E-09	3.80E-08	no
RAB33A	-0.216569098	-5.856580776	7.28E-09	3.80E-08	no
ABLIM1	-0.216495284	-5.854486468	7.37E-09	3.85E-08	no
CCNI2	-0.216490545	-5.854352019	7.37E-09	3.85E-08	no
RAD51L1	0.216472774	5.853847827	7.40E-09	3.86E-08	no
PRKD2	0.216437116	5.852836164	7.44E-09	3.88E-08	no
C1orf83	0.216397992	5.851726217	7.49E-09	3.91E-08	no

FDPSL2A	0.21639349	5.851598496	7.49E-09	3.91E-08	no
PRSS27	-0.2163781	-5.851161882	7.51E-09	3.92E-08	no
ASAM	0.216364702	5.850781796	7.53E-09	3.92E-08	no
TTC39C	0.216337238	5.850002642	7.56E-09	3.94E-08	no
GAL3ST3	-0.216293108	-5.848750743	7.61E-09	3.97E-08	no
RFESD	0.216226524	5.846861947	7.70E-09	4.01E-08	no
PI15	0.216177815	5.845480265	7.76E-09	4.04E-08	no
GTF3C1	-0.216154245	-5.844811669	7.79E-09	4.06E-08	no
PPME1	-0.216101912	-5.843327266	7.86E-09	4.09E-08	no
OPLAH	0.216080024	5.842706442	7.88E-09	4.10E-08	no
PCDH11X	-0.216041456	-5.841612516	7.93E-09	4.13E-08	no
TRPM8	0.216007781	5.840657401	7.98E-09	4.15E-08	no
CUL9	-0.215957184	-5.839222345	8.04E-09	4.18E-08	no
MGC12916	0.215909246	5.837862791	8.10E-09	4.21E-08	no
C13orf33	0.215899576	5.837588543	8.12E-09	4.22E-08	no
KBTBD11	-0.215872397	-5.836817752	8.15E-09	4.24E-08	no
THSD1P1	-0.2158722	-5.836812145	8.15E-09	4.24E-08	no
TRIM8	-0.215853516	-5.836282287	8.18E-09	4.25E-08	no
MYCBPAP	0.215748331	5.833299403	8.32E-09	4.32E-08	no
CXCL14	0.215674938	5.83121821	8.42E-09	4.37E-08	no
LRP8	-0.215661813	-5.830846036	8.44E-09	4.38E-08	no
SLC7A1	-0.215661774	-5.830844935	8.44E-09	4.38E-08	no
PDZD8	-0.21548741	-5.825900995	8.68E-09	4.50E-08	no
PA2G4	-0.215475565	-5.825565154	8.69E-09	4.51E-08	no
EPM2AIP1	-0.215444403	-5.824681645	8.74E-09	4.53E-08	no
UQCRB	-0.215436518	-5.824458088	8.75E-09	4.53E-08	no
EXT2	0.215424808	5.824126109	8.77E-09	4.54E-08	no
YARS2	0.215409064	5.823679733	8.79E-09	4.55E-08	no
TMEM111	0.215353607	5.822107514	8.87E-09	4.59E-08	no
FAM181B	-0.21532149	-5.821197008	8.91E-09	4.62E-08	no
PLEKHO1	0.215308877	5.82083944	8.93E-09	4.62E-08	no
RCN1	0.215297062	5.820504488	8.95E-09	4.63E-08	no
ANAPC2	-0.215293682	-5.820408665	8.95E-09	4.63E-08	no
C15orf51	0.2152541	5.819286605	9.01E-09	4.66E-08	no
COL12A1	0.215226957	5.818517159	9.05E-09	4.68E-08	no
RND2	-0.215211126	-5.818068396	9.07E-09	4.69E-08	no
SVEP1	0.215171672	5.816950003	9.13E-09	4.72E-08	no
ANO10	0.215161324	5.816656675	9.15E-09	4.73E-08	no
HERC5	0.215133918	5.81587981	9.19E-09	4.75E-08	no
TMIGD2	0.215093533	5.814735082	9.25E-09	4.78E-08	no
LAT	0.215078567	5.814310885	9.27E-09	4.79E-08	no
LOC645166	0.215068699	5.814031183	9.29E-09	4.79E-08	no
GIP	0.215039204	5.813195181	9.33E-09	4.81E-08	no
TMEM67	0.215008073	5.812312792	9.38E-09	4.84E-08	no
CAPNS1	0.215003753	5.812190351	9.38E-09	4.84E-08	no
GPR137C	-0.214980073	-5.811519193	9.42E-09	4.86E-08	no
CCDC94	-0.214956978	-5.810864643	9.46E-09	4.87E-08	no
DUSP8	-0.214951	-5.810695199	9.46E-09	4.88E-08	no
LRP1B	-0.214928169	-5.810048139	9.50E-09	4.89E-08	no
C10orf54	0.21487006	5.808401247	9.59E-09	4.94E-08	no
RBM10	-0.214857671	-5.808050125	9.61E-09	4.95E-08	no

CUX2	-0.214851751	-5.807882361	9.62E-09	4.95E-08	no
RBP1	0.214805829	5.806580936	9.69E-09	4.99E-08	no
MMD2	-0.214792004	-5.806189138	9.71E-09	5.00E-08	no
C11orf68	-0.214771086	-5.805596364	9.74E-09	5.01E-08	no
FLJ10661	0.214750435	5.805011127	9.78E-09	5.03E-08	no
DNAJB11	0.214749698	5.804990242	9.78E-09	5.03E-08	no
WDR81	0.214749362	5.804980732	9.78E-09	5.03E-08	no
LAMP2	0.214742936	5.804798642	9.79E-09	5.03E-08	no
TOX	-0.214710527	-5.803880242	9.84E-09	5.06E-08	no
RAB18	-0.214708872	-5.803833317	9.84E-09	5.06E-08	no
LOC148824	0.214631476	5.801640168	9.96E-09	5.12E-08	no
HTR3C	0.214593711	5.80057007	1.00E-08	5.15E-08	no
SEC11A	0.214574148	5.800015743	1.01E-08	5.16E-08	no
C1orf70	-0.21451339	-5.798294221	1.02E-08	5.21E-08	no
AADAT	-0.214510457	-5.798211105	1.02E-08	5.21E-08	no
BEX2	-0.214495239	-5.797779922	1.02E-08	5.22E-08	no
PSENEN	0.214482586	5.797421442	1.02E-08	5.23E-08	no
PTMA	0.214466883	5.796976522	1.02E-08	5.25E-08	no
DPP10	-0.214439134	-5.796190314	1.03E-08	5.27E-08	no
SERPINB2	0.214437734	5.796150645	1.03E-08	5.27E-08	no
NEU4	-0.214411139	-5.795397148	1.03E-08	5.29E-08	no
C22orf34	0.214396017	5.794968734	1.04E-08	5.30E-08	no
LRSAM1	-0.214388666	-5.79476046	1.04E-08	5.31E-08	no
DOK4	-0.214354636	-5.793796343	1.04E-08	5.33E-08	no
BIN3	0.214324901	5.792953941	1.05E-08	5.36E-08	no
PELI2	-0.214290452	-5.791978019	1.05E-08	5.39E-08	no
FLJ42627	-0.214271909	-5.79145271	1.06E-08	5.40E-08	no
KIF7	-0.214262148	-5.791176194	1.06E-08	5.41E-08	no
GNL1	-0.214249331	-5.790813102	1.06E-08	5.42E-08	no
TCEAL4	-0.214239639	-5.790538522	1.06E-08	5.42E-08	no
MTL5	0.214226721	5.790172589	1.06E-08	5.43E-08	no
USP49	-0.214223053	-5.790068664	1.06E-08	5.44E-08	no
ABCA13	0.214222708	5.790058906	1.06E-08	5.44E-08	no
SUPT16H	-0.214192818	-5.78921217	1.07E-08	5.46E-08	no
USP53	0.214179029	5.788821578	1.07E-08	5.47E-08	no
NAA20	0.214174441	5.788691601	1.07E-08	5.47E-08	no
EGFL6	0.214109988	5.786865858	1.08E-08	5.53E-08	no
LOC285696	-0.214099786	-5.786576891	1.09E-08	5.54E-08	no
FZD2	0.214094289	5.786421166	1.09E-08	5.54E-08	no
RPTOR	-0.21405457	-5.785296111	1.09E-08	5.57E-08	no
BFAR	0.21402645	5.784499619	1.10E-08	5.60E-08	no
LOC643763	-0.214024193	-5.784435701	1.10E-08	5.60E-08	no
SF4	-0.214022134	-5.784377375	1.10E-08	5.60E-08	no
MGAT4C	-0.213981825	-5.783235666	1.11E-08	5.63E-08	no
RNPS1	-0.213965254	-5.782766313	1.11E-08	5.65E-08	no
NR2F2	0.213957939	5.782559122	1.11E-08	5.65E-08	no
ERP29	0.213931121	5.781799558	1.12E-08	5.67E-08	no
PIK3R2	-0.213930848	-5.781791821	1.12E-08	5.67E-08	no
AGAP4	-0.213906656	-5.781106643	1.12E-08	5.70E-08	no
TRIM58	0.21390265	5.780993187	1.12E-08	5.70E-08	no
DCX	-0.213897129	-5.78083683	1.12E-08	5.70E-08	no

FBXO4	0.213892382	5.780702382	1.12E-08	5.70E-08	no
IGF2R	0.213874452	5.780194565	1.13E-08	5.72E-08	no
DDB1	-0.213855239	-5.779650434	1.13E-08	5.74E-08	no
AP2S1	0.213798535	5.778044544	1.14E-08	5.79E-08	no
GPR158	-0.213788798	-5.777768794	1.14E-08	5.79E-08	no
LRRC4B	-0.213768657	-5.777198411	1.14E-08	5.81E-08	no
ZSCAN2	-0.213751968	-5.776725779	1.15E-08	5.83E-08	no
POMT1	-0.213719937	-5.775818695	1.15E-08	5.85E-08	no
UBXN4	0.213666171	5.774296148	1.16E-08	5.90E-08	no
RPL3	-0.213626399	-5.773169913	1.17E-08	5.94E-08	no
POLR2F	-0.213586885	-5.772051015	1.18E-08	5.98E-08	no
DCK	0.213573278	5.771665714	1.18E-08	5.99E-08	no
POMT2	-0.213571637	-5.771619255	1.18E-08	5.99E-08	no
SUSD2	0.213555526	5.771163058	1.18E-08	6.00E-08	no
PRUNE	0.213550908	5.771032297	1.19E-08	6.00E-08	no
PIH1D2	0.213549178	5.770983302	1.19E-08	6.00E-08	no
UBA6	0.213538191	5.770672195	1.19E-08	6.01E-08	no
ZFP37	-0.213533202	-5.770530928	1.19E-08	6.02E-08	no
ZFP1	-0.213447894	-5.768115506	1.21E-08	6.10E-08	no
AKTIP	-0.21342297	-5.767409831	1.21E-08	6.12E-08	no
BHLHE40	0.213349146	5.765319678	1.22E-08	6.19E-08	no
LOC150568	-0.213341536	-5.765104252	1.23E-08	6.20E-08	no
MIER1	0.213307344	5.764136227	1.23E-08	6.23E-08	no
GBF1	-0.213289611	-5.763634193	1.24E-08	6.25E-08	no
ZNF23	-0.213223936	-5.761774941	1.25E-08	6.31E-08	no
ANKRD23	-0.21320776	-5.761317017	1.25E-08	6.33E-08	no
WISP1	0.213202999	5.761182239	1.25E-08	6.33E-08	no
SMAD9	-0.213145912	-5.759566189	1.27E-08	6.39E-08	no
POLR3H	-0.213143866	-5.759508284	1.27E-08	6.39E-08	no
FAM5B	-0.213078018	-5.757644317	1.28E-08	6.45E-08	no
PRKACA	0.213060745	5.757155383	1.28E-08	6.47E-08	no
IL28RA	0.212989118	5.755127952	1.30E-08	6.54E-08	no
R3HDML	0.212983119	5.754958138	1.30E-08	6.55E-08	no
SUOX	-0.212925594	-5.75332994	1.31E-08	6.61E-08	no
RPUSD4	-0.212919345	-5.753153082	1.31E-08	6.61E-08	no
PPP1R13B	-0.212874818	-5.751892831	1.32E-08	6.66E-08	no
TMED7	0.212860454	5.751486298	1.32E-08	6.67E-08	no
DCTN6	-0.212852712	-5.751267168	1.33E-08	6.68E-08	no
NIPAL2	0.21282584	5.750506645	1.33E-08	6.70E-08	no
RAP1GAP	-0.212825619	-5.75050038	1.33E-08	6.70E-08	no
TBX15	0.212750851	5.748384375	1.35E-08	6.78E-08	no
ERN1	0.212736909	5.747989819	1.35E-08	6.79E-08	no
ZNF768	-0.212731441	-5.747835092	1.35E-08	6.80E-08	no
ZNF142	-0.212725993	-5.747680901	1.35E-08	6.80E-08	no
C12orf26	0.212689014	5.746634427	1.36E-08	6.84E-08	no
TAB2	0.212657795	5.745750958	1.37E-08	6.87E-08	no
PPID	0.212627647	5.744897813	1.37E-08	6.91E-08	no
CYP19A1	0.21258878	5.743797973	1.38E-08	6.95E-08	no
AXIN1	-0.212541444	-5.74245851	1.39E-08	7.00E-08	no
MS4A2	0.212529607	5.742123591	1.40E-08	7.01E-08	no
HMG20A	-0.212500892	-5.741311057	1.40E-08	7.04E-08	no

IL13RA2	0.212476562	5.740622642	1.41E-08	7.07E-08	no
ALCAM	-0.212436475	-5.739488397	1.42E-08	7.11E-08	no
CSK	0.21238988	5.738170024	1.43E-08	7.16E-08	no
TECTA	-0.212377234	-5.737812232	1.43E-08	7.17E-08	no
DZIP1	-0.21235791	-5.737265501	1.43E-08	7.19E-08	no
PPP2R5B	-0.212332095	-5.736535132	1.44E-08	7.22E-08	no
CRYZ	0.212307118	5.735828467	1.45E-08	7.25E-08	no
SLITRK2	-0.212306502	-5.735811041	1.45E-08	7.25E-08	no
MN1	-0.212253353	-5.734307383	1.46E-08	7.31E-08	no
OR51E1	0.212251258	5.734248118	1.46E-08	7.31E-08	no
ECSCR	0.212232046	5.733704615	1.46E-08	7.33E-08	no
MCAM	0.212218952	5.733334162	1.47E-08	7.34E-08	no
PPM1A	-0.212174076	-5.732064649	1.48E-08	7.39E-08	no
AGXT2L1	-0.212129333	-5.730798916	1.49E-08	7.44E-08	no
PLD1	0.211967589	5.726223646	1.53E-08	7.64E-08	no
CCDC122	0.211966238	5.726185445	1.53E-08	7.64E-08	no
LOC283761	-0.21193599	-5.725329877	1.53E-08	7.67E-08	no
CDR2	0.211872873	5.723544643	1.55E-08	7.75E-08	no
ITGA10	0.211754054	5.720184142	1.58E-08	7.89E-08	no
LOC100128977	-0.211667696	-5.71774186	1.60E-08	8.00E-08	no
THNSL1	-0.21165174	-5.717290655	1.61E-08	8.02E-08	no
LOC168474	-0.211591437	-5.71558532	1.62E-08	8.09E-08	no
GPLD1	-0.211433522	-5.71111997	1.66E-08	8.30E-08	no
MRPS25	-0.211417016	-5.710653263	1.67E-08	8.32E-08	no
C21orf62	0.211377994	5.709549932	1.68E-08	8.36E-08	no
SLC43A2	0.211351911	5.708812468	1.68E-08	8.40E-08	no
SERHL2	0.211343914	5.708586345	1.69E-08	8.41E-08	no
SPPL3	-0.211263152	-5.706302955	1.71E-08	8.51E-08	no
NF2	-0.211240423	-5.705660369	1.71E-08	8.54E-08	no
MAPK8	-0.211230318	-5.705374686	1.72E-08	8.55E-08	no
SNURF	-0.21114753	-5.703034194	1.74E-08	8.66E-08	no
ETV1	-0.211114968	-5.702113689	1.75E-08	8.71E-08	no
PRR24	0.211111627	5.702019225	1.75E-08	8.71E-08	no
IER5L	0.211072369	5.700909431	1.76E-08	8.76E-08	no
DLG5	-0.211064147	-5.700677011	1.76E-08	8.77E-08	no
RSF1	-0.211057971	-5.700502428	1.76E-08	8.78E-08	no
GLCCI1	-0.211056844	-5.700470584	1.76E-08	8.78E-08	no
HHIPL2	0.211013931	5.699257508	1.78E-08	8.83E-08	no
LOC729020	0.210964577	5.697862401	1.79E-08	8.90E-08	no
ARRDC3	0.21094525	5.697316118	1.80E-08	8.93E-08	no
SMAP1	-0.210908707	-5.696283186	1.81E-08	8.98E-08	no
KIAA1467	-0.210869844	-5.695184718	1.82E-08	9.03E-08	no
PLCXD2	-0.210843266	-5.694433502	1.83E-08	9.06E-08	no
ZNF512B	-0.21081133	-5.693530857	1.83E-08	9.11E-08	no
C8orf44	-0.210798505	-5.693168369	1.84E-08	9.12E-08	no
ALG14	0.210778097	5.692591582	1.84E-08	9.15E-08	no
IGFBP5	0.210772736	5.692440043	1.85E-08	9.16E-08	no
KIAA1211	-0.210767994	-5.692306027	1.85E-08	9.16E-08	no
C2orf58	0.210616603	5.688027487	1.89E-08	9.38E-08	no
SLC35A3	0.210609858	5.687836858	1.89E-08	9.39E-08	no
COL13A1	0.210592259	5.687339531	1.90E-08	9.41E-08	no

KDM4D	-0.210577123	-5.686911785	1.90E-08	9.43E-08	no
CHRM4	-0.210569069	-5.686684188	1.91E-08	9.44E-08	no
RPL4	-0.210472806	-5.683963961	1.94E-08	9.59E-08	no
SUPT3H	-0.210437238	-5.682958897	1.95E-08	9.64E-08	no
DKFZP586I142	0.210425041	5.682614257	1.95E-08	9.65E-08	no
PNLIPRP3	0.210424885	5.682609846	1.95E-08	9.65E-08	no
ADCK1	-0.210419838	-5.682467224	1.95E-08	9.66E-08	no
DOK6	-0.21041651	-5.68237319	1.95E-08	9.66E-08	no
PPP1R9B	-0.210385422	-5.681494762	1.96E-08	9.70E-08	no
IPW	-0.210355767	-5.680656843	1.97E-08	9.75E-08	no
SH3D20	0.210269469	5.678218553	2.00E-08	9.88E-08	no
FAM45A	-0.210243016	-5.677471152	2.01E-08	9.92E-08	no
RRM2B	0.21023753	5.677316178	2.01E-08	9.92E-08	no
ZNF517	-0.210223206	-5.676911472	2.01E-08	9.94E-08	no
KIAA1161	-0.210223176	-5.676910627	2.01E-08	9.94E-08	no
VCAM1	0.210207403	5.67646498	2.02E-08	9.96E-08	no
SDF2L1	0.210189086	5.675947484	2.02E-08	9.99E-08	no
PRKCE	-0.210186127	-5.675863911	2.02E-08	9.99E-08	no
CPLX1	-0.210158671	-5.675088226	2.03E-08	1.00E-07	no
TTC22	0.210148757	5.674808135	2.04E-08	1.00E-07	no
CBLN3	0.210142709	5.674637262	2.04E-08	1.01E-07	no
CPN2	0.210091235	5.673183072	2.06E-08	1.01E-07	no
VANGL1	0.210030588	5.671469809	2.08E-08	1.02E-07	no
SF3A1	-0.210027399	-5.67137973	2.08E-08	1.02E-07	no
MPV17	0.209999497	5.670591522	2.09E-08	1.03E-07	no
PLXNB1	-0.209961385	-5.669514921	2.10E-08	1.03E-07	no
ARMC10	0.209955938	5.669361066	2.10E-08	1.03E-07	no
AKAP11	-0.209916817	-5.668256002	2.11E-08	1.04E-07	no
ERP27	0.20991247	5.668133199	2.11E-08	1.04E-07	no
TTF1	-0.209846063	-5.666257441	2.14E-08	1.05E-07	no
ERBB4	-0.20984325	-5.666177983	2.14E-08	1.05E-07	no
MGAT3	-0.209733071	-5.663066021	2.17E-08	1.07E-07	no
NEURL2	0.209726795	5.662888756	2.18E-08	1.07E-07	no
HECW1	-0.209697573	-5.662063427	2.19E-08	1.07E-07	no
C12orf72	0.209672832	5.661364683	2.20E-08	1.08E-07	no
RNF166	0.209661342	5.661040163	2.20E-08	1.08E-07	no
BOLA3	0.209660864	5.661026674	2.20E-08	1.08E-07	no
PPP2R5A	-0.209659545	-5.660989415	2.20E-08	1.08E-07	no
RNF157	-0.209598689	-5.659270753	2.22E-08	1.09E-07	no
MCOLN1	0.209571105	5.658491749	2.23E-08	1.09E-07	no
SGMS2	0.209552129	5.657955845	2.24E-08	1.10E-07	no
USP21	-0.209547216	-5.65781712	2.24E-08	1.10E-07	no
FLJ16779	-0.20954362	-5.65771556	2.24E-08	1.10E-07	no
RRAGD	-0.209522631	-5.657122826	2.25E-08	1.10E-07	no
GNG11	0.209465336	5.655504874	2.27E-08	1.11E-07	no
UGGT1	0.209463281	5.655446829	2.27E-08	1.11E-07	no
ZNF629	-0.209461598	-5.655399293	2.27E-08	1.11E-07	no
ZNF701	0.209429549	5.654494285	2.28E-08	1.12E-07	no
MAT1A	0.20942842	5.654462399	2.28E-08	1.12E-07	no
UBQLN2	-0.209403611	-5.653761866	2.29E-08	1.12E-07	no
PSMA1	0.209387037	5.653293846	2.30E-08	1.12E-07	no

TUBGCP4	-0.209363762	-5.652636641	2.30E-08	1.13E-07	no
ASB8	-0.20935994	-5.652528702	2.31E-08	1.13E-07	no
ATP5S	-0.209351983	-5.652304031	2.31E-08	1.13E-07	no
TIGD4	0.209330717	5.65170356	2.32E-08	1.13E-07	no
BIVM	-0.209282207	-5.650333854	2.33E-08	1.14E-07	no
RETN	0.209266856	5.649900406	2.34E-08	1.14E-07	no
EIF3L	-0.209229537	-5.648846714	2.35E-08	1.15E-07	no
CLCN1	0.209171564	5.64720992	2.38E-08	1.16E-07	no
MEX3A	-0.209163305	-5.646976737	2.38E-08	1.16E-07	no
CACNG4	-0.209153947	-5.64671253	2.38E-08	1.16E-07	no
PODNL1	0.209135649	5.646195919	2.39E-08	1.17E-07	no
ENY2	0.209128342	5.645989639	2.39E-08	1.17E-07	no
EGFLAM	0.209120844	5.645777956	2.39E-08	1.17E-07	no
KCTD15	-0.209080415	-5.644636558	2.41E-08	1.18E-07	no
TTC15	-0.209068482	-5.644299668	2.41E-08	1.18E-07	no
KCNK3	-0.209048106	-5.643724448	2.42E-08	1.18E-07	no
SLC23A2	-0.208998318	-5.642318897	2.44E-08	1.19E-07	no
SULF2	-0.208977675	-5.641736149	2.45E-08	1.19E-07	no
NEGR1	-0.20897626	-5.641696203	2.45E-08	1.19E-07	no
GALNT1	0.208966118	5.641409896	2.45E-08	1.20E-07	no
SLC10A7	0.208960814	5.641260156	2.46E-08	1.20E-07	no
PCDHGC3	-0.208957625	-5.641170152	2.46E-08	1.20E-07	no
PDLIM4	0.20894584	5.640837455	2.46E-08	1.20E-07	no
LRRC8A	-0.20892875	-5.640355022	2.47E-08	1.20E-07	no
SRXN1	0.208916349	5.640004977	2.47E-08	1.20E-07	no
TM4SF1	0.208911875	5.639878664	2.47E-08	1.20E-07	no
ZC3H18	-0.208886876	-5.639173007	2.48E-08	1.21E-07	no
CADM4	-0.208870731	-5.638717265	2.49E-08	1.21E-07	no
SF3A2	-0.208801422	-5.636760876	2.52E-08	1.22E-07	no
PLK5P	-0.208759019	-5.635563997	2.53E-08	1.23E-07	no
STK17A	0.20867478	5.633186364	2.57E-08	1.25E-07	no
TMEM169	-0.208673966	-5.633163392	2.57E-08	1.25E-07	no
NSMAF	0.208668078	5.632997219	2.57E-08	1.25E-07	no
SLC39A7	0.208659382	5.632751787	2.57E-08	1.25E-07	no
TMX3	0.208640477	5.632218219	2.58E-08	1.25E-07	no
FBXL4	0.208634436	5.632047722	2.58E-08	1.25E-07	no
WDR17	-0.208606559	-5.631260943	2.60E-08	1.26E-07	no
CCNDBP1	0.208599285	5.631055639	2.60E-08	1.26E-07	no
ZBTB3	-0.208595721	-5.630955064	2.60E-08	1.26E-07	no
PDHX	-0.208595024	-5.630935386	2.60E-08	1.26E-07	no
MEMO1	0.208586511	5.630695139	2.60E-08	1.26E-07	no
VAT1	0.208579596	5.63049996	2.61E-08	1.26E-07	no
ZNF653	-0.208571849	-5.630281332	2.61E-08	1.26E-07	no
SLC26A11	-0.208526997	-5.62901553	2.63E-08	1.27E-07	no
CBR4	-0.208464904	-5.627263217	2.65E-08	1.28E-07	no
PTPRN2	-0.208442863	-5.626641235	2.66E-08	1.29E-07	no
PTBP2	-0.208406956	-5.625627933	2.68E-08	1.30E-07	no
MEGF11	-0.208385795	-5.625030803	2.69E-08	1.30E-07	no
MYH10	-0.208320653	-5.623192606	2.71E-08	1.31E-07	no
GOPC	-0.208294006	-5.62244072	2.73E-08	1.32E-07	no
NOTCH3	0.208273896	5.621873263	2.73E-08	1.32E-07	no

HIST1H4H	0.208260811	5.621504058	2.74E-08	1.32E-07	no
LAMC3	0.208102116	5.617026491	2.81E-08	1.36E-07	no
CUX1	-0.208013878	-5.614537058	2.85E-08	1.38E-07	no
LRFN1	-0.207998945	-5.614115778	2.85E-08	1.38E-07	no
PARP6	-0.207981664	-5.61362823	2.86E-08	1.38E-07	no
CREBZF	-0.207865705	-5.610357008	2.91E-08	1.41E-07	no
KLB	-0.207724535	-5.606374908	2.98E-08	1.44E-07	no
PTGER1	0.207702047	5.605740596	2.99E-08	1.44E-07	no
VIPR2	-0.207702043	-5.605740504	2.99E-08	1.44E-07	no
NFIL3	0.207688676	5.605363455	3.00E-08	1.44E-07	no
ADCY3	0.207660741	5.604575535	3.01E-08	1.45E-07	no
TAF3	-0.207637421	-5.603917804	3.02E-08	1.46E-07	no
LOC152217	-0.207634819	-5.603844407	3.02E-08	1.46E-07	no
HSPB11	0.207625551	5.603583008	3.02E-08	1.46E-07	no
HSPA4	0.207603273	5.602954658	3.04E-08	1.46E-07	no
CPZ	0.207519582	5.60059427	3.08E-08	1.48E-07	no
PKNOX2	-0.207512411	-5.600392029	3.08E-08	1.48E-07	no
SERPINA12	0.207505163	5.60018763	3.08E-08	1.48E-07	no
CYLD	0.207490489	5.59977379	3.09E-08	1.49E-07	no
SFMBT2	0.207484892	5.599615931	3.09E-08	1.49E-07	no
C9orf86	-0.207481002	-5.599506235	3.09E-08	1.49E-07	no
TNKS1BP1	0.207366232	5.596269587	3.15E-08	1.52E-07	no
UQCRC2	-0.207345363	-5.595681088	3.16E-08	1.52E-07	no
LPAR1	0.207327501	5.595177393	3.17E-08	1.52E-07	no
ASB3	-0.207320688	-5.594985274	3.17E-08	1.52E-07	no
FAM66C	-0.207283161	-5.593927047	3.19E-08	1.53E-07	no
POPDC2	0.207275034	5.593697877	3.19E-08	1.53E-07	no
CAMK2G	-0.207229314	-5.592408676	3.22E-08	1.55E-07	no
IMP5	-0.207213679	-5.591967815	3.23E-08	1.55E-07	no
KIF2A	-0.207209477	-5.591849335	3.23E-08	1.55E-07	no
DUSP5	0.207196584	5.591485772	3.23E-08	1.55E-07	no
ECM2	0.207156488	5.590355221	3.25E-08	1.56E-07	no
HDAC3	0.207141654	5.589936953	3.26E-08	1.56E-07	no
TAOK3	-0.207132992	-5.589692723	3.27E-08	1.57E-07	no
RPS2P32	0.207125405	5.589478814	3.27E-08	1.57E-07	no
C17orf51	-0.207104797	-5.588897762	3.28E-08	1.57E-07	no
CUEDC1	-0.207094236	-5.588599973	3.29E-08	1.57E-07	no
FADS2	-0.207075826	-5.588080908	3.30E-08	1.58E-07	no
TBX18	0.207065937	5.587802088	3.30E-08	1.58E-07	no
CTNND2	-0.207006018	-5.58611275	3.33E-08	1.60E-07	no
TCF4	-0.207004198	-5.586061435	3.33E-08	1.60E-07	no
KCNJ11	-0.206987751	-5.585597739	3.34E-08	1.60E-07	no
NCRNA00152	0.20694377	5.584357805	3.36E-08	1.61E-07	no
ZNF319	-0.206937138	-5.584170817	3.37E-08	1.61E-07	no
IL17D	-0.206920415	-5.58369936	3.38E-08	1.61E-07	no
LPIN3	0.206905487	5.583278521	3.38E-08	1.62E-07	no
INTS10	0.206874413	5.5824025	3.40E-08	1.63E-07	no
CNN3	0.206856059	5.581885107	3.41E-08	1.63E-07	no
LOC728264	0.206850216	5.581720392	3.41E-08	1.63E-07	no
EYA1	-0.206822945	-5.580951589	3.43E-08	1.64E-07	no
C10orf88	-0.20682072	-5.580888883	3.43E-08	1.64E-07	no

TAS2R40	0.206778995	5.579712659	3.45E-08	1.65E-07	no
EEF1A1P9	-0.206773503	-5.579557865	3.45E-08	1.65E-07	no
PNPLA4	0.206766547	5.579361761	3.46E-08	1.65E-07	no
BCAP31	0.20674228	5.578677722	3.47E-08	1.66E-07	no
FOLR3	0.206734312	5.578453104	3.47E-08	1.66E-07	no
PSIP1	-0.206711584	-5.577812454	3.49E-08	1.66E-07	no
ATOH8	-0.206674519	-5.576767658	3.51E-08	1.67E-07	no
TRIM9	-0.2066551	-5.576220306	3.52E-08	1.68E-07	no
NPHP1	0.206654719	5.576209547	3.52E-08	1.68E-07	no
GTF2IP1	-0.206644948	-5.575934135	3.52E-08	1.68E-07	no
ANXA5	0.206635706	5.575673652	3.53E-08	1.68E-07	no
USP34	-0.20663565	-5.575672071	3.53E-08	1.68E-07	no
RIMS4	-0.206625115	-5.57537512	3.53E-08	1.68E-07	no
LOC646762	-0.20661501	-5.575090302	3.54E-08	1.69E-07	no
ZNF830	-0.206611736	-5.574998027	3.54E-08	1.69E-07	no
PSMA3	0.206597427	5.5745947	3.55E-08	1.69E-07	no
AKT3	-0.206590872	-5.574409954	3.55E-08	1.69E-07	no
GEM	0.206565874	5.573705354	3.57E-08	1.70E-07	no
FAM196B	-0.20653437	-5.572817423	3.58E-08	1.70E-07	no
AFF2	-0.206517242	-5.572334687	3.59E-08	1.71E-07	no
ARAF	0.206505223	5.571995929	3.60E-08	1.71E-07	no
EPS15L1	-0.206453937	-5.570550481	3.63E-08	1.72E-07	no
GNA14	0.20644851	5.570397544	3.63E-08	1.73E-07	no
DIS3L2	-0.206437753	-5.570094382	3.64E-08	1.73E-07	no
RHOF	0.206437505	5.570087387	3.64E-08	1.73E-07	no
CCNG1	0.206417661	5.569528115	3.65E-08	1.73E-07	no
C8orf48	0.206413699	5.569416452	3.65E-08	1.73E-07	no
TREM1	0.20638341	5.568562847	3.67E-08	1.74E-07	no
HEATR2	0.206353986	5.567733632	3.69E-08	1.75E-07	no
GUCA1B	-0.206324826	-5.566911866	3.70E-08	1.76E-07	no
RTN4R	-0.206298162	-5.56616045	3.72E-08	1.76E-07	no
CDAN1	-0.206276011	-5.565536233	3.73E-08	1.77E-07	no
DIAPH1	0.206255576	5.56496037	3.74E-08	1.77E-07	no
DYX1C1	0.206209396	5.56365907	3.77E-08	1.79E-07	no
HINFP	-0.206158818	-5.56223384	3.80E-08	1.80E-07	no
DDX6	-0.206150125	-5.561988909	3.80E-08	1.80E-07	no
SEC14L3	0.206147216	5.56190693	3.81E-08	1.80E-07	no
GINS4	0.206141261	5.561739147	3.81E-08	1.80E-07	no
PLXNC1	0.206079787	5.560006987	3.84E-08	1.82E-07	no
NXPH1	-0.20606959	-5.559719672	3.85E-08	1.82E-07	no
CREBBP	-0.20606099	-5.559477347	3.86E-08	1.82E-07	no
LIPC	0.206026292	5.558499704	3.88E-08	1.83E-07	no
ASCC1	-0.205995925	-5.557644092	3.90E-08	1.84E-07	no
SYT3	-0.205950983	-5.556377879	3.92E-08	1.85E-07	no
4-Mar	-0.205870293	-5.554104579	3.97E-08	1.88E-07	no
ZBTB44	-0.205807892	-5.552346598	4.01E-08	1.90E-07	no
KCNC1	-0.205752766	-5.550793639	4.04E-08	1.91E-07	no
RUNX1T1	-0.205743914	-5.550544275	4.05E-08	1.91E-07	no
C21orf7	0.205743909	5.55054414	4.05E-08	1.91E-07	no
QSOX1	0.205738101	5.550380539	4.05E-08	1.91E-07	no
PRKCA	-0.205633277	-5.54742771	4.12E-08	1.94E-07	no

SLC39A4	0.205626363	5.547232942	4.12E-08	1.95E-07	no
GSTO1	0.205571526	5.545688318	4.16E-08	1.96E-07	no
FAM22D	-0.205569789	-5.545639383	4.16E-08	1.96E-07	no
GNAI3	0.20556249	5.5454338	4.16E-08	1.96E-07	no
C5orf36	0.205545499	5.544955202	4.18E-08	1.97E-07	no
PSMD12	0.205453739	5.542370698	4.23E-08	2.00E-07	no
ODF3L1	0.205451931	5.542319769	4.24E-08	2.00E-07	no
PSMA5	0.20543282	5.541781518	4.25E-08	2.00E-07	no
CAMK2D	0.205432558	5.54177413	4.25E-08	2.00E-07	no
FAM129B	0.205432318	5.541767376	4.25E-08	2.00E-07	no
FAM7A2	0.205413181	5.541228401	4.26E-08	2.01E-07	no
TMC4	0.205400583	5.540873568	4.27E-08	2.01E-07	no
CYYR1	0.205348958	5.539419624	4.30E-08	2.03E-07	no
PPP1R10	-0.205348567	-5.539408629	4.30E-08	2.03E-07	no
SSTR5	-0.205311564	-5.538366495	4.33E-08	2.04E-07	no
RGS11	-0.205310861	-5.538346717	4.33E-08	2.04E-07	no
RP1-177G6.2	-0.205272951	-5.537279083	4.35E-08	2.05E-07	no
TGDS	0.205269672	5.537186735	4.36E-08	2.05E-07	no
NCRNA00081	-0.205253001	-5.536717254	4.37E-08	2.05E-07	no
SCN3B	-0.205211975	-5.535561912	4.40E-08	2.07E-07	no
C19orf76	0.205172697	5.534455837	4.42E-08	2.08E-07	no
MVD	-0.205168164	-5.534328182	4.43E-08	2.08E-07	no
TCP10L	-0.205150478	-5.533830155	4.44E-08	2.08E-07	no
SLC25A32	0.205117715	5.532907558	4.46E-08	2.09E-07	no
CLPTM1L	0.205080932	5.531871793	4.49E-08	2.10E-07	no
LPPR5	-0.205072169	-5.531625061	4.49E-08	2.11E-07	no
OPCML	-0.205063288	-5.531374983	4.50E-08	2.11E-07	no
GRID2	-0.20505964	-5.531272257	4.50E-08	2.11E-07	no
GPATCH8	-0.205002117	-5.529652566	4.54E-08	2.13E-07	no
GLCE	0.204990599	5.529328241	4.55E-08	2.13E-07	no
SOX4	-0.204935301	-5.52777126	4.59E-08	2.15E-07	no
C10orf50	0.204919052	5.527313752	4.60E-08	2.15E-07	no
RBM7	0.204896781	5.526686708	4.61E-08	2.16E-07	no
GPR125	-0.204874097	-5.526048049	4.63E-08	2.17E-07	no
C1orf212	0.204857815	5.525589642	4.64E-08	2.17E-07	no
LSS	-0.204854232	-5.525488767	4.64E-08	2.17E-07	no
ODF3B	0.204827631	5.52473982	4.66E-08	2.18E-07	no
LOC645676	-0.204823619	-5.524626868	4.67E-08	2.18E-07	no
CER1	0.204811188	5.524276891	4.68E-08	2.19E-07	no
ZCCHC18	-0.204798353	-5.523915562	4.68E-08	2.19E-07	no
PSEN2	0.204796703	5.523869099	4.69E-08	2.19E-07	no
SEC31B	-0.204788494	-5.523637981	4.69E-08	2.19E-07	no
CCDC23	0.204787965	5.52362311	4.69E-08	2.19E-07	no
FAM22A	-0.204738487	-5.522230166	4.73E-08	2.21E-07	no
TMEM45A	0.20471067	5.521447066	4.75E-08	2.22E-07	no
GUF1	-0.204702862	-5.521227252	4.75E-08	2.22E-07	no
C1orf97	0.204695426	5.521017906	4.76E-08	2.22E-07	no
JAZF1	0.204692184	5.520926661	4.76E-08	2.22E-07	no
SRC	-0.204678283	-5.520535317	4.77E-08	2.23E-07	no
TAF1C	-0.204674867	-5.520439153	4.77E-08	2.23E-07	no
RAB41	-0.204670369	-5.520312521	4.78E-08	2.23E-07	no

PDCD6IP	0.204668061	5.520247551	4.78E-08	2.23E-07	no
CKAP5	-0.204664935	-5.520159548	4.78E-08	2.23E-07	no
VAMP7	0.204638601	5.519418252	4.80E-08	2.24E-07	no
FGD1	-0.204584987	-5.51790901	4.84E-08	2.26E-07	no
ADAM32	0.204544336	5.51676473	4.87E-08	2.27E-07	no
HSF2BP	-0.20448537	-5.515104931	4.91E-08	2.29E-07	no
IL3RA	0.204442747	5.51390523	4.95E-08	2.30E-07	no
CNFN	0.204407104	5.512901993	4.97E-08	2.32E-07	no
ZXDC	-0.204380922	-5.512165063	4.99E-08	2.32E-07	no
KIF13A	-0.204377978	-5.512082216	5.00E-08	2.33E-07	no
DOCK3	-0.204367314	-5.511782067	5.00E-08	2.33E-07	no
NOP10	0.204360614	5.511593507	5.01E-08	2.33E-07	no
C3orf18	-0.204307852	-5.510108513	5.05E-08	2.35E-07	no
C14orf119	0.204301044	5.509916907	5.06E-08	2.35E-07	no
CMTM5	-0.204272067	-5.509101374	5.08E-08	2.36E-07	no
C18orf8	0.204261317	5.508798844	5.09E-08	2.36E-07	no
DNAJC1	0.204232436	5.507986017	5.11E-08	2.37E-07	no
ZER1	-0.204127683	-5.505038051	5.19E-08	2.41E-07	no
RIMS2	-0.20412096	-5.504848854	5.20E-08	2.41E-07	no
FMR1	-0.204115547	-5.504696532	5.20E-08	2.42E-07	no
FGF9	-0.204089611	-5.503966683	5.22E-08	2.42E-07	no
KIAA1143	-0.204032856	-5.502369584	5.27E-08	2.44E-07	no
LOC80154	-0.204032399	-5.502356739	5.27E-08	2.44E-07	no
PHF6	-0.204022051	-5.502065537	5.28E-08	2.45E-07	no
SCARA5	0.204016221	5.501901489	5.28E-08	2.45E-07	no
TPM1	0.20394064	5.499774741	5.34E-08	2.48E-07	no
SMS	0.203920099	5.49919677	5.36E-08	2.48E-07	no
TAGLN	0.20390201	5.498687797	5.37E-08	2.49E-07	no
LRRC8E	0.20386735	5.497712587	5.40E-08	2.50E-07	no
DNASE1L2	-0.203798229	-5.495767803	5.46E-08	2.53E-07	no
SNX17	0.203783619	5.495356734	5.47E-08	2.53E-07	no
NIP7	0.203782455	5.495323993	5.47E-08	2.53E-07	no
RHOJ	0.203769612	5.494962659	5.48E-08	2.54E-07	no
KIR2DL3	0.203754304	5.494531963	5.50E-08	2.54E-07	no
DZIP3	-0.203740807	-5.494152244	5.51E-08	2.55E-07	no
CUBN	0.20369914	5.49297998	5.54E-08	2.56E-07	no
OAT	-0.203689995	-5.492722713	5.55E-08	2.57E-07	no
UBE2A	0.203662915	5.491960877	5.57E-08	2.58E-07	no
LPPR3	-0.203637207	-5.491237625	5.60E-08	2.59E-07	no
GRRP1	0.203624903	5.490891481	5.61E-08	2.59E-07	no
MAGED1	-0.203614289	-5.49059289	5.62E-08	2.60E-07	no
ANKRD28	-0.203606952	-5.490386489	5.62E-08	2.60E-07	no
CUL1	0.203603311	5.490284056	5.62E-08	2.60E-07	no
KIAA0182	-0.203580816	-5.489651237	5.64E-08	2.61E-07	no
UPRT	0.203528634	5.488183327	5.69E-08	2.63E-07	no
CKMT1B	-0.203527033	-5.488138274	5.69E-08	2.63E-07	no
POFUT1	0.203487497	5.487026125	5.73E-08	2.64E-07	no
EPHA3	0.203477916	5.48675662	5.73E-08	2.65E-07	no
WDR77	0.203380067	5.484004275	5.82E-08	2.68E-07	no
OTUD4	0.20336356	5.483539974	5.83E-08	2.69E-07	no
ITGA3	0.203327961	5.482538672	5.87E-08	2.70E-07	no

ENDOD1	0.203281061	5.481219519	5.91E-08	2.72E-07	no
SERF2	0.203257059	5.480544445	5.93E-08	2.73E-07	no
SC5DL	-0.203252811	-5.480424968	5.93E-08	2.73E-07	no
DNAJB7	-0.203227433	-5.479711224	5.96E-08	2.74E-07	no
C12orf24	-0.20318664	-5.478563923	5.99E-08	2.76E-07	no
SLC16A14	-0.203142842	-5.477332164	6.03E-08	2.78E-07	no
CD101	0.203073628	5.475385644	6.10E-08	2.81E-07	no
SH2D1B	0.203049113	5.474696256	6.12E-08	2.82E-07	no
MTERFD3	-0.203028955	-5.474129359	6.14E-08	2.82E-07	no
APPBP2	-0.203015146	-5.473741035	6.15E-08	2.83E-07	no
AGPHD1	0.202965844	5.472354633	6.20E-08	2.85E-07	no
USP47	-0.202965098	-5.472333656	6.20E-08	2.85E-07	no
LOC100129387	-0.202952976	-5.471992764	6.21E-08	2.86E-07	no
CCRN4L	0.202929602	5.471335496	6.23E-08	2.86E-07	no
SLC25A18	-0.202857451	-5.469306672	6.30E-08	2.90E-07	no
STK32C	-0.202843939	-5.468926755	6.31E-08	2.90E-07	no
PYROXD2	0.20280688	5.467884716	6.35E-08	2.92E-07	no
CCNB1IP1	-0.202786859	-5.467321782	6.37E-08	2.92E-07	no
SFRS8	-0.202778148	-5.46707687	6.38E-08	2.93E-07	no
LOC100128640	-0.202717576	-5.465373777	6.44E-08	2.95E-07	no
PON1	-0.20267526	-5.464184061	6.48E-08	2.97E-07	no
ATG9A	-0.202639195	-5.463170096	6.51E-08	2.99E-07	no
SPATA12	0.202597124	5.461987302	6.56E-08	3.01E-07	no
ZSCAN29	-0.202553712	-5.460766831	6.60E-08	3.03E-07	no
ANXA2P3	0.202540807	5.460404051	6.61E-08	3.03E-07	no
ATG2A	-0.202508572	-5.459497845	6.64E-08	3.05E-07	no
PGGT1B	0.202498723	5.45922097	6.65E-08	3.05E-07	no
NOLC1	-0.202492325	-5.459041092	6.66E-08	3.05E-07	no
IGJ	0.20248207	5.458752821	6.67E-08	3.06E-07	no
P4HA1	0.202469435	5.458397623	6.68E-08	3.06E-07	no
TMEM69	0.202457467	5.458061187	6.70E-08	3.07E-07	no
C5orf43	0.2024521	5.45791032	6.70E-08	3.07E-07	no
GPRC5D	0.202427999	5.457232807	6.73E-08	3.08E-07	no
ENTHD1	0.202423397	5.457103436	6.73E-08	3.08E-07	no
MCTP2	0.202417417	5.456935334	6.74E-08	3.08E-07	no
MYO9B	0.202389384	5.456147318	6.77E-08	3.09E-07	no
IMMT	0.202343841	5.454867133	6.81E-08	3.12E-07	no
FAM69C	-0.202326541	-5.454380828	6.83E-08	3.12E-07	no
ME2	0.202324125	5.454312922	6.83E-08	3.12E-07	no
SSR1	0.202302068	5.453692941	6.86E-08	3.13E-07	no
DNAJB5	-0.202287594	-5.453286081	6.87E-08	3.14E-07	no
SLC25A39	0.202274924	5.45292997	6.88E-08	3.14E-07	no
ACVR2A	-0.202267617	-5.452724575	6.89E-08	3.15E-07	no
USP46	-0.202255249	-5.452376935	6.91E-08	3.15E-07	no
KCNIP2	-0.202252445	-5.452298128	6.91E-08	3.15E-07	no
HORMAD2	0.202224992	5.451526487	6.94E-08	3.17E-07	no
XPNPEP2	0.202204042	5.450937652	6.96E-08	3.17E-07	no
BHLHE41	0.202196493	5.450725475	6.97E-08	3.18E-07	no
ADAMTS13	-0.202183288	-5.450354326	6.98E-08	3.18E-07	no
TUBGCP6	-0.202181577	-5.450306221	6.98E-08	3.18E-07	no
ABCC8	-0.202138167	-5.449086153	7.03E-08	3.20E-07	no

BTBD17	-0.202126253	-5.448751302	7.04E-08	3.21E-07	no
CEP110	0.202094249	5.447851831	7.08E-08	3.22E-07	no
NAV1	-0.202093233	-5.447823281	7.08E-08	3.22E-07	no
DPT	0.202083201	5.447541321	7.09E-08	3.23E-07	no
RDH14	0.202054189	5.446725966	7.12E-08	3.24E-07	no
NAA50	0.202026959	5.445960687	7.15E-08	3.25E-07	no
SLC47A2	0.201960948	5.444105571	7.22E-08	3.29E-07	no
RAB3C	-0.201938372	-5.443471136	7.24E-08	3.30E-07	no
FEZ2	0.201928044	5.443180909	7.26E-08	3.30E-07	no
TBRG1	-0.201869101	-5.44152451	7.32E-08	3.33E-07	no
PLEKHG7	0.201853591	5.441088695	7.34E-08	3.34E-07	no
SLC2A13	-0.201797949	-5.439525127	7.40E-08	3.36E-07	no
PAIP1	-0.201757109	-5.438377572	7.45E-08	3.38E-07	no
ZMYND15	0.201719392	5.437317765	7.49E-08	3.40E-07	no
SPACA3	0.201688081	5.43643799	7.52E-08	3.42E-07	no
NUDT18	0.201629698	5.434797609	7.59E-08	3.45E-07	no
SSTR2	-0.20158607	-5.433571827	7.64E-08	3.47E-07	no
OXA1L	0.201574551	5.433248184	7.65E-08	3.47E-07	no
FOXO3	-0.201555822	-5.432721978	7.68E-08	3.48E-07	no
FBXW12	-0.20154713	-5.432477789	7.69E-08	3.49E-07	no
PHF17	-0.201531482	-5.43203814	7.70E-08	3.50E-07	no
CYP2E1	-0.20152406	-5.431829642	7.71E-08	3.50E-07	no
WBSCR27	0.201515522	5.431589754	7.72E-08	3.50E-07	no
NFKBIL1	-0.201515386	-5.431585941	7.72E-08	3.50E-07	no
C17orf68	-0.20149961	-5.431142732	7.74E-08	3.51E-07	no
IARS2	0.201453918	5.429859044	7.80E-08	3.53E-07	no
R3HDM2	-0.201422247	-5.428969326	7.83E-08	3.55E-07	no
FAM123A	-0.201383851	-5.427890683	7.88E-08	3.57E-07	no
TAF4	-0.201362213	-5.427282809	7.90E-08	3.58E-07	no
BATF3	0.201317792	5.426034973	7.96E-08	3.60E-07	no
ACSL6	-0.201306356	-5.425713705	7.97E-08	3.61E-07	no
CIAO1	-0.201284134	-5.425089484	8.00E-08	3.62E-07	no
KLC1	-0.201274923	-5.424830741	8.01E-08	3.62E-07	no
MED18	0.201247555	5.424061964	8.04E-08	3.64E-07	no
PPP1CA	0.201207879	5.422947501	8.09E-08	3.66E-07	no
SLC41A2	0.201173817	5.421990734	8.13E-08	3.68E-07	no
ZDHHC19	0.201165857	5.421767154	8.14E-08	3.68E-07	no
ATAD1	-0.201157407	-5.421529809	8.15E-08	3.68E-07	no
SLC2A11	-0.201157129	-5.421522	8.15E-08	3.68E-07	no
MCF2	-0.201151888	-5.421374797	8.16E-08	3.69E-07	no
C1orf112	0.20112917	5.420736671	8.19E-08	3.70E-07	no
CAPN5	0.201050111	5.418516142	8.29E-08	3.74E-07	no
ZNF33A	-0.200881084	-5.41376904	8.50E-08	3.84E-07	no
PDXP	-0.200834674	-5.412465714	8.56E-08	3.86E-07	no
AADAC	0.200828789	5.412300456	8.57E-08	3.86E-07	no
TNRC6B	-0.200802707	-5.411568023	8.60E-08	3.88E-07	no
CHRAC1	0.200794811	5.411346276	8.61E-08	3.88E-07	no
EPHX3	0.200777261	5.410853454	8.63E-08	3.89E-07	no
TNKS	-0.200724946	-5.409384374	8.70E-08	3.92E-07	no
MANEA	0.200710343	5.408974334	8.72E-08	3.93E-07	no
POLL	-0.200658707	-5.407524393	8.79E-08	3.96E-07	no

SOS2	-0.200620168	-5.40644224	8.84E-08	3.98E-07	no
SVOP	-0.200604766	-5.40600978	8.86E-08	3.99E-07	no
CLGN	-0.200563352	-5.404846959	8.92E-08	4.01E-07	no
PTCH1	-0.200530756	-5.403931744	8.96E-08	4.03E-07	no
GLRX5	-0.200522902	-5.403711209	8.97E-08	4.04E-07	no
APLNR	0.200511785	5.403399087	8.98E-08	4.04E-07	no
ADAM10	0.200484711	5.402638955	9.02E-08	4.06E-07	no
MYH7B	-0.20047953	-5.402493492	9.03E-08	4.06E-07	no
FKBP7	0.200475676	5.402385265	9.03E-08	4.06E-07	no
ELL2	0.200468843	5.402193438	9.04E-08	4.07E-07	no
RHBDL1	-0.200357297	-5.399061748	9.20E-08	4.13E-07	no
FAM18B	0.200351641	5.398902968	9.20E-08	4.14E-07	no
LOC541471	0.200348291	5.398808934	9.21E-08	4.14E-07	no
TXNDC9	0.200311477	5.397775408	9.26E-08	4.16E-07	no
LOC441666	-0.200305004	-5.397593702	9.27E-08	4.16E-07	no
TDRD3	-0.200295803	-5.397335407	9.28E-08	4.17E-07	no
SLC27A3	0.200295304	5.397321386	9.28E-08	4.17E-07	no
C16orf52	-0.20027845	-5.396848248	9.30E-08	4.18E-07	no
CPT1C	-0.200243783	-5.395875073	9.35E-08	4.20E-07	no
RPRD1A	-0.200224819	-5.395342718	9.38E-08	4.21E-07	no
RAI1	-0.200194235	-5.394484175	9.42E-08	4.23E-07	no
GALNT6	0.200177735	5.394020997	9.45E-08	4.24E-07	no
SPON1	-0.200173074	-5.393890151	9.45E-08	4.24E-07	no
RER1	0.200166703	5.393711301	9.46E-08	4.24E-07	no
ACSL4	0.200157945	5.393465462	9.47E-08	4.25E-07	no
IGFALS	-0.200153635	-5.393344499	9.48E-08	4.25E-07	no
MRPL10	-0.200135537	-5.392836459	9.51E-08	4.26E-07	no
SLC22A11	0.200125801	5.392563185	9.52E-08	4.26E-07	no
AMDHD1	0.200074748	5.391130142	9.59E-08	4.30E-07	no
ITGA2	0.200069663	5.390987406	9.60E-08	4.30E-07	no
HIC2	-0.200059555	-5.390703674	9.62E-08	4.30E-07	no
RMND5A	-0.200040222	-5.390161019	9.64E-08	4.31E-07	no
C3orf38	0.200019295	5.389573651	9.67E-08	4.33E-07	no
GPR157	0.200013416	5.38940863	9.68E-08	4.33E-07	no
AKAP7	-0.199940221	-5.38735423	9.79E-08	4.38E-07	no
CDC42SE1	0.199927926	5.387009154	9.81E-08	4.38E-07	no
TSPAN14	0.199881718	5.385712269	9.88E-08	4.41E-07	no
SYCE2	-0.199871427	-5.385423453	9.89E-08	4.42E-07	no
REXO1	-0.199867312	-5.385307945	9.90E-08	4.42E-07	no
10-Sep	0.199805349	5.383568957	9.99E-08	4.46E-07	no
TBC1D23	0.199795189	5.383283821	1.00E-07	4.47E-07	no
CLCA3P	0.199768707	5.382540625	1.00E-07	4.48E-07	no
CFHR3	0.199765321	5.382445626	1.00E-07	4.48E-07	no
LARGE	-0.199761746	-5.382345281	1.01E-07	4.49E-07	no
WDR48	-0.199751108	-5.382046756	1.01E-07	4.49E-07	no
KIDINS220	-0.199740634	-5.381752819	1.01E-07	4.50E-07	no
FAM115C	0.199717472	5.381102819	1.01E-07	4.51E-07	no
ASTE1	0.199672499	5.379840765	1.02E-07	4.54E-07	no
C9orf140	-0.199638429	-5.378884689	1.02E-07	4.56E-07	no
RSAD2	0.199518123	5.37550884	1.04E-07	4.65E-07	no
PSRC1	0.19951239	5.375347987	1.04E-07	4.65E-07	no

DTX1	-0.199490315	-5.374728572	1.05E-07	4.66E-07	no
CWC27	-0.199431535	-5.373079296	1.06E-07	4.70E-07	no
GPKOW	-0.199422312	-5.372820512	1.06E-07	4.71E-07	no
FZR1	-0.199378939	-5.371603592	1.06E-07	4.74E-07	no
PRMT5	-0.199374105	-5.371467957	1.07E-07	4.74E-07	no
SPATA13	0.199320207	5.369955761	1.07E-07	4.78E-07	no
ASXL3	-0.199313807	-5.369776208	1.08E-07	4.78E-07	no
RDH5	0.199301626	5.369434464	1.08E-07	4.79E-07	no
ZNF133	-0.199298052	-5.369334191	1.08E-07	4.79E-07	no
NCRNA00171	-0.199279441	-5.368812056	1.08E-07	4.80E-07	no
NHP2L1	-0.199264586	-5.36839531	1.08E-07	4.81E-07	no
C14orf93	-0.199252653	-5.368060523	1.08E-07	4.82E-07	no
LRRC24	-0.199236156	-5.367597716	1.09E-07	4.83E-07	no
HRH3	-0.199222996	-5.36722851	1.09E-07	4.84E-07	no
ENOX1	-0.199213473	-5.366961343	1.09E-07	4.85E-07	no
DNAJC13	0.19919019	5.366308174	1.10E-07	4.86E-07	no
GBE1	0.199188446	5.366259236	1.10E-07	4.86E-07	no
PAPD4	0.199083948	5.363327795	1.11E-07	4.94E-07	no
PLEKHA3	0.199053316	5.362468514	1.12E-07	4.96E-07	no
ANGEL2	-0.199031563	-5.361858324	1.12E-07	4.97E-07	no
NAP1L2	-0.199010491	-5.361267228	1.12E-07	4.99E-07	no
VSTM1	0.198992367	5.360758854	1.13E-07	5.00E-07	no
LRGUK	0.198979946	5.360410419	1.13E-07	5.01E-07	no
LOC100128288	-0.19894749	-5.359500055	1.14E-07	5.03E-07	no
RSRC2	-0.198940356	-5.359299951	1.14E-07	5.04E-07	no
CNTNAP2	-0.198939195	-5.359267385	1.14E-07	5.04E-07	no
GRLF1	-0.198895708	-5.358047627	1.14E-07	5.07E-07	no
HFM1	-0.198884666	-5.357737912	1.15E-07	5.08E-07	no
ELP3	-0.198861044	-5.35707536	1.15E-07	5.09E-07	no
MEX3B	-0.198858584	-5.357006362	1.15E-07	5.09E-07	no
RNF7	0.198854047	5.356879107	1.15E-07	5.10E-07	no
GOLGA7B	-0.198737333	-5.353605641	1.17E-07	5.18E-07	no
CBR1	0.198695653	5.352436719	1.18E-07	5.21E-07	no
PCDH17	-0.198669796	-5.351711549	1.18E-07	5.23E-07	no
NCAM2	-0.19864339	-5.350971004	1.19E-07	5.25E-07	no
ANKRD24	-0.198622575	-5.350387269	1.19E-07	5.27E-07	no
GPR85	-0.198618817	-5.35028189	1.19E-07	5.27E-07	no
DHPS	-0.198598473	-5.349711355	1.20E-07	5.28E-07	no
UBC	0.198593987	5.349585562	1.20E-07	5.29E-07	no
MAGOH	0.198554091	5.348466767	1.20E-07	5.32E-07	no
SEMA6A	-0.198543658	-5.348174186	1.21E-07	5.32E-07	no
STMN4	-0.198521432	-5.347550927	1.21E-07	5.34E-07	no
LRRC49	-0.19836495	-5.343163057	1.24E-07	5.47E-07	no
EIF4EBP2	-0.198358933	-5.342994357	1.24E-07	5.47E-07	no
ZNRF1	-0.198352748	-5.342820928	1.24E-07	5.47E-07	no
RSC1A1	0.198350019	5.342744399	1.24E-07	5.47E-07	no
CST2	0.198326751	5.342091994	1.25E-07	5.49E-07	no
DGCR5	-0.198306091	-5.341512718	1.25E-07	5.51E-07	no
FKBP14	0.198270707	5.340520628	1.26E-07	5.54E-07	no
ZNF454	-0.198251158	-5.339972519	1.26E-07	5.55E-07	no
DNAJB6	0.198215949	5.338985373	1.27E-07	5.58E-07	no

OPA1	-0.198146825	-5.337047389	1.28E-07	5.63E-07	no
DTX4	-0.198114229	-5.336133569	1.29E-07	5.66E-07	no
CHST4	0.19810767	5.335949679	1.29E-07	5.66E-07	no
BZW1	0.198088969	5.335425396	1.29E-07	5.68E-07	no
KLC2	-0.197992562	-5.332722765	1.31E-07	5.76E-07	no
DLGAP3	-0.197970373	-5.332100741	1.31E-07	5.78E-07	no
PQBP1	-0.197883081	-5.329653796	1.33E-07	5.85E-07	no
NTM	-0.197874096	-5.329401947	1.33E-07	5.86E-07	no
BAI1	-0.197852192	-5.328787943	1.34E-07	5.88E-07	no
CELF6	-0.197842022	-5.328502877	1.34E-07	5.88E-07	no
FTSJ2	0.197803568	5.327425016	1.35E-07	5.91E-07	no
SUCNR1	0.197803511	5.32742343	1.35E-07	5.91E-07	no
NANS	0.197803323	5.327418145	1.35E-07	5.91E-07	no
ACADSB	-0.197758282	-5.326155696	1.36E-07	5.95E-07	no
VAX2	-0.197739252	-5.325622302	1.36E-07	5.97E-07	no
RB1CC1	-0.197712022	-5.324859102	1.36E-07	5.99E-07	no
CPM	0.197684151	5.324077947	1.37E-07	6.01E-07	no
FBLN7	0.197635048	5.322701709	1.38E-07	6.06E-07	no
CR1L	0.197624106	5.322395062	1.38E-07	6.06E-07	no
KBTBD7	-0.197588805	-5.321405702	1.39E-07	6.10E-07	no
RFX7	-0.197584622	-5.32128846	1.39E-07	6.10E-07	no
BCL2L14	0.197562425	5.320666372	1.39E-07	6.12E-07	no
RPS6KA5	-0.197539409	-5.320021362	1.40E-07	6.14E-07	no
KCNJ10	-0.197521452	-5.319518099	1.40E-07	6.15E-07	no
ANKRD6	-0.197504797	-5.319051353	1.41E-07	6.16E-07	no
SEMA4B	-0.197483578	-5.318456719	1.41E-07	6.18E-07	no
ERRFI1	0.197477367	5.318282657	1.41E-07	6.19E-07	no
TICAM1	0.197473901	5.318185535	1.41E-07	6.19E-07	no
NFYB	-0.197386946	-5.315748787	1.43E-07	6.27E-07	no
SYNC	0.197375051	5.315415451	1.43E-07	6.28E-07	no
SGPP1	0.197288767	5.312997645	1.45E-07	6.36E-07	no
BICD1	0.197241907	5.311684604	1.46E-07	6.40E-07	no
HLF	-0.197223777	-5.311176616	1.47E-07	6.42E-07	no
RNF141	-0.197202799	-5.310588817	1.47E-07	6.43E-07	no
ARHGEF15	0.197191519	5.310272765	1.47E-07	6.44E-07	no
KIAA1267	-0.197179372	-5.309932432	1.48E-07	6.45E-07	no
EEA1	0.197171025	5.309698543	1.48E-07	6.46E-07	no
TMEM41B	-0.197161767	-5.309439142	1.48E-07	6.47E-07	no
CRB1	-0.19713198	-5.308604572	1.49E-07	6.49E-07	no
GPR97	0.197125475	5.30842232	1.49E-07	6.50E-07	no
ANKRD45	0.197065755	5.306749125	1.50E-07	6.56E-07	no
ABCB5	0.197058643	5.306549868	1.50E-07	6.56E-07	no
PDLIM5	-0.196972474	-5.304135762	1.52E-07	6.64E-07	no
C9orf47	0.196947106	5.303425061	1.53E-07	6.67E-07	no
EPT1	-0.196946242	-5.303400866	1.53E-07	6.67E-07	no
MREG	0.196942268	5.303289539	1.53E-07	6.67E-07	no
RPAIN	-0.196847187	-5.300625938	1.55E-07	6.76E-07	no
TREML3	0.196846602	5.300609545	1.55E-07	6.76E-07	no
FMNL3	0.196811582	5.299628536	1.56E-07	6.79E-07	no
HNF1A	-0.196811557	-5.299627862	1.56E-07	6.79E-07	no
ATG3	0.196807203	5.299505884	1.56E-07	6.80E-07	no

SERINC2	0.196782132	5.298803603	1.57E-07	6.82E-07	no
ELOVL1	0.196774662	5.298594331	1.57E-07	6.83E-07	no
IER5	0.196767222	5.298385943	1.57E-07	6.83E-07	no
C22orf15	0.196765887	5.298348524	1.57E-07	6.83E-07	no
PTK6	-0.196764872	-5.298320102	1.57E-07	6.83E-07	no
LOC644165	-0.196763058	-5.298269304	1.57E-07	6.83E-07	no
PCBP1	0.19676044	5.298195951	1.57E-07	6.83E-07	no
C4orf12	-0.196730527	-5.297358061	1.58E-07	6.86E-07	no
ARL1	0.196719524	5.297049855	1.58E-07	6.87E-07	no
RPE	0.196716095	5.296953801	1.58E-07	6.87E-07	no
LOC285033	0.196671476	5.295704014	1.59E-07	6.92E-07	no
C10orf91	0.19663104	5.294571407	1.60E-07	6.96E-07	no
KERA	0.196630912	5.294567824	1.60E-07	6.96E-07	no
IER2	0.196610078	5.293984276	1.61E-07	6.97E-07	no
TCEA3	0.196573546	5.29296106	1.61E-07	7.01E-07	no
POLRMT	-0.196519738	-5.291454015	1.63E-07	7.07E-07	no
PHACTR1	-0.196517212	-5.291383251	1.63E-07	7.07E-07	no
EPS8L1	0.196495738	5.290781844	1.63E-07	7.09E-07	no
VLDLR	0.196491199	5.290654722	1.63E-07	7.09E-07	no
TRIM62	-0.196481332	-5.290378364	1.64E-07	7.10E-07	no
C14orf45	0.196462953	5.289863619	1.64E-07	7.12E-07	no
GJD3	0.196456443	5.289681308	1.64E-07	7.12E-07	no
KCNN1	-0.19645493	-5.289638946	1.64E-07	7.12E-07	no
BRAP	-0.196443527	-5.289319568	1.65E-07	7.13E-07	no
SNN	-0.196418456	-5.288617434	1.65E-07	7.16E-07	no
ZNF804A	-0.196414738	-5.288513314	1.65E-07	7.16E-07	no
ATP6V1C2	0.196397277	5.288024325	1.66E-07	7.18E-07	no
FGFRL1	0.196373117	5.287347722	1.66E-07	7.20E-07	no
PHLDA3	0.196367289	5.287184505	1.66E-07	7.21E-07	no
ATP5EP2	0.196357009	5.286896605	1.67E-07	7.21E-07	no
TNFSF4	0.196339839	5.286415768	1.67E-07	7.23E-07	no
PIWIL4	0.196326286	5.286036232	1.67E-07	7.24E-07	no
IL1RAPL1	-0.196303535	-5.28539912	1.68E-07	7.27E-07	no
POU3F3	-0.196275939	-5.284626327	1.69E-07	7.30E-07	no
CD34	0.196249703	5.283891629	1.69E-07	7.32E-07	no
SLC9A2	-0.196240101	-5.283622748	1.70E-07	7.33E-07	no
BAT3	-0.196220247	-5.283066789	1.70E-07	7.35E-07	no
MYL4	0.196205977	5.282667201	1.70E-07	7.36E-07	no
TRIM13	-0.196203261	-5.282591139	1.70E-07	7.37E-07	no
RPS27L	0.196184207	5.282057612	1.71E-07	7.38E-07	no
PRDX4	0.196181071	5.281969792	1.71E-07	7.39E-07	no
CS	-0.196149883	-5.28109647	1.72E-07	7.42E-07	no
ATF3	0.196140358	5.280829758	1.72E-07	7.43E-07	no
GRWD1	0.196125627	5.280417287	1.72E-07	7.44E-07	no
WWOX	-0.196120462	-5.280272664	1.73E-07	7.45E-07	no
ASH1L	-0.19610967	-5.279970484	1.73E-07	7.46E-07	no
TADA2B	-0.196070093	-5.27886233	1.74E-07	7.50E-07	no
TAF1D	-0.196045634	-5.27817748	1.74E-07	7.52E-07	no
C15orf54	0.196021236	5.277494359	1.75E-07	7.55E-07	no
BRP44	0.195957953	5.275722555	1.77E-07	7.62E-07	no
TIGD1	-0.195920093	-5.274662558	1.78E-07	7.66E-07	no

LOC153328	-0.195899641	-5.274089961	1.78E-07	7.68E-07	no
MTHFR	0.195898799	5.274066386	1.78E-07	7.68E-07	no
NUP54	0.195895246	5.273966912	1.78E-07	7.68E-07	no
MLLT4	-0.195844785	-5.27255419	1.80E-07	7.74E-07	no
KAT2A	-0.19579693	-5.271214468	1.81E-07	7.79E-07	no
SPTLC2	0.195760178	5.270185618	1.82E-07	7.83E-07	no
LGALS9B	0.195722953	5.269143521	1.83E-07	7.87E-07	no
PUM2	-0.195719989	-5.269060538	1.83E-07	7.87E-07	no
MBD5	-0.195673117	-5.267748455	1.84E-07	7.93E-07	no
ZNF202	-0.195672502	-5.267731224	1.84E-07	7.93E-07	no
SLC1A1	-0.195627373	-5.266467952	1.85E-07	7.98E-07	no
RANBP17	-0.195576511	-5.26504424	1.87E-07	8.03E-07	no
RNF5P1	-0.195565031	-5.26472289	1.87E-07	8.05E-07	no
DLG4	-0.195469542	-5.262050144	1.90E-07	8.16E-07	no
PTGDR	0.195445722	5.261383415	1.91E-07	8.18E-07	no
RAB11FIP4	-0.195436173	-5.261116156	1.91E-07	8.19E-07	no
RAB2A	-0.195422903	-5.26074476	1.91E-07	8.21E-07	no
SS18	0.195404304	5.26022419	1.92E-07	8.23E-07	no
MATN2	0.195385313	5.259692679	1.92E-07	8.25E-07	no
APOD	0.19537945	5.259528561	1.92E-07	8.26E-07	no
FLJ36031	0.195362212	5.259046109	1.93E-07	8.27E-07	no
CACNA1A	-0.19532525	-5.25801166	1.94E-07	8.32E-07	no
C9orf45	-0.195309479	-5.257570281	1.94E-07	8.34E-07	no
TSPAN1	0.195223667	5.255168721	1.97E-07	8.44E-07	no
POLR3C	0.195200629	5.254524022	1.97E-07	8.47E-07	no
GNL3L	0.195198207	5.254456225	1.98E-07	8.47E-07	no
ZMYM3	-0.195187356	-5.254152579	1.98E-07	8.48E-07	no
WDR37	-0.195184838	-5.254082116	1.98E-07	8.48E-07	no
SLC31A2	0.195143352	5.252921153	1.99E-07	8.53E-07	no
TMEFF1	-0.195113709	-5.252091641	2.00E-07	8.56E-07	no
PABPC5	-0.195097073	-5.251626089	2.00E-07	8.58E-07	no
F3	0.195080264	5.251155748	2.01E-07	8.60E-07	no
DNAH9	0.195041491	5.25007077	2.02E-07	8.65E-07	no
FLT3LG	0.195006556	5.249093223	2.03E-07	8.69E-07	no
HACE1	-0.195001525	-5.248952433	2.03E-07	8.70E-07	no
ZNF618	0.194946973	5.247426024	2.05E-07	8.76E-07	no
CD83	0.19494179	5.247281015	2.05E-07	8.77E-07	no
CCDC62	-0.194922979	-5.246754671	2.06E-07	8.79E-07	no
TMEM30A	-0.194900432	-5.246123809	2.06E-07	8.82E-07	no
SSFA2	0.194885974	5.245719256	2.07E-07	8.84E-07	no
LRPPRC	-0.19487865	-5.245514339	2.07E-07	8.84E-07	no
GLT8D2	0.194856367	5.244890885	2.08E-07	8.87E-07	no
KIF5C	-0.194842689	-5.244508201	2.08E-07	8.89E-07	no
DNAJC9	-0.194739812	-5.241629915	2.11E-07	9.02E-07	no
PIGV	0.194687159	5.24015684	2.13E-07	9.09E-07	no
UBR3	-0.194682799	-5.24003489	2.13E-07	9.09E-07	no
ROBLD3	0.194665149	5.239541095	2.14E-07	9.11E-07	no
RPA3	0.194659948	5.239395593	2.14E-07	9.12E-07	no
CBX4	-0.194654603	-5.239246067	2.14E-07	9.12E-07	no
MAGEE1	-0.1946381	-5.238784394	2.14E-07	9.14E-07	no
SELS	0.194629301	5.238538247	2.15E-07	9.15E-07	no

C16orf57	0.194602563	5.237790253	2.15E-07	9.19E-07	no
BET1	0.194600515	5.237732938	2.16E-07	9.19E-07	no
RNF148	0.194577759	5.237096362	2.16E-07	9.21E-07	no
MLLT11	-0.194565474	-5.236752705	2.17E-07	9.23E-07	no
MYO1A	0.194560328	5.236608737	2.17E-07	9.23E-07	no
P2RY2	0.194545811	5.236202644	2.17E-07	9.25E-07	no
SYTL2	0.194537453	5.235968831	2.18E-07	9.26E-07	no
SETDB2	0.19451211	5.23525992	2.18E-07	9.29E-07	no
PLD4	0.194483866	5.234469836	2.19E-07	9.33E-07	no
YPEL3	-0.194437216	-5.233164924	2.21E-07	9.39E-07	no
PAR5	-0.194411488	-5.232445276	2.22E-07	9.42E-07	no
SCN3A	-0.194393756	-5.231949284	2.22E-07	9.45E-07	no
UBAP2	-0.194357304	-5.230929703	2.23E-07	9.50E-07	no
LOC157627	-0.194331214	-5.230199963	2.24E-07	9.53E-07	no
NCAPH2	-0.194274323	-5.228608742	2.26E-07	9.61E-07	no
IFI27	0.194269262	5.228467182	2.26E-07	9.61E-07	no
CMYA5	0.194241699	5.227696279	2.27E-07	9.65E-07	no
KLHL11	-0.19420848	-5.226767186	2.28E-07	9.69E-07	no
INHBA	0.194205361	5.226679967	2.28E-07	9.70E-07	no
RIMBP3	0.194196341	5.226427683	2.29E-07	9.70E-07	no
SEC61B	0.194196012	5.226418496	2.29E-07	9.70E-07	no
LMF2	0.194160796	5.225433585	2.30E-07	9.75E-07	no
FBXO39	0.194123506	5.2243907	2.31E-07	9.80E-07	no
RPS10P7	0.194102471	5.223802425	2.32E-07	9.83E-07	no
TBC1D8B	0.194053314	5.222427715	2.33E-07	9.90E-07	no
GK	0.194010362	5.221226561	2.35E-07	9.96E-07	no
NR1D2	-0.193985534	-5.220532261	2.36E-07	9.99E-07	no
GCDH	-0.193879944	-5.217579589	2.39E-07	1.01E-06	no
RNF13	0.193850184	5.216747444	2.40E-07	1.02E-06	no
COL29A1	0.193721482	5.21314882	2.45E-07	1.04E-06	no
FAM100A	-0.19368958	-5.212256861	2.46E-07	1.04E-06	no
ACTA2	0.19367497	5.211848358	2.47E-07	1.04E-06	no
WNT5A	0.193666851	5.211621379	2.47E-07	1.05E-06	no
OBSL1	-0.193647697	-5.21108584	2.48E-07	1.05E-06	no
RFFL	0.193588798	5.209439138	2.50E-07	1.06E-06	no
MLST8	-0.193515105	-5.207378912	2.52E-07	1.07E-06	no
UGP2	0.193513485	5.207333622	2.52E-07	1.07E-06	no
MTMR3	-0.193503222	-5.207046708	2.53E-07	1.07E-06	no
C14orf147	0.19349688	5.206869408	2.53E-07	1.07E-06	no
RAB6C	-0.193487531	-5.206608052	2.53E-07	1.07E-06	no
LOC145837	-0.193468448	-5.206074583	2.54E-07	1.07E-06	no
C10orf137	-0.193460608	-5.205855413	2.54E-07	1.08E-06	no
RPL23AP82	0.193458152	5.205786755	2.54E-07	1.08E-06	no
PNMA6A	-0.193451485	-5.20560036	2.55E-07	1.08E-06	no
EXD3	-0.193444831	-5.20541434	2.55E-07	1.08E-06	no
ZBTB5	-0.193432598	-5.205072392	2.55E-07	1.08E-06	no
KHDC1	-0.193286061	-5.200976137	2.61E-07	1.10E-06	no
YDJC	0.193270285	5.200535144	2.61E-07	1.10E-06	no
ZNF738	-0.193261847	-5.200299284	2.62E-07	1.10E-06	no
KIR2DL1	0.193230819	5.199431992	2.63E-07	1.11E-06	no
CRELD2	0.193157653	5.197386956	2.66E-07	1.12E-06	no

PDK2	-0.193123199	-5.196423966	2.67E-07	1.13E-06	no
DIP2A	-0.193088895	-5.195465177	2.68E-07	1.13E-06	no
NKD1	-0.193083308	-5.195309029	2.69E-07	1.13E-06	no
SNORA7B	0.193055925	5.194543702	2.70E-07	1.14E-06	no
IDI2	-0.193027374	-5.193745758	2.71E-07	1.14E-06	no
ICK	-0.193008724	-5.193224532	2.72E-07	1.14E-06	no
ANKH	0.193000382	5.192991377	2.72E-07	1.15E-06	no
SRGAP2	0.192944143	5.191419649	2.74E-07	1.15E-06	no
SEZ6L2	-0.192909547	-5.19045281	2.75E-07	1.16E-06	no
CXorf40A	0.192875026	5.189488104	2.77E-07	1.17E-06	no
TTLL1	-0.192828538	-5.188188982	2.79E-07	1.17E-06	no
KRT80	0.192822125	5.188009791	2.79E-07	1.17E-06	no
LOC338799	-0.192805612	-5.187548324	2.80E-07	1.18E-06	no
GGA1	-0.192760162	-5.18627829	2.82E-07	1.18E-06	no
ORAI2	0.192759637	5.186263609	2.82E-07	1.18E-06	no
WDR45L	-0.192725776	-5.185317408	2.83E-07	1.19E-06	no
AQP3	0.192724128	5.185271368	2.83E-07	1.19E-06	no
TPSB2	0.192713889	5.18498526	2.83E-07	1.19E-06	no
UPK2	-0.192713074	-5.184962479	2.83E-07	1.19E-06	no
TCTN3	0.19271062	5.184893915	2.84E-07	1.19E-06	no
SOX21	-0.192665242	-5.183625953	2.85E-07	1.20E-06	no
LRRN4	-0.192655746	-5.183360604	2.86E-07	1.20E-06	no
SNED1	0.192614756	5.182215271	2.87E-07	1.21E-06	no
OCIAD2	0.192613841	5.182189723	2.88E-07	1.21E-06	no
TMCO1	0.192541283	5.180162418	2.91E-07	1.22E-06	no
WDR53	0.192538271	5.180078248	2.91E-07	1.22E-06	no
NFYA	-0.192536915	-5.180040373	2.91E-07	1.22E-06	no
ZNF219	-0.19250625	-5.179183596	2.92E-07	1.22E-06	no
OAS3	0.192482476	5.17851937	2.93E-07	1.23E-06	no
ZC3H6	-0.19245682	-5.177802564	2.94E-07	1.23E-06	no
CHST6	0.192434551	5.177180402	2.95E-07	1.24E-06	no
C1orf96	-0.192427546	-5.17698472	2.95E-07	1.24E-06	no
HS1BP3	0.192424733	5.176906117	2.95E-07	1.24E-06	no
TBPL1	-0.192392981	-5.176019047	2.97E-07	1.24E-06	no
STOX1	-0.19238038	-5.175667012	2.97E-07	1.25E-06	no
PHLDA2	0.192367668	5.175311858	2.98E-07	1.25E-06	no
CNTN1	-0.192366821	-5.175288211	2.98E-07	1.25E-06	no
NRG3	-0.19225708	-5.172222466	3.03E-07	1.27E-06	no
C2orf40	0.192254575	5.172152478	3.03E-07	1.27E-06	no
QPCT	0.192246344	5.171922543	3.03E-07	1.27E-06	no
CDH22	-0.192210181	-5.170912355	3.05E-07	1.27E-06	no
REEP6	-0.19214338	-5.169046327	3.08E-07	1.29E-06	no
ATXN7L2	-0.192143073	-5.169037752	3.08E-07	1.29E-06	no
KIF26A	-0.192102675	-5.167909324	3.09E-07	1.29E-06	no
BSN	-0.192091073	-5.167585252	3.10E-07	1.30E-06	no
KIAA1486	-0.191961793	-5.163974285	3.16E-07	1.32E-06	no
PTGES	0.191935241	5.163232696	3.17E-07	1.32E-06	no
SSRP1	-0.191923659	-5.16290922	3.18E-07	1.33E-06	no
DLC1	0.191896156	5.162141066	3.19E-07	1.33E-06	no
GOLPH3	0.191890061	5.161970845	3.19E-07	1.33E-06	no
GGCT	0.191860549	5.161146599	3.20E-07	1.34E-06	no

PRRX2	0.191859736	5.161123889	3.20E-07	1.34E-06	no
LOC284440	-0.191819343	-5.159995788	3.22E-07	1.34E-06	no
UBN2	-0.191793911	-5.159285524	3.24E-07	1.35E-06	no
PRKCZ	-0.191788124	-5.159123928	3.24E-07	1.35E-06	no
MYCNOS	-0.191775598	-5.158774097	3.24E-07	1.35E-06	no
ATRN	-0.19175883	-5.158305834	3.25E-07	1.36E-06	no
ENO2	-0.191724418	-5.157344822	3.27E-07	1.36E-06	no
TM7SF2	-0.191706896	-5.156855478	3.28E-07	1.37E-06	no
C1orf88	0.191703585	5.156763023	3.28E-07	1.37E-06	no
RHOD	0.191702827	5.156741857	3.28E-07	1.37E-06	no
OSTCL	0.191669099	5.155799992	3.29E-07	1.37E-06	no
WDR41	0.191660829	5.155569048	3.30E-07	1.37E-06	no
CLDN9	-0.191645977	-5.155154307	3.30E-07	1.38E-06	no
ANKRD36BP1	0.191639175	5.154964344	3.31E-07	1.38E-06	no
GZF1	-0.191626867	-5.15462066	3.31E-07	1.38E-06	no
HN1L	0.191618416	5.154384669	3.32E-07	1.38E-06	no
DVL2	-0.19160561	-5.154027062	3.32E-07	1.38E-06	no
CTNNBIP1	-0.191592982	-5.153674431	3.33E-07	1.38E-06	no
BIRC2	0.191565369	5.152903366	3.34E-07	1.39E-06	no
SLC16A4	0.191531771	5.151965206	3.36E-07	1.40E-06	no
PTAR1	0.191501453	5.151118635	3.37E-07	1.40E-06	no
PTPN11	-0.191492107	-5.150857676	3.38E-07	1.40E-06	no
SYBU	-0.191458051	-5.149906754	3.40E-07	1.41E-06	no
LETMD1	-0.191458014	-5.149905729	3.40E-07	1.41E-06	no
SCN1A	-0.191404059	-5.14839922	3.42E-07	1.42E-06	no
SMC3	-0.191403647	-5.148387728	3.42E-07	1.42E-06	no
RELT	0.191392756	5.148083642	3.43E-07	1.42E-06	no
COPG	0.191386556	5.147910536	3.43E-07	1.42E-06	no
TNFAIP8L3	0.191383573	5.147827239	3.43E-07	1.42E-06	no
NCOA6	-0.191370334	-5.147457605	3.44E-07	1.43E-06	no
IMPDH2	-0.191366023	-5.147337238	3.44E-07	1.43E-06	no
CA10	-0.191362365	-5.147235111	3.44E-07	1.43E-06	no
LETM1	-0.191354384	-5.147012272	3.45E-07	1.43E-06	no
SPATA16	0.191342234	5.146673055	3.45E-07	1.43E-06	no
CASQ1	-0.191331502	-5.146373419	3.46E-07	1.43E-06	no
CCL20	0.191328895	5.146300611	3.46E-07	1.43E-06	no
OR2A9P	0.191326844	5.146243354	3.46E-07	1.43E-06	no
TMEM200B	0.191310263	5.145780411	3.47E-07	1.44E-06	no
TIGD7	-0.191293019	-5.145298976	3.48E-07	1.44E-06	no
RANBP9	0.191286945	5.145129406	3.48E-07	1.44E-06	no
MORC2	-0.191276634	-5.144841529	3.48E-07	1.44E-06	no
CDK5R2	-0.191256621	-5.144282789	3.49E-07	1.45E-06	no
MGC42105	-0.191242631	-5.143892218	3.50E-07	1.45E-06	no
CES1	0.19124077	5.14384027	3.50E-07	1.45E-06	no
ZNF195	-0.191237957	-5.143761739	3.50E-07	1.45E-06	no
FAM35B	-0.191193078	-5.142508795	3.53E-07	1.46E-06	no
TBL3	-0.191176517	-5.142046452	3.53E-07	1.46E-06	no
C5orf58	0.191164262	5.141704352	3.54E-07	1.46E-06	no
PADI6	0.191151302	5.141342532	3.55E-07	1.47E-06	no
ZNF613	0.191146167	5.141199197	3.55E-07	1.47E-06	no
EYA4	0.191140572	5.141042982	3.55E-07	1.47E-06	no

BRSK1	-0.191126485	-5.140649743	3.56E-07	1.47E-06	no
AMN1	-0.191108934	-5.140159779	3.57E-07	1.47E-06	no
NCKIPSD	-0.191029715	-5.137948336	3.61E-07	1.49E-06	no
LYPLA1	0.191017336	5.137602797	3.62E-07	1.49E-06	no
SOCS7	-0.191011642	-5.137443837	3.62E-07	1.49E-06	no
OLFM1	-0.191006317	-5.137295199	3.62E-07	1.49E-06	no
GDF15	0.1909788	5.136527076	3.64E-07	1.50E-06	no
TTC9B	-0.190961658	-5.136048594	3.65E-07	1.50E-06	no
SFMBT1	0.190945744	5.135604372	3.65E-07	1.51E-06	no
VGLL3	0.190934916	5.135302131	3.66E-07	1.51E-06	no
MORN3	0.190882369	5.133835391	3.69E-07	1.52E-06	no
C14orf142	0.190870111	5.133493262	3.69E-07	1.52E-06	no
UBA3	0.190847713	5.132868067	3.71E-07	1.53E-06	no
NUDT21	-0.190845233	-5.132798871	3.71E-07	1.53E-06	no
C8orf76	0.190844077	5.132766595	3.71E-07	1.53E-06	no
ATF6	0.190838012	5.132597312	3.71E-07	1.53E-06	no
PI4K2B	0.190803581	5.131636291	3.73E-07	1.53E-06	no
C12orf65	-0.190758205	-5.130369814	3.75E-07	1.54E-06	no
SNCAIP	-0.190741084	-5.129891969	3.76E-07	1.55E-06	no
RNMT	-0.190709954	-5.129023159	3.78E-07	1.55E-06	no
CEND1	-0.190676439	-5.128087777	3.80E-07	1.56E-06	no
NECAB2	-0.190661088	-5.127659346	3.81E-07	1.56E-06	no
LRRIQ3	0.190655552	5.127504846	3.81E-07	1.57E-06	no
FAM48B2	0.190614395	5.126356226	3.83E-07	1.57E-06	no
NDUFS2	-0.190593906	-5.125784413	3.84E-07	1.58E-06	no
KIAA0040	0.190534873	5.124136976	3.87E-07	1.59E-06	no
GNAI1	-0.190524161	-5.123838044	3.88E-07	1.59E-06	no
RHOC	0.190523422	5.123817427	3.88E-07	1.59E-06	no
MOSPD2	0.190505105	5.123306281	3.89E-07	1.60E-06	no
ZNF280B	-0.190501259	-5.123198949	3.89E-07	1.60E-06	no
KCNH8	-0.190476342	-5.122503624	3.91E-07	1.60E-06	no
AXIN2	-0.190455566	-5.121923859	3.92E-07	1.61E-06	no
LRRC37A4	-0.190452162	-5.121828856	3.92E-07	1.61E-06	no
PLXNB3	-0.19045211	-5.121827415	3.92E-07	1.61E-06	no
AATF	-0.190422231	-5.120993647	3.94E-07	1.61E-06	no
TM4SF18	0.190406678	5.120559642	3.95E-07	1.62E-06	no
FBXO44	-0.190397652	-5.120307793	3.95E-07	1.62E-06	no
NKX3-1	0.190382544	5.119886205	3.96E-07	1.62E-06	no
GOSR2	0.190381092	5.1198457	3.96E-07	1.62E-06	no
AGBL2	0.190374611	5.119664852	3.96E-07	1.62E-06	no
SMC4	0.190357855	5.119197278	3.97E-07	1.63E-06	no
DDX47	0.190345328	5.118847748	3.98E-07	1.63E-06	no
PDLIM3	0.190327105	5.118339247	3.99E-07	1.63E-06	no
GPRC5B	-0.190319009	-5.118113365	4.00E-07	1.64E-06	no
CCBP2	0.190284043	5.117137707	4.02E-07	1.64E-06	no
CMPK1	0.19028202	5.11708127	4.02E-07	1.64E-06	no
ANKRD1	0.19021625	5.11524617	4.05E-07	1.66E-06	no
TSPAN9	0.190199866	5.114789039	4.06E-07	1.66E-06	no
RBM14	-0.190186625	-5.114419604	4.07E-07	1.67E-06	no
EYA3	0.190147335	5.113323374	4.09E-07	1.67E-06	no
C10orf10	0.190139246	5.113097708	4.10E-07	1.68E-06	no

SLC22A18AS	0.190131238	5.112874267	4.10E-07	1.68E-06	no
C2CD4C	-0.190099223	-5.111981066	4.12E-07	1.68E-06	no
PSPC1	-0.190066528	-5.111068901	4.14E-07	1.69E-06	no
SPATA17	0.190058966	5.110857945	4.15E-07	1.69E-06	no
AFF3	-0.189992013	-5.108990073	4.19E-07	1.71E-06	no
SERTAD1	0.189989532	5.108920857	4.19E-07	1.71E-06	no
FAM188A	-0.189974175	-5.108492445	4.20E-07	1.71E-06	no
NTRK2	-0.18995526	-5.107964758	4.21E-07	1.72E-06	no
LYPLA2P1	0.189858629	5.105269133	4.27E-07	1.74E-06	no
C2orf28	0.189831375	5.104508886	4.28E-07	1.75E-06	no
FAM171B	-0.18981047	-5.103925738	4.30E-07	1.75E-06	no
RPIA	0.189799878	5.10363029	4.30E-07	1.75E-06	no
TRAPPC6B	-0.189770629	-5.102814417	4.32E-07	1.76E-06	no
ARMC2	0.189757444	5.102446637	4.33E-07	1.76E-06	no
SYS1	0.189738478	5.101917589	4.34E-07	1.77E-06	no
ANKRD36	-0.189700773	-5.100865888	4.36E-07	1.78E-06	no
METT10D	-0.189685844	-5.100449481	4.37E-07	1.78E-06	no
NRD1	0.189680157	5.100290851	4.38E-07	1.78E-06	no
LGALS3	0.189669371	5.099989999	4.38E-07	1.78E-06	no
SLC5A12	-0.18964888	-5.099418479	4.40E-07	1.79E-06	no
HMGCLL1	-0.189604595	-5.098183301	4.42E-07	1.80E-06	no
NCOA1	-0.189567481	-5.097148141	4.45E-07	1.81E-06	no
PNMA3	-0.189441138	-5.093624515	4.53E-07	1.84E-06	no
ADCY2	-0.189421517	-5.093077315	4.54E-07	1.85E-06	no
LOC100130148	-0.189417553	-5.09296675	4.54E-07	1.85E-06	no
PSAT1	-0.189410521	-5.092770659	4.55E-07	1.85E-06	no
CHI3L1	0.189369811	5.091635353	4.57E-07	1.86E-06	no
CNNM2	-0.189366547	-5.091544319	4.58E-07	1.86E-06	no
LRRC42	0.189362651	5.09143566	4.58E-07	1.86E-06	no
EMR3	0.189314763	5.090100232	4.61E-07	1.87E-06	no
C20orf118	0.189274928	5.088989392	4.63E-07	1.88E-06	no
MRPS15	0.18927273	5.088928091	4.64E-07	1.88E-06	no
SSTR1	-0.189266093	-5.088743016	4.64E-07	1.88E-06	no
UBA5	-0.189255948	-5.088460114	4.65E-07	1.89E-06	no
TATDN1	-0.18924472	-5.088147019	4.65E-07	1.89E-06	no
TMEM170A	-0.189219826	-5.087452843	4.67E-07	1.90E-06	no
PTDSS1	0.189202436	5.086967934	4.68E-07	1.90E-06	no
COL5A3	0.189177473	5.086271869	4.70E-07	1.91E-06	no
PABPN1	-0.189143282	-5.085318472	4.72E-07	1.92E-06	no
SOX12	-0.189136626	-5.085132897	4.73E-07	1.92E-06	no
DCAF8	-0.189124035	-5.084781816	4.74E-07	1.92E-06	no
EFHC2	0.189102578	5.084183517	4.75E-07	1.93E-06	no
ERICH1	0.189076259	5.083449683	4.77E-07	1.93E-06	no
APH1A	0.189006718	5.081510737	4.81E-07	1.95E-06	no
LGALS14	0.189004723	5.081455105	4.82E-07	1.95E-06	no
FAM19A2	-0.189002585	-5.08139549	4.82E-07	1.95E-06	no
EVL	-0.188995217	-5.081190063	4.82E-07	1.95E-06	no
PLAC8	0.188965729	5.080367929	4.84E-07	1.96E-06	no
EPC1	-0.188964258	-5.080326919	4.84E-07	1.96E-06	no
FAM124B	0.188951459	5.079970059	4.85E-07	1.96E-06	no
RPRD2	-0.188923811	-5.079199244	4.87E-07	1.97E-06	no

TCAP	-0.188918331	-5.079046451	4.87E-07	1.97E-06	no
ZNF691	0.188879943	5.077976211	4.90E-07	1.98E-06	no
KIAA2022	-0.188859376	-5.077402808	4.92E-07	1.99E-06	no
DNAJC27	-0.188846954	-5.077056502	4.92E-07	1.99E-06	no
IFT20	0.188829447	5.076568439	4.94E-07	2.00E-06	no
CDK7	0.188815181	5.07617071	4.95E-07	2.00E-06	no
USP4	0.188778078	5.075136369	4.97E-07	2.01E-06	no
LOC100128239	-0.188767349	-5.074837278	4.98E-07	2.01E-06	no
TMEM196	-0.188745455	-5.074226914	5.00E-07	2.02E-06	no
PITRM1	-0.188743605	-5.074175344	5.00E-07	2.02E-06	no
HERC1	-0.188734029	-5.073908409	5.00E-07	2.02E-06	no
UBL3	-0.188697587	-5.072892532	5.03E-07	2.03E-06	no
WBP5	0.188695614	5.072837524	5.03E-07	2.03E-06	no
LOC25845	-0.188694163	-5.072797072	5.03E-07	2.03E-06	no
TMX1	0.188623722	5.070833482	5.08E-07	2.05E-06	no
C20orf24	0.188603412	5.070267344	5.10E-07	2.06E-06	no
KIAA1244	-0.188572024	-5.069392431	5.12E-07	2.06E-06	no
STT3B	0.188561183	5.069090224	5.13E-07	2.07E-06	no
PPP1R1A	-0.188557703	-5.068993241	5.13E-07	2.07E-06	no
DCXR	-0.188533384	-5.068315378	5.15E-07	2.07E-06	no
PL-5283	0.188524901	5.068078928	5.15E-07	2.08E-06	no
CDH18	-0.188477156	-5.066748126	5.19E-07	2.09E-06	no
WLS	0.18847265	5.066622525	5.19E-07	2.09E-06	no
MXRA7	0.188471956	5.066603192	5.19E-07	2.09E-06	no
OSTalpha	0.188451353	5.066028932	5.21E-07	2.10E-06	no
MYO5A	-0.188429293	-5.065414067	5.22E-07	2.10E-06	no
WDFY3	-0.188422122	-5.065214194	5.23E-07	2.10E-06	no
FBXL5	0.18841812	5.065102662	5.23E-07	2.11E-06	no
UBXN1	-0.188406206	-5.064770599	5.24E-07	2.11E-06	no
GREB1	-0.188396458	-5.064498913	5.25E-07	2.11E-06	no
KCNN2	-0.188383294	-5.064132003	5.26E-07	2.11E-06	no
RGS7	-0.188344822	-5.063059744	5.29E-07	2.13E-06	no
LOXL1	0.18832145	5.062408384	5.30E-07	2.13E-06	no
BAHCC1	-0.188301948	-5.061864852	5.32E-07	2.14E-06	no
SLC44A3	0.188300888	5.061835297	5.32E-07	2.14E-06	no
SLC12A6	-0.18829678	-5.061720828	5.32E-07	2.14E-06	no
GHRLOS	0.188264377	5.060817768	5.35E-07	2.15E-06	no
HNRNPH2	-0.188256246	-5.060591163	5.35E-07	2.15E-06	no
RPL7	-0.188234705	-5.059990848	5.37E-07	2.16E-06	no
RPL15	-0.188225488	-5.059733968	5.38E-07	2.16E-06	no
LOC100190938	-0.188194813	-5.058879116	5.40E-07	2.17E-06	no
UCN2	0.188193241	5.058835295	5.40E-07	2.17E-06	no
ACBD7	-0.188181193	-5.058499538	5.41E-07	2.17E-06	no
SFRS2B	-0.188147019	-5.057547187	5.44E-07	2.18E-06	no
WDR92	0.188097385	5.05616406	5.47E-07	2.20E-06	no
MOBKL2B	-0.188095061	-5.056099301	5.48E-07	2.20E-06	no
PIM2	0.188073512	5.055498792	5.49E-07	2.20E-06	no
RND3	0.188046736	5.054752657	5.51E-07	2.21E-06	no
LUC7L	-0.18803794	-5.054507571	5.52E-07	2.21E-06	no
POLR2L	0.187928015	5.051444546	5.61E-07	2.25E-06	no
ABHD6	-0.187920716	-5.05124116	5.61E-07	2.25E-06	no

MYL9	0.187811049	5.048185541	5.70E-07	2.28E-06	no
ASGR1	-0.187790036	-5.047600071	5.72E-07	2.29E-06	no
NUCKS1	-0.187785997	-5.047487554	5.72E-07	2.29E-06	no
ANO8	-0.187765994	-5.046930236	5.74E-07	2.30E-06	no
SESTD1	-0.187737502	-5.046136417	5.76E-07	2.30E-06	no
CIT	-0.187728845	-5.045895244	5.77E-07	2.31E-06	no
PTOV1	-0.18769641	-5.044991604	5.79E-07	2.32E-06	no
PDE5A	0.187678776	5.044500304	5.81E-07	2.32E-06	no
SLC25A20	0.187667642	5.044190121	5.82E-07	2.32E-06	no
FOXS1	0.187646353	5.043597017	5.83E-07	2.33E-06	no
NEK8	0.187594984	5.042165956	5.87E-07	2.35E-06	no
SEC24C	-0.187568997	-5.041442008	5.90E-07	2.36E-06	no
NCRNA00176	-0.18754778	-5.04085095	5.91E-07	2.36E-06	no
NDN	-0.187543346	-5.040727414	5.92E-07	2.36E-06	no
SCAMP1	-0.187516483	-5.039979085	5.94E-07	2.37E-06	no
TRIM25	0.187502388	5.039586454	5.95E-07	2.38E-06	no
GNB5	-0.187484666	-5.039092768	5.97E-07	2.38E-06	no
PPP2R5D	-0.187456985	-5.038321686	5.99E-07	2.39E-06	no
LYVE1	0.18745365	5.038228782	5.99E-07	2.39E-06	no
BASP1	-0.187443314	-5.037940854	6.00E-07	2.39E-06	no
ORC5L	0.187406881	5.036926009	6.03E-07	2.41E-06	no
PFKP	-0.187387231	-5.036378665	6.05E-07	2.41E-06	no
IFRD2	0.187387158	5.036376614	6.05E-07	2.41E-06	no
DNAJB4	0.187363995	5.035731425	6.07E-07	2.42E-06	no
FRMD6	0.187326396	5.034684138	6.10E-07	2.43E-06	no
LOC100129637	-0.18732508	-5.034647484	6.10E-07	2.43E-06	no
ST7L	0.187320922	5.034531641	6.11E-07	2.43E-06	no
SNUPN	0.187247508	5.032486824	6.17E-07	2.46E-06	no
CHST12	0.18724094	5.032303899	6.17E-07	2.46E-06	no
GPAT2	0.187191503	5.030926973	6.22E-07	2.48E-06	no
LTB4R	0.187172232	5.030390244	6.23E-07	2.48E-06	no
XKR7	-0.18714793	-5.029713388	6.26E-07	2.49E-06	no
TRAPPC2	-0.187085464	-5.027973691	6.31E-07	2.51E-06	no
OTUB1	-0.187083352	-5.02791486	6.31E-07	2.51E-06	no
RABL5	0.187069932	5.027541128	6.32E-07	2.52E-06	no
RAPGEF2	-0.187064572	-5.02739184	6.33E-07	2.52E-06	no
TEAD2	0.18705272	5.027061761	6.34E-07	2.52E-06	no
SGK269	-0.187047377	-5.026912974	6.34E-07	2.52E-06	no
LIMS3-LOC440	0.187024793	5.026284015	6.36E-07	2.53E-06	no
C3orf66	0.187018536	5.026109788	6.37E-07	2.53E-06	no
HNRNPA0	-0.187014342	-5.025992979	6.37E-07	2.53E-06	no
C2CD2	0.18700291	5.02567461	6.38E-07	2.54E-06	no
FAAH	-0.186947035	-5.024118586	6.43E-07	2.56E-06	no
CDK2	0.18688486	5.022387185	6.49E-07	2.58E-06	no
C16orf7	-0.18686443	-5.021818271	6.51E-07	2.58E-06	no
BTNL9	-0.186857822	-5.021634258	6.51E-07	2.59E-06	no
ACVR1B	-0.186846266	-5.021312478	6.53E-07	2.59E-06	no
PLEKHM3	-0.186833691	-5.020962298	6.54E-07	2.59E-06	no
ZNF827	-0.18679261	-5.019818371	6.57E-07	2.61E-06	no
POU2F1	-0.186784335	-5.019587968	6.58E-07	2.61E-06	no
CNIH3	0.186782284	5.019530862	6.58E-07	2.61E-06	no

KIAA1755	-0.186774144	-5.019304185	6.59E-07	2.61E-06	no
CENPT	-0.186765623	-5.019066919	6.60E-07	2.62E-06	no
CRIP3	-0.186753145	-5.018719466	6.61E-07	2.62E-06	no
C16orf63	0.186748435	5.018588338	6.61E-07	2.62E-06	no
RASSF2	-0.186734102	-5.018189246	6.63E-07	2.63E-06	no
MARK4	-0.186676471	-5.016584551	6.68E-07	2.65E-06	no
SAMHD1	0.186613883	5.01484194	6.74E-07	2.67E-06	no
REEP1	-0.186584745	-5.014030658	6.77E-07	2.68E-06	no
ZFP36L1	0.18655264	5.013136811	6.80E-07	2.69E-06	no
SNIP1	0.186541647	5.012830749	6.81E-07	2.69E-06	no
CNKSR1	-0.186540489	-5.012798492	6.81E-07	2.69E-06	no
MRPS2	-0.186518776	-5.012193985	6.83E-07	2.70E-06	no
LIMK1	0.186492915	5.011474001	6.86E-07	2.71E-06	no
PCOTH	0.186483046	5.011199235	6.86E-07	2.71E-06	no
HAR1A	-0.186456149	-5.010450432	6.89E-07	2.72E-06	no
FBXO17	0.186447296	5.010203975	6.90E-07	2.73E-06	no
VSTM2B	-0.186406188	-5.00905953	6.94E-07	2.74E-06	no
ITGB4	0.186359234	5.007752399	6.98E-07	2.76E-06	no
SYP	-0.186358369	-5.007728315	6.99E-07	2.76E-06	no
CHGA	-0.186353955	-5.007605436	6.99E-07	2.76E-06	no
SYNGR1	-0.186329807	-5.006933227	7.01E-07	2.77E-06	no
SULT1E1	0.186289069	5.005799164	7.05E-07	2.78E-06	no
TTC7B	-0.186175851	-5.002647588	7.17E-07	2.83E-06	no
NCRNA00158	-0.186138093	-5.001596601	7.20E-07	2.84E-06	no
ZNF341	-0.186117125	-5.00101298	7.22E-07	2.85E-06	no
GPR4	0.186094645	5.000387263	7.25E-07	2.86E-06	no
LOC100286844	-0.186082545	-5.000050472	7.26E-07	2.86E-06	no
PM20D2	-0.186055604	-4.999300596	7.29E-07	2.87E-06	no
DPEP3	0.186015548	4.998185711	7.33E-07	2.89E-06	no
ZBTB4	-0.185996171	-4.997646414	7.35E-07	2.90E-06	no
KLRC2	-0.185981751	-4.997245055	7.36E-07	2.90E-06	no
CHST1	-0.18595711	-4.996559261	7.39E-07	2.91E-06	no
KCTD2	-0.185944983	-4.996221746	7.40E-07	2.92E-06	no
RAI2	-0.185939443	-4.996067545	7.41E-07	2.92E-06	no
RBM6	-0.18584192	-4.993353409	7.51E-07	2.96E-06	no
HSD17B1	-0.185834492	-4.993146701	7.51E-07	2.96E-06	no
SLC2A3	0.185814589	4.992592806	7.54E-07	2.97E-06	no
NBR1	-0.185715528	-4.989836061	7.64E-07	3.01E-06	no
UBE2QL1	-0.185700293	-4.98941208	7.66E-07	3.01E-06	no
EPDR1	0.185678211	4.988797613	7.68E-07	3.02E-06	no
MMP11	0.185672912	4.988650136	7.69E-07	3.02E-06	no
YEATS2	-0.185648427	-4.9879688	7.71E-07	3.03E-06	no
FLOT1	0.18563719	4.987656104	7.72E-07	3.04E-06	no
LMBRD1	0.185621505	4.987219654	7.74E-07	3.04E-06	no
ARHGEF17	-0.185611741	-4.986947943	7.75E-07	3.05E-06	no
MGAT5B	-0.185599842	-4.986616855	7.76E-07	3.05E-06	no
ETS2	0.185569554	4.985774074	7.80E-07	3.06E-06	no
SEZ6	-0.185552104	-4.985288497	7.82E-07	3.07E-06	no
DIP2B	-0.185479477	-4.983267676	7.89E-07	3.10E-06	no
NCRNA00093	-0.185429221	-4.981869355	7.95E-07	3.12E-06	no
MEOX2	0.185420143	4.981616781	7.96E-07	3.12E-06	no

ASPHD1	-0.185412206	-4.98139594	7.97E-07	3.13E-06	no
TNFAIP8L1	0.18537797	4.980443405	8.01E-07	3.14E-06	no
LOC283070	-0.185368523	-4.980180567	8.02E-07	3.15E-06	no
LOC550643	0.185367807	4.98016065	8.02E-07	3.15E-06	no
GLT8D1	0.185356502	4.97984612	8.03E-07	3.15E-06	no
ZNF525	0.185322478	4.978899517	8.07E-07	3.16E-06	no
C9orf128	-0.18528284	-4.977796713	8.11E-07	3.18E-06	no
DENR	0.185277435	4.977646351	8.12E-07	3.18E-06	no
MGC23270	0.185251976	4.976938078	8.15E-07	3.19E-06	no
NEDD1	0.185134335	4.973665333	8.28E-07	3.25E-06	no
SFT2D1	0.185090087	4.972434433	8.33E-07	3.26E-06	no
FAM76A	0.185077487	4.972083931	8.35E-07	3.27E-06	no
CPNE8	0.185066804	4.971786753	8.36E-07	3.27E-06	no
ADARB2	-0.185064103	-4.971711628	8.36E-07	3.27E-06	no
C5orf22	0.18501103	4.970235278	8.42E-07	3.30E-06	no
SNRNP200	-0.184987793	-4.96958891	8.45E-07	3.31E-06	no
LOC729991-ME	-0.184978014	-4.969316898	8.46E-07	3.31E-06	no
GSS	0.184925603	4.967859047	8.53E-07	3.34E-06	no
ATP11C	0.184865116	4.966176607	8.60E-07	3.36E-06	no
LOC678655	-0.184847456	-4.965685417	8.62E-07	3.37E-06	no
TCF7L2	-0.184827094	-4.965119072	8.64E-07	3.38E-06	no
KAAG1	0.184806126	4.964535878	8.67E-07	3.39E-06	no
ZNF578	-0.184786849	-4.963999727	8.69E-07	3.40E-06	no
SNX7	0.184765902	4.963417129	8.72E-07	3.41E-06	no
TNNT3	0.18473515	4.962561828	8.75E-07	3.42E-06	no
ZNF474	0.184729227	4.962397092	8.76E-07	3.42E-06	no
TBC1D4	0.184718869	4.962109008	8.77E-07	3.43E-06	no
C2orf80	-0.18469263	-4.96137927	8.80E-07	3.44E-06	no
CASP9	-0.184689016	-4.961278755	8.81E-07	3.44E-06	no
AHSA1	-0.184654234	-4.960311427	8.85E-07	3.45E-06	no
LOC100270804	-0.184624811	-4.959493159	8.89E-07	3.47E-06	no
DAGLA	-0.184603861	-4.958910532	8.91E-07	3.48E-06	no
PPP2R1A	-0.184602671	-4.95887743	8.91E-07	3.48E-06	no
BCL7B	0.184596446	4.95870432	8.92E-07	3.48E-06	no
FAM180A	0.184540442	4.957146856	8.99E-07	3.51E-06	no
FLJ40292	-0.184529462	-4.956841519	9.01E-07	3.51E-06	no
SPATA6	0.18452807	4.956802808	9.01E-07	3.51E-06	no
KTI12	0.184500462	4.956035049	9.04E-07	3.52E-06	no
DNAJC16	0.184499713	4.956014237	9.04E-07	3.52E-06	no
USP14	0.184496564	4.955926671	9.05E-07	3.52E-06	no
CTPS2	-0.184484264	-4.955584628	9.06E-07	3.53E-06	no
ENDOG	-0.184482954	-4.955548205	9.06E-07	3.53E-06	no
C9orf139	0.184480898	4.955491011	9.07E-07	3.53E-06	no
HMX1	-0.184475077	-4.955329149	9.07E-07	3.53E-06	no
PSMB2	0.184406154	4.95341258	9.16E-07	3.56E-06	no
TIAL1	-0.184380536	-4.952700203	9.19E-07	3.58E-06	no
LOC154761	0.18425681	4.949259945	9.35E-07	3.64E-06	no
TFB2M	0.18424859	4.949031375	9.36E-07	3.64E-06	no
FKBP8	-0.184222496	-4.948305866	9.40E-07	3.65E-06	no
APBA1	-0.184215558	-4.948112958	9.40E-07	3.66E-06	no
IL17A	0.184211309	4.947994801	9.41E-07	3.66E-06	no

ADAM22	-0.184207528	-4.947889691	9.41E-07	3.66E-06	no
ZNF778	-0.184203026	-4.947764496	9.42E-07	3.66E-06	no
USP19	-0.184199038	-4.947653641	9.43E-07	3.66E-06	no
ACCN3	-0.184174243	-4.946964233	9.46E-07	3.67E-06	no
IPO4	0.184160394	4.946579198	9.48E-07	3.68E-06	no
BRD1	-0.184103232	-4.944989945	9.55E-07	3.71E-06	no
YY1AP1	-0.184097576	-4.9448327	9.56E-07	3.71E-06	no
AEN	0.184089183	4.94459935	9.57E-07	3.71E-06	no
MPP4	0.184065895	4.943951897	9.60E-07	3.73E-06	no
HARBI1	0.184049292	4.943490321	9.62E-07	3.73E-06	no
TMEM106C	0.184044841	4.94336656	9.63E-07	3.73E-06	no
SCRT2	-0.184003199	-4.942208889	9.68E-07	3.76E-06	no
NR3C2	-0.183967348	-4.941212197	9.73E-07	3.77E-06	no
DUSP14	0.183963743	4.941111994	9.74E-07	3.77E-06	no
UNC5B	0.183961083	4.941038053	9.74E-07	3.78E-06	no
FDX1	0.183918649	4.939858399	9.80E-07	3.80E-06	no
KRT7	0.183918543	4.939855449	9.80E-07	3.80E-06	no
WIPF3	0.183882688	4.938858721	9.85E-07	3.81E-06	no
KHDRBS2	-0.18387607	-4.938674742	9.85E-07	3.82E-06	no
KIAA1984	-0.183873961	-4.938616128	9.86E-07	3.82E-06	no
GNL2	0.183839842	4.937667658	9.90E-07	3.83E-06	no
PRR14	-0.1838358	-4.937555297	9.91E-07	3.84E-06	no
KANK1	-0.183821466	-4.937156846	9.93E-07	3.84E-06	no
SLC26A1	-0.183804811	-4.936693883	9.95E-07	3.85E-06	no
MAFK	0.183802083	4.936618041	9.96E-07	3.85E-06	no
ACO2	-0.18379325	-4.936372525	9.97E-07	3.85E-06	no
AQP1	0.183792898	4.936362733	9.97E-07	3.85E-06	no
RAB9B	-0.183785753	-4.936164125	9.98E-07	3.86E-06	no
ZNF358	-0.183776861	-4.935916958	9.99E-07	3.86E-06	no
GPN2	0.183774858	4.935861276	9.99E-07	3.86E-06	no
PRR5	0.183764829	4.935582509	1.00E-06	3.87E-06	no
EEF1DP3	-0.183753216	-4.935259698	1.00E-06	3.87E-06	no
PAQR7	0.183752215	4.935231861	1.00E-06	3.87E-06	no
SPAG4	0.183736702	4.934800652	1.00E-06	3.88E-06	no
CBX5	-0.183723897	-4.934444729	1.01E-06	3.89E-06	no
GRIK5	-0.183718275	-4.934288451	1.01E-06	3.89E-06	no
SLC30A1	0.183671989	4.933001908	1.01E-06	3.91E-06	no
ADH4	0.183667887	4.932887889	1.01E-06	3.91E-06	no
FCRL4	0.183660561	4.932684268	1.02E-06	3.92E-06	no
MX1	0.183640127	4.932116297	1.02E-06	3.93E-06	no
PKIA	-0.183638169	-4.932061874	1.02E-06	3.93E-06	no
TMEM180	-0.183634267	-4.931953426	1.02E-06	3.93E-06	no
JRK	-0.1835423	-4.929397281	1.03E-06	3.98E-06	no
SLC7A8	0.183516352	4.928676123	1.04E-06	3.99E-06	no
SELM	0.183504146	4.928336876	1.04E-06	4.00E-06	no
ZNF253	-0.183494497	-4.928068691	1.04E-06	4.00E-06	no
ATP6AP1L	-0.183486981	-4.927859816	1.04E-06	4.01E-06	no
SLC35B4	0.183476717	4.927574557	1.04E-06	4.01E-06	no
GNLY	0.183467267	4.927311905	1.04E-06	4.01E-06	no
CERKL	0.183393465	4.925260852	1.05E-06	4.05E-06	no
LOC728743	-0.183368375	-4.924563591	1.06E-06	4.07E-06	no

GOLGA8A	-0.183361343	-4.924368168	1.06E-06	4.07E-06	no
LOC401397	0.183356471	4.924232759	1.06E-06	4.07E-06	no
SUMO3	0.183322193	4.923280185	1.06E-06	4.09E-06	no
FBXL17	-0.183310299	-4.922949644	1.07E-06	4.10E-06	no
SCAP	-0.183296966	-4.922579129	1.07E-06	4.10E-06	no
LOC146336	-0.183277562	-4.922039893	1.07E-06	4.11E-06	no
MRM1	-0.183248712	-4.921238187	1.07E-06	4.13E-06	no
RPAP2	0.183136792	4.918128171	1.09E-06	4.19E-06	no
GABBR2	-0.183121202	-4.917694984	1.09E-06	4.20E-06	no
AOX1	0.18304591	4.91560293	1.10E-06	4.24E-06	no
KIAA0913	-0.183044563	-4.915565502	1.10E-06	4.24E-06	no
SLU7	-0.182999473	-4.914312675	1.11E-06	4.27E-06	no
FLJ37453	-0.182994808	-4.914183058	1.11E-06	4.27E-06	no
TPM2	0.182987077	4.913968254	1.11E-06	4.27E-06	no
MAPKBP1	-0.182985815	-4.913933173	1.11E-06	4.27E-06	no
ZNF254	-0.182977161	-4.913692732	1.11E-06	4.28E-06	no
ARPP19	-0.182971722	-4.913541622	1.12E-06	4.28E-06	no
HLA-J	0.182951385	4.912976577	1.12E-06	4.29E-06	no
IAH1	0.182928514	4.912341139	1.12E-06	4.31E-06	no
SAPS3	-0.182917977	-4.912048378	1.12E-06	4.31E-06	no
WBP4	-0.182892269	-4.911334126	1.13E-06	4.33E-06	no
RASA2	0.182856009	4.910326712	1.13E-06	4.35E-06	no
SGTA	-0.182848254	-4.910111256	1.13E-06	4.35E-06	no
PCBD1	0.182831287	4.909639899	1.14E-06	4.36E-06	no
SIAH2	0.18282233	4.90939103	1.14E-06	4.36E-06	no
RFPL4A	0.182813637	4.909149538	1.14E-06	4.37E-06	no
FAM43A	0.182798575	4.908731098	1.14E-06	4.38E-06	no
TMEM179	-0.182777538	-4.908146653	1.15E-06	4.39E-06	no
ABCB6	-0.182774489	-4.908061935	1.15E-06	4.39E-06	no
PGPEP1	0.18275546	4.907533288	1.15E-06	4.40E-06	no
SMCR7L	-0.182728404	-4.906781679	1.15E-06	4.41E-06	no
RARG	0.18271064	4.906288184	1.16E-06	4.42E-06	no
C20orf177	-0.182671983	-4.905214287	1.16E-06	4.45E-06	no
NECAP1	-0.182656671	-4.90478893	1.16E-06	4.46E-06	no
CRHR1	-0.182655668	-4.904761073	1.17E-06	4.46E-06	no
SMARCD3	-0.182631013	-4.904076167	1.17E-06	4.47E-06	no
FAM72A	0.182577991	4.902603302	1.18E-06	4.50E-06	no
XPA	-0.182566516	-4.902284546	1.18E-06	4.51E-06	no
KGFLP2	-0.182534888	-4.901405994	1.18E-06	4.53E-06	no
NELF	-0.182518925	-4.900962592	1.19E-06	4.54E-06	no
MLLT3	-0.182508807	-4.90068154	1.19E-06	4.54E-06	no
ELL	0.18250402	4.900548584	1.19E-06	4.54E-06	no
C13orf15	-0.182480856	-4.899905141	1.19E-06	4.56E-06	no
FBXW2	0.182460246	4.899332695	1.20E-06	4.57E-06	no
BBS2	-0.18243755	-4.898702277	1.20E-06	4.58E-06	no
EFHA2	-0.182422228	-4.898276688	1.20E-06	4.59E-06	no
ZNF337	-0.182380327	-4.897112874	1.21E-06	4.62E-06	no
S100A16	0.18229745	4.894811041	1.22E-06	4.67E-06	no
PYROXD1	0.182284358	4.894447411	1.23E-06	4.68E-06	no
AZI2	0.182280215	4.894332364	1.23E-06	4.68E-06	no
NT5M	-0.182232512	-4.893007488	1.23E-06	4.71E-06	no

ABCA6	0.182195101	4.891968513	1.24E-06	4.73E-06	no
KLHL32	-0.182191449	-4.891867073	1.24E-06	4.73E-06	no
RPL39L	0.182163815	4.891099639	1.25E-06	4.75E-06	no
PABPC3	0.182109395	4.889588333	1.26E-06	4.78E-06	no
SLC23A3	0.182101549	4.889370446	1.26E-06	4.79E-06	no
OR2C1	-0.182093178	-4.889137977	1.26E-06	4.79E-06	no
PNCK	-0.182081231	-4.888806216	1.26E-06	4.80E-06	no
SIGIRR	0.182020458	4.887118551	1.27E-06	4.84E-06	no
TLR9	0.182001416	4.886589779	1.27E-06	4.85E-06	no
CEP57	-0.18198887	-4.886241393	1.28E-06	4.86E-06	no
ZNF205	-0.181981707	-4.886042465	1.28E-06	4.86E-06	no
PREX1	0.181964603	4.885567525	1.28E-06	4.87E-06	no
ITGA7	0.181949254	4.885141293	1.28E-06	4.88E-06	no
NETO1	-0.181907793	-4.883990017	1.29E-06	4.91E-06	no
FYTTD1	0.181892735	4.883571901	1.29E-06	4.92E-06	no
DSG2	0.181889679	4.883487048	1.29E-06	4.92E-06	no
ZNF561	0.181887743	4.883433282	1.29E-06	4.92E-06	no
KANK3	-0.181881954	-4.883272534	1.29E-06	4.92E-06	no
ADRB2	0.181859819	4.882657922	1.30E-06	4.93E-06	no
BAIAP2L2	-0.181841022	-4.882135993	1.30E-06	4.95E-06	no
XYLB	0.181829071	4.881804138	1.30E-06	4.95E-06	no
SCP2	0.181772877	4.880243877	1.31E-06	4.99E-06	no
ADCY5	-0.181767595	-4.880097221	1.32E-06	4.99E-06	no
SCRN2	-0.181767223	-4.880086879	1.32E-06	4.99E-06	no
ACSF2	0.18172275	4.878852102	1.32E-06	5.02E-06	no
SNORA8	-0.181710517	-4.878512456	1.33E-06	5.03E-06	no
NOX4	0.181694418	4.878065478	1.33E-06	5.04E-06	no
ZNF708	-0.181693986	-4.87805349	1.33E-06	5.04E-06	no
PLA2G5	0.181652127	4.876891311	1.34E-06	5.07E-06	no
CHD6	-0.181643881	-4.876662365	1.34E-06	5.07E-06	no
LPPR2	-0.181615166	-4.875865166	1.34E-06	5.09E-06	no
AZI1	-0.181589479	-4.875152016	1.35E-06	5.11E-06	no
PRRT1	-0.181575749	-4.874770845	1.35E-06	5.12E-06	no
HECTD3	0.181559565	4.874321533	1.35E-06	5.13E-06	no
MAGEL2	-0.181535442	-4.873651844	1.36E-06	5.14E-06	no
ZNF414	-0.181497273	-4.872592208	1.36E-06	5.17E-06	no
KIAA2013	0.181480677	4.87213147	1.37E-06	5.18E-06	no
ALG8	0.181470818	4.87185778	1.37E-06	5.18E-06	no
SH3GLB2	-0.181470008	-4.871835307	1.37E-06	5.18E-06	no
VPS29	0.181442586	4.871074043	1.37E-06	5.20E-06	no
MARK3	-0.181417191	-4.870369082	1.38E-06	5.22E-06	no
NCKAP1	-0.181415299	-4.870316555	1.38E-06	5.22E-06	no
C6orf170	-0.181400284	-4.869899756	1.38E-06	5.23E-06	no
ZNF770	-0.181367193	-4.868981181	1.39E-06	5.25E-06	no
CN5H6.4	-0.181346668	-4.868411404	1.39E-06	5.27E-06	no
UPP2	-0.181341049	-4.868255445	1.39E-06	5.27E-06	no
HIST1H2BC	0.181326686	4.867856731	1.40E-06	5.28E-06	no
LOC348926	0.181306707	4.867302154	1.40E-06	5.29E-06	no
UNC45A	0.18129765	4.867050767	1.40E-06	5.30E-06	no
LDHD	-0.181288427	-4.866794748	1.40E-06	5.30E-06	no
LHFPL3	-0.181254101	-4.865841943	1.41E-06	5.33E-06	no

KIAA2026	-0.181228856	-4.865141203	1.42E-06	5.34E-06	no
GLYR1	-0.181228601	-4.865134134	1.42E-06	5.34E-06	no
AKIRIN1	0.18117919	4.863762659	1.42E-06	5.38E-06	no
PPP4R1L	0.181169619	4.863496987	1.43E-06	5.38E-06	no
EIF2AK2	0.181141806	4.862725038	1.43E-06	5.40E-06	no
C9orf7	-0.181128724	-4.862361937	1.43E-06	5.41E-06	no
CPE	-0.181112531	-4.861912489	1.44E-06	5.42E-06	no
E4F1	-0.181096879	-4.861478055	1.44E-06	5.43E-06	no
TXNRD2	-0.181084807	-4.861143008	1.44E-06	5.44E-06	no
KIAA1875	-0.181071042	-4.860760966	1.45E-06	5.45E-06	no
CEP72	-0.181043828	-4.860005651	1.45E-06	5.47E-06	no
CLEC18B	0.181015804	4.859227898	1.46E-06	5.49E-06	no
UBQLNL	0.18100355	4.858887809	1.46E-06	5.50E-06	no
ADAT1	0.180980926	4.858259898	1.46E-06	5.51E-06	no
SLC47A1	0.180944084	4.857237452	1.47E-06	5.54E-06	no
PFN4	0.180927287	4.856771297	1.47E-06	5.55E-06	no
TRIM17	-0.180907908	-4.856233471	1.48E-06	5.56E-06	no
GPR98	-0.180870704	-4.855201012	1.49E-06	5.59E-06	no
ACAD8	-0.180812255	-4.853579007	1.50E-06	5.63E-06	no
GSK3B	-0.180788451	-4.85291842	1.50E-06	5.65E-06	no
TBX4	0.180758842	4.85209677	1.51E-06	5.67E-06	no
EXOSC3	0.180724335	4.851139243	1.52E-06	5.70E-06	no
WDR83	-0.180722203	-4.851080061	1.52E-06	5.70E-06	no
KLF17	0.180690644	4.85020435	1.52E-06	5.72E-06	no
IL17RC	0.180687217	4.850109242	1.52E-06	5.72E-06	no
GRIN2C	-0.180652718	-4.849151981	1.53E-06	5.75E-06	no
LRP5	-0.180588476	-4.847369406	1.54E-06	5.80E-06	no
COL10A1	0.180553894	4.84640987	1.55E-06	5.83E-06	no
GEMIN6	0.180524047	4.845581736	1.56E-06	5.85E-06	no
BOP1	-0.180522801	-4.84554717	1.56E-06	5.85E-06	no
TECPR2	-0.180521085	-4.84549957	1.56E-06	5.85E-06	no
FAM86B1	0.180510635	4.845209616	1.56E-06	5.86E-06	no
TBC1D5	-0.180496602	-4.844820252	1.56E-06	5.87E-06	no
FOXD4L2	-0.180482815	-4.84443773	1.57E-06	5.87E-06	no
MT1L	0.180473685	4.844184417	1.57E-06	5.88E-06	no
ELMO1	-0.180428148	-4.842921008	1.58E-06	5.92E-06	no
CYP4V2	0.18041637	4.842594228	1.58E-06	5.92E-06	no
TMEM121	-0.180414823	-4.842551317	1.58E-06	5.92E-06	no
RAB6A	-0.180414663	-4.842546864	1.58E-06	5.92E-06	no
ARHGAP31	-0.180340917	-4.840500868	1.60E-06	5.98E-06	no
SCNN1D	-0.180327776	-4.84013631	1.60E-06	5.99E-06	no
NPEPPS	-0.1803256	-4.840075951	1.60E-06	5.99E-06	no
ADAMTS4	0.180320975	4.83994762	1.60E-06	5.99E-06	no
LGR5	-0.180319857	-4.839916604	1.60E-06	5.99E-06	no
GPN3	0.180286371	4.838987631	1.61E-06	6.02E-06	no
SH3BP2	0.180272582	4.838605084	1.61E-06	6.03E-06	no
GPR179	-0.18020436	-4.836712518	1.63E-06	6.09E-06	no
OR6K3	0.180193013	4.836397721	1.63E-06	6.09E-06	no
NKPD1	-0.180192621	-4.83638686	1.63E-06	6.09E-06	no
PMS2	-0.180166378	-4.835658859	1.63E-06	6.11E-06	no
SNHG7	-0.180121463	-4.834412917	1.64E-06	6.15E-06	no

MMRN2	0.180105245	4.833963037	1.65E-06	6.16E-06	no
PPM1F	0.180071894	4.833037905	1.65E-06	6.19E-06	no
IL6	0.180067086	4.832904523	1.66E-06	6.19E-06	no
TRPC3	-0.180059865	-4.83270424	1.66E-06	6.20E-06	no
CYS1	-0.180056259	-4.832604222	1.66E-06	6.20E-06	no
WSCD2	-0.180046545	-4.832334749	1.66E-06	6.20E-06	no
HDDC2	-0.18002261	-4.831670838	1.67E-06	6.22E-06	no
GCHFR	0.179983959	4.83059874	1.67E-06	6.26E-06	no
CSNK1G2	0.179967435	4.830140412	1.68E-06	6.27E-06	no
IDH3G	-0.179964373	-4.830055489	1.68E-06	6.27E-06	no
WHSC1	-0.179962293	-4.829997785	1.68E-06	6.27E-06	no
HRH4	0.179935453	4.829253314	1.69E-06	6.29E-06	no
TMPRSS7	0.179932518	4.829171907	1.69E-06	6.29E-06	no
KRCC1	0.179926726	4.829011257	1.69E-06	6.30E-06	no
SUPV3L1	-0.179914489	-4.828671855	1.69E-06	6.31E-06	no
CDA	0.179911719	4.828595006	1.69E-06	6.31E-06	no
C6orf162	-0.179878882	-4.827684254	1.70E-06	6.33E-06	no
RCBTB1	-0.179862192	-4.82722134	1.70E-06	6.35E-06	no
ARRDC4	0.179829324	4.826309733	1.71E-06	6.37E-06	no
PIGZ	-0.179764407	-4.824509265	1.72E-06	6.43E-06	no
BATF2	0.179750048	4.824111044	1.73E-06	6.44E-06	no
TGFB1I1	0.179721687	4.823324489	1.73E-06	6.46E-06	no
HAS3	0.179714439	4.823123477	1.74E-06	6.47E-06	no
GABRG2	-0.179695533	-4.822599131	1.74E-06	6.48E-06	no
KCNK17	0.179692398	4.822512206	1.74E-06	6.49E-06	no
CSTF2T	-0.179689433	-4.822429976	1.74E-06	6.49E-06	no
D2HGDH	-0.179684056	-4.822280843	1.74E-06	6.49E-06	no
TAS2R19	-0.179664157	-4.82172899	1.75E-06	6.51E-06	no
C10orf111	-0.179656835	-4.821525947	1.75E-06	6.51E-06	no
SEC23IP	0.179641955	4.821113283	1.75E-06	6.52E-06	no
EFNB1	0.179582815	4.819473218	1.77E-06	6.57E-06	no
ZNF24	-0.179570991	-4.819145316	1.77E-06	6.58E-06	no
TMEM184C	0.179562943	4.818922131	1.77E-06	6.59E-06	no
TTC4	0.1795413	4.818321969	1.78E-06	6.61E-06	no
CYP2D6	-0.179523087	-4.81781691	1.78E-06	6.62E-06	no
FAM136A	0.179518711	4.817695567	1.78E-06	6.63E-06	no
PCSK2	-0.179515428	-4.817604514	1.78E-06	6.63E-06	no
KIAA1370	-0.179509787	-4.817448095	1.79E-06	6.63E-06	no
TUBGCP3	-0.179504469	-4.817300632	1.79E-06	6.63E-06	no
MCM5	0.179483641	4.816723072	1.79E-06	6.65E-06	no
PEX16	-0.179376331	-4.81374746	1.82E-06	6.75E-06	no
RPL21	-0.179364623	-4.813422825	1.82E-06	6.76E-06	no
EPN2	-0.179319089	-4.81216027	1.83E-06	6.80E-06	no
THBS4	0.179277525	4.81100781	1.84E-06	6.83E-06	no
VWA5B2	-0.179269259	-4.810778638	1.84E-06	6.84E-06	no
ZNF652	-0.179259603	-4.810510894	1.85E-06	6.85E-06	no
GNS	0.179259552	4.810509497	1.85E-06	6.85E-06	no
MAP4K5	-0.179258686	-4.810485468	1.85E-06	6.85E-06	no
TFRC	0.179256867	4.810435055	1.85E-06	6.85E-06	no
MACROD1	-0.179198173	-4.808807705	1.86E-06	6.90E-06	no
SENP3	-0.179195697	-4.808739049	1.86E-06	6.90E-06	no

CISD2	0.179148043	4.807417839	1.87E-06	6.94E-06	no
EIF4G2	0.179120823	4.80666317	1.88E-06	6.97E-06	no
PLEKHG1	0.179110636	4.806380745	1.88E-06	6.98E-06	no
OSBPL7	-0.179088275	-4.80576082	1.89E-06	7.00E-06	no
FLJ43390	-0.17906601	-4.80514357	1.89E-06	7.01E-06	no
PCDHB19P	-0.179065304	-4.805123987	1.89E-06	7.01E-06	no
FCN3	0.17904328	4.804513395	1.90E-06	7.03E-06	no
ARCN1	0.179018865	4.803836559	1.91E-06	7.06E-06	no
TRIOBP	0.178960934	4.802230564	1.92E-06	7.11E-06	no
SLCO1A2	-0.178954128	-4.802041898	1.92E-06	7.11E-06	no
NAP1L1	-0.178929376	-4.801355733	1.93E-06	7.14E-06	no
ZNF124	0.178888125	4.800212212	1.94E-06	7.17E-06	no
CLVS2	-0.178863873	-4.799539928	1.95E-06	7.20E-06	no
TRAPPC1	0.178808229	4.797997495	1.96E-06	7.25E-06	no
LTA	0.178807344	4.797972976	1.96E-06	7.25E-06	no
HNRNPA3	-0.178784066	-4.797327705	1.97E-06	7.27E-06	no
SP2	-0.178768663	-4.796900754	1.97E-06	7.28E-06	no
L1CAM	-0.178753468	-4.796479569	1.98E-06	7.30E-06	no
NUP133	-0.17873338	-4.795922778	1.98E-06	7.32E-06	no
PTCD2	0.178724439	4.795674932	1.98E-06	7.32E-06	no
DPM1	0.178695251	4.794865906	1.99E-06	7.35E-06	no
SUPT5H	-0.178651555	-4.793654762	2.00E-06	7.39E-06	no
KIAA1210	0.178620905	4.792805249	2.01E-06	7.42E-06	no
VAPA	-0.178620417	-4.792791717	2.01E-06	7.42E-06	no
C15orf56	0.178617802	4.792719247	2.01E-06	7.42E-06	no
C20orf132	-0.178601145	-4.792257574	2.02E-06	7.44E-06	no
SPARCL1	-0.178593567	-4.792047535	2.02E-06	7.44E-06	no
WNT7B	-0.178574984	-4.791532489	2.02E-06	7.46E-06	no
HBQ1	-0.178572127	-4.791453292	2.02E-06	7.46E-06	no
S100A13	0.178556064	4.791008093	2.03E-06	7.48E-06	no
NRG2	-0.178548415	-4.790796104	2.03E-06	7.48E-06	no
SPATS2	-0.178541706	-4.790610176	2.03E-06	7.49E-06	no
WDR66	0.178534134	4.790400321	2.03E-06	7.49E-06	no
GLT25D2	-0.178491873	-4.789229047	2.05E-06	7.54E-06	no
POLK	0.17848081	4.788922442	2.05E-06	7.54E-06	no
OSR2	0.178469319	4.788603978	2.05E-06	7.56E-06	no
LOC150786	-0.178464584	-4.788472735	2.05E-06	7.56E-06	no
HDHD2	-0.178461576	-4.788389392	2.05E-06	7.56E-06	no
TAF5	-0.178439057	-4.7877653	2.06E-06	7.58E-06	no
ZNF141	-0.178418359	-4.787191666	2.07E-06	7.60E-06	no
ULBP3	0.178413045	4.787044418	2.07E-06	7.61E-06	no
CEACAM1	0.178401655	4.786728757	2.07E-06	7.62E-06	no
SEC23A	0.178391631	4.786450957	2.07E-06	7.62E-06	no
EID3	0.178307845	4.784129056	2.10E-06	7.71E-06	no
PNMA2	-0.178296137	-4.783804603	2.10E-06	7.72E-06	no
LOC339047	-0.178288043	-4.783580285	2.10E-06	7.73E-06	no
NAT8B	0.178267579	4.783013215	2.11E-06	7.75E-06	no
TBC1D2	0.178256465	4.78270522	2.11E-06	7.76E-06	no
IPO7	-0.178212986	-4.781500394	2.12E-06	7.80E-06	no
WDR33	-0.178187374	-4.780790677	2.13E-06	7.83E-06	no
YJEFN3	-0.178183596	-4.78068601	2.13E-06	7.83E-06	no

SFXN2	0.178171914	4.780362303	2.14E-06	7.84E-06	no
RGS2	0.178146837	4.779667429	2.14E-06	7.86E-06	no
ANKRD32	0.178119469	4.778909066	2.15E-06	7.89E-06	no
C3orf58	0.178099808	4.778364289	2.16E-06	7.91E-06	no
ZNF702P	0.178091393	4.77813113	2.16E-06	7.92E-06	no
TGFB2	0.178083807	4.777920943	2.16E-06	7.92E-06	no
ZNF704	-0.178083629	-4.777915991	2.16E-06	7.92E-06	no
WDR20	-0.178076552	-4.777719921	2.16E-06	7.93E-06	no
ATN1	-0.178073358	-4.777631406	2.16E-06	7.93E-06	no
ADIPOR1	0.178069816	4.777533254	2.16E-06	7.93E-06	no
LOC84931	0.178055599	4.777139339	2.17E-06	7.95E-06	no
TTC30B	0.178027714	4.776366723	2.18E-06	7.97E-06	no
RIMKLB	-0.177994254	-4.775439619	2.19E-06	8.01E-06	no
LOC400043	0.177978207	4.77499501	2.19E-06	8.02E-06	no
TUG1	-0.177972939	-4.774849049	2.19E-06	8.03E-06	no
LRP11	-0.177951632	-4.774258712	2.20E-06	8.05E-06	no
C5orf42	-0.177951167	-4.774245833	2.20E-06	8.05E-06	no
GK3P	0.177949289	4.774193804	2.20E-06	8.05E-06	no
TMEM97	-0.177912083	-4.77316296	2.21E-06	8.09E-06	no
CD47	0.177890482	4.772564502	2.22E-06	8.11E-06	no
NEURL3	0.177867947	4.771940169	2.22E-06	8.13E-06	no
GCLC	-0.177859378	-4.771702745	2.23E-06	8.14E-06	no
LOC440957	0.177810575	4.77035068	2.24E-06	8.19E-06	no
ENPP1	0.177808731	4.770299606	2.24E-06	8.19E-06	no
RPL17	-0.177807614	-4.770268652	2.24E-06	8.19E-06	no
ZFAND6	0.177749124	4.768648266	2.26E-06	8.26E-06	no
THSD7B	0.177692863	4.767089694	2.28E-06	8.32E-06	no
TNR	-0.177649251	-4.765881542	2.29E-06	8.36E-06	no
AP3D1	-0.177637674	-4.765560834	2.29E-06	8.37E-06	no
LRRC59	0.177583639	4.764063998	2.31E-06	8.43E-06	no
MAP1D	-0.17757137	-4.763724136	2.31E-06	8.45E-06	no
INPP5B	0.177551658	4.763178117	2.32E-06	8.47E-06	no
PPM1L	-0.177548979	-4.763103903	2.32E-06	8.47E-06	no
UNC13D	0.177507871	4.761965216	2.33E-06	8.51E-06	no
CCDC153	0.177495583	4.761624847	2.34E-06	8.52E-06	no
DCAF6	0.177436948	4.760000734	2.36E-06	8.59E-06	no
C6orf62	0.177430192	4.759813588	2.36E-06	8.60E-06	no
TXLNB	0.177371526	4.758188662	2.38E-06	8.66E-06	no
ESRRB	0.177368156	4.758095318	2.38E-06	8.66E-06	no
CCDC18	0.177362484	4.757938219	2.38E-06	8.67E-06	no
REXO4	-0.177358078	-4.757816193	2.38E-06	8.67E-06	no
KHDRBS3	-0.177303539	-4.756305632	2.40E-06	8.73E-06	no
CPLX2	-0.177292545	-4.756001152	2.40E-06	8.75E-06	no
TNIP3	0.177289652	4.75592102	2.40E-06	8.75E-06	no
OXCT1	-0.177231549	-4.7543118	2.42E-06	8.81E-06	no
THEMIS	0.177206	4.753604224	2.43E-06	8.84E-06	no
EIF4B	-0.177195291	-4.753307646	2.43E-06	8.85E-06	no
ALPK2	0.177163647	4.752431278	2.44E-06	8.89E-06	no
LPHN2	0.177161409	4.752369295	2.44E-06	8.89E-06	no
PSMD11	0.177127293	4.751424491	2.45E-06	8.93E-06	no
TRIM61	0.177119053	4.751196284	2.46E-06	8.94E-06	no

JDP2	0.177107032	4.750863372	2.46E-06	8.95E-06	no
NDUFA5	-0.177106929	-4.750860534	2.46E-06	8.95E-06	no
LOC390595	-0.177088039	-4.750337403	2.47E-06	8.97E-06	no
HUWE1	-0.177075714	-4.749996079	2.47E-06	8.98E-06	no
KPNA5	-0.177068819	-4.749805134	2.47E-06	8.99E-06	no
LOC442454	-0.177056358	-4.749460055	2.48E-06	9.00E-06	no
NSUN7	0.177053404	4.749378251	2.48E-06	9.00E-06	no
PEX6	-0.177030155	-4.748734422	2.49E-06	9.03E-06	no
SLC25A13	0.17694268	4.746312093	2.51E-06	9.13E-06	no
RBBP6	-0.176940381	-4.746248442	2.52E-06	9.13E-06	no
PZP	0.17690584	4.745291986	2.53E-06	9.17E-06	no
CDC5L	-0.176902427	-4.745197483	2.53E-06	9.18E-06	no
STAR	-0.176850008	-4.743746	2.55E-06	9.24E-06	no
LOC284749	0.176827468	4.743121861	2.55E-06	9.26E-06	no
KEAP1	-0.176782297	-4.741871131	2.57E-06	9.32E-06	no
RILPL1	-0.17677728	-4.741732222	2.57E-06	9.32E-06	no
PSMC5	-0.176775512	-4.741683267	2.57E-06	9.32E-06	no
KIAA1644	-0.176770751	-4.741551458	2.57E-06	9.33E-06	no
FZD7	0.176742406	4.740766614	2.58E-06	9.36E-06	no
LRRC58	-0.176739404	-4.740683517	2.58E-06	9.36E-06	no
RCCD1	-0.176735749	-4.740582298	2.58E-06	9.37E-06	no
CYB5D1	0.176668907	4.738731624	2.61E-06	9.45E-06	no
OR2H2	-0.176637561	-4.737863777	2.62E-06	9.49E-06	no
NRP2	0.176624904	4.737513357	2.62E-06	9.50E-06	no
RRP7B	-0.176617583	-4.73731064	2.63E-06	9.51E-06	no
C19orf39	0.176609495	4.73708672	2.63E-06	9.52E-06	no
ATP6V0B	0.176523018	4.734692579	2.66E-06	9.62E-06	no
UPF2	-0.176521034	-4.734637672	2.66E-06	9.62E-06	no
VARS2	-0.17651641	-4.734509642	2.66E-06	9.63E-06	no
NARFL	-0.176511747	-4.73438056	2.66E-06	9.63E-06	no
RGS22	0.17650563	4.734211222	2.66E-06	9.64E-06	no
RNF115	0.176505007	4.734193966	2.66E-06	9.64E-06	no
SNORD115-26	-0.176500177	-4.73406026	2.67E-06	9.64E-06	no
BRPF3	-0.176481132	-4.733533011	2.67E-06	9.66E-06	no
ATAD3C	0.176474308	4.733344101	2.68E-06	9.67E-06	no
IL1F8	0.176415578	4.731718235	2.70E-06	9.75E-06	no
YIPF2	0.176396298	4.731184509	2.70E-06	9.77E-06	no
ADH1C	0.176336189	4.72952056	2.72E-06	9.84E-06	no
ZNF28	0.176249729	4.727127244	2.76E-06	9.96E-06	no
AMMECR1	0.176247529	4.727066349	2.76E-06	9.96E-06	no
DNAH11	0.176139633	4.724079834	2.80E-06	1.01E-05	no
METTL7B	0.17610888	4.723228637	2.81E-06	1.01E-05	no
RAD9B	0.176074488	4.722276726	2.82E-06	1.02E-05	no
PPP1R12B	-0.176062808	-4.721953443	2.82E-06	1.02E-05	no
OTUD7A	-0.17605892	-4.721845849	2.83E-06	1.02E-05	no
DCAKD	-0.17602899	-4.721017471	2.84E-06	1.02E-05	no
C10orf58	-0.175992526	-4.720008248	2.85E-06	1.03E-05	no
ARHGEF3	0.175953904	4.71893934	2.87E-06	1.03E-05	no
PTPRD	-0.175942911	-4.718635085	2.87E-06	1.03E-05	no
ERAS	-0.175941455	-4.718594786	2.87E-06	1.03E-05	no
ATL1	-0.175926392	-4.718177919	2.88E-06	1.04E-05	no

ABCA7	0.175919457	4.717985982	2.88E-06	1.04E-05	no
MT1A	0.17588884	4.717138654	2.89E-06	1.04E-05	no
SUN3	0.175864129	4.716454772	2.90E-06	1.04E-05	no
CXorf36	0.175856957	4.716256303	2.90E-06	1.05E-05	no
TRIM3	-0.17585679	-4.716251684	2.90E-06	1.05E-05	no
CEP250	-0.175854839	-4.716197688	2.90E-06	1.05E-05	no
C2orf77	0.175850269	4.716071201	2.91E-06	1.05E-05	no
CDH26	0.175843581	4.715886119	2.91E-06	1.05E-05	no
CMC1	0.175834856	4.71564466	2.91E-06	1.05E-05	no
LRRC6	0.17582202	4.715289431	2.92E-06	1.05E-05	no
SNTG1	-0.17581174	-4.715004949	2.92E-06	1.05E-05	no
CEP55	0.175786628	4.71431001	2.93E-06	1.05E-05	no
ST6GAL2	-0.175784606	-4.714254054	2.93E-06	1.05E-05	no
FEM1C	0.17577753	4.714058232	2.93E-06	1.05E-05	no
SERTAD2	0.175775776	4.714009702	2.93E-06	1.05E-05	no
IL17RD	-0.175744974	-4.713157309	2.95E-06	1.06E-05	no
NADSYN1	0.175724382	4.712587486	2.95E-06	1.06E-05	no
ARF3	-0.175701606	-4.711957207	2.96E-06	1.06E-05	no
CHPT1	0.17568829	4.711588737	2.97E-06	1.07E-05	no
LOC100133612	0.175685273	4.711505244	2.97E-06	1.07E-05	no
NACAP1	-0.175679991	-4.711359078	2.97E-06	1.07E-05	no
EXD2	-0.175652493	-4.710598155	2.98E-06	1.07E-05	no
TP53RK	0.175634875	4.710110656	2.99E-06	1.07E-05	no
ULBP2	0.175631584	4.71001959	2.99E-06	1.07E-05	no
ECHDC2	0.175628577	4.709936378	2.99E-06	1.07E-05	no
RAF1	-0.175624069	-4.709811629	2.99E-06	1.07E-05	no
EHD3	-0.175624018	-4.709810234	2.99E-06	1.07E-05	no
LONRF2	-0.17562262	-4.709771541	2.99E-06	1.07E-05	no
TCTEX1D1	0.175607185	4.709344423	3.00E-06	1.08E-05	no
SCO2	0.175570832	4.708338536	3.01E-06	1.08E-05	no
CACNA1D	-0.175521869	-4.706983741	3.03E-06	1.09E-05	no
LOC134466	-0.175506383	-4.70655525	3.04E-06	1.09E-05	no
PHC2	0.175482043	4.705881763	3.05E-06	1.09E-05	no
GATA6	0.175464837	4.705405699	3.06E-06	1.10E-05	no
STXBP1	-0.175457059	-4.705190504	3.06E-06	1.10E-05	no
CKMT1A	-0.175440565	-4.704734145	3.07E-06	1.10E-05	no
RASGEF1C	-0.175423517	-4.704262451	3.07E-06	1.10E-05	no
ERO1LB	-0.175412373	-4.70395413	3.08E-06	1.10E-05	no
LYPD3	0.175358197	4.702455205	3.10E-06	1.11E-05	no
DKFZp6860241	-0.175356518	-4.702408756	3.10E-06	1.11E-05	no
LRFN4	-0.175274109	-4.700128796	3.13E-06	1.12E-05	no
KIF21A	-0.175235394	-4.699057719	3.15E-06	1.13E-05	no
HDAC6	-0.175229838	-4.698904023	3.15E-06	1.13E-05	no
RBM23	-0.175219873	-4.69862834	3.16E-06	1.13E-05	no
C14orf1	-0.17519282	-4.697879914	3.17E-06	1.13E-05	no
UACA	0.175177359	4.697452189	3.17E-06	1.13E-05	no
RAB24	-0.175150783	-4.696716985	3.19E-06	1.14E-05	no
GLOD4	-0.175148924	-4.696665552	3.19E-06	1.14E-05	no
MRPL34	0.175138133	4.696367043	3.19E-06	1.14E-05	no
LOC440040	-0.175135159	-4.696284773	3.19E-06	1.14E-05	no
INSM2	-0.175128838	-4.696109897	3.19E-06	1.14E-05	no

TPT1	0.17512184	4.695916319	3.20E-06	1.14E-05	no
MDN1	-0.175101803	-4.69536204	3.21E-06	1.14E-05	no
BMPR2	-0.17507625	-4.694655164	3.22E-06	1.15E-05	no
MST1	-0.175051593	-4.693973073	3.23E-06	1.15E-05	no
CPSF6	-0.175036118	-4.693544997	3.23E-06	1.15E-05	no
ZMYM2	-0.175012357	-4.692887718	3.24E-06	1.16E-05	no
HRSP12	-0.174990967	-4.692296031	3.25E-06	1.16E-05	no
FGFBP3	-0.174985244	-4.692137742	3.25E-06	1.16E-05	no
MMP25	0.174976385	4.691892691	3.26E-06	1.16E-05	no
NECAB3	-0.174962355	-4.691504601	3.26E-06	1.16E-05	no
IVD	-0.174929545	-4.690597044	3.28E-06	1.17E-05	no
BEST3	-0.174918103	-4.690280536	3.28E-06	1.17E-05	no
LOC283174	-0.17486928	-4.68893011	3.30E-06	1.18E-05	no
C8orf55	-0.174861539	-4.688715989	3.31E-06	1.18E-05	no
LSM10	0.17483055	4.687858848	3.32E-06	1.18E-05	no
C4orf33	0.17481998	4.687566492	3.33E-06	1.18E-05	no
ZHX3	-0.174819019	-4.687539914	3.33E-06	1.18E-05	no
NPTXR	-0.174810726	-4.687310544	3.33E-06	1.19E-05	no
PMAIP1	0.17477635	4.686359746	3.35E-06	1.19E-05	no
MOXD1	0.174738956	4.685325511	3.36E-06	1.20E-05	no
RAD21	-0.17470382	-4.684353737	3.38E-06	1.20E-05	no
LOC729467	0.174680967	4.683721677	3.39E-06	1.21E-05	no
HRASLS2	0.174661067	4.683171301	3.40E-06	1.21E-05	no
RWDD2A	-0.174640256	-4.682595759	3.41E-06	1.21E-05	no
MVK	-0.174633655	-4.682413198	3.41E-06	1.21E-05	no
MT1H	0.174630353	4.682321868	3.41E-06	1.21E-05	no
C1orf69	-0.174629988	-4.682311764	3.41E-06	1.21E-05	no
PNN	-0.174604396	-4.681604009	3.42E-06	1.22E-05	no
RAET1K	0.174580204	4.680934965	3.43E-06	1.22E-05	no
SACS	-0.174579456	-4.68091427	3.43E-06	1.22E-05	no
SHANK3	-0.174548996	-4.680071892	3.45E-06	1.22E-05	no
TMEM55B	-0.174533928	-4.679655198	3.45E-06	1.23E-05	no
MTRF1L	-0.174533213	-4.67963542	3.45E-06	1.23E-05	no
IPP	0.174505206	4.678860894	3.47E-06	1.23E-05	no
VSX2	-0.174499969	-4.678716078	3.47E-06	1.23E-05	no
MMADHC	0.174491361	4.678478021	3.47E-06	1.23E-05	no
FAM168A	-0.174472641	-4.677960349	3.48E-06	1.24E-05	no
TSNAX	0.174469	4.677859665	3.48E-06	1.24E-05	no
ZNF700	0.174376303	4.675296317	3.52E-06	1.25E-05	no
RNF38	-0.174362247	-4.674907613	3.53E-06	1.25E-05	no
WNK2	-0.174322927	-4.673820354	3.55E-06	1.26E-05	no
CCDC116	-0.174317305	-4.67366491	3.55E-06	1.26E-05	no
ESM1	0.17430712	4.67338327	3.56E-06	1.26E-05	no
ATP2B2	-0.174247011	-4.671721203	3.58E-06	1.27E-05	no
PEG3	-0.174240951	-4.671553636	3.59E-06	1.27E-05	no
LENG9	0.174237299	4.671452669	3.59E-06	1.27E-05	no
BPTF	-0.174225833	-4.671135635	3.59E-06	1.27E-05	no
NEIL2	-0.174168391	-4.669547371	3.62E-06	1.28E-05	no
ENOSF1	0.174127912	4.66842816	3.64E-06	1.29E-05	no
SIRT3	-0.174121744	-4.668257635	3.64E-06	1.29E-05	no
EGR2	0.174073336	4.666919249	3.67E-06	1.30E-05	no

PSMA4	0.174053491	4.666370568	3.68E-06	1.30E-05	no
RPP30	-0.174051294	-4.666309839	3.68E-06	1.30E-05	no
CCDC88A	-0.174008279	-4.665120593	3.70E-06	1.31E-05	no
DACH2	-0.173994553	-4.664741099	3.70E-06	1.31E-05	no
TM4SF20	-0.173982767	-4.664415253	3.71E-06	1.31E-05	no
AFP	0.173965439	4.663936208	3.72E-06	1.32E-05	no
TSPAN17	0.173961019	4.663814008	3.72E-06	1.32E-05	no
TSLP	0.173945775	4.663392571	3.73E-06	1.32E-05	no
COG4	-0.173942165	-4.663292763	3.73E-06	1.32E-05	no
EGLN1	-0.173920811	-4.662702414	3.74E-06	1.32E-05	no
ZXDB	-0.173852693	-4.660819295	3.77E-06	1.33E-05	no
PANK4	-0.17384743	-4.66067379	3.78E-06	1.33E-05	no
NME6	0.173835562	4.660345713	3.78E-06	1.34E-05	no
PFAS	-0.173784061	-4.658922039	3.81E-06	1.34E-05	no
MMAB	-0.173714507	-4.65699934	3.84E-06	1.36E-05	no
C1QTNF9	0.173702394	4.65666452	3.85E-06	1.36E-05	no
DAB2IP	-0.173665271	-4.655638355	3.87E-06	1.37E-05	no
PPY	0.173662423	4.655559628	3.87E-06	1.37E-05	no
MIOS	0.173652391	4.655282324	3.87E-06	1.37E-05	no
ZMAT1	-0.173645295	-4.655086193	3.88E-06	1.37E-05	no
WDR27	-0.173645108	-4.655081014	3.88E-06	1.37E-05	no
CAMTA1	-0.173626958	-4.654579341	3.89E-06	1.37E-05	no
TP53TG1	0.173621251	4.654421586	3.89E-06	1.37E-05	no
PIRT	0.173596027	4.653724377	3.90E-06	1.38E-05	no
ATG2B	-0.173592812	-4.653635509	3.90E-06	1.38E-05	no
ARID1B	-0.173592647	-4.653630934	3.90E-06	1.38E-05	no
DNALI1	0.173591112	4.653588514	3.90E-06	1.38E-05	no
PAK3	-0.173577032	-4.653199345	3.91E-06	1.38E-05	no
CACNA1E	-0.173575781	-4.65316475	3.91E-06	1.38E-05	no
HSPA12A	-0.173574797	-4.653137567	3.91E-06	1.38E-05	no
ECHDC1	0.173554495	4.652576398	3.92E-06	1.38E-05	no
MFSD6	-0.173527751	-4.651837198	3.94E-06	1.39E-05	no
SEC61A2	-0.173493836	-4.650899807	3.95E-06	1.39E-05	no
DUSP9	-0.173470321	-4.650249882	3.97E-06	1.40E-05	no
ARID4A	-0.173467654	-4.650176164	3.97E-06	1.40E-05	no
WBSCR16	0.173441145	4.649443482	3.98E-06	1.40E-05	no
FAT3	-0.173416659	-4.64876674	3.99E-06	1.40E-05	no
LASS3	0.173381046	4.647782484	4.01E-06	1.41E-05	no
FAM40A	-0.173331715	-4.646419098	4.04E-06	1.42E-05	no
CCDC103	0.173320801	4.646117487	4.04E-06	1.42E-05	no
AGPAT4	-0.173288979	-4.645238038	4.06E-06	1.43E-05	no
ABHD11	0.173282141	4.64504906	4.06E-06	1.43E-05	no
PRLHR	-0.173263705	-4.644539547	4.07E-06	1.43E-05	no
CYB5R1	0.17325345	4.644256154	4.08E-06	1.43E-05	no
EGOT	0.1732504	4.644171857	4.08E-06	1.43E-05	no
LYST	0.173239981	4.64388392	4.09E-06	1.44E-05	no
SRP19	0.173238581	4.643845247	4.09E-06	1.44E-05	no
PVR	0.173180279	4.642234066	4.12E-06	1.45E-05	no
FBXO34	-0.173129892	-4.640841646	4.15E-06	1.46E-05	no
IGSF21	-0.173116897	-4.640482546	4.15E-06	1.46E-05	no
ACTN2	-0.173111566	-4.640335226	4.16E-06	1.46E-05	no

SUMO1P3	0.173068625	4.639148649	4.18E-06	1.47E-05	no
PLA2G2C	0.173068248	4.63913821	4.18E-06	1.47E-05	no
ESPNL	0.173066577	4.639092061	4.18E-06	1.47E-05	no
NAE1	-0.173023511	-4.637902032	4.20E-06	1.47E-05	no
DDHD2	-0.173019929	-4.637803043	4.21E-06	1.47E-05	no
C4orf36	0.173000446	4.637264689	4.22E-06	1.48E-05	no
PLAGL1	0.17298321	4.636788435	4.23E-06	1.48E-05	no
PDE4A	-0.172982638	-4.636772632	4.23E-06	1.48E-05	no
SMARCD1	-0.172961904	-4.636199719	4.24E-06	1.48E-05	no
IGSF8	-0.172948054	-4.63581702	4.24E-06	1.49E-05	no
TTYH3	0.172907428	4.634694487	4.27E-06	1.49E-05	no
MRPL36	0.172902143	4.634548465	4.27E-06	1.50E-05	no
GRB2	0.172883807	4.634041837	4.28E-06	1.50E-05	no
SDAD1	0.172851831	4.633158346	4.30E-06	1.50E-05	no
TNFRSF8	0.172844526	4.632956506	4.30E-06	1.51E-05	no
PTX3	0.172838801	4.63279832	4.31E-06	1.51E-05	no
SLC38A1	-0.172826886	-4.632469107	4.31E-06	1.51E-05	no
DHX9	-0.172797962	-4.63166998	4.33E-06	1.51E-05	no
C7orf28A	0.172745396	4.630217647	4.36E-06	1.52E-05	no
CHPF	0.172722301	4.629579587	4.37E-06	1.53E-05	no
LOC730811	-0.172673874	-4.628241663	4.40E-06	1.54E-05	no
MYLPF	0.172657058	4.627777091	4.41E-06	1.54E-05	no
ZNF720	-0.17265598	-4.62774731	4.41E-06	1.54E-05	no
WWP2	-0.172639017	-4.627278672	4.42E-06	1.54E-05	no
MYL6	0.172621492	4.626794507	4.43E-06	1.55E-05	no
DDIT4L	0.172614527	4.626602094	4.43E-06	1.55E-05	no
HCFC1	-0.172590846	-4.625947895	4.45E-06	1.55E-05	no
CABLES1	-0.172577281	-4.625573133	4.45E-06	1.56E-05	no
5-Sep	-0.172571547	-4.62541473	4.46E-06	1.56E-05	no
PKD1	-0.172557992	-4.625040278	4.46E-06	1.56E-05	no
SYN1	-0.172542832	-4.624621475	4.47E-06	1.56E-05	no
A4GNT	0.172542337	4.624607798	4.47E-06	1.56E-05	no
FAM149A	-0.172519454	-4.623975661	4.49E-06	1.57E-05	no
PSMC4	0.172484857	4.623019945	4.51E-06	1.57E-05	no
SLC25A44	-0.172456187	-4.622227939	4.52E-06	1.58E-05	no
SRFBP1	0.172454021	4.622168107	4.53E-06	1.58E-05	no
SPTLC3	0.172433263	4.621594701	4.54E-06	1.58E-05	no
ANKRD53	0.172407061	4.620870919	4.55E-06	1.59E-05	no
CSRNP3	-0.172390522	-4.62041405	4.56E-06	1.59E-05	no
MMP12	0.172353272	4.619385121	4.58E-06	1.60E-05	no
NUP62	0.172353182	4.619382629	4.58E-06	1.60E-05	no
ZNF418	-0.172330163	-4.618746799	4.60E-06	1.60E-05	no
MGMT	0.17228698	4.617553999	4.62E-06	1.61E-05	no
HIST2H2BF	0.172261159	4.616840791	4.64E-06	1.62E-05	no
TMEM194A	0.172233491	4.616076591	4.66E-06	1.62E-05	no
LOC100303728	-0.172225716	-4.615861832	4.66E-06	1.62E-05	no
NRL	-0.172178348	-4.614553515	4.69E-06	1.63E-05	no
S100A5	0.172175766	4.614482205	4.69E-06	1.63E-05	no
PKM2	0.172170723	4.61434293	4.69E-06	1.63E-05	no
PCGF6	-0.172155118	-4.61391193	4.70E-06	1.64E-05	no
EPHA2	0.172132135	4.61327713	4.72E-06	1.64E-05	no

ADAM29	-0.172106243	-4.612562041	4.73E-06	1.65E-05	no
ZWILCH	0.172063965	4.61139439	4.76E-06	1.65E-05	no
C20orf108	0.172060894	4.611309566	4.76E-06	1.65E-05	no
CRKL	-0.172032431	-4.610523467	4.78E-06	1.66E-05	no
SRRM2	-0.172029365	-4.610438788	4.78E-06	1.66E-05	no
CHD9	-0.172012616	-4.609976249	4.79E-06	1.66E-05	no
CENPL	0.172011419	4.609943186	4.79E-06	1.66E-05	no
CCDC42	0.171990987	4.609378891	4.80E-06	1.67E-05	no
LOC342346	-0.171977887	-4.609017128	4.81E-06	1.67E-05	no
C7orf29	0.171970485	4.608812695	4.82E-06	1.67E-05	no
MITD1	0.171962674	4.608596982	4.82E-06	1.67E-05	no
CHCHD6	-0.171944395	-4.608092187	4.83E-06	1.68E-05	no
ZNF483	-0.171942663	-4.608044344	4.83E-06	1.68E-05	no
BCHE	-0.171933882	-4.60780185	4.84E-06	1.68E-05	no
OR5B21	0.171925475	4.607569691	4.84E-06	1.68E-05	no
TBC1D14	-0.171906943	-4.607057899	4.86E-06	1.68E-05	no
FOLR4	0.171874465	4.606161011	4.88E-06	1.69E-05	no
TMEM48	0.171872702	4.606112317	4.88E-06	1.69E-05	no
NXN	-0.171872045	-4.606094179	4.88E-06	1.69E-05	no
LPCAT3	0.171861673	4.605807743	4.88E-06	1.69E-05	no
C3orf67	0.171822809	4.604734538	4.91E-06	1.70E-05	no
RGR	-0.171819425	-4.604641075	4.91E-06	1.70E-05	no
LCA5L	0.171795512	4.603980752	4.93E-06	1.71E-05	no
DCTN1	-0.171762083	-4.603057635	4.95E-06	1.71E-05	no
SFRS13A	0.171754414	4.602845854	4.95E-06	1.72E-05	no
CYB5R3	0.171743878	4.602554936	4.96E-06	1.72E-05	no
FAM46B	0.171724048	4.602007361	4.97E-06	1.72E-05	no
HEXDC	-0.171665676	-4.600395541	5.01E-06	1.73E-05	no
DAAM2	-0.171631525	-4.599452565	5.03E-06	1.74E-05	no
LRP3	-0.171619943	-4.599132762	5.04E-06	1.74E-05	no
KLRG1	0.171594287	4.598424376	5.06E-06	1.75E-05	no
NHEDC1	-0.171578327	-4.597983711	5.07E-06	1.75E-05	no
TRIAP1	0.171573621	4.597853752	5.07E-06	1.75E-05	no
TRIM15	0.171551852	4.597252694	5.08E-06	1.76E-05	no
MRPL32	0.171544429	4.597047756	5.09E-06	1.76E-05	no
STARD13	0.171523903	4.596481017	5.10E-06	1.76E-05	no
RANBP3	-0.171520438	-4.596385353	5.10E-06	1.76E-05	no
SLC25A21	-0.171465324	-4.594863666	5.14E-06	1.78E-05	no
EPC2	-0.171426888	-4.593802493	5.17E-06	1.78E-05	no
ASMTL	-0.17138214	-4.592567059	5.20E-06	1.79E-05	no
C17orf64	0.17137879	4.592474579	5.20E-06	1.80E-05	no
CLYBL	0.171320146	4.59085554	5.24E-06	1.81E-05	no
GSPT2	-0.171308882	-4.590544584	5.24E-06	1.81E-05	no
GRIN3A	-0.171298891	-4.590268766	5.25E-06	1.81E-05	no
C16orf87	-0.171296576	-4.590204845	5.25E-06	1.81E-05	no
LIN54	-0.171284499	-4.589871452	5.26E-06	1.82E-05	no
PIR	0.171259455	4.589180072	5.28E-06	1.82E-05	no
TOLLIP	-0.171254375	-4.589039832	5.28E-06	1.82E-05	no
DDAH1	-0.171246103	-4.588811459	5.29E-06	1.82E-05	no
FHDC1	-0.171206577	-4.587720318	5.31E-06	1.83E-05	no
PNP	0.171197028	4.587456718	5.32E-06	1.83E-05	no

GFER	-0.17119482	-4.587395769	5.32E-06	1.83E-05	no
FLJ37201	-0.17119257	-4.587333661	5.32E-06	1.83E-05	no
ZNF37B	-0.171132951	-4.585687877	5.36E-06	1.85E-05	no
FAM126B	-0.171121582	-4.585374025	5.37E-06	1.85E-05	no
CST6	0.171106424	4.584955622	5.38E-06	1.85E-05	no
EFNA3	-0.171085585	-4.584380368	5.40E-06	1.86E-05	no
MTERFD1	0.171052405	4.583464492	5.42E-06	1.87E-05	no
C5orf25	0.171050003	4.58339819	5.42E-06	1.87E-05	no
NSUN4	0.170995849	4.581903385	5.46E-06	1.88E-05	no
CCDC8	0.170981252	4.581500454	5.47E-06	1.88E-05	no
ENOPH1	-0.170972813	-4.581267535	5.48E-06	1.88E-05	no
SIPA1L1	0.170956145	4.580807458	5.49E-06	1.89E-05	no
UTP6	0.170940851	4.580385313	5.50E-06	1.89E-05	no
LRRTM1	-0.170921783	-4.579859007	5.51E-06	1.90E-05	no
SHCBP1	0.170919306	4.579790645	5.51E-06	1.90E-05	no
ADAM28	0.170896379	4.579157828	5.53E-06	1.90E-05	no
FBXL16	-0.170891254	-4.579016362	5.53E-06	1.90E-05	no
SAP30L	-0.170873801	-4.578534653	5.55E-06	1.91E-05	no
KIR3DL2	0.170865168	4.578296372	5.55E-06	1.91E-05	no
RRP12	-0.17083933	-4.57758325	5.57E-06	1.91E-05	no
TRIM43	0.170820763	4.577070802	5.58E-06	1.92E-05	no
ZNF655	0.170819752	4.577042882	5.58E-06	1.92E-05	no
CHTF8	0.170806739	4.576683719	5.59E-06	1.92E-05	no
GRAPL	0.170788116	4.576169739	5.61E-06	1.93E-05	no
C5orf35	0.170743007	4.574924774	5.64E-06	1.94E-05	no
TRNAU1AP	0.170738572	4.57480236	5.64E-06	1.94E-05	no
SMCR8	0.170735147	4.574707848	5.64E-06	1.94E-05	no
DCAF5	-0.170728856	-4.574534211	5.65E-06	1.94E-05	no
PHTF2	0.170723228	4.574378893	5.65E-06	1.94E-05	no
FAM5C	-0.170669648	-4.572900185	5.69E-06	1.95E-05	no
GABRD	-0.170667759	-4.572848047	5.69E-06	1.95E-05	no
C13orf34	0.170656793	4.572545428	5.70E-06	1.96E-05	no
CAPN6	0.170653759	4.57246169	5.70E-06	1.96E-05	no
WT1	0.170653627	4.572458061	5.70E-06	1.96E-05	no
MYT1L	-0.170638274	-4.572034357	5.71E-06	1.96E-05	no
TSPAN11	-0.170598832	-4.570945853	5.74E-06	1.97E-05	no
ACOX1	-0.17056969	-4.570141653	5.77E-06	1.98E-05	no
MYO16	-0.170521206	-4.568803683	5.80E-06	1.99E-05	no
ZNF212	-0.170468754	-4.567356275	5.84E-06	2.00E-05	no
GCAT	-0.170463016	-4.567197915	5.84E-06	2.00E-05	no
HECTD1	-0.170372081	-4.564688646	5.91E-06	2.02E-05	no
SLC7A14	-0.170354695	-4.564208914	5.93E-06	2.03E-05	no
HSF4	-0.170333949	-4.563636474	5.94E-06	2.03E-05	no
PRELID2	0.170258059	4.56154251	6.00E-06	2.05E-05	no
ZNF714	-0.170247364	-4.561247407	6.01E-06	2.06E-05	no
FAM117A	0.1702391	4.561019395	6.01E-06	2.06E-05	no
ZMIZ2	-0.170136585	-4.55819092	6.09E-06	2.08E-05	no
NUBP1	0.170127437	4.557938521	6.10E-06	2.09E-05	no
ARPC1A	0.170067081	4.556273342	6.15E-06	2.10E-05	no
LRRC70	0.170062499	4.556146939	6.15E-06	2.10E-05	no
CDK5RAP1	-0.17005316	-4.555889275	6.16E-06	2.10E-05	no

PRIC285	0.169982371	4.553936334	6.21E-06	2.12E-05	no
SULF1	0.169981902	4.553923404	6.21E-06	2.12E-05	no
ADAM15	0.169924781	4.552347589	6.26E-06	2.14E-05	no
SRRM3	-0.169911012	-4.551967746	6.27E-06	2.14E-05	no
ZNF396	0.169907472	4.551870086	6.27E-06	2.14E-05	no
WDR69	0.169889038	4.551361537	6.29E-06	2.15E-05	no
ERAL1	-0.1698862	-4.551283253	6.29E-06	2.15E-05	no
LDOC1	-0.169877606	-4.551046187	6.30E-06	2.15E-05	no
COBRA1	-0.1698762	-4.551007394	6.30E-06	2.15E-05	no
SAR1B	0.169872208	4.550897275	6.30E-06	2.15E-05	no
SLC30A9	-0.169845155	-4.550150998	6.32E-06	2.16E-05	no
PAWR	0.169786006	4.548519348	6.37E-06	2.17E-05	no
WDR25	-0.169780843	-4.548376927	6.38E-06	2.17E-05	no
SNRNP40	0.169764059	4.547913937	6.39E-06	2.18E-05	no
LHFP	0.169650229	4.544774076	6.48E-06	2.21E-05	no
ANKRA2	-0.169648264	-4.544719884	6.48E-06	2.21E-05	no
FAM81A	-0.169610107	-4.543667406	6.52E-06	2.22E-05	no
PDZK1P1	-0.169609503	-4.543650737	6.52E-06	2.22E-05	no
ZNF280A	0.169592439	4.54318008	6.53E-06	2.22E-05	no
DOLPP1	-0.169587001	-4.54303009	6.53E-06	2.23E-05	no
DKFZp434J022	-0.169584053	-4.542948781	6.54E-06	2.23E-05	no
FAM169A	-0.169575737	-4.542719394	6.54E-06	2.23E-05	no
POLE4	0.169566892	4.542475445	6.55E-06	2.23E-05	no
CARM1	0.169561083	4.542315229	6.56E-06	2.23E-05	no
C1orf21	-0.169550819	-4.542032131	6.56E-06	2.23E-05	no
DUSP3	0.169524648	4.541310299	6.59E-06	2.24E-05	no
LOC158376	0.169522041	4.54123838	6.59E-06	2.24E-05	no
C14orf174	0.169521051	4.541211075	6.59E-06	2.24E-05	no
KRTAP5-7	-0.169516715	-4.541091505	6.59E-06	2.24E-05	no
CPT2	0.169510203	4.540911888	6.60E-06	2.24E-05	no
PPCDC	0.169498737	4.540595646	6.61E-06	2.25E-05	no
RGS9	-0.169459251	-4.539506612	6.64E-06	2.26E-05	no
PGRMC2	0.169429821	4.538694911	6.67E-06	2.27E-05	no
USH1C	-0.169426718	-4.538609345	6.67E-06	2.27E-05	no
C11orf42	-0.169417634	-4.538358821	6.68E-06	2.27E-05	no
SOCS6	0.169410432	4.538160189	6.68E-06	2.27E-05	no
IL1RL2	0.169409861	4.53814443	6.68E-06	2.27E-05	no
POTEF	0.169362265	4.53683179	6.72E-06	2.28E-05	no
LRRC55	0.169348747	4.536458955	6.74E-06	2.29E-05	no
IQSEC3	-0.169322857	-4.535744953	6.76E-06	2.29E-05	no
ARHGAP5	-0.169298604	-4.535076119	6.78E-06	2.30E-05	no
C11orf41	-0.169288277	-4.534791308	6.79E-06	2.30E-05	no
TFAP2E	-0.1692845	-4.534687149	6.79E-06	2.30E-05	no
HMGB3	-0.169262841	-4.534089862	6.81E-06	2.31E-05	no
C16orf89	0.169233073	4.533268951	6.83E-06	2.32E-05	no
CCDC65	0.169226195	4.53307926	6.84E-06	2.32E-05	no
SFRS5	-0.169202158	-4.532416395	6.86E-06	2.33E-05	no
MIPOL1	-0.169188794	-4.53204786	6.87E-06	2.33E-05	no
UBAP1	-0.169178654	-4.531768242	6.88E-06	2.33E-05	no
TTL	0.169172878	4.531608958	6.89E-06	2.33E-05	no
SYT4	-0.16915728	-4.531178853	6.90E-06	2.34E-05	no

PKN2	0.16910204	4.529655578	6.95E-06	2.35E-05	no
AQP5	0.169080641	4.529065488	6.97E-06	2.36E-05	no
MAP1A	-0.169076512	-4.528951655	6.97E-06	2.36E-05	no
LOC283392	-0.169055037	-4.528359482	6.99E-06	2.37E-05	no
KRT23	0.169046853	4.528133799	7.00E-06	2.37E-05	no
CYP1A1	-0.169033627	-4.527769126	7.01E-06	2.37E-05	no
DUSP11	0.169013898	4.527225111	7.03E-06	2.38E-05	no
C21orf125	-0.168927591	-4.524845365	7.10E-06	2.40E-05	no
SH3BGR	0.168904806	4.524217127	7.13E-06	2.41E-05	no
CCL26	0.168894753	4.523939922	7.13E-06	2.41E-05	no
THAP1	0.168893969	4.52391833	7.14E-06	2.41E-05	no
ARHGDIG	-0.168875108	-4.523398291	7.15E-06	2.42E-05	no
MME	0.168831674	4.522200753	7.19E-06	2.43E-05	no
CTSF	-0.168815483	-4.521754332	7.21E-06	2.44E-05	no
TMED4	0.168787431	4.520980939	7.23E-06	2.44E-05	no
SEC24A	0.168778755	4.520741722	7.24E-06	2.45E-05	no
RIMS1	-0.168751033	-4.519977434	7.27E-06	2.46E-05	no
NEK11	0.168735131	4.519539	7.28E-06	2.46E-05	no
SYN3	-0.168715276	-4.518991622	7.30E-06	2.47E-05	no
KCTD16	-0.168687481	-4.518225321	7.32E-06	2.47E-05	no
C14orf149	0.168684295	4.518137499	7.33E-06	2.47E-05	no
PRMT6	0.168629535	4.516627823	7.38E-06	2.49E-05	no
WDR55	0.168544668	4.514288237	7.46E-06	2.52E-05	no
ARMCX2	0.168539054	4.514133497	7.46E-06	2.52E-05	no
ATP1A1	0.168528263	4.513836026	7.47E-06	2.52E-05	no
TFAP2C	0.168453786	4.511782961	7.54E-06	2.55E-05	no
CALN1	-0.168441277	-4.511438157	7.56E-06	2.55E-05	no
PARN	0.168439993	4.511402756	7.56E-06	2.55E-05	no
SCHIP1	-0.168403532	-4.51039769	7.59E-06	2.56E-05	no
H3F3C	-0.168387647	-4.509959823	7.61E-06	2.56E-05	no
KIFC2	-0.168386109	-4.509917423	7.61E-06	2.56E-05	no
PI4KAP1	-0.16837792	-4.509691708	7.62E-06	2.57E-05	no
PNMAL2	-0.168369966	-4.509472466	7.62E-06	2.57E-05	no
KIAA0146	0.168346197	4.508817289	7.65E-06	2.58E-05	no
KIAA0415	0.168300914	4.507569098	7.69E-06	2.59E-05	no
B4GALT4	0.168273652	4.506817668	7.72E-06	2.60E-05	no
DLK2	-0.168256401	-4.506342193	7.73E-06	2.60E-05	no
GRAMD1B	-0.168247614	-4.506099992	7.74E-06	2.61E-05	no
SLC8A2	-0.168212955	-4.505144688	7.78E-06	2.62E-05	no
DNAH5	0.168204268	4.50490526	7.78E-06	2.62E-05	no
RBM46	0.168150433	4.503421487	7.84E-06	2.64E-05	no
ZNF624	-0.168107455	-4.502236957	7.88E-06	2.65E-05	no
SHROOM3	0.16809547	4.501906638	7.89E-06	2.66E-05	no
ZNF76	-0.168079333	-4.501461898	7.91E-06	2.66E-05	no
CELF2	-0.168077446	-4.501409884	7.91E-06	2.66E-05	no
AMH	-0.168075751	-4.501363174	7.91E-06	2.66E-05	no
CD177	0.168075199	4.50134797	7.91E-06	2.66E-05	no
CELF4	-0.168064767	-4.501060456	7.92E-06	2.66E-05	no
LOC150622	-0.168019576	-4.499815009	7.97E-06	2.68E-05	no
11-Mar	-0.168009252	-4.499530462	7.98E-06	2.68E-05	no
ADPRHL2	0.167966372	4.498348738	8.02E-06	2.70E-05	no

TMEM22	0.167965834	4.498333909	8.02E-06	2.70E-05	no
PTPRJ	0.167956576	4.498078777	8.03E-06	2.70E-05	no
APOM	-0.167946907	-4.497812324	8.04E-06	2.70E-05	no
MAPK14	0.167906256	4.496692043	8.08E-06	2.71E-05	no
FAM3D	0.16787983	4.495963803	8.11E-06	2.72E-05	no
ADCY10	0.167866708	4.495602188	8.12E-06	2.73E-05	no
SPRY2	0.167852337	4.495206175	8.14E-06	2.73E-05	no
C8orf46	-0.167823393	-4.494408572	8.17E-06	2.74E-05	no
ANKS3	-0.167805246	-4.493908482	8.19E-06	2.75E-05	no
RAB8B	0.167779456	4.493197813	8.21E-06	2.75E-05	no
JMJD1C	-0.167773473	-4.493032959	8.22E-06	2.76E-05	no
C16orf62	-0.16768721	-4.490655944	8.31E-06	2.79E-05	no
MGRN1	-0.167665465	-4.490056765	8.33E-06	2.79E-05	no
UHRF1BP1L	-0.167662233	-4.489967702	8.33E-06	2.79E-05	no
BEND4	-0.167631848	-4.489130487	8.37E-06	2.80E-05	no
GIGYF1	-0.167617954	-4.488747653	8.38E-06	2.81E-05	no
SYAP1	0.167591934	4.488030702	8.41E-06	2.82E-05	no
LRTM2	-0.167566031	-4.487316978	8.44E-06	2.83E-05	no
PLCB4	-0.167561239	-4.48718494	8.44E-06	2.83E-05	no
PCCB	-0.167555932	-4.487038738	8.45E-06	2.83E-05	no
DEGS2	-0.167538375	-4.486554991	8.47E-06	2.83E-05	no
OMD	0.167524267	4.48616628	8.48E-06	2.84E-05	no
ATXN10	-0.167415516	-4.483169989	8.60E-06	2.88E-05	no
ACSS3	0.167394171	4.482581935	8.62E-06	2.88E-05	no
GUSBL2	-0.167367159	-4.481837739	8.65E-06	2.89E-05	no
GRIN2D	-0.167360958	-4.481666906	8.66E-06	2.90E-05	no
BTG3	0.167307006	4.480180536	8.71E-06	2.92E-05	no
FAM178A	-0.167291222	-4.479745709	8.73E-06	2.92E-05	no
AVEN	0.167289747	4.479705071	8.73E-06	2.92E-05	no
SR140	-0.167282139	-4.479495479	8.74E-06	2.92E-05	no
GRPR	0.167237308	4.478260447	8.79E-06	2.94E-05	no
TFCP2	-0.167236113	-4.478227507	8.79E-06	2.94E-05	no
PINK1	-0.167205782	-4.477391963	8.83E-06	2.95E-05	no
TSTD1	0.167192137	4.477016078	8.84E-06	2.95E-05	no
RNF217	0.167118925	4.47499929	8.92E-06	2.98E-05	no
OLAH	0.167076139	4.47382067	8.97E-06	3.00E-05	no
LOC388428	-0.167029533	-4.472536886	9.02E-06	3.01E-05	no
NARS2	-0.166966638	-4.470804438	9.09E-06	3.04E-05	no
ZNF610	-0.166945277	-4.470216076	9.12E-06	3.04E-05	no
HIST1H2BJ	0.166935403	4.469944101	9.13E-06	3.05E-05	no
PES1	-0.166923513	-4.469616598	9.14E-06	3.05E-05	no
HCG18	-0.166911396	-4.469282855	9.16E-06	3.06E-05	no
EP400	-0.166890368	-4.468703661	9.18E-06	3.06E-05	no
B3GNT9	0.166874874	4.468276922	9.20E-06	3.07E-05	no
NEK5	0.166871265	4.4681775	9.20E-06	3.07E-05	no
FSTL5	-0.166866929	-4.468058074	9.21E-06	3.07E-05	no
TBC1D10B	-0.166854264	-4.467709248	9.22E-06	3.08E-05	no
PEPD	0.166813397	4.466583654	9.27E-06	3.09E-05	no
ATP13A4	-0.166800613	-4.466231559	9.29E-06	3.09E-05	no
DVL3	-0.166779421	-4.465647884	9.31E-06	3.10E-05	no
ZBTB43	-0.166778196	-4.46561416	9.31E-06	3.10E-05	no

QPRT	0.166769367	4.465370999	9.32E-06	3.11E-05	no
MYCL1	-0.166767381	-4.465316289	9.32E-06	3.11E-05	no
CNTD1	-0.166721348	-4.464048466	9.38E-06	3.12E-05	no
MYH7	-0.166699019	-4.463433522	9.40E-06	3.13E-05	no
SPTBN1	-0.166698389	-4.463416163	9.40E-06	3.13E-05	no
GALNT9	-0.166675689	-4.462790995	9.43E-06	3.14E-05	no
CTRB1	0.16667085	4.462657732	9.44E-06	3.14E-05	no
C9orf4	-0.166668885	-4.462603617	9.44E-06	3.14E-05	no
CACNB1	-0.166653059	-4.46216777	9.46E-06	3.15E-05	no
SLC25A26	-0.166650309	-4.462092035	9.46E-06	3.15E-05	no
GPR108	0.166596245	4.460603119	9.53E-06	3.17E-05	no
GNPDA2	-0.166571993	-4.459935251	9.55E-06	3.18E-05	no
PQLC2	0.166557474	4.459535399	9.57E-06	3.18E-05	no
CASP3	0.166539832	4.459049578	9.59E-06	3.19E-05	no
SLC25A33	-0.166539213	-4.459032524	9.59E-06	3.19E-05	no
NID2	0.16653776	4.458992513	9.60E-06	3.19E-05	no
CETN2	0.166507981	4.458172437	9.63E-06	3.20E-05	no
CXorf57	-0.166495258	-4.457822092	9.65E-06	3.20E-05	no
HNF4G	0.166468023	4.45707211	9.68E-06	3.22E-05	no
FGD4	0.166450208	4.45658153	9.70E-06	3.22E-05	no
ORAI1	0.166449718	4.456568033	9.70E-06	3.22E-05	no
TMEM167B	0.166424807	4.455882049	9.73E-06	3.23E-05	no
ZNF131	-0.16641262	-4.455546455	9.75E-06	3.24E-05	no
C2orf72	-0.166398082	-4.455146137	9.76E-06	3.24E-05	no
LRRC37B2	-0.166376575	-4.45455391	9.79E-06	3.25E-05	no
ZSCAN5A	0.166361432	4.454136934	9.81E-06	3.25E-05	no
ACOX2	0.166356862	4.454011105	9.81E-06	3.26E-05	no
CDH5	0.16635136	4.453859595	9.82E-06	3.26E-05	no
RANGAP1	-0.166344926	-4.453682418	9.83E-06	3.26E-05	no
SDHAP3	-0.166260862	-4.451367709	9.93E-06	3.29E-05	no
PRAC	0.166244532	4.450918079	9.95E-06	3.30E-05	no
C2CD2L	-0.166226281	-4.450415547	9.98E-06	3.31E-05	no
SHPRH	-0.166183838	-4.44924694	1.00E-05	3.32E-05	no
FGD6	0.166176098	4.449033832	1.00E-05	3.33E-05	no
SRPRB	0.166165909	4.448753293	1.01E-05	3.33E-05	no
AGRP	0.166164334	4.448709935	1.01E-05	3.33E-05	no
COMMD1	0.166159454	4.448575555	1.01E-05	3.33E-05	no
DET1	-0.166153488	-4.448411317	1.01E-05	3.33E-05	no
BBS5	0.166122236	4.447550861	1.01E-05	3.35E-05	no
CLVS1	-0.166108264	-4.44716618	1.01E-05	3.35E-05	no
BTBD9	-0.166106285	-4.447111691	1.01E-05	3.35E-05	no
TMEM17	0.166095341	4.446810374	1.01E-05	3.36E-05	no
ITM2B	0.165994487	4.444033699	1.03E-05	3.40E-05	no
MORC4	0.165981078	4.443664551	1.03E-05	3.40E-05	no
C2orf69	-0.165977664	-4.44357055	1.03E-05	3.40E-05	no
TPSAB1	0.165976983	4.443551807	1.03E-05	3.40E-05	no
RNF150	-0.165967382	-4.443287494	1.03E-05	3.41E-05	no
PHEX	0.165948634	4.442771358	1.03E-05	3.41E-05	no
TACSTD2	0.165918293	4.441936061	1.04E-05	3.43E-05	no
VARS	-0.165883812	-4.440986836	1.04E-05	3.44E-05	no
MAZ	-0.165877855	-4.440822847	1.04E-05	3.44E-05	no

SEMA5B	-0.165859281	-4.440311505	1.04E-05	3.45E-05	no
FFAR3	0.165859185	4.440308861	1.04E-05	3.45E-05	no
FBXO8	0.165846699	4.439965132	1.05E-05	3.45E-05	no
COL5A1	0.165830611	4.439522276	1.05E-05	3.46E-05	no
DHRSX	0.165829565	4.43949347	1.05E-05	3.46E-05	no
8-Mar	-0.165825543	-4.439382741	1.05E-05	3.46E-05	no
GRK4	-0.165779269	-4.438108916	1.05E-05	3.48E-05	no
FAM163B	-0.165764205	-4.437694244	1.06E-05	3.49E-05	no
C21orf2	-0.165758552	-4.437538621	1.06E-05	3.49E-05	no
C10orf57	-0.165728265	-4.436704918	1.06E-05	3.50E-05	no
SNORD116-4	-0.165691127	-4.435682616	1.07E-05	3.52E-05	no
EFCAB10	0.165634655	4.434128179	1.07E-05	3.54E-05	no
DCC	-0.1656296	-4.433989037	1.07E-05	3.54E-05	no
BACH2	-0.165625086	-4.433864789	1.08E-05	3.54E-05	no
PRTG	-0.165586961	-4.432815373	1.08E-05	3.56E-05	no
MECOM	0.165561452	4.432113249	1.08E-05	3.57E-05	no
PDGFD	0.165554716	4.431927839	1.08E-05	3.57E-05	no
APIP	0.165505347	4.430569001	1.09E-05	3.59E-05	no
USP18	0.16550248	4.430490095	1.09E-05	3.60E-05	no
ZC3H4	-0.165446978	-4.428962476	1.10E-05	3.62E-05	no
DUS3L	-0.165431909	-4.428547734	1.10E-05	3.63E-05	no
ERO1L	0.165425411	4.428368891	1.10E-05	3.63E-05	no
MESDC2	0.165389293	4.427374832	1.11E-05	3.64E-05	no
C12orf43	-0.165389061	-4.427368443	1.11E-05	3.64E-05	no
CNTNAP5	-0.165379041	-4.427092668	1.11E-05	3.65E-05	no
ULK1	-0.165347287	-4.426218735	1.11E-05	3.66E-05	no
ZNF462	-0.165229485	-4.422976732	1.13E-05	3.71E-05	no
MSL2	-0.165224511	-4.422839844	1.13E-05	3.72E-05	no
ZNF236	-0.165209676	-4.422431606	1.13E-05	3.72E-05	no
TMEM41A	0.165189934	4.421888295	1.13E-05	3.73E-05	no
SIX5	0.165174	4.421449817	1.14E-05	3.74E-05	no
TMEM108	-0.165171023	-4.421367893	1.14E-05	3.74E-05	no
DCLRE1C	0.165168128	4.421288207	1.14E-05	3.74E-05	no
SP1	0.165165671	4.421220599	1.14E-05	3.74E-05	no
C6orf153	0.165147789	4.420728497	1.14E-05	3.75E-05	no
BBS9	0.165144751	4.420644915	1.14E-05	3.75E-05	no
RNF114	0.165135309	4.420385075	1.14E-05	3.75E-05	no
ZFYVE9	-0.165119293	-4.419944336	1.14E-05	3.76E-05	no
PPP3CB	-0.165107769	-4.419627211	1.15E-05	3.76E-05	no
PTHLH	0.165107559	4.419621447	1.15E-05	3.76E-05	no
POM121L10P	-0.165105902	-4.419575834	1.15E-05	3.76E-05	no
C7orf41	-0.165104078	-4.419525647	1.15E-05	3.76E-05	no
SPCS2	-0.16510249	-4.41948194	1.15E-05	3.76E-05	no
FLYWCH2	-0.165065667	-4.418468644	1.15E-05	3.78E-05	no
THUMPD1	-0.165062023	-4.418368375	1.15E-05	3.78E-05	no
PTPN14	0.165050697	4.418056706	1.15E-05	3.79E-05	no
LOC100302640	-0.165049502	-4.418023829	1.15E-05	3.79E-05	no
ITGB3BP	0.165031622	4.417531829	1.16E-05	3.79E-05	no
PKIB	0.165018971	4.417183699	1.16E-05	3.80E-05	no
RSAD1	-0.165006747	-4.416847332	1.16E-05	3.80E-05	no
SMPD1	0.164982366	4.416176455	1.16E-05	3.82E-05	no

GUCY2C	0.164974333	4.415955413	1.17E-05	3.82E-05	no
KCNT2	-0.164903043	-4.413993802	1.18E-05	3.85E-05	no
CCT3	-0.16478448	-4.410731596	1.19E-05	3.91E-05	no
C2orf50	0.164783555	4.410706145	1.19E-05	3.91E-05	no
CCDC73	-0.164766997	-4.410250569	1.20E-05	3.91E-05	no
LOC285954	-0.164761215	-4.410091476	1.20E-05	3.92E-05	no
CSDE1	0.164756757	4.409968818	1.20E-05	3.92E-05	no
OMP	0.164746605	4.409689507	1.20E-05	3.92E-05	no
C8ORFK29	-0.164742796	-4.409584716	1.20E-05	3.92E-05	no
WASH7P	-0.164717773	-4.408896251	1.20E-05	3.94E-05	no
PPIL5	0.164692289	4.408195125	1.21E-05	3.95E-05	no
ZNF408	-0.164660418	-4.407318287	1.21E-05	3.96E-05	no
C6orf58	-0.16460884	-4.405899282	1.22E-05	3.99E-05	no
RASEF	0.164585351	4.405253057	1.22E-05	4.00E-05	no
CLU	0.164581117	4.405136576	1.22E-05	4.00E-05	no
ARSE	0.164574623	4.404957936	1.22E-05	4.00E-05	no
SETD4	-0.164544354	-4.404125216	1.23E-05	4.02E-05	no
FAM164C	0.164480554	4.402370071	1.24E-05	4.05E-05	no
OGN	0.164473772	4.402183508	1.24E-05	4.05E-05	no
AP4S1	-0.16447355	-4.402177407	1.24E-05	4.05E-05	no
MRPL13	0.164458419	4.401761158	1.24E-05	4.06E-05	no
IMPACT	0.16442141	4.40074308	1.25E-05	4.07E-05	no
LOC286238	0.164418079	4.400651458	1.25E-05	4.08E-05	no
C8orf84	0.164391552	4.39992172	1.25E-05	4.09E-05	no
ARHGEF35	0.164389269	4.399858933	1.25E-05	4.09E-05	no
FAM131B	-0.164375797	-4.399488344	1.25E-05	4.09E-05	no
NPAS2	0.164373514	4.399425548	1.26E-05	4.10E-05	no
CHD2	-0.164371031	-4.399357224	1.26E-05	4.10E-05	no
UROS	-0.164357553	-4.398986477	1.26E-05	4.10E-05	no
S100PBP	0.164307943	4.397621828	1.27E-05	4.13E-05	no
B9D2	0.164296398	4.397304259	1.27E-05	4.13E-05	no
SLC25A22	-0.164286307	-4.397026708	1.27E-05	4.14E-05	no
LCMT1	-0.164265417	-4.396452083	1.27E-05	4.15E-05	no
ACTR3B	-0.164258322	-4.396256923	1.27E-05	4.15E-05	no
WASH3P	-0.16422909	-4.395452855	1.28E-05	4.16E-05	no
ZNF100	-0.164208472	-4.394885732	1.28E-05	4.17E-05	no
GLDN	0.164190781	4.394399152	1.28E-05	4.18E-05	no
IQCH	0.164183059	4.394186737	1.29E-05	4.19E-05	no
PARG	-0.164167593	-4.393761343	1.29E-05	4.19E-05	no
TPPP2	-0.164154191	-4.39339273	1.29E-05	4.20E-05	no
ZNF263	-0.164147307	-4.393203374	1.29E-05	4.20E-05	no
EIF2AK3	0.164072298	4.391140298	1.30E-05	4.24E-05	no
ZNF765	0.164033941	4.390085361	1.31E-05	4.26E-05	no
ELF3	0.164018868	4.389670802	1.31E-05	4.27E-05	no
CYB5RL	0.164009407	4.389410586	1.31E-05	4.27E-05	no
CYP4F11	0.16398945	4.388861708	1.32E-05	4.28E-05	no
LRRC39	0.163969254	4.388306271	1.32E-05	4.29E-05	no
RORC	0.163963643	4.388151964	1.32E-05	4.29E-05	no
CLUL1	0.163915899	4.3868389	1.33E-05	4.32E-05	no
SLC15A2	-0.163915162	-4.386818643	1.33E-05	4.32E-05	no
MSI1	-0.16386014	-4.385305466	1.34E-05	4.35E-05	no

THAP9	-0.163847803	-4.384966189	1.34E-05	4.35E-05	no
FAM115A	-0.163843287	-4.384841977	1.34E-05	4.35E-05	no
TCF7L1	-0.163827585	-4.38441018	1.34E-05	4.36E-05	no
LRRC69	0.163780518	4.383115828	1.35E-05	4.39E-05	no
TTYH2	-0.163702766	-4.380977717	1.36E-05	4.43E-05	no
C4A	0.163701875	4.380953205	1.36E-05	4.43E-05	no
WDR89	-0.163665233	-4.379945603	1.37E-05	4.45E-05	no
STRC	-0.163641434	-4.379291186	1.37E-05	4.46E-05	no
SIPA1L2	0.163566848	4.377240269	1.39E-05	4.50E-05	no
RBM5	-0.163472339	-4.374641624	1.40E-05	4.55E-05	no
MAPK11	-0.163472208	-4.374638035	1.40E-05	4.55E-05	no
GATSL3	-0.16346854	-4.374537165	1.40E-05	4.55E-05	no
SENP7	-0.163457988	-4.374247047	1.40E-05	4.56E-05	no
MAT2B	0.163457896	4.37424451	1.40E-05	4.56E-05	no
RABL3	0.163449194	4.374005252	1.41E-05	4.56E-05	no
LOC100190939	-0.163428944	-4.373448465	1.41E-05	4.57E-05	no
FAM98B	-0.163424722	-4.373332383	1.41E-05	4.57E-05	no
MYCN	-0.163392178	-4.372437615	1.42E-05	4.59E-05	no
SOX1	-0.16339155	-4.372420327	1.42E-05	4.59E-05	no
ATP8B2	0.163348326	4.371231944	1.42E-05	4.61E-05	no
NUDT16	0.163341308	4.37103899	1.43E-05	4.62E-05	no
SLC16A11	0.163337435	4.370932496	1.43E-05	4.62E-05	no
CDC14B	-0.163332223	-4.370789208	1.43E-05	4.62E-05	no
PPP2R3B	-0.163314025	-4.370288876	1.43E-05	4.63E-05	no
UBAC1	-0.163291506	-4.369669757	1.43E-05	4.64E-05	no
SLFN13	0.163270482	4.369091758	1.44E-05	4.65E-05	no
FAM182B	-0.163251817	-4.368578588	1.44E-05	4.66E-05	no
GPX7	0.163250652	4.368546558	1.44E-05	4.66E-05	no
PRR22	-0.163250343	-4.368538071	1.44E-05	4.66E-05	no
PCDH10	-0.163241009	-4.36828146	1.44E-05	4.67E-05	no
KLHL38	0.163195213	4.36702243	1.45E-05	4.69E-05	no
HNRNPU	-0.163180453	-4.366616659	1.45E-05	4.70E-05	no
KIAA0196	0.163178444	4.366561429	1.45E-05	4.70E-05	no
MAP3K10	-0.163158627	-4.366016639	1.46E-05	4.71E-05	no
BAZ2B	-0.163132341	-4.365294009	1.46E-05	4.72E-05	no
HS3ST3A1	0.163128113	4.365177791	1.46E-05	4.73E-05	no
ELL3	0.163124973	4.365091456	1.46E-05	4.73E-05	no
IDE	0.163102816	4.364482348	1.47E-05	4.74E-05	no
LUZP6	0.163061649	4.363350661	1.47E-05	4.76E-05	no
PRDM10	-0.163000388	-4.361666624	1.49E-05	4.80E-05	no
HAPLN1	-0.16298929	-4.36136157	1.49E-05	4.80E-05	no
MMEL1	0.162969266	4.360811134	1.49E-05	4.81E-05	no
C11orf20	-0.16286641	-4.357983838	1.51E-05	4.87E-05	no
VPS13D	-0.162854425	-4.357654413	1.51E-05	4.88E-05	no
ARF1	0.162841912	4.357310475	1.51E-05	4.89E-05	no
IRF3	0.162841641	4.35730301	1.51E-05	4.89E-05	no
PAR1	-0.162819664	-4.356698941	1.52E-05	4.90E-05	no
PTGES2	-0.162814118	-4.356546501	1.52E-05	4.90E-05	no
PITPNB	-0.162797602	-4.356092532	1.52E-05	4.91E-05	no
QSER1	-0.162787783	-4.355822646	1.52E-05	4.92E-05	no
HSBP1L1	0.162706614	4.353591694	1.54E-05	4.96E-05	no

TRIM39	-0.16269565	-4.353290337	1.54E-05	4.97E-05	no
MYH3	-0.162675775	-4.352744066	1.55E-05	4.98E-05	no
TBC1D13	-0.162671512	-4.352626905	1.55E-05	4.98E-05	no
GNA11	-0.162650397	-4.352046588	1.55E-05	5.00E-05	no
GJC1	0.16263565	4.351641285	1.55E-05	5.00E-05	no
YTHDF3	0.16260471	4.350790922	1.56E-05	5.02E-05	no
NUP205	0.162562159	4.349621492	1.57E-05	5.05E-05	no
SNAPC2	0.162550515	4.349301486	1.57E-05	5.05E-05	no
FBXO41	-0.162504203	-4.348028701	1.58E-05	5.08E-05	no
FAM106C	-0.162473511	-4.347185228	1.58E-05	5.10E-05	no
EPB41L5	-0.162430485	-4.346002822	1.59E-05	5.13E-05	no
POLR3A	-0.162420357	-4.345724495	1.59E-05	5.13E-05	no
E2F7	0.162401764	4.345213552	1.60E-05	5.14E-05	no
SYCP2L	-0.162387786	-4.344829424	1.60E-05	5.15E-05	no
TJP1	-0.162308096	-4.342639525	1.62E-05	5.20E-05	no
TSSK4	-0.162284856	-4.342000918	1.62E-05	5.21E-05	no
SRPX	0.162269207	4.341570904	1.62E-05	5.22E-05	no
GHITM	-0.162261481	-4.341358582	1.63E-05	5.23E-05	no
ZNF157	-0.162219221	-4.34019735	1.63E-05	5.25E-05	no
C20orf117	-0.162142644	-4.338093181	1.65E-05	5.30E-05	no
GPR21	-0.162141465	-4.338060785	1.65E-05	5.30E-05	no
SASH1	-0.162099038	-4.336895027	1.66E-05	5.33E-05	no
NME5	0.162072637	4.336169619	1.66E-05	5.34E-05	no
CELSR2	-0.162071001	-4.336124672	1.66E-05	5.34E-05	no
LOC653786	0.162040888	4.335297298	1.67E-05	5.36E-05	no
RABEP1	-0.16203222	-4.335059147	1.67E-05	5.37E-05	no
GADD45A	0.16201997	4.334722555	1.67E-05	5.37E-05	no
IGBP1	-0.162019542	-4.334710794	1.67E-05	5.37E-05	no
PAQR3	0.161993832	4.334004408	1.68E-05	5.39E-05	no
GCK	0.161993226	4.333987759	1.68E-05	5.39E-05	no
BAG1	-0.161982592	-4.333695578	1.68E-05	5.40E-05	no
LOC284837	0.16193724	4.332449566	1.69E-05	5.42E-05	no
SMAD5OS	0.161856299	4.330225808	1.71E-05	5.48E-05	no
RUNDC3B	-0.161853005	-4.330135314	1.71E-05	5.48E-05	no
TSGA10IP	0.16183267	4.329576628	1.71E-05	5.49E-05	no
CCDC40	0.161813341	4.329045616	1.72E-05	5.50E-05	no
FST	0.161799797	4.328673535	1.72E-05	5.51E-05	no
C6orf186	0.161792027	4.328460074	1.72E-05	5.52E-05	no
SPSB1	0.161768535	4.327814706	1.73E-05	5.53E-05	no
GPR180	0.161749659	4.327296128	1.73E-05	5.54E-05	no
PXDN	0.161730789	4.326777743	1.73E-05	5.55E-05	no
PSMA2	0.161660624	4.324850245	1.75E-05	5.60E-05	no
TSKU	0.161641998	4.324338562	1.75E-05	5.61E-05	no
PRRT2	-0.161631122	-4.324039818	1.76E-05	5.62E-05	no
PCDHB9	-0.161626452	-4.323911531	1.76E-05	5.62E-05	no
SHANK1	-0.161623981	-4.323843636	1.76E-05	5.62E-05	no
ARHGAP6	0.16162062	4.323751317	1.76E-05	5.62E-05	no
POU2F3	0.16161994	4.323732639	1.76E-05	5.62E-05	no
YWHAE	-0.161581275	-4.322670521	1.77E-05	5.65E-05	no
UTF1	0.161576759	4.322546456	1.77E-05	5.65E-05	no
VN1R1	-0.161565409	-4.32223468	1.77E-05	5.66E-05	no

TMEM147	0.161561929	4.322139087	1.77E-05	5.66E-05	no
MED13	-0.161516464	-4.32089023	1.78E-05	5.69E-05	no
RAB7A	0.161442491	4.318858328	1.80E-05	5.74E-05	no
TERF1	-0.161441112	-4.318820436	1.80E-05	5.74E-05	no
LUZP1	0.161386147	4.3173107	1.81E-05	5.78E-05	no
CNKSR2	-0.161354735	-4.316447932	1.82E-05	5.80E-05	no
RG9MTD3	-0.161347943	-4.316261388	1.82E-05	5.80E-05	no
DCLRE1B	0.161299479	4.314930264	1.83E-05	5.84E-05	no
C1orf220	-0.16127983	-4.3143906	1.83E-05	5.85E-05	no
LGALS9C	0.161275285	4.314265774	1.83E-05	5.85E-05	no
COL21A1	0.161251454	4.313611248	1.84E-05	5.87E-05	no
IL22RA1	0.161244745	4.313426988	1.84E-05	5.87E-05	no
UST	-0.161239049	-4.31327054	1.84E-05	5.87E-05	no
TMPRSS5	-0.16121579	-4.312631751	1.85E-05	5.89E-05	no
TAGLN3	-0.161195956	-4.312087017	1.85E-05	5.90E-05	no
HMGXB3	0.161189702	4.311915262	1.85E-05	5.91E-05	no
SEMA6C	-0.161169846	-4.311369923	1.86E-05	5.92E-05	no
SPA17	0.161169002	4.311346759	1.86E-05	5.92E-05	no
C1orf59	0.161110913	4.309751413	1.87E-05	5.96E-05	no
MASP1	-0.161105934	-4.309614689	1.87E-05	5.96E-05	no
MBLAC2	-0.1610703	-4.308636087	1.88E-05	5.99E-05	no
ZSCAN5B	-0.161058994	-4.308325578	1.88E-05	6.00E-05	no
PPFIA2	-0.161041977	-4.307858256	1.89E-05	6.01E-05	no
GOLT1B	0.161035918	4.307691874	1.89E-05	6.01E-05	no
PCDH20	-0.160988054	-4.306377454	1.90E-05	6.04E-05	no
SECISBP2L	-0.160977587	-4.306090004	1.90E-05	6.05E-05	no
NCRNA00119	0.16096369	4.30570837	1.90E-05	6.06E-05	no
ZFR2	-0.160945581	-4.305211083	1.91E-05	6.07E-05	no
CDK13	0.160940344	4.305067285	1.91E-05	6.07E-05	no
SPIC	0.160911942	4.304287333	1.92E-05	6.09E-05	no
SLC22A6	-0.160907419	-4.304163141	1.92E-05	6.10E-05	no
PYGB	-0.160890749	-4.303705377	1.92E-05	6.11E-05	no
KDM2B	-0.160869624	-4.30312529	1.92E-05	6.12E-05	no
APOL4	0.160850929	4.302611926	1.93E-05	6.14E-05	no
MIMT1	-0.160840833	-4.302334694	1.93E-05	6.14E-05	no
BAT4	-0.160810497	-4.301501704	1.94E-05	6.16E-05	no
FAM155B	-0.160797342	-4.301140473	1.94E-05	6.17E-05	no
ZNF467	0.16077192	4.300442428	1.95E-05	6.19E-05	no
HBEGF	0.160771321	4.300425981	1.95E-05	6.19E-05	no
KDR	0.160746683	4.299749461	1.95E-05	6.21E-05	no
LDB1	-0.160736873	-4.299480102	1.96E-05	6.21E-05	no
RAB35	0.160693512	4.298289508	1.97E-05	6.25E-05	no
CLSTN1	-0.160673158	-4.297730642	1.97E-05	6.26E-05	no
INSIG2	0.160670781	4.297665374	1.97E-05	6.26E-05	no
GOLGA8B	-0.160670064	-4.297645695	1.97E-05	6.26E-05	no
CABIN1	-0.160643776	-4.296923889	1.98E-05	6.28E-05	no
CLIP2	-0.160635764	-4.296703925	1.98E-05	6.28E-05	no
STOX2	-0.160631834	-4.296596002	1.98E-05	6.29E-05	no
PSMA7	0.160630098	4.296548352	1.98E-05	6.29E-05	no
ZNF383	0.160625248	4.296415168	1.98E-05	6.29E-05	no
SLC16A10	0.160594602	4.295573755	1.99E-05	6.31E-05	no

ACSM3	0.160582347	4.295237293	1.99E-05	6.32E-05	no
FAM149B1	-0.160569444	-4.294883034	2.00E-05	6.33E-05	no
C9orf46	0.16055465	4.294476854	2.00E-05	6.34E-05	no
SHFM1	0.160540182	4.294079614	2.00E-05	6.35E-05	no
BCAS3	-0.160504241	-4.293092844	2.01E-05	6.38E-05	no
LTF	0.160497919	4.292919301	2.01E-05	6.38E-05	no
GPT	-0.160496546	-4.292881593	2.01E-05	6.38E-05	no
TTLL13	-0.16046171	-4.291925186	2.02E-05	6.41E-05	no
IFI6	0.160460344	4.29188769	2.02E-05	6.41E-05	no
SOX15	-0.160450519	-4.29161794	2.02E-05	6.41E-05	no
PIP5KL1	-0.160421999	-4.290834964	2.03E-05	6.43E-05	no
PPP1R2P3	0.160391786	4.290005498	2.04E-05	6.46E-05	no
NCOR2	-0.160356091	-4.289025576	2.05E-05	6.48E-05	no
CPA5	0.160332876	4.288388263	2.05E-05	6.50E-05	no
MLF2	-0.160326828	-4.288222221	2.06E-05	6.50E-05	no
SRPK3	-0.160312783	-4.287836651	2.06E-05	6.51E-05	no
ANKRD12	-0.160306939	-4.287676227	2.06E-05	6.52E-05	no
EGLN2	0.160305861	4.287646625	2.06E-05	6.52E-05	no
C22orf29	-0.160293539	-4.287308376	2.06E-05	6.53E-05	no
KCTD6	-0.160283401	-4.28703007	2.07E-05	6.53E-05	no
TCF19	0.160282434	4.28700351	2.07E-05	6.53E-05	no
PDS5A	0.160255973	4.286277112	2.07E-05	6.55E-05	no
RICH2	-0.160231527	-4.285606048	2.08E-05	6.57E-05	no
NOL7	-0.160211742	-4.285062921	2.08E-05	6.59E-05	no
CSNK2A1	-0.160175448	-4.284066645	2.09E-05	6.61E-05	no
COL27A1	0.160175321	4.284063144	2.09E-05	6.61E-05	no
CORIN	0.160170013	4.283917453	2.09E-05	6.62E-05	no
PRKG2	-0.160167164	-4.283839236	2.10E-05	6.62E-05	no
C11orf71	-0.160158555	-4.283602935	2.10E-05	6.62E-05	no
BTBD18	-0.160147321	-4.283294546	2.10E-05	6.63E-05	no
CPEB2	0.160095096	4.281860992	2.11E-05	6.67E-05	no
SPTA1	0.160092841	4.281799088	2.11E-05	6.67E-05	no
MAK	0.16007952	4.281433451	2.12E-05	6.68E-05	no
TJP2	-0.160074517	-4.281296104	2.12E-05	6.68E-05	no
FLVCR2	0.160029703	4.280066022	2.13E-05	6.72E-05	no
MRPL43	-0.160004762	-4.279381433	2.14E-05	6.74E-05	no
SYCE1	-0.15999646	-4.279153558	2.14E-05	6.74E-05	no
YTHDF2	0.15996166	4.278198396	2.15E-05	6.77E-05	no
MPP3	-0.159937893	-4.277546051	2.15E-05	6.79E-05	no
HNRNPA3P1	-0.159933937	-4.277437464	2.15E-05	6.79E-05	no
CYB5A	0.159925649	4.277209985	2.16E-05	6.80E-05	no
PROM2	-0.159925097	-4.277194847	2.16E-05	6.80E-05	no
PTBP1	0.159891071	4.276260928	2.17E-05	6.82E-05	no
H1F0	-0.15987954	-4.275944441	2.17E-05	6.83E-05	no
KIAA0090	0.159863196	4.275495872	2.17E-05	6.84E-05	no
CLTCL1	-0.159807896	-4.273978113	2.19E-05	6.89E-05	no
ITIH5L	0.159799814	4.273756312	2.19E-05	6.89E-05	no
IQUB	0.159789488	4.273472904	2.19E-05	6.90E-05	no
FAM53A	0.159785984	4.27337673	2.19E-05	6.90E-05	no
GPR61	-0.159748382	-4.27234477	2.20E-05	6.93E-05	no
TOP3B	-0.159682983	-4.270549925	2.22E-05	6.99E-05	no

GNE	-0.159677036	-4.270386736	2.22E-05	6.99E-05	no
PCDHB1	0.159663645	4.270019239	2.23E-05	7.00E-05	no
SETBP1	-0.15960033	-4.268281692	2.24E-05	7.05E-05	no
SHISA4	-0.159586179	-4.267893345	2.25E-05	7.06E-05	no
FAM181A	0.159585683	4.267879737	2.25E-05	7.06E-05	no
SLC1A2	-0.159580487	-4.267737138	2.25E-05	7.07E-05	no
GPR56	-0.159568422	-4.26740606	2.25E-05	7.08E-05	no
SAP130	-0.15956725	-4.267373885	2.25E-05	7.08E-05	no
ADCY8	-0.159545515	-4.266777437	2.26E-05	7.09E-05	no
LIFR	-0.159523953	-4.266185731	2.26E-05	7.11E-05	no
SH3BGRL2	-0.159521446	-4.266116949	2.26E-05	7.11E-05	no
ZC3HAV1L	0.159521127	4.266108175	2.26E-05	7.11E-05	no
CLEC4F	-0.159452716	-4.264230906	2.28E-05	7.17E-05	no
RFT1	0.159444886	4.264016053	2.28E-05	7.17E-05	no
PRDX6	0.159363493	4.261782638	2.31E-05	7.24E-05	no
SDHC	0.159362171	4.261746361	2.31E-05	7.24E-05	no
TMEM175	-0.159336656	-4.261046243	2.31E-05	7.26E-05	no
RPS25	-0.159327387	-4.260791915	2.32E-05	7.27E-05	no
USP13	-0.159322739	-4.260664389	2.32E-05	7.27E-05	no
C9orf30	0.159278708	4.259456238	2.33E-05	7.31E-05	no
CRISPLD2	0.159266258	4.259114616	2.33E-05	7.32E-05	no
PLSCR2	0.159262066	4.258999605	2.34E-05	7.32E-05	no
BAHD1	-0.159259769	-4.258936584	2.34E-05	7.32E-05	no
PRCP	0.159232801	4.258196633	2.34E-05	7.35E-05	no
ARL10	-0.15921637	-4.257745807	2.35E-05	7.36E-05	no
MRPL17	0.159191611	4.257066498	2.36E-05	7.38E-05	no
NFKBIE	0.15919117	4.257054391	2.36E-05	7.38E-05	no
KATNAL2	0.159177186	4.256670719	2.36E-05	7.39E-05	no
DMAP1	-0.159156643	-4.256107084	2.36E-05	7.41E-05	no
RTN4RL2	-0.159154607	-4.256051236	2.37E-05	7.41E-05	no
TDRD7	0.159148826	4.255892602	2.37E-05	7.41E-05	no
KCNQ5	-0.159138292	-4.25560359	2.37E-05	7.42E-05	no
CHRNE	0.159131297	4.255411676	2.37E-05	7.43E-05	no
BCL2L2	-0.159050859	-4.253204797	2.39E-05	7.50E-05	no
NCRNA00169	0.15903805	4.252853383	2.40E-05	7.51E-05	no
CNNM1	-0.158987644	-4.251470522	2.41E-05	7.55E-05	no
GPR3	0.158985004	4.251398078	2.41E-05	7.55E-05	no
PDZK1IP1	0.158983624	4.251360233	2.41E-05	7.55E-05	no
MOCS1	-0.158953392	-4.250530851	2.42E-05	7.58E-05	no
PDZK1	0.158952195	4.250498	2.42E-05	7.58E-05	no
JUP	0.158950783	4.250459257	2.42E-05	7.58E-05	no
PRSS37	-0.158902765	-4.249141977	2.44E-05	7.62E-05	no
ALAS1	0.15888946	4.248776953	2.44E-05	7.63E-05	no
C6orf208	-0.158886738	-4.248702288	2.44E-05	7.63E-05	no
VCP	0.158886377	4.248692387	2.44E-05	7.63E-05	no
ALS2CR8	-0.158834368	-4.247265638	2.46E-05	7.68E-05	no
MAP2K7	-0.158833875	-4.247252128	2.46E-05	7.68E-05	no
C17orf28	-0.158832174	-4.247205455	2.46E-05	7.68E-05	no
SETDB1	-0.158806993	-4.246514679	2.47E-05	7.70E-05	no
CLEC14A	0.158799153	4.246299623	2.47E-05	7.71E-05	no
SLC7A6OS	-0.15878297	-4.245855691	2.47E-05	7.72E-05	no

SLC22A2	0.158778382	4.245729843	2.47E-05	7.72E-05	no
SAP18	-0.158775437	-4.245649058	2.47E-05	7.72E-05	no
GGN	0.158772202	4.24556031	2.48E-05	7.73E-05	no
PUF60	-0.158697853	-4.243520857	2.50E-05	7.79E-05	no
ZNF407	-0.158650101	-4.242211015	2.51E-05	7.84E-05	no
CXCL3	0.158625586	4.241538569	2.52E-05	7.86E-05	no
PDCD10	0.158597462	4.240767137	2.53E-05	7.88E-05	no
LOXL4	0.158586869	4.240476595	2.53E-05	7.89E-05	no
PLEKHG3	-0.158562916	-4.239819592	2.54E-05	7.91E-05	no
LOC648740	-0.15855534	-4.23961178	2.54E-05	7.92E-05	no
SGIP1	-0.158555003	-4.239602542	2.54E-05	7.92E-05	no
LAD1	0.158528401	4.238872904	2.55E-05	7.94E-05	no
CACNB4	-0.158508742	-4.238333681	2.55E-05	7.96E-05	no
SPRED3	0.158498274	4.23804658	2.56E-05	7.97E-05	no
APC	-0.158487134	-4.237741025	2.56E-05	7.98E-05	no
ZNF594	-0.158479145	-4.237521921	2.56E-05	7.98E-05	no
WDR63	0.158478578	4.237506349	2.56E-05	7.98E-05	no
CYR61	0.158472115	4.237329108	2.57E-05	7.99E-05	no
LRRC17	0.15844776	4.236661109	2.57E-05	8.01E-05	no
COQ10A	-0.158435333	-4.236320283	2.58E-05	8.02E-05	no
GRIP1	-0.158413236	-4.235714232	2.58E-05	8.04E-05	no
MAPKAP1	0.158382405	4.234868648	2.59E-05	8.07E-05	no
TMF1	0.158366378	4.234429071	2.60E-05	8.08E-05	no
SLC25A28	-0.158363164	-4.234340936	2.60E-05	8.09E-05	no
ST20	0.158345647	4.233860511	2.60E-05	8.10E-05	no
KRI1	-0.158345332	-4.233851875	2.61E-05	8.10E-05	no
GABPB2	0.158287777	4.232273391	2.62E-05	8.15E-05	no
RNF40	-0.158280722	-4.232079935	2.63E-05	8.16E-05	no
EEF1G	-0.158275624	-4.231940103	2.63E-05	8.16E-05	no
OR7E91P	0.158268115	4.231734164	2.63E-05	8.17E-05	no
LOC400940	-0.158241661	-4.231008682	2.64E-05	8.19E-05	no
CCDC144C	-0.158235244	-4.230832694	2.64E-05	8.20E-05	no
CAPN2	0.158231147	4.230720344	2.64E-05	8.20E-05	no
DST	-0.158230727	-4.230708827	2.64E-05	8.20E-05	no
KLHDC4	-0.158214473	-4.230263092	2.65E-05	8.22E-05	no
DOPEY2	0.158189371	4.229574684	2.65E-05	8.24E-05	no
LOC220930	-0.158178125	-4.229266276	2.66E-05	8.25E-05	no
RPL22	-0.158148425	-4.228451807	2.67E-05	8.28E-05	no
MKRN1	-0.158100382	-4.227134329	2.68E-05	8.32E-05	no
WDR93	0.158095821	4.22700925	2.68E-05	8.33E-05	no
ZNF845	0.158088667	4.226813084	2.69E-05	8.33E-05	no
CYP39A1	0.158057567	4.225960266	2.70E-05	8.36E-05	no
GPC2	-0.158046956	-4.225669272	2.70E-05	8.37E-05	no
BCAT1	0.158027372	4.225132251	2.71E-05	8.39E-05	no
TXNIP	0.158016801	4.224842392	2.71E-05	8.40E-05	no
HDLBP	0.157947082	4.222930636	2.73E-05	8.47E-05	no
NONO	-0.157945359	-4.222883391	2.73E-05	8.47E-05	no
JOSD2	0.157939239	4.222715573	2.73E-05	8.47E-05	no
CCDC82	-0.157935256	-4.22260635	2.74E-05	8.47E-05	no
CSNK1A1	0.157929528	4.222449297	2.74E-05	8.48E-05	no
DNER	-0.157901291	-4.221675045	2.75E-05	8.51E-05	no

KDM3A	-0.157878909	-4.221061313	2.75E-05	8.53E-05	no
C6orf97	0.157872891	4.220896307	2.76E-05	8.53E-05	no
UTS2	0.157868245	4.220768913	2.76E-05	8.54E-05	no
ZNF330	0.157857283	4.220468351	2.76E-05	8.55E-05	no
RPL23AP7	0.157849054	4.220242712	2.76E-05	8.55E-05	no
GGT7	-0.157801616	-4.218942034	2.78E-05	8.60E-05	no
RPL6	-0.157745192	-4.217394991	2.80E-05	8.66E-05	no
PIKFYVE	-0.157743622	-4.217351953	2.80E-05	8.66E-05	no
C17orf39	-0.157728922	-4.21694889	2.80E-05	8.67E-05	no
RNF5	-0.157724952	-4.216840047	2.80E-05	8.67E-05	no
C9orf122	-0.157693241	-4.215970632	2.81E-05	8.70E-05	no
ZSCAN23	-0.157690814	-4.215904083	2.82E-05	8.71E-05	no
ARHGEF5	0.157687638	4.215817013	2.82E-05	8.71E-05	no
C14orf86	0.157672388	4.215398905	2.82E-05	8.72E-05	no
TBC1D10A	-0.157638837	-4.214479038	2.83E-05	8.76E-05	no
GPRASP2	-0.157612836	-4.213766195	2.84E-05	8.78E-05	no
RTN2	-0.157600125	-4.213417732	2.85E-05	8.79E-05	no
SNX11	0.157597162	4.213336491	2.85E-05	8.79E-05	no
UBOX5	-0.157570448	-4.212604098	2.86E-05	8.82E-05	no
KCNJ8	0.15756802	4.212537546	2.86E-05	8.82E-05	no
PYCR1	-0.157494552	-4.210523435	2.88E-05	8.90E-05	no
OSGIN2	-0.157482763	-4.210200231	2.89E-05	8.91E-05	no
HIST1H2BD	0.157457719	4.209513676	2.89E-05	8.93E-05	no
ERBB2	0.157441958	4.209081611	2.90E-05	8.95E-05	no
ANKFN1	-0.157418688	-4.208443702	2.91E-05	8.97E-05	no
C2orf39	0.157414066	4.208316982	2.91E-05	8.98E-05	no
ALG10	0.157412945	4.208286252	2.91E-05	8.98E-05	no
DUS2L	-0.157409785	-4.208199635	2.91E-05	8.98E-05	no
BMS1P5	-0.157384373	-4.207503003	2.92E-05	9.00E-05	no
MCC	0.157378671	4.207346701	2.92E-05	9.01E-05	no
LGTN	0.157377356	4.207310663	2.92E-05	9.01E-05	no
MUC12	0.157358813	4.206802343	2.93E-05	9.03E-05	no
CACYBP	-0.157353302	-4.20665128	2.93E-05	9.03E-05	no
GOLGA6L10	-0.157352016	-4.206616017	2.93E-05	9.03E-05	no
CECR5	-0.157344747	-4.206416768	2.93E-05	9.04E-05	no
CHD1	0.157322507	4.205807101	2.94E-05	9.06E-05	no
DIS3L	-0.157305401	-4.205338195	2.95E-05	9.08E-05	no
SERPINB7	0.157237598	4.203479631	2.97E-05	9.15E-05	no
IGLON5	-0.157236697	-4.203454922	2.97E-05	9.15E-05	no
FGF11	-0.157200883	-4.202473231	2.98E-05	9.19E-05	no
DNAJC22	0.157182335	4.201964839	2.99E-05	9.20E-05	no
C2orf66	0.157178094	4.201848574	2.99E-05	9.21E-05	no
ZNF19	-0.157151315	-4.20111456	3.00E-05	9.24E-05	no
GPR172A	0.157126668	4.20043899	3.01E-05	9.26E-05	no
DOCK7	0.157124811	4.200388102	3.01E-05	9.26E-05	no
C15orf58	0.157114455	4.200104245	3.01E-05	9.27E-05	no
1-Mar	0.157114125	4.200095214	3.01E-05	9.27E-05	no
ARG1	0.157102514	4.199776952	3.02E-05	9.28E-05	no
PCYT2	-0.157094694	-4.199562602	3.02E-05	9.29E-05	no
C11orf70	0.157094362	4.199553524	3.02E-05	9.29E-05	no
UNC5CL	-0.157043033	-4.19814664	3.04E-05	9.34E-05	no

NQO2	0.157039553	4.198051267	3.04E-05	9.35E-05	no
UHMK1	0.15703251	4.197858222	3.04E-05	9.35E-05	no
C16orf93	0.157013846	4.197346686	3.05E-05	9.37E-05	no
CYP46A1	-0.157012529	-4.197310601	3.05E-05	9.37E-05	no
MKNK2	0.157008714	4.197206034	3.05E-05	9.37E-05	no
ZNF835	-0.156997052	-4.196886403	3.06E-05	9.38E-05	no
AK3L1	0.156974889	4.19627897	3.06E-05	9.41E-05	no
UBE2G2	-0.156972604	-4.196216332	3.07E-05	9.41E-05	no
KRTAP5-1	-0.156962858	-4.195949218	3.07E-05	9.42E-05	no
ARFGAP1	-0.15688745	-4.193882523	3.10E-05	9.50E-05	no
C4orf3	0.156866753	4.193315286	3.10E-05	9.52E-05	no
ZNF664	-0.156846401	-4.192757541	3.11E-05	9.54E-05	no
LOC653113	0.156833188	4.192395432	3.12E-05	9.56E-05	no
NCAN	-0.156823406	-4.192127352	3.12E-05	9.57E-05	no
MYH6	-0.156799964	-4.191484901	3.13E-05	9.59E-05	no
AAAS	-0.156799576	-4.191474271	3.13E-05	9.59E-05	no
SLCO3A1	0.156749224	4.19009439	3.15E-05	9.65E-05	no
TRUB1	-0.156733463	-4.189662446	3.15E-05	9.66E-05	no
MYBBP1A	-0.156712753	-4.189094909	3.16E-05	9.69E-05	no
FLJ36777	0.15669224	4.188532783	3.17E-05	9.71E-05	no
S100P	0.156644711	4.187230335	3.19E-05	9.76E-05	no
SLC6A8	-0.156622128	-4.18661148	3.19E-05	9.79E-05	no
EXOC5	-0.156590917	-4.185756212	3.21E-05	9.82E-05	no
MLYCD	-0.156554461	-4.184757243	3.22E-05	9.86E-05	no
CXXC1	-0.156544584	-4.184486598	3.22E-05	9.87E-05	no
DKFZp779M065	-0.156537814	-4.184301073	3.23E-05	9.88E-05	no
SNHG6	-0.156513281	-4.183628852	3.24E-05	9.90E-05	no
IFT80	0.156500347	4.183274436	3.24E-05	9.92E-05	no
HSP90AB2P	-0.156455624	-4.182048971	3.26E-05	9.97E-05	no
SLC16A9	-0.156451986	-4.181949285	3.26E-05	9.97E-05	no
PLEKHG2	0.156425824	4.181232433	3.27E-05	0.000100001	no
GPR44	-0.156414918	-4.180933628	3.27E-05	0.000100114	no
MSRB3	0.156390167	4.180255431	3.28E-05	0.000100391	no
SFTPA2	-0.156378254	-4.179929024	3.29E-05	0.000100516	no
NME1-NME2	-0.156365156	-4.179570152	3.29E-05	0.00010065	no
ZNF318	-0.156364329	-4.179547487	3.29E-05	0.00010065	no
RASA4P	-0.156349636	-4.179144912	3.30E-05	0.000100809	no
NAT6	-0.156345004	-4.179018001	3.30E-05	0.000100848	no
ZDHHC13	0.156313501	4.178154836	3.31E-05	0.000101207	no
C9orf135	0.156301407	4.177823485	3.32E-05	0.000101336	no
WTIP	0.156297752	4.177723343	3.32E-05	0.000101364	no
ACP6	0.156276761	4.177148225	3.33E-05	0.000101599	no
RRN3P2	0.156263533	4.176785807	3.33E-05	0.000101741	no
ZNF563	0.156258365	4.176644211	3.33E-05	0.000101781	no
FLJ39582	-0.156257645	-4.176624482	3.33E-05	0.000101781	no
JAKMIP3	-0.156232855	-4.175945299	3.34E-05	0.000102062	no
GABARAPL3	-0.156217435	-4.175522815	3.35E-05	0.000102232	no
C20orf123	0.156205971	4.175208726	3.35E-05	0.000102354	no
CXCL1	0.156185682	4.174652873	3.36E-05	0.000102583	no
KIF9	0.156178703	4.174461652	3.37E-05	0.000102651	no
RDX	0.156156238	4.173846199	3.37E-05	0.000102907	no

UEVLD	0.15613856	4.173361884	3.38E-05	0.000103105	no
CLUAP1	-0.156136531	-4.173306284	3.38E-05	0.000103114	no
APRT	0.156070764	4.171504545	3.41E-05	0.000103897	no
MGC23284	-0.156063851	-4.171315154	3.41E-05	0.000103966	no
DSN1	0.156030622	4.17040484	3.42E-05	0.000104356	no
DEXI	-0.156012582	-4.169910626	3.43E-05	0.000104561	no
MRPS6	0.156002631	4.169638028	3.44E-05	0.000104668	no
TAS2R31	-0.155982487	-4.169086191	3.44E-05	0.000104899	no
DCDC2	0.155958182	4.168420374	3.45E-05	0.000105183	no
LOC619207	-0.15595343	-4.168290197	3.46E-05	0.000105225	no
SNCB	-0.155927407	-4.167577319	3.47E-05	0.000105531	no
KPNA4	0.155918643	4.167337247	3.47E-05	0.000105624	no
C14orf178	-0.155907419	-4.167029782	3.47E-05	0.000105747	no
COG5	0.155802246	4.164148806	3.52E-05	0.000107038	no
C5orf45	-0.155801214	-4.164120521	3.52E-05	0.000107038	no
TNFRSF19	0.155771832	4.163315691	3.53E-05	0.000107391	no
ANK1	-0.155760085	-4.16299392	3.53E-05	0.000107523	no
DSTYK	-0.155755659	-4.162872701	3.54E-05	0.000107562	no
GTF2H2	0.155734528	4.162293888	3.55E-05	0.000107812	no
ST6GALNAC4	0.155722969	4.161977283	3.55E-05	0.000107942	no
SPEN	-0.155600615	-4.158625974	3.60E-05	0.000109482	no
AIM1L	0.155593034	4.15841833	3.60E-05	0.000109563	no
SLC2A14	0.155473299	4.155138973	3.66E-05	0.000111091	no
KIAA0087	-0.15544886	-4.154469631	3.67E-05	0.000111392	no
PLEKHF1	0.155423175	4.153766216	3.68E-05	0.00011171	no
CYTH3	0.155386289	4.152756024	3.69E-05	0.000112175	no
C14orf21	-0.15535153	-4.15180408	3.71E-05	0.000112615	no
SLC39A6	-0.15532015	-4.150944706	3.72E-05	0.000113011	no
EED	0.155291934	4.150172011	3.73E-05	0.000113367	no
KCTD5	-0.155264279	-4.149414658	3.75E-05	0.000113717	no
GPS1	-0.155260033	-4.149298377	3.75E-05	0.000113756	no
MPST	-0.155229103	-4.148451386	3.76E-05	0.00011415	no
THAP11	-0.15522635	-4.148375988	3.76E-05	0.00011417	no
TNIK	-0.155211127	-4.147959119	3.77E-05	0.000114356	no
GRIP2	-0.155160143	-4.146562958	3.79E-05	0.000115021	no
MDC1	-0.155131691	-4.145783864	3.80E-05	0.000115386	no
H19	0.155060719	4.14384044	3.84E-05	0.000116327	no
GPAM	-0.155059089	-4.1437958	3.84E-05	0.000116332	no
APCDD1L	0.155055454	4.143696267	3.84E-05	0.000116364	no
GYPE	0.154992981	4.141985647	3.87E-05	0.000117196	no
LOC728758	0.154988635	4.141866641	3.87E-05	0.000117238	no
NAA16	-0.154950103	-4.140811606	3.89E-05	0.000117748	no
LLGL1	-0.154946288	-4.140707141	3.89E-05	0.000117783	no
INTS2	-0.154923262	-4.140076666	3.90E-05	0.000118081	no
GTF2B	0.154916751	4.139898394	3.90E-05	0.000118153	no
COX15	-0.15488079	-4.138913781	3.92E-05	0.000118631	no
TCTA	0.154878165	4.138841893	3.92E-05	0.00011865	no
TNMD	0.154876044	4.138783828	3.92E-05	0.000118661	no
ZNF667	-0.154870359	-4.138628186	3.92E-05	0.000118722	no
PCP2	0.154853923	4.13817816	3.93E-05	0.00011892	no
LOC441294	0.154853464	4.138165586	3.93E-05	0.00011892	no

LOC100289341	-0.154842502	-4.137865468	3.93E-05	0.000119054	no
ELOVL6	0.154803951	4.136809962	3.95E-05	0.000119572	no
PLA2G2A	0.154800885	4.13672602	3.95E-05	0.000119597	no
RASGRF1	-0.154778104	-4.136102284	3.96E-05	0.000119896	no
SSPO	-0.15476731	-4.135806779	3.97E-05	0.000120029	no
DNAJC5	-0.154735075	-4.13492422	3.98E-05	0.000120454	no
RPGR	0.1547334	4.134878364	3.98E-05	0.000120454	no
ZNF682	-0.154733094	-4.134869997	3.98E-05	0.000120454	no
AQP7	-0.154718856	-4.134480185	3.99E-05	0.000120636	no
SOX30	0.154690813	4.133712428	4.00E-05	0.000121012	no
FMN2	-0.154674824	-4.133274688	4.01E-05	0.000121219	no
HCN2	-0.154671067	-4.133171842	4.01E-05	0.000121254	no
LOC284551	0.154651689	4.132641318	4.02E-05	0.000121509	no
ANKK1	0.154646467	4.132498371	4.02E-05	0.000121565	no
ZNF286A	-0.154637545	-4.1322541	4.03E-05	0.000121673	no
JMJD7-PLA2G4	-0.154636038	-4.132212858	4.03E-05	0.000121676	no
TTC23L	0.154628108	4.131995753	4.03E-05	0.00012177	no
CNTROB	-0.154567893	-4.130347274	4.06E-05	0.000122607	no
CHRNA9	0.154538179	4.129533845	4.08E-05	0.000123013	no
INPP5K	-0.154513902	-4.128869238	4.09E-05	0.000123342	no
SPEF2	0.154474709	4.127796337	4.11E-05	0.000123886	no
CCL8	0.15446044	4.127405723	4.11E-05	0.000124073	no
AP3S2	-0.15444882	-4.127087651	4.12E-05	0.000124222	no
PUS7	0.154447199	4.127043271	4.12E-05	0.000124227	no
P704P	0.154421191	4.126331317	4.13E-05	0.000124584	no
SBF2	-0.154414615	-4.126151308	4.13E-05	0.000124661	no
AP4M1	0.154362168	4.124715632	4.16E-05	0.000125404	no
ZNF675	-0.154350977	-4.124409302	4.17E-05	0.000125548	no
C8orf77	-0.154340063	-4.124110559	4.17E-05	0.000125688	no
SF3A3	0.154333944	4.123943067	4.17E-05	0.000125743	no
FAM186B	0.154333724	4.123937034	4.17E-05	0.000125743	no
UTP20	0.15430743	4.123217304	4.19E-05	0.000126109	no
NRAS	0.154300822	4.123036432	4.19E-05	0.000126187	no
ACAT1	-0.154277803	-4.122406345	4.20E-05	0.000126505	no
KLHL17	-0.154255205	-4.121787783	4.21E-05	0.000126818	no
SRPK2	-0.15424702	-4.121563754	4.22E-05	0.00012692	no
OXNAD1	0.154242642	4.121443932	4.22E-05	0.000126966	no
ANKRD7	0.15422071	4.120843613	4.23E-05	0.00012727	no
PPP4R4	-0.154214298	-4.12066809	4.23E-05	0.000127346	no
ADAMTS20	-0.154199024	-4.120250029	4.24E-05	0.000127553	no
RSPH4A	0.154194783	4.120133956	4.24E-05	0.000127596	no
SETD8	0.154142734	4.11870932	4.27E-05	0.00012835	no
RFC2	0.154121374	4.118124692	4.28E-05	0.000128649	no
C14orf28	-0.154089727	-4.117258528	4.29E-05	0.000129102	no
FBXL15	-0.154083792	-4.117096084	4.30E-05	0.000129172	no
ACIN1	-0.154054373	-4.116290914	4.31E-05	0.000129593	no
SRA1	0.15405106	4.116200233	4.31E-05	0.000129624	no
HTR1A	-0.154034712	-4.115752811	4.32E-05	0.00012985	no
AKR7L	-0.154008658	-4.115039729	4.33E-05	0.000130224	no
DLG2	-0.153968301	-4.113935222	4.35E-05	0.000130814	no
FAM164A	-0.153955136	-4.113574921	4.36E-05	0.000130994	no

C19orf57	-0.153941422	-4.113199594	4.37E-05	0.000131183	no
TMPPE	0.153931983	4.112941267	4.37E-05	0.000131303	no
MSL3L2	0.153930974	4.112913665	4.37E-05	0.000131303	no
CLEC18A	0.15392297	4.112694607	4.38E-05	0.000131405	no
CLEC18C	0.153898249	4.112018071	4.39E-05	0.000131751	no
NUDT22	0.153897052	4.111985311	4.39E-05	0.000131751	no
DNASE1	-0.153896374	-4.111966741	4.39E-05	0.000131751	no
GTF3A	0.15386519	4.111113347	4.41E-05	0.000132208	no
CCDC34	0.153845861	4.110584375	4.42E-05	0.000132484	no
P2RY11	0.153837401	4.110352852	4.42E-05	0.000132595	no
ALKBH2	-0.153834292	-4.110267769	4.42E-05	0.000132623	no
UGDH	0.153823281	4.10996643	4.43E-05	0.000132772	no
GJA5	0.153821753	4.109924604	4.43E-05	0.000132776	no
GOLGA6L9	-0.153812831	-4.109680464	4.43E-05	0.000132893	no
EXT1	0.15378356	4.108879436	4.45E-05	0.000133324	no
ADH5	0.153762318	4.108298132	4.46E-05	0.000133632	no
C7orf30	0.153753782	4.108064532	4.46E-05	0.000133744	no
ZNF451	-0.153726544	-4.107319147	4.48E-05	0.000134146	no
SENP5	-0.153715551	-4.107018348	4.48E-05	0.000134297	no
ADAMTS15	0.153703626	4.106692008	4.49E-05	0.000134463	no
IGFBP2	0.153694697	4.10644768	4.49E-05	0.000134581	no
FNTA	0.15368974	4.106312014	4.50E-05	0.000134639	no
CLSTN2	-0.153684828	-4.10617761	4.50E-05	0.000134695	no
MAML2	-0.153661269	-4.10553292	4.51E-05	0.000135042	no
BNIP3	-0.153659463	-4.105483519	4.51E-05	0.000135047	no
POLR3B	-0.153658388	-4.105454103	4.51E-05	0.000135047	no
SAP30	0.153636578	4.104857289	4.52E-05	0.000135368	no
LLPH	0.153631838	4.104727585	4.53E-05	0.000135401	no
PPM1K	-0.153631335	-4.104713811	4.53E-05	0.000135401	no
XKR4	-0.15363066	-4.104695349	4.53E-05	0.000135401	no
SCN9A	0.153628803	4.10464452	4.53E-05	0.00013541	no
NDNL2	0.153592908	4.103662326	4.55E-05	0.000135952	no
LMAN2L	0.153572328	4.10309918	4.56E-05	0.000136256	no
LPAL2	-0.15354399	-4.102323771	4.57E-05	0.000136682	no
PTPRH	0.153526883	4.101855674	4.58E-05	0.000136932	no
EXTL2	0.153512217	4.1014544	4.59E-05	0.000137144	no
S100A2	0.153503596	4.101218504	4.59E-05	0.000137261	no
ZNF282	-0.153495688	-4.101002131	4.60E-05	0.000137366	no
CLK4	-0.153488297	-4.100799888	4.60E-05	0.000137463	no
PER3	-0.153466051	-4.100191209	4.61E-05	0.000137796	no
ELN	0.153402092	4.098441203	4.65E-05	0.000138796	no
YSK4	0.153372337	4.097627091	4.66E-05	0.000139253	no
EEF2K	0.15335997	4.097288718	4.67E-05	0.000139431	no
ZNF684	0.153339923	4.096740217	4.68E-05	0.000139733	no
POMP	0.153319947	4.096193685	4.69E-05	0.000140035	no
ARL8A	-0.153312774	-4.095997442	4.70E-05	0.00014013	no
PRPF38B	0.153292234	4.095435461	4.71E-05	0.000140442	no
FGD5	0.15327933	4.09508243	4.72E-05	0.00014063	no
CCNY	-0.153252891	-4.09435908	4.73E-05	0.000141039	no
NACA	-0.153249474	-4.094265601	4.73E-05	0.000141073	no
WDR86	-0.153245844	-4.094166272	4.73E-05	0.000141112	no

ADAM19	0.153237401	4.09393528	4.74E-05	0.000141228	no
BBS4	0.153202716	4.092986361	4.76E-05	0.000141773	no
TSPYL1	-0.153199826	-4.092907288	4.76E-05	0.000141799	no
TMBIM6	0.153134462	4.091119066	4.79E-05	0.00014285	no
TCF3	-0.153119756	-4.090716757	4.80E-05	0.000143072	no
NGLY1	0.153105501	4.090326771	4.81E-05	0.000143286	no
ADCY4	0.153084222	4.089744647	4.82E-05	0.000143616	no
TXNDC15	0.153078575	4.089590171	4.83E-05	0.000143688	no
CCDC111	0.15306252	4.089150962	4.83E-05	0.000143933	no
TMEM57	-0.153051337	-4.088845034	4.84E-05	0.000144097	no
TXNDC17	0.152991002	4.08719453	4.87E-05	0.00014508	no
EIF3I	0.15297427	4.086736806	4.88E-05	0.000145338	no
TBC1D26	-0.15294808	-4.086020376	4.90E-05	0.000145756	no
EVI2A	0.152943549	4.085896417	4.90E-05	0.00014581	no
C12orf63	0.152906499	4.08488294	4.92E-05	0.000146411	no
LIMCH1	-0.152903252	-4.08479411	4.92E-05	0.000146445	no
IFIT2	0.15282968	4.08278163	4.97E-05	0.000147667	no
MED7	0.152811789	4.082292257	4.98E-05	0.00014795	no
IL29	0.152780661	4.081440809	4.99E-05	0.000148458	no
MLXIP	0.152776648	4.081331028	5.00E-05	0.000148505	no
SYNM	0.152752245	4.080663542	5.01E-05	0.0001489	no
PIGO	0.152736061	4.08022087	5.02E-05	0.000149155	no
TTPA	-0.152718963	-4.079753224	5.03E-05	0.000149427	no
C1orf113	0.152705548	4.079386294	5.04E-05	0.000149635	no
LOC150776	-0.152677732	-4.078625472	5.05E-05	0.000150092	no
RAD54B	0.152665375	4.078287486	5.06E-05	0.000150283	no
FAM104B	-0.152609154	-4.076749781	5.09E-05	0.000151234	no
ATRNL1	-0.152603549	-4.076596483	5.10E-05	0.00015131	no
HAGH	-0.152596928	-4.076415389	5.10E-05	0.000151402	no
VEPH1	-0.152591542	-4.076268083	5.10E-05	0.000151474	no
ARAP3	0.152589904	4.076223281	5.10E-05	0.00015148	no
NOL10	0.15256302	4.075488	5.12E-05	0.000151926	no
DNAJC6	-0.152561391	-4.075443467	5.12E-05	0.000151932	no
RNF103	-0.15254345	-4.074952783	5.13E-05	0.000152223	no
DNAJB2	-0.152516172	-4.074206731	5.15E-05	0.000152678	no
ANAPC10	0.152511642	4.074082846	5.15E-05	0.000152735	no
KIAA0284	-0.152508542	-4.07399806	5.15E-05	0.000152767	no
RNF121	-0.152485002	-4.073354264	5.17E-05	0.000153158	no
TPMT	0.152480425	4.073229078	5.17E-05	0.000153216	no
C5orf56	0.152467506	4.072875759	5.18E-05	0.00015342	no
RPL7A	-0.152454176	-4.072511187	5.18E-05	0.000153633	no
BEND5	-0.152436914	-4.072039096	5.19E-05	0.000153914	no
R3HCC1	-0.152424496	-4.071699503	5.20E-05	0.000154111	no
H1FOO	0.152408936	4.071273947	5.21E-05	0.000154364	no
FMN1	0.152384137	4.070595745	5.23E-05	0.000154781	no
OR2A7	0.152358734	4.069901039	5.24E-05	0.00015521	no
JAG1	0.152354588	4.069787667	5.24E-05	0.00015526	no
NPLOC4	0.152353363	4.069754151	5.24E-05	0.00015526	no
GATSL1	-0.15232396	-4.068950079	5.26E-05	0.000155761	no
ZNF526	0.152318464	4.06879978	5.27E-05	0.000155836	no
PRRG2	0.152260662	4.067219075	5.30E-05	0.000156849	no

STC1	0.15225027	4.066934904	5.31E-05	0.000157013	no
GTF2IRD2P1	-0.15222786	-4.06632209	5.32E-05	0.000157394	no
WDSUB1	0.152218193	4.066057739	5.33E-05	0.000157545	no
PHRF1	-0.152181468	-4.065053479	5.35E-05	0.000158186	no
NTN4	-0.152179685	-4.065004708	5.35E-05	0.000158195	no
NAA15	0.152149179	4.064170539	5.37E-05	0.000158725	no
AFAP1	-0.152131644	-4.063691033	5.38E-05	0.000159021	no
ST8SIA1	-0.15207021	-4.062011182	5.42E-05	0.00016012	no
NIT2	0.1520626	4.061803096	5.42E-05	0.000160236	no
LIPH	0.152057332	4.061659037	5.43E-05	0.000160309	no
KIAA1257	0.152049855	4.061454598	5.43E-05	0.000160423	no
LOC284276	0.152031133	4.060942677	5.44E-05	0.000160743	no
GFRA1	-0.152025259	-4.060782062	5.45E-05	0.000160828	no
PAX8	0.152022463	4.060705608	5.45E-05	0.000160856	no
TMEM132B	-0.152006612	-4.0602722	5.46E-05	0.000161124	no
MKLN1	-0.151988598	-4.059779652	5.47E-05	0.000161433	no
CDH9	-0.151977521	-4.059476765	5.48E-05	0.000161614	no
ANKRD36B	-0.151974836	-4.059403354	5.48E-05	0.00016164	no
PPP2R2C	-0.151934648	-4.058304531	5.50E-05	0.000162361	no
ZCCHC5	0.151912566	4.057700748	5.52E-05	0.000162748	no
PHAX	-0.151908111	-4.057578946	5.52E-05	0.000162807	no
HTR2A	-0.151885005	-4.056947208	5.53E-05	0.000163214	no
C11orf57	-0.151865594	-4.056416488	5.55E-05	0.000163552	no
MAK16	0.151825669	4.055324906	5.57E-05	0.000164276	no
CAPZA2	0.151793153	4.054435898	5.59E-05	0.000164864	no
CASP2	0.151780427	4.054087974	5.60E-05	0.000165079	no
TIMP2	0.151766228	4.053699775	5.61E-05	0.000165323	no
CNOT6	-0.151762113	-4.053587255	5.61E-05	0.000165377	no
VTA1	-0.151756107	-4.053423069	5.62E-05	0.000165466	no
TFE3	0.151751653	4.053301273	5.62E-05	0.000165526	no
BAI2	-0.151746198	-4.053152138	5.62E-05	0.000165605	no
C6orf124	-0.151738893	-4.052952433	5.63E-05	0.000165719	no
PTK2	-0.151725828	-4.052595246	5.64E-05	0.000165943	no
GTPBP4	-0.151720269	-4.052443252	5.64E-05	0.000166024	no
CPNE2	-0.151687635	-4.05155108	5.66E-05	0.000166619	no
ZNF345	0.151673324	4.051159837	5.67E-05	0.000166868	no
FAM110A	-0.151624038	-4.049812421	5.70E-05	0.000167784	no
TTLL2	-0.151605958	-4.049318158	5.71E-05	0.000168106	no
IFT27	-0.15153955	-4.047502749	5.76E-05	0.00016936	no
GRIN1	-0.151530828	-4.047264295	5.76E-05	0.000169504	no
PAR4	-0.151529366	-4.047224333	5.76E-05	0.000169508	no
NMNAT3	0.151527236	4.047166112	5.76E-05	0.000169524	no
SSBP1	0.151518522	4.046927893	5.77E-05	0.000169668	no
SLC34A2	0.151474612	4.045727547	5.80E-05	0.000170495	no
DPEP1	0.151459412	4.045312041	5.81E-05	0.000170766	no
FLJ32063	-0.151452187	-4.045114556	5.81E-05	0.000170851	no
FEZF2	-0.151451473	-4.04509503	5.81E-05	0.000170851	no
HTR1D	0.151451199	4.045087545	5.81E-05	0.000170851	no
C6orf141	0.151421964	4.044288383	5.83E-05	0.000171397	no
MYST3	-0.15141881	-4.044202178	5.84E-05	0.000171427	no
ZNF347	0.151417883	4.044176813	5.84E-05	0.000171427	no

C7orf57	0.151399512	4.043674653	5.85E-05	0.000171761	no
PLAG1	0.151385621	4.043294928	5.86E-05	0.000172008	no
CYGB	0.151376681	4.043050566	5.86E-05	0.000172158	no
ZNF876P	-0.151374616	-4.042994122	5.87E-05	0.000172174	no
TCF21	0.151341274	4.042082731	5.89E-05	0.000172804	no
SCN11A	-0.151325041	-4.041639009	5.90E-05	0.000173099	no
GPR148	-0.151321105	-4.04153141	5.90E-05	0.000173151	no
HS3ST3B1	0.151277524	4.040340173	5.93E-05	0.000173988	no
ZNF786	-0.151256657	-4.039769809	5.95E-05	0.000174376	no
ANK3	-0.151247362	-4.039515746	5.95E-05	0.000174535	no
FBXO31	-0.151237352	-4.03924214	5.96E-05	0.000174709	no
SERINC5	-0.151236097	-4.039207828	5.96E-05	0.000174709	no
FAM70A	0.151234307	4.03915892	5.96E-05	0.000174719	no
ZNF286B	-0.151210661	-4.038512583	5.98E-05	0.000175165	no
CHRM1	-0.151192787	-4.03802405	5.99E-05	0.000175496	no
C14orf143	0.151180359	4.037684362	6.00E-05	0.000175719	no
CTDSP1	0.151172694	4.03747487	6.00E-05	0.000175847	no
GOLM1	-0.151162675	-4.037201018	6.01E-05	0.000176022	no
GAP43	0.151157497	4.037059487	6.01E-05	0.0001761	no
ADIG	0.151146162	4.036749673	6.02E-05	0.000176302	no
C20orf29	0.151126194	4.036203916	6.03E-05	0.000176677	no
NDE1	0.15112366	4.036134665	6.04E-05	0.000176702	no
FAM177A1	-0.151082977	-4.035022735	6.06E-05	0.000177496	no
CIB1	0.151040643	4.033865713	6.09E-05	0.000178327	no
DYNC1LI2	-0.15100814	-4.032977388	6.12E-05	0.000178945	no
BCKDK	0.151007675	4.032964669	6.12E-05	0.000178945	no
DEPDC5	-0.150986331	-4.032381346	6.13E-05	0.000179354	no
KNDC1	-0.150951393	-4.031426487	6.16E-05	0.000180041	no
MLF1	0.150946051	4.03128051	6.16E-05	0.000180124	no
ZNF605	-0.150909746	-4.030288327	6.18E-05	0.000180843	no
GXYLT1	0.150863308	4.029019219	6.22E-05	0.000181773	no
C13orf1	-0.150783901	-4.026849186	6.27E-05	0.000183393	no
E2F8	0.150761299	4.026231541	6.29E-05	0.000183837	no
EARS2	-0.150756014	-4.026087116	6.29E-05	0.000183921	no
INO80D	-0.150734894	-4.025509989	6.31E-05	0.000184336	no
TDRKH	-0.150693557	-4.024380384	6.34E-05	0.000185174	no
PKP4	-0.150692341	-4.024347152	6.34E-05	0.000185174	no
C3orf19	-0.150670152	-4.02374081	6.35E-05	0.000185614	no
KDELC1	0.150660838	4.023486305	6.36E-05	0.000185784	no
WEE1	0.150609056	4.022071338	6.40E-05	0.000186851	no
ZNF169	-0.150602983	-4.021905371	6.40E-05	0.000186953	no
HCG11	0.150565413	4.020878781	6.43E-05	0.000187724	no
TAS2R3	-0.150550359	-4.020467432	6.44E-05	0.000188017	no
C5orf38	-0.150542491	-4.020252452	6.45E-05	0.000188158	no
DNAJB12	-0.150531499	-4.019952106	6.46E-05	0.000188365	no
C1QTNF2	0.150508307	4.0193184	6.47E-05	0.00018882	no
ELOVL4	-0.15050769	-4.019301554	6.47E-05	0.00018882	no
RAB40AL	-0.150499482	-4.019077285	6.48E-05	0.000188953	no
C10orf18	-0.150498922	-4.019061968	6.48E-05	0.000188953	no
CSGALNACT2	0.150455445	4.01787402	6.51E-05	0.000189858	no
ZMYM1	0.150439652	4.017442525	6.52E-05	0.000190171	no

ABCA10	0.150422527	4.016974612	6.54E-05	0.000190513	no
INTS9	0.150413046	4.016715556	6.54E-05	0.00019069	no
MYO18A	-0.150381969	-4.015866448	6.57E-05	0.000191335	no
TSPAN32	0.150335892	4.014607537	6.60E-05	0.000192308	no
RNF181	0.150297332	4.013554027	6.63E-05	0.000193121	no
LOC100302401	0.150288664	4.013317205	6.64E-05	0.000193283	no
GPR19	-0.150276038	-4.012972253	6.65E-05	0.000193531	no
KLF5	0.15026427	4.012650741	6.65E-05	0.000193761	no
KRT222	-0.150225512	-4.01159185	6.68E-05	0.000194585	no
SCN2A	-0.150220926	-4.011466565	6.69E-05	0.000194657	no
P2RX6	-0.150204276	-4.011011658	6.70E-05	0.000194969	no
C19orf55	0.150204253	4.01101105	6.70E-05	0.000194969	no
RPF1	0.150186529	4.010526828	6.71E-05	0.000195332	no
PTPN13	0.150173509	4.010171113	6.72E-05	0.000195592	no
BPI	0.150149989	4.009528565	6.74E-05	0.000196084	no
SENP2	-0.150135193	-4.009124343	6.75E-05	0.000196384	no
TAF5L	-0.150121769	-4.008757613	6.76E-05	0.000196654	no
RBM9	-0.150119737	-4.008702103	6.76E-05	0.000196671	no
SYNE1	-0.150114651	-4.008563149	6.77E-05	0.000196756	no
LOC283856	-0.150110167	-4.008440661	6.77E-05	0.000196827	no
EDNRB	-0.150105235	-4.008305929	6.77E-05	0.000196909	no
LOC643387	-0.150039056	-4.006498021	6.83E-05	0.000198359	no
SARDH	0.149977252	4.004809676	6.87E-05	0.00019972	no
SLC25A1	-0.149954703	-4.004193703	6.89E-05	0.0002002	no
TTC27	0.149952758	4.004140564	6.89E-05	0.000200215	no
SNX3	0.149950544	4.004080093	6.89E-05	0.000200237	no
MNT	-0.149944933	-4.003926817	6.90E-05	0.000200335	no
PLAC2	-0.149908473	-4.002930865	6.93E-05	0.000201132	no
SLC35D3	-0.14989917	-4.002676747	6.93E-05	0.000201315	no
CAPS	0.149894188	4.002540647	6.94E-05	0.000201399	no
NCRNA00032	-0.149886107	-4.002319896	6.94E-05	0.000201554	no
UNK	-0.149861749	-4.001654539	6.96E-05	0.00020208	no
AMDHD2	0.149827812	4.000727539	6.99E-05	0.000202826	no
NPDC1	-0.149772708	-3.999222359	7.03E-05	0.000204062	no
CDYL	-0.149746993	-3.998519962	7.05E-05	0.000204625	no
RRP1	-0.149728479	-3.998014289	7.07E-05	0.000205023	no
C18orf10	0.149722411	3.997848541	7.07E-05	0.000205134	no
NCRNA00094	-0.149709943	-3.997507982	7.08E-05	0.000205393	no
RIBC2	0.149698532	3.997196318	7.09E-05	0.000205628	no
TOP1P1	0.149689343	3.996945325	7.10E-05	0.000205812	no
SLC19A3	0.149672595	3.99648788	7.11E-05	0.000206171	no
STXBP3	0.149661171	3.996175852	7.12E-05	0.000206407	no
CAMP	0.149655871	3.9960311	7.13E-05	0.000206501	no
MYO6	-0.149652531	-3.995939855	7.13E-05	0.000206549	no
PRDX3	0.149636408	3.995499502	7.14E-05	0.000206895	no
FOXJ1	0.149565147	3.99355318	7.20E-05	0.000208533	no
TPPP3	0.149563011	3.993494839	7.20E-05	0.000208554	no
RNF20	-0.14955087	-3.993163237	7.21E-05	0.000208809	no
FLJ44606	0.14952844	3.992550645	7.23E-05	0.000209247	no
TEKT1	0.149527789	3.992532875	7.23E-05	0.000209247	no
ABCA17P	-0.149527219	-3.992517285	7.23E-05	0.000209247	no

AJAP1	-0.149526425	-3.992495619	7.23E-05	0.000209247	no
COMMD10	0.149525923	3.992481896	7.23E-05	0.000209247	no
STRA6	0.149498599	3.991735643	7.25E-05	0.000209862	no
G3BP1	0.149479685	3.991219081	7.27E-05	0.000210279	no
PPIA	0.14946917	3.990931898	7.28E-05	0.000210498	no
SPNS2	-0.149426017	-3.989753374	7.31E-05	0.000211493	no
NR2C2	-0.149363453	-3.988044749	7.37E-05	0.000212956	no
HIF3A	-0.14933388	-3.987237148	7.39E-05	0.000213635	no
FGF17	-0.149331475	-3.987171449	7.39E-05	0.000213663	no
TLE3	-0.149299152	-3.986288762	7.42E-05	0.00021441	no
OTOA	0.149288133	3.985987825	7.43E-05	0.000214645	no
ZNF385D	0.149244261	3.984789775	7.47E-05	0.000215676	no
ZBTB32	0.149214647	3.983981071	7.49E-05	0.000216364	no
CA2	0.149204538	3.983705027	7.50E-05	0.000216579	no
DCDC1	0.149169564	3.982749981	7.53E-05	0.000217401	no
OSBPL9	0.149110923	3.981148676	7.58E-05	0.000218807	no
INS-IGF2	0.149076742	3.980215326	7.61E-05	0.000219617	no
CACNA1I	-0.149069135	-3.98000761	7.61E-05	0.000219743	no
BRCC3	0.149069099	3.980006614	7.61E-05	0.000219743	no
CAMK4	-0.149067295	-3.979957361	7.62E-05	0.000219756	no
CACNA1B	-0.149059977	-3.979757548	7.62E-05	0.000219905	no
TPP2	-0.149031596	-3.978982576	7.65E-05	0.000220576	no
SFRS2IP	0.149020038	3.978666985	7.66E-05	0.000220831	no
SNX22	-0.149013196	-3.978480164	7.66E-05	0.000220969	no
GPSM2	-0.149010453	-3.978405262	7.66E-05	0.000221005	no
SMU1	-0.148990159	-3.977851131	7.68E-05	0.000221449	no
APOC1P1	0.148990064	3.977848553	7.68E-05	0.000221449	no
EREG	0.148984005	3.977683108	7.69E-05	0.000221568	no
CANX	0.148973005	3.977382755	7.70E-05	0.00022181	no
TAF6L	-0.148960872	-3.977051459	7.71E-05	0.00022208	no
ELK4	0.148944354	3.976600466	7.72E-05	0.000222461	no
NME2	0.148939441	3.976466305	7.73E-05	0.000222552	no
MAPRE3	-0.148933865	-3.976314065	7.73E-05	0.000222659	no
HPSE2	-0.148924412	-3.976055951	7.74E-05	0.000222864	no
S100A3	0.148910211	3.975668214	7.75E-05	0.000223187	no
C12orf48	0.14885164	3.974069016	7.80E-05	0.000224626	no
MRPS10	0.14883164	3.973522947	7.82E-05	0.000225099	no
MGC16703	-0.148748748	-3.971259816	7.89E-05	0.000227168	no
GPR27	-0.148722206	-3.970535177	7.92E-05	0.000227812	no
MACROD2	-0.14865219	-3.968623644	7.98E-05	0.000229573	no
TADA2A	-0.148598929	-3.96716961	8.03E-05	0.000230913	no
MAP4K4	-0.148563552	-3.966203827	8.06E-05	0.000231797	no
KCNQ4	0.148546625	3.965741721	8.07E-05	0.000232203	no
EFNA2	-0.148543899	-3.965667319	8.08E-05	0.000232241	no
MRO	-0.148510529	-3.964756336	8.11E-05	0.000233077	no
KIAA1107	-0.148492721	-3.964270219	8.12E-05	0.000233509	no
CALM1	-0.148457092	-3.963297584	8.15E-05	0.000234408	no
LEMD3	-0.14836696	-3.960837204	8.24E-05	0.000236749	no
UBL4A	0.148339385	3.960084479	8.26E-05	0.000237446	no
CCDC154	-0.148316038	-3.959447195	8.28E-05	0.000238033	no
OAZ3	0.148294192	3.958850883	8.30E-05	0.000238571	no

COPS2	0.148293279	3.958825939	8.30E-05	0.000238571	no
H1FX	-0.148287899	-3.958679112	8.31E-05	0.000238681	no
C14orf50	0.148282948	3.958543961	8.31E-05	0.000238779	no
CCDC28B	-0.148270283	-3.958198257	8.33E-05	0.000239083	no
C17orf106	0.148265545	3.958068913	8.33E-05	0.000239176	no
UBE2R2	-0.148195755	-3.956163977	8.40E-05	0.000241013	no
PFN2	-0.148190844	-3.956029943	8.40E-05	0.000241111	no
ACTL6A	0.148185448	3.955882651	8.41E-05	0.000241222	no
CTBP1	-0.148177413	-3.955663323	8.41E-05	0.000241404	no
POLR3F	-0.148169524	-3.955447999	8.42E-05	0.000241583	no
EIF3A	-0.148136848	-3.954556123	8.45E-05	0.000242431	no
RAB3A	-0.14810838	-3.953779139	8.48E-05	0.000243153	no
C20orf3	0.148107677	3.95375994	8.48E-05	0.000243153	no
C6orf221	-0.148102936	-3.953630541	8.48E-05	0.000243247	no
FARS2	0.14805977	3.952452385	8.52E-05	0.000244387	no
NFKBID	0.148045068	3.952051119	8.54E-05	0.000244754	no
ZSCAN1	-0.148040586	-3.951928782	8.54E-05	0.000244841	no
ZNF788	0.148028158	3.951589598	8.55E-05	0.000245146	no
DEPDC6	0.148025277	3.951510967	8.56E-05	0.00024519	no
ZNF620	-0.14800399	-3.950929993	8.58E-05	0.000245738	no
TRPV1	-0.147995866	-3.950708254	8.59E-05	0.000245926	no
MT1DP	0.147982405	3.950340882	8.60E-05	0.000246261	no
RRN3P1	-0.147927071	-3.948830705	8.65E-05	0.00024775	no
LDHAL6B	0.147901211	3.948124956	8.68E-05	0.00024843	no
WBSCR28	0.147854953	3.946862507	8.72E-05	0.000249679	no
RETSAT	0.147849919	3.946725121	8.73E-05	0.000249784	no
MRPS12	0.147841675	3.946500142	8.73E-05	0.000249978	no
ALG6	0.147811034	3.945663945	8.76E-05	0.000250792	no
ALOX12B	-0.147809937	-3.945633996	8.76E-05	0.000250792	no
ADIPOR2	-0.147806389	-3.945537168	8.77E-05	0.000250856	no
C7orf61	-0.147800111	-3.945365853	8.77E-05	0.000250996	no
OR8J1	0.147755016	3.944135225	8.82E-05	0.000252224	no
MARVELD3	-0.147748524	-3.943958049	8.83E-05	0.000252371	no
MRVI1	-0.147746107	-3.943892091	8.83E-05	0.000252403	no
CCDC126	0.147730937	3.943478108	8.84E-05	0.000252794	no
KIF20B	0.14772416	3.943293184	8.85E-05	0.000252949	no
PBX3	0.147719103	3.943155188	8.85E-05	0.000253056	no
TRIM16L	-0.147678832	-3.942056239	8.89E-05	0.000254157	no
CDH19	0.147673166	3.941901622	8.90E-05	0.000254281	no
MFSD10	0.14764251	3.94106505	8.93E-05	0.000255114	no
ELANE	0.147632592	3.940794408	8.94E-05	0.00025536	no
MMP8	0.147621738	3.940498223	8.95E-05	0.000255632	no
FAM98A	0.147584721	3.939488119	8.99E-05	0.000256651	no
INTS4	-0.147564109	-3.938925681	9.01E-05	0.000257204	no
RYK	0.147533738	3.938096963	9.04E-05	0.000258037	no
PDE7A	0.147493219	3.936991328	9.08E-05	0.000259166	no
GEMIN7	0.147482993	3.936712295	9.09E-05	0.000259424	no
TEKT3	0.147463688	3.936185537	9.11E-05	0.000259945	no
ERG	0.147462247	3.93614621	9.11E-05	0.00025995	no
PGK1	0.147444877	3.935672281	9.13E-05	0.000260415	no
KIAA0232	-0.147436887	-3.935454255	9.14E-05	0.00026061	no

RGS14	0.147401788	3.934496566	9.17E-05	0.000261592	no
KCNH4	-0.147395157	-3.934315628	9.18E-05	0.000261747	no
RCVRN	0.147386783	3.934087147	9.19E-05	0.000261954	no
IRGQ	-0.147376064	-3.933794692	9.20E-05	0.000262229	no
KALRN	-0.147368097	-3.933577318	9.21E-05	0.000262424	no
WISP2	0.147363934	3.933463734	9.21E-05	0.000262509	no
BCLAF1	-0.147344104	-3.932922672	9.23E-05	0.000263051	no
DNAJC15	0.147316774	3.932176985	9.26E-05	0.000263811	no
C17orf46	0.147315572	3.932144187	9.26E-05	0.000263811	no
C3orf70	-0.147290544	-3.931461344	9.29E-05	0.000264508	no
TSHZ2	0.147276848	3.931087651	9.30E-05	0.000264873	no
ACMSD	0.147268346	3.930855701	9.31E-05	0.000265086	no
CDK8	-0.147232647	-3.929881691	9.35E-05	0.000266101	no
SMPDL3B	0.147221992	3.929590997	9.36E-05	0.000266378	no
ZBTB42	0.147215975	3.929426838	9.36E-05	0.000266519	no
C6orf165	0.147192369	3.928782809	9.39E-05	0.00026718	no
ATG16L1	0.147189896	3.928715327	9.39E-05	0.000267216	no
AHSG	-0.147169086	-3.928147578	9.41E-05	0.000267778	no
UROD	0.147168414	3.928129258	9.41E-05	0.000267778	no
LMTK2	-0.147150795	-3.927648561	9.43E-05	0.000268264	no
RHOBTB3	-0.147119657	-3.926799058	9.46E-05	0.000269155	no
FUT8	0.147117967	3.926752958	9.47E-05	0.000269157	no
ZNF16	-0.147117053	-3.926728028	9.47E-05	0.000269157	no
IGSF10	0.147111549	3.926577863	9.47E-05	0.000269283	no
ACOX3	-0.147096933	-3.926179112	9.49E-05	0.000269682	no
LEO1	-0.147072346	-3.925508346	9.51E-05	0.000270363	no
METTL4	0.147071661	3.925489683	9.51E-05	0.000270363	no
ASPDH	-0.147041056	-3.924654731	9.55E-05	0.000271244	no
GCC1	0.147037515	3.924558132	9.55E-05	0.000271304	no
RGAG1	-0.147036265	-3.924524029	9.55E-05	0.000271304	no
ZNF808	0.147035259	3.924496588	9.55E-05	0.000271304	no
PEBP1	-0.146982222	-3.923049729	9.61E-05	0.000272865	no
TSTA3	0.146974618	3.922842299	9.62E-05	0.000273057	no
THSD4	-0.146951348	-3.922207499	9.64E-05	0.000273723	no
PKHD1L1	0.146931172	3.921657098	9.66E-05	0.000274297	no
OXSR1	0.14692824	3.921577127	9.67E-05	0.000274348	no
ABI1	-0.146882949	-3.920341615	9.72E-05	0.000275689	no
CDNF	-0.146871886	-3.92003985	9.73E-05	0.000275988	no
FAM36A	-0.146860984	-3.919742438	9.74E-05	0.000276282	no
MRPS27	-0.146849355	-3.919425218	9.75E-05	0.0002766	no
EIF3F	-0.146833004	-3.918979191	9.77E-05	0.000277062	no
ATP11B	0.14678915	3.917782952	9.82E-05	0.000278371	no
HOXB7	0.14670447	3.915473136	9.91E-05	0.000280952	no
RBM45	0.146700079	3.915353352	9.91E-05	0.000281049	no
BRE	0.146694033	3.915188457	9.92E-05	0.000281198	no
ANKZF1	-0.146681113	-3.914836043	9.93E-05	0.00028156	no
KLRC4	-0.146676075	-3.914698624	9.94E-05	0.000281678	no
PRMT2	0.14666651	3.914437721	9.95E-05	0.000281937	no
RNF19A	0.146598013	3.912569408	0.000100267	0.00028404	no
PPP1R11	-0.146594079	-3.912462121	0.00010031	0.000284124	no
SKIL	0.146555462	3.911408837	0.000100739	0.000285299	no

ST6GALNAC2	0.146509304	3.910149906	0.000101255	0.000286718	no
C17orf42	0.146499487	3.909882147	0.000101364	0.000286952	no
PMS2L2	0.146499386	3.909879397	0.000101366	0.000286952	no
AQP2	0.146485354	3.909496688	0.000101523	0.000287357	no
MIAT	-0.146473653	-3.909177564	0.000101654	0.000287688	no
RERE	-0.146458597	-3.908766937	0.000101823	0.000288126	no
SMG7	-0.146453893	-3.908638632	0.000101876	0.000288236	no
TNFRSF25	0.146436518	3.908164745	0.000102072	0.000288749	no
MRPL37	0.146429247	3.907966462	0.000102154	0.00028894	no
APLP2	0.14642649	3.907891261	0.000102185	0.000288988	no
C6orf48	-0.146410451	-3.907453821	0.000102366	0.00028946	no
ZNF586	0.146401595	3.9072123	0.000102466	0.000289702	no
C12orf74	0.146388103	3.906844321	0.000102619	0.000290094	no
EFHD2	0.146383838	3.906728012	0.000102667	0.00029019	no
RGS7BP	-0.146382547	-3.906692805	0.000102682	0.00029019	no
COG2	-0.146357466	-3.906008787	0.000102967	0.000290954	no
MECP2	-0.146325197	-3.905128738	0.000103334	0.000291951	no
TGM1	-0.146319383	-3.904970189	0.0001034	0.000292098	no
GAS2L1	-0.146302274	-3.904503582	0.000103596	0.000292609	no
C14orf159	-0.146292104	-3.904226231	0.000103712	0.000292897	no
SMAD4	-0.146282026	-3.90395139	0.000103827	0.000293182	no
GRM5	-0.146275084	-3.903762078	0.000103907	0.000293365	no
WDR38	0.146191843	3.901492018	0.000104865	0.000296029	no
GUCY2D	0.146187624	3.901376968	0.000104914	0.000296088	no
COLEC11	0.146187425	3.901371559	0.000104916	0.000296088	no
C21orf33	-0.146186232	-3.901339012	0.00010493	0.000296088	no
ZNF22	-0.146173578	-3.900993945	0.000105076	0.00029646	no
NXPH3	-0.146153247	-3.90043952	0.000105312	0.000297084	no
SMARCD2	0.146144205	3.900192935	0.000105417	0.00029731	no
HEXIM2	-0.146143833	-3.900182798	0.000105422	0.00029731	no
VAV3	0.146118168	3.899482933	0.00010572	0.00029811	no
RBMX	-0.146061106	-3.897926888	0.000106387	0.000299948	no
C22orf45	-0.146052947	-3.897704408	0.000106483	0.000300176	no
ACAT2	-0.146047657	-3.89756016	0.000106545	0.000300292	no
ZNF45	0.146046919	3.897540046	0.000106553	0.000300292	no
MEOX1	0.146002152	3.896319321	0.00010708	0.000301734	no
C10orf32	-0.145975937	-3.895604488	0.000107389	0.000302564	no
TLX1NB	-0.14597455	-3.895566683	0.000107406	0.000302568	no
C15orf26	0.145955368	3.89504362	0.000107633	0.000303166	no
LEPREL2	0.145928128	3.894300862	0.000107956	0.000304029	no
HPCAL4	-0.145926996	-3.894269992	0.00010797	0.000304029	no
CD70	0.145909746	3.893799641	0.000108175	0.000304565	no
SYNJ1	-0.145894375	-3.893380532	0.000108358	0.000305038	no
DOCK5	0.145886202	3.893157663	0.000108455	0.00030527	no
ZNF513	-0.145884383	-3.893108077	0.000108477	0.000305288	no
KCNH7	-0.145786592	-3.890441705	0.00010965	0.000308547	no
PTDSS2	-0.145772201	-3.89004934	0.000109824	0.000308993	no
MYL6B	-0.14574445	-3.889292715	0.00011016	0.000309894	no
TOE1	0.145740858	3.889194775	0.000110203	0.000309949	no
HDAC11	-0.145740309	-3.889179811	0.00011021	0.000309949	no
HMGN4	0.145719353	3.888608444	0.000110464	0.00031062	no

FNDC5	-0.14570618	-3.888249283	0.000110624	0.000311027	no
EPM2A	-0.145686327	-3.887708019	0.000110866	0.000311663	no
ATE1	-0.145678916	-3.887505951	0.000110956	0.000311874	no
SGSM3	-0.145668631	-3.887225541	0.000111081	0.000312183	no
PMVK	-0.145660691	-3.887009059	0.000111178	0.000312412	no
GCNT3	0.145639872	3.88644147	0.000111433	0.000313084	no
SLAIN1	-0.145620464	-3.885912349	0.000111671	0.000313708	no
FAM154B	0.145617519	3.885832063	0.000111707	0.000313766	no
SH3BP4	-0.145612148	-3.885685616	0.000111773	0.000313908	no
SLC12A4	-0.145599624	-3.885344169	0.000111927	0.000314296	no
CTSG	0.145594625	3.885207894	0.000111988	0.000314425	no
OR2A4	0.145589374	3.885064716	0.000112053	0.000314563	no
MEIS2	-0.145579907	-3.884806618	0.000112169	0.000314847	no
REV3L	-0.145569272	-3.884516699	0.0001123	0.000315171	no
CPEB4	-0.145562524	-3.884332726	0.000112384	0.000315361	no
IFRD1	0.14554978	3.883985301	0.000112541	0.000315758	no
THRB	-0.145538756	-3.883684753	0.000112677	0.000316095	no
MAP4K3	-0.145537539	-3.883651573	0.000112692	0.000316095	no
SFRS9	0.145532912	3.883525427	0.00011275	0.000316212	no
ITGAD	0.145517431	3.883103391	0.000112941	0.000316706	no
ANKRD11	-0.145501529	-3.882669868	0.000113139	0.000317215	no
ANGPTL2	-0.145451353	-3.881301976	0.000113763	0.000318923	no
PRSS21	0.145443987	3.881101178	0.000113855	0.000319136	no
EIF1AX	-0.14542547	-3.880596375	0.000114087	0.000319741	no
TRIM67	-0.145406376	-3.880075868	0.000114326	0.000320367	no
TAF15	-0.145386052	-3.87952182	0.000114581	0.000321037	no
CD99	0.145384143	3.879469769	0.000114605	0.00032106	no
PPIF	-0.145368225	-3.879035843	0.000114806	0.000321577	no
CTU2	-0.145354698	-3.878667103	0.000114976	0.000322009	no
PIAS3	0.145344436	3.878387351	0.000115106	0.000322327	no
GTF2H2C	0.145330312	3.878002337	0.000115284	0.000322782	no
CDK9	-0.145294098	-3.877015151	0.000115743	0.000324021	no
VMAC	0.145287932	3.876847075	0.000115821	0.000324196	no
CHKA	-0.145260523	-3.876099924	0.000116169	0.000325126	no
C6orf72	0.145247779	3.875752558	0.000116332	0.000325535	no
TP53BP2	-0.145245919	-3.875701834	0.000116355	0.000325557	no
KIAA1731	-0.145243271	-3.875629677	0.000116389	0.000325566	no
ZMYND10	0.145243136	3.875625972	0.000116391	0.000325566	no
STUB1	-0.145234671	-3.875395232	0.000116499	0.000325823	no
UFSP1	0.145229331	3.87524967	0.000116567	0.000325968	no
NGF	0.145200085	3.874452493	0.000116941	0.000326969	no
UNC50	0.145183547	3.874001696	0.000117153	0.000327517	no
TRAK2	0.145182288	3.873967358	0.000117169	0.000327517	no
DARS	-0.145151317	-3.873123158	0.000117567	0.000328584	no
TACR2	0.145139728	3.872807269	0.000117717	0.000328956	no
LOC388955	0.145117071	3.872189708	0.000118009	0.000329728	no
DEPDC1	0.145038536	3.870049103	0.000119028	0.000332528	no
METTL3	-0.145035577	-3.869968454	0.000119067	0.00033259	no
TRIM46	-0.144992964	-3.868806997	0.000119623	0.000334098	no
DNAJC2	0.14498275	3.86852859	0.000119757	0.000334426	no
KIF22	-0.144949911	-3.867633548	0.000120188	0.000335583	no

ITGA11	0.144911978	3.866599696	0.000120687	0.000336931	no
SFXN4	-0.144885043	-3.865865588	0.000121043	0.000337878	no
LOC642826	-0.144839531	-3.864625211	0.000121647	0.000339517	no
ARHGAP19	-0.144837042	-3.864557381	0.00012168	0.000339538	no
DHX33	-0.144836438	-3.864540907	0.000121688	0.000339538	no
LOC100129534	0.144833214	3.864453045	0.000121731	0.00033961	no
BDP1	-0.144767302	-3.862656719	0.000122611	0.000342018	no
G6PC3	0.144757978	3.862402601	0.000122736	0.000342319	no
CAMK1D	-0.144743192	-3.861999655	0.000122934	0.000342825	no
CSRP1	0.144735383	3.861786842	0.000123039	0.00034307	no
CYTH1	-0.144708639	-3.861057992	0.000123399	0.000344027	no
FMOD	0.144692709	3.860623877	0.000123614	0.000344552	no
RBM3	0.144692125	3.860607952	0.000123622	0.000344552	no
B3GALNT2	0.144671441	3.860044261	0.000123901	0.000345284	no
HAUS1	0.144660088	3.859734885	0.000124055	0.000345664	no
C8orf51	0.144653953	3.859567687	0.000124138	0.000345848	no
FMNL2	-0.144641318	-3.859223377	0.000124309	0.000346278	no
ADAM20	-0.144638654	-3.859150776	0.000124345	0.000346331	no
AARS2	-0.144609222	-3.858348717	0.000124746	0.000347397	no
ACBD3	0.144605529	3.858248068	0.000124796	0.00034749	no
SLC22A4	0.144586327	3.857724809	0.000125058	0.00034817	no
ADAM11	-0.144581992	-3.857606668	0.000125117	0.000348287	no
KCNC2	-0.144539945	-3.856460878	0.000125692	0.00034984	no
STMN2	-0.144532819	-3.856266694	0.00012579	0.000350024	no
SAA4	0.144532613	3.856261073	0.000125793	0.000350024	no
NAB2	0.144489773	3.85509368	0.000126382	0.000351615	no
ITIH2	0.144479648	3.854817795	0.000126521	0.000351955	no
SDHD	0.144469857	3.854550975	0.000126656	0.000352282	no
TMEM132C	-0.144460541	-3.854297122	0.000126785	0.000352586	no
GCKR	0.144459426	3.854266756	0.000126801	0.000352586	no
FOXD4	-0.144423373	-3.853284339	0.0001273	0.000353926	no
TET2	-0.144409459	-3.852905208	0.000127493	0.000354414	no
PLEKHG6	0.144371422	3.851868758	0.000128023	0.000355838	no
KCNT1	-0.144352581	-3.851355364	0.000128286	0.00035652	no
NACC1	-0.144329048	-3.850714139	0.000128615	0.000357386	no
LOC84989	-0.144321504	-3.85050859	0.000128721	0.000357631	no
OR4F15	0.14428738	3.849578812	0.0001292	0.000358913	no
ZNF33B	-0.144276376	-3.849278976	0.000129355	0.000359294	no
H3F3B	-0.144270227	-3.849111432	0.000129442	0.000359485	no
TOX4	-0.144263363	-3.848924397	0.000129538	0.000359705	no
ITIH5	0.144244919	3.848421867	0.000129799	0.000360378	no
TLE4	0.144224973	3.847878399	0.000130081	0.000361112	no
SENP6	-0.144223162	-3.84782907	0.000130107	0.000361134	no
PRTFDC1	-0.144213931	-3.847577557	0.000130237	0.000361447	no
VPS54	0.144193117	3.847010463	0.000130533	0.000362217	no
CX3CL1	-0.144138721	-3.845528393	0.000131308	0.000364318	no
TECPR1	-0.14413627	-3.845461609	0.000131343	0.000364365	no
IL1B	0.144134461	3.84541233	0.000131369	0.000364386	no
ACADM	0.144115602	3.844898506	0.000131639	0.000365085	no
POLD3	0.144102663	3.84454598	0.000131824	0.000365549	no
ANGPT2	0.144071102	3.843686101	0.000132277	0.000366755	no

CYP2D7P1	-0.144064088	-3.843495014	0.000132378	0.000366985	no
EXOC3	-0.144046966	-3.84302854	0.000132625	0.000367618	no
EPS8L2	0.144039785	3.842832906	0.000132728	0.000367855	no
ZNF668	-0.143994835	-3.84160828	0.000133379	0.000369606	no
TUFT1	0.143980437	3.841216023	0.000133588	0.000370135	no
METTL8	0.143975903	3.841092505	0.000133653	0.000370266	no
STAT5B	-0.143971522	-3.840973154	0.000133717	0.000370392	no
MAPK1	-0.143945336	-3.840259762	0.000134098	0.000371397	no
KIF5B	-0.143940524	-3.840128679	0.000134168	0.00037154	no
PCDP1	0.143913593	3.839394989	0.000134561	0.000372578	no
MPPED2	-0.143903661	-3.839124416	0.000134707	0.000372929	no
TAS2R5	-0.143878469	-3.838438134	0.000135076	0.000373899	no
LOC643008	0.143833284	3.837207194	0.00013574	0.000375687	no
ZFAND1	0.14383046	3.837130278	0.000135782	0.000375738	no
C14orf102	-0.143829526	-3.837104814	0.000135796	0.000375738	no
LOC100216001	0.143817436	3.836775483	0.000135974	0.00037618	no
IFNB1	0.143788689	3.835992357	0.000136399	0.000377304	no
RINT1	0.143785565	3.835907273	0.000136445	0.00037738	no
PRMT8	-0.143763199	-3.835297978	0.000136777	0.000378229	no
MTSS1	-0.143762339	-3.835274551	0.00013679	0.000378229	no
USPL1	-0.143750486	-3.834951686	0.000136966	0.000378664	no
GADD45G	-0.143701018	-3.83360414	0.000137703	0.00038065	no
STOM	0.143689538	3.833291427	0.000137875	0.000381073	no
PYGO1	-0.143670635	-3.832776523	0.000138158	0.000381803	no
SERAC1	0.143662367	3.832551308	0.000138281	0.000382093	no
DNTTIP2	0.143655305	3.832358929	0.000138387	0.000382334	no
GLDC	-0.143642701	-3.832015604	0.000138577	0.000382804	no
FLJ42709	-0.143629086	-3.831644735	0.000138781	0.000383317	no
FAM183B	0.14360676	3.831036609	0.000139118	0.000384194	no
KAZ	-0.143567001	-3.829953621	0.000139719	0.000385801	no
ZBED5	-0.143561791	-3.829811703	0.000139798	0.000385966	no
DDX43	0.143557073	3.829683185	0.000139869	0.000386111	no
DNAJC14	0.143553107	3.829575154	0.000139929	0.000386224	no
MOGS	0.143541835	3.82926812	0.0001401	0.000386643	no
TMEM14C	0.14353682	3.829131522	0.000140177	0.000386801	no
MYH14	-0.143513435	-3.828494558	0.000140532	0.000387729	no
FAM73B	-0.14349697	-3.828046087	0.000140783	0.000388369	no
HSP90AB1	-0.143482867	-3.827661966	0.000140998	0.00038891	no
USP40	0.143469104	3.827287086	0.000141209	0.000389437	no
PCDHGC4	-0.143434075	-3.82633301	0.000141746	0.000390864	no
CLCNKA	0.143413373	3.825769161	0.000142064	0.000391688	no
C15orf41	0.143398507	3.825364259	0.000142293	0.000392265	no
PET112L	-0.143379445	-3.82484507	0.000142587	0.000393022	no
ITK	0.143370559	3.824603038	0.000142724	0.000393347	no
TMEM5	0.143364057	3.824425969	0.000142824	0.00039357	no
SDPR	-0.143344909	-3.823904448	0.000143121	0.000394333	no
HBXIP	0.143300491	3.822694695	0.00014381	0.000396179	no
EIF4A2	-0.143180158	-3.819417475	0.000145694	0.000401314	no
ATG5	0.143105463	3.817383251	0.000146875	0.000404513	no
CALML6	0.143084901	3.816823292	0.000147202	0.000405357	no
EXOC2	-0.143048438	-3.81583033	0.000147783	0.000406902	no

PRKAR1B	-0.143044921	-3.815734531	0.000147839	0.000407001	no
TMEM50B	0.143006813	3.814696796	0.000148449	0.000408624	no
C1orf203	0.142987748	3.814177598	0.000148755	0.000409395	no
CCDC71	0.142986848	3.814153109	0.00014877	0.000409395	no
CALM3	-0.142975037	-3.813831468	0.000148959	0.000409862	no
MGC15885	0.142952017	3.813204613	0.00014933	0.000410826	no
NCL	-0.142928281	-3.812558252	0.000149713	0.000411824	no
DMXL2	-0.14290352	-3.811883982	0.000150114	0.000412869	no
C1orf183	-0.14289216	-3.811574656	0.000150298	0.00041332	no
TRMT5	-0.14288102	-3.811271315	0.000150479	0.000413761	no
ARHGEF11	-0.142863073	-3.810782611	0.000150771	0.000414506	no
HHLA3	0.142849248	3.810406149	0.000150996	0.000415069	no
KCNAB3	-0.142810773	-3.809358485	0.000151624	0.000416738	no
NCRNA00087	-0.142797783	-3.809004758	0.000151836	0.000417266	no
SCAND2	-0.142791453	-3.808832415	0.00015194	0.000417494	no
PXDNL	0.142788235	3.808744789	0.000151993	0.000417582	no
IER3IP1	0.14277891	3.808490859	0.000152146	0.000417946	no
C14orf129	0.142738252	3.80738381	0.000152814	0.000419725	no
DYDC1	0.142734817	3.807290266	0.000152871	0.000419823	no
LOC202781	-0.142733215	-3.807246647	0.000152897	0.000419839	no
MAP3K11	0.142690924	3.806095124	0.000153596	0.0004217	no
IFT122	0.14266472	3.805381649	0.00015403	0.000422835	no
C4orf47	0.142656772	3.805165249	0.000154162	0.00042314	no
GABARAPL2	-0.142639487	-3.804694608	0.000154449	0.000423871	no
PRRC1	0.142617936	3.804107848	0.000154808	0.000424799	no
ASAH2B	-0.142593849	-3.80345202	0.000155211	0.000425844	no
ERCC3	-0.142554038	-3.802368111	0.000155877	0.000427575	no
C1orf74	0.142553677	3.802358281	0.000155883	0.000427575	no
DKK2	0.142546339	3.802158497	0.000156007	0.000427855	no
SNAPC3	-0.142542002	-3.802040404	0.00015608	0.000427996	no
CBX7	-0.142536258	-3.801884019	0.000156176	0.000428203	no
C20orf111	0.142533856	3.801818612	0.000156217	0.000428256	no
ANKS1B	-0.142528895	-3.801683564	0.0001563	0.000428426	no
ZNF541	0.14251991	3.801438926	0.000156451	0.000428783	no
PKN1	-0.142511563	-3.801211681	0.000156592	0.00042911	no
ZNF98	-0.142486736	-3.800535742	0.000157011	0.0004302	no
BCAP29	0.142477718	3.800290214	0.000157163	0.00043056	no
SDK1	0.142475947	3.800242001	0.000157193	0.000430583	no
KAT2B	-0.142463117	-3.799892695	0.000157411	0.00043112	no
SPRYD3	-0.142418186	-3.798669443	0.000158173	0.000433151	no
DIRAS1	-0.142390927	-3.797927333	0.000158638	0.000434364	no
STXBP5L	-0.142375219	-3.79749969	0.000158906	0.000435039	no
LOC145845	0.142355987	3.796976103	0.000159235	0.000435881	no
PEMT	-0.142315941	-3.795885897	0.000159922	0.000437703	no
LCAT	-0.142306909	-3.795640012	0.000160078	0.000438069	no
WFDC8	0.142302575	3.795522027	0.000160152	0.000438214	no
PDCL3	0.142294782	3.795309894	0.000160287	0.000438521	no
CSMD2	-0.14227322	-3.794722901	0.000160658	0.000439479	no
C1orf35	-0.142264164	-3.79447636	0.000160815	0.000439848	no
LOC100268168	-0.142257533	-3.794295846	0.00016093	0.000440102	no
GDE1	0.142250734	3.794110745	0.000161047	0.000440364	no

UBLCP1	0.142240588	3.793834568	0.000161223	0.000440785	no
SEPX1	0.142226974	3.793463941	0.000161459	0.000441371	no
KCTD4	-0.14221809	-3.793222104	0.000161613	0.000441732	no
RNASEH2C	-0.142202622	-3.792801038	0.000161882	0.000442407	no
SYMPK	-0.142193377	-3.792549345	0.000162043	0.000442787	no
LOC440944	-0.14219026	-3.792464492	0.000162097	0.000442875	no
ADD3	-0.14218313	-3.792270417	0.000162221	0.000443155	no
C20orf152	-0.142175004	-3.792049199	0.000162363	0.000443482	no
BEST4	0.142166726	3.791823858	0.000162507	0.000443816	no
C16orf72	0.14215817	3.791590947	0.000162657	0.000444164	no
LOC158696	-0.142134159	-3.790937324	0.000163077	0.000445251	no
CDH6	0.142130793	3.790845702	0.000163136	0.000445352	no
GJA9	0.142125528	3.790702375	0.000163228	0.000445544	no
PHKA2	0.142108061	3.790226916	0.000163534	0.00044632	no
TRIM2	-0.142081683	-3.789508867	0.000163998	0.000447525	no
PRH2	-0.14207223	-3.789251548	0.000164165	0.000447919	no
NSMCE2	0.142067855	3.789132466	0.000164242	0.000448069	no
ATP2B3	-0.142042601	-3.788445044	0.000164687	0.000449224	no
GABARAPL1	-0.142035136	-3.788241845	0.000164819	0.000449524	no
TAS1R1	0.142019658	3.787820512	0.000165093	0.00045021	no
MSRA	0.142016546	3.787735804	0.000165149	0.0004503	no
DDR1	-0.142012604	-3.787628523	0.000165218	0.00045043	no
NCRNA00086	-0.141998081	-3.787233211	0.000165476	0.000451071	no
TUBB4	-0.141994439	-3.787134073	0.000165541	0.000451187	no
FASTKD2	0.141973073	3.786552501	0.000165921	0.000452161	no
SPINLW1	0.141971682	3.786514629	0.000165945	0.000452168	no
C5orf28	0.141954367	3.786043309	0.000166254	0.000452947	no
ALOXE3	-0.141952391	-3.785989542	0.000166289	0.000452963	no
CCDC144A	-0.141951533	-3.785966194	0.000166304	0.000452963	no
B3GNT7	0.141929631	3.785370026	0.000166696	0.000453967	no
FAM23A	0.141910751	3.784856145	0.000167033	0.000454826	no
PHACTR4	0.14189904	3.784537384	0.000167243	0.000455336	no
ZNF692	-0.141827766	-3.782597421	0.000168526	0.000458766	no
TXN2	-0.141810901	-3.782138394	0.000168831	0.000459534	no
FAM116A	0.141790195	3.781574818	0.000169206	0.000460493	no
WDR13	-0.141782816	-3.781373993	0.00016934	0.000460795	no
OR5K2	-0.141771807	-3.781074348	0.000169539	0.000461277	no
CTNNA3	-0.141769195	-3.781003252	0.000169587	0.000461344	no
C6orf154	-0.141761957	-3.780806265	0.000169718	0.000461639	no
FGF13	-0.141758933	-3.780723962	0.000169773	0.000461727	no
TAF10	0.141733532	3.780032627	0.000170236	0.000462923	no
GBA2	-0.141703531	-3.779216095	0.000170784	0.00046435	no
LTB4R2	0.141697736	3.779058377	0.00017089	0.000464576	no
RTDR1	0.141684122	3.778687871	0.000171139	0.000465191	no
PLCH2	-0.141657292	-3.777957667	0.000171631	0.000466466	no
CHADL	-0.1416189	-3.776912807	0.000172338	0.000468324	no
TSR2	-0.141603585	-3.776495982	0.000172621	0.000469029	no
GLTSCR2	-0.141589678	-3.776117508	0.000172878	0.000469664	no
LOC493754	0.141584224	3.775969074	0.000172979	0.000469876	no
THAP2	-0.14157234	-3.775645658	0.000173199	0.00047041	no
RAB11FIP2	-0.141533989	-3.774601961	0.000173911	0.000472281	no

ZDHHC6	0.141521698	3.77426746	0.00017414	0.000472838	no
AKAP14	0.141456716	3.772499044	0.000175354	0.000476021	no
OR51E2	0.141456451	3.772491837	0.000175359	0.000476021	no
G6PC	-0.141448531	-3.772276313	0.000175507	0.00047636	no
EN1	0.141422662	3.771572327	0.000175993	0.000477615	no
LRRC7	-0.141402328	-3.771018982	0.000176376	0.00047859	no
DLL4	0.141400238	3.770962099	0.000176416	0.000478633	no
GGH	0.141394736	3.770812389	0.000176519	0.00047885	no
UBL7	-0.141391013	-3.770711073	0.00017659	0.000478976	no
EEFSEC	-0.141383998	-3.770520181	0.000176722	0.000479271	no
SLC5A3	0.141355507	3.769744855	0.000177261	0.000480668	no
GMPS	0.141350062	3.769596688	0.000177364	0.000480883	no
DDX20	0.141341948	3.769375883	0.000177518	0.000481235	no
GPR113	-0.141314793	-3.76863695	0.000178033	0.000482569	no
GRM1	-0.141282679	-3.767763065	0.000178645	0.00048415	no
KIN	-0.141281656	-3.767735248	0.000178665	0.00048415	no
PRPF8	-0.141257445	-3.767076416	0.000179127	0.000485338	no
FGF14	-0.141246084	-3.766767282	0.000179344	0.000485862	no
DGKG	-0.141218901	-3.766027619	0.000179866	0.000487208	no
SIRT4	-0.141204871	-3.765645833	0.000180135	0.000487831	no
CLPX	-0.141204419	-3.765633536	0.000180144	0.000487831	no
STK38L	0.141199714	3.765505531	0.000180234	0.000488011	no
NSUN3	0.141193316	3.765331439	0.000180357	0.000488279	no
MTMR4	-0.141169473	-3.764682665	0.000180817	0.000489457	no
CCDC85A	-0.141138473	-3.763839148	0.000181416	0.000491013	no
IK	-0.14113487	-3.763741126	0.000181486	0.000491136	no
NAB1	-0.14112293	-3.763416224	0.000181717	0.000491696	no
CPSF1	-0.141107613	-3.762999452	0.000182014	0.000492434	no
SCMH1	0.141100883	3.76281634	0.000182145	0.000492722	no
ZBTB8A	-0.141095223	-3.76266235	0.000182255	0.000492954	no
C9orf171	0.141083738	3.762349847	0.000182478	0.000493492	no
FRAT1	-0.141081625	-3.762292362	0.000182519	0.000493496	no
LMCD1	0.141081155	3.762279566	0.000182528	0.000493496	no
RAPGEF3	-0.141059207	-3.761682373	0.000182956	0.000494586	no
MBOAT7	0.141056014	3.761595492	0.000183018	0.000494688	no
ELF5	0.14103487	3.761020206	0.000183432	0.000495738	no
TTC23	0.141028804	3.76085515	0.00018355	0.000495993	no
KIAA1586	-0.140988783	-3.759766258	0.000184335	0.000498047	no
NTNG1	0.140962691	3.759056349	0.000184848	0.000499367	no
RFPL1S	-0.140948635	-3.758673908	0.000185125	0.000500048	no
BCO2	0.140938941	3.758410162	0.000185317	0.000500498	no
EFHC1	0.140921991	3.757949019	0.000185651	0.000501336	no
CACNA2D3	-0.140919403	-3.757878602	0.000185703	0.000501367	no
PA2G4P4	0.140918891	3.75786467	0.000185713	0.000501367	no
OR4A15	0.140912347	3.757686617	0.000185842	0.00050165	no
CBFB	0.140906789	3.757535408	0.000185952	0.00050188	no
SPAG7	-0.140902718	-3.757424637	0.000186033	0.000502031	no
CAMTA2	-0.140893605	-3.757176708	0.000186214	0.000502451	no
PGAP1	-0.140890191	-3.757083831	0.000186281	0.000502567	no
NDST4	-0.140860382	-3.756272836	0.000186874	0.000504098	no
C1orf43	0.140840929	3.755743581	0.000187261	0.000505076	no

HOXB2	0.140834997	3.755582191	0.000187379	0.000505327	no
STXBP6	-0.140811615	-3.754946052	0.000187846	0.000506519	no
ARHGEF10L	-0.140785022	-3.754222573	0.000188379	0.000507887	no
ESYT2	0.140779814	3.75408089	0.000188483	0.000508101	no
PDGFA	0.14077654	3.753991815	0.000188549	0.00050821	no
FAM113A	-0.140765504	-3.753691573	0.000188771	0.00050874	no
ZNF428	-0.140757539	-3.753474898	0.000188931	0.000509103	no
ABI3BP	0.140719735	3.752446442	0.000189692	0.000511087	no
POR	0.140714963	3.752316608	0.000189789	0.000511279	no
CHD4	-0.140695539	-3.751788194	0.000190181	0.000512268	no
BTBD12	-0.140684512	-3.751488201	0.000190404	0.000512801	no
CRLF2	0.140663981	3.750929663	0.000190821	0.000513853	no
C1QTNF9B	0.14058196	3.748698398	0.000192492	0.000518285	no
NEURL	-0.140567467	-3.748304145	0.000192789	0.000519015	no
TRMT1	-0.140543021	-3.747639157	0.00019329	0.000520296	no
WDTC1	0.140499499	3.746455239	0.000194186	0.000522637	no
GPR107	0.140496053	3.746361508	0.000194257	0.000522759	no
WBP11	-0.140491718	-3.746243578	0.000194347	0.00052293	no
NEDD9	0.140489408	3.74618075	0.000194394	0.000522989	no
SEMA3D	-0.140487844	-3.746138196	0.000194427	0.000523003	no
LOC149837	0.140486655	3.746105852	0.000194451	0.000523003	no
CRABP2	0.140481429	3.745963687	0.000194559	0.000523224	no
FN3KRP	-0.140412399	-3.744085982	0.000195991	0.000527003	no
MAPK9	-0.14040867	-3.743984535	0.000196068	0.000527142	no
RAB26	-0.140406429	-3.743923571	0.000196115	0.000527197	no
TPRN	-0.140387754	-3.743415605	0.000196504	0.000528173	no
SH3BP5L	-0.140343384	-3.742208701	0.000197432	0.000530596	no
GDPD2	0.140322403	3.741638012	0.000197872	0.000531708	no
C12orf61	0.140315984	3.74146342	0.000198007	0.000532	no
POC5	0.140312951	3.74138092	0.00019807	0.0005321	no
ERGIC3	0.14030053	3.741043081	0.000198331	0.000532731	no
FDPS	-0.140289086	-3.740731787	0.000198572	0.000533282	no
PDZD7	-0.140288278	-3.740709816	0.000198589	0.000533282	no
RPH3A	-0.140278928	-3.740455493	0.000198787	0.00053374	no
MTF1	0.140275645	3.740366213	0.000198856	0.000533855	no
SGK494	-0.140256285	-3.73983963	0.000199265	0.000534882	no
GK5	-0.140232943	-3.739204728	0.000199758	0.000536136	no
SPINK8	0.140216727	3.738763668	0.000200102	0.000536988	no
MRPS18C	0.140207669	3.738517307	0.000200294	0.000537432	no
FNTB	0.140203749	3.738410693	0.000200378	0.000537585	no
TTC16	0.140167844	3.737434123	0.000201142	0.000539563	no
UNC13C	-0.140165158	-3.737361072	0.000201199	0.000539645	no
KPNA2	0.14014763	3.736884351	0.000201573	0.000540577	no
RWDD2B	0.140141116	3.736707167	0.000201713	0.000540879	no
FUT6	-0.140128327	-3.736359329	0.000201986	0.00054154	no
STMN1	-0.140111925	-3.735913231	0.000202337	0.00054241	no
MYADML2	-0.140090189	-3.735322082	0.000202804	0.000543589	no
GCOM1	0.140055004	3.73436515	0.000203561	0.000545546	no
KCNB2	-0.140050833	-3.734251699	0.000203651	0.000545655	no
NT5C1A	-0.140050623	-3.734245998	0.000203656	0.000545655	no
ERGIC2	0.140045758	3.734113661	0.000203761	0.000545864	no

PRDX2	-0.140010449	-3.733153393	0.000204524	0.000547836	no
ASPSCR1	-0.139994953	-3.73273194	0.00020486	0.000548663	no
HADHB	-0.139989492	-3.732583437	0.000204978	0.000548908	no
CLDN4	0.139969812	3.732048203	0.000205406	0.00054998	no
C7orf63	0.13995115	3.731540669	0.000205812	0.000550994	no
IL9	-0.139946313	-3.731409138	0.000205918	0.000551204	no
UBE2W	0.139932631	3.731037043	0.000206216	0.000551929	no
MED12	-0.139921683	-3.7307393	0.000206455	0.000552496	no
C5orf49	0.139916563	3.730600058	0.000206567	0.000552722	no
EHHADH	0.139911881	3.730472737	0.000206669	0.000552923	no
LOC729156	0.139841641	3.72856257	0.000208211	0.000556973	no
NOXO1	-0.139758688	-3.726306727	0.000210045	0.000561806	no
MKRN2	-0.139754831	-3.72620183	0.000210131	0.000561961	no
COBLL1	0.139725663	3.725408658	0.00021078	0.000563622	no
GP6	-0.139709515	-3.724969549	0.00021114	0.000564443	no
SPRY4	0.139708867	3.724951921	0.000211154	0.000564443	no
SOX3	-0.139708136	-3.724932055	0.00021117	0.000564443	no
RBM4	-0.13970264	-3.724782595	0.000211293	0.000564696	no
GAS2L3	0.13967169	3.72394098	0.000211985	0.000566471	no
NCBP2	-0.139664227	-3.723738036	0.000212152	0.000566803	no
ANKRD42	0.139663634	3.723721917	0.000212166	0.000566803	no
ATP6AP1	0.13965281	3.723427598	0.000212408	0.000567377	no
GALNT14	-0.139649791	-3.723345493	0.000212476	0.000567483	no
GDF5	0.139645723	3.723234872	0.000212568	0.000567652	no
UBE2D1	0.139596781	3.721904045	0.000213669	0.000570518	no
PPHLN1	0.139541837	3.720410038	0.000214912	0.000573762	no
YY1	0.139512672	3.719617025	0.000215575	0.000575455	no
SCN8A	-0.139503456	-3.719366436	0.000215785	0.000575938	no
GOLGA2B	0.13950112	3.719302917	0.000215838	0.000576004	no
IFT140	-0.139481019	-3.718756369	0.000216296	0.000577151	no
BCKDHA	-0.139469795	-3.718451185	0.000216552	0.000577759	no
LONP1	-0.139463706	-3.718285602	0.000216692	0.000577983	no
KDM6B	-0.139463625	-3.718283423	0.000216693	0.000577983	no
ZMYND17	-0.139455758	-3.718069514	0.000216873	0.000578386	no
HADHA	-0.139405356	-3.716699075	0.00021803	0.000581393	no
DND1	-0.139398022	-3.716499691	0.000218198	0.000581766	no
KCNAB1	-0.139368412	-3.715694613	0.000218881	0.000583508	no
DNAJB9	0.139338729	3.714887553	0.000219567	0.000585246	no
STON2	-0.139337709	-3.714859841	0.00021959	0.000585246	no
LY6K	-0.13932896	-3.714621948	0.000219793	0.000585709	no
DMRTC1	-0.139319611	-3.714367779	0.00022001	0.000586209	no
GLI3	0.139301815	3.713883921	0.000220423	0.000587232	no
PRKY	0.139293176	3.713649044	0.000220623	0.000587689	no
SH3BP5	0.139280277	3.713298355	0.000220923	0.000588411	no
ERCC5	-0.139277934	-3.71323464	0.000220978	0.000588479	no
PTH1R	-0.13927486	-3.713151058	0.00022105	0.000588592	no
ATOH7	-0.139271962	-3.713072285	0.000221117	0.000588694	no
MLXIPL	0.139221275	3.71169422	0.000222301	0.000591737	no
KIAA1109	-0.139220524	-3.711673806	0.000222319	0.000591737	no
CAPNS2	0.139212505	3.711455795	0.000222506	0.00059216	no
XIRP2	0.139205793	3.71127331	0.000222664	0.0005925	no

F7	-0.139190236	-3.710850363	0.000223029	0.000593394	no
NRF1	-0.139182573	-3.710642031	0.000223209	0.000593795	no
GPR1	0.139179947	3.710570621	0.000223271	0.000593881	no
ST3GAL5	-0.139168287	-3.710253634	0.000223545	0.000594466	no
NAP1L4	-0.139168089	-3.710248258	0.00022355	0.000594466	no
PLCL1	-0.139132899	-3.709291563	0.000224379	0.000596595	no
PIGY	0.139124698	3.709068584	0.000224573	0.000597031	no
TMX4	0.139102424	3.708463051	0.0002251	0.000598354	no
DKFZp761E198	0.139094361	3.708243849	0.000225292	0.000598783	no
MED13L	-0.139087048	-3.708045035	0.000225465	0.000599166	no
CACNG1	-0.139076493	-3.707758105	0.000225716	0.000599753	no
DUSP4	0.139066813	3.707494955	0.000225946	0.000600285	no
DLGAP2	-0.139042103	-3.706823184	0.000226534	0.000601769	no
PSMB4	0.139033708	3.706594961	0.000226734	0.000602221	no
C22orf32	-0.139010825	-3.705972883	0.00022728	0.000603593	no
TYSND1	-0.139003144	-3.705764089	0.000227464	0.000604002	no
PAPPA	0.13899219	3.705466311	0.000227726	0.000604619	no
FGF1	0.138947196	3.70424318	0.000228806	0.000607407	no
DMP1	0.138940431	3.704059276	0.000228969	0.000607759	no
SLC45A1	-0.13892846	-3.703733852	0.000229258	0.000608444	no
ZFYVE16	-0.138896499	-3.702865032	0.000230029	0.000610412	no
C20orf20	0.138873839	3.702249059	0.000230578	0.000611787	no
PAX6	-0.138840373	-3.701339332	0.00023139	0.000613862	no
LNPEP	0.13881968	3.700776846	0.000231893	0.000615117	no
CLTB	-0.138816928	-3.700702032	0.000231961	0.000615214	no
SLCO2A1	0.138774171	3.699539807	0.000233005	0.000617902	no
URGCP	-0.138766833	-3.699340333	0.000233184	0.000618298	no
NUCB2	0.138759704	3.699146558	0.000233359	0.000618679	no
YKT6	0.138742434	3.69867711	0.000233782	0.000619721	no
ABCC4	0.138739479	3.698596806	0.000233855	0.000619832	no
PCDHB10	-0.138715419	-3.697942803	0.000234446	0.000621318	no
ADSSL1	0.138703542	3.697619973	0.000234739	0.000621973	no
LOC388692	0.138702894	3.697602358	0.000234755	0.000621973	no
HTT	-0.138686496	-3.697156652	0.000235159	0.000622963	no
TIPARP	0.138670748	3.696728599	0.000235548	0.000623911	no
UHRF2	0.138648633	3.696127486	0.000236096	0.000625279	no
JUB	0.138629625	3.695610846	0.000236567	0.000626445	no
FEM1B	-0.138525125	-3.692770546	0.000239174	0.000633266	no
OVCA2	0.138497936	3.69203157	0.000239857	0.000634989	no
RNASE10	0.138496712	3.691998292	0.000239888	0.000634989	no
RING1	-0.138495456	-3.69196417	0.000239919	0.00063499	no
PGLYRP1	0.138492677	3.691888644	0.000239989	0.000635068	no
TK1	0.138491779	3.691864217	0.000240012	0.000635068	no
C9orf172	-0.13848919	-3.691793864	0.000240077	0.000635157	no
ATP6V1F	0.138466227	3.691169762	0.000240655	0.000636605	no
C6orf81	-0.138442956	-3.690537288	0.000241243	0.000638076	no
SMURF1	0.13842401	3.690022364	0.000241722	0.00063926	no
CYP7A1	0.138414423	3.689761812	0.000241965	0.000639819	no
TIPIN	0.138401933	3.689422364	0.000242282	0.000640537	no
NAGPA	0.13840081	3.689391837	0.000242311	0.000640537	no
GATSL2	-0.138399973	-3.689369095	0.000242332	0.000640537	no

PANX2	-0.138386356	-3.688998999	0.000242678	0.000641367	no
GALE	0.138381774	3.688874466	0.000242794	0.000641537	no
ZBTB17	-0.138381326	-3.68886229	0.000242806	0.000641537	no
TFPI2	0.138362371	3.688347141	0.000243288	0.000642728	no
KIAA0513	-0.138358383	-3.688238772	0.00024339	0.000642913	no
ZNF300	-0.138330394	-3.687478096	0.000244104	0.000644716	no
CCDC30	0.138324915	3.687329188	0.000244244	0.000644918	no
HOOK3	-0.1383249	-3.687328794	0.000244245	0.000644918	no
CENPJ	-0.138321992	-3.687249764	0.000244319	0.00064503	no
EIF2A	-0.138319987	-3.687195268	0.00024437	0.000645082	no
PDPK1	-0.138310532	-3.686938308	0.000244612	0.000645636	no
SEC63	-0.138280111	-3.686111581	0.000245393	0.000647611	no
KIF5A	-0.138277276	-3.686034535	0.000245465	0.000647719	no
GRB10	0.138257691	3.685502285	0.000245969	0.000648963	no
C7orf52	-0.138211075	-3.684235448	0.000247172	0.000652051	no
PPM1H	-0.138180684	-3.68340954	0.000247959	0.000654043	no
ZFPM2	-0.138162847	-3.682924823	0.000248422	0.000655178	no
SLC39A14	0.138160374	3.682857625	0.000248486	0.000655262	no
PAQR6	-0.13812704	-3.681951772	0.000249354	0.000657465	no
LY6G5B	-0.138122981	-3.681841464	0.00024946	0.000657658	no
SLC36A4	0.138114146	3.68160139	0.000249691	0.00065818	no
DGKQ	-0.13811191	-3.681540607	0.000249749	0.000658248	no
DLL1	-0.138102478	-3.681284301	0.000249995	0.000658812	no
SLC4A10	-0.138097789	-3.681156875	0.000250118	0.000659049	no
JMJD5	-0.138092847	-3.681022597	0.000250247	0.000659304	no
SSBP2	0.138043924	3.679693155	0.00025153	0.000662598	no
NXT1	0.1380169	3.678958808	0.000252241	0.000664385	no
ARMC3	0.138000115	3.678502709	0.000252684	0.000665464	no
BBS1	-0.13799465	-3.678354222	0.000252829	0.000665758	no
DIRAS2	-0.13797611	-3.677850425	0.000253319	0.000666962	no
CCDC160	-0.137960379	-3.677422958	0.000253735	0.000667971	no
SYT16	-0.137904389	-3.675901582	0.000255223	0.000671801	no
PPP3CA	-0.137897682	-3.675719324	0.000255402	0.000672184	no
FLOT2	0.137841016	3.674179612	0.000256917	0.000676084	no
EIF4E	0.137832615	3.673951335	0.000257143	0.000676589	no
ESRP2	0.137818248	3.67356098	0.000257528	0.000677516	no
C19orf51	0.137797943	3.673009272	0.000258075	0.000678865	no
PRDM8	-0.137782126	-3.672579495	0.000258501	0.000679897	no
INTS1	-0.137769716	-3.672242322	0.000258836	0.00068069	no
VRK3	0.137755107	3.67184539	0.000259231	0.000681579	no
FRMD3	0.137754701	3.671834363	0.000259242	0.000681579	no
KRTCAP2	0.137749749	3.671699809	0.000259375	0.000681843	no
TP53I11	0.137713984	3.670728059	0.000260345	0.000684302	no
PYGO2	0.137702257	3.670409448	0.000260663	0.00068505	no
FAM172A	0.137700934	3.670373494	0.000260699	0.000685056	no
ZNF32	-0.137696672	-3.670257699	0.000260815	0.000685271	no
SLMAP	0.137648758	3.6689559	0.000262121	0.000688613	no
ASB14	-0.137646071	-3.668882902	0.000262195	0.000688716	no
LAMC2	0.137644283	3.668834307	0.000262243	0.000688755	no
SETD3	-0.137621209	-3.668207407	0.000262875	0.000690324	no
HOXA4	0.137616364	3.668075774	0.000263008	0.000690558	no

AREG	0.137615469	3.668051468	0.000263032	0.000690558	no
ACTG1	0.137572078	3.666872598	0.000264224	0.000693597	no
TMEM20	-0.137569078	-3.666791071	0.000264307	0.000693723	no
CASC1	0.137567001	3.666734645	0.000264364	0.000693783	no
PLEKHM1	0.137547817	3.666213467	0.000264893	0.000695081	no
R3HDM1	-0.137546141	-3.666167933	0.000264939	0.000695112	no
MRPL22	0.137532156	3.665787973	0.000265325	0.000695953	no
CYP17A1	-0.137532047	-3.665785027	0.000265328	0.000695953	no
CROCCL2	-0.137519963	-3.665456736	0.000265662	0.000696677	no
GABPB1	0.137519567	3.665445969	0.000265673	0.000696677	no
TAF9B	-0.137495852	-3.664801675	0.00026633	0.000698309	no
C4orf10	-0.1373947	-3.662053709	0.000269149	0.000705609	no
KIAA0226	-0.137391156	-3.661957425	0.000269248	0.000705778	no
NBL1	0.137380516	3.661668358	0.000269547	0.000706468	no
ABCA1	0.137344776	3.66069745	0.000270551	0.000709008	no
PTPN4	-0.13732773	-3.660234399	0.000271031	0.000710174	no
LOC400891	0.137302735	3.659555391	0.000271736	0.00071193	no
MFAP2	0.137285693	3.659092429	0.000272218	0.0007131	no
FZD4	0.137280821	3.658960091	0.000272356	0.000713289	no
ZNF846	0.13728066	3.65895572	0.000272361	0.000713289	no
PPFIA4	-0.137274592	-3.658790874	0.000272533	0.000713646	no
SYT13	-0.137267914	-3.658609485	0.000272722	0.00071405	no
ACOT13	0.137234473	3.657701047	0.000273672	0.000716443	no
NR2F1	-0.137197875	-3.656706903	0.000274715	0.000719081	no
ABHD3	0.137193564	3.65658979	0.000274838	0.00071931	no
STARD3NL	0.137186518	3.656398396	0.000275039	0.000719743	no
TNXB	0.137169416	3.655933836	0.000275528	0.00072093	no
TMEM104	0.137150821	3.65542874	0.000276061	0.000722231	no
RORA	-0.137111389	-3.654357633	0.000277194	0.000725101	no
LHX5	-0.137103505	-3.654143483	0.000277421	0.000725601	no
WHSC1L1	-0.13709686	-3.653962963	0.000277612	0.000726008	no
NICN1	-0.137067928	-3.653177097	0.000278448	0.000728098	no
ARHGEF10	0.137064742	3.653090568	0.00027854	0.000728245	no
LOC148145	-0.13704763	-3.652625753	0.000279035	0.000729446	no
C1orf129	0.137033141	3.652232208	0.000279455	0.000730449	no
C9orf130	-0.137030066	-3.652148681	0.000279544	0.000730588	no
SELENBP1	0.137019833	3.651870728	0.000279841	0.00073127	no
CCDC41	-0.137014083	-3.651714542	0.000280008	0.000731612	no
PPA2	0.136965867	3.650404921	0.000281413	0.000735186	no
RAB14	-0.13696041	-3.65025668	0.000281572	0.000735508	no
C22orf23	-0.136956505	-3.65015062	0.000281686	0.000735711	no
TDH	-0.136940871	-3.649725977	0.000282144	0.00073681	no
UBL4B	0.136914171	3.649000797	0.000282926	0.000738758	no
SLC2A4	-0.136911979	-3.64894124	0.000282991	0.00073883	no
ZNF174	-0.136846483	-3.647162355	0.000284919	0.000743769	no
LTV1	0.136813511	3.646266844	0.000285894	0.000746219	no
CHFR	0.136796329	3.645800171	0.000286404	0.000747453	no
ONECUT2	-0.136734183	-3.644112341	0.000288254	0.000752184	no
ARMC6	-0.136705579	-3.643335501	0.00028911	0.000754319	no
VAMP4	-0.136697807	-3.643124432	0.000289342	0.000754794	no
WDR44	0.136697013	3.64310285	0.000289366	0.000754794	no

GRHPR	-0.136686087	-3.642806126	0.000289694	0.000755551	no
PTGS2	0.136682614	3.642711805	0.000289798	0.000755725	no
LOC441204	-0.136655761	-3.641982521	0.000290605	0.000757731	no
CRISPLD1	0.13664536	3.64170006	0.000290918	0.00075845	no
SPINT2	0.136612651	3.640811743	0.000291905	0.000760924	no
FHOD3	-0.136587333	-3.64012418	0.000292671	0.000762823	no
FUT5	-0.136567981	-3.639598638	0.000293257	0.000764253	no
TTBK2	-0.136552518	-3.639178708	0.000293727	0.000765378	no
KCTD14	0.136532009	3.63862177	0.000294351	0.000766905	no
EBF2	0.136522499	3.638363507	0.00029464	0.000767544	no
PRG2	0.136520857	3.638318917	0.000294691	0.000767544	no
C2orf68	-0.136520225	-3.638301743	0.00029471	0.000767544	no
FOXD4L6	-0.136505499	-3.637901848	0.000295159	0.000768615	no
FAM19A1	-0.136503255	-3.637840905	0.000295228	0.000768694	no
TM9SF3	-0.136472167	-3.636996673	0.000296178	0.00077107	no
ASPN	0.136449537	3.636382147	0.000296872	0.000772777	no
C15orf50	-0.136447938	-3.636338716	0.000296921	0.000772806	no
CWF19L2	-0.13644003	-3.636123978	0.000297164	0.000773338	no
SF3B3	-0.136428525	-3.635811562	0.000297518	0.000774159	no
ACER2	0.136407008	3.635227258	0.00029818	0.000775783	no
EPHX2	0.136362655	3.634022846	0.00029955	0.000779247	no
DGKD	-0.13632111	-3.632894737	0.000300839	0.000782498	no
LRFN2	-0.136316954	-3.632781873	0.000300968	0.000782706	no
ZNF790	0.136316053	3.632757401	0.000300996	0.000782706	no
C6orf191	0.136309803	3.632587686	0.00030119	0.00078311	no
PHLDA1	-0.13629545	-3.632197955	0.000301637	0.000784171	no
CD36	0.136292628	3.632121322	0.000301725	0.000784299	no
DLEU7	0.136284389	3.631897601	0.000301982	0.000784866	no
AHNAK2	0.136280685	3.63179704	0.000302098	0.000785065	no
RLN2	0.136270526	3.631521184	0.000302415	0.000785788	no
FAM103A1	0.136262389	3.631300223	0.000302669	0.000786348	no
C7orf33	0.136250432	3.630975545	0.000303043	0.000787218	no
TRIM10	0.136241589	3.630735423	0.00030332	0.000787836	no
MDFI	-0.136237996	-3.630637885	0.000303432	0.000788027	no
ROR2	0.136226809	3.630334111	0.000303783	0.000788836	no
SNX5	0.136222772	3.630224481	0.000303909	0.000789017	no
TSPAN6	0.136222102	3.630206304	0.00030393	0.000789017	no
CDK17	-0.136199337	-3.629588166	0.000304646	0.000790772	no
RAET1G	0.136196949	3.629523329	0.000304721	0.000790865	no
CDX1	0.136192268	3.62939622	0.000304868	0.000791146	no
TMTC1	0.136154615	3.628373856	0.000306055	0.000794124	no
TYW1	0.136152038	3.628303874	0.000306136	0.000794234	no
JUN	0.136142597	3.628047534	0.000306435	0.000794906	no
CTGF	0.136131162	3.627737052	0.000306796	0.000795742	no
C20orf106	0.136126176	3.627601665	0.000306954	0.000796049	no
C19orf26	-0.136106316	-3.627062429	0.000307584	0.00079758	no
SNAP25	-0.136056252	-3.625703132	0.000309176	0.000801606	no
WBP1	-0.136042182	-3.625321104	0.000309625	0.000802667	no
PCNA	0.136011351	3.624484022	0.000310611	0.000805119	no
PGM5	0.13601	3.624447343	0.000310654	0.000805128	no
PARD3	-0.135994692	-3.624031715	0.000311144	0.000806296	no

PTPDC1	0.135971001	3.623388497	0.000311905	0.000808163	no
FZD3	0.135954189	3.622932045	0.000312446	0.000809461	no
C14orf139	0.135936525	3.622452493	0.000313015	0.000810832	no
SRCAP	-0.135932887	-3.622353695	0.000313133	0.000811032	no
FGFR1OP	0.13592705	3.622195237	0.000313321	0.000811416	no
FKBP4	-0.135918654	-3.621967279	0.000313592	0.000812014	no
SPTB	-0.135914777	-3.62186202	0.000313717	0.000812234	no
F2RL1	0.135885383	3.62106401	0.000314669	0.000814593	no
HAUS2	0.135852587	3.620173626	0.000315733	0.000817244	no
SVOPL	0.13583002	3.619560959	0.000316468	0.000819041	no
PLEKHB1	-0.135807526	-3.618950275	0.000317202	0.000820835	no
SH3GL3	-0.135790708	-3.618493709	0.000317751	0.000822152	no
ZNF572	-0.135777903	-3.618146065	0.00031817	0.000823131	no
CDSN	0.135762748	3.617734647	0.000318667	0.00082431	no
UTP11L	0.13575016	3.617392911	0.00031908	0.000825273	no
LRRC8B	-0.135748461	-3.617346778	0.000319136	0.000825312	no
ZNF333	-0.135726987	-3.616763799	0.000319842	0.000826959	no
ARHGAP10	0.135726599	3.61675329	0.000319854	0.000826959	no
ALDH1L2	-0.135723309	-3.616663954	0.000319963	0.000827133	no
OR2L13	-0.135719507	-3.616560746	0.000320088	0.000827351	no
PCGF5	0.13571507	3.616440293	0.000320234	0.000827623	no
PBOV1	-0.13569682	-3.61594485	0.000320836	0.000829072	no
ZIM2	-0.135685966	-3.615650212	0.000321194	0.000829892	no
MNS1	0.13565496	3.614808502	0.00032222	0.000832436	no
OXER1	0.135628753	3.614097065	0.00032309	0.000834477	no
SNPH	-0.135628666	-3.614094708	0.000323093	0.000834477	no
DCLRE1A	-0.135622147	-3.613917735	0.000323309	0.000834861	no
KCNN3	-0.135621704	-3.613905712	0.000323324	0.000834861	no
NAPB	-0.135597632	-3.613252249	0.000324125	0.000836823	no
ICAM2	0.135590237	3.613051511	0.000324372	0.000837352	no
CHD5	-0.135586395	-3.612947216	0.0003245	0.000837576	no
GPC4	0.135580488	3.612786868	0.000324697	0.000837887	no
TMEM39B	0.135580306	3.612781928	0.000324703	0.000837887	no
SRCIN1	-0.135570484	-3.612515292	0.000325031	0.000838573	no
VASH1	0.135569212	3.612480762	0.000325073	0.000838573	no
CYP11A1	0.135568619	3.612464652	0.000325093	0.000838573	no
SFT2D3	-0.135546819	-3.611872901	0.000325822	0.000840346	no
BAT1	-0.135512525	-3.610941966	0.000326972	0.000843205	no
C11orf30	-0.135498988	-3.610574501	0.000327427	0.000844271	no
C12orf12	0.135496604	3.610509799	0.000327508	0.00084437	no
ATP5G3	0.135450213	3.60925052	0.000329072	0.000848295	no
MYCBP2	-0.135446406	-3.609147194	0.000329201	0.000848518	no
SIKE1	0.135428083	3.608649818	0.000329821	0.000850008	no
ENPP7	0.135396448	3.60779114	0.000330894	0.000852657	no
GIGYF2	-0.13539529	-3.607759712	0.000330933	0.000852657	no
BREA2	-0.13538566	-3.607498314	0.00033126	0.000853392	no
GLI4	-0.135382522	-3.607413127	0.000331367	0.000853558	no
JAKMIP1	-0.135380056	-3.607346186	0.000331451	0.000853665	no
PPIAL4C	-0.135370679	-3.60709166	0.00033177	0.000854379	no
UBE2MP1	0.135366493	3.606978061	0.000331913	0.000854637	no
TRIM31	0.135358503	3.60676116	0.000332185	0.000855229	no

KIAA0664P3	-0.135325479	-3.605864792	0.000333313	0.000858023	no
LOC646999	-0.135310442	-3.605456649	0.000333827	0.000859226	no
C1orf201	0.135309339	3.605426709	0.000333865	0.000859226	no
H1FNT	-0.135294274	-3.605017813	0.000334381	0.000860446	no
BTD	0.135277282	3.604556607	0.000334965	0.000861837	no
FAM48B1	0.135271956	3.60441204	0.000335148	0.000862143	no
AURKA	0.135271347	3.604395514	0.000335169	0.000862143	no
PCSK1N	-0.135253149	-3.603901578	0.000335795	0.000863643	no
SPRED2	0.135230247	3.603279967	0.000336584	0.000865564	no
CDKN3	0.135211339	3.602766769	0.000337238	0.000867133	no
CCL7	0.135196598	3.602366691	0.000337748	0.000868334	no
C1orf182	0.135187758	3.602126752	0.000338054	0.000869011	no
UBE2B	0.13513435	3.6006772	0.000339909	0.00087367	no
GIN1	-0.135122498	-3.60035552	0.000340323	0.000874621	no
RCOR3	-0.135058148	-3.598609056	0.000342573	0.000880294	no
ZNF501	-0.135051452	-3.598427331	0.000342808	0.000880785	no
HEPACAM	-0.134984071	-3.596598636	0.000345182	0.000886771	no
ZNF432	0.134961319	3.595981166	0.000345987	0.000888725	no
PLCE1	0.134952322	3.595737011	0.000346305	0.000889431	no
ELMO2	-0.134942956	-3.595482826	0.000346637	0.000890171	no
C9orf96	-0.134911037	-3.594616584	0.000347772	0.00089297	no
FLJ11235	0.134901961	3.59437027	0.000348095	0.000893686	no
SLC4A1AP	-0.134871703	-3.593549137	0.000349174	0.000896343	no
LRTM1	0.134863106	3.593315838	0.000349481	0.000897018	no
ATP5A1	-0.134826969	-3.592335179	0.000350775	0.000900225	no
NKX2-2	-0.134817097	-3.592067258	0.000351129	0.00090102	no
IL1RAP	0.134790229	3.591338154	0.000352095	0.000903383	no
BSDC1	-0.134788415	-3.591288919	0.000352161	0.000903436	no
PERP	0.134778862	3.591029674	0.000352505	0.000904204	no
MRPL42	0.134754711	3.590374305	0.000353376	0.000906324	no
THAP6	0.134750416	3.590257758	0.000353531	0.000906607	no
IGFN1	-0.134739475	-3.589960864	0.000353927	0.000907506	no
ARHGAP39	-0.134686968	-3.588536015	0.000355831	0.000912273	no
ZNF608	-0.134679079	-3.588321944	0.000356118	0.000912893	no
LOC389705	-0.134672152	-3.588133964	0.00035637	0.000913423	no
CATSPER2	-0.134652919	-3.587612092	0.00035707	0.000915103	no
OSGEPL1	-0.134650603	-3.587549238	0.000357155	0.000915119	no
ATP8A2	-0.134650272	-3.587540243	0.000357167	0.000915119	no
FUT11	0.134645589	3.587413186	0.000357338	0.00091544	no
NTSR2	-0.134589911	-3.58590236	0.000359375	0.000920543	no
SLC4A4	-0.134586294	-3.585804206	0.000359508	0.000920767	no
FAM190A	-0.134582994	-3.585714656	0.000359629	0.00092096	no
FAM122A	0.134581317	3.585669167	0.000359691	0.000921001	no
PHF5A	0.134482671	3.58299249	0.00036333	0.000930202	no
ZNF429	-0.134453464	-3.582200003	0.000364414	0.00093286	no
TMTC2	0.134451305	3.582141432	0.000364495	0.000932947	no
MPHOSPH8	-0.134437085	-3.581755591	0.000365024	0.000934183	no
FAM72B	0.13437642	3.580109582	0.000367289	0.000939861	no
SUFU	-0.134367063	-3.579855723	0.000367639	0.000940639	no
SYNGR3	-0.134352586	-3.579462921	0.000368182	0.000941909	no
GPR115	0.13432896	3.578821913	0.00036907	0.00094406	no

SPRED1	0.134304624	3.578161635	0.000369986	0.000946284	no
EEF1A2	-0.134302495	-3.578103878	0.000370066	0.00094637	no
C1orf95	-0.134297798	-3.577976426	0.000370243	0.000946703	no
TULP2	0.134239584	3.576397026	0.000372446	0.000952214	no
SLC17A6	-0.134225513	-3.576015256	0.00037298	0.000953459	no
LOC388387	-0.134180666	-3.574798542	0.000374687	0.000957702	no
KCNK10	-0.13417371	-3.574609823	0.000374952	0.000958259	no
REV1	-0.134171297	-3.574544364	0.000375044	0.000958291	no
ZNF389	-0.134170897	-3.574533507	0.00037506	0.000958291	no
CA11	-0.134097476	-3.572541637	0.000377873	0.000965357	no
SPINK2	0.134068254	3.571748857	0.000378998	0.000968109	no
NDUFA4L2	0.133935734	3.568153827	0.00038414	0.000981119	no
NMRAL1	0.133928681	3.56796252	0.000384415	0.000981698	no
AATK	-0.133906674	-3.567365527	0.000385276	0.000983772	no
ZMAT2	-0.133904941	-3.567318497	0.000385344	0.000983821	no
ADORA2B	0.133879231	3.566621069	0.000386352	0.00098627	no
NFRKB	-0.133861195	-3.566131811	0.00038706	0.000987954	no
MDH1B	0.133857142	3.566021873	0.00038722	0.000988236	no
HIST2H2AA3	0.133843261	3.565645333	0.000387766	0.000989506	no
LRRC36	0.133823695	3.565114587	0.000388537	0.000991349	no
SDCCAG1	-0.133821254	-3.565048375	0.000388634	0.00099147	no
ANGPTL6	0.133786532	3.564106497	0.000390006	0.000994846	no
SNX33	0.133773472	3.563752227	0.000390524	0.000996041	no
KLHL35	-0.133765293	-3.563530375	0.000390848	0.000996742	no
TRIM78P	0.13375068	3.563134003	0.000391429	0.000998096	no
MYH15	-0.13374403	-3.562953619	0.000391693	0.000998644	no
C9orf37	-0.133729633	-3.562563091	0.000392266	0.000999958	no
CLRN3	0.133728596	3.562534948	0.000392307	0.000999958	no
HVCN1	0.133709671	3.562021605	0.000393061	0.001001754	no
PCF11	-0.133699318	-3.561740795	0.000393474	0.00100268	no
WDR73	0.133693625	3.561586366	0.000393702	0.001003133	no
RWDD4A	0.133649413	3.560387151	0.000395472	0.001007516	no
GREB1L	-0.13361338	-3.559409768	0.00039692	0.001011078	no
KHDC1L	-0.133596865	-3.558961828	0.000397585	0.001012645	no
TMCO3	0.133578345	3.558459501	0.000398332	0.00101442	no
NPM2	-0.133565855	-3.558120729	0.000398837	0.001015578	no
ANO5	-0.133538057	-3.557366756	0.000399963	0.001018315	no
PUS3	-0.133528282	-3.557101635	0.000400359	0.001019197	no
LOC100131434	0.133508708	3.556570728	0.000401154	0.001021092	no
MAS1L	0.133485626	3.555944697	0.000402093	0.001023354	no
CDKN2D	-0.133471262	-3.555555112	0.000402679	0.001024716	no
FSD1L	-0.133452797	-3.555054292	0.000403433	0.001026505	no
TEAD4	0.133448161	3.554928569	0.000403623	0.001026858	no
ALS2CR12	0.13343063	3.554453086	0.00040434	0.001028554	no
SEC22A	0.133414331	3.554011023	0.000405008	0.001030123	no
DGAT1	-0.133408204	-3.553844846	0.000405259	0.001030633	no
SUMO4	0.133405181	3.55376287	0.000405383	0.001030819	no
MAPK3	-0.133391294	-3.553386229	0.000405954	0.00103214	no
IGFL4	-0.133373926	-3.552915184	0.000406668	0.001033827	no
PHF3	-0.133337563	-3.551928988	0.000408168	0.001037509	no
C1orf87	0.133327236	3.551648899	0.000408595	0.001038463	no

CABP1	-0.13330388	-3.551015468	0.000409562	0.00104079	no
TMEM182	0.133281906	3.550419527	0.000410474	0.001042976	no
NOTCH2NL	0.133272276	3.550158361	0.000410874	0.001043862	no
DIO3OS	0.133266314	3.549996672	0.000411122	0.00104436	no
TYMS	0.133245596	3.549434804	0.000411984	0.001046365	no
PDZD2	-0.133244877	-3.549415301	0.000412014	0.001046365	no
GNASAS	0.133192703	3.54800038	0.000414194	0.001051769	no
C2orf70	0.133158445	3.547071325	0.000415632	0.001055286	no
DHX32	0.133144563	3.546694887	0.000416215	0.001056635	no
MRPL28	-0.133138208	-3.546522536	0.000416483	0.001057181	no
NKTR	-0.133124553	-3.546152219	0.000417058	0.001058508	no
PCDH19	-0.13311642	-3.545931664	0.000417401	0.001059246	no
BARD1	0.133083541	3.545040054	0.00041879	0.001062638	no
FAM110C	0.133028799	3.543555581	0.000421113	0.001068397	no
SLC39A9	-0.133022268	-3.543378469	0.00042139	0.001068968	no
PTF1A	-0.133011982	-3.543099564	0.000421828	0.001069902	no
RABL2B	0.133011137	3.543076641	0.000421864	0.001069902	no
IL12A	-0.133003602	-3.542872306	0.000422186	0.001070582	no
GNB4	0.132986232	3.54240129	0.000422927	0.001072327	no
C1orf161	0.132929138	3.540853098	0.000425372	0.001078391	no
SIRT2	-0.132860646	-3.538995879	0.000428322	0.001085734	no
C17orf95	0.132848958	3.538678941	0.000428827	0.001086879	no
SCN2B	-0.132838841	-3.538404628	0.000429265	0.001087852	no
C6orf132	0.132834561	3.538288573	0.000429451	0.001088185	no
LOC100129055	-0.132830434	-3.538176647	0.000429629	0.001088502	no
DYRK1A	-0.132826279	-3.538063982	0.00042981	0.001088757	no
CDHR4	0.132825633	3.538046468	0.000429838	0.001088757	no
TMEM62	0.132800555	3.537366473	0.000430926	0.001091377	no
HSD17B7P2	-0.13278745	-3.537011154	0.000431496	0.001092683	no
SLCO5A1	-0.132739807	-3.535719339	0.000433574	0.001097807	no
ZNF317	-0.132734907	-3.535586483	0.000433788	0.001098212	no
C9orf117	0.132726281	3.53535259	0.000434165	0.00109903	no
KCTD13	-0.132723085	-3.535265927	0.000434305	0.001099246	no
CDON	-0.132720479	-3.535195275	0.000434419	0.001099398	no
GPR161	-0.132717805	-3.535122767	0.000434536	0.001099556	no
ATP5J2	0.1327162	3.53507925	0.000434607	0.001099597	no
NAT15	-0.132710107	-3.534914056	0.000434874	0.001100134	no
ADAMTS6	-0.132686263	-3.534267536	0.00043592	0.001102643	no
ZC3H3	-0.132683667	-3.534197145	0.000436034	0.001102794	no
CES4	0.132676707	3.534008438	0.00043634	0.001103429	no
C1QTNF4	-0.132664446	-3.53367601	0.000436879	0.001104655	no
XKRX	0.13263472	3.532870039	0.000438189	0.00110783	no
PLA2G7	0.132626934	3.532658949	0.000438533	0.00110856	no
ADAMTS7	0.132618434	3.532428476	0.000438909	0.001109371	no
FNDC7	0.132589534	3.531644919	0.000440188	0.001112466	no
ENDOU	-0.132582727	-3.531460353	0.00044049	0.001113089	no
ARHGEF12	-0.132580266	-3.531393652	0.000440599	0.001113226	no
SUSD5	-0.132570688	-3.531133971	0.000441024	0.001114161	no
EIF4E1B	-0.132556499	-3.530749251	0.000441655	0.001115614	no
ERCC6L	0.132542579	3.530371871	0.000442274	0.001117039	no
UBXN10	0.132505305	3.529361288	0.000443937	0.001121098	no

FNDC8	-0.132495829	-3.529104386	0.00044436	0.001122028	no
RSU1	0.132456735	3.528044508	0.000446112	0.00112631	no
DAPK1	-0.132421784	-3.52709695	0.000447683	0.001130135	no
ZNF200	0.132412635	3.526848901	0.000448095	0.001131035	no
KCNMB2	-0.13239715	-3.526429107	0.000448794	0.001132656	no
NAPG	-0.132376394	-3.525866388	0.000449731	0.001134816	no
CPSF4	0.132375724	3.525848232	0.000449762	0.001134816	no
ZNF862	-0.132341873	-3.524930546	0.000451295	0.001138543	no
PDE4DIP	-0.132299427	-3.523779849	0.000453224	0.001143268	no
C16orf71	0.132279694	3.523244904	0.000454124	0.001145394	no
PPBP	0.132243684	3.522268698	0.00045577	0.001149403	no
UBAC2	0.132236884	3.522084367	0.000456082	0.001150045	no
DFNA5	0.132224348	3.521744519	0.000456656	0.00115135	no
NME7	0.132214988	3.521490778	0.000457086	0.00115229	no
DDA1	-0.13221283	-3.521432297	0.000457185	0.001152396	no
BAT2L1	-0.132209468	-3.52134114	0.000457339	0.001152641	no
ABL1	-0.132186512	-3.520718864	0.000458395	0.001155158	no
PSMC6	-0.132165201	-3.520141152	0.000459377	0.001157488	no
ACVR1C	-0.132162098	-3.520057035	0.00045952	0.001157704	no
MRPL47	0.132135251	3.519329286	0.00046076	0.001160684	no
CAMK2B	-0.132111777	-3.518692959	0.000461847	0.001163278	no
C17orf50	-0.132104291	-3.518490031	0.000462194	0.001164007	no
GMDS	0.132089767	3.518096318	0.000462869	0.001165517	no
C9orf11	-0.132088888	-3.518072507	0.000462909	0.001165517	no
RAB27B	-0.132085117	-3.517970289	0.000463085	0.001165813	no
LOC90586	0.132065724	3.517444599	0.000463987	0.001167939	no
ZNF732	-0.132056988	-3.51720779	0.000464394	0.001168817	no
KIAA0947	-0.132023906	-3.516311043	0.000465937	0.001172557	no
DNM2	0.132019825	3.516200422	0.000466128	0.001172891	no
NUP62CL	0.131998414	3.515620064	0.00046713	0.001175194	no
FBXO18	-0.131997784	-3.515602969	0.00046716	0.001175194	no
DNM1L	-0.131990192	-3.515397194	0.000467516	0.001175943	no
KIF20A	0.131966808	3.514763348	0.000468613	0.001178557	no
CCDC108	0.131944376	3.514155316	0.000469668	0.001181064	no
AMPH	-0.131934567	-3.513889427	0.00047013	0.001182079	no
SGMS1	-0.131907178	-3.513147063	0.000471423	0.001185181	no
PDS5B	-0.13190325	-3.513040594	0.000471608	0.0011855	no
RALYL	-0.131881521	-3.512451626	0.000472637	0.001187937	no
NDUFS7	-0.131843483	-3.511420617	0.000474441	0.001192325	no
TLE6	-0.131841088	-3.511355689	0.000474555	0.001192463	no
ABLIM2	-0.131826159	-3.510951064	0.000475265	0.001194085	no
RNF126	-0.131825038	-3.510920687	0.000475319	0.001194085	no
GYS1	0.131791875	3.510021822	0.0004769	0.001197909	no
CBLN2	-0.131783876	-3.509805031	0.000477282	0.00119872	no
MRPL33	0.131774666	3.509555386	0.000477723	0.001199677	no
NT5C1B	0.131693877	3.507365754	0.000481602	0.001209269	no
BHMT2	0.131691861	3.507311111	0.000481699	0.001209363	no
PIP5K1A	0.131679718	3.506982	0.000482285	0.001210683	no
COL19A1	0.131674635	3.506844248	0.00048253	0.001211149	no
MAEA	0.131651955	3.506229556	0.000483627	0.00121375	no
PLAGL2	0.131565059	3.503874518	0.000487848	0.001224193	no

SPRR2G	-0.131553944	-3.503573287	0.000488391	0.00122538	no
PSMD8	0.131552885	3.503544583	0.000488443	0.00122538	no
CEP97	-0.13154187	-3.503246065	0.000488981	0.001226578	no
POMC	0.131524741	3.502781863	0.000489819	0.001228414	no
IL8	0.131524424	3.502773258	0.000489834	0.001228414	no
SNCG	-0.131514238	-3.502497213	0.000490333	0.001229513	no
EXOSC2	-0.13151129	-3.502417311	0.000490478	0.001229613	no
ACSS1	-0.131510557	-3.50239747	0.000490514	0.001229613	no
WIPF2	-0.131509703	-3.502374321	0.000490556	0.001229613	no
FAM156A	-0.131507842	-3.502323884	0.000490647	0.001229689	no
C2CD3	-0.131490495	-3.501853778	0.000491498	0.00123167	no
LRIG3	0.13147792	3.501512978	0.000492116	0.001233066	no
GPIHBP1	-0.131472758	-3.501373084	0.00049237	0.001233549	no
ZNF154	-0.131460432	-3.501039061	0.000492977	0.001234833	no
SYTL4	-0.131458899	-3.500997506	0.000493053	0.001234833	no
STIL	0.131458632	3.500990271	0.000493066	0.001234833	no
ST6GALNAC1	-0.131425323	-3.500087582	0.000494709	0.001238795	no
LOC442421	-0.131351116	-3.498076634	0.000498389	0.001247855	no
BTAF1	-0.13130046	-3.496703899	0.000500916	0.001254026	no
C1orf158	0.131292381	3.496484979	0.00050132	0.001254882	no
SAMD8	-0.131274445	-3.495998953	0.000502218	0.001256975	no
ZYG11B	-0.131262518	-3.495675742	0.000502816	0.001258316	no
ITGA6	0.131249671	3.495327607	0.000503461	0.001259774	no
TRIM26	0.131240566	3.4950809	0.000503919	0.001260763	no
ZNF781	-0.131227359	-3.494723014	0.000504583	0.001262269	no
CAMKV	-0.131224332	-3.494640992	0.000504735	0.001262494	no
RNF144A	-0.131196995	-3.493900225	0.000506113	0.001265784	no
SLC2A2	0.131186794	3.493623802	0.000506629	0.001266916	no
GPR12	-0.131179601	-3.493428881	0.000506992	0.001267669	no
PPA1	-0.131167403	-3.493098371	0.000507609	0.001269054	no
CAB39	0.131162711	3.492971223	0.000507847	0.001269491	no
AK7	0.13115511	3.492765248	0.000508232	0.001270297	no
ZNF230	0.131152704	3.492700054	0.000508354	0.001270445	no
UTP14C	-0.131134264	-3.492200402	0.000509289	0.001272625	no
C15orf44	0.131131426	3.492123499	0.000509433	0.001272769	no
CBWD6	0.131130649	3.49210245	0.000509473	0.001272769	no
RRM2	0.131122014	3.491868458	0.000509911	0.001273586	no
HTR6	-0.131121732	-3.491860822	0.000509926	0.001273586	no
HP	0.131120097	3.491816522	0.000510009	0.001273636	no
MTIF2	0.131080729	3.490749795	0.000512014	0.001278425	no
MAN2C1	-0.131079966	-3.490729131	0.000512053	0.001278425	no
NAA11	-0.131074762	-3.490588122	0.000512319	0.00127893	no
PSORS1C1	0.131052512	3.489985219	0.000513456	0.001281611	no
ZNF503	0.13101378	3.488935752	0.000515441	0.001286407	no
FANCE	-0.131011138	-3.488864165	0.000515576	0.001286586	no
CLEC2L	-0.131009768	-3.488827042	0.000515647	0.001286603	no
KCNA4	-0.130972994	-3.487830664	0.000517539	0.001291164	no
C21orf121	0.130938588	3.486898438	0.000519315	0.001295435	no
ANKRD49	0.130924333	3.486512214	0.000520052	0.001297115	no
UBXN6	-0.130918771	-3.486361502	0.00052034	0.001297673	no
UBXN11	0.130897128	3.485775099	0.000521462	0.001300311	no

LOC147804	0.130887621	3.485517518	0.000521956	0.001301382	no
CCNB3	-0.130880743	-3.485331157	0.000522313	0.001302112	no
SNHG8	-0.130872951	-3.485120058	0.000522718	0.001302962	no
PEX26	-0.130862928	-3.484848482	0.00052324	0.001304101	no
WDR61	0.130839073	3.484202165	0.000524483	0.001307039	no
GK2	-0.130824283	-3.483801466	0.000525256	0.001308802	no
GSTM2	-0.130814784	-3.483544097	0.000525752	0.001309878	no
ZNF800	0.130805668	3.483297121	0.000526229	0.001310905	no
PTK7	0.130802318	3.483206358	0.000526405	0.00131118	no
LZIC	0.130792733	3.48294666	0.000526907	0.001312147	no
SLC35E1	0.130792424	3.482938313	0.000526923	0.001312147	no
MED21	0.130786375	3.482774405	0.00052724	0.001312775	no
WDR18	-0.130777729	-3.482540167	0.000527693	0.001313743	no
MAGI2	-0.130763161	-3.482145472	0.000528458	0.001315485	no
SPATA2	-0.130740548	-3.481532847	0.000529648	0.001318284	no
SBK2	0.130720615	3.480992805	0.000530698	0.001320736	no
SERINC1	-0.130716835	-3.480890414	0.000530898	0.00132107	no
KBTBD10	0.130709056	3.480679649	0.000531309	0.001321929	no
SNAP47	-0.130669858	-3.47961771	0.000533382	0.001326926	no
FAM123B	-0.130660183	-3.479355592	0.000533895	0.001328039	no
TP53I3	0.130650474	3.479092556	0.000534411	0.001329023	no
FER1L4	0.130650253	3.479086577	0.000534422	0.001329023	no
LRRC46	0.130645975	3.47897067	0.00053465	0.001329424	no
AIP	-0.130620868	-3.47829051	0.000535985	0.001332571	no
MPI	-0.1306197	-3.478258866	0.000536047	0.001332571	no
BANF2	0.130613707	3.478096503	0.000536366	0.0013332	no
OVOL1	-0.130606159	-3.477892004	0.000536769	0.001334036	no
EIF3CL	-0.130596266	-3.477624006	0.000537296	0.001335184	no
HARS	-0.130591967	-3.477507548	0.000537526	0.00133559	no
C19orf29	-0.130588927	-3.477425186	0.000537688	0.001335829	no
SUCLA2	-0.130583317	-3.477273206	0.000537988	0.001336409	no
TCEAL6	-0.130575773	-3.477068844	0.000538391	0.001337246	no
IRX2	-0.130565345	-3.476786339	0.000538949	0.001338467	no
UBB	0.130495693	3.47489944	0.000542688	0.001347589	no
APCDD1	-0.130484193	-3.474587908	0.000543308	0.001348962	no
PROC	0.130445431	3.473537869	0.000545402	0.001353994	no
ZNF397	-0.130413166	-3.472663856	0.00054715	0.001358168	no
LOC401010	-0.130382234	-3.471825944	0.000548831	0.001362094	no
NR4A3	0.130381592	3.471808555	0.000548866	0.001362094	no
CCDC81	0.130354503	3.471074749	0.000550343	0.00136559	no
DDX54	-0.130345445	-3.470829386	0.000550837	0.001366649	no
ADI1	0.130319994	3.470139959	0.000552229	0.001369934	no
CHMP2B	0.130317639	3.470076184	0.000552358	0.001370085	no
WDR67	0.130313639	3.469967832	0.000552577	0.00137046	no
LRRC48	0.130301823	3.469647751	0.000553224	0.001371898	no
ZNF322B	-0.130296459	-3.469502457	0.000553518	0.001372459	no
FANCB	0.13027871	3.469021679	0.000554493	0.001374707	no
LOC100009676	0.130240175	3.467977867	0.000556614	0.001379797	no
SPATA20	0.130233984	3.467810175	0.000556956	0.001380474	no
LOR	-0.130227945	-3.467646608	0.000557289	0.00138103	no
TCHP	-0.130227445	-3.467633061	0.000557316	0.00138103	no

SLC41A3	-0.130226002	-3.467593967	0.000557396	0.001381058	no
DDX17	-0.130194862	-3.466750493	0.000559118	0.001385155	no
LY6G6D	0.1301869	3.466534826	0.000559559	0.001385883	no
IGDCC3	-0.130186312	-3.466518891	0.000559592	0.001385883	no
PCDHA1	-0.130185846	-3.466506277	0.000559618	0.001385883	no
TET3	-0.13017296	-3.466157256	0.000560332	0.001387482	no
LOC100133985	-0.130153407	-3.465627618	0.000561418	0.001389846	no
C14orf109	0.130153296	3.465624617	0.000561424	0.001389846	no
RIBC1	0.13014981	3.465530189	0.000561618	0.001390156	no
KLHL33	0.13012616	3.464889623	0.000562935	0.001393244	no
MIDN	-0.130098259	-3.46413391	0.000564491	0.001396926	no
GLRA3	-0.130042267	-3.462617342	0.000567628	0.001404516	no
TUBGCP5	-0.130008642	-3.461706627	0.000569519	0.001409023	no
SLC13A3	0.130006318	3.461643692	0.00056965	0.001409174	no
NT5C2	-0.129995935	-3.461362453	0.000570235	0.00141045	no
TOB1	0.129934483	3.45969809	0.000573711	0.001418825	no
ADCYAP1R1	-0.129933586	-3.4596738	0.000573762	0.001418825	no
RAD52	-0.129930452	-3.459588905	0.000573939	0.001419091	no
GAS2L2	0.129926165	3.459472805	0.000574183	0.001419519	no
RALBP1	0.129917606	3.459241016	0.000574669	0.001420547	no
VCX3B	0.129863336	3.457771184	0.000577759	0.001428012	no
GAN	-0.129858878	-3.457650448	0.000578014	0.001428466	no
C1orf31	0.129854711	3.457537591	0.000578252	0.00142888	no
TDG	0.129841023	3.457166901	0.000579034	0.001430639	no
ZNF717	-0.129839197	-3.45711744	0.000579139	0.001430722	no
AGPS	0.129836706	3.457049973	0.000579281	0.001430899	no
PRPF39	-0.129822544	-3.456666443	0.000580092	0.001432727	no
BCORL2	0.129779207	3.455492767	0.000582581	0.001438697	no
GAL3ST1	-0.12971645	-3.453793189	0.000586202	0.001447462	no
EIF5AL1	0.129714404	3.453737785	0.00058632	0.001447578	no
HERC6	0.129704875	3.453479737	0.000586872	0.001448763	no
PNKP	0.129701364	3.453384665	0.000587075	0.001449088	no
KLK1	0.129691335	3.453113057	0.000587656	0.001450346	no
ASF1B	0.12965159	3.452036727	0.000589966	0.001455868	no
ACOT12	0.129603803	3.450742623	0.000592753	0.001462569	no
SEL1L2	0.129600373	3.45064974	0.000592954	0.001462885	no
SCARNA9	-0.129593421	-3.450461476	0.000593361	0.00146371	no
C12orf60	0.129584326	3.450215185	0.000593893	0.001464845	no
C21orf59	0.129569476	3.44981305	0.000594763	0.001466812	no
FAM35B2	-0.129544882	-3.449147067	0.000596207	0.001470194	no
CHML	-0.129530464	-3.44875663	0.000597056	0.001472106	no
TWIST2	0.129523815	3.448576577	0.000597447	0.001472892	no
STAC	0.12950766	3.44813912	0.000598399	0.001475059	no
KCNA2	-0.129480624	-3.447407001	0.000599996	0.001478814	no
CALCRL	-0.129457646	-3.446784781	0.000601356	0.001481937	no
COX7B	0.129456743	3.446760342	0.000601409	0.001481937	no
TMEM130	-0.129450338	-3.446586895	0.000601789	0.001482692	no
ALG1L	-0.129431968	-3.446089454	0.000602879	0.001485197	no
MEPCE	-0.129423875	-3.445870322	0.00060336	0.0014862	no
HRNBP3	-0.129413985	-3.445602513	0.000603948	0.001487467	no
CSF2	0.129386273	3.444852131	0.000605599	0.001491351	no

IVL	-0.129374679	-3.444538195	0.00060629	0.001492873	no
BRCA1	0.12934958	3.443858559	0.000607791	0.001496385	no
NR2E3	-0.129346695	-3.44378044	0.000607963	0.001496628	no
NR4A2	0.129334447	3.443448806	0.000608697	0.001498251	no
SETD6	-0.129322397	-3.443122527	0.000609419	0.001499847	no
WBP11P1	-0.129304817	-3.442646509	0.000610475	0.001502261	no
KCNK7	-0.12929908	-3.442491177	0.00061082	0.001502927	no
RBM22	-0.129296543	-3.442422479	0.000610972	0.001503119	no
FAM76B	-0.129241337	-3.440927679	0.0006143	0.001511123	no
STAP2	0.12920684	3.439993646	0.000616388	0.001516075	no
MYOM2	-0.129173306	-3.439085691	0.000618424	0.001520898	no
UBE2E2	-0.129164601	-3.438849998	0.000618954	0.001522015	no
FUT2	-0.129162198	-3.438784914	0.0006191	0.00152219	no
TMEM189	0.129146107	3.438349268	0.00062008	0.001524414	no
PLD3	0.129083538	3.436655215	0.000623906	0.001533632	no
SCARB1	-0.129076759	-3.436471662	0.000624321	0.001534468	no
C11orf17	0.129069109	3.43626454	0.000624791	0.001535435	no
C10orf107	0.129051002	3.435774321	0.000625903	0.001537982	no
C6orf195	0.129043124	3.435561004	0.000626388	0.001538986	no
LARP4B	-0.12903145	-3.435244959	0.000627107	0.001540565	no
TGS1	0.129027939	3.435149908	0.000627323	0.001540909	no
CCDC146	0.129015366	3.434809502	0.000628098	0.001542626	no
ARSI	0.129010042	3.434665347	0.000628427	0.001543113	no
RPS23	-0.129009679	-3.434655523	0.000628449	0.001543113	no
PPP1R3D	0.128991416	3.434161086	0.000629578	0.001545695	no
GRM2	-0.128953775	-3.433142004	0.000631909	0.001551231	no
SLC37A1	0.128930618	3.432515064	0.000633347	0.001554573	no
CLIP4	-0.128906741	-3.431868657	0.000634833	0.001558031	no
TUBA8	-0.128893263	-3.431503768	0.000635674	0.001559904	no
MFSD4	-0.128871323	-3.430909803	0.000637044	0.001563076	no
TBC1D3B	-0.128864674	-3.430729796	0.000637459	0.001563831	no
RNF6	0.128863929	3.430709631	0.000637506	0.001563831	no
SFTPD	-0.128854122	-3.430444122	0.00063812	0.001565147	no
SLC9A11	0.128846547	3.430239071	0.000638594	0.00156612	no
HPGD	0.128831589	3.429834102	0.000639532	0.00156823	no
NDUFB3	0.128812918	3.429328659	0.000640704	0.001570914	no
DNMT3A	0.128799164	3.428956318	0.000641569	0.001572844	no
CYP4A11	-0.12879225	-3.428769132	0.000642004	0.00157372	no
PACSIN1	-0.128790653	-3.428725909	0.000642105	0.001573776	no
SLC9A8	0.128759536	3.427883527	0.000644067	0.001578394	no
MED28	0.128741304	3.427389967	0.000645219	0.001581026	no
PRKX	-0.128734583	-3.427208029	0.000645645	0.001581876	no
RAVER2	-0.128719577	-3.426801797	0.000646595	0.001584013	no
GALNT8	-0.128704377	-3.426390336	0.000647559	0.001586183	no
TRAF3IP1	0.128701786	3.426320177	0.000647724	0.001586393	no
IL11	0.128695969	3.426162704	0.000648093	0.001587106	no
OGFOD1	0.128675555	3.425610093	0.000649391	0.001590091	no
MFSD3	-0.128671574	-3.425502328	0.000649644	0.001590519	no
ATP6V1A	-0.128654076	-3.425028672	0.000650759	0.001593055	no
SNORD115-13	-0.128642178	-3.424706575	0.000651518	0.00159472	no
STX2	0.128624687	3.424233112	0.000652635	0.001597262	no

PMP2	0.128607335	3.423763394	0.000653745	0.001599785	no
ATP5F1	0.128594993	3.42342929	0.000654536	0.001601526	no
LOC401127	-0.12859182	-3.423343417	0.000654739	0.00160183	no
CCDC158	0.12858575	3.423179085	0.000655129	0.001602588	no
PCMT1	-0.128548755	-3.422177679	0.000657506	0.001608209	no
NRIP2	-0.128542362	-3.422004625	0.000657918	0.001609022	no
CYP2A6	-0.128531604	-3.421713403	0.000658611	0.001610522	no
TMEM132D	-0.12851793	-3.421343291	0.000659493	0.001612484	no
USP35	-0.128493187	-3.420673517	0.000661092	0.001616199	no
FOXD4L1	-0.128488702	-3.420552122	0.000661382	0.001616713	no
ZNF85	-0.128482675	-3.420388973	0.000661773	0.001617471	no
METTL6	-0.128478512	-3.420276292	0.000662042	0.001617934	no
C18orf32	-0.128476872	-3.420231905	0.000662148	0.001617998	no
IARS	0.128454085	3.419615095	0.000663626	0.001621414	no
CLEC4G	0.128433474	3.419057204	0.000664966	0.00162449	no
NSUN5	0.128402469	3.418217987	0.000666986	0.001629227	no
MPND	-0.128399941	-3.418149561	0.00066715	0.001629433	no
MATN3	0.128370357	3.417348785	0.000669083	0.001633957	no
MGC16275	-0.128351901	-3.416849261	0.000670292	0.00163671	no
SLC3A1	-0.128342667	-3.416599313	0.000670897	0.00163799	no
DDX24	-0.1283405	-3.416540655	0.000671039	0.00163814	no
KRTAP1-5	0.128328683	3.416220824	0.000671815	0.001639835	no
PDXK	-0.128326143	-3.416152071	0.000671982	0.001640044	no
TMEM84	0.128303263	3.415532778	0.000673486	0.001643517	no
NEUROD1	-0.128298282	-3.415397954	0.000673814	0.001644119	no
ALG5	0.128277264	3.414829084	0.000675199	0.001647191	no
ATG4D	-0.128276	-3.414794886	0.000675283	0.001647191	no
ACN9	0.128275476	3.414780696	0.000675317	0.001647191	no
MRPS16	-0.128268049	-3.41457968	0.000675808	0.001648188	no
PRKAA2	-0.128259719	-3.414354205	0.000676358	0.001649331	no
NRM	0.128256007	3.414253752	0.000676603	0.00164973	no
FXYD7	-0.1282507	-3.414110119	0.000676954	0.00165028	no
ID4	-0.128250131	-3.414094721	0.000676992	0.00165028	no
RPL13P5	0.128235138	3.413688913	0.000677984	0.001652499	no
IRGM	0.128231285	3.413584619	0.000678239	0.001652922	no
KLHL21	0.128222915	3.413358102	0.000678794	0.001654075	no
TCF20	-0.128213021	-3.413090304	0.00067945	0.001655474	no
AFAP1L2	-0.128170432	-3.411937635	0.000682282	0.001662173	no
CTNNAL1	0.128148874	3.41135417	0.00068372	0.001665475	no
STH	-0.128132086	-3.4108998	0.000684841	0.001668006	no
TNNI3K	-0.12812729	-3.410770011	0.000685162	0.001668586	no
WASH2P	-0.128121249	-3.410606499	0.000685566	0.001669369	no
LCNL1	-0.128109329	-3.410283907	0.000686364	0.001671111	no
MST4	0.128086483	3.409665601	0.000687896	0.001674639	no
SYTL5	0.128077171	3.409413564	0.000688521	0.00167596	no
AKR1B10	0.12806037	3.408958875	0.000689651	0.001678507	no
C10orf114	0.128053858	3.408782623	0.000690089	0.001679372	no
LIMS3	0.128045896	3.408567145	0.000690625	0.001680475	no
LDLRAP1	0.128022084	3.407922713	0.000692231	0.00168418	no
OR10J3	0.128006505	3.407501104	0.000693284	0.001686538	no
OR52K1	0.128003744	3.407426379	0.000693471	0.001686707	no

ZNF774	-0.128003008	-3.40740647	0.00069352	0.001686707	no
AKR1C4	0.127969976	3.406512506	0.000695758	0.001691945	no
RBM26	-0.127964263	-3.406357911	0.000696146	0.001692684	no
BCAS2	0.127955233	3.40611353	0.000696759	0.001693972	no
C14orf167	-0.127946756	-3.40588412	0.000697335	0.001695168	no
SLC32A1	-0.127944799	-3.405831167	0.000697468	0.001695288	no
CD200	-0.127912196	-3.404948844	0.000699688	0.001700479	no
PI4KA	-0.127906245	-3.404787804	0.000700094	0.001701067	no
SFTPC	-0.127906187	-3.404786217	0.000700098	0.001701067	no
THSD1	0.127896165	3.404515001	0.000700782	0.001702524	no
OR2T35	0.127893959	3.404455311	0.000700932	0.001702686	no
NAT10	-0.127892074	-3.404404306	0.000701061	0.001702794	no
EFHB	-0.127874056	-3.403916685	0.000702293	0.001705581	no
TRAPPC2P1	0.127872034	3.40386197	0.000702432	0.001705712	no
ZCCHC8	0.127868869	3.403776327	0.000702648	0.001706034	no
ARHGAP21	-0.127848052	-3.403212983	0.000704075	0.001709292	no
ZNF496	-0.127838909	-3.402965574	0.000704702	0.001710609	no
EXOC7	-0.127818278	-3.402407252	0.00070612	0.001713845	no
CDC6	0.127677648	3.398601744	0.000715853	0.00173726	no
FLJ45445	0.127675492	3.398543418	0.000716003	0.001737416	no
SAMM50	-0.127647753	-3.397792789	0.000717938	0.001741902	no
STRBP	-0.127637351	-3.397511324	0.000718665	0.001743456	no
B4GALT7	0.127623914	3.397147734	0.000719605	0.001745432	no
PNMA5	-0.127623241	-3.397129517	0.000719652	0.001745432	no
ASAP3	0.127619807	3.397036607	0.000719892	0.001745806	no
RPL23P8	-0.127612427	-3.396836907	0.000720409	0.00174685	no
C9orf116	0.127581172	3.395991173	0.000722602	0.001751957	no
CDC73	0.127571522	3.395730075	0.00072328	0.001753391	no
EIF4G1	0.127560914	3.395443033	0.000724027	0.00175499	no
RPS24	-0.127555554	-3.395298002	0.000724404	0.001755622	no
GMEB2	-0.127554146	-3.395259885	0.000724503	0.001755622	no
ADRBK2	-0.127552946	-3.395227421	0.000724588	0.001755622	no
ARHGAP12	-0.127552281	-3.395209431	0.000724635	0.001755622	no
REEP3	0.127524969	3.394470404	0.000726561	0.001760078	no
CSNK1A1P	0.127496372	3.393696634	0.000728583	0.001764765	no
COMMD7	0.12749201	3.393578604	0.000728892	0.001765302	no
SHROOM1	0.127477728	3.39319217	0.000729904	0.001767541	no
TTC14	-0.127472109	-3.393040143	0.000730303	0.001768295	no
ACSBG1	-0.12745609	-3.392606709	0.00073144	0.001770837	no
SC65	0.127443246	3.392259194	0.000732353	0.001772835	no
SULT4A1	-0.127431407	-3.391938848	0.000733196	0.001774663	no
SNRK	-0.127417433	-3.391560746	0.000734192	0.00177686	no
PTPRQ	0.127389642	3.390808818	0.000736176	0.001781449	no
DHRS7B	0.127387599	3.39075354	0.000736322	0.001781589	no
PRH1	-0.127386172	-3.390714946	0.000736424	0.001781623	no
RASA4	-0.127371119	-3.390307661	0.000737501	0.001784015	no
WDR16	0.127368689	3.390241896	0.000737675	0.001784175	no
RALGAPA2	0.12736773	3.390215964	0.000737744	0.001784175	no
CCS	-0.127340741	-3.38948573	0.000739679	0.001788642	no
HNRNPM	-0.127318079	-3.388872587	0.000741308	0.001792366	no
LOC91316	-0.127302869	-3.388461067	0.000742403	0.001794799	no

SYN2	-0.127301267	-3.388417717	0.000742518	0.001794864	no
ARG2	0.127278217	3.387794102	0.000744181	0.001798667	no
CCT7	-0.127264567	-3.387424772	0.000745167	0.001800836	no
RBP3	-0.127259349	-3.387283615	0.000745545	0.001801532	no
ORMDL3	-0.127252718	-3.387104216	0.000746024	0.001802476	no
GALNTL1	-0.127243119	-3.386844514	0.000746719	0.001803939	no
MRPL30	-0.127223718	-3.386319618	0.000748126	0.001807121	no
RHOT2	-0.127203423	-3.385770533	0.0007496	0.001810465	no
OR2A1	0.127181139	3.385167643	0.000751221	0.001814165	no
SOHLH1	-0.127169493	-3.384852566	0.00075207	0.001815998	no
RAN	0.127161999	3.384649837	0.000752616	0.0018171	no
TCEB1	0.127157936	3.384539915	0.000752913	0.001817599	no
ZNF761	0.127154246	3.384440085	0.000753182	0.001818032	no
KRT73	0.12712119	3.383545772	0.000755599	0.001823649	no
FAM195B	-0.127117112	-3.383435443	0.000755898	0.001824152	no
NFIC	0.127110249	3.38324978	0.000756401	0.001825148	no
KIAA1310	0.127086652	3.382611395	0.000758132	0.001829108	no
LGR4	0.127080409	3.382442487	0.000758591	0.001829996	no
RIMKLA	-0.127016282	-3.38070766	0.000763318	0.001841179	no
INSIG1	-0.12700807	-3.380485512	0.000763925	0.001842424	no
LEKR1	0.127001985	3.380320888	0.000764376	0.00184329	no
SYT14	-0.126984425	-3.379845844	0.000765676	0.001846207	no
TRIM50	0.126982992	3.379807076	0.000765783	0.001846243	no
ST3GAL4	0.126980417	3.379737415	0.000765974	0.001846483	no
SLC19A1	-0.126949882	-3.37891137	0.000768241	0.001851729	no
TYRO3	-0.126939348	-3.378626413	0.000769025	0.001853397	no
ADAMTS1	0.126893701	3.377391586	0.00077243	0.00186138	no
YWHAZ	0.126869053	3.376724811	0.000774274	0.001865602	no
SLITRK3	-0.126843026	-3.376020764	0.000776226	0.001870082	no
TRABD	0.126825459	3.375545554	0.000777546	0.001873039	no
HES4	-0.126815037	-3.375263647	0.00077833	0.00187451	no
PABPC1L2A	-0.126814877	-3.375259311	0.000778342	0.00187451	no
MMD	0.126791054	3.374614889	0.000780137	0.001878609	no
CTH	-0.126778881	-3.374285611	0.000781055	0.001880598	no
TM7SF3	0.1267583	3.373728877	0.000782611	0.001884119	no
C19orf73	-0.126756685	-3.373685204	0.000782733	0.001884188	no
C9orf102	-0.126752039	-3.373559513	0.000783085	0.00188481	no
NIPBL	-0.126743577	-3.373330611	0.000783726	0.001886128	no
ZNF285	-0.126733897	-3.373068794	0.000784459	0.001887669	no
RPLP0P2	0.126722742	3.37276705	0.000785305	0.00188948	no
LAMB4	0.12671024	3.37242886	0.000786255	0.001891539	no
NIN	-0.126705424	-3.372298597	0.000786621	0.001892195	no
C10orf67	0.126704021	3.372260642	0.000786727	0.001892226	no
GARS	0.126697997	3.372097689	0.000787185	0.001893073	no
C17orf72	0.126696193	3.372048914	0.000787323	0.001893073	no
SOX2OT	-0.126695699	-3.372035547	0.00078736	0.001893073	no
C1QTNF1	0.126692209	3.371941131	0.000787626	0.001893353	no
NUFIP2	0.126691703	3.371927452	0.000787664	0.001893353	no
SLPI	0.126678121	3.371560065	0.000788698	0.001895614	no
ZNF514	-0.126659166	-3.371047362	0.000790144	0.001898863	no
ADPRHL1	-0.126640532	-3.370543314	0.000791567	0.001902057	no

KCNJ4	-0.126633833	-3.370362122	0.00079208	0.001903062	no
TPTE2	-0.126561757	-3.368412592	0.000797611	0.001916125	no
C16orf42	-0.126550067	-3.368096382	0.000798512	0.00191806	no
SLC6A17	-0.126548505	-3.368054143	0.000798632	0.001918121	no
ZNF646	-0.126529281	-3.367534177	0.000800115	0.001921455	no
SFN	0.126502649	3.366813855	0.000802174	0.001926171	no
SETD2	-0.126478764	-3.366167821	0.000804025	0.001930386	no
COL6A6	0.126438748	3.365085506	0.000807134	0.001937621	no
PPP2R2A	0.126402176	3.364096372	0.000809986	0.001944236	no
NAT9	-0.126374115	-3.363337416	0.00081218	0.001949271	no
SAA2	0.126348868	3.362654591	0.000814159	0.001953789	no
MED16	-0.126343461	-3.36250836	0.000814584	0.001954575	no
LOC100134259	0.126309567	3.361591666	0.000817249	0.001960537	no
THOC5	0.126309395	3.361587028	0.000817262	0.001960537	no
ELF2	0.126282882	3.360869972	0.000819353	0.001965319	no
C19orf25	-0.126270636	-3.360538782	0.00082032	0.001967405	no
KDM5C	-0.126260335	-3.360260181	0.000821134	0.001969125	no
CBX6	-0.126258473	-3.36020983	0.000821281	0.001969212	no
SLED1	0.126257413	3.36018116	0.000821365	0.001969212	no
EFHD1	-0.126248923	-3.359951559	0.000822037	0.001970463	no
KIAA1462	-0.126248358	-3.359936273	0.000822082	0.001970463	no
HEATR6	-0.126222707	-3.359242553	0.000824115	0.001975102	no
APEX1	-0.126189437	-3.358342786	0.000826759	0.001981205	no
LOC646982	0.12616428	3.357662442	0.000828764	0.001985773	no
NOB1	-0.1261298	-3.356729974	0.000831519	0.001992138	no
HSCB	0.126071148	3.355143825	0.000836225	0.002003174	no
GIYD2	0.126067335	3.355040702	0.000836531	0.002003671	no
SKP2	0.126062622	3.354913249	0.000836911	0.002004343	no
DOT1L	-0.126055095	-3.35470971	0.000837517	0.002005557	no
7-Mar	0.126052729	3.354645718	0.000837707	0.002005775	no
NUDCD1	0.126030069	3.354032928	0.000839535	0.002009913	no
N6AMT2	0.126015433	3.35363715	0.000840717	0.002012506	no
NFATC4	0.125975128	3.35254722	0.000843981	0.00202008	no
POM121L1P	0.125959147	3.352115054	0.000845279	0.002022946	no
NDUFC2	-0.125944363	-3.351715282	0.000846481	0.002025582	no
AMAC1L3	-0.125908234	-3.350738302	0.000849425	0.002032386	no
AFG3L2	-0.125875364	-3.349849465	0.000852111	0.002038573	no
CNR1	0.125872991	3.349785283	0.000852305	0.002038797	no
SRD5A1	0.125828572	3.348584168	0.000855949	0.002047271	no
MGC45800	0.125822714	3.348425761	0.000856431	0.00204818	no
KIAA0831	-0.125820492	-3.348365689	0.000856613	0.002048375	no
LOC256880	-0.125811391	-3.348119599	0.000857362	0.002049923	no
HSPB7	0.125800754	3.34783197	0.000858238	0.002051754	no
TOMM70A	-0.125799632	-3.347801623	0.000858331	0.002051754	no
AGR3	0.125772586	3.347070315	0.000860562	0.002056844	no
DNAJC11	-0.125734589	-3.346042873	0.000863706	0.002064115	no
PVRL4	0.125659381	3.344009318	0.00086996	0.002078816	no
TSSK3	-0.125657554	-3.343959934	0.000870113	0.002078934	no
NFE2L1	0.125644364	3.343603277	0.000871214	0.00208132	no
C12orf49	0.125640923	3.343510251	0.000871502	0.002081761	no
CXCL5	0.125626712	3.343126005	0.00087269	0.002084353	no

C20orf195	0.125620794	3.342965985	0.000873186	0.00208529	no
ROPN1L	0.125602131	3.342461383	0.000874749	0.002088778	no
LAMB3	0.125588053	3.342080734	0.000875931	0.002091352	no
RBM25	-0.125565297	-3.341465459	0.000877843	0.002095671	no
LOC285627	0.12554862	3.341014563	0.000879248	0.002098775	no
TMEM195	0.125546114	3.340946815	0.000879459	0.002099031	no
ICAM5	-0.12554432	-3.340898307	0.00087961	0.002099145	no
FAM131C	-0.125506316	-3.339870776	0.000882819	0.002106554	no
LPIN1	-0.125484476	-3.339280282	0.000884668	0.002110717	no
GALNTL6	-0.125458769	-3.338585247	0.000886849	0.00211567	no
ZSCAN21	-0.125448768	-3.33831487	0.000887698	0.002117448	no
MAGI3	-0.125424773	-3.337666127	0.00088974	0.002122068	no
PACSIN2	0.125415247	3.337408574	0.000890552	0.002123604	no
LYPD6	0.125414752	3.337395199	0.000890595	0.002123604	no
KIF18A	0.125361391	3.335952547	0.000895155	0.00213402	no
OIT3	0.125361172	3.335946628	0.000895174	0.00213402	no
XIAP	-0.125352628	-3.335715626	0.000895906	0.002135453	no
CA12	0.125350943	3.335670062	0.000896051	0.002135453	no
KIAA1671	-0.125350462	-3.335657071	0.000896092	0.002135453	no
RGN	0.125335416	3.335250304	0.000897383	0.002138278	no
BCAM	0.125313732	3.334664079	0.000899247	0.002142466	no
SPSB2	0.125302862	3.334370191	0.000900182	0.002144443	no
TBC1D3	-0.125299739	-3.334285771	0.000900451	0.002144831	no
PDCD11	-0.125291556	-3.334064534	0.000901156	0.002146258	no
CBFA2T3	-0.125199361	-3.331572097	0.000909136	0.002165008	no
CCDC90B	0.125194392	3.331437777	0.000909568	0.002165663	no
FADS3	0.125193733	3.331419967	0.000909625	0.002165663	no
PPP4C	0.125186819	3.331233059	0.000910227	0.002166839	no
ZNF90	0.125158027	3.330454685	0.000912735	0.002172554	no
OSR1	0.125121908	3.329478289	0.00091589	0.002179809	no
SDC4	0.125115712	3.329310778	0.000916433	0.002180843	no
CDC123	-0.125112451	-3.329222639	0.000916718	0.002181265	no
LOC100130691	-0.125101235	-3.328919437	0.000917701	0.002183347	no
FAM189A2	-0.125056677	-3.327714919	0.000921615	0.0021924	no
UNG	0.125044288	3.327379994	0.000922706	0.002194737	no
ZNF727	-0.125033959	-3.327100781	0.000923616	0.002196645	no
PCSK1	0.12499295	3.325992244	0.000927239	0.002205002	no
SEC11C	0.124983606	3.32573966	0.000928067	0.002206653	no
SPAG17	0.124982643	3.325713622	0.000928152	0.002206653	no
MESP1	-0.124973331	-3.325461909	0.000928977	0.002208355	no
C21orf122	0.124950233	3.32483753	0.000931027	0.002212968	no
TTC21B	-0.124947194	-3.324755379	0.000931297	0.00221335	no
PBLD	-0.124934555	-3.324413737	0.000932421	0.00221576	no
MPDZ	-0.124929054	-3.324265029	0.00093291	0.002216607	no
LRBA	0.124928083	3.324238796	0.000932997	0.002216607	no
PPM1J	0.124919055	3.323994762	0.000933801	0.002218257	no
RHPN1	-0.124912676	-3.323822315	0.000934369	0.002219346	no
SET	-0.124894738	-3.32333744	0.000935969	0.002222787	no
ZNF207	0.124893974	3.323316796	0.000936038	0.002222787	no
MMP15	-0.124869634	-3.322658871	0.000938213	0.002227691	no
PDE1C	0.124849611	3.322117637	0.000940006	0.002231687	no

PI3	0.12482338	3.321408595	0.00094236	0.002237012	no
C11orf16	0.124807625	3.320982745	0.000943776	0.002240112	no
LANCL1	-0.124800219	-3.320782567	0.000944443	0.00224143	no
LOC100288778	-0.124788657	-3.320470046	0.000945484	0.002243639	no
TNRC6A	-0.124783247	-3.32032383	0.000945972	0.002244532	no
PIK3CB	-0.124773751	-3.320067147	0.000946828	0.002246301	no
ISPD	-0.124766896	-3.31988186	0.000947447	0.002247505	no
HSD17B3	0.124750309	3.31943353	0.000948946	0.002250796	no
WSB2	-0.12474214	-3.31921272	0.000949685	0.002252285	no
LOC415056	-0.124732668	-3.318956688	0.000950542	0.002254054	no
CORO6	-0.124722009	-3.318668609	0.000951508	0.002256079	no
HIP1	-0.124703513	-3.318168678	0.000953186	0.002259792	no
ZNF804B	-0.124689949	-3.317802065	0.000954418	0.002262449	no
HIST1H1C	0.12466685	3.317177722	0.00095652	0.002267165	no
SKI	0.124633593	3.316278832	0.000959554	0.002274089	no
SEL1L3	0.124611235	3.315674548	0.000961598	0.002278667	no
SNHG4	0.124581049	3.314858704	0.000964365	0.002284956	no
POFUT2	0.12457324	3.31464764	0.000965082	0.002286386	no
CPXM1	0.124564862	3.314421193	0.000965851	0.002287942	no
CCDC96	0.124561683	3.314335279	0.000966144	0.002288226	no
UGT2B11	0.124561094	3.314319371	0.000966198	0.002288226	no
TRIM59	0.124546653	3.313929052	0.000967526	0.002290905	no
MS4A6E	0.124546336	3.3139205	0.000967555	0.002290905	no
TMEM135	-0.124532244	-3.313539631	0.000968854	0.00229371	no
ATP11A	0.124503331	3.312758202	0.000971522	0.002299758	no
NOV	0.124473724	3.311958031	0.000974261	0.002305972	no
UMPS	0.124462144	3.311645072	0.000975335	0.002308242	no
LOC92659	-0.124459801	-3.311581737	0.000975552	0.002308487	no
GABRG1	-0.12444721	-3.311241447	0.000976721	0.002310981	no
HTR5A	-0.124436434	-3.310950209	0.000977722	0.00231308	no
HMCN1	0.124417257	3.310431945	0.000979506	0.002317029	no
CALY	-0.124415576	-3.310386502	0.000979662	0.002317128	no
SCML1	0.124400389	3.309976073	0.000981077	0.002320204	no
HSPB9	-0.124388547	-3.309656023	0.000982182	0.002322545	no
GORAB	0.124380861	3.309448322	0.0009829	0.00232397	no
PPRC1	-0.1243778	-3.309365576	0.000983186	0.002324375	no
USP31	-0.124298951	-3.307234703	0.000990578	0.002341577	no
WSB1	-0.12428303	-3.306804447	0.000992077	0.002344846	no
TUFM	-0.1242773	-3.30664958	0.000992617	0.002345848	no
VCAN	-0.124210469	-3.304843536	0.000998935	0.002360503	no
TESK1	-0.124205462	-3.304708229	0.00099941	0.002361349	no
MPO	0.124181101	3.304049897	0.001001723	0.002366538	no
GORASP1	-0.124172061	-3.303805615	0.001002583	0.002368293	no
NCRNA00164	-0.124166796	-3.303663327	0.001003084	0.002369199	no
RNF34	0.124142704	3.303012289	0.001005379	0.002374343	no
CCBL2	0.12412912	3.302645218	0.001006675	0.002377127	no
SURF2	-0.124120392	-3.30240935	0.001007509	0.002378818	no
ZNF648	0.124094161	3.301700523	0.001010019	0.002384465	no
C19orf36	-0.12408757	-3.301522432	0.00101065	0.002385677	no
EIF3C	-0.124078661	-3.301281695	0.001011504	0.002387415	no
POT1	0.124077231	3.301243029	0.001011642	0.00238746	no

MTUS2	-0.124069268	-3.301027859	0.001012406	0.002388984	no
GLIPR2	0.124007182	3.299350175	0.001018382	0.002402613	no
PIAS4	-0.124006795	-3.299339712	0.001018419	0.002402613	no
IFI44L	0.124003957	3.299263038	0.001018693	0.002402979	no
TOP1P2	-0.123999708	-3.299148215	0.001019103	0.002403419	no
ATP6V1B1	-0.123999564	-3.299144317	0.001019117	0.002403419	no
UBE2H	0.123994199	3.298999362	0.001019635	0.002404361	no
CD99L2	-0.123988478	-3.298844771	0.001020188	0.002405384	no
THG1L	0.123986899	3.2988021	0.001020341	0.002405464	no
WWP1	0.123984889	3.298747796	0.001020535	0.0024055	no
IKBKAP	-0.123984277	-3.298731253	0.001020595	0.0024055	no
ENTPD6	-0.123971496	-3.298385879	0.001021831	0.002408135	no
DPY30	0.123961759	3.298122777	0.001022774	0.002410076	no
KIF13B	0.123940504	3.297548456	0.001024836	0.002414653	no
CRYGA	-0.123938158	-3.297485055	0.001025064	0.002414908	no
CEP120	0.123934775	3.297393655	0.001025392	0.0024154	no
ERVFRDE1	0.123929821	3.297259775	0.001025874	0.002416253	no
RAB11A	0.123927988	3.297210259	0.001026052	0.002416391	no
SYT5	-0.123915029	-3.296860083	0.001027312	0.002419077	no
DYNC1I1	-0.123906972	-3.296642388	0.001028096	0.002420641	no
COL11A2	-0.12389834	-3.296409144	0.001028937	0.002422339	no
PLA2G16	0.123881667	3.295958638	0.001030563	0.002425884	no
DYDC2	0.123868005	3.295589492	0.001031897	0.002428532	no
DOLK	0.123867682	3.295580764	0.001031928	0.002428532	no
LOC283663	0.123858133	3.295322727	0.001032862	0.002430446	no
SPCS1	0.123849757	3.295096419	0.001033681	0.002432091	no
PHYHIP	-0.123830644	-3.294579994	0.001035553	0.002436212	no
TELO2	-0.123817919	-3.294236172	0.001036801	0.002438864	no
MTHFD2	-0.123811304	-3.294057417	0.00103745	0.002440107	no
MANSC1	0.123788156	3.29343198	0.001039725	0.002445174	no
ZBTB10	-0.123782203	-3.293271126	0.001040311	0.002446267	no
KIAA1045	-0.123777011	-3.293130846	0.001040822	0.002447184	no
PRSS35	-0.123722046	-3.291645743	0.001046248	0.002459656	no
SPINK1	0.12369583	3.290937417	0.001048845	0.002465475	no
RALGDS	-0.123677372	-3.290438715	0.001050677	0.002469494	no
FAM69A	-0.12366381	-3.290072297	0.001052025	0.002472375	no
SYT7	-0.123657145	-3.289892222	0.001052688	0.002473646	no
SMAD6	0.123631673	3.289204027	0.001055226	0.00247906	no
GLS2	-0.123631555	-3.289200833	0.001055238	0.00247906	no
DTYMK	0.123618149	3.288838632	0.001056575	0.002481691	no
NEUROD2	-0.123617875	-3.288831235	0.001056603	0.002481691	no
PRKAR2A	0.123612837	3.288695102	0.001057106	0.002482584	no
ZNF491	-0.123605662	-3.28850125	0.001057823	0.00248398	no
ARHGEF18	-0.123586941	-3.287995477	0.001059696	0.002488088	no
PURA	-0.123559382	-3.287250903	0.001062459	0.002494285	no
DYNLL1	0.123554351	3.287114973	0.001062964	0.002495181	no
SYNPO	0.123544274	3.286842732	0.001063976	0.002497268	no
MGC72080	0.123529343	3.286439348	0.001065477	0.002500502	no
RBM8A	-0.123508287	-3.285870484	0.001067598	0.002505188	no
TMEM178	-0.123475533	-3.284985584	0.001070905	0.002512656	no
KCNK12	-0.123456973	-3.284484161	0.001072783	0.00251672	no

PIGX	-0.123455954	-3.284456623	0.001072886	0.00251672	no
TSPAN10	0.12344982	3.28429092	0.001073508	0.002517886	no
MRPS5	-0.123440182	-3.284030533	0.001074485	0.002519885	no
C20orf112	-0.123408905	-3.283185568	0.001077661	0.002527041	no
RDH11	-0.123389427	-3.282659364	0.001079643	0.002531396	no
DNAH6	0.123367793	3.282074914	0.001081849	0.002536274	no
HDC	0.12335084	3.281616916	0.001083581	0.002539893	no
NOMO1	-0.123350219	-3.281600138	0.001083644	0.002539893	no
LOC728875	0.123348137	3.281543891	0.001083857	0.002540097	no
XKR9	0.123343588	3.281421023	0.001084322	0.002540893	no
CLCC1	0.123337397	3.281253757	0.001084955	0.002542082	no
CELF1	-0.123329944	-3.281052415	0.001085718	0.002543575	no
SRCRB4D	0.123320983	3.28081034	0.001086636	0.00254543	no
PABPC1L2B	-0.123309567	-3.280501948	0.001087806	0.002547877	no
LOC283332	-0.123292495	-3.280040764	0.001089559	0.002551686	no
GSC	0.123272541	3.279501702	0.001091611	0.002556194	no
ACR	-0.123262174	-3.279221645	0.001092678	0.002558397	no
TADA1	-0.123248004	-3.278838876	0.001094138	0.00256152	no
TCF23	0.123202861	3.277619377	0.001098803	0.002572143	no
TEX264	0.123149113	3.276167494	0.00110438	0.0025849	no
GUSBL1	-0.123110627	-3.275127856	0.00110839	0.002593985	no
FREM1	0.123051378	3.273527418	0.00111459	0.002608192	no
ATP2A2	-0.123032312	-3.273012402	0.001116591	0.002612573	no
INSL5	0.123027101	3.272871659	0.001117139	0.002613552	no
HSPC072	-0.12298685	-3.271784406	0.001121378	0.002623165	no
SRI	-0.122973879	-3.271434056	0.001122747	0.002625841	no
XPO7	-0.122973551	-3.271425185	0.001122782	0.002625841	no
RGAG4	-0.122955378	-3.270934327	0.001124702	0.002630029	no
GOLGA6B	-0.122947504	-3.270721645	0.001125536	0.002631673	no
NXF4	-0.122941348	-3.270555347	0.001126188	0.002632893	no
C3orf54	0.122938305	3.270473157	0.00112651	0.002633342	no
CXorf26	0.122932852	3.270325881	0.001127088	0.002634388	no
SLC35F3	-0.122929729	-3.270241515	0.001127419	0.002634857	no
UBXN8	0.122873703	3.268728242	0.001133373	0.002648466	no
TUBA1B	0.122855293	3.268230983	0.001135336	0.002652746	no
KIAA0101	0.122816768	3.267190437	0.001139453	0.00266206	no
UGT8	-0.122776724	-3.266108873	0.001143748	0.002671784	no
ZFYVE21	-0.122766289	-3.265827014	0.001144869	0.002674095	no
PPP1R7	-0.122761489	-3.265697379	0.001145386	0.002674992	no
AKAP5	-0.122735764	-3.265002575	0.001148156	0.002681152	no
ABCG4	-0.122708883	-3.264276567	0.001151057	0.002687618	no
FANK1	0.122692279	3.263828117	0.001152853	0.002691499	no
JKAMP	0.122684499	3.263617978	0.001153695	0.002693155	no
ANGPT4	-0.122672063	-3.263282119	0.001155043	0.002695818	no
CXorf22	0.12267151	3.263267176	0.001155103	0.002695818	no
SEC13	0.122668614	3.263188954	0.001155417	0.002696239	no
CYTL1	0.122659224	3.262935352	0.001156435	0.00269811	no
BFSP1	0.122658765	3.262922965	0.001156485	0.00269811	no
LOC144486	-0.122643455	-3.262509472	0.001158148	0.002701677	no
FAM71F1	0.122631675	3.262191314	0.001159429	0.002704353	no
LOC652276	-0.12261359	-3.261702883	0.001161398	0.002708633	no

MED12L	-0.122608722	-3.261571412	0.001161928	0.002709558	no
TNPO1	0.122594555	3.261188806	0.001163474	0.002712849	no
NMB	-0.122579165	-3.260773172	0.001165154	0.002716454	no
SAA1	0.122562079	3.260311709	0.001167023	0.002720497	no
AMIGO1	-0.122556954	-3.260173301	0.001167584	0.002721491	no
DOCK4	0.122553177	3.260071288	0.001167998	0.002722142	no
GYG2	0.122551896	3.260036694	0.001168138	0.002722155	no
TRERF1	-0.122537747	-3.25965459	0.001169689	0.002725264	no
INPP5J	-0.122537263	-3.259641527	0.001169742	0.002725264	no
SOX18	0.122534801	3.25957503	0.001170012	0.002725579	no
SYNE2	-0.122533032	-3.259527247	0.001170206	0.002725718	no
C16orf88	-0.122513939	-3.259011617	0.001172303	0.002730287	no
SELO	-0.122483314	-3.258184548	0.001175673	0.002737617	no
SNRPD2	0.12248288	3.258172822	0.001175721	0.002737617	no
KCNH3	-0.12247713	-3.258017548	0.001176355	0.002738636	no
DBP	-0.122476452	-3.257999239	0.001176429	0.002738636	no
LOC100129726	-0.122473502	-3.257919558	0.001176755	0.002739078	no
C2orf73	0.122463856	3.257659059	0.001177819	0.002741239	no
HIST4H4	0.122460963	3.257580934	0.001178138	0.002741667	no
ZNF844	-0.122399394	-3.255918228	0.001184954	0.002757211	no
LOC389458	-0.122389368	-3.255647468	0.001186068	0.002759484	no
SCRIB	-0.122382103	-3.255451257	0.001186875	0.002761044	no
DNAJB1	0.12236938	3.25510767	0.00118829	0.002764018	no
RPRD1B	0.122365765	3.25501007	0.001188692	0.002764636	no
HEATR1	0.12236452	3.254976443	0.001188831	0.00276464	no
RYR1	-0.122347802	-3.254524963	0.001190694	0.002768399	no
AQP11	-0.122347556	-3.254518323	0.001190721	0.002768399	no
RNF41	-0.122331553	-3.254086184	0.001192506	0.002772231	no
ABCG8	-0.122308882	-3.253473937	0.00119504	0.002777802	no
PPARA	-0.122300465	-3.253246646	0.001195982	0.002779672	no
SYT9	-0.12229457	-3.253087472	0.001196642	0.002780886	no
CDCA2	0.12228958	3.252952714	0.001197201	0.002781865	no
TTC29	0.122276568	3.252601324	0.00119866	0.002784935	no
ZNF99	-0.12227292	-3.252502833	0.001199069	0.002785566	no
FAM90A7	0.122259097	3.252129547	0.001200621	0.002788794	no
HCN1	-0.122258087	-3.252102272	0.001200735	0.002788794	no
C16orf79	-0.122252279	-3.251945423	0.001201388	0.00278999	no
MTUS1	0.122236131	3.251509389	0.001203204	0.002793888	no
CYP8B1	0.12222252	3.25114183	0.001204737	0.002797126	no
CHTF18	-0.122211756	-3.250851159	0.001205951	0.002799623	no
SYT1	-0.122192598	-3.250333829	0.001208114	0.002804322	no
UTP3	0.122182171	3.250052282	0.001209293	0.002806736	no
LOC646214	-0.122147604	-3.249118847	0.001213208	0.0028155	no
NELL1	-0.122145396	-3.249059238	0.001213458	0.002815758	no
ANKFY1	-0.122126364	-3.248545331	0.001215619	0.002820449	no
MPPED1	-0.122114723	-3.248230981	0.001216943	0.002823196	no
CDC34	-0.122099961	-3.247832369	0.001218623	0.00282677	no
PSMD4	-0.122095771	-3.247719236	0.001219101	0.002827552	no
FAM154A	0.122081309	3.247328719	0.001220749	0.002831052	no
PGD	0.122039818	3.2462084	0.001225491	0.002841723	no
SCAF1	-0.122015482	-3.245551266	0.001228281	0.002847864	no

HSD17B7	0.122008489	3.245362458	0.001229083	0.002849398	no
LOC341056	-0.121996754	-3.245045601	0.001230431	0.002852196	no
NCRNA00188	-0.121994684	-3.244989699	0.001230669	0.00285242	no
B4GALT6	-0.12199011	-3.244866203	0.001231195	0.002853312	no
CYP3A43	-0.121973377	-3.244414403	0.00123312	0.002857446	no
GUSBP3	0.121951702	3.243829151	0.001235618	0.002862907	no
TSSC4	-0.121940368	-3.243523112	0.001236926	0.002865492	no
SERBP1	0.121939582	3.243501881	0.001237017	0.002865492	no
EMID1	0.121924187	3.243086207	0.001238796	0.002869284	no
HERC2P2	-0.121917676	-3.242910428	0.001239549	0.0028707	no
ZC3H13	-0.12188289	-3.24197118	0.00124358	0.002879705	no
LOC647309	0.121838946	3.24078471	0.001248689	0.002891205	no
FAM135B	-0.121806057	-3.239896697	0.001252526	0.002899756	no
DCP2	0.121797099	3.23965484	0.001253573	0.002901847	no
TMC6	0.121789756	3.239456583	0.001254432	0.00290334	no
SGCE	0.121789131	3.239439719	0.001254505	0.00290334	no
TMEM181	-0.121735039	-3.237979301	0.001260847	0.002917685	no
CCDC115	-0.121690277	-3.2367708	0.001266118	0.002929547	no
LOC100132215	0.121686455	3.236667607	0.00126657	0.002930255	no
CHST9	-0.121644923	-3.235546323	0.00127148	0.00294128	no
LPIN2	0.121624512	3.234995267	0.0012739	0.00294654	no
SLC4A11	0.121619562	3.234861631	0.001274487	0.002947561	no
TM4SF19	0.121601061	3.234362144	0.001276685	0.002952307	no
CRYZL1	-0.121586121	-3.233958811	0.001278462	0.002956079	no
SHISA9	-0.12157601	-3.233685851	0.001279667	0.002958525	no
BAIAP2	-0.121563446	-3.233346652	0.001281164	0.002961649	no
TRMT61A	-0.121537885	-3.232656579	0.001284217	0.002968299	no
C11orf90	-0.121536898	-3.232629934	0.001284335	0.002968299	no
EAPP	-0.121526273	-3.232343095	0.001285605	0.002970896	no
C5orf39	0.121513652	3.232002386	0.001287116	0.002974048	no
NDRG1	0.121498335	3.231588886	0.001288952	0.002977949	no
DCP1B	0.121490551	3.231378737	0.001289886	0.002979767	no
USHBP1	-0.121466779	-3.230736983	0.001292742	0.002986023	no
CHRNB4	0.121450819	3.230306143	0.001294663	0.002990118	no
PROCA1	-0.121448211	-3.230235736	0.001294977	0.002990502	no
AMY2A	-0.121443553	-3.230109983	0.001295538	0.002991457	no
LBP	0.121440978	3.23004047	0.001295849	0.002991832	no
THSD7A	-0.121438291	-3.229967917	0.001296173	0.002992238	no
HRK	-0.121412648	-3.229275674	0.001299268	0.00299904	no
PEX7	-0.121390574	-3.228679776	0.001301937	0.003004859	no
FAM160A2	-0.121383768	-3.228496067	0.001302761	0.003006418	no
ANXA13	0.121372471	3.228191103	0.00130413	0.003009234	no
C9orf84	0.121361813	3.227903376	0.001305423	0.003011873	no
HSPA1L	-0.121322266	-3.226835814	0.001310231	0.00302262	no
L3MBTL4	0.121318272	3.22672799	0.001310717	0.003023397	no
DDN	-0.121284644	-3.225820227	0.001314819	0.003032513	no
SLC12A5	-0.121266141	-3.225320769	0.001317081	0.003037252	no
U2AF1	-0.121264835	-3.225285514	0.001317241	0.003037252	no
CHAF1B	0.121264152	3.225267071	0.001317324	0.003037252	no
DDI2	0.121255422	3.225031402	0.001318393	0.00303937	no
CDH7	-0.12124793	-3.224829184	0.001319311	0.003041139	no

LOC644669	-0.121246009	-3.22477732	0.001319546	0.003041335	no
ELOVL5	0.121213318	3.223894865	0.001323559	0.003050235	no
MBOAT4	0.121198504	3.223494982	0.001325381	0.003054085	no
SP3	0.121192584	3.223335181	0.001326109	0.003055416	no
GABRA1	-0.121170981	-3.222752056	0.001328772	0.003061201	no
NUDC	-0.121151753	-3.222233047	0.001331145	0.00306632	no
MADCAM1	-0.121136995	-3.221834681	0.00133297	0.003070174	no
BUB3	-0.121133498	-3.221740279	0.001333403	0.00307082	no
OR13C2	0.121128934	3.221617098	0.001333967	0.003071771	no
CARKD	-0.121124693	-3.221502621	0.001334492	0.003072631	no
SHC3	-0.121091183	-3.220598104	0.001338648	0.003081848	no
SLC44A2	-0.121069571	-3.220014748	0.001341335	0.003087682	no
ZDHHC11	-0.121048193	-3.219437704	0.001343997	0.003093458	no
EPB41L1	-0.121044663	-3.219342421	0.001344437	0.003094119	no
SLITRK4	-0.121041989	-3.219270243	0.001344771	0.003094534	no
EDEM3	0.121032905	3.21902506	0.001345904	0.00309679	no
HES5	-0.12100269	-3.21820952	0.00134968	0.003105125	no
SNORD116-28	-0.120993715	-3.217967266	0.001350804	0.003107356	no
TMEM44	0.120975966	3.217488194	0.001353028	0.003112119	no
ZNF592	-0.120973604	-3.217424442	0.001353325	0.003112447	no
TYW3	0.120967894	3.217270325	0.001354041	0.00311374	no
SLC25A12	-0.120964652	-3.217182811	0.001354448	0.003114322	no
SNORD1C	0.120949899	3.21678462	0.001356301	0.003118229	no
CCDC102B	0.120937474	3.216449261	0.001357864	0.003121466	no
ISG15	0.120935673	3.21640066	0.001358091	0.003121632	no
ASPRV1	-0.12092065	-3.215995158	0.001359983	0.003125626	no
OR10AD1	0.120905006	3.215572929	0.001361955	0.003129804	no
FAM18A	0.120884317	3.215014527	0.001364568	0.003135452	no
PTPN5	-0.120882604	-3.214968292	0.001364785	0.003135593	no
MYOZ2	-0.120853194	-3.214174517	0.001368508	0.003143789	no
SPDYA	0.120851597	3.214131416	0.00136871	0.003143897	no
CENPN	0.120813848	3.213112579	0.001373503	0.003154271	no
ZFPL1	-0.120813569	-3.213105042	0.001373538	0.003154271	no
SERINC4	-0.120807079	-3.212929902	0.001374364	0.003155808	no
PSD2	-0.120802677	-3.21281107	0.001374924	0.003156736	no
ZNF365	-0.12078255	-3.212267856	0.001377488	0.003162264	no
DLEC1	0.120781099	3.212228711	0.001377673	0.003162329	no
C2orf60	-0.120770046	-3.211930388	0.001379084	0.003165207	no
MIB2	-0.120758973	-3.211631556	0.001380497	0.003168093	no
MRPS14	0.120748016	3.211335835	0.001381898	0.003170947	no
C19orf24	0.120731384	3.21088694	0.001384027	0.003175471	no
DHX15	0.120711532	3.210351166	0.001386571	0.003180669	no
KLK13	0.120711252	3.210343614	0.001386607	0.003180669	no
BLOC1S3	0.120676627	3.209409147	0.001391055	0.003190272	no
MAB21L1	0.120676213	3.209397972	0.001391109	0.003190272	no
NSMCE4A	-0.120674588	-3.20935413	0.001391318	0.003190389	no
ZNF524	0.120668658	3.209194088	0.001392081	0.003191778	no
TTC13	-0.120649554	-3.208678511	0.001394543	0.003196736	no
KIF23	0.120649422	3.20867493	0.00139456	0.003196736	no
DDO	0.120642702	3.208493594	0.001395427	0.00319836	no
BIRC7	0.12059815	3.207291232	0.001401186	0.003211197	no

DNAJB13	0.120593541	3.207166839	0.001401783	0.003212202	no
LOC100131496	0.120590673	3.207089433	0.001402155	0.003212689	no
GPR75	-0.120527568	-3.205386425	0.001410356	0.003231113	no
UQCR10	0.120485491	3.204250926	0.001415849	0.003243329	no
SFRP2	-0.120472442	-3.203898773	0.001417556	0.003246873	no
FAM66E	-0.120459596	-3.203552107	0.001419239	0.003250359	no
EIF4A1	0.120454045	3.203402311	0.001419966	0.003251657	no
QARS	0.120438822	3.202991516	0.001421964	0.003255862	no
SLC17A7	-0.120437445	-3.202954348	0.001422144	0.003255907	no
MYL3	-0.120414519	-3.20233567	0.001425158	0.003262437	no
CDV3	0.120389294	3.201654963	0.00142848	0.003269672	no
DYNLT1	0.120339513	3.20031162	0.001435057	0.003284354	no
ENAH	-0.120303314	-3.199334826	0.001439857	0.003294967	no
TCEAL1	-0.120296733	-3.199157222	0.001440732	0.003296595	no
LRFN5	-0.120281726	-3.198752279	0.001442727	0.003300787	no
AUP1	0.120240945	3.19765183	0.001448163	0.003312848	no
WNT7A	-0.120226527	-3.197262787	0.001450089	0.003316879	no
ADM	0.120211163	3.196848203	0.001452144	0.003321204	no
MAGED4	-0.12019464	-3.196402364	0.001454357	0.00332589	no
SMAD7	-0.120191865	-3.196327473	0.001454729	0.003326364	no
ZZEF1	-0.120187001	-3.196196225	0.001455381	0.00332748	no
FOS	0.120182408	3.196072296	0.001455998	0.003328199	no
AP4E1	0.1201822	3.196066702	0.001456025	0.003328199	no
C13orf16	-0.120163986	-3.195575215	0.001458471	0.00333336	no
NIPA1	-0.120162932	-3.195546772	0.001458613	0.00333336	no
ZNRF4	-0.120158448	-3.195425783	0.001459216	0.003334361	no
CTSH	0.120151445	3.195236818	0.001460158	0.003336136	no
MTCH2	0.120142745	3.19500207	0.001461328	0.003338434	no
DDX1	-0.12013454	-3.194780684	0.001462433	0.003340581	no
STX1A	-0.120129207	-3.194636774	0.001463152	0.003341845	no
HLX	0.120120776	3.19440928	0.001464289	0.003344063	no
NCOA5	-0.120108559	-3.194079645	0.001465938	0.00334745	no
HRCT1	0.120103463	3.193942148	0.001466626	0.003348644	no
SAPS1	0.120095384	3.193724156	0.001467717	0.003350758	no
AVPI1	-0.120091238	-3.193612289	0.001468278	0.003351659	no
IMPG2	0.12005412	3.192610765	0.001473305	0.003362754	no
MTCH1	-0.120004145	-3.191262346	0.001480098	0.003377878	no
ITGB1BP1	0.11998665	3.190790314	0.001482483	0.003382939	no
NCRNA00029	0.119969939	3.190339431	0.001484764	0.003387763	no
GRIN2B	-0.119960431	-3.190082873	0.001486064	0.003390345	no
GORASP2	0.119917618	3.188927758	0.001491928	0.00340334	no
CRIPAK	-0.119905984	-3.18861386	0.001493525	0.003406599	no
UBR4	-0.119897934	-3.188396673	0.001494631	0.003408592	no
GPR35	0.11989717	3.188376046	0.001494736	0.003408592	no
RPS27A	-0.119888827	-3.188150948	0.001495884	0.003410824	no
MUS81	-0.119883231	-3.187999983	0.001496653	0.003412194	no
ZNF251	-0.119878055	-3.187860332	0.001497366	0.003413434	no
MAP3K7	0.119873292	3.187731819	0.001498022	0.003414544	no
SYCP2	-0.119844975	-3.186967831	0.001501927	0.003423059	no
DNM1	-0.119815602	-3.186175349	0.001505987	0.003431569	no
FSTL4	-0.11981551	-3.18617286	0.001506	0.003431569	no

GPR26	-0.119793751	-3.185585821	0.001509014	0.003437947	no
POLDIP2	-0.11979285	-3.185561523	0.001509139	0.003437947	no
FBXL12	0.119790338	3.185493733	0.001509488	0.003438353	no
CBX3	0.119773383	3.185036312	0.001511841	0.003443326	no
C20orf194	-0.119761525	-3.184716387	0.001513489	0.003446691	no
ZMYND19	-0.119751624	-3.184449274	0.001514866	0.003449439	no
APPL1	-0.119739015	-3.184109081	0.001516622	0.003453048	no
FRMPD4	-0.119734919	-3.183998585	0.001517193	0.003453958	no
CHCHD3	0.119728853	3.183834918	0.001518038	0.003455494	no
CAB39L	-0.119680504	-3.182530542	0.001524793	0.003470413	no
GBX2	-0.119679484	-3.182503033	0.001524936	0.003470413	no
PCDH8	-0.119662789	-3.182052614	0.001527275	0.003475247	no
OR4N4	-0.119661872	-3.182027887	0.001527404	0.003475247	no
SUB1	-0.119650666	-3.181725556	0.001528976	0.003478352	no
CRYBA1	0.119649691	3.181699273	0.001529112	0.003478352	no
SNAPIN	0.119647076	3.181628718	0.001529479	0.003478796	no
S1PR1	-0.119641133	-3.181468381	0.001530314	0.003480303	no
LIPT1	0.119639502	3.181424397	0.001530543	0.003480432	no
C17orf70	-0.119637375	-3.181366993	0.001530842	0.00348072	no
COPS8	0.119614028	3.180737167	0.001534126	0.003487794	no
EPB41L4B	-0.119585832	-3.179976509	0.0015381	0.003496436	no
PPIL6	0.119581337	3.179855238	0.001538734	0.003497485	no
C3orf31	-0.119579294	-3.179800129	0.001539023	0.003497747	no
ULBP1	0.119550833	3.179032311	0.001543046	0.003506498	no
UBE2Q2P1	-0.119543691	-3.178839653	0.001544057	0.003508401	no
CNIH2	-0.119535744	-3.178625271	0.001545183	0.003510565	no
PLA2G12A	-0.119479816	-3.177116523	0.001553128	0.003528218	no
POLE3	0.119437818	3.17598357	0.001559118	0.003541428	no
HTR1E	-0.11940544	-3.175110162	0.001563751	0.003551552	no
JTB	0.119388139	3.174643453	0.001566232	0.003556588	no
C11orf10	0.119387524	3.174626852	0.00156632	0.003556588	no
CSF3	0.119339941	3.173343291	0.001573162	0.003571722	no
TAS2R60	0.119336126	3.173240378	0.001573712	0.003572569	no
RNF185	-0.119307201	-3.172460138	0.001577885	0.003581642	no
RIMS3	-0.119298872	-3.172235484	0.001579089	0.003583971	no
GPR22	-0.119290496	-3.172009544	0.0015803	0.003585999	no
AP3M1	-0.119290242	-3.172002688	0.001580337	0.003585999	no
C1orf106	-0.119281054	-3.171754833	0.001581667	0.003588614	no
NANOS3	-0.119277694	-3.171664219	0.001582153	0.003589315	no
CSNK2A2	-0.11926193	-3.171238973	0.001584438	0.003594095	no
RARB	0.119252587	3.170986964	0.001585793	0.003596766	no
XYLT1	-0.11924853	-3.170877526	0.001586382	0.003597698	no
C14orf80	-0.119230633	-3.170394768	0.001588983	0.003603191	no
HFE2	-0.119186848	-3.169213732	0.001595361	0.003617249	no
MYOD1	-0.119181407	-3.169066982	0.001596155	0.003618643	no
PCGF1	0.119177278	3.168955605	0.001596758	0.003619605	no
GSTM4	0.119165461	3.168636841	0.001598485	0.003623113	no
CRYL1	-0.119162546	-3.168558217	0.001598911	0.003623673	no
LOC100132111	0.119156504	3.168395266	0.001599795	0.003625269	no
PCDH11Y	-0.119144872	-3.168081514	0.001601497	0.003628721	no
TOX2	-0.119142578	-3.168019639	0.001601833	0.003629075	no

GJB2	0.119137745	3.167889274	0.001602542	0.003629879	no
N4BP2L2	-0.119137708	-3.167888275	0.001602547	0.003629879	no
A2BP1	-0.119124958	-3.167544357	0.001604417	0.003633706	no
NCKAP5	-0.119064393	-3.165910777	0.001613324	0.00365347	no
ANGPTL4	0.119034901	3.165115298	0.001617678	0.00366292	no
FAM53C	-0.119026511	-3.164889016	0.001618918	0.003665318	no
RSRC1	0.118997131	3.164096587	0.00162327	0.003674758	no
SFRP1	0.118990558	3.163919309	0.001624244	0.003676553	no
IGSF11	-0.118983109	-3.163718396	0.00162535	0.003678644	no
ZDHHC4	0.118977324	3.163562361	0.001626209	0.003680176	no
PSMD3	-0.118966087	-3.163259282	0.001627879	0.003683543	no
TGIF2	0.118946249	3.16272422	0.001630831	0.003689809	no
NCRNA00092	0.118922199	3.162075586	0.001634416	0.003697506	no
LOC81691	-0.118867472	-3.160599546	0.001642601	0.003715608	no
SAMD12	-0.1188581	-3.160346784	0.001644006	0.003718371	no
GRHL3	-0.118852578	-3.160197871	0.001644835	0.003719828	no
LOC388588	0.118822533	3.159387554	0.00164935	0.003729622	no
C9orf40	0.118800044	3.158781009	0.001652737	0.003736864	no
CORT	-0.118784416	-3.158359526	0.001655094	0.003741776	no
CYP27C1	-0.118742453	-3.157227812	0.00166144	0.003755701	no
C17orf104	-0.118700689	-3.156101493	0.001667777	0.003769606	no
6-Mar	-0.118648408	-3.154691548	0.001675742	0.003787184	no
SPSB4	-0.118641317	-3.154500324	0.001676825	0.003789208	no
ZMYND8	-0.118631044	-3.154223269	0.001678395	0.003792333	no
PTPLA	0.118622944	3.154004827	0.001679634	0.003794708	no
NPY	-0.118591532	-3.15315773	0.001684446	0.003805155	no
ZNF793	-0.118586739	-3.153028474	0.001685182	0.003806391	no
PLXNA1	0.118584344	3.152963878	0.001685549	0.003806796	no
P2RX5	-0.118579288	-3.15282753	0.001686326	0.003808124	no
TAF1B	0.118568156	3.152527327	0.001688036	0.00381156	no
ZNF35	0.118560049	3.152308712	0.001689282	0.003813949	no
HOXB13	0.118494836	3.150550111	0.00169934	0.003836228	no
AGPAT1	-0.11846782	-3.149821568	0.001703523	0.003845241	no
NUDT3	0.118441926	3.149123321	0.001707541	0.00385388	no
DENND2C	0.118433692	3.14890128	0.00170882	0.003856337	no
GDF11	-0.118428122	-3.148751074	0.001709686	0.003857861	no
LHFPL5	-0.118419533	-3.148519458	0.001711022	0.003860445	no
EXOC6	-0.118389679	-3.147714427	0.001715674	0.003870162	no
RASGRP3	0.118389438	3.147707921	0.001715712	0.003870162	no
EIF2S3	0.118336636	3.146284084	0.001723968	0.003888352	no
OLFM4	-0.118332032	-3.146159936	0.00172469	0.003889545	no
QPCTL	0.118324138	3.145947076	0.001725928	0.003891903	no
TRPC6	0.118322908	3.145913924	0.00172612	0.003891904	no
RNF170	-0.118319682	-3.145826915	0.001726627	0.003892611	no
FTSJ3	-0.118297036	-3.145216283	0.001730184	0.003900196	no
SLC35F1	-0.118260601	-3.144233831	0.001735921	0.003912692	no
ACBD6	-0.118245347	-3.143822498	0.001738328	0.003917388	no
ANP32A	-0.118244943	-3.143811603	0.001738392	0.003917388	no
NTHL1	-0.118233541	-3.143504177	0.001740193	0.003920639	no
ATP10A	-0.118233356	-3.143499174	0.001740222	0.003920639	no
RPL10	-0.118198186	-3.142550858	0.00174579	0.003932388	no

ZBTB8B	-0.118197886	-3.14254276	0.001745837	0.003932388	no
BCL6B	0.118196727	3.142511508	0.001746021	0.003932388	no
ZNF43	-0.118175001	-3.141925692	0.001749469	0.003939459	no
LOC285359	-0.118174487	-3.141911849	0.00174955	0.003939459	no
PDE6C	-0.118165922	-3.141680914	0.001750911	0.003942084	no
AMBRA1	-0.118117592	-3.140377767	0.001758609	0.003958974	no
PDZRN4	-0.118101078	-3.139932489	0.001761246	0.00396447	no
ESAM	0.118081873	3.13941468	0.001764318	0.003970942	no
COG7	-0.118076732	-3.139276073	0.001765141	0.003972352	no
TAS2R14	-0.118059297	-3.138805972	0.001767935	0.003978197	no
GGTLC1	-0.118027697	-3.137953949	0.001773009	0.003989172	no
CHIC1	-0.118024513	-3.137868102	0.001773521	0.003989516	no
PMS1	-0.118024292	-3.137862157	0.001773557	0.003989516	no
PCLO	-0.117989285	-3.136918289	0.001779195	0.004001754	no
CUL4A	0.11796761	3.13633389	0.001782695	0.004009179	no
C6orf118	0.117964116	3.136239695	0.001783259	0.004009607	no
KHK	-0.117963977	-3.136235959	0.001783282	0.004009607	no
TSC22D3	0.1179586	3.136090986	0.001784151	0.004011116	no
MAP7D2	-0.117910667	-3.134798655	0.001791918	0.00402813	no
ROGDI	-0.117900201	-3.134516461	0.001793618	0.004031503	no
C1orf131	0.117865155	3.133571592	0.001799322	0.004043873	no
ZBTB6	-0.11786037	-3.133442604	0.001800102	0.004045176	no
HOXB4	0.11785891	3.133403228	0.00180034	0.004045262	no
C12orf42	0.117852345	3.133226245	0.00180141	0.004047218	no
PDE6B	0.117842533	3.132961702	0.001803012	0.004050366	no
C3orf16	0.11782663	3.132532942	0.00180561	0.004055753	no
GRM7	-0.117803471	-3.131908571	0.001809401	0.004063815	no
SMYD2	0.117799783	3.131809151	0.001810005	0.00406472	no
PIGF	0.117786465	3.131450094	0.001812188	0.004069172	no
PSMD14	0.117783661	3.131374503	0.001812648	0.004069658	no
TTC32	-0.117782691	-3.131348361	0.001812807	0.004069658	no
FABP4	0.117763914	3.130842142	0.001815891	0.004076128	no
LOC643677	-0.117747275	-3.130393555	0.001818628	0.004081818	no
PDRG1	0.117722528	3.129726388	0.001822705	0.004090515	no
KRT18	0.117719655	3.129648934	0.001823179	0.004091124	no
NUDT7	-0.117718017	-3.129604783	0.001823449	0.004091276	no
PDE11A	-0.117664958	-3.128174347	0.001832222	0.004110504	no
TFB1M	0.117643784	3.12760355	0.001835734	0.004117925	no
ECE2	-0.117623619	-3.127059911	0.001839084	0.004124983	no
NRGN	-0.117617574	-3.126896948	0.001840089	0.004126781	no
GOT1	-0.117616286	-3.126862233	0.001840304	0.004126803	no
TUBB2A	-0.117596438	-3.126327175	0.001843609	0.004133756	no
TEAD1	-0.117577771	-3.125823941	0.001846722	0.004139929	no
TLX1	-0.117577476	-3.125815996	0.001846771	0.004139929	no
MCM6	0.11756775	3.125553813	0.001848395	0.00414311	no
HIST3H2BB	-0.117544723	-3.124933043	0.001852246	0.00415128	no
GNRHR	-0.117525865	-3.124424692	0.001855404	0.004157899	no
GDPD5	0.117517555	3.124200665	0.001856798	0.00416056	no
LSM5	0.117496516	3.123633521	0.00186033	0.004168013	no
NRXN3	-0.117489799	-3.123452447	0.001861459	0.004170081	no
CYP2B7P1	-0.117487047	-3.12337827	0.001861922	0.004170655	no

BBS10	-0.117484137	-3.123299812	0.001862411	0.004171289	no
CAMK2A	-0.117451889	-3.122430531	0.001867843	0.004182991	no
BTNL8	0.117439269	3.122090339	0.001869972	0.004187297	no
KCTD3	-0.117433441	-3.121933218	0.001870957	0.004189037	no
SMARCC1	-0.11742277	-3.121645561	0.00187276	0.00419261	no
SPNS3	0.117420029	3.121571692	0.001873223	0.004193003	no
DGKK	-0.117419277	-3.121551425	0.00187335	0.004193003	no
C15orf29	-0.117410112	-3.121304365	0.001874901	0.004195666	no
BTBD11	0.117409794	3.121295789	0.001874955	0.004195666	no
SCML2	-0.117401439	-3.121070574	0.001876369	0.004198366	no
DUSP2	0.117395346	3.120906326	0.001877402	0.004200211	no
TRIM29	0.117388525	3.120722453	0.001878558	0.004202333	no
EXOSC7	-0.117385816	-3.120649434	0.001879017	0.004202895	no
PPP3R2	-0.11738218	-3.120551429	0.001879634	0.004203809	no
CCNB1	0.117379646	3.120483118	0.001880064	0.004204306	no
C3orf47	-0.117374689	-3.120349506	0.001880905	0.004205722	no
KIF24	0.117366995	3.120142094	0.001882211	0.004208178	no
CAMK1G	-0.117345602	-3.119565435	0.001885848	0.004215842	no
ATXN2L	-0.117339815	-3.119409437	0.001886833	0.004217578	no
RTBDN	-0.117313334	-3.118695623	0.001891346	0.004227198	no
C20orf46	-0.117253419	-3.117080633	0.001901593	0.00424963	no
DBR1	0.117232171	3.116507888	0.001905239	0.004257308	no
KCNRG	-0.11720399	-3.115748282	0.001910085	0.004267665	no
IKZF2	0.117188613	3.115333814	0.001912734	0.004273111	no
PCSK6	-0.11717507	-3.114968773	0.00191507	0.004277856	no
PGBD2	0.117169599	3.114821309	0.001916014	0.004279493	no
OR2L3	-0.117166494	-3.114737632	0.00191655	0.004280217	no
DCBLD1	0.11712697	3.11367231	0.001923386	0.00429501	no
ORC3L	-0.117125502	-3.113632741	0.001923641	0.004295104	no
C7orf13	-0.117118484	-3.113443583	0.001924857	0.004297345	no
ZBTB16	-0.117114149	-3.11332675	0.001925609	0.004298549	no
C8orf34	0.117091176	3.112707534	0.001929597	0.004306976	no
RYR2	-0.117088348	-3.112631329	0.001930088	0.004307597	no
LMO3	-0.11707206	-3.112192309	0.001932921	0.004313443	no
MESP2	0.117060291	3.111875102	0.00193497	0.00431754	no
DAD1L	0.117056111	3.111762442	0.001935699	0.004318688	no
GABRB1	-0.117050913	-3.11162232	0.001936605	0.004320233	no
ZNF626	-0.116999632	-3.110240193	0.001945564	0.004339741	no
C14orf166	0.116988924	3.109951571	0.00194744	0.004343445	no
HOXA3	0.116952605	3.108972701	0.001953813	0.004357181	no
MEST	0.116949077	3.108877623	0.001954434	0.004358083	no
LHPP	-0.116935387	-3.108508663	0.001956842	0.004362972	no
FAM183A	0.11692114	3.108124663	0.001959351	0.004368084	no
HOXC4	0.116859123	3.106453228	0.001970307	0.004391444	no
CASR	0.116858941	3.10644833	0.001970339	0.004391444	no
UFM1	0.116858142	3.106426809	0.001970481	0.004391444	no
NUDT15	0.116841019	3.105965311	0.001973516	0.004397724	no
TPRA1	0.116793558	3.10468622	0.001981951	0.004416034	no
CCDC109A	-0.116789193	-3.104568585	0.001982729	0.004417279	no
MAP3K12	-0.116785698	-3.104474384	0.001983352	0.00441818	no
PFDN2	-0.116782321	-3.104383385	0.001983953	0.004418329	no

LIMS2	-0.116782192	-3.1043799	0.001983976	0.004418329	no
ZNF441	-0.116781645	-3.104365149	0.001984074	0.004418329	no
CCDC74A	0.116758408	3.103738914	0.00198822	0.004427074	no
MTHFSD	-0.116755992	-3.103673804	0.001988651	0.004427505	no
NSF	-0.116754872	-3.103643618	0.001988852	0.004427505	no
TMEM80	-0.116687326	-3.101823288	0.002000952	0.004453952	no
ATP6V1H	-0.11667532	-3.101499735	0.00200311	0.004458264	no
FAM108B1	-0.116654447	-3.100937238	0.002006866	0.004466133	no
DHX57	-0.116645757	-3.100703033	0.002008432	0.004469022	no
SSTR3	-0.116644789	-3.100676945	0.002008607	0.004469022	no
FAM125B	-0.116632968	-3.100358396	0.002010738	0.004473274	no
WDR7	-0.116621542	-3.100050477	0.002012801	0.00447737	no
UCP1	0.116608496	3.099698918	0.002015159	0.004482121	no
MPHOSPH10	-0.116592876	-3.099277972	0.002017985	0.004487896	no
CYTSB	-0.116591692	-3.099246068	0.002018199	0.004487896	no
SYT15	-0.116580389	-3.098941465	0.002020247	0.004491955	no
CCDC48	0.116562418	3.098457167	0.002023506	0.004498707	no
IGDCC4	0.116532362	3.097647222	0.002028968	0.004510354	no
UPF3B	-0.116529542	-3.097571237	0.002029481	0.004510998	no
MRPL15	0.116514324	3.097161144	0.002032252	0.004516661	no
C1orf63	0.11651152	3.097085581	0.002032763	0.0045173	no
PHF12	-0.116503187	-3.096861031	0.002034282	0.00452018	no
ATP1A2	-0.11647933	-3.096218144	0.002038637	0.004529359	no
TBR1	-0.116460331	-3.095706183	0.002042112	0.00453658	no
REC8	-0.116456862	-3.09561271	0.002042747	0.004537492	no
LRRC45	-0.11643143	-3.094927395	0.002047407	0.004547345	no
HIST3H2A	-0.116416813	-3.094533498	0.002050091	0.004552805	no
ALS2	-0.116414058	-3.094459259	0.002050597	0.004553428	no
CDH12	-0.116395041	-3.093946817	0.002054093	0.004560208	no
OR52K2	0.116394999	3.093945696	0.002054101	0.004560208	no
AFF4	-0.116359303	-3.092983819	0.002060679	0.004574309	no
LOC100134368	-0.116352878	-3.092810681	0.002061865	0.004576439	no
ASAH2	-0.116330015	-3.09219461	0.00206609	0.004585076	no
GALNTL2	0.11632937	3.092177236	0.00206621	0.004585076	no
RDM1	0.116300224	3.091391882	0.002071608	0.00459655	no
MLL3	-0.11626838	-3.09053382	0.002077521	0.004609164	no
GPR83	-0.11626475	-3.09043601	0.002078196	0.004610156	no
LOC80054	0.116255435	3.090185023	0.002079929	0.004613494	no
C12orf47	-0.116237614	-3.089704843	0.002083248	0.00462035	no
YAF2	-0.116235794	-3.089655793	0.002083587	0.004620595	no
LQK1	0.116226231	3.08939811	0.002085371	0.004624043	no
ZIC3	-0.116179212	-3.088131207	0.00209416	0.004643023	no
HNRNPK	-0.116169723	-3.087875524	0.002095938	0.004646455	no
HPCA	-0.116166337	-3.08778429	0.002096572	0.004647352	no
STAG2	-0.116164261	-3.08772836	0.002096962	0.004647706	no
AKAP12	0.116160963	3.087639493	0.00209758	0.004648567	no
C21orf131	-0.116110636	-3.086283486	0.002107039	0.004669018	no
EPHA6	-0.116097875	-3.085939666	0.002109444	0.004673834	no
NACA2	-0.116075476	-3.085336162	0.002113671	0.004682686	no
C1orf141	0.116064914	3.085051578	0.002115667	0.004686594	no
C8orf40	0.11604827	3.084603137	0.002118815	0.004693055	no

MOGAT2	0.116023127	3.083925708	0.00212358	0.004703016	no
CDR1	-0.116022085	-3.083897616	0.002123777	0.004703016	no
PDGFC	0.116010465	3.083584565	0.002125983	0.004707384	no
MAP3K9	-0.115983598	-3.082860695	0.00213109	0.004718176	no
CIR1	-0.115956372	-3.082127159	0.002136277	0.004729142	no
LOC283867	0.115949763	3.081949086	0.002137538	0.004731415	no
ITFG3	0.115939847	3.081681926	0.002139431	0.004735087	no
AZIN1	-0.115925354	-3.081291461	0.002142201	0.004740698	no
SMCP	-0.115923956	-3.081253795	0.002142468	0.00474077	no
ZUFSP	-0.11591452	-3.080999556	0.002144273	0.004744246	no
RXFP1	-0.115911512	-3.080918523	0.002144849	0.004745	no
HOXA5	0.115906245	3.080776618	0.002145858	0.004746712	no
CCDC135	0.115894242	3.080453234	0.002148157	0.00475128	no
CREG2	-0.115886206	-3.080236748	0.002149698	0.004754168	no
PADI1	0.115881049	3.080097785	0.002150688	0.004755836	no
CST4	0.115873627	3.079897824	0.002152113	0.004758063	no
PKD1L3	0.115873351	3.079890408	0.002152166	0.004758063	no
TMEM27	-0.115870954	-3.079825815	0.002152626	0.00475856	no
WNK4	0.115869542	3.079787768	0.002152897	0.00475864	no
NDUFA10	-0.115865674	-3.079683569	0.00215364	0.004759762	no
CEP350	-0.11584865	-3.079224914	0.002156914	0.004766475	no
LPCAT4	-0.115834921	-3.078855038	0.002159557	0.004771795	no
CDH24	-0.115828619	-3.078685257	0.002160771	0.004773956	no
HHAT	0.115817408	3.07838323	0.002162933	0.00477821	no
OCEL1	0.115793632	3.07774269	0.002167524	0.004787828	no
TRMT2A	-0.115781074	-3.077404351	0.002169952	0.004792669	no
POLR3G	0.115734859	3.076159317	0.002178911	0.004811929	no
EEF1D	-0.115710838	-3.075512185	0.00218358	0.004821715	no
NCS1	-0.115694799	-3.075080098	0.002186703	0.004828084	no
SELT	0.115679287	3.074662198	0.002189728	0.004834234	no
TMPRSS3	0.115673427	3.07450434	0.002190871	0.00483623	no
QRICH1	-0.115665068	-3.074279131	0.002192503	0.004839305	no
LOC642597	-0.115657161	-3.074066124	0.002194048	0.004842186	no
CCT8L2	-0.1156364	-3.073506833	0.002198109	0.004850619	no
GNG2	-0.11556627	-3.071617621	0.002211878	0.00488047	no
FAM134B	0.11556406	3.071558071	0.002212313	0.004880898	no
ODF3	-0.115551553	-3.071221166	0.002214778	0.004885802	no
SAT2	-0.115546846	-3.071094362	0.002215706	0.004887316	no
UHRF1	-0.115527038	-3.070560768	0.002219615	0.004895405	no
POLR2K	0.115513109	3.070185537	0.002222368	0.004900536	no
CPNE9	-0.115512813	-3.070177575	0.002222426	0.004900536	no
FAM50B	0.115488323	3.069517857	0.002227274	0.00491069	no
H2BFXP	-0.115475301	-3.069167073	0.002229856	0.004915846	no
RPL41	0.115446859	3.068400923	0.002235504	0.004927761	no
KRTAP5-6	-0.115441987	-3.068269676	0.002236473	0.00492918	no
NPPC	0.11544117	3.068247676	0.002236635	0.00492918	no
PFKFB3	0.115436761	3.068128891	0.002237513	0.004930576	no
FLJ34503	-0.115403323	-3.067228175	0.002244175	0.004944718	no
ZNF7	-0.115398349	-3.067094198	0.002245168	0.004946366	no
ZNF214	0.115379519	3.066586972	0.002248929	0.004954113	no
CYP4Z2P	-0.115362911	-3.066139611	0.002252251	0.004960891	no

CNST	-0.115354745	-3.065919635	0.002253887	0.004963489	no
MLNR	0.115354568	3.065914876	0.002253922	0.004963489	no
FAM92B	0.115352059	3.065847283	0.002254425	0.004963654	no
AGPAT5	-0.115351744	-3.0658388	0.002254488	0.004963654	no
C12orf50	0.115347778	3.065731966	0.002255283	0.004964863	no
ITGA8	0.115323996	3.065091361	0.002260054	0.004974825	no
TRIM45	0.115315948	3.064874582	0.00226167	0.004977842	no
LIX1	-0.115310468	-3.064726975	0.002262772	0.004979724	no
DHX8	0.115304059	3.064554342	0.002264061	0.004982017	no
IDH3B	-0.115274685	-3.063763109	0.002269976	0.004994491	no
SNRPB2	0.115246615	3.06300702	0.002275642	0.005006412	no
SLC22A7	-0.115237994	-3.062774819	0.002277384	0.005009701	no
GPR89C	0.115234583	3.062682945	0.002278074	0.005010673	no
MYO5C	0.115222656	3.062361695	0.002280488	0.005015436	no
APOB	0.115210197	3.062026094	0.002283012	0.005020441	no
HEPN1	-0.115194308	-3.061598112	0.002286234	0.005026981	no
USP36	-0.115170258	-3.060950337	0.00229112	0.005037175	no
PSMD6	0.115158225	3.060626226	0.002293568	0.005042008	no
ABL2	-0.115136789	-3.060048869	0.002297934	0.005051058	no
FADS6	-0.115117108	-3.059518754	0.00230195	0.005059336	no
C18orf55	-0.115114908	-3.059459503	0.0023024	0.005059773	no
PLCXD1	-0.115110323	-3.059336	0.002303336	0.005061282	no
SHE	0.115100923	3.05908283	0.002305258	0.005064953	no
SIRT5	-0.115085651	-3.058671483	0.002308382	0.005071268	no
VWC2L	-0.115075591	-3.058400539	0.002310443	0.005075243	no
AGTR1	0.115029608	3.05716204	0.002319882	0.005094925	no
PABPC1L	0.115029487	3.057158787	0.002319907	0.005094925	no
IGF2BP3	0.115012442	3.056699688	0.002323415	0.005102076	no
MESTIT1	-0.115010228	-3.056640068	0.002323871	0.005102523	no
XKRY2	0.114998936	3.056335938	0.002326198	0.005107078	no
SYT6	-0.114993886	-3.056199926	0.00232724	0.005108809	no
KDM5B	-0.114974786	-3.055685503	0.002331182	0.005116909	no
ERN2	0.114965717	3.055441254	0.002333057	0.005120467	no
LRRC37A	-0.114953523	-3.055112841	0.002335579	0.005125445	no
C3orf15	0.114949088	3.054993384	0.002336497	0.005126904	no
EHF	0.114940819	3.054770685	0.002338209	0.005130104	no
NEFM	-0.114927995	-3.054425282	0.002340867	0.005135379	no
RPUSD2	-0.114922321	-3.054272475	0.002342044	0.005137403	no
FBXW7	-0.114920118	-3.054213146	0.002342501	0.005137848	no
MATK	-0.114905268	-3.053813184	0.002345584	0.00514401	no
COL4A6	0.114904136	3.053782705	0.002345819	0.00514401	no
ZNF676	-0.114886124	-3.053297602	0.002349564	0.005151664	no
UBN1	0.114883581	3.053229127	0.002350093	0.005152265	no
EDA2R	0.114874858	3.052994182	0.002351909	0.005155687	no
CTNNA2	-0.114870349	-3.05287274	0.002352849	0.005157187	no
BSND	0.114862802	3.052669487	0.002354421	0.005160075	no
RAET1E	0.114849466	3.052310329	0.002357203	0.005165611	no
GAD2	-0.114821824	-3.05156587	0.002362978	0.005177706	no
BAGE2	-0.114798784	-3.050945371	0.002367801	0.005187712	no
ALKBH4	-0.114780114	-3.050442577	0.002371716	0.005195727	no
RPA2	0.114777887	3.050382595	0.002372184	0.005196188	no

MGST3	0.114747357	3.049560406	0.0023786	0.005209677	no
SELE	0.114727877	3.049035785	0.002382702	0.005218097	no
HMGB1	-0.114703934	-3.048390981	0.002387753	0.005228314	no
CD160	-0.11470331	-3.048374183	0.002387885	0.005228314	no
PPIAL4E	0.11469819	3.048236287	0.002388966	0.005230116	no
EMX2OS	-0.114685331	-3.047890004	0.002391684	0.005235499	no
SLC25A35	0.114680956	3.047772169	0.002392609	0.005236958	no
SDCBP2	-0.114650642	-3.046955818	0.00239903	0.005250443	no
HIST1H2BH	0.114604344	3.04570901	0.002408867	0.005271401	no
LBH	0.114598933	3.045563317	0.002410019	0.005273351	no
CSTF1	0.114588203	3.045274364	0.002412305	0.005277781	no
RHBDF1	0.114552452	3.044311601	0.002419936	0.005293904	no
TRHDE	-0.114497882	-3.042842086	0.002431626	0.005318903	no
LRRC31	0.114480911	3.042385099	0.002435272	0.005326302	no
TMEM161B	-0.114479004	-3.042333732	0.002435682	0.00532646	no
ZNF37A	-0.114478123	-3.042310027	0.002435872	0.00532646	no
LOC100144603	0.114471688	3.042136723	0.002437256	0.005328911	no
TRAM1L1	-0.114436207	-3.041181306	0.0024449	0.005345047	no
DGKZ	-0.114418833	-3.040713444	0.002448652	0.005352338	no
CACNG3	-0.114418309	-3.040699356	0.002448765	0.005352338	no
FAM182A	-0.114374435	-3.039517915	0.002458262	0.005372246	no
CCDC138	0.114373779	3.039500265	0.002458404	0.005372246	no
ABCB7	0.114358523	3.039089446	0.002461715	0.005378899	no
NPHS1	-0.114311111	-3.037812793	0.002472029	0.005400851	no
RNF44	-0.114302729	-3.03758709	0.002473856	0.00540426	no
STRAP	-0.114300139	-3.037517355	0.002474421	0.00540491	no
MOSPD1	0.114280409	3.036986094	0.002478728	0.005413733	no
LRRC18	0.114278421	3.036932549	0.002479163	0.005414098	no
CEBPZ	-0.114258145	-3.036386589	0.002483597	0.005423196	no
POMGNT1	0.114230829	3.035651087	0.002489583	0.005435679	no
VAC14	-0.114224812	-3.035489061	0.002490903	0.005437974	no
DOC2A	-0.114191965	-3.03460463	0.002498122	0.005452872	no
PLD6	-0.114191305	-3.034586874	0.002498267	0.005452872	no
SHOX2	0.114162105	3.033800646	0.0025047	0.005466324	no
ADRBK1	0.11414885	3.033443737	0.002507626	0.005472118	no
NUPL1	-0.114142581	-3.033274949	0.00250901	0.005474452	no
SYS1-DBNDD2	-0.114141557	-3.033247363	0.002509237	0.005474452	no
FAM175A	0.114132346	3.03299936	0.002511272	0.005478302	no
ANGEL1	-0.114130873	-3.032959713	0.002511598	0.005478422	no
SLMO1	-0.114124099	-3.032777323	0.002513096	0.005481099	no
CXorf59	0.114116843	3.032581942	0.002514702	0.005483503	no
ZZZ3	0.114116668	3.032577227	0.002514741	0.005483503	no
ABCA5	0.114074956	3.031454142	0.002523991	0.005503079	no
GABRA4	-0.114038227	-3.030465251	0.002532162	0.005520298	no
DIABLO	-0.114019313	-3.029955988	0.002536379	0.005528896	no
DHRS4	-0.114016538	-3.029881273	0.002536998	0.005529649	no
C15orf52	0.11401477	3.029833693	0.002537393	0.005529913	no
PITPNM3	-0.114006465	-3.029610087	0.002539247	0.005533358	no
PTRH1	0.113995064	3.029303125	0.002541795	0.005538095	no
NHEJ1	0.113994287	3.029282193	0.002541968	0.005538095	no
PRR25	-0.113992204	-3.029226105	0.002542434	0.005538513	no

CA7	-0.113966903	-3.028544917	0.002548097	0.005550252	no
KPNA7	0.113954017	3.028197993	0.002550986	0.005555945	no
SETX	0.113951718	3.028136086	0.002551502	0.00555647	no
IGF2BP2	0.113947654	3.028026677	0.002552414	0.005557857	no
SLC9A5	-0.113924413	-3.027400941	0.002557634	0.005568547	no
ZNF449	0.113923346	3.02737222	0.002557874	0.005568547	no
SMARCE1	0.113911678	3.02705809	0.002560499	0.005573661	no
VWA3B	0.113887457	3.026405982	0.002565955	0.005584938	no
KCNJ12	-0.113874962	-3.026069573	0.002568775	0.005590472	no
C9orf5	-0.113864603	-3.025790679	0.002571114	0.005594961	no
RYR3	0.113861645	3.025711035	0.002571782	0.005595813	no
FAM180B	0.113840819	3.025150357	0.002576492	0.005605457	no
C4orf39	-0.113827585	-3.024794066	0.002579489	0.005611374	no
ANKRD27	0.113816146	3.024486117	0.002582082	0.00561641	no
THOC7	0.113807846	3.024262659	0.002583965	0.005619509	no
GAB2	-0.113807418	-3.02425112	0.002584062	0.005619509	no
EHMT1	-0.113800984	-3.024077905	0.002585523	0.00562208	no
WDR5	-0.113768843	-3.023212623	0.00259283	0.005637363	no
ABCB10	0.113762261	3.023035413	0.002594329	0.005639735	no
ACBD4	-0.113761604	-3.023017741	0.002594479	0.005639735	no
ANO3	-0.113738203	-3.022387743	0.002599814	0.005650287	no
PPP1R12A	-0.113737861	-3.022378519	0.002599892	0.005650287	no
TMLHE	-0.11373171	-3.022212927	0.002601296	0.005652731	no
CUL3	-0.113722722	-3.021970957	0.002603349	0.005656585	no
LOC339788	-0.113716012	-3.021790315	0.002604883	0.005659309	no
ENO1	0.113690601	3.021106219	0.002610698	0.005671333	no
SERPINF1	0.113685628	3.020972343	0.002611838	0.005673199	no
AP1AR	0.113682558	3.020889708	0.002612541	0.005674118	no
BRCA2	0.113677274	3.020747452	0.002613753	0.005676139	no
KRT75	0.113670169	3.020556194	0.002615383	0.005679069	no
BEX5	-0.113653717	-3.020113287	0.00261916	0.005686661	no
PLEKHG4	0.113622117	3.019262587	0.00262643	0.005701832	no
CYB5B	0.113616188	3.019102994	0.002627796	0.005704185	no
FAM13B	0.113592882	3.018475577	0.002633172	0.005715241	no
C9orf106	0.113584819	3.018258519	0.002635034	0.005718669	no
PAQR9	-0.113582999	-3.018209527	0.002635455	0.005718968	no
SDHB	0.113557702	3.017528546	0.002641306	0.005731049	no
C9orf110	0.113552345	3.017384315	0.002642546	0.005733126	no
C10orf82	-0.113547495	-3.017253774	0.00264367	0.005734948	no
PRDX1	0.113518483	3.016472787	0.0026504	0.005748932	no
ENGASE	-0.113501741	-3.016022101	0.002654292	0.005756755	no
GLT1D1	-0.113432196	-3.014150024	0.002670511	0.00579131	no
DDX55	-0.11340191	-3.013334766	0.002677602	0.005806066	no
CCDC110	0.113392319	3.013076578	0.002679852	0.00581032	no
ADA	0.113361189	3.012238626	0.002687164	0.00582555	no
TWF1	0.113355103	3.012074805	0.002688596	0.005828029	no
GCFC1	-0.113339812	-3.011663215	0.002692196	0.005835208	no
KLF8	0.113278772	3.010020162	0.002706612	0.005865825	no
AHCYL1	-0.113263366	-3.009605478	0.002710262	0.005873105	no
C6orf138	0.113237846	3.008918559	0.002716317	0.005885596	no
ZNF177	-0.113232035	-3.008762153	0.002717697	0.005887956	no

SLC30A3	-0.113199221	-3.007878906	0.002725505	0.005904058	no
C14orf128	-0.113198346	-3.007855363	0.002725714	0.005904058	no
EML3	0.113191384	3.007667955	0.002727373	0.00590702	no
RCC1	0.113189004	3.007603896	0.002727941	0.005907224	no
GGT6	0.113188537	3.007591338	0.002728052	0.005907224	no
APBB3	-0.113155026	-3.006689337	0.002736053	0.005923916	no
PPIP5K2	0.113146278	3.006453872	0.002738146	0.005927812	no
ZNF518A	-0.11312916	-3.005993123	0.002742244	0.005936049	no
ZNF138	-0.113106206	-3.005375308	0.002747749	0.005947327	no
NOC4L	-0.113101597	-3.005251247	0.002748855	0.005949085	no
MURC	-0.113099442	-3.005193261	0.002749373	0.005949568	no
LOC91149	-0.113094972	-3.005072935	0.002750446	0.005951255	no
CLCNKB	0.113071987	3.004454292	0.002755973	0.005962576	no
ZSCAN18	-0.113069775	-3.004394752	0.002756506	0.00596309	no
TUBE1	-0.113065504	-3.004279788	0.002757534	0.005964676	no
RPS6KB1	-0.113049479	-3.003848483	0.002761395	0.005972389	no
C14orf162	-0.113035841	-3.003481427	0.002764685	0.005978558	no
CORO1C	-0.113035205	-3.003464297	0.002764839	0.005978558	no
TENC1	-0.113021319	-3.003090568	0.002768193	0.005985169	no
LIN7B	-0.113018652	-3.003018771	0.002768837	0.005985923	no
CDH8	-0.11301452	-3.002907572	0.002769836	0.005987442	no
RFK	0.11299789	3.002459972	0.00277386	0.005995499	no
HEYL	0.112986651	3.002157489	0.002776582	0.006000741	no
TPPP	-0.112976376	-3.001880935	0.002779073	0.006005482	no
C10orf108	0.112970061	3.001710969	0.002780605	0.006008151	no
SPATA4	0.112960032	3.001441037	0.002783039	0.006012768	no
BRD2	-0.112942949	-3.000981262	0.00278719	0.006021094	no
TMEM155	-0.1129398	-3.000896508	0.002787956	0.006022104	no
KLF1	-0.112923809	-3.000466143	0.002791848	0.006029867	no
EIF2B3	0.11292231	3.000425801	0.002792213	0.006030011	no
UTP18	0.112908217	3.000046501	0.002795647	0.006036783	no
HMMR	0.112883655	2.999385455	0.002801642	0.006049082	no
EHBP1	0.112877342	2.999215527	0.002803185	0.006051767	no
CWC15	-0.112869877	-2.999014635	0.00280501	0.006055061	no
RNF128	0.112866956	2.998936012	0.002805725	0.006055957	no
KIAA0528	-0.112856417	-2.998652386	0.002808304	0.006060877	no
PLEKHA7	0.112850489	2.99849283	0.002809756	0.006063055	no
TDRD6	-0.112849847	-2.998475562	0.002809913	0.006063055	no
DIS3	-0.112826346	-2.997843059	0.002815676	0.006074841	no
GJA3	0.112803898	2.997238926	0.00282119	0.006086088	no
CTAGE6	0.112790645	2.996882264	0.00282445	0.006092471	no
HOXD13	0.112763676	2.996156438	0.002831095	0.006106153	no
STXBP5	-0.112757021	-2.995977348	0.002832737	0.006109042	no
PROK1	0.112751377	2.99582545	0.00283413	0.006111001	no
CCT6B	-0.112750893	-2.995812439	0.002834249	0.006111001	no
VSNL1	-0.112748586	-2.995750339	0.002834819	0.006111578	no
MORN2	0.112741872	2.995569649	0.002836478	0.006114501	no
C11orf74	-0.112738056	-2.995466952	0.002837421	0.006115882	no
NEU3	-0.112723659	-2.995079502	0.002840981	0.006122903	no
TREX2	0.112711323	2.994747508	0.002844035	0.006128831	no
PP14571	-0.112676093	-2.99379942	0.002852773	0.006147006	no

PTPRZ1	-0.112666004	-2.993527897	0.00285528	0.006151752	no
RPL13A	-0.112664481	-2.99348691	0.002855658	0.006151912	no
ZFP14	-0.112635773	-2.992714342	0.002862803	0.006166214	no
AGAP6	-0.112635356	-2.992703125	0.002862907	0.006166214	no
PMM1	-0.112629537	-2.992546519	0.002864357	0.006168681	no
SST	-0.112620308	-2.992298163	0.002866659	0.00617298	no
ROBO1	-0.112616024	-2.992182864	0.002867728	0.006174624	no
SH2B2	-0.112609482	-2.992006823	0.002869361	0.006177482	no
MAP2K4	-0.112601916	-2.991803203	0.002871251	0.006180893	no
IGFBP3	0.112584086	2.991323405	0.002875708	0.006189792	no
PCNP	0.112582932	2.99129233	0.002875997	0.006189792	no
ZSWIM6	-0.112581562	-2.991255477	0.00287634	0.006189871	no
SV2B	-0.112578167	-2.991164122	0.002877189	0.00619104	no
FNBP4	-0.112574935	-2.991077144	0.002877998	0.006192122	no
SLC6A7	-0.112569308	-2.990925696	0.002879408	0.006194495	no
10-Mar	0.112540574	2.990152458	0.002886613	0.006209336	no
HAGHL	-0.112491693	-2.988837076	0.002898909	0.006235121	no
SLBP	0.112468535	2.988213877	0.002904751	0.006247022	no
OGDH	0.112462559	2.988053071	0.00290626	0.006249603	no
9-Sep	0.112459201	2.987962702	0.002907108	0.006250762	no
FOXD4L5	-0.112441168	-2.987477463	0.002911668	0.0062599	no
AP3M2	-0.112439185	-2.98742409	0.00291217	0.006260314	no
C7orf46	0.112424751	2.987035687	0.002915825	0.006267504	no
NFS1	-0.112387899	-2.986044028	0.002925176	0.006286935	no
SLC5A9	0.112384135	2.98594275	0.002926132	0.006288323	no
ENPP5	0.112361337	2.985329266	0.002931932	0.006300118	no
ACCN1	-0.112359653	-2.985283956	0.002932361	0.00630037	no
VSIG1	0.112344446	2.984874755	0.002936236	0.006307437	no
FANCF	0.112344296	2.984870731	0.002936274	0.006307437	no
OVGP1	-0.112251604	-2.982376545	0.002959996	0.006357648	no
ZFP106	0.112250508	2.982347063	0.002960278	0.006357648	no
DNAJC8	0.112225454	2.981672907	0.00296672	0.006370807	no
DCUN1D5	0.112213175	2.981342525	0.002969882	0.00637692	no
GABRB2	-0.112194566	-2.980841798	0.00297468	0.006386544	no
AGAP2	-0.112164596	-2.980035411	0.002982422	0.006402486	no
ARMC7	0.112160279	2.979919253	0.002983539	0.006404203	no
FUT3	-0.112143121	-2.979457581	0.002987981	0.006413057	no
C9orf131	-0.112138636	-2.979336907	0.002989143	0.006414221	no
LRPAP1	0.11213858	2.979335378	0.002989158	0.006414221	no
ZFP90	-0.112133728	-2.979204838	0.002990415	0.006416239	no
USP5	-0.112127738	-2.979043674	0.002991969	0.00641889	no
TOMM5	0.112123372	2.978926186	0.002993102	0.006420639	no
PRSS3	-0.112115017	-2.978701379	0.00299527	0.00642461	no
ABCF2	0.112094687	2.978154385	0.003000553	0.006435258	no
RDH16	-0.112054537	-2.977074117	0.003011011	0.006457002	no
LOC100130522	-0.112045962	-2.976843383	0.003013249	0.006461116	no
DKKL1	0.112034193	2.976526721	0.003016323	0.006467022	no
LOC151009	-0.1120153	-2.976018401	0.003021263	0.006476398	no
LOC284798	-0.112015019	-2.976010832	0.003021337	0.006476398	no
TIMM17A	0.112001548	2.975648405	0.003024864	0.006483271	no
GPR20	0.111998582	2.975568601	0.003025641	0.00648425	no

DTNBP1	0.111977054	2.974989381	0.003031287	0.006495661	no
LOC399959	-0.111948005	-2.97420782	0.003038921	0.006511329	no
EGR1	0.111938182	2.973943537	0.003041506	0.006516178	no
PAIP2B	-0.111923929	-2.973560056	0.003045261	0.006523531	no
FRAT2	-0.111910815	-2.97320723	0.003048719	0.006530248	no
ADCK4	0.111909106	2.973161254	0.00304917	0.006530522	no
CD300LD	0.111902835	2.972992518	0.003050826	0.006533375	no
LOC647946	0.111884237	2.97249215	0.00305574	0.006543205	no
AGPAT3	-0.111863554	-2.971935698	0.003061213	0.006554231	no
VEZF1	-0.111845317	-2.971445059	0.003066046	0.006563884	no
LRRIQ1	0.111835508	2.971181147	0.003068649	0.00656876	no
IQCK	-0.111823287	-2.97085235	0.003071894	0.006575011	no
SPATA9	-0.11181963	-2.970753968	0.003072866	0.006576395	no
HOXA6	0.111815886	2.97065323	0.003073861	0.006577829	no
GINS3	-0.111794436	-2.97007615	0.003079568	0.006589344	no
DDX19A	-0.11179294	-2.970035906	0.003079966	0.006589499	no
ZNF518B	0.111780333	2.96969673	0.003083326	0.006595988	no
N6AMT1	-0.111760573	-2.969165138	0.003088597	0.006606566	no
MYO9A	-0.111725201	-2.968213521	0.003098055	0.006626094	no
MXD4	-0.111717544	-2.968007517	0.003100105	0.006629779	no
FOXC2	0.111691287	2.96730113	0.003107147	0.006644135	no
SPDEF	-0.111666043	-2.966622009	0.003113931	0.006657937	no
SUMO2	-0.11163783	-2.965863005	0.003121528	0.006673476	no
HPR	-0.111623784	-2.965485139	0.003125317	0.006680869	no
SOCS2	0.111592824	2.96465227	0.003133683	0.006698044	no
NAV2	-0.111566328	-2.963939485	0.003140858	0.006712672	no
PACS2	-0.111551702	-2.963546019	0.003144826	0.006720441	no
ADRA1B	-0.111440369	-2.960551088	0.003175176	0.006784581	no
LOC401431	-0.11142875	-2.960238519	0.003178359	0.006790664	no
CD9	0.111356002	2.958281619	0.003198352	0.006832658	no
SLC35E2	-0.111347492	-2.958052687	0.003200698	0.006836948	no
PIPOX	0.111342869	2.957928336	0.003201973	0.00683895	no
GRK5	0.111334509	2.957703456	0.003204281	0.006843155	no
LOC284578	-0.111320681	-2.957331486	0.0032081	0.006850589	no
SCUBE2	0.111306607	2.956952909	0.003211992	0.006858175	no
C12orf45	0.111304191	2.956887928	0.003212661	0.006858878	no
IRF4	0.111292243	2.956566541	0.003215969	0.006865215	no
PRKCG	-0.111275098	-2.956105363	0.003220721	0.006874634	no
C1orf110	0.111263462	2.955792364	0.00322395	0.0068808	no
AOC2	-0.111225017	-2.954758256	0.003234639	0.006902885	no
TIGD5	-0.111221976	-2.954676462	0.003235486	0.006903963	no
GOLGA6L5	-0.111220567	-2.954638563	0.003235878	0.006904072	no
CKS1B	0.111218254	2.95457635	0.003236523	0.006904718	no
SULT2B1	0.111197855	2.954027653	0.00324221	0.006916123	no
PLXNA2	-0.111195759	-2.953971268	0.003242795	0.006916641	no
SLC26A6	0.111193771	2.953917782	0.00324335	0.006917095	no
PNPLA5	-0.111154175	-2.952852739	0.00325442	0.006939971	no
DAZL	0.111149012	2.952713866	0.003255866	0.006942323	no
C14orf73	0.111121064	2.951962142	0.003263703	0.0069583	no
FUT10	0.11109833	2.951350658	0.003270091	0.006971183	no
ZMAT4	-0.111083735	-2.950958093	0.003274198	0.006979203	no

CACNA2D1	-0.111067745	-2.950528012	0.003278703	0.006988068	no
MAP3K5	-0.111048131	-2.950000449	0.003284236	0.006999124	no
SCARA3	0.111031182	2.949544566	0.003289025	0.007008591	no
CHST5	-0.111029398	-2.949496607	0.003289529	0.007008926	no
RAB39	0.11101867	2.949208051	0.003292564	0.007014245	no
MORN5	0.111018121	2.94919329	0.003292719	0.007014245	no
SLC4A2	0.111015824	2.949131494	0.003293369	0.007014891	no
NPNT	0.110992751	2.948510933	0.003299906	0.007028073	no
LOC388242	0.1109787	2.948133015	0.003303892	0.007035822	no
FAM122B	0.110941949	2.947144548	0.00331434	0.007057328	no
CASD1	-0.11093688	-2.947008221	0.003315783	0.007059658	no
SH2D5	-0.110930909	-2.94684761	0.003317484	0.00706225	no
ZNF287	-0.110930153	-2.946827291	0.0033177	0.00706225	no
ST7OT1	-0.110912976	-2.946365312	0.003322598	0.007071931	no
LOC143666	-0.110905657	-2.946168459	0.003324686	0.007075633	no
ARL15	0.110878315	2.945433085	0.003332501	0.007091517	no
LOC654433	0.110861123	2.944970701	0.003337423	0.007101243	no
RLTPR	-0.110859226	-2.944919685	0.003337966	0.007101652	no
PPP2R1B	0.110852374	2.944735406	0.00333993	0.007105083	no
HOXB1	0.110839642	2.944392968	0.003343582	0.007112103	no
HNRNPD	-0.110807854	-2.943538034	0.003352716	0.007130781	no
NR1D1	-0.110799306	-2.943308144	0.003355175	0.007135262	no
LYPLAL1	0.110785177	2.942928161	0.003359245	0.007143165	no
HS6ST1	0.110776235	2.942687658	0.003361823	0.007147895	no
MAP3K13	-0.110756588	-2.942159291	0.003367493	0.007159198	no
MGC16025	-0.110745427	-2.941859117	0.003370718	0.007165301	no
BUD31	0.11072238	2.941239282	0.003377387	0.007178722	no
TLE1	-0.110716215	-2.941073497	0.003379173	0.007181763	no
DTWD1	0.110711804	2.940954868	0.003380451	0.007183724	no
RCC2	0.110673632	2.93992829	0.003391531	0.007206514	no
FOXR1	-0.110610283	-2.938224641	0.003409992	0.00724498	no
VIPR1	-0.110603355	-2.938038313	0.003412017	0.00724852	no
HIST1H4F	0.11057077	2.93716202	0.003421554	0.007268016	no
PROSC	0.110555474	2.936750675	0.003426039	0.007276778	no
C16orf92	-0.110546017	-2.936496363	0.003428814	0.007281908	no
ABCF1	-0.110541226	-2.936367518	0.003430221	0.007284131	no
LEFTY2	0.110525024	2.935931811	0.003434983	0.007293477	no
GSG1L	-0.110515586	-2.935678008	0.00343776	0.007298606	no
LOC649330	-0.110505477	-2.935406148	0.003440736	0.007304158	no
FAM161A	-0.110493194	-2.935075854	0.003444355	0.007311074	no
LCN12	-0.110472393	-2.934516475	0.003450493	0.007323332	no
C6orf52	-0.110432001	-2.933430291	0.003462439	0.007347915	no
SGPP2	-0.110421336	-2.933143473	0.0034656	0.007353568	no
ADAT3	0.110420558	2.933122577	0.00346583	0.007353568	no
SEMA4A	-0.110408042	-2.932785999	0.003469543	0.007360673	no
ABHD10	-0.110399237	-2.932549209	0.003472157	0.007365446	no
ACCN5	0.110397461	2.932501455	0.003472684	0.007365792	no
FMO9P	0.110386595	2.932209277	0.003475913	0.007371867	no
INO80B	-0.110379174	-2.932009713	0.00347812	0.007375773	no
CG030	-0.11035078	-2.931246171	0.003486575	0.007392927	no
COX10	-0.110345114	-2.931093808	0.003488264	0.007395734	no

C19orf69	0.110318152	2.930368816	0.003496313	0.007412022	no
PCDHA5	-0.110313561	-2.93024536	0.003497685	0.007414154	no
MED10	0.110278954	2.929314781	0.003508045	0.007435334	no
ZNF598	-0.110274995	-2.929208305	0.003509232	0.007437071	no
FEZ1	-0.110266173	-2.928971081	0.003511878	0.007441899	no
TIGD6	0.110239858	2.92826349	0.003519782	0.007457535	no
HECW2	-0.110239151	-2.928244478	0.003519995	0.007457535	no
GPD1	0.110223952	2.927835798	0.003524568	0.007466441	no
GREM2	-0.110216813	-2.927643843	0.003526717	0.007470212	no
PSMG1	0.110210978	2.927486934	0.003528476	0.007472886	no
TAS2R50	-0.11021017	-2.927465207	0.003528719	0.007472886	no
GFOD1	-0.110180075	-2.926655972	0.003537799	0.007491331	no
NSDHL	-0.110176614	-2.926562909	0.003538845	0.00749276	no
RHOV	-0.11016269	-2.926188507	0.003543054	0.007500887	no
TMEM114	0.110149113	2.925823453	0.003547163	0.007508799	no
KRT86	0.110131508	2.925350068	0.003552498	0.007519304	no
C10orf79	0.110129836	2.925305119	0.003553004	0.00751959	no
C14orf181	0.110114068	2.924881144	0.003557789	0.007528928	no
PAFAH1B3	-0.110091457	-2.924273172	0.00356466	0.007542679	no
C11orf88	0.110081532	2.924006304	0.00356768	0.007548279	no
SOX11	-0.11007794	-2.923909726	0.003568773	0.007549802	no
RPSAP52	0.110057885	2.923370479	0.003574884	0.007561939	no
LOC338651	-0.110046858	-2.923074009	0.003578248	0.007568263	no
KRT32	0.110009057	2.922057625	0.003589802	0.007591906	no
PLEKHN1	0.109975314	2.921150373	0.003600144	0.007612982	no
OSBPL11	-0.109921164	-2.919694446	0.003616798	0.007647398	no
LOC220729	-0.10991012	-2.919397503	0.003620203	0.007653311	no
ACOT11	0.109909637	2.919384528	0.003620352	0.007653311	no
KCTD11	0.109897082	2.919046955	0.003624226	0.007660702	no
PIGR	0.10987658	2.91849575	0.003630561	0.00767329	no
VSTM2L	-0.109872502	-2.918386117	0.003631823	0.007675153	no
ANKHD1	-0.109868758	-2.918285437	0.003632981	0.007676799	no
NGEF	-0.109864313	-2.918165922	0.003634357	0.007678904	no
TMEM190	0.109832976	2.917323416	0.003644069	0.007698619	no
MMP27	0.109816599	2.916883108	0.003649154	0.007708556	no
HIPK4	-0.109815076	-2.916842165	0.003649627	0.00770875	no
SLTM	-0.109782829	-2.915975181	0.003659659	0.007729133	no
PTGIS	0.109763665	2.91545996	0.003665633	0.007740941	no
PIK3C3	0.109761137	2.915391993	0.003666422	0.007741798	no
GLP1R	-0.109746246	-2.914991637	0.003671071	0.007750805	no
SLC15A1	0.109730342	2.914564067	0.003676042	0.00776049	no
SLC12A8	0.109707304	2.913944688	0.003683254	0.007774903	no
TRIM33	-0.109705511	-2.91389649	0.003683816	0.007775277	no
KCNU1	0.109688119	2.913428911	0.003689269	0.007785975	no
CGNL1	0.109674157	2.913053556	0.003693653	0.007794413	no
RQCD1	0.109665113	2.912810416	0.003696494	0.007799596	no
HRAS	-0.109656413	-2.91257651	0.00369923	0.007804554	no
SMG6	-0.109642722	-2.912208452	0.003703539	0.00781219	no
SRF	0.109642457	2.912201329	0.003703622	0.00781219	no
SH2D3C	0.109629622	2.911856269	0.003707666	0.007819903	no
OLFM3	-0.109620156	-2.911601779	0.003710651	0.007825382	no

METAP1	0.109610823	2.911350867	0.003713596	0.007830776	no
C9orf24	0.109609274	2.911309243	0.003714085	0.00783099	no
PARVA	0.109598369	2.911016057	0.003717529	0.007837435	no
FAM71A	0.109595876	2.910949037	0.003718317	0.007838279	no
ORM1	0.109588073	2.910739276	0.003720783	0.00784266	no
DKK4	0.109552195	2.909774734	0.003732144	0.007865787	no
KRTAP13-2	0.109529149	2.909155206	0.003739458	0.00788038	no
DHCR7	-0.109512803	-2.908715765	0.003744654	0.007890507	no
PEF1	0.109511429	2.90867884	0.003745091	0.007890605	no
ACPL2	0.109509812	2.908635358	0.003745605	0.007890867	no
CENPW	0.109499059	2.908346302	0.003749028	0.007897253	no
SELK	-0.109468619	-2.907527986	0.003758731	0.007916868	no
AGBL3	0.109461047	2.907324434	0.003761148	0.007921134	no
S100B	0.109458286	2.907250208	0.00376203	0.007922166	no
RBM33	-0.109448824	-2.906995842	0.003765053	0.007927707	no
MELK	0.109444259	2.906873113	0.003766512	0.007929954	no
ABCB11	0.109404976	2.905817121	0.003779091	0.007955609	no
BVES	0.109389065	2.905389401	0.003784197	0.007965529	no
ZNF283	0.109382435	2.90521118	0.003786327	0.007969181	no
LOC221442	-0.109329874	-2.903798261	0.003803247	0.00800396	no
MLH1	0.109314993	2.903398239	0.00380805	0.008013233	no
IRF9	0.109298943	2.902966804	0.003813236	0.008023312	no
HSF5	0.109296226	2.902893759	0.003814114	0.008024325	no
NKX2-5	0.109284383	2.90257541	0.003817946	0.008031551	no
NAA38	0.109244153	2.901493988	0.003830989	0.008058149	no
ZSCAN22	0.109235296	2.901255913	0.003833865	0.008063361	no
STK24	0.109233662	2.901211992	0.003834396	0.008063639	no
SKINTL	0.109206968	2.900494441	0.00384308	0.008081059	no
SIRT6	-0.10915935	-2.899214484	0.003858614	0.008112879	no
ANKRD35	0.109137403	2.898624537	0.003865793	0.008127128	no
CYP2R1	0.109130455	2.898437778	0.003868068	0.008131066	no
TSGA10	0.109090544	2.897364999	0.00388116	0.00815774	no
PRR11	0.109082801	2.897156872	0.003883705	0.00816224	no
ANKRD20B	-0.109056571	-2.896451856	0.003892336	0.00817953	no
SLC38A11	0.10905432	2.896391336	0.003893078	0.008180238	no
MAFF	0.109043866	2.896110358	0.003896524	0.008186627	no
HOOK1	-0.109036317	-2.895907444	0.003899013	0.008191007	no
MUT	-0.10902188	-2.895519395	0.003903779	0.008200167	no
ALS2CL	0.108989308	2.89464393	0.00391455	0.008221938	no
C5orf47	-0.108986493	-2.894568265	0.003915483	0.008223042	no
CHRD	-0.108919916	-2.892778805	0.003937588	0.008268608	no
LARS2	-0.108917511	-2.892714169	0.003938389	0.008269168	no
UBE2I	0.108916657	2.892691209	0.003938673	0.008269168	no
KIAA0564	-0.108890055	-2.891976209	0.003947539	0.008286923	no
GRHL2	0.108874223	2.891550693	0.003952825	0.008297156	no
ZNF20	0.108862922	2.891246951	0.003956601	0.008304222	no
NPTX1	-0.108853982	-2.891006684	0.003959591	0.008309634	no
VWDE	0.108844924	2.890763224	0.003962623	0.008315133	no
ZNF41	-0.108831129	-2.890392452	0.003967244	0.008323966	no
MAL2	-0.108829574	-2.890350658	0.003967765	0.008324195	no
SYT14L	-0.108795669	-2.889439409	0.003979144	0.008347202	no

ASCC2	0.10879081	2.889308826	0.003980777	0.008349762	no
ESPNP	-0.108780312	-2.889026675	0.003984308	0.008355982	no
C4orf44	-0.108779535	-2.88900578	0.003984569	0.008355982	no
CECR4	-0.108770974	-2.888775688	0.003987451	0.008361158	no
MTA2	-0.10876452	-2.888602236	0.003989624	0.008364848	no
CREB3L4	-0.108748751	-2.888178434	0.003994939	0.008374754	no
MAP2K2	-0.108748044	-2.88815943	0.003995178	0.008374754	no
KLHL30	0.108744901	2.888074947	0.003996238	0.008376109	no
EMCN	0.108710323	2.887145638	0.004007919	0.008399721	no
ARPM1	0.108695873	2.886757268	0.00401281	0.008409099	no
FLJ26850	0.108677157	2.88625427	0.004019152	0.008421517	no
C1orf190	0.108667118	2.885984486	0.004022557	0.008427779	no
CRAT	-0.108663465	-2.885886303	0.004023797	0.008429503	no
TMEM218	0.108634507	2.885108057	0.004033639	0.008449244	no
CXCR7	0.108623691	2.884817373	0.00403732	0.00845608	no
TBX6	-0.108614679	-2.884575161	0.00404039	0.008461633	no
TRIM63	0.108605026	2.884315733	0.00404368	0.008467647	no
WNT10B	-0.108582448	-2.88370898	0.004051386	0.008482903	no
ZNF559	-0.108568605	-2.88333695	0.004056117	0.00849193	no
CXorf30	0.10853518	2.882438671	0.004067561	0.008515007	no
DDX41	-0.10850059	-2.881509099	0.004079434	0.008538979	no
WNK3	-0.108497257	-2.881419519	0.00408058	0.008540493	no
CTNND1	0.108495432	2.881370469	0.004081208	0.008540923	no
C9orf170	0.108418118	2.879292786	0.004107871	0.008595832	no
LLGL2	0.108415674	2.879227104	0.004108716	0.008596711	no
LOC100192379	-0.108381834	-2.878317705	0.004120439	0.008620346	no
C21orf70	-0.10837567	-2.878152067	0.004122577	0.008623928	no
PANX1	0.108368167	2.87795045	0.004125181	0.008628483	no
PFDN6	-0.10833049	-2.876937972	0.004138282	0.008654989	no
CAMKK1	-0.108313195	-2.8764732	0.004144308	0.008665873	no
THUMPD3	0.108313094	2.876470509	0.004144343	0.008665873	no
COIL	-0.108310078	-2.876389441	0.004145395	0.008667176	no
PGBD5	-0.108298217	-2.876070726	0.004149533	0.008674931	no
ACTR5	-0.108295487	-2.875997364	0.004150486	0.008676027	no
COG3	-0.108264403	-2.875162067	0.004161352	0.00869784	no
SERP1	0.108255739	2.874929247	0.004164385	0.00870328	no
NOC2L	-0.108250411	-2.874786093	0.004166251	0.008705544	no
AP4B1	0.108250187	2.874780069	0.004166329	0.008705544	no
GNRH1	0.10821806	2.87391675	0.004177599	0.00872819	no
MMP10	0.108204102	2.873541683	0.004182503	0.008737534	no
ETNK2	0.108197552	2.873365693	0.004184807	0.008741442	no
RSPH1	0.108195186	2.873302102	0.004185639	0.008742278	no
PEX10	-0.108191311	-2.873197974	0.004187003	0.008744222	no
TAS2R20	-0.10818559	-2.873044261	0.004189016	0.008747524	no
AEBP2	0.108180953	2.872919653	0.004190649	0.008749578	no
ANKRD30B	-0.108180339	-2.872903143	0.004190865	0.008749578	no
OR4N3P	-0.10817517	-2.872764264	0.004192686	0.008752476	no
TBCCD1	0.108145866	2.871976836	0.004203023	0.008773148	no
KLHL24	-0.108118022	-2.871228633	0.004212866	0.008792787	no
TCTEX1D2	0.10808528	2.870348842	0.004224467	0.00881609	no
LOC730668	0.108084003	2.870314536	0.00422492	0.008816126	no

SPTLC1	0.108053533	2.869495803	0.004235744	0.008837798	no
ZBTB24	-0.10801969	-2.868586446	0.004247794	0.008862028	no
NOP14	0.108015794	2.868481754	0.004249184	0.008864011	no
HOXA10	0.108010612	2.868342536	0.004251032	0.008866952	no
DONSON	0.107996741	2.867969833	0.004255984	0.008876364	no
IFIT3	0.107977438	2.867451174	0.004262883	0.008889837	no
ABCC11	-0.10795797	-2.866928067	0.004269852	0.008903451	no
ELAC1	0.107915917	2.865798141	0.00428494	0.008933991	no
LOC643719	-0.107904004	-2.865478057	0.004289223	0.008941999	no
MORF4	0.107902086	2.865426523	0.004289913	0.008942515	no
C4orf49	0.107892366	2.865165358	0.00429341	0.008948884	no
NAA35	-0.107881306	-2.864868194	0.004297394	0.008956263	no
RHPN2	0.107867528	2.86449801	0.00430236	0.00896569	no
AKR1C3	-0.107860218	-2.864301616	0.004304998	0.008970261	no
TMEM38B	0.107849588	2.864015988	0.004308835	0.008977333	no
NARS	-0.107846578	-2.863935109	0.004309923	0.008978673	no
RASGEF1B	0.10783662	2.863667578	0.004313521	0.008985243	no
CISD1	-0.107825376	-2.863365461	0.004317588	0.008992788	no
SRBD1	0.107815783	2.863107731	0.00432106	0.008999093	no
BMP5	0.107794576	2.862537935	0.004328746	0.00901417	no
C3orf37	-0.107780402	-2.862157104	0.004333889	0.009023952	no
AS3MT	-0.10777075	-2.861897779	0.004337395	0.009030321	no
C1orf53	0.107760176	2.861613681	0.004341238	0.009037392	no
MCOLN3	0.107749661	2.861331179	0.004345063	0.009044424	no
FABP7	0.107742302	2.86113346	0.004347742	0.009049068	no
NKAIN3	-0.107732583	-2.860872351	0.004351282	0.009055504	no
PGBD4	-0.107723537	-2.860629298	0.00435458	0.009060074	no
SSU72	0.107722086	2.860590317	0.004355109	0.009060074	no
ACSF3	-0.107721912	-2.860585649	0.004355172	0.009060074	no
EFR3A	0.107721642	2.860578382	0.004355271	0.009060074	no
SELL	-0.107697644	-2.859933638	0.00436403	0.009077363	no
WDR85	-0.107694587	-2.859851495	0.004365147	0.009078752	no
CYSLTR2	0.107666831	2.859105805	0.004375301	0.009098934	no
ITPKA	-0.10766384	-2.859025427	0.004376397	0.009100276	no
SCT	-0.107659361	-2.8589051	0.004378037	0.009102752	no
RNASE13	-0.107647827	-2.858595227	0.004382265	0.009110606	no
STYK1	-0.107635796	-2.858272005	0.004386679	0.009118845	no
SNRNP70	-0.10761822	-2.8577998	0.004393135	0.009131326	no
INTS8	0.107608766	2.857545803	0.004396612	0.009137612	no
CNN1	0.107606809	2.85749323	0.004397331	0.009138169	no
AMOT	-0.107595706	-2.857194943	0.004401418	0.00914572	no
CXADR	0.107574379	2.856621962	0.004409276	0.009161109	no
TRIM41	-0.107572782	-2.85657907	0.004409865	0.009161391	no
TRIM47	0.107551023	2.855994491	0.004417897	0.009177134	no
TKTL2	-0.107523145	-2.855245548	0.004428207	0.009197605	no
CAPN10	-0.107462667	-2.85362079	0.004450648	0.009243266	no
CCR10	0.107419537	2.852462129	0.004466714	0.00927568	no
ZNF530	0.10734676	2.850507035	0.004493943	0.009331267	no
EFNA1	0.107319718	2.849780579	0.004504099	0.009351394	no
ESX1	-0.107249132	-2.84788443	0.004530706	0.009405669	no
psiTPTE22	-0.107240605	-2.847655362	0.00453393	0.009411396	no

LPL	-0.107233926	-2.847475935	0.004536457	0.009414761	no
SLC25A29	-0.107233857	-2.84747409	0.004536483	0.009414761	no
VTN	-0.107231939	-2.84742258	0.004537208	0.009415301	no
ARHGAP1	0.107229653	2.847361167	0.004538073	0.00941613	no
NANOS2	-0.107227374	-2.847299952	0.004538936	0.009416953	no
AGAP8	-0.107207649	-2.84677009	0.004546409	0.009431489	no
ABTB2	-0.107205009	-2.846699169	0.00454741	0.009432598	no
FAM162B	0.107189587	2.846284887	0.004553262	0.009443767	no
GCN1L1	-0.107181631	-2.846071161	0.004556283	0.009449065	no
WFDC6	0.107156192	2.845387822	0.004565956	0.009468153	no
VWA2	-0.107100935	-2.843903525	0.004587031	0.009510879	no
FAM48A	-0.107092467	-2.843676058	0.004590268	0.009516616	no
LOC100189589	-0.107066623	-2.84298186	0.004600161	0.009536149	no
BMPR1B	-0.107047841	-2.842477345	0.004607363	0.0095501	no
SFRS16	-0.107027192	-2.841922676	0.004615293	0.009564732	no
WDFY2	0.107026995	2.84191739	0.004615369	0.009564732	no
MFHAS1	0.107021854	2.841779294	0.004617345	0.009567847	no
CCDC11	0.10701134	2.841496876	0.004621389	0.009575245	no
KCNJ3	-0.10700551	-2.841340276	0.004623633	0.009578913	no
UCN	-0.106983081	-2.840737835	0.004632274	0.009595831	no
C11orf31	0.106977752	2.840594696	0.004634329	0.009599105	no
RASAL1	-0.10695358	-2.839945429	0.004643662	0.009617451	no
CEBPG	0.106943147	2.839665193	0.004647695	0.009624502	no
C11orf46	-0.106942312	-2.839642746	0.004648019	0.009624502	no
C19orf30	-0.106940497	-2.839593998	0.004648721	0.00962497	no
KIAA0391	0.106939006	2.839553971	0.004649297	0.009625178	no
LOC286002	-0.106936834	-2.839495614	0.004650138	0.009625933	no
C6orf163	-0.106920842	-2.839066067	0.004656329	0.009637763	no
CDK10	-0.106912448	-2.838840605	0.004659582	0.009643509	no
MYO19	0.1068832	2.838055006	0.004670933	0.00966601	no
ZFR	-0.106876169	-2.837866149	0.004673665	0.009670675	no
FAM102B	0.10686702	2.837620414	0.004677223	0.009677046	no
RPP40	0.106859985	2.837431454	0.00467996	0.009681718	no
NCRNA00115	-0.106814965	-2.836222253	0.00469751	0.009717032	no
LUZP4	0.106789694	2.835543505	0.004707388	0.009736468	no
FLJ40504	-0.106765123	-2.834883566	0.00471701	0.009755371	no
HELZ	-0.106763627	-2.834843374	0.004717597	0.009755586	no
KCTD1	-0.106694546	-2.83298798	0.004744747	0.009810408	no
FAM13A	-0.106693707	-2.832965448	0.004745078	0.009810408	no
SLC16A1	0.106688332	2.832821092	0.004747196	0.009813784	no
TMTC3	0.106682613	2.832667481	0.004749451	0.009817435	no
ZBTB39	-0.106681391	-2.832634653	0.004749933	0.009817435	no
NEFH	-0.106675596	-2.832479028	0.004752219	0.009821156	no
GNG13	-0.106651858	-2.83184146	0.004761595	0.009839527	no
PTPRN	-0.106643969	-2.831629578	0.004764715	0.009844967	no
INHBC	-0.106640465	-2.831535486	0.004766101	0.009846824	no
LOC84856	-0.106630771	-2.83127511	0.004769938	0.009853745	no
ATXN7L3	-0.106589963	-2.830179122	0.00478612	0.009885391	no
C2orf62	0.106589672	2.830171323	0.004786235	0.009885391	no
RPS6KA6	-0.106586367	-2.830082557	0.004787548	0.009887093	no
CCDC124	-0.106553239	-2.829192832	0.004800724	0.009913292	no

PPIH	0.106537846	2.828779413	0.004806858	0.009924945	no
C1orf64	-0.106536326	-2.828738605	0.004807464	0.009925182	no
FAM40B	-0.106530347	-2.828578016	0.004809849	0.009929092	no
AK5	-0.106512802	-2.828106822	0.004816853	0.009942126	no
LOC391322	-0.106512066	-2.828087069	0.004817146	0.009942126	no
GPR156	0.106502554	2.827831603	0.004820948	0.009948956	no
C6orf108	-0.106492602	-2.827564337	0.004824927	0.009956121	no
DTHD1	0.106491409	2.82753229	0.004825405	0.009956121	no
EIF3H	-0.106489737	-2.827487393	0.004826074	0.009956485	no
CHRNA2	-0.106487047	-2.827415151	0.00482715	0.00995769	no
SERINC3	0.106476203	2.827123904	0.004831492	0.00996563	no
GTF2IRD1	-0.106445785	-2.826307016	0.004843689	0.00998977	no
SERPINA5	0.106443986	2.82625868	0.004844412	0.009990241	no
ARNTL	0.106441827	2.826200707	0.004845279	0.009991009	no
NUDT9	0.10643521	2.826023013	0.004847937	0.00999547	no
FLJ42289	-0.106432534	-2.825951136	0.004849012	0.009996668	no
C7orf34	0.106430613	2.825899538	0.004849784	0.00999724	no
DSCC1	0.106414542	2.825467937	0.004856247	0.010009543	no
NINJ2	0.106369261	2.824251905	0.004874499	0.010046138	no
MRPL38	-0.106328267	-2.823151023	0.004891076	0.010079275	no
KLHDC8A	0.106316165	2.822826011	0.00489598	0.010088353	no
TAAR5	-0.106310246	-2.822667072	0.004898379	0.010092269	no
HINT1	0.106306857	2.822576051	0.004899754	0.010094072	no
C3orf50	-0.106293422	-2.822215253	0.004905207	0.010104276	no
GAB1	-0.106272756	-2.82166029	0.004913605	0.010120544	no
ATP5SL	0.106263788	2.821419458	0.004917253	0.010127027	no
DYNC1I2	-0.106254808	-2.821178294	0.004920909	0.010133524	no
MYCT1	0.106248064	2.820997191	0.004923656	0.010138148	no
HOMER3	0.106231012	2.820539286	0.004930608	0.010151428	no
VWA5A	0.10620688	2.819891231	0.004940462	0.01017068	no
CACNG8	-0.106203223	-2.81979303	0.004941956	0.010172722	no
TPI1	0.106197737	2.819645708	0.0049442	0.010176303	no
SLC7A5P1	-0.106142877	-2.818172542	0.004966681	0.010221535	no
SLC6A12	-0.106115772	-2.817444685	0.004977823	0.010243423	no
ZNF187	-0.106084088	-2.81659388	0.004990876	0.010269237	no
MRPL35	-0.106060541	-2.81596158	0.005000596	0.01028746	no
PREPL	-0.10606017	-2.815951603	0.00500075	0.01028746	no
SBF1P1	-0.106019283	-2.81485369	0.00501767	0.010321217	no
CORO7	-0.105985146	-2.813937041	0.005031836	0.010349304	no
PRKACB	-0.105982621	-2.813869257	0.005032885	0.010350408	no
SPDYE6	0.105937247	2.812650875	0.005051773	0.010388197	no
TMEM132E	0.105930426	2.81246772	0.005054618	0.010392991	no
CHMP1A	-0.105887927	-2.811326581	0.005072377	0.010428444	no
RANBP3L	-0.105881687	-2.81115902	0.005074989	0.010432754	no
HCFC2	-0.105796195	-2.808863504	0.0051109	0.010505509	no
PIK3IP1	0.10579006	2.808698774	0.005113486	0.010509756	no
RAB15	-0.105778175	-2.808379661	0.005118499	0.010518989	no
SPATA5L1	-0.105773388	-2.808251135	0.005120519	0.010522071	no
KCNH2	-0.105767519	-2.80809356	0.005122997	0.010526093	no
EGFL8	-0.105762459	-2.807957678	0.005125134	0.010529414	no
GDF2	-0.105759096	-2.807867377	0.005126555	0.01053063	no

C2orf63	-0.105758593	-2.807853878	0.005126768	0.01053063	no
TST	-0.105745788	-2.807510068	0.005132181	0.010540679	no
ISCU	0.10574251	2.807422065	0.005133568	0.010542455	no
SLC5A10	0.105718425	2.806775373	0.005143767	0.010562328	no
CCNA2	0.105712932	2.806627891	0.005146095	0.010566036	no
PRPF3	-0.105709312	-2.806530691	0.005147631	0.010568115	no
TBC1D3H	-0.105675197	-2.805614738	0.005162118	0.010596781	no
LRRC41	0.105651712	2.80498419	0.005172112	0.010616219	no
CDH13	-0.105649713	-2.804930506	0.005172964	0.01061689	no
LSM1	0.105628114	2.804350595	0.005182172	0.01063347	no
GNG3	-0.105627593	-2.804336611	0.005182394	0.01063347	no
CARD14	-0.105627064	-2.804322419	0.00518262	0.01063347	no
NPR2	0.10561338	2.803955015	0.005188462	0.010644377	no
LIPE	-0.10561178	-2.803912057	0.005189146	0.010644699	no
KIAA1704	-0.105591834	-2.803376525	0.005197673	0.01066111	no
ERCC1	0.105588125	2.803276939	0.00519926	0.010663283	no
ANKRD56	-0.105585039	-2.803194093	0.005200581	0.01066491	no
TMEM133	-0.105579708	-2.803050964	0.005202863	0.010668508	no
PI4KAP2	-0.105573542	-2.802885424	0.005205504	0.010672553	no
SNX31	-0.105572638	-2.802861161	0.005205892	0.010672553	no
TRUB2	-0.105567803	-2.802731343	0.005207964	0.010675718	no
RAB25	-0.105554576	-2.802376213	0.005213635	0.010686261	no
C3orf45	-0.105529945	-2.801714905	0.005224213	0.010706855	no
XPNPEP3	0.105500017	2.800911418	0.00523709	0.010732159	no
DUSP27	0.105493003	2.800723105	0.005240112	0.010737264	no
CNGA3	0.105489567	2.800630862	0.005241593	0.01073921	no
SGK196	-0.105478514	-2.8003341	0.00524636	0.010747888	no
SLC22A23	0.105465622	2.799988	0.005251925	0.010758198	no
SLC7A5	-0.105442952	-2.799379366	0.005261723	0.010777177	no
DDX46	-0.105435319	-2.79917444	0.005265026	0.01078285	no
PDK1	0.105414285	2.798609726	0.005274137	0.010800416	no
CYP4X1	-0.105396728	-2.798138387	0.005281753	0.010814917	no
NPEPL1	0.105389076	2.797932946	0.005285076	0.010820624	no
DAB1	-0.105337031	-2.796535732	0.005307723	0.010865892	no
CCDC66	-0.105320207	-2.796084066	0.005315063	0.010879816	no
FUNDC1	0.105301129	2.795571896	0.005323397	0.010895772	no
HIVEP2	-0.10529776	-2.795481458	0.00532487	0.010897684	no
CCDC19	0.10527158	2.794778613	0.005336329	0.01092003	no
PLAT	0.105263109	2.794551218	0.005340041	0.01092652	no
ARID2	-0.105255461	-2.794345896	0.005343395	0.010932276	no
NEIL1	-0.105233807	-2.793764579	0.0053529	0.010950616	no
DTNB	-0.10523112	-2.793692447	0.005354081	0.010951923	no
NLK	-0.105226875	-2.793578492	0.005355947	0.010954614	no
C2orf52	-0.105225662	-2.793545919	0.00535648	0.010954614	no
FAM106A	-0.105218106	-2.793343091	0.005359802	0.0109603	no
NEIL3	0.105200806	2.792878668	0.005367417	0.01097476	no
ZNF828	-0.105196081	-2.792751808	0.005369498	0.010977906	no
HSPA9	-0.105181162	-2.792351321	0.005376074	0.010990239	no
SAMD11	-0.105176246	-2.792219355	0.005378243	0.010993561	no
KCMF1	0.105157616	2.791719228	0.005386468	0.011009262	no
IPCEF1	-0.105140763	-2.791266807	0.005393919	0.011023375	no

TMCO7	-0.105107682	-2.79037877	0.005408571	0.011052202	no
TBCA	0.105094147	2.790015433	0.005414576	0.011063354	no
CHM	0.105068664	2.789331353	0.005425898	0.011085369	no
EBAG9	0.105011002	2.787783497	0.005451597	0.011136747	no
OR1J2	0.105004106	2.787598366	0.005454678	0.011141915	no
TNKS2	-0.104997542	-2.787422168	0.005457612	0.011146782	no
PLA2G2F	0.104991175	2.787251276	0.005460458	0.01115147	no
ERLEC1	0.104989481	2.787205807	0.005461216	0.01115189	no
CBR3	0.104979132	2.786927991	0.005465848	0.011160221	no
SLAMF9	0.104972766	2.786757119	0.005468698	0.011164913	no
IRF2BP1	-0.104935258	-2.785750274	0.005485521	0.011198128	no
PNKD	0.104932096	2.785665403	0.005486941	0.011199897	no
ZFP3	-0.104927266	-2.78553575	0.005489111	0.011203195	no
PABPN1L	-0.104924436	-2.78545979	0.005490383	0.01120466	no
VSIG8	0.104911163	2.78510349	0.005496352	0.01121571	no
PSMD1	0.104906304	2.784973062	0.005498539	0.011219039	no
FLJ14107	0.104873882	2.784102788	0.005513149	0.011247714	no
RPS12	-0.104867551	-2.78393285	0.005516006	0.011252407	no
NCALD	-0.104862509	-2.783797519	0.005518282	0.011255915	no
TMEM53	0.104851802	2.783510104	0.005523119	0.011264644	no
USE1	-0.104847123	-2.783384504	0.005525234	0.011267821	no
C18orf34	-0.104808589	-2.78235019	0.005542678	0.011302255	no
WASH5P	-0.104768061	-2.781262344	0.005561078	0.011338633	no
TTC30A	0.104766149	2.781211028	0.005561948	0.011339262	no
FBXO40	-0.10474625	-2.780676922	0.005571004	0.011356579	no
DNASE2B	0.104730579	2.780256281	0.005578145	0.01136999	no
HAR1B	-0.104711494	-2.779744013	0.005586853	0.011386592	no
EPHB3	-0.104698439	-2.77939361	0.005592817	0.011397598	no
C7orf31	0.104686338	2.779068812	0.00559835	0.011406814	no
RAVER1	-0.10468608	-2.779061894	0.005598467	0.011406814	no
CYTH2	-0.104681778	-2.778946419	0.005600436	0.011409675	no
GOLGA8F	0.104676696	2.778810017	0.005602762	0.011413263	no
PRSS1	0.104673532	2.778725099	0.00560421	0.011415063	no
RGPD4	-0.104663051	-2.778443774	0.005609011	0.011423691	no
PLAC8L1	-0.104629896	-2.777553863	0.005624222	0.011453518	no
ANKRD43	-0.104624013	-2.777395973	0.005626925	0.011457868	no
TTC25	0.104607011	2.776939649	0.005634743	0.011472631	no
LOC26102	-0.104603169	-2.776836525	0.005636511	0.011475076	no
STAM2	0.104589914	2.776480745	0.005642615	0.011486345	no
ADRB1	-0.104579064	-2.776189546	0.005647615	0.011494939	no
ELOVL3	0.104578287	2.776168674	0.005647973	0.011494939	no
RRAD	0.104571477	2.775985897	0.005651114	0.011500174	no
RARRES1	0.10456567	2.775830049	0.005653793	0.011504468	no
KBTBD12	0.104556513	2.77558427	0.005658021	0.011511912	no
ANKRD34C	-0.104535667	-2.775024776	0.005667656	0.011529635	no
CNTN6	-0.104535199	-2.775012207	0.005667872	0.011529635	no
TM2D1	0.104529031	2.774846681	0.005670726	0.011533281	no
FKBP9L	0.104528858	2.77484204	0.005670806	0.011533281	no
ALMS1	-0.104526346	-2.774774617	0.005671968	0.011534485	no
RNF182	-0.104501188	-2.774099395	0.005683623	0.011557025	no
SLC27A6	0.10448811	2.773748388	0.005689691	0.011568198	no

ZNF492	-0.104485318	-2.773673445	0.005690987	0.01156967	no
SKA1	0.104481589	2.773573373	0.005692718	0.011572026	no
FAM109A	-0.104476665	-2.773441217	0.005695005	0.011575511	no
RSPH10B2	0.104466291	2.773162774	0.005699826	0.011584146	no
PRCD	-0.104448326	-2.772680612	0.005708184	0.011599214	no
CNOT7	0.104447888	2.772668857	0.005708388	0.011599214	no
ZNF595	-0.104434859	-2.772319182	0.005714456	0.011610377	no
KIAA0895	0.104432971	2.772268505	0.005715336	0.011610998	no
KLF4	0.104403505	2.771477687	0.005729083	0.011637757	no
NGB	-0.104383483	-2.770940345	0.00573844	0.011655594	no
RCBTB2	0.104365912	2.770468753	0.005746664	0.011671126	no
LOC286367	0.104357195	2.770234806	0.005750748	0.011678247	no
PRR12	-0.104354329	-2.770157891	0.005752091	0.011679802	no
MRPL20	0.104316223	2.769135225	0.005769978	0.011714943	no
KIAA1609	0.104305645	2.768851341	0.005774952	0.011723865	no
GTDC1	-0.104294745	-2.768558794	0.005780081	0.011733101	no
KCNV1	-0.104288778	-2.768398666	0.005782891	0.011736479	no
RPS6KB2	0.104288745	2.768397763	0.005782907	0.011736479	no
CDC40	-0.104274944	-2.768027384	0.00578941	0.011748499	no
NHS	0.104269655	2.767885452	0.005791904	0.01175238	no
RASD2	-0.104233027	-2.766902472	0.005809203	0.011786298	no
YME1L1	0.104218036	2.766500149	0.005816296	0.011799506	no
CITED4	0.104210128	2.766287939	0.005820041	0.011805918	no
SPOCK2	-0.104184632	-2.765603703	0.00583213	0.011828447	no
DDRGK1	0.104184238	2.765593122	0.005832317	0.011828447	no
PCDH1	-0.104181385	-2.765516573	0.005833671	0.011830006	no
CLSTN3	-0.104177635	-2.765415935	0.005835451	0.01183243	no
NNT	-0.10416217	-2.765000904	0.005842799	0.011845955	no
TMPRSS6	0.10416113	2.764972981	0.005843294	0.011845955	no
SYNPR	-0.104145067	-2.76454192	0.005850935	0.011860256	no
ROCK1	0.104134283	2.764252532	0.005856069	0.011869475	no
IDS	-0.104129841	-2.764133301	0.005858186	0.011872575	no
SAV1	0.104113797	2.76370276	0.005865835	0.011886885	no
LRP2BP	-0.104099221	-2.763311604	0.005872792	0.011899791	no
TLK1	0.104095185	2.763203278	0.005874721	0.011902505	no
REM2	-0.10409038	-2.763074345	0.005877016	0.011905963	no
FAM10A4	-0.104035785	-2.761609263	0.005903158	0.011957725	no
CPNE7	-0.104025817	-2.761341762	0.005907942	0.011966217	no
RAB23	0.104022654	2.761256877	0.005909461	0.011968095	no
DBF4B	-0.104016625	-2.761095101	0.005912357	0.01197276	no
PDCL	-0.104000899	-2.760673081	0.005919918	0.01198687	no
SUGT1P1	-0.10399073	-2.760400209	0.005924811	0.011995576	no
RIMBP2	-0.10398924	-2.760360233	0.005925528	0.011995827	no
FBXO10	-0.103987222	-2.760306065	0.0059265	0.011996593	no
MCHR2	-0.103980853	-2.760135166	0.005929567	0.0120016	no
CNNM4	0.103975065	2.759979846	0.005932356	0.012006043	no
CDCA4	0.103969303	2.75982521	0.005935133	0.012010462	no
CPOX	0.103927169	2.758694566	0.005955478	0.012049915	no
TULP1	0.103926459	2.758675532	0.005955821	0.012049915	no
HOXA7	0.103922487	2.758568923	0.005957743	0.012052597	no
GRM4	-0.103916886	-2.758418638	0.005960453	0.012056873	no

ALG9	-0.10388948	-2.757683206	0.005973731	0.012082521	no
LIPT2	0.103882335	2.757491489	0.005977196	0.012088322	no
ABLIM3	-0.103859825	-2.756887462	0.005988127	0.012109217	no
CARHSP1	0.103852814	2.756699328	0.005991535	0.012114897	no
C3orf10	0.103820358	2.755828406	0.006007336	0.012145631	no
CTSO	0.103809621	2.755540305	0.006012571	0.012155	no
KCNA1	-0.10380011	-2.755285103	0.006017211	0.012162988	no
INSR	-0.103799057	-2.755256844	0.006017725	0.012162988	no
RBM38	0.103750791	2.753961721	0.006041328	0.012209474	no
CTXN2	-0.10373451	-2.753524856	0.006049309	0.01222438	no
CCDC114	0.103727564	2.753338462	0.006052716	0.012230044	no
VPS39	-0.103702405	-2.752663383	0.006065073	0.012253788	no
LRRC50	0.103694876	2.752461383	0.006068775	0.012260042	no
RPL13AP20	-0.103686951	-2.752248734	0.006072675	0.012266693	no
CCK	-0.103684445	-2.752181485	0.006073908	0.01226796	no
RPSA	-0.103676935	-2.751979971	0.006077606	0.012274202	no
FAM18B2	0.103669177	2.751771801	0.006081428	0.012280695	no
EEF1B2	-0.103663574	-2.75162147	0.00608419	0.012285044	no
GABRR3	0.103638722	2.750954651	0.006096453	0.012308576	no
NOXA1	-0.103613123	-2.75026778	0.006109108	0.012332895	no
TMEM177	-0.103611736	-2.750230568	0.006109794	0.012333048	no
SERPINI1	-0.103604525	-2.750037072	0.006113364	0.012339022	no
WDR47	-0.103600946	-2.749941039	0.006115136	0.012340787	no
LOC554202	0.103600294	2.749923549	0.006115459	0.012340787	no
TDRD5	-0.103596365	-2.749818137	0.006117405	0.012343482	no
C8orf86	-0.10358675	-2.749560144	0.00612217	0.01235091	no
RHEBL1	0.103586472	2.74955268	0.006122308	0.01235091	no
MTX3	-0.103557136	-2.748765569	0.006136867	0.012379046	no
C9orf44	0.10353691	2.748222865	0.006146924	0.012398095	no
MIXL1	0.103501247	2.747266	0.006164691	0.012432692	no
CCDC12	-0.103499726	-2.747225204	0.00616545	0.012432981	no
CYB561D1	0.103494822	2.747093621	0.006167897	0.012436676	no
TNF	0.103487614	2.746900213	0.006171496	0.01244203	no
SLC37A4	0.103485873	2.746853517	0.006172365	0.01244203	no
GLRA1	-0.103485805	-2.746851693	0.006172399	0.01244203	no
PLEKHJ1	-0.103460031	-2.746160168	0.006185283	0.012466758	no
TMEM87A	-0.103433396	-2.74544553	0.006198623	0.0124924	no
PROX1	-0.103431431	-2.745392821	0.006199608	0.01249314	no
CCDC45	-0.103418957	-2.745058137	0.006205865	0.012504503	no
ZNRF3	-0.1034177	-2.745024401	0.006206496	0.012504528	no
CDCA8	0.103412146	2.744875389	0.006209284	0.012508899	no
SMNDC1	0.103405771	2.744704348	0.006212486	0.012514102	no
CIDEA	-0.103403775	-2.744650807	0.006213488	0.012514874	no
MED31	0.103396401	2.744452958	0.006217194	0.012521091	no
MOSC2	0.103394334	2.744397499	0.006218233	0.012521937	no
LOC154822	-0.103390172	-2.744285833	0.006220326	0.012524903	no
CEP170L	-0.103381032	-2.744040601	0.006224924	0.012532914	no
SPHK2	-0.103379576	-2.744001542	0.006225657	0.012533141	no
PTP4A3	0.103376809	2.743927297	0.00622705	0.012534697	no
RABEP2	0.103367659	2.743681819	0.006231657	0.012542722	no
C2orf43	0.10335827	2.743429908	0.006236388	0.012550995	no

C17orf63	-0.103330426	-2.742682868	0.006250438	0.012578019	no
RPS15AP10	0.103313793	2.742236618	0.006258844	0.012593681	no
DUSP7	-0.10328728	-2.741525294	0.006272265	0.012619429	no
XRCC4	0.103272593	2.741131279	0.00627971	0.012633151	no
CDK14	-0.103240346	-2.740266132	0.006296085	0.012664834	no
C4BPA	0.103238754	2.740223408	0.006296894	0.012665202	no
KLHL3	-0.103224277	-2.739835021	0.006304259	0.012678754	no
CCDC6	-0.103202554	-2.739252218	0.006315325	0.012699745	no
NUP85	0.103191127	2.738945671	0.006321152	0.0127102	no
IL19	0.103183419	2.738738871	0.006325086	0.012716845	no
ZIC5	0.103169746	2.738372057	0.00633207	0.01272962	no
STOML3	0.103159986	2.738110221	0.006337059	0.012738246	no
ARSB	0.103158888	2.738080744	0.006337621	0.012738246	no
ZBTB22	-0.103154512	-2.737963365	0.006339859	0.012741477	no
ITCH	0.103152759	2.737916314	0.006340756	0.012742014	no
LRRCC1	0.103135747	2.73745994	0.006349465	0.012758247	no
SDF2	0.10311784	2.736979544	0.006358644	0.012775421	no
RPL13AP3	-0.103094929	-2.736364889	0.006370406	0.01279778	no
C16orf86	-0.103066433	-2.735600412	0.006385062	0.012825949	no
CXorf41	0.103043013	2.734972122	0.00639713	0.012848914	no
SLC26A8	-0.103024092	-2.734464534	0.006406894	0.012867248	no
INPP5F	-0.10301856	-2.734316124	0.006409752	0.012871709	no
LOC121952	-0.103012042	-2.734141293	0.006413119	0.012877192	no
CTCFL	-0.103007367	-2.734015877	0.006415536	0.012880766	no
ZSWIM1	-0.103001665	-2.733862906	0.006418485	0.012885407	no
AURKAPS1	0.102998246	2.733771182	0.006420254	0.012887678	no
RPL12	-0.102995308	-2.733692372	0.006421774	0.01288945	no
VIP	-0.102988898	-2.733520401	0.006425092	0.01289483	no
BECN1	0.102954255	2.732591066	0.006443051	0.012929589	no
MLLT1	-0.102951175	-2.732508432	0.00644465	0.012931514	no
7-Sep	0.102947962	2.732422237	0.006446318	0.012932737	no
CASP8AP2	-0.102947537	-2.732410844	0.006446539	0.012932737	no
CENPA	0.102941298	2.732243488	0.006449779	0.012937954	no
ATP6V1C1	-0.102933133	-2.732024449	0.006454022	0.012945181	no
UQCRQ	0.102911627	2.731447539	0.006465211	0.012966336	no
PIN1	-0.102909992	-2.731403672	0.006466062	0.012966757	no
C1QL2	-0.102905463	-2.731282165	0.006468421	0.012970201	no
GTF2H3	0.102887502	2.730800367	0.006477782	0.012987683	no
CENPE	0.102879538	2.730586714	0.006481937	0.012994726	no
DNTTIP1	0.102864426	2.730181332	0.006489828	0.01300873	no
FAM54A	0.102863695	2.730161724	0.00649021	0.01300873	no
EXOC3L	0.102861743	2.730109357	0.00649123	0.013009485	no
RAP1GAP2	-0.102846381	-2.729697267	0.006499261	0.01302429	no
BAZ2A	-0.102842138	-2.729583438	0.006501481	0.013027448	no
RARS2	0.102833041	2.729339421	0.006506243	0.013035696	no
CNTN4	-0.10281525	-2.728862172	0.006515564	0.013053079	no
ZNF14	-0.102806604	-2.728630245	0.006520099	0.013060869	no
WASL	-0.102786852	-2.728100402	0.006530468	0.013080345	no
PCP4L1	-0.102783618	-2.72801365	0.006532167	0.013082452	no
TSC22D4	-0.102777862	-2.727859256	0.006535192	0.013087215	no
CDK15	0.102768104	2.727597498	0.006540324	0.013096194	no

SNX19	-0.102680745	-2.725254151	0.006586426	0.013187202	no
F8A1	0.102679409	2.725218314	0.006587134	0.013187312	no
NDST1	-0.102677422	-2.725165036	0.006588185	0.013188112	no
OXCT2	-0.102610885	-2.723380264	0.006623503	0.013257498	no
DOM3Z	-0.102576235	-2.722450854	0.006641962	0.013293129	no
PRKCB	-0.102571531	-2.722324674	0.006644472	0.013296836	no
MT2A	0.10255177	2.721794633	0.006655023	0.013316633	no
GRK7	0.102543182	2.72156427	0.006659614	0.0133245	no
WSCD1	-0.102534755	-2.72133824	0.006664121	0.0133321	no
PDSS1	-0.102533309	-2.721299443	0.006664895	0.0133321	no
MAGOHB	0.102532382	2.721274584	0.00666539	0.0133321	no
B3GNT4	-0.102496388	-2.720309124	0.006684676	0.013369351	no
PSMG2	0.102482047	2.719924484	0.006692373	0.013383422	no
TRPV6	-0.102478773	-2.71983667	0.006694131	0.013385614	no
XPO4	-0.102473292	-2.719689651	0.006697076	0.013390179	no
NEFL	-0.102463966	-2.719439496	0.00670209	0.013398877	no
ZNF485	-0.102459927	-2.71933118	0.006704261	0.013401894	no
IL1F9	0.102450723	2.719084301	0.006709214	0.013410468	no
C10orf131	-0.102427429	-2.718459505	0.006721762	0.013434222	no
C3orf49	-0.102422763	-2.718334373	0.006724278	0.013437922	no
PAX5	0.102407706	2.717930514	0.006732403	0.01345283	no
DPP3	0.102394067	2.71756468	0.006739771	0.013466221	no
CADPS	-0.102389305	-2.717436957	0.006742345	0.013470033	no
FAF2	0.102385524	2.717335557	0.006744389	0.013472785	no
KIAA1524	0.102376269	2.717087313	0.006749396	0.013481455	no
OR4M2	-0.102357937	-2.716595643	0.006759322	0.013499948	no
ZNF480	0.102331584	2.715888834	0.006773615	0.013527158	no
SYDE2	-0.102322702	-2.715650593	0.006778439	0.013535454	no
C1orf77	-0.102320272	-2.715585428	0.006779759	0.013536753	no
EPB49	-0.10231449	-2.715430359	0.0067829	0.013541689	no
NCDN	-0.10230562	-2.715192446	0.006787723	0.01354998	no
KRT81	0.102296466	2.714946931	0.006792704	0.013558583	no
RPL18A	-0.102282029	-2.714559708	0.006800565	0.013572935	no
PEX2	0.102231561	2.71320614	0.006828111	0.013626566	no
CLCN4	-0.102204939	-2.712492142	0.006842681	0.013654296	no
HOXB3	0.102187818	2.712032969	0.006852066	0.013671674	no
C7orf28B	0.102178003	2.71176973	0.006857452	0.01368107	no
SLC7A4	-0.102166653	-2.71146534	0.006863684	0.013692152	no
ZBBX	0.102149381	2.711002092	0.006873179	0.01370974	no
AACS	-0.102059849	-2.708600936	0.006922582	0.013806921	no
RNF123	-0.102028501	-2.707760228	0.006939954	0.013840205	no
C22orf13	0.102026093	2.707695642	0.006941291	0.013841504	no
ZNF81	-0.102023171	-2.707617301	0.006942912	0.013843372	no
C2orf64	-0.102002873	-2.70707292	0.006954186	0.01386403	no
NAF1	0.102002049	2.707050837	0.006954644	0.01386403	no
SLC6A16	0.102000709	2.707014908	0.006955389	0.013864148	no
ARL2BP	0.101993699	2.706826902	0.006959287	0.01387055	no
CLLU1OS	0.101982019	2.706513671	0.006965786	0.013882135	no
NEUROD6	-0.10196478	-2.706051356	0.006975388	0.013899901	no
COMMD5	0.101951581	2.705697376	0.006982748	0.013913196	no
ATP5E	0.101926206	2.705016893	0.006996917	0.013940054	no

FAM81B	0.101916061	2.704744818	0.007002589	0.01394998	no
TFAM	-0.101911126	-2.704612474	0.00700535	0.013954104	no
C2orf84	-0.101905311	-2.704456523	0.007008604	0.013959211	no
DPP7	0.101901209	2.704346523	0.0070109	0.013962409	no
CDK5RAP3	-0.101883994	-2.703884868	0.007020545	0.013980239	no
ADAR	0.101874086	2.703619157	0.007026101	0.013989926	no
DOCK1	-0.101862818	-2.703316977	0.007032425	0.014001139	no
C12orf4	0.101853269	2.703060916	0.007037787	0.014010436	no
CENPK	0.101848163	2.702923984	0.007040657	0.014014768	no
INTS5	-0.101845642	-2.702856386	0.007042073	0.014016208	no
NEUROD4	-0.101837932	-2.702649625	0.007046409	0.014023456	no
DHODH	-0.101803224	-2.701718876	0.007065954	0.01406097	no
CBWD1	0.101800027	2.701633125	0.007067757	0.014063174	no
LOC202181	0.101778713	2.701061563	0.007079786	0.014085723	no
NRSN2	-0.101765072	-2.70069577	0.007087494	0.014099671	no
C10orf105	0.10174314	2.700107634	0.007099904	0.014122969	no
PGP	-0.10174018	-2.700028259	0.00710158	0.014124914	no
VIPAR	-0.101732116	-2.699812004	0.007106149	0.014132611	no
AFAP1L1	0.101730436	2.699766957	0.007107101	0.014133114	no
SERF1A	-0.101716983	-2.699406202	0.007114729	0.014146892	no
BPGM	0.101698803	2.698918704	0.00712505	0.01416602	no
COASY	-0.101686073	-2.69857733	0.007132284	0.014179009	no
FLAD1	0.101661796	2.697926338	0.007146099	0.014205077	no
BOD1L	-0.101648785	-2.69757743	0.007153514	0.014218417	no
MBD3	-0.101642346	-2.69740479	0.007157185	0.014224315	no
PEX11G	0.101630551	2.697088505	0.007163915	0.014236291	no
ST3GAL2	0.101621075	2.696834404	0.007169326	0.014244837	no
ADSL	0.101620553	2.696820401	0.007169624	0.014244837	no
HESRG	-0.101617508	-2.696738754	0.007171364	0.014246893	no
KLHL4	0.101614747	2.696664705	0.007172942	0.014248628	no
B9D1	0.101611784	2.696585261	0.007174635	0.014250591	no
RASSF9	0.101597215	2.69619459	0.007182967	0.014265739	no
SSR4	0.101587702	2.695939513	0.007188412	0.01427515	no
LARP7	0.101544845	2.694790315	0.007212988	0.014322549	no
STK11	-0.101539757	-2.694653878	0.007215911	0.014326945	no
TP53INP2	0.101513471	2.69394903	0.007231027	0.014355549	no
PCDH9	-0.101510346	-2.693865235	0.007232826	0.014357711	no
LRRC10B	-0.101507977	-2.693801715	0.00723419	0.014357787	no
LOC285780	-0.101507812	-2.693797304	0.007234285	0.014357787	no
PHACTR2	0.101475938	2.692942616	0.007252661	0.014392844	no
ACP1	0.101459884	2.692512144	0.007261932	0.014409828	no
KLHL9	-0.101456371	-2.692417947	0.007263962	0.014412442	no
C1orf115	-0.101439404	-2.691963002	0.007273774	0.014430494	no
RBM34	-0.101386733	-2.690550723	0.00730431	0.014489652	no
ACADL	0.101381816	2.690418898	0.007307166	0.014493896	no
NHSL1	-0.101350148	-2.689569786	0.007325587	0.014529009	no
GCNT2	-0.101339573	-2.689286218	0.007331748	0.014539803	no
UQCRC1	-0.101317619	-2.688697597	0.007344552	0.014563766	no
ECH1	-0.101294077	-2.688066371	0.007358305	0.014589607	no
CLK3	-0.101274199	-2.6875334	0.007369936	0.014611234	no
COQ9	-0.101265228	-2.687292849	0.00737519	0.014620218	no

USP2	-0.101236807	-2.686530841	0.007391858	0.014651822	no
ACOT7	-0.101234534	-2.686469885	0.007393193	0.014653031	no
IMP4	-0.101219313	-2.686061787	0.007402135	0.014669316	no
SAP30BP	-0.101216174	-2.68597764	0.00740398	0.014671534	no
TPI1P2	0.101207789	2.685752803	0.007408911	0.014679868	no
DNAH12	0.101199262	2.68552419	0.007413929	0.01468837	no
SFTA2	0.101132708	2.683739791	0.007453197	0.014764721	no
HSPE1	0.10112773	2.683606313	0.007456142	0.014769108	no
RAPGEF6	0.101114163	2.683242589	0.007464172	0.014783566	no
DGCR8	-0.101105149	-2.683000894	0.007469513	0.014792694	no
CHMP5	0.101093335	2.682684156	0.007476516	0.014805113	no
KIAA0240	-0.101090834	-2.682617103	0.007478	0.0148066	no
KNTC1	0.10107978	2.682320745	0.007484559	0.014818137	no
SPEM1	-0.101055717	-2.681675612	0.007498856	0.014844989	no
EFCAB4A	0.101040826	2.681276374	0.007507716	0.014859845	no
WBSCR17	-0.101040633	-2.68127121	0.007507831	0.014859845	no
CORO2A	0.101032694	2.681058356	0.007512558	0.014867747	no
FAM13AOS	-0.101030431	-2.680997689	0.007513906	0.014868959	no
CCDC64	-0.101028562	-2.680947576	0.00751502	0.014869707	no
IGSF9B	-0.101010363	-2.680459644	0.00752587	0.01488972	no
ADCK5	-0.101001042	-2.680209752	0.007531433	0.014899267	no
COQ7	-0.10098038	-2.679655806	0.007543776	0.014922226	no
METTL2A	-0.100976729	-2.679557927	0.007545959	0.014925084	no
C8orf73	0.100973605	2.679474186	0.007547827	0.014927318	no
ANO4	-0.100959613	-2.679099049	0.007556201	0.014942417	no
CCDC7	0.100954223	2.678954555	0.007559429	0.014947338	no
ITIH4	0.100939208	2.678552001	0.007568427	0.014963667	no
ZNF12	0.100934825	2.678434505	0.007571055	0.014967399	no
SEMA3F	0.100923457	2.678129722	0.007577877	0.01497942	no
LRRC43	0.100911496	2.677809059	0.007585059	0.014992153	no
DAND5	-0.100899521	-2.677488038	0.007592256	0.015004911	no
FOXL2	0.100895775	2.67738759	0.007594509	0.015007897	no
MICALCL	0.100892326	2.677295148	0.007596584	0.015010528	no
WDR34	0.100888151	2.677183217	0.007599096	0.015014025	no
FAM104A	-0.100859064	-2.676403409	0.007616618	0.015047174	no
SUV420H2	-0.100857797	-2.676369459	0.007617381	0.015047212	no
CTAGE4	0.100854342	2.676276827	0.007619465	0.015049859	no
LOC374491	-0.100844359	-2.676009193	0.00762549	0.015060286	no
LOC389333	0.100789652	2.674542599	0.007658576	0.015124109	no
APOBEC4	0.100787909	2.674495856	0.007659633	0.015124109	no
DMWD	-0.100787222	-2.674477439	0.007660049	0.015124109	no
FLJ39609	-0.100761488	-2.673787555	0.007675661	0.015153453	no
HMSD	0.100750315	2.67348803	0.007682448	0.015165371	no
KIF4A	0.100748142	2.673429781	0.007683768	0.015166496	no
KPNA6	0.100740473	2.673224203	0.00768843	0.015173226	no
KTELC1	0.100740065	2.673213248	0.007688678	0.015173226	no
SGK223	0.100717493	2.67260816	0.007702415	0.015198852	no
CCDC123	0.100692386	2.671935086	0.007717722	0.015227568	no
C5orf4	-0.100668541	-2.671295865	0.007732283	0.015254135	no
KCNK1	-0.100667866	-2.671277791	0.007732695	0.015254135	no
SLC5A5	0.100656978	2.670985916	0.007739353	0.015264439	no

AFG3L1	-0.100656855	-2.670982606	0.007739428	0.015264439	no
MOB2	-0.100652797	-2.670873835	0.007741911	0.015267845	no
SCG5	-0.100634552	-2.670384725	0.007753082	0.015288385	no
NRBP2	-0.100627625	-2.670199036	0.007757327	0.015295263	no
PTH2R	-0.10061898	-2.669967304	0.007762627	0.015304222	no
EYA2	0.100596588	2.669367031	0.007776372	0.015329826	no
MAP1S	-0.100591862	-2.669240343	0.007779276	0.015334055	no
MRPL45	-0.100588616	-2.669153341	0.007781271	0.015334998	no
TMEM205	0.100588615	2.669153304	0.007781271	0.015334998	no
MRRF	0.100584133	2.669033155	0.007784027	0.015338933	no
SCN7A	0.100568806	2.668622308	0.007793455	0.015356016	no
KIAA0802	-0.100532534	-2.667649972	0.00781581	0.015398563	no
ZFAND3	-0.100523084	-2.667396655	0.007821644	0.015408554	no
C9orf72	0.100515786	2.667201027	0.007826151	0.015415932	no
OR52H1	0.100469872	2.665970282	0.007854564	0.015470392	no
ZNF593	0.100464538	2.665827283	0.007857871	0.015475398	no
DDX23	-0.100452398	-2.665501876	0.007865401	0.015488202	no
CD300LG	-0.100451587	-2.665480141	0.007865905	0.015488202	no
DKK3	0.100448993	2.66541059	0.007867515	0.015489865	no
RASSF4	-0.1004465	-2.665343779	0.007869062	0.015490614	no
ADCY1	-0.100445911	-2.665327989	0.007869428	0.015490614	no
SGEF	-0.100436439	-2.665074092	0.007875311	0.015500685	no
UMODL1	0.100410539	2.664379826	0.007891417	0.015530875	no
TSPO2	-0.10040434	-2.664213663	0.007895277	0.015536958	no
STAM	-0.100398555	-2.664058598	0.00789888	0.015541253	no
RRP8	0.100397877	2.664040427	0.007899302	0.015541253	no
C4orf50	-0.100395966	-2.663989189	0.007900493	0.015541253	no
MYOM1	-0.100395899	-2.663987412	0.007900534	0.015541253	no
HOMER1	-0.100381056	-2.663589538	0.007909787	0.015557942	no
PART1	0.100373866	2.66339682	0.007914273	0.01556525	no
C17orf67	0.100285262	2.661021837	0.007969738	0.01567281	no
CHRND	0.100254564	2.660199009	0.007989035	0.015709232	no
TAAR1	0.100252956	2.660155888	0.007990048	0.015709695	no
TTC37	0.10023426	2.65965477	0.008001822	0.015731315	no
PPIE	0.100220094	2.659275069	0.008010754	0.015747344	no
RMND1	-0.100213568	-2.659100152	0.008014872	0.015753906	no
MFSD8	0.100207384	2.658934403	0.008018776	0.015760047	no
VWCE	-0.100200285	-2.658744124	0.008023259	0.015765701	no
EFNB3	-0.100199965	-2.658735555	0.008023461	0.015765701	no
SLC25A40	0.100199125	2.658713028	0.008023992	0.015765701	no
OPRM1	-0.100174685	-2.658057974	0.008039445	0.015794529	no
HCRTR1	-0.100165222	-2.65780433	0.008045436	0.015804509	no
SMN1	0.100163443	2.657756649	0.008046562	0.015804509	no
KATNA1	0.100162957	2.657743625	0.00804687	0.015804509	no
KRTCAP3	-0.100144062	-2.657237181	0.008058846	0.015826493	no
EFCAB1	0.100137168	2.657052386	0.008063219	0.015833544	no
TXLNG	-0.100126085	-2.656755324	0.008070255	0.01584582	no
THAP7	-0.100081935	-2.655572003	0.008098334	0.015898123	no
C22orf46	-0.100081729	-2.655566477	0.008098465	0.015898123	no
SLC38A7	0.100052856	2.654792612	0.008116876	0.015932121	no
PLD2	0.100052098	2.654772291	0.00811736	0.015932121	no

C1orf51	-0.100041015	-2.654475263	0.008124437	0.015943096	no
ACTG2	0.100040872	2.654471414	0.008124528	0.015943096	no
1-Dec	0.100033651	2.654277891	0.008129142	0.015950601	no
POU3F4	-0.100015567	-2.653793195	0.008140708	0.015971745	no
WDR82	-0.099997794	-2.653316838	0.00815209	0.015992523	no
PRB2	-0.099995623	-2.653258654	0.008153481	0.015993701	no
PDCD7	-0.099975081	-2.652708098	0.008166655	0.016017988	no
HERPUD2	0.099968874	2.652541734	0.008170639	0.016024249	no
CDIPT	-0.099967462	-2.652503902	0.008171546	0.016024323	no
ADAM33	0.099965166	2.652442349	0.00817302	0.016024323	no
C17orf101	0.099965113	2.652440929	0.008173054	0.016024323	no
LYPLA2	0.099961936	2.652355807	0.008175094	0.016026768	no
EPHB6	-0.099952155	-2.652093654	0.00818138	0.016037535	no
C12orf10	-0.099948874	-2.65200571	0.008183489	0.016040115	no
LOC162632	-0.099933115	-2.651583342	0.008193628	0.01605843	no
INPP5E	-0.09992804	-2.651447336	0.008196895	0.016063276	no
IRX5	0.099920593	2.651247737	0.008201691	0.016071118	no
CXorf48	-0.099890827	-2.650449981	0.008220888	0.016107173	no
RPL13	-0.099888922	-2.650398938	0.008222118	0.016108021	no
KIAA0317	-0.099856788	-2.649537717	0.008242889	0.01614715	no
OSCP1	0.099840892	2.649111699	0.008253182	0.016165746	no
HMGN2	0.099820067	2.648553568	0.008266684	0.016190624	no
TMEM158	0.099796343	2.647917775	0.008282088	0.016219223	no
ZNF295	0.099790163	2.647752147	0.008286105	0.016225519	no
NOC3L	0.099774909	2.647343331	0.008296028	0.016243376	no
SFRS2	0.09974962	2.646665588	0.008312503	0.0162731	no
RAC1	0.099749134	2.646652572	0.008312819	0.0162731	no
PPP4R2	0.099738977	2.646380365	0.008319444	0.016284493	no
CRB3	-0.099733435	-2.646231847	0.008323061	0.016289995	no
SULT1A1	0.099731591	2.646182421	0.008324265	0.016290774	no
MPP7	-0.099702925	-2.6454142	0.008342998	0.016325854	no
SYT2	-0.099696375	-2.645238658	0.008347283	0.01633266	no
HMHB1	0.09967925	2.644779712	0.008358498	0.01635302	no
VAMP1	-0.099666414	-2.644435729	0.008366912	0.016367898	no
C1QL3	-0.099661843	-2.644313216	0.00836991	0.01637218	no
SPIN2A	-0.099659064	-2.644238761	0.008371733	0.016374161	no
NPAT	-0.099656466	-2.64416913	0.008373438	0.016375912	no
VDAC1	0.099638743	2.643694181	0.008385076	0.016397086	no
MGC87042	0.09962212	2.643248705	0.008396006	0.01641687	no
MGC2889	-0.09961542	-2.64306913	0.008400415	0.016423143	no
ACTR1B	-0.099614777	-2.643051904	0.008400838	0.016423143	no
C11orf86	0.099595242	2.642528391	0.008413704	0.016446706	no
STAU2	-0.099592433	-2.64245312	0.008415556	0.016447294	no
MYO5B	0.099592317	2.642450003	0.008415633	0.016447294	no
EMX1	-0.099584431	-2.642238691	0.008420832	0.016455866	no
SKIV2L2	-0.099551697	-2.641361458	0.008442448	0.016496513	no
CPT1B	-0.099521538	-2.640553259	0.008462407	0.016533915	no
MFN1	0.099518656	2.640476022	0.008464317	0.016534954	no
C12orf62	0.099518267	2.640465602	0.008464575	0.016534954	no
LOC728024	0.099505853	2.64013294	0.008472804	0.016549431	no
SLC6A15	-0.099487006	-2.639627887	0.008485312	0.01657226	no

DMRTA2	0.099482401	2.639504475	0.008488371	0.016576633	no
TMEM126B	0.099479428	2.639424815	0.008490346	0.016578889	no
STYXL1	0.099475617	2.639322679	0.008492879	0.016582233	no
QTRT1	-0.099453867	-2.63873984	0.008507345	0.016608875	no
CRYM	-0.09943701	-2.638288115	0.008518572	0.016629188	no
EIF2C3	0.099427263	2.638026939	0.00852507	0.016640265	no
PCOLCE2	0.099421052	2.637860506	0.008529213	0.016646744	no
TGM5	0.099419509	2.637819136	0.008530243	0.016647147	no
ZAR1	0.099391571	2.637070503	0.008548901	0.01668195	no
GRASP	-0.099385049	-2.636895719	0.008553263	0.016688851	no
WDR65	0.099361307	2.636259527	0.008569155	0.016718246	no
GTF2H2B	0.099357307	2.636152319	0.008571836	0.016721862	no
FOXP4	-0.099349855	-2.635952642	0.008576831	0.016729992	no
RNF215	-0.099343434	-2.635780585	0.008581137	0.016736777	no
PTPRO	0.099296267	2.634516692	0.008612828	0.016796966	no
C3orf24	0.099255146	2.633414816	0.008640541	0.016849389	no
MRPS26	-0.099244712	-2.633135211	0.008647586	0.016861501	no
SDR16C5	-0.099234827	-2.632870351	0.008654265	0.016872896	no
ARL6IP6	-0.099222547	-2.63254129	0.008662569	0.016887456	no
ANKRD2	-0.099216143	-2.632369686	0.008666902	0.016894275	no
LOC100128811	-0.099198316	-2.631892027	0.008678973	0.016916175	no
CIDECP	-0.099194412	-2.631787407	0.008681619	0.016919701	no
P2RY1	0.099187118	2.631591976	0.008686564	0.016927706	no
FOXK1	-0.099169877	-2.631129983	0.008698263	0.01694887	no
ADAMTS9	0.099161994	2.630918776	0.008703616	0.016957667	no
GIPC3	0.099158961	2.630837505	0.008705677	0.016960047	no
UNC5C	0.099151211	2.63062984	0.008710944	0.016968674	no
STAG3	0.099145986	2.630489842	0.008714497	0.016973959	no
GMFB	-0.099088843	-2.628958701	0.008753436	0.017048161	no
C13orf23	-0.099073854	-2.628557093	0.008763675	0.017066459	no
TSPYL6	-0.099063969	-2.628292226	0.008770434	0.017077976	no
HOXD11	0.099052216	2.627977322	0.008778475	0.017091989	no
SUCLG1	-0.099040773	-2.627670713	0.008786312	0.017105599	no
PALM3	0.099011593	2.626888867	0.008806322	0.017142906	no
DDX49	-0.099005976	-2.626738353	0.008810179	0.017148763	no
MTTP	0.098991333	2.626346027	0.00882024	0.017166693	no
LOC100133893	-0.098977681	-2.62598023	0.008829629	0.017183313	no
C10orf95	-0.098972509	-2.625841668	0.008833188	0.017188585	no
INHA	-0.098962992	-2.625586667	0.008839741	0.017199681	no
BMPR1A	-0.098953376	-2.625329024	0.008846367	0.017210916	no
KCTD17	-0.098947272	-2.625165478	0.008850575	0.017217446	no
INSL4	0.098945491	2.625117764	0.008851803	0.017218178	no
ARID4B	-0.098930161	-2.624707027	0.00886238	0.017237095	no
C1orf228	0.098903326	2.62398801	0.008880924	0.0172715	no
CRYBA4	0.098887163	2.623554958	0.008892109	0.017291589	no
RAB3GAP2	0.098858223	2.62277958	0.008912167	0.017328929	no
MGC12982	0.098835568	2.622172601	0.008927898	0.017357846	no
CCNE1	0.098825703	2.62190829	0.008934755	0.017369508	no
OSBPL8	-0.098819692	-2.621747265	0.008938936	0.017375964	no
LOC727924	-0.098813867	-2.621591183	0.008942989	0.017382172	no
UBE2D4	0.098802098	2.621275873	0.008951183	0.017396426	no

ZNF439	-0.098775651	-2.620567309	0.00896962	0.017430583	no
POLE2	0.098753964	2.619986258	0.008984765	0.017458336	no
NDUFB9	-0.098729647	-2.619334785	0.009001773	0.017487141	no
LOC100130264	-0.098729623	-2.619334124	0.00900179	0.017487141	no
PAPD7	-0.098729059	-2.619319033	0.009002184	0.017487141	no
CWF19L1	-0.098724785	-2.619204519	0.009005177	0.017491274	no
LASS4	-0.098702778	-2.618614911	0.0090206	0.017519547	no
PCDHAC2	-0.098698068	-2.618488728	0.009023903	0.01752428	no
COPS4	0.098680302	2.618012754	0.009036375	0.017546814	no
LOC642846	-0.098651914	-2.617252213	0.009056335	0.017583883	no
BIRC6	-0.098649814	-2.617195952	0.009057813	0.017585064	no
GJD2	-0.098625114	-2.61653423	0.009075214	0.017617155	no
C1orf192	0.098619076	2.616372448	0.009079472	0.01762373	no
OR14I1	-0.098594411	-2.61571166	0.009096886	0.017655836	no
RNF169	-0.098575543	-2.615206187	0.009110227	0.017680031	no
OGDHL	-0.098550991	-2.614548427	0.009127613	0.017712072	no
DNAJC4	0.098544451	2.614373241	0.009132249	0.017719367	no
EPN3	-0.098541047	-2.614282049	0.009134662	0.01772235	no
HIST1H4J	0.098534467	2.614105749	0.009139331	0.017729705	no
DUSP19	-0.098518861	-2.613687677	0.00915041	0.017749494	no
SHISA6	-0.098501652	-2.613226654	0.00916264	0.017771514	no
C9orf68	0.098485329	2.612789367	0.009174255	0.017792335	no
ST6GALNAC6	-0.098467304	-2.612306471	0.009187097	0.01781553	no
TRIM11	-0.098459302	-2.612092123	0.009192802	0.017824884	no
MAB21L2	0.098455147	2.611980805	0.009195766	0.017828922	no
WDR45	0.098438184	2.611526378	0.009207875	0.017850688	no
GRM3	-0.098434278	-2.611421744	0.009210666	0.017854385	no
KDM2A	0.09840534	2.610646524	0.009231362	0.017892788	no
MYO1D	0.0983988	2.61047132	0.009236045	0.017900149	no
PPIAL4G	0.098384484	2.610087807	0.009246303	0.017918313	no
ZNF416	0.098370127	2.609703199	0.009256602	0.017936551	no
FA2H	-0.098368288	-2.609653936	0.009257921	0.017937389	no
HIST2H4A	0.098364151	2.609543106	0.009260891	0.017941424	no
UBTD1	0.098355038	2.609299003	0.009267435	0.017952382	no
OR10R2	0.098329249	2.608608144	0.009285979	0.01798658	no
NOMO3	-0.098324723	-2.608486905	0.009289236	0.017991166	no
CA3	0.098309493	2.608078914	0.009300206	0.018010687	no
TCP11L1	-0.098299791	-2.607819027	0.0093072	0.018022412	no
TMEM186	-0.098298015	-2.60777143	0.009308482	0.018022412	no
EIF3K	0.098297386	2.607754595	0.009308935	0.018022412	no
GLT6D1	-0.0982942	-2.60766925	0.009311233	0.018022452	no
KBTBD2	0.098293741	2.607656949	0.009311564	0.018022452	no
MAX	0.09829365	2.607654515	0.00931163	0.018022452	no
GNB2	0.098291123	2.607586808	0.009313453	0.018024256	no
TRIM4	0.098282183	2.607347324	0.009319906	0.018035018	no
FAM83D	0.098253063	2.606567263	0.009340952	0.018074014	no
PRDM15	-0.098219457	-2.605667057	0.009365293	0.018119377	no
MON1A	-0.098205611	-2.605296154	0.009375338	0.018137077	no
TMEM164	0.098175837	2.604498586	0.009396971	0.018177188	no
LHX3	-0.09816657	-2.60425035	0.009403714	0.01818849	no
WBSCR22	0.09813696	2.603457191	0.009425286	0.01822847	no

KCNH6	-0.098124172	-2.60311465	0.009434615	0.018244769	no
FOXN3	-0.098093252	-2.602286403	0.009457209	0.018286711	no
EPGN	0.098089831	2.602194755	0.009459712	0.018289801	no
GRIK3	-0.098086955	-2.602117721	0.009461816	0.018292121	no
CCDC140	0.098056583	2.601304164	0.009484065	0.018333381	no
GTPBP8	0.098046456	2.601032908	0.009491494	0.018345988	no
CDC20	0.09804404	2.600968197	0.009493267	0.01834766	no
CDC42BPG	0.098012761	2.600130365	0.009516249	0.01839032	no
CEP78	-0.09799087	-2.599543987	0.009532363	0.0184197	no
EXOSC10	0.09793562	2.598064105	0.00957314	0.018496726	no
IGFBPL1	-0.097923655	-2.597743608	0.009581991	0.01851206	no
ABCA12	0.09791386	2.597481241	0.009589243	0.018524147	no
IGF1R	-0.097912729	-2.597450951	0.00959008	0.018524147	no
ZBTB20	-0.097900131	-2.597113531	0.009599414	0.018540405	no
KLK4	0.097883908	2.596679001	0.009611446	0.01856187	no
TPH2	-0.097878987	-2.59654719	0.009615099	0.018567151	no
ATG4B	-0.097847805	-2.595711991	0.009638271	0.01861012	no
PDIA2	-0.097839008	-2.595476368	0.009644817	0.018620981	no
DNAI2	0.097822216	2.595026602	0.009657324	0.018643347	no
NKX6-1	0.097819082	2.594942667	0.00965966	0.018646076	no
ZNF384	-0.09779094	-2.5941889	0.009680657	0.018684823	no
FXN	-0.097773276	-2.593715776	0.009693857	0.018708515	no
MLL5	-0.097769779	-2.593622124	0.009696472	0.018711776	no
ATIC	-0.097741347	-2.592860614	0.009717757	0.018751061	no
NCRNA00120	-0.097736839	-2.592739868	0.009721136	0.018755791	no
FGF8	-0.097730975	-2.592582806	0.009725533	0.018762483	no
ZNF257	-0.097723071	-2.592371099	0.009731462	0.018772131	no
CRHR2	-0.097720795	-2.592310142	0.00973317	0.018773634	no
GIPR	-0.097714622	-2.592144816	0.009737803	0.018780779	no
TJAP1	-0.097691503	-2.591525609	0.009755174	0.018812486	no
ZNF707	-0.097650467	-2.59042653	0.009786074	0.018869176	no
LYNX1	-0.097649985	-2.590413644	0.009786437	0.018869176	no
ZNF528	0.09764415	2.590257356	0.009790838	0.018875862	no
ABCF3	-0.097636016	-2.590039505	0.009796976	0.018885894	no
SATL1	-0.097627013	-2.589798373	0.009803774	0.018897197	no
NENF	0.097624588	2.589733424	0.009805605	0.018898926	no
C22orf41	0.097615136	2.589480291	0.009812747	0.018910888	no
PTH	0.097611859	2.589392511	0.009815225	0.01891386	no
PCDHGA10	-0.097597981	-2.589020824	0.009825723	0.018932284	no
ABCC5	-0.097593848	-2.588910145	0.00982885	0.018936506	no
NDUFB8	-0.097563899	-2.588108033	0.009851545	0.018978422	no
FSCN2	0.097557777	2.587944076	0.00985619	0.01898556	no
TUBB3	-0.09754339	-2.587558753	0.009867113	0.019004791	no
MAGEA12	0.097522292	2.586993718	0.00988315	0.019033867	no
IFNE	0.097498936	2.586368195	0.009900932	0.019066296	no
GPC1	0.097481158	2.585892076	0.009914485	0.019090578	no
DSP	0.097458791	2.585293042	0.009931562	0.019121637	no
C5orf13	-0.097455379	-2.585201666	0.009934169	0.019124835	no
NCKAP5L	-0.097440332	-2.584798677	0.009945674	0.019145162	no
CDRT4	0.097435103	2.584658637	0.009949675	0.01915104	no
LOC729603	0.097422673	2.584325768	0.00995919	0.01916753	no

PRPF4	0.09742125	2.584287637	0.009960281	0.019167805	no
NEK4	-0.097415815	-2.5841421	0.009964444	0.019173992	no
FAM43B	0.097403475	2.583811623	0.009973904	0.019189635	no
MIF	0.097402735	2.583791785	0.009974472	0.019189635	no
STK39	-0.097394143	-2.583561684	0.009981063	0.019200489	no
SPIN1	-0.097354975	-2.582512751	0.010011161	0.019256555	no
ARHGAP20	0.097334158	2.581955256	0.01002719	0.019285553	no
ANKRD55	-0.097299241	-2.581020163	0.010054128	0.019335524	no
MYLIP	0.097273988	2.580343887	0.01007365	0.019371225	no
C1orf229	-0.097265341	-2.58011233	0.010080342	0.01938225	no
PASK	0.097242403	2.579498055	0.010098114	0.019414576	no
MYEOV	0.097240603	2.579449843	0.01009951	0.019415413	no
C15orf37	0.097238468	2.579392669	0.010101166	0.01941675	no
MRPL46	-0.097236119	-2.579329771	0.010102988	0.019417438	no
HUNK	-0.09723553	-2.579314001	0.010103445	0.019417438	no
ZDHHC3	0.097231192	2.579197833	0.010106811	0.01942206	no
AIPL1	0.097199239	2.578342128	0.010131633	0.019467911	no
PCSK7	0.097187228	2.578020502	0.010140977	0.019484014	no
CHCHD10	0.097185686	2.577979208	0.010142178	0.019484468	no
NDUFS1	-0.097160025	-2.577292026	0.01016217	0.019521021	no
PRPF38A	-0.097126371	-2.57639081	0.010188442	0.01956794	no
KCNA6	-0.097126258	-2.576387786	0.01018853	0.01956794	no
NPR1	0.097123813	2.576322297	0.010190442	0.019569752	no
ADSS	0.097122253	2.576280534	0.010191661	0.019570234	no
RUFY4	0.097098458	2.575643334	0.010210278	0.019604122	no
C7orf44	0.097083607	2.575245641	0.010221913	0.019624598	no
INVS	0.097044542	2.574199551	0.010252574	0.019681594	no
GPR111	0.097030403	2.573820929	0.010263692	0.019701066	no
PRX	-0.097023863	-2.573645796	0.010268838	0.019709073	no
IL24	0.097020779	2.573563231	0.010271265	0.01971186	no
FGF7	0.097019416	2.573526726	0.010272338	0.019712048	no
ATHL1	0.096996017	2.572900143	0.010290775	0.019745552	no
LOC402377	0.096974025	2.57231124	0.010308129	0.019776974	no
PCGF3	-0.096967644	-2.572140378	0.010313169	0.019784766	no
PLCD4	-0.096961272	-2.571969747	0.010318205	0.019791054	no
ANKRD34A	-0.096960666	-2.571953527	0.010318683	0.019791054	no
ANKRD52	0.09695978	2.571929819	0.010319383	0.019791054	no
DOCK9	-0.096953785	-2.571769266	0.010324124	0.019798212	no
RASGRP1	-0.096952583	-2.571737085	0.010325074	0.019798212	no
ZNF274	-0.096950288	-2.571675621	0.010326889	0.019799815	no
NCRNA00204B	0.096930965	2.571158218	0.010342183	0.019827258	no
RGS17	0.096909752	2.570590201	0.010358996	0.019857607	no
AHCTF1	-0.096890466	-2.570073759	0.010374304	0.019885066	no
N4BP2L1	-0.096877889	-2.569736996	0.010384297	0.019902333	no
RAB40B	-0.096868125	-2.569475536	0.010392061	0.019915325	no
PCDHA6	-0.096864621	-2.569381732	0.010394848	0.019915888	no
GOLGA8DP	-0.096864503	-2.569378575	0.010394941	0.019915888	no
NUP98	0.096864041	2.569366187	0.010395309	0.019915888	no
PPP1R16B	-0.096860217	-2.569263808	0.010398352	0.019919829	no
EIF2B5	-0.096855747	-2.569144102	0.01040191	0.019924758	no
SLC35B1	0.096842844	2.568798614	0.010412186	0.019942552	no

CSPP1	0.096824154	2.568298148	0.010427088	0.019967368	no
COPA	0.096823964	2.56829307	0.010427239	0.019967368	no
HIST1H4I	0.096822878	2.568263993	0.010428106	0.019967368	no
CCDC155	0.096813662	2.568017218	0.010435461	0.01997956	no
CAMKK2	-0.096786513	-2.567290272	0.010457156	0.0200192	no
CCDC53	0.096766233	2.566747246	0.010473388	0.020048377	no
GPR17	-0.096740154	-2.566048971	0.010494293	0.020086493	no
SMTN	0.096726194	2.565675168	0.0105055	0.020106039	no
ALMS1P	-0.096711213	-2.565274056	0.010517537	0.020127172	no
RERG	0.096709846	2.565237459	0.010518636	0.02012737	no
MSTO1	0.096703614	2.565070574	0.010523648	0.020135055	no
TRAPPC5	0.09669574	2.564859756	0.010529983	0.020141916	no
ADCY9	0.096695702	2.564858743	0.010530014	0.020141916	no
C14orf37	-0.096695442	-2.564851771	0.010530223	0.020141916	no
TCP11L2	0.096663083	2.563985351	0.010556294	0.020189873	no
FKRP	0.096658315	2.56385769	0.01056014	0.020195319	no
MCM8	0.096652039	2.563689651	0.010565205	0.020203094	no
C2orf56	0.096627537	2.563033611	0.010584999	0.02023903	no
TARBP1	-0.096615955	-2.562723494	0.010594367	0.020255026	no
EHD1	-0.096611788	-2.562611943	0.010597738	0.020259557	no
CCL28	0.096558905	2.561196027	0.010640617	0.020339604	no
KIAA0174	-0.096550479	-2.560970403	0.010647464	0.020350269	no
GJA8	0.096549561	2.56094584	0.010648209	0.020350269	no
POLR1E	-0.096546446	-2.560862431	0.010650742	0.020353185	no
GTPBP2	0.096534833	2.560551513	0.010660186	0.020369307	no
PRICKLE4	-0.096530075	-2.560424127	0.010664058	0.02037478	no
DLGAP5	0.096500321	2.559627472	0.010688298	0.020419165	no
IGFL3	0.09648155	2.559124905	0.010703616	0.020446495	no
MYOZ1	-0.096459148	-2.558525116	0.010721922	0.02047953	no
SLC17A3	0.096441907	2.558063529	0.010736029	0.020504538	no
C17orf102	-0.096419188	-2.557455256	0.010754645	0.020538151	no
C19orf12	0.096377116	2.556328874	0.010789192	0.020602181	no
BRDT	-0.096375542	-2.55628673	0.010790487	0.020602707	no
FASTKD3	0.096373568	2.556233862	0.010792111	0.020603862	no
LOC349114	-0.096321058	-2.554828029	0.010835379	0.020684514	no
BCMO1	0.096311997	2.554585461	0.01084286	0.020696841	no
SLC29A4	-0.09630632	-2.554433469	0.01084755	0.020703839	no
YPEL2	0.096298664	2.554228496	0.010853878	0.02071396	no
LRRC67	0.096287497	2.553929547	0.010863112	0.020729628	no
CCDC86	0.096267716	2.553399966	0.010879489	0.020758919	no
CBWD2	0.096257205	2.553118561	0.0108882	0.020773579	no
EVI5	0.096243641	2.552755427	0.010899449	0.020793081	no
ZCCHC10	0.096240228	2.552664039	0.010902282	0.020796523	no
FAM200B	-0.096231502	-2.552430433	0.010909527	0.020808378	no
PLEKHG4B	0.096212449	2.551920356	0.010925359	0.020836611	no
C2orf79	0.096209259	2.551834964	0.010928012	0.020839705	no
POLR3GL	-0.096166991	-2.550703379	0.010963217	0.020903557	no
POLA2	0.096166576	2.550692276	0.010963563	0.020903557	no
ARFIP2	-0.096164984	-2.550649636	0.010964892	0.020903913	no
CDKL1	-0.096163874	-2.550619929	0.010965817	0.020903913	no
IQGAP3	0.096153577	2.550344281	0.01097441	0.020918321	no

ZBTB7C	0.096131141	2.549743642	0.010993155	0.020952075	no
SLC22A9	-0.096109823	-2.549172942	0.011010992	0.020984092	no
ADH1B	0.096084717	2.548500835	0.011032031	0.021022206	no
DSTN	0.096072524	2.548174415	0.011042262	0.021039719	no
MTVR2	0.096065416	2.547984133	0.01104823	0.021049106	no
PRRT4	-0.09605794	-2.547783999	0.01105451	0.021059086	no
PEG10	-0.096053605	-2.547667941	0.011058153	0.021064042	no
RBL1	0.096017143	2.546691833	0.011088837	0.0211205	no
FAM83H	0.096004316	2.546348467	0.011099649	0.021139101	no
SPOCK1	-0.095996392	-2.546136343	0.011106332	0.021149838	no
TMEM131	0.095989382	2.545948693	0.011112248	0.02115911	no
FAT4	0.095987609	2.545901223	0.011113745	0.021159546	no
YPEL5	0.095986632	2.545875061	0.01111457	0.021159546	no
TUBB2B	-0.095933311	-2.544447667	0.011159669	0.021243404	no
FAM167A	-0.095923804	-2.544193178	0.011167727	0.021256742	no
HSPB6	0.0959215	2.544131497	0.011169681	0.021258459	no
FAM47E	-0.095903236	-2.5436426	0.011185178	0.021285949	no
FAM127A	-0.095872159	-2.542810692	0.011211591	0.021334207	no
CHDH	-0.095864529	-2.542606443	0.011218084	0.021344554	no
DOHH	-0.095839217	-2.541928874	0.011239649	0.021383573	no
WDR11	0.095832399	2.541746356	0.011245465	0.021392624	no
PARD3B	-0.095816118	-2.541310546	0.011259361	0.021417045	no
MPZL1	0.09579654	2.540786463	0.011276093	0.021446852	no
CDKN2C	0.09577599	2.540236384	0.011293678	0.021478278	no
ANKS6	-0.095774641	-2.540200272	0.011294833	0.021478455	no
CRLS1	-0.095752773	-2.539614897	0.011313575	0.021512072	no
HYOU1	0.095746875	2.539457031	0.011318634	0.021519668	no
C19orf34	0.095740996	2.539299644	0.01132368	0.021527237	no
BOLA1	-0.095727972	-2.538951044	0.011334863	0.021546471	no
C1orf50	0.095707968	2.538415565	0.011352061	0.021577133	no
LGI3	-0.095691553	-2.537976175	0.011366189	0.021601957	no
LIG3	-0.095667759	-2.537339275	0.011386697	0.021638898	no
RBM11	-0.095652148	-2.536921412	0.01140017	0.021662465	no
C17orf103	0.095636409	2.536500124	0.011413767	0.021686265	no
ISCA2	0.09562914	2.536305556	0.011420052	0.021696167	no
LOC400752	-0.095590002	-2.535257947	0.011453944	0.021758511	no
ARSF	0.095576192	2.534888292	0.011465924	0.021779223	no
PLEC	-0.095563177	-2.534539927	0.011477224	0.02179864	no
NDFIP2	-0.095556214	-2.534353539	0.011483274	0.021808082	no
FIZ1	-0.095525304	-2.533526214	0.011510164	0.021857095	no
LOC100132724	0.095517934	2.533328929	0.011516584	0.021867233	no
RAB12	0.095515194	2.533255586	0.011518972	0.021869713	no
OR51B5	0.095513197	2.533202155	0.011520712	0.021870962	no
ANKRD39	-0.09549326	-2.532668495	0.0115381	0.021901916	no
SLC39A5	-0.095456731	-2.531690778	0.011570019	0.021958995	no
DLX4	0.095456362	2.531680887	0.011570342	0.021958995	no
VPS37B	-0.09545502	-2.531644987	0.011571515	0.021959161	no
IL20	0.09541477	2.530567663	0.011606781	0.022024016	no
PADI2	0.095407398	2.530370341	0.01161325	0.022034224	no
CKS2	0.095400531	2.530186555	0.011619279	0.022043593	no
HOXC6	0.095391956	2.52995704	0.011626811	0.022055814	no

TMEM229B	0.095390061	2.529906331	0.011628476	0.022056903	no
USP25	0.095360443	2.529113601	0.011654529	0.022104247	no
CDC27	0.095356484	2.529007633	0.011658016	0.022108786	no
GATM	0.095304961	2.527628617	0.011703474	0.022192912	no
FAM19A3	0.095299343	2.527478274	0.011708439	0.022200246	no
NT5DC3	-0.095289095	-2.527203988	0.011717503	0.022215348	no
CCT8	0.095275532	2.526840982	0.011729508	0.022236023	no
ADAMTSL2	-0.095272638	-2.526763516	0.011732071	0.022238797	no
ZNF766	0.095271358	2.526729262	0.011733205	0.022238861	no
RBM12B	-0.095268536	-2.526653723	0.011735705	0.022241515	no
EXOSC5	-0.095259056	-2.526399998	0.011744106	0.022255351	no
SCAMP4	0.095251282	2.526191944	0.011750999	0.022266327	no
LCE1D	-0.095227814	-2.525563833	0.011771831	0.02230371	no
ARL9	0.095203308	2.524907968	0.011793619	0.022342896	no
MGAM	0.095194962	2.524684594	0.011801048	0.022354875	no
ERC2	-0.095178317	-2.524239121	0.011815875	0.022380865	no
CTNNBL1	-0.095169679	-2.52400792	0.011823577	0.022392348	no
MTFMT	0.095169033	2.523990651	0.011824152	0.022392348	no
TCP1	-0.095149918	-2.523479047	0.011841211	0.022422554	no
ZFP92	-0.095126971	-2.522864928	0.011861718	0.022459282	no
RSL24D1	-0.095090994	-2.521902078	0.011893933	0.02251817	no
CLDN1	0.095085927	2.521766462	0.011898476	0.022524663	no
NPY5R	0.095081369	2.52164447	0.011902565	0.022530293	no
TCERG1L	-0.095079307	-2.521589286	0.011904415	0.022531685	no
LOC550112	0.095074307	2.521455474	0.011908902	0.022538067	no
DPCR1	0.095020469	2.520014639	0.011957308	0.02262756	no
CHURC1	0.095008434	2.519692538	0.011968153	0.022645964	no
SDSL	0.094990339	2.519208283	0.011984475	0.022674725	no
DLD	0.094988474	2.519158363	0.011986158	0.022675788	no
UCN3	-0.094947365	-2.518058199	0.012023317	0.022743957	no
DEFB1	0.094942962	2.517940371	0.012027302	0.022749368	no
TOPBP1	-0.094933058	-2.517675339	0.012036272	0.022764204	no
SGOL2	0.094928652	2.517557419	0.012040264	0.022769625	no
HNRNPUL1	-0.094924076	-2.517434967	0.012044412	0.022775338	no
ZNF718	-0.094861477	-2.515759727	0.012101278	0.022879104	no
OR1F2P	-0.094861179	-2.51575174	0.01210155	0.022879104	no
BARHL1	-0.094858543	-2.515681213	0.012103949	0.022881501	no
IL1F5	0.094844308	2.515300265	0.012116917	0.022903872	no
C14orf48	0.094816592	2.514558559	0.012142199	0.022949517	no
PPPDE1	0.094809819	2.514377296	0.012148385	0.022959062	no
VPS35	0.094804491	2.514234717	0.012153253	0.022966115	no
INMT	0.094802055	2.514169542	0.012155478	0.022966197	no
C11orf65	-0.094801957	-2.514166906	0.012155568	0.022966197	no
MIA	0.094791394	2.513884227	0.012165226	0.022982296	no
LOC148696	0.094767865	2.513254583	0.012186761	0.02302083	no
HNRNPL	-0.094763334	-2.513133339	0.012190912	0.023026519	no
SEC62	-0.094721122	-2.512003744	0.012229644	0.023097519	no
SLC24A4	-0.094717418	-2.511904608	0.012233048	0.023101791	no
GTPBP10	0.094715315	2.511848334	0.012234981	0.023103283	no
IMPG1	-0.094699674	-2.511429795	0.012249366	0.023128285	no
EIF3J	-0.094687493	-2.511103817	0.012260579	0.023147296	no

PPP1R14C	-0.094677728	-2.510842515	0.012269575	0.023162116	no
ZNF642	-0.094670729	-2.510655227	0.012276026	0.02317213	no
NCRNA00235	-0.09466887	-2.510605467	0.01227774	0.023173203	no
C9orf82	0.094647931	2.510045163	0.01229706	0.0232075	no
LDHAL6A	-0.09464654	-2.51000794	0.012298344	0.023207758	no
CCL15	0.094635886	2.509722847	0.012308185	0.023224162	no
TAS2R10	-0.094633334	-2.509654548	0.012310544	0.023226445	no
PLK2	0.09461746	2.509229757	0.012325224	0.023251971	no
STRADA	-0.094610226	-2.50903618	0.012331918	0.023262429	no
HOXB6	0.094585187	2.508366165	0.012355114	0.023302441	no
TMEM216	0.094584842	2.508356926	0.012355434	0.023302441	no
EMID2	-0.094567592	-2.507895334	0.012371438	0.023330447	no
RAB4B	0.094561783	2.507739892	0.012376831	0.023338441	no
C7orf47	0.094558149	2.507642674	0.012380205	0.023342627	no
TOB2	-0.094555247	-2.50756501	0.012382901	0.023344627	no
MTMR9L	-0.094554522	-2.507545598	0.012383575	0.023344627	no
CST1	0.094552147	2.507482044	0.012385782	0.02334661	no
P2RX7	-0.094522036	-2.506676326	0.012413789	0.023397221	no
TANC1	0.094519051	2.506596463	0.012416568	0.023400278	no
MRPL39	0.094514537	2.506475657	0.012420773	0.02340602	no
LCN15	-0.094512214	-2.506413495	0.012422937	0.023407917	no
RNF8	-0.09451006	-2.506355871	0.012424944	0.023409516	no
LOC400657	-0.094492866	-2.505895773	0.012440975	0.023437536	no
FAM74A4	0.094484727	2.505678	0.012448569	0.023449658	no
RPL19	-0.094474423	-2.505402289	0.01245819	0.023465594	no
C16orf75	0.0944631	2.505099304	0.01246877	0.023483334	no
INO80	-0.094443992	-2.504588005	0.012486642	0.023514803	no
LY6H	-0.094441927	-2.504532747	0.012488575	0.023516253	no
C15orf57	0.094437861	2.504423955	0.012492381	0.023521229	no
SNRPG	0.094432832	2.504289388	0.01249709	0.023527905	no
RTN4RL1	-0.094425454	-2.504091962	0.012504002	0.023538726	no
LOC151162	0.094423131	2.504029797	0.01250618	0.023540633	no
RET	-0.09440872	-2.503644202	0.012519692	0.023563873	no
C16orf48	-0.09439939	-2.503394545	0.012528447	0.023576113	no
FXR1	-0.094399305	-2.503392268	0.012528527	0.023576113	no
TSC22D2	0.094387481	2.503075895	0.01253963	0.02359481	no
C4orf22	0.094385458	2.503021758	0.01254153	0.02359619	no
SGCG	-0.094374575	-2.502730554	0.012551759	0.023613238	no
JOSD1	0.09436521	2.502479978	0.012560567	0.023627609	no
EIF3E	-0.094353334	-2.502162199	0.012571744	0.023646434	no
KIAA1468	-0.094348996	-2.502046138	0.012575829	0.023651917	no
C4orf52	0.09434622	2.50197187	0.012578443	0.023654633	no
SLC38A6	0.09434108	2.501834323	0.012583286	0.02366154	no
CRHBP	-0.094337424	-2.501736511	0.012586731	0.023665817	no
RASA1	0.094316003	2.501163331	0.012606936	0.023701602	no
CMTM2	0.094296469	2.500640663	0.012625385	0.023734081	no
CCDC78	-0.094283305	-2.500288454	0.012637831	0.023755268	no
GRINL1A	-0.094250484	-2.499410255	0.012668911	0.023811475	no
SPTBN5	0.09424698	2.49931651	0.012672233	0.023815504	no
CCDC136	-0.094238947	-2.499101565	0.012679852	0.023825539	no
POLR2E	-0.094238865	-2.499099394	0.012679929	0.023825539	no

STX19	-0.094206764	-2.498240481	0.012710415	0.023880603	no
GPATCH3	-0.094198456	-2.498018176	0.012718316	0.023891821	no
NLRP7	0.094198	2.498005993	0.012718749	0.023891821	no
SORBS3	-0.094110071	-2.495653389	0.012802635	0.024047164	no
USP29	0.094107493	2.495584396	0.012805102	0.024049564	no
UPF0639	-0.094095408	-2.495261073	0.012816671	0.024069056	no
ARMC8	-0.094079478	-2.494834861	0.012831936	0.024095484	no
CELA3B	0.09407747	2.494781123	0.012833861	0.024096334	no
MLF1IP	0.09407652	2.494755711	0.012834772	0.024096334	no
ODF2	0.09407193	2.49463292	0.012839174	0.02410236	no
PLCD3	-0.094062019	-2.494367729	0.012848684	0.024117974	no
ARHGDIA	0.094055114	2.494182991	0.012855313	0.024128177	no
SILV	0.094049428	2.494030879	0.012860774	0.024136185	no
ZW10	0.094039991	2.49377839	0.012869842	0.024150962	no
LRCH4	0.094025011	2.493377591	0.012884249	0.024175753	no
PPOX	-0.09402112	-2.493273503	0.012887992	0.024180533	no
C19orf54	0.093989533	2.492428394	0.012918424	0.024235381	no
FAM46C	0.093976007	2.492066517	0.012931475	0.024257613	no
CDK12	0.093971785	2.491953567	0.012935551	0.024263008	no
TSHR	-0.093954101	-2.49148044	0.012952635	0.024291868	no
ZNF444	-0.093953372	-2.491460941	0.01295334	0.024291868	no
IZUMO1	-0.093943456	-2.491195624	0.01296293	0.024307598	no
FAM120B	-0.093929762	-2.490829259	0.012976183	0.024330192	no
PDF	0.093923858	2.490671308	0.0129819	0.024338655	no
LOC100132288	-0.093919599	-2.490557367	0.012986026	0.024344133	no
MARVELD2	0.093913581	2.490396352	0.012991858	0.024352808	no
RNF207	-0.093909535	-2.490288101	0.01299578	0.024355742	no
DUSP5P	-0.093909481	-2.490286656	0.012995833	0.024355742	no
ACYP1	-0.093907066	-2.490222049	0.012998174	0.024357873	no
NARF	-0.093897115	-2.489955836	0.013007826	0.024373701	no
ZNF488	-0.093890146	-2.48976938	0.01301459	0.024384115	no
SNAPC5	0.093886974	2.489684512	0.01301767	0.024387625	no
PCBP2	-0.093878955	-2.489469981	0.013025458	0.024399954	no
SEC61G	0.093849313	2.488676957	0.013054282	0.024451684	no
PPM1N	0.09380151	2.487398059	0.013100886	0.024536703	no
TCTE3	-0.093789978	-2.487089543	0.013112151	0.024555526	no
ATP6V1G1	0.093761769	2.486334888	0.013139741	0.024604916	no
NUP210	0.093752712	2.486092574	0.013148611	0.024619245	no
PPAN-P2RY11	-0.093743724	-2.485852118	0.013157418	0.024633454	no
PHF20	-0.093731688	-2.485530143	0.013169219	0.024652117	no
TMEM232	0.093731071	2.48551363	0.013169824	0.024652117	no
MSI2	-0.093725909	-2.485375526	0.013174889	0.024659314	no
LRAT	0.093704543	2.484803925	0.013195871	0.024696299	no
NAT8L	-0.093664455	-2.483731501	0.013235316	0.024767828	no
GFRA4	-0.093659458	-2.483597821	0.01324024	0.02477475	no
ALDOB	-0.093647877	-2.483288003	0.013251659	0.024793822	no
EP300	-0.093633381	-2.482900194	0.013265964	0.02481829	no
OSBP	-0.093613788	-2.482376065	0.01328532	0.024852202	no
GALNTL5	-0.093582236	-2.481532002	0.013316543	0.024908305	no
CUL2	-0.093575512	-2.481352114	0.013323206	0.024918462	no
CD200R1L	0.093493554	2.479159659	0.013404648	0.025068465	no

NOP56	-0.093479026	-2.478771033	0.01341913	0.025093227	no
ALKBH5	0.093457594	2.478197702	0.01344052	0.025130902	no
RECQL5	-0.093451358	-2.478030885	0.01344675	0.025140225	no
GGT3P	-0.093449973	-2.477993844	0.013448133	0.025140486	no
VPS36	-0.09344641	-2.477898538	0.013451694	0.025144818	no
POP5	0.093435302	2.477601387	0.0134628	0.025163252	no
ORM2	0.093433508	2.477553397	0.013464595	0.02516428	no
PER1	-0.093431436	-2.477497969	0.013466668	0.025165827	no
FAM9B	0.093423103	2.477275054	0.013475007	0.025179084	no
ASB2	0.093407356	2.476853839	0.013490777	0.025204342	no
NECAB1	-0.093407117	-2.476847424	0.013491017	0.025204342	no
DDX4	-0.093405809	-2.47681244	0.013492328	0.025204461	no
PROM1	0.093384428	2.476240489	0.01351377	0.025242183	no
CPA2	0.093374248	2.475968186	0.013523988	0.025258938	no
NKX1-2	-0.093365964	-2.475746605	0.013532309	0.025272143	no
CENPI	0.09333436	2.474901187	0.013564097	0.025329168	no
TMEM31	-0.093332806	-2.474859624	0.013565661	0.02532975	no
GGA3	-0.093322429	-2.474582057	0.013576113	0.025346925	no
ZBTB49	-0.093300565	-2.473997198	0.01359816	0.025385743	no
CETP	0.093290099	2.473717256	0.013608724	0.025403118	no
PLSCR4	0.093271019	2.473206859	0.013628002	0.025436757	no
MBIP	-0.093269539	-2.473167292	0.013629498	0.0254372	no
SPEF1	0.093245266	2.472518008	0.01365406	0.025480689	no
RBBP8	0.093232418	2.472174338	0.013667077	0.025502627	no
CDK3	-0.093224495	-2.471962398	0.01367511	0.025515261	no
PSD3	-0.093196193	-2.471205357	0.013703837	0.025566502	no
ATP13A2	-0.093186276	-2.470940115	0.013713915	0.025582943	no
SNHG10	-0.09316625	-2.470404441	0.013734288	0.025618583	no
NKX6-3	-0.09315079	-2.469990903	0.013750033	0.025645588	no
C10orf122	0.093137666	2.469639881	0.013763412	0.025668172	no
KRT10	0.093114987	2.469033249	0.013786559	0.025708969	no
RANBP1	-0.093112886	-2.468977062	0.013788704	0.025710599	no
C6orf114	-0.093096588	-2.468541121	0.013805362	0.025737382	no
PVALB	-0.093096341	-2.468534514	0.013805614	0.025737382	no
LOC100272217	-0.093094611	-2.46848823	0.013807384	0.025738308	no
MFRP	-0.093079927	-2.468095481	0.013822408	0.025763939	no
CWH43	0.093071484	2.467869654	0.013831054	0.025777677	no
C6	0.093016399	2.466396269	0.013887577	0.025880638	no
DNHD1	-0.093014713	-2.46635118	0.01388931	0.025881482	no
XYLT2	0.093008075	2.466173637	0.013896136	0.025891814	no
KCNH1	-0.093005707	-2.466110281	0.013898572	0.025893968	no
BTNL3	0.09298905	2.465664757	0.013915716	0.025923519	no
CEP192	-0.092929601	-2.464074704	0.013977055	0.026035387	no
EPCAM	-0.092894547	-2.463137127	0.014013335	0.026100563	no
KRTAP5-2	-0.092885495	-2.462895027	0.014022717	0.026115631	no
EDN3	0.092849266	2.461926056	0.014060322	0.026183253	no
AMY2B	-0.092846464	-2.461851119	0.014063234	0.026186264	no
C11orf87	-0.092837959	-2.461623625	0.014072077	0.026200318	no
CFL1	0.092775342	2.459948904	0.01413733	0.026319386	no
C15orf63	-0.09276239	-2.459602493	0.01415086	0.02634122	no
FLT4	0.092761621	2.459581939	0.014151664	0.02634122	no

BLVRA	0.09268043	2.457410476	0.014236746	0.026497149	no
SMYD4	0.092674596	2.457254472	0.014242876	0.026506117	no
ILDR2	-0.092662803	-2.456939056	0.014255276	0.026526754	no
CYP4F3	0.092658779	2.456831454	0.014259509	0.026532188	no
SFRS6	-0.092614934	-2.455658831	0.014305708	0.026615699	no
WHAMM	0.092608318	2.4554819	0.01431269	0.026626239	no
CHN1	-0.092604701	-2.455385155	0.014316509	0.026630893	no
ACTBL2	0.092560197	2.454194958	0.014363567	0.026715168	no
GDF10	-0.092559356	-2.454172478	0.014364457	0.026715168	no
C13orf30	0.092533643	2.453484795	0.01439171	0.026763391	no
CNIH	0.092520982	2.453146196	0.014405145	0.026785913	no
PSMB5	-0.092511784	-2.452900232	0.014414912	0.026801609	no
CCAR1	-0.092509283	-2.452833328	0.01441757	0.026802315	no
OR1J4	0.092508931	2.452823936	0.014417943	0.026802315	no
AADACL3	0.092503332	2.452674182	0.014423893	0.026810441	no
MAMDC4	-0.092502322	-2.452647172	0.014424967	0.026810441	no
HOXB5	0.092489751	2.452310986	0.014438334	0.026832819	no
PF4	0.092484319	2.452165734	0.014444113	0.026841091	no
FAM166B	0.092465943	2.451674294	0.014463679	0.026874981	no
ZNF468	0.092461163	2.45154647	0.014468773	0.026881974	no
PRINS	0.09240377	2.450011611	0.014530053	0.026993349	no
KLF16	0.092402404	2.449975094	0.014531514	0.026993582	no
GLP2R	-0.092386642	-2.44955357	0.014548385	0.027022439	no
GRHL1	-0.092368269	-2.449062237	0.014568072	0.027056521	no
SLC35F2	0.09235784	2.448783342	0.014579258	0.027074808	no
F2RL3	0.092350491	2.448586813	0.014587144	0.027086966	no
C11orf73	-0.092331266	-2.448072698	0.014607793	0.027121643	no
LOC100188947	-0.092330607	-2.448055065	0.014608502	0.027121643	no
GHRH	-0.092327611	-2.447974955	0.014611722	0.027125131	no
C14orf104	-0.092321796	-2.447819443	0.014617975	0.027134247	no
OR2L2	-0.092319874	-2.447768043	0.014620042	0.027135592	no
C15orf24	0.09231757	2.447706424	0.01462252	0.027137701	no
BOD1	0.092295182	2.447107743	0.01464662	0.027179932	no
ITIH1	0.09224641	2.445803497	0.014699243	0.027275083	no
RAB40A	-0.092226952	-2.445283156	0.014720284	0.027311619	no
PRAMEF9	0.092204019	2.444669908	0.014745116	0.027355181	no
SGTB	-0.092193146	-2.444379165	0.014756902	0.027374535	no
ZGPAT	-0.09218657	-2.444203302	0.014764035	0.027385254	no
SP4	-0.092177314	-2.443955801	0.014774079	0.02740137	no
CPA1	0.092142096	2.44301403	0.014812353	0.027469835	no
C8orf58	0.092140708	2.442976937	0.014813862	0.027470115	no
CCNT2	-0.092137815	-2.442899569	0.01481701	0.027473433	no
FXYD2	-0.092135076	-2.44282632	0.014819992	0.027476441	no
TTC28	-0.092129837	-2.442686218	0.014825695	0.027484495	no
CES2	-0.092090948	-2.441646312	0.014868092	0.027560565	no
NPB	0.092070186	2.441091142	0.01489077	0.027600073	no
SRRM4	-0.092058977	-2.440791425	0.014903026	0.027620256	no
MFAP5	0.092048579	2.440513363	0.014914404	0.027638811	no
ESR2	0.092046332	2.440453285	0.014916864	0.027640835	no
OR13J1	-0.092026815	-2.439931407	0.014938242	0.027677913	no
FAM74A3	0.092006957	2.439400405	0.014960023	0.027715728	no

TRIM52	-0.091990977	-2.438973117	0.01497757	0.027745694	no
GPR6	-0.091964848	-2.438274455	0.0150063	0.027796369	no
DDX31	-0.091958932	-2.438116259	0.015012812	0.027805884	no
SULT1C4	-0.091944449	-2.437728983	0.015028764	0.02783288	no
ISCA1	-0.09192209	-2.437131146	0.015053419	0.027875987	no
ZNF582	0.0918924	2.436337258	0.015086214	0.027934159	no
DAD1	0.091887977	2.43621898	0.015091106	0.027940657	no
WFDC10B	0.091881402	2.436043188	0.015098378	0.027951563	no
GAL	0.091872986	2.435818147	0.015107693	0.027966246	no
KIAA1012	0.091852293	2.435264855	0.015130615	0.028006114	no
ZNF643	-0.091821495	-2.434441372	0.015164788	0.028066797	no
TFIP11	-0.09181349	-2.434227328	0.015173682	0.028080687	no
ZNF259	0.091812026	2.434188183	0.015175309	0.028081127	no
IPO5	-0.091801461	-2.433905682	0.015187055	0.02809512	no
TRIP13	0.091801284	2.433900966	0.015187251	0.02809512	no
THYN1	-0.091800673	-2.433884639	0.01518793	0.02809512	no
CDK19	-0.091800225	-2.433872653	0.015188429	0.02809512	no
UBE2V2	-0.09178861	-2.433562077	0.015201353	0.028116453	no
LOC100101266	-0.091774836	-2.433193801	0.01521669	0.028142247	no
POLR2D	0.091747276	2.432456907	0.01524742	0.028196501	no
GSDMB	-0.091737699	-2.432200852	0.015258111	0.02821369	no
FLVCR1	-0.091718397	-2.431684743	0.01527968	0.028250989	no
PMCHL2	-0.091714089	-2.431569578	0.015284496	0.028257311	no
SP7	-0.091710357	-2.43146979	0.015288671	0.028262444	no
CDKN1A	0.091669508	2.430377605	0.015334427	0.028344436	no
C10orf62	-0.091668068	-2.430339086	0.015336043	0.028344832	no
ZXDA	-0.091662242	-2.430183324	0.015342579	0.02835432	no
ZKSCAN4	0.091651468	2.429895258	0.015354673	0.028374077	no
UXT	0.091642774	2.42966282	0.015364438	0.028389527	no
GGTLC2	-0.091630676	-2.429339351	0.015378036	0.028412056	no
BAT5	0.091615947	2.428945529	0.015394606	0.028440071	no
SPACA5	-0.091602657	-2.428590223	0.015409568	0.028465112	no
ZNF678	-0.091593034	-2.428332917	0.015420412	0.028482541	no
CCDC144B	-0.091585001	-2.428118144	0.015429469	0.028496666	no
VIL1	0.091538749	2.426881551	0.015481704	0.028590528	no
ATP5G2	-0.091537423	-2.426846085	0.015483205	0.028590687	no
ORC2L	0.091510217	2.426118702	0.015514006	0.028644947	no
JAGN1	0.09150031	2.425853845	0.015525235	0.028663062	no
DFFA	0.091498797	2.425813379	0.015526951	0.028663613	no
DGKH	-0.091484065	-2.425419519	0.015543664	0.028691847	no
NCRNA00173	-0.091468813	-2.425011757	0.015560983	0.028721194	no
HS6ST3	-0.09145073	-2.424528291	0.015581541	0.028756512	no
THEG	-0.091420901	-2.423730809	0.015615502	0.028816559	no
PTTG1	0.09141249	2.42350592	0.015625091	0.028831622	no
C21orf57	-0.091410373	-2.423449343	0.015627504	0.028833444	no
MRPL49	0.091408483	2.423398807	0.01562966	0.02883479	no
NDUFC1	0.09137933	2.422619401	0.015662941	0.028893553	no
CLDN11	0.091372382	2.422433647	0.015670882	0.028903616	no
DFNB59	-0.091372056	-2.422424924	0.015671255	0.028903616	no
RPL24	-0.091367664	-2.422307504	0.015676277	0.02891024	no
RRS1	-0.091354444	-2.421954063	0.015691401	0.028935493	no

BTF3	-0.091350889	-2.421859027	0.01569547	0.028940357	no
NOTUM	-0.09133102	-2.421327849	0.015718229	0.028979678	no
KCTD8	-0.091306914	-2.420683386	0.015745881	0.029027888	no
ZNF215	0.091305183	2.420637091	0.015747869	0.029027888	no
FLJ22536	-0.091304472	-2.4206181	0.015748685	0.029027888	no
TMEM45B	-0.091277176	-2.41988836	0.015780052	0.029083054	no
PPP2R4	0.091271824	2.419745265	0.01578621	0.02909175	no
GCNT7	0.091240431	2.418906006	0.015822366	0.029155723	no
MAST3	-0.091218691	-2.418324824	0.015847446	0.029199277	no
ITGB1BP2	-0.09120496	-2.417957754	0.015863305	0.029225834	no
PURB	-0.091176924	-2.417208228	0.01589573	0.029282905	no
TLN2	-0.091170927	-2.417047918	0.015902673	0.029293025	no
SORT1	0.09116497	2.416888675	0.015909572	0.029303064	no
F2R	0.091161234	2.416788807	0.015913901	0.029308366	no
PSMB3	0.091153804	2.416590178	0.015922512	0.029321555	no
ABHD12	0.091152532	2.416556152	0.015923988	0.029321601	no
NMT1	0.091150317	2.416496939	0.015926556	0.029323659	no
ACY3	-0.091149001	-2.416461759	0.015928082	0.029323799	no
AOC3	0.091142385	2.416284907	0.015935755	0.029335254	no
RNF222	-0.091136995	-2.416140807	0.015942009	0.029344095	no
WNT8A	-0.091131911	-2.416004892	0.015947911	0.029352285	no
PRKRIP1	-0.091124449	-2.41580542	0.015956575	0.02936274	no
OPRD1	-0.091123359	-2.415776281	0.015957841	0.02936274	no
DEK	0.091123267	2.415773823	0.015957948	0.02936274	no
OTUB2	-0.091069686	-2.41434144	0.016020291	0.029471607	no
SRP68	-0.091068909	-2.414320666	0.016021197	0.029471607	no
CLCN5	0.091068445	2.414308278	0.016021737	0.029471607	no
KIAA1217	0.091067409	2.414280571	0.016022945	0.029471607	no
WDR87	-0.091028929	-2.413251903	0.016067856	0.029551525	no
VILL	0.091016291	2.412914073	0.01608263	0.029576006	no
GPRASP1	-0.091014209	-2.412858408	0.016085065	0.029577794	no
ZNF780B	-0.091002729	-2.412551525	0.016098498	0.029599802	no
KIAA0892	-0.091001247	-2.412511906	0.016100233	0.0296003	no
COPS3	0.090996507	2.412385189	0.016105783	0.029607811	no
LOC100130274	-0.090958181	-2.411360676	0.016150715	0.029687714	no
PAPOLA	0.090943648	2.410972165	0.016167784	0.029716386	no
LRRC61	0.090923078	2.410422303	0.016191967	0.029758131	no
LMF1	-0.09090365	-2.409902967	0.016214838	0.029797454	no
CEP63	-0.090867819	-2.408945149	0.016257093	0.029872389	no
COLEC10	0.090860899	2.408760178	0.016265264	0.029884688	no
VPS11	-0.090858409	-2.408693629	0.016268205	0.029887375	no
GYG1	0.090855023	2.40860311	0.016272205	0.029892009	no
CAPN8	0.090848478	2.408428155	0.016279941	0.029900826	no
MAS1	-0.090848459	-2.408427644	0.016279963	0.029900826	no
SEMA3G	-0.090844521	-2.408322377	0.016284619	0.02990666	no
SKA2	-0.090838973	-2.408174074	0.01629118	0.029913962	no
C16orf46	-0.09083809	-2.408150482	0.016292224	0.029913962	no
ZFC3H1	-0.090837406	-2.408132188	0.016293033	0.029913962	no
PRAP1	-0.090835475	-2.408080571	0.016295317	0.029915439	no
NHP2	-0.09081549	-2.407546365	0.016318975	0.029956151	no
AACSL	-0.090803571	-2.407227747	0.0163331	0.029979357	no

ATPAF1	-0.09080163	-2.407175877	0.016335401	0.029980858	no
C9orf114	-0.090795876	-2.40702206	0.016342224	0.029990659	no
IL6ST	0.090783189	2.40668293	0.016357277	0.03001556	no
FAM71D	-0.090775404	-2.406474828	0.016366521	0.030029795	no
GUSBP1	-0.090770339	-2.406339438	0.016372537	0.030038108	no
C10orf47	0.090765781	2.406217593	0.016377953	0.030045318	no
RSPH9	0.09073095	2.405286572	0.016419388	0.030118597	no
RFPL2	-0.090724261	-2.405107763	0.016427356	0.03013048	no
F8	0.090715403	2.404870977	0.016437913	0.030147109	no
ELMO3	-0.090692538	-2.404259806	0.016465191	0.030194397	no
ANKS4B	-0.090672917	-2.403735354	0.016488629	0.030234637	no
HAPLN4	-0.090665192	-2.403528864	0.016497866	0.03024883	no
C14orf101	0.090660823	2.403412085	0.016503091	0.030254624	no
ZFP91	-0.090660049	-2.403391373	0.016504018	0.030254624	no
LOC387647	-0.090639358	-2.402838325	0.016528787	0.030297282	no
NIT1	0.090633865	2.402691491	0.016535369	0.030306598	no
TNNT2	-0.090622344	-2.40238354	0.01654918	0.030329162	no
PEX5L	-0.090618915	-2.402291884	0.016553292	0.030333949	no
HCN4	-0.090611745	-2.40210025	0.016561894	0.03034649	no
LOC100130987	-0.090610709	-2.402072543	0.016563137	0.03034649	no
ST8SIA2	-0.090604014	-2.401893605	0.016571173	0.030357556	no
RAB3IP	-0.090603175	-2.401871179	0.01657218	0.030357556	no
LECT1	0.090553571	2.400545306	0.016631832	0.030464067	no
FLJ45983	0.09051339	2.39947132	0.016680289	0.030550057	no
ZNF224	0.090504149	2.399224334	0.01669145	0.03056773	no
H2AFY	0.090500868	2.399136635	0.016695415	0.030572222	no
VN1R2	-0.090496095	-2.399009066	0.016701184	0.030580016	no
SCYL1	-0.09046857	-2.398273367	0.016734486	0.030638218	no
NCOA2	-0.090411756	-2.396754821	0.01680341	0.030761621	no
C21orf128	-0.090389871	-2.396169887	0.016830025	0.030807556	no
SLC22A10	-0.090358924	-2.39534276	0.016867724	0.030873769	no
SLC14A2	-0.090344595	-2.394959783	0.016885205	0.030902967	no
ST6GALNAC3	0.090330249	2.394576353	0.016902722	0.030932227	no
ARRB1	-0.090317389	-2.394232639	0.016918439	0.030958186	no
GAS5	-0.090300911	-2.393792228	0.016938595	0.030992264	no
ZNF417	0.090286008	2.393393918	0.016956843	0.031022845	no
MRPS33	0.090272018	2.393019999	0.01697399	0.031047791	no
ECT2	0.090271787	2.393013847	0.016974272	0.031047791	no
ZNF70	-0.090271124	-2.392996106	0.016975086	0.031047791	no
RBX1	0.090260712	2.392717835	0.016987857	0.031068339	no
COL11A1	0.090242895	2.39224165	0.01700973	0.031105528	no
C3orf36	0.090229769	2.391890838	0.01702586	0.03113221	no
SPRY3	-0.090220764	-2.391650165	0.017036934	0.031149642	no
MMP16	-0.090183134	-2.390644466	0.017083277	0.031231549	no
LOC285847	-0.090177449	-2.390492525	0.017090289	0.031241542	no
INTU	0.090157251	2.389952694	0.017115219	0.031284287	no
PNPLA2	0.09015139	2.389796078	0.017122458	0.031294689	no
ZNF8	-0.090110792	-2.388711041	0.017172682	0.031383647	no
ACOT4	0.090107503	2.388623165	0.017176755	0.031388254	no
METT5D1	-0.090098886	-2.388392856	0.017187435	0.031404931	no
CTXN3	-0.090091084	-2.388184341	0.017197109	0.031419767	no

TMUB1	0.090089384	2.38813891	0.017199217	0.03142078	no
PCDHB2	-0.09004385	-2.386922007	0.017255776	0.031521257	no
LOC116437	-0.090035874	-2.386708837	0.0172657	0.031536537	no
ARHGAP11A	0.090019024	2.386258524	0.017286682	0.031572009	no
NCOA4	0.089986589	2.385391712	0.017327133	0.031643028	no
COX4I1	-0.089976651	-2.385126119	0.017339543	0.031662833	no
SERPINA6	0.089960311	2.384689436	0.017359966	0.031697263	no
CSRP3	0.089954299	2.384528767	0.017367485	0.031708129	no
KLHL26	0.089925435	2.383757412	0.017403625	0.031771241	no
DENND4B	-0.08992028	-2.383619645	0.017410087	0.031780168	no
ERBB2IP	0.089899204	2.383056403	0.017436526	0.031825557	no
AGL	0.089896463	2.38298316	0.017439967	0.031828963	no
RFX1	0.089881487	2.382582923	0.01745878	0.031857769	no
TSPAN19	0.089880252	2.382549938	0.017460331	0.031857769	no
FAM111B	0.089880136	2.382546818	0.017460478	0.031857769	no
HS3ST4	-0.089868353	-2.382231945	0.017475292	0.031881921	no
SNCA	-0.089850216	-2.381747241	0.017498117	0.031920683	no
CDCA7L	0.089830337	2.381216004	0.017523164	0.031963491	no
ZCWPW1	-0.089824324	-2.381055331	0.017530746	0.031974436	no
TSPAN33	0.08981826	2.380893275	0.017538396	0.031985503	no
PER4	0.089793827	2.380240337	0.017569247	0.032038878	no
MPV17L	0.089791746	2.38018472	0.017571878	0.032040785	no
STRN3	-0.089784044	-2.379978903	0.017581614	0.032055647	no
AQP6	-0.089782449	-2.379936271	0.017583631	0.032056434	no
CAPSL	0.089777416	2.379801784	0.017589996	0.032065146	no
KLF13	-0.089771681	-2.379648516	0.017597252	0.032075482	no
CATSPERB	0.089768438	2.379561868	0.017601356	0.03208007	no
ACSM4	0.089766003	2.379496785	0.017604439	0.032080525	no
MRPL42P5	-0.089764877	-2.379466696	0.017605864	0.032080525	no
MTP18	0.08976448	2.379456101	0.017606366	0.032080525	no
SYNRG	-0.089738336	-2.37875745	0.017639492	0.032137987	no
DNAH1	-0.089725368	-2.378410905	0.017655943	0.032165062	no
PCM1	-0.089716119	-2.37816375	0.017667684	0.032183552	no
ME1	0.089692179	2.377524014	0.017698108	0.032236066	no
C5orf51	-0.089675458	-2.377077181	0.017719384	0.032270053	no
FOXD4L3	-0.089675007	-2.37706512	0.017719959	0.032270053	no
C17orf78	-0.089667172	-2.376855748	0.017729936	0.032285315	no
TTC1	0.089658553	2.376625437	0.017740918	0.032302402	no
MBL2	0.089650595	2.37641279	0.017751062	0.032317962	no
SHC4	0.089618604	2.3755579	0.017791895	0.032389388	no
NDC80	0.089616739	2.375508059	0.017794279	0.03239081	no
MMP13	0.089581261	2.374560045	0.017839662	0.032470498	no
ARHGEF33	-0.089573966	-2.374365092	0.017849008	0.032484583	no
HOOK2	-0.089565817	-2.374147343	0.017859451	0.032500664	no
HJURP	0.089547436	2.373656176	0.017883027	0.032540639	no
ARHGEF2	-0.089527201	-2.373115462	0.017909013	0.032584991	no
LAMA3	0.089511221	2.372688467	0.017929557	0.032619435	no
C7orf43	0.089501942	2.372440508	0.017941497	0.03263822	no
ZNF764	-0.089469215	-2.371566004	0.017983661	0.032709078	no
MCRS1	-0.089468334	-2.371542482	0.017984797	0.032709078	no
C19orf6	-0.089467943	-2.371532029	0.017985301	0.032709078	no

SNX12	-0.089464832	-2.371448889	0.017989315	0.032713375	no
F5	-0.089463603	-2.371416048	0.0179909	0.032713375	no
DYNC1LI1	-0.089458124	-2.371269652	0.01799797	0.032723287	no
PTPRR	-0.089450743	-2.37107242	0.018007498	0.032737667	no
CDC26	-0.089438039	-2.370732965	0.018023907	0.032764553	no
SLC25A31	0.089435846	2.370674352	0.018026742	0.03276676	no
IRS2	0.089420548	2.370265591	0.018046522	0.032799765	no
UBAP2L	-0.089414805	-2.370112134	0.018053953	0.032810321	no
PEX11A	-0.089410182	-2.36998861	0.018059936	0.032818244	no
BCL9L	-0.089396959	-2.369635292	0.01807706	0.032846409	no
CSRNP2	-0.089388119	-2.36939907	0.018088516	0.032864272	no
WNT9B	-0.089358301	-2.368602328	0.018127204	0.032931603	no
ZEB2	-0.08935072	-2.368399755	0.018137052	0.032946533	no
RNF183	-0.089345422	-2.368258208	0.018143936	0.032956077	no
KLHL34	-0.089340287	-2.36812098	0.018150612	0.032965242	no
AKAP10	0.089307949	2.367256914	0.018192699	0.033038712	no
CHRNG	-0.089303111	-2.36712766	0.018199002	0.03304719	no
HTR3B	-0.089300103	-2.367047278	0.018202922	0.033051341	no
ZNF148	-0.089294976	-2.366910279	0.018209607	0.033060509	no
FAM72D	0.089291565	2.36681914	0.018214054	0.033065615	no
POLD1	0.089271723	2.366288989	0.018239946	0.033109645	no
PDDC1	-0.08926488	-2.366106135	0.018248883	0.033122895	no
SRPK1	-0.089258866	-2.365945437	0.018256741	0.033134183	no
GTF2H4	-0.089251632	-2.365752156	0.018266196	0.033148367	no
LOC153684	0.089231757	2.365221122	0.018292196	0.033192571	no
C1orf198	-0.089219553	-2.364895018	0.018308178	0.03321859	no
PLCL2	-0.089207837	-2.364581984	0.018323532	0.033243464	no
TM9SF4	0.089196124	2.364269039	0.018338892	0.033268346	no
TSG101	-0.089183196	-2.363923616	0.018355859	0.033296139	no
MCART6	-0.089176103	-2.363734094	0.018365175	0.0333084	no
POLG2	-0.089175541	-2.363719062	0.018365914	0.0333084	no
SEC24B	0.089152162	2.363094413	0.018396647	0.033361145	no
PMS2L1	0.089150735	2.363056295	0.018398523	0.033361556	no
SP6	0.089148144	2.362987057	0.018401933	0.033364746	no
C14orf176	-0.089143356	-2.362859144	0.018408234	0.033373177	no
CCR9	0.089136827	2.362684693	0.018416829	0.033384583	no
RABAC1	0.089136069	2.362664444	0.018417827	0.033384583	no
C1orf104	-0.089128168	-2.362453337	0.018428235	0.033400452	no
RBM19	-0.089081116	-2.36119617	0.018490318	0.033509214	no
RAB5C	0.089080178	2.361171108	0.018491557	0.033509214	no
VOPP1	0.089066123	2.36079559	0.018510138	0.033539879	no
LRDD	-0.089046634	-2.360274891	0.01853593	0.033583604	no
ZBTB34	0.089043706	2.360196649	0.018539808	0.03358762	no
ALOX15	0.089042326	2.360159791	0.018541635	0.033587921	no
FAF1	0.089020093	2.359565771	0.018571105	0.033638292	no
SLC9A10	0.089016703	2.359475194	0.018575603	0.033643423	no
GLRA4	-0.089005758	-2.35918277	0.018590128	0.033666716	no
ANXA6	-0.088994203	-2.358874044	0.018605475	0.03369149	no
KRT33A	-0.088972077	-2.358282903	0.01863489	0.033741735	no
SNTN	0.088970442	2.358239222	0.018637065	0.033742651	no
KIFAP3	-0.088936507	-2.357332564	0.018682265	0.033821457	no

C17orf108	-0.088930908	-2.357182983	0.018689732	0.033831945	no
SLC35C2	0.088921692	2.356936749	0.018702028	0.033851173	no
ZNF516	-0.08890332	-2.356445904	0.018726561	0.033892544	no
SERPIND1	0.088895249	2.35623028	0.018737347	0.03390903	no
C9orf78	-0.088888549	-2.356051285	0.018746305	0.033922205	no
CHMP1B	0.088885181	2.355961293	0.01875081	0.033925129	no
ATP5D	-0.088884832	-2.355951962	0.018751277	0.033925129	no
RXRG	-0.088833154	-2.354571307	0.018820515	0.034047349	no
EFHA1	-0.088786193	-2.353316697	0.018883626	0.034158465	no
HMGB2	0.088772241	2.352943956	0.018902412	0.034189387	no
STL	-0.088765417	-2.352761647	0.018911607	0.034202957	no
HOXD10	0.088760044	2.352618086	0.018918849	0.034212996	no
PRSS42	0.0887537	2.352448623	0.018927402	0.034225402	no
BCL6	-0.088733213	-2.351901278	0.01895505	0.03427233	no
ADORA1	0.088715941	2.351439859	0.018978384	0.034311453	no
ASB15	0.088711948	2.351333172	0.018983783	0.034318145	no
ZNF350	0.088709725	2.351273785	0.018986789	0.034320192	no
IL5RA	0.088708363	2.351237405	0.018988631	0.034320192	no
MT1M	0.088707344	2.351210191	0.018990008	0.034320192	no
C16orf90	-0.088694477	-2.350866428	0.019007419	0.034348587	no
ZNF18	0.08868988	2.350743621	0.019013642	0.034356762	no
CHRNA5	0.088667236	2.350138688	0.019044322	0.034409125	no
ETS1	0.088661922	2.349996713	0.019051529	0.03441907	no
CYP4Z1	-0.088648285	-2.349632409	0.019070032	0.034449421	no
JRKL	-0.088636427	-2.349315613	0.019086136	0.03447543	no
C7orf68	0.088628946	2.349115761	0.019096301	0.03449071	no
PGAM1	-0.088620611	-2.348893094	0.019107632	0.034508092	no
DAAM1	0.088595827	2.348230999	0.019141359	0.034565819	no
AVPR2	0.088594611	2.348198509	0.019143015	0.034565819	no
EXOSC6	-0.088589318	-2.348057122	0.019150225	0.034575749	no
ZNF581	0.08858656	2.347983434	0.019153983	0.034579447	no
CSNK1D	-0.088574895	-2.347671816	0.019169884	0.034605064	no
WWC1	-0.08856402	-2.347381288	0.019184719	0.034628752	no
TMEM213	-0.088560634	-2.347290825	0.019189341	0.034634002	no
C4orf42	-0.088558983	-2.347246718	0.019191594	0.034634977	no
BTG2	0.088553923	2.34711156	0.019198501	0.03464435	no
CLEC4GP1	-0.088551292	-2.347041255	0.019202095	0.034647743	no
TMUB2	0.088536974	2.346658783	0.019221656	0.034675571	no
DMD	0.088536657	2.346650303	0.01922209	0.034675571	no
COL6A4P2	-0.088535601	-2.346622097	0.019223533	0.034675571	no
SEPT7L	-0.088534983	-2.346605587	0.019224378	0.034675571	no
GABRE	0.088532786	2.346546902	0.019227382	0.034677895	no
LCE1F	0.088482165	2.345194595	0.019296702	0.034799814	no
PATE2	-0.088465713	-2.344755112	0.019319277	0.034837419	no
PIGW	0.088455918	2.344493453	0.019332729	0.034858567	no
PUM1	-0.088433768	-2.343901726	0.01936318	0.034910359	no
DCAF13	0.088412578	2.343335679	0.019392348	0.03495983	no
RNF4	0.088391966	2.342785053	0.019420759	0.035007927	no
NAGS	0.088366079	2.342093553	0.01945649	0.035069209	no
PTGER3	-0.08833825	-2.341350147	0.019494968	0.03513543	no
SLC48A1	-0.088307221	-2.34052129	0.019537947	0.035209752	no

KLK2	0.088296165	2.340225939	0.019553281	0.035234246	no
KIAA1191	-0.088291132	-2.340091501	0.019560265	0.03524369	no
SNRPC	0.088278153	2.339744801	0.019578285	0.035273015	no
FAM65A	-0.088235125	-2.338595439	0.019638128	0.035377678	no
FAM186A	-0.088202361	-2.337720225	0.019683805	0.035456804	no
KBTBD5	-0.088199889	-2.337654193	0.019687255	0.035459859	no
C13orf38	0.088191262	2.337423753	0.019699299	0.035478391	no
AHCYL2	-0.08818858	-2.337352122	0.019703044	0.035481975	no
FGF18	0.088181517	2.337163461	0.01971291	0.035496582	no
PHF20L1	-0.088097024	-2.334906533	0.019831279	0.035706546	no
NPBWR2	-0.088087725	-2.334658151	0.019844344	0.035726888	no
LOC728606	-0.088082942	-2.334530371	0.019851068	0.035735811	no
KRTAP13-4	0.08807458	2.334307019	0.019862826	0.035753795	no
HIF1A	0.088058199	2.333869481	0.019885878	0.035792102	no
CHL1	0.088036806	2.333298039	0.019916019	0.035843161	no
MYEOV2	0.088027289	2.333043838	0.01992944	0.035864123	no
ID2B	-0.088020222	-2.332855073	0.019939411	0.035878873	no
TULP4	-0.088005219	-2.332454332	0.019960594	0.035913793	no
ZNF30	-0.087980929	-2.331805558	0.01999493	0.03597237	no
LOC440925	0.087886391	2.329280438	0.020129062	0.036206327	no
PCDHB18	-0.087885959	-2.329268897	0.020129677	0.036206327	no
MED1	-0.087885489	-2.329256356	0.020130345	0.036206327	no
RTEL1	-0.087840969	-2.328067238	0.020193786	0.0363172	no
RPP25	0.087838807	2.328009493	0.020196871	0.036319518	no
ADRA2C	-0.087811792	-2.327287964	0.020235455	0.036385667	no
ATRIP	-0.08777861	-2.326401687	0.020282938	0.036467803	no
MORF4L2	0.087762844	2.325980608	0.020305532	0.036505179	no
ADAP1	-0.087747876	-2.32558082	0.020327004	0.036540531	no
KCNE2	-0.087746416	-2.325541822	0.020329099	0.03654105	no
RAET1L	0.087744688	2.325495675	0.020331579	0.036542258	no
RBM18	0.08773716	2.325294618	0.020342387	0.036558434	no
CCDC39	0.087691269	2.324068944	0.020408382	0.036673778	no
KCNS1	-0.087687773	-2.323975583	0.020413417	0.036679565	no
SQLE	-0.087684928	-2.32389958	0.020417516	0.03668367	no
MCM7	-0.087678691	-2.323732997	0.020426504	0.036696557	no
TLR4	0.087641738	2.32274606	0.020479822	0.036787173	no
CCDC59	0.087641211	2.322731993	0.020480582	0.036787173	no
TMEM219	0.087634216	2.32254516	0.02049069	0.036802058	no
TMEM56	-0.087618485	-2.322125023	0.020513435	0.036839636	no
ZNF343	-0.087602983	-2.321711014	0.020535869	0.03687665	no
LOC728989	-0.087598466	-2.32159036	0.020542411	0.036885122	no
ATOX1	0.087579076	2.321072516	0.020570511	0.036932296	no
ZNF606	-0.087570931	-2.320854987	0.020582324	0.036950225	no
STAMBP	-0.087513862	-2.319330825	0.020665265	0.03709583	no
C1orf194	0.087476915	2.318344078	0.020719117	0.037189196	no
LCOR	-0.087473545	-2.318254092	0.020724034	0.03719472	no
TTC5	0.087463067	2.317974243	0.020739332	0.037218873	no
KRAS	-0.087461023	-2.317919654	0.020742318	0.037220926	no
USP44	-0.087443129	-2.317441771	0.020768468	0.037264544	no
ZNF709	-0.087440566	-2.317373337	0.020772215	0.03726796	no
G6PC2	-0.087425786	-2.316978597	0.02079384	0.037303448	no

BMS1	-0.087409145	-2.316534177	0.020818211	0.037343855	no
KCNAB2	-0.087372818	-2.315564034	0.020871498	0.03743612	no
KRT33B	-0.087341881	-2.314737831	0.020916972	0.037514357	no
CACNB2	-0.087334904	-2.31455151	0.020927239	0.037529441	no
MMP23A	0.087332786	2.31449495	0.020930357	0.037531703	no
CLN3	0.087326963	2.31433943	0.020938931	0.037543749	no
FHL1	-0.087321577	-2.314195611	0.020946863	0.037554631	no
GPR153	-0.08732032	-2.314162028	0.020948716	0.037554631	no
TDRD12	-0.087317964	-2.314099119	0.020952186	0.037557523	no
SLC25A4	-0.087287147	-2.313276148	0.020997636	0.037635656	no
NBPF7	-0.087285769	-2.313239343	0.02099967	0.037635966	no
MTBP	0.087278941	2.313056979	0.021009754	0.037650701	no
SNRNP48	-0.087272455	-2.312883768	0.021019335	0.037664533	no
ZBTB26	-0.087193039	-2.310762977	0.021136961	0.037871949	no
CA5BP	-0.087188823	-2.310650377	0.021143222	0.037879811	no
FAM128A	-0.087180183	-2.310419667	0.021156056	0.037899445	no
GLYCTK	0.08713615	2.309243791	0.021221572	0.038013445	no
KLHL10	0.087129775	2.309073534	0.021231073	0.038027094	no
OR1F1	-0.087121209	-2.308844786	0.021243844	0.038046597	no
LRP1	0.087110043	2.308546621	0.0212605	0.038073055	no
ANKRD57	0.087076535	2.307651809	0.021310555	0.038159313	no
PRRT3	-0.087073527	-2.307571486	0.021315054	0.038162775	no
ZC3H12C	-0.087072718	-2.307549883	0.021316264	0.038162775	no
PGBD3	-0.087064345	-2.307326311	0.021328789	0.038181818	no
H2AFV	-0.087062086	-2.307265965	0.021332171	0.038184491	no
ALG10B	-0.087033503	-2.306502696	0.021374987	0.038257744	no
TCN1	0.087013301	2.305963226	0.021405294	0.038308598	no
FRMPD2	-0.087011562	-2.305916794	0.021407904	0.038309878	no
WNT1	0.087008833	2.305843927	0.021412001	0.038313818	no
GATC	0.08700596	2.305767208	0.021416315	0.038318147	no
DEFA4	0.087001955	2.305660264	0.02142233	0.038324472	no
TGFBRAP1	-0.087000502	-2.305621452	0.021424514	0.038324472	no
PREX2	-0.08699982	-2.305603261	0.021425537	0.038324472	no
ZKSCAN1	-0.086977656	-2.305011388	0.021458858	0.038380679	no
C5orf54	-0.086974858	-2.304936671	0.021463068	0.038384813	no
C14orf4	0.086972085	2.304862621	0.021467241	0.03838888	no
CCNC	0.086966013	2.304700488	0.02147638	0.038401826	no
CCDC121	0.086958995	2.304513086	0.021486947	0.038417324	no
PKIG	0.086944959	2.304138295	0.021508095	0.038451734	no
ENC1	-0.08693299	-2.303818682	0.021526143	0.038480598	no
C16orf80	-0.086927913	-2.303683104	0.021533803	0.038490884	no
C22orf24	-0.086926653	-2.30364946	0.021535705	0.038490884	no
ADM2	0.086904249	2.303051225	0.021569535	0.038547942	no
VPS4B	0.086899949	2.302936396	0.021576035	0.038556149	no
ZNF193	-0.086860878	-2.301893106	0.02163516	0.038658389	no
CCDC33	0.086846662	2.301513489	0.021656709	0.038693474	no
C3orf34	0.086841938	2.30138736	0.021663873	0.038702853	no
UBXN2A	0.086827301	2.300996521	0.021686085	0.038739112	no
WNT2	0.086819009	2.300775091	0.021698678	0.038758183	no
PRICKLE2	-0.08681073	-2.300554042	0.021711256	0.038773802	no
USP3	-0.086810695	-2.300553104	0.021711309	0.038773802	no

DHRS7C	-0.086809467	-2.300520306	0.021713176	0.038773802	no
AP2A1	-0.086790955	-2.300026003	0.021741327	0.038820643	no
C19orf53	0.086787573	2.299935709	0.021746472	0.038826031	no
CYB5R2	0.086786448	2.299905666	0.021748185	0.038826031	no
C12orf11	0.086778973	2.299706063	0.021759564	0.038842917	no
HEPH	0.086736852	2.298581354	0.021823783	0.038954114	no
GATAD1	0.086731709	2.298444032	0.021831635	0.038964689	no
IPMK	-0.086727893	-2.298342134	0.021837463	0.038971651	no
MSX2P1	-0.086712726	-2.297937151	0.02186064	0.039009569	no
PCDHGB7	-0.086696438	-2.297502254	0.021885553	0.039050579	no
PGLYRP2	0.086691185	2.297361995	0.021893593	0.039061477	no
EIF5B	-0.086687554	-2.297265034	0.021899152	0.039067948	no
KIF12	-0.086677263	-2.296990257	0.021914914	0.039092617	no
PKD2L1	0.086665888	2.296686527	0.021932348	0.039120265	no
AHCY	0.086643505	2.296088877	0.021966688	0.03917806	no
RBPMS2	0.086616747	2.295374416	0.022007801	0.039247925	no
GADD45B	0.086591897	2.294710905	0.022046043	0.039312656	no
TEX2	0.086590053	2.294661672	0.022048882	0.039314252	no
TAF1	-0.086573405	-2.294217161	0.022074537	0.039356525	no
FAM160B1	-0.086553217	-2.293678137	0.022105682	0.039408577	no
RAD51C	-0.086548366	-2.293548626	0.02211317	0.039418452	no
CGA	0.086544908	2.293456293	0.022118511	0.039424495	no
CENPP	0.086527938	2.293003185	0.022144734	0.039467756	no
12-Sep	-0.086517142	-2.292714947	0.022161429	0.039493778	no
FAM193B	-0.086515971	-2.292683678	0.022163241	0.039493778	no
ADRM1	0.086497988	2.292203538	0.022191079	0.039539898	no
DNAJC30	0.086493652	2.292087772	0.022197795	0.03954838	no
TCEB3	0.086460067	2.291191059	0.02224988	0.039636931	no
ZNF584	0.086459076	2.291164604	0.022251418	0.039636931	no
PPP2R3A	0.086453965	2.291028122	0.022259355	0.039647576	no
DSG1	-0.086440237	-2.290661598	0.022280683	0.039682069	no
SLC4A3	0.086428976	2.290360938	0.022298191	0.03970643	no
KIAA1239	-0.086428913	-2.290359262	0.022298289	0.03970643	no
ADAMTS3	0.086417574	2.290056514	0.022315931	0.039734093	no
ZC3H15	0.086416299	2.290022466	0.022317916	0.039734093	no
ADH1A	0.08641514	2.289991521	0.02231972	0.039734093	no
NANOS1	-0.086411944	-2.289906205	0.022324695	0.03973945	no
XPO5	-0.086383112	-2.289136394	0.022369624	0.039815923	no
HNMT	0.086356275	2.288419888	0.022411514	0.039886971	no
PPP1R8	0.086341663	2.288029777	0.02243435	0.039924099	no
ANKRD44	0.086329717	2.287710826	0.022453035	0.039953835	no
LOC407835	0.086297594	2.286853186	0.022503347	0.040039837	no
UBE4A	-0.086286492	-2.28655677	0.022520758	0.040067291	no
PRB1	-0.086268892	-2.286086881	0.022548384	0.04011291	no
RC3H1	-0.086263213	-2.285935255	0.022557304	0.040125248	no
ITGAE	0.086260593	2.285865322	0.022561419	0.040129038	no
YIF1B	0.086249407	2.285566685	0.022579001	0.040156777	no
UBE2J2	0.086239118	2.285291983	0.022595184	0.040182023	no
OR2C3	0.086218887	2.284751849	0.022627032	0.040235122	no
FTCD	-0.086215112	-2.284651064	0.02263298	0.040242158	no
RFWD3	0.086179773	2.283707583	0.022688718	0.040337716	no

ISLR2	-0.086177631	-2.283650412	0.0226921	0.04034018	no
RAB19	0.086173649	2.2835441	0.022698389	0.040347813	no
RPA4	0.086151789	2.282960488	0.02273294	0.040400354	no
CNTF	0.086151389	2.282949822	0.022733572	0.040400354	no
ZDHHC8	-0.086151156	-2.282943577	0.022733942	0.040400354	no
MED9	-0.086113917	-2.281949397	0.022792907	0.040501581	no
NRIP3	-0.086093212	-2.281396652	0.022825748	0.040556374	no
AIFM2	0.086088818	2.281279329	0.022832724	0.040565204	no
ASXL2	0.086083377	2.281134075	0.022841363	0.040576987	no
SFPQ	-0.086075294	-2.28091828	0.022854204	0.040593077	no
FLJ35024	-0.086075147	-2.280914372	0.022854436	0.040593077	no
C7orf53	0.086063285	2.280597677	0.022873292	0.040622696	no
JHDM1D	-0.086062128	-2.280566801	0.022875131	0.040622696	no
TAOK1	-0.086050363	-2.280252718	0.022893845	0.04065236	no
SLC5A8	-0.08603647	-2.279881826	0.022915962	0.040685849	no
C15orf39	-0.086035988	-2.279868956	0.02291673	0.040685849	no
TMC7	0.086014253	2.279288708	0.022951369	0.040743769	no
NFIA	-0.086005361	-2.279051307	0.022965555	0.040764249	no
PCDHGA1	-0.086004494	-2.279028155	0.022966939	0.040764249	no
BAZ1A	0.085992978	2.278720723	0.022985321	0.040793295	no
NCRNA00181	0.085984474	2.278493709	0.022998903	0.040813817	no
DSEL	-0.085963474	-2.277933082	0.023032475	0.040869806	no
ATP2C2	-0.085957987	-2.277786616	0.023041252	0.040880751	no
BCS1L	-0.085957091	-2.277762686	0.023042687	0.040880751	no
FOXP1	0.085932975	2.277118882	0.023081307	0.040945675	no
POLR1B	-0.085930352	-2.277048856	0.023085511	0.04094954	no
SLC30A10	-0.085925822	-2.27692792	0.023092773	0.040958828	no
MAP1B	0.085918704	2.276737905	0.023104187	0.040975478	no
BCL2L1	0.085915149	2.276643006	0.023109889	0.040981997	no
MON1B	-0.085911719	-2.27655144	0.023115393	0.040988161	no
SLC5A6	0.085882309	2.275766318	0.023162627	0.041068314	no
FBXW9	0.085866994	2.275357486	0.023187256	0.041108378	no
LOC151658	0.085842703	2.274709022	0.023226368	0.041174109	no
C8orf39	-0.085832959	-2.2744489	0.023242073	0.041198338	no
MGAT5	0.085798144	2.273519507	0.023298263	0.041294317	no
NR1H4	-0.085795452	-2.273447649	0.023302612	0.041298406	no
CT62	-0.085773147	-2.272852214	0.023338679	0.0413587	no
SOCS5	0.085758959	2.27247347	0.023361646	0.041395771	no
MICAL1	-0.085735735	-2.271853508	0.023399282	0.041458827	no
UOX	0.08573262	2.271770358	0.023404334	0.041464145	no
SSR2	0.085729865	2.27169683	0.023408802	0.041468427	no
SLC38A8	-0.085716575	-2.27134204	0.023430371	0.041503001	no
PHYHD1	-0.085712845	-2.271242472	0.023436428	0.041510077	no
RBM24	0.085711586	2.271208865	0.023438472	0.041510077	no
ZNF182	-0.085707376	-2.271096481	0.02344531	0.04151855	no
SIN3B	-0.085698865	-2.270869287	0.02345914	0.041539401	no
CNPY2	0.08569714	2.270823235	0.023461944	0.041540728	no
SIX4	-0.085679165	-2.270343415	0.023491176	0.041588844	no
C7orf69	0.085668247	2.270051962	0.023508948	0.041616664	no
DPYSL3	-0.085606826	-2.268412408	0.023609142	0.041790373	no
MMP7	0.085598172	2.268181391	0.02362329	0.041808878	no

NPTX2	0.085597901	2.268174161	0.023623732	0.041808878	no
HSP90B3P	0.085591621	2.268006523	0.023634003	0.041823394	no
NUBP2	-0.085575161	-2.267567156	0.023660941	0.041867399	no
ABT1	0.0855611	2.267191825	0.023683974	0.041904487	no
PIAS1	0.085545603	2.266778163	0.023709381	0.04194577	no
C19orf75	0.085516991	2.266014416	0.023756353	0.042025195	no
KIAA1522	0.085508909	2.265798681	0.023769636	0.042045013	no
IQSEC2	-0.085505958	-2.265719919	0.023774487	0.042049915	no
DULLARD	-0.085468497	-2.264719967	0.02383615	0.04215529	no
PRPS1	-0.085457074	-2.264415049	0.02385498	0.042184902	no
CCL14-CCL15	0.085450713	2.264245252	0.023865472	0.042199765	no
RUNDC1	-0.08544683	-2.264141621	0.023871877	0.042207399	no
C6orf147	0.085436664	2.263870274	0.023888656	0.042233372	no
KLK5	-0.085434409	-2.263810074	0.023892379	0.042236262	no
SOS1	-0.08542982	-2.263687571	0.023899959	0.042245966	no
ZNF532	-0.085396717	-2.262803981	0.023954688	0.042339006	no
TRAF6	0.085376281	2.262258497	0.02398853	0.042395114	no
MBLAC1	-0.085355912	-2.261714798	0.024022302	0.042451089	no
MGC70857	-0.085350567	-2.261572145	0.02403117	0.042463048	no
BHLHB9	-0.085307661	-2.260426902	0.024102466	0.042585306	no
CHST7	0.085290613	2.259971863	0.024130845	0.042631722	no
NOP16	0.085278666	2.259652982	0.02415075	0.042663159	no
FGF5	0.08526272	2.259227379	0.024177338	0.042706397	no
MSRB2	-0.085249395	-2.258871702	0.024199577	0.042741946	no
LOC645752	0.085244576	2.258743082	0.024207624	0.042752423	no
RNASEH2B	0.085234144	2.258464639	0.024225052	0.042779465	no
ZNF419	-0.085226298	-2.258255219	0.024238166	0.042798886	no
ZSCAN10	-0.085189629	-2.2572765	0.02429954	0.04290351	no
DYNLRB2	0.085170849	2.256775253	0.024331024	0.042955348	no
HMGA1	0.085156509	2.256392491	0.02435509	0.042994081	no
LOC100134713	0.08514813	2.25616885	0.024369161	0.043015164	no
PDE10A	-0.085135085	-2.25582068	0.02439108	0.043050097	no
LYAR	0.085111577	2.255193249	0.024430625	0.043116129	no
PAQR8	-0.085098704	-2.254849667	0.024452303	0.043150621	no
CREB3L1	0.085092899	2.254694712	0.024462086	0.043164117	no
TBC1D3G	-0.085063478	-2.253909467	0.024511711	0.043247907	no
SSBP4	0.085046586	2.253458638	0.024540241	0.043294467	no
ABR	-0.085022369	-2.252812287	0.024581195	0.043362935	no
MST1P9	-0.08502059	-2.252764819	0.024584205	0.043364462	no
MED26	-0.08499762	-2.252151739	0.02462311	0.043429298	no
ZDHHC9	0.084956176	2.251045633	0.024693437	0.043549538	no
MON2	-0.084949808	-2.250875668	0.024704259	0.043564824	no
NCEH1	0.084942958	2.250692847	0.024715904	0.043581558	no
FNDC3A	0.08490609	2.24970888	0.024778661	0.043688408	no
RPL7L1	-0.084856229	-2.248378158	0.024863754	0.043833742	no
ULK2	-0.08485525	-2.248352051	0.024865426	0.043833742	no
GRAMD3	-0.084834257	-2.247791757	0.024901332	0.043893211	no
SFRS7	0.084827487	2.247611088	0.02491292	0.043909808	no
PRPF18	-0.084824057	-2.247519561	0.024918792	0.04391633	no
IL11RA	-0.084820281	-2.247418777	0.024925259	0.043921837	no
CPLX3	-0.084819696	-2.247403156	0.024926262	0.043921837	no

TDRD10	0.084789513	2.246597637	0.024978006	0.044005983	no
ZNF410	0.084789302	2.246592002	0.024978369	0.044005983	no
NDUFB1	0.084787239	2.246536943	0.024981909	0.044008385	no
CRISP1	0.084785225	2.246483207	0.024985365	0.044010638	no
TMEM88	-0.084771426	-2.246114933	0.025009059	0.044048537	no
CA5B	0.084769065	2.246051924	0.025013115	0.044050461	no
USP48	0.084767615	2.246013211	0.025015607	0.044050461	no
C13orf36	-0.084766986	-2.245996426	0.025016688	0.044050461	no
TP63	0.084746683	2.245454582	0.025051594	0.044108083	no
FAM27A	0.084691652	2.243985957	0.025146416	0.044271181	no
VPS25	0.084684857	2.243804614	0.025158146	0.044287976	no
EZR	-0.084636587	-2.242516411	0.02524161	0.044431035	no
PCBD2	-0.084624524	-2.242194491	0.025262505	0.044463944	no
LOC344595	0.084621321	2.242109022	0.025268055	0.044469841	no
RPL10L	-0.084599946	-2.241538593	0.025305124	0.044531203	no
CCDC37	0.084582101	2.24106236	0.025336107	0.044581847	no
VCX3A	0.084575213	2.240878531	0.025348076	0.044599026	no
OR4D1	0.084570495	2.240752635	0.025356276	0.044609571	no
CTAGE9	0.084549245	2.240185551	0.025393239	0.044670713	no
CLIC5	-0.084525213	-2.239544214	0.025435098	0.044740456	no
HEATR5B	-0.084521561	-2.239446751	0.025441464	0.044747762	no
NUDCD3	-0.084507352	-2.239067577	0.025466246	0.044787453	no
PGM1	0.08449335	2.238693915	0.025490688	0.044826539	no
DCLK3	0.084490688	2.238622871	0.025495337	0.044829619	no
RHCE	0.084489808	2.238599395	0.025496873	0.044829619	no
FAM116B	-0.084459474	-2.237789897	0.025549905	0.044918954	no
SPPL2B	-0.084452665	-2.237608218	0.02556182	0.044935995	no
ZNF362	-0.084450046	-2.237538304	0.025566407	0.04494015	no
ISYNA1	0.084443433	2.237361852	0.025577985	0.044956297	no
FOXH1	-0.08444226	-2.237330551	0.02558004	0.044956297	no
STK32B	0.084413689	2.236568116	0.025630127	0.045040408	no
CCDC148	0.084410824	2.236491665	0.025635154	0.045045327	no
C15orf21	-0.084404943	-2.236334718	0.025645476	0.04505955	no
LOC389332	-0.084402459	-2.236268429	0.025649837	0.045063296	no
MSTN	-0.084399793	-2.23619728	0.025654519	0.045067604	no
CARS2	0.084383749	2.235769136	0.025682705	0.0451132	no
SEPSECS	0.084359989	2.235135122	0.025724495	0.04518268	no
UBE2M	-0.084355354	-2.235011416	0.025732655	0.045193088	no
NCRNA00114	0.084341338	2.234637403	0.025757342	0.045232514	no
SESN2	0.084328124	2.234284799	0.025780634	0.045265747	no
ZNF192	-0.084328062	-2.234283139	0.025780744	0.045265747	no
TNFSF9	0.084320918	2.234092501	0.025793344	0.045283938	no
EIF2S2	0.084308607	2.233763979	0.025815071	0.045318148	no
GNMT	-0.084263351	-2.232556354	0.025895075	0.045452128	no
OR10Q1	-0.084262892	-2.2325441	0.025895888	0.045452128	no
PECR	0.084258076	2.232415584	0.025904415	0.045463148	no
GAPDH	0.084245887	2.232090327	0.025926006	0.045497092	no
EDDM3A	0.084233034	2.231747348	0.025948791	0.045533124	no
LOC613037	0.084229067	2.231641495	0.025955826	0.045538521	no
TOPORS	-0.08422876	-2.231633294	0.025956371	0.045538521	no
CD22	0.08421198	2.231185556	0.025986149	0.045586808	no

ALKBH6	0.08420088	2.230889352	0.026005865	0.045617437	no
C9orf80	0.084197627	2.230802557	0.026011645	0.045623617	no
COCH	0.084189073	2.230574298	0.02602685	0.045646326	no
RUVBL1	0.084179643	2.230322654	0.026043621	0.045671779	no
NDUFB5	0.084174894	2.230195956	0.026052069	0.045682631	no
HSD17B10	0.084169397	2.230049255	0.026061854	0.045695825	no
TRPS1	0.084139504	2.229251606	0.02611511	0.045785232	no
KIAA1529	-0.084111063	-2.228492694	0.026165867	0.045870243	no
SHROOM2	-0.084104783	-2.228325132	0.026177086	0.04588593	no
C4orf45	-0.084089633	-2.227920879	0.026204168	0.045929421	no
RNF220	-0.08408637	-2.227833801	0.026210005	0.045935669	no
TMEM82	0.084069844	2.227392839	0.02623958	0.045983516	no
MED22	-0.084066355	-2.227299736	0.026245828	0.045990478	no
SNORA31	0.084044368	2.226713063	0.026285228	0.046055528	no
MEG3	-0.084040821	-2.226618416	0.026291589	0.046062682	no
FABP5L3	0.084017579	2.225998259	0.026333303	0.046131766	no
TNFAIP1	-0.083981415	-2.225033299	0.026398322	0.046239872	no
PSMG3	0.083980713	2.225014561	0.026399586	0.046239872	no
NUP214	-0.083975989	-2.224888522	0.02640809	0.046250759	no
FAM73A	-0.083967051	-2.224650029	0.026424186	0.046274941	no
FNIP2	0.083960637	2.224478908	0.026435741	0.046291166	no
MGC3771	-0.083951208	-2.224227307	0.026452737	0.046316917	no
PRKAB2	-0.083942136	-2.223985255	0.026469098	0.046341551	no
MUC20	-0.08393545	-2.22380685	0.026481162	0.046358658	no
RNF32	-0.083909266	-2.223108202	0.026528453	0.046437425	no
OGG1	-0.083897526	-2.222794952	0.02654968	0.046470559	no
LCN10	0.083892964	2.222673236	0.026557932	0.046480979	no
UCMA	0.083886872	2.222510692	0.026568955	0.046496247	no
PRIM2	0.083883232	2.22241357	0.026575544	0.046503751	no
OIP5	0.083878059	2.222275549	0.026584909	0.046516114	no
POLR3E	-0.083870064	-2.222062233	0.026599389	0.046537422	no
DNAJC24	-0.083843939	-2.221365174	0.026646754	0.046616257	no
NLRP2	0.083837725	2.221199369	0.026658031	0.04663195	no
LINS1	0.083809169	2.220437438	0.026709907	0.046718652	no
FOXA1	0.083805354	2.220335652	0.026716844	0.046726743	no
SLC22A15	0.08375863	2.219088991	0.026801929	0.046869438	no
GGPS1	0.083758005	2.219072316	0.026803069	0.046869438	no
FLCN	0.08373928	2.218572737	0.026837233	0.046925121	no
ST7OT3	-0.083732156	-2.218382651	0.026850241	0.046943807	no
ZNF616	0.083712739	2.217864593	0.026885724	0.047001778	no
TAF9	0.083705064	2.217659811	0.02689976	0.047022251	no
CD1A	0.083680472	2.217003676	0.026944778	0.047096872	no
CCKBR	-0.083617883	-2.215333793	0.027059643	0.047293556	no
C18orf19	0.083597534	2.214790892	0.027097078	0.04735489	no
CLSPN	0.083583047	2.214404365	0.027123758	0.047397419	no
IFT52	0.083570113	2.214059286	0.027147596	0.047434976	no
ITPA	0.083560763	2.213809849	0.027164839	0.047461001	no
RS1	-0.083512715	-2.212527949	0.0272536	0.047611967	no
XDH	0.083510924	2.212480154	0.027256915	0.047613643	no
INTS4L1	-0.083507613	-2.212391816	0.027263041	0.04762023	no
EEF1E1	-0.083468945	-2.211360188	0.027334677	0.047741232	no

CBLL1	-0.08346117	-2.211152748	0.027349101	0.047762298	no
CHODL	0.083456848	2.211037459	0.02735712	0.047772177	no
C2orf88	-0.083451136	-2.210885055	0.027367725	0.047786567	no
EIF4EBP3	0.083447942	2.210799844	0.027373655	0.047789612	no
ARHGAP8	0.08344765	2.210792072	0.027374196	0.047789612	no
ZNF560	-0.083442162	-2.210645657	0.027384389	0.047803279	no
FABP2	0.083407462	2.209719888	0.027448914	0.047911779	no
DUSP28	-0.083396093	-2.20941659	0.027470082	0.047944588	no
BRWD3	-0.083366836	-2.20863606	0.027524622	0.048035633	no
CHMP2A	0.083353589	2.208282643	0.027549348	0.048074634	no
AKAP9	-0.083348095	-2.208136068	0.027559608	0.048088389	no
TARBP2	0.083337088	2.207842412	0.027580175	0.048120122	no
C19orf50	0.083317063	2.207308203	0.027617622	0.0481813	no
CCDC70	0.083306134	2.20701663	0.027638079	0.048210803	no
ODF1	-0.083305481	-2.206999204	0.027639303	0.048210803	no
GSC2	-0.083286467	-2.206491968	0.027674924	0.048268746	no
RPUSD1	-0.083285202	-2.206458213	0.027677296	0.048268746	no
SLIT3	0.083271229	2.206085439	0.027703502	0.048310281	no
CTTN	0.083258084	2.20573476	0.027728174	0.048349135	no
ANKRD33	0.083244833	2.205381267	0.027753063	0.048386963	no
CBLC	0.083243987	2.205358675	0.027754655	0.048386963	no
ORMDL1	0.083242656	2.205323184	0.027757155	0.048387149	no
QRICH2	-0.083238827	-2.205221041	0.027764351	0.048394588	no
MASP2	-0.083237839	-2.20519467	0.027766209	0.048394588	no
DGKE	-0.083222455	-2.204784261	0.027795142	0.04844084	no
TMEM204	0.083216197	2.20461731	0.027806919	0.048457188	no
NCAPG	0.083214255	2.20456552	0.027810574	0.048459379	no
MSH3	0.083206386	2.204355583	0.027825391	0.04848102	no
C6orf222	-0.083194245	-2.204031696	0.027848264	0.048516691	no
PLEKHH3	-0.083184543	-2.203772874	0.027866554	0.048544373	no
PAK6	-0.08317271	-2.203457212	0.027888875	0.04857907	no
VSIG10	-0.083142368	-2.202647776	0.027946181	0.048674697	no
KIAA1383	0.083137587	2.202520232	0.02795522	0.048686246	no
CXCL2	0.083135198	2.202456493	0.027959738	0.048689921	no
B4GALT3	0.083113736	2.201883967	0.02800035	0.048756444	no
JAM3	0.083084419	2.201101873	0.02805591	0.048848982	no
OSTBETA	0.083078826	2.20095269	0.028066518	0.048863246	no
GSK3A	-0.083063875	-2.200553832	0.028094899	0.048908444	no
PPIP5K1	-0.083032135	-2.199707138	0.028155228	0.049009246	no
SNX13	0.082984251	2.198429786	0.028246454	0.049163808	no
PDE7B	0.082974032	2.198157198	0.028265954	0.049193515	no
SLN	0.082922815	2.196790931	0.028363871	0.049359678	no
ANXA9	-0.082915486	-2.196595441	0.028377905	0.04937985	no
OTC	-0.082889877	-2.19591231	0.028426994	0.049461012	no
PUSL1	0.082879396	2.195632735	0.028447104	0.049491744	no
PPAN	-0.082871174	-2.195413403	0.028462891	0.049514948	no
RFWD2	0.082857723	2.195054611	0.02848873	0.049555635	no
NR2C1	-0.082847692	-2.194787018	0.028508015	0.049584915	no
P2RY14	0.082835783	2.194469341	0.028530924	0.049620492	no
C14orf135	-0.082812606	-2.193851121	0.028575552	0.049693834	no
OR1D2	-0.082805753	-2.193668322	0.028588759	0.049712526	no

C19orf56	0.082781709	2.193026952	0.02863514	0.049788895	no
PTS	0.082766829	2.192630021	0.028663877	0.049834575	no
OR2B11	0.082744137	2.192024727	0.028707747	0.049906555	no
PDZD11	0.082728856	2.191617118	0.028737322	0.049953673	no
MCM2	0.082714679	2.191238972	0.028764783	0.049995459	no
ZNF335	-0.082713893	-2.191218001	0.028766306	0.049995459	no
LGI2	0.082710362	2.191123816	0.02877315	0.050003055	no
PSMD2	0.082701597	2.190890028	0.028790143	0.050028286	no
NPM1	-0.082636358	-2.189149846	0.028916902	0.050244235	no
HSF1	-0.082619686	-2.188705144	0.028949372	0.050296331	no
SFTA1P	0.082614051	2.188554851	0.028960353	0.050311085	no
FAM3C	0.082586083	2.187808851	0.029014911	0.050401535	no
ABCA8	0.082580633	2.187663493	0.029025552	0.050415688	no
ITLN2	0.082569746	2.18737309	0.029046821	0.050448297	no
A1BG	0.082552037	2.186900731	0.029081445	0.050504094	no
COPZ1	0.082550023	2.186847023	0.029085384	0.050506596	no
RBMX2	-0.082519987	-2.186045882	0.029144197	0.050600768	no
PDLIM2	0.082519771	2.186040111	0.029144621	0.050600768	no
CFC1B	-0.082505638	-2.185663161	0.029172329	0.050644526	no
IQCJ	-0.082491198	-2.185277991	0.029200665	0.050689366	no
COG8	-0.082480138	-2.184983009	0.029222382	0.050721739	no
RABGGTB	-0.082479146	-2.184956532	0.029224332	0.050721739	no
GABRA5	-0.082473784	-2.184813537	0.029234865	0.050735665	no
HIATL2	0.082469021	2.184686477	0.029244227	0.050747556	no
FLJ23867	-0.082460289	-2.184453588	0.029261393	0.050772988	no
POLM	0.082455455	2.184324648	0.029270901	0.050785127	no
AMAC1	-0.08243939	-2.183896164	0.029302517	0.050835618	no
CTR9	0.08241911	2.183355245	0.02934247	0.050896734	no
MID1IP1	-0.082418953	-2.183351056	0.02934278	0.050896734	no
ART1	-0.082393867	-2.182681955	0.029392267	0.050978198	no
TMEM120A	0.082385859	2.18246837	0.029408079	0.051000825	no
DHDPSL	-0.082384705	-2.182437591	0.029410358	0.051000825	no
MAP3K1	0.082376186	2.182210392	0.029427187	0.051021456	no
WDR4	0.082375547	2.18219334	0.02942845	0.051021456	no
FAM160B2	-0.082374851	-2.182174775	0.029429826	0.051021456	no
LOC100128164	-0.082371027	-2.182072786	0.029437384	0.051030183	no
LRRC37A2	-0.082351915	-2.181563028	0.029475184	0.051091329	no
EIF6	0.082347545	2.181446485	0.029483832	0.051101938	no
ADAMTS8	-0.082337403	-2.181175984	0.029503912	0.051132358	no
NDP	0.082331948	2.18103049	0.029514718	0.051146701	no
WHAMML1	-0.082327646	-2.180915741	0.029523242	0.051157089	no
CST3	0.08230245	2.180243732	0.029573208	0.051239276	no
GNPNAT1	0.082289227	2.179891053	0.029599459	0.051280366	no
DOC2B	-0.082271549	-2.179419559	0.029634586	0.051336823	no
TUBB2C	0.082240547	2.178592719	0.029696274	0.051439279	no
SIM2	-0.082215056	-2.17791284	0.02974708	0.05152287	no
FBXW8	-0.082188973	-2.177217198	0.029799142	0.051608621	no
WDR88	-0.08217177	-2.176758389	0.029833522	0.051663738	no
TIMP3	-0.082166807	-2.176626002	0.029843448	0.051676502	no
MUC5B	-0.082161548	-2.176485765	0.029853967	0.051690288	no
C12orf29	-0.082136045	-2.175805587	0.029905028	0.051774263	no

ANKLE2	0.082133547	2.175738972	0.029910032	0.051778494	no
IFNA21	-0.08212795	-2.1755897	0.02992125	0.051793479	no
VPRBP	-0.082117329	-2.175306432	0.029942547	0.051825907	no
ZNF763	-0.082109237	-2.17509061	0.029958782	0.051849568	no
TMEM54	0.082106425	2.175015622	0.029964425	0.051854895	no
SLC43A1	0.082100903	2.174868339	0.02997551	0.051869639	no
NUP160	0.082087016	2.174497967	0.030003402	0.05190998	no
BEND2	0.082086739	2.174490588	0.030003958	0.05190998	no
UBE2CBP	-0.082072923	-2.174122122	0.030031729	0.051953307	no
FBP2	0.082071724	2.174090143	0.03003414	0.051953307	no
TRMT12	0.082036136	2.173141017	0.030105782	0.052072779	no
PCDHGA2	-0.082021008	-2.172737559	0.030136281	0.052117128	no
NEK2	0.08202086	2.172733625	0.030136578	0.052117128	no
C1QTNF7	0.082018394	2.172667853	0.030141553	0.052121272	no
DHRS11	-0.082001447	-2.172215893	0.030175754	0.052175952	no
PCNXL2	-0.081999925	-2.172175295	0.030178828	0.052176804	no
ACPT	-0.081992647	-2.171981189	0.030193528	0.052197756	no
MMP17	-0.081978699	-2.171609207	0.030221717	0.052240806	no
OLFM2	-0.081977768	-2.171584399	0.030223598	0.052240806	no
XPR1	0.08197573	2.171530033	0.03022772	0.052243464	no
LRRIQ4	0.081947518	2.170777651	0.030284814	0.052337668	no
C6orf27	-0.081939347	-2.17055975	0.030301367	0.052361798	no
C17orf77	0.081926484	2.170216693	0.030327443	0.052402379	no
RAB3B	-0.081910766	-2.169797527	0.03035933	0.052452994	no
C4orf29	0.081902332	2.169572581	0.030376454	0.052478095	no
SMPX	-0.081900985	-2.169536675	0.030379188	0.052478334	no
KIAA0748	-0.081888492	-2.169203504	0.030404568	0.05251769	no
C1orf133	0.081881616	2.169020121	0.030418546	0.052537345	no
PDE1A	-0.081880131	-2.168980515	0.030421565	0.052538072	no
TMEM30B	0.081862239	2.168503371	0.030457962	0.052596436	no
HTRA2	-0.081858674	-2.168408313	0.030465218	0.052604473	no
RNF160	-0.081847503	-2.168110387	0.030487967	0.052632044	no
ABHD4	-0.081847214	-2.168102671	0.030488557	0.052632044	no
RPL30	-0.081846998	-2.168096933	0.030488995	0.052632044	no
GRIA1	-0.081833262	-2.167730611	0.030516988	0.05267587	no
GDNF	-0.081823252	-2.167463648	0.030537403	0.052706608	no
ACER1	0.081814747	2.167236839	0.030554756	0.052732058	no
ZNF93	-0.081802692	-2.16691535	0.030579368	0.052770029	no
RPL5	-0.081793116	-2.166659996	0.030598929	0.052799278	no
MPL	0.081760284	2.165784441	0.03066608	0.05290581	no
PROKR2	-0.081759434	-2.165761761	0.030667822	0.05290581	no
OR2J2	0.081759094	2.165752694	0.030668518	0.05290581	no
ZBED3	-0.081740623	-2.165260121	0.030706355	0.052966562	no
MKL2	0.081730976	2.165002845	0.030726133	0.052996157	no
FARP1	-0.081725696	-2.164862056	0.030736961	0.053010311	no
LOC157381	0.081713339	2.164532527	0.030762318	0.053049518	no
PHOSPHO2	0.081691004	2.1639369	0.030808196	0.053124103	no
MPV17L2	0.081677496	2.163576699	0.030835969	0.05316746	no
ARGLU1	-0.081654263	-2.162957139	0.030883791	0.053245372	no
SLC22A12	-0.081652233	-2.162902991	0.030887973	0.053248043	no
HMOX2	-0.081641293	-2.162611258	0.030910516	0.05328236	no

LOC222699	0.081631396	2.162347347	0.030930921	0.05330945	no
NEK10	0.081631113	2.162339799	0.030931504	0.05330945	no
TMEM151A	-0.081621387	-2.162080429	0.03095157	0.053339485	no
KBTBD4	-0.081609538	-2.161764463	0.030976028	0.053377085	no
FBXL18	-0.081578205	-2.160928905	0.031040789	0.05348412	no
INO80E	-0.081530585	-2.159659067	0.031139431	0.053649511	no
CSN3	0.081527578	2.159578884	0.031145669	0.053655686	no
PICK1	0.081524102	2.159486169	0.031152883	0.053663541	no
ATP2C1	0.081517368	2.15930661	0.031166858	0.053683041	no
KLF9	-0.081507754	-2.159050237	0.031186821	0.05371285	no
OR4C6	0.081493573	2.158672091	0.031216286	0.053759018	no
CPS1	-0.08148632	-2.158478674	0.031231367	0.053780408	no
KRBA2	-0.081474721	-2.158169389	0.031255494	0.053817372	no
NFYC	0.08147084	2.158065887	0.031263572	0.053826697	no
CCDC24	0.081458206	2.157728991	0.031289877	0.0538674	no
TTC35	-0.08145449	-2.157629914	0.031297617	0.053876137	no
CDC20B	0.081445421	2.157388077	0.031316516	0.05390408	no
DYRK4	0.081436764	2.157157239	0.031334564	0.053930555	no
NDUFS5	0.081416161	2.156607855	0.031377555	0.053999949	no
TUSC3	0.081404205	2.156289044	0.031402526	0.054038324	no
DES	0.081383266	2.155730695	0.0314463	0.054109046	no
ELAVL1	-0.081373596	-2.155472837	0.031466533	0.054135746	no
ABCG5	-0.081373291	-2.155464694	0.031467172	0.054135746	no
TNFRSF13C	-0.081365286	-2.15525126	0.031483929	0.054159965	no
OVOL2	-0.081342884	-2.154653895	0.031530868	0.054236097	no
RNF24	0.081340813	2.15459868	0.031535209	0.054236816	no
WDR76	0.081339667	2.154568122	0.031537612	0.054236816	no
VSTM2A	-0.081337786	-2.154517959	0.031541558	0.054236816	no
RBM16	-0.081337567	-2.154512124	0.031542016	0.054236816	no
ABHD5	0.081320348	2.154052995	0.031578145	0.054294322	no
BEAN	0.081316481	2.153949879	0.031586264	0.054303664	no
MYL5	0.081289576	2.153232453	0.031642802	0.054396239	no
ZNF574	-0.081288289	-2.153198141	0.031645508	0.054396266	no
BCL9	-0.081285668	-2.153128253	0.031651021	0.054401117	no
PSMB1	0.08128329	2.153064847	0.031656023	0.054405089	no
INCA1	-0.081275323	-2.152852416	0.031672786	0.054429273	no
HMG20B	0.0812724	2.152774458	0.03167894	0.054435221	no
CLTC	0.081265934	2.152602056	0.031692553	0.054453984	no
C20orf165	-0.081260333	-2.152452691	0.031704351	0.054469626	no
HABP2	0.081251365	2.152213579	0.031723245	0.054497457	no
LOC154449	-0.081244713	-2.15203622	0.031737266	0.054516911	no
PAPD5	-0.081239898	-2.151907819	0.03174742	0.05452972	no
HS6ST2	-0.08122896	-2.151616158	0.031770495	0.054564719	no
CLK2P	0.08122645	2.15154925	0.03177579	0.054569178	no
C6orf103	0.081201907	2.150894819	0.031827626	0.054653554	no
GJB6	-0.081168806	-2.150012228	0.031897649	0.054769143	no
C2orf47	0.081157658	2.149714961	0.031921263	0.054805035	no
CCDC74B	0.081121158	2.148741746	0.031998678	0.054933282	no
DPY19L2	-0.081098457	-2.14813646	0.032046907	0.055011408	no
MEGF10	0.081091056	2.147939136	0.032062643	0.055032912	no
GPRC5C	0.081090006	2.14791112	0.032064878	0.055032912	no

GALNTL4	-0.081079646	-2.147634909	0.032086918	0.055066064	no
DIAPH3	0.081051788	2.146892107	0.032146253	0.055159329	no
C6orf120	0.081051569	2.146886274	0.032146719	0.055159329	no
NTN3	-0.081047388	-2.146774783	0.032155634	0.055169942	no
LCE1E	-0.081010132	-2.145781441	0.032235149	0.055301676	no
MDM1	0.080988135	2.145194937	0.032282177	0.055377657	no
HAO1	0.08096343	2.144536245	0.032335064	0.055459782	no
TFDP3	0.080963209	2.144530344	0.032335538	0.055459782	no
LSM14A	0.080881616	2.142354901	0.032510736	0.055755541	no
REP15	0.080866961	2.141964169	0.03254229	0.055804921	no
CRYGS	0.080834631	2.141102187	0.032611992	0.055919706	no
OR1N1	0.080819066	2.140687214	0.032645594	0.055972576	no
WIT1	0.080810141	2.140449265	0.032664875	0.056000885	no
LOC284009	-0.080806831	-2.140360996	0.032672029	0.056008402	no
ZNF382	-0.080779258	-2.139625859	0.03273167	0.056105884	no
ITM2A	0.080777351	2.139575028	0.032735797	0.056108202	no
KHDRBS1	-0.080759235	-2.139092025	0.032775038	0.056168858	no
MS4A10	0.080758448	2.139071052	0.032776743	0.056168858	no
SALL3	-0.080752121	-2.138902369	0.032790457	0.056187597	no
NCRNA00175	-0.080730069	-2.138314445	0.032838295	0.056264801	no
LOC284688	0.080716614	2.137955712	0.032867514	0.056310092	no
GTF3C4	-0.08071382	-2.137881222	0.032873584	0.05631572	no
GGNBP2	-0.080697114	-2.137435818	0.032909899	0.056373154	no
CLRN2	-0.080688972	-2.137218746	0.03292761	0.056398714	no
BSPRY	-0.08068637	-2.13714939	0.03293327	0.056403631	no
FOXO1	0.080664781	2.136573818	0.032980278	0.056479354	no
FLJ43663	-0.080653311	-2.136268017	0.033005276	0.056517377	no
ZNF576	0.080639883	2.135910009	0.033034563	0.056548936	no
GPR68	0.080639815	2.135908201	0.033034711	0.056548936	no
USP32	-0.080639798	-2.135907743	0.033034749	0.056548936	no
CADPS2	0.080639731	2.135905967	0.033034894	0.056548936	no
RPL29P2	-0.080626528	-2.135553961	0.033063712	0.056593475	no
ACRC	-0.080621145	-2.135410442	0.033075468	0.056608804	no
KCNC4	-0.080595131	-2.134716928	0.033132324	0.056701315	no
KIF4B	0.080589317	2.13456192	0.033145044	0.056718281	no
C17orf58	0.080586038	2.134474501	0.033152219	0.056725759	no
IL17B	0.080578676	2.134278224	0.033168334	0.05674853	no
IL4	0.08054734	2.13344281	0.033236999	0.056861199	no
HSPB2	0.080537043	2.133168289	0.03325959	0.056895032	no
HOXA2	0.080531436	2.133018803	0.033271897	0.05691127	no
CCDC91	0.080525098	2.132849841	0.033285811	0.056928433	no
KIAA0355	0.080523986	2.132820204	0.033288253	0.056928433	no
CRYAA	-0.080523018	-2.132794403	0.033290378	0.056928433	no
SNRPB	0.080510869	2.13247051	0.03331707	0.056969258	no
EPS8L3	0.080498422	2.132138681	0.033344434	0.057011227	no
TDP1	0.080467158	2.131305208	0.033413251	0.057121278	no
C1orf56	-0.080466614	-2.1312907	0.03341445	0.057121278	no
CSAG1	0.080464168	2.131225491	0.03341984	0.057125661	no
CSRP2	0.080443979	2.130687266	0.033464351	0.057196911	no
NCOA3	0.080429434	2.130299496	0.033496452	0.057246937	no
CHKB	0.080419666	2.130039113	0.033518022	0.05727896	no

TIPRL	0.080407322	2.129710031	0.0335453	0.05732073	no
CCNI	-0.080403302	-2.129602863	0.033554187	0.057331071	no
AKR1B15	0.080362411	2.128512744	0.033644705	0.057480873	no
TROVE2	-0.080320794	-2.127403306	0.033737042	0.057633757	no
CDC42SE2	0.080312305	2.127177012	0.033755903	0.057661105	no
CCDC9	0.080305588	2.126997949	0.033770833	0.057681736	no
HBB	0.080289466	2.12656816	0.033806693	0.057733146	no
SIN3A	-0.080287825	-2.126524417	0.033810344	0.057733146	no
HIST1H2BE	0.08028781	2.126524026	0.033810377	0.057733146	no
IL1RAPL2	-0.080286922	-2.12650034	0.033812354	0.057733146	no
PLA2G10	-0.080263931	-2.125887441	0.033863556	0.057815687	no
MCM3AP	-0.080256853	-2.125698757	0.033879332	0.057833561	no
SCEL	-0.080256667	-2.125693807	0.033879746	0.057833561	no
IWS1	-0.080244321	-2.125364686	0.033907279	0.057875675	no
RAB30	-0.080225809	-2.124871194	0.033948599	0.057941312	no
SMG1	-0.08022142	-2.124754188	0.033958402	0.057953151	no
USP1	0.08019692	2.124101095	0.034013166	0.05804171	no
ARL14	0.080174692	2.123508544	0.034062918	0.058121704	no
P2RX1	0.080173394	2.123473947	0.034065825	0.058121759	no
HN1	-0.080166959	-2.123302398	0.034080241	0.058141449	no
PPP1R3G	0.080141286	2.122618042	0.034137804	0.058234738	no
PNLIP	0.080135553	2.122465205	0.034150671	0.058251773	no
CDKL2	-0.08012818	-2.122268652	0.034167224	0.058275092	no
MPHOSPH6	-0.080111573	-2.121825964	0.034204532	0.058333802	no
SPRR4	-0.080093932	-2.12135573	0.034244199	0.058396526	no
ADAL	-0.080090228	-2.12125697	0.034252535	0.058405815	no
TEPP	0.080088526	2.12121162	0.034256363	0.058407417	no
RAB21	0.080078904	2.120955113	0.034278025	0.058439422	no
RPS28	-0.080055755	-2.120338047	0.034330182	0.058523408	no
TIMM50	-0.080048449	-2.120143282	0.034346658	0.058544064	no
C9orf53	0.080047814	2.120126357	0.03434809	0.058544064	no
HERC2P4	-0.080039438	-2.119903083	0.034366988	0.058571336	no
ZNF767	-0.08002421	-2.119497175	0.034401365	0.058624984	no
ELOVL7	0.080005958	2.119010632	0.034442611	0.058690326	no
BUB1	0.079988936	2.118556895	0.034481114	0.058750983	no
DDX53	0.07991969	2.11671107	0.034638126	0.059013536	no
FAM75C1	-0.07991083	-2.116474896	0.03465826	0.059042863	no
GAPDHS	-0.079873262	-2.115473507	0.034743739	0.059183497	no
SNORD115-7	-0.079847697	-2.114792057	0.034802012	0.059277765	no
MOBKL1A	0.079837202	2.114512327	0.034825956	0.059313553	no
HLTF	-0.079833577	-2.11441569	0.034834232	0.059322649	no
C10orf68	-0.079825112	-2.114190067	0.034853559	0.059350565	no
TMC2	-0.079819804	-2.114048562	0.034865685	0.059366214	no
KDM1A	-0.07980347	-2.113613192	0.034903017	0.059424774	no
IFNA14	0.079798492	2.113480508	0.034914401	0.059439151	no
TP53BP1	-0.079741021	-2.111948616	0.035046065	0.059658275	no
CCNG2	-0.079712836	-2.111197377	0.035110788	0.059760441	no
IFNG	0.079712312	2.111183393	0.035111993	0.059760441	no
WDR74	-0.07970671	-2.111034082	0.03512487	0.059777325	no
RBCK1	0.079693604	2.110684746	0.035155012	0.059823586	no
RAD18	0.079690836	2.110610962	0.035161381	0.059829389	no

LOC100125556	0.079669862	2.110051926	0.035209671	0.059906515	no
LZTS2	-0.079665507	-2.109935854	0.035219704	0.059918544	no
TRPM5	-0.079660583	-2.109804597	0.035231053	0.059932808	no
EXTL1	-0.079644942	-2.1093877	0.03526712	0.059989116	no
PGBD1	-0.079616412	-2.108627281	0.035332988	0.060096101	no
EIF4EBP1	0.07959833	2.108145307	0.035374791	0.06016214	no
DHRS4L2	-0.079593124	-2.108006566	0.035386832	0.060177557	no
C1orf94	-0.079584393	-2.107773845	0.035407038	0.060206854	no
GRB14	0.079578615	2.107619854	0.035420414	0.060224533	no
NR1H2	0.079576097	2.107552732	0.035426245	0.060229383	no
FAM20B	-0.079474808	-2.10485305	0.035661472	0.060624203	no
APOBEC2	-0.079469827	-2.104720303	0.035673073	0.060638825	no
SETD1B	-0.079461695	-2.104503547	0.035692022	0.060665936	no
APLP1	-0.079459938	-2.104456716	0.035696117	0.060667796	no
RASSF6	0.079455116	2.104328215	0.035707356	0.060681796	no
HAUS3	-0.079449592	-2.104180965	0.035720239	0.060698587	no
RPA1	0.079368796	2.102027551	0.035909091	0.06101437	no
FAM71E2	0.079354517	2.101647006	0.035942552	0.061066094	no
ZNF623	-0.079349349	-2.101509266	0.035954671	0.061076669	no
GTF2A1	-0.079349286	-2.101507591	0.035954818	0.061076669	no
FMO3	0.079331513	2.101033905	0.035996519	0.06114237	no
RGPD6	-0.079329859	-2.100989803	0.036000404	0.061143831	no
METTL12	-0.079324201	-2.100839026	0.036013688	0.061161254	no
BRI3BP	0.079312795	2.100535016	0.036040484	0.061201621	no
CTPS	0.079311148	2.100491123	0.036044354	0.061203053	no
B3GALT5	0.079308029	2.100408007	0.036051684	0.061210358	no
SYCE1L	0.079289944	2.099926004	0.036094215	0.061277424	no
HCG4P6	-0.079283061	-2.099742572	0.036110412	0.061299775	no
NUDT14	0.079276559	2.099569258	0.036125722	0.061320615	no
DIDO1	-0.079268655	-2.099358612	0.036144336	0.061347061	no
PRMT3	-0.079253585	-2.098956969	0.036179852	0.061402186	no
CGN	-0.079244206	-2.098707006	0.03620197	0.061434567	no
PTPRK	-0.079239527	-2.098582312	0.036213008	0.06144814	no
MRS2	0.079235911	2.098485942	0.036221541	0.061457461	no
AQP10	0.079227708	2.098267325	0.036240903	0.061485154	no
ZNF772	-0.079213422	-2.097886584	0.036274647	0.061537238	no
RHOXF1	-0.079205661	-2.097679739	0.036292989	0.061558411	no
SERPINC1	0.079205426	2.097673491	0.036293544	0.061558411	no
PITPNA	-0.079204277	-2.097642852	0.036296261	0.061558411	no
TUBA3D	-0.079190796	-2.097283574	0.036328143	0.061607314	no
FAM153B	-0.079184707	-2.097121302	0.036342551	0.061626578	no
FBXL20	-0.079166774	-2.09664337	0.036385013	0.061693408	no
RXRA	-0.079125535	-2.095544321	0.036482821	0.06185406	no
DYNC2LI1	0.079121769	2.095443948	0.036491764	0.061864036	no
PRKAG2	-0.079113463	-2.095222596	0.036511494	0.061892294	no
LOC100130238	-0.07910939	-2.095114027	0.036521175	0.061903514	no
OPN1MW	0.079107103	2.095053094	0.036526609	0.061907534	no
SMAD3	-0.079089046	-2.094571855	0.03656955	0.061975119	no
SGCD	-0.079069189	-2.094042671	0.036616819	0.062043802	no
JPH2	0.079068446	2.094022852	0.036618591	0.062043802	no
STXBP4	0.079068154	2.094015092	0.036619284	0.062043802	no

RAB1B	0.07906511	2.093933966	0.036626536	0.062050888	no
C6orf164	-0.079032625	-2.09306822	0.036703998	0.062176912	no
OR10P1	0.079016602	2.092641211	0.036742257	0.062236507	no
KCNV2	-0.079013962	-2.092570863	0.036748563	0.062241974	no
RABIF	0.078990026	2.091932979	0.036805786	0.062333672	no
ZNF619	-0.078979731	-2.091658602	0.036830423	0.062370173	no
C21orf49	-0.078971947	-2.091451158	0.03684906	0.062396506	no
BCL2L10	-0.078938589	-2.090562203	0.036929013	0.062526655	no
RAB11FIP5	0.078874994	2.088867431	0.037081852	0.062780179	no
GTPBP5	-0.078853308	-2.088289526	0.037134093	0.062863359	no
NPRL2	-0.078837721	-2.087874145	0.037171681	0.062921721	no
CA9	0.078833243	2.087754804	0.037182486	0.062934742	no
CSAD	-0.078815853	-2.087291382	0.037224469	0.062995728	no
PRCC	-0.078815737	-2.087288304	0.037224748	0.062995728	no
RGMA	-0.078794884	-2.086732603	0.037275145	0.063075736	no
UBXN7	-0.078791307	-2.086637279	0.037283796	0.063085095	no
NOMO2	-0.07877566	-2.086220305	0.037321658	0.063143873	no
ZNF404	-0.078766538	-2.085977224	0.037343745	0.063175955	no
MAP1LC3B2	0.078756055	2.08569786	0.037369143	0.063210412	no
PKHD1	0.078754887	2.08566675	0.037371972	0.063210412	no
GAST	0.07875426	2.085650033	0.037373492	0.063210412	no
C11orf1	-0.078748728	-2.085502618	0.037386901	0.063227802	no
PRKAG1	0.078733128	2.085086895	0.037424738	0.063281915	no
RACGAP1P	-0.078731762	-2.085050504	0.037428052	0.063281915	no
HCRTR2	-0.078731665	-2.085047905	0.037428289	0.063281915	no
NMBR	-0.078726676	-2.084914977	0.037440395	0.063297091	no
EML1	0.07871301	2.084550806	0.037473579	0.063347894	no
DDX11	-0.078708429	-2.084428728	0.037484708	0.06336141	no
CCND2	-0.078700528	-2.084218161	0.037503911	0.063388571	no
B3GALTL	0.07869432	2.08405274	0.037519004	0.063408778	no
SH3GL1	-0.07868508	-2.083806514	0.037541477	0.063441457	no
EXOC6B	-0.078679643	-2.083661631	0.037554707	0.063457667	no
SGCA	0.078678558	2.083632721	0.037557347	0.063457667	no
MCHR1	0.078668177	2.083356083	0.037582619	0.063495061	no
FBXO47	0.078666557	2.083312926	0.037586563	0.063496418	no
PCSK4	-0.078659434	-2.083123119	0.037603913	0.06352042	no
ROMO1	0.078634181	2.082450176	0.03766548	0.063619104	no
HAS2AS	0.07862535	2.082214855	0.03768703	0.063650185	no
ZDHHC23	0.078611069	2.081834294	0.037721902	0.063700024	no
GPR149	-0.078610685	-2.081824063	0.03772284	0.063700024	no
KCNC3	-0.078570321	-2.080748469	0.037821552	0.063861378	no
MUM1L1	-0.078544042	-2.080048215	0.037885936	0.063961076	no
DHX40	0.078543638	2.080037464	0.037886925	0.063961076	no
FGF22	-0.078538221	-2.079893096	0.037900211	0.063978163	no
CP110	-0.078534446	-2.079792528	0.037909468	0.063988447	no
ZNF334	-0.0785322	-2.079732667	0.037914979	0.063992407	no
OR2T4	-0.078511769	-2.07918825	0.037965132	0.064071706	no
YOD1	-0.078507022	-2.079061749	0.037976793	0.064086037	no
FAM100B	0.078501816	2.078923033	0.037989585	0.064102272	no
PPP1R1C	0.078498469	2.078833842	0.037997811	0.064110802	no
CHP2	-0.078494905	-2.078738874	0.038006572	0.064119446	no

ANXA3	-0.078493805	-2.078709551	0.038009277	0.064119446	no
CDK2AP2	0.078478061	2.078290046	0.038048	0.064179414	no
RAB31	-0.078459474	-2.077794764	0.03809376	0.064251242	no
ATP5B	-0.07843187	-2.077059214	0.038161806	0.064360644	no
SUMF2	0.07842297	2.076822056	0.038183768	0.064388144	no
NRTN	-0.078422681	-2.076814352	0.038184481	0.064388144	no
COMP	0.078414453	2.07659513	0.038204792	0.06441702	no
RNF138P1	0.078411419	2.076514284	0.038212285	0.06442428	no
LOC727896	-0.078408717	-2.076442285	0.038218958	0.064430159	no
C10orf120	-0.078397036	-2.076131016	0.038247822	0.064473442	no
RPTN	0.078391947	2.075995435	0.0382604	0.064489268	no
ZNF471	-0.078389365	-2.075926616	0.038266786	0.064494654	no
ARRDC1	0.078378812	2.075645415	0.038292888	0.064533268	no
MIIP	-0.078367409	-2.075341583	0.038321108	0.064575443	no
PSTPIP1	-0.078360282	-2.075151673	0.038338756	0.064599798	no
ZC3H14	-0.078340972	-2.074637143	0.038386605	0.064675032	no
ZMYM5	0.078315002	2.073945162	0.038451037	0.06477819	no
MEIS1	0.078293238	2.073365252	0.038505104	0.064863872	no
GGNBP1	-0.078279561	-2.073000814	0.038539115	0.064915491	no
CAPRIN2	0.078278333	2.072968117	0.038542168	0.064915491	no
HEY2	-0.078267313	-2.072674474	0.038569593	0.06495627	no
GNA13	0.078264957	2.072611701	0.038575457	0.064960736	no
PPP3R1	-0.078253179	-2.07229787	0.03860479	0.065004717	no
LOC100131551	0.078245281	2.072087429	0.038624469	0.065032438	no
SUN1	0.078229087	2.071655951	0.038664846	0.065095	no
CRYBG3	0.07822745	2.071612318	0.038668931	0.065096457	no
OR4N2	-0.078216538	-2.071321571	0.038696161	0.065136643	no
CACNA1H	-0.078215302	-2.071288642	0.038699246	0.065136643	no
TMED6	0.078204603	2.071003568	0.038725963	0.065176185	no
ERLIN2	0.078185902	2.070505294	0.038772698	0.065249409	no
MCM3	0.078177963	2.070293751	0.038792553	0.06527739	no
CAPN12	0.078176428	2.070252845	0.038796394	0.06527842	no
TOMM6	0.078166016	2.069975435	0.038822447	0.065316821	no
ESRRG	-0.078116944	-2.068667938	0.038945445	0.065518306	no
HSPA14	-0.078085835	-2.067839046	0.039023591	0.06564431	no
EIF2B1	0.078066579	2.067325999	0.039072027	0.065715418	no
SYNPO2L	-0.078066445	-2.067322434	0.039072363	0.065715418	no
TSEN34	0.078044562	2.066739383	0.039127471	0.065802629	no
MCART1	0.078024809	2.066213085	0.039177271	0.0658809	no
HSPA6	0.077991398	2.065322906	0.039261625	0.06601726	no
C5orf24	-0.077977822	-2.06496118	0.039295947	0.066069476	no
CLPS	0.077967962	2.064698482	0.039320888	0.066103933	no
TRIM72	-0.077967135	-2.064676461	0.03932298	0.066103933	no
FOXG1	-0.077954669	-2.064344325	0.039354535	0.066151479	no
NEBL	-0.077946517	-2.06412712	0.039375182	0.066180683	no
WDHD1	0.077938782	2.063921047	0.03939478	0.066208119	no
IFIT5	-0.077921969	-2.063473092	0.03943741	0.066274255	no
C15orf40	0.07790575	2.063040949	0.039478572	0.066337914	no
FNBP1L	0.077902407	2.062951901	0.039487059	0.06634666	no
SLC39A2	0.077900953	2.062913145	0.039490753	0.066347352	no
ALOX12P2	0.077886479	2.062527528	0.039527524	0.066403613	no

TNRC18	-0.077881434	-2.0623931	0.039540349	0.06641964	no
C14orf19	0.077879037	2.062329246	0.039546443	0.066423096	no
PPIL2	-0.07787804	-2.062302681	0.039548978	0.066423096	no
B3GALNT1	0.077872961	2.062167376	0.039561893	0.066439268	no
SFRS12IP1	0.077857137	2.061745769	0.039602159	0.066497464	no
CDKL5	-0.077856758	-2.061735668	0.039603124	0.066497464	no
SLC4A9	-0.077847609	-2.061491926	0.039626419	0.066531054	no
PIN1L	-0.077831736	-2.061069037	0.039666864	0.066593428	no
TMED1	0.077829066	2.060997884	0.039673672	0.066599328	no
CCDC104	-0.077802777	-2.060297501	0.039740743	0.06670638	no
BET3L	0.077792258	2.060017243	0.039767608	0.066745933	no
FAM55B	-0.077711632	-2.057869196	0.039974032	0.067086824	no
ANO7	0.077703955	2.057664681	0.039993733	0.067114317	no
GPR87	0.077668862	2.056729738	0.040083901	0.067260047	no
NDUFA2	0.077655191	2.056365531	0.040119073	0.067313478	no
SLC6A18	-0.077633717	-2.05579343	0.040174374	0.067400671	no
PDE6D	-0.077632126	-2.055751034	0.040178475	0.067401958	no
PRSS50	-0.077628229	-2.055647221	0.040188517	0.067413212	no
BEGAIN	-0.077623686	-2.055526181	0.040200229	0.067427263	no
KIAA0319	-0.077618703	-2.055393447	0.040213076	0.067443216	no
PHF13	0.077616448	2.055333372	0.040218892	0.067447375	no
C1orf92	0.077611046	2.05518944	0.040232828	0.06746515	no
BAG2	0.077602684	2.054966684	0.040254404	0.067495733	no
C9orf9	0.077589732	2.054621621	0.040287847	0.067546206	no
PXT1	-0.077585279	-2.054502998	0.040299349	0.067559888	no
C17orf59	-0.077576921	-2.054280339	0.040320947	0.06759049	no
LIPF	0.077563879	2.053932864	0.040354671	0.067641414	no
PLCB3	-0.077556904	-2.053747053	0.040372714	0.067666048	no
ZNF473	0.077555393	2.053706796	0.040376624	0.067666992	no
ITPKB	0.077540744	2.05331655	0.040414546	0.067723812	no
PLIN1	-0.077539709	-2.053288957	0.040417228	0.067723812	no
C1orf128	-0.07752803	-2.052977821	0.040447485	0.067768895	no
ZNF430	-0.077509443	-2.052482656	0.040495678	0.06784402	no
PRSS22	0.077497186	2.052156125	0.040527486	0.067891682	no
STOML2	0.077467159	2.051356196	0.040605496	0.068016729	no
ZNF813	0.077464009	2.051272298	0.040613685	0.068024811	no
ZNF670	-0.077455737	-2.051051935	0.040635202	0.068055211	no
TMEM150C	0.077446625	2.050809174	0.040658916	0.068089287	no
CCDC75	-0.077440025	-2.050633349	0.040676099	0.068109037	no
GRIN3B	0.077439507	2.050619561	0.040677447	0.068109037	no
C12orf73	-0.077432441	-2.050431309	0.040695851	0.06813421	no
RHBDL2	0.077430841	2.050388702	0.040700018	0.068135544	no
LOC730101	-0.077409628	-2.049823584	0.040755315	0.068222467	no
GOLGA6L6	-0.077379285	-2.049015247	0.040834522	0.068349398	no
C3orf20	-0.077369228	-2.048747344	0.040860802	0.068387724	no
PCDHA4	-0.077322958	-2.047514739	0.040981901	0.068584725	no
TINF2	0.077314899	2.047300028	0.041003026	0.0686144	no
PIP5K1C	-0.077312434	-2.047234367	0.041009488	0.068619535	no
CETN4P	0.077273952	2.046209236	0.041110493	0.068782849	no
MAPK15	0.077259149	2.045814921	0.0411494	0.068842248	no
RAD51	0.07724624	2.04547104	0.041183357	0.068893356	no

NT5C3L	-0.077229694	-2.045030256	0.041226917	0.06896052	no
NCRNA00202	-0.077219077	-2.044747443	0.041254886	0.069001595	no
CTRC	0.077195135	2.044109661	0.04131802	0.069101474	no
OOEP	0.07718418	2.043817835	0.041346935	0.069139269	no
USP39	0.077183982	2.043812557	0.041347458	0.069139269	no
KCTD19	0.077168498	2.043400103	0.041388355	0.069201932	no
FAM66A	-0.077114316	-2.041956791	0.041531737	0.06943324	no
MOS	-0.077113627	-2.041938421	0.041533565	0.06943324	no
C1orf25	0.077102009	2.041628957	0.041564363	0.069478983	no
ITGA2B	-0.077096359	-2.041478447	0.04157935	0.069498288	no
ANK2	-0.077087563	-2.041244151	0.041602688	0.069531548	no
KLK7	-0.077074072	-2.040884756	0.041638508	0.069585664	no
ADAMTS5	-0.077058428	-2.040468052	0.041680073	0.06964937	no
HIST1H2AG	0.077026028	2.039604987	0.041766274	0.069787647	no
BCL11A	-0.07702351	-2.039537906	0.04177298	0.069793084	no
CDK11B	-0.077013926	-2.039282617	0.04179851	0.069829968	no
RDBP	-0.076997343	-2.038840887	0.041842716	0.069898045	no
IL17REL	0.076989458	2.038630853	0.041863749	0.069927403	no
PGAP3	-0.076984661	-2.038503094	0.041876548	0.069940372	no
KLHL1	-0.076983011	-2.03845914	0.041880952	0.069940372	no
CUTA	-0.076982659	-2.038449757	0.041881892	0.069940372	no
C12orf75	0.076971963	2.038164845	0.041910448	0.069982279	no
CEACAM19	-0.076967399	-2.038043273	0.041922638	0.069993369	no
TK2	0.076966884	2.038029559	0.041924013	0.069993369	no
STK32A	-0.076956582	-2.037755127	0.041951542	0.070029852	no
EMG1	0.076956114	2.037742662	0.041952793	0.070029852	no
DSCR3	0.07695131	2.037614706	0.041965634	0.070045504	no
ENPP4	0.076932132	2.037103879	0.042016932	0.070125337	no
RNASEH1	0.076918579	2.036742844	0.04205322	0.070180108	no
ALDH7A1	-0.076915825	-2.036669497	0.042060595	0.070186622	no
ZNF566	-0.076900147	-2.036251886	0.042102609	0.070248823	no
FERMT2	0.076899322	2.036229925	0.042104819	0.070248823	no
DACT1	0.076895498	2.036128059	0.042115073	0.070260133	no
MAGEC3	-0.076892573	-2.036050149	0.042122917	0.070267421	no
MRPS34	-0.076879652	-2.035705959	0.042157585	0.070319451	no
DEFA1B	0.076862151	2.035239792	0.042204578	0.070392027	no
CLDN20	0.076847187	2.034841231	0.042244791	0.070449277	no
LOC90834	-0.076846785	-2.034830521	0.042245872	0.070449277	no
AMPD2	-0.076839731	-2.034642623	0.042264841	0.070475098	no
SOLH	0.076825942	2.034275342	0.042301941	0.070531144	no
OR56B4	0.076820695	2.034135574	0.042316067	0.070548878	no
ORAOV1	-0.076811361	-2.033886951	0.042341204	0.070584966	no
DALRD3	0.076790156	2.033322136	0.042398357	0.070674415	no
NOL12	-0.076768267	-2.032739087	0.042457423	0.070767039	no
POLR2A	-0.076758447	-2.032477539	0.042483942	0.070805403	no
ART5	0.076750296	2.032260421	0.042505967	0.0708293	no
PRDM6	0.076750276	2.0322599	0.04250602	0.0708293	no
C16orf68	-0.076749251	-2.032232596	0.042508791	0.0708293	no
FAM184A	-0.076734391	-2.03183677	0.042548971	0.070890407	no
PDXDC2	-0.076716085	-2.031349183	0.04259851	0.070967096	no
CHIC2	0.076712228	2.031246471	0.042608952	0.070978643	no

ISOC2	0.076709475	2.03117314	0.042616408	0.070985216	no
SRR	-0.076696018	-2.030814684	0.042652872	0.0710401	no
CLEC3B	0.076693596	2.030750183	0.042659436	0.07104518	no
ATAD2B	-0.076682689	-2.030459665	0.042689012	0.07108858	no
TIMM13	-0.076670991	-2.030148083	0.042720752	0.071135576	no
PREP	0.076646058	2.029484001	0.042788466	0.071242461	no
LSM12	0.076639013	2.029296356	0.042807616	0.071268477	no
TRPC5	-0.076612583	-2.028592387	0.042879524	0.071382315	no
MIS12	0.076603575	2.028352459	0.042904055	0.071417273	no
KCNK15	0.076590668	2.028008687	0.042939225	0.07146993	no
FAM89A	0.076571055	2.027486309	0.042992713	0.071553068	no
PGPEP1L	-0.076567211	-2.027383922	0.043003203	0.071564636	no
PIGQ	-0.076557182	-2.027116799	0.043030582	0.071604306	no
SNX4	0.076553643	2.027022543	0.043040247	0.071614493	no
ZNF557	0.076551647	2.026969375	0.043045699	0.071617672	no
EFCAB6	0.076546054	2.026820414	0.043060978	0.071637197	no
CYLC1	0.076518622	2.026089783	0.043135985	0.071756076	no
PRR5L	0.076496197	2.02549251	0.043197384	0.071852301	no
OTOS	0.076486934	2.025245781	0.043222769	0.071888611	no
JUND	-0.076478825	-2.025029812	0.043245	0.071919668	no
C22orf30	-0.076454337	-2.0243776	0.043312194	0.072025492	no
FNDC4	0.076451361	2.024298332	0.043320366	0.072033157	no
FRMPD2L1	-0.076430365	-2.023739131	0.043378057	0.072123154	no
SPINK6	0.076423752	2.023563001	0.043396241	0.072147455	no
NDUFA9	0.076414379	2.023313361	0.043422026	0.072184387	no
SEC1	-0.076398221	-2.022883024	0.043466505	0.072249935	no
EIF1B	-0.076397459	-2.022862737	0.043468602	0.072249935	no
PKP2	0.076381316	2.022432785	0.043513082	0.072317919	no
NDUFA3	0.076371245	2.022164553	0.04354085	0.072358122	no
C6orf146	-0.076367485	-2.022064405	0.043551222	0.07236941	no
ATP4A	-0.076364166	-2.021976014	0.043560378	0.072378675	no
LOC388789	0.076361222	2.02189762	0.0435685	0.072386221	no
RNF214	-0.076310464	-2.02054575	0.043708755	0.072613279	no
FER1L5	0.076306811	2.020448474	0.043718862	0.072624103	no
FAM63A	-0.076299011	-2.020240724	0.043740454	0.072649255	no
NUMBL	0.076298745	2.020233644	0.04374119	0.072649255	no
LOC646627	-0.076292955	-2.02007944	0.043757223	0.072669914	no
C6orf125	-0.076290779	-2.020021487	0.04376325	0.072673953	no
RGL1	0.076285978	2.019893634	0.043776548	0.072690067	no
ZFAND5	-0.076256451	-2.019107231	0.04385842	0.072820032	no
CACNB3	-0.076244117	-2.01877875	0.043892656	0.072870892	no
OR10G9	0.076231946	2.018454597	0.043926463	0.072919491	no
ADAM7	0.076230982	2.018428919	0.043929143	0.072919491	no
DCTN5	0.076216163	2.018034252	0.043970336	0.072981878	no
IGSF22	-0.076214577	-2.01799201	0.043974747	0.072983207	no
LHFPL1	-0.076201469	-2.017642916	0.044011214	0.073037735	no
ATP5C1	-0.07619414	-2.017447737	0.044031614	0.07305975	no
LRRC26	-0.076194107	-2.017446845	0.044031707	0.07305975	no
LRRC19	-0.076187321	-2.017266118	0.044050604	0.073085106	no
IKZF4	-0.076177393	-2.017001723	0.044078261	0.073124992	no
GSPT1	-0.07617485	-2.016934004	0.044085347	0.073130747	no

UBR5	-0.076160495	-2.016551688	0.044125371	0.073191135	no
SMOX	-0.076153542	-2.016366511	0.044144767	0.073217302	no
GALK1	0.076142452	2.016071163	0.044175719	0.073262627	no
TRIM44	0.076119453	2.015458656	0.044239967	0.073363161	no
RPS13	-0.076108673	-2.015171581	0.044270107	0.07340712	no
KIAA1328	-0.076103037	-2.015021482	0.044285872	0.07342724	no
DDX19B	-0.076097697	-2.014879264	0.044300814	0.073445991	no
C21orf91	0.076093965	2.014779867	0.04431126	0.073454238	no
FGF10	0.076093324	2.014762789	0.044313055	0.073454238	no
IFI27L1	0.076088745	2.014640851	0.044325873	0.073469462	no
USP6NL	0.076073105	2.014224326	0.04436968	0.073531651	no
XIST	-0.076072753	-2.014214948	0.044370667	0.073531651	no
LOC55908	-0.076055232	-2.013748362	0.044419784	0.073607015	no
GLIPR1L1	-0.076044821	-2.01347109	0.044448994	0.073649382	no
DBX1	0.076037402	2.013273513	0.044469818	0.073677849	no
GMPR	0.076035609	2.013225772	0.044474851	0.07368015	no
FBXW10	-0.076021551	-2.012851371	0.044514339	0.073739525	no
DNAH3	0.075979757	2.011738351	0.044631902	0.073928216	no
RAD23A	-0.075974602	-2.011601064	0.044646422	0.073946207	no
CLEC2D	0.075963319	2.01130059	0.044678213	0.073992801	no
LYPD5	-0.075949157	-2.01092345	0.044718143	0.074052864	no
DEFB131	-0.075947827	-2.010888039	0.044721894	0.07405301	no
SLC22A1	0.075926379	2.010316862	0.04478243	0.074147176	no
C7orf59	0.075900106	2.009617186	0.044856679	0.07426403	no
ATP6V1B2	0.075896366	2.009517595	0.044867256	0.074275459	no
CLDN8	0.075839697	2.008008479	0.045027789	0.07453511	no
LOC285456	-0.075833941	-2.007855181	0.045044123	0.074556044	no
CDH2	0.075790179	2.006689815	0.045168459	0.074755722	no
CCNJ	-0.075785592	-2.006567658	0.045181509	0.074771199	no
KCP	0.075780741	2.006438466	0.045195314	0.074787924	no
ZNF671	-0.075760955	-2.005911584	0.045251652	0.074873344	no
MCPH1	-0.075760011	-2.005886446	0.045254341	0.074873344	no
FBXO3	-0.075746183	-2.005518203	0.045293753	0.07493242	no
TBPL2	0.075741187	2.005385158	0.045308	0.074949856	no
LOC100128822	-0.075724827	-2.004949502	0.045354677	0.075020933	no
ZNF630	0.075712096	2.004610476	0.045391029	0.075070285	no
PPP1R2	0.075711777	2.004601987	0.04539194	0.075070285	no
CDC14A	0.075706812	2.004469773	0.045406123	0.0750876	no
GNAS	-0.075680132	-2.003759318	0.045482403	0.075207592	no
MCART3P	-0.07567692	-2.003673783	0.045491594	0.075216638	no
GSTO2	-0.075672352	-2.003552127	0.045504669	0.075232104	no
TTC8	0.075668919	2.00346072	0.045514495	0.075242197	no
LOC494141	0.075656142	2.003120475	0.045551087	0.075295784	no
DCP1A	0.075655	2.003090056	0.045554359	0.075295784	no
APOE	-0.075645213	-2.002829456	0.045582404	0.07533598	no
ELMOD3	0.075640853	2.002713343	0.045594904	0.07535048	no
CCNJL	0.075625429	2.002302614	0.045639145	0.075417427	no
GPR78	-0.075600497	-2.001638719	0.045710731	0.075529549	no
SLC34A3	-0.075598565	-2.001587255	0.045716284	0.075532551	no
F2	-0.075594477	-2.001478409	0.045728031	0.075545786	no
OR2T6	-0.075589919	-2.00135702	0.045741135	0.07556126	no

SERTAD4	0.075580631	2.001109692	0.045767843	0.075599203	no
HIBCH	-0.075579062	-2.00106792	0.045772355	0.075600479	no
ANUBL1	0.075555855	2.000449941	0.045839151	0.07570462	no
TRAFD1	0.075550329	2.000302817	0.045855065	0.075724717	no
FAM198A	0.07552334	1.999584135	0.045932873	0.075847013	no
UQCR11	0.07551735	1.999424627	0.045950157	0.075869357	no
LOC100132832	-0.075491121	-1.998726214	0.046025901	0.075988214	no
TSEN54	-0.07548004	-1.998431148	0.046057933	0.076034889	no
VIT	0.075476745	1.998343406	0.046067462	0.076044411	no
PYDC1	-0.075469522	-1.998151073	0.046088355	0.076072688	no
PTCHD1	0.075458941	1.997869319	0.046118976	0.076117017	no
BAG5	-0.075446017	-1.997525187	0.0461564	0.076172566	no
ZNF544	0.075444535	1.997485715	0.046160695	0.076172795	no
NOM1	0.075443368	1.997454647	0.046164075	0.076172795	no
CDK6	0.075426384	1.997002401	0.046213302	0.0762478	no
GRINA	-0.075412398	-1.99662998	0.046253873	0.076308512	no
CENPM	0.075400789	1.99632087	0.046287571	0.076357874	no
DDX3X	0.075396346	1.996202544	0.046300475	0.076372931	no
PDE12	0.075381692	1.995812347	0.046343052	0.076436367	no
ECD	-0.075380507	-1.995780812	0.046346494	0.076436367	no
CROCCL1	-0.075371956	-1.995553104	0.046371357	0.076471134	no
MYRIP	-0.075354661	-1.995092585	0.046421675	0.076542618	no
SDR42E1	0.075353503	1.995061748	0.046425046	0.076542618	no
LIPG	0.075353154	1.995052455	0.046426062	0.076542618	no
C9orf123	0.075341865	1.994751882	0.046458932	0.076590564	no
TIMM8B	0.075321256	1.9942031	0.046518994	0.076683329	no
LOC220594	-0.075310588	-1.993919069	0.046550106	0.076728359	no
SPATS1	0.075307822	1.993845393	0.04655818	0.07673541	no
GSTA2	0.07528083	1.993126687	0.046636995	0.076859045	no
ATXN2	-0.075269967	-1.99283746	0.046668744	0.0769051	no
RELL2	-0.075264413	-1.992689549	0.046684988	0.07692287	no
F11	0.075263678	1.992669981	0.046687137	0.07692287	no
CHRNB1	0.075258027	1.992519536	0.046703665	0.076943831	no
BRPF1	-0.075230824	-1.991795196	0.046783308	0.077068762	no
TPTE2P3	-0.075220728	-1.99152639	0.046812893	0.077111216	no
IFIT1	0.075205699	1.991126207	0.046856967	0.077177528	no
RHOB	-0.075186014	-1.990602064	0.046914745	0.0772664	no
FNBP1	0.075174513	1.99029586	0.046948528	0.077315739	no
OPRK1	-0.075164719	-1.990035079	0.046977315	0.077356845	no
MAN1B1	0.075148884	1.989613452	0.047023888	0.077427232	no
PKNOX1	-0.075114951	-1.988709945	0.047123823	0.07758546	no
TCEAL8	0.075105685	1.988463228	0.047151143	0.077624119	no
DENND4A	0.075101226	1.988344516	0.047164293	0.077639446	no
LCMT2	-0.075065453	-1.987392048	0.047269913	0.077802521	no
WDR5B	-0.075065068	-1.987381773	0.047271054	0.077802521	no
LOC653501	-0.07504836	-1.986936933	0.047320452	0.077877484	no
TUBBP5	0.075034587	1.986570225	0.047361206	0.077938211	no
BCAT2	0.07501223	1.985974955	0.047427425	0.07804083	no
HSD17B2	0.075006667	1.985826848	0.047443913	0.078061607	no
GLYAT	0.074997232	1.985575643	0.047471889	0.078101282	no
EMX2	-0.074984287	-1.98523096	0.047510298	0.078157512	no

TBC1D29	-0.074983107	-1.985199559	0.047513798	0.078157512	no
LOC650368	0.074976868	1.985033455	0.047532318	0.078179949	no
PHLDB1	-0.074975907	-1.985007866	0.047535172	0.078179949	no
CHRM2	-0.074961495	-1.984624144	0.047577981	0.078243991	no
VWA3A	0.074953107	1.984400814	0.047602911	0.078278622	no
PEX3	0.074935249	1.983925353	0.047656023	0.078359587	no
NDUFB2	0.074922061	1.983574219	0.047695279	0.078417757	no
ARFRP1	-0.074914352	-1.983368955	0.04771824	0.078444772	no
ATAD3A	-0.074913939	-1.983357961	0.04771947	0.078444772	no
ZNF146	-0.074897659	-1.982924522	0.047767986	0.078518141	no
PCDHGA8	-0.074864595	-1.982044213	0.047866649	0.078673921	no
HIST1H2BN	0.074850031	1.981656466	0.047910161	0.078739037	no
OCLN	-0.074845218	-1.981528324	0.047924548	0.07875628	no
ODZ2	-0.07482709	-1.981045667	0.047978771	0.078838979	no
KCNIP1	-0.0748156	-1.980739775	0.048013163	0.07888908	no
ANKLE1	-0.074804	-1.980430937	0.048047906	0.078939751	no
TRNP1	-0.07479952	-1.98031166	0.048061331	0.07895539	no
SIX3	0.074764288	1.979373641	0.048167011	0.079117147	no
ZNF184	-0.074764085	-1.979368229	0.048167621	0.079117147	no
AGK	0.074759197	1.979238104	0.048182297	0.079134824	no
SC4MOL	-0.074738735	-1.978693338	0.048243778	0.079229365	no
KIAA0922	0.074720677	1.978212555	0.048298094	0.079312123	no
DACH1	-0.074709195	-1.977906867	0.048332655	0.079362431	no
LIAS	-0.074687991	-1.977342345	0.048396534	0.079460868	no
RHOBTB2	-0.074686648	-1.97730661	0.04840058	0.079461058	no
GPR37	0.074662608	1.976666573	0.048473096	0.079573648	no
CCDC105	-0.074660822	-1.976619041	0.048478485	0.079576033	no
ZNF134	0.074655405	1.976474808	0.04849484	0.079596418	no
DDX39	0.074648252	1.97628438	0.048516441	0.079625409	no
UNC5D	-0.074636018	-1.975958672	0.048553407	0.079679608	no
WIZ	-0.074623487	-1.97562507	0.048591292	0.079735309	no
PCDHGB4	-0.074617589	-1.975468056	0.048609132	0.079758111	no
LOC375190	-0.074604569	-1.975121408	0.048648538	0.07981629	no
DENND2A	0.074593229	1.974819513	0.048682879	0.07986615	no
PER2	-0.074574255	-1.974314381	0.048740383	0.079953092	no
ZNF69	0.074573134	1.974284524	0.048743783	0.079953092	no
VPS37A	0.074571097	1.974230296	0.04874996	0.079956737	no
TOMM40L	0.074552061	1.973723511	0.04880772	0.080044977	no
TINAGL1	0.074526486	1.97304266	0.048885408	0.080165884	no
PPIG	0.074518787	1.972837691	0.048908816	0.080197765	no
TBCEL	-0.074515371	-1.972746741	0.048919206	0.080208297	no
ETV2	0.074508833	1.9725727	0.048939093	0.080234397	no
RYBP	0.074504364	1.972453707	0.048952694	0.080250188	no
LOC727677	-0.074492254	-1.972131311	0.04898956	0.080304113	no
GPR52	-0.074488636	-1.972035001	0.049000578	0.080315661	no
OR9A4	0.074486764	1.971985157	0.049006281	0.080318497	no
NDUFA1	0.074483624	1.971901564	0.049015846	0.080327663	no
REG4	0.074475792	1.97169308	0.049039709	0.080360256	no
TEX10	-0.074453594	-1.971102114	0.049107405	0.080464666	no
SFRS15	-0.07444983	-1.971001923	0.04911889	0.080476962	no
OR52E2	-0.074437647	-1.970677577	0.049156084	0.080531376	no

TULP3	0.074429749	1.970467314	0.049180209	0.080564371	no
WDR19	0.074383385	1.969233048	0.049322025	0.080790141	no
NSMCE1	-0.074365662	-1.968761229	0.049376328	0.080869178	no
CNOT1	0.074365026	1.968744303	0.049378277	0.080869178	no
RAB4A	-0.074360376	-1.968620502	0.049392534	0.080885975	no
C4orf46	0.074355997	1.96850393	0.049405962	0.080901412	no
MTO1	0.074292373	1.966810198	0.049601409	0.081214876	no
C1orf168	-0.074289355	-1.966729869	0.049610695	0.081223502	no
STX6	-0.074282047	-1.966535324	0.049633189	0.081253751	no
FAM194A	0.074233554	1.965244404	0.049782671	0.081486089	no
ACRV1	-0.074233392	-1.96524008	0.049783172	0.081486089	no
OR2AE1	0.074188223	1.964037673	0.049922745	0.08170558	no
DDX10	-0.074186931	-1.964003282	0.049926742	0.08170558	no
ILKAP	-0.074186075	-1.963980488	0.049929391	0.08170558	no
FAM153A	-0.074182468	-1.963884479	0.049940551	0.081717228	no
GPR155	-0.074163393	-1.963376691	0.049999611	0.081807246	no
MYB	0.074157128	1.963209908	0.050019022	0.081832382	no
PCDHA12	-0.074148395	-1.962977444	0.050046088	0.081870038	no
SPATA21	-0.07414324	-1.962840216	0.050062071	0.081889559	no
TRPA1	0.074138089	1.9627031	0.050078045	0.081909062	no
LSM6	0.074131126	1.96251775	0.050099646	0.081934637	no
NXPH2	-0.074130436	-1.962499384	0.050101787	0.081934637	no
RPS4Y2	0.074112577	1.962023989	0.050157227	0.082018668	no
C6orf94	-0.074110951	-1.961980686	0.050162279	0.082020296	no
SLC17A8	-0.074105543	-1.96183674	0.050179078	0.082041128	no
APOLD1	-0.074092416	-1.961487307	0.050219876	0.082101193	no
PAN2	-0.074088513	-1.961383397	0.050232013	0.082114396	no
KNG1	-0.07408444	-1.96127498	0.05024468	0.082128462	no
HIVEP1	-0.074078482	-1.961116378	0.050263215	0.082152116	no
CCDC151	0.074071596	1.960933075	0.050284643	0.082180496	no
LOC150197	0.074049761	1.960351841	0.050352641	0.082284973	no
IQCD	0.074023349	1.959648771	0.050434995	0.082412894	no
FAM35A	-0.074019424	-1.959544278	0.050447245	0.082422536	no
OR2D3	0.074018845	1.959528885	0.050449049	0.082422536	no
LOC729678	-0.074009918	-1.959291253	0.050476917	0.082461401	no
MMP28	-0.073980899	-1.958518796	0.050567592	0.082602859	no
NBPF15	0.073976711	1.958407297	0.050580692	0.082617582	no
DYTN	0.073974637	1.958352092	0.050587179	0.082621503	no
VPS13B	-0.073970106	-1.958231492	0.050601353	0.082637976	no
AKAP3	-0.073962987	-1.958041981	0.050623632	0.082667684	no
NDST3	-0.073946079	-1.957591932	0.050676574	0.082747453	no
CCDC3	0.073935289	1.957304692	0.050710387	0.082795979	no
CSNK2B	-0.073901321	-1.956400521	0.05081695	0.082963267	no
KITLG	0.073893109	1.956181925	0.050842741	0.082998671	no
PDE1B	-0.07388963	-1.956089334	0.050853669	0.083009808	no
ZPLD1	0.073877193	1.95575828	0.050892757	0.083066905	no
ZNF776	0.07387243	1.955631488	0.050907734	0.083084643	no
MRPS23	0.073861133	1.955330793	0.050943268	0.083135926	no
GRM8	-0.073856559	-1.955209019	0.050957664	0.083152708	no
CYCS	0.073844954	1.954900126	0.050994197	0.083205606	no
NAA10	0.073837599	1.954704353	0.051017362	0.083236687	no

C4orf19	0.073825826	1.954390977	0.051054462	0.083290495	no
LOC100129550	-0.073812117	-1.954026054	0.051097692	0.083354296	no
PAH	-0.073808313	-1.95392482	0.051109691	0.083367142	no
UFC1	0.07379864	1.953667323	0.05114022	0.08341021	no
MAEL	0.073786751	1.953350885	0.051177758	0.083462296	no
HIGD2B	0.073785911	1.953328526	0.051180411	0.083462296	no
C6orf204	-0.073780706	-1.953189983	0.051196854	0.083482377	no
INCENP	-0.073767361	-1.952834763	0.051239033	0.083544417	no
LOC729991	0.073762479	1.95270482	0.05125447	0.083562848	no
ALS2CR4	0.073740628	1.952123177	0.051323615	0.083668832	no
RNFT2	-0.073731362	-1.951876557	0.051352957	0.083709916	no
ZNF323	-0.073711593	-1.951350339	0.051415611	0.08380529	no
NTN5	-0.073708211	-1.951260323	0.051426335	0.083809395	no
ODF4	-0.073708184	-1.951259614	0.05142642	0.083809395	no
C15orf27	-0.073690166	-1.950780028	0.051483587	0.083895798	no
MGC2752	0.073677091	1.950431991	0.051525108	0.083956193	no
CDC37L1	-0.07367588	-1.950399757	0.051528954	0.083956193	no
MRPS28	-0.073665893	-1.950133939	0.051560687	0.084001125	no
NEK9	0.073655912	1.949868265	0.051592418	0.084046049	no
GPR128	0.073636664	1.949355944	0.051653656	0.084136104	no
GOLGA1	-0.07363592	-1.949336152	0.051656022	0.084136104	no
ZNF589	-0.073623003	-1.948992349	0.051697152	0.084196313	no
SULT1C2	0.073616307	1.948814113	0.051718486	0.084224273	no
SCCPDH	-0.073580477	-1.947860432	0.051832761	0.084403573	no
SLC16A6	0.073575403	1.947725369	0.051848962	0.084423154	no
FBXO2	-0.073571333	-1.947617048	0.051861958	0.084437516	no
APPL2	-0.073547841	-1.946991766	0.051937033	0.084552937	no
CPXCR1	0.073511122	1.946014448	0.052054557	0.084737442	no
ZNF135	-0.073507648	-1.945921977	0.052065688	0.084748739	no
ADAMTS19	-0.073487892	-1.945396163	0.052129022	0.084844998	no
KRT31	-0.073471073	-1.944948512	0.052182992	0.084926002	no
ZNF579	-0.073468845	-1.944889194	0.052190147	0.08493081	no
OR4K2	-0.07345766	-1.944591506	0.052226067	0.084979889	no
ACOT1	-0.073456837	-1.944569587	0.052228712	0.084979889	no
RFNG	-0.073443137	-1.944204956	0.05227274	0.085044682	no
C5orf34	0.073433726	1.94395447	0.052303003	0.085085534	no
MSMP	0.073431734	1.943901451	0.052309411	0.085085534	no
ZNF669	-0.073431403	-1.943892643	0.052310475	0.085085534	no
FAM8A1	-0.073425824	-1.943744156	0.052328424	0.085105726	no
FKTN	-0.073424928	-1.943720303	0.052331308	0.085105726	no
SLC29A2	-0.073417072	-1.943511221	0.052356592	0.085139996	no
EIF4E2	0.073415247	1.943462638	0.052362468	0.085142704	no
INHBB	0.07340786	1.943266045	0.052386253	0.085174528	no
OR8D1	0.073390698	1.942809274	0.05244155	0.08525758	no
UTS2D	0.073381103	1.942553893	0.052472488	0.085301019	no
HIST1H3H	0.073364923	1.942123252	0.052524693	0.08537902	no
DPY19L2P1	0.073356498	1.941899014	0.052551894	0.085416367	no
MLL2	-0.073343233	-1.941545974	0.052594742	0.08547914	no
RTP3	0.0733327	1.941265625	0.052628789	0.085527599	no
NCBP1	0.073328305	1.941148654	0.052643	0.085543817	no
PMPCA	-0.073317035	-1.940848702	0.052679456	0.085596178	no

POPDC3	0.073312424	1.940725969	0.052694379	0.085613546	no
UNC119B	0.073308938	1.940633199	0.052705662	0.085624995	no
FAM7A3	0.073240051	1.938799775	0.052929049	0.085980999	no
GPI	0.073225925	1.938423823	0.052974954	0.086048656	no
TMEM206	-0.073217312	-1.938194594	0.053002959	0.08608654	no
KIAA0907	-0.073216133	-1.938163226	0.053006793	0.08608654	no
ATXN7	0.073213956	1.938105267	0.053013876	0.086090115	no
ETAA1	0.073212838	1.938075535	0.05301751	0.086090115	no
ROPN1B	0.073197452	1.937666037	0.053067582	0.086164502	no
LYRM2	-0.073193868	-1.937570661	0.05307925	0.086173513	no
SNX30	-0.07319313	-1.937550996	0.053081656	0.086173513	no
HSPA13	-0.073190624	-1.937484313	0.053089816	0.086179839	no
ATP5H	0.073174921	1.937066376	0.053140978	0.086255965	no
MLC1	-0.073167163	-1.936859906	0.053166269	0.086272052	no
DDX52	-0.073166767	-1.936849375	0.053167559	0.086272052	no
FAM107A	-0.073166651	-1.936846294	0.053167937	0.086272052	no
HOXC10	0.073166645	1.936846122	0.053167958	0.086272052	no
ZNF233	-0.073157275	-1.936596742	0.053198519	0.086314714	no
PAG1	-0.073141073	-1.936165555	0.053251395	0.086393573	no
FOXN2	-0.073130829	-1.935892913	0.053284851	0.086440915	no
ZGLP1	-0.073124661	-1.935728754	0.053305004	0.08646667	no
ZNF391	-0.07311722	-1.935530722	0.053329324	0.086494894	no
OR4F6	0.073116719	1.935517402	0.05333096	0.086494894	no
LUC7L2	-0.073110132	-1.935342089	0.053352498	0.086522884	no
IGFL1	-0.073076808	-1.934455217	0.053461565	0.086692807	no
SPATA24	0.073056087	1.933903754	0.053529478	0.086795973	no
GDA	-0.073036252	-1.933375875	0.053594554	0.086894522	no
ISOC1	0.0730028	1.9324856	0.053704456	0.087065727	no
CCNB2	0.073000505	1.932424537	0.053712001	0.087066715	no
TBC1D9B	0.072999995	1.932410966	0.053713678	0.087066715	no
C3orf23	-0.072991299	-1.932179518	0.053742284	0.0871061	no
ARF5	0.07296951	1.931599647	0.053814011	0.087215364	no
CHRDL2	0.072956918	1.931264556	0.053855496	0.087275603	no
LOC340017	-0.072940384	-1.930824523	0.053910014	0.087356951	no
C6orf130	-0.072936794	-1.930728988	0.053921857	0.087369138	no
PATL1	0.072931468	1.930587251	0.053939431	0.087390609	no
WDR60	-0.072927459	-1.930480561	0.053952662	0.087405042	no
DPM3	0.072926063	1.930443398	0.053957271	0.087405506	no
FAM118B	0.072914796	1.930143563	0.053994474	0.087458762	no
ARHGAP24	0.072906877	1.92993282	0.054020634	0.087491475	no
TYK2	0.072906063	1.929911152	0.054023325	0.087491475	no
FAM82B	0.07290272	1.92982218	0.054034373	0.087502359	no
HDAC8	-0.072895532	-1.92963089	0.054058133	0.087533825	no
C4orf6	0.072890641	1.929500734	0.054074305	0.087549646	no
C21orf34	-0.072889958	-1.929482548	0.054076565	0.087549646	no
ZNF280D	-0.072884065	-1.929325739	0.054096054	0.087574187	no
ASIP	0.07287822	1.929170169	0.054115395	0.087598484	no
GNAQ	-0.07285934	-1.928667741	0.054177899	0.087692639	no
ABCC6	0.072857003	1.928605528	0.054185642	0.087698152	no
CNOT8	-0.072848754	-1.928386019	0.054212972	0.087735362	no
RSPH3	0.072836286	1.928054226	0.054254304	0.087795223	no

NBPF3	0.072827951	1.927832396	0.054281952	0.087832933	no
MKS1	-0.072811161	-1.927385592	0.054337676	0.087911303	no
EYS	-0.072810738	-1.927374325	0.054339082	0.087911303	no
DCTN4	-0.072791492	-1.926862148	0.054403019	0.0880077	no
GOLT1A	0.07276983	1.926285673	0.054475058	0.088117187	no
CAPN14	-0.072753297	-1.925845708	0.054530091	0.088199151	no
DCHS1	0.07273265	1.92529627	0.054598884	0.088303354	no
B4GALT2	-0.07272397	-1.925065268	0.054627828	0.088343099	no
CDH23	0.072719621	1.924949524	0.054642336	0.088359252	no
PGM2L1	0.072718355	1.92491585	0.054646557	0.088359252	no
SCGB2A1	0.072705689	1.924578796	0.054688825	0.088420524	no
XG	0.072694775	1.92428835	0.054725269	0.088472373	no
NXNL2	0.072675537	1.923776409	0.054789556	0.088569221	no
AKT2	0.07266834	1.923584883	0.054813623	0.088593999	no
PITX1	0.072668333	1.923584688	0.054813648	0.088593999	no
C1orf156	0.072663858	1.923465609	0.054828616	0.088611108	no
ZKSCAN3	-0.072662273	-1.923423418	0.05483392	0.088612596	no
CLK2	-0.07265395	-1.92320195	0.054861769	0.088650516	no
TAS2R43	-0.072642385	-1.922894173	0.054900491	0.088705996	no
FIGLA	-0.072634359	-1.922680611	0.054927373	0.088742339	no
C6orf167	0.072601085	1.921795155	0.055038948	0.088915497	no
COMMD3	0.072589992	1.921499941	0.055076189	0.088968551	no
IGFBP1	0.072584588	1.921356152	0.055094336	0.088990755	no
POU5F1B	-0.072573847	-1.921070314	0.055130424	0.089041933	no
SLC39A10	-0.07257147	-1.921007059	0.055138413	0.089047722	no
PIGA	0.072564002	1.920808332	0.055163518	0.089081151	no
TP53TG5	-0.072553922	-1.920540092	0.055197419	0.089128778	no
FOXN4	-0.0725395	-1.920156321	0.055245953	0.089200022	no
SERPINA10	-0.072526017	-1.919797531	0.055291359	0.089266206	no
GSX2	0.072508407	1.919328925	0.05535071	0.089354891	no
C13orf27	-0.072496259	-1.919005661	0.055391684	0.089413897	no
BGLAP	-0.072494199	-1.91895085	0.055398633	0.089417976	no
TMEM212	0.072488773	1.918806453	0.055416946	0.089440394	no
GPR63	-0.072474666	-1.918431077	0.055464575	0.089510119	no
ASCL4	0.072439692	1.917500408	0.055582808	0.089693768	no
POLG	-0.072420995	-1.9170029	0.055646099	0.089788733	no
OTX1	0.072411122	1.916740164	0.055679547	0.089835534	no
GTSE1	0.072395232	1.916317344	0.05573341	0.089915264	no
ACTR8	-0.072383504	-1.916005254	0.055773196	0.089972271	no
CRYAB	-0.072354433	-1.915231689	0.055871912	0.090124327	no
NOP58	-0.072348146	-1.915064413	0.055893277	0.090151598	no
PIAS2	0.072342391	1.914911266	0.055912844	0.090175964	no
DMXL1	-0.072335967	-1.914740342	0.05593469	0.090204	no
SON	-0.0723328	-1.914656067	0.055945463	0.090214178	no
POU5F1	0.072324579	1.914437297	0.055973438	0.090252091	no
ZNF487	0.072320861	1.914338363	0.055986093	0.090265297	no
NME2P1	0.072314315	1.91416418	0.056008378	0.090294027	no
NCRNA00160	0.072297064	1.913705152	0.056067145	0.09038156	no
SNRNP25	-0.072291448	-1.913555721	0.056086286	0.090400246	no
SNRNP35	-0.07229104	-1.913544852	0.056087679	0.090400246	no
GAS8	0.072275329	1.913126801	0.05614126	0.090479394	no

ALDH3A2	0.072256382	1.912622639	0.056205935	0.090576406	no
AQP4	0.072249616	1.912442613	0.056229044	0.090598104	no
C10orf27	0.072249603	1.912442265	0.056229088	0.090598104	no
ATP13A5	-0.072248504	-1.912413027	0.056232842	0.090598104	no
C3orf51	-0.072242347	-1.912249198	0.05625388	0.090624777	no
CKAP2L	0.0722323	1.911981849	0.056288226	0.090672883	no
ZNF529	-0.072213366	-1.911478041	0.056352996	0.090769987	no
PLGLB2	-0.072200943	-1.911147479	0.056395528	0.090831258	no
DNAJA1	0.072189725	1.91084898	0.056433957	0.090878059	no
ZRANB3	0.072189638	1.910846663	0.056434255	0.090878059	no
CPNE6	-0.072188524	-1.910817035	0.056438071	0.090878059	no
RLIM	-0.072184698	-1.910715228	0.056451183	0.090891934	no
ZFAND2A	0.072182228	1.910649505	0.056459649	0.090898327	no
H2BFM	-0.072154003	-1.909898478	0.056556471	0.091046956	no
HDAC2	-0.072126506	-1.909166851	0.056650924	0.091180297	no
CNGB3	-0.072123309	-1.909081772	0.056661916	0.091180297	no
ATG9B	0.072123117	1.909076674	0.056662575	0.091180297	no
TLL1	-0.072122772	-1.909067485	0.056663763	0.091180297	no
MED29	-0.072122693	-1.909065377	0.056664035	0.091180297	no
RNF167	0.072122017	1.909047397	0.056666358	0.091180297	no
TNNI3	-0.072113524	-1.908821403	0.056695567	0.09121967	no
AURKC	0.072112278	1.908788269	0.056699851	0.09121967	no
CLDND2	-0.07210363	-1.908558153	0.056729608	0.091260281	no
PPP1R12C	-0.072090729	-1.908214878	0.056774021	0.091324462	no
KIAA1407	0.07208096	1.907954962	0.056807669	0.091371317	no
ADNP	-0.072070989	-1.907689646	0.056842033	0.091419316	no
MNAT1	-0.072063224	-1.90748303	0.056868806	0.091455101	no
VMA21	-0.072054058	-1.907239145	0.056900422	0.091493122	no
LOC100130557	-0.072053746	-1.907230837	0.056901499	0.091493122	no
ANKDD1A	-0.072050765	-1.907151542	0.056911782	0.091502379	no
MT1X	-0.072045862	-1.907021072	0.056928704	0.091522308	no
C9orf109	0.072041871	1.906914869	0.056942482	0.09153718	no
FSHR	-0.072039116	-1.906841572	0.056951993	0.091545189	no
PDPR	-0.072000951	-1.905826103	0.05708389	0.091747411	no
NHEDC2	-0.072000088	-1.905803145	0.057086875	0.091747411	no
CALCOCO2	0.071997161	1.905725255	0.057097003	0.091756395	no
ARID5B	0.071978425	1.905226734	0.05716186	0.091853321	no
ENAM	0.071975137	1.905139259	0.057173247	0.091864317	no
KCNS2	-0.071966412	-1.904907101	0.057203476	0.091905585	no
CCDC80	0.071953606	1.904566375	0.057247867	0.091967877	no
SCGB1D4	-0.07195246	-1.904535883	0.057251841	0.091967877	no
ICA1	-0.071951291	-1.904504786	0.057255894	0.091967877	no
KLHL15	-0.071925865	-1.903828288	0.057344125	0.092102282	no
HOXD9	0.071890604	1.902890092	0.057466675	0.092291782	no
HIF1AN	-0.071839335	-1.901526002	0.057645246	0.092571214	no
CSTF3	-0.071820223	-1.901017504	0.057711931	0.092670227	no
AG2	-0.071819038	-1.900985964	0.057716069	0.092670227	no
C6orf64	-0.071806459	-1.900651295	0.057759996	0.092733391	no
GPR89B	0.071804643	1.900602975	0.05776634	0.092736213	no
CHP	-0.071797271	-1.900406828	0.057792101	0.092770202	no
PRDX5	-0.071789479	-1.900199506	0.057819339	0.092806557	no

RGS4	-0.071786712	-1.9001259	0.057829012	0.092814715	no
PPDPF	0.07177839	1.899904482	0.057858118	0.092854059	no
EVPL	-0.071744482	-1.899002309	0.057976838	0.093037202	no
POM121C	0.071739787	1.898877417	0.057993289	0.093056215	no
MRPL21	-0.071724202	-1.898462759	0.058047936	0.093136511	no
MEGF8	-0.071705887	-1.897975481	0.058112208	0.093232235	no
GNG8	-0.071703759	-1.897918863	0.05811968	0.093236824	no
OR2L1P	-0.071692636	-1.89762293	0.058158747	0.093292094	no
TPR	-0.071690617	-1.897569202	0.058165842	0.093296073	no
WIBG	0.071676087	1.897182629	0.058216913	0.093370581	no
RSL1D1	-0.071654752	-1.896614982	0.058291973	0.09348355	no
MRPL11	0.071652933	1.896566593	0.058298375	0.093485931	no
RIOK1	-0.071651703	-1.896533866	0.058302706	0.093485931	no
RBL2	-0.071645321	-1.896364083	0.058325175	0.093514543	no
NEO1	-0.071643752	-1.896322333	0.058330702	0.093515988	no
AGER	-0.071635463	-1.896101793	0.058359901	0.093555383	no
ZNF837	0.071626951	1.895875344	0.058389897	0.093596046	no
ENTPD4	0.071617867	1.895633648	0.058421925	0.093635684	no
FEZF1	0.07161731	1.895618844	0.058423888	0.093635684	no
GRTP1	0.071594977	1.895024679	0.058502689	0.093748208	no
EPHB2	0.071594369	1.895008481	0.058504838	0.093748208	no
C10orf84	-0.071593471	-1.8949846	0.058508007	0.093748208	no
FRYL	0.071562355	1.894156759	0.058617956	0.093913018	no
POLQ	0.071561733	1.894140214	0.058620155	0.093913018	no
TMEM208	0.071539651	1.893552731	0.058698287	0.09403074	no
TMOD4	0.071493413	1.892322593	0.058862169	0.094285798	no
FAM131A	-0.07146969	-1.891691455	0.058946399	0.094413238	no
RGSL1	0.071456851	1.891349868	0.058992028	0.094478836	no
FGFBP2	0.071452262	1.891227786	0.059008343	0.0944914	no
RHD	0.071452015	1.891221222	0.05900922	0.0944914	no
NARG2	-0.071431662	-1.890679728	0.05908163	0.094599858	no
FAM92A1	-0.071420372	-1.890379384	0.059121825	0.09465672	no
BIRC5	0.071413622	1.890199812	0.059145868	0.094687716	no
SMCR7	-0.07140934	-1.890085894	0.059161125	0.094704641	no
KLF2	0.071390279	1.889578783	0.05922908	0.094805917	no
SORD	-0.071375322	-1.889180869	0.059282448	0.094883829	no
ODAM	0.071366332	1.888941706	0.059314543	0.094927685	no
ZNF229	-0.071350319	-1.888515695	0.05937175	0.095011717	no
EXOSC4	-0.071331823	-1.888023647	0.059437881	0.095110018	no
COQ6	-0.071310278	-1.887450458	0.059514994	0.095225875	no
CPSF3L	-0.07130504	-1.88731112	0.059533753	0.095248351	no
SPDYE7P	-0.071300136	-1.887180663	0.05955132	0.095264875	no
NELL2	-0.071299526	-1.887164441	0.059553505	0.095264875	no
SMCHD1	-0.07126594	-1.886270918	0.059673945	0.095449985	no
HSD17B6	-0.071242345	-1.885643225	0.059758674	0.09557795	no
ZNF836	0.07123789	1.885524715	0.059774682	0.095592822	no
TMEM128	0.071237126	1.88550439	0.059777428	0.095592822	no
HIST2H2BE	0.071221557	1.885090202	0.059833407	0.095674772	no
PRPH2	-0.071213897	-1.884886437	0.059860962	0.095709027	no
RADIL	-0.071212971	-1.884861784	0.059864297	0.095709027	no
TUBB4Q	0.071195569	1.884398866	0.05992694	0.095801603	no

OPN4	-0.071177592	-1.883920627	0.059991713	0.095897569	no
BAZ1B	0.071159726	1.883445326	0.060056146	0.095989162	no
CCDC85B	-0.071157916	-1.883397177	0.060062677	0.095989162	no
SLC28A2	-0.071157756	-1.883392916	0.060063255	0.095989162	no
KRT39	0.071124305	1.882503047	0.060184055	0.096174614	no
KCNQ1OT1	-0.071112707	-1.882194524	0.060225984	0.096234011	no
CEP70	-0.071097373	-1.881786603	0.060281459	0.096314854	no
FAM74A1	0.071096089	1.881752447	0.060286105	0.096314854	no
NAA25	-0.071091383	-1.881627243	0.060303142	0.09633446	no
C9orf150	0.07107828	1.881278674	0.060350593	0.096402646	no
SNORA28	0.071072902	1.881135612	0.060370077	0.096426151	no
DBF4	0.071071256	1.881091833	0.060376041	0.096428059	no
DNA2	-0.07105864	-1.880756213	0.060421775	0.096493478	no
MRPL50	0.071036236	1.880160218	0.060503059	0.096615659	no
ITM2C	0.071032734	1.880067069	0.060515772	0.096628327	no
PGLS	0.071030081	1.879996494	0.060525405	0.096636076	no
CCDC92	-0.07102541	-1.879872244	0.060542367	0.096655526	no
KRTAP5-4	-0.07094647	-1.877772304	0.060829647	0.097106499	no
C8orf31	-0.070939621	-1.877590124	0.060854623	0.0971387	no
NPHS2	0.070934966	1.877466281	0.060871607	0.097158139	no
TAF1A	0.070931509	1.877374317	0.060884221	0.097170601	no
RHBDD2	0.070921258	1.877101635	0.060921635	0.09722264	no
SBDSP1	0.070899074	1.876511525	0.06100267	0.097344276	no
IFT46	0.07088728	1.87619779	0.061045788	0.097405394	no
HAP1	0.070884923	1.876135076	0.06105441	0.097411464	no
FZD8	0.070876788	1.875918688	0.061084168	0.097451252	no
FAM189A1	-0.070872015	-1.875791711	0.061101636	0.097471428	no
VBP1	-0.070844443	-1.875058285	0.061202611	0.097624804	no
TCP11	-0.070823413	-1.874498871	0.061279722	0.097740093	no
LYZL2	0.070821874	1.874457925	0.061285369	0.097741389	no
CCDC36	0.070802345	1.873938455	0.061357051	0.097847994	no
CD81	0.070786317	1.873512105	0.061415936	0.097934175	no
ID1	-0.070772101	-1.87313393	0.061468207	0.098009795	no
COL16A1	0.07076666	1.872989214	0.061488219	0.098033972	no
TUSC1	0.070747357	1.872475756	0.061559266	0.098139507	no
TIAF1	-0.070717291	-1.871675997	0.061670064	0.098308393	no
C10orf93	0.070713717	1.871580913	0.061683248	0.098321657	no
SLC12A9	0.070704426	1.871333771	0.061717526	0.098368541	no
PRKAR2B	-0.070702882	-1.871292706	0.061723223	0.098369866	no
KLHL31	-0.070691093	-1.870979129	0.061766742	0.098426471	no
PSMB11	-0.070690529	-1.870964125	0.061768825	0.098426471	no
DRD4	-0.070689305	-1.870931567	0.061773345	0.098426471	no
PPP3CC	-0.070681766	-1.870731033	0.061801192	0.09846308	no
ADAMTS18	0.070668372	1.870374752	0.061850691	0.098531652	no
MYC	-0.070667483	-1.870351102	0.061853978	0.098531652	no
CHST15	-0.070656555	-1.870060428	0.061894388	0.098581615	no
UBE2C	0.070656365	1.870055351	0.061895094	0.098581615	no
PLK1	0.070651305	1.869920765	0.061913812	0.09860366	no
GATA5	-0.070645255	-1.869759843	0.0619362	0.098627582	no
C9orf119	0.070644609	1.869742665	0.06193859	0.098627582	no
IAPP	-0.070635928	-1.869511741	0.061970728	0.098670986	no

SLC39A13	0.070579291	1.868005239	0.062180732	0.098997563	no
ZNF324B	-0.070563126	-1.867575256	0.06224078	0.099085361	no
RGPD3	-0.070550669	-1.867243911	0.062287085	0.09915127	no
VPS13C	0.070536293	1.86686153	0.062340558	0.09922682	no
DDX5	0.070535271	1.866834341	0.062344361	0.09922682	no
FAM89B	-0.070532411	-1.866758257	0.062355006	0.09923595	no
LAS1L	-0.070517998	-1.866374903	0.062408663	0.099312609	no
CAMK2N1	-0.070516834	-1.866343939	0.062412999	0.099312609	no
MUSK	0.070506024	1.866056398	0.062453273	0.099368873	no
INE1	-0.070504254	-1.866009314	0.06245987	0.099371549	no
TNK1	-0.070469076	-1.86507362	0.062591087	0.099572476	no
GRPEL2	0.070455142	1.864703018	0.062643122	0.099642056	no
LGR6	0.070454725	1.864691912	0.062644682	0.099642056	no
GEMIN4	-0.070444488	-1.864419634	0.062682935	0.099695058	no
HCG2P7	-0.07044301	-1.864380321	0.06268846	0.099696002	no
KIAA0406	0.070422332	1.863830301	0.062765798	0.099811146	no
C9orf41	0.070416407	1.863672726	0.062787969	0.09983855	no
CCDC68	-0.070402718	-1.86330861	0.062839226	0.099912195	no
USP37	-0.070394932	-1.86310151	0.062868395	0.099950713	no
LCN2	0.070377155	1.862628678	0.062935033	0.100043662	no
RASAL2	-0.070376696	-1.86261646	0.062936756	0.100043662	no
SCARNA20	0.070334229	1.861486942	0.063096185	0.100289203	no
LHX1	-0.070315629	-1.860992226	0.063166118	0.100392467	no
TRMT112	0.070302062	1.860631373	0.063217168	0.100465706	no
CALCA	0.070297091	1.860499155	0.063235882	0.100487547	no
ZDHHC21	-0.070291148	-1.860341083	0.063258261	0.100515209	no
CC2D2B	0.07028884	1.860279692	0.063266954	0.100521122	no
LZTFL1	0.070281543	1.860085617	0.063294443	0.100556895	no
PDHB	-0.070279998	-1.860044507	0.063300267	0.100558245	no
ZNF354C	-0.070257076	-1.859434849	0.063386688	0.100687622	no
PSMD7	0.070241056	1.85900875	0.063447147	0.100775742	no
COL20A1	-0.070239133	-1.858957618	0.063454405	0.100779353	no
ISG20L2	0.070230324	1.858723312	0.063487674	0.100824271	no
FAM21B	-0.070221273	-1.858482573	0.063521872	0.100868417	no
DHX34	0.070220326	1.858457387	0.06352545	0.100868417	no
C20orf199	-0.07021577	-1.858336219	0.063542669	0.100887835	no
ENTPD5	0.070188387	1.857607896	0.06364625	0.101044357	no
LEMD1	0.070184157	1.857495404	0.063662261	0.10106184	no
IMMP2L	0.070167218	1.857044865	0.06372642	0.101149607	no
C6orf1	0.070166918	1.857036899	0.063727554	0.101149607	no
CCDC76	0.070162876	1.856929391	0.063742872	0.101165978	no
PKD1L2	0.070140907	1.856345075	0.063826178	0.101290241	no
OR2T8	-0.07013406	-1.856162976	0.063852159	0.101323518	no
C4orf23	0.070126386	1.855958866	0.06388129	0.101361789	no
TSNAX-DISC1	0.070096079	1.855152817	0.063996438	0.101534051	no
TMEM115	0.070092465	1.855056688	0.064010183	0.101534051	no
EXO1	0.070091418	1.855028844	0.064014164	0.101534051	no
HNRNPC	-0.070091417	-1.855028806	0.064014169	0.101534051	no
SPR	0.070091208	1.855023249	0.064014964	0.101534051	no
SPRYD4	0.070083349	1.854814246	0.064044857	0.101573495	no
EPB41L2	0.070074248	1.854572188	0.064079491	0.101620453	no

KDM5A	0.070069715	1.854451612	0.064096749	0.101639849	no
HTRA1	-0.070065098	-1.854328829	0.064114327	0.10165975	no
GHR	0.070036123	1.85355819	0.064224746	0.101826844	no
GSTA3	0.070030428	1.853406737	0.064246465	0.101853292	no
ASCL3	-0.070028603	-1.853358193	0.064253428	0.101856344	no
ORC4L	-0.070026318	-1.853297418	0.064262146	0.101862177	no
GNPTG	-0.070004063	-1.85270554	0.064347099	0.101988841	no
ETFDH	0.069980194	1.852070704	0.064438321	0.102122271	no
H2AFZ	0.069979393	1.852049394	0.064441385	0.102122271	no
PPL	0.06994469	1.851126443	0.064574204	0.102324733	no
C17orf79	0.069942458	1.851067078	0.064582755	0.102330262	no
GALK2	0.069920878	1.850493165	0.064665468	0.10245329	no
NSFL1C	-0.069913735	-1.850303183	0.064692868	0.10248867	no
SUV39H2	-0.069879206	-1.84938486	0.064825446	0.102690659	no
LRP12	0.069854369	1.848724334	0.064920946	0.102833883	no
FRMD4A	-0.069846121	-1.848504978	0.064952686	0.102876099	no
C18orf1	-0.069839476	-1.848328241	0.064978269	0.102908557	no
EPR1	0.069835144	1.848213024	0.064994951	0.102926915	no
LCN8	-0.069800976	-1.847304344	0.065126642	0.103127385	no
MS4A3	0.069797999	1.847225175	0.065138126	0.103137492	no
TMEM223	0.069788168	1.846963711	0.065176066	0.103189483	no
SAR1A	-0.069784443	-1.846864647	0.065190445	0.103204167	no
GOLGA6A	-0.069781587	-1.846788686	0.065201473	0.103213543	no
OR5K1	-0.069772654	-1.846551114	0.065235972	0.103260071	no
GFRA2	0.069761867	1.846264243	0.065277651	0.103317823	no
RAB9A	0.069760566	1.84622965	0.065282678	0.103317823	no
LOC100287718	0.069746187	1.845847243	0.065338274	0.103397717	no
LATS1	-0.06973486	-1.845545993	0.065382099	0.103458972	no
RHO	-0.069708274	-1.844838956	0.065485053	0.103613773	no
COX6B2	0.069706645	1.844795648	0.065491363	0.103615649	no
TMCO6	0.069705075	1.844753881	0.065497449	0.10361717	no
MLL4	-0.069669374	-1.843804448	0.065635931	0.103828123	no
MIER3	-0.069662308	-1.843616522	0.06566337	0.103863402	no
C1orf109	0.069653736	1.843388567	0.065696666	0.103907939	no
OR12D2	0.069625341	1.842633434	0.065807064	0.104067331	no
PCNXL3	-0.069625168	-1.842628833	0.065807737	0.104067331	no
FMO5	0.069623051	1.842572533	0.065815974	0.104072217	no
NPY2R	0.069509294	1.839547328	0.066259837	0.104765886	no
HPRT1	0.069491108	1.839063708	0.066331023	0.10487024	no
CCDC14	-0.069484667	-1.838892414	0.066356252	0.104901924	no
DHRS4L1	-0.069481758	-1.838815044	0.06636765	0.104911739	no
LOC144438	0.069452411	1.838034629	0.066482708	0.105085403	no
C1orf89	-0.069435111	-1.837574563	0.066550614	0.105184514	no
HSPA8	0.069431241	1.837471661	0.06656581	0.105200308	no
ZSWIM4	-0.069429879	-1.837435448	0.066571158	0.105200537	no
NUDT2	-0.069405735	-1.836793382	0.066666047	0.105342254	no
FOXP2	-0.069376905	-1.836026714	0.066779496	0.105513274	no
INSL3	0.069363373	1.835666863	0.066832801	0.105589245	no
SCLT1	0.069337474	1.834978161	0.066934916	0.105742313	no
CRCP	0.069333635	1.834876059	0.066950066	0.105757983	no
COPS6	0.069323443	1.834605047	0.066990292	0.105813259	no

ATXN8OS	-0.069316797	-1.834428316	0.067016535	0.105846441	no
GLRX3	0.069294276	1.833829438	0.067105525	0.105978715	no
HGFAC	-0.069285931	-1.83360751	0.067138528	0.106022553	no
BLOC1S1	0.069279999	1.833449758	0.067161995	0.106051328	no
C1orf65	-0.069275373	-1.83332675	0.067180298	0.106069927	no
ZNF639	-0.06927437	-1.833300086	0.067184266	0.106069927	no
PPIL4	0.069271577	1.83322581	0.067195321	0.106079097	no
ZFHX4	-0.06926891	-1.833154897	0.067205876	0.106087477	no
RNMTL1	-0.069248601	-1.832614829	0.067286311	0.106206154	no
SEMA5A	-0.069209242	-1.831568227	0.067442411	0.106444236	no
IDH3A	-0.069206812	-1.83150361	0.067452059	0.106451153	no
IL20RB	0.069203069	1.831404056	0.067466924	0.106466303	no
SSTR4	-0.069188732	-1.831022837	0.067523874	0.106547857	no
PARP1	-0.069183739	-1.83089004	0.067543722	0.106570858	no
ARHGAP11B	0.069176714	1.830703257	0.067571647	0.106606598	no
IPO11	0.069154502	1.830112606	0.067660013	0.106729695	no
BUD13	-0.069154448	-1.830111165	0.067660229	0.106729695	no
NSL1	0.069142416	1.829791215	0.067708136	0.106796934	no
CXorf40B	0.069127259	1.829388174	0.067768525	0.106883847	no
RNF2	0.069104519	1.828783496	0.067859208	0.107018523	no
TNFRSF21	-0.069087295	-1.828325491	0.067927962	0.107118597	no
FAM101B	0.069054054	1.8274416	0.06806081	0.107311742	no
SLC11A2	-0.069053844	-1.827435995	0.068061653	0.107311742	no
FAM199X	-0.069052175	-1.827391636	0.068068326	0.107311742	no
ZNF79	0.069051339	1.827369383	0.068071673	0.107311742	no
SNTB1	0.069049768	1.827327614	0.068077957	0.107313281	no
AIMP1	0.069026377	1.826705633	0.068171588	0.107452496	no
ZNF782	-0.069015915	-1.826427438	0.068213501	0.107510178	no
OR52D1	0.068979859	1.825468702	0.068358107	0.107729691	no
N4BP3	-0.06895805	-1.824888809	0.068445694	0.107859318	no
AVPR1A	0.068948007	1.824621751	0.068486062	0.107906788	no
PPP1R3C	-0.0689479	-1.824618903	0.068486493	0.107906788	no
TUBA4A	-0.068942881	-1.824485457	0.068506671	0.107930171	no
LOC285205	0.068940049	1.824410144	0.068518062	0.107939705	no
TEX13A	0.068931491	1.824182594	0.068552486	0.107985522	no
C1orf213	-0.068914097	-1.823720083	0.0686225	0.108087388	no
MT1G	0.068910858	1.823633962	0.068635544	0.108099511	no
UBR7	-0.0689034	-1.823435664	0.068665585	0.108138401	no
ERBB3	-0.068894834	-1.823207894	0.068700103	0.108184336	no
CATSPER4	-0.068891643	-1.823123044	0.068712966	0.108196165	no
TSIX	-0.068886287	-1.822980629	0.06873456	0.108221738	no
COX7A2L	0.068866676	1.822459178	0.068813673	0.108337864	no
CLMN	0.068856385	1.822185538	0.068855219	0.108394832	no
COX8C	-0.068852679	-1.822086996	0.068870186	0.108409951	no
FBXL7	0.06884755	1.821950612	0.068890904	0.108434122	no
SLC4A5	-0.068840662	-1.821767478	0.068918732	0.108469479	no
RAB3IL1	0.068838571	1.821711873	0.068927183	0.108474336	no
ZNF583	0.068833578	1.821579113	0.068947365	0.108497651	no
C1orf185	0.068812926	1.821029974	0.069030893	0.108620639	no
SLC35A1	0.068803394	1.820776538	0.069069471	0.108672884	no
MMACHC	0.068801193	1.820718016	0.069078381	0.108678447	no

FAU	-0.0687826	-1.820223622	0.069153697	0.108788472	no
HBG1	0.068770514	1.819902278	0.069202686	0.108857069	no
ASPM	0.068760547	1.819637252	0.069243111	0.108912184	no
KIF14	0.068758073	1.819571478	0.069253146	0.108919495	no
ZNF180	0.068754342	1.819472288	0.069268283	0.108931227	no
C17orf47	0.068753579	1.819451989	0.069271381	0.108931227	no
C18orf45	0.068740651	1.819108234	0.069323861	0.109005275	no
FLJ33360	-0.068722941	-1.81863736	0.069395802	0.109109909	no
CCDC42B	0.068691928	1.817812755	0.069521933	0.109299723	no
AKR7A2	0.068677493	1.817428952	0.069580704	0.109383615	no
ZFPM1	-0.068673475	-1.817322124	0.069597069	0.109400836	no
LIMD1	0.068669537	1.817217405	0.069613115	0.109417551	no
CHMP6	-0.068663406	-1.817054392	0.069638099	0.109448311	no
TRPM6	0.068657489	1.816897075	0.069662216	0.109477706	no
ESCO2	0.068645354	1.816574416	0.069711704	0.109546963	no
PCDHGB5	-0.068637098	-1.816354893	0.069745389	0.109590468	no
IL20RA	0.068635546	1.816313638	0.069751721	0.109590468	no
B3GAT2	-0.068634583	-1.81628804	0.06975565	0.109590468	no
ING4	-0.068624781	-1.816027428	0.069795663	0.109644811	no
PITPNM2	-0.068602259	-1.815428604	0.069887674	0.109780825	no
PLEKHA1	-0.06859858	-1.815330767	0.069902717	0.109795923	no
EIF2C2	0.068588003	1.815049556	0.069945968	0.109855323	no
RALY	0.068577734	1.814776522	0.069987982	0.109912772	no
PVRL3	0.068567838	1.814513401	0.070028491	0.109967847	no
LOC441177	0.068557893	1.814248985	0.070069218	0.110023257	no
LASP1	0.068549893	1.814036293	0.070101993	0.110066172	no
RAD51AP1	0.068547857	1.813982143	0.070110339	0.110070728	no
CPSF4L	0.068536837	1.813689141	0.070155514	0.110133099	no
CYP3A4	-0.068502485	-1.812775814	0.070296485	0.110345833	no
GPS2	-0.068492785	-1.8125179	0.070336335	0.110399816	no
OTUD3	-0.06848038	-1.8121881	0.07038732	0.110471266	no
SCUBE3	0.068470918	1.811936524	0.070426233	0.110523758	no
CC2D1A	0.068455177	1.81151801	0.070491006	0.110616823	no
PYDC2	0.068435787	1.811002487	0.07057086	0.110729959	no
SND1	0.068435012	1.810981867	0.070574055	0.110729959	no
MED25	-0.068420496	-1.810595932	0.070633887	0.110815235	no
C10orf53	-0.068396065	-1.80994639	0.07073468	0.110964755	no
ASB6	0.068392673	1.809856197	0.070748686	0.110974879	no
MMP20	0.068391843	1.809834135	0.070752112	0.110974879	no
WNT16	0.068386032	1.809679634	0.070776109	0.111003907	no
LOC441208	-0.068376295	-1.809420763	0.070816331	0.111058376	no
GINS1	0.068374311	1.809367993	0.070824533	0.111062624	no
GLB1L2	-0.068363999	-1.80909384	0.070867155	0.111120842	no
UPK3A	0.068360614	1.809003833	0.070881153	0.111134172	no
ALDH8A1	-0.068359097	-1.808963522	0.070887423	0.111135384	no
C9orf129	-0.068352317	-1.808783262	0.070915465	0.111170728	no
VPS28	-0.068344515	-1.808575825	0.070947747	0.111212711	no
PGAM2	0.06834004	1.80845684	0.070966269	0.111233121	no
TOMM34	0.06833759	1.808391711	0.070976409	0.111240391	no
SCNN1G	0.068324487	1.80804335	0.071030667	0.111316799	no
LOC100128842	0.06832017	1.807928561	0.071048553	0.111336199	no

C3orf1	0.068314977	1.807790494	0.071070071	0.111361288	no
HES2	0.068311605	1.807700855	0.071084044	0.111374551	no
UBE2D2	0.068307919	1.80760285	0.071099324	0.11138986	no
BMP8A	0.068290639	1.807143428	0.071170989	0.111493496	no
IFLTD1	0.068272231	1.806654029	0.071247395	0.111604543	no
LHB	0.068245646	1.805947233	0.07135786	0.11176892	no
SSX2IP	0.068243481	1.805889687	0.071366861	0.111774358	no
FBXO46	-0.068241816	-1.805845412	0.071373786	0.111776545	no
TXNL1	0.068231127	1.805561227	0.071418249	0.111837515	no
FAM38B	0.06821909	1.805241209	0.071468345	0.111907296	no
HOPX	0.068215681	1.805150573	0.071482539	0.111920853	no
KRTAP5-10	-0.068170054	-1.803937536	0.071672726	0.11220994	no
RAB5A	-0.068162571	-1.803738594	0.071703956	0.112250142	no
ELK1	0.068138684	1.803103556	0.071803722	0.11239762	no
LOC100128573	-0.068127028	-1.802793673	0.071852447	0.112465183	no
UBE3A	-0.068125347	-1.802748973	0.071859478	0.112467481	no
CYorf15A	0.068099861	1.802071401	0.071966119	0.112625667	no
ANKRD20A3	-0.068098246	-1.802028473	0.07197288	0.11262753	no
LOC100134868	0.068075413	1.801421452	0.072068535	0.112768489	no
SLITRK6	-0.068061879	-1.80106164	0.072125284	0.112848552	no
DHX36	-0.068054217	-1.80085796	0.072157424	0.112890103	no
DNAI1	0.068041846	1.80052907	0.072209348	0.112962596	no
PPARD	0.068021271	1.799982063	0.072295773	0.113089048	no
LOC338758	-0.068009882	-1.799679305	0.072343645	0.113149678	no
LOC255167	-0.068009387	-1.79966615	0.072345726	0.113149678	no
TMIE	0.067993182	1.799235327	0.072413893	0.113243669	no
FAM185A	0.067991084	1.799179567	0.07242272	0.113243669	no
NFAT5	-0.06798997	-1.799149927	0.072427412	0.113243669	no
NUS1	0.067989776	1.799144783	0.072428226	0.113243669	no
PCMTD2	-0.067987895	-1.799094784	0.072436142	0.113247288	no
SIAE	0.067972445	1.798684038	0.072501198	0.113340233	no
DNAH17	-0.067970038	-1.798620046	0.072511338	0.11334732	no
KBTBD13	-0.067966565	-1.798527711	0.072525971	0.113361429	no
GP2	0.067943242	1.797907696	0.072624291	0.113505527	no
UPK3BL	0.067942034	1.797875561	0.072629389	0.113505527	no
ZNF502	-0.06793373	-1.79765481	0.072664424	0.113545455	no
OR2M4	-0.067933316	-1.797643805	0.072666171	0.113545455	no
SLC38A10	0.067917476	1.797222702	0.072733042	0.113641162	no
GUCY1A2	-0.067900725	-1.796777399	0.07280381	0.113742944	no
NUSAP1	0.067870831	1.795982686	0.072930249	0.113931677	no
SFRS12	0.067868597	1.795923287	0.072939706	0.113937648	no
LPGAT1	0.067849864	1.795425281	0.073019039	0.11405276	no
MPPE1	0.067834673	1.795021461	0.073083419	0.114144502	no
DENND1B	0.067817185	1.794556559	0.073157596	0.114251528	no
CREB3L3	-0.067811087	-1.794394431	0.073183479	0.114283121	no
POLR2G	0.067803486	1.794192383	0.073215744	0.114324677	no
APOH	0.067782208	1.793626728	0.073306138	0.114456986	no
MRPS9	-0.067767859	-1.793245281	0.073367147	0.114543396	no
ZFP64	-0.067763504	-1.79312951	0.073385672	0.114563471	no
FAM90A1	-0.067761367	-1.793072688	0.073394765	0.114568821	no
KLHL5	-0.067710953	-1.791732526	0.073609506	0.114895159	no

ATF2	-0.067687166	-1.791100186	0.073711008	0.115044709	no
GBP7	-0.067679513	-1.79089675	0.073743688	0.11508683	no
RNASEK	0.067648163	1.790063345	0.073877688	0.115287056	no
LRRC34	0.06763443	1.789698285	0.073936447	0.115369846	no
TAAR8	-0.067614886	-1.789178755	0.074020136	0.11549152	no
HGD	0.067601909	1.788833798	0.074075746	0.115569368	no
HMGB3L1	-0.067590448	-1.788529115	0.074124893	0.11563712	no
OR7A5	0.067587305	1.78844559	0.07413837	0.115642816	no
SIVA1	0.067586929	1.788435595	0.074139983	0.115642816	no
PCDHA3	-0.067584136	-1.78836133	0.074151968	0.115652588	no
PIGL	0.067575542	1.788132898	0.074188844	0.115701176	no
SNORA30	0.067574171	1.788096437	0.074194731	0.115701432	no
AKD1	-0.067562588	-1.787788537	0.074244462	0.115770054	no
TCEA1	-0.067554078	-1.78756232	0.074281017	0.115818122	no
MKL1	0.067547484	1.787387032	0.074309352	0.115853368	no
PRKAB1	0.067516139	1.786553815	0.074444163	0.116054598	no
ODZ4	0.067513453	1.786482398	0.074455727	0.116063677	no
CCDC54	0.067508957	1.786362881	0.074475083	0.1160849	no
ZBTB45	-0.067505703	-1.786276391	0.074489093	0.116097787	no
NDUFAF1	-0.067480868	-1.785616234	0.074596099	0.116255602	no
SMO	0.067474579	1.785449052	0.074623218	0.116288902	no
ANKRD13C	-0.067467669	-1.785265378	0.074653021	0.116326379	no
TMEM101	0.067455558	1.784943426	0.074705285	0.116398847	no
AGFG2	0.067436325	1.784432174	0.07478834	0.116519277	no
CIAPIN1	-0.067432596	-1.784333072	0.074804448	0.116535393	no
SCYL3	0.067411992	1.783785366	0.074893525	0.116665173	no
DTNA	0.067389522	1.783188081	0.074990764	0.116807648	no
UBL5	0.067374122	1.782778719	0.075057468	0.116902542	no
MTCP1NB	0.06736014	1.782407064	0.075118071	0.116987254	no
RELN	-0.067358904	-1.782374207	0.075123431	0.116987254	no
OR52N4	0.067347344	1.782066926	0.075173569	0.117056317	no
CLDN12	0.067335476	1.781751459	0.075225072	0.117127494	no
PEAR1	0.067334114	1.781715255	0.075230985	0.117127679	no
TRIM69	0.06732438	1.781456507	0.075273252	0.117184462	no
CXorf58	0.067290452	1.780554655	0.075420724	0.117405004	no
GSG1	0.067283148	1.780360528	0.075452498	0.117445424	no
LY6G6F	0.067275369	1.780153753	0.075486355	0.117489079	no
BCL2L13	0.067271964	1.780063227	0.075501182	0.117503109	no
C9orf152	0.067267612	1.779947544	0.075520132	0.117523555	no
PATE4	-0.067262511	-1.779811972	0.075542345	0.117549075	no
ZCRB1	-0.067258348	-1.779701294	0.075560484	0.117568252	no
C3orf57	0.067256797	1.779660085	0.075567238	0.117569713	no
AGAP7	-0.067203031	-1.778230947	0.075801788	0.117925198	no
ZNF143	-0.067201749	-1.778196862	0.07580739	0.117925198	no
UNC119	-0.067153052	-1.776902473	0.076020351	0.118247381	no
TTTY1B	0.067131814	1.77633795	0.076113383	0.118382982	no
TMEM89	0.06712792	1.776234446	0.076130451	0.118400419	no
TMEM52	0.067088602	1.775189391	0.076302951	0.118659569	no
ZCCHC4	0.067082228	1.77501996	0.076330947	0.118693977	no
C2CD4B	0.067079148	1.774938089	0.076344479	0.118705889	no
LOC100128554	-0.067057091	-1.774351823	0.076441433	0.118847499	no

MPP5	-0.06704767	-1.774101404	0.076482876	0.118902791	no
SHMT2	-0.067035143	-1.773768452	0.076538007	0.118979351	no
CD5L	0.067025436	1.773510434	0.076580753	0.119036647	no
GOLGA7	0.067019155	1.773343503	0.076608419	0.119070496	no
HEMK1	0.066996529	1.772742115	0.076708156	0.119211786	no
PLCH1	-0.066995858	-1.772724277	0.076711115	0.119211786	no
HHATL	-0.066993123	-1.772651566	0.076723182	0.119215864	no
PHYH	0.06699259	1.772637399	0.076725533	0.119215864	no
LHX6	-0.066951523	-1.771545869	0.076906861	0.119488429	no
RPL34	-0.066943419	-1.771330484	0.076942683	0.119534898	no
ACACB	0.066937544	1.771174316	0.076968665	0.119566075	no
UBE2Q2	0.066931711	1.771019292	0.076994463	0.119589427	no
ELP2	0.066931471	1.771012899	0.076995527	0.119589427	no
CHST10	-0.066918218	-1.770660649	0.077054174	0.119671323	no
TPX2	0.066914838	1.770570816	0.077069136	0.119685367	no
ITIH3	0.066910539	1.770456561	0.07708817	0.119705731	no
LYG2	0.066888184	1.769862389	0.077187213	0.119850324	no
FAM71B	0.06688228	1.769705441	0.077213392	0.119881766	no
NCOR1	-0.066878757	-1.769611821	0.077229011	0.119891819	no
FAM179B	-0.066878145	-1.769595546	0.077231727	0.119891819	no
SHH	0.066873611	1.769475047	0.077251835	0.119913827	no
LONP2	-0.066854892	-1.76897751	0.077334907	0.120033559	no
C11orf82	0.066835516	1.768462527	0.077420968	0.120151185	no
C19orf61	0.066835154	1.768452908	0.077422576	0.120151185	no
GRSF1	0.066806653	1.767695385	0.077549313	0.120338631	no
HOTAIR	0.066796237	1.767418538	0.077595674	0.12040133	no
RBP4	-0.06679073	-1.767272167	0.077620194	0.120430134	no
C1orf105	-0.066778231	-1.766939979	0.077675865	0.120507263	no
C16orf58	-0.066763575	-1.766550453	0.077741188	0.120599351	no
CDADC1	-0.066756178	-1.766353854	0.077774174	0.120637947	no
BCOR	-0.06675532	-1.766331045	0.077778002	0.120637947	no
RG9MTD1	0.066750139	1.766193337	0.077801115	0.12066454	no
PRIMA1	0.066732527	1.765725237	0.077879723	0.12077719	no
MGC27382	0.066729531	1.765645604	0.077893102	0.120788674	no
PTX4	0.06671169	1.76517143	0.077972807	0.120903	no
SPERT	0.06670954	1.765114306	0.077982414	0.120908622	no
BAALC	-0.066707619	-1.765063249	0.077991001	0.120912664	no
KRIT1	0.06669082	1.76461676	0.078066127	0.121019856	no
C19orf47	0.066661426	1.763835527	0.07819772	0.12121456	no
LNX2	0.066658263	1.763751452	0.078211893	0.121227235	no
C16orf81	-0.066645233	-1.763405155	0.07827029	0.12130845	no
PTPRU	0.066610346	1.762477957	0.078426823	0.121541738	no
SUPT4H1	0.066593754	1.762036975	0.078501361	0.121647928	no
CRNKL1	0.06657105	1.76143357	0.078603446	0.121796786	no
LOC286094	-0.066553665	-1.760971506	0.078681692	0.121908686	no
ZNHIT6	0.066523551	1.760171182	0.078817369	0.122109545	no
C6orf218	-0.066490327	-1.75928818	0.078967284	0.12233243	no
OR8D4	-0.066474863	-1.75887721	0.079037137	0.122431262	no
ANAPC16	0.066472603	1.758817147	0.07904735	0.122437702	no
TGM4	0.066463314	1.758570257	0.079089343	0.122493361	no
ADRA1A	-0.066447864	-1.758159663	0.07915922	0.122592195	no

ARID3B	0.066437042	1.757872039	0.079208199	0.122658653	no
NGFR	0.06642996	1.757683844	0.07924026	0.122698903	no
TLE2	-0.066414934	-1.757284501	0.079308327	0.122794897	no
TTC9C	0.066411378	1.757190001	0.079324442	0.122810442	no
THAP3	-0.066398929	-1.756859145	0.079380881	0.122888411	no
KIAA2018	-0.066394577	-1.75674347	0.079400621	0.122909559	no
MTMR6	0.066392383	1.756685179	0.07941057	0.122914615	no
C16orf13	-0.066391176	-1.756653098	0.079416046	0.122914615	no
SRM	0.06637538	1.756233299	0.079487731	0.123016146	no
LOC401052	0.066353489	1.755651515	0.079587163	0.123160601	no
LRIT3	-0.066342052	-1.755347563	0.079639151	0.123231621	no
ZIC1	0.066329251	1.755007375	0.07969737	0.12331227	no
ID3	0.066320574	1.754776772	0.079736855	0.123363922	no
TMEM184A	0.066316133	1.754658759	0.079757068	0.123385752	no
IL12RB2	0.066304907	1.754360414	0.079808185	0.123455386	no
RGS9BP	-0.06629912	-1.754206608	0.079834549	0.123486719	no
LOC641367	0.066294749	1.754090439	0.079854465	0.123508077	no
MSX1	0.066283273	1.753785473	0.07990677	0.123579521	no
FAM21C	-0.066281637	-1.753741998	0.079914229	0.123581603	no
HSPA4L	0.066246934	1.752819726	0.080072589	0.123817025	no
FBXL22	-0.066224069	-1.752212086	0.080177064	0.123969095	no
PLCD1	0.066222627	1.752173759	0.080183658	0.123969809	no
HAUS6	-0.066218646	-1.752067972	0.080201859	0.123988468	no
TUBD1	0.066194543	1.75142743	0.080312139	0.124149463	no
WDR43	0.066189994	1.751306536	0.080332967	0.124172166	no
ZNHIT3	-0.066185852	-1.751196466	0.080351934	0.124191988	no
ZNF331	0.06616305	1.750590497	0.080456418	0.124343973	no
DOCK6	0.06613855	1.749939399	0.080568806	0.12450815	no
CDC45	0.066124772	1.749573268	0.080632061	0.124596378	no
CCDC93	-0.066123169	-1.749530669	0.080639424	0.124598232	no
ZFYVE28	0.066108745	1.749147348	0.080705697	0.124688098	no
C1QL1	-0.066107827	-1.749122937	0.080709919	0.124688098	no
C15orf28	-0.066072127	-1.748174231	0.080874141	0.124932257	no
PCDHA7	-0.066052055	-1.747640823	0.080966594	0.12506552	no
HIPK1	0.06603214	1.747111597	0.081058407	0.125197774	no
RAB2B	-0.066026827	-1.746970414	0.081082914	0.12522606	no
LY6D	-0.066017602	-1.746725261	0.081125484	0.125282235	no
C12orf23	0.066005921	1.746414852	0.081179411	0.12535594	no
CYP26C1	-0.065961599	-1.745237017	0.0813843	0.125662729	no
DDIT4	-0.065932377	-1.744460477	0.081519612	0.125862048	no
CTRB2	0.065900855	1.743622833	0.081665777	0.126078091	no
CENPBD1	-0.065898364	-1.743556619	0.08167734	0.126086314	no
CCDC147	0.065889295	1.743315638	0.081719434	0.126133234	no
PRDM14	0.065889128	1.74331119	0.081720211	0.126133234	no
NPIPL3	-0.065886871	-1.743251214	0.081730691	0.126139779	no
CNPY1	0.065873976	1.742908544	0.081790585	0.126222582	no
FBXL6	-0.065857538	-1.742471751	0.081866982	0.126330839	no
GPR182	-0.065849142	-1.74224864	0.081906028	0.126381445	no
DPP8	-0.065832204	-1.741798518	0.081984848	0.126493411	no
GSG2	0.06581474	1.741334464	0.082066172	0.126609223	no
CACHD1	0.065804595	1.741064876	0.082113447	0.126672491	no

RFPL3S	-0.065795258	-1.740816755	0.082156977	0.126729973	no
KIF2C	0.06579305	1.740758084	0.082167273	0.126736185	no
C3orf77	-0.065791603	-1.740719631	0.082174021	0.126736926	no
ERC1	-0.065789586	-1.740666019	0.082183431	0.12674177	no
LOC100270746	-0.0657775	-1.740344858	0.082239817	0.126819054	no
NCRNA00107	-0.06577449	-1.740264871	0.082253865	0.126831044	no
ACTRT1	-0.06577206	-1.740200314	0.082265205	0.126838855	no
PBK	0.065767446	1.740077705	0.082286745	0.126862392	no
ARL5B	-0.065726396	-1.738986895	0.082478583	0.127148455	no
IL1RL1	0.065712703	1.738623015	0.082542658	0.127237531	no
ZNF570	0.065697263	1.738212744	0.08261495	0.127339259	no
SLC22A3	-0.065689172	-1.73799775	0.082652854	0.127387971	no
PTER	-0.065681275	-1.737787909	0.082689863	0.127427958	no
C17orf88	0.065680946	1.737779174	0.082691404	0.127427958	no
RPL28	0.065677645	1.737691447	0.082706881	0.127434802	no
AQP12A	-0.06567731	-1.737682544	0.082708452	0.127434802	no
NUDT13	-0.065644507	-1.736810902	0.082862353	0.1276622	no
CDKL3	-0.065625589	-1.736308203	0.082951218	0.127789372	no
SGCZ	-0.065620143	-1.736163487	0.082976815	0.127819066	no
C2orf78	-0.065611482	-1.735933351	0.083017533	0.127872046	no
LRP6	-0.065588	-1.735309371	0.083128016	0.12803247	no
RASGRF2	-0.065572716	-1.73490325	0.083199989	0.128133561	no
C2orf74	-0.065559544	-1.734553253	0.083262057	0.128219381	no
LOC729082	-0.06555726	-1.734492544	0.083272826	0.1282262	no
STK19	-0.065550921	-1.734324123	0.08330271	0.128262447	no
SOST	0.065549303	1.734281131	0.08331034	0.128264427	no
C5orf33	-0.065541948	-1.734085691	0.083345031	0.128308066	no
HEXIM1	0.065540596	1.734049771	0.083351408	0.128308114	no
CHST3	-0.065526053	-1.73366333	0.083420042	0.12840399	no
LDB2	-0.065522565	-1.733570647	0.083436509	0.128419561	no
RAB17	0.065510283	1.733244297	0.083494515	0.128499057	no
CYMP	-0.06550861	-1.73319985	0.083502418	0.128501438	no
PLEKHH1	-0.065461286	-1.73194236	0.08372625	0.128836085	no
TMEM126A	0.065441316	1.731411743	0.083820845	0.12897183	no
FLJ10213	0.065428754	1.731077949	0.083880397	0.129053638	no
PSMA6	0.065366599	1.729426441	0.084175543	0.129497879	no
RASSF10	0.065354676	1.729109623	0.084232258	0.129575272	no
SLC25A34	-0.06534516	-1.728856771	0.084277545	0.129635074	no
SAC3D1	-0.065330463	-1.728466271	0.084347525	0.129732845	no
PSMB7	-0.0653235	-1.728281262	0.084380695	0.129773992	no
FBXL14	-0.065315524	-1.728069315	0.084418709	0.129821363	no
PSORS1C3	0.065314342	1.728037931	0.084424339	0.129821363	no
FBXO16	0.06529864	1.72762071	0.084499214	0.129926618	no
C17orf74	-0.06529623	-1.727556688	0.084510708	0.12993441	no
DMRTA1	0.065289276	1.72737191	0.08454389	0.129969985	no
ZMYM4	0.065288687	1.727356246	0.084546703	0.129969985	no
LOC100272146	-0.065281178	-1.727156732	0.084582543	0.130015195	no
KCNK5	0.065277931	1.727070476	0.084598042	0.130029133	no
SPRR1A	-0.065265664	-1.72674453	0.084656629	0.130109292	no
KLKP1	0.065248138	1.726278852	0.08474039	0.130228125	no
DHFRL1	-0.065236748	-1.725976214	0.084794862	0.130300003	no

TRIM16	-0.065235663	-1.725947388	0.084800051	0.130300003	no
STK36	0.065219228	1.725510718	0.084878701	0.130410941	no
INSM1	-0.065191377	-1.724770719	0.085012118	0.130606003	no
CALCB	0.065178787	1.724436189	0.085072487	0.130688819	no
RPL13AP6	-0.065173852	-1.724305075	0.085096158	0.13071525	no
PMF1	-0.065118226	-1.72282709	0.085363352	0.131114861	no
NCRNA00203	-0.065116994	-1.722794359	0.085369276	0.131114861	no
PRSS55	-0.065115414	-1.722752393	0.085376874	0.131116569	no
LOC100130932	0.065104845	1.722471581	0.085427723	0.131184695	no
ACY1	0.065101786	1.722390291	0.085442447	0.131191705	no
RNF219	-0.0651012	-1.722374734	0.085445265	0.131191705	no
TFAP2A	-0.065096263	-1.722243566	0.08546903	0.131218228	no
GPD2	0.065092096	1.72213285	0.085489092	0.131239064	no
PDCD2	0.065087408	1.72200827	0.085511672	0.131263761	no
LOC100133957	-0.065080525	-1.721825399	0.085544826	0.131304684	no
VPS24	0.065073779	1.721646157	0.085577332	0.131344606	no
GTF2A2	0.065069666	1.7215369	0.085597151	0.131365052	no
KLHL29	0.065022555	1.7202852	0.085824471	0.13170392	no
EIF2B4	-0.064994109	-1.719529411	0.085961965	0.131904903	no
KPTN	-0.064970935	-1.718913698	0.086074109	0.13206696	no
SNORD89	0.06495094	1.718382454	0.086170962	0.132205533	no
NTS	0.064909547	1.717282701	0.086371744	0.132503522	no
PRELID1	0.06489553	1.716910301	0.086439819	0.13259474	no
KILLIN	0.064894429	1.716881053	0.086445167	0.13259474	no
PDZRN3	-0.064893254	-1.71684982	0.086450879	0.13259474	no
OR5C1	-0.064883118	-1.716580529	0.086500137	0.132660227	no
TACC1	0.064859569	1.715954874	0.08661467	0.132825803	no
RGS20	-0.064853846	-1.71580283	0.086642521	0.132858437	no
RIPPLY1	-0.064841562	-1.715476461	0.08670233	0.132940067	no
CHAC1	0.064833054	1.71525041	0.086743775	0.132993528	no
RPRML	-0.0648229	-1.714980652	0.086793255	0.133059298	no
MYBPH	0.064818219	1.714856268	0.086816077	0.133084195	no
CARTPT	-0.064811475	-1.714677111	0.086848958	0.133124506	no
WIF1	-0.064805962	-1.71453062	0.086875851	0.133155633	no
NEK7	0.064791553	1.714147828	0.086946156	0.133253289	no
DSCR6	0.064785666	1.713991402	0.086974899	0.133287237	no
ZNF281	-0.064780621	-1.713857385	0.086999531	0.133314879	no
DPPA5	-0.064775839	-1.713730325	0.087022889	0.133340566	no
NTF4	0.06477417	1.713685981	0.087031042	0.133342954	no
PAX1	-0.064769096	-1.713551182	0.087055831	0.133362319	no
LARP1	-0.064768882	-1.713545506	0.087056875	0.133362319	no
SRP54	0.064755749	1.713196573	0.087121067	0.133450545	no
C9orf71	0.064742356	1.712840759	0.087186566	0.133540756	no
LOC100128292	-0.064727968	-1.712458499	0.087256976	0.133638478	no
GJD4	-0.064716863	-1.712163472	0.08731135	0.133711625	no
CCDC21	0.064711705	1.712026441	0.087336615	0.133740186	no
SPAG8	0.064697631	1.71165254	0.087405581	0.133835659	no
BRD7P3	-0.064689612	-1.711439485	0.087444899	0.133885722	no
PPAP2B	-0.064666151	-1.710816185	0.087560007	0.134051811	no
ACER3	0.064663135	1.710736075	0.08757481	0.134064322	no
FASTKD5	0.064658103	1.71060238	0.087599519	0.134091996	no

C11orf53	-0.064653983	-1.710492931	0.087619752	0.134112812	no
ACTL8	-0.064642592	-1.710190291	0.087675717	0.134188315	no
SMC1A	-0.064633563	-1.709950412	0.087720096	0.13423739	no
ATP9B	-0.064633367	-1.709945202	0.08772106	0.13423739	no
UCHL5	-0.064614518	-1.709444443	0.087813764	0.134369082	no
COX17	0.064605906	1.709215658	0.087856145	0.134423758	no
ARL17B	-0.064601535	-1.709099534	0.087877662	0.134446506	no
PRAMEF13	0.06458407	1.708635552	0.087963679	0.134567922	no
VPREB1	0.064576321	1.708429666	0.088001869	0.134616161	no
NEK1	-0.064553595	-1.707825931	0.088113936	0.134777391	no
FAM45B	-0.064531481	-1.707238439	0.088223098	0.134934155	no
CCDC43	-0.064520318	-1.706941864	0.088278246	0.135008289	no
ABHD13	0.064512813	1.706742478	0.088315338	0.135054799	no
HBZ	-0.06450637	-1.70657133	0.088347186	0.135093284	no
ZNF622	-0.064501737	-1.706448228	0.088370099	0.135118102	no
C16orf55	0.064486575	1.706045434	0.088445107	0.135222562	no
VEGFA	0.064479913	1.70586845	0.08847808	0.135262747	no
ANKRD29	0.064478417	1.705828707	0.088485486	0.135263841	no
ORC1L	0.064471958	1.705657117	0.088517467	0.135302498	no
MRPS30	0.064443258	1.704894677	0.088659681	0.135509633	no
C4orf37	0.064438674	1.704772901	0.088682412	0.135525066	no
PAF1	-0.064438519	-1.704768765	0.088683184	0.135525066	no
ROBO4	0.064436039	1.704702891	0.088695482	0.135533615	no
POLI	-0.06441548	-1.704156715	0.088797505	0.135679259	no
PPP2CB	-0.064404577	-1.703867074	0.088851646	0.135751725	no
TM4SF5	0.064398264	1.703699367	0.088883007	0.135789379	no
METTL13	0.06439481	1.703607604	0.088900171	0.135805338	no
SCRN3	-0.064391148	-1.703510312	0.088918371	0.135822879	no
NBAS	-0.064387726	-1.703419415	0.088935378	0.135838594	no
ATF7IP	-0.064348861	-1.702386927	0.089128741	0.13612365	no
C19orf45	-0.064339041	-1.702126071	0.089177647	0.136188056	no
TBP	-0.06432252	-1.701687173	0.089259983	0.136303499	no
PGAP2	0.064315932	1.701512155	0.089292832	0.136343364	no
NRN1L	-0.064259995	-1.70002617	0.089572135	0.13675951	no
SCARNA9L	-0.064254337	-1.699875875	0.089600424	0.136792371	no
SLC9A3R2	-0.064235856	-1.699384913	0.089692882	0.136923188	no
KIT	-0.064234214	-1.699341296	0.0897011	0.136925394	no
SETD7	0.064231636	1.699272815	0.089714004	0.136934752	no
LOC728855	0.064205742	1.698584945	0.089843699	0.13712236	no
CYP3A5	0.064194282	1.698280525	0.089901144	0.137199677	no
LEAP2	-0.0641856	-1.698049886	0.089944686	0.137255768	no
ZNF826	-0.064180795	-1.697922233	0.089968793	0.137282193	no
FAHD2B	0.064173825	1.697737076	0.090003769	0.137325198	no
CNGB1	-0.06415513	-1.697240446	0.090097635	0.137458042	no
USP6	-0.06414003	-1.696839343	0.090173503	0.137563411	no
LOC729668	0.064111751	1.696088102	0.090315739	0.137770002	no
SNORA15	0.06410949	1.696028054	0.090327116	0.137776961	no
KDM4DL	0.064072406	1.69504294	0.090513924	0.138051488	no
PNPLA3	-0.064063952	-1.694818376	0.090556552	0.138106085	no
UTRN	0.064047164	1.694372409	0.090641256	0.138219919	no
UBE2T	0.064045843	1.694337328	0.090647922	0.138219919	no

MT1E	0.064045094	1.69431743	0.090651703	0.138219919	no
MYSM1	0.064025841	1.693805982	0.090748932	0.138357733	no
MAP2K1	-0.06402109	-1.693679796	0.090772933	0.138383891	no
KCNMB3	0.064009298	1.693366554	0.090832536	0.138453985	no
PAX3	0.064009284	1.693366178	0.090832608	0.138453985	no
GOLGA6L1	-0.063996769	-1.693033717	0.090895902	0.138540018	no
SIP1	-0.063993598	-1.692949492	0.090911942	0.138554022	no
ADH7	0.063990663	1.692871531	0.090926792	0.138566208	no
C5orf46	0.063987397	1.692784771	0.09094332	0.13858095	no
SMARCA2	-0.06398178	-1.692635561	0.09097175	0.138613825	no
FKBP1AP1	-0.06395362	-1.691887535	0.091114386	0.138820698	no
HIBADH	0.063945	1.691658544	0.091158087	0.138876815	no
LRRC16A	0.063936499	1.69143274	0.091201196	0.13892286	no
EAF2	0.06393633	1.69142825	0.091202054	0.13892286	no
EDAR	-0.063899519	-1.690450414	0.091388927	0.139197026	no
SLC17A2	0.063884527	1.690052188	0.09146512	0.139302583	no
KRTAP12-3	0.06386673	1.689579441	0.091555637	0.13942994	no
SNHG12	0.063861304	1.689435319	0.091583247	0.139461481	no
NBPF14	0.063847696	1.68907385	0.091652524	0.139556464	no
C7orf11	0.063843906	1.688973156	0.091671829	0.139575348	no
ANKRD50	-0.063840364	-1.688879089	0.091689868	0.1395923	no
ME3	-0.063837929	-1.688814398	0.091702274	0.139600676	no
MMP23B	0.063835953	1.688761905	0.091712343	0.139605492	no
RPL37	-0.063796194	-1.687705783	0.091915099	0.139902227	no
IGSF1	-0.063795014	-1.687674458	0.091921119	0.139902227	no
HTR1B	-0.063773017	-1.68709016	0.092033455	0.140062656	no
RAPH1	0.063770967	1.687035709	0.092043929	0.140068053	no
CCL16	0.063758157	1.686695439	0.092109406	0.140157143	no
IFNA7	0.063739122	1.686189813	0.09220677	0.140294737	no
NDUFA4	0.06373595	1.686105552	0.092223003	0.140308878	no
CAPS2	-0.063728836	-1.685916591	0.092259416	0.140353716	no
DGAT2L6	0.063724373	1.685798036	0.092282268	0.140368665	no
CABYR	-0.063724205	-1.68579357	0.092283129	0.140368665	no
DNAL4	-0.063718643	-1.685645846	0.09231161	0.140401424	no
KIAA0562	0.063704265	1.685263927	0.092385275	0.140502895	no
ZNF155	0.063698716	1.685116548	0.092413714	0.140534873	no
DUS1L	0.063697451	1.685082925	0.092420204	0.140534873	no
MND1	0.063688828	1.684853878	0.092464419	0.140591533	no
ADNP2	-0.063686156	-1.68478291	0.092478122	0.140601795	no
ARMCX3	-0.063679492	-1.684605899	0.092512308	0.140643194	no
TOMM7	0.063675281	1.684494047	0.092533915	0.140665465	no
FAM175B	-0.063658971	-1.684060825	0.092617641	0.140782157	no
CDR2L	0.063654348	1.683938017	0.092641387	0.140796971	no
OR1G1	0.063653874	1.683925428	0.092643821	0.140796971	no
OGFRL1	0.063653006	1.683902377	0.092648279	0.140796971	no
LOC145474	0.063604281	1.682608152	0.092898836	0.14116713	no
NDUFB4	0.063582026	1.682017012	0.093013459	0.141330687	no
CUL5	-0.063577835	-1.681905706	0.093035055	0.141352877	no
DISC2	0.063563907	1.681535759	0.09310686	0.141451344	no
RNF180	-0.063560121	-1.681435191	0.093126387	0.141470182	no
CDKN2A	0.06355879	1.681399833	0.093133253	0.141470182	no

SCARNA8	0.063555871	1.681322299	0.093148311	0.141482425	no
CDK16	-0.063550606	-1.681182442	0.093175478	0.141513057	no
CDK1	0.06353937	1.680884024	0.093233467	0.141590493	no
DRD5	-0.063529791	-1.680629567	0.093282935	0.141654979	no
ZNF438	-0.063518802	-1.680337685	0.093339706	0.141725821	no
PON3	-0.063518047	-1.680317636	0.093343607	0.141725821	no
XAGE3	-0.063514673	-1.680228036	0.09336104	0.141741646	no
CDCP2	-0.063490567	-1.679587739	0.093485697	0.141920245	no
FAM54B	0.0634757	1.679192857	0.093562642	0.14202639	no
GPAA1	0.06346221	1.678834538	0.093632506	0.142121772	no
APBA3	0.063440656	1.678262054	0.093744215	0.142280649	no
FAM46D	-0.063438124	-1.678194794	0.093757346	0.142289898	no
CILP2	0.063426019	1.677873264	0.09382014	0.14237451	no
LOC148709	-0.063419884	-1.677710338	0.093851972	0.142412127	no
PUS1	0.063413872	1.677550643	0.093883182	0.142448793	no
C5orf40	-0.063412427	-1.677512267	0.093890682	0.142449484	no
PARP16	0.063406418	1.677352671	0.093921883	0.142486128	no
ABCC13	0.063391365	1.676952828	0.094000086	0.142594069	no
FNIP1	-0.063361702	-1.676164975	0.094154333	0.142817338	no
C2orf15	0.063357502	1.676053428	0.094176188	0.142839772	no
EBNA1BP2	0.063327643	1.675260357	0.09433169	0.143064895	no
KIAA0100	0.063321808	1.675105371	0.094362103	0.143100285	no
SREBF1	-0.063303617	-1.674622224	0.094456962	0.143233396	no
FGF6	-0.063298472	-1.674485566	0.094483807	0.143263358	no
RIT2	-0.063294108	-1.674369642	0.094506584	0.143287147	no
C7orf16	0.063276144	1.673892532	0.094600372	0.143409311	no
C4orf26	0.063275958	1.673887589	0.094601344	0.143409311	no
ZNF660	-0.063259207	-1.673442675	0.094688872	0.143531235	no
PTGER2	0.063250569	1.673213242	0.094734034	0.143588927	no
MTHFD1	-0.063241208	-1.672964626	0.094782991	0.143652362	no
MYOZ3	0.063238535	1.672893617	0.094796978	0.143662791	no
RGL3	-0.063220368	-1.672411115	0.094892061	0.143796109	no
DYM	0.063169334	1.671055678	0.095159576	0.144190684	no
AGFG1	0.063166216	1.670972855	0.095175941	0.144204675	no
RBM27	-0.063158975	-1.670780535	0.095213953	0.144251457	no
ASPH	0.063150132	1.670545674	0.095260389	0.144300992	no
ANKRD31	-0.063150031	-1.670542975	0.095260923	0.144300992	no
GJB3	0.063146189	1.670440935	0.095281103	0.144320748	no
ZNHIT1	0.063135797	1.670164935	0.095335706	0.144392636	no
NUPR1	0.063097969	1.669160257	0.095534679	0.144683157	no
C1orf86	0.063093062	1.669029934	0.095560514	0.144711442	no
STS	0.06308987	1.668945158	0.095577322	0.144726056	no
SLC35E4	0.063069859	1.668413683	0.095682752	0.14487485	no
C2orf3	0.063065938	1.668309539	0.095703422	0.144895296	no
KCNG3	-0.063059403	-1.668135985	0.095737876	0.144936606	no
LOC643486	-0.063045537	-1.667767708	0.09581102	0.145036478	no
FAM174B	-0.063020588	-1.667105082	0.095942738	0.145224996	no
GTPBP3	-0.062978134	-1.665977558	0.096167203	0.145533874	no
PCDHA2	-0.062977168	-1.665951919	0.096172312	0.145533874	no
MCM9	0.062976932	1.665945656	0.09617356	0.145533874	no
ZNF497	-0.062976549	-1.665935468	0.09617559	0.145533874	no

NKAPL	0.062965601	1.665644717	0.096233545	0.145610673	no
CLEC1B	0.062961843	1.665544895	0.096253448	0.14562989	no
LOC100286793	0.062947634	1.665167541	0.096328719	0.145732868	no
MYOT	-0.062925927	-1.664591037	0.096443806	0.145896062	no
C4orf7	0.062922855	1.664509441	0.096460104	0.145909799	no
TMEM14B	0.062882974	1.663450262	0.096671862	0.146219174	no
NKAIN2	-0.062853616	-1.662670579	0.096827979	0.146439222	no
COL4A5	0.062852892	1.662651356	0.096831831	0.146439222	no
SUPT6H	-0.062842219	-1.662367889	0.096888642	0.146514178	no
UCK2	0.06283465	1.662166871	0.096928945	0.146564162	no
C2orf81	0.062821001	1.661804401	0.097001653	0.146663133	no
NDUFB10	-0.062800006	-1.661246817	0.097113584	0.146821389	no
ZNF3	-0.06279759	-1.661182643	0.097126473	0.146829896	no
BTF3L1	-0.062770572	-1.66046512	0.097270677	0.147036901	no
PTPRM	-0.062742099	-1.659708953	0.097422834	0.147255896	no
OTUD6A	-0.062739355	-1.659636086	0.097437507	0.147267064	no
INADL	0.062735411	1.659531319	0.097458605	0.147287942	no
C11orf35	0.062712707	1.658928385	0.097580101	0.147460534	no
RPF2	0.062640017	1.656997944	0.097969914	0.148038545	no
DNAJC5G	-0.062625431	-1.656610587	0.098048283	0.148145893	no
ACSM2A	-0.062621109	-1.656495798	0.098071517	0.148169924	no
NR2E1	0.062613475	1.65629308	0.098112558	0.148220854	no
SLC39A11	-0.062604286	-1.656049036	0.098161984	0.148273411	no
KCND3	-0.06260428	-1.656048885	0.098162014	0.148273411	no
RBM15B	-0.062591664	-1.655713848	0.098229901	0.148357758	no
VTI1A	-0.062591176	-1.655700875	0.098232531	0.148357758	no
INE2	-0.062589413	-1.655654055	0.098242021	0.148361008	no
RFPL4B	-0.062579707	-1.65539631	0.098294277	0.148428837	no
CGB7	0.06257421	1.655250322	0.098323886	0.148462458	no
KIRREL2	0.062549604	1.654596874	0.0984565	0.148651596	no
TBC1D25	-0.06254098	-1.654367838	0.098503016	0.148710722	no
SNORA36A	-0.062516533	-1.65371862	0.098634964	0.148898806	no
DCTPP1	0.062508406	1.653502802	0.098678859	0.148953947	no
MNX1	-0.06249374	-1.653113336	0.09875811	0.149062447	no
PPARGC1A	-0.06248233	-1.652810315	0.098819807	0.149135427	no
ALB	-0.062482069	-1.652803401	0.098821215	0.149135427	no
QDPR	-0.062480571	-1.652763597	0.098829322	0.14913653	no
NUF2	0.062472536	1.652550235	0.098872785	0.149190981	no
KIF25	-0.062459113	-1.65219377	0.098945433	0.14928946	no
SLC4A8	-0.062433	-1.651500306	0.099086885	0.149491728	no
MAP6	-0.062428134	-1.651371087	0.099113261	0.149520363	no
MAP7	-0.062422793	-1.65122925	0.099142219	0.14955289	no
GLMN	0.06241792	1.651099839	0.099168645	0.149581593	no
WFDC11	0.062410686	1.650907755	0.099207881	0.149629611	no
LOC100271836	-0.062396955	-1.650543103	0.0992824	0.149730834	no
SLIT2	0.062393107	1.65044093	0.099303288	0.149751165	no
C1orf27	-0.062379719	-1.650085391	0.099375999	0.149849639	no
HSPD1	-0.062377417	-1.650024277	0.099388502	0.149857315	no
SLMO2	0.062354822	1.649424249	0.099511324	0.150031316	no
C1orf123	0.062348469	1.649255531	0.099545881	0.150072226	no
P4HTM	-0.062296779	-1.64788289	0.099827385	0.150485392	no

CDYL2	-0.062256467	-1.6468124	0.100047365	0.150805758	no
C6orf145	0.062238437	1.646333612	0.100145879	0.150942999	no
SLC10A5	-0.062236193	-1.646274024	0.100158145	0.150950233	no
PRKRIR	-0.062224684	-1.645968409	0.100221074	0.151033816	no
TMEM141	0.062211268	1.645612145	0.100294472	0.151132332	no
SPAG16	-0.062210002	-1.645578545	0.100301396	0.151132332	no
C6orf176	0.062207047	1.645500055	0.100317574	0.151145443	no
LOC100130386	-0.06220233	-1.645374815	0.100343391	0.151173075	no
DEPDC4	0.062196075	1.645208709	0.10037764	0.151213406	no
KNCN	0.062170091	1.644518719	0.100520009	0.151416595	no
FRK	0.062162852	1.644326475	0.100559704	0.151465104	no
KLKB1	0.062158333	1.644206468	0.10058449	0.151491151	no
ADRA1D	0.062152128	1.64404171	0.100618526	0.151531126	no
RPP21	-0.062145723	-1.643871627	0.100653673	0.151572766	no
GRIN2A	-0.062134992	-1.643586669	0.100712579	0.151650176	no
SRGAP1	-0.062112322	-1.642984674	0.100837113	0.151826389	no
LASS5	0.062110942	1.642948033	0.100844697	0.151826501	no
GCM2	-0.062103888	-1.642760727	0.100883472	0.15187357	no
PFDN5	0.062095106	1.642527535	0.100931763	0.151934956	no
ATP6V0D1	-0.062078668	-1.642091031	0.101022207	0.152059781	no
HOMEZ	0.062063327	1.641683672	0.10110667	0.152170815	no
C21orf56	-0.062062137	-1.64165206	0.101113227	0.152170815	no
THRSP	0.06206117	1.641626387	0.101118552	0.152170815	no
GAS7	0.06200421	1.640113883	0.101432684	0.152632182	no
CTAGE1	0.061995298	1.639877226	0.101481905	0.152694884	no
CALM2	0.061991669	1.639780858	0.101501954	0.152713685	no
PDCD5	0.061973368	1.639294899	0.101603103	0.152854493	no
APOA1BP	0.061963465	1.639031948	0.101657868	0.152920057	no
NFX1	-0.061962752	-1.63901302	0.101661811	0.152920057	no
CCRL1	0.061949902	1.638671817	0.10173291	0.153015621	no
CH25H	0.061925967	1.638036257	0.101865454	0.153203581	no
DOK5	0.061837807	1.635695319	0.102354835	0.153928151	no
MGC14436	-0.061818381	-1.635179513	0.102462918	0.154079232	no
TIMM17B	-0.061798635	-1.634655188	0.102572879	0.154233115	no
ZNF44	0.061792216	1.634484757	0.102608642	0.154275416	no
PACSIN3	0.061772418	1.633959062	0.102719016	0.154429882	no
C17orf89	-0.061758956	-1.633601609	0.102794119	0.154527603	no
PCSK9	-0.061758027	-1.633576958	0.1027993	0.154527603	no
FAM153C	-0.061752045	-1.633418106	0.102832692	0.154566305	no
MECR	-0.061748455	-1.633322785	0.102852733	0.154584936	no
KIAA1614	0.061714003	1.632408009	0.103045222	0.15486273	no
TAAR6	-0.061708861	-1.632271482	0.103073975	0.154894427	no
C9orf156	-0.061698782	-1.632003864	0.103130354	0.154967633	no
ATOH1	0.06169611	1.631932895	0.10314531	0.154978587	no
POLR2J3	-0.061665163	-1.631111191	0.103318593	0.155227414	no
HAX1	0.0616523	1.630769657	0.103390685	0.155324184	no
FIGF	0.06164883	1.630677515	0.103410141	0.15534187	no
GTF2I	-0.061635387	-1.630320581	0.103485538	0.155443581	no
FECH	0.061566061	1.628479856	0.103875057	0.156017079	no
UPF1	-0.061528378	-1.62747932	0.104087272	0.156324206	no
DPY19L4	-0.061512455	-1.627056533	0.104177049	0.156447417	no

AKAP8	-0.061441089	-1.625161694	0.104580168	0.157041135	no
GPRIN2	-0.061408861	-1.624306007	0.104762618	0.157303425	no
PKN3	0.061403738	1.624170005	0.10479164	0.157335317	no
L1TD1	0.061401	1.624097305	0.104807156	0.157346928	no
RAPGEF1	0.061388963	1.6237777	0.104875391	0.157437678	no
C4orf43	0.061363808	1.623109815	0.105018096	0.157633307	no
WEE2	-0.061363243	-1.623094825	0.1050213	0.157633307	no
LOC285768	-0.061351058	-1.622771307	0.105090483	0.157725438	no
TNNI1	0.061310859	1.621704016	0.105318973	0.158045885	no
IFITM5	-0.061310745	-1.621700968	0.105319626	0.158045885	no
EPS15	0.061291227	1.621182771	0.105430707	0.158200834	no
LTBP3	0.061284644	1.621007983	0.105468196	0.158245342	no
DNAH2	0.061280546	1.620899184	0.105491536	0.158268617	no
C16orf59	-0.061264843	-1.620482261	0.105581017	0.158391111	no
DNMBP	-0.061253195	-1.620173002	0.105647429	0.158478982	no
ZNF746	-0.061245272	-1.619962667	0.105692617	0.158535005	no
CYP2C18	-0.061237978	-1.619768995	0.105734239	0.15858567	no
IPO9	-0.06122955	-1.619545242	0.105782341	0.158646048	no
BMP8B	0.061227699	1.619496086	0.105792911	0.158650132	no
LOC100131691	0.061225348	1.619433679	0.105806331	0.158658489	no
CES3	0.061200749	1.618780572	0.105946861	0.158854446	no
TIMM44	-0.061199723	-1.618753333	0.105952725	0.158854446	no
ABCA2	-0.061186065	-1.618390727	0.106030815	0.158949888	no
NAPRT1	-0.061184509	-1.618349401	0.106039718	0.158949888	no
GOLGA6C	-0.061184465	-1.618348238	0.106039968	0.158949888	no
4-Sep	-0.061181796	-1.618277371	0.106055237	0.158960988	no
SNORA24	0.061157308	1.617627243	0.106195386	0.159159253	no
COL25A1	0.061144973	1.617299737	0.106266043	0.159253344	no
TUBA3C	-0.061141309	-1.617202458	0.106287038	0.159261549	no
BTG4	0.061141268	1.617201371	0.106287273	0.159261549	no
ATP1B1	-0.061134752	-1.617028383	0.106324614	0.159303703	no
LY6G6E	-0.061133609	-1.616998047	0.106331164	0.159303703	no
PSME3	0.06111771	1.616575939	0.10642233	0.159428473	no
NPC1	0.061103045	1.616186593	0.106506475	0.159542707	no
HSDL2	-0.061097369	-1.616035885	0.106539061	0.159579695	no
BCL11B	-0.061095115	-1.615976043	0.106552001	0.159587256	no
RPS4X	-0.061092672	-1.615911184	0.106566029	0.159596442	no
GSTA1	0.06104928	1.614759178	0.10681542	0.159948403	no
TTN	-0.06104903	-1.61475252	0.106816863	0.159948403	no
TBC1D22B	0.061041698	1.614557861	0.10685905	0.159999724	no
CRH	-0.061035377	-1.61439005	0.106895429	0.160042341	no
FLJ39653	-0.061033055	-1.614328419	0.106908792	0.160050496	no
PSMD13	0.061030707	1.614266079	0.10692231	0.160058882	no
TMEM68	0.060995971	1.613343872	0.107122446	0.160346607	no
ATF7	0.060986815	1.613100797	0.107175248	0.160413766	no
LAGE3	0.060980076	1.612921877	0.107214126	0.160460079	no
CCDC61	0.060970273	1.612661611	0.107270701	0.160532867	no
LOC284100	-0.060933695	-1.611690515	0.107482	0.160837176	no
CYC1	-0.06092821	-1.611544916	0.107513709	0.160872719	no
SLC8A1	0.060924247	1.611439701	0.107536628	0.160895105	no
HSD11B1	0.060920389	1.611337265	0.107558945	0.160916587	no

MRE11A	0.060917198	1.611252549	0.107577405	0.160932295	no
ATP8B5P	-0.060914315	-1.611176005	0.107594086	0.16094534	no
OPALIN	-0.060905582	-1.610944159	0.107644623	0.161009025	no
VGLL1	-0.060896113	-1.610692793	0.107699437	0.161079096	no
RNASE7	0.060890026	1.610531176	0.107734692	0.161119905	no
TRAF7	0.060879074	1.610240417	0.10779814	0.161202869	no
LOC285375	-0.060874239	-1.610112081	0.107826154	0.161231761	no
GSX1	-0.060872988	-1.610078848	0.107833409	0.161231761	no
C11orf48	-0.060867385	-1.609930114	0.107865885	0.161258491	no
SCARNA21	0.060867152	1.609923915	0.107867239	0.161258491	no
STX5	-0.060857783	-1.609675189	0.107921566	0.16132778	no
SLC45A4	-0.060849504	-1.609455395	0.107969592	0.161387639	no
NUP88	0.060830796	1.60895874	0.108078175	0.161538001	no
POLR2J	0.060826458	1.60884357	0.108103367	0.16156371	no
SERPINE2	-0.060820167	-1.60867656	0.108139906	0.161606374	no
NR0B1	-0.060816872	-1.608589074	0.108159051	0.161623037	no
OR2J3	0.060788817	1.607844277	0.108322144	0.161854787	no
PPP1CC	0.060786883	1.607792932	0.108333395	0.161859635	no
MAPK6	-0.060783015	-1.607690264	0.108355894	0.161879047	no
CDCA7	0.060781897	1.607660573	0.108362401	0.161879047	no
KCNK18	-0.060770208	-1.607350259	0.10843043	0.161968706	no
RANGRF	-0.060763753	-1.60717889	0.108468014	0.162012877	no
CMA1	0.060751739	1.606859955	0.108537987	0.162105417	no
MAPRE2	-0.060741977	-1.606600803	0.108594871	0.162166914	no
PCDHA8	-0.06074192	-1.606599284	0.108595205	0.162166914	no
HSP90AA1	-0.060739469	-1.606534221	0.10860949	0.162176267	no
NAT8	-0.060715285	-1.605892188	0.108750532	0.162374881	no
NXF1	-0.060696111	-1.60538318	0.108862455	0.16252999	no
CKMT2	0.060688592	1.60518355	0.108906376	0.162583556	no
MAP4K2	0.060685763	1.605108465	0.108922899	0.162596217	no
BRP44L	-0.060674435	-1.604807721	0.1089891	0.162683028	no
DCI	-0.060669957	-1.604688843	0.109015276	0.162710088	no
CHID1	0.060659951	1.604423219	0.109073784	0.162785396	no
FUK	-0.06061941	-1.603346972	0.109311099	0.163127531	no
PPY2	-0.060606873	-1.603014161	0.109384567	0.163217757	no
CCDC107	0.060606338	1.602999953	0.109387704	0.163217757	no
ATP1B4	0.060603599	1.602927252	0.109403759	0.163229665	no
TARDBP	-0.060595918	-1.602723336	0.109448799	0.163284815	no
POLR1A	-0.060588596	-1.602528956	0.109491747	0.163336835	no
ZACN	-0.060573248	-1.602121512	0.109581813	0.163459134	no
BCYRN1	-0.060562462	-1.601835191	0.10964514	0.163541531	no
FOXD2	0.060539906	1.601236398	0.109777673	0.163727131	no
PDGFRA	-0.060533562	-1.601067999	0.109814968	0.163770673	no
GRIK1	0.060528269	1.600927487	0.109846094	0.16380501	no
ZNF167	-0.060506521	-1.600350146	0.109974061	0.163983743	no
NOL8	0.060501793	1.600224647	0.110001894	0.164013148	no
USP12	-0.06048439	-1.599762643	0.110104402	0.164153883	no
C7orf70	0.060477065	1.5995682	0.110147567	0.164206129	no
OR2AG2	-0.060475542	-1.599527759	0.110156547	0.164207407	no
COX6B1	0.060470742	1.599400358	0.110184838	0.164237471	no
CYP2J2	-0.060469324	-1.599362708	0.1101932	0.164237827	no

POC1B	0.060464573	1.599236591	0.110221214	0.164263413	no
TIMELESS	0.060463657	1.599212272	0.110226616	0.164263413	no
TUBA4B	0.060427278	1.598246551	0.110441321	0.164567484	no
ACADVL	0.060426327	1.598221305	0.110446938	0.164567484	no
RNF122	0.060414402	1.597904747	0.110517392	0.16464737	no
BMP15	-0.060413397	-1.597878056	0.110523334	0.16464737	no
H3F3A	0.060413118	1.59787065	0.110524983	0.16464737	no
DNAL1	-0.060400573	-1.597537628	0.110599144	0.164739238	no
ZNF536	-0.060399929	-1.597520545	0.110602949	0.164739238	no
AAK1	-0.060393429	-1.597348001	0.110641389	0.164784354	no
DLEU1	0.060389235	1.59723667	0.110666198	0.164809162	no
C6orf123	0.060364314	1.596575125	0.110813705	0.165016681	no
FAM165B	-0.06036264	-1.596530671	0.110823622	0.165019295	no
ATP6V1D	-0.060350934	-1.596219943	0.110892965	0.165110388	no
APOL5	0.060344361	1.596045458	0.110931918	0.165156224	no
TMEM90B	0.060341279	1.595963644	0.110950187	0.16517126	no
LCORL	-0.060335522	-1.595810819	0.110984318	0.165209906	no
LOC253724	0.060314675	1.595257427	0.111107979	0.16538181	no
NDST2	0.060289474	1.594588455	0.111257614	0.165592347	no
RAD17	-0.060276039	-1.594231801	0.111337455	0.165698982	no
KIR3DP1	0.060268057	1.594019925	0.111384907	0.165757402	no
CETN3	0.060264962	1.593937762	0.111403313	0.16577259	no
XPNPEP1	-0.060244429	-1.593392706	0.111525474	0.165931866	no
NDUFA6	0.060244212	1.593386952	0.111526765	0.165931866	no
NOTCH2	0.060239449	1.593260524	0.111555116	0.165961835	no
ZNF446	-0.060236184	-1.593173839	0.111574558	0.165978546	no
ZNF688	-0.060217148	-1.592668538	0.111687944	0.166134994	no
CNOT4	0.060208894	1.592449447	0.111737134	0.166195937	no
GABRA2	-0.060196973	-1.592132988	0.111808216	0.166278776	no
C21orf81	-0.060196795	-1.592128265	0.111809277	0.166278776	no
MUSTN1	-0.060186964	-1.591867313	0.111867919	0.166350974	no
MAP1LC3B	-0.060185898	-1.591839011	0.11187428	0.166350974	no
YTHDC2	0.060177771	1.591623277	0.111922781	0.166410853	no
FAM197Y2	0.060163683	1.591249312	0.112006893	0.166521397	no
ALG13	0.06016256	1.591219496	0.112013602	0.166521397	no
GPR88	0.060155151	1.591022834	0.112057857	0.16657494	no
ING1	-0.060148874	-1.590856222	0.112095362	0.16661844	no
BRIP1	0.060146078	1.590782004	0.112112071	0.16662236	no
PCDHB8	-0.060145675	-1.590771299	0.112114482	0.16662236	no
PTPLB	0.060143492	1.590713341	0.112127532	0.166629507	no
ZNF677	-0.060134993	-1.590487738	0.112178342	0.166682142	no
AGAP1	-0.060134809	-1.590482868	0.112179439	0.166682142	no
TTTY20	0.060126136	1.590252653	0.112231308	0.166746956	no
EAF1	0.060118088	1.590039013	0.112279458	0.166806238	no
SCAND3	0.060104685	1.589683228	0.112359683	0.166913157	no
POC1A	0.060100206	1.589564349	0.112386498	0.166940726	no
GRK6	0.060095126	1.589429488	0.112416924	0.166973654	no
C9orf173	-0.060078567	-1.588989962	0.112516133	0.167108733	no
OR52W1	-0.060065873	-1.588653009	0.112592237	0.167209478	no
PRPF4B	-0.060058102	-1.58844672	0.112638848	0.167266415	no
CHMP4A	0.060042318	1.588027737	0.112733566	0.167394774	no

PCDHA9	-0.060025304	-1.587576134	0.112835729	0.167534168	no
MAD2L1	0.060023	1.587514958	0.112849574	0.167535099	no
PVRIG	0.060022441	1.587500133	0.112852929	0.167535099	no
INGX	0.060005127	1.587040549	0.112956985	0.167677262	no
SPG11	0.059985462	1.586518571	0.113075259	0.16784051	no
C11orf84	-0.059977976	-1.586319858	0.113120311	0.167895055	no
PRSS54	-0.059963345	-1.585931487	0.113208402	0.168013468	no
TH1L	-0.059950836	-1.585599466	0.113283755	0.16811296	no
LSM4	0.05994042	1.585322985	0.113346534	0.168193777	no
DEFB124	-0.05993343	-1.585137434	0.113388681	0.168243971	no
ALX1	0.05988753	1.583919083	0.11366573	0.168642675	no
TRAPPC6A	-0.059884735	-1.583844891	0.113682618	0.168655355	no
FOXD1	0.059879601	1.583708626	0.113713641	0.168687054	no
SEC16A	-0.059878438	-1.58367775	0.113720672	0.168687054	no
ZNF284	0.059872473	1.583519431	0.113756726	0.168717358	no
SFXN3	0.059871722	1.583499484	0.113761269	0.168717358	no
TOR1AIP2	0.059870917	1.583478118	0.113766136	0.168717358	no
SNORA14A	0.059858549	1.583149837	0.113840929	0.168815895	no
LOC145783	0.05984725	1.58284993	0.113909292	0.168904882	no
RPL14	-0.059831959	-1.582444037	0.114001866	0.169029754	no
ADIPOQ	0.059825781	1.582280077	0.114039278	0.169072825	no
SGK1	0.059811237	1.581894023	0.114127405	0.169191074	no
NLRP5	0.059786284	1.581231706	0.114278721	0.169402975	no
TCL6	0.059781952	1.581116703	0.114305011	0.169429525	no
ERCC4	0.059773211	1.580884713	0.11435806	0.169494254	no
ZNF641	0.059771995	1.580852414	0.114365447	0.169494254	no
CLPP	0.059765946	1.580691869	0.114402172	0.169536254	no
LBR	0.059753407	1.580359034	0.114478337	0.169636692	no
UNC93A	-0.059711845	-1.579255881	0.114731068	0.169998734	no
OR2B6	0.05970651	1.579114267	0.114763543	0.170034392	no
HIST2H2BA	0.059668039	1.578093173	0.114997918	0.170369159	no
DENND4C	-0.059647216	-1.577540467	0.11512494	0.170544845	no
SDHAF2	0.059636593	1.577258504	0.115189783	0.1706284	no
TIE1	-0.059633732	-1.577182574	0.115207249	0.17064177	no
GEFT	-0.059612754	-1.576625784	0.115335394	0.17081906	no
C7orf64	0.059586349	1.57592494	0.115496851	0.171045659	no
ASXL1	-0.059550409	-1.574971018	0.115716898	0.171358987	no
BEND3	-0.059529366	-1.574412505	0.115845887	0.171537435	no
NANOG	-0.059525474	-1.574309188	0.11586976	0.171560221	no
ENTPD3	-0.059512172	-1.573956132	0.115951371	0.171661112	no
SPIN4	0.0595116	1.573940944	0.115954882	0.171661112	no
C10orf26	0.059505091	1.573768183	0.115994834	0.171704143	no
OR2T10	-0.059504097	-1.573741809	0.116000934	0.171704143	no
OR2W3	-0.059501763	-1.57367987	0.116015261	0.171712778	no
GPHB5	0.059482783	1.573176107	0.116131836	0.171872737	no
DPPA4	0.059471219	1.572869168	0.11620291	0.171965337	no
CCT6P1	-0.059465822	-1.572725944	0.116236086	0.172001844	no
MFSD9	0.059464375	1.572687538	0.116244984	0.172002421	no
EXTL3	0.059460335	1.572580305	0.116269829	0.172016398	no
KIF6	-0.059459802	-1.572566158	0.116273108	0.172016398	no
CYHR1	-0.059458689	-1.572536611	0.116279954	0.172016398	no

C8orf59	0.059450392	1.572316407	0.116330992	0.172079309	no
C5	0.059443476	1.572132834	0.116373553	0.172129672	no
KIAA1712	-0.0594405	-1.572053861	0.116391866	0.172144166	no
CD164L2	0.059433797	1.571875939	0.116433133	0.172192604	no
KRTAP27-1	0.059429057	1.571750149	0.116462316	0.172223165	no
ZNF227	-0.059427079	-1.571697636	0.116474501	0.172228586	no
TRIM28	-0.059404519	-1.571098872	0.116613503	0.172421515	no
OR1J1	0.059402148	1.57103595	0.116628117	0.172430513	no
FAM162A	0.059399823	1.570974227	0.116642455	0.172439101	no
UBIAD1	-0.059397296	-1.570907176	0.116658032	0.172439774	no
MICALL1	0.059396982	1.570898843	0.116659968	0.172439774	no
AUTS2	-0.059394471	-1.570832187	0.116675455	0.172450058	no
HBS1L	-0.059376091	-1.570344358	0.116788847	0.172605037	no
ZYG11A	0.059350935	1.569676701	0.116944179	0.172821973	no
ARHGAP36	-0.059349299	-1.569633275	0.116954288	0.17282428	no
LRRK2	0.059344896	1.569516419	0.116981493	0.172851848	no
KIF27	0.059330356	1.569130508	0.117071374	0.172972013	no
CTDSP2	0.059310867	1.568613264	0.117191926	0.173137477	no
C6orf70	-0.059301239	-1.568357747	0.117251515	0.173212855	no
KIAA1324L	-0.059292823	-1.568134357	0.117303632	0.173277184	no
SNORA67	0.059278838	1.567763194	0.117390263	0.173392485	no
ZNF71	0.05927661	1.567704069	0.117404068	0.173400208	no
FAIM	0.059254508	1.567117469	0.117541098	0.173589914	no
PSCA	-0.05924947	-1.566983757	0.117572351	0.173623388	no
RAD54L	0.059241312	1.566767237	0.117622973	0.173685457	no
TMEM146	0.059213725	1.566035067	0.11779428	0.173916638	no
FH	0.059213329	1.566024569	0.117796737	0.173916638	no
LRRC14B	-0.059189366	-1.565388583	0.117945701	0.174123854	no
SLC7A9	0.059178502	1.565100262	0.118013282	0.174205583	no
ZNF585A	0.059177697	1.565078883	0.118018294	0.174205583	no
MFGE8	0.059169181	1.564852867	0.118071294	0.174271092	no
ZDHHC16	-0.059160035	-1.564610127	0.118128236	0.174342411	no
DLG1	-0.059150674	-1.564361699	0.118186535	0.174415721	no
ZBTB46	0.059135124	1.56394901	0.118283432	0.174545977	no
KLK15	-0.059132497	-1.563879272	0.118299812	0.174557408	no
C4orf41	0.059129349	1.563795745	0.118319433	0.174573619	no
TSEN15	0.05911877	1.563514969	0.118385409	0.174658217	no
FGFR4	-0.059114761	-1.563408569	0.118410418	0.174682366	no
DCST1	-0.059106773	-1.563196581	0.118460258	0.174743141	no
SNORA78	0.059085539	1.562633028	0.118592832	0.174925073	no
FAM173B	0.059084248	1.562598768	0.118600896	0.174925073	no
EIF2B2	-0.059072954	-1.562299024	0.118671461	0.175016383	no
C1orf216	-0.059070626	-1.562237244	0.118686009	0.17502201	no
SAMD4A	0.059069573	1.562209301	0.11869259	0.17502201	no
ZNF567	0.05904958	1.561678697	0.118817602	0.175192243	no
FAM170B	0.059048339	1.561645766	0.118825365	0.175192243	no
SNORA77	0.059032444	1.561223929	0.11892483	0.175315872	no
FAM107B	-0.059032168	-1.561216597	0.118926559	0.175315872	no
CCDC63	0.059007591	1.560564342	0.119080486	0.175529984	no
C17orf82	0.059002398	1.560426523	0.11911303	0.175565156	no
LOC646471	-0.058984634	-1.559955072	0.11922441	0.175715112	no

C3orf62	-0.0589834	-1.559922318	0.119232151	0.175715112	no
CNTLN	0.058968667	1.559531318	0.119324591	0.175833455	no
TAS2R1	0.058967829	1.559509095	0.119329847	0.175833455	no
DCPS	0.058951705	1.559081158	0.119431087	0.175969809	no
C10orf72	0.058939439	1.558755643	0.119508142	0.17607051	no
BTBD8	-0.058928313	-1.558460368	0.119578072	0.176160702	no
C10orf46	0.058917065	1.558161859	0.119648801	0.176252056	no
RPS18	-0.058909036	-1.557948793	0.119699305	0.176313606	no
CHCHD1	-0.058898853	-1.557678532	0.119763391	0.176395151	no
MRPL9	0.058882941	1.557256263	0.119863575	0.176529848	no
MTERFD2	-0.058880647	-1.557195378	0.119878025	0.17653827	no
AR	0.058874853	1.557041604	0.119914528	0.176579165	no
SLC16A12	0.058816217	1.555485505	0.120284407	0.177110926	no
OR11H12	-0.058787765	-1.554730428	0.120464207	0.177362755	no
STX8	0.058781265	1.55455793	0.120505313	0.177410355	no
SSSCA1	-0.058771548	-1.554300073	0.120566779	0.177487923	no
PEX11B	0.058763451	1.554085186	0.120618021	0.17755043	no
TARS2	-0.058761625	-1.55403673	0.120629578	0.177554515	no
SLC25A17	0.05875508	1.553863029	0.120671015	0.177602576	no
NLRP10	0.058699979	1.552400758	0.121020284	0.178103661	no
KLRK1	-0.058677244	-1.551797421	0.121164624	0.178303105	no
KDM4A	0.058661914	1.551390621	0.121262021	0.178433446	no
CXorf42	0.058659884	1.551336724	0.12127493	0.178439454	no
THUMPD2	-0.058652575	-1.551142769	0.121321393	0.178494827	no
SMAD5	-0.05864353	-1.550902743	0.121378911	0.178566458	no
KIAA0494	0.058615774	1.55016616	0.121555556	0.178813317	no
OR2L8	-0.058599404	-1.549731746	0.12165983	0.178953688	no
DMRTB1	-0.058592006	-1.549535435	0.121706974	0.17901001	no
KLC3	-0.058585097	-1.549352079	0.12175102	0.179061768	no
PRR23A	-0.058568561	-1.548913267	0.121856483	0.179203839	no
SORBS1	-0.058556801	-1.548601181	0.121931532	0.179301165	no
SLC2A7	0.05854568	1.548306075	0.122002532	0.179392523	no
SLC22A13	-0.058543102	-1.54823766	0.122018996	0.179403685	no
C7orf60	-0.058513455	-1.547450909	0.122208458	0.179669184	no
LHCGR	0.058505904	1.547250534	0.122256749	0.17972711	no
HIST1H2AE	0.058498384	1.547050967	0.122304859	0.179784764	no
C11orf92	-0.058477187	-1.546488476	0.122440541	0.179971127	no
FGF2	-0.058466331	-1.546200378	0.12251008	0.18006025	no
MEF2B	-0.058458507	-1.545992777	0.122560209	0.180120833	no
RPL37A	-0.058450186	-1.545771968	0.122613545	0.18018612	no
CCDC52	0.058446494	1.545673994	0.122637216	0.180207808	no
PCID2	0.058442949	1.545579917	0.122659949	0.180228113	no
PHPT1	0.058373116	1.543726781	0.123108416	0.180873916	no
ABCA11P	-0.058348567	-1.54307536	0.123266368	0.181092822	no
IFNA1	-0.058326553	-1.542491185	0.123408148	0.181287942	no
RNF113A	-0.058318207	-1.542269715	0.123461933	0.181353775	no
HBG2	0.058311425	1.542089764	0.123505648	0.181404809	no
PLDN	-0.058309111	-1.542028355	0.123520569	0.181413546	no
C12orf68	-0.058307238	-1.541978651	0.123532647	0.181418105	no
RNF31	-0.058300482	-1.541799369	0.123576219	0.18145111	no
LDB3	-0.058299754	-1.541780046	0.123580916	0.18145111	no

LOC441869	0.058299579	1.541775402	0.123582045	0.18145111	no
BHMT	-0.058285181	-1.54139333	0.123674946	0.181574327	no
COX16	-0.058282991	-1.541335222	0.12368908	0.181581892	no
SPATA3	0.05826196	1.540777162	0.123824883	0.18176806	no
WFDC3	0.058257293	1.540653305	0.12385504	0.181797958	no
HS3ST2	-0.058256025	-1.540619652	0.123863234	0.181797958	no
TCFL5	-0.058253489	-1.540552378	0.123879617	0.181808805	no
PIN4	-0.058246241	-1.540360048	0.123926464	0.181864357	no
NUP50	0.058233349	1.540017944	0.124009825	0.181973483	no
TMEM211	-0.05822773	-1.539868834	0.124046173	0.18201361	no
SRMS	-0.058216822	-1.539579392	0.124116753	0.182103955	no
CHMP7	-0.058214822	-1.539526329	0.124129696	0.18210973	no
ZC3H10	0.058212233	1.539457622	0.124146456	0.182121103	no
PRODH	-0.058209064	-1.539373513	0.124166975	0.182137989	no
TMSB4Y	-0.058196163	-1.539031196	0.124250515	0.18224731	no
MYH2	0.058168745	1.538303634	0.124428218	0.182494719	no
TAF7L	0.058162846	1.538147111	0.124466473	0.182534132	no
FOXA3	0.058161817	1.538119811	0.124473147	0.182534132	no
PRRG3	-0.058143701	-1.537639095	0.124590701	0.182683181	no
SSX6	-0.058143369	-1.537630281	0.124592857	0.182683181	no
GNA12	0.058139088	1.537516677	0.124620651	0.182710683	no
OR7E37P	-0.058127813	-1.5372175	0.124693869	0.182804774	no
MAN1A2	0.058105626	1.536628759	0.124838051	0.183002879	no
OLA1	-0.058071698	-1.535728483	0.125058779	0.183313158	no
SFXN5	-0.058065915	-1.535575029	0.125096433	0.183355059	no
MRAP	0.058058317	1.535373442	0.125145911	0.183414283	no
HRG	-0.058037328	-1.534816485	0.125282692	0.18360144	no
FLJ40330	-0.058035712	-1.534773622	0.125293223	0.183603565	no
SNRNP27	-0.057981401	-1.533332494	0.125647709	0.184109681	no
RNF10	0.057973655	1.533126967	0.125698328	0.184170505	no
VCX	0.057966231	1.532929962	0.125746863	0.184228266	no
CCNA1	0.057948062	1.532447862	0.125865697	0.184389005	no
ARL6IP1	-0.05793527	-1.532108427	0.125949417	0.184498284	no
TCOF1	-0.057897773	-1.531113473	0.12619507	0.184844739	no
TRPC4AP	0.05788458	1.530763413	0.126281589	0.184958067	no
CES7	-0.057873079	-1.530458246	0.126357049	0.185055185	no
LMBR1	-0.057861181	-1.530142558	0.126435149	0.185156152	no
TSPAN15	-0.057832943	-1.5293933	0.126620661	0.185414393	no
COX18	0.057821703	1.529095042	0.126694567	0.185509181	no
STON1-GTF2A1	0.057803974	1.528624648	0.126811196	0.185666504	no
PNLIPRP2	0.057792232	1.528313074	0.126888493	0.185766224	no
PRMT10	0.057790794	1.52827493	0.126897959	0.185766631	no
SMAD1	0.057782703	1.528060241	0.126951245	0.185831181	no
ZNF165	0.057779983	1.527988063	0.126969163	0.185843955	no
LIN52	-0.057776654	-1.527899733	0.126991094	0.185862599	no
ZNF507	-0.057764672	-1.527581822	0.127070051	0.185964698	no
ANKMY2	0.057760172	1.527462414	0.127099718	0.185994651	no
CCDC56	-0.057747204	-1.527118323	0.127185236	0.186106325	no
LOC286359	-0.057744933	-1.527058078	0.127200213	0.186114771	no
SLC28A1	0.057737198	1.526852832	0.127251249	0.186175972	no
KLK12	-0.0577317	-1.526706968	0.127287529	0.186215577	no

LEPR	0.057724793	1.526523682	0.127333129	0.186268808	no
NASP	-0.057715738	-1.526283421	0.127392922	0.186342794	no
RP9	0.057686307	1.525502525	0.127587414	0.186613784	no
LOC285548	0.057680618	1.525351576	0.127625036	0.186644373	no
CCNL1	0.057680352	1.525344538	0.127626791	0.186644373	no
TREML2P1	0.05766695	1.524988942	0.127715454	0.186760528	no
CUTC	-0.057651561	-1.524580608	0.127817327	0.18689598	no
ST3GAL6	-0.057625496	-1.523889047	0.127990004	0.187129721	no
CHEK1	0.057624638	1.523866276	0.127995693	0.187129721	no
OSBP2	-0.057590437	-1.52295882	0.128222561	0.187447847	no
NIPSNAP1	-0.05757662	-1.522592222	0.1283143	0.187568399	no
NCAPD3	-0.057574916	-1.522547013	0.128325617	0.18757138	no
NCAPD2	0.057572853	1.52249227	0.128339322	0.187573831	no
SH2D4B	0.05757187	1.522466198	0.128345849	0.187573831	no
ZCCHC2	0.057567406	1.522347759	0.128375505	0.187603611	no
CATSPER2P1	0.057561496	1.52219095	0.128414776	0.187647437	no
KLK8	0.057550875	1.521909157	0.128485372	0.187737027	no
FLJ40852	0.057546926	1.52180437	0.128511631	0.187761826	no
FLJ42875	-0.057538635	-1.521584401	0.128566768	0.187828811	no
FMR1NB	-0.057510441	-1.520836327	0.128754416	0.188089363	no
ZNF706	-0.057498389	-1.520516577	0.128834688	0.188193029	no
ZNF296	-0.057486623	-1.520204383	0.128913101	0.188293965	no
LIM2	0.057459474	1.519484085	0.129094157	0.188544799	no
FLJ46361	-0.057430498	-1.518715275	0.129287625	0.188801019	no
LONRF1	-0.057430402	-1.518712741	0.129288263	0.188801019	no
RBM15	0.057399147	1.517883491	0.129497195	0.189092466	no
MT4	-0.057395427	-1.517784795	0.129522079	0.189104608	no
RAX2	-0.057395108	-1.517776318	0.129524217	0.189104608	no
CHN2	0.057376439	1.517281011	0.129649156	0.189273351	no
HNRNPA2B1	-0.057371279	-1.517144089	0.129683711	0.189310128	no
IL17C	-0.057333091	-1.51613092	0.129939624	0.189670011	no
ZPBP2	0.057324152	1.515893742	0.129999589	0.189743842	no
INPP4B	0.057318688	1.515748779	0.13003625	0.18978365	no
CCDC117	0.057313716	1.51561686	0.13006962	0.189818649	no
ALX4	0.057298937	1.515224767	0.13016884	0.189949737	no
ATP6V0A2	0.0572925	1.515053973	0.130212078	0.189999119	no
CLN8	-0.05727573	-1.514609058	0.130324766	0.190149824	no
PLAC9	0.057272104	1.514512837	0.130349146	0.190171672	no
NAPA	0.057263147	1.514275209	0.130409372	0.19024581	no
RESP18	-0.057235616	-1.513544773	0.130594634	0.190502331	no
CDC25B	0.057219157	1.51310811	0.130705483	0.190650274	no
SELV	-0.057213636	-1.512961639	0.130742682	0.190690776	no
ATXN7L1	0.057209492	1.512851683	0.130770612	0.190717755	no
LOC100134229	0.057189518	1.512321757	0.130905287	0.190900396	no
LOC100132247	0.057160803	1.511559918	0.131099089	0.19116923	no
EIF5A2	0.057150035	1.511274235	0.13117182	0.19124695	no
EDF1	-0.057148871	-1.511243359	0.131179683	0.19124695	no
FKBP3	-0.057148711	-1.51123911	0.131180765	0.19124695	no
LOC100133331	0.057127397	1.510673648	0.131324824	0.191443168	no
MTX1	-0.057109786	-1.510206424	0.131443949	0.191603012	no
PHB2	-0.057078671	-1.509380914	0.131654629	0.191882467	no

TUBB	0.057078669	1.509380858	0.131654644	0.191882467	no
CPEB1	-0.057069122	-1.509127575	0.131719337	0.191962918	no
PRO0611	-0.057066084	-1.50904699	0.131739925	0.191979084	no
DUT	-0.057064047	-1.508992946	0.131753734	0.191985369	no
TRA2B	0.057054863	1.508749298	0.131816002	0.192062261	no
LHX2	-0.05705341	-1.50871074	0.131825859	0.192062781	no
CBX2	-0.057034512	-1.508209372	0.131954071	0.192235726	no
TUBA1A	0.057028104	1.508039367	0.131997567	0.192285238	no
SNORA3	0.057025832	1.507979088	0.132012993	0.192293854	no
PLCZ1	0.057022576	1.507892702	0.132035101	0.192304606	no
LOC151174	0.057021943	1.507875916	0.132039398	0.192304606	no
PYY2	0.057007756	1.507499531	0.13213576	0.192431089	no
RPS26	0.057003818	1.507395065	0.132162516	0.19245619	no
STX18	0.056997281	1.507221649	0.13220694	0.192507015	no
CARD18	-0.056992912	-1.50710573	0.132236641	0.192536397	no
WDR75	-0.056985548	-1.506910354	0.132286713	0.192584709	no
LOC100128076	0.05698523	1.506901924	0.132288873	0.192584709	no
GMNN	-0.05696597	-1.506390954	0.132419899	0.192760242	no
COPS5	0.056964703	1.506357357	0.132428517	0.192760242	no
CRLF1	-0.056934914	-1.505567051	0.132631379	0.193041626	no
SLC22A14	-0.056928531	-1.505397715	0.132674877	0.193091035	no
GON4L	-0.056915424	-1.505049994	0.132764232	0.193207172	no
HSFYL1	-0.056906239	-1.504806323	0.132826876	0.193284424	no
OR4F17	0.056898605	1.504603787	0.132878963	0.193346303	no
DERA	0.056883294	1.504197603	0.132983471	0.193484442	no
MINPP1	-0.056873213	-1.503930162	0.133052315	0.193570678	no
PGR	-0.056861679	-1.503624165	0.133131119	0.19367139	no
CDC42BPA	-0.05685677	-1.503493946	0.133164666	0.193706254	no
GPC3	0.056850516	1.50332803	0.133207418	0.193754502	no
OXTR	0.056838396	1.503006494	0.1332903	0.193861109	no
GET4	-0.05682342	-1.502609196	0.133392765	0.193996183	no
YRDC	0.056816089	1.502414695	0.133442951	0.19405521	no
HDDC3	0.056811266	1.502286741	0.133475974	0.194089272	no
OR3A3	-0.05680443	-1.502105405	0.133522784	0.194143377	no
AZU1	-0.05678802	-1.501670045	0.133635222	0.194292889	no
ZFP28	-0.056774732	-1.50131754	0.133726314	0.194411348	no
C9orf153	-0.0567648	-1.501054043	0.133794437	0.1944964	no
KIRREL3	-0.056756085	-1.500822854	0.13385423	0.194569331	no
OXR1	-0.056747677	-1.500599809	0.133911936	0.194639218	no
DBT	-0.056744609	-1.500518408	0.133933001	0.194655841	no
SLC36A3	-0.056733187	-1.500215398	0.134011435	0.194755836	no
2-Sep	-0.056706642	-1.499511189	0.134193859	0.195006931	no
MED27	-0.056694258	-1.499182657	0.13427903	0.195116676	no
RPS7	-0.056683227	-1.498890041	0.134354925	0.195212926	no
AMY1A	-0.056661333	-1.498309205	0.134505674	0.195417916	no
SYT10	-0.056653912	-1.498112335	0.134556799	0.195478146	no
SRRM1	0.056645527	1.497889901	0.13461458	0.195548037	no
FSCN1	0.056643243	1.497829305	0.134630325	0.195556858	no
CLK1	-0.05663507	-1.497612507	0.134686666	0.195624641	no
ATXN3	-0.05660588	-1.496838148	0.134888055	0.195903074	no
TRAPPC4	-0.056591517	-1.496457129	0.134987232	0.196033031	no

NSUN5P2	-0.056587388	-1.496347575	0.135015759	0.196060376	no
SEBOX	-0.056551465	-1.495394619	0.135264098	0.196392608	no
GOLGA2	0.056551339	1.495391279	0.135264969	0.196392608	no
COX11	0.056549951	1.495354453	0.135274573	0.196392608	no
CHRDL1	-0.056548675	-1.495320594	0.135283403	0.196392608	no
ZSWIM3	-0.056546093	-1.49525211	0.135301266	0.196404437	no
UBA1	-0.056542898	-1.49516735	0.135323376	0.19641394	no
MRPL24	-0.056542339	-1.495152529	0.135327242	0.19641394	no
BMP4	-0.056537369	-1.495020679	0.135361643	0.196449766	no
SNORD116-20	-0.056531669	-1.494869474	0.135401101	0.196492927	no
C4orf14	-0.056528044	-1.494773319	0.135426198	0.196502415	no
FATE1	0.056527917	1.49476995	0.135427077	0.196502415	no
NHLH2	0.056521103	1.494589168	0.135474272	0.196556788	no
LCE3A	0.056510436	1.494306219	0.135548165	0.196638711	no
C1orf101	0.056510144	1.494298473	0.135550189	0.196638711	no
OR4M1	-0.056501974	-1.494081726	0.135606814	0.196706742	no
DEPDC1B	0.056484557	1.493619694	0.135727583	0.1968678	no
EIF3G	-0.056482993	-1.493578204	0.135738431	0.196869412	no
ATAD3B	-0.056461549	-1.493009356	0.135887244	0.197068708	no
C4orf21	-0.056459457	-1.492953866	0.135901766	0.197068708	no
WDFY1	0.056458979	1.492941189	0.135905085	0.197068708	no
KTN1	-0.056457162	-1.492892965	0.135917707	0.197072877	no
SEC14L5	-0.056427903	-1.492116816	0.136120988	0.19735347	no
HOXC13	0.056400119	1.491379773	0.136314245	0.197616476	no
LOC344967	-0.056399013	-1.49135044	0.13632194	0.197616476	no
CCKAR	0.05637516	1.490717687	0.136488028	0.197843056	no
CEP152	0.05636623	1.490480807	0.136550246	0.197919053	no
RAE1	0.056358787	1.490283357	0.136602123	0.197980052	no
IFT74	0.056352694	1.490121716	0.136644604	0.198027425	no
ADAM21	0.056333987	1.489625495	0.136775079	0.198202305	no
RPS19	0.056329611	1.489509416	0.136805615	0.198232346	no
LOC285401	-0.056322008	-1.489307713	0.136858687	0.198295036	no
KRT6A	-0.056309497	-1.48897585	0.136946041	0.198407385	no
RPL26	-0.056268301	-1.487883051	0.137233997	0.198810329	no
DEFB132	-0.056246287	-1.487299098	0.137388062	0.199019261	no
AES	-0.056233398	-1.486957202	0.137478326	0.19913575	no
ADAMTS17	0.056173413	1.485366002	0.137899027	0.19973082	no
CTRL	-0.056153273	-1.484831765	0.138040497	0.199921402	no
ZP1	0.056147724	1.484684579	0.138079493	0.199963555	no
PCDHGA5	-0.056142433	-1.484544227	0.138116686	0.200003091	no
PMS2CL	-0.056140207	-1.484485171	0.138132338	0.200011431	no
MORC3	-0.056132595	-1.484283262	0.138185862	0.200074603	no
SPRR2A	-0.056111629	-1.483727132	0.138333369	0.200273831	no
ITGB8	0.056108433	1.483642343	0.138355869	0.200292063	no
UBE2J1	0.056106181	1.483582601	0.138371724	0.200300674	no
C5orf60	0.056092547	1.483220966	0.138467729	0.200425297	no
DGAT2	-0.056083068	-1.482969508	0.138534515	0.200507612	no
RAD23B	0.056075821	1.482777297	0.138585582	0.200561731	no
LGSN	0.056074025	1.482729653	0.138598243	0.200561731	no
SLCO4C1	0.05607354	1.482716768	0.138601667	0.200561731	no
C1orf180	0.056069045	1.482597547	0.138633352	0.200593224	no

IMPA1	0.056064995	1.482490114	0.138661909	0.200620186	no
SBDS	0.056056078	1.482253585	0.138724798	0.200696813	no
IL17RE	-0.056039572	-1.481815768	0.138841263	0.200850934	no
ERI2	-0.056034991	-1.481694239	0.138873604	0.200883346	no
KLK3	0.056013674	1.481128785	0.139024161	0.201086743	no
ATPBD4	0.055991387	1.480537612	0.139181701	0.201300209	no
TMEM134	0.05597348	1.480062634	0.139308375	0.201469008	no
TIA1	-0.055930619	-1.47892573	0.139611945	0.201886154	no
RBM44	0.055929936	1.478907611	0.139616787	0.201886154	no
LOC100131726	0.055872781	1.477391575	0.140022394	0.202458182	no
TMEM185A	-0.055869899	-1.477315128	0.140042871	0.20247331	no
ANKRD10	0.055865654	1.477202548	0.140073031	0.202502434	no
C6orf227	-0.055854067	-1.476895212	0.14015539	0.202597016	no
PLEKHG5	0.055853631	1.476883627	0.140158496	0.202597016	no
KLHL14	0.055842164	1.476579483	0.140240038	0.202700393	no
PAPLN	-0.055826016	-1.476151166	0.140354934	0.202851959	no
SHBG	-0.055817182	-1.475916836	0.140417824	0.202928346	no
DMTF1	-0.055781027	-1.474957849	0.140675423	0.203285472	no
MRGPRE	0.055779677	1.474922041	0.140685049	0.203285472	no
SNTG2	0.055775621	1.474814462	0.140713971	0.203312732	no
ABHD8	-0.055772527	-1.474732386	0.140736039	0.203330087	no
C12orf52	0.055767382	1.474595904	0.140772742	0.203345812	no
PFDN4	-0.05576737	-1.474595594	0.140772826	0.203345812	no
MIP	-0.055766771	-1.474579715	0.140777097	0.203345812	no
FIGNL1	0.055740527	1.4738836	0.140964417	0.203601841	no
FAM53B	0.055719821	1.4733344	0.141112338	0.203800933	no
ZNF367	0.055712816	1.473148586	0.141162413	0.20385869	no
RPL32	-0.055705157	-1.472945438	0.141217174	0.203923207	no
GSTM2P1	-0.055699012	-1.472782461	0.141261118	0.203972096	no
DYNC2H1	-0.05569403	-1.472650311	0.141296758	0.204008988	no
FOXB1	0.055687424	1.472475088	0.141344026	0.204062661	no
VPS8	-0.055685345	-1.472419949	0.141358902	0.204069567	no
ZNF221	-0.055672708	-1.472084771	0.141449359	0.204168574	no
POLR2H	0.055671756	1.472059506	0.14145618	0.204168574	no
PDX1	0.055671532	1.472053579	0.14145778	0.204168574	no
FAT1	-0.055667508	-1.471946827	0.141486601	0.204195595	no
PRAMEF5	0.055657229	1.471674193	0.141560226	0.20428727	no
PAOX	0.055634998	1.471084539	0.141719565	0.204502617	no
ART3	0.055617765	1.470627477	0.14184317	0.204666372	no
PELI1	-0.055611338	-1.470456995	0.141889295	0.204718316	no
LOC646498	0.055595628	1.470040298	0.142002084	0.204866428	no
LRRC3B	-0.05558728	-1.469818899	0.142062039	0.204938302	no
KIAA1715	-0.055568914	-1.469331762	0.142194024	0.205114068	no
PSORS1C2	0.05556489	1.469225036	0.142222953	0.205141162	no
HPD	0.055561867	1.469144843	0.142244693	0.205157883	no
IL28B	-0.055533702	-1.468397809	0.142447334	0.205435495	no
ASAP2	-0.055527148	-1.468223988	0.142494517	0.205488884	no
GTF3C2	-0.055525114	-1.468170025	0.142509167	0.205495353	no
SLC38A2	0.055496071	1.467399725	0.142718422	0.205782418	no
TNNC1	0.055486129	1.467136013	0.142790115	0.205871107	no
MIR17HG	-0.05547611	-1.466870294	0.142862381	0.205960612	no

SPINK9	-0.055468555	-1.466669916	0.142916895	0.206024513	no
ZNF366	0.055464163	1.466553425	0.142948595	0.206055518	no
PSPN	-0.055455359	-1.466319911	0.143012155	0.206132441	no
TESC	0.055443776	1.466012704	0.143095807	0.206238311	no
SH3RF3	-0.055437922	-1.46585742	0.143138105	0.206284568	no
ARIH2	-0.055431605	-1.465689895	0.143183748	0.206335639	no
NCRNA00167	-0.055424672	-1.465506001	0.143233864	0.206393148	no
NR6A1	-0.055413832	-1.4652185	0.143312242	0.20649137	no
ZNF558	0.055404779	1.464978386	0.143377727	0.206571002	no
MXD1	0.055395862	1.464741879	0.14344225	0.206649239	no
ABTB1	0.055387589	1.464522449	0.143502135	0.20671253	no
OR52A4	-0.055386965	-1.464505914	0.143506648	0.20671253	no
AASS	-0.055385555	-1.464468521	0.143516855	0.20671253	no
CLCA1	0.055381826	1.464369592	0.143543863	0.206736701	no
NIPSNAP3B	0.055375093	1.464191035	0.143592618	0.206792189	no
KARS	-0.055359992	-1.463790516	0.143702026	0.206935011	no
GPR143	0.055348564	1.463487399	0.14378487	0.207039562	no
IQSEC1	-0.055341127	-1.463290157	0.143838797	0.207102464	no
ZNF611	0.055337945	1.463205777	0.143861872	0.207120937	no
ZNF326	-0.055334087	-1.463103441	0.14388986	0.207133075	no
OR2M1P	-0.055333959	-1.463100035	0.143890792	0.207133075	no
PRKACG	-0.055328132	-1.462945511	0.143933063	0.207179173	no
TCTE1	-0.055326219	-1.462894754	0.14394695	0.207182688	no
UTP14A	0.055324971	1.462861676	0.143956	0.207182688	no
GAK	-0.055321187	-1.462761298	0.143983467	0.207207469	no
CPLX4	0.055277478	1.461602043	0.144300974	0.207649615	no
PEX1	0.055232883	1.460419279	0.144625474	0.20810176	no
ZNF302	-0.055224458	-1.460195842	0.144686838	0.208175242	no
MSH6	0.055220431	1.460089025	0.144716182	0.208202644	no
GOLGA9P	0.055216198	1.459976775	0.144747022	0.208232197	no
HECTD2	-0.055194994	-1.459414382	0.144901615	0.208439762	no
RHOT1	-0.055179034	-1.458991111	0.145018049	0.20859241	no
UQCRHL	0.05517259	1.458820198	0.145065084	0.208645221	no
DDI1	0.055135952	1.457848506	0.145332717	0.209015285	no
C16orf73	-0.055130125	-1.457693956	0.145375319	0.209061683	no
HAND2	0.055113012	1.457240106	0.145500481	0.209226794	no
AMOTL1	-0.055107249	-1.457087245	0.145542655	0.209272555	no
FTSJ1	0.055099852	1.456891079	0.145596791	0.209335508	no
TMEM117	-0.055095559	-1.456777214	0.145628221	0.209365809	no
NOX3	-0.055087492	-1.456563259	0.145687294	0.209435843	no
PALM2	-0.055059289	-1.455815275	0.145893954	0.209718021	no
C1orf130	0.055057345	1.455763727	0.145908205	0.209723595	no
THRAP3	0.055048642	1.455532911	0.145972027	0.209800416	no
GAL3ST2	-0.05503434	-1.455153608	0.146076954	0.209936299	no
KRTAP10-4	-0.055020136	-1.454776912	0.146181217	0.210071209	no
MST1R	-0.055015249	-1.454647284	0.146217109	0.210107854	no
LRRC23	0.054999319	1.454224822	0.146334128	0.210261062	no
SNORD88B	0.054989935	1.453975928	0.146403105	0.210345222	no
LACE1	-0.054982753	-1.45378546	0.146455906	0.210406132	no
UBA52	0.0549812	1.453744274	0.146467325	0.210407587	no
PRDM13	0.054952805	1.45299121	0.146676244	0.210692739	no

ARMC4	0.054928108	1.45233624	0.146858135	0.210939029	no
HDHD1A	0.054922614	1.452190526	0.146898624	0.210982197	no
ZAR1L	-0.054918132	-1.452071678	0.146931655	0.211014647	no
PTPLAD1	-0.054912747	-1.451928862	0.146971354	0.211056669	no
FAM174A	-0.05491123	-1.451888616	0.146982543	0.211057745	no
CNP	-0.054900553	-1.451605451	0.147061285	0.211155817	no
ZCCHC12	-0.054878793	-1.451028362	0.147221861	0.211371366	no
RPL9	-0.054873797	-1.450895876	0.147258744	0.211409308	no
SPDYE3	0.054844244	1.450112112	0.147477084	0.211707731	no
MMP3	0.054833345	1.449823069	0.147557668	0.211808373	no
PRDM4	-0.054815218	-1.449342355	0.147691764	0.211985807	no
TAS2R13	-0.054809977	-1.44920334	0.147730559	0.212012693	no
COL17A1	-0.054809854	-1.449200086	0.147731468	0.212012693	no
PMPCB	0.054791494	1.448713186	0.147867412	0.212192727	no
UGT3A2	0.054787202	1.448599352	0.147899209	0.212223293	no
PPFIBP2	-0.054785098	-1.448543555	0.147914796	0.212230597	no
IL3	0.054782455	1.448473473	0.147934376	0.212243628	no
FCRLA	0.054757098	1.447800987	0.148122359	0.212498251	no
C10orf96	-0.054747563	-1.447548132	0.148193088	0.212584636	no
RPL29	-0.054740512	-1.447361148	0.148245408	0.212644602	no
CDC25C	0.05470718	1.446477194	0.148492939	0.212984552	no
KRT2	0.054701439	1.446324928	0.14853561	0.213030642	no
FAM150A	-0.054686275	-1.445922798	0.148648347	0.213177208	no
SNORA16A	0.054663673	1.44532339	0.148816512	0.213403237	no
RAD51L3	0.054656728	1.445139214	0.148868212	0.213462235	no
DNLZ	-0.054650585	-1.444976297	0.148913956	0.213512684	no
C9orf142	-0.054642879	-1.444771942	0.14897135	0.213567725	no
OSGIN1	0.054642594	1.444764395	0.14897347	0.213567725	no
C9orf85	-0.05463709	-1.444618422	0.149014478	0.213611367	no
SOX5	-0.054630993	-1.444456741	0.149059909	0.213661343	no
KCNH5	-0.054625185	-1.444302705	0.149103202	0.213708247	no
MET	0.054620375	1.444175149	0.149139059	0.213744489	no
SCNN1B	0.054618417	1.444123227	0.149153657	0.213750258	no
KIAA1024	0.054593171	1.443453719	0.149341987	0.214004982	no
FRG1B	-0.054588483	-1.443329406	0.149376976	0.21403995	no
NR1I3	-0.054571476	-1.442878381	0.149503972	0.21420674	no
ACOT8	-0.054562803	-1.442648393	0.149568762	0.214284384	no
TPI1P3	-0.05454973	-1.442301693	0.149666472	0.214409178	no
HMGXB4	-0.054543833	-1.442145315	0.14971056	0.214457142	no
C1orf114	0.054538541	1.44200498	0.149750133	0.214498632	no
KLHL12	0.054532581	1.441846921	0.149794713	0.214547288	no
C14orf126	-0.054526681	-1.441690459	0.149838854	0.214595307	no
RPS3	-0.054516809	-1.441428665	0.149912732	0.214685905	no
LIN7A	0.054492405	1.440781496	0.150095482	0.214932393	no
SNORA80	0.054472802	1.440261653	0.150242401	0.21512754	no
B3GNT6	0.054470168	1.440191814	0.150262148	0.215140578	no
C1orf230	-0.054449644	-1.439647537	0.150416105	0.215334583	no
FBXW5	0.054449265	1.439637487	0.150418949	0.215334583	no
ZNF607	-0.054435505	-1.439272583	0.150522237	0.21546719	no
C5orf27	-0.054422151	-1.438918459	0.150622526	0.215595485	no
DHRS1	0.054415201	1.438734174	0.150674736	0.215654949	no

FAM122C	0.054363393	1.437360293	0.151064408	0.216197366	no
BOC	0.054325858	1.436364934	0.1513472	0.216586756	no
ASPG	-0.054306711	-1.435857199	0.151491609	0.21677807	no
SLC26A3	0.054277367	1.435079073	0.151713126	0.217079687	no
SUPT7L	0.054272276	1.434944053	0.151751588	0.217119355	no
NPPA	-0.054267424	-1.434815382	0.151788249	0.217156441	no
CARD10	0.05425448	1.434472141	0.151886078	0.217281025	no
FRMD7	0.054250248	1.434359918	0.151918074	0.217311421	no
MRPL40	0.054241024	1.434115335	0.151987825	0.217395815	no
PPIL3	0.054238485	1.434047987	0.152007035	0.217407912	no
STRN4	-0.054229829	-1.433818461	0.152072521	0.217486187	no
TNP1	0.054219617	1.433547664	0.152149808	0.217581329	no
DFNB31	0.054213597	1.433388028	0.152195384	0.217619744	no
C7orf4	-0.054213225	-1.433378171	0.152198199	0.217619744	no
SPOCK3	-0.054201841	-1.433076286	0.152284415	0.217727622	no
C6orf47	-0.054195535	-1.432909074	0.152332186	0.217780522	no
G3BP2	-0.054189162	-1.432740076	0.152380479	0.21783416	no
PPP1R14D	-0.054177244	-1.43242404	0.152470821	0.217947897	no
OR1K1	-0.054174365	-1.432347684	0.152492654	0.217963696	no
CSTF2	-0.054142834	-1.43151157	0.152731887	0.218290209	no
TIMP4	-0.054139561	-1.431424792	0.152756733	0.218310287	no
HIST1H3D	0.05410391	1.430479425	0.153027605	0.218681942	no
ATP4B	0.054099401	1.430359841	0.153061895	0.218715485	no
TXNRD3IT1	0.054095621	1.430259606	0.153090641	0.218741101	no
FLJ40434	-0.054081617	-1.429888272	0.153197171	0.218877847	no
KY	-0.054074512	-1.429699858	0.153251246	0.218939633	no
C8orf41	0.054050546	1.42906436	0.153433741	0.219184864	no
CA14	-0.054032897	-1.428596381	0.153568236	0.219361494	no
OR1B1	-0.054018244	-1.428207811	0.153679977	0.219505601	no
TRPV4	0.054013214	1.428074453	0.153718341	0.219543653	no
EGR3	0.054011904	1.428039713	0.153728336	0.219543653	no
RPL32P3	-0.054008161	-1.427940446	0.153756899	0.219568935	no
FKBPL	0.05397702	1.427114697	0.153994657	0.219892928	no
GYS2	-0.053968046	-1.426876734	0.154063225	0.219975302	no
HNRNPR	-0.053949501	-1.426384991	0.154204993	0.220162174	no
CELP	0.053945735	1.426285133	0.154233794	0.220187745	no
FAM150B	0.053943478	1.426225282	0.154251058	0.220196843	no
ZNF697	-0.053940985	-1.426159179	0.154270127	0.220208517	no
ULK3	-0.053913327	-1.425425795	0.154481813	0.220495114	no
FAM86A	0.053904148	1.425182383	0.15455212	0.220579893	no
ZNF546	0.053893554	1.424901489	0.154633285	0.220680155	no
CXorf64	0.05387992	1.42453997	0.154737794	0.220813715	no
EIF2C1	-0.053876074	-1.424437977	0.154767288	0.220840216	no
ANP32E	-0.053829236	-1.423196028	0.155126775	0.221337554	no
LOC100133469	0.053819655	1.422941963	0.155200393	0.221426967	no
PPP1R14B	-0.053804476	-1.422539477	0.155317073	0.2215778	no
KDM4C	0.053778116	1.421840541	0.155519851	0.221847682	no
RNU4ATAC	0.053777032	1.421811797	0.155528195	0.221847682	no
C20orf4	0.053714388	1.420150758	0.15601093	0.222520563	no
LOC100133161	0.053667217	1.418899994	0.156375181	0.223024366	no
TFCP2L1	0.053664807	1.418836095	0.156393807	0.223035198	no

ANAPC13	-0.053661233	-1.418741317	0.156421438	0.223058868	no
ASCC3	0.053653039	1.418524058	0.156484789	0.223133469	no
C2orf82	-0.053642925	-1.418255894	0.15656301	0.223229262	no
CEACAM22P	0.053639667	1.418169497	0.156588218	0.223249459	no
SNORA71B	0.053619465	1.417633854	0.156744568	0.223456613	no
ATP5L	0.053616701	1.417560565	0.15676597	0.223471366	no
C1orf116	-0.053590977	-1.416878492	0.156965255	0.223739673	no
C19orf63	0.05358408	1.416695601	0.157018724	0.22380011	no
GHRHR	0.053571608	1.416364927	0.157115433	0.223922165	no
RIC8B	-0.053567178	-1.416247464	0.157149798	0.223952742	no
SNORA5B	0.053565987	1.41621587	0.157159041	0.223952742	no
TAX1BP1	0.053564225	1.416169157	0.15717271	0.223956434	no
ZNF585B	0.053545792	1.415680413	0.15731577	0.224144485	no
GPR144	0.053515281	1.414871419	0.157552789	0.224466373	no
NUDCD2	0.053512268	1.414791547	0.157576205	0.224483915	no
ZBTB11	-0.053503686	-1.414564002	0.157642927	0.224563144	no
PLRG1	0.053500771	1.414486691	0.157665602	0.224579621	no
DSG3	-0.053485465	-1.414080875	0.157784665	0.224733382	no
SLC37A3	0.053478703	1.41390157	0.157837293	0.224792504	no
C20orf201	-0.053461454	-1.413444228	0.157971589	0.224967921	no
C21orf82	-0.053458382	-1.413362781	0.157995514	0.224986146	no
PTPMT1	-0.053408042	-1.412028052	0.15838799	0.225529149	no
TF	-0.053400986	-1.411840968	0.158443061	0.225591677	no
ATP1A4	-0.053393799	-1.411650422	0.158499166	0.225653701	no
RGPD8	-0.053392547	-1.411617215	0.158508945	0.225653701	no
TFF3	0.0533752	1.411157274	0.15864444	0.22583069	no
OR5B12	-0.053372911	-1.411096593	0.158662323	0.225840246	no
GPR112	0.053358639	1.410718192	0.158773872	0.225983115	no
AQP7P3	-0.05335087	-1.410512203	0.158834621	0.226053665	no
FUT1	-0.053344771	-1.410350485	0.158882326	0.226105643	no
OR51Q1	-0.053323312	-1.409781541	0.159050245	0.226328678	no
FAM59A	0.053320845	1.409716128	0.15906956	0.226340232	no
FBXO27	-0.05331693	-1.40961232	0.159100215	0.22636792	no
PLK4	0.053309766	1.409422381	0.159156317	0.226431808	no
ZNF431	-0.053308277	-1.409382882	0.159167986	0.226432475	no
VTI1B	-0.053303042	-1.409244095	0.159208991	0.226474873	no
LOC100127888	0.053263516	1.408196108	0.159518881	0.22689174	no
GNG7	-0.053261376	-1.408139382	0.159535668	0.22689174	no
METTL11A	0.053261371	1.408139242	0.15953571	0.22689174	no
RTKN2	-0.053256239	-1.408003181	0.15957598	0.226928617	no
FAM19A4	-0.053255206	-1.407975791	0.159584087	0.226928617	no
NWD1	0.053232737	1.407380074	0.1597605	0.227157271	no
RP1L1	-0.053231865	-1.407356933	0.159767355	0.227157271	no
ENPP6	0.05318355	1.406075964	0.160147207	0.227681333	no
SNTA1	-0.053180581	-1.405997237	0.160170575	0.227698543	no
C11orf54	-0.053178592	-1.405944499	0.16018623	0.227704787	no
SLCO6A1	-0.053169728	-1.405709498	0.160256003	0.227787954	no
C10orf35	-0.053153773	-1.405286473	0.160381659	0.227950537	no
TSTD2	-0.053126793	-1.404571165	0.160594306	0.228230175	no
MYBL2	0.053125947	1.404548711	0.160600984	0.228230175	no
KLK14	-0.053121108	-1.404420415	0.160639148	0.228268364	no

RPUSD3	-0.053116413	-1.404295937	0.160676182	0.228304679	no
ABCE1	0.053115005	1.404258615	0.160687287	0.228304679	no
LOC284232	0.053079102	1.40330672	0.160970722	0.228691311	no
XRN1	0.053066993	1.40298569	0.161066396	0.228811158	no
SLC6A4	0.053064413	1.402917297	0.161086785	0.228824043	no
NUDT4	-0.053052609	-1.402604325	0.161180108	0.228940525	no
DIRC3	0.053039751	1.402263433	0.161281803	0.22906888	no
NMUR2	-0.05303337	-1.402094247	0.161332293	0.229124495	no
VTCN1	-0.05301961	-1.401729444	0.161441201	0.229263062	no
SGOL1	0.052980795	1.400700359	0.161748723	0.229672881	no
CDK2AP1	-0.052980318	-1.400687713	0.161752505	0.229672881	no
OR13F1	0.052964553	1.400269747	0.161877535	0.22983427	no
NCRNA00116	0.052958077	1.400098059	0.161928915	0.229891075	no
OR8B2	-0.052954081	-1.399992124	0.161960623	0.229919947	no
ELP4	-0.052933168	-1.399437666	0.16212666	0.230139494	no
ATL2	0.05290024	1.398564679	0.162388344	0.230494773	no
MTERF	0.052888483	1.398252987	0.162481853	0.23061131	no
ATXN1L	-0.052878545	-1.397989501	0.162560932	0.230707352	no
MTG1	-0.052871937	-1.397814311	0.162613528	0.230755763	no
TTK	0.052871391	1.397799845	0.162617871	0.230755763	no
PLAC4	0.052853861	1.39733508	0.162757467	0.230937642	no
HCG9	0.05284841	1.397190576	0.162800888	0.230983043	no
GSTM1	0.052842091	1.397023035	0.162851243	0.231038273	no
STAG3L4	0.052839323	1.396949664	0.162873298	0.23105335	no
NPFFR1	0.052836516	1.396875248	0.16289567	0.231068874	no
ACAP2	0.052828502	1.39666277	0.16295956	0.231143286	no
PLG	-0.052826246	-1.39660296	0.162977549	0.231152583	no
KCTD18	-0.052823028	-1.396517659	0.163003205	0.231172756	no
LOC441046	0.05281284	1.39624756	0.163084466	0.231271777	no
FAM24B	-0.052804976	-1.396039053	0.163147217	0.231342794	no
MEPE	-0.052803696	-1.396005127	0.163157429	0.231342794	no
G2E3	-0.052796754	-1.395821086	0.163212835	0.231397688	no
FHL5	0.052795978	1.395800498	0.163219034	0.231397688	no
NBPF10	-0.052787818	-1.395584186	0.163284175	0.231473808	no
TOM1	-0.052760692	-1.394865035	0.163500884	0.231764768	no
PRKD1	-0.05275272	-1.394653677	0.163564617	0.231838854	no
SRY	0.052744061	1.394424118	0.163633858	0.231920739	no
MST1P2	-0.052717502	-1.393720016	0.163846374	0.232205662	no
ARL8B	0.052696719	1.39316903	0.16401282	0.232425261	no
EVX2	-0.052690727	-1.393010162	0.164060836	0.23247701	no
ARMC1	-0.052680516	-1.392739463	0.164142676	0.232576678	no
C1orf187	0.052678721	1.39269189	0.164157062	0.232580762	no
MED19	-0.052672789	-1.392534605	0.164204631	0.232631857	no
SPDYE8P	0.052665116	1.392331186	0.164266168	0.232702732	no
RNF152	0.052658488	1.392155484	0.164319334	0.232761739	no
KRT6C	-0.052634591	-1.391521961	0.164511142	0.233017113	no
TMEM106B	-0.052613758	-1.390969657	0.164678498	0.233230297	no
ADAD2	-0.052612984	-1.390949123	0.164684722	0.233230297	no
LOC348021	-0.05260821	-1.390822567	0.16472309	0.233263916	no
SNORA57	0.052607159	1.390794709	0.164731536	0.233263916	no
IKBKG	0.05259801	1.390552146	0.164805095	0.233351733	no

SLC16A2	-0.052562046	-1.389598719	0.165094466	0.233745092	no
IGLL3	-0.052556028	-1.389439187	0.165142922	0.233797325	no
SCLY	0.05254221	1.389072874	0.165254227	0.233938521	no
HELLS	0.052513316	1.388306868	0.165487162	0.234245499	no
MDS2	0.052512437	1.388283579	0.165494248	0.234245499	no
CBY1	0.052484688	1.387547938	0.16571819	0.234546052	no
ZNF443	-0.052462872	-1.386969583	0.165894411	0.234779029	no
LOC100128675	0.052441538	1.386404036	0.166066867	0.235006643	no
MED17	-0.052422085	-1.385888338	0.166224239	0.235212883	no
ZNF83	0.052418531	1.385794108	0.166253007	0.235237126	no
TOR2A	0.052414326	1.385682628	0.166287046	0.235268824	no
CSPG4	-0.052400449	-1.385314767	0.166399405	0.235411319	no
GBA3	0.052383084	1.384854407	0.166540097	0.235593876	no
C17orf53	0.05236878	1.384475213	0.16665605	0.235741414	no
ZDHHC15	0.052367022	1.384428611	0.166670305	0.235745084	no
SOD1	-0.052360405	-1.384253209	0.166723965	0.235804486	no
PROKR1	0.052358223	1.384195368	0.166741663	0.235813021	no
DPY19L2P4	-0.05233095	-1.383472354	0.166963008	0.23610954	no
GLRA2	-0.052328976	-1.383420025	0.166979037	0.236115692	no
OR4Q3	-0.052313312	-1.383004774	0.167106272	0.236279082	no
DNM1P35	0.052309463	1.382902765	0.167137539	0.236306767	no
MGC34034	0.052281076	1.382150217	0.167368342	0.236616539	no
PRC1	0.052270994	1.381882969	0.167450363	0.236715945	no
GNGT1	0.05226002	1.381592058	0.167539681	0.23682565	no
CITED1	0.052253825	1.381427816	0.167590124	0.236880392	no
PCDHGA6	-0.052238701	-1.381026885	0.167713308	0.237021958	no
RNF138	0.052238601	1.381024257	0.167714116	0.237021958	no
OFD1	-0.05223721	-1.380987379	0.16772545	0.237021958	no
EIF5A	0.052234779	1.380922941	0.167745256	0.237033379	no
C12orf71	-0.05223148	-1.380835466	0.167772145	0.237054807	no
KRTAP8-1	-0.05221448	-1.380384826	0.16791072	0.237234028	no
PTN	0.052212366	1.380328771	0.167927964	0.237241812	no
KIAA1958	-0.052195009	-1.379868654	0.168069553	0.237425252	no
HIST1H1T	0.052190739	1.37975548	0.168104393	0.23744525	no
SNORA63	0.052190396	1.37974637	0.168107197	0.23744525	no
AUH	0.052185774	1.37962384	0.168144924	0.237481947	no
SORBS2	-0.052167651	-1.379143438	0.168292901	0.237674342	no
MYNN	0.052162749	1.379013488	0.168332946	0.237714291	no
PMS2L5	-0.05216044	-1.378952261	0.168351816	0.237724334	no
GLI2	0.052119865	1.377876679	0.168683567	0.238169671	no
RNF26	0.052117983	1.377826797	0.168698965	0.238169671	no
PWWP2B	-0.052117547	-1.377815231	0.168702535	0.238169671	no
TBC1D21	-0.05211529	-1.377755394	0.168721007	0.238179118	no
SNORA40	0.05210511	1.37748555	0.168804329	0.238280103	no
PIWIL3	-0.052096397	-1.377254583	0.16887567	0.238364165	no
MEA1	0.05209185	1.377134046	0.168912911	0.238400086	no
MAPKSP1	0.052085434	1.376963967	0.168965469	0.238457619	no
TRPC7	0.052078183	1.376771737	0.169024886	0.238524824	no
TMEM42	0.052066682	1.376466881	0.169119148	0.238641188	no
PPAPDC1B	0.05205195	1.37607636	0.169239955	0.23879499	no
PTMS	-0.052041602	-1.375802039	0.169324855	0.238898111	no

HIST2H2AC	0.052003186	1.374783691	0.169640304	0.239326472	no
GLE1	0.051996449	1.374605112	0.169695667	0.239387874	no
ZNF542	0.051964488	1.373757887	0.169958509	0.239741934	no
PAQR4	-0.051961904	-1.373689386	0.169979774	0.239755203	no
DBNDD1	0.05195596	1.373531818	0.170028697	0.239807477	no
OR52I2	0.051916936	1.372497376	0.170350135	0.240228984	no
TSN	-0.051916792	-1.37249357	0.170351318	0.240228984	no
EIF1	0.051908942	1.372285467	0.170416039	0.240303492	no
TDRD9	-0.051901607	-1.372091045	0.170476521	0.240343707	no
FOSB	0.051901488	1.372087885	0.170477504	0.240343707	no
CHRNA1	0.051901158	1.372079152	0.170480221	0.240343707	no
TPD52	0.051898756	1.372015459	0.170500039	0.240354887	no
RBM39	-0.051894529	-1.371903435	0.170534899	0.240373564	no
PTGFR	0.051894267	1.371896472	0.170537066	0.240373564	no
GRAMD1A	0.051884023	1.371624942	0.170621585	0.24046432	no
FANCI	0.05188358	1.371613199	0.170625241	0.24046432	no
FAM26D	0.051864578	1.371109492	0.170782116	0.240668629	no
PHF23	0.051852735	1.370795549	0.170879945	0.24077407	no
C20orf186	0.051852636	1.370792947	0.170880757	0.24077407	no
NLRP9	0.0518417	1.37050306	0.170971127	0.240884617	no
SLC6A2	0.051821394	1.369964783	0.171139028	0.241104373	no
CT45A3	-0.051773275	-1.368689268	0.171537382	0.241648745	no
MCCD1	-0.051766466	-1.36850878	0.171593806	0.241711389	no
C11orf61	-0.051761938	-1.368388762	0.171631334	0.241747409	no
SNORA41	0.051759547	1.368325376	0.171651156	0.241758487	no
ASAP1	0.051754058	1.36817989	0.171696659	0.24180573	no
OR2M2	-0.051750823	-1.368094141	0.171723483	0.241826662	no
PRG1	-0.051734965	-1.367673791	0.171855022	0.241995043	no
NDUFS3	-0.051733062	-1.367623347	0.171870812	0.242000423	no
MAP2K6	0.051703603	1.366842474	0.172115384	0.242327914	no
ASB5	0.051701318	1.366781898	0.172134368	0.242337766	no
MEN1	-0.051687325	-1.366410982	0.172250641	0.242481522	no
TSPY1	-0.051686053	-1.366377286	0.172261207	0.242481522	no
NT5E	0.051683576	1.366311618	0.172281799	0.242481522	no
KRTAP5-11	-0.051683257	-1.366303157	0.172284453	0.242481522	no
FSCB	-0.051679105	-1.366193109	0.172318967	0.242497402	no
OR10A3	0.051679014	1.366190696	0.172319723	0.242497402	no
FAM99B	0.051663396	1.365776713	0.172449606	0.242663288	no
OR51B6	0.051645379	1.365299136	0.172599531	0.242857354	no
ZNF354A	0.051640934	1.36518133	0.172636529	0.242892508	no
SLC9A4	0.051618878	1.364596681	0.17282023	0.243134048	no
KLHL36	0.051608136	1.364311945	0.172909749	0.24323999	no
ZDHHC2	0.051606954	1.364280633	0.172919595	0.24323999	no
SMEK3P	-0.051590475	-1.363843828	0.173056997	0.243416333	no
LOC148189	-0.051578655	-1.363530521	0.173155602	0.243538084	no
ITPR2	0.051573813	1.363402158	0.173196012	0.243577976	no
KCNG1	0.051558007	1.362983205	0.173327955	0.243746581	no
TIAM2	-0.051547712	-1.362710326	0.173413934	0.24385053	no
DCTN3	-0.051540219	-1.362511726	0.17347653	0.243921586	no
ARHGAP23	-0.051483764	-1.361015307	0.173948721	0.244568514	no
B3GAT3	0.051460627	1.360402048	0.17414251	0.244823954	no

OPTC	-0.051454097	-1.360228942	0.174197241	0.244869274	no
IFNA10	0.05145389	1.360223476	0.174198969	0.244869274	no
HSPB3	-0.051428588	-1.359552806	0.174411138	0.245150472	no
PRDM7	0.05142246	1.35939039	0.174462547	0.245205685	no
TRIML2	0.051412411	1.359124038	0.174546881	0.245307163	no
C20orf103	-0.05139739	-1.3587259	0.174672998	0.245467343	no
LMBRD2	-0.051350317	-1.357478189	0.175068672	0.246006284	no
ECT2L	-0.051334861	-1.357068506	0.175198737	0.246171941	no
LOC146481	-0.051331945	-1.356991236	0.175223277	0.246189313	no
DHRS2	-0.051315717	-1.356561094	0.175359929	0.246364189	no
POU3F1	0.051301115	1.35617406	0.175482954	0.246519897	no
DRD2	-0.05129146	-1.355918154	0.175564334	0.246608517	no
PRSS16	0.051290736	1.355898981	0.175570432	0.246608517	no
AIF1L	-0.051287944	-1.355824979	0.175593971	0.246624446	no
BBC3	0.051274234	1.355461579	0.175709597	0.246769702	no
KIFC3	0.051268016	1.355296775	0.175762053	0.246826226	no
C17orf49	0.051262742	1.355156974	0.175806559	0.246861507	no
SDHAP2	-0.051262145	-1.355141156	0.175811596	0.246861507	no
ZIC4	0.051255103	1.354954519	0.175871028	0.246927808	no
XPO6	-0.051234776	-1.354415741	0.176042678	0.247151647	no
FXC1	0.051224805	1.35415145	0.176126925	0.247252754	no
VWA5B1	0.051211023	1.353786166	0.176243414	0.247399107	no
NCRNA00183	0.051195313	1.353369788	0.176376267	0.24756841	no
SV2C	-0.051182182	-1.353021737	0.176487377	0.247707171	no
CRCT1	-0.051171705	-1.352744055	0.17657606	0.247814437	no
C6orf89	-0.051148374	-1.352125668	0.176773674	0.248074556	no
STK11IP	0.051130815	1.351660283	0.176922502	0.248264257	no
SPRR1B	-0.051129357	-1.351621628	0.176934868	0.248264257	no
QRFPR	-0.051127663	-1.35157672	0.176949235	0.248264257	no
TRY6	0.051126633	1.351549423	0.176957968	0.248264257	no
TMEM65	-0.051097196	-1.350769219	0.177207722	0.2485974	no
CCDC113	-0.051078151	-1.350264444	0.177369447	0.248795314	no
ZNF674	-0.051077684	-1.350252072	0.177373413	0.248795314	no
SESN3	0.051063238	1.349869188	0.17749616	0.248950217	no
SNAPC1	0.051053799	1.349619011	0.177576399	0.249045479	no
NAV3	-0.051035355	-1.349130148	0.177733267	0.249248193	no
FSD2	0.051023413	1.348813633	0.177834887	0.249373405	no
UQCRFS1	0.051018461	1.348682394	0.177877035	0.249415209	no
ERF	0.051015139	1.348594356	0.177905313	0.249437561	no
CTAGE5	0.050999719	1.348185663	0.178036631	0.249604369	no
LOC728323	-0.050994543	-1.348048459	0.178080732	0.249633419	no
LRFN3	-0.050994388	-1.348044373	0.178082046	0.249633419	no
NR3C1	-0.05095801	-1.347080207	0.178392188	0.250050836	no
CUL4B	0.050945268	1.346742498	0.178500914	0.25018589	no
MED14	0.050940395	1.34661334	0.178542509	0.25022064	no
NCRNA00051	-0.050939464	-1.34658866	0.178550458	0.25022064	no
C7orf36	0.050927886	1.346281796	0.178649317	0.250341828	no
UBE4B	-0.050921782	-1.346120027	0.178701448	0.250397524	no
BOLA2	0.050902819	1.34561743	0.178863487	0.250607206	no
REN	-0.050900401	-1.345553346	0.178884155	0.250618797	no
MEF2D	-0.050896158	-1.34544088	0.178920433	0.250652253	no

NEUROG3	-0.050891216	-1.345309904	0.178962688	0.250694078	no
CT45A5	-0.050883914	-1.345116363	0.179025141	0.250763715	no
TTC21A	-0.050882503	-1.345078981	0.179037206	0.250763715	no
FAM173A	-0.050880536	-1.345026836	0.179054036	0.250769915	no
PBRM1	-0.050878846	-1.344982045	0.179068493	0.250772792	no
OR13C9	-0.050875258	-1.34488695	0.179099191	0.250798409	no
COX19	0.050848751	1.344184419	0.179326095	0.251098759	no
PAGE2B	-0.050844987	-1.344084673	0.179358328	0.251126501	no
KRT40	-0.050840429	-1.343963877	0.17939737	0.251163771	no
ZNF77	-0.050829144	-1.343664791	0.179494062	0.251281744	no
SOX17	0.050815826	1.343311804	0.179608231	0.251424164	no
PRKCQ	-0.050809927	-1.343155471	0.179658811	0.251477558	no
MAN2A2	-0.050803155	-1.342975993	0.179716894	0.251541444	no
CALML3	-0.050769575	-1.342086004	0.180005117	0.251927417	no
TUBB1	-0.050759157	-1.341809903	0.180094603	0.252019505	no
CCL3	0.050759013	1.341806068	0.180095846	0.252019505	no
MYLK2	-0.050741314	-1.341337003	0.180247948	0.252214895	no
NFKBIB	0.050738664	1.341266762	0.180270733	0.252229321	no
CLDN15	-0.050710396	-1.340517577	0.180513891	0.252552062	no
C2orf34	0.050704336	1.340356974	0.180566048	0.252600641	no
C5orf48	-0.050702781	-1.340315751	0.180579438	0.252600641	no
LMNB1	0.05070147	1.340281002	0.180590725	0.252600641	no
BZW2	0.050700556	1.340256793	0.180598589	0.252600641	no
C21orf54	-0.050688309	-1.339932198	0.180704053	0.252730668	no
YWHAG	-0.05066618	-1.339345737	0.180894716	0.252979827	no
NUDT17	-0.050657767	-1.339122769	0.180967244	0.253063753	no
SIK1	0.050652168	1.338974383	0.181015523	0.25311376	no
NUDT12	-0.050650578	-1.338932236	0.181029238	0.253115431	no
MDGA1	-0.050647348	-1.338846631	0.181057097	0.253136877	no
SCRN1	-0.050638952	-1.338624114	0.181129526	0.253220631	no
RGP1	0.050619871	1.338118432	0.181294206	0.253433331	no
PHIP	-0.050614587	-1.337978368	0.181339839	0.253479596	no
SNORA55	0.050603989	1.337697504	0.18143137	0.253590007	no
DNAJC28	-0.050597267	-1.337519354	0.181489446	0.253653644	no
KRT85	0.050587485	1.337260108	0.181573983	0.253754253	no
HHIPL1	-0.050581961	-1.337113699	0.181621738	0.253803448	no
EDN1	-0.050557095	-1.336454693	0.181836805	0.254086427	no
GLIS1	-0.050531721	-1.335782243	0.182056454	0.254372099	no
SORCS1	-0.050530572	-1.335751784	0.182066408	0.254372099	no
C22orf33	-0.050528015	-1.335684017	0.182088555	0.254385462	no
HAPLN2	-0.050496661	-1.334853071	0.182360284	0.254732734	no
CROCC	-0.050495203	-1.334814433	0.182372927	0.254732734	no
FAM189B	0.050494972	1.334808311	0.18237493	0.254732734	no
FANCC	0.050493138	1.334759697	0.182390837	0.254737355	no
VPS37C	0.050486727	1.33458981	0.182446436	0.254797406	no
D4S234E	-0.050463092	-1.33396342	0.182651544	0.255066233	no
FAM47C	0.050441082	1.333380129	0.182842693	0.255315531	no
NCAPG2	0.05043748	1.33328466	0.182873993	0.255334797	no
DDX12	-0.050436588	-1.333261008	0.182881749	0.255334797	no
SNORA54	0.050434495	1.333205555	0.182899932	0.255342551	no
CD38	-0.050432851	-1.333161983	0.18291422	0.255344866	no

MOCS2	-0.050422912	-1.332898587	0.183000611	0.255447828	no
SCARNA22	0.050414307	1.332670544	0.183075431	0.255534626	no
ALDH1A1	0.050397029	1.332212626	0.18322574	0.255726771	no
PON2	-0.050378667	-1.331726019	0.183385567	0.255932173	no
HSD17B14	-0.050367415	-1.331427819	0.183483562	0.256051261	no
ARTN	0.050360672	1.331249126	0.183542304	0.256115556	no
C18orf21	0.050344486	1.330820153	0.183683376	0.25629472	no
PITX3	-0.050326494	-1.330343365	0.183840267	0.25649593	no
CCDC17	-0.050317888	-1.330115275	0.183915357	0.25658299	no
SNRPA	-0.050289038	-1.329350733	0.18416722	0.256916639	no
GUCY1B3	-0.05026721	-1.328772263	0.184357955	0.257164974	no
PIK3C2A	-0.050242231	-1.328110278	0.184576407	0.257451935	no
XKR3	0.050204439	1.32710875	0.184907271	0.257895641	no
PEX19	-0.050198456	-1.326950197	0.184959691	0.257950958	no
KLHDC9	0.050196669	1.326902848	0.184975347	0.257954999	no
GNB2L1	-0.05019434	-1.326841127	0.184995757	0.257965668	no
PCDHA11	-0.050183287	-1.326548218	0.18509264	0.258082966	no
GART	0.050181321	1.326496117	0.185109877	0.2580892	no
GYPA	-0.050159933	-1.325929309	0.185297475	0.258323356	no
TOMM20L	0.05015926	1.325911487	0.185303375	0.258323356	no
CHRNA7	-0.050150454	-1.325678117	0.185380657	0.258413274	no
KRT35	-0.05014508	-1.325535696	0.185427832	0.258461214	no
ANP32B	-0.050138381	-1.32535816	0.185486651	0.258525376	no
ZNF239	-0.05010947	-1.324592009	0.185740641	0.258861535	no
LOC283999	-0.050082593	-1.32387975	0.185976997	0.259168221	no
IBTK	-0.050081531	-1.323851623	0.185986335	0.259168221	no
ANP32C	-0.050078554	-1.323772725	0.186012531	0.259186861	no
PRDXDD1P	0.050055127	1.323151902	0.186218755	0.259456329	no
PLXNA4	-0.050036989	-1.322671234	0.186378539	0.25966106	no
OR6T1	-0.050032108	-1.322541889	0.186421553	0.259703091	no
E2F5	-0.05002738	-1.322416594	0.186463227	0.25974325	no
VHL	-0.050005926	-1.321848078	0.186652408	0.259988866	no
TIAM1	-0.049988219	-1.321378828	0.186808664	0.26018859	no
SLC27A1	0.049985952	1.321318742	0.186828679	0.260198543	no
CIC	-0.049974182	-1.321006838	0.186932603	0.260325345	no
LOC100126784	0.049967656	1.32083391	0.186990239	0.260387675	no
ZNF470	0.049966035	1.320790961	0.187004556	0.260389677	no
ROM1	0.049961994	1.320683879	0.187040254	0.260421449	no
C9orf91	-0.04993102	-1.319863065	0.187314061	0.260784719	no
SLC17A1	0.049925566	1.319718519	0.18736231	0.260833931	no
ANKRD26P1	0.049892103	1.318831767	0.187658502	0.261212544	no
MTMR12	0.049891921	1.318826943	0.187660115	0.261212544	no
SFRS11	0.04987411	1.318354966	0.187817906	0.261414184	no
CHUK	0.049835909	1.317342656	0.188156674	0.261867669	no
ASB18	-0.049793714	-1.316224529	0.188531377	0.262371102	no
PPARGC1B	0.049791981	1.316178597	0.188546781	0.26237448	no
IDUA	0.04975871	1.315296945	0.188842644	0.262768106	no
BAAT	-0.049753038	-1.315146636	0.188893119	0.262820252	no
FAM119A	0.049746027	1.314960864	0.188955516	0.262888978	no
HYI	-0.049738609	-1.314764289	0.189021558	0.262962765	no
C9orf23	-0.049734704	-1.314660801	0.189056334	0.262993048	no

EXOSC8	-0.049729012	-1.314509974	0.189107025	0.263033866	no
TATDN3	0.049728488	1.314496076	0.189111696	0.263033866	no
NCRNA00182	-0.049710875	-1.314029363	0.189268618	0.263234018	no
ZNF92	-0.049702437	-1.313805753	0.189343837	0.263320518	no
SNORA51	0.049680529	1.31322523	0.189539217	0.263574104	no
CRK	0.049644929	1.312281857	0.189857035	0.263997905	no
C6orf15	0.049629339	1.311868761	0.189996329	0.264173426	no
USP51	-0.049606835	-1.311272434	0.190197541	0.264435008	no
DBH	-0.049603259	-1.311177668	0.190229531	0.264461298	no
SCARF2	0.049585162	1.310698127	0.190391471	0.264668232	no
PRSS41	-0.049579191	-1.310539915	0.190444922	0.264724333	no
AMBP	0.049577257	1.310488655	0.190462242	0.264730208	no
LIPI	-0.049566812	-1.310211874	0.190555782	0.264842016	no
TH	0.049556605	1.309941428	0.190647214	0.264948899	no
ERCC2	-0.049555302	-1.309906886	0.190658894	0.264948899	no
KLRG2	0.049547534	1.309701063	0.190728504	0.265027415	no
TIMM22	0.049539658	1.309492355	0.190799108	0.265102098	no
ZNF440	-0.049538613	-1.309464671	0.190808474	0.265102098	no
C14orf43	-0.049534962	-1.309367914	0.190841214	0.265129367	no
GNL3	-0.04952022	-1.308977285	0.190973434	0.265294825	no
FAM22G	0.049510829	1.308728448	0.191057696	0.265393644	no
GAR1	0.049508622	1.308669962	0.191077504	0.265402925	no
FAM83F	0.049472532	1.307713634	0.191401617	0.265834848	no
BTBD10	0.049460987	1.307407728	0.191505378	0.26596069	no
SLC30A8	0.049448406	1.307074352	0.191618504	0.26609952	no
ZIC2	0.049439055	1.306826557	0.191702621	0.266198049	no
HHIP	0.049429481	1.306572877	0.191788764	0.266299378	no
CENPO	0.049424999	1.306454113	0.191829104	0.266333779	no
GAGE10	-0.049423801	-1.306422371	0.191839886	0.266333779	no
ADAT2	0.049393907	1.305630241	0.19210911	0.266656732	no
SLC38A3	-0.049393631	-1.305622921	0.192111599	0.266656732	no
GP5	0.049393578	1.305621517	0.192112076	0.266656732	no
PIK3R4	-0.049390861	-1.305549539	0.192136554	0.2666724	no
ARID3C	-0.049361276	-1.304765599	0.192403296	0.267024289	no
LOC654342	0.049356884	1.304649238	0.192442912	0.267050024	no
FLJ12825	0.049356292	1.304633543	0.192448256	0.267050024	no
C17orf99	-0.04935118	-1.304498097	0.192494378	0.267095693	no
PMS2L4	-0.049341921	-1.304252755	0.192577943	0.267185479	no
HEMGN	0.049339277	1.304182675	0.192601818	0.267185479	no
SLC35A5	0.049338526	1.304162786	0.192608594	0.267185479	no
GLB1L3	-0.049338155	-1.304152943	0.192611947	0.267185479	no
XRCC5	0.049316403	1.303576571	0.192808394	0.267439634	no
TMEM200A	0.049314802	1.303534149	0.192822858	0.267441349	no
LYZL4	-0.049309675	-1.303398305	0.192869182	0.26748725	no
FRG2C	-0.049306465	-1.303313245	0.192898193	0.267509134	no
CPA6	0.049302114	1.303197965	0.192937515	0.267545314	no
GLIPR1L2	0.049287457	1.302809583	0.193070037	0.267710719	no
NT5DC1	0.049284379	1.302728044	0.193097867	0.267730948	no
SMTNL1	0.049275436	1.302491057	0.193178772	0.267824756	no
ETFB	-0.049269294	-1.302328316	0.193234345	0.267883433	no
LOC723972	-0.049252599	-1.301885965	0.193385458	0.268074541	no

NBR2	-0.049249191	-1.301795665	0.193416316	0.268098936	no
LOC441454	-0.049235586	-1.301435158	0.193539549	0.268231093	no
TRIM40	-0.049235285	-1.301427178	0.193542277	0.268231093	no
WRB	-0.049233382	-1.301376752	0.193559519	0.268231093	no
TMC3	-0.049232807	-1.301361522	0.193564727	0.268231093	no
ZNF649	-0.049220873	-1.301045304	0.193672878	0.268362569	no
ZNF385B	-0.049216825	-1.300938058	0.193709568	0.268395014	no
PRKG1	0.049213864	1.300859592	0.193736415	0.268413817	no
PITX2	0.049211065	1.300785421	0.193761795	0.268430586	no
RBM12	-0.04920834	-1.300713219	0.193786504	0.268446422	no
LOC100192378	-0.049195703	-1.30037837	0.193901125	0.268586801	no
OR5H1	0.049190797	1.300248382	0.193945634	0.26863005	no
OR2B3	0.04917793	1.29990744	0.194062412	0.268773383	no
FAM71C	-0.049176059	-1.299857865	0.194079396	0.268778494	no
NEDD4L	-0.049174033	-1.299804206	0.194097781	0.268785544	no
SYT12	-0.049172304	-1.299758378	0.194113484	0.268788879	no
OR2M5	-0.049160849	-1.299454855	0.194217509	0.268914505	no
TMX2	-0.049103398	-1.297932593	0.194739845	0.269619269	no
PTPN20B	-0.049088713	-1.297543501	0.19487352	0.269785869	no
USP50	0.049081839	1.297361365	0.194936117	0.269854051	no
HOXC9	0.049079251	1.297292781	0.194959692	0.269868209	no
GDF7	-0.049067315	-1.296976536	0.195068425	0.270000234	no
NSUN2	0.049059529	1.296770219	0.195139386	0.270079964	no
C20orf43	-0.04900202	-1.295246459	0.195664059	0.270787593	no
KRT6B	-0.048996315	-1.295095301	0.195716163	0.270841163	no
SPDYE5	-0.048994269	-1.295041098	0.19573485	0.270848483	no
ARL13A	0.048945507	1.293749094	0.196180652	0.271415605	no
TMEM120B	-0.048945263	-1.293742609	0.196182891	0.271415605	no
RPGRIP1L	0.04894399	1.293708894	0.196194534	0.271415605	no
PYGM	0.048943569	1.293697727	0.196198391	0.271415605	no
ARHGEF6	-0.048922368	-1.293135998	0.196392459	0.271665486	no
HSD17B12	-0.048919703	-1.293065398	0.19641686	0.271680651	no
TMEM215	-0.048897594	-1.292479594	0.196619415	0.271942217	no
ARHGEF38	0.048893015	1.292358277	0.196661382	0.271981655	no
XKR5	-0.04888717	-1.292203389	0.196714971	0.272022209	no
TTLL10	0.048886881	1.292195754	0.196717614	0.272022209	no
C6orf217	0.048864872	1.291612605	0.196919476	0.272282722	no
RPL26L1	0.048857911	1.291428165	0.196983353	0.27235242	no
PGF	-0.048844733	-1.291079005	0.19710432	0.272501035	no
ACSM1	0.048841877	1.291003323	0.197130547	0.272518659	no
DHCR24	0.048829155	1.290666253	0.197247388	0.27266154	no
ZNF280C	-0.048809499	-1.290145468	0.197428011	0.272892564	no
KPNA1	0.048790734	1.289648283	0.197600562	0.27310652	no
PRDM12	-0.048789728	-1.289621625	0.197609817	0.27310652	no
PCGEM1	0.048763643	1.288930494	0.197849871	0.273419596	no
TTC36	-0.04875092	-1.28859341	0.197967029	0.273562805	no
ASNA1	0.048739191	1.288282634	0.198075089	0.273693422	no
PCA3	0.048717606	1.287710741	0.198274054	0.273949622	no
NOS1AP	-0.048713258	-1.28759555	0.198314147	0.273982467	no
DCAF4L1	0.048712089	1.287564578	0.198324929	0.273982467	no
WFDC5	0.048710421	1.287520386	0.198340312	0.273984998	no

C17orf81	-0.048704464	-1.287362536	0.198395269	0.274042191	no
PDYN	-0.048684093	-1.286822809	0.198583262	0.274283125	no
PWP2	-0.048650543	-1.285933914	0.198893159	0.274692389	no
SIK2	-0.048647986	-1.285866165	0.198916792	0.274706264	no
VGLL4	0.04864392	1.285758438	0.198954377	0.274739402	no
NCRNA00105	-0.048633981	-1.285495103	0.199046273	0.27484753	no
NPFFR2	-0.048628084	-1.285338864	0.19910081	0.274900199	no
APOF	0.048626916	1.285307929	0.199111609	0.274900199	no
ADARB1	-0.048612879	-1.284936025	0.199241476	0.275060714	no
SLC41A1	-0.048603184	-1.284679152	0.199331211	0.275165808	no
CALML4	0.048589034	1.284304249	0.199462231	0.275310437	no
HBE1	0.048588927	1.284301434	0.199463215	0.275310437	no
DEFA5	-0.048581704	-1.284110061	0.19953012	0.275383983	no
PRR18	-0.048574097	-1.283908495	0.199600606	0.275462461	no
C3orf26	0.048551358	1.283306062	0.199811382	0.275734525	no
DPCD	0.048547763	1.283210796	0.199844728	0.275761719	no
BEST1	-0.048543507	-1.283098053	0.199884197	0.275797358	no
EME2	-0.048516958	-1.282394639	0.200130575	0.276106837	no
IL1F7	0.048516394	1.282379715	0.200135804	0.276106837	no
C20orf151	-0.048514032	-1.282317124	0.200157739	0.276118256	no
MFAP3L	0.048478253	1.281369196	0.200490145	0.276532563	no
PHLDB3	0.0484773	1.281343946	0.200499005	0.276532563	no
TCEB3C	0.048477009	1.281336228	0.200501713	0.276532563	no
HSN2	-0.048475819	-1.281304691	0.200512779	0.276532563	no
RAD51AP2	0.048466397	1.281055083	0.200600383	0.276634509	no
MUC4	-0.04846207	-1.280940429	0.200640631	0.276671142	no
ARSH	-0.048449285	-1.280601713	0.200759571	0.276816272	no
TIMM8A	-0.048441928	-1.280406781	0.200828044	0.276891803	no
NLRP4	0.048385559	1.278913365	0.201353199	0.27759693	no
KRT5	-0.048382286	-1.278826646	0.201383724	0.277620083	no
CLCN3	-0.048380022	-1.278766653	0.201404844	0.277630267	no
PPAPDC3	-0.048371588	-1.278543215	0.201483516	0.27771978	no
VENTXP1	0.048355436	1.27811529	0.201634251	0.277901791	no
C9orf103	-0.048353541	-1.278065083	0.201651941	0.277901791	no
RBPJ	-0.04835302	-1.278051295	0.2016568	0.277901791	no
MAGEE2	-0.048343895	-1.277809525	0.201742005	0.278000263	no
ZIM3	0.048341815	1.277754415	0.201761431	0.278008084	no
YIPF7	-0.048305343	-1.276788151	0.202102253	0.278458727	no
OR7D4	-0.048266783	-1.275766556	0.202463049	0.278936826	no
C14orf179	0.048262279	1.275647245	0.202505216	0.278975911	no
ELSPBP1	0.048254992	1.275454196	0.202573458	0.279050908	no
PRAMEF12	0.04823619	1.274956054	0.202749627	0.279274558	no
SHROOM4	0.048234281	1.274905492	0.202767515	0.27928017	no
SSNA1	0.048225635	1.27467643	0.202848566	0.279372773	no
POLR2J2	-0.0482049	-1.274127086	0.203043041	0.27961672	no
CLC	0.048203801	1.27409797	0.203053352	0.27961672	no
OR3A1	0.048195537	1.273879037	0.203130899	0.279704455	no
FAM196A	-0.048173384	-1.273292134	0.203338886	0.279971779	no
ALDH3A1	0.048166164	1.273100865	0.203406702	0.280046081	no
PPAPDC1A	-0.048155768	-1.272825432	0.203504388	0.280161495	no
PCDHB7	0.048143353	1.272496534	0.203621081	0.280303058	no

WNT5B	-0.048131195	-1.272174424	0.203735412	0.280441351	no
FRG2	0.04812526	1.272017187	0.20379124	0.280499101	no
C11orf58	0.048115577	1.271760663	0.203882344	0.280605393	no
PSMC3	-0.048104418	-1.271465046	0.203987368	0.280730829	no
TBL1X	0.048089937	1.271081393	0.204123728	0.280899369	no
WDR31	-0.048069939	-1.270551593	0.204312142	0.281125007	no
NDUFB6	0.048069582	1.270542143	0.204315503	0.281125007	no
LOC100132287	-0.048053529	-1.27011687	0.204466838	0.281314088	no
TAL2	-0.048044952	-1.269889623	0.204547737	0.281406243	no
CST5	-0.048034382	-1.269609601	0.204647457	0.281524276	no
FBXO30	0.048031238	1.269526312	0.204677124	0.28154593	no
CCDC67	0.048009862	1.268960019	0.204878918	0.281804338	no
LOC149620	0.048000954	1.26872403	0.204963054	0.281900886	no
MRP63	-0.047999402	-1.268682911	0.204977716	0.281901875	no
GPR146	-0.047996702	-1.268611369	0.205003229	0.281917785	no
STIP1	-0.047987964	-1.268379884	0.205085795	0.282012147	no
ODF3L2	-0.047955014	-1.267506971	0.205397363	0.282421375	no
HAUS8	0.047944538	1.267229446	0.205496492	0.282538462	no
C1orf163	0.047942227	1.267168225	0.205518365	0.28254932	no
DEM1	0.047931699	1.266889314	0.205618032	0.282656792	no
PRAMEF4	0.047931016	1.266871222	0.205624498	0.282656792	no
LOC728554	0.047917746	1.266519674	0.205750174	0.282810322	no
FAM49A	-0.047903179	-1.266133766	0.205888198	0.282980802	no
LOC282997	-0.047877402	-1.26545088	0.206132605	0.283295945	no
MID1	0.047876042	1.265414837	0.206145511	0.283295945	no
RFPL1	-0.047864809	-1.265117265	0.206252084	0.283423138	no
LIN37	-0.047843907	-1.264563537	0.206450503	0.283676517	no
MSH2	0.047832815	1.264269686	0.206555856	0.283801991	no
C19orf48	0.047824346	1.264045347	0.206636314	0.283893245	no
ZNF805	0.047817861	1.263873533	0.206697949	0.283958628	no
GTF2H1	0.047811655	1.263709124	0.206756941	0.284007164	no
DBX2	0.047811188	1.263696771	0.206761373	0.284007164	no
MEX3D	0.047802723	1.263472497	0.206841866	0.284098427	no
PLAA	0.047781622	1.2629135	0.207042591	0.284354806	no
ZDHHC14	0.047766364	1.262509308	0.207187816	0.284534932	no
FSHB	-0.047763253	-1.262426898	0.207217435	0.28455628	no
LOC285593	0.047760462	1.262352947	0.207244016	0.284573453	no
C20orf202	-0.047758069	-1.262289568	0.207266799	0.284579122	no
CCDC157	-0.047757072	-1.262263152	0.207276296	0.284579122	no
DUPD1	0.047731203	1.261577838	0.207522774	0.284898176	no
WDR3	0.047727167	1.26147093	0.207561243	0.284931642	no
SPRR2E	-0.047716306	-1.26118321	0.207664801	0.285039551	no
MUTYH	0.047715966	1.261174191	0.207668048	0.285039551	no
CCT5	0.047713409	1.261106454	0.207692434	0.285053671	no
LAMA5	0.047688364	1.26044298	0.207931402	0.285362279	no
CABLES2	-0.047684448	-1.26033926	0.207968778	0.2853942	no
FUNDC2P2	-0.047672577	-1.260024784	0.208082129	0.285526478	no
CDS1	0.047671396	1.259993481	0.208093414	0.285526478	no
ANAPC7	0.047648044	1.259374877	0.208316527	0.285813216	no
RNGTT	-0.047644455	-1.259279792	0.208350837	0.285840892	no
DUSP21	0.047618449	1.258590877	0.208599544	0.28616268	no

APOOL	-0.047604211	-1.258213697	0.208735801	0.286330174	no
DUSP15	0.047592792	1.257911207	0.208845124	0.286460699	no
DQX1	-0.047581356	-1.257608266	0.20895465	0.286591488	no
BTBD3	0.047579713	1.257564742	0.20897039	0.286593633	no
ZNF597	0.047563528	1.257135973	0.209125489	0.286786891	no
MCEE	0.047558782	1.257010246	0.209170985	0.286829827	no
PDP1	-0.047544709	-1.256637443	0.209305928	0.286995406	no
LINGO2	-0.047537521	-1.256447044	0.209374872	0.28707047	no
OR13C4	-0.047534338	-1.256362721	0.20940541	0.287092872	no
ZWINT	0.047532255	1.256307546	0.209425394	0.287100801	no
HOXA9	0.047514688	1.255842171	0.209594005	0.287312468	no
MAGIX	-0.047504339	-1.255568031	0.209693375	0.287429197	no
ARL6	0.04749342	1.255278788	0.209798257	0.287540778	no
ANKS1A	0.04749289	1.255264735	0.209803353	0.287540778	no
OXSM	0.047491423	1.255225883	0.209817445	0.287540778	no
KRTAP4-8	0.047469186	1.254636817	0.210031176	0.287814174	no
ZNRD1	0.047433596	1.253694047	0.210373571	0.288263834	no
IFNA4	0.047390494	1.252552273	0.210788783	0.288813202	no
CCL27	0.047381899	1.252324576	0.210871657	0.288907174	no
C1orf189	0.047377312	1.252203076	0.210915888	0.288942371	no
SNORA14B	0.047376271	1.252175496	0.21092593	0.288942371	no
ZNF433	-0.047368522	-1.251970221	0.211000677	0.289025183	no
RPS10	-0.047362107	-1.251800305	0.211062564	0.289090368	no
SNORA48	0.047357495	1.251678117	0.211107075	0.289131747	no
C16orf70	0.047355299	1.25161994	0.21112827	0.289141189	no
GATA4	0.04734851	1.251440114	0.211193795	0.289211335	no
RPL19P12	-0.04734185	-1.251263677	0.2112581	0.289279801	no
SNF8	0.047336917	1.251133011	0.211305732	0.289325429	no
UBE2N	0.047313905	1.250523431	0.211528046	0.289603582	no
STAMBPL1	0.047312924	1.250497447	0.211537526	0.289603582	no
CCM2	0.047303081	1.250236701	0.211632675	0.289714227	no
FBXO33	-0.04728055	-1.249639873	0.21185058	0.289992893	no
WRAP53	-0.047266147	-1.24925835	0.211989962	0.290164041	no
DGUOK	0.047262802	1.249169733	0.212022345	0.29018872	no
PPP2R5E	-0.047250134	-1.248834158	0.212145009	0.290336952	no
DTL	0.047223201	1.248120733	0.21240596	0.290674408	no
KRT24	-0.047206863	-1.24768796	0.212564369	0.2908715	no
SLC5A2	-0.047204346	-1.247621274	0.212588786	0.290885225	no
FAM169B	0.047202642	1.247576125	0.212605319	0.290888159	no
PCDHGA12	-0.047135133	-1.245787889	0.213260871	0.291746705	no
C10orf40	-0.04713505	-1.245785698	0.213261675	0.291746705	no
TMEM209	0.047125763	1.245539693	0.213351973	0.291850487	no
TARP	0.047105253	1.244996406	0.213551489	0.292103646	no
IFNA8	-0.047095933	-1.244749508	0.213642204	0.292207961	no
TRMU	-0.04708404	-1.244434488	0.213757988	0.292346547	no
CPT1A	0.047070759	1.244082687	0.213887345	0.292503675	no
STAG3L2	0.047068564	1.244024556	0.213908725	0.292513128	no
SNRPA1	-0.047031292	-1.243037252	0.214272084	0.292990193	no
C16orf74	0.047029444	1.242988303	0.214290111	0.292995026	no
SPINK5	-0.047017335	-1.242667573	0.214408253	0.293136736	no
KLHL20	-0.047000514	-1.242221992	0.214572462	0.293338361	no

SNHG11	0.046999256	1.242188674	0.214584744	0.293338361	no
SNORA32	0.046989942	1.241941975	0.214675702	0.29344286	no
PRNP	-0.046972598	-1.241482547	0.214845169	0.293654653	no
DEFB119	-0.046970523	-1.241427573	0.214865453	0.293662525	no
C14orf145	0.046968407	1.241371529	0.214886134	0.293670938	no
SP5	0.046966602	1.241323712	0.21490378	0.293675202	no
CLDN6	-0.046939998	-1.240619038	0.215163947	0.293992459	no
FRG2B	-0.046939889	-1.240616128	0.215165022	0.293992459	no
C1orf122	0.046936082	1.240515298	0.215202268	0.294020883	no
PIK3R3	0.04693479	1.240481076	0.21521491	0.294020883	no
ZNF208	-0.046912627	-1.239894025	0.215431861	0.29429739	no
NAA40	-0.046885887	-1.239185724	0.215693832	0.294635356	no
HADH	0.04687869	1.238995103	0.215764374	0.294703161	no
CPNE3	0.04687707	1.238952183	0.21578026	0.294703161	no
HES7	-0.046876362	-1.238933435	0.215787199	0.294703161	no
SERPINA7	0.046867592	1.238701142	0.215873192	0.294800688	no
GP9	-0.046863638	-1.238596392	0.215911977	0.29483374	no
HES1	0.046859271	1.238480735	0.215954807	0.294872309	no
GOLIM4	0.046844614	1.238092498	0.216098622	0.295048754	no
APOO	0.046837786	1.23791163	0.216165646	0.295120334	no
PDCD4	-0.046836019	-1.237864825	0.216182992	0.295124088	no
AP1G1	-0.046818462	-1.23739979	0.216355396	0.295339504	no
OR11H4	-0.046804251	-1.237023369	0.21649502	0.295510148	no
FAM47B	0.04679743	1.236842691	0.216562061	0.295581702	no
SH3PXD2A	0.04678419	1.236491994	0.216692231	0.295730675	no
PPP5C	-0.046780718	-1.236400021	0.216726378	0.295730675	no
SLC25A11	-0.046780449	-1.236392912	0.216729018	0.295730675	no
PLP1	-0.046780378	-1.236391031	0.216729717	0.295730675	no
CLTA	0.046769177	1.236094336	0.2168399	0.295851171	no
SCFD1	-0.046768426	-1.236074442	0.21684729	0.295851171	no
TNFSF18	-0.046755313	-1.235727106	0.216976335	0.296001503	no
SPHAR	-0.046754254	-1.235699056	0.216986758	0.296001503	no
HOXC5	0.046738589	1.235284125	0.217140994	0.29618657	no
CDH15	-0.046737248	-1.235248601	0.217154202	0.29618657	no
NTSR1	0.046736012	1.235215871	0.217166372	0.29618657	no
HMGN3	-0.046726648	-1.234967857	0.217258608	0.296292381	no
C12orf32	0.04669408	1.234105213	0.217579644	0.296710188	no
IQCF6	-0.046670599	-1.233483252	0.21781132	0.296996966	no
ZP4	0.046669788	1.233461785	0.21781932	0.296996966	no
UCKL1AS	-0.046662234	-1.233261687	0.217893895	0.297078614	no
CXADRP2	0.046652703	1.233009236	0.217988008	0.297186887	no
FITM1	-0.046642263	-1.232732704	0.218091132	0.29730743	no
ADRA2A	0.046637302	1.232601311	0.218140143	0.297337217	no
ASNSD1	-0.046637074	-1.232595273	0.218142395	0.297337217	no
VSIG2	0.046621217	1.232175263	0.218299118	0.297530778	no
TMEM231	0.046613646	1.231974734	0.218373972	0.297612738	no
PABPC1	-0.046567352	-1.230748536	0.218832094	0.29821699	no
NME4	-0.046559287	-1.23053493	0.21891197	0.298305737	no
OR6V1	0.046544519	1.230143764	0.219058298	0.298465025	no
ABHD12B	0.04654451	1.230143522	0.219058389	0.298465025	no
C3orf33	-0.046541197	-1.230055775	0.219091223	0.298489646	no

PAEP	0.046528726	1.229725471	0.219214852	0.298637954	no
LOC392196	-0.046526231	-1.22965938	0.219239595	0.298651538	no
SHISA2	0.04652314	1.229577507	0.219270249	0.298673172	no
ZP3	0.046519752	1.229487765	0.219303853	0.298698821	no
TROAP	0.046518039	1.2294424	0.219320842	0.298701838	no
ARID1A	-0.0465077	-1.229168564	0.219423409	0.298821399	no
C19orf60	0.046502786	1.229038405	0.219472173	0.298867677	no
TMEM81	-0.046496876	-1.228881854	0.219530835	0.29891613	no
SKIV2L	-0.046496222	-1.22886454	0.219537324	0.29891613	no
JMJD6	-0.046492457	-1.22876483	0.219574693	0.298946879	no
LANCL3	0.046488338	1.228655721	0.219615591	0.298982426	no
FAM179A	0.046467457	1.228102664	0.219822978	0.299244611	no
STRN	0.04646459	1.228026726	0.219851464	0.29926324	no
ZCCHC3	-0.046459586	-1.227894192	0.219901188	0.299310773	no
FANCA	0.046453413	1.227730692	0.21996254	0.299374126	no
ZFYVE1	-0.046433753	-1.227209962	0.220158023	0.299620013	no
METTL9	-0.046405265	-1.226455412	0.220441504	0.299985619	no
SLC7A6	0.046398458	1.226275125	0.220509276	0.300057649	no
IQCF3	-0.046394004	-1.226157153	0.220553631	0.300097807	no
TADA3	0.046387519	1.225985396	0.22061822	0.300165489	no
FAM184B	-0.046372585	-1.22558985	0.220767015	0.300329992	no
NMU	-0.046372402	-1.22558501	0.220768836	0.300329992	no
ETV3L	0.046359118	1.225233162	0.220901255	0.300489912	no
GDF1	-0.046322408	-1.224260866	0.221267476	0.300967831	no
LCT	0.046303276	1.223754141	0.221458511	0.301207411	no
RLF	0.046293952	1.223507169	0.221551661	0.301302353	no
NCRNA00189	0.046293305	1.223490047	0.22155812	0.301302353	no
GPHA2	0.046289349	1.223385254	0.221597655	0.301335847	no
CCDC55	-0.046281194	-1.223169263	0.221679155	0.301426401	no
PFKFB4	0.046276697	1.22305015	0.22172411	0.301467253	no
FGF19	0.046239188	1.222056713	0.222099299	0.301957073	no
IPO13	-0.046220215	-1.221554193	0.222289259	0.302195013	no
SLC6A19	-0.046212813	-1.221358148	0.222363398	0.302275477	no
TMED10P1	0.046187909	1.220698546	0.222612973	0.302592293	no
RSPRY1	-0.04618657	-1.2206631	0.22262639	0.302592293	no
TAC3	-0.046182933	-1.220566768	0.222662858	0.302621515	no
CHRM3	-0.046175117	-1.220359765	0.222741236	0.302707691	no
TLCD1	0.046171691	1.220269023	0.2227756	0.302734042	no
CINP	-0.046164003	-1.220065402	0.222852726	0.302818496	no
AQP8	-0.04614885	-1.219664062	0.223004797	0.303004771	no
UQCRH	-0.046137935	-1.219374963	0.223114386	0.303133301	no
NCLN	0.046122386	1.21896316	0.223270554	0.303325095	no
CEACAM8	0.046110189	1.218640103	0.223393122	0.303471218	no
OR13G1	0.046107208	1.218561174	0.223423075	0.303491516	no
CCNK	-0.046093282	-1.218192329	0.223563088	0.303661304	no
TMEM129	-0.046087695	-1.218044371	0.22361927	0.30371721	no
C17orf37	0.046077364	1.217770747	0.223723196	0.30381383	no
CEACAM18	-0.046076303	-1.21774264	0.223733873	0.30381383	no
C3orf72	0.046076142	1.217738386	0.223735489	0.30381383	no
KRT74	-0.046052815	-1.21712055	0.223970288	0.304112242	no
OR7C1	0.04602856	1.216478169	0.224214603	0.304420394	no

MTRF1	0.046027295	1.216444672	0.224227347	0.304420394	no
NCR2	0.045992581	1.215525272	0.224577366	0.304875121	no
C10orf28	0.045981666	1.215236183	0.224687504	0.305004159	no
FICD	0.045970996	1.214953608	0.224795197	0.305129862	no
PRR23C	-0.045965825	-1.214816652	0.224847406	0.30518024	no
FLJ45340	-0.045958134	-1.214612945	0.224925078	0.305265169	no
RFC4	0.045942665	1.214203259	0.225081345	0.305456748	no
JAK1	0.045932484	1.213933625	0.225184235	0.305575868	no
PSIMCT-1	-0.045919913	-1.213600695	0.225311324	0.305727809	no
LOC440354	0.045918266	1.213557061	0.225327984	0.305729896	no
HSD3B2	-0.045907969	-1.213284356	0.225432127	0.305847926	no
MTMR2	-0.045906674	-1.213250055	0.225445229	0.305847926	no
RGS5	-0.045900024	-1.21307393	0.225512511	0.30591694	no
NRG1	0.045898475	1.213032925	0.225528177	0.30591694	no
LMAN1L	0.045897159	1.212998071	0.225541493	0.30591694	no
SNHG3	0.045893504	1.212901263	0.225578485	0.305946589	no
KEL	0.045860149	1.212017879	0.225916233	0.306384116	no
GTF2IRD2B	-0.045825052	-1.21108837	0.226272007	0.306846029	no
LINGO3	-0.045813737	-1.210788698	0.226386793	0.306981099	no
TBCC	-0.045810233	-1.210695884	0.226422353	0.307008728	no
PKD1L1	0.045808298	1.210644642	0.226441988	0.30701476	no
CALB2	-0.045790866	-1.210182988	0.226618932	0.307223075	no
GATA2	-0.045790168	-1.210164491	0.226626023	0.307223075	no
GPATCH4	-0.045775346	-1.209771953	0.226776558	0.307406533	no
LOC100272228	-0.045750241	-1.209107058	0.227031701	0.30773176	no
ZNF564	0.045745023	1.208968869	0.227084754	0.307783037	no
MARK2	-0.045723745	-1.208405345	0.227301195	0.308055742	no
PARL	-0.045693769	-1.207611479	0.227606356	0.308448643	no
FBXL8	0.045681208	1.207278802	0.227734324	0.308601378	no
NT5C3	0.045679046	1.207221561	0.227756347	0.308610537	no
C3orf48	0.045658008	1.206664398	0.227970795	0.308880414	no
PRAF2	0.045639078	1.206163059	0.22816388	0.309121311	no
GJB5	-0.045622216	-1.205716502	0.228335965	0.309333727	no
DEFA6	-0.045614291	-1.205506612	0.22841688	0.309422612	no
ACOXL	-0.045611693	-1.205437827	0.228443402	0.309437806	no
KRT38	0.045597498	1.205061872	0.2285884	0.30961347	no
UAP1L1	0.045575147	1.204469963	0.22881682	0.309902095	no
DUSP16	0.045573383	1.204423238	0.228834858	0.309905765	no
ZSCAN20	0.045555325	1.203945009	0.229019538	0.310135099	no
PSKH1	-0.045542177	-1.203596814	0.229154069	0.310296495	no
GPX3	-0.045540364	-1.203548781	0.229172632	0.310300848	no
CHKB-CPT1B	0.045529176	1.203252496	0.229287158	0.310435127	no
TP53AIP1	0.04546301	1.201500241	0.229965307	0.311332434	no
EXOSC9	0.04545927	1.201401191	0.230003683	0.31135793	no
CNO	0.04545719	1.201346089	0.230025034	0.31135793	no
C3orf75	0.04545539	1.201298431	0.230043502	0.31135793	no
DPYS	0.045455172	1.201292657	0.230045739	0.31135793	no
MYL1	0.045436554	1.200799589	0.230236869	0.311595757	no
ROPN1	0.045421615	1.200403966	0.230390308	0.311767106	no
KCNIP4	-0.045421224	-1.200393628	0.230394318	0.311767106	no
TTLL11	-0.045418601	-1.200324158	0.230421269	0.311782708	no

GPX5	0.045416818	1.200276947	0.230439586	0.311786626	no
KRT76	0.045410229	1.200102443	0.2305073	0.311857373	no
CXorf23	-0.045391689	-1.199611462	0.230697892	0.312094345	no
VGLL2	0.045359746	1.198765534	0.231026534	0.312518029	no
SLC26A9	-0.045356599	-1.198682175	0.231058937	0.31254095	no
CPSF3	0.045354108	1.198616226	0.231084574	0.312554718	no
MRPL51	0.045346723	1.198420652	0.231160615	0.312636653	no
C9orf100	0.045323895	1.197816106	0.231395782	0.312933773	no
FANCD2	0.045295633	1.197067661	0.23168716	0.313305021	no
NOL11	0.045294263	1.197031385	0.231701289	0.313305021	no
TMEM93	0.045290528	1.19693247	0.231739819	0.313336165	no
CEP135	0.045270711	1.196407673	0.231944318	0.313591696	no
FIBIN	0.045266894	1.196306603	0.231983717	0.313623991	no
FBXO43	0.045251113	1.195888667	0.232146686	0.313823328	no
LOC100302650	0.045245526	1.195740716	0.232204397	0.313880357	no
FAM132A	-0.045242404	-1.195658057	0.232236645	0.31390296	no
KIAA1009	0.045240861	1.195617199	0.232252585	0.313903521	no
NLRP13	-0.045215992	-1.194958596	0.232509648	0.314229951	no
GINS2	0.045147295	1.193139394	0.233220763	0.315169934	no
RRP9	-0.045145655	-1.193095956	0.233237761	0.315171839	no
RC3H2	-0.045137539	-1.192881026	0.233321882	0.31526444	no
SNHG9	0.045121103	1.192445777	0.2334923	0.315473624	no
RANBP2	0.045109969	1.192150941	0.23360779	0.315598448	no
KRTAP5-5	-0.045109187	-1.192130227	0.233615905	0.315598448	no
UBE2DNL	-0.045103998	-1.191992817	0.233669746	0.315647272	no
GPR37L1	-0.045102695	-1.191958302	0.233683271	0.315647272	no
PCDHB13	-0.045064898	-1.190957379	0.234075735	0.316156271	no
EIF4A3	0.045051439	1.190600978	0.234215594	0.31632404	no
ALDH3B2	0.045013837	1.189605214	0.234606666	0.316831045	no
PATE3	-0.044976116	-1.188606324	0.234999431	0.317340268	no
NLRX1	-0.044958707	-1.188145321	0.235180855	0.31756405	no
GMCL1L	0.044957003	1.188100204	0.235198616	0.317566823	no
TSPY2	-0.044938953	-1.187622209	0.235386842	0.317799745	no
DMPK	-0.044936034	-1.187544915	0.235417289	0.317819628	no
LANCL2	0.044922796	1.187194355	0.235555414	0.317984868	no
FLJ25328	-0.044913094	-1.186937434	0.235656681	0.318100332	no
SNX16	0.044901648	1.186634359	0.235776179	0.318224184	no
AQP12B	-0.044901291	-1.186624891	0.235779913	0.318224184	no
FBL	-0.044898645	-1.186554814	0.23580755	0.318224553	no
MGC16142	0.04489825	1.186544378	0.235811666	0.318224553	no
C16orf53	-0.044891499	-1.186365583	0.235882189	0.318297587	no
PNPLA7	-0.044890055	-1.186327351	0.235897272	0.318297587	no
ABHD2	0.044880607	1.186077165	0.235995985	0.318409531	no
GPX4	0.044860884	1.185554884	0.23620215	0.318666427	no
ZNF277	-0.044854347	-1.185381794	0.236270503	0.318737375	no
TAF1L	-0.044846832	-1.18518279	0.236349108	0.318822142	no
ZNF580	-0.044844931	-1.185132435	0.236369001	0.318827704	no
DCAF15	0.044833019	1.184817013	0.236493634	0.318974536	no
ABCB8	0.044808657	1.184171892	0.236748688	0.319297243	no
HSPBP1	-0.044790976	-1.183703676	0.236933923	0.31952575	no
FLJ30679	0.04477645	1.183319032	0.237086172	0.319705524	no

ARX	0.04477524	1.183286999	0.237098854	0.319705524	no
SPANXN1	0.044767071	1.183070668	0.237184516	0.319797296	no
FPGS	0.044765733	1.183035235	0.237198548	0.319797296	no
OCLM	-0.044761022	-1.182910492	0.237247955	0.319842579	no
PSG3	-0.044750957	-1.182643959	0.237353545	0.319963594	no
TMEM116	0.044748185	1.182570572	0.237382624	0.319981458	no
NUBPL	-0.044746551	-1.18252731	0.237399768	0.319983233	no
C3orf42	0.04473159	1.182131126	0.237556804	0.320173552	no
ZNF599	0.044722515	1.18189082	0.23765209	0.320280627	no
SPRR2D	-0.044712618	-1.181628733	0.237756044	0.320399366	no
GBAS	0.044706636	1.18147035	0.23781888	0.320462685	no
VCX2	0.044698886	1.181265125	0.237900318	0.320551058	no
UBA2	-0.044696149	-1.181192635	0.237929088	0.32056846	no
CA5A	0.044694274	1.181142996	0.237948791	0.320573642	no
E2F6	0.044684655	1.180888284	0.238049908	0.320675896	no
TACR1	0.044684037	1.180871906	0.238056411	0.320675896	no
DPPA3	-0.044654974	-1.180102328	0.238362114	0.321066304	no
ZFAT	-0.044653455	-1.180062109	0.238378098	0.321066444	no
AK3	0.044629541	1.179428862	0.238629866	0.321384135	no
RWDD3	0.044613276	1.178998174	0.238801208	0.321593473	no
AP2A2	-0.044610741	-1.17893106	0.238827915	0.321602628	no
RNF111	0.044609612	1.178901149	0.238839819	0.321602628	no
RGS13	0.044604652	1.17876981	0.238892093	0.321651594	no
PRODH2	-0.044538618	-1.177021264	0.239588801	0.322555879	no
HNRNPAB	0.044537973	1.177004175	0.239595617	0.322555879	no
SYVN1	-0.044519646	-1.176518901	0.239789233	0.32279504	no
PCDHGA11	-0.044505204	-1.176136472	0.239941893	0.322979041	no
MAGEB3	-0.044496853	-1.175915356	0.24003019	0.32306658	no
PACRGL	-0.044496031	-1.175893587	0.240038884	0.32306658	no
KRT83	-0.0444817	-1.175514114	0.240190476	0.323249087	no
AGBL4	-0.044465227	-1.175077904	0.240364816	0.323462183	no
GFAP	0.044448011	1.174622055	0.2405471	0.323685941	no
LMX1A	-0.044429682	-1.174136709	0.240741287	0.323925685	no
SNORD9	0.044409267	1.173596136	0.240957701	0.324195301	no
RALA	0.044397045	1.173272497	0.241087332	0.324348129	no
NOP2	0.044388446	1.173044806	0.241178562	0.324449276	no
MTX2	-0.044380411	-1.172832053	0.241263829	0.324542388	no
PPEF1	0.044372362	1.172618923	0.241349268	0.324635719	no
SRP14	0.044367472	1.172489435	0.241401187	0.324683954	no
PCDHB5	-0.04436461	-1.172413677	0.241431566	0.324703213	no
LOC728640	-0.044361609	-1.1723342	0.24146344	0.324724479	no
POU4F3	-0.044356684	-1.172203803	0.241515741	0.324773212	no
BHLHE22	-0.044347448	-1.171959229	0.241613859	0.324883546	no
TRIM74	-0.044330027	-1.171497938	0.241798996	0.325097384	no
C19orf70	0.044329457	1.171482863	0.241805048	0.325097384	no
GRAMD2	0.044319349	1.17121522	0.241912513	0.32522024	no
CEACAM20	0.044311464	1.171006417	0.241996376	0.325311351	no
DCLK1	-0.044308655	-1.17093205	0.242026249	0.325329879	no
C20orf12	0.044298224	1.170655841	0.242137226	0.325457415	no
OR1L1	0.044296073	1.170598894	0.242160111	0.325466537	no
C9orf50	-0.04427673	-1.170086715	0.242366005	0.325721609	no

LSMD1	0.044255661	1.169528834	0.242590412	0.326001525	no
NOX1	-0.044249835	-1.169374584	0.242652485	0.326063268	no
AMMECR1L	-0.044230255	-1.168856125	0.242861203	0.326322044	no
NIF3L1	-0.044203856	-1.168157128	0.243142801	0.326678705	no
TNPO3	-0.044194281	-1.167903581	0.243245002	0.326794302	no
STX4	0.044191212	1.167822322	0.243277763	0.326816598	no
MYPOP	-0.044185992	-1.167684104	0.243333494	0.326855215	no
SNORA34	0.044185491	1.167670844	0.243338841	0.326855215	no
C6orf182	0.04417974	1.167518568	0.243400253	0.326915985	no
IVNS1ABP	-0.044172087	-1.16731593	0.243481992	0.327004046	no
TYW1B	0.04416396	1.167100745	0.243568813	0.327098921	no
CT45A1	-0.044119378	-1.165920298	0.244045477	0.327717286	no
SNORA68	0.044097397	1.165338275	0.24428074	0.328011423	no
PARS2	0.04407935	1.164860446	0.244474004	0.328249131	no
GABRR1	0.044071195	1.164644499	0.244561383	0.328344647	no
ZNF75A	-0.04406679	-1.164527872	0.244608583	0.32838621	no
ADAM18	0.044064207	1.164459491	0.24463626	0.32840156	no
C1QTNF8	-0.044062574	-1.164416253	0.244653761	0.32840325	no
LRRN3	-0.044057685	-1.164286801	0.244706166	0.328451786	no
C8orf85	0.044044741	1.163944073	0.244844946	0.328589675	no
C17orf107	-0.044043627	-1.163914568	0.244856896	0.328589675	no
REEP5	-0.044043556	-1.163912697	0.244857654	0.328589675	no
C14orf165	-0.044041344	-1.163854124	0.244881378	0.328599701	no
SCNM1	0.044030274	1.163561011	0.245000124	0.328737225	no
IFNA2	-0.04401161	-1.163066832	0.245200417	0.328984142	no
LOC389493	-0.044004379	-1.162875371	0.245278049	0.329066462	no
MRPS17	0.043987537	1.162429432	0.24545893	0.329287283	no
F9	-0.043983708	-1.162328068	0.245500058	0.329320606	no
ILVBL	-0.043976252	-1.162130648	0.245580174	0.32940622	no
C20orf166	0.043964818	1.161827898	0.245703071	0.329549203	no
TMEM200C	-0.043954953	-1.161566709	0.245809131	0.329669587	no
GAMT	-0.043951628	-1.16147865	0.245844896	0.329695683	no
LOH3CR2A	0.043940298	1.161178658	0.245966766	0.32983724	no
MAP1LC3A	0.043921946	1.160692755	0.24616425	0.33008017	no
OR6F1	0.043912724	1.160448579	0.246263532	0.330191397	no
OR51B4	0.04391052	1.160390215	0.246287266	0.330201323	no
ARRDC2	0.043903688	1.160209347	0.24636083	0.33027805	no
ZNF789	0.043886675	1.159758873	0.246544117	0.330501854	no
GTF2H5	-0.043879322	-1.159564185	0.246623361	0.330586164	no
ZNF484	-0.04386883	-1.159286404	0.246736456	0.330715836	no
PKLR	-0.043857723	-1.158992297	0.246856239	0.330840045	no
SDC3	-0.043856588	-1.158962264	0.246868473	0.330840045	no
PABPC1P2	-0.043855685	-1.158938347	0.246878215	0.330840045	no
CLDN16	-0.043853254	-1.158873971	0.246904441	0.33085326	no
TBC1D20	-0.043845565	-1.158670402	0.246987385	0.330942471	no
LBX2	0.043833072	1.158339611	0.247122207	0.331101178	no
JPH1	0.043825872	1.158148991	0.247199922	0.331177165	no
FCAMR	0.043824783	1.15812015	0.247211682	0.331177165	no
DDIT3	0.043816238	1.157893908	0.247303945	0.331278815	no
CABP5	-0.043809591	-1.157717926	0.247375729	0.33135302	no
HS3ST1	0.043798161	1.157415281	0.247499213	0.331496461	no

TARS	0.043790883	1.157222576	0.247577862	0.331579837	no
FOXJ2	-0.043777117	-1.1568581	0.247726665	0.331757152	no
EGR4	-0.043771502	-1.156709446	0.247787373	0.331796831	no
THAP5	-0.043771341	-1.156705179	0.247789116	0.331796831	no
CREB3	0.043759955	1.156403707	0.247912266	0.331939748	no
RPL36A	0.043758269	1.156359073	0.247930502	0.331942182	no
MRPS18A	0.043742793	1.155949329	0.248097958	0.332142889	no
LRRC8D	0.043741379	1.155911877	0.248113268	0.332142889	no
TXNDC2	-0.043729752	-1.155604027	0.248239139	0.332289387	no
GCM1	0.043725178	1.155482941	0.248288659	0.332333672	no
TSPAN5	-0.043723326	-1.155433895	0.24830872	0.332338521	no
ST3GAL3	-0.043670393	-1.154032418	0.248882421	0.333084318	no
DHRS9	-0.043661929	-1.153808307	0.248974248	0.333185157	no
RPL27	0.043643795	1.153328204	0.249171045	0.333426447	no
CYP21A2	0.043623691	1.152795921	0.249389358	0.333696495	no
TRAF3IP2	0.043620113	1.152701182	0.249428229	0.333726419	no
C6orf155	-0.043611635	-1.152476708	0.249520345	0.333785213	no
GYPB	-0.043611542	-1.15247426	0.24952135	0.333785213	no
FBXO42	0.04361151	1.15247341	0.249521699	0.333785213	no
ZFP42	0.043609249	1.15241355	0.249546268	0.333795993	no
CAPN13	-0.043600131	-1.152172136	0.24964537	0.333906463	no
MYBPHL	0.043594742	1.152029461	0.249703952	0.333962724	no
FAM22F	-0.043579997	-1.151639074	0.249864294	0.334155067	no
IDH2	-0.043571804	-1.15142215	0.249953421	0.334252151	no
MIA3	-0.043556401	-1.151014343	0.250121037	0.334454175	no
XRCC3	-0.043553582	-1.150939705	0.250151723	0.334473086	no
HTR1F	0.043548667	1.150809556	0.250205237	0.334522516	no
CMBL	-0.04354705	-1.150766747	0.250222841	0.33452393	no
XRCC2	0.043530781	1.150336024	0.250400011	0.334738656	no
TRHR	-0.043498846	-1.149490512	0.250748052	0.33518176	no
SRP9	0.043493518	1.149349439	0.250806156	0.335237264	no
MOCS3	0.043490798	1.149277426	0.250835819	0.335254749	no
NAALAD2	0.043482796	1.149065567	0.250923101	0.335349238	no
IP6K3	0.043479866	1.148988003	0.250955062	0.335369783	no
CDKN2B	0.043472634	1.14879653	0.251033971	0.335452281	no
C22orf28	0.043471167	1.148757695	0.251049978	0.335452281	no
NAT2	0.04346926	1.148707195	0.251070794	0.335457924	no
NTN1	0.043452435	1.148261734	0.251254462	0.335681141	no
MOGAT3	-0.043441758	-1.147979064	0.251371058	0.335814725	no
B4GALNT1	-0.043439022	-1.147906613	0.251400949	0.335832467	no
ZBTB2	0.043427603	1.147604292	0.251525703	0.33597692	no
POLR2C	0.043423995	1.147508776	0.251565127	0.336007382	no
OR10A6	-0.043417194	-1.147328717	0.251639458	0.33608446	no
BNC1	0.043409763	1.147131977	0.251720693	0.336170749	no
HSPA12B	-0.043367749	-1.146019617	0.252180335	0.336762352	no
SERGEF	-0.043343145	-1.145368231	0.252449768	0.337099889	no
C6orf127	-0.043262449	-1.143231783	0.25333488	0.338259451	no
SDS	0.043255937	1.143059387	0.253406397	0.338319835	no
GOLGA8C	-0.043255284	-1.143042095	0.253413571	0.338319835	no
ANKRD19	-0.043231016	-1.142399612	0.253680227	0.338653472	no
LOC286467	-0.043223039	-1.142188414	0.253767926	0.338748179	no

ESF1	-0.043217797	-1.142049625	0.253825568	0.338802756	no
SERPINI2	-0.043180872	-1.141072038	0.254231843	0.339322644	no
LOC388796	0.043178206	1.141001463	0.254261191	0.339333765	no
CLOCK	0.043177066	1.14097128	0.254273743	0.339333765	no
C15orf42	-0.043167524	-1.140718653	0.254378818	0.339451584	no
BIN1	-0.043145149	-1.140126298	0.254625315	0.339758092	no
SCN1B	-0.043142464	-1.140055214	0.254654906	0.339775153	no
HAS1	0.043132685	1.139796319	0.254762701	0.339890713	no
MAP2K5	-0.043131557	-1.139766452	0.254775139	0.339890713	no
COPS7A	0.043105325	1.139071964	0.255064467	0.340254248	no
LOC572558	0.043103111	1.139013338	0.255088901	0.340264393	no
C17orf93	0.043089891	1.138663357	0.255234802	0.340436549	no
ZNF695	-0.04308054	-1.138415789	0.255338043	0.340537114	no
OR56B1	0.043080011	1.138401785	0.255343884	0.340537114	no
MINK1	-0.04307212	-1.138192867	0.255431032	0.340630869	no
CENPB	0.043049952	1.137605994	0.25567595	0.340934992	no
CEP170	-0.043047412	-1.137538752	0.255704022	0.340949939	no
PXMP2	-0.043041795	-1.137390048	0.255766111	0.341010237	no
CRIP2	-0.043038238	-1.137295875	0.255805437	0.341011849	no
C8orf47	-0.043036458	-1.137248764	0.255825112	0.341011849	no
FLJ44635	0.043035928	1.137234711	0.255830981	0.341011849	no
MRPS11	0.043035583	1.137225597	0.255834787	0.341011849	no
APOA5	-0.043025152	-1.13694944	0.255950141	0.341143118	no
C6orf10	0.043017341	1.13674264	0.256036548	0.34123579	no
PHB	0.042983358	1.135842976	0.256412689	0.341711243	no
LOC253039	-0.042982057	-1.135808539	0.256427094	0.341711243	no
APEH	0.042976048	1.135649439	0.256493655	0.341777414	no
C14orf53	-0.042919687	-1.134157347	0.257118468	0.342587398	no
LOC347376	-0.042916072	-1.134061648	0.257158578	0.342618262	no
C10orf110	-0.042903721	-1.133734677	0.257295653	0.342778302	no
HIST1H2BG	0.042901847	1.133685059	0.257316459	0.342783433	no
FAM83E	-0.04288636	-1.133275054	0.257488425	0.342963531	no
CAGE1	0.042885606	1.133255096	0.257496798	0.342963531	no
PEX14	-0.042885089	-1.133241405	0.257502542	0.342963531	no
C9orf169	-0.042877101	-1.133029935	0.257591273	0.34305911	no
TAS1R2	-0.042862384	-1.132640325	0.257754805	0.343254291	no
CARNS1	-0.042843987	-1.132153281	0.257959335	0.343504039	no
KREMEN2	-0.042841282	-1.132081661	0.25798942	0.343521476	no
LOC728819	0.042826033	1.131677989	0.258159038	0.343724691	no
LOC642587	-0.042815122	-1.131389118	0.258280465	0.34386372	no
NCRNA00110	-0.042812559	-1.131321266	0.258308992	0.343879055	no
FKBP2	-0.042806146	-1.131151507	0.258380375	0.343951437	no
DCDC2B	0.042803028	1.131068956	0.258415092	0.343975004	no
TARM1	0.042794552	1.130844561	0.258509478	0.344077987	no
ADRB3	0.042789219	1.130703391	0.25856887	0.344134383	no
CRBN	-0.042786154	-1.130622231	0.258603019	0.344157178	no
NACAD	-0.042769824	-1.130189925	0.258784972	0.344376658	no
MYH4	-0.042764137	-1.130039377	0.258848356	0.344438335	no
NCRNA00201	-0.042755021	-1.129798052	0.258949983	0.344550889	no
FABP12	0.042751679	1.129709562	0.258987255	0.344577804	no
LST-3TM12	-0.042749835	-1.129660757	0.259007813	0.344582481	no

PATE1	-0.042729685	-1.129127324	0.259232585	0.344858824	no
WRN	0.042726555	1.129044463	0.259267512	0.344882595	no
C20orf173	-0.042712039	-1.128660177	0.259429537	0.34507542	no
KRR1	0.042710461	1.128618412	0.259447151	0.345076146	no
ZBTB9	-0.042708154	-1.128557339	0.259472909	0.345087703	no
TRDMT1	0.042678908	1.127783103	0.259799598	0.345491368	no
REXO1L1	0.042677924	1.127757035	0.259810602	0.345491368	no
ALKBH3	0.042675125	1.127682937	0.259841884	0.345510241	no
LSM2	0.042673292	1.127634415	0.25986237	0.345514757	no
TRIM36	0.042671032	1.127574602	0.259887624	0.345525612	no
OR10G7	-0.042667838	-1.127490044	0.259923329	0.34555036	no
AAA1	0.042664724	1.127407593	0.259958148	0.345551926	no
MRPS18B	-0.042664401	-1.127399038	0.259961761	0.345551926	no
UNC13B	-0.042663147	-1.12736584	0.259975781	0.345551926	no
ZNF322A	-0.042645512	-1.126899008	0.260172992	0.345791319	no
TMEM143	0.042635191	1.126625772	0.260288467	0.345922055	no
STX16	0.042632981	1.126567281	0.260313191	0.345932174	no
C16orf61	0.042628596	1.126451193	0.260362267	0.345974651	no
PLS1	-0.042623258	-1.126309886	0.260422011	0.346031298	no
BEND6	-0.042618241	-1.126177065	0.260478177	0.346083182	no
BCL7C	0.042591914	1.125480109	0.260773035	0.346452177	no
OR10W1	-0.04257557	-1.125047447	0.260956196	0.346672737	no
RASGRP2	0.042568091	1.124849439	0.26104005	0.346761349	no
AGAP5	-0.042559124	-1.124612064	0.261140599	0.346872126	no
KRTAP10-10	0.042512321	1.123373071	0.261665858	0.347546992	no
KRTAP9-8	0.042506149	1.123209692	0.261735175	0.347616223	no
ZNF527	0.042469578	1.122241555	0.262146191	0.348139234	no
PPPDE2	-0.04246765	-1.122190532	0.262167865	0.348145149	no
NHLRC2	0.042459459	1.121973679	0.262259995	0.34824462	no
FUBP1	0.042451916	1.121773999	0.262344849	0.348320301	no
OR1E2	-0.042451329	-1.121758475	0.262351447	0.348320301	no
DPH2	0.042427869	1.12113743	0.262615486	0.348631874	no
STAU1	0.042427414	1.121125387	0.262620608	0.348631874	no
PROP1	-0.0424163	-1.120831182	0.262745755	0.348775109	no
TRIM49L	-0.042412035	-1.120718275	0.262793794	0.348815975	no
LOC143188	0.042407531	1.120599028	0.262844538	0.348860425	no
RBBP7	-0.04238292	-1.119947544	0.263121882	0.349205607	no
CHCHD5	0.042367139	1.119529785	0.263299834	0.349386281	no
C11orf67	-0.042366556	-1.119514338	0.263306415	0.349386281	no
RARA	-0.04236625	-1.119506252	0.263309861	0.349386281	no
RXFP3	-0.042341896	-1.11886155	0.263584653	0.349727951	no
UBR2	0.042336118	1.118708594	0.263649877	0.349791536	no
MAP6D1	-0.042322258	-1.118341704	0.263806372	0.349976196	no
ZCCHC17	0.04231037	1.118026995	0.263940661	0.350131375	no
PAPSS1	-0.042308824	-1.117986068	0.263958129	0.350131573	no
HIST1H2AM	0.042305616	1.117901146	0.263994375	0.350149799	no
IFT88	-0.042304542	-1.117872727	0.264006506	0.350149799	no
UTY	0.04229355	1.11758175	0.264130732	0.35029158	no
COL28A1	-0.042285677	-1.117373337	0.264219734	0.350386631	no
COPE	0.042272701	1.117029843	0.264366467	0.350558223	no
ARMCX5	-0.042261033	-1.116720957	0.264498464	0.350676483	no

MRI1	0.042260863	1.11671646	0.264500386	0.350676483	no
TOR1AIP1	-0.042260218	-1.116699383	0.264507685	0.350676483	no
ENO3	-0.042247281	-1.116356915	0.264654089	0.350847576	no
INTS4L2	-0.042228643	-1.115863551	0.2648651	0.351104287	no
C20orf54	-0.042190877	-1.114863826	0.265293037	0.351648503	no
MAPKAPK5	0.042182127	1.114632198	0.265392254	0.351756955	no
PEX12	-0.042174775	-1.114437572	0.265475642	0.351844413	no
GGT8P	0.042162723	1.114118537	0.265612371	0.351998227	no
METRN	-0.042159965	-1.114045554	0.265643657	0.351998227	no
ACTL9	0.042159941	1.11404492	0.265643928	0.351998227	no
SKA3	0.042061305	1.111433906	0.266764855	0.353460374	no
NUP188	0.042046886	1.111052228	0.266928985	0.353654667	no
PPFIA1	0.04202426	1.110453286	0.267186683	0.353972896	no
KRTDAP	-0.042015304	-1.110216201	0.267288738	0.354084897	no
SMEK2	-0.042003176	-1.109895175	0.267426968	0.354244804	no
SLC7A11	0.041999275	1.109791919	0.26747144	0.354280501	no
PPP6C	-0.041942961	-1.108301245	0.268114026	0.355108378	no
HAO2	0.041939067	1.10819816	0.268158503	0.35514402	no
KIAA1826	0.041935161	1.108094759	0.26820312	0.355179844	no
SPINK13	0.041933588	1.108053135	0.268221083	0.355180368	no
UGT3A1	-0.041905755	-1.107316377	0.268539157	0.355578275	no
TOP1MT	-0.041826909	-1.105229304	0.269441602	0.356749857	no
MYF6	0.041819922	1.105044347	0.269521678	0.356825435	no
SH3RF1	-0.041818848	-1.10501593	0.269533982	0.356825435	no
SPARC	-0.041811699	-1.1048267	0.269615927	0.356899513	no
POF1B	0.041810887	1.104805189	0.269625243	0.356899513	no
CDH1	-0.04178908	-1.104227951	0.269875324	0.357207156	no
THOC1	-0.041776444	-1.103893501	0.270020294	0.357375641	no
ETNK1	0.041738311	1.102884111	0.270458143	0.357931711	no
SPC24	0.041724706	1.102523979	0.270614478	0.358115167	no
C2orf89	0.041721271	1.102433066	0.270653954	0.358143965	no
TRAF2	0.041706358	1.102038316	0.270825405	0.358347385	no
DMGDH	0.04169223	1.101664353	0.270987897	0.358538924	no
DEFB134	0.041688941	1.101577287	0.271025738	0.358565526	no
SLC44A1	-0.041682141	-1.101397312	0.27110397	0.358645558	no
GPR139	0.041676303	1.101242781	0.271171155	0.358696016	no
ZNF385A	-0.041675744	-1.101227972	0.271177595	0.358696016	no
MED30	0.041673155	1.101159446	0.271207392	0.358711961	no
SNORA6	0.041664135	1.100920686	0.271311228	0.358812193	no
INHBE	0.041663489	1.100903584	0.271318667	0.358812193	no
CTAG1B	-0.041650672	-1.100564318	0.271466264	0.358983905	no
HAND1	0.041645739	1.100433757	0.271523079	0.359035553	no
IP6K2	-0.041638553	-1.100243543	0.271605867	0.359121536	no
CD3EAP	0.041634715	1.100141943	0.271650094	0.359156526	no
MRPL19	-0.041622701	-1.099823946	0.271788552	0.359298624	no
ZNF705A	-0.041622305	-1.099813473	0.271793113	0.359298624	no
ZNF235	0.04160819	1.099439857	0.271955852	0.359471138	no
EFCAB3	0.041607903	1.099432238	0.271959171	0.359471138	no
DSCR8	0.04160443	1.099340323	0.271999218	0.359500568	no
WNT10A	0.041582455	1.098758663	0.272252737	0.359805978	no
KCNJ1	0.041581316	1.098728508	0.272265884	0.359805978	no

LMO1	-0.041557957	-1.0981102	0.272535563	0.360138824	no
MYO15A	-0.041553304	-1.09798706	0.272589293	0.360186284	no
FOXA2	0.041545252	1.097773922	0.27268231	0.360285645	no
ABCC10	0.041525408	1.097248654	0.272911636	0.360565083	no
COL9A1	-0.041523819	-1.097206616	0.272929996	0.360565777	no
MEP1B	-0.041517594	-1.097041845	0.273001964	0.360637288	no
KDSR	0.041514936	1.096971476	0.273032703	0.360654331	no
NDEL1	0.041508284	1.096795416	0.273109622	0.360732367	no
URB1	0.041486811	1.096227043	0.273358041	0.3610369	no
SNORA5A	0.041481676	1.096091113	0.273417475	0.361091809	no
RHOU	-0.041476332	-1.095949653	0.273479336	0.361149916	no
ANKRD37	0.041474587	1.095903483	0.273499529	0.361152993	no
PSMD10	-0.041472948	-1.095860089	0.273518508	0.361154467	no
USP28	0.041451978	1.095305029	0.273761355	0.361451516	no
C12orf41	0.041444301	1.09510183	0.273850294	0.361545334	no
RPS14	0.041433194	1.094807851	0.273979003	0.361691641	no
SMARCAD1	-0.041430435	-1.094734807	0.274010989	0.361710249	no
HBM	0.041413217	1.094279085	0.274210609	0.361950127	no
RNASEH2A	0.041405551	1.094076166	0.274299526	0.362040068	no
ZBTB37	-0.041404255	-1.094041857	0.274314561	0.362040068	no
ATRX	-0.041399691	-1.09392105	0.274367509	0.362086312	no
SLC6A20	0.041380715	1.093418799	0.274587712	0.362353264	no
GMEB1	0.041367333	1.093064591	0.27474308	0.36253463	no
YBX2	-0.041353979	-1.09271113	0.274898181	0.362715619	no
MMP21	0.041337421	1.092272865	0.275090578	0.362945791	no
SLC17A4	-0.041332073	-1.092131317	0.275152737	0.363004113	no
SNORA25	-0.041312024	-1.091600625	0.275385868	0.363287973	no
DCAF8L1	0.041295042	1.091151145	0.275583428	0.363501272	no
NMD3	0.041295034	1.091150949	0.275583514	0.363501272	no
TALDO1	-0.041280014	-1.09075338	0.275758339	0.363708142	no
C7orf45	0.041273528	1.090581708	0.275833853	0.36378369	no
LOC284788	0.041272003	1.090541355	0.275851605	0.36378369	no
C1orf100	0.041268722	1.090454502	0.275889816	0.363810351	no
SLC35F4	0.041261265	1.09025713	0.275976664	0.363901141	no
ANGPTL3	-0.041249284	-1.089940003	0.276116246	0.364061449	no
RPLP0	-0.041225586	-1.089312776	0.276392458	0.364401872	no
LOC285796	-0.041219779	-1.089159057	0.27646018	0.364447325	no
RPL36AL	-0.041219538	-1.089152691	0.276462985	0.364447325	no
RRP15	-0.041206758	-1.088814417	0.276612055	0.364620063	no
EXOC8	-0.041201429	-1.088673368	0.276674229	0.364678242	no
COMMD4	0.041194895	1.088500433	0.27675047	0.364754955	no
PLIN5	-0.041187772	-1.088311904	0.276833603	0.36484074	no
OR1C1	-0.041160992	-1.087603086	0.277146313	0.365229055	no
SLC25A25	-0.041128815	-1.086751429	0.277522359	0.365700779	no
C10orf99	-0.041095265	-1.085863442	0.277914816	0.366194067	no
MBD3L5	-0.041086558	-1.085632974	0.278016737	0.366304489	no
LOC126536	0.041073057	1.085275623	0.278174819	0.366488889	no
SIAH3	-0.041054312	-1.084779492	0.278394395	0.366731038	no
BCL2L11	0.041054269	1.084778365	0.278394894	0.366731038	no
DUOXA2	0.041049832	1.08466092	0.27844689	0.366759972	no
UGGT2	0.041049299	1.084646805	0.27845314	0.366759972	no

PLA2G1B	0.041029829	1.084131482	0.278681368	0.367036668	no
LECT2	0.041011387	1.083643385	0.278897656	0.367297603	no
NFASC	0.041008678	1.083571666	0.278929446	0.367309551	no
C19orf44	0.041007517	1.083540947	0.278943063	0.367309551	no
UBE2G1	-0.041000535	-1.083356143	0.279024993	0.367393508	no
CPO	0.040983226	1.082898029	0.279228162	0.367637079	no
RIPK2	-0.040952616	-1.082087851	0.279587715	0.368086502	no
ZSCAN12	-0.040946724	-1.081931921	0.279656952	0.368153682	no
PCDHB3	-0.040943307	-1.081841474	0.279697118	0.368182586	no
FANCM	-0.040919898	-1.081221893	0.279972371	0.368510565	no
CLEC4M	-0.040919017	-1.081198589	0.279982728	0.368510565	no
IRF2BP2	-0.040913433	-1.081050785	0.280048419	0.368573033	no
SUMO1	0.040875891	1.080057163	0.280490309	0.369130577	no
CCDC77	0.040859571	1.07962522	0.280682553	0.369359532	no
PTPRVP	-0.040855837	-1.079526398	0.280726548	0.369393384	no
GFM2	0.040851057	1.079399887	0.280782877	0.36944346	no
RDH12	0.040847608	1.07930859	0.280823532	0.369472907	no
PECI	0.04083463	1.07896512	0.280976516	0.36965013	no
NUAK1	-0.040826458	-1.078748832	0.281072881	0.369752847	no
C2orf24	0.040823977	1.078683164	0.281102143	0.369767283	no
HOXA11	0.040806428	1.078218683	0.28130918	0.370015548	no
PIGM	-0.040803741	-1.078147581	0.281340881	0.370033173	no
TMC5	0.040794367	1.077899488	0.281451516	0.370154606	no
CALB1	-0.040787527	-1.077718444	0.28153227	0.370236727	no
KRTAP13-1	-0.04076085	-1.077012386	0.281847355	0.37062698	no
TRPV3	-0.04074433	-1.076575165	0.282042588	0.370859589	no
OR1Q1	0.040739834	1.076456155	0.282095746	0.370905365	no
MRPL3	0.040729898	1.076193191	0.282213227	0.371035702	no
C6orf225	-0.040723071	-1.076012509	0.282293968	0.371117722	no
TRIP11	0.040719183	1.075909598	0.282339962	0.371154054	no
SMEK1	-0.040678844	-1.074841984	0.282817415	0.371757526	no
KRTAP9-4	-0.040674027	-1.074714496	0.282874466	0.371808345	no
RNF139	-0.040668844	-1.074577309	0.282935866	0.371864873	no
PCYOX1L	0.040660968	1.074368857	0.28302918	0.371963336	no
SNORA1	0.040640534	1.073828055	0.283271367	0.372257426	no
SNORD10	0.04062349	1.073376958	0.283473488	0.372498829	no
CASQ2	0.040618963	1.073257132	0.283527195	0.37254519	no
STIM2	0.040584838	1.072353978	0.283932215	0.373053128	no
RPL27A	-0.040579736	-1.072218951	0.283992802	0.373108485	no
MIA2	0.040562498	1.071762723	0.284197576	0.373353255	no
POLE	-0.040556616	-1.071607072	0.284267462	0.37340546	no
SNX1	-0.040556045	-1.071591949	0.284274253	0.37340546	no
CCDC84	-0.040538752	-1.071134267	0.284479818	0.373651203	no
FGF21	0.040533893	1.071005679	0.284537591	0.373702807	no
GNB3	-0.040507735	-1.070313382	0.284848767	0.374087195	no
OTP	0.040490048	1.069845274	0.285059305	0.374339375	no
NPHP4	-0.040476422	-1.069484647	0.285221573	0.374515862	no
KCNK2	-0.040474755	-1.06944053	0.285241428	0.374515862	no
MUDENG	-0.040474096	-1.069423104	0.285249271	0.374515862	no
CSN2	0.040469499	1.069301427	0.285304039	0.374563445	no
RTTN	0.040459459	1.069035717	0.285423662	0.374683984	no

ISM1	-0.040458682	-1.069015161	0.285432917	0.374683984	no
JMJD8	0.040452931	1.068862944	0.285501462	0.374749631	no
TTPAL	0.040436985	1.068440938	0.285691554	0.374974802	no
TDGF1	-0.040425738	-1.068143254	0.285825698	0.375126514	no
C4orf31	0.04041889	1.06796203	0.285907382	0.375209363	no
SARS2	-0.040397948	-1.067407786	0.286157299	0.375498056	no
IQCB1	0.040397344	1.067391801	0.286164509	0.375498056	no
OR2H1	0.040394491	1.067316294	0.286198568	0.375518376	no
DHX35	-0.040384933	-1.067063334	0.286312692	0.375643738	no
SLC25A15	-0.040380302	-1.066940786	0.286367991	0.375691911	no
ADRA2B	0.040361854	1.066452533	0.286588384	0.375956653	no
TOP2A	0.040355002	1.066271196	0.286670267	0.376039671	no
ZNF680	-0.040352905	-1.066215713	0.286695323	0.376048141	no
TMEM174	0.040345884	1.066029903	0.286779248	0.376133819	no
C19orf33	0.040336474	1.065780849	0.286891763	0.376256984	no
ACOT2	-0.040321005	-1.065371461	0.287076778	0.376475209	no
FAM99A	-0.04031166	-1.065124136	0.28718859	0.376597413	no
LOH12CR1	0.040305319	1.064956334	0.287264468	0.376672483	no
ATG12	0.040276292	1.064188113	0.287612021	0.377103752	no
LETM2	-0.040260864	-1.06377981	0.287796859	0.377321633	no
THAP8	-0.040245159	-1.063364181	0.287985095	0.377521606	no
C11orf59	0.040245022	1.063360569	0.287986731	0.377521606	no
VN1R5	-0.04024213	-1.063284033	0.288021404	0.377542579	no
SLC12A2	-0.040240101	-1.063230326	0.288045735	0.377549995	no
STEAP2	-0.040218791	-1.062666353	0.288301325	0.377860508	no
SPOPL	0.040197025	1.062090318	0.28856254	0.378178351	no
QRFP	0.040187368	1.06183475	0.288678483	0.37830578	no
MYO3A	0.040173994	1.061480808	0.288839108	0.378491742	no
PRSS53	-0.040161434	-1.061148424	0.288990004	0.378664932	no
KRTAP9-2	0.040155473	1.060990649	0.28906165	0.378734265	no
GSTP1	0.040141751	1.060627502	0.289226601	0.378891633	no
VPS45	-0.040141064	-1.060609323	0.28923486	0.378891633	no
SLCO1B3	0.040140804	1.060602458	0.289237979	0.378891633	no
ZFP30	-0.040118681	-1.060016978	0.28950406	0.379215619	no
C6orf122	0.040105056	1.059656387	0.289668018	0.379405804	no
PODN	-0.040099058	-1.059497652	0.289740214	0.379475783	no
PARK7	0.040068362	1.058685298	0.290109879	0.379935324	no
GATAD2A	0.040065502	1.058609603	0.29014434	0.379955843	no
FOLR1	0.040049734	1.058192322	0.290334364	0.380180063	no
C11orf93	-0.040004658	-1.0569994	0.290878067	0.38086735	no
OR10C1	0.039987826	1.056553954	0.291081266	0.381108731	no
SEL1L	0.039968117	1.056032394	0.291319307	0.381379646	no
BCORL1	0.039967571	1.056017929	0.29132591	0.381379646	no
TMEM199	0.039964985	1.055949488	0.291357157	0.381395856	no
TACR3	-0.039946648	-1.055464214	0.291578774	0.381661249	no
OAZ2	0.039944342	1.055403194	0.291606649	0.381673026	no
DMBX1	0.039942596	1.055356986	0.291627759	0.381675947	no
BMS1P4	0.039939445	1.055273613	0.29166585	0.381701091	no
NIPAL3	0.039913571	1.054588863	0.291978821	0.382047775	no
C2orf7	0.039913307	1.054581888	0.29198201	0.382047775	no
KLF14	0.039912858	1.054569996	0.291987447	0.382047775	no

RAPGEF5	-0.03990775	-1.054434831	0.292049254	0.382103916	no
TP73	0.039905901	1.054385891	0.292071634	0.38210847	no
SNAR-B2	-0.03988758	-1.053901043	0.292293422	0.382373886	no
RPL35A	0.039871907	1.053486287	0.292483237	0.382597444	no
TTC24	0.039856688	1.053083522	0.292667644	0.382813899	no
PPBPL2	-0.039854188	-1.05301736	0.292697944	0.382828764	no
C19orf41	0.03983263	1.052446871	0.292959296	0.383145808	no
UCKL1	-0.039827941	-1.052322783	0.293016164	0.383195394	no
TMEM40	0.039819821	1.052107896	0.293114661	0.383299413	no
SPACA1	0.039817757	1.052053279	0.293139699	0.383307363	no
C1orf124	0.039797349	1.051513205	0.293387364	0.383606398	no
NCRNA00028	0.039794819	1.051446252	0.293418078	0.383613643	no
FAM58B	0.039793767	1.051418402	0.293430853	0.383613643	no
ANTXR1	-0.03978395	-1.051158618	0.293550046	0.383744655	no
NXT2	0.039746992	1.050180567	0.293999083	0.384306811	no
PDE4C	-0.039744519	-1.050115146	0.294029135	0.384321247	no
TRPC4	0.039740221	1.050001393	0.294081394	0.384364705	no
PSG9	-0.039719929	-1.049464403	0.294328176	0.384662383	no
NDUFAF3	0.039717597	1.049402693	0.294356544	0.384674592	no
METTL1	0.039703984	1.049042434	0.294522196	0.384866194	no
ZNF587	0.039664316	1.047992707	0.295005229	0.385472482	no
DRG1	-0.039658528	-1.047839535	0.295075756	0.38553972	no
NUDT6	0.039652384	1.047676933	0.295150637	0.385612638	no
LOC339524	0.039650177	1.047618521	0.29517754	0.385622867	no
ACTA1	-0.039646053	-1.04750939	0.295227807	0.385663617	no
PHKG2	-0.039585419	-1.045904832	0.295967548	0.38660498	no
ZNF705D	0.039577157	1.045686197	0.29606844	0.386711785	no
LRRC56	-0.039571699	-1.045541771	0.296135101	0.386770239	no
C14orf183	-0.03957036	-1.045506347	0.296151452	0.386770239	no
ZNF543	-0.039567713	-1.045436288	0.296183793	0.38678749	no
EBPL	-0.039552875	-1.045043638	0.296365092	0.386999253	no
GNB1L	-0.039551289	-1.045001674	0.296384473	0.386999566	no
GFI1B	-0.039545648	-1.04485238	0.296453429	0.387064607	no
PPP1R13L	0.039508265	1.043863129	0.29691062	0.387636505	no
FOLH1B	0.039497868	1.043587998	0.297037858	0.387775258	no
MSLNL	-0.039496447	-1.043550382	0.297055257	0.387775258	no
NKX3-2	0.039475825	1.043004676	0.297307744	0.388079797	no
PADI3	0.039466276	1.042752002	0.2974247	0.388207398	no
ENTPD8	0.039459875	1.042582598	0.29750313	0.388284699	no
ZNF490	-0.039454066	-1.042428883	0.297574308	0.388352528	no
C17orf76	0.039441453	1.042095103	0.297728905	0.388529207	no
HIST1H2AK	0.0394322	1.041850241	0.297842352	0.388652167	no
UFSP2	-0.039428497	-1.041752276	0.297887748	0.388686318	no
LPCAT1	0.039420172	1.041531964	0.297989856	0.388794457	no
BHLHA15	-0.039417161	-1.041452296	0.298026786	0.388817549	no
RMST	0.039404533	1.041118115	0.298181726	0.388994588	no
GAPVD1	-0.039392521	-1.040800252	0.29832915	0.389161801	no
NDUFB11	-0.039386846	-1.040650091	0.298398812	0.38922756	no
IPO8	0.039381387	1.040505626	0.298465841	0.389289876	no
HCG27	0.039378566	1.040430962	0.298500488	0.38930729	no
ZNF189	-0.039377164	-1.040393866	0.298517703	0.38930729	no

OR2S2	-0.039371021	-1.040231309	0.298593147	0.389380563	no
MTNR1A	-0.039360243	-1.039946104	0.298725545	0.389528092	no
C10orf129	0.039355104	1.039810124	0.298788683	0.389581197	no
KLHL13	-0.039353792	-1.039775399	0.298804808	0.389581197	no
C12orf36	-0.039325527	-1.039027448	0.299152269	0.390009065	no
OR4X2	-0.039294643	-1.038210175	0.299532244	0.390479264	no
LOC285074	-0.039285614	-1.037971257	0.299643384	0.390598965	no
ZNF614	-0.039280493	-1.037835742	0.299706436	0.39063821	no
GLTSCR1	-0.039279698	-1.037814705	0.299716224	0.39063821	no
LOC91450	-0.039278461	-1.037781976	0.299731454	0.39063821	no
SPACA4	-0.039254587	-1.037150238	0.300025519	0.390996258	no
TINAG	0.039248059	1.036977492	0.300105963	0.391075886	no
MTMR10	-0.039226081	-1.036395902	0.300376904	0.391403728	no
SNORA26	0.039222082	1.036290077	0.300426221	0.391442762	no
AURKB	0.039218766	1.03620233	0.300467118	0.391470821	no
SLC25A38	-0.039214579	-1.03609154	0.30051876	0.391512873	no
LOC254312	0.039207875	1.035914141	0.300601462	0.391595384	no
TSPAN13	0.039203274	1.035792389	0.30065823	0.391644101	no
HIST1H1D	0.039192011	1.035494344	0.300797228	0.39179992	no
RPL31P11	0.03917288	1.034988128	0.301033408	0.392082294	no
C4orf11	-0.03914298	-1.034196919	0.301402803	0.392538126	no
ZNF232	-0.039139636	-1.034108414	0.301444142	0.392566677	no
SHARPIN	-0.039119244	-1.033568826	0.301696258	0.392869698	no
PRELP	0.039098277	1.033014017	0.301955632	0.393182132	no
WAPAL	-0.039091574	-1.032836624	0.302038595	0.393264832	no
ZNF883	-0.039048512	-1.031697164	0.302571858	0.39393379	no
MYOCD	0.038999233	1.030393166	0.303182894	0.394703912	no
LILRA4	0.038987285	1.03007702	0.30333116	0.394849527	no
INF2	-0.038987072	-1.030071381	0.303333804	0.394849527	no
MRPL48	-0.038962187	-1.029412911	0.303642769	0.395226261	no
SARS	-0.038958089	-1.029304455	0.303693679	0.395267078	no
MCCC1	-0.038948622	-1.029053957	0.303811285	0.395394693	no
ROBO3	-0.038946544	-1.028998967	0.303837106	0.395402845	no
SLC9A7	-0.038942238	-1.028885041	0.303890606	0.395436319	no
SNORD97	0.038941326	1.028860895	0.303901945	0.395436319	no
SNX29	0.038928474	1.028520815	0.30406169	0.395618717	no
FXYD3	0.038892557	1.027570426	0.304508407	0.396174451	no
LCE5A	-0.038851855	-1.026493438	0.305015157	0.396808213	no
NCAPH	0.038827342	1.0258448	0.305320628	0.397180058	no
C1orf172	-0.038824938	-1.025781187	0.305350597	0.397193487	no
ZFP57	0.038806567	1.025295087	0.305579671	0.39745796	no
BCAR4	-0.03880548	-1.025266314	0.305593233	0.39745796	no
LOC729609	0.038796811	1.025036942	0.305701367	0.397573024	no
C20orf185	0.038767689	1.024266337	0.306064843	0.398020132	no
ADAMTS10	-0.038763747	-1.02416204	0.306114059	0.398058532	no
LOC100133050	-0.038746269	-1.023699575	0.306332354	0.398316775	no
C19orf42	0.038739953	1.023532437	0.306411273	0.398393769	no
NFATC2IP	-0.038736938	-1.023452656	0.306448948	0.398417133	no
MPHOSPH9	0.038723518	1.023097563	0.306616673	0.398609561	no
GOT1L1	0.0387186	1.022967437	0.306678152	0.398663851	no
XKR6	-0.038716138	-1.022902288	0.306708936	0.398678235	no

TEKT4	0.03871117	1.022770842	0.306771051	0.39873334	no
AP2M1	0.038707231	1.02266662	0.306820307	0.398771726	no
MLLT10	0.038700493	1.022488334	0.306904579	0.398855614	no
TMEM105	-0.038695868	-1.022365937	0.306962442	0.398905172	no
DKFZp566F094	-0.038644888	-1.021016999	0.307600634	0.399708825	no
TRIM24	-0.038608474	-1.020053497	0.308057011	0.400276135	no
MRPL16	0.03859546	1.019709159	0.308220221	0.400462466	no
VWC2	-0.038585338	-1.019441321	0.308347211	0.400601717	no
GDEP	-0.038570842	-1.01905776	0.308529129	0.400812307	no
KDM1B	-0.038548015	-1.018453766	0.30881574	0.401158868	no
EMD	0.038537861	1.018185095	0.308943288	0.401298772	no
TSPAN8	-0.038532725	-1.018049194	0.309007818	0.401338324	no
DCAF10	-0.038532278	-1.018037359	0.309013438	0.401338324	no
RPL18	-0.038526788	-1.017892107	0.309082419	0.40140213	no
RPS15	-0.038497842	-1.017126203	0.309446322	0.401848914	no
GTF2IRD2	-0.038483438	-1.01674507	0.309627515	0.402058388	no
PCDHB15	-0.038466427	-1.01629497	0.309841586	0.402283834	no
GRPEL1	0.038465616	1.016273524	0.309851788	0.402283834	no
PI16	0.038464898	1.016254528	0.309860825	0.402283834	no
COX6A2	0.038462939	1.016202679	0.309885492	0.402290026	no
HOXB8	0.03845403	1.015966973	0.309997644	0.402409783	no
SLC26A10	-0.038451424	-1.015898001	0.310030468	0.402426553	no
HIST1H2AL	0.038444569	1.015716627	0.310116793	0.402512764	no
OR51I2	0.038440607	1.015611804	0.310166691	0.402551685	no
TNNT1	-0.03843661	-1.015506037	0.310217043	0.402591192	no
OR6C2	0.038425788	1.01521969	0.310353392	0.40274229	no
C10orf25	-0.038424111	-1.015175335	0.310374516	0.402743852	no
WDR8	0.038380601	1.014024081	0.310923129	0.403415312	no
C1D	0.038379907	1.014005722	0.310931883	0.403415312	no
LOC100128788	-0.038366289	-1.013645395	0.311103725	0.403612366	no
IL1F10	0.038350662	1.013231917	0.311300994	0.403842381	no
MEGF9	-0.038346487	-1.013121448	0.311353713	0.403884856	no
USH2A	0.038331991	1.012737903	0.311536795	0.404096421	no
LOC100271722	-0.038327456	-1.012617916	0.311594084	0.404132018	no
CNTN5	-0.038326654	-1.012596699	0.311604215	0.404132018	no
RPS4Y1	0.038321174	1.012451709	0.311673454	0.404195888	no
YWHAQ	0.038317616	1.01235756	0.311718419	0.404203202	no
GALNT11	-0.038317564	-1.01235618	0.311719078	0.404203202	no
CDKN1C	-0.038306609	-1.012066316	0.311857543	0.404356814	no
SNORA45	0.0383018	1.011939078	0.311918336	0.404409704	no
CHD1L	0.038297224	1.011818001	0.311976193	0.404458779	no
DYNLRB1	-0.0382867	-1.011539559	0.312109273	0.404605366	no
ITGB1BP3	-0.038243602	-1.010399234	0.31265468	0.405286421	no
PHOSPHO1	0.038233437	1.010130276	0.312783411	0.405427298	no
GPR32	-0.038230394	-1.010049772	0.31282195	0.405451258	no
FBXO5	0.038214665	1.009633595	0.31302123	0.405667603	no
HNRPLL	0.038214052	1.009617376	0.313028998	0.405667603	no
DISC1	-0.038210681	-1.00952817	0.313071724	0.405673634	no
TRAF3	0.038210518	1.009523875	0.313073781	0.405673634	no
ZNF268	0.038201264	1.009279026	0.313191076	0.40578887	no
TRDN	0.038200335	1.009254441	0.313202855	0.40578887	no

MAPK4	0.038198217	1.009198403	0.313229705	0.405797653	no
PPP2CA	-0.038194127	-1.00909017	0.313281567	0.405838836	no
PIGU	0.038190746	1.009000715	0.313324436	0.405868365	no
GAGE4	0.038173346	1.00854035	0.313545113	0.406128201	no
AMAC1L2	-0.038168511	-1.008412428	0.313606451	0.406181629	no
PRSS38	0.038154421	1.008039618	0.313785257	0.406387184	no
ZNF223	0.038149313	1.00790446	0.313850098	0.406445124	no
OBFC2B	-0.038143644	-1.007754466	0.313922066	0.406512287	no
ZNF833	-0.038137574	-1.007593871	0.313999133	0.406586042	no
EIF3M	0.038128668	1.007358222	0.31411224	0.406686968	no
GPR116	0.038128269	1.007347668	0.314117306	0.406686968	no
CBX8	-0.038120911	-1.00715299	0.314210769	0.406781925	no
ESPL1	0.038108393	1.006821792	0.314369815	0.40696177	no
ATP5J	0.038105604	1.006747993	0.314405262	0.406981598	no
ADAM5P	0.038068221	1.005758916	0.314880583	0.407568943	no
SPINK7	-0.038066748	-1.005719945	0.314899321	0.407568943	no
MCM4	0.038062986	1.005620398	0.314947188	0.407604804	no
GDI2	0.038054868	1.005405606	0.315050488	0.407706801	no
SNORA70	0.038053623	1.005372669	0.31506633	0.407706801	no
CDHR2	-0.038050319	-1.005285243	0.315108383	0.407735123	no
FAM170A	0.038038197	1.004964534	0.315262681	0.407908671	no
SCGBL	-0.03801733	-1.00441243	0.315528425	0.408226383	no
CA1	0.037978508	1.003385276	0.316023214	0.408833966	no
LOC100132354	-0.037977311	-1.003353594	0.316038483	0.408833966	no
FAM27B	0.037965504	1.003041199	0.316189073	0.409002602	no
USP17	-0.037961186	-1.002926962	0.316244152	0.409047679	no
LPAR4	0.037938697	1.00233196	0.316531135	0.409392688	no
NKX2-8	-0.037935396	-1.002244609	0.316573281	0.409421007	no
CHORDC1	-0.03793377	-1.002201595	0.316594037	0.40942166	no
RPL8	-0.037926697	-1.002014449	0.316684349	0.409512258	no
INTS6	-0.037921077	-1.001865772	0.316756108	0.409542315	no
PTPRF	-0.037920998	-1.001863664	0.316757126	0.409542315	no
FDX1L	-0.037920118	-1.001840388	0.316768361	0.409542315	no
CENPF	0.037910238	1.00157899	0.316894556	0.409679271	no
GALR1	0.037908328	1.001528452	0.316918958	0.409684622	no
KPNB1	0.037871977	1.000566686	0.317383575	0.410244449	no
IPPK	-0.037871271	-1.000548005	0.317392604	0.410244449	no
MRPS24	0.037863414	1.000340139	0.317493082	0.410348088	no
SEH1L	-0.037845074	-0.999854893	0.317727722	0.410603799	no
OR11H1	-0.03784416	-0.999830726	0.31773941	0.410603799	no
BTC	0.037843188	0.999804994	0.317751857	0.410603799	no
DEFB103B	-0.037831754	-0.999502488	0.317898197	0.410766649	no
ACAD11	-0.037826798	-0.999371375	0.317961639	0.410822368	no
DAK	-0.037824432	-0.999308758	0.31799194	0.410835263	no
PRR19	-0.03781159	-0.998968985	0.318156394	0.411021468	no
PDC	0.037807043	0.998848697	0.318214628	0.411070433	no
OSBPL6	0.037803482	0.998754482	0.318260244	0.411103093	no
C10orf140	0.0378008	0.998683508	0.318294611	0.411121219	no
AGBL1	-0.037790033	-0.998398665	0.31843256	0.411273125	no
HAUS4	0.037746868	0.997256621	0.318986046	0.411961666	no
ENPP2	0.037723113	0.99662813	0.319290911	0.412329051	no

C14orf64	-0.037709343	-0.996263804	0.319467723	0.412531035	no
MAGEA5	0.037697139	0.995940934	0.319624469	0.412707084	no
RNF168	-0.037662345	-0.995020389	0.320071652	0.413258104	no
FIBCD1	-0.037654358	-0.994809073	0.320174363	0.413364321	no
CAMK1	0.037650209	0.994699309	0.320227722	0.413399063	no
PMCH	0.037649086	0.994669599	0.320242166	0.413399063	no
ZFX	0.037644614	0.99455128	0.320299693	0.413446925	no
DUOX2	0.037624504	0.994019227	0.320558462	0.413739161	no
TIMM16	-0.037623839	-0.99400164	0.320567017	0.413739161	no
RMRP	0.037616398	0.993804775	0.320662801	0.413836366	no
RAD54L2	-0.037589364	-0.993089524	0.321010959	0.414259243	no
ART4	0.037581442	0.992879931	0.321113028	0.414364514	no
ADCY6	-0.037544227	-0.991895366	0.321592783	0.414957105	no
SPANXN3	-0.037539921	-0.991781421	0.321648336	0.4150023	no
MGC26647	-0.037516701	-0.991167112	0.321947944	0.415344989	no
SSX1	0.037515062	0.991123756	0.321969097	0.415344989	no
CCND1	-0.037514561	-0.991110495	0.321975567	0.415344989	no
YES1	-0.037504817	-0.990852697	0.322101361	0.415480752	no
SUV39H1	-0.037502814	-0.990799697	0.322127226	0.415487608	no
CDH17	0.037497932	0.990670558	0.322190256	0.415542396	no
KCNJ16	-0.037490906	-0.990484661	0.322281002	0.41563292	no
TMPRSS11BNL	0.03747722	0.990122588	0.322457796	0.4158344	no
SFRS17A	-0.037466063	-0.989827405	0.322601976	0.415993797	no
MRGPRX3	0.037450298	0.989410324	0.322805768	0.416230039	no
RCAN2	0.037441988	0.989190476	0.322913223	0.41634204	no
DNAH7	-0.037434271	-0.988986289	0.323013044	0.416444186	no
ANKRD40	-0.037423771	-0.98870851	0.323148874	0.41659274	no
CD82	-0.037412569	-0.988412138	0.323293838	0.416753048	no
ZFP112	-0.037398109	-0.988029576	0.323481022	0.416967759	no
FCRLB	0.037384897	0.987680042	0.323652107	0.417152583	no
PPM1G	0.037383849	0.987652332	0.323665673	0.417152583	no
CCDC22	-0.037376143	-0.987448463	0.32376549	0.417254633	no
KCNK9	-0.037360813	-0.987042892	0.323964124	0.417484011	no
TRIB3	0.03734747	0.986689877	0.324137082	0.417680275	no
TMSB15A	-0.037338368	-0.986449061	0.324255103	0.417805728	no
PRSS48	-0.037335557	-0.986374691	0.324291557	0.41782607	no
FLJ37307	-0.037331805	-0.986275433	0.324340214	0.417862132	no
ACAA1	0.037325255	0.98610215	0.32442517	0.417944952	no
POTEB	-0.037314446	-0.985816199	0.324565396	0.418071652	no
GADD45GIP1	-0.037313367	-0.985787634	0.324579405	0.418071652	no
SNORA11B	0.037312893	0.985775099	0.324585554	0.418071652	no
ESRRA	-0.037311217	-0.985730768	0.324607297	0.418073024	no
USP33	0.037307735	0.985638633	0.324652491	0.418104597	no
HIST1H4K	0.037295963	0.985327193	0.324805288	0.418274733	no
PIP5K1B	-0.037279227	-0.984884436	0.325022592	0.418527913	no
GOSR1	-0.03726216	-0.984432913	0.325244295	0.41877525	no
FOXJ3	0.037261252	0.984408885	0.325256096	0.41877525	no
AFTPH	0.037251083	0.984139873	0.325388235	0.418918704	no
C21orf90	0.037247783	0.984052564	0.325431128	0.418947249	no
PPTC7	0.037234324	0.983696505	0.325606093	0.419145803	no
SPDYE2	0.037224045	0.983424554	0.325739769	0.419291185	no

WNT9A	-0.037218893	-0.983288252	0.325806781	0.419350744	no
C20orf160	-0.037216791	-0.983232647	0.325834121	0.419359237	no
ZC3H8	-0.037192143	-0.982580569	0.326154852	0.419745309	no
CNTNAP4	-0.037179459	-0.982245004	0.326319983	0.419931094	no
ZNF324	-0.037152412	-0.981529448	0.32667229	0.420352441	no
C8orf75	0.03715113	0.98149555	0.326688985	0.420352441	no
KIAA1751	0.037148168	0.981417186	0.326727584	0.420375353	no
KLHL18	0.037140431	0.981212489	0.326828424	0.420478338	no
C18orf22	-0.037138421	-0.981159307	0.326854626	0.420485291	no
ARV1	0.037133548	0.981030402	0.326918143	0.420540244	no
LYZL1	-0.037125674	-0.9808221	0.327020798	0.42062638	no
LOC100271831	0.037125221	0.9808101	0.327026712	0.42062638	no
OR11A1	0.037117051	0.980593973	0.327133247	0.420718323	no
C15orf43	0.037116548	0.980580651	0.327139814	0.420718323	no
SMUG1	-0.037114652	-0.980530505	0.327164536	0.420723356	no
LOC349196	0.037086365	0.979782165	0.32753361	0.421120695	no
PRAMEF6	0.037086298	0.979780378	0.327534491	0.421120695	no
AIFM3	-0.037086182	-0.979777312	0.327536004	0.421120695	no
PCDHB17	0.037078992	0.979587099	0.327629859	0.42121458	no
CCDC47	0.037066181	0.979248173	0.327797135	0.42140284	no
NDUFA12	0.037045419	0.978698924	0.328068333	0.421700657	no
RCN2	-0.037045252	-0.97869451	0.328070513	0.421700657	no
UGT2A3	-0.037035624	-0.978439788	0.328196335	0.421816607	no
KISS1R	0.037034896	0.978420533	0.328205847	0.421816607	no
TRAF4	-0.03703356	-0.978385188	0.328223309	0.421816607	no
BCAS1	-0.037028734	-0.978257528	0.328286383	0.42187085	no
C17orf71	-0.037012178	-0.977819531	0.328502847	0.422122191	no
OR2F1	0.037010369	0.97777166	0.328526511	0.42212577	no
ASAP1IT1	0.036999895	0.97749457	0.328663507	0.422274961	no
WWC3	0.036996057	0.977393047	0.32871371	0.422312626	no
ACTR3C	0.036979273	0.976949022	0.328933339	0.422567941	no
SPRR2B	-0.036976495	-0.976875527	0.328969702	0.422587803	no
LOC148413	0.036969052	0.976678624	0.329067134	0.422686106	no
EIF1AD	0.036958896	0.976409947	0.329200112	0.422830053	no
TAC1	-0.036956598	-0.976349163	0.329230201	0.422841837	no
PSPH	0.036922431	0.975445268	0.329677854	0.423389878	no
KRTAP1-3	-0.036916596	-0.975290916	0.329754336	0.423461202	no
OCM2	-0.036878021	-0.97427041	0.330260291	0.424083999	no
GUK1	0.036855208	0.973666903	0.330559739	0.42444156	no
HIST1H2BO	0.036847045	0.973450962	0.330666927	0.424552228	no
LOC144776	0.036837096	0.97318776	0.330797606	0.42469304	no
FBXO38	-0.036832797	-0.973074043	0.330854076	0.424713962	no
BYSL	-0.036831135	-0.973030062	0.330875918	0.424713962	no
KPRP	0.036831058	0.97302804	0.330876923	0.424713962	no
FOXM1	0.036827362	0.972930262	0.330925485	0.42474933	no
TLL2	0.036814992	0.972603008	0.331088052	0.424931012	no
SCGB1D2	0.036811173	0.972501979	0.33113825	0.424968461	no
SNORA22	0.036805033	0.97233954	0.33121897	0.425045074	no
KRT27	0.036802307	0.97226743	0.331254808	0.425064084	no
ZNF461	0.036797425	0.972138273	0.331319003	0.425117046	no
WFDC10A	-0.03679597	-0.972099784	0.331338135	0.425117046	no

ZCCHC16	-0.036791227	-0.971974326	0.331400502	0.425170084	no
SF3B14	0.036763168	0.971232029	0.331769664	0.425616692	no
KCNMA1	-0.036759546	-0.971136206	0.331817339	0.425650844	no
TCEANC	-0.036742111	-0.970674992	0.332046867	0.425918257	no
MAFG	-0.036737913	-0.970563941	0.332102148	0.425962141	no
CSNK1A1L	-0.0367334	-0.970444553	0.332161586	0.425998126	no
SCARNA12	0.036732584	0.970422945	0.332172344	0.425998126	no
FGF3	0.036724996	0.970222228	0.33227229	0.426099274	no
TMEM14E	0.03670255	0.969628432	0.33256808	0.426443214	no
PFKL	0.036701443	0.969599134	0.332582679	0.426443214	no
FLJ42393	0.036687198	0.969222307	0.332770483	0.426656962	no
ZNF204P	-0.036682059	-0.969086365	0.332838251	0.426716789	no
CALHM3	-0.036680423	-0.969043067	0.332859838	0.426717405	no
PRDM5	0.03666978	0.968761514	0.333000228	0.426848096	no
JAM2	0.036669493	0.968753936	0.333004007	0.426848096	no
AGGF1	0.036665538	0.968649296	0.333056194	0.426882028	no
SERPINE3	-0.036664286	-0.968616187	0.333072707	0.426882028	no
DPPA2	0.036651227	0.968270734	0.333245037	0.427075821	no
ATP6V0A4	-0.036636487	-0.967880795	0.333439627	0.427298116	no
CDKL4	0.036611831	0.967228528	0.333765291	0.427688342	no
FAM95B1	-0.036607605	-0.967116741	0.333821125	0.427732778	no
STRA8	-0.036603945	-0.96701993	0.333869483	0.427767631	no
FLJ25363	-0.036601884	-0.966965415	0.333896716	0.427775414	no
ELFN1	-0.036599641	-0.966906072	0.333926363	0.427786289	no
LMNB2	0.0365865	0.96655845	0.334100062	0.427974603	no
NYNRIN	0.036585318	0.966527169	0.334115695	0.427974603	no
PSAPL1	-0.036583709	-0.966484599	0.334136971	0.427974741	no
LASS1	0.036569985	0.966121559	0.334318449	0.428180059	no
CYP20A1	0.036559265	0.965837971	0.334460255	0.428334543	no
L3MBTL3	0.036544986	0.965460247	0.334649193	0.428538115	no
TMEM63A	0.036543146	0.965411553	0.334673554	0.428538115	no
HBD	0.036542447	0.965393075	0.334682799	0.428538115	no
LOC340074	0.036524853	0.964927653	0.334915714	0.428809189	no
KDM6A	0.036522512	0.964865718	0.334946716	0.428821726	no
RAP1GDS1	0.03650567	0.964420182	0.33516979	0.429080151	no
CDH4	0.036501455	0.964308674	0.335225636	0.429124471	no
SLC10A1	-0.036497614	-0.964207084	0.335276519	0.429162435	no
TRIM7	-0.036477273	-0.963668991	0.335546118	0.429480338	no
OTUD7B	0.036467693	0.963415544	0.335673151	0.429615735	no
RNF113B	0.036450436	0.962959041	0.335902036	0.429881464	no
NEUROG2	-0.036440542	-0.962697328	0.336033302	0.430022236	no
C6orf26	-0.036438473	-0.96264258	0.336060766	0.430030162	no
SSX3	0.036436259	0.962584005	0.336090151	0.430040546	no
CFLP1	-0.036430984	-0.962444482	0.336160152	0.430098844	no
PCDHGB1	-0.03642962	-0.962408395	0.336178259	0.430098844	no
ADORA2A	0.036413302	0.961976731	0.336394898	0.430348776	no
SYCP3	-0.036409862	-0.961885722	0.336440584	0.430379989	no
CCDC141	-0.036402001	-0.961677778	0.336544986	0.430486305	no
RIC8A	0.036399303	0.961606398	0.336580829	0.430504915	no
SPDYC	-0.036391202	-0.9613921	0.33668845	0.430594134	no
BCDIN3D	-0.036390846	-0.961382689	0.336693177	0.430594134	no

TGM7	0.036385437	0.961239604	0.336765048	0.430658807	no
C1QBP	0.036342894	0.960114213	0.337330672	0.43135485	no
GJC3	-0.03632161	-0.959551164	0.337613891	0.431689707	no
PRMT1	0.03631485	0.959372348	0.337703869	0.431777451	no
SLC39A12	0.036301001	0.959005997	0.337888261	0.43198589	no
EPHX4	-0.036290343	-0.958724067	0.338030207	0.432140039	no
ACTN3	-0.036277022	-0.958371693	0.338207673	0.432339576	no
NBLA00301	0.036267276	0.958113891	0.338337548	0.432478254	no
BBX	-0.036253488	-0.957749135	0.338521358	0.432685853	no
PCDHGA7	-0.036225385	-0.957005755	0.338896167	0.433120161	no
SLC13A1	-0.036224799	-0.956990237	0.338903994	0.433120161	no
POLR3D	-0.036218131	-0.956813853	0.338992966	0.433206485	no
CCDC99	0.036207291	0.956527105	0.339137641	0.433363976	no
EXOSC1	0.036202528	0.956401122	0.339201216	0.433417822	no
TSPAN31	0.036194344	0.956184634	0.339310482	0.433530038	no
METT11D1	-0.036187805	-0.95601165	0.339397807	0.433614209	no
TRIM66	-0.036171149	-0.955571044	0.339620295	0.433871043	no
RPL3L	0.036163622	0.955371945	0.339720863	0.433972099	no
SMYD1	-0.036158156	-0.95522734	0.339793917	0.434037997	no
CACNA1C	-0.036143268	-0.954833524	0.339992924	0.434264763	no
DHX37	0.036133493	0.954574955	0.340123627	0.434404263	no
LOC399815	-0.036128747	-0.954449412	0.340187099	0.434457884	no
PLA2G4D	-0.036119877	-0.954214777	0.340305745	0.434581958	no
C22orf9	0.036110635	0.953970304	0.340429395	0.434691729	no
PPP1R14A	0.036110238	0.953959809	0.340434704	0.434691729	no
ATP13A1	-0.036096103	-0.953585904	0.340623875	0.434905811	no
OR1L3	-0.036092703	-0.953495956	0.340669393	0.434936461	no
SLC44A5	0.036082231	0.953218938	0.340809602	0.435087992	no
BRF2	0.036075873	0.953050767	0.340894737	0.435139435	no
DTWD2	0.03607566	0.953045133	0.34089759	0.435139435	no
DLAT	0.0360744	0.953011802	0.340914465	0.435139435	no
SYNCRIP	-0.03606175	-0.952677164	0.34108392	0.435328243	no
VCPIP1	0.036036363	0.952005631	0.341424136	0.435734956	no
OR4D2	-0.036030789	-0.951858195	0.34149886	0.435802812	no
CLLU1	0.036026818	0.951753154	0.341552104	0.435843249	no
HIGD1A	0.036011402	0.951345364	0.341758857	0.436079556	no
CIB3	0.036003485	0.951135942	0.341865067	0.436166658	no
BNIP1	0.036002842	0.951118937	0.341873692	0.436166658	no
NVL	-0.03600149	-0.95108316	0.341891839	0.436166658	no
ZNF273	-0.035983989	-0.950620223	0.342126707	0.436438751	no
PIK3C2G	-0.035964492	-0.950104481	0.342388488	0.436745138	no
LRP2	0.035952743	0.94979369	0.342546301	0.436918877	no
OR10G2	0.035924228	0.949039415	0.342929501	0.437380056	no
LOC100132831	0.03591823	0.948880764	0.343010136	0.437455304	no
SNX14	-0.035905883	-0.948554158	0.343176175	0.437639454	no
MTHFD2L	-0.035897525	-0.948333088	0.34328859	0.437755201	no
LHX4	-0.03589316	-0.948217611	0.34334732	0.43780248	no
RACGAP1	0.035885515	0.9480154	0.343450178	0.437906016	no
FAM9A	-0.035881566	-0.947910923	0.343503329	0.437946166	no
LIMA1	-0.035871804	-0.947652722	0.343634708	0.438086042	no
RAB37	0.035863985	0.947445871	0.343739983	0.438192621	no

KLRA1	-0.035858218	-0.947293328	0.343817631	0.438263972	no
CADM1	0.035846146	0.946974014	0.343980206	0.438443563	no
DBI	-0.035842399	-0.946874883	0.344030687	0.438480264	no
RPS6KA2	-0.035840191	-0.946816502	0.344060419	0.438490517	no
FOXE1	0.035837778	0.946752656	0.344092937	0.438504317	no
YIPF3	-0.035835591	-0.946694813	0.344122398	0.438514222	no
ST7	-0.035816533	-0.946190687	0.344379236	0.438787189	no
PRR7	0.035816475	0.94618916	0.344380014	0.438787189	no
HSD17B13	0.035813611	0.9461134	0.344418622	0.438808727	no
NDOR1	-0.035803487	-0.945845613	0.344555112	0.438954962	no
NCRNA00207	-0.035791718	-0.945534301	0.344713829	0.439129494	no
APAF1	0.035776189	0.945123536	0.344923323	0.439354825	no
DDT	0.035775385	0.945102263	0.344934174	0.439354825	no
OR2D2	-0.035760039	-0.944696338	0.345141284	0.439590933	no
PKDREJ	-0.035739149	-0.944143778	0.345423336	0.439901227	no
LCE1A	-0.035738772	-0.944133812	0.345428425	0.439901227	no
GABRQ	-0.03573676	-0.944080569	0.345455611	0.43990814	no
GRXCR2	0.035733035	0.943982044	0.345505921	0.439944496	no
C14orf118	-0.035731305	-0.943936277	0.345529293	0.439946548	no
ZNF26	0.035722167	0.943694578	0.345652738	0.440076011	no
COX7C	-0.035716653	-0.943548714	0.345727249	0.44014316	no
AP1G2	0.035714474	0.943491071	0.345756698	0.440152936	no
SLC14A1	-0.035710059	-0.943374313	0.345816352	0.440182115	no
NUP155	0.035709556	0.943360984	0.345823163	0.440182115	no
SNX25	-0.035687779	-0.942784963	0.346117565	0.440528847	no
RFPL3	-0.035686183	-0.94274275	0.346139146	0.440528847	no
SNORA64	0.035682664	0.942649662	0.34618674	0.440561686	no
NEDD8	0.03561348	0.940819665	0.347123225	0.441725665	no
GSTM3	-0.035604647	-0.94058603	0.347242902	0.441850147	no
SERPINB10	0.035596142	0.940361073	0.347358159	0.44196899	no
CIB2	0.0355661	0.939566424	0.347765492	0.442459424	no
PGM5P2	0.035562845	0.939480331	0.34780964	0.442487749	no
RBP2	-0.035544464	-0.93899412	0.34805904	0.442777177	no
ACSBG2	-0.035505965	-0.937975796	0.348581753	0.443414237	no
XPO1	0.035498707	0.937783827	0.348680348	0.443511751	no
AADACL4	-0.035495568	-0.937700794	0.348722999	0.443538098	no
PDE4D	0.035490947	0.93757856	0.348785793	0.443590059	no
FAM63B	-0.035488071	-0.937502504	0.348824867	0.443611848	no
IQCE	-0.035480433	-0.937300468	0.34892868	0.44371596	no
LOC650293	-0.035476272	-0.937190395	0.348985246	0.443719034	no
C1orf175	0.035475917	0.937181005	0.348990072	0.443719034	no
LOC84740	-0.035475412	-0.937167648	0.348996937	0.443719034	no
GJA10	0.035464513	0.936879357	0.349145122	0.443879524	no
KCNG2	-0.035460438	-0.936771588	0.349200527	0.443922048	no
SLC35B2	0.035436571	0.936140295	0.349525191	0.444306842	no
GGTA1	-0.03539747	-0.935106053	0.350057501	0.444955523	no
SULT1A3	-0.035392816	-0.934982948	0.350120896	0.445008127	no
C21orf99	-0.035385221	-0.934782043	0.350224371	0.445111662	no
PRR21	-0.035371966	-0.934431449	0.350404988	0.445313222	no
CDC42EP4	-0.035359289	-0.934096134	0.35057779	0.445504824	no
HCG22	0.035335211	0.933459281	0.350906135	0.44589405	no

EIF5	0.035330186	0.933326355	0.350974693	0.445953138	no
CGB1	0.03531943	0.933041858	0.351121453	0.446111578	no
ZNF137	0.035317774	0.932998058	0.351144051	0.446112255	no
MRPL12	-0.035307949	-0.93273818	0.351278152	0.446254582	no
TGFA	-0.035298578	-0.932490312	0.351406085	0.446389057	no
IL17F	0.035295851	0.932418181	0.35144332	0.446408308	no
RRAGA	-0.035284074	-0.932106691	0.351604144	0.446573942	no
CENPH	0.035283067	0.932080063	0.351617894	0.446573942	no
RPL23AP64	-0.035276969	-0.931918751	0.3517012	0.446651687	no
CDH3	-0.035259649	-0.931460635	0.351937854	0.446898382	no
SNORA52	0.035259517	0.931457141	0.351939659	0.446898382	no
PDE8A	0.03524444	0.93105835	0.35214575	0.447131996	no
DRD1	-0.035239877	-0.930937669	0.352208131	0.447183119	no
RAB10	0.035234513	0.930795798	0.352281475	0.447248154	no
HOXD8	0.035217396	0.930343053	0.352515598	0.44750173	no
CLEC2A	-0.035216674	-0.930323958	0.352525475	0.44750173	no
CCDC97	0.035208128	0.930097898	0.352642414	0.44762207	no
ARMS2	0.035205678	0.930033108	0.352675933	0.447636513	no
CDKN2BAS	0.03517876	0.929321136	0.353044414	0.44807608	no
ANKRD20A4	-0.035164045	-0.928931929	0.35324595	0.448303724	no
OR2V2	-0.035161758	-0.928871439	0.35327728	0.448315343	no
ABCD2	0.035149338	0.928542917	0.353447459	0.448503152	no
MAF1	-0.035111556	-0.927543595	0.353965442	0.449132253	no
HHLA1	-0.035106232	-0.927402777	0.354038472	0.449196726	no
ANAPC1	0.03509555	0.927120248	0.354185023	0.449354468	no
C3orf74	0.035083957	0.926813626	0.354344115	0.449528099	no
RPS6KC1	-0.035073562	-0.926538665	0.354486817	0.449680918	no
APOBEC1	-0.035059229	-0.926159562	0.354683628	0.449902353	no
AGXT	0.035041682	0.925695478	0.354924652	0.450158319	no
LSM3	0.035041297	0.925685275	0.354929952	0.450158319	no
FABP9	0.035032152	0.925443409	0.355055608	0.450289442	no
CYP26A1	0.03498577	0.924216633	0.355693385	0.45106999	no
TFEB	-0.034976442	-0.923969907	0.35582174	0.451204462	no
KRT14	-0.034972661	-0.923869917	0.355873767	0.451228991	no
ZNF469	-0.034970633	-0.923816264	0.355901685	0.451228991	no
OR1L6	-0.034970171	-0.923804055	0.355908039	0.451228991	no
C6orf142	0.034955948	0.923427864	0.356103833	0.451448915	no
ZRSR2	-0.034940229	-0.92301211	0.356320297	0.451695013	no
HYAL1	-0.034931819	-0.922789656	0.356436153	0.451813551	no
MRPL4	-0.034894371	-0.921799194	0.356952282	0.452439423	no
KLF7	0.034891958	0.921735375	0.356985554	0.452453231	no
PRAMEF16	-0.034882246	-0.921478504	0.357119495	0.452573172	no
FOXB2	-0.03488185	-0.921468029	0.357124957	0.452573172	no
TMEM139	0.034863746	0.920989201	0.35737472	0.452861303	no
KRTAP10-3	-0.034828394	-0.920054164	0.357862764	0.453451326	no
CC2D2A	-0.0348244	-0.919948541	0.35791792	0.453492794	no
ZFP82	-0.034800761	-0.919323303	0.35824453	0.453878175	no
OR8U1	-0.034790947	-0.919063741	0.358380175	0.454021578	no
SMC1B	0.03478277	0.918847465	0.358493223	0.45413634	no
HHLA2	-0.034773834	-0.91861113	0.358616782	0.454264401	no
LGALS4	-0.034771954	-0.918561401	0.358642784	0.454268877	no

SCG2	0.034734683	0.917575648	0.359158462	0.454893553	no
ATP1B2	-0.034729849	-0.917447779	0.359225388	0.454949818	no
AP1S1	0.034722143	0.917243976	0.359332074	0.455056428	no
BAD	-0.03470091	-0.916682391	0.359626155	0.455400325	no
ADC	-0.034690339	-0.916402795	0.359772625	0.45555727	no
GPR89A	0.034680128	0.916132736	0.359914135	0.455707914	no
MC1R	0.034646056	0.915231588	0.360386585	0.456260243	no
OR2M3	-0.034645414	-0.915214605	0.360395493	0.456260243	no
RNU6ATAC	0.034624712	0.914667067	0.360682749	0.456590258	no
GPR151	-0.034623373	-0.914631649	0.360701335	0.456590258	no
WHAMML2	0.034620849	0.914564903	0.360736363	0.45660601	no
TBL1Y	-0.03460453	-0.914133307	0.360962915	0.456864168	no
CALML5	-0.034595463	-0.913893486	0.36108884	0.45699494	no
WFDC1	-0.034589076	-0.913724558	0.361177557	0.457078608	no
YIF1A	-0.034576702	-0.913397289	0.36134947	0.457256713	no
GAS2	0.03457569	0.913370535	0.361363526	0.457256713	no
MICAL2	0.03456734	0.913149676	0.361479574	0.457374931	no
OR10H2	-0.034550309	-0.912699261	0.361716312	0.457645832	no
FOLH1	0.034533047	0.912242691	0.361956385	0.457920918	no
OCM	-0.034530845	-0.91218446	0.36198701	0.457931009	no
SCAND1	-0.034491219	-0.911136437	0.362538488	0.458599959	no
RHCG	0.034488843	0.911073584	0.362571578	0.458613123	no
OR4E2	-0.034484982	-0.910971491	0.362625332	0.458638482	no
PAGE4	-0.034484145	-0.910949339	0.362636996	0.458638482	no
SLC10A4	0.034477124	0.910763651	0.362734777	0.458733454	no
ZNF699	0.034464718	0.910435529	0.362907604	0.458912293	no
PTTG1IP	0.034463714	0.910408986	0.362921587	0.458912293	no
TAF11	0.034458552	0.910272475	0.362993507	0.458974529	no
PRAMEF10	-0.03445674	-0.910224541	0.363018762	0.458977759	no
LSG1	0.034452445	0.910110952	0.363078615	0.459024727	no
LYRM5	0.034442158	0.90983888	0.363222001	0.459177291	no
LOC648691	0.034434667	0.909640752	0.36332644	0.459280603	no
PITPNM1	0.03442785	0.90946047	0.363421489	0.459372032	no
OR10G4	-0.034367768	-0.90787144	0.364259929	0.460403054	no
DAO	0.034364497	0.907784919	0.364305616	0.460432016	no
HARS2	-0.034351518	-0.907441661	0.364486907	0.460632347	no
C2orf51	-0.034342593	-0.907205606	0.364611612	0.460761146	no
TMPRSS15	0.034325241	0.906746687	0.36485413	0.4610388	no
SEMA4F	0.034301594	0.906121295	0.365184783	0.461427782	no
GRXCR1	-0.034286788	-0.905729716	0.365391912	0.461660647	no
C1orf204	-0.034262039	-0.905075166	0.365738305	0.462069427	no
NUPL2	-0.034255607	-0.904905049	0.365828366	0.46215433	no
OR5AC2	-0.034237697	-0.904431397	0.366079193	0.462405678	no
PLA2G4C	-0.034236671	-0.904404252	0.366093571	0.462405678	no
PWWP2A	-0.034236502	-0.904399783	0.366095938	0.462405678	no
ZHX1	-0.034232784	-0.904301459	0.366148022	0.462416131	no
WDR54	0.034232646	0.904297804	0.366149958	0.462416131	no
SLC25A5	0.034229598	0.904217188	0.366192665	0.462439148	no
CHRNA3	-0.03422808	-0.904177052	0.366213928	0.462439148	no
HIST1H3G	0.034211468	0.903737715	0.366446733	0.462704225	no
UFD1L	0.03420602	0.90359362	0.366523109	0.462771762	no

ASB7	0.034187638	0.903107467	0.366780863	0.463068283	no
LRRN2	0.034185147	0.903041595	0.366815797	0.46308347	no
LIN7C	-0.034151938	-0.902163299	0.367281776	0.463642791	no
ALAS2	0.03414391	0.901950991	0.367394472	0.463756098	no
SULT2A1	-0.034139118	-0.901824273	0.367461745	0.463812059	no
CHAD	0.034130133	0.901586643	0.367587922	0.463942356	no
LCE2A	0.034127147	0.901507659	0.367629866	0.463966332	no
C1orf210	0.034124977	0.901450283	0.367660338	0.463975827	no
GLUL	-0.034116376	-0.901222816	0.367781159	0.464083108	no
PXK	0.034115658	0.901203806	0.367791257	0.464083108	no
SPATA8	0.034100744	0.900809396	0.368000813	0.464318549	no
CCL4	0.034098538	0.900751055	0.368031816	0.46432869	no
ESD	-0.034095994	-0.900683761	0.36806758	0.464344835	no
TG	-0.034090958	-0.900550589	0.368138361	0.464366808	no
CPXM2	0.03409061	0.900541373	0.36814326	0.464366808	no
RSBN1	-0.034089853	-0.900521353	0.368153901	0.464366808	no
BFSP2	0.034086649	0.900436623	0.368198941	0.464394647	no
S100A7A	-0.034051936	-0.899518566	0.368687169	0.464981423	no
TRIM71	-0.034043556	-0.899296935	0.368805095	0.465101136	no
RRAS2	0.034037021	0.899124129	0.368897058	0.465170906	no
CDC14C	0.034036355	0.89910651	0.368906435	0.465170906	no
PRKRA	-0.034012894	-0.898486034	0.369236759	0.46555839	no
SNORA38B	0.034010876	0.898432676	0.369265173	0.465565183	no
SULT6B1	0.034007443	0.89834189	0.369313523	0.465597108	no
KRTAP4-1	-0.033995884	-0.898036181	0.369476363	0.465773357	no
COMMD6	0.033993537	0.897974121	0.369509426	0.465785994	no
ZBTB1	0.033989957	0.897879426	0.369559878	0.46582055	no
LOC100240726	-0.033952365	-0.896885264	0.370089815	0.46645944	no
COX5A	0.033938935	0.896530075	0.370279263	0.466669127	no
PITPNC1	-0.033932707	-0.89636538	0.370367126	0.466750768	no
C20orf56	0.033928685	0.896259015	0.370423878	0.466793192	no
SMARCA5	-0.033924443	-0.89614682	0.370483748	0.466839539	no
LOC146880	0.033904886	0.895629611	0.370759815	0.467158291	no
TSFM	0.033900386	0.895510588	0.370823363	0.467209245	no
ARAP2	-0.033882403	-0.895035007	0.371077351	0.467497521	no
VPS18	0.033880912	0.894995578	0.371098413	0.467497521	no
RCOR1	-0.033878455	-0.894930599	0.371133125	0.46751212	no
YIPF6	0.033864262	0.894555251	0.371333678	0.467735612	no
DNMT3B	0.033860394	0.89445295	0.37138835	0.467775334	no
PHC3	-0.033833767	-0.893748777	0.371764814	0.468220333	no
LOXHD1	-0.033823765	-0.893484272	0.371906284	0.468369332	no
HNF4A	-0.033820864	-0.893407537	0.371947332	0.468387579	no
PCDHGB8P	-0.033818367	-0.893341491	0.371982664	0.468387579	no
IL5	-0.033817829	-0.893327268	0.371990274	0.468387579	no
C20orf200	0.033801542	0.892896549	0.372220747	0.468648591	no
NCOA7	0.033791715	0.892636664	0.372359852	0.468794539	no
C9orf69	0.03377429	0.892175846	0.372606587	0.469075964	no
CYP3A7	0.033767211	0.891988617	0.372706864	0.469172989	no
HDX	-0.033733476	-0.891096486	0.373184905	0.469745511	no
CADM3	-0.033684301	-0.889796013	0.373882433	0.470594225	no
ZNF658	0.033681539	0.88972297	0.373921634	0.470614268	no

WFDC2	0.033650916	0.888913103	0.374356455	0.471132201	no
AIG1	0.033630091	0.888362383	0.374652318	0.471432097	no
KIF1C	-0.033629224	-0.88833946	0.374664636	0.471432097	no
BSG	-0.03362922	-0.888339339	0.374664701	0.471432097	no
LOC339568	-0.03361867	-0.888060354	0.374814638	0.471578097	no
CEACAM5	-0.033617774	-0.888036644	0.374827383	0.471578097	no
SYNJ2	0.033584818	0.887165101	0.375296032	0.472138334	no
STOML1	-0.033581652	-0.887081398	0.37534106	0.472165602	no
CTAG2	-0.033550659	-0.886261755	0.375782165	0.472691086	no
OR6X1	-0.033547715	-0.886183905	0.375824079	0.472714397	no
USP9X	0.033524059	0.885558326	0.376160984	0.473093822	no
KCNA3	0.033523249	0.885536886	0.376172534	0.473093822	no
ZNF140	0.033518699	0.885416573	0.37623735	0.473145907	no
OR5E1P	0.033516685	0.885363303	0.376266051	0.47315257	no
OR6M1	-0.033491753	-0.884703969	0.376621399	0.47356575	no
AHI1	-0.033490345	-0.884666738	0.376641471	0.47356575	no
C14orf148	0.033445183	0.88347242	0.377285697	0.47434626	no
PIP4K2A	-0.033437286	-0.883263598	0.377398407	0.474444894	no
PDE3A	0.033436399	0.88324012	0.377411081	0.474444894	no
PNPT1	0.033432012	0.883124111	0.377473706	0.474490185	no
RAB3GAP1	-0.033430587	-0.883086435	0.377494046	0.474490185	no
UGT1A9	-0.033421606	-0.882848925	0.377622286	0.474621869	no
SAG	-0.033389761	-0.88200679	0.378077201	0.475164098	no
CHIA	-0.033387341	-0.881942792	0.378111786	0.475178026	no
LCE3C	0.033375709	0.881635169	0.378278055	0.475357431	no
CYorf15B	0.033369299	0.881465665	0.378369691	0.475443033	no
HKR1	-0.033356087	-0.881116287	0.378558611	0.475650861	no
ALDH18A1	-0.033349549	-0.880943381	0.378652129	0.475738798	no
TERC	0.033334903	0.880556082	0.378861655	0.475972469	no
OR2Z1	-0.033330713	-0.880445268	0.378921617	0.476018221	no
KRT12	0.033305017	0.879765751	0.379289439	0.476450692	no
GUCA2B	-0.033290275	-0.879375884	0.379500573	0.476669032	no
LOC100130331	-0.033289588	-0.879357729	0.379510407	0.476669032	no
RABEPK	-0.033279875	-0.879100867	0.379649553	0.47681418	no
C6orf35	-0.03325283	-0.878385673	0.380037151	0.477255607	no
TSNAXIP1	-0.033252057	-0.878365223	0.380048238	0.477255607	no
MRPL18	-0.03324976	-0.878304495	0.380081161	0.477267308	no
TSR1	0.033245907	0.878202611	0.380136401	0.477307027	no
ATP6V1E1	-0.033224386	-0.877633484	0.380445062	0.477664923	no
FOXQ1	-0.033210761	-0.877273168	0.380640556	0.477880698	no
CPNE1	0.033207832	0.877195718	0.380682585	0.477903788	no
RLN1	0.03320439	0.877104706	0.380731978	0.477936119	no
C11orf51	-0.033192712	-0.876795895	0.380899601	0.478116852	no
CDRT15	-0.03318767	-0.876662552	0.380971994	0.478178035	no
UPB1	-0.033170285	-0.876202801	0.381221661	0.478461702	no
LDHC	0.033162131	0.875987185	0.381338786	0.478578993	no
DCT	-0.033154355	-0.87578156	0.381450504	0.478689485	no
LOC100130776	0.033146666	0.875578232	0.381560994	0.478798422	no
UGT2B15	0.033142132	0.875458337	0.381626155	0.478828602	no
OR4C3	0.033141697	0.875446826	0.381632412	0.478828602	no
PCDHAC1	-0.033136449	-0.875308044	0.381707846	0.47889353	no

SNORA11	0.033132085	0.875192651	0.381770576	0.478942511	no
LCE4A	-0.033126164	-0.875036068	0.381855706	0.479013642	no
ANXA8L2	-0.033124846	-0.875001208	0.38187466	0.479013642	no
SORCS2	-0.033110033	-0.874609486	0.382087688	0.479251124	no
COL23A1	0.033099089	0.874320078	0.382245121	0.47941885	no
MYL7	0.033092415	0.874143604	0.38234114	0.479509532	no
DSC1	-0.033080473	-0.87382781	0.382512999	0.479695312	no
LOC440905	0.03305835	0.87324277	0.382831511	0.480064969	no
KLHL8	0.033047614	0.872958863	0.382986137	0.480203487	no
PSMF1	0.033047382	0.872952732	0.382989476	0.480203487	no
SLC6A9	0.033025856	0.872383501	0.383299616	0.480562547	no
C20orf27	0.033024108	0.872337293	0.383324799	0.48056432	no
GNPTAB	0.033011301	0.871998609	0.383509408	0.480746696	no
ZNF75D	-0.033010717	-0.871983163	0.383517829	0.480746696	no
WNT8B	-0.03299409	-0.871543493	0.383757568	0.481017391	no
KCNE1L	0.032984352	0.871285973	0.383898028	0.481163619	no
TNP2	-0.032968474	-0.870866104	0.384127108	0.481420894	no
LCA5	0.032949318	0.870359549	0.384403594	0.481737548	no
LOC653566	0.032938818	0.870081871	0.384555207	0.481897681	no
ZNF645	0.032910097	0.869322391	0.384970074	0.482383797	no
PPP1R2P9	-0.032908659	-0.869284375	0.384990847	0.482383797	no
LOC732275	0.032900851	0.869077898	0.385103686	0.482491275	no
PRSS30P	0.032899238	0.86903523	0.385127007	0.482491275	no
OPN1SW	0.03289777	0.868996421	0.385148219	0.482491275	no
GRM6	-0.03288494	-0.868657158	0.385333682	0.482693704	no
NBPF22P	0.032880049	0.868527806	0.385404409	0.482752391	no
TMEM74	0.032875844	0.868416629	0.385465205	0.482798632	no
KIAA1908	0.032863948	0.868102055	0.385637256	0.482984207	no
FLJ44054	0.032851568	0.867774683	0.385816358	0.483178588	no
GPR101	-0.032846433	-0.867638895	0.38589066	0.483241708	no
LMOD2	0.03282193	0.866990942	0.38624534	0.483655908	no
UVRAG	0.032814625	0.866797771	0.386351117	0.4837584	no
MCTP1	0.032808912	0.866646686	0.386433861	0.483832042	no
C3orf21	0.032807146	0.866600006	0.386459428	0.48383409	no
OR10H5	-0.03280034	-0.866420023	0.386558017	0.483927554	no
BOLL	-0.032796426	-0.866316522	0.386614719	0.48394506	no
AVPR1B	-0.03279607	-0.866307113	0.386619874	0.48394506	no
C7orf66	0.032787368	0.866076991	0.386745962	0.484072918	no
REG1A	0.032769862	0.865614087	0.386999671	0.484321892	no
NBPF9	0.032769677	0.865609179	0.387002362	0.484321892	no
FKBP6	0.032768685	0.865582946	0.387016742	0.484321892	no
ADAM2	0.032756048	0.865248792	0.387199954	0.484521178	no
CT45A6	-0.032713803	-0.864131701	0.387812824	0.485258057	no
ISX	-0.032691559	-0.863543511	0.388135761	0.485632082	no
IFNW1	0.032683498	0.863330337	0.388252842	0.485748512	no
NOTO	0.032680116	0.863240897	0.388301971	0.485779917	no
EBP	0.032671509	0.863013321	0.388426995	0.48590626	no
SASS6	0.032669469	0.862959381	0.388456632	0.48591327	no
CDX4	-0.032663198	-0.862793546	0.388547756	0.485997187	no
OR8H1	0.032656478	0.862615852	0.388645412	0.486089263	no
STX12	0.032648985	0.862417712	0.388754322	0.486195402	no

UTP23	-0.03264583	-0.862334291	0.388800181	0.486222679	no
TAS2R7	0.032636059	0.862075917	0.388942237	0.486370247	no
C1orf103	0.032632781	0.861989217	0.388989913	0.486399781	no
PIP5K1P1	-0.032630316	-0.861924039	0.389025756	0.486414517	no
RBMS3	0.032613762	0.861486314	0.389266525	0.486685461	no
PRAMEF20	-0.032599623	-0.861112432	0.389472249	0.486912561	no
LMO4	0.032587281	0.860786071	0.38965188	0.487107012	no
SERPINB13	0.03257951	0.860580595	0.389765001	0.4872183	no
HIST1H2AH	0.032571971	0.860381233	0.389874775	0.48732539	no
KCNJ14	0.032543512	0.859628711	0.390289303	0.487802573	no
CACNA1F	-0.03254245	-0.859600609	0.390304789	0.487802573	no
CSNK2A1P	-0.032532889	-0.859347792	0.390444117	0.487922972	no
C17orf90	-0.032532527	-0.859338225	0.39044939	0.487922972	no
ARPC5L	0.032522749	0.859079665	0.390591916	0.488062462	no
C14orf49	-0.032521557	-0.859048144	0.390609293	0.488062462	no
LOC728276	0.032497827	0.858420677	0.390955313	0.488464622	no
TPRG1L	-0.032495884	-0.858369281	0.390983663	0.488469858	no
CNOT10	0.032491036	0.858241098	0.391054377	0.488528014	no
PSG5	-0.032483785	-0.858049369	0.39116016	0.488602496	no
RBBP4	0.032482152	0.858006172	0.391183995	0.488602496	no
LOC285194	-0.03248198	-0.858001638	0.391186497	0.488602496	no
AFMID	0.032446895	0.85707389	0.391698634	0.489211946	no
KRTAP10-5	-0.032424301	-0.85647645	0.392028649	0.489593874	no
LIPJ	-0.032407098	-0.856021548	0.392280042	0.489877572	no
TAS2R9	-0.032403213	-0.855918831	0.392336821	0.489918216	no
CNGA4	-0.032395168	-0.855706091	0.392454431	0.490034813	no
CYP2C9	0.032387178	0.85549484	0.39257124	0.490150394	no
DUOX1	-0.032378518	-0.855265845	0.392697884	0.49027824	no
GRB7	0.032371125	0.855070337	0.392806028	0.490382974	no
ANO2	0.032358912	0.85474741	0.392984692	0.490575729	no
SLCO1C1	-0.032345544	-0.854393919	0.393180323	0.490789639	no
C12orf39	-0.032336293	-0.854149313	0.393315729	0.490928349	no
TMEM191A	-0.032325482	-0.853863435	0.393474017	0.491073297	no
TPTE	-0.032325043	-0.853851847	0.393480434	0.491073297	no
BRMS1	0.03231538	0.853596335	0.393621943	0.491217262	no
BLMH	-0.032313848	-0.853555828	0.39364438	0.491217262	no
RPPH1	0.032311721	0.853499584	0.393675535	0.49122582	no
C19orf62	-0.032309886	-0.853451057	0.393702416	0.491229045	no
RANBP6	-0.032298563	-0.853151646	0.393868298	0.491405692	no
LOC641298	0.032286355	0.852828837	0.39404719	0.491598549	no
PNLIPRP1	-0.032261185	-0.852163307	0.394416164	0.492028507	no
ZNF568	0.032230933	0.851363373	0.39485993	0.492551707	no
CT45A4	-0.032226475	-0.8512455	0.394925346	0.492602915	no
HYALP1	0.032206084	0.850706311	0.395224664	0.492945851	no
C20orf196	-0.032164107	-0.849596383	0.395841245	0.49368443	no
ANTXRL	-0.032150529	-0.849237355	0.396040815	0.493902862	no
SLC44A4	0.032137191	0.848884662	0.396236923	0.49411695	no
ADAM3A	0.032124418	0.848546914	0.396424776	0.494320718	no
CCDC57	-0.032119702	-0.848422223	0.396494142	0.494376723	no
TMEM63B	-0.032115273	-0.848305128	0.396559289	0.494427461	no
LOC100271832	0.032107498	0.848099529	0.396673691	0.494530245	no

EN2	0.032106346	0.84806906	0.396690647	0.494530245	no
PCDHGB2	-0.032096048	-0.847796766	0.396842196	0.494688669	no
FERD3L	0.032093116	0.847719237	0.396885352	0.494711964	no
RANBP10	-0.03208159	-0.847414479	0.397055022	0.494892943	no
CMAS	-0.032079821	-0.847367709	0.397081064	0.494894893	no
PHKB	0.032061928	0.846894597	0.39734456	0.49519277	no
MRPS35	0.032053514	0.846672098	0.397468515	0.495316718	no
NPW	-0.032044262	-0.846427465	0.397604828	0.49545605	no
LOC651250	-0.03204177	-0.846361576	0.397641548	0.495471269	no
SPRR2F	-0.031999028	-0.845231436	0.398271681	0.496225849	no
LOC390858	0.031978394	0.844685827	0.398576113	0.496573899	no
CGB8	0.031976765	0.84464276	0.398600149	0.496573899	no
NOL6	0.03197286	0.844539507	0.398657778	0.496615092	no
OR4N5	-0.03195522	-0.844073095	0.398918164	0.496892281	no
COMMD9	0.031954456	0.844052893	0.398929444	0.496892281	no
LRIG2	-0.031939724	-0.843663351	0.399146997	0.497132631	no
BCL8	-0.031924284	-0.84325509	0.399375082	0.497386067	no
DICER1	-0.031919038	-0.84311638	0.399452594	0.497428711	no
TBCK	0.031918636	0.843105757	0.39945853	0.497428711	no
MAGED2	-0.031912857	-0.842952964	0.399543922	0.497504405	no
CASC2	-0.031901355	-0.842648833	0.399713926	0.497685439	no
PRR23B	-0.031897726	-0.842552881	0.399767571	0.497721581	no
BMP3	-0.031893864	-0.842450758	0.399824671	0.49776202	no
C19orf23	0.03188887	0.842318706	0.399898511	0.497823293	no
FAM200A	0.031887181	0.842274043	0.399923488	0.497823734	no
GP1BA	0.03188424	0.842196301	0.399966965	0.497847202	no
MT1B	-0.031867985	-0.841766489	0.400207389	0.498115796	no
CCDC79	-0.031862186	-0.841613166	0.400293175	0.498191899	no
ZNF398	-0.03184177	-0.841073349	0.400595294	0.498537219	no
FARSB	-0.031839244	-0.841006549	0.40063269	0.49855307	no
AIFM1	0.031824103	0.840606219	0.400856844	0.498801309	no
PDE3B	0.0318057	0.840119617	0.401129405	0.499109749	no
CST11	-0.031797116	-0.839892659	0.40125657	0.499218203	no
MUC15	0.031796483	0.839875916	0.401265952	0.499218203	no
LOC100240734	0.031779132	0.839417146	0.401523078	0.499507359	no
OR4D6	-0.031771496	-0.839215232	0.401636276	0.499617439	no
EML6	-0.031758796	-0.83887943	0.401824578	0.499820925	no
CHCHD4	-0.031733213	-0.838203005	0.402204045	0.500262159	no
KRTAP25-1	0.031718773	0.837821205	0.402418326	0.500436322	no
OR5R1	0.031718773	0.837821205	0.402418326	0.500436322	no
OR6Y1	0.031718773	0.837821205	0.402418326	0.500436322	no
PBX2	0.031696632	0.83723578	0.402747023	0.500814276	no
PIPSL	-0.031694643	-0.837183185	0.402776561	0.500820204	no
ARHGAP42	-0.031689415	-0.837044962	0.402854196	0.500885931	no
SLC9A3R1	-0.031686352	-0.836963973	0.402899688	0.500895439	no
HBA2	0.031685564	0.836943137	0.402911392	0.500895439	no
RBPJL	-0.031681682	-0.836840486	0.402969058	0.500936327	no
SH3RF2	0.031677608	0.836732783	0.403029567	0.500980744	no
SENP1	0.03164443	0.835855519	0.403522631	0.501562803	no
CSTT	0.03163719	0.835664096	0.403630268	0.50166575	no
RNF212	0.031613383	0.835034642	0.40398433	0.502074944	no

LOC283404	-0.031555141	-0.833494706	0.404851315	0.503121512	no
PKDCC	0.031542207	0.833152719	0.405044005	0.503330037	no
EIF4E3	-0.031538599	-0.833057332	0.40509776	0.503365897	no
OR10K2	-0.03152051	-0.832579059	0.405367353	0.503659564	no
LRRC10	-0.031519399	-0.832549675	0.40538392	0.503659564	no
C3orf43	0.031502776	0.832110153	0.40563177	0.503936533	no
C19orf77	-0.031498758	-0.832003934	0.405691682	0.503979995	no
SFRS1	-0.031481678	-0.831552336	0.405946459	0.504265514	no
LOC150527	0.031473833	0.83134491	0.406063515	0.504379931	no
CCBE1	-0.031454454	-0.830832522	0.406352754	0.504708194	no
C6orf129	-0.031446178	-0.830613696	0.406476317	0.504830652	no
SLC6A10P	-0.03143869	-0.830415718	0.406588127	0.504938499	no
FZD9	-0.031424962	-0.830052752	0.406793164	0.505162103	no
CTDP1	0.031422494	0.829987501	0.406830031	0.505176856	no
ISY1	0.031410271	0.829664327	0.407012651	0.505372584	no
PRG3	0.031399617	0.829382629	0.407171875	0.505511036	no
KIAA0556	0.031398135	0.829343448	0.407194024	0.505511036	no
LIN9	-0.031397791	-0.829334352	0.407199166	0.505511036	no
FAM25A	-0.031389663	-0.829119454	0.407320662	0.505630819	no
C21orf130	0.031385483	0.829008936	0.407383154	0.505677345	no
OR51G2	-0.031383377	-0.828953251	0.407414642	0.505685385	no
SEMA3E	0.031380119	0.828867117	0.407463353	0.505703767	no
GOLGA4	0.031379041	0.828838605	0.407479477	0.505703767	no
TBC1D12	-0.031370765	-0.828619794	0.407603236	0.505826309	no
RNF25	0.031339204	0.827785323	0.408075419	0.506381195	no
ALDH4A1	-0.031329323	-0.827524073	0.408223313	0.506533629	no
PRAMEF11	0.031308321	0.826968782	0.408537771	0.506892707	no
RBM42	0.031297729	0.826688741	0.408696412	0.507058423	no
FAM27C	-0.03128552	-0.82636595	0.408879315	0.507254219	no
COL4A4	-0.031275157	-0.826091951	0.40903461	0.507415741	no
ZNF521	-0.03125731	-0.825620082	0.409302134	0.507716458	no
FOXE3	0.031238976	0.82513535	0.409577059	0.508026318	no
SCGN	-0.031230841	-0.824920253	0.409699092	0.508121563	no
NYX	-0.031230506	-0.824911406	0.409704111	0.508121563	no
LOC150381	0.031222715	0.82470541	0.409821001	0.508235355	no
BTBD1	0.031218284	0.824588254	0.409887488	0.508286631	no
C7orf71	-0.031207982	-0.824315888	0.410042084	0.508447155	no
CACNG6	-0.031203608	-0.824200226	0.410107745	0.508497386	no
SFI1	-0.031196128	-0.824002465	0.410220027	0.508605415	no
TRIM53	-0.031191934	-0.823891583	0.41028299	0.508652287	no
ADAMTS16	0.031184169	0.823686286	0.410399581	0.508765634	no
C15orf32	0.031179258	0.823556443	0.410473331	0.508796583	no
FLJ37543	-0.031179155	-0.823553722	0.410474877	0.508796583	no
C8orf79	-0.031171661	-0.823355588	0.410587431	0.508904897	no
RPE65	-0.03116293	-0.82312474	0.410718592	0.509036258	no
NUDT5	0.03114456	0.822639041	0.410994633	0.509332731	no
PLEKHA6	-0.031143658	-0.822615199	0.411008186	0.509332731	no
RERGL	0.031124285	0.822102995	0.411299417	0.509662393	no
C11orf36	-0.031116996	-0.821910274	0.411409026	0.509766973	no
ANGPTL7	0.031107648	0.821663124	0.411549618	0.509909926	no
HULC	0.03108497	0.821063539	0.41189081	0.510301391	no

MRAS	-0.031076967	-0.820851956	0.412011252	0.510403838	no
OR1L4	-0.031076121	-0.82082958	0.41202399	0.510403838	no
EPB41L3	0.031072951	0.820745778	0.4120717	0.510431665	no
C14orf156	-0.031054964	-0.820270214	0.41234251	0.510735824	no
SNAP29	0.03104212	0.81993063	0.41253595	0.510944121	no
FBXL3	0.03102927	0.819590892	0.412729532	0.511136407	no
TCP10L2	-0.031027639	-0.819547769	0.412754107	0.511136407	no
HIST1H1B	0.03102678	0.819525067	0.412767045	0.511136407	no
LY6G6C	-0.031016622	-0.819256501	0.41292012	0.511294646	no
NSA2	0.030990361	0.818562177	0.41331602	0.511753524	no
OR10H3	-0.030980339	-0.81829721	0.413467162	0.511909313	no
ASB4	-0.030973949	-0.81812827	0.413563546	0.511997292	no
CELA2B	-0.030968489	-0.817983905	0.41364592	0.512067916	no
CASP12	0.030955532	0.817641334	0.413841428	0.512278577	no
PAGE3	0.030948812	0.817463662	0.413942848	0.51237275	no
HIST1H4L	0.030933547	0.817060074	0.414173283	0.512626595	no
VHLL	-0.030920322	-0.81671042	0.414372985	0.512842373	no
SERPINB11	-0.030916869	-0.816619153	0.41442512	0.512875502	no
MUC7	-0.030909085	-0.816413344	0.414542702	0.512989616	no
SNORA71A	0.030904696	0.816297304	0.414609006	0.513040264	no
RRN3P3	0.030900627	0.816189719	0.414670484	0.513084935	no
SNORA71D	0.030869876	0.815376707	0.415135246	0.513628567	no
SLC7A3	0.030857994	0.815062577	0.415314903	0.513819404	no
NTF3	0.03085391	0.814954605	0.415376664	0.51386437	no
DRGX	-0.030842795	-0.814660725	0.415544796	0.514040914	no
CST9	-0.030834537	-0.814442411	0.415669722	0.514163991	no
DHFR	-0.030825213	-0.814195877	0.415810823	0.514301802	no
DECR1	0.030823813	0.814158864	0.415832009	0.514301802	no
MBTPS2	-0.030813866	-0.81389588	0.41598256	0.514456533	no
RAMP2	-0.030810351	-0.813802959	0.416035763	0.514490859	no
SPANXE	0.030794159	0.813374881	0.416280914	0.51476254	no
ALOX12	0.030784438	0.813117858	0.416428147	0.51491311	no
ZFYVE26	0.030762546	0.812539085	0.416759803	0.515291689	no
C2orf57	0.030739885	0.811939958	0.417103286	0.515684843	no
OR2M7	-0.030721919	-0.811464958	0.417375726	0.515990121	no
MED23	-0.03070963	-0.81114008	0.417562123	0.516188994	no
C17orf80	-0.030697071	-0.810808049	0.417752674	0.516392979	no
STRADB	-0.030680055	-0.810358165	0.418010943	0.516680641	no
PAGE2	-0.030664728	-0.809952949	0.41824365	0.516936674	no
SGK3	0.030660059	0.809829502	0.418314557	0.516992709	no
CATSPER3	-0.030656044	-0.809723367	0.418375528	0.517036456	no
HILS1	0.030643367	0.809388194	0.418568103	0.517236467	no
MORC1	0.030642021	0.809352633	0.418588538	0.517236467	no
CDC42EP2	0.030635081	0.809169128	0.418693997	0.517335161	no
MTF2	0.030624811	0.808897623	0.418850059	0.517496363	no
TBX2	0.030622367	0.808833016	0.4188872	0.517510627	no
CCDC149	-0.030608493	-0.808466213	0.419098103	0.517733436	no
COBL	-0.030607134	-0.808430288	0.419118763	0.517733436	no
DLEU2L	-0.030601962	-0.808293531	0.419197414	0.517798956	no
OVCH2	-0.030595401	-0.80812009	0.419297174	0.517890541	no
ZNF311	-0.030591989	-0.808029887	0.419349063	0.51792299	no

EZH2	0.030588446	0.807936205	0.419402957	0.517957913	no
S100G	-0.030584447	-0.807830483	0.419463783	0.51800139	no
EIF4G3	-0.030571206	-0.807480418	0.419665224	0.5182185	no
ERCC8	0.030562786	0.807257816	0.419793348	0.518345054	no
RAG2	-0.030551588	-0.806961771	0.41996378	0.518523829	no
GOLGA8G	0.030547798	0.806861579	0.420021469	0.518563389	no
DCAF17	0.030542922	0.806732643	0.420095716	0.518623385	no
ZNF271	-0.030533394	-0.806480768	0.420240777	0.518770792	no
PRIM1	0.030528729	0.806357419	0.420311829	0.518826824	no
TRMT6	0.03051352	0.805955327	0.420543489	0.51908109	no
PLXNA3	0.030502559	0.805665557	0.420710482	0.519255512	no
SNORA16B	0.030497189	0.805523576	0.42079232	0.519324816	no
PPP1R15B	0.03049425	0.805445887	0.420837104	0.519348384	no
S100A7	-0.030487973	-0.805279944	0.420932771	0.519434739	no
GPR135	-0.03048553	-0.80521534	0.420970019	0.519446224	no
TRYX3	-0.030483992	-0.805174681	0.420993463	0.519446224	no
FAM83A	-0.030480367	-0.805078859	0.421048715	0.519482695	no
ZSWIM7	-0.030461681	-0.804584832	0.421333647	0.519802519	no
RXFP2	-0.030444178	-0.804122095	0.421600634	0.520100166	no
COX6C	-0.030421579	-0.803524644	0.421945496	0.520463057	no
GPR137B	-0.030421528	-0.803523279	0.421946284	0.520463057	no
AOX2P	-0.03041822	-0.803435824	0.421996779	0.520493587	no
KRTAP4-7	-0.030409266	-0.803199103	0.422133475	0.520630427	no
LGI4	0.030400674	0.802971954	0.422264668	0.520760465	no
TRAPPC10	0.030397707	0.802893524	0.422309972	0.520784569	no
RPS5	0.03037075	0.80218086	0.422721762	0.521230414	no
RPSAP9	0.030370664	0.802178592	0.422723073	0.521230414	no
C2orf44	-0.030366064	-0.802056974	0.42279337	0.521272077	no
SNORA37	0.030365079	0.802030923	0.422808428	0.521272077	no
FAM55D	0.030335465	0.801248023	0.423261128	0.521798384	no
RBMXL2	0.03033041	0.801114362	0.423338444	0.521861878	no
RSPO2	-0.030315711	-0.800725778	0.423563266	0.52207307	no
PNPLA8	-0.030314974	-0.800706297	0.423574539	0.52207307	no
OR2AT4	0.030312885	0.800651052	0.423606509	0.52207307	no
KIAA0586	-0.030311765	-0.800621449	0.423623639	0.52207307	no
ASNS	-0.030309547	-0.800562816	0.423657572	0.52207307	no
RNF186	-0.030309081	-0.800550503	0.423664698	0.52207307	no
SEPT7P2	0.030301075	0.800338841	0.423787205	0.522192206	no
GAGE12J	-0.030295079	-0.80018031	0.423878975	0.522273455	no
LARP1B	0.030292007	0.800099098	0.423925991	0.522299554	no
HIST1H3F	0.030272409	0.799580992	0.42422601	0.522637345	no
SLC24A5	-0.030265627	-0.799401689	0.424329867	0.522733442	no
RAB28	-0.030260901	-0.799276752	0.424402244	0.522790749	no
OR5A1	-0.030257241	-0.79917999	0.424458303	0.52282795	no
WDR36	0.030249631	0.798978804	0.424574874	0.522939677	no
ADAM23	0.030231461	0.798498457	0.424853273	0.523250699	no
GULP1	0.030225342	0.79833669	0.424947054	0.523304805	no
IFNK	0.030225217	0.798333385	0.42494897	0.523304805	no
ABHD1	0.030223127	0.798278122	0.424981011	0.523312387	no
UGT2B28	-0.03021709	-0.798118523	0.425073551	0.523372354	no
TAZ	-0.030216573	-0.798104844	0.425081483	0.523372354	no

PMCHL1	-0.030213997	-0.798036745	0.425120973	0.523389102	no
NPY6R	-0.030198208	-0.797619336	0.42536307	0.523655273	no
OR5AN1	-0.030187642	-0.797340018	0.42552512	0.523822874	no
FBN2	0.030183316	0.797225632	0.425591493	0.523870993	no
C14orf115	0.030181716	0.797183345	0.425616031	0.523870993	no
WISP3	-0.030179965	-0.797137048	0.425642898	0.523872168	no
SCARNA2	0.030175562	0.797020649	0.42571045	0.523923416	no
FCHO1	-0.030166369	-0.796777613	0.425851515	0.524065125	no
SCARNA5	0.030164582	0.796730376	0.425878936	0.524066971	no
OR5P2	0.030157318	0.796538338	0.425990425	0.52417226	no
LOC389791	-0.030154917	-0.796474861	0.42602728	0.524185708	no
BCAR3	0.030150395	0.796355323	0.426096691	0.524239207	no
GABRG3	-0.030143625	-0.796176342	0.426200629	0.524335178	no
MDP1	0.030133284	0.795902959	0.426359417	0.524439249	no
KIF3B	-0.030133143	-0.795899238	0.426361579	0.524439249	no
OR8S1	-0.030133048	-0.795896723	0.42636304	0.524439249	no
EPN1	-0.030121597	-0.795593994	0.426538915	0.524623663	no
ZBED1	-0.030116175	-0.795450657	0.426622203	0.524694185	no
NCRNA00161	-0.030112742	-0.795359897	0.426674946	0.524727132	no
GNAT3	-0.030105046	-0.795156435	0.426793196	0.524840632	no
NDUFAF2	-0.030101281	-0.795056909	0.426851046	0.524879848	no
GLTPD1	0.030095869	0.794913825	0.426934223	0.524950199	no
EPHA5	-0.030093632	-0.794854681	0.426968608	0.524960551	no
OR5AK2	-0.030086892	-0.79467649	0.427072211	0.525049461	no
DPH5	0.030085548	0.794640979	0.42709286	0.525049461	no
SGK2	-0.030049905	-0.793698691	0.427640982	0.525691334	no
CAPN9	0.030037482	0.793370272	0.427832117	0.525894318	no
NLRP14	-0.030035765	-0.793324879	0.427858539	0.525894823	no
OR52B6	0.030029297	0.79315389	0.427958075	0.52598519	no
CCDC127	0.030022173	0.792965543	0.428067732	0.526087983	no
C22orf40	0.030003441	0.792470356	0.428356111	0.526410398	no
OR4C13	-0.029960747	-0.791341663	0.429013845	0.527155407	no
PLEKHH2	-0.029960172	-0.791326467	0.429022704	0.527155407	no
SNORA10	0.029959013	0.791295825	0.429040569	0.527155407	no
LRWD1	0.029951719	0.791102997	0.429153	0.527261506	no
KCNK16	0.029945362	0.790934936	0.429251004	0.52734987	no
ZNF673	0.029938372	0.790750171	0.429358765	0.527450207	no
FBXO36	0.029928568	0.790490985	0.429509956	0.527603882	no
ANAPC5	-0.029917833	-0.790207174	0.429675548	0.527775227	no
P2RY12	0.029912777	0.790073524	0.42975354	0.527838957	no
RFX2	0.029909739	0.789993202	0.429800416	0.527864464	no
ALDOA	-0.029898734	-0.789702286	0.42997022	0.528040935	no
RPS20	-0.029895713	-0.789622411	0.430016849	0.528066123	no
BRS3	-0.029889529	-0.789458935	0.430112292	0.528151249	no
THPO	-0.029885331	-0.789347961	0.430177089	0.528198736	no
C11orf83	-0.029877883	-0.789151057	0.430292074	0.528307836	no
XRCC6	0.029865758	0.788830532	0.430479287	0.5285056	no
FSCN3	0.02986323	0.7887637	0.430518329	0.528521438	no
FRAS1	-0.029849671	-0.788405229	0.430727773	0.528746455	no
LYPD1	0.029846575	0.788323399	0.430775592	0.528773051	no
CDK4	0.029821049	0.787648582	0.431170054	0.529225119	no

C14orf138	-0.029802627	-0.787161576	0.431454862	0.529542549	no
OR2A5	-0.029794303	-0.786941541	0.431583578	0.529668374	no
ARHGAP26	0.029768696	0.786264594	0.431979717	0.530122362	no
PBX4	0.029763106	0.786116819	0.43206622	0.530196337	no
EDDM3B	0.029760051	0.786036055	0.432113502	0.530222175	no
LCE3D	-0.029756368	-0.785938677	0.432170513	0.530259949	no
PIWIL2	-0.029722483	-0.785042898	0.432695168	0.530845428	no
HIST1H3A	0.029720502	0.784990529	0.432725852	0.530845428	no
SNORD67	-0.029720463	-0.784989497	0.432726456	0.530845428	no
HAUS7	-0.029711495	-0.784752443	0.432865366	0.530983616	no
FKSG73	-0.029686309	-0.784086633	0.433255656	0.531430131	no
SIPA1L3	0.029680156	0.783923956	0.433351047	0.531514891	no
OTOF	-0.029670619	-0.783671844	0.433498904	0.531663988	no
IGSF5	-0.029663644	-0.783487452	0.433607064	0.531764383	no
OR7E156P	-0.029659277	-0.783372005	0.433674791	0.531815183	no
OR52I1	-0.029649333	-0.783109141	0.433829021	0.531972049	no
HBA1	0.029644234	0.782974337	0.433908127	0.532036783	no
LCN6	0.029639023	0.782836577	0.433988976	0.532103646	no
OR13H1	-0.029637099	-0.782785722	0.434018825	0.532107974	no
KCNQ1DN	-0.029628613	-0.782561384	0.434150509	0.532237145	no
HTA	0.029616077	0.782230004	0.434345069	0.532443376	no
C6orf25	0.029613924	0.782173075	0.434378499	0.532452073	no
DLEU2	0.02960519	0.781942182	0.434514096	0.532585995	no
PFDN1	0.029588722	0.781506843	0.434769827	0.532867141	no
IFNA13	-0.029582363	-0.781338751	0.434868592	0.532955882	no
SIGMAR1	-0.029577497	-0.781210119	0.434944181	0.533014792	no
MYO15B	0.029575875	0.781167229	0.434969386	0.533014792	no
IL34	-0.029568732	-0.780978404	0.435080364	0.533118472	no
NXNL1	0.02956099	0.780773746	0.435200666	0.533233565	no
SNRPD1	-0.029556075	-0.780643826	0.435277045	0.533268419	no
SCARNA6	0.029555765	0.780635629	0.435281864	0.533268419	no
LCE3E	-0.029549314	-0.780465075	0.435382144	0.533358955	no
NPAS4	-0.029544489	-0.780337532	0.435457144	0.533418512	no
PTPRE	-0.029540857	-0.780241521	0.435513607	0.533455356	no
GTSF1	0.029522491	0.77975601	0.435799195	0.533772831	no
OR10A2	-0.029511801	-0.77947341	0.435965476	0.533944148	no
FAM119B	0.029507671	0.779364234	0.436029725	0.533990489	no
DMBT1	-0.029497919	-0.779106455	0.436181446	0.534143942	no
FETUB	0.02949252	0.778963708	0.436265475	0.53419892	no
WNT3A	0.029491639	0.77894042	0.436279186	0.53419892	no
RPS19BP1	-0.029489938	-0.778895454	0.436305658	0.534198982	no
PAK4	0.029487201	0.778823123	0.436348242	0.53421877	no
SPANXN2	-0.029484652	-0.77875573	0.436387921	0.534234998	no
NME1	0.029482736	0.778705072	0.436417749	0.534239165	no
ZFP91-CNTF	-0.029477117	-0.77855655	0.436505206	0.534313874	no
EGFL7	-0.029436893	-0.777493197	0.437131655	0.535048299	no
TAS1R3	-0.029424571	-0.777167486	0.437323644	0.535250889	no
DDX18	-0.029417521	-0.776981108	0.437433526	0.535330255	no
OR6B2	-0.029412684	-0.776853253	0.437508913	0.535330255	no
LOC100131193	-0.029412617	-0.776851461	0.43750997	0.535330255	no
TEX19	-0.029412186	-0.776840079	0.437516682	0.535330255	no

AVP	0.029411596	0.776824476	0.437525883	0.535330255	no
C17orf56	-0.029410218	-0.776788059	0.437547357	0.535330255	no
FAM178B	0.029407375	0.776712899	0.43759168	0.535352086	no
HEATR5A	0.029382549	0.776056628	0.4379788	0.535758338	no
TPO	0.029380404	0.77599992	0.43801226	0.535758338	no
WWC2	0.02937966	0.775980264	0.438023858	0.535758338	no
GJC2	-0.029379283	-0.775970292	0.438029742	0.535758338	no
CYP11B2	-0.029373471	-0.775816657	0.438120403	0.53583681	no
UBTD2	0.029361542	0.775501295	0.438306533	0.536032028	no
FAM133B	0.029354399	0.77531247	0.438418001	0.536128764	no
CCDC25	0.029353074	0.775277465	0.438438667	0.536128764	no
ZNF121	0.029331483	0.77470669	0.43877572	0.536508467	no
CEL	-0.029326976	-0.774587562	0.438846085	0.536562056	no
VKORC1L1	0.029324647	0.774525998	0.438882452	0.536574071	no
DECR2	0.029322724	0.77447516	0.438912485	0.536578342	no
CAND1	0.029304356	0.773989606	0.439199381	0.536896613	no
OR4F29	0.029266899	0.772999426	0.439784777	0.537577334	no
HIST1H3I	0.029265323	0.772957775	0.439809411	0.537577334	no
ALS2CR11	0.0292586	0.772780063	0.439914526	0.53767331	no
RTP2	0.029245046	0.772421752	0.440126506	0.537899879	no
CLDN25	-0.02923956	-0.77227674	0.440212314	0.537972229	no
NRADDP	0.029230738	0.772043524	0.440350334	0.538108374	no
MC5R	0.029222788	0.771833386	0.440474717	0.53822784	no
ABCG1	-0.029211006	-0.771521916	0.440659118	0.538420623	no
SCARNA23	0.029204986	0.771362783	0.440753347	0.538503213	no
FASTKD1	-0.029188437	-0.770925314	0.44101245	0.53878722	no
C3orf71	-0.02917485	-0.770566147	0.441225242	0.539014618	no
FAM60A	-0.02917213	-0.770494245	0.441267848	0.539034097	no
FAM138B	0.029159199	0.770152421	0.44147043	0.539248983	no
DHX29	0.029141772	0.769691754	0.44174353	0.539532932	no
NLGN4X	-0.02914096	-0.769670277	0.441756264	0.539532932	no
C14orf153	0.029137792	0.769586552	0.441805911	0.539560972	no
ELOVL2	0.02912893	0.769352281	0.441944843	0.539698046	no
LHX8	0.029123109	0.769198401	0.442036114	0.539706054	no
KRT82	0.029121854	0.76916523	0.44205579	0.539706054	no
MAST4	-0.029121842	-0.769164914	0.442055977	0.539706054	no
DENND1A	0.029121702	0.769161204	0.442058179	0.539706054	no
KLK10	-0.029104818	-0.768714886	0.442322972	0.53999673	no
LRCH3	0.029102705	0.768659054	0.442356102	0.540004569	no
CBFA2T2	-0.029092476	-0.768388659	0.442516575	0.540167851	no
XRCC1	-0.029084351	-0.768173864	0.442644074	0.540290865	no
EPRS	-0.029071516	-0.767834594	0.442845502	0.540504096	no
GLYATL1	0.029064202	0.767641239	0.442960322	0.540611602	no
ZNF799	0.029044364	0.767116835	0.443271816	0.54095911	no
ZNF442	-0.029039889	-0.76699854	0.4433421	0.541012227	no
HIST1H2AB	0.029021605	0.766515229	0.443629322	0.541330052	no
C15orf62	-0.029016414	-0.766378001	0.443710893	0.541396912	no
ZNF549	-0.029004712	-0.766068668	0.443894798	0.54158862	no
PIK3C2B	-0.028988547	-0.765641358	0.444148915	0.541865965	no
KAZALD1	0.02897835	0.76537181	0.444309256	0.542028874	no
DEFB123	-0.028964716	-0.765011427	0.444523681	0.54225774	no

GNRH2	0.028962763	0.764959804	0.444554401	0.542262497	no
SRP72	0.028960745	0.764906445	0.444586156	0.542268515	no
NOL3	0.028944756	0.764483793	0.444837726	0.542542629	no
TP53	0.028916163	0.763727963	0.445287813	0.543058813	no
DDX3Y	0.028907491	0.763498726	0.445424372	0.543163956	no
FAM138D	-0.028907276	-0.763493045	0.445427757	0.543163956	no
EFTUD2	0.028902595	0.763369324	0.445501469	0.543221078	no
ZC3H11A	0.028868057	0.762456343	0.446045633	0.543851804	no
TMCO2	0.028864943	0.762374022	0.446094718	0.543878852	no
NPM3	-0.028860812	-0.762264823	0.446159833	0.543925441	no
ZNF829	0.028838246	0.761668322	0.446515622	0.544299371	no
GLI1	0.028837943	0.761660321	0.446520396	0.544299371	no
KCNA10	0.028833686	0.761547785	0.446587538	0.544348396	no
AVL9	-0.028823281	-0.761272755	0.446751652	0.544483816	no
ZNF132	0.028822828	0.761260783	0.446758796	0.544483816	no
LOC400931	-0.028821521	-0.761226209	0.44677943	0.544483816	no
KIAA1804	0.028818231	0.761139268	0.446831318	0.544514229	no
FGF4	0.028807765	0.760862611	0.446996454	0.544682637	no
HOXC11	0.028799593	0.760646585	0.447125424	0.544806957	no
OTUD6B	-0.028795032	-0.760526026	0.447197408	0.54486183	no
CYP4B1	-0.028755727	-0.759487057	0.447818038	0.545585122	no
CNOT6L	-0.02875139	-0.759372399	0.44788656	0.545635723	no
TMEM11	0.028732357	0.758869307	0.448187285	0.545969182	no
RPL23	-0.028728665	-0.758771693	0.448245647	0.54600738	no
ANKMY1	-0.028722168	-0.758599972	0.448348328	0.546099554	no
HIST1H4B	0.028714245	0.758390537	0.448473578	0.546219205	no
SNORA38	0.028697059	0.757936241	0.448745332	0.546450694	no
FAM75A6	-0.028696471	-0.7579207	0.448754631	0.546450694	no
VEZT	0.028695833	0.75790383	0.448764724	0.546450694	no
SP8	0.028695388	0.757892079	0.448771754	0.546450694	no
TRMT61B	0.028684591	0.757606691	0.448942524	0.546625712	no
NDUFA8	-0.028667679	-0.75715965	0.449210097	0.546918568	no
INTS12	0.028659409	0.75694103	0.449340984	0.547037392	no
GPR62	-0.028658093	-0.756906251	0.449361807	0.547037392	no
HIST1H4A	0.02865616	0.756855151	0.449392404	0.547041702	no
IFT172	-0.028647847	-0.756635415	0.449523989	0.547168935	no
ST5	0.028628462	0.756123	0.449830925	0.547498601	no
COX7B2	-0.028627321	-0.756092856	0.449848984	0.547498601	no
TMPO	0.028625284	0.75603901	0.449881246	0.547504908	no
TFDP1	-0.028612505	-0.755701219	0.45008366	0.547718276	no
ANKRD30A	-0.028610578	-0.755650269	0.450114195	0.547722467	no
CABP2	0.028604579	0.755491704	0.450209233	0.547805144	no
CASC5	0.028599894	0.755367849	0.450283475	0.547862508	no
GNB1	0.028592336	0.755168089	0.450403232	0.54797524	no
LOC100190940	-0.028586238	-0.755006877	0.450499891	0.54805986	no
C17orf73	0.028548535	0.754010274	0.451097699	0.548754108	no
HK1	0.028543502	0.75387724	0.451177533	0.548807328	no
SCGB2A2	-0.028542354	-0.753846907	0.451195737	0.548807328	no
C20orf94	0.028534918	0.753650339	0.451313715	0.548917806	no
TMPRSS11F	0.02852804	0.753468546	0.45142284	0.549017504	no
GPR81	0.028525927	0.753412677	0.451456381	0.549025269	no

FIGN	0.028498452	0.752686434	0.451892494	0.549522581	no
IFI27L2	-0.028493589	-0.752557881	0.451969715	0.549583431	no
OR8B3	-0.028481288	-0.752232742	0.452165059	0.549787898	no
LOC100128023	0.028465068	0.751803991	0.452422726	0.550068115	no
DEFB126	-0.028450987	-0.751431799	0.452646471	0.550307057	no
TTC17	-0.028440568	-0.751156399	0.452812069	0.550475282	no
TMEM95	0.02842742	0.75080885	0.453021099	0.550696283	no
OR11H6	-0.028417261	-0.750540314	0.453182645	0.550859539	no
FLJ45079	-0.02840714	-0.750272806	0.453343604	0.551022063	no
ADAM21P1	0.028376506	0.749463054	0.453831029	0.55158135	no
OR2G2	-0.028374038	-0.749397836	0.4538703	0.55159592	no
ERCC6	-0.028366114	-0.749188367	0.453996443	0.55171606	no
PHF14	0.028358156	0.748978028	0.45412313	0.551836846	no
POTEH	-0.028348167	-0.748713993	0.454282186	0.551996949	no
SPATA2L	-0.02832128	-0.748003305	0.454710465	0.552484146	no
HPX	0.028308577	0.747667526	0.454912893	0.552696886	no
OR5D14	-0.028292781	-0.747250002	0.455164673	0.552969557	no
NUDT8	-0.028287573	-0.747112324	0.455247715	0.55303721	no
TM6SF2	0.0282713	0.746682194	0.455507205	0.55331582	no
BMP6	-0.028269758	-0.746641442	0.455531795	0.55331582	no
EVX1	-0.028261261	-0.746416842	0.45566733	0.553447199	no
SRRT	-0.028248227	-0.746072326	0.455875272	0.553637001	no
ZNF815	-0.028248033	-0.746067202	0.455878365	0.553637001	no
PRAMEF17	0.028240344	0.745863956	0.456001066	0.553752751	no
CIDEC	0.02821494	0.745192479	0.456406572	0.554211896	no
TCF15	-0.028191117	-0.744562767	0.45678704	0.554640585	no
EPAS1	-0.028183289	-0.744355853	0.456912096	0.554749987	no
PDE8B	-0.028182042	-0.744322893	0.456932018	0.554749987	no
OGFR	0.028168485	0.743964551	0.457148645	0.554963022	no
FKSG29	0.028167625	0.743941823	0.457162387	0.554963022	no
HSD17B8	0.028126119	0.74284473	0.457825977	0.555735207	no
VAT1L	-0.02812131	-0.742717606	0.457902904	0.55576267	no
IGLL1	-0.028119785	-0.742677312	0.457927289	0.55576267	no
IRS4	-0.028119392	-0.742666931	0.457933572	0.55576267	no
KIAA0114	-0.028117831	-0.742625649	0.457958556	0.55576267	no
TEK	-0.02809995	-0.742153039	0.458244633	0.556076466	no
STAG3L3	-0.02809711	-0.742077975	0.45829008	0.556098238	no
COX4I2	0.028093982	0.741995282	0.458340148	0.556125615	no
ZNF460	0.028082982	0.741704539	0.45851621	0.556281955	no
AKR7A3	-0.028082494	-0.741691631	0.458524027	0.556281955	no
C2orf76	0.028075717	0.741512501	0.458632521	0.556350104	no
SNORA44	0.028075547	0.74150802	0.458635235	0.556350104	no
USP17L2	-0.02806529	-0.741236893	0.458799476	0.556515947	no
NDUFV3	0.028062368	0.741159664	0.458846265	0.556539312	no
HAUS5	0.0280546	0.74095434	0.458970674	0.556656814	no
BRIX1	0.028051066	0.740860939	0.459027273	0.556674209	no
C1QL4	-0.028049079	-0.740808404	0.45905911	0.556674209	no
FAM64A	0.028048548	0.740794366	0.459067617	0.556674209	no
SLC27A5	-0.028045615	-0.740716839	0.459114602	0.556697795	no
MYADML	-0.028035107	-0.740439112	0.459282941	0.556868515	no
SSX2	0.028028015	0.740251639	0.459396593	0.556972914	no

LASS6	-0.02801023	-0.739781564	0.459681637	0.557285084	no
LOC440563	-0.028001828	-0.739559485	0.459816336	0.557409834	no
CHCHD2	0.028000373	0.739521004	0.459839678	0.557409834	no
PAIP2	0.027995485	0.739391821	0.459918045	0.557471405	no
PAPL	0.027980156	0.738986648	0.460163885	0.557735952	no
HSPA2	-0.027976259	-0.738883627	0.460226404	0.55777829	no
ACTRT2	-0.027939441	-0.737910466	0.460817217	0.558428044	no
ARL5C	0.027939407	0.737909571	0.460817761	0.558428044	no
ABCB9	0.027933636	0.737757032	0.460910407	0.558498146	no
DHRS12	-0.027932362	-0.737723365	0.460930857	0.558498146	no
ZNF780A	0.027928856	0.737630708	0.46098714	0.558532869	no
PHOX2B	0.027907839	0.737075192	0.461324657	0.558908313	no
HSBP1	0.027890754	0.736623593	0.46159914	0.559207346	no
GSTM5	0.027880747	0.736359103	0.46175994	0.559368632	no
C12orf69	0.027878911	0.73631056	0.461789455	0.559370871	no
ANXA7	0.027873396	0.736164804	0.461878086	0.559444713	no
CDT1	-0.027865335	-0.735951737	0.462007664	0.559568139	no
HIST1H1A	0.027857863	0.735754231	0.462127796	0.559680112	no
IRGC	-0.027852511	-0.73561278	0.462213845	0.559750794	no
C1orf107	0.027842529	0.735348926	0.462374377	0.559911665	no
CLCN7	-0.027825523	-0.734899455	0.462647913	0.56020935	no
HIST1H2BL	0.027796484	0.734131914	0.463115228	0.560741627	no
FKBP1B	0.027786503	0.733868078	0.463275925	0.560858783	no
ZSCAN16	0.027786211	0.733860378	0.463280615	0.560858783	no
MTOR	-0.027783751	-0.733795348	0.463320228	0.560858783	no
ASTN2	-0.027783582	-0.733790881	0.463322949	0.560858783	no
CDC25A	-0.027757586	-0.733103763	0.463741627	0.561331989	no
RAD9A	-0.027743924	-0.732742666	0.463961737	0.561564798	no
HIST2H2AB	0.027739458	0.732624634	0.464033697	0.561618275	no
NPBWR1	-0.027735512	-0.732520331	0.464097292	0.561661622	no
C14orf166B	0.027724715	0.732234961	0.464271311	0.561838593	no
NHLRC1	0.027714128	0.731955123	0.464441993	0.562011504	no
OR1N2	0.027702739	0.73165411	0.464625629	0.562200069	no
HOXD4	0.027697859	0.731525114	0.464704336	0.562261656	no
NEU2	-0.027694964	-0.731448595	0.464751028	0.5622845	no
CCDC142	-0.027685318	-0.731193631	0.464906626	0.562439095	no
DEFB136	-0.027666554	-0.730697703	0.465209361	0.562771667	no
C7orf10	0.027658408	0.730482394	0.465340829	0.562884708	no
ZDHHC20	0.027657314	0.730453477	0.465358488	0.562884708	no
CCND3	0.027634893	0.729860852	0.465720459	0.56328884	no
CDK20	0.027623683	0.729564574	0.465901483	0.563474079	no
HTR4	0.027619766	0.729461046	0.465964747	0.563516884	no
HOXD12	0.027616909	0.729385508	0.46601091	0.563539002	no
C11orf94	-0.027614512	-0.729322177	0.466049615	0.5635521	no
C19orf21	-0.027604995	-0.729070626	0.466203369	0.563704306	no
TTLL6	-0.027589871	-0.728670889	0.466447755	0.563966075	no
C20orf79	-0.027567368	-0.728076096	0.466811524	0.564372146	no
SNORA23	0.02755516	0.727753446	0.46700892	0.564577035	no
PRNT	-0.02755302	-0.727696883	0.467043529	0.564585117	no
SUDS3	-0.027536428	-0.727258345	0.46731191	0.564875774	no
GGT1	-0.027512631	-0.726629358	0.467696993	0.565307455	no

SLC26A4	0.027501823	0.726343686	0.467871947	0.565485116	no
MBD6	0.027497972	0.726241922	0.467934279	0.56551892	no
GCNT4	0.027496639	0.726206694	0.467955858	0.56551892	no
ZNF878	0.027493571	0.726125598	0.468005535	0.56554515	no
SATB2	-0.027485341	-0.72590808	0.468138795	0.565672374	no
REG1B	0.027478618	0.725730367	0.468247685	0.565770136	no
CPB1	-0.027461418	-0.725275758	0.4685263	0.566072949	no
LOC728613	-0.027455679	-0.725124077	0.468619281	0.566151456	no
PLEKHM1P	0.02745131	0.725008619	0.468690064	0.566203138	no
BNIP3L	0.027447461	0.724906865	0.46875245	0.56624467	no
TFAMP1	-0.027437614	-0.724646615	0.468912033	0.566403602	no
MRPL1	-0.027420416	-0.724192067	0.46919083	0.566685753	no
OR1A1	-0.027419012	-0.724154951	0.4692136	0.566685753	no
POTED	-0.027418019	-0.724128697	0.469229706	0.566685753	no
ESRP1	-0.027411823	-0.723964942	0.469330172	0.56677323	no
POLH	0.027399777	0.723646559	0.469525539	0.566975294	no
OR1L8	0.027391843	0.723436871	0.469654233	0.567091406	no
RPP38	-0.027390391	-0.723398489	0.469677792	0.567091406	no
ZFY	0.02737276	0.72293248	0.469963878	0.567371622	no
DUOXA1	0.027372629	0.722929027	0.469965998	0.567371622	no
LOC728392	0.027364414	0.722711891	0.470099333	0.567498705	no
C1orf125	-0.027360986	-0.722621308	0.470154963	0.567507105	no
SEC14L2	0.027360526	0.722609149	0.47016243	0.567507105	no
SCNN1A	0.027341772	0.722113467	0.47046691	0.567840725	no
XAGE1D	0.027337467	0.721999671	0.470536826	0.56789121	no
OR9G9	0.027324978	0.721669589	0.470739661	0.568102099	no
IHH	0.027312742	0.721346196	0.470938432	0.568308059	no
CRIM1	0.027309288	0.721254891	0.470994561	0.568318301	no
COL7A1	0.02730765	0.721211601	0.471021174	0.568318301	no
SVIP	0.02730703	0.721195215	0.471031248	0.568318301	no
MIER2	-0.027293399	-0.720834938	0.471252768	0.568541891	no
NUP35	-0.027292166	-0.720802358	0.471272804	0.568541891	no
NUP153	0.027283839	0.720582266	0.471408161	0.568671254	no
BAMBI	0.027268986	0.720189708	0.47164964	0.568928117	no
COX7A1	0.027267281	0.720144634	0.471677371	0.568928117	no
CHRNB3	-0.027259271	-0.719932944	0.471807623	0.569051275	no
TUBA3E	-0.027252542	-0.719755078	0.471917078	0.569149337	no
CPSF2	-0.027250536	-0.719702072	0.4719497	0.569154729	no
FGFBP1	-0.027233147	-0.719242481	0.4722326	0.569461929	no
IL33	0.027226024	0.719054199	0.472348523	0.569567748	no
C1orf52	-0.027212666	-0.718701157	0.47256593	0.569795919	no
ST6GALNAC5	-0.027206402	-0.718535594	0.472667904	0.569884887	no
FAM108C1	-0.027199134	-0.718343494	0.472786238	0.569993569	no
SCGB1A1	-0.027191063	-0.718130194	0.472917651	0.570118005	no
ANKIB1	0.027179778	0.717831917	0.473101451	0.570305577	no
ZNF703	0.027151389	0.717081602	0.473563974	0.570829096	no
TREX1	0.027142838	0.716855613	0.473703331	0.570935664	no
OR6Q1	0.027142499	0.716846646	0.473708861	0.570935664	no
SIRPA	-0.027135662	-0.716665938	0.473820313	0.571035949	no
OR2A12	0.027132928	0.716593685	0.473864879	0.571055619	no
DSC3	0.027126687	0.716428736	0.473966629	0.571137394	no

C2orf27B	-0.027125301	-0.716392093	0.473989234	0.571137394	no
PCDHGC5	-0.027119454	-0.716237559	0.474084573	0.571180162	no
OBSCN	-0.02711835	-0.716208394	0.474102567	0.571180162	no
PDK4	0.027117926	0.716197189	0.474109481	0.571180162	no
COPS7B	-0.027110117	-0.71599078	0.474236843	0.571299559	no
PRR4	0.027098653	0.715687792	0.474423834	0.571490769	no
GFRAL	-0.027090504	-0.715472427	0.474556772	0.571616849	no
PSMC1	0.027083092	0.71527651	0.474677723	0.571728477	no
TMEM14A	-0.027071932	-0.714981552	0.47485985	0.57191377	no
TAS2R16	-0.027052833	-0.714476776	0.475171623	0.572255174	no
RBM41	0.027049803	0.714396687	0.4752211	0.572280671	no
SHOX	0.027040883	0.714160953	0.475366747	0.57242197	no
MOV10L1	-0.027024863	-0.713737534	0.475628415	0.572702954	no
SNORD17	0.027019814	0.713604088	0.475710899	0.572768161	no
RHEB	-0.027004016	-0.713186565	0.475969026	0.573029666	no
SLCO4A1	-0.027003052	-0.713161093	0.475984776	0.573029666	no
C9orf79	0.026994099	0.712924462	0.476131106	0.573171701	no
OGT	-0.026983715	-0.712650017	0.476300851	0.573341903	no
C9orf93	-0.026932226	-0.711289184	0.47714302	0.574288264	no
THOC4	-0.026932176	-0.711287852	0.477143845	0.574288264	no
ZNF556	-0.026929137	-0.711207553	0.477193565	0.574313917	no
CNBP	-0.026923372	-0.711055187	0.477287914	0.57436795	no
GOLGA3	-0.026922923	-0.711043297	0.477295277	0.57436795	no
DNMT3L	0.026905441	0.710581263	0.477581449	0.574678117	no
TPCN2	-0.026894064	-0.710280592	0.477767727	0.574868053	no
C3orf30	-0.026863684	-0.709477649	0.478265378	0.575426858	no
BMX	0.026862238	0.709439439	0.478289066	0.575426858	no
CCDC38	-0.026840377	-0.708861672	0.478647343	0.575823633	no
LOC389634	0.02683503	0.708720357	0.478734995	0.575894813	no
LSR	-0.026831348	-0.708623045	0.478795359	0.575933161	no
KCTD21	-0.026819677	-0.708314585	0.47898673	0.57612908	no
SNORA59B	0.026814669	0.708182211	0.479068868	0.576168804	no
NRK	0.026814188	0.708169505	0.479076752	0.576168804	no
EXPH5	-0.026807574	-0.707994702	0.47918523	0.576264988	no
SLC26A5	0.026798418	0.707752713	0.479335424	0.576411325	no
HIST1H2AJ	0.026784909	0.707395675	0.479557073	0.576643565	no
FEN1	-0.026768594	-0.706964487	0.479824829	0.576931215	no
MAP9	-0.02676491	-0.706867127	0.479885297	0.576940169	no
OR52N1	0.026764663	0.706860606	0.479889348	0.576940169	no
MDH1	-0.02676048	-0.706750061	0.479958011	0.576988409	no
GOLGB1	-0.026756897	-0.706655363	0.480016836	0.577024816	no
LOC100216545	-0.026751916	-0.706523708	0.480098624	0.577088821	no
OSGEP	0.026741723	0.706254309	0.480266006	0.577255699	no
ITFG1	0.026738332	0.706164691	0.480321695	0.577288314	no
SNORA84	0.02673151	0.705984402	0.480433737	0.577388652	no
DKC1	0.026697343	0.705081397	0.48099513	0.578028978	no
PINX1	0.02668103	0.704650257	0.481263293	0.578316866	no
MBP	-0.026648897	-0.703801033	0.481791738	0.578917471	no
KAL1	-0.026641061	-0.703593933	0.481920658	0.579037967	no
MYO18B	-0.026637744	-0.703506248	0.481975248	0.579069144	no
CD24	-0.026618497	-0.702997583	0.482291991	0.579415263	no

CTDSPL	0.026615202	0.702910511	0.482346221	0.579445983	no
DNAJB14	0.026611533	0.70281354	0.482406621	0.57948411	no
ZNF434	-0.026608288	-0.702727768	0.482460049	0.579513859	no
ARL6IP4	0.026600091	0.70251114	0.482595002	0.579641523	no
SLC22A5	-0.026592877	-0.70232048	0.482713795	0.579749763	no
HIST1H4D	0.026587326	0.702173768	0.482805216	0.579825118	no
TEX12	0.026585524	0.70212614	0.482834897	0.579826321	no
BCL2L15	-0.026576509	-0.701887896	0.482983381	0.579970184	no
PPWD1	0.026558439	0.701410335	0.48328109	0.580293211	no
SNORA2A	0.02655553	0.701333434	0.48332904	0.58031632	no
AKR1C2	0.026545888	0.701078617	0.483487941	0.580472636	no
COQ5	0.026539887	0.700920006	0.483586864	0.580556927	no
APOA2	-0.026529911	-0.700656375	0.48375131	0.580713515	no
HIST1H3J	0.02652849	0.700618814	0.483774742	0.580713515	no
LOC646851	-0.026514004	-0.700235953	0.484013621	0.580965767	no
NIPAL4	0.026500767	0.699886126	0.484231945	0.581160653	no
ATP2A1	-0.026500674	-0.699883677	0.484233473	0.581160653	no
TACO1	0.026484913	0.699467128	0.484493509	0.581438224	no
CAPN11	0.026480024	0.699337925	0.48457418	0.581500521	no
HSFY2	-0.026473337	-0.699161182	0.484684547	0.581598443	no
SCARNA3	0.026462849	0.698884011	0.484857653	0.581771634	no
LTK	0.026458086	0.698758136	0.484936278	0.581831445	no
LELP1	-0.026452976	-0.698623089	0.485020641	0.581898132	no
C14orf184	0.026447478	0.698477769	0.48511143	0.58197252	no
GPC5	0.026436824	0.69819621	0.485287361	0.582149036	no
PCTP	0.026421033	0.697778878	0.485548192	0.582427372	no
GCG	0.02641723	0.697678379	0.485611015	0.582436163	no
TRIM54	-0.026416177	-0.697650538	0.48562842	0.582436163	no
RPS15A	0.026415358	0.697628903	0.485641945	0.582436163	no
C17orf75	-0.026407217	-0.697413733	0.485776469	0.582562943	no
C2orf71	-0.026402753	-0.697295761	0.485850234	0.58261517	no
NODAL	0.026401094	0.697251913	0.485877652	0.58261517	no
RRP1B	0.026392249	0.69701816	0.486023834	0.582755894	no
OR6C1	-0.026378083	-0.696643793	0.486258002	0.583002092	no
GSTCD	0.026332054	0.695427311	0.487019336	0.583880273	no
DEFB4A	0.026328874	0.695343281	0.48707195	0.583908727	no
LOC729799	0.026320717	0.695127683	0.487206957	0.584003476	no
OR4X1	-0.026320608	-0.69512481	0.487208756	0.584003476	no
FJX1	0.026308113	0.694794583	0.487415583	0.584216757	no
KRTAP26-1	-0.026296719	-0.694493481	0.48760421	0.584408199	no
SEC22C	0.026290149	0.694319849	0.487713	0.58447396	no
INPPL1	0.026289914	0.694313635	0.487716895	0.58447396	no
PHGR1	0.026286721	0.694229243	0.487769776	0.584502687	no
COX5B	0.026278742	0.694018385	0.487901917	0.584626382	no
IL17RB	0.026273443	0.69387834	0.487989691	0.584694673	no
KCNA5	-0.02627181	-0.693835173	0.488016748	0.584694673	no
OR2T5	-0.026267859	-0.69373076	0.488082197	0.584738437	no
PCDHB12	-0.026255089	-0.693393274	0.488293777	0.58492527	no
CCDC64B	-0.026254954	-0.693389713	0.488296009	0.58492527	no
C1orf146	-0.026243546	-0.693088213	0.48848507	0.585117077	no
KRTAP10-2	-0.026239144	-0.692971865	0.488558038	0.585169811	no

ETV4	0.026231981	0.69278257	0.488676769	0.58527601	no
TSG1	0.026230302	0.692738201	0.4887046	0.58527601	no
MLH3	0.026225225	0.69260403	0.488788767	0.585335682	no
RUVBL2	-0.026223804	-0.692566475	0.488812328	0.585335682	no
MBNL2	0.026214104	0.692310125	0.488973166	0.58548533	no
DTX3	-0.026212775	-0.692274983	0.488995216	0.58548533	no
ZBED4	-0.026209169	-0.692179698	0.489055008	0.585522245	no
RGS6	-0.026195639	-0.691822111	0.489279432	0.58575625	no
DLST	0.026181638	0.691452112	0.489511704	0.585999623	no
TRPV5	0.026178635	0.69137275	0.489561532	0.586024574	no
CNGA2	0.026165145	0.691016243	0.489785403	0.586254846	no
AK1	0.026163549	0.690974063	0.489811893	0.586254846	no
MBD3L1	0.026161529	0.690920678	0.489845422	0.586260271	no
LOC100190986	-0.026158149	-0.690831333	0.48990154	0.586292727	no
ORC6L	0.026156061	0.690776163	0.489936194	0.586299495	no
MAD2L1BP	-0.026151252	-0.690649066	0.490016031	0.586360329	no
CPAMD8	-0.026147787	-0.690557488	0.490073562	0.586394465	no
RNH1	0.026138885	0.690322229	0.490221371	0.586536612	no
EPB42	0.026126118	0.689984823	0.4904334	0.586755576	no
RNF43	0.026121928	0.689874094	0.490502993	0.586804113	no
SLC18A1	0.02611824	0.689776621	0.49056426	0.586817179	no
ASPA	-0.026117776	-0.689764365	0.490571964	0.586817179	no
SNORA12	0.026100691	0.689312857	0.490855816	0.587121983	no
CHRFAM7A	-0.026081916	-0.68881668	0.491167851	0.58746046	no
SPRYD5	-0.026065082	-0.688371778	0.491447732	0.58776044	no
MYL10	0.026059115	0.688214091	0.491546951	0.587844331	no
ACSS2	0.026052526	0.688039964	0.491656526	0.587940596	no
PCNX	-0.026040013	-0.687709261	0.49186467	0.588154714	no
PPAPDC2	-0.026036048	-0.687604478	0.49193063	0.588198798	no
C1orf55	-0.026003153	-0.686735162	0.492478039	0.588818509	no
PBXIP1	0.025999496	0.686638501	0.492538927	0.588856485	no
TMEM167A	-0.025981128	-0.686153084	0.492844757	0.589175191	no
CDHR5	-0.025979985	-0.686122888	0.492863785	0.589175191	no
HIST1H2AD	0.025970187	0.685863939	0.493026977	0.589335428	no
ARFGEF1	0.025957977	0.685541254	0.493230377	0.589543704	no
AGXT2	-0.025934127	-0.68491097	0.493627798	0.58998385	no
UBE2NL	-0.025929157	-0.684779633	0.493710633	0.590047974	no
METAP2	-0.025923307	-0.684625014	0.493808162	0.59012965	no
C22orf31	-0.025919676	-0.684529056	0.493868694	0.590167106	no
SMPD4	-0.025914196	-0.68438423	0.493960061	0.590235006	no
DPRX	-0.025911509	-0.684313241	0.49400485	0.590235006	no
TTTY23	-0.025911015	-0.684300172	0.494013096	0.590235006	no
AMT	-0.025900877	-0.684032265	0.494182145	0.590386673	no
LDLR	0.025898418	0.683967265	0.494223165	0.590386673	no
SIX2	0.025898149	0.683960173	0.49422764	0.590386673	no
LSM7	-0.025895494	-0.683889998	0.494271928	0.590404693	no
CDC16	0.025874988	0.68334807	0.494614013	0.590778408	no
PTRH2	0.025844355	0.682538538	0.495125256	0.591354113	no
C10orf119	0.025833625	0.682254962	0.495304409	0.59152106	no
OR51G1	-0.025832478	-0.682224666	0.495323551	0.59152106	no
MFSD2B	-0.025819808	-0.681889821	0.495535143	0.591738794	no

BASE	-0.025815965	-0.681788263	0.495599328	0.591780488	no
ABCG2	-0.025813679	-0.68172786	0.495637505	0.591791122	no
TEX9	0.025811248	0.681663611	0.495678115	0.59180466	no
PANK3	-0.025780664	-0.680855356	0.496189138	0.592379805	no
DSCR9	-0.025776358	-0.680741575	0.496261099	0.592410195	no
HIST1H3C	0.025774527	0.680693169	0.496291715	0.592410195	no
CXorf49B	-0.025773136	-0.680656414	0.496314963	0.592410195	no
C2orf55	-0.025772129	-0.680629798	0.496331798	0.592410195	no
ENPP3	-0.025761824	-0.680357465	0.496504072	0.592580834	no
LOC285370	-0.025741142	-0.679810908	0.496849912	0.592958593	no
EIF2AK1	-0.025737987	-0.67972753	0.496902682	0.592986567	no
PGK2	0.025733694	0.679614092	0.496974481	0.593037245	no
PSMC3IP	0.025728132	0.679467087	0.497067535	0.593080882	no
RUNDC2C	0.025726787	0.679431547	0.497090032	0.593080882	no
ITFG2	-0.025726248	-0.6794173	0.497099051	0.593080882	no
BTNL2	-0.025715446	-0.679131832	0.497279784	0.593261502	no
HSPB8	-0.025695712	-0.678610336	0.497610039	0.593620472	no
PSMB6	-0.02569088	-0.678482628	0.497690932	0.593681944	no
CNGA1	-0.025676348	-0.6780986	0.497934226	0.59393712	no
NPFF	-0.025673143	-0.678013907	0.497987891	0.593966089	no
KRT4	-0.025670009	-0.677931077	0.498040377	0.593993037	no
CTXN1	-0.025667913	-0.677875681	0.498075482	0.593993037	no
CAPN7	-0.025666531	-0.677839166	0.498098622	0.593993037	no
SLC46A1	0.025664517	0.677785948	0.498132348	0.593993228	no
FAM101A	-0.025663013	-0.677746197	0.498157541	0.593993228	no
PM20D1	0.025655384	0.677544581	0.498285327	0.594110559	no
CRNN	-0.025639619	-0.677127979	0.498549429	0.594390397	no
SHISA3	0.025589134	0.675793827	0.499395708	0.595364256	no
CCDC130	-0.025579585	-0.675541469	0.499555869	0.595520081	no
SFRS4	0.025572213	0.675346647	0.499679534	0.595629336	no
NAPSA	-0.025569857	-0.67528439	0.499719055	0.595629336	no
OR5AP2	-0.025568853	-0.675257855	0.4997359	0.595629336	no
HIST1H3B	0.025560203	0.675029269	0.499881026	0.595767188	no
LCE2C	-0.025555609	-0.674907849	0.499958123	0.595823951	no
NCRNA00095	-0.02555205	-0.674813798	0.500017846	0.595860003	no
RETNLB	-0.025533324	-0.674318937	0.500332148	0.596199409	no
OR6B3	-0.025527951	-0.674176956	0.500422344	0.596271744	no
STAB2	-0.025526159	-0.674129587	0.500452438	0.596272461	no
CD207	0.025514694	0.673826609	0.500644945	0.596466677	no
SLC28A3	0.025488779	0.673141783	0.501080218	0.596950083	no
C10orf90	0.02548461	0.673031592	0.501150274	0.596998364	no
SPZ1	-0.025480818	-0.672931402	0.501213976	0.597039072	no
SPAG11A	0.025449183	0.672095393	0.501745687	0.597637228	no
STK33	0.025423702	0.671422011	0.502174184	0.59811238	no
NPSR1	0.025406174	0.670958807	0.502469049	0.598428324	no
DEFB104A	-0.02538833	-0.670487264	0.502769317	0.598750666	no
BSPH1	-0.025381884	-0.670316934	0.502877803	0.598844588	no
COX6A1	-0.025374885	-0.670131956	0.502995632	0.598909919	no
HDAC10	0.025373867	0.670105051	0.503012771	0.598909919	no
POM121L2	0.025373346	0.6700913	0.503021532	0.598909919	no
UCP3	-0.025370158	-0.670007048	0.503075206	0.598938553	no

CSGALNACT1	0.02536753	0.669937588	0.503119459	0.598955969	no
CEP164	-0.02533535	-0.669087199	0.503661407	0.599565846	no
OR2AK2	-0.02532116	-0.668712222	0.503900476	0.599815121	no
RSBN1L	-0.025311683	-0.668461769	0.504060188	0.599969909	no
TDRG1	0.02530743	0.668349387	0.504131861	0.600019895	no
TBCE	0.025301025	0.668180125	0.504239821	0.600113062	no
SCN4A	0.025295368	0.66803064	0.504335176	0.600191218	no
NFKBIL2	-0.025287255	-0.66781624	0.504471958	0.600301717	no
C15orf23	0.025286339	0.667792021	0.504487411	0.600301717	no
FRMD1	-0.025266546	-0.667268991	0.504821177	0.600663522	no
ABCD3	0.025262712	0.667167671	0.504885847	0.600705117	no
CDH16	-0.025250263	-0.666838682	0.505095862	0.600919627	no
AP1M1	0.02523973	0.666560346	0.505273578	0.601095687	no
OR2T12	-0.025222918	-0.666116066	0.505557317	0.601397849	no
TEDDM1	-0.025217805	-0.665980951	0.505643625	0.601465129	no
HOXD1	-0.025206444	-0.665680722	0.50583543	0.601657885	no
MRPL23	0.025193246	0.665331958	0.506058291	0.601887554	no
ICT1	0.025188261	0.665200232	0.506142478	0.601952272	no
WDR64	0.025165982	0.664611494	0.506518834	0.602343766	no
PRKAR1A	0.025165248	0.664592083	0.506531245	0.602343766	no
ZNF329	-0.02516267	-0.664523952	0.506574808	0.602360141	no
RGS8	-0.025142749	-0.663997522	0.506911477	0.60272502	no
WDR26	-0.02513453	-0.663780345	0.507050403	0.602837567	no
HSPA1B	0.025133622	0.663756331	0.507065766	0.602837567	no
WNT6	0.025118677	0.663361411	0.507318448	0.603102509	no
C10orf71	0.025114535	0.663251942	0.507388501	0.603150324	no
THOC6	0.02510361	0.662963256	0.507573267	0.603334488	no
FAM135A	0.025095199	0.66274098	0.507715552	0.603468139	no
MYBL1	0.02508623	0.66250397	0.507867293	0.603613012	no
GAGE12D	0.025081434	0.662377231	0.507948445	0.603640503	no
PRR5-ARHGAP8	0.025081334	0.662374588	0.507950137	0.603640503	no
TSPY3	0.025075311	0.66221544	0.508052051	0.603723466	no
MMP26	-0.025073679	-0.662172312	0.50807967	0.603723466	no
CDY2B	-0.02504173	-0.661328044	0.508620507	0.604330596	no
TMEM85	0.025014308	0.660603383	0.509084965	0.604846907	no
HEATR7A	-0.025010854	-0.660512114	0.509143478	0.604880882	no
SH2D6	-0.025006681	-0.660401844	0.509214177	0.604929328	no
STC2	-0.024999425	-0.660210108	0.509337121	0.60503983	no
TRIM27	-0.024981945	-0.659748187	0.509633373	0.60535618	no
KIAA0408	0.024970242	0.659438916	0.509831775	0.60552572	no
C3P1	-0.024969992	-0.659432325	0.509836004	0.60552572	no
PSG4	0.024955692	0.659054436	0.51007848	0.60577812	no
ELOF1	-0.024945097	-0.658774457	0.510258171	0.605930521	no
PTPRB	0.024943236	0.658725281	0.510289735	0.605930521	no
MAGEA10	0.024942825	0.658714408	0.510296715	0.605930521	no
TSHB	0.024929516	0.658362725	0.510522481	0.606133732	no
C21orf84	0.024929202	0.65835442	0.510527813	0.606133732	no
SESN1	0.024914839	0.657974864	0.510771532	0.606387483	no
NCRNA00162	0.024908302	0.65780213	0.510882468	0.606449658	no
XAGE5	-0.024908218	-0.65779991	0.510883894	0.606449658	no
TMOD1	0.024905095	0.657717391	0.510936896	0.606473443	no

IMPAD1	-0.024903503	-0.65767531	0.510963925	0.606473443	no
BMI1	-0.024888283	-0.657273128	0.51122229	0.606744482	no
DNAJA3	-0.024883872	-0.657156568	0.511297182	0.606797747	no
PRO0628	0.024872156	0.656846967	0.511496135	0.606998231	no
OR5D16	-0.024868071	-0.656739028	0.511565507	0.607041458	no
MED6	-0.024866476	-0.656696858	0.51159261	0.607041458	no
GSTZ1	-0.024861112	-0.656555131	0.511683709	0.607113921	no
USMG5	-0.024857757	-0.656466454	0.511740712	0.607145925	no
FTMT	-0.024855946	-0.656418611	0.511771467	0.607146786	no
LOC339240	0.02485093	0.656286055	0.511856685	0.607172004	no
NT5C	-0.02484962	-0.656251434	0.511878943	0.607172004	no
PISRT1	-0.024849392	-0.656245408	0.511882818	0.607172004	no
PPP1R1B	-0.024844307	-0.65611104	0.511969211	0.607218531	no
LOH12CR2	-0.024843548	-0.656090979	0.51198211	0.607218531	no
DDX21	0.024830342	0.655742007	0.512206522	0.607449054	no
FUZ	-0.024778078	-0.654360946	0.513095142	0.608467217	no
KRT19	-0.024765748	-0.654035121	0.513304906	0.608635186	no
NCCRP1	0.024763956	0.653987752	0.513335405	0.608635186	no
FLJ39739	-0.02476363	-0.653979154	0.513340942	0.608635186	no
TFR2	0.024762675	0.653953907	0.513357198	0.608635186	no
SNORD15B	0.024750438	0.653630556	0.513565424	0.608846355	no
PLGLA	-0.024747733	-0.653559065	0.513611467	0.608865238	no
PAN3	-0.024735741	-0.653242175	0.513815585	0.609047017	no
CXXC5	0.024735185	0.653227475	0.513825055	0.609047017	no
UBQLN3	0.024719439	0.652811385	0.514093137	0.609329057	no
CMIP	-0.024706278	-0.652463621	0.514317253	0.609558956	no
EGFR	0.024671568	0.651546406	0.514908597	0.610224033	no
IMP3	-0.024669501	-0.651491801	0.514943813	0.610229998	no
OR8G5	-0.02465088	-0.650999726	0.515261218	0.61057035	no
OR5M8	-0.024629905	-0.650445476	0.515618851	0.610930396	no
THAP4	0.024629516	0.65043518	0.515625496	0.610930396	no
MACF1	-0.024621727	-0.650229364	0.515758333	0.611024398	no
S100A14	0.02462132	0.650218606	0.515765277	0.611024398	no
DGCR6L	-0.024613179	-0.650003495	0.515904134	0.61115309	no
FAHD1	-0.024584341	-0.649241445	0.516396203	0.611687182	no
GTF2A1L	0.02458321	0.649211573	0.516415497	0.611687182	no
SLC18A2	0.024574208	0.64897368	0.516569162	0.611833351	no
NLRP11	-0.024544501	-0.648188704	0.517076377	0.612364487	no
IL25	-0.024544396	-0.648185923	0.517078174	0.612364487	no
GLO1	0.024538802	0.6480381	0.51717372	0.612441766	no
ZFYVE19	0.024524921	0.647671293	0.517410846	0.612686686	no
FER1L6	0.024515413	0.647420052	0.517573295	0.612843155	no
SPNS1	-0.024503902	-0.647115888	0.517769999	0.613040164	no
CREB1	0.024466351	0.646123611	0.518411979	0.613764325	no
GMPR2	-0.024454487	-0.645810112	0.518614891	0.613949651	no
WDR35	-0.024453648	-0.645787936	0.518629246	0.613949651	no
AGT	-0.024429406	-0.645147367	0.519043988	0.614404646	no
HSD17B4	-0.024420995	-0.644925106	0.519187932	0.614539056	no
SYT8	-0.024415095	-0.644769207	0.519288911	0.614622597	no
BANF1	-0.024405753	-0.644522336	0.519448834	0.61477589	no
ATP2B4	0.024397402	0.644301691	0.519591791	0.614909086	no

SNORA49	0.024391711	0.644151296	0.519689243	0.614988418	no
HIST3H3	-0.024387443	-0.644038513	0.51976233	0.615038908	no
KRTAP3-2	-0.024381789	-0.643889122	0.519859148	0.615117473	no
LCE2B	-0.024375074	-0.643711685	0.519974155	0.615217548	no
PTH2	-0.02437266	-0.643647901	0.5200155	0.615230463	no
OTOL1	-0.024357643	-0.643251062	0.520272771	0.615497541	no
PCDHA10	-0.024355929	-0.643205782	0.520302131	0.615497541	no
FAM124A	-0.024352151	-0.643105942	0.52036687	0.61553811	no
MTNR1B	0.024339443	0.642770155	0.520584633	0.615759674	no
HIST1H4E	0.024333698	0.642618335	0.520683106	0.615840121	no
WNT11	-0.024295605	-0.641611766	0.521336227	0.616576533	no
HOXB9	0.024282831	0.641274229	0.521555335	0.616793435	no
RGS21	-0.024281356	-0.641235252	0.521580639	0.616793435	no
DAG1	0.024277206	0.641125585	0.52165184	0.616841555	no
PI4KB	-0.024264793	-0.640797603	0.521864812	0.6170573	no
LOC100133669	-0.02424429	-0.640255815	0.522216714	0.617437283	no
ZNF354B	-0.02423594	-0.640035164	0.522360066	0.617562807	no
PILRB	-0.024234547	-0.639998373	0.522383971	0.617562807	no
ZNF160	-0.024224197	-0.639724867	0.522561694	0.617720078	no
IQCC	0.024221465	0.639652694	0.522608597	0.617720078	no
RFC1	-0.024221363	-0.63964999	0.522610355	0.617720078	no
FLJ13224	0.024218318	0.639569537	0.522662641	0.617720078	no
ZFAND2B	-0.024217903	-0.639558571	0.522669768	0.617720078	no
OR5H6	-0.024212808	-0.639423945	0.522757269	0.617787378	no
AKIRIN2	-0.024203058	-0.63916631	0.52292474	0.617949172	no
PPYR1	-0.024197577	-0.639021459	0.523018909	0.618006211	no
INO80C	-0.024196029	-0.638980566	0.523045497	0.618006211	no
DYRK1B	-0.024194911	-0.638951016	0.523064709	0.618006211	no
RAB22A	-0.024185436	-0.638700652	0.523227503	0.618162429	no
SLC24A2	-0.024183343	-0.63864536	0.523263459	0.618168786	no
GPD1L	-0.024177073	-0.638479677	0.523371209	0.618259953	no
LRIT1	0.024158232	0.63798183	0.523695046	0.618599781	no
KIAA0754	-0.024156776	-0.63794336	0.523720074	0.618599781	no
ZNF555	-0.02414909	-0.637740267	0.523852213	0.618719713	no
RNPC3	-0.024144579	-0.637621057	0.523929784	0.61874757	no
OR10H4	-0.024143719	-0.637598328	0.523944574	0.61874757	no
STX17	0.024142379	0.637562929	0.52396761	0.61874757	no
OR6K2	0.024108195	0.636659653	0.524555589	0.619405729	no
ASB17	0.02407935	0.635897467	0.52505199	0.619955681	no
TTLL5	-0.024060224	-0.635392082	0.525381273	0.620308256	no
C11orf85	0.024057957	0.635332173	0.525420313	0.620318125	no
TRIM55	-0.024055763	-0.63527421	0.525458087	0.620326498	no
XIRP1	0.024049503	0.635108791	0.525565896	0.620417545	no
DAZAP2	-0.024045686	-0.635007936	0.525631633	0.620458919	no
NDUFS4	-0.024031551	-0.634634425	0.525875121	0.620710094	no
TATDN2	-0.024028031	-0.634541426	0.525935755	0.620745423	no
OR5V1	0.024024866	0.634457795	0.525990285	0.620773544	no
KIAA0776	-0.024012237	-0.634124078	0.526207905	0.620994129	no
OR10G3	-0.024009807	-0.634059879	0.526249775	0.621007293	no
ENTPD2	0.02400307	0.63388185	0.526365893	0.621108067	no
ZKSCAN5	-0.023972711	-0.633079661	0.526889276	0.621689372	no

PTTG2	0.023954782	0.632605926	0.527198487	0.622017917	no
ADCYAP1	-0.023948078	-0.632428777	0.527314138	0.622118062	no
GPR142	0.023943079	0.632296685	0.527400381	0.622176402	no
SOX9	-0.023941644	-0.632258776	0.527425134	0.622176402	no
COG6	0.023923063	0.631767786	0.527745776	0.622510961	no
GHDC	0.02392164	0.631730204	0.527770323	0.622510961	no
RPL39	0.023902246	0.631217743	0.5281051	0.622860147	no
SLC7A13	0.023900921	0.631182726	0.52812798	0.622860147	no
FLJ41941	0.023884632	0.630752307	0.528409251	0.623155521	no
TRIM42	-0.023875844	-0.630520102	0.528561025	0.623298151	no
VPS16	-0.023862768	-0.630174605	0.528786889	0.623528131	no
OR2A25	0.023844915	0.62970286	0.529095367	0.623855493	no
GALR2	0.0238379	0.629517502	0.529216599	0.623962048	no
GDPD4	0.0238133	0.628867481	0.529641853	0.624427021	no
OR2T1	-0.023802008	-0.628569114	0.529837107	0.624620795	no
ZNF823	0.023799746	0.628509346	0.529876224	0.624630488	no
IGF2	0.023796439	0.628421979	0.529933407	0.624636182	no
IL31RA	0.023795894	0.628407561	0.529942845	0.624636182	no
PAFAH1B2	-0.023773278	-0.62780998	0.530334058	0.625060859	no
IRX6	-0.023764044	-0.627565997	0.530493826	0.625212718	no
HOXA13	-0.023760022	-0.62745972	0.530563428	0.625258299	no
DMKN	0.023754728	0.627319845	0.53065504	0.625329812	no
GLG1	0.023752538	0.627261957	0.530692956	0.625338045	no
MALAT1	-0.023748889	-0.627165544	0.530756109	0.625376014	no
GNAT2	0.023741507	0.62697049	0.530883887	0.625490118	no
OGFOD2	0.023730138	0.626670081	0.531080712	0.625685557	no
PWP1	0.023718511	0.626362868	0.531282034	0.62588627	no
RDH13	0.023709876	0.626134703	0.531431579	0.626025967	no
ZNF880	0.023674477	0.625199351	0.532044857	0.626659805	no
PFKFB1	0.02367352	0.62517407	0.532061438	0.626659805	no
CNOT2	0.023673451	0.625172255	0.532062628	0.626659805	no
C2orf48	-0.023662502	-0.624882932	0.532252403	0.626846804	no
OR51F2	-0.02365085	-0.624575075	0.532454372	0.627048142	no
FRMD5	-0.023638115	-0.62423856	0.532675187	0.627271649	no
TNIP2	0.023584611	0.622824842	0.533603351	0.628299418	no
CAPRIN1	0.023584221	0.622814544	0.533610115	0.628299418	no
ZNF57	-0.023575221	-0.622576743	0.533766323	0.62842095	no
PNPO	0.023574693	0.622562779	0.533775496	0.62842095	no
INTS3	-0.023571056	-0.622466685	0.533838626	0.628458678	no
SIX1	-0.023560104	-0.622177297	0.534028764	0.628645913	no
ZNF181	0.023555756	0.622062423	0.53410425	0.628650451	no
BAG4	-0.023554751	-0.622035866	0.534121702	0.628650451	no
C11orf66	0.02355451	0.622029478	0.5341259	0.628650451	no
C3orf79	-0.023551096	-0.621939293	0.534185167	0.628683608	no
VGF	-0.023544328	-0.621760453	0.534302706	0.628785338	no
OR6C74	-0.023529755	-0.621375384	0.534555829	0.629046607	no
CRYBB2	-0.023495665	-0.620474638	0.535148166	0.629703485	no
PARM1	-0.023493552	-0.620418823	0.535184881	0.629703485	no
TMEM33	0.023491022	0.620351956	0.535228869	0.629703485	no
OR10H1	-0.023490461	-0.620337134	0.535238619	0.629703485	no
ST8SIA5	0.023473462	0.619887974	0.535534138	0.630014501	no

TMEM125	-0.02346734	-0.619726232	0.535640576	0.630103052	no
PMS2L3	-0.023464848	-0.619660375	0.535683916	0.630117374	no
NDUFAF4	-0.023455024	-0.619400808	0.535854758	0.630281662	no
NRAP	0.02344604	0.619163414	0.536011029	0.630416861	no
SLC36A2	0.02344483	0.619131463	0.536032063	0.630416861	no
HIST1H4C	0.02343931	0.618985587	0.536128103	0.630493136	no
ZC3H7A	-0.023430915	-0.61876377	0.536274158	0.630628216	no
RRH	-0.023427103	-0.618663066	0.536340473	0.630643117	no
EPB41	0.023426601	0.618649793	0.536349213	0.630643117	no
IFNA16	-0.023420865	-0.618498229	0.536449028	0.630723799	no
FAM50A	-0.023405848	-0.618101444	0.536710382	0.630994389	no
SLC27A4	0.02340345	0.618038092	0.536752117	0.631006762	no
CCDC58	-0.023399963	-0.617945945	0.536812823	0.631041436	no
SLC5A4	0.023392023	0.617736161	0.536951043	0.631135413	no
PTPN21	0.023391784	0.617729851	0.536955201	0.631135413	no
CLDN22	-0.023382927	-0.617495815	0.537109421	0.631279983	no
TMEM55A	0.023380132	0.617421977	0.537158082	0.631300476	no
NPY1R	0.023376748	0.617332564	0.53721701	0.631333033	no
KRTAP4-12	-0.023373432	-0.617244928	0.53727477	0.631364214	no
OR4F21	0.023368165	0.617105768	0.537366495	0.631435301	no
EPHA4	-0.023363265	-0.616976307	0.537451835	0.631498878	no
KIAA1530	-0.0233515	-0.616665448	0.537656778	0.631693689	no
STARD3	-0.02335016	-0.616630045	0.537680122	0.631693689	no
C8A	-0.023344735	-0.616486699	0.537774642	0.631768025	no
C1orf26	0.023332043	0.616151358	0.537995795	0.631991109	no
OR6N1	-0.023329596	-0.616086697	0.538038443	0.632004487	no
GLTPD2	-0.023326552	-0.61600626	0.538091499	0.632030089	no
MAGEB6	-0.023308042	-0.615517189	0.538414145	0.632372324	no
FLT1	-0.023263301	-0.614335035	0.539194428	0.633251986	no
FREM2	0.023251357	0.614019442	0.539402831	0.633435364	no
REG3G	0.023250761	0.614003698	0.539413229	0.633435364	no
EPOR	0.023236911	0.613637755	0.539654935	0.633682394	no
TTTY16	0.023233212	0.61354	0.539719511	0.633705713	no
RPS11	0.023230934	0.613479807	0.539759276	0.633705713	no
PCDHB6	-0.023228128	-0.613405668	0.539808257	0.633705713	no
C1orf9	0.023226997	0.613375786	0.539827999	0.633705713	no
MC4R	0.023226795	0.61337047	0.539831511	0.633705713	no
UTS2R	-0.023210097	-0.612929271	0.540123043	0.634011129	no
MAST2	-0.023206738	-0.612840512	0.540181702	0.634043173	no
HMGCS2	-0.02320359	-0.612757323	0.540236683	0.634070896	no
SPDYE4	0.023179641	0.612124539	0.540654991	0.634525025	no
OR13C3	-0.023176413	-0.612039261	0.540711378	0.634554367	no
TMEM161A	0.023161472	0.611644486	0.540972443	0.634823893	no
RPLP1	-0.023137021	-0.610998456	0.541399801	0.635288519	no
IGSF3	-0.02312864	-0.610776992	0.541546341	0.635423593	no
OR5M10	-0.023111588	-0.610326466	0.541844511	0.635736555	no
LOC283731	-0.023103214	-0.610105188	0.541990988	0.635871514	no
TOP1	0.023099492	0.610006868	0.542056078	0.635910978	no
USH1G	-0.023073531	-0.609320912	0.542510307	0.636406927	no
CDK18	0.023054537	0.608819067	0.542842741	0.636759953	no
CHST8	0.023049986	0.608698824	0.542922408	0.636816456	no

AMN	-0.023043446	-0.60852602	0.54303691	0.636913809	no
PNPLA1	0.02303762	0.608372081	0.543138921	0.636996503	no
RUSC1	0.023031138	0.608200807	0.543252431	0.637092671	no
PLUNC	-0.023027403	-0.608102127	0.543317836	0.637132417	no
HIST1H1E	0.023017721	0.607846312	0.543487406	0.637294304	no
ACAN	-0.023007404	-0.607573724	0.543668125	0.637469243	no
RHOXF2B	0.0229875	0.607047816	0.544016872	0.637841169	no
LINGO4	-0.022979924	-0.606847665	0.544149629	0.637959825	no
TBC1D3C	-0.022964064	-0.606428616	0.544427628	0.63824874	no
MAD1L1	-0.022938117	-0.605743036	0.544882597	0.638745077	no
NACC2	-0.022935252	-0.605667343	0.544932841	0.638756378	no
LOC90246	0.022933964	0.605633321	0.544955424	0.638756378	no
SNORA46	0.022921944	0.605315716	0.545166274	0.638966477	no
SNHG5	-0.022908104	-0.604950065	0.545409069	0.639213991	no
HEPACAM2	-0.022892798	-0.604545637	0.545677677	0.639477538	no
NCRNA00230B	0.022891685	0.604516248	0.545697198	0.639477538	no
RECQL4	-0.022871836	-0.603991806	0.546045618	0.639848749	no
FAM32A	0.02286753	0.603878037	0.546121217	0.639900248	no
KCTD20	-0.022855556	-0.603561657	0.546331476	0.640109515	no
TUSC5	-0.022853713	-0.603512954	0.546363847	0.640110347	no
SNORA74B	0.022849987	0.603414511	0.54642928	0.640149912	no
GUCY1A3	-0.022831934	-0.602937537	0.546746371	0.640484276	no
OCA2	-0.022822576	-0.60269028	0.546910782	0.640639756	no
KCNQ3	-0.022820407	-0.602632978	0.546948888	0.640639798	no
SPAG6	0.022818168	0.602573801	0.546988243	0.640639798	no
LYPD4	-0.022817164	-0.602547285	0.547005877	0.640639798	no
OR14A16	-0.022805214	-0.60223155	0.547215876	0.64084862	no
KRTAP11-1	0.022774953	0.601432003	0.547747843	0.641434457	no
RPL22L1	0.022770571	0.601316227	0.547824894	0.641487531	no
OR3A2	0.022768754	0.601268219	0.547856846	0.641487793	no
OR1E1	-0.022763976	-0.601141975	0.547940872	0.641549025	no
DAPK3	0.022757071	0.600959561	0.548062296	0.641654033	no
OR6C65	0.022753848	0.600874383	0.548118999	0.641683262	no
CDC42EP1	0.022745334	0.600649439	0.548268759	0.641821422	no
LOC729234	0.022733265	0.600330558	0.548481093	0.642002679	no
RPL23AP53	0.022732165	0.600301504	0.548500442	0.642002679	no
TLX3	-0.022731118	-0.600273849	0.548518858	0.642002679	no
ZNF2	-0.022728996	-0.600217769	0.548556206	0.642009226	no
APOC3	-0.022720012	-0.59998039	0.548714308	0.642157089	no
CST8	-0.022717226	-0.599906796	0.548763328	0.64215725	no
DAPL1	-0.022716394	-0.599884817	0.548777969	0.64215725	no
LOC29034	0.022710121	0.599719075	0.548888377	0.642249274	no
TNN	0.022686879	0.599104981	0.549297551	0.642690849	no
MATN1	-0.022680635	-0.598940004	0.549407501	0.642782296	no
KLHL2	0.02267691	0.598841592	0.549473094	0.642821839	no
LIG4	0.022653303	0.598217867	0.549888905	0.643271068	no
CHST11	0.022644797	0.597993149	0.550038752	0.643409135	no
CARS	0.02264274	0.597938779	0.550075011	0.643414323	no
EIF2S1	-0.022637989	-0.597813264	0.550158719	0.643475008	no
MDM2	0.022633243	0.597687868	0.550242354	0.6435356	no
GALT	-0.022596595	-0.596719586	0.550888378	0.64421959	no

PTPN20A	-0.022596451	-0.596715787	0.550890914	0.64421959	no
ENSA	0.022581318	0.59631595	0.551157789	0.644494401	no
CLRN1OS	-0.022572634	-0.596086508	0.551310961	0.644636228	no
PDIK1L	-0.022569245	-0.595996988	0.551370729	0.64466883	no
SIX6	0.022553151	0.595571765	0.551654673	0.644963521	no
C16orf78	-0.022542843	-0.595299423	0.551836567	0.645138876	no
C5orf52	0.022528409	0.594918063	0.552091323	0.645399386	no
FBXL2	-0.022525736	-0.594847442	0.552138506	0.645417225	no
BACE1	-0.022515487	-0.594576651	0.552319443	0.645591403	no
TRNT1	-0.022511925	-0.594482541	0.552382332	0.645627587	no
LOC151300	-0.022497607	-0.594104246	0.552635163	0.645885758	no
CEACAM6	-0.022487676	-0.593841856	0.552810564	0.646053409	no
PQLC1	-0.022484428	-0.593756042	0.552867934	0.646063991	no
SLC1A3	-0.022483545	-0.593732718	0.552883528	0.646063991	no
LOC442459	-0.022471609	-0.593417345	0.553094396	0.646273045	no
SIDT1	-0.022447449	-0.59277903	0.553521314	0.646734507	no
HOXC8	0.022436324	0.592485103	0.553717952	0.646926874	no
DDX27	-0.022404354	-0.591640418	0.554283241	0.6475499	no
EXOG	-0.022384154	-0.59110672	0.554640554	0.647929896	no
NEDD4	0.022379186	0.590975481	0.554728436	0.647995119	no
C14orf68	-0.022376208	-0.590896802	0.554781126	0.648019227	no
KCNA7	0.022372234	0.590791803	0.554851445	0.648063924	no
AZGP1	0.022330943	0.58970086	0.555582323	0.6488801	no
KRTAP4-9	-0.022323397	-0.589501492	0.55571594	0.648998664	no
EPHX1	-0.022317117	-0.589335578	0.555827149	0.649091047	no
FAM158A	-0.022314092	-0.58925565	0.555880726	0.649116122	no
CRY1	0.022310226	0.589153503	0.555949202	0.649158589	no
SIDT2	0.022306365	0.589051511	0.556017578	0.649200935	no
UGT1A10	0.022303488	0.588975502	0.556068537	0.649222942	no
FAM157A	0.022279871	0.588351529	0.556486959	0.649673942	no
NRN1	-0.022267316	-0.588019801	0.55670947	0.64987953	no
HERC3	-0.022266307	-0.587993156	0.556727345	0.64987953	no
SLC39A3	0.022247593	0.587498716	0.557059083	0.650229233	no
C3orf65	0.022244026	0.587404468	0.557122329	0.650265514	no
FBXL21	-0.022240896	-0.587321783	0.557177818	0.650292739	no
RMI1	-0.022226116	-0.586931283	0.557439915	0.650561083	no
WDR46	0.022210627	0.586522075	0.557714633	0.650806035	no
S1PR2	0.02220954	0.586493347	0.557733921	0.650806035	no
ACLY	-0.022208838	-0.586474802	0.557746373	0.650806035	no
EEF1A1	-0.022205557	-0.586388116	0.557804579	0.650816	no
UBE2E3	0.022204728	0.586366204	0.557819293	0.650816	no
MDH2	0.022198596	0.586204194	0.557928085	0.650876975	no
DVWA	-0.022195949	-0.586134265	0.557975047	0.650876975	no
OR9G4	-0.022195675	-0.586127033	0.557979904	0.650876975	no
C20orf71	-0.022194524	-0.586096622	0.558000327	0.650876975	no
MAP7D1	-0.02218344	-0.585803783	0.558197011	0.651068834	no
POLR3K	0.022174754	0.585574278	0.558351181	0.651211086	no
DDX26B	0.022172905	0.585525447	0.558383986	0.651211781	no
CCNE2	0.022167539	0.585383677	0.558479233	0.651285293	no
NXF2B	-0.022158788	-0.585152471	0.558634584	0.651428886	no
AMIGO3	0.02215694	0.585103629	0.558667404	0.651429585	no

KRTAP1-1	-0.022144941	-0.584786633	0.558880439	0.651640411	no
NSUN5P1	-0.02212985	-0.584387911	0.559148453	0.651915312	no
TAS2R39	-0.022117767	-0.584068696	0.559363069	0.652127928	no
CCDC163P	0.022114366	0.583978836	0.559423491	0.652137337	no
AHSA2	-0.022113682	-0.583960755	0.55943565	0.652137337	no
SLC30A4	-0.022099346	-0.583581996	0.559690367	0.652396647	no
OR52E4	-0.022095314	-0.583475485	0.559762006	0.652442536	no
LOC402644	-0.022086365	-0.583239046	0.55992105	0.652590292	no
LPO	-0.022082416	-0.583134696	0.55999125	0.652634488	no
LOC100170939	0.022078328	0.583026709	0.560063901	0.652681535	no
MEG8	0.022064793	0.582669114	0.560304515	0.652910581	no
VPS13A	-0.022063639	-0.582638626	0.560325031	0.652910581	no
GUCA1A	0.022052954	0.582356329	0.560515018	0.65309432	no
SCARNA16	0.022034318	0.581863952	0.560846465	0.653442853	no
GPATCH2	-0.022026511	-0.581657683	0.560985344	0.653558975	no
MUC21	0.022025081	0.581619904	0.561010783	0.653558975	no
OR2A14	-0.022018553	-0.581447442	0.561126916	0.653656602	no
RAPSN	0.022016549	0.581394511	0.561162561	0.653660463	no
SLC7A2	0.022008952	0.581193775	0.561297752	0.653780271	no
POP7	-0.021988031	-0.580641048	0.561670082	0.654176261	no
TBL1XR1	-0.021985898	-0.580584694	0.561708049	0.654182796	no
AMZ2P1	-0.021980469	-0.580441279	0.56180468	0.654257648	no
FGFR1OP2	0.021973801	0.580265092	0.561923402	0.654358216	no
MASTL	0.021971015	0.580191491	0.561973002	0.654378285	no
CSH1	-0.021957188	-0.579826178	0.562219216	0.654627281	no
FAM65B	0.021951145	0.579666539	0.562326825	0.654714872	no
SNORA42	0.021940479	0.579384736	0.562516808	0.654898354	no
ZNF547	-0.021937444	-0.579304547	0.562570875	0.654923587	no
FOXF1	0.021923388	0.578933194	0.562821288	0.655177384	no
LGALS7	0.021913342	0.578667774	0.563000301	0.655348039	no
RPLP2	-0.021908704	-0.578545251	0.563082946	0.655406506	no
KIAA0319L	0.021898453	0.578274423	0.563265648	0.655581421	no
PPAP2C	0.021894404	0.57816745	0.56333782	0.655590253	no
SCARNA10	0.021894389	0.578167053	0.563338088	0.655590253	no
RBBP9	0.021887696	0.577990233	0.563457395	0.655674622	no
CCNF	0.021886334	0.577954246	0.563481677	0.655674622	no
HIGD2A	0.021884865	0.577915422	0.563507875	0.655674622	no
RASA3	-0.021876371	-0.577691017	0.563659313	0.655780647	no
CFHR1	0.021876116	0.577684268	0.563663868	0.655780647	no
ZNF226	0.021859248	0.577238645	0.563964652	0.656092832	no
TCEB2	-0.021831847	-0.576514713	0.564453453	0.6566237	no
IQCF2	-0.021823467	-0.57629331	0.564602986	0.656759863	no
LOC149134	-0.021819036	-0.576176254	0.564682052	0.656814045	no
OR7A10	-0.021807335	-0.575867122	0.564890881	0.657019147	no
KGFLP1	-0.021794628	-0.575531408	0.56511771	0.65724516	no
RAGE	-0.021785316	-0.575285373	0.565283975	0.657400713	no
APH1B	-0.021767681	-0.574819475	0.565598882	0.657729103	no
C15orf55	0.021762475	0.574681937	0.565691861	0.657799394	no
OR51L1	-0.021717705	-0.573499119	0.566491786	0.658691679	no
DGCR11	-0.021711437	-0.57333353	0.566603815	0.658784055	no
C13orf39	-0.021686864	-0.572684337	0.567043129	0.659256928	no

TMEM8C	-0.021677566	-0.572438675	0.567209413	0.659412334	no
ZCCHC13	0.021667948	0.572184574	0.567381433	0.659572108	no
YBX1	0.021666234	0.572139291	0.567412091	0.659572108	no
OR6C68	0.021659232	0.571954307	0.567537341	0.659679772	no
ALPL	-0.021653813	-0.571811134	0.567634289	0.659754531	no
COMMD2	0.021651283	0.571744304	0.567679546	0.659769203	no
SPO11	-0.021649269	-0.571691084	0.567715587	0.659773164	no
EFNA5	-0.021627492	-0.571115768	0.568105265	0.660188082	no
CCDC50	-0.021625223	-0.57105581	0.568145884	0.660197338	no
MOSPD3	0.021621681	0.570962242	0.568209275	0.660233053	no
LOC285735	-0.021616575	-0.570827349	0.568300669	0.660301038	no
PIBF1	0.021614763	0.57077948	0.568333102	0.660301038	no
ZNF654	-0.021600104	-0.57039219	0.568595548	0.660543712	no
DEPDC7	0.021597657	0.570327549	0.568639358	0.660543712	no
MAPRE1	0.021597622	0.570326616	0.56863999	0.660543712	no
MDM4	0.021595428	0.57026864	0.568679284	0.660551405	no
DPYSL2	-0.021546178	-0.568967517	0.569561472	0.66147769	no
KCNS3	0.021545525	0.568950242	0.569573189	0.66147769	no
OR13A1	-0.02154543	-0.568947735	0.569574889	0.66147769	no
OR1M1	0.021535917	0.56869643	0.569745357	0.661604991	no
FAM55C	-0.021535661	-0.568689658	0.569749951	0.661604991	no
NCRNA00085	-0.021529808	-0.568535014	0.569854863	0.661688813	no
NKD2	-0.021524669	-0.568399256	0.56994697	0.661738748	no
HIST1H2BI	0.021523756	0.568375147	0.569963328	0.661738748	no
OR5J2	-0.021515509	-0.568157264	0.570111171	0.661872389	no
MAL	-0.021482711	-0.56729076	0.570699313	0.662449958	no
SNX21	0.021482648	0.567289088	0.570700448	0.662449958	no
LIME1	0.021482284	0.567279492	0.570706962	0.662449958	no
KRTAP13-3	-0.021474482	-0.567073363	0.570846917	0.66257437	no
TJP3	-0.021464417	-0.56680745	0.571027486	0.662724253	no
AHDC1	-0.02146363	-0.566786656	0.571041607	0.662724253	no
OR52A5	0.021444324	0.566276619	0.571388031	0.66305687	no
USP17L6P	-0.021442718	-0.566234185	0.571416856	0.66305687	no
SLC7A5P2	-0.021442091	-0.566217635	0.571428099	0.66305687	no
POLR2I	0.021440348	0.566171574	0.57145939	0.66305687	no
SAE1	0.0214315	0.565937809	0.571618209	0.663203085	no
TIRAP	0.021427997	0.565845283	0.571681077	0.663237964	no
FAM151A	-0.021409357	-0.565352814	0.572015745	0.663588153	no
PKMYT1	-0.02139535	-0.56498277	0.572267277	0.663841862	no
SKP1	-0.021386873	-0.564758818	0.572419531	0.663980383	no
SH3TC2	-0.0213797	-0.56456932	0.572548376	0.664068563	no
OR5W2	-0.021378984	-0.564550399	0.572561242	0.664068563	no
SMR3A	0.021347116	0.563708495	0.573133851	0.664671038	no
OR5B2	-0.021346415	-0.563689979	0.573146447	0.664671038	no
LOC400794	-0.021340004	-0.563520601	0.573261682	0.664766543	no
FOXF2	0.021337542	0.563455569	0.573305928	0.664779723	no
PKP3	0.021333745	0.563355248	0.573374188	0.664820745	no
MYO3B	-0.021331615	-0.563298982	0.573412474	0.664827009	no
SEMA7A	0.021326535	0.563164776	0.573503798	0.664894764	no
FOXR2	-0.021315899	-0.562883784	0.57369503	0.665048475	no
SOSTDC1	-0.021315502	-0.562873295	0.573702169	0.665048475	no

SLC13A5	-0.021313091	-0.562809595	0.573745525	0.665060602	no
HEPHL1	0.02131066	0.562745365	0.573789244	0.665073149	no
FBXO25	-0.02130631	-0.562630445	0.573867469	0.665114104	no
C1orf49	-0.021305036	-0.562596799	0.573890372	0.665114104	no
C1orf157	-0.021281652	-0.56197902	0.574310982	0.66556342	no
RIN2	-0.021277523	-0.561869919	0.574385278	0.665583233	no
FLG2	-0.021273238	-0.561756735	0.574462359	0.665583233	no
AKR1D1	0.021273071	0.561752323	0.574465363	0.665583233	no
CBLN4	-0.021272623	-0.561740487	0.574473424	0.665583233	no
PRM2	-0.021271553	-0.561712214	0.57449268	0.665583233	no
RBMY2FP	-0.021269114	-0.561647765	0.574536574	0.665595947	no
KRTAP12-2	-0.02126383	-0.561508184	0.574631645	0.665667942	no
BOK	-0.021259662	-0.561398076	0.574706646	0.665716682	no
USP8	0.021256032	0.561302175	0.574771974	0.665754212	no
PRAMEF22	-0.021253964	-0.561247537	0.574809195	0.665759183	no
RAD50	-0.021215512	-0.560231677	0.575501438	0.66649749	no
TTTY5	-0.021214893	-0.560215343	0.575512572	0.66649749	no
PPP4R1	-0.021194091	-0.559665768	0.575887239	0.666893189	no
RFX6	0.021179504	0.559280396	0.576150031	0.667133851	no
HIST1H2BM	0.021178892	0.559264236	0.576161053	0.667133851	no
CYP4F2	0.021174806	0.559156288	0.576234675	0.667180888	no
SPATA7	-0.021170103	-0.55903205	0.576319414	0.667240789	no
ZNF879	0.021168008	0.558976697	0.57635717	0.667246293	no
C2orf61	-0.021155119	-0.558636183	0.576589459	0.667476993	no
MAP4	-0.021138436	-0.558195452	0.57689018	0.66778688	no
AFM	0.021124737	0.557833536	0.577137178	0.668034549	no
CYP4F22	0.021109885	0.557441174	0.577405012	0.668306305	no
YTHDF1	0.02109756	0.557115571	0.57762732	0.668525339	no
PRKCSH	-0.021091494	-0.556955302	0.577736759	0.668613726	no
NEB	-0.021069078	-0.556363109	0.578141222	0.669043514	no
FAM24A	0.021067202	0.556313571	0.578175062	0.66904438	no
XK	-0.021049414	-0.555843632	0.57849613	0.66934838	no
VDAC3	-0.021048979	-0.555832136	0.578503986	0.66934838	no
SCARNA18	0.02104361	0.55569031	0.5786009	0.669422203	no
TPRXL	-0.021033331	-0.555418748	0.578786489	0.669592146	no
RNASE8	0.021031647	0.555374263	0.578816893	0.669592146	no
BDH1	0.021029972	0.555330005	0.578847143	0.669592146	no
FAM128B	-0.021020297	-0.555074413	0.579021851	0.669755924	no
C4orf17	0.021017269	0.554994419	0.579076536	0.669780859	no
FAM194B	0.0210143	0.554915966	0.57913017	0.669804575	no
UBE2E1	0.021010044	0.554803543	0.579207031	0.669855151	no
DEFB112	-0.020994269	-0.554386791	0.579491996	0.670146381	no
ZNF222	-0.020987947	-0.554219767	0.579606221	0.670240138	no
CTDSPL2	-0.02098396	-0.554114448	0.579678253	0.670285097	no
TRIO	-0.020980525	-0.554023696	0.579740325	0.670318535	no
ZNF415	-0.020973758	-0.553844944	0.579862597	0.670395049	no
SNRPF	-0.020971782	-0.553792735	0.579898311	0.670395049	no
SCARNA1	0.020971358	0.553781531	0.579905975	0.670395049	no
BDH2	0.020962778	0.553554851	0.580061054	0.670535985	no
DLX6	-0.020957421	-0.553413344	0.580157872	0.670609563	no
OR4A47	-0.020950961	-0.553242671	0.580274657	0.670706209	no

ZNF266	-0.020945722	-0.553104266	0.580369369	0.670777334	no
FEV	-0.020942217	-0.553011677	0.580432734	0.670812222	no
FAM195A	-0.020934652	-0.552811825	0.580569515	0.67093195	no
ST7OT4	0.020925477	0.552569446	0.580735423	0.671085322	no
IYD	-0.020913322	-0.55224833	0.580955261	0.671300992	no
FIG4	0.020906964	0.552080368	0.581070264	0.671376407	no
OR5K4	-0.020906042	-0.552056014	0.58108694	0.671376407	no
CSRNP1	-0.020896531	-0.551804739	0.581259009	0.671511638	no
C6orf59	-0.020895901	-0.55178809	0.581270411	0.671511638	no
GPX6	-0.020887801	-0.551574106	0.581416965	0.671642566	no
OR5D13	0.020880791	0.551388907	0.581543818	0.671750724	no
FER	0.020869658	0.551094796	0.581745298	0.671945066	no
CCDC129	0.020861764	0.550886276	0.581888163	0.672071687	no
LOC100287227	0.020851002	0.550601963	0.582082985	0.672236064	no
VEGFB	0.020850229	0.550581537	0.582096982	0.672236064	no
ZBTB40	-0.020847353	-0.550505543	0.582149061	0.672257809	no
LENG8	-0.02084465	-0.550434141	0.582197996	0.67227592	no
OR7G1	-0.02083946	-0.550297045	0.582291959	0.672346021	no
TTTY13	-0.020836303	-0.550213626	0.582349135	0.67237364	no
HS3ST5	-0.020832458	-0.550112059	0.582418755	0.672396963	no
PRM3	0.020830727	0.55006634	0.582450095	0.672396963	no
FBXO7	-0.020829678	-0.550038604	0.582469107	0.672396963	no
TRIML1	-0.020798766	-0.549221988	0.583029024	0.673004899	no
FIP1L1	-0.020789315	-0.548972311	0.583200267	0.673164135	no
ZNF565	0.02078293	0.548803631	0.58331597	0.67325925	no
FASTK	-0.020780357	-0.548735648	0.583362605	0.673274641	no
KIAA1430	0.020777564	0.548661881	0.58341321	0.67329461	no
SCGB3A1	-0.020754038	-0.548040361	0.583839658	0.673748299	no
TCP10	0.020741458	0.547708039	0.584067736	0.673973032	no
TMEM66	0.020732887	0.547481608	0.584223163	0.674113909	no
ANKRD13A	0.020729307	0.547387017	0.584288098	0.674150361	no
NPAS1	-0.020725555	-0.547287907	0.584356139	0.674190391	no
PCDHGB6	-0.020715638	-0.547025916	0.584536019	0.674359443	no
DHX40P1	-0.02071086	-0.546899693	0.584622691	0.674420949	no
SLC6A5	-0.020701908	-0.546663218	0.584785085	0.674569798	no
TEX101	-0.020693622	-0.546444309	0.584935435	0.674702801	no
CCDC72	0.020691875	0.546398167	0.584967128	0.674702801	no
IRF6	0.020686825	0.546264763	0.585058763	0.674750445	no
ZDBF2	-0.020684135	-0.546193681	0.585107591	0.674750445	no
TEKT2	0.020684082	0.546192275	0.585108557	0.674750445	no
WNK1	-0.020681469	-0.546123255	0.585155971	0.674766636	no
C10orf113	-0.020675335	-0.545961207	0.585267299	0.674856522	no
SSX7	-0.020670161	-0.545824513	0.585361216	0.674876351	no
ZNF260	-0.020670038	-0.545821284	0.585363435	0.674876351	no
SNRPE	0.02066887	0.545790428	0.585384636	0.674876351	no
MOBP	-0.02066622	-0.545720409	0.585432747	0.674893333	no
CATSPERG	0.020663203	0.545640716	0.585487507	0.674917978	no
OR51A7	-0.020659826	-0.545551504	0.585548811	0.674950163	no
UGT2B4	0.020655343	0.545433076	0.585630196	0.675005491	no
KRBA1	-0.020652087	-0.545347039	0.585689325	0.67503516	no
OR2Y1	-0.020645441	-0.545171488	0.585809981	0.67511585	no

C6orf203	-0.020644553	-0.54514801	0.585826119	0.67511585	no
NHSL2	0.020639738	0.545020832	0.585913536	0.675149275	no
RFC3	0.020639277	0.54500865	0.58592191	0.675149275	no
KRTAP21-1	-0.020627377	-0.544694283	0.586138023	0.675359808	no
ROS1	0.020619007	0.544473154	0.58629006	0.675496492	no
C1orf152	0.020614242	0.544347274	0.586376618	0.675557722	no
RPS27	-0.020610771	-0.544255573	0.586439677	0.675591874	no
OR4D11	-0.020602039	-0.544024915	0.586598304	0.675736113	no
AMFR	0.020591635	0.543750042	0.586787366	0.675915392	no
LOC729375	0.020561735	0.542960166	0.58733081	0.676479286	no
TTTY22	-0.020560328	-0.54292299	0.587356394	0.676479286	no
ZNF662	-0.020559179	-0.542892639	0.587377281	0.676479286	no
RNF11	-0.020550013	-0.542650507	0.587543924	0.676605451	no
RRM1	0.020549472	0.542636217	0.587553759	0.676605451	no
SYNPO2	0.020543078	0.542467291	0.587670033	0.676668617	no
SALL4	0.020542774	0.542459278	0.587675549	0.676668617	no
KLHL7	-0.020536506	-0.542293676	0.587789546	0.676761335	no
RBMY1J	-0.020530298	-0.542129694	0.587902438	0.676852769	no
LOC100133991	0.02051989	0.541854722	0.588091762	0.677032186	no
WDYHV1	-0.020511783	-0.541640555	0.58823924	0.677132653	no
OR2T33	-0.02051141	-0.541630718	0.588246014	0.677132653	no
LRRC29	0.020485746	0.540952731	0.588713001	0.677631622	no
TBRG4	0.020482375	0.540863677	0.588774353	0.677637978	no
OR52E8	-0.020481759	-0.540847417	0.588785555	0.677637978	no
TMEM132A	0.020476282	0.540702717	0.58888525	0.677714138	no
C22orf42	0.020467383	0.540467633	0.589047234	0.677861972	no
OR4D5	-0.0204538	-0.54010881	0.58929452	0.678107945	no
LCLAT1	-0.020434576	-0.539600957	0.589644593	0.678472163	no
C7orf72	-0.020419781	-0.539210118	0.589914071	0.678743608	no
KLHDC10	-0.020403953	-0.538791991	0.590202427	0.679036742	no
KIAA1377	-0.020395074	-0.538557423	0.590364223	0.679184241	no
RNASE12	0.020363905	0.537734024	0.590932331	0.679799139	no
SAMD4B	0.020359477	0.537617052	0.591013057	0.679853322	no
DYRK2	0.020354906	0.537496309	0.591096391	0.679910498	no
OR8H2	-0.02033449	-0.536956958	0.591468703	0.680300047	no
GUCA1C	-0.020327445	-0.536770858	0.591597193	0.680409127	no
C1orf135	0.020317742	0.536514522	0.591774196	0.680573987	no
HEATR4	0.020288787	0.535749627	0.592302511	0.681141265	no
KRTAP5-3	-0.020287016	-0.535702837	0.592334836	0.681141265	no
CLPB	-0.020276304	-0.535419862	0.592530348	0.681327337	no
USF1	0.020269279	0.535234273	0.59265859	0.681436042	no
DUSP12	-0.020261561	-0.535030401	0.59279948	0.681559278	no
ZNF569	-0.020251022	-0.534751978	0.592991916	0.681741758	no
UGT1A3	-0.020241235	-0.534493429	0.593170641	0.681908456	no
LAPTM4B	-0.020236951	-0.53438026	0.593248879	0.681933977	no
OR56A1	-0.020232587	-0.534265001	0.593328566	0.681933977	no
ZCWPW2	-0.020231778	-0.534243615	0.593343352	0.681933977	no
SLC27A2	-0.020230998	-0.534223022	0.593357589	0.681933977	no
TAS2R46	-0.020230785	-0.534217387	0.593361486	0.681933977	no
SLC25A2	-0.020202922	-0.533481329	0.593870505	0.682480184	no
NR2C2AP	0.020189423	0.533124738	0.594117177	0.682724854	no

PXMP4	-0.020167041	-0.532533454	0.594526301	0.683156167	no
RBM20	0.02016242	0.532411385	0.59461078	0.683214409	no
L3MBTL2	0.020106263	0.5309279	0.595637873	0.684355659	no
SLC6A3	0.020102822	0.530836994	0.595700839	0.68438911	no
PAX9	-0.020096328	-0.530665463	0.595819657	0.684452229	no
DAOA	-0.020096119	-0.530659935	0.595823486	0.684452229	no
GCGR	0.020093292	0.530585243	0.595875228	0.684472778	no
BBOX1	-0.020076863	-0.530151252	0.596175911	0.684779262	no
LOC388946	-0.020063363	-0.529794618	0.59642305	0.685024213	no
CYP2U1	0.020060454	0.529717777	0.596476305	0.685046463	no
PSKH2	-0.020055971	-0.529599361	0.596558378	0.685101805	no
C12orf54	-0.020048698	-0.529407235	0.59669155	0.685215822	no
CFHR2	0.020045971	0.52933518	0.596741498	0.68523426	no
TMPRSS12	-0.020042775	-0.529250752	0.596800026	0.685262547	no
SLC23A1	0.020023737	0.528747829	0.59714872	0.685623989	no
SAMD5	-0.020019228	-0.528628733	0.597231307	0.685679873	no
OR4S1	-0.020016418	-0.528554502	0.597282785	0.685700037	no
KRTAP10-1	0.020014418	0.528501672	0.597319423	0.685703163	no
ZBTB41	-0.020010543	-0.528399294	0.597390426	0.685745735	no
SNORA18	0.020006874	0.528302369	0.59745765	0.685783966	no
BAIAP3	-0.020001666	-0.528164792	0.597553076	0.685854561	no
FDXACB1	0.019997672	0.528059276	0.597626268	0.68589963	no
C18orf25	0.019995802	0.52800988	0.597660534	0.685900021	no
MARS	0.019976428	0.527498107	0.598015598	0.686268552	no
C10orf118	-0.019964574	-0.527184965	0.5982329	0.686478958	no
LGI1	-0.019961997	-0.527116872	0.598280157	0.686494224	no
PDSS2	0.019934332	0.526386052	0.598787462	0.687037336	no
ATF4	0.019931035	0.526298979	0.598847917	0.68706771	no
POTEG	-0.01992376	-0.526106792	0.598981364	0.687181821	no
CDY1B	-0.019910494	-0.525756346	0.599224734	0.68742202	no
FTHL17	-0.019897812	-0.525421343	0.599457422	0.687649938	no
LOC100130015	-0.019892696	-0.525286176	0.599551318	0.687718629	no
ITPR1	-0.019888213	-0.525167765	0.59963358	0.687773968	no
DEFB108B	-0.019878237	-0.524904241	0.599816673	0.687944945	no
PANX3	0.019872629	0.524756078	0.599919626	0.688007912	no
CNDP1	0.019871203	0.524718408	0.599945803	0.688007912	no
PCNAP1	0.019869685	0.524678318	0.599973661	0.688007912	no
GSTA5	-0.019862027	-0.524476033	0.60011424	0.688130088	no
OR4P4	-0.019858714	-0.524388501	0.600175075	0.688160816	no
SUGT1	-0.019847978	-0.524104894	0.600372203	0.688347806	no
STIM1	0.019830729	0.52364923	0.600688984	0.68863276	no
TMPRSS11A	-0.019828087	-0.523579455	0.600737499	0.68863276	no
OR5AR1	-0.019827386	-0.523560938	0.600750375	0.68863276	no
MORF4L1	-0.019827027	-0.523551438	0.60075698	0.68863276	no
TRRAP	-0.01982454	-0.52348576	0.600802649	0.688646066	no
WDR62	0.019806477	0.523008601	0.601134483	0.688987357	no
EID1	-0.019802584	-0.522905753	0.601206019	0.689023802	no
ASB12	-0.019801037	-0.522864892	0.60123444	0.689023802	no
KRTAP10-11	-0.019798118	-0.522787772	0.601288084	0.689046222	no
KRT72	0.019773442	0.522135937	0.601741581	0.689499509	no
C6orf57	0.019772884	0.522121185	0.601751847	0.689499509	no

FCF1	0.01976907	0.522020442	0.60182195	0.689540757	no
RTN4IP1	0.019761677	0.521825138	0.601957867	0.6896574	no
POU3F2	-0.019756517	-0.521688832	0.602052733	0.689727003	no
LRRC66	0.019744742	0.521377788	0.602269238	0.689935943	no
ZNF548	0.019724077	0.520831888	0.602649303	0.690332216	no
IQCA1	0.019714832	0.520587669	0.602819368	0.690487903	no
C8orf83	-0.019712456	-0.52052491	0.602863074	0.690498846	no
E2F1	0.019708577	0.520422436	0.602934442	0.690541468	no
DLGAP4	-0.019704629	-0.520318165	0.603007065	0.690585524	no
FAM102A	-0.019681281	-0.519701396	0.603436715	0.691020057	no
BEYLA	-0.019680296	-0.51967537	0.603454848	0.691020057	no
ALDH9A1	-0.019669861	-0.519399709	0.603646925	0.691200857	no
ERH	-0.019663709	-0.519237199	0.603760173	0.691291379	no
DOCK10	0.01965585	0.519029593	0.60390486	0.691417886	no
FGFR3	-0.019649242	-0.518855034	0.604026528	0.691518026	no
LIN28A	-0.019631084	-0.518375375	0.604360907	0.691861663	no
NBEAL1	0.01961472	0.517943114	0.604662317	0.692167519	no
C12orf56	0.01956986	0.516758095	0.605488959	0.693074549	no
PHOX2A	0.019560048	0.516498893	0.60566984	0.693242348	no
C2orf86	0.019552895	0.516309945	0.605801711	0.693354033	no
RAX	-0.019543213	-0.516054193	0.605980225	0.693519087	no
ZNF426	0.019539661	0.515960368	0.606045721	0.693554785	no
MCM10	-0.019531388	-0.515741833	0.606198284	0.693665659	no
SOX7	0.019529427	0.515690015	0.606234461	0.693665659	no
OR5M11	-0.019528827	-0.515674156	0.606245534	0.693665659	no
UGT1A5	-0.019515462	-0.515321114	0.606492044	0.693908446	no
HPN	0.01950697	0.51509681	0.606648687	0.69404839	no
ABCC2	0.019494568	0.514769194	0.60687751	0.694270893	no
TFPT	-0.019482983	-0.514463156	0.607091297	0.694476171	no
FAM163A	-0.019475332	-0.514261058	0.607232494	0.694598391	no
PRDM16	-0.019473373	-0.514209304	0.607268654	0.694600455	no
HRNR	-0.019469965	-0.514119283	0.607331554	0.694633102	no
TMEM222	0.019455842	0.513746221	0.607592253	0.694891966	no
TEKT5	-0.019448832	-0.513561046	0.607721673	0.695000667	no
PAGE1	0.019441619	0.513370513	0.607854852	0.695113653	no
SERPINB12	0.01943713	0.513251915	0.607937755	0.695169138	no
NOTCH4	0.019427674	0.513002142	0.608112371	0.695329483	no
LENEP	-0.019415582	-0.51268272	0.608335712	0.695545519	no
SPAG11B	-0.019403033	-0.512351223	0.608567533	0.695771227	no
KRTAP3-1	-0.019390131	-0.512010416	0.608805906	0.6960044	no
ATP10B	0.01938404	0.511849518	0.608918459	0.696062377	no
AQP7P1	-0.019383209	-0.511827566	0.608933816	0.696062377	no
HTR2C	-0.019381798	-0.511790275	0.608959903	0.696062377	no
ARHGEF37	-0.019378283	-0.511697424	0.609024862	0.696097273	no
C14orf33	-0.019364836	-0.511342215	0.609273392	0.69634197	no
ST8SIA6	-0.019346541	-0.510858958	0.609611588	0.696689112	no
UGT1A6	-0.019327408	-0.510353555	0.609965371	0.697022224	no
FAM138F	-0.019327049	-0.510344064	0.609972015	0.697022224	no
ETFA	0.019321671	0.510202004	0.610071475	0.697096478	no
TNNC2	-0.019311005	-0.509920244	0.610268762	0.6972825	no
SLC5A7	-0.019302908	-0.509706362	0.610418541	0.697414221	no

ANXA8L1	0.019298445	0.509588474	0.610501103	0.697469136	no
GLYCAM1	-0.019282416	-0.509165064	0.610797677	0.69776853	no
KRT8	0.019276534	0.509009679	0.610906531	0.697853452	no
TAF4B	-0.01927303	-0.508917123	0.610971375	0.697888094	no
OR1I1	-0.019260247	-0.508579458	0.611207965	0.698118899	no
MRPS22	0.019250172	0.508313319	0.611394468	0.698292473	no
CYP1A2	0.019202649	0.507057975	0.612274521	0.699258107	no
UPK3B	-0.019199887	-0.506985015	0.612325686	0.699277041	no
LYSMD4	0.019189102	0.50670012	0.612525496	0.699465715	no
GUCY1B2	0.019186348	0.506627377	0.612576518	0.699484471	no
OTOP1	-0.019181899	-0.506509851	0.612658956	0.699539096	no
CCT6A	0.019171921	0.506246289	0.612843848	0.699710691	no
ZSCAN12P1	0.019168279	0.506150087	0.61291134	0.699748234	no
COL4A3BP	-0.019159372	-0.5059148	0.613076425	0.699897186	no
GOLGA8E	0.01911718	0.504800277	0.613858679	0.70071378	no
SLC45A2	0.019117052	0.504796916	0.613861038	0.70071378	no
C2orf14	0.019109074	0.504586177	0.614009001	0.700843108	no
TAS2R38	-0.019076922	-0.503736874	0.614605464	0.701484321	no
SLC2A8	0.019071689	0.503598628	0.614702578	0.701555559	no
NOX5	-0.019038355	-0.502718105	0.615321279	0.702209905	no
KLRC1	0.019037057	0.502683814	0.615345379	0.702209905	no
PARD6G	0.019025138	0.502368973	0.615566674	0.702422791	no
GOT2	-0.019017429	-0.502165346	0.615709817	0.70254648	no
ARL17A	-0.019013091	-0.502050772	0.615790365	0.702598736	no
CES8	-0.019010166	-0.501973491	0.615844698	0.702621077	no
RASGEF1A	-0.019008074	-0.501918227	0.615883553	0.702625758	no
C21orf45	0.019001619	0.501747721	0.61600344	0.702722877	no
TKT	0.018981482	0.501215796	0.616377513	0.703109939	no
LCE2D	-0.018971384	-0.500949078	0.616565119	0.703284264	no
THEM5	0.018967166	0.500837655	0.6166435	0.703289221	no
UPK1A	-0.01896652	-0.500820576	0.616655515	0.703289221	no
JMJD4	-0.018965535	-0.500794554	0.616673821	0.703289221	no
SETMAR	0.018957433	0.500580563	0.616824369	0.703385968	no
HYDIN	0.018957226	0.50057507	0.616828233	0.703385968	no
CAV3	0.018947276	0.500312261	0.617013149	0.703543416	no
PTEN	-0.018946052	-0.500279927	0.617035901	0.703543416	no
SPRR2C	-0.018942508	-0.500186295	0.617101789	0.703578862	no
PGC	-0.018934294	-0.499969328	0.617254477	0.703713264	no
SPINK4	-0.018915954	-0.499484882	0.617595461	0.704062308	no
KCNJ6	0.018911806	0.499375306	0.617672599	0.704110544	no
PSG2	0.018906292	0.499229672	0.617775127	0.704187716	no
DDX56	0.01889228	0.498859527	0.618035748	0.704445076	no
OR6K6	-0.018887709	-0.498738787	0.618120772	0.70450227	no
DEDD	0.018885136	0.498670841	0.618168621	0.70451709	no
GEMIN8	-0.018882155	-0.498592083	0.618224086	0.70452492	no
EPHA8	-0.018881021	-0.498562126	0.618245184	0.70452492	no
LOC283922	0.018874923	0.498401044	0.618358634	0.704614488	no
RABL2A	0.018861963	0.49805873	0.618599757	0.704826688	no
SMC6	0.018859033	0.497981325	0.618654286	0.704826688	no
GEN1	0.018858944	0.497978968	0.618655947	0.704826688	no
WDR49	0.01885742	0.497938717	0.618684303	0.704826688	no

NTRK1	0.018842149	0.497535342	0.618968507	0.705110732	no
CCL25	-0.01882691	-0.497132801	0.619252179	0.705394138	no
SLC20A2	0.018820075	0.496952267	0.619379421	0.705499331	no
STAG1	-0.018813602	-0.496781259	0.619499959	0.705596877	no
SCTR	0.018811492	0.496725535	0.619539239	0.705601867	no
METTL7A	0.018801386	0.496458576	0.619727436	0.705776449	no
TECRL	-0.018790384	-0.496167973	0.619932329	0.705970025	no
CTSE	-0.018772036	-0.495683314	0.62027411	0.706319457	no
CYP51A1	-0.018767827	-0.49557214	0.620352522	0.706368962	no
OR6B1	-0.018765752	-0.495517313	0.620391193	0.706373212	no
CLDN10	0.018757319	0.495294583	0.620548302	0.706512308	no
PFN3	-0.018732325	-0.494634369	0.621014106	0.707002827	no
DPF3	0.018728866	0.494543006	0.621078578	0.707036412	no
C10orf12	0.018711709	0.4940898	0.621398433	0.707360707	no
DCAF4	-0.018688417	-0.493474541	0.621832774	0.707815281	no
C1orf126	0.018675738	0.493139625	0.622069262	0.708044606	no
ZNF234	0.018666158	0.492886582	0.622247966	0.708208138	no
BTBD6	0.018642671	0.492266197	0.622686186	0.708649688	no
SMR3B	0.018640031	0.492196461	0.622735454	0.708649688	no
ZNHIT2	-0.01863973	-0.492188507	0.622741073	0.708649688	no
CRTC3	0.018628256	0.491885427	0.622955216	0.708814765	no
MUC16	0.018628201	0.491883957	0.622956254	0.708814765	no
PLAC1	-0.018614921	-0.491533181	0.623204138	0.709039994	no
ROBO2	-0.018611112	-0.491432577	0.623275239	0.709039994	no
ZNF644	-0.018610767	-0.491423457	0.623281685	0.709039994	no
TYRP1	-0.018610081	-0.491405353	0.623294481	0.709039994	no
CD320	0.018594585	0.490996027	0.623583811	0.709321153	no
CERK	-0.018593086	-0.490956426	0.623611806	0.709321153	no
DNAH10	-0.018558562	-0.4900445	0.62425662	0.71000762	no
OR52R1	-0.018557013	-0.49000358	0.624285561	0.71000762	no
TAF8	0.018545996	0.489712584	0.624491386	0.710179856	no
DPY19L3	0.018545147	0.489690151	0.624507254	0.710179856	no
SF3B5	0.018543126	0.489636753	0.624545027	0.710182866	no
OR5B17	-0.018537586	-0.489490415	0.624648549	0.710238355	no
OR10S1	-0.018536754	-0.489468457	0.624664083	0.710238355	no
MSH4	0.018521987	0.489078381	0.624940068	0.710512191	no
RPL38	-0.018510014	-0.488762119	0.625163867	0.710714505	no
DMRT2	0.018508285	0.488716449	0.625196187	0.710714505	no
CCDC112	-0.018506825	-0.488677895	0.625223473	0.710714505	no
SPRR3	-0.018502851	-0.488572919	0.625297768	0.710758997	no
TSGA14	0.018499711	0.488489974	0.625356475	0.710785766	no
OR5T2	-0.018489988	-0.488233169	0.625538249	0.710952405	no
APCS	-0.018471204	-0.487736995	0.625889521	0.711311655	no
DSCR10	0.018463532	0.487534338	0.626033019	0.711434747	no
SLC25A6	-0.018452132	-0.487233207	0.626246271	0.71163709	no
OR51S1	-0.018440775	-0.486933219	0.626458745	0.711809172	no
XRCC6BP1	0.018440274	0.486919986	0.626468118	0.711809172	no
DRG2	0.01842747	0.486581785	0.626707695	0.71204137	no
TP53TG3B	-0.018422733	-0.486456667	0.626796338	0.712059898	no
LOC285740	-0.018422186	-0.486442229	0.626806567	0.712059898	no
MXD3	0.018420953	0.486409636	0.62682966	0.712059898	no

C7orf50	0.018406599	0.486030503	0.627098302	0.712304808	no
OR1S1	-0.018405669	-0.486005926	0.627115718	0.712304808	no
OR5H14	0.018391268	0.485625531	0.627385308	0.712570988	no
ANKRD34B	0.018386105	0.485489164	0.627481965	0.712640736	no
DIAPH2	0.018380889	0.485351386	0.627579629	0.712678294	no
C14orf105	-0.018380573	-0.485343054	0.627585535	0.712678294	no
SCARNA4	0.018367165	0.484988877	0.627836624	0.712923384	no
MYH1	-0.018341189	-0.484302757	0.628323161	0.713402108	no
DEFB129	-0.018340026	-0.484272044	0.628344943	0.713402108	no
VENTXP7	-0.018339006	-0.484245078	0.628364069	0.713402108	no
SURF1	-0.018331477	-0.484046213	0.628505121	0.713522182	no
LOC144742	0.018282154	0.482743395	0.629429527	0.714531509	no
ANGPTL1	-0.018276276	-0.482588138	0.629539727	0.71458304	no
DDX59	0.018275962	0.482579853	0.629545608	0.71458304	no
C8B	0.018259129	0.482135213	0.629861258	0.714901191	no
SCN5A	-0.018251136	-0.481924093	0.630011156	0.715022024	no
VPS41	-0.018249681	-0.481885655	0.630038449	0.715022024	no
TMEM183A	0.018244386	0.481745801	0.630137757	0.715076835	no
TRIM64	-0.018243335	-0.481718024	0.630157482	0.715076835	no
GPR137	-0.018229115	-0.481342417	0.630424232	0.715339383	no
HOMER2	0.018225245	0.481240202	0.630496831	0.715374288	no
SERPINA13	-0.018223703	-0.481199475	0.63052576	0.715374288	no
FABP1	-0.018218895	-0.481072479	0.630615967	0.715436486	no
CRX	-0.018207795	-0.480779296	0.630824241	0.715632618	no
SLC22A20	0.018199401	0.480557566	0.630981775	0.715771168	no
POLB	0.018195558	0.480456055	0.631053902	0.715812825	no
LIPK	-0.018189722	-0.480301917	0.631163429	0.715896898	no
C20orf70	-0.01818184	-0.48009371	0.631311389	0.716024552	no
CXorf27	0.018159329	0.47949912	0.631734009	0.716429418	no
MRTO4	0.018159052	0.479491781	0.631739226	0.716429418	no
IL22	0.018155677	0.479402641	0.631802595	0.716452424	no
POU6F2	0.018154197	0.479363551	0.631830385	0.716452424	no
ZPBP	0.018151693	0.47929742	0.6318774	0.716465553	no
ASH2L	0.018145806	0.479141923	0.631987954	0.716550721	no
SLC25A16	-0.018132098	-0.478779832	0.632245423	0.716802442	no
HIGD1B	0.01812812	0.478674757	0.632320146	0.71682932	no
IREB2	0.018127061	0.478646783	0.63234004	0.71682932	no
LOC348840	-0.018114698	-0.478320247	0.63257228	0.717052386	no
WFDC12	0.018083148	0.477486887	0.63316515	0.717666938	no
LOC340094	-0.01808207	-0.477458399	0.633185421	0.717666938	no
C2CD4A	0.0180675	0.477073557	0.633459289	0.717937098	no
C19orf22	0.018064669	0.476998784	0.633512506	0.717957166	no
OS9	0.01804678	0.47652626	0.633848853	0.718298083	no
PRDM9	0.018040856	0.476369787	0.633960248	0.718384054	no
SNORA39	0.018037506	0.47628131	0.63402324	0.718415169	no
LYPD6B	0.018034245	0.476195176	0.634084566	0.718444394	no
SNORA19	-0.018032032	-0.476136711	0.634126194	0.718451297	no
TMPRSS9	-0.018013006	-0.475634186	0.634484045	0.718816453	no
IGF2BP1	0.018002555	0.475358116	0.634680673	0.718979859	no
C17orf98	-0.018001559	-0.475331821	0.634699403	0.718979859	no
MKI67	0.017990125	0.475029796	0.634914549	0.719183279	no

GREM1	0.017972432	0.474562481	0.6352475	0.719520109	no
POMZP3	-0.017957666	-0.474172438	0.635525453	0.719730248	no
LOC647288	0.017957124	0.47415813	0.63553565	0.719730248	no
CYP2C19	-0.017956902	-0.474152276	0.635539822	0.719730248	no
PLA2G4E	-0.017952898	-0.474046519	0.635615196	0.719775291	no
NIPAL1	0.017949532	0.4739576	0.635678573	0.719806742	no
OR56A5	-0.01794671	-0.473883052	0.635731708	0.719826595	no
OR10T2	0.017924519	0.473296925	0.636149549	0.720227565	no
KRTAP6-3	-0.017924119	-0.473286361	0.636157081	0.720227565	no
CRTC2	-0.017921127	-0.473207319	0.636213437	0.720251038	no
HCFC1R1	-0.01791527	-0.47305263	0.636323737	0.720335572	no
RD3	-0.017900709	-0.472668008	0.636598023	0.720605723	no
UBE2K	0.017886686	0.472297621	0.636862204	0.720864407	no
SORL1	-0.017867458	-0.471789736	0.637224532	0.721234149	no
PCDHGA9	-0.01783553	-0.470946427	0.637826346	0.721874892	no
PAXIP1	-0.017819478	-0.470522427	0.638129017	0.722142376	no
CCDC150	0.017819207	0.470515276	0.638134122	0.722142376	no
CELA3A	0.017815913	0.470428256	0.638196249	0.72217226	no
SLC12A1	0.017803738	0.470106677	0.638425858	0.72239165	no
HGC6.3	-0.01777675	-0.469393828	0.638934958	0.722852761	no
RPS6	-0.017775431	-0.469359002	0.638959835	0.722852761	no
OR5T1	-0.017774755	-0.46934115	0.638972587	0.722852761	no
BBS7	-0.017774554	-0.469335833	0.638976385	0.722852761	no
SLK	-0.017772119	-0.469271517	0.639022327	0.722864288	no
TBX3	-0.017751974	-0.468739432	0.639402464	0.723253833	no
OR8I2	-0.017740419	-0.468434226	0.639620554	0.723460048	no
UQCC	-0.01773732	-0.468352372	0.63967905	0.723485737	no
CNKSR3	-0.017731131	-0.4681889	0.639795879	0.723577395	no
C9orf163	-0.017711492	-0.46767017	0.640166661	0.723956234	no
CHCHD8	-0.017699153	-0.467344265	0.64039966	0.724179223	no
DNAJB3	0.017680998	0.466864728	0.64074256	0.724526458	no
CSE1L	0.017676721	0.466751765	0.640823347	0.724555618	no
SNORA21	0.017675839	0.466728449	0.640840022	0.724555618	no
KRT20	-0.017670994	-0.46660048	0.640931547	0.724618577	no
OPN1LW	-0.017666922	-0.466492942	0.641008464	0.724665014	no
MBD3L2	-0.017664648	-0.466432871	0.641051431	0.724673069	no
FFAR1	0.017658504	0.466270585	0.641167517	0.724763774	no
C11orf64	-0.017653032	-0.466126041	0.641270919	0.724838709	no
SPANXA2	0.017650294	0.46605373	0.64132265	0.724838709	no
SNORA36B	0.017649304	0.466027575	0.641341362	0.724838709	no
ASB11	0.017630867	0.465540615	0.641689785	0.725191954	no
TSPYL5	0.017625979	0.465411506	0.641782176	0.725246108	no
CRABP1	0.017624537	0.465373401	0.641809445	0.725246108	no
OSBPL1A	-0.017619703	-0.46524574	0.641900808	0.72530881	no
GLS	-0.017614439	-0.465106688	0.642000329	0.725375008	no
OR2F2	-0.017612809	-0.465063627	0.642031149	0.725375008	no
TMCO5A	-0.017598301	-0.464680439	0.642305439	0.725641207	no
OR13C8	0.01759655	0.464634194	0.642338544	0.725641207	no
SPDYE1	-0.017577942	-0.464142689	0.642690448	0.725998182	no
PAK2	0.017565562	0.463815696	0.642924609	0.726222121	no
GDF3	0.017554699	0.463528783	0.643130099	0.72641365	no

ZFP161	-0.017550351	-0.463413941	0.643212358	0.726435383	no
ATP5G1	-0.017549884	-0.463401597	0.6432212	0.726435383	no
FANCL	-0.017546844	-0.463321311	0.643278709	0.726459752	no
KRTAP4-5	-0.01753889	-0.463111228	0.643429204	0.726589123	no
CDKN2AIPNL	-0.017531164	-0.462907158	0.643575406	0.726713631	no
C1orf177	0.017513455	0.462439392	0.643910579	0.727051496	no
KRTAP19-6	-0.017510193	-0.462353246	0.643972315	0.727080598	no
TOMM40	-0.017503909	-0.462187254	0.644091276	0.727174303	no
MUC6	-0.017490041	-0.461820984	0.644353805	0.727417775	no
DBNDD2	-0.017488717	-0.461785993	0.644378888	0.727417775	no
SERHL	0.017486686	0.461732369	0.644417328	0.727420553	no
MTR	0.017484248	0.461667967	0.644463496	0.727432054	no
RELA	-0.017472125	-0.461347767	0.644693055	0.727650544	no
SNW1	0.017464514	0.461146733	0.644837199	0.727772608	no
SNAR-A3	0.017454401	0.460879625	0.64502874	0.727948148	no
ZMAT5	-0.017440513	-0.460512789	0.645291834	0.728204415	no
RHBDD3	-0.017436546	-0.460408019	0.645366982	0.728247583	no
PAK1IP1	0.017434691	0.460359024	0.645402126	0.728247583	no
CBWD3	-0.017420833	-0.459992997	0.645664702	0.728499663	no
HSPA1A	0.017419097	0.45994715	0.645697594	0.728499663	no
TIMM10	0.017409402	0.459691067	0.64588133	0.728666298	no
CFL2	0.017406228	0.459607237	0.645941482	0.728693498	no
ZNF17	0.017402153	0.459499597	0.646018721	0.728739971	no
CEMP1	-0.017382691	-0.458985557	0.646387636	0.729115444	no
C7orf27	-0.017369517	-0.458637594	0.64663741	0.729161434	no
CLDN18	0.017367818	0.458592729	0.646669617	0.729161434	no
EDA	0.01736777	0.458591444	0.64667054	0.729161434	no
DEFB106A	-0.017367225	-0.458577065	0.646680862	0.729161434	no
KRTAP19-7	-0.017367225	-0.458577065	0.646680862	0.729161434	no
OR5M3	-0.017367225	-0.458577065	0.646680862	0.729161434	no
OR8B4	-0.017367225	-0.458577065	0.646680862	0.729161434	no
LOC284023	-0.017364437	-0.458503413	0.646733737	0.729180387	no
NHLH1	0.017359567	0.458374794	0.646826078	0.729243833	no
KRT13	-0.017352406	-0.458185644	0.646961887	0.729298873	no
TTTY8	-0.017351353	-0.45815784	0.646981851	0.729298873	no
MYH16	-0.017351287	-0.458156081	0.646983114	0.729298873	no
NXPH4	0.017337818	0.457800331	0.647238575	0.729546162	no
DKFZp686A162	-0.01732062	-0.457346091	0.647564822	0.729873206	no
CCDC144NL	-0.017316141	-0.457227788	0.647649801	0.729928295	no
TRIM60	-0.017303863	-0.456903496	0.647882771	0.73015016	no
SNORA53	0.017300854	0.456824028	0.647939865	0.730173803	no
VN1R4	-0.017266683	-0.455921477	0.648588457	0.730863974	no
DLX1	-0.017256648	-0.455656429	0.648778977	0.731037918	no
MBTPS1	-0.017241911	-0.455267195	0.649058804	0.731312467	no
FAM136B	-0.017219988	-0.454688154	0.64947518	0.731728163	no
C3orf35	0.017216841	0.454605015	0.649534972	0.731728163	no
C16orf91	-0.01721677	-0.454603139	0.649536322	0.731728163	no
DCST2	-0.017209989	-0.454424042	0.649665134	0.731832498	no
CREB5	0.017207055	0.454346549	0.649720872	0.73185451	no
PDE6H	-0.017203552	-0.454254039	0.649787415	0.731888688	no
SNORD94	0.017181255	0.453665102	0.650211103	0.732325111	no

OR8B8	-0.01717808	-0.453581249	0.650271437	0.732352267	no
NUDT1	0.017175364	0.453509513	0.650323054	0.732369604	no
TMIGD1	-0.017156875	-0.453021176	0.65067448	0.732687137	no
PRAMEF14	0.01715529	0.452979316	0.650704607	0.732687137	no
FAM98C	-0.017153283	-0.452926314	0.650742755	0.732687137	no
OR1S2	0.017152904	0.452916288	0.650749971	0.732687137	no
MUC13	0.01713618	0.452474573	0.651067928	0.732996109	no
FAM134C	-0.017134656	-0.452434329	0.6510969	0.732996109	no
C3orf32	-0.017123042	-0.452127564	0.651317759	0.733203922	no
WDR91	0.017104329	0.451633301	0.651673672	0.733563738	no
C21orf129	-0.0170945	-0.451373711	0.651860632	0.733701794	no
NUDT9P1	-0.017094066	-0.451362238	0.651868896	0.733701794	no
UBE2S	0.017073196	0.450811005	0.652265975	0.734107854	no
PNOC	-0.017067877	-0.450670529	0.652367183	0.734180891	no
PDAP1	-0.017048237	-0.4501518	0.652740962	0.734523615	no
SNORA74A	0.017048058	0.450147063	0.652744375	0.734523615	no
DNAJC17	-0.017034452	-0.44978771	0.653003365	0.734748063	no
EME1	0.017033388	0.449759599	0.653023627	0.734748063	no
NR0B2	0.017031853	0.449719044	0.653052859	0.734748063	no
FGB	-0.017024759	-0.449531681	0.653187915	0.734834334	no
ABO	-0.017024007	-0.449511824	0.653202229	0.734834334	no
LOC653545	-0.017019695	-0.449397942	0.653284323	0.734885798	no
MUC2	-0.017017334	-0.449335568	0.65332929	0.734895492	no
NCRNA00185	-0.017013702	-0.449239652	0.653398438	0.734932385	no
OR6N2	-0.017006333	-0.449045022	0.653538762	0.735049327	no
PRDM2	-0.016999552	-0.448865917	0.653667904	0.735153679	no
KRTAP24-1	-0.016996396	-0.448782554	0.653728016	0.735180389	no
KRT26	-0.016970172	-0.448089912	0.654227555	0.735701247	no
GOLGA5	-0.016953331	-0.447645127	0.65454842	0.736007804	no
PRPF31	0.016950395	0.447567566	0.654604378	0.736007804	no
MUCL1	0.016950133	0.447560641	0.654609374	0.736007804	no
ESCO1	-0.016943905	-0.447396145	0.654728061	0.736100314	no
TPRX1	-0.016933696	-0.447126525	0.654922616	0.736278106	no
IRX4	0.01692879	0.446996944	0.655016128	0.736288222	no
NR4A1	0.016928543	0.446990415	0.65502084	0.736288222	no
NKX2-1	0.016925571	0.446911904	0.655077501	0.736288222	no
KRTAP12-1	-0.016923187	-0.446848949	0.655122936	0.736288222	no
UBE3B	-0.016922386	-0.446827793	0.655138205	0.736288222	no
C1orf93	-0.016921761	-0.446811284	0.65515012	0.736288222	no
UIMC1	-0.01690676	-0.446415089	0.655436093	0.736568669	no
PRICKLE1	-0.016889704	-0.445964587	0.655761325	0.736810949	no
LOC644936	-0.016888714	-0.445938443	0.655780201	0.736810949	no
STK17B	-0.016888614	-0.445935797	0.655782112	0.736810949	no
LOC728410	0.016886228	0.445872786	0.655827608	0.736810949	no
QRSL1	-0.016885898	-0.445864068	0.655833902	0.736810949	no
FHIT	-0.016883307	-0.445795638	0.655883312	0.736825516	no
RRP7A	0.016875707	0.445594896	0.656028267	0.736947411	no
MANBAL	-0.016867353	-0.445374249	0.656187609	0.737085454	no
THOC3	-0.016864782	-0.445306361	0.656236639	0.737099576	no
PIGS	-0.016850229	-0.444921971	0.656514276	0.737370459	no
MYO1H	-0.016829199	-0.444366539	0.656915538	0.737716226	no

TMPRSS2	0.016828396	0.444345338	0.656930857	0.737716226	no
TAF7	-0.016828358	-0.44434432	0.656931592	0.737716226	no
LOC121838	0.016782342	0.443128957	0.657809963	0.738661585	no
LOC283914	-0.016775626	-0.442951571	0.657938204	0.738764556	no
IL26	0.016772614	0.442872024	0.657995715	0.738784263	no
CYP2A7	-0.01677088	-0.442826214	0.658028836	0.738784263	no
DDTL	0.01675271	0.442346313	0.658375849	0.739132818	no
KATNAL1	0.016746541	0.44218337	0.658493689	0.739224065	no
NBPF16	-0.016731795	-0.441793915	0.658775376	0.739499225	no
HPVC1	-0.016729757	-0.441740078	0.658814319	0.739501882	no
ARSG	-0.016720985	-0.44150839	0.658981922	0.739648948	no
RPL31	-0.016711793	-0.441265631	0.659157552	0.739805007	no
TTC31	0.016708074	0.441167393	0.65922863	0.739830791	no
TTTY15	-0.016706762	-0.44113273	0.659253711	0.739830791	no
ARHGAP22	0.016700317	0.440962526	0.659376868	0.73992793	no
NUP107	0.016688981	0.440663103	0.659593549	0.740034615	no
RBMY1B	-0.016687561	-0.440625604	0.659620687	0.740034615	no
OR2G3	-0.016687169	-0.440615244	0.659628185	0.740034615	no
ARL16	-0.016686278	-0.440591723	0.659645207	0.740034615	no
HIST1H2BB	0.016685768	0.440578259	0.659654952	0.740034615	no
C1orf223	0.016681852	0.440474823	0.659729814	0.740077533	no
BLOC1S2	-0.016679105	-0.440402276	0.659782322	0.740095372	no
PASD1	0.01667074	0.440181326	0.659942251	0.7402337	no
FAM41C	0.0166566	0.439807862	0.66021261	0.74049587	no
ALKBH7	-0.016648344	-0.439589813	0.66037048	0.740605327	no
MED4	0.016647665	0.439571885	0.660383462	0.740605327	no
OR2T29	-0.016620333	-0.43884999	0.660906236	0.741150496	no
FBXO9	-0.016612444	-0.438641649	0.661057142	0.74126914	no
LOC440173	-0.016610969	-0.438602689	0.661085362	0.74126914	no
SPAG5	0.016607291	0.438505532	0.661155741	0.741306942	no
OPTN	0.016596191	0.438212361	0.661368127	0.741503953	no
KRTAP6-1	-0.016593316	-0.438136427	0.661423141	0.741524512	no
WDR52	-0.016576311	-0.437687307	0.661748566	0.741848212	no
PJA2	-0.016570965	-0.437546099	0.661850896	0.741919256	no
SLC6A11	-0.016569166	-0.437498584	0.661885331	0.741919256	no
ZNF256	-0.01656054	-0.437270773	0.662050437	0.742063185	no
RBMY1A1	-0.016553413	-0.437082541	0.662186871	0.742170782	no
OR6P1	-0.016551691	-0.437037045	0.662219849	0.742170782	no
GFRA3	0.01651701	0.436121083	0.662883927	0.742873856	no
OSTN	-0.016497681	-0.435610549	0.663254183	0.743247593	no
PIP4K2C	0.0164946	0.435529184	0.663313199	0.74327253	no
HNF1B	-0.016487945	-0.435353414	0.663440697	0.743374197	no
PEBP4	-0.016481042	-0.43517109	0.663572959	0.743481191	no
FAM55A	-0.016440992	-0.434113324	0.664340495	0.744299907	no
SFTA3	0.016416878	0.433476442	0.664802799	0.744776583	no
MAPK1IP1L	0.016404948	0.433161355	0.665031563	0.744991586	no
IQCF1	0.01638974	0.432759681	0.665323239	0.745277037	no
TMEM9	-0.016377248	-0.432429745	0.66556286	0.745504149	no
NPC1L1	-0.016371826	-0.432286539	0.665666876	0.745579353	no
C7orf26	-0.016357428	-0.431906273	0.665943109	0.745847429	no
C2orf53	0.016348902	0.431681082	0.666106715	0.74598934	no

TBX1	0.016345522	0.431591813	0.666171574	0.746020654	no
BPIL1	0.016342731	0.431518097	0.666225136	0.746039313	no
C21orf94	-0.01633163	-0.431224915	0.666438176	0.746236544	no
GATA1	-0.01632221	-0.430976109	0.666618991	0.746397672	no
OR51B2	0.01630624	0.430554335	0.666925553	0.74669957	no
LGALS7B	0.016298302	0.430344669	0.667077968	0.746828858	no
MAGEB4	-0.0162798	-0.429856009	0.667433247	0.747185237	no
OR13D1	-0.016276679	-0.429773594	0.667493174	0.747210951	no
CRISP2	-0.016262918	-0.429410139	0.66775748	0.747465438	no
ASPHD2	-0.016239228	-0.428784467	0.66821257	0.747832451	no
MZF1	-0.016238977	-0.428777836	0.668217394	0.747832451	no
HSPC159	-0.016238313	-0.42876029	0.668230158	0.747832451	no
LOC100192426	-0.016238149	-0.428755957	0.66823331	0.747832451	no
FLJ43950	-0.016229619	-0.428530669	0.668397208	0.74797447	no
TFDP2	0.016190295	0.42749208	0.669152989	0.748778787	no
ANKRD9	0.016179463	0.427205988	0.669361238	0.748970364	no
MYOG	0.016169431	0.426941044	0.669554114	0.74914472	no
EIF3IP1	0.016136659	0.426075488	0.670184385	0.749808417	no
ZNF735	-0.016134055	-0.426006727	0.670234464	0.749822952	no
PLCXD3	0.016128128	0.425850173	0.67034849	0.749909023	no
PAGE5	0.016123687	0.425732892	0.670433916	0.749929109	no
IQCF5	-0.016123338	-0.425723676	0.67044063	0.749929109	no
RP1	0.016120627	0.425652066	0.670492792	0.749945965	no
TEX13B	0.016113693	0.425468947	0.670626187	0.750053673	no
CCL3L3	0.016110922	0.425395768	0.670679498	0.750071805	no
GBX1	-0.016107107	-0.42529501	0.670752903	0.750083736	no
NR5A1	-0.016106512	-0.425279277	0.670764366	0.750083736	no
VCY	-0.016099664	-0.425098424	0.670896131	0.75018959	no
P2RX6P	0.016078415	0.424537225	0.671305073	0.750605351	no
TRIM68	-0.016042423	-0.423586626	0.67199799	0.75133857	no
GRK1	-0.016034469	-0.423376571	0.672151143	0.75141169	no
HYAL4	0.016033762	0.423357884	0.672164768	0.75141169	no
OR4K1	-0.016033237	-0.423344009	0.672174885	0.75141169	no
GUCY2F	-0.016025378	-0.423136447	0.672326236	0.751539328	no
FAM47A	-0.016010027	-0.422731034	0.672621893	0.751828253	no
EML2	-0.016007323	-0.42265962	0.672673979	0.751844906	no
LEMD2	-0.015999165	-0.422444134	0.672831153	0.751979008	no
SYNJ2BP	-0.015994157	-0.422311885	0.672927621	0.752045252	no
LOC440356	-0.015989329	-0.422184357	0.673020652	0.752081612	no
POU4F1	-0.015988607	-0.422165301	0.673034554	0.752081612	no
PARP3	0.015981174	0.421968985	0.673177774	0.752162799	no
FGA	-0.015980302	-0.421945951	0.67319458	0.752162799	no
OR51A2	-0.015979044	-0.421912732	0.673218816	0.752162799	no
ECEL1	0.015967078	0.42159669	0.673449412	0.752358688	no
ARFGEF2	-0.015966084	-0.421570436	0.673468569	0.752358688	no
ZNF395	0.015958632	0.421373648	0.673612171	0.752477533	no
ADK	-0.015946335	-0.421048852	0.673849209	0.752673847	no
BAGE	-0.015945652	-0.421030827	0.673862365	0.752673847	no
FOXI3	0.01593257	0.420685326	0.674114552	0.752813971	no
C16orf11	-0.015931976	-0.420669625	0.674126013	0.752813971	no
OR4K14	-0.01593156	-0.42065863	0.674134039	0.752813971	no

LPAR3	0.015931419	0.420654907	0.674136757	0.752813971	no
CYLC2	0.015923126	0.420435906	0.67429663	0.752950915	no
C9orf70	0.015917115	0.420277144	0.674412537	0.753038751	no
URB2	0.015912311	0.420150261	0.674505176	0.753052801	no
OR7G3	-0.015911555	-0.420130291	0.674519757	0.753052801	no
EFCAB7	-0.015910443	-0.420100925	0.674541198	0.753052801	no
HMGA2	0.015908737	0.420055856	0.674574106	0.753052801	no
SCUBE1	-0.015899741	-0.41981828	0.674747584	0.753204872	no
CLPTM1	-0.015896639	-0.419736341	0.674807419	0.753230078	no
OTOP2	-0.015883379	-0.419386149	0.675063169	0.75346979	no
ATP12A	0.015881641	0.419340228	0.675096708	0.75346979	no
FAM127C	-0.015874332	-0.419147212	0.67523769	0.753585538	no
TMEM91	0.015864374	0.418884218	0.675429802	0.753749418	no
CDO1	0.015862856	0.418844122	0.675459093	0.753749418	no
GC	-0.015857631	-0.418706133	0.675559902	0.753820304	no
PEG3AS	-0.015840512	-0.418253989	0.675890259	0.75414731	no
SLC5A1	-0.015836019	-0.418135321	0.675976973	0.754202441	no
ZNF628	-0.015824944	-0.417842823	0.676190729	0.754394149	no
SLC2A6	-0.01582325	-0.417798089	0.676223423	0.754394149	no
ZNF114	0.015805046	0.417317318	0.676574831	0.754744534	no
CCNT1	0.015786673	0.416832065	0.676929588	0.755098616	no
ZNF107	0.015777969	0.416602193	0.677097666	0.755244434	no
TRIM77	-0.015775111	-0.416526712	0.67715286	0.755264331	no
ATPIF1	-0.015769997	-0.416391654	0.677251623	0.755332818	no
ZNF252	-0.015763991	-0.416233033	0.677367624	0.755420521	no
FTHL3	-0.01576158	-0.416169347	0.6774142	0.755430794	no
AASDH	-0.015758372	-0.416084616	0.677476169	0.755439274	no
ATXN3L	-0.015757317	-0.416056773	0.677496533	0.755439274	no
CT45A2	-0.015751384	-0.415900067	0.677611149	0.755525408	no
SLC45A3	0.015744548	0.41571953	0.677743205	0.755630976	no
MSX2	-0.015733757	-0.415434538	0.677951686	0.755792235	no
CNTD2	0.015732686	0.415406244	0.677972385	0.755792235	no
FGFR2	-0.015731257	-0.415368512	0.677999989	0.755792235	no
CSN1S2A	-0.015728931	-0.415307068	0.678044941	0.755800674	no
OR5M1	-0.015725225	-0.415209191	0.678116552	0.755838825	no
CXorf51	-0.015721165	-0.415101966	0.678195003	0.755884596	no
EDIL3	0.015716722	0.41498463	0.678280857	0.755931234	no
PRSS33	0.01571513	0.414942581	0.678311626	0.755931234	no
ZBTB48	0.015709424	0.41479188	0.678421901	0.756009416	no
MSH5	-0.01570763	-0.414744509	0.678456565	0.756009416	no
SNORA71C	0.015704763	0.41466879	0.678511977	0.756029493	no
HYLS1	0.015689934	0.414277131	0.678798619	0.756307201	no
PRAMEF18	-0.015668877	-0.413721012	0.679205704	0.756719067	no
C10orf76	0.015666436	0.413656556	0.679252892	0.756729942	no
CGB	-0.015656634	-0.413397664	0.679442441	0.756867318	no
TRIM35	-0.015656187	-0.413385873	0.679451075	0.756867318	no
CKAP2	0.015636787	0.412873514	0.679826262	0.757243532	no
OR8B12	-0.015633415	-0.412784448	0.679891491	0.757274469	no
PRSS8	-0.015629607	-0.412683876	0.679965149	0.75731479	no
SLC5A11	-0.015615366	-0.412307757	0.680240644	0.757496395	no
SP9	-0.015614795	-0.412292676	0.680251691	0.757496395	no

RNU11	-0.015613817	-0.412266853	0.680270607	0.757496395	no
TUBB8	-0.015613431	-0.412256663	0.680278072	0.757496395	no
EBF3	0.015610717	0.412184987	0.680330577	0.757513141	no
CDCA3	0.015604008	0.412007805	0.680460379	0.757615945	no
LOC646813	-0.015601607	-0.411944395	0.680506835	0.757625946	no
EVPLL	-0.015596021	-0.411796862	0.680614925	0.757704563	no
EPPK1	0.015581329	0.41140884	0.680899243	0.757963854	no
USF2	0.015580111	0.411376673	0.680922815	0.757963854	no
XAGE2	-0.015574411	-0.411226123	0.681033142	0.758044927	no
OR10X1	-0.015561423	-0.410883106	0.68128454	0.758283007	no
KIAA0368	0.015555044	0.410714644	0.681408019	0.758349479	no
SOCS4	0.015554463	0.410699282	0.681419279	0.758349479	no
SI	0.015520302	0.40979708	0.682080724	0.759043817	no
CNOT3	0.015505091	0.409395363	0.682375319	0.759291222	no
RAD1	0.015504945	0.409391499	0.682378153	0.759291222	no
MTAP	-0.015496812	-0.409176718	0.682535681	0.759424709	no
DNAJC19	0.01548187	0.408782101	0.682825142	0.759693705	no
RPS2	-0.015480454	-0.408744683	0.682852592	0.759693705	no
SCN4B	-0.015475594	-0.408616337	0.682946749	0.759756651	no
LDLRAD3	-0.015473616	-0.408564108	0.682985066	0.759757473	no
SMYD3	0.015468155	0.408419874	0.683090887	0.75983052	no
NRCAM	0.015466348	0.408372158	0.683125896	0.75983052	no
ING2	0.015459936	0.408202807	0.683250155	0.759926924	no
OR4L1	-0.015453967	-0.408045171	0.683365825	0.760013766	no
SLC22A8	-0.015440064	-0.407677975	0.683635296	0.76027164	no
OR5L2	-0.015421144	-0.407178315	0.684002042	0.760637659	no
ONECUT1	0.015389813	0.406350834	0.68460957	0.761271383	no
OR8H3	-0.015381085	-0.40612034	0.684778833	0.761398871	no
ANAPC11	0.015380017	0.406092146	0.684799539	0.761398871	no
ZMAT3	-0.015359489	-0.405549974	0.685197749	0.761799729	no
NUDT16L1	0.015355245	0.405437902	0.685280073	0.761804752	no
UCA1	-0.015354359	-0.405414492	0.68529727	0.761804752	no
ESYT3	-0.015353429	-0.405389942	0.685315305	0.761804752	no
MLANA	-0.015350454	-0.405311383	0.685373014	0.7618101	no
DPH3B	-0.015349297	-0.405280809	0.685395475	0.7618101	no
CA6	0.015338786	0.405003225	0.685599409	0.761945318	no
ZNF117	-0.015338659	-0.404999855	0.685601885	0.761945318	no
HYMAI	-0.0153372	-0.404961331	0.68563019	0.761945318	no
NOS2	0.015326883	0.404688869	0.685830386	0.762026425	no
CASP14	0.015326619	0.404681897	0.685835509	0.762026425	no
FLJ25758	-0.015325993	-0.404665352	0.685847667	0.762026425	no
ID2	0.01532567	0.404656822	0.685853935	0.762026425	no
MT3	0.015321832	0.404555472	0.68592841	0.762030985	no
LOC728190	-0.015320013	-0.404507414	0.685963726	0.762030985	no
DNAH14	0.015319632	0.404497364	0.685971111	0.762030985	no
OR8J3	-0.015316424	-0.404412633	0.686033378	0.762050475	no
SYF2	0.015311343	0.404278456	0.686131987	0.762050475	no
MAGEA4	-0.015311015	-0.404269778	0.686138364	0.762050475	no
MAGEB16	0.01531096	0.404268337	0.686139423	0.762050475	no
SLC13A2	-0.015306831	-0.404159294	0.686219564	0.762097619	no
SNORA9	0.015299588	0.403967995	0.686360168	0.762211901	no

NUTF2	0.015297139	0.403903316	0.686407709	0.762216157	no
ZNF420	0.015295506	0.403860202	0.6864394	0.762216157	no
C19orf18	0.015288181	0.403666741	0.686581611	0.762332199	no
ATXN1	-0.015282445	-0.403515262	0.686692968	0.762413972	no
TMEM189-UBE2	0.015245271	0.402533472	0.687414882	0.763173581	no
NBPF1	0.015235936	0.402286959	0.687596189	0.763332953	no
RORB	0.015228937	0.402102096	0.687732165	0.763414808	no
TAT	-0.015228253	-0.402084048	0.687745441	0.763414808	no
FRG1	-0.01522408	-0.40197384	0.68782651	0.763462881	no
WFS1	0.01519984	0.401333665	0.688297493	0.763943715	no
METTL2B	-0.015185707	-0.400960408	0.688572157	0.764206613	no
PADI4	0.015170636	0.400562388	0.68886509	0.764489757	no
HCG4	0.015163853	0.400383238	0.688996955	0.764594129	no
OR14C36	0.015153242	0.400103013	0.689203236	0.764781066	no
CSN1S1	0.015148059	0.399966126	0.689304011	0.764850913	no
BLID	0.015137497	0.39968719	0.689509377	0.7650368	no
APP	-0.015125333	-0.399365933	0.68974593	0.765257269	no
PALB2	-0.015118326	-0.399180893	0.689882196	0.765332569	no
ZCCHC7	-0.015117951	-0.399170965	0.689889507	0.765332569	no
NBPF4	0.015108008	0.398908389	0.69008289	0.765505095	no
EBF1	-0.015100291	-0.39870459	0.690232998	0.765629602	no
VPS33A	0.015098191	0.398649125	0.690273853	0.765632915	no
IFNA6	-0.015089816	-0.398427951	0.690436776	0.765771614	no
HIST1H2BF	0.015082241	0.398227879	0.690584167	0.765893072	no
MUC17	-0.015076793	-0.398083992	0.690690175	0.765968622	no
C2orf67	-0.015064929	-0.397770687	0.690921021	0.766163012	no
C1orf227	0.015063757	0.397739728	0.690943833	0.766163012	no
ITGB6	-0.015061942	-0.397691805	0.690979146	0.766163012	no
CHMP4B	0.01505031	0.397384583	0.691205545	0.766372016	no
MYLK3	0.015039415	0.397096867	0.691417594	0.766541753	no
CYP2B6	-0.015038549	-0.397073981	0.691434462	0.766541753	no
GLTP	-0.015029728	-0.39684104	0.691606162	0.766690063	no
TTLL3	0.015016393	0.396488852	0.691865787	0.766935823	no
OR2AG1	-0.015000215	-0.396061604	0.692180793	0.767242943	no
CENPC1	-0.01498712	-0.39571577	0.692435813	0.767452507	no
PCDHGB3	0.014986609	0.395702265	0.692445773	0.767452507	no
CORO2B	0.014982253	0.395587222	0.692530614	0.767504465	no
BLM	0.014977059	0.395450068	0.692631767	0.767555573	no
OR51T1	-0.014975854	-0.395418237	0.692655244	0.767555573	no
NUP93	0.014974038	0.39537027	0.692690622	0.767555573	no
NDUFB7	0.014971861	0.395312774	0.692733029	0.767560496	no
ABCC6P2	-0.014965167	-0.395135984	0.692863429	0.767586807	no
KRTAP4-4	-0.014964425	-0.395116388	0.692877883	0.767586807	no
TMEM72	-0.014960336	-0.395008407	0.692957535	0.767586807	no
LCN9	-0.014959823	-0.39499485	0.692967536	0.767586807	no
MS4A13	-0.014958948	-0.394971762	0.692984567	0.767586807	no
S100A7L2	-0.014958948	-0.394971762	0.692984567	0.767586807	no
C13orf35	-0.014950227	-0.394741444	0.693154474	0.767732944	no
MYLK4	-0.014946472	-0.394642272	0.693227639	0.76777192	no
KRTAP20-1	-0.014942298	-0.394532043	0.693308965	0.76781993	no
MRPL55	0.014936835	0.394387748	0.693415428	0.767895773	no

OBP2B	-0.014928053	-0.394155835	0.693586552	0.768043209	no
LCE1B	-0.014919821	-0.39393842	0.693746992	0.768178798	no
OCIAD1	0.014912817	0.393753446	0.693883503	0.768287878	no
HTR3E	-0.014908139	-0.393629907	0.69397468	0.768346754	no
DMRT1	0.014895845	0.393305235	0.694214324	0.768569992	no
DAZ2	-0.014887673	-0.393089417	0.694373638	0.768704278	no
C3orf17	0.014883482	0.392978723	0.694455357	0.768721512	no
NPR3	0.014882974	0.392965323	0.694465249	0.768721512	no
RALGPS2	-0.014878573	-0.392849074	0.694551073	0.768774423	no
NSD1	-0.014858694	-0.392324076	0.694938714	0.769115011	no
UHRF1BP1	-0.014858593	-0.392321433	0.694940666	0.769115011	no
RPS26P11	0.01485694	0.392277779	0.694972903	0.769115011	no
LOC284233	-0.014850978	-0.392120318	0.695089184	0.769176962	no
TMEM47	-0.014850169	-0.392098943	0.69510497	0.769176962	no
MRGPRG	0.014845599	0.391978248	0.695194106	0.769233496	no
FILIP1	0.014838665	0.391795124	0.695329356	0.769341045	no
SLC25A3	-0.014832156	-0.391623233	0.695456319	0.769405421	no
AGRN	0.01483178	0.391613307	0.69546365	0.769405421	no
ARL2	0.014828468	0.391525826	0.695528269	0.769434808	no
RNF151	0.014822419	0.391366081	0.695646273	0.769523244	no
SLC16A7	-0.014809601	-0.391027573	0.695896352	0.769757765	no
DAPK2	-0.01480189	-0.390823918	0.696046822	0.769882086	no
MRPL27	0.014790769	0.390530215	0.696263845	0.770063593	no
KRTAP6-2	-0.014788836	-0.390479174	0.696301563	0.770063593	no
TMEM98	0.014787625	0.390447207	0.696325186	0.770063593	no
PGM3	0.014774296	0.390095181	0.696585346	0.770309169	no
LOC221122	0.014743485	0.389281492	0.69718683	0.770932144	no
KLHDC8B	0.014738575	0.389151827	0.697282696	0.770995983	no
PGA3	-0.014735714	-0.389076264	0.697338566	0.771015592	no
FABP3	-0.014721732	-0.388707006	0.697611608	0.771275304	no
ZNF562	0.014715664	0.388546759	0.697730112	0.771358825	no
RCHY1	-0.014713957	-0.388501672	0.697763456	0.771358825	no
CLCN2	0.014708201	0.388349653	0.697875884	0.771440931	no
ALPI	-0.014697052	-0.38805522	0.698093657	0.771639471	no
AWAT2	-0.014691657	-0.387912736	0.698199052	0.771676654	no
OR2T27	-0.014690373	-0.387878844	0.698224123	0.771676654	no
LOC401387	0.014689469	0.387854947	0.6982418	0.771676654	no
C15orf61	0.014686991	0.387789511	0.698290206	0.771687968	no
COMTD1	0.014683174	0.3876887	0.698364782	0.771728201	no
PROX2	0.014677165	0.387530029	0.698482168	0.771815733	no
HTR3A	-0.014668354	-0.387297331	0.698654331	0.771963781	no
NKX2-6	0.014664027	0.387183058	0.698738884	0.772015014	no
UGT2A1	-0.014660094	-0.387079181	0.698815746	0.772057746	no
CYP2F1	-0.014647146	-0.386737238	0.699068786	0.77222618	no
SPATA5	0.014647078	0.386735439	0.699070117	0.77222618	no
NEK3	0.014644617	0.386670454	0.69911821	0.77222618	no
DHX16	0.014643514	0.38664131	0.699139779	0.77222618	no
LONRF3	-0.014642521	-0.386615101	0.699159176	0.77222618	no
C3orf27	-0.014627974	-0.386230926	0.699443521	0.772498039	no
ABCC12	-0.014603874	-0.385594457	0.699914692	0.772976197	no
GCC2	0.014596216	0.385392226	0.700064427	0.773099332	no

ANXA10	0.014589856	0.385224255	0.700188803	0.77317291	no
COX4NB	0.014586328	0.385131081	0.700257798	0.77317291	no
DNMT1	-0.014585811	-0.385117417	0.700267916	0.77317291	no
WFDC9	-0.014584952	-0.38509475	0.700284702	0.77317291	no
POU4F2	-0.014583032	-0.385044029	0.700322262	0.77317291	no
MC2R	0.014568165	0.384651419	0.700613027	0.773451686	no
DDX28	0.014565213	0.384573459	0.700670769	0.773473197	no
AMELY	-0.014541371	-0.383943813	0.701137187	0.773945821	no
DKFZp434L192	0.014528248	0.383597242	0.701393962	0.774186991	no
SEMA3B	0.014524924	0.383509462	0.701459004	0.774216516	no
SNORD8	-0.014503355	-0.382939842	0.701881124	0.774640132	no
TTR	-0.014499637	-0.382841641	0.701953906	0.774661662	no
OR4F5	0.014498443	0.382810126	0.701977264	0.774661662	no
FTSJD2	-0.014491259	-0.382620381	0.702117903	0.774717132	no
C14orf70	0.014490919	0.382611417	0.702124547	0.774717132	no
CLDN19	-0.014490003	-0.38258722	0.702142483	0.774717132	no
GIF	-0.014484163	-0.382432988	0.702256809	0.774800991	no
RBP7	0.014474729	0.382183843	0.702441504	0.774944204	no
C1orf170	0.014473617	0.382154482	0.702463272	0.774944204	no
REM1	-0.0144664	-0.381963877	0.702604584	0.775057806	no
TIGD2	0.01444958	0.381519681	0.702933945	0.775378826	no
SLC25A46	0.014443899	0.381369652	0.703045201	0.77545924	no
IFITM4P	0.014435176	0.381139283	0.703216046	0.775575922	no
SPANXC	0.01443458	0.381123559	0.703227708	0.775575922	no
ZBTB33	-0.014427666	-0.380940965	0.703363134	0.775682968	no
SEMA4D	-0.014424287	-0.380851736	0.703429317	0.775713643	no
BMP10	0.014415727	0.380625656	0.703597014	0.775856255	no
SNHG3-RCC1	0.014411646	0.3805179	0.703676948	0.775885675	no
TYR	0.014410447	0.380486224	0.703700447	0.775885675	no
DUX4	-0.014401803	-0.380257936	0.703869805	0.776030087	no
AHSP	0.014372562	0.379485721	0.704442794	0.776619467	no
KRTAP19-1	-0.014369771	-0.379412002	0.704497502	0.776637432	no
BPIL3	-0.014364699	-0.37927808	0.704596894	0.776704651	no
OR4A16	-0.014361391	-0.379190713	0.704661736	0.776733779	no
DSCR4	0.014319862	0.378093984	0.705475896	0.777577213	no
TAC4	-0.014318438	-0.378056366	0.705503828	0.777577213	no
OR7E5P	-0.01431122	-0.377865739	0.705645377	0.777690827	no
PRKDC	0.014300546	0.377583866	0.705854699	0.777879118	no
ZNF264	0.014297753	0.377510097	0.705909484	0.777883963	no
RTP1	0.014296399	0.377474333	0.705936045	0.777883963	no
PLN	-0.014286937	-0.377224459	0.706121631	0.778046058	no
METTL5	0.014276799	0.376956718	0.706320506	0.778185823	no
CWC22	-0.014276514	-0.376949207	0.706326085	0.778185823	no
TRIM65	-0.014274584	-0.376898241	0.706363944	0.778185823	no
PPIL1	0.014272185	0.376834886	0.706411008	0.778191428	no
LOC642929	-0.014270401	-0.376787766	0.706446012	0.778191428	no
PHKA1	-0.01426088	-0.376536331	0.706632805	0.778354784	no
OR5T3	-0.014257049	-0.376435162	0.70670797	0.778395169	no
NES	0.014233686	0.375818171	0.70716643	0.778857705	no
APOA1	0.014227989	0.375667704	0.707278252	0.77893843	no
SNORA76	0.014224259	0.375569206	0.707351456	0.778976618	no

SDK2	-0.014214806	-0.375319578	0.707536992	0.779138502	no
CRYGB	-0.014187855	-0.37460783	0.708066094	0.779678683	no
KLK11	-0.014175964	-0.374293803	0.708299581	0.779863691	no
STAT4	0.01417537	0.374278104	0.708311255	0.779863691	no
C4orf40	-0.014161678	-0.373916532	0.708580129	0.780117243	no
SNORD23	0.014137818	0.373286409	0.709048791	0.780590714	no
PAK1	-0.014130991	-0.373106118	0.709182906	0.78069585	no
CRISP3	0.014125257	0.372954695	0.709295553	0.780777344	no
COL9A3	0.014113302	0.372638987	0.709530436	0.780993377	no
OR4K17	-0.014109612	-0.372541541	0.709602939	0.781030662	no
OR51A4	-0.014106658	-0.372463528	0.709660987	0.781052032	no
RNF216L	0.014101812	0.372335537	0.709756224	0.781114329	no
SLC9A3	-0.014068486	-0.371455442	0.710411224	0.781792626	no
DEFB115	-0.014050111	-0.370970198	0.710772452	0.782147577	no
UCHL1	-0.014046587	-0.370877118	0.710841751	0.782181263	no
RALGAPB	0.014043593	0.370798072	0.710900603	0.782183715	no
CPB2	-0.014041037	-0.370730566	0.710950864	0.782183715	no
DEFB125	0.01404057	0.37071824	0.710960042	0.782183715	no
C6orf41	0.014023353	0.370263545	0.711298619	0.782485777	no
POU5F2	0.014022672	0.370245572	0.711312004	0.782485777	no
SCARNA17	-0.014016427	-0.370080653	0.711434821	0.782578303	no
DDAH2	0.014012797	0.369984792	0.711506215	0.782614256	no
RPL10A	-0.014008173	-0.369862679	0.711597162	0.782671712	no
STX10	0.013993829	0.369483856	0.711879329	0.78293947	no
ATAD5	-0.013987287	-0.369311107	0.712008014	0.783038405	no
GNAT1	0.01398178	0.369165661	0.712116368	0.78311497	no
BRD4	-0.013977939	-0.369064232	0.712191932	0.783155471	no
ZNF511	-0.013972986	-0.368933436	0.71228938	0.78322003	no
OR10Z1	-0.013940389	-0.368072603	0.71293085	0.783882746	no
PPM1D	-0.013931255	-0.367831387	0.713110635	0.784031294	no
ARL4A	0.013929585	0.36778728	0.71314351	0.784031294	no
MEX3C	-0.013923943	-0.367638271	0.71325458	0.784110766	no
MAD2L2	0.013881881	0.366527488	0.714082739	0.784939426	no
TTC9	-0.013881715	-0.366523108	0.714086005	0.784939426	no
LOC645431	-0.013879012	-0.366451724	0.714139239	0.784940853	no
SNORA72	0.013877706	0.366417245	0.714164951	0.784940853	no
SMARCA1	0.013874436	0.366330885	0.714229355	0.784968966	no
NFU1	-0.01386822	-0.366166728	0.714351781	0.785060842	no
MTMR8	0.013859475	0.36593578	0.714524033	0.785207463	no
PRPS1L1	-0.01385492	-0.365815489	0.714613758	0.78525045	no
UBQLN1	-0.013851718	-0.365730925	0.714676836	0.78525045	no
RFC5	-0.013850142	-0.365689328	0.714707864	0.78525045	no
LOC91948	0.013849074	0.365661115	0.71472891	0.78525045	no
GFPT1	-0.013846663	-0.365597443	0.714776407	0.78525045	no
PIK3CA	-0.013845659	-0.365570928	0.714796186	0.78525045	no
CHAC2	-0.013839361	-0.365404619	0.714920253	0.785344073	no
LOC284379	-0.013831476	-0.365196378	0.715075611	0.785472058	no
SCARNA11	0.013821108	0.364922565	0.715279908	0.785653782	no
LARP6	0.013802979	0.364443809	0.715637164	0.786003487	no
C8G	0.01379229	0.364161531	0.715847835	0.786192163	no
TPD52L1	-0.013782237	-0.363896058	0.716045983	0.786367066	no

MOG	0.013769248	0.363553052	0.71630203	0.786605532	no
SPANXB2	0.013742101	0.362836145	0.71683729	0.787150572	no
SUN5	-0.013735447	-0.362660421	0.716968511	0.787251907	no
B3GNTL1	-0.013714261	-0.362100938	0.717386358	0.787667937	no
POTEA	-0.013710249	-0.361994989	0.717465495	0.787712049	no
BRAF	0.013704156	0.361834077	0.717585692	0.787801234	no
SCGB1C1	-0.013695709	-0.361611005	0.717752332	0.787941393	no
LACRT	0.013685283	0.361335665	0.717958036	0.78812442	no
UBE2V1	-0.013682696	-0.361267349	0.718009077	0.788137658	no
MYBPC1	0.013678853	0.361165877	0.718084893	0.788178087	no
ABRA	0.013674613	0.361053912	0.718168552	0.78822712	no
RNASE11	0.013663804	0.360768459	0.718381854	0.788418431	no
SYPL2	-0.013651098	-0.360432909	0.718632619	0.788650833	no
RBMXL1	-0.013647526	-0.360338578	0.718703121	0.788685394	no
MS4A15	-0.013639581	-0.360128784	0.718859927	0.788814654	no
AHRR	-0.01363452	-0.359995119	0.718959837	0.788881471	no
AADACL2	0.013620388	0.359621917	0.71923882	0.78909737	no
DEFB114	-0.013618976	-0.359584643	0.719266686	0.78909737	no
YEATS4	0.013617201	0.359537771	0.719301728	0.78909737	no
DLK1	0.013616645	0.359523071	0.719312718	0.78909737	no
CXorf56	0.013610673	0.359365365	0.719430625	0.789183896	no
NKAP	0.013601597	0.359125687	0.71960983	0.789337649	no
SCN10A	-0.013597283	-0.359011753	0.719695023	0.789353487	no
MYF5	-0.013596911	-0.35900195	0.719702353	0.789353487	no
LYRM4	0.013593874	0.358921734	0.719762336	0.789376453	no
SERPINA4	-0.013583774	-0.358655004	0.719961801	0.78955238	no
PKP1	-0.013575513	-0.358436862	0.720124945	0.78968846	no
OR7E24	0.01356933	0.358273568	0.720247077	0.789758907	no
FGF16	-0.013568305	-0.358246516	0.720267311	0.789758907	no
STARD7	-0.01354602	-0.357658003	0.720707541	0.790181238	no
TBX22	-0.01354485	-0.357627118	0.720730646	0.790181238	no
NXF5	-0.013533649	-0.357331323	0.72095195	0.790381007	no
LRRC40	0.013530909	0.357258942	0.721006107	0.79039752	no
LOC200030	-0.013526449	-0.357141179	0.721094221	0.790451256	no
CYP2W1	-0.013518683	-0.356936096	0.721247682	0.790576613	no
CCHCR1	-0.013516177	-0.356869912	0.721297209	0.790588039	no
DYSFIP1	0.013512944	0.356784538	0.721361097	0.790615204	no
SNORA62	-0.013497648	-0.356380591	0.721663413	0.790874637	no
LOC100133920	0.013496645	0.356354111	0.721683233	0.790874637	no
OR6A2	-0.01349503	-0.356311457	0.721715158	0.790874637	no
CCDC106	-0.013482045	-0.355968563	0.721971822	0.791113018	no
PRR15L	0.013464519	0.355505742	0.722318304	0.791449787	no
NXF2	-0.013452881	-0.355198388	0.72254843	0.791638041	no
GLOD5	0.013451531	0.355162752	0.722575113	0.791638041	no
OR4C45	-0.013449889	-0.355119393	0.72260758	0.791638041	no
C4orf48	-0.01344589	-0.355013766	0.722686675	0.791681794	no
tAKR	-0.013420325	-0.354338666	0.723192266	0.792192731	no
OR2G6	-0.013412147	-0.354122706	0.723354027	0.792326998	no
OR56A3	-0.013392407	-0.353601398	0.723744554	0.792674118	no
HOXD3	-0.013392165	-0.353595006	0.723749343	0.792674118	no
LYZL6	-0.013386421	-0.353443314	0.723862994	0.792755647	no

C9orf98	0.013353987	0.35258681	0.724504818	0.793415579	no
AKT1S1	0.01335023	0.352487586	0.724579185	0.793454041	no
KIAA0753	-0.013335338	-0.352094341	0.72487394	0.793690063	no
C7orf65	-0.013335014	-0.352085778	0.724880359	0.793690063	no
DDC	-0.013333391	-0.352042915	0.724912489	0.793690063	no
ZNF621	0.01332975	0.351946762	0.724984567	0.793716895	no
BNIPL	-0.013328187	-0.351905484	0.725015512	0.793716895	no
LOC100129066	0.013318283	0.351643954	0.725211576	0.79388855	no
KRT17	0.01331565	0.351574422	0.725263706	0.793902631	no
SNORD22	-0.013309389	-0.351409069	0.725387681	0.793991401	no
TMEM144	0.013306551	0.351334144	0.725443859	0.793991401	no
WIPI2	-0.013305604	-0.351309128	0.725462615	0.793991401	no
AGBL5	0.013297976	0.351107684	0.725613665	0.794113731	no
LOC100133308	-0.013289659	-0.350888056	0.725778361	0.794250982	no
ILF2	-0.013279517	-0.350620236	0.725979213	0.794427783	no
GH2	-0.013269286	-0.350350039	0.726181866	0.794606536	no
C19orf2	0.013262191	0.350162678	0.726322402	0.794717303	no
UBE2U	-0.013238638	-0.349540716	0.726788991	0.795151306	no
NOSTRIN	0.013238198	0.349529093	0.726797712	0.795151306	no
MSMB	-0.013230739	-0.349332123	0.726945498	0.795269958	no
DIO2	0.013228034	0.349260683	0.726999102	0.795285567	no
FAM84A	-0.01322282	-0.349122999	0.727102415	0.79535555	no
PAPOLB	-0.013206575	-0.348693986	0.727424361	0.795664669	no
ACTC1	-0.013202302	-0.348581166	0.727509034	0.795714236	no
PLA2G4F	-0.013193516	-0.348349127	0.727683191	0.795861666	no
KRTAP10-6	-0.013165058	-0.347597638	0.72824732	0.796435566	no
C1orf14	-0.013161826	-0.347512291	0.728311397	0.796462561	no
YARS	-0.013139999	-0.346935867	0.728744221	0.796890269	no
ZBTB25	0.013138127	0.346886442	0.728781337	0.796890269	no
PRB3	0.013135125	0.346807158	0.728840877	0.796912274	no
HDAC9	-0.013129172	-0.346649961	0.728958933	0.796998254	no
FDXR	0.013126266	0.346573213	0.729016574	0.797018174	no
C17orf85	0.013118998	0.346381282	0.729160728	0.79713267	no
ODZ3	0.01311442	0.346260396	0.729251527	0.797188827	no
SIRT7	-0.01310815	-0.346094812	0.729375905	0.797235471	no
ZSCAN4	-0.013107555	-0.346079116	0.729387696	0.797235471	no
TECTB	-0.013106305	-0.346046107	0.729412491	0.797235471	no
HRASLS	-0.013101676	-0.345923861	0.729504323	0.79729274	no
ANXA8	-0.013089767	-0.345609376	0.729740581	0.797507842	no
RSPO4	-0.013087415	-0.345547269	0.729787243	0.797515727	no
SFRP5	-0.013076892	-0.345269384	0.729996032	0.797700777	no
NOL9	0.013065085	0.344957593	0.73023032	0.797913668	no
CXADRP3	0.01304423	0.344406846	0.730644227	0.798322794	no
ASTL	0.013028232	0.343984392	0.730961771	0.798622244	no
C8orf71	-0.013026443	-0.343937139	0.730997292	0.798622244	no
AMELX	-0.013021854	-0.343815966	0.731088383	0.798641103	no
ACSL3	-0.013019036	-0.343741551	0.731144327	0.798641103	no
SNORA69	-0.013016556	-0.343676067	0.731193557	0.798641103	no
IGFL2	0.013016475	0.343673923	0.731195169	0.798641103	no
PRL	-0.013013376	-0.343592067	0.731256709	0.798641103	no
CDKN2AIP	-0.013012734	-0.343575131	0.731269442	0.798641103	no

RLBP1	-0.013011645	-0.343546371	0.731291064	0.798641103	no
SERPINB3	-0.012993675	-0.343071816	0.731647879	0.798920503	no
C14orf39	-0.012993135	-0.343057555	0.731658603	0.798920503	no
MYO7B	-0.01299279	-0.343048451	0.731665449	0.798920503	no
OR6C3	-0.012984004	-0.342816453	0.73183991	0.799067845	no
AKR1E2	0.012980675	0.342728522	0.731906037	0.799096892	no
PGA5	0.012975121	0.342581871	0.732016327	0.799174151	no
LOC645332	-0.012968672	-0.34241156	0.732144419	0.799270835	no
C20orf191	-0.012962961	-0.342260752	0.732257848	0.799351502	no
ARHGEF16	-0.012956657	-0.342094279	0.732383066	0.799403365	no
OR4C15	0.012956588	0.342092458	0.732384436	0.799403365	no
AFARP1	-0.012952253	-0.341977984	0.732470546	0.799435789	no
TMEM79	0.012951112	0.341947838	0.732493223	0.799435789	no
NKX6-2	0.012948835	0.341887718	0.732538449	0.799441993	no
CACNA1G	-0.01294652	-0.341826591	0.732584433	0.799449024	no
PGLYRP3	0.012937768	0.341595461	0.732758313	0.799595616	no
MOGAT1	0.012932284	0.341450637	0.732867272	0.799671353	no
TBC1D28	0.012924829	0.34125378	0.733015387	0.799789806	no
TPCN1	0.012920884	0.341149596	0.733093779	0.799832174	no
PACRG	0.012916314	0.34102893	0.733184576	0.799888072	no
RGPD1	0.012912028	0.340915746	0.733269746	0.799937826	no
CCDC27	0.012902481	0.340663631	0.733459474	0.800101632	no
LGALS13	0.012894735	0.340459086	0.733613415	0.800226384	no
TAF6	-0.012870291	-0.339813575	0.734099297	0.800713185	no
RPL23AP32	-0.012868204	-0.339758475	0.734140777	0.80071523	no
MYL2	-0.012863138	-0.33962469	0.734241493	0.80075477	no
LOC723809	0.012862396	0.3396051	0.734256241	0.80075477	no
CENPQ	0.012852603	0.339346476	0.734450955	0.800923916	no
FAM166A	-0.01284636	-0.339181635	0.734575069	0.800989644	no
EFTUD1	0.012845586	0.33916119	0.734590463	0.800989644	no
NPS	0.012839454	0.338999263	0.734712391	0.80107939	no
TTTY19	0.012835812	0.338903088	0.734784812	0.801115149	no
H2AFB1	-0.01282622	-0.338649787	0.734975562	0.801279909	no
AANAT	0.012815497	0.338366627	0.735188818	0.801469186	no
TBC1D9	0.012804089	0.338065375	0.735415721	0.801673319	no
LOC100144604	-0.01280111	-0.337986705	0.73547498	0.801694691	no
CSAG3	0.012797723	0.337897249	0.735542364	0.801711744	no
KRTAP19-5	-0.012796337	-0.337860655	0.73556993	0.801711744	no
PHKG1	0.01278489	0.337558378	0.735797644	0.801916704	no
LOC284661	-0.012782815	-0.337503572	0.735838934	0.801918476	no
KRTAP20-4	-0.012754821	-0.336764325	0.73639594	0.802482246	no
POM121L12	-0.012744321	-0.336487061	0.736604888	0.802666682	no
CYTSA	0.012741153	0.336403386	0.73666795	0.802692137	no
BAG3	0.012737631	0.33631038	0.736738046	0.802715708	no
MAVS	-0.012736076	-0.336269326	0.736768988	0.802715708	no
HOXC12	-0.012731058	-0.336136805	0.736868871	0.802779694	no
PLEKHA5	-0.012729135	-0.336086047	0.736907129	0.802779694	no
SEMA6D	0.012725156	0.335980955	0.736986343	0.80282273	no
MGA	0.012720014	0.335845189	0.737088681	0.802890952	no
ABCC6P1	0.01270789	0.335525009	0.737330048	0.803090473	no
ZNF787	0.012706823	0.335496828	0.737351293	0.803090473	no

VAV2	0.012687497	0.334986486	0.737736069	0.803466029	no
TECR	0.012685512	0.334934076	0.737775587	0.803466029	no
SLC34A1	0.012672942	0.334602136	0.738025896	0.803660002	no
GANC	0.012672575	0.334592449	0.738033201	0.803660002	no
OR4D9	-0.012662855	-0.33433578	0.738226769	0.803786057	no
DSG4	0.01266277	0.334333519	0.738228474	0.803786057	no
ZNF304	0.012646423	0.333901858	0.738554051	0.804066606	no
C18orf20	-0.012645839	-0.333886439	0.738565681	0.804066606	no
SMN2	0.012640027	0.333732938	0.73868147	0.804116064	no
RPS29	0.012638642	0.333696367	0.738709057	0.804116064	no
ADAMTSL3	-0.012637569	-0.333668039	0.738730427	0.804116064	no
THEM4	0.01262278	0.333277505	0.739025047	0.804356017	no
ERI3	0.012622509	0.333270361	0.739030436	0.804356017	no
USP16	0.012620152	0.33320811	0.739077403	0.804363833	no
GUCA2A	0.012606684	0.332852453	0.739345751	0.804612574	no
DPY19L2P2	-0.01260285	-0.332751208	0.739422148	0.804652402	no
NCRNA00200	-0.012594954	-0.332542699	0.739579491	0.80478031	no
SOX10	0.012583391	0.332237365	0.73980992	0.804987727	no
GSTT1	-0.012572956	-0.331961799	0.740017903	0.805170701	no
KCNJ13	-0.012562546	-0.331686895	0.740225405	0.805352833	no
ALDH1B1	-0.012560561	-0.331634489	0.740264964	0.805352833	no
ABCA4	-0.012548355	-0.331312164	0.74050829	0.805574205	no
FGF23	-0.012539437	-0.33107667	0.740686082	0.805724266	no
GKN1	-0.012531781	-0.330874505	0.740838723	0.805824402	no
ZNF479	-0.012530822	-0.330849175	0.740857848	0.805824402	no
ZFATAS	-0.012526377	-0.330731803	0.740946473	0.805848724	no
CCNH	-0.012525703	-0.330713987	0.740959926	0.805848724	no
TEX11	0.012518828	0.330532445	0.741097012	0.805954461	no
CACNA1S	0.012511145	0.330329573	0.741250214	0.806077713	no
OR51F1	-0.012497098	-0.329958626	0.741530368	0.806338997	no
UGT1A8	0.012477823	0.32944962	0.741914844	0.806713688	no
C6orf201	-0.012472938	-0.32932063	0.742012287	0.806776253	no
SSX4	-0.012465692	-0.329129294	0.742156835	0.806806519	no
CYP4F8	0.012465615	0.329127259	0.742158373	0.806806519	no
DCHS2	0.012465542	0.329125317	0.74215984	0.806806519	no
TKTL1	0.01245564	0.32886384	0.742357392	0.806977889	no
CNTNAP1	-0.012433052	-0.328267365	0.742808108	0.807393153	no
PHF15	0.012432493	0.328252593	0.742819271	0.807393153	no
PSG6	-0.012420125	-0.327925997	0.743066096	0.807618016	no
MRAP2	-0.01241415	-0.327768211	0.743185353	0.807704212	no
ADAMTSL5	-0.012406455	-0.327565009	0.743338944	0.807827713	no
DNAJA2	-0.012400179	-0.327399289	0.743464211	0.807920421	no
GTF3C6	-0.012398074	-0.327343701	0.743506232	0.807922659	no
LOC731779	0.012395583	0.327277922	0.743555957	0.807933269	no
ACCSL	-0.012387372	-0.32706111	0.743719862	0.808067937	no
NCRNA00111	-0.012362495	-0.326404186	0.744216554	0.808564151	no
MFN2	-0.012359106	-0.326314687	0.744284231	0.808594228	no
TMEM202	0.012355938	0.32623103	0.744347493	0.808619505	no
C9orf144	0.012351019	0.326101135	0.744445722	0.808682764	no
PARP11	-0.012307701	-0.324957254	0.745310935	0.809579138	no
ACYP2	-0.012301852	-0.324802797	0.745427789	0.809662568	no

MS4A8B	-0.012296353	-0.324657579	0.745537658	0.809738403	no
CEP290	-0.012270757	-0.323981679	0.746049099	0.810246375	no
SHPK	0.012268935	0.32393357	0.746085506	0.810246375	no
KRT34	-0.01226671	-0.323874814	0.746129971	0.810251142	no
RASSF7	0.012261524	0.323737862	0.746233618	0.810320172	no
SNORA11D	-0.012257569	-0.323633414	0.746312668	0.810358329	no
COL24A1	0.012255755	0.323585517	0.746348919	0.810358329	no
LOC127841	0.012237676	0.323108121	0.746710269	0.810707133	no
FLJ46321	-0.012233814	-0.323006141	0.746787468	0.81074741	no
ARHGAP28	-0.012226613	-0.322815977	0.746931427	0.810860158	no
OR4K15	-0.012224157	-0.322751113	0.746980532	0.810863303	no
C21orf15	0.012222456	0.3227062	0.747014535	0.810863303	no
MEGF6	0.012219488	0.322627826	0.747073871	0.810884174	no
UBXN2B	-0.012213577	-0.322471729	0.747192054	0.810968915	no
OTX2	-0.012206286	-0.322279196	0.747337832	0.811083595	no
OR51D1	-0.012204183	-0.322223684	0.747379865	0.811085674	no
BCKDHB	-0.012201758	-0.322159643	0.747428357	0.811094763	no
MAGEA6	-0.012194993	-0.321981005	0.747563627	0.811198015	no
CSNK1G3	-0.012188239	-0.321802654	0.747698689	0.81130103	no
NLRP8	0.012181172	0.32161603	0.747840023	0.811409001	no
TMEM30C	-0.01217925	-0.321565276	0.747878461	0.811409001	no
FAM125A	0.012176596	0.321495204	0.747931531	0.8114198	no
C20orf96	0.01217439	0.321436933	0.747975665	0.8114198	no
LOC285629	-0.012172732	-0.321393163	0.748008816	0.8114198	no
TDRD1	0.012156107	0.320954148	0.748341348	0.811736969	no
EPHA7	-0.01212024	-0.320007031	0.749058902	0.812471721	no
WFIKKN2	-0.012112555	-0.319804082	0.749212689	0.812594933	no
SALL1	-0.012106913	-0.319655112	0.749325579	0.812673777	no
OR9K2	0.012096961	0.319392317	0.749524739	0.812815708	no
C5orf44	0.012096249	0.319373498	0.749539001	0.812815708	no
KRTAP20-2	-0.012094347	-0.319323289	0.749577054	0.812815708	no
CDC37	0.012088165	0.319160027	0.749700794	0.812906288	no
TFAP2D	-0.012084915	-0.319074221	0.74976583	0.812933209	no
IFIT1B	0.012081424	0.318982038	0.749835703	0.812965371	no
BTN1A1	0.012062621	0.318485504	0.750212097	0.813235454	no
ITSN1	-0.012061836	-0.318464781	0.750227807	0.813235454	no
ZNF596	-0.012060965	-0.31844177	0.750245252	0.813235454	no
C10orf116	-0.01206087	-0.318439275	0.750247143	0.813235454	no
H2AFX	-0.012057657	-0.318354427	0.750311469	0.813235454	no
SAGE1	0.012056924	0.318335058	0.750326153	0.813235454	no
SOX14	0.012053072	0.318233354	0.75040326	0.813249523	no
PYY	-0.012052257	-0.318211827	0.750419582	0.813249523	no
LZTR1	0.012045353	0.318029509	0.750557813	0.813355731	no
LRRC37A3	0.012037689	0.317827139	0.750711258	0.813478412	no
ZC3HC1	0.012013344	0.317184266	0.751198774	0.813950629	no
CIRH1A	-0.012010629	-0.317112572	0.751253148	0.813950629	no
RASIP1	0.012009896	0.317093234	0.751267815	0.813950629	no
SPATA22	-0.01200253	-0.316898709	0.751415355	0.814066855	no
USP26	-0.011999504	-0.316818814	0.751475955	0.814088885	no
DCUN1D4	-0.011987469	-0.316501	0.751717031	0.814306416	no
DPM2	0.011973843	0.316141202	0.751989983	0.814522388	no

PLEKHM2	0.011973494	0.316131982	0.751996978	0.814522388	no
ZNF625	-0.01196697	-0.315959695	0.75212769	0.814620327	no
KRT3	0.011951512	0.315551497	0.752437415	0.81491213	no
OR5L1	0.011938928	0.315219209	0.752689572	0.815141559	no
PRTN3	-0.011923565	-0.314813516	0.752997468	0.815427919	no
ZNF225	-0.011921709	-0.314764518	0.753034656	0.815427919	no
TGFBR3	-0.011913656	-0.314551877	0.753196056	0.815559012	no
KRTAP4-2	-0.011908	-0.314402512	0.753309434	0.815596562	no
C11orf40	-0.011907606	-0.314392098	0.753317339	0.815596562	no
KIF17	-0.011905889	-0.314346759	0.753351756	0.815596562	no
CCDC60	-0.011900691	-0.31420952	0.753455935	0.815636076	no
RPS17	-0.011900043	-0.314192391	0.753468939	0.815636076	no
LYSMD2	0.011890842	0.313949427	0.753653389	0.815767144	no
KRTAP21-2	0.011889978	0.313926607	0.753670713	0.815767144	no
LOC100130093	0.011865986	0.313293083	0.754151731	0.816244095	no
PGAM4	-0.011848965	-0.312843623	0.754493053	0.816569806	no
FBRS	0.011839567	0.312595441	0.754681543	0.816730085	no
GABARAP	-0.011835636	-0.312491656	0.754760371	0.816771674	no
POLR2B	0.011831861	0.312391973	0.754836086	0.81680989	no
PRY2	-0.011819035	-0.312053281	0.755093357	0.817044554	no
KC6	-0.011810195	-0.311819833	0.755270701	0.817192713	no
MAGEC2	-0.011787113	-0.311210336	0.755733779	0.817592645	no
EIF1AY	-0.011786247	-0.311187458	0.755751164	0.817592645	no
CYP2A13	-0.011785227	-0.311160518	0.755771634	0.817592645	no
TSSK6	-0.011783709	-0.311120445	0.755802084	0.817592645	no
LOC401463	0.011781506	0.311062268	0.755846291	0.817596721	no
OBP2A	-0.011750097	-0.310232867	0.756476617	0.818234765	no
SPINT3	-0.011726967	-0.309622106	0.756940885	0.818693137	no
CSHL1	0.011714793	0.309300639	0.757185282	0.818901152	no
KRTAP15-1	0.011713352	0.309262582	0.757214217	0.818901152	no
EIF3D	-0.011697518	-0.30884447	0.75753213	0.819201145	no
LOC285045	-0.011693869	-0.308748104	0.757605408	0.81923657	no
ATAD2	0.011670708	0.308136529	0.758070509	0.819695665	no
ACE2	-0.011667805	-0.308059855	0.758128825	0.819714882	no
TMEM184B	0.011654254	0.307702041	0.758400989	0.819965304	no
KIAA1429	0.011639905	0.307323119	0.75868924	0.820233092	no
DFFB	0.011596396	0.306174232	0.759563421	0.821117655	no
FAM83B	-0.01159514	-0.30614107	0.759588659	0.821117655	no
NAP1L6	-0.011592572	-0.30607324	0.75964028	0.821129554	no
CT47B1	0.011581846	0.305790013	0.75985584	0.821318651	no
FZD10	0.011576527	0.305649567	0.759962738	0.821351065	no
IFT81	0.011576311	0.305643866	0.759967077	0.821351065	no
CDS2	-0.011561161	-0.305243807	0.760271601	0.821636264	no
MRGPRX1	-0.011558656	-0.305177654	0.760321961	0.821646769	no
SNORD15A	0.011548393	0.304906669	0.760528259	0.82182578	no
AP1M2	-0.011537866	-0.304628677	0.76073991	0.822010555	no
FAM9C	0.011529369	0.304404299	0.760910754	0.822127067	no
SPC25	0.011528458	0.304380254	0.760929063	0.822127067	no
CXorf66	-0.011517772	-0.30409808	0.761143933	0.822315274	no
BTBD7	-0.011512742	-0.303965261	0.761245077	0.822380602	no
MRGPRX2	-0.011508563	-0.303854905	0.76132912	0.822427449	no

GJB7	0.011489977	0.303364126	0.761702909	0.822787274	no
APOA4	-0.011486558	-0.303273836	0.761771682	0.822817601	no
KL	-0.011459451	-0.302558064	0.762316946	0.823362573	no
STK35	0.011456914	0.302491057	0.762367996	0.823373726	no
LALBA	0.011445482	0.302189183	0.762597999	0.823559576	no
OR51M1	0.011444312	0.302158283	0.762621544	0.823559576	no
TRIM49	-0.01143082	-0.301802031	0.762893008	0.823749205	no
TMEM192	0.011430755	0.30180031	0.76289432	0.823749205	no
HES3	0.011428893	0.301751146	0.762931784	0.823749205	no
OR4C12	-0.011427485	-0.301713969	0.762960116	0.823749205	no
ZNF575	0.011418867	0.301486385	0.763133555	0.823892466	no
RPL21P44	-0.011416581	-0.301426044	0.763179542	0.823898119	no
FAM138E	-0.011410568	-0.301267247	0.763300568	0.823984777	no
GABRA6	-0.011402302	-0.301048984	0.763466926	0.824120358	no
ACSM2B	-0.011393575	-0.300818528	0.763642589	0.824265969	no
PIGH	-0.011390964	-0.300749592	0.763695137	0.824278683	no
SIRPB1	0.011385655	0.3006094	0.763802006	0.824350022	no
OR2A2	0.011373657	0.300292586	0.764043529	0.824559115	no
RBMY1F	-0.011371979	-0.30024828	0.764077308	0.824559115	no
CDC7	0.011359591	0.299921172	0.764326707	0.824784232	no
9-Mar	-0.011356831	-0.299848281	0.764382285	0.824800183	no
KBTBD3	0.011348373	0.299624941	0.764552585	0.824939917	no
OR11G2	-0.011343004	-0.29948316	0.764660701	0.825002508	no
DAZ3	-0.011339991	-0.299403615	0.76472136	0.825002508	no
CAP2	-0.011339413	-0.299388339	0.76473301	0.825002508	no
NUP43	-0.011330414	-0.299150708	0.764914233	0.825119721	no
GPER	0.011329964	0.299138842	0.764923282	0.825119721	no
ZNF773	-0.011320718	-0.298894689	0.765109493	0.825276554	no
ARR3	-0.011311038	-0.298639082	0.765304454	0.825442809	no
C2orf16	0.011280078	0.297821559	0.765928109	0.826071402	no
RABGEF1	0.011277004	0.297740389	0.765990039	0.826094127	no
C8orf37	0.011261756	0.297337749	0.76629726	0.826381374	no
LOC90784	-0.01125155	-0.297068248	0.766502914	0.826559065	no
NEURL1B	0.011242221	0.29682192	0.766690899	0.826717685	no
C12orf57	0.011238856	0.296733057	0.766758718	0.826746721	no
PPP1R2P1	0.011219069	0.296210564	0.767157516	0.827132608	no
ZAN	-0.011206549	-0.29587996	0.767409884	0.827360585	no
GJA1	0.011200497	0.295720147	0.767531888	0.827437164	no
AWAT1	0.011198965	0.2956797	0.767562766	0.827437164	no
OPRL1	-0.01119378	-0.295542788	0.767667292	0.827485753	no
FLJ36000	-0.011192669	-0.295513443	0.767689696	0.827485753	no
TAS2R8	-0.011189961	-0.295441953	0.767744278	0.827500469	no
CHRM5	0.011162993	0.29472984	0.768288027	0.828039718	no
APTX	0.011161085	0.294679464	0.768326497	0.828039718	no
OTOP3	-0.011157949	-0.294596647	0.768389742	0.828051573	no
KCND1	0.011156478	0.2945578	0.768419409	0.828051573	no
BARX2	0.01113364	0.29395476	0.768879986	0.828503734	no
C6orf223	-0.011130723	-0.293877736	0.76893882	0.828522973	no
CNTN2	-0.011121414	-0.293631927	0.769126587	0.82864386	no
LEFTY1	-0.011121097	-0.293623553	0.769132984	0.82864386	no
URM1	0.011096799	0.29298195	0.769623151	0.829122818	no

C13orf37	0.011094995	0.292934294	0.769659563	0.829122818	no
LOC389033	0.011090234	0.292808575	0.769755621	0.829182117	no
BTBD16	-0.011082571	-0.292606232	0.769910233	0.82930448	no
ESPN	0.011077534	0.29247324	0.770011858	0.829367879	no
C19orf43	-0.011074599	-0.292395745	0.770071077	0.829367879	no
ZBTB7A	-0.011073555	-0.292368163	0.770092155	0.829367879	no
TBC1D15	0.011065755	0.292162201	0.770249552	0.829493205	no
LOC100128191	-0.011057405	-0.291941724	0.770418052	0.829630476	no
STAG3L1	0.011046914	0.291664689	0.770629793	0.829794251	no
HP1BP3	0.011045802	0.291635342	0.770652224	0.829794251	no
KCNMB4	0.011028792	0.291186178	0.770995563	0.83011973	no
OR5D18	-0.011026555	-0.291127098	0.771040727	0.83012415	no
MRPL53	0.01102041	0.290964842	0.771164768	0.830189053	no
ARMCX6	0.0110195	0.290940819	0.771183134	0.830189053	no
METTL11B	0.01101585	0.290844432	0.771256823	0.830224175	no
SLC13A4	-0.011012219	-0.290748551	0.771330127	0.830258878	no
CPN1	-0.011008668	-0.290654798	0.771401806	0.830291829	no
OR5AS1	-0.010993576	-0.290256264	0.771706529	0.830575598	no
UPK1B	-0.010968941	-0.289605775	0.772203975	0.831037489	no
ADAM30	-0.010968252	-0.28958758	0.77221789	0.831037489	no
PDXDC1	-0.010960861	-0.28939243	0.772367146	0.831153872	no
PCDHGA3	0.010944103	0.288949926	0.772705615	0.831473848	no
OR2T11	-0.010939589	-0.288830732	0.772796793	0.831527704	no
PRB4	0.010936759	0.288755998	0.772853963	0.831544964	no
ZNF750	-0.010926562	-0.288486738	0.773059951	0.831722333	no
C20orf107	0.010901527	0.287825684	0.773565736	0.832222213	no
LOC150185	0.010899271	0.287766102	0.773611328	0.832226978	no
MRGPRD	0.010891654	0.287564961	0.773765246	0.832310949	no
OR14J1	-0.01089016	-0.287525517	0.773795431	0.832310949	no
TGM6	-0.010889296	-0.28750271	0.773812885	0.832310949	no
PRAMEF1	0.01088267	0.287327739	0.773946787	0.832400628	no
FTLP10	-0.010881095	-0.287286165	0.773978604	0.832400628	no
DEFB110	-0.010874551	-0.287113371	0.774110848	0.83249857	no
LRP5L	0.010869154	0.286970858	0.774219922	0.832571586	no
ZNF747	-0.01085425	-0.2865773	0.774521161	0.83285123	no
NDUFA7	-0.010844014	-0.286307024	0.774728056	0.833029401	no
TSSK1B	-0.010833711	-0.286034952	0.774936342	0.833202663	no
LMX1B	-0.010831966	-0.285988882	0.774971613	0.833202663	no
GSTT2	-0.010819256	-0.285653273	0.775228566	0.833434603	no
SNORA4	0.010807167	0.28533405	0.775472996	0.833653057	no
NR2F6	0.010797021	0.285066163	0.775678136	0.833787021	no
CDRT1	0.010796925	0.28506361	0.775680091	0.833787021	no
CLCA4	-0.010782567	-0.284684492	0.775970434	0.834054771	no
MEAF6	0.010774876	0.284481391	0.77612599	0.834177623	no
LOC401093	-0.010771164	-0.284383392	0.776201051	0.834213951	no
MYH8	-0.010755824	-0.283978326	0.776511328	0.834503058	no
LCE6A	-0.010753613	-0.283919947	0.776556049	0.834506761	no
BUB1B	0.010743035	0.283640629	0.776770028	0.834692343	no
T	0.010734473	0.283414536	0.776943246	0.834834106	no
SUN2	-0.010714782	-0.282894599	0.777341628	0.835196164	no
IRX1	-0.010713735	-0.282866949	0.777362816	0.835196164	no

LOC340357	0.010710735	0.282787737	0.777423515	0.835216995	no
GHSR	0.010703806	0.282604787	0.777563712	0.835323227	no
FAM58A	-0.010697437	-0.282436614	0.777692592	0.835417291	no
E2F2	-0.010685409	-0.282119003	0.777936011	0.835634378	no
CCT2	0.010681661	0.282020032	0.778011867	0.835644098	no
VSX1	0.010680878	0.281999345	0.778027722	0.835644098	no
TGIF2LY	0.010654897	0.281313312	0.778553594	0.836164492	no
SLC25A14	-0.010640671	-0.280937678	0.778841574	0.836390305	no
OR8K5	-0.010640423	-0.280931142	0.778846586	0.836390305	no
FXYD4	-0.010629743	-0.280649113	0.779062825	0.836578085	no
KIFC1	0.010624356	0.280506877	0.779171887	0.83661127	no
KIF2B	0.010624129	0.280500878	0.779176487	0.83661127	no
OR6C4	-0.010614761	-0.280253525	0.779366161	0.836750492	no
TAS2R30	-0.010613637	-0.280223841	0.779388924	0.836750492	no
PDHA2	-0.010599663	-0.27985485	0.779671899	0.837009847	no
PDE6A	-0.010596855	-0.2797807	0.779728767	0.837026452	no
PRSS12	-0.0105886	-0.279562732	0.779895941	0.837161462	no
MIOX	0.010573138	0.279154468	0.780209093	0.837453145	no
FKSG83	-0.010559419	-0.278792194	0.780487	0.837706968	no
GLYATL2	0.010549982	0.278543015	0.780678165	0.837867669	no
NF1P1	-0.01053793	-0.278224789	0.780922322	0.838085222	no
C14orf180	-0.010535188	-0.278152385	0.780977876	0.838100356	no
KRTAP9-9	0.010531724	0.278060913	0.781048063	0.838131189	no
ACAD9	-0.010514986	-0.277618942	0.781387213	0.838450625	no
SLC1A7	-0.010504901	-0.277352655	0.781591571	0.83858201	no
TFF2	-0.01050485	-0.277351301	0.78159261	0.83858201	no
CCDC13	0.010502208	0.27728153	0.781646157	0.838594959	no
OR7C2	-0.010497252	-0.277150688	0.781746577	0.838614138	no
GBAP1	-0.010497232	-0.27715015	0.78174699	0.838614138	no
C17orf65	0.010490482	0.276971911	0.781883793	0.83871639	no
SERPINA11	0.01045731	0.276096006	0.78255617	0.839393105	no
SH2D7	0.010450579	0.275918277	0.782692621	0.83949493	no
TTLL4	0.010445161	0.275775209	0.782802466	0.839568207	no
LYG1	0.010442584	0.275707165	0.782854711	0.839579703	no
OR7G2	0.010434929	0.275505018	0.783009927	0.839664296	no
LOC653544	0.010434597	0.275496262	0.78301665	0.839664296	no
CCDC51	0.010428402	0.275332698	0.783142247	0.839710573	no
MRGPRX4	0.010428372	0.275331884	0.783142871	0.839710573	no
GOLGA2P3	-0.010413266	-0.274933024	0.78344917	0.839932011	no
C7orf40	-0.010411892	-0.274896726	0.783477046	0.839932011	no
CYCSP52	0.01041166	0.27489061	0.783481743	0.839932011	no
EIF4H	-0.010408494	-0.274807022	0.783545938	0.839932011	no
CCDC137	0.010407164	0.274771902	0.783572911	0.839932011	no
SCGB3A2	-0.010405895	-0.274738382	0.783598655	0.839932011	no
OR6C75	-0.010402382	-0.274645628	0.783669892	0.839941858	no
CYP11B1	0.010401345	0.274618237	0.783690929	0.839941858	no
OR2W1	-0.010394907	-0.274448244	0.783821494	0.840037263	no
ATP5L2	-0.010386968	-0.274238621	0.783982506	0.840165287	no
CXCL17	-0.010357622	-0.273463736	0.784577777	0.840758652	no
OR11L1	0.010354038	0.273369089	0.784650493	0.840792011	no
GADL1	0.010347298	0.273191144	0.784787213	0.840893946	no

ASB16	-0.010330524	-0.272748205	0.785127562	0.841214046	no
ECHS1	-0.010321271	-0.272503882	0.785315315	0.841370624	no
C4orf27	-0.010310026	-0.272206967	0.7855435	0.841492288	no
SNX18	-0.01030702	-0.272127584	0.785604511	0.841492288	no
ARL4D	-0.010306668	-0.272118288	0.785611656	0.841492288	no
RCE1	0.010306112	0.272103627	0.785622923	0.841492288	no
OPN5	-0.01030542	-0.27208534	0.785636978	0.841492288	no
TREH	-0.010298829	-0.27191131	0.785770736	0.84159097	no
PDE4B	0.010295406	0.27182092	0.785840213	0.841620798	no
IMMP1L	0.010292486	0.271743812	0.785899481	0.84163969	no
HMBOX1	-0.010287146	-0.271602811	0.786007863	0.841705921	no
NNAT	0.010282661	0.271484394	0.786098889	0.841705921	no
TUSC2	-0.01028209	-0.271469328	0.78611047	0.841705921	no
MB	0.010281234	0.271446717	0.786127851	0.841705921	no
LRRC52	-0.010278777	-0.271381824	0.786177735	0.841714756	no
IFNA5	0.010269016	0.271124095	0.786375865	0.8418823	no
C5orf55	-0.010260562	-0.270900858	0.786547489	0.842021452	no
GPX2	-0.010255494	-0.270767049	0.786650366	0.842086996	no
OR8G2	-0.010241823	-0.270406072	0.786927917	0.842339508	no
MKX	-0.010238971	-0.270330754	0.786985832	0.842356903	no
RFXANK	0.010236366	0.270261973	0.787038721	0.842368918	no
ACTL7A	0.010231243	0.270126714	0.787142731	0.842373803	no
CTU1	0.010229576	0.270082687	0.787176587	0.842373803	no
POP1	-0.010229512	-0.270081008	0.787177879	0.842373803	no
KIAA1632	-0.010227933	-0.270039312	0.787209943	0.842373803	no
KIAA1324	-0.01022569	-0.269980081	0.787255492	0.842377959	no
FLJ44082	0.010214127	0.269674749	0.787490306	0.84258117	no
MKI67IP	0.010210651	0.269582963	0.787560898	0.84258117	no
REG3A	-0.010210181	-0.269570569	0.78757043	0.84258117	no
SAA3P	-0.010206317	-0.269468534	0.787648907	0.842620541	no
OR51V1	-0.010176409	-0.268678809	0.788256365	0.843225778	no
EPO	-0.010171237	-0.26854226	0.788361412	0.843270812	no
STON1	-0.01017023	-0.268515652	0.788381882	0.843270812	no
PAX7	0.01013601	0.267612088	0.789077095	0.843969777	no
EI24	0.010122287	0.267249736	0.78935594	0.844188755	no
EXOC1	0.010121824	0.267237524	0.789365338	0.844188755	no
LOC220429	0.010096111	0.266558576	0.789887892	0.84470292	no
BSX	0.010085628	0.266281767	0.790100966	0.844873568	no
GAS1	0.010084148	0.266242695	0.790131043	0.844873568	no
ZNF573	-0.010080192	-0.26613824	0.790211452	0.844914863	no
COL2A1	-0.010072291	-0.265929593	0.790372074	0.845041914	no
OR10AG1	-0.010052141	-0.265397538	0.790781707	0.845399132	no
ROCK2	0.010051742	0.265387014	0.79078981	0.845399132	no
CLDN3	-0.010039213	-0.265056185	0.791044547	0.845626746	no
PCP4	0.010033012	0.264892446	0.791170633	0.845716816	no
CFHR5	0.010027206	0.264739143	0.791288689	0.845798293	no
KIAA0467	0.010024091	0.264656885	0.791352036	0.845821287	no
B4GALNT2	0.010007042	0.264206708	0.791698743	0.846147127	no
FAM71F2	-0.01000302	-0.264100528	0.791780525	0.846189801	no
PYCR2	0.010000718	0.264039728	0.791827355	0.846195119	no
SNORA70B	0.009991296	0.263790953	0.792018976	0.846355162	no

LDLRAD1	0.009980955	0.263517901	0.792229313	0.846506635	no
XRRA1	0.00998021	0.263498235	0.792244463	0.846506635	no
KIF18B	-0.009977688	-0.263431643	0.792295762	0.846516711	no
OTUD1	0.009968027	0.263176547	0.792492284	0.846681939	no
SFTPA1	0.009959061	0.262939807	0.792674677	0.846832054	no
TESK2	-0.00994028	-0.26244389	0.793056788	0.84719078	no
GML	0.009938438	0.262395251	0.793094267	0.84719078	no
HPYR1	-0.009926619	-0.262083173	0.793334755	0.847402899	no
PCMTD1	0.00991353	0.261737552	0.793601114	0.847627417	no
OR10A7	-0.009912169	-0.261701635	0.793628796	0.847627417	no
C3orf63	-0.009895655	-0.261265586	0.793964883	0.847941577	no
SNAR-G1	-0.009855365	-0.260201741	0.794785008	0.848772623	no
KRT9	0.009848993	0.260033494	0.794914731	0.84886632	no
SSX5	0.009845959	0.259953384	0.794976501	0.848887446	no
SDHAF1	-0.009842676	-0.259866701	0.79504334	0.848913981	no
NOS1	0.00983775	0.259736635	0.795143633	0.848962552	no
LRRC57	-0.009836318	-0.259698799	0.795172808	0.848962552	no
C6orf126	0.009799503	0.258726737	0.795922474	0.849686183	no
MAT2A	0.009798906	0.25871096	0.795934643	0.849686183	no
MLN	-0.009794572	-0.258596528	0.796022907	0.84973554	no
GLYATL3	-0.009781243	-0.258244567	0.796294399	0.849967142	no
OR2T2	-0.009779445	-0.258197103	0.796331014	0.849967142	no
C2orf49	-0.009777728	-0.258151763	0.796365989	0.849967142	no
INPP4A	0.009774561	0.258068149	0.796430492	0.849991115	no
CST9L	-0.009771627	-0.257990677	0.796490258	0.850010031	no
SLC19A2	-0.009766011	-0.257842393	0.796604655	0.850087243	no
ZNF814	-0.009758434	-0.257642313	0.796759017	0.850207094	no
ZDHHC8P1	0.009756193	0.257583143	0.796804669	0.850210935	no
AKR1C1	-0.009748322	-0.257375327	0.796965011	0.850337147	no
C20orf85	-0.009741352	-0.257191283	0.797107019	0.850406117	no
TAAR2	-0.009741021	-0.257182525	0.797113776	0.850406117	no
OR6C76	0.009726255	0.256792656	0.797414621	0.850682187	no
IGF2AS	-0.009720475	-0.256640024	0.797532408	0.850762952	no
FLJ13197	-0.009715144	-0.256499255	0.797641046	0.850833948	no
ZNF486	0.009693601	0.255930426	0.798080073	0.851257342	no
OR8K1	0.009690542	0.255849672	0.798142404	0.851278916	no
MSGN1	0.009680481	0.255584021	0.798347463	0.851426311	no
PNMT	-0.00967963	-0.255561531	0.798364823	0.851426311	no
DKFZP434K028	-0.009674179	-0.25541761	0.798475924	0.85149988	no
BANP	-0.009665462	-0.255187452	0.798653603	0.851644438	no
DAZAP1	0.009648029	0.254727128	0.799008999	0.851978479	no
C5orf23	0.009629898	0.254248392	0.799378655	0.85232769	no
HYAL3	0.009621205	0.254018858	0.799555906	0.852471724	no
TAS2R42	-0.009611247	-0.253755926	0.79975896	0.852633644	no
PRSS45	-0.009609621	-0.25371299	0.799792119	0.852633644	no
KRT78	-0.00960083	-0.253480875	0.799971387	0.85277979	no
TCL1B	0.009584586	0.253051964	0.800302673	0.853046679	no
OR51I1	0.009584417	0.253047485	0.800306133	0.853046679	no
KRTAP19-3	-0.009574444	-0.252784151	0.800509547	0.853218516	no
C1orf58	0.009568655	0.252631294	0.800627629	0.853299389	no
C15orf60	0.009558562	0.252364811	0.800833498	0.853464475	no

RPSAP58	-0.009556922	-0.252321509	0.800866951	0.853464475	no
CTSL3	0.009551693	0.252183446	0.800973618	0.853533158	no
FAM75A5	0.009541305	0.25190915	0.801185546	0.853713996	no
HIST1H2BA	0.009524281	0.251459627	0.801532892	0.854000802	no
LFNG	-0.009523972	-0.251451484	0.801539184	0.854000802	no
KRTAP5-9	-0.009521545	-0.251387405	0.801588701	0.854008556	no
TBX5	-0.009506454	-0.250988917	0.801896651	0.854291626	no
LOC100129935	-0.009474365	-0.25014163	0.802551533	0.854906182	no
ADAD1	-0.009474044	-0.250133155	0.802558084	0.854906182	no
ALPP	0.009471591	0.250068405	0.802608136	0.854914456	no
RPH3AL	0.009453002	0.249577567	0.802987582	0.855273572	no
ALAD	0.009450917	0.24952251	0.803030148	0.855273852	no
LOC158572	-0.00944221	-0.249292609	0.803207894	0.855418099	no
MYH11	0.009420621	0.24872256	0.803648665	0.855811513	no
THOC2	-0.00941997	-0.248705375	0.803661954	0.855811513	no
PRO1768	-0.009414984	-0.248573718	0.803763762	0.855874848	no
SHB	0.009411672	0.248486274	0.803831384	0.855887334	no
PHF7	-0.009410263	-0.248449071	0.803860154	0.855887334	no
FLJ43860	-0.00940113	-0.248207927	0.804046642	0.856040811	no
ZRANB2	0.009391834	0.247962479	0.80423647	0.856197828	no
EIF3B	0.009383801	0.247750366	0.804400526	0.856327392	no
C9orf144B	0.009379537	0.24763778	0.804487608	0.856356469	no
TUBG1	0.009378316	0.247605531	0.804512552	0.856356469	no
RUNDC2A	0.009375019	0.247518491	0.804579877	0.856383046	no
TCAM1P	0.009357361	0.247052235	0.804940548	0.856721837	no
HDGFL1	-0.009349456	-0.246843503	0.805102026	0.856848595	no
LOC441601	-0.009317557	-0.24600125	0.805753688	0.857497003	no
TAS2R41	-0.009297569	-0.245473464	0.806162112	0.857886498	no
CISD3	0.009291961	0.245325396	0.806276703	0.857933264	no
HTN3	-0.009291265	-0.245307018	0.806290927	0.857933264	no
LPPR4	-0.009283983	-0.245114761	0.806439723	0.858046433	no
DAZ1	-0.009277944	-0.244955302	0.80656314	0.858100563	no
TRAPPC2L	-0.00927734	-0.244939356	0.806575482	0.858100563	no
CDK5RAP2	-0.009270851	-0.244767999	0.806708115	0.858164686	no
DHDH	0.009270237	0.244751809	0.806720646	0.858164686	no
DCAF12L1	-0.009254921	-0.244347401	0.807033686	0.85845252	no
C11orf80	-0.009243842	-0.24405485	0.80726016	0.858648249	no
RG9MTD2	-0.009230424	-0.243700556	0.807534453	0.858894816	no
TBCB	0.009218921	0.243396852	0.807769599	0.859077333	no
RPP14	0.009217873	0.243369164	0.807791037	0.859077333	no
C17orf55	0.009206188	0.243060625	0.808029945	0.859286211	no
SLCO1B1	-0.009195526	-0.242779126	0.808247932	0.859472818	no
MPG	0.009191987	0.242685682	0.808320296	0.859504562	no
HKDC1	0.009188103	0.242583108	0.808399733	0.859543823	no
CHAF1A	0.009175471	0.242249585	0.808658036	0.859773253	no
DSPP	0.009170715	0.242124005	0.808755301	0.85981631	no
SERPINB4	-0.009169332	-0.242087482	0.808783589	0.85981631	no
MED15	0.009162026	0.241894573	0.808933007	0.859897471	no
OR5F1	-0.00916144	-0.241879096	0.808944995	0.859897471	no
SGPL1	0.009157946	0.241786861	0.809016438	0.859928203	no
RBMXL3	-0.009133464	-0.241140436	0.809517192	0.860370357	no

ZNF615	0.009133448	0.241140007	0.809517524	0.860370357	no
C17orf54	0.009117636	0.240722495	0.809840992	0.860668901	no
NDUFV2	0.009105228	0.240394893	0.810094825	0.860893412	no
AGTR2	-0.009091553	-0.240033819	0.810374615	0.861145483	no
SNORA65	-0.009084723	-0.239853478	0.810514367	0.861248724	no
HSP90AB4P	-0.009068275	-0.239419187	0.81085094	0.861561083	no
RNASE9	0.009065484	0.239345487	0.81090806	0.861576496	no
OR52N2	-0.009055567	-0.239083622	0.811111023	0.861746856	no
NPVF	-0.009049821	-0.238931912	0.811228615	0.861826501	no
PCDHB11	-0.009043532	-0.238765872	0.811357319	0.861854351	no
RBAK	0.009043222	0.238757679	0.81136367	0.861854351	no
GKN2	-0.009042292	-0.23873311	0.811382714	0.861854351	no
NPHP3	0.009032862	0.238484131	0.811575719	0.862014074	no
C6orf192	-0.009028349	-0.238364961	0.811668102	0.862066909	no
KRT15	-0.009012037	-0.237934269	0.812002003	0.862353741	no
DPYSL5	0.009010988	0.23790658	0.81202347	0.862353741	no
CGGBP1	0.009006279	0.237782238	0.812119876	0.862410823	no
MED20	-0.00900351	-0.237709115	0.812176571	0.862425731	no
PAICS	0.008989312	0.237334241	0.812467242	0.862689076	no
OR7D2	-0.008980363	-0.237097951	0.812650471	0.862834915	no
TTTY2	-0.008976623	-0.236999209	0.812727042	0.862834915	no
ANKRD13D	0.008975223	0.23696223	0.812755719	0.862834915	no
KLHDC7A	0.008974267	0.236936984	0.812775297	0.862834915	no
OR6C70	0.008965951	0.23671741	0.812945579	0.862970373	no
ERMP1	0.008958303	0.236515487	0.813102181	0.863091295	no
KLK9	-0.00895369	-0.236393676	0.813196654	0.863110111	no
HSFX1	-0.008950665	-0.236313804	0.813258603	0.863110111	no
KRTAP19-4	-0.008950665	-0.236313804	0.813258603	0.863110111	no
HTR3D	0.008949099	0.236272463	0.813290668	0.863110111	no
SMTNL2	-0.008941373	-0.236068446	0.81344891	0.863232734	no
GH1	0.008934799	0.235894887	0.813583533	0.863330283	no
CYP4A22	-0.008931997	-0.2358209	0.813640925	0.863331339	no
DIXDC1	-0.008930581	-0.235783507	0.813669931	0.863331339	no
DRD3	0.008918013	0.235451671	0.813927346	0.863559146	no
RPL36	-0.008912993	-0.23531911	0.814030184	0.863622935	no
DCUN1D1	-0.008909599	-0.235229511	0.814099695	0.863651361	no
MAGEA2	-0.008906539	-0.235148697	0.814162391	0.863672555	no
PHF8	0.008903098	0.235057844	0.814232877	0.86370201	no
C20orf114	-0.00889054	-0.234726278	0.814490128	0.863929563	no
SPATA19	-0.008887568	-0.234647798	0.814551021	0.863948827	no
PRAMEF8	0.008869293	0.234165261	0.814925447	0.86428187	no
NME3	0.008864914	0.234049631	0.815015178	0.86428187	no
OVCH1	0.008864593	0.234041162	0.81502175	0.86428187	no
GPR176	0.008858593	0.233882749	0.815144684	0.86428187	no
ACTR10	-0.008856627	-0.23383084	0.815184968	0.86428187	no
ZNF627	-0.008852198	-0.233713898	0.815275723	0.86428187	no
N4BP1	0.008851796	0.233703266	0.815283975	0.86428187	no
C21orf67	0.008851032	0.2336831	0.815299625	0.86428187	no
OR5P3	0.008849764	0.233649624	0.815325605	0.86428187	no
GABRR2	0.008848428	0.233614338	0.815352991	0.86428187	no
NCRNA00052	0.008848093	0.233605503	0.815359847	0.86428187	no

LMO7	-0.008847207	-0.233582114	0.815377999	0.86428187	no
MRPL52	-0.008831494	-0.233167232	0.815700007	0.864577864	no
SERPINB5	-0.008828749	-0.233094749	0.815756268	0.864592169	no
ST18	-0.008820078	-0.232865792	0.815933987	0.864735197	no
NETO2	0.008815531	0.232745742	0.816027175	0.864788627	no
LEUTX	-0.008812655	-0.23266981	0.816086118	0.864805762	no
SAAL1	0.008800853	0.23235819	0.816328028	0.865016775	no
SNORA20	0.008797136	0.232260049	0.816404218	0.865052171	no
DMRT3	0.008788001	0.232018856	0.816591472	0.86520524	no
FAM57A	-0.00878011	-0.231810501	0.81675324	0.86533129	no
TBC1D3P2	0.008777595	0.231744096	0.8168048	0.86534057	no
WFDC13	0.008774681	0.231667146	0.816864547	0.865358523	no
CCDC90A	-0.008764781	-0.231405746	0.817067517	0.865528191	no
CDX2	-0.008744371	-0.230866835	0.817486006	0.865914113	no
ACSM5	-0.008742835	-0.230826295	0.817517489	0.865914113	no
ZNF397OS	-0.008730576	-0.230502607	0.817768876	0.866135006	no
NINL	-0.008722925	-0.230300586	0.817925781	0.86624173	no
BEST2	0.008721484	0.230262541	0.81795533	0.86624173	no
LEPROTL1	-0.008706785	-0.229874427	0.818256793	0.8665156	no
BPY2	-0.008694632	-0.229553561	0.818506042	0.866734152	no
PCDHB4	-0.008678782	-0.229135045	0.818831173	0.867026596	no
ALKBH1	-0.008676987	-0.229087667	0.818867982	0.867026596	no
KRT79	0.008673245	0.228988857	0.818944749	0.867052207	no
PTCH2	0.008671138	0.228933211	0.818987983	0.867052207	no
LOC286135	-0.008669537	-0.228890939	0.819020825	0.867052207	no
KPNA3	0.00863621	0.228010979	0.819704578	0.867730622	no
FAM66D	-0.008632614	-0.227916037	0.819778358	0.86776329	no
RFX3	-0.008627577	-0.227783044	0.819881711	0.867827257	no
TRIM48	-0.008622617	-0.227652097	0.819983478	0.867889538	no
SCXB	0.008604974	0.227186233	0.820345551	0.868227313	no
PPP2R3C	0.008600629	0.227071515	0.820434717	0.868271639	no
C17orf97	-0.008598748	-0.227021846	0.820473324	0.868271639	no
CEP76	-0.008593751	-0.22688992	0.820575869	0.868334707	no
SLC38A5	0.008589923	0.226788844	0.820654436	0.868372397	no
SNORA36C	-0.008584994	-0.226658698	0.820755603	0.868433995	no
LCN1	0.008580018	0.22652731	0.820857737	0.86849661	no
MPDU1	-0.008559087	-0.225974664	0.821287373	0.868905709	no
NKIRAS2	0.008555684	0.225884805	0.821357236	0.868934153	no
RABGGTA	-0.008553547	-0.22582837	0.821401113	0.868935104	no
FAM25B	-0.008546383	-0.225639215	0.821548182	0.869045213	no
LOC100287704	-0.0085441	-0.225578958	0.821595034	0.869049304	no
FLJ46111	0.008540411	0.225481554	0.82167077	0.869083946	no
PWRN2	-0.008505568	-0.224561565	0.822386182	0.869795136	no
MPZ	0.008494836	0.224278207	0.82260656	0.869982708	no
KRT77	0.008484522	0.224005866	0.822818382	0.870158412	no
UNCX	-0.008480798	-0.223907531	0.822894869	0.870158412	no
ASMT	0.008480459	0.223898604	0.822901812	0.870158412	no
HMGB4	-0.00847487	-0.223751036	0.823016597	0.870234274	no
DUXA	-0.008463788	-0.223458431	0.823244208	0.870429422	no
GEMIN8P4	0.008459093	0.223334446	0.823340658	0.870449312	no
LOC729176	0.008456928	0.223277295	0.823385118	0.870449312	no

CUZD1	-0.008456584	-0.223268217	0.82339218	0.870449312	no
KRT37	-0.008448735	-0.223060957	0.823553419	0.870535354	no
SEMA4C	0.00844843	0.223052903	0.823559685	0.870535354	no
FAM96B	-0.008441866	-0.222879591	0.82369452	0.870632361	no
INSC	-0.008435764	-0.222718491	0.823819859	0.870661693	no
LIG1	0.008434096	0.22267445	0.823854124	0.870661693	no
A2ML1	-0.008432029	-0.222619856	0.823896601	0.870661693	no
PRAMEF2	0.008430556	0.222580977	0.823926851	0.870661693	no
NAT14	-0.008430033	-0.222567176	0.823937589	0.870661693	no
BCL2	-0.008427489	-0.222499998	0.823989857	0.870671419	no
SRRM5	-0.008421534	-0.22234276	0.824112202	0.870755187	no
SSX8	-0.008416527	-0.222210553	0.824215073	0.870818371	no
GRP	-0.008413284	-0.222124933	0.824281696	0.870843253	no
PSG1	0.00839455	0.221630285	0.824666621	0.871204397	no
FIBP	0.008386894	0.221428135	0.824823942	0.871325067	no
CEACAM16	-0.008384053	-0.221353143	0.824882305	0.871341194	no
KIR3DX1	-0.008375073	-0.221116015	0.82506686	0.87149061	no
MS4A12	-0.008367344	-0.220911943	0.825225695	0.871612846	no
PTENP1	-0.008362428	-0.220782145	0.825326724	0.871665733	no
HPCAL1	-0.008359058	-0.220693179	0.825395973	0.871665733	no
S100A1	-0.008358614	-0.220681444	0.825405107	0.871665733	no
RXFP4	-0.008346959	-0.220373706	0.825644654	0.871873165	no
HELT	-0.008320636	-0.219678703	0.826185712	0.872398951	no
LNX1	-0.008309731	-0.21939077	0.826409891	0.872589293	no
KRT28	-0.008307669	-0.219336317	0.826452289	0.872589293	no
FAM75A2	-0.008297613	-0.219070817	0.826659017	0.872761985	no
RPS21	-0.008293537	-0.218963178	0.826742832	0.872804897	no
PLA2G12B	-0.008279289	-0.218586993	0.827035772	0.873068569	no
SCAMP3	0.008271556	0.218382812	0.82719478	0.873190835	no
MC3R	-0.008255037	-0.217946655	0.827534466	0.873503802	no
SRRD	0.008251134	0.217843612	0.827614722	0.873542911	no
MT1IP	0.00824672	0.217727062	0.827705501	0.87359312	no
NDUFAB1	0.008242097	0.217605007	0.827800569	0.873647495	no
SCARB2	0.008240013	0.217549965	0.827843442	0.873647495	no
CCL1	-0.008232086	-0.217340678	0.828006463	0.873773928	no
AGTPBP1	-0.008222083	-0.217076566	0.828212202	0.873945422	no
SDC4P	0.008219525	0.217009018	0.828264822	0.873955335	no
SEC23B	0.008200551	0.216508031	0.828655118	0.87432153	no
CYP26B1	0.008192242	0.216288647	0.828826043	0.87445624	no
LIPM	0.008180052	0.215966787	0.829076823	0.874651112	no
HMX2	-0.008179058	-0.215940548	0.829097268	0.874651112	no
OR7A17	-0.008170246	-0.21570788	0.829278566	0.874796726	no
FAHD2A	0.00815588	0.215328568	0.82957415	0.875062877	no
ETV5	0.008139356	0.214892273	0.829914168	0.875375868	no
PPP1R16A	-0.008135283	-0.214784731	0.829997984	0.875418605	no
TMEM86B	-0.008123731	-0.214479731	0.830235705	0.875579727	no
FOXI2	0.008122295	0.21444181	0.830265262	0.875579727	no
ARGFXP2	-0.008121177	-0.214412291	0.83028827	0.875579727	no
KRT25	-0.008119442	-0.214366484	0.830323974	0.875579727	no
CLDN2	-0.008109847	-0.214113149	0.830521443	0.875742283	no
EIF2C4	-0.008089544	-0.213577069	0.830939341	0.876137241	no

THY1	-0.008080686	-0.213343207	0.831121661	0.876283779	no
FLG	-0.008066328	-0.212964091	0.831417243	0.876541996	no
PCDHB16	-0.008064577	-0.212917872	0.831453279	0.876541996	no
IL13	-0.008061293	-0.212831165	0.831520885	0.87654532	no
CXorf61	0.008060212	0.21280262	0.831543142	0.87654532	no
FAM71E1	-0.008056199	-0.212696654	0.831625766	0.876586713	no
STK31	-0.008050663	-0.2125505	0.83173973	0.876661133	no
FAM187B	-0.008040211	-0.212274531	0.831954926	0.87684224	no
PLLP	-0.00803127	-0.212038443	0.832139032	0.876990562	no
HINT2	-0.008024691	-0.211864756	0.832274484	0.877087595	no
MKKS	-0.008013483	-0.211568824	0.83250528	0.877285091	no
LOC100130933	-0.007979086	-0.210660618	0.833213678	0.877985831	no
SGCB	-0.007973559	-0.210514689	0.833327514	0.878015453	no
C2orf83	0.007973504	0.210513241	0.833328644	0.878015453	no
TTLL8	-0.007964937	-0.210287044	0.833505104	0.878155612	no
SLC22A25	-0.00795573	-0.210043934	0.833694766	0.878309665	no
SEPW1	-0.007941125	-0.209658328	0.833995618	0.878580836	no
NCRNA00159	0.007927486	0.209298204	0.83427661	0.878831058	no
NDUFA13	-0.007894867	-0.208436972	0.834948685	0.879493202	no
C19orf20	-0.007891749	-0.208354635	0.835012945	0.879515067	no
SPESP1	-0.00788639	-0.208213139	0.835123377	0.879585561	no
RFX4	-0.00788211	-0.208100144	0.835211567	0.879632623	no
H2BFWT	-0.007866091	-0.207677188	0.835541694	0.879912349	no
CSPG4PY2	0.007864999	0.207648343	0.83556421	0.879912349	no
ZP2	-0.007848551	-0.207214084	0.835903192	0.880223475	no
SAMD13	0.007840482	0.207001023	0.836069518	0.880352769	no
B4GALNT3	-0.007837513	-0.206922639	0.836130711	0.880371352	no
CYP24A1	0.007828337	0.206680361	0.836319857	0.880523402	no
OR5B3	0.007826282	0.206626097	0.836362223	0.880523402	no
PLA2G3	0.007821746	0.206506344	0.836455718	0.880575981	no
C1orf57	-0.007819439	-0.206445413	0.83650329	0.88058021	no
C19orf52	-0.007804853	-0.2060603	0.836803982	0.880850883	no
UGT1A4	-0.007799832	-0.205927726	0.8369075	0.880913985	no
LENG1	-0.007787838	-0.20561105	0.837154782	0.881128397	no
E2F3	0.007777544	0.205339261	0.837367026	0.88130591	no
RHBG	-0.007773972	-0.205244954	0.837440674	0.881337543	no
DAZ4	-0.007770652	-0.205157291	0.837509135	0.881363715	no
TMPRSS4	0.007768016	0.205087699	0.837563485	0.881375034	no
LOC731789	0.007765316	0.205016417	0.837619155	0.881387742	no
TMEM9B	-0.007762407	-0.204939603	0.837679146	0.881404995	no
KRTAP4-3	-0.007759608	-0.204865703	0.837736862	0.881419852	no
LYPD2	0.007731184	0.204115219	0.838323045	0.881990702	no
GAB4	0.007725084	0.203954167	0.83844885	0.882077159	no
ASFMR1	0.00771224	0.203615044	0.838713768	0.882309951	no
PIWIL1	0.007709392	0.20353983	0.838772526	0.882325855	no
STAC2	0.007695547	0.203174275	0.839058117	0.882580355	no
GTF3C3	0.007687173	0.202953195	0.839230846	0.88271612	no
CLDND1	0.007681656	0.202807519	0.839344668	0.882789913	no
C17orf105	-0.00767275	-0.202572382	0.839528394	0.882937217	no
WBP2NL	-0.007667545	-0.202434955	0.839635778	0.883004221	no
TMEM99	0.007665167	0.202372155	0.83968485	0.883009898	no

P2RX3	0.007652155	0.202028614	0.839953306	0.883217347	no
FAM160A1	0.007651371	0.202007904	0.83996949	0.883217347	no
AKT1	0.007647603	0.201908423	0.840047231	0.883253155	no
LRRFIP2	-0.007641695	-0.201752422	0.840169145	0.883335402	no
KCNF1	-0.007639344	-0.201690346	0.840217659	0.883340472	no
PTGDS	-0.007629773	-0.201437658	0.840415144	0.883502151	no
MPP6	-0.007621063	-0.201207675	0.840594893	0.88364517	no
OR8K3	0.007617785	0.201121131	0.840662535	0.883670331	no
CDRT15P	0.007611171	0.200946504	0.840799028	0.883767858	no
LOC643923	-0.00760871	-0.200881519	0.840849823	0.883775302	no
NAPEPLD	-0.007604874	-0.200780242	0.840928986	0.883812561	no
CCT4	-0.007578769	-0.200091	0.841467776	0.884332857	no
TTTY10	-0.00757082	-0.199881099	0.841631874	0.88445934	no
FAT2	-0.007568519	-0.199820353	0.841679365	0.884463275	no
DCD	-0.007556537	-0.199504002	0.8419267	0.884677201	no
LOC401588	0.007552634	0.199400948	0.842007275	0.884715886	no
CASC4	0.007549359	0.199314469	0.842074891	0.884740953	no
OR8D2	-0.007530862	-0.198826109	0.842456753	0.885041016	no
RBMY2EP	-0.007530862	-0.198826109	0.842456753	0.885041016	no
ZNF679	-0.007529164	-0.198781279	0.842491809	0.885041016	no
BMPER	0.00751659	0.198449274	0.842751437	0.885254998	no
PRPF40A	0.007515058	0.198408819	0.842783074	0.885254998	no
RASD1	0.007504754	0.198136767	0.842995833	0.885432477	no
ASB10	0.007459346	0.196937869	0.843933571	0.886371373	no
SLC10A2	0.007445402	0.196569709	0.844221577	0.886601983	no
PPEF2	0.00744447	0.196545082	0.844240844	0.886601983	no
INS	-0.007435949	-0.196320121	0.844416839	0.88674075	no
ABCC9	-0.007400884	-0.195394286	0.845141235	0.887455359	no
COL9A2	-0.007392467	-0.19517207	0.845315122	0.887591854	no
KLK6	-0.007382739	-0.194915226	0.845516115	0.887756793	no
CLCA2	0.007335389	0.193665035	0.846494596	0.888738004	no
PCK1	0.0073249	0.193388108	0.846711368	0.888912901	no
HSD11B2	-0.007323074	-0.193339892	0.846749112	0.888912901	no
SLC30A2	0.007315684	0.193144762	0.846901865	0.889027098	no
GAGE2A	-0.007307647	-0.192932559	0.847067989	0.889155319	no
FXYD1	0.007303629	0.192826469	0.847151045	0.889196336	no
MAGEA8	-0.007299528	-0.192718203	0.847235806	0.889239138	no
STRA13	0.00728421	0.192313769	0.847552451	0.889525304	no
OR2K2	0.007280193	0.192207708	0.847635493	0.889566281	no
TMEM225	0.007268535	0.191899906	0.847876503	0.889773027	no
FBXO15	-0.007257917	-0.191619564	0.848096024	0.889957203	no
DCAF8L2	-0.007250726	-0.191429678	0.84824472	0.890067042	no
PCDHA13	-0.007247255	-0.19133806	0.848316468	0.890096131	no
OR4A5	-0.007236806	-0.191062172	0.848532524	0.890276625	no
INSL6	-0.007227413	-0.19081416	0.84872676	0.890434209	no
TMEM171	0.007219079	0.190594126	0.848899091	0.890568796	no
SPAM1	-0.007216311	-0.190521027	0.848956345	0.890582648	no
CCNL2	-0.007210532	-0.190368469	0.849075835	0.890661784	no
TMEM203	-0.007187347	-0.189756294	0.849555353	0.891118554	no
SHOC2	0.007181498	0.189601879	0.849676315	0.891154039	no
OC90	0.007181449	0.189600574	0.849677338	0.891154039	no

RGNEF	0.00717757	0.189498166	0.849757562	0.891191949	no
TTTY6	-0.007147194	-0.188696157	0.850385892	0.891804656	no
USP9Y	0.00713043	0.188253547	0.850732693	0.892122074	no
ARIH1	0.007124275	0.188091044	0.850860028	0.892209328	no
RGL2	0.007117248	0.187905499	0.851005422	0.892315508	no
HMGN1	0.007112414	0.187777856	0.851105447	0.892333712	no
STX7	-0.007110223	-0.187720021	0.851150769	0.892333712	no
GRAMD1C	-0.007110009	-0.187714379	0.85115519	0.892333712	no
LOC93432	-0.00710778	-0.187655518	0.851201317	0.8923358	no
MRPL44	-0.007103714	-0.187548161	0.85128545	0.892377727	no
SCARNA14	-0.007095104	-0.18732083	0.851463607	0.892518208	no
DRAP1	0.007071856	0.186707034	0.851944671	0.892944004	no
CABP7	0.007071205	0.18668984	0.851958148	0.892944004	no
LOC100124692	-0.007063529	-0.186487164	0.852117008	0.893032071	no
SSPN	0.007062876	0.186469934	0.852130513	0.893032071	no
TGIF2LX	0.007058896	0.186364853	0.85221288	0.893072099	no
RAB9BP1	0.007042995	0.185945024	0.852541975	0.893370667	no
GAGE2D	-0.007027889	-0.185546186	0.852854639	0.893651987	no
TMEM160	-0.007022866	-0.185413547	0.852958625	0.893675602	no
TRIT1	0.007022231	0.185396798	0.852971757	0.893675602	no
GIPC1	0.007020114	0.185340907	0.853015575	0.893675602	no
DCAF4L2	0.007018259	0.185291916	0.853053983	0.893675602	no
MFSD2A	-0.007007882	-0.185017939	0.853268788	0.893854319	no
MEP1A	-0.007003343	-0.184898083	0.853362761	0.893906445	no
FOXD3	-0.006999306	-0.184791512	0.85344632	0.893947658	no
CFHR4	0.006993849	0.184647432	0.853559292	0.893995846	no
POM121	-0.006992812	-0.184620053	0.85358076	0.893995846	no
OR1A2	-0.006982171	-0.184339091	0.853801068	0.894180264	no
SNORA47	0.006965825	0.183907514	0.854139499	0.894488366	no
LAMA1	0.006960961	0.1837791	0.854240204	0.894547492	no
PRAME	0.006953427	0.183580169	0.854396213	0.894664524	no
C8orf12	-0.006949022	-0.183463887	0.854487408	0.894713678	no
KRT16	-0.006943171	-0.183309382	0.854608584	0.894794218	no
EGLN3	-0.00693447	-0.183079665	0.854788752	0.894936513	no
SRL	-0.006923613	-0.182793009	0.85501359	0.895125557	no
AURKAIP1	-0.00691611	-0.182594908	0.855168976	0.895241876	no
CLEC3A	0.006903418	0.182259811	0.855431832	0.895470683	no
C18orf56	0.006892167	0.181962761	0.855664856	0.895668241	no
FITM2	-0.006886722	-0.181818996	0.85577764	0.895739921	no
LOC338588	-0.006868748	-0.181344425	0.856149959	0.896083236	no
MAG	-0.006863881	-0.181215927	0.856250776	0.896142364	no
KRTAP19-8	0.006854631	0.180971704	0.856442395	0.896274456	no
RIPK4	0.006853509	0.180942068	0.856465649	0.896274456	no
CWC25	-0.006846794	-0.180764778	0.856604758	0.896373635	no
GAGE13	-0.006840988	-0.180611495	0.856725034	0.896453097	no
C9orf43	-0.006837885	-0.180529557	0.856789329	0.896473977	no
TAF2	0.006823904	0.180160423	0.857078994	0.896730651	no
RASL12	-0.006818102	-0.180007242	0.857199203	0.896743009	no
OR52M1	0.006816844	0.179974019	0.857225275	0.896743009	no
CGB2	0.006816637	0.179968575	0.857229548	0.896743009	no
OR3A4	-0.006813654	-0.179889795	0.857291372	0.896743009	no

MAGEA1	-0.00681263	-0.179862779	0.857312574	0.896743009	no
PAX4	-0.00680791	-0.179738138	0.857410391	0.896798928	no
NBN	-0.006799176	-0.179507556	0.857591356	0.896941155	no
EXD1	-0.006796997	-0.179450018	0.857636514	0.896941155	no
UMOD	-0.006794925	-0.179395296	0.857679462	0.896941155	no
ATPAF2	0.006788054	0.179213905	0.857821828	0.897043639	no
MGLL	-0.006778446	-0.178960226	0.858020939	0.897205449	no
OR6C6	-0.006773401	-0.178827028	0.858125489	0.897268366	no
KRTAP10-12	-0.006739683	-0.177936791	0.858824318	0.897932484	no
TMEM38A	0.006738471	0.177904766	0.858849459	0.897932484	no
XPOT	-0.006711139	-0.177183132	0.85941602	0.898375145	no
ZNF710	0.006710353	0.177162387	0.859432308	0.898375145	no
NAIF1	-0.006710114	-0.177156082	0.859437258	0.898375145	no
PENK	-0.006709471	-0.177139101	0.859450591	0.898375145	no
ELAC2	-0.006704921	-0.177018969	0.859544916	0.898427292	no
CGB5	-0.006701985	-0.176941449	0.859605784	0.898444465	no
MRPS7	0.006697241	0.176816197	0.859704131	0.898500808	no
SNAR-F	0.006683694	0.176458519	0.859984993	0.898747885	no
OR9Q2	-0.006667845	-0.176040076	0.860313592	0.8990401	no
C1orf173	-0.006664225	-0.175944495	0.860388654	0.8990401	no
MATN4	-0.006663774	-0.175932586	0.860398006	0.8990401	no
PDCD6	0.006657798	0.175774815	0.86052191	0.8991231	no
LRRC30	-0.006650009	-0.175569161	0.860683424	0.899233628	no
PLSCR5	-0.006648407	-0.17552686	0.860716647	0.899233628	no
RPL23A	0.00663011	0.17504378	0.861096069	0.899583544	no
MAPK7	0.006622249	0.174836212	0.861259107	0.899645694	no
RBBP5	-0.006622233	-0.174835811	0.861259422	0.899645694	no
SOX13	0.006620805	0.174798089	0.861289052	0.899645694	no
ST7OT2	0.00661205	0.174566955	0.861470609	0.89975775	no
MRPL41	0.00661134	0.174548207	0.861485336	0.89975775	no
RPS16	-0.006598887	-0.1742194	0.861743629	0.899981026	no
HPS6	-0.006578901	-0.173691726	0.862158172	0.900367455	no
LOC442308	0.006572785	0.17353026	0.862285028	0.900453422	no
GEMIN5	-0.006569033	-0.173431177	0.862362875	0.900488204	no
DNAJB8	0.006554646	0.173051341	0.862661313	0.900753314	no
SEC14L4	-0.006543838	-0.172765988	0.862885528	0.900940901	no
HIST1H2AA	-0.006538424	-0.172623048	0.862997847	0.901011643	no
TMSB15B	0.0065308	0.17242174	0.863156035	0.901130265	no
AQPEP	-0.006526414	-0.172305931	0.86324704	0.90117874	no
OR10G8	0.006520829	0.172158493	0.863362903	0.901253158	no
RGPD5	0.006515144	0.172008385	0.863480866	0.901329761	no
HNRNPH1	0.006502121	0.171664559	0.863751077	0.901562508	no
P2RY4	-0.0065001	-0.171611186	0.863793023	0.901562508	no
FAM118A	0.006475761	0.170968586	0.864298086	0.902018821	no
LOC643955	-0.006474732	-0.170941407	0.864319449	0.902018821	no
OXT	-0.00647	-0.17081649	0.864417636	0.902074728	no
SYCP1	-0.006445395	-0.170166838	0.864928311	0.902561063	no
TTC18	0.006432395	0.169823616	0.865198131	0.902796027	no
C14orf23	0.006429694	0.169752308	0.865254192	0.902807929	no
PARD6B	0.006426955	0.169679982	0.865311053	0.902820666	no
C6orf226	-0.006406609	-0.169142805	0.865733391	0.903214702	no

SNORA56	-0.006398403	-0.168926136	0.865903751	0.903345823	no
C18orf62	-0.006357453	-0.167844977	0.866753926	0.904186104	no
ALKBH8	-0.006347067	-0.167570764	0.866969579	0.904364408	no
SLC22A24	0.006336684	0.167296605	0.8671852	0.90454266	no
LMOD3	0.006331149	0.167150488	0.867300122	0.904615862	no
SS18L2	-0.006319397	-0.166840189	0.867544185	0.904801669	no
GALP	0.006318261	0.166810205	0.867567769	0.904801669	no
MOBKL3	0.006297436	0.166260373	0.868000266	0.905206034	no
HTN1	-0.006280836	-0.165822112	0.868345031	0.905518869	no
VRK1	-0.006276272	-0.165701598	0.868439839	0.905571028	no
TTTY18	-0.006271292	-0.165570127	0.868543269	0.905632172	no
KIAA0141	0.006267638	0.165473637	0.868619181	0.905664618	no
OR4C46	0.006260654	0.165289248	0.868764249	0.905769161	no
TPSD1	-0.006254494	-0.165126601	0.868892215	0.905855865	no
C1orf111	0.00624773	0.16494802	0.869032722	0.905955634	no
PRRG1	-0.006241854	-0.164792877	0.869154791	0.906036172	no
TFAP2B	-0.006231264	-0.164513281	0.869374789	0.906218782	no
ZIK1	0.006226514	0.164387862	0.869473478	0.906251714	no
EID2	-0.006225428	-0.1643592	0.869496031	0.906251714	no
KIF15	0.006221236	0.164248529	0.869583117	0.906279726	no
CAPZA3	-0.006219819	-0.164211116	0.869612557	0.906279726	no
TNS3	-0.006200487	-0.163700691	0.870014229	0.906651599	no
ARGFX	-0.006195311	-0.163564037	0.870121772	0.906670648	no
UNC45B	0.006194371	0.163539212	0.870141309	0.906670648	no
TMCC3	0.006193132	0.163506514	0.870167042	0.906670648	no
TACC3	0.006181027	0.163186921	0.870418567	0.906849024	no
CCNO	-0.006180576	-0.163175006	0.870427944	0.906849024	no
OSBPL5	-0.006170682	-0.162913771	0.87063355	0.907016494	no
KIAA1683	0.006165028	0.162764507	0.870751032	0.907078113	no
GPATCH1	-0.006163517	-0.16272462	0.870782426	0.907078113	no
ZNF136	-0.006161127	-0.162661495	0.870832112	0.907083134	no
ANP32D	0.006134152	0.161949306	0.87139271	0.907620308	no
FAM21A	-0.006119791	-0.161570135	0.871691199	0.907884435	no
FLJ33630	0.00610361	0.161142928	0.872027526	0.908187942	no
RSPO3	0.00609047	0.160795991	0.872300675	0.908425623	no
MAMSTR	-0.006087319	-0.160712814	0.872366164	0.908447031	no
ZNF175	0.006084643	0.160642158	0.872421795	0.908458172	no
SCRG1	0.00607891	0.160490807	0.872540964	0.90853547	no
CSAG2	-0.006075303	-0.160395568	0.872615955	0.908566761	no
PWRN1	-0.00606879	-0.160223603	0.872751359	0.90866095	no
PRKAG3	0.006066007	0.160150128	0.872809215	0.908674393	no
PTTG3P	-0.006056957	-0.159911199	0.872997356	0.90879983	no
PHF19	-0.006055888	-0.15988295	0.873019601	0.90879983	no
CCDC87	-0.006053619	-0.159823051	0.873066769	0.908802139	no
FAM27L	0.006046403	0.159632547	0.873216786	0.9089115	no
C11orf52	-0.006043005	-0.159542817	0.873287448	0.908938256	no
C8orf33	-0.006030071	-0.159201348	0.873556361	0.909171343	no
POLA1	0.006024959	0.15906636	0.873662671	0.909235182	no
WDR70	-0.006005933	-0.158564041	0.874058292	0.909559365	no
USP24	0.006005651	0.158556608	0.874064146	0.909559365	no
USP38	0.005990987	0.158169435	0.874369101	0.909829876	no

C1QTNF3	0.005974601	0.157736802	0.874709884	0.910137638	no
HIST2H3C	-0.005971455	-0.157653741	0.874775314	0.910144657	no
SUZ12	-0.005969947	-0.157613944	0.874806663	0.910144657	no
CGRRF1	0.005962293	0.157411842	0.87496587	0.910263454	no
ZNF689	0.005954854	0.157215453	0.875120581	0.910377561	no
RCL1	-0.00594972	-0.157079903	0.875227366	0.910441803	no
ASZ1	0.005938953	0.156795636	0.875451318	0.910605354	no
LOC340508	0.005937831	0.156765996	0.87547467	0.910605354	no
TRPT1	-0.005932576	-0.156627265	0.875583969	0.910672189	no
IRAK1BP1	0.00592941	0.156543679	0.875649824	0.910693834	no
METTL14	0.005918675	0.156260231	0.875873149	0.910879241	no
LOC200726	0.005914844	0.156159083	0.875952845	0.910915267	no
FBN3	-0.005908514	-0.155991979	0.876084511	0.910972778	no
OR13C5	-0.005907853	-0.155974525	0.876098264	0.910972778	no
ZNF665	0.005898793	0.155735323	0.876286744	0.911121903	no
NDUFS6	-0.00589606	-0.155663166	0.876343602	0.911134164	no
FLNB	-0.005867823	-0.154917643	0.876931094	0.911698095	no
VASH2	-0.005847875	-0.154390973	0.877346165	0.912082721	no
MORN1	-0.005842618	-0.154252184	0.87745555	0.912149536	no
TCEB3B	0.005823586	0.153749696	0.877851603	0.912514329	no
IL31	0.005804413	0.153243478	0.878250626	0.912882173	no
LOC255025	-0.005801887	-0.153176777	0.878303205	0.912889893	no
ODC1	-0.005793391	-0.152952473	0.878480023	0.913026736	no
KIAA1797	0.005788531	0.152824156	0.878581178	0.913067431	no
KRTAP10-8	-0.00578717	-0.152788229	0.8786095	0.913067431	no
SH3D19	-0.005781254	-0.152632038	0.878732631	0.913098024	no
LRTOMT	-0.005779992	-0.152598725	0.878758894	0.913098024	no
MRPL54	0.005779246	0.152579022	0.878774427	0.913098024	no
ZNF749	-0.005776447	-0.152505119	0.878832689	0.913111635	no
USP42	-0.005771763	-0.152381444	0.878930191	0.913122634	no
C16orf3	-0.005771599	-0.152377116	0.878933603	0.913122634	no
TXNL4B	0.005761942	0.152122163	0.879134606	0.913284527	no
TTTY4C	-0.005756804	-0.151986515	0.879241554	0.913321572	no
DUSP13	-0.005755889	-0.151962341	0.879260613	0.913321572	no
ATP6V1E2	-0.005738437	-0.151501588	0.879623897	0.913651988	no
LYRM1	0.00572642	0.151184301	0.879874079	0.913864899	no
MEIG1	-0.005720563	-0.151029668	0.879996012	0.913944592	no
TRAIP	0.005705865	0.150641605	0.880302024	0.914215447	no
TTTY9B	0.005703681	0.150583954	0.880347487	0.914215702	no
TAAR9	-0.005701321	-0.150521637	0.88039663	0.914219778	no
LBX1	0.005688955	0.150195163	0.880654095	0.914440167	no
LTBP4	-0.005682013	-0.150011865	0.880798653	0.914543301	no
PCDHGA4	-0.005646718	-0.149080009	0.881533623	0.915259425	no
CRYGC	-0.005641034	-0.148929948	0.881651988	0.915335313	no
C9orf57	0.00563687	0.148819996	0.881738718	0.915378352	no
GAGE2C	-0.005630628	-0.148655203	0.881868708	0.915466294	no
FAM83C	-0.005611513	-0.148150521	0.882266827	0.915832557	no
PDILT	-0.005607971	-0.148057001	0.882340604	0.915862118	no
PTP4A1	0.005603929	0.147950307	0.882424775	0.91589417	no
TBC1D7	-0.005602138	-0.147903014	0.882462085	0.91589417	no
OR4F4	0.005591778	0.147629494	0.882677872	0.916071106	no

CA8	-0.005589301	-0.147564073	0.882729485	0.916077648	no
AQR	0.005580617	0.147334805	0.88291037	0.916179493	no
KIF11	0.005580238	0.147324812	0.882918253	0.916179493	no
ANKAR	-0.005576234	-0.147219095	0.883001663	0.916219021	no
PRR20A	0.005539625	0.146252537	0.883764321	0.916963309	no
FAR1	-0.005537261	-0.14619013	0.883813567	0.916967347	no
LOC113230	0.005534845	0.146126349	0.883863897	0.916972509	no
NHEG1	0.005527755	0.145939157	0.884011615	0.917078703	no
FAM75A3	-0.005524846	-0.145862348	0.884072228	0.917094526	no
C13orf28	-0.005522432	-0.145798623	0.884122517	0.917099638	no
C21orf58	-0.005514192	-0.145581057	0.884294213	0.917230679	no
ELMOD1	0.005504149	0.145315907	0.884503468	0.917400663	no
FBXO24	0.005500521	0.145220114	0.884579069	0.917432011	no
SCARNA27	-0.005494306	-0.145056038	0.884708563	0.917519247	no
TMEM90A	-0.005488295	-0.144897341	0.884833815	0.917602075	no
PTCD1	-0.005471388	-0.144450953	0.885186141	0.917857899	no
CTSL2	-0.005470265	-0.144421312	0.885209537	0.917857899	no
COPG2	0.005469922	0.14441224	0.885216697	0.917857899	no
TMEM229A	0.005456803	0.144065889	0.885490084	0.918084465	no
LOC441089	0.005455079	0.144020357	0.885526024	0.918084465	no
SYNGR4	-0.005449048	-0.143861126	0.885651716	0.918136458	no
AMTN	-0.005448315	-0.143841768	0.885666997	0.918136458	no
MYH13	-0.005441537	-0.143662829	0.885808249	0.918235807	no
ONECUT3	-0.005435179	-0.143494956	0.885940769	0.918326095	no
DHRS13	0.005428778	0.143325953	0.886074185	0.918417301	no
POTEC	-0.005422358	-0.143156462	0.886207989	0.918508901	no
SLC6A13	-0.005419352	-0.143077094	0.886270647	0.918526755	no
PCDHB14	-0.005407191	-0.142756015	0.886524133	0.918742372	no
SENP8	0.005400859	0.142588854	0.886656108	0.918813258	no
OR2T3	-0.005397899	-0.142510689	0.886717821	0.918813258	no
TTTY11	0.005397369	0.142496696	0.886728869	0.918813258	no
LOC647859	0.00539174	0.142348084	0.886846204	0.918887745	no
MAFA	0.00537554	0.141920378	0.887183909	0.919190544	no
RTN4	-0.00534824	-0.141199605	0.887753057	0.919733093	no
RPL13AP17	-0.005338334	-0.140938057	0.887959598	0.919895605	no
NKX2-4	0.005336352	0.140885737	0.888000916	0.919895605	no
AKAP4	0.005327133	0.140642347	0.888193127	0.920047579	no
TMPRSS11B	-0.005318964	-0.140426666	0.888363461	0.920176877	no
NRG4	0.005314298	0.140303486	0.888460745	0.920230499	no
AKNAD1	0.005303618	0.140021511	0.888683446	0.920414012	no
LOC360030	-0.005276094	-0.139294809	0.88925743	0.920931279	no
LOC440896	-0.005274016	-0.13923995	0.889300762	0.920931279	no
HUS1B	-0.005273116	-0.139216188	0.889319532	0.920931279	no
OR5K3	-0.00526678	-0.139048924	0.889451655	0.921020925	no
OR9Q1	0.005263642	0.138966052	0.889517116	0.921041537	no
OR9I1	-0.005259544	-0.138857875	0.889602568	0.921082846	no
AMHR2	0.005249584	0.138594903	0.889810303	0.921250754	no
OR10J5	-0.005226579	-0.137987527	0.890290129	0.921690829	no
FLRT2	0.005222779	0.137887205	0.890369387	0.921690829	no
OR52B4	0.005220089	0.137816175	0.890425504	0.921690829	no
POLD2	-0.00521771	-0.137753374	0.890475119	0.921690829	no

ANKRD17	-0.005216639	-0.137725108	0.890497451	0.921690829	no
TRIL	0.005216091	0.137710635	0.890508886	0.921690829	no
LOC643837	-0.00520619	-0.13744922	0.890715424	0.921857406	no
PDCL2	-0.005190293	-0.13702952	0.891047033	0.922097519	no
LHX9	0.005188892	0.136992516	0.891076271	0.922097519	no
SNORA5C	-0.005188509	-0.136982421	0.891084248	0.922097519	no
B3GALT6	-0.005178241	-0.136711318	0.891298461	0.922271983	no
C2CD4D	0.005160559	0.136244487	0.891667348	0.922606471	no
DEFB109P1B	0.005156066	0.136125855	0.891761094	0.922656251	no
A1CF	-0.005145199	-0.13583895	0.891987821	0.922843607	no
OR52N5	0.005141109	0.135730963	0.892073159	0.922843741	no
KRTAP5-8	0.005140817	0.135723268	0.89207924	0.922843741	no
C5orf41	0.005129806	0.135432544	0.892308997	0.923034193	no
PTPN3	-0.005118504	-0.135134158	0.892544818	0.923230898	no
MRPL14	-0.005112218	-0.134968194	0.892675988	0.923304793	no
ABCB1	-0.005110703	-0.134928206	0.892707592	0.923304793	no
ZG16	-0.005104957	-0.134776487	0.892827506	0.923381581	no
MGC16384	0.005085414	0.134260524	0.893235326	0.923756104	no
OR10A4	-0.005062149	-0.133646293	0.893720854	0.924210947	no
SPOP	-0.005057391	-0.133520673	0.893820157	0.924266363	no
TEX14	-0.005050984	-0.133351502	0.893953889	0.924329041	no
FARP2	0.005050106	0.133328332	0.893972206	0.924329041	no
MAGEA3	-0.005034801	-0.132924238	0.894291663	0.924612061	no
SMURF2	-0.005021979	-0.13258572	0.894559293	0.92484147	no
POLN	-0.005012374	-0.132332126	0.89475979	0.925001454	no
TLX2	0.00500488	0.132134287	0.89491621	0.925087374	no
WDR12	-0.005004008	-0.132111266	0.894934412	0.925087374	no
UBE2Q1	-0.004982673	-0.131547976	0.895379798	0.925500448	no
CRYGN	0.004974871	0.131341988	0.895542679	0.925621486	no
PRPH	0.004972474	0.131278713	0.895592714	0.925625881	no
HMBS	-0.004957921	-0.130894486	0.895896547	0.925886197	no
SNAR-D	0.004956023	0.130844376	0.895936173	0.925886197	no
RLN3	-0.004952643	-0.130755141	0.896006739	0.925894449	no
ISCA1P1	0.004951254	0.130718456	0.89603575	0.925894449	no
OR12D3	-0.004935791	-0.130310206	0.896358604	0.926180726	no
TPH1	-0.004920338	-0.129902219	0.896681267	0.926466776	no
TEX15	0.004914859	0.129757564	0.896795675	0.926537634	no
ZNF211	0.004910794	0.129650236	0.896880561	0.926577986	no
TPRKB	0.00490573	0.129516542	0.896986303	0.926639879	no
CYP7B1	0.004896488	0.129272547	0.897179287	0.926791888	no
GFM1	-0.004893473	-0.129192946	0.897242249	0.926809574	no
DCAF12	0.004863269	0.128395502	0.897873028	0.927389799	no
PACS1	-0.004862183	-0.128366837	0.897895703	0.927389799	no
KRTAP10-9	-0.004854441	-0.128162441	0.898057392	0.927472719	no
NKX2-3	-0.004853102	-0.128127076	0.898085367	0.927472719	no
C19orf71	0.004849934	0.128043434	0.898151535	0.927472719	no
C4orf35	0.004849553	0.12803339	0.89815948	0.927472719	no
RIOK2	-0.004842481	-0.127846664	0.898307197	0.927577881	no
LMBR1L	0.00483562	0.127665516	0.898450504	0.927678479	no
AKR1CL1	-0.004825849	-0.127407542	0.898654595	0.927841825	no
LOC400927	-0.004814853	-0.127117238	0.898884271	0.928031568	no

C12orf27	0.004808398	0.126946822	0.899019101	0.928096798	no
KANK4	-0.004807433	-0.126921341	0.899039261	0.928096798	no
BHLHE23	-0.004799443	-0.126710389	0.899206167	0.928221703	no
RNF175	0.004794945	0.12659164	0.899300123	0.928253076	no
DUS4L	0.004791575	0.126502656	0.899370529	0.928253076	no
PTK2B	-0.004791394	-0.126497896	0.899374296	0.928253076	no
KIF19	-0.004764367	-0.125784318	0.899938928	0.928788425	no
CACNG5	0.004762083	0.125724027	0.899986637	0.928790252	no
CAPN3	-0.004756113	-0.125566416	0.900111358	0.928848914	no
PRM1	-0.004754964	-0.12553608	0.900135363	0.928848914	no
LOC728723	0.004743545	0.125234602	0.900373936	0.929007406	no
LOC153910	-0.004742873	-0.125216852	0.900387983	0.929007406	no
LOC100130581	0.004741015	0.125167795	0.900426804	0.929007406	no
PIP	-0.004712492	-0.12441474	0.901022775	0.929546117	no
KIF26B	-0.004711625	-0.124391846	0.901040894	0.929546117	no
CHAT	0.004708985	0.124322159	0.901096047	0.929555584	no
LOC100101938	0.004701323	0.124119862	0.901256157	0.929673316	no
FCER2	-0.004692821	-0.123895392	0.901433821	0.929809142	no
RPL35	0.00469032	0.123829371	0.901486077	0.929815606	no
TBX20	0.004666187	0.123192216	0.901990404	0.930230961	no
MAGEB18	0.004664929	0.123158991	0.902016703	0.930230961	no
SLC26A7	0.004664445	0.123146226	0.902026808	0.930230961	no
CBL	-0.004652334	-0.122826479	0.902279913	0.930444521	no
SNORA11E	0.004649805	0.122759712	0.902332765	0.930451566	no
TRPM3	-0.004644263	-0.122613394	0.902448592	0.930518458	no
C1orf150	0.004641098	0.122529835	0.90251474	0.930518458	no
BAT2L2	0.004640095	0.122503345	0.902535709	0.930518458	no
SSB	-0.00462103	-0.121999995	0.902934187	0.930881821	no
TERT	-0.004582741	-0.120989106	0.903734533	0.931659433	no
MTM1	0.004579575	0.120905526	0.90380071	0.931680149	no
SEC14L1	0.004577091	0.120839935	0.903852644	0.931686181	no
GPR150	-0.004570929	-0.120677243	0.903981463	0.931741993	no
SBNO1	0.004570092	0.120655148	0.903998958	0.931741993	no
PLEKHA8	0.004547865	0.120068326	0.904463624	0.932173398	no
TMEM163	0.00454196	0.119912434	0.90458707	0.932241005	no
SNORA29	-0.004538398	-0.119818374	0.904661554	0.932241005	no
DDX50	-0.004538111	-0.119810803	0.904667549	0.932241005	no
FLRT3	0.004531985	0.119649056	0.904795636	0.932306941	no
FLJ35390	0.004529744	0.11958991	0.904842473	0.932306941	no
ACOT6	-0.00452744	-0.11952907	0.904890654	0.932306941	no
SLC4A1	0.004526228	0.119497081	0.904915986	0.932306941	no
C14orf79	0.004505849	0.118959041	0.905342082	0.932698406	no
SLC25A10	-0.004501882	-0.1188543	0.905425034	0.932736336	no
ADH6	-0.004488034	-0.118488697	0.905714589	0.932987088	no
STATH	-0.004480619	-0.118292922	0.905869647	0.933099273	no
NOSIP	0.004471344	0.118048042	0.906063603	0.933205136	no
DKFZP434L187	-0.00447129	-0.118046612	0.906064735	0.933205136	no
CHRNA10	0.00446121	0.117780483	0.906275527	0.933374693	no
ACO1	0.004453025	0.117564395	0.906446687	0.933503419	no
PGS1	-0.004434123	-0.117065349	0.906841992	0.933840004	no
MBL1P	0.00443298	0.117035176	0.906865894	0.933840004	no

FAM127B	0.004428103	0.116906411	0.906967895	0.933897474	no
C8orf30A	0.004421076	0.116720902	0.907114849	0.934001223	no
SNORA81	0.004408029	0.116376448	0.907387723	0.934217262	no
PRND	-0.004403348	-0.11625285	0.907485638	0.934217262	no
NPTN	0.004402892	0.116240806	0.90749518	0.934217262	no
POLR1C	-0.004402207	-0.116222733	0.907509498	0.934217262	no
OR4K13	0.004397201	0.116090568	0.907614203	0.934277479	no
CECR7	-0.004380256	-0.115643178	0.90796865	0.934594754	no
UBE2L3	-0.004376317	-0.115539179	0.908051047	0.934631984	no
LMOD1	-0.004353784	-0.114944285	0.908522388	0.935069519	no
ANLN	0.004345147	0.114716247	0.908703074	0.935162355	no
RSPO1	0.00434505	0.114713695	0.908705096	0.935162355	no
FAM108A1	0.004342296	0.114640978	0.908762714	0.935174049	no
C10orf78	-0.004306287	-0.11369029	0.909516049	0.935867574	no
COLQ	-0.004305657	-0.113673657	0.90952923	0.935867574	no
CT47A2	0.004302552	0.113591682	0.909594192	0.935886787	no
XPC	0.004283688	0.113093638	0.909988884	0.936245242	no
NDUFA11	0.004252519	0.112270739	0.910641067	0.936868568	no
LY6G5C	-0.004246879	-0.112121847	0.910759077	0.9369423	no
SNORA58	-0.00424387	-0.11204238	0.910822062	0.936959421	no
LIN28B	-0.004226768	-0.111590882	0.911179929	0.937272556	no
SULT1A2	-0.004224893	-0.111541364	0.911219179	0.937272556	no
TIMM9	-0.004207192	-0.111074028	0.91158962	0.9375151	no
C6orf105	-0.00420697	-0.111068179	0.911594256	0.9375151	no
CBLB	-0.004205417	-0.111027185	0.911626752	0.9375151	no
OR4C11	-0.004202669	-0.110954615	0.911684278	0.9375151	no
DIMT1L	-0.004202547	-0.110951392	0.911686833	0.9375151	no
PPIAL4B	-0.004197017	-0.110805403	0.911802558	0.937586417	no
CABC1	-0.004188672	-0.110585082	0.911977211	0.937718317	no
NUP210L	0.004183977	0.110461118	0.912075482	0.937747225	no
SNRPD3	-0.004182896	-0.110432599	0.91209809	0.937747225	no
PRUNE2	-0.004165047	-0.109961342	0.912471686	0.938012046	no
MTIF3	-0.004163594	-0.109922987	0.912502093	0.938012046	no
C8orf22	0.00416231	0.109889099	0.912528959	0.938012046	no
FBXO28	-0.004161723	-0.109873599	0.912541247	0.938012046	no
F10	0.004157472	0.109761352	0.912630236	0.938052512	no
C19orf46	0.004155409	0.109706897	0.912673408	0.938052512	no
CDHR3	0.004147703	0.109503442	0.91283471	0.938124465	no
PNLDC1	-0.004147631	-0.109501544	0.912836216	0.938124465	no
HMX3	-0.004140164	-0.109304405	0.912992513	0.938152466	no
SF3B1	-0.004139525	-0.109287543	0.913005883	0.938152466	no
3-Mar	-0.004138055	-0.109248725	0.913036659	0.938152466	no
C18orf54	-0.004137462	-0.109233073	0.913049068	0.938152466	no
MAGEB1	0.00412919	0.109014663	0.913222236	0.93828271	no
GUCY2GP	0.004126728	0.108949675	0.913273764	0.93828797	no
F13B	-0.004123011	-0.108851542	0.913351571	0.938320227	no
ZNF320	-0.004119694	-0.108763973	0.913421003	0.938336088	no
NDUFS8	-0.004116916	-0.108690628	0.913479157	0.938336088	no
KRTAP2-1	0.004112067	0.108562605	0.913580667	0.938336088	no
OLIG3	-0.004111259	-0.108541273	0.913597582	0.938336088	no
LOC339535	0.004111188	0.108539402	0.913599065	0.938336088	no

MCCC2	-0.004090473	-0.107992485	0.914032732	0.93873381	no
CSH2	-0.004078624	-0.107679659	0.914280793	0.938940878	no
PRPSAP1	0.004069728	0.107444784	0.914467047	0.939084456	no
CDK5	-0.004054716	-0.107048455	0.914781343	0.9393595	no
PLAC1L	-0.0040515	-0.106963538	0.914848686	0.939380941	no
SNAR-E	0.004044323	0.106774062	0.91499895	0.939487521	no
SNORA50	0.00403656	0.106569117	0.915161485	0.939606688	no
OR2B2	-0.004030086	-0.106398182	0.915297051	0.939686933	no
CCDC88C	0.004028388	0.106353361	0.915332597	0.939686933	no
KRT71	-0.00401825	-0.106085709	0.915544873	0.939857134	no
IL28A	-0.004011029	-0.105895047	0.915696092	0.939879646	no
TRIM32	0.004010659	0.105885293	0.915703828	0.939879646	no
UBR1	0.004008972	0.105840756	0.915739153	0.939879646	no
SCGB1D1	0.004008323	0.105823609	0.915752752	0.939879646	no
PRKCI	0.004000743	0.105623503	0.915911466	0.939994823	no
YWHAB	-0.003997914	-0.105548793	0.915970723	0.940007922	no
UBP1	-0.00397184	-0.104860424	0.91651673	0.940503938	no
RNF17	-0.003968219	-0.104764818	0.916592567	0.940503938	no
HNRNPCL1	-0.003968169	-0.104763504	0.916593609	0.940503938	no
CRYGD	0.003963295	0.104634816	0.916695689	0.940560946	no
CMTM1	-0.003951682	-0.104328205	0.916938908	0.940762755	no
PPIAL4D	0.003943253	0.104105682	0.91711543	0.940896116	no
C2orf54	-0.003937915	-0.103964747	0.917227232	0.94096307	no
MAGEB10	0.003932072	0.103810489	0.917349605	0.94104086	no
SPANXN4	-0.003906288	-0.103129743	0.917889664	0.941547094	no
MRPS21	-0.003900982	-0.102989664	0.918000798	0.941613319	no
MRPS36	-0.003898088	-0.102913263	0.918061413	0.941627722	no
SDR9C7	-0.003891765	-0.102746328	0.918193857	0.941709426	no
SLC2A12	-0.003889838	-0.102695444	0.918234228	0.941709426	no
DIRC2	-0.003886301	-0.102602054	0.918308323	0.941737646	no
RNF216	-0.003881402	-0.102472718	0.91841094	0.94179511	no
GJB4	-0.003859831	-0.101903231	0.918862793	0.942210679	no
PSG11	-0.003851885	-0.101693449	0.919029249	0.942333571	no
DKFZP686I152	-0.003846257	-0.101544863	0.91914715	0.942406668	no
FAM92A3	-0.00384117	-0.101410557	0.919253721	0.942440907	no
NDFIP1	0.003840213	0.101385288	0.919273772	0.942440907	no
MEF2C	-0.003836078	-0.101276114	0.919360403	0.94248193	no
C11orf9	-0.003829659	-0.101106642	0.919494882	0.942560099	no
HOXA11AS	-0.003824088	-0.100959559	0.919611597	0.942560099	no
KRT1	0.003822175	0.100909065	0.919651666	0.942560099	no
ANKRD54	0.003821632	0.10089471	0.919663058	0.942560099	no
PPP1R3A	0.003821312	0.100886273	0.919669753	0.942560099	no
DNAH8	-0.003808497	-0.100547946	0.919938235	0.942787471	no
C8orf74	-0.003801341	-0.100359018	0.920088164	0.942859437	no
CDCA5	0.003799373	0.100307054	0.920129402	0.942859437	no
EXOC4	-0.003797131	-0.100247854	0.920176382	0.942859437	no
PRLH	-0.003796243	-0.100224401	0.920194995	0.942859437	no
SNORA79	0.003783875	0.099897875	0.920454129	0.94307716	no
FCN2	-0.003781223	-0.099827866	0.92050969	0.943086293	no
ATP6V1G3	0.003767913	0.099476451	0.920788588	0.943315166	no
RAMP1	0.003766108	0.099428811	0.920826398	0.943315166	no

SNORA2B	0.003760649	0.099284687	0.920940784	0.943384545	no
ARC	-0.003756793	-0.099182892	0.921021576	0.943419507	no
YWHAH	-0.003735466	-0.098619817	0.921468489	0.943829471	no
GPRC6A	0.003721144	0.098241711	0.921768607	0.944089042	no
PUS7L	-0.003709565	-0.09793599	0.922011278	0.944289753	no
PROL1	-0.00370537	-0.097825244	0.922099186	0.944331949	no
PCYOX1	0.003687713	0.097359089	0.922469221	0.944663056	no
HS3ST6	0.003685427	0.097298716	0.922517147	0.944664287	no
LOC152024	0.00365992	0.096625316	0.923051728	0.94516383	no
ZNF791	-0.003648646	-0.096327671	0.923288024	0.945357909	no
C20orf144	0.003632432	0.095899602	0.923627876	0.945657993	no
BPESC1	-0.003625367	-0.095713074	0.923775968	0.945761724	no
C12orf40	-0.003614102	-0.09541565	0.924012111	0.945955586	no
PLIN4	0.003599301	0.095024895	0.924322364	0.946225294	no
NOBOX	0.003594425	0.094896157	0.924424583	0.946282022	no
TMTC4	-0.003565623	-0.094135747	0.925028379	0.946852155	no
PGA4	0.003560708	0.094005985	0.925131419	0.946909686	no
COX7A2	0.003556952	0.093906834	0.925210153	0.946942334	no
EFCAB2	-0.003549922	-0.093721211	0.925357554	0.947045256	no
SOHLH2	0.003539141	0.09343658	0.925583583	0.947228633	no
TMEM233	-0.003525632	-0.093079935	0.925866806	0.947460975	no
ATM	0.003523842	0.093032672	0.92590434	0.947460975	no
VAPB	0.003518021	0.092879007	0.926026374	0.947537893	no
RPS8	0.00351375	0.092766241	0.926115928	0.947581571	no
KRT84	-0.003508641	-0.092631362	0.926223046	0.947643213	no
C16orf82	-0.003501344	-0.092438709	0.926376048	0.947751793	no
COX8A	-0.003498629	-0.092367023	0.92643298	0.94776208	no
AVIL	-0.003484388	-0.091991054	0.92673158	0.947965662	no
AGPAT6	0.003482585	0.091943448	0.92676939	0.947965662	no
C7orf55	-0.00348243	-0.091939353	0.926772642	0.947965662	no
SAMD7	0.003475849	0.091765605	0.926910639	0.948047644	no
SDHAP1	-0.003474135	-0.091720362	0.926946573	0.948047644	no
FBF1	0.003471452	0.091649522	0.927002837	0.948057229	no
FAM3A	0.003460975	0.091372913	0.927222537	0.948233953	no
ITLN1	0.00345689	0.09126505	0.92730821	0.948273602	no
PSG10	-0.003448761	-0.091050434	0.927478677	0.948399954	no
JAG2	0.003446104	0.090980296	0.927534387	0.948408954	no
NCRNA00157	-0.003442493	-0.090884968	0.927610107	0.948438411	no
DNAJC25-GNG1	0.003431126	0.090584848	0.927848497	0.94863418	no
C21orf29	0.003422266	0.090350937	0.928034301	0.948742911	no
HSD3B1	-0.003421579	-0.090332815	0.928048696	0.948742911	no
NFXL1	0.00340842	0.089985381	0.928324684	0.948977068	no
DAXX	0.003393611	0.089594429	0.92863525	0.94914881	no
ZNF771	-0.00339289	-0.089575374	0.928650387	0.94914881	no
PISD	-0.003391899	-0.089549229	0.928671157	0.94914881	no
ERMN	-0.003391455	-0.089537505	0.92868047	0.94914881	no
LOC644145	-0.003385018	-0.089367545	0.928815488	0.949238818	no
PKD2L2	0.003370659	0.088988455	0.929116648	0.949498605	no
LCE1C	0.003343314	0.08826652	0.929690202	0.95003672	no
ETV3	0.003316738	0.08756489	0.930247659	0.950519343	no
UGT2B10	0.003316316	0.087553744	0.930256516	0.950519343	no

C3orf22	0.003298056	0.087071657	0.930639563	0.950862678	no
C6orf106	-0.003292311	-0.086919979	0.930760083	0.950937761	no
HPDL	-0.003289687	-0.086850691	0.930815138	0.950945956	no
HIGD1C	-0.003282335	-0.086656599	0.930969363	0.951055459	no
ALK	-0.003269126	-0.086307872	0.931246468	0.951290476	no
MRS2P2	0.00325519	0.085939924	0.931538854	0.951541078	no
KCTD7	-0.003250668	-0.085820563	0.931633706	0.951570939	no
OR10K1	-0.003249309	-0.085784683	0.931662218	0.951570939	no
OR52E6	0.00324134	0.085574289	0.931829413	0.951693628	no
DEFB105A	-0.003236277	-0.085440616	0.93193564	0.951754042	no
ATP6V0C	-0.00322143	-0.085048641	0.932247144	0.952024081	no
11-Sep	0.003213336	0.084834939	0.932416977	0.952149424	no
CCDC15	-0.003199705	-0.084475069	0.932702981	0.95236722	no
SLC6A14	0.003198681	0.084448031	0.93272447	0.95236722	no
RIF1	0.00318322	0.084039833	0.933048893	0.952650365	no
HDGF	-0.003167741	-0.083631191	0.93337368	0.952933852	no
MAOA	-0.003156076	-0.083323203	0.933618475	0.953135645	no
KCNG4	-0.00315368	-0.083259956	0.933668746	0.953138839	no
CA4	-0.003141729	-0.082944441	0.933919532	0.953346718	no
CELA2A	0.003129074	0.082610332	0.934185103	0.953569668	no
DNAJA4	0.003117717	0.082310487	0.934423447	0.953764804	no
FRS2	-0.003092571	-0.081646596	0.934951186	0.954225727	no
REG1P	-0.003091702	-0.081623663	0.934969416	0.954225727	no
CLDN5	0.003087968	0.081525096	0.935047772	0.954257526	no
C18orf2	-0.003071435	-0.081088598	0.935394771	0.954563471	no
SFRS3	0.003066822	0.080966806	0.935491594	0.954580278	no
HSPH1	0.003066151	0.0809491	0.935505669	0.954580278	no
MAP3K15	0.003044736	0.080383714	0.935955154	0.954984793	no
LOC285692	0.003042763	0.08033162	0.93599657	0.954984793	no
KIAA1841	-0.003029136	-0.079971862	0.93628259	0.95522841	no
SYCN	-0.003022131	-0.079786925	0.936429626	0.955330213	no
SLFNL1	0.003017712	0.079670245	0.936522394	0.955376646	no
PYCRL	0.00301458	0.079587564	0.936588131	0.955395501	no
PTGES3	-0.003007271	-0.079394594	0.936741557	0.955503799	no
GOLGA6D	-0.002994381	-0.079054284	0.937012136	0.955731579	no
BARX1	0.002976001	0.078569036	0.937397967	0.956076886	no
STARD4	-0.002967111	-0.078334311	0.937584607	0.956219007	no
WDR72	-0.002963365	-0.078235422	0.93766324	0.956250965	no
SEMG2	-0.002959034	-0.078121071	0.937754167	0.956295458	no
KISS1	0.00294912	0.077859344	0.937962286	0.956459449	no
C12orf66	-0.002943909	-0.077721772	0.938071681	0.956518873	no
GSDMC	-0.002941837	-0.077667067	0.938115181	0.956518873	no
CLEC9A	-0.002935489	-0.077499472	0.938248452	0.956606516	no
OR6W1P	-0.002929474	-0.077340656	0.938374745	0.956682556	no
MGC4473	-0.002927429	-0.077286677	0.93841767	0.956682556	no
MST02P	-0.002908407	-0.07678446	0.938817048	0.95704145	no
HECA	-0.002905979	-0.07672037	0.938868016	0.957045153	no
TDGF3	-0.002899474	-0.076548631	0.939004592	0.957136115	no
CASZ1	-0.00288184	-0.076083075	0.939374838	0.957465239	no
NEUROG1	-0.002862478	-0.075571909	0.939781371	0.957822013	no
CCDC159	0.002860657	0.075523816	0.93981962	0.957822013	no

SMC2	-0.002855391	-0.075384785	0.939930195	0.95788642	no
IRS1	-0.002838371	-0.074935444	0.940287577	0.95820233	no
PIH1D1	-0.002820563	-0.074465303	0.940661515	0.958535079	no
GRAMD4	-0.002805237	-0.074060678	0.940983353	0.958814707	no
SLC18A3	-0.002798736	-0.07388905	0.941119869	0.958888712	no
NPPB	-0.002797262	-0.073850118	0.941150836	0.958888712	no
C15orf38	-0.002793611	-0.073753728	0.941227507	0.958918505	no
CSTL1	0.002785047	0.073527645	0.941407342	0.959053393	no
SDCCAG8	0.002781454	0.07343278	0.941482802	0.959081941	no
TMEM207	-0.002774464	-0.07324824	0.941629594	0.95916898	no
CCDC132	0.002772868	0.073206085	0.941663126	0.95916898	no
TSPAN18	0.002766344	0.073033854	0.941800131	0.959206124	no
PROZ	-0.002765323	-0.073006896	0.941821575	0.959206124	no
CRELD1	0.002764354	0.072981318	0.941841922	0.959206124	no
RABGAP1L	-0.002757491	-0.072800122	0.94198606	0.959231958	no
SARNP	0.002756591	0.072776374	0.942004951	0.959231958	no
ATP2B1	0.002756369	0.072770502	0.942009621	0.959231958	no
CYB5D2	0.002741265	0.072371747	0.942326831	0.959460334	no
GUCY2E	-0.002741171	-0.07236926	0.942328809	0.959460334	no
MAGEA9B	-0.002715094	-0.07168079	0.942876507	0.959946379	no
COQ4	0.002711345	0.071581838	0.942955228	0.959946379	no
FOXI1	-0.002709652	-0.071537118	0.942990806	0.959946379	no
TBX10	0.0027094	0.07153047	0.942996095	0.959946379	no
ZSWIM2	0.002702071	0.071336976	0.943150031	0.960054744	no
ING3	0.002696786	0.071197443	0.94326104	0.960119403	no
SLC35E3	0.002688713	0.070984313	0.943430603	0.960201615	no
CFTR	0.002688418	0.070976532	0.943436793	0.960201615	no
CCL24	0.0026853	0.070894207	0.94350229	0.960217857	no
ODZ1	0.002683136	0.070837083	0.943547738	0.960217857	no
C15orf2	0.002673865	0.070592313	0.943742478	0.960367698	no
NLGN4Y	0.002656144	0.070124454	0.944114717	0.960660874	no
SIM1	-0.002655625	-0.070110764	0.94412561	0.960660874	no
RAD21L1	0.002643963	0.069802874	0.944370581	0.960861779	no
MIF4GD	-0.002637605	-0.06963502	0.944504136	0.960889413	no
ALLC	0.002636948	0.069617657	0.944517951	0.960889413	no
BPNT1	-0.002635883	-0.069589543	0.94454032	0.960889413	no
SMG5	-0.002625607	-0.069318256	0.944756176	0.961060648	no
LOC541473	0.002621079	0.069198722	0.944851288	0.961109043	no
KRTAP10-7	-0.002616354	-0.069073959	0.944950561	0.961161666	no
LPA	-0.002611595	-0.068948321	0.945050531	0.961214993	no
MED11	0.002600647	0.068659296	0.945280511	0.961400542	no
LOC441455	0.002595505	0.068523524	0.945388547	0.961441185	no
DCAF12L2	0.002594218	0.068489551	0.94541558	0.961441185	no
GABRP	0.002577389	0.068045255	0.945769125	0.961701495	no
C8orf38	0.002576424	0.068019773	0.945789402	0.961701495	no
SCARNA15	0.002575241	0.067988545	0.945814251	0.961701495	no
ZNF498	-0.002550608	-0.067338204	0.946331775	0.962179322	no
RWDD1	-0.002541088	-0.067086864	0.94653179	0.962330809	no
UBFD1	-0.002538986	-0.067031359	0.946575961	0.962330809	no
ATG16L2	-0.002523312	-0.066617569	0.946905262	0.962617186	no
STK16	-0.002516814	-0.06644602	0.947041787	0.96270757	no

BTF3L4	0.002511516	0.066306121	0.947153124	0.962772341	no
TMEM201	-0.002502944	-0.066079819	0.947333227	0.962907002	no
MT1F	0.00249607	0.065898347	0.947477653	0.962991372	no
TOMM22	-0.002492658	-0.065808252	0.947549357	0.962991372	no
PPIEL	0.002492029	0.065791659	0.947562563	0.962991372	no
TSPAN16	0.002489926	0.065736134	0.947606754	0.962991372	no
MCART2	-0.002479753	-0.065467551	0.947820514	0.963160191	no
OR52A1	0.002473031	0.06529009	0.947961754	0.963255302	no
DDX11L2	-0.002468923	-0.065181635	0.948048073	0.9632946	no
TRIB2	-0.002457739	-0.064886371	0.948283077	0.963484962	no
C15orf33	0.002451022	0.064709019	0.948424236	0.96357996	no
AGAP11	-0.002440324	-0.064426591	0.94864903	0.963759916	no
MYPN	0.002423913	0.063993327	0.948993888	0.964061824	no
FMO6P	0.002413812	0.063726653	0.949206153	0.964229011	no
UROC1	-0.002407256	-0.063553558	0.949343934	0.964320521	no
AIMP2	-0.002389876	-0.063094724	0.949709165	0.964643049	no
ALX3	0.00238608	0.062994496	0.949788947	0.964675622	no
RBM28	-0.002380512	-0.062847501	0.949905959	0.964746002	no
TCHHL1	-0.002370666	-0.062587564	0.950112876	0.964907681	no
P2RX2	-0.002363842	-0.062407405	0.95025629	0.965004855	no
SPP2	-0.002360085	-0.06230821	0.950335253	0.965036572	no
LOC284632	0.00235544	0.062185567	0.950432884	0.965082845	no
TSPAN12	-0.002353375	-0.062131042	0.950476289	0.965082845	no
KIR3DL3	-0.002341064	-0.061806036	0.950735015	0.965259883	no
GABPA	0.002337422	0.061709879	0.950811563	0.965259883	no
CDK11A	-0.002336817	-0.061693899	0.950824285	0.965259883	no
USP45	-0.002335992	-0.061672126	0.950841618	0.965259883	no
ATF6B	-0.002330677	-0.061531796	0.950953333	0.965324822	no
PPAT	-0.002322695	-0.061321064	0.951121095	0.965405995	no
UGT1A7	-0.002322328	-0.061311389	0.951128797	0.965405995	no
SLC2A1	0.0023136	0.061080967	0.951312237	0.965543715	no
GNPAT	-0.002310965	-0.061011396	0.951367623	0.965551458	no
PGAM5	-0.002303731	-0.060820398	0.95151968	0.965657308	no
BIRC8	-0.002288642	-0.060422043	0.951836823	0.965930678	no
GAGE1	-0.002276187	-0.060093215	0.95209862	0.966147856	no
CYP2C8	-0.002253276	-0.059488335	0.952580208	0.966588038	no
OR4C16	-0.00224994	-0.059400277	0.952650319	0.966610667	no
SF3B4	0.002246699	0.059314701	0.952718453	0.966631289	no
FLJ10357	-0.00223532	-0.059014276	0.952957652	0.966825462	no
B3GALT1	-0.002228555	-0.058835681	0.953099852	0.966884337	no
CCL4L2	0.002228009	0.058821268	0.953111329	0.966884337	no
PSEN1	0.002207621	0.058283007	0.953539908	0.967270576	no
C12orf77	-0.002190081	-0.057819931	0.953908634	0.967555302	no
ZNF672	-0.002189359	-0.057800864	0.953923816	0.967555302	no
ISM2	-0.002187439	-0.057750197	0.953964161	0.967555302	no
C11orf34	-0.002183481	-0.057645681	0.954047384	0.967591172	no
VPS26A	-0.002162965	-0.057104059	0.954478669	0.967980023	no
API5	0.002156164	0.056924492	0.954621658	0.968065307	no
OR9A2	-0.002153714	-0.056859817	0.954673159	0.968065307	no
TMEM187	-0.002152133	-0.056818064	0.954706408	0.968065307	no
TTLL7	0.00214954	0.056749623	0.954760909	0.968072019	no

HACL1	0.002130158	0.056237915	0.955168394	0.968436618	no
FMO2	0.002109507	0.055692698	0.955602577	0.968777473	no
TCEAL7	0.002108744	0.055672556	0.955618617	0.968777473	no
OR4B1	-0.002107331	-0.055635247	0.955648329	0.968777473	no
MAGEA11	0.002094939	0.055308108	0.955908852	0.968880475	no
TGM3	-0.002094848	-0.055305701	0.95591077	0.968880475	no
LOC595101	0.002093678	0.055274819	0.955935363	0.968880475	no
CYP4F12	-0.002093336	-0.055265781	0.95594256	0.968880475	no
C17orf61	0.002091102	0.055206785	0.955989544	0.968880475	no
CRYBA2	0.002083001	0.054992925	0.95615986	0.969004514	no
TM4SF4	-0.002077391	-0.054844805	0.956277821	0.969050082	no
HCRT	0.002076303	0.054816097	0.956300684	0.969050082	no
PRHOXNB	0.002056237	0.054286331	0.956722596	0.969398629	no
GYLTL1B	-0.002055384	-0.054263799	0.956740541	0.969398629	no
NFKBIA	0.002052132	0.05417794	0.956808921	0.969419331	no
ATP5I	-0.002035068	-0.05372744	0.957167716	0.96973426	no
ACHE	-0.002025492	-0.053474625	0.957369071	0.969889656	no
ANAPC4	-0.001995927	-0.05269408	0.957990754	0.970470842	no
MAP3K4	0.001987068	0.052460213	0.958177029	0.970610911	no
NBPF6	0.00197536	0.052151097	0.958423241	0.970811678	no
BARHL2	-0.001972402	-0.052072999	0.958485447	0.97082605	no
SLURP1	-0.001963316	-0.05183314	0.9586765	0.970928172	no
HERC4	-0.00196304	-0.051825836	0.958682318	0.970928172	no
UGT1A1	0.001960751	0.051765419	0.958730442	0.970928275	no
SPG20	0.001935885	0.051108919	0.959253372	0.971392767	no
TSGA13	-0.001932159	-0.05101056	0.959331721	0.971392767	no
OTOR	0.001932087	0.051008657	0.959333236	0.971392767	no
C17orf48	0.001923659	0.050786144	0.959510483	0.971523585	no
ATP5O	0.001917937	0.050635077	0.959630818	0.971596769	no
ALG1L2	-0.001914512	-0.05054465	0.95970285	0.971621043	no
C1orf68	-0.001894222	-0.050008973	0.960129565	0.972004384	no
VAX1	-0.001882023	-0.049686913	0.96038612	0.972215431	no
AHNAK	-0.001866303	-0.049271894	0.960716733	0.972501423	no
ZCCHC11	0.00186142	0.049142981	0.96081943	0.972556685	no
TXNDC6	0.001857929	0.04905081	0.960892856	0.972582316	no
SNORA75	0.001853913	0.048944791	0.960977315	0.972619111	no
SNORA66	-0.001851471	-0.048880328	0.961028669	0.972622397	no
OR1D4	0.001837153	0.04850231	0.961329818	0.972878479	no
FUNDC2	-0.001832521	-0.048380019	0.961427243	0.972928374	no
PFKFB2	0.001826284	0.048215371	0.961558413	0.972974969	no
F12	0.001825756	0.048201409	0.961569535	0.972974969	no
SNORA27	0.001804566	0.047641999	0.962015207	0.973377212	no
CT47A9	-0.001794162	-0.047367315	0.962234048	0.9735012	no
SNAR-G2	-0.001794162	-0.047367315	0.962234048	0.9735012	no
CDY1	0.001772701	0.046800734	0.96268545	0.973909153	no
KDM5D	0.001755534	0.046347495	0.96304656	0.974185289	no
TAL1	0.001755144	0.046337189	0.963054771	0.974185289	no
BPHL	0.001745819	0.046091009	0.963250914	0.97433495	no
DLX6AS	-0.001726059	-0.04556932	0.963666575	0.97470663	no
DEFB118	0.001704516	0.045000569	0.964119745	0.975031137	no
CPNE4	-0.001702231	-0.044940265	0.964167795	0.975031137	no

TTTY6B	0.001701729	0.04492699	0.964178372	0.975031137	no
SCOC	0.001701636	0.044924557	0.96418031	0.975031137	no
CCDC83	-0.001684649	-0.044476069	0.964537666	0.975343107	no
C15orf5	0.001678048	0.044301812	0.964676516	0.975343107	no
C3orf14	0.001676477	0.044260319	0.964709579	0.975343107	no
ZNF841	-0.001676158	-0.044251903	0.964716284	0.975343107	no
C14orf2	0.001673543	0.044182874	0.964771288	0.975343107	no
KRTAP7-1	-0.001673212	-0.044174128	0.964778257	0.975343107	no
CCL3L1	-0.001662036	-0.043879075	0.965013362	0.975532008	no
SRD5A2	-0.001657445	-0.043757876	0.965109937	0.975580857	no
RDH8	0.001653275	0.043647771	0.965197673	0.975620766	no
RPS3A	-0.001645928	-0.043453811	0.965352228	0.975728208	no
DMRTC2	-0.001639783	-0.04329156	0.965481518	0.975810105	no
PTPRG	-0.00162508	-0.042903387	0.965790836	0.976033211	no
C8orf56	-0.0016247	-0.042893375	0.965798814	0.976033211	no
MMGT1	-0.001608838	-0.042474592	0.966132531	0.976321663	no
C18orf26	-0.001595279	-0.042116639	0.966417778	0.976561107	no
ACTL7B	-0.001589676	-0.041968711	0.966535661	0.976631415	no
WDR90	0.001585936	0.04186996	0.966614355	0.97666212	no
CAD	0.001572476	0.041514615	0.966897531	0.976899419	no
SLC16A8	0.001568951	0.041421539	0.966971704	0.97692554	no
KRTAP3-3	-0.0015596	-0.041174671	0.967168438	0.977008211	no
NCRNA00099	0.001557167	0.041110432	0.967219631	0.977008211	no
OR10V1	0.001556125	0.041082926	0.967241552	0.977008211	no
MARS2	0.001555874	0.041076306	0.967246827	0.977008211	no
UGT2B7	0.001532172	0.040450555	0.96774551	0.977463092	no
OR5AU1	-0.001523165	-0.040212764	0.967935017	0.977605664	no
14-Sep	-0.001519814	-0.040124291	0.968005526	0.977628039	no
HIST1H4G	0.0015118	0.03991271	0.968174148	0.977749496	no
FLJ43859	0.001494497	0.039455904	0.968538208	0.978057411	no
ENKUR	-0.001492711	-0.039408738	0.968575798	0.978057411	no
OR52B2	-0.001488074	-0.039286317	0.968673365	0.978107082	no
GPR110	0.001481219	0.039105349	0.968817594	0.978203861	no
CLDN17	0.001461097	0.038574109	0.969240991	0.978582489	no
SCFD2	0.001442979	0.038095765	0.969622236	0.978918524	no
GPR64	0.001439541	0.038005006	0.969694573	0.978942671	no
SNORA61	-0.001435191	-0.037890168	0.969786101	0.978986189	no
OCRL	0.001430926	0.037777556	0.969875856	0.979027913	no
C4orf51	-0.001427434	-0.037685377	0.969949326	0.979053194	no
GPR119	0.001412747	0.037297628	0.970258376	0.979277625	no
TTTY14	0.001412264	0.037284874	0.970268542	0.979277625	no
CBWD5	-0.001407261	-0.037152787	0.970373821	0.979334993	no
MGC16121	-0.001399803	-0.036955886	0.970530761	0.979444491	no
LRRC3	0.001395794	0.036850049	0.970615119	0.979457515	no
BPIL2	0.001394585	0.036818134	0.970640557	0.979457515	no
OR4S2	0.001382141	0.0364896	0.970902419	0.97967286	no
DEFB135	0.001378759	0.036400307	0.970973591	0.979695781	no
ZNF551	-0.001368202	-0.03612159	0.971195749	0.979857872	no
TOP3A	-0.001366518	-0.036077155	0.971231168	0.979857872	no
C1orf159	-0.001355019	-0.035773557	0.971473161	0.98005311	no
SLC29A1	0.001352239	0.035700168	0.971531658	0.980063221	no

C9	0.001342092	0.035432265	0.971745202	0.980191581	no
EDN2	-0.001341585	-0.035418889	0.971755864	0.980191581	no
PLEKHB2	-0.001337499	-0.035311008	0.971841856	0.980229416	no
MFAP1	-0.001328069	-0.035062071	0.972040284	0.9803506	no
HRC	0.001326774	0.03502788	0.972067539	0.9803506	no
GSTTP1	0.001324877	0.034977785	0.97210747	0.9803506	no
GDF6	-0.001315747	-0.03473674	0.97229961	0.980495462	no
OR4D10	-0.001302469	-0.034386184	0.972579045	0.980722902	no
OR4K5	0.00130042	0.034332092	0.972622164	0.980722902	no
RSPH6A	0.001293377	0.034146165	0.972770372	0.980823428	no
SULT1C3	0.001289978	0.034056435	0.972841899	0.980846632	no
AMBN	-0.001283135	-0.033875774	0.97298591	0.980911773	no
CDKAL1	0.001279096	0.033769126	0.973070924	0.980911773	no
IFNA17	0.001278044	0.033741359	0.973093058	0.980911773	no
IFFO2	-0.001277036	-0.033714746	0.973114273	0.980911773	no
CLIC6	-0.001275382	-0.033671065	0.973149092	0.980911773	no
ZNF571	-0.001254589	-0.033122125	0.973586681	0.98130393	no
GPR50	-0.001236714	-0.032650224	0.973962865	0.981634158	no
CRYBB3	-0.001210754	-0.031964859	0.974509226	0.982096118	no
CNBD1	0.00121032	0.031953394	0.974518366	0.982096118	no
GSTTP2	0.001204337	0.031795444	0.974644283	0.982130834	no
BICD2	0.001204067	0.031788312	0.974649968	0.982130834	no
CRIPT	-0.001186411	-0.03132218	0.975021569	0.982411509	no
TRA2A	-0.001183485	-0.031244929	0.975083154	0.982411509	no
TTTY7	-0.001180316	-0.031161263	0.975149853	0.982411509	no
ALPPL2	0.001179977	0.031152304	0.975156995	0.982411509	no
N4BP2	-0.00117929	-0.03113416	0.97517146	0.982411509	no
TMC1	-0.001145973	-0.03025458	0.975872681	0.98299908	no
C2orf42	-0.001145375	-0.03023878	0.975885277	0.98299908	no
KRTAP4-11	-0.001144649	-0.030219609	0.975900561	0.98299908	no
SMAD2	0.001121988	0.029621345	0.976377521	0.983375735	no
KLRAQ1	0.001118502	0.029529328	0.976450882	0.983375735	no
GMCL1	0.001116694	0.029481579	0.97648895	0.983375735	no
FOXN1	0.001116642	0.029480204	0.976490046	0.983375735	no
CENPV	-0.001115328	-0.029445531	0.976517689	0.983375735	no
BSCL2	-0.001109407	-0.029289213	0.976642314	0.983452252	no
TRIM6-TRIM34	-0.001103181	-0.029124835	0.976773366	0.983535231	no
POU1F1	0.001088321	0.028732526	0.97708614	0.983801174	no
C22orf43	0.001079177	0.028491115	0.97727861	0.983945966	no
NCRNA00174	0.001072727	0.02832082	0.977414382	0.984033661	no
TCHH	-0.001063711	-0.028082795	0.977604155	0.984175711	no
PSG8	-0.001059632	-0.027975097	0.977690021	0.984213147	no
FAM41AY1	0.001055595	0.027868532	0.977774984	0.98424967	no
FIGNL2	0.001052985	0.027799626	0.977829922	0.984255967	no
DLX5	-0.001044133	-0.027565919	0.978016254	0.984376928	no
ACAD10	0.00104265	0.027526768	0.97804747	0.984376928	no
MID2	0.00103952	0.02744412	0.978113365	0.984394246	no
OR52L1	0.001032019	0.027246086	0.978271257	0.984504145	no
CEACAM7	-0.001019237	-0.026908648	0.978540298	0.984725885	no
OR2T34	0.001007085	0.026587818	0.9787961	0.984911273	no
DNASE1L3	-0.001005857	-0.026555395	0.978821952	0.984911273	no

TRPM1	0.000997174	0.026326152	0.979004732	0.985011486	no
AMZ2	0.000996497	0.026308276	0.979018984	0.985011486	no
OR6S1	-0.000992596	-0.026205312	0.97910108	0.985045065	no
C18orf16	0.000980804	0.02589397	0.979349321	0.985245787	no
LOC285501	-0.000962132	-0.025401015	0.979742372	0.985592162	no
C20orf7	0.000946112	0.024978086	0.980079592	0.985851231	no
S1PR5	-0.000944949	-0.024947368	0.980104085	0.985851231	no
EMILIN3	0.000942949	0.024894566	0.980146187	0.985851231	no
ISL2	-0.000937255	-0.024744265	0.98026603	0.985893699	no
ZNF577	0.000931048	0.024580387	0.980396699	0.985893699	no
COMT	0.000930959	0.024578032	0.980398576	0.985893699	no
RPS9	0.000929466	0.024538622	0.980430001	0.985893699	no
OR5M9	-0.000929361	-0.024535833	0.980432224	0.985893699	no
PAQR5	-0.000903764	-0.023860065	0.980971058	0.986386474	no
OR10A5	-0.000886982	-0.023416997	0.981324349	0.986663419	no
LOC285733	-0.000886044	-0.023392248	0.981344084	0.986663419	no
PDCD2L	-0.000879787	-0.023227048	0.981475811	0.98674679	no
PSG7	0.000873947	0.023072877	0.981598745	0.986821313	no
LPAR2	-0.000856876	-0.022622192	0.981958116	0.987130502	no
ISL1	-0.000854699	-0.022564717	0.982003947	0.987130502	no
PDZD3	0.000849266	0.022421277	0.982118325	0.987196395	no
OR52J3	-0.000838873	-0.022146892	0.98233712	0.987367233	no
DOK7	0.000832313	0.021973694	0.982475228	0.987456958	no
LOC729121	0.000814124	0.021493508	0.982858134	0.9877927	no
ABCD4	-0.000808379	-0.021341823	0.98297909	0.987865157	no
PNPLA6	-0.000801741	-0.021166564	0.983118844	0.987956499	no
AIRE	-0.000782667	-0.02066302	0.983520382	0.988244953	no
RTL1	-0.000781375	-0.020628901	0.98354759	0.988244953	no
CRADD	0.000781141	0.020622712	0.983552525	0.988244953	no
MAGEB2	-0.000771885	-0.02037836	0.983747379	0.988368347	no
COL4A3	0.000770663	0.020346099	0.983773104	0.988368347	no
DACT2	-0.000763965	-0.020169255	0.983914126	0.988460909	no
C20orf135	0.000751808	0.019848309	0.984170061	0.9886689	no
SLC7A10	0.00073336	0.019361264	0.984558452	0.989009926	no
INTS7	0.000724707	0.019132813	0.984740631	0.989143783	no
KRTAP2-2	-0.000717107	-0.01893218	0.984900626	0.989255346	no
SNORA13	0.000709795	0.018739138	0.985054569	0.989360819	no
OR2W5	-0.000690073	-0.01821845	0.985469798	0.989728696	no
OR5A2	0.000675523	0.017834324	0.985776126	0.989976367	no
U2AF1L4	0.000673708	0.017786413	0.985814334	0.989976367	no
SLC3A2	-0.000658862	-0.017394456	0.98612691	0.990241078	no
FANCG	-0.000649815	-0.0171556	0.986317393	0.990383166	no
CLRN1	0.000644022	0.017002679	0.986439344	0.990422597	no
TPD52L3	0.000641241	0.016929254	0.9864979	0.990422597	no
RPL11	-0.000639784	-0.016890788	0.986528576	0.990422597	no
SPEG	-0.000638643	-0.01686065	0.98655261	0.990422597	no
SFTPB	-0.000629349	-0.016615281	0.986748289	0.990569856	no
CDC23	0.000622387	0.016431494	0.986894857	0.990667802	no
CYP27B1	0.000617575	0.016304464	0.986996162	0.990705538	no
ZNF550	-0.000615947	-0.016261469	0.987030451	0.990705538	no
PIF1	-0.000590539	-0.015590691	0.987565394	0.991193265	no

DLX2	-0.000588043	-0.01552477	0.987617966	0.991196825	no
RP9P	0.000583536	0.015405808	0.987712839	0.991237891	no
PSMG4	0.000581442	0.015350511	0.987756938	0.991237891	no
PRLR	-0.000577478	-0.015245852	0.987840404	0.991272449	no
FGG	-0.000565765	-0.014936618	0.988087021	0.991470713	no
IGSF9	-0.000558032	-0.014732468	0.988249832	0.991584869	no
OR8A1	0.000542448	0.014321052	0.988577943	0.991864862	no
TAB3	-0.000539323	-0.014238545	0.988643743	0.991881659	no
DLX3	0.000534434	0.014109457	0.988746694	0.991935724	no
TTLL9	0.000508806	0.013432858	0.989286296	0.992427822	no
PLD5	-0.00050476	-0.013326044	0.989371484	0.992464036	no
U2AF2	0.000489774	0.012930412	0.989687011	0.992680805	no
MRPL2	-0.000485866	-0.012827236	0.989769297	0.992680805	no
TPSG1	0.000485198	0.0128096	0.989783363	0.992680805	no
FAAH2	-0.000485169	-0.012808836	0.989783972	0.992680805	no
MCAT	-0.000458852	-0.012114051	0.990338089	0.993113294	no
C9orf16	-0.0004577	-0.012083628	0.990362353	0.993113294	no
SLC38A4	0.00045769	0.012083367	0.99036256	0.993113294	no
C21orf119	0.000427722	0.011292195	0.990993555	0.993649904	no
GAGE8	0.000425912	0.011244389	0.991031682	0.993649904	no
AGR2	0.000420798	0.011109394	0.991139348	0.993649904	no
IRX3	-0.000420393	-0.011098703	0.991147874	0.993649904	no
ALG11	0.000418308	0.011043654	0.991191779	0.993649904	no
CALR3	-0.000418271	-0.011042669	0.991192564	0.993649904	no
UBTFL1	0.000412025	0.010877785	0.991324067	0.993684318	no
GDPD3	0.000411972	0.010876377	0.99132519	0.993684318	no
TXNL4A	-0.000403641	-0.010656428	0.991500611	0.993782566	no
MCTS1	0.000402648	0.010630223	0.991521511	0.993782566	no
TFF1	-0.000387434	-0.010228553	0.991841864	0.99405437	no
TMEM88B	-0.0003718	-0.009815794	0.992171064	0.994335014	no
TMEM18	0.000356202	0.009403997	0.992499497	0.994614862	no
FIS1	0.0003416	0.0090185	0.992806956	0.994818766	no
SDCCAG3	0.000340172	0.008980792	0.992837031	0.994818766	no
KRTAP17-1	0.000339528	0.008963802	0.992850582	0.994818766	no
HBBP1	0.000335269	0.008851353	0.992940267	0.994859325	no
TNS1	-0.000322927	-0.008525511	0.993200149	0.995070397	no
CRP	-0.000320544	-0.008462597	0.993250327	0.99507136	no
PLCG1	-0.000303155	-0.008003531	0.993616464	0.995328139	no
FABP6	-0.000301713	-0.007965459	0.99364683	0.995328139	no
HIST2H3D	0.000301357	0.007956061	0.993654325	0.995328139	no
RASL11B	0.000279678	0.007383717	0.994110813	0.995736062	no
SCARNA7	0.0002731	0.007210036	0.994249337	0.995825477	no
PGRMC1	0.000258813	0.006832848	0.994550173	0.996077446	no
SEMG1	-0.000254209	-0.006711316	0.994647105	0.996125182	no
MAGEC1	0.000250508	0.006613602	0.99472504	0.996153889	no
OR5H2	-0.00024307	-0.00641723	0.994881662	0.996261389	no
ZNF713	-0.00023495	-0.006202867	0.995052635	0.996383249	no
LOC440461	-0.000230032	-0.006073032	0.995156189	0.99643759	no
DNTT	0.000223255	0.005894108	0.995298896	0.996531128	no
C12orf44	0.000198221	0.005233182	0.995826042	0.99700412	no
C9orf27	0.000195635	0.005164902	0.9958805	0.99700412	no

BDNF	0.000193795	0.00511633	0.995919241	0.99700412	no
POLR2J4	-0.000189417	-0.005000749	0.996011427	0.997047038	no
KIAA1598	0.000164742	0.004349318	0.996531002	0.997517764	no
ZNF784	0.00015725	0.004151527	0.996688758	0.997626284	no
NT5DC2	-0.000154314	-0.004074003	0.996750591	0.997638784	no
OR56A4	-0.00015185	-0.00400895	0.996802477	0.997641328	no
STARD6	0.000139545	0.00368409	0.997061582	0.997851256	no
CIB4	-0.000121344	-0.003203567	0.997444844	0.998161769	no
DCTN2	-0.000120121	-0.003171288	0.99747059	0.998161769	no
ZNF716	-0.00011705	-0.003090219	0.99753525	0.998177069	no
GJB1	-0.000113004	-0.002983378	0.997620466	0.998212936	no
C14orf177	-8.45E-05	-0.002231192	0.998220407	0.998750141	no
CERCAM	-8.20E-05	-0.002165685	0.998272654	0.998750141	no
TSPY4	8.05E-05	0.002124442	0.998305549	0.998750141	no
GPCPD1	-7.24E-05	-0.001911726	0.998475211	0.998870451	no
MYOC	5.88E-05	0.001552357	0.998761843	0.999012986	no
MAML1	5.72E-05	0.001510173	0.998795489	0.999012986	no
PAX2	-5.66E-05	-0.001495291	0.998807359	0.999012986	no
EP400NL	-5.60E-05	-0.001477143	0.998821834	0.999012986	no
DEFB109P1	5.39E-05	0.001423337	0.998864749	0.999012986	no
ALDH1L1	1.41E-05	0.00037115	0.999703972	0.999802874	no
RHAG	-7.33E-06	-0.000193537	0.999845635	0.999895091	no
OXGR1	1.51E-06	3.99E-05	0.999968196	0.999968196	no
