# Supplementary material for: CD96, a new immune checkpoint, correlates with immune profile and clinical outcome of glioma
Source: Sci Rep. 2020 Jul 1;10:10768. doi: 10.1038/s41598-020-66806-z (PMC7330044; doi:10.1038/s41598-020-66806-z)
Supplement: Supplementary file 6 — Supplementary Information. [file 41598_2020_66806_MOESM6_ESM.pdf]

# CD96, a new immune checkpoint, correlates with immune profile and clinical outcome of glioma

**Fangkun Liu[1, 2 †], Jing Huang[3, 4], Fengqiong He[1, 2], Xiaodong Ma[5], Fan Fan[1, 2], Ming Meng[1, 2], Yang Zhuo[1, 2], and Liyang Zhang[1, 2 *]**

[1] Department of Neurosurgery, Xiangya Hospital, Central South University, Central South University; 87 Xiangya Road; Changsha, Hunan, 410008. China;

[2] Clinical Diagnosis and Therapy Center for Glioma of Xiangya Hospital, Central South University; 87 Xiangya Road; Changsha, Hunan, 410008. China;

[3] Department of Psychiatry, The Second Xiangya Hospital, Central South University, Changsha, Hunan 410011, China;

[4] Mental Health Institute of the Second Xiangya Hospital, Central South University, Chinese National Clinical Research Center on Mental Disorders (xiangya), Chinese National Technology Institute on Mental Disorders, Hunan Key Laboratory of Psychiatry and Mental Health, Changsha, Hunan 410011, China;

[5] Director and Training and Exchange Cooperation Center, Orient Science & Technology College, Hunan Agricultural University, Changsha, Hunan 410000, China.

[†]The first author;

[*]Corresponding Author:

Dr. Liyang Zhang MD, Ph. D

Department of Neurosurgery, Xiangya Hospital, Central South University

87 Xiangya Rd, Changsha, Hunan, 410008, China,

Email: zhangliyang@csu.edu.cn

GENE
GRAP2
NCF4
C1RL
GZMM
LAX1
HCST
C5AR1
TLR1
CST7
SERPINB9
TNFRSF9
NPC2
LAIR1
C3
LAMP3
SERPING1
TNFRSF10A
SAMSN1
CD6
HLA-DRA
GMFG
PIK3AP1
SLAMF1
MAN2B1
CD300A
MCOLN2
LTBR
BTK
MILR1
KLHL6
HLA-DOA
SECTM1
B2M
IGLL5
IL18
GCH1
CD79B
ITGB2
PSMB8
TRIM14
PIK3CD
IL10RB
THEMIS2
CD86
HLA-DQA1
COLEC12
PTGER4
CD68
CCR7
RGS1

PTPRC
CCR4
XCL1
SLA2
C1S
FCGR2A
EBI3
RIPK3
SAMHD1
CTLA4
TLR7
CD28
HLA-DPB1
BIN2
PLSCR1
TNFSF8
GZMB
FCGR3B
CYBB
C2
ARPC1B
FUCA1
CD247
TNFRSF14
LCP2
ICAM3
CCL5
PDCD1
LYZ
LCP1
PRKCH
ICOS
PDCD1LG2
FCER1G
LY9
C1QA
NOD2
GBP5
TLR2
IFITM1
PARP9
HAVCR2
CD3E
LY96
IL1R1
ITGAL
KLRB1
IL2RG
ZAP70
IL12RB1
VAMP8

FGL2
C1R
CD300C
CD4
DOCK2
FPR3
CD1D
SH2D1A
TRIM22
RNASE6
HLA-H
RAC2
CARD11
OSTF1
HLA-E
SWAP70
C3AR1
CTSW
TLR6
B4GALT1
CD40
LPXN
SERPINA1
RUNX1
PARP14
MNDA
TRAT1
FTL
STAP1
ITGB7
IFITM2
CD180
CTSS
LCK
CCR2
LTB
TLR8
HLA-DMB
CD7
CD27
S1PR4
CD226
HEXB
SIT1
CD3D
UBD
CMTM6
AIF1
MR1
NFAM1
TLR3

BATF
GAPT
FASLG
NBEAL2
TYROBP
GPR65
PECAM1
PML
SKAP1
IFITM3
PLCG2
CAPZA1
CCR1
ARHGAP9
BST2
LYN
SP100
CD80
TXK
NOD1
TRIM21
CFI
GZMA
LY75
CD79A
DOK3
IFNG
CREG1
TBC1D10C
SASH3
TMEM173
IFI30
CORO1A
CXCL9
TNFRSF1B
TRIM38
CD300LB
TEC
TCIRG1
GRN
C1QB
CD48
APOBEC3C
CD3G
IL7R
IFI35
NMI
IL16
CD96
HLA-DMA
HLA-DRB1

HLA-B
CD300LF
JAK3
APOBEC3G
MYO1G
UNC93B1
CTSZ
SIGLEC7
PSMB10
CD40LG
PSMB9
CTSC
