# Supplementary material for: CD96, a new immune checkpoint, correlates with immune profile and clinical outcome of glioma
Source: Sci Rep. 2020 Jul 1;10:10768. doi: 10.1038/s41598-020-66806-z (PMC7330044; doi:10.1038/s41598-020-66806-z)
Supplement: Supplementary file 7 — Supplementary Information. [file 41598_2020_66806_MOESM7_ESM.pdf]

CD96, a new immune checkpoint, correlates with immune profile and clinical outcome of glioma

Fangkun Liu[1, 2 †], Jing Huang[3, 4], Fengqiong He[1, 2], Xiaodong Ma[5], Fan Fan[1, 2], Ming Meng[1, 2], Yang Zhuo[1, 2], and Liyang Zhang[1, 2 *]

[1] Department of Neurosurgery, Xiangya Hospital, Central South University, Central South University; 87 Xiangya Road; Changsha, Hunan, 410008. China;

[2] Clinical Diagnosis and Therapy Center for Glioma of Xiangya Hospital, Central South University; 87 Xiangya Road; Changsha, Hunan, 410008. China;

[3] Department of Psychiatry, The Second Xiangya Hospital, Central South University, Changsha, Hunan 410011, China;

[4] Mental Health Institute of the Second Xiangya Hospital, Central South University, Chinese National Clinical Research Center on Mental Disorders (xiangya), Chinese National Technology Institute on Mental Disorders, Hunan Key Laboratory of Psychiatry and Mental Health, Changsha, Hunan 410011, China;

[5] Director and Training and Exchange Cooperation Center, Orient Science & Technology College, Hunan Agricultural University, Changsha, Hunan 410000, China.

[†]The first author;

[*]Corresponding Author:

Dr. Liyang Zhang MD, Ph. D

Department of Neurosurgery, Xiangya Hospital, Central South University

87 Xiangya Rd, Changsha, Hunan, 410008, China,

Email: zhangliyang@csu.edu.cn

GENE
GRAP2
NCF4
C1RL
LILRB2
GZMM
SLC2A5
FGR
HLA-DRB5
SPN
LAX1
PTPN22
HCST
A2M
SIGLEC9
TLR1
FOXP3
CST7
CCL19
NPC2
LAIR1
C3
BTN3A1
LAMP3
SERPING1
TICAM2
TNFRSF10A
SAMSN1
FCN1
CD6
HLA-DRA
GMFG
PIK3AP1
SLAMF1
MAN2B1
PILRA
IL6R
CD53
CIITA
MCOLN2
LTBR
LILRA6
PTPN2
GFI1
HK3
GAA
BTK
HCK
IFI16
KLHL6
CARD9

CTSB
ATP8B4
SLA
ITGB1
NAIP
HLA-DOA
JAK2
CASP1
CD8A
B2M
NCF1
ELF4
TLR5
ELF1
FCGR3A
IL18
CD93
TRPM2
ESR1
PLEKHO2
GCH1
CD79B
ITGB2
TGFB1
EOMES
IL10RB
DNASE1L1
APOBEC3F
CD86
SLC11A1
POU2F2
TNFAIP3
TREML2
HLA-DQA1
TNFSF13
SLAMF6
PTGER4
CD68
CCR7
PRDM1
PTPRC
CCR4
CD14
XCL1
SLA2
C1S
FCGR2A
CLEC4D
EBI3
FCGRT
CCR6

SPPL2A
IL10
TRIM34
RIPK3
LILRB3
TLR7
CLEC7A
CD28
HLA-DPB1
BIN2
CD74
TNFSF8
NCKAP1L
HLA-DQA2
CYBB
C2
ARPC1B
FUCA1
CD247
TNFRSF14
PTPN6
IRF2
SLAMF7
CXCL10
LCP2
TNFRSF13B
ICAM3
IL4R
CCL5
NLRP1
SIGLEC5
PDCD1
CD8B
CD84
LYZ
LCP1
LRG1
PRKCH
FCGR1A
ICOS
NFKB1
MANBA
CTSA
IGSF6
HLA-DOB
CD200R1
WIPF1
PDCD1LG2
IL12B
FCER1G
LY9

C1QA
NOD2
FUT7
GBP5
PIK3R6
TLR2
CASP4
CLEC10A
HAVCR2
CD3E
LY96
IL1R1
PYCARD
ITGAL
KLRB1
CD1C
IL2RG
ACTR3
ZAP70
RNF135
IL12RB1
VAMP8
FGL2
CYBA
C1R
CD300C
APOBEC3D
C1QC
BTLA
NLRC4
CD4
DOCK2
FPR3
CD1D
SH2D1A
ALOX5
TRIM22
RNASE6
CD33
FCGR2B
RAC2
ADAMDEC1
CARD11
OSTF1
HLA-E
MYO1C
LILRB4
BTN3A2
POU2AF1
CLEC2B
TNFSF10

C3AR1
TNFSF15
NCF2
AICDA
CTSW
TLR6
B4GALT1
CD40
CASP8
FCRL3
PPARG
PRKCD
LPXN
SERPINA1
RUNX1
HLA-DQB1
PARP14
MNDA
TRAT1
FTL
ITGB7
CLEC4C
CD180
FES
CTSS
LCK
NCR3
HLA-DPA1
CCR2
LTB
TLR8
HLA-DMB
CD7
CD27
S1PR4
CD226
KYNU
IFNGR2
CAP1
IRAK3
HEXB
WAS
IL15
LAT2
VSIG4
SIT1
CD3D
ACPP
STAT6
CMTM6
AIF1

MR1
CLEC6A
S100A11
RAP2B
NFAM1
TLR3
BATF
MVP
XCL2
GAPT
TNFSF12-TNFSF13
CXCR5
ITGA4
ITGAM
ARPC2
FASLG
HLA-F
NBEAL2
TYROBP
GPR65
STXBP2
OSCAR
PECAM1
MS4A1
MYD88
CCR8
CNR2
CD244
PLCG2
CAPZA1
CCR1
ARHGAP9
LYN
SP100
LGMN
GPR183
LILRB1
CD80
TXK
TRIM21
CFI
GZMA
PIK3CG
LY75
SNAP23
CD79A
VNN1
SERPINB1
SYK
DOK3
IQGAP1

CREG1
TBC1D10C
APOBEC3H
IRF5
SASH3
TMEM173
IFI30
FCGR2C
CORO1A
NCR1
CXCL9
CD19
TNFRSF1B
LGALS9
TRIM38
ISG20
TEC
TCIRG1
BTN3A3
IKBKE
GRN
C1QB
TNFRSF11A
SIGLEC10
NFATC2
CD48
APOBEC3C
INPP5D
CLEC12A
CD3G
IL7R
NMI
CCL21
IL16
HFE
CD96
CD1E
PTAFR
HLA-DMA
HLA-DRB1
HLA-B
CD300LF
JAK3
APOBEC3G
CCR5
MYO1G
UNC93B1
RAB27A
HLA-DQB2
CLEC4A
IRF8

CTSZ
ALDH3B1
SIGLEC7
CSF1R
APBB1IP
CXCL16
ZNF683
TNFAIP8L2
CD40LG
KLRD1
VAV1
CTSC
